# FAK suppresses antigen processing and presentation to promote immune evasion in pancreatic cancer

**DOI:** 10.1136/gutjnl-2022-327927

**Published:** 2023-03-28

**Authors:** Marta Canel, Aleksandra Dominika Sławińska, David W Lonergan, Ashwin Adrian Kallor, Rosie Upstill-Goddard, Catherine Davidson, Alex von Kriegsheim, Andrew V Biankin, Adam Byron, Javier Alfaro, Alan Serrels

**Affiliations:** 1 Cancer Research UK Scotland Centre, Institute of Genetics and Cancer, University of Edinburgh, Edinburgh, UK; 2 International Centre for Cancer Vaccine Science, University of Gdansk, Gdansk, Poland; 3 The Wolfson Wohl Cancer Research Centre, Institute of Cancer Sciences, University of Glasgow, Glasgow, UK; 4 Centre for Inflammation Research, Institute for Regeneration and Repair, University of Edinburgh, Edinburgh, UK; 5 Division of Molecular and Cellular Function, School of Biological Sciences, Faculty of Biology, Medicine and Health, Manchester Academic Health Science Centre, University of Manchester, Manchester, UK

**Keywords:** pancreatic cancer, antigen presentation, antigen processing, immune response, cell adhesion molecules

## Abstract

**Objective:**

Immunotherapy for the treatment of pancreatic ductal adenocarcinoma (PDAC) has shown limited efficacy. Poor CD8 T-cell infiltration, low neoantigen load and a highly immunosuppressive tumour microenvironment contribute to this lack of response. Here, we aimed to further investigate the immunoregulatory function of focal adhesion kinase (FAK) in PDAC, with specific emphasis on regulation of the type-II interferon response that is critical in promoting T-cell tumour recognition and effective immunosurveillance.

**Design:**

We combined CRISPR, proteogenomics and transcriptomics with mechanistic experiments using a Kras^G12D^p53^R172H^ mouse model of pancreatic cancer and validated findings using proteomic analysis of human patient-derived PDAC cell lines and analysis of publicly available human PDAC transcriptomics datasets.

**Results:**

Loss of PDAC cell-intrinsic FAK signalling promotes expression of the immunoproteasome and Major Histocompatibility Complex class-I (MHC-I), resulting in increased antigen diversity and antigen presentation by FAK-/- PDAC cells. Regulation of the immunoproteasome by FAK is a critical determinant of this response, optimising the physicochemical properties of the peptide repertoire for high affinity binding to MHC-I. Expression of these pathways can be further amplified in a STAT1-dependent manner via co-depletion of FAK and STAT3, resulting in extensive infiltration of tumour-reactive CD8 T-cells and further restraint of tumour growth. FAK-dependent regulation of antigen processing and presentation is conserved between mouse and human PDAC, but is lost in cells/tumours with an extreme squamous phenotype.

**Conclusion:**

Therapies aimed at FAK degradation may unlock additional therapeutic benefit for the treatment of PDAC through increasing antigen diversity and promoting antigen presentation.

**WHAT IS ALREADY KNOWN ON THIS TOPIC**

- Focal adhesion kinase (FAK) kinase activity is elevated in human pancreatic ductal adenocarcinoma (PDAC) and FAK kinase inhibitors in combination with immunotherapy and chemotherapy can impair tumour growth in a mouse model of PDAC.
- FAK kinase inhibitors can reprogram the immunosuppressive tumour microenvironment in a mouse model of PDAC.
- Tumour neoantigens are rare in pancreatic cancer and there is no clear association between neoantigen load, effector CD8 T-cell infiltration and pancreatic cancer patient survival.
- Interferon-γ signalling is often downregulated in tumours resulting in immune evasion.

**WHAT THIS STUDY ADDS**

- FAK regulates antigen processing and presentation in a kinase-independent manner that requires FAK nuclear translocation.
- Expression of the immunoproteasome subunit Psmb8 in response to FAK loss optimises the physicochemical properties of the antigen repertoire for high affinity binding to Major Histocompatibility Complex class-I (MHC-I) and is necessary for effective immunosurveillance in a PDAC mouse model.
- Co-depletion of FAK and STAT3 promotes STAT1-dependent hyperactivation of Psmb8 and MHC-I, resulting in improved tumour control.
- STAT3 depletion promotes CD8 T-cell infiltration into PDAC tumours independent of FAK, but CD8 T-cell engagement does not occur without co-deletion of FAK.

**HOW THIS STUDY MIGHT AFFECT RESEARCH, PRACTICE OR POLICY**

- Next generation FAK targeted therapies aimed at protein degradation rather than simply kinase inhibition may offer additional therapeutic benefit for the treatment of PDAC.
- PDAC patients may benefit from co-targeting FAK and STAT3 via increased cancer cell antigenicity and tumour-reactive CD8 T-cell infiltration.
- The lack of tumour neoantigens in PDAC does not preclude effective immunosurveillance and greater attention should be given to the potential of immunogenic self-antigens in this regard.
- PDAC cell heterogeneity impacts FAK immunoregulatory function.

## Introduction

The contribution of pancreatic cancer to global cancer-related mortality continues to increase, with an almost uniformly fatal outcome.^1 2^ Current chemotherapy regimens are minimally effective^3^ and immune checkpoint inhibitors that have shown promise in the treatment of other cancer types have generally failed to show patient benefit for pancreatic cancer.^4 5^ With pancreatic cancer set to become the second-leading cause of cancer mortality within the next decade,^6^ there is an urgent need to identify new therapeutic strategies for the treatment of this disease.

Effective T-cell responses require the presence of immunogenic tumour antigens. Mutations or gene rearrangements can give rise to tumour ‘neoantigens’ which are recognised by the immune system as non-self. In contrast, proteins differentially expressed in cancer can give rise to non-mutated tumour-associated antigens (TAAs), which despite being classed as self-antigens, can still be recognised by the immune system.^4^ Neoantigens have been identified as T-cell targets in rare long-term survivors of pancreatic ductal adenocarcinoma (PDAC)^7^ and are associated with increased expression of an antitumour immunity gene signature in human pancreatic cancer patients with double-strand break repair and mismatch repair signatures.^8^ However, outside of these rare cases, no clear association between neoantigen load, effector T-cell infiltration and pancreatic cancer patient survival has been identified.^9 10^ Studies using the *Pdx-1 Cre; LSL-Kras^G12D/+^; LSL-Trp53^R172H/+^* (KPC) mouse model of PDAC have only identified a very low number of somatic mutations with even fewer predicted neoantigens.^11 12^ Despite this, some tumours can exhibit high T-cell infiltration^12^ and combination therapies can unlock effective antitumour CD8 T-cell responses.^13–15^ Such observations suggest that non-mutated TAAs may also be important in T-cell-mediated immunity against pancreatic tumours.

Interferon-γ (IFNγ) is an essential mediator of immunity, promoting T-cell tumour recognition through regulating multiple pathways, including those involved in antigen processing and presentation.^16^ Expression of Major Histocompatibility Complex class-I (MHC-I), which is induced in response to IFNγ, is often downregulated in tumours, resulting in immune evasion due to a lack of antigen presentation.^17^ The composition of the proteasome that degrades ubiquitinated proteins to generate peptide antigens is also regulated by IFNγ, with the catalytic β subunits of the constitutive proteasome, β1, β2 and β5, being replaced by Psmb8 (β5i), Psmb9 (β1i) and Psmb10 (β2i) to form the immunoproteasome.^18 19^ Immunoproteasome deficiency has been linked to a reduction in the diversity of the antigen repertoire and poor prognosis in patients with non-small cell lung cancer.^20^ Similar findings have been reported in the context of melanoma, where immunoproteasome expression has also been identified as a better predictor of response to immune checkpoint therapy than mutational burden.^21^ Therefore, deregulated IFNγ signalling is an important mechanism of immune evasion in cancer.

Here, we identify a novel kinase-independent, nuclear-dependent role for the non-receptor protein tyrosine kinase focal adhesion kinase (FAK) in suppressing antigen processing and presentation to promote immune evasion in PDAC. We show that FAK deletion in cancer cells derived from mouse KPC tumours reprogrammes the cellular response to IFNγ, increasing both antigen diversity through activation of the immunoproteasome and surface presentation through upregulation of MHC-I to promote immunosurveillance. Mechanistically, we find that expression of the immunoproteasome and MHC-I is dependent on interferon regulatory factor 1 (IRF-1) and that FAK loss leads to upregulation of the class-I transcriptional co-activator NLRC5. In addition, we also find that FAK stabilises the STAT1/STAT3 heterodimer and show that co-depletion of FAK and STAT3 leads to STAT1-dependent hyperactivation of these pathways, further amplifying the effector CD8 T-cell response. In human PDAC, proteomic analysis of 13 patient-derived PDAC cell lines and bioinformatic analysis of International Cancer Genome Consortium and The Cancer Genome Atlas (TCGA) transcriptomics data from PDAC tumours also identified FAK-dependent suppression of antigen processing/presentation and IFNγ signalling. Lastly, we identify a previously unappreciated role for PDAC molecular subtype in impacting FAK function with respect to regulation of antigen processing and presentation pathways. These findings highlight the need to develop a new generation of FAK targeted therapies aimed at protein degradation in order to fully harness the therapeutic potential of targeting FAK for the treatment of PDAC.

## Results

### FAK regulates antigen processing and presentation pathways

**Figure 1:**
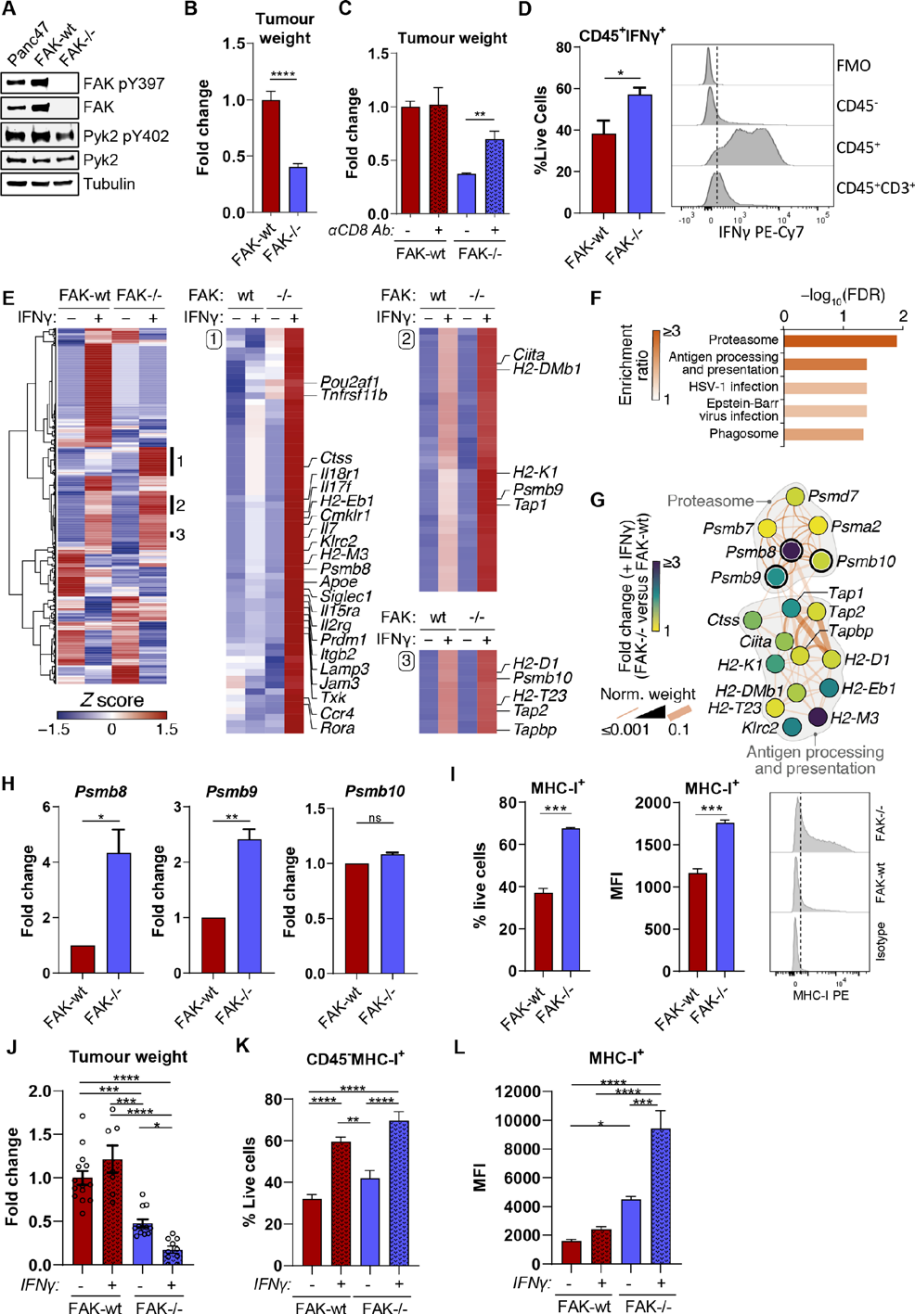
FAK regulates antigen processing and presentation pathways in *Kras^+/G12D^p53^+/R172H^PDXcre* pancreatic cancer cells. (A) Representative anti-FAK, FAK pY397, Pyk2 and Pyk2 pY402 western blot using whole cell lysates from parental Panc47 cells and FAK-wt and FAK-/- clonal cell lines. Anti-tubulin antibody used as a loading control. (B) Fold-change in tumour weight relative to FAK-wt tumours 2 weeks postimplantation into the pancreas of C57BL/6 mice. n=13–16 tumours per group. (C) Fold-change in tumour weight relative to FAK-wt tumours 2 weeks postimplantation into the pancreas of C57BL/6 mice. Mice were either treated with isotype control or anti-CD8 T-cell-depleting antibodies. n=4–6 mice per group. (D) Flow cytometry analysis of IFNγ expression in FAK-wt and FAK-/- tumours. Left, quantification of the frequency of CD45^+^IFNγ^+^ cells as a percentage of live cells; *right*, representative histograms of IFNγ staining in CD45^-^, CD45^+^ and CD45^+^CD3^+^ cells. FMO=fully stained sample minus IFNγ antibody. n=4. (E) Cluster analysis of NanoString nCounter gene expression data acquired using the mouse PanCancer Immune Profiling panel. Clusters 1–3 are annotated with the top 20 most over-expressed genes in IFNγ-stimulated FAK-/- cells compared with FAK-wt cells and further annotated with immunoproteasome and antigen processing and presentation components identified in (F, G). (F) KEGG pathway enrichment analysis of genes in clusters 1–3 in E. (G) Functional association network analysis of hits in the top two most enriched pathways in (F). Network edges (connecting lines) represent reported physical (dark orange) or predicted (light orange) interactions. Pathway membership is delineated in grey. Nodes (circles) representing immunoproteasome components have thick node borders. (H) Relative quantification of *Psmb8*, *Psmb9* and *Psmb10* gene expression using qRT-PCR following stimulation with IFNγ. n=3. (I) Flow cytometry quantification of MHC-I surface expression on FAK-wt and FAK-/- cells following IFNγ stimulation. n=3. (J) Fold-change in tumour weight relative to untreated FAK-wt tumours 2 weeks postimplantation into the pancreas of C57BL/6 mice. Mice were treated daily with PBS or IFNγ by intraperitoneal injection from day 8 until day 14. n=7–14 mice per group. (K) Flow cytometry quantification of the frequency of CD45^-^MHC-I^+^ cells in FAK-wt and FAK-/- tumours±IFNγ as a percentage of live cells. n=4–8 tumours per group. (L) Flow cytometry quantification of the median fluorescence intensity of MHC-I expression in CD45^-^MHC-I^+^ cells in FAK-wt and FAK-/- tumours±IFNγ. n=4–8 tumours per group. IFNγ treatment was 10 ng/mL for 24 hours (E–H) or 72 hours (I). Unless otherwise stated, all data are represented as mean±SEM. Statistical significance in B, D, H and I was calculated using an unpaired t-test. Statistical significance in (C, J, K, L) was calculated using a one-way ANOVA with Tukey’s multiple comparison test. *p≤0.05, **p≤0.01, ***p≤0.001, ****p≤0.0001. ANOVA, analysis of variance; FAK, focal adhesion kinase; MHC-I, Major Histocompatibility Complex class-I.

FAK kinase activity is elevated in human PDAC^14^ and FAK inhibitors, either alone or in combination with immunotherapies, can impair tumour growth in mouse models of PDAC.^14 22 23^ Clinical trials are now testing FAK inhibitors in combination with immune checkpoint inhibitors in patients with advanced pancreatic cancer (ClinicalTrials.gov; NCT02546531, NCT02758587, NCT03727880). However, our understanding of FAK as an immune modulator in PDAC is limited to the effects of FAK inhibitors on the tumour stroma and immune microenvironment in murine models of pancreatic cancer.^14 23^ Therefore, we set out to better understand how FAK regulates the antitumour immune response with particular focus on cancer cell-intrinsic mechanisms of immune evasion. We first used CRISPR-Cas9 gene editing to delete *ptk2* (FAK gene) expression in Panc47 cells, a syngeneic cell line isolated from PDAC arising in fully back-crossed C57BL/6 KPC mice, and reconstituted wild-type FAK (FAK-wt) expression into a clonal Panc47 FAK-/- (herein termed FAK-/-) cell line (figure 1A). No increase in Pyk2 expression or phosphorylation on tyrosine-402 were observed following FAK loss. 0.5×10^6^ FAK-wt or FAK-/- cells were then implanted into the pancreas of C57BL/6 mice and tumours harvested and weighed after 2 weeks. FAK loss resulted in a tumour growth delay (figure 1B). We have previously shown using a murine model of skin squamous cell carcinoma that cancer cell-intrinsic deletion of FAK expression can promote an antitumour CD8 T-cell response via modulation of the effector CD8 T-cell: regulatory T-cell ratio in tumours.^24^ To determine whether the observed delay in FAK-/- tumour growth was also dependent on CD8 T-cells, C57BL/6 mice were treated with either isotype control or anti-CD8 T-cell-depleting antibodies and 0.5×10^6^ FAK-wt or FAK-/- cells were implanted into the pancreas. Tumours were harvested and weighed 2 weeks postimplantation. CD8 T-cell depletion restored a significant proportion of the delay in FAK-/- tumour growth, but had no effect on the growth of FAK-wt tumours (figure 1C). Thus, FAK-loss was sufficient to promote an antitumour CD8 T-cell response that could restrain tumour growth.

IFNγ signalling plays an important role in promoting T-cell tumour recognition,^16^ and IFNγ is secreted by multiple cell types present within the tumour microenvironment (TME).^17^ We, therefore, sought to determine whether IFNγ was present within the TME of FAK-wt and FAK-/- tumours, and whether FAK deletion altered the response of PDAC cells to this important proinflammatory cytokine. 0.5×10^6^ FAK-wt or FAK-/- cells were implanted into the pancreas of C57BL/6 mice and tumours harvested 2 weeks later for flow cytometry analysis. FAK-/- tumours exhibited a significant increase in the number of cells positive for IFNγ expression when compared with FAK-wt tumours (figure 1D, left), with almost all of the IFNγ being secreted by immune (CD45^+^) cells (figure 1D, right). Interestingly, while T-cells were a source of IFNγ, they did not appear to be the major source in these tumours. To investigate whether FAK expression had any impact on the cancer cell response to IFNγ, we treated FAK-wt and FAK-/- cells in vitro with 10 ng/mL IFNγ and measured (1) the secretion of chemokines and cytokines, and (2) the expression of 770 immune-related genes. Forward-phase array profiling of tumour cell-secreted proteins identified a number of IFNγ-induced chemokines and cytokines likely to mediate paracrine signalling with the TME, several of which were downregulated in response to FAK loss and are protumourigenic, such as interleukin-6 (IL-6),^25 26^ in the context of pancreatic cancer (online supplemental figure 1A). We performed NanoString nCounter digital gene expression analysis using the PanCancer Immune Profiling Panel, which identified subsets of IFNγ-induced genes that were upregulated in FAK-/- cells compared with FAK-wt cells (figure 1E and online supplemental table 1). Pathway enrichment analysis of these gene clusters identified the proteasome and antigen processing and presentation as the two most significantly enriched pathways (figure 1F). Network analysis of these pathway hits showed direct association of the immunoproteasome components Psmb8, 9 and 10 with the antigen presentation module and enrichment in IFNγ-treated FAK-/- cells of several key pathway components, including *Psmb8*, *Psmb9*, *Tap1* and *H2-K1* (figure 1G and online supplemental figure 1B). Similar findings were observed when FAK-wt and FAK-/- cells were cultured in conditioned media from activated CD8 T-cells (online supplemental figure 1C and D and online supplemental table 2). qRT-PCR confirmed the upregulation of IFNγ-induced *Psmb8* and *Psmb9* expression in FAK-/- cells when compared with FAK-wt cells (figure 1H). Expression of *Psmb10* was not regulated by FAK. Flow cytometry analysis confirmed the upregulation of IFNγ-induced MHC-I (including H2-K) expression on FAK-/- cells when compared with FAK-wt cells, with both an increase in the proportion of cells positive for expression of MHC-I and the level of MHC-I expression (figure 1I). To determine whether similar modulation of the IFNγ response also occurred in vivo, 0.5×10^6^ FAK-wt or FAK-/- cells were implanted into the pancreas of C57BL/6 mice and tumours allowed to develop for 7 days. On day 8, half of each cohort was dosed by intraperitoneal injection with vehicle (PBS) and the other half with IFNγ. Treatment was administered daily for a total of 7 days, and on day 14 tumours were harvested, weighed and analysed by flow cytometry. IFNγ treatment resulted in a small increase in the average weight of FAK-wt tumours; however, this was not statistically significant (figure 1J). In contrast, IFNγ treatment resulted in a significant decrease in the growth of FAK-/- tumours (figure 1J). Flow cytometry analysis of MHC-I expression showed a greater proportion of CD45^-^ cells (including tumour cells) positive for expression of MHC-I in both FAK-wt and FAK-/- tumours in response to IFNγ treatment (figure 1K). However, the median fluorescence intensity (MFI) of MHC-I-positive staining was significantly lower in FAK-wt tumours when compared with FAK-/- tumours, irrespective of IFNγ treatment (figure 1L), supporting in vitro findings that FAK loss upregulates MHC-I expression on the cell surface. Overall, these findings imply that FAK deletion reprogrammes the response of PDAC cells to IFNγ, resulting in increased expression of pathways important for T-cell tumour recognition.

10.1136/gutjnl-2022-327927.supp1 Supplementary data

10.1136/gutjnl-2022-327927.supp11 Supplementary data

10.1136/gutjnl-2022-327927.supp12 Supplementary data

### FAK-dependent regulation of antigen processing and presentation is kinase-independent but requires FAK nuclear translocation

**Figure 2:**
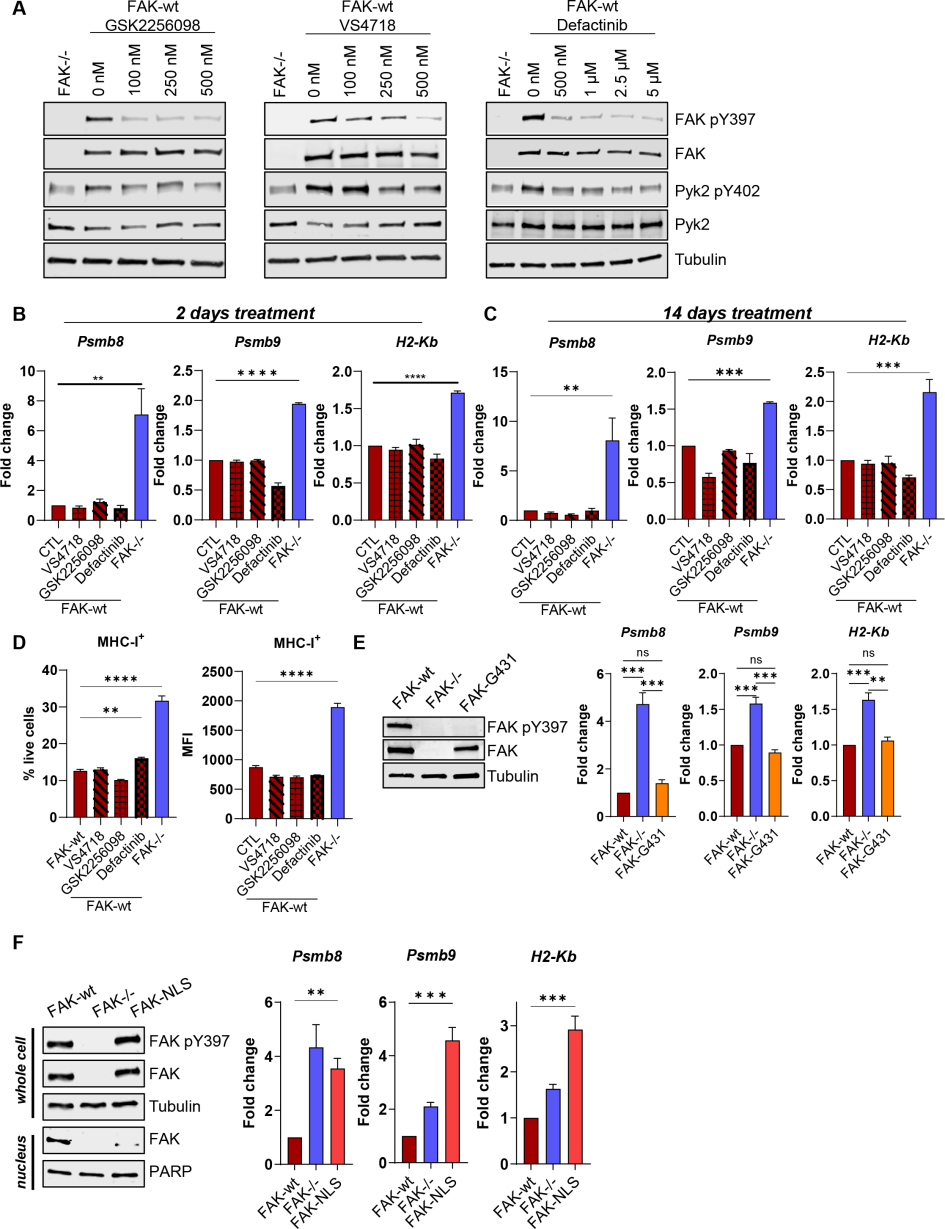
FAK-dependent suppression of antigen processing and presentation is independent of kinase activity but requires nuclear translocation. (A) Anti-FAK, FAK pY397, Pyk2 and Pyk2 pY402 western blots of FAK-wt cells treated with a range of concentrations of GSK2256098, VS4718 and Defactinib. Anti-tubulin used as a loading control. (B, C) Relative quantification of *Psmb8, Psmb9* and *H2-Kb* expression in IFNγ stimulated FAK-wt cells using qRT-PCR following either 2 days or 14 days of treatment with FAK kinase inhibitors. n=3. (D) Relative quantification of the percentage of cells positive for MHC-I expression and the median fluorescence intensity (MFI) of MHC-I expression using flow cytometry. FAK-wt cells were treated with FAK kinase inhibitors for 2 days prior to IFNγ stimulation in the presence of inhibitor. n=3. (E) Left—Representative anti-FAK and anti-FAK pY397 western blot using whole cell lysates isolated from FAK-wt, FAK-/- and FAK-G431 cells. Anti-tubulin used as a loading control. Right—Relative quantification of *Psmb8*, Ps*mb9* and *H2-Kb* expression using qRT-PCR following IFNγ stimulation. n=3. (F) Left—Representative anti-FAK and anti-FAK pY397 western blot from whole cell and nuclear lysates isolated from FAK-wt, FAK-/- and FAK-NLS cells. Right—Relative quantification of *Psmb8, Psmb9* and *H2-Kb* expression using qRT-PCR following IFNγ stimulation. n=3. IFNγ treatment was 10 ng/mL for 24 hours (B, C, E, F) or 72 hours (D). Data in (B–F) represented as mean±SEM. Statistical significance in (B–F) was calculated using a one-way ANOVA with Tukey’s multiple comparison test. **p≤0.01, ***p≤0.001, ****p≤0.0001. ANOVA, analysis of variance; FAK, focal adhesion kinase.

A number of FAK kinase inhibitors are currently in clinical development and are being tested in combination with immunotherapies for the treatment of PDAC.^27^ Therefore, to determine whether regulation of antigen processing and presentation pathways was dependent on FAK kinase activity we used three different FAK kinase inhibitors: GSK2256098, VS4718 and Defactinib. To identify the lowest dose required to achieve maximum inhibition of FAK phosphorylation on tyrosine-397, the autophosphorylation site used as a surrogate readout of FAK kinase activity, we first treated FAK-wt cells with a range of drug concentrations. This identified 100 nM of GSK2256098 and 500 nM of both VS4718 and Defactinib as optimal concentrations (figure 2A). We next treated FAK-wt cells with these concentrations of each inhibitor for either 2 days or 14 days and then stimulated cells with IFNγ in the presence of inhibitor. qRT-PCR showed that neither regulation of *Psmb8, Psmb9* nor *H2-Kb* transcription was dependent on FAK kinase activity (figure 2B,C). Flow cytometry analysis also confirmed that regulation of MHC-I surface expression on FAK-wt cells was unchanged following treatment with FAK kinase inhibitors (figure 2D). To further support these findings, we also re-expressed a FAK kinase-deficient mutant, FAK-G431,^28^ into FAK-/- cells at similar levels to FAK-wt cells (figure 2E—left). FAK-wt, FAK-/- and FAK-G431 cells were stimulated with IFNγ and qRT-PCR used to quantify *Psmb8, Psmb9* and *H2-Kb* gene expression (figure 2E—right). FAK-G431 cells expressed comparable levels of *Psmb8, Psmb9* and *H2-Kb* to FAK-wt cells. Thus, FAK-dependent regulation of *Psmb8, Psmb9* and *H2-Kb* is independent of FAK kinase activity.

We have previously shown that FAK can localise to the nucleus where it can interact with transcription factors and transcriptional regulators to control chemokine and cytokine expression.^24^ We therefore re-expressed a FAK mutant deficient in nuclear translocation (FAK-NLS) into FAK-/- cells at comparable levels to FAK-wt cells (figure 2F—left) and stimulated FAK-wt, FAK-/- and FAK-NLS cells with IFNγ. qRT-PCR showed that FAK-dependent suppression of *Psmb8, Psmb9* and *H2-Kb* transcription required nuclear FAK (figure 2F—right). Thus, FAK-dependent regulation of immunoproteasome and MHC-I genes is independent of FAK kinase activity but requires FAK nuclear translocation.

### Psmb8 deletion restores FAK-/- tumour growth

**Figure 3:**
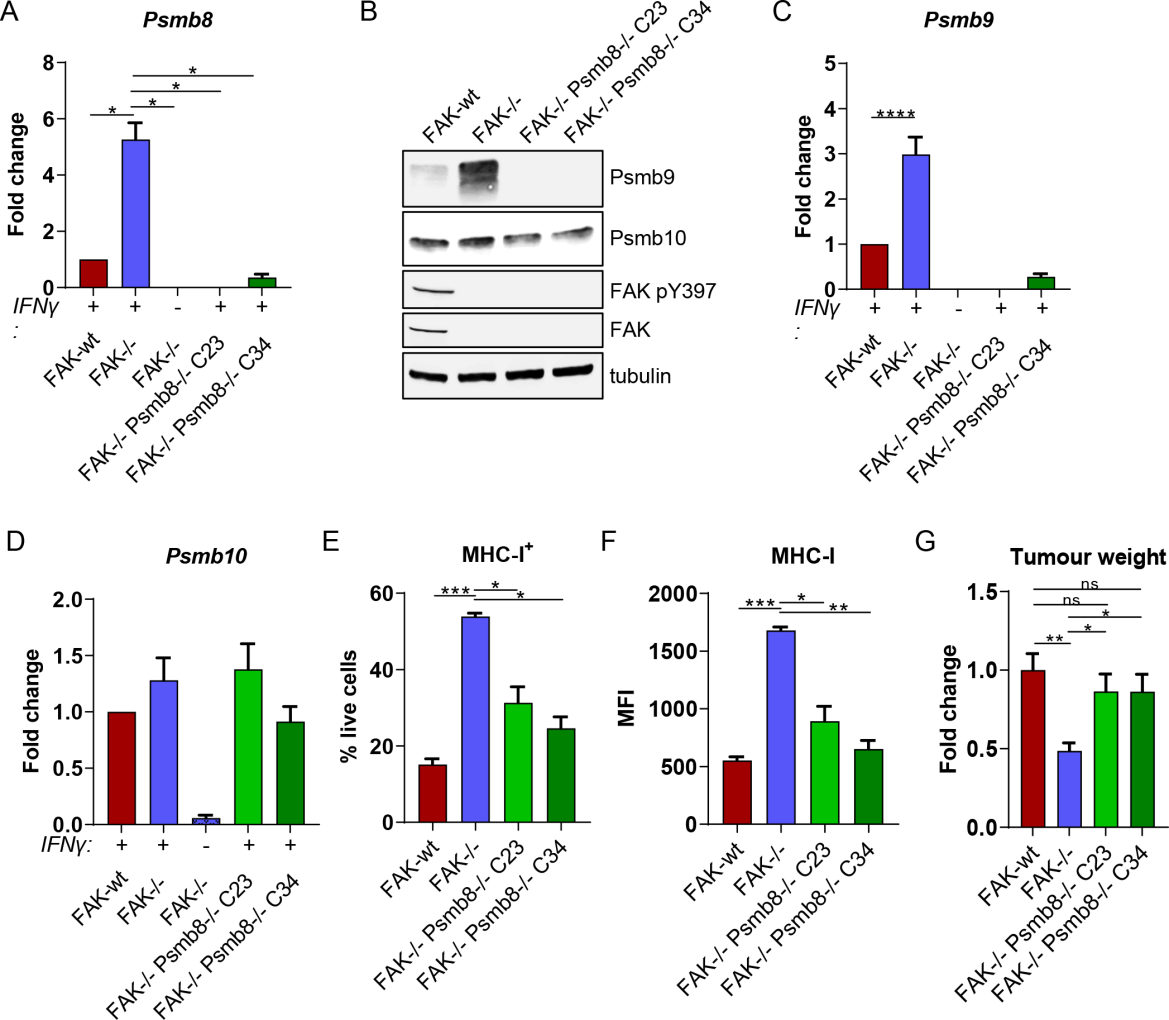
*Psmb8* deletion restores FAK-/- tumour growth. (A) Relative quantification of *Psmb8* expression using qRT-PCR following IFNγ stimulation. n=3. (B) Representative western blot showing expression of Psmb9, Psmb10, FAK pY397 and FAK in FAK-wt, FAK-/-, FAK-/-Psmb8-/-C23 and FAK-/-Psmb8-/-C34 cells following IFNγ stimulation. Anti-tubulin was used as a loading control. (C) Relative quantification of *Psmb9* expression using qRT-PCR following IFNγ stimulation. n=3. (D) Relative quantification of *Psmb10* expression using qRT-PCR following IFNγ stimulation. n=3. (E) Flow cytometry quantification of the frequency of cells positive for MHC-I expression as a percentage of live cells following IFNγ stimulation. n=3. (F) Flow cytometry quantification of the median fluorescence intensity of MHC-I expression following IFNγ stimulation. n=3. (G) Fold change in tumour weight relative to FAK-wt tumours 2 weeks postimplantation into the pancreas of C57BL/6 mice. n=6–9 tumours per group. IFNγ treatment was 10 ng/mL for 24 hours (A–D) or 72 hours (E and F). All data represented as mean±SEM. Statistical significance was calculated using a one-way ANOVA with Tukey’s multiple comparison test. *p≤0.05, **p≤0.01, ***p≤0.001, ****p≤0.0001. ANOVA, analysis of variance; FAK, focal adhesion kinase; MHC-I, Major Histocompatibility Complex class-I.

Psmb8 is a key member of the immunoproteasome and is essential for maturation of the preproteasome containing Psmb9 and Psmb10, and subsequent acquisition of catalytic activity.^29 30^ Little is known about the immunoproteasome in the context of pancreatic cancer and whether it can contribute to the induction of antitumour T-cell responses. We, therefore, used CRISPR-Cas9 gene editing to delete *Psmb8* expression in FAK-/- cells, generating two independent *Psmb8* knockout clones termed C23 and C34 (figure 3A). Western blotting of whole cell lysates from IFNγ-stimulated FAK-wt, FAK-/-, FAK-/-Psmb8-/-C23 and FAK-/-Psmb8-/-C34 cells identified that *Psmb8* knockout also resulted in loss of Psmb9 expression, but had no effect on Psmb10 (figure 3B). qRT-PCR further confirmed these results (figure 3C,D), suggesting that loss of *Psmb9* expression was due to transcriptional downregulation. *Psmb8* knockout also resulted in downregulation of MHC-I expression (figure 3E,F), implying that the activity of Psmb8 was important for sustaining the elevated MHC-I expression observed in FAK-/- cells. To determine whether *Psmb8* upregulation contributed to the growth defect of FAK-/- tumours, 0.5×10^6^ FAK-wt, FAK-/-, FAK-/-Psmb8-/-C23 or FAK-/-Psmb8-/-C34 cells were implanted into the pancreas of C57BL/6 mice and tumours harvested and weighed after 2 weeks. *Psmb8* knockout was sufficient to rescue the FAK-/- tumour growth delay (figure 3G), suggesting that *Psmb8* upregulation was critical in restraining FAK-/- tumour growth. These findings were further validated using two independent shRNAs to deplete *Psmb8* expression in FAK-/- cells (online supplemental figure 2A), both of which promoted the growth of FAK-/- tumours (online supplemental figure 2B).

10.1136/gutjnl-2022-327927.supp2 Supplementary data

In addition to the constitutive and immunoproteasomes, two further proteasomes have been identified and termed intermediate proteasomes.^19^ These contain either Psmb8 or Psmb8 and Psmb9 from the immunoproteasome, with the remaining catalytic subunits coming from the constitutive proteasome. Therefore, we also investigated the requirement for Psmb9 in regulating the growth of FAK-/- tumours. CRISPR-Cas9 gene editing was used to delete *Psmb9* expression, generating two *Psmb9* knockout clones termed C2 and C6 (online supplemental figure 3A). *Psmb9* deletion resulted in a reduction in *Psmb8* expression. However, *Psmb8* expression was still significantly higher in FAK-/-Psmb9-/-C2 and C6 cells when compared with FAK-wt cells (online supplemental figure 3B). The percentage of cells positive for expression of MHC-I was variable between *Psmb9-/-* clones but remained significantly higher than FAK-wt cells (online supplemental figure 3C). A small decrease in the MFI of MHC-I expression was observed in FAK-/-Psmb9-/-C2 and C6 cells when compared with FAK-/- cells; however, MHC-I expression was still significantly higher than in FAK-wt cells (online supplemental figure 3D). To determine whether *Psmb9* upregulation in FAK-/- cells contributed to the defect in tumour growth, 0.5×10^6^ FAK-wt, FAK-/-, FAK-/-Psmb9C2 or FAK-/-Psmb9C6 cells were implanted into the pancreas of C57BL/6 mice and tumours harvested and weighed after 2 weeks (online supplemental figure 3E). *Psmb9* depletion did not rescue FAK-/- tumour growth, implying that an intermediate proteasome containing Psmb8 is sufficient to restrain FAK-/- tumour growth.

10.1136/gutjnl-2022-327927.supp3 Supplementary data

### FAK regulates the antigen repertoire in a Psmb8-dependent manner

**Figure 4:**
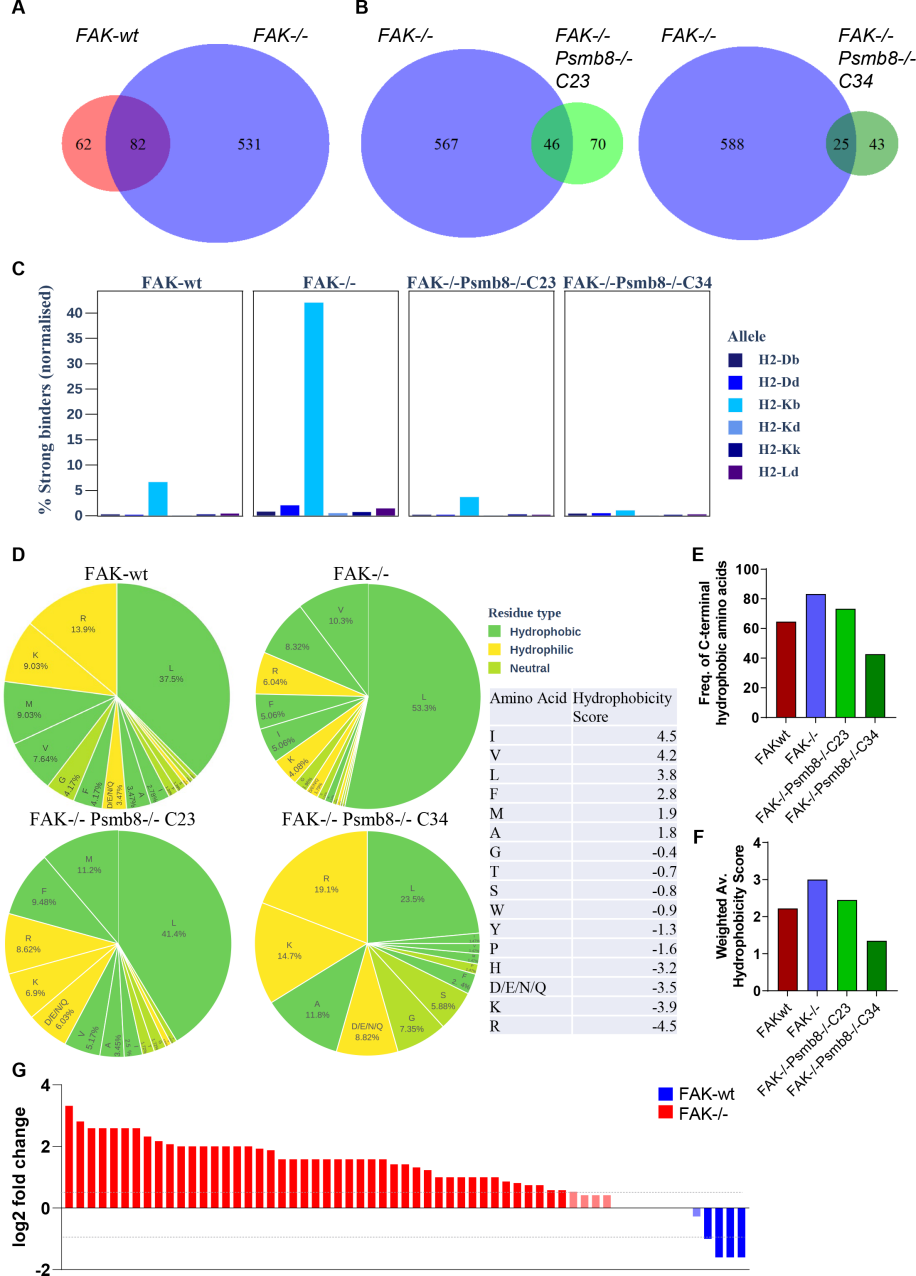
FAK regulates the antigen repertoire in a Psmb8-dependent manner. (A) Venn diagram showing common and unique antigen peptides identified from IFNγ treated FAK-wt and FAK-/- cells using MS. (B) Venn diagram showing common and unique peptides from IFNγ treated FAK-/- vs FAK-/-Psmb8-/-C23 cells (left) and FAK-/- vs FAK-/-Psmb8-/-C34 cells (right). (C) The percentage of peptide antigens predicted to bind strongly to MHC-I molecules normalised to sample size. (D) Analysis of amino acid frequency and hydrophobicity of the C-terminal residue of each peptide antigen identified from FAK-wt, FAK-/-, FAK-/-Psmb8-/-C23 and FAK-/-Psmb8-/-C34 cells. (E) Bar chart showing the frequency of peptides containing a hydrophobic C-terminal residue as a proportion of the total peptide number. (F) Weighted average hydrophobicity score representing all hydrophobic residues in each sample. (G) Log_2_ fold change in the expression of antigen peptides common between FAK-wt and FAK-/- cells grouped by protein of origin. Blue, enriched in FAK-wt cells; red, enriched in FAK-/- cells. IFNγ treatment was 10 ng/mL for 24 hours. FAK, focal adhesion kinase.

To better understand how FAK and Psmb8 might act to restrain tumour growth, we next profiled the antigen repertoire using mass spectrometry (MS)-based immunopeptidomics. 1×10^8^ FAK-wt, FAK-/-, FAK-/-Psmb8-/-C23 and FAK-/-Psmb8-/-C34 cells were stimulated with IFNγ for 24 hours, then lysed as detailed in materials and methods. MHC-I (specifically, H2-Kb) was immunoprecipitated using 20 mg of protein lysate and bound peptides eluted for analysis by MS. A total of 144 peptide antigens were identified from FAK-wt cells, 613 from FAK-/- cells, 116 from FAK-/-Psmb8-/-C23 cells and 68 from FAK-/-Psmb8-/-C34 cells. Comparison of peptide antigens from FAK-wt and FAK-/- cells identified 62 peptides specific to FAK-wt cells, 82 common to both FAK-wt and FAK-/- cells and 531 peptides specific to FAK-/- cells (figure 4A and online supplemental table 3). Further comparisons between FAK-/- and FAK-/-Psmb8-/-C23 and C34 cells suggested that a substantial proportion of the unique peptides presented by FAK-/- cells were dependent on *Psmb8* expression (figure 4B and online supplemental table 3). Thus, FAK deletion increases the diversity of the antigen repertoire presented by Panc47 cells in a Psmb8-dependent manner.

10.1136/gutjnl-2022-327927.supp13 Supplementary data

Assembly of the immunoproteasome results in increased tryptic and chymotryptic activity, with a concomitant decrease in caspase-like activity. As a consequence, there is a preference for C-terminal cleavage at basic and hydrophobic residues. MHC-I preferentially binds peptides with basic or hydrophobic C-termini, suggesting that the immunoproteasome likely yields peptides with a higher affinity for binding MHC-I.^19^ Our data identifying an important role for Psmb8 but not Psmb9 in regulating FAK-/- tumour growth implied that an intermediate proteasome containing β1, β2 and Psmb8 may be sufficient to increase antigen diversity. However, this intermediate proteasome retains caspase-like activity via the β1 subunit, rendering it difficult to predict whether Psmb8 expression could influence the MHC-I binding affinity of the antigen repertoire presented by FAK-/- cells. To understand the binding affinity landscape in this model, we used NetMHCpan-4.0 to predict the binding affinity of each peptide in the immunopeptidomics profiles gathered. Peptides were classified based on their predicted percentage rank,^31^ which ranks stronger binders in lower percentiles. Strong binders are those peptides in the top 0.5% percentile, while weak binders are in the top 2%. The number of strong binders was then normalised to the number of peptides in the sample. Forty percent of all peptides identified from FAK-/- cells were predicted to bind H2-Kb strongly, compared with fewer than 10% of all peptides identified from FAK-wt, FAK-/-Psmb8-/-C23 and FAK-/-Psmb8-/-C34 (figure 4C and online supplemental table 4). Hence, Psmb8 expression in response to FAK loss resulted in an antigen repertoire predicted to bind H2-Kb with higher affinity.

10.1136/gutjnl-2022-327927.supp14 Supplementary data

To better understand why peptide antigens presented by FAK-/- cells might have higher affinity binding to H2-Kb, we next looked at important physicochemical properties of the peptides. Typical peptide binding lengths for MHC-I are 8–9 amino acids.^32^ Peptides identified from FAK-/- cells were consistently shorter than those from FAK-wt, FAK-/-Psmb8-/-C23 and FAK-/-Psmb8-/-C34 cells when comparing their length distributions (pairwise permutation test, 95% CI, p<0.05). Indeed, 53.5% of all peptides identified from FAK-/- cells were 8 amino acids long and 22.5% were 9 amino acids long (online supplemental figure 4 and online supplemental table 3). In contrast, only 37.5% of all peptides identified from FAK-wt cells were 8 amino acids long and 15.3% were 9 amino acids long. An even more pronounced reduction in the frequency of 8 and 9mer peptides was evident in FAK-/-Psmb8-/-C23 and C34 cells, suggesting that Psmb8 expression was important in the generation of peptide antigens with optimal length for binding MHC-I.

10.1136/gutjnl-2022-327927.supp4 Supplementary data

MHC-I preferentially binds peptides with either hydrophobic or basic C-termini.^19^ We, therefore, calculated the hydrophobicity score of the C-terminal amino acid for each peptide according to the Kyte-Doolittle scale.^33^ The C-terminal hydrophobicity of peptides differed significantly between samples (pairwise permutation test, 95% CI, p<0.05). Peptides identified from FAK-/- cells displayed a greater proportion of hydrophobic residues at their C-termini than those identified from any of the other samples, with leucine and valine residues dominating (53.3% and 10.3%, respectively) (figure 4D–F and online supplemental table 5). Notably, these are among the most hydrophobic of amino acids according to the assigned hydrophobicity score. While leucine was the most common C-terminal residue for peptides in all samples, the proportion of peptides containing a C-terminal leucine residue was lower in all other samples when compared with FAK-/-. Furthermore, the second most common hydrophobic residue in the FAK-/- sample was valine with a hydrophobicity score of 4.2, while in all other samples it was either methionine or alanine which have notably lower hydrophobicity scores than valine (4.2 vs 1.9 for methionine and 1.8 for alanine). These samples also showed an increase in peptides containing hydrophilic/polar C-terminal residues when compared with FAK-/- (figure 4D and online supplemental table 5). Hydrophobic C-terminal residues are generated through chymotryptic activity, a function of Psmb8.^19^ These data suggest that increased expression of Psmb8 as a consequence of FAK loss results in a preference for cleavage at hydrophobic C-terminal residues, further optimising peptide antigens for high affinity binding to MHC-I.

10.1136/gutjnl-2022-327927.supp15 Supplementary data

Having assessed potential differences in the physicochemical properties of the antigen peptides present in each sample, we next evaluated differences in the expression of those peptides that were commonly expressed between FAK-/- and FAK-wt cells. The number of unique spectra mapping onto each peptide was calculated and normalised as described in the methods section (online supplemental figure 5 and online supplemental table 6). There were significant differences in the expression of peptides common to the two samples (Welch’s two-sample t-test, 95% CI, p<0.05), with 58 out of 82 peptides being upregulated in the FAK-/- sample, 6 downregulated and 18 remaining unchanged in expression, taking 1.5-fold (log_2_ fold change=0.58) as the cut-off for the upregulated peptides and 0.5-fold (log_2_ fold change = −1) as the cut-off for downregulated peptides. In some cases, multiple peptides were found to have originated from the same protein, resulting in a total of 61 proteins being represented by the peptides common to both samples. We, therefore, also grouped peptides based on the protein from which they originated, and summed the unique spectra mapping onto each peptide as a total measure of protein expression (figure 4G and online supplemental table 7). In total, 45 proteins were upregulated in the FAK-/- sample, 4 were downregulated and 12 were unchanged using the same cut-offs as those applied to the peptide analysis.

10.1136/gutjnl-2022-327927.supp5 Supplementary data

10.1136/gutjnl-2022-327927.supp16 Supplementary data

10.1136/gutjnl-2022-327927.supp17 Supplementary data

In order to determine whether any of the peptides detected were neoantigens, whole genome sequencing datasets derived from FAK-/- cells were converted to protein FASTA files (online supplemental table 8) and used to search for the presence of mutated peptides in the immunopeptidomics datasets. No mutated peptides were identified.

10.1136/gutjnl-2022-327927.supp18 Supplementary data

### FAK and STAT3 impair STAT1-dependent expression of Psmb8 and MHC-I

**Figure 5:**
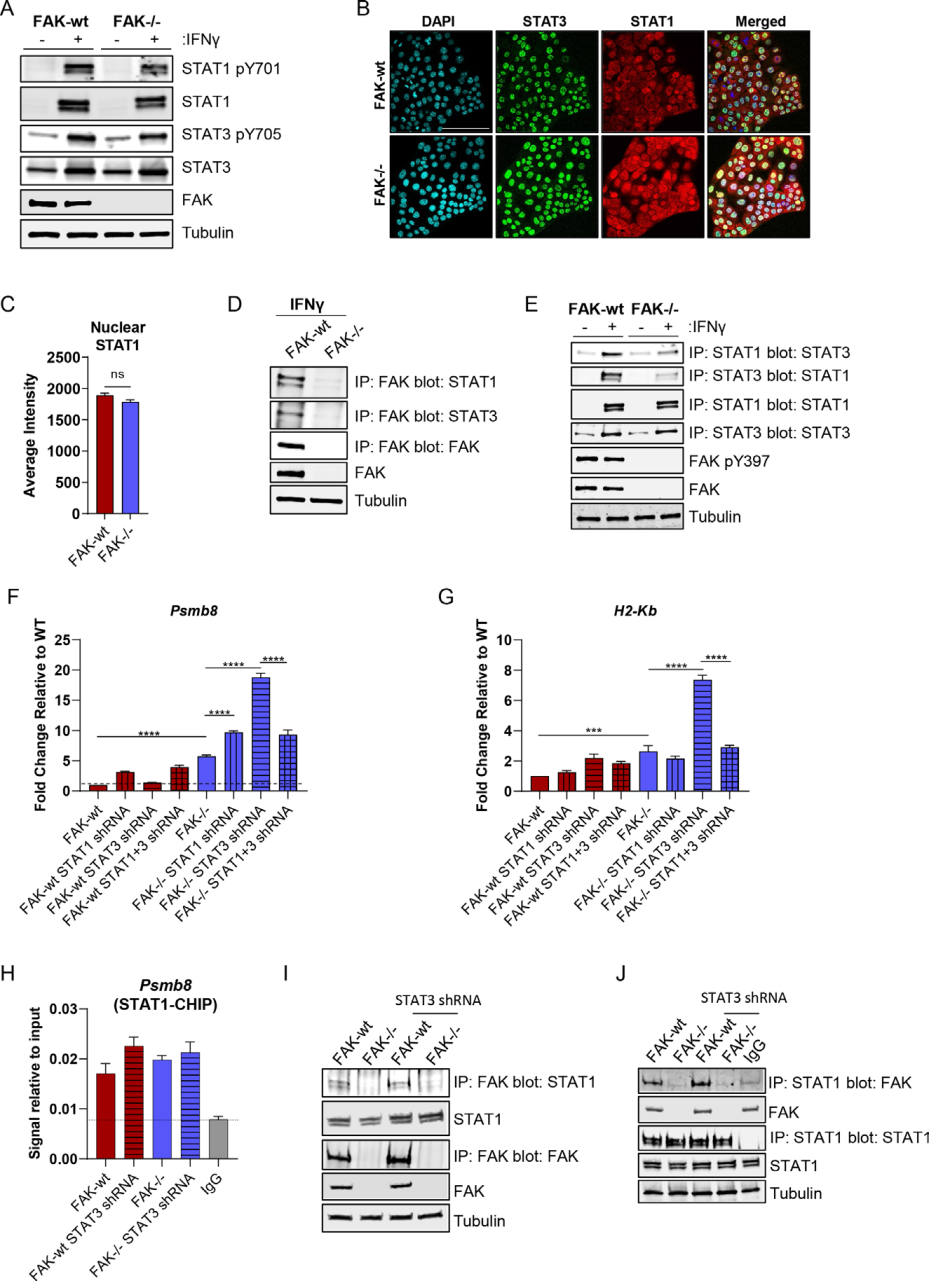
FAK and STAT3 impair STAT1-dependent expression of *Psmb8* and *H2-Kb*. (A) Representative western blot of STAT1 pY701, STAT1, STAT3 pY705, STAT3 and FAK expression in FAK-wt and FAK-/- cells±stimulation with IFNγ. Anti-tubulin used as a loading control. (B) Representative confocal fluorescence images of IFNγ treated FAK-wt and FAK-/- cells stained with DAPI, anti-STAT3 antibody and anti-STAT1 antibody. Scale bar=100 µm. (C) Quantification of nuclear anti-STAT1 immunofluorescence staining from 5000 FAK-wt and FAK-/- cells treated with IFNγ. (D) Representative western blot showing anti-FAK IP blotted with either anti-STAT1, anti-STAT3 or anti-FAK antibodies. Anti-tubulin used as a loading control. Cells were stimulated with IFNγ. (E) Representative western blot showing anti-STAT1 and anti-STAT3 IPs blotted with either anti-STAT1 or anti-STAT3 antibodies. Anti-FAK and anti-FAK pY397 were used to confirm FAK expression and activation, respectively, and anti-tubulin used as a loading control. Cells±stimulation with IFNγ. (F) Relative quantification of *Psmb8* gene expression using qRT-PCR following stimulation with IFNγ. n=3. (G) Relative quantification of *H2-Kb* gene expression using qRT-PCR following stimulation with IFNγ. n=3. (H) Relative quantification of STAT1-*Psmb8* promoter binding using anti-STAT1 chromatin immunoprecipitation (CHIP) and *Psmb8* promoter-specific qRT-PCR from cells treated with IFNγ. n=3. (I) Representative western blot showing anti-FAK IP blotted with anti-STAT1 antibody in whole cell lysates from cells treated with IFNγ. (J) Representative western blot showing anti-STAT1 IP blotted with an anti-FAK antibody from whole cell lysates isolated from cells treated with IFNγ. IFNγ treatment was 10 ng/mL for 24 hours. Data in (C, F, G, H) represented as mean±SEM. Statistical significance in (F, G, H) calculated using a one-way ANOVA with Tukey’s multiple comparison test. ***p≤0.001, ****p≤0.0001. Statistical significance in C calculated using an unpaired two-tailed t-test. ANOVA, analysis of variance; FAK, focal adhesion kinase; IP, immunoprecipitation.

To explore the mechanism underpinning FAK-dependent regulation of these pathways, we first used flow cytometry to quantify IFNγ receptor 1 and 2 (IFNγR1 and IFNγR2) cell-surface expression on FAK-wt and FAK-/- cells untreated or treated with 10 ng/mL IFNγ for 24 hours (online supplemental figure 6). No difference in the expression of IFNγR1 or IFNγR2 was observed, suggesting that regulation of IFNγ receptor expression was not contributing to the observed phenotype following FAK loss. Downstream of IFNγ receptor activation, the Signal Transducer and Activation of Transcription family member STAT1 plays an important role in driving gene expression. Reciprocal regulation of STAT1 by STAT3 can impair STAT1 activity, suggesting that STAT3 controls the balance between activation of these transcription factors and subsequent downstream signalling.^34^ Tyrosine phosphorylation of STATs is crucial for IFN-mediated signalling and translocation to the nucleus.^35^ We, therefore, asked whether FAK could regulate STAT1/3 expression or tyrosine phosphorylation as a mechanism of controlling MHC-I and Psmb8 expression. Western blotting using whole cell lysates from FAK-wt and FAK-/- cells either untreated or treated with 10 ng/mL IFNγ identified that STAT1 was not constitutively expressed in either cell type, but that expression and phosphorylation on tyrosine-701 was induced in response to IFNγ. Little to no difference was observed in either the expression or phosphorylation of STAT1 when comparing FAK-wt and FAK-/- cells (figure 5A). In contrast, STAT3 was constitutively expressed and phosphorylated on tyrosine-705 in both FAK-wt and FAK-/- cells, with both expression and phosphorylation increasing in response to IFNγ. No difference in either expression or phosphorylation of STAT3 was observed between FAK-wt and FAK-/- cells. Thus, FAK does not regulate STAT1/3 expression or phosphorylation in these cells. To investigate whether FAK might regulate STAT1 or STAT3 subcellular localisation we performed confocal immunofluorescence studies in FAK-wt and FAK-/- cells treated with IFNγ (figure 5B). STAT3 localisation was restricted to the nucleus whereas STAT1 showed both nuclear and cytoplasmic staining. Quantification of STAT1 nuclear staining did not identify any difference between FAK-wt and FAK-/- cells (figure 5C). These findings show that FAK does not regulate the subcellular localisation of either STAT1 or STAT3.

10.1136/gutjnl-2022-327927.supp6 Supplementary data

We next checked whether FAK could interact with STAT1 and/or STAT3 as a potential mechanism of regulation. Immunoprecipitation (IP) of FAK from IFNγ-stimulated FAK-wt or FAK-/- whole cell lysates, followed by western blotting identified that FAK was in complex with both STAT1 and STAT3 (figure 5D). STAT1 and STAT3 are known to form a heterodimer and STAT3 can impair STAT1 transcriptional activity.^34 35^ We, therefore, performed further IP studies to determine whether FAK-expression status could influence STAT1/3 heterodimer formation. Both STAT1 and STAT3 IPs confirmed the presence of a STAT1/3 heterodimer following IFNγ-stimulation and showed a reduction in heterodimer formation in FAK-/- cells when compared with FAK-wt cells (figure 5E). Collectively, these findings imply that FAK stabilises the STAT1/3 heterodimer in the nucleus.

To investigate the role of STAT1 and STAT3 in regulating expression of MHC-I and Psmb8, we next used shRNA to deplete either STAT1, STAT3 or STAT1 and STAT3 in FAK-wt and FAK-/- cells. Successful knockdown of expression was confirmed using western blotting (online supplemental figure 7). The resulting panel of cell lines were stimulated with IFNγ for 24 hours and RNA extracted for qRT-PCR analysis. qRT-PCR using a primer set specific for *Psmb8* identified a significant STAT1-dependent induction of *Psmb8* expression only in response to co-depletion of FAK and STAT3 (figure 5F). Similar findings were observed for *H2-Kb* expression (figure 5G). Therefore, while the induction of these genes in response to FAK depletion alone was not fully dependent on STAT1, the co-depletion of FAK and STAT3 appeared to hyperactivate both *Psmb8* and *H2-Kb* expression in a STAT1-dependent manner. Given that STAT1-dependent hyperactivation of these pathways only occurred in the absence of FAK and STAT3 we next performed chromatin IP using an anti-STAT1 antibody and qRT-PCR using primers specific to the promoter of *Psmb8*.^36^ STAT1 was observed to bind to the *Psmb8* promoter in all cell lines tested when compared with IgG control and neither FAK nor STAT3 expression status significantly altered the extent of promoter binding (figure 5H). Therefore, changes in STAT1-*Psmb8* promoter binding did not explain the STAT1-dependent hyperactivation observed. We next asked whether the FAK-STAT1 interaction was dependent on STAT3. IPs using both anti-FAK and anti-STAT1 antibodies identified that FAKs interaction with STAT1 was independent of STAT3 (figure 5I,J). Thus, the only scenario in which STAT1 was not in complex with FAK and / or STAT3 was in the FAK-/- STAT3 shRNA cells, implying that both FAK and STAT3 can exert inhibitory effects on the transcriptional function of STAT1, at least in the context of *Psmb8* expression.

10.1136/gutjnl-2022-327927.supp7 Supplementary data

**Figure 6:**
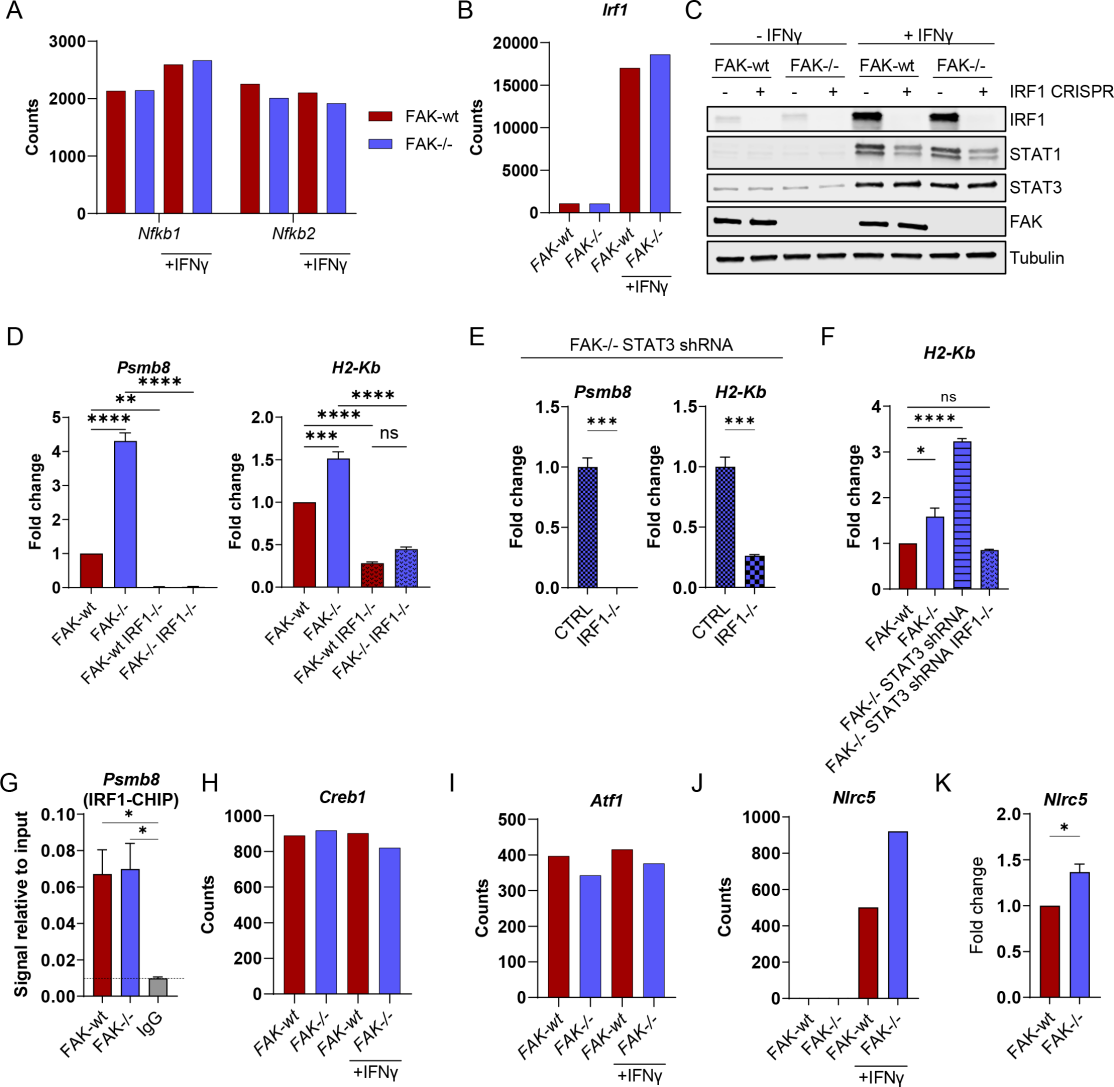
Expression of *Psmb8* and *H2-Kb* requires IRF1. (A, B) NanoString nCounter gene expression data showing the expression of *Nfkb1*, *Nfkb2* and *Irf1* in FAK-wt and FAK-/- cells±IFNγ stimulation. (C) Anti-IRF1, STAT1, STAT3 and FAK western blot from whole cell lysates isolated from FAK-wt and FAK-/- cells±IRF1 CRISPR-Cas9±IFNγ stimulation. Anti-tubulin used as a loading control. (D, E) Relative quantification of *Psmb8* and *H2-Kb* expression using qRT-PCR following IFNγ stimulation. n=3. (F) Relative quantification of *H2-Kb* expression using qRT-PCR following IFNγ stimulation. (G) Relative quantification of IRF1-*Psmb8* promoter binding using anti-IRF1 chromatin immunoprecipitation (CHIP) and *Psmb8* promoter-specific qRT-PCR from cells treated with IFNγ. n=3. (H, I, J) NanoString nCounter gene expression data showing the expression of *Creb1, Atf1 and Nlrc5* in FAK-wt and FAK-/- cells±IFNγ stimulation. (K) Relative quantification of *Nlrc5* expression using qRT-PCR following IFNγ stimulation. Cells were treated with 10 ng/mL IFNγ for 24 hours. n=3. Data in (D–G, K) represented as mean±SEM. Statistical significance was calculated using a one-way ANOVA with Tukey’s multiple comparison test. *p≤0.05, **p≤0.01, ***p≤0.001, ****p≤0.0001. ANOVA, analysis of variance; FAK, focal adhesion kinase.

Given that depletion of STAT1 alone or in combination with STAT3 in FAK-/- cells did not revert *Psmb8* and *H2-Kb* expression to levels comparable with FAK-wt cells, we next investigated other potential regulators of MHC-I gene expression. The promoters of MHC-I genes contain a number of cis-regulatory elements including an enhancer A element which contains nuclear factor-κ B (NF-κB) binding sites, an interferon-stimulated response element which contains binding sites for IRF1 and an SXY module that is required for assembly of the NLRC5-enhanceosome.^37^ Further analysis of NanoString gene expression data from figure 1E identified that the NF-κB genes *Nfkb1* and *Nfkb2* were expressed in FAK-wt and FAK-/- cells, but poorly stimulated by IFNγ (figure 6A). In contrast, *Irf1* was highly stimulated by IFNγ in both FAK-wt and FAK-/- cells (figure 6B), leading us to focus on IRF1 as a candidate transcription factor responsible for expression of both *Psmb8* and *H2-Kb*. Western blotting of protein lysates from FAK-wt and FAK-/- cells±IFNγ confirmed a substantial induction of IRF1 protein expression following IFNγ stimulation, but no difference was observed between FAK-wt and FAK-/- cells (figure 6C). Additionally, CRISPR-Cas9 mediated deletion of IRF1 in FAK-wt and FAK-/- cells identified that expression of STAT1 in response to IFNγ was partially dependent on IRF1 in both cell types, but that STAT3 expression was independent of IRF1 (figure 6C). To investigate whether IRF1 was important for *Psmb8* and *H2-Kb* expression we next treated FAK-wt, FAK-/-, FAK-wt IRF-/- and FAK-/- IRF1-/- cells with IFNγ and quantified *Psmb8* and *H2-Kb* expression using qRT-PCR (figure 6D). IRF1-deletion in both FAK-wt and FAK-/- cells completely abolished *Psmb8* expression and significantly reduced the expression of *H2-Kb*. IRF1-deletion also abolished the STAT1-dependent hyperactivation of *Psmb8* and *H2-Kb* in FAK-/- STAT3 shRNA cells (figure 6E,F). Collectively, these findings imply that IRF1 expression is critical for downstream expression of *Psmb8* and *H2-Kb*. To better understand how FAK may impact IRF1-dependent transcription, we next performed chromatin IP using an anti-IRF1 antibody and qRT-PCR using primers specific for the promoter of *Psmb8* (figure 6G). IRF1 was observed to bind the *Psmb8* promoter at similar levels in both FAK-wt and FAK-/- cells, suggesting a mechanism based on enhancing IRF1 transcriptional function rather than differential promoter binding. Further analysis of NanoString gene expression data from figure 1E also identified expression of several genes encoding proteins belonging to the NLRC5-enhanceosome. *Creb1* and *Atf1*, two transcription factors belonging to the NLRC5-enhancesome, were both expressed in FAK-wt and FAK-/- cells, but not stimulated by IFNγ or regulated by FAK (figure 6H,I). In contrast, *Nlrc5* was only expressed in response to IFNγ stimulation and was upregulated in FAK-/- cells when compared with FAK-wt cells (figure 6J,K). NLRC5 is a transcriptional co-activator that has been shown to induce the expression of MHC-I genes, including H2-K, and also MHC-I accessory genes including Psmb8, Psmb9 and Tap 1.^37 38^ Thus, FAK likely regulates IRF1-dependent transcription of MHC-I and MHC-I accessory genes through regulation of the NLRC5-enhanceosome in the context of IFNγ stimulation.

### Co-depletion of FAK and STAT3 further impairs tumour growth

**Figure 7:**
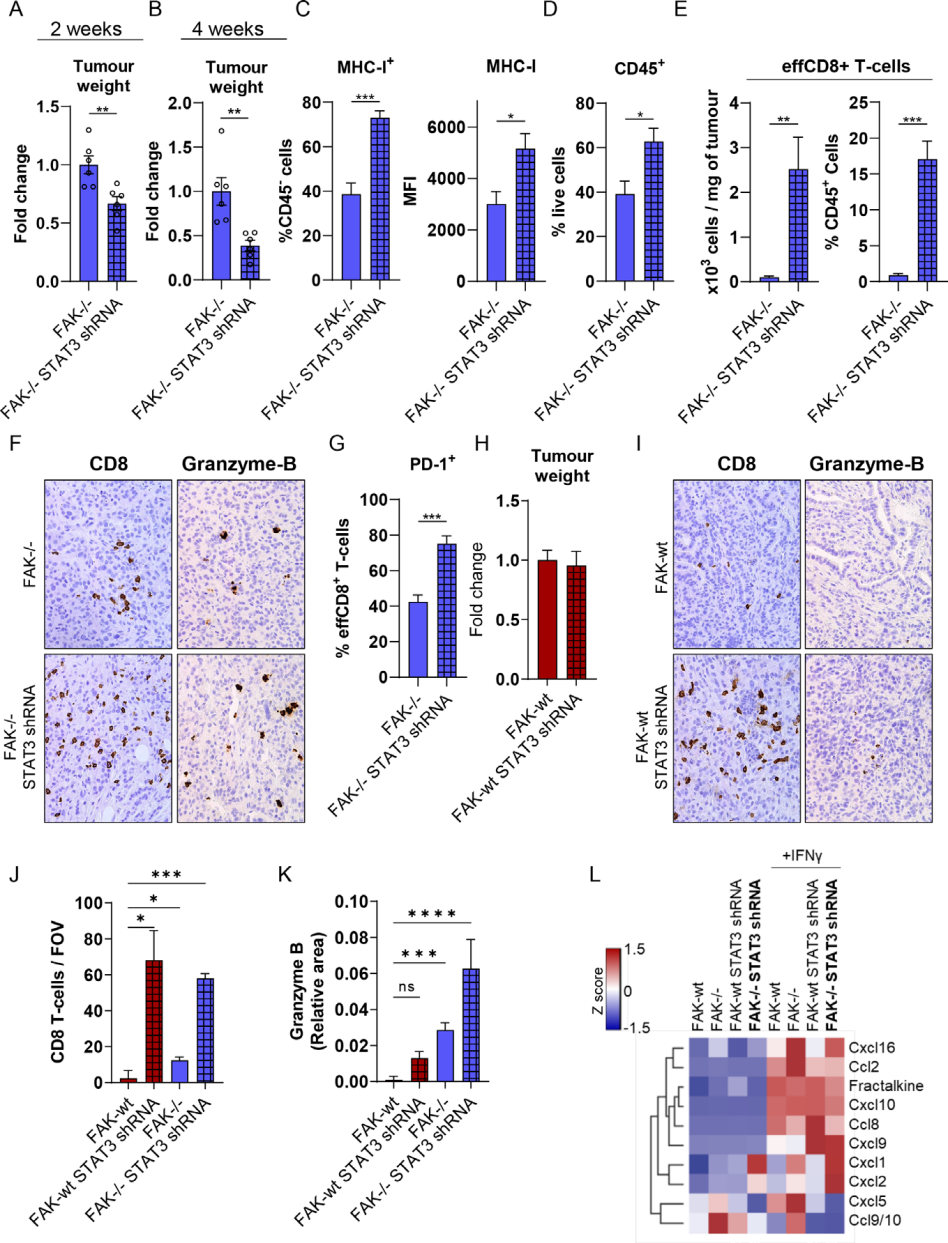
Co-depletion of FAK and STAT3 potentiates antitumour immunity. (A) Fold change in tumour weight relative to FAK-/- tumours 2 weeks postimplantation into the pancreas of C57BL/6 mice. n=6 tumours per group. (B) Fold-change in tumour weight relative to FAK-/- tumours 4 weeks postimplantation into the pancreas of C57BL/6 mice. n=6 tumours per group. (C) Left, flow cytometry quantification of MHC-I-expressing cells as a percentage of CD45^-^ cells from FAK-/- and FAK-/-STAT3shRNA tumours. *Right*, flow cytometry quantification of the median fluorescence intensity of MHC-I expression in CD45^-^ cells from FAK-/- and FAK-/-STAT3shRNA tumours. n=5 tumours per group. (D) Flow cytometry quantification of CD45^+^ cells as a percentage of live cells from FAK-/- and FAK-/-STAT3shRNA tumours. n=5 tumours per group. (E) Flow cytometry quantification of effCD8^+^ T-cells represented as number of cells per mg of tumour tissue (left) and as a percentage of CD45^+^ cells (right) from FAK-/- and FAK-/-STAT3shRNA tumours. n=5 tumours per group. (F) Representative images of FAK-/- and FAK-/- STAT3shRNA tumour sections stained with anti-CD8 or anti-granzyme-B antibodies. (G) Flow cytometry quantification of PD-1^+^ cells as a percentage of effCD8^+^ T-cells from FAK-/- and FAK-/-STAT3shRNA tumours. n=5 tumours per group. (H) Fold change in tumour weight relative to FAK-wt tumours 2 weeks postimplantation into the pancreas of C57BL/6 mice. n=6 tumours per group. (I) Representative images of FAK-wt and FAK-wt STAT3shRNA tumour sections stained with anti-CD8 or anti-granzyme-B antibodies. (J) Quantification of the number of CD8 T-cells per field of view (FOV) from F and I. (K) Quantification of the relative area positive for Granzyme B staining per FOV from (F, I). (L) Proteome profiler array analysis of chemokine secretion from FAK-wt, FAK-/-, FAK-wt STAT3shRNA and FAK-/- STAT3shRNA cells±10 ng/mL IFNγ stimulation for 24 hours. Data in (A–E, G, H, J, K) represented as mean±SEM. Statistical significance in (A–E, G and H) calculated using a two-tailed unpaired t-test. Statistical significance in J and K calculated using a Kruskal-Wallis test with Dunn’s multiple comparison. *p≤0.05, **p≤0.01, ***p≤0.001, ****p≤0.0001. FAK, focal adhesion kinase; MHC-I, Major Histocompatibility Complex class-I.

Having identified that Psmb8 was important for regulating the growth of FAK-/- tumours (figure 3G) and that the FAK-/- tumour growth defect was CD8 T-cell-dependent (figure 1C), we postulated that increased expression of both *Psmb8* and *H2-Kb* in response to co-depletion of FAK and STAT3 might further restrain tumour growth and potentiate the antitumour CD8 T-cell response. To address this, 0.5×10^6^ FAK-/- or FAK-/-STAT3shRNA cells were implanted into the pancreas of C57BL/6 mice and mice sacrificed either 2 weeks or 4 weeks later (figure 7A,B). At both time points, mice bearing FAK-/-STAT3shRNA tumours were found to have a significant reduction in tumour burden when compared with those bearing FAK-/- tumours. Flow cytometry analysis of dissociated tissue from 4-week-old tumours also confirmed in vitro observations, identifying an increase in MHC-I expression on CD45^-^ cells in FAK-/-STAT3shRNA tumours when compared with FAK-/- tumours (figure 7C and online supplemental figure 8). The frequency of immune cells (figure 7D and online supplemental figure 8) and effector CD8 T-cells (effCD8) (figure 7E and online supplemental figure 8) infiltrating into FAK-/-STAT3shRNA tumours was also significantly increased. Immunohistochemical staining of FAK-/- and FAK-/-STAT3shRNA tumour sections using an anti-CD8 antibody also confirmed the infiltration of CD8 T-cells within the tumour mass (figure 7F—left and J), while an anti-granzyme-B antibody showed positive intratumoural staining indicative of ongoing CD8 T-cell activity (figure 7F—right and K). Further, a greater proportion of effCD8 T-cells in FAK-/-STAT3shRNA tumours were positive for expression of the receptor programmed death receptor 1 (PD-1) (figure 7G and online supplemental figure 8), which has been shown to be a marker of tumour-reactive T-cells that have encountered antigen.^39^ Thus, co-depletion of FAK and STAT3 promoted extensive CD8 T-cell infiltration and further restrained pancreatic tumour growth, likely via increased CD8 T-cell engagement. In contrast, STAT3-depletion in FAK-wt cells had no impact on tumour growth (figure 7H) despite driving a notable influx of CD8 T-cells into the tumour mass (figure 7I—left and J). This was associated with a reduction in granzyme-B positive intratumoural staining when compared with FAK-/- and FAK-/- STAT3 shRNA tumours (figure 7I—right and K), implying a lack of effective CD8 T-cell engagement in the context of FAK-wt tumours. To better understand how STAT3 could promote CD8 T-cell infiltration independent of FAK, we next profiled chemokine secretion from FAK-wt, FAK-/-, FAK-wt STAT3shRNA and FAK-/-STAT3shRNA cells±IFNγ using Proteome Profiler Chemokine Antibody Arrays (figure 7L). In the presence of IFNγ, which we have shown is secreted by immune cells within the FAK-wt and FAK-/- TMEs (figure 1D), STAT3-depletion resulted in upregulation of Cxcl9 independent of FAK expression status. CXCL9 is a ligand for the receptor CXCR3 and has been shown to promote the infiltration of CD8 T-cells into tumours.^40^ In addition, the blood plasma levels of CXCL9 in PDAC patients receiving chemotherapy has been shown to correlate with longer overall survival and a longer time to progression.^41^ Thus, STAT3-depletion reprograms the chemokine secretory profile of FAK-wt and FAK-/- cells in favour of CD8 T-cell infiltration, complementing FAK-dependent reprogramming of antigen processing and presentation pathways required to promote T-cell tumour recognition.

10.1136/gutjnl-2022-327927.supp8 Supplementary data

### PDAC cell heterogeneity impacts FAK function

**Figure 8:**
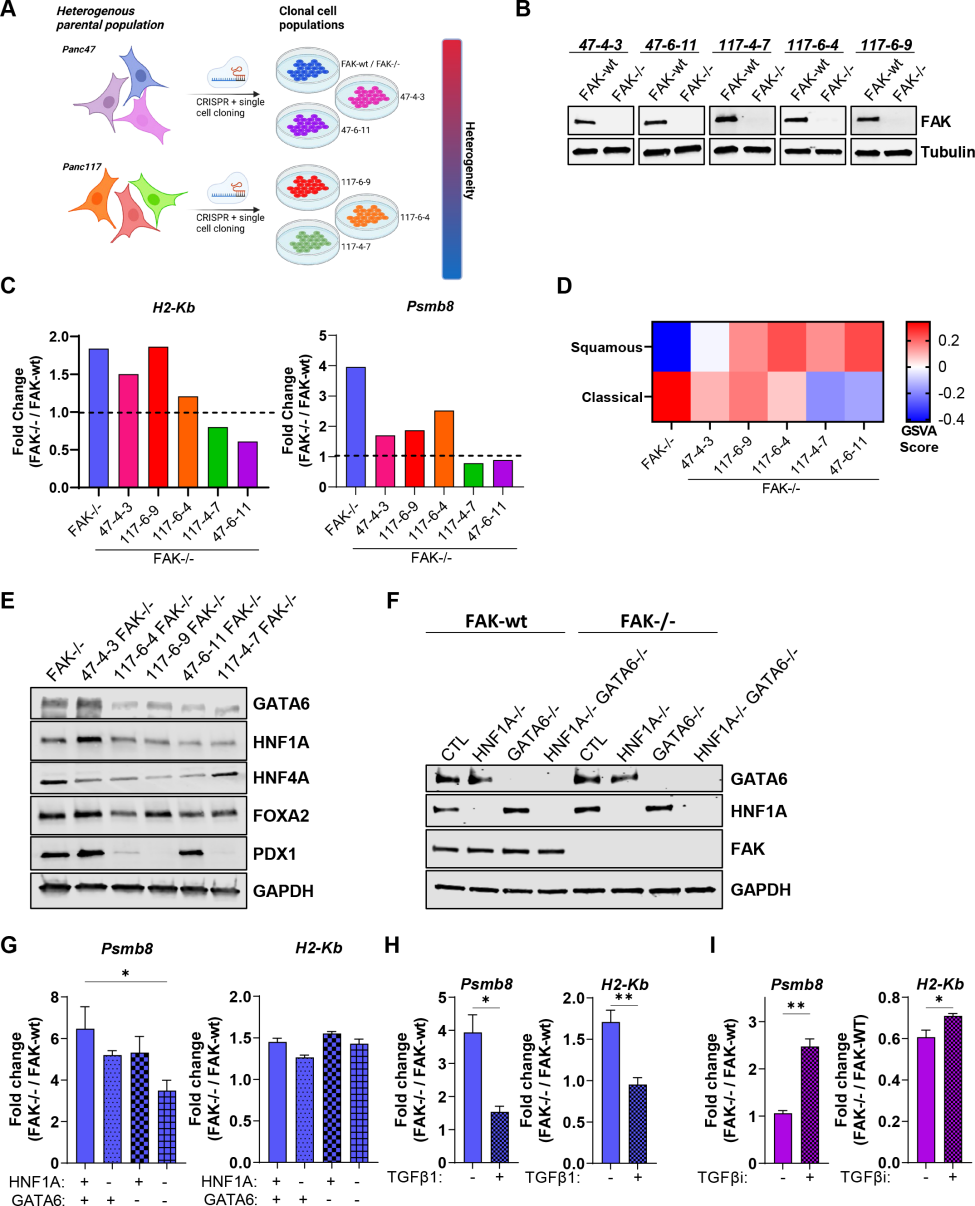
PDAC heterogeneity and transcriptional subtype impact FAK function. (A) Graphical summary showing that single-cell cloning can result in a panel of cell lines broadly representing the heterogeneity of the original parental population. (B) Representative anti-FAK western blots showing FAK re-expression in a panel of clonal cell lines in which CRISPR-Cas9 has been used to delete FAK expression. (C) NanoString nCounter gene expression data from IFNγ stimulated cells generated using a mouse PanCancer Immune Profiling panel. Data represented as fold change in gene expression relative to FAK-wt counterpart. (D) Heat map of FAK-/- GSVA scores for expression of genes associated with classical and squamous subtypes of pancreatic cancer. (E) Representative anti-GATA6, HNF1A, HNF4A, FOXA2 and PDX1 western blot from whole cell lysates isolated from six FAK-/- clonal cell lines. Anti-GAPDH used as a loading control. (F) Western blot showing CRISPR-Cas9 deletion of HNF1A, GATA6 and HNF1A+GATA6 in FAK-wt and FAK-/- cells. Anti-GAPDH used as a loading control. (G) Relative quantification of *Psmb8* and *H2-Kb* gene expression using qRT-PCR following stimulation with IFNγ. n=3. (H) Relative quantification of *Psmb8* and *H2-Kb* expression using qRT-PCR in FAK-wt and FAK-/- cells±treatment with TGFβ1 for 9 days. Cells were stimulated for 24 hours with IFNγ ± TGFβ1 prior to RNA extraction. n=3 (I) Relative quantification of *Psmb8* and *H2-Kb* expression using qRT-PCR in FAK-wt and FAK-/- cells±treatment with TGFβ inhibitor for 2 weeks. Cells were stimulated for 24 hours with IFNγ ± TGFβ inhibitor prior to RNA extraction. n=3. IFNγ treatment was 10 ng/mL for 24 hours (C, G, H, I). Data in G–I represented as mean±SEM. Statistical significance calculated using a one-way ANOVA with Tukey’s multiple comparison test. *p≤0.05, **p≤0.01. ANOVA, analysis of variance; FAK, focal adhesion kinase; GSVA, gene set variation analysis; PDAC, pancreatic ductal adenocarcinoma.

Transcriptomic analysis of human pancreatic cancer has identified two major molecular subtypes, here termed ‘classical’ and ‘squamous’.^42–44^ However, single-cell RNA sequencing (scRNAseq) studies have identified an intermediate transitional phenotype,^45^ suggesting that the terms ‘classical’ and ‘squamous’ should be considered as two poles of a continuum rather than as a binary classification. The process of generating FAK-/- cells using CRISPR-Cas9 gene editing required single-cell cloning, resulting in the isolation of a further five FAK-knockout clonal cell populations (figure 8A). Two of these additional cell clones originated from parental Panc47 cells and three from an independently established additional parental cell line from KPC tumours termed Panc117. Therefore, we sought to use these to better understand whether cancer cell heterogeneity could impact FAK function with respect to regulation of Psmb8 and MHC-I. All five FAK-knockout cell lines were reconstituted with FAK-wt and expression confirmed using western blotting (figure 8B). Cells were treated with IFNγ for 24 hours and RNA isolated for NanoString nCounter digital gene expression analysis using the PanCancer Immune Profiling Panel. *H2-Kb* expression was upregulated in 47-4-3 FAK-/-, 117-6-9 FAK-/- and 117-6-4 FAK-/- cells (figure 8C, left), with all other cell lines showing either unaltered or reduced expression when compared with FAK-wt counterparts. *Psmb8* expression was also upregulated in 47-4-3 FAK-/-, 117-6-9 FAK-/- and 117-6-4 FAK-/- cells when compared with FAK-wt counterparts, although the fold-increase in expression was less than that observed in FAK-/- cells (figure 8C, right). No change in *Psmb8* expression was observed in 117-4-7 FAK-/- and 47-6-11 FAK-/- cells when compared with FAK-wt counterparts. Thus, FAK-dependent regulation of Psmb8 and MHC-I is not universal across PDAC cell clones, even from the same tumour, suggesting that pancreatic cancer cell heterogeneity has the potential to impact FAK function. Notably, *Nlrc5* expression mirrored that of *Psmb8* and *H2-Kb* across the clones, further supporting a role for the *Nlrc5* enhanceosome in the regulation of these genes (online supplemental figure 9).

10.1136/gutjnl-2022-327927.supp9 Supplementary data

To characterise features that define pancreatic cancer cells in which FAK loss leads to upregulation of Psmb8 and MHC-I expression in response to IFNγ, we next performed RNA sequencing of all six FAK-/- cell populations and compared this with characterised gene sets that define classical and squamous molecular subtypes^42^ using gene set variation analysis (GSVA) (figure 8D). Based on GSVA scores, FAK-/- cells were the most enriched in genes associated with the classical subtype, with the 47-4-3 FAK-/-, 117-6-9 FAK-/- and 117-6-4 FAK-/- cells showing some enrichment of both classical-associated and squamous-associated genes, perhaps suggesting that these cell clones have an intermediate transitional phenotype. 117-4-7 FAK-/- and 47-6-11 FAK-/- cells, in which FAK loss did not positively regulate Psmb8 and MHC-I expression, were found to be enriched in genes associated with the squamous subtype, implying that FAK-dependent regulation of Psmb8 and MHC-I is lost as cells differentiate towards a more extreme squamous phenotype.

Emerging evidence suggests that PDAC cells can interconvert between subtypes, implying a degree of plasticity.^45^ Therefore, to further determine whether transition between subtypes could impact FAK-dependent regulation of Psmb8 and MHC-I, we next set out to switch FAK-/- cells (the most classical-like clone) towards a squamous phenotype. Loss of GATA6 expression together with HNF1A and HNF4A has been shown to drive differentiation towards the squamous subtype.^46^ Notably, western blotting using lysates from all 6 FAK-/- cell clones identified downregulation of GATA6 and HNF1A in both 117-4-7 FAK-/- and 47-6-11 FAK-/- cells when compared with the more classical-like cell clones, FAK-/- or 47-4-3 FAK-/- (figure 8E). We, therefore, used CRISPR-Cas9 gene editing to delete expression of HNF1A, GATA6 or HNF1A and GATA6 (figure 8F) and determined the effects on *Psmb8* and *H2-Kb* expression using qRT-PCR (figure 8G). Co-deletion of GATA6 and HNF1A attenuated the upregulation of *Psmb8* expression in FAK-/- cells when compared with mock transfected controls, but had no impact on the regulation of H2-Kb expression by FAK. Therefore, the loss of GATA6 and HNF1A during subtype transition may contribute to altering FAK function but is not sufficient. TGFβ^45^ and expression of the transcription factor *Gli2*,^47^ a TGFβ-induced gene,^48^ have also been reported to drive squamous differentiation in culture. Treatment of FAK-wt and FAK-/- cells with TGFβ1 for 9 days almost completely abolished the upregulation of both *Psmb8* and *H2-Kb* expression in response to FAK loss (figure 8H), suggesting an important role for TGFβ driven squamous differentiation in the regulation of FAK function. Given the profound impact of TGFβ treatment, we next treated the most squamous-like cell clone, 47-6-11, with a TGFβ inhibitor (TGFβi) for 2 weeks to promote differentiation towards a more classical-like phenotype. Treatment of 47-6-11 FAK-wt and FAK-/- cells with TGFβi restored FAK-dependent regulation of both *Psmb8* and *H2-Kb* (figure 8I), further supporting the conclusion that TGFβ signalling plays an important role in regulating FAK function in PDAC.

### FAK regulates antigen processing and presentation in human PDCLs

**Figure 9:**
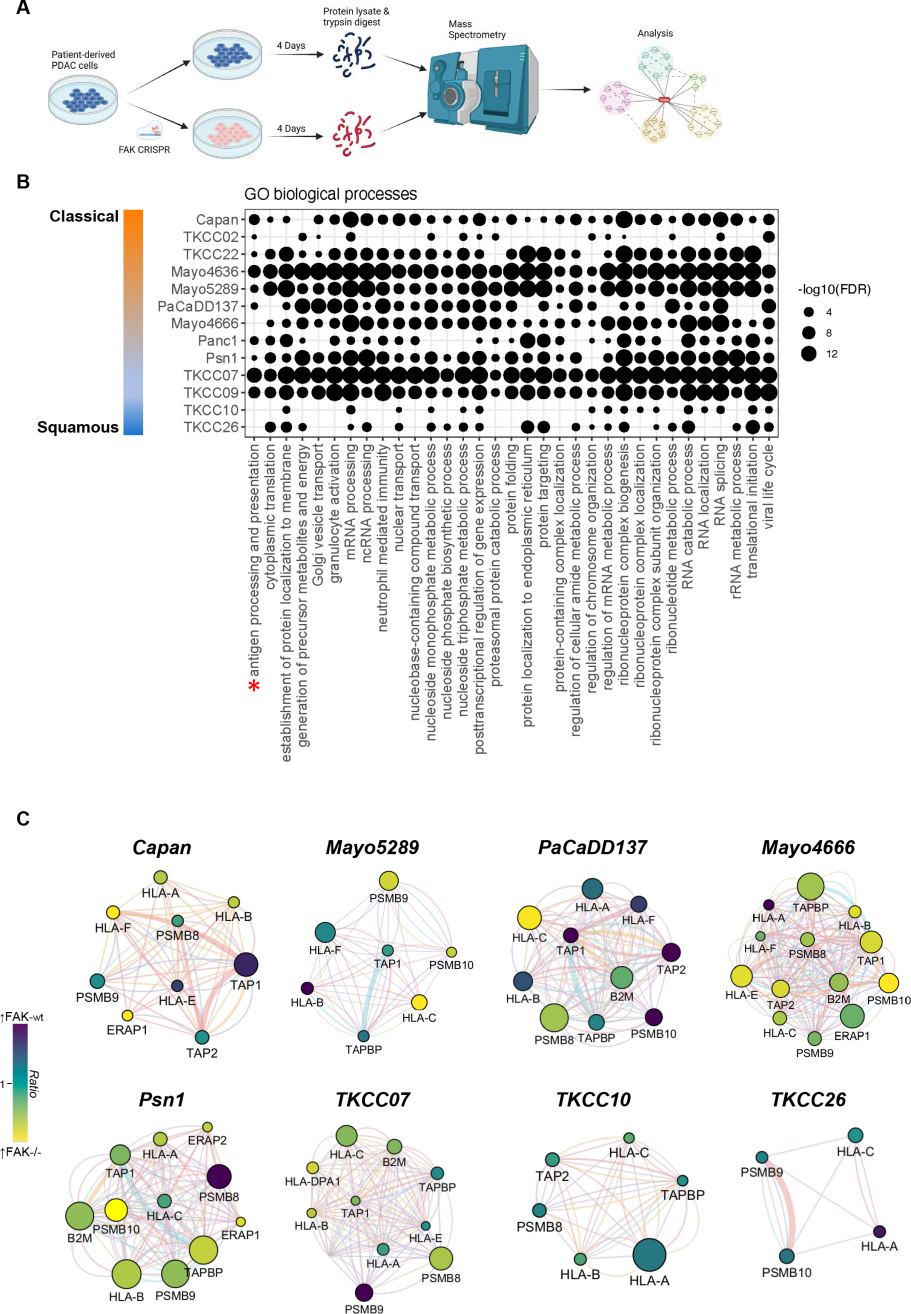
FAK regulates antigen processing and presentation in human PDCLs. (A) Graphical summary of experimental setup used for proteomic analysis of FAK function in human PDCLs. (B) Gene ontology biological process enrichment analysis of differentially expressed proteins in 13 human PDCLs following FAK-deletion using CRISPR-Cas9. (C) Functional association network analysis of proteins related to antigen processing and presentation from proteomics expression analysis of human PDCLs in which FAK expression has been deleted using CRISPR-Cas9. FAK, focal adhesion kinase; FDR, false discovery rate; GO, gene ontology; PDCLs, patient-derived cell lines.

Having established a role for FAK in regulating antigen processing and presentation in mouse models of PDAC, we next sought to determine whether similar pathways were also regulated by FAK in human pancreatic cancer cells. To address this, we used a panel of 13 (Capan-1, TKCC22, TKCC02, Mayo4636, Mayo5289, PacaDD137, Mayo4666, TKCC07, TKCC26, TKCC09, Panc1, Psn1 and TKCC10) human patient-derived pancreatic cancer cell lines (PDCLs) for which genomic and transcriptomic characterisation is available, ensuring that a broad range of both the classical and squamous subtypes were represented. To maintain the heterogeneity of the parental cell populations, FAK expression was deleted in PDCLs using CRISPR-Cas9 gene editing and 4 days later protein lysates were isolated for analysis using mass spectrometry (figure 9A and online supplemental figure 10A). To confirm the molecular subtype of each cell line, proteomic and transcriptomic data relating to genes previously reported to define the classical and squamous subtypes^47 49^ was used to cluster the cell lines, confirming two main clusters representing classical and squamous (online supplemental figure 10B,C). Based on this classification, we next used western blotting to determine the expression of common classical markers including GATA6, HNF1A, HNF4A, FOXA2 and PDX1 in protein lysates from 8 of the cell lines (online supplemental figure 10D). While Capan, TKCC02 and TKCC22 cells were positive for expression of all five markers, TKCC10 cells were negative for all markers suggesting that these cells represented the most extreme of each subtype. Notably, TKCC07, TKCC09, Panc1 and Psn1 cells retained expression of at least some classical makers implying that these represent different points of progression from classical to squamous. The list of significantly differentially expressed proteins (q-value≤0.05; online supplemental table 9) from each cell line was then analysed using the WEB-based Gene Set Analysis Toolkit (www.webgesalt.org) to identify Gene Ontology (GO) biological processes overrepresented within each dataset. GO biological processes enriched in ≥10 cell lines are shown in figure 9B. Notably, only 3 cell lines from 13 lacked significant overrepresentation of antigen processing and presentation, 2 of which were classified as the most extreme squamous from the panel of cell lines analysed. Such findings are in broad agreement with our observations from mouse models suggesting that FAK's role in regulating antigen processing and presentation is diminished as cells differentiate towards an extreme squamous phenotype. Network analysis of proteins relating specifically to the antigen processing and presentation pathways regulated by FAK in our mouse models identified significant (q-value≤0.05) regulation of proteins including Human Leucocyte Antigen (HLA)-A, HLA-B, HLA-C, HLA-E, HLA-F, beta-2-microglobulin (B2M), TAP1, TAP2, tapasin (TAPBP), ERAP1 and the immunoproteasome in response to FAK loss (figure 9C) in the majority of human PDCLs tested, including the Mayo4666 which did not show significant overrepresentation of antigen processing and presentation in GO analysis. Notably, regulation of these proteins was not evident in TKCC10 and TKCC26 cells, further confirming our observations linking the extreme squamous phenotype with loss of FAK function in relation to the regulation of antigen processing and presentation pathways.

10.1136/gutjnl-2022-327927.supp10 Supplementary data

10.1136/gutjnl-2022-327927.supp19 Supplementary data

**Figure 10:**
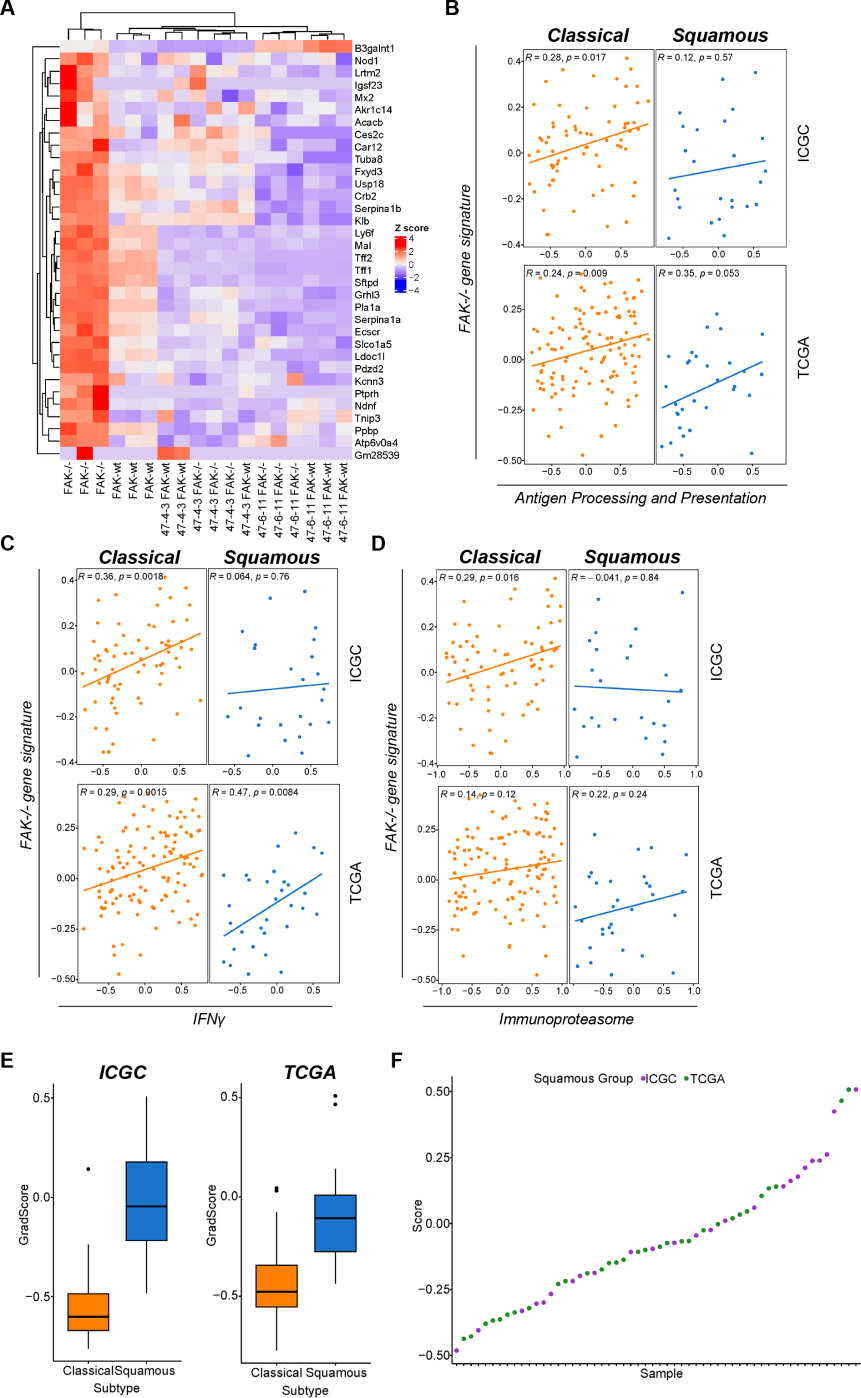
A FAK loss gene signature positively correlates with antigen processing and presentation gene signatures in human PDAC tumours. (A) Cluster analysis showing a gene signature of FAK loss specific to the most classical FAK-/- clonal cell line. (B–D) Analysis of ICGC and TCGA bulk RNA sequencing datasets from human PDAC examining the correlation between a gene signature of FAK loss and gene signatures associated with antigen processing and presentation (B), interferon-γ signalling (C) and the immunoproteasome (D). (E) Box and whiskers plot of classical and squamous scores for all tumours in ICGC and TCGA datasets. (F) Graph representing the squamous score for each tumour classified as squamous in ICGC and TCGA datasets. FAK, focal adhesion kinase; ICGC, International Cancer Genome Consortium; PDAC, pancreatic ductal adenocarcinoma; TCGA, The Cancer Genome Atlas.

To further interrogate the link between FAK expression and regulation of antigen processing and presentation we next analysed publicly available bulk RNAseq datasets from human PDAC, acknowledging that such analysis may be confounded by the expression of genes within the TME. Using RNA sequencing data from Panc47 FAK-wt and FAK-/- murine cell clones, we first identified a unique gene signature relating to FAK loss in the most classical-like cell clone (figure 10A). We next subdivided the International Cancer Genome Consortium (ICGC)^42^ and TCGA^50^ datasets into classical and squamous transcriptional subtypes, and investigated the association of this gene signature with three gene signatures (detailed in Methods) based on genes we had observed to be regulated by FAK in our mouse models: (1) a refined gene set based on the antigen processing and presentation Kegg pathway, (2) a refined gene set based on the IFNγ Reactome pathway and (3) the immunoproteasome genes Psmb8, Psmb9 and Psmb10. In classical tumours from both the ICGC and TCGA datasets, the gene signature relating to FAK loss showed a statistically significant positive correlation with all three other gene signatures, with the exception of the immunoproteasome signature in the TCGA dataset (figure 10B-D). In squamous tumours from the ICGC dataset no significant correlation was identified. In contrast, a statistically significant positive correlation between FAK loss and both antigen processing and presentation and IFNγ was observed in squamous tumours from the TCGA dataset, implying potential differences between the squamous tumours represented within each dataset. To better understand the reason for these differences we next plotted the classical and squamous score for each tumour from both datasets (figure 10E). Comparison of the score distribution suggested that the ICGC dataset contained more tumours of an extreme squamous phenotype. Plotting the score for each individual squamous tumour further confirmed this observation (figure 10F), showing that the ICGC dataset was skewed towards tumours of the extreme squamous phenotype when compared with TCGA. Thus, the differences in correlation observed are likely the consequence of the squamous datasets representing different points of differentiation within the squamous subtype. This observation further supports our conclusions from both murine and human cell line models implying that FAK-dependent regulation of antigen processing and presentation is diminished in cells and tumours of the extreme squamous phenotype.

## Discussion

PDAC is generally regarded as an immunologically ‘cold’ tumour type, devoid of CD8 T-cell infiltration and unresponsive to single-agent immunotherapy. Immunogenic tumour antigens are critical for CD8 T-cell responses. However, with a relatively low somatic mutational burden, only a small number of potentially actionable neoantigens have been identified in human PDAC.^9 10^ Similar observations have been made using cell lines isolated from PDAC arising in KPC mice, where poor antigenicity is thought to preclude the process of immunoediting.^11^ Despite this, numerous studies using GEM models of PDAC, or cancer cell lines derived from them, suggest that effective CD8 T-cell immunity can be mounted in response to combination immunotherapies.^13–15^ Such observations support a potentially important role for self-antigens in promoting antitumour immunity in PDAC. Our findings identify a novel role for FAK in regulating the antigenicity of PDAC in favour of an antitumour T-cell response. This was not simply the consequence of increased antigen presentation, but rather an extensive reprogramming of the antigen repertoire mediated via regulation of the immunoproteasome. Comparison of immunopeptidomics data with neoantigens predicted from whole genome sequencing failed to identify any neoantigens presented by H2-Kb on FAK-/- cells. While this does not exclude the possibility that neoantigens may be presented by MHC-I molecules, it is not supportive of neoantigens as a major factor promoting immunosurveillance in response to FAK loss. The immunogenicity of tumour self-antigens can be influenced by the stability of the MHC-I peptide antigen complex,^51^ suggesting that antigens with higher affinity binding to MHC-I may elicit stronger immune responses. In this regard, FAK loss increased the frequency of peptides with strong predicted binding to H2-Kb, potentially via fine-tuning their physicochemical properties for optimised binding. It is also possible that Psmb8 incorporation into the proteasome could result in presentation of an antigen repertoire more likely to mediate a successful encounter between T-cells and tumour cells. Mature dendritic cells that prime antitumour T-cell responses also express the immunoproteasome,^52^ and therefore, generate peptide antigens for presentation to T-cells via this proteolytic pathway. Thus, the resulting synergism may also promote more effective immunosurveillance. While we did not profile the antigen repertoire of cells following co-depletion of FAK and STAT3, we found that STAT3-depletion resulted in substantial infiltration of CD8 T-cells into tumours independent of FAK expression status. However, only in the context of FAK deletion did we observe inhibition of tumour growth and elevated granzyme-B expression indicative of CD8 T-cell engagement, implying that co-depletion of FAK and STAT3 is complementary through enhancing both CD8 T-cell infiltration and PDAC cell immunogenicity.

Deep molecular profiling of human cancers has uncovered extensive heterogeneity and highlighted the need to develop a greater understanding of how to define patient populations most likely to benefit from a given therapeutic regimen. Although KRAS mutations are present in the majority of pancreatic cancers, the nature of the KRAS mutation, the presence of additional mutations with lower prevalence and the molecular subtype all have the potential to alter dependency on cell signalling pathways. For example, human patient-derived PDAC cells have been shown to have different metabolic dependencies based on molecular subtype.^49^ Further, in non-small cell lung cancer, the KRAS G12D mutation has been shown to preferentially drive the PI3K/AKT pathway, whereas G12V and G12C drive the Raf/Ral pathway.^53^ Using a panel of mouse PDAC tumour cells clones, we identified that FAK-dependent regulation of antigen processing and presentation occurs in cells that have a transcriptional signature aligning to the classical and intermediate transitional subtypes, but is lost as cells differentiate to an extreme squamous phenotype. All cell lines harboured the same KRAS G12D and p53 R172H mutations (online supplemental table 10). Therefore, it will be interesting in the future to determine whether KRAS mutational status may also impact FAK function. Notably, cell clones isolated from the same tumour mass displayed heterogeneity in subtype specification, implying that FAK function may differ even within the same tumour mass or between different tumours with distinct molecular signatures. Proteomic studies using a panel of 13 human PDCLs broadly aligned with findings from mouse cell lines, not only identifying conservation of function across species, but also supporting the conclusion that FAK-dependent regulation of antigen processing and presentation occurs broadly in PDAC but is lost as cells differentiate towards an extreme squamous phenotype. Similarly, analysis of human PDAC bulk RNAseq data from two independent cohorts showed a reproducible correlation between a gene signature of FAK loss and pathways related to antigen processing and presentation, with the loss of correlation again being associated with tumours of a more extreme squamous phenotype. Thus, a broad population of PDAC patients may benefit from FAK targeted therapies aimed at protein degradation. Emerging evidence suggests that PDAC cells can interconvert between subtypes, implying a degree of plasticity.^45 47^ Identifying regulators of PDAC cell state may yield opportunities for reprogramming subtype specification in order to bolster therapeutic response. In this regard, both TGFβ signalling and the transcription factor Gli2 represent potential candidates. Treatment of tumour organoids from surgically resected metastases from pancreatic cancer patients with TGFβ was found to shift gene expression programmes in favour of the squamous subtype.^45^ Similarly, expression of Gli2 can drive squamous differentiation.^47^ We found that treatment of FAK-wt and FAK-/- cells, the most classical-like cell clone, with TGFβ resulted in loss of FAK function with respect to regulation of *Psmb8* and *H2-Kb* expression, while treatment with a TGFβ inhibitor could restore FAK-dependent regulation of *Psmb8* and *H2-Kb* in the most squamous-like cell clone. These findings imply that PDAC cells in which FAK does not regulate antigen processing and presentation may be reprogrammed to respond to FAK-targeted therapy in this way and highlight the TGFβ pathway as a candidate target in this regard.

10.1136/gutjnl-2022-327927.supp20 Supplementary data

FAK kinase inhibitors are currently in clinical testing in combination with immunotherapies in patients with advanced pancreatic cancer (ClinicalTrials.gov; NCT02546531, NCT02758587, NCT03727880). Our findings imply that targeting FAK degradation could bring additional therapeutic benefit. Proteolysis-targeting chimeric molecules based on FAK kinase inhibitors are in early preclinical testing^54 55^ and our data strongly support the continued development of these next generation FAK degraders to fully harness the potential of targeting FAK for the treatment of PDAC.

## Materials and methods

### Materials

Recombinant mouse IFNγ was purchased from R&D Systems and used in vitro at a final concentration of 10 ng/mL. Recombinant mouse IFNγ for use in vivo was purchased from Peprotech, and mice were dosed with 250 µL of 5 µg/mL IFNγ or PBS using intraperitoneal injection beginning at day 8 (with tumours being implanted at day 0). Mice were treated daily for 7 days (days 7–14) and culled on day 14. Recombinant mouse TGFβ was purchased from Biolegend and used in vitro at a final concentration of 5 ng/mL. TGFβ inhibitor SB 431542 (Catalogue # 1614 Bio-techne (Tocris)) was used at 10 µM for 2 weeks. The FAK kinase inhibitors GSK2256098, VS4718 and Defactinib (VS6063) were obtained from Selleckchem. All flow cytometry antibodies used are listed in online supplemental table 11. All IP, western blotting and immunohistochemistry antibodies are listed in online supplemental table 12. Schematics were created in BioRender.com.

10.1136/gutjnl-2022-327927.supp21 Supplementary data

10.1136/gutjnl-2022-327927.supp22 Supplementary data

### Cell lines

Panc47 and Panc117 cell lines were a generous gift from Dr Jen Morton (CRUK Beatson Institute, Glasgow, UK). These cell lines were originally derived from PDAC arising on *LSL-Kras^G12D/+^;LSL-Trp53^R172H/+^; Pdx1 Cre* (KPC) mice. All cell lines were cultured at 37°C with 5% CO_2_ in high-glucose Dulbecco’s Minimum Essential Medium (Sigma) supplemented with 10% fetal bovine serum (Life Technologies). Panc47 and Panc117 cells were pathogen tested in September 2016 using the ImpactIII test (Idex Bioresearch) and were negative for all pathogens. TKCC cell lines were obtained from the Australian Pancreatic Cancer Genome Initiative. Psn-1, Panc-1 and Capan-1 cell lines were obtained from Eric O’Neill at the University of Oxford and have been validated by Eurofins Genomics. PacaDD137 cells were a generous gift from Christian Pilarsky at the Friedrich-Alexander-Universitat, Erlangen, Germany. Mayo cell lines were a generous gift from Debabrata Mukhopadhyay at the Mayo Foundation for Medical Education and Research. TKCC10, TKCC07 and TKCC09 cells were cultured in 5% CO_2_ at 37°C in a 1:1 mix of M199 and HAM’s F12 medium (Life technologies, UK) supplemented with 15 mM HEPES (Life Technologies, UK), 20 mM L-Glutamine (Life technologies, UK), 20 ng/mL EGF (Invitrogen, UK), 40 ng/mL hydrocortisone (Sigma, UK), 25 µg/mL apo-transferrin (Sigma, UK), 0.21 U/mL Insulin (Life technologies UK), 0.06% Glucose (Sigma, UK), 7.5% heat-inactivated FBS (Thermo scientific, UK), 0.5 pg/mL Tri-iodo-L-thyronine (Sigma, UK), 1x MEM vitamins (Life technologies, UK), 2 µg/mL O-phosphoryl ethanolamine, 5 mL Pen/Strep (Life technologies, UK) and 250 µL of Gentamicin (Life Technologies, UK). TKCC26 were cultured in 5% CO_2_, 5% O_2_ at 37°C using the same growth media as above. TKCC02 cells were cultured in 5% CO_2_, 5% O_2_ at 37°C in RPMI1640 medium (Life Technologies, UK) supplemented with 10% heat inactivated FBS, 20 ng/mL EGF, 5 mL Pen/Strep and 250 µL of Gentamicin. TKCC22 were cultured in 5% CO_2_, 5% O_2_ at 37°C in IMDM medium (Life Technologies, UK) supplemented with 20% heat inactivated FBS, MEM vitamins, 20 ng/mL EGF, 12.5 µg/mL Apo-transferrin, 0.21 U/mL Insulin, 5 mL Pen/Strep and 250 µL of Gentamicin. Psn-1 and Panc-1 cells were cultured in 5% CO_2_ at 37°C in high glucose DMEM (Sigma, UK) supplemented with 10% heat-inactivated FBS. Capan-1 cells were cultured in 5% CO_2_ at 37°C in IMDM medium supplemented with 20% heat inactivated FBS. Mayo cell lines were cultured in DMEM/F12 (Life Technologies, UK) supplemented with 10% FBS (ThermoFisher Scientific) and 15 mM HEPES (Life Technologies) on collagen coated plates. PaCaDD137 cells were cultured in DMEM (Life Technologies) supplemented with 10% FBS and KSFM formulation (Life Technologies). Cell lines were routinely tested for mycoplasma every 2–3 months in-house and were mycoplasma-negative. Cell lines are cultured for no more than 3 months following thawing.

### CRISPR-Cas9

#### Deletion of FAK using CRISPR

Type II CRISPR-Cas9 genome editing technology was used to deplete FAK expression in Panc47 and Panc117 cells as described in the protocol published by Ran *et al*.^56^ Briefly, guide RNAs (gFAK4: forward oligo: p5'-CAC CGT TAC TCT AAT ACT TCA TAG T-3'; reverse oligo: p5'-AAA CAC TAT GAA GTA TTA GAG TAA C-3'; gFAK6: forward oligo: p5'-CAC CGC ATA GTT GGA CTT CTT CTC T-3'; reverse oligo: p5'-AAA CAG AGA AGA AGT CCA ACT ATG C-3') were cloned into the target vector pSPCas9(BB)−2A-GFP (PX458). To generate FAK-depleted Panc47 cell clones, cells were transfected with the expression plasmids containing either the gFAK4 or gFAK6 guide sequences using Lipofectamine 2000 (Thermo Fisher Scientific). Seven days post-transfection, cells positive for GFP expression were single-cell sorted using a FACSAria II (BD Biosciences) into 96-well plates containing normal pancreatic culture media supplemented with penicillin–streptomycin (Gibco Life Technologies; 10 000 U/mL, diluted 1:100). Resulting cell colonies were tested for successful depletion of FAK expression using anti-FAK western blotting. A pWZL-FAK-wt construct was used to re-express FAK-wt into individual Panc47 and Panc117 FAK-/- clones using retroviral transduction and selection with 0.25 mg/mL hygromycin. Mutations resulting in loss of FAK expression following CRISPR-Cas9 were identified from whole genome sequencing datasets (online supplemental table 13).

10.1136/gutjnl-2022-327927.supp23 Supplementary data

#### Deletion of Psmb8, Psmb9, IRF1, HNF1A and GATA6 using CRISPR

Gene deletion was performed using the Alt-R CRISPR-Cas9 system (Integrated DNA Technologies). Cells were trypsinised and 1×10^6^ cells resuspended in 20 µL Amaxa SE 4D-Nucleofector solution (Lonza). To generate RNP complexes for transfection, 1.1 µL of 100 µM crRNA specific for the target gene was mixed with 1.1 µL of 100 µM tracrRNA, incubated at 95°C for 5 min and allowed to cool at room temperature for 30 min. 1 µL of a 61 µM stock of recombinant Cas9 was added to the crRNA/tracrRNA mix and incubated at room temperature for 10 min. The resulting RNP complex plus 1 µL of 100 µM Nucleofector enhancer was added to cells in SE buffer and nucleofected using an Amaxa 4D X Unit set to programme EN-150. Cells were allowed to recover for 48 hours, after which they were trypsinised and seeded at 1 cell per well of a 96-well plate for single-cell dilution cloning. Resulting colonies were tested for loss of *Psmb8* using PCR (as detailed in the qRT-PCR method, below) and loss of Psmb9 using western blotting. crRNAs used: *Psmb8* (ATGGCGTTACTGGATCTGTG), *Psmb9* (CGGTGTGGACTTCTTCCGTC), IRF1 (CACTGATCTGTATAACCTAC, GCACGGCTGGGACATCAACA), HNF1A (GCGTAGGAACCGGTTCAAGT, AGGCTCCAACCTTGTCACGG) and GATA6 (CGTGGTGGGCACGTAGACCG, ACAGGTCCTCCCAAGTCGAC). IRF1, HNF1A and GATA6 CRISPR-Cas9 transfected cells were not subjected to single cell cloning.

### shRNA

*Stat1*, *Stat3* and *Psmb8* knockdown cells were generated as previously described^24^ by lentiviral transduction of pLKO shRNA constructs (*Stat1*, TRCN0000054923-7; *Stat3*, TRCN0000071453-7; *Psmb8*, TRCN0000031874-8; Dharmacon), or non-targeting control pLKO-NTCO and selection with 1 mg/mL puromycin.

### IP and immunoblotting

For IP experiments, 0.25 to 1 mg of cell lysate was immunoprecipitated with either 5 µL of STAT1/3 antibody+15 µL of either anti-rabbit or anti-mouse Sepharose-conjugated antibody, 10 µL of agarose-conjugated mouse FAK antibody, or 10 µL of agarose-conjugated control IgG, and immune complexes collected. Beads were washed three times with lysis buffer, once with 0.6 mol/L lithium chloride, and then added to SDS sample buffer (Tris-HCl, pH 6.8, 20% glycerol, 5% SDS, β-mercaptoethanol and bromophenol blue). Samples were separated by SDS-PAGE, transferred to nitrocellulose and immunoblotted with specific antibodies at 1:1000 dilution.

Cell lysates prepared using RIPA buffer (50 mmol/L Tris-HCl, pH 7.6, 150 mmol/L sodium chloride, 1% Triton X-100, 0.5% deoxycholate, 0.1% SDS) supplemented with phosphatase inhibitor cocktail (Roche) and protease inhibitor cocktail (Roche) (10–20 µg protein, as measured by Micro BCA Protein Assay kit (Pierce)) were supplemented with SDS sample buffer, separated by SDS–PAGE, transferred to nitrocellulose and immunoblotted with specific antibodies (online supplemental table 12). Fluorescent detection was carried out following incubation with DyLight 680/800-conjugated secondary antibodies using a LI-COR Odyssey CLx scanner (LI-COR Biosciences).

### Immunofluorescence

Cells seeded on glass coverslips were washed twice with PBS then incubated in ice-cold 100% methanol for 10 min at −20°C. Following a 5 min PBS wash, coverslips were incubated in blocking buffer (1X PBS, 5% FBS, 0.3% Triton X-100) for 1 hour at room temperature. Coverslips were incubated with anti-STAT1 (Cell Signalling Technology, clone D1K9Y, 1:400) and anti-STAT3 (Cell Signalling Technology, clone 124H6, 1:800) antibodies overnight at 4°C. Following 3x PBS washes, coverslips were incubated with anti-Rabbit AF594 (Invitrogen, A11012, 1:200) and anti-Mouse AF488 (Invitrogen, A11029, 1:200) for 1 hour at room temperature in the dark. Following 3x PBS washes, coverslips were mounted using Prolong Gold Antifade Reagent with DAPI. Images were acquired using an Olympus Fluoview FV3000 confocal microscope equipped with a 60x PlanSApo 1.35 NA Oil objective lens. For quantification of nuclear staining, cells were seeded on glass-bottom 96-well plates and stained as above. Images were acquired using an ImageExpress High Content confocal microscope and analysed using IN Carta image analysis software (Molecular Devices).

### Chemokine secretion analysis

#### Forward-phase protein arrays

Conditioned medium was collected after 48 hours incubation. Microarrays were generated using the in-house Aushon BioSystems 2470 array printing platform. Microarrays were blocked for 1 hour with SuperG Blocking Buffer (Grace Bio Labs) at room temperature on a rocker. Media from samples were centrifuged at 1000×g for 5 min at 4°C. Supernatants were added to microarrays for 12 hours at 4°C. Microarrays were washed three times for 5 min in TBST and blocked for 10 min with SuperG Blocking Buffer at room temperature on an orbital shaker, then washed again washed three times for 5 min in TBST. Detection antibody mixtures (1:500 antibody diluted in 5% bovine serum albumin/phosphate buffered saline tween-20 (BSA/PBST), 1% SuperG Blocking Buffer) were made in plates. Microarrays were clamped and 50 µL of each antibody was added to corresponding microarray wells. Microarrays were incubated for 1 hour on a flat surface. Clamps were removed and microarrays were washed three times for 5 min in TBST. Microarrays were then blocked for 10 min with SuperG Blocking Buffer at room temperature on a rocker and again washed three times for 5 min in PBST. 3 mL of IRDye 800CW Streptavidin (LI-COR Biosciences) diluted 1 in 5000 in PBST supplemented with 5% BSA, 1% SuperG Blocking Buffer. Microarrays were covered and incubated on a rocker at room temperature for 45 min then washed for 5 min, three times in PBST followed by three 5 min PBS washes and then washed with distilled water. Microarrays were dried then scanned on the InnoScan 710 high-resolution microarray scanner (Innopsys Life Sciences). Data were normalised for protein concentration and background fluorescence in Microsoft Excel.

#### Proteome Profiler mouse chemokine arrays

Conditioned medium was collected after 48 hours incubation. Proteome Profiler Mouse Chemokine Array kit (R&D Systems, Catalogue # ARY020) was used following manufacturer’s instructions using fluorescence detection.

### Orthotopic implantation of cancer cells into the pancreas

Female C57BL/6 mice (Envigo) were supplied as age-matched, 5-week-old females and isolated for 1 week after delivery. Mice were anaethestised with inhalational isoflurane anaesthetic in oxygen, and received perioperative analgesia: buprenorphine (Vetergesic, 0.1 mg/kg s.c.) and carprofen (Rimadyl, 10 mg/kg s.c.) and also postsurgery, once daily for 48 hours. Cell lines were propagated to subconfluency to ensure they were in their exponential growth phase. Once detached from the flask and washed with PBS, 0.5×10^6^ cells of the appropriate cell line were suspended in growth factor-reduced matrigel basement membrane matrix (Scientific Laboratory Supplies), at a concentration of 0.5×10^6^ cells in 25 µL. Using aseptic technique, a 3 mm skin incision was made in the left lateral flank and lateral abdominal muscles in order to visualise the pancreas. 0.5×10^6^ cells in 25 µL matrigel were injected into the pancreas in a sterile manner. The abdominal wall was closed with Polyglactin 910 (Vicryl, 2M, Henryschein), with a single cruciate suture. Skin was closed with skin clips. Mice were monitored in a heat box set to 37°C postsurgery for 1 hour. Mice were closely monitored daily with twice-weekly weight checks following implantation. If any single terminal symptom caused by pancreatic tumour growth including weight loss equal to or exceeding 10% of the starting weight, signs of abdominal pain or abdominal distension became apparent, the animal was humanely euthanised. After either two or 4 weeks, the animals were culled (cervical dislocation) and the pancreatic tumours were harvested for analysis. Tumour weights were recorded and agreed by two observers. All tumour weights are provided in online supplemental table 14.

10.1136/gutjnl-2022-327927.supp24 Supplementary data

### CD8^+^ T-cell depletion

Anti-mouse CD8-depleting antibody (clone 53–5.8) and isotype control were purchased from BioXcell. Mice were treated with 150 µg of antibody administered by intraperitoneal injection for three consecutive days, followed by a rest period of 3 days. Following this, cells were surgically implanted into the pancreas and T-cell depletion maintained by further administration of 150 µg depleting antibody at 3-day intervals for the remainder of the experiment. Mice were culled 2 weeks after surgery and pancreatic tumours harvested for analysis as described above.

### FACS analysis

Tumours established following intrapancreatic injections of cells into mice were removed at day 14 into DMEM (Sigma-Aldrich). Tumour tissue was mashed using a scalpel and resuspended in DMEM (Sigma-Aldrich) supplemented with 2 mg/mL collagenase D (Roche) and 40 units/mL DNase1 (Roche). Samples were incubated for 30 min at 37°C, 5% CO_2_ on an orbital shaker set at 120 rpm, and then pelleted by centrifugation at 1300 rpm for 5 min at 4°C. Samples were resuspended in 5 mL of red blood cell lysis buffer (Pharm Lysis Buffer, Becton Dickinson) for 10 min at 37°C, pelleted by centrifugation at 1300 rpm for 5 min at 4°C, resuspended in PBS and mashed through a 70 µm cell strainer using the plunger from a 5 mL syringe. The cell strainer was further washed with PBS. The resulting single cell suspension was pelleted by centrifugation at 1300 rpm for 5 min at 4°C and resuspended in PBS. This step was repeated twice. The resulting cell pellet was resuspended in PBS containing Zombie NIR viability dye (1:1000 dilution (BioLegend)) and incubated at 4°C for 30 min then pelleted by centrifugation at 1300 rpm for 5 min at 4°C. Cells were resuspended in FACS buffer and pelleted by centrifugation at 1300 rpm for 5 min at 4°C. This step was repeated twice. Cell pellets were resuspended in 100 µL of Fc block (1:200 dilution of Fc antibody (eBioscience) in FACS buffer) and incubated for 15 min. A 100 µL of antibody mixture (diluted in FACS buffer (antibody details listed in online supplemental table 11)) was added to each well and the samples incubated for 30 min in the dark. The cells were then pelleted by centrifugation at 1300 rpm for 5 min at 4°C and washed twice with FACS buffer as above. Finally, cells were resuspended in FACS buffer and analysed using a BD Fortessa. For calculation of absolute numbers, tumours were weighed following removal and 123count eBeads (eBioscience) added prior to data acquisition on the flow cytometer. Data analysis was performed using FlowJo software. Statistics and graphs were calculated using Prism (GraphPad).

For flow cytometry analysis of cell lines, growth medium was removed and cells washed twice in PBS. Adhered cells were dissociated from tissue culture flasks by incubating them in enzyme-free cell dissociation solution (Millipore) for 10 min at 37°C, 5% CO2, and then scraping with a cell scraper. Cells were pelleted by centrifugation at 1300 rpm for 5 min at 4°C and washed with PBS. This step was repeated twice. Cells were then resuspended in viability dye and stained as above.

### Immunohistochemistry

Tumours were formalin fixed paraffin embedded. 5 µm sections were cut and stained as per the manufacturer’s optimised protocol for Leica BOND. Anti-CD8 antibody was used at a final concentration of 1:800 and anti-granzyme-B was used at a final concentration of 1:50 (online supplemental table 12). For quantification of CD8 staining, random intratumoural images were acquired and the number of positively stained cells per field of view manually counted using QuPath software. For quantification of Granzyme B staining, regions displaying positive staining were selected for analysis. Within these regions the area covered by positive staining was quantified using ImageJ. All images were acquired using an Olympus BX51 microscope equipped with a 20 × lens from a minimum of 3 tumours per group.

### NanoString nCounter and gene network analyses

RNA extracts were obtained using a RNeasy kit (Qiagen), following manufacturer’s instructions. A 100 ng of RNA was analysed using a mouse nCounter PanCancer Immune Profiling panel (NanoString Technologies) as per the manufacturer’s instructions. Hybridisation was performed for 18 hours at 65°C and samples processed using the NanoString Prep Station set on high sensitivity. Images were analysed at maximum (555 fields of view). Data were normalised using nSolver V.4.0 software (NanoString Technologies). Hierarchical cluster analysis of *Z*-transformed RNA abundance was performed using Cluster V.3.0 (C Clustering Library, V.1.54). A Euclidean distance matrix was computed using average linkage, and clustering results were visualised using Java TreeView (V.1.1.5r2).

Pathway over-representation analysis was performed using WebGestalt (V.2019), computing enrichment for selected gene clusters against a background of the total PanCancer Immune Profiling panel. Composite functional association networks were constructed for selected enriched pathways using GeneMANIA (V.3.5.1, mouse interactions) in Cytoscape (V.3.8.0). Network edges were weighted according to evidence of co-functionality using GeneMANIA. Connected networks were clustered using the force-directed algorithm in the Prefuse toolkit.

### Quantitative PCR

RNA extracts were obtained using an RNeasy kit (Qiagen), following the manufacturer’s instructions. cDNA was made from 5 µg of RNA using a First-Strand cDNA Synthesis Kit (Invitrogen). cDNA samples were diluted one in six using sterile water to make a working solution. To make cDNA dilutions for a standard curve, stock cDNA (normally FAK-/-+IFNγ) was initially diluted one in four using sterile water and three further one in five serial dilutions were prepared to make a total of 4 standards. For each gene target, a PCR master mix was prepared as follows (volumes provided per sample): 10 µL of 2×SYBR Select Master Mix (Applied Biosystems), 0.8 µL of forward primer (10 µM stock concentration), 0.8 µL of reverse primer (10 µM stock concentration), 4.4 µL of sterile water. A 16 µL of PCR master mix was added to each well a PCR plate (Applied Biosystems) and a further 4 µL of template cDNA (diluted cDNA or standard curve cDNA) added to make a total volume of 20 µL. Plates were sealed, pulse spun in a centrifuge and loaded into a StepOnePlus Real Time PCR machine (Applied Biosystems). PCR cycle conditions were as follows: 94°C for 10 min, 40× (94°C for 10 min, 57°C for 20 s, 72°C for 20 s). Primer sequences were as follows: *Eef1g* (housekeeping gene) forward primer GGCAAGGTTCCAGCATTTGA, *Eef1g* reverse primer GGAACGATGTCACTGTCAGC; *Psmb8* forward primer CGCATTCCTGAGGTCCTTTG, *Psmb8* reverse primer CAACAGCCTCTCCCAGTACT; *Psmb9* forward primer GAACCATGGGAGGGATGCTA, *Psmb9* reverse primer GACCAGGTAGATGACACCCC; *Psmb10* forward primer AAATCTACTGCTGTGGGGCT, *Psmb10* reverse primer AGAGCTGAGGTCCGTTCAAA; *H2-Kb* forward primer TGAATGGGGAGGAGCTGATC, *H2-Kb* reverse primer GCTCCAGTGACTATTGCAGC; *Nlrc5* forward primer ATCTGGCATTTGGTCTGGGA, *Nlrc5* reverse primer TGAATGAGCAAGGCCAGAGA.

### Chromatin IP

Cells were treated with 10 ng/mL IFNγ for 24 hours and Chromatin IP performed using the SimpleChIP Plus Enzymatic Chromatin IP Kit (Magnetic Beads, Cell Signalling Technologies, Catalogue # 9005) as per the manufacturer’s instructions. ChIP grade antibodies were obtained from Cell Signalling Technologies: STAT1 (Catalogue # 9172), IRF1 (Catalogue # 8478). Previously validated primer sequences specific to the promoter of mouse Psmb8 were obtained from ChIPprimersDB.^36^

### RNAseq analysis

Prior to transcript quantification, rRNA reads were removed from sequencing reads using SortMeRNA^57^ (V.2.1) and fastq files were processed with fastp^58^ (V.0.20.0) using default settings to trim adaptors. Quantification was performed against GRCm38 using Salmon (V.1.0.0).^59^ All analyses were performed in R using R and Bioconductor packages. Transcript-level abundances from Salmon were imported using the package tximport^60^ and transcripts were mapped to genes using the package EnsDb.Mmusculus.V.79.^61^ Read counts were normalised using the package DESeq2^62^ and genes without a normalised read count of at least five in at least three samples were removed. For downstream analysis, counts were transformed using varianceStabilizingTransformation (DESeq2). Samples were scored for expression of genes associated with ‘squamous’ and ‘classical’ subtypes of pancreatic cancer using the package GSVA.^63^ Squamous and classical genes were previously identified as differentially expressed between squamous and classical PDCLs^49^ and genes with adjusted p≤0.05 and log fold change ≥1 (squamous) or log fold change ≤−2 (classical) were selected. Human genes were converted to mouse homologs using biomaRt^64^ and each sample was scored using GSVA for the squamous and classical gene sets independently.

### Antigen profiling

#### Sample processing

Cells were lysed in mild detergent buffer (50 mM Tris-HCl, pH 8.5, 150 mM NaCl, 1% Triton X-100) with protease and phosphatase inhibitors (Roche). Lysates were cleared by centrifugation at 13 000 rpm for 15 min at 4°C. MHC-I antibody (anti-mouse MHC-1 (H-2Kb) clone Y-3 (2BScientific/BioXCell)) was conjugated to Dynabeads (Invitrogen) as per manufacturer’s protocol, and 50 µg of conjugated MHC-I antibody incubated with 20 mg of total protein overnight at 4°C. Dynabeads were washed 3×with mild detergent buffer, transferred to a fresh tube and washed a further 3×with detergent-free TBS. Peptides were eluted from Dynabeads by incubating them for 5 min with 0.1% trifluoroacetic acid (TFA) and then desalted using stage-tips.

#### MS/MS analysis

Desalted peptides were loaded onto a 15 cm emitter packed with 1.9 µm ReproSil-Pur 200 C18-AQ (Dr Maisch, Germany) using a RSLC-nano uHPLC systems connected to a Fusion Lumos mass spectrometer (both Thermo Fisher Scientific). Peptides were separated by a 40 min linear gradient from 5% to 30% acetonitrile, 0.5% acetic acid. The mass spectrometer was operated in DDA mode, acquiring an MS in the range 350–1650 Da at 120 k resolution followed by MS/MS at 30 k resolution with a NCE setting of 28. Data were analysed using MaxQuant searching against the murine UniProt database using unspecific protein digest.

#### Closed database search

Raw files obtained from the MS/MS experiments were converted to mzML format with Trans-Proteomic Pipeline compatibility using MSConvert (V. 3.0.21).^65^ The murine UniProt database (UP000000589), including isoforms, was downloaded from https://www.uniprot.org/proteomes/UP000000589 containing only reviewed sequences. The database was customised to include contaminants and reverse target decoy sequences using Philosopher (V.4.0.0).^66^ mzML files were subjected to a closed proteomic search against the customised target decoy mouse database using MS-GF+ (V.20210322).^67^ The spectra obtained from the closed search were validated at a false discovery rate (FDR) of 1% (peptide spectrum match (PSM) level) using Scavager (V.0.2.4).^68^ The validation was performed against the target decoy database. A validated list of PSMs was obtained from all four samples and merged with the search results obtained from MaxQuant. The final, consolidated dataset was used for downstream analysis.

#### Computational characterisation of physicochemical properties of peptides

Two physicochemical properties of each peptide that were important for their binding to HLA molecules, length and C-terminal hydrophobicity, were evaluated and statistically analysed for significant differences using in-house developed Python (V.3.9) scripts. The C-terminal hydrophobicity was calculated using the Kyte-Doolittle index.^33^

#### HLA binding analysis

The binding of each peptide to MHC-I molecules was analysed using NetMHCpan (V.4.0),^31^ at a cut-off threshold of 0.5 for strong binders and two for weak binders and against all six mouse MHC-I alleles, namely H2-Db, Dd, Kb, Kd, Kk and Ld. Strong and weak binders were identified and sorted based on their predicted affinity scores (measured in nM) and strong binders in each sample were selected. To correct for possible overestimation in the number of binders due to imbalanced sample sizes, a scaled binding score was derived for strong binders as follows:

$$scaledbindingscore=\frac{n_{strong}\times 100}{\sum s}$$

where *n_strong_* =number of strong binders and *s*=total peptide number of FAK-/-, FAK-wt, FAK-/-Psmb8-/-C23 or FAK-/-Psmb8-/-C34. The scaled frequency of strong binders was compared across samples to identify the sample(s) with the maximum number of strong binders.

### Expression analysis of common peptides between FAK-wt and FAK-/- samples

To undertake expression analysis of label-free proteomic data, the number of unique spectra mapping onto each peptide were calculated to obtain the expression of peptides common between FAK-wt and FAK-/- samples.^69^ The raw spectral counts obtained were then normalised to obtain a Normalised Expression Score (NES) as follows. For FAK-/- samples,

$$NES_{-/-}=\frac{SC\times 1000}{n_{peptide}}$$

For FAK-WT samples,

$$NES_{wt}=\frac{SC\times 1000}{n_{peptide}}\times \frac{s_{1}}{s_{2}}$$

where *SC*=number of raw spectral counts for each peptide, *n_peptide_* =total number of peptides in that sample, 1000=scaling factor to express the results up to two decimal places and *s*1/*s*2=correction factor equal to (total peptide number of FAK-wt)/(total peptide number of FAK-/-) to correct for overestimation due to a smaller denominator. The fold change of the common peptides was determined by computing NES_-/-_/NES_wt_, which was then binary-logarithm transformed. To assess the significance of the fold change, a Wilcoxon rank sum test was performed, which was corrected for multiple comparisons using Bonferroni correction.

#### Mutational analysis of FAK-/- peptides

To identify the presence of mutations in peptides identified from FAK-/- cells, whole genome sequencing was performed using Illumina SeqLab. Variant data in the form of variant calling files were converted to a FASTA file by an in-house-developed Python (V.3.9) script, using mouse gene sets (Mus_musculus.GRCm39.104.gtf) and CDS sequences (Mus_musculus.GRCm39.cds.all.fa), both downloaded from the ENSEMBL genome browser (release 104). This FASTA file was then appended to the mouse database downloaded previously and converted to a target decoy database using Philosopher. This target decoy database was then used to search the mzML files from the FAK-/- sample and validated at 1% FDR using Scavager. The validated PSMs were then searched for the presence of mutants by evaluating the coverage of these peptide sequences over the mutated sequences that were identified by the vcf-to-FASTA conversion step.

### Proteomic analysis of human PDCLs

Cells were plated 24 hours before nucleofection to allow 100% confluency. On the day of nucleofection, cells were washed with PBS and incubated with TrypLE (Thermofisher, UK) at 37°C to detach cells from the plate. Cells were washed x2 with PBS and centrifuged at 800 g for 5 min at 4°C. Supernatant was removed and the cell pellet resuspended in 20 µL of Amaxa SE buffer (Lonza, Switzerland). To generate RNP complexes for transfection, 1.1 µL of 100 µM crRNA was mixed with 1.1 µL of 100 µM tracrRNA, incubated at 95°C for 5 min and allowed to cool at room temperature for 30 min. A 1 µL of a 61 µM stock of recombinant HiFi Cas9 Nuclease V3 was added to the crRNA/tracrRNA mix and incubated at room temperature for 10 min. Cas9 RNP complex formation was performed separately for two different cRNAs (cRNA 1: Hs.Cas9.PTK.1.AX and cRNA 2: Hs.Cas9.PTK.1.AA, IDT technologies, Belgium) and the two annealed Cas9 RNP complexes mixed prior to adding 1 µL of 100 µM Nucleofector enhancer. The resulting mix containing two cRNAs was added to cells in SE buffer and nucleofected using an Amaxa 4D X Unit set to programme EN-150. Cells were resuspended in pre-heated medium, plated and cultured under normal growth conditions for 3 days or until reaching 60% confluence. Following this, medium was removed and replaced with high glucose DMEM containing L-Glutamine (Sigma, UK) supplemented with 10% heat inactivated FBS and cells were cultured in 5% CO_2_ at 37°C for 24 hours.

For proteomic analysis, cells were lysed using 6M GuHCl, 200 mM Tris (pH=8.5) supplemented with 1 mg/mL Chloracetonitrille and 1.5 mg/mL Tris(2- carboxyethyl)phosphine. Samples were boiled for 5 min at 95°C then snap frozen for storage. Snap-frozen samples were thawed, sonicated and boiled for 5 min at 95°C. Lysyl endopeptidase solution at a ratio of 50:200 enzyme to protein concentration was added to the lysates to aid digestion. Samples were incubated for 4 hours at 37°C and pulse spun in a centrifuge. The alkaline pH of the solution was checked using pH paper. 5 µg of trypsin was added per sample and lysates were incubated at 37°C overnight. The following day, stage tips were prepared using a C18 membrane. A 15 µL of methanol was added onto each stage tip in order to activate them and the stage tips centrifuged at 200 g for 2 min. A 50 µL of 10% TFA was then added to each stage tip and centrifuged at 200 g for 2 min. Stage tips were then washed using TFA and centrifuged for a further 3 min. A 20 µL of 10% TFA was added to the cell lysates and samples vortexed. The acidic pH was checked using pH paper to ensure correct digestion. Samples were then centrifuged at 17 000 rpm for 5 min. The volume of sample relating to 10 µg of protein was calculated and added onto the stage tips. The loaded stage tips were centrifuged at 500 g for 5 min then washed twice with 0.1% TFA for 3 min at 300 g. The stage tips were then washed using 50% acetonitrile (ACN) and 0.5% TFA elution buffer, centrifuged at 300 g for 3 min and the eluted solution vacuum dried. The dried samples were reconstituted in 15 µg of equilibration buffer (0.1% TFA) and the yield checked using NanoDrop. Samples were then diluted to a final concentration of 0.5 mg/mL and analysed using an Orbitrap Fusion Lumos Tribrid mass spectrometer (Thermo Scientific). The sequence was randomised and imported into Xcalibur software. The Xcalibur file was imported into Spectronaut software and analysis performed using the data-independent acquisition method.

The Spectronaut output file with LFQ values was filtered for Homo sapiens proteins and a Q-value equal to or less than 0.05. The resulting gene list was uploaded to the WEB-based GEne SeT AnaLysis Toolkit (available as both, R-script and online platform at http://www.webgestalt.org) and the following parameters selected: Organism—Homo sapiens, Method—Over representation analysis, Functional database—geneontology—Biological process noRedundant . The gene list was uploaded using Gene Symbol ID. The reference set was ‘genome-protein coding’. The significance level was set up as the FDR and the number of categories expected from the set cover was selected as max value. Graphs plotted using R-studio V.4.1.2 and ggplot2 package. Pathway analysis was performed using Cytoscape V.3.9.1 software with GeneMANIA V.3.5.2 plugin. Significantly differentiated proteins of interest (Q value≤0.05) were exported from the proteomic dataset. The network was drawn using Homo sapiens database as well as the predicted and physical interaction networks. The colour of nodes corresponds to the ratio of differentially expressed proteins and the size of the node corresponds to the -log10 of Q-value. Hierarchical clustering to confirm subtype specification based on protein expression was performed using Morpheus software (https://software.broadinstitute.org/morpheus). Proteins present within the proteomics datasets were selected based on previously reported gene sets associated with classical and squamous subtypes.^47 49^ Normalised raw intensities exported from Spectronaut software were converted to a robust Z score and hierarchical clustering performed using one minus Pearson correlation, average linkage. Colouring is relative within each row.

### Analysis of ICGC and TCGA bulk RNAseq datasets

Transcriptomic profiling and molecular subtyping of the ICGC PACA-AU cohort was performed as previously described.^42^ Individual tumours were classified as squamous, pancreatic progenitor, aberrantly differentiated endocrine exocrine (ADEX) or immunogenic. Expression values were processed and normalised as previously described.^42^

TCGA PAAD gene expression data were downloaded, preprocessed and normalised using the R/Bioconductor packages ‘TCGAWorkflow’^70^ and ‘TCGAbiolinks’.^70–72^ Normalised values underwent a log2(n+1) transformation. TCGA PAAD samples underwent molecular subtyping previously and were classified as squamous, pancreatic progenitor, ADEX or immunogenic.^50^

Gene lists were obtained from the curated gene-sets (C2) from the Molecular Signatures Database (MSigDB, V.7.5.1) through the R package ‘msigdbr’.^73^ The following gene-sets were used for analysis: ‘KEGG_ANTIGEN_PROCESSING_AND_PRESENTATION’ and ‘REACTOME_INTERFERON_GAMMA_SIGNALING’. Gene sets were modified to include only genes that were regulated by FAK in the mouse models (AP&P - B2M, CD74, CIITA, HLA-A, HLA-B, HLA-C, IFI30, TAP1, TAP2, TAPBP; IFNγ signalling—CIITA, HLA-A, HLA-B, HLA-C, IFI30, IRF7, MT2A, OAS2, SOCS1, SOCS3). ‘Immunoproteasome’ represents the genes ‘PSMB8’, ‘PSMB9’ and ‘PSMB10. Gene-set enrichment scores were obtained for each gene-set in each sample using the R/Bioconductor package GSVA.^63^

Differential expression analysis was performed with the R/Bioconductor package DESeq2^62^ for the following comparisons: 47–1 FAK-/- vs 47-4-3 FAK-/- and 47-6-11 FAK-/-; 47–1 FAK-/- vs 47–1 FAK-wt. The up-regulated genes from both comparisons were overlapped to define a 34 gene signature of FAK loss. 32 of these genes had human homologs; these genes were used as the FAK-/- signature. A score for this signature was obtained for each ICGC and TCGA sample using GSVA.^63^ FAK-/- scores were correlated with the three gene-set scores (Antigen processing and presentation, IFNγ signalling, immunoproteasome) and scatter plots were created using the R package ‘ggpubr’.^62^

ICGC and TCGA samples were assigned a ‘gradient score’ using genes upregulated and downregulated in squamous samples versus non-squamous samples from ICGC PACA-AU.^42^ Genes were filtered to retain only those with adjusted p<1×10^−5^. Genes with logFC>3 were used as squamous markers (26 genes) and genes with logFC < −3 were used as classical markers (22 genes). All ICGC and TCGA samples were assigned a score for these gene-sets using the R package ‘singscore’^74 75^ with the squamous markers used as the ‘upSet’ and classical markers used as the ‘downSet’. Box plots and dotcharts were created using the R package ‘ggpubr’.^76^

### Transcriptomic subtyping of human PDCLs

TKCC, Mayo and PaCaDD cell lines have been previously subtyped as squamous or classical through transcriptomic analysis.^49 77^ Genes differentially expressed between the squamous and classical cell lines in these studies (adjusted p<0.05 and absolute logFC>2; 406 genes) were used to subtype 3 cell lines from CCLE: Capan, Panc1 and PSN1. Expression data for the CCLE project were downloaded from the depmap portal and expression of the 406 differentially expressed genes was used to classify the cell lines as classical or squamous. Heatmaps of gene expression data were created using the R/Bioconductor package ‘ComplexHeatmap’^78^ and colours were defined using the ‘colorRamp2’ function from the R package ‘circlize’.^79^

## Statistics

Statistical analysis was carried out using GraphPad Prism V.8 for Windows (GraphPad Software), R (V.4.0.5) and Python (V.3.9). All error bars on graphs represent SEM. Statistical tests are detailed in the figure legends.

## Data Availability

All data relevant to the study are included in the article or uploaded as online supplemental information.
