# Supplementary figures and images for: FAK suppresses antigen processing and presentation to promote immune evasion in pancreatic cancer
Source: Gut. 2023 Mar 28;73(1):131–55. doi: 10.1136/gutjnl-2022-327927 (PMC10715489; doi:10.1136/gutjnl-2022-327927)

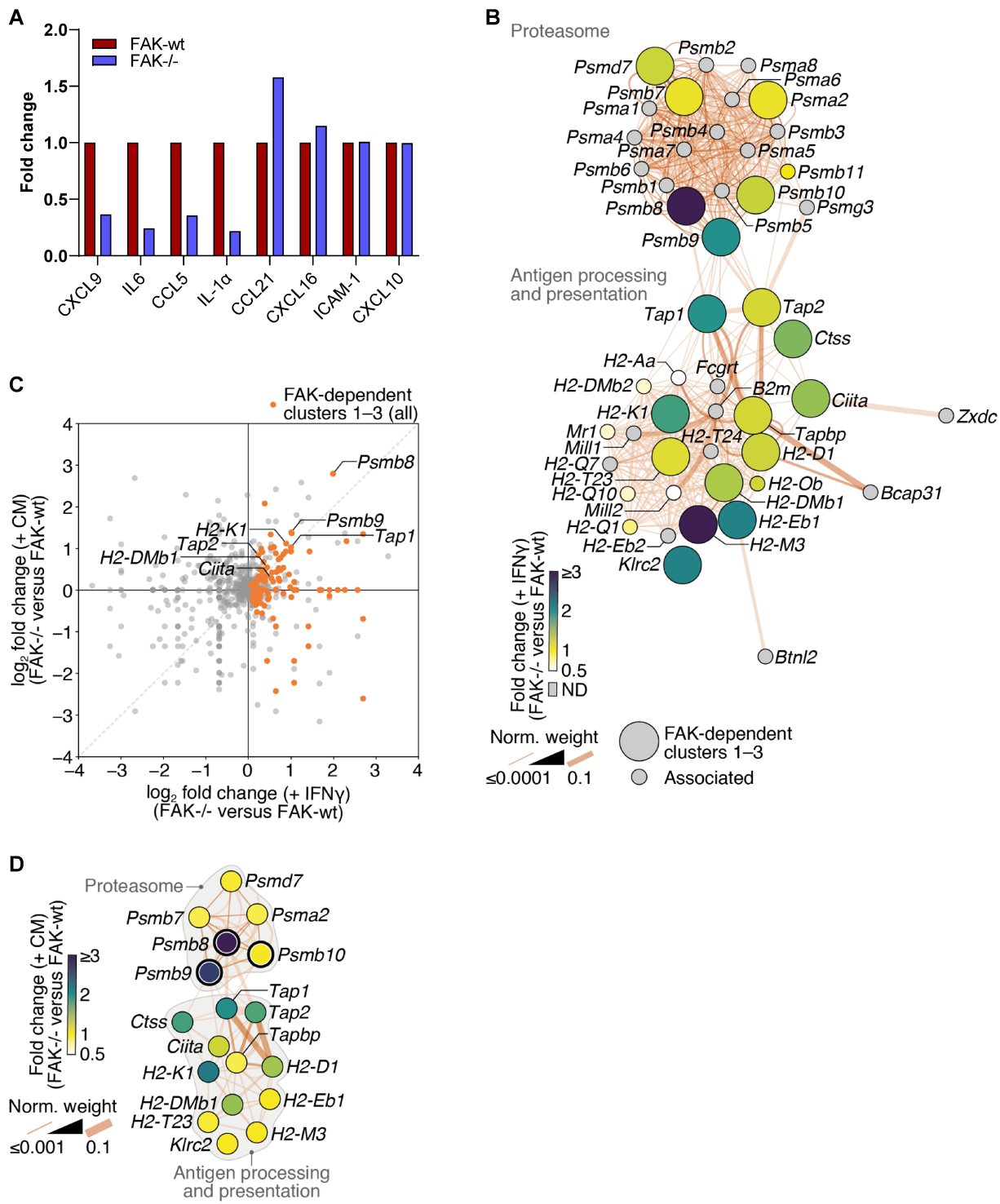

Supplement: Supplementary data [file gutjnl-2022-327927supp001.pdf]

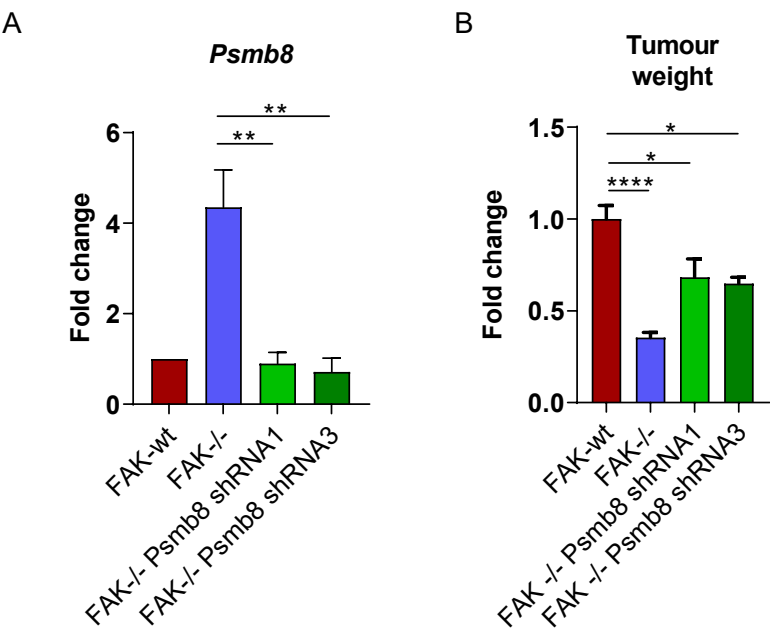

**Supplementary Figure 2. *Psmb8* depletion promotes growth of Panc47 FAK<sup>-/-</sup> tumours.**

Supplement: Supplementary data [file gutjnl-2022-327927supp002.pdf]

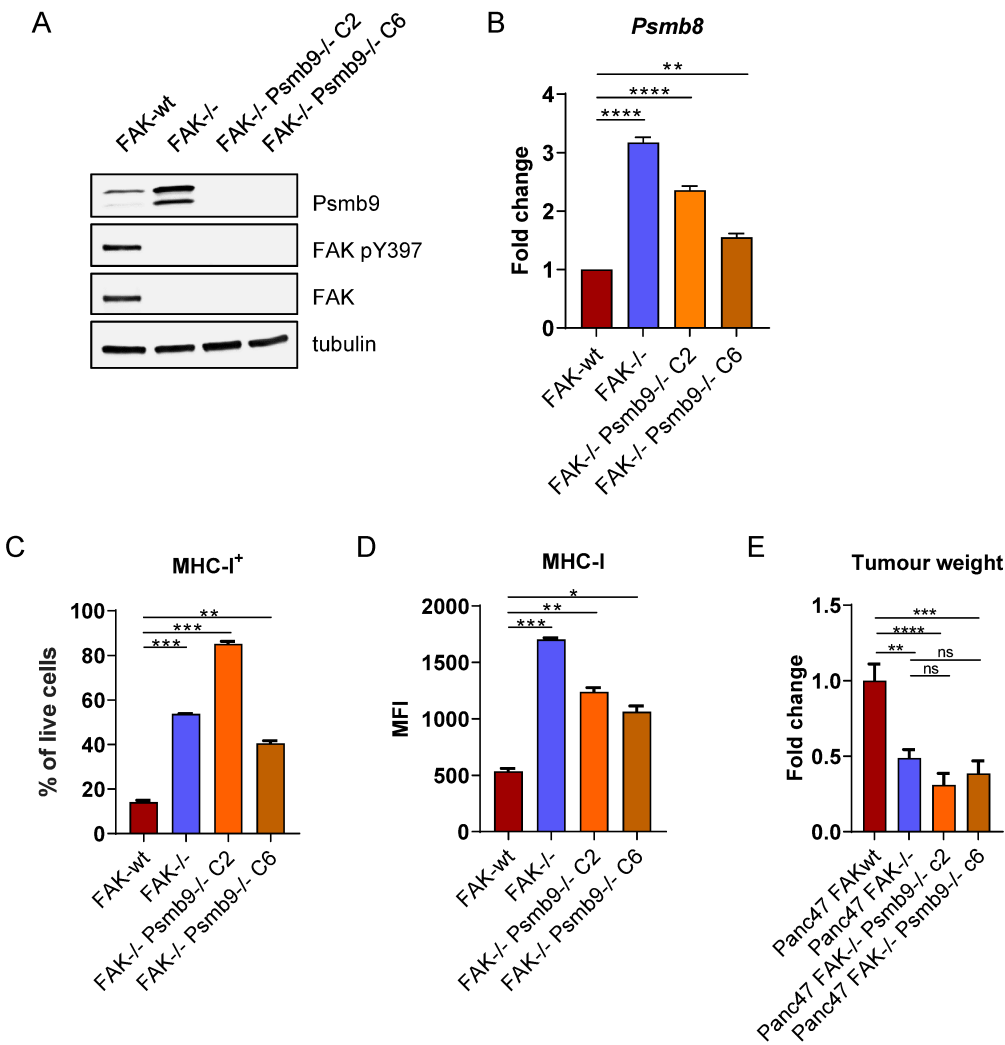

Supplementary Figure 3. Psmb9 deletion has no effect on the growth of Panc47 FAK-/- tumours.

Supplement: Supplementary data [file gutjnl-2022-327927supp003.pdf]

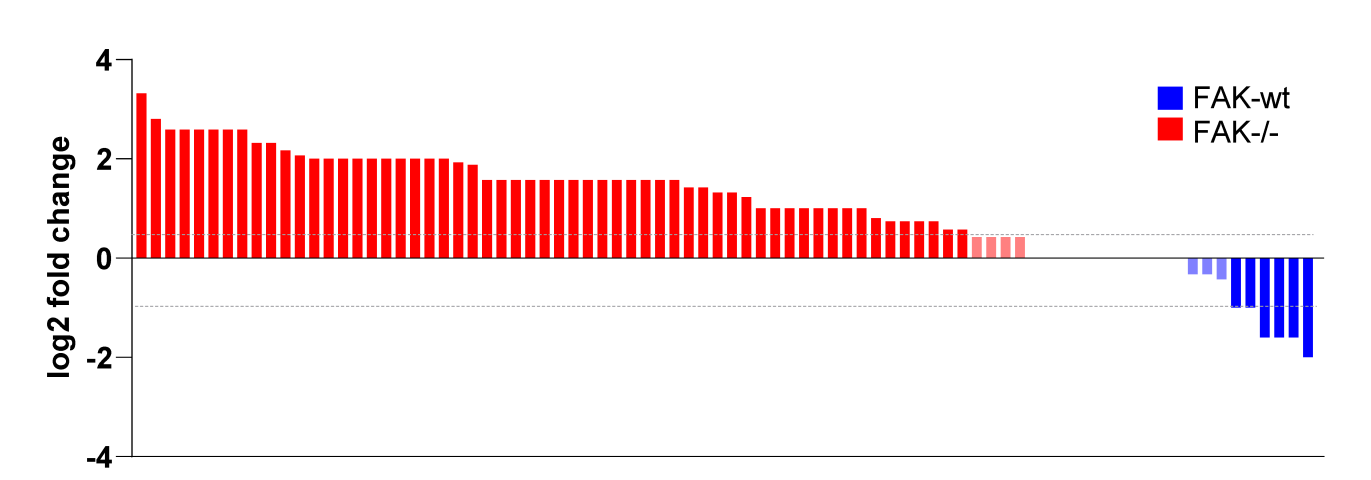

Supplement: Supplementary data [file gutjnl-2022-327927supp005.pdf]

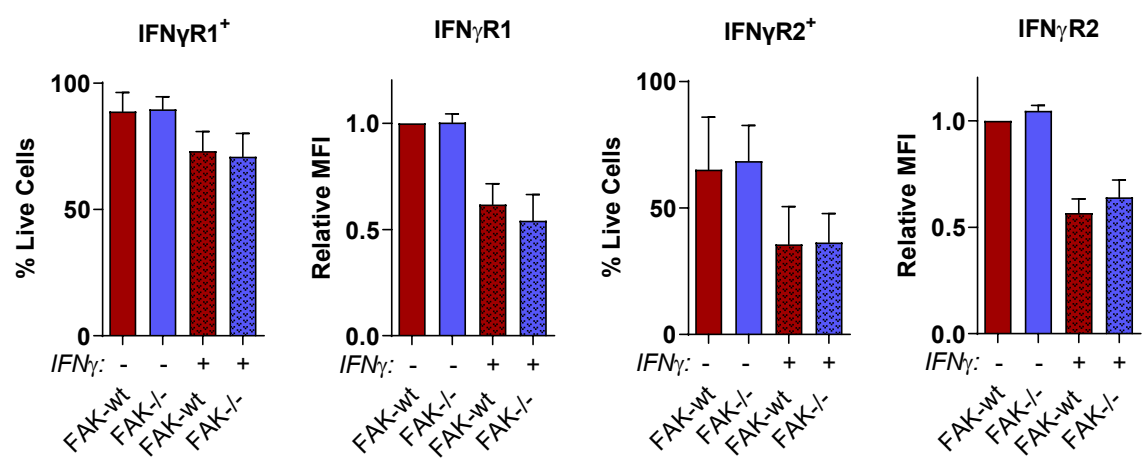

Supplementary Figure 6. FAK does not regulate surface expression of the IFNγ receptors.

Supplement: Supplementary data [file gutjnl-2022-327927supp006.pdf]

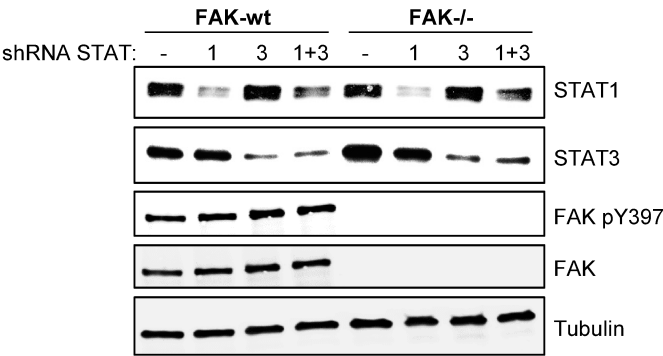

Supplementary Figure 7. STAT1 and STAT3 shRNA in FAK-wt and FAK-/- cells.

Supplement: Supplementary data [file gutjnl-2022-327927supp007.pdf]

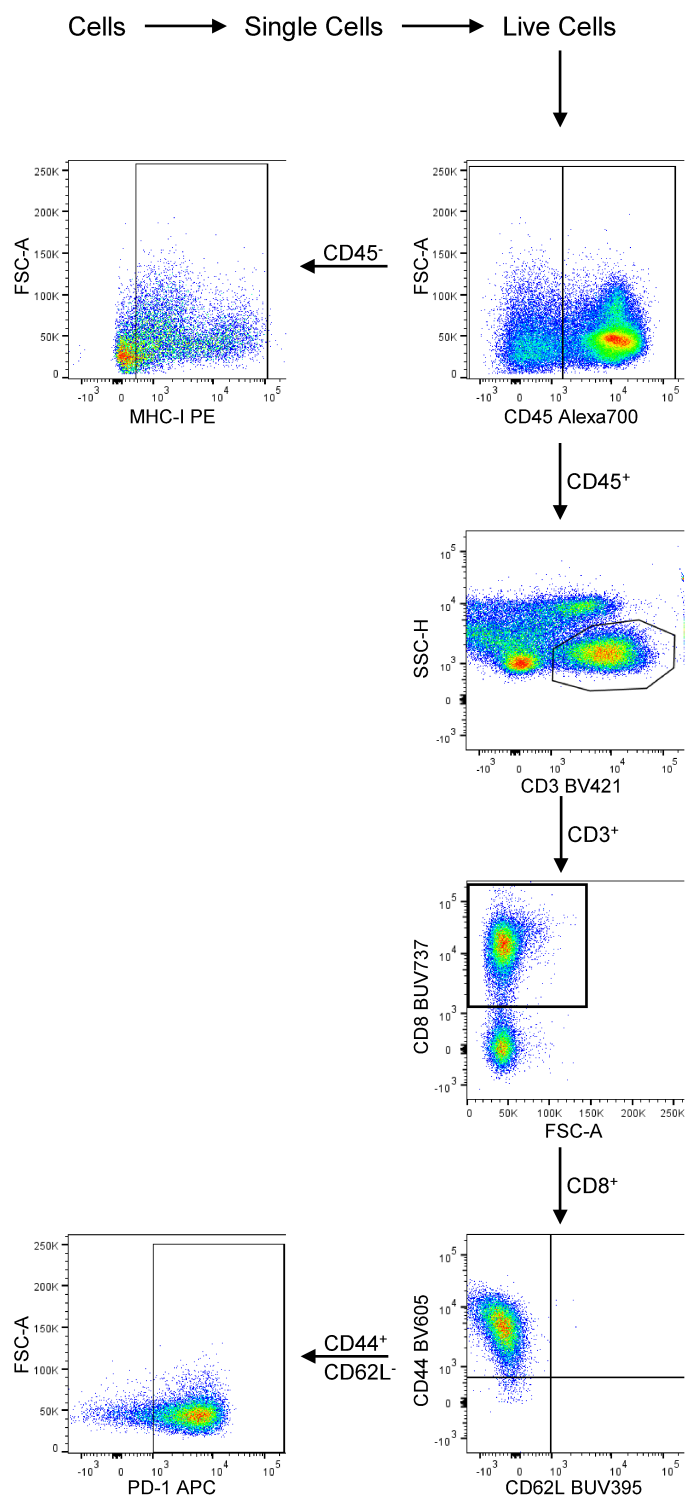

Supplementary Figure 8. Flow cytometry gating strategy for data in Figure 5.

Supplement: Supplementary data [file gutjnl-2022-327927supp008.pdf]

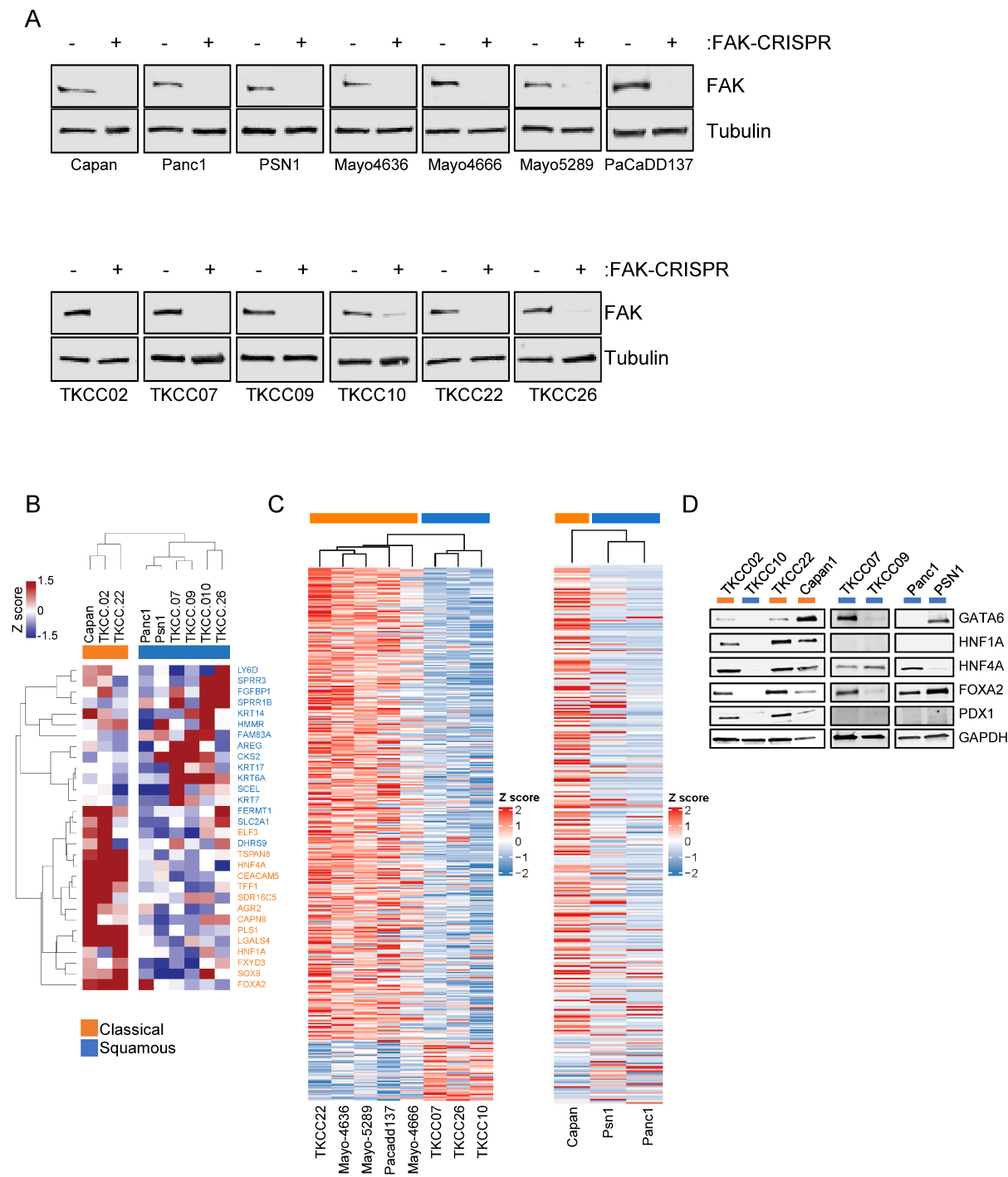

Supplementary Figure 10. Proteomic analysis of FAK function in human PDCLs.

Supplement: Supplementary data [file gutjnl-2022-327927supp010.pdf]
