# Supplementary material for: FAK suppresses antigen processing and presentation to promote immune evasion in pancreatic cancer
Source: Gut. 2023 Mar 28;73(1):131–55. doi: 10.1136/gutjnl-2022-327927 (PMC10715489; doi:10.1136/gutjnl-2022-327927)
Supplement: Supplementary data [file gutjnl-2022-327927supp015.pdf]

FAK -/- hydrophobicity data

S.No.	Score	Frequency	Hydrophobocity	Residues	Percentage
1	3.8	327	Hydrophobic	L	53.30
2	4.2	63	Hydrophobic	V	10.30
3	1.9	51	Hydrophobic	M	8.32
4	-4.5	37	Hydrophilic	R	6.04
5	2.8	31	Hydrophobic	F	5.06
6	4.5	31	Hydrophobic	I	5.06
7	-3.9	25	Hydrophilic	K	4.08
8	-0.4	12	Neutral	G	1.96
9	-3.5	11	Hydrophilic	D/E/N/Q	1.79
10	-0.8	7	Neutral	S	1.14
11	1.8	7	Hydrophobic	A	1.14
12	-0.9	4	Neutral	W	0.63
13	-3.2	3	Hydrophilic	H	0.49
14	-1.6	2	Neutral	P	0.33
15	-1.3	2	Neutral	Y	0.33

FAK WT hydrophobicity data

S.No.	Score	Frequency	Hydrophobicity	Residues	Percentage
1	3.8	54	Hydrophobic	L	37.50
2	-4.5	20	Hydrophilic	R	13.90
3	-3.9	13	Hydrophilic	K	9.03
4	1.9	13	Hydrophobic	M	9.03
5	4.2	11	Hydrophobic	V	7.64
6	-0.4	6	Neutral	G	4.17
7	2.8	6	Hydrophobic	F	4.17
8	1.8	5	Hydrophobic	A	3.47
9	-3.5	5	Hydrophilic	D/E/N/Q	3.47
10	4.5	4	Hydrophobic	I	2.78
11	-1.6	2	Neutral	P	1.40
12	-0.8	2	Neutral	S	1.40
13	-3.2	1	Hydrophilic	H	0.69
14	-1.3	1	Neutral	Y	0.69
15	-0.7	1	Neutral	T	0.69

Clone 23 hydrophobicity data

S.No.	Score	Frequency	Hydrophobicity	Residues	Percentage
11	3.8	48	Hydrophobic	L	41.40
9	1.9	13	Hydrophobic	M	11.20
10	2.8	11	Hydrophobic	F	9.48
1	-4.5	10	Hydrophilic	R	8.62
2	-3.9	8	Hydrophilic	K	6.90
3	-3.5	7	Hydrophilic	D/E/N/Q	6.03
12	4.2	6	Hydrophobic	V	5.17
8	1.8	4	Hydrophobic	A	3.45
13	4.5	3	Hydrophobic	I	2.59
6	-0.7	2	Neutral	T	1.72
7	-0.4	2	Neutral	G	1.72
4	-3.2	1	Hydrophilic	H	0.90
5	-0.8	1	Neutral	S	0.90

Clone 34 hydrophobicity data

S.No.	Score	Frequency	Hydrophobicity	Residues	Percentage
1	3.8	16	Hydrophobic	L	23.50
2	-4.5	13	Hydrophilic	R	19.10
3	-3.9	10	Hydrophilic	K	14.70
4	1.8	8	Hydrophobic	A	11.80
5	-3.5	6	Hydrophilic	D/E/N/Q	8.82
6	-0.4	5	Neutral	G	7.35
7	-0.8	4	Neutral	S	5.88
8	2.8	2	Hydrophobic	F	2.94
9	1.9	1	Hydrophobic	M	1.47
10	-1.6	1	Neutral	P	1.47
11	4.2	1	Hydrophobic	V	1.47
12	4.5	1	Hydrophobic	I	1.47
