# Supplementary material for: FAK suppresses antigen processing and presentation to promote immune evasion in pancreatic cancer
Source: Gut. 2023 Mar 28;73(1):131–55. doi: 10.1136/gutjnl-2022-327927 (PMC10715489; doi:10.1136/gutjnl-2022-327927)
Supplement: Supplementary data [file gutjnl-2022-327927supp020.pdf]

	Kras: chr6:145246771 C>T c.35G>A p.Gly12Asp				Trp53: chr11:69588512 G>A c.506G>A p.Arg172His			
Sample	Ref	Alt	Total	Alt AF	Ref	Alt	Total	Alt AF
FAK-/-	17	44	61	0.72	0	44	44	1.00
47_4_3_FAK-/-	14	20	34	0.59	0	42	42	1.00
47_6_11_FAK-/-	21	36	57	0.63	0	37	37	1.00
117_4_7_FAK-/-	15	58	73	0.79	0	44	44	1.00
117_6_4_FAK-/-	17	38	55	0.69	0	37	37	1.00
117_6_9_FAK-/-	24	68	92	0.74	0	41	41	1.00

Supplementary Table 10. Kras and Trp53 mutational status of pancreatic cell lines.
