# Supplementary material for: FAK suppresses antigen processing and presentation to promote immune evasion in pancreatic cancer
Source: Gut. 2023 Mar 28;73(1):131–55. doi: 10.1136/gutjnl-2022-327927 (PMC10715489; doi:10.1136/gutjnl-2022-327927)
Supplement: Supplementary data [file gutjnl-2022-327927supp021.pdf]

**Antigen**	**Fluorophore**	**Clone**	**Supplier**
CD45	Alexa700	30-F11	Biolegend
MHC-I	PE	34-1-2S	Invitrogen
IFNγ	PE-Cy7	XMG1.2	Biolegend
CD3	BV412	KT3.1.1	Biolegend
CD8	BUV737	53-6.7	BD Biosciences
CD44	BV605	IM7	Biolegend
CD62L	BUV395	MEL-14	BD Biosciences
PD-1	APC	J43	eBioscience
IFNγR1	PE	200	eBioscience
IFNγR2	PE	MOB-47	Biolegend
Live/Dead	Zombie NIR		Biolegend

Supplementary Table 11. Flow cytometry antibodies.
