# Supplementary material for: FAK suppresses antigen processing and presentation to promote immune evasion in pancreatic cancer
Source: Gut. 2023 Mar 28;73(1):131–55. doi: 10.1136/gutjnl-2022-327927 (PMC10715489; doi:10.1136/gutjnl-2022-327927)
Supplement: Supplementary data [file gutjnl-2022-327927supp022.pdf]

Antibody	Supplier	Cat. No.
FAK clone 4.47	Merck-Millipore	05-537
phosphoTyr397 FAK	Cell Signaling Technologies	3283
FAK clone 4.47 agarose conjugated	Merck-Millipore	16-173
PSMB9	Abcam	ab184172
PSMB10	Abcam	ab183506
phosphoTyr701 STAT1	Cell Signaling Technologies	7649
STAT1	Cell Signaling Technologies	9172
phosphoTyr705 STAT3	Cell Signaling Technologies	4113
STAT3	Cell Signaling Technologies	9139
tubulin	Cell Signaling Technologies	2144
HNF1A	Cell Signaling Technologies	89670
GATA6	Cell Signaling Technologies	5851
HNF4A	Abcam	ab181604
FOXA2	Cell Signaling Technologies	8186
PDX1	Cell Signaling Technologies	5679
IRF1	Cell Signaling Technologies	8478
Pyk2	BD	610549
Pyk2 pY402	Cell Signaling Technologies	3291
GAPDH	Cell Signaling Technologies	5174
CD8	Cell Signaling Technologies	98941
Granzyme B	Cell Signaling Technologies	46890

Supplementary Table 12. IP / Western blotting and immunohistochemistry antibodies.
