# Supplementary material for: FAK suppresses antigen processing and presentation to promote immune evasion in pancreatic cancer
Source: Gut. 2023 Mar 28;73(1):131–55. doi: 10.1136/gutjnl-2022-327927 (PMC10715489; doi:10.1136/gutjnl-2022-327927)
Supplement: Supplementary data [file gutjnl-2022-327927supp023.pdf]

gFAK oligo	Sample	Chr	Pos	Ref	Alt	Depth	Alt depth	Alt AF
4	117_4_7_FAK-/-	chr15	73320799	TTC	T	50	36	*0.72
6	117_6_4_FAK-/-	chr15	73320774	CTCTAATACTTCATAGTTGGACTTCTTCTCTAGGGCATTACCCCTCA	C	51	12	0.24
6	117_6_4_FAK-/-	chr15	73320786	A	AT	34	32	*0.94
6	117_6_9_FAK-/-	chr15	73320786	AT	A	16	16	*1.00
4	FAK-/-	chr15	73320759	CACGTGCAGTACTTACTCTAATACTTCATAGTTGG	C	32	9	0.28
4	FAK-/-	chr15	73320786	AT	A	14	14	*1.00
4	47_4_3_FAK-/-	chr15	73320785	CA	C	41	17	*0.41
4	47_4_3_FAK-/-	chr15	73320785	CAT	C	41	12	0.29
4	47_4_3_FAK-/-	chr15	73320787	T	TA	39	12	0.31

Supplementary Table 13. FAK indels detected in pancreatic cell lines following CRISPR.
