# Supplementary material for: FAK suppresses antigen processing and presentation to promote immune evasion in pancreatic cancer
Source: Gut. 2023 Mar 28;73(1):131–55. doi: 10.1136/gutjnl-2022-327927 (PMC10715489; doi:10.1136/gutjnl-2022-327927)
Supplement: Supplementary data [file gutjnl-2022-327927supp018.pdf]

>ENSMUSP00000043594_c.3228:A>G_p.I1077V ENSMUST00000041447.5|Trpa1-201|ENSMUSG00000032769|-|chr1:g.14943879:T>C
MKRGLRRILLPEERKEVQGVVYRGVGEDMDCSKESFKVDIEGDMCRLEDFIKNRRKLSKYEDENLCPLHHAAA
EGQVELMELIINGSSCEVLNIMDGYGNTPLHCAAEKNQVESVKFLLSQGANPNLRNRNMMSPLHIAVHGMYNE
VIKVLTEHKATNINLEGENGNTALMSTCAKDNSEALQILLEKGAKLCKSNKWGDYPVHQAAFSGAKKCMELIL
AYGEKNGYSRETHINFVNHKKASPLHLAVQSGDLDMIKMCLDNGAHIDMMENAKCMALHFAATQGATDIVKLM
ISSYTGSSDIVNAVDGNQETLLHRASLFDHHDLAEYLISVGADINSTDSEGRSPLILATASASWNIVNLLLCK
GAKVDIKDHLGRNFLHLTVQQPYGLRNLRPEFMQMQHIKELVMDEDNDGCTPLHYACRQGVPVSVNNLLGFNV
SIHSKSKDKKSPLHFAASYGRINTCQRLLQDISDTRLLNEGDLHGMTPLHLAAKNGHDKVVQLLLKKGALFLS
DHNGWTALHHASMGGYTQTMKVILDTNLKCTDRLDEEGNTALHFAAREGHAKAVAMLLSYNADILLNKKQASF
LHIALHNKRKEVVLTTIRNKRWDECLQVFTHNSPSNRCPIMEMVEYLPECMKVLLDFCMIPSTEDKSCQDYHI
EYNFKYLQCPLSMTKKVAPTQDVVYEPLTILNVMVQHNRIELLNHPVCREYLLMKWCAYGFRAHMMNLGSYCL
GLIPMTLLVVKIQPGMAFNSTGIINGTSSTHEERIDTLNSFPIKICMILVFLSSIFGYCKEVIQIFQQKRNYF
LDYNNALEWVIYTTSIIFVLPLFLNIPAYMQWQCGAIAIFFYWMNFLLYLQRFENCGIFIVMLEVIFKTLLRS
TGVFIFLLLAFGLSFYVLLNFQDAFSTPLLSLIQTFSMMLGDINYRDAFLEPLFRNELAYPVLTFGQLIAFTM
FVPIVLMNLLIGLAVGDIAEVQKHASLKRIAMQVELHTNLEKKLPLWYLRKVDQRSTIVYPNRPRHGRMLRFF
HYFLNMQETRQEVPNIDTCLEMEILKQKYRLKDLTSLLEKQHELIKLIIQKMEIVSETEDEDNHCSFQDRFKK
ERLEQMHSKWNFVLNAVKTKTHCSISHPDF*
>ENSMUSP00000157371_c.3138:A>G_p.I1047V ENSMUST00000235071.2|Trpa1-202|ENSMUSG00000032769|-|chr1:g.14943879:T>C
MKRGLRRILLPEERKEVQGVVYRGVGEDMDCSKESFKVDIEGDMCRLEDFIKNRRKLSKYEDENLCPLHHAAA
EGQVELMELIINGSSCEVLNIMDGYGNTPLHCAAEKNQVESVKFLLSQGANPNLRNRNMMSPLHIAVHGMYNE
VIKVLTEHKATNINLEGENGNTALMSTCAKDNSEALQILLEKGAKLCKSNKWGDYPVHQAAFSGAKKCMELIL
AYGEKNGYSRETHINFVNHKKASPLHLAVQSGDLDMIKMCLDNGAHIDMMENAKCMALHFAATQGATDIVKLM
ISSYTGSSDIVNAVDGNQETLLHRASLFDHHDLAEYLISVGADINSTDSEGRSPLILATASASWNIVNLLLCK
GAKVDIKDHLGRNFLHLTVQQPYGLRNLRPEFMQMQHIKELVMDEDNDGCTPLHYACRQGVPVSVNNLLGFNV
SIHSKSKDKKSPLHFAASYGRINTCQRLLQDISDTRLLNEGDLHGMTPLHLAAKNGHDKVVQLLLKKGALFLS
DHNGWTALHHASMGGYTQTMKVILDTNLKCTDRLDEEGNTALHFAAREGHAKAVAMLLSYNADILLNKKQASF
LHIALHNKRKEVVLTTIRNKRWDECLQVFTHNSPSNRCPIMEMVEYLPECMKVLLDFCMIPSTEDKSCQDYHI
EYNFKYLQCPLSMTKKVAPTQDVVYEPLTILNVMVQHNRIELLNHPVCREYLLMKWCAYGFRAHMMNLGSYCL
GLIPMTLLVVKIQPGMAFNSTGIINGTSSTHEERIDTLKRNYFLDYNNALEWVIYTTSIIFVLPLFLNIPAYM
QWQCGAIAIFFYWMNFLLYLQRFENCGIFIVMLEVIFKTLLRSTGVFIFLLLAFGLSFYVLLNFQDAFSTPLL
SLIQTFSMMLGDINYRDAFLEPLFRNELAYPVLTFGQLIAFTMFVPIVLMNLLIGLAVGDIAEVQKHASLKRI
AMQVELHTNLEKKLPLWYLRKVDQRSTIVYPNRPRHGRMLRFFHYFLNMQETRQEVPNIDTCLEMEILKQKYR
LKDLTSLLEKQHELIKLIIQKMEIVSETEDEDNHCSFQDRFKKERLEQMHSKWNFVLNAVKTKTHCSISHPDF
*
>ENSMUSP00000043594_c.505:C>A_p.A169D ENSMUST00000041447.5|Trpa1-201|ENSMUSG00000032769|-|chr1:g.14976196:G>T
MKRGLRRILLPEERKEVQGVVYRGVGEDMDCSKESFKVDIEGDMCRLEDFIKNRRKLSKYEDENLCPLHHAAA
EGQVELMELIINGSSCEVLNIMDGYGNTPLHCAAEKNQVESVKFLLSQGANPNLRNRNMMSPLHIAVHGMYNE
VIKVLTEHKATNINLEGENGNTDLMSTCAKDNSEALQILLEKGAKLCKSNKWGDYPVHQAAFSGAKKCMELIL
AYGEKNGYSRETHINFVNHKKASPLHLAVQSGDLDMIKMCLDNGAHIDMMENAKCMALHFAATQGATDIVKLM
ISSYTGSSDIVNAVDGNQETLLHRASLFDHHDLAEYLISVGADINSTDSEGRSPLILATASASWNIVNLLLCK
GAKVDIKDHLGRNFLHLTVQQPYGLRNLRPEFMQMQHIKELVMDEDNDGCTPLHYACRQGVPVSVNNLLGFNV
SIHSKSKDKKSPLHFAASYGRINTCQRLLQDISDTRLLNEGDLHGMTPLHLAAKNGHDKVVQLLLKKGALFLS
DHNGWTALHHASMGGYTQTMKVILDTNLKCTDRLDEEGNTALHFAAREGHAKAVAMLLSYNADILLNKKQASF
LHIALHNKRKEVVLTTIRNKRWDECLQVFTHNSPSNRCPIMEMVEYLPECMKVLLDFCMIPSTEDKSCQDYHI
EYNFKYLQCPLSMTKKVAPTQDVVYEPLTILNVMVQHNRIELLNHPVCREYLLMKWCAYGFRAHMMNLGSYCL
GLIPMTLLVVKIQPGMAFNSTGIINGTSSTHEERIDTLNSFPIKICMILVFLSSIFGYCKEVIQIFQQKRNYF
LDYNNALEWVIYTTSIIFVLPLFLNIPAYMQWQCGAIAIFFYWMNFLLYLQRFENCGIFIVMLEVIFKTLLRS
TGVFIFLLLAFGLSFYVLLNFQDAFSTPLLSLIQTFSMMLGDINYRDAFLEPLFRNELAYPVLTFGQLIAFTM
FVPIVLMNLLIGLAVGDIAEVQKHASLKRIAMQVELHTNLEKKLPLWYLRKVDQRSTIVYPNRPRHGRMLRFF
HYFLNMQETRQEVPNIDTCLEMEILKQKYRLKDLTSLLEKQHELIKLIIQKMEIISETEDEDNHCSFQDRFKK
ERLEQMHSKWNFVLNAVKTKTHCSISHPDF*
>ENSMUSP00000157371_c.505:C>A_p.A169D ENSMUST00000235071.2|Trpa1-202|ENSMUSG00000032769|-|chr1:g.14976196:G>T
MKRGLRRILLPEERKEVQGVVYRGVGEDMDCSKESFKVDIEGDMCRLEDFIKNRRKLSKYEDENLCPLHHAAA
EGQVELMELIINGSSCEVLNIMDGYGNTPLHCAAEKNQVESVKFLLSQGANPNLRNRNMMSPLHIAVHGMYNE

VIKVLTEHKATNINLEGENGNTDLMSTCAKDNSEALQILLEKGAKLCKSNKWGDYPVHQAAFSGAKKCMELIL
AYGEKNGYSRETHINFVNHKKASPLHLAVQSGDLDMIKMCLDNGAHIDMMENAKCMALHFAATQGATDIVKLM
ISSYTGSSDIVNAVDGNQETLLHRASLFDHHDLAEYLISVGADINSTDSEGRSPLILATASASWNIVNLLLCK
GAKVDIKDHLGRNFLHLTVQQPYGLRNLRPEFMQMQHIKELVMDEDNDGCTPLHYACRQGVPVSVNNLLGFNV
SIHSKSKDKKSPLHFAASYGRINTCQRLLQDISDTRLLNEGDLHGMTPLHLAAKNGHDKVVQLLLKKGALFLS
DHNGWTALHHASMGGYTQTMKVILDTNLKCTDRLDEEGNTALHFAAREGHAKAVAMLLSYNADILLNKKQASF
LHIALHNKRKEVVLTTIRNKRWDECLQVFTHNSPSNRCPIMEMVEYLPECMKVLLDFCMIPSTEDKSCQDYHI
EYNFKYLQCPLSMTKKVAPTQDVVYEPLTILNVMVQHNRIELLNHPVCREYLLMKWCAYGFRAHMMNLGSYCL
GLIPMTLLVVKIQPGMAFNSTGIINGTSSTHEERIDTLKRNYFLDYNNALEWVIYTTSIIFVLPLFLNIPAYM
QWQCGAIAIFFYWMNFLLYLQRFENCGIFIVMLEVIFKTLLRSTGVFIFLLLAFGLSFYVLLNFQDAFSTPLL
SLIQTFSMMLGDINYRDAFLEPLFRNELAYPVLTFGQLIAFTMFVPIVLMNLLIGLAVGDIAEVQKHASLKRI
AMQVELHTNLEKKLPLWYLRKVDQRSTIVYPNRPRHGRMLRFFHYFLNMQETRQEVPNIDTCLEMEILKQKYR
LKDLTSLLEKQHELIKLIIQKMEIISETEDEDNHCSFQDRFKKERLEQMHSKWNFVLNAVKTKTHCSISHPDF
*
>ENSMUSP00000140422_c.162:GA>CT_p.N55L ENSMUST00000188641.7|Eloc-208|ENSMUSG00000079658|-|chr1:g.16713688:TC>AG
MDGEEKTYGGCEGPDAMYVKLISSDGHEFIVKREHALTSGTIKAMLSGPGQFAEL*NQRGQF*RDPLTCAIKS
VHVFYLQGPLY*QLH*NS*IPNCT*NCTGTADGCKLPRLL
>ENSMUSP00000111009_c.162:GA>CT_p.N55L ENSMUST00000115352.10|Eloc-201|ENSMUSG00000079658|-|chr1:g.16713688:TC>AG
MDGEEKTYGGCEGPDAMYVKLISSDGHEFIVKREHALTSGTIKAMLSGPGQFAEL*NQRGQF*RDPLTCAIKS
VHVFYLQGPLY*QLH*NS*IPNCT*NCTGTADGCKLPRLL
>ENSMUSP00000140962_c.162:GA>CT_p.N55L ENSMUST00000186948.7|Eloc-206|ENSMUSG00000079658|-|chr1:g.16713688:TC>AG
MDGEEKTYGGCEGPDAMYVKLISSDGHEFIVKREHALTSGTIKAMLSGPGQFAEL*NQRGQF*RDPLTCAIKS
VHVFYLQGPLY*QLH*NS*IPNCT*NCTGTADGCKLPRLL
>ENSMUSP00000139480_c.228:GA>CT_p.N77L ENSMUST00000187910.7|Eloc-207|ENSMUSG00000079658|-|chr1:g.16713688:TC>AG
MDSLELEEEEEEGGGSGRRGRRHDGEEKTYGGCEGPDAMYVKLISSDGHEFIVKREHALTSGTIKAMLSGPGQ
FAEL*NQRGQF*RDPLTCAIKSVHVFYLQGPLY*QLH*NS*IPNCT*NCTGTADGCKLPRLL
>ENSMUSP00000139675_c.162:GA>CT_p.N55L ENSMUST00000185771.7|Eloc-203|ENSMUSG00000079658|-|chr1:g.16713688:TC>AG
MDGEEKTYGGCEGPDAMYVKLISSDGHEFIVKREHALTSGTIKAMLSGPGQFAEL*NQRGQF*RDPLTCAIKS
VHVFYLQGPLY*QLH*NS*IPNCT*NCTGTADGCKLPRLL
>ENSMUSP00000140161_c.162:GA>CT_p.N55L ENSMUST00000185393.7|Eloc-202|ENSMUSG00000079658|-|chr1:g.16713688:TC>AG
MDGEEKTYGGCEGPDAMYVKLISSDGHEFIVKREHALTSGTIKAMLSGPGQFAEL*NQRGQF*RDPLTCAIKS
VHVFYLQGPLY*QLH*NS*IPN
>ENSMUSP00000158584_c.3647:C>G_p.H1216Q ENSMUST00000238425.2|Vwa3b-211|ENSMUSG00000050122|+|chr1:g.37226023:C>G
MAEQSLLSSERWLQLHGLKSKKLTLKQILSQIGFPQSEDYVTSLRRLVASRYADGLFPQIYTAEDGRLYNLTA
KTELICQFVDYLTQAVESYKQRMDWLTSGSRQIFGVILEQRVTIVLDFGDVLMEELNLCRDALKMVLQEQVAL
ITRFNMIWVSAEPVKWQEYAILVTEHSIAAAISWIEKMTFEPKPVREVSGLDALLEAGKDKTIEAIYYFVVGD
VPQEFQKLLLTGVLEIPYPVCTVSFNARGEETIAFLKDLSTKTHSRFHAFAERTECEEFSAFSTEGVDGSMTW
NSRKLKGRLPPGAGVREDVFLIWREMEEACRILAQVRSLVDKAPQSDVSPADCKSRPAESKENAEDTWESQKW
LQKYGLKAQKLTFYDVLAHCSFRHADGVVDIKAKPENESVQTSADTNKKTIHAKYCSRFIHIPWKDGSLVHVN
ITKEKYKWYRERIHTALAMIQRRIKWLQNGSHILFGKARGECIYVLIDTSCSMKSRLNLVKDKIIQFIQEQLR
YKSKFNFVTFDGQAIAWREKLAEVNEDNLKQAQSWIRGIKVGSSTNTLNALKIAFADEETQVIYLLTDGRPDQ
PPEMVMDHVKLFQNIPICAISFSYNDEVANEFLKELAALTGGEFRAYNFGCKDPIQDTQDEDLNLLLQEMEQG
HSDLEKMQELYMESLVVDWWYNADKDTDSKHQKEILSMVSTPEERANSQPDADSLASSPPNVSTGSWKFLEEK
TQKKKILHAESTKTSLLRSHISNFKSSSNKDSSNSSSRSHTSALSNREMSVLLTNKCLDDKLSESASGNGSQV
SGHTSSDTSSKTWLQTHGLVAKKLTIADALAGAMVPHSSTYVPILNKNVVSKVFDEVFPLAHVCNNSNKMVLI
NPHGINLNIYKQNVEQAIKSYEKCLNKIVWQALSEEEKEKLDAIKPIQYLENKDVLNKALERLNWPISLKELS
VLENEILVGKMYIQQALELQETSRKNYQSKTLQEQQKLQGYPTKKIKSRKLDPLKGQKVIARCEENGFYFPGV
VKKCVSSSHALVDFRYGDTKVVPISFIMPVGGAMPCPQLQVGDYVFAKIMTSKGFDFYVPAIVIALPNHNVAS
EKFYTVLKCNNRREFCPRSALIKISQNKYALSCSMIHSSLVEKNGAGEEVDGSNSSNIWTVTEEATGNLKQPR
QENLRKKRKKRTKRLPLQELMCSDFDGSSRSTRFSTRGLGRRQLRPRQTATRSSPALTESSHPQRKGLRTHR*

```
>ENSMUSP00000158612_c.6691:A>C_p.H2231P ENSMUST00000238212.2|Dnah7b-
207|ENSMUSG00000041144|+|chr1:g.46272697:A>C
MSSKKDKLSTKGTSKPPARFLPQLPMDKLSSREKDKLPTTVLPQLTSTGVKPQWQQIAPSFHLNMKQENPILE
PYPVKNEQSFADYMEYFQQKGKQLYQIDDKRSGPSTSRSKVKSPQKERENFRSTLVKLIMQQDGDLESDVIDE
SGIPKATTTAREKDISRYYYYIRHGLDTDHVAPMEDSWLEHVLQLVPQHLKILNNSIMVLSDEIREDYLLSVK
KSIVDFVLKDPRKKDDDGKITELPPHRAEMEVLPKPWRRSFLSACSYIRDHLNAMNPMMLAVLDLWHSTFKKL
RLVDIEEFHNRQDSLELSEFQNIVIKHMESAKEMLLKTWFPEVQNIYYKGNKKKQLPTGKSSAKLDSFFNCAA
TLMTLQLQDLILVSMQDFTDLIAQPPESTRAFEHPGFIMRLILDKKTIKFAPDFNDYIDILINVYEIMIKMVS
FVPRVETKLYSQWESKSKPTTLKPIILNEIIDTHKEKIREVVLRESVAPTEHLKMYDKYQFLITGKAERDIDE
FLFQSQNYERLIEEIRKYQKLEEEIQYTSRKAVRLGMFEMHCEELIKSLVKRADVICGKLIAKMFRDHQEVNT
MLCDEFEKIAEKALSMPLNTAELMEMKAHVQKLETTDMLELRQRLVDSKNCLAFLIECVNFSPADIRLNNSVF
QWYGRMGEIFDEHRKIIKDKTEQYQEALKFRCERFVEELESYAKQVEEFHTFGDLLDVQRYLKKAQVLNGKLD
AAADKIEQFNAEEEAYGWVPSVYPQRKKIQDGLNPYLRLYETAVEFSTKHRGWTEGPYHKVNPDQVEADVGNY
WRGLYKLEKVFHDSPNALAMTKKVQSMVEEFKQYIPLIQVICNPGLRPRHWEAMSTIVGFPLLPSDDSTVFSF
IDMNLEPFLDRFEGISEAASKEYSLEKAMDKMMTEWDSMEFVILPYRESGTYILSSVDDIQMLLDDHIIKTQT
MRGSPFIKPYEKQMREWEGKLLLLQEILDEWLKVQATWLYLEPIFSSPDIMSQMPEEGRRFKAVDKTWRDVMK
TVVQDKRVLAVVTIERMLERLKKSNELLELILKGLNEYLEKKRLFFPRFFFLSNDELLEILSETKDPTRVQPH
LKKCFEGIAKVEFTETLDITHMKSSEGEVVELVDTISTAKARGQVEKWLVELERTMIKSIHKVIGDAIAAYTK
NSRISWVRDWPGQTVLCVSQTFWTVEVQTAIPKGHRALEGYLAKCNHQIDDIVTLVRGKLSKQNRVTLGALVV
LDVHARDVLASLVDKKISDDSDFQWLSQLRYYWQENNLETKMINAGLRYGYEYLGNSPRLVITPLTDRCYRTL
FGALHLHLGGAPEGPAGTGKTETTKDLAKAVAKQCVVFNCSDGLDYLALGKFFKGLLSCGAWACFDEFNRIDL
EVLSVVAQQILTIQRGINAGTELLVFEGTELKLDPTCAVFITMNPGYAGRSELPDNLKALFRTVAMMVPDYAM
IAEIVLYSCGFVTARPLSIKIVATYRLCSEQLSSQHHYNYGMRAVKSVLTAAGNLKLKYPNENEEILLLRSII
DVNLPKFLSHDLPLFEGITSDLFPGVKLPKPDYNELLAAIRENCHTMNLQMTDFFSEKILQIYEMMIVRHGFM
IVGEPFGGKTSAYRVLAGALNDICEKGLMEENKVQITVLNPKSVTMGQLYGQFDLVSHEWSDGILAVSFRAFA
ASSTPDRKWLIFDGPVDAVWIENMNTVLDDNKKLCLMSGEIIQMSPQMNLIFEPMDLEVASPATVSRCGMIYM
EPHMLGWRPLMVSWINTLPQSVSIIQKEFIEGLFDRMVPLSVEFIRRHTKELSPTSDTNLVQSLMNLIDCFMD
DFADENKQKERNDRESFSLLEGIFMFSLAWSVGATCTDDDRLKFDKILRELMEGPISDLTRNKFKLLSGTEQT
SSKVFIVPFPEKGTIYEYQFIPEGLGRWDKWIKKLADTPPIPKDVQFIEIIVPTLDTIRYSALMHLLTTHQKP
SIFVGPTGTGKSVYIINFLLNQLNKDIYKPLIVNFSAQTTAAQTQNIIMSKLDKRRKGVFGPPLGKRMIVFVD
DVNMPAREVYGAQPPIELLRQWLDHWNWYDLKDCSVIKLVDIQIMCAMGPPGGGRNPITPRYMRHFNIVTINE
FSDKSMFTIFSRILAWHLRTCYKFPDDFLDLTTQIVNGTMALYKDAMKNLLPTPAKSHYLFNLRDFSRVIQGV
CLSRPETAENKEAIKRLWVHEVLRVYYDRLVDNADRSWLIPYIQEILKNYMQEDFHDLFKNLDFNQDGTVEED
DLRSLMFCDFHDPKREDFGYREIANVDALRMIVEGHLDEYNNMSKKPMNLVLFRFAIEHISRISRILKQPRSH
ALLVGVGGSGRQSVTRLAAHMADYSLFQVEISKGYGSHEWHEDLKVILRKCAENDMQGVFLFTDTQIKRESFL
EDVNNLLNAGEVPNLFALDEKQEICEKMRQLDRQRDKTRQTDGSPIALFNMFIDRCRNQLHVVLAMSPIGDAF
RIRLRKFPALVNCCTIDWFQSWPEDALEAVASRFLEDIEMSEEIQEGCIDMCKRFHTSTINLSTSFHNELQRY
NYVTPTSYLELISTFKLLLEKKRNEVMKMKRRYEVGLDKLDSASSQVATMQSELEALHPQLKVASREVDEMMI
IIERESMEVAKTEKIVKADETVANDQAMAAKAIKDECDADLAEALPILESALAALDTLTAQDITVVKSMKSPP
AGVKLVMEAICILKGIKADKIPDPTGSGKKIEDFWGPAKRLLGDIRFLQSLHEYDKDNIPPAYMNIIRKSYIP
NPDFVPEKIRNASTAAEGLCKWVIAMDSYDKVAKIVAPKKIKLAAAEGKLRIAMEGLRKKQAALYEVQDKLAK
LQDTLELNKQKKADLENQVDLCSKKLERAEQLIGGLGGEKTRWSNSALELGHLYINLTGDILISSGVVAYLGA
FTSNYRQNQTQEWSQSCKERDIPCSDDYSLMGTLGEAVTIRAWNIAGLPSDSFSIDNGIIIMNARRWPLMIDP
QGQANKWIKNMEKTNSLQLIKLSDPDYVRTLENCIQFGTPVLLENVGEELDPILEPLLLKQTFKQGGSTCIRL
GDSTIEYAPDFRFYITTKLRNPHYLPETSVKVTLLNFMITPEGMQDQLLGIVVARERPDLEEEKQSLIVQGAD
NKRQLKEIEDKILEVLSLSEGNILEDETAIKILSSSKSLANEISQKQEVAEETEKKIDNTRMGYRIIAIHSSI
LFFSIADLANIEPMYQYSLTWFINLFILSIENSEKSDILSKRLQILRDHFTYSLYVNICRSLFEKDKLLFSFC
LTVNLLIHENAINKTEWRFLLTGGIGLDNPYTNPCTWLPQKSWDEICRLDDLPAFKTIRREFMRLKDGWKKVY
DSMEPHHEMFPEDWENKANDFQRMLIIRCLRPDKVIPMLQEFIIKKLGRPFIEPPPFDLAKAFGDSNCCAPLI
FVLSPGADPMNALLKFADDQGYGGSKLSSLSLGQGQGPIAMKMLEKAVKDGTWVVLQNCHLATSWMPTLEKVC
EELSAESTHPDFRIWLTSYPSPNFPVSVLQNGVKMTNEAPKGLRANIIRSYLMDPISDPEFFGSCKKPEEFKK
LLYGLCFFHALVQERRKFGPLGWNIPYEFNETDLRISVQQLHMFLDQYEELPYDALRYMTGECNYGGRVTDDW
DRRTLRSILNKFFCTELVENPQYKFDSSGIYFVPPSGDHKSYINYTKTLPLIPAPEVFGMNANADITKDQSET
QLLFDNILLTQSQSSGSGTKSSDEVVNEVAGDILSKLPNNFDVEAAMRRYPTTYTQSMNTVLVQEMGRFNKLL
ITIRESCINIQKAIKGLVVMSTELEEVVSSILNVKIPVMWMGKSYPSLKPLGSYVNDFLERLKFLQQWYEVGP
PPVFWLSGFFFTQAFLTGAQQNFARKFTIPIDLLGFDYEVMDDKEYKNAPEDGVYIHGLFLDGASWNRKTKKL
AESHPKVLYDTVPVMWLKPCKKSDIPKRPSYVAPLYKTSERRGTLSTTGHSTNFVIAMILPSDQPKEHWIGRG
VALLCQLNS*
```

```
>ENSMUSP00000068738_c.6823:A>C_p.H2275P ENSMUST00000069293.10|Dnah7b-
201|ENSMUSG00000041144|+|chr1:g.46272697:A>C
MPQRATLLDLSESSAPNLADADDRKWIPPQRSRPILPGPQVLAAMSSKKDKLSTKGTSKPPARFLPQLPMDKL
SSREKDKLPTTVLPQLTSTGVKPQWQQIAPSFHLNMKQENPILEPYPVKNEQSFADYMEYFQQKGKQLYQIDD
KRSGPSTSRSKVKSPQKERENFRSTLVKLIMQQDGDLESDVIDESGIPKATTTAREKDISRYYYYIRHGLDTD
HVAPMEDSWLEHVLQLVPQHLKILNNSIMVLSDEIREDYLLSVKKSIVDFVLKDPRKKDDDGKITELPPHRAE
MEVLPKPWRRSFLSACSYIRDHLNAMNPMMLAVLDLWHSTFKKLRLVDIEEFHNRQDSLELSEFQNIVIKHME
SAKEMLLKTWFPEVQNIYYKGNKKKQLPTGKSSAKLDSFFNCAATLMTLQLQDLILVSMQDFTDLIAQPPEST
RAFEHPGFIMRLILDKKTIKFAPDFNDYIDILINVYEIMIKMVSFVPRVETKLYSQWESKSKPTTLKPIILNE
IIDTHKEKIREVVLRESVAPTEHLKMYDKYQFLITGKAERDIDEFLFQSQNYERLIEEIRKYQKLEEEIQYTS
RKAVRLGMFEMHCEELIKSLVKRADVICGKLIAKMFRDHQEVNTMLCDEFEKIAEKALSMPLNTAELMEMKAH
VQKLETTDMLELRQRLVDSKNCLAFLIECVNFSPADIRLNNSVFQWYGRMGEIFDEHRKIIKDKTEQYQEALK
FRCERFVEELESYAKQVEEFHTFGDLLDVQRYLKKAQVLNGKLDAAADKIEQFNAEEEAYGWVPSVYPQRKKI
QDGLNPYLRLYETAVEFSTKHRGWTEGPYHKVNPDQVEADVGNYWRGLYKLEKVFHDSPNALAMTKKVQSMVE
EFKQYIPLIQVICNPGLRPRHWEAMSTIVGFPLLPSDDSTVFSFIDMNLEPFLDRFEGISEAASKEYSLEKAM
DKMMTEWDSMEFVILPYRESGTYILSSVDDIQMLLDDHIIKTQTMRGSPFIKPYEKQMREWEGKLLLLQEILD
EWLKVQATWLYLEPIFSSPDIMSQMPEEGRRFKAVDKTWRDVMKTVVQDKRVLAVVTIERMLERLKKSNELLE
LILKGLNEYLEKKRLFFPRFFFLSNDELLEILSETKDPTRVQPHLKKCFEGIAKVEFTETLDITHMKSSEGEV
VELVDTISTAKARGQVEKWLVELERTMIKSIHKVIGDAIAAYTKNSRISWVRDWPGQTVLCVSQTFWTVEVQT
AIPKGHRALEGYLAKCNHQIDDIVTLVRGKLSKQNRVTLGALVVLDVHARDVLASLVDKKISDDSDFQWLSQL
RYYWQENNLETKMINAGLRYGYEYLGNSPRLVITPLTDRCYRTLFGALHLHLGGAPEGPAGTGKTETTKDLAK
AVAKQCVVFNCSDGLDYLALGKFFKGLLSCGAWACFDEFNRIDLEVLSVVAQQILTIQRGINAGTELLVFEGT
ELKLDPTCAVFITMNPGYAGRSELPDNLKALFRTVAMMVPDYAMIAEIVLYSCGFVTARPLSIKIVATYRLCS
EQLSSQHHYNYGMRAVKSVLTAAGNLKLKYPNENEEILLLRSIIDVNLPKFLSHDLPLFEGITSDLFPGVKLP
KPDYNELLAAIRENCHTMNLQMTDFFSEKILQIYEMMIVRHGFMIVGEPFGGKTSAYRVLAGALNDICEKGLM
EENKVQITVLNPKSVTMGQLYGQFDLVSHEWSDGILAVSFRAFAASSTPDRKWLIFDGPVDAVWIENMNTVLD
DNKKLCLMSGEIIQMSPQMNLIFEPMDLEVASPATVSRCGMIYMEPHMLGWRPLMVSWINTLPQSVSIIQKEF
IEGLFDRMVPLSVEFIRRHTKELSPTSDTNLVQSLMNLIDCFMDDFADENKQKERNDRESFSLLEGIFMFSLA
WSVGATCTDDDRLKFDKILRELMEGPISDLTRNKFKLLSGTEQTSSKVFIVPFPEKGTIYEYQFIPEGLGRWD
KWIKKLADTPPIPKDVQFIEIIVPTLDTIRYSALMHLLTTHQKPSIFVGPTGTGKSVYIINFLLNQLNKDIYK
PLIVNFSAQTTAAQTQNIIMSKLDKRRKGVFGPPLGKRMIVFVDDVNMPAREVYGAQPPIELLRQWLDHWNWY
DLKDCSVIKLVDIQIMCAMGPPGGGRNPITPRYMRHFNIVTINEFSDKSMFTIFSRILAWHLRTCYKFPDDFL
DLTTQIVNGTMALYKDAMKNLLPTPAKSHYLFNLRDFSRVIQGVCLSRPETAENKEAIKRLWVHEVLRVYYDR
LVDNADRSWLIPYIQEILKNYMQEDFHDLFKNLDFNQDGTVEEDDLRSLMFCDFHDPKREDFGYREIANVDAL
RMIVEGHLDEYNNMSKKPMNLVLFRFAIEHISRISRILKQPRSHALLVGVGGSGRQSVTRLAAHMADYSLFQV
EISKGYGSHEWHEDLKVILRKCAENDMQGVFLFTDTQIKRESFLEDVNNLLNAGEVPNLFALDEKQEICEKMR
QLDRQRDKTRQTDGSPIALFNMFIDRCRNQLHVVLAMSPIGDAFRIRLRKFPALVNCCTIDWFQSWPEDALEA
VASRFLEDIEMSEEIQEGCIDMCKRFHTSTINLSTSFHNELQRYNYVTPTSYLELISTFKLLLEKKRNEVMKM
KRRYEVGLDKLDSASSQVATMQSELEALHPQLKVASREVDEMMIIIERESMEVAKTEKIVKADETVANDQAMA
AKAIKDECDADLAEALPILESALAALDTLTAQDITVVKSMKSPPAGVKLVMEAICILKGIKADKIPDPTGSGK
KIEDFWGPAKRLLGDIRFLQSLHEYDKDNIPPAYMNIIRKSYIPNPDFVPEKIRNASTAAEGLCKWVIAMDSY
DKVAKIVAPKKIKLAAAEGKLRIAMEGLRKKQAALYEVQDKLAKLQDTLELNKQKKADLENQVDLCSKKLERA
EQLIGGLGGEKTRWSNSALELGHLYINLTGDILISSGVVAYLGAFTSNYRQNQTQEWSQSCKERDIPCSDDYS
LMGTLGEAVTIRAWNIAGLPSDSFSIDNGIIIMNARRWPLMIDPQGQANKWIKNMEKTNSLQLIKLSDPDYVR
TLENCIQFGTPVLLENVGEELDPILEPLLLKQTFKQGGSTCIRLGDSTIEYAPDFRFYITTKLRNPHYLPETS
VKVTLLNFMITPEGMQDQLLGIVVARERPDLEEEKQSLIVQGADNKRQLKEIEDKILEVLSLSEGNILEDETA
IKILSSSKSLANEISQKQEVAEETEKKIDNTRMGYRIIAIHSSILFFSIADLANIEPMYQYSLTWFINLFILS
IENSEKSDILSKRLQILRDHFTYSLYVNICRSLFEKDKLLFSFCLTVNLLIHENAINKTEWRFLLTGGIGLDN
PYTNPCTWLPQKSWDEICRLDDLPAFKTIRREFMRLKDGWKKVYDSMEPHHEMFPEDWENKANDFQRMLIIRC
LRPDKVIPMLQEFIIKKLGRPFIEPPPFDLAKAFGDSNCCAPLIFVLSPGADPMNALLKFADDQGYGGSKLSS
LSLGQGQGPIAMKMLEKAVKDGTWVVLQNCHLATSWMPTLEKVCEELSAESTHPDFRIWLTSYPSPNFPVSVL
QNGVKMTNEAPKGLRANIIRSYLMDPISDPEFFGSCKKPEEFKKLLYGLCFFHALVQERRKFGPLGWNIPYEF
NETDLRISVQQLHMFLDQYEELPYDALRYMTGECNYGGRVTDDWDRRTLRSILNKFFCTELVENPQYKFDSSG
IYFVPPSGDHKSYINYTKTLPLIPAPEVFGMNANADITKDQSETQLLFDNILLTQSQSSGSGTKSSDEVVNEV
AGDILSKLPNNFDVEAAMRRYPTTYTQSMNTVLVQEMGRFNKLLITIRESCINIQKAIKGLVVMSTELEEVVS
SILNVKIPVMWMGKSYPSLKPLGSYVNDFLERLKFLQQWYEVGPPPVFWLSGFFFTQAFLTGAQQNFARKFTI
PIDLLGFDYEVMDDKEYKNAPEDGVYIHGLFLDGASWNRKTKKLAESHPKVLYDTVPVMWLKPCKKSDIPKRP
SYVAPLYKTSERRGTLSTTGHSTNFVIAMILPSDQPKEHWIGRGVALLCQLNS*
```

>ENSMUSP00000092571_c.11792:T>G_p.D3931E ENSMUST00000094964.7|Dnah7a-
201|ENSMUSG00000096141|-|chr1:g.53444910:A>C
MSSKKDKLSTKGKSKTPVRFLPQLPMDKLSSKEKVKLPTTVLPQLSLTGVKPQWQQTAPSFHLNIKQENPVLE
PYTVKNEQSFAEYMEHCQKKGKLLDQIDDDRSAPSTSRSKIKSPQKERENLRSTLAKVIMQKDGGLESDVIDE
SGIPKETTSATEKDISRYYYYIRQGLDTDHVAPMEDSWLEHVLQLVPEHLKVLTNSIKVLSDEIREDYLLSVK
KSIVDFVLKDPREKDDDGKITELPPHRAEMEVLPKPWRRSFLSACSYIRDHLNAMNPMMLAVLDLWHSTFKKL
RLVDIEEFHSCQDALELSEFQNIVIKHMESAKETLLKTWFPEVQNIFNQGNKKKQLPTGKSTAKLDSFFNCAA
TLMTLQLQDLILVSMQDFTDLIAQPLESIRVFEHPGFIMRLVLDKEAIKFEPEFNEYIDSLINIYEIMIKAVS
FVPRVETKLYSQWESKSKPTTLKPIILNEIIDTHKEKIREVILRESVAPTEHLKMYDKYQFLITGKAERDIDE
LLFQNQNYERLIEEIRKYQKLGEEIQYTSQKTVCLGMFEMHCEELIRSLVKRADVICGKLIAKMFRDHQEVNT
MLCDEFEKIAEKALSTPLNTAELMEMKAHVQKLETTDMLELGQRLVDSKICLAFLIECVNFSPADIRLNKSVF
QWYGRMGEIFDEHRKIIKEKTELYQEFLKFRCERFVEELESYAKQVEEFHAFGDLLDVQRYLQKAQVLNGKLD
AAADKIDQFNAEEEAFGWVPSAYPQRKKIQDALNPYLHLYETAVEFSTKHRGWTEGPYHKVNPDQVEADVGNY
WRGLYKLEKVFHDSPNALAMTKKVHSMVEEFKQYIPLIQVFCNPGLRPRHWEAMSTIVGYPLQPSDDSTVFSF
IDMNLEPFLDRFGSISEAASKEYSLEKAMDKMMTEWDSMEFVILPYRESGTYILSSVDDIQMLLDDHIIKTQT
MRGSPFIKPYEKQMREWEGKLLLLQEILDEWLKVQATWLYLEPIFSSRDIMSQMPEEGRRFTAVDKTWRDVMK
TVVQDKQVLAVVTIERMLERLKKSNELLELILKGLNEYLEKKRLFFPRFFFLSNDELLEILSETKDPTRVQPH
LKKCFEGIAKVEFTETLDITHMKSSEGEVVELVDTISTAKARGQVEKWLVELERTMIKSIHKVIRDAIVAYTK
TSRISWVRDWPGQTVLCVSQTFWTVEVQIAIPEGHRALEGYLAKCNHQIDDIVTLVRGKLSKQNRVTLGALVV
LDVHARDVLASLVDKKISDDSDFQWLSQLRYYWQENNLETKMINAGLRYGYEYLGNSPRLVITPLTDRCYRTL
FGALHLHLGGAPEGPAGTGKTETTKDLAKAVAKQCVVFNCSDGLDYLALGKFFKGLLSCGAWACFDEFNRIDL
EVLSVVAQQILTIQRGINAGTDLLVFEGTELKLDPTCAVFITMNPGYAGRSELPDNLKALFRTVAMMVPDYAM
IAEIVLYSCGFVTARPLSIKIVATYRLCSEQLSSQHHYDYGMRAVKSVLTAAGNLKLKYPNENEEILLLRSII
DVNLPKFLSHDLPLFEGITSDLFPGVKLPKPDYNDLLAAIRDNCHSMNLQMTDFFSEKILQIYEMMIVRHGFM
IVGEPFGGKTSAYRVLAGALNDICEKGLMEENKVQITVLNPKSVTMGQLYGQFDLVSHEWSDGVLAVSFRAFA
ASSTPDRKWLIFDGPVDAVWIENMNTVLDDNKKLCLMSGEIIQMSPQMNLIFEPMDLEVASPATVSRCGMIYM
EPHMLGWRPLMVSWINTLPQSVSIIQKEFIEGLFDRMVPLSVEFIRRHTKELSPTSDTNLVRSLMNLIDCFMD
DFADENKQKERNDRESFSLLEGIFLFSLIWSVGATCTDDDRLKFDKILRELLEGPISNLTRNKFKLLSGTEQT
SSKVFIVPFPEKGTIYDYQFIPEGLGRWDKWIKDLADTPPIPKDVQFNEIIVPTLDTIRYSALMNLLTTHQKP
SIFVGPTGTGKSVYIINFLLNQLNKDIYKPLIVNFSAQTTAAQTQNIIMSKLDKRRKGVFGPPLGKRMIVFVD
DVNMPAREVYGAQPPIELLRQWLDHWNWYDLKDCSVIKLVDIQIMCAMGPPGGGRNPITPRYMRHFNIVTINE
FSNKSMFTIFSRILAWHLRTCYKFPDDFLDLTTQIVNGTMALYKDAMKNLLPTPAKSHYLFNLRDFSRVIQGV
CLSRPETAENKEAIKRLWVHEVLRVYYDRLVDNADRSWLINYIQEILKNYMQEDFHDLFKNLDFNHDGTVEED
DLRSLMFCDFHDPKREDFGYREIANVDALRMIVEGHLDEYNNMSKKPMNLVLFRFAIEHISRISRILKQPRSH
ALLVGVGGSGRQSVTRLAAHMADYSLFQVEISKGYGSHEWHEDLKVILRKCAESDMQGVFLFTDTQIKRESFL
EDVNNLLNAGEVPNLFALDEKQEICEKMRQIDRQRDKSKQTDGSPIALFNMFIDCCRNQLHVVLAMSPIGDAF
RIRLRKFPALVNCCTIDWFQSWPEDALEAVASRFLEDIEMSEEIREGCIDMCKRFHTSTINLSTSFHNELQRY
NYVTPTSYLELISTFKLLLEKKRNEVMKMKRRYEVGLDKLDSASSQVATMQSELEALHPQLKVASREVDEMMI
IIERESMEVAKTEKIVKADETVANDQAMAAKAIKDECDADLAGALPILESALAALDTLTAQDITVVKSMKSPP
AGVKLVMEAVCILKGIKADKIPDPTGSGKKTEDFWGPAKRLLGDIRFLQSLHEYDKDNIPPAYMNIIRKSYIP
NPDFVPEKIRNASTAAEGLCKWVIAMDSYDKVAKIVAPKKIKLAAAEGKLKVAMEGLRKKQAALHEVQDKLAK
LQDTLELNKQKKADLEHQVDLCSKKLERAEQLIGGLGGEKTRWSNSALELGHLYINLTGDILISSGVVAYLGA
FTSNYRQNQTKQWSQSCKERDIPCSDDYSLMGTLGEAVTIRAWNIAGLPSDSFSIDNGIIIMNARRWPLMIDP
QGQANKWIKNMEKTNSLQLIKLSDPSYVRTLENCIQFGTPVLLENVGEELDPILEPLLLKQTFKQGGSTCIRL
GDSTIEYSPDFRFYITTKLRNPHYLPETSVKVTLLNFMITPEGMQDQLLGIVVARERPDLEEEKQALILQGAD
NKRQLKEIEDKILEVLSLSEGNILEDETAIKILSSSKSLANEISQKQEVAEETEKKIDITRMGYRPIAIHSSI
LFFSIADLANIEPMYQYSLTWFINLFILSIENSEKSDILSKRLQILRDHFTYSLYVNICRSLFEKDKLLFSFC
LTVNLLIHDNAINKTEWRFLLTGGIGLDNPYTNPCTWLPQKSWDEICRLDDLPAFKTIREEFMRLKDGWKKVY
DSMEPHHEMFPEDWGNKANDFQRMLIIRCLRPDKVIPMLQEFIIKKLGRSFIEPPPFDLAKAFGDSNCCAPLI
FVLSPGADPMNALLKFADDQGYGGSKLSSLSLGQGQGPIAMKMLEKAVKDGTWVVLQNCHLATSWMPTLEKVC
EELSAESTHPDFRIWLTSYPSPNFPVSVLQNGVKMTNEAPKGLRANIIRSYLMDPISDPEFFGSCKKPEEFKK
LLYGLCFFHALVQERRKFGPLGWNIPYEFNETDLRISVQQLHMFLDQYEELPYDALRYMTGECNYGGRVTDDW
DRRTLRSILNKFFCTELVENPQYKFDSSGIYFVPPSGDHKSYIDYTKTLPLIPAPEVFGMNANADITKDQSET
QLLFDNILLTQSHSSGSGTKSSDEVVNEVAGDILGKLPNNFDVEAAMRRYPTTYTQSMNTVLVQEMGRFNKLL
ITIRESCINIQKAVKGLVVMSTELEEVVSSILNVKIPAMWMGKSYPSLKPLGSYVNDFLERLKFLQQWYEVGP
PPVFWLSGFFFTQAFLTGAQQNYARKFTIPIDLLGFDYEVMDDKEYKKAPEDGVYIHGLFLEGASWDRKTKKL
AESHPKVLYDTVPVMWLKPCKKSDIPRRPSYVAPLYKTSERRGTLSTTGHSTNFVIAMILPSVHPKEHWIGRG
VALLCQLNS*

>ENSMUSP00000109881_c.801:A>G_p.T268A ENSMUST00000114243.8|Gm973-201|ENSMUSG00000047361|+|chr1:g.59590296:A>G
MKAVAREPDATSRPLQQRRNTSTEARSAGDYLNWKSPEDYILVSKAQNGDSASQHSWNLFLPKTFSTRKGALI
LYSEGLAVSAWTPRDRKRGPRKKQDLGLHTLRDLKEAILAYGRRKRKQDSAWRPYLYFRSKPESQTQRQIQPG
YSAKRYLRGFLRTWPPDIMHRLQCAGHIKDSVLLQDSQLGIPKNLRPQQDLSGVPPKYHLLPVIPSFRIQQRS
SYAQGLDEWEIDTYMDQGSVAQNHSNPDTHLMSVRRQLWQEDELQAEDALRESHLRVHASEQSHNGKSQQASR
EVLGRASFSYFQLPTDKSLFSFYCGAFPDKQRNMKPHKARGGCLSQEPPVERCLFPPLASAVGSEQNTLGETK
KKKALRTLKLPPVSEEPPGVLKPRRSRFKDSEPPKELFIIPMEIHFHAAQPPKEKASRRGARHPESEPEEATP
LWWPPMKHLYLERPRGITVHLPVDSSQDTPSPQEDGDILAQSVNQPLGLLPPMKGKKSSEIQRDMDRTRTSGF
SSPTGCPNERAPPAGPEDSRDLTLKHFLLSPDGENVCLTLPRLMGTEVLPLGQANAGAGASLHGNLYETSSRL
AQAAESQERGSELDSHEEPGGPLRGNAKDSQDPELRNKTLACLSTSLAEYNQPSEADTLKSMDGDYNVHHLHR
GPLKREPAFPKKLASETPRKKKKRRSKLLNQKTGVGINHGKDLVDKAKRKKRTKTHQAKALKKEREERGLGQA
EAAGGKPKHSKIKKKSELTPKKEKLGRKMKRTHKERNMEMAAGLSKSDITNSKEAGGTSHQGLLRSHSAAGQL
SLELDALESQVAIDGRLSSIQATDVASDMECEEERSHEDPSKALQDKKQQEKASRDRIRIEKAEMRWLKVEQR
RREQEELTWLHKEQLEKAEKMKEELELEQQRRTEENRLRKQRLEEERQQQEEAEKKRRLQLQAARERARQQQE
ELRRKLQEIQRKKQQEAAERAEAEKQRQKELEMQLAEEQKRLMEMAEEERLEYQQQKLAAEEKARQEAEERRK
QEEEAAKLALEEATKLAQEQIRYLDRGTTAVRACSEA*
>ENSMUSP00000141102_c.798:A>G_p.T267A ENSMUST00000186434.3|Gm973-205|ENSMUSG00000047361|+|chr1:g.59590296:A>G
MFTLSLLSRGHGKLAQNKQKLEVYFEPEDYLNWKSPEDYILVSKAQNGDSASQHSWNLFLPKTFSTRKGALIL
YSEGLAVSAWTPRDRKRGPRKKQDLGLHTLRDLKEAILAYGRRKRKQDSAWRPYLYFRSKPESQTQRQIQPGY
SAKRYLRGFLRTWPPDIMHRLQCAGHIKDSVLLQDSQLGIPKNLRPQQDLSGVPPKYHLLPVIPSFRIQQRSS
YAQGLDEWEIDTYMDQGSVAQNHSNPDTHLMSVRRQLWQEDELQAEDALRESHLRVHASEQSHNGKSQQASRE
VLGRASFSYFQLPTDKSLFSFYCGAFPDKQRNMKPHKARGGCLSQEPPVERCLFPPLASAVGS
>ENSMUSP00000006462_c.1213:T>C_p.L405P ENSMUST00000006462.14|Aamp-201|ENSMUSG00000006299|-|chr1:g.74319960:A>G
MESESESGAAADTPPLETLSFHGDEEIIEVVELDPGPPDPDDLAQEMEDVDFEEEEEEEEGNDEGWVLEPQEG
VVGSMEGPDDSEVTFALHSASVFCVSLDPKTNTLAVTGGEDDKAFVWRLGDGELLFECAGHKDSVTCAGFSHD
STLVATGDMSGLLKVWQVDTKEEVWSFEAGDLEWMEWHPRAPVLLAGTADGNTWMWKVPNGDCKTFQGPNCPA
TCGRVLPDGKRAVVGYEDGTIRIWDLKQGNPIHVLKGTEGHQGPLTCVATNQDGSLILTGSVDCQAKLVSATT
GKVVGVFRPETVASQPSLGEGEESESNSVESLGFCSVMPLAAVGYLDGTLAIYDLSTQTLRHQCQHQSGIVQL
LWEAGTAVVYTCSLDGVVRLWDARTGRLLTDYRGHTAEIPDFALSKDASLVVTTSGDHKAKVFCVQRPDR*
>ENSMUSP00000136644_c.1216:T>C_p.L406P ENSMUST00000178235.8|Aamp-202|ENSMUSG00000006299|-|chr1:g.74319960:A>G
MESESESGAAADTPPLETLSFHGDEEIIEVVELDPGPPDPADDLAQEMEDVDFEEEEEEEEGNDEGWVLEPQE
GVVGSMEGPDDSEVTFALHSASVFCVSLDPKTNTLAVTGGEDDKAFVWRLGDGELLFECAGHKDSVTCAGFSH
DSTLVATGDMSGLLKVWQVDTKEEVWSFEAGDLEWMEWHPRAPVLLAGTADGNTWMWKVPNGDCKTFQGPNCP
ATCGRVLPDGKRAVVGYEDGTIRIWDLKQGNPIHVLKGTEGHQGPLTCVATNQDGSLILTGSVDCQAKLVSAT
TGKVVGVFRPETVASQPSLGEGEESESNSVESLGFCSVMPLAAVGYLDGTLAIYDLSTQTLRHQCQHQSGIVQ
LLWEAGTAVVYTCSLDGVVRLWDARTGRLLTDYRGHTAEIPDFALSKDASLVVTTSGDHKAKVFCVQRPDR*
>ENSMUSP00000159251_c.811:T>G_p.L271W ENSMUST00000239185.2|Gm7582-201|ENSMUSG00000118640|-|chr1:g.85005754:A>C
MNSSSEHCNVSDWLRLEATVKASVYVVAFSITTSLTIVIIAIVSQSLQLRKEVRLVLLCHHLLCISSYCGLGV
VFQGMWALLANSPLLLCWLVFGAQLSVGEGILLTLTFMALNTYLAICYPLNSPSFVDSAKYRILAGTWTTVIL
KNVGLFLIEGTSPTPASVFQSAPLCPVILNGMPARVIGMFSLAFLLSVILVSYCLIYREGKRAGHFNKSNIKA
RRTVLVHLLQISLHVIPTLIIIGLGKRCGVFFFALNLALFGIFAFAQCFNPWFYGLHNRDVQSKLYPFLCCRK
KLLP*
>ENSMUSP00000159251_c.793:C>T_p.A265V ENSMUST00000239185.2|Gm7582-201|ENSMUSG00000118640|-|chr1:g.85005772:G>A
MNSSSEHCNVSDWLRLEATVKASVYVVAFSITTSLTIVIIAIVSQSLQLRKEVRLVLLCHHLLCISSYCGLGV
VFQGMWALLANSPLLLCWLVFGAQLSVGEGILLTLTFMALNTYLAICYPLNSPSFVDSAKYRILAGTWTTVIL
KNVGLFLIEGTSPTPASVFQSAPLCPVILNGMPARVIGMFSLAFLLSVILVSYCLIYREGKRAGHFNKSNIKA
RRTVLVHLLQISLHVIPTLIIIGLGKRCGVFFFALNLALFGIFAFVQCFNPLFYGLHNRDVQSKLYPFLCCRK
KLLP*
>ENSMUSP00000159251_c.399:T>A_p.Y134N ENSMUST00000239185.2|Gm7582-201|ENSMUSG00000118640|-|chr1:g.85006166:A>T
MNSSSEHCNVSDWLRLEATVKASVYVVAFSITTSLTIVIIAIVSQSLQLRKEVRLVLLCHHLLCISSYCGLGV
VFQGMWALLANSPLLLCWLVFGAQLSVGEGILLTLTFMALNTYLAICYPLNSPSFVDSAKNRILAGTWTTVIL

KNVGLFLIEGTSPTPASVFQSAPLCPVILNGMPARVIGMFSLAFLLSVILVSYCLIYREGKRAGHFNKSNIKA
RRTVLVHLLQISLHVIPTLIIIGLGKRCGVFFFALNLALFGIFAFAQCFNPLFYGLHNRDVQSKLYPFLCCRK
KLLP*
>ENSMUSP00000123750_c.450:A>G_p.N151D ENSMUST00000161724.8|A530032D15Rik-203|ENSMUSG00000089844|-|chr1:g.85068890:T>C
MAGGDNELSSRTIPEDQNEEESDDYQLMFKHFKENKVEIASAITKPFPFLMSLRDRDFISEQKFQEYQETCKN
LAPVERVVYDILSNVQKKFSRDLLKVIFSKTHLKVYPDLKETLKHFFLNASKTNDEQAEEMLSLPQCNGGVLA
QEHSDPHVKRSVPVSCVPQHMCQKTWKQGWEAAKEKVWAPGGTFLQENPRSREEKRKATTGQNQNGESQ*
>ENSMUSP00000125122_c.450:A>G_p.N151D ENSMUST00000160792.8|A530032D15Rik-201|ENSMUSG00000089844|-|chr1:g.85068890:T>C
MAGGDNELSSRTIPEDQNEEESDDYQLMFKHFKENKVEIASAITKPFPFLMSLRDRDFISEQKFQEYQETCKN
LAPVERVVYDILSNVQKKFSRDLLKVIFSKTHLKVYPDLKETLKHFFLNASKTNDEQAEEMLSLPQCNGGVLA
QEHSDPHVKRSVPVSCVPQHMCQKTWKQGWEAAKEKVWAPGGTFLQENPRSRESWQVWSLHCRPW*
>ENSMUSP00000123750_c.75:C>T_p.Q26* ENSMUST00000161724.8|A530032D15Rik-203|ENSMUSG00000089844|-|chr1:g.85078372:G>A
MAGGDNELSSRTIPEDQNEEESDDY*LMFKHFKENKVEIASAITKPFPFLMSLRDRDFISEQKFQEYQETCKN
LAPVERVVYDILSNVQKKFSRDLLKVIFSKTHLKVYPDLKETLKHFFLNASKTNDEQAEEMLSLPQCNGGVLA
QEHSNPHVKRSVPVSCVPQHMCQKTWKQGWEAAKEKVWAPGGTFLQENPRSREEKRKATTGQNQNGESQ*
>ENSMUSP00000125122_c.75:C>T_p.Q26* ENSMUST00000160792.8|A530032D15Rik-201|ENSMUSG00000089844|-|chr1:g.85078372:G>A
MAGGDNELSSRTIPEDQNEEESDDY*LMFKHFKENKVEIASAITKPFPFLMSLRDRDFISEQKFQEYQETCKN
LAPVERVVYDILSNVQKKFSRDLLKVIFSKTHLKVYPDLKETLKHFFLNASKTNDEQAEEMLSLPQCNGGVLA
QEHSNPHVKRSVPVSCVPQHMCQKTWKQGWEAAKEKVWAPGGTFLQENPRSRESWQVWSLHCRPW*
>ENSMUSP00000123812_c.75:C>T_p.Q26* ENSMUST00000161675.3|A530032D15Rik-202|ENSMUSG00000089844|-|chr1:g.85078372:G>A
MAGGDNELSSRTIPEDQNEEESDDY*LMFKHFKENKVEIASAITKPFPFLMSLRDRDFISEQKFQEYQETCKN
LAPVERVVYDILSNVQKKFSRDLLKVIFSKTHLKVYPDLKETLKHFFLN
>ENSMUSP00000123750_c.60:G>A_p.E21K ENSMUST00000161724.8|A530032D15Rik-203|ENSMUSG00000089844|-|chr1:g.85078387:C>T
MAGGDNELSSRTIPEDQNEEKSDDYQLMFKHFKENKVEIASAITKPFPFLMSLRDRDFISEQKFQEYQETCKN
LAPVERVVYDILSNVQKKFSRDLLKVIFSKTHLKVYPDLKETLKHFFLNASKTNDEQAEEMLSLPQCNGGVLA
QEHSNPHVKRSVPVSCVPQHMCQKTWKQGWEAAKEKVWAPGGTFLQENPRSREEKRKATTGQNQNGESQ*
>ENSMUSP00000125122_c.60:G>A_p.E21K ENSMUST00000160792.8|A530032D15Rik-201|ENSMUSG00000089844|-|chr1:g.85078387:C>T
MAGGDNELSSRTIPEDQNEEKSDDYQLMFKHFKENKVEIASAITKPFPFLMSLRDRDFISEQKFQEYQETCKN
LAPVERVVYDILSNVQKKFSRDLLKVIFSKTHLKVYPDLKETLKHFFLNASKTNDEQAEEMLSLPQCNGGVLA
QEHSNPHVKRSVPVSCVPQHMCQKTWKQGWEAAKEKVWAPGGTFLQENPRSRESWQVWSLHCRPW*
>ENSMUSP00000123812_c.60:G>A_p.E21K ENSMUST00000161675.3|A530032D15Rik-202|ENSMUSG00000089844|-|chr1:g.85078387:C>T
MAGGDNELSSRTIPEDQNEEKSDDYQLMFKHFKENKVEIASAITKPFPFLMSLRDRDFISEQKFQEYQETCKN
LAPVERVVYDILSNVQKKFSRDLLKVIFSKTHLKVYPDLKETLKHFFLN
>ENSMUSP00000124435_c.222:G>C_p.V75L ENSMUST00000161267.8|C130026I21Rik-205|ENSMUSG00000052477|-|chr1:g.85090303:C>G
MAGGNNELSSRTIPEDQNEEESDDYQLMFKHFKENKVEIASAITKPFPFLMSLRDRDFISEQKFQEYQETCKN
LLPLERVVYDILSNVQKKFSRDLLKVIFSKTHLKAYPEIRNQVHQL*
>ENSMUSP00000125215_c.270:G>C_p.V91L ENSMUST00000162421.2|C130026I21Rik-207|ENSMUSG00000052477|-|chr1:g.85090303:C>G
MDEPHMSHTCLSPGSTALPLQMSWKKRTIPEDQNEEESDDYQLMFKHFKENKVEIASAITKPFPFLMSLRDRD
FISEQKFQEYQETCKNLLPLERVVYDILSNVQKKFSRDLLKVIFSKTHLKAYPEIRNQVHQL*
>ENSMUSP00000159243_c.88:C>A_p.S30* ENSMUST00000161424.5|Gm7609-202|ENSMUSG00000079457|+|chr1:g.85190459:C>A
MNSSSEHCNVSDWLRLEATVKASVYVVAF*ITTSLTVVIIAIVSQSLQLRKEVPLVLLCHHLLCISSYCGLGV
VFQGMRALLANSPLLLCWLVFGAQLSVGEGILLTLTLMALNTYLAICYPLNSPSFVDSAKYRILAGTWTTVIL
KNVGLFLIEGTSPTPASVFQSAPLCPVILNGMPARVIGMFSLAFLLSVILVSYCLIYREGKWAGHFNKSNIKA
RRTVLVHLLQISLHVIPTLIIIGLGKRCGVFFFALNLALFGIFAFAQCFNHLVYGLHNRDLQSKLYPWLCCRK
KLLP*
>ENSMUSP00000159200_c.88:C>A_p.S30* ENSMUST00000113402.4|Gm7609-201|ENSMUSG00000079457|+|chr1:g.85190459:C>A

MNSSSEHCNVSDWLRLEATVKASVYVVAF*ITTSLTVVIIAIVSQSLQLRKEVPLVLLCHHLLCISSYCGLGV
VFQGMRALLANSPLLLCWLVFGAQLSVGEGILLTLTLMALNTYLAICYPLNSPSFVDSAKYRILAGTWTTVIL
KNVGLFLIEGTSPTPASVFQSAPLCPVILNGMPARVIGMFSLAFLLSVILVSYCLIYREGKWAGHFNKSNIKA
RRTVLVHLLQISLHVIPTLIIIGLGKRCGVFFFALNLALFGIFAFAQCFNHLVYGLHNRDLQSKLYPWLCCRK
KLLP*
>rs107861961_ENSMUSP00000159243_c.558:T>C_p.S187P
ENSMUST00000161424.5|Gm7609-202|ENSMUSG00000079457|+|chr1:g.85190929:T>C
MNSSSEHCNVSDWLRLEATVKASVYVVAFSITTSLTVVIIAIVSQSLQLRKEVPLVLLCHHLLCISSYCGLGV
VFQGMRALLANSPLLLCWLVFGAQLSVGEGILLTLTLMALNTYLAICYPLNSPSFVDSAKYRILAGTWTTVIL
KNVGLFLIEGTSPTPASVFQSAPLCPVILNGMPARVIGMFPLAFLLSVILVSYCLIYREGKWAGHFNKSNIKA
RRTVLVHLLQISLHVIPTLIIIGLGKRCGVFFFALNLALFGIFAFAQCFNHLVYGLHNRDLQSKLYPWLCCRK
KLLP*
>rs107861961_ENSMUSP00000159200_c.558:T>C_p.S187P
ENSMUST00000113402.4|Gm7609-201|ENSMUSG00000079457|+|chr1:g.85190929:T>C
MNSSSEHCNVSDWLRLEATVKASVYVVAFSITTSLTVVIIAIVSQSLQLRKEVPLVLLCHHLLCISSYCGLGV
VFQGMRALLANSPLLLCWLVFGAQLSVGEGILLTLTLMALNTYLAICYPLNSPSFVDSAKYRILAGTWTTVIL
KNVGLFLIEGTSPTPASVFQSAPLCPVILNGMPARVIGMFPLAFLLSVILVSYCLIYREGKWAGHFNKSNIKA
RRTVLVHLLQISLHVIPTLIIIGLGKRCGVFFFALNLALFGIFAFAQCFNHLVYGLHNRDLQSKLYPWLCCRK
KLLP*
>ENSMUSP00000125160_c.359:C>A_p.F120L ENSMUST00000159582.8|C130026I21Rik-204|ENSMUSG00000052477|-|chr1:g.85237423:G>T
MAGGNNELSSRTIPEDQNEEESDDYQLMFKHFKENKVEIASAITKPFPFLMSLRDRDFISEQKFQEYQETCKN
LVPVERVVYDVLSNVQKKFSRDLLKVIFSKTHLKAYPDLKETLKHFLLNASKTNDEQAEEMLSLPQCNGGEGS
SSCEQTCDEQEPQDDLPSSLRQEAARRKKRPNWSNSKRRLQKKKPRQDEMMEISKEASKTSGIKRPSTARRTT
Q
>ENSMUSP00000091224_c.359:C>A_p.F120L
ENSMUST00000093506.12|C130026I21Rik-202|ENSMUSG00000052477|-|chr1:g.85237423:G>T
MAGGNNELSSRTIPEDQNEEESDDYQLMFKHFKENKVEIASAITKPFPFLMSLRDRDFISEQKFQEYQETCKN
LVPVERVVYDVLSNVQKKFSRDLLKVIFSKTHLKAYPDLKETLKHFLLNVSDSHRTHQRINGRNVEERPRLPS
VVREASKTNDEQAEEMLSLPQCNGGEGSSSCEQTCDEQEPQDDLPSSLRQEAARRKKRPNWSNSKRRLQKKKP
RQDEMMGVPSPGHGVQEKLKAVSRRTLWKDDSSTNVKEVTKTLRARMRCGTCHWKGSAWANLSELVQGSRDT*
>ENSMUSP00000066587_c.359:C>A_p.F120L ENSMUST00000064341.9|C130026I21Rik-201|ENSMUSG00000052477|-|chr1:g.85237423:G>T
MAGGNNELSSRTIPEDQNEEESDDYQLMFKHFKENKVEIASAITKPFPFLMSLRDRDFISEQKFQEYQETCKN
LVPVERVVYDVLSNVQKKFSRDLLKVIFSKTHLKAYPDLKETLKHFLLNASKTNDEQAEEMLSLPQCNGGEGS
SSCEQTCDEQEPQDDLPSSLRQEAARRKKRPNWSNSKRRLQKKKPRQDEMMGVPSPGHGVQEKLKAVSRRTLW
KDDSSTNVKEVTKTLRARMRCGTCHWKGSAWANLSELVQGSRDT*
>ENSMUSP00000159221_c.477:A>C_p.T160P ENSMUST00000113407.11|Gm7592-201|ENSMUSG00000118607|+|chr1:g.85477885:A>C
MNSSSEHCNVSDWLRLEATVKASVYVVAFSITTSLTVVIIAIVSQSLQLRKEVRLVLLCHHLLCISSYCGLGV
VFQGMRALLANSPLLLCWLVFGAQLSVGEGILLTLTLMALNTYLAICYPLNSPSFVDSAKYRILAGTWTMVIL
KNVGLFLIEGTSPPPASVFQSAPLCPVILNGMPARVIGMFSLAFLLSVILVSYYLIYQEGKRAGHFNKSNIKA
QRTVLVHLLQISLHVIPTLIIIGLGKRCGVFFFALNLALFGIFAFAQCFNPLVYGLHNRDLQSKLYPWLCCRK
KLLP*
>ENSMUSP00000159207_c.585:A>C_p.T196P ENSMUST00000239236.2|Gm7592-202|ENSMUSG00000118607|+|chr1:g.85477885:A>C
MTVLCELFNEVNMEKYPDLNLIRRSFECGAKPALKTMNSSSEHCNVSDWLRLEATVKASVYVVAFSITTSLTV
VIIAIVSQSLQLRKEVRLVLLCHHLLCISSYCGLGVVFQGMRALLANSPLLLCWLVFGAQLSVGEGILLTLTL
MALNTYLAICYPLNSPSFVDSAKYRILAGTWTMVILKNVGLFLIEGTSPPPASVFQSAPLCPVILNGMPARVI
GMFSLAFLLSVILVSYYLIYQEGKRAGHFNKSNIKAQRTVLVHLLQISLHVIPTLIIIGLGKRCGVFFFALNL
ALFGIFAFAQCFNPLVYGLHNRDLQSKLYPWLCCRKKLLP*
>ENSMUSP00000091226_c.1322:A>T_p.Q441H ENSMUST00000093508.7|Sp110-201|ENSMUSG00000070034|-|chr1:g.85505028:T>A
MFTLTKALEKALLQHFIYMKVNIAYAINKPFPFFEALRDNSFITERMYKESLEACQNLVPLSKVVHNILTSLE
QTFHPSVLLTLFSKVNLREYPSLVAIFRSFRNVGYTYEEKNRPPLTLLEDLANPAEGCSLQTLLPPPRPQISL
PSHLSSAPRVCDPRATAQPIIEILDEQPSPSPRAVPLLGCIQEGKTTPVSSRDHQRKDKEDSREMPHSPSGPE
SVVKDDSPAANDLEMAREVPCTPANKKARRKKRPNWSNSKRRRQKKKPRQDEMMGVASPGHGVQEKLKAVSRR

TLWKDDSSTNVKEVTKTQRTRMRRAQTSNSQEISKEASKTSGRKRPSTARRTTQVPEKTKNDAVDFSPTLPVT
CGKAKGTLFQEKLKQGASKKCIQNEAGDWLTVKEFLNEGGRATSKDWKGVIRCNGETLRHLEQKGLLFFTSKS
KPHKKGA*
>rs108615523_ENSMUSP00000091226_c.1047:A>G_p.K350E
ENSMUST00000093508.7|Sp110-201|ENSMUSG00000070034|-|chr1:g.85507458:T>C
MFTLTKALEKALLQHFIYMKVNIAYAINKPFPFFEALRDNSFITERMYKESLEACQNLVPLSKVVHNILTSLE
QTFHPSVLLTLFSKVNLREYPSLVAIFRSFRNVGYTYEEKNRPPLTLLEDLANPAEGCSLQTLLPPPRPQISL
PSHLSSAPRVCDPRATAQPIIEILDEQPSPSPRAVPLLGCIQEGKTTPVSSRDHQRKDKEDSREMPHSPSGPE
SVVKDDSPAANDLEMAREVPCTPANKKARRKKRPNWSNSKRRRQKKKPRQDEMMGVASPGHGVQEKLKAVSRR
TLWKDDSSTNVKEVTKTQRTRMRRAQTSNSQEISKEASKTSGRKRPSTARRTTQVPEETKNDAVDFSPTLPVT
CGKAKGTLFQEKLKQGASKKCIQNEAGDWLTVKEFLNEGGRATSKDWKGVIRCNGETLRHLEQKGLLFFTSKS
KPQKKGA*
>ENSMUSP00000091226_c.138:A>G_p.M47V ENSMUST00000093508.7|Sp110-
201|ENSMUSG00000070034|-|chr1:g.85523691:T>C
MFTLTKALEKALLQHFIYMKVNIAYAINKPFPFFEALRDNSFITERVYKESLEACQNLVPLSKVVHNILTSLE
QTFHPSVLLTLFSKVNLREYPSLVAIFRSFRNVGYTYEEKNRPPLTLLEDLANPAEGCSLQTLLPPPRPQISL
PSHLSSAPRVCDPRATAQPIIEILDEQPSPSPRAVPLLGCIQEGKTTPVSSRDHQRKDKEDSREMPHSPSGPE
SVVKDDSPAANDLEMAREVPCTPANKKARRKKRPNWSNSKRRRQKKKPRQDEMMGVASPGHGVQEKLKAVSRR
TLWKDDSSTNVKEVTKTQRTRMRRAQTSNSQEISKEASKTSGRKRPSTARRTTQVPEKTKNDAVDFSPTLPVT
CGKAKGTLFQEKLKQGASKKCIQNEAGDWLTVKEFLNEGGRATSKDWKGVIRCNGETLRHLEQKGLLFFTSKS
KPQKKGA*
>rs48330068_ENSMUSP00000079095_c.25:G>A_p.S9N
ENSMUST00000080204.11|Sp140-201|ENSMUSG00000070031|+|chr1:g.85528906:G>A
MAGGYNELNSRTIPEDQNEEESDDYQLMFKHFKENKVEIASAITKPFPFLMSLRDRDFISEQKFQEYQETCKN
LVPVERVVYDILSNVQKKFSRDLLKVIFSKTHLKAYPDLKETLKHFFLNASKTNDEQAEEMQCNGGEGSNSCE
QTRQTRQEPQDDLPSSLRQEAGAQQPTREKKCSCVICSPTYVPEDLEARMGSSQGECVGTRRNIPARKPKKQR
RKKKGHNWSKPRWRKPMSVQRKRLRRRRKENANFSAELLPVTCGNLKGVLHKEKFKQGISVKSIQCQNGNWFT
PSEFEMMGGYGKSKNWKLSLRCHNWPLKLLIQRNFLPNPPRIHRKRKEQRTQNLHRSPADPSIQNSDKCEVCR
DGGLLFCCDTCFRAFHEDCHIPTVEAEITPWSCIFCRMQSLGSQPSLPESEILQREMAPQEQLKCEYVLLRVY
CCSESSFFSKMPYYYYFREMTVSVQEPMWLDIIKKKLSDQAYCQVEDFVQDMRLIFRNHKITFKDPKFGEMGF
RLESKFEKSFKEVFAIQETNEKS*
>ENSMUSP00000079095_c.26:C>A_p.S9R ENSMUST00000080204.11|Sp140-
201|ENSMUSG00000070031|+|chr1:g.85528907:C>A
MAGGYNELRSRTIPEDQNEEESDDYQLMFKHFKENKVEIASAITKPFPFLMSLRDRDFISEQKFQEYQETCKN
LVPVERVVYDILSNVQKKFSRDLLKVIFSKTHLKAYPDLKETLKHFFLNASKTNDEQAEEMQCNGGEGSNSCE
QTRQTRQEPQDDLPSSLRQEAGAQQPTREKKCSCVICSPTYVPEDLEARMGSSQGECVGTRRNIPARKPKKQR
RKKKGHNWSKPRWRKPMSVQRKRLRRRRKENANFSAELLPVTCGNLKGVLHKEKFKQGISVKSIQCQNGNWFT
PSEFEMMGGYGKSKNWKLSLRCHNWPLKLLIQRNFLPNPPRIHRKRKEQRTQNLHRSPADPSIQNSDKCEVCR
DGGLLFCCDTCFRAFHEDCHIPTVEAEITPWSCIFCRMQSLGSQPSLPESEILQREMAPQEQLKCEYVLLRVY
CCSESSFFSKMPYYYYFREMTVSVQEPMWLDIIKKKLSDQAYCQVEDFVQDMRLIFRNHKITFKDPKFGEMGF
RLESKFEKSFKEVFAIQETNEKS*
>rs108167277_ENSMUSP00000079095_c.1598:G>C_p.K533N
ENSMUST00000080204.11|Sp140-201|ENSMUSG00000070031|+|chr1:g.85572397:G>C
MAGGYNELSSRTIPEDQNEEESDDYQLMFKHFKENKVEIASAITKPFPFLMSLRDRDFISEQKFQEYQETCKN
LVPVERVVYDILSNVQKKFSRDLLKVIFSKTHLKAYPDLKETLKHFFLNASKTNDEQAEEMQCNGGEGSNSCE
QTRQTRQEPQDDLPSSLRQEAGAQQPTREKKCSCVICSPTYVPEDLEARMGSSQGECVGTRRNIPARKPKKQR
RKKKGHNWSKPRWRKPMSVQRKRLRRRRKENANFSAELLPVTCGNLKGVLHKEKFKQGISVKSIQCQNGNWFT
PSEFEMMGGYGKSKNWKLSLRCHNWPLKLLIQRNFLPNPPRIHRKRKEQRTQNLHRSPADPSIQNSDKCEVCR
DGGLLFCCDTCFRAFHEDCHIPTVEAEITPWSCIFCRMQSLGSQPSLPESEILQREMAPQEQLKCEYVLLRVY
CCSESSFFSKMPYYYYFREMTVSVQEPMWLDIIKKKLSDQAYCQVEDFVQDMRLIFRNHKITFKDPKFGEMGF
RLESKFEKSFKEVFAIQETNENS*
>rs108167277_ENSMUSP00000125026_c.548:G>C_p.K183N
ENSMUST00000161497.2|Sp140-205|ENSMUSG00000070031|+|chr1:g.85572397:G>C
XISVKSIQCQNGNWFTPSEFEMMGGYGKSKNWKLSLRCHNWPLKLLIQRNFLPNPPRIHRKRKEQRTQNLHRS
PADPSIQNSDKCEVCRDGGLLFCCDTCFRAFHEDCHIPTVEAEITPWSCIFCRMQSLGSQPSLPESEILQREM
APQEQLDPKFGEMGFRLESKFEKSFKEVFAIQETNENS*
>ENSMUSP00000118481_c.1272:G>A_p.D425N ENSMUST00000155094.8|Sp100-
213|ENSMUSG00000026222|+|chr1:g.85627381:G>A

MEGSDGSPRMSTEQENTEMHLIECMLKHFKTQKVAISNAIRSTFPFLESLRDHEFITGKMYEDLLDSCRSLVP
VDKVIYRALEELEKKFDMTVLCELFNEVNMEKYPDLNLIRRSFGCVFPNELCFQGIDGGNPNSQLSLEQGPGA
SYSQGSPNGSSLDLSASEGWRSNDRRNSNLMQANQTENHQLAESPGHLDSCELQVQLNNRDATPESCSLLPQN
EERAVQLNYELQINPCFVQLVDVKKENSSFSLAGNQQTRARTNQNEDSEIIELSSGDSDNGENFSEATTTVPS
QPAPAYSRKPPTLRRDRGGDTSDTESSIIIRRRKRTGRKKRERLGSYLIRNIKIPMKPSWKTAFLARSANPSS
QRRRKRGPRIPREENADFGGAELPVVCGNAQGFLDKEKFKQGIYVRSIRGKTGRLFTPMNFEIEGNCEKAKNW
RQSIRCKGWTLRELIQKGVLQDPPRKKKETPRNPRQTRRQVNAL*
>ENSMUSP00000116942_c.1218:G>A_p.D407N ENSMUST00000147552.8|Sp100-
210|ENSMUSG00000026222|+|chr1:g.85627381:G>A
MEGSDGSPRMSTEQENTEMHLIECMLKHFKTQKVAISNAIRSTFPFLESLRDHEFITGKMYEDLLDSCRSLVP
VDKVIYRALEELEKKFDMTVLCELFNEVNMEKYPDLNLIRRSFGCVFPNELCFQGIDGGNPNSQLSLEQGPGA
SYSQGSPNGSSLDLSASEGWRSNDRRNSNLMQANQTENHQLAESPGHLDSCELQVQLNNRDATPESCSLLPQN
EERAVQLNYELQINPCFVQLVDVKKENSSFSLAGNQQTRARTNQNEDSEIIELSSGDSDNGENFSEATTTVPS
QPAPAYSRKPPTLRRDRGGGKKRERLGSYLIRNIKIPMKPSWKTAFLARSANPSSQRRRKRGPRIPREENADF
GGAELPVVCGNAQGFLDKEKFKQGIYVRSIRGKTGRLFTPMNFEIEGNCEKAKNWRQSIRCKGWTLRELIQKG
VLQDPPRKKKETPRNPRQTRRQVNAL*
>ENSMUSP00000122670_c.1197:G>A_p.D400N ENSMUST00000153574.8|Sp100-
212|ENSMUSG00000026222|+|chr1:g.85627381:G>A
MEGSDGSPRMSTEQENTEMHLIECMLKHFKTQKVAISNAIRSTFPFLESLRDHEFITGKMYEDLLDSCRSLVP
VDKVIYRALEELEKKFDMTVLCELFNEVNMEKYPDLNLIRRSFGCVFPNELCFQGIDGGNPNSQLSLEQGPGA
SYSQGSPNGSSLDLSASEGWRSNDRRNSNLMQANQTENHQLAESPGHLDSCELQVQLNNRDATPESCSLLPQN
EERAVQLNYELQINPCFVQLVDVKKENSSFSLAGNQQTRARTNQNEDSEIIELSSGDSDNGENFSEATTTVPS
QPAPAYSRKPPTLRRDRGGDTSDTESSIIIRRRKRTGPFLARSANPSSQRRRKRGPRIPREENADFGGAELPV
VCGNAQGFLDKEKFKQGIYVRSIRGKTGRLFTPMNFEIEGNCEKAKNWRQSIRCKGWTLRELIQKGVLQDPPR
KKKETPRNPRQTRRQKRFKNLGIIVGKKFEKNFKRIFSIEDTSEQHEPCNHTVLLT*
>ENSMUSP00000122899_c.1143:G>A_p.D382N ENSMUST00000150967.8|Sp100-
211|ENSMUSG00000026222|+|chr1:g.85627381:G>A
MEGSDGSPRMSTEQENTEMHLIECMLKHFKTQKVAISNAIRSTFPFLESLRDHEFITGKMYEDLLDSCRSLVP
VDKVIYRALEELEKKFDMTVLCELFNEVNMEKYPDLNLIRRSFGCVFPNELCFQGIDGGNPNSQLSLEQGPGA
SYSQGSPNGSSLDLSASEGWRSNDRRNSNLMQANQTENHQLAESPGHLDSCELQVQLNNRDATPESCSLLPQN
EERAVQLNYELQINPCFVQLVDVKKENSSFSLAGNQQTRARTNQNEDSEIIELSSGDSDNGENFSEATTTVPS
QPAPAYSRKPPTLRRDRGGAFLARSANPSSQRRRKRGPRIPREENADFGGAELPVVCGNAQGFLDKEKFKQGI
YVRSIRGKTGRLFTPMNFEIEGNCEKAKNWRQSIRCKGWTLRELIQKGVLQDP
>ENSMUSP00000066399_c.1272:G>A_p.D425N ENSMUST00000066427.11|Sp100-
202|ENSMUSG00000026222|+|chr1:g.85627381:G>A
MEGSDGSPRMSTEQENTEMHLIECMLKHFKTQKVAISNAIRSTFPFLESLRDHEFITGKMYEDLLDSCRSLVP
VDKVIYRALEELEKKFDMTVLCELFNEVNMEKYPDLNLIRRSFGCVFPNELCFQGIDGGNPNSQLSLEQGPGA
SYSQGSPNGSSLDLSASEGWRSNDRRNSNLMQANQTENHQLAESPGHLDSCELQVQLNNRDATPESCSLLPQN
EERAVQLNYELQINPCFVQLVDVKKENSSFSLAGNQQTRARTNQNEDSEIIELSSGDSDNGENFSEATTTVPS
QPAPAYSRKPPTLRRDRGGDTSDTESSIIIRRRKRTGRKKRERLGSYLIRNIKIPMKPSWKTAFLARSANPSS
QRRRKRGPRIPREENADFGGAELPVVCGNAQGFLDKEKFKQGIYVRSIRGKTGRLFTPMNFEIEGNCEKAKNW
RQSIRCKGWTLRELIQKGVLQDPPRKKKETPRNPRQTRRQKCELLLLTIYCYPKSGFFIPKPKQRKEKFPDLR
EHMWLNKIKNRLNKKAYHSVRRFVEDMRLIFHNHSIFYKKRFKNLGIIVGKKFEKNFKRIFSIEDTSEQHEPC
NHTVLLT*
>ENSMUSP00000120267_c.171:G>A_p.D58N ENSMUST00000132641.8|Sp100-
204|ENSMUSG00000026222|+|chr1:g.85627381:G>A
RRKRGPRIPREENADFGGAELPVVCGNAQGFLDKEKFKQGIYVRSIRGKTGRLFTPMNFEIEGNCEKAKNWRQ
SIRCKGWTLRELIQKGVLQDPPRKKKETPRNPRQTRRQRGNPKACKVCGQPRKVHPCTACKEFYHKNCHIPPV
EDKSRSWHCAFCKRKNQLRGQKTQACHKEAEVLKRKMSPEEQLKCELLLLTIYCYPKSGFFIPKPKQRKEKFP
DLREHMWLNKIKNRLNKKAYHSVRRFVEDMRLIFHNHSIFYKKRFKNLGIIVGKKFEKNFKRIFSIEDTSEQH
EPCNHTVLLT*
>ENSMUSP00000120267_c.558:G>A_p.E187K ENSMUST00000132641.8|Sp100-
204|ENSMUSG00000026222|+|chr1:g.85634667:G>A
RRKRGPRIPREENADFGGAELPVVCGNAQGFLDKEKFKQGIYVRSIRGKTGRLFTPMDFEIEGNCEKAKNWRQ
SIRCKGWTLRELIQKGVLQDPPRKKKETPRNPRQTRRQRGNPKACKVCGQPRKVHPCTACKEFYHKNCHIPPV
EDKSRSWHCAFCKRKNQLRGQKTQACHKEAEVLKRKMSPEKQLKCELLLLTIYCYPKSGFFIPKPKQRKEKFP
DLREHMWLNKIKNRLNKKAYHSVRRFVEDMRLIFHNHSIFYKKRFKNLGIIVGKKFEKNFKRIFSIEDTSEQH
EPCNHTVLLT*

```
>ENSMUSP00000119301_c.315:G>A_p.E106K ENSMUST00000141709.2|Sp100-
207|ENSMUSG00000026222|+|chr1:g.85634667:G>A
XHPGRRLRQLPRMQNWTLGAFPLFGGFLVRRGNPKACKVCGQPRKVHPCTACKEFYHKNCHIPPVEDKSRSWH
CAFCKRKNQLRGQKTQACHKEAEVLKRKMSPEKQLKCELLLLTIYCYPKSGFFIPKPKQRKEK
>rs107748797_ENSMUSP00000122670_c.1380:A>G_p.I461V
ENSMUST00000153574.8|Sp100-212|ENSMUSG00000026222|+|chr1:g.85636735:A>G
MEGSDGSPRMSTEQENTEMHLIECMLKHFKTQKVAISNAIRSTFPFLESLRDHEFITGKMYEDLLDSCRSLVP
VDKVIYRALEELEKKFDMTVLCELFNEVNMEKYPDLNLIRRSFGCVFPNELCFQGIDGGNPNSQLSLEQGPGA
SYSQGSPNGSSLDLSASEGWRSNDRRNSNLMQANQTENHQLAESPGHLDSCELQVQLNNRDATPESCSLLPQN
EERAVQLNYELQINPCFVQLVDVKKENSSFSLAGNQQTRARTNQNEDSEIIELSSGDSDNGENFSEATTTVPS
QPAPAYSRKPPTLRRDRGGDTSDTESSIIIRRRKRTGPFLARSANPSSQRRRKRGPRIPREENADFGGAELPV
VCGNAQGFLDKEKFKQGIYVRSIRGKTGRLFTPMDFEIEGNCEKAKNWRQSIRCKGWTLRELIQKGVLQDPPR
KKKETPRNPRQTRRQKRFKNLGVIVGKKFEKNFKRIFSIEDTSEQHEPCNHTVLLT*
>rs107748797_ENSMUSP00000066399_c.1671:A>G_p.I558V
ENSMUST00000066427.11|Sp100-202|ENSMUSG00000026222|+|chr1:g.85636735:A>G
MEGSDGSPRMSTEQENTEMHLIECMLKHFKTQKVAISNAIRSTFPFLESLRDHEFITGKMYEDLLDSCRSLVP
VDKVIYRALEELEKKFDMTVLCELFNEVNMEKYPDLNLIRRSFGCVFPNELCFQGIDGGNPNSQLSLEQGPGA
SYSQGSPNGSSLDLSASEGWRSNDRRNSNLMQANQTENHQLAESPGHLDSCELQVQLNNRDATPESCSLLPQN
EERAVQLNYELQINPCFVQLVDVKKENSSFSLAGNQQTRARTNQNEDSEIIELSSGDSDNGENFSEATTTVPS
QPAPAYSRKPPTLRRDRGGDTSDTESSIIIRRRKRTGRKKRERLGSYLIRNIKIPMKPSWKTAFLARSANPSS
QRRRKRGPRIPREENADFGGAELPVVCGNAQGFLDKEKFKQGIYVRSIRGKTGRLFTPMDFEIEGNCEKAKNW
RQSIRCKGWTLRELIQKGVLQDPPRKKKETPRNPRQTRRQKCELLLLTIYCYPKSGFFIPKPKQRKEKFPDLR
EHMWLNKIKNRLNKKAYHSVRRFVEDMRLIFHNHSIFYKKRFKNLGVIVGKKFEKNFKRIFSIEDTSEQHEPC
NHTVLLT*
>rs107748797_ENSMUSP00000120267_c.804:A>G_p.I269V
ENSMUST00000132641.8|Sp100-204|ENSMUSG00000026222|+|chr1:g.85636735:A>G
RRKRGPRIPREENADFGGAELPVVCGNAQGFLDKEKFKQGIYVRSIRGKTGRLFTPMDFEIEGNCEKAKNWRQ
SIRCKGWTLRELIQKGVLQDPPRKKKETPRNPRQTRRQRGNPKACKVCGQPRKVHPCTACKEFYHKNCHIPPV
EDKSRSWHCAFCKRKNQLRGQKTQACHKEAEVLKRKMSPEEQLKCELLLLTIYCYPKSGFFIPKPKQRKEKFP
DLREHMWLNKIKNRLNKKAYHSVRRFVEDMRLIFHNHSIFYKKRFKNLGVIVGKKFEKNFKRIFSIEDTSEQH
EPCNHTVLLT*
>ENSMUSP00000124137_c.523:G>T_p.S175I ENSMUST00000159122.8|A630001G21Rik-
202|ENSMUSG00000052760|-|chr1:g.85646080:C>A
MFGVIQKEERFNEVFLNYFKENKIEIAHAITKLFPFLESLRDHSFITDKIYADSYEACRNSVPIVEVVYNVLS
HLERLSDSSFLWALFNRVNLKEYPALREIHKSLGTELQDGYSPQKCKQEEQKKLATVHPSYEQGTNLCTHEKW
AQPCFVKQDNLQEPEIQQNQVDEIILISIESSECSDEDEEDQALGTGLRRQMNKEAEDSSPPGFQNIELPAGS
HTSCVWTNHS*
>ENSMUSP00000070374_c.523:G>T_p.S175I
ENSMUST00000064788.14|A630001G21Rik-201|ENSMUSG00000052760|-
|chr1:g.85646080:C>A
MFGVIQKEERFNEVFLNYFKENKIEIAHAITKLFPFLESLRDHSFITDKIYADSYEACRNSVPIVEVVYNVLS
HLERLSDSSFLWALFNRVNLKEYPALREIHKSLGTELQDGYSPQKCKQEEQKKLATVHPSYEQGTNLCTHEKW
AQPCFVKQDNLQEPEIQQNQVDEIILISIESSECSDEDEEDQALGTGLRRQMNKEAEDSSPPGFQNIELPAGS
HTSCVWTNHS*
>ENSMUSP00000124137_c.445:C>A_p.P149H ENSMUST00000159122.8|A630001G21Rik-
202|ENSMUSG00000052760|-|chr1:g.85646814:G>T
MFGVIQKEERFNEVFLNYFKENKIEIAHAITKLFPFLESLRDHSFITDKIYADSYEACRNSVPIVEVVYNVLS
HLERLSDSSFLWALFNRVNLKEYPALREIHKSLGTELQDGYSPQKCKQEEQKKLATVHPSYEQGTNLCTHEKW
AQHCFVKQDNLQEPEIQQNQVDEIILISSESSECSDEDEEDQALGTGLRRQMNKEAEDSSPPGFQNIELPAGS
HTSCVWTNHS*
>ENSMUSP00000070374_c.445:C>A_p.P149H
ENSMUST00000064788.14|A630001G21Rik-201|ENSMUSG00000052760|-
|chr1:g.85646814:G>T
MFGVIQKEERFNEVFLNYFKENKIEIAHAITKLFPFLESLRDHSFITDKIYADSYEACRNSVPIVEVVYNVLS
HLERLSDSSFLWALFNRVNLKEYPALREIHKSLGTELQDGYSPQKCKQEEQKKLATVHPSYEQGTNLCTHEKW
AQHCFVKQDNLQEPEIQQNQVDEIILISSESSECSDEDEEDQALGTGLRRQMNKEAEDSSPPGFQNIELPAGS
HTSCVWTNHS*
```

>ENSMUSP00000072983_c.481:T>A_p.F161Y ENSMUST00000073252.9|Chrnd-201|ENSMUSG00000026251|+|chr1:g.87120327:T>A
MAGPVLTLGLLAALVVCALPGSWGLNEEQRLIQHLFNEKGYDKDLRPVARKEDKVDVALSLTLSNLISLKEVE
ETLTTNVWIDHAWVDSRLQWDANDFGNITVLRLPPDMVWLPEIVLENNNDGSFQISYACNVLVYDSGYVTWLP
PAIFRSSCPISVTYYPFDWQNCSLKFSSLKYTAKEITLSLKQEEENNRSYPIEWIIIDPEGFTENGEWEIVHR
AAKLNVDPSVPMDSTNHQDVTFYLIIRRKPLFYIINILVPCVLISFMINLVFYLPGDCGEKTSVAISVLLAQS
VFLLLISKRLPATSMAIPLVGKFLLFGMVLVTMVVVICVIVLNIHFRTPSTHVLSEGVKKFFLETLPKLLHMS
RPAEEDPGPRALIRRSSSLGYICKAEEYFSLKSRSDLMFEKQSERHGLARRLTTARRPPASSEQVQQELFNEM
KPAVDGANFIVNHMRDQNSYNEEKDNWNQVARTVDRLCLFVVTPVMVVGTAWIFLQGVYNQPPLQPFPGDPFS
YSEQDKRFI*
>ENSMUSP00000139537_c.154:T>A_p.F52Y ENSMUST00000186373.2|Chrnd-202|ENSMUSG00000026251|+|chr1:g.87120327:T>A
MVWLPEIVLENNNDGSFQISYACNVLVYDSGYVTWLPPAIFRSSCPISVTYYPFDWQNCSLKFSSLKYTAKEI
TLSLKQEEENNRSYPIEWIIIDPEGFTENGEWEIVHRAAKLNVDPSVPMDSTNHQDVTFYLIIRRKPLFYIIN
ILVPCVLISFMINLVFYLPGDCGEKTSVAISVLLAQSVFLLLISKRLPATSMAIPLVGKFLLFGMVLVTMVVV
ICVIVLNIHFRTPSTHVLSEGVKKFFLETLPKLLHMSRPAEEDPGPRALIRRSSSLGYICKAEEYFSLKSRSD
LMFEKQSERHGLARRLTTARRPPASSEQVQQELFNEMKPAVDGANFIVNHMRDQNSYNEEKDNWNQVARTVDR
LCLFVVTPVMVVGTAWIFLQGVYNQPPLQPFPGDPFSYSEQDKRFI*
>ENSMUSP00000108739_c.106:A>C_p.Y36S ENSMUST00000113114.10|Trpm8-202|ENSMUSG00000036251|+|chr1:g.88246798:A>C
MSFEGARLSMRSRRNGTMGSTRTLYSSVSRSTDVSSSDSDLVNFIQANFKKRECVFFTRDSKAMENICKCGYA
QSQHIEGTQINQNEKWNYKKHTKEFPTDAFGDIQFETLGKKGKYLRLSCDTDSETLYELLTQHWHLKTPNLVI
SVTGGAKNFALKPRMRKIFSRLIYIAQSKGAWILTGGTHYGLMKYIGEVVRDNTISRNSEENIVAIGIAAWGM
VSNRDTLIRSCDDEGHFSAQYIMDDFTRDPLYILDNNHTHLLLVDNGCHGHPTVEAKLRNQLEKYISERTSQD
SNYGGKIPIVCFAQGGGRETLKAINTSVKSKIPCVVVEGSGQIADVIASLVEVEDVLTSSMVKEKLVRFLPRT
VSRLPEEEIESWIKWLKEILESSHLLTVIKMEEAGDEIVSNAISYALYKAFSTNEQDKDNWNGQLKLLLEWNQ
LDLASDEIFTNDRRWESADLQEVMFTALIKDRPKFVRLFLENGLNLQKFLTNEVLTELFSTHFSTLVYRNLQI
AKNSYNDALLTFVWKLVANFRRSFWKEDRSSREDLDVELHDASLTTRHPLQALFIWAILQNKKELSKVIWEQT
KGCTLAALGASKLLKTLAKVKNDINAAGESEELANEYETRAVELFTECYSNDEDLAEQLLVYSCEAWGGSNCL
ELAVEATDQHFIAQPGVQNFLSKQWYGEISRDTKNWKIILCLFIIPLVGCGLVSFRKKPIDKHKKLLWYYVAF
FTSPFVVFSWNVVFYIAFLLLFAYVLLMDFHSVPHTPELILYALVFVLFCDEVRQWYMNGVNYFTDLWNVMDT
LGLFYFIAGIVFRLHSSNKSSLYSGRVIFCLDYIIFTLRLIHIFTVSRNLGPKIIMLQRMLIDVFFFLFLFAV
WMVAFGVARQGILRQNEQRWRWIFRSVIYEPYLAMFGQVPSDVDSTTYDFSHCTFSGNESKPLCVELDEHNLP
RFPEWITIPLVCIYMLSTNILLVNLLVAMFGYTVGIVQENNDQVWKFQRYFLVQEYCNRLNIPFPFVVFAYFY
MVVKKCFKCCCKEKNMESNACCFRNEDNETLAWEGVMKENYLVKINTKANDNSEEMRHRFRQLDSKLNDLKSL
LKEIANNIK*
>ENSMUSP00000036991_c.106:A>C_p.Y36S ENSMUST00000040210.14|Trpm8-201|ENSMUSG00000036251|+|chr1:g.88246798:A>C
MSFEGARLSMRSRRNGTMGSTRTLYSSVSRSTDVSSSDSDLVNFIQANFKKRECVFFTRDSKAMENICKCGYA
QSQHIEGTQINQNEKWNYKKHTKEFPTDAFGDIQFETLGKKGKYLRLSCDTDSETLYELLTQHWHLKTPNLVI
SVTGGAKNFALKPRMRKIFSRLIYIAQSKGAWILTGGTHYGLMKYIGEVVRDNTISRNSEENIVAIGIAAWGM
VSNRDTLIRSCDDEGHFSAQYIMDDFTRDPLYILDNNHTHLLLVDNGCHGHPTVEAKLRNQLEKYISERTSQD
SNYGGKIPIVCFAQGGGRETLKAINTSVKSKIPCVVVEGSGQIADVIASLVEVEDVLTSSMVKEKLVRFLPRT
VSRLPEEEIESWIKWLKEILESSHLLTVIKMEEAGDEIVSNAISYALYKAFSTNEQDKDNWNGQLKLLLEWNQ
LDLASDEIFTNDRRWESADLQEVMFTALIKDRPKFVRLFLENGLNLQKFLTNEVLTELFSTHFSTLVYRNLQI
AKNSYNDALLTFVWKLVANFRRSFWKEDRSSREDLDVELHDASLTTRHPLQALFIWAILQNKKELSKVIWEQT
KGCTLAALGASKLLKTLAKVKNDINAAGESEELANEYETRAVELFTECYSNDEDLAEQLLVYSCEAWGGSNCL
ELAVEATDQHFIAQPGVQNFLSKQWYGEISRDTKNWKIILCLFIIPLVGCGLVSFRKKPIDKHKKLLWYYVAF
FTSPFVVFSWNVVFYIAFLLLFAYVLLMDFHSVPHTPELILYALVFVLFCDEVRQWYMNGVNYFTDLWNVMDT
LGLFYFIAGIVFRLHSSNKSSLYSGRVIFCLDYIIFTLRLIHIFTVSRNLGPKIIMLQRMLIDVFFFLFLFAV
WMVAFGVARQGILRQNEQRWRWIFRSVIYEPYLAMFGQVPSDVDSTTYDFSHCTFSGNESKPLCVELDEHNLP
RFPEWITIPLVCIYMLSTNILLVNLLVAMFGYTVGIVQENNDQVWKFQRYFLVQEYCNRLNIPFPFVVFAYFY
MVVKKCFKCCCKEKNMESNACCFRNEDNETLAWEGVMKENYLVKINTKANDNSEEMRHRFRQLDSKLNDLKSL
LKEIANNIK*
>ENSMUSP00000131209_c.106:A>C_p.Y36S ENSMUST00000171176.3|Trpm8-204|ENSMUSG00000036251|+|chr1:g.88246798:A>C
MSFEGARLSMRSRRNGTMGSTRTLYSSVSRSTDVSSSDSDLVNFIQANFKKRECVFFTRDSKAMENICKCGYA
QSQHIEGTQINQNEKWNYKKHTKEFPTDAFGDIQFETLGKKGKYLRLSCDTDSETLYELLTQHWHLKTPNLVI

SVTGGAKNFALKPRMRKIFSRLIYIAQSKGAWILTGGTHYGLMKYIGEVVRDNTISRNSEENIVAIGIAAWGM
VSNRDTLIRSCDDEGHFSAQYIMDDFTRDPLYILDNNHTHLLLVDNGCHGHPTVEAKLRNQLEKYISERTSQD
SNYGGKIPIVCFAQGGGRETLKAINTSVKSKIPCVVVEGSGQIADVIASLVEVEDVLTSSMVKEKLVRFLPRT
VSRLPEEEIESWIKWLKEILESSHLLTVIKMEEAGDEIVSNAISYALYKAFSTNEQDKDNWNGQLKLLLEWNQ
LDLASDEIFTNDRRWESADLQEVMFTALIKDRPKFVRLFLENGLNLQKFLTNEVLTELFSTHFSTLVYRNLQI
AKNSYNDALLTFVWKLVANFRRSFWKEDRSSREDLDVELHDASLTTRHPLQALFIWAILQNKKELSKVIWEQT
KGCTLAALGASKLLKTLAKVKNDINAAGESEELANEYETRAVELFTECYSNDEDLAEQLLVYSCEAWGGSNCL
ELAVEATDQHFIAQPGVQNFLSKQWYGEISRDTKNWKIILCLFIIPLVGCGLVSFRKKPIDKHKKLLWYYVAF
FTSPFVVFSWNVVFYIAFLLLFAYVLLMDFHSVPHTPELILYALVFVLFCDEVRQWYMNGVNYFTDLWNVMDT
LGLFYFIAGIVFRLHSSNKSSLYSGRVIFCLDYIIFTLRLIHIFTVSRNLGPKIIMLQRMLIDVFFFLFLFAV
WMVAFGVARQGILRQNEQRWRWIFRSVIYEPYLAMFGQVPSDVDSTTYDFSHCTFSGNESKPLCVELDEHNLP
RFPEWITIPLVCIYMLSTNILLVNLLVAMFGYTVGIVQENNDQVWKFQRYFLVQEYCNRLNIPFPFVVFAYFY
MVVKKCFKCCCKEKNMESNACCFRNEDNETLAWEGVMKENYLVKINTKANDNSEEMRHRFRQLDSKLNDLKSL
LKEIANNIK*
>rs50202562_ENSMUSP00000108739_c.145:T>C_p.F49S
ENSMUST00000113114.10|Trpm8-202|ENSMUSG00000036251|+|chr1:g.88248614:T>C
MSFEGARLSMRSRRNGTMGSTRTLYSSVSRSTDVSYSDSDLVNFIQANSKKRECVFFTRDSKAMENICKCGYA
QSQHIEGTQINQNEKWNYKKHTKEFPTDAFGDIQFETLGKKGKYLRLSCDTDSETLYELLTQHWHLKTPNLVI
SVTGGAKNFALKPRMRKIFSRLIYIAQSKGAWILTGGTHYGLMKYIGEVVRDNTISRNSEENIVAIGIAAWGM
VSNRDTLIRSCDDEGHFSAQYIMDDFTRDPLYILDNNHTHLLLVDNGCHGHPTVEAKLRNQLEKYISERTSQD
SNYGGKIPIVCFAQGGGRETLKAINTSVKSKIPCVVVEGSGQIADVIASLVEVEDVLTSSMVKEKLVRFLPRT
VSRLPEEEIESWIKWLKEILESSHLLTVIKMEEAGDEIVSNAISYALYKAFSTNEQDKDNWNGQLKLLLEWNQ
LDLASDEIFTNDRRWESADLQEVMFTALIKDRPKFVRLFLENGLNLQKFLTNEVLTELFSTHFSTLVYRNLQI
AKNSYNDALLTFVWKLVANFRRSFWKEDRSSREDLDVELHDASLTTRHPLQALFIWAILQNKKELSKVIWEQT
KGCTLAALGASKLLKTLAKVKNDINAAGESEELANEYETRAVELFTECYSNDEDLAEQLLVYSCEAWGGSNCL
ELAVEATDQHFIAQPGVQNFLSKQWYGEISRDTKNWKIILCLFIIPLVGCGLVSFRKKPIDKHKKLLWYYVAF
FTSPFVVFSWNVVFYIAFLLLFAYVLLMDFHSVPHTPELILYALVFVLFCDEVRQWYMNGVNYFTDLWNVMDT
LGLFYFIAGIVFRLHSSNKSSLYSGRVIFCLDYIIFTLRLIHIFTVSRNLGPKIIMLQRMLIDVFFFLFLFAV
WMVAFGVARQGILRQNEQRWRWIFRSVIYEPYLAMFGQVPSDVDSTTYDFSHCTFSGNESKPLCVELDEHNLP
RFPEWITIPLVCIYMLSTNILLVNLLVAMFGYTVGIVQENNDQVWKFQRYFLVQEYCNRLNIPFPFVVFAYFY
MVVKKCFKCCCKEKNMESNACCFRNEDNETLAWEGVMKENYLVKINTKANDNSEEMRHRFRQLDSKLNDLKSL
LKEIANNIK*
>rs50202562_ENSMUSP00000036991_c.145:T>C_p.F49S
ENSMUST00000040210.14|Trpm8-201|ENSMUSG00000036251|+|chr1:g.88248614:T>C
MSFEGARLSMRSRRNGTMGSTRTLYSSVSRSTDVSYSDSDLVNFIQANSKKRECVFFTRDSKAMENICKCGYA
QSQHIEGTQINQNEKWNYKKHTKEFPTDAFGDIQFETLGKKGKYLRLSCDTDSETLYELLTQHWHLKTPNLVI
SVTGGAKNFALKPRMRKIFSRLIYIAQSKGAWILTGGTHYGLMKYIGEVVRDNTISRNSEENIVAIGIAAWGM
VSNRDTLIRSCDDEGHFSAQYIMDDFTRDPLYILDNNHTHLLLVDNGCHGHPTVEAKLRNQLEKYISERTSQD
SNYGGKIPIVCFAQGGGRETLKAINTSVKSKIPCVVVEGSGQIADVIASLVEVEDVLTSSMVKEKLVRFLPRT
VSRLPEEEIESWIKWLKEILESSHLLTVIKMEEAGDEIVSNAISYALYKAFSTNEQDKDNWNGQLKLLLEWNQ
LDLASDEIFTNDRRWESADLQEVMFTALIKDRPKFVRLFLENGLNLQKFLTNEVLTELFSTHFSTLVYRNLQI
AKNSYNDALLTFVWKLVANFRRSFWKEDRSSREDLDVELHDASLTTRHPLQALFIWAILQNKKELSKVIWEQT
KGCTLAALGASKLLKTLAKVKNDINAAGESEELANEYETRAVELFTECYSNDEDLAEQLLVYSCEAWGGSNCL
ELAVEATDQHFIAQPGVQNFLSKQWYGEISRDTKNWKIILCLFIIPLVGCGLVSFRKKPIDKHKKLLWYYVAF
FTSPFVVFSWNVVFYIAFLLLFAYVLLMDFHSVPHTPELILYALVFVLFCDEVRQWYMNGVNYFTDLWNVMDT
LGLFYFIAGIVFRLHSSNKSSLYSGRVIFCLDYIIFTLRLIHIFTVSRNLGPKIIMLQRMLIDVFFFLFLFAV
WMVAFGVARQGILRQNEQRWRWIFRSVIYEPYLAMFGQVPSDVDSTTYDFSHCTFSGNESKPLCVELDEHNLP
RFPEWITIPLVCIYMLSTNILLVNLLVAMFGYTVGIVQENNDQVWKFQRYFLVQEYCNRLNIPFPFVVFAYFY
MVVKKCFKCCCKEKNMESNACCFRNEDNETLAWEGVMKENYLVKINTKANDNSEEMRHRFRQLDSKLNDLKSL
LKEIANNIK*
>rs50202562_ENSMUSP00000131209_c.145:T>C_p.F49S
ENSMUST00000171176.3|Trpm8-204|ENSMUSG00000036251|+|chr1:g.88248614:T>C
MSFEGARLSMRSRRNGTMGSTRTLYSSVSRSTDVSYSDSDLVNFIQANSKKRECVFFTRDSKAMENICKCGYA
QSQHIEGTQINQNEKWNYKKHTKEFPTDAFGDIQFETLGKKGKYLRLSCDTDSETLYELLTQHWHLKTPNLVI
SVTGGAKNFALKPRMRKIFSRLIYIAQSKGAWILTGGTHYGLMKYIGEVVRDNTISRNSEENIVAIGIAAWGM
VSNRDTLIRSCDDEGHFSAQYIMDDFTRDPLYILDNNHTHLLLVDNGCHGHPTVEAKLRNQLEKYISERTSQD
SNYGGKIPIVCFAQGGGRETLKAINTSVKSKIPCVVVEGSGQIADVIASLVEVEDVLTSSMVKEKLVRFLPRT
VSRLPEEEIESWIKWLKEILESSHLLTVIKMEEAGDEIVSNAISYALYKAFSTNEQDKDNWNGQLKLLLEWNQ

LDLASDEIFTNDRRWESADLQEVMFTALIKDRPKFVRLFLENGLNLQKFLTNEVLTELFSTHFSTLVYRNLQI
AKNSYNDALLTFVWKLVANFRRSFWKEDRSSREDLDVELHDASLTTRHPLQALFIWAILQNKKELSKVIWEQT
KGCTLAALGASKLLKTLAKVKNDINAAGESEELANEYETRAVELFTECYSNDEDLAEQLLVYSCEAWGGSNCL
ELAVEATDQHFIAQPGVQNFLSKQWYGEISRDTKNWKIILCLFIIPLVGCGLVSFRKKPIDKHKKLLWYYVAF
FTSPFVVFSWNVVFYIAFLLLFAYVLLMDFHSVPHTPELILYALVFVLFCDEVRQWYMNGVNYFTDLWNVMDT
LGLFYFIAGIVFRLHSSNKSSLYSGRVIFCLDYIIFTLRLIHIFTVSRNLGPKIIMLQRMLIDVFFFLFLFAV
WMVAFGVARQGILRQNEQRWRWIFRSVIYEPYLAMFGQVPSDVDSTTYDFSHCTFSGNESKPLCVELDEHNLP
RFPEWITIPLVCIYMLSTNILLVNLLVAMFGYTVGIVQENNDQVWKFQRYFLVQEYCNRLNIPFPFVVFAYFY
MVVKKCFKCCCKEKNMESNACCFRNEDNETLAWEGVMKENYLVKINTKANDNSEEMRHRFRQLDSKLNDLKSL
LKEIANNIK*
>rs51885729_ENSMUSP00000108739_c.186:G>A_p.A63T
ENSMUST00000113114.10|Trpm8-202|ENSMUSG00000036251|+|chr1:g.88248655:G>A
MSFEGARLSMRSRRNGTMGSTRTLYSSVSRSTDVSYSDSDLVNFIQANFKKRECVFFTRDSKTMENICKCGYA
QSQHIEGTQINQNEKWNYKKHTKEFPTDAFGDIQFETLGKKGKYLRLSCDTDSETLYELLTQHWHLKTPNLVI
SVTGGAKNFALKPRMRKIFSRLIYIAQSKGAWILTGGTHYGLMKYIGEVVRDNTISRNSEENIVAIGIAAWGM
VSNRDTLIRSCDDEGHFSAQYIMDDFTRDPLYILDNNHTHLLLVDNGCHGHPTVEAKLRNQLEKYISERTSQD
SNYGGKIPIVCFAQGGGRETLKAINTSVKSKIPCVVVEGSGQIADVIASLVEVEDVLTSSMVKEKLVRFLPRT
VSRLPEEEIESWIKWLKEILESSHLLTVIKMEEAGDEIVSNAISYALYKAFSTNEQDKDNWNGQLKLLLEWNQ
LDLASDEIFTNDRRWESADLQEVMFTALIKDRPKFVRLFLENGLNLQKFLTNEVLTELFSTHFSTLVYRNLQI
AKNSYNDALLTFVWKLVANFRRSFWKEDRSSREDLDVELHDASLTTRHPLQALFIWAILQNKKELSKVIWEQT
KGCTLAALGASKLLKTLAKVKNDINAAGESEELANEYETRAVELFTECYSNDEDLAEQLLVYSCEAWGGSNCL
ELAVEATDQHFIAQPGVQNFLSKQWYGEISRDTKNWKIILCLFIIPLVGCGLVSFRKKPIDKHKKLLWYYVAF
FTSPFVVFSWNVVFYIAFLLLFAYVLLMDFHSVPHTPELILYALVFVLFCDEVRQWYMNGVNYFTDLWNVMDT
LGLFYFIAGIVFRLHSSNKSSLYSGRVIFCLDYIIFTLRLIHIFTVSRNLGPKIIMLQRMLIDVFFFLFLFAV
WMVAFGVARQGILRQNEQRWRWIFRSVIYEPYLAMFGQVPSDVDSTTYDFSHCTFSGNESKPLCVELDEHNLP
RFPEWITIPLVCIYMLSTNILLVNLLVAMFGYTVGIVQENNDQVWKFQRYFLVQEYCNRLNIPFPFVVFAYFY
MVVKKCFKCCCKEKNMESNACCFRNEDNETLAWEGVMKENYLVKINTKANDNSEEMRHRFRQLDSKLNDLKSL
LKEIANNIK*
>rs51885729_ENSMUSP00000036991_c.186:G>A_p.A63T
ENSMUST00000040210.14|Trpm8-201|ENSMUSG00000036251|+|chr1:g.88248655:G>A
MSFEGARLSMRSRRNGTMGSTRTLYSSVSRSTDVSYSDSDLVNFIQANFKKRECVFFTRDSKTMENICKCGYA
QSQHIEGTQINQNEKWNYKKHTKEFPTDAFGDIQFETLGKKGKYLRLSCDTDSETLYELLTQHWHLKTPNLVI
SVTGGAKNFALKPRMRKIFSRLIYIAQSKGAWILTGGTHYGLMKYIGEVVRDNTISRNSEENIVAIGIAAWGM
VSNRDTLIRSCDDEGHFSAQYIMDDFTRDPLYILDNNHTHLLLVDNGCHGHPTVEAKLRNQLEKYISERTSQD
SNYGGKIPIVCFAQGGGRETLKAINTSVKSKIPCVVVEGSGQIADVIASLVEVEDVLTSSMVKEKLVRFLPRT
VSRLPEEEIESWIKWLKEILESSHLLTVIKMEEAGDEIVSNAISYALYKAFSTNEQDKDNWNGQLKLLLEWNQ
LDLASDEIFTNDRRWESADLQEVMFTALIKDRPKFVRLFLENGLNLQKFLTNEVLTELFSTHFSTLVYRNLQI
AKNSYNDALLTFVWKLVANFRRSFWKEDRSSREDLDVELHDASLTTRHPLQALFIWAILQNKKELSKVIWEQT
KGCTLAALGASKLLKTLAKVKNDINAAGESEELANEYETRAVELFTECYSNDEDLAEQLLVYSCEAWGGSNCL
ELAVEATDQHFIAQPGVQNFLSKQWYGEISRDTKNWKIILCLFIIPLVGCGLVSFRKKPIDKHKKLLWYYVAF
FTSPFVVFSWNVVFYIAFLLLFAYVLLMDFHSVPHTPELILYALVFVLFCDEVRQWYMNGVNYFTDLWNVMDT
LGLFYFIAGIVFRLHSSNKSSLYSGRVIFCLDYIIFTLRLIHIFTVSRNLGPKIIMLQRMLIDVFFFLFLFAV
WMVAFGVARQGILRQNEQRWRWIFRSVIYEPYLAMFGQVPSDVDSTTYDFSHCTFSGNESKPLCVELDEHNLP
RFPEWITIPLVCIYMLSTNILLVNLLVAMFGYTVGIVQENNDQVWKFQRYFLVQEYCNRLNIPFPFVVFAYFY
MVVKKCFKCCCKEKNMESNACCFRNEDNETLAWEGVMKENYLVKINTKANDNSEEMRHRFRQLDSKLNDLKSL
LKEIANNIK*
>rs51885729_ENSMUSP00000131209_c.186:G>A_p.A63T
ENSMUST00000171176.3|Trpm8-204|ENSMUSG00000036251|+|chr1:g.88248655:G>A
MSFEGARLSMRSRRNGTMGSTRTLYSSVSRSTDVSYSDSDLVNFIQANFKKRECVFFTRDSKTMENICKCGYA
QSQHIEGTQINQNEKWNYKKHTKEFPTDAFGDIQFETLGKKGKYLRLSCDTDSETLYELLTQHWHLKTPNLVI
SVTGGAKNFALKPRMRKIFSRLIYIAQSKGAWILTGGTHYGLMKYIGEVVRDNTISRNSEENIVAIGIAAWGM
VSNRDTLIRSCDDEGHFSAQYIMDDFTRDPLYILDNNHTHLLLVDNGCHGHPTVEAKLRNQLEKYISERTSQD
SNYGGKIPIVCFAQGGGRETLKAINTSVKSKIPCVVVEGSGQIADVIASLVEVEDVLTSSMVKEKLVRFLPRT
VSRLPEEEIESWIKWLKEILESSHLLTVIKMEEAGDEIVSNAISYALYKAFSTNEQDKDNWNGQLKLLLEWNQ
LDLASDEIFTNDRRWESADLQEVMFTALIKDRPKFVRLFLENGLNLQKFLTNEVLTELFSTHFSTLVYRNLQI
AKNSYNDALLTFVWKLVANFRRSFWKEDRSSREDLDVELHDASLTTRHPLQALFIWAILQNKKELSKVIWEQT
KGCTLAALGASKLLKTLAKVKNDINAAGESEELANEYETRAVELFTECYSNDEDLAEQLLVYSCEAWGGSNCL
ELAVEATDQHFIAQPGVQNFLSKQWYGEISRDTKNWKIILCLFIIPLVGCGLVSFRKKPIDKHKKLLWYYVAF

FTSPFVVFSWNVVFYIAFLLLFAYVLLMDFHSVPHTPELILYALVFVLFCDEVRQWYMNGVNYFTDLWNVMDT
LGLFYFIAGIVFRLHSSNKSSLYSGRVIFCLDYIIFTLRLIHIFTVSRNLGPKIIMLQRMLIDVFFFLFLFAV
WMVAFGVARQGILRQNEQRWRWIFRSVIYEPYLAMFGQVPSDVDSTTYDFSHCTFSGNESKPLCVELDEHNLP
RFPEWITIPLVCIYMLSTNILLVNLLVAMFGYTVGIVQENNDQVWKFQRYFLVQEYCNRLNIPFPFVVFAYFY
MVVKKCFKCCCKEKNMESNACCFRNEDNETLAWEGVMKENYLVKINTKANDNSEEMRHRFRQLDSKLNDLKSL
LKEIANNIK*
>rs51130807_ENSMUSP00000108739_c.210:G>C_p.G71R
ENSMUST00000113114.10|Trpm8-202|ENSMUSG00000036251|+|chr1:g.88253123:G>C
MSFEGARLSMRSRRNGTMGSTRTLYSSVSRSTDVSYSDSDLVNFIQANFKKRECVFFTRDSKAMENICKCRYA
QSQHIEGTQINQNEKWNYKKHTKEFPTDAFGDIQFETLGKKGKYLRLSCDTDSETLYELLTQHWHLKTPNLVI
SVTGGAKNFALKPRMRKIFSRLIYIAQSKGAWILTGGTHYGLMKYIGEVVRDNTISRNSEENIVAIGIAAWGM
VSNRDTLIRSCDDEGHFSAQYIMDDFTRDPLYILDNNHTHLLLVDNGCHGHPTVEAKLRNQLEKYISERTSQD
SNYGGKIPIVCFAQGGGRETLKAINTSVKSKIPCVVVEGSGQIADVIASLVEVEDVLTSSMVKEKLVRFLPRT
VSRLPEEEIESWIKWLKEILESSHLLTVIKMEEAGDEIVSNAISYALYKAFSTNEQDKDNWNGQLKLLLEWNQ
LDLASDEIFTNDRRWESADLQEVMFTALIKDRPKFVRLFLENGLNLQKFLTNEVLTELFSTHFSTLVYRNLQI
AKNSYNDALLTFVWKLVANFRRSFWKEDRSSREDLDVELHDASLTTRHPLQALFIWAILQNKKELSKVIWEQT
KGCTLAALGASKLLKTLAKVKNDINAAGESEELANEYETRAVELFTECYSNDEDLAEQLLVYSCEAWGGSNCL
ELAVEATDQHFIAQPGVQNFLSKQWYGEISRDTKNWKIILCLFIIPLVGCGLVSFRKKPIDKHKKLLWYYVAF
FTSPFVVFSWNVVFYIAFLLLFAYVLLMDFHSVPHTPELILYALVFVLFCDEVRQWYMNGVNYFTDLWNVMDT
LGLFYFIAGIVFRLHSSNKSSLYSGRVIFCLDYIIFTLRLIHIFTVSRNLGPKIIMLQRMLIDVFFFLFLFAV
WMVAFGVARQGILRQNEQRWRWIFRSVIYEPYLAMFGQVPSDVDSTTYDFSHCTFSGNESKPLCVELDEHNLP
RFPEWITIPLVCIYMLSTNILLVNLLVAMFGYTVGIVQENNDQVWKFQRYFLVQEYCNRLNIPFPFVVFAYFY
MVVKKCFKCCCKEKNMESNACCFRNEDNETLAWEGVMKENYLVKINTKANDNSEEMRHRFRQLDSKLNDLKSL
LKEIANNIK*
>rs51130807_ENSMUSP00000036991_c.210:G>C_p.G71R
ENSMUST00000040210.14|Trpm8-201|ENSMUSG00000036251|+|chr1:g.88253123:G>C
MSFEGARLSMRSRRNGTMGSTRTLYSSVSRSTDVSYSDSDLVNFIQANFKKRECVFFTRDSKAMENICKCRYA
QSQHIEGTQINQNEKWNYKKHTKEFPTDAFGDIQFETLGKKGKYLRLSCDTDSETLYELLTQHWHLKTPNLVI
SVTGGAKNFALKPRMRKIFSRLIYIAQSKGAWILTGGTHYGLMKYIGEVVRDNTISRNSEENIVAIGIAAWGM
VSNRDTLIRSCDDEGHFSAQYIMDDFTRDPLYILDNNHTHLLLVDNGCHGHPTVEAKLRNQLEKYISERTSQD
SNYGGKIPIVCFAQGGGRETLKAINTSVKSKIPCVVVEGSGQIADVIASLVEVEDVLTSSMVKEKLVRFLPRT
VSRLPEEEIESWIKWLKEILESSHLLTVIKMEEAGDEIVSNAISYALYKAFSTNEQDKDNWNGQLKLLLEWNQ
LDLASDEIFTNDRRWESADLQEVMFTALIKDRPKFVRLFLENGLNLQKFLTNEVLTELFSTHFSTLVYRNLQI
AKNSYNDALLTFVWKLVANFRRSFWKEDRSSREDLDVELHDASLTTRHPLQALFIWAILQNKKELSKVIWEQT
KGCTLAALGASKLLKTLAKVKNDINAAGESEELANEYETRAVELFTECYSNDEDLAEQLLVYSCEAWGGSNCL
ELAVEATDQHFIAQPGVQNFLSKQWYGEISRDTKNWKIILCLFIIPLVGCGLVSFRKKPIDKHKKLLWYYVAF
FTSPFVVFSWNVVFYIAFLLLFAYVLLMDFHSVPHTPELILYALVFVLFCDEVRQWYMNGVNYFTDLWNVMDT
LGLFYFIAGIVFRLHSSNKSSLYSGRVIFCLDYIIFTLRLIHIFTVSRNLGPKIIMLQRMLIDVFFFLFLFAV
WMVAFGVARQGILRQNEQRWRWIFRSVIYEPYLAMFGQVPSDVDSTTYDFSHCTFSGNESKPLCVELDEHNLP
RFPEWITIPLVCIYMLSTNILLVNLLVAMFGYTVGIVQENNDQVWKFQRYFLVQEYCNRLNIPFPFVVFAYFY
MVVKKCFKCCCKEKNMESNACCFRNEDNETLAWEGVMKENYLVKINTKANDNSEEMRHRFRQLDSKLNDLKSL
LKEIANNIK*
>rs51130807_ENSMUSP00000131209_c.210:G>C_p.G71R
ENSMUST00000171176.3|Trpm8-204|ENSMUSG00000036251|+|chr1:g.88253123:G>C
MSFEGARLSMRSRRNGTMGSTRTLYSSVSRSTDVSYSDSDLVNFIQANFKKRECVFFTRDSKAMENICKCRYA
QSQHIEGTQINQNEKWNYKKHTKEFPTDAFGDIQFETLGKKGKYLRLSCDTDSETLYELLTQHWHLKTPNLVI
SVTGGAKNFALKPRMRKIFSRLIYIAQSKGAWILTGGTHYGLMKYIGEVVRDNTISRNSEENIVAIGIAAWGM
VSNRDTLIRSCDDEGHFSAQYIMDDFTRDPLYILDNNHTHLLLVDNGCHGHPTVEAKLRNQLEKYISERTSQD
SNYGGKIPIVCFAQGGGRETLKAINTSVKSKIPCVVVEGSGQIADVIASLVEVEDVLTSSMVKEKLVRFLPRT
VSRLPEEEIESWIKWLKEILESSHLLTVIKMEEAGDEIVSNAISYALYKAFSTNEQDKDNWNGQLKLLLEWNQ
LDLASDEIFTNDRRWESADLQEVMFTALIKDRPKFVRLFLENGLNLQKFLTNEVLTELFSTHFSTLVYRNLQI
AKNSYNDALLTFVWKLVANFRRSFWKEDRSSREDLDVELHDASLTTRHPLQALFIWAILQNKKELSKVIWEQT
KGCTLAALGASKLLKTLAKVKNDINAAGESEELANEYETRAVELFTECYSNDEDLAEQLLVYSCEAWGGSNCL
ELAVEATDQHFIAQPGVQNFLSKQWYGEISRDTKNWKIILCLFIIPLVGCGLVSFRKKPIDKHKKLLWYYVAF
FTSPFVVFSWNVVFYIAFLLLFAYVLLMDFHSVPHTPELILYALVFVLFCDEVRQWYMNGVNYFTDLWNVMDT
LGLFYFIAGIVFRLHSSNKSSLYSGRVIFCLDYIIFTLRLIHIFTVSRNLGPKIIMLQRMLIDVFFFLFLFAV
WMVAFGVARQGILRQNEQRWRWIFRSVIYEPYLAMFGQVPSDVDSTTYDFSHCTFSGNESKPLCVELDEHNLP
RFPEWITIPLVCIYMLSTNILLVNLLVAMFGYTVGIVQENNDQVWKFQRYFLVQEYCNRLNIPFPFVVFAYFY

MVVKKCFKCCCKEKNMESNACCFRNEDNETLAWEGVMKENYLVKINTKANDNSEEMRHRFRQLDSKLNDLKSL
LKEIANNIK*
>ENSMUSP00000108739_c.302:C>CAGTGA_p.D101ins-fs
ENSMUST00000113114.10|Trpm8-
202|ENSMUSG00000036251|+|chr1:g.88253215:C>CAGTGA
MSFEGARLSMRSRRNGTMGSTRTLYSSVSRSTDVSYSDSDLVNFIQANFKKRECVFFTRDSKAMENICKCGYA
QSQHIEGTQINQNEKWNYKKHTKEFPTDSEPSGTFSLRLWGRKASTYACPVTPTLKLSTNC
>ENSMUSP00000036991_c.302:C>CAGTGA_p.D101ins-fs
ENSMUST00000040210.14|Trpm8-
201|ENSMUSG00000036251|+|chr1:g.88253215:C>CAGTGA
MSFEGARLSMRSRRNGTMGSTRTLYSSVSRSTDVSYSDSDLVNFIQANFKKRECVFFTRDSKAMENICKCGYA
QSQHIEGTQINQNEKWNYKKHTKEFPTDSEPSGTFSLRLWGRKASTYACPVTPTLKLSTNC
>ENSMUSP00000131209_c.302:C>CAGTGA_p.D101ins-fs
ENSMUST00000171176.3|Trpm8-
204|ENSMUSG00000036251|+|chr1:g.88253215:C>CAGTGA
MSFEGARLSMRSRRNGTMGSTRTLYSSVSRSTDVSYSDSDLVNFIQANFKKRECVFFTRDSKAMENICKCGYA
QSQHIEGTQINQNEKWNYKKHTKEFPTDSEPSGTFSLRLWGRKASTYACPVTPTLKLSTNC
>rs45725458_ENSMUSP00000108739_c.480:A>T_p.M161L
ENSMUST00000113114.10|Trpm8-202|ENSMUSG00000036251|+|chr1:g.88254259:A>T
MSFEGARLSMRSRRNGTMGSTRTLYSSVSRSTDVSYSDSDLVNFIQANFKKRECVFFTRDSKAMENICKCGYA
QSQHIEGTQINQNEKWNYKKHTKEFPTDAFGDIQFETLGKKGKYLRLSCDTDSETLYELLTQHWHLKTPNLVI
SVTGGAKNFALKPRLRKIFSRLIYIAQSKGAWILTGGTHYGLMKYIGEVVRDNTISRNSEENIVAIGIAAWGM
VSNRDTLIRSCDDEGHFSAQYIMDDFTRDPLYILDNNHTHLLLVDNGCHGHPTVEAKLRNQLEKYISERTSQD
SNYGGKIPIVCFAQGGGRETLKAINTSVKSKIPCVVVEGSGQIADVIASLVEVEDVLTSSMVKEKLVRFLPRT
VSRLPEEEIESWIKWLKEILESSHLLTVIKMEEAGDEIVSNAISYALYKAFSTNEQDKDNWNGQLKLLLEWNQ
LDLASDEIFTNDRRWESADLQEVMFTALIKDRPKFVRLFLENGLNLQKFLTNEVLTELFSTHFSTLVYRNLQI
AKNSYNDALLTFVWKLVANFRRSFWKEDRSSREDLDVELHDASLTTRHPLQALFIWAILQNKKELSKVIWEQT
KGCTLAALGASKLLKTLAKVKNDINAAGESEELANEYETRAVELFTECYSNDEDLAEQLLVYSCEAWGGSNCL
ELAVEATDQHFIAQPGVQNFLSKQWYGEISRDTKNWKIILCLFIIPLVGCGLVSFRKKPIDKHKKLLWYYVAF
FTSPFVVFSWNVVFYIAFLLLFAYVLLMDFHSVPHTPELILYALVFVLFCDEVRQWYMNGVNYFTDLWNVMDT
LGLFYFIAGIVFRLHSSNKSSLYSGRVIFCLDYIIFTLRLIHIFTVSRNLGPKIIMLQRMLIDVFFFLFLFAV
WMVAFGVARQGILRQNEQRWRWIFRSVIYEPYLAMFGQVPSDVDSTTYDFSHCTFSGNESKPLCVELDEHNLP
RFPEWITIPLVCIYMLSTNILLVNLLVAMFGYTVGIVQENNDQVWKFQRYFLVQEYCNRLNIPFPFVVFAYFY
MVVKKCFKCCCKEKNMESNACCFRNEDNETLAWEGVMKENYLVKINTKANDNSEEMRHRFRQLDSKLNDLKSL
LKEIANNIK*
>rs45725458_ENSMUSP00000036991_c.480:A>T_p.M161L
ENSMUST00000040210.14|Trpm8-201|ENSMUSG00000036251|+|chr1:g.88254259:A>T
MSFEGARLSMRSRRNGTMGSTRTLYSSVSRSTDVSYSDSDLVNFIQANFKKRECVFFTRDSKAMENICKCGYA
QSQHIEGTQINQNEKWNYKKHTKEFPTDAFGDIQFETLGKKGKYLRLSCDTDSETLYELLTQHWHLKTPNLVI
SVTGGAKNFALKPRLRKIFSRLIYIAQSKGAWILTGGTHYGLMKYIGEVVRDNTISRNSEENIVAIGIAAWGM
VSNRDTLIRSCDDEGHFSAQYIMDDFTRDPLYILDNNHTHLLLVDNGCHGHPTVEAKLRNQLEKYISERTSQD
SNYGGKIPIVCFAQGGGRETLKAINTSVKSKIPCVVVEGSGQIADVIASLVEVEDVLTSSMVKEKLVRFLPRT
VSRLPEEEIESWIKWLKEILESSHLLTVIKMEEAGDEIVSNAISYALYKAFSTNEQDKDNWNGQLKLLLEWNQ
LDLASDEIFTNDRRWESADLQEVMFTALIKDRPKFVRLFLENGLNLQKFLTNEVLTELFSTHFSTLVYRNLQI
AKNSYNDALLTFVWKLVANFRRSFWKEDRSSREDLDVELHDASLTTRHPLQALFIWAILQNKKELSKVIWEQT
KGCTLAALGASKLLKTLAKVKNDINAAGESEELANEYETRAVELFTECYSNDEDLAEQLLVYSCEAWGGSNCL
ELAVEATDQHFIAQPGVQNFLSKQWYGEISRDTKNWKIILCLFIIPLVGCGLVSFRKKPIDKHKKLLWYYVAF
FTSPFVVFSWNVVFYIAFLLLFAYVLLMDFHSVPHTPELILYALVFVLFCDEVRQWYMNGVNYFTDLWNVMDT
LGLFYFIAGIVFRLHSSNKSSLYSGRVIFCLDYIIFTLRLIHIFTVSRNLGPKIIMLQRMLIDVFFFLFLFAV
WMVAFGVARQGILRQNEQRWRWIFRSVIYEPYLAMFGQVPSDVDSTTYDFSHCTFSGNESKPLCVELDEHNLP
RFPEWITIPLVCIYMLSTNILLVNLLVAMFGYTVGIVQENNDQVWKFQRYFLVQEYCNRLNIPFPFVVFAYFY
MVVKKCFKCCCKEKNMESNACCFRNEDNETLAWEGVMKENYLVKINTKANDNSEEMRHRFRQLDSKLNDLKSL
LKEIANNIK*
>rs45725458_ENSMUSP00000131209_c.480:A>T_p.M161L
ENSMUST00000171176.3|Trpm8-204|ENSMUSG00000036251|+|chr1:g.88254259:A>T
MSFEGARLSMRSRRNGTMGSTRTLYSSVSRSTDVSYSDSDLVNFIQANFKKRECVFFTRDSKAMENICKCGYA
QSQHIEGTQINQNEKWNYKKHTKEFPTDAFGDIQFETLGKKGKYLRLSCDTDSETLYELLTQHWHLKTPNLVI
SVTGGAKNFALKPRLRKIFSRLIYIAQSKGAWILTGGTHYGLMKYIGEVVRDNTISRNSEENIVAIGIAAWGM

VSNRDTLIRSCDDEGHFSAQYIMDDFTRDPLYILDNNHTHLLLVDNGCHGHPTVEAKLRNQLEKYISERTSQD
SNYGGKIPIVCFAQGGGRETLKAINTSVKSKIPCVVVEGSGQIADVIASLVEVEDVLTSSMVKEKLVRFLPRT
VSRLPEEEIESWIKWLKEILESSHLLTVIKMEEAGDEIVSNAISYALYKAFSTNEQDKDNWNGQLKLLLEWNQ
LDLASDEIFTNDRRWESADLQEVMFTALIKDRPKFVRLFLENGLNLQKFLTNEVLTELFSTHFSTLVYRNLQI
AKNSYNDALLTFVWKLVANFRRSFWKEDRSSREDLDVELHDASLTTRHPLQALFIWAILQNKKELSKVIWEQT
KGCTLAALGASKLLKTLAKVKNDINAAGESEELANEYETRAVELFTECYSNDEDLAEQLLVYSCEAWGGSNCL
ELAVEATDQHFIAQPGVQNFLSKQWYGEISRDTKNWKIILCLFIIPLVGCGLVSFRKKPIDKHKKLLWYYVAF
FTSPFVVFSWNVVFYIAFLLLFAYVLLMDFHSVPHTPELILYALVFVLFCDEVRQWYMNGVNYFTDLWNVMDT
LGLFYFIAGIVFRLHSSNKSSLYSGRVIFCLDYIIFTLRLIHIFTVSRNLGPKIIMLQRMLIDVFFFLFLFAV
WMVAFGVARQGILRQNEQRWRWIFRSVIYEPYLAMFGQVPSDVDSTTYDFSHCTFSGNESKPLCVELDEHNLP
RFPEWITIPLVCIYMLSTNILLVNLLVAMFGYTVGIVQENNDQVWKFQRYFLVQEYCNRLNIPFPFVVFAYFY
MVVKKCFKCCCKEKNMESNACCFRNEDNETLAWEGVMKENYLVKINTKANDNSEEMRHRFRQLDSKLNDLKSL
LKEIANNIK*
>rs47939991_ENSMUSP00000108739_c.702:C>A_p.H235N
ENSMUST00000113114.10|Trpm8-202|ENSMUSG00000036251|+|chr1:g.88258421:C>A
MSFEGARLSMRSRRNGTMGSTRTLYSSVSRSTDVSYSDSDLVNFIQANFKKRECVFFTRDSKAMENICKCGYA
QSQHIEGTQINQNEKWNYKKHTKEFPTDAFGDIQFETLGKKGKYLRLSCDTDSETLYELLTQHWHLKTPNLVI
SVTGGAKNFALKPRMRKIFSRLIYIAQSKGAWILTGGTHYGLMKYIGEVVRDNTISRNSEENIVAIGIAAWGM
VSNRDTLIRSCDDEGNFSAQYIMDDFTRDPLYILDNNHTHLLLVDNGCHGHPTVEAKLRNQLEKYISERTSQD
SNYGGKIPIVCFAQGGGRETLKAINTSVKSKIPCVVVEGSGQIADVIASLVEVEDVLTSSMVKEKLVRFLPRT
VSRLPEEEIESWIKWLKEILESSHLLTVIKMEEAGDEIVSNAISYALYKAFSTNEQDKDNWNGQLKLLLEWNQ
LDLASDEIFTNDRRWESADLQEVMFTALIKDRPKFVRLFLENGLNLQKFLTNEVLTELFSTHFSTLVYRNLQI
AKNSYNDALLTFVWKLVANFRRSFWKEDRSSREDLDVELHDASLTTRHPLQALFIWAILQNKKELSKVIWEQT
KGCTLAALGASKLLKTLAKVKNDINAAGESEELANEYETRAVELFTECYSNDEDLAEQLLVYSCEAWGGSNCL
ELAVEATDQHFIAQPGVQNFLSKQWYGEISRDTKNWKIILCLFIIPLVGCGLVSFRKKPIDKHKKLLWYYVAF
FTSPFVVFSWNVVFYIAFLLLFAYVLLMDFHSVPHTPELILYALVFVLFCDEVRQWYMNGVNYFTDLWNVMDT
LGLFYFIAGIVFRLHSSNKSSLYSGRVIFCLDYIIFTLRLIHIFTVSRNLGPKIIMLQRMLIDVFFFLFLFAV
WMVAFGVARQGILRQNEQRWRWIFRSVIYEPYLAMFGQVPSDVDSTTYDFSHCTFSGNESKPLCVELDEHNLP
RFPEWITIPLVCIYMLSTNILLVNLLVAMFGYTVGIVQENNDQVWKFQRYFLVQEYCNRLNIPFPFVVFAYFY
MVVKKCFKCCCKEKNMESNACCFRNEDNETLAWEGVMKENYLVKINTKANDNSEEMRHRFRQLDSKLNDLKSL
LKEIANNIK*
>rs47939991_ENSMUSP00000036991_c.702:C>A_p.H235N
ENSMUST00000040210.14|Trpm8-201|ENSMUSG00000036251|+|chr1:g.88258421:C>A
MSFEGARLSMRSRRNGTMGSTRTLYSSVSRSTDVSYSDSDLVNFIQANFKKRECVFFTRDSKAMENICKCGYA
QSQHIEGTQINQNEKWNYKKHTKEFPTDAFGDIQFETLGKKGKYLRLSCDTDSETLYELLTQHWHLKTPNLVI
SVTGGAKNFALKPRMRKIFSRLIYIAQSKGAWILTGGTHYGLMKYIGEVVRDNTISRNSEENIVAIGIAAWGM
VSNRDTLIRSCDDEGNFSAQYIMDDFTRDPLYILDNNHTHLLLVDNGCHGHPTVEAKLRNQLEKYISERTSQD
SNYGGKIPIVCFAQGGGRETLKAINTSVKSKIPCVVVEGSGQIADVIASLVEVEDVLTSSMVKEKLVRFLPRT
VSRLPEEEIESWIKWLKEILESSHLLTVIKMEEAGDEIVSNAISYALYKAFSTNEQDKDNWNGQLKLLLEWNQ
LDLASDEIFTNDRRWESADLQEVMFTALIKDRPKFVRLFLENGLNLQKFLTNEVLTELFSTHFSTLVYRNLQI
AKNSYNDALLTFVWKLVANFRRSFWKEDRSSREDLDVELHDASLTTRHPLQALFIWAILQNKKELSKVIWEQT
KGCTLAALGASKLLKTLAKVKNDINAAGESEELANEYETRAVELFTECYSNDEDLAEQLLVYSCEAWGGSNCL
ELAVEATDQHFIAQPGVQNFLSKQWYGEISRDTKNWKIILCLFIIPLVGCGLVSFRKKPIDKHKKLLWYYVAF
FTSPFVVFSWNVVFYIAFLLLFAYVLLMDFHSVPHTPELILYALVFVLFCDEVRQWYMNGVNYFTDLWNVMDT
LGLFYFIAGIVFRLHSSNKSSLYSGRVIFCLDYIIFTLRLIHIFTVSRNLGPKIIMLQRMLIDVFFFLFLFAV
WMVAFGVARQGILRQNEQRWRWIFRSVIYEPYLAMFGQVPSDVDSTTYDFSHCTFSGNESKPLCVELDEHNLP
RFPEWITIPLVCIYMLSTNILLVNLLVAMFGYTVGIVQENNDQVWKFQRYFLVQEYCNRLNIPFPFVVFAYFY
MVVKKCFKCCCKEKNMESNACCFRNEDNETLAWEGVMKENYLVKINTKANDNSEEMRHRFRQLDSKLNDLKSL
LKEIANNIK*
>rs47939991_ENSMUSP00000131209_c.702:C>A_p.H235N
ENSMUST00000171176.3|Trpm8-204|ENSMUSG00000036251|+|chr1:g.88258421:C>A
MSFEGARLSMRSRRNGTMGSTRTLYSSVSRSTDVSYSDSDLVNFIQANFKKRECVFFTRDSKAMENICKCGYA
QSQHIEGTQINQNEKWNYKKHTKEFPTDAFGDIQFETLGKKGKYLRLSCDTDSETLYELLTQHWHLKTPNLVI
SVTGGAKNFALKPRMRKIFSRLIYIAQSKGAWILTGGTHYGLMKYIGEVVRDNTISRNSEENIVAIGIAAWGM
VSNRDTLIRSCDDEGNFSAQYIMDDFTRDPLYILDNNHTHLLLVDNGCHGHPTVEAKLRNQLEKYISERTSQD
SNYGGKIPIVCFAQGGGRETLKAINTSVKSKIPCVVVEGSGQIADVIASLVEVEDVLTSSMVKEKLVRFLPRT
VSRLPEEEIESWIKWLKEILESSHLLTVIKMEEAGDEIVSNAISYALYKAFSTNEQDKDNWNGQLKLLLEWNQ
LDLASDEIFTNDRRWESADLQEVMFTALIKDRPKFVRLFLENGLNLQKFLTNEVLTELFSTHFSTLVYRNLQI

AKNSYNDALLTFVWKLVANFRRSFWKEDRSSREDLDVELHDASLTTRHPLQALFIWAILQNKKELSKVIWEQT
KGCTLAALGASKLLKTLAKVKNDINAAGESEELANEYETRAVELFTECYSNDEDLAEQLLVYSCEAWGGSNCL
ELAVEATDQHFIAQPGVQNFLSKQWYGEISRDTKNWKIILCLFIIPLVGCGLVSFRKKPIDKHKKLLWYYVAF
FTSPFVVFSWNVVFYIAFLLLFAYVLLMDFHSVPHTPELILYALVFVLFCDEVRQWYMNGVNYFTDLWNVMDT
LGLFYFIAGIVFRLHSSNKSSLYSGRVIFCLDYIIFTLRLIHIFTVSRNLGPKIIMLQRMLIDVFFFLFLFAV
WMVAFGVARQGILRQNEQRWRWIFRSVIYEPYLAMFGQVPSDVDSTTYDFSHCTFSGNESKPLCVELDEHNLP
RFPEWITIPLVCIYMLSTNILLVNLLVAMFGYTVGIVQENNDQVWKFQRYFLVQEYCNRLNIPFPFVVFAYFY
MVVKKCFKCCCKEKNMESNACCFRNEDNETLAWEGVMKENYLVKINTKANDNSEEMRHRFRQLDSKLNDLKSL
LKEIANNIK*
>ENSMUSP00000108739_c.702:C>G_p.H235D ENSMUST00000113114.10|Trpm8-
202|ENSMUSG00000036251|+|chr1:g.88258421:C>G
MSFEGARLSMRSRRNGTMGSTRTLYSSVSRSTDVSYSDSDLVNFIQANFKKRECVFFTRDSKAMENICKCGYA
QSQHIEGTQINQNEKWNYKKHTKEFPTDAFGDIQFETLGKKGKYLRLSCDTDSETLYELLTQHWHLKTPNLVI
SVTGGAKNFALKPRMRKIFSRLIYIAQSKGAWILTGGTHYGLMKYIGEVVRDNTISRNSEENIVAIGIAAWGM
VSNRDTLIRSCDDEGDFSAQYIMDDFTRDPLYILDNNHTHLLLVDNGCHGHPTVEAKLRNQLEKYISERTSQD
SNYGGKIPIVCFAQGGGRETLKAINTSVKSKIPCVVVEGSGQIADVIASLVEVEDVLTSSMVKEKLVRFLPRT
VSRLPEEEIESWIKWLKEILESSHLLTVIKMEEAGDEIVSNAISYALYKAFSTNEQDKDNWNGQLKLLLEWNQ
LDLASDEIFTNDRRWESADLQEVMFTALIKDRPKFVRLFLENGLNLQKFLTNEVLTELFSTHFSTLVYRNLQI
AKNSYNDALLTFVWKLVANFRRSFWKEDRSSREDLDVELHDASLTTRHPLQALFIWAILQNKKELSKVIWEQT
KGCTLAALGASKLLKTLAKVKNDINAAGESEELANEYETRAVELFTECYSNDEDLAEQLLVYSCEAWGGSNCL
ELAVEATDQHFIAQPGVQNFLSKQWYGEISRDTKNWKIILCLFIIPLVGCGLVSFRKKPIDKHKKLLWYYVAF
FTSPFVVFSWNVVFYIAFLLLFAYVLLMDFHSVPHTPELILYALVFVLFCDEVRQWYMNGVNYFTDLWNVMDT
LGLFYFIAGIVFRLHSSNKSSLYSGRVIFCLDYIIFTLRLIHIFTVSRNLGPKIIMLQRMLIDVFFFLFLFAV
WMVAFGVARQGILRQNEQRWRWIFRSVIYEPYLAMFGQVPSDVDSTTYDFSHCTFSGNESKPLCVELDEHNLP
RFPEWITIPLVCIYMLSTNILLVNLLVAMFGYTVGIVQENNDQVWKFQRYFLVQEYCNRLNIPFPFVVFAYFY
MVVKKCFKCCCKEKNMESNACCFRNEDNETLAWEGVMKENYLVKINTKANDNSEEMRHRFRQLDSKLNDLKSL
LKEIANNIK*
>ENSMUSP00000036991_c.702:C>G_p.H235D ENSMUST00000040210.14|Trpm8-
201|ENSMUSG00000036251|+|chr1:g.88258421:C>G
MSFEGARLSMRSRRNGTMGSTRTLYSSVSRSTDVSYSDSDLVNFIQANFKKRECVFFTRDSKAMENICKCGYA
QSQHIEGTQINQNEKWNYKKHTKEFPTDAFGDIQFETLGKKGKYLRLSCDTDSETLYELLTQHWHLKTPNLVI
SVTGGAKNFALKPRMRKIFSRLIYIAQSKGAWILTGGTHYGLMKYIGEVVRDNTISRNSEENIVAIGIAAWGM
VSNRDTLIRSCDDEGDFSAQYIMDDFTRDPLYILDNNHTHLLLVDNGCHGHPTVEAKLRNQLEKYISERTSQD
SNYGGKIPIVCFAQGGGRETLKAINTSVKSKIPCVVVEGSGQIADVIASLVEVEDVLTSSMVKEKLVRFLPRT
VSRLPEEEIESWIKWLKEILESSHLLTVIKMEEAGDEIVSNAISYALYKAFSTNEQDKDNWNGQLKLLLEWNQ
LDLASDEIFTNDRRWESADLQEVMFTALIKDRPKFVRLFLENGLNLQKFLTNEVLTELFSTHFSTLVYRNLQI
AKNSYNDALLTFVWKLVANFRRSFWKEDRSSREDLDVELHDASLTTRHPLQALFIWAILQNKKELSKVIWEQT
KGCTLAALGASKLLKTLAKVKNDINAAGESEELANEYETRAVELFTECYSNDEDLAEQLLVYSCEAWGGSNCL
ELAVEATDQHFIAQPGVQNFLSKQWYGEISRDTKNWKIILCLFIIPLVGCGLVSFRKKPIDKHKKLLWYYVAF
FTSPFVVFSWNVVFYIAFLLLFAYVLLMDFHSVPHTPELILYALVFVLFCDEVRQWYMNGVNYFTDLWNVMDT
LGLFYFIAGIVFRLHSSNKSSLYSGRVIFCLDYIIFTLRLIHIFTVSRNLGPKIIMLQRMLIDVFFFLFLFAV
WMVAFGVARQGILRQNEQRWRWIFRSVIYEPYLAMFGQVPSDVDSTTYDFSHCTFSGNESKPLCVELDEHNLP
RFPEWITIPLVCIYMLSTNILLVNLLVAMFGYTVGIVQENNDQVWKFQRYFLVQEYCNRLNIPFPFVVFAYFY
MVVKKCFKCCCKEKNMESNACCFRNEDNETLAWEGVMKENYLVKINTKANDNSEEMRHRFRQLDSKLNDLKSL
LKEIANNIK*
>ENSMUSP00000131209_c.702:C>G_p.H235D ENSMUST00000171176.3|Trpm8-
204|ENSMUSG00000036251|+|chr1:g.88258421:C>G
MSFEGARLSMRSRRNGTMGSTRTLYSSVSRSTDVSYSDSDLVNFIQANFKKRECVFFTRDSKAMENICKCGYA
QSQHIEGTQINQNEKWNYKKHTKEFPTDAFGDIQFETLGKKGKYLRLSCDTDSETLYELLTQHWHLKTPNLVI
SVTGGAKNFALKPRMRKIFSRLIYIAQSKGAWILTGGTHYGLMKYIGEVVRDNTISRNSEENIVAIGIAAWGM
VSNRDTLIRSCDDEGDFSAQYIMDDFTRDPLYILDNNHTHLLLVDNGCHGHPTVEAKLRNQLEKYISERTSQD
SNYGGKIPIVCFAQGGGRETLKAINTSVKSKIPCVVVEGSGQIADVIASLVEVEDVLTSSMVKEKLVRFLPRT
VSRLPEEEIESWIKWLKEILESSHLLTVIKMEEAGDEIVSNAISYALYKAFSTNEQDKDNWNGQLKLLLEWNQ
LDLASDEIFTNDRRWESADLQEVMFTALIKDRPKFVRLFLENGLNLQKFLTNEVLTELFSTHFSTLVYRNLQI
AKNSYNDALLTFVWKLVANFRRSFWKEDRSSREDLDVELHDASLTTRHPLQALFIWAILQNKKELSKVIWEQT
KGCTLAALGASKLLKTLAKVKNDINAAGESEELANEYETRAVELFTECYSNDEDLAEQLLVYSCEAWGGSNCL
ELAVEATDQHFIAQPGVQNFLSKQWYGEISRDTKNWKIILCLFIIPLVGCGLVSFRKKPIDKHKKLLWYYVAF
FTSPFVVFSWNVVFYIAFLLLFAYVLLMDFHSVPHTPELILYALVFVLFCDEVRQWYMNGVNYFTDLWNVMDT

LGLFYFIAGIVFRLHSSNKSSLYSGRVIFCLDYIIFTLRLIHIFTVSRNLGPKIIMLQRMLIDVFFFLFLFAV
WMVAFGVARQGILRQNEQRWRWIFRSVIYEPYLAMFGQVPSDVDSTTYDFSHCTFSGNESKPLCVELDEHNLP
RFPEWITIPLVCIYMLSTNILLVNLLVAMFGYTVGIVQENNDQVWKFQRYFLVQEYCNRLNIPFPFVVFAYFY
MVVKKCFKCCCKEKNMESNACCFRNEDNETLAWEGVMKENYLVKINTKANDNSEEMRHRFRQLDSKLNDLKSL
LKEIANNIK*
>rs236575408_ENSMUSP00000108739_c.702:C>CA_p.H235ins-fs
ENSMUST00000113114.10|Trpm8-202|ENSMUSG00000036251|+|chr1:g.88258421:C>CA
MSFEGARLSMRSRRNGTMGSTRTLYSSVSRSTDVSYSDSDLVNFIQANFKKRECVFFTRDSKAMENICKCGYA
QSQHIEGTQINQNEKWNYKKHTKEFPTDAFGDIQFETLGKKGKYLRLSCDTDSETLYELLTQHWHLKTPNLVI
SVTGGAKNFALKPRMRKIFSRLIYIAQSKGAWILTGGTHYGLMKYIGEVVRDNTISRNSEENIVAIGIAAWGM
VSNRDTLIRSCDDEGQFFSSIHHG
>rs236575408_ENSMUSP00000036991_c.702:C>CA_p.H235ins-fs
ENSMUST00000040210.14|Trpm8-201|ENSMUSG00000036251|+|chr1:g.88258421:C>CA
MSFEGARLSMRSRRNGTMGSTRTLYSSVSRSTDVSYSDSDLVNFIQANFKKRECVFFTRDSKAMENICKCGYA
QSQHIEGTQINQNEKWNYKKHTKEFPTDAFGDIQFETLGKKGKYLRLSCDTDSETLYELLTQHWHLKTPNLVI
SVTGGAKNFALKPRMRKIFSRLIYIAQSKGAWILTGGTHYGLMKYIGEVVRDNTISRNSEENIVAIGIAAWGM
VSNRDTLIRSCDDEGQFFSSIHHG
>rs236575408_ENSMUSP00000131209_c.702:C>CA_p.H235ins-fs
ENSMUST00000171176.3|Trpm8-204|ENSMUSG00000036251|+|chr1:g.88258421:C>CA
MSFEGARLSMRSRRNGTMGSTRTLYSSVSRSTDVSYSDSDLVNFIQANFKKRECVFFTRDSKAMENICKCGYA
QSQHIEGTQINQNEKWNYKKHTKEFPTDAFGDIQFETLGKKGKYLRLSCDTDSETLYELLTQHWHLKTPNLVI
SVTGGAKNFALKPRMRKIFSRLIYIAQSKGAWILTGGTHYGLMKYIGEVVRDNTISRNSEENIVAIGIAAWGM
VSNRDTLIRSCDDEGQFFSSIHHG
>rs243361112_ENSMUSP00000108739_c.766:A>G_p.N256S
ENSMUST00000113114.10|Trpm8-202|ENSMUSG00000036251|+|chr1:g.88258485:A>G
MSFEGARLSMRSRRNGTMGSTRTLYSSVSRSTDVSYSDSDLVNFIQANFKKRECVFFTRDSKAMENICKCGYA
QSQHIEGTQINQNEKWNYKKHTKEFPTDAFGDIQFETLGKKGKYLRLSCDTDSETLYELLTQHWHLKTPNLVI
SVTGGAKNFALKPRMRKIFSRLIYIAQSKGAWILTGGTHYGLMKYIGEVVRDNTISRNSEENIVAIGIAAWGM
VSNRDTLIRSCDDEGHFSAQYIMDDFTRDPLYILDNSHTHLLLVDNGCHGHPTVEAKLRNQLEKYISERTSQD
SNYGGKIPIVCFAQGGGRETLKAINTSVKSKIPCVVVEGSGQIADVIASLVEVEDVLTSSMVKEKLVRFLPRT
VSRLPEEEIESWIKWLKEILESSHLLTVIKMEEAGDEIVSNAISYALYKAFSTNEQDKDNWNGQLKLLLEWNQ
LDLASDEIFTNDRRWESADLQEVMFTALIKDRPKFVRLFLENGLNLQKFLTNEVLTELFSTHFSTLVYRNLQI
AKNSYNDALLTFVWKLVANFRRSFWKEDRSSREDLDVELHDASLTTRHPLQALFIWAILQNKKELSKVIWEQT
KGCTLAALGASKLLKTLAKVKNDINAAGESEELANEYETRAVELFTECYSNDEDLAEQLLVYSCEAWGGSNCL
ELAVEATDQHFIAQPGVQNFLSKQWYGEISRDTKNWKIILCLFIIPLVGCGLVSFRKKPIDKHKKLLWYYVAF
FTSPFVVFSWNVVFYIAFLLLFAYVLLMDFHSVPHTPELILYALVFVLFCDEVRQWYMNGVNYFTDLWNVMDT
LGLFYFIAGIVFRLHSSNKSSLYSGRVIFCLDYIIFTLRLIHIFTVSRNLGPKIIMLQRMLIDVFFFLFLFAV
WMVAFGVARQGILRQNEQRWRWIFRSVIYEPYLAMFGQVPSDVDSTTYDFSHCTFSGNESKPLCVELDEHNLP
RFPEWITIPLVCIYMLSTNILLVNLLVAMFGYTVGIVQENNDQVWKFQRYFLVQEYCNRLNIPFPFVVFAYFY
MVVKKCFKCCCKEKNMESNACCFRNEDNETLAWEGVMKENYLVKINTKANDNSEEMRHRFRQLDSKLNDLKSL
LKEIANNIK*
>rs243361112_ENSMUSP00000036991_c.766:A>G_p.N256S
ENSMUST00000040210.14|Trpm8-201|ENSMUSG00000036251|+|chr1:g.88258485:A>G
MSFEGARLSMRSRRNGTMGSTRTLYSSVSRSTDVSYSDSDLVNFIQANFKKRECVFFTRDSKAMENICKCGYA
QSQHIEGTQINQNEKWNYKKHTKEFPTDAFGDIQFETLGKKGKYLRLSCDTDSETLYELLTQHWHLKTPNLVI
SVTGGAKNFALKPRMRKIFSRLIYIAQSKGAWILTGGTHYGLMKYIGEVVRDNTISRNSEENIVAIGIAAWGM
VSNRDTLIRSCDDEGHFSAQYIMDDFTRDPLYILDNSHTHLLLVDNGCHGHPTVEAKLRNQLEKYISERTSQD
SNYGGKIPIVCFAQGGGRETLKAINTSVKSKIPCVVVEGSGQIADVIASLVEVEDVLTSSMVKEKLVRFLPRT
VSRLPEEEIESWIKWLKEILESSHLLTVIKMEEAGDEIVSNAISYALYKAFSTNEQDKDNWNGQLKLLLEWNQ
LDLASDEIFTNDRRWESADLQEVMFTALIKDRPKFVRLFLENGLNLQKFLTNEVLTELFSTHFSTLVYRNLQI
AKNSYNDALLTFVWKLVANFRRSFWKEDRSSREDLDVELHDASLTTRHPLQALFIWAILQNKKELSKVIWEQT
KGCTLAALGASKLLKTLAKVKNDINAAGESEELANEYETRAVELFTECYSNDEDLAEQLLVYSCEAWGGSNCL
ELAVEATDQHFIAQPGVQNFLSKQWYGEISRDTKNWKIILCLFIIPLVGCGLVSFRKKPIDKHKKLLWYYVAF
FTSPFVVFSWNVVFYIAFLLLFAYVLLMDFHSVPHTPELILYALVFVLFCDEVRQWYMNGVNYFTDLWNVMDT
LGLFYFIAGIVFRLHSSNKSSLYSGRVIFCLDYIIFTLRLIHIFTVSRNLGPKIIMLQRMLIDVFFFLFLFAV
WMVAFGVARQGILRQNEQRWRWIFRSVIYEPYLAMFGQVPSDVDSTTYDFSHCTFSGNESKPLCVELDEHNLP
RFPEWITIPLVCIYMLSTNILLVNLLVAMFGYTVGIVQENNDQVWKFQRYFLVQEYCNRLNIPFPFVVFAYFY

MVVKKCFKCCCKEKNMESNACCFRNEDNETLAWEGVMKENYLVKINTKANDNSEEMRHRFRQLDSKLNDLKSL
LKEIANNIK*
>rs243361112_ENSMUSP00000131209_c.766:A>G_p.N256S
ENSMUST00000171176.3|Trpm8-204|ENSMUSG00000036251|+|chr1:g.88258485:A>G
MSFEGARLSMRSRRNGTMGSTRTLYSSVSRSTDVSYSDSDLVNFIQANFKKRECVFFTRDSKAMENICKCGYA
QSQHIEGTQINQNEKWNYKKHTKEFPTDAFGDIQFETLGKKGKYLRLSCDTDSETLYELLTQHWHLKTPNLVI
SVTGGAKNFALKPRMRKIFSRLIYIAQSKGAWILTGGTHYGLMKYIGEVVRDNTISRNSEENIVAIGIAAWGM
VSNRDTLIRSCDDEGHFSAQYIMDDFTRDPLYILDNSHTHLLLVDNGCHGHPTVEAKLRNQLEKYISERTSQD
SNYGGKIPIVCFAQGGGRETLKAINTSVKSKIPCVVVEGSGQIADVIASLVEVEDVLTSSMVKEKLVRFLPRT
VSRLPEEEIESWIKWLKEILESSHLLTVIKMEEAGDEIVSNAISYALYKAFSTNEQDKDNWNGQLKLLLEWNQ
LDLASDEIFTNDRRWESADLQEVMFTALIKDRPKFVRLFLENGLNLQKFLTNEVLTELFSTHFSTLVYRNLQI
AKNSYNDALLTFVWKLVANFRRSFWKEDRSSREDLDVELHDASLTTRHPLQALFIWAILQNKKELSKVIWEQT
KGCTLAALGASKLLKTLAKVKNDINAAGESEELANEYETRAVELFTECYSNDEDLAEQLLVYSCEAWGGSNCL
ELAVEATDQHFIAQPGVQNFLSKQWYGEISRDTKNWKIILCLFIIPLVGCGLVSFRKKPIDKHKKLLWYYVAF
FTSPFVVFSWNVVFYIAFLLLFAYVLLMDFHSVPHTPELILYALVFVLFCDEVRQWYMNGVNYFTDLWNVMDT
LGLFYFIAGIVFRLHSSNKSSLYSGRVIFCLDYIIFTLRLIHIFTVSRNLGPKIIMLQRMLIDVFFFLFLFAV
WMVAFGVARQGILRQNEQRWRWIFRSVIYEPYLAMFGQVPSDVDSTTYDFSHCTFSGNESKPLCVELDEHNLP
RFPEWITIPLVCIYMLSTNILLVNLLVAMFGYTVGIVQENNDQVWKFQRYFLVQEYCNRLNIPFPFVVFAYFY
MVVKKCFKCCCKEKNMESNACCFRNEDNETLAWEGVMKENYLVKINTKANDNSEEMRHRFRQLDSKLNDLKSL
LKEIANNIK*
>rs212640068_ENSMUSP00000108739_c.819:G>C_p.E274Q
ENSMUST00000113114.10|Trpm8-202|ENSMUSG00000036251|+|chr1:g.88258538:G>C
MSFEGARLSMRSRRNGTMGSTRTLYSSVSRSTDVSYSDSDLVNFIQANFKKRECVFFTRDSKAMENICKCGYA
QSQHIEGTQINQNEKWNYKKHTKEFPTDAFGDIQFETLGKKGKYLRLSCDTDSETLYELLTQHWHLKTPNLVI
SVTGGAKNFALKPRMRKIFSRLIYIAQSKGAWILTGGTHYGLMKYIGEVVRDNTISRNSEENIVAIGIAAWGM
VSNRDTLIRSCDDEGHFSAQYIMDDFTRDPLYILDNNHTHLLLVDNGCHGHPTVQAKLRNQLEKYISERTSQD
SNYGGKIPIVCFAQGGGRETLKAINTSVKSKIPCVVVEGSGQIADVIASLVEVEDVLTSSMVKEKLVRFLPRT
VSRLPEEEIESWIKWLKEILESSHLLTVIKMEEAGDEIVSNAISYALYKAFSTNEQDKDNWNGQLKLLLEWNQ
LDLASDEIFTNDRRWESADLQEVMFTALIKDRPKFVRLFLENGLNLQKFLTNEVLTELFSTHFSTLVYRNLQI
AKNSYNDALLTFVWKLVANFRRSFWKEDRSSREDLDVELHDASLTTRHPLQALFIWAILQNKKELSKVIWEQT
KGCTLAALGASKLLKTLAKVKNDINAAGESEELANEYETRAVELFTECYSNDEDLAEQLLVYSCEAWGGSNCL
ELAVEATDQHFIAQPGVQNFLSKQWYGEISRDTKNWKIILCLFIIPLVGCGLVSFRKKPIDKHKKLLWYYVAF
FTSPFVVFSWNVVFYIAFLLLFAYVLLMDFHSVPHTPELILYALVFVLFCDEVRQWYMNGVNYFTDLWNVMDT
LGLFYFIAGIVFRLHSSNKSSLYSGRVIFCLDYIIFTLRLIHIFTVSRNLGPKIIMLQRMLIDVFFFLFLFAV
WMVAFGVARQGILRQNEQRWRWIFRSVIYEPYLAMFGQVPSDVDSTTYDFSHCTFSGNESKPLCVELDEHNLP
RFPEWITIPLVCIYMLSTNILLVNLLVAMFGYTVGIVQENNDQVWKFQRYFLVQEYCNRLNIPFPFVVFAYFY
MVVKKCFKCCCKEKNMESNACCFRNEDNETLAWEGVMKENYLVKINTKANDNSEEMRHRFRQLDSKLNDLKSL
LKEIANNIK*
>rs212640068_ENSMUSP00000036991_c.819:G>C_p.E274Q
ENSMUST00000040210.14|Trpm8-201|ENSMUSG00000036251|+|chr1:g.88258538:G>C
MSFEGARLSMRSRRNGTMGSTRTLYSSVSRSTDVSYSDSDLVNFIQANFKKRECVFFTRDSKAMENICKCGYA
QSQHIEGTQINQNEKWNYKKHTKEFPTDAFGDIQFETLGKKGKYLRLSCDTDSETLYELLTQHWHLKTPNLVI
SVTGGAKNFALKPRMRKIFSRLIYIAQSKGAWILTGGTHYGLMKYIGEVVRDNTISRNSEENIVAIGIAAWGM
VSNRDTLIRSCDDEGHFSAQYIMDDFTRDPLYILDNNHTHLLLVDNGCHGHPTVQAKLRNQLEKYISERTSQD
SNYGGKIPIVCFAQGGGRETLKAINTSVKSKIPCVVVEGSGQIADVIASLVEVEDVLTSSMVKEKLVRFLPRT
VSRLPEEEIESWIKWLKEILESSHLLTVIKMEEAGDEIVSNAISYALYKAFSTNEQDKDNWNGQLKLLLEWNQ
LDLASDEIFTNDRRWESADLQEVMFTALIKDRPKFVRLFLENGLNLQKFLTNEVLTELFSTHFSTLVYRNLQI
AKNSYNDALLTFVWKLVANFRRSFWKEDRSSREDLDVELHDASLTTRHPLQALFIWAILQNKKELSKVIWEQT
KGCTLAALGASKLLKTLAKVKNDINAAGESEELANEYETRAVELFTECYSNDEDLAEQLLVYSCEAWGGSNCL
ELAVEATDQHFIAQPGVQNFLSKQWYGEISRDTKNWKIILCLFIIPLVGCGLVSFRKKPIDKHKKLLWYYVAF
FTSPFVVFSWNVVFYIAFLLLFAYVLLMDFHSVPHTPELILYALVFVLFCDEVRQWYMNGVNYFTDLWNVMDT
LGLFYFIAGIVFRLHSSNKSSLYSGRVIFCLDYIIFTLRLIHIFTVSRNLGPKIIMLQRMLIDVFFFLFLFAV
WMVAFGVARQGILRQNEQRWRWIFRSVIYEPYLAMFGQVPSDVDSTTYDFSHCTFSGNESKPLCVELDEHNLP
RFPEWITIPLVCIYMLSTNILLVNLLVAMFGYTVGIVQENNDQVWKFQRYFLVQEYCNRLNIPFPFVVFAYFY
MVVKKCFKCCCKEKNMESNACCFRNEDNETLAWEGVMKENYLVKINTKANDNSEEMRHRFRQLDSKLNDLKSL
LKEIANNIK*
>rs212640068_ENSMUSP00000131209_c.819:G>C_p.E274Q
ENSMUST00000171176.3|Trpm8-204|ENSMUSG00000036251|+|chr1:g.88258538:G>C

MSFEGARLSMRSRRNGTMGSTRTLYSSVSRSTDVSYSDSDLVNFIQANFKKRECVFFTRDSKAMENICKCGYA
QSQHIEGTQINQNEKWNYKKHTKEFPTDAFGDIQFETLGKKGKYLRLSCDTDSETLYELLTQHWHLKTPNLVI
SVTGGAKNFALKPRMRKIFSRLIYIAQSKGAWILTGGTHYGLMKYIGEVVRDNTISRNSEENIVAIGIAAWGM
VSNRDTLIRSCDDEGHFSAQYIMDDFTRDPLYILDNNHTHLLLVDNGCHGHPTVQAKLRNQLEKYISERTSQD
SNYGGKIPIVCFAQGGGRETLKAINTSVKSKIPCVVVEGSGQIADVIASLVEVEDVLTSSMVKEKLVRFLPRT
VSRLPEEEIESWIKWLKEILESSHLLTVIKMEEAGDEIVSNAISYALYKAFSTNEQDKDNWNGQLKLLLEWNQ
LDLASDEIFTNDRRWESADLQEVMFTALIKDRPKFVRLFLENGLNLQKFLTNEVLTELFSTHFSTLVYRNLQI
AKNSYNDALLTFVWKLVANFRRSFWKEDRSSREDLDVELHDASLTTRHPLQALFIWAILQNKKELSKVIWEQT
KGCTLAALGASKLLKTLAKVKNDINAAGESEELANEYETRAVELFTECYSNDEDLAEQLLVYSCEAWGGSNCL
ELAVEATDQHFIAQPGVQNFLSKQWYGEISRDTKNWKIILCLFIIPLVGCGLVSFRKKPIDKHKKLLWYYVAF
FTSPFVVFSWNVVFYIAFLLLFAYVLLMDFHSVPHTPELILYALVFVLFCDEVRQWYMNGVNYFTDLWNVMDT
LGLFYFIAGIVFRLHSSNKSSLYSGRVIFCLDYIIFTLRLIHIFTVSRNLGPKIIMLQRMLIDVFFFLFLFAV
WMVAFGVARQGILRQNEQRWRWIFRSVIYEPYLAMFGQVPSDVDSTTYDFSHCTFSGNESKPLCVELDEHNLP
RFPEWITIPLVCIYMLSTNILLVNLLVAMFGYTVGIVQENNDQVWKFQRYFLVQEYCNRLNIPFPFVVFAYFY
MVVKKCFKCCCKEKNMESNACCFRNEDNETLAWEGVMKENYLVKINTKANDNSEEMRHRFRQLDSKLNDLKSL
LKEIANNIK*
>rs49882804_ENSMUSP00000108739_c.1126:G>A_p.S376N
ENSMUST00000113114.10|Trpm8-202|ENSMUSG00000036251|+|chr1:g.88265019:G>A
MSFEGARLSMRSRRNGTMGSTRTLYSSVSRSTDVSYSDSDLVNFIQANFKKRECVFFTRDSKAMENICKCGYA
QSQHIEGTQINQNEKWNYKKHTKEFPTDAFGDIQFETLGKKGKYLRLSCDTDSETLYELLTQHWHLKTPNLVI
SVTGGAKNFALKPRMRKIFSRLIYIAQSKGAWILTGGTHYGLMKYIGEVVRDNTISRNSEENIVAIGIAAWGM
VSNRDTLIRSCDDEGHFSAQYIMDDFTRDPLYILDNNHTHLLLVDNGCHGHPTVEAKLRNQLEKYISERTSQD
SNYGGKIPIVCFAQGGGRETLKAINTSVKSKIPCVVVEGSGQIADVIASLVEVEDVLTSSMVKEKLVRFLPRT
VSRLPEEEIENWIKWLKEILESSHLLTVIKMEEAGDEIVSNAISYALYKAFSTNEQDKDNWNGQLKLLLEWNQ
LDLASDEIFTNDRRWESADLQEVMFTALIKDRPKFVRLFLENGLNLQKFLTNEVLTELFSTHFSTLVYRNLQI
AKNSYNDALLTFVWKLVANFRRSFWKEDRSSREDLDVELHDASLTTRHPLQALFIWAILQNKKELSKVIWEQT
KGCTLAALGASKLLKTLAKVKNDINAAGESEELANEYETRAVELFTECYSNDEDLAEQLLVYSCEAWGGSNCL
ELAVEATDQHFIAQPGVQNFLSKQWYGEISRDTKNWKIILCLFIIPLVGCGLVSFRKKPIDKHKKLLWYYVAF
FTSPFVVFSWNVVFYIAFLLLFAYVLLMDFHSVPHTPELILYALVFVLFCDEVRQWYMNGVNYFTDLWNVMDT
LGLFYFIAGIVFRLHSSNKSSLYSGRVIFCLDYIIFTLRLIHIFTVSRNLGPKIIMLQRMLIDVFFFLFLFAV
WMVAFGVARQGILRQNEQRWRWIFRSVIYEPYLAMFGQVPSDVDSTTYDFSHCTFSGNESKPLCVELDEHNLP
RFPEWITIPLVCIYMLSTNILLVNLLVAMFGYTVGIVQENNDQVWKFQRYFLVQEYCNRLNIPFPFVVFAYFY
MVVKKCFKCCCKEKNMESNACCFRNEDNETLAWEGVMKENYLVKINTKANDNSEEMRHRFRQLDSKLNDLKSL
LKEIANNIK*
>rs49882804_ENSMUSP00000036991_c.1126:G>A_p.S376N
ENSMUST00000040210.14|Trpm8-201|ENSMUSG00000036251|+|chr1:g.88265019:G>A
MSFEGARLSMRSRRNGTMGSTRTLYSSVSRSTDVSYSDSDLVNFIQANFKKRECVFFTRDSKAMENICKCGYA
QSQHIEGTQINQNEKWNYKKHTKEFPTDAFGDIQFETLGKKGKYLRLSCDTDSETLYELLTQHWHLKTPNLVI
SVTGGAKNFALKPRMRKIFSRLIYIAQSKGAWILTGGTHYGLMKYIGEVVRDNTISRNSEENIVAIGIAAWGM
VSNRDTLIRSCDDEGHFSAQYIMDDFTRDPLYILDNNHTHLLLVDNGCHGHPTVEAKLRNQLEKYISERTSQD
SNYGGKIPIVCFAQGGGRETLKAINTSVKSKIPCVVVEGSGQIADVIASLVEVEDVLTSSMVKEKLVRFLPRT
VSRLPEEEIENWIKWLKEILESSHLLTVIKMEEAGDEIVSNAISYALYKAFSTNEQDKDNWNGQLKLLLEWNQ
LDLASDEIFTNDRRWESADLQEVMFTALIKDRPKFVRLFLENGLNLQKFLTNEVLTELFSTHFSTLVYRNLQI
AKNSYNDALLTFVWKLVANFRRSFWKEDRSSREDLDVELHDASLTTRHPLQALFIWAILQNKKELSKVIWEQT
KGCTLAALGASKLLKTLAKVKNDINAAGESEELANEYETRAVELFTECYSNDEDLAEQLLVYSCEAWGGSNCL
ELAVEATDQHFIAQPGVQNFLSKQWYGEISRDTKNWKIILCLFIIPLVGCGLVSFRKKPIDKHKKLLWYYVAF
FTSPFVVFSWNVVFYIAFLLLFAYVLLMDFHSVPHTPELILYALVFVLFCDEVRQWYMNGVNYFTDLWNVMDT
LGLFYFIAGIVFRLHSSNKSSLYSGRVIFCLDYIIFTLRLIHIFTVSRNLGPKIIMLQRMLIDVFFFLFLFAV
WMVAFGVARQGILRQNEQRWRWIFRSVIYEPYLAMFGQVPSDVDSTTYDFSHCTFSGNESKPLCVELDEHNLP
RFPEWITIPLVCIYMLSTNILLVNLLVAMFGYTVGIVQENNDQVWKFQRYFLVQEYCNRLNIPFPFVVFAYFY
MVVKKCFKCCCKEKNMESNACCFRNEDNETLAWEGVMKENYLVKINTKANDNSEEMRHRFRQLDSKLNDLKSL
LKEIANNIK*
>rs49882804_ENSMUSP00000131209_c.1126:G>A_p.S376N
ENSMUST00000171176.3|Trpm8-204|ENSMUSG00000036251|+|chr1:g.88265019:G>A
MSFEGARLSMRSRRNGTMGSTRTLYSSVSRSTDVSYSDSDLVNFIQANFKKRECVFFTRDSKAMENICKCGYA
QSQHIEGTQINQNEKWNYKKHTKEFPTDAFGDIQFETLGKKGKYLRLSCDTDSETLYELLTQHWHLKTPNLVI
SVTGGAKNFALKPRMRKIFSRLIYIAQSKGAWILTGGTHYGLMKYIGEVVRDNTISRNSEENIVAIGIAAWGM
VSNRDTLIRSCDDEGHFSAQYIMDDFTRDPLYILDNNHTHLLLVDNGCHGHPTVEAKLRNQLEKYISERTSQD

SNYGGKIPIVCFAQGGGRETLKAINTSVKSKIPCVVVEGSGQIADVIASLVEVEDVLTSSMVKEKLVRFLPRT
VSRLPEEEIENWIKWLKEILESSHLLTVIKMEEAGDEIVSNAISYALYKAFSTNEQDKDNWNGQLKLLLEWNQ
LDLASDEIFTNDRRWESADLQEVMFTALIKDRPKFVRLFLENGLNLQKFLTNEVLTELFSTHFSTLVYRNLQI
AKNSYNDALLTFVWKLVANFRRSFWKEDRSSREDLDVELHDASLTTRHPLQALFIWAILQNKKELSKVIWEQT
KGCTLAALGASKLLKTLAKVKNDINAAGESEELANEYETRAVELFTECYSNDEDLAEQLLVYSCEAWGGSNCL
ELAVEATDQHFIAQPGVQNFLSKQWYGEISRDTKNWKIILCLFIIPLVGCGLVSFRKKPIDKHKKLLWYYVAF
FTSPFVVFSWNVVFYIAFLLLFAYVLLMDFHSVPHTPELILYALVFVLFCDEVRQWYMNGVNYFTDLWNVMDT
LGLFYFIAGIVFRLHSSNKSSLYSGRVIFCLDYIIFTLRLIHIFTVSRNLGPKIIMLQRMLIDVFFFLFLFAV
WMVAFGVARQGILRQNEQRWRWIFRSVIYEPYLAMFGQVPSDVDSTTYDFSHCTFSGNESKPLCVELDEHNLP
RFPEWITIPLVCIYMLSTNILLVNLLVAMFGYTVGIVQENNDQVWKFQRYFLVQEYCNRLNIPFPFVVFAYFY
MVVKKCFKCCCKEKNMESNACCFRNEDNETLAWEGVMKENYLVKINTKANDNSEEMRHRFRQLDSKLNDLKSL
LKEIANNIK*
>rs49585932_ENSMUSP00000108739_c.1135:A>G_p.K379R
ENSMUST00000113114.10|Trpm8-202|ENSMUSG00000036251|+|chr1:g.88265028:A>G
MSFEGARLSMRSRRNGTMGSTRTLYSSVSRSTDVSYSDSDLVNFIQANFKKRECVFFTRDSKAMENICKCGYA
QSQHIEGTQINQNEKWNYKKHTKEFPTDAFGDIQFETLGKKGKYLRLSCDTDSETLYELLTQHWHLKTPNLVI
SVTGGAKNFALKPRMRKIFSRLIYIAQSKGAWILTGGTHYGLMKYIGEVVRDNTISRNSEENIVAIGIAAWGM
VSNRDTLIRSCDDEGHFSAQYIMDDFTRDPLYILDNNHTHLLLVDNGCHGHPTVEAKLRNQLEKYISERTSQD
SNYGGKIPIVCFAQGGGRETLKAINTSVKSKIPCVVVEGSGQIADVIASLVEVEDVLTSSMVKEKLVRFLPRT
VSRLPEEEIESWIRWLKEILESSHLLTVIKMEEAGDEIVSNAISYALYKAFSTNEQDKDNWNGQLKLLLEWNQ
LDLASDEIFTNDRRWESADLQEVMFTALIKDRPKFVRLFLENGLNLQKFLTNEVLTELFSTHFSTLVYRNLQI
AKNSYNDALLTFVWKLVANFRRSFWKEDRSSREDLDVELHDASLTTRHPLQALFIWAILQNKKELSKVIWEQT
KGCTLAALGASKLLKTLAKVKNDINAAGESEELANEYETRAVELFTECYSNDEDLAEQLLVYSCEAWGGSNCL
ELAVEATDQHFIAQPGVQNFLSKQWYGEISRDTKNWKIILCLFIIPLVGCGLVSFRKKPIDKHKKLLWYYVAF
FTSPFVVFSWNVVFYIAFLLLFAYVLLMDFHSVPHTPELILYALVFVLFCDEVRQWYMNGVNYFTDLWNVMDT
LGLFYFIAGIVFRLHSSNKSSLYSGRVIFCLDYIIFTLRLIHIFTVSRNLGPKIIMLQRMLIDVFFFLFLFAV
WMVAFGVARQGILRQNEQRWRWIFRSVIYEPYLAMFGQVPSDVDSTTYDFSHCTFSGNESKPLCVELDEHNLP
RFPEWITIPLVCIYMLSTNILLVNLLVAMFGYTVGIVQENNDQVWKFQRYFLVQEYCNRLNIPFPFVVFAYFY
MVVKKCFKCCCKEKNMESNACCFRNEDNETLAWEGVMKENYLVKINTKANDNSEEMRHRFRQLDSKLNDLKSL
LKEIANNIK*
>rs49585932_ENSMUSP00000036991_c.1135:A>G_p.K379R
ENSMUST00000040210.14|Trpm8-201|ENSMUSG00000036251|+|chr1:g.88265028:A>G
MSFEGARLSMRSRRNGTMGSTRTLYSSVSRSTDVSYSDSDLVNFIQANFKKRECVFFTRDSKAMENICKCGYA
QSQHIEGTQINQNEKWNYKKHTKEFPTDAFGDIQFETLGKKGKYLRLSCDTDSETLYELLTQHWHLKTPNLVI
SVTGGAKNFALKPRMRKIFSRLIYIAQSKGAWILTGGTHYGLMKYIGEVVRDNTISRNSEENIVAIGIAAWGM
VSNRDTLIRSCDDEGHFSAQYIMDDFTRDPLYILDNNHTHLLLVDNGCHGHPTVEAKLRNQLEKYISERTSQD
SNYGGKIPIVCFAQGGGRETLKAINTSVKSKIPCVVVEGSGQIADVIASLVEVEDVLTSSMVKEKLVRFLPRT
VSRLPEEEIESWIRWLKEILESSHLLTVIKMEEAGDEIVSNAISYALYKAFSTNEQDKDNWNGQLKLLLEWNQ
LDLASDEIFTNDRRWESADLQEVMFTALIKDRPKFVRLFLENGLNLQKFLTNEVLTELFSTHFSTLVYRNLQI
AKNSYNDALLTFVWKLVANFRRSFWKEDRSSREDLDVELHDASLTTRHPLQALFIWAILQNKKELSKVIWEQT
KGCTLAALGASKLLKTLAKVKNDINAAGESEELANEYETRAVELFTECYSNDEDLAEQLLVYSCEAWGGSNCL
ELAVEATDQHFIAQPGVQNFLSKQWYGEISRDTKNWKIILCLFIIPLVGCGLVSFRKKPIDKHKKLLWYYVAF
FTSPFVVFSWNVVFYIAFLLLFAYVLLMDFHSVPHTPELILYALVFVLFCDEVRQWYMNGVNYFTDLWNVMDT
LGLFYFIAGIVFRLHSSNKSSLYSGRVIFCLDYIIFTLRLIHIFTVSRNLGPKIIMLQRMLIDVFFFLFLFAV
WMVAFGVARQGILRQNEQRWRWIFRSVIYEPYLAMFGQVPSDVDSTTYDFSHCTFSGNESKPLCVELDEHNLP
RFPEWITIPLVCIYMLSTNILLVNLLVAMFGYTVGIVQENNDQVWKFQRYFLVQEYCNRLNIPFPFVVFAYFY
MVVKKCFKCCCKEKNMESNACCFRNEDNETLAWEGVMKENYLVKINTKANDNSEEMRHRFRQLDSKLNDLKSL
LKEIANNIK*
>rs49585932_ENSMUSP00000131209_c.1135:A>G_p.K379R
ENSMUST00000171176.3|Trpm8-204|ENSMUSG00000036251|+|chr1:g.88265028:A>G
MSFEGARLSMRSRRNGTMGSTRTLYSSVSRSTDVSYSDSDLVNFIQANFKKRECVFFTRDSKAMENICKCGYA
QSQHIEGTQINQNEKWNYKKHTKEFPTDAFGDIQFETLGKKGKYLRLSCDTDSETLYELLTQHWHLKTPNLVI
SVTGGAKNFALKPRMRKIFSRLIYIAQSKGAWILTGGTHYGLMKYIGEVVRDNTISRNSEENIVAIGIAAWGM
VSNRDTLIRSCDDEGHFSAQYIMDDFTRDPLYILDNNHTHLLLVDNGCHGHPTVEAKLRNQLEKYISERTSQD
SNYGGKIPIVCFAQGGGRETLKAINTSVKSKIPCVVVEGSGQIADVIASLVEVEDVLTSSMVKEKLVRFLPRT
VSRLPEEEIESWIRWLKEILESSHLLTVIKMEEAGDEIVSNAISYALYKAFSTNEQDKDNWNGQLKLLLEWNQ
LDLASDEIFTNDRRWESADLQEVMFTALIKDRPKFVRLFLENGLNLQKFLTNEVLTELFSTHFSTLVYRNLQI
AKNSYNDALLTFVWKLVANFRRSFWKEDRSSREDLDVELHDASLTTRHPLQALFIWAILQNKKELSKVIWEQT

KGCTLAALGASKLLKTLAKVKNDINAAGESEELANEYETRAVELFTECYSNDEDLAEQLLVYSCEAWGGSNCL
ELAVEATDQHFIAQPGVQNFLSKQWYGEISRDTKNWKIILCLFIIPLVGCGLVSFRKKPIDKHKKLLWYYVAF
FTSPFVVFSWNVVFYIAFLLLFAYVLLMDFHSVPHTPELILYALVFVLFCDEVRQWYMNGVNYFTDLWNVMDT
LGLFYFIAGIVFRLHSSNKSSLYSGRVIFCLDYIIFTLRLIHIFTVSRNLGPKIIMLQRMLIDVFFFLFLFAV
WMVAFGVARQGILRQNEQRWRWIFRSVIYEPYLAMFGQVPSDVDSTTYDFSHCTFSGNESKPLCVELDEHNLP
RFPEWITIPLVCIYMLSTNILLVNLLVAMFGYTVGIVQENNDQVWKFQRYFLVQEYCNRLNIPFPFVVFAYFY
MVVKKCFKCCCKEKNMESNACCFRNEDNETLAWEGVMKENYLVKINTKANDNSEEMRHRFRQLDSKLNDLKSL
LKEIANNIK*
>ENSMUSP00000108739_c.1702:C>G_p.A568G ENSMUST00000113114.10|Trpm8-202|ENSMUSG00000036251|+|chr1:g.88277618:C>G
MSFEGARLSMRSRRNGTMGSTRTLYSSVSRSTDVSYSDSDLVNFIQANFKKRECVFFTRDSKAMENICKCGYA
QSQHIEGTQINQNEKWNYKKHTKEFPTDAFGDIQFETLGKKGKYLRLSCDTDSETLYELLTQHWHLKTPNLVI
SVTGGAKNFALKPRMRKIFSRLIYIAQSKGAWILTGGTHYGLMKYIGEVVRDNTISRNSEENIVAIGIAAWGM
VSNRDTLIRSCDDEGHFSAQYIMDDFTRDPLYILDNNHTHLLLVDNGCHGHPTVEAKLRNQLEKYISERTSQD
SNYGGKIPIVCFAQGGGRETLKAINTSVKSKIPCVVVEGSGQIADVIASLVEVEDVLTSSMVKEKLVRFLPRT
VSRLPEEEIESWIKWLKEILESSHLLTVIKMEEAGDEIVSNAISYALYKAFSTNEQDKDNWNGQLKLLLEWNQ
LDLASDEIFTNDRRWESADLQEVMFTALIKDRPKFVRLFLENGLNLQKFLTNEVLTELFSTHFSTLVYRNLQI
AKNSYNDALLTFVWKLVANFRRSFWKEDRSSREDLDVELHDASLTTRHPLQALFIWGILQNKKELSKVIWEQT
KGCTLAALGASKLLKTLAKVKNDINAAGESEELANEYETRAVELFTECYSNDEDLAEQLLVYSCEAWGGSNCL
ELAVEATDQHFIAQPGVQNFLSKQWYGEISRDTKNWKIILCLFIIPLVGCGLVSFRKKPIDKHKKLLWYYVAF
FTSPFVVFSWNVVFYIAFLLLFAYVLLMDFHSVPHTPELILYALVFVLFCDEVRQWYMNGVNYFTDLWNVMDT
LGLFYFIAGIVFRLHSSNKSSLYSGRVIFCLDYIIFTLRLIHIFTVSRNLGPKIIMLQRMLIDVFFFLFLFAV
WMVAFGVARQGILRQNEQRWRWIFRSVIYEPYLAMFGQVPSDVDSTTYDFSHCTFSGNESKPLCVELDEHNLP
RFPEWITIPLVCIYMLSTNILLVNLLVAMFGYTVGIVQENNDQVWKFQRYFLVQEYCNRLNIPFPFVVFAYFY
MVVKKCFKCCCKEKNMESNACCFRNEDNETLAWEGVMKENYLVKINTKANDNSEEMRHRFRQLDSKLNDLKSL
LKEIANNIK*
>ENSMUSP00000036991_c.1702:C>G_p.A568G ENSMUST00000040210.14|Trpm8-201|ENSMUSG00000036251|+|chr1:g.88277618:C>G
MSFEGARLSMRSRRNGTMGSTRTLYSSVSRSTDVSYSDSDLVNFIQANFKKRECVFFTRDSKAMENICKCGYA
QSQHIEGTQINQNEKWNYKKHTKEFPTDAFGDIQFETLGKKGKYLRLSCDTDSETLYELLTQHWHLKTPNLVI
SVTGGAKNFALKPRMRKIFSRLIYIAQSKGAWILTGGTHYGLMKYIGEVVRDNTISRNSEENIVAIGIAAWGM
VSNRDTLIRSCDDEGHFSAQYIMDDFTRDPLYILDNNHTHLLLVDNGCHGHPTVEAKLRNQLEKYISERTSQD
SNYGGKIPIVCFAQGGGRETLKAINTSVKSKIPCVVVEGSGQIADVIASLVEVEDVLTSSMVKEKLVRFLPRT
VSRLPEEEIESWIKWLKEILESSHLLTVIKMEEAGDEIVSNAISYALYKAFSTNEQDKDNWNGQLKLLLEWNQ
LDLASDEIFTNDRRWESADLQEVMFTALIKDRPKFVRLFLENGLNLQKFLTNEVLTELFSTHFSTLVYRNLQI
AKNSYNDALLTFVWKLVANFRRSFWKEDRSSREDLDVELHDASLTTRHPLQALFIWGILQNKKELSKVIWEQT
KGCTLAALGASKLLKTLAKVKNDINAAGESEELANEYETRAVELFTECYSNDEDLAEQLLVYSCEAWGGSNCL
ELAVEATDQHFIAQPGVQNFLSKQWYGEISRDTKNWKIILCLFIIPLVGCGLVSFRKKPIDKHKKLLWYYVAF
FTSPFVVFSWNVVFYIAFLLLFAYVLLMDFHSVPHTPELILYALVFVLFCDEVRQWYMNGVNYFTDLWNVMDT
LGLFYFIAGIVFRLHSSNKSSLYSGRVIFCLDYIIFTLRLIHIFTVSRNLGPKIIMLQRMLIDVFFFLFLFAV
WMVAFGVARQGILRQNEQRWRWIFRSVIYEPYLAMFGQVPSDVDSTTYDFSHCTFSGNESKPLCVELDEHNLP
RFPEWITIPLVCIYMLSTNILLVNLLVAMFGYTVGIVQENNDQVWKFQRYFLVQEYCNRLNIPFPFVVFAYFY
MVVKKCFKCCCKEKNMESNACCFRNEDNETLAWEGVMKENYLVKINTKANDNSEEMRHRFRQLDSKLNDLKSL
LKEIANNIK*
>ENSMUSP00000131209_c.1702:C>G_p.A568G ENSMUST00000171176.3|Trpm8-204|ENSMUSG00000036251|+|chr1:g.88277618:C>G
MSFEGARLSMRSRRNGTMGSTRTLYSSVSRSTDVSYSDSDLVNFIQANFKKRECVFFTRDSKAMENICKCGYA
QSQHIEGTQINQNEKWNYKKHTKEFPTDAFGDIQFETLGKKGKYLRLSCDTDSETLYELLTQHWHLKTPNLVI
SVTGGAKNFALKPRMRKIFSRLIYIAQSKGAWILTGGTHYGLMKYIGEVVRDNTISRNSEENIVAIGIAAWGM
VSNRDTLIRSCDDEGHFSAQYIMDDFTRDPLYILDNNHTHLLLVDNGCHGHPTVEAKLRNQLEKYISERTSQD
SNYGGKIPIVCFAQGGGRETLKAINTSVKSKIPCVVVEGSGQIADVIASLVEVEDVLTSSMVKEKLVRFLPRT
VSRLPEEEIESWIKWLKEILESSHLLTVIKMEEAGDEIVSNAISYALYKAFSTNEQDKDNWNGQLKLLLEWNQ
LDLASDEIFTNDRRWESADLQEVMFTALIKDRPKFVRLFLENGLNLQKFLTNEVLTELFSTHFSTLVYRNLQI
AKNSYNDALLTFVWKLVANFRRSFWKEDRSSREDLDVELHDASLTTRHPLQALFIWGILQNKKELSKVIWEQT
KGCTLAALGASKLLKTLAKVKNDINAAGESEELANEYETRAVELFTECYSNDEDLAEQLLVYSCEAWGGSNCL
ELAVEATDQHFIAQPGVQNFLSKQWYGEISRDTKNWKIILCLFIIPLVGCGLVSFRKKPIDKHKKLLWYYVAF
FTSPFVVFSWNVVFYIAFLLLFAYVLLMDFHSVPHTPELILYALVFVLFCDEVRQWYMNGVNYFTDLWNVMDT
LGLFYFIAGIVFRLHSSNKSSLYSGRVIFCLDYIIFTLRLIHIFTVSRNLGPKIIMLQRMLIDVFFFLFLFAV

WMVAFGVARQGILRQNEQRWRWIFRSVIYEPYLAMFGQVPSDVDSTTYDFSHCTFSGNESKPLCVELDEHNLP
RFPEWITIPLVCIYMLSTNILLVNLLVAMFGYTVGIVQENNDQVWKFQRYFLVQEYCNRLNIPFPFVVFAYFY
MVVKKCFKCCCKEKNMESNACCFRNEDNETLAWEGVMKENYLVKINTKANDNSEEMRHRFRQLDSKLNDLKSL
LKEIANNIK*
>ENSMUSP00000108739_c.1745:G>T_p.E582D ENSMUST00000113114.10|Trpm8-202|ENSMUSG00000036251|+|chr1:g.88277661:G>T
MSFEGARLSMRSRRNGTMGSTRTLYSSVSRSTDVSYSDSDLVNFIQANFKKRECVFFTRDSKAMENICKCGYA
QSQHIEGTQINQNEKWNYKKHTKEFPTDAFGDIQFETLGKKGKYLRLSCDTDSETLYELLTQHWHLKTPNLVI
SVTGGAKNFALKPRMRKIFSRLIYIAQSKGAWILTGGTHYGLMKYIGEVVRDNTISRNSEENIVAIGIAAWGM
VSNRDTLIRSCDDEGHFSAQYIMDDFTRDPLYILDNNHTHLLLVDNGCHGHPTVEAKLRNQLEKYISERTSQD
SNYGGKIPIVCFAQGGGRETLKAINTSVKSKIPCVVVEGSGQIADVIASLVEVEDVLTSSMVKEKLVRFLPRT
VSRLPEEEIESWIKWLKEILESSHLLTVIKMEEAGDEIVSNAISYALYKAFSTNEQDKDNWNGQLKLLLEWNQ
LDLASDEIFTNDRRWESADLQEVMFTALIKDRPKFVRLFLENGLNLQKFLTNEVLTELFSTHFSTLVYRNLQI
AKNSYNDALLTFVWKLVANFRRSFWKEDRSSREDLDVELHDASLTTRHPLQALFIWAILQNKKELSKVIWDQT
KGCTLAALGASKLLKTLAKVKNDINAAGESEELANEYETRAVELFTECYSNDEDLAEQLLVYSCEAWGGSNCL
ELAVEATDQHFIAQPGVQNFLSKQWYGEISRDTKNWKIILCLFIIPLVGCGLVSFRKKPIDKHKKLLWYYVAF
FTSPFVVFSWNVVFYIAFLLLFAYVLLMDFHSVPHTPELILYALVFVLFCDEVRQWYMNGVNYFTDLWNVMDT
LGLFYFIAGIVFRLHSSNKSSLYSGRVIFCLDYIIFTLRLIHIFTVSRNLGPKIIMLQRMLIDVFFFLFLFAV
WMVAFGVARQGILRQNEQRWRWIFRSVIYEPYLAMFGQVPSDVDSTTYDFSHCTFSGNESKPLCVELDEHNLP
RFPEWITIPLVCIYMLSTNILLVNLLVAMFGYTVGIVQENNDQVWKFQRYFLVQEYCNRLNIPFPFVVFAYFY
MVVKKCFKCCCKEKNMESNACCFRNEDNETLAWEGVMKENYLVKINTKANDNSEEMRHRFRQLDSKLNDLKSL
LKEIANNIK*
>ENSMUSP00000036991_c.1745:G>T_p.E582D ENSMUST00000040210.14|Trpm8-201|ENSMUSG00000036251|+|chr1:g.88277661:G>T
MSFEGARLSMRSRRNGTMGSTRTLYSSVSRSTDVSYSDSDLVNFIQANFKKRECVFFTRDSKAMENICKCGYA
QSQHIEGTQINQNEKWNYKKHTKEFPTDAFGDIQFETLGKKGKYLRLSCDTDSETLYELLTQHWHLKTPNLVI
SVTGGAKNFALKPRMRKIFSRLIYIAQSKGAWILTGGTHYGLMKYIGEVVRDNTISRNSEENIVAIGIAAWGM
VSNRDTLIRSCDDEGHFSAQYIMDDFTRDPLYILDNNHTHLLLVDNGCHGHPTVEAKLRNQLEKYISERTSQD
SNYGGKIPIVCFAQGGGRETLKAINTSVKSKIPCVVVEGSGQIADVIASLVEVEDVLTSSMVKEKLVRFLPRT
VSRLPEEEIESWIKWLKEILESSHLLTVIKMEEAGDEIVSNAISYALYKAFSTNEQDKDNWNGQLKLLLEWNQ
LDLASDEIFTNDRRWESADLQEVMFTALIKDRPKFVRLFLENGLNLQKFLTNEVLTELFSTHFSTLVYRNLQI
AKNSYNDALLTFVWKLVANFRRSFWKEDRSSREDLDVELHDASLTTRHPLQALFIWAILQNKKELSKVIWDQT
KGCTLAALGASKLLKTLAKVKNDINAAGESEELANEYETRAVELFTECYSNDEDLAEQLLVYSCEAWGGSNCL
ELAVEATDQHFIAQPGVQNFLSKQWYGEISRDTKNWKIILCLFIIPLVGCGLVSFRKKPIDKHKKLLWYYVAF
FTSPFVVFSWNVVFYIAFLLLFAYVLLMDFHSVPHTPELILYALVFVLFCDEVRQWYMNGVNYFTDLWNVMDT
LGLFYFIAGIVFRLHSSNKSSLYSGRVIFCLDYIIFTLRLIHIFTVSRNLGPKIIMLQRMLIDVFFFLFLFAV
WMVAFGVARQGILRQNEQRWRWIFRSVIYEPYLAMFGQVPSDVDSTTYDFSHCTFSGNESKPLCVELDEHNLP
RFPEWITIPLVCIYMLSTNILLVNLLVAMFGYTVGIVQENNDQVWKFQRYFLVQEYCNRLNIPFPFVVFAYFY
MVVKKCFKCCCKEKNMESNACCFRNEDNETLAWEGVMKENYLVKINTKANDNSEEMRHRFRQLDSKLNDLKSL
LKEIANNIK*
>ENSMUSP00000131209_c.1745:G>T_p.E582D ENSMUST00000171176.3|Trpm8-204|ENSMUSG00000036251|+|chr1:g.88277661:G>T
MSFEGARLSMRSRRNGTMGSTRTLYSSVSRSTDVSYSDSDLVNFIQANFKKRECVFFTRDSKAMENICKCGYA
QSQHIEGTQINQNEKWNYKKHTKEFPTDAFGDIQFETLGKKGKYLRLSCDTDSETLYELLTQHWHLKTPNLVI
SVTGGAKNFALKPRMRKIFSRLIYIAQSKGAWILTGGTHYGLMKYIGEVVRDNTISRNSEENIVAIGIAAWGM
VSNRDTLIRSCDDEGHFSAQYIMDDFTRDPLYILDNNHTHLLLVDNGCHGHPTVEAKLRNQLEKYISERTSQD
SNYGGKIPIVCFAQGGGRETLKAINTSVKSKIPCVVVEGSGQIADVIASLVEVEDVLTSSMVKEKLVRFLPRT
VSRLPEEEIESWIKWLKEILESSHLLTVIKMEEAGDEIVSNAISYALYKAFSTNEQDKDNWNGQLKLLLEWNQ
LDLASDEIFTNDRRWESADLQEVMFTALIKDRPKFVRLFLENGLNLQKFLTNEVLTELFSTHFSTLVYRNLQI
AKNSYNDALLTFVWKLVANFRRSFWKEDRSSREDLDVELHDASLTTRHPLQALFIWAILQNKKELSKVIWDQT
KGCTLAALGASKLLKTLAKVKNDINAAGESEELANEYETRAVELFTECYSNDEDLAEQLLVYSCEAWGGSNCL
ELAVEATDQHFIAQPGVQNFLSKQWYGEISRDTKNWKIILCLFIIPLVGCGLVSFRKKPIDKHKKLLWYYVAF
FTSPFVVFSWNVVFYIAFLLLFAYVLLMDFHSVPHTPELILYALVFVLFCDEVRQWYMNGVNYFTDLWNVMDT
LGLFYFIAGIVFRLHSSNKSSLYSGRVIFCLDYIIFTLRLIHIFTVSRNLGPKIIMLQRMLIDVFFFLFLFAV
WMVAFGVARQGILRQNEQRWRWIFRSVIYEPYLAMFGQVPSDVDSTTYDFSHCTFSGNESKPLCVELDEHNLP
RFPEWITIPLVCIYMLSTNILLVNLLVAMFGYTVGIVQENNDQVWKFQRYFLVQEYCNRLNIPFPFVVFAYFY
MVVKKCFKCCCKEKNMESNACCFRNEDNETLAWEGVMKENYLVKINTKANDNSEEMRHRFRQLDSKLNDLKSL
LKEIANNIK*

>rs51912375_ENSMUSP00000108739_c.1788:C>T_p.L597F
ENSMUST00000113114.10|Trpm8-202|ENSMUSG00000036251|+|chr1:g.88278617:C>T
MSFEGARLSMRSRRNGTMGSTRTLYSSVSRSTDVSYSDSDLVNFIQANFKKRECVFFTRDSKAMENICKCGYA
QSQHIEGTQINQNEKWNYKKHTKEFPTDAFGDIQFETLGKKGKYLRLSCDTDSETLYELLTQHWHLKTPNLVI
SVTGGAKNFALKPRMRKIFSRLIYIAQSKGAWILTGGTHYGLMKYIGEVVRDNTISRNSEENIVAIGIAAWGM
VSNRDTLIRSCDDEGHFSAQYIMDDFTRDPLYILDNNHTHLLLVDNGCHGHPTVEAKLRNQLEKYISERTSQD
SNYGGKIPIVCFAQGGGRETLKAINTSVKSKIPCVVVEGSGQIADVIASLVEVEDVLTSSMVKEKLVRFLPRT
VSRLPEEEIESWIKWLKEILESSHLLTVIKMEEAGDEIVSNAISYALYKAFSTNEQDKDNWNGQLKLLLEWNQ
LDLASDEIFTNDRRWESADLQEVMFTALIKDRPKFVRLFLENGLNLQKFLTNEVLTELFSTHFSTLVYRNLQI
AKNSYNDALLTFVWKLVANFRRSFWKEDRSSREDLDVELHDASLTTRHPLQALFIWAILQNKKELSKVIWEQT
KGCTLAALGASKFLKTLAKVKNDINAAGESEELANEYETRAVELFTECYSNDEDLAEQLLVYSCEAWGGSNCL
ELAVEATDQHFIAQPGVQNFLSKQWYGEISRDTKNWKIILCLFIIPLVGCGLVSFRKKPIDKHKKLLWYYVAF
FTSPFVVFSWNVVFYIAFLLLFAYVLLMDFHSVPHTPELILYALVFVLFCDEVRQWYMNGVNYFTDLWNVMDT
LGLFYFIAGIVFRLHSSNKSSLYSGRVIFCLDYIIFTLRLIHIFTVSRNLGPKIIMLQRMLIDVFFFLFLFAV
WMVAFGVARQGILRQNEQRWRWIFRSVIYEPYLAMFGQVPSDVDSTTYDFSHCTFSGNESKPLCVELDEHNLP
RFPEWITIPLVCIYMLSTNILLVNLLVAMFGYTVGIVQENNDQVWKFQRYFLVQEYCNRLNIPFPFVVFAYFY
MVVKKCFKCCCKEKNMESNACCFRNEDNETLAWEGVMKENYLVKINTKANDNSEEMRHRFRQLDSKLNDLKSL
LKEIANNIK*
>rs51912375_ENSMUSP00000036991_c.1788:C>T_p.L597F
ENSMUST00000040210.14|Trpm8-201|ENSMUSG00000036251|+|chr1:g.88278617:C>T
MSFEGARLSMRSRRNGTMGSTRTLYSSVSRSTDVSYSDSDLVNFIQANFKKRECVFFTRDSKAMENICKCGYA
QSQHIEGTQINQNEKWNYKKHTKEFPTDAFGDIQFETLGKKGKYLRLSCDTDSETLYELLTQHWHLKTPNLVI
SVTGGAKNFALKPRMRKIFSRLIYIAQSKGAWILTGGTHYGLMKYIGEVVRDNTISRNSEENIVAIGIAAWGM
VSNRDTLIRSCDDEGHFSAQYIMDDFTRDPLYILDNNHTHLLLVDNGCHGHPTVEAKLRNQLEKYISERTSQD
SNYGGKIPIVCFAQGGGRETLKAINTSVKSKIPCVVVEGSGQIADVIASLVEVEDVLTSSMVKEKLVRFLPRT
VSRLPEEEIESWIKWLKEILESSHLLTVIKMEEAGDEIVSNAISYALYKAFSTNEQDKDNWNGQLKLLLEWNQ
LDLASDEIFTNDRRWESADLQEVMFTALIKDRPKFVRLFLENGLNLQKFLTNEVLTELFSTHFSTLVYRNLQI
AKNSYNDALLTFVWKLVANFRRSFWKEDRSSREDLDVELHDASLTTRHPLQALFIWAILQNKKELSKVIWEQT
KGCTLAALGASKFLKTLAKVKNDINAAGESEELANEYETRAVELFTECYSNDEDLAEQLLVYSCEAWGGSNCL
ELAVEATDQHFIAQPGVQNFLSKQWYGEISRDTKNWKIILCLFIIPLVGCGLVSFRKKPIDKHKKLLWYYVAF
FTSPFVVFSWNVVFYIAFLLLFAYVLLMDFHSVPHTPELILYALVFVLFCDEVRQWYMNGVNYFTDLWNVMDT
LGLFYFIAGIVFRLHSSNKSSLYSGRVIFCLDYIIFTLRLIHIFTVSRNLGPKIIMLQRMLIDVFFFLFLFAV
WMVAFGVARQGILRQNEQRWRWIFRSVIYEPYLAMFGQVPSDVDSTTYDFSHCTFSGNESKPLCVELDEHNLP
RFPEWITIPLVCIYMLSTNILLVNLLVAMFGYTVGIVQENNDQVWKFQRYFLVQEYCNRLNIPFPFVVFAYFY
MVVKKCFKCCCKEKNMESNACCFRNEDNETLAWEGVMKENYLVKINTKANDNSEEMRHRFRQLDSKLNDLKSL
LKEIANNIK*
>rs51912375_ENSMUSP00000131209_c.1788:C>T_p.L597F
ENSMUST00000171176.3|Trpm8-204|ENSMUSG00000036251|+|chr1:g.88278617:C>T
MSFEGARLSMRSRRNGTMGSTRTLYSSVSRSTDVSYSDSDLVNFIQANFKKRECVFFTRDSKAMENICKCGYA
QSQHIEGTQINQNEKWNYKKHTKEFPTDAFGDIQFETLGKKGKYLRLSCDTDSETLYELLTQHWHLKTPNLVI
SVTGGAKNFALKPRMRKIFSRLIYIAQSKGAWILTGGTHYGLMKYIGEVVRDNTISRNSEENIVAIGIAAWGM
VSNRDTLIRSCDDEGHFSAQYIMDDFTRDPLYILDNNHTHLLLVDNGCHGHPTVEAKLRNQLEKYISERTSQD
SNYGGKIPIVCFAQGGGRETLKAINTSVKSKIPCVVVEGSGQIADVIASLVEVEDVLTSSMVKEKLVRFLPRT
VSRLPEEEIESWIKWLKEILESSHLLTVIKMEEAGDEIVSNAISYALYKAFSTNEQDKDNWNGQLKLLLEWNQ
LDLASDEIFTNDRRWESADLQEVMFTALIKDRPKFVRLFLENGLNLQKFLTNEVLTELFSTHFSTLVYRNLQI
AKNSYNDALLTFVWKLVANFRRSFWKEDRSSREDLDVELHDASLTTRHPLQALFIWAILQNKKELSKVIWEQT
KGCTLAALGASKFLKTLAKVKNDINAAGESEELANEYETRAVELFTECYSNDEDLAEQLLVYSCEAWGGSNCL
ELAVEATDQHFIAQPGVQNFLSKQWYGEISRDTKNWKIILCLFIIPLVGCGLVSFRKKPIDKHKKLLWYYVAF
FTSPFVVFSWNVVFYIAFLLLFAYVLLMDFHSVPHTPELILYALVFVLFCDEVRQWYMNGVNYFTDLWNVMDT
LGLFYFIAGIVFRLHSSNKSSLYSGRVIFCLDYIIFTLRLIHIFTVSRNLGPKIIMLQRMLIDVFFFLFLFAV
WMVAFGVARQGILRQNEQRWRWIFRSVIYEPYLAMFGQVPSDVDSTTYDFSHCTFSGNESKPLCVELDEHNLP
RFPEWITIPLVCIYMLSTNILLVNLLVAMFGYTVGIVQENNDQVWKFQRYFLVQEYCNRLNIPFPFVVFAYFY
MVVKKCFKCCCKEKNMESNACCFRNEDNETLAWEGVMKENYLVKINTKANDNSEEMRHRFRQLDSKLNDLKSL
LKEIANNIK*
>rs48579533_ENSMUSP00000108739_c.2109:T>A_p.L704I
ENSMUST00000113114.10|Trpm8-202|ENSMUSG00000036251|+|chr1:g.88282215:T>A
MSFEGARLSMRSRRNGTMGSTRTLYSSVSRSTDVSYSDSDLVNFIQANFKKRECVFFTRDSKAMENICKCGYA
QSQHIEGTQINQNEKWNYKKHTKEFPTDAFGDIQFETLGKKGKYLRLSCDTDSETLYELLTQHWHLKTPNLVI

SVTGGAKNFALKPRMRKIFSRLIYIAQSKGAWILTGGTHYGLMKYIGEVVRDNTISRNSEENIVAIGIAAWGM
VSNRDTLIRSCDDEGHFSAQYIMDDFTRDPLYILDNNHTHLLLVDNGCHGHPTVEAKLRNQLEKYISERTSQD
SNYGGKIPIVCFAQGGGRETLKAINTSVKSKIPCVVVEGSGQIADVIASLVEVEDVLTSSMVKEKLVRFLPRT
VSRLPEEEIESWIKWLKEILESSHLLTVIKMEEAGDEIVSNAISYALYKAFSTNEQDKDNWNGQLKLLLEWNQ
LDLASDEIFTNDRRWESADLQEVMFTALIKDRPKFVRLFLENGLNLQKFLTNEVLTELFSTHFSTLVYRNLQI
AKNSYNDALLTFVWKLVANFRRSFWKEDRSSREDLDVELHDASLTTRHPLQALFIWAILQNKKELSKVIWEQT
KGCTLAALGASKLLKTLAKVKNDINAAGESEELANEYETRAVELFTECYSNDEDLAEQLLVYSCEAWGGSNCL
ELAVEATDQHFIAQPGVQNFLSKQWYGEISRDTKNWKIILCLFIIPIVGCGLVSFRKKPIDKHKKLLWYYVAF
FTSPFVVFSWNVVFYIAFLLLFAYVLLMDFHSVPHTPELILYALVFVLFCDEVRQWYMNGVNYFTDLWNVMDT
LGLFYFIAGIVFRLHSSNKSSLYSGRVIFCLDYIIFTLRLIHIFTVSRNLGPKIIMLQRMLIDVFFFLFLFAV
WMVAFGVARQGILRQNEQRWRWIFRSVIYEPYLAMFGQVPSDVDSTTYDFSHCTFSGNESKPLCVELDEHNLP
RFPEWITIPLVCIYMLSTNILLVNLLVAMFGYTVGIVQENNDQVWKFQRYFLVQEYCNRLNIPFPFVVFAYFY
MVVKKCFKCCCKEKNMESNACCFRNEDNETLAWEGVMKENYLVKINTKANDNSEEMRHRFRQLDSKLNDLKSL
LKEIANNIK*
>rs48579533_ENSMUSP00000036991_c.2109:T>A_p.L704I
ENSMUST00000040210.14|Trpm8-201|ENSMUSG00000036251|+|chr1:g.88282215:T>A
MSFEGARLSMRSRRNGTMGSTRTLYSSVSRSTDVSYSDSDLVNFIQANFKKRECVFFTRDSKAMENICKCGYA
QSQHIEGTQINQNEKWNYKKHTKEFPTDAFGDIQFETLGKKGKYLRLSCDTDSETLYELLTQHWHLKTPNLVI
SVTGGAKNFALKPRMRKIFSRLIYIAQSKGAWILTGGTHYGLMKYIGEVVRDNTISRNSEENIVAIGIAAWGM
VSNRDTLIRSCDDEGHFSAQYIMDDFTRDPLYILDNNHTHLLLVDNGCHGHPTVEAKLRNQLEKYISERTSQD
SNYGGKIPIVCFAQGGGRETLKAINTSVKSKIPCVVVEGSGQIADVIASLVEVEDVLTSSMVKEKLVRFLPRT
VSRLPEEEIESWIKWLKEILESSHLLTVIKMEEAGDEIVSNAISYALYKAFSTNEQDKDNWNGQLKLLLEWNQ
LDLASDEIFTNDRRWESADLQEVMFTALIKDRPKFVRLFLENGLNLQKFLTNEVLTELFSTHFSTLVYRNLQI
AKNSYNDALLTFVWKLVANFRRSFWKEDRSSREDLDVELHDASLTTRHPLQALFIWAILQNKKELSKVIWEQT
KGCTLAALGASKLLKTLAKVKNDINAAGESEELANEYETRAVELFTECYSNDEDLAEQLLVYSCEAWGGSNCL
ELAVEATDQHFIAQPGVQNFLSKQWYGEISRDTKNWKIILCLFIIPIVGCGLVSFRKKPIDKHKKLLWYYVAF
FTSPFVVFSWNVVFYIAFLLLFAYVLLMDFHSVPHTPELILYALVFVLFCDEVRQWYMNGVNYFTDLWNVMDT
LGLFYFIAGIVFRLHSSNKSSLYSGRVIFCLDYIIFTLRLIHIFTVSRNLGPKIIMLQRMLIDVFFFLFLFAV
WMVAFGVARQGILRQNEQRWRWIFRSVIYEPYLAMFGQVPSDVDSTTYDFSHCTFSGNESKPLCVELDEHNLP
RFPEWITIPLVCIYMLSTNILLVNLLVAMFGYTVGIVQENNDQVWKFQRYFLVQEYCNRLNIPFPFVVFAYFY
MVVKKCFKCCCKEKNMESNACCFRNEDNETLAWEGVMKENYLVKINTKANDNSEEMRHRFRQLDSKLNDLKSL
LKEIANNIK*
>rs48579533_ENSMUSP00000131209_c.2109:T>A_p.L704I
ENSMUST00000171176.3|Trpm8-204|ENSMUSG00000036251|+|chr1:g.88282215:T>A
MSFEGARLSMRSRRNGTMGSTRTLYSSVSRSTDVSYSDSDLVNFIQANFKKRECVFFTRDSKAMENICKCGYA
QSQHIEGTQINQNEKWNYKKHTKEFPTDAFGDIQFETLGKKGKYLRLSCDTDSETLYELLTQHWHLKTPNLVI
SVTGGAKNFALKPRMRKIFSRLIYIAQSKGAWILTGGTHYGLMKYIGEVVRDNTISRNSEENIVAIGIAAWGM
VSNRDTLIRSCDDEGHFSAQYIMDDFTRDPLYILDNNHTHLLLVDNGCHGHPTVEAKLRNQLEKYISERTSQD
SNYGGKIPIVCFAQGGGRETLKAINTSVKSKIPCVVVEGSGQIADVIASLVEVEDVLTSSMVKEKLVRFLPRT
VSRLPEEEIESWIKWLKEILESSHLLTVIKMEEAGDEIVSNAISYALYKAFSTNEQDKDNWNGQLKLLLEWNQ
LDLASDEIFTNDRRWESADLQEVMFTALIKDRPKFVRLFLENGLNLQKFLTNEVLTELFSTHFSTLVYRNLQI
AKNSYNDALLTFVWKLVANFRRSFWKEDRSSREDLDVELHDASLTTRHPLQALFIWAILQNKKELSKVIWEQT
KGCTLAALGASKLLKTLAKVKNDINAAGESEELANEYETRAVELFTECYSNDEDLAEQLLVYSCEAWGGSNCL
ELAVEATDQHFIAQPGVQNFLSKQWYGEISRDTKNWKIILCLFIIPIVGCGLVSFRKKPIDKHKKLLWYYVAF
FTSPFVVFSWNVVFYIAFLLLFAYVLLMDFHSVPHTPELILYALVFVLFCDEVRQWYMNGVNYFTDLWNVMDT
LGLFYFIAGIVFRLHSSNKSSLYSGRVIFCLDYIIFTLRLIHIFTVSRNLGPKIIMLQRMLIDVFFFLFLFAV
WMVAFGVARQGILRQNEQRWRWIFRSVIYEPYLAMFGQVPSDVDSTTYDFSHCTFSGNESKPLCVELDEHNLP
RFPEWITIPLVCIYMLSTNILLVNLLVAMFGYTVGIVQENNDQVWKFQRYFLVQEYCNRLNIPFPFVVFAYFY
MVVKKCFKCCCKEKNMESNACCFRNEDNETLAWEGVMKENYLVKINTKANDNSEEMRHRFRQLDSKLNDLKSL
LKEIANNIK*
>ENSMUSP00000108739_c.2505:T>A_p.Y836N ENSMUST00000113114.10|Trpm8-
202|ENSMUSG00000036251|+|chr1:g.88288365:T>A
MSFEGARLSMRSRRNGTMGSTRTLYSSVSRSTDVSYSDSDLVNFIQANFKKRECVFFTRDSKAMENICKCGYA
QSQHIEGTQINQNEKWNYKKHTKEFPTDAFGDIQFETLGKKGKYLRLSCDTDSETLYELLTQHWHLKTPNLVI
SVTGGAKNFALKPRMRKIFSRLIYIAQSKGAWILTGGTHYGLMKYIGEVVRDNTISRNSEENIVAIGIAAWGM
VSNRDTLIRSCDDEGHFSAQYIMDDFTRDPLYILDNNHTHLLLVDNGCHGHPTVEAKLRNQLEKYISERTSQD
SNYGGKIPIVCFAQGGGRETLKAINTSVKSKIPCVVVEGSGQIADVIASLVEVEDVLTSSMVKEKLVRFLPRT
VSRLPEEEIESWIKWLKEILESSHLLTVIKMEEAGDEIVSNAISYALYKAFSTNEQDKDNWNGQLKLLLEWNQ

LDLASDEIFTNDRRWESADLQEVMFTALIKDRPKFVRLFLENGLNLQKFLTNEVLTELFSTHFSTLVYRNLQI
AKNSYNDALLTFVWKLVANFRRSFWKEDRSSREDLDVELHDASLTTRHPLQALFIWAILQNKKELSKVIWEQT
KGCTLAALGASKLLKTLAKVKNDINAAGESEELANEYETRAVELFTECYSNDEDLAEQLLVYSCEAWGGSNCL
ELAVEATDQHFIAQPGVQNFLSKQWYGEISRDTKNWKIILCLFIIPLVGCGLVSFRKKPIDKHKKLLWYYVAF
FTSPFVVFSWNVVFYIAFLLLFAYVLLMDFHSVPHTPELILYALVFVLFCDEVRQWYMNGVNYFTDLWNVMDT
LGLFYFIAGIVFRLHSSNKSSLYSGRVIFCLDNIIFTLRLIHIFTVSRNLGPKIIMLQRMLIDVFFFLFLFAV
WMVAFGVARQGILRQNEQRWRWIFRSVIYEPYLAMFGQVPSDVDSTTYDFSHCTFSGNESKPLCVELDEHNLP
RFPEWITIPLVCIYMLSTNILLVNLLVAMFGYTVGIVQENNDQVWKFQRYFLVQEYCNRLNIPFPFVVFAYFY
MVVKKCFKCCCKEKNMESNACCFRNEDNETLAWEGVMKENYLVKINTKANDNSEEMRHRFRQLDSKLNDLKSL
LKEIANNIK*
>ENSMUSP00000036991_c.2505:T>A_p.Y836N ENSMUST00000040210.14|Trpm8-201|ENSMUSG00000036251|+|chr1:g.88288365:T>A
MSFEGARLSMRSRRNGTMGSTRTLYSSVSRSTDVSYSDSDLVNFIQANFKKRECVFFTRDSKAMENICKCGYA
QSQHIEGTQINQNEKWNYKKHTKEFPTDAFGDIQFETLGKKGKYLRLSCDTDSETLYELLTQHWHLKTPNLVI
SVTGGAKNFALKPRMRKIFSRLIYIAQSKGAWILTGGTHYGLMKYIGEVVRDNTISRNSEENIVAIGIAAWGM
VSNRDTLIRSCDDEGHFSAQYIMDDFTRDPLYILDNNHTHLLLVDNGCHGHPTVEAKLRNQLEKYISERTSQD
SNYGGKIPIVCFAQGGGRETLKAINTSVKSKIPCVVVEGSGQIADVIASLVEVEDVLTSSMVKEKLVRFLPRT
VSRLPEEEIESWIKWLKEILESSHLLTVIKMEEAGDEIVSNAISYALYKAFSTNEQDKDNWNGQLKLLLEWNQ
LDLASDEIFTNDRRWESADLQEVMFTALIKDRPKFVRLFLENGLNLQKFLTNEVLTELFSTHFSTLVYRNLQI
AKNSYNDALLTFVWKLVANFRRSFWKEDRSSREDLDVELHDASLTTRHPLQALFIWAILQNKKELSKVIWEQT
KGCTLAALGASKLLKTLAKVKNDINAAGESEELANEYETRAVELFTECYSNDEDLAEQLLVYSCEAWGGSNCL
ELAVEATDQHFIAQPGVQNFLSKQWYGEISRDTKNWKIILCLFIIPLVGCGLVSFRKKPIDKHKKLLWYYVAF
FTSPFVVFSWNVVFYIAFLLLFAYVLLMDFHSVPHTPELILYALVFVLFCDEVRQWYMNGVNYFTDLWNVMDT
LGLFYFIAGIVFRLHSSNKSSLYSGRVIFCLDNIIFTLRLIHIFTVSRNLGPKIIMLQRMLIDVFFFLFLFAV
WMVAFGVARQGILRQNEQRWRWIFRSVIYEPYLAMFGQVPSDVDSTTYDFSHCTFSGNESKPLCVELDEHNLP
RFPEWITIPLVCIYMLSTNILLVNLLVAMFGYTVGIVQENNDQVWKFQRYFLVQEYCNRLNIPFPFVVFAYFY
MVVKKCFKCCCKEKNMESNACCFRNEDNETLAWEGVMKENYLVKINTKANDNSEEMRHRFRQLDSKLNDLKSL
LKEIANNIK*
>ENSMUSP00000131209_c.2505:T>A_p.Y836N ENSMUST00000171176.3|Trpm8-204|ENSMUSG00000036251|+|chr1:g.88288365:T>A
MSFEGARLSMRSRRNGTMGSTRTLYSSVSRSTDVSYSDSDLVNFIQANFKKRECVFFTRDSKAMENICKCGYA
QSQHIEGTQINQNEKWNYKKHTKEFPTDAFGDIQFETLGKKGKYLRLSCDTDSETLYELLTQHWHLKTPNLVI
SVTGGAKNFALKPRMRKIFSRLIYIAQSKGAWILTGGTHYGLMKYIGEVVRDNTISRNSEENIVAIGIAAWGM
VSNRDTLIRSCDDEGHFSAQYIMDDFTRDPLYILDNNHTHLLLVDNGCHGHPTVEAKLRNQLEKYISERTSQD
SNYGGKIPIVCFAQGGGRETLKAINTSVKSKIPCVVVEGSGQIADVIASLVEVEDVLTSSMVKEKLVRFLPRT
VSRLPEEEIESWIKWLKEILESSHLLTVIKMEEAGDEIVSNAISYALYKAFSTNEQDKDNWNGQLKLLLEWNQ
LDLASDEIFTNDRRWESADLQEVMFTALIKDRPKFVRLFLENGLNLQKFLTNEVLTELFSTHFSTLVYRNLQI
AKNSYNDALLTFVWKLVANFRRSFWKEDRSSREDLDVELHDASLTTRHPLQALFIWAILQNKKELSKVIWEQT
KGCTLAALGASKLLKTLAKVKNDINAAGESEELANEYETRAVELFTECYSNDEDLAEQLLVYSCEAWGGSNCL
ELAVEATDQHFIAQPGVQNFLSKQWYGEISRDTKNWKIILCLFIIPLVGCGLVSFRKKPIDKHKKLLWYYVAF
FTSPFVVFSWNVVFYIAFLLLFAYVLLMDFHSVPHTPELILYALVFVLFCDEVRQWYMNGVNYFTDLWNVMDT
LGLFYFIAGIVFRLHSSNKSSLYSGRVIFCLDNIIFTLRLIHIFTVSRNLGPKIIMLQRMLIDVFFFLFLFAV
WMVAFGVARQGILRQNEQRWRWIFRSVIYEPYLAMFGQVPSDVDSTTYDFSHCTFSGNESKPLCVELDEHNLP
RFPEWITIPLVCIYMLSTNILLVNLLVAMFGYTVGIVQENNDQVWKFQRYFLVQEYCNRLNIPFPFVVFAYFY
MVVKKCFKCCCKEKNMESNACCFRNEDNETLAWEGVMKENYLVKINTKANDNSEEMRHRFRQLDSKLNDLKSL
LKEIANNIK*
>rs30294597_ENSMUSP00000108739_c.2541:A>G_p.T848A ENSMUST00000113114.10|Trpm8-202|ENSMUSG00000036251|+|chr1:g.88288401:A>G
MSFEGARLSMRSRRNGTMGSTRTLYSSVSRSTDVSYSDSDLVNFIQANFKKRECVFFTRDSKAMENICKCGYA
QSQHIEGTQINQNEKWNYKKHTKEFPTDAFGDIQFETLGKKGKYLRLSCDTDSETLYELLTQHWHLKTPNLVI
SVTGGAKNFALKPRMRKIFSRLIYIAQSKGAWILTGGTHYGLMKYIGEVVRDNTISRNSEENIVAIGIAAWGM
VSNRDTLIRSCDDEGHFSAQYIMDDFTRDPLYILDNNHTHLLLVDNGCHGHPTVEAKLRNQLEKYISERTSQD
SNYGGKIPIVCFAQGGGRETLKAINTSVKSKIPCVVVEGSGQIADVIASLVEVEDVLTSSMVKEKLVRFLPRT
VSRLPEEEIESWIKWLKEILESSHLLTVIKMEEAGDEIVSNAISYALYKAFSTNEQDKDNWNGQLKLLLEWNQ
LDLASDEIFTNDRRWESADLQEVMFTALIKDRPKFVRLFLENGLNLQKFLTNEVLTELFSTHFSTLVYRNLQI
AKNSYNDALLTFVWKLVANFRRSFWKEDRSSREDLDVELHDASLTTRHPLQALFIWAILQNKKELSKVIWEQT
KGCTLAALGASKLLKTLAKVKNDINAAGESEELANEYETRAVELFTECYSNDEDLAEQLLVYSCEAWGGSNCL
ELAVEATDQHFIAQPGVQNFLSKQWYGEISRDTKNWKIILCLFIIPLVGCGLVSFRKKPIDKHKKLLWYYVAF

FTSPFVVFSWNVVFYIAFLLLFAYVLLMDFHSVPHTPELILYALVFVLFCDEVRQWYMNGVNYFTDLWNVMDT
LGLFYFIAGIVFRLHSSNKSSLYSGRVIFCLDYIIFTLRLIHIFAVSRNLGPKIIMLQRMLIDVFFFLFLFAV
WMVAFGVARQGILRQNEQRWRWIFRSVIYEPYLAMFGQVPSDVDSTTYDFSHCTFSGNESKPLCVELDEHNLP
RFPEWITIPLVCIYMLSTNILLVNLLVAMFGYTVGIVQENNDQVWKFQRYFLVQEYCNRLNIPFPFVVFAYFY
MVVKKCFKCCCKEKNMESNACCFRNEDNETLAWEGVMKENYLVKINTKANDNSEEMRHRFRQLDSKLNDLKSL
LKEIANNIK*
>rs30294597_ENSMUSP00000036991_c.2541:A>G_p.T848A
ENSMUST00000040210.14|Trpm8-201|ENSMUSG00000036251|+|chr1:g.88288401:A>G
MSFEGARLSMRSRRNGTMGSTRTLYSSVSRSTDVSYSDSDLVNFIQANFKKRECVFFTRDSKAMENICKCGYA
QSQHIEGTQINQNEKWNYKKHTKEFPTDAFGDIQFETLGKKGKYLRLSCDTDSETLYELLTQHWHLKTPNLVI
SVTGGAKNFALKPRMRKIFSRLIYIAQSKGAWILTGGTHYGLMKYIGEVVRDNTISRNSEENIVAIGIAAWGM
VSNRDTLIRSCDDEGHFSAQYIMDDFTRDPLYILDNNHTHLLLVDNGCHGHPTVEAKLRNQLEKYISERTSQD
SNYGGKIPIVCFAQGGGRETLKAINTSVKSKIPCVVVEGSGQIADVIASLVEVEDVLTSSMVKEKLVRFLPRT
VSRLPEEEIESWIKWLKEILESSHLLTVIKMEEAGDEIVSNAISYALYKAFSTNEQDKDNWNGQLKLLLEWNQ
LDLASDEIFTNDRRWESADLQEVMFTALIKDRPKFVRLFLENGLNLQKFLTNEVLTELFSTHFSTLVYRNLQI
AKNSYNDALLTFVWKLVANFRRSFWKEDRSSREDLDVELHDASLTTRHPLQALFIWAILQNKKELSKVIWEQT
KGCTLAALGASKLLKTLAKVKNDINAAGESEELANEYETRAVELFTECYSNDEDLAEQLLVYSCEAWGGSNCL
ELAVEATDQHFIAQPGVQNFLSKQWYGEISRDTKNWKIILCLFIIPLVGCGLVSFRKKPIDKHKKLLWYYVAF
FTSPFVVFSWNVVFYIAFLLLFAYVLLMDFHSVPHTPELILYALVFVLFCDEVRQWYMNGVNYFTDLWNVMDT
LGLFYFIAGIVFRLHSSNKSSLYSGRVIFCLDYIIFTLRLIHIFAVSRNLGPKIIMLQRMLIDVFFFLFLFAV
WMVAFGVARQGILRQNEQRWRWIFRSVIYEPYLAMFGQVPSDVDSTTYDFSHCTFSGNESKPLCVELDEHNLP
RFPEWITIPLVCIYMLSTNILLVNLLVAMFGYTVGIVQENNDQVWKFQRYFLVQEYCNRLNIPFPFVVFAYFY
MVVKKCFKCCCKEKNMESNACCFRNEDNETLAWEGVMKENYLVKINTKANDNSEEMRHRFRQLDSKLNDLKSL
LKEIANNIK*
>rs30294597_ENSMUSP00000131209_c.2541:A>G_p.T848A
ENSMUST00000171176.3|Trpm8-204|ENSMUSG00000036251|+|chr1:g.88288401:A>G
MSFEGARLSMRSRRNGTMGSTRTLYSSVSRSTDVSYSDSDLVNFIQANFKKRECVFFTRDSKAMENICKCGYA
QSQHIEGTQINQNEKWNYKKHTKEFPTDAFGDIQFETLGKKGKYLRLSCDTDSETLYELLTQHWHLKTPNLVI
SVTGGAKNFALKPRMRKIFSRLIYIAQSKGAWILTGGTHYGLMKYIGEVVRDNTISRNSEENIVAIGIAAWGM
VSNRDTLIRSCDDEGHFSAQYIMDDFTRDPLYILDNNHTHLLLVDNGCHGHPTVEAKLRNQLEKYISERTSQD
SNYGGKIPIVCFAQGGGRETLKAINTSVKSKIPCVVVEGSGQIADVIASLVEVEDVLTSSMVKEKLVRFLPRT
VSRLPEEEIESWIKWLKEILESSHLLTVIKMEEAGDEIVSNAISYALYKAFSTNEQDKDNWNGQLKLLLEWNQ
LDLASDEIFTNDRRWESADLQEVMFTALIKDRPKFVRLFLENGLNLQKFLTNEVLTELFSTHFSTLVYRNLQI
AKNSYNDALLTFVWKLVANFRRSFWKEDRSSREDLDVELHDASLTTRHPLQALFIWAILQNKKELSKVIWEQT
KGCTLAALGASKLLKTLAKVKNDINAAGESEELANEYETRAVELFTECYSNDEDLAEQLLVYSCEAWGGSNCL
ELAVEATDQHFIAQPGVQNFLSKQWYGEISRDTKNWKIILCLFIIPLVGCGLVSFRKKPIDKHKKLLWYYVAF
FTSPFVVFSWNVVFYIAFLLLFAYVLLMDFHSVPHTPELILYALVFVLFCDEVRQWYMNGVNYFTDLWNVMDT
LGLFYFIAGIVFRLHSSNKSSLYSGRVIFCLDYIIFTLRLIHIFAVSRNLGPKIIMLQRMLIDVFFFLFLFAV
WMVAFGVARQGILRQNEQRWRWIFRSVIYEPYLAMFGQVPSDVDSTTYDFSHCTFSGNESKPLCVELDEHNLP
RFPEWITIPLVCIYMLSTNILLVNLLVAMFGYTVGIVQENNDQVWKFQRYFLVQEYCNRLNIPFPFVVFAYFY
MVVKKCFKCCCKEKNMESNACCFRNEDNETLAWEGVMKENYLVKINTKANDNSEEMRHRFRQLDSKLNDLKSL
LKEIANNIK*
>ENSMUSP00000150858_c.910:T>A_p.V304E ENSMUST00000213247.2|Olfr12-
202|ENSMUSG00000061616|+|chr1:g.92548540:T>A
MATAVHRNGSLTPVSLRVFVLVGFGGGALTQALLFAVFLVLYVVTVLGNLTMIVVITLDARLHSPMYFFLKNL
SFVDLCYSSAIAPNALANFLSTSKVISFEACATQFFFFSLLATTETFLLAVMAYDRFMAICSPLRYPVTMCPT
TCTRLVLGTFCVGCLNSIVQTSLTFQLPFCSSNRIDHFYCDVPPLLQLACASTALNELFLFGLCGFIIVSTTL
AVLVSYGYITVTILRMHSGSGRHKVFSTCGSHLTAVSLFYGTLFVMYAQPGALTSMEQGKVVSIFYTLVIPML
NPLIYSLRNKDEKDALQRLGQRHSLVKAVRGCPAAGGNASV*
>ENSMUSP00000080027_c.910:T>A_p.V304E ENSMUST00000081274.6|Olfr12-
201|ENSMUSG00000061616|+|chr1:g.92548540:T>A
MATAVHRNGSLTPVSLRVFVLVGFGGGALTQALLFAVFLVLYVVTVLGNLTMIVVITLDARLHSPMYFFLKNL
SFVDLCYSSAIAPNALANFLSTSKVISFEACATQFFFFSLLATTETFLLAVMAYDRFMAICSPLRYPVTMCPT
TCTRLVLGTFCVGCLNSIVQTSLTFQLPFCSSNRIDHFYCDVPPLLQLACASTALNELFLFGLCGFIIVSTTL
AVLVSYGYITVTILRMHSGSGRHKVFSTCGSHLTAVSLFYGTLFVMYAQPGALTSMEQGKVVSIFYTLVIPML
NPLIYSLRNKDEKDALQRLGQRHSLVKAVRGCPAAGGNASV*
>ENSMUSP00000036916_c.331:T>G_p.L111R ENSMUST00000049027.10|Slc26a9-
201|ENSMUSG00000042268|+|chr1:g.131680924:T>G

MNQPRPRYVVDRAAYSLSLFDDEFEKKDRAYPVGEKLRNTFRCSSAKFKAFVFGLLPVLSWLPKYKIKDYIIP
DLLGGLSGGCIQVPQGMAFALLANLPAVNGLYSSFFPRLTYFFLGGIHQMVPGTFAVISILVGNICLQLAPES
KFQIFNNVTNETYVDTAAMEAERLHVSATLACLTAVIQMALGFMQFGFVAIYLSESFIRGFMTAAGLQILISV
LKYIFGLTIPSYTGPGSIVFTFIDICKNLPHTNIASLIFALVSGVFLVLVKELNARYMHKIHFPIPTEMIVVV
VATAISGSCKMPKKYHMQIVGEIRQGFPTPVAPMVSQWKDMVGTAFSLAIVGYVINLAMGRTLASKHGYDVDS
NQEMIALGCSNFFGSFFKIHVICCALSVTLAVDGAGGKSQVASLCVSLVVMITMLVLGSYLYPLPKAVLGALI
AVNLKNSLKQLTDPYYLWRKSKLDCCVWVVSFLSSFFLSLPYGVAVGVAFSILVVIFQTQFRNGSTLAQVMDT
DIYVNPKTYNRAQEIAGVKIVTYCSPLYFANSEIFRQKVIAKTGMDPQKVLLAKQKYLRKQEKRTAIPTQQRK
SLFMKTKTVSLQELQQDFESAPSTDPNNNQAPAAEAHISYITFSPDASTAAACELPASTRSPQEASDTLASVP
PFVTFHTLILDMSGVSFVDLMGIKALAKLSSTYEKIGVQIFLVNIHAQVYNDISHGGVFEDGCVQRSHVFPSI
HDAVLFAQANAREAPDRNFHGAPGDTEFSLYDSEEEGPSYWDLEQEMFGTMFHTETLTAL*
>ENSMUSP00000122075_c.331:T>G_p.L111R ENSMUST00000147800.2|Slc26a9-203|ENSMUSG00000042268|+|chr1:g.131680924:T>G
MNQPRPRYVVDRAAYSLSLFDDEFEKKDRAYPVGEKLRNTFRCSSAKFKAFVFGLLPVLSWLPKYKIKDYIIP
DLLGGLSGGCIQVPQGMAFALLANLPAVNGLYSSFFPRLTYFFLGGIHQMVPGTFAVISILVGNICLQLAPES
KFQIFNNVTNETYVDTAAMEAERLHVSATLACL
>rs239134730_ENSMUSP00000140443_c.423:G>A_p.V142I
ENSMUST00000188145.7|Edem3-203|ENSMUSG00000043019|+|chr1:g.151651683:G>A
MSKAGGCRGCGCRVPQRASWSLVAATAALCLVLATSVCTAGAAPMSREEKQKLGNQVLEMFDHAYGNYMEHAY
PADELMPLTCRGRVRGQEPSRGDVDDALGKFSLTLIDSLDTLVVLNKTKEFEDAVRKVLRDVNLDNDVIVSVF
ETNIRVLGGLLGGHSLAIMLKEKGEHMQWYNDELLHMAKQLGYKLLPAFNTTSGLPYPRINLKFGIRKPEART
GTETDTCTACAGTLILEFAALSRFTGATIFEEYARKALDFLWEKRQRSSNLVGVTINIHTGDWVRKDSGVGAG
IDSYYEYLLKAYVLLGDDSFLERFNTHYDAIMRYISQPPLLLDVHIHKPMLNARTWMDALLAFFPGLQVLKGD
IRPAIETHEMLYQVIKKHNFLPEAFTTDFRVHWAQHPLRPEFAESTYFLYKATGDPYYLEVGKTLIENLNKYA
RVPCGFAAMKDVRTGSHEDRMDSFFLAEMFKYLYLLFADKEDIIFDIEDYIFTTEAHLLPLWLSTTNRSISKK
NTTSEYTELDDSNFDWTCPNTQILFPNDPLYAQSIREPLKNVVDKSCPRGIIRVEESFRSGAKPPLRARDFMA
TNPEHLEILKKMGVSLIHLKDGRVQLVQHAIQAASSIDAEDGLRFMQEMIELSSQQQKEQQLPPRAVQIISHP
FFGRVVLTAGPAQFGLDLSKHKETRGFVASSKPYNGCSELTNPEAVMGKIALIQRGQCMFAEKARNIQNAGAI
GGIVIDDNEGSSSDTAPLFQMAGDGKDTDDIKIPMLFLFSKEGSIILDAIREHEQVEVLLSDKARDRAAILKG
KMIPSYIINSNPEMENEDQPSSENDSQNQSAEQMLSLSQTVDLADKESPEHPADSHSEASPSDSEEAAGFAPS
EQISGSTENHETTSLDGECTDLDNQVQEQSETEEDSSPNVSWGTKAQPIDSILADWNEDIEAFEMMEKDEL*
>rs239134730_ENSMUSP00000058941_c.423:G>A_p.V142I
ENSMUST00000059498.12|Edem3-201|ENSMUSG00000043019|+|chr1:g.151651683:G>A
MSKAGGCRGCGCRVPQRASWSLVAATAALCLVLATSVCTAGAAPMSREEKQKLGNQVLEMFDHAYGNYMEHAY
PADELMPLTCRGRVRGQEPSRGDVDDALGKFSLTLIDSLDTLVVLNKTKEFEDAVRKVLRDVNLDNDVIVSVF
ETNIRVLGGLLGGHSLAIMLKEKGEHMQWYNDELLHMAKQLGYKLLPAFNTTSGLPYPRINLKFGIRKPEART
GTETDTCTACAGTLILEFAALSRFTGATIFEEYARKALDFLWEKRQRSSNLVGVTINIHTGDWVRKDSGVGAG
IDSYYEYLLKAYVLLGDDSFLERFNTHYDAIMRYISQPPLLLDVHIHKPMLNARTWMDALLAFFPGLQVLKGD
IRPAIETHEMLYQVIKKHNFLPEAFTTDFRVHWAQHPLRPEFAESTYFLYKATGDPYYLEVGKTLIENLNKYA
RVPCGFAAMKDVRTGSHEDRMDSFFLAEMFKYLYLLFADKEDIIFDIEDYIFTTEAHLLPLWLSTTNRSISKK
NTTSEYTELDDSNFDWTCPNTQILFPNDPLYAQSIREPLKNVVDKSCPRGIIRVEESFRSGAKPPLRARDFMA
TNPEHLEILKKMGVSLIHLKDGRVQLVQHAIQAASSIDAEDGLRFMQEMIELSSQQQKEQQLPPRAVQIISHP
FFGRVVLTAGPAQFGLDLSKHKETRGFVASSKPYNGCSELTNPEAVMGKIALIQRGQCMFAEKARNIQNAGAI
GGIVIDDNEGSSSDTAPLFQMAGDGKDTDDIKIPMLFLFSKEGSIILDAIREHEQVEVLLSDKARDRDPEMEN
EDQPSSENDSQNQSAEQMLSLSQTVDLADKESPEHPADSHSEASPSDSEEAAGFAPSEQISGSTENHETTSLD
GECTDLDNQVQEQSETEEDSSPNVSWGTKAQPIDSILADWNEDIEAFEMMEKDEL*
>rs239134730_ENSMUSP00000140775_c.423:G>A_p.V142I
ENSMUST00000187951.7|Edem3-202|ENSMUSG00000043019|+|chr1:g.151651683:G>A
MSKAGGCRGCGCRVPQRASWSLVAATAALCLVLATSVCTAGAAPMSREEKQKLGNQVLEMFDHAYGNYMEHAY
PADELMPLTCRGRVRGQEPSRGDVDDALGKFSLTLIDSLDTLVVLNKTKEFEDAVRKVLRDVNLDNDVIVSVF
ETNIRVLGGLLGGHSLAIMLKEKGEHMQWYNDELLHMAKQLGYKLLPAFNTTSGLPYPRINLKFGIRKPEART
GTETDTCTACAGTLILEFAALSRFTGATIFEEYARKALDFLWEKRQRSSNLVGVTINIHTGDWVRKDSGVGAG
IDSYYEYLLKAYVLLGDDSFLERFNTHYDAIMRYISQPPLLLDVHIHKPMLNARTWMDALLAFFPGLQVLKGD
IRPAIETHEMLYQVIKKHNFLPEAFTTDFRVHWAQHPLRPEFAESTYFLYKATGDPYYLEVGKTLIENLNKYA
RVPCGFAAMKDVRTGSHEDRMDSFFLAEMFKYLYLLFADKEDIIFDIEDYIFTTEAHLLPLWLSTTNRSISKK
NTTSEYTELDDSNFDWTCPNTQILFPNDPLYAQSIREPLKNVVDKSCPRGIIRVREESFRSGAKPPLRARDFM
ATNPEHLEILKKMGAASSIDAEDGLRFMQEMIELSSQQQKEQQLPPRAVQIISHPFFGRVVLTAGPAQFGLDL
SKHKETRGFVASSKPYNGCSELTNPEAVMGKIALIQRGQCMFAEKARNIQNAGAIGGIVIDDNEGSSSDTAPL

FQMAGDGKDTDDIKIPMLFLFSKEGSIILDAIREHEQVEVLLSDKARDRDPEMENEDQPSSENDSQNQSAEQM
LSLSQTVDLADKESPEHPADSHSEASPSDSEEAAGFAPSEQISGSTENHETTSLDGECTDLDNQVQEQSETEE
DSSPNVSWGTKAQPIDSILADWNEDIEAFEMMEKDEL*
>rs239134730_ENSMUSP00000140234_c.423:G>A_p.V142I
ENSMUST00000191070.2|Edem3-204|ENSMUSG00000043019|+|chr1:g.151651683:G>A
MSKAGGCRGCGCRVPQRASWSLVAATAALCLVLATSVCTAGAAPMSREEKQKLGNQVLEMFDHAYGNYMEHAY
PADELMPLTCRGRVRGQEPSRGDVDDALGKFSLTLIDSLDTLVVLNKTKEFEDAVRKVLRDVNLDNDVIVSVF
ETNIRVLGGLLGGHSLAIMLKEKGEHMQWYNDELLHMAKQLGYKLLPAFNTTSGLPYPRINLKFGIRKPEART
GTETDTCTACAGTLILEFAALSRFTGATIFEEYARKALDFLWEKRQRSSNLVGVTINIHTGDWVRKDSGVGAG
IDSYYEYLLKAYVLLGDDSFLERFNTHYDAIMRYISQPPLLLDVHIHKPMLNARTWMDALLAFFPGLQVLKGD
IRPAIETHEMLYQVIKKHNFLPEAFTTDFRVHWAQHPLRPEFAESTYFLYKATGDPYYLEVGKTLIENLNKYA
RVPCGFAAMKDVRTGSHEDRMDSFFLAEMFKYLYLLFADKEDIIFDIEDYIFTTEAHLLPLWLSTTNRSISKK
NTTSEYTELDDSNFDWTCPNTQILFPNDPLYAQSIREPLKNVVDKSCPRGIIRVEESFRSGAKPPLRARDFMA
TNPEHLEILKKMGAASSIDAEDGLRFMQEMIELSSQQQKEQQLPPRAVQIISHPFFGRVVLTAGPAQFGLDLS
KHKETRGFVASSKPYNGCSELTNPEAVMGKIALIQRGQCMFAEKARNIQNAGAIGGIVIDDNEGSSSDTAPLF
QMAGDGKDTDDIKIPMLFLFSKEGSIILDAIREHEQVEVLLSDKARDRAAILKDPEMENEDQPSSENDSQNQS
AEQMLSLSQTVDLADKESPEHPADSHSEASPSDSEEAAGFAPSEQISGSTENHETTSLDGECTDLDNQVQEQS
ETEEDSSPNVSWGTKAQPIDSILADWNEDIEAFEMMEKDEL*
>ENSMUSP00000073144_c.549:T>G_p.S184A ENSMUST00000073441.13|Smg7-
202|ENSMUSG00000042772|-|chr1:g.152736207:A>C
MSLQSAQYLRQAEVLKAEMTDSKLGPAEVWTSRQALQDLYQKMLVTDLEYALDKKVEQDLWNHAFKNQITTLQ
GQAKNRANPNRSEVQANLSLFLEAASGFYTQLLQELCTVFNVDLPCRVKSSQLGIISNKQTHSSTIVKPQSSS
CSYICQHCLVHLGDIARYRNQTSQAESYYRHAAQLVPANGQPYNQLAILASSKGDHLTTIFYYCRSIAVKFPF
PAASTNLQKALSKALESRDELKTKWGVSDFIKAFIKFHGHVYLSKSLEKLSPLREKLEEQFKRLLFQKAFNSQ
QLVHVTVINLFQLHHLRDFSNETEQHSYSQDEQLCWTQLLALFMSFLGILCKCPLQNDSQESNNAYPLPAVKV
SMDWLRLRPRVFQEAVVDERQYIWPWLISLLNSFHPREDDLSNTNATPLPEEFELQGFLALRPSFRNLDFSKG
HQGITGDKEGQQRRIRQQRLISIGKWIADNQPRLIQCENEVGKLLFITEIPELILEDPSEAKENLILQETSVV
ESLATDGSPGLKSVLSTGRNPSNSCDSGEKPVVTFKENIKPREVNQGRSFPPKEVRRDCSKGVTVTQEDGQKD
SSKRRAETKRCTLGKLQETGKQSVAVQVKSQTELRKTPVSEARKTPVTQTPSQTSNSQFIPIHHPGAFPPLPS
RPGFPPPTYVIPPPVAFSMGSGYTFPAGVSVPGTFLQSTAHSPAGNQVQAGKQSHIPYSQQRPSGPGPMNQGP
QQSQPPSQPPLTSLPAQPTAQSTSQLQVQALAQQQQSPTKVIPALGKSPPHHSGFQQYQQADASKQLWNPPQV
QSPLGKIMPVKQSYYLQTQDPIKLFEPSLQPPVIQQQPLEKKMKPFPMEPYNHNPSEVKVPEFYWDSSYSMAD
NRAVMAQQPNMDRRSKRSPGVFRPEQDPVPRMPFEKSLLEKPSELMSHSSSFLSLTGFSVNQERYPNSSMFNE
VYGKNLTTSSKAELNPSVASQETSLYSLFEGTPWSPSLPASSDHSTPASQSPHSSNPSSLPSSPPTHNHNSAP
FSNFGPIGTPDNRDRRPADRWKTDKPAMGGFGVDYLSATSSSESSWHQASTPSGTWTGHGPSMEDSSAVLMES
LKSIWSSSMMHPGPSALEQLLMQQKQKQQRGQGAMNPPH*
>ENSMUSP00000041241_c.573:T>G_p.S192A ENSMUST00000043560.15|Smg7-
201|ENSMUSG00000042772|-|chr1:g.152736207:A>C
MRTENLKSEEHLKSSNIRQAEVLKAEMTDSKLGPAEVWTSRQALQDLYQKMLVTDLEYALDKKVEQDLWNHAF
KNQITTLQGQAKNRANPNRSEVQANLSLFLEAASGFYTQLLQELCTVFNVDLPCRVKSSQLGIISNKQTHSST
IVKPQSSSCSYICQHCLVHLGDIARYRNQTSQAESYYRHAAQLVPANGQPYNQLAILASSKGDHLTTIFYYCR
SIAVKFPFPAASTNLQKALSKALESRDELKTKWGVSDFIKAFIKFHGHVYLSKSLEKLSPLREKLEEQFKRLL
FQKAFNSQQLVHVTVINLFQLHHLRDFSNETEQHSYSQDEQLCWTQLLALFMSFLGILCKCPLQNDSQESNNA
YPLPAVKVSMDWLRLRPRVFQEAVVDERQYIWPWLISLLNSFHPREDDLSNTNATPLPEEFELQGFLALRPSF
RNLDFSKGHQGITGDKEGQQRRIRQQRLISIGKWIADNQPRLIQCENEVGKLLFITEIPELILEDPSEAKENL
ILQETSVVESLATDGSPGLKSVLSTGRNPSNSCDSGEKPVVTFKENIKPREVNQGRSFPPKEVKSQTELRKTP
VSEARKTPVTQTPSQTSNSQFIPIHHPGAFPPLPSRPGFPPPTYVIPPPVAFSMGSGYTFPAGVSVPGTFLQS
TAHSPAGNQVQAGKQSHIPYSQQRPSGPGPMNQGPQQSQPPSQPPLTSLPAQPTAQSTSQLQVQALAQQQQSP
TKVIPALGKSPPHHSGFQQYQQADASKQLWNPPQVQSPLGKIMPVKQSYYLQTQDPIKLFEPSLQPPVIQQQP
LEKKMKPFPMEPYNHNPSEVKVPEFYWDSSYSMADNRAVMAQQPNMDRRSKRSPGVFRPEQDPVPRMPFEDPK
SSPLLPPDLLKSLAALEEEEELIFSNPPDLYPALLGPLASLPGRSLFKSLLEKPSELMSHSSSFLSLTGFSVN
QERYPNSSMFNEVYGKNLTTSSKAELNPSVASQETSLYSLFEGTPWSPSLPASSDHSTPASQSPHSSNPSSLP
SSPPTHNHNSAPFSNFGPIGTPDNRDRRPADRWKTDKPAMGGFGVDYLSATSSSESSWHQASTPSGTWTGHGP
SMEDSSAVLMESLKSIWSSSMMHPGPSALEQLLMQQKQKQQRGQGAMNPPH*
>ENSMUSP00000107467_c.549:T>G_p.S184A ENSMUST00000111836.4|Smg7-
203|ENSMUSG00000042772|-|chr1:g.152736207:A>C
MSLQSAQYLRQAEVLKAEMTDSKLGPAEVWTSRQALQDLYQKMLVTDLEYALDKKVEQDLWNHAFKNQITTLQ
GQAKNRANPNRSEVQANLSLFLEAASGFYTQLLQELCTVFNVDLPCRVKSSQLGIISNKQTHSSTIVKPQSSS

CSYICQHCLVHLGDIARYRNQTSQAESYYRHAAQLVPANGQPYNQLAILASSKGDHLTTIFYYCRSIAVKFPF
PAASTNLQKALSKALESRDELKTKWGVSDFIKAFIKFHGHVYLSKSLEKLSPLREKLEEQFKRLLFQKAFNSQ
QLVHVTVINLFQLHHLRDFSNETEQHSYSQDEQLCWTQLLALFMSFLGILCKCPLQNDSQESNNAYPLPAVKV
SMDWLRLRPRVFQEAVVDERQYIWPWLISLLNSFHPREDDLSNTNATPLPEEFELQGFLALRPSFRNLDFSKG
HQGITGDKEGQQRRIRQQRLISIGKWIADNQPRLIQCENEVGKLLFITEIPELILEDPSEAKENLILQETSVV
ESLATDGSPGLKSVLSTGRNPSNSCDSGEKPVVTFKENIKPREVNQGRSFPPKEVKSQTELRKTPVSEARKTP
VTQTPSQTSNSQFIPIHHPGAFPPLPSRPGFPPPTYVIPPPVAFSMGSGYTFPAGVSVPGTFLQSTAHSPAGN
QVQAGKQSHIPYSQQRPSGPGPMNQGPQQSQPPSQPPLTSLPAQPTAQSTSQLQVQALAQQQQSPTKVIPALG
KSPPHHSGFQQYQQADASKQLWNPPQVQSPLGKIMPVKQSYYLQTQDPIKLFEPSLQPPVIQQQPLEKKMKPF
PMEPYNHNPSEVKVPEFYWDSSYSMADNRAVMAQQPNMDRRSKRSPGVFRPEQDPVPRMPFEDPKSSPLLPPD
LLKSLAALEEEEELIFSNPPDLYPALLGPLASLPGRSLFKSLLEKPSELMSHSSSFLSLTGFSVNQERYPNSS
MFNEVYGKNLTTSSKAELNPSVASQETSLYSLFEGTPWSPSLPASSDHSTPASQSPHSSNPSSLPSSPPTHNH
NSAPFSNFGPIGTPDNRDRRPADRWKTDKPAMGGFGVDYLSATSSSESSWHQASTPSGTWTGHGPSMEDSSAV
LMESLKSIWSSSMMHPGPSALEQLLMQQKQKQQRGQGAMNPPH*
>ENSMUSP00000106947_c.2008:A>T_p.K670M ENSMUST00000111315.9|Adamts4-202|ENSMUSG00000006403|+|chr1:g.171085397:A>T
MSQMGLHPRRGLTGHWLQRFQPCLPLHTVQWRRLLLLAFLLSLAWPASPLPREEEIVFPEKLNGSSILPGSGV
PARLLYRLPAFGEMLLLELEQDPGVQVEGLTVQYLGQAPEMLGGAEPGTYLTGTINGDPESVASLHWDGGALL
GVLQYRGAELHLQPLEGGALNSAGGPGAHILRRKSPASSQGPMCTVKAPSGSPSPISRRTKRFASLSRFVETL
VVADDKMAAFHGTGLKRYLLTVMAAAAKAFKHPSIRNPVNLVVTRLVILGSGQEGPQVGPSAAQTLRSFCTWQ
RGLNTPNDSDPDHFDTAILFTRQDLCGVSTCDTLGMADVGTVCDPARSCAIVEDDGLQSAFTAAHELGHVFNM
LHDNSKPCTNLNGQGGSSRHVMAPVMAHVDPEEPWSPCSARFITDFLDNGYGHCLLDKPEAPLHLPATFPGKD
YDADRQCQLTFGPDSSHCPQLPPPCAALWCSGHLNGHAMCQTKHSPWADGTPCGSSQACMGGRCLHVDQLKDF
NVPQAGGWGPWGPWGDCSRTCGGGVQFSSRDCTRPVPRNGGKYCEGRRTRFRSCNTENCPHGSALTFREEQCA
AYNHRTDLFKSFPGPMDWVPRYTGVAPRDQCKLTCQARALGYYYVLEPRVADGTPCSPDTSSVCVQGRCIHAG
CDRIIGSKKKFDMCMVCGGDGSRCSKQSGSFKKFRYGYSDVVTIPAGATHILVRQQGGSGLKSIYLALKLSDG
SYALNGEYTLMPSPTDVVLPGAVSLRYSGATAASETLSGHGPLAQPLTLQVLVAGNPQNARLRYSFFVPRPVP
STPRPPPQDWLQRRAEILKILRKRPWAGRK*
>ENSMUSP00000106946_c.1453:A>T_p.K485M ENSMUST00000111314.2|Adamts4-201|ENSMUSG00000006403|+|chr1:g.171085397:A>T
MKGGERDLRTLLSLIFFLLLLKRFASLSRFVETLVVADDKMAAFHGTGLKRYLLTVMAAAAKAFKHPSIRNPV
NLVVTRLVILGSGQEGPQVGPSAAQTLRSFCTWQRGLNTPNDSDPDHFDTAILFTRQDLCGVSTCDTLGMADV
GTVCDPARSCAIVEDDGLQSAFTAAHELGHVFNMLHDNSKPCTNLNGQGGSSRHVMAPVMAHVDPEEPWSPCS
ARFITDFLDNGYGHCLLDKPEAPLHLPATFPGKDYDADRQCQLTFGPDSSHCPQLPPPCAALWCSGHLNGHAM
CQTKHSPWADGTPCGSSQACMGGRCLHVDQLKDFNVPQAGGWGPWGPWGDCSRTCGGGVQFSSRDCTRPVPRN
GGKYCEGRRTRFRSCNTENCPHGSALTFREEQCAAYNHRTDLFKSFPGPMDWVPRYTGVAPRDQCKLTCQARA
LGYYYVLEPRVADGTPCSPDTSSVCVQGRCIHAGCDRIIGSKKKFDMCMVCGGDGSRCSKQSGSFKKFRYGYS
DVVTIPAGATHILVRQQGGSGLKSIYLALKLSDGSYALNGEYTLMPSPTDVVLPGAVSLRYSGATAASETLSG
HGPLAQPLTLQVLVAGNPQNARLRYSFFVPRPVPSTPRPPPQDWLQRRAEILKILRKRPWAGRK*
>ENSMUSP00000151387_c.2044:A>T_p.K682M ENSMUST00000219033.2|Adamts4-203|ENSMUSG00000006403|+|chr1:g.171085397:A>T
MASIHPSCSPGTMSQMGLHPRRGLTGHWLQRFQPCLPLHTVQWRRLLLLAFLLSLAWPASPLPREEEIVFPEK
LNGSSILPGSGVPARLLYRLPAFGEMLLLELEQDPGVQVEGLTVQYLGQAPEMLGGAEPGTYLTGTINGDPES
VASLHWDGGALLGVLQYRGAELHLQPLEGGALNSAGGPGAHILRRKSPASSQGPMCTVKAPSGSPSPISRRTK
RFASLSRFVETLVVADDKMAAFHGTGLKRYLLTVMAAAAKAFKHPSIRNPVNLVVTRLVILGSGQEGPQVGPS
AAQTLRSFCTWQRGLNTPNDSDPDHFDTAILFTRQDLCGVSTCDTLGMADVGTVCDPARSCAIVEDDGLQSAF
TAAHELGHVFNMLHDNSKPCTNLNGQGGSSRHVMAPVMAHVDPEEPWSPCSARFITDFLDNGYGHCLLDKPEA
PLHLPATFPGKDYDADRQCQLTFGPDSSHCPQLPPPCAALWCSGHLNGHAMCQTKHSPWADGTPCGSSQACMG
GRCLHVDQLKDFNVPQAGGWGPWGPWGDCSRTCGGGVQFSSRDCTRPVPRNGGKYCEGRRTRFRSCNTENCPH
GSALTFREEQCAAYNHRTDLFKSFPGPMDWVPRYTGVAPRDQCKLTCQARALGYYYVLEPRVADGTPCSPDTS
SVCVQGRCIHAGCDRIIGSKKKFDMCMVCGGDGSRCSKQSGSFKKFRYGYSDVVTIPAGATHILVRQQGGSGL
KSIYLALKLSDGSYALNGEYTLMPSPTDVVLPGAVSLRYSGATAASETLSGHGPLAQPLTLQVLVAGNPQNAR
LRYSFFVPRPVPSTPRPPPQDWLQRRAEILKILRKRPWAGRK*
>ENSMUSP00000134646_c.892:A>T_p.Q298L ENSMUST00000160565.4|Ifi206-201|ENSMUSG00000037849|-|chr1:g.173309102:T>A
MVNYYKQIVLLSGLEYMNDYNFRALKSLLNHDLKLTKNMQDDYDRIKIADLMEEKFPEDAGLSKLIEVCEDIP
ELADHVDILRKKKEKVKNKTKIKSESSPPPLTSSLMEAWEVEPAMVTASSEESKDTIPESPDTMTTQFLEEKP
KFPLLSTTSTSQAEGEPLTPQRFPTTASSSLQTPLEPPEISSTILATSQGSSVPYSTCDKSSRVPPVTASSST

QTIQTCLATSTLPCSHQATLKSPKTEPSSVQATQMTQAIKASGHNCPQVPAAPVSSSFIKPQVTPAMLLSGVQ
APLMPLATVPSCAQTFEMNPATMTNGCNSPQMSAATVHSSYSNPRVTPVTVASSGQSFRLTPSRWSSGFNSPH
VSAATISSSYSKPHVTLAIVPHNAQTLQQTPAAMTSGCNSPQMSAATISSSYNNPQVTPATVPRSAQSLLIPQ
VAVPSSAFQMIFQMTPAAMAHGCNSPQISAATVHSSYSNPRVTPATVASSGQSFQLTPSRWSSGFNSPHVSAA
TISHSYSKPHVTLAIVPHNAQTLRQTPAAMTSGCNSPQMSAATISSSYNNPQVTPATVPRSAQSLLIPQVAVP
SSAFQMIFQMTPAAMANGCNSPQISAATVHSNYSNPRVTPATVASSDQSYRLTPSRWSSGFNSPHVSAATIPS
SYQNSPQVSSATMRRSFPAMSLSPATPPKKPRLKKVPKQPSEENGHQQGFKQVMVLKVTEPFTYDIRKDKRMF
HATVATETEFFRVKVFDIALEEKFIPRKVIVISDYIGYNGFLEIYTASCVSEVNDSNVMNIPTSLRQKANATP
KISTLCTQRAGTFVNGTFTVYEKTVRSEFIYYGIEDRTGRMEVVVYGQFTKLYCEPGDKLSLFCFELSSSLDM
WQLRSVRHSYMQVIQARKRDYELPNVHAIIETAQSSLSWIPEQQW*
>ENSMUSP00000030039_c.1048:A>T_p.E350V ENSMUST00000030039.13|Fmn2-201|ENSMUSG00000028354|+|chr1:g.174330660:A>T
MGNQDGKLKRSAGDASHEGGGAEDAAGPRDAEITKKASGSKKALGKHGKGGGGSGETSKKKSKSDSRASVFSN
LRIRKNLTKGKGACDSREDVLDSQALPIGELDSAHSIVTKTPDLSLSAEETGLSDTECADPFEVIHPGASRPA
EAGVGIQATAEDLETAAGAQDGQRTSSGSDTDIYSFHSATEQEDLLSDIQQAIRLQQQQQKLLLQDSEEPAA
PPTAISPQPGAFLGLDQFLLGPRSEAEKDTVQALPVRPDLPETTKSLVPEHPPSSGSHLTSETPGYATAPSAV
TDSLSSPAFTFPEAGPGEGAAGVPVAGTGDTDEECEEDAFEDAPRGSPGEEWVPEVEVASQRLEKEPEEGMRE
SITSAVVSLPGSPAPSPRCFKPYPLITPCYIKTTTRQLSSPNHSPSQSPNQSPRIKKRPDPSVSRSSRTALAS
AAAPAKKHRLEGGLTGGLSRSADWTEELGVRTPGAGGSVHLLGRGATADDSGGGSPVLAAKAPGAPATADGFQ
NVFTGRTLLEKLFSQQENGPPEEAEKFCSRIIAMGLLLPFSDCFREPCNQNAGSSSAPFDQDQLYTWAAVSQP
THSMDYSEGQFPRREPSMWPSSKLPEEEPSPKDVDTEPKSSILESPKKCSNGVQQEVFDVKSEGQATVIQQLE
QTIEDLRTKIAELEKQYPALDLEGPRGLSGLENGLTASADVSLDALVLHGKVAQPPRTLEAKSIQTSPTEEGR
ILTLPPPKAPPEGLLGSPAAASGESALLTSPSGPQTKFCSEISLIVSPRRISVQLDAQQIQSASQLPPPPPLL
GSDSQGQPSQPSLHTESETSHEHSVSSSFGNNCNVPPAPPLPCTESSSFMPGLGMAIPPPPCLSDITVPALPS
PTAPALQFSNLQGPEMLPAPPQPPPLPGLGVPPPPPAPPLPGMGIPPPPPLPGMGIPPPPPLPGMGISPLPPL
PGMGIPPPPPLPGVGIPPPPPLPGVGIPPPPPLPGVGIPPPPPLPGVGIPPPPPLPGVGIPPPPPLPGVGIPP
PPPLPGVGIPPPPPLPGVGIPPPPPLPGSGIPPPPALPGVAIPPPPPLPGMGVPPPAPPPPGAGIPPPPLLPG
SGPPHSSQVGSSTLPAAPQGCGFLFPPLPTGLFGLGMNQDRVARKQLIEPCRPMKPLYWTRIQLHSKRDSSPS
LIWEKIEEPSIDCHEFEELFSKTAVKERKKPISDTISKTKAKQVVKLLSNKRSQAVGILMSSLHLDMKDIQHA
VVNLDNSVVDLETLQALYENRAQSDELEKIEKHSRSSKDKENAKSLDKPEQFLYELSLIPNFSERVFCILFQS
TFSESICSIRRKLELLQKLCETLKNGPGVMQVLGLVLAFGNYMNAGNKTRGQADGFGLDILPKLKDVKSSDNS
RSLLSYIVSYYLRNFDEDAGKEQCVFPLAEPQELFQASQMKFEDFQKDLRKLKKDLKACEAEAGKVYQVSSAE
HMQPFKENMEQFISQAKIDQESQEAALTETHKCFLETTAYYFMKPKLGEKEVSPNVFFSVWHEFSSDFKDAWK
KENKLILQERVKEAEEVCRQKKGKSLYKVKPRHDSGIKAKISMKT*
>rs265450187_ENSMUSP00000046686_c.1444:T>GT_p.L482ins-fs ENSMUST00000044190.12|Hhat-201|ENSMUSG00000037375|-|chr1:g.192196247:A>AC
MLPGWELTLCLLVSLGFHFRSFYEVYKVSREHEEELDQEFELEMDTLFGGLKKDPTDFEWNFWMEWGKRRLVW
LFIGHMAVSQLATLLTKKHRPWIVMVYGMWACWCVLGAPGVVMVLLHSTIAFCVAQFRSVLLSWLCSLLLLST
LRLQSVEEVKRRWYKTENEYYLLQFTLTVRCLYYTSFSLELCRQPPSAQPTPSAQGASHSYPWLLTYVFYYPV
FHNGPILNFPEFFRQMQQPELNSLQHSLCIVAKGLGRLLCWWWLAELMVHLMYMHALYSSAPLLESVSCWTLG
GLALAQVLFFYVKYLVLFGVPALLMRLDGLTPPPLPRCVSTMFSFTGMWRYFDVGLHNFLIRYVYIPLGGSQH
GLLGTLLSTATTFAFVSYWHGSYEDLWCWAALNWLGVTVESGVRRLLETPCVRETLARHLSPQAHHRLHALLA
ACSTSMLILFNLVFLGGIQVGKTYWNRIFLQGWPWVTLSVLGFRVLLFSRGHCLGSDIHSSL
>ENSMUSP00000014290_c.880:A>T_p.N294I ENSMUST00000014290.15|Apbb1ip-201|ENSMUSG00000026786|+|chr2:g.22739607:A>T
MGESNEDIDQMFSTLLGEMDLLTQSLGVDTLPPPDPNPPREEFNYTVGFKDLNESLNALEDQDLDALMADLVA
DISEAEQRTIQAQKESSQNQDRFALLRASDGQGTASGGYGASAAAIDVSHHEEALPPPPVEPMLDLLPPPPPP
PPPELLSKEEEEAKAKADKIKLALEKLKEAKVKKLVVKVHMDDSSTKSLMVDERQLARDVLDNLFEKTHCDCN
VDWCLYEIYPELQIERVFEDHENVVEVLSDWTRDTENKVLFLEKEERYAVFKNPQNFYLDNKGKKENKETNEK
MIAKNKEYLLEESFCGTSIIVPELEGALYLKEDGKKSWKRRYFLLRASGIYYVPKGKTKTSRDLACFIQFENV
NIYYGIQCKMKYKAPTDHCFVLKHPQIQKESQYIKYLCCDDARTLSQWVMGIRIAKYGKTLYDNYQRAVARAG
LASRWTNLGTVGTPMPAQPSTVSSGLKTGTSQPNGQMPQAIPSAGPPLQEAQTQIETTKDEKQGLGNHSPGAT
RENHRPKSSLPPPPPPVRRSSDTCGSPALPSKVKGPGTCTFPHPPENFLPPPPPPPPEEDNSGLLPPPPPPY
LEEPPDFVPPPPPPAAVEDSALPPPPPPPPCLSQEITKSSPLPPKKPLVPPKRQENQGLPGAPGNSEQDFMSD
LMKALQKKRGNIP*
>rs228911540_ENSMUSP00000109957_c.1728:C>T_p.Q577*
ENSMUST00000114318.10|Grin1-208|ENSMUSG00000026959|-|chr2:g.25187693:G>A

MSTMHLLTFALLFSCSFARAACDPKIVNIGAVLSTRKHEQMFREAVNQANKRHGSWKIQLNATSVTHKPNAIQ
MALSVCEDLISSQVYAILVSHPPTPNDHFTPTPVSYTAGFYRIPVLGLTTRMSIYSDKSIHLSFLRTVPPYSH
QSSVWFEMMRVYNWNHIILLVSDDHEGRAAQKRLETLLEERESKSKKRNYENLDQLSYDNKRGPKAEKVLQFD
PGTKNVTALLMEARDLEARVIILSASEDDAATVYRAAAMLNMTGSGYVWLVGEREISGNALRYAPDGIIGLQL
INGKNESAHISDAVGVVAQAVHELLEKENITDPPRGCVGNTNIWKTGPLFKRVLMSSKYADGVTGRVEFNEDG
DRKFANYSIMNLQNRKLVQVGIYNGTHVIPNDRKIIWPGGETEKPRGYQMSTRLKIVTIHQEPFVYVKPTMSD
GTCKEEFTVNGDPVKKVICTGPNDTSPGSPRHTVPQCCYGFCVDLLIKLARTMNFTYEVHLVADGKFGTQERV
NNSNKKEWNGMMGELLSGQADMIVAPLTINNERAQYIEFSKPFKYQGLTILVKKEIPRSTLDSFM*PFQSTLW
LLVGLSVHVVAVMLYLLDRFSPFGRFKVNSEEEEEDALTLSSAMWFSWGVLLNSGIGEGAPRSFSARILGMVW
AGFAMIIVASYTANLAAFLVLDRPEERITGINDPRLRNPSDKFIYATVKQSSVDIYFRRQVELSTMYRHMEKH
NYESAAEAIQAVRDNKLHAFIWDSAVLEFEASQKCDLVTTGELFFRSGFGIGMRKDSPWKQNVSLSILKSHEN
GFMEDLDKTWVRYQECDSRSNAPATLTFENMAGVFMLVAGGIVAGIFLIFIEIAYKRHKDARRKQMQLAFAAV
NVWRKNLQDRKSGRAEPDPKKKATFRAITSTLASSFKRRRSSKDTQYHPTDITGPLNLSDPSVSTVV*
>rs228911540_ENSMUSP00000109949_c.1728:C>T_p.Q577*
ENSMUST00000114310.10|Grin1-204|ENSMUSG00000026959|-|chr2:g.25187693:G>A
MSTMHLLTFALLFSCSFARAACDPKIVNIGAVLSTRKHEQMFREAVNQANKRHGSWKIQLNATSVTHKPNAIQ
MALSVCEDLISSQVYAILVSHPPTPNDHFTPTPVSYTAGFYRIPVLGLTTRMSIYSDKSIHLSFLRTVPPYSH
QSSVWFEMMRVYNWNHIILLVSDDHEGRAAQKRLETLLEERESKSKKRNYENLDQLSYDNKRGPKAEKVLQFD
PGTKNVTALLMEARDLEARVIILSASEDDAATVYRAAAMLNMTGSGYVWLVGEREISGNALRYAPDGIIGLQL
INGKNESAHISDAVGVVAQAVHELLEKENITDPPRGCVGNTNIWKTGPLFKRVLMSSKYADGVTGRVEFNEDG
DRKFANYSIMNLQNRKLVQVGIYNGTHVIPNDRKIIWPGGETEKPRGYQMSTRLKIVTIHQEPFVYVKPTMSD
GTCKEEFTVNGDPVKKVICTGPNDTSPGSPRHTVPQCCYGFCVDLLIKLARTMNFTYEVHLVADGKFGTQERV
NNSNKKEWNGMMGELLSGQADMIVAPLTINNERAQYIEFSKPFKYQGLTILVKKEIPRSTLDSFM*PFQSTLW
LLVGLSVHVVAVMLYLLDRFSPFGRFKVNSEEEEEDALTLSSAMWFSWGVLLNSGIGEGAPRSFSARILGMVW
AGFAMIIVASYTANLAAFLVLDRPEERITGINDPRLRNPSDKFIYATVKQSSVDIYFRRQVELSTMYRHMEKH
NYESAAEAIQAVRDNKLHAFIWDSAVLEFEASQKCDLVTTGELFFRSGFGIGMRKDSPWKQNVSLSILKSHEN
GFMEDLDKTWVRYQECDSRSNAPATLTFENMAGVFMLVAGGIVAGIFLIFIEIAYKRHKDARRKQMQLAFAAV
NVWRKNLQDRKSGRAEPDPKKKATFRAITSTLASSFKRRRSSKDTSTGGGRGALQNQKDTVLPRRAIEREEGQ
LQLCSRHRES*
>rs228911540_ENSMUSP00000109947_c.1728:C>T_p.Q577*
ENSMUST00000114308.10|Grin1-203|ENSMUSG00000026959|-|chr2:g.25187693:G>A
MSTMHLLTFALLFSCSFARAACDPKIVNIGAVLSTRKHEQMFREAVNQANKRHGSWKIQLNATSVTHKPNAIQ
MALSVCEDLISSQVYAILVSHPPTPNDHFTPTPVSYTAGFYRIPVLGLTTRMSIYSDKSIHLSFLRTVPPYSH
QSSVWFEMMRVYNWNHIILLVSDDHEGRAAQKRLETLLEERESKSKKRNYENLDQLSYDNKRGPKAEKVLQFD
PGTKNVTALLMEARDLEARVIILSASEDDAATVYRAAAMLNMTGSGYVWLVGEREISGNALRYAPDGIIGLQL
INGKNESAHISDAVGVVAQAVHELLEKENITDPPRGCVGNTNIWKTGPLFKRVLMSSKYADGVTGRVEFNEDG
DRKFANYSIMNLQNRKLVQVGIYNGTHVIPNDRKIIWPGGETEKPRGYQMSTRLKIVTIHQEPFVYVKPTMSD
GTCKEEFTVNGDPVKKVICTGPNDTSPGSPRHTVPQCCYGFCVDLLIKLARTMNFTYEVHLVADGKFGTQERV
NNSNKKEWNGMMGELLSGQADMIVAPLTINNERAQYIEFSKPFKYQGLTILVKKEIPRSTLDSFM*PFQSTLW
LLVGLSVHVVAVMLYLLDRFSPFGRFKVNSEEEEEDALTLSSAMWFSWGVLLNSGIGEGAPRSFSARILGMVW
AGFAMIIVASYTANLAAFLVLDRPEERITGINDPRLRNPSDKFIYATVKQSSVDIYFRRQVELSTMYRHMEKH
NYESAAEAIQAVRDNKLHAFIWDSAVLEFEASQKCDLVTTGELFFRSGFGIGMRKDSPWKQNVSLSILKSHEN
GFMEDLDKTWVRYQECDSRSNAPATLTFENMAGVFMLVAGGIVAGIFLIFIEIAYKRHKDARRKQMQLAFAAV
NVWRKNLQSTGGGRGALQNQKDTVLPRRAIEREEGQLQLCSRHRES*
>rs228911540_ENSMUSP00000109956_c.1728:C>T_p.Q577*
ENSMUST00000114317.10|Grin1-207|ENSMUSG00000026959|-|chr2:g.25187693:G>A
MSTMHLLTFALLFSCSFARAACDPKIVNIGAVLSTRKHEQMFREAVNQANKRHGSWKIQLNATSVTHKPNAIQ
MALSVCEDLISSQVYAILVSHPPTPNDHFTPTPVSYTAGFYRIPVLGLTTRMSIYSDKSIHLSFLRTVPPYSH
QSSVWFEMMRVYNWNHIILLVSDDHEGRAAQKRLETLLEERESKSKKRNYENLDQLSYDNKRGPKAEKVLQFD
PGTKNVTALLMEARDLEARVIILSASEDDAATVYRAAAMLNMTGSGYVWLVGEREISGNALRYAPDGIIGLQL
INGKNESAHISDAVGVVAQAVHELLEKENITDPPRGCVGNTNIWKTGPLFKRVLMSSKYADGVTGRVEFNEDG
DRKFANYSIMNLQNRKLVQVGIYNGTHVIPNDRKIIWPGGETEKPRGYQMSTRLKIVTIHQEPFVYVKPTMSD
GTCKEEFTVNGDPVKKVICTGPNDTSPGSPRHTVPQCCYGFCVDLLIKLARTMNFTYEVHLVADGKFGTQERV
NNSNKKEWNGMMGELLSGQADMIVAPLTINNERAQYIEFSKPFKYQGLTILVKKEIPRSTLDSFM*PFQSTLW
LLVGLSVHVVAVMLYLLDRFSPFGRFKVNSEEEEEDALTLSSAMWFSWGVLLNSGIGEGAPRSFSARILGMVW
AGFAMIIVASYTANLAAFLVLDRPEERITGINDPRLRNPSDKFIYATVKQSSVDIYFRRQVELSTMYRHMEKH
NYESAAEAIQAVRDNKLHAFIWDSAVLEFEASQKCDLVTTGELFFRSGFGIGMRKDSPWKQNVSLSILKSHEN

GFMEDLDKTWVRYQECDSRSNAPATLTFENMAGVFMLVAGGIVAGIFLIFIEIAYKRHKDARRKQMQLAFAAV
NVWRKNLQQYHPTDITGPLNLSDPSVSTVV*
>rs228911540_ENSMUSP00000028335_c.1665:C>T_p.Q556*
ENSMUST00000028335.13|Grin1-201|ENSMUSG00000026959|-|chr2:g.25187693:G>A
MSTMHLLTFALLFSCSFARAACDPKIVNIGAVLSTRKHEQMFREAVNQANKRHGSWKIQLNATSVTHKPNAIQ
MALSVCEDLISSQVYAILVSHPPTPNDHFTPTPVSYTAGFYRIPVLGLTTRMSIYSDKSIHLSFLRTVPPYSH
QSSVWFEMMRVYNWNHIILLVSDDHEGRAAQKRLETLLEERESKAEKVLQFDPGTKNVTALLMEARDLEARVI
ILSASEDDAATVYRAAAMLNMTGSGYVWLVGEREISGNALRYAPDGIIGLQLINGKNESAHISDAVGVVAQAV
HELLEKENITDPPRGCVGNTNIWKTGPLFKRVLMSSKYADGVTGRVEFNEDGDRKFANYSIMNLQNRKLVQVG
IYNGTHVIPNDRKIIWPGGETEKPRGYQMSTRLKIVTIHQEPFVYVKPTMSDGTCKEEFTVNGDPVKKVICTG
PNDTSPGSPRHTVPQCCYGFCVDLLIKLARTMNFTYEVHLVADGKFGTQERVNNSNKKEWNGMMGELLSGQAD
MIVAPLTINNERAQYIEFSKPFKYQGLTILVKKEIPRSTLDSFM*PFQSTLWLLVGLSVHVVAVMLYLLDRFS
PFGRFKVNSEEEEEDALTLSSAMWFSWGVLLNSGIGEGAPRSFSARILGMVWAGFAMIIVASYTANLAAFLVL
DRPEERITGINDPRLRNPSDKFIYATVKQSSVDIYFRRQVELSTMYRHMEKHNYESAAEAIQAVRDNKLHAFI
WDSAVLEFEASQKCDLVTTGELFFRSGFGIGMRKDSPWKQNVSLSILKSHENGFMEDLDKTWVRYQECDSRSN
APATLTFENMAGVFMLVAGGIVAGIFLIFIEIAYKRHKDARRKQMQLAFAAVNVWRKNLQDRKSGRAEPDPKK
KATFRAITSTLASSFKRRRSSKDTSTGGGRGALQNQKDTVLPRRAIEREEGQLQLCSRHRES*
>rs228911540_ENSMUSP00000109953_c.1665:C>T_p.Q556*
ENSMUST00000114314.10|Grin1-206|ENSMUSG00000026959|-|chr2:g.25187693:G>A
MSTMHLLTFALLFSCSFARAACDPKIVNIGAVLSTRKHEQMFREAVNQANKRHGSWKIQLNATSVTHKPNAIQ
MALSVCEDLISSQVYAILVSHPPTPNDHFTPTPVSYTAGFYRIPVLGLTTRMSIYSDKSIHLSFLRTVPPYSH
QSSVWFEMMRVYNWNHIILLVSDDHEGRAAQKRLETLLEERESKAEKVLQFDPGTKNVTALLMEARDLEARVI
ILSASEDDAATVYRAAAMLNMTGSGYVWLVGEREISGNALRYAPDGIIGLQLINGKNESAHISDAVGVVAQAV
HELLEKENITDPPRGCVGNTNIWKTGPLFKRVLMSSKYADGVTGRVEFNEDGDRKFANYSIMNLQNRKLVQVG
IYNGTHVIPNDRKIIWPGGETEKPRGYQMSTRLKIVTIHQEPFVYVKPTMSDGTCKEEFTVNGDPVKKVICTG
PNDTSPGSPRHTVPQCCYGFCVDLLIKLARTMNFTYEVHLVADGKFGTQERVNNSNKKEWNGMMGELLSGQAD
MIVAPLTINNERAQYIEFSKPFKYQGLTILVKKEIPRSTLDSFM*PFQSTLWLLVGLSVHVVAVMLYLLDRFS
PFGRFKVNSEEEEEDALTLSSAMWFSWGVLLNSGIGEGAPRSFSARILGMVWAGFAMIIVASYTANLAAFLVL
DRPEERITGINDPRLRNPSDKFIYATVKQSSVDIYFRRQVELSTMYRHMEKHNYESAAEAIQAVRDNKLHAFI
WDSAVLEFEASQKCDLVTTGELFFRSGFGIGMRKDSPWKQNVSLSILKSHENGFMEDLDKTWVRYQECDSRSN
APATLTFENMAGVFMLVAGGIVAGIFLIFIEIAYKRHKDARRKQMQLAFAAVNVWRKNLQDRKSGRAEPDPKK
KATFRAITSTLASSFKRRRSSKDTQYHPTDITGPLNLSDPSVSTVV*
>rs228911540_ENSMUSP00000109946_c.1665:C>T_p.Q556*
ENSMUST00000114307.8|Grin1-202|ENSMUSG00000026959|-|chr2:g.25187693:G>A
MSTMHLLTFALLFSCSFARAACDPKIVNIGAVLSTRKHEQMFREAVNQANKRHGSWKIQLNATSVTHKPNAIQ
MALSVCEDLISSQVYAILVSHPPTPNDHFTPTPVSYTAGFYRIPVLGLTTRMSIYSDKSIHLSFLRTVPPYSH
QSSVWFEMMRVYNWNHIILLVSDDHEGRAAQKRLETLLEERESKAEKVLQFDPGTKNVTALLMEARDLEARVI
ILSASEDDAATVYRAAAMLNMTGSGYVWLVGEREISGNALRYAPDGIIGLQLINGKNESAHISDAVGVVAQAV
HELLEKENITDPPRGCVGNTNIWKTGPLFKRVLMSSKYADGVTGRVEFNEDGDRKFANYSIMNLQNRKLVQVG
IYNGTHVIPNDRKIIWPGGETEKPRGYQMSTRLKIVTIHQEPFVYVKPTMSDGTCKEEFTVNGDPVKKVICTG
PNDTSPGSPRHTVPQCCYGFCVDLLIKLARTMNFTYEVHLVADGKFGTQERVNNSNKKEWNGMMGELLSGQAD
MIVAPLTINNERAQYIEFSKPFKYQGLTILVKKEIPRSTLDSFM*PFQSTLWLLVGLSVHVVAVMLYLLDRFS
PFGRFKVNSEEEEEDALTLSSAMWFSWGVLLNSGIGEGAPRSFSARILGMVWAGFAMIIVASYTANLAAFLVL
DRPEERITGINDPRLRNPSDKFIYATVKQSSVDIYFRRQVELSTMYRHMEKHNYESAAEAIQAVRDNKLHAFI
WDSAVLEFEASQKCDLVTTGELFFRSGFGIGMRKDSPWKQNVSLSILKSHENGFMEDLDKTWVRYQECDSRSN
APATLTFENMAGVFMLVAGGIVAGIFLIFIEIAYKRHKDARRKQMQLAFAAVNVWRKNLQSTGGGRGALQNQK
DTVLPRRAIEREEGQLQLCSRHRES*
>rs228911540_ENSMUSP00000109951_c.1665:C>T_p.Q556*
ENSMUST00000114312.2|Grin1-205|ENSMUSG00000026959|-|chr2:g.25187693:G>A
MSTMHLLTFALLFSCSFARAACDPKIVNIGAVLSTRKHEQMFREAVNQANKRHGSWKIQLNATSVTHKPNAIQ
MALSVCEDLISSQVYAILVSHPPTPNDHFTPTPVSYTAGFYRIPVLGLTTRMSIYSDKSIHLSFLRTVPPYSH
QSSVWFEMMRVYNWNHIILLVSDDHEGRAAQKRLETLLEERESKAEKVLQFDPGTKNVTALLMEARDLEARVI
ILSASEDDAATVYRAAAMLNMTGSGYVWLVGEREISGNALRYAPDGIIGLQLINGKNESAHISDAVGVVAQAV
HELLEKENITDPPRGCVGNTNIWKTGPLFKRVLMSSKYADGVTGRVEFNEDGDRKFANYSIMNLQNRKLVQVG
IYNGTHVIPNDRKIIWPGGETEKPRGYQMSTRLKIVTIHQEPFVYVKPTMSDGTCKEEFTVNGDPVKKVICTG
PNDTSPGSPRHTVPQCCYGFCVDLLIKLARTMNFTYEVHLVADGKFGTQERVNNSNKKEWNGMMGELLSGQAD
MIVAPLTINNERAQYIEFSKPFKYQGLTILVKKEIPRSTLDSFM*PFQSTLWLLVGLSVHVVAVMLYLLDRFS
PFGRFKVNSEEEEEDALTLSSAMWFSWGVLLNSGIGEGAPRSFSARILGMVWAGFAMIIVASYTANLAAFLVL

DRPEERITGINDPRLRNPSDKFIYATVKQSSVDIYFRRQVELSTMYRHMEKHNYESAAEAIQAVRDNKLHAFI
WDSAVLEFEASQKCDLVTTGELFFRSGFGIGMRKDSPWKQNVSLSILKSHENGFMEDLDKTWVRYQECDSRSN
APATLTFENMAGVFMLVAGGIVAGIFLIFIEIAYKRHKDARRKQMQLAFAAVNVWRKNLQQYHPTDITGPLNL
SDPSVSTVV*
>ENSMUSP00000028170_c.549:C>T_p.R184C ENSMUST00000028170.15|Ralgds-201|ENSMUSG00000026821|+|chr2:g.28433696:C>T
MMVDCQSSTQEIGEELINGVIYSISLRKVQLHQGATKGQRWLGCENESALNLYETCKVRTVKAGTLEKLVEHL
VPAFQGSDLSYVTVFLCTYRAFTTTQQVLDLLFKRYGRCDALTASSRYGCILPYSSEDGGPQDQLKNAISSIL
GTWLDQYSEDFCQPPDFPCLKQLVAYVQLNMPGSDLECRAHLLLAQLEDLEPSEAESEALSPAPVLSLKPASQ
LEPALLLTPSQVVTSTPVREPAAAPVPVLASSPVVAPAPELEPVPEPPQEPEPSLALAPELEPAVSQSLELES
APVPTPALEPSWSLPEATENGLTEKPHLLLFPPDLVAEQFTLMDAELFKKVVPYHCLGSIWSQRDKKGKEHLA
PTIRATVAQFNNVANCVITTCLGDQSMKAPDRARVVEHWIEVARECRALKNFSSLYAILSALQSNAIHRLKKT
WEEVSRDSFRVFQKLSEIFSDENNYSLSRELLIKEGTSKFATLEMNPRRAQRRQKETGVIQGTVPYLGTFLTD
LVMLDTAMKDYLYGRLINFEKRRKEFEVIAQIKLLQSACNNYSIAPEEHFGTWFRAMERLSEAESYTLSCELE
PPSESASNTLRSKKSTAIVKRWSDRQAPSTELSTSSSAHSKSCDQLRCSPYLGSGDITDALSVHSAGSSSSDV
EEINMSFVPESPDGQEKKFWESASQSSPETSGISSASSSTSSSSASTTPVSTTRTHKRSVSGVCSYSSSLPLY
NQQVGDCCIIRVSLDVDNGNMYKSILVTSQDKAPTVIRKAMDKHNLDEDEPEDYELVQIISEDHKLKIPENAN
VFYAMNSTANYDFILKKRTFTKGAKVKHGASSTLPRMKQKGLRIAKGIF*
>ENSMUSP00000158697_c.717:C>T_p.R240C ENSMUST00000238699.2|Ralgds-211|ENSMUSG00000026821|+|chr2:g.28433696:C>T
MAREADRVHARPAVPRGRKGSVFFACVSVVTARRWAIARRTTAHSPTPPTPCPAPLLAPTAESSTQEIGEELI
NGVIYSISLRKVQLHQGATKGQRWLGCENESALNLYETCKVRTVKAGTLEKLVEHLVPAFQGSDLSYVTVFLC
TYRAFTTTQQVLDLLFKRYGRCDALTASSRYGCILPYSSEDGGPQDQLKNAISSILGTWLDQYSEDFCQPPDF
PCLKQLVAYVQLNMPGSDLECRAHLLLAQLEDLEPSEAESEALSPAPVLSLKPASQLEPALLLTPSQVVTSTP
VREPAAAPVPVLASSPVVAPAPELEPVPEPPQEPEPSLALAPELEPAVSQSLELESAPVPTPALEPSWSLPEA
TENGLTEKPHLLLFPPDLVAEQFTLMDAELFKKVVPYHCLGSIWSQRDKKGKEHLAPTIRATVAQFNNVANCV
ITTCLGDQSMKAPDRARVVEHWIEVARECRALKNFSSLYAILSALQSNAIHRLKKTWEEVSRDSFRVFQKLSE
IFSDENNYSLSRELLIKEGTSKFATLEMNPRRAQRRQKETGVIQGTVPYLGTFLTDLVMLDTAMKDYLYGRLI
NFEKRRKEFEVIAQIKLLQSACNNYSIAPEEHFGTWFRAMERLSEAESYTLSCELEPPSESASNTLRSKKSTA
IVKRWSDRQAPSTELSTSSSAHSKSCDQLRCSPYLGSGDITDALSVHSAGSSSSDVEEINMSFVPESPDGQEK
KFWESASQSSPETSGISSASSSTSSSSASTTPVSTTRTHKRSVSGVCSYSSSLPLYNQQVGDCCIIRVSLDVD
NGNMYKSILVTSQDKAPTVIRKAMDKHNLDEDEPEDYELVQIISEDHTEDSRKRQCVLCHELYRQL*
>ENSMUSP00000109526_c.678:C>T_p.R227C ENSMUST00000113893.8|Ralgds-203|ENSMUSG00000026821|+|chr2:g.28433696:C>T
MVQRMWAEAAGPIGGTEPLFPGSRRSRSVWDAVRLEVGVPDSCPVVLHSFTQLDPDLPRLESSTQEIGEELIN
GVIYSISLRKVQLHQGATKGQRWLGCENESALNLYETCKVRTVKAGTLEKLVEHLVPAFQGSDLSYVTVFLCT
YRAFTTTQQVLDLLFKRYGCILPYSSEDGGPQDQLKNAISSILGTWLDQYSEDFCQPPDFPCLKQLVAYVQLN
MPGSDLECRAHLLLAQLEDLEPSEAESEALSPAPVLSLKPASQLEPALLLTPSQVVTSTPVREPAAAPVPVLA
SSPVVAPAPELEPVPEPPQEPEPSLALAPELEPAVSQSLELESAPVPTPALEPSWSLPEATENGLTEKPHLLL
FPPDLVAEQFTLMDAELFKKVVPYHCLGSIWSQRDKKGKEHLAPTIRATVAQFNNVANCVITTCLGDQSMKAP
DRARVVEHWIEVARECRALKNFSSLYAILSALQSNAIHRLKKTWEEVSRDSFRVFQKLSEIFSDENNYSLSRE
LLIKEGTSKFATLEMNPRRAQRRQKETGVIQGTVPYLGTFLTDLVMLDTAMKDYLYGRLINFEKRRKEFEVIA
QIKLLQSACNNYSIAPEEHFGTWFRAMERLSEAESYTLSCELEPPSESASNTLRSKKSTAIVKRWSDRQAPST
ELSTSSSAHSKSCDQLRCSPYLGSGDITDALSVHSAGSSSSDVEEINMSFVPESPDGQEKKFWESASQSSPET
SGISSASSSTSSSSASTTPVSTTRTHKRSVSGVCSYSSSLPLYNQQVGDCCIIRVSLDVDNGNMYKSILVTSQ
DKAPTVIRKAMDKHNLDEDEPEDYELVQIISEDHKLKIPENANVFYAMNSTANYDFILKKRTFTKGAKVKHGA
SSTLPRMKQKGLRIAKGIF*
>ENSMUSP00000097812_c.714:C>T_p.R239C ENSMUST00000100241.10|Ralgds-202|ENSMUSG00000026821|+|chr2:g.28433696:C>T
MVQRMWAEAAGPIGGTEPLFPGSRRSRSVWDAVRLEVGVPDSCPVVLHSFTQLDPDLPRLESSTQEIGEELIN
GVIYSISLRKVQLHQGATKGQRWLGCENESALNLYETCKVRTVKAGTLEKLVEHLVPAFQGSDLSYVTVFLCT
YRAFTTTQQVLDLLFKRYGRCDALTASSRYGCILPYSSEDGGPQDQLKNAISSILGTWLDQYSEDFCQPPDFP
CLKQLVAYVQLNMPGSDLECRAHLLLAQLEDLEPSEAESEALSPAPVLSLKPASQLEPALLLTPSQVVTSTPV
REPAAAPVPVLASSPVVAPAPELEPVPEPPQEPEPSLALAPELEPAVSQSLELESAPVPTPALEPSWSLPEAT
ENGLTEKPHLLLFPPDLVAEQFTLMDAELFKKVVPYHCLGSIWSQRDKKGKEHLAPTIRATVAQFNNVANCVI
TTCLGDQSMKAPDRARVVEHWIEVARECRALKNFSSLYAILSALQSNAIHRLKKTWEEVSRDSFRVFQKLSEI
FSDENNYSLSRELLIKEGTSKFATLEMNPRRAQRRQKETGVIQGTVPYLGTFLTDLVMLDTAMKDYLYGRLIN
FEKRRKEFEVIAQIKLLQSACNNYSIAPEEHFGTWFRAMERLSEAESYTLSCELEPPSESASNTLRSKKSTAI

VKRWSDRQAPSTELSTSSSAHSKSCDQLRCSPYLGSGDITDALSVHSAGSSSSDVEEINMSFVPESPDGQEKK
FWESASQSSPETSGISSASSSTSSSSASTTPVSTTRTHKRSVSGVCSYSSSLPLYNQQVGDCCIIRVSLDVDN
GNMYKSILVTSQDKAPTVIRKAMDKHNLDEDEPEDYELVQIISEDHKLKIPENANVFYAMNSTANYDFILKKR
TFTKGAKVKHGASSTLPRMKQKGLRIAKGIF*
>ENSMUSP00000066492_c.6232:A>T_p.Q2078L ENSMUST00000065398.13|Nup214-201|ENSMUSG00000001855|+|chr2:g.31942139:A>T
MGDEMDAMIPEREMKDFQFRALKKVRIFDSPEELPKERSSVLTISNKYGMLFAGGTNGLNVFPTKSLLIQNKP
GDDPNKIVDTIQGLNVPMKFPVHHLALSCDSLTLSACMMSSEYGSIIAFFDVRTFSNQAKPLKRPFTYHKVSN
DASGMVNDMKWNPTVPSMVAVCLADGSISVLQVTDVVKVCATLPPSTGVTCVCWSPKGKQLAVGKQNGTVVQY
LPTLQEKKVIPCPPFYESDHPVRVLDVLWIGTYVFTIVYAGADGTLETCPDVVMALLPKKEEKHPEIFVNFME
PCYSSCTERQHHYYLSYIEEWDLVLAASAASTEVSILARQNDQTNWESWLLEDSSRAELPVTDKSDDSLPMGV
AIDYTNEVEVTINEEKTLPPAPVLLLLSTDGVLCPFYMINQNPGVRSLIKTLELISTEGERQPKSSGSFPGTP
SSPQAPQNLDAPATASSPLPPVSAAPTSTFPMPSAAGSPSVFSFGPSSFKSSASVTGEPPLYPTGSDSSRAAP
GSGTSTFSFAPPSKGSLASTPAVAPVATSAAPFTFGFKPTLESTPMSSTPNTGMKPSFPPSASSVKVNLNEKF
TAVASSAPVHSSTSTPSVLPFSSSPKPTASGPLSHPTPLPASSSSMPLKSSVSPSPAAGRSTQTAPSSAPSTG
QKSPRVNPPVPKSGSSQAKALQPPVTEKQRPQWKDSDPVLAGIGEEIAHFQKELEELKARTAKACLQVGTSEE
MKMLRTESDDLHTFLFEIRETTESLHGDISTLKTTLLEGFAGVEEAREQHGRNHDSGYLHLLYKRPLDPKSEA
QLQEIRRLHQYVKFAVQDVNDVLDLEWDRHLEQKKRQRRLIVPERETLFNTLANNREIINQQRKRLNQLVDSL
QQLRLYNHTAPWSLPSALSTQSNSHSFDSDLECLLKTTIESHTKPSPRVPGKLSPAKQAQLRNFLAKRKTPPV
RSTAPASLSRSAFLSQRYYEDLDEGSSASSVAQPLEGEDARPTCTSVAQPLEGEDAQPICKEEEAVVPVPRHA
PVVRTPSIQPSLLPQSMPFAKPHLIHSSSPAVMSSAVSTSATKVIPQGADSTMLATKTVKHGAPGPSHTVAAP
QAAAAAALRRQMASQAPAMSTLTESTLKTVPQVVNVQELRSNPSPPSAAMGSAVQHSAAKTPHAVLTPVANSQ
AKQGSLINSFKPSGPTAASCQLSSGDKAVGQGTAKTESAATSTPSAAGQLNKPFSFASPGTFTFGTITPTPSS
SFTATPGAGPPTKEPTQLEAFSFGGGGKPFSEAIPGNSPATGATSAPSTSVTAASLEDSAPSSSKPAAPPETT
VSSASSKLETPPSKLGELLFPSSLAGETLGSFSGLRVGQAEDSTKPVSKASSTNLAGAQPAKPSGVSFPNTSV
LGKPVEPAVTSSVSPAPAAPASALNVSTSSSSATVFGSVPLTSAGPPGLISFGGAALASSKASFSFGNQQTSS
STASSATPTSTSVAPSLPASFPTLSFGGLLSSPSASSLPVSSGKSTEEAAPPAVPDKSDSSEVSATTPSLPVQ
PQSTQASLQTSDPVKKEPVLVQTTDSSPSRPASSASFVASTESMPVTLGAPDSKIEAVSPASTFAGPGQAAVA
TAVLPGAGSAATEASGTPTTSTVSSSSPTSATETAVFGTATSGSSVFTQPPAASSSSAFSQLSSNTATAPSAT
PVFGQVAASITSTAAATPQASSSGFGSPAFGASAPGVFGQTAFGQTPAFGQATSSPASGFSFSQPGFSSVPAF
GQSVSSTPASTSANVFGATSSTSSPGSFSFGQASTNTGGTLFGQNNPPAFGQSPGFGQGSSVFGGTSATTSTA
APSGFSFCQASGFGSSNTGSVFGQAANTGGSVFGQSSTSSGGVFGSGNATRGGGFFSGLGGKPSQDAANKNPF
SSAGGGFGSTAAPNTSNLFGNSGAKTFGGFGSSSFGEQKPAGTFSSGGGSVASQGFGFSTPNKTGGFGAAPVF
GSPPTFGGSPGFGGVPAFGSAPAFTSPLGSTGGKVFGEGTAAASAGGFGFGSSGNTASFGTLASQNAPTFGSL
SQQTSGFGTPSSGFAGFGSGTGAFTFGSSNSSVLGFGGWRS*
>ENSMUSP00000115665_c.1720:A>T_p.Q574L ENSMUST00000138012.2|Nup214-208|ENSMUSG00000001855|+|chr2:g.31942139:A>T
TEEAAPPAVPDKSDSSEVSATTPSLPVQPQSTQASLQTSDPVKKEPVLVQTTDSSPSRPASSASFVASTESMP
VTLGAPDSKIEAVSPASTFAGPGQAAVATAVLPGAGSAATEASGTPTTSTVSSSSPTSATETAVFGTATSGSS
VFTQPPAASSSSAFSQLSSNTATAPSATPVFGQVAASITSTAAATPQASSSGFGSPAFGASAPGVFGQTAFGQ
TPAFGQATSSPASGFSFSQPGFSSVPAFGQSVSSTPASTSANVFGATSSTSSPGSFSFGQASTNTGGTLFGQN
NPPAFGQSPGFGQGSSVFGGTSATTSTAAPSGFSFCQASGFGSSNTGSVFGQAANTGGSVFGQQSSTSSGGVF
GSGNATRGGGFFSGLGGKPSQDAANKNPFSSAGGGFGSTAAPNTSNLFGNSGAKTFGGFGSSSFGEQKPAGTF
SSGGGSVASQGFGFSTPNKTGGFGAAPVFGSPPTFGGSPGFGGVPAFGSAPAFTSPLGSTGGKVFGEGTAAAS
AGGFGFGSSGNTASFGTLASQNAPTFGSLSQQTSGFGTPSSGFAGFGSGTGAFTFGSSNSSVLGFGGWRS*
>ENSMUSP00000142255_c.225:A>T_p.R76W ENSMUST00000152791.4|Nup214-210|ENSMUSG00000001855|+|chr2:g.31942139:A>T
GGSVASQGFGFSTPNKTGSGAAAILHPSVPSPVRTHPPSGPCPSRPQALGPRAVDSLGLDRAQEPSPLDHLTR
LSWASVAGEAELMSAFPQVPVSSCAHPSTATDCQKNFLMKTLSQEETLETKAQQCLTNCLFPAFKFYIKFFVC
Y*
>ENSMUSP00000050219_c.1608:T>C_p.W537R ENSMUST00000053087.4|Klhl23-201|ENSMUSG00000042155|+|chr2:g.69664261:T>C
MALKGQEDYIFHFKDSSHPVDFLDAFRTFYMDGLFTDITLQCPSGIIFHCHRAVLAACSNYFKAMFTADMKEK
FKSKIKLSGIHHDILEGLVNYAYTSQIEITKRNVQSLLEAADLLQFLSVKKACEQFLVRHLDIDNCIGMHSFA
EFHVCSELEKESRRILCSRFKEVWQQEEFLEISLEKFLFILSRKNLSVWKEEAILEPVIKWTAHDVENRIECI
YNLLSYINIDIDPVYLKTALGLQRSCLLTENKIRSLIYNALNPMHKEISQRSTATMYIIGGYYWHPLSEVHIW
DPLTNVWIQGAEIPDYTRESYGVTCLGPNIYVTGGYRTDNIDALDTVWIYNSEGDEWTEGLPMLNARYYHCAV
TLGGCVYALGGYRKGAPAEEAEFYDPLKEKWLPIANMIKGVGNATACVLHEVIYVIGGHCGYRGSCTYDKVQS

YNSDINEWSLITASPHPEYGLCSVPFENKLYLVGGQTTITECYDPEQNEWRETAPMMERRMECGAVIMNGCIY
VTGGYSYSKGTYLQSIEKYDPDLNKREIVGNLPSAMRSHGCVCVYNV*
>ENSMUSP00000011934_c.57582:A>C_p.K19195Q ENSMUST00000011934.13|Ttn-201|ENSMUSG00000051747|-|chr2:g.76568328:T>G
MTTQAPMFTQPLQSVVVLEGSTATFEAHVSGSPVPEVSWFRDGQVISTSTLPGVQISFSDGRARLMIPAVTKA
NSGRYSLRATNGSGQATSTAELLVTAETAPPNFSQRLQSMTVRQGSQVRLQVRVTGIPTPVVKFYRDGAEIQS
SLDFQISQEGDLYSLLIAEAYPEDSGTYSVNATNSVGRATSTAELVVQGEEVVPAKKTKTIVSTAQISETRQT
RIEKKIEAHFDARSIATVEMVIDGATGQLPHKTPPRIPPKPKSRSPTPPSIAAKAQLARQQSPSPIRHSPSPV
RHVRAPTPSPVRSVSPAGRISTSPIRSVKSPLLIRKTQTTTMATGPEVPPPWKQEGYVASSTEAEMRETTMTS
STQIRREERWEGRYGVQEQVTISGAAAAAASASVSSSFTAGAITTGTKEVKQETDKSAAVATVVAAVDMARVR
EPAISAVEQTAQRTTTTAVHIQPAQEQARKEAEKTAVTKVVVAADKAKEQELKSRTREVMVTTQEQTHISHEQ
IRKETEKAFVPKVVISATKAKEQETRITGEITTKQEQKRITQETMMKETRKTVVPKVIVATPKIKEQDLVSRS
REAITTKRDQVQITQEKKRKEVETTALSTIAVATAKAKEQETVLRSREAMATRQEHIQVTHGQVGVGKKAEAV
ATVVAAVDQARVREPREPTHVEESHSQQTTLEYGYKEHISTTKVPEQPRRPASEPHVVPQAVKPAVIQAPSET
HIKTTDQMGMHISSQVKKTTDISTERLVHVDKRPRTASPHFTVSKISVPKTEHGYEASIAGSAIATLQKELSA
TSSTQKITKSVKAPTVKPGETRVRAEPTPSPQFPFADMPPPDTYKSQAGIEVKKEVGVSISGSTVREEHFEVL
RGREAKVTETARVPAPAEVPVTPPTLVSGLKNVTVIEGESVTLECHISGYPSPKVTWYREDYQIESSIDFQIT
FQGGIARLMIREAFAEDSGRFTCSAVNEAGTVSTSCYLAVQVSEEFDKETTLTEKFATEEKRFVESRDVVMTD
TSITEEQAGPGEPAAPFFISKPVVQKLVEGGSVVFECQIGGNPKPHVYWKKSGVPLTTGYRYKVSYNKQTGEC
RLVISMTFADDAGEYTIVIRNKHGETSASASLLEEADYEALVKTQQEMLYQTQMSTFIQEPKVGEIAPGFAYS
EYEKEYEKEQALIRKKMAKDTVMVRTFVEDQEFHISSFEERLIKEIEYRIIKTTLEELLEEDGEEKMAVDISE
SEAIESGFDIRIKNYRILEGMGVTFHCKMSGYPLPKIAWYKDGKRIRHGERYQMDFLQDGRASLRIPVVLPED
EGIYTAFASNIKGNAICSGKLYVEPAAPFSAPTYMPTPEAVSRIRSVSPRSLSRSPIRMSPAMSPARMSPARM
SPARMSPARMSPGRRLEETDESQLERLYKPVFVLKPASFKCLEGQTARFDLKVVGRPMPETFWFHNGQQIVND
YTHKVVIKEDGTQSLIIVPASPSDSGEWTVVAQNRAGKSTISVTLTVEAVEHQIKPAFVEKLKNVNIKEGSRL
EMKVRATGNPNPDIVWLKNSDIIVPHKYPRIRIEGTRGEAALKIDSIISQDSAWYTATAINKAGRDTTRCKVN
VEVEFAEPEPERKLIIPRGTYRAKEIAAPELEPLHLRYGQEQWEEGDLYDKEKQQKPFFKKKLTSLRLKRFGP
AHFECRLTPIGDPTMVVEWLHDGKPLEAANRLRMINEFGYCSLDYGAAYSRDSGVITCRATNKYGTDHTSATL
IVKDEKSLVEESQLPDGKKGLQRIEELERMAHEGALTGVTTDQKEKQKPDIVLFPEPVRVLEGETARFRCRVT
GYPQPKVNWYLNGQLIRKSKRFRVRYDGIHYLDIVDCKSYDTGEVKVTAENPEGVTEHKVKLEIQQREDFRSV
LRRAPEPKPEFHVHEPGKLQFEVQKVDRPVDTSETKEVVKLKRAERITHEKVSEESEELRSKFKRRTEEGYYE
AITAVELKSRKKDESYEELLKKTKDELLHWTKELTEEEKKALAEEGKITIPTFKPERIELSPSMEAPKIFERI
QSQTVGQGSDAHFRVRVVGKPDPECEWYKNGVKIERSDRIYWYWPEDNVCELVIRDVTAEDSASIMVKAINIA
GETSSHAFLLVQAKQLITFTQELQDVVAKEKDTMATFECETSEPFIKVKWYKDGIEVHAGDKYRMHSDRKVHF
LSVLTIDTSDAEDYSCVLVEDENIKTTAKLIVEGAVVEFVKELQDIEVPESYSGELECIISPENIEGKWYHND
VELKSNGKYSITSRRGRQNLTVKDVTKEDQGEYSFVVDGKKTTCKLKMKPRPIAILQGLSDQKVCEGDIVQLE
VKVSLENVEGVWMKDGQEVQHSDRVHIVIDKQSHMLLIEDMTKEDAGNYSFTIPALGLSTSGNVSVYSVDVIT
PLKDVNVIEGTKAVLECKVSVPDVTSVKWYLNDEQIKPDDRVQSIVKGTKQRLVINRTHASDEGPYKLMVGRV
ETSCNLSVEKIKIIRGLRDLTCTETQNVVFEVELSHSGIDVVWNFKGKEIKPSSKYKIEAHGKIYKLTVLNMM
KDDEGEYAFYAGENTTSGKLTVAGGAISTPLTDQTVAESQEAVFECEVANPESEGEWLKDGKHLALSNNFRGE
SDGHKRRLVIAAAKLDDAGEYTYKVATSKTSAKLKVEAVKIKKTLRNLTVTETQDAVFSVELTHPDVKGVQWI
KNGVVLDSNDKYEISVKGTLYSLKIKNCAMADESVYGFKLGRLGASARLHVETVKIIKKPKDVTALENATVTF
EVSVSHDTVPVKWFHKNVEIKPSDKHRLVSERKVHKLMLQSISPSDAGEYTAMVGQLECKAKLFVETLHITKT
MKSIEVPETKAASFECEVSHFNVPSMWLKNGVEIEMSEKFKIVVQGKLHQLIIMNTSTEDSAEYTFVCGNDQV
SATLTVTPIMITSMLKDINAEEKDTITFEVTVNYEGISYKWLKNGVEIKSTDRCQMRTKKLTHSLNIRNVHFG
DAADYTFVAGKATSTATLYVEARHIEFRKHIKDIKVLEKKRAMFECEVSEPDITVQWMKDGQELQIADRIKIQ
KEKYVHRLLIPSTRMSDAGKYTVVAGGNMSTANLFVEGRDVRIRSIKKEVQVIEKQRAVVEFEVNEDDVDAHW
YKDGIEINFQVQERHQYVVERRIHRMFISETRHSDAGEYTFVAGRNRSSVTLYVNAPEPPQVLQELQPVTVQS
GKPARFCAVISGRPQPKISWYKEEQLLSTGFKCKFLHDGQEYTLLLIEAFPEDAAVYTCEAKNDYGVATTSAS
LSVEVPEVVSPDQEMPVYPPAIVTPLQDTVTSEGRPARFQCQVSGTDLKVSWYCKDKKIKPSRFFRMTQFEDT
YQLEIAEAYPEDEGTYAFVANNAVGQVSSTATLRLEAPESILHERIGQQIEMEMKEIASLLSAEEDFQTYSSD
LRLPNANETLELLSEPPARSTQFDSRQEGAAPVFIREISDVEISVEDVAKLSVTVTGCPKPKIQWFFNGMLLT
PSADYKFVFDGDTHSLIILFTRFQDEGEYTCLASNEYGKAVCSAHLRISPRGERSTEMESGEKKALEKPKGPC
PPYFFKELKPVHCGPGIPAVFEYSVHGEPAPTVLWFKEDMPLYTSVCYTIIHSPDGSGTFIVNDPQRGDSGLY
LCKAQNLWGESTCAAELLVLPEDTDVPDASCKEESTLGVPGDFLETSARGPLVQGVDSRQEITAFAEGTISKA
ALIAEETLQLSYERSVDDSEVGTGVTIGAQKLPPVVLSTPQGTGELPSIDGAVHTQPGRGPPPTLNLQAVQAQ
TTLPKEATLQFEEPEGVFPGASSAAQVSPVTIKPLITLTAEPKGNYPQSSTAAPDHALLSSVAAETLQLGEKK
IPEVDKAQRALLLSQSLAEGCVESLEVPDVAVSNMRSEPQVPFQHTCTEGKILMASADTLKSTGQDVALRTEE

GKSLSFPLALEEKQVLLKEEQSEVVAVPTSQTSKSEKEPEAIKGVKEVREQELLSKETLFPSMPEEQRLHLKT
QVRRALQAAVAREQANLFSEWLRNIDKVEVTAVNFTQEPKRILCTYLITSVSSLTEELTVTIEDIDPQMANLE
TGLKDALCSIVCEERNILMAEDPRIHEEDKIDVQGGRDHLSDAQKVETVIEAEADSKYLVSKEEVSWSKVESQ
LKDGDTNEVPQAETLKLAEESGTQKTSTEMSQEEAEGTLADLCPAVLKHLVDTISEEGDTVHLTSSISNAKEV
HWYFKGNLVPSDGKFKCLKEQNAYTLVIEAVKTEDEGEYVCEASNDSGKAKTSAKLTVGERAAPVIKRRIEPL
EVALGHLAKFTCEIQGAPNVRFQWFKAGREIYESDKCSIRSSNYVSSLEILRTQVVDCGEYTCKASNEYGSVS
CTATLTVTVPTAEKKVRKLLPEPKPQPKEEVVLKSVLRKKPEEEEPKVEPKKVEKAKKPEEPQPPPKAVEVEA
PPEPTPKERKVPEPAKVPEIKPAIPLPGPEPKPKPEPEVKTMKAPPIEPAPTPIAAPVTAPVVGKKAEAKPKD
EAAKPKGPIKGVAKKTPSPIEAERKKLRPGSGGEKPPDEAPFTYQLKAVPLKFVKEIKDIVLTEAESVGSSAI
FECLVSPSTAITTWMKDGSNIRESPKHRFIADGKDRKLHIIDVQLSDAGEYTCVLRLGNKEKTSTAKLIVEEL
PVRFVKTLEEEVTVVKGQPLYLSCELNKERDVVWRKDGKIVVEKPGRIVPGVIGLMRALTINDADDTDAGTYT
VTVENANNLECSSCVKVVEIIREWLVKPIRDQHVKPKGTAVFTCDIAKDTPNIKWFKGYDEIPLEPNDKTEIL
KEGNHLFLKVKNAMPEDIDEYAVEIEGKRYPAKLTLGEREVELLKPIEDVTIYEKESASFDAEISEEDIPGEW
KLKGELLRPSPTCEIKAEGGKRFLTLHKVKLDQAGEVLYQACNAITTAILTVKEIELDFAVPLKDVTVPEKRQ
ARFECVLTREANVIWSKGPDIIKASDKFDIIADGKKHILVINDSQFDDEGVYTAEVEGKKTSAQLFVTGIRLK
FISPLEDQTVKEGQTATFVCELSHEKMHVVWFKNDVKLHTTRTVLMSSEGKTYKLEIRETTLDDISQIKAQVK
NLSSTANLKVLEADPYFTVKLHDKTGVEKDEIILKCEVSKDVPVKWFKDGEEIVPSPKHSVKTDGLRRILKIK
KAELKDKGEYVCDCGTDTTKANVTVEARLIKVEKPLYGVEVFVGETARFEIELSEPDVHGQWKLKGEPLTASP
DCEIIEDGKKHVLVLYNCQLDMTGEISFQAANAKSAANLKVKELPLIFITPLSDVKVFEKDEAKFECEVSREP
KTFRWLKGTQEITGDDRFELIKDGTRHSLVIKSAAFEDEAKYMFEAEDKRTSGKLIIEGIRLKFLTPLKDVTA
KERENAVFTVELSHDNIPVSWFKNDQRLHTSKRVSMHDEGKTHSITFKDLSIDDTSQIRVEAMGISSEAKLTV
LEGDPYFTGKLQDYTGVEKDEVILQCEISKADAPVKWFKDGKEIKPSKNVVIKADGKKRMLILKKALKSDIGQ
YTCDCGTDQTSGKLDIEDREIKLVRPLYSVEVMETETARFETEISEDDIHANWKLKGEALLQTPECEIKEEGK
IHVLILHNCRLDQTGGVDFQAANVKSSAHLRVKPRVIGLLRPLKDVTVTAGETATFDCELSYEDIPVEWYLKG
KKLEPNDKVVTRSEGRVHTLTLRDVKLEDAGEVQLTAKDFKTQANLFVKEPPVEFTKPLEDQTVEEEATAVLE
CEVSRENAKVKWFKNGTEILKSKKYEIVADGRVRKLIIHGCTPEDIKTYTCDAKDFKTSCNLNVVPPHVEFLR
PLTDLQVKEKETARFECEISKENEKVQWFKDGAEIKKGKKYDIISKGAVRILVINKCLLNDEAEYSCEVRTAR
TSGMLTVLEEEAVFTKNLANLEVSEGDTIKLVCEVSKPGAEVIWYKGDEEIIETGRFEILTDGRKRILIIQNA
QLEDAGSYNCRLPSSRTDSKVKVHELAAEFISKPQNLEILEGEKAEFVCTISKESFEVQWKRDDQTLESGDKY
DIIADGKKRVLVVKDATLQDMGTYVVMVGAARAAAHLTVIEKLRIIVPLKDTKVKEQQEVVFNCEVNTEGAKA
KWFRNEEAIFDSSKYIILQKDLVYTLRIRDARLDDQANFNVSLTNHRGENVKSAANLIVEEEDLRIVEPLKDI
ETMEKKSVTFWCKVNRLNVTLKWTKNGEEVAFDNRISYRIDKYKHSLIIKDCGFPDEGEYVVTAGQDKSVAEL
LIIEAPTEFVEHLEDQTVTEFDDAVFSCQLSREKANVKWYRNGREIKEGKKYKFEKDGSIHRLIIKDCRLEDE
CEYACGVEDRKSRARLFVEEIPVEIIRPPQDILEAPGADVIFLAELNKDKVEVQWLRNNMIVVQGDKHQMMSE
GKIHRLQICDIKPRDQGEYRFIAKDKEARAKLELAAAPKIKTADQDLVVDAGQPLTMVVPYDAYPKAEAEWFK
ENEPLSTKTVDTTAEQTSFRISEAKKDDKGRYKIVLQNKHGKAEGFINLQVIDVPGPVRNLEVTETFDGEVSL
AWEEPLTDGGSKIIGYVVERRDIKRKTWVLVTDRADSCEFTVTGLQKGGVEYLFRVSARNRVGTGEPVETDSP
VEARSKYDVPGPPLNVTITDVNRFGVSLTWEPPEYDGGAEITNYVIELRDKTSIRWDTAMTVRAEDLSATVTD
VVEGQEYSFRVRAQNRIGVGKPSAATPFVKVADPIERPSPPVNLNASEQTQSSVQLTWEPPLKDGGSPILGYI
IERREEGKDNWIRCNMKPVPELTYKVTGLQKGNKYLYRVSAENAAGVSDPSEILGPLTADDAFVEPTMDLSAF
KDGLEVIVPNPIKILVPSTGYPRPKATWTFGDQVLEEGDRVKMKTISAYAELVISPSERTDKGIYTLTLENPV
KSISGEINVNVIAPPSAPKELKFSDITKDSVHLTWEPPDDDGGSPLTGYVVEKRDMSRKTWTKVMDFVTDLEF
TVPDLVQGKEYLFKVCARNKCGPGEPAYTDEPVNMSAPATVPDPPENVKWRDRTANSIFLTWDPPKNDGGSRI
KGYIVEKCPRGSDKWVACGEPVPDTKMEVTGLEEGKWYAYRVKALNRQGASKPSKPTEEIQAVDTQEAPEIFL
DVKLLAGITVKAGTKIELPATVTGKPEPKITWTKADTLLKPDQRITIENVPKKSTVTITDSKRSDTGTYIIEA
VNVCGRATAVVEVNVLDKPGPPAAFDITDVTNESCLLTWNPPRDDGGSKITNYVVERKATDSDVWHKLSSTVK
DTNFKATKLTPNKEYIFRVAAENMYGVGEPVQAAPIIAKYQFDPPGPPTRLEPSDITKDAVTLTWCEPDDDGG
SPITGYWVERLDPDTDKWVRCNKMPVKDTTYRVKGLTNKKKYRFRVLAENLAGPGKPSRSTEPILIKDPIDPP
WPPGKPTVKDIGKTSLVLNWTKPEHDGGAKIESYVIEMLKTGTDEWVRVAEGVPTTEHLLTGLMEGQEYSFRV
RAVNKAGESEPSEPSDPVLCREKLYPPSPPRWLEVINITKNTADLKWTVPEKDGGSPITNYIVEKRDVRRKGW
QTVDTTVKDTKCTVTPLTEGSLYVFRVAAENAIGQSDYTEIGDSVLAKDTFTTPGPPYALTVVDVTKRHVDLK
WEPPKNDGGRPIQRYIIEKKEKLGTRWVKAGKTSGPDCNFRVTDVIEGTEVQFQVRAENEAGVGHPSEPTEIL
SIEDPTSPPSPPLDLHVTDAGRKHIAIAWKPPEKNGGSPIIGYHVEMCPVGTEKWMRVNSRPIKDLKFKVEEG
IVPDKEYVLRVRAVNAVGVSEPSEISENVVAKDPDCKPTIDLETHDIVVIEGEKLNIPVPFRAVPVPTVSWHK
DGKEVKASDRLTMKNDHISAHLEVPKSVHADAGVYTITLENKLGSATASINVKVIGLPGPCKDIKASDITKSS
CKLTWEPPEFDGGSPILHYVLERREAGRRTYIPVMSGENKLSWTVKDLIPNGEYFFRVKAVNKIGGGEYIELK
NPVIAQDPKQPPDPPVDVEVHNPTAKAMTITWKPPLYDGGSKIMGYIIEKITKGEDRWKRCNEHLVPVLTYTA
KGLEEGKEYQFRVRAENAAGIGEPSRATPPTKAVDPIDAPKVILRTSLEVKRGDEIALDATISGSPYPTITWI

KDENVIVPEEIKKRAAPPVRRKKGEAEEEEPFSLPLTERLSINNSKQGESQLRIRDSLRPDHGQYMIKVENDH
GVAKAPCSVSVLDTPGPPINFVFEDIRKDSVLCKWEPPLDDGGSEIINYTLEKKDKTKPDSDWIVITSTLRNC
KYSVTKLIEGKEYLFRVRAENRFGPGPPCVSKPLLAKDPFEPPDAPDKPIVEDVTSNSMLVKWNEPKDNGSPI
LGYWLEKREVNSTHWSRVNKTLLSSLKTKVDGLLEGLTYVFRVCAENAAGPGKFSPPSDPKTARDPISPPGPP
VPRVADTSSTTIELEWEPPAFNGGGEIMGYFVDKQLVGTNEWSRCTEKMIKVRQFTVKEIREGADYKLRVSAV
NAAGEGPPGETEPVTVAEPQEPPTVELDVSVKGGIQIMAGKTLRIPAEVTGRPVPTKVWTIEEGELDKERVII
ENVGTKSELIIKNALRKDHGRYVITATNSCGSKFAAVRVEVFDVPGPVLDLKPVVTNRKMCLLNWSDPADDGG
SDITGFIIERKDAKMHTWRQPIETERSKCDITGLIEGQEYKFRVIAKNKFGCGPPVEIGPILAVDPLGPPTSP
ERLTYTERTKSTITLDWKEPRSDGGSPIQGYIIEKRRHDKPDFERVNKRLCPTTSFLVENLDEHQMYEFRVKA
VNDVGESEPSLPLNVVIQDDEVPPTIKLRLAVRGDTIKVKAGEPVNIPADVTGLPMPKIEWSKNEKVIDKPTD
TLNITKEEVSRSEAKTELSIPKAAREDKGTYTITASNRLGSVFRNVHVEVYDRPSPPRNLAVTDIKAESCYLT
WDAPLDNGGSEITHYIIDKRDASRKKSEWEEVTNTAVERRYGIWKLIPNGQYEFRVRAVNKYGISDECKSDKV
VIQDPYRLPGPPGKPKVLERTKGSMLVSWTPPLDNGGSPITGYWLEKREEGGTYWSRVSRAPITKVGLKGVEF
NVPRLIEGVKYQFRAMAINAAGIGPPSEPSDPAVAGDPIYPPGPPSCPEVKDKTKSSISLAWKPPAKDGGSPI
KGYIVEMQEEGTTDWKPVNEPDKLLTACECVVPNLKELRKYRFRVKAVNEAGESEPSDTTGEIPATDIQEVPE
VFIDIGAQDCLVCKAGSQVKIPAVIKGRPTPKSSWEFDGKAKKAMKDGVHDIPEDAQLETAENSSVIIIPECT
RAHSGKYSITAKNKAGQKTANCRVKVMDAPGPPKDLKVSDITRGSCRLSWKMPDDDGGDRIKGYVIEKKTIDG
KAWTKVNPNCGSTTFVVPDLISEQQYFFRVRAENRFGIGPPAETIQRTTARDPIYPPDPPIKLKIGLITKNTV
HLSWKPPKNDGGSPVTHYIVECLAWDPTGKKKEAWRQCNRRDVEELEFTVEDLIEGGEYEFRVKAVNEAGVSK
PSATVGPVIVKDQTCPPAIELKEFMEVEEGTDVNIVAKIKGVPFPTLTWFKAPPKKPDSKEPVVYDTHVNKQV
VDDTCTLVIPQSRRSDTGLYSITAVNNLGTASKEMRLNVLGRPGPPVGPIKFESISADQMTLSWLPPKDDGGS
KITNYVIEKREANRKTWVRVSSEPKECMYTIPKLLEGHEYVFRIMAQNKYGIGEPLDSEPETARNLFSVPGAP
DKPTVSSVTRNSMTVNWEEPEYDGGSPVTGYWLEMKDTTSKRWKRVNRDPIKAMTLGVSYKVTGLIEGSDYQF
RVYAINAAGVGPASLPSDPVTARDPVAPPGPPFPKVTDWTKSSVDLEWSPPLKDGGSKITGYIVEYKEEGKEE
WEKGKDKEVRGTKLVVTGLKEGAFYKFRVRAVNVAGVGEPGEVTDVIEMKDRIVSPDLQLDASVRDRIVVHAG
GVIRIIAYVSGKPPPTVTWSMNERALPQEAAIETTAISSSMVIKNCQRSHQGVYSLLAKNEGGERKKTIIVDV
LDVPGPVGIPFLSDNLTNDSCKLTWFSPEDDGGSPITNYVIQKREADRRAWTPVTYTVTRQNATVQGLIQGKS
YFFRIAAENSIGMGPFVETPNALVIRDPITVPERPEDLEVKEVTKNTVSLTWNPPKYDGGSEIINYVLESRLI
GTEKFHKVTNDNLLSRKYTVKGLKEGDTYEYRVSAVNIVGQGKPSFCTKPITCKDELAPPTLDLDFRDKLTVR
VGESFALTGRYSGKPKPKIDWFKDEADVLEDDRTHIKTTPTTLALEKTKAKRSDSGKYCVVVENSTGSRKGFC
QVNVVDRPGPPVGPVIFDEVTKEYMVISWKPPLDDGGSEITNYIIEKKELGKDIWMPVTSASAKTTCKVPKLL
EGKDYIFRIHAENLYGISDPLVSDSMKAKDRFRVPDAPEQPVVTEVTKDSALVTWNKPNDGGKPITNYILEKR
ETMSKRWVRVTKEPIHPYTKYRVPDLLEGCQYEFRVSAENEIGIGDPSPPSKPVFARDPIAKPSPPINPEAID
TTCNSVELTWQPPRHDGGSKILGYIVEYQKVGDEEWRRANHTPESCPETKYKVTGLRDGQSYKFRVLAVNEAG
ESDPAHVPEPVLVKDRLEPPELILDANMAREQHIRVGDTLRLSAIIKGVPFPKVTWKKEDREAPTKAQIDVTP
VGSKLEIRNAAHEDGGIYSLTVENPAGTKTVSVKVLVLDKPGPPRDLEVSEIRKDSCYLTWKEPLDDGGSVVT
NYVVERKDVATAQWSPLSTTSKKKSHMAKHLTEGNQYLFRVAAENQYGRGPFVETPKPIKALDPLHPPGPPKD
LHHVDVDKTEVSLVWNKPDRDGGSPITGYLVEYQEEGDKDWIKFKTVKNLDCVVTGLQQGKTYRFRVKAENII
GLGLPDTTIPIECQEKLVPPSVELDVKLIEGLVVKAGTTVRFPAIIRGVPVPTAKWTTDGTEIKTDDHYTVET
DSFSSVLTIKNCLRKDTGEYQLTVSNAAGTKTVAVHLTVLDVPGPPTGPINILDVTPEYMTISWQPPKDDGGS
PVINYIVEKQDTRKGTWGVVSAGSSKLKLKVPHLQKGCEYVFRVKAENKMGVGPPLDSIPTVAKHKFSPPSPP
GKPVVTDITENAATVSWTLPKSDGGSPITGYYVERREITGKWVRVNKTPIADLKFRVTGLYEGNTYEFRVFAE
NLAGLSNPSPSSDPIKACRPIKPPGPPINPKLKDKSKESADLVWTKPLSDGGSPILGYVVEYQKPGTAQWDRI
NKDELIRQCAFRVPGLIEGNEYRFRIRAANIVGEGEPRELAESVIAKDILHPPEVELDVTCRDVITVRVGQTI
RILARVKGRPEPDITWSKEGKVLVKDKRVDLIHDLPRVELQIKEAVRADHGKYIISAKNSSGHAQGSAIVNVL
DRPGPCQNLKVSNVTKENCTISWENPLDNGGSEITNFIVEYRKPNQKGWSIVASDVTKRLVKANLLANNEYYF
RVCAENKVGVGPTIETKTPILAINPIDRPGEPENLHIADKGKTFVYLKWRRPDYDGGSPNLSYHVERRLKGSA
DWERVHKGSIKETHYMVDKCVENQIYEFRVQTKNEGGESDWVRTEEVVVKEDLQKPVLDLKLSGVLTVKAGDT
IRLEAGVRGKPFPEVAWTKDKDATDLTRSPRVKIDTSAESSKFSLTKAKRSDGGKYVITATNPAGSFVAYATV
NVLDKPGPVRNLKITDVSSDRCTIRWDPPEDDGGCEIQNYILEKCESKRMVWSTYSANVLTPSATVTRLIEGN
EYIFRVRAENKIGTGPPTESKPVIAKTKYDRPGRPDPPEVTKVSKEEMTVVWNAPEYDGGKSITGYYLEKKEK
HAVRWVPVNKSAIPERRLKVQNLLPGHEYQFRVKAENEVGIGEPSLPSRPVVAKDPIEPPGPPTNFKVVDTTK
SSITLAWGKPVYDGGAPIIGYVVEMRPKIADASPDEGWKRCNAAAQLIRMEFTVTSLDENQEYEFRVCAQNQV
GIGRPAELKEAIKPKEILEPPEIDLDASMRKLVVVRAGCPIRLFAIVRGRPAPKVTWRKVGIDNVVRKGQVDL
VDTMAFLVIPNSTRDDSGKYSLTLVNPAGEKAVFVNVKVLDTPGPVADLKVSDVTKTSCHVSWAPPENDGGSQ
VTHYIVEKREAERKTWSTVTPEVKKTSFNVTNLVPGNEYFFRVTAVNEYGPGVPTDIPKPVLASDPLSEPDPP
RKLEVTEMTKNSATLAWLPPLRDGGAKIDGYIISYREEDQPADRWTEYSVVKDLSLIVTGLKEGKKYKFRVAA
RNAVGVSMPREAEGVYEAKEQLLPPKILMPEQITIKAGKKLRVEAHVYGKPNPICKWKKGDDEVVTSSHLAIH

KADGSSVLIIKDVTRKDSGYYSLTAENSSGSDTQKIKVTVMDAPGPPQPPFDISEIDADACSLSWHIPLEDGG
SNITNYIVEKCDVSRGDWVTALASVTKTSCRVGKLIPGQEYIFRVRAENRFGISEPLTSPKMLAKFPFDVPSE
PKNARVTKVNKDCIFVAWDRPDSDGGSPITGYLIERKERNSLLWVKANDTIVRSTEYPCAGLVEGLEYSFRIY
ALNKAGSSPPSKPTEYVTARMPVDPPGKPEVVDVTKNSASLIWARPKHDGGSRIIGYFVEACKLPGDKWVRCN
TTPHQIPQEEYTATGLEENAQYQFRAIAKTAVNISQPSEPSDPVTILAENVPPRIELSVEMKSLLTVKAGTNV
CLDATVFGKPMPTVSWKKDTTPIKQAEGIKMAMKRNLCTLELFSVNRKDSGDYTITAENSSGSKSATIKLKVL
DKPGPPASVKINKMYADRAMLSWEPPLEDGGSEITNYIIDKRETSRPNWAQVSATVPITSCTVEKLIEGHEYQ
FRICAENKYGVGDPILTEPAIAKNPYDPPGRCDPPVISNITKDHMTVSWKAPADDGGSPITGYLVEKRETQAV
NWTKVNRKPVIERTLKATGLQEGTEYEFRVTAINKAGPGKPSDASKAVYAQDPLYPPGPPAFPKVYDTTRSSV
SLSWGKPAYDGGSPIIGYLVEVKRADSDHWVRCNLPEKLQKTRFEVTGLMENTEYQFRVYAVNKIGYSDPSDV
PDKHCPKDILIPPEGELDAELRKTLILRAGVTMRLYVPVKGRPPPKITWSKPNVNLRERIGLDIKSTDFDTFL
RCENVNKYDAGKYILTLENSCGKKEYTIVVKVLDTPGPPVNVTVKEVSKDSAYVTWDPPIIDGGSPIINYVVE
KRDAERKSWSTVTTECSKTSFRVSNLEEGKSYFFRVFAENEYGIGDPGETRDAVKASETPGPVVDLKALAITK
SSCTIGWKKPRSDGGSRITGYVVDFLTEENKWQRVMKSLSLQYSTKDLKEGKEYTFRVSAENENGEGTPSEIV
VVAKDDVVAPDLDLKDLPDLCYLAKENSNFRLKIPIKGKPAPSVSWKKGEDPLATDTRVSVESTAVNTTLVVY
DCQKSDAGKYTITLKNVAGTKEGTLSIKVVGKPGIPTGPIKFDEVTAEAMTLKWGPPKDDGGSEITNYVLEKR
DSVNNKWVTCASAVQKTTFRVTRLHEGIEYTFRVSAENKYGVGEGLKSEPIVAKHPFDVPDAPPPPNIVDVRH
DSVSLTWTDPKKTGGSPITGYHIEFKERNSLLWKRANKTPIRMKDFKVTGLTEGLEYEFRVMAINLAGVGKPS
LPSEPVVALDPIDPPGKPEVISVTRNSVTLIWTEPKYDGGHKLTGYIVEKRDLPSKSWMKANHVNVPDCAFTV
TDLVEGGKYEFRIRAKNTAGAISAPSESTGTIICKDEYEAPTIVLDPTIKDGLTVKAGDSIVLSAISILGKPL
PKSSWSRAGKDIRPSDIAQITSTPTSSMLTVKYATRKDAGEYTITATNPFGTKEEHVKVSVLDVPGPPGPIEI
SNVSAEKATLTWTPPLEDGGSPIKAYVLEKRETSRLLWTVVSEDIQACRHVVTKLIQGNEYLFRVSAVNHYGK
GEPVQSEPVKMVDRFGPPGPPGKPEISNVTKNTATVSWKRPTDDGGSEITGYHVERREKKGLRWVRATKTPVS
DLRCKVTGLQEGNTYEFRVSAENRAGIGPPSDASNPVLMKDVAYPPGPPSNAHVTDTTKKSASLAWGKPHYDG
GLEITGYVVEHQKVGDDAWIKDTTGTALRITQFVVPDLQTKEKYNFRISAINDAGVGEPAVIPNVEIVEKEVA
PDFELDAELRRTLVVRAGLSIRIFVPIKGRPAPEVTWTKDNINLKHRANIENTESFTLLIIPECNRYDTGKFV
MTIENPAGKKSGFVNVRVLDTPGPVLNLRPTDITKDSVTLHWDLPLIDGGSRITNYIVEKREATRKSYSTVTT
KCHKCTYKVTGLTEGCEYFFRVMAENEYGVGEPTETTEPVRASEAPLPPDSLNIMDITKNTVSLAWPKPRHDG
GSKITGYVIEAQRKGSDQWTHISTVKGLECVVRNLTEGEEYTFQVMAVNSAGRSAPRESRPVIVKEQTMLPEL
DLRGIYQKLVIARAGDNIKVEIPVLGRPKPTVTWKKGDQILKQTQRVNVENTATSTILNINECVRSDSGPYPL
TAKNTVGEVGDVITIQVHDIPGPPTGPIKFDEVSSDFVTFSWEPPENDGGVPISNYVVEMRQTDSTTWVELAT
TVIRTTYKATRLTTGVEYQFRVRAQNRYGVGPGITSASVVANYPFKVPGPPGTPQVTAVTKDSMTISWHEPLS
DGGSPILGYHIERKERNGILWQTVSKALVPGNIFKSTGLTDGIAYEFRVIAENMAGKSKPSKPSEPMFALDPI
DPPGKPVPLNITRHTVALKWAKPEYTGGFKITSYVVEKRDLPNGRWLKANFSNILENEFTVSGLTEDAAYEFR
VIAKNAAGAISPPSEPSDAITCRDDLEAPRIMVDVRFKDTITLKAGEAFKLEADVSGRPPPTMEWTKDGKELE
GTGKLEIKIADFSTHLINKDSSRTDSGAYILTATNPGGFAKHIFNVKVLDRPGPPEGPLAVSDVTSEKCVLSW
LPPLDDGGAKIDHYIVQKRETSRLAWTNVATEVQVTKLKVTKLLKGNEYIFRVMAVNKYGVGEPLESEPVLAV
DPYGPPDPPKNPEVTTITKDSMVVCWGHPDSDGGSEIINYIVERRDKAGQRWVKCNKKALTDLRFKVSGLTEG
HEYEFRIMAENAAGISAPSATSPFYKACDTVFKPGPPGNPRVLDTSRSSISIAWNKPIYDGGSEITGYMVEIA
LPEEDEWQVVTPPAGLKATSYTITSLIENQEYKIRIYAMNSEGLGEPALVPGTPKAEERMLPPEIELDADLRK
VVTIRACCTLRLFVPIKGRPAPEVKWAREHGESLDKASIESTSSYTLLVVGNVNRFDSGKYILTVENSSGSKS
AFVNVRVLDTPGPPQNLKIKEVTKTSVTLTWEPPLLDGGSKIKNYIVEKRESTRKAYSTVATNCHKTSWKVDQ
LQEGCSYYFRVLAENEYGIGLPAETAESVKASERPLPPGKITLTDVTRNSVSLSWEKPEHDGGSRILGYIVEM
QSKGSDRWATCATVKVTEATITGLIQGEEYSFRVSAQNEKGISDPRQLSVPVIAKDLVIPPAFKLLFNTFTVL
AGEDLKIDVPFIGRPPPAVTWHKDDIPLKQTTRVNAESTENNSLLTIKEACREDVGHYTVKLTNSAGEATETL
NVIVLDKPGPPTGPVKMDEVTADSVTLSWEPPKYDGGSSINNYIVEKRDTSTTAWQIVSATVARTTIKACRLK
TGCEYQFRIAAENRYGKSTYLNSEPVVAQYPFKVPGPPGTPFVTLASKDSMEVQWHEPVSDGGSKVIGYHLER
KERNSILWVKLNKTPIPQTKFKTTGLEEGIEYEFRVSAENIVGIGKPSKPSECYAAHDPCDPPGRPEAIIVTR
NSVTLQWKKPTYDGGSKITGYIVEKKELPDGRWMKASFTNIIDTQFEVTGLLEDHRYEFRVIARNAAGVFSEP
SESTGAITARDEVEPPRISMDPKYRDTVVVQAGESFKIDADIYGKPIPTTQWVKGDQELSSTARLEIKSTDFA
TSLSVKDAVRVDSGNYILKAKNVAGEKSVTINVKVLDRPGPPEGPVAISGVTAEKCTLAWKPPLQDGGSDITN
YIVERRETSRLVWTLVDANVQTLSCKVLKLLEGNEYIFRIMAVNKYGVGEPLESESLIAKNPFVVPDAPKAPE
VTAVTKDSMIVVWERPASDGGSEILGYVLEKRDKEGIRWTRCHKRLIGELRLRVTGLLENHNYEFRVSAENAA
GLSEPSPPSAYQKACDPIYKPGPPNNPKVMDVTRSSVFLSWTKPIYDGGCEIQGYIVEKCDVSVGEWTMCTPP
TGINKTNLEVEKLLEKHEYNFRICAINKAGVGEHADVPGPVMVEEKLEAPDIDLDLELRKVINIRAGGSLRLF
VPIKGRPTPEVKWGKVDGDIRDAAIIDVTSSFTSLVLDNVNRYDSGKYTLTLENSSGTKSAFVTVRVLDTPSP
PVNLKVTEITKDSVSITWEPPLLDGGSKIKNYIVEKREATRKSYAAVVTNCHKNSWKIDQLQEGCSYYFRVTA
ENEYGIGLPARTADPIKVAEVPQPPGKITVDDVTRNSVSLSWTKPEHDGGSKIIQYIVEMQAKNTDKWSECAR

VKSLDAVITNLTQGEEYLFRVIAVNEKGRSDPRSLAVPIIAKDLVIEPDVRPAFSSYSVQVGQDLKIEVPISG
RPKPSISWTKDGMPLKQTTRINVTDSLDLTTLSIKETHKDDGGQYGITVSNVVGQKTAAIEIITLDKPDPPKG
PVKFDEISAESITLSWNPPLYTGGCQITNYIVQKRDTTTTVWDVVSATVARTTLKVTKLKTGTEYQFRIFAEN
RYGQSFALESEPVVAQYPYKEPGPPGTPFVTAISKESMVVQWHEPINNGGSPVIGYHLERKERNSILWTKVNK
TIIHDTQFKALNLEEGIEYEFRVYAENIVGVGKASKNSECYVARDPCDPPGTPEAIIVKRNEITLQWTKPVYD
GGSMITGYIVEKRDLPEGRWMKASFTNVIETQFTVSGLTEDQRYEFRVIAKNAAGAISKPSDSTGPITAKDEV
ELPRISMDPKFRDTIVVNAGETFRLEADVHGKPLPTIEWLRGDKEIEESARCEIKNTDFKALLIVKDAIRIDG
GQYILRASNVAGSKSFPVNVKVLDRPGPPEGPVQVTGVTAEKCTLAWSPPLQDGGSDISHYVVEKRETSRLAW
TVVASEVVTNSLKVTKLLEGNKYIFRIMAVNKYGVGEPLESAPVLMKNPFVLPGPPKSLEVTNIAKDSMTVCW
NRPDSDGGSEIIGYIVEKRDRSGIRWIKCNKRRITDLRLRVTGLTEDHEYEFRVSAENAAGVGEPSPATVYYK
ACDPVFKPGPPTNAHVVDTTKNSITLAWSKPIYDGGSEILGYVVEICKADEEEWQIVTPQTGLRVTRFEIAKL
IEHQEYKIRVCALNKVGLGEAASVPGTVKPEDKLEAPELDLDSELRKGIVVRAGGSARIHIPFKGRPTPEITW
SKEEGEFTDKVQIEKGINFTQLSIDNCDRNDAGKYILKLENSSGSKSAFVTVKVLDTPGPPQNLAVKEVRKDS
VLLVWEPPIIDGGAKVKNYVIDKRESTRKAYANVSSKCNKTSFRVENLTEGAIYYFRVMAENEFGVGVPTETS
DAVKASEPPSPPGKVTLTDVSQTSASLMWEKPEHDGGSRILGYVVEMQPKGTEKWSVVAESKVCNAVVSGLSS
GQEYQFRVKAYNEKGKSDPRVLGIPVIAKDLTIQPSFKLPFNTYSVQAGEDLKIEIPVIGRPRPKISWVKDGE
PLKQTTRVNVEETATSTILHIKESSKDDFGKYSVTATNSAGTATENLSVIVLEKPGPPVGPVKFDEVSADFVV
ISWEPPAYTGGCQISNYIVEKRDTTTTNWQMVSATVARTTIKISKLKTGTEYQFRIFAENRYGKSTPLDSKPV
VVQYPFKEPGPPGTPFVTSISKDQMLVQWHEPVNDGGSKVTGYHLEQKEKNSILWVKLNKIPIQDTKFKTTGL
DEGLEYEFRVSAENIVGIGKPSKVSECYVARDPCDPPGRPEAIVITRNSVTLKWKKPVYDGGSKITGYIVEKK
DLPDGRWMKASFTNVVETEFTVTGLVEDQRYEFRVIARNAADNFSEPSESSGAITARDEIDAPNASLDPKYRD
VIIVHAGETFVLEADIRGKPIPDIIWSKDGNELEETAARMEIKSTLQKTTLIVKDCIRTDGGQYTLKLSNVGG
TKTIPITVKVLDRPGPPEGPLKVTGVTAEKCYLAWNPPLQDGGASISHYIIEKRETSRLSWTQVSNEVQALNY
KVTKLLPGNEYIFRVMAVNKYGIGEALESEPVIACNPYKRPGPPSTPEASAITKDSMVLTWTRPVDDGGAEIE
GYILEKRDKEGIRWTKCNKKTLTDLRFRVTGLTEGHSYEFRVAAENAAGVGEPSEPSVFYRACDALYPPGPPS
NPKVTDTSRSSVSLAWNKPIYDGGAPVRGYVIELKKAAADEWTTCTPPSGLQGKQFTVTKLKENTEYNFRICA
FNTEGVGEPATIPGSVVAQERMEAPEIELDADLRKVVTLRASATLRLFVTIKGRPEPEVKWEKAEGILTERAQ
IEVTSSYTMLVIDNVTRFDSGRYNLTLENNSGSKTAFVNVRVLDSPSAPVNLTIREVKKDSVTLSWEPPLIDG
GAKITNYIVEKRETTRKAYATITNNCTKNTFKIENLQEGCSYYFRVLASNEYGIGLPAETAEPVKVSEPPLPP
GRVTLVDVTRNTATIKWEKPESDGGSKITGYVVEMQTKGSEKWSACTQVKTLETTISGLTAGEEYVFRVAAVN
EKGRSDPRQLGVPVIAKDIEIKPSVELPFNTFNVKANDQLKIDIPFKGRPQATVAWKKDGQVLRETTRVNVAS
SKTVTTLSIKEASREDVGTYELCVSNTAGSITVPITVIVLDRPGPPGPIRIDEVSCDNVSISWNPPEYDGGCQ
ISNYIVEKRETTSTTWQVVSQAVARTSIKIVRLTTGSEYQFRVCAENRYGKSSYSESSAVVAEYPFSPPGPPG
TPKVVHATKSTMVVSWQVPVNDGGSQVIGYHLEYKERSSILWSKANKVLIADTQMKVSGLDEGLMYEYRVYAE
NIAGIGKCSKACEPVPARDPCDPPGQPEVTNITRKSVSLKWSKPRYDGGAKITGYIVERRELPDGRWLQCNFT
NVQETYFEVTELTEDQRYEFRVFARNAADSVSEPSESTGPITVKDDVEAPRIMMDVKFRDVIIVKAGEVLKIN
ADIAGRPLPVISWAKDGVEIEERAKTEIVSTDYTTTLTVKDCVRRDTGQYVLTLKNVAGTRTMAVNCKVLDKP
GPPAGPLEINGLTAEKCSLSWGRPQEDGGADIDYYIVEKRETSRLAWTICEAELRTTSCKVTKLLKGNEYIFR
VTGVNKYGVGEPLESMAVKALDPFTTPSPPTSLEITSVTKDSMTLCWSRPETDGGSDISGYIIERREKNSLRW
MRVNKKPVYDLRVKSTGLREGCEYEYRVFAENAAGLSLPSETSPLVRAEDPVFLPSPPSKPKIVDSGKTTITI
GWVKPLFDGGAPITGYTVEYKKSEETDWKVAIQSFRGTEYTMSGLTTGDEYVFRVRSLNKMGASDPSDSSDPQ
VAKEREEEPVFDVDSEMRKTLIVKAGSSFTMTVPFRGRPIPNVSWSKPDTDLRTRAYIDSTDSRTLLTIENAN
RNDSGKYTLTIQNVLSAASMTFVVKVLDSPGPPANITVREVTKETAMLSWDVPENDGGAPVKNYHIEKREASK
KAWVSVTNNCNRLSYKVTNLQEGAIYYFRVSGENEFGVGVPAETKEGVKITEKPSPPEKLGVTSVSKDSVSLS
WLKPEHDGGSRIIHYVVEALEKGQKTWVKCAVVKTTHHVVSGLRESHEYFFRVFAENQAGLSDPRELLLPVLI
KDQLEPPEIDMKNFPSHTVYVRAGSNLKVDIPISGKPLPKVTLSRDGVPLKATMRFNTEITAENLTINLKESV
TTDAGRYEITAANSSGTTKTFINIIVLDRPGPPTGPVAISDITEESVTLKWEPPKYDGGSHVTNYIVLKRETS
TAVWSEVSATVARTMIKVMKLTTGEEYQFRIKAENRFGISDHIDSVCVVVKLPYTTPGPPSTPWVSNVTRESI
TVGWHEPVSNGGSAVTGYHLEMKDRNSILWQKANKMIIRTTHFKVTTISAGLIYEFRVYAENAAGIGKPSHPS
EPVLAIDACEPPRNVRITDISKNSVNLSWQQPAFDGGSKITGYIVERRDLPDGRWTKASFTNVIETQFTVSGL
TQNSQYEFRVFARNAVGSVSNPSEVVGPITCIDSYGGPVIDLPLEYTEVVKYRAGTSVKLRAGISGKPEPTIE
WYKDDKELQTNALVCVENSTDLASILIKDANRLNSGSYELKLRNAMGSASATIRVQILDKPGPPGGPIEFKTV
TAEKITLLWRPPADDGGAKITHYIVEKRETSRVVWSMVAENLEECIVTTTKIIKGNEYVFRVRAVNKYGIGEP
LESEPVVAKNAFVTPGPPSIPEVTKITKNSMTVVWDRPTVDGGSEINGYFLERRDKKSLAWLKVLKETIRDTR
QKVTGLTENSDYQYRVCAVNAAGVGPFSEPSDFYKAADPIDPPGPPAKIRIADSTKSSITLGWSKPVYDGGSD
VTGYVVEMKQGDEEEWTIVSTRGEVRTTEYVVSNLKPGVNYYFQVSAVNCAGQGEPITMTEPAQAKDVLEEPE
IDLDVALRTSVIAKAGEDVQLLIPFKGRPPPTVTWRKDEKNLGSDTRYSIQNTDSSSLLVIPQVTRNDTGKYI
LTIENGVGQPKSSTVSVKVLDTPAACQKLQVKHVSLGTVTLLWDPPLIDGGSPIINYVIEKRDATKRTWSVVS

HKCSGTSFKVTDLSEKTPFFFRVLAENEIGIGEPCETTEPVKAAEVPAPIRDLSMKDSTKTSVVLSWTKPDFD
GGSIITDYLVERKGKGEQAWSHAGISKTCEIEIGQLKEQSVLEFRVSARNEKGQSDPVTIGPLTVKELVITPE
VDLSEIPGAQISVRIGHNVHLELPYKGKPKPSISWLKDGLPLKESEYVRFSKTENKITLSIKNSKKEHGGKYT
VILDNAVCRNSFPITIITLGPPSKPKGPIRFDEIKADSAIMSWDIPEDDGGGEITCYSIEKREASQTNWKMVC
SSVARTTFKVSNLVKDSEYQFRVRAENRYGVSEPLASNIIVAKHQFRIPGPPGKPVIYNVTSDGMSLTWDAPV
YDGGSEVTGFHVEKKERNSILWQRVNTSPISGREYRATGLIEGLDYQFRVYAENSAGLSSPSDPSKFTLAVSP
VDPPGTPDYIDVTRETITLKWNPPLRDGGSKIVAYSIEKRQGSDRWVRCNFTDVSECQYTVTGLSPGDRYEFR
IIARNAVGTISPPSQSSGLIMTRDENVPPTVEFGPEYFDGLVIKSGDSLRIKALVQGRPVPRVTWFKDGVEIE
RRMNMEITDVLGSTSLFVRDATRDHRGVYTVEAKNVSGSTKAEVTVKVQDTPGKVVGPIRFTNITGEKMTLWW
EAPLNDGCAPVTHYIIEKRETSRLAWALIEDNCEALSYTAIKLITGNEYQFRISAVNKFGVGRPLESDPVVAQ
IQYTIPDAPGVPEPSNVTGNSITLTWTRPESDGGSEIQHYILERREKKSTRWVKVISKRPISETRFKVTGLVE
GNEYEFHVMAENAAGIGPASGISRLIKCREPVNPPSAPSVVKVTDTSKTTVSLEWARPVFDGGMEIIGYIIEM
CKADLGDWHKVNTEPCVKTRYTVTDLQAGEEYKFRVSAVNGAGKGDSCEVTGTIKAVDRLSAPELDIDANFKQ
THIVRAGVSIRLFIAYQGRPTPTAVWSKPDSNLSIRADIHTTDSFSTLTVENCNRNDAGKYTLTVENNSGKKS
ITFTVKVLDSPGPPGPITFKDVTRGSATLMWDAPLLDGGARIHHYVIEKREASRRSWQVVSEKCTRQILKVSE
LTEGVPYYFRVSAENEYGVGEPYEMPEPIVATEQPAPPRRLDVVDTSKSSAVLAWLKPDHDGGSRITSYLLEM
RQKGSDFWVEAGHTKQLTFTVERLVENTEYEFRVKAKNDAGYSEPREAFSSVIIKEPQIEPTADLTGITNQLI
TCKAGSTFTIDVPISGRPAPKVTWKLEEMRLKETDRMSIATTKDRTTLTVKDSMRGDSGRYFLTLENTAGVKT
FTITVVVIGRPGPVTGPIEVSSVSAESCVLSWTEPKDDGGTEITNYIVEKRESGTTAWQLINSSVKRTQIKVT
HLTKYKEYCFRVSSENRFGVSKPLESVPIVAEHPFVPPSAPTRPEVYYVSANAMSIRWEEPYHDGGSKIVGYW
VEKKERNTILWVKENKVPCLECNYKVTGLVEGLEYQFRTYALNAAGVSKASEASRPIMAQNPVDPPGRPEVTD
VTRSTVSLIWSAPVYDGGSKVVGYIIERKPVSEVGDGRWLKCNYTIVSDNFFTVTALSEGDTYEFRVLAKNAA
GVISKGSESTGPVTCRDEYAPPKAELDARLQGDLVTIRAGSDLVLDAAVGGKPEPKIIWTKGDKELDLCEKIS
LQYTGKRATAVIKYCDRSDSGKYTLTVKNASGTKSVSVMVKVLDSPGPCGKLTVSRVTEEKCTLAWSLPQEDG
GAEITHYIVERRETSRLNWVIVEGECLTASYVVTRLIKNNEYTFRVRAVNKYGLGVPVESEPIVARNSFTIPS
QPGIPEEVGAGKEHIIIQWTKPESDGGNEISNYLVDKREKKSLRWTRVNKDYVVYDTRLKVTSLMEGCDYQFR
VTAVNAAGNSEPSEASNFISCREPSYTPGPPSAPRVVDTTKRSISLAWTKPMYDGGTDIIGYVLEMQEKDTDQ
WCRVHTNATIRNNEFTVPDLKMGQKYSFRVAAVNAKGMSDYSETTAEIEPVERLEIPDLELADDLKKTVIVRA
GASLRLMVSVSGRPSPVITWSKKGIDLANRAIIDNTESYSLLIVDKVNRYDAGKYTIEAENQSGKKSATVLVK
VYDTPGPCPSVSVKEVSRDSVTITWEIPTIDGGAPVNNYIIEKREAAMRAFKTVTTKCSKTLYRISGLVEGTM
YYFRVLPENIYGIGEPCETSDAVLVSEVPLVPTKLEVVDVTKSTVTLAWEKPLYDGGSRLTGYVLEACKAGTE
RWMKVVTLKPTVLEHTVISLNEGEQYLFRVRAQNEKGVSEPREIVTPVTVQDLRVLPTIDLSTMPQKTIHVPA
GRPIELVIPITGRPPPTASWFFAGSKLRESERVTVETHTKVTKLTIRETTIRDTGEYTLELKNVTGTTSETIK
VIILDKPGPPTGPIKIDEIDATSVTISWEPPELDGGAPLSGYVVEQRDAHRPGWLPVSESVTRPTFKFTRLTE
GNEYVFRVAATNRFGIGSYLQSEVIECRSSISIPGPPETLQIFDVSRDGMTLTWYPPEDDGGSQVTGYIVERK
EVRADRWVRVNKVPVTMTRYRSTGLIEGLEYEHRVTAINARGTGKPSRPSKPTVAMDPIAPPGKPQNPRVTDT
TRTSVSLAWSVPEDEGGSKVTGYLIEMQKVDQREWTKCNTTPTKIREYTLTHLPQGAEYRFRVLACNAGGPGE
PAEVPGTVKVTEMLEYPDYELDERYQEGVFVRQGGVIRLTIPIKGKPFPVCKWTKEGQDISKRAMIATSETHT
ELVIKEADRNDSGTYDLVLENKCGKKTVYIKVKVIGSPNTPEGPLEYDDIQARSVRVSWRPPADDGGADILGY
ILERREVPKAAWYTIDSRVRGTSLVVKGLKENVEYHFRVSAENQFGISKPLKSEEPVIPKTPLNPPEPPSNPP
EVLDVTKSSVSLSWSRPKDDGGSRVTGYYIERKETSTDKWVRHNKTQITTTMYTVTGLVPDAEYQFRIIAQND
VGLSETSPASEPVVCKDPFDKPSQPGELEILSISKDSVTLQWEKPECDGGKEILGYWVEYRQSGDSAWKKSNK
ERIKDRQFTIGGLLEATEYEFRVFAENETGLSRPRRTAMSVKTKLTSGEAPGVRKEMADVTTKLGEAAQLSCQ
IVGRPLPDIKWYRFGKELIQSRKYKMSSDGRTHTLTVMTDEQEDEGVYTCVATNEVGEVESSSKLLLQAAPQF
HPGYPLKEKYYGAVGSTLRLHVMYIGRPVPAMTWFHGQKLLQNSEKITIENTEHYTHLVMKNVQRKTHAGKYK
VQLSNAFGTVDATLDVEIQDKPDKPTGPIVIEALLKNSVVISWKPPADDGGSWITNYVVEKCEAKEGAEWQLV
SSAISVTTCRIVNLTENAGYYFRVSAQNTFGISEPLEVASIVIIKSPFEKPGVPGKPTITAVTKDSCVVAWKP
PASDGGAKIRNYYLERREKKQNKWIAVTTEEIRETVFSVQNLIEGLEYEFRVKCENLGGESEWSEISEPVTPK
SDVPIQAPHFKEELRNLNVRYQSNATLVCKVTGHPKPIVKWYRQGKEIIADGLKYRIQEFKGGYHQLIIASVT
DDDATVYQVRATNQGGSVSGTASLEVEVPAKIHLPKTLEGMGAVHALRGEVVSIKIPFSGKPDPVITWQKGQD
LIDNNGHYQVIVTRSFTSLVFSNGVERKDAGFYVVCAKNRFGIDQKTVELDVADVPDPPRGVKVSDVSRDSVN
LTWTEPASDGGSKVTNYIVEKCATTAERWLRVGQARETRYTVINLFGKTSYQFRVIAENKFGLSKPSEPSEPT
VTKEDKTRAMNYDDEVDETREVTTTKASHSKTKELYEKYMIAEDLGRGEFGIVHRCVETSSKRTFMAKFVKVK
GTDQVLVKKEISILNIARHRNILYLHESFESMEELVMIFEFISGLDIFERINTSAFELNEREIVSYVRQVCEA
LEFLHSQNIGHFDIRPENIIYQTRKNSTIKIIEFGQARQLKPGDNFRLLFTAPEYYAPEVHQHDVVSTATDMW
SLGTLVYVLLSGINPFLAETNQQMIENIMNAEYTFDEEAFKEISLEAMDFVDRLLVKERKSRMTASEALQHPW
LKQRIDRVSTKVIRTLKHRRYYHTLIKKDLNMVVSAARISCGGAIRSQRGVSVAKVKVASIEIGPVSGQIMHA
IGEEGGYVKYVCKIENYDQSTQVTWYFGVRQLENSEKYEITYEDGVATMYVKDITKFDDGTYRCKVVNDYGED

SSYAELFVKGVREVYDYYCRRTKKVKRRTDAMRLLERPPEFTLPLYNKTAYVGENVRFGVTITVHPEPRVTWY
KSGQKIKPGDDEKKYTFESDKGLYQLTINSVTTDDDAEYTVVARNKHGEDSCKAKLTVTLHPPPTETTLRPMF
KRLLANAECHEGQSVCFEIRVSGIPAPTLKWEKDGQPLSLGPHIEIVHEGLDYYALHIRDTLPEDTGYYRVTA
TNTAGSTSCQAHLQVERLRYVKQEFQSKEERERHVQKQIDKTLRMAEILSGTETVPLTPVAQEALREAAILYK
PAVSTKTVKGEYRLQTEEKKEERKLRMPYEVPEPRRFKQATVEEDQRIKQFVPMSDMKWYKKIRDQYEMPGKL
DRVVQKRPKRIRLSRWEQFYVMPLPRITDQYRPKWRIPKLTQDDLEMVRPARRRTPSPDYDLYYYRRRRRSLG
DMSDEELLLPIDDYLAMKRTEEERLRLEEELELGFSASPPSRSPPRFELSSLRYSSPPAHVKVEDRRRDFRYS
TYHVPTKEETSTSYAELRERHAQASYRQPKLRQRIMAEKEEEELLRPVTTTQRLSEYKSELDYMSKEEKSKKK
SKRQRQVTEITEIEEEYEISRRAQRESSSSVSRLLRRRRSLSPTYIELMRPVSELIRSHPRPAEEYEDDAERR
SPTPERTRPRSPSPVSSERSLSRFERSARFDIFSRYESMKAALKTQKTSERKYEVLSQQPFTLDHAPRITLRM
RSHRVPCGQNTRFILNVQSKPTAEVKWYHNGVELQESSKIHYTNTSGVLTLEILDCQTEDGGTYRAVCTNYKG
EASDYATLDVTGGAYTTYASQRRDEEVPKSVFPELTKTEAYAVSSFKRTSELEAASSVREVKSQMTETRESLS
TYEHYASAEMKSATSEEKSLEEKATVRKIKTTLAARILTKPRSITVHEGESARFSCDTDGEPVPTVTWLREGQ
VVSTSARHQVTTTKYKSTFEISSVQASDEGNYSVVVENSDGKQEAQFTLTVQKARVIEKAVTSPPRVKSPEPR
VKSPETVKSPKRVKSPEPVTSHPKAVSPTETKPTEKGQHLPVSAPPKITQSLKAEASKDIAKLTCAVESSALC
AKEVAWYKDGKKLKENGHFQFHYSADGTYELKIHNLSESDCGEYVCEVSGEGGTSKTSFQFTGQSFKSIHEQV
SSISETTKSVQKTAESAEAKKSAQKTAESVEDKKAAQKTVESAEAKKPAQKTAESPEAKKPAQKTAESPEAKK
QEPIAPESISSKPVIVTGLRDTTVSSDSVAKFTIKVTGEPQPTITWTKDGKAIAQGSKYKLSSNKEEFILEIL
KTETSDGGLYACTVTNSAGSVSSSCKLTIKAVKDTEAQKVSTQKTSEVTSQKKASAQEEISQKALTSEEIKMS
EVKSHETLAIKEEASKVLIAEEVKRSAAASLEKSIVHEEVTKTSQASEEVKTHAEIKTLSTQMNITKGQRATL
KANIAGATDVKWVLNGTELPNSEEYRYGVSGSDQTLTIKQASHREEGILSCIGKTSQGVVKCQFDLTLSEELS
DAPSFITQPRSQNINEGQNVLFSCEVSGEPSPEIEWFKNNLPISISSNISVSRSRNVYTLEIRNAAVSDSGKY
TIKAKNFHGQCSATASLTVLPLVEEPPREVVLKTSSDVSLHGSVSSQSVQMSASKQEASFSSFSSSSASSMTE
MKFASMSAQSMSSMQESFVEMSSSSFMGKSSMTQLESSTSRMLKAGGRGIPPKIEALPSDISIDEGKVLTVAC
AFTGEPTPEITWSCGGRKIQNQEQQGRFHIENTDDLTTLIIMDVQKQDGGLYTLSLGNEFGSDSATVNINIRS
M*
>ENSMUSP00000097561_c.82938:A>C_p.K27647Q ENSMUST00000099981.10|Ttn-
203|ENSMUSG00000051747|-|chr2:g.76568328:T>G
MTTQAPMFTQPLQSVVVLEGSTATFEAHVSGSPVPEVSWFRDGQVISTSTLPGVQISFSDGRARLMIPAVTKA
NSGRYSLRATNGSGQATSTAELLVTAETAPPNFSQRLQSMTVRQGSQVRLQVRVTGIPTPVVKFYRDGAEIQS
SLDFQISQEGDLYSLLIAEAYPEDSGTYSVNATNSVGRATSTAELVVQGEEVVPAKKTKTIVSTAQISETRQT
RIEKKIEAHFDARSIATVEMVIDGATGQLPHKTPPRIPPKPKSRSPTPPSIAAKAQLARQQSPSPIRHSPSPV
RHVRAPTPSPVRSVSPAGRISTSPIRSVKSPLLIRKTQTTTMATGPEVPPPWKQEGYVASSTEAEMRETTMTS
STQIRREERWEGRYGVQEQVTISGAAAAAASASVSSSFTAGAITTGTKEVKQETDKSAAVATVVAAVDMARVR
EPAISAVEQTAQRTTTTAVHIQPAQEQARKEAEKTAVTKVVVAADKAKEQELKSRTREVMVTTQEQTHISHEQ
IRKETEKAFVPKVVISATKAKEQETRITGEITTKQEQKRITQETIRQETEEIAASMVVVATAKSTKLEAAVGV
QEETAIQQDQMHLTHEQMMKETRKTVVPKVIVATPKIKEQDLVSRSREAITTKRDQVQITQEKKRKEVETTAL
STIAVATAKAKEQETVLRSREAMATRQEHIQVTHGQVGVGKKAEAVATVVAAVDQARVREPREPTHVEESHSQ
QTTLEYGYKEHISTTKVPEQPRRPASEPHVVPQAVKPAVIQAPSETHIKTTDQMGMHISSQVKKTTDISTERL
VHVDKRPRTASPHFTVSKISVPKTEHGYEASIAGSAIATLQKELSATSSTQKITKSVKAPTVKPGETRVRAEP
TPSPQFPFADMPPPDTYKSQAGIEVKKEVGVSISGSTVREEHFEVLRGREAKVTETARVPAPAEVPVTPPTLV
SGLKNVTVIEGESVTLECHISGYPSPKVTWYREDYQIESSIDFQITFQGGIARLMIREAFAEDSGRFTCSAVN
EAGTVSTSCYLAVQVSEEFDKETTLTEKFATEEKRFVESRDVVMTDTSITEEQAGPGEPAAPFFISKPVVQKL
VEGGSVVFECQIGGNPKPHVYWKKSGVPLTTGYRYKVSYNKQTGECRLVISMTFADDAGEYTIVIRNKHGETS
ASASLLEEADYEALVKTQQEMLYQTQMSTFIQEPKVGEIAPGFAYSEYEKEYEKEQALIRKKMAKDTVMVRTF
VEDQEFHISSFEERLIKEIEYRIIKTTLEELLEEDGEEKMAVDISESEAIESGFDIRIKNYRILEGMGVTFHC
KMSGYPLPKIAWYKDGKRIRHGERYQMDFLQDGRASLRIPVVLPEDEGIYTAFASNIKGNAICSGKLYVEPAA
PFSAPTYMPTPEAVSRIRSVSPRSLSRSPIRMSPAMSPARMSPARMSPARMSPARMSPGRRLEETDESQLERL
YKPVFVLKPASFKCLEGQTARFDLKVVGRPMPETFWFHNGQQIVNDYTHKVVIKEDGTQSLIIVPASPSDSGE
WTVVAQNRAGKSTISVTLTVEAVEHQIKPAFVEKLKNVNIKEGSRLEMKVRATGNPNPDIVWLKNSDIIVPHK
YPRIRIEGTRGEAALKIDSIISQDSAWYTATAINKAGRDTTRCKVNVEVEFAEPEPERKLIIPRGTYRAKEIA
APELEPLHLRYGQEQWEEGDLYDKEKQQKPFFKKKLTSLRLKRFGPAHFECRLTPIGDPTMVVEWLHDGKPLE
AANRLRMINEFGYCSLDYGAAYSRDSGVITCRATNKYGTDHTSATLIVKDEKSLVEESQLPDGKKGLQRIEEL
ERMAHEGALTGVTTDQKEKQKPDIVLFPEPVRVLEGETARFRCRVTGYPQPKVNWYLNGQLIRKSKRFRVRYD
GIHYLDIVDCKSYDTGEVKVTAENPEGVTEHKVKLEIQQREDFRSVLRRAPEPKPEFHVHEPGKLQFEVQKVD
RPVDTSETKEVVKLKRAERITHEKVSEESEELRSKFKRRTEEGYYEAITAVELKSRKKDESYEELLKKTKDEL
LHWTKELTEEEKKALAEEGKITIPTFKPERIELSPSMEAPKIFERIQSQTVGQGSDAHFRVRVVGKPDPECEW
YKNGVKIERSDRIYWYWPEDNVCELVIRDVTAEDSASIMVKAINIAGETSSHAFLLVQAKQLITFTQELQDVV

AKEKDTMATFECETSEPFIKVKWYKDGIEVHAGDKYRMHSDRKVHFLSVLTIDTSDAEDYSCVLVEDENIKTT
AKLIVEGAVVEFVKELQDIEVPESYSGELECIISPENIEGKWYHNDVELKSNGKYSITSRRGRQNLTVKDVTK
EDQGEYSFVVDGKKTTCKLKMKPRPIAILQGLSDQKVCEGDIVQLEVKVSLENVEGVWMKDGQEVQHSDRVHI
VIDKQSHMLLIEDMTKEDAGNYSFTIPALGLSTSGNVSVYSVDVITPLKDVNVIEGTKAVLECKVSVPDVTSV
KWYLNDEQIKPDDRVQSIVKGTKQRLVINRTHASDEGPYKLMVGRVETSCNLSVEKIKIIRGLRDLTCTETQN
VVFEVELSHSGIDVVWNFKGKEIKPSSKYKIEAHGKIYKLTVLNMMKDDEGEYAFYAGENTTSGKLTVAGGAI
STPLTDQTVAESQEAVFECEVANPESEGEWLKDGKHLALSNNFRGESDGHKRRLVIAAAKLDDAGEYTYKVAT
SKTSAKLKVEAVKIKKTLRNLTVTETQDAVFSVELTHPDVKGVQWIKNGVVLDSNDKYEISVKGTLYSLKIKN
CAMADESVYGFKLGRLGASARLHVETVKIIKKPKDVTALENATVTFEVSVSHDTVPVKWFHKNVEIKPSDKHR
LVSERKVHKLMLQSISPSDAGEYTAMVGQLECKAKLFVETLHITKTMKSIEVPETKAASFECEVSHFNVPSMW
LKNGVEIEMSEKFKIVVQGKLHQLIIMNTSTEDSAEYTFVCGNDQVSATLTVTPIMITSMLKDINAEEKDTIT
FEVTVNYEGISYKWLKNGVEIKSTDRCQMRTKKLTHSLNIRNVHFGDAADYTFVAGKATSTATLYVEARHIEF
RKHIKDIKVLEKKRAMFECEVSEPDITVQWMKDGQELQIADRIKIQKEKYVHRLLIPSTRMSDAGKYTVVAGG
NMSTANLFVEGRDVRIRSIKKEVQVIEKQRAVVEFEVNEDDVDAHWYKDGIEINFQVQERHQYVVERRIHRMF
ISETRHSDAGEYTFVAGRNRSSVTLYVNAPEPPQVLQELQPVTVQSGKPARFCAVISGRPQPKISWYKEEQLL
STGFKCKFLHDGQEYTLLLIEAFPEDAAVYTCEAKNDYGVATTSASLSVEVPEVVSPDQEMPVYPPAIVTPLQ
DTVTSEGRPARFQCQVSGTDLKVSWYCKDKKIKPSRFFRMTQFEDTYQLEIAEAYPEDEGTYAFVANNAVGQV
SSTATLRLEGFSKSEEVTSKSQWHTSPSVSLKKEALGQKPIFIQPLSSLRVHSGETVRFHARVSGVPKPAIQW
FHNHQPILPSKDVVFHFEESTGMALMLIVDAYPEHAGKYSCRAANSAGEATCAATLTVTAKAPESILHERIGQ
QIEMEMKEIASLLSAEEDFQTYSSDLRLPNANETLELLSEPPARSTQFDSRQEGAAPVFIREISDVEISVEDV
AKLSVTVTGCPKPKIQWFFNGMLLTPSADYKFVFDGDTHSLIILFTRFQDEGEYTCLASNEYGKAVCSAHLRI
SPRGERSTEMESGEKKALEKPKGPCPPYFFKELKPVHCGPGIPAVFEYSVHGEPAPTVLWFKEDMPLYTSVCY
TIIHSPDGSGTFIVNDPQRGDSGLYLCKAQNLWGESTCAAELLVLPEDTDVPDASCKEESTLGVPGDFLETSA
RGPLVQGVDSRQEITAFAEGTISKAALIAEETLQLSYERSVDDSEVGTGVTIGAQKLPPVVLSTPQGTGELPS
IDGAVHTQPGRGPPPTLNLQAVQAQTTLPKEATLQFEEPEGVFPGASSAAQVSPVTIKPLITLTAEPKGNYPQ
SSTAAPDHALLSSVAAETLQLGEKKIPEVDKAQRALLLSQSLAEGCVESLEVPDVAVSNMRSEPQVPFQHTCT
EGKILMASADTLKSTGQDVALRTEEGKSLSFPLALEEKQVLLKEEQSEVVAVPTSQTSKSEKEPEAIKGVKEV
REQELLSKETLFPSMPEEQRLHLKTQVRRALQAAVAREQANLFSEWLRNIDKVEVTAVNFTQEPKRILCTYLI
TSVSSLTEELTVTIEDIDPQMANLETGLKDALCSIVCEERNILMAEDPRIHEEDKIDVQGGRDHLSDAQKVET
VIEAEADSKYLVSKEEVSWSKVESQLKDGDTNEVPQAETLKLAEESGTQKTSTEMSQEEAEGTLADLCPAVLK
HLVDTISEEGDTVHLTSSISNAKEVHWYFKGNLVPSDGKFKCLKEQNAYTLVIEAVKTEDEGEYVCEASNDSG
KAKTSAKLTVGERAAPVIKRRIEPLEVALGHLAKFTCEIQGAPNVRFQWFKAGREIYESDKCSIRSSNYVSSL
EILRTQVVDCGEYTCKASNEYGSVSCTATLTVTEAYPPTFLSRPKALTTFVGKAAKFLCTVSGTPVIEIIWQK
DGAALSPSPDCRVTDADNKHSLELSNLTVQDRGIYSCKASNKFGADICQAELTIIDKPHFIKELEAVQSAINK
KIHLECQVDEDRKVTITWSKDGQKLPAGKDYKIYFEDKIASLEIPLAKLKDSGTYTCTASNEAGSSSSSAAVA
VREPPSFVKKVDPSYLMLPGESARLHCKLKGSPVIQVTWFKNNKELSESNTVRMSFVNSEAILDITDVKVDDS
GTYSCEATNDVGSDSCSTEVVIKEPPSFIKTLEPADIVRGANALLQCEIAGTGPFEVNWFKDKKQIRSSKKYR
LFTQKTFVYLEISSFNSADVGDYECVVANEVGKCGCVATHLLKEPPTFVKKVDDFTALAGQTVTLQAAVRGSE
PISVMWMKGQEVIKEDGKIKMSFSNGVAVLTIPDVQISLGGKYTCLAENEAGSQTSVGELIVKEPAKIIERAE
LIQVTAGDPATLEYTVSGTPELKPKWYKDGRPLVASKKYRISFKNNIAQLKFYSAELHDSGQYTFEISNEVGS
SSCETTFTVLDRDIAPLFTKPLRNVDSVVGGACRLDCKIAGSLPMRVSWFKDGKELTASDRYQIAFVEGTASL
EISRVDMNDAGNFTCRATNSVGSKDSSGALIVQEPPSFVTKPGSRDVLPGSAVCLKSAFQGSAPLTIKWFKGD
KELVSGGSCYITKETSESSLELYAVKTSDSGTYTCKVSNVAGSVECSADLFVKEPATFIEKLEPSQLLKKGDG
TQLACKVTGTPPIKITWFANDRELRESSKHKMSFAESTAVLRLTDVAIEDSGEYMCEAQNEAGSDHCTGIVIV
KESPYFTKEFKSIEVLKEYDVMLLAEVAGTPPFEITWFKDNTTLRSGRKYKTFLQDQLVSLQVLKFVAADAGE
YQCRVTNEVGSSTCSARVTLREPPSFIKKIEATSSLRGGTAAFQATLKGSLPITVTWLKDNDEITEDDNIRMT
FENNVASLYLSGIEVKHDGKYVCQAKNDAGIQRCSALLSVKEPATIMEEAVSIDVTQGDPATLQVKFSGTKEI
SAKWFKDGQELTLGPKYKISVTDTVSILKIISTEKKDSGEYTFEVQNDVGRSSCKASINVLDLIIPPSFTKKL
RKMDSIKGSFIDLECIVAGSHPISIQWFKDDQEISASDKHKFSFHDNTAFLEISQLEGTDSGTYTCSATNKAG
HSQCSGHLTVKEPPYFVEKPQSQDVNPGTRVQLKALVGGTAPMTIKWFKDNKELHPGAARSVWKDDTSTILEL
FSAKAADSGTYICQLSNDVGTTSSKATIFVKEPPQFIKKPSPVLVLRNGQSTTFECQVTGTPEIRVSWYLDGN
EITDLRKYGISFVDGLATFQISNARVENSGTYVCEARNDAGTASCSIELKVKEPPIFIRELEPVEVVKDSDVE
LECEVMGTTPFEVTWLKNNKEIRSGKKYTMSEKMSVFYLHITKCDPSDVGEYQCIIANEGGSCACSARVALKE
PPSFIKKIENVTTVLKSSATFQSTVAGSPPISITWLKDDQILEENDNVHISFEDSVATLQVRSVDNGHSGRYT
CQAKNESGIERCYAFLLVQEPAQIIEKAKSVDVTEKDPVTLECVVAGTPELKVKWLKDGKQIVPSRYFSMSFE
NNVASFRIQSVMKQDSGQYTFKVENDFGSSSCDAYLRVLDQDIPPSFTKKLTKMDKVLGSSIHMECKVSGSLP
ISAQWFKDGKEISTSAKYRLVCHENTVSLEVSNLELEDTANYTCKVSNVAGDNACSGILTVKEPPSFLVKPER
QQAIPDSTVEFKAVLKGTPPFKIKWLKDDVELVSGPKCFIGLEGSTSFLNLYSVDSSKTGQYTCQVTNDVGSD

SCTTMLLVTEPPKFVKKLEASKIIKAGDSARLECKITGSPEIQVVWYRNEHELTASDKYQMTFIDSVAVIQMN
SLGTEDSGDFICEAQNPAGSTSCSTKVIVKEPPVFSSFPPIVETLKNTEVSLECELSGTPPFEVVWYKDKRQL
RSSKKYKVASKNFHASIHILNVESTDIGEYHCKAQNEVGSDACVCAVKLKEPPKFISKLNSLTVVAGEPAELQ
ASIEGAQPISVQWLKEKEEVIRESENIRISFVNNVATLQFAKVEPANAGKYICQVKNDGGVRENMATLTVLEP
AVIIEKAGSMTVTVGETCALECKVAGTPELSVEWYKDGKLLTSSQKHKFSFYNKISSLKILSVEKEDAGTYTF
QVQNNVGKSSCTAVVDVSDRMVPPSFTRRLKDTGGVLGTSCILECKVAGSSPISIAWFHEKTKIVSGAKYQTT
FSDNVCTLQLNSLDSSDMGSYTCVAANVAGSDECRALLTVQEPPSFVKEPEPLEVLPGKNITFTSVIRGTPPF
KVGWFRGARELVKGNRCNIYFEDTVAELELFNIDISQSGEYTCVVSNNAGQASCTTRLFVKEPATFVKKLSDH
SVEPGKSIILEGTYTGTLPISVTWKKDGVSITPSERCNIVTTEKTCILEILSSTKGDAGHYSCEIENEAGRDA
CDALVSTLEPPYFVTELEPLEASVGDSVSLQCQVAGTPEITVSWFKGDTKLRSTPEYRTYFTNNVATLVFNKV
SINDSGEYTCMAENSIGTAASKTIFRIQERQLPPSFARQLKDIEQTVGLPVTLTCRLNGSAPIQVCWYRDGVL
LRDDENLQMSFVDNVATLKILQTDLSHSGQYSCSASNPLGTASSTARLTAREPKKSPFFDIKPVSIDVIAGES
ADFECHVTGAQPMRVTWSKDNKEIRPGGNYTITCVGNTPHLRILKVGKGDSGQYTCQATNDVGKDMCSAQLSV
KEPPKFIKKLDASKVAKQGESIQLECKISGSPEIKVVWFRNDSELHESWKYNMSFVNSVALLTINEASAEDTG
DYICEAHNGVGDASCSTALKVKAPPVFTQKPPPVGALKGSDVILQCEISGTPPFEVVWVKDRKQVRSSKKFKI
TSKNFDTSLHIFNLEAPDIGEYHCKATNEVGSDTCACTVKFKEPPRFVKKLSDASTLIGDPVELQAVVEGFQP
ISVVWLKDKGEVIRESENVRISFVDNIATLQLGSPEASQSGKYVCQIKNDAGMRECSAVLTVLEPATIVEKPE
PMTVTTGNPFTLECVVAGTPELSAKWFKDGRELSSGSRHHITFVRNLASLKIPSAEMNDKGLYTFEVENRVGK
SSCTVSVHVSDRVVPPSFVRRLKDTSATLGASVVLECRVSGSAPISVGWFLDGNEIISSPKCQSSFADNVCTL
TLSSLEPSDTGAYTCVAANVAGQDESSAVLTVQEPPSFEQTPDSVEVLPGMSLTFTSVIRGTPPFKVKWFKGS
RELVSGEACTISLEDFVTELELLEVEPGQSGDYSCLVTNDAGSASCTTHLFVKEPATFVKRLADTSVETGSPI
VLEATYSGTPPISVSWMKNEYPLSQSPNCGITTTEKSSILEILESTIEDYAQYACLIENEAGQDICEALVSVL
EPPYFIEPLEHVEAAIGEPITLQCKVDGTPEIRISWYKEHTKLRSAPAYKMQFKNNVASLVINKVDHSDVGEY
TCKAENSVGAVASSAVLVIKERKLPPSFARKLKDVHETLGFPVAFECRINGSEPLQVSWYKDGELLKDDANLQ
MSFVHHVATLQILQTDQSHVGQYNCSASNPLGTASSSAKLILSEHEVPPFFDLKPVSVDLALGESGSFKCHVT
GTAPIKITWAKDNREIRPGGNYKMTLVENTATLTVLKVAKGDAGQYTCYASNVAGKDSCSAQLGVQEPPRFIK
KLDQSRIVKQDEYTRYECKIGGSPEIKVLWYKDEVEIQESSKFRMSFEDSVAILEMHSLSVEDSGDYTCEARN
AAGSASSSTSLKVKEPPVFRKKPFPVETLKGADVHLECELQGTPPFQVSWHKDKRELRSGKKYKIMSENLLTS
IHILNVDTADIGEYQCKATNDVGSDTCVGSVTMKAPPQFVKKLTDISTIIGKEVQLQTTIEGAEPISVAWFKD
KGEIVRESDNIWISYSENIATLQFSRAEPANAGKYTCQIKNDAGMQECYATLSVLEPAAIVEKPESIKVTTGD
TCTLECTVSGTPELSTKWFKDGKELTSDNKYKISFFNKVSGLKIINVVPGDSGVYSFEVQNPVGKDSCKVSIQ
VSDRIIPPSFTRKLKETNGLSGSSVVMECKVYGSPPISVLWFHDGNEISSGRKYQTTLTDNTCALTVNMLEEA
DAGDYTCIATNVAGSDECSAPLTVREPPSFVQKPDPMDVLTGSNVTFTSIVKGSPPFTVSWFKGSTELVPGAR
CNVSLQDSVGELELFDVDTSQSGEYTCIVSNEAGRASCTTRLFVKAPAIFVKRLNDYSIEKGKPLILEGTFSG
TPPISVTWKKNGINVIASQRCNITTTEKSAILEILSSTVEDSGQYNCYIENASGKDSCSAQILILEPPYFVKQ
LEPVKVTVGDSASLQCQLAGTPEIGVSWYKGDTKLRPTATCKMHFKNNVATLVFTQVDSSDSGEYICRAENSV
GEVSSSTFLTVQEQKLPPSFSRQLRDVQETVGLPVVFECAVSGSEPISVSWYKDGKPLKDSPNIQTSFLDNIA
TLNIFKTDRSLSGQYSCTATNPIGSASSGAKLILTEGKNPPFFDIPLAPMDAVVGESADLECHVTGTQPIKVT
WAKDNREIRSGGNYQISYLENSAHLTIVKVDKGDSGQYTCYAVNEVGKDSCTAQLNIKERLIPPSFTKKLSET
VEETEGNSFKLEGRVAGSQPITVAWYKNNVEIHPTSNCEIMFKNNALLLQVKRASMADAGLYTCKATNDAGSA
LCTSSIVIKEPKKPPVFDQHLAPVTASEGDSVQLSCHVQGSEPIRIQWLKAGREVKPSDRCSFSFASGTAMLE
LKETAKADSGDYVCKASNVAGSDTSKCKVTIKEKPAAAPAAKKAAVDGKLFFVSEPQSIRVVEKTTATFIAKV
GGDPIPNVKWTKGKWRQLNQGGRILIHQKGDEAKLEIRDTTKTDSGLYRCVAFNKHGEIESNVNLQVDERKKQ
EKIEGDLRAMLKKTPALKKGSGEEEEIDIMELLKNVDPKEYEKYARMYGITDFRGLLQAFELLKQSQEEETHR
LEIEELEKSERDEKEFEELVAFIQQRLTQTEPVTLIKDIENQTVLKDNDAIFEIDIKINYPEIKLSWYKGTEK
LEPSNKYEISIDGDRHTLRVKNCQPKDQGNYRLVCGPHIASAKLTVIEPAWERHLQDVTLKEGQTCTMTCQFS
VPNVKSEWFRNGRVLKPQGRVKTEVEHKVHKLTIADVRAEDQGQYTCKHEDLETSAELRIEAEPIQFTKRIQN
IVVSEHQSATFECEVSFDDAIVTWYKGPTELTESQKYNFRNDGRCHYMTIHNVTPDDEGVYSVIARLEPRGEA
RSTAELYLTTKEIKLEMKPPDIPDSRVPIPTMPIRAVPPEEIPPAVAPSIPLLLPLPEEKKPPAKRIEVTKKG
VKKDTKKVVTKPKEEAPPPPVAKKPPPPTPMIPAKASEIIDVSSKAEEVKITTITRKKEVHKEKEAVYEREEA
VYEKKVHIEPWEEPYEELETEPYTEPYEEPYYEEPDEDYEEIKVEAKKQVHEEWEEDFEEGQEYYEREEGYDE
GEEEWEEIYHEREIIQVQKEVHEELHEKKIPAKVPEKKVPPPKVVKKPVVEKVEKTTRRMEEEKVQVIKVPEV
SKKIVPQKPSRTPVQEEIIEVKVPAVHTKKMVISEEKMFFASHTEEEVSVSVPEVQKKTVTEEKIHVAVSKKI
EPPPKVPEPPKKPVPEEVVPVPIPKKVEPPAAKVPEAPKKPVPEEKKPVPIPKKKEPAAPPQVPEAPKKPAPE
EKIPVPVTKKKEAPPAKVPEVQKKVVTEEKIAIITQREESPPPAVPEIPKKKVPEEKRPVPRKEEVPPPKVPV
PPKKAVPEAVVPAPIPKKAPPRAEVSKKTVVEEKRFAAEEKLSVAVPQRVELMRHEEEEWTYSEEEERVSVSV
YREEERDEEEAEITEYEVLEEPEEYVVEEKMHFISKKVEVEPAKVPEKKIIPKPKVPAKIEEPPPTKVPEPPK
KIVPEKKVPAPAPKKVPPAKAPEESKRPVPEKRAPAEEVGIEEPPPTKVAERHMKITQEEKVLVAVTKKEAPP

RARVPEEPKKVAPEERFPKLKPRREEEPPAKVTEVRKRAVKEEKVSIEVPKREPRPTKEVTVTEEKKWSYTRE
EETVSEHREEEYEDYEDYEEYKEFEEYEPTEEYDQYDEYAEREVEHYEEHEEYVTEPKKPVPVKPAQEPVPAK
PKAPPPKVLKKAVPEEKAPLPIQKKLKPLPPKAPEEPKKVVEEKIQISITKREKQQVTEPVAKVPMKPKRVVP
EAKIPAPTKEVAVPVRVPGVPKKRELEEVVVFKEEVEAHEEYIVEEEEEYVHEEEYVHKEEYVHEEEYVHKEE
YIHEEEEHLHEEEETIAEEEVVPVAPVKVPVVPKKPVPEEKKPVPVPKKKEAPPAKVPEIPKKPEEKVPVPIP
KKEKAPPAKVPEVPKKPVPEEKPPVPVPKKVEPPPAKVPEVPKKPVPEKKVPAPTPKKVEAPPAKVPEVPKKP
IPEEKKPTALLKKMEAPPPKAPKKREVVPVPVALPREEEEEEVPFEEVPEEEILPEEEVPSEEEAPPEEVPPE
EEEVLPEEEEVLPEEEEVLPEEEEVQPEEEALPEIKPKVPKPAPVPKKTVPEKKVPVPVPKKVEPPPPPKVPE
IKKKVPEKKVVVPKKEEAPPTKVPEVSKKVEERRIIPPKEEEVPPAEVYEEAEEPTPEEIPEEPPSIEEEEIV
EEEEEEEEVLPPRAPEVVKKAVPEAPTPVPKKAEAPPAKVPKKIPEEKVPVPVQKKEAPPAKVPEKKKIPEKK
VPVPKKEAVPPAKGKAVFEEKISVAYQQEELVQERIELELVEAKVEEAFEEEEFHEVQEYFEEEEFHEVEEFI
RVEERRFQEEHKVEEVHRVIEFLEAEEVEVYEKPKIPPKKGPEVSEKVIPPKKPPTKVIPRKEPPAKVPEVTK
KTVVEEKIRAPEEPKVPAPKAPEVPKKITPEEKVREAVPKKPEVPPPKVPEVPKKIIQEEKLPVVLPEDTEIY
MYEASEETVIEEEHVTLPQKARLKVAKVPAPPQTVVTEEKTYVTIRKTRETLALKESETTREAFPELKSYKAV
PEIPEPPSPEDLEIIEDVLPEKRPPASKRRKTQLPTAPEAPREMPPEMNTFEEISVEPEMLPTQVLDTYQEAT
VEKKTLRISRKKPELPSDEEVPEAPREVVAKKKVLPPQVPEVVPVKVPGAPKEVVSERKSLEEPPKKPAVRPV
TVPEEPKEVIPEKKVSLVPPKKPAAPPVTVPEAPEEVFSEDEETLAPPQEPEAPPAKVPEAPKEVVPEKKVSV
VPPKKPEAPPAKVPEAPKEAAPEKKVPVAPKKKPEAPPVKVPEAPKKVVPEKKLPVAAPKKPEAPAAEVPEVP
KTAVPQKKIPEAIPPKPESPPLEVPEVPPKEVTPEKKVPAAPPTKPEIPPPKVPEAPQAAVVEEKTPEALPKK
AEAAPVPVPQVQETVPEKTRPVGPPKKPEATTVPVPKVQKTIPEKTRPEAPPKRPEARTVPETVPEKTRPMAP
PKKPEATTLPVPEVQETVPEKTRPVGPPKKPEATTVPVPEVQETIPEKTRPPKKPEATPVPVPEVQETVPEKT
RPVGPPKKPEATTVSVPEVQETIPEKTRPAAPPKKPEATAVPETIPEKTRPEAPPKRPEATTVPVPEADQAVV
PEKKVPRVPPKKVEAPPITVPEEPKEVIPEKKVSLVPPKKPAAPPVTVPEAPEEVFSEDEETLAPPQEPEAPP
AKVPEAPKEVVPEKKVSVVPPKKPEAPPAKVPEAPKEAAPEKKVPVAPKKKPEAPPVKVPEAPKKVVPEKKLP
VAAPKKPEAPAAEVPEVPKAAVPQKKIPEAIPPKPESPPPEVYEEPEEEIVPEEPPEEAVEEPVPAPPPKVTE
PPKKPVPEKKAPPAVVKKPEPPPAKVPEVPKEAPPEKKVPPKKPEAPPAKVPEVPKEVVTEKKVAVPKKPEVP
PAKVPEVPKKPVIEEKPAIPVVEKVASPPAEVYEEPEEVTAEEEEPAPAVEEEEYEAPPPPAPVPEEPKKVVP
EKKFPVIKKPEAPPPKVPEVPKKAVPVKKVPVVKKPEPPEAEVPEVPKKLVPVKKEPVPVTKKTEVLPEKVPE
APKKITPEKKESVPVPEEPEAPPASVEETPEETIYEEKATITIGRKETPPVEEREIEKFIQPEEPELEPEPEE
IPVQEPEPEKKVIEKPKLKPRPPARPPSPPKEDVKEKMFQLKAVSKKKVPEKPEVVEKVEPAPLKVPTAEKKV
RKLLPEPKPQPKEEVVLKSVLRKKPEEEEPKVEPKKVEKAKKPEEPQPPPKAVEVEAPPEPTPKERKVPEPAK
VPEIKPAIPLPGPEPKPKPEPEVKTMKAPPIEPAPTPIAAPVTAPVVGKKAEAKPKDEAAKPKGPIKGVAKKT
PSPIEAERKKLRPGSGGEKPPDEAPFTYQLKAVPLKFVKEIKDIVLTEAESVGSSAIFECLVSPSTAITTWMK
DGSNIRESPKHRFIADGKDRKLHIIDVQLSDAGEYTCVLRLGNKEKTSTAKLIVEELPVRFVKTLEEEVTVVK
GQPLYLSCELNKERDVVWRKDGKIVVEKPGRIVPGVIGLMRALTINDADDTDAGTYTVTVENANNLECSSCVK
VVEIIREWLVKPIRDQHVKPKGTAVFTCDIAKDTPNIKWFKGYDEIPLEPNDKTEILKEGNHLFLKVKNAMPE
DIDEYAVEIEGKRYPAKLTLGEREVELLKPIEDVTIYEKESASFDAEISEEDIPGEWKLKGELLRPSPTCEIK
AEGGKRFLTLHKVKLDQAGEVLYQACNAITTAILTVKEIELDFAVPLKDVTVPEKRQARFECVLTREANVIWS
KGPDIIKASDKFDIIADGKKHILVINDSQFDDEGVYTAEVEGKKTSAQLFVTGIRLKFISPLEDQTVKEGQTA
TFVCELSHEKMHVVWFKNDVKLHTTRTVLMSSEGKTYKLEIRETTLDDISQIKAQVKNLSSTANLKVLEADPY
FTVKLHDKTGVEKDEIILKCEVSKDVPVKWFKDGEEIVPSPKHSVKTDGLRRILKIKKAELKDKGEYVCDCGT
DTTKANVTVEARLIKVEKPLYGVEVFVGETARFEIELSEPDVHGQWKLKGEPLTASPDCEIIEDGKKHVLVLY
NCQLDMTGEISFQAANAKSAANLKVKELPLIFITPLSDVKVFEKDEAKFECEVSREPKTFRWLKGTQEITGDD
RFELIKDGTRHSLVIKSAAFEDEAKYMFEAEDKRTSGKLIIEGIRLKFLTPLKDVTAKERENAVFTVELSHDN
IPVSWFKNDQRLHTSKRVSMHDEGKTHSITFKDLSIDDTSQIRVEAMGISSEAKLTVLEGDPYFTGKLQDYTG
VEKDEVILQCEISKADAPVKWFKDGKEIKPSKNVVIKADGKKRMLILKKALKSDIGQYTCDCGTDQTSGKLDI
EDREIKLVRPLYSVEVMETETARFETEISEDDIHANWKLKGEALLQTPECEIKEEGKIHVLILHNCRLDQTGG
VDFQAANVKSSAHLRVKPRVIGLLRPLKDVTVTAGETATFDCELSYEDIPVEWYLKGKKLEPNDKVVTRSEGR
VHTLTLRDVKLEDAGEVQLTAKDFKTQANLFVKEPPVEFTKPLEDQTVEEEATAVLECEVSRENAKVKWFKNG
TEILKSKKYEIVADGRVRKLIIHGCTPEDIKTYTCDAKDFKTSCNLNVVPPHVEFLRPLTDLQVKEKETARFE
CEISKENEKVQWFKDGAEIKKGKKYDIISKGAVRILVINKCLLNDEAEYSCEVRTARTSGMLTVLEEEAVFTK
NLANLEVSEGDTIKLVCEVSKPGAEVIWYKGDEEIIETGRFEILTDGRKRILIIQNAQLEDAGSYNCRLPSSR
TDSKVKVHELAAEFISKPQNLEILEGEKAEFVCTISKESFEVQWKRDDQTLESGDKYDIIADGKKRVLVVKDA
TLQDMGTYVVMVGAARAAAHLTVIEKLRIIVPLKDTKVKEQQEVVFNCEVNTEGAKAKWFRNEEAIFDSSKYI
ILQKDLVYTLRIRDARLDDQANFNVSLTNHRGENVKSAANLIVEEEDLRIVEPLKDIETMEKKSVTFWCKVNR
LNVTLKWTKNGEEVAFDNRISYRIDKYKHSLIIKDCGFPDEGEYVVTAGQDKSVAELLIIEAPTEFVEHLEDQ
TVTEFDDAVFSCQLSREKANVKWYRNGREIKEGKKYKFEKDGSIHRLIIKDCRLEDECEYACGVEDRKSRARL
FVEEIPVEIIRPPQDILEAPGADVIFLAELNKDKVEVQWLRNNMIVVQGDKHQMMSEGKIHRLQICDIKPRDQ

GEYRFIAKDKEARAKLELAAAPKIKTADQDLVVDAGQPLTMVVPYDAYPKAEAEWFKENEPLSTKTVDTTAEQ
TSFRISEAKKDDKGRYKIVLQNKHGKAEGFINLQVIDVPGPVRNLEVTETFDGEVSLAWEEPLTDGGSKIIGY
VVERRDIKRKTWVLVTDRADSCEFTVTGLQKGGVEYLFRVSARNRVGTGEPVETDSPVEARSKYDVPGPPLNV
TITDVNRFGVSLTWEPPEYDGGAEITNYVIELRDKTSIRWDTAMTVRAEDLSATVTDVVEGQEYSFRVRAQNR
IGVGKPSAATPFVKVADPIERPSPPVNLNASEQTQSSVQLTWEPPLKDGGSPILGYIIERREEGKDNWIRCNM
KPVPELTYKVTGLQKGNKYLYRVSAENAAGVSDPSEILGPLTADDAFVEPTMDLSAFKDGLEVIVPNPIKILV
PSTGYPRPKATWTFGDQVLEEGDRVKMKTISAYAELVISPSERTDKGIYTLTLENPVKSISGEINVNVIAPPS
APKELKFSDITKDSVHLTWEPPDDDGGSPLTGYVVEKRDMSRKTWTKVMDFVTDLEFTVPDLVQGKEYLFKVC
ARNKCGPGEPAYTDEPVNMSAPATVPDPPENVKWRDRTANSIFLTWDPPKNDGGSRIKGYIVEKCPRGSDKWV
ACGEPVPDTKMEVTGLEEGKWYAYRVKALNRQGASKPSKPTEEIQAVDTQEAPEIFLDVKLLAGITVKAGTKI
ELPATVTGKPEPKITWTKADTLLKPDQRITIENVPKKSTVTITDSKRSDTGTYIIEAVNVCGRATAVVEVNVL
DKPGPPAAFDITDVTNESCLLTWNPPRDDGGSKITNYVVERKATDSDVWHKLSSTVKDTNFKATKLTPNKEYI
FRVAAENMYGVGEPVQAAPIIAKYQFDPPGPPTRLEPSDITKDAVTLTWCEPDDDGGSPITGYWVERLDPDTD
KWVRCNKMPVKDTTYRVKGLTNKKKYRFRVLAENLAGPGKPSRSTEPILIKDPIDPPWPPGKPTVKDIGKTSL
VLNWTKPEHDGGAKIESYVIEMLKTGTDEWVRVAEGVPTTEHLLTGLMEGQEYSFRVRAVNKAGESEPSEPSD
PVLCREKLYPPSPPRWLEVINITKNTADLKWTVPEKDGGSPITNYIVEKRDVRRKGWQTVDTTVKDTKCTVTP
LTEGSLYVFRVAAENAIGQSDYTEIGDSVLAKDTFTTPGPPYALTVVDVTKRHVDLKWEPPKNDGGRPIQRYI
IEKKEKLGTRWVKAGKTSGPDCNFRVTDVIEGTEVQFQVRAENEAGVGHPSEPTEILSIEDPTSPPSPPLDLH
VTDAGRKHIAIAWKPPEKNGGSPIIGYHVEMCPVGTEKWMRVNSRPIKDLKFKVEEGIVPDKEYVLRVRAVNA
VGVSEPSEISENVVAKDPDCKPTIDLETHDIVVIEGEKLNIPVPFRAVPVPTVSWHKDGKEVKASDRLTMKND
HISAHLEVPKSVHADAGVYTITLENKLGSATASINVKVIGLPGPCKDIKASDITKSSCKLTWEPPEFDGGSPI
LHYVLERREAGRRTYIPVMSGENKLSWTVKDLIPNGEYFFRVKAVNKIGGGEYIELKNPVIAQDPKQPPDPPV
DVEVHNPTAKAMTITWKPPLYDGGSKIMGYIIEKITKGEDRWKRCNEHLVPVLTYTAKGLEEGKEYQFRVRAE
NAAGIGEPSRATPPTKAVDPIDAPKVILRTSLEVKRGDEIALDATISGSPYPTITWIKDENVIVPEEIKKRAA
PPVRRKKGEAEEEEPFSLPLTERLSINNSKQGESQLRIRDSLRPDHGQYMIKVENDHGVAKAPCSVSVLDTPG
PPINFVFEDIRKDSVLCKWEPPLDDGGSEIINYTLEKKDKTKPDSDWIVITSTLRNCKYSVTKLIEGKEYLFR
VRAENRFGPGPPCVSKPLLAKDPFEPPDAPDKPIVEDVTSNSMLVKWNEPKDNGSPILGYWLEKREVNSTHWS
RVNKTLLSSLKTKVDGLLEGLTYVFRVCAENAAGPGKFSPPSDPKTARDPISPPGPPVPRVADTSSTTIELEW
EPPAFNGGGEIMGYFVDKQLVGTNEWSRCTEKMIKVRQFTVKEIREGADYKLRVSAVNAAGEGPPGETEPVTV
AEPQEPPTVELDVSVKGGIQIMAGKTLRIPAEVTGRPVPTKVWTIEEGELDKERVIIENVGTKSELIIKNALR
KDHGRYVITATNSCGSKFAAVRVEVFDVPGPVLDLKPVVTNRKMCLLNWSDPADDGGSDITGFIIERKDAKMH
TWRQPIETERSKCDITGLIEGQEYKFRVIAKNKFGCGPPVEIGPILAVDPLGPPTSPERLTYTERTKSTITLD
WKEPRSDGGSPIQGYIIEKRRHDKPDFERVNKRLCPTTSFLVENLDEHQMYEFRVKAVNDVGESEPSLPLNVV
IQDDEVPPTIKLRLAVRGDTIKVKAGEPVNIPADVTGLPMPKIEWSKNEKVIDKPTDTLNITKEEVSRSEAKT
ELSIPKAAREDKGTYTITASNRLGSVFRNVHVEVYDRPSPPRNLAVTDIKAESCYLTWDAPLDNGGSEITHYI
IDKRDASRKKSEWEEVTNTAVERRYGIWKLIPNGQYEFRVRAVNKYGISDECKSDKVVIQDPYRLPGPPGKPK
VLERTKGSMLVSWTPPLDNGGSPITGYWLEKREEGGTYWSRVSRAPITKVGLKGVEFNVPRLIEGVKYQFRAM
AINAAGIGPPSEPSDPAVAGDPIYPPGPPSCPEVKDKTKSSISLAWKPPAKDGGSPIKGYIVEMQEEGTTDWK
PVNEPDKLLTACECVVPNLKELRKYRFRVKAVNEAGESEPSDTTGEIPATDIQEVPEVFIDIGAQDCLVCKAG
SQVKIPAVIKGRPTPKSSWEFDGKAKKAMKDGVHDIPEDAQLETAENSSVIIIPECTRAHSGKYSITAKNKAG
QKTANCRVKVMDAPGPPKDLKVSDITRGSCRLSWKMPDDDGGDRIKGYVIEKKTIDGKAWTKVNPNCGSTTFV
VPDLISEQQYFFRVRAENRFGIGPPAETIQRTTARDPIYPPDPPIKLKIGLITKNTVHLSWKPPKNDGGSPVT
HYIVECLAWDPTGKKKEAWRQCNRRDVEELEFTVEDLIEGGEYEFRVKAVNEAGVSKPSATVGPVIVKDQTCP
PAIELKEFMEVEEGTDVNIVAKIKGVPFPTLTWFKAPPKKPDSKEPVVYDTHVNKQVVDDTCTLVIPQSRRSD
TGLYSITAVNNLGTASKEMRLNVLGRPGPPVGPIKFESISADQMTLSWLPPKDDGGSKITNYVIEKREANRKT
WVRVSSEPKECMYTIPKLLEGHEYVFRIMAQNKYGIGEPLDSEPETARNLFSVPGAPDKPTVSSVTRNSMTVN
WEEPEYDGGSPVTGYWLEMKDTTSKRWKRVNRDPIKAMTLGVSYKVTGLIEGSDYQFRVYAINAAGVGPASLP
SDPVTARDPVAPPGPPFPKVTDWTKSSVDLEWSPPLKDGGSKITGYIVEYKEEGKEEWEKGKDKEVRGTKLVV
TGLKEGAFYKFRVRAVNVAGVGEPGEVTDVIEMKDRIVSPDLQLDASVRDRIVVHAGGVIRIIAYVSGKPPPT
VTWSMNERALPQEAAIETTAISSSMVIKNCQRSHQGVYSLLAKNEGGERKKTIIVDVLDVPGPVGIPFLSDNL
TNDSCKLTWFSPEDDGGSPITNYVIQKREADRRAWTPVTYTVTRQNATVQGLIQGKSYFFRIAAENSIGMGPF
VETPNALVIRDPITVPERPEDLEVKEVTKNTVSLTWNPPKYDGGSEIINYVLESRLIGTEKFHKVTNDNLLSR
KYTVKGLKEGDTYEYRVSAVNIVGQGKPSFCTKPITCKDELAPPTLDLDFRDKLTVRVGESFALTGRYSGKPK
PKIDWFKDEADVLEDDRTHIKTTPTTLALEKTKAKRSDSGKYCVVVENSTGSRKGFCQVNVVDRPGPPVGPVI
FDEVTKEYMVISWKPPLDDGGSEITNYIIEKKELGKDIWMPVTSASAKTTCKVPKLLEGKDYIFRIHAENLYG
ISDPLVSDSMKAKDRFRVPDAPEQPVVTEVTKDSALVTWNKPNDGGKPITNYILEKRETMSKRWVRVTKEPIH
PYTKYRVPDLLEGCQYEFRVSAENEIGIGDPSPPSKPVFARDPIAKPSPPINPEAIDTTCNSVELTWQPPRHD
GGSKILGYIVEYQKVGDEEWRRANHTPESCPETKYKVTGLRDGQSYKFRVLAVNEAGESDPAHVPEPVLVKDR

LEPPELILDANMAREQHIRVGDTLRLSAIIKGVPFPKVTWKKEDREAPTKAQIDVTPVGSKLEIRNAAHEDGG
IYSLTVENPAGTKTVSVKVLVLDKPGPPRDLEVSEIRKDSCYLTWKEPLDDGGSVVTNYVVERKDVATAQWSP
LSTTSKKKSHMAKHLTEGNQYLFRVAAENQYGRGPFVETPKPIKALDPLHPPGPPKDLHHVDVDKTEVSLVWN
KPDRDGGSPITGYLVEYQEEGDKDWIKFKTVKNLDCVVTGLQQGKTYRFRVKAENIIGLGLPDTTIPIECQEK
LVPPSVELDVKLIEGLVVKAGTTVRFPAIIRGVPVPTAKWTTDGTEIKTDDHYTVETDSFSSVLTIKNCLRKD
TGEYQLTVSNAAGTKTVAVHLTVLDVPGPPTGPINILDVTPEYMTISWQPPKDDGGSPVINYIVEKQDTRKGT
WGVVSAGSSKLKLKVPHLQKGCEYVFRVKAENKMGVGPPLDSIPTVAKHKFSPPSPPGKPVVTDITENAATVS
WTLPKSDGGSPITGYYVERREITGKWVRVNKTPIADLKFRVTGLYEGNTYEFRVFAENLAGLSNPSPSSDPIK
ACRPIKPPGPPINPKLKDKSKESADLVWTKPLSDGGSPILGYVVEYQKPGTAQWDRINKDELIRQCAFRVPGL
IEGNEYRFRIRAANIVGEGEPRELAESVIAKDILHPPEVELDVTCRDVITVRVGQTIRILARVKGRPEPDITW
SKEGKVLVKDKRVDLIHDLPRVELQIKEAVRADHGKYIISAKNSSGHAQGSAIVNVLDRPGPCQNLKVSNVTK
ENCTISWENPLDNGGSEITNFIVEYRKPNQKGWSIVASDVTKRLVKANLLANNEYYFRVCAENKVGVGPTIET
KTPILAINPIDRPGEPENLHIADKGKTFVYLKWRRPDYDGGSPNLSYHVERRLKGSADWERVHKGSIKETHYM
VDKCVENQIYEFRVQTKNEGGESDWVRTEEVVVKEDLQKPVLDLKLSGVLTVKAGDTIRLEAGVRGKPFPEVA
WTKDKDATDLTRSPRVKIDTSAESSKFSLTKAKRSDGGKYVITATNPAGSFVAYATVNVLDKPGPVRNLKITD
VSSDRCTIRWDPPEDDGGCEIQNYILEKCESKRMVWSTYSANVLTPSATVTRLIEGNEYIFRVRAENKIGTGP
PTESKPVIAKTKYDRPGRPDPPEVTKVSKEEMTVVWNAPEYDGGKSITGYYLEKKEKHAVRWVPVNKSAIPER
RLKVQNLLPGHEYQFRVKAENEVGIGEPSLPSRPVVAKDPIEPPGPPTNFKVVDTTKSSITLAWGKPVYDGGA
PIIGYVVEMRPKIADASPDEGWKRCNAAAQLIRMEFTVTSLDENQEYEFRVCAQNQVGIGRPAELKEAIKPKE
ILEPPEIDLDASMRKLVVVRAGCPIRLFAIVRGRPAPKVTWRKVGIDNVVRKGQVDLVDTMAFLVIPNSTRDD
SGKYSLTLVNPAGEKAVFVNVKVLDTPGPVADLKVSDVTKTSCHVSWAPPENDGGSQVTHYIVEKREAERKTW
STVTPEVKKTSFNVTNLVPGNEYFFRVTAVNEYGPGVPTDIPKPVLASDPLSEPDPPRKLEVTEMTKNSATLA
WLPPLRDGGAKIDGYIISYREEDQPADRWTEYSVVKDLSLIVTGLKEGKKYKFRVAARNAVGVSMPREAEGVY
EAKEQLLPPKILMPEQITIKAGKKLRVEAHVYGKPNPICKWKKGDDEVVTSSHLAIHKADGSSVLIIKDVTRK
DSGYYSLTAENSSGSDTQKIKVTVMDAPGPPQPPFDISEIDADACSLSWHIPLEDGGSNITNYIVEKCDVSRG
DWVTALASVTKTSCRVGKLIPGQEYIFRVRAENRFGISEPLTSPKMLAKFPFDVPSEPKNARVTKVNKDCIFV
AWDRPDSDGGSPITGYLIERKERNSLLWVKANDTIVRSTEYPCAGLVEGLEYSFRIYALNKAGSSPPSKPTEY
VTARMPVDPPGKPEVVDVTKNSASLIWARPKHDGGSRIIGYFVEACKLPGDKWVRCNTTPHQIPQEEYTATGL
EENAQYQFRAIAKTAVNISQPSEPSDPVTILAENVPPRIELSVEMKSLLTVKAGTNVCLDATVFGKPMPTVSW
KKDTTPIKQAEGIKMAMKRNLCTLELFSVNRKDSGDYTITAENSSGSKSATIKLKVLDKPGPPASVKINKMYA
DRAMLSWEPPLEDGGSEITNYIIDKRETSRPNWAQVSATVPITSCTVEKLIEGHEYQFRICAENKYGVGDPIL
TEPAIAKNPYDPPGRCDPPVISNITKDHMTVSWKAPADDGGSPITGYLVEKRETQAVNWTKVNRKPVIERTLK
ATGLQEGTEYEFRVTAINKAGPGKPSDASKAVYAQDPLYPPGPPAFPKVYDTTRSSVSLSWGKPAYDGGSPII
GYLVEVKRADSDHWVRCNLPEKLQKTRFEVTGLMENTEYQFRVYAVNKIGYSDPSDVPDKHCPKDILIPPEGE
LDAELRKTLILRAGVTMRLYVPVKGRPPPKITWSKPNVNLRERIGLDIKSTDFDTFLRCENVNKYDAGKYILT
LENSCGKKEYTIVVKVLDTPGPPVNVTVKEVSKDSAYVTWDPPIIDGGSPIINYVVEKRDAERKSWSTVTTEC
SKTSFRVSNLEEGKSYFFRVFAENEYGIGDPGETRDAVKASETPGPVVDLKALAITKSSCTIGWKKPRSDGGS
RITGYVVDFLTEENKWQRVMKSLSLQYSTKDLKEGKEYTFRVSAENENGEGTPSEIVVVAKDDVVAPDLDLKD
LPDLCYLAKENSNFRLKIPIKGKPAPSVSWKKGEDPLATDTRVSVESTAVNTTLVVYDCQKSDAGKYTITLKN
VAGTKEGTLSIKVVGKPGIPTGPIKFDEVTAEAMTLKWGPPKDDGGSEITNYVLEKRDSVNNKWVTCASAVQK
TTFRVTRLHEGIEYTFRVSAENKYGVGEGLKSEPIVAKHPFDVPDAPPPPNIVDVRHDSVSLTWTDPKKTGGS
PITGYHIEFKERNSLLWKRANKTPIRMKDFKVTGLTEGLEYEFRVMAINLAGVGKPSLPSEPVVALDPIDPPG
KPEVISVTRNSVTLIWTEPKYDGGHKLTGYIVEKRDLPSKSWMKANHVNVPDCAFTVTDLVEGGKYEFRIRAK
NTAGAISAPSESTGTIICKDEYEAPTIVLDPTIKDGLTVKAGDSIVLSAISILGKPLPKSSWSRAGKDIRPSD
IAQITSTPTSSMLTVKYATRKDAGEYTITATNPFGTKEEHVKVSVLDVPGPPGPIEISNVSAEKATLTWTPPL
EDGGSPIKAYVLEKRETSRLLWTVVSEDIQACRHVVTKLIQGNEYLFRVSAVNHYGKGEPVQSEPVKMVDRFG
PPGPPGKPEISNVTKNTATVSWKRPTDDGGSEITGYHVERREKKGLRWVRATKTPVSDLRCKVTGLQEGNTYE
FRVSAENRAGIGPPSDASNPVLMKDVAYPPGPPSNAHVTDTTKKSASLAWGKPHYDGGLEITGYVVEHQKVGD
DAWIKDTTGTALRITQFVVPDLQTKEKYNFRISAINDAGVGEPAVIPNVEIVEKEVAPDFELDAELRRTLVVR
AGLSIRIFVPIKGRPAPEVTWTKDNINLKHRANIENTESFTLLIIPECNRYDTGKFVMTIENPAGKKSGFVNV
RVLDTPGPVLNLRPTDITKDSVTLHWDLPLIDGGSRITNYIVEKREATRKSYSTVTTKCHKCTYKVTGLTEGC
EYFFRVMAENEYGVGEPTETTEPVRASEAPLPPDSLNIMDITKNTVSLAWPKPRHDGGSKITGYVIEAQRKGS
DQWTHISTVKGLECVVRNLTEGEEYTFQVMAVNSAGRSAPRESRPVIVKEQTMLPELDLRGIYQKLVIARAGD
NIKVEIPVLGRPKPTVTWKKGDQILKQTQRVNVENTATSTILNINECVRSDSGPYPLTAKNTVGEVGDVITIQ
VHDIPGPPTGPIKFDEVSSDFVTFSWEPPENDGGVPISNYVVEMRQTDSTTWVELATTVIRTTYKATRLTTGV
EYQFRVRAQNRYGVGPGITSASVVANYPFKVPGPPGTPQVTAVTKDSMTISWHEPLSDGGSPILGYHIERKER
NGILWQTVSKALVPGNIFKSTGLTDGIAYEFRVIAENMAGKSKPSKPSEPMFALDPIDPPGKPVPLNITRHTV
ALKWAKPEYTGGFKITSYVVEKRDLPNGRWLKANFSNILENEFTVSGLTEDAAYEFRVIAKNAAGAISPPSEP

SDAITCRDDLEAPRIMVDVRFKDTITLKAGEAFKLEADVSGRPPPTMEWTKDGKELEGTGKLEIKIADFSTHL
INKDSSRTDSGAYILTATNPGGFAKHIFNVKVLDRPGPPEGPLAVSDVTSEKCVLSWLPPLDDGGAKIDHYIV
QKRETSRLAWTNVATEVQVTKLKVTKLLKGNEYIFRVMAVNKYGVGEPLESEPVLAVDPYGPPDPPKNPEVTT
ITKDSMVVCWGHPDSDGGSEIINYIVERRDKAGQRWVKCNKKALTDLRFKVSGLTEGHEYEFRIMAENAAGIS
APSATSPFYKACDTVFKPGPPGNPRVLDTSRSSISIAWNKPIYDGGSEITGYMVEIALPEEDEWQVVTPPAGL
KATSYTITSLIENQEYKIRIYAMNSEGLGEPALVPGTPKAEERMLPPEIELDADLRKVVTIRACCTLRLFVPI
KGRPAPEVKWAREHGESLDKASIESTSSYTLLVVGNVNRFDSGKYILTVENSSGSKSAFVNVRVLDTPGPPQN
LKIKEVTKTSVTLTWEPPLLDGGSKIKNYIVEKRESTRKAYSTVATNCHKTSWKVDQLQEGCSYYFRVLAENE
YGIGLPAETAESVKASERPLPPGKITLTDVTRNSVSLSWEKPEHDGGSRILGYIVEMQSKGSDRWATCATVKV
TEATITGLIQGEEYSFRVSAQNEKGISDPRQLSVPVIAKDLVIPPAFKLLFNTFTVLAGEDLKIDVPFIGRPP
PAVTWHKDDIPLKQTTRVNAESTENNSLLTIKEACREDVGHYTVKLTNSAGEATETLNVIVLDKPGPPTGPVK
MDEVTADSVTLSWEPPKYDGGSSINNYIVEKRDTSTTAWQIVSATVARTTIKACRLKTGCEYQFRIAAENRYG
KSTYLNSEPVVAQYPFKVPGPPGTPFVTLASKDSMEVQWHEPVSDGGSKVIGYHLERKERNSILWVKLNKTPI
PQTKFKTTGLEEGIEYEFRVSAENIVGIGKPSKPSECYAAHDPCDPPGRPEAIIVTRNSVTLQWKKPTYDGGS
KITGYIVEKKELPDGRWMKASFTNIIDTQFEVTGLLEDHRYEFRVIARNAAGVFSEPSESTGAITARDEVEPP
RISMDPKYRDTVVVQAGESFKIDADIYGKPIPTTQWVKGDQELSSTARLEIKSTDFATSLSVKDAVRVDSGNY
ILKAKNVAGEKSVTINVKVLDRPGPPEGPVAISGVTAEKCTLAWKPPLQDGGSDITNYIVERRETSRLVWTLV
DANVQTLSCKVLKLLEGNEYIFRIMAVNKYGVGEPLESESLIAKNPFVVPDAPKAPEVTAVTKDSMIVVWERP
ASDGGSEILGYVLEKRDKEGIRWTRCHKRLIGELRLRVTGLLENHNYEFRVSAENAAGLSEPSPPSAYQKACD
PIYKPGPPNNPKVMDVTRSSVFLSWTKPIYDGGCEIQGYIVEKCDVSVGEWTMCTPPTGINKTNLEVEKLLEK
HEYNFRICAINKAGVGEHADVPGPVMVEEKLEAPDIDLDLELRKVINIRAGGSLRLFVPIKGRPTPEVKWGKV
DGDIRDAAIIDVTSSFTSLVLDNVNRYDSGKYTLTLENSSGTKSAFVTVRVLDTPSPPVNLKVTEITKDSVSI
TWEPPLLDGGSKIKNYIVEKREATRKSYAAVVTNCHKNSWKIDQLQEGCSYYFRVTAENEYGIGLPARTADPI
KVAEVPQPPGKITVDDVTRNSVSLSWTKPEHDGGSKIIQYIVEMQAKNTDKWSECARVKSLDAVITNLTQGEE
YLFRVIAVNEKGRSDPRSLAVPIIAKDLVIEPDVRPAFSSYSVQVGQDLKIEVPISGRPKPSISWTKDGMPLK
QTTRINVTDSLDLTTLSIKETHKDDGGQYGITVSNVVGQKTAAIEIITLDKPDPPKGPVKFDEISAESITLSW
NPPLYTGGCQITNYIVQKRDTTTTVWDVVSATVARTTLKVTKLKTGTEYQFRIFAENRYGQSFALESEPVVAQ
YPYKEPGPPGTPFVTAISKESMVVQWHEPINNGGSPVIGYHLERKERNSILWTKVNKTIIHDTQFKALNLEEG
IEYEFRVYAENIVGVGKASKNSECYVARDPCDPPGTPEAIIVKRNEITLQWTKPVYDGGSMITGYIVEKRDLP
EGRWMKASFTNVIETQFTVSGLTEDQRYEFRVIAKNAAGAISKPSDSTGPITAKDEVELPRISMDPKFRDTIV
VNAGETFRLEADVHGKPLPTIEWLRGDKEIEESARCEIKNTDFKALLIVKDAIRIDGGQYILRASNVAGSKSF
PVNVKVLDRPGPPEGPVQVTGVTAEKCTLAWSPPLQDGGSDISHYVVEKRETSRLAWTVVASEVVTNSLKVTK
LLEGNKYIFRIMAVNKYGVGEPLESAPVLMKNPFVLPGPPKSLEVTNIAKDSMTVCWNRPDSDGGSEIIGYIV
EKRDRSGIRWIKCNKRRITDLRLRVTGLTEDHEYEFRVSAENAAGVGEPSPATVYYKACDPVFKPGPPTNAHV
VDTTKNSITLAWSKPIYDGGSEILGYVVEICKADEEEWQIVTPQTGLRVTRFEIAKLIEHQEYKIRVCALNKV
GLGEAASVPGTVKPEDKLEAPELDLDSELRKGIVVRAGGSARIHIPFKGRPTPEITWSKEEGEFTDKVQIEKG
INFTQLSIDNCDRNDAGKYILKLENSSGSKSAFVTVKVLDTPGPPQNLAVKEVRKDSVLLVWEPPIIDGGAKV
KNYVIDKRESTRKAYANVSSKCNKTSFRVENLTEGAIYYFRVMAENEFGVGVPTETSDAVKASEPPSPPGKVT
LTDVSQTSASLMWEKPEHDGGSRILGYVVEMQPKGTEKWSVVAESKVCNAVVSGLSSGQEYQFRVKAYNEKGK
SDPRVLGIPVIAKDLTIQPSFKLPFNTYSVQAGEDLKIEIPVIGRPRPKISWVKDGEPLKQTTRVNVEETATS
TILHIKESSKDDFGKYSVTATNSAGTATENLSVIVLEKPGPPVGPVKFDEVSADFVVISWEPPAYTGGCQISN
YIVEKRDTTTTNWQMVSATVARTTIKISKLKTGTEYQFRIFAENRYGKSTPLDSKPVVVQYPFKEPGPPGTPF
VTSISKDQMLVQWHEPVNDGGSKVTGYHLEQKEKNSILWVKLNKIPIQDTKFKTTGLDEGLEYEFRVSAENIV
GIGKPSKVSECYVARDPCDPPGRPEAIVITRNSVTLKWKKPVYDGGSKITGYIVEKKDLPDGRWMKASFTNVV
ETEFTVTGLVEDQRYEFRVIARNAADNFSEPSESSGAITARDEIDAPNASLDPKYRDVIIVHAGETFVLEADI
RGKPIPDIIWSKDGNELEETAARMEIKSTLQKTTLIVKDCIRTDGGQYTLKLSNVGGTKTIPITVKVLDRPGP
PEGPLKVTGVTAEKCYLAWNPPLQDGGASISHYIIEKRETSRLSWTQVSNEVQALNYKVTKLLPGNEYIFRVM
AVNKYGIGEALESEPVIACNPYKRPGPPSTPEASAITKDSMVLTWTRPVDDGGAEIEGYILEKRDKEGIRWTK
CNKKTLTDLRFRVTGLTEGHSYEFRVAAENAAGVGEPSEPSVFYRACDALYPPGPPSNPKVTDTSRSSVSLAW
NKPIYDGGAPVRGYVIELKKAAADEWTTCTPPSGLQGKQFTVTKLKENTEYNFRICAFNTEGVGEPATIPGSV
VAQERMEAPEIELDADLRKVVTLRASATLRLFVTIKGRPEPEVKWEKAEGILTERAQIEVTSSYTMLVIDNVT
RFDSGRYNLTLENNSGSKTAFVNVRVLDSPSAPVNLTIREVKKDSVTLSWEPPLIDGGAKITNYIVEKRETTR
KAYATITNNCTKNTFKIENLQEGCSYYFRVLASNEYGIGLPAETAEPVKVSEPPLPPGRVTLVDVTRNTATIK
WEKPESDGGSKITGYVVEMQTKGSEKWSACTQVKTLETTISGLTAGEEYVFRVAAVNEKGRSDPRQLGVPVIA
KDIEIKPSVELPFNTFNVKANDQLKIDIPFKGRPQATVAWKKDGQVLRETTRVNVASSKTVTTLSIKEASRED
VGTYELCVSNTAGSITVPITVIVLDRPGPPGPIRIDEVSCDNVSISWNPPEYDGGCQISNYIVEKRETTSTTW
QVVSQAVARTSIKIVRLTTGSEYQFRVCAENRYGKSSYSESSAVVAEYPFSPPGPPGTPKVVHATKSTMVVSW
QVPVNDGGSQVIGYHLEYKERSSILWSKANKVLIADTQMKVSGLDEGLMYEYRVYAENIAGIGKCSKACEPVP

ARDPCDPPGQPEVTNITRKSVSLKWSKPRYDGGAKITGYIVERRELPDGRWLQCNFTNVQETYFEVTELTEDQ
RYEFRVFARNAADSVSEPSESTGPITVKDDVEAPRIMMDVKFRDVIIVKAGEVLKINADIAGRPLPVISWAKD
GVEIEERAKTEIVSTDYTTTLTVKDCVRRDTGQYVLTLKNVAGTRTMAVNCKVLDKPGPPAGPLEINGLTAEK
CSLSWGRPQEDGGADIDYYIVEKRETSRLAWTICEAELRTTSCKVTKLLKGNEYIFRVTGVNKYGVGEPLESM
AVKALDPFTTPSPPTSLEITSVTKDSMTLCWSRPETDGGSDISGYIIERREKNSLRWMRVNKKPVYDLRVKST
GLREGCEYEYRVFAENAAGLSLPSETSPLVRAEDPVFLPSPPSKPKIVDSGKTTITIGWVKPLFDGGAPITGY
TVEYKKSEETDWKVAIQSFRGTEYTMSGLTTGDEYVFRVRSLNKMGASDPSDSSDPQVAKEREEEPVFDVDSE
MRKTLIVKAGSSFTMTVPFRGRPIPNVSWSKPDTDLRTRAYIDSTDSRTLLTIENANRNDSGKYTLTIQNVLS
AASMTFVVKVLDSPGPPANITVREVTKETAMLSWDVPENDGGAPVKNYHIEKREASKKAWVSVTNNCNRLSYK
VTNLQEGAIYYFRVSGENEFGVGVPAETKEGVKITEKPSPPEKLGVTSVSKDSVSLSWLKPEHDGGSRIIHYV
VEALEKGQKTWVKCAVVKTTHHVVSGLRESHEYFFRVFAENQAGLSDPRELLLPVLIKDQLEPPEIDMKNFPS
HTVYVRAGSNLKVDIPISGKPLPKVTLSRDGVPLKATMRFNTEITAENLTINLKESVTTDAGRYEITAANSSG
TTKTFINIIVLDRPGPPTGPVAISDITEESVTLKWEPPKYDGGSHVTNYIVLKRETSTAVWSEVSATVARTMI
KVMKLTTGEEYQFRIKAENRFGISDHIDSVCVVVKLPYTTPGPPSTPWVSNVTRESITVGWHEPVSNGGSAVT
GYHLEMKDRNSILWQKANKMIIRTTHFKVTTISAGLIYEFRVYAENAAGIGKPSHPSEPVLAIDACEPPRNVR
ITDISKNSVNLSWQQPAFDGGSKITGYIVERRDLPDGRWTKASFTNVIETQFTVSGLTQNSQYEFRVFARNAV
GSVSNPSEVVGPITCIDSYGGPVIDLPLEYTEVVKYRAGTSVKLRAGISGKPEPTIEWYKDDKELQTNALVCV
ENSTDLASILIKDANRLNSGSYELKLRNAMGSASATIRVQILDKPGPPGGPIEFKTVTAEKITLLWRPPADDG
GAKITHYIVEKRETSRVVWSMVAENLEECIVTTTKIIKGNEYVFRVRAVNKYGIGEPLESEPVVAKNAFVTPG
PPSIPEVTKITKNSMTVVWDRPTVDGGSEINGYFLERRDKKSLAWLKVLKETIRDTRQKVTGLTENSDYQYRV
CAVNAAGVGPFSEPSDFYKAADPIDPPGPPAKIRIADSTKSSITLGWSKPVYDGGSDVTGYVVEMKQGDEEEW
TIVSTRGEVRTTEYVVSNLKPGVNYYFQVSAVNCAGQGEPITMTEPAQAKDVLEEPEIDLDVALRTSVIAKAG
EDVQLLIPFKGRPPPTVTWRKDEKNLGSDTRYSIQNTDSSSLLVIPQVTRNDTGKYILTIENGVGQPKSSTVS
VKVLDTPAACQKLQVKHVSLGTVTLLWDPPLIDGGSPIINYVIEKRDATKRTWSVVSHKCSGTSFKVTDLSEK
TPFFFRVLAENEIGIGEPCETTEPVKAAEVPAPIRDLSMKDSTKTSVVLSWTKPDFDGGSIITDYLVERKGKG
EQAWSHAGISKTCEIEIGQLKEQSVLEFRVSARNEKGQSDPVTIGPLTVKELVITPEVDLSEIPGAQISVRIG
HNVHLELPYKGKPKPSISWLKDGLPLKESEYVRFSKTENKITLSIKNSKKEHGGKYTVILDNAVCRNSFPITI
ITLGPPSKPKGPIRFDEIKADSAIMSWDIPEDDGGGEITCYSIEKREASQTNWKMVCSSVARTTFKVSNLVKD
SEYQFRVRAENRYGVSEPLASNIIVAKHQFRIPGPPGKPVIYNVTSDGMSLTWDAPVYDGGSEVTGFHVEKKE
RNSILWQRVNTSPISGREYRATGLIEGLDYQFRVYAENSAGLSSPSDPSKFTLAVSPVDPPGTPDYIDVTRET
ITLKWNPPLRDGGSKIVAYSIEKRQGSDRWVRCNFTDVSECQYTVTGLSPGDRYEFRIIARNAVGTISPPSQS
SGLIMTRDENVPPTVEFGPEYFDGLVIKSGDSLRIKALVQGRPVPRVTWFKDGVEIERRMNMEITDVLGSTSL
FVRDATRDHRGVYTVEAKNVSGSTKAEVTVKVQDTPGKVVGPIRFTNITGEKMTLWWEAPLNDGCAPVTHYII
EKRETSRLAWALIEDNCEALSYTAIKLITGNEYQFRISAVNKFGVGRPLESDPVVAQIQYTIPDAPGVPEPSN
VTGNSITLTWTRPESDGGSEIQHYILERREKKSTRWVKVISKRPISETRFKVTGLVEGNEYEFHVMAENAAGI
GPASGISRLIKCREPVNPPSAPSVVKVTDTSKTTVSLEWARPVFDGGMEIIGYIIEMCKADLGDWHKVNTEPC
VKTRYTVTDLQAGEEYKFRVSAVNGAGKGDSCEVTGTIKAVDRLSAPELDIDANFKQTHIVRAGVSIRLFIAY
QGRPTPTAVWSKPDSNLSIRADIHTTDSFSTLTVENCNRNDAGKYTLTVENNSGKKSITFTVKVLDSPGPPGP
ITFKDVTRGSATLMWDAPLLDGGARIHHYVIEKREASRRSWQVVSEKCTRQILKVSELTEGVPYYFRVSAENE
YGVGEPYEMPEPIVATEQPAPPRRLDVVDTSKSSAVLAWLKPDHDGGSRITSYLLEMRQKGSDFWVEAGHTKQ
LTFTVERLVENTEYEFRVKAKNDAGYSEPREAFSSVIIKEPQIEPTADLTGITNQLITCKAGSTFTIDVPISG
RPAPKVTWKLEEMRLKETDRMSIATTKDRTTLTVKDSMRGDSGRYFLTLENTAGVKTFTITVVVIGRPGPVTG
PIEVSSVSAESCVLSWTEPKDDGGTEITNYIVEKRESGTTAWQLINSSVKRTQIKVTHLTKYKEYCFRVSSEN
RFGVSKPLESVPIVAEHPFVPPSAPTRPEVYYVSANAMSIRWEEPYHDGGSKIVGYWVEKKERNTILWVKENK
VPCLECNYKVTGLVEGLEYQFRTYALNAAGVSKASEASRPIMAQNPVDPPGRPEVTDVTRSTVSLIWSAPVYD
GGSKVVGYIIERKPVSEVGDGRWLKCNYTIVSDNFFTVTALSEGDTYEFRVLAKNAAGVISKGSESTGPVTCR
DEYAPPKAELDARLQGDLVTIRAGSDLVLDAAVGGKPEPKIIWTKGDKELDLCEKISLQYTGKRATAVIKYCD
RSDSGKYTLTVKNASGTKSVSVMVKVLDSPGPCGKLTVSRVTEEKCTLAWSLPQEDGGAEITHYIVERRETSR
LNWVIVEGECLTASYVVTRLIKNNEYTFRVRAVNKYGLGVPVESEPIVARNSFTIPSQPGIPEEVGAGKEHII
IQWTKPESDGGNEISNYLVDKREKKSLRWTRVNKDYVVYDTRLKVTSLMEGCDYQFRVTAVNAAGNSEPSEAS
NFISCREPSYTPGPPSAPRVVDTTKRSISLAWTKPMYDGGTDIIGYVLEMQEKDTDQWCRVHTNATIRNNEFT
VPDLKMGQKYSFRVAAVNAKGMSDYSETTAEIEPVERLEIPDLELADDLKKTVIVRAGASLRLMVSVSGRPSP
VITWSKKGIDLANRAIIDNTESYSLLIVDKVNRYDAGKYTIEAENQSGKKSATVLVKVYDTPGPCPSVSVKEV
SRDSVTITWEIPTIDGGAPVNNYIIEKREAAMRAFKTVTTKCSKTLYRISGLVEGTMYYFRVLPENIYGIGEP
CETSDAVLVSEVPLVPTKLEVVDVTKSTVTLAWEKPLYDGGSRLTGYVLEACKAGTERWMKVVTLKPTVLEHT
VISLNEGEQYLFRVRAQNEKGVSEPREIVTPVTVQDLRVLPTIDLSTMPQKTIHVPAGRPIELVIPITGRPPP
TASWFFAGSKLRESERVTVETHTKVTKLTIRETTIRDTGEYTLELKNVTGTTSETIKVIILDKPGPPTGPIKI
DEIDATSVTISWEPPELDGGAPLSGYVVEQRDAHRPGWLPVSESVTRPTFKFTRLTEGNEYVFRVAATNRFGI

GSYLQSEVIECRSSISIPGPPETLQIFDVSRDGMTLTWYPPEDDGGSQVTGYIVERKEVRADRWVRVNKVPVT
MTRYRSTGLIEGLEYEHRVTAINARGTGKPSRPSKPTVAMDPIAPPGKPQNPRVTDTTRTSVSLAWSVPEDEG
GSKVTGYLIEMQKVDQREWTKCNTTPTKIREYTLTHLPQGAEYRFRVLACNAGGPGEPAEVPGTVKVTEMLEY
PDYELDERYQEGVFVRQGGVIRLTIPIKGKPFPVCKWTKEGQDISKRAMIATSETHTELVIKEADRNDSGTYD
LVLENKCGKKTVYIKVKVIGSPNTPEGPLEYDDIQARSVRVSWRPPADDGGADILGYILERREVPKAAWYTID
SRVRGTSLVVKGLKENVEYHFRVSAENQFGISKPLKSEEPVIPKTPLNPPEPPSNPPEVLDVTKSSVSLSWSR
PKDDGGSRVTGYYIERKETSTDKWVRHNKTQITTTMYTVTGLVPDAEYQFRIIAQNDVGLSETSPASEPVVCK
DPFDKPSQPGELEILSISKDSVTLQWEKPECDGGKEILGYWVEYRQSGDSAWKKSNKERIKDRQFTIGGLLEA
TEYEFRVFAENETGLSRPRRTAMSVKTKLTSGEAPGVRKEMADVTTKLGEAAQLSCQIVGRPLPDIKWYRFGK
ELIQSRKYKMSSDGRTHTLTVMTDEQEDEGVYTCVATNEVGEVESSSKLLLQAAPQFHPGYPLKEKYYGAVGS
TLRLHVMYIGRPVPAMTWFHGQKLLQNSEKITIENTEHYTHLVMKNVQRKTHAGKYKVQLSNAFGTVDATLDV
EIQDKPDKPTGPIVIEALLKNSVVISWKPPADDGGSWITNYVVEKCEAKEGAEWQLVSSAISVTTCRIVNLTE
NAGYYFRVSAQNTFGISEPLEVASIVIIKSPFEKPGVPGKPTITAVTKDSCVVAWKPPASDGGAKIRNYYLER
REKKQNKWIAVTTEEIRETVFSVQNLIEGLEYEFRVKCENLGGESEWSEISEPVTPKSDVPIQAPHFKEELRN
LNVRYQSNATLVCKVTGHPKPIVKWYRQGKEIIADGLKYRIQEFKGGYHQLIIASVTDDDATVYQVRATNQGG
SVSGTASLEVEVPAKIHLPKTLEGMGAVHALRGEVVSIKIPFSGKPDPVITWQKGQDLIDNNGHYQVIVTRSF
TSLVFSNGVERKDAGFYVVCAKNRFGIDQKTVELDVADVPDPPRGVKVSDVSRDSVNLTWTEPASDGGSKVTN
YIVEKCATTAERWLRVGQARETRYTVINLFGKTSYQFRVIAENKFGLSKPSEPSEPTVTKEDKTRAMNYDDEV
DETREVTTTKASHSKTKELYEKYMIAEDLGRGEFGIVHRCVETSSKRTFMAKFVKVKGTDQVLVKKEISILNI
ARHRNILYLHESFESMEELVMIFEFISGLDIFERINTSAFELNEREIVSYVRQVCEALEFLHSQNIGHFDIRP
ENIIYQTRKNSTIKIIEFGQARQLKPGDNFRLLFTAPEYYAPEVHQHDVVSTATDMWSLGTLVYVLLSGINPF
LAETNQQMIENIMNAEYTFDEEAFKEISLEAMDFVDRLLVKERKSRMTASEALQHPWLKQRIDRVSTKVIRTL
KHRRYYHTLIKKDLNMVVSAARISCGGAIRSQRGVSVAKVKVASIEIGPVSGQIMHAIGEEGGYVKYVCKIEN
YDQSTQVTWYFGVRQLENSEKYEITYEDGVATMYVKDITKFDDGTYRCKVVNDYGEDSSYAELFVKGVREVYD
YYCRRTKKVKRRTDAMRLLERPPEFTLPLYNKTAYVGENVRFGVTITVHPEPRVTWYKSGQKIKPGDDEKKYT
FESDKGLYQLTINSVTTDDDAEYTVVARNKHGEDSCKAKLTVTLHPPPTETTLRPMFKRLLANAECHEGQSVC
FEIRVSGIPAPTLKWEKDGQPLSLGPHIEIVHEGLDYYALHIRDTLPEDTGYYRVTATNTAGSTSCQAHLQVE
RLRYVKQEFQSKEERERHVQKQIDKTLRMAEILSGTETVPLTPVAQEALREAAILYKPAVSTKTVKGEYRLQT
EEKKEERKLRMPYEVPEPRRFKQATVEEDQRIKQFVPMSDMKWYKKIRDQYEMPGKLDRVVQKRPKRIRLSRW
EQFYVMPLPRITDQYRPKWRIPKLTQDDLEMVRPARRRTPSPDYDLYYYRRRRRSLGDMSDEELLLPIDDYLA
MKRTEEERLRLEEELELGFSASPPSRSPPRFELSSLRYSSPPAHVKVEDRRRDFRYSTYHVPTKEETSTSYAE
LRERHAQASYRQPKLRQRIMAEKEEEELLRPVTTTQRLSEYKSELDYMSKEEKSKKKSKRQRQVTEITEIEEE
YEISRRAQRESSSSVSRLLRRRRSLSPTYIELMRPVSELIRSHPRPAEEYEDDAERRSPTPERTRPRSPSPVS
SERSLSRFERSARFDIFSRYESMKAALKTQKTSERKYEVLSQQPFTLDHAPRITLRMRSHRVPCGQNTRFILN
VQSKPTAEVKWYHNGVELQESSKIHYTNTSGVLTLEILDCQTEDGGTYRAVCTNYKGEASDYATLDVTGGAYT
TYASQRRDEEVPKSVFPELTKTEAYAVSSFKRTSELEAASSVREVKSQMTETRESLSTYEHYASAEMKSATSE
EKSLEEKATVRKIKTTLAARILTKPRSITVHEGESARFSCDTDGEPVPTVTWLREGQVVSTSARHQVTTTKYK
STFEISSVQASDEGNYSVVVENSDGKQEAQFTLTVQKARVIEKAVTSPPRVKSPEPRVKSPETVKSPKRVKSP
EPVTSHPKAVSPTETKPTEKGQHLPVSAPPKITQSLKAEASKDIAKLTCAVESSALCAKEVAWYKDGKKLKEN
GHFQFHYSADGTYELKIHNLSESDCGEYVCEVSGEGGTSKTSFQFTGQSFKSIHEQVSSISETTKSVQKTAES
AEAKKSAQKTAESVEDKKAAQKTVESAEAKKPAQKTAESPEAKKPAQKTAESPEAKKQEPIAPESISSKPVIV
TGLRDTTVSSDSVAKFTIKVTGEPQPTITWTKDGKAIAQGSKYKLSSNKEEFILEILKTETSDGGLYACTVTN
SAGSVSSSCKLTIKAVKDTEAQKVSTQKTSEVTSQKKASAQEEISQKALTSEEIKMSEVKSHETLAIKEEASK
VLIAEEVKRSAAASLEKSIVHEEVTKTSQASEEVKTHAEIKTLSTQMNITKGQRATLKANIAGATDVKWVLNG
TELPNSEEYRYGVSGSDQTLTIKQASHREEGILSCIGKTSQGVVKCQFDLTLSEELSDAPSFITQPRSQNINE
GQNVLFSCEVSGEPSPEIEWFKNNLPISISSNISVSRSRNVYTLEIRNAAVSDSGKYTIKAKNFHGQCSATAS
LTVLPLVEEPPREVVLKTSSDVSLHGSVSSQSVQMSASKQEASFSSFSSSSASSMTEMKFASMSAQSMSSMQE
SFVEMSSSSFMGKSSMTQLESSTSRMLKAGGRGIPPKIEALPSDISIDEGKVLTVACAFTGEPTPEITWSCGG
RKIQNQEQQGRFHIENTDDLTTLIIMDVQKQDGGLYTLSLGNEFGSDSATVNINIRSM*
>ENSMUSP00000107477_c.77325:A>C_p.K25776Q ENSMUST00000111846.9|Ttn-204|ENSMUSG00000051747|-|chr2:g.76568328:T>G
MTTQAPMFTQPLQSVVVLEGSTATFEAHVSGSPVPEVSWFRDGQVISTSTLPGVQISFSDGRARLMIPAVTKA
NSGRYSLRATNGSGQATSTAELLVTAETAPPNFSQRLQSMTVRQGSQVRLQVRVTGIPTPVVKFYRDGAEIQS
SLDFQISQEGDLYSLLIAEAYPEDSGTYSVNATNSVGRATSTAELVVQGEEVVPAKKTKTIVSTAQISETRQT
RIEKKIEAHFDARSIATVEMVIDGATGQLPHKTPPRIPPKPKSRSPTPPSIAAKAQLARQQSPSPIRHSPSPV
RHVRAPTPSPVRSVSPAGRISTSPIRSVKSPLLIRKTQTTTMATGPEVPPPWKQEGYVASSTEAEMRETTMTS
STQIRREERWEGRYGVQEQVTISGAAAAAASASVSSSFTAGAITTGTKEVKQETDKSAAVATVVAAVDMARVR
EPAISAVEQTAQRTTTTAVHIQPAQEQARKEAEKTAVTKVVVAADKAKEQELKSRTREVMVTTQEQTHISHEQ

IRKETEKAFVPKVVISATKAKEQETRITGEITTKQEQKRITQETIRQETEEIAASMVVVATAKSTKLEAAVGV
QEETAIQQDQMHLTHEQMMKETRKTVVPKVIVATPKIKEQDLVSRSREAITTKRDQVQITQEKKRKEVETTAL
STIAVATAKAKEQETVLRSREAMATRQEHIQVTHGQVGVGKKAEAVATVVAAVDQARVREPREPTHVEESHSQ
QTTLEYGYKEHISTTKVPEQPRRPASEPHVVPQAVKPAVIQAPSETHIKTTDQMGMHISSQVKKTTDISTERL
VHVDKRPRTASPHFTVSKISVPKTEHGYEASIAGSAIATLQKELSATSSTQKITKSVKAPTVKPGETRVRAEP
TPSPQFPFADMPPPDTYKSQAGIEVKKEVGVSISGSTVREEHFEVLRGREAKVTETARVPAPAEVPVTPPTLV
SGLKNVTVIEGESVTLECHISGYPSPKVTWYREDYQIESSIDFQITFQGGIARLMIREAFAEDSGRFTCSAVN
EAGTVSTSCYLAVQVSEEFDKETTLTEKFATEEKRFVESRDVVMTDTSITEEQAGPGEPAAPFFISKPVVQKL
VEGGSVVFECQIGGNPKPHVYWKKSGVPLTTGYRYKVSYNKQTGECRLVISMTFADDAGEYTIVIRNKHGETS
ASASLLEEADYEALVKTQQEMLYQTQMSTFIQEPKVGEIAPGFAYSEYEKEYEKEQALIRKKMAKDTVMVRTF
VEDQEFHISSFEERLIKEIEYRIIKTTLEELLEEDGEEKMAVDISESEAIESGFDIRIKNYRILEGMGVTFHC
KMSGYPLPKIAWYKDGKRIRHGERYQMDFLQDGRASLRIPVVLPEDEGIYTAFASNIKGNAICSGKLYVEPAA
PFSAPTYMPTPEAVSRIRSVSPRSLSRSPIRMSPAMSPARMSPARMSPARMSPARMSPGRRLEETDESQLERL
YKPVFVLKPASFKCLEGQTARFDLKVVGRPMPETFWFHNGQQIVNDYTHKVVIKEDGTQSLIIVPASPSDSGE
WTVVAQNRAGKSTISVTLTVEAVEHQIKPAFVEKLKNVNIKEGSRLEMKVRATGNPNPDIVWLKNSDIIVPHK
YPRIRIEGTRGEAALKIDSIISQDSAWYTATAINKAGRDTTRCKVNVEVEFAEPEPERKLIIPRGTYRAKEIA
APELEPLHLRYGQEQWEEGDLYDKEKQQKPFFKKKLTSLRLKRFGPAHFECRLTPIGDPTMVVEWLHDGKPLE
AANRLRMINEFGYCSLDYGAAYSRDSGVITCRATNKYGTDHTSATLIVKDEKSLVEESQLPDGKKGLQRIEEL
ERMAHEGALTGVTTDQKEKQKPDIVLFPEPVRVLEGETARFRCRVTGYPQPKVNWYLNGQLIRKSKRFRVRYD
GIHYLDIVDCKSYDTGEVKVTAENPEGVTEHKVKLEIQQREDFRSVLRRAPEPKPEFHVHEPGKLQFEVQKVD
RPVDTSETKEVVKLKRAERITHEKVSEESEELRSKFKRRTEEGYYEAITAVELKSRKKDESYEELLKKTKDEL
LHWTKELTEEEKKALAEEGKITIPTFKPERIELSPSMEAPKIFERIQSQTVGQGSDAHFRVRVVGKPDPECEW
YKNGVKIERSDRIYWYWPEDNVCELVIRDVTAEDSASIMVKAINIAGETSSHAFLLVQAKQLITFTQELQDVV
AKEKDTMATFECETSEPFIKVKWYKDGIEVHAGDKYRMHSDRKVHFLSVLTIDTSDAEDYSCVLVEDENIKTT
AKLIVEGAVVEFVKELQDIEVPESYSGELECIISPENIEGKWYHNDVELKSNGKYSITSRRGRQNLTVKDVTK
EDQGEYSFVVDGKKTTCKLKMKPRPIAILQGLSDQKVCEGDIVQLEVKVSLENVEGVWMKDGQEVQHSDRVHI
VIDKQSHMLLIEDMTKEDAGNYSFTIPALGLSTSGNVSVYSVDVITPLKDVNVIEGTKAVLECKVSVPDVTSV
KWYLNDEQIKPDDRVQSIVKGTKQRLVINRTHASDEGPYKLMVGRVETSCNLSVEKIKIIRGLRDLTCTETQN
VVFEVELSHSGIDVVWNFKGKEIKPSSKYKIEAHGKIYKLTVLNMMKDDEGEYAFYAGENTTSGKLTVAGGAI
STPLTDQTVAESQEAVFECEVANPESEGEWLKDGKHLALSNNFRGESDGHKRRLVIAAAKLDDAGEYTYKVAT
SKTSAKLKVEAVKIKKTLRNLTVTETQDAVFSVELTHPDVKGVQWIKNGVVLDSNDKYEISVKGTLYSLKIKN
CAMADESVYGFKLGRLGASARLHVETVKIIKKPKDVTALENATVTFEVSVSHDTVPVKWFHKNVEIKPSDKHR
LVSERKVHKLMLQSISPSDAGEYTAMVGQLECKAKLFVETLHITKTMKSIEVPETKAASFECEVSHFNVPSMW
LKNGVEIEMSEKFKIVVQGKLHQLIIMNTSTEDSAEYTFVCGNDQVSATLTVTPIMITSMLKDINAEEKDTIT
FEVTVNYEGISYKWLKNGVEIKSTDRCQMRTKKLTHSLNIRNVHFGDAADYTFVAGKATSTATLYVEARHIEF
RKHIKDIKVLEKKRAMFECEVSEPDITVQWMKDGQELQIADRIKIQKEKYVHRLLIPSTRMSDAGKYTVVAGG
NMSTANLFVEGRDVRIRSIKKEVQVIEKQRAVVEFEVNEDDVDAHWYKDGIEINFQVQERHQYVVERRIHRMF
ISETRHSDAGEYTFVAGRNRSSVTLYVNAPEPPQVLQELQPVTVQSGKPARFCAVISGRPQPKISWYKEEQLL
STGFKCKFLHDGQEYTLLLIEAFPEDAAVYTCEAKNDYGVATTSASLSVEVPEVVSPDQEMPVYPPAIVTPLQ
DTVTSEGRPARFQCQVSGTDLKVSWYCKDKKIKPSRFFRMTQFEDTYQLEIAEAYPEDEGTYAFVANNAVGQV
SSTATLRLEAPESILHERIGQQIEMEMKAAPVIKRRIEPLEVALGHLAKFTCEIQGAPNVRFQWFKAGREIYE
SDKCSIRSSNYVSSLEILRTQVVDCGEYTCKASNEYGSVSCTATLTVTEAYPPTFLSRPKALTTFVGKAAKFL
CTVSGTPVIEIIWQKDGAALSPSPDCRVTDADNKHSLELSNLTVQDRGIYSCKASNKFGADICQAELTIIDKP
HFIKELEAVQSAINKKIHLECQVDEDRKVTITWSKDGQKLPAGKDYKIYFEDKIASLEIPLAKLKDSGTYTCT
ASNEAGSSSSSAAVAVREPPSFVKKVDPSYLMLPGESARLHCKLKGSPVIQVTWFKNNKELSESNTVRMSFVN
SEAILDITDVKVDDSGTYSCEATNDVGSDSCSTEVVIKEPPSFIKTLEPADIVRGANALLQCEIAGTGPFEVN
WFKDKKQIRSSKKYRLFTQKTFVYLEISSFNSADVGDYECVVANEVGKCGCVATHLLKEPPTFVKKVDDFTAL
AGQTVTLQAAVRGSEPISVMWMKGQEVIKEDGKIKMSFSNGVAVLTIPDVQISLGGKYTCLAENEAGSQTSVG
ELIVKEPAKIIERAELIQVTAGDPATLEYTVSGTPELKPKWYKDGRPLVASKKYRISFKNNIAQLKFYSAELH
DSGQYTFEISNEVGSSSCETTFTVLDRDIAPLFTKPLRNVDSVVGGACRLDCKIAGSLPMRVSWFKDGKELTA
SDRYQIAFVEGTASLEISRVDMNDAGNFTCRATNSVGSKDSSGALIVQEPPSFVTKPGSRDVLPGSAVCLKSA
FQGSAPLTIKWFKGDKELVSGGSCYITKETSESSLELYAVKTSDSGTYTCKVSNVAGSVECSADLFVKEPATF
IEKLEPSQLLKKGDGTQLACKVTGTPPIKITWFANDRELRESSKHKMSFAESTAVLRLTDVAIEDSGEYMCEA
QNEAGSDHCTGIVIVKESPYFTKEFKSIEVLKEYDVMLLAEVAGTPPFEITWFKDNTTLRSGRKYKTFLQDQL
VSLQVLKFVAADAGEYQCRVTNEVGSSTCSARVTLREPPSFIKKIEATSSLRGGTAAFQATLKGSLPITVTWL
KDNDEITEDDNIRMTFENNVASLYLSGIEVKHDGKYVCQAKNDAGIQRCSALLSVKEPATIMEEAVSIDVTQG
DPATLQVKFSGTKEISAKWFKDGQELTLGPKYKISVTDTVSILKIISTEKKDSGEYTFEVQNDVGRSSCKASI
NVLDLIIPPSFTKKLRKMDSIKGSFIDLECIVAGSHPISIQWFKDDQEISASDKHKFSFHDNTAFLEISQLEG

TDSGTYTCSATNKAGHSQCSGHLTVKEPPYFVEKPQSQDVNPGTRVQLKALVGGTAPMTIKWFKDNKELHPGA
ARSVWKDDTSTILELFSAKAADSGTYICQLSNDVGTTSSKATIFVKEPPQFIKKPSPVLVLRNGQSTTFECQV
TGTPEIRVSWYLDGNEITDLRKYGISFVDGLATFQISNARVENSGTYVCEARNDAGTASCSIELKVKEPPIFI
RELEPVEVVKDSDVELECEVMGTTPFEVTWLKNNKEIRSGKKYTMSEKMSVFYLHITKCDPSDVGEYQCIIAN
EGGSCACSARVALKEPPSFIKKIENVTTVLKSSATFQSTVAGSPPISITWLKDDQILEENDNVHISFEDSVAT
LQVRSVDNGHSGRYTCQAKNESGIERCYAFLLVQEPAQIIEKAKSVDVTEKDPVTLECVVAGTPELKVKWLKD
GKQIVPSRYFSMSFENNVASFRIQSVMKQDSGQYTFKVENDFGSSSCDAYLRVLDQDIPPSFTKKLTKMDKVL
GSSIHMECKVSGSLPISAQWFKDGKEISTSAKYRLVCHENTVSLEVSNLELEDTANYTCKVSNVAGDNACSGI
LTVKEPPSFLVKPERQQAIPDSTVEFKAVLKGTPPFKIKWLKDDVELVSGPKCFIGLEGSTSFLNLYSVDSSK
TGQYTCQVTNDVEPPKFVKKLEASKIIKAGDSARLECKITGSPEIQVVWYRNEHELTASDKYQMTFIDSVAVI
QMNSLGTEDSGDFICEAQNPAGSTSCSTKVIVKEPPVFSSFPPIVETLKNTEVSLECELSGTPPFEVVWYKDK
RQLRSSKKYKVASKNFHASIHILNVESTDIGEYHCKAQNEVGSDACVCAVKLKEPPKFISKLNSLTVVAGEPA
ELQASIEGAQPISVQWLKEKEEVIRESENIRISFVNNVATLQFAKVEPANAGKYICQVKNDGGVRENMATLTV
LEPAVIIEKAGSMTVTVGETCALECKVAGTPELSVEWYKDGKLLTSSQKHKFSFYNKISSLKILSVEKEDAGT
YTFQVQNNVGKSSCTAVVDVSDRMVPPSFTRRLKDTGGVLGTSCILECKVAGSSPISIAWFHEKTKIVSGAKY
QTTFSDNVCTLQLNSLDSSDMGSYTCVAANVAGSDECRALLTVQEPPSFVKEPEPLEVLPGKNITFTSVIRGT
PPFKVGWFRGARELVKGNRCNIYFEDTVAELELFNIDISQSGEYTCVVSNNAGQASCTTRLFVKEPATFVKKL
SDHSVEPGKSIILEGTYTGTLPISVTWKKDGVSITPSERCNIVTTEKTCILEILSSTKGDAGHYSCEIENEAG
RDACDALVSTLEPPYFVTELEPLEASVGDSVSLQCQVAGTPEITVSWFKGDTKLRSTPEYRTYFTNNVATLVF
NKVSINDSGEYTCMAENSIGTAASKTIFRIQERQLPPSFARQLKDIEQTVGLPVTLTCRLNGSAPIQVCWYRD
GVLLRDDENLQMSFVDNVATLKILQTDLSHSGQYSCSASNPLGTASSTARLTAREPKKSPFFDIKPVSIDVIA
GESADFECHVTGAQPMRVTWSKDNKEIRPGGNYTITCVGNTPHLRILKVGKGDSGQYTCQATNDVGKDMCSAQ
LSVKEPPKFIKKLDASKVAKQGESIQLECKISGSPEIKVVWFRNDSELHESWKYNMSFVNSVALLTINEASAE
DTGDYICEAHNGVGDASCSTALKVKAPPVFTQKPPPVGALKGSDVILQCEISGTPPFEVVWVKDRKQVRSSKK
FKITSKNFDTSLHIFNLEAPDIGEYHCKATNEVGSDTCACTVKFKEPPRFVKKLSDASTLIGDPVELQAVVEG
FQPISVVWLKDKGEVIRESENVRISFVDNIATLQLGSPEASQSGKYVCQIKNDAGMRECSAVLTVLEPATIVE
KPEPMTVTTGNPFTLECVVAGTPELSAKWFKDGRELSSGSRHHITFVRNLASLKIPSAEMNDKGLYTFEVENR
VGKSSCTVSVHVSDRVVPPSFVRRLKDTSATLGASVVLECRVSGSAPISVGWFLDGNEIISSPKCQSSFADNV
CTLTLSSLEPSDTGAYTCVAANVAGQDESSAVLTVQEPPSFEQTPDSVEVLPGMSLTFTSVIRGTPPFKVKWF
KGSRELVSGEACTISLEDFVTELELLEVEPGQSGDYSCLVTNDAGSASCTTHLFVKEPATFVKRLADTSVETG
SPIVLEATYSGTPPISVSWMKNEYPLSQSPNCGITTTEKSSILEILESTIEDYAQYACLIENEAGQDICEALV
SVLEPPYFIEPLEHVEAAIGEPITLQCKVDGTPEIRISWYKEHTKLRSAPAYKMQFKNNVASLVINKVDHSDV
GEYTCKAENSVGAVASSAVLVIKERKLPPSFARKLKDVHETLGFPVAFECRINGSEPLQVSWYKDGELLKDDA
NLQMSFVHHVATLQILQTDQSHVGQYNCSASNPLGTASSSAKLILSEHEVPPFFDLKPVSVDLALGESGSFKC
HVTGTAPIKITWAKDNREIRPGGNYKMTLVENTATLTVLKVAKGDAGQYTCYASNVAGKDSCSAQLGVQEPPR
FIKKLDQSRIVKQDEYTRYECKIGGSPEIKVLWYKDEVEIQESSKFRMSFEDSVAILEMHSLSVEDSGDYTCE
ARNAAGSASSSTSLKVKEPPVFRKKPFPVETLKGADVHLECELQGTPPFQVSWHKDKRELRSGKKYKIMSENL
LTSIHILNVDTADIGEYQCKATNDVGSDTCVGSVTMKAPPQFVKKLTDISTIIGKEVQLQTTIEGAEPISVAW
FKDKGEIVRESDNIWISYSENIATLQFSRAEPANAGKYTCQIKNDAGMQECYATLSVLEPAAIVEKPESIKVT
TGDTCTLECTVSGTPELSTKWFKDGKELTSDNKYKISFFNKVSGLKIINVVPGDSGVYSFEVQNPVGKDSCKV
SIQVSDRIIPPSFTRKLKETNGLSGSSVVMECKVYGSPPISVLWFHDGNEISSGRKYQTTLTDNTCALTVNML
EEADAGDYTCIATNVAGSDECSAPLTVREPPSFVQKPDPMDVLTGSNVTFTSIVKGSPPFTVSWFKGSTELVP
GARCNVSLQDSVGELELFDVDTSQSGEYTCIVSNEAGRASCTTRLFVKAPAIFVKRLNDYSIEKGKPLILEGT
FSGTPPISVTWKKNGINVIASQRCNITTTEKSAILEILSSTVEDSGQYNCYIENASGKDSCSAQILILEPPYF
VKQLEPVKVTVGDSASLQCQLAGTPEIGVSWYKGDTKLRPTATCKMHFKNNVATLVFTQVDSSDSGEYICRAE
NSVGEVSSSTFLTVQEQKLPPSFSRQLRDVQETVGLPVVFECAVSGSEPISVSWYKDGKPLKDSPNIQTSFLD
NIATLNIFKTDRSLSGQYSCTATNPIGSASSGAKLILTEGKNPPFFDIPLAPMDAVVGESADLECHVTGTQPI
KVTWAKDNREIRSGGNYQISYLENSAHLTIVKVDKGDSGQYTCYAVNEVGKDSCTAQLNIKERLIPPSFTKKL
SETVEETEGNSFKLEGRVAGSQPITVAWYKNNVEIHPTSNCEIMFKNNALLLQVKRASMADAGLYTCKATNDA
GSALCTSSIVIKEPKKPPVFDQHLAPVTASEGDSVQLSCHVQGSEPIRIQWLKAGREVKPSDRCSFSFASGTA
MLELKETAKADSGDYVCKASNVAGSDTSKCKVTIKEKPAAAPAAKKAAVDGKLFFVSEPQSIRVVEKTTATFI
AKVGGDPIPNVKWTKGKWRQLNQGGRILIHQKGDEAKLEIRDTTKTDSGLYRCVAFNKHGEIESNVNLQVDER
KKQEKIEGDLRAMLKKTPALKKGSGEEEEIDIMELLKNVDPKEYEKYARMYGITDFRGLLQAFELLKQSQEEE
THRLEIEELEKSERDEKEFEELVAFIQQRLTQTEPVTLIKDIENQTVLKDNDAIFEIDIKINYPEIKLSWYKG
TEKLEPSNKYEISIDGDRHTLRVKNCQPKDQGNYRLVCGPHIASAKLTVIEPAWERHLQDVTLKEGQTCTMTC
QFSVPNVKSEWFRNGRVLKPQGRVKTEVEHKVHKLTIADVRAEDQGQYTCKHEDLETSAELRIEAEPIQFTKR
IQNIVVSEHQSATFECEVSFDDAIVTWYKGPTELTESQKYNFRNDGRCHYMTIHNVTPDDEGVYSVIARLEPR
GEARSTAELYLTTKEIKLEMKPPDIPDSRVPIPTMPIRAVPPEEIPPAVAPSIPLLLPLPEEKKPPAKRIEVT

KKGVKKDTKKVVTKPKEEAPPPPVPEVAKKPPPPTPMIPAKASEIIDVSSKAEEVKITTITRKKEVHKEKEAV
YEREEAVYEKKVHIEPWEEPYEELETEPYTEPYEEPYYEEPDEDYEEIKVEAKKQVHEEWEEDFEEGQEYYER
EEGYDEGEEEWEEIYHEREIIQVQKEVHEELHEKKIPAKVPEKKVPPPKVVKKPVVEKVEKTTRRMEEEKVQV
IKVPEVSKKIVPQKPSRTPVQEEIIEVKVPAVHTKKMVISEEKMFFASHTEEEVSVSVPEVQKKTVTEEKIHV
AVSKKIEPPPKVPEPPKKPVPEEVVPVPIPKKVEPPAAKVPEAPKKPVPEEKKPVPIPKKKEPAAPPQVPEAP
KKPAPEEKIPVPVTKKKEAPPAKVPEVQKKVVTEEKIAIITQREESPPPAVPEIPKKKVPEEKRPVPRKEEVP
PPKVPVPPKKAVPEAVVPAPIPKKAPPRAEVSKKTVVEEKRFAAEEKLSVAVPQRVELMRHEEEEWTYSEEEE
RVSVSVYREEERDEEEAEITEYEVLEEPEEYVVEEKMHFISKKVEVEPAKVPEKKIIPKPKVPAKIEEPPPTK
VPEPPKKIVPEKKVPAPAPKKVPPAKAPEESKRPVPEKRAPAEEVGIEEPPPTKVAERHMKITQEEKVLVAVT
KKEAPPRARVPEEPKKVAPEERFPKLKPRREEEPPAKVTEVRKRAVKEEKVSIEVPKREPRPTKEVTVTEEKK
WSYTREEETVSEHREEEYEDYEDYEEYKEFEEYEPTEEYDQYDEYAEREVEHYEEHEEYVTEPKKPVPVKPAQ
EPVPAKPKAPPPKVLKKAVPEEKAPLPIQKKLKPLPPKAPEEPKKVVEEKIQISITKREKQQVTEPVAKVPMK
PKRVVPEAKIPAPTKEVAVPVRESLSFVICGSVLLKCLNFIEELENSQLKDDIFKVPGVPKKRELEEVVVFKE
EVEAHEEYIVEEEEEYVHEEEYVHKEEYVHEEEYVHKEEYIHEEEEHLHEEEETIAEEEVVPVAPVKVPVVPK
KPVPEEKKPVPVPKKKEAPPAKVPEIPKKPEEKVPVPIPKKEKAPPAKVPEVPKKPVPEEKPPVPVPKKVEPP
PAKVPEVPKKPVPEKKVPAPTPKKVEAPPAKVPEVPKKPIPEEKKPTALLKKMEAPPPKAPKKREVVPVPVAL
PREEEEEEVPFEEVPEEEILPEEEVPSEEEAPPEEVPPEEEEVLPEEEEVLPEEEEVLPEEEEVQPEEEALPE
IKPKVPKPAPVPEIKKKVPEKKVVVPKKEEAPPTKVPEVSKKVEERRIIPPKEEEVPPAEVYEEAEEPTPEEI
PEEPPSIEEEEIVEEEEEEEEVLPPRAPEVVKKAVPEAPTPVPKKAEAPPAKVPKKIPEEKVPVPVQKKEAPP
AKVPEVPKKVPEKKKIPEKKVPVPKKEAVPPAKGKAVFEEKISVAYQQEELVQERIELELVEAKVEEAFEEEE
FHEVQEYFEEEEFHEVEEFIRVEERRFQEEHKVEEVHRVIEFLEAEEVEVYEKPKIPPKKGPEVSEKVIPPKK
PPTKVIPRKEPPAKVPEVTKKTVVEEKIRAPEEPKVPAPKEVVPEKKVSVVPPKKPEAPPAKVPEAPKEAAPE
KKVPVAPKKKPEAPPVKVPEVPKTAVPQKKIPEAIPPKPESPPLEVPEAPKEVVPEKKVSVVPPKKPEAPPAK
VPEAPKEAAPEKKVPVAPKKKPEAPPVKVPEVPKAAVPQKKIPEAIPPKPESPPPEVYEEPEEEIVPEEPPEE
AVEEPVPAPPPKVTEPPKKPVPEKKAPPAVVKKPEPPPAKVPEVPKEAPPEKKVPPKKPEAPPAKVPEVPKEV
VTEKKVAVPKKPEVPPAKVPEVPKKPVIEEKPAIPVVEKVASPPAEVYEEPEEVTAEEEEPAPAVEEEEYEAP
PPPAPVPEEPKKVVPEKKFPVIKKPEAPPPKEPEPEKKVIEKPKLKPRPPARPPSPPKEDVKEKMFQLKAVSK
KKVPEKPEVVEKVEPAPLKVPTAEKKVRKLLPEPKPQPKEEVVLKSVLRKKPEEEEPKVEPKKVEKAKKPEEP
QPPPKAVEVEAPPEPTPKERKVPEPAKVPEIKPAIPLPGPEPKPKPEPEVKTMKAPPIEPAPTPIAAPVTAPV
VGKKAEAKPKDEAAKPKGPIKGVAKKTPSPIEAERKKLRPGSGGEKPPDEAPFTYQLKAVPLKFVKEIKDIVL
TEAESVGSSAIFECLVSPSTAITTWMKDGSNIRESPKHRFIADGKDRKLHIIDVQLSDAGEYTCVLRLGNKEK
TSTAKLIVEELPVRFVKTLEEEVTVVKGQPLYLSCELNKERDVVWRKDGKIVVEKPGRIVPGVIGLMRALTIN
DADDTDAGTYTVTVENANNLECSSCVKVVEIIREWLVKPIRDQHVKPKGTAVFTCDIAKDTPNIKWFKGYDEI
PLEPNDKTEILKEGNHLFLKVKNAMPEDIDEYAVEIEGKRYPAKLTLGEREVELLKPIEDVTIYEKESASFDA
EISEEDIPGEWKLKGELLRPSPTCEIKAEGGKRFLTLHKVKLDQAGEVLYQACNAITTAILTVKEIELDFAVP
LKDVTVPEKRQARFECVLTREANVIWSKGPDIIKASDKFDIIADGKKHILVINDSQFDDEGVYTAEVEGKKTS
AQLFVTGIRLKFISPLEDQTVKEGQTATFVCELSHEKMHVVWFKNDVKLHTTRTVLMSSEGKTYKLEIRETTL
DDISQIKAQVKNLSSTANLKVLEADPYFTVKLHDKTGVEKDEIILKCEVSKDVPVKWFKDGEEIVPSPKHSVK
TDGLRRILKIKKAELKDKGEYVCDCGTDTTKANVTVEARLIKVEKPLYGVEVFVGETARFEIELSEPDVHGQW
KLKGEPLTASPDCEIIEDGKKHVLVLYNCQLDMTGEISFQAANAKSAANLKVKELPLIFITPLSDVKVFEKDE
AKFECEVSREPKTFRWLKGTQEITGDDRFELIKDGTRHSLVIKSAAFEDEAKYMFEAEDKRTSGKLIIEGIRL
KFLTPLKDVTAKERENAVFTVELSHDNIPVSWFKNDQRLHTSKRVSMHDEGKTHSITFKDLSIDDTSQIRVEA
MGISSEAKLTVLEGDPYFTGKLQDYTGVEKDEVILQCEISKADAPVKWFKDGKEIKPSKNVVIKADGKKRMLI
LKKALKSDIGQYTCDCGTDQTSGKLDIEDREIKLVRPLYSVEVMETETARFETEISEDDIHANWKLKGEALLQ
TPECEIKEEGKIHVLILHNCRLDQTGGVDFQAANVKSSAHLRVKPRVIGLLRPLKDVTVTAGETATFDCELSY
EDIPVEWYLKGKKLEPNDKVVTRSEGRVHTLTLRDVKLEDAGEVQLTAKDFKTQANLFVKEPPVEFTKPLEDQ
TVEEEATAVLECEVSRENAKVKWFKNGTEILKSKKYEIVADGRVRKLIIHGCTPEDIKTYTCDAKDFKTSCNL
NVVPPHVEFLRPLTDLQVKEKETARFECEISKENEKVQWFKDGAEIKKGKKYDIISKGAVRILVINKCLLNDE
AEYSCEVRTARTSGMLTVLEEEAVFTKNLANLEVSEGDTIKLVCEVSKPGAEVIWYKGDEEIIETGRFEILTD
GRKRILIIQNAQLEDAGSYNCRLPSSRTDSKVKVHELAAEFISKPQNLEILEGEKAEFVCTISKESFEVQWKR
DDQTLESGDKYDIIADGKKRVLVVKDATLQDMGTYVVMVGAARAAAHLTVIEKLRIIVPLKDTKVKEQQEVVF
NCEVNTEGAKAKWFRNEEAIFDSSKYIILQKDLVYTLRIRDARLDDQANFNVSLTNHRGENVKSAANLIVEEE
DLRIVEPLKDIETMEKKSVTFWCKVNRLNVTLKWTKNGEEVAFDNRISYRIDKYKHSLIIKDCGFPDEGEYVV
TAGQDKSVAELLIIEAPTEFVEHLEDQTVTEFDDAVFSCQLSREKANVKWYRNGREIKEGKKYKFEKDGSIHR
LIIKDCRLEDECEYACGVEDRKSRARLFVEEIPVEIIRPPQDILEAPGADVIFLAELNKDKVEVQWLRNNMIV
VQGDKHQMMSEGKIHRLQICDIKPRDQGEYRFIAKDKEARAKLELAAAPKIKTADQDLVVDAGQPLTMVVPYD
AYPKAEAEWFKENEPLSTKTVDTTAEQTSFRISEAKKDDKGRYKIVLQNKHGKAEGFINLQVIDVPGPVRNLE
VTETFDGEVSLAWEEPLTDGGSKIIGYVVERRDIKRKTWVLVTDRADSCEFTVTGLQKGGVEYLFRVSARNRV

GTGEPVETDSPVEARSKYDVPGPPLNVTITDVNRFGVSLTWEPPEYDGGAEITNYVIELRDKTSIRWDTAMTV
RAEDLSATVTDVVEGQEYSFRVRAQNRIGVGKPSAATPFVKVADPIERPSPPVNLNASEQTQSSVQLTWEPPL
KDGGSPILGYIIERREEGKDNWIRCNMKPVPELTYKVTGLQKGNKYLYRVSAENAAGVSDPSEILGPLTADDA
FVEPTMDLSAFKDGLEVIVPNPIKILVPSTGYPRPKATWTFGDQVLEEGDRVKMKTISAYAELVISPSERTDK
GIYTLTLENPVKSISGEINVNVIAPPSAPKELKFSDITKDSVHLTWEPPDDDGGSPLTGYVVEKRDMSRKTWT
KVMDFVTDLEFTVPDLVQGKEYLFKVCARNKCGPGEPAYTDEPVNMSAPATVPDPPENVKWRDRTANSIFLTW
DPPKNDGGSRIKGYIVEKCPRGSDKWVACGEPVPDTKMEVTGLEEGKWYAYRVKALNRQGASKPSKPTEEIQA
VDTQEAPEIFLDVKLLAGITVKAGTKIELPATVTGKPEPKITWTKADTLLKPDQRITIENVPKKSTVTITDSK
RSDTGTYIIEAVNVCGRATAVVEVNVLDKPGPPAAFDITDVTNESCLLTWNPPRDDGGSKITNYVVERKATDS
DVWHKLSSTVKDTNFKATKLTPNKEYIFRVAAENMYGVGEPVQAAPIIAKYQFDPPGPPTRLEPSDITKDAVT
LTWCEPDDDGGSPITGYWVERLDPDTDKWVRCNKMPVKDTTYRVKGLTNKKKYRFRVLAENLAGPGKPSRSTE
PILIKDPIDPPWPPGKPTVKDIGKTSLVLNWTKPEHDGGAKIESYVIEMLKTGTDEWVRVAEGVPTTEHLLTG
LMEGQEYSFRVRAVNKAGESEPSEPSDPVLCREKLYPPSPPRWLEVINITKNTADLKWTVPEKDGGSPITNYI
VEKRDVRRKGWQTVDTTVKDTKCTVTPLTEGSLYVFRVAAENAIGQSDYTEIGDSVLAKDTFTTPGPPYALTV
VDVTKRHVDLKWEPPKNDGGRPIQRYIIEKKEKLGTRWVKAGKTSGPDCNFRVTDVIEGTEVQFQVRAENEAG
VGHPSEPTEILSIEDPTSPPSPPLDLHVTDAGRKHIAIAWKPPEKNGGSPIIGYHVEMCPVGTEKWMRVNSRP
IKDLKFKVEEGIVPDKEYVLRVRAVNAVGVSEPSEISENVVAKDPDCKPTIDLETHDIVVIEGEKLNIPVPFR
AVPVPTVSWHKDGKEVKASDRLTMKNDHISAHLEVPKSVHADAGVYTITLENKLGSATASINVKVIGLPGPCK
DIKASDITKSSCKLTWEPPEFDGGSPILHYVLERREAGRRTYIPVMSGENKLSWTVKDLIPNGEYFFRVKAVN
KIGGGEYIELKNPVIAQDPKQPPDPPVDVEVHNPTAKAMTITWKPPLYDGGSKIMGYIIEKITKGEDRWKRCN
EHLVPVLTYTAKGLEEGKEYQFRVRAENAAGIGEPSRATPPTKAVDPIDAPKVILRTSLEVKRGDEIALDATI
SGSPYPTITWIKDENVIVPEEIKKRAAPPVRRKKGEAEEEEPFSLPLTERLSINNSKQGESQLRIRDSLRPDH
GQYMIKVENDHGVAKAPCSVSVLDTPGPPINFVFEDIRKDSVLCKWEPPLDDGGSEIINYTLEKKDKTKPDSD
WIVITSTLRNCKYSVTKLIEGKEYLFRVRAENRFGPGPPCVSKPLLAKDPFEPPDAPDKPIVEDVTSNSMLVK
WNEPKDNGSPILGYWLEKREVNSTHWSRVNKTLLSSLKTKVDGLLEGLTYVFRVCAENAAGPGKFSPPSDPKT
ARDPISPPGPPVPRVADTSSTTIELEWEPPAFNGGGEIMGYFVDKQLVGTNEWSRCTEKMIKVRQFTVKEIRE
GADYKLRVSAVNAAGEGPPGETEPVTVAEPQEPPTVELDVSVKGGIQIMAGKTLRIPAEVTGRPVPTKVWTIE
EGELDKERVIIENVGTKSELIIKNALRKDHGRYVITATNSCGSKFAAVRVEVFDVPGPVLDLKPVVTNRKMCL
LNWSDPADDGGSDITGFIIERKDAKMHTWRQPIETERSKCDITGLIEGQEYKFRVIAKNKFGCGPPVEIGPIL
AVDPLGPPTSPERLTYTERTKSTITLDWKEPRSDGGSPIQGYIIEKRRHDKPDFERVNKRLCPTTSFLVENLD
EHQMYEFRVKAVNDVGESEPSLPLNVVIQDDEVPPTIKLRLAVRGDTIKVKAGEPVNIPADVTGLPMPKIEWS
KNEKVIDKPTDTLNITKEEVSRSEAKTELSIPKAAREDKGTYTITASNRLGSVFRNVHVEVYDRPSPPRNLAV
TDIKAESCYLTWDAPLDNGGSEITHYIIDKRDASRKKSEWEEVTNTAVERRYGIWKLIPNGQYEFRVRAVNKY
GISDECKSDKVVIQDPYRLPGPPGKPKVLERTKGSMLVSWTPPLDNGGSPITGYWLEKREEGGTYWSRVSRAP
ITKVGLKGVEFNVPRLIEGVKYQFRAMAINAAGIGPPSEPSDPAVAGDPIYPPGPPSCPEVKDKTKSSISLAW
KPPAKDGGSPIKGYIVEMQEEGTTDWKPVNEPDKLLTACECVVPNLKELRKYRFRVKAVNEAGESEPSDTTGE
IPATDIQEVPEVFIDIGAQDCLVCKAGSQVKIPAVIKGRPTPKSSWEFDGKAKKAMKDGVHDIPEDAQLETAE
NSSVIIIPECTRAHSGKYSITAKNKAGQKTANCRVKVMDAPGPPKDLKVSDITRGSCRLSWKMPDDDGGDRIK
GYVIEKKTIDGKAWTKVNPNCGSTTFVVPDLISEQQYFFRVRAENRFGIGPPAETIQRTTARDPIYPPDPPIK
LKIGLITKNTVHLSWKPPKNDGGSPVTHYIVECLAWDPTGKKKEAWRQCNRRDVEELEFTVEDLIEGGEYEFR
VKAVNEAGVSKPSATVGPVIVKDQTCPPAIELKEFMEVEEGTDVNIVAKIKGVPFPTLTWFKAPPKKPDSKEP
VVYDTHVNKQVVDDTCTLVIPQSRRSDTGLYSITAVNNLGTASKEMRLNVLGRPGPPVGPIKFESISADQMTL
SWLPPKDDGGSKITNYVIEKREANRKTWVRVSSEPKECMYTIPKLLEGHEYVFRIMAQNKYGIGEPLDSEPET
ARNLFSVPGAPDKPTVSSVTRNSMTVNWEEPEYDGGSPVTGYWLEMKDTTSKRWKRVNRDPIKAMTLGVSYKV
TGLIEGSDYQFRVYAINAAGVGPASLPSDPVTARDPVAPPGPPFPKVTDWTKSSVDLEWSPPLKDGGSKITGY
IVEYKEEGKEEWEKGKDKEVRGTKLVVTGLKEGAFYKFRVRAVNVAGVGEPGEVTDVIEMKDRIVSPDLQLDA
SVRDRIVVHAGGVIRIIAYVSGKPPPTVTWSMNERALPQEAAIETTAISSSMVIKNCQRSHQGVYSLLAKNEG
GERKKTIIVDVLDVPGPVGIPFLSDNLTNDSCKLTWFSPEDDGGSPITNYVIQKREADRRAWTPVTYTVTRQN
ATVQGLIQGKSYFFRIAAENSIGMGPFVETPNALVIRDPITVPERPEDLEVKEVTKNTVSLTWNPPKYDGGSE
IINYVLESRLIGTEKFHKVTNDNLLSRKYTVKGLKEGDTYEYRVSAVNIVGQGKPSFCTKPITCKDELAPPTL
DLDFRDKLTVRVGESFALTGRYSGKPKPKIDWFKDEADVLEDDRTHIKTTPTTLALEKTKAKRSDSGKYCVVV
ENSTGSRKGFCQVNVVDRPGPPVGPVIFDEVTKEYMVISWKPPLDDGGSEITNYIIEKKELGKDIWMPVTSAS
AKTTCKVPKLLEGKDYIFRIHAENLYGISDPLVSDSMKAKDRFRVPDAPEQPVVTEVTKDSALVTWNKPNDGG
KPITNYILEKRETMSKRWVRVTKEPIHPYTKYRVPDLLEGCQYEFRVSAENEIGIGDPSPPSKPVFARDPIAK
PSPPINPEAIDTTCNSVELTWQPPRHDGGSKILGYIVEYQKVGDEEWRRANHTPESCPETKYKVTGLRDGQSY
KFRVLAVNEAGESDPAHVPEPVLVKDRLEPPELILDANMAREQHIRVGDTLRLSAIIKGVPFPKVTWKKEDRE
APTKAQIDVTPVGSKLEIRNAAHEDGGIYSLTVENPAGTKTVSVKVLVLDKPGPPRDLEVSEIRKDSCYLTWK
EPLDDGGSVVTNYVVERKDVATAQWSPLSTTSKKKSHMAKHLTEGNQYLFRVAAENQYGRGPFVETPKPIKAL

DPLHPPGPPKDLHHVDVDKTEVSLVWNKPDRDGGSPITGYLVEYQEEGDKDWIKFKTVKNLDCVVTGLQQGKT
YRFRVKAENIIGLGLPDTTIPIECQEKLVPPSVELDVKLIEGLVVKAGTTVRFPAIIRGVPVPTAKWTTDGTE
IKTDDHYTVETDSFSSVLTIKNCLRKDTGEYQLTVSNAAGTKTVAVHLTVLDVPGPPTGPINILDVTPEYMTI
SWQPPKDDGGSPVINYIVEKQDTRKGTWGVVSAGSSKLKLKVPHLQKGCEYVFRVKAENKMGVGPPLDSIPTV
AKHKFSPPSPPGKPVVTDITENAATVSWTLPKSDGGSPITGYYVERREITGKWVRVNKTPIADLKFRVTGLYE
GNTYEFRVFAENLAGLSNPSPSSDPIKACRPIKPPGPPINPKLKDKSKESADLVWTKPLSDGGSPILGYVVEY
QKPGTAQWDRINKDELIRQCAFRVPGLIEGNEYRFRIRAANIVGEGEPRELAESVIAKDILHPPEVELDVTCR
DVITVRVGQTIRILARVKGRPEPDITWSKEGKVLVKDKRVDLIHDLPRVELQIKEAVRADHGKYIISAKNSSG
HAQGSAIVNVLDRPGPCQNLKVSNVTKENCTISWENPLDNGGSEITNFIVEYRKPNQKGWSIVASDVTKRLVK
ANLLANNEYYFRVCAENKVGVGPTIETKTPILAINPIDRPGEPENLHIADKGKTFVYLKWRRPDYDGGSPNLS
YHVERRLKGSADWERVHKGSIKETHYMVDKCVENQIYEFRVQTKNEGGESDWVRTEEVVVKEDLQKPVLDLKL
SGVLTVKAGDTIRLEAGVRGKPFPEVAWTKDKDATDLTRSPRVKIDTSAESSKFSLTKAKRSDGGKYVITATN
PAGSFVAYATVNVLDKPGPVRNLKITDVSSDRCTIRWDPPEDDGGCEIQNYILEKCESKRMVWSTYSANVLTP
SATVTRLIEGNEYIFRVRAENKIGTGPPTESKPVIAKTKYDRPGRPDPPEVTKVSKEEMTVVWNAPEYDGGKS
ITGYYLEKKEKHAVRWVPVNKSAIPERRLKVQNLLPGHEYQFRVKAENEVGIGEPSLPSRPVVAKDPIEPPGP
PTNFKVVDTTKSSITLAWGKPVYDGGAPIIGYVVEMRPKIADASPDEGWKRCNAAAQLIRMEFTVTSLDENQE
YEFRVCAQNQVGIGRPAELKEAIKPKEILEPPEIDLDASMRKLVVVRAGCPIRLFAIVRGRPAPKVTWRKVGI
DNVVRKGQVDLVDTMAFLVIPNSTRDDSGKYSLTLVNPAGEKAVFVNVKVLDTPGPVADLKVSDVTKTSCHVS
WAPPENDGGSQVTHYIVEKREAERKTWSTVTPEVKKTSFNVTNLVPGNEYFFRVTAVNEYGPGVPTDIPKPVL
ASDPLSEPDPPRKLEVTEMTKNSATLAWLPPLRDGGAKIDGYIISYREEDQPADRWTEYSVVKDLSLIVTGLK
EGKKYKFRVAARNAVGVSMPREAEGVYEAKEQLLPPKILMPEQITIKAGKKLRVEAHVYGKPNPICKWKKGDD
EVVTSSHLAIHKADGSSVLIIKDVTRKDSGYYSLTAENSSGSDTQKIKVTVMDAPGPPQPPFDISEIDADACS
LSWHIPLEDGGSNITNYIVEKCDVSRGDWVTALASVTKTSCRVGKLIPGQEYIFRVRAENRFGISEPLTSPKM
LAKFPFDVPSEPKNARVTKVNKDCIFVAWDRPDSDGGSPITGYLIERKERNSLLWVKANDTIVRSTEYPCAGL
VEGLEYSFRIYALNKAGSSPPSKPTEYVTARMPVDPPGKPEVVDVTKNSASLIWARPKHDGGSRIIGYFVEAC
KLPGDKWVRCNTTPHQIPQEEYTATGLEENAQYQFRAIAKTAVNISQPSEPSDPVTILAENVPPRIELSVEMK
SLLTVKAGTNVCLDATVFGKPMPTVSWKKDTTPIKQAEGIKMAMKRNLCTLELFSVNRKDSGDYTITAENSSG
SKSATIKLKVLDKPGPPASVKINKMYADRAMLSWEPPLEDGGSEITNYIIDKRETSRPNWAQVSATVPITSCT
VEKLIEGHEYQFRICAENKYGVGDPILTEPAIAKNPYDPPGRCDPPVISNITKDHMTVSWKAPADDGGSPITG
YLVEKRETQAVNWTKVNRKPVIERTLKATGLQEGTEYEFRVTAINKAGPGKPSDASKAVYAQDPLYPPGPPAF
PKVYDTTRSSVSLSWGKPAYDGGSPIIGYLVEVKRADSDHWVRCNLPEKLQKTRFEVTGLMENTEYQFRVYAV
NKIGYSDPSDVPDKHCPKDILIPPEGELDAELRKTLILRAGVTMRLYVPVKGRPPPKITWSKPNVNLRERIGL
DIKSTDFDTFLRCENVNKYDAGKYILTLENSCGKKEYTIVVKVLDTPGPPVNVTVKEVSKDSAYVTWDPPIID
GGSPIINYVVEKRDAERKSWSTVTTECSKTSFRVSNLEEGKSYFFRVFAENEYGIGDPGETRDAVKASETPGP
VVDLKALAITKSSCTIGWKKPRSDGGSRITGYVVDFLTEENKWQRVMKSLSLQYSTKDLKEGKEYTFRVSAEN
ENGEGTPSEIVVVAKDDVVAPDLDLKDLPDLCYLAKENSNFRLKIPIKGKPAPSVSWKKGEDPLATDTRVSVE
STAVNTTLVVYDCQKSDAGKYTITLKNVAGTKEGTLSIKVVGKPGIPTGPIKFDEVTAEAMTLKWGPPKDDGG
SEITNYVLEKRDSVNNKWVTCASAVQKTTFRVTRLHEGIEYTFRVSAENKYGVGEGLKSEPIVAKHPFDVPDA
PPPPNIVDVRHDSVSLTWTDPKKTGGSPITGYHIEFKERNSLLWKRANKTPIRMKDFKVTGLTEGLEYEFRVM
AINLAGVGKPSLPSEPVVALDPIDPPGKPEVISVTRNSVTLIWTEPKYDGGHKLTGYIVEKRDLPSKSWMKAN
HVNVPDCAFTVTDLVEGGKYEFRIRAKNTAGAISAPSESTGTIICKDEYEAPTIVLDPTIKDGLTVKAGDSIV
LSAISILGKPLPKSSWSRAGKDIRPSDIAQITSTPTSSMLTVKYATRKDAGEYTITATNPFGTKEEHVKVSVL
DVPGPPGPIEISNVSAEKATLTWTPPLEDGGSPIKAYVLEKRETSRLLWTVVSEDIQACRHVVTKLIQGNEYL
FRVSAVNHYGKGEPVQSEPVKMVDRFGPPGPPGKPEISNVTKNTATVSWKRPTDDGGSEITGYHVERREKKGL
RWVRATKTPVSDLRCKVTGLQEGNTYEFRVSAENRAGIGPPSDASNPVLMKDVAYPPGPPSNAHVTDTTKKSA
SLAWGKPHYDGGLEITGYVVEHQKVGDDAWIKDTTGTALRITQFVVPDLQTKEKYNFRISAINDAGVGEPAVI
PNVEIVEKEVAPDFELDAELRRTLVVRAGLSIRIFVPIKGRPAPEVTWTKDNINLKHRANIENTESFTLLIIP
ECNRYDTGKFVMTIENPAGKKSGFVNVRVLDTPGPVLNLRPTDITKDSVTLHWDLPLIDGGSRITNYIVEKRE
ATRKSYSTVTTKCHKCTYKVTGLTEGCEYFFRVMAENEYGVGEPTETTEPVRASEAPLPPDSLNIMDITKNTV
SLAWPKPRHDGGSKITGYVIEAQRKGSDQWTHISTVKGLECVVRNLTEGEEYTFQVMAVNSAGRSAPRESRPV
IVKEQTMLPELDLRGIYQKLVIARAGDNIKVEIPVLGRPKPTVTWKKGDQILKQTQRVNVENTATSTILNINE
CVRSDSGPYPLTAKNTVGEVGDVITIQVHDIPGPPTGPIKFDEVSSDFVTFSWEPPENDGGVPISNYVVEMRQ
TDSTTWVELATTVIRTTYKATRLTTGVEYQFRVRAQNRYGVGPGITSASVVANYPFKVPGPPGTPQVTAVTKD
SMTISWHEPLSDGGSPILGYHIERKERNGILWQTVSKALVPGNIFKSTGLTDGIAYEFRVIAENMAGKSKPSK
PSEPMFALDPIDPPGKPVPLNITRHTVALKWAKPEYTGGFKITSYVVEKRDLPNGRWLKANFSNILENEFTVS
GLTEDAAYEFRVIAKNAAGAISPPSEPSDAITCRDDLEAPRIMVDVRFKDTITLKAGEAFKLEADVSGRPPPT
MEWTKDGKELEGTGKLEIKIADFSTHLINKDSSRTDSGAYILTATNPGGFAKHIFNVKVLDRPGPPEGPLAVS
DVTSEKCVLSWLPPLDDGGAKIDHYIVQKRETSRLAWTNVATEVQVTKLKVTKLLKGNEYIFRVMAVNKYGVG

EPLESEPVLAVDPYGPPDPPKNPEVTTITKDSMVVCWGHPDSDGGSEIINYIVERRDKAGQRWVKCNKKALTD
LRFKVSGLTEGHEYEFRIMAENAAGISAPSATSPFYKACDTVFKPGPPGNPRVLDTSRSSISIAWNKPIYDGG
SEITGYMVEIALPEEDEWQVVTPPAGLKATSYTITSLIENQEYKIRIYAMNSEGLGEPALVPGTPKAEERMLP
PEIELDADLRKVVTIRACCTLRLFVPIKGRPAPEVKWAREHGESLDKASIESTSSYTLLVVGNVNRFDSGKYI
LTVENSSGSKSAFVNVRVLDTPGPPQNLKIKEVTKTSVTLTWEPPLLDGGSKIKNYIVEKRESTRKAYSTVAT
NCHKTSWKVDQLQEGCSYYFRVLAENEYGIGLPAETAESVKASERPLPPGKITLTDVTRNSVSLSWEKPEHDG
GSRILGYIVEMQSKGSDRWATCATVKVTEATITGLIQGEEYSFRVSAQNEKGISDPRQLSVPVIAKDLVIPPA
FKLLFNTFTVLAGEDLKIDVPFIGRPPPAVTWHKDDIPLKQTTRVNAESTENNSLLTIKEACREDVGHYTVKL
TNSAGEATETLNVIVLDKPGPPTGPVKMDEVTADSVTLSWEPPKYDGGSSINNYIVEKRDTSTTAWQIVSATV
ARTTIKACRLKTGCEYQFRIAAENRYGKSTYLNSEPVVAQYPFKVPGPPGTPFVTLASKDSMEVQWHEPVSDG
GSKVIGYHLERKERNSILWVKLNKTPIPQTKFKTTGLEEGIEYEFRVSAENIVGIGKPSKPSECYAAHDPCDP
PGRPEAIIVTRNSVTLQWKKPTYDGGSKITGYIVEKKELPDGRWMKASFTNIIDTQFEVTGLLEDHRYEFRVI
ARNAAGVFSEPSESTGAITARDEVEPPRISMDPKYRDTVVVQAGESFKIDADIYGKPIPTTQWVKGDQELSST
ARLEIKSTDFATSLSVKDAVRVDSGNYILKAKNVAGEKSVTINVKVLDRPGPPEGPVAISGVTAEKCTLAWKP
PLQDGGSDITNYIVERRETSRLVWTLVDANVQTLSCKVLKLLEGNEYIFRIMAVNKYGVGEPLESESLIAKNP
FVVPDAPKAPEVTAVTKDSMIVVWERPASDGGSEILGYVLEKRDKEGIRWTRCHKRLIGELRLRVTGLLENHN
YEFRVSAENAAGLSEPSPPSAYQKACDPIYKPGPPNNPKVMDVTRSSVFLSWTKPIYDGGCEIQGYIVEKCDV
SVGEWTMCTPPTGINKTNLEVEKLLEKHEYNFRICAINKAGVGEHADVPGPVMVEEKLEAPDIDLDLELRKVI
NIRAGGSLRLFVPIKGRPTPEVKWGKVDGDIRDAAIIDVTSSFTSLVLDNVNRYDSGKYTLTLENSSGTKSAF
VTVRVLDTPSPPVNLKVTEITKDSVSITWEPPLLDGGSKIKNYIVEKREATRKSYAAVVTNCHKNSWKIDQLQ
EGCSYYFRVTAENEYGIGLPARTADPIKVAEVPQPPGKITVDDVTRNSVSLSWTKPEHDGGSKIIQYIVEMQA
KNTDKWSECARVKSLDAVITNLTQGEEYLFRVIAVNEKGRSDPRSLAVPIIAKDLVIEPDVRPAFSSYSVQVG
QDLKIEVPISGRPKPSISWTKDGMPLKQTTRINVTDSLDLTTLSIKETHKDDGGQYGITVSNVVGQKTAAIEI
ITLDKPDPPKGPVKFDEISAESITLSWNPPLYTGGCQITNYIVQKRDTTTTVWDVVSATVARTTLKVTKLKTG
TEYQFRIFAENRYGQSFALESEPVVAQYPYKEPGPPGTPFVTAISKESMVVQWHEPINNGGSPVIGYHLERKE
RNSILWTKVNKTIIHDTQFKALNLEEGIEYEFRVYAENIVGVGKASKNSECYVARDPCDPPGTPEAIIVKRNE
ITLQWTKPVYDGGSMITGYIVEKRDLPEGRWMKASFTNVIETQFTVSGLTEDQRYEFRVIAKNAAGAISKPSD
STGPITAKDEVELPRISMDPKFRDTIVVNAGETFRLEADVHGKPLPTIEWLRGDKEIEESARCEIKNTDFKAL
LIVKDAIRIDGGQYILRASNVAGSKSFPVNVKVLDRPGPPEGPVQVTGVTAEKCTLAWSPPLQDGGSDISHYV
VEKRETSRLAWTVVASEVVTNSLKVTKLLEGNKYIFRIMAVNKYGVGEPLESAPVLMKNPFVLPGPPKSLEVT
NIAKDSMTVCWNRPDSDGGSEIIGYIVEKRDRSGIRWIKCNKRRITDLRLRVTGLTEDHEYEFRVSAENAAGV
GEPSPATVYYKACDPVFKPGPPTNAHVVDTTKNSITLAWSKPIYDGGSEILGYVVEICKADEEEWQIVTPQTG
LRVTRFEIAKLIEHQEYKIRVCALNKVGLGEAASVPGTVKPEDKLEAPELDLDSELRKGIVVRAGGSARIHIP
FKGRPTPEITWSKEEGEFTDKVQIEKGINFTQLSIDNCDRNDAGKYILKLENSSGSKSAFVTVKVLDTPGPPQ
NLAVKEVRKDSVLLVWEPPIIDGGAKVKNYVIDKRESTRKAYANVSSKCNKTSFRVENLTEGAIYYFRVMAEN
EFGVGVPTETSDAVKASEPPSPPGKVTLTDVSQTSASLMWEKPEHDGGSRILGYVVEMQPKGTEKWSVVAESK
VCNAVVSGLSSGQEYQFRVKAYNEKGKSDPRVLGIPVIAKDLTIQPSFKLPFNTYSVQAGEDLKIEIPVIGRP
RPKISWVKDGEPLKQTTRVNVEETATSTILHIKESSKDDFGKYSVTATNSAGTATENLSVIVLEKPGPPVGPV
KFDEVSADFVVISWEPPAYTGGCQISNYIVEKRDTTTTNWQMVSATVARTTIKISKLKTGTEYQFRIFAENRY
GKSTPLDSKPVVVQYPFKEPGPPGTPFVTSISKDQMLVQWHEPVNDGGSKVTGYHLEQKEKNSILWVKLNKIP
IQDTKFKTTGLDEGLEYEFRVSAENIVGIGKPSKVSECYVARDPCDPPGRPEAIVITRNSVTLKWKKPVYDGG
SKITGYIVEKKDLPDGRWMKASFTNVVETEFTVTGLVEDQRYEFRVIARNAADNFSEPSESSGAITARDEIDA
PNASLDPKYRDVIIVHAGETFVLEADIRGKPIPDIIWSKDGNELEETAARMEIKSTLQKTTLIVKDCIRTDGG
QYTLKLSNVGGTKTIPITVKVLDRPGPPEGPLKVTGVTAEKCYLAWNPPLQDGGASISHYIIEKRETSRLSWT
QVSNEVQALNYKVTKLLPGNEYIFRVMAVNKYGIGEALESEPVIACNPYKRPGPPSTPEASAITKDSMVLTWT
RPVDDGGAEIEGYILEKRDKEGIRWTKCNKKTLTDLRFRVTGLTEGHSYEFRVAAENAAGVGEPSEPSVFYRA
CDALYPPGPPSNPKVTDTSRSSVSLAWNKPIYDGGAPVRGYVIELKKAAADEWTTCTPPSGLQGKQFTVTKLK
ENTEYNFRICAFNTEGVGEPATIPGSVVAQERMEAPEIELDADLRKVVTLRASATLRLFVTIKGRPEPEVKWE
KAEGILTERAQIEVTSSYTMLVIDNVTRFDSGRYNLTLENNSGSKTAFVNVRVLDSPSAPVNLTIREVKKDSV
TLSWEPPLIDGGAKITNYIVEKRETTRKAYATITNNCTKNTFKIENLQEGCSYYFRVLASNEYGIGLPAETAE
PVKVSEPPLPPGRVTLVDVTRNTATIKWEKPESDGGSKITGYVVEMQTKGSEKWSACTQVKTLETTISGLTAG
EEYVFRVAAVNEKGRSDPRQLGVPVIAKDIEIKPSVELPFNTFNVKANDQLKIDIPFKGRPQATVAWKKDGQV
LRETTRVNVASSKTVTTLSIKEASREDVGTYELCVSNTAGSITVPITVIVLDRPGPPGPIRIDEVSCDNVSIS
WNPPEYDGGCQISNYIVEKRETTSTTWQVVSQAVARTSIKIVRLTTGSEYQFRVCAENRYGKSSYSESSAVVA
EYPFSPPGPPGTPKVVHATKSTMVVSWQVPVNDGGSQVIGYHLEYKERSSILWSKANKVLIADTQMKVSGLDE
GLMYEYRVYAENIAGIGKCSKACEPVPARDPCDPPGQPEVTNITRKSVSLKWSKPRYDGGAKITGYIVERREL
PDGRWLQCNFTNVQETYFEVTELTEDQRYEFRVFARNAADSVSEPSESTGPITVKDDVEAPRIMMDVKFRDVI
IVKAGEVLKINADIAGRPLPVISWAKDGVEIEERAKTEIVSTDYTTTLTVKDCVRRDTGQYVLTLKNVAGTRT

MAVNCKVLDKPGPPAGPLEINGLTAEKCSLSWGRPQEDGGADIDYYIVEKRETSRLAWTICEAELRTTSCKVT
KLLKGNEYIFRVTGVNKYGVGEPLESMAVKALDPFTTPSPPTSLEITSVTKDSMTLCWSRPETDGGSDISGYI
IERREKNSLRWMRVNKKPVYDLRVKSTGLREGCEYEYRVFAENAAGLSLPSETSPLVRAEDPVFLPSPPSKPK
IVDSGKTTITIGWVKPLFDGGAPITGYTVEYKKSEETDWKVAIQSFRGTEYTMSGLTTGDEYVFRVRSLNKMG
ASDPSDSSDPQVAKEREEEPVFDVDSEMRKTLIVKAGSSFTMTVPFRGRPIPNVSWSKPDTDLRTRAYIDSTD
SRTLLTIENANRNDSGKYTLTIQNVLSAASMTFVVKVLDSPGPPANITVREVTKETAMLSWDVPENDGGAPVK
NYHIEKREASKKAWVSVTNNCNRLSYKVTNLQEGAIYYFRVSGENEFGVGVPAETKEGVKITEKPSPPEKLGV
TSVSKDSVSLSWLKPEHDGGSRIIHYVVEALEKGQKTWVKCAVVKTTHHVVSGLRESHEYFFRVFAENQAGLS
DPRELLLPVLIKDQLEPPEIDMKNFPSHTVYVRAGSNLKVDIPISGKPLPKVTLSRDGVPLKATMRFNTEITA
ENLTINLKESVTTDAGRYEITAANSSGTTKTFINIIVLDRPGPPTGPVAISDITEESVTLKWEPPKYDGGSHV
TNYIVLKRETSTAVWSEVSATVARTMIKVMKLTTGEEYQFRIKAENRFGISDHIDSVCVVVKLPYTTPGPPST
PWVSNVTRESITVGWHEPVSNGGSAVTGYHLEMKDRNSILWQKANKMIIRTTHFKVTTISAGLIYEFRVYAEN
AAGIGKPSHPSEPVLAIDACEPPRNVRITDISKNSVNLSWQQPAFDGGSKITGYIVERRDLPDGRWTKASFTN
VIETQFTVSGLTQNSQYEFRVFARNAVGSVSNPSEVVGPITCIDSYGGPVIDLPLEYTEVVKYRAGTSVKLRA
GISGKPEPTIEWYKDDKELQTNALVCVENSTDLASILIKDANRLNSGSYELKLRNAMGSASATIRVQILDKPG
PPGGPIEFKTVTAEKITLLWRPPADDGGAKITHYIVEKRETSRVVWSMVAENLEECIVTTTKIIKGNEYVFRV
RAVNKYGIGEPLESEPVVAKNAFVTPGPPSIPEVTKITKNSMTVVWDRPTVDGGSEINGYFLERRDKKSLAWL
KVLKETIRDTRQKVTGLTENSDYQYRVCAVNAAGVGPFSEPSDFYKAADPIDPPGPPAKIRIADSTKSSITLG
WSKPVYDGGSDVTGYVVEMKQGDEEEWTIVSTRGEVRTTEYVVSNLKPGVNYYFQVSAVNCAGQGEPITMTEP
AQAKDVLEEPEIDLDVALRTSVIAKAGEDVQLLIPFKGRPPPTVTWRKDEKNLGSDTRYSIQNTDSSSLLVIP
QVTRNDTGKYILTIENGVGQPKSSTVSVKVLDTPAACQKLQVKHVSLGTVTLLWDPPLIDGGSPIINYVIEKR
DATKRTWSVVSHKCSGTSFKVTDLSEKTPFFFRVLAENEIGIGEPCETTEPVKAAEVPAPIRDLSMKDSTKTS
VVLSWTKPDFDGGSIITDYLVERKGKGEQAWSHAGISKTCEIEIGQLKEQSVLEFRVSARNEKGQSDPVTIGP
LTVKELVITPEVDLSEIPGAQISVRIGHNVHLELPYKGKPKPSISWLKDGLPLKESEYVRFSKTENKITLSIK
NSKKEHGGKYTVILDNAVCRNSFPITIITLGPPSKPKGPIRFDEIKADSAIMSWDIPEDDGGGEITCYSIEKR
EASQTNWKMVCSSVARTTFKVSNLVKDSEYQFRVRAENRYGVSEPLASNIIVAKHQFRIPGPPGKPVIYNVTS
DGMSLTWDAPVYDGGSEVTGFHVEKKERNSILWQRVNTSPISGREYRATGLIEGLDYQFRVYAENSAGLSSPS
DPSKFTLAVSPVDPPGTPDYIDVTRETITLKWNPPLRDGGSKIVAYSIEKRQGSDRWVRCNFTDVSECQYTVT
GLSPGDRYEFRIIARNAVGTISPPSQSSGLIMTRDENVPPTVEFGPEYFDGLVIKSGDSLRIKALVQGRPVPR
VTWFKDGVEIERRMNMEITDVLGSTSLFVRDATRDHRGVYTVEAKNVSGSTKAEVTVKVQDTPGKVVGPIRFT
NITGEKMTLWWEAPLNDGCAPVTHYIIEKRETSRLAWALIEDNCEALSYTAIKLITGNEYQFRISAVNKFGVG
RPLESDPVVAQIQYTIPDAPGVPEPSNVTGNSITLTWTRPESDGGSEIQHYILERREKKSTRWVKVISKRPIS
ETRFKVTGLVEGNEYEFHVMAENAAGIGPASGISRLIKCREPVNPPSAPSVVKVTDTSKTTVSLEWARPVFDG
GMEIIGYIIEMCKADLGDWHKVNTEPCVKTRYTVTDLQAGEEYKFRVSAVNGAGKGDSCEVTGTIKAVDRLSA
PELDIDANFKQTHIVRAGVSIRLFIAYQGRPTPTAVWSKPDSNLSIRADIHTTDSFSTLTVENCNRNDAGKYT
LTVENNSGKKSITFTVKVLDSPGPPGPITFKDVTRGSATLMWDAPLLDGGARIHHYVIEKREASRRSWQVVSE
KCTRQILKVSELTEGVPYYFRVSAENEYGVGEPYEMPEPIVATEQPAPPRRLDVVDTSKSSAVLAWLKPDHDG
GSRITSYLLEMRQKGSDFWVEAGHTKQLTFTVERLVENTEYEFRVKAKNDAGYSEPREAFSSVIIKEPQIEPT
ADLTGITNQLITCKAGSTFTIDVPISGRPAPKVTWKLEEMRLKETDRMSIATTKDRTTLTVKDSMRGDSGRYF
LTLENTAGVKTFTITVVVIGRPGPVTGPIEVSSVSAESCVLSWTEPKDDGGTEITNYIVEKRESGTTAWQLIN
SSVKRTQIKVTHLTKYKEYCFRVSSENRFGVSKPLESVPIVAEHPFVPPSAPTRPEVYYVSANAMSIRWEEPY
HDGGSKIVGYWVEKKERNTILWVKENKVPCLECNYKVTGLVEGLEYQFRTYALNAAGVSKASEASRPIMAQNP
VDPPGRPEVTDVTRSTVSLIWSAPVYDGGSKVVGYIIERKPVSEVGDGRWLKCNYTIVSDNFFTVTALSEGDT
YEFRVLAKNAAGVISKGSESTGPVTCRDEYAPPKAELDARLQGDLVTIRAGSDLVLDAAVGGKPEPKIIWTKG
DKELDLCEKISLQYTGKRATAVIKYCDRSDSGKYTLTVKNASGTKSVSVMVKVLDSPGPCGKLTVSRVTEEKC
TLAWSLPQEDGGAEITHYIVERRETSRLNWVIVEGECLTASYVVTRLIKNNEYTFRVRAVNKYGLGVPVESEP
IVARNSFTIPSQPGIPEEVGAGKEHIIIQWTKPESDGGNEISNYLVDKREKKSLRWTRVNKDYVVYDTRLKVT
SLMEGCDYQFRVTAVNAAGNSEPSEASNFISCREPSYTPGPPSAPRVVDTTKRSISLAWTKPMYDGGTDIIGY
VLEMQEKDTDQWCRVHTNATIRNNEFTVPDLKMGQKYSFRVAAVNAKGMSDYSETTAEIEPVERLEIPDLELA
DDLKKTVIVRAGASLRLMVSVSGRPSPVITWSKKGIDLANRAIIDNTESYSLLIVDKVNRYDAGKYTIEAENQ
SGKKSATVLVKVYDTPGPCPSVSVKEVSRDSVTITWEIPTIDGGAPVNNYIIEKREAAMRAFKTVTTKCSKTL
YRISGLVEGTMYYFRVLPENIYGIGEPCETSDAVLVSEVPLVPTKLEVVDVTKSTVTLAWEKPLYDGGSRLTG
YVLEACKAGTERWMKVVTLKPTVLEHTVISLNEGEQYLFRVRAQNEKGVSEPREIVTPVTVQDLRVLPTIDLS
TMPQKTIHVPAGRPIELVIPITGRPPPTASWFFAGSKLRESERVTVETHTKVTKLTIRETTIRDTGEYTLELK
NVTGTTSETIKVIILDKPGPPTGPIKIDEIDATSVTISWEPPELDGGAPLSGYVVEQRDAHRPGWLPVSESVT
RPTFKFTRLTEGNEYVFRVAATNRFGIGSYLQSEVIECRSSISIPGPPETLQIFDVSRDGMTLTWYPPEDDGG
SQVTGYIVERKEVRADRWVRVNKVPVTMTRYRSTGLIEGLEYEHRVTAINARGTGKPSRPSKPTVAMDPIAPP
GKPQNPRVTDTTRTSVSLAWSVPEDEGGSKVTGYLIEMQKVDQREWTKCNTTPTKIREYTLTHLPQGAEYRFR

VLACNAGGPGEPAEVPGTVKVTEMLEYPDYELDERYQEGVFVRQGGVIRLTIPIKGKPFPVCKWTKEGQDISK
RAMIATSETHTELVIKEADRNDSGTYDLVLENKCGKKTVYIKVKVIGSPNTPEGPLEYDDIQARSVRVSWRPP
ADDGGADILGYILERREVPKAAWYTIDSRVRGTSLVVKGLKENVEYHFRVSAENQFGISKPLKSEEPVIPKTP
LNPPEPPSNPPEVLDVTKSSVSLSWSRPKDDGGSRVTGYYIERKETSTDKWVRHNKTQITTTMYTVTGLVPDA
EYQFRIIAQNDVGLSETSPASEPVVCKDPFDKPSQPGELEILSISKDSVTLQWEKPECDGGKEILGYWVEYRQ
SGDSAWKKSNKERIKDRQFTIGGLLEATEYEFRVFAENETGLSRPRRTAMSVKTKLTSGEAPGVRKEMADVTT
KLGEAAQLSCQIVGRPLPDIKWYRFGKELIQSRKYKMSSDGRTHTLTVMTDEQEDEGVYTCVATNEVGEVESS
SKLLLQAAPQFHPGYPLKEKYYGAVGSTLRLHVMYIGRPVPAMTWFHGQKLLQNSEKITIENTEHYTHLVMKN
VQRKTHAGKYKVQLSNAFGTVDATLDVEIQDKPDKPTGPIVIEALLKNSVVISWKPPADDGGSWITNYVVEKC
EAKEGAEWQLVSSAISVTTCRIVNLTENAGYYFRVSAQNTFGISEPLEVASIVIIKSPFEKPGVPGKPTITAV
TKDSCVVAWKPPASDGGAKIRNYYLERREKKQNKWIAVTTEEIRETVFSVQNLIEGLEYEFRVKCENLGGESE
WSEISEPVTPKSDVPIQAPHFKEELRNLNVRYQSNATLVCKVTGHPKPIVKWYRQGKEIIADGLKYRIQEFKG
GYHQLIIASVTDDDATVYQVRATNQGGSVSGTASLEVEVPAKIHLPKTLEGMGAVHALRGEVVSIKIPFSGKP
DPVITWQKGQDLIDNNGHYQVIVTRSFTSLVFSNGVERKDAGFYVVCAKNRFGIDQKTVELDVADVPDPPRGV
KVSDVSRDSVNLTWTEPASDGGSKVTNYIVEKCATTAERWLRVGQARETRYTVINLFGKTSYQFRVIAENKFG
LSKPSEPSEPTVTKEDKTRAMNYDDEVDETREVTTTKASHSKTKELYEKYMIAEDLGRGEFGIVHRCVETSSK
RTFMAKFVKVKGTDQVLVKKEISILNIARHRNILYLHESFESMEELVMIFEFISGLDIFERINTSAFELNERE
IVSYVRQVCEALEFLHSQNIGHFDIRPENIIYQTRKNSTIKIIEFGQARQLKPGDNFRLLFTAPEYYAPEVHQ
HDVVSTATDMWSLGTLVYVLLSGINPFLAETNQQMIENIMNAEYTFDEEAFKEISLEAMDFVDRLLVKERKSR
MTASEALQHPWLKQRIDRVSTKVIRTLKHRRYYHTLIKKDLNMVVSAARISCGGAIRSQRGVSVAKVKVASIE
IGPVSGQIMHAIGEEGGYVKYVCKIENYDQSTQVTWYFGVRQLENSEKYEITYEDGVATMYVKDITKFDDGTY
RCKVVNDYGEDSSYAELFVKGVREVYDYYCRRTKKVKRRTDAMRLLERPPEFTLPLYNKTAYVGENVRFGVTI
TVHPEPRVTWYKSGQKIKPGDDEKKYTFESDKGLYQLTINSVTTDDDAEYTVVARNKHGEDSCKAKLTVTLHP
PPTETTLRPMFKRLLANAECHEGQSVCFEIRVSGIPAPTLKWEKDGQPLSLGPHIEIVHEGLDYYALHIRDTL
PEDTGYYRVTATNTAGSTSCQAHLQVERLRYVKQEFQSKEERERHVQKQIDKTLRMAEILSGTETVPLTPVAQ
EALREAAILYKPAVSTKTVKGEYRLQTEEKKEERKLRMPYEVPEPRRFKQATVEEDQRIKQFVPMSDMKWYKK
IRDQYEMPGKLDRVVQKRPKRIRLSRWEQFYVMPLPRITDQYRPKWRIPKLTQDDLEMVRPARRRTPSPDYDL
YYYRRRRRSLGDMSDEELLLPIDDYLAMKRTEEERLRLEEELELGFSASPPSRSPPRFELSSLRYSSPPAHVK
VEDRRRDFRYSTYHVPTKEETSTSYAELRERHAQASYRQPKLRQRIMAEKEEEELLRPVTTTQRLSEYKSELD
YMSKEEKSKKKSKRQRQVTEITEIEEEYEISRRAQRESSSSVSRLLRRRRSLSPTYIELMRPVSELIRSHPRP
AEEYEDDAERRSPTPERTRPRSPSPVSSERSLSRFERSARFDIFSRYESMKAALKTQKTSERKYEVLSQQPFT
LDHAPRITLRMRSHRVPCGQNTRFILNVQSKPTAEVKWYHNGVELQESSKIHYTNTSGVLTLEILDCQTEDGG
TYRAVCTNYKGEASDYATLDVTGGAYTTYASQRRDEEVPKSVFPELTKTEAYAVSSFKRTSELEAASSVREVK
SQMTETRESLSTYEHYASAEMKSATSEEKSLEEKATVRKIKTTLAARILTKPRSITVHEGESARFSCDTDGEP
VPTVTWLREGQVVSTSARHQVTTTKYKSTFEISSVQASDEGNYSVVVENSDGKQEAQFTLTVQKARVIEKAVT
SPPRVKSPEPRVKSPETVKSPKRVKSPEPVTSHPKAVSPTETKPTEKGQHLPVSAPPKITQSLKAEASKDIAK
LTCAVESSALCAKEVAWYKDGKKLKENGHFQFHYSADGTYELKIHNLSESDCGEYVCEVSGEGGTSKTSFQFT
GQSFKSIHEQVSSISETTKSVQKTAESAEAKKSEPIAPESISSKPVIVTGLRDTTVSSDSVAKFTIKVTGEPQ
PTITWTKDGKAIAQGSKYKLSSNKEEFILEILKTETSDGGLYACTVTNSAGSVSSSCKLTIKAVKDTEAQKVS
TQKTSEVTSQKKASAQEEISQKALTSEEIKMSEVKSHETLAIKEEASKVLIAEEVKRSAAASLEKSIVHEEVT
KTSQASEEVKTHAEIKTLSTQMNITKGQRATLKANIAGATDVKWVLNGTELPNSEEYRYGVSGSDQTLTIKQA
SHREEGILSCIGKTSQGVVKCQFDLTLSEELSDAPSFITQPRSQNINEGQNVLFSCEVSGEPSPEIEWFKNNL
PISISSNISVSRSRNVYTLEIRNAAVSDSGKYTIKAKNFHGQCSATASLTVLPLVEEPPREVVLKTSSDVSLH
GSVSSQSVQMSASKQEASFSSFSSSSASSMTEMKFASMSAQSMSSMQESFVEMSSSSFMGKSSMTQLESSTSR
MLKAGGRGIPPKIEALPSDISIDEGKVLTVACAFTGEPTPEITWSCGGRKIQNQEQQGRFHIENTDDLTTLII
MDVQKQDGGLYTLSLGNEFGSDSATVNINIRSM*
>ENSMUSP00000107513_c.77325:A>C_p.K25776Q ENSMUST00000111882.9|Ttn-205|ENSMUSG00000051747|-|chr2:g.76568328:T>G
MTTQAPMFTQPLQSVVVLEGSTATFEAHVSGSPVPEVSWFRDGQVISTSTLPGVQISFSDGRARLMIPAVTKA
NSGRYSLRATNGSGQATSTAELLVTAETAPPNFSQRLQSMTVRQGSQVRLQVRVTGIPTPVVKFYRDGAEIQS
SLDFQISQEGDLYSLLIAEAYPEDSGTYSVNATNSVGRATSTAELVVQGEEVVPAKKTKTIVSTAQISETRQT
RIEKKIEAHFDARSIATVEMVIDGATGQLPHKTPPRIPPKPKSRSPTPPSIAAKAQLARQQSPSPIRHSPSPV
RHVRAPTPSPVRSVSPAGRISTSPIRSVKSPLLIRKTQTTTMATGPEVPPPWKQEGYVASSTEAEMRETTMTS
STQIRREERWEGRYGVQEQVTISGAAAAAASASVSSSFTAGAITTGTKEVKQETDKSAAVATVVAAVDMARVR
EPAISAVEQTAQRTTTTAVHIQPAQEQARKEAEKTAVTKVVVAADKAKEQELKSRTREVMVTTQEQTHISHEQ
IRKETEKAFVPKVVISATKAKEQETRITGEITTKQEQKRITQETIRQETEEIAASMVVVATAKSTKLEAAVGV
QEETAIQQDQMHLTHEQMMKETRKTVVPKVIVATPKIKEQDLVSRSREAITTKRDQVQITQEKKRKEVETTAL
STIAVATAKAKEQETVLRSREAMATRQEHIQVTHGQVGVGKKAEAVATVVAAVDQARVREPREPTHVEESHSQ

QTTLEYGYKEHISTTKVPEQPRRPASEPHVVPQAVKPAVIQAPSETHIKTTDQMGMHISSQVKKTTDISTERL
VHVDKRPRTASPHFTVSKISVPKTEHGYEASIAGSAIATLQKELSATSSTQKITKSVKAPTVKPGETRVRAEP
TPSPQFPFADMPPPDTYKSQAGIEVKKEVGVSISGSTVREEHFEVLRGREAKVTETARVPAPAEVPVTPPTLV
SGLKNVTVIEGESVTLECHISGYPSPKVTWYREDYQIESSIDFQITFQGGIARLMIREAFAEDSGRFTCSAVN
EAGTVSTSCYLAVQVSEEFDKETTLTEKFATEEKRFVESRDVVMTDTSITEEQAGPGEPAAPFFISKPVVQKL
VEGGSVVFECQIGGNPKPHVYWKKSGVPLTTGYRYKVSYNKQTGECRLVISMTFADDAGEYTIVIRNKHGETS
ASASLLEEADYEALVKTQQEMLYQTQMSTFIQEPKVGEIAPGFAYSEYEKEYEKEQALIRKKMAKDTVMVRTF
VEDQEFHISSFEERLIKEIEYRIIKTTLEELLEEDGEEKMAVDISESEAIESGFDIRIKNYRILEGMGVTFHC
KMSGYPLPKIAWYKDGKRIRHGERYQMDFLQDGRASLRIPVVLPEDEGIYTAFASNIKGNAICSGKLYVEPAA
PFSAPTYMPTPEAVSRIRSVSPRSLSRSPIRMSPAMSPARMSPARMSPARMSPARMSPGRRLEETDESQLERL
YKPVFVLKPASFKCLEGQTARFDLKVVGRPMPETFWFHNGQQIVNDYTHKVVIKEDGTQSLIIVPASPSDSGE
WTVVAQNRAGKSTISVTLTVEAVEHQIKPAFVEKLKNVNIKEGSRLEMKVRATGNPNPDIVWLKNSDIIVPHK
YPRIRIEGTRGEAALKIDSIISQDSAWYTATAINKAGRDTTRCKVNVEVEFAEPEPERKLIIPRGTYRAKEIA
APELEPLHLRYGQEQWEEGDLYDKEKQQKPFFKKKLTSLRLKRFGPAHFECRLTPIGDPTMVVEWLHDGKPLE
AANRLRMINEFGYCSLDYGAAYSRDSGVITCRATNKYGTDHTSATLIVKDEKSLVEESQLPDGKKGLQRIEEL
ERMAHEGALTGVTTDQKEKQKPDIVLFPEPVRVLEGETARFRCRVTGYPQPKVNWYLNGQLIRKSKRFRVRYD
GIHYLDIVDCKSYDTGEVKVTAENPEGVTEHKVKLEIQQREDFRSVLRRAPEPKPEFHVHEPGKLQFEVQKVD
RPVDTSETKEVVKLKRAERITHEKVSEESEELRSKFKRRTEEGYYEAITAVELKSRKKDESYEELLKKTKDEL
LHWTKELTEEEKKALAEEGKITIPTFKPERIELSPSMEAPKIFERIQSQTVGQGSDAHFRVRVVGKPDPECEW
YKNGVKIERSDRIYWYWPEDNVCELVIRDVTAEDSASIMVKAINIAGETSSHAFLLVQAKQLITFTQELQDVV
AKEKDTMATFECETSEPFIKVKWYKDGIEVHAGDKYRMHSDRKVHFLSVLTIDTSDAEDYSCVLVEDENIKTT
AKLIVEGAVVEFVKELQDIEVPESYSGELECIISPENIEGKWYHNDVELKSNGKYSITSRRGRQNLTVKDVTK
EDQGEYSFVVDGKKTTCKLKMKPRPIAILQGLSDQKVCEGDIVQLEVKVSLENVEGVWMKDGQEVQHSDRVHI
VIDKQSHMLLIEDMTKEDAGNYSFTIPALGLSTSGNVSVYSVDVITPLKDVNVIEGTKAVLECKVSVPDVTSV
KWYLNDEQIKPDDRVQSIVKGTKQRLVINRTHASDEGPYKLMVGRVETSCNLSVEKIKIIRGLRDLTCTETQN
VVFEVELSHSGIDVVWNFKGKEIKPSSKYKIEAHGKIYKLTVLNMMKDDEGEYAFYAGENTTSGKLTVAGGAI
STPLTDQTVAESQEAVFECEVANPESEGEWLKDGKHLALSNNFRGESDGHKRRLVIAAAKLDDAGEYTYKVAT
SKTSAKLKVEAVKIKKTLRNLTVTETQDAVFSVELTHPDVKGVQWIKNGVVLDSNDKYEISVKGTLYSLKIKN
CAMADESVYGFKLGRLGASARLHVETVKIIKKPKDVTALENATVTFEVSVSHDTVPVKWFHKNVEIKPSDKHR
LVSERKVHKLMLQSISPSDAGEYTAMVGQLECKAKLFVETLHITKTMKSIEVPETKAASFECEVSHFNVPSMW
LKNGVEIEMSEKFKIVVQGKLHQLIIMNTSTEDSAEYTFVCGNDQVSATLTVTPIMITSMLKDINAEEKDTIT
FEVTVNYEGISYKWLKNGVEIKSTDRCQMRTKKLTHSLNIRNVHFGDAADYTFVAGKATSTATLYVEARHIEF
RKHIKDIKVLEKKRAMFECEVSEPDITVQWMKDGQELQIADRIKIQKEKYVHRLLIPSTRMSDAGKYTVVAGG
NMSTANLFVEGRDVRIRSIKKEVQVIEKQRAVVEFEVNEDDVDAHWYKDGIEINFQVQERHQYVVERRIHRMF
ISETRHSDAGEYTFVAGRNRSSVTLYVNAPEPPQVLQELQPVTVQSGKPARFCAVISGRPQPKISWYKEEQLL
STGFKCKFLHDGQEYTLLLIEAFPEDAAVYTCEAKNDYGVATTSASLSVEVPEVVSPDQEMPVYPPAIVTPLQ
DTVTSEGRPARFQCQVSGTDLKVSWYCKDKKIKPSRFFRMTQFEDTYQLEIAEAYPEDEGTYAFVANNAVGQV
SSTATLRLEAPESILHERIGQQIEMEMKAAPVIKRRIEPLEVALGHLAKFTCEIQGAPNVRFQWFKAGREIYE
SDKCSIRSSNYVSSLEILRTQVVDCGEYTCKASNEYGSVSCTATLTVTEAYPPTFLSRPKALTTFVGKAAKFL
CTVSGTPVIEIIWQKDGAALSPSPDCRVTDADNKHSLELSNLTVQDRGIYSCKASNKFGADICQAELTIIDKP
HFIKELEAVQSAINKKIHLECQVDEDRKVTITWSKDGQKLPAGKDYKIYFEDKIASLEIPLAKLKDSGTYTCT
ASNEAGSSSSSAAVAVREPPSFVKKVDPSYLMLPGESARLHCKLKGSPVIQVTWFKNNKELSESNTVRMSFVN
SEAILDITDVKVDDSGTYSCEATNDVGSDSCSTEVVIKEPPSFIKTLEPADIVRGANALLQCEIAGTGPFEVN
WFKDKKQIRSSKKYRLFTQKTFVYLEISSFNSADVGDYECVVANEVGKCGCVATHLLKEPPTFVKKVDDFTAL
AGQTVTLQAAVRGSEPISVMWMKGQEVIKEDGKIKMSFSNGVAVLTIPDVQISLGGKYTCLAENEAGSQTSVG
ELIVKEPAKIIERAELIQVTAGDPATLEYTVSGTPELKPKWYKDGRPLVASKKYRISFKNNIAQLKFYSAELH
DSGQYTFEISNEVGSSSCETTFTVLDRDIAPLFTKPLRNVDSVVGGACRLDCKIAGSLPMRVSWFKDGKELTA
SDRYQIAFVEGTASLEISRVDMNDAGNFTCRATNSVGSKDSSGALIVQEPPSFVTKPGSRDVLPGSAVCLKSA
FQGSAPLTIKWFKGDKELVSGGSCYITKETSESSLELYAVKTSDSGTYTCKVSNVAGSVECSADLFVKEPATF
IEKLEPSQLLKKGDGTQLACKVTGTPPIKITWFANDRELRESSKHKMSFAESTAVLRLTDVAIEDSGEYMCEA
QNEAGSDHCTGIVIVKESPYFTKEFKSIEVLKEYDVMLLAEVAGTPPFEITWFKDNTTLRSGRKYKTFLQDQL
VSLQVLKFVAADAGEYQCRVTNEVGSSTCSARVTLREPPSFIKKIEATSSLRGGTAAFQATLKGSLPITVTWL
KDNDEITEDDNIRMTFENNVASLYLSGIEVKHDGKYVCQAKNDAGIQRCSALLSVKEPATIMEEAVSIDVTQG
DPATLQVKFSGTKEISAKWFKDGQELTLGPKYKISVTDTVSILKIISTEKKDSGEYTFEVQNDVGRSSCKASI
NVLDLIIPPSFTKKLRKMDSIKGSFIDLECIVAGSHPISIQWFKDDQEISASDKHKFSFHDNTAFLEISQLEG
TDSGTYTCSATNKAGHSQCSGHLTVKEPPYFVEKPQSQDVNPGTRVQLKALVGGTAPMTIKWFKDNKELHPGA
ARSVWKDDTSTILELFSAKAADSGTYICQLSNDVGTTSSKATIFVKEPPQFIKKPSPVLVLRNGQSTTFECQV
TGTPEIRVSWYLDGNEITDLRKYGISFVDGLATFQISNARVENSGTYVCEARNDAGTASCSIELKVKEPPIFI

RELEPVEVVKDSDVELECEVMGTTPFEVTWLKNNKEIRSGKKYTMSEKMSVFYLHITKCDPSDVGEYQCIIAN
EGGSCACSARVALKEPPSFIKKIENVTTVLKSSATFQSTVAGSPPISITWLKDDQILEENDNVHISFEDSVAT
LQVRSVDNGHSGRYTCQAKNESGIERCYAFLLVQEPAQIIEKAKSVDVTEKDPVTLECVVAGTPELKVKWLKD
GKQIVPSRYFSMSFENNVASFRIQSVMKQDSGQYTFKVENDFGSSSCDAYLRVLDQDIPPSFTKKLTKMDKVL
GSSIHMECKVSGSLPISAQWFKDGKEISTSAKYRLVCHENTVSLEVSNLELEDTANYTCKVSNVAGDNACSGI
LTVKEPPSFLVKPERQQAIPDSTVEFKAVLKGTPPFKIKWLKDDVELVSGPKCFIGLEGSTSFLNLYSVDSSK
TGQYTCQVTNDVEPPKFVKKLEASKIIKAGDSARLECKITGSPEIQVVWYRNEHELTASDKYQMTFIDSVAVI
QMNSLGTEDSGDFICEAQNPAGSTSCSTKVIVKEPPVFSSFPPIVETLKNTEVSLECELSGTPPFEVVWYKDK
RQLRSSKKYKVASKNFHASIHILNVESTDIGEYHCKAQNEVGSDACVCAVKLKEPPKFISKLNSLTVVAGEPA
ELQASIEGAQPISVQWLKEKEEVIRESENIRISFVNNVATLQFAKVEPANAGKYICQVKNDGGVRENMATLTV
LEPAVIIEKAGSMTVTVGETCALECKVAGTPELSVEWYKDGKLLTSSQKHKFSFYNKISSLKILSVEKEDAGT
YTFQVQNNVGKSSCTAVVDVSDRMVPPSFTRRLKDTGGVLGTSCILECKVAGSSPISIAWFHEKTKIVSGAKY
QTTFSDNVCTLQLNSLDSSDMGSYTCVAANVAGSDECRALLTVQEPPSFVKEPEPLEVLPGKNITFTSVIRGT
PPFKVGWFRGARELVKGNRCNIYFEDTVAELELFNIDISQSGEYTCVVSNNAGQASCTTRLFVKEPATFVKKL
SDHSVEPGKSIILEGTYTGTLPISVTWKKDGVSITPSERCNIVTTEKTCILEILSSTKGDAGHYSCEIENEAG
RDACDALVSTLEPPYFVTELEPLEASVGDSVSLQCQVAGTPEITVSWFKGDTKLRSTPEYRTYFTNNVATLVF
NKVSINDSGEYTCMAENSIGTAASKTIFRIQERQLPPSFARQLKDIEQTVGLPVTLTCRLNGSAPIQVCWYRD
GVLLRDDENLQMSFVDNVATLKILQTDLSHSGQYSCSASNPLGTASSTARLTAREPKKSPFFDIKPVSIDVIA
GESADFECHVTGAQPMRVTWSKDNKEIRPGGNYTITCVGNTPHLRILKVGKGDSGQYTCQATNDVGKDMCSAQ
LSVKEPPKFIKKLDASKVAKQGESIQLECKISGSPEIKVVWFRNDSELHESWKYNMSFVNSVALLTINEASAE
DTGDYICEAHNGVGDASCSTALKVKAPPVFTQKPPPVGALKGSDVILQCEISGTPPFEVVWVKDRKQVRSSKK
FKITSKNFDTSLHIFNLEAPDIGEYHCKATNEVGSDTCACTVKFKEPPRFVKKLSDASTLIGDPVELQAVVEG
FQPISVVWLKDKGEVIRESENVRISFVDNIATLQLGSPEASQSGKYVCQIKNDAGMRECSAVLTVLEPATIVE
KPEPMTVTTGNPFTLECVVAGTPELSAKWFKDGRELSSGSRHHITFVRNLASLKIPSAEMNDKGLYTFEVENR
VGKSSCTVSVHVSDRVVPPSFVRRLKDTSATLGASVVLECRVSGSAPISVGWFLDGNEIISSPKCQSSFADNV
CTLTLSSLEPSDTGAYTCVAANVAGQDESSAVLTVQEPPSFEQTPDSVEVLPGMSLTFTSVIRGTPPFKVKWF
KGSRELVSGEACTISLEDFVTELELLEVEPGQSGDYSCLVTNDAGSASCTTHLFVKEPATFVKRLADTSVETG
SPIVLEATYSGTPPISVSWMKNEYPLSQSPNCGITTTEKSSILEILESTIEDYAQYACLIENEAGQDICEALV
SVLEPPYFIEPLEHVEAAIGEPITLQCKVDGTPEIRISWYKEHTKLRSAPAYKMQFKNNVASLVINKVDHSDV
GEYTCKAENSVGAVASSAVLVIKERKLPPSFARKLKDVHETLGFPVAFECRINGSEPLQVSWYKDGELLKDDA
NLQMSFVHHVATLQILQTDQSHVGQYNCSASNPLGTASSSAKLILSEHEVPPFFDLKPVSVDLALGESGSFKC
HVTGTAPIKITWAKDNREIRPGGNYKMTLVENTATLTVLKVAKGDAGQYTCYASNVAGKDSCSAQLGVQEPPR
FIKKLDQSRIVKQDEYTRYECKIGGSPEIKVLWYKDEVEIQESSKFRMSFEDSVAILEMHSLSVEDSGDYTCE
ARNAAGSASSSTSLKVKEPPVFRKKPFPVETLKGADVHLECELQGTPPFQVSWHKDKRELRSGKKYKIMSENL
LTSIHILNVDTADIGEYQCKATNDVGSDTCVGSVTMKAPPQFVKKLTDISTIIGKEVQLQTTIEGAEPISVAW
FKDKGEIVRESDNIWISYSENIATLQFSRAEPANAGKYTCQIKNDAGMQECYATLSVLEPAAIVEKPESIKVT
TGDTCTLECTVSGTPELSTKWFKDGKELTSDNKYKISFFNKVSGLKIINVVPGDSGVYSFEVQNPVGKDSCKV
SIQVSDRIIPPSFTRKLKETNGLSGSSVVMECKVYGSPPISVLWFHDGNEISSGRKYQTTLTDNTCALTVNML
EEADAGDYTCIATNVAGSDECSAPLTVREPPSFVQKPDPMDVLTGSNVTFTSIVKGSPPFTVSWFKGSTELVP
GARCNVSLQDSVGELELFDVDTSQSGEYTCIVSNEAGRASCTTRLFVKAPAIFVKRLNDYSIEKGKPLILEGT
FSGTPPISVTWKKNGINVIASQRCNITTTEKSAILEILSSTVEDSGQYNCYIENASGKDSCSAQILILEPPYF
VKQLEPVKVTVGDSASLQCQLAGTPEIGVSWYKGDTKLRPTATCKMHFKNNVATLVFTQVDSSDSGEYICRAE
NSVGEVSSSTFLTVQEQKLPPSFSRQLRDVQETVGLPVVFECAVSGSEPISVSWYKDGKPLKDSPNIQTSFLD
NIATLNIFKTDRSLSGQYSCTATNPIGSASSGAKLILTEGKNPPFFDIPLAPMDAVVGESADLECHVTGTQPI
KVTWAKDNREIRSGGNYQISYLENSAHLTIVKVDKGDSGQYTCYAVNEVGKDSCTAQLNIKERLIPPSFTKKL
SETVEETEGNSFKLEGRVAGSQPITVAWYKNNVEIHPTSNCEIMFKNNALLLQVKRASMADAGLYTCKATNDA
GSALCTSSIVIKEPKKPPVFDQHLAPVTASEGDSVQLSCHVQGSEPIRIQWLKAGREVKPSDRCSFSFASGTA
MLELKETAKADSGDYVCKASNVAGSDTSKCKVTIKEKPAAAPAAKKAAVDGKLFFVSEPQSIRVVEKTTATFI
AKVGGDPIPNVKWTKGKWRQLNQGGRILIHQKGDEAKLEIRDTTKTDSGLYRCVAFNKHGEIESNVNLQVDER
KKQEKIEGDLRAMLKKTPALKKGSGEEEEIDIMELLKNVDPKEYEKYARMYGITDFRGLLQAFELLKQSQEEE
THRLEIEELEKSERDEKEFEELVAFIQQRLTQTEPVTLIKDIENQTVLKDNDAIFEIDIKINYPEIKLSWYKG
TEKLEPSNKYEISIDGDRHTLRVKNCQPKDQGNYRLVCGPHIASAKLTVIEPAWERHLQDVTLKEGQTCTMTC
QFSVPNVKSEWFRNGRVLKPQGRVKTEVEHKVHKLTIADVRAEDQGQYTCKHEDLETSAELRIEAEPIQFTKR
IQNIVVSEHQSATFECEVSFDDAIVTWYKGPTELTESQKYNFRNDGRCHYMTIHNVTPDDEGVYSVIARLEPR
GEARSTAELYLTTKEIKLEMKPPDIPDSRVPIPTMPIRAVPPEEIPPAVAPSIPLLLPLPEEKKPPAKRIEVT
KKGVKKDTKKVVTKPKEEAPPPPVPEVAKKPPPPTPMIPAKASEIIDVSSKAEEVKITTITRKKEVHKEKEAV
YEREEAVYEKKVHIEPWEEPYEELETEPYTEPYEEPYYEEPDEDYEEIKVEAKKQVHEEWEEDFEEGQEYYER
EEGYDEGEEEWEEIYHEREIIQVQKEVHEELHEKKIPAKVPEKKVPPPKVVKKPVVEKVEKTTRRMEEEKVQV

IKVPEVSKKIVPQKPSRTPVQEEIIEVKVPAVHTKKMVISEEKMFFASHTEEEVSVSVPEVQKKTVTEEKIHV
AVSKKIEPPPKVPEPPKKPVPEEVVPVPIPKKVEPPAAKVPEAPKKPVPEEKKPVPIPKKKEPAAPPQVPEAP
KKPAPEEKIPVPVTKKKEAPPAKVPEVQKKVVTEEKIAIITQREESPPPAVPEIPKKKVPEEKRPVPRKEEVP
PPKVPVPPKKAVPEAVVPAPIPKKAPPRAEVSKKTVVEEKRFAAEEKLSVAVPQRVELMRHEEEEWTYSEEEE
RVSVSVYREEERDEEEAEITEYEVLEEPEEYVVEEKMHFISKKVEVEPAKVPEKKIIPKPKVPAKIEEPPPTK
VPEPPKKIVPEKKVPAPAPKKVPPAKAPEESKRPVPEKRAPAEEVGIEEPPPTKVAERHMKITQEEKVLVAVT
KKEAPPRARVPEEPKKVAPEERFPKLKPRREEEPPAKVTEVRKRAVKEEKVSIEVPKREPRPTKEVTVTEEKK
WSYTREEETVSEHREEEYEDYEDYEEYKEFEEYEPTEEYDQYDEYAEREVEHYEEHEEYVTEPKKPVPVKPAQ
EPVPAKPKAPPPKVLKKAVPEEKAPLPIQKKLKPLPPKAPEEPKKVVEEKIQISITKREKQQVTEPVAKVPMK
PKRVVPEAKIPAPTKEVAVPVRESLSFVICGSVLLKCLNFIEELENSQLKDDIFKVPGVPKKRELEEVVVFKE
EVEAHEEYIVEEEEEYVHEEEYVHKEEYVHEEEYVHKEEYIHEEEEHLHEEEETIAEEEVVPVAPVKVPVVPK
KPVPEEKKPVPVPKKKEAPPAKVPEIPKKPEEKVPVPIPKKEKAPPAKVPEVPKKPVPEEKPPVPVPKKVEPP
PAKVPEVPKKPVPEKKVPAPTPKKVEAPPAKVPEVPKKPIPEEKKPTALLKKMEAPPPKAPKKREVVPVPVAL
PREEEEEEVPFEEVPEEEILPEEEVPSEEEAPPEEVPPEEEEVLPEEEEVLPEEEEVLPEEEEVQPEEEALPE
IKPKVPKPAPVPEIKKKVPEKKVVVPKKEEAPPTKVPEVSKKVEERRIIPPKEEEVPPAEVYEEAEEPTPEEI
PEEPPSIEEEEIVEEEEEEEEVLPPRAPEVVKKAVPEAPTPVPKKAEAPPAKVPKKIPEEKVPVPVQKKEAPP
AKVPEVPKKVPEKKKIPEKKVPVPKKEAVPPAKGKAVFEEKISVAYQQEELVQERIELELVEAKVEEAFEEEE
FHEVQEYFEEEEFHEVEEFIRVEERRFQEEHKVEEVHRVIEFLEAEEVEVYEKPKIPPKKGPEVSEKVIPPKK
PPTKVIPRKEPPAKVPEVTKKTVVEEKIRAPEEPKVPAPKEVVPEKKVSVVPPKKPEAPPAKVPEAPKEAAPE
KKVPVAPKKKPEAPPVKVPEVPKTAVPQKKIPEAIPPKPESPPLEVPEAPKEVVPEKKVSVVPPKKPEAPPAK
VPEAPKEAAPEKKVPVAPKKKPEAPPVKVPEVPKAAVPQKKIPEAIPPKPESPPPEVYEEPEEEIVPEEPPEE
AVEEPVPAPPPKVTEPPKKPVPEKKAPPAVVKKPEPPPAKVPEVPKEAPPEKKVPPKKPEAPPAKVPEVPKEV
VTEKKVAVPKKPEVPPAKVPEVPKKPVIEEKPAIPVVEKVASPPAEVYEEPEEVTAEEEEPAPAVEEEEYEAP
PPPAPVPEEPKKVVPEKKFPVIKKPEAPPPKEPEPEKKVIEKPKLKPRPPARPPSPPKEDVKEKMFQLKAVSK
KKVPEKPEVVEKVEPAPLKVPTAEKKVRKLLPEPKPQPKEEVVLKSVLRKKPEEEEPKVEPKKVEKAKKPEEP
QPPPKAVEVEAPPEPTPKERKVPEPAKVPEIKPAIPLPGPEPKPKPEPEVKTMKAPPIEPAPTPIAAPVTAPV
VGKKAEAKPKDEAAKPKGPIKGVAKKTPSPIEAERKKLRPGSGGEKPPDEAPFTYQLKAVPLKFVKEIKDIVL
TEAESVGSSAIFECLVSPSTAITTWMKDGSNIRESPKHRFIADGKDRKLHIIDVQLSDAGEYTCVLRLGNKEK
TSTAKLIVEELPVRFVKTLEEEVTVVKGQPLYLSCELNKERDVVWRKDGKIVVEKPGRIVPGVIGLMRALTIN
DADDTDAGTYTVTVENANNLECSSCVKVVEIIREWLVKPIRDQHVKPKGTAVFTCDIAKDTPNIKWFKGYDEI
PLEPNDKTEILKEGNHLFLKVKNAMPEDIDEYAVEIEGKRYPAKLTLGEREVELLKPIEDVTIYEKESASFDA
EISEEDIPGEWKLKGELLRPSPTCEIKAEGGKRFLTLHKVKLDQAGEVLYQACNAITTAILTVKEIELDFAVP
LKDVTVPEKRQARFECVLTREANVIWSKGPDIIKASDKFDIIADGKKHILVINDSQFDDEGVYTAEVEGKKTS
AQLFVTGIRLKFISPLEDQTVKEGQTATFVCELSHEKMHVVWFKNDVKLHTTRTVLMSSEGKTYKLEIRETTL
DDISQIKAQVKNLSSTANLKVLEADPYFTVKLHDKTGVEKDEIILKCEVSKDVPVKWFKDGEEIVPSPKHSVK
TDGLRRILKIKKAELKDKGEYVCDCGTDTTKANVTVEARLIKVEKPLYGVEVFVGETARFEIELSEPDVHGQW
KLKGEPLTASPDCEIIEDGKKHVLVLYNCQLDMTGEISFQAANAKSAANLKVKELPLIFITPLSDVKVFEKDE
AKFECEVSREPKTFRWLKGTQEITGDDRFELIKDGTRHSLVIKSAAFEDEAKYMFEAEDKRTSGKLIIEGIRL
KFLTPLKDVTAKERENAVFTVELSHDNIPVSWFKNDQRLHTSKRVSMHDEGKTHSITFKDLSIDDTSQIRVEA
MGISSEAKLTVLEGDPYFTGKLQDYTGVEKDEVILQCEISKADAPVKWFKDGKEIKPSKNVVIKADGKKRMLI
LKKALKSDIGQYTCDCGTDQTSGKLDIEDREIKLVRPLYSVEVMETETARFETEISEDDIHANWKLKGEALLQ
TPECEIKEEGKIHVLILHNCRLDQTGGVDFQAANVKSSAHLRVKPRVIGLLRPLKDVTVTAGETATFDCELSY
EDIPVEWYLKGKKLEPNDKVVTRSEGRVHTLTLRDVKLEDAGEVQLTAKDFKTQANLFVKEPPVEFTKPLEDQ
TVEEEATAVLECEVSRENAKVKWFKNGTEILKSKKYEIVADGRVRKLIIHGCTPEDIKTYTCDAKDFKTSCNL
NVVPPHVEFLRPLTDLQVKEKETARFECEISKENEKVQWFKDGAEIKKGKKYDIISKGAVRILVINKCLLNDE
AEYSCEVRTARTSGMLTVLEEEAVFTKNLANLEVSEGDTIKLVCEVSKPGAEVIWYKGDEEIIETGRFEILTD
GRKRILIIQNAQLEDAGSYNCRLPSSRTDSKVKVHELAAEFISKPQNLEILEGEKAEFVCTISKESFEVQWKR
DDQTLESGDKYDIIADGKKRVLVVKDATLQDMGTYVVMVGAARAAAHLTVIEKLRIIVPLKDTKVKEQQEVVF
NCEVNTEGAKAKWFRNEEAIFDSSKYIILQKDLVYTLRIRDARLDDQANFNVSLTNHRGENVKSAANLIVEEE
DLRIVEPLKDIETMEKKSVTFWCKVNRLNVTLKWTKNGEEVAFDNRISYRIDKYKHSLIIKDCGFPDEGEYVV
TAGQDKSVAELLIIEAPTEFVEHLEDQTVTEFDDAVFSCQLSREKANVKWYRNGREIKEGKKYKFEKDGSIHR
LIIKDCRLEDECEYACGVEDRKSRARLFVEEIPVEIIRPPQDILEAPGADVIFLAELNKDKVEVQWLRNNMIV
VQGDKHQMMSEGKIHRLQICDIKPRDQGEYRFIAKDKEARAKLELAAAPKIKTADQDLVVDAGQPLTMVVPYD
AYPKAEAEWFKENEPLSTKTVDTTAEQTSFRISEAKKDDKGRYKIVLQNKHGKAEGFINLQVIDVPGPVRNLE
VTETFDGEVSLAWEEPLTDGGSKIIGYVVERRDIKRKTWVLVTDRADSCEFTVTGLQKGGVEYLFRVSARNRV
GTGEPVETDSPVEARSKYDVPGPPLNVTITDVNRFGVSLTWEPPEYDGGAEITNYVIELRDKTSIRWDTAMTV
RAEDLSATVTDVVEGQEYSFRVRAQNRIGVGKPSAATPFVKVADPIERPSPPVNLNASEQTQSSVQLTWEPPL
KDGGSPILGYIIERREEGKDNWIRCNMKPVPELTYKVTGLQKGNKYLYRVSAENAAGVSDPSEILGPLTADDA

FVEPTMDLSAFKDGLEVIVPNPIKILVPSTGYPRPKATWTFGDQVLEEGDRVKMKTISAYAELVISPSERTDK
GIYTLTLENPVKSISGEINVNVIAPPSAPKELKFSDITKDSVHLTWEPPDDDGGSPLTGYVVEKRDMSRKTWT
KVMDFVTDLEFTVPDLVQGKEYLFKVCARNKCGPGEPAYTDEPVNMSAPATVPDPPENVKWRDRTANSIFLTW
DPPKNDGGSRIKGYIVEKCPRGSDKWVACGEPVPDTKMEVTGLEEGKWYAYRVKALNRQGASKPSKPTEEIQA
VDTQEAPEIFLDVKLLAGITVKAGTKIELPATVTGKPEPKITWTKADTLLKPDQRITIENVPKKSTVTITDSK
RSDTGTYIIEAVNVCGRATAVVEVNVLDKPGPPAAFDITDVTNESCLLTWNPPRDDGGSKITNYVVERKATDS
DVWHKLSSTVKDTNFKATKLTPNKEYIFRVAAENMYGVGEPVQAAPIIAKYQFDPPGPPTRLEPSDITKDAVT
LTWCEPDDDGGSPITGYWVERLDPDTDKWVRCNKMPVKDTTYRVKGLTNKKKYRFRVLAENLAGPGKPSRSTE
PILIKDPIDPPWPPGKPTVKDIGKTSLVLNWTKPEHDGGAKIESYVIEMLKTGTDEWVRVAEGVPTTEHLLTG
LMEGQEYSFRVRAVNKAGESEPSEPSDPVLCREKLYPPSPPRWLEVINITKNTADLKWTVPEKDGGSPITNYI
VEKRDVRRKGWQTVDTTVKDTKCTVTPLTEGSLYVFRVAAENAIGQSDYTEIGDSVLAKDTFTTPGPPYALTV
VDVTKRHVDLKWEPPKNDGGRPIQRYIIEKKEKLGTRWVKAGKTSGPDCNFRVTDVIEGTEVQFQVRAENEAG
VGHPSEPTEILSIEDPTSPPSPPLDLHVTDAGRKHIAIAWKPPEKNGGSPIIGYHVEMCPVGTEKWMRVNSRP
IKDLKFKVEEGIVPDKEYVLRVRAVNAVGVSEPSEISENVVAKDPDCKPTIDLETHDIVVIEGEKLNIPVPFR
AVPVPTVSWHKDGKEVKASDRLTMKNDHISAHLEVPKSVHADAGVYTITLENKLGSATASINVKVIGLPGPCK
DIKASDITKSSCKLTWEPPEFDGGSPILHYVLERREAGRRTYIPVMSGENKLSWTVKDLIPNGEYFFRVKAVN
KIGGGEYIELKNPVIAQDPKQPPDPPVDVEVHNPTAKAMTITWKPPLYDGGSKIMGYIIEKITKGEDRWKRCN
EHLVPVLTYTAKGLEEGKEYQFRVRAENAAGIGEPSRATPPTKAVDPIDAPKVILRTSLEVKRGDEIALDATI
SGSPYPTITWIKDENVIVPEEIKKRAAPPVRRKKGEAEEEEPFSLPLTERLSINNSKQGESQLRIRDSLRPDH
GQYMIKVENDHGVAKAPCSVSVLDTPGPPINFVFEDIRKDSVLCKWEPPLDDGGSEIINYTLEKKDKTKPDSD
WIVITSTLRNCKYSVTKLIEGKEYLFRVRAENRFGPGPPCVSKPLLAKDPFEPPDAPDKPIVEDVTSNSMLVK
WNEPKDNGSPILGYWLEKREVNSTHWSRVNKTLLSSLKTKVDGLLEGLTYVFRVCAENAAGPGKFSPPSDPKT
ARDPISPPGPPVPRVADTSSTTIELEWEPPAFNGGGEIMGYFVDKQLVGTNEWSRCTEKMIKVRQFTVKEIRE
GADYKLRVSAVNAAGEGPPGETEPVTVAEPQEPPTVELDVSVKGGIQIMAGKTLRIPAEVTGRPVPTKVWTIE
EGELDKERVIIENVGTKSELIIKNALRKDHGRYVITATNSCGSKFAAVRVEVFDVPGPVLDLKPVVTNRKMCL
LNWSDPADDGGSDITGFIIERKDAKMHTWRQPIETERSKCDITGLIEGQEYKFRVIAKNKFGCGPPVEIGPIL
AVDPLGPPTSPERLTYTERTKSTITLDWKEPRSDGGSPIQGYIIEKRRHDKPDFERVNKRLCPTTSFLVENLD
EHQMYEFRVKAVNDVGESEPSLPLNVVIQDDEVPPTIKLRLAVRGDTIKVKAGEPVNIPADVTGLPMPKIEWS
KNEKVIDKPTDTLNITKEEVSRSEAKTELSIPKAAREDKGTYTITASNRLGSVFRNVHVEVYDRPSPPRNLAV
TDIKAESCYLTWDAPLDNGGSEITHYIIDKRDASRKKSEWEEVTNTAVERRYGIWKLIPNGQYEFRVRAVNKY
GISDECKSDKVVIQDPYRLPGPPGKPKVLERTKGSMLVSWTPPLDNGGSPITGYWLEKREEGGTYWSRVSRAP
ITKVGLKGVEFNVPRLIEGVKYQFRAMAINAAGIGPPSEPSDPAVAGDPIYPPGPPSCPEVKDKTKSSISLAW
KPPAKDGGSPIKGYIVEMQEEGTTDWKPVNEPDKLLTACECVVPNLKELRKYRFRVKAVNEAGESEPSDTTGE
IPATDIQEVPEVFIDIGAQDCLVCKAGSQVKIPAVIKGRPTPKSSWEFDGKAKKAMKDGVHDIPEDAQLETAE
NSSVIIIPECTRAHSGKYSITAKNKAGQKTANCRVKVMDAPGPPKDLKVSDITRGSCRLSWKMPDDDGGDRIK
GYVIEKKTIDGKAWTKVNPNCGSTTFVVPDLISEQQYFFRVRAENRFGIGPPAETIQRTTARDPIYPPDPPIK
LKIGLITKNTVHLSWKPPKNDGGSPVTHYIVECLAWDPTGKKKEAWRQCNRRDVEELEFTVEDLIEGGEYEFR
VKAVNEAGVSKPSATVGPVIVKDQTCPPAIELKEFMEVEEGTDVNIVAKIKGVPFPTLTWFKAPPKKPDSKEP
VVYDTHVNKQVVDDTCTLVIPQSRRSDTGLYSITAVNNLGTASKEMRLNVLGRPGPPVGPIKFESISADQMTL
SWLPPKDDGGSKITNYVIEKREANRKTWVRVSSEPKECMYTIPKLLEGHEYVFRIMAQNKYGIGEPLDSEPET
ARNLFSVPGAPDKPTVSSVTRNSMTVNWEEPEYDGGSPVTGYWLEMKDTTSKRWKRVNRDPIKAMTLGVSYKV
TGLIEGSDYQFRVYAINAAGVGPASLPSDPVTARDPVAPPGPPFPKVTDWTKSSVDLEWSPPLKDGGSKITGY
IVEYKEEGKEEWEKGKDKEVRGTKLVVTGLKEGAFYKFRVRAVNVAGVGEPGEVTDVIEMKDRIVSPDLQLDA
SVRDRIVVHAGGVIRIIAYVSGKPPPTVTWSMNERALPQEAAIETTAISSSMVIKNCQRSHQGVYSLLAKNEG
GERKKTIIVDVLDVPGPVGIPFLSDNLTNDSCKLTWFSPEDDGGSPITNYVIQKREADRRAWTPVTYTVTRQN
ATVQGLIQGKSYFFRIAAENSIGMGPFVETPNALVIRDPITVPERPEDLEVKEVTKNTVSLTWNPPKYDGGSE
IINYVLESRLIGTEKFHKVTNDNLLSRKYTVKGLKEGDTYEYRVSAVNIVGQGKPSFCTKPITCKDELAPPTL
DLDFRDKLTVRVGESFALTGRYSGKPKPKIDWFKDEADVLEDDRTHIKTTPTTLALEKTKAKRSDSGKYCVVV
ENSTGSRKGFCQVNVVDRPGPPVGPVIFDEVTKEYMVISWKPPLDDGGSEITNYIIEKKELGKDIWMPVTSAS
AKTTCKVPKLLEGKDYIFRIHAENLYGISDPLVSDSMKAKDRFRVPDAPEQPVVTEVTKDSALVTWNKPNDGG
KPITNYILEKRETMSKRWVRVTKEPIHPYTKYRVPDLLEGCQYEFRVSAENEIGIGDPSPPSKPVFARDPIAK
PSPPINPEAIDTTCNSVELTWQPPRHDGGSKILGYIVEYQKVGDEEWRRANHTPESCPETKYKVTGLRDGQSY
KFRVLAVNEAGESDPAHVPEPVLVKDRLEPPELILDANMAREQHIRVGDTLRLSAIIKGVPFPKVTWKKEDRE
APTKAQIDVTPVGSKLEIRNAAHEDGGIYSLTVENPAGTKTVSVKVLVLDKPGPPRDLEVSEIRKDSCYLTWK
EPLDDGGSVVTNYVVERKDVATAQWSPLSTTSKKKSHMAKHLTEGNQYLFRVAAENQYGRGPFVETPKPIKAL
DPLHPPGPPKDLHHVDVDKTEVSLVWNKPDRDGGSPITGYLVEYQEEGDKDWIKFKTVKNLDCVVTGLQQGKT
YRFRVKAENIIGLGLPDTTIPIECQEKLVPPSVELDVKLIEGLVVKAGTTVRFPAIIRGVPVPTAKWTTDGTE
IKTDDHYTVETDSFSSVLTIKNCLRKDTGEYQLTVSNAAGTKTVAVHLTVLDVPGPPTGPINILDVTPEYMTI

SWQPPKDDGGSPVINYIVEKQDTRKGTWGVVSAGSSKLKLKVPHLQKGCEYVFRVKAENKMGVGPPLDSIPTV
AKHKFSPPSPPGKPVVTDITENAATVSWTLPKSDGGSPITGYYVERREITGKWVRVNKTPIADLKFRVTGLYE
GNTYEFRVFAENLAGLSNPSPSSDPIKACRPIKPPGPPINPKLKDKSKESADLVWTKPLSDGGSPILGYVVEY
QKPGTAQWDRINKDELIRQCAFRVPGLIEGNEYRFRIRAANIVGEGEPRELAESVIAKDILHPPEVELDVTCR
DVITVRVGQTIRILARVKGRPEPDITWSKEGKVLVKDKRVDLIHDLPRVELQIKEAVRADHGKYIISAKNSSG
HAQGSAIVNVLDRPGPCQNLKVSNVTKENCTISWENPLDNGGSEITNFIVEYRKPNQKGWSIVASDVTKRLVK
ANLLANNEYYFRVCAENKVGVGPTIETKTPILAINPIDRPGEPENLHIADKGKTFVYLKWRRPDYDGGSPNLS
YHVERRLKGSADWERVHKGSIKETHYMVDKCVENQIYEFRVQTKNEGGESDWVRTEEVVVKEDLQKPVLDLKL
SGVLTVKAGDTIRLEAGVRGKPFPEVAWTKDKDATDLTRSPRVKIDTSAESSKFSLTKAKRSDGGKYVITATN
PAGSFVAYATVNVLDKPGPVRNLKITDVSSDRCTIRWDPPEDDGGCEIQNYILEKCESKRMVWSTYSANVLTP
SATVTRLIEGNEYIFRVRAENKIGTGPPTESKPVIAKTKYDRPGRPDPPEVTKVSKEEMTVVWNAPEYDGGKS
ITGYYLEKKEKHAVRWVPVNKSAIPERRLKVQNLLPGHEYQFRVKAENEVGIGEPSLPSRPVVAKDPIEPPGP
PTNFKVVDTTKSSITLAWGKPVYDGGAPIIGYVVEMRPKIADASPDEGWKRCNAAAQLIRMEFTVTSLDENQE
YEFRVCAQNQVGIGRPAELKEAIKPKEILEPPEIDLDASMRKLVVVRAGCPIRLFAIVRGRPAPKVTWRKVGI
DNVVRKGQVDLVDTMAFLVIPNSTRDDSGKYSLTLVNPAGEKAVFVNVKVLDTPGPVADLKVSDVTKTSCHVS
WAPPENDGGSQVTHYIVEKREAERKTWSTVTPEVKKTSFNVTNLVPGNEYFFRVTAVNEYGPGVPTDIPKPVL
ASDPLSEPDPPRKLEVTEMTKNSATLAWLPPLRDGGAKIDGYIISYREEDQPADRWTEYSVVKDLSLIVTGLK
EGKKYKFRVAARNAVGVSMPREAEGVYEAKEQLLPPKILMPEQITIKAGKKLRVEAHVYGKPNPICKWKKGDD
EVVTSSHLAIHKADGSSVLIIKDVTRKDSGYYSLTAENSSGSDTQKIKVTVMDAPGPPQPPFDISEIDADACS
LSWHIPLEDGGSNITNYIVEKCDVSRGDWVTALASVTKTSCRVGKLIPGQEYIFRVRAENRFGISEPLTSPKM
LAKFPFDVPSEPKNARVTKVNKDCIFVAWDRPDSDGGSPITGYLIERKERNSLLWVKANDTIVRSTEYPCAGL
VEGLEYSFRIYALNKAGSSPPSKPTEYVTARMPVDPPGKPEVVDVTKNSASLIWARPKHDGGSRIIGYFVEAC
KLPGDKWVRCNTTPHQIPQEEYTATGLEENAQYQFRAIAKTAVNISQPSEPSDPVTILAENVPPRIELSVEMK
SLLTVKAGTNVCLDATVFGKPMPTVSWKKDTTPIKQAEGIKMAMKRNLCTLELFSVNRKDSGDYTITAENSSG
SKSATIKLKVLDKPGPPASVKINKMYADRAMLSWEPPLEDGGSEITNYIIDKRETSRPNWAQVSATVPITSCT
VEKLIEGHEYQFRICAENKYGVGDPILTEPAIAKNPYDPPGRCDPPVISNITKDHMTVSWKAPADDGGSPITG
YLVEKRETQAVNWTKVNRKPVIERTLKATGLQEGTEYEFRVTAINKAGPGKPSDASKAVYAQDPLYPPGPPAF
PKVYDTTRSSVSLSWGKPAYDGGSPIIGYLVEVKRADSDHWVRCNLPEKLQKTRFEVTGLMENTEYQFRVYAV
NKIGYSDPSDVPDKHCPKDILIPPEGELDAELRKTLILRAGVTMRLYVPVKGRPPPKITWSKPNVNLRERIGL
DIKSTDFDTFLRCENVNKYDAGKYILTLENSCGKKEYTIVVKVLDTPGPPVNVTVKEVSKDSAYVTWDPPIID
GGSPIINYVVEKRDAERKSWSTVTTECSKTSFRVSNLEEGKSYFFRVFAENEYGIGDPGETRDAVKASETPGP
VVDLKALAITKSSCTIGWKKPRSDGGSRITGYVVDFLTEENKWQRVMKSLSLQYSTKDLKEGKEYTFRVSAEN
ENGEGTPSEIVVVAKDDVVAPDLDLKDLPDLCYLAKENSNFRLKIPIKGKPAPSVSWKKGEDPLATDTRVSVE
STAVNTTLVVYDCQKSDAGKYTITLKNVAGTKEGTLSIKVVGKPGIPTGPIKFDEVTAEAMTLKWGPPKDDGG
SEITNYVLEKRDSVNNKWVTCASAVQKTTFRVTRLHEGIEYTFRVSAENKYGVGEGLKSEPIVAKHPFDVPDA
PPPPNIVDVRHDSVSLTWTDPKKTGGSPITGYHIEFKERNSLLWKRANKTPIRMKDFKVTGLTEGLEYEFRVM
AINLAGVGKPSLPSEPVVALDPIDPPGKPEVISVTRNSVTLIWTEPKYDGGHKLTGYIVEKRDLPSKSWMKAN
HVNVPDCAFTVTDLVEGGKYEFRIRAKNTAGAISAPSESTGTIICKDEYEAPTIVLDPTIKDGLTVKAGDSIV
LSAISILGKPLPKSSWSRAGKDIRPSDIAQITSTPTSSMLTVKYATRKDAGEYTITATNPFGTKEEHVKVSVL
DVPGPPGPIEISNVSAEKATLTWTPPLEDGGSPIKAYVLEKRETSRLLWTVVSEDIQACRHVVTKLIQGNEYL
FRVSAVNHYGKGEPVQSEPVKMVDRFGPPGPPGKPEISNVTKNTATVSWKRPTDDGGSEITGYHVERREKKGL
RWVRATKTPVSDLRCKVTGLQEGNTYEFRVSAENRAGIGPPSDASNPVLMKDVAYPPGPPSNAHVTDTTKKSA
SLAWGKPHYDGGLEITGYVVEHQKVGDDAWIKDTTGTALRITQFVVPDLQTKEKYNFRISAINDAGVGEPAVI
PNVEIVEKEVAPDFELDAELRRTLVVRAGLSIRIFVPIKGRPAPEVTWTKDNINLKHRANIENTESFTLLIIP
ECNRYDTGKFVMTIENPAGKKSGFVNVRVLDTPGPVLNLRPTDITKDSVTLHWDLPLIDGGSRITNYIVEKRE
ATRKSYSTVTTKCHKCTYKVTGLTEGCEYFFRVMAENEYGVGEPTETTEPVRASEAPLPPDSLNIMDITKNTV
SLAWPKPRHDGGSKITGYVIEAQRKGSDQWTHISTVKGLECVVRNLTEGEEYTFQVMAVNSAGRSAPRESRPV
IVKEQTMLPELDLRGIYQKLVIARAGDNIKVEIPVLGRPKPTVTWKKGDQILKQTQRVNVENTATSTILNINE
CVRSDSGPYPLTAKNTVGEVGDVITIQVHDIPGPPTGPIKFDEVSSDFVTFSWEPPENDGGVPISNYVVEMRQ
TDSTTWVELATTVIRTTYKATRLTTGVEYQFRVRAQNRYGVGPGITSASVVANYPFKVPGPPGTPQVTAVTKD
SMTISWHEPLSDGGSPILGYHIERKERNGILWQTVSKALVPGNIFKSTGLTDGIAYEFRVIAENMAGKSKPSK
PSEPMFALDPIDPPGKPVPLNITRHTVALKWAKPEYTGGFKITSYVVEKRDLPNGRWLKANFSNILENEFTVS
GLTEDAAYEFRVIAKNAAGAISPPSEPSDAITCRDDLEAPRIMVDVRFKDTITLKAGEAFKLEADVSGRPPPT
MEWTKDGKELEGTGKLEIKIADFSTHLINKDSSRTDSGAYILTATNPGGFAKHIFNVKVLDRPGPPEGPLAVS
DVTSEKCVLSWLPPLDDGGAKIDHYIVQKRETSRLAWTNVATEVQVTKLKVTKLLKGNEYIFRVMAVNKYGVG
EPLESEPVLAVDPYGPPDPPKNPEVTTITKDSMVVCWGHPDSDGGSEIINYIVERRDKAGQRWVKCNKKALTD
LRFKVSGLTEGHEYEFRIMAENAAGISAPSATSPFYKACDTVFKPGPPGNPRVLDTSRSSISIAWNKPIYDGG
SEITGYMVEIALPEEDEWQVVTPPAGLKATSYTITSLIENQEYKIRIYAMNSEGLGEPALVPGTPKAEERMLP

PEIELDADLRKVVTIRACCTLRLFVPIKGRPAPEVKWAREHGESLDKASIESTSSYTLLVVGNVNRFDSGKYI
LTVENSSGSKSAFVNVRVLDTPGPPQNLKIKEVTKTSVTLTWEPPLLDGGSKIKNYIVEKRESTRKAYSTVAT
NCHKTSWKVDQLQEGCSYYFRVLAENEYGIGLPAETAESVKASERPLPPGKITLTDVTRNSVSLSWEKPEHDG
GSRILGYIVEMQSKGSDRWATCATVKVTEATITGLIQGEEYSFRVSAQNEKGISDPRQLSVPVIAKDLVIPPA
FKLLFNTFTVLAGEDLKIDVPFIGRPPPAVTWHKDDIPLKQTTRVNAESTENNSLLTIKEACREDVGHYTVKL
TNSAGEATETLNVIVLDKPGPPTGPVKMDEVTADSVTLSWEPPKYDGGSSINNYIVEKRDTSTTAWQIVSATV
ARTTIKACRLKTGCEYQFRIAAENRYGKSTYLNSEPVVAQYPFKVPGPPGTPFVTLASKDSMEVQWHEPVSDG
GSKVIGYHLERKERNSILWVKLNKTPIPQTKFKTTGLEEGIEYEFRVSAENIVGIGKPSKPSECYAAHDPCDP
PGRPEAIIVTRNSVTLQWKKPTYDGGSKITGYIVEKKELPDGRWMKASFTNIIDTQFEVTGLLEDHRYEFRVI
ARNAAGVFSEPSESTGAITARDEVEPPRISMDPKYRDTVVVQAGESFKIDADIYGKPIPTTQWVKGDQELSST
ARLEIKSTDFATSLSVKDAVRVDSGNYILKAKNVAGEKSVTINVKVLDRPGPPEGPVAISGVTAEKCTLAWKP
PLQDGGSDITNYIVERRETSRLVWTLVDANVQTLSCKVLKLLEGNEYIFRIMAVNKYGVGEPLESESLIAKNP
FVVPDAPKAPEVTAVTKDSMIVVWERPASDGGSEILGYVLEKRDKEGIRWTRCHKRLIGELRLRVTGLLENHN
YEFRVSAENAAGLSEPSPPSAYQKACDPIYKPGPPNNPKVMDVTRSSVFLSWTKPIYDGGCEIQGYIVEKCDV
SVGEWTMCTPPTGINKTNLEVEKLLEKHEYNFRICAINKAGVGEHADVPGPVMVEEKLEAPDIDLDLELRKVI
NIRAGGSLRLFVPIKGRPTPEVKWGKVDGDIRDAAIIDVTSSFTSLVLDNVNRYDSGKYTLTLENSSGTKSAF
VTVRVLDTPSPPVNLKVTEITKDSVSITWEPPLLDGGSKIKNYIVEKREATRKSYAAVVTNCHKNSWKIDQLQ
EGCSYYFRVTAENEYGIGLPARTADPIKVAEVPQPPGKITVDDVTRNSVSLSWTKPEHDGGSKIIQYIVEMQA
KNTDKWSECARVKSLDAVITNLTQGEEYLFRVIAVNEKGRSDPRSLAVPIIAKDLVIEPDVRPAFSSYSVQVG
QDLKIEVPISGRPKPSISWTKDGMPLKQTTRINVTDSLDLTTLSIKETHKDDGGQYGITVSNVVGQKTAAIEI
ITLDKPDPPKGPVKFDEISAESITLSWNPPLYTGGCQITNYIVQKRDTTTTVWDVVSATVARTTLKVTKLKTG
TEYQFRIFAENRYGQSFALESEPVVAQYPYKEPGPPGTPFVTAISKESMVVQWHEPINNGGSPVIGYHLERKE
RNSILWTKVNKTIIHDTQFKALNLEEGIEYEFRVYAENIVGVGKASKNSECYVARDPCDPPGTPEAIIVKRNE
ITLQWTKPVYDGGSMITGYIVEKRDLPEGRWMKASFTNVIETQFTVSGLTEDQRYEFRVIAKNAAGAISKPSD
STGPITAKDEVELPRISMDPKFRDTIVVNAGETFRLEADVHGKPLPTIEWLRGDKEIEESARCEIKNTDFKAL
LIVKDAIRIDGGQYILRASNVAGSKSFPVNVKVLDRPGPPEGPVQVTGVTAEKCTLAWSPPLQDGGSDISHYV
VEKRETSRLAWTVVASEVVTNSLKVTKLLEGNKYIFRIMAVNKYGVGEPLESAPVLMKNPFVLPGPPKSLEVT
NIAKDSMTVCWNRPDSDGGSEIIGYIVEKRDRSGIRWIKCNKRRITDLRLRVTGLTEDHEYEFRVSAENAAGV
GEPSPATVYYKACDPVFKPGPPTNAHVVDTTKNSITLAWSKPIYDGGSEILGYVVEICKADEEEWQIVTPQTG
LRVTRFEIAKLIEHQEYKIRVCALNKVGLGEAASVPGTVKPEDKLEAPELDLDSELRKGIVVRAGGSARIHIP
FKGRPTPEITWSKEEGEFTDKVQIEKGINFTQLSIDNCDRNDAGKYILKLENSSGSKSAFVTVKVLDTPGPPQ
NLAVKEVRKDSVLLVWEPPIIDGGAKVKNYVIDKRESTRKAYANVSSKCNKTSFRVENLTEGAIYYFRVMAEN
EFGVGVPTETSDAVKASEPPSPPGKVTLTDVSQTSASLMWEKPEHDGGSRILGYVVEMQPKGTEKWSVVAESK
VCNAVVSGLSSGQEYQFRVKAYNEKGKSDPRVLGIPVIAKDLTIQPSFKLPFNTYSVQAGEDLKIEIPVIGRP
RPKISWVKDGEPLKQTTRVNVEETATSTILHIKESSKDDFGKYSVTATNSAGTATENLSVIVLEKPGPPVGPV
KFDEVSADFVVISWEPPAYTGGCQISNYIVEKRDTTTTNWQMVSATVARTTIKISKLKTGTEYQFRIFAENRY
GKSTPLDSKPVVVQYPFKEPGPPGTPFVTSISKDQMLVQWHEPVNDGGSKVTGYHLEQKEKNSILWVKLNKIP
IQDTKFKTTGLDEGLEYEFRVSAENIVGIGKPSKVSECYVARDPCDPPGRPEAIVITRNSVTLKWKKPVYDGG
SKITGYIVEKKDLPDGRWMKASFTNVVETEFTVTGLVEDQRYEFRVIARNAADNFSEPSESSGAITARDEIDA
PNASLDPKYRDVIIVHAGETFVLEADIRGKPIPDIIWSKDGNELEETAARMEIKSTLQKTTLIVKDCIRTDGG
QYTLKLSNVGGTKTIPITVKVLDRPGPPEGPLKVTGVTAEKCYLAWNPPLQDGGASISHYIIEKRETSRLSWT
QVSNEVQALNYKVTKLLPGNEYIFRVMAVNKYGIGEALESEPVIACNPYKRPGPPSTPEASAITKDSMVLTWT
RPVDDGGAEIEGYILEKRDKEGIRWTKCNKKTLTDLRFRVTGLTEGHSYEFRVAAENAAGVGEPSEPSVFYRA
CDALYPPGPPSNPKVTDTSRSSVSLAWNKPIYDGGAPVRGYVIELKKAAADEWTTCTPPSGLQGKQFTVTKLK
ENTEYNFRICAFNTEGVGEPATIPGSVVAQERMEAPEIELDADLRKVVTLRASATLRLFVTIKGRPEPEVKWE
KAEGILTERAQIEVTSSYTMLVIDNVTRFDSGRYNLTLENNSGSKTAFVNVRVLDSPSAPVNLTIREVKKDSV
TLSWEPPLIDGGAKITNYIVEKRETTRKAYATITNNCTKNTFKIENLQEGCSYYFRVLASNEYGIGLPAETAE
PVKVSEPPLPPGRVTLVDVTRNTATIKWEKPESDGGSKITGYVVEMQTKGSEKWSACTQVKTLETTISGLTAG
EEYVFRVAAVNEKGRSDPRQLGVPVIAKDIEIKPSVELPFNTFNVKANDQLKIDIPFKGRPQATVAWKKDGQV
LRETTRVNVASSKTVTTLSIKEASREDVGTYELCVSNTAGSITVPITVIVLDRPGPPGPIRIDEVSCDNVSIS
WNPPEYDGGCQISNYIVEKRETTSTTWQVVSQAVARTSIKIVRLTTGSEYQFRVCAENRYGKSSYSESSAVVA
EYPFSPPGPPGTPKVVHATKSTMVVSWQVPVNDGGSQVIGYHLEYKERSSILWSKANKVLIADTQMKVSGLDE
GLMYEYRVYAENIAGIGKCSKACEPVPARDPCDPPGQPEVTNITRKSVSLKWSKPRYDGGAKITGYIVERREL
PDGRWLQCNFTNVQETYFEVTELTEDQRYEFRVFARNAADSVSEPSESTGPITVKDDVEAPRIMMDVKFRDVI
IVKAGEVLKINADIAGRPLPVISWAKDGVEIEERAKTEIVSTDYTTTLTVKDCVRRDTGQYVLTLKNVAGTRT
MAVNCKVLDKPGPPAGPLEINGLTAEKCSLSWGRPQEDGGADIDYYIVEKRETSRLAWTICEAELRTTSCKVT
KLLKGNEYIFRVTGVNKYGVGEPLESMAVKALDPFTTPSPPTSLEITSVTKDSMTLCWSRPETDGGSDISGYI
IERREKNSLRWMRVNKKPVYDLRVKSTGLREGCEYEYRVFAENAAGLSLPSETSPLVRAEDPVFLPSPPSKPK

IVDSGKTTITIGWVKPLFDGGAPITGYTVEYKKSEETDWKVAIQSFRGTEYTMSGLTTGDEYVFRVRSLNKMG
ASDPSDSSDPQVAKEREEEPVFDVDSEMRKTLIVKAGSSFTMTVPFRGRPIPNVSWSKPDTDLRTRAYIDSTD
SRTLLTIENANRNDSGKYTLTIQNVLSAASMTFVVKVLDSPGPPANITVREVTKETAMLSWDVPENDGGAPVK
NYHIEKREASKKAWVSVTNNCNRLSYKVTNLQEGAIYYFRVSGENEFGVGVPAETKEGVKITEKPSPPEKLGV
TSVSKDSVSLSWLKPEHDGGSRIIHYVVEALEKGQKTWVKCAVVKTTHHVVSGLRESHEYFFRVFAENQAGLS
DPRELLLPVLIKDQLEPPEIDMKNFPSHTVYVRAGSNLKVDIPISGKPLPKVTLSRDGVPLKATMRFNTEITA
ENLTINLKESVTTDAGRYEITAANSSGTTKTFINIIVLDRPGPPTGPVAISDITEESVTLKWEPPKYDGGSHV
TNYIVLKRETSTAVWSEVSATVARTMIKVMKLTTGEEYQFRIKAENRFGISDHIDSVCVVVKLPYTTPGPPST
PWVSNVTRESITVGWHEPVSNGGSAVTGYHLEMKDRNSILWQKANKMIIRTTHFKVTTISAGLIYEFRVYAEN
AAGIGKPSHPSEPVLAIDACEPPRNVRITDISKNSVNLSWQQPAFDGGSKITGYIVERRDLPDGRWTKASFTN
VIETQFTVSGLTQNSQYEFRVFARNAVGSVSNPSEVVGPITCIDSYGGPVIDLPLEYTEVVKYRAGTSVKLRA
GISGKPEPTIEWYKDDKELQTNALVCVENSTDLASILIKDANRLNSGSYELKLRNAMGSASATIRVQILDKPG
PPGGPIEFKTVTAEKITLLWRPPADDGGAKITHYIVEKRETSRVVWSMVAENLEECIVTTTKIIKGNEYVFRV
RAVNKYGIGEPLESEPVVAKNAFVTPGPPSIPEVTKITKNSMTVVWDRPTVDGGSEINGYFLERRDKKSLAWL
KVLKETIRDTRQKVTGLTENSDYQYRVCAVNAAGVGPFSEPSDFYKAADPIDPPGPPAKIRIADSTKSSITLG
WSKPVYDGGSDVTGYVVEMKQGDEEEWTIVSTRGEVRTTEYVVSNLKPGVNYYFQVSAVNCAGQGEPITMTEP
AQAKDVLEEPEIDLDVALRTSVIAKAGEDVQLLIPFKGRPPPTVTWRKDEKNLGSDTRYSIQNTDSSSLLVIP
QVTRNDTGKYILTIENGVGQPKSSTVSVKVLDTPAACQKLQVKHVSLGTVTLLWDPPLIDGGSPIINYVIEKR
DATKRTWSVVSHKCSGTSFKVTDLSEKTPFFFRVLAENEIGIGEPCETTEPVKAAEVPAPIRDLSMKDSTKTS
VVLSWTKPDFDGGSIITDYLVERKGKGEQAWSHAGISKTCEIEIGQLKEQSVLEFRVSARNEKGQSDPVTIGP
LTVKELVITPEVDLSEIPGAQISVRIGHNVHLELPYKGKPKPSISWLKDGLPLKESEYVRFSKTENKITLSIK
NSKKEHGGKYTVILDNAVCRNSFPITIITLGPPSKPKGPIRFDEIKADSAIMSWDIPEDDGGGEITCYSIEKR
EASQTNWKMVCSSVARTTFKVSNLVKDSEYQFRVRAENRYGVSEPLASNIIVAKHQFRIPGPPGKPVIYNVTS
DGMSLTWDAPVYDGGSEVTGFHVEKKERNSILWQRVNTSPISGREYRATGLIEGLDYQFRVYAENSAGLSSPS
DPSKFTLAVSPVDPPGTPDYIDVTRETITLKWNPPLRDGGSKIVAYSIEKRQGSDRWVRCNFTDVSECQYTVT
GLSPGDRYEFRIIARNAVGTISPPSQSSGLIMTRDENVPPTVEFGPEYFDGLVIKSGDSLRIKALVQGRPVPR
VTWFKDGVEIERRMNMEITDVLGSTSLFVRDATRDHRGVYTVEAKNVSGSTKAEVTVKVQDTPGKVVGPIRFT
NITGEKMTLWWEAPLNDGCAPVTHYIIEKRETSRLAWALIEDNCEALSYTAIKLITGNEYQFRISAVNKFGVG
RPLESDPVVAQIQYTIPDAPGVPEPSNVTGNSITLTWTRPESDGGSEIQHYILERREKKSTRWVKVISKRPIS
ETRFKVTGLVEGNEYEFHVMAENAAGIGPASGISRLIKCREPVNPPSAPSVVKVTDTSKTTVSLEWARPVFDG
GMEIIGYIIEMCKADLGDWHKVNTEPCVKTRYTVTDLQAGEEYKFRVSAVNGAGKGDSCEVTGTIKAVDRLSA
PELDIDANFKQTHIVRAGVSIRLFIAYQGRPTPTAVWSKPDSNLSIRADIHTTDSFSTLTVENCNRNDAGKYT
LTVENNSGKKSITFTVKVLDSPGPPGPITFKDVTRGSATLMWDAPLLDGGARIHHYVIEKREASRRSWQVVSE
KCTRQILKVSELTEGVPYYFRVSAENEYGVGEPYEMPEPIVATEQPAPPRRLDVVDTSKSSAVLAWLKPDHDG
GSRITSYLLEMRQKGSDFWVEAGHTKQLTFTVERLVENTEYEFRVKAKNDAGYSEPREAFSSVIIKEPQIEPT
ADLTGITNQLITCKAGSTFTIDVPISGRPAPKVTWKLEEMRLKETDRMSIATTKDRTTLTVKDSMRGDSGRYF
LTLENTAGVKTFTITVVVIGRPGPVTGPIEVSSVSAESCVLSWTEPKDDGGTEITNYIVEKRESGTTAWQLIN
SSVKRTQIKVTHLTKYKEYCFRVSSENRFGVSKPLESVPIVAEHPFVPPSAPTRPEVYYVSANAMSIRWEEPY
HDGGSKIVGYWVEKKERNTILWVKENKVPCLECNYKVTGLVEGLEYQFRTYALNAAGVSKASEASRPIMAQNP
VDPPGRPEVTDVTRSTVSLIWSAPVYDGGSKVVGYIIERKPVSEVGDGRWLKCNYTIVSDNFFTVTALSEGDT
YEFRVLAKNAAGVISKGSESTGPVTCRDEYAPPKAELDARLQGDLVTIRAGSDLVLDAAVGGKPEPKIIWTKG
DKELDLCEKISLQYTGKRATAVIKYCDRSDSGKYTLTVKNASGTKSVSVMVKVLDSPGPCGKLTVSRVTEEKC
TLAWSLPQEDGGAEITHYIVERRETSRLNWVIVEGECLTASYVVTRLIKNNEYTFRVRAVNKYGLGVPVESEP
IVARNSFTIPSQPGIPEEVGAGKEHIIIQWTKPESDGGNEISNYLVDKREKKSLRWTRVNKDYVVYDTRLKVT
SLMEGCDYQFRVTAVNAAGNSEPSEASNFISCREPSYTPGPPSAPRVVDTTKRSISLAWTKPMYDGGTDIIGY
VLEMQEKDTDQWCRVHTNATIRNNEFTVPDLKMGQKYSFRVAAVNAKGMSDYSETTAEIEPVERLEIPDLELA
DDLKKTVIVRAGASLRLMVSVSGRPSPVITWSKKGIDLANRAIIDNTESYSLLIVDKVNRYDAGKYTIEAENQ
SGKKSATVLVKVYDTPGPCPSVSVKEVSRDSVTITWEIPTIDGGAPVNNYIIEKREAAMRAFKTVTTKCSKTL
YRISGLVEGTMYYFRVLPENIYGIGEPCETSDAVLVSEVPLVPTKLEVVDVTKSTVTLAWEKPLYDGGSRLTG
YVLEACKAGTERWMKVVTLKPTVLEHTVISLNEGEQYLFRVRAQNEKGVSEPREIVTPVTVQDLRVLPTIDLS
TMPQKTIHVPAGRPIELVIPITGRPPPTASWFFAGSKLRESERVTVETHTKVTKLTIRETTIRDTGEYTLELK
NVTGTTSETIKVIILDKPGPPTGPIKIDEIDATSVTISWEPPELDGGAPLSGYVVEQRDAHRPGWLPVSESVT
RPTFKFTRLTEGNEYVFRVAATNRFGIGSYLQSEVIECRSSISIPGPPETLQIFDVSRDGMTLTWYPPEDDGG
SQVTGYIVERKEVRADRWVRVNKVPVTMTRYRSTGLIEGLEYEHRVTAINARGTGKPSRPSKPTVAMDPIAPP
GKPQNPRVTDTTRTSVSLAWSVPEDEGGSKVTGYLIEMQKVDQREWTKCNTTPTKIREYTLTHLPQGAEYRFR
VLACNAGGPGEPAEVPGTVKVTEMLEYPDYELDERYQEGVFVRQGGVIRLTIPIKGKPFPVCKWTKEGQDISK
RAMIATSETHTELVIKEADRNDSGTYDLVLENKCGKKTVYIKVKVIGSPNTPEGPLEYDDIQARSVRVSWRPP
ADDGGADILGYILERREVPKAAWYTIDSRVRGTSLVVKGLKENVEYHFRVSAENQFGISKPLKSEEPVIPKTP

LNPPEPPSNPPEVLDVTKSSVSLSWSRPKDDGGSRVTGYYIERKETSTDKWVRHNKTQITTTMYTVTGLVPDA
EYQFRIIAQNDVGLSETSPASEPVVCKDPFDKPSQPGELEILSISKDSVTLQWEKPECDGGKEILGYWVEYRQ
SGDSAWKKSNKERIKDRQFTIGGLLEATEYEFRVFAENETGLSRPRRTAMSVKTKLTSGEAPGVRKEMADVTT
KLGEAAQLSCQIVGRPLPDIKWYRFGKELIQSRKYKMSSDGRTHTLTVMTDEQEDEGVYTCVATNEVGEVESS
SKLLLQAAPQFHPGYPLKEKYYGAVGSTLRLHVMYIGRPVPAMTWFHGQKLLQNSEKITIENTEHYTHLVMKN
VQRKTHAGKYKVQLSNAFGTVDATLDVEIQDKPDKPTGPIVIEALLKNSVVISWKPPADDGGSWITNYVVEKC
EAKEGAEWQLVSSAISVTTCRIVNLTENAGYYFRVSAQNTFGISEPLEVASIVIIKSPFEKPGVPGKPTITAV
TKDSCVVAWKPPASDGGAKIRNYYLERREKKQNKWIAVTTEEIRETVFSVQNLIEGLEYEFRVKCENLGGESE
WSEISEPVTPKSDVPIQAPHFKEELRNLNVRYQSNATLVCKVTGHPKPIVKWYRQGKEIIADGLKYRIQEFKG
GYHQLIIASVTDDDATVYQVRATNQGGSVSGTASLEVEVPAKIHLPKTLEGMGAVHALRGEVVSIKIPFSGKP
DPVITWQKGQDLIDNNGHYQVIVTRSFTSLVFSNGVERKDAGFYVVCAKNRFGIDQKTVELDVADVPDPPRGV
KVSDVSRDSVNLTWTEPASDGGSKVTNYIVEKCATTAERWLRVGQARETRYTVINLFGKTSYQFRVIAENKFG
LSKPSEPSEPTVTKEDKTRAMNYDDEVDETREVTTTKASHSKTKELYEKYMIAEDLGRGEFGIVHRCVETSSK
RTFMAKFVKVKGTDQVLVKKEISILNIARHRNILYLHESFESMEELVMIFEFISGLDIFERINTSAFELNERE
IVSYVRQVCEALEFLHSQNIGHFDIRPENIIYQTRKNSTIKIIEFGQARQLKPGDNFRLLFTAPEYYAPEVHQ
HDVVSTATDMWSLGTLVYVLLSGINPFLAETNQQMIENIMNAEYTFDEEAFKEISLEAMDFVDRLLVKERKSR
MTASEALQHPWLKQRIDRVSTKVIRTLKHRRYYHTLIKKDLNMVVSAARISCGGAIRSQRGVSVAKVKVASIE
IGPVSGQIMHAIGEEGGYVKYVCKIENYDQSTQVTWYFGVRQLENSEKYEITYEDGVATMYVKDITKFDDGTY
RCKVVNDYGEDSSYAELFVKGVREVYDYYCRRTKKVKRRTDAMRLLERPPEFTLPLYNKTAYVGENVRFGVTI
TVHPEPRVTWYKSGQKIKPGDDEKKYTFESDKGLYQLTINSVTTDDDAEYTVVARNKHGEDSCKAKLTVTLHP
PPTETTLRPMFKRLLANAECHEGQSVCFEIRVSGIPAPTLKWEKDGQPLSLGPHIEIVHEGLDYYALHIRDTL
PEDTGYYRVTATNTAGSTSCQAHLQVERLRYVKQEFQSKEERERHVQKQIDKTLRMAEILSGTETVPLTPVAQ
EALREAAILYKPAVSTKTVKGEYRLQTEEKKEERKLRMPYEVPEPRRFKQATVEEDQRIKQFVPMSDMKWYKK
IRDQYEMPGKLDRVVQKRPKRIRLSRWEQFYVMPLPRITDQYRPKWRIPKLTQDDLEMVRPARRRTPSPDYDL
YYYRRRRRSLGDMSDEELLLPIDDYLAMKRTEEERLRLEEELELGFSASPPSRSPPRFELSSLRYSSPPAHVK
VEDRRRDFRYSTYHVPTKEETSTSYAELRERHAQASYRQPKLRQRIMAEKEEEELLRPVTTTQRLSEYKSELD
YMSKEEKSKKKSKRQRQVTEITEIEEEYEISRRAQRESSSSVSRLLRRRRSLSPTYIELMRPVSELIRSHPRP
AEEYEDDAERRSPTPERTRPRSPSPVSSERSLSRFERSARFDIFSRYESMKAALKTQKTSERKYEVLSQQPFT
LDHAPRITLRMRSHRVPCGQNTRFILNVQSKPTAEVKWYHNGVELQESSKIHYTNTSGVLTLEILDCQTEDGG
TYRAVCTNYKGEASDYATLDVTGGAYTTYASQRRDEEVPKSVFPELTKTEAYAVSSFKRTSELEAASSVREVK
SQMTETRESLSTYEHYASAEMKSATSEEKSLEEKATVRKIKTTLAARILTKPRSITVHEGESARFSCDTDGEP
VPTVTWLREGQVVSTSARHQVTTTKYKSTFEISSVQASDEGNYSVVVENSDGKQEAQFTLTVQKARVIEKAVT
SPPRVKSPEPRVKSPETVKSPKRVKSPEPVTSHPKAVSPTETKPTEKGQHLPVSAPPKITQSLKAEASKDIAK
LTCAVESSALCAKEVAWYKDGKKLKENGHFQFHYSADGTYELKIHNLSESDCGEYVCEVSGEGGTSKTSFQFT
GQSFKSIHEQVSSISETTKSVQKTAESAEAKKQEPIAPESISSKPVIVTGLRDTTVSSDSVAKFTIKVTGEPQ
PTITWTKDGKAIAQGSKYKLSSNKEEFILEILKTETSDGGLYACTVTNSAGSVSSSCKLTIKAVKDTEAQKVS
TQKTSEVTSQKKASAQEEISQKALTSEEIKMSEVKSHETLAIKEEASKVLIAEEVKRSAAASLEKSIVHEEVT
KTSQASEEVKTHAEIKTLSTQMNITKGQRATLKANIAGATDVKWVLNGTELPNSEEYRYGVSGSDQTLTIKQA
SHREEGILSCIGKTSQGVVKCQFDLTLSEELSDAPSFITQPRSQNINEGQNVLFSCEVSGEPSPEIEWFKNNL
PISISSNISVSRSRNVYTLEIRNAAVSDSGKYTIKAKNFHGQCSATASLTVLPLVEEPPREVVLKTSSDVSLH
GSVSSQSVQMSASKQEASFSSFSSSSASSMTEMKFASMSAQSMSSMQESFVEMSSSSFMGKSSMTQLESSTSR
MLKAGGRGIPPKIEALPSDISIDEGKVLTVACAFTGEPTPEITWSCGGRKIQNQEQQGRFHIENTDDLTTLII
MDVQKQDGGLYTLSLGNEFGSDSATVNINIRSM*
>rs218903976_ENSMUSP00000150405_c.751:T>C_p.L251P
ENSMUST00000215548.3|Olfr1002-202|ENSMUSG00000075214|-
|chr2:g.85477912:A>G
MMHRNQTVVTEFFFTGLTSSFHLQIVLFLTFLCVYLATLLGNLGMIILIHQDTRLHIPMYFFLSHLSFVDACS
SSVISPKMLSDIFVDKKVISFLGCAIQFCLFSQFVVTECFLLASMAYDRYVAICKPLLYTLIMSQRVCVQLVI
GPYSIGLISTVVHTTSAFILPYCGPNLINHFFCDLLPVLSLACADTQMNKHLLFIMAGILGVFSGIIILVSYV
YIAITILKINSADGRRKAFSTCSSHLTAVSIPYGTLFFIYVRPSSSFSLDINKVVSLFYTAVIPMLNPFIYSL
RNKEVKDALIRTFEKKFCYSLQDKIL*
>rs218903976_ENSMUSP00000097504_c.751:T>C_p.L251P
ENSMUST00000099920.2|Olfr1002-201|ENSMUSG00000075214|-
|chr2:g.85477912:A>G
MMHRNQTVVTEFFFTGLTSSFHLQIVLFLTFLCVYLATLLGNLGMIILIHQDTRLHIPMYFFLSHLSFVDACS
SSVISPKMLSDIFVDKKVISFLGCAIQFCLFSQFVVTECFLLASMAYDRYVAICKPLLYTLIMSQRVCVQLVI
GPYSIGLISTVVHTTSAFILPYCGPNLINHFFCDLLPVLSLACADTQMNKHLLFIMAGILGVFSGIIILVSYV

YIAITILKINSADGRRKAFSTCSSHLTAVSIPYGTLFFIYVRPSSSFSLDINKVVSLFYTAVIPMLNPFIYSL
RNKEVKDALIRTFEKKFCYSLQDKIL*
>ENSMUSP00000150405_c.282:CT>AA_p.L95N ENSMUST00000215548.3|Olfr1002-202|ENSMUSG00000075214|-|chr2:g.85478381:AG>TT
MMHRNQTVVTEFFFTGLTSSFHLQIVLFLTFLCVYLATLLGNLGMIILIHQDTRLHIPMYFFLSHLSFVDACS
SSVISPKMLSDIFVDKKVISFNRLCYPVLFIQPVCSDRMFPPGLHGL**ICCHL*AFAVYSHHVPASLCTVSD
WPLQHRPYKHCGPYYFCIYSSILWSKSHQSLFL*PSSCPLTGMCRYSDE*TFAFHHGWDPRCIQWYNHIGVLC
LHCHHHPKDQFC*WEAQSLLHLLFTPDSSLYSLRDSLLYLCTSKLQLLSGYQ*SCVTILHCCNPYVEPIHL*L
EK*GSQRCINQDI*KEVLLQLAR*NII
>ENSMUSP00000097504_c.282:CT>AA_p.L95N ENSMUST00000099920.2|Olfr1002-201|ENSMUSG00000075214|-|chr2:g.85478381:AG>TT
MMHRNQTVVTEFFFTGLTSSFHLQIVLFLTFLCVYLATLLGNLGMIILIHQDTRLHIPMYFFLSHLSFVDACS
SSVISPKMLSDIFVDKKVISFNRLCYPVLFIQPVCSDRMFPPGLHGL**ICCHL*AFAVYSHHVPASLCTVSD
WPLQHRPYKHCGPYYFCIYSSILWSKSHQSLFL*PSSCPLTGMCRYSDE*TFAFHHGWDPRCIQWYNHIGVLC
LHCHHHPKDQFC*WEAQSLLHLLFTPDSSLYSLRDSLLYLCTSKLQLLSGYQ*SCVTILHCCNPYVEPIHL*L
EK*GSQRCINQDI*KEVLLQLAR*NII
>ENSMUSP00000150405_c.52:C>T_p.T18I ENSMUST00000215548.3|Olfr1002-202|ENSMUSG00000075214|-|chr2:g.85478611:G>A
MMHRNQTVVTEFFFTGLISSFHLQIVLFLTFLCVYLATLLGNLGMIILIHQDTRLHIPMYFFLSHLSFVDACS
SSVISPKMLSDIFVDKKVISFLGCAIQFCLFSQFVVTECFLLASMAYDRYVAICKPLLYTLIMSQRVCVQLVI
GPYSIGLISTVVHTTSAFILPYCGPNLINHFFCDLLPVLSLACADTQMNKHLLFIMAGILGVFSGIIILVSYV
YIAITILKINSADGRRKAFSTCSSHLTAVSILYGTLFFIYVRPSSSFSLDINKVVSLFYTAVIPMLNPFIYSL
RNKEVKDALIRTFEKKFCYSLQDKIL*
>ENSMUSP00000097504_c.52:C>T_p.T18I ENSMUST00000099920.2|Olfr1002-201|ENSMUSG00000075214|-|chr2:g.85478611:G>A
MMHRNQTVVTEFFFTGLISSFHLQIVLFLTFLCVYLATLLGNLGMIILIHQDTRLHIPMYFFLSHLSFVDACS
SSVISPKMLSDIFVDKKVISFLGCAIQFCLFSQFVVTECFLLASMAYDRYVAICKPLLYTLIMSQRVCVQLVI
GPYSIGLISTVVHTTSAFILPYCGPNLINHFFCDLLPVLSLACADTQMNKHLLFIMAGILGVFSGIIILVSYV
YIAITILKINSADGRRKAFSTCSSHLTAVSILYGTLFFIYVRPSSSFSLDINKVVSLFYTAVIPMLNPFIYSL
RNKEVKDALIRTFEKKFCYSLQDKIL*
>ENSMUSP00000149830_c.474:C>A_p.Q159K ENSMUST00000216566.3|Olfr1039-203|ENSMUSG00000075204|-|chr2:g.85961531:G>T
MAEGNSSTVFQFILEGLTDDPELEVTLFAVFLVIYLTTVLGNLGLIMLIQVSPQLHTPMYFFLCHLAFVDFCY
TSSVTPNTIINFLREIKSITFYACATQVCCFITFAVCEMYLLSVMAYDRYVAIWNPLLYVVLMPKKLCLQVIT
STYIYGFTVGLAKAVATFRLSFCGSNVINHFYCDDVPLVALACSDTHVKELMLLIIAGFNTLCSLVIVVISYI
CILFAILRIHSAEGRRKAFSTCASHLTSITIFYGTIIFMYLQPKSSHSLNTDKFVSVFYVVVIPMLNPLIYSL
RNKEVKNALKRFTEKLSLTIHDSRKSE*
>ENSMUSP00000150584_c.474:C>A_p.Q159K ENSMUST00000214364.2|Olfr1039-202|ENSMUSG00000075204|-|chr2:g.85961531:G>T
MAEGNSSTVFQFILEGLTDDPELEVTLFAVFLVIYLTTVLGNLGLIMLIQVSPQLHTPMYFFLCHLAFVDFCY
TSSVTPNTIINFLREIKSITFYACATQVCCFITFAVCEMYLLSVMAYDRYVAIWNPLLYVVLMPKKLCLQVIT
STYIYGFTVGLAKAVATFRLSFCGSNVINHFYCDDVPLVALACSDTHVKELMLLIIAGFNTLCSLVIVVISYI
CILFAILRIHSAEGRRKAFSTCASHLTSITIFYGTIIFMYLQPKSSHSLNTDKFVSVFYVVVIPMLNPLIYSL
RNKEVKNALKRFTEKLSLTIHDSRKSE*
>ENSMUSP00000097494_c.474:C>A_p.Q159K ENSMUST00000099910.2|Olfr1039-201|ENSMUSG00000075204|-|chr2:g.85961531:G>T
MAEGNSSTVFQFILEGLTDDPELEVTLFAVFLVIYLTTVLGNLGLIMLIQVSPQLHTPMYFFLCHLAFVDFCY
TSSVTPNTIINFLREIKSITFYACATQVCCFITFAVCEMYLLSVMAYDRYVAIWNPLLYVVLMPKKLCLQVIT
STYIYGFTVGLAKAVATFRLSFCGSNVINHFYCDDVPLVALACSDTHVKELMLLIIAGFNTLCSLVIVVISYI
CILFAILRIHSAEGRRKAFSTCASHLTSITIFYGTIIFMYLQPKSSHSLNTDKFVSVFYVVVIPMLNPLIYSL
RNKEVKNALKRFTEKLSLTIHDSRKSE*
>ENSMUSP00000149099_c.118:C>G_p.T40R ENSMUST00000217575.3|Olfr1166-202|ENSMUSG00000101078|-|chr2:g.87955209:G>C
MAYEVMNQTSATTFILVGFSEYPQLQKPLFLLFLAIYSVRLVGNLGILVVIKVNPKLHTPMYFFLSHLSFLDI
CYSSVFTPKLLQILIMEDRTVSFKACMVQFFFICTFVITEMFMLAVMAYDRFVAVCNPLLYTVVMPFQFCALL
VAGTYMIGGLCTVILLYTLLQLSYCEYGIINHFGCEYSAVISVSCSDSSFSQLTSLVIAIVSESSSVLITLAS
YVFIVVTIIKMPSKGGLRKAFSTCTSHLTAISIFHGIILLLYCVPNSNSSRLFVKVATALYTIMIPMLNPLIY
SLRNKDVKDTVRRLISSKLHSHLT*

>ENSMUSP00000097421_c.118:C>G_p.T40R ENSMUST00000099833.6|Olfr1166-201|ENSMUSG00000101078|-|chr2:g.87955209:G>C
MAYEVMNQTSATTFILVGFSEYPQLQKPLFLLFLAIYSVRLVGNLGILVVIKVNPKLHTPMYFFLSHLSFLDI
CYSSVFTPKLLQILIMEDRTVSFKACMVQFFFICTFVITEMFMLAVMAYDRFVAVCNPLLYTVVMPFQFCALL
VAGTYMIGGLCTVILLYTLLQLSYCEYGIINHFGCEYSAVISVSCSDSSFSQLTSLVIAIVSESSSVLITLAS
YVFIVVTIIKMPSKGGLRKAFSTCTSHLTAISIFHGIILLLYCVPNSNSSRLFVKVATALYTIMIPMLNPLIY
SLRNKDVKDTVRRLISSKLHSHLT*
>ENSMUSP00000149507_c.520:A>C_p.H174P ENSMUST00000216587.2|Olfr1254-203|ENSMUSG00000075074|-|chr2:g.89619174:T>G
MGKNNNVTEFILLGLTQDPVGQKALFVLFLLMYIVTMAGNLIIVVTIIASPSLSSPMYFFLAYLSLMDAIYST
AISPKLIMDLLCNKKTISFRACMGQLFVEHLFGATEIFLLVAMAYDRYVAICKPLHYLTIMNHRVCILLVMAT
WVGGFAHSMAQVLFVYDLPFCGPNVIDPFACDMYPLLVLVCSDTYFLGLTVIANDGAICMVVFVILLASYGII
LNSLKTHSQEGRRKALSTCSSHIMVVILFFVPCIFMYVRPVSNFPVDKSVTIFYTVVTPMLNPLIYTLRNSEI
KHSMLKLWSKILHSDRLRKSCC*
>ENSMUSP00000107148_c.520:A>C_p.H174P ENSMUST00000111523.3|Olfr1254-202|ENSMUSG00000075074|-|chr2:g.89619174:T>G
MGKNNNVTEFILLGLTQDPVGQKALFVLFLLMYIVTMAGNLIIVVTIIASPSLSSPMYFFLAYLSLMDAIYST
AISPKLIMDLLCNKKTISFRACMGQLFVEHLFGATEIFLLVAMAYDRYVAICKPLHYLTIMNHRVCILLVMAT
WVGGFAHSMAQVLFVYDLPFCGPNVIDPFACDMYPLLVLVCSDTYFLGLTVIANDGAICMVVFVILLASYGII
LNSLKTHSQEGRRKALSTCSSHIMVVILFFVPCIFMYVRPVSNFPVDKSVTIFYTVVTPMLNPLIYTLRNSEI
KHSMLKLWSKILHSDRLRKSCC*
>ENSMUSP00000097352_c.520:A>C_p.H174P ENSMUST00000099764.2|Olfr1254-201|ENSMUSG00000075074|-|chr2:g.89619174:T>G
MGKNNNVTEFILLGLTQDPVGQKALFVLFLLMYIVTMAGNLIIVVTIIASPSLSSPMYFFLAYLSLMDAIYST
AISPKLIMDLLCNKKTISFRACMGQLFVEHLFGATEIFLLVAMAYDRYVAICKPLHYLTIMNHRVCILLVMAT
WVGGFAHSMAQVLFVYDLPFCGPNVIDPFACDMYPLLVLVCSDTYFLGLTVIANDGAICMVVFVILLASYGII
LNSLKTHSQEGRRKALSTCSSHIMVVILFFVPCIFMYVRPVSNFPVDKSVTIFYTVVTPMLNPLIYTLRNSEI
KHSMLKLWSKILHSDRLRKSCC*
>ENSMUSP00000149507_c.234:T>G_p.L79V ENSMUST00000216587.2|Olfr1254-203|ENSMUSG00000075074|-|chr2:g.89619460:A>C
MGKNNNVTEFILLGLTQDPVGQKALFVLFLLMYIVTMAGNLIIVVTIIASPSLSSPMYFFLAYLSLMDAIYST
AISPKVIMDLLCNKKTISFRACMGQLFVEHLFGATEIFLLVAMAYDRYVAICKPLHYLTIMNHRVCILLVMAT
WVGGFAHSMAQVLFVYDLPFCGPNVIDHFACDMYPLLVLVCSDTYFLGLTVIANDGAICMVVFVILLASYGII
LNSLKTHSQEGRRKALSTCSSHIMVVILFFVPCIFMYVRPVSNFPVDKSVTIFYTVVTPMLNPLIYTLRNSEI
KHSMLKLWSKILHSDRLRKSCC*
>ENSMUSP00000107148_c.234:T>G_p.L79V ENSMUST00000111523.3|Olfr1254-202|ENSMUSG00000075074|-|chr2:g.89619460:A>C
MGKNNNVTEFILLGLTQDPVGQKALFVLFLLMYIVTMAGNLIIVVTIIASPSLSSPMYFFLAYLSLMDAIYST
AISPKVIMDLLCNKKTISFRACMGQLFVEHLFGATEIFLLVAMAYDRYVAICKPLHYLTIMNHRVCILLVMAT
WVGGFAHSMAQVLFVYDLPFCGPNVIDHFACDMYPLLVLVCSDTYFLGLTVIANDGAICMVVFVILLASYGII
LNSLKTHSQEGRRKALSTCSSHIMVVILFFVPCIFMYVRPVSNFPVDKSVTIFYTVVTPMLNPLIYTLRNSEI
KHSMLKLWSKILHSDRLRKSCC*
>ENSMUSP00000097352_c.234:T>G_p.L79V ENSMUST00000099764.2|Olfr1254-201|ENSMUSG00000075074|-|chr2:g.89619460:A>C
MGKNNNVTEFILLGLTQDPVGQKALFVLFLLMYIVTMAGNLIIVVTIIASPSLSSPMYFFLAYLSLMDAIYST
AISPKVIMDLLCNKKTISFRACMGQLFVEHLFGATEIFLLVAMAYDRYVAICKPLHYLTIMNHRVCILLVMAT
WVGGFAHSMAQVLFVYDLPFCGPNVIDHFACDMYPLLVLVCSDTYFLGLTVIANDGAICMVVFVILLASYGII
LNSLKTHSQEGRRKALSTCSSHIMVVILFFVPCIFMYVRPVSNFPVDKSVTIFYTVVTPMLNPLIYTLRNSEI
KHSMLKLWSKILHSDRLRKSCC*
>ENSMUSP00000028672_c.345:A>G_p.T116A ENSMUST00000028672.13|Mdk-201|ENSMUSG00000027239|-|chr2:g.91761214:T>C
MQHRGFFLLALLALLVVTSAVAKKKEKVKKGSECSEWTWGPCTPSSKDCGMGFREGTCGAQTQRVHCKVPCNW
KKEFGADCKYKFESWGACDGSTGTKARQGTLKKARYNAQCQEAIRVTKPCTSKTKSKTKAKKGKGKD*
>ENSMUSP00000068413_c.345:A>G_p.T116A ENSMUST00000069423.13|Mdk-202|ENSMUSG00000027239|-|chr2:g.91761214:T>C
MQHRGFFLLALLALLVVTSAVAKKKEKVKKGSECSEWTWGPCTPSSKDCGMGFREGTCGAQTQRVHCKVPCNW
KKEFGADCKYKFESWGACDGSTGTKARQGTLKKARYNAQCQEAIRVTKPCTSKTKSKTKAKKGKGKD*

>ENSMUSP00000088090_c.345:A>G_p.T116A ENSMUST00000090602.6|Mdk-
203|ENSMUSG00000027239|-|chr2:g.91761214:T>C
MQHRGFFLLALLALLVVTSAVAKKKEKVKKGSECSEWTWGPCTPSSKDCGMGFREGTCGAQTQRVHCKVPCNW
KKEFGADCKYKFESWGACDGSTGTKARQGTLKKARYNAQCQEAIRVTKPCTSKTKSKTKAKKGKGKD*
>ENSMUSP00000106941_c.345:A>G_p.T116A ENSMUST00000111309.8|Mdk-
204|ENSMUSG00000027239|-|chr2:g.91761214:T>C
MQHRGFFLLALLALLVVTSAVAKKKEKVKKGSECSEWTWGPCTPSSKDCGMGFREGTCGAQTQRVHCKVPCNW
KKEFGADCKYKFESWGACDGSTGTKARQGTLKKARYNAQCQEAIRVTKPCTSKTKSKTKAKKGKGKD*
>ENSMUSP00000028612_c.326:G>T_p.K109N ENSMUST00000028612.8|Pamr1-
201|ENSMUSG00000027188|+|chr2:g.102417016:G>T
MELDRWAQLGLVFLQLLLISSLPREYTVINEACPGAEWNIMCRECCEYDQIECLCPGKKEVVGYTIPCCRNED
NECDSCLIHPGCTIFENCKSCRNGSWGGTLDDFYVNGFYCAECRAGWYGGDCMRCGQVLRASKGQILLESYPL
NAHCEWTIHARPGFIIQLRFGMLSLEFDYMCQYDYVEVRDGDNSDSPIIKRFCGNERPAPIRSTGSSLHVLFH
SDGSKNFDGFHAVFEEITACSSSPCFHDGTCLLDTTGSFKCACLAGYTGQRCENLLEERNCSDLGGPVNGYKK
ITEGPGLLNERHVKIGTVVSFFCNGSYVLSGNEKRTCQQNGEWSGKQPVCMKACREPKISDLVRRRVLSMQVQ
SRETPLHQLYSTAFSKQKLQDASTKKPALPFGDLPPGYQHLHTQVQYECISPFYRRLGSSRRTCLRTGKWSGR
APSCIPICGKIESTPSPKTQGTRWPWQAAIYRRTSGVHDGGLHKGAWFLVCSGALVNERTVVVAAHCVTELGK
ATIIKTADLKVVLGKFYRDDDRDEKSIQNLRVSAIILHPNYDPILLDTDIAVLKLLDKARISTRVQPICLATT
RDLSTSFQESHITVAGWNILADVRSPGFKNDTLHYGMVRVVDPMLCEEQHEDHGIPVSVTDNMFCASKDPSTP
SDICTAETGGIAALSFPGRASPEPRWHLVGLVSWSYDKTCSNGLSTAFTKVLPFKDWIERNMK*
>ENSMUSP00000155023_c.262:C>T_p.T88I ENSMUST00000136156.9|D430041D05Rik-
202|ENSMUSG00000068373|-|chr2:g.104088826:G>A
MALQEAKPQGPSEAEPAAREPRDRLREQGLCGAGGIAARCQGVRSSEARRSEDVLLRRPLLLGLLLLAALSGH
CWADSGTDNLQIHVIPTSESFPLGKLLPISPTWPFLEVKSSSAADRFKNVLGQPPDNTTLLKLSQTPLPKAHR
RTRAHVDFSSSPYQGSGHSASLDLAKDPTETLVQTRPEGGREAADMSVETSVSSKGELSATAAATATTTLFLR
RSSPAPLSAALTAKGTGSSSSNLAKSSLTTALAKNVTNKAASGPKVTAGTIHTAFPFTPTYMLARTAHSLSTH
TAMQGATGSVSGLLSTTHLPKKPQAMHTGLPNPTRPDTPRASTPRPLTITAALTSMTASVKATRLPSLQTGNT
DAAFPAVSTAMATTGRMASNLDCQMSHKFLVKTVFFLTQRRIQSSESLKLGVTKGLTQALRKAFHQNDVSAHV
DILEYSHNVTVGYYATKGRLVYLPAVVMDVLGVYGVSNVTADLKQHTPNLQSVAVLASPWKPQPAGAFQLKTV
LQFVSQSDNIQSCRFAQTMEQRLQKAFQDAERKVLSTRSNLTVQIVSTSNASQAVTLVYAVGNQSSFLNGTVA
SSLLSQLSAELVGFYLTYPPLTIAEPLEYPNLDTSETTRDYWVITVLQGVDNSLVGLHNQSFARVMEQRLAQL
FMMSQQQGRRFKRATTLGSYTVQMVKMQRVPGPKDPAELTYYTLYNGKPLLGTAAAKILSTIDSQRMALTLHH
VVLLQADPVVKNPPNNLWIIAAVLAPIAVVTIIIIIITAVLCRKNKNDFKPDTMINLPQRAKPVQGFDYAKQH
LGQQGADEEVIPVTQETVVLPIPVRDTPQERDTAQDGSAIKTAKSTETRRSRSPSENGSVISNESGKPSSGRR
SPQNGMTQQKVTKEESRKRNVTASDEEEGAGLFDSAGKAAADPFDTSSGSVQLIAIKPSALPVVHPASDRGQE
SSAALNGEVNKALKQKSDIEHYRNKLRLKAKRKGYYDFPPVEAGKGLAERKMYEKAPKEVEHVLDADPELCAP
FAESKNRQQMKNSVYRSRQSLNSPSPGETEMDLLVTRERPRRGIRNSGYDTEPEIIEETNVDRVHEPRGYGRA
RQAKGHSETSTLSSQPSIDEVRQQMHMLLEEAFSLASAGHAGQSRHQETYGSTQHLPYSEVVTSAPGTMTRPR
AGVQWVPTYRPEMYQYSLPRPAYRFSQLPEMVMGSPPPPVPPRTGPVAVASLRRSTSDIGSKTRMAESTGPEP
TQLHDSASFAQVSRGPVSVTQLDQSALNYSGNTVPAVFAIPAANRPGFTGYFIPTPPSSYRSQAWMSYAGENE
LPSQWADSVPLPGYIEAYPRSRYQQSSPSRLPRQYSQPANLHPSLEQAPVPSAAASQQSLTENEPPDTPLTNI
STAALVKAIREEVAKLAKKQTDMFEFQV*
>ENSMUSP00000106756_c.262:C>T_p.T88I ENSMUST00000089726.10|D430041D05Rik-
201|ENSMUSG00000068373|-|chr2:g.104088826:G>A
MALQEAKPQGPSEAEPAAREPRDRLREQGLCGAGGIAARCQGVRSSEARRSEDVLLRRPLLLGLLLLAALSGH
CWADSGTDNLQIHVIPTSESFPLGKLLPISPTWPFLEVKSSSAADRFKNVLGQPPDNTTLLKLSQTPLPKAHR
RTRAHVDFSSSPYQGSGHSASLDLAKDPTETLVQTRPEGGREAADMSVETSVSSKGELSATAAATATTTLFLR
RSSPAPLSAALTAKGTGSSSSNLAKSSLTTALAKNVTNKAASGPKVTAGTIHTAFPFTPTYMLARTAHSLSTH
TAMQGATGSVSGLLSTTHLPKKPQAMHTGLPNPTRPDTPRASTPRPLTITAALTSMTASVKATRLPSLQTGNT
DAAFPAVSTAMATTGRMASNLDCQMSHKFLVKTVFFLTQRRIQSSESLKLGVTKGLTQALRKAFHQNDVSAHV
DILEYSHNVTVGYYATKGRLVYLPAVVMDVLGVYGVSNVTADLKQHTPNLQSVAVLASPWKPQPAGAFQLKTV
LQFVSQSDNIQSCRFAQTMEQRLQKAFQDAERKVLSTRSNLTVQIVSTSNASQAVTLVYAVGNQSSFLNGTVA
SSLLSQLSAELVGFYLTYPPLTIAEPLEYPNLDTSETTRDYWVITVLQGVDNSLVGLHNQSFARVMEQRLAQL
FMMSQQQGRRFKRATTLGSYTVQMVKMQRVPGPKDPAELTYYTLYNGKPLLGTAAAKILSTIDSQRMALTLHH
VVLLQADPVVKNPPNNLWIIAAVLAPIAVVTIIIIIITAVLCRKNKNDFKPDTMINLPQRAKPVQGFDYAKQH
LGQQGADEEVIPVTQETVVLPIPVRDTPQERDTAQDGSAIKTAKSTETRRRSPSENGSVISNESGKPSSGRRS
PQNGMTQQKVTKEESRKRNVTASDEEEGAGLFDSAGKAAADPFDTSSGSVQLIAIKPSALPVVHPASDRGQES
SAALNGEVNKALKQKSDIEHYRNKLRLKAKRKGYYDFPPVEAGKGLAERKMYEKAPKEVEHVLDADPELCAPF

AESKNRQQMKNSVYRSRQSLNSPSPGETEMDLLVTRERPRRGIRNSGYDTEPEIIEETNVDRVHEPRGYGRAR
QAKGHSETSTLSSQPSIDEVRQQMHMLLEEAFSLASAGHAGQSRHQETYGSTQHLPYSEVVTSAPGTMTRPRA
GVQWVPTYRPEMYQYSLPRPAYRFSQLPEMVMGSPPPPVPPRTGPVAVASLRRSTSDIGSKTRMAESTGPEPT
QLHDSASFAQVSRGPVSVTQLDQSALNYSGNTVPAVFAIPAANRPGFTGYFIPTPPSSYRSQAWMSYAGENEL
PSQWADSVPLPGYIEAYPRSRYQQSSPSRLPRQYSQPANLHPSLEQAPVPSAAASQQSLTENEPPDTPLTNIS
TAALVKAIREEVAKLAKKQTDMFEFQV*
>ENSMUSP00000155485_c.148:C>T_p.T50I ENSMUST00000230671.2|D430041D05Rik-207|ENSMUSG00000068373|-|chr2:g.104088826:G>A
MCCCGGRCCWDSCCWLPSPGTAGPTPCILIPHLMWMLSFTGTDNLQIHVIPTSESFPLGKLLPISPTWPFLEV
KSSSAADRFKNVLGQPPDNTTLLKLSQTPLPKAHRRTRAHVDFSSSPYQGSGHSASLDLAKDPTETLVQTRPE
GGREAADMSGLPRTSMLPPLPTAPSATSSAVSPSQPLVGMPFLSKHPLRSDSSLLLPPPSSSSLAPDSPHSIP
TPKPVQHPTKVPLFPQMTPADFSSGQSPVNVLNPFFHKTNLFPSRDAQTLIGTPRLDFSTSSTKQYSEPSLSE
SPRPAASPTPGVLSSIAEQDHLSLPSLAPGGLPGVTSSRSMWEEALSPVSTQPFRTSGPVRGRGAESLPIYLR
TLAASYGAWAPRFQTAESVMTSRELAPVPPTNSFANWPHLSEVPESSRKPTLSPENKPSSLVPPPQPLTTLSQ
GQMVHPEAAPGSPLKGTGTPLSLLTVRQPNKDHASPAPVPEAPLSREGAHDEYLPDAATQLLSSKLPNPPPTI
WTFPQQKEDRAAAIFWTNEKANGTTSLWALPTKEAPSRRAVTGSTSDFNTGRLSPLIMTALRTDLSPPGSPPS
SALTTQAASPRSSGTKLESLAAVTSQSELPASASKQVTELPSSTDVHGLPTMGGTRKPAATDVFWSFLSAETA
SLATQSGISGSQQQTNHDVNTHTINSTHRELRPASVALPGGTTSAANALQSRNFKEIAGQLVAADSFNFQDPV
LSPRTSQPLQPSEEAVVESHTDFPPTGKHARDFDTEMVHYYSSTSQNPMSQLPIRPAHPFWPTWPSLASAASL
HQMLSDGTDAGSHISSDIYLSPGENGSPQFQSVLEYHSSTASPSVPTGAFSRTPSKVLRTSRRPKKWTGAATH
TVETSVSSKGELSATAAATATTTLFLRRSSPAPLSAALTAKGTGSSSSNLAKSSLTTALAKNVTNKAASGPKV
TAGTIHTAFPFTPTYMLARTAHSLSTHTAMQGATGSVSGLLSTTHLPKKPQAMHTGLPNPTRPDTPRASTPRP
LTITAALTSMTASVKATRLPSLQTGNTDAAFPAVSTAMATTGRMASNLDCQMSHKFLVKTVFFLTQRRIQSSE
SLKLGVTKGLTQALRKAFHQNDVSAHVDILEYSHNVTVGYYATKGRLVYLPAVVMDVLGVYGVSNVTADLKQH
TPNLQSVAVLASPWKPQPAGAFQLKTVLQFVSQSDNIQSCRFAQTMEQRLQKAFQDAERKVLSTRSNLTVQIV
STSNASQAVTLVYAVGNQSSFLNGTVASSLLSQLSAELVGFYLTYPPLTIAEPLEYPNLDTSETTRDYWVITV
LQGVDNSLVGLHNQSFARVMEQRLAQLFMMSQQQGRRFKRATTLGSYTVQMVKMQRVPGPKDPAELTYYTLYN
GKPLLGTAAAKILSTIDSQRMALTLHHVVLLQADPVVKNPPNNLWIIAAVLAPIAVVTIIIIIITAVLCRKNK
NDFKPDTMINLPQRAKPVQGFDYAKQHLGQQGADEEVIPVTQETVVLPIPVRDTPQERDTAQDGSAIKTAKST
ETRRSRSPSENGSVISNESGKPSSGRRSPQNGMTQQKVTKEESRKRNVTASDEEEGAGLFDSAGKAAADPFDT
SSGSVQLIAIKPSALPVVHPASDRGQESSAALNGEVNKALKQKSDIEHYRNKLRLKAKRKGYYDFPPVEAGKG
LAERKMYEKAPKEVEHVLDADPELCAPFAESKNRQQMKNSVYRSRQSLNSPSPGETEMDLLVTRERPRRGIRN
SGYDTEPEIIEETNVDRVHEPRGYGRARQAKGHSETSTLSSQPSIDEVRQQMHMLLEEAFSLASAGHAGQSRH
QETYGSTQHLPYSEVVTSAPGTMTRPRAGVQWVPTYRPEMYQYSLPRPAYRFSQLPEMVMGSPPPPVPPRTGP
VAVASLRRSTSDIGSKTRMAESTGPEPTQLHDSASFAQVSRGPVSVTQLDQSALNYSGNTVPAVFAIPAANRP
GFTGYFIPTPPSSYRSQAWMSYAGENELPSQWADSVPLPGYIEAYPRSRYQQSSPSRLPRQYSQPANLHPSLE
QAPVPSAAASQQSLTENEPPDTPLTNISTAALVKAIREEVAKLAKKQTDMFEFQV*
>ENSMUSP00000150073_c.843:A>G_p.M282V ENSMUST00000214801.3|Olfr1317-203|ENSMUSG00000050776|+|chr2:g.111973135:A>G
MDGGNHSVVSEFLLLGLTNSWRIQILLFLFFTVFYVASMLGNLLIVLTIISDHHLHSPMYFLLANLSFIDTGV
SSIATPKMIYDLFRKHKVISLNGCITQMFFIHTVGGTEMVLLIVMAYDRYIAICKPLHYLTIMSLRMCIVLLA
LAWIIGLIHSVAQLAFVVNLPFCGANKMDSFYCDFPRFIKLACTDTYRLEFLVTANSGFISMATFFILIVSYI
FILVTVRKHSSGASSKALSTLSAHITVVVFFFGPCIIVYVWPFPTLPIDKFLAIFDAIITPSVNPVIYTLRNK
EMKVAMRRLFARALSFIDSLRDSN*
>ENSMUSP00000149091_c.843:A>G_p.M282V ENSMUST00000216988.2|Olfr1317-204|ENSMUSG00000050776|+|chr2:g.111973135:A>G
MDGGNHSVVSEFLLLGLTNSWRIQILLFLFFTVFYVASMLGNLLIVLTIISDHHLHSPMYFLLANLSFIDTGV
SSIATPKMIYDLFRKHKVISLNGCITQMFFIHTVGGTEMVLLIVMAYDRYIAICKPLHYLTIMSLRMCIVLLA
LAWIIGLIHSVAQLAFVVNLPFCGANKMDSFYCDFPRFIKLACTDTYRLEFLVTANSGFISMATFFILIVSYI
FILVTVRKHSSGASSKALSTLSAHITVVVFFFGPCIIVYVWPFPTLPIDKFLAIFDAIITPSVNPVIYTLRNK
EMKVAMRRLFARALSFIDSLRDSN*
>ENSMUSP00000058587_c.843:A>G_p.M282V ENSMUST00000060098.2|Olfr1317-201|ENSMUSG00000050776|+|chr2:g.111973135:A>G
MDGGNHSVVSEFLLLGLTNSWRIQILLFLFFTVFYVASMLGNLLIVLTIISDHHLHSPMYFLLANLSFIDTGV
SSIATPKMIYDLFRKHKVISLNGCITQMFFIHTVGGTEMVLLIVMAYDRYIAICKPLHYLTIMSLRMCIVLLA
LAWIIGLIHSVAQLAFVVNLPFCGANKMDSFYCDFPRFIKLACTDTYRLEFLVTANSGFISMATFFILIVSYI
FILVTVRKHSSGASSKALSTLSAHITVVVFFFGPCIIVYVWPFPTLPIDKFLAIFDAIITPSVNPVIYTLRNK
EMKVAMRRLFARALSFIDSLRDSN*

>ENSMUSP00000106076_c.114:A>C_p.I39L ENSMUST00000110446.9|Fam227b-201|ENSMUSG00000027209|-|chr2:g.125968958:T>G
MELPVPQKLPDPPRSFDEFLKSQNWDYWPRDVHFRDSDLWEDTLKKLEEAISYTSIYSYLWTNVPRLYDIVDS
LESKLKESSHLLQHHASRLFESDKMISKKRSYTNLERYKAFVKEHYRPKKIALSDRMETEKNIEGCTFLFNQN
EVTQLPRHLDAKQIYLYVLKTHNFEEKVFKVWKTHVLSDCSIALLHDSFWWWFLHKFKPDKRDEDFLFDRIAE
SYVTLFIKIPLRRKDAFFKMYPDCLTQAVYTTFQESFPESCSLFNDKFKEDLGNTIFLWLSGLKPETGFWTHW
KLQDLCTTTIHGSRRVPVKLRRSVIPSQEHIPGIRDLKIEDILKNPRAYAMPKLMKESVASKAATKPSHYRSL
GPEFHKVLFDFGGQSPLILYYLKMHELGGISVTHNPKGTKFTKILREPSPAPTYCDIIRDAKRKFADNKKDFK
RVKQRIKDDIKFLREQQELIDKELDRIQAKASKNLQEVKNEFENFLHKQRVEAKLKEEYGGSTSASESPQSMQ
SPQSSSSFPTISEDFNNVEEG*
>ENSMUSP00000136349_c.114:A>C_p.I39L ENSMUST00000178118.8|Fam227b-204|ENSMUSG00000027209|-|chr2:g.125968958:T>G
MELPVPQKLPDPPRSFDEFLKSQNWDYWPRDVHFRDSDLWEDTLKKLEEAISYTSIYSYLWTNVPRLYDIVDS
LESKLKESSHLLQHHASRLFESDKMISKKRSYTNLERYKAFVKEHYRPKKIALSDRMETEKNIEGCTFLFNQN
EVTQLPRHLDAKQIYLYVLKTHNFEEKVFKVWKTHVLSDCSIALLHDSFWWWFLHKFKPDKRDEDFLFDRIAE
SYVTLFIKIPLRRKDAFFKMYPDCLTQAVYTTFQESFPESCSLFNDKFKEDLGNTIFLWLSGLKPETGFWTHW
KLQDLCTTTIHGSRRVPVKLRRSVIPSQEHIPGIRDLKIEDILKNPRAYAMPKLMKESVASKAATKPSHYRSL
GPEFHKVLFDFGGQSPLILYYLKMHELGGISVTHNPKGTKFTKILREPSPAPTYCDIIRDAKRKFADNKKDFK
RVKQRIKDDIKFLREQQELIDKELDRIQAKASKNLQEVKNEFENFLHKQRVEAKLKEEYGGSTSASESPQSMQ
SPQSSSSFPTISEDFNNVEEG*
>ENSMUSP00000106078_c.114:A>C_p.I39L ENSMUST00000110448.3|Fam227b-202|ENSMUSG00000027209|-|chr2:g.125968958:T>G
MELPVPQKLPDPPRSFDEFLKSQNWDYWPRDVHFRDSDLWEDTLKKLEEAISYTSIYSYLWTNVPRLYDIVDS
LESKLKESSHLLQHHASRLFESDKMISKKRSYTNLERYKAFVKEHYRPKKIALSDRMETEKNIEGCTFLFNQN
EVTQLPRHLDAKQIYLYVLKTHNFEEKVFKVWKTHVLSDCSIALLHDSFWWWFLHKFKPDKRDEDFLFDRIAE
SYVTLFIKIPLRRKDAFFKMYPDCLTQAVYTTFQESFPESCSLFNDKFKEDLGNTIFLWLSGLKPETGFWTHW
KLQDLCTTTIHGSRRVPVKLRRSVIPSQEHIPGIRDLKIEDILKNPRAYAMPKLMKESVASKAATKPSHYRSL
GPEFHKVLFDFGGQSPLILYYLKMHELGGISVTHNPKGTKFTKILREPYPFPTYCDIIRDAKRKFADNKKDFK
RVKQRIKDDIKFLREQQELIDKELDRIQAKASKNLQEVKNEFENFLHKQRVEAKLKEEYGGSTSASESPQSMQ
SPQSSSSFPTISEDFNNVEEG*
>rs262016965_ENSMUSP00000028897_c.448:A>G_p.D150G
ENSMUST00000028897.8|Cpxm1-201|ENSMUSG00000027408|-|chr2:g.130237860:T>C
MWGLLLAVTAFAPSVGLGLGAPSASVPGLAPGSTLAPHSSVAQPSTKANETSERHVRLRVIKKKKIVVKKRKK
LRHPGPLGTARPVVPTHPAKTLTLPEKQEPGCPPLGLESLRVSDSQLEASSSQSFGLGAHRGRLNIQSGLEDG
DLYGGAWCAEQQDTEPWLQVDAKNPVRFAGIVTQGRNSVWRYDWVTSFKVQFSNDSQTWWKSRNSTGMDIVFP
ANSDAETPVLNLLPEPQVARFIRLLPQTWFQGGAPCLRAEILACPVSDPNDLFPEAHTLGSSNSLDFRHHNYK
AMRKLMKQVNEQCPNITRIYSIGKSHQGLKLYVMEMSDHPGEHELGEPEVRYVAGMHGNEALGRELLLLLMQF
LCHEFLRGDPRVTRLLTETRIHLLPSMNPDGYETAYHRGSELVGWAEGRWTHQGIDLNHNFADLNTQLWYAED
DGLVPDTVPNHHLPLPTYYTLPNATVAPETWAVIKWMKRIPFVLSANLHGGELVVSYPFDMTRTPWAARELTP
TPDDAVFRWLSTVYAGTNRAMQDTDRRPCHSQDFSLHGNVINGADWHTVPGSMNDFSYLHTNCFEVTVELSCD
KFPHEKELPQEWENNKDALLTYLEQVRMGITGVVRDKDTELGIADAVIAVEGINHDVTTAWGGDYWRLLTPGD
YVVTASAEGYHTVRQHCQVTFEEGPVPCNFLLTKTPKERLRELLATRGKLPPDLRRKLERLRGQK*
>ENSMUSP00000119606_c.166:G>C_p.S56T ENSMUST00000150843.9|Pank2-204|ENSMUSG00000037514|+|chr2:g.131104616:G>C
LGGWLGRQRRLLRMGAGRFGAPMERQGRAAATSAAVGESADSEARRRDPLRRRASTAAPSGSGEAESVRRERP
GSLGGSTSAGRPRAEGLRKRRPLFPWFGLDIGGTLVKLVYFEPKDITAEEEKEEVESLKSIRKYLTSNVAYGS
TGIRDVHLELKDLTLCGRKGNLHFIRFPTHDMPAFIQMGRDKNFSSLHTVFCATGGGSYKFEQDFLTIGDLQL
RKLDELDCLIKGILYIDSVGFNGRSQCYYFENPADSEKCQKLPFDLKNPYPLLLVNIGSGVSILAVYSKDNYK
RVTGTSLGGGTFFGLCCLLTGCSTFEEALEMASRGDSTKVDKLVRDIYGGDYERFGLPGWAVASSFGNMMSKE
KREAASKEDLARATLITITNNIGSIARMCALNENINQVVFVGNFLRVNTIAMRLLAYALDYWSKGQLKALFSE
HEGYFGAVGALLELLKIP*
>ENSMUSP00000138992_c.166:G>C_p.S56T ENSMUST00000184105.9|Pank2-207|ENSMUSG00000037514|+|chr2:g.131104616:G>C
LGGWLGRQRRLLRMGAGRFGAPMERQGRAAATSAAVGESADSEARRRDPLRRRASTAAPSGSGEAESVRRERP
GSLGGSTSAGRPRAEGLRKRRPLFPWFGLDIGGTLVKLVYFEPKDITAEEEKEEVESLKSIRKYLTSNVAYGS
TGIRDVHLELKDLTLCGRKDR*
>ENSMUSP00000139259_c.130:G>C_p.S44T ENSMUST00000184932.9|Pank2-208|ENSMUSG00000037514|+|chr2:g.131104616:G>C

XMGAGRFGAPMERQGRAAATSAAVGESADSEARRRDPLRRRASTAAPSGSGEAESVRRERPGSLGGSTSAGRP
RAEGLRKRRPLFPWFGLDIGGTLVKLVYFEPKDITAEEEKEEVESLKSIRKYLTSNVAYGSTGIRDVHLELKD
LTLCGRKVLET*
>ENSMUSP00000000369_c.182:T>TC_p.P61ins-fs ENSMUST00000000369.4|Rem1-201|ENSMUSG00000000359|+|chr2:g.152470198:T>TC
MTLNTQQEAKTTLRRRASTPLPLSSRGHQPGRLCTAPSAPSQHPRLGQSVSLNPPVRKPSPRPGWVVL
>ENSMUSP00000119532_c.182:T>TC_p.P61ins-fs ENSMUST00000150913.2|Rem1-203|ENSMUSG00000000359|+|chr2:g.152470198:T>TC
MTLNTQQEAKTTLRRRASTPLPLSSRGHQPGRLCTAPSAPSQHPRLGQSVSLNPPVRKPSPRPGWVVL
>ENSMUSP00000105198_c.484:T>TG_p.L162ins-fs ENSMUST00000109570.8|Aar2-202|ENSMUSG00000027628|+|chr2:g.156393097:T>TG
MSSMQMDPELAKQLFFEGATVVILNMPKGTEFGIDYNSWEVGPKFRGVKMIPPGIHFLYYSSVDKANPREVGP
RMGFFLSLKQRGLTVLRWNAVQEEVDLSPAPEAEVEAMRANLPDLDQFLGPYPYATLKKWISLTNFISEATME
KLQPESRQICAFSDVLACAFHEAHQGQGGAEPAPLRHRVQKLPRGPSPAARDEAQGWDRDPLLRVAHSDVPSR
SHTSRDHQAQHGLELCPRNCAQQAVPWQPPGCAW
>ENSMUSP00000029158_c.484:T>TG_p.L162ins-fs ENSMUST00000029158.4|Aar2-201|ENSMUSG00000027628|+|chr2:g.156393097:T>TG
MSSMQMDPELAKQLFFEGATVVILNMPKGTEFGIDYNSWEVGPKFRGVKMIPPGIHFLYYSSVDKANPREVGP
RMGFFLSLKQRGLTVLRWNAVQEEVDLSPAPEAEVEAMRANLPDLDQFLGPYPYATLKKWISLTNFISEATME
KLQPESRQICAFSDVLACAFHEAHQGQGGAEPAPLRHRVQKLPRGPSPAARDEAQGWDRDPLLRVAHSDVPSR
SHTSRDHQAQHGLELCPRNCAQQAVPWQPPGCAW
>ENSMUSP00000070052_c.132:A>C_p.T45P ENSMUST00000069600.13|Ndrg3-201|ENSMUSG00000027634|-|chr2:g.156792020:T>G
MDELQDVQLTEIKPLLNDKNGTRNFQDFDCQEHDIETPHGMVHVPIRGLPKGNRPVILTYHDIGLNHKSCFNT
FFNFEDMQEITQHFAVCHVDAPGQQEAAPSFPTGYQYPTMDELAEMLPPVLTHLSMKSIIGIGVGAGAYILSR
FALNHPELVEGLVLINIDPCAKGWIDWAASKLSGFTTNIVDIILAHHFGQEELQANLDLIQTYRLHIAQDINQ
ENLQLFLGSYNGRRDLEIERPILGQNDNRLKTLKCSTLLVVGDNSPAVEAVVECNSRLDPINTTLLKMADCGG
LPQVVQPGKLTEAFKYFLQGMGYIPYVQLSHLSSESVPSASMTRLARSRTHSTSSSIGSGESPFSRSVTSNQS
DGTQESCESPDVLDRHQTMEVSC*
>ENSMUSP00000072144_c.132:A>C_p.T45P ENSMUST00000072298.13|Ndrg3-202|ENSMUSG00000027634|-|chr2:g.156792020:T>G
MDELQDVQLTEIKPLLNDKNGTRNFQDFDCQEHDIETPHGMVHVPIRGLPKGNRPVILTYHDIGLNHKSCFNT
FFNFEDMQEITQHFAVCHVDAPGQQEAAPSFPTGYQYPTMDELAEMLPPVLTHLSMKSIIGIGVGAGAYILSR
FALNHPELVEGLVLINIDPCAKGWIDWAASKLSGFTTNIVDIILAHHFGQEELQANLDLIQTYRLHIAQDINQ
ENLQLFLGSYNGRRDLEIERPILGQNDNRLKTLKCSTLLVVGDNSPAVEAVVECNSRLDPINTTLLKMADCGG
LPQVVQPGKLTEAFKYFLQGMGYIPSASMTRLARSRTHSTSSSIGSGESPFSRSVTSNQSDGTQESCESPDVL
DRHQTMEVSC*
>ENSMUSP00000105185_c.132:A>C_p.T45P ENSMUST00000109558.2|Ndrg3-203|ENSMUSG00000027634|-|chr2:g.156792020:T>G
MDELQDVQLTEIKPLLNDKNGTRNFQDFDCQEHDIETPHGMVHVPIRGLPKGNRPVILTYHDIGLNHKSCFNT
FFNFEDMQEITQHFAVCHVDAPGQQEAAPSFPTGYQYPTMDELAEMLPPVLTHLSMKSIIGIGVGAGAYILSR
FALNHPELVEGLVLINIDPCAKGWIDWAASKLSGFTTNIVDIILAHHFGQEELQANLDLIQTYRLHIAQDINQ
ENLQLFLGSYNGRRDLEIERPILGQNDNRLKTLK*
>ENSMUSP00000105038_c.6:A>C_p.M3L ENSMUST00000109411.8|Hnf4a-202|ENSMUSG00000017950|+|chr2:g.163389258:A>C
MDLADYSAALDPAYTTLEFENVQVLTMGNDTSPSEGANLNSSNSLGVSALCAICGDRATGKHYGASSCDGCKG
FFRRSVRKNHMYSCRFSRQCVVDKDKRNQCRYCRLKKCFRAGMKKEAVQNERDRISTRRSSYEDSSLPSINAL
LQAEVLSQQITSPISGINGDIRAKKIANITDVCESMKEQLLVLVEWAKYIPAFCELLLDDQVALLRAHAGEHL
LLGATKRSMVFKDVLLLGNDYIVPRHCPELAEMSRVSIRILDELVLPFQELQIDDNEYACLKAIIFFDPDAKG
LSDPGKIKRLRSQVQVSLEDYINDRQYDSRGRFGELLLLLPTLQSITWQMIEQIQFIKLFGMAKIDNLLQEML
LGGSASDAPHTHHPLHPHLMQEHMGTNVIVANTMPSHLSNGQMCEWPRPRGQAATPETPQPSPPSGSGSESYK
LLPGAITTIVKPPSAIPQPTITKQEAI*
>ENSMUSP00000018094_c.33:A>C_p.M12L ENSMUST00000018094.13|Hnf4a-201|ENSMUSG00000017950|+|chr2:g.163389258:A>C
MRLSKTLAGMDLADYSAALDPAYTTLEFENVQVLTMGNDTSPSEGANLNSSNSLGVSALCAICGDRATGKHYG
ASSCDGCKGFFRRSVRKNHMYSCRFSRQCVVDKDKRNQCRYCRLKKCFRAGMKKEAVQNERDRISTRRSSYED
SSLPSINALLQAEVLSQQITSPISGINGDIRAKKIANITDVCESMKEQLLVLVEWAKYIPAFCELLLDDQVAL
LRAHAGEHLLLGATKRSMVFKDVLLLGNDYIVPRHCPELAEMSRVSIRILDELVLPFQELQIDDNEYACLKAI

IFFDPDAKGLSDPGKIKRLRSQVQVSLEDYINDRQYDSRGRFGELLLLLPTLQSITWQMIEQIQFIKLFGMAK
IDNLLQEMLLGGSASDAPHTHHPLHPHLMQEHMGTNVIVANTMPSHLSNGQMCEWPRPRGQAATPETPQPSPP
SGSGSESYKLLPGAITTIVKPPSAIPQPTITKQEAI*
>ENSMUSP00000041806_c.430:A>T_p.E144V ENSMUST00000042775.5|Neurl2-201|ENSMUSG00000039873|-|chr2:g.164674930:T>A
MAAPSEHVGLGGPRSPARPEPPPTRFHQVHGANIRMDPSGTRATRVESFAHGVCFSREPLAPGQVFLVEIEEK
ELGWCGHLRLGLTALDPASLAAVPEFSLPDLVSLGHSWVFAITRHHNRVPREGQPEAEAAVPSGPQALLVVPY
LRIEQFRIPRDRLVGRSRPGLYSHLLDQLYEQNVLPPTARRSRLGVLFCPREDGTADMHIIINGEDMGPSARG
LPAAQPLYAVVDVFASTKSVRLVQLEYGLPSLQTLCRLVIQKRVVHRLAIDVLHLPKGLKDFCKYE*
>ENSMUSP00000074198_c.2630:A>T_p.R877S ENSMUST00000074618.10|Nfatc2-202|ENSMUSG00000027544|-|chr2:g.168346462:T>A
MDVPEPQPDPDGGDGPGHEPGGSPQDELDFSILFDYDYLNPIEEEPIAHKAISSPSGLAYPDDVLDYGLKPCN
PLASLSGEPPGRFGEPDSIGFQNFLSPVKPAGASGPSPRIEITPSHELMQAGGALRGRDAGLSPEQPALALAG
VAASPRFTLPVPGYEGYREPLCLSPASSGSSASFISDTFSPYTSPCVSPNNAGPDDLCPQFQNIPAHYSPRTS
PIMSPRTSLAEDSCLGRHSPVPRPASRSSSPGAKRRHSCAEALVAPLPAASPQRSRSPSPQPSPHVALQDDSI
PAGYPPTAGSAVLMDALNTLATDSPCGIPSKIWKTSPDPTPVSTAPSKAGLARHIYPTVEFLGPCEQEERRNS
APESILLVPPTWPKQLVPAIPICSIPVTASLPPLEWPLSNQSGSYELRIEVQPKPHHRAHYETEGSRGAVKAP
TGGHPVVQLHGYMENKPLGLQIFIGTADERILKPHAFYQVHRITGKTVTTTSYEKIVGNTKVLEIPLEPKNNM
RATIDCAGILKLRNADIELRKGETDIGRKNTRVRLVFRVHVPEPSGRIVSLQAASNPIECSQRSAHELPMVER
QDMDSCLVYGGQQMILTGQNFTAESKVVFMEKTTDGQQIWEMEATVDKDKSQPNMLFVEIPEYRNKHIRVPVK
VNFYVINGKRKRSQPQHFTYHPVPAIKTEPSDEYEPSLICSPAHGGLGSQPYYPQHPMLAESPSCLVATMAPC
QQFRSGLSSPDARYQQQSPAAALYQRSKSLSPGLLGYQQPSLLAAPLGLADAHRSVLVHAGSQGQGQGSTLPH
TSSASQQASPVIHYSPTNQQLRGGGHQEFQHIMYCENFGPSSARPGPPPINQGQRLSPGAYPTVIQQQTAPSQ
SAAKNGPSDQKEALPTGVTVKQEQNLDQTYLDDVNEIIRKEFSGPPSRNQT*
>ENSMUSP00000104812_c.2630:A>T_p.R877S ENSMUST00000109184.8|Nfatc2-204|ENSMUSG00000027544|-|chr2:g.168346462:T>A
MDVPEPQPDPDGGDGPGHEPGGSPQDELDFSILFDYDYLNPIEEEPIAHKAISSPSGLAYPDDVLDYGLKPCN
PLASLSGEPPGRFGEPDSIGFQNFLSPVKPAGASGPSPRIEITPSHELMQAGGALRGRDAGLSPEQPALALAG
VAASPRFTLPVPGYEGYREPLCLSPASSGSSASFISDTFSPYTSPCVSPNNAGPDDLCPQFQNIPAHYSPRTS
PIMSPRTSLAEDSCLGRHSPVPRPASRSSSPGAKRRHSCAEALVAPLPAASPQRSRSPSPQPSPHVALQDDSI
PAGYPPTAGSAVLMDALNTLATDSPCGIPSKIWKTSPDPTPVSTAPSKAGLARHIYPTVEFLGPCEQEERRNS
APESILLVPPTWPKQLVPAIPICSIPVTASLPPLEWPLSNQSGSYELRIEVQPKPHHRAHYETEGSRGAVKAP
TGGHPVVQLHGYMENKPLGLQIFIGTADERILKPHAFYQVHRITGKTVTTTSYEKIVGNTKVLEIPLEPKNNM
RATIDCAGILKLRNADIELRKGETDIGRKNTRVRLVFRVHVPEPSGRIVSLQAASNPIECSQRSAHELPMVER
QDMDSCLVYGGQQMILTGQNFTAESKVVFMEKTTDGQQIWEMEATVDKDKSQPNMLFVEIPEYRNKHIRVPVK
VNFYVINGKRKRSQPQHFTYHPVPAIKTEPSDEYEPSLICSPAHGGLGSQPYYPQHPMLAESPSCLVATMAPC
QQFRSGLSSPDARYQQQSPAAALYQRSKSLSPGLLGYQQPSLLAAPLGLADAHRSVLVHAGSQGQGQGSTLPH
TSSASQQASPVIHYSPTNQQLRGGGHQEFQHIMYCENFGPSSARPGPPPINQGQRLSPGAYPTVIQQQTAPSQ
SAAKNGPSDQKEALPTGVTVKQEQNLDQTYLDDELIDTHLSWIQNIL*
>ENSMUSP00000116617_c.558:T>A_p.Y187N ENSMUST00000128657.8|Gm14418-202|ENSMUSG00000078867|-|chr2:g.177079435:A>T
MDLVTYDDVHVNFTQEEWALLHPSQKSLYKGVMLETYRNLTAIGYIWEEHTIEDHFQTSRSHGRHERSCSAEQ
PPEFIQCGKAFAYESGRQRHQIKHTGGKHHDCNQCGKDFRTRNAPQIHKRTHTGEKPYDCNQYGKAFARISHL
RVHKRTHTGEKPYECNQCGKAFASSSDLQKHKRTHTGEKPNECNQCGKAFAQSSHLRIHKRTHAGDKRYECNQ
CGKAFTQSNNLQIHKGTHAGEKRYECNQCGKAFASSSDLQKHKRTHTGEKPYECNQCGKAFAKSCDLQKHKRT
HTGEKPYECKQCGKAFAISYTLQIHNRTHTGEKPYECKQCGKAFAQSSNLRIHKQTHTGERLYECN*
>ENSMUSP00000116617_c.388:C>T_p.P130L ENSMUST00000128657.8|Gm14418-202|ENSMUSG00000078867|-|chr2:g.177079605:G>A
MDLVTYDDVHVNFTQEEWALLHPSQKSLYKGVMLETYRNLTAIGYIWEEHTIEDHFQTSRSHGRHERSCSAEQ
PPEFIQCGKAFAYESGRQRHQIKHTGGKHHDCNQCGKDFRTRNAPQIHKRTHTGEKLYDCNQYGKAFARISHL
RVHKRTHTGEKPYECNQCGKAFASSSDLQKHKRTHTGEKPYECNQCGKAFAQSSHLRIHKRTHAGDKRYECNQ
CGKAFTQSNNLQIHKGTHAGEKRYECNQCGKAFASSSDLQKHKRTHTGEKPYECNQCGKAFAKSCDLQKHKRT
HTGEKPYECKQCGKAFAISYTLQIHNRTHTGEKPYECKQCGKAFAQSSNLRIHKQTHTGERLYECN*
>rs107973180_ENSMUSP00000079909_c.504:C>A_p.Q169K ENSMUST00000081134.10|Sycp2-201|ENSMUSG00000060445|-|chr2:g.178037639:G>T
MPVRPDLQQLEKCIDDALRKNDFKPLLALLQIDICEDVKIKCSKQFLRKLDDLICRELNKKDIQTVSSILISI
GRCSKNIFILGQAGLQTMIKQGLVQKMVSWFENSKEIILNQQQSKDEAVMNMIEDLFDLLMVIYDISDEGKNQ
VLESFIPQICALVIDSRVNFCIKQEALKKMNLMLDRIPQDANKILSNQEMLTLMSNMGERILDVGDYELQVGI

VEALCRMTTEKRRQELAYEWFSMDFIANAFKEIKDCEFETDCRIFLNLVNGILGDKRRVYTFPCLSAFLGKYE
LQIPSDEKLEEFWIDFNLGSHTLSFYIAGDEEDHQWEAVTVPEEKVQMYNIEVRESKKLLTLTLKNIVKISKK
EGKELLFYFDESLEITNVTKKVFGGNKYKEFTRKQGISVAKTSIHVLFDASGSQILVPESQPSPVKENLIHLK
EKSDIQKKLVNPLELGNSSSQDEITTPSRKKMSEASMIVPDTDRYTVRSPILLINTSTPRRSREPLQAINSVE
KAVSKTSESGMDYAASPKSRQSDGRKRWNNRANHNKTTAVIQNKQYEDNESPDQNFNEIEDTLSNVSSAVGKV
DKPVLPGVLDISKNTTHSRWACWTPVTTIKLCNNQRSRALPGDTCTQDTGVNKKCTKQKSVSDDDSEETQKGK
YSKDVIKCNKSDEAEFCERNIQEQNHPKYSQKKNTANAKKSDWHIESETTYKSVLLNKTTEESLIYKKTCVLS
KDVNTTICDKSPSRKSKRNHTKSRKELMSELTSCELEEIPVRENSKGKRFTGASESLINQISRRYNPSDSMMS
TRKLKEPQDGSGFSKKPDLQFNKVQRKSYRKLKATVVNVTSECPLDDVYNFSLNGADEPVIKLGIQEFQATTR
EASMDNSLKLVKNHDEHDPFLKTKDKRMLSYEKKTLLSDTETECGCDDSKTDISWLKEPKTKRLMDYSRNKNT
TKYKSRKSRSSMEKGQPRPTMVLNKNSMKNDYEVVVDGRTRLPRRATKTKKNYKDLSTSESESESEKECSYLF
KDKLPTKEETIHSRAQTKKLPEKQQKVFNSEALKGQPSEEQKNSSRLREGREDSLCLSSASVSRSSSSVEVMR
CTEKITERDFTQDYDYITKSLSPYPKAPSPEFLNGNNSVVGRGQSPRISETSAMCVRKSYSPASGPPFSPRHT
PTKNNSVVNMKKANSVINNQRTQHCNSYSDVSSNSSEKLYMEPESPESCDNHMQNKREGNHAASPLSLSSEKI
EKMWFDMPSENTHVSGPSQRGSKRRMYLEDDELSNSNEAEVEEAEEREHLLSKKRCQWENSDQHTFKTSLSTP
DFSVPKDWQQELQGAGMFYDNISSDYKRKTDSQHKIMDDFTTKTLKLTQQHLMAMTSQAQGRRDENVEKFQVT
LLDELEKVEKDSQTLRDLEKELVDIEEKLVQKMRAYHRCERERFRVLKTSLDKSFLVYNSVYEESVFTSEMCL
MKANMKMLQDKLLKEMHEEEVLNIRRGLQSLFKAHEGNDA*
>rs107973180_ENSMUSP00000116917_c.504:C>A_p.Q169K
ENSMUST00000138175.2|Sycp2-204|ENSMUSG00000060445|-|chr2:g.178037639:G>T
MPVRPDLQQLEKCIDDALRKNDFKPLLALLQIDICEDVKIKCSKQFLRKLDDLICRELNKKDIQTVSSILISI
GRCSKNIFILGQAGLQTMIKQGLVQKMVSWFENSKEIILNQQQSKDEAVMNMIEDLFDLLMVIYDISDEGKNQ
VLESFIPQICALVIDSRVNFCIKQEALKKMNLMLDRIPQ
>ENSMUSP00000015791_c.1006:G>A_p.G336D ENSMUST00000015791.6|Lama5-
201|ENSMUSG00000015647|-|chr2:g.179844265:C>T
MAKRGGQLCAGSAPGALGPRSPAPRPLLLLLAGLALVGEARTPGGDGFSLHPPYFNLAEGARITASATCGEEA
PTRSVSRPTEDLYCKLVGGPVAGGDPNQTIQGQYCDICTAANSNKAHPVSNAIDGTERWWQSPPLSRGLEYNE
VNVTLDLGQVFHVAYVLIKFANSPRPDLWVLERSTDFGHTYQPWQFFASSKRDCLERFGPRTLERITQDDDVI
CTTEYSRIVPLENGEIVVSLVNGRPGALNFSYSPLLRDFTKATNIRLRFLRTNTLLGHLMGKALRDPTVTRRY
YYSIKDISIGGRCVCHGHADVCDAKDPLDPFRLQCACQHNTCGDSCDRCCPGFNQQPWKPATTDSANECQSCN
CHGHAYDCYYDPEVDRRNASQNQDNVYQGGGVCLDCQHHTTGINCERCLPGFFRAPDQPLDSPHVCRPCDCES
DFTDGTCEDLTGRCYCRPNFTGELCAACAEGYTDFPHCYPLPSFPHNDTREQVLPAGQIVNCDCNAAGTQGNA
CRKDPRLGRCVCKPNFRGAHCELCAPGFHGPSCHPCQCSSPGVANSLCDPESGQCMCRTGFEGDRCDHCALGY
FHFPLCQLCGCSPAGTLPEGCDEAGRCQCRPGFDGPHCDRCLPGYHGYPDCHACACDPRGALDQQCGVGGLCH
CRPGYTGATCQECSPGFYGFPSCIPCHCSADGSLHTTCDPTTGQCRCRPRVTGLHCDMCVPGAYNFPYCEAGS
CHPAGLAPANPALPETQAPCMCRAHVEGPSCDRCKPGYWGLSASNPEGCTRCSCDPRGTLGGVTECQGNGQCF
CKAHVCGKTCAACKDGFFGLDYADYFGCRSCRCDVGGALGQGCEPKTGACRCRPNTQGPTCSEPAKDHYLPDL
HHMRLELEEAATPEGHAVRFGFNPLEFENFSWRGYAHMMAIQPRIVARLNVTSPDLFRLVFRYVNRGSTSVNG
QISVREEGKLSSCTNCTEQSQPVAFPPSTEPAFVTVPQRGFGEPFVLNPGIWALLVEAEGVLLDYVVLLPSTY
YEAALLQHRVTEACTYRPSALHSTENCLVYAHLPLDGFPSAAGTEALCRHDNSLPRPCPTEQLSPSHPPLATC
FGSDVDIQLEMAVPQPGQYVLVVEYVGEDSHQEMGVAVHTPQRAPQQGVLNLHPCPYSSLCRSPARDTQHHLA
IFYLDSEASIRLTAEQAHFFLHSVTLVPVEEFSTEFVEPRVFCVSSHGTFNPSSAACLASRFPKPPQPIILKD
CQVLPLPPDLPLTQSQELSPGAPPEGPQPRPPTAVDPNAEPTLLRHPQGTVVFTTQVPTLGRYAFLLHGYQPV
HPSFPVEVLINGGRIWQGHANASFCPHGYGCRTLVLCEGQTMLDVTDNELTVTVRVPEGRWLWLDYVLIVPED
AYSSSYLQEEPLDKSYDFISHCATQGYHISPSSSSPFCRNAATSLSLFYNNGALPCGCHEVGAVSPTCEPFGG
QCPCRGHVIGRDCSRCATGYWGFPNCRPCDCGARLCDELTGQCICPPRTVPPDCLVCQPQSFGCHPLVGCEEC
NCSGPGVQELTDPTCDMDSGQCRCRPNVAGRRCDTCAPGFYGYPSCRPCDCHEAGTMASVCDPLTGQCHCKEN
VQGSRCDQCRVGTFSLDAANPKGCTRCFCFGATERCGNSNLARHEFVDMEGWVLLSSDRQVVPHEHRPEIELL
HADLRSVADTFSELYWQAPPSYLGDRVSSYGGTLHYELHSETQRGDIFIPYESRPDVVLQGNQMSIAFLELAY
PPPGQVHRGQLQLVEGNFRHLETHNPVSREELMMVLAGLEQLQIRALFSQTSSSVSLRRVVLEVASEAGRGPP
ASNVELCMCPANYRGDSCQECAPGYYRDTKGLFLGRCVPCQCHGHSDRCLPGSGICVGCQHNTEGDQCERCRP
GFVSSDPSNPASPCVSCPCPLAVPSNNFADGCVLRNGRTQCLCRPGYAGASCERCAPGFFGNPLVLGSSCQPC
DCSGNGDPNMIFSDCDPLTGACRGCLRHTTGPHCERCAPGFYGNALLPGNCTRCDCSPCGTETCDPQSGRCLC
KAGVTGQRCDRCLEGYFGFEQCQGCRPCACGPAAKGSECHPQSGQCHCQPGTTGPQCLECAPGYWGLPEKGCR
RCQCPRGHCDPHTGHCTCPPGLSGERCDTCSQQHQVPVPGKPGGHGIHCEVCDHCVVLLLDDLERAGALLPAI
REQLQGINASSAAWARLHRLNASIADLQSKLRSPPGPRYQAAQQLQTLEQQSISLQQDTERLGSQATGVQGQA
GQLLDTTESTLGRAQKLLESVRAVGRALNELASRMGQGSPGDALVPSGEQLRWALAEVERLLWDMRTRDLGAQ
GAVAEAELAEAQRLMARVQEQLTSFWEENQSLATHIRDQLAQYESGLMDLREALNQAVNTTREAEELNSRNQE

RLKEALQWKQELSQDNATLKATLQAASLILGHVSELLQGIDQAKEDLEHLAASLDGAWTPLLKRMQAFSPASS
KVDLVEAAEAHAQKLNQLAINLSGIILGINQDRFIQRAVEASNAYSSILQAVQAAEDAAGQALRQASRTWEMV
VQRGLAAGARQLLANSSALEETILGHQGRLGLAQGRLQAAGIQLHNVWARKNQLAAQIQEAQAMLAMDTSETS
EKIAHAKAVAAEALSTATHVQSQLQGMQKNVERWQSQLGGLQGQDLSQVERDASSSVSTLEKTLPQLLAKLSR
LENRGVHNASLALSANIGRVRKLIAQARSAASKVKVSMKFNGRSGVRLRTPRDLADLAAYTALKFHIQSPVPA
PEPGKNTGDHFVLYMGSRQATGDYMGVSLRNQKVHWVYRLGKAGPTTLSIDENIGEQFAAVSIDRTLQFGHMS
VTVEKQMVHEIKGDTVAPGSEGLLNLHPDDFVFYVGGYPSNFTPPEPLRFPGYLGCIEMETLNEEVVSLYNFE
QTFMLDTAVDKPCARSKATGDPWLTDGSYLDGSGFARISFEKQFSNTKRFDQELRLVSYNGIIFFLKQESQFL
CLAVQEGTLVLFYDFGSGLKKADPLQPPQALTAASKAIQVFLLAGNRKRVLVRVERATVFSVDQDNMLEMADA
YYLGGVPPEQLPLSLRQLFPSGGSVRGCIKGIKALGKYVDLKRLNTTGISFGCTADLLVGRTMTFHGHGFLPL
ALPDVAPITEVVYSGFGFRGTQDNNLLYYRTSPDGPYQVSLREGHVTLRFMNQEVETQRVFADGAPHYVAFYS
NVTGVWLYVDDQLQLVKSHERTTPMLQLQPEEPSRLLLGGLPVSGTFHNFSGCISNVFVQRLRGPQRVFDLHQ
NMGSVNVSVGCTPAQLIETSRATAQKVSRRSRQPSQDLACTTPWLPGTIQDAYQFGGPLPSYLQFVGISPSHR
NRLHLSMLVRPHAASQGLLLSTAPMSGRSPSLVLFLNHGHFVAQTEGPGPRLQVQSRQHSRAGQWHRVSVRWG
MQQIQLVVDGSQTWSQKALHHRVPRAERPQPYTLSVGGLPASSYSSKLPVSVGFSGCLKKLQLDKRPLRTPTQ
MVGVTPCVSGPLEDGLFFPGSEGVVTLELPKAKMPYVSLELEMRPLAAAGLIFHLGQALATPYMQLKVLTEQV
LLQANDGAGEFSTWVTYPKLCDGRWHRVAVIMGRDTLRLEVDTQSNHTTGRLPESLAGSPALLHLGSLPKSST
ARPELPAYRGCLRKLLINGAPVNVTASVQIQGAVGMRGCPSGTLALSKQGKALTQRQAKPSVSPLLWH*
>ENSMUSP00000139512_c.157:C>GC_p.A53ins-fs ENSMUST00000185384.7|Rbis-202|ENSMUSG00000078784|-|chr3:g.14672310:G>GC
MAKNKLKGQKSRNVFHIASHKTFKAKNKAKPVTTNLKKINIMNHEKVNRMNRGFCKYTERTCKLLKKPFS
>ENSMUSP00000104002_c.283:C>GC_p.A95ins-fs ENSMUST00000108365.4|Rbis-201|ENSMUSG00000078784|-|chr3:g.14672310:G>GC
MLAAAAPRALFCQLLVLYRFLVGDRTPGVPRPETLRPPLSRTMAKNKLKGQKSRNVFHIASHKTFKAKNKAKP
VTTNLKKINIMNHEKVNRMNRGFCKYTERTCKLLKKPFS
>ENSMUSP00000139464_c.157:C>GC_p.A53ins-fs ENSMUST00000186870.7|Rbis-204|ENSMUSG00000078784|-|chr3:g.14672310:G>GC
MAKNKLKGQKSRNVFHIASHKTFKAKNKAKPVTTNLKKINIMNHEKVNRMNRGFCKYTERTCKLLKKPFS
>ENSMUSP00000096807_c.87:G>A_p.E30K ENSMUST00000099201.9|Sirpb1a-201|ENSMUSG00000095788|-|chr3:g.15482239:C>T
MLLLDAWTHIPHSVLLLILLLGFKGAAVRKLKVIQPVKSFFVGAGGSATLNCTVTSLLPVGPIRWYRGVGQSR
LLIYPFTGEHSPRITNVSDVTKRNNMDFSIRISNVTPADSGTYYCVKFQRGSSEPDIEIQSGGGTELLVLAKP
SSPMVSGPAARAVPQQTVTFTCRSHGFFPRNLTLKWFKNGDEISHLETSVEPEETSVSYRVSSTVQVVLEPRD
VRSQIICEVDHVTLDRAPLRGIAHISEFIQVPPTLEIRQQPTMVWNVINVTCQIQKFYPPSFQLTWLENGNIS
RREVPFTLIVNKDGTYNWISCLLVNISALEENMVVTCKVEHDEQAEVIETHTVLVTEHQRVKGTATKSELKTA
GIAKIPVAVLLGSKILLLLIAATVIYMHKKQNA*
>ENSMUSP00000141504_c.87:G>A_p.E30K ENSMUST00000192700.3|Sirpb1a-203|ENSMUSG00000095788|-|chr3:g.15482239:C>T
MLLLDAWTHIPHSVLLLILLLGFKGAAVRKLKVIQPVKSFFVGAGGSATLNCTVTSLLPVGPIRWYRGVGQSR
LLIYPFTGEHSPRITNVSDVTKRNNMDFSIRISNVTPADSGTYYCVKFQRGSSEPDIEIQSGGGTELLVLAKP
SSPMVSGPAARAVPQQTVTFTCRSHGFFPRNLTLKWFKNGDEISHLETSVEPEETSVSYRVSSTVQVVLEPRD
VRSQIICEVDHVTLDRAPLRGIAHISEFIQVPPTLEIRQQPTMVWNVINVTCQIQKFYPPSFQLTWLENGNIS
RREVPFTLIVNKDGTYNWISCLLVNISALEENMVVTCKVEHDEQAEVIETHTVLVTEHQRVKELKTAGIAKIP
VAVLLGSKILLLLIAATVIYMHKKQNA*
>rs49659380_ENSMUSP00000103989_c.1177:A>G_p.K393R ENSMUST00000108352.9|Sirpb1c-204|ENSMUSG00000074677|-|chr3:g.15856671:T>C
MLLLDAWTHIPHCVLLLILLLGLKGAAMRELKVIQPVKSFFVGAGGSATLNCTVTSLLPVGPIRWFKGVGQSR
LLIYSFTGERFPRITNVSDVTKRSNLDFSIRISNVTPADSGTYYCVKFQRGPSEPDMEIQSGGGTELSVFAKP
SSPMVSGPAARAVPQQTVAFTCRSHGFFPRNLTLKWFKNGNEISHLETSVEPEETSVSYRVSSTVQVVLEPRD
VRSQIICEVDHVTLDRAPLRGIAHISEFIQVPPTLEISQQPTMVWNVIIVTCQIQKFYPPRFQVTWLENGNIS
RREVPFTLIVNKDGTYNWISCLLVNISALEENMVVTCQVEHDGQAEVIETHTVVVTEHQRVKGTATKSELKTA
GIAKIPVAVLLGSKILLLLIAATVIYMRRKQNA*
>rs49659380_ENSMUSP00000061216_c.1159:A>G_p.K387R ENSMUST00000050623.11|Sirpb1c-201|ENSMUSG00000074677|-|chr3:g.15856671:T>C
MLLLDAWTHIPHCVLLLILLLGLKGAAMRELKVIQPVKSFFVGAGGSATLNCTVTSLLPVGPIRWFKGVGQSR
LLIYSFTGERFPRITNVSDVTKRSNLDFSIRISNVTPADSGTYYCVKFQRGPSEPDMEIQSGGGTELSVFAKP
SSPMVSGPAARAVPQQTVAFTCRSHGFFPRNLTLKWFKNGNEISHLETSVEPEETSVSYRVSSTVQVVLEPRD

VRSQIICEVDHVTLDRAPLRGIAHISEFIQVPPTLEISQQPTMVWNVIIVTCQIQKFYPPRFQVTWLENGNIS
RREVPFTLIVNKDGTYNWISCLLVNISALEENMVVTCQVEHDGQAEVIETHTVVVTEHQRVKELKTAGIAKIP
VAVLLGSKILLLIAATVIYMRRKQNA*
>ENSMUSP00000103989_c.1164:A>T_p.I389F ENSMUST00000108352.9|Sirpb1c-204|ENSMUSG00000074677|-|chr3:g.15856684:T>A
MLLLDAWTHIPHCVLLLILLLGLKGAAMRELKVIQPVKSFFVGAGGSATLNCTVTSLLPVGPIRWFKGVGQSR
LLIYSFTGERFPRITNVSDVTKRSNLDFSIRISNVTPADSGTYYCVKFQRGPSEPDMEIQSGGGTELSVFAKP
SSPMVSGPAARAVPQQTVAFTCRSHGFFPRNLTLKWFKNGNEISHLETSVEPEETSVSYRVSSTVQVVLEPRD
VRSQIICEVDHVTLDRAPLRGIAHISEFIQVPPTLEISQQPTMVWNVIIVTCQIQKFYPPRFQVTWLENGNIS
RREVPFTLIVNKDGTYNWISCLLVNISALEENMVVTCQVEHDGQAEVIETHTVVVTEHQRVKGTATKSELKTA
GIAKIPVAVLLGSKILLLIAATVFYMRKKQNA*
>ENSMUSP00000061216_c.1146:A>T_p.I383F ENSMUST00000050623.11|Sirpb1c-201|ENSMUSG00000074677|-|chr3:g.15856684:T>A
MLLLDAWTHIPHCVLLLILLLGLKGAAMRELKVIQPVKSFFVGAGGSATLNCTVTSLLPVGPIRWFKGVGQSR
LLIYSFTGERFPRITNVSDVTKRSNLDFSIRISNVTPADSGTYYCVKFQRGPSEPDMEIQSGGGTELSVFAKP
SSPMVSGPAARAVPQQTVAFTCRSHGFFPRNLTLKWFKNGNEISHLETSVEPEETSVSYRVSSTVQVVLEPRD
VRSQIICEVDHVTLDRAPLRGIAHISEFIQVPPTLEISQQPTMVWNVIIVTCQIQKFYPPRFQVTWLENGNIS
RREVPFTLIVNKDGTYNWISCLLVNISALEENMVVTCQVEHDGQAEVIETHTVVVTEHQRVKELKTAGIAKIP
VAVLLGSKILLLIAATVFYMRKKQNA*
>rs219323197_ENSMUSP00000120527_c.232:G>A_p.S78N
ENSMUST00000148194.8|Sirpb1c-207|ENSMUSG00000074677|-|chr3:g.15887212:C>T
XQSGGGTELSVFAKPSSPMVSGPAARAVPQQTVAFTCRSHGFFPRNLTLKWFKNGNEISHLETSVEPEETSVS
YRVSNTVQVVLEPRDVRSQIICEVDHVTLDRAPLRGIAHISEFIQVPPTLEISQQPTMVWNVIIVTCQIQKFY
PPRFQVTWLENGNISRREVPFTLIVNKDGTYNWISCLLVNISALEENMVVTCQVEHDGQAEVIETHTVVVTEH
QRVKGTATKSEAPNGNKC*
>rs219323197_ENSMUSP00000103991_c.625:G>A_p.S209N
ENSMUST00000108354.8|Sirpb1c-205|ENSMUSG00000074677|-|chr3:g.15887212:C>T
MLLLDAWTHIPHCVLLLILLLGLKGAAMRELKVIQPVKSFFVGAGGSATLNCTVTSLLPVGPIRWFKGVGQSR
LLIYSFTGERFPRITNVSDVTKRSNLDFSIRISNVTPADSGTYYCVKFQRGPSEPDMEIQSGGGTELSVFAKP
SSPMVSGPAARAVPQQTVAFTCRSHGFFPRNLTLKWFKNGNEISHLETSVEPEETSVSYRVSNTVQVVLEPRD
VRSQIICEVDHVTLDRAPLRGIAHISEFIQVPPTLEISQQPTMVWNVIIVTCQIQKFYPPRFQVTWLENGNIS
RREVPFTLIVNKDGTYNWISCLLVNISALEENMVVTCQVEHDGQAEVIETHTVVVTEHQRVKEAPNGNKC*
>rs219323197_ENSMUSP00000103989_c.625:G>A_p.S209N
ENSMUST00000108352.9|Sirpb1c-204|ENSMUSG00000074677|-|chr3:g.15887212:C>T
MLLLDAWTHIPHCVLLLILLLGLKGAAMRELKVIQPVKSFFVGAGGSATLNCTVTSLLPVGPIRWFKGVGQSR
LLIYSFTGERFPRITNVSDVTKRSNLDFSIRISNVTPADSGTYYCVKFQRGPSEPDMEIQSGGGTELSVFAKP
SSPMVSGPAARAVPQQTVAFTCRSHGFFPRNLTLKWFKNGNEISHLETSVEPEETSVSYRVSNTVQVVLEPRD
VRSQIICEVDHVTLDRAPLRGIAHISEFIQVPPTLEISQQPTMVWNVIIVTCQIQKFYPPRFQVTWLENGNIS
RREVPFTLIVNKDGTYNWISCLLVNISALEENMVVTCQVEHDGQAEVIETHTVVVTEHQRVKGTATKSELKTA
GIAKIPVAVLLGSKILLLIAATVIYMRKKQNA*
>rs219323197_ENSMUSP00000061216_c.625:G>A_p.S209N
ENSMUST00000050623.11|Sirpb1c-201|ENSMUSG00000074677|-|chr3:g.15887212:C>T
MLLLDAWTHIPHCVLLLILLLGLKGAAMRELKVIQPVKSFFVGAGGSATLNCTVTSLLPVGPIRWFKGVGQSR
LLIYSFTGERFPRITNVSDVTKRSNLDFSIRISNVTPADSGTYYCVKFQRGPSEPDMEIQSGGGTELSVFAKP
SSPMVSGPAARAVPQQTVAFTCRSHGFFPRNLTLKWFKNGNEISHLETSVEPEETSVSYRVSNTVQVVLEPRD
VRSQIICEVDHVTLDRAPLRGIAHISEFIQVPPTLEISQQPTMVWNVIIVTCQIQKFYPPRFQVTWLENGNIS
RREVPFTLIVNKDGTYNWISCLLVNISALEENMVVTCQVEHDGQAEVIETHTVVVTEHQRVKELKTAGIAKIP
VAVLLGSKILLLIAATVIYMRKKQNA*
>rs219323197_ENSMUSP00000103987_c.625:G>A_p.S209N
ENSMUST00000108350.8|Sirpb1c-203|ENSMUSG00000074677|-|chr3:g.15887212:C>T
MLLLDAWTHIPHCVLLLILLLGLKGAAMRELKVIQPVKSFFVGAGGSATLNCTVTSLLPVGPIRWFKGVGQSR
LLIYSFTGERFPRITNVSDVTKRSNLDFSIRISNVTPADSGTYYCVKFQRGPSEPDMEIQSGGGTELSVFAKP
SSPMVSGPAARAVPQQTVAFTCRSHGFFPRNLTLKWFKNGNEISHLETSVEPEETSVSYRVSNTVQVVLEPRD
VRSQIICEVDHVTLDRAPLRGIAHISEFIQVPPTLEISQQPTMVWNVIIVTCQIQKFYPPRFQVTWLENGNIS
RREVPFTLIVNKDGTYNWISCLLVNISALEENMVVTCQVEHDGQAEVIETHTVVVTEHQRVKGTATKSGEVFT
PPLCLNVNWASFFMYKVTFLIIVALS*

>rs219323197_ENSMUSP00000103986_c.619:G>A_p.S207N
ENSMUST00000108349.2|Sirpb1c-202|ENSMUSG00000074677|-|chr3:g.15887212:C>T
MLLLDAWTHIPHCVLLLILLLGLKAMRELKVIQPVKSFFVGAGGSATLNCTVTSLLPVGPIRWFKGVGQSRLL
IYSFTGERFPRITNVSDVTKRSNLDFSIRISNVTPADSGTYYCVKFQRGPSEPDMEIQSGGGTELSVFAKPSS
PMVSGPAARAVPQQTVAFTCRSHGFFPRNLTLKWFKNGNEISHLETSVEPEETSVSYRVSNTVQVVLEPRDVR
SQIICEVDHVTLDRAPLRGIAHISEFIQVPPTLEISQQPTMVWNVIIVTCQIQKFYPPRFQVTWLENGNISRR
EVPFTLIVNKDGTYNWISCLLVNISALEENMVVTCQVEHDGQAEVIETHTVVVTEHQRVKGTATKSGEVFTPP
LCLNVNWASFFMYKVTFLIIVALS*
>ENSMUSP00000120527_c.94:C>T_p.T32I ENSMUST00000148194.8|Sirpb1c-
207|ENSMUSG00000074677|-|chr3:g.15887350:G>A
XQSGGGTELSVFAKPSSPMVSGPAARAVPQQIVAFTCRSHGFFPRNLTLKWFKNGNEISHLETSVEPEETSVS
YRVSSTVQVVLEPRDVRSQIICEVDHVTLDRAPLRGIAHISEFIQVPPTLEISQQPTMVWNVIIVTCQIQKFY
PPRFQVTWLENGNISRREVPFTLIVNKDGTYNWISCLLVNISALEENMVVTCQVEHDGQAEVIETHTVVVTEH
QRVKGTATKSEAPNGNKC*
>ENSMUSP00000103991_c.487:C>T_p.T163I ENSMUST00000108354.8|Sirpb1c-
205|ENSMUSG00000074677|-|chr3:g.15887350:G>A
MLLLDAWTHIPHCVLLLILLLGLKGAAMRELKVIQPVKSFFVGAGGSATLNCTVTSLLPVGPIRWFKGVGQSR
LLIYSFTGERFPRITNVSDVTKRSNLDFSIRISNVTPADSGTYYCVKFQRGPSEPDMEIQSGGGTELSVFAKP
SSPMVSGPAARAVPQQIVAFTCRSHGFFPRNLTLKWFKNGNEISHLETSVEPEETSVSYRVSSTVQVVLEPRD
VRSQIICEVDHVTLDRAPLRGIAHISEFIQVPPTLEISQQPTMVWNVIIVTCQIQKFYPPRFQVTWLENGNIS
RREVPFTLIVNKDGTYNWISCLLVNISALEENMVVTCQVEHDGQAEVIETHTVVVTEHQRVKEAPNGNKC*
>ENSMUSP00000103989_c.487:C>T_p.T163I ENSMUST00000108352.9|Sirpb1c-
204|ENSMUSG00000074677|-|chr3:g.15887350:G>A
MLLLDAWTHIPHCVLLLILLLGLKGAAMRELKVIQPVKSFFVGAGGSATLNCTVTSLLPVGPIRWFKGVGQSR
LLIYSFTGERFPRITNVSDVTKRSNLDFSIRISNVTPADSGTYYCVKFQRGPSEPDMEIQSGGGTELSVFAKP
SSPMVSGPAARAVPQQIVAFTCRSHGFFPRNLTLKWFKNGNEISHLETSVEPEETSVSYRVSSTVQVVLEPRD
VRSQIICEVDHVTLDRAPLRGIAHISEFIQVPPTLEISQQPTMVWNVIIVTCQIQKFYPPRFQVTWLENGNIS
RREVPFTLIVNKDGTYNWISCLLVNISALEENMVVTCQVEHDGQAEVIETHTVVVTEHQRVKGTATKSELKTA
GIAKIPVAVLLGSKILLLIAATVIYMRKKQNA*
>ENSMUSP00000061216_c.487:C>T_p.T163I ENSMUST00000050623.11|Sirpb1c-
201|ENSMUSG00000074677|-|chr3:g.15887350:G>A
MLLLDAWTHIPHCVLLLILLLGLKGAAMRELKVIQPVKSFFVGAGGSATLNCTVTSLLPVGPIRWFKGVGQSR
LLIYSFTGERFPRITNVSDVTKRSNLDFSIRISNVTPADSGTYYCVKFQRGPSEPDMEIQSGGGTELSVFAKP
SSPMVSGPAARAVPQQIVAFTCRSHGFFPRNLTLKWFKNGNEISHLETSVEPEETSVSYRVSSTVQVVLEPRD
VRSQIICEVDHVTLDRAPLRGIAHISEFIQVPPTLEISQQPTMVWNVIIVTCQIQKFYPPRFQVTWLENGNIS
RREVPFTLIVNKDGTYNWISCLLVNISALEENMVVTCQVEHDGQAEVIETHTVVVTEHQRVKELKTAGIAKIP
VAVLLGSKILLLIAATVIYMRKKQNA*
>ENSMUSP00000103987_c.487:C>T_p.T163I ENSMUST00000108350.8|Sirpb1c-
203|ENSMUSG00000074677|-|chr3:g.15887350:G>A
MLLLDAWTHIPHCVLLLILLLGLKGAAMRELKVIQPVKSFFVGAGGSATLNCTVTSLLPVGPIRWFKGVGQSR
LLIYSFTGERFPRITNVSDVTKRSNLDFSIRISNVTPADSGTYYCVKFQRGPSEPDMEIQSGGGTELSVFAKP
SSPMVSGPAARAVPQQIVAFTCRSHGFFPRNLTLKWFKNGNEISHLETSVEPEETSVSYRVSSTVQVVLEPRD
VRSQIICEVDHVTLDRAPLRGIAHISEFIQVPPTLEISQQPTMVWNVIIVTCQIQKFYPPRFQVTWLENGNIS
RREVPFTLIVNKDGTYNWISCLLVNISALEENMVVTCQVEHDGQAEVIETHTVVVTEHQRVKGTATKSGEVFT
PPLCLNVNWASFFMYKVTFLIIVALS*
>ENSMUSP00000103986_c.481:C>T_p.T161I ENSMUST00000108349.2|Sirpb1c-
202|ENSMUSG00000074677|-|chr3:g.15887350:G>A
MLLLDAWTHIPHCVLLLILLLGLKAMRELKVIQPVKSFFVGAGGSATLNCTVTSLLPVGPIRWFKGVGQSRLL
IYSFTGERFPRITNVSDVTKRSNLDFSIRISNVTPADSGTYYCVKFQRGPSEPDMEIQSGGGTELSVFAKPSS
PMVSGPAARAVPQQIVAFTCRSHGFFPRNLTLKWFKNGNEISHLETSVEPEETSVSYRVSSTVQVVLEPRDVR
SQIICEVDHVTLDRAPLRGIAHISEFIQVPPTLEISQQPTMVWNVIIVTCQIQKFYPPRFQVTWLENGNISRR
EVPFTLIVNKDGTYNWISCLLVNISALEENMVVTCQVEHDGQAEVIETHTVVVTEHQRVKGTATKSGEVFTPP
LCLNVNWASFFMYKVTFLIIVALS*
>ENSMUSP00000043492_c.1044:G>T_p.A349S ENSMUST00000037141.9|Setd7-
201|ENSMUSG00000037111|-|chr3:g.51428784:C>A
MDSDDEVVEEAVEGHLDDDGLPHGFCTVTYSSTDRFEGNFVHGEKNGRGKFFFFDGSTLEGYYVDDALQGQGV
YTYEDGGVLQGTYVDGELNGPAQEYDSDGRLIFKGQYKDNNRHGVCWIHYPDGGSLVGEVNEDGEMTGEKIAY
VYPDQRTALYGKFIDGEMLEGKLATLMATEEGRPHFEVTSGSSVYHFDKSTSSCISSDALLPDPYESERVYVA

DSLISSAGEGLFSKVAVGPNTVMSFYNGVRITHQEVDSRDWALNGNTLSLDEETVIDVPEPYNHVSKYCASLG
HKANHSFTPNCVYDLFVHPRFGPIKCIRTLRAVEAEEELTVAYGYDHSPPGKSGPESPEWYQVELKAFQATQQ
K*
>ENSMUSP00000029419_c.814:T>G_p.F272C ENSMUST00000029419.8|Veph1-201|ENSMUSG00000027831|-|chr3:g.66112988:A>C
MHQLFRLVLGQKDLSKAGDLFSLDDAEIEDSLTEALEQIKVISSSLDYQTNNNDQAVVEICITRITTAIRETE
SIEKHARALVGLWDSCLEHNLRPAGKDEDTPHAKIASDIMSCILQNYNRTPVMVLAVPIAVKFLHRGSKELCR
NMSNYLSLAAITKADLLADHTEGIIKSILQGNAMLLRVLPAVYEKQPQPINRHLAELLALMSQLEQTEQYHLL
RLLHVAAKRKDVEVVQKCVPFLIRNLKDSTYNDIILNILIEIAGHEPLALNSCLPMLKEIAEQFPYLTGQMAR
IFGAVGHVDEERARSCLRYLVSQLANMEHPFHHILLLEIKSITDAFSSILGPHSRDIFRMSNSFTNIAKLLSR
QLENSKADSSRRKTSTEVSIPEKLRELNSMEPESEDHEKLQVKIQAFEDKINAESNTPGSGRRYSLDHISKEE
RKSIRFSRSRSLALNTVLTNGVSVEDNEVEEKAGMHASISLSQIDPLSHGIGKLPFKTDTHGSPLRNSSASHP
SIIHTEPETMPETFKENIQEEILEAATSPIEYQDKLYLHLRENLSKVKAYALEIAKKVPIPDQCTIEDTMRSC
VAKLFFTCSLKGHYCLYSKSSFILVSQAPQPWIQVMFLSQQSLFPEPLSIQSGSVQFLKALWEKTQDTGAHSF
EVAMTESTFPQQKDLEQLQLHLEEVRFFDVFGFSETAGAWQCFMCNNPEKATVVNQDGQPLIEGKLKEKQVRW
KFIKRWKTHYFTLAGNQLLFQKGKSKDDPDDSPIELSKVQSVKAVAKKRRDRSLPRAFEIFTDSKTYVFKAKD
EKNAEEWLQCINVALAQAKERESREVTTYL*
>ENSMUSP00000056728_c.123:C>G_p.P42A ENSMUST00000054825.5|Rarres1-201|ENSMUSG00000049404|-|chr3:g.67422625:G>C
MQPRQPPMPALLLSLWLLPSLALAAAVTEPADLEYTEVPRQAAAVGLPRGILQLAARAALHFFNFRAGSPSAL
RVLATVQEGRAWVNPQEGCEVDLVFSTEQYNPEQEGERLGKCSARVFFKNEKPRPAVNVTCARLFDKVKRQEK
DYRLYKQMKQLKTPLHSISIPDSHGHIDNSLRPLWDLAFLGSSYVMWEKTTQVLHYYLVQLSSVERLKTDDDS
IDFDFTVLLHEFSTQEIIPCRIHLVWYPGKPLKVNYHCQEQQSPEEASGTMEASAMAPPEFGNF*
>ENSMUSP00000135168_c.1170:A>C_p.I391L ENSMUST00000175903.9|Arhgef2-207|ENSMUSG00000028059|+|chr3:g.88540665:A>C
MNRSVPGEPFIGEDPVPHGLPQCCPAAVESREQLEEVEGREENQEDNKDGFHTSLLRRSGAFRARSYTTDSFK
RHSWGPGKEFQDPISRRRLQSSECPGSQEAPFLQSLEELGTPLGAQQQEQQQGQEQQRGQPFHRGQSFCYPSA
ASSVPSVTGMLSKAVSMSDINLLSDCSDPNGNSPQSYVTDLSKCGKQLEEDSGTWAGSSLRRTFSFLLGMTGR
DKTREKEKMKEAKDARYTNGHLFTTISVSGMTMCYACNKSITAKEALICPTCNVTIHNRCKDTLANCTKVKQK
QQKAALLRNNTALQSVSLRSKTTTRERPTSAIYPSDSFRQSLLGSRRGLSSLSLAKSVSTTNIAGHFNDESPL
GLRQILSQSTDSLNMRNRTLSVESLLDEEVFYNELMSDFEMDEKDFEADSWSLAVDSSFLQQHKKEVMKKQDV
IYELIQTELHHVRTLKIMTRLFRTGMLEELQMEPEVVQGLFPCVDELSDIHTRFLNQLLERRRQALCPGSTRN
FVIHRLGDLLISQFSGSNAEQMRKTYSEFCSRHTKALKLYKELYARDKRFQQFIRKMTRSAVLKRHGVQECIL
LVTQRITKYPVLINRILQNSHGVEEEYQDLASALGLVKELLSNVDQDVHELEKEARLQEIYNRMDPRAQTPVP
GKGPFGRDELLRRKLIHEGCLLWKTATGRFKDVLLLLMTDVLVFLQEKDQKYIFTSLDKPSVVSLQNLIVRDI
ANQAKGMFLISSGPPEMYEVHAASRDDRTTWIRVIQQSVRLCPSREDFPLIETEDKAYLRRIKTKLQQKNQAL
VELLQKNVELFAEMVHFQALKAGFVGMPPPALPRGLFRLESFESLRGERLLKDALREVEGLKDLLLGPCVDLP
MTSREPALPLDSDSGSCPGVTANGEARTFNGSIELCRADSDSSQKDRNGNQLRSPQEEVLQPLINLYGLLHGL
QAVVVQQERLMEALFPEGPERWEKLSRANSRDGEAGRAAVASVTPEKQATELALLQRQHTLLQEELRRCQRLG
EERATEAGSLEARLRESEQARALLEREAEEIRRQLAALGQNEPLPAEAPWARRPLDPRRRSLPAGDALYLSFN
PPQPSRGHDRLDLPVTVRSLHRPFDDREAQELGSPEDRLQDSSDPDTGSEEEVSSRLSPPHSPRDFTRMQDIP
EETESRDGEPTASES*
>ENSMUSP00000134859_c.519:A>C_p.I174L ENSMUST00000177023.8|Arhgef2-225|ENSMUSG00000028059|+|chr3:g.88540665:A>C
MTGRDKTREKEKMKEAKDARYTNGHLFTTISVSGMTMCYACNKSITAKEALICPTCNVTIHNRCKDTLANCTK
VKQKQQKAALLRNNTALQSVSLRTTTRERPTSAIYPSDSFRQSLLGSRRGLSSLSLAKSVSTTNIAGHFNDES
PLGLRQILSQSTDSLNMRNRTLSVESLLDEGVEVFYNELMSDFEMDEKDFEADSWSLAVDSS
>ENSMUSP00000135612_c.525:A>C_p.I176L ENSMUST00000176539.8|Arhgef2-222|ENSMUSG00000028059|+|chr3:g.88540665:A>C
MTGRDKTREKEKMKEAKDARYTNGHLFTTISVSGMTMCYACNKSITAKEALICPTCNVTIHNRCKDTLANCTK
VKQKQQKAALLRNNTALQSVSLRSKTTTRERPTSAIYPSDSFRQSLLGSRRGLSSLSLAKSVSTTNIAGHFND
ESPLGLRQILSQSTDSLNMRNRTLSVESLLDEGVEVFYNELMSDFEMDEKDFEADSWSLAVDSSFLQQHKKEV
MKKQDVIYELIQTELHHVRTLKIMTRLFRTGMLEELQM
>ENSMUSP00000134840_c.519:A>C_p.I174L ENSMUST00000177498.8|Arhgef2-231|ENSMUSG00000028059|+|chr3:g.88540665:A>C
MTGRDKTREKEKMKEAKDARYTNGHLFTTISVSGMTMCYACNKSITAKEALICPTCNVTIHNRCKDTLANCTK
VKQKQQKAALLRNNTALQSVSLRTTTRERPTSAIYPSDSFRQSLLGSRRGLSSLSLAKSVSTTNIAGHFNDES
PLGLRQILSQSTDSLNMRNRTLSVESLLDEGVEVFYNELMSDFEMDEKDFEADSWSLAVDSSFLQQHKKEVMK

KQDVIYELIQTELHHVRTLKIMTRLFRTGMLEELQMEPEVVQGLFPCVDELSDIHTRFLNQLLERRRQALCPG
STRNFVIHRLGDLLISQFSGSNAEQMRKTYSEFCSRHTKALKLYKELYARDKRFQQFIRKMTRSAVLKRHGVQ
ECILLVTQRITKYPVLINRILQNSHGVEEEYQDLASALGLVKELLSNVDQDVHELEKEARLQEIYNRMDPRAQ
TPVPGKGPFGRDELLRRKLIHEGCLLWKTATGRFKDVLLLLMTDVLVFLQEKDQKYIFTSLDKPSVVSLQNLI
VRDIANQAKGMFLISSGPPEMYEVHAASRDDRTTWIRVIQQSVRLCPSREDFPLIETEDKAYLRRIKTKLQQK
NQALVELLQKNVELFAEMVHFQALKAGFVGMPPPALPRGLFRLESFESLRGERLLKDALREVEGLKDLLLGPC
VDLPMTSREPALPLDSDSGSCPGVTANGEARTFNGSIELCRADSDSSQKDRNGNQLRSPQEEVLQPLINLYGL
LHGLQAVVVQQERLMEALFPEGPERWEKLSRANSRDGEAGRAAVASVTPEKQATELALLQRQHTLLQEELRRC
QRLGEERATEAGSLEARLRESEQARALLEREAEEIRRQLAALGQNEPLPAEAPWARRPLDPRRRSLPAGDALY
LSFNPPQPSRGHDRLDLPVTVRSLHRPFDDREAQELGSPEDRLQDSSDPDTGSEEEVSSRLSPPHSPRDFTRM
QDIPEETESRDGEPTASES*
>ENSMUSP00000134834_c.525:A>C_p.I176L ENSMUST00000176500.8|Arhgef2-
219|ENSMUSG00000028059|+|chr3:g.88540665:A>C
MTGRDKTREKEKMKEAKDARYTNGHLFTTISVSGMTMCYACNKSITAKEALICPTCNVTIHNRCKDTLANCTK
VKQKQQKAALLRNNTALQSVSLRSKTTTRERPTSAIYPSDSFRQSLLGSRRGLSSLSLAKSVSTTNIAGHFND
ESPLGLRQILSQSTDSLNMRNRTLSVESLLDEGVEVFYNELMSDFEMDEKDFEADSWSLAVDSSFLQQHKKEV
MKKQDVIYELIQTELHHVRTLKIMTRLFRTGMLEELQMEPEVVQGLFPCVDELSDIHTRFLNQLLERRRQALC
PGSTRNFVIHRLGDLLISQFSGSNAEQMRKTYSEFCSRHTKALKLYKELYARDKRFQQFIRKMTRSAVLKRHG
VQECILLVTQRITKYPVLINRILQNSHGVEEEYQDLASALGLVKELLSNVDQDVHELEKEARLQEIYNRMDPR
AQTPVPGKGPFGRDELLRRKLIHEGCLLWKTATGRFKDVLLLLMTDVLVFLQEKDQKYIFTSLDKPSVVSLQN
LIVRDIANQAKGMFLISSGPPEMYEVHAASRDDRTTWIRVIQQSVRLCPSREDFPLIETEDKAYLRRIKTKLQ
QKNQALVELLQKNVELFAEMVHFQALKAGFVGMPPPALPRGLFRLESFESLRGERLLKDALREVEGLKDLLLG
PCVDLPMTSREPALPLDSDSGSCPGVTANGEARTFNGSIELCRADSDSSQKDRNGNQLRSPQEEVLQPLINLY
GLLHGLQAVVVQQERLMEALFPEGPERWEKLSRANSRDGEAGRAAVASVTPEKQATELALLQRQHTLLQEELR
RCQRLGEERATEAGSLEARLRESEQARALLEREAEEIRRQLAALGQNEPLPAEAPWARRPLDPRRRSLPAGDA
LYLSFNPPQPSRGHDRLDLPVTVRSLHRPFDDREAQELGSPEDRLQDSSDPDTGSEEEVSSRLSPPHSPRDFT
RMQDIPEETESRDGEPTASES*
>ENSMUSP00000135177_c.525:A>C_p.I176L ENSMUST00000175779.8|Arhgef2-
206|ENSMUSG00000028059|+|chr3:g.88540665:A>C
MTGRDKTREKEKMKEAKDARYTNGHLFTTISVSGMTMCYACNKSITAKEALICPTCNVTIHNRCKDTLANCTK
VKQKQQKAALLRNNTALQSVSLRSKTTTRERPTSAIYPSDSFRQSLLGSRRGLSSLSLAKSVSTTNIAGHFND
ESPLGLRQILSQSTDSLNMRNRTLSVESLLDEGVEVFYNELMSDFEMDEKDFEADSWSLAVDSSFLQQHKKEV
MKKQDVIYELIQTELHHVRTLKIMTRLFRTGMLEELQMEPEVVQGLFPCVDELSDIHTRFLNQLLERRRQALC
PGSTRNFVIHRLGDLLISQFSGSNAEQMRKTYSEFCSRHTKALKLYKELYARDKRFQQFIRKMTRSAVLKRHG
VQECILLVTQRITKYPVLINRILQNSHGVEEEYQDLASALGLVKELLSNVDQDVHELEKEARLQEIYNRMDPR
AQTPVPGKGPFGRDELLRRKLIHEGCLLWKTATGRFKDVLLLLMTDVLVFLQEKDQKYIFTSLDKPSVVSLQN
LIVRDIANQAKGMFLISSGPPEMYEVHAASRDDRTTWIRVIQQSVRLCPSREDFPLIETEDKAYLRRIKTKLQ
QKNQALVELLQKNVELFAEMVHFQALKAGFVGMPPPALPRGLFRLESFESLRGERLLKDALREVEGLKDLLLG
PCVDLPMTSREPALPLDSDSGSCPGVTANGEARTFNGSIELCRADSDSSQKDRNGNQLRSPQEEVLQPLINLY
GLLHGLQAVVVQQERLMEALFPEGPERWEKLSRANSRDGEAGRAAVASVTPEKQATELALLQRQHTLLQEELR
RCQRLGEERATEAGSLEARLRESEQARALLEREAEEIRRQLAALGQNEPLPAEAPWARRPLDPRRRSLPAGDA
LYLSFNPPQPSRGHDRLDLPVTVRSLHRPFDDREAQELGSPEDRLQDSSDPDTGSEEEVSSRLSPPHSPRDFT
RMQDIPEETESRDGEPTASES*
>ENSMUSP00000029694_c.570:A>C_p.I191L ENSMUST00000029694.14|Arhgef2-
201|ENSMUSG00000028059|+|chr3:g.88540665:A>C
MSRIESLTRARIDRSKEQATKTREKEKMKEAKDARYTNGHLFTTISVSGMTMCYACNKSITAKEALICPTCNV
TIHNRCKDTLANCTKVKQKQQKAALLRNNTALQSVSLRSKTTTRERPTSAIYPSDSFRQSLLGSRRGLSSLSL
AKSVSTTNIAGHFNDESPLGLRQILSQSTDSLNMRNRTLSVESLLDEGVEVFYNELMSDFEMDEKDFEADSWS
LAVDSSFLQQHKKEVMKKQDVIYELIQTELHHVRTLKIMTRLFRTGMLEELQMEPEVVQGLFPCVDELSDIHT
RFLNQLLERRRQALCPGSTRNFVIHRLGDLLISQFSGSNAEQMRKTYSEFCSRHTKALKLYKELYARDKRFQQ
FIRKMTRSAVLKRHGVQECILLVTQRITKYPVLINRILQNSHGVEEEYQDLASALGLVKELLSNVDQDVHELE
KEARLQEIYNRMDPRAQTPVPGKGPFGRDELLRRKLIHEGCLLWKTATGRFKDVLLLLMTDVLVFLQEKDQKY
IFTSLDKPSVVSLQNLIVRDIANQAKGMFLISSGPPEMYEVHAASRDDRTTWIRVIQQSVRLCPSREDFPLIE
TEDKAYLRRIKTKLQQKNQALVELLQKNVELFAEMVHFQALKAGFVGMPPPALPRGLFRLESFESLRGERLLK
DALREVEGLKDLLLGPCVDLPMTSREPALPLDSDSGSCPGVTANGEARTFNGSIELCRADSDSSQKDRNGNQL
RSPQEEVLQPLINLYGLLHGLQAVVVQQERLMEALFPEGPERWEKLSRANSRDGEAGRAAVASVTPEKQATEL
ALLQRQHTLLQEELRRCQRLGEERATEAGSLEARLRESEQARALLEREAEEIRRQLAALGQNEPLPAEAPWAR

RPLDPRRRSLPAGDALYLSFNPPQPSRGHDRLDLPVTVRSLHRPFDDREAQELGSPEDRLQDSSDPDTGSEEE
VSSRLSPPHSPRDFTRMQDIPEETESRDGEPTASES*
>ENSMUSP00000135397_c.570:A>C_p.I191L ENSMUST00000176804.8|Arhgef2-223|ENSMUSG00000028059|+|chr3:g.88540665:A>C
MSRIESLTRARIDRSKEQATKTREKEKMKEAKDARYTNGHLFTTISVSGMTMCYACNKSITAKEALICPTCNV
TIHNRCKDTLANCTKVKQKQQKAALLRNNTALQSVSLRSKTTTRERPTSAIYPSDSFRQSLLGSRRGLSSLSL
AKSVSTTNIAGHFNDESPLGLRQILSQSTDSLNMRNRTLSVESLLDEEVFYNELMSDFEMDEKDFEADSWSLA
VDSSFLQQHKKEVMKKQDVIYELIQTELHHVRTLKIMTRLFRTGMLEELQMEPEVVQGLFPCVDELSDIHTRF
LNQLLERRRQALCPGSTRNFVIHRLGDLLISQFSGSNAEQMRKTYSEFCSRHTKALKLYKELYARDKRFQQFI
RKMTRSAVLKRHGVQECILLVTQRITKYPVLINRILQNSHGVEEEYQDLASALGLVKELLSNVDQDVHELEKE
ARLQEIYNRMDPRAQTPVPGKGPFGRDELLRRKLIHEGCLLWKTATGRFKDVLLLLMTDVLVFLQEKDQKYIF
TSLDKPSVVSLQNLIVRDIANQAKGMFLISSGPPEMYEVHAASRDDRTTWIRVIQQSVRLCPSREDFPLIETE
DKAYLRRIKTKLQQKNQALVELLQKNVELFAEMVHFQALKAGFVGMPPPALPRGLFRLESFESLRGERLLKDA
LREVEGLKDLLLGPCVDLPMTSREPALPLDSDSGSCPGVTANGEARTFNGSIELCRADSDSSQKDRNGNQLRS
PQEEVLQPLINLYGLLHGLQAVVVQQERLMEALFPEGPERWEKLSRANSRDGEAGRAAVASVTPEKQATELAL
LQRQHTLLQEELRRCQRLGEERATEAGSLEARLRESEQARALLEREAEEIRRQLAALGQNEPLPAEAPWARRP
LDPRRRSLPAGDALYLSFNPPQPSRGHDRLDLPVTVRSLHRPFDDREAQELGSPEDRLQDSSDPDTGSEEEVS
SRLSPPHSPRDFTRMQDIPEETESRDGEPTASES*
>ENSMUSP00000103134_c.489:A>C_p.I164L ENSMUST00000107510.10|Arhgef2-202|ENSMUSG00000028059|+|chr3:g.88540665:A>C
MKEAKDARYTNGHLFTTISVSGMTMCYACNKSITAKEALICPTCNVTIHNRCKDTLANCTKVKQKQQKAALLR
NNTALQSVSLRSKTTTRERPTSAIYPSDSFRQSLLGSRRGLSSLSLAKSVSTTNIAGHFNDESPLGLRQILSQ
STDSLNMRNRTLSVESLLDEGVEVFYNELMSDFEMDEKDFEADSWSLAVDSSFLQQHKKEVMKKQDVIYELIQ
TELHHVRTLKIMTRLFRTGMLEELQMEPEVVQGLFPCVDELSDIHTRFLNQLLERRRQALCPGSTRNFVIHRL
GDLLISQFSGSNAEQMRKTYSEFCSRHTKALKLYKELYARDKRFQQFIRKMTRSAVLKRHGVQECILLVTQRI
TKYPVLINRILQNSHGVEEEYQDLASALGLVKELLSNVDQDVHELEKEARLQEIYNRMDPRAQTPVPGKGPFG
RDELLRRKLIHEGCLLWKTATGRFKDVLLLLMTDVLVFLQEKDQKYIFTSLDKPSVVSLQNLIVRDIANQAKG
MFLISSGPPEMYEVHAASRDDRTTWIRVIQQSVRLCPSREDFPLIETEDKAYLRRIKTKLQQKNQALVELLQK
NVELFAEMVHFQALKAGFVGMPPPALPRGLFRLESFESLRGERLLKDALREVEGLKDLLLGPCVDLPMTSREP
ALPLDSDSGSCPGVTANGEARTFNGSIELCRADSDSSQKDRNGNQLRSPQEEVLQPLINLYGLLHGLQAVVVQ
QERLMEALFPEGPERWEKLSRANSRDGEAGRAAVASVTPEKQATELALLQRQHTLLQEELRRCQRLGEERATE
AGSLEARLRESEQARALLEREAEEIRRQLAALGQNEPLPAEAPWARRPLDPRRRSLPAGDALYLSFNPPQPSR
GHDRLDLPVTVRSLHRPFDDREAQELGSPEDRLQDSSDPDTGSEEEVSSRLSPPHSPRDFTRMQDIPEETESR
DGEPTASES*
>ENSMUSP00000127843_c.483:A>C_p.I162L ENSMUST00000170653.9|Arhgef2-203|ENSMUSG00000028059|+|chr3:g.88540665:A>C
MKEAKDARYTNGHLFTTISVSGMTMCYACNKSITAKEALICPTCNVTIHNRCKDTLANCTKVKQKQQKAALLR
NNTALQSVSLRTTTRERPTSAIYPSDSFRQSLLGSRRGLSSLSLAKSVSTTNIAGHFNDESPLGLRQILSQST
DSLNMRNRTLSVESLLDEGVEVFYNELMSDFEMDEKDFEADSWSLAVDSSFLQQHKKEVMKKQDVIYELIQTE
LHHVRTLKIMTRLFRTGMLEELQMEPEVVQGLFPCVDELSDIHTRFLNQLLERRRQALCPGSTRNFVIHRLGD
LLISQFSGSNAEQMRKTYSEFCSRHTKALKLYKELYARDKRFQQFIRKMTRSAVLKRHGVQECILLVTQRITK
YPVLINRILQNSHGVEEEYQDLASALGLVKELLSNVDQDVHELEKEARLQEIYNRMDPRAQTPVPGKGPFGRD
ELLRRKLIHEGCLLWKTATGRFKDVLLLLMTDVLVFLQEKDQKYIFTSLDKPSVVSLQNLIVRDIANQAKGMF
LISSGPPEMYEVHAASRDDRTTWIRVIQQSVRLCPSREDFPLIETEDKAYLRRIKTKLQQKNQALVELLQKNV
ELFAEMVHFQALKAGFVGMPPPALPRGLFRLESFESLRGERLLKDALREVEGLKDLLLGPCVDLPMTSREPAL
PLDSDSGSCPGVTANGEARTFNGSIELCRADSDSSQKDRNGNQLRSPQEEVLQPLINLYGLLHGLQAVVVQQE
RLMEALFPEGPERWEKLSRANSRDGEAGRAAVASVTPEKQATELALLQRQHTLLQEELRRCQRLGEERATEAG
SLEARLRESEQARALLEREAEEIRRQLAALGQNEPLPAEAPWARRPLDPRRRSLPAGDALYLSFNPPQPSRGH
DRLDLPVTVRSLHRPFDDREAQELGSPEDRLQDSSDPDTGSEEEVSSRLSPPHSPRDFTRMQDIPEETESRDG
EPTASES*
>ENSMUSP00000135131_c.489:A>C_p.I164L ENSMUST00000177303.8|Arhgef2-229|ENSMUSG00000028059|+|chr3:g.88540665:A>C
MKEAKDARYTNGHLFTTISVSGMTMCYACNKSITAKEALICPTCNVTIHNRCKDTLANCTKVKQKQQKAALLR
NNTALQSVSLRSKTTTRERPTSAIYPSDSFRQSLLGSRRGLSSLSLAKSVSTTNIAGHFNDESPLGLRQILSQ
STDSLNMRNRTLSVESLLDEEVFYNELMSDFEMDEKDFEADSWSLAVDSSFLQQHKKEVMKKQDVIYELIQTE
LHHVRTLKIMTRLFRTGMLEELQMEPEVVQGLFPCVDELSDIHTRFLNQLLERRRQALCPGSTRNFVIHRLGD
LLISQFSGSNAEQMRKTYSEFCSRHTKALKLYKELYARDKRFQQFIRKMTRSAVLKRHGVQECILLVTQRITK
YPVLINRILQNSHGVEEEYQDLASALGLVKELLSNVDQDVHELEKEARLQEIYNRMDPRAQTPVPGKGPFGRD

ELLRRKLIHEGCLLWKTATGRFKDVLLLLMTDVLVFLQEKDQKYIFTSLDKPSVVSLQNLIVRDIANQAKGMF
LISSGPPEMYEVHAASRDDRTTWIRVIQQSVRLCPSREDFPLIETEDKAYLRRIKTKLQQKNQALVELLQKNV
ELFAEMVHFQALKAGFVGMPPPALPRGLFRLESFESLRGERLLKDALREVEGLKDLLLGPCVDLPMTSREPAL
PLDSDSGSCPGVTANGEARTFNGSIELCRADSDSSQKDRNGNQLRSPQEEVLQPLINLYGLLHGLQAVVVQQE
RLMEALFPEGPERWEKLSRANSRDGEAGRAAVASVTPEKQATELALLQRQHTLLQEELRRCQRLGEERATEAG
SLEARLRESEQARALLEREAEEIRRQLAALGQNEPLPAEAPWARRPLDPRRRSLPAGDALYLSFNPPQPSRGH
DRLDLPVTVRSLHRPFDDREAQELGSPEDRLQDSSDPDTGSEEEVSSRLSPPHSPRDFTRMQDIPEETESRDG
EPTASES*
>ENSMUSP00000135428_c.546:A>C_p.I183L ENSMUST00000175911.8|Arhgef2-208|ENSMUSG00000028059|+|chr3:g.88540665:A>C
MSGNRRQPSRRGQTREKEKMKEAKDARYTNGHLFTTISVSGMTMCYACNKSITAKEALICPTCNVTIHNRCKD
TLANCTKVKQKQQKAALLRNNTALQSVSLRSKTTTRERPTSAIYPSDSFRQSLLGSRRGLSSLSLAKSVSTTN
IAGHFNDESPLGLRQILSQSTDSLNMRNRTLSVESLLDEGVEVFYNELMSDFEMDEKDFEADSWSLAVDSSFL
QQHKKEVMKKQDVIYELIQTELHHVRTLKIMTRLFRTGMLEELQMEPEVVQGLFPCVDELSDIHTRFLNQLLE
RRRQALCPGSTRNFVIHRLGDLLISQFSGSNAEQMRKTYSEFCSRHTKALKLYKELYARDKRFQQFIRKMTRS
AVLKRHGVQECILLVTQRITKYPVLINRILQNSHGVEEEYQDLASALGLVKELLSNVDQDVHELEKEARLQEI
YNRMDPRAQTPVPGKGPFGRDELLRRKLIHEGCLLWKTATGRFKDVLLLLMTDVLVFLQEKDQKYIFTSLDKP
SVVSLQNLIVRDIANQAKGMFLISSGPPEMYEVHAASRDDRTTWIRVIQQSVRLCPSREDFPLIETEDKAYLR
RIKS*
>ENSMUSP00000135771_c.483:A>C_p.I162L ENSMUST00000176243.8|Arhgef2-212|ENSMUSG00000028059|+|chr3:g.88540665:A>C
MKEAKDARYTNGHLFTTISVSGMTMCYACNKSITAKEALICPTCNVTIHNRCKDTLANCTKVKQKQQKAALLR
NNTALQSVSLRTTTRERPTSAIYPSDSFRQSLLGSRRGLSSLSLAKSVSTTNIAGHFNDESPLGLRQILSQST
DSLNMRNRTLSVESLLDEGVEVFYNELMSDFEMDEKDFEADSWSLAVDSSFLQQHKK
>ENSMUSP00000140251_c.876:G>A_p.A293T ENSMUST00000186583.7|Ash1l-202|ENSMUSG00000028053|+|chr3:g.88888999:G>A
MDPRNTAMLGLGSDSEGFSRKSPSTINPGTLASKREAEIEGATEEEDPRKRNRERGTEAGKEDGSTDAQQQFS
VKETNFSEGNLKLKIGLQAKRTKKPPKNLENYVCRPAIKTTIKHSRKALKSGKMTDEKNEHCPSKWDSSKLFK
KAGDATAIDCQAEESIHLHSQGESNPLSKKLSPVHSQMADYISAAPSLVGSRDPDIKDRALLNGGTSVTEKLA
QLIATCPPSKSSKAKPKKLGTGTTVGLVSKDLIRKPGVGSIAGIIHKDLIKKPALSTAVGLVTKDPGKKPMFN
TAVGLINKDSVKKLGTGTTAVFINKDLGKKPGAITTVGLLSKESGKKLGIGIVPGLVNKESGKKLGLGTVVGL
VNKELGKKLSSTVGLVAKDVTKKIVASSAMGLVNKDIGKKLLNCPMAGQLGSKDALNLKSEALLPTQEQLKAS
CSANISNHDSQELPESLKDSATGKAFEKSVMRHSKESMLEKFSVRKEITNLEKEMFNEGTCIQQDNFSSSERG
AFETSKHEKQPPVYCTSPDFQIGGASDASTAKSPFSAVGESNLPSSSPTVSVNPVTRSPPEASSQLVPNPLLL
NSTAEQMEEISESIGKSQFTAESTHLNVGHRSLGHSLSIECKGIDKELNESKNTHLDIPRISSSLGKKPSLTS
DSGIHAITPSVVNFTSLFSNKPFLKLGAVTAPDKHCQVAESLSSSFQSKPLKKRKGRKPRWTKVVARSTCRSP
KGLDLERSELFKNVSCSSLSNSSEPAKFMKTIGASSFVDHDFLKRRLPKLSKSSAPSLALLTDSEKPSHKSFI
THKLSSSMCVTSDLLSDIYKPKRGRPKSKEMPQLEGPPKRTLKIPASKVFSLQSKEEQEPPILQPEIEIPSFK
QSLSVSPFPKKRGRPKRQMRSPVKMKPPVLSVAPFVATESPSKLESESENHRSSSDFFESEDQLQDTDDLDDS
HRQSVCSMSDLEMEPDKKISKRNNGQLMKTIIRKINKMKTLKRKKLLNQILSSSVESSNKGKVQSKLHNTVSS
LAATFGSKLGQQINVSKKGTIYIGKRRGRKPKTVLNGLLSGSPASLAVLEQTAQQAAGSALGQILPPLLPSPA
SSSEILPSPICSQSSGTSGGQSPVSSDAGFVEPSSVPYLHVHSRQGSMIQTLAMKKASKGRRRLSPPTLLPNS
PSHLSELTSLKEATPSPVSESHSDETIPSDSGIGTDNNSTSDRAEKFCGQKKRRHSFEHISLIPPETSTVLNS
LKEKHKHKCKRRSHDYLSYDKMKRQKRKRKKKYPQLRNRQDPDFIAELEELISRLSEIRITHRSHHFIPRDLL
PTIFRINFNSFYTHPSFPLDPLHYIRKPDLKKKRGRPPKMREAMAEMPFMHSLSFPLSSTGFYPSYGMPYSPS
PLTAAPIGLGYYGRYPPTLYPPPPSPSFTTPLPPPSYMHAGHLLLNPTKYHKKKHKLLRQEAFLTTSRTPLLS
MSTYPSVPPEMAYGWMVEHKHRHRHKHREHRSEQPQVSMDSGSSRSVLESLKRYRFGKDTVGDRYKHKEKHRC
HMSCPHLSPSKNLINREEQWVSREPSESSSLALGLQTPLQIDCSESSPSLSLGGFTPNSEPASSDEHMNLFTS
AIGSCRVSNPNSSCRKKLTDSPGLFPVQDTALNRPHRKEPLPSSERAIQSLAGSQSASDKPSQRSSESTNCSP
TRKRSSSESTSSTVNGVPSRSPRLVASMDDSVDSLLQRIVHHDEQESMEKNGDASITTVSAPPSSSPGHSYSK
ERALGKSDSLLVPAVPNDSCSNIPLLSEKSASRCSPHHIKRSVVEAMQRQARKMCNYDKILATKKNLDHVNKI
LKAKKLQRQARTGNNFVKRRPGRPRKCPLQAVVSMQAFQAAQFVSPELNEGEDMSLHLSPDTVTDVIEAVVQS
VNLTSEHKKGVKRKNWLLEEQTRKKQKTVPEEEEQENNKSFIETPVEIPSPLETPAEPSEPENTLQPVLALIP
REKKAPRPPKKKYQRAGLYSDVYKTIDPKSRLIQLKKEKLEYTPGEHEYGLFPAPIHVGKYLRQKRIDFQLPY
DILWQWKHNQLYKKPDVPLYKKIRSNVYVDVKPLSGYEATTCNCKKPDDDTRKGCGDDCLNRMIFAECSPNTC
PCGEQCCNQRIQRHEWVQCLERFRAEEKGWGIRTKEPLKAGQFIIEYLGEVVSEQEFRNRMIEQYHNHSDHYC
LNLDSGMVIDSYRMGNEARFINHSCDPNCEMQKWSVNGVYRIGLYALKDMPAGTELTYDYNFHSFNVEKQQLC
KCGFEKCRGIIGGKSQRMNGLPSHKGSQSSSTHRKSARAKEKRKSKHKLKKRKGHPSEEPSENINTPTRLTPQ

LQMKPMSNRERNFVLKHHVFLVRNWEKIHQKQEEVKHTRDIHSASLYTRWNGLCRDDGNIKSDVFMTQFSALQ
TARSVRTRRLAAAEENLEVARAARLAQIFKEICDGIISYRDSSQQTLAAPLLNLPPKKKNADYYEKISDPLDL
STIEKQILIGYYKTVEAFDADMLKVFRNAEKYYGRKSPIGRDVCRLRKAYYSARHEASAQIDEIVGETASEAD
SSETSVSEKESGHEKDDDVIRCICGLYKDEGLMIQCDKCMVWQHCDCMGVNTDVEHYLCEQCDPRPVDREVPM
IPRPHYAQPGCVYFICLLRDDLLLRQGDCVYLMRDSRRTPDGHPVRQSYRLLSHINRDKLDIFRIEKLWKNEK
EERFAFGHHYFRPHETHHSPSRRFYHNELFRVPLYEIIPLEAVVGTCCVLDLYTYCKGRPKGIKEQDVYICDY
RLDKSAHLFYKIHRNRYPVCTKPYAFDHFPKKLTPKRDFSPHYVPDNYKRNGGRSSWKSERSKPPLKDLGQED
DALPLIEEVLASQEQAAREVPSPEEPDQERATGDIGDAEKKPEESSQEAQLASTPEERRHSQRERLNQILLNL
LEKIPGKNAIDVTYLLEEGSGRKLRRRTLFIPENSFRK*
>ENSMUSP00000088451_c.876:G>A_p.A293T ENSMUST00000090933.5|Ash1l-201|ENSMUSG00000028053|+|chr3:g.88888999:G>A
MDPRNTAMLGLGSDSEGFSRKSPSTINPGTLASKREAEIEGATEEEDPRKRNRERGTEAGKEDGSTDAQQQFS
VKETNFSEGNLKLKIGLQAKRTKKPPKNLENYVCRPAIKTTIKHSRKALKSGKMTDEKNEHCPSKWDSSKLFK
KAGDATAIDCQAEESIHLHSQGESNPLSKKLSPVHSQMADYISAAPSLVGSRDPDIKDRALLNGGTSVTEKLA
QLIATCPPSKSSKAKPKKLGTGTTVGLVSKDLIRKPGVGSIAGIIHKDLIKKPALSTAVGLVTKDPGKKPMFN
TAVGLINKDSVKKLGTGTTAVFINKDLGKKPGAITTVGLLSKESGKKLGIGIVPGLVNKESGKKLGLGTVVGL
VNKELGKKLSSTVGLVAKDVTKKIVASSAMGLVNKDIGKKLLNCPMAGQLGSKDALNLKSEALLPTQEQLKAS
CSANISNHDSQELPESLKDSATGKAFEKSVMRHSKESMLEKFSVRKEITNLEKEMFNEGTCIQQDNFSSSERG
AFETSKHEKQPPVYCTSPDFQIGGASDASTAKSPFSAVGESNLPSSSPTVSVNPVTRSPPEASSQLVPNPLLL
NSTAEQMEEISESIGKSQFTAESTHLNVGHRSLGHSLSIECKGIDKELNESKNTHLDIPRISSSLGKKPSLTS
DSGIHAITPSVVNFTSLFSNKPFLKLGAVTAPDKHCQVAESLSSSFQSKPLKKRKGRKPRWTKVVARSTCRSP
KGLDLERSELFKNVSCSSLSNSSEPAKFMKTIGASSFVDHDFLKRRLPKLSKSSAPSLALLTDSEKPSHKSFI
THKLSSSMCVTSDLLSDIYKPKRGRPKSKEMPQLEGPPKRTLKIPASKVFSLQSKEEQEPPILQPEIEIPSFK
QSLSVSPFPKKRGRPKRQMRSPVKMKPPVLSVAPFVATESPSKLESESENHRSSSDFFESEDQLQDTDDLDDS
HRQSVCSMSDLEMEPDKKISKRNNGQLMKTIIRKINKMKTLKRKKLLNQILSSSVESSNKGKVQSKLHNTVSS
LAATFGSKLGQQINVSKKGTIYIGKRRGRKPKTVLNGLLSGSPASLAVLEQTAQQAAGSALGQILPPLLPSPA
SSSEILPSPICSQSSGTSGGQSPVSSDAGFVEPSSVPYLHVHSRQGSMIQTLAMKKASKGRRRLSPPTLLPNS
PSHLSELTSLKEATPSPVSESHSDETIPSDSGIGTDNNSTSDRAEKFCGQKKRRHSFEHISLIPPETSTVLNS
LKEKHKHKCKRRSHDYLSYDKMKRQKRKRKKKYPQLRNRQDPDFIAELEELISRLSEIRITHRSHHFIPRDLL
PTIFRINFNSFYTHPSFPLDPLHYIRKPDLKKKRGRPPKMREAMAEMPFMHSLSFPLSSTGFYPSYGMPYSPS
PLTAAPIGLGYYGRYPPTLYPPPPSPSFTTPLPPPSYMHAGHLLLNPTKYHKKKHKLLRQEAFLTTSRTPLLS
MSTYPSVPPEMAYGWMVEHKHRHRHKHREHRSEQPQVSMDSGSSRSVLESLKRYRFGKDTVGDRYKHKEKHRC
HMSCPHLSPSKNLINREEQWVSREPSESSSLALGLQTPLQIDCSESSPSLSLGGFTPNSEPASSDEHMNLFTS
AIGSCRVSNPNSSCRKKLTDSPGLFPVQDTALNRPHRKEPLPSSERAIQSLAGSQSASDKPSQRSSESTNCSP
TRKRSSSESTSSTVNGVPSRSPRLVASMDDSVDSLLQRIVHHDEQESMEKNGDASITTVSAPPSSSPGHSYSK
ERALGKSDSLLVPAVPNDSCSNIPLLSEKSASRCSPHHIKRSVVEAMQRQARKMCNYDKILATKKNLDHVNKI
LKAKKLQRQARTGNNFVKRRPGRPRKCPLQAVVSMQAFQAAQFVSPELNEGEDMSLHLSPDTVTDVIEAVVQS
VNLTSEHKKGVKRKNWLLEEQTRKKQKTVPEEEEQENNKSFIETPVEIPSPLETPAEPSEPENTLQPVLALIP
REKKAPRPPKKKYQRAGLYSDVYKTIDPKSRLIQLKKEKLEYTPGEHEYGLFPAPIHVGKYLRQKRIDFQLPY
DILWQWKHNQLYKKPDVPLYKKIRSNVYVDVKPLSGYEATTCNCKKPDDDTRKGCGDDCLNRMIFAECSPNTC
PCGEQCCNQRIQRHEWVQCLERFRAEEKGWGIRTKEPLKAGQFIIEYLGEVVSEQEFRNRMIEQYHNHSDHYC
LNLDSGMVIDSYRMGNEARFINHSCDPNCEMQKWSVNGVYRIGLYALKDMPAGTELTYDYNFHSFNVEKQQLC
KCGFEKCRGIIGGKSQRMNGLPSHKGSQSSSTHRKSARAKEKRKSKHKLKKRKGHPSEEPSENINTPTRLTPQ
LQMKPMSNRERNFVLKHHVFLVRNWEKIHQKQEEVKHTRDIHSASLYTRWNGLCRDDGNIKSDVFMTQFSALQ
TARSVRTRRLAAAEENLEVARAARLAQIFKEICDGIISYRDSSQQTLAAPLLNLPPKKKNADYYEKISDPLDL
STIEKQILIGYYKTVEAFDADMLKVFRNAEKYYGRKSPIGRDVCRLRKAYYSARHEASAQIDEIVGETASEAD
SSETSVSEKESGHEKDDDVIRCICGLYKDEGLMIQCDKCMVWQHCDCMGVNTDVEHYLCEQCDPRPVDREVPM
IPRPHYAQPGCVYFICLLRDDLLLRQGDCVYLMRDSRRTPDGHPVRQSYRLLSHINRDKLDIFRIEKLWKNEK
EERFAFGHHYFRPHETHHSPSRRFYHNELFRVPLYEIIPLEAVVGTCCVLDLYTYCKGRPKGIKEQDVYICDY
RLDKSAHLFYKIHRNRYPVCTKPYAFDHFPKKLTPKRDFSPHYVPDNYKRNGGRSSWKSERSKPPLKDLGQED
DALPLIEEVLASQEQAAREVPSPEEPDQERATGDIGDAEKKPEESSQEAQLASTPEERRHSQRERLNQILLNL
LEKIPGKNAIDVTYLLEEGSGRKLRRRTLFIPENSFRK*
>ENSMUSP00000142428_c.186:G>A_p.G63R ENSMUST00000199538.5|S100a14-203|ENSMUSG00000042306|+|chr3:g.90435394:G>A
MGQCRSANAEDAQEFSDVERAIETLIKNFHKYSVAGKKETLTPAELRDLVTQQLPHLMPSNCRLEEKIANLGN
CNDSKLEFGSFWELIGEAAKSVKMERPVTRS*
>ENSMUSP00000129862_c.39:G>A_p.G14R ENSMUST00000164481.7|S100a14-201|ENSMUSG00000042306|+|chr3:g.90435394:G>A

MGQCRSANAESNCRLEEKIANLGNCNDSKLEFGSFWELIGEAAKSVKMERPVTRS*
>ENSMUSP00000126821_c.186:G>A_p.G63R ENSMUST00000167598.6|S100a14-202|ENSMUSG00000042306|+|chr3:g.90435394:G>A
MGQCRSANAEDAQEFSDVERAIETLIKNFHKYSVAGKKETLTPAELRDLVTQQLPHLMPSNCRLEEKIANLGN
CNDSKLEFGSFWELIGEAAKSVKMERPVTRS*
>ENSMUSP00000088366_c.650:T>A_p.F217L ENSMUST00000090853.5|Gm5773-201|ENSMUSG00000068879|+|chr3:g.93680980:T>A
MSGDMEAKIWGSTQISVKEFCYEWTISNFSFCMGGIRRKIKSPVFSLVANEEVAWCLRVHPNGFDEESKDYLS
VYLVLVNCPKRQVRAKFEFWIKNSQGEKYQYTQSLNVPSFQRKQNWGFSKFILRDSLLSHRNWLLPKDKLTLC
CKVSIVGAILNMPGQNMIPAIKDPRHMLTDDLGKLWENPLFTDCSLLVAGHEIRAHKAILAARSPVFRAMLEH
QMEERLANCFEIQELDFQVFKEMMDFIYTGKAPTLHSHSMACDVLAAAEKYGLEGLKVICEDSLCRNLSVENA
AHTLIVADLHSTEQLKTRALHFIAVHASEVSKSSGWKSMVESHPHLVDERFHSLASAQSVFLESSFKCLKGF*
>ENSMUSP00000089793_c.4394:A>T_p.Q1465H ENSMUST00000092155.12|Col11a1-201|ENSMUSG00000027966|+|chr3:g.113999472:A>T
MEPWSRWKTKRWIWDLTISTLALTFLFQAREVRGAAPVDILKALDFHNSPVGISKTTGFCTNRKNSKDPDVAY
RVTEEAQISAPTKQLFPGGIFPQDFSILFTIKPKKGTQAFLLSLYNEHGIQQLGVEVGRSPVFLFEDHTGKPT
PENYPLFSTVNIADGKWHRVAISVEKKTVTMIVDCKKKITKPLDRSERSIVDTNGIMVFGTRILETDVFQGDI
QQFLITGDPKAAYDYCDHYSPDCDLTSKAAQAQEPHIDEYAPEDIIEYDYEYGETDYKEAESVTEMPTFTEET
VAQTEANIVDDFQDYNYGTMEPYQTETPRRVSGSNEPNPVEEGFTEEYLTGEDYDVQRNTSEDILYGNKGVDG
RDSDLLVDGDLGEYDFYEYKEYEERTTTSPNEEFGPGVPAETDFTETSINGHGAYGEKGQKGEPAVVEPGMLV
EGPPGPAGPAGLMGPPGLQGPSGLPGDPGDRGPPGRPGLPGADGLPGPPGTMLMLPFRYGGDGSKGPTISAQE
AQAQAILQQARIALRGPPGPMGLTGRPGPVGGPGSAGAKGESGDPGPQGPRGVQGPPGPTGKPGKRGRPGADG
GRGMPGESGSKGDRGFDGLPGLPGDKGHRGERGPQGPPGLPGDDGMRGEDGEIGPRGLPGEAGPRGLLGPRGT
PGPPGQPGIGGIDGPQGPKGNMGPQGEPGPPGQQGNPGPQGLPGPQGPIGPPGEKGPQGKPGLAGLPGADGPP
GHPGKEGQSGEKGALGPPGPQGPIGYPGPRGVKGADGVRGLKGSKGEKGEDGFPGFKGDMGLKGDRGEVGQVG
PRGEDGPEGPKGRAGPTGDPGPSGQAGEKGKLGVPGLPGYPGRQGPKGSTGFPGFPGANGEKGARGIAGKPGP
RGQRGPTGPRGSRGARGPTGKPGPKGTSGGDGPPGPPGERGPQGPQGPVGFPGPKGPPGPAGKDGLPGHPGQR
GETGFQGKTGPPGPGGVVGPQGPTGETGPIGERGHPGPPGPPGEQGLPGAAGKEGAKGDPGPQGISGKDGPAG
IRGFPGERGLPGAQGAPGLKGGEGPQGPQGPVGSPGERGSAGTAGPIGLPGRPGPQGPPGPAGEKGAPGEKGP
QGPAGRDGVQGPVGLPGPAGPAGSPGEDGDKGEIGEPGQKGSKGDKGENGPPGPPGLQGPVGAPGIAGGDGEP
GPRGQQGMFGQKGDEGARGFPGLPGPIGLQGLPGPPGEKGENGDVGPMGPPGPPGPRGPQGPNGADGPQGPPG
SIGSVGVVGDKGEPGEAGNPGPPGEAGSGGLKGERGEKGEAGPPGAAGPAGIKGPPGDDGPKGNPGPVGFPGD
PGPPGEPGPAGQDGVGGDKGEDGDPGQPGPPGPSGEAGPPGPPGKRGPPGASGSEGRQGEKGAKGEAGAEGPP
GKTGPVGPQGPSGKPGPEGLRGIPGPVGEQGLPGAAGQDGPPGPLGPPGLPGLKGDPGSKGEKGHPGLIGLIG
PPGEHGEKGDRGLPGTQGSPGAKGDGGIPGPAGPIGPPGPPGLPGPAGPKGNKGSSGPTGQKGDSGMPGPPGP
PGPPGEVIQPLPILSPKKTRRHTESIQGDAGDNILDYSDGMEEIFGSLNSLKQDIEHMKFPMGTQTNPARTCK
DLQLSHPDFPDGEYWIDPNQGCSGDSFKVYCNFTAGGETCIYPDKKSEGVRISSWPKEKPGSWYSEFKRGKLL
SYLDVEGNSINMVQMTFLKLLTASARQNFTYNCHQSAAWYDVLSGSYDKALRFLGSNDEEMSYENNPHIKALY
DGCASRKGYEKTVIEINTPKIDQVPIIDVMINDFGDQNQKFGFEVGPACFLG*
>ENSMUSP00000138879_c.974:A>T_p.Q325H ENSMUST00000184978.2|Col11a1-204|ENSMUSG00000027966|+|chr3:g.113999472:A>T
KGENGPPGPPGLQGPVGAPGIAGGDGEPGPRGQQGMFGQKGDEGARGFPGLPGPIGLQGLPGPPGEKGENGDV
GPMGPPGPPGPRGPQGPNGADGPQGPPGSIGSVGVVGDKGEPGEAGNPGPPGEAGSGGLKGERGEKGEAGPPG
AAGPAGIKGPPGDDGPKGNPGPVGFPGDPGPPGEPGPAGQDGVGGDKGEDGDPGQPGPPGPSGEAGPPGPPGK
RGPPGASGSEGRQGEKGAKGEAGAEGPPGKTGPVGPQGPSGKPGPEGLRGIPGPVGEQGLPGAAGQDGPPGPL
GPPGLPGLKGDPGSKGEKGHPGLIGLIGPPGEHGEKGDRGLPGTQGSPGAKGDGGIPGPAGPIGPPGPPGLPG
PAGPKGNKGSSGPTGQKGDSGMPGPPGPPGPPGEVIQPLPILSPKKTRRHTESIQGDAGDNILDYSDGMEEIF
GSLNSLKQDIEHMKFPMGTQTNPARTCKDLQLSHPDFPDGEYWIDPNQGCSGDSFKVYCNFTAGGETCIYPDK
KSEGMYSLGTCLLSKILWLSMTQHS*
>ENSMUSP00000029569_c.867:T>G_p.L290V ENSMUST00000029569.9|Slc35a3-201|ENSMUSG00000027957|-|chr3:g.116468842:A>C
MSANLKYLSLGILVFQTTSLVLTMRYSRTLKEEGPRYLSSTAVVVAEFLKIMACIFLVYKDSKCSVRALNRVL
HDEILNKPMETLKLAIPSGIYTLQNNLLYVALSNLDAATYQVTYQLKILTTALFSVSMLGKKLGVYQWLSLVI
LMAGVAFVQWPSDSQELNSKDLSTGSQFVGLMAVLTACFSSGFAGVYFEKILKETKQSVWIRNIQLGFFGSIF
GLMGVYVYDGELVSKNGFFQGYNQLTWIVVALQALGGLVIAAVIKYADNILKGFATSLSIILSTIISYFWVQD
FVPTSVFFLGAILVIAATFLYGYDPKPAGNPTKA*
>ENSMUSP00000112674_c.867:T>G_p.L290V ENSMUST00000120120.8|Slc35a3-202|ENSMUSG00000027957|-|chr3:g.116468842:A>C

MSANLKYLSLGILVFQTTSLVLTMRYSRTLKEEGPRYLSSTAVVVAEFLKIMACIFLVYKDSKCSVRALNRVL
HDEILNKPMETLKLAIPSGIYTLQNNLLYVALSNLDAATYQVTYQLKILTTALFSVSMLGKKLGVYQWLSLVI
LMAGVAFVQWPSDSQELNSKDLSTGSQFVGLMAVLTACFSSGFAGVYFEKILKETKQSVWIRNIQLGFFGSIF
GLMGVYVYDGELVSKNGFFQGYNQLTWIVVALQALGGLVIAAVIKYADNILKGFATSLSIILSTIISYFWVQD
FVPTSVFFLGAILVIAATFLYGYDPKPAGNPTKA*
>ENSMUSP00000096149_c.617:C>A_p.S206R ENSMUST00000098549.4|Clca4b-201|ENSMUSG00000074195|-|chr3:g.144631886:G>T
MMAFSRGPVFLLFLLYLLWGSDTSLIKLNGNGYEDIIIAIDPAVPEDTTIIEHIKGMVTKASTYLFEATEKRF
FFKNVSILIPESWKNSSQYRRPKQESYKHADIKVAPPAFEGRDEPYTRQFTQCEEKAEYIHFTPDFVLGRKQV
EYGDSGKLLVHEWAHLRWGVFDEYNEDQPFYSASSKKIEATRCSTGITGMNRVHTCQGGRCITRRCRTNSTTK
LYEKDCQFFPDKVQSEKASIMFMQSIDSVTEFCKKENHNREAPTLHNEKCQWRSTWEVISSSEDFNSSTPMET
PPAPPFFSLLRISERIVCLVLDVSGSMSSSDRLNRMNQAAKYFLSQIIENRSWVGMVHFSSQATIVHELIQMN
SDIERNKLLQTLPTSAIGGTSICSGIKTAFQVFKNGEYQTDGTEILLLSDGEDSTAKDCIDEVKDSGSIVHFI
ALGPSADLAVTNMSILTGGNHKLATDEAQNNGLIDAFGALASENTDITQKSLQLESKGAILNNSLWLNDTVVI
DSTVGRDTFFLVTWSKQAPAIYLRDPKGTQTTNFTMDFVSKMAYLSIPGTAEVGVWTYNLEAKENSEILTITV
TSRAANSSVPPISVNAKVNTDTNTFPSPMIVYAEVLQGYTPIIGARVTATIESNSGKTEELVLLDNGAGADAF
KDDGVYSRFFTAYSVNGRYSLKVRADGGTNSARRSLRHPSSRAAYIPGWVVDGEIQGNPPRPEMTEATQPVLE
NFSRTASGGAFVMSNVPIGPLPDQYPPNRITDLQATLDGEEISLTWTAPGDDYDVGRVQQYIIRTSENIIDLR
DNFNNSLRVDTTKLTPKEANSKETFAFKPENISEENATYIFIAIESVDKNNLSSGPSNIAQVAMFTPQAEPVP
DESPRSSGVSISTIVLSVVGSVVLVCIIVSTTICILKNKRSSSGAATTF*
>ENSMUSP00000029671_c.165:A>T_p.S56C ENSMUST00000029671.9|Ifi44-201|ENSMUSG00000028037|-|chr3:g.151455058:T>A
MAMRTRLTWQQEKCLQNYFGGKRFCLLYKASVQKFSHQNLLCTCENQGPTMIVVYCEKCVIGMYLKEGFQGKD
VSITIFALQETGFSLCAKGPDSPYLLFHKRKTNDFSILLDEKAVIVSSAICKMLQLTARNNVIPIQECEAFRC
EELLDERKTRGIAVLHSNLLQALRDYKPYGDLVQQTRVLLLGPIGAGKSSFVNSVKSVFKGSITHQILVGCDE
DGISDKYRTYSIKAKDDSDPLPFILCDSLGLGENAGLHTDDVWHILKGHTPDRYQFDSMKPITSNHPNYTHDP
LLKDRIHCVVFVFDINSFEMHSSELVAKIKKIRRDLIKHGILHLALLTHVDSLDLITKEDMTDIYNYSPVKSK
LEAFHGVFGFALSDILVVSNYVSEWQLDPVKDMLILSALKEILYTANEFLEDLPLNK*
>ENSMUSP00000036227_c.840:A>T_p.M281L ENSMUST00000044278.6|St6galnac5-201|ENSMUSG00000039037|-|chr3:g.152527315:T>A
MKTLMRHGLAVCLVLTTMCTSLLLVYSSLGSQKERPPQQQQQQQQQQAATATGSTQLVESSPQPRRTAPAGP
RQLEGYLGVADHKPLKMHCKDCALVTSSGHLLRSQQGPHIDQTECVIRMNDAPTRGYGLDVGNRTSLRVIAHS
SIQRILRNRHDLLNVSQGTVFIFWGPSSYMRRDGKGQAYNNLQLLSQVLPRLKAFMITRHRMLQFDELFKQET
GKDRKISNTWLSTGWFTMTIALELCDRIDVYGMVPPDFCRDPKHPSVPYHYYEPSGPDECTLYLSHERGRKGS
HHRFITEKRVFKNWARTFNIHFFQPDWKPESPAVNHAEGKPVF*
>ENSMUSP00000143365_c.4563:G>T_p.D1522Y ENSMUST00000200196.5|Lrrc7-206|ENSMUSG00000028176|-|chr3:g.157790108:C>A
XIGRLVPCRCFRGEEEIISVLDYSHCSLQQVPKEVFNFERTLEELYLDANQIEELPKQLFNCQALRKLSIPDN
DLSSLPTSIASLVNLKELDISKNGVQEFPENIKCCKCLTIIEASVNPISKLPDGFTQLLNLTQLYLNDAFLEF
LPANFGRLVKLRILELRENHLKTLPKSMHKLAQLERLDLGNNEFSELPEVLDQIQNLRELWMDNNALQVLPGS
IGKLKMLVYLDMSKNRIETVDMDISGCEALEDLLLSSNMLQQLPDSIGLLKKLTTLKVDDNQLTMLPNTIGNL
SLLEEFDCSCNELESLPPTIGYLHSLRTLAVDENFLPELPREIGSCKNVTVMSLRSNKLEFLPEEIGQMQRLR
VLNLSDNRLKNLPFSFTKLKELAALWLSDNQSKALIPLQTEAHPETKQRVLTNYMFPQQPRGDEDFQSDSDSF
NPTLWEEQRQQRMTVAFEFEDKKEDDESAGKVKALSCQAPWDRGQRGITLQPARLSGDCCTPWARCDQQIQDM
PVPQSDPQLAWGCISGLQQERSMCAPLPVAAQSTTLPSLSGRQVEINLKRYPTPYPEDLKNMVKSVQNLVGKP
SHGVRVENSNPTANTEQTVKEKFEHKWPVAPKEITVEDSFVHPANEMRIGELHPSLAETPLYPPKLVLLGKDK
KESTDESEVDKTHCLNNSVSSGTYSDYSPSQASSASSNTRMKVGSLQATAKDAVHNSLWGNRIAPPFPQPLDA
KPLLSQREAVPPGNIPQRPDRLPMSDAFPDNWTDGSHYDNTGFVSEEAAGENANNNPLLSSKARSVPAHGRRP
LIRQERIVGVPLELEQSTHRHTPETEVPPSNPWQNWTRTPSPFEDRTAFPSKLETTPTTSPLPERKDHMKEPT
ETPGPFSPGVPWEYHDPTPNRSLGNVFSQIHCRPDSSKGVIAISKSTERLSPLMKDIKSNKFKKSQSIDEIDV
GTYKVYNIPLENYASGSDHLGSHERPDKFLGPEHGMSSMSRSQSVPMLDDEMLMYGSSKGPPQQKASMTKKVY
QFDQSFNPQGAVEVKAEKRIPPPFAHNSEYVQQPSKNIAKDLVSPRAYRGYPPMEQMFSFSQPSVNEDAMVNA
QFASQGPRAGFLRRADSLASSTEMAMFRRVSEPHELPPGDRYGRATYRGGLEGQSSISMTDPQFLKRNGRYED
EHPSYQEVKAQAGSFPAKNLTQRRPLSARSYSTESYGASQTRPVSARPTMAALLEKIPSDYNLGNYGDKTSDN
SDIKTRPTPVKGEESCGKMPADWRQQLLRHIEARRLDRNAAYKHNTVNLGMLPYGGISAMHAGRSMTLNLQTK
SKFDLQDLPLQKTPSQQSNILDNGQEDVSPSGQWNPYPLGRRDVPPDTITKKAGSHIQTLMGSQSLQHRSREQ
QPYEGNINKVTIQQFQSPLPIQIPSSQATRGPQPGRCLIQTKGQRSMDGYPEQFCVRIEKNPGLGFSISGGIS
GQGNPFKPSDKGIFVTRVQPDGPASNLLQPGDKILQANGHSFVHMEHEKAVLLLKSFQNTVYLVIQRELTV*

>ENSMUSP00000142498_c.4455:G>T_p.D1486Y ENSMUST00000200137.5|Lrrc7-205|ENSMUSG00000028176|-|chr3:g.157790108:C>A
MQCLEMTTKRKLIGRLVPCRCFRGEEEIISVLDYSHCSLQQVPKEVFNFERTLEELYLDANQIEELPKQLFNC
QALRKLSIPDNDLSSLPTSIASLVNLKELDISKNGVQEFPENIKCCKCLTIIEASVNPISKLPDGFTQLLNLT
QLYLNDAFLEFLPANFGRLVKLRILELRENHLKTLPKSMHKLAQLERLDLGNNEFSELPEVLDQIQNLRELWM
DNNALQVLPGSIGKLKMLVYLDMSKNRIETVDMDISGCEALEDLLLSSNMLQQLPDSIGLLKKLTTLKVDDNQ
LTMLPNTIGNLSLLEEFDCSCNELESLPPTIGYLHSLRTLAVDENFLPELPREIGSCKNVTVMSLRSNKLEFL
PEEIGQMQRLRVLNLSDNRLKNLPFSFTKLKELAALWLSDNQSKALIPLQTEAHPETKQRVLTNYMFPQQPRG
DEDFQSDSDSFNPTLWEEQRQQRMTVAFEFEDKKEDDESAGKVKALSCQAPWDRGQRGITLQPARLSGDCCTP
WARCDQQIQDMPVPQSDPQLAWGCISGLQQERSMCAPLPVAAQSTTLPSLSGRQVEINLKRYPTPYPEDLKNM
VKSVQNLVGKPSHGVRVENSNPTANTEQTVKEKFEHKWPVAPKEITVEDSFVHPANEMRIGELHPSLAETPLY
PPKLVLLGKDKKESTDESEVDKTHCLNNSVSSGTYSDYSPSQASSASSNTRMKVGSLQATAKDAVHNSLWGNR
IAPPFPQPLDAKPLLSQREAVPPGNIPQRPDRLPMSDAFPDNWTDGSHYDNTGFVSEEAAGENANNNPLLSSK
ARSVPAHGRRPLIRQERIVGVPLELEQSTHRHTPETEVPPSNPWQNWTRTPSPFEDRTAFPSKLETTPTTSPL
PERKDHMKEPTETPGPFSPGVPWEYHDPTPNRSLGNVFSQIHCRPDSSKGVIAISKSTERLSPLMKDIKSNKF
KKSQSIDEIDVGTYKVYNIPLENYASGSDHLGSHERPDKFLGPEHGMSSMSRSQSVPMLDDEMLMYGSSKGPP
QQKASMTKKVYQFDQSFNPQGAVEVKAEKRIPPPFAHNSEYVQQPSKNIAKDLVSPRAYRGYPPMEQMFSFSQ
PSVNEDAMVNAQFASQGPRAGFLRRADSLASSTEMAMFRRVSEPHELPPGDRYGRATYRGGLEGQSSISMTDP
QFLKRNGRYEDEHPSYQEVKAQAGSFPAKNLTQRRPLSARSYSTESYGASQTRPVSARPTMAALLEKIPSDYN
LGNYGDKTSDNSDIKTRPTPVKGEESCGKMPADWRQQLLRHIEARRLDRTPSQQSNILDNGQEDVSPSGQWNP
YPLGRRDVPPDTITKKAGSHIQTLMGSQSLQHRSREQQPYEGNINKVTIQQFQSPLPIQIPSSQATRGPQPGR
CLIQTKGQRSMDGYPEQFCVRIEKNPGLGFSISGGISGQGNPFKPSDKGIFVTRVQPDGPASNLLQPGDKILQ
ANGHSFVHMEHEKAVLLLKSFQNTVYLVIQRELTV*
>ENSMUSP00000158628_c.4434:G>T_p.D1479Y ENSMUST00000238317.3|Lrrc7-207|ENSMUSG00000028176|-|chr3:g.157790108:C>A
MMESLIRGRNPPQYQRSPCKEARAALRKRPEEELQCLEMTTKRKLIGRLVPCRCFRGEEEIISVLDYSHCSLQ
QVPKEVFNFERTLEELYLDANQIEELPKQLFNCQALRKLSIPDNDLSSLPTSIASLVNLKELDISKNGVQEFP
ENIKCCKCLTIIEASVNPISKLPDGFTQLLNLTQLYLNDAFLEFLPANFGRLVKLRILELRENHLKTLPKSMH
KLAQLERLDLGNNEFSELPEVLDQIQNLRELWMDNNALQVLPGSIGKLKMLVYLDMSKNRIETVDMDISGCEA
LEDLLLSSNMLQQLPDSIGLLKKLTTLKVDDNQLTMLPNTIGNLSLLEEFDCSCNELESLPPTIGYLHSLRTL
AVDENFLPELPREIGSCKNVTVMSLRSNKLEFLPEEIGQMQRLRVLNLSDNRLKNLPFSFTKLKELAALWLSD
NQSKALIPLQTEAHPETKQRVLTNYMFPQQPRGDEDFQSDSDSFNPTLWEEQRQQRMTVAFEFEDKKEDDESA
GKVKALSCQAPWDRGQRGITLQPARLSGDCCTPWARCDQQIQDMPVPQSDPQLAWGCISGLQQERSMCAPLPV
AAQSTTLPSLSGRQVEINLKRYPTPYPEDLKNMVKSVQNLVGKPSHGVRVENSNPTANTEQTVKEKFEHKWPV
APKEITVEDSFVHPANEMRIGELHPSLAETPLYPPKLVLLGKDKKESTDESEVDKTHCLNNSVSSGTYSDYSP
SQASSASSNTRMKVGSLQATAKDAVHNSLWGNRIAPPFPQPLDAKPLLSQREAVPPGNIPQRPDRLPMSDAFP
DNWTDGSHYDNTGFVSEEAAGENANNNPLLSSKARSVPAHGRRPLIRQERIVGVPLELEQSTHRHTPETEVPP
SNPWQNWTRTPSPFEDRTAFPSKLETTPTTSPLPERKDHMKEPTETPGPFSPGVPWEYHDPTPNRSLGNVFSQ
IHCRPDSSKGVIAISKSTERLSPLMKDIKSNKFKKSQSIDEIDVGTYKVYNIPLENYASGSDHLGSHERPDKF
LGPEHGMSSMSRSQSVPMLDDEMLMYGSSKGPPQQKASMTKKVYQFDQSFNPQGAVEVKAEKRIPPPFAHNSE
YVQQPSKNIAKDLVSPRAYRGYPPMEQMFSFSQPSVNEDAMVNAQFASQGPRAGFLRRADSLASSTEMAMFRR
VSEPHELPPGDRYGRATYRGGLEGQSSISMTDPQFLKRNGRYEDEHPSYQEVKAQAGSFPAKNLTQRRPLSAR
SYSTESYGASQTRPVSARPTMAALLEKIPSDYNLGNYGDKTSDNSDIKTRPTPVKGEESCGKMPADWRQQLLR
HIEARRLDRAGSHIQTLMGSQSLQHRSREQQPYEGNINKVTIQQFQSPLPIQIPSSQATRGPQPGRCLIQTKG
QRSMDGYPEQFCVRIEKNPGLGFSISGGISGQGNPFKPSDKGIFVTRVQPDGPASNLLQPGDKILQANGHSFV
HMEHEKAVLLLKSFQNTVYLVIQRELTV*
>ENSMUSP00000101659_c.4596:G>T_p.D1533Y ENSMUST00000106044.6|Lrrc7-201|ENSMUSG00000028176|-|chr3:g.157790108:C>A
MQCLEMTTKRKLIGRLVPCRCFRGEEEIISVLDYSHCSLQQVPKEVFNFERTLEELYLDANQIEELPKQLFNC
QALRKLSIPDNDLSSLPTSIASLVNLKELDISKNGVQEFPENIKCCKCLTIIEASVNPISKLPDGFTQLLNLT
QLYLNDAFLEFLPANFGRLVKLRILELRENHLKTLPKSMHKLAQLERLDLGNNEFSELPEVLDQIQNLRELWM
DNNALQVLPGSIGKLKMLVYLDMSKNRIETVDMDISGCEALEDLLLSSNMLQQLPDSIGLLKKLTTLKVDDNQ
LTMLPNTIGNLSLLEEFDCSCNELESLPPTIGYLHSLRTLAVDENFLPELPREIGSCKNVTVMSLRSNKLEFL
PEEIGQMQRLRVLNLSDNRLKNLPFSFTKLKELAALWLSDNQSKALIPLQTEAHPETKQRVLTNYMFPQQPRG
DEDFQSDSDSFNPTLWEEQRQQRMTVAFEFEDKKEDDESAGKVKALSCQAPWDRGQRGITLQPARLSGDCCTP
WARCDQQIQDMPVPQSDPQLAWGCISGLQQERSMCAPLPVAAQSTTLPSLSGRQVEINLKRYPTPYPEDLKNM
VKSVQNLVGKPSHGVRVENSNPTANTEQTVKEKFEHKWPVAPKEITVEDSFVHPANEMRIGELHPSLAETPLY
PPKLVLLGKDKKESTDESEVDKTHCLNNSVSSGTYSDYSPSQASSASSNTRMKVGSLQATAKDAVHNSLWGNR

IAPPFPQPLDAKPLLSQREAVPPGNIPQRPDRLPMSDAFPDNWTDGSHYDNTGFVSEEAAGENANNNPLLSSK
ARSVPAHGRRPLIRQERIVGVPLELEQSTHRHTPETEVPPSNPWQNWTRTPSPFEDRTAFPSKLETTPTTSPL
PERKDHMKEPTETPGPFSPGVPWEYHDPTPNRSLGNVFSQIHCRPDSSKGVIAISKSTERLSPLMKDIKSNKF
KKSQSIDEIDVGTYKVYNIPLENYASGSDHLGSHERPDKFLGPEHGMSSMSRSQSVPMLDDEMLMYGSSKGPP
QQKASMTKKVYQFDQSFNPQGAVEVKAEKRIPPPFAHNSEYVQQPSKNIAKDLVSPRAYRGYPPMEQMFSFSQ
PSVNEDAMVNAQFASQGPRAGFLRRADSLASSTEMAMFRRVSEPHELPPGDRYGRATYRGGLEGQSSISMTDP
QFLKRNGRYEDEHPSYQEVKAQAGSFPAKNLTQRRPLSARSYSTESYGASQTRPVSARPTMAALLEKIPSDYN
LGNYGDKTSDNSDIKTRPTPVKGEESCGKMPADWRQQLLRHIEARRLDRNAAYKHNTVNLGMLPYGGISAMHA
GRSMTLNLQTKSKFDLQDLPLQKTPSQQSNILDNGQEDVSPSGQWNPYPLGRRDVPPDTITKKAGSHIQTLMG
SQSLQHRSREQQPYEGNINKVTIQQFQSPLPIQIPSSQATRGPQPGRCLIQTKGQRSMDGYPEQFCVRIEKNP
GLGFSISGGISGQGNPFKPSDKGIFVTRVQPDGPASNLLQPGDKILQANGHSFVHMEHEKAVLLLKSFQNTVY
LVIQRELTV*
>ENSMUSP00000154142_c.823:C>T_p.S275F ENSMUST00000226198.2|Vmn1r2-202|ENSMUSG00000115072|+|chr4:g.3172906:C>T
MKMDPGNFAIETFLFCQITVGMFGNSSILFYYIILIFTEKHLTSKDLIIEHLTFANYLTVILRGFPQTMSYFG
FKNFLDDMGCKLIVYISRITRGVSLYALCLLSCFQAITTSPNNSRWIKLKYRATKYIGPSCSVSCLVHLLLNI
LTPLRESGTNYKKNATNRLSYGYCSLFASDSVATTLYISLVCFSDVFCLGLMACSSISMVSILYRHERQVKHI
HSAQYFLKVYPEDRATQTILILLCTFVISYSFSSIVAIIRTYLNYPVIWGVNIFTFLEIFFPMFCPFVLITNM
RSSSNLFLPCLGKR*
>ENSMUSP00000100791_c.823:C>T_p.S275F ENSMUST00000105160.5|Vmn1r2-201|ENSMUSG00000115072|+|chr4:g.3172906:C>T
MKMDPGNFAIETFLFCQITVGMFGNSSILFYYIILIFTEKHLTSKDLIIEHLTFANYLTVILRGFPQTMSYFG
FKNFLDDMGCKLIVYISRITRGVSLYALCLLSCFQAITTSPNNSRWIKLKYRATKYIGPSCSVSCLVHLLLNI
LTPLRESGTNYKKNATNRLSYGYCSLFASDSVATTLYISLVCFSDVFCLGLMACSSISMVSILYRHERQVKHI
HSAQYFLKVYPEDRATQTILILLCTFVISYSFSSIVAIIRTYLNYPVIWGVNIFTFLEIFFPMFCPFVLITNM
RSSSNLFLPCLGKR*
>ENSMUSP00000154142_c.840:C>T_p.P281S ENSMUST00000226198.2|Vmn1r2-202|ENSMUSG00000115072|+|chr4:g.3172923:C>T
MKMDPGNFAIETFLFCQITVGMFGNSSILFYYIILIFTEKHLTSKDLIIEHLTFANYLTVILRGFPQTMSYFG
FKNFLDDMGCKLIVYISRITRGVSLYALCLLSCFQAITTSPNNSRWIKLKYRATKYIGPSCSVSCLVHLLLNI
LTPLRESGTNYKKNATNRLSYGYCSLFASDSVATTLYISLVCFSDVFCLGLMACSSISMVSILYRHERQVKHI
HSAQYFLKVYPEDRATQTILILLCTFVISYSFSSIVAIIRTYLNYPVIWGVNIFTSLEIFFSMFCPFVLITNM
RSSSNLFLPCLGKR*
>ENSMUSP00000100791_c.840:C>T_p.P281S ENSMUST00000105160.5|Vmn1r2-201|ENSMUSG00000115072|+|chr4:g.3172923:C>T
MKMDPGNFAIETFLFCQITVGMFGNSSILFYYIILIFTEKHLTSKDLIIEHLTFANYLTVILRGFPQTMSYFG
FKNFLDDMGCKLIVYISRITRGVSLYALCLLSCFQAITTSPNNSRWIKLKYRATKYIGPSCSVSCLVHLLLNI
LTPLRESGTNYKKNATNRLSYGYCSLFASDSVATTLYISLVCFSDVFCLGLMACSSISMVSILYRHERQVKHI
HSAQYFLKVYPEDRATQTILILLCTFVISYSFSSIVAIIRTYLNYPVIWGVNIFTSLEIFFSMFCPFVLITNM
RSSSNLFLPCLGKR*
>ENSMUSP00000154142_c.882:A>T_p.S295C ENSMUST00000226198.2|Vmn1r2-202|ENSMUSG00000115072|+|chr4:g.3172965:A>T
MKMDPGNFAIETFLFCQITVGMFGNSSILFYYIILIFTEKHLTSKDLIIEHLTFANYLTVILRGFPQTMSYFG
FKNFLDDMGCKLIVYISRITRGVSLYALCLLSCFQAITTSPNNSRWIKLKYRATKYIGPSCSVSCLVHLLLNI
LTPLRESGTNYKKNATNRLSYGYCSLFASDSVATTLYISLVCFSDVFCLGLMACSSISMVSILYRHERQVKHI
HSAQYFLKVYPEDRATQTILILLCTFVISYSFSSIVAIIRTYLNYPVIWGVNIFTSLEIFFPMFCPFVLITNM
RSCSNLFLPCLGKR*
>ENSMUSP00000100791_c.882:A>T_p.S295C ENSMUST00000105160.5|Vmn1r2-201|ENSMUSG00000115072|+|chr4:g.3172965:A>T
MKMDPGNFAIETFLFCQITVGMFGNSSILFYYIILIFTEKHLTSKDLIIEHLTFANYLTVILRGFPQTMSYFG
FKNFLDDMGCKLIVYISRITRGVSLYALCLLSCFQAITTSPNNSRWIKLKYRATKYIGPSCSVSCLVHLLLNI
LTPLRESGTNYKKNATNRLSYGYCSLFASDSVATTLYISLVCFSDVFCLGLMACSSISMVSILYRHERQVKHI
HSAQYFLKVYPEDRATQTILILLCTFVISYSFSSIVAIIRTYLNYPVIWGVNIFTSLEIFFPMFCPFVLITNM
RSCSNLFLPCLGKR*
>ENSMUSP00000100790_c.882:A>T_p.S295C ENSMUST00000105159.5|Vmn1r3-201|ENSMUSG00000115466|-|chr4:g.3184423:T>A
MKMGPGNFAIETFLFCQITVGMFGNSSIPFYYIILIFTEKHLTPKDLIIEHLTFANYLTVILRGFPQTMSYFG
FKNFLDDMGCKLLVYISRITRGVSLYAMCLLSCFQAITTSPNNSRWIKLKYRATKYIGPSCSVSCLVHLLLNI

LTPLRESGTNYKKNATNRLSYGYCSLFASDSVATTLYISLVCFSDVFCLGLMACSSISMVSILYRHKRQVKHI
HSAQHFLKVSPEDRATQTILILLCTFVISYSFSSIVAIIRTYLNYPVIWGVNIFTSLEIFFPMFCPFVLITNM
RSCSNLFLPCLGKR*
>ENSMUSP00000100790_c.840:C>T_p.P281S ENSMUST00000105159.5|Vmn1r3-201|ENSMUSG00000115466|-|chr4:g.3184465:G>A
MKMGPGNFAIETFLFCQITVGMFGNSSIPFYYIILIFTEKHLTPKDLIIEHLTFANYLTVILRGFPQTMSYFG
FKNFLDDMGCKLLVYISRITRGVSLYAMCLLSCFQAITTSPNNSRWIKLKYRATKYIGPSCSVSCLVHLLLNI
LTPLRESGTNYKKNATNRLSYGYCSLFASDSVATTLYISLVCFSDVFCLGLMACSSISMVSILYRHKRQVKHI
HSAQHFLKVSPEDRATQTILILLCTFVISYSFSSIVAIIRTYLNYPVIWGVNIFTSLEIFFSMFCPFVLITNM
RSSSNLFLPCLGKR*
>ENSMUSP00000100790_c.823:C>T_p.S275F ENSMUST00000105159.5|Vmn1r3-201|ENSMUSG00000115466|-|chr4:g.3184482:G>A
MKMGPGNFAIETFLFCQITVGMFGNSSIPFYYIILIFTEKHLTPKDLIIEHLTFANYLTVILRGFPQTMSYFG
FKNFLDDMGCKLLVYISRITRGVSLYAMCLLSCFQAITTSPNNSRWIKLKYRATKYIGPSCSVSCLVHLLLNI
LTPLRESGTNYKKNATNRLSYGYCSLFASDSVATTLYISLVCFSDVFCLGLMACSSISMVSILYRHKRQVKHI
HSAQHFLKVSPEDRATQTILILLCTFVISYSFSSIVAIIRTYLNYPVIWGVNIFTFLEIFFPMFCPFVLITNM
RSSSNLFLPCLGKR*
>ENSMUSP00000100790_c.614:G>A_p.M205I ENSMUST00000105159.5|Vmn1r3-201|ENSMUSG00000115466|-|chr4:g.3184691:C>T
MKMGPGNFAIETFLFCQITVGMFGNSSIPFYYIILIFTEKHLTPKDLIIEHLTFANYLTVILRGFPQTMSYFG
FKNFLDDMGCKLLVYISRITRGVSLYAMCLLSCFQAITTSPNNSRWIKLKYRATKYIGPSCSVSCLVHLLLNI
LTPLRESGTNYKKNATNRLSYGYCSLFASDSVATTLYISLVCFSDVFCLGLMACSSISIVSILYRHKRQVKHI
HSAQHFLKVSPEDRATQTILILLCTFVISYSFSSIVAIIRTYLNYPVIWGVNIFTSLEIFFPMFCPFVLITNM
RSSSNLFLPCLGKR*
>ENSMUSP00000100790_c.531:G>A_p.V178I ENSMUST00000105159.5|Vmn1r3-201|ENSMUSG00000115466|-|chr4:g.3184774:C>T
MKMGPGNFAIETFLFCQITVGMFGNSSIPFYYIILIFTEKHLTPKDLIIEHLTFANYLTVILRGFPQTMSYFG
FKNFLDDMGCKLLVYISRITRGVSLYAMCLLSCFQAITTSPNNSRWIKLKYRATKYIGPSCSVSCLVHLLLNI
LTPLRESGTNYKKNATNRLSYGYCSLFASDSIATTLYISLVCFSDVFCLGLMACSSISMVSILYRHKRQVKHI
HSAQHFLKVSPEDRATQTILILLCTFVISYSFSSIVAIIRTYLNYPVIWGVNIFTSLEIFFPMFCPFVLITNM
RSSSNLFLPCLGKR*
>ENSMUSP00000100790_c.334:C>T_p.T112I ENSMUST00000105159.5|Vmn1r3-201|ENSMUSG00000115466|-|chr4:g.3184971:G>A
MKMGPGNFAIETFLFCQITVGMFGNSSIPFYYIILIFTEKHLTPKDLIIEHLTFANYLTVILRGFPQTMSYFG
FKNFLDDMGCKLLVYISRITRGVSLYAMCLLSCFQAITISPNNSRWIKLKYRATKYIGPSCSVSCLVHLLLNI
LTPLRESGTNYKKNATNRLSYGYCSLFASDSVATTLYISLVCFSDVFCLGLMACSSISMVSILYRHKRQVKHI
HSAQHFLKVSPEDRATQTILILLCTFVISYSFSSIVAIIRTYLNYPVIWGVNIFTSLEIFFPMFCPFVLITNM
RSSSNLFLPCLGKR*
>ENSMUSP00000100790_c.283:G>A_p.G95E ENSMUST00000105159.5|Vmn1r3-201|ENSMUSG00000115466|-|chr4:g.3185022:C>T
MKMGPGNFAIETFLFCQITVGMFGNSSIPFYYIILIFTEKHLTPKDLIIEHLTFANYLTVILRGFPQTMSYFG
FKNFLDDMGCKLLVYISRITREVSLYAMCLLSCFQAITTSPNNSRWIKLKYRATKYIGPSCSVSCLVHLLLNI
LTPLRESGTNYKKNATNRLSYGYCSLFASDSVATTLYISLVCFSDVFCLGLMACSSISMVSILYRHKRQVKHI
HSAQHFLKVSPEDRATQTILILLCTFVISYSFSSIVAIIRTYLNYPVIWGVNIFTSLEIFFPMFCPFVLITNM
RSSSNLFLPCLGKR*
>ENSMUSP00000100790_c.255:C>A_p.L86I ENSMUST00000105159.5|Vmn1r3-201|ENSMUSG00000115466|-|chr4:g.3185050:G>T
MKMGPGNFAIETFLFCQITVGMFGNSSIPFYYIILIFTEKHLTPKDLIIEHLTFANYLTVILRGFPQTMSYFG
FKNFLDDMGCKLIVYISRITRGVSLYAMCLLSCFQAITTSPNNSRWIKLKYRATKYIGPSCSVSCLVHLLLNI
LTPLRESGTNYKKNATNRLSYGYCSLFASDSVATTLYISLVCFSDVFCLGLMACSSISMVSILYRHKRQVKHI
HSAQHFLKVSPEDRATQTILILLCTFVISYSFSSIVAIIRTYLNYPVIWGVNIFTSLEIFFPMFCPFVLITNM
RSSSNLFLPCLGKR*
>ENSMUSP00000100790_c.85:C>T_p.P29L ENSMUST00000105159.5|Vmn1r3-201|ENSMUSG00000115466|-|chr4:g.3185220:G>A
MKMGPGNFAIETFLFCQITVGMFGNSSILFYYIILIFTEKHLTPKDLIIEHLTFANYLTVILRGFPQTMSYFG
FKNFLDDMGCKLLVYISRITRGVSLYAMCLLSCFQAITTSPNNSRWIKLKYRATKYIGPSCSVSCLVHLLLNI
LTPLRESGTNYKKNATNRLSYGYCSLFASDSVATTLYISLVCFSDVFCLGLMACSSISMVSILYRHKRQVKHI

HSAQHFLKVSPEDRATQTILILLCTFVISYSFSSIVAIIRTYLNYPVIWGVNIFTSLEIFFPMFCPFVLITNM
RSSSNLFLPCLGKR*
>ENSMUSP00000062039_c.547:G>A_p.G183D ENSMUST00000058183.9|Ndufaf6-
201|ENSMUSG00000050323|-|chr4:g.11062082:C>T
MATSMLGSVRGPRPFGLANLFHRQPPRDAWERVRRLPGPSAVRRSVAAASGPGIPGSHLYCLELLRKRDYESY
LCSLLFPAECQRSASALRAFNVELAQVKDSVSEKTIGLMRMQFWKKAVEDMYCDNPPHQPVAIELWKAVKKHN
LTKRWLMRIIDEREKNLDDKAYRSMQELENYAENTQDSLLYLTLEVLGVKDLHADHAASHIGKAQGIVTCLRA
TPYHSSRRQVFLPMDVCVQHGVSQEDFLRRNQDKNVRDVVYDIASQAHLHLKHARSFHRSVPAEAFPAFLQTV
SLEDYLKKIQRVDFDIFHPSLQQKNMLLPLSLYIQSWRKRY*
>ENSMUSP00000115154_c.267:A>G_p.N90D ENSMUST00000131145.9|Tmem67-
203|ENSMUSG00000049488|-|chr4:g.12085959:T>C
XMVTRTRPVAAMAVRSRSSSRTGTAYLLLVLCEVSWAQIFSFPFRRPETCDFNQYFDISALSCAPCGANQRRD
ALGTSCVCLPGYHMISDNGGPSIICKKCPENMKGVTKDGWDCISCPSGLTAEGKCHCPTGHILVERNVSGSLL
AQATCELCDESENSFTKANALGTRCVRCEPTFVNTSRSCSCSEPHTLVDYVSATQGIFINV*
>ENSMUSP00000103928_c.471:A>G_p.N158D ENSMUST00000108293.3|Tmem67-
202|ENSMUSG00000049488|-|chr4:g.12085959:T>C
METTLPSGPDKPMHRLSAHAQSRPNQPPLGALNARAQTYCFGYSDWRVSADSPRRLLGSWRLRTQDMGTMVTR
TRPVAAMAVRSRSSSRTGTAYLLLVLCEVSWAQIFSFPFRRPETCDFNQYFDISALSCAPCGANQRRDALGTS
CVCLPGYHMISDNGGPSIICKKCPENMKGVTKDGWDCISCPSGLTAEGKCHCPTGHILVERNVSGSLLAQATC
ELCDESENSFTKANALGTRCVRCEPTFVNTSRSCSCSEPHTLTGGLCFSNTGNFHQRVISTARYGELGMSLNS
EWFAKYLQATAAACWTHANLTSCQALGNMCVMNMNSYDSTTLDACRLFHYIFESTAGLISVHSVPFWRQNLPW
LFYGDQPGLAPQVLSTTPLPTNFSFKGQNQLKFVAASYDIRGNFIKWQPLEGGVLQLCPDTERRLDAAYAFGT
TYQQNCEISLSKLLVDFSSPVFYDVYLEYTDEEQHRYLWPIPVLNLNLQHNKLFVNQDSSSSKWLLTRRIFLV
DAVSGRENDLGNQPRVIRVATQISLSIRLVPNTKNGNIYTPLLTIAYSDIDIKNAHSQSAKISFSVKYEMNQG
DASVHTDIALGVLGGLAVLSSLLKTAGWKRRIGSPMIDLQTVMKFLLYYAGDLANVFFIITVGTGLYWLIFFK
AQKSVSVLLPMPVQEERFVTYVGCAFAMKALQFLHKFISQISIDIFFIDWERPKGKVLKAVEGEGGVRSATVP
VSIWRTYFVANEWNEIQTVRKINPLFQVLTTLFFLEVVGFKNLALMDSSSSLSRNPSDYTAPYSRILRYAVAT
AIWLVIGIIQVVFFAAFYERFIEDKIRQFVDLCSMSNVSVFLLSHRCFGYYIHGRSVHGHADTNMEEMNMNLK
REAENLCSQRGLVPNTDGQTFQIAVSSQMRQHYDRIHETLTRRNGPARLLSSSGSTFEQSIKAYHAMNKFLGS
FIDHVHKEMDYFIKDKLLLERILGMEFMEPMEKSIFYNDEGHSFSSVLYYGNEATLLIFDLLFFCVVDLACQD
FVLASFLTYLQQEIFRFIRNTVGQKNLATKTLVDERFLI*
>ENSMUSP00000052644_c.273:A>G_p.N92D ENSMUST00000050686.10|Tmem67-
201|ENSMUSG00000049488|-|chr4:g.12085959:T>C
MGTMVTRTRPVAAMAVRSRSSSRTGTAYLLLVLCEVSWAQIFSFPFRRPETCDFNQYFDISALSCAPCGANQR
RDALGTSCVCLPGYHMISDNGGPSIICKKCPENMKGVTKDGWDCISCPSGLTAEGKCHCPTGHILVERNVSGS
LLAQATCELCDESENSFTKANALGTRCVRCEPTFVNTSRSCSCSEPHTLTGGLCFSNTGNFHQRVISTARYGE
LGMSLNSEWFAKYLQATAAACWTHANLTSCQALGNMCVMNMNSYDSTTLDACRLFHYIFESTAGLISVHSVPF
WRQNLPWLFYGDQPGLAPQVLSTTPLPTNFSFKGQNQLKFVAASYDIRGNFIKWQPLEGGVLQLCPDTERRLD
AAYAFGTTYQQNCEISLSKLLVDFSSPVFYDVYLEYTDEEQHRYLWPIPVLNLNLQHNKLFVNQDSSSSKWLL
TRRIFLVDAVSGRENDLGNQPRVIRVATQISLSIRLVPNTKNGNIYTPLLTIAYSDIDIKNAHSQSAKISFSV
KYEMNQGDASVHTDIALGVLGGLAVLSSLLKTAGWKRRIGSPMIDLQTVMKFLLYYAGDLANVFFIITVGTGL
YWLIFFKAQKSVSVLLPMPVQEERFVTYVGCAFAMKALQFLHKFISQISIDIFFIDWERPKGKVLKAVEGEGG
VRSATVPVSIWRTYFVANEWNEIQTVRKINPLFQVLTTLFFLEVVGFKNLALMDSSSSLSRNPSDYTAPYSRI
LRYAVATAIWLVIGIIQVVFFAAFYERFIEDKIRQFVDLCSMSNVSVFLLSHRCFGYYIHGRSVHGHADTNME
EMNMNLKREAENLCSQRGLVPNTDGQTFQIAVSSQMRQHYDRIHETLTRRNGPARLLSSSGSTFEQSIKAYHA
MNKFLGSFIDHVHKEMDYFIKDKLLLERILGMEFMEPMEKSIFYNDEGHSFSSVLYYGNEATLLIFDLLFFCV
VDLACQDFVLASFLTYLQQEIFRFIRNTVGQKNLATKTLVDERFLI*
>ENSMUSP00000041789_c.217:C>G_p.S73C ENSMUST00000042221.14|Slc26a7-
201|ENSMUSG00000040569|-|chr4:g.14593896:G>C
MTGAKRKKRSVLWGKMHTPHREDIKQWCKRRLPILEWAPQYNLKENLLPDTVSGIMLAVQQVAQGLSFAMLSC
VHPVFGLYGSLFPAIIYAIFGMGRHVATGTFALTSLISANAVERLVPQSSRNLTTQSNSSVLGLSEFELQRIG
VAAAVSFLGGVIQLVMFVLQLGSATFLLTEPVISAMTTGAATHVVTSQVKYLLGIKMPYISGPLGFFYIYAYV
FENIKSVQLEALLFSLLSIIVLVLVKELNEQFKRKIKVVLPVDLVLIIAASFACYCTNMENTYGLEVVGHIPN
GIPPPRAPPMNILSAVLTEAFGVALVGYVASLALAQGSAKKFKYSVDDNQEFLAHGLSNVIPSFLFCIPSAAA
MGRTAGLYSTGAKTQVACLISCIFVLIVIYAIGPLLYWLPMCVLASIIVVGLKGMLIQFRDLKKYWNVDKIDW
GIWISTYIFTICFAANVGLLFGVICTIAIVLGRFPRAKTLSITDMKEMELKVKTEMHDETSQQIKIISINNPL
VFLNAKKFSADLMKIILKESDSNQPLDDVSKCEQNTLLSSLSNGNCNEEASQPCSSEKCSLVLNCSGLTFFDY
TGVSTLVELYLDCKSRSVDVFLANCTASLIKAMTYYGDLDTEKPIFFDSVPAAISIIQSNKNLSKASDHSEV*

```
>ENSMUSP00000116157_c.217:C>G_p.S73C ENSMUST00000149633.2|Slc26a7-204|ENSMUSG00000040569|-|chr4:g.14593896:G>C
MTGAKRKKRSVLWGKMHTPHREDIKQWCKRRLPILEWAPQYNLKENLLPDTVSGIMLAVQQVAQGLSFAMLSC
VHPVFGLYGSLFPAIIYAIFGMGRHVATGTFALTSLISANAVERLVPQSSRNLTTQSNSSVLGLSEFELQRIG
VAAAVSFLGGVIQLVMFVLQLGSATFLLTEPVISAMTTGAATHVVTSQVKYLLG
>ENSMUSP00000122870_c.217:C>G_p.S73C ENSMUST00000143105.2|Slc26a7-203|ENSMUSG00000040569|-|chr4:g.14593896:G>C
MTGAKRKKRSVLWGKMHTPHREDIKQWCKRRLPILEWAPQYNLKENLLPDTVSGIMLAVQQVAQGLSFAMLSC
VHPVFGLYGSLFPAIIYAIFGMGRHV
>ENSMUSP00000055293_c.105:C>T_p.R36C ENSMUST00000054538.13|Arhgef39-201|ENSMUSG00000051517|-|chr4:g.43499502:G>A
MESSGSAACCPVLQQRARWERKRVCTARELLETERCYQEQLGLVATYFLRILKAKGTLRPPELQTLFGTWELI
YAASLELLPYLEEGQWGLGLQGFCPHLELYAQFAANAERSQTTLQAQLKKNKRFRRFVKLQEGRPEFRGLQLQ
DLLPLPLQRLQQYENLVVALAENTVPNSPDYQQLTRAARLVSETAQKVHAIGQSQKNDQHLLRVQALLSGRKA
KGLTSGRWFLRQGWLLVVPPTGEPRPRMFFLFSDVLLMAKPRPPLHLLKSGTFVCRALYPMSQCHLSRVFGHS
GGPCGGLLSLSFPHEKLLLMSTDQEELSQWHHSLTLAIRSQKSSTHRSVTAT*
>ENSMUSP00000103497_c.324:C>A_p.H109N ENSMUST00000107865.9|Tmem8b-202|ENSMUSG00000078716|+|chr4:g.43685342:C>A
MNMPQSLGTQPLPPEPPSLGTPIEGSGAIAPTEHCWPVRPTLRNELDTFSVHFYIFFGPSVALPPERPAVFAL
RLLPVLDSGGVLSLELQLNASSLRQENVTVFGCLTNEVPLSLGDAAVTCSKESLAGFLLSVSATSRVARLRIP
FPQTGTWFLTLRSLCGVGPRFVRCRNATAEVRLRTFLSPCVDDCGPYGQCKLLRTHNYLYAACECKAGWRGWG
CTDSADALTYGFQLLSTLLLCLSNLMFLPPVVLAIRSRYVLEAAVYTFTMFFSTFYHACDQPGIVVFCIMDYD
VLQFCDFLGSLMSVWVTVIAMARLQPVIKQVLYLLGAMLLSMALQLDRHGLWNLLGPSLFALGILATAWTVRS
VRRRHCYPPTWRRWLFYLCPGSLIAGSAVLLYAFVETRDNYFYIHSIWHMLIAGSVGFLLPPRAKTDRRVPSG
ARARGCGYQLCINEQEELGLVGPGGTTVSSICVS*
>ENSMUSP00000129760_c.324:C>A_p.H109N ENSMUST00000167153.8|Tmem8b-209|ENSMUSG00000078716|+|chr4:g.43685342:C>A
MNMPQSLGTQPLPPEPPSLGTPIEGSGAIAPTEHCWPVRPTLRNELDTFSVHFYIFFGPSVALPPERPAVFAL
RLLPVLDSGGVLSLELQLNASSLRQENVTVFGCLTNEVPLSLGDAAVTCSKESLAGFLLSVSATSRVARLRIP
FPQTGTWFLTLRSLCGVGPRFVRCRNATAEVRLRTFLSPCVDDCGPYGQCKLLRTHNYLYAACECKAGWRGWG
CTDSADALTYGFQLLSTLLLCLSNLMFLPPVVLAIRSRYVLEAAVYTFTMFFSTFYHACDQPGIVVFCIMDYD
VLQFCDFLGSLMSVWVTVIAMARLQPVIKQVLYLLGAMLLSMALQLDRHGLWNLLGPSLFALGILATAWTVRS
VRRRHCYPPTWRRWLFYLCPGSLIAGSAVLLYAFVETRDNYFYIHSIWHMLIAGSVGFLLPPRAKTDRRVPSG
ARARGCGYQLCINEQEELGLVGPGGTTVSSICVS*
>ENSMUSP00000103496_c.324:C>A_p.H109N ENSMUST00000107864.8|Tmem8b-201|ENSMUSG00000078716|+|chr4:g.43685342:C>A
MNMPQSLGTQPLPPEPPSLGTPIEGSGAIAPTEHCWPVRPTLRNELDTFSVHFYIFFGPSVALPPERPAVFAL
RLLPVLDSGGVLSLELQLNASSLRQENVTVFGCLTNEVPLSLGDAAVTCSKESLAGFLLSVSATSRVARLRIP
FPQTGTWFLTLRSLCGVGPRFVRCRNATAEVRLRTFLSPCVDDCGPYGQCKLLRTHNYLYAACECKAGWRGWG
CTDSADALTYGFQLLSTLLLCLSNLMFLPPVVLAIRSRYVLEAAVYTFTMFFSTFYHACDQPGIVVFCIMDYD
VLQFCDFLGSLMSVWVTVIAMARLQPVIKQVLYLLGAMLLSMALQLDRHGLWNLLGPSLFALGILATAWTVRS
VRRRHCYPPTWRRWLFYLCPGSLIAGSAVLLYAFVETRDNYFYIHSIWHMLIAGSVGFLLPPRAKTDRRVPSG
ARARGCGYQLCINEQEELGLVGPGGTTVSSICVS*
>ENSMUSP00000103498_c.1701:C>A_p.H568N ENSMUST00000107866.9|Tmem8b-203|ENSMUSG00000078716|+|chr4:g.43685342:C>A
MAQPLSRPLILSQFQPWPPATPSPRFPNLSRPRPVSQRMSRSRSPLQPRSLPPAWPLSPPRPLFHPLSQFPAQ
HLTLSHSPRLLKPLAQPQPLLQSPSHPLLPSHLLPLFEPRCSVQRHPKKAQSLPPSLCLPKSLPLGPRLSHTL
PLSQPRLRSGLQLPPALLLLLLFSVLGPGAGGLFLTDYSTCSPRKLSPFRSFASTELFHFHVPEDTFLAVWNL
IIFKEQGGTFGDHCPDQSVTVYFRSGAPPVINPLHTHFPADTAVPGVFSLTLSWTLPNRTSGIFNVSSPLPGD
WFLAAHLPQAHGHISVKGLQDECQYLLQPQLIVRRLLDVAVLVPGRPSEQTLSSHNRSALYKVFVPSFTYRVS
AQLVCVGGRGASVCPLTLRLRPKAPPLHNSSSVACGGASVCQLELALPPWGHWVYVRVETPSRGPGRTISFQL
CVRLQECPQPSLSRALVPGAAMNMPQSLGTQPLPPEPPSLGTPIEGSGAIAPTEHCWPVRPTLRNELDTFSVH
FYIFFGPSVALPPERPAVFALRLLPVLDSGGVLSLELQLNASSLRQENVTVFGCLTNEVPLSLGDAAVTCSKE
SLAGFLLSVSATSRVARLRIPFPQTGTWFLTLRSLCGVGPRFVRCRNATAEVRLRTFLSPCVDDCGPYGQCKL
LRTHNYLYAACECKAGWRGWGCTDSADALTYGFQLLSTLLLCLSNLMFLPPVVLAIRSRYVLEAAVYTFTMFF
STFYHACDQPGIVVFCIMDYDVLQFCDFLGSLMSVWVTVIAMARLQPVIKQVLYLLGAMLLSMALQLDRHGLW
NLLGPSLFALGILATAWTVRSVRRRHCYPPTWRRWLFYLCPGSLIAGSAVLLYAFVETRDNYFYIHSIWHMLI
AGSVGFLLPPRAKTDRRVPSGARARGCGYQLCINEQEELGLVGPGGTTVSSICVS*
```

>ENSMUSP00000130133_c.324:C>A_p.H109N ENSMUST00000143339.8|Tmem8b-206|ENSMUSG00000078716|+|chr4:g.43685342:C>A
MNMPQSLGTQPLPPEPPSLGTPIEGSGAIAPTEHCWPVRPTLRNELDTFSVHFYIFFGPSVALPPERPAVFAL
RLLPVLDSGGVLSLELQLNASSLRQENVTVFGCLTNEVPLSLGDAAVTCSKVPGRLPSLGQCHLQSGQASNPI
PTDGDLVP
>ENSMUSP00000080908_c.440:C>G_p.D147E ENSMUST00000082287.3|Mup5-201|ENSMUSG00000058523|-|chr4:g.61750865:G>C
MKLLLLLCLELTLVYVHAEEASSEGQNLNVEKINGKWFSILLASDKREKIEEHGTMRVFVEHIDVLENSLAFK
FHTVIDEECTEIYLVADKTEKAGEYSVTYDGFNTFTILKTDYDNYIMFHLINKKDEENFQLMELFGREPDLSS
EIKEKFAKLCEEHGIVRENIIDLSNANRCLQARE*
>ENSMUSP00000056520_c.3685:A>T_p.H1229L ENSMUST00000053414.13|Ccdc171-201|ENSMUSG00000052407|+|chr4:g.83736316:A>T
MSLNTSSNATPGDTQRLKNASLDVKQMLKNETESDIIADLRKKLHRAKKEKLEMTTKHNAELSSYESQIARLR
SEVEKGEALRQRLEYDLAVARKEAGLGRRAAEERLAEAQRIQERLCAQNSELQGKANEIEKTFQISQEKWREE
CRRFEHDLEERDNIIQNCNQEYESLMQEKTRLQKTLQEILEKHEQEKTELESRVRETALGEFRLQTEEWEAER
RELQLIVQEQDSAVQSMQKKVEQLEAEHMDCSDLLRRQTSELEFSTQREERLRKEFEATTLRVRKLEENIEAE
RAAHLESKFNSEIIQLRIRDLEGALQVEKASQAEAVADLEMIKNEFKEVESAYEREKQNTQESCAKLNLLERE
YFSQNKKLNEEIEDQKKVIIDLSKRLQYNEKSCGELQEELVMAKKHQAFLVETCENNVRELESILGSFSVSAQ
WTSGVHKDKDKPPSFSVVLETLRRTLTDYQNKLEDASNEEKTSNELDSTKQKIETHIKNTKELQDKLTEVHKE
LSHLRAKCADREALITSLKVELQNVLHCWEKEKACAAQCESELQKLSQAFQKDSEEKLTFLHTLYQHLVAGCV
LIKQPEGMLDKFSWSELCAVLQENVDALIADLNRANEKISHLEYICKNKSDTMRELQQTQEDTFNKVAEQIKA
QESCWQKQKKELEFQYSELLLEVQRRAQKFQEIAEKNSEKLNRIETSHEQLVRENSHFKTTLSRTQREQTCLL
AACALMAGALCPLYSRSCALSTQRDFLQEQVNSLELFKLEIRTLAQALSAVDEKKQEEAKTKKKTFKGLVRVF
RKGVIAILAANRLKLLGQSCAFLFTWMESCKEGIGMLVCTGEPKDKRQFPKHQREQLRCLQALAWLTSSDLLG
TVISSMTELQEVISKTDPNSRICGHLLIGAAKNSFAKLMDKLSSAMASIPLHSSRSITYVEKDSLVQRLARGL
HKVNTLALKYGLCSHIPIMKSTAALQKQIFGFTQRLHAAEVERRSLRLEVTEYKRTVHEMKKELDKSQSLQTQ
LNEFKHSKLITHEKFESACEELNNALLREQQAQMLLNEQAQQLQELNYRLELHSSEEADKNQTLGEAVKSLSE
AKMELRRKDQSLRQLNRHLTQLEQDKRRLEENIRDAESALRMAAKDKECVANHMRTIENMLHKVRDQISLSRT
AATRNDFTLQLPKLHLETFAMEGLQGGPEVVACQAMIKSFMDVYQLASARISTLEKEMTSLRSHIATLKSELH
TACLRENESLQSMGSRDHSNLSVPSRAAAPMDTVGDLLPLQAELDTTYTFLKETFVNTAPHSLSSQSSPGVPT
NAKRPSQIGL*
>ENSMUSP00000155912_c.3709:A>T_p.H1237L ENSMUST00000231339.2|Ccdc171-206|ENSMUSG00000052407|+|chr4:g.83736316:A>T
MSLNTSSNATPGDTQRLKNASLDVKQMLKNETESDIIADLRKKLHRAKKEKLEMTTKHNAELSSYESQIARLR
SEVEKGEALRQRLEYDLAVARKEAGLGRRAAEERLAEAQRIQERLCAQNSELQGKANEIEKTFQISQEKWREE
CRRFEHDLEERDNIIQNCNQEYESLMQEKTRLQKTLQEILEKHEQEKTELESRVRETALGEFRLQTEEWEAER
RELQLIVQEQDSAVQSMQKKVEQLEAEHMDCSDLLRRQTSELEFSTQREERLRKEFEATTLRVRKLEENIEAE
RAAHLESKFNSEIIQLRIRDLEGALQVEKASQAEAVADLEMIKNEFKEVESAYEREKQNTQESCAKLNLLERE
YFSQNKKLNEEIEDQKKVIIDLSKRLQYNEKSCGELQEELVMAKKHQAFLVETCENNVRELESILGSFSVSAQ
WTSGVHKDKDKPPSFSVVLETLRRTLTDYQNKLEDASNELNNINDAKEKTSNELDSTKQKIETHIKNTKELQD
KLTEVHKELSHLRAKCADREALITSLKVELQNVLHCWEKEKACAAQCESELQKLSQAFQKDSEEKLTFLHTLY
QHLVAGCVLIKQPEGMLDKFSWSELCAVLQENVDALIADLNRANEKISHLEYICKNKSDTMRELQQTQEDTFN
KVAEQIKAQESCWQKQKKELEFQYSELLLEVQRRAQKFQEIAEKNSEKLNRIETSHEQLVRENSHFKTTLSRT
QREQTCLLAACALMAGALCPLYSRSCALSTQRDFLQEQVNSLELFKLEIRTLAQALSAVDEKKQEEAKTKKKT
FKGLVRVFRKGVIAILAANRLKLLGQSCAFLFTWMESCKEGIGMLVCTGEPKDKRQFPKHQREQLRCLQALAW
LTSSDLLGTVISSMTELQEVISKTDPNSRICGHLLIGAAKNSFAKLMDKLSSAMASIPLHSSRSITYVEKDSL
VQRLARGLHKVNTLALKYGLCSHIPIMKSTAALQKQIFGFTQRLHAAEVERRSLRLEVTEYKRTVHEMKKELD
KSQSLQTQLNEFKHSKLITHEKFESACEELNNALLREQQAQMLLNEQAQQLQELNYRLELHSSEEADKNQTLG
EAVKSLSEAKMELRRKDQSLRQLNRHLTQLEQDKRRLEENIRDAESALRMAAKDKECVANHMRTIENMLHKVR
DQISLSRTAATRNDFTLQLPKLHLETFAMEGLQGGPEVVACQAMIKSFMDVYQLASARISTLEKEMTSLRSHI
ATLKSELHTACLRENESLQSMGSRDHSNLSVPSRAAAPMDTVGDLLPLQAELDTTYTFLKETFVNTAPHSLSS
QSSPGVPTNAKRPSQIGL*
>ENSMUSP00000092521_c.402:A>C_p.S135R ENSMUST00000094916.5|Ldlrad1-201|ENSMUSG00000070877|+|chr4:g.107073369:A>C
MSVGCERAEFCPACFWFLGQADAESNITTRRKARPGDEGCGPLCCSRRGACLSVGLLLLLAMLAALLAVATIL
GHPPRTPETHSCVTLANRTGFLCHDRRHCIPAHAVCDGIRTCPHGEDEAESLCRDVPQSLPRFLLTTCGDPDS
WIYSDQKCDGVNNCGDCSDELSPVTTCPPCGPGWWRCSPTVFKYCSCVPRDLCRDSVQHCSDWSDEYSCPGP*

>ENSMUSP00000118617_c.282:C>T_p.H95Y ENSMUST00000152717.8|Hspb11-204|ENSMUSG00000063172|+|chr4:g.107135132:C>T
MRKVDLCSVTEGTEVILATSSDEKHPPENIIDGNPETFWTTTGMFPQEFIICFHKHVKIEKLVIQSYLVRTLR
IEKTTSKEPLDFEQWVEKDLVYTEGQLQNEEIVARDGYATFLRFIIVSAFDHFASVHSISAEGLTVSSLP
>ENSMUSP00000102360_c.282:C>T_p.H95Y ENSMUST00000106749.8|Hspb11-202|ENSMUSG00000063172|+|chr4:g.107135132:C>T
MRKVDLCSVTEGTEVILATSSDEKHPPENIIDGNPETFWTTTGMFPQEFIICFHKHVKIEKLVIQSYLVRTLR
IEKTTSKEPLDFEQWVEKDLVYTEGQLQNEEIVARDGYATFLRFIIVSAFDHFASVHSISAEGLTVSSLP*
>ENSMUSP00000048810_c.282:C>T_p.H95Y ENSMUST00000046558.8|Hspb11-201|ENSMUSG00000063172|+|chr4:g.107135132:C>T
MRKVDLCSVTEGTEVILATSSDEKHPPENIIDGNPETFWTTTGMFPQEFIICFHKHVKIEKLVIQSYLVRTLR
IEKTTSKEPLDFEQWVEKDLVYTEGQLQNEEIVARDGYATFLRFIIVSAFDHFASVHSISAEGLTVSSLP*
>ENSMUSP00000049474_c.2941:T>A_p.I981N ENSMUST00000044823.4|Zswim5-201|ENSMUSG00000033948|+|chr4:g.116843905:T>A
MAEGGEREELLSPPPISPAKRLCSWPSPQAHHPRGTPGAAGGGAGGGGGGCLAPGARPHLQPESLLDCAAKTV
AEKWAYERVEERFERIPEPVQRRIVYWSFPRNEREICMYSSFQYRGGPGAGAAAGAAGASPVEEGPPPPPGAA
APAGSAPGAAGAGSSPGLGAGTGTASGGCGGGEGLPFRRGIRLLDSGSVENVLQVGFHLSGTVTEPAMASEPA
VTYKVAISFDRCKITSVSCGCGNKDIFYCAHVVALSLYRIRKPDQVKLRLPISETLFQMNRDQLQKFIQYLIT
AHHTEVLPTAQKLADEILSSNSEINQVNGAPDPTAGASIDDENCWHLDEEQVKEQVKLFLSQGGYYGSGKQLN
SMFAKVREMLRMRDSNGARMLTLITEQFMADPRLTLWRQQGTSMTDKCRQLWDELGALWVCIILNPHCKLEEK
SCWLQQLQKWSDLDICPLEDGNYGHELPNITNALSQSASHSPDSLSRPRRTVFTRAIEGRELHWQDSHLQRII
SSGIYTAPACQRENERLLFNSHGQPLWLEHVPTACARVDALRSHGYPKEALRLTVAIINTLRLQQQRQLEIYK
HQKKELLQRGTTTITNLEGWVGHPLDPIGCLFLTLTEACRLNDDSYMEMSDMNESRLPVYQHVPVASGCPNSN
ESYLSLALEVALMGMGQQRVMPEGLYAQDKVCRNEEQLLSQLQELQLDDELVQTLRKQCILLLEGGPFSGLGE
VIHRESVPMHTFAKYLFSALLPHDPDLSYKLALRAMRLPVLENSSSAGDTSHPHHMVSVVPSRYPRWFTLGHL
ESQQCELASTMLTAAKGDTLRLRTLLEAIQKHIHSPSLIFKLAQDAFKIATPTDSSTDGTLLNVALELGLQVM
RMTLSTLNWRRREMVRWLVTCATEVGVRALVSILQSWYTLFTPTEATSIVAATAVSHTTILRLSLDYPQREEL
ASCARTLALQCAMKDPQSCALSALTLCEKDHNAFEAAYQIAIDAAAGGMTHSQLFTIARYMELRGYPLRAFKL
ASLAMSHLNLAYNQDTHPAINDVLWACALSHSLGKNELAALIPLVVKSVHCATVLSDILRRCTVTAPGLAGIP
GRRSSGKLMSTDKAPLRQLLDATINAYINTTHSRLTHISPRHYGEFIEFLSKARETFLLPQDGHLQFAQFIDN
LKQIYKGKKKLMLLVRERFG*
>rs46360603_ENSMUSP00000149966_c.412:T>C_p.L138P
ENSMUST00000216226.2|Olfr1342-202|ENSMUSG00000043383|-|chr4:g.118547235:A>G
MQTLRKDNCSSVSEFLLLGFSSESQVRVALFIFFLLLYMITLLGNGLIITLIYLDSRLHTPMYFFLSILSLVD
MSYVTTTVPQMLVNMVCPRRTISWGACVAQMFIFLLLGIAECVLYAIMAYDRYVAICFPLHYSVPMSRLVCIK
MVTVCWSISITGALIYTVFTMRLPYCGPYKINHFFCEVPAVLKLACADTSFNDRLDFILGFIFLLVPLSLILA
SYACIFASILRIRSSQGRLKSFSTCASHITVVTMFYGPAMIMYMRPGSWYDPERDKKLALFYNVVSAFLNPII
YSLRNKDVKGAFLKVLGDRGAAK*
>rs46360603_ENSMUSP00000053925_c.412:T>C_p.L138P
ENSMUST00000060562.6|Olfr1342-201|ENSMUSG00000043383|-|chr4:g.118547235:A>G
MQTLRKDNCSSVSEFLLLGFSSESQVRVALFIFFLLLYMITLLGNGLIITLIYLDSRLHTPMYFFLSILSLVD
MSYVTTTVPQMLVNMVCPRRTISWGACVAQMFIFLLLGIAECVLYAIMAYDRYVAICFPLHYSVPMSRLVCIK
MVTVCWSISITGALIYTVFTMRLPYCGPYKINHFFCEVPAVLKLACADTSFNDRLDFILGFIFLLVPLSLILA
SYACIFASILRIRSSQGRLKSFSTCASHITVVTMFYGPAMIMYMRPGSWYDPERDKKLALFYNVVSAFLNPII
YSLRNKDVKGAFLKVLGDRGAAK*
>rs213024301_ENSMUSP00000138958_c.2263:G>A_p.G755D
ENSMUST00000184063.3|Csmd2-205|ENSMUSG00000028804|+|chr4:g.128266349:G>A
MPRSRGPEQGCCGCPAVRARGEIGIWALVPGTGSRWGRPPPPPPPPPPPPPLLLLLGWGLLSASAAAGQNCTF
HLQGPNGTVESPGFPYGYPNYANCTWTITAEDQHRIQLVFQSFALEEDFDVLSVFDGPPQAENLRTRLTGFQL
PATIVSAASTLSLSLISDYAVSAQGFRASYEVLPSHTCGNPGRLPNGIQQGSTFNLGDKVRYSCNPGFFLEGH
AVLTCHASSENSATWDFPLPSCRADDACGGTLRGQSGIISSPHFPAEYHNNADCTWTILAELGDTIALVFIDF
QLEDGYDFLEVTGTEGSSLWFTGASLPAPVISSKNWLRLHFTSDGNHRQRGFSAQYQVKKQIELKSRGVKLMP
SKDNNQKTSVLTQVGVSQGHNMCPDPGIPEKGKRLGSDFRLGSSVQFTCNEGYDLQGSKRITCMKVSDMFAAW
SDHRPVCRARMCDAHLRGPSGIITSPNFPIQYDNNAHCVWIITALNPAKVIKLAFEEFDLERGYDTLTVGDGG
QDGDQKTVLYILTGTSVPDLIVSTHHQMWLLFQSDSSGSSLGFKASYEEIEQGSCGDPGIPAYGQREGSRFRH
GDTLEFECQPAFELVGQKSITCQKNNQWSAKKPGCVFSCFFNFTSPSGIVLSPNYPEDYGNHLHCVWLILARP

ESRIHLAFNDIDVEPQFDFLVIKDGATAEAPVLGTFSGNQLPSSITSSGHVARLEFQTDHSTGKRGFNITFTT
FRHNECPDPGVPVNGKRFGDSLQLDSSISFLCDEGFLGTQGSETITCVLKEGSVVWNSAVLRCEAPCGGHLTS
PSGTILSPGWPGFYKDALSCAWVIEAQPGYPIKITFDRFKTEVNYDTLEVRDGRTYSAPLIGVYHGTQVPQFL
ISTSNYLYLLFSTDKSHSDIGFQLRYETITLQSDHCLDPGIPVNGQRHGNDFYVGALVTFSCDSGYTLSDGEP
LECEPNFQWSHALPSCEALCGGFIQGSSGTILSPGFPDFYPNNLNCTWVIETSHGKGVFFTFHTFHLESGHDY
LLITENGSFSQPLRQLTGSRLPAPISAGLYGNFTAQVRFISDFSMSYEGFNITFSEYDLEPCEEPEVPAYSIR
KGLQFGVGDTLTFSCFPGYRLEGTARITCLGGRRRLWSSPLPRCVAECGNSVTGTQGTLLSPNFPVNYNNNHE
CIYSIQTQPGKGIQLKAKAFELAEGDLLKVYDGTNNSARLLGVFSRSEMLGVTLNSTSSSLWLDFITDAENTS
KGFELQFSSFELIRCEDPGTPQFGYKVHDGGHFAGSSVTFSCDPGYSLRGSEELMCLSGERRTWDRPLPTCVA
ECGGTVRGEVSGQVLSPGYPAPYEHNLNCIWTIEADAGCTIGLHFLVFDTEEVHDVLRIWDGPVESGVLLKEL
SGPMLPKDLHSTFNSVVLQFSTDFFTSKQGFAIQFSVSTATSCNDPGVPQNGSRSGDSWEAGDSTVFQCDPGY
ALQGSAEISCVKIQNRFFWQPSPPTCIAPCGGDLTGPSGVILSPNYPEPYPPGKECDWKVTVSPDYVIALVFN
TFNLEPGYDFLHIYDGRDSLSPLIGSFYGSQLPSRIESSSNSLFLAFRSDASVSNAGFVIDYTENPRESCFDP
GSIKNGTRVGSDLKLGSSVTYYCHGGYEVEGASTLSCILGPDGKPMWNNPRPVCTAPCGGQYVGSDGVVLSPN
YPQNYTSGQTCLYFVTVPKDYVVFGQFAFFHTALNDIVEVHDGYSQHSRLLSSLSGSHTGESLPLATSNQVLI
KFSAKGQVSARGFHFVYQAVPRTSATQCSSVPEPRYGKRLGSDFSVGAIIRFECNSGYALQGSPEIECLPVPG
ALAQWNVSAPTCVVPCGGNLTERRGTILSPGFPEPYLNSLNCVWKIMVPEGAGIQIQVISFVTEQNWDSLEVF
DGADNTVTMLGSFSGTTVPALLNSTSNQLYLHFYSDISVSAAGFHLEYKTVGLSSCPEPAVPSNGVKTGERYL
VNDVVSFQCEPGYALQGHAHISCMPGTVRRWNYPPPLCIAQCGGAVEEMEGVILSPGFPGNYPSNMDCSWKIS
LPVGFGAHIQFLNFSTEPNHDFLEIRSGPSETSRMMGRFSGSELPGSLLSTSHDSIVYFHSDHSQNRPGFKLE
YQAYELQECPDPEPFANGIVRGAGYNVGQSVTFECLPGYQLTGQPVLTCQHGTNRNWDHPLPRCEVPCGGNIT
SFNGTVYSPGYPSPYSSSQDCVWQITVPIGHGVHLNLSLLQIEPFGDYITVWDGPQQTSLQLGVFTRSLSKKI
AHSSSNQVLLKFHRDTATGGIFAIAFSAYPLTKCPPPTILPNAEVVTENEEFNIGDIVRYRCLPGFTLVGSEI
LTCKLGTYLQFEGPPPICEVHCPTNELLTDSTGVILSQSYPGSYPQFQTCSWLVRVEPEYNISITVEYFLSEK
QYDEFEIFDGPSGQSPLLKALSGNYSAPLIVTSSSNSVYLRWSSDHAYNRKGFKIRYSAPYCSLPKAPLHGFI
LGQTTTQPGGSIHFGCNTGYRLVGHSMAICTRHPQGYHLWSEAIPLCQALSCGLPDAPKNGIVFGKEYTVGTK
AVYSCNEGYHLQAGAEATAECLDTGLWSNSNVPPQCVPVTCPDISSISVEHGRWRLIFETQYQFQAQLMLICD
PGYYYTGQRVIRCQANGRWSLGESMPTCQIISCGELPTPPSGHRIGTMSVYGATAIFSCNSGYTLVGSRVREC
MANGLWSGSEVRCLAGHCGTPEPIVNGHINGENFNYRGSVVYQCRAGFRLIGMSVRICQQDHHWSGKTPFCVP
ITCGHPGNPVNGLTQGSQFNLNDVVKFICNPGYIAEGAARSQCLASGQWSDTLPTCRIINCTDPGHQENSVRQ
VHTSGPHRFSFGTTVSYQCNHGFYLLGTPALSCQGDGTWDRPRPQCLLVSCGHPGSPPHAQMSGDSYIVGAVV
RYSCTGKRTLVGNSTRMCGLDGHWTGSLPHCSGTSTGVCGDPGIPAHGIRLGDSFAPGSVMRFSCDAGHVLRG
SSERMCQANGSWSGSQPECGVISCGNPGTPSNARVVFSDGLVFSSSVVYECREGYYATGLLSRHCSVNGTWTG
SDPECTVINCGDPGIPANGIRLGNDFRYNKTVTYQCVPGYVMESHRVSVLSCTKDRTWNGTKPVCKAIMCKPP
QLIPNGKVVGSDFTWGSSVSYACLEGYQLSLPAVLTCEGNGSWTGELPQCFPVFCGDPGVPPRGRREDRGFSY
RSSVSFSCHAPLVLVGSPRRFCQSDGTWSGTQPSCIDPTLTTCADPGMPQFGIQNSSQGYQVGSTVLFRCQKG
YLLQGSTTRTCLPNLTWSGTPPDCVPHHCKQPETPTHANVGALDLPSMGYTLIYSCQEGFSLRGGSEHRTCKA
DGSWTGKPPVCLEVRPSGRPINTAREPMLTQASVPGDVFAKNSLWKGAYEYQGKKQPAMLRVTGFQVANSKVN
ATMIDHSGVELHLAGNYKKEDFRLLLQVYQVTGPVESFVNKFKDDHWALDGHVSSESSGGTFVYQGSVKGQGF
GQFGFQRLDLRLLESDPESIGRHFASNSSSVAAAILVPFIALIIAGFVLYLYKHRRRPKVPFNGYAGHENTNV
RATFENPMYDRNIQPTDIMSNEAEFTVSTVCTAV*
>ENSMUSP00000101487_c.2030:G>T_p.E677D ENSMUST00000105861.8|Srrm1-
203|ENSMUSG00000028809|-|chr4:g.135052315:C>A
MDAGFFRGTSAEQDNRFSNKQKKLLKQLKFAECLEKKVDMSKVNLEVIKPWITKRVTEILGFEDDVVIEFIFN
QLEVKNPDSKMMQINLTGFLNGKNAREFMGELWPLLLSAQENIAGIPSAFLELKKEEIKQRQIEQEKLASLKK
QDEDKDKRDKEEKESSREKRERSRSPRRRKSRSPSPRRRSSPVRRERKRSHSRSPRHRTKSRSPSPAPEKKEK
SPELPEPSVRMKDSSVQEATSTSDILKAPKPEPVPEPKEPSPEKNSKKEKEKTRPRSRSRSKSRSRTRSRSPS
HTRPRRRHRSRSRSYSPRRRPSPRRRPSPRRRTPPRRMPPPPRHRRSRSPGRRRRRSSASLSGSSSSSSSSRS
RSPPKKPPKRTSSPPRKTRRLSPSASPPRRRHRPSSPATPPPKTRHSPTPQQSNRTRKSRVSVSPGRTSGKVT
KHKGTEKRESPSPAPKPRKVELSESEEDKGSKMAAADSVQQRRQYRRQNQQSSSDSGSSSTSEDERPKRSHVK
NGEVGRRRRHSPSRSASPSPRKRQKETSPRSRRRRSPSPPPARRRRSPSPAPPPPPPPPPPRRRRSPTPPPRR
RTPSPPPRRRSPSPRRYSPPIQRRYSPSPPPKRRTASPPPPPKRRASPSPPPKRRVSHSPPPKQRSPTVTKRR
SPSLSSKHRKGSSPGRSTRDARSPQPNKRHSPSPRPRAPQTSSPPPVRRGASASPQGRQSPSPSTRPIRRVSR
TPEPKKIKKAASPSPQSVRRVSSSRSVSGSPEPAAKKPPAPPSPVQSQSPSTNWSPAVPAKKAKSPTPSLSPA
RNSDQEGGGKKKKKKKDKKHKKDKKHKKHKKHKKEKAVTIATPATAAPAAVSAATTTSAQEEPAAAPEPRKET
ESEAEDDNLDDLERHLREKALRSMRKAQVSPQS*
>ENSMUSP00000081906_c.2057:G>T_p.E686D ENSMUST00000084846.12|Srrm1-
202|ENSMUSG00000028809|-|chr4:g.135052315:C>A

MDAGFFRGTSAEQDNRFSNKQKKLLKQLKFAECLEKKVDMSKVNLEVIKPWITKRVTEILGFEDDVVIEFIFN
QLEVKNPDSKMMQINLTGFLNGKNAREFMGELWPLLLSAQENIAGIPSAFLELKKEEIKQRQIEQEKLASLKK
QDEDKDKRDKEEKESSREKRERSRSPRRRKSRSPSPRRRSSPVRRERKRSHSRSPRHRTKSRSPSPAPEKKEK
SPELPEPSVRMKDSSVQEATSTSDILKAPKPEPVPEPKEPSPEKNSKKEKEKTRPRSRSRSKSRSRTRSRSPS
HTRPRRRHRSRSRRRPSPRRRPSPRRRTPPRRMPPPPRHRRSRSPGRRRRRSSASLSGSSSSSSSSRSRSPPK
KPPKRTSSPPRKTRRLSPSASPPRRRHRPSSPATPPPKTRHSPTPQQSNRTRKSRVSVSPGRTSGKVTKHKGT
EKRESPSPAPKPRKVELSESEEDKGSKMAAADSVQQRRQYRRQNQQSSSDSGSSSTSEDERPKRSHVKNGEVG
RRRRHSPSRSASPSPRKRQKETSPRMQMGKRWQSPVTKSSRRRRSPSPPPARRRRSPSPAPPPPPPPPPPRRR
RSPTPPPRRRTPSPPPRRRSPSPRRYSPPIQRRYSPSPPPKRRTASPPPPPKRRASPSPPPKRRVSHSPPPKQ
RSPTVTKRRSPSLSSKHRKGSSPGRSTRDARSPQPNKRHSPSPRPRAPQTSSPPPVRRGASASPQGRQSPSPS
TRPIRRVSRTPEPKKIKKAASPSPQSVRRVSSSRSVSGSPEPAAKKPPAPPSPVQSQSPSTNWSPAVPAKKAK
SPTPSLSPARNSDQEGGGKKKKKKKDKKHKKDKKHKKHKKHKKEKAVTIATPATAAPAAVSAATTTSAQEEPA
AAPEPRKETESEAEDDNLDDLERHLREKALRSMRKAQVSPQS*
>ENSMUSP00000125003_c.2072:G>T_p.E691D ENSMUST00000136342.9|Srrm1-208|ENSMUSG00000028809|-|chr4:g.135052315:C>A
MDAGFFRGTSAEQDNRFSNKQKKLLKQLKFAECLEKKVDMSKVNLEVIKPWITKRVTEILGFEDDVVIEFIFN
QLEVKNPDSKMMQINLTGFLNGKNAREFMGELWPLLLSAQENIAGIPSAFLELKKEEIKQRQIEQEKLASLKK
QDEDKDKRDKEEKESSREKRERSRSPRRRKSRSPSPRRRSSPVRRERKRSHSRSPRHRTKSRSPSPAPEKKEK
SPELPEPSVRMKDSSVQEATSTSDILKAPKPEPVPEPKEPSPEKNSKKEKEKTRPRSRSRSKSRSRTRSRSPS
HTRPRRRHRSRSRSYSPRRRPSPRRRPSPRRRTPPRRMPPPPRHRRSRSPGRRRRRSSASLSGSSSSSSSSRS
RSPPKKPPKRTSSPPRKTRRLSPSASPPRRRHRPSSPATPPPKTRHSPTPQQSNRTRKSRVSVSPGRTSGKVT
KHKGTEKRESPSPAPKPRKVELSESEEDKGSKMAAADSVQQRRQYRRQNQQSSSDSGSSSTSEDERPKRSHVK
NGEVGRRRRHSPSRSASPSPRKRQKETSPRMQMGKRWQSPVTKSSRRRRSPSPPPARRRRSPSPAPPPPPPPP
PPRRRRSPTPPPRRRTPSPPPRRRSPSPRRYSPPIQRRYSPSPPPKRRTASPPPPPKRRASPSPPPKRRVSHS
PPPKQRSPTVTKRRSPSLSSKHRKGSSPGRSTRDARSPQPNKRHSPSPRPRAPQTSSPPPVRRGASASPQGRQ
SPSPSTRPIRRVSRTPEPKKIKKAASPSPQSVRRVSSSRSVSGSPEPAAKKPPAPPSPVQSQSPSTNWSPAVP
AKKAKSPTPSLSPARNSDQEGGGKKKKKKKDKKHKKDKKHKKHKKHKKEKAVTIATPATAAPAAVSAATTTSA
QEEPAAAPEPRKETESEAEDDNLDDLERHLREKALRSMRKAQVSPQS*
>ENSMUSP00000030613_c.2072:G>T_p.E691D ENSMUST00000030613.11|Srrm1-201|ENSMUSG00000028809|-|chr4:g.135052315:C>A
MDAGFFRGTSAEQDNRFSNKQKKLLKQLKFAECLEKKVDMSKVNLEVIKPWITKRVTEILGFEDDVVIEFIFN
QLEVKNPDSKMMQINLTGFLNGKNAREFMGELWPLLLSAQENIAGIPSAFLELKKEEIKQRQIEQEKLASLKK
QDEDKDKRDKEEKESSREKRERSRSPRRRKSRSPSPRRRSSPVRRERKRSHSRSPRHRTKSRSPSPAPEKKEK
SPELPEPSVRMKDSSVQEATSTSDILKAPKPEPVPEPKEPSPEKNSKKEKEKTRPRSRSRSKSRSRTRSRSPS
HTRPRRRHRSRSRSYSPRRRPSPRRRPSPRRRTPPRRMPPPPRHRRSRSPGRRRRRSSASLSGSSSSSSSSRS
RSPPKKPPKRTSSPPRKTRRLSPSASPPRRRHRPSSPATPPPKTRHSPTPQQSNRTRKSRVSVSPGRTSGKVT
KHKGTEKRESPSPAPKPRKVELSESEEDKGSKMAAADSVQQRRQYRRQNQQSSSDSGSSSTSEDERPKRSHVK
NGEVGRRRRHSPSRSASPSPRKRQKETSPRMQMGKRWQSPVTKSSRRRRSPSPPPARRRRSPSPAPPPPPPPP
PPRRRRSPTPPPRRRTPSPPPRRRSPSPRRYSPPIQRRYSPSPPPKRRTASPPPPPKRRASPSPPPKRRVSHS
PPPKQRSPTVTKRRSPSLSSKHRKGSSPGRSTRDARSPQPNKRHSPSPRPRAPQTSSPPPVRRGASASPQGRQ
SPSPSTRPIRRVSRTPEPKKIKKAASPSPQSVRRVSSSRSVSGSPEPAAKKPPAPPSPVQSQSPSTNWSPAVP
AKKAKSPTPSLSPARNSDQEGGGKKKKKKKDKKHKKDKKHKKHKKHKKEKAVTIATPATAAPAAVSAATTTSA
QEEPAAAPEPRKVFTPPLPAV*
>ENSMUSP00000120952_c.410:G>T_p.E137D ENSMUST00000140050.3|Srrm1-211|ENSMUSG00000028809|-|chr4:g.135052315:C>A
XSRRRRSPSPPPARRRRSPSPAPPPPPPPPPPPRRRRSPTPPPRRRTPSPPPRRRSPSPRRYSPPIQRRYSPSP
PPKRRTASPPPPPKRRASPSPPPKRRVSHSPPPKQRSPTVTKRRSPSLSSKHRKGSSPGRSTRDARSPQPNKR
HSPSPRPRAPQTSSPPPVRRGASASPQGRQSPSPSTRPIRRVSRTPEPKKIKKSVSGSPEPAAKKPPAPPSPV
QSQSPSTNWSPAVPAKKAKSPTPSLSPARNSDQEGGGKKKKKKKDKKHKKDKKHKKHKKHKKEKAVTIATPAT
AAPAAVSAATTTSAQEEPAAAPEPRK
>ENSMUSP00000101442_c.42:G>TAAAG_p.V15ins-fs ENSMUST00000105816.8|Pink1-202|ENSMUSG00000028756|-|chr4:g.138043014:C>CTTTA
MAPEVSTAHSGPSA
>ENSMUSP00000030536_c.1278:G>TAAAG_p.V427ins-fs ENSMUST00000030536.13|Pink1-201|ENSMUSG00000028756|-|chr4:g.138043014:C>CTTTA
MAVRQALGRGLQLGRALLLRFAPKPGPLFGWGKPGPAAAWGRGERPGQVVSPGAQPRPVGLPLPDRYRFFRQS
VAGLAARIQRQFMVRARGGAGPCGRAVFLAFGLGLGLIEEKQAEGRRAASACQEIQAIFTQKTKRVSDPLDTR

CWQGFRLEDYLIGQAIGKGCNAAVYEATMPTLPQHLEKAKHLGLIGKGPDVVLKGADGEQAPGTPTFPFAIKM
MWNISAGSSSEAILSKMSQELVPASRVALAGEYGAVTYRRSRDGPKQLAPHPNIIRVFRAFTSSVPLLPGALA
DYPDMLPPHYYPEGLGHGRTLFLVMKNYPCTLRQYLEEQTPSSRLATMMTLQLLEGVDHLVQQGIAHRDLKSD
NILVEWDSDGCPWLVISDFGCCLADQHVGLRLPFNSSSVERGGNGSLMAPEVSTAHSGPSA
>ENSMUSP00000101443_c.1188:G>TAAAG_p.V397ins-fs
ENSMUST00000105817.4|Pink1-203|ENSMUSG00000028756|-
|chr4:g.138043014:C>CTTTA
MAVRQALGRGLQLERPGQVVSPGAQPRPVGLPLPDRYRFFRQSVAGLAARIQRQFMVRARGGAGPCGRAVFLA
FGLGLGLIEEKQAEGRRAASACQEIQAIFTQKTKRVSDPLDTRCWQGFRLEDYLIGQAIGKGCNAAVYEATMP
TLPQHLEKAKHLGLIGKGPDVVLKGADGEQAPGTPTFPFAIKMMWNISAGSSSEAILSKMSQELVPASRVALA
GEYGAVTYRRSRDGPKQLAPHPNIIRVFRAFTSSVPLLPGALADYPDMLPPHYYPEGLGHGRTLFLVMKNYPC
TLRQYLEEQTPSSRLATMMTLQLLEGVDHLVQQGIAHRDLKSDNILVEWDSDGCPWLVISDFGCCLADQHVGL
RLPFNSSSVERGGNGSLMAPEVSTAHSGPSA
>rs50525384_ENSMUSP00000030536_c.1155:T>C_p.C386R
ENSMUST00000030536.13|Pink1-201|ENSMUSG00000028756|-|chr4:g.138043407:A>G
MAVRQALGRGLQLGRALLLRFAPKPGPLFGWGKPGPAAAWGRGERPGQVVSPGAQPRPVGLPLPDRYRFFRQS
VAGLAARIQRQFMVRARGGAGPCGRAVFLAFGLGLGLIEEKQAEGRRAASACQEIQAIFTQKTKRVSDPLDTR
CWQGFRLEDYLIGQAIGKGCNAAVYEATMPTLPQHLEKAKHLGLIGKGPDVVLKGADGEQAPGTPTFPFAIKM
MWNISAGSSSEAILSKMSQELVPASRVALAGEYGAVTYRRSRDGPKQLAPHPNIIRVFRAFTSSVPLLPGALA
DYPDMLPPHYYPEGLGHGRTLFLVMKNYPCTLRQYLEEQTPSSRLATMMTLQLLEGVDHLVQQGIAHRDLKSD
NILVEWDSDGCPWLVISDFGRCLADQHVGLRLPFNSSSVERGGNGSLMAPEVSTAHSGPSAVIDYSKADTWAV
GAIAYEIFGLANPFYGQGSAHLESRSYQEAQLPEMPESVPPEARRLVRSLLQREASKRPSARLAANVLHLSLW
GEHLLALKNLKLDKMIAWLLQQSAATLLADRLREKSCVETKLQMLFLANLECEALCQAALLLSSWRAAP*
>rs50525384_ENSMUSP00000101443_c.1065:T>C_p.C356R
ENSMUST00000105817.4|Pink1-203|ENSMUSG00000028756|-|chr4:g.138043407:A>G
MAVRQALGRGLQLERPGQVVSPGAQPRPVGLPLPDRYRFFRQSVAGLAARIQRQFMVRARGGAGPCGRAVFLA
FGLGLGLIEEKQAEGRRAASACQEIQAIFTQKTKRVSDPLDTRCWQGFRLEDYLIGQAIGKGCNAAVYEATMP
TLPQHLEKAKHLGLIGKGPDVVLKGADGEQAPGTPTFPFAIKMMWNISAGSSSEAILSKMSQELVPASRVALA
GEYGAVTYRRSRDGPKQLAPHPNIIRVFRAFTSSVPLLPGALADYPDMLPPHYYPEGLGHGRTLFLVMKNYPC
TLRQYLEEQTPSSRLATMMTLQLLEGVDHLVQQGIAHRDLKSDNILVEWDSDGCPWLVISDFGRCLADQHVGL
RLPFNSSSVERGGNGSLMAPEVSTAHSGPSAVIDYSKADTWAVGAIAYEIFGLANPFYGQGSAHLESRSYQEA
QLPEMPESVPPEARRLVRSLLQREASKRPSARLAANVLHLSLWGEHLLALKNLKLDKMIAWLLQQSAATLLAD
RLREKSCVETKLQMLFLANLECEALCQAALLLSSWRAAP*
>ENSMUSP00000115949_c.53:G>T_p.L18F ENSMUST00000148204.8|Szrd1-
205|ENSMUSG00000040842|-|chr4:g.140847667:C>A
VAESWEEAADSGEIDRRFEKKLKITQKERKSKSPPKVPIVIQDDSLPTGPPPQIRILKRPTSNGVVSSPNSTS
RPALPVKSLAQREAEYAEARRRILGSASPEEEQEKPILDRPTRISQPEDSRQPSNVIRQPLGPDGSQGFKQRR
*
>ENSMUSP00000099545_c.68:G>T_p.L23F ENSMUST00000102487.4|Szrd1-
203|ENSMUSG00000040842|-|chr4:g.140847667:C>A
MEDEEVAESWEEAADSGEIDRRFEKKLKITQKESRKSKSPPKVPIVIQDDSLPTGPPPQIRILKRPTSNGVVS
SPNSTSRPALPVKSLAQREAEYAEARRRILGSASPEEEQEKPILDRPTRISQPEDSRQPSNVIRQPLGPDGSQ
GFKQRR*
>ENSMUSP00000077761_c.646:G>C_p.W216S ENSMUST00000078695.5|Pramel24-
201|ENSMUSG00000046435|+|chr4:g.143453540:G>C
MSFKDPPTLQQLARRSLLKDEALTISALPNLPMQLFPPLFKDAFTNKHPNILRQMVVAWPFPTLPVGSLMKIP
HLETLKAVLDGLDLLMSQKDRPSRWNLQVLDLRDAHKDFWDGWAGLLHEVCSQDVFGKNQPVGNHPILGGKQT
MTIKMNLSLMSRRPSKYLKYLYRWAKERKNGIQVICEKLEFGAIPAYDPLDVLKLCDAASIQELAINTCSDIY
TLASLALGVGQMKNLQKLLFKEICISWDRPWDLEKEARIVTEIFSEFSKLHKLQHLYLNDVYFLTERLDQMLR
CFESPLETLAITHCTLSESDMSYLSQCPSIHQLKHLDLSGVTLILSHPFLQSLLERLTATLQTLKLKGCKLMD
WQISDLLPALSQCSQLTEVDFVENFFSMDSLKKLLQNTANLTQLTLEKYPAPDEVYDDSDGVIPDRFLQLCSE
LIDTLKVVRQPKQVYFVSKRCLYCRNFCIYSFVGNI*
>rs108334209_ENSMUSP00000092103_c.849:C>T_p.L284F
ENSMUST00000094526.5|Pramel31-
201|ENSMUSG00000070619|+|chr4:g.144089533:C>T
MSFQAPPTLLSLAVQSLAKNEALAINALQELPRELFPPLFKAAFTGRHTKILKEMVGAWPLPCLPVGALMNIP
DMTVLQAVLDGVDMQLTGNFHPSKRKLQVLDLRNVHHHFWDVSAGPEDEDSSAETVCQKQKATHPHRYALRRR
LKVVTDLHLQFDLEEHQTYFLQWAQERRASLNLCCVKLQIRGLAMHTVRKILQIFQPYCIEELELNTGWTLST

LACFAPCLGQMKNLRKLHLTLVHEKLFTFLHTSTDIQEKSVTKFISQFSKLNSLQHLALAGLYFFTGHMNELL
GCLKTPLEFLSMALCKYSQSDMESFAQCQSLHHLKHLHLAGIILHDLSLVPLGIFLENVANTLKTLELEHCRM
TDSQVIAFIPALNKCSQLIEVNFCDNDISTSVLMNLLHHTANLSQLIMELYPAPLECYETGFVLRAERFVQLS
SELMNILRTVRQPHSICFASQLCLICSQRCTYGLETRLCHCCQ*
>rs46446139_ENSMUSP00000101364_c.430:T>C_p.V144A
ENSMUST00000105738.9|Zfp980-201|ENSMUSG00000058186|+|chr4:g.145427703:T>C
MFAPHQESGRRTTKRMSASLENTAQCLLTFKDVFLDFSSEEWECLNFAQRTLYMDVMLENYNNLLFVENHCIC
GNYEKEKVLEQDTQHIVNEHGHNRKKSSKCNELRNVIHESSESTPYNTNHRDATLQSSNLKRHKTGTTKEACT
YKDCVNCLKGSSIISLNQGTHIEKKEHNRNKNLDEVLVSRQNNSEMSTYTCGEFDKCFTQSDNLQSQQRIYPG
KKSYIYSESDKCFTQLSHLSIHHTIHSGEKPYKCSECDKCFTDKFTLRKHQRIHTGEKPYKCNECDKCFTDKG
SLRVHQRIHTGEKPYKCSECDKCFTQPSHLSIHRRIHSGEKPYKCSECDKCFTDKSSLRVHQRIHTGEKPYQC
SECDKCFTDKGSLRVHHRIHAGEKPYQCSECDKCFTDKGSLRVHHRIHAGEKPYKCSECDKCFTHKDSLRVHH
RIHAGEKPYKCSECDKCFTEKNGLRRHQRIHTGEKPYKCSECDKCFTDKSSLRVHQRIHTGEKPYQCSECDKC
FTDKGSLRVHHRIHAGEKPYKCSECDKCFTDKGYLRVHHRIHAGEKPYKCSECDKCFTHKGSLRVHHRIHAGE
KPYKCSECDKCFTEKNGLRRHQRIHTGEKPYKCSECDKCFTRKSHLSIHQNSYRRETVQMQ*
>rs46446139_ENSMUSP00000137589_c.430:T>C_p.V144A
ENSMUST00000180014.2|Zfp980-202|ENSMUSG00000058186|+|chr4:g.145427703:T>C
MFAPHQESGRRTTKRMSASLENTAQCLLTFKDVFLDFSSEEWECLNFAQRTLYMDVMLENYNNLLFVENHCIC
GNYEKEKVLEQDTQHIVNEHGHNRKKSSKCNELRNVIHESSESTPYNTNHRDATLQSSNLKRHKTGTTKEACT
YKDCVNCLKGSSIISLNQGTHIEKKEHNRNKNLDEVLVSRQNNSEMSTYTCGEFDKCFTQSDNLQSQQRIYPG
KKSYIYSESDKCFTQLSHLSIHHTIHSGEKPYKCSECDKCFTDKFTLRKHQRIHTGEKPYKCNECDKCFTDKG
SLRVHQRIHTGEKPYKCSECDKCFTQPSHLSIHRRIHSGEKPYKCSECDKCFTDKSSLRVHQRIHTGEKPYQC
SECDKCFTDKGSLRVHHRIHAGEKPYQCSECDKCFTDKGSLRVHHRIHAGEKPYKCSECDKCFTHKDSLRVHH
RIHAGEKPYKCSECDKCFTEKNGLRRHQRIHTGEKPYKCSECDKCFTDKSSLRVHQRIHTGEKPYQCSECDKC
FTDKGSLRVHHRIHAGEKPYKCSECDKCFTDKGYLRVHHRIHAGEKPYKCSECDKCFTHKGSLRVHHRIHAGE
KPYKCSECDKCFTEKNGLRRHQRIHTGEKPYKCSECDKCFTRKSHLSIHQNSYRRETVQMQ*
>ENSMUSP00000101364_c.816:C>G_p.H273D ENSMUST00000105738.9|Zfp980-
201|ENSMUSG00000058186|+|chr4:g.145428089:C>G
MFAPHQESGRRTTKRMSASLENTAQCLLTFKDVFLDFSSEEWECLNFAQRTLYMDVMLENYNNLLFVENHCIC
GNYEKEKVLEQDTQHIVNEHGHNRKKSSKCNELRNVIHESSESTPYNTNHRDATLQSSNLKRHKTGTTKEVCT
YKDCVNCLKGSSIISLNQGTHIEKKEHNRNKNLDEVLVSRQNNSEMSTYTCGEFDKCFTQSDNLQSQQRIYPG
KKSYIYSESDKCFTQLSHLSIHHTIHSGEKPYKCSECDKCFTDKFTLRKHQRIDTGEKPYKCNECDKCFTDKG
SLRVHQRIHTGEKPYKCSECDKCFTQPSHLSIHRRIHSGEKPYKCSECDKCFTDKSSLRVHQRIHTGEKPYQC
SECDKCFTDKGSLRVHHRIHAGEKPYQCSECDKCFTDKGSLRVHHRIHAGEKPYKCSECDKCFTHKDSLRVHH
RIHAGEKPYKCSECDKCFTEKNGLRRHQRIHTGEKPYKCSECDKCFTDKSSLRVHQRIHTGEKPYQCSECDKC
FTDKGSLRVHHRIHAGEKPYKCSECDKCFTDKGYLRVHHRIHAGEKPYKCSECDKCFTHKGSLRVHHRIHAGE
KPYKCSECDKCFTEKNGLRRHQRIHTGEKPYKCSECDKCFTRKSHLSIHQNSYRRETVQMQ*
>ENSMUSP00000137589_c.816:C>G_p.H273D ENSMUST00000180014.2|Zfp980-
202|ENSMUSG00000058186|+|chr4:g.145428089:C>G
MFAPHQESGRRTTKRMSASLENTAQCLLTFKDVFLDFSSEEWECLNFAQRTLYMDVMLENYNNLLFVENHCIC
GNYEKEKVLEQDTQHIVNEHGHNRKKSSKCNELRNVIHESSESTPYNTNHRDATLQSSNLKRHKTGTTKEVCT
YKDCVNCLKGSSIISLNQGTHIEKKEHNRNKNLDEVLVSRQNNSEMSTYTCGEFDKCFTQSDNLQSQQRIYPG
KKSYIYSESDKCFTQLSHLSIHHTIHSGEKPYKCSECDKCFTDKFTLRKHQRIDTGEKPYKCNECDKCFTDKG
SLRVHQRIHTGEKPYKCSECDKCFTQPSHLSIHRRIHSGEKPYKCSECDKCFTDKSSLRVHQRIHTGEKPYQC
SECDKCFTDKGSLRVHHRIHAGEKPYQCSECDKCFTDKGSLRVHHRIHAGEKPYKCSECDKCFTHKDSLRVHH
RIHAGEKPYKCSECDKCFTEKNGLRRHQRIHTGEKPYKCSECDKCFTDKSSLRVHQRIHTGEKPYQCSECDKC
FTDKGSLRVHHRIHAGEKPYKCSECDKCFTDKGYLRVHHRIHAGEKPYKCSECDKCFTHKGSLRVHHRIHAGE
KPYKCSECDKCFTEKNGLRRHQRIHTGEKPYKCSECDKCFTRKSHLSIHQNSYRRETVQMQ*
>rs30953777_ENSMUSP00000101364_c.902:T>A_p.H301Q
ENSMUST00000105738.9|Zfp980-201|ENSMUSG00000058186|+|chr4:g.145428175:T>A
MFAPHQESGRRTTKRMSASLENTAQCLLTFKDVFLDFSSEEWECLNFAQRTLYMDVMLENYNNLLFVENHCIC
GNYEKEKVLEQDTQHIVNEHGHNRKKSSKCNELRNVIHESSESTPYNTNHRDATLQSSNLKRHKTGTTKEVCT
YKDCVNCLKGSSIISLNQGTHIEKKEHNRNKNLDEVLVSRQNNSEMSTYTCGEFDKCFTQSDNLQSQQRIYPG
KKSYIYSESDKCFTQLSHLSIHHTIHSGEKPYKCSECDKCFTDKFTLRKHQRIHTGEKPYKCNECDKCFTDKG
SLRVHQRIQTGEKPYKCSECDKCFTQPSHLSIHRRIHSGEKPYKCSECDKCFTDKSSLRVHQRIHTGEKPYQC
SECDKCFTDKGSLRVHHRIHAGEKPYQCSECDKCFTDKGSLRVHHRIHAGEKPYKCSECDKCFTHKDSLRVHH
RIHAGEKPYKCSECDKCFTEKNGLRRHQRIHTGEKPYKCSECDKCFTDKSSLRVHQRIHTGEKPYQCSECDKC

FTDKGSLRVHHRIHAGEKPYKCSECDKCFTDKGYLRVHHRIHAGEKPYKCSECDKCFTHKGSLRVHHRIHAGE
KPYKCSECDKCFTEKNGLRRHQRIHTGEKPYKCSECDKCFTRKSHLSIHQNSYRRETVQMQ*
>rs30953777_ENSMUSP00000137589_c.902:T>A_p.H301Q
ENSMUST00000180014.2|Zfp980-202|ENSMUSG00000058186|+|chr4:g.145428175:T>A
MFAPHQESGRRTTKRMSASLENTAQCLLTFKDVFLDFSSEEWECLNFAQRTLYMDVMLENYNNLLFVENHCIC
GNYEKEKVLEQDTQHIVNEHGHNRKKSSKCNELRNVIHESSESTPYNTNHRDATLQSSNLKRHKTGTTKEVCT
YKDCVNCLKGSSIISLNQGTHIEKKEHNRNKNLDEVLVSRQNNSEMSTYTCGEFDKCFTQSDNLQSQQRIYPG
KKSYIYSESDKCFTQLSHLSIHHTIHSGEKPYKCSECDKCFTDKFTLRKHQRIHTGEKPYKCNECDKCFTDKG
SLRVHQRIQTGEKPYKCSECDKCFTQPSHLSIHRRIHSGEKPYKCSECDKCFTDKSSLRVHQRIHTGEKPYQC
SECDKCFTDKGSLRVHHRIHAGEKPYQCSECDKCFTDKGSLRVHHRIHAGEKPYKCSECDKCFTHKDSLRVHH
RIHAGEKPYKCSECDKCFTEKNGLRRHQRIHTGEKPYKCSECDKCFTDKSSLRVHQRIHTGEKPYQCSECDKC
FTDKGSLRVHHRIHAGEKPYKCSECDKCFTDKGYLRVHHRIHAGEKPYKCSECDKCFTHKGSLRVHHRIHAGE
KPYKCSECDKCFTEKNGLRRHQRIHTGEKPYKCSECDKCFTRKSHLSIHQNSYRRETVQMQ*
>rs108218830_ENSMUSP00000101364_c.1317:A>G_p.I440V
ENSMUST00000105738.9|Zfp980-201|ENSMUSG00000058186|+|chr4:g.145428590:A>G
MFAPHQESGRRTTKRMSASLENTAQCLLTFKDVFLDFSSEEWECLNFAQRTLYMDVMLENYNNLLFVENHCIC
GNYEKEKVLEQDTQHIVNEHGHNRKKSSKCNELRNVIHESSESTPYNTNHRDATLQSSNLKRHKTGTTKEVCT
YKDCVNCLKGSSIISLNQGTHIEKKEHNRNKNLDEVLVSRQNNSEMSTYTCGEFDKCFTQSDNLQSQQRIYPG
KKSYIYSESDKCFTQLSHLSIHHTIHSGEKPYKCSECDKCFTDKFTLRKHQRIHTGEKPYKCNECDKCFTDKG
SLRVHQRIHTGEKPYKCSECDKCFTQPSHLSIHRRIHSGEKPYKCSECDKCFTDKSSLRVHQRIHTGEKPYQC
SECDKCFTDKGSLRVHHRIHAGEKPYQCSECDKCFTDKGSLRVHHRIHAGEKPYKCSECDKCFTHKDSLRVHH
RVHAGEKPYKCSECDKCFTEKNGLRRHQRIHTGEKPYKCSECDKCFTDKSSLRVHQRIHTGEKPYQCSECDKC
FTDKGSLRVHHRIHAGEKPYKCSECDKCFTDKGYLRVHHRIHAGEKPYKCSECDKCFTHKGSLRVHHRIHAGE
KPYKCSECDKCFTEKNGLRRHQRIHTGEKPYKCSECDKCFTRKSHLSIHQNSYRRETVQMQ*
>rs108218830_ENSMUSP00000137589_c.1317:A>G_p.I440V
ENSMUST00000180014.2|Zfp980-202|ENSMUSG00000058186|+|chr4:g.145428590:A>G
MFAPHQESGRRTTKRMSASLENTAQCLLTFKDVFLDFSSEEWECLNFAQRTLYMDVMLENYNNLLFVENHCIC
GNYEKEKVLEQDTQHIVNEHGHNRKKSSKCNELRNVIHESSESTPYNTNHRDATLQSSNLKRHKTGTTKEVCT
YKDCVNCLKGSSIISLNQGTHIEKKEHNRNKNLDEVLVSRQNNSEMSTYTCGEFDKCFTQSDNLQSQQRIYPG
KKSYIYSESDKCFTQLSHLSIHHTIHSGEKPYKCSECDKCFTDKFTLRKHQRIHTGEKPYKCNECDKCFTDKG
SLRVHQRIHTGEKPYKCSECDKCFTQPSHLSIHRRIHSGEKPYKCSECDKCFTDKSSLRVHQRIHTGEKPYQC
SECDKCFTDKGSLRVHHRIHAGEKPYQCSECDKCFTDKGSLRVHHRIHAGEKPYKCSECDKCFTHKDSLRVHH
RVHAGEKPYKCSECDKCFTEKNGLRRHQRIHTGEKPYKCSECDKCFTDKSSLRVHQRIHTGEKPYQCSECDKC
FTDKGSLRVHHRIHAGEKPYKCSECDKCFTDKGYLRVHHRIHAGEKPYKCSECDKCFTHKGSLRVHHRIHAGE
KPYKCSECDKCFTEKNGLRRHQRIHTGEKPYKCSECDKCFTRKSHLSIHQNSYRRETVQMQ*
>ENSMUSP00000101364_c.1398:A>G_p.R467G ENSMUST00000105738.9|Zfp980-
201|ENSMUSG00000058186|+|chr4:g.145428671:A>G
MFAPHQESGRRTTKRMSASLENTAQCLLTFKDVFLDFSSEEWECLNFAQRTLYMDVMLENYNNLLFVENHCIC
GNYEKEKVLEQDTQHIVNEHGHNRKKSSKCNELRNVIHESSESTPYNTNHRDATLQSSNLKRHKTGTTKEVCT
YKDCVNCLKGSSIISLNQGTHIEKKEHNRNKNLDEVLVSRQNNSEMSTYTCGEFDKCFTQSDNLQSQQRIYPG
KKSYIYSESDKCFTQLSHLSIHHTIHSGEKPYKCSECDKCFTDKFTLRKHQRIHTGEKPYKCNECDKCFTDKG
SLRVHQRIHTGEKPYKCSECDKCFTQPSHLSIHRRIHSGEKPYKCSECDKCFTDKSSLRVHQRIHTGEKPYQC
SECDKCFTDKGSLRVHHRIHAGEKPYQCSECDKCFTDKGSLRVHHRIHAGEKPYKCSECDKCFTHKDSLRVHH
RIHAGEKPYKCSECDKCFTEKNGLRRHQGIHTGEKPYKCSECDKCFTDKSSLRVHQRIHTGEKPYQCSECDKC
FTDKGSLRVHHRIHAGEKPYKCSECDKCFTDKGYLRVHHRIHAGEKPYKCSECDKCFTHKGSLRVHHRIHAGE
KPYKCSECDKCFTEKNGLRRHQRIHTGEKPYKCSECDKCFTRKSHLSIHQNSYRRETVQMQ*
>ENSMUSP00000137589_c.1398:A>G_p.R467G ENSMUST00000180014.2|Zfp980-
202|ENSMUSG00000058186|+|chr4:g.145428671:A>G
MFAPHQESGRRTTKRMSASLENTAQCLLTFKDVFLDFSSEEWECLNFAQRTLYMDVMLENYNNLLFVENHCIC
GNYEKEKVLEQDTQHIVNEHGHNRKKSSKCNELRNVIHESSESTPYNTNHRDATLQSSNLKRHKTGTTKEVCT
YKDCVNCLKGSSIISLNQGTHIEKKEHNRNKNLDEVLVSRQNNSEMSTYTCGEFDKCFTQSDNLQSQQRIYPG
KKSYIYSESDKCFTQLSHLSIHHTIHSGEKPYKCSECDKCFTDKFTLRKHQRIHTGEKPYKCNECDKCFTDKG
SLRVHQRIHTGEKPYKCSECDKCFTQPSHLSIHRRIHSGEKPYKCSECDKCFTDKSSLRVHQRIHTGEKPYQC
SECDKCFTDKGSLRVHHRIHAGEKPYQCSECDKCFTDKGSLRVHHRIHAGEKPYKCSECDKCFTHKDSLRVHH
RIHAGEKPYKCSECDKCFTEKNGLRRHQGIHTGEKPYKCSECDKCFTDKSSLRVHQRIHTGEKPYQCSECDKC
FTDKGSLRVHHRIHAGEKPYKCSECDKCFTDKGYLRVHHRIHAGEKPYKCSECDKCFTHKGSLRVHHRIHAGE
KPYKCSECDKCFTEKNGLRRHQRIHTGEKPYKCSECDKCFTRKSHLSIHQNSYRRETVQMQ*

>ENSMUSP00000101364_c.1702:T>A_p.F568Y ENSMUST00000105738.9|Zfp980-201|ENSMUSG00000058186|+|chr4:g.145428975:T>A
MFAPHQESGRRTTKRMSASLENTAQCLLTFKDVFLDFSSEEWECLNFAQRTLYMDVMLENYNNLLFVENHCIC
GNYEKEKVLEQDTQHIVNEHGHNRKKSSKCNELRNVIHESSESTPYNTNHRDATLQSSNLKRHKTGTTKEVCT
YKDCVNCLKGSSIISLNQGTHIEKKEHNRNKNLDEVLVSRQNNSEMSTYTCGEFDKCFTQSDNLQSQQRIYPG
KKSYIYSESDKCFTQLSHLSIHHTIHSGEKPYKCSECDKCFTDKFTLRKHQRIHTGEKPYKCNECDKCFTDKG
SLRVHQRIHTGEKPYKCSECDKCFTQPSHLSIHRRIHSGEKPYKCSECDKCFTDKSSLRVHQRIHTGEKPYQC
SECDKCFTDKGSLRVHHRIHAGEKPYQCSECDKCFTDKGSLRVHHRIHAGEKPYKCSECDKCFTHKDSLRVHH
RIHAGEKPYKCSECDKCFTEKNGLRRHQRIHTGEKPYKCSECDKCFTDKSSLRVHQRIHTGEKPYQCSECDKC
FTDKGSLRVHHRIHAGEKPYKCSECDKCFTDKGYLRVHHRIHAGEKPYKCSECDKCYTHKGSLRVHHRIHAGE
KPYKCSECDKCFTEKNGLRRHQRIHTGEKPYKCSECDKCFTRKSHLSIHQNSYRRETVQMQ*
>ENSMUSP00000137589_c.1702:T>A_p.F568Y ENSMUST00000180014.2|Zfp980-202|ENSMUSG00000058186|+|chr4:g.145428975:T>A
MFAPHQESGRRTTKRMSASLENTAQCLLTFKDVFLDFSSEEWECLNFAQRTLYMDVMLENYNNLLFVENHCIC
GNYEKEKVLEQDTQHIVNEHGHNRKKSSKCNELRNVIHESSESTPYNTNHRDATLQSSNLKRHKTGTTKEVCT
YKDCVNCLKGSSIISLNQGTHIEKKEHNRNKNLDEVLVSRQNNSEMSTYTCGEFDKCFTQSDNLQSQQRIYPG
KKSYIYSESDKCFTQLSHLSIHHTIHSGEKPYKCSECDKCFTDKFTLRKHQRIHTGEKPYKCNECDKCFTDKG
SLRVHQRIHTGEKPYKCSECDKCFTQPSHLSIHRRIHSGEKPYKCSECDKCFTDKSSLRVHQRIHTGEKPYQC
SECDKCFTDKGSLRVHHRIHAGEKPYQCSECDKCFTDKGSLRVHHRIHAGEKPYKCSECDKCFTHKDSLRVHH
RIHAGEKPYKCSECDKCFTEKNGLRRHQRIHTGEKPYKCSECDKCFTDKSSLRVHQRIHTGEKPYQCSECDKC
FTDKGSLRVHHRIHAGEKPYKCSECDKCFTDKGYLRVHHRIHAGEKPYKCSECDKCYTHKGSLRVHHRIHAGE
KPYKCSECDKCFTEKNGLRRHQRIHTGEKPYKCSECDKCFTRKSHLSIHQNSYRRETVQMQ*
>ENSMUSP00000101364_c.1771:A>G_p.E591G ENSMUST00000105738.9|Zfp980-201|ENSMUSG00000058186|+|chr4:g.145429044:A>G
MFAPHQESGRRTTKRMSASLENTAQCLLTFKDVFLDFSSEEWECLNFAQRTLYMDVMLENYNNLLFVENHCIC
GNYEKEKVLEQDTQHIVNEHGHNRKKSSKCNELRNVIHESSESTPYNTNHRDATLQSSNLKRHKTGTTKEVCT
YKDCVNCLKGSSIISLNQGTHIEKKEHNRNKNLDEVLVSRQNNSEMSTYTCGEFDKCFTQSDNLQSQQRIYPG
KKSYIYSESDKCFTQLSHLSIHHTIHSGEKPYKCSECDKCFTDKFTLRKHQRIHTGEKPYKCNECDKCFTDKG
SLRVHQRIHTGEKPYKCSECDKCFTQPSHLSIHRRIHSGEKPYKCSECDKCFTDKSSLRVHQRIHTGEKPYQC
SECDKCFTDKGSLRVHHRIHAGEKPYQCSECDKCFTDKGSLRVHHRIHAGEKPYKCSECDKCFTHKDSLRVHH
RIHAGEKPYKCSECDKCFTEKNGLRRHQRIHTGEKPYKCSECDKCFTDKSSLRVHQRIHTGEKPYQCSECDKC
FTDKGSLRVHHRIHAGEKPYKCSECDKCFTDKGYLRVHHRIHAGEKPYKCSECDKCFTHKGSLRVHHRIHAGE
KPYKCSGCDKCFTEKNGLRRHQRIHTGEKPYKCSECDKCFTRKSHLSIHQNSYRRETVQMQ*
>ENSMUSP00000137589_c.1771:A>G_p.E591G ENSMUST00000180014.2|Zfp980-202|ENSMUSG00000058186|+|chr4:g.145429044:A>G
MFAPHQESGRRTTKRMSASLENTAQCLLTFKDVFLDFSSEEWECLNFAQRTLYMDVMLENYNNLLFVENHCIC
GNYEKEKVLEQDTQHIVNEHGHNRKKSSKCNELRNVIHESSESTPYNTNHRDATLQSSNLKRHKTGTTKEVCT
YKDCVNCLKGSSIISLNQGTHIEKKEHNRNKNLDEVLVSRQNNSEMSTYTCGEFDKCFTQSDNLQSQQRIYPG
KKSYIYSESDKCFTQLSHLSIHHTIHSGEKPYKCSECDKCFTDKFTLRKHQRIHTGEKPYKCNECDKCFTDKG
SLRVHQRIHTGEKPYKCSECDKCFTQPSHLSIHRRIHSGEKPYKCSECDKCFTDKSSLRVHQRIHTGEKPYQC
SECDKCFTDKGSLRVHHRIHAGEKPYQCSECDKCFTDKGSLRVHHRIHAGEKPYKCSECDKCFTHKDSLRVHH
RIHAGEKPYKCSECDKCFTEKNGLRRHQRIHTGEKPYKCSECDKCFTDKSSLRVHQRIHTGEKPYQCSECDKC
FTDKGSLRVHHRIHAGEKPYKCSECDKCFTDKGYLRVHHRIHAGEKPYKCSECDKCFTHKGSLRVHHRIHAGE
KPYKCSGCDKCFTEKNGLRRHQRIHTGEKPYKCSECDKCFTRKSHLSIHQNSYRRETVQMQ*
>ENSMUSP00000101364_c.1786:T>C_p.F596S ENSMUST00000105738.9|Zfp980-201|ENSMUSG00000058186|+|chr4:g.145429059:T>C
MFAPHQESGRRTTKRMSASLENTAQCLLTFKDVFLDFSSEEWECLNFAQRTLYMDVMLENYNNLLFVENHCIC
GNYEKEKVLEQDTQHIVNEHGHNRKKSSKCNELRNVIHESSESTPYNTNHRDATLQSSNLKRHKTGTTKEVCT
YKDCVNCLKGSSIISLNQGTHIEKKEHNRNKNLDEVLVSRQNNSEMSTYTCGEFDKCFTQSDNLQSQQRIYPG
KKSYIYSESDKCFTQLSHLSIHHTIHSGEKPYKCSECDKCFTDKFTLRKHQRIHTGEKPYKCNECDKCFTDKG
SLRVHQRIHTGEKPYKCSECDKCFTQPSHLSIHRRIHSGEKPYKCSECDKCFTDKSSLRVHQRIHTGEKPYQC
SECDKCFTDKGSLRVHHRIHAGEKPYQCSECDKCFTDKGSLRVHHRIHAGEKPYKCSECDKCFTHKDSLRVHH
RIHAGEKPYKCSECDKCFTEKNGLRRHQRIHTGEKPYKCSECDKCFTDKSSLRVHQRIHTGEKPYQCSECDKC
FTDKGSLRVHHRIHAGEKPYKCSECDKCFTDKGYLRVHHRIHAGEKPYKCSECDKCFTHKGSLRVHHRIHAGE
KPYKCSECDKCSTEKNGLRRHQRIHTGEKPYKCSECDKCFTRKSHLSIHQNSYRRETVQMQ*
>ENSMUSP00000137589_c.1786:T>C_p.F596S ENSMUST00000180014.2|Zfp980-202|ENSMUSG00000058186|+|chr4:g.145429059:T>C

MFAPHQESGRRTTKRMSASLENTAQCLLTFKDVFLDFSSEEWECLNFAQRTLYMDVMLENYNNLLFVENHCIC
GNYEKEKVLEQDTQHIVNEHGHNRKKSSKCNELRNVIHESSESTPYNTNHRDATLQSSNLKRHKTGTTKEVCT
YKDCVNCLKGSSIISLNQGTHIEKKEHNRNKNLDEVLVSRQNNSEMSTYTCGEFDKCFTQSDNLQSQQRIYPG
KKSYIYSESDKCFTQLSHLSIHHTIHSGEKPYKCSECDKCFTDKFTLRKHQRIHTGEKPYKCNECDKCFTDKG
SLRVHQRIHTGEKPYKCSECDKCFTQPSHLSIHRRIHSGEKPYKCSECDKCFTDKSSLRVHQRIHTGEKPYQC
SECDKCFTDKGSLRVHHRIHAGEKPYQCSECDKCFTDKGSLRVHHRIHAGEKPYKCSECDKCFTHKDSLRVHH
RIHAGEKPYKCSECDKCFTEKNGLRRHQRIHTGEKPYKCSECDKCFTDKSSLRVHQRIHTGEKPYQCSECDKC
FTDKGSLRVHHRIHAGEKPYKCSECDKCFTDKGYLRVHHRIHAGEKPYKCSECDKCFTHKGSLRVHHRIHAGE
KPYKCSECDKCSTEKNGLRRHQRIHTGEKPYKCSECDKCFTRKSHLSIHQNSYRRETVQMQ*
>ENSMUSP00000101364_c.1798:A>G_p.N600S ENSMUST00000105738.9|Zfp980-201|ENSMUSG00000058186|+|chr4:g.145429071:A>G
MFAPHQESGRRTTKRMSASLENTAQCLLTFKDVFLDFSSEEWECLNFAQRTLYMDVMLENYNNLLFVENHCIC
GNYEKEKVLEQDTQHIVNEHGHNRKKSSKCNELRNVIHESSESTPYNTNHRDATLQSSNLKRHKTGTTKEVCT
YKDCVNCLKGSSIISLNQGTHIEKKEHNRNKNLDEVLVSRQNNSEMSTYTCGEFDKCFTQSDNLQSQQRIYPG
KKSYIYSESDKCFTQLSHLSIHHTIHSGEKPYKCSECDKCFTDKFTLRKHQRIHTGEKPYKCNECDKCFTDKG
SLRVHQRIHTGEKPYKCSECDKCFTQPSHLSIHRRIHSGEKPYKCSECDKCFTDKSSLRVHQRIHTGEKPYQC
SECDKCFTDKGSLRVHHRIHAGEKPYQCSECDKCFTDKGSLRVHHRIHAGEKPYKCSECDKCFTHKDSLRVHH
RIHAGEKPYKCSECDKCFTEKNGLRRHQRIHTGEKPYKCSECDKCFTDKSSLRVHQRIHTGEKPYQCSECDKC
FTDKGSLRVHHRIHAGEKPYKCSECDKCFTDKGYLRVHHRIHAGEKPYKCSECDKCFTHKGSLRVHHRIHAGE
KPYKCSECDKCFTEKSGLRRHQRIHTGEKPYKCSECDKCFTRKSHLSIHQNSYRRETVQMQ*
>ENSMUSP00000137589_c.1798:A>G_p.N600S ENSMUST00000180014.2|Zfp980-202|ENSMUSG00000058186|+|chr4:g.145429071:A>G
MFAPHQESGRRTTKRMSASLENTAQCLLTFKDVFLDFSSEEWECLNFAQRTLYMDVMLENYNNLLFVENHCIC
GNYEKEKVLEQDTQHIVNEHGHNRKKSSKCNELRNVIHESSESTPYNTNHRDATLQSSNLKRHKTGTTKEVCT
YKDCVNCLKGSSIISLNQGTHIEKKEHNRNKNLDEVLVSRQNNSEMSTYTCGEFDKCFTQSDNLQSQQRIYPG
KKSYIYSESDKCFTQLSHLSIHHTIHSGEKPYKCSECDKCFTDKFTLRKHQRIHTGEKPYKCNECDKCFTDKG
SLRVHQRIHTGEKPYKCSECDKCFTQPSHLSIHRRIHSGEKPYKCSECDKCFTDKSSLRVHQRIHTGEKPYQC
SECDKCFTDKGSLRVHHRIHAGEKPYQCSECDKCFTDKGSLRVHHRIHAGEKPYKCSECDKCFTHKDSLRVHH
RIHAGEKPYKCSECDKCFTEKNGLRRHQRIHTGEKPYKCSECDKCFTDKSSLRVHQRIHTGEKPYQCSECDKC
FTDKGSLRVHHRIHAGEKPYKCSECDKCFTDKGYLRVHHRIHAGEKPYKCSECDKCFTHKGSLRVHHRIHAGE
KPYKCSECDKCFTEKSGLRRHQRIHTGEKPYKCSECDKCFTRKSHLSIHQNSYRRETVQMQ*
>ENSMUSP00000101364_c.1836:A>G_p.K613E ENSMUST00000105738.9|Zfp980-201|ENSMUSG00000058186|+|chr4:g.145429109:A>G
MFAPHQESGRRTTKRMSASLENTAQCLLTFKDVFLDFSSEEWECLNFAQRTLYMDVMLENYNNLLFVENHCIC
GNYEKEKVLEQDTQHIVNEHGHNRKKSSKCNELRNVIHESSESTPYNTNHRDATLQSSNLKRHKTGTTKEVCT
YKDCVNCLKGSSIISLNQGTHIEKKEHNRNKNLDEVLVSRQNNSEMSTYTCGEFDKCFTQSDNLQSQQRIYPG
KKSYIYSESDKCFTQLSHLSIHHTIHSGEKPYKCSECDKCFTDKFTLRKHQRIHTGEKPYKCNECDKCFTDKG
SLRVHQRIHTGEKPYKCSECDKCFTQPSHLSIHRRIHSGEKPYKCSECDKCFTDKSSLRVHQRIHTGEKPYQC
SECDKCFTDKGSLRVHHRIHAGEKPYQCSECDKCFTDKGSLRVHHRIHAGEKPYKCSECDKCFTHKDSLRVHH
RIHAGEKPYKCSECDKCFTEKNGLRRHQRIHTGEKPYKCSECDKCFTDKSSLRVHQRIHTGEKPYQCSECDKC
FTDKGSLRVHHRIHAGEKPYKCSECDKCFTDKGYLRVHHRIHAGEKPYKCSECDKCFTHKGSLRVHHRIHAGE
KPYKCSECDKCFTEKNGLRRHQRIHTGEEPYKCSECDKCFTRKSHLSIHQNSYRRETVQMQ*
>ENSMUSP00000137589_c.1836:A>G_p.K613E ENSMUST00000180014.2|Zfp980-202|ENSMUSG00000058186|+|chr4:g.145429109:A>G
MFAPHQESGRRTTKRMSASLENTAQCLLTFKDVFLDFSSEEWECLNFAQRTLYMDVMLENYNNLLFVENHCIC
GNYEKEKVLEQDTQHIVNEHGHNRKKSSKCNELRNVIHESSESTPYNTNHRDATLQSSNLKRHKTGTTKEVCT
YKDCVNCLKGSSIISLNQGTHIEKKEHNRNKNLDEVLVSRQNNSEMSTYTCGEFDKCFTQSDNLQSQQRIYPG
KKSYIYSESDKCFTQLSHLSIHHTIHSGEKPYKCSECDKCFTDKFTLRKHQRIHTGEKPYKCNECDKCFTDKG
SLRVHQRIHTGEKPYKCSECDKCFTQPSHLSIHRRIHSGEKPYKCSECDKCFTDKSSLRVHQRIHTGEKPYQC
SECDKCFTDKGSLRVHHRIHAGEKPYQCSECDKCFTDKGSLRVHHRIHAGEKPYKCSECDKCFTHKDSLRVHH
RIHAGEKPYKCSECDKCFTEKNGLRRHQRIHTGEKPYKCSECDKCFTDKSSLRVHQRIHTGEKPYQCSECDKC
FTDKGSLRVHHRIHAGEKPYKCSECDKCFTDKGYLRVHHRIHAGEKPYKCSECDKCFTHKGSLRVHHRIHAGE
KPYKCSECDKCFTEKNGLRRHQRIHTGEEPYKCSECDKCFTRKSHLSIHQNSYRRETVQMQ*
>ENSMUSP00000116281_c.628:A>G_p.K210R ENSMUST00000123460.2|Zfp986-201|ENSMUSG00000078500|+|chr4:g.145625970:A>G
MSVCLVNTPQGLLTFKDVALDFSLEEWECLSFAQRSLYMDVMLENYNNLLFVENHCIRGKNGKVLDQDSQYIV
HEHMNIQEKSSKWDKLSNVILESLQCTPYKTNHSSDALQFSNQKRLKPRNTKEVCKYNDSVNSLSLFSTISLN
QGINMQKKKHNRNAELEKVFVSKHKVMVKKDNIAGNPYKCSEFHKYLTQREKLQSQQRIYHGKRPYESSKSDK

CFTHQIHLSIHQEIHTEEKIYKCSECDKCFKNKFNLTMHQRIHTGEKPYKCSECDKCFTKQSNLSIHQRIHTG
EKPYKCSECDKCFTQQSCLSIHQRIHTGEKPYKCSECGKCFTEKGSLRIHQRIHTGEKPYKCSECDKCFTQQS
NLTIHQRIHTGEKPYKCPECGKCFTEKSSLRIHQRI
>ENSMUSP00000089493_c.1225:A>G_p.K409R ENSMUST00000091878.6|Zfp987-
201|ENSMUSG00000066009|+|chr4:g.146061795:A>G
MSVCLVNTPQGLLTFKDVALDFSLEEWECLSFAQRSLYMDVMLENYNNLLFVENHCIRGKNGKVLDQDSQYIV
HEHMNIQEKSSKWDKLSNVILESLQCTPYKTNHSSDALQFSNQKRLKPRNTKEVCKYNDSVNSLSLFSTISLN
QGINMQKKKHNRNAELDKIFVSKHKVMVKKDNIAGNPYKCSEFHKYLTQRENLQSQQRIYHGKKPYKSSKSDK
CFTHQIHLSIHQEIHTEEKIYKCSECDKCFKNKFNLTMHQRIHTGEKPYKCSECDKCFTKQSNLSIHQRIHTG
EKPYKCSECDKCFTQESYLSIHQRIHTGEKPYKCSECGKCFTEKSSLRIHQRIHTGEKPYKCSECDKCFTQQS
HLSIHQRIHTGEKPYKCSECDKCFTQQSNLSIHQRIHTGEKPYRCSECGKCFTEKSSLRIHQRIHTGEKPYKC
SECGKCFTEKSSLRIHQRIHTGEKPYKCNECGKCFTEKSSLRIHQRIHTGEKPYKCSECGKCFTVKSSLRIHQ
KIHTGEKPYKCSECDKCFTKPSHLSIHQRIHTGEKPYKCSECEKCFNEKNILKIHQRIHTGERPYKCSECDKC
FSRKFHLGIHQRIHTGKKPYKCSECDKCFTQKSHLSIHQKIHTGEKPYK*
>ENSMUSP00000090425_c.1121:C>A_p.D374E ENSMUST00000092750.12|Zfp600-
201|ENSMUSG00000066007|+|chr4:g.146132455:C>A
MFAPHQESGRRTTKRMSASLENTAQCLLTFKDVFLDFSSEEWECLNFAQRTLYMDVMLENYNNLLFVENHCIC
GNYEKEKVLEQETQHIVNEHGHNRKKSSKCNEVRNVIHESSESTPYNTNHRDATLQSSNLKRHKTGTTKGVCT
YKDCVNCLKGSSIISLNQGTHIEKKEHNRNKNLDEVLVSRQNNSEMNTYTCGEFDKCFTQSDNLQSQQRIYPG
KKSYIYSESDKCFTQPSHLSIHHTIHSGEKPYKCSECDKCFTDKCTLRKHQRIHTGEKPYKCSECDKCFTDKC
TLRKHQRIHTGEKPYKCNECDKCFTDKGSLRVHQRIHTGEKPYKCSECDKCFTQPSHLSIHRRIHSGEKPYKC
SECDKCFTEKCTLRKHQRIHTGEKPYKCSECDKCFTEKFTLRKHQRIHTGEKPYKCNECDKCFTDKGSLRVHQ
RIHTGEKPYKCSECDKCFTKPSHLSIHRRIHSGEKPYKCSECDKCFTQPSPLSIHRRIHSGEKPYKCSECDKC
FTDKGSLRVHQRIHIGEKPYKCSECDKCFTQPSHLSIHCRIHSGEKPYKCSECDKCFTDKSSLRVHQRIHTGE
KPYQCSECDKCFTDKGSLRVHHRIHAGEKPYKCSECGKCFTDKGYLRVHYRIHAGEKPYKCSECDKCFTHKGS
LRVHHRIHTGEKPYKCSECDKCFTRKSHLSIHQRIHTGEKLYKCNECDKCFSDKHSLRRHQRIHTGETPYKCN
ECDKCFT*
>ENSMUSP00000126879_c.1121:C>A_p.D374E ENSMUST00000168483.9|Zfp600-
204|ENSMUSG00000066007|+|chr4:g.146132455:C>A
MFAPHQESGRRTTKRMSASLENTAQCLLTFKDVFLDFSSEEWECLNFAQRTLYMDVMLENYNNLLFVENHCIC
GNYEKEKVLEQETQHIVNEHGHNRKKSSKCNEVRNVIHESSESTPYNTNHRDATLQSSNLKRHKTGTTKGVCT
YKDCVNCLKGSSIISLNQGTHIEKKEHNRNKNLDEVLVSRQNNSEMNTYTCGEFDKCFTQSDNLQSQQRIYPG
KKSYIYSESDKCFTQPSHLSIHHTIHSGEKPYKCSECDKCFTDKCTLRKHQRIHTGEKPYKCSECDKCFTDKC
TLRKHQRIHTGEKPYKCNECDKCFTDKGSLRVHQRIHTGEKPYKCSECDKCFTQPSHLSIHRRIHSGEKPYKC
SECDKCFTEKCTLRKHQRIHTGEKPYKCSECDKCFTEKFTLRKHQRIHTGEKPYKCNECDKCFTDKGSLRVHQ
RIHTGEKPYKCSECDKCFTKPSHLSIHRRIHSGEKPYKCSECDKCFTQPSPLSIHRRIHSGEKPYKCSECDKC
FTDKGSLRVHQRIHIGEKPYKCSECDKCFTQPSHLSIHCRIHSGEKPYKCSECDKCFTDKSSLRVHQRIHTGE
KPYQCSECDKCFTDKGSLRVHHRIHAGEKPYKCSECGKCFTDKGYLRVHYRIHAGEKPYKCSECDKCFTHKGS
LRVHHRIHTGEKPYKCSECDKCFTRKSHLSIHQRIHTGEKLYKCNECDKCFSDKHSLRRHQRIHTGETPYKCN
ECDKCFT*
>ENSMUSP00000101359_c.391:A>ATT_p.N131ins-fs ENSMUST00000105733.3|Zfp992-
201|ENSMUSG00000070605|+|chr4:g.146550672:A>ATT
MSVCLVNTPQGLLTFKDVALDFSLEEWESLSFAQRTLYMDVMLENYNNLLFVENHCICGKYEKVLDQDSQYIV
HEHMNIQEKSSKWDKLSNVILEAPQCTPYKTNHTSDALQFSNQKRLKPRNTKEVCKYNLTL
>rs240941694_ENSMUSP00000101359_c.565:T>G_p.F189C
ENSMUST00000105733.3|Zfp992-201|ENSMUSG00000070605|+|chr4:g.146550846:T>G
MSVCLVNTPQGLLTFKDVALDFSLEEWESLSFAQRTLYMDVMLENYNNLLFVENHCICGKYEKVLDQDSQYIV
HEHMNIQEKSSKWDKLSNVILEAPQCTPYKTNHTSDALQFSNQKRLKPRNTKEVCKYNDSVNSLTLFSTISLN
QGIHMQKKKHNGNAESEKAFISKHKVMVKRNNTGVNPYKCSKCDKYLTQRDKLQSQQRIYHEKKHFRSSKSDK
CFTHQIDLSIHQGIHAEEKIYKCSECDKCFTHKFHLNIHQRIHTGEKPYKCSECDKCFTQKSHLNIHQRIHTG
EKPYKCIVCEKCFTEKGNLRIHQRIHTGDKPYKCSECDKCFTQKSHLNIHQRIHTGEKPYKCSKCDKSFTENG
HLRIHQRNHTGEKPYKCSQCDKSFTENGHLRIHQRIHTGEKPYKCSECDKCFTKKFSLRIHQRIHRGEKPYKC
SECDKCFTFKGSLRIHQRIHTGEKPYKCSECDKSFTENGCLRIHQRIHTGEKPYKCTECDKCFTFKGSLRIHQ
RIHTGEKPYKCTECDKSFTEKGKLRIHQRIHTGEKPYKCSECDKCFTEKGHQRIHTGEIPYK*
>ENSMUSP00000101359_c.649:G>C_p.S217T ENSMUST00000105733.3|Zfp992-
201|ENSMUSG00000070605|+|chr4:g.146550930:G>C
MSVCLVNTPQGLLTFKDVALDFSLEEWESLSFAQRTLYMDVMLENYNNLLFVENHCICGKYEKVLDQDSQYIV
HEHMNIQEKSSKWDKLSNVILEAPQCTPYKTNHTSDALQFSNQKRLKPRNTKEVCKYNDSVNSLTLFSTISLN

QGIHMQKKKHNGNAESEKAFISKHKVMVKRNNTGVNPYKCSKFDKYLTQRDKLQSQQRIYHEKKHFRSSKTDK
CFTHQIDLSIHQGIHAEEKIYKCSECDKCFTHKFHLNIHQRIHTGEKPYKCSECDKCFTQKSHLNIHQRIHTG
EKPYKCIVCEKCFTEKGNLRIHQRIHTGDKPYKCSECDKCFTQKSHLNIHQRIHTGEKPYKCSKCDKSFTENG
HLRIHQRNHTGEKPYKCSQCDKSFTENGHLRIHQRIHTGEKPYKCSECDKCFTKKFSLRIHQRIHRGEKPYKC
SECDKCFTFKGSLRIHQRIHTGEKPYKCSECDKSFTENGCLRIHQRIHTGEKPYKCTECDKCFTFKGSLRIHQ
RIHTGEKPYKCTECDKSFTEKGKLRIHQRIHTGEKPYKCSECDKCFTEKGHQRIHTGEIPYK*
>ENSMUSP00000101359_c.700:A>G_p.H234R ENSMUST00000105733.3|Zfp992-201|ENSMUSG00000070605|+|chr4:g.146550981:A>G
MSVCLVNTPQGLLTFKDVALDFSLEEWESLSFAQRTLYMDVMLENYNNLLFVENHCICGKYEKVLDQDSQYIV
HEHMNIQEKSSKWDKLSNVILEAPQCTPYKTNHTSDALQFSNQKRLKPRNTKEVCKYNDSVNSLTLFSTISLN
QGIHMQKKKHNGNAESEKAFISKHKVMVKRNNTGVNPYKCSKFDKYLTQRDKLQSQQRIYHEKKHFRSSKSDK
CFTHQIDLSIHQGIRAEEKIYKCSECDKCFTHKFHLNIHQRIHTGEKPYKCSECDKCFTQKSHLNIHQRIHTG
EKPYKCIVCEKCFTEKGNLRIHQRIHTGDKPYKCSECDKCFTQKSHLNIHQRIHTGEKPYKCSKCDKSFTENG
HLRIHQRNHTGEKPYKCSQCDKSFTENGHLRIHQRIHTGEKPYKCSECDKCFTKKFSLRIHQRIHRGEKPYKC
SECDKCFTFKGSLRIHQRIHTGEKPYKCSECDKSFTENGCLRIHQRIHTGEKPYKCTECDKCFTFKGSLRIHQ
RIHTGEKPYKCTECDKSFTEKGKLRIHQRIHTGEKPYKCSECDKCFTEKGHQRIHTGEIPYK*
>ENSMUSP00000121301_c.285:G>A_p.A96T ENSMUST00000130825.8|Zfp993-202|ENSMUSG00000063245|+|chr4:g.146741963:G>A
MSVCLVNTPQGLLTFKDVALDFSLEEWESLSFAQRTLYMDVMLENYNNLLFVENHCICGKYEKVLDQDSQYIV
HEHMNIQEKSSKWDKLSNVILETPQCTPYKTNHTSDALQFSNQKRLKPRNTKEVCKYNDSVNSLTLFSTVSLN
QGIHMQKKKHNGNAESEKAFISKHKVMVKRNNTGVNPYKCSKFDKYLTQRDKLQSQQRIYHEKKHYRSSKSDK
CFTHQIDLSIHQGIHAEEKIYKCSECDKCFTHKFHLNIHQRIHTGEKPYKCSECDKCFTQKSHLNIHQRIHTG
EKPYKCIVCEKCFTEKGNLRI
>ENSMUSP00000065923_c.285:G>A_p.A96T ENSMUST00000070932.4|Zfp993-201|ENSMUSG00000063245|+|chr4:g.146741963:G>A
MSVCLVNTPQGLLTFKDVALDFSLEEWESLSFAQRTLYMDVMLENYNNLLFVENHCICGKYEKVLDQDSQYIV
HEHMNIQEKSSKWDKLSNVILETPQCTPYKTNHTSDALQFSNQKRLKPRNTKEVCK
>ENSMUSP00000158852_c.997:T>C_p.F333S ENSMUST00000238583.2|Gm21411-202|ENSMUSG00000045699|-|chr4:g.146976275:A>G
MSVCLVNTPQGLLTFKDVALDFSLEEWECLSFAQRTLYMDVMLENYNNLLFVENYCIRGKNGKVLDQDSQYIV
HEHMNIQEKSSKWDKLSNVILESLQCTPYKTNHSSDALQFSNQKRLKPRNTKEVCKYNHFVNSLSLFSTISLN
QGINMQKKKHNRNAELEKVFVSKHKVVVKKNNIAGNPYKCSEFHKYLTQREKLQSQQRIYHGKKPYESSKSDK
CFTHQIHLSIHQGIHTEEKIYKCSECDKCFKHKFNLTMHQRIHTGETPNKCSECDKCFTKKSSLRIHQRIHTG
EKPYKCSECDKCFTQQSNLSIHQRIHTGEKPYKCRECDKCSTGKGSLRIHQRIHTGEKPYKCSECGKCFTEKS
SLRIHQRI
>ENSMUSP00000075179_c.592:G>A_p.G198D ENSMUST00000075775.6|Rex2-201|ENSMUSG00000067919|+|chr4:g.147142106:G>A
MFAPHQESGRRTTKRMSASLENTAQCLLTFKDVFLDFSSEEWECLNFAQRTLYMDVMLENYNNLLFVENHCIC
GNYEKEKVLEQDTQHIVNEHGHNRKKSSKCNELRNVIHESSESTPYNTNHRDATLQSSNLKRHKTGTTKGVCT
YKDCVNCLKGSSIISLNQGTHIEKKEHNRNKNLDEVLVSRQNNSEMNTYTCDEFDKCFTQSDNLQSQQRIYPG
KKSYKYSESDKCFTQTSHLSIHHTIHSGEKPYKCSECDKCFTDKCTLRKHQRIHTGEKPYKCSECDKCFTDKC
TLRKHQRIHTGEKPYKCNECDKCFTDKGSLRVHQRIHTGEKPYKCSECDKCFTQPSHLSIHRRIHSGEKPYKC
SECDKCFTDKCTLRKHQRIHTGEKPYKCSECDKCFTEKFTLRKHQRIHTGEKPYKCNECDKCFTDKGSLRVHQ
RIHTGEKPYKCSECDKCFTKPSHLSIHRRIHSGEKPYKCSECDKCFTQPSPLSIHRRIHSGEKPYKCSECDKC
FTDKGSLRVHQRIHIGEKPYKCSECDKCFTQPSHLSIHCRIHSGEKPYKCSECDKCFTHKGSLRVHHRIHAGE
KPYKCSECDKCFTEKNGLRRHQRIHTGEKPYKCSECDKCFTRKSHLSIHQRIHTGEKLYKCSECDKCFTQQCH
LSIHQKIHSGENLTNAMNATNALVTNTV*
>ENSMUSP00000075179_c.695:T>A_p.F232L ENSMUST00000075775.6|Rex2-201|ENSMUSG00000067919|+|chr4:g.147142209:T>A
MFAPHQESGRRTTKRMSASLENTAQCLLTFKDVFLDFSSEEWECLNFAQRTLYMDVMLENYNNLLFVENHCIC
GNYEKEKVLEQDTQHIVNEHGHNRKKSSKCNELRNVIHESSESTPYNTNHRDATLQSSNLKRHKTGTTKGVCT
YKDCVNCLKGSSIISLNQGTHIEKKEHNRNKNLDEVLVSRQNNSEMNTYTCGEFDKCFTQSDNLQSQQRIYPG
KKSYKYSESDKCLTQTSHLSIHHTIHSGEKPYKCSECDKCFTDKCTLRKHQRIHTGEKPYKCSECDKCFTDKC
TLRKHQRIHTGEKPYKCNECDKCFTDKGSLRVHQRIHTGEKPYKCSECDKCFTQPSHLSIHRRIHSGEKPYKC
SECDKCFTDKCTLRKHQRIHTGEKPYKCSECDKCFTEKFTLRKHQRIHTGEKPYKCNECDKCFTDKGSLRVHQ
RIHTGEKPYKCSECDKCFTKPSHLSIHRRIHSGEKPYKCSECDKCFTQPSPLSIHRRIHSGEKPYKCSECDKC
FTDKGSLRVHQRIHIGEKPYKCSECDKCFTQPSHLSIHCRIHSGEKPYKCSECDKCFTHKGSLRVHHRIHAGE

KPYKCSECDKCFTEKNGLRRHQRIHTGEKPYKCSECDKCFTRKSHLSIHQRIHTGEKLYKCSECDKCFTQQCH
LSIHQKIHSGENLTNAMNATNALVTNTV*
>ENSMUSP00000081167_c.133:A>T_p.N45I ENSMUST00000084149.10|Zfp991-201|ENSMUSG00000067916|+|chr4:g.147261307:A>T
MSVCLVNTPQGLLTFKDVALDFSLEEWESLSFAQRTLYMDVMLEIYNNLLFVENHCICGKYEKVLDQDSQYIV
HEHMNIQEKSSKWDKLSNVILEAPQCTPYKTNHTSDALQFSNQKRLKPRNTKEVCKYNDSVNSLTLFSTISLN
QGIHMQKKKHNGNAESEKAFISKHKVMVKRNNTGVNPYKCSKFDKYLTQRDKLQSQQRIYHEKKHYRSSKSDK
CFTHQIDLSIHQGIHAEEKIYKCSECDKCFTHKFHLNIHQRIHTGEKPYKCSECDKCFTQKSHLNIHQRIHTG
EKPYKCIVCEKCFTEKGNLRIHQRIHTGDKPYKCSECDKCFTQKSHLNIHQRIHTGEKPYKCSQCDKSFTENG
HLRIHQRNHTGEKPYKCSRCDKSFTENGHLRIHQRIHTGEKPYKCSECDKCFTKKFSLRIHQRIHTGEKPYKC
SECDKCFTFKGSLRIHQRIHTGEKPYKCSECDKSFTENGCLRIHQRIHTGEKPYKCTECDKCFTFKGSLRIHQ
RIHTGEKPYKCTECDKSFTEKGKLRIHQRIHKGEKPYKCSECDKCFTEKGHQRIHTGEIAYK*
>rs49128380_ENSMUSP00000081167_c.1183:T>C_p.L395P
ENSMUST00000084149.10|Zfp991-201|ENSMUSG00000067916|+|chr4:g.147263808:T>C
MSVCLVNTPQGLLTFKDVALDFSLEEWESLSFAQRTLYMDVMLENYNNLLFVENHCICGKYEKVLDQDSQYIV
HEHMNIQEKSSKWDKLSNVILEAPQCTPYKTNHTSDALQFSNQKRLKPRNTKEVCKYNDSVNSLTLFSTISLN
QGIHMQKKKHNGNAESEKAFISKHKVMVKRNNTGVNPYKCSKFDKYLTQRDKLQSQQRIYHEKKHYRSSKSDK
CFTHQIDLSIHQGIHAEEKIYKCSECDKCFTHKFHLNIHQRIHTGEKPYKCSECDKCFTQKSHLNIHQRIHTG
EKPYKCIVCEKCFTEKGNLRIHQRIHTGDKPYKCSECDKCFTQKSHLNIHQRIHTGEKPYKCSQCDKSFTENG
HLRIHQRNHTGEKPYKCSRCDKSFTENGHPRIHQRIHTGEKPYKCSECDKCFTKKFSLRIHQRIHTGEKPYKC
SECDKCFTFKGSLRIHQRIHTGEKPYKCSECDKSFTENGCLRIHQRIHTGEKPYKCTECDKCFTFKGSLRIHQ
RIHTGEKPYKCTECDKSFTEKGKLRIHQRIHKGEKPYKCSECDKCFTEKGHQRIHTGEIAYK*
>ENSMUSP00000081167_c.1315:G>A_p.S439N ENSMUST00000084149.10|Zfp991-201|ENSMUSG00000067916|+|chr4:g.147263940:G>A
MSVCLVNTPQGLLTFKDVALDFSLEEWESLSFAQRTLYMDVMLENYNNLLFVENHCICGKYEKVLDQDSQYIV
HEHMNIQEKSSKWDKLSNVILEAPQCTPYKTNHTSDALQFSNQKRLKPRNTKEVCKYNDSVNSLTLFSTISLN
QGIHMQKKKHNGNAESEKAFISKHKVMVKRNNTGVNPYKCSKFDKYLTQRDKLQSQQRIYHEKKHYRSSKSDK
CFTHQIDLSIHQGIHAEEKIYKCSECDKCFTHKFHLNIHQRIHTGEKPYKCSECDKCFTQKSHLNIHQRIHTG
EKPYKCIVCEKCFTEKGNLRIHQRIHTGDKPYKCSECDKCFTQKSHLNIHQRIHTGEKPYKCSQCDKSFTENG
HLRIHQRNHTGEKPYKCSRCDKSFTENGHLRIHQRIHTGEKPYKCSECDKCFTKKFSLRIHQRIHTGEKPYKC
NECDKCFTFKGSLRIHQRIHTGEKPYKCSECDKSFTENGCLRIHQRIHTGEKPYKCTECDKCFTFKGSLRIHQ
RIHTGEKPYKCTECDKSFTEKGKLRIHQRIHKGEKPYKCSECDKCFTEKGHQRIHTGEIAYK*
>ENSMUSP00000081167_c.1362:C>T_p.Q455* ENSMUST00000084149.10|Zfp991-201|ENSMUSG00000067916|+|chr4:g.147263987:C>T
MSVCLVNTPQGLLTFKDVALDFSLEEWESLSFAQRTLYMDVMLENYNNLLFVENHCICGKYEKVLDQDSQYIV
HEHMNIQEKSSKWDKLSNVILEAPQCTPYKTNHTSDALQFSNQKRLKPRNTKEVCKYNDSVNSLTLFSTISLN
QGIHMQKKKHNGNAESEKAFISKHKVMVKRNNTGVNPYKCSKFDKYLTQRDKLQSQQRIYHEKKHYRSSKSDK
CFTHQIDLSIHQGIHAEEKIYKCSECDKCFTHKFHLNIHQRIHTGEKPYKCSECDKCFTQKSHLNIHQRIHTG
EKPYKCIVCEKCFTEKGNLRIHQRIHTGDKPYKCSECDKCFTQKSHLNIHQRIHTGEKPYKCSQCDKSFTENG
HLRIHQRNHTGEKPYKCSRCDKSFTENGHLRIHQRIHTGEKPYKCSECDKCFTKKFSLRIHQRIHTGEKPYKC
SECDKCFTFKGSLRIH*RIHTGEKPYKCSECDKSFTENGCLRIHQRIHTGEKPYKCTECDKCFTFKGSLRIHQ
RIHTGEKPYKCTECDKSFTEKGKLRIHQRIHKGEKPYKCSECDKCFTEKGHQRIHTGEIAYK*
>rs252974414_ENSMUSP00000120413_c.109:T>G_p.L37W
ENSMUST00000148762.4|Zfp988-201|ENSMUSG00000078498|+|chr4:g.147413518:T>G
MSVCLVNTPQGLLTFKDVALDFSLEEWECLSFAQRSWYMDVMLENYNNLLFVENHCIRGKNGKVLDQDSQYIV
HEHMNIQEKSSKWDKLSNVILESLQCTPYKTNHSSDALQFSNQKRLKPRNTKEVCKYNDSVNSLSLFSTISLN
QGINMQKKKHNRNAELDNIFVSKHKVMVKKDNIAGNPYKCSEFHKYLTQRENLQSQQRIYHGKKPYKSSKSDK
CFTHQIHLSIHQGIHTEEIIYKCSECDKCFKNKFNLTMHQRIHTGEKPYKCSECGKCFTEKSSLRIHQRIHTG
EKPYKCSECDKCFTKQSNLSIHQRIHTGEKPYKCSECDKCFTQQSCLSIHQRIHTGEKPYKCSECGKCFTEKS
SLRIHQRIHTGEKTYKCSECDKCFTRKFHLGIHQRIHTGEKPYKCSECDKCFTQQSNLTIHQRIHTGEKPYKC
PECGKCFTEKSSLRIHQRIHTGEKPYKCSECGKCFTEKSSLRIHQRIHTGEKPYKCNECGKCFTEKSSLRIHQ
RIHTGEKPYKCSECGKCFTVKSSLRIHQRIHTGEKPYKCSECDKCFTKQSNLSIHQRIHTGEKPYKCSECEKC
FSRKLHLGIHQRIHTGERPYKCSECEKCFSRKFHLGIHQRIHTGKKPYK*
>ENSMUSP00000120413_c.751:A>G_p.N251S ENSMUST00000148762.4|Zfp988-201|ENSMUSG00000078498|+|chr4:g.147416319:A>G
MSVCLVNTPQGLLTFKDVALDFSLEEWECLSFAQRSLYMDVMLENYNNLLFVENHCIRGKNGKVLDQDSQYIV
HEHMNIQEKSSKWDKLSNVILESLQCTPYKTNHSSDALQFSNQKRLKPRNTKEVCKYNDSVNSLSLFSTISLN

QGINMQKKKHNRNAELDNIFVSKHKVMVKKDNIAGNPYKCSEFHKYLTQRENLQSQQRIYHGKKPYKSSKSDK
CFTHQIHLSIHQGIHTEEIIYKCSECDKCFKSKFNLTMHQRIHTGEKPYKCSECGKCFTEKSSLRIHQRIHTG
EKPYKCSECDKCFTKQSNLSIHQRIHTGEKPYKCSECDKCFTQQSCLSIHQRIHTGEKPYKCSECGKCFTEKS
SLRIHQRIHTGEKTYKCSECDKCFTRKFHLGIHQRIHTGEKPYKCSECDKCFTQQSNLTIHQRIHTGEKPYKC
PECGKCFTEKSSLRIHQRIHTGEKPYKCSECGKCFTEKSSLRIHQRIHTGEKPYKCNECGKCFTEKSSLRIHQ
RIHTGEKPYKCSECGKCFTVKSSLRIHQRIHTGEKPYKCSECDKCFTKQSNLSIHQRIHTGEKPYKCSECEKC
FSRKLHLGIHQRIHTGERPYKCSECEKCFSRKFHLGIHQRIHTGKKPYK*
>rs48329995_ENSMUSP00000120413_c.838:A>T_p.K280I
ENSMUST00000148762.4|Zfp988-201|ENSMUSG00000078498|+|chr4:g.147416406:A>T
MSVCLVNTPQGLLTFKDVALDFSLEEWECLSFAQRSLYMDVMLENYNNLLFVENHCIRGKNGKVLDQDSQYIV
HEHMNIQEKSSKWDKLSNVILESLQCTPYKTNHSSDALQFSNQKRLKPRNTKEVCKYNDSVNSLSLFSTISLN
QGINMQKKKHNRNAELDNIFVSKHKVMVKKDNIAGNPYKCSEFHKYLTQRENLQSQQRIYHGKKPYKSSKSDK
CFTHQIHLSIHQGIHTEEIIYKCSECDKCFKNKFNLTMHQRIHTGEKPYKCSECGKCFTEISSLRIHQRIHTG
EKPYKCSECDKCFTKQSNLSIHQRIHTGEKPYKCSECDKCFTQQSCLSIHQRIHTGEKPYKCSECGKCFTEKS
SLRIHQRIHTGEKTYKCSECDKCFTRKFHLGIHQRIHTGEKPYKCSECDKCFTQQSNLTIHQRIHTGEKPYKC
PECGKCFTEKSSLRIHQRIHTGEKPYKCSECGKCFTEKSSLRIHQRIHTGEKPYKCNECGKCFTEKSSLRIHQ
RIHTGEKPYKCSECGKCFTVKSSLRIHQRIHTGEKPYKCSECDKCFTKQSNLSIHQRIHTGEKPYKCSECEKC
FSRKLHLGIHQRIHTGERPYKCSECEKCFSRKFHLGIHQRIHTGKKPYK*
>ENSMUSP00000120413_c.1106:TCAT>C_p.H370del ENSMUST00000148762.4|Zfp988-
201|ENSMUSG00000078498|+|chr4:g.147416674:TCAT>C
MSVCLVNTPQGLLTFKDVALDFSLEEWECLSFAQRSLYMDVMLENYNNLLFVENHCIRGKNGKVLDQDSQYIV
HEHMNIQEKSSKWDKLSNVILESLQCTPYKTNHSSDALQFSNQKRLKPRNTKEVCKYNDSVNSLSLFSTISLN
QGINMQKKKHNRNAELDNIFVSKHKVMVKKDNIAGNPYKCSEFHKYLTQRENLQSQQRIYHGKKPYKSSKSDK
CFTHQIHLSIHQGIHTEEIIYKCSECDKCFKNKFNLTMHQRIHTGEKPYKCSECGKCFTEKSSLRIHQRIHTG
EKPYKCSECDKCFTKQSNLSIHQRIHTGEKPYKCSECDKCFTQQSCLSIHQRIHTGEKPYKCSECGKCFTEKS
SLRIQRIHTGEKTYKCSECDKCFTRKFHLGIHQRIHTGEKPYKCSECDKCFTQQSNLTIHQRIHTGEKPYKCP
ECGKCFTEKSSLRIHQRIHTGEKPYKCSECGKCFTEKSSLRIHQRIHTGEKPYKCNECGKCFTEKSSLRIHQR
IHTGEKPYKCSECGKCFTVKSSLRIHQRIHTGEKPYKCSECDKCFTKQSNLSIHQRIHTGEKPYKCSECEKCF
SRKLHLGIHQRIHTGERPYKCSECEKCFSRKFHLGIHQRIHTGKKPYK*
>rs47993660_ENSMUSP00000120413_c.1218:C>G_p.P407A
ENSMUST00000148762.4|Zfp988-201|ENSMUSG00000078498|+|chr4:g.147416786:C>G
MSVCLVNTPQGLLTFKDVALDFSLEEWECLSFAQRSLYMDVMLENYNNLLFVENHCIRGKNGKVLDQDSQYIV
HEHMNIQEKSSKWDKLSNVILESLQCTPYKTNHSSDALQFSNQKRLKPRNTKEVCKYNDSVNSLSLFSTISLN
QGINMQKKKHNRNAELDNIFVSKHKVMVKKDNIAGNPYKCSEFHKYLTQRENLQSQQRIYHGKKPYKSSKSDK
CFTHQIHLSIHQGIHTEEIIYKCSECDKCFKNKFNLTMHQRIHTGEKPYKCSECGKCFTEKSSLRIHQRIHTG
EKPYKCSECDKCFTKQSNLSIHQRIHTGEKPYKCSECDKCFTQQSCLSIHQRIHTGEKPYKCSECGKCFTEKS
SLRIHQRIHTGEKTYKCSECDKCFTRKFHLGIHQRIHTGEKAYKCSECDKCFTQQSNLTIHQRIHTGEKPYKC
PECGKCFTEKSSLRIHQRIHTGEKPYKCSECGKCFTEKSSLRIHQRIHTGEKPYKCNECGKCFTEKSSLRIHQ
RIHTGEKPYKCSECGKCFTVKSSLRIHQRIHTGEKPYKCSECDKCFTKQSNLSIHQRIHTGEKPYKCSECEKC
FSRKLHLGIHQRIHTGERPYKCSECEKCFSRKFHLGIHQRIHTGKKPYK*
>rs108848453_ENSMUSP00000120413_c.1618:G>A_p.R540K
ENSMUST00000148762.4|Zfp988-201|ENSMUSG00000078498|+|chr4:g.147417186:G>A
MSVCLVNTPQGLLTFKDVALDFSLEEWECLSFAQRSLYMDVMLENYNNLLFVENHCIRGKNGKVLDQDSQYIV
HEHMNIQEKSSKWDKLSNVILESLQCTPYKTNHSSDALQFSNQKRLKPRNTKEVCKYNDSVNSLSLFSTISLN
QGINMQKKKHNRNAELDNIFVSKHKVMVKKDNIAGNPYKCSEFHKYLTQRENLQSQQRIYHGKKPYKSSKSDK
CFTHQIHLSIHQGIHTEEIIYKCSECDKCFKNKFNLTMHQRIHTGEKPYKCSECGKCFTEKSSLRIHQRIHTG
EKPYKCSECDKCFTKQSNLSIHQRIHTGEKPYKCSECDKCFTQQSCLSIHQRIHTGEKPYKCSECGKCFTEKS
SLRIHQRIHTGEKTYKCSECDKCFTRKFHLGIHQRIHTGEKPYKCSECDKCFTQQSNLTIHQRIHTGEKPYKC
PECGKCFTEKSSLRIHQRIHTGEKPYKCSECGKCFTEKSSLRIHQRIHTGEKPYKCNECGKCFTEKSSLRIHQ
RIHTGEKPYKCSECGKCFTVKSSLRIHQKIHTGEKPYKCSECDKCFTKQSNLSIHQRIHTGEKPYKCSECEKC
FSRKLHLGIHQRIHTGERPYKCSECEKCFSRKFHLGIHQRIHTGKKPYK*
>ENSMUSP00000120413_c.1819:G>A_p.S607N ENSMUST00000148762.4|Zfp988-
201|ENSMUSG00000078498|+|chr4:g.147417387:G>A
MSVCLVNTPQGLLTFKDVALDFSLEEWECLSFAQRSLYMDVMLENYNNLLFVENHCIRGKNGKVLDQDSQYIV
HEHMNIQEKSSKWDKLSNVILESLQCTPYKTNHSSDALQFSNQKRLKPRNTKEVCKYNDSVNSLSLFSTISLN
QGINMQKKKHNRNAELDNIFVSKHKVMVKKDNIAGNPYKCSEFHKYLTQRENLQSQQRIYHGKKPYKSSKSDK
CFTHQIHLSIHQGIHTEEIIYKCSECDKCFKNKFNLTMHQRIHTGEKPYKCSECGKCFTEKSSLRIHQRIHTG
EKPYKCSECDKCFTKQSNLSIHQRIHTGEKPYKCSECDKCFTQQSCLSIHQRIHTGEKPYKCSECGKCFTEKS

SLRIHQRIHTGEKTYKCSECDKCFTRKFHLGIHQRIHTGEKPYKCSECDKCFTQQSNLTIHQRIHTGEKPYKC
PECGKCFTEKSSLRIHQRIHTGEKPYKCSECGKCFTEKSSLRIHQRIHTGEKPYKCNECGKCFTEKSSLRIHQ
RIHTGEKPYKCSECGKCFTVKSSLRIHQRIHTGEKPYKCSECDKCFTKQSNLSIHQRIHTGEKPYKCSECEKC
FSRKLHLGIHQRIHTGERPYKCNECEKCFSRKFHLGIHQRIHTGKKPYK*
>rs48702416_ENSMUSP00000115135_c.170:G>A_p.M57I
ENSMUST00000133078.8|Zfp978-201|ENSMUSG00000078497|+|chr4:g.147472874:G>A
MFAPHQESGRRTTKRMSASLENTAQCLLTFKDVFLDFSSEEWECLNFAQRTLYMDVILENYNNLLFVENHCIC
GNYEKEKVVEQDTQHIFNEHGHIQ
>rs48702416_ENSMUSP00000114248_c.170:G>A_p.M57I
ENSMUST00000154154.2|Zfp978-202|ENSMUSG00000078497|+|chr4:g.147472874:G>A
MFAPHQESGRRTTKRMSASLENTAQCLLTFKDVFLDFSSEEWECLNFAQRTLYMDVILENYNNLLFVENHCIC
GNYEKEKVVEQDTQHIFNEHGHIQEKSFKCNESSNIIHESSESTPHKTNHRDATLQSSNLKRHKTGTTKQVCT
YKDCVNCLKGSSIISLNQGTHVEKKEHNRNKNLDEVLVSKHKPIVRQNNSEMNTYTCGEFDKCFTQSDNLQSQ
QRIYPGKKSYKYSESDKCFTQPSHLSIHHTIHSGEKPYKCSECDKCFTDKCTLRKHQRIHTGEKPYKCNECDK
CFTDKGSLRVHQRIHTGEKPYKCSECDKCFTQTSHLSIHRRIH
>rs52171009_ENSMUSP00000114248_c.403:G>A_p.R135K
ENSMUST00000154154.2|Zfp978-202|ENSMUSG00000078497|+|chr4:g.147474858:G>A
MFAPHQESGRRTTKRMSASLENTAQCLLTFKDVFLDFSSEEWECLNFAQRTLYMDVMLENYNNLLFVENHCIC
GNYEKEKVVEQDTQHIFNEHGHIQEKSFKCNESSNIIHESSESTPHKTNHRDATLQSSNLKKHKTGTTKQVCT
YKDCVNCLKGSSIISLNQGTHVEKKEHNRNKNLDEVLVSKHKPIVRQNNSEMNTYTCGEFDKCFTQSDNLQSQ
QRIYPGKKSYKYSESDKCFTQPSHLSIHHTIHSGEKPYKCSECDKCFTDKCTLRKHQRIHTGEKPYKCNECDK
CFTDKGSLRVHQRIHTGEKPYKCSECDKCFTQTSHLSIHRRIH
>rs108687733_ENSMUSP00000114248_c.801:G>T_p.D268Y
ENSMUST00000154154.2|Zfp978-202|ENSMUSG00000078497|+|chr4:g.147475256:G>T
MFAPHQESGRRTTKRMSASLENTAQCLLTFKDVFLDFSSEEWECLNFAQRTLYMDVMLENYNNLLFVENHCIC
GNYEKEKVVEQDTQHIFNEHGHIQEKSFKCNESSNIIHESSESTPHKTNHRDATLQSSNLKRHKTGTTKQVCT
YKDCVNCLKGSSIISLNQGTHVEKKEHNRNKNLDEVLVSKHKPIVRQNNSEMNTYTCGEFDKCFTQSDNLQSQ
QRIYPGKKSYKYSESDKCFTQPSHLSIHHTIHSGEKPYKCSECDKCFTYKCTLRKHQRIHTGEKPYKCNECDK
CFTDKGSLRVHQRIHTGEKPYKCSECDKCFTQTSHLSIHRRIH
>ENSMUSP00000101346_c.579:T>G_p.C194G ENSMUST00000105721.9|Zfp982-
202|ENSMUSG00000078496|+|chr4:g.147597224:T>G
MSVFLVNTPQGLLTFKDVAVEFSLEEWERLSFAQRSLYIDVMLENYNNLLFVENHFICAKCGKALDEDSQYIV
HEHMNIQEKSSKWNKLSNVILESPHCVPHKCSEWDKCFSQRSHLSIHQKIHTGEKPYKCSECDKCFTEKGILR
IHQKIHSGEKSYKYHECDKCFPKKSHLSIHLRIHKGEKPYKCSDCDKGFTEKRKLRRHQKIHTGEKPYKCSEC
DKCFIQKYSLSDHQRIHTGEKPYKCSECNKCFIQKSSLNNHQSIHTGEKPYKCSECDKCFTDKRNLRRHQKIH
TGEKPYKCSECEKCFTDKGSLRRHQKIHTGEKPYKCSECEKCYTDKGSLRRHQKIHKGEKLTNAVNVTNALLK
KAI*
>ENSMUSP00000068021_c.579:T>G_p.C194G ENSMUST00000063704.8|Zfp982-
201|ENSMUSG00000078496|+|chr4:g.147597224:T>G
MSVFLVNTPQGLLTFKDVAVEFSLEEWERLSFAQRSLYIDVMLENYNNLLFVENHFICAKCGKALDEDSQYIV
HEHMNIQEKSSKWNKLSNVILESPHCVPHKCSEWDKCFSQRSHLSIHQKIHTGEKPYKCSECDKCFTEKGILR
IHQKIHSGEKSYKYHECDKCFPKKSHLSIHLRIHKGEKPYKCSDCDKGFTEKRKLRRHQKIHTGEKPYKCSEC
DKCFIQKYSLSDHQRIHTGEKPYKCSECNKCFIQKSSLNNHQSIHTGEKPYKCSECDKCFTDKRNLRRHQKIH
TGEKPYKCSECEKCFTDKGSLRRHQKIHTGEKPYKCSECEKCYTDKGSLRRHQKIHKGEKLTNAVNVTNALLK
KAI*
>ENSMUSP00000101346_c.950:G>T_p.Q317H ENSMUST00000105721.9|Zfp982-
202|ENSMUSG00000078496|+|chr4:g.147597595:G>T
MSVFLVNTPQGLLTFKDVAVEFSLEEWERLSFAQRSLYIDVMLENYNNLLFVENHFICAKCGKALDEDSQYIV
HEHMNIQEKSSKWNKLSNVILESPHCVPHKCSEWDKCFSQRSHLSIHQKIHTGEKPYKCSECDKCFTEKGILR
IHQKIHSGEKSYKYHECDKCFPKKSHLSIHLRIHKGEKPYKCSDCDKCFTEKRKLRRHQKIHTGEKPYKCSEC
DKCFIQKYSLSDHQRIHTGEKPYKCSECNKCFIQKSSLNNHQSIHTGEKPYKCSECDKCFTDKRNLRRHQKIH
TGEKPYKCSECEKCFTDKGSLRRHHKIHTGEKPYKCSECEKCYTDKGSLRRHQKIHKGEKLTNAVNVTNALLK
KAI*
>ENSMUSP00000068021_c.950:G>T_p.Q317H ENSMUST00000063704.8|Zfp982-
201|ENSMUSG00000078496|+|chr4:g.147597595:G>T
MSVFLVNTPQGLLTFKDVAVEFSLEEWERLSFAQRSLYIDVMLENYNNLLFVENHFICAKCGKALDEDSQYIV
HEHMNIQEKSSKWNKLSNVILESPHCVPHKCSEWDKCFSQRSHLSIHQKIHTGEKPYKCSECDKCFTEKGILR
IHQKIHSGEKSYKYHECDKCFPKKSHLSIHLRIHKGEKPYKCSDCDKCFTEKRKLRRHQKIHTGEKPYKCSEC

```
DKCFIQKYSLSDHQRIHTGEKPYKCSECNKCFIQKSSLNNHQSIHTGEKPYKCSECDKCFTDKRNLRRHQKIH
TGEKPYKCSECEKCFTDKGSLRRHHKIHTGEKPYKCSECEKCYTDKGSLRRHQKIHKGEKLTNAVNVTNALLK
KAI*
>ENSMUSP00000101346_c.1051:A>G_p.E351G ENSMUST00000105721.9|Zfp982-
202|ENSMUSG00000078496|+|chr4:g.147597696:A>G
MSVFLVNTPQGLLTFKDVAVEFSLEEWERLSFAQRSLYIDVMLENYNNLLFVENHFICAKCGKALDEDSQYIV
HEHMNIQEKSSKWNKLSNVILESPHCVPHKCSEWDKCFSQRSHLSIHQKIHTGEKPYKCSECDKCFTEKGILR
IHQKIHSGEKSYKYHECDKCFPKKSHLSIHLRIHKGEKPYKCSDCDKCFTEKRKLRRHQKIHTGEKPYKCSEC
DKCFIQKYSLSDHQRIHTGEKPYKCSECNKCFIQKSSLNNHQSIHTGEKPYKCSECDKCFTDKRNLRRHQKIH
TGEKPYKCSECEKCFTDKGSLRRHQKIHTGEKPYKCSECEKCYTDKGSLRRHQKIHKGGKLTNAVNVTNALLK
KAI*
>ENSMUSP00000068021_c.1051:A>G_p.E351G ENSMUST00000063704.8|Zfp982-
201|ENSMUSG00000078496|+|chr4:g.147597696:A>G
MSVFLVNTPQGLLTFKDVAVEFSLEEWERLSFAQRSLYIDVMLENYNNLLFVENHFICAKCGKALDEDSQYIV
HEHMNIQEKSSKWNKLSNVILESPHCVPHKCSEWDKCFSQRSHLSIHQKIHTGEKPYKCSECDKCFTEKGILR
IHQKIHSGEKSYKYHECDKCFPKKSHLSIHLRIHKGEKPYKCSDCDKCFTEKRKLRRHQKIHTGEKPYKCSEC
DKCFIQKYSLSDHQRIHTGEKPYKCSECNKCFIQKSSLNNHQSIHTGEKPYKCSECDKCFTDKRNLRRHQKIH
TGEKPYKCSECEKCFTDKGSLRRHQKIHTGEKPYKCSECEKCYTDKGSLRRHQKIHKGGKLTNAVNVTNALLK
KAI*
>ENSMUSP00000101346_c.1066:C>A_p.A356E ENSMUST00000105721.9|Zfp982-
202|ENSMUSG00000078496|+|chr4:g.147597711:C>A
MSVFLVNTPQGLLTFKDVAVEFSLEEWERLSFAQRSLYIDVMLENYNNLLFVENHFICAKCGKALDEDSQYIV
HEHMNIQEKSSKWNKLSNVILESPHCVPHKCSEWDKCFSQRSHLSIHQKIHTGEKPYKCSECDKCFTEKGILR
IHQKIHSGEKSYKYHECDKCFPKKSHLSIHLRIHKGEKPYKCSDCDKCFTEKRKLRRHQKIHTGEKPYKCSEC
DKCFIQKYSLSDHQRIHTGEKPYKCSECNKCFIQKSSLNNHQSIHTGEKPYKCSECDKCFTDKRNLRRHQKIH
TGEKPYKCSECEKCFTDKGSLRRHQKIHTGEKPYKCSECEKCYTDKGSLRRHQKIHKGEKLTNEVNVTNALLK
KAI*
>ENSMUSP00000068021_c.1066:C>A_p.A356E ENSMUST00000063704.8|Zfp982-
201|ENSMUSG00000078496|+|chr4:g.147597711:C>A
MSVFLVNTPQGLLTFKDVAVEFSLEEWERLSFAQRSLYIDVMLENYNNLLFVENHFICAKCGKALDEDSQYIV
HEHMNIQEKSSKWNKLSNVILESPHCVPHKCSEWDKCFSQRSHLSIHQKIHTGEKPYKCSECDKCFTEKGILR
IHQKIHSGEKSYKYHECDKCFPKKSHLSIHLRIHKGEKPYKCSDCDKCFTEKRKLRRHQKIHTGEKPYKCSEC
DKCFIQKYSLSDHQRIHTGEKPYKCSECNKCFIQKSSLNNHQSIHTGEKPYKCSECDKCFTDKRNLRRHQKIH
TGEKPYKCSECEKCFTDKGSLRRHQKIHTGEKPYKCSECEKCYTDKGSLRRHQKIHKGEKLTNEVNVTNALLK
KAI*
>ENSMUSP00000123296_c.412:T>C_p.F138S ENSMUST00000139784.8|Zfp985-
202|ENSMUSG00000065999|+|chr4:g.147667546:T>C
MSESLVNTPQGLLTFKDVALDFSLEEWERLSFAQRTLYIDVMLENYNNLLFVENHCIRGKYRKVLDQDSQYIV
HEHMNIQEKSSKWDKLSKVLLDSPQYTPYKTYHSSLQYWNQKRLKPRNTKEVCKYNDSVNSVSLSSTISLNQG
IHMQ
>ENSMUSP00000080438_c.412:T>C_p.F138S ENSMUST00000081742.7|Zfp985-
201|ENSMUSG00000065999|+|chr4:g.147667546:T>C
MSESLVNTPQGLLTFKDVALDFSLEEWERLSFAQRTLYIDVMLENYNNLLFVENHCIRGKYRKVLDQDSQYIV
HEHMNIQEKSSKWDKLSKVLLDSPQYTPYKTYHSSLQYWNQKRLKPRNTKEVCKYNDSVNSVSLSSTISLNQG
IHMQKKKHKRNAEFEKVFVSKHKDMVKQKNTGVNPYKCSEFHKYLTQREKLQTQQRIYHGKKPYKSSKSDKCF
THQIHLRSHQGIHTGEKPHKCSECDKCFTHKFNLRAHQRIHTGEKLYKCSECDKCFTHKSRLNYHQRIHTGEK
PHKCSECDKCFTHKFNLRAHQIIHTGEKPHKCSECDKCFTHKFNLRAHQRIHTGEKPYKCSECDKCFTRKFHL
GIHHRIHTGEKPYKCSECDKCFTGKGSLRIHQRIHIGEKPYKCSECDKCFTGKGSLRIHQRIHTGERPHKCSE
CDKCFTGKGSLRIHQRMHTGEKPYKCSECDKCFTQKSHLNIHQGIHTGEKPYKCSECDKCFTHKSHLINHQKI
HTGEKPYK*
>ENSMUSP00000080438_c.598:A>G_p.Q200R ENSMUST00000081742.7|Zfp985-
201|ENSMUSG00000065999|+|chr4:g.147667732:A>G
MSESLVNTPQGLLTFKDVALDFSLEEWERLSFAQRTLYIDVMLENYNNLLFVENHCIRGKYRKVLDQDSQYIV
HEHMNIQEKSSKWDKLSKVLLDSPQYTPYKTYHSSLQYWNQKRLKPRNTKEVCKYNDSVNSVSLFSTISLNQG
IHMQKKKHKRNAEFEKVFVSKHKDMVKQKNTGVNPYKCSEFHKYLTQREKLQTRQRIYHGKKPYKSSKSDKCF
THQIHLRSHQGIHTGEKPHKCSECDKCFTHKFNLRAHQRIHTGEKLYKCSECDKCFTHKSRLNYHQRIHTGEK
PHKCSECDKCFTHKFNLRAHQIIHTGEKPHKCSECDKCFTHKFNLRAHQRIHTGEKPYKCSECDKCFTRKFHL
GIHHRIHTGEKPYKCSECDKCFTGKGSLRIHQRIHIGEKPYKCSECDKCFTGKGSLRIHQRIHTGERPHKCSE
```

CDKCFTGKGSLRIHQRMHTGEKPYKCSECDKCFTQKSHLNIHQGIHTGEKPYKCSECDKCFTHKSHLINHQKI
HTGEKPYK*
>ENSMUSP00000080438_c.865:C>T_p.T289I ENSMUST00000081742.7|Zfp985-201|ENSMUSG00000065999|+|chr4:g.147667999:C>T
MSESLVNTPQGLLTFKDVALDFSLEEWERLSFAQRTLYIDVMLENYNNLLFVENHCIRGKYRKVLDQDSQYIV
HEHMNIQEKSSKWDKLSKVLLDSPQYTPYKTYHSSLQYWNQKRLKPRNTKEVCKYNDSVNSVSLFSTISLNQG
IHMQKKKHKRNAEFEKVFVSKHKDMVKQKNTGVNPYKCSEFHKYLTQREKLQTQQRIYHGKKPYKSSKSDKCF
THQIHLRSHQGIHTGEKPHKCSECDKCFTHKFNLRAHQRIHTGEKLYKCSECDKCFTHKSRLNYHQRIHIGEK
PHKCSECDKCFTHKFNLRAHQIIHTGEKPHKCSECDKCFTHKFNLRAHQRIHTGEKPYKCSECDKCFTRKFHL
GIHHRIHTGEKPYKCSECDKCFTGKGSLRIHQRIHIGEKPYKCSECDKCFTGKGSLRIHQRIHTGERPHKCSE
CDKCFTGKGSLRIHQRMHTGEKPYKCSECDKCFTQKSHLNIHQGIHTGEKPYKCSECDKCFTHKSHLINHQKI
HTGEKPYK*
>ENSMUSP00000080438_c.1506:T>C_p.S503P ENSMUST00000081742.7|Zfp985-201|ENSMUSG00000065999|+|chr4:g.147668640:T>C
MSESLVNTPQGLLTFKDVALDFSLEEWERLSFAQRTLYIDVMLENYNNLLFVENHCIRGKYRKVLDQDSQYIV
HEHMNIQEKSSKWDKLSKVLLDSPQYTPYKTYHSSLQYWNQKRLKPRNTKEVCKYNDSVNSVSLFSTISLNQG
IHMQKKKHKRNAEFEKVFVSKHKDMVKQKNTGVNPYKCSEFHKYLTQREKLQTQQRIYHGKKPYKSSKSDKCF
THQIHLRSHQGIHTGEKPHKCSECDKCFTHKFNLRAHQRIHTGEKLYKCSECDKCFTHKSRLNYHQRIHTGEK
PHKCSECDKCFTHKFNLRAHQIIHTGEKPHKCSECDKCFTHKFNLRAHQRIHTGEKPYKCSECDKCFTRKFHL
GIHHRIHTGEKPYKCSECDKCFTGKGSLRIHQRIHIGEKPYKCSECDKCFTGKGSLRIHQRIHTGERPHKCSE
CDKCFTGKGSLRIHQRMHTGEKPYKCSECDKCFTQKSHLNIHQGIHTGEKPYKCSECDKCFTHKPHLINHQKI
HTGEKPYK*
>rs47730106_ENSMUSP00000113561_c.1179:A>G_p.N394D
ENSMUST00000117638.2|Zfp534-201|ENSMUSG00000062518|-|chr4:g.147759488:T>C
MSVCLVNTPQGLLTFKDVALDFSLEEWECLSFAQRSLYMDVMLENYNNLLFVENHCIRGKNGKVLDQDSQYIV
HEHMNIQEKSSKWDKLSNVILESLQCTPYKTNHSSDALQFSTQKRLKPRNTKEVCKYNDSVNSLSLFSTISLN
QGINMQKKKHNRNAELDKIFVSKHKVMVKKDNIAGNPYKCSEFHKYLTQREKLQSQQRIYHGKKPYESSKSDK
CFTHQIHLSIHQGIHTEEKIYKCSECDKCFKNKFNLTMHQRIHKGEKPYKCSECGKCFTDKTSLRIHQRIHTG
EKPYKCSECGKCFTEKSSLRIHQRIHTGEKPYKCSECGKCFTEKSSLRIHQRIHTGEKPYKCSECDKCFTQQS
YLSIHQRIHTGEKPYKCSECDKCFTKQSDLSIHQRIHTGEKPYKCSECDKCFTQQSHLSIHQRIHTGEKPYKC
SECDKCFTQQSYLSIHQRIHAGEKPYKCSECDKCFTTKGNLIIHQRIHTREKPYKCSECGKCFTEKSSLRIHQ
RIHTGEKPYKCSECDKCFTKQSKLSIHQRIHTGEKPYKCSECEKCFNEKNSLKIHQRIHTGERPYKCSECDKC
FTQKSYLNIHQRIHAGEKPYKCSECDKCFTTKGNLIIHQRIHTREKPYKCSECDKCFTQKSQLSIHQKIHTGE
KPYK*
>rs107953444_ENSMUSP00000113561_c.729:G>A_p.E244K
ENSMUST00000117638.2|Zfp534-201|ENSMUSG00000062518|-|chr4:g.147759938:C>T
MSVCLVNTPQGLLTFKDVALDFSLEEWECLSFAQRSLYMDVMLENYNNLLFVENHCIRGKNGKVLDQDSQYIV
HEHMNIQEKSSKWDKLSNVILESLQCTPYKTNHSSDALQFSTQKRLKPRNTKEVCKYNDSVNSLSLFSTISLN
QGINMQKKKHNRNAELDKIFVSKHKVMVKKDNIAGNPYKCSEFHKYLTQREKLQSQQRIYHGKKPYESSKSDK
CFTHQIHLSIHQGIHTEEKIYKCSKCDKCFKNKFNLTMHQRIHKGEKPYKCSECGKCFTDKTSLRIHQRIHTG
EKPYKCSECGKCFTEKSSLRIHQRIHTGEKPYKCSECGKCFTEKSSLRIHQRIHTGEKPYKCSECDKCFTQQS
YLSIHQRIHTGEKPYKCSECDKCFTKQSNLSIHQRIHTGEKPYKCSECDKCFTQQSHLSIHQRIHTGEKPYKC
SECDKCFTQQSYLSIHQRIHAGEKPYKCSECDKCFTTKGNLIIHQRIHTREKPYKCSECGKCFTEKSSLRIHQ
RIHTGEKPYKCSECDKCFTKQSKLSIHQRIHTGEKPYKCSECEKCFNEKNSLKIHQRIHTGERPYKCSECDKC
FTQKSYLNIHQRIHAGEKPYKCSECDKCFTTKGNLIIHQRIHTREKPYKCSECDKCFTQKSQLSIHQKIHTGE
KPYK*
>ENSMUSP00000114023_c.452:G>C_p.K151N ENSMUST00000122309.2|Zfp984-202|ENSMUSG00000078495|-|chr4:g.147840397:C>G
MSVSLVNTPQGLLTFKDVALYFSLEEWECLSFAQRTLYMDVMLENYNNLLFVENHCIHGKYGKVLGEESQYIV
HEHVNIQEKSSKWDKLIKVILESPQCTPYKTNHSSLQYSNQKRLKPRNTKEVCKYNDSVNSVSLFSTISLNQG
IHLQNKKHNRNAEFEKVFVSKHKVMVKRNNTGVNPYKCSEFDKYLTQREKLQSQQRIYHGKKPYRSSKSDKCF
THQIHLSIHQGIHAEEKIYKCSECDKCFTHKSHLNIHQRIHTGENPYKCSECDKCFKHKFSFSMHQRIHTGEK
PYKCSECDKCFTQKSHLSVHQRIHTGEKPYKCSECDKCFTHKSHLNSHQRIHTGEKPYKCSECDKCFTKNGSL
RIHQRIHTGENPYKCSECDKCFTHKSNLNIHQRIHTGEKPYKCSECDKCFTHKSHLNSHQRIHTGEKPYKCSE
CDKCFTRKFHLSIHQRIHTGENPYKCSECDKCFTQKSNLNIHQRIHTGEKPYKCSECDKCFTEKGSLRIHQRI
HTGENPYKCSECDKCFTRKFHLSIHQKIHTGEKPYK*
>ENSMUSP00000101360_c.452:G>C_p.K151N ENSMUST00000105734.10|Zfp984-201|ENSMUSG00000078495|-|chr4:g.147840397:C>G

BMJ Publishing Group Limited (BMJ) disclaims all liability and responsibility arising from any reliance placed on this supplemental material which has been supplied by the author(s)

MSVSLVNTPQGLLTFKDVALYFSLEEWECLSFAQRTLYMDVMLENYNNLLFVENHCIHGKYGKVLGEESQYIV
HEHVNIQEKSSKWDKLIKVILESPQCTPYKTNHSSLQYSNQKRLKPRNTKEVCKYNDSVNSVSLFSTISLNQG
IHLQNKKHNRNAEFEKVFVSKHKVMVKRNNTGVNPYKCSEFDKYLTQREKLQSQQRIYHGKKPYRSSKSDKCF
THQIHLSIHQGIHAEEKIYKCSECDKCFTHKSHLNIHQRIHTGENPYKCSECDKCFKHKFSFSMHQRIHTGEK
PYKCSECDKCFTQKSHLSVHQRIHTGEKPYKCSECDKCFTHKSHLNSHQRIHTGEKPYKCSECDKCFTKNGSL
RIHQRIHTGENPYKCSECDKCFTHKSNLNIHQRIHTGEKPYKCSECDKCFTHKSHLNSHQRIHTGEKPYKCSE
CDKCFTRKFHLSIHQRIHTGENPYKCSECDKCFTQKSNLNIHQRIHTGEKPYKCSECDKCFTEKGSLRIHQRI
HTGENPYKCSECDKCFTRKFHLSIHQKIHTGEKPYK*
>ENSMUSP00000095395_c.1668:G>T_p.A557S ENSMUST00000097788.11|Mthfr-
202|ENSMUSG00000029009|+|chr4:g.148139481:G>T
MVNEARGSGSPNPRSEGSSSGSESSKDSSRCSTPSLDPERHERLREKMRRRMDSGDKWFSLEFFPPRTAEGAV
NLISRFDRMAAGGPLFVDVTWHPAGDPGSDKETSSMMIASTAVNYCGLETILHMTCCQQRPEEITGHLHRAKQ
LGLKNIMALRGDPVGDHWEAEEGGFSYATDLVKHIRTEFADYFDICVAGYPRGHPDAESFEDDLKHLKEKVSA
GADFIITQLFFEASTFFSFVKACTEIGISCPILPGIFPIQGYTSLRQLVKLSKLEVPQKIKDVIEPIKDNDAA
IRNYGIELAVSLCRELLDSGLVPGLHFYTLNREVATMEVLKQLGMWTEDPRRPLPWALSAHPKRREEDVRPIF
WASRPKSYIYRTQDWDEFPNGRWGNSSSPAFGELKDYYLFYLKSKSPREELLKMWGEELTSEESVFEVFEHYL
SGEPNRHGYRVTCLPWNDEPLAAETSLMKEELLRVNRLGILTINSQPNINAKPSSDPVVGWGPSGGYVFQKAY
LEFFTSRETVEALLQVLKTYELRVNYHIVDVKGENITNAPELQPNSVTWGIFPGREIIQPTVVDPISFMFWKD
EAFALWIEQWGKLYEEESPSRMIIQYIHDNYFLVNLVDNEFPLDSCLWQVVEDTFELLNRHPTERETQAP*
>ENSMUSP00000069774_c.1791:G>T_p.A598S ENSMUST00000069604.15|Mthfr-
201|ENSMUSG00000029009|+|chr4:g.148139481:G>T
MNHQKAKVLPAGHCYPSLGMWASEAGCVRLSVPPSISRNPAMVNEARGSGSPNPRSEGSSSGSESSKDSSRCS
TPSLDPERHERLREKMRRRMDSGDKWFSLEFFPPRTAEGAVNLISRFDRMAAGGPLFVDVTWHPAGDPGSDKE
TSSMMIASTAVNYCGLETILHMTCCQQRPEEITGHLHRAKQLGLKNIMALRGDPVGDHWEAEEGGFSYATDLV
KHIRTEFADYFDICVAGYPRGHPDAESFEDDLKHLKEKVSAGADFIITQLFFEASTFFSFVKACTEIGISCPI
LPGIFPIQGYTSLRQLVKLSKLEVPQKIKDVIEPIKDNDAAIRNYGIELAVSLCRELLDSGLVPGLHFYTLNR
EVATMEVLKQLGMWTEDPRRPLPWALSAHPKRREEDVRPIFWASRPKSYIYRTQDWDEFPNGRWGNSSSPAFG
ELKDYYLFYLKSKSPREELLKMWGEELTSEESVFEVFEHYLSGEPNRHGYRVTCLPWNDEPLAAETSLMKEEL
LRVNRLGILTINSQPNINAKPSSDPVVGWGPSGGYVFQKAYLEFFTSRETVEALLQVLKTYELRVNYHIVDVK
GENITNAPELQPNSVTWGIFPGREIIQPTVVDPISFMFWKDEAFALWIEQWGKLYEEESPSRMIIQYIHDNYF
LVNLVDNEFPLDSCLWQVVEDTFELLNRHPTERETQAP*
>ENSMUSP00000117095_c.1716:G>T_p.A573S ENSMUST00000152498.8|Mthfr-
206|ENSMUSG00000029009|+|chr4:g.148139481:G>T
GCVRLSVPPSISRNPAMVNEARGSGSPNPRSEGSSSGSESSKDSSRCSTPSLDPERHERLREKMRRRMDSGDK
WFSLEFFPPRTAEGAVNLISRFDRMAAGGPLFVDVTWHPAGDPGSDKETSSMMIASTAVNYCGLETILHMTCC
QQRPEEITGHLHRAKQLGLKNIMALRGDPVGDHWEAEEGGFSYATDLVKHIRTEFADYFDICVAGYPRGHPDA
ESFEDDLKHLKEKVSAGADFIITQLFFEASTFFSFVKACTEIGISCPILPGIFPIQGYTSLRQLVKLSKLEVP
QKIKDVIEPIKDNDAAIRNYGIELAVSLCRELLDSGLVPGLHFYTLNREVATMEVLKQLGMWTEDPRRPLPWA
LSAHPKRREEDVRPIFWASRPKSYIYRTQDWDEFPNGRWGNSSSPAFGELKDYYLFYLKSKSPREELLKMWGE
ELTSEESVFEVFEHYLSGEPNRHGYRVTCLPWNDEPLAAETSLMKEELLRVNRLGILTINSQPNINAKPSSDP
VVGWGPSGGYVFQKAYLEFFTSRETVEALLQVLKTYELRVNYHIVDVKGENITNAPELQPNSVTWGIFPGREI
IQPTVVDPISFMFWKDEAFALWIEQWGKLYEEESPSRMIIQYIHDNYFLVNLVDNEFPLDSCLWQVVEDTFEL
LNRHPTERETQAP*
>ENSMUSP00000116840_c.162:G>T_p.A55S ENSMUST00000156113.2|Mthfr-
207|ENSMUSG00000029009|+|chr4:g.148139481:G>T
XGYVFQKAYLEFFTSRETVEALLQVLKTYELRVNYHIVDVKGENITNAPELQPNSVTWGIFPGREIIQPTVVD
PISFMFWKDEAFALWIEQWGKLYEEESPSRMIIQYIHDNYFLVNLVDNEFPLDSCLWQVVEDTFELLNRHPTE
RETQAP*
>ENSMUSP00000030949_c.328:T>C_p.M110T ENSMUST00000030949.4|Tas1r3-
201|ENSMUSG00000029072|-|chr4:g.155947355:A>G
MPALAIMGLSLAAFLELGMGASLCLSQQFKAQGDYILGGLFPLGSTEEATLNQRTQPNSIPCNRFSPLGLFLA
MAMKMAVEEINNGSALLPGLRLGYDLFDTCSEPVVTTKSSLMFLAKVGSQSIAAYCNYTQYQPRVLAVIGPHS
SELALITGKFFSFFLMPQVSYSASMDRLSDRETFPSFFRTVPSDRVQLQAVVTLLQNFSWNWVAALGSDDDYG
REGLSIFSSLANARGICIAHEGLVPQHDTSGQQLGKVLDVLRQVNQSKVQVVVLFASARAVYSLFSYSIHHGL
SPKVWVASESWLTSDLVMTLPNIARVGTVLGFLQRGALLPEFSHYVETHLALAADPAFCASLNAELDLEEHVM
GQRCPRCDDIMLQNLSSGLLQNLSAGQLHHQIFATYAAVYSVAQALHNTLQCNVSHCHVSEHVLPWQLLENMY
NMSFHARDLTLQFDAEGNVDMEYDLKMWVWQSPTPVLHTVGTFNGTLQLQQSKMYWPGNQVPVSQCSRQCKDG
QVRRVKGFHSCCYDCVDCKAGSYRKHPDDFTCTPCNQDQWSPEKSTACLPRRPKFLAWGEPVVLSLLLLLCLV

LGLALAALGLSVHHWDSPLVQASGGSQFCFGLICLGLFCLSVLLFPGRPSSASCLAQQPMAHLPLTGCLSTLF
LQAAETFVESELPLSWANWLCSYLRGLWAWLVVLLATFVEAALCAWYLIAFPPEVVTDWSVLPTEVLEHCHVR
SWVSLGLVHITNAMLAFLCFLGTFLVQSQPGRYNRARGLTFAMLAYFITWVSFVPLLANVQVAYQPAVQMGAI
LVCALGILVTFHLPKCYVLLWLPKLNTQEFFLGRNAKKAADENSGGGEAAQGHNE*
>ENSMUSP00000101194_c.1180:G>T_p.G394V ENSMUST00000105569.5|Klhl17-201|ENSMUSG00002076083|-|chr4:g.156316259:C>A
MQPRGERPAGRTQSPEHSSPGPGPEAPPPPQPPAPEAERARPRQARPAAPMEGAMQLLSREGHSVAHNSKRHY
HDAFVAMSRMRQRGLLCDIVLHVAAKEIRAHKVVLASCSPYFHAMFTNEMSESRQTHVTLHDIDPQALDQLVQ
FAYTAEIVVGEGNVQTLLPAASLLQLNGVRDACCKFLLSQLDPSNCLGIRGFADTHSCSDLLKAAHRYVLQHF
VDVAKTEEFMLLPLKQVLELVSSDSLNVPSEEDVYRAVLSWVKHDVDTRRQHVPRLMKCVRLPLLSRDFLLGH
VDAESLVRHHPDCKDLLIEALKFHLLPEQRGVLGTSRTRPRRCEGAGPVLFAVGGGSLFAIHGDCEAYDTRTD
RWHVVASMSTRRARVGVAAVGNRLYAVGVYDGTSDLATVESYDPVTNTWQPEVSMGTRRSCLGVAALHGLLYA
AGGYDGASCLNSAERYDPLTGTWTSIAAMSTRRRYVRVATLDGNLYAVGGYDSSSHLATVEKYEPQVNSWTPV
ASMLSRRSSAGVAVLEGALYVAGGNDGTSCLNSVERYSTKAGAWESVAPMNIRRSTHDLVAMDGWLYAVGGND
GSSSLNSIEKYNPRTNKWVAASCMFTRRSSVGVAVLELLNFPPPSSPTLSVSSTSL*
>ENSMUSP00001091659_c.52:G>T_p.G18V ENSMUST00020183129.1|Klhl17-204|ENSMUSG00002076083|-|chr4:g.156316259:C>A
XARVGVAAVGNRLYAVGVYDGTSDLATVESYDPVTNTWQPEVSMGTRRSCLGVAALHGLLYAAGGYDGASCLN
SAERYDPLTGTWTSIAAMSTRRRYVRVATLDGNLYAVGGYDSSSHLATVEKYEPQEHT*
>ENSMUSP00000101194_c.1161:C>T_p.R388* ENSMUST00000105569.5|Klhl17-201|ENSMUSG00002076083|-|chr4:g.156316278:G>A
MQPRGERPAGRTQSPEHSSPGPGPEAPPPPQPPAPEAERARPRQARPAAPMEGAMQLLSREGHSVAHNSKRHY
HDAFVAMSRMRQRGLLCDIVLHVAAKEIRAHKVVLASCSPYFHAMFTNEMSESRQTHVTLHDIDPQALDQLVQ
FAYTAEIVVGEGNVQTLLPAASLLQLNGVRDACCKFLLSQLDPSNCLGIRGFADTHSCSDLLKAAHRYVLQHF
VDVAKTEEFMLLPLKQVLELVSSDSLNVPSEEDVYRAVLSWVKHDVDTRRQHVPRLMKCVRLPLLSRDFLLGH
VDAESLVRHHPDCKDLLIEALKFHLLPEQRGVLGTSRTRPRRCEGAGPVLFAVGGGSLFAIHGDCEAYDTRTD
RWHVVASMSTRRARVGVAAVGN*LYAVGGYDGTSDLATVESYDPVTNTWQPEVSMGTRRSCLGVAALHGLLYA
AGGYDGASCLNSAERYDPLTGTWTSIAAMSTRRRYVRVATLDGNLYAVGGYDSSSHLATVEKYEPQVNSWTPV
ASMLSRRSSAGVAVLEGALYVAGGNDGTSCLNSVERYSTKAGAWESVAPMNIRRSTHDLVAMDGWLYAVGGND
GSSSLNSIEKYNPRTNKWVAASCMFTRRSSVGVAVLELLNFPPPSSPTLSVSSTSL*
>ENSMUSP00001091659_c.33:C>T_p.R12* ENSMUST00020183129.1|Klhl17-204|ENSMUSG00002076083|-|chr4:g.156316278:G>A
XARVGVAAVGN*LYAVGGYDGTSDLATVESYDPVTNTWQPEVSMGTRRSCLGVAALHGLLYAAGGYDGASCLN
SAERYDPLTGTWTSIAAMSTRRRYVRVATLDGNLYAVGGYDSSSHLATVEKYEPQEHT*
>ENSMUSP00000101194_c.1038:G>C_p.G347R ENSMUST00000105569.5|Klhl17-201|ENSMUSG00002076083|-|chr4:g.156316401:C>G
MQPRGERPAGRTQSPEHSSPGPGPEAPPPPQPPAPEAERARPRQARPAAPMEGAMQLLSREGHSVAHNSKRHY
HDAFVAMSRMRQRGLLCDIVLHVAAKEIRAHKVVLASCSPYFHAMFTNEMSESRQTHVTLHDIDPQALDQLVQ
FAYTAEIVVGEGNVQTLLPAASLLQLNGVRDACCKFLLSQLDPSNCLGIRGFADTHSCSDLLKAAHRYVLQHF
VDVAKTEEFMLLPLKQVLELVSSDSLNVPSEEDVYRAVLSWVKHDVDTRRQHVPRLMKCVRLPLLSRDFLLGH
VDAESLVRHHPDCKDLLIEALKFHLLPEQRGVLGTSRTRPRRCEGAGPVLFAVGRGSLFAIHGDCEAYDTRTD
RWHVVASMSTRRARVGVAAVGNRLYAVGGYDGTSDLATVESYDPVTNTWQPEVSMGTRRSCLGVAALHGLLYA
AGGYDGASCLNSAERYDPLTGTWTSIAAMSTRRRYVRVATLDGNLYAVGGYDSSSHLATVEKYEPQVNSWTPV
ASMLSRRSSAGVAVLEGALYVAGGNDGTSCLNSVERYSTKAGAWESVAPMNIRRSTHDLVAMDGWLYAVGGND
GSSSLNSIEKYNPRTNKWVAASCMFTRRSSVGVAVLELLNFPPPSSPTLSVSSTSL*
>ENSMUSP00000137253_c.180:C>A_p.P61T ENSMUST00000179543.8|Noc2l-201|ENSMUSG00000095567|+|chr4:g.156320766:C>A
MAASRAPRRRLEDLSVDEFLASGFESGSESELEGAAEAAAEERRARGAAWNRERRGARTSTGPAGRPRKGRAS
EHKDQLSRLKDRDPEFYKFLQENDRSLLDFSDSDSSAEEEEPFHSLPDTLEEASETEEDGGEDSDALPRGLRS
KKNEPVPVTLAMVERWRQGSRHHLSPRLFHEVVQAFRAAVATTQGEQEAAETCRFQVADSAVFNALVTFCIRD
LCGCLQKLLFGKTPKDSNRLLPSSSPLWGKLRVDVKTYLSAVVQLVACLAEATVSAAVLQHISSLVPYYLTFP
KQCRMLLKRMVVLWSTGEESLRVLAFLVLIRVCRHKKEAFLGPILKQMYIMYVRNCKFTSPSTLPLISFMQRT
LTEMLALDPSVSYQHAFLYIRQLAVHLRNAMTAGKKETHQSVYNWQYVHCLYLWCRVLSTLGSSEILQPLLYP
LSQIIIGCIKLLPTARFYPLRMHCVRALTLLSQTIGTFIPVLPFILEIFQQVDFNRRPGRMSSKPINFSVILK
LSSTNLQEKAYRDGLLEQLCDLTLEYLHSQAHSIAFPELVLPTVLQLKSFLRECKVANYCRQVRQLLEKVQEN
AQHIQSLRQSATFSVSDQMAVDAWEKQVREEGTPLTRYYGHWKKLRDREIQLEISGKERLEDLNFPEIKRRKV
EDRKDEDRKELKDLFELDSSEGEDSTDFFERGVPRLPEAHQGLKEDQEEEDKEEGDSDSEDGDTDTGVDLSEL
WQLAQGPQDELEDLQLSEED*

>ENSMUSP00000137253_c.1791:A>G_p.S598G ENSMUST00000179543.8|Noc2l-201|ENSMUSG00000095567|+|chr4:g.156329744:A>G
MAASRAPRRRLEDLSVDEFLASGFESGSESELEGAAEAAAEERRARGAAWNRERRGARTSPGPAGRPRKGRAS
EHKDQLSRLKDRDPEFYKFLQENDRSLLDFSDSDSSAEEEPFHSLPDTLEEASETEEDGGEDSDALPRGLRS
KKNEPVPVTLAMVERWRQGSRHHLSPRLFHEVVQAFRAAVATTQGEQEAAETCRFQVADSAVFNALVTFCIRD
LCGCLQKLLFGKTPKDSNRLLPSSSPLWGKLRVDVKTYLSAVVQLVACLAEATVSAAVLQHISSLVPYYLTFP
KQCRMLLKRMVVLWSTGEESLRVLAFLVLIRVCRHKKEAFLGPILKQMYIMYVRNCKFTSPSTLPLISFMQRT
LTEMLALDPSVSYQHAFLYIRQLAVHLRNAMTAGKKETHQSVYNWQYVHCLYLWCRVLSTLGSSEILQPLLYP
LSQIIIGCIKLLPTARFYPLRMHCVRALTLLSQTIGTFIPVLPFILEIFQQVDFNRRPGRMSSKPINFSVILK
LSSTNLQEKAYRDGLLEQLCDLTLEYLHSQAHSIAFPELVLPTVLQLKSFLRECKVANYCRQVRQLLEKVQEN
AQHIQSLRQSATFGVSDQMAVDAWEKQVREEGTPLTRYYGHWKKLRDREIQLEISGKERLEDLNFPEIKRRKV
EDRKDEDRKELKDLFELDSSEGEDSTDFFERGVPRLPEAHQGLKEDQEEEDKEEGDSDSEDGDTDTGVDLSEL
WQLAQGPQDELEDLQLSEED*
>rs30778148_ENSMUSP00000137253_c.1843:A>G_p.E615G
ENSMUST00000179543.8|Noc2l-201|ENSMUSG00000095567|+|chr4:g.156329915:A>G
MAASRAPRRRLEDLSVDEFLASGFESGSESELEGAAEAAAEERRARGAAWNRERRGARTSPGPAGRPRKGRAS
EHKDQLSRLKDRDPEFYKFLQENDRSLLDFSDSDSSAEEEPFHSLPDTLEEASETEEDGGEDSDALPRGLRS
KKNEPVPVTLAMVERWRQGSRHHLSPRLFHEVVQAFRAAVATTQGEQEAAETCRFQVADSAVFNALVTFCIRD
LCGCLQKLLFGKTPKDSNRLLPSSSPLWGKLRVDVKTYLSAVVQLVACLAEATVSAAVLQHISSLVPYYLTFP
KQCRMLLKRMVVLWSTGEESLRVLAFLVLIRVCRHKKEAFLGPILKQMYIMYVRNCKFTSPSTLPLISFMQRT
LTEMLALDPSVSYQHAFLYIRQLAVHLRNAMTAGKKETHQSVYNWQYVHCLYLWCRVLSTLGSSEILQPLLYP
LSQIIIGCIKLLPTARFYPLRMHCVRALTLLSQTIGTFIPVLPFILEIFQQVDFNRRPGRMSSKPINFSVILK
LSSTNLQEKAYRDGLLEQLCDLTLEYLHSQAHSIAFPELVLPTVLQLKSFLRECKVANYCRQVRQLLEKVQEN
AQHIQSLRQSATFSVSDQMAVDAWEKQVREGGTPLTRYYGHWKKLRDREIQLEISGKERLEDLNFPEIKRRKV
EDRKDEDRKELKDLFELDSSEGEDSTDFFERGVPRLPEAHQGLKEDQEEEDKEEGDSDSEDGDTDTGVDLSEL
WQLAQGPQDELEDLQLSEED*
>ENSMUSP00000137253_c.2188:T>C_p.L730P ENSMUST00000179543.8|Noc2l-201|ENSMUSG00000095567|+|chr4:g.156331512:T>C
MAASRAPRRRLEDLSVDEFLASGFESGSESELEGAAEAAAEERRARGAAWNRERRGARTSPGPAGRPRKGRAS
EHKDQLSRLKDRDPEFYKFLQENDRSLLDFSDSDSSAEEEPFHSLPDTLEEASETEEDGGEDSDALPRGLRS
KKNEPVPVTLAMVERWRQGSRHHLSPRLFHEVVQAFRAAVATTQGEQEAAETCRFQVADSAVFNALVTFCIRD
LCGCLQKLLFGKTPKDSNRLLPSSSPLWGKLRVDVKTYLSAVVQLVACLAEATVSAAVLQHISSLVPYYLTFP
KQCRMLLKRMVVLWSTGEESLRVLAFLVLIRVCRHKKEAFLGPILKQMYIMYVRNCKFTSPSTLPLISFMQRT
LTEMLALDPSVSYQHAFLYIRQLAVHLRNAMTAGKKETHQSVYNWQYVHCLYLWCRVLSTLGSSEILQPLLYP
LSQIIIGCIKLLPTARFYPLRMHCVRALTLLSQTIGTFIPVLPFILEIFQQVDFNRRPGRMSSKPINFSVILK
LSSTNLQEKAYRDGLLEQLCDLTLEYLHSQAHSIAFPELVLPTVLQLKSFLRECKVANYCRQVRQLLEKVQEN
AQHIQSLRQSATFSVSDQMAVDAWEKQVREEGTPLTRYYGHWKKLRDREIQLEISGKERLEDLNFPEIKRRKV
EDRKDEDRKELKDLFELDSSEGEDSTDFFERGVPRLPEAHQGLKEDQEEEDKEEGDSDSEDGDTDTGVDLSEP
WQLAQGPQDELEDLQLSEED*
>ENSMUSP00000136611_c.219:G>A_p.A74T ENSMUST00000179919.3|Samd11-201|ENSMUSG00000096351|-|chr4:g.156336502:C>T
MPEHPRHCDFQRGNVEIGLGPGGDLLGKRLSCPCITSHCSSEKKARSKDPQGESPTLEKDAATSLLPELESTM
TPEDHYHQLMSALSEASSFEETQRLYHLGIPSHDLLRVRQEVATATLRGPSGLEVHLPSSTADHRRKQGLVQR
REGAVPAAATSFSEREMSQPPPLLSPQNAAHITMSSHLRPPFLGMPTAVCQTPGFSFLPSAQAEMLARQQELL
RKQSLARLEMSELLRQKELGSVHRPLLPAPEVALHIPEGPDELQRRGSMLVLKHSSAPLLALPPQGPPGPGPP
IPPKESARSRSEKGSLGVQPSQPKETTGAGLWAQEVSEEPSKDSDGEDPETAAAREGTSTPSQVPAGGTRAEG
RGLLSGSTLPPPLPLGFPCGAVSPYFHTGTMGGLFTDEETTTLEDVNKWTVDDVCNFVGGLSGCGEYARVFGE
QGIDGETLPLLTEEHLLNTMGLKLGPALKIRAQVAKRLGRVFYMASFPVALPLQPPSLQAPELSPGHQPLSPA
TTTSPYEGTHLPTGQASPKQENGSGTIALLSGAPDPSQLLQ*
>ENSMUSP00000151756_c.189:G>A_p.A64T ENSMUST00000217934.2|Samd11-202|ENSMUSG00000096351|-|chr4:g.156336502:C>T
MPEHPRHCDFQRGNVEIGLGPGGDLLGKRLSCPCITSHCSSEKKARSKDPQATSLLPELESTMTPEDHYHQLM
SALSEASSFEETQRLYHLGIPSHDLLRVRQEVATATLRGPSGLEVHLPSSTADHRRKQGLVQRREGAVPAAAT
SFSEREMSQPPPLLSPQNAAHITMSSHLRPPFLGMPTAVCQTPGFSFLPSAQAEMLARQQELLRKQSLARLEM
SELLRQKELGSVHRPLLPAPEVALHIPEGPDELQRRGSMLVLKHSSAPLLALPPQGPPGPGPPIPPKESARSR
SEKGSLGVQPSQPKETTGAGLWAQEVSEEPSKDSDGEDPETAAAREGTSTPSQVPAGGTRAEGRGLLSGSTLP
PPLPLGFPCGAVSPYFHTGTMGGLFTDEETTTLEDVNKWTVDDVCNFVGGLSGCGEYARVFGEQGIDGETLPL

LTEEHLLNTMGLKLGPALKIRAQVAKRLGRVFYMASFPVALPLQPPSLQAPELSPGHQPLSPATTTSPYEGTH
LPTGQASPKQENGSGTIALLSGAPDPSQLLQ*
>ENSMUSP00000151442_c.189:G>A_p.A64T ENSMUST00000218788.2|Samd11-204|ENSMUSG00000096351|-|chr4:g.156336502:C>T
MPEHPRHCDFQRGNVEIGLGPGGDLLGKRLSCPCITSHCSSEKKARSKDPQATSLLPELESTMTPEDHYHQLM
SALSEASSFEETQRLYHLGIPSHDLLRVRQEVATATLRGPSGLEVHLPSSTADHRRKQGLVQRREGAVPAAAT
SFSEREMSQPPPLLSPQNAAHITMSSHLRPPFLGMPTAVCQTPGFSFLPSAQAEMLARQQELLRKQSLARLEM
SELLRQKELGSVHRPLLPAPEVALHIPEGPDELQRRGSMLVLKHSSAPLLALPPQGPPGPGPPIPPKESARSR
SEKGSLGVQPSQPKETTGAGLWAQEVSEEPSKDSDGEDPETAAAREGTSTPSQVPAGGTRAEGRGLLSGSTLP
PPLPLGFPCGAVSPYFHTGTMGGLFTDEETTTLEDVNKWTVDDVCNFVGGLSGCGEYARVFGEQGIDGETLPL
LTEEHLLNTMGLKLGPALKIRAQVAKRLGRVFYMASFPVALPLQPPSLQAPELSPGHQPLSPATTTSPYEGTH
LPTGQASPKQENGSGTIALLSGAPDPSQLLQ*
>ENSMUSP00000151261_c.219:G>A_p.A74T ENSMUST00000220228.2|Samd11-209|ENSMUSG00000096351|-|chr4:g.156336502:C>T
MPEHPRHCDFQRGNVEIGLGPGGDLLGKRLSCPCITSHCSSEKKARSKDPQGESPTLEKDAATSLLPELESTM
TPEDHYHQLMSALSEASSFEETQRLYHLGIPSHADHRRKQGLVQRREGAVPAAATSFSEREMSQPPPLLSPQN
AAHITMSSHLRPPFLGMPTAVCQTPGFSFLPSAQAEMLARQQELLRKQSLARLEMSELLRQKELGSVHRPLLP
APEVALHIPEGPDELQRRGSMLVLKH
>ENSMUSP00000151817_c.189:G>A_p.A64T ENSMUST00000219393.2|Samd11-206|ENSMUSG00000096351|-|chr4:g.156336502:C>T
MPEHPRHCDFQRGNVEIGLGPGGDLLGKRLSCPCITSHCSSEKKARSKDPQATSLLPELESTMTPEDHYHQLM
SALSEASSFEETQRLYHLGIPSHDLLRVRQEVATATLRGPSGLEVHLPSSTADHRRKQGLVQR
>ENSMUSP00000041597_c.748:A>T_p.D250V ENSMUST00000035980.9|Gm9758-201|ENSMUSG00000033219|-|chr5:g.14960484:T>A
MALFARLCRLFQRANVDGRENREGRKDADIPSERNEGRRRGTWRMWMALRQTSSPVPVISKKQFEKEEKKLIK
EIQLTTEETNELRDRLTYVTEGSMNKRPYHRQNPLYEKLKLKEKEIMTFLHNLEMENMEAQENKQELKKETHF
YRNLHSRLLMEENLIKKKSMTLQQESKEVQADWAIIHQRLVELNLSGKDEQENSNLETPEYQVSEAARELGLA
TAEEDSILQNELPGQEAPAEHHLQHPQSSSVESSSI*
>ENSMUSP00000041597_c.748:A>G_p.D250G ENSMUST00000035980.9|Gm9758-201|ENSMUSG00000033219|-|chr5:g.14960484:T>C
MALFARLCRLFQRANVDGRENREGRKDADIPSERNEGRRRGTWRMWMALRQTSSPVPVISKKQFEKEEKKLIK
EIQLTTEETNELRDRLTYVTEGSMNKRPYHRQNPLYEKLKLKEKEIMTFLHNLEMENMEAQENKQELKKETHF
YRNLHSRLLMEENLIKKKSMTLQQESKEVQADWAIIHQRLVELNLSGKDEQENSNLETPEYQVSEAARELGLA
TAEEDSILQNELPGQEAPAEHHLQHPQSSSGESSSI*
>ENSMUSP00000125587_c.232:C>G_p.T78S ENSMUST00000159973.3|Speer4e-201|ENSMUSG00000091255|-|chr5:g.14987171:G>C
MALFARLCRLFQRANVDGSDTREGRKDADLPSERNEGRSRGTWRMWMALRQTSSPVPVISKKQFEKEEKKLIK
EIQLSTEETNELRDRLIYVTEGSMNKRPYHRQNPLYEKLKLKEKEIMTFLHNLEMENMEAKETKQELKKETHF
YRNLHSRLLMEENLIKKKLMTLQQESKEVQADWAIIHQCLLELNLSGKDEQENSNLETPEYQVSEAARELGLA
TAEEDSILQNELPGQEAPAEHHLQHPQSSSDESSSI*
>ENSMUSP00000094716_c.703:A>G_p.Q235R ENSMUST00000096953.5|Gm10354-201|ENSMUSG00000072188|-|chr5:g.15024578:T>C
MALFARLCRLFQRANVDGRETREGRKDADLPSESNEGRRRGTWRMCMALRQTSSPVPVISKKQFEKEEKKLIK
EIQLTMEETNELRDRLIYVTEGSMNKRPYHRQNPLYEKLKLKEKEIMTFLHNLEMENMEAKETKQELKKETHF
YHNLHSRLLMEENLIKKKLMPLQQESKEVQADWAIIHQRLVELNLSGKDEQEENSNLETPEYQVSETARELGL
ATAEEDSILQNELPGREAPAEHHLQHPQSSSDESSSI*
>rs49800592_ENSMUSP00000094716_c.516:A>G_p.K173E
ENSMUST00000096953.5|Gm10354-201|ENSMUSG00000072188|-|chr5:g.15025509:T>C
MALFARLCRLFQRANVDGRETREGRKDADLPSESNEGRRRGTWRMCMALRQTSSPVPVISKKQFEKEEKKLIK
EIQLTMEETNELRDRLIYVTEGSMNKRPYHRQNPLYEKLKLKEKEIMTFLHNLEMENMEAKETKQELKKETHF
YHNLHSRLLMEENLIKKKLMPLQQESEEVQADWAIIHQRLVELNLSGKDEQEENSNLETPEYQVSETARELGL
ATAEEDSILQNELPGQEAPAEHHLQHPQSSSDESSSI*
>ENSMUSP00000094716_c.262:G>A_p.R88H ENSMUST00000096953.5|Gm10354-201|ENSMUSG00000072188|-|chr5:g.15027612:C>T
MALFARLCRLFQRANVDGRETREGRKDADLPSESNEGRRRGTWRMCMALRQTSSPVPVISKKQFEKEEKKLIK
EIQLTMEETNELRDHLIYVTEGSMNKRPYHRQNPLYEKLKLKEKEIMTFLHNLEMENMEAKETKQELKKETHF
YHNLHSRLLMEENLIKKKLMPLQQESKEVQADWAIIHQRLVELNLSGKDEQEENSNLETPEYQVSETARELGL
ATAEEDSILQNELPGQEAPAEHHLQHPQSSSDESSSI*

>ENSMUSP00000143356_c.462:CC>TT_p.L155F ENSMUST00000197995.2|Gm21149-201|ENSMUSG00000099762|-|chr5:g.15678133:GG>AA
MALFARLCRLFQRANVDRRETREGRKDADLPPDSNEGRRRGTWRMWMALRQTSSPVPVISKKQFEKEEKKLIK
EIQLTTEETNELRDRLIYVTEGSMNKRPYHRQNPLYEKLKLKEKEIMTFLHNLEMENMEAQENKQELKKETHF
YRNLHSRLFDGGESYKEEVDDIAAGEQGSTG*LGHHSPTFGGIEPEW*RRTGEQQS*DSRISGLRNCKRAGIG
HS*RRQHPAE*VARPGGPC*APSPAPTIFLG*IFFHI
>ENSMUSP00000143356_c.168:C>T_p.P57S ENSMUST00000197995.2|Gm21149-201|ENSMUSG00000099762|-|chr5:g.15680276:G>A
MALFARLCRLFQRANVDRRETREGRKDADLPPDSNEGRRRGTWRMWMALRQTSSPVSVISKKQFEKEEKKLIK
EIQLTTEETNELRDRLIYVTEGSMNKRPYHRQNPLYEKLKLKEKEIMTFLHNLEMENMEAQENKQELKKETHF
YRNLHSRLLMEENLIKKKSMTLQQESKEVQADWAIIHQRLVELNLSGKDEQENSNLETPEYQVSETARELGLA
TAEEDSILQNELPGQEAPAEHHLQHPQSSSDESSSI*
>ENSMUSP00000158761_c.1020:T>G_p.C341G ENSMUST00000238335.2|Tmub1-206|ENSMUSG00000028958|-|chr5:g.24650653:A>C
MALIEGVGDEVTVLFAVLACLLVLALAWVSTHTTESTDPQPQPPGTTTPAQPSEAMSASDSIREEAPGAESPS
LRHRGPSAQPEPDTGVTASTPPDSPQEPLLLRLKFLNDSEQVARAWPQDTIGSLKRSLSPQDPVSRPGTAGSA
HLPRSTARRRHSDTRQSPPAPQLRSPLPRVHESRSPTSSLPTGVRARPLRAGNRQPSVAPAASAAAPALVLPD
PVPALLSPDRYPGSGRLHPAPQSPGLCHVSAVVPPRAFGSVASPSGPHSPRRKGSCCLPRPTSPAACLFPLPW
SPALRRRGLRVLAEAAPCDLHSSRPSPGAAGPQPSLAVGSSGSGTCCRGLGRQGTPMGPS*
>rs387511256_ENSMUSP00000030795_c.1139:T>GGT_p.V380ins-fs ENSMUST00000030795.10|Abcf2-201|ENSMUSG00000028953|-|chr5:g.24773748:A>ACC
MPSDLAKKKAAKKKEAAKARQRPRKGHEENGDAVTEPQVAEEKIEEANGRETTGDGEVDLLTKELEDFEMKKA
AARAVTGVLASHPNSTDVHIINLSLTFHGQELLSDTKLELNSGRRYGLIGLNGIGKSMLLSAIGKREVPIPEH
IDIYHLTREMPPSEKTPLQCVMEVDTERAMLEREAERLAHEDAECEKLMELYERLEELDADKAEMRASRILHG
LGFTPAMQRKKLKDFSGGWRMRVALARALFIRPFMLLLDEPTNHLDLDACVWLEEELKTFKRILVLVSHSQDF
LNGVCTNIIHMHNKKLKYYTGNYDQYVKTRLELEENQMKRFHWEQDQIAHMKNYIARFGHGSAKLARQAQSKE
KTLQKMMASGLTERVV
>ENSMUSP00000085578_c.103:T>G_p.I35S ENSMUST00000088244.6|Actr3b-201|ENSMUSG00000056367|+|chr5:g.26014786:T>G
MAGSLPPCVVDCGTGYTKLGYAGNTEPQFIIPSCSAIRESAKVVDQAQRRVLRGVDDLDFFIGDEAIDKPTYA
TKWPIRHGIVEDWDLMERFMEQVVFKYLRAEPEDHYFLMTEPPLNTPENREYLAEIMFESFNVPGLYIAVQAV
LALAASWTSRQVGERTLTGIVIDSGDGVTHVIPVAEGYVIGSCIKHIPIAGRDITYFIQQLLREREVGIPPEQ
SLETAKAIKEKYCYICPDIVREFAKYDVDPRKWIKQYTGINAINQKKFIIDVGYERFLGPEIFFHPEFANPDF
MESISDVVDEVIQSCPIDVRRPLYKNVVLSGGSTMFRDFGRRLQRDLKRVVDARLKLSQELSGGRIKPKPVEV
QVVTHHMQRYAVWFGGSMLASTPEFFQVCHTKKDYEEYGPSICRHNPVFGVMS*
>ENSMUSP00000085578_c.226:G>A_p.W76* ENSMUST00000088244.6|Actr3b-201|ENSMUSG00000056367|+|chr5:g.26016916:G>A
MAGSLPPCVVDCGTGYTKLGYAGNTEPQFIIPSCIAIRESAKVVDQAQRRVLRGVDDLDFFIGDEAIDKPTYA
TK*PIRHGIVEDWDLMERFMEQVVFKYLRAEPEDHYFLMTEPPLNTPENREYLAEIMFESFNVPGLYIAVQAV
LALAASWTSRQVGERTLTGIVIDSGDGVTHVIPVAEGYVIGSCIKHIPIAGRDITYFIQQLLREREVGIPPEQ
SLETAKAIKEKYCYICPDIVREFAKYDVDPRKWIKQYTGINAINQKKFIIDVGYERFLGPEIFFHPEFANPDF
MESISDVVDEVIQSCPIDVRRPLYKNVVLSGGSTMFRDFGRRLQRDLKRVVDARLKLSQELSGGRIKPKPVEV
QVVTHHMQRYAVWFGGSMLASTPEFFQVCHTKKDYEEYGPSICRHNPVFGVMS*
>ENSMUSP00000085578_c.240:G>T_p.G81C ENSMUST00000088244.6|Actr3b-201|ENSMUSG00000056367|+|chr5:g.26016930:G>T
MAGSLPPCVVDCGTGYTKLGYAGNTEPQFIIPSCIAIRESAKVVDQAQRRVLRGVDDLDFFIGDEAIDKPTYA
TKWPIRHCIVEDWDLMERFMEQVVFKYLRAEPEDHYFLMTEPPLNTPENREYLAEIMFESFNVPGLYIAVQAV
LALAASWTSRQVGERTLTGIVIDSGDGVTHVIPVAEGYVIGSCIKHIPIAGRDITYFIQQLLREREVGIPPEQ
SLETAKAIKEKYCYICPDIVREFAKYDVDPRKWIKQYTGINAINQKKFIIDVGYERFLGPEIFFHPEFANPDF
MESISDVVDEVIQSCPIDVRRPLYKNVVLSGGSTMFRDFGRRLQRDLKRVVDARLKLSQELSGGRIKPKPVEV
QVVTHHMQRYAVWFGGSMLASTPEFFQVCHTKKDYEEYGPSICRHNPVFGVMS*
>ENSMUSP00000121629_c.119:A>T_p.E40D ENSMUST00000128727.8|Actr3b-202|ENSMUSG00000056367|+|chr5:g.26027450:A>T
MERFMEQVVFKYLRAEPEDHYFLMTEPPLNTPENREYLADIMFESFNVPGLYIAVQAVLALAASWTSRQVGER
TLTGIVIDSGDGVTHVIPVAEGYVIGSCIKHIPIAGRDITYFIQQLLREREVGIPPEQSLETAKAIKEKYCYI
CPDIVREFAKYDVDPRKWIKQYTGINAINQKKFIIDVGYERFLGPEIFFHPEFANPDFMESISDVVDEVIQSC

PIDVRRPLYKNVVLSGGSTMFRDFGRRLQRDLKRVVDARLKLSQELSGGRIKPKPVEVQVVTHHMQRYAVWFG
GSMLASTPEFFQVCHTKKDYEEYGPSICRHNPVFGVMS*
>ENSMUSP00000085578_c.383:A>T_p.E128D ENSMUST00000088244.6|Actr3b-201|ENSMUSG00000056367|+|chr5:g.26027450:A>T
MAGSLPPCVVDCGTGYTKLGYAGNTEPQFIIPSCIAIRESAKVVDQAQRRVLRGVDDLDFFIGDEAIDKPTYA
TKWPIRHGIVEDWDLMERFMEQVVFKYLRAEPEDHYFLMTEPPLNTPENREYLADIMFESFNVPGLYIAVQAV
LALAASWTSRQVGERTLTGIVIDSGDGVTHVIPVAEGYVIGSCIKHIPIAGRDITYFIQQLLREREVGIPPEQ
SLETAKAIKEKYCYICPDIVREFAKYDVDPRKWIKQYTGINAINQKKFIIDVGYERFLGPEIFFHPEFANPDF
MESISDVVDEVIQSCPIDVRRPLYKNVVLSGGSTMFRDFGRRLQRDLKRVVDARLKLSQELSGGRIKPKPVEV
QVVTHHMQRYAVWFGGSMLASTPEFFQVCHTKKDYEEYGPSICRHNPVFGVMS*
>rs50914511_ENSMUSP00000121629_c.456:G>T_p.E153*
ENSMUST00000128727.8|Actr3b-202|ENSMUSG00000056367|+|chr5:g.26036645:G>T
MERFMEQVVFKYLRAEPEDHYFLMTEPPLNTPENREYLAEIMFESFNVPGLYIAVQAVLALAASWTSRQVGER
TLTGIVIDSGDGVTHVIPVAEGYVIGSCIKHIPIAGRDITYFIQQLLREREVGIPPEQSLETAKAIKEKYCYI
CPDIVR*FAKYDVDPRKWIKQYTGINAINQKKFIIDVGYERFLGPEIFFHPEFANPDFMESISDVVDEVIQSC
PIDVRRPLYKNVVLSGGSTMFRDFGRRLQRDLKRVVDARLKLSQELSGGRIKPKPVEVQVVTHHMQRYAVWFG
GSMLASTPEFFQVCHTKKDYEEYGPSICRHNPVFGVMS*
>rs50914511_ENSMUSP00000085578_c.720:G>T_p.E241*
ENSMUST00000088244.6|Actr3b-201|ENSMUSG00000056367|+|chr5:g.26036645:G>T
MAGSLPPCVVDCGTGYTKLGYAGNTEPQFIIPSCIAIRESAKVVDQAQRRVLRGVDDLDFFIGDEAIDKPTYA
TKWPIRHGIVEDWDLMERFMEQVVFKYLRAEPEDHYFLMTEPPLNTPENREYLAEIMFESFNVPGLYIAVQAV
LALAASWTSRQVGERTLTGIVIDSGDGVTHVIPVAEGYVIGSCIKHIPIAGRDITYFIQQLLREREVGIPPEQ
SLETAKAIKEKYCYICPDIVR*FAKYDVDPRKWIKQYTGINAINQKKFIIDVGYERFLGPEIFFHPEFANPDF
MESISDVVDEVIQSCPIDVRRPLYKNVVLSGGSTMFRDFGRRLQRDLKRVVDARLKLSQELSGGRIKPKPVEV
QVVTHHMQRYAVWFGGSMLASTPEFFQVCHTKKDYEEYGPSICRHNPVFGVMS*
>ENSMUSP00000121629_c.723:G>C_p.D242H ENSMUST00000128727.8|Actr3b-202|ENSMUSG00000056367|+|chr5:g.26053396:G>C
MERFMEQVVFKYLRAEPEDHYFLMTEPPLNTPENREYLAEIMFESFNVPGLYIAVQAVLALAASWTSRQVGER
TLTGIVIDSGDGVTHVIPVAEGYVIGSCIKHIPIAGRDITYFIQQLLREREVGIPPEQSLETAKAIKEKYCYI
CPDIVREFAKYDVDPRKWIKQYTGINAINQKKFIIDVGYERFLGPEIFFHPEFANPDFMESISDVVDEVIQSC
PIDVRRPLYKNVVLSGGSTMFRHFGRRLQRDLKRVVDARLKLSQELSGGRIKPKPVEVQVVTHHMQRYAVWFG
GSMLASTPEFFQVCHTKKDYEEYGPSICRHNPVFGVMS*
>ENSMUSP00000085578_c.987:G>C_p.D330H ENSMUST00000088244.6|Actr3b-201|ENSMUSG00000056367|+|chr5:g.26053396:G>C
MAGSLPPCVVDCGTGYTKLGYAGNTEPQFIIPSCIAIRESAKVVDQAQRRVLRGVDDLDFFIGDEAIDKPTYA
TKWPIRHGIVEDWDLMERFMEQVVFKYLRAEPEDHYFLMTEPPLNTPENREYLAEIMFESFNVPGLYIAVQAV
LALAASWTSRQVGERTLTGIVIDSGDGVTHVIPVAEGYVIGSCIKHIPIAGRDITYFIQQLLREREVGIPPEQ
SLETAKAIKEKYCYICPDIVREFAKYDVDPRKWIKQYTGINAINQKKFIIDVGYERFLGPEIFFHPEFANPDF
MESISDVVDEVIQSCPIDVRRPLYKNVVLSGGSTMFRHFGRRLQRDLKRVVDARLKLSQELSGGRIKPKPVEV
QVVTHHMQRYAVWFGGSMLASTPEFFQVCHTKKDYEEYGPSICRHNPVFGVMS*
>ENSMUSP00000129503_c.952:G>A_p.G318E ENSMUST00000165512.4|Mnx1-202|ENSMUSG00000001566|-|chr5:g.29679129:C>T
MEKSKNFRIDALLAVDPPRAASTQSAPLALVTSLATTVSGPGRGGSGGGTSSGASRSCSPASSEATAAPGDR
LRAESPSPPRLLAAHCALLPKPGFLGAGGGGGAAGGPGTPHHHAHPGAAAAAAAAAAAAAAGGLALGLHPGGA
QGGAGLPAQAALYGHPVYSYSAAAAAAALAGQHPALSYSYPQVQGAHPAHPADPIKLGASTFQLDQWLRASTA
GMILPKMPDFSSQAQSNLLGKCRRPRTAFTSQQLLELEHQFKLNKYLSRPKRFEVATSLMLTETQVKIWFQNR
RMKWKRSKKAKEQAAQEAEKQKGGGEGTGKGGSEEKTEEELMGPPVSGDKASGRRLRDLRDSDPDEDEDDEEE
DNFPYSNGAGAHAASSDCSSEDDSPPPRLGGPGHQPLPQ*
>ENSMUSP00000001608_c.952:G>A_p.G318E ENSMUST00000001608.8|Mnx1-201|ENSMUSG00000001566|-|chr5:g.29679129:C>T
MEKSKNFRIDALLAVDPPRAASTQSAPLALVTSLATTVSGPGRGGSGGGTSSGASRSCSPASSEATAAPGDR
LRAESPSPPRLLAAHCALLPKPGFLGAGGGGGAAGGPGTPHHHAHPGAAAAAAAAAAAAAAGGLALGLHPGGA
QGGAGLPAQAALYGHPVYSYSAAAAAAALAGQHPALSYSYPQVQGAHPAHPADPIKLGASTFQLDQWLRASTA
GMILPKMPDFSCEAQSNLLGKCRRPRTAFTSQQLLELEHQFKLNKYLSRPKRFEVATSLMLTETQVKIWFQNR
RMKWKRSKKAKEQAAQEAEKQKGGGEGTGKGGSEEKTEEELMGPPVSGDKASGRRLRDLRDSDPDEDEDDEEE
DNFPYSNGAGAHAASSDCSSEDDSPPPRLGGPGHQPLPQ*

>rs218645019_ENSMUSP00000144615_c.613:TG>T_p.L205del-fs ENSMUST00000201203.4|Tmem214-202|ENSMUSG00000038828|+|chr5:g.31029176:TG>T
MAARSAGSGGWEVVKRGRRPGASSGGRGGGGGSDRRALGEANGVLKYDLSSPIQTTSTLYERGFEKIMKRQNK
EQVPPPAAESKKPINKKQPKKVTAVPSQNQKQGPFRRLEDALKALDVAALQKELDKSQSVFTGNPSVWLKDLA
SYLNYKLQTPRMEPTLSQYPHDYPYSLVSRELRGIIRGLLTKAAGSVELFFDHCLFTMLKSWIKHQGSRYMGT
ASVFKPFCKTSLRLSPQTWTSS
>ENSMUSP00000143938_c.204:C>T_p.P69S ENSMUST00000202520.4|Slc5a6-205|ENSMUSG00000006641|-|chr5:g.31200479:G>A
MSVASTAAPFHTTSGSSGAISTFSVVDYVVFGLLLVLSLVIGLYHACRGWGHHTVGELLMADRKMGCLSVALS
LLATFQSAVAILGAPAEIFRFGTQYWFLGCSYFLGLLIPAHIFIPVFYRLHLTSAYEYLELRFNKAVRICGTV
TFIFQMVIYMGVALYAPSLALNAVTGFDLWLSVLALGIVCNIYTALGGLKAVIWTDVFQTLVMFLGQLVVIIV
GAARVGGLGHVWNVTSQHGLISGINLDPDPFVRHTFWTLAFGGVFMMLSLYGVNQAQVQRYLSSHSERAAVLS
CYAVFPCQQVALCMSCLIGLVMFAYYNMYSMSPELKQAAPDQLVLYFVMDLLKDMPGLPGLFVACLFSGSLST
ISSAFNSLATVTMEDLIQPWFPQLTETRAIMLSRGLAFAYGLVCLGMAYISSHLGSVLQAALSIFGMVGGPLL
GLFCLGLFFPCANPLGAIVGLLTGLTMAFWIGIGSIVSRMSSAVAPPPLNGSSSFLPANVTVAAVTTVMPSTL
SKPTGLQHFYSLSYLWYSAHNSTTVIVVGLIVSLLTGGMRGRSLNPGTIYPVLPKLLALLPLSCQKRLCWRSH
SQDIPVIPNLFPEKMRNGVLQDSTDKERMAEDGLVHQPCSPTYVVQETSL*
>ENSMUSP00000110316_c.204:C>T_p.P69S ENSMUST00000114668.7|Slc5a6-202|ENSMUSG00000006641|-|chr5:g.31200479:G>A
MSVASTAAPFHTTSGSSGAISTFSVVDYVVFGLLLVLSLVIGLYHACRGWGHHTVGELLMADRKMGCLSVALS
LLATFQSAVAILGAPAEIFRFGTQYWFLGCSYFLGLLIPAHIFIPVFYRLHLTSAYEYLELRFNKAVRICGTV
TFIFQMVIYMGVALYAPSLALNAVTGFDLWLSVLALGIVCNIYTALGGLKAVIWTDVFQTLVMFLGQLVVIIV
GAARVGGLGHVWNVTSQHGLISGINLDPDPFVRHTFWTLAFGGVFMMLSLYGVNQAQVQRYLSSHSERAAVLS
CYAVFPCQQVALCMSCLIGLVMFAYYNMYSMSPELKQAAPDQLVLYFVMDLLKDMPGLPGLFVACLFSGSLST
ISSAFNSLATVTMEDLIQPWFPQLTETRAIMLSRGLAFAYGLVCLGMAYISSHLGSVLQAALSIFGMVGGPLL
GLFCLGLFFPCANPLGAIVGLLTGLTMAFWIGIGSIVSRMSSAVAPPPLNGSSSFLPANVTVAAVTTVMPSTL
SKPTGLQHFYSLSYLWYSAHNSTTVIVVGLIVSLLTGGMRGRSLNPGTIYPVLPKLLALLPLSCQKRLCWRSH
SQDIPVIPNLFPEKMRNGVLQDSTDKERMAEDGLVHQPCSPTYVVQETSL*
>ENSMUSP00000079291_c.204:C>T_p.P69S ENSMUST00000080431.8|Slc5a6-201|ENSMUSG00000006641|-|chr5:g.31200479:G>A
MSVASTAAPFHTTSGSSGAISTFSVVDYVVFGLLLVLSLVIGLYHACRGWGHHTVGELLMADRKMGCLSVALS
LLATFQSAVAILGAPAEIFRFGTQYWFLGCSYFLGLLIPAHIFIPVFYRLHLTSAYEYLELRFNKAVRICGTV
TFIFQMVIYMGVALYAPSLALNAVTGFDLWLSVLALGIVCNIYTALGGLKAVIWTDVFQTLVMFLGQLVVIIV
GAARVGGLGHVWNVTSQHGLISGINLDPDPFVRHTFWTLAFGGVFMMLSLYGVNQAQVQRYLSSHSERAAVLS
CYAVFPCQQVALCMSCLIGLVMFAYYNMYSMSPELKQAAPDQLVLYFVMDLLKDMPGLPGLFVACLFSGSLST
ISSAFNSLATVTMEDLIQPWFPQLTETRAIMLSRGLAFAYGLVCLGMAYISSHLGSVLQAALSIFGMVGGPLL
GLFCLGLFFPCANPLGAIVGLLTGLTMAFWIGIGSIVSRMSSAVAPPPLNGSSSFLPANVTVAAVTTVMPSTL
SKPTGLQHFYSLSYLWYSAHNSTTVIVVGLIVSLLTGGMRGRSLNPGTIYPVLPKLLALLPLSCQKRLCWRSH
SQDIPVIPNLFPEKMRNGVLQDSTDKERMAEDGLVHQPCSPTYVVQETSL*
>ENSMUSP00000143993_c.204:C>T_p.P69S ENSMUST00000202556.4|Slc5a6-206|ENSMUSG00000006641|-|chr5:g.31200479:G>A
MSVASTAAPFHTTSGSSGAISTFSVVDYVVFGLLLVLSLVIGLYHACRGWGHHTVGELLMADRKMGCLSVALS
LLATFQSAVAILGAPAEIFRFGTQYWFLGCSYFLGLLIPAHIFIPVFYRLHLTSAYEYLELRFNKAVRICGTV
TFIFQMVIYMGVALYAPSLALNAVTGFDLWLSVLALGIVCNIYTALGGLKAVIWTDVFQTLVMFLGQLVVIIV
GAARVGGLGHVWNVTSQHGLISGINLDPDPFVRHTFWTLAFGGVFMMLSLYGVNQAQVQRYLSSHSERAAVLS
CYAVFPCQQVALCMSCLIGLVMFAYYNMYSMSPELKQAAPDQLVLYFVMDLLKDMPGLPGLFVACLFSGSLST
ISSAFNSLATVTMEDLIQPWFPQLTETRAIMLSRGLAFAYGLVCLGMAYISSHLGSVLQAALSIFGMVGGPLL
GLFCLGLFFPCANPLGAIVGLLTGLTMAFWIGIGSIVSRMSSAVAPPPLNGSSSFLPANVTVAAVTTVMPSTL
SKPTGLQHFYSLSYLWYSAHNSTTVIVVGLIVSLLTGGMRGRSLNPGTIYPVLPKLLALLPLSCQKRLCWRSH
SQDIPVIPNLFPEKMRNGVLQDSTDKERMAEDGLVHQPCSPTYVVQETSL*
>ENSMUSP00000144665_c.204:C>T_p.P69S ENSMUST00000200816.2|Slc5a6-203|ENSMUSG00000006641|-|chr5:g.31200479:G>A
MSVASTAAPFHTTSGSSGAISTFSVVDYVVFGLLLVLSLVIGLYHACRGWGHHTVGELLMADRKMGCLSVALS
LLATFQSA
>ENSMUSP00000142442_c.238:T>G_p.I80S ENSMUST00000199534.5|Ldb2-207|ENSMUSG00000039706|-|chr5:g.44699198:A>C

MSSTPHDPFYSSPFGPFYRRHTPYMVQPEYRIYEMNKRLQSRTEDSDNLWWDAFATEFFEDDATLTLSFCLED
GPKRYTSGRTLIPRYFSTVFEGGVTDLYYILKHSKESYHNSSITVDCDQCAMVTQHGKPMFTKVCTEGRLILE
FTFDDLMRIKTWHFTIRQYRELVPRSILAMHAQDPQVLDQLSKNITRMGLTNFTLNYLRLCVILEPMQELMSR
HKTYNLSPRDCLKTCLFQKWQRMVAPPAEPTRQPTTKRRKRKNSTSSTSNSSAGNTTNSAGSKKKTPAASLSL
ATQVPGLGAIPNCSLNPGRDGDLCHSTAVTPSGQFKEKH*
>ENSMUSP00000067737_c.238:T>G_p.I80S ENSMUST00000070748.10|Ldb2-201|ENSMUSG00000039706|-|chr5:g.44699198:A>C
MSSTPHDPFYSSPFGPFYRRHTPYMVQPEYRIYEMNKRLQSRTEDSDNLWWDAFATEFFEDDATLTLSFCLED
GPKRYTSGRTLIPRYFSTVFEGGVTDLYYILKHSKESYHNSSITVDCDQCAMVTQHGKPMFTKVCTEGRLILE
FTFDDLMRIKTWHFTIRQYRELVPRSILAMHAQDPQVLDQLSKNITRMGLTNFTLNYLRLCVILEPMQELMSR
HKTYNLSPRDCLKTCLFQKWQRMVAPPAEPTRQPTTKRRKRKNSTSSTSNSSAGNTTNSAGSKKKTPAASLSL
ATQVPDVMVVGEPTLMGGEFGDEDERLITRLENTQYDAANGMDDEEDFNNSPALGNNSPWNSKPPATQETKSE
NAPPQASQ*
>ENSMUSP00000143775_c.238:T>G_p.I80S ENSMUST00000199256.5|Ldb2-204|ENSMUSG00000039706|-|chr5:g.44699198:A>C
MSSTPHDPFYSSPFGPFYRRHTPYMVQPEYRIYEMNKRLQSRTEDSDNLWWDAFATEFFEDDATLTLSFCLED
GPKRYTSGRTLIPRYFSTVFEGGVTDLYYILKHSKESYHNSSITVDCDQCAMVTQHGKPMFTKVCTEGRLILE
FTFDDLMRIKTWHFTIRQYRELVPRSILAMHAQDPQVLDQLSKNITRMGLTNFTLNYLRLCVILEPMQELMSR
HKTYNLSPRDCLKTCLFQKWQRMVAPPAEPTRQPTTKRRKRKNSTSSTSNSSAGNTTNSAGSKKKTPAASLSL
ATQGLGAIPNCSLNPGRDGDLCHSTAVTPSGQFKEKH*
>ENSMUSP00000143289_c.238:T>G_p.I80S ENSMUST00000199261.2|Ldb2-205|ENSMUSG00000039706|-|chr5:g.44699198:A>C
MSSTPHDPFYSSPFGPFYRRHTPYMVQPEYRIYEMNKRLQSRTEDSDNLWWDAFATEFFEDDATLTLSFCLED
GPKRYTSGRTLIPRYFSTVFEGGVTDLYYILKHSKESYHNSSITVDCDQCAMVTQHGKPMFTKVCTEGRLILE
FTFDDLMRIKTWHFTIRQYRELVPRSILAMHAQDPQVLDQLSKNITRMGLTNFTLNYLRLCVILEPMQELMSR
HKTYNLSPRDCLKTCLFQKWQRMVAPPAEPTRQPTTKRRKRKNSTSSTSNSSAGNTTNSAGSKKKTPAASLSL
ATQDVMVVGEPTLMGGEFGDEDERLITRLENTQYDAANGMDDEEDFNNSPALGNNSPWNSKPPATQETKSENA
PPQASQ*
>rs230178563_ENSMUSP00000133840_c.1389:G>GA_p.A464ins-fs
ENSMUST00000173107.8|Slit2-208|ENSMUSG00000031558|+|chr5:g.48377374:G>GA
MSGIGWQTLSLSLGLVLSILNKVAPQACPAQCSCSGSTVDCHGLALRSVPRNIPRNTERLDLNGNNITRITKI
DFAGLRHLRVLQLMENRISTIERGAFQDLKELERLRLNRNNLQLFPELLFLGTAKLYRLDLSENQIQAIPRKA
FRGAVDIKNLQLDYNQISCIEDGAFRALRDLEVLTLNNNNITRLSVASFNHMPKLRTFRLHSNNLYCDCHLAW
LSDWLRQRPRVGLYTQCMGPSHLRGHNVAEVQKREFVCSGHQSFMAPSCSVLHCPAACTCSNNIVDCRGKGLT
EIPTNLPETITEIRLEQNSIRVIPPGAFSPYKKLRRLDLSNNQISELAPDAFQGLRSLNSLVLYGNKITELPK
SLFEGLFSLQLLLLNANKINCLRVDAFQDLHNLNLLSLYDNKLQTVAKGTFSALRAIQTMHLAQNPFICDCHL
KWLADYLHTNPIETSGARCTSPRRLDKQKNWTDQKQEIPLFRYRRLSIKIKWRLLCRLGLS
>rs230178563_ENSMUSP00000033967_c.1389:G>GA_p.A464ins-fs
ENSMUST00000033967.15|Slit2-201|ENSMUSG00000031558|+|chr5:g.48377374:G>GA
MSGIGWQTLSLSLGLVLSILNKVAPQACPAQCSCSGSTVDCHGLALRSVPRNIPRNTERLDLNGNNITRITKI
DFAGLRHLRVLQLMENRISTIERGAFQDLKELERLRLNRNNLQLFPELLFLGTAKLYRLDLSENQIQAIPRKA
FRGAVDIKNLQLDYNQISCIEDGAFRALRDLEVLTLNNNNITRLSVASFNHMPKLRTFRLHSNNLYCDCHLAW
LSDWLRQRPRVGLYTQCMGPSHLRGHNVAEVQKREFVCSGHQSFMAPSCSVLHCPAACTCSNNIVDCRGKGLT
EIPTNLPETITEIRLEQNSIRVIPPGAFSPYKKLRRLDLSNNQISELAPDAFQGLRSLNSLVLYGNKITELPK
SLFEGLFSLQLLLLNANKINCLRVDAFQDLHNLNLLSLYDNKLQTVAKGTFSALRAIQTMHLAQNPFICDCHL
KWLADYLHTNPIETSGARCTSPRRLDKQKNWTDQKQEIPLFRYRRLSIKIKWRLLCRLGLS
>rs230178563_ENSMUSP00000133912_c.1401:G>GA_p.A468ins-fs
ENSMUST00000174313.8|Slit2-216|ENSMUSG00000031558|+|chr5:g.48377374:G>GA
MSGIGWQTLSLSLGLVLSILNKVAPQACPAQCSCSGSTVDCHGLALRSVPRNIPRNTERLDLNGNNITRITKI
DFAGLRHLRVLQLMENRISTIERGAFQDLKELERLRLNRNNLQLFPELLFLGTAKLYRLDLSENQIQAIPRKA
FRGAVDIKNLQLDYNQISCIEDGAFRALRDLEVLTLNNNNITRLSVASFNHMPKLRTFRLHSNNLYCDCHLAW
LSDWLRQRPRVGLYTQCMGPSHLRGHNVAEVQKREFVCSDEEEGHQSFMAPSCSVLHCPAACTCSNNIVDCRG
KGLTEIPTNLPETITEIRLEQNSIRVIPPGAFSPYKKLRRLDLSNNQISELAPDAFQGLRSLNSLVLYGNKIT
ELPKSLFEGLFSLQLLLLNANKINCLRVDAFQDLHNLNLLSLYDNKLQTVAKGTFSALRAIQTMHLAQNPFIC
DCHLKWLADYLHTNPIETSGARCTSPRRLDKQKNWTDQKQEIPLFRYRRLSIKIKWRLLCRLGLS
>rs230178563_ENSMUSP00000134263_c.1401:G>GA_p.A468ins-fs
ENSMUST00000174421.8|Slit2-217|ENSMUSG00000031558|+|chr5:g.48377374:G>GA

MSGIGWQTLSLSLGLVLSILNKVAPQACPAQCSCSGSTVDCHGLALRSVPRNIPRNTERLDLNGNNITRITKI
DFAGLRHLRVLQLMENRISTIERGAFQDLKELERLRLNRNNLQLFPELLFLGTAKLYRLDLSENQIQAIPRKA
FRGAVDIKNLQLDYNQISCIEDGAFRALRDLEVLTLNNNNITRLSVASFNHMPKLRTFRLHSNNLYCDCHLAW
LSDWLRQRPRVGLYTQCMGPSHLRGHNVAEVQKREFVCSDEEEGHQSFMAPSCSVLHCPAACTCSNNIVDCRG
KGLTEIPTNLPETITEIRLEQNSIRVIPPGAFSPYKKLRRLDLSNNQISELAPDAFQGLRSLNSLVLYGNKIT
ELPKSLFEGLFSLQLLLLNANKINCLRVDAFQDLHNLNLLSLYDNKLQTVAKGTFSALRAIQTMHLAQNPFIC
DCHLKWLADYLHTNPIETSGARCTSPRRLDKQKNWTDQKQEIPLFS
>rs230178563_ENSMUSP00000127615_c.1401:G>GA_p.A468ins-fs
ENSMUST00000170109.9|Slit2-202|ENSMUSG00000031558|+|chr5:g.48377374:G>GA
MSGIGWQTLSLSLGLVLSILNKVAPQACPAQCSCSGSTVDCHGLALRSVPRNIPRNTERLDLNGNNITRITKI
DFAGLRHLRVLQLMENRISTIERGAFQDLKELERLRLNRNNLQLFPELLFLGTAKLYRLDLSENQIQAIPRKA
FRGAVDIKNLQLDYNQISCIEDGAFRALRDLEVLTLNNNNITRLSVASFNHMPKLRTFRLHSNNLYCDCHLAW
LSDWLRQRPRVGLYTQCMGPSHLRGHNVAEVQKREFVCSDEEEGHQSFMAPSCSVLHCPAACTCSNNIVDCRG
KGLTEIPTNLPETITEIRLEQNSIRVIPPGAFSPYKKLRRLDLSNNQISELAPDAFQGLRSLNSLVLYGNKIT
ELPKSLFEGLFSLQLLLLNANKINCLRVDAFQDLHNLNLLSLYDNKLQTVAKGTFSALRAIQTMHLAQNPFIC
DCHLKWLADYLHTNPIETSGARCTSPRRLDKQKNWTDQKQEIPLFS
>ENSMUSP00000031121_c.1506:C>A_p.P503T ENSMUST00000031121.10|Gabra4-
201|ENSMUSG00000029211|-|chr5:g.71729194:G>T
MVSVQKVPAIALCSGVSLALLHFLCLAACLNESPGQNSKDEKLCPENFTRILDSLLDGYDNRLRPGFGGPVTE
VKTDIYVTSFGPVSDVEMEYTMDVFFRQTWIDKRLKYDGPIEILRLNNMMVTKVWTPDTFFRNGKKSVSHNMT
APNKLFRIMRNGTILYTMRLTISAECPMRLVDFPMDGHACPLKFGSYAYPKSEMIYTWTKGPEKSVEVPKESS
SLVQYDLIGQTVSSETIKSITGEYIVMTVYFHLRRKMGYFMIQTYIPCIMTVILSQVSFWINKESVPARTVFG
ITTVLTMTTLSISARHSLPKVSYATAMDWFIAVCFAFVFSALIEFAAVNYFTNIQMQKAKKKISKPPPEVPAA
PVLKEKHTETSLQNTHANLNMRKRTNALVHSESDVKSRTEVGNHSSKTSAVQESSEATPKAHLASSPNPFSRA
NAAETMSAAARGLSSAASPSPHGTLRPASLGSASTRPAFGSRLGRIKTTVNTTGAAGNVSATPPTPAPPPSGS
GTSKIDKYARILFPVTFGAFNMVYWVVYLSKDTMEKSESLM*
>ENSMUSP00000143675_c.1584:C>A_p.P529T ENSMUST00000199357.2|Gabra4-
205|ENSMUSG00000029211|-|chr5:g.71729194:G>T
MVSVQKVPAIALCSGVSLALLHFLCLAACLNESPGQNSKDEKLCPENFTRILDSLLDGYDNRLRPGFGGTDCF
KTCKCALSVRLWSACLFVEILGPVTEVKTDIYVTSFGPVSDVEMEYTMDVFFRQTWIDKRLKYDGPIEILRLN
NMMVTKVWTPDTFFRNGKKSVSHNMTAPNKLFRIMRNGTILYTMRLTISAECPMRLVDFPMDGHACPLKFGSY
AYPKSEMIYTWTKGPEKSVEVPKESSSLVQYDLIGQTVSSETIKSITGEYIVMTVYFHLRRKMGYFMIQTYIP
CIMTVILSQVSFWINKESVPARTVFGITTVLTMTTLSISARHSLPKVSYATAMDWFIAVCFAFVFSALIEFAA
VNYFTNIQMQKAKKKISKPPPEVPAAPVLKEKHTETSLQNTHANLNMRKRTNALVHSESDVKSRTEVGNHSSK
TSAVQESSEATPKAHLASSPNPFSRANAAETMSAAARGLSSAASPSPHGTLRPASLGSASTRPAFGSRLGRIK
TTVNTTGAAGNVSATPPTPAPPPSGSGTSKIDKYARILFPVTFGAFNMVYWVVYLSKDTMEKSESLM*
>ENSMUSP00000079231_c.804:A>G_p.K269E ENSMUST00000080359.12|Rest-
201|ENSMUSG00000029249|+|chr5:g.77416592:A>G
MATQVMGQSSGGGSLFNNSANMGMALTNDMYDLHELSKAELAAPQLIMLANVALTGEASGSCCDYLVGEERQM
AELMPVGDNHFSESEGEGLEESADLKGLENMELGSLELSAVEPQPVFEASAAPEIYSANKDPAPETPVAEDKC
RSSKAKPFRCKPCQYEAESEEQFVHHIRIHSAKKFFVEESAEKQAKAWESGSSPAEEGEFSKGPIRCDRCGYN
TNRYDHYMAHLKHHLRAGENERIYKCIICTYTTVSEYHWRKHLRNHFPREVYTCSKCNYFSDRKNNYVQHVRT
HTGERPYKCELCPYSSSQKTHLTRHMRTHSGEKPFKCDQCNYVASNQHEVTRHARQVHNGPKPLNCPHCDYKT
ADRSNFKKHVELHVNPRQFNCPVCDYAASKKCNLQYHFKSKHPTCPSKTMDVSKVKLKKTKKREADLLNNAVS
NEKMENEQTKTKGDVSGKKNEKPVKAVGKDASKEKKPGSSVSVVQVTTRTRKSAVAAETKAAEVKHTDGQTGN
NPEKPCKAKKNKRKKDAEAHPSEEPVNEGPVTKKKKKSECKSKIGTNVPKGGGRAEERPGVKKQSASLKKGTK
KTPPKTKTSKKGGKLAPKGMGQTEPSSGALAQVGVSPDPALIQAEVTGSGSSQTELPSPMDIAKSEPAQMEVS
LTGPPPVEPAQMEPSPAKPPQVEAPTYPQPPQRGPAPPTGPAPPTGPAPPTEPAPPTGLAEMEPSPTEPSQKE
PPPSMEPPCPEELPQAEPPPMEDCQKELPSPVEPAQIEVAQTAPTQVQEEPPPVSEPPRVKPTKRSSLRKDRA
EKELSLLSEMARQEQVLMGVGLVPVRDSKLLKGNKSAQDPPAPPSPSPKGNSREETPKDQEMVSDGEGTIVFP
LKKGGPEEAGESPAELAALKESARVSSSEQNSAMPEGGASHSKCQTGSSGLCDVDTEQKTDTVPMKDSAAEPV
SPPTPTVDRDAGSPAVVASPPITLAENESQEIDEDEGIHSHDGSDLSDNMSEGSDDSGLHGARPTPPEATSKN
GKAGLAGKVTEGEFVCIFCDRSFRKEKDYSKHLNRHLVNVYFLEEAAEEQEEQEEREEQE*
>ENSMUSP00000109076_c.804:A>G_p.K269E ENSMUST00000113449.2|Rest-
202|ENSMUSG00000029249|+|chr5:g.77416592:A>G
MATQVMGQSSGGGSLFNNSANMGMALTNDMYDLHELSKAELAAPQLIMLANVALTGEASGSCCDYLVGEERQM
AELMPVGDNHFSESEGEGLEESADLKGLENMELGSLELSAVEPQPVFEASAAPEIYSANKDPAPETPVAEDKC
RSSKAKPFRCKPCQYEAESEEQFVHHIRIHSAKKFFVEESAEKQAKAWESGSSPAEEGEFSKGPIRCDRCGYN

TNRYDHYMAHLKHHLRAGENERIYKCIICTYTTVSEYHWRKHLRNHFPREVYTCSKCNYFSDRKNNYVQHVRT
HTGERPYKCELCPYSSSQKTHLTRHMRTHSGEKPFKCDQCNYVASNQHEVTRHARQVHNGPKPLNCPHCDYKT
ADRSNFKKHVELHVNPRQFNCPVCDYAASKKCNLQYHFKSKHPTCPSKTMDVSKVKLKKTKKREADLLNNAVS
NEKMENEQTKTKGDVSGKKNEKPVKAVGKDASKEKKPGSSVSVVQVTTRTRKSAVAAETKAAEVKHTDGQTGN
NPEKPCKAKKNKRKKDAEAHPSEEPVNEGPVTKKKKKSECKSKIGTNVPKGGGRAEERPGVKKQSASLKKGTK
KTPPKTKTSKKGGKLAPKGMGQTEPSSGALAQVGVSPDPALIQAEVTGSGSSQTELPSPMDIAKSEPAQMEVS
LTGPPPVEPAQMEPSPAKPPQVEAPTYPQPPQRGPAPPTGPAPPTGPAPPTEPAPPTGLAEMEPSPTEPSQKE
PPPSMEPPCPEELPQAEPPPMEDCQKELPSPVEPAQIEVAQTAPTQVQEEPPPVSEPPRVKPTKRSSLRKDRA
EKELSLLSEMARQEQVLMGVGLVPVRDSKLLKGNKSAQDPPAPPSPSPKGNSREETPKDQEMVSDGEGTIVFP
LKKGGPEEAGESPAELAALKESARVSSSEQNSAMPEGGASHSKCQTGSSGLCDVDTEQKTDTVPMKDSAAEPV
SPPTPTVDRDAGSPAVVASPPITLAENESQEIDEDEGIHSHDGSDLSDNMSEGSDDSGLHGARPTPPEATSKN
GKAGLAGKVTEGEFVCIFCDRSFRKEKDYSKHLNRHLVNVYFLEEAAEEQEEQEEREEQE*
>ENSMUSP00000079231_c.834:T>A_p.F279I ENSMUST00000080359.12|Rest-
201|ENSMUSG00000029249|+|chr5:g.77416622:T>A
MATQVMGQSSGGGSLFNNSANMGMALTNDMYDLHELSKAELAAPQLIMLANVALTGEASGSCCDYLVGEERQM
AELMPVGDNHFSESEGEGLEESADLKGLENMELGSLELSAVEPQPVFEASAAPEIYSANKDPAPETPVAEDKC
RSSKAKPFRCKPCQYEAESEEQFVHHIRIHSAKKFFVEESAEKQAKAWESGSSPAEEGEFSKGPIRCDRCGYN
TNRYDHYMAHLKHHLRAGENERIYKCIICTYTTVSEYHWRKHLRNHFPRKVYTCSKCNYISDRKNNYVQHVRT
HTGERPYKCELCPYSSSQKTHLTRHMRTHSGEKPFKCDQCNYVASNQHEVTRHARQVHNGPKPLNCPHCDYKT
ADRSNFKKHVELHVNPRQFNCPVCDYAASKKCNLQYHFKSKHPTCPSKTMDVSKVKLKKTKKREADLLNNAVS
NEKMENEQTKTKGDVSGKKNEKPVKAVGKDASKEKKPGSSVSVVQVTTRTRKSAVAAETKAAEVKHTDGQTGN
NPEKPCKAKKNKRKKDAEAHPSEEPVNEGPVTKKKKKSECKSKIGTNVPKGGGRAEERPGVKKQSASLKKGTK
KTPPKTKTSKKGGKLAPKGMGQTEPSSGALAQVGVSPDPALIQAEVTGSGSSQTELPSPMDIAKSEPAQMEVS
LTGPPPVEPAQMEPSPAKPPQVEAPTYPQPPQRGPAPPTGPAPPTGPAPPTEPAPPTGLAEMEPSPTEPSQKE
PPPSMEPPCPEELPQAEPPPMEDCQKELPSPVEPAQIEVAQTAPTQVQEEPPPVSEPPRVKPTKRSSLRKDRA
EKELSLLSEMARQEQVLMGVGLVPVRDSKLLKGNKSAQDPPAPPSPSPKGNSREETPKDQEMVSDGEGTIVFP
LKKGGPEEAGESPAELAALKESARVSSSEQNSAMPEGGASHSKCQTGSSGLCDVDTEQKTDTVPMKDSAAEPV
SPPTPTVDRDAGSPAVVASPPITLAENESQEIDEDEGIHSHDGSDLSDNMSEGSDDSGLHGARPTPPEATSKN
GKAGLAGKVTEGEFVCIFCDRSFRKEKDYSKHLNRHLVNVYFLEEAAEEQEEQEEREEQE*
>ENSMUSP00000109076_c.834:T>A_p.F279I ENSMUST00000113449.2|Rest-
202|ENSMUSG00000029249|+|chr5:g.77416622:T>A
MATQVMGQSSGGGSLFNNSANMGMALTNDMYDLHELSKAELAAPQLIMLANVALTGEASGSCCDYLVGEERQM
AELMPVGDNHFSESEGEGLEESADLKGLENMELGSLELSAVEPQPVFEASAAPEIYSANKDPAPETPVAEDKC
RSSKAKPFRCKPCQYEAESEEQFVHHIRIHSAKKFFVEESAEKQAKAWESGSSPAEEGEFSKGPIRCDRCGYN
TNRYDHYMAHLKHHLRAGENERIYKCIICTYTTVSEYHWRKHLRNHFPRKVYTCSKCNYISDRKNNYVQHVRT
HTGERPYKCELCPYSSSQKTHLTRHMRTHSGEKPFKCDQCNYVASNQHEVTRHARQVHNGPKPLNCPHCDYKT
ADRSNFKKHVELHVNPRQFNCPVCDYAASKKCNLQYHFKSKHPTCPSKTMDVSKVKLKKTKKREADLLNNAVS
NEKMENEQTKTKGDVSGKKNEKPVKAVGKDASKEKKPGSSVSVVQVTTRTRKSAVAAETKAAEVKHTDGQTGN
NPEKPCKAKKNKRKKDAEAHPSEEPVNEGPVTKKKKKSECKSKIGTNVPKGGGRAEERPGVKKQSASLKKGTK
KTPPKTKTSKKGGKLAPKGMGQTEPSSGALAQVGVSPDPALIQAEVTGSGSSQTELPSPMDIAKSEPAQMEVS
LTGPPPVEPAQMEPSPAKPPQVEAPTYPQPPQRGPAPPTGPAPPTGPAPPTEPAPPTGLAEMEPSPTEPSQKE
PPPSMEPPCPEELPQAEPPPMEDCQKELPSPVEPAQIEVAQTAPTQVQEEPPPVSEPPRVKPTKRSSLRKDRA
EKELSLLSEMARQEQVLMGVGLVPVRDSKLLKGNKSAQDPPAPPSPSPKGNSREETPKDQEMVSDGEGTIVFP
LKKGGPEEAGESPAELAALKESARVSSSEQNSAMPEGGASHSKCQTGSSGLCDVDTEQKTDTVPMKDSAAEPV
SPPTPTVDRDAGSPAVVASPPITLAENESQEIDEDEGIHSHDGSDLSDNMSEGSDDSGLHGARPTPPEATSKN
GKAGLAGKVTEGEFVCIFCDRSFRKEKDYSKHLNRHLVNVYFLEEAAEEQEEQEEREEQE*
>ENSMUSP00000079231_c.1713:G>T_p.V572F ENSMUST00000080359.12|Rest-
201|ENSMUSG00000029249|+|chr5:g.77429296:G>T
MATQVMGQSSGGGSLFNNSANMGMALTNDMYDLHELSKAELAAPQLIMLANVALTGEASGSCCDYLVGEERQM
AELMPVGDNHFSESEGEGLEESADLKGLENMELGSLELSAVEPQPVFEASAAPEIYSANKDPAPETPVAEDKC
RSSKAKPFRCKPCQYEAESEEQFVHHIRIHSAKKFFVEESAEKQAKAWESGSSPAEEGEFSKGPIRCDRCGYN
TNRYDHYMAHLKHHLRAGENERIYKCIICTYTTVSEYHWRKHLRNHFPRKVYTCSKCNYFSDRKNNYVQHVRT
HTGERPYKCELCPYSSSQKTHLTRHMRTHSGEKPFKCDQCNYVASNQHEVTRHARQVHNGPKPLNCPHCDYKT
ADRSNFKKHVELHVNPRQFNCPVCDYAASKKCNLQYHFKSKHPTCPSKTMDVSKVKLKKTKKREADLLNNAVS
NEKMENEQTKTKGDVSGKKNEKPVKAVGKDASKEKKPGSSVSVVQVTTRTRKSAVAAETKAAEVKHTDGQTGN
NPEKPCKAKKNKRKKDAEAHPSEEPVNEGPVTKKKKKSECKSKIGTNVPKGGGRAEERPGFKKQSASLKKGTK
KTPPKTKTSKKGGKLAPKGMGQTEPSSGALAQVGVSPDPALIQAEVTGSGSSQTELPSPMDIAKSEPAQMEVS
LTGPPPVEPAQMEPSPAKPPQVEAPTYPQPPQRGPAPPTGPAPPTGPAPPTEPAPPTGLAEMEPSPTEPSQKE

PPPSMEPPCPEELPQAEPPPMEDCQKELPSPVEPAQIEVAQTAPTQVQEEPPPVSEPPRVKPTKRSSLRKDRA
EKELSLLSEMARQEQVLMGVGLVPVRDSKLLKGNKSAQDPPAPPSPSPKGNSREETPKDQEMVSDGEGTIVFP
LKKGGPEEAGESPAELAALKESARVSSSEQNSAMPEGGASHSKCQTGSSGLCDVDTEQKTDTVPMKDSAAEPV
SPPTPTVDRDAGSPAVVASPPITLAENESQEIDEDEGIHSHDGSDLSDNMSEGSDDSGLHGARPTPPEATSKN
GKAGLAGKVTEGEFVCIFCDRSFRKEKDYSKHLNRHLVNVYFLEEAAEEQEEQEEREEQE*
>ENSMUSP00000109076_c.1713:G>T_p.V572F ENSMUST00000113449.2|Rest-202|ENSMUSG00000029249|+|chr5:g.77429296:G>T
MATQVMGQSSGGGSLFNNSANMGMALTNDMYDLHELSKAELAAPQLIMLANVALTGEASGSCCDYLVGEERQM
AELMPVGDNHFSESEGEGLEESADLKGLENMELGSLELSAVEPQPVFEASAAPEIYSANKDPAPETPVAEDKC
RSSKAKPFRCKPCQYEAESEEQFVHHIRIHSAKKFFVEESAEKQAKAWESGSSPAEEGEFSKGPIRCDRCGYN
TNRYDHYMAHLKHHLRAGENERIYKCIICTYTTVSEYHWRKHLRNHFPRKVYTCSKCNYFSDRKNNYVQHVRT
HTGERPYKCELCPYSSSQKTHLTRHMRTHSGEKPFKCDQCNYVASNQHEVTRHARQVHNGPKPLNCPHCDYKT
ADRSNFKKHVELHVNPRQFNCPVCDYAASKKCNLQYHFKSKHPTCPSKTMDVSKVKLKKTKKREADLLNNAVS
NEKMENEQTKTKGDVSGKKNEKPVKAVGKDASKEKKPGSSVSVVQVTTRTRKSAVAAETKAAEVKHTDGQTGN
NPEKPCKAKKNKRKKDAEAHPSEEPVNEGPVTKKKKKSECKSKIGTNVPKGGGRAEERPGFKKQSASLKKGTK
KTPPKTKTSKKGGKLAPKGMGQTEPSSGALAQVGVSPDPALIQAEVTGSGSSQTELPSPMDIAKSEPAQMEVS
LTGPPPVEPAQMEPSPAKPPQVEAPTYPQPPQRGPAPPTGPAPPTGPAPPTEPAPPTGLAEMEPSPTEPSQKE
PPPSMEPPCPEELPQAEPPPMEDCQKELPSPVEPAQIEVAQTAPTQVQEEPPPVSEPPRVKPTKRSSLRKDRA
EKELSLLSEMARQEQVLMGVGLVPVRDSKLLKGNKSAQDPPAPPSPSPKGNSREETPKDQEMVSDGEGTIVFP
LKKGGPEEAGESPAELAALKESARVSSSEQNSAMPEGGASHSKCQTGSSGLCDVDTEQKTDTVPMKDSAAEPV
SPPTPTVDRDAGSPAVVASPPITLAENESQEIDEDEGIHSHDGSDLSDNMSEGSDDSGLHGARPTPPEATSKN
GKAGLAGKVTEGEFVCIFCDRSFRKEKDYSKHLNRHLVNVYFLEEAAEEQEEQEEREEQE*
>ENSMUSP00000031181_c.1518:G>A_p.V507M ENSMUST00000031181.16|Ugt2b34-201|ENSMUSG00000029260|-|chr5:g.87039141:C>T
MPVKMTAALLLLLLQLSGFFGSGTGGKVLVWPMEFSHWLNLKTILDELLKKGHEVMVLRPSASLSYEVDNTSA
IEFETYPTSYSLSELEEIFWESLKKYIYELPKQSFWGYFLMLQEMVWVDSKYFESLCKDVVFNKELMTKLQKS
RFDVILADPFIPCGDLLAEVLKIPLVYSLRFFPGSTYEKYSGGLPLPPSYVPVVMSELSDRMTFMERVRNVIY
MLCFDFWFQTFNEKNWNQLYTEVLGRPTTLSETMAKADIWLIRTYWDLEFPHPVLPNFDFIGGLHCRPAKPLP
KEIEDFVQSSGEHGVVVFSLGSMVGSITEERANVIAAGLAQIPQKVLWRFEGKKPETLGSNTRLYKWIPQNDL
LGHSKTRAFITHGGTNGIYEAIYHGIPVVGIPLFGDQYDNIVHLKAKGAAVRLDFLTMSSTDLHTALKTVTND
PSYKENAMRLSRIHHDQPVKPLDRAVFWIEFVMRHKGAKHLRVAAHDLSWVQYHSLDVLGFLLACVLTMMFIL
KKCCLFCCQKLTKAGRKKKGE*
>ENSMUSP00000046636_c.985:C>A_p.P329Q ENSMUST00000049209.13|Gc-201|ENSMUSG00000035540|-|chr5:g.89586153:G>T
MKRVLVLLLALAFGHALERGRDYEKDKVCNELAMLGKEDFRSLSLILYSRKFSSSTFEQVNQLVKEVVSLTEE
CCAEGADPTCYDTRTSELSVKSCESDAPFPVHPGTPECCTKEGLERKLCMAALSHQPQEFPTYVEPTNDEICE
AFRRDPKGFADQFLYEYSSNYGQAPLPLLVAYTKNYLSMVGSCCTSANPTVCFVKERLQMKHLSLLTTMSNRV
CSQYAAYGKEKSRLSHLIKLAQKVPTANLENVLPLAEDFTEILSRCCESTSEDCMASELPEHTIKICQNLSKK
NSKFEECCQENTPMNIFMCTYFMPAAEPLQLPAIKLQTGKDLCGQSTTQAMDQYTFELSRRTQVPEVFLSKVL
EPTLKTLRECCDTQDSVACFSTQSPLLKRQLTSFIEKGQEMCADYSENTFTEYKKKLAERLRTKTPNTSPAEL
KDMVEKHSDFASKCCSINSPPLYCSSQIDAEMIDTLQS*
>ENSMUSP00000043764_c.1034:T>G_p.N345K ENSMUST00000049146.12|Ephx4-201|ENSMUSG00000033805|+|chr5:g.107577712:T>G
MAPPRPPRLLPALRALLYWSLVYGYCGLCASVHLLKLLWSIGRAPAQTFRRAARANPPACLNDPSLGTHCYVR
IKDSGLRFHYVAAGERGKPLMLLLHGFPEFWYSWRHQLREFKSEYRVVALDLRGYGESDAPAHQESYKLDCLI
ADIKDILDSLGYSKCVLIGHDWGGMIAWLIAVCYPEMIMKLIVINFPHPSVFTEYILRHPAQLFRSSFYYFFQ
IPRFPEFMFSINDFKALKHLFTSQSTGIGRKGRQLTTEDLEAYVYVFSQPGALSGPINHYRNIFSCLPLKHHM
VTTPTLLLWGEEDAFMEVEMAEVTKIYVKNYFRLTILSEGSHWLQQDQPDIVKGLIWAFLKEETRRD*
>ENSMUSP00000107978_c.430:C>T_p.T144I ENSMUST00000112359.9|Hps4-202|ENSMUSG00000042328|+|chr5:g.112511458:C>T
MATTTPPETKSAAWWNYFFLYDGSKVKGEGDPTRAGICYFYPPQTLLDQQELLCGQLAGVVRCLWDLSGTPPM
LIRMRNLKFAIRADGDYLWALGCGVEISDASCRQFLDQLIGFFHFYMGPVSLAYKSHPQEELSLQWDTSIIQV
LRSTSESHRIFNALWNLDRTKVEPLLLLKAALILQTCQRSPHVLAGCILYKGLIVNSQLLPSLTAKVLLHQTV
PADQRLPGAGAAPQETGAALPPDVQITSVFLSEEEVASLHEFPVEHETRLQGSSVQYPPWDQSSPTQAEDAWA
SAAIPEPTPHDGACPSGSGADERLPRLEQECAGPTGLCTTACGQGSGLSSRLQKELCLSREELDSSEMHVSEA
QEAFPPLPALGDLETLHSSHSAPTLPEDTAICSCLHPCPLERLPESGRLGQLADLPLTNGQTQVPGTDPLPSS
MPVALPPQHPVGVEPSVEPYGNGAQESHSALPRSSRSPDSPGPSPSADRTGFKPSPSGRHAGLVPMNLYTHSV
NGLVLSLLAEETLLSDTAAIEEVYHSSLASLNGLEVHLKETLPRDEASLTSSTYNFLHYDRIQSVLSANLPLV

TAPQDRRFLQAVNLMHSDFALLPMLYEMTIRNASTAVYACSSPAQETYFQQLAPTARSSGFPNPQDCAFSLAG
KAKQKLLKHGVNLL*
>ENSMUSP00000047920_c.430:C>T_p.T144I ENSMUST00000035279.4|Hps4-201|ENSMUSG00000042328|+|chr5:g.112511458:C>T
MATTTPPETKSAAWWNYFFLYDGSKVKGEGDPTRAGICYFYPPQTLLDQQELLCGQLAGVVRCLWDLSGTPPM
LIRMRNLKFAIRADGDYLWALGCGVEISDASCRQFLDQLIGFFHFYMGPVSLAYKSHPQEELSLQWDTSIIQV
LRSTSESHRIFNALWNLDRTKVEPLLLLKAALILQTCQRSPHVLAGCILYKGLIVNSQLLPSLTAKVLLHQTV
PADQRLPGAGAAPQETGAALPPDVQITSVFLSEEEVASLHEFPVEHETRLQGSSVQYPPWDQSSPTQAEDAWA
SAAIPEPTPHDGACPSGSGADERLPRLEQECAGPTGLCTTACGQGSGLSSRLQKELCLSREELDSSEMHVSEA
QEAFPPLPALGDLETLHSSHSAPTLPEDTAICSCLHPCPLERLPESGRLGQLADLPLTNGQTQVPGTDPLPSS
MPVALPPQHPVGVEPSVEPYGNGAQESHSALPRSSRSPDSPGPSPSADRTGFKPSPSGRHAGLVPMNLYTHSV
NGLVLSLLAEETLLSDTAAIEEVYHSSLASLNGLEVHLKETLPRDEASLTSSTYNFLHYDRIQSVLSANLPLV
TAPQDRRFLQAVNLMHSDFALLPMLYEMTIRNASTAVYACSSPAQETYFQQLAPTARSSGFPNPQDCAFSLAG
KAKQKLLKHGVNLL*
>ENSMUSP00000031590_c.2342:A>C_p.K781N ENSMUST00000031590.12|Rbm19-201|ENSMUSG00000029594|+|chr5:g.120279677:A>C
MSRLIVKNLPNGMKEERFRQLFAAFGTLTDCSLKFTKDGKFRKFGFIGFKSEEEAQAALNHFHRSFIDTTRIT
VEFCKSFGDPSKPRAWSKHAQKSSQPKQPSQDSVPSDTKKDKKKKGPSDLEKLKEDAKFQEFLSIHQKRTQVA
TWANDALEAKLPKAKTKASSDYLNFDSDSNSDSGQESEEEPAREDPEEEQGLQPKAAVQKELSDMDYLKSKMV
RAEVSSEDEDEEDSEDEAVNCEEGSEEEEEEGSPASPAKQGGVSRGAVPGVLRPQEAAGKVEKPVSQKEPTTP
YTVKLRGAPFNVTEKNVIEFLAPLKPVAIRIVRNAHGNKTGYVFVDLSSEEEVKKALKCNRDYMGGRYIEVFR
EKQAPTARGPPKSTTPWQGRTLGENEEEEDLADSGRLFVRNLSYTSSEEDLEKLFSAYGPLSELHYPIDSLTK
KPKGFAFVTFMFPEHAVKAYAEVDGQVFQGRMLHVLPSTIKKEASQEANAPGSSYKKKKEAMDKANSSSSHNW
NTLFMGPNAVADAIAQKYNATKSQVFDHETRGSVAVRVALGETQLVQEVRSFLIDNGVCLDSFSQAAAERSKT
VILAKNLPAGTLAAEIQETFSRFGSLGRVLLPEGGITAIVEFLEPLEARKAFRHLAYSKFHHVPLYLEWAPIG
VFGAAPQKKDSQHEQPAEKAEVEQETVLDPEGEKASVEGAEASTGKMEEEEEEEEEEEEEESIPGCTLFIKNLN
FSTTEETLKGVFSKVGAIKSCTISKKKNKAGVLLSMGFGFVEYKKPEQAQNALKQLQGHTVDGHKLEVRISER
ATKPALTSTRKKQVPKKQTTSKILVRNIPFQANQREIRELFSTFGELKTVRLPKKMTGTGAHRGFGFVDFITK
QDAKKAFNALCHSTHLYGRRLVLEWADSEVTVQTLRRKTARHFQEPPKKKRSAVLDGILEQLEDEDNSDGEQA
LQL*
>ENSMUSP00000144339_c.2342:A>C_p.K781N ENSMUST00000202777.2|Rbm19-209|ENSMUSG00000029594|+|chr5:g.120279677:A>C
MSRLIVKNLPNGMKEERFRQLFAAFGTLTDCSLKFTKDGKFRKFGFIGFKSEEEAQAALNHFHRSFIDTTRIT
VEFCKSFGDPSKPRAWSKHAQKSSQPKQPSQDSVPSDTKKDKKKKGPSDLEKLKEDAKFQEFLSIHQKRTQVA
TWANDALEAKLPKAKTKASSDYLNFDSDSNSDSGQESEEEPAREDPEEEQGLQPKAAVQKELSDMDYLKSKMV
RAEVSSEDEDEEDSEDEAVNCEEGSEEEEEEGSPASPAKQGGVSRGAVPGVLRPQEAAGKVEKPVSQKEPTTP
YTVKLRGAPFNVTEKNVIEFLAPLKPVAIRIVRNAHGNKTGYVFVDLSSEEEVKKALKCNRDYMGGRYIEVFR
EKQAPTARGPPKSTTPWQGRTLGENEEEEDLADSGRLFVRNLSYTSSEEDLEKLFSAYGPLSELHYPIDSLTK
KPKGFAFVTFMFPEHAVKAYAEVDGQVFQGRMLHVLPSTIKKEASQEANAPGSSYKKKKEAMDKANSSSSHNW
NTLFMGPNAVADAIAQKYNATKSQVFDHETRGSVAVRVALGETQLVQEVRSFLIDNGVCLDSFSQAAAERSKT
VILAKNLPAGTLAAEIQETFSRFGSLGRVLLPEGGITAIVEFLEPLEARKAFRHLAYSKFHHVPLYLEWAPIG
VFGAAPQKKDSQHEQPAEKAEVEQETVLDPEGEKASVEGAEASTGKMEEEEEEEEEEEEEESIPGCTLFIKNLN
FSTTEETLKGVFSKVGAIKSCTISKKKNKAGVLLSMGFGFVEYKKPEQAQNALKQLQGHTVDGHKLEVRISER
ATKPALTSTRKKQVPKKQTTSKILVRNIPFQANQREIRELFSTFGELKTVRLPKKMTGTESLQCAVS*
>ENSMUSP00000042188_c.2354:T>G_p.H785Q ENSMUST00000046426.10|Tpcn1-201|ENSMUSG00000032741|-|chr5:g.120674272:A>C
MAVSLDDDVPLILTLDEAESAPLPPSNSLGQEQLPSKNGGSHSIHNSQVPSLVSGADSPPSSPTGHNWEMNYQ
EAAIYLQEGQNNDKFFTHPKDARALAAYLFVHNHFFYMMELLTALLLLLLSLCESPAVPVLKLHTYVHATLEL
FALMVVVFELCMKLRWLGFHTFVRHKRTMVKTSVLVVQFIEAIVVLVRQTSHVRVTRALRCIFLVDCRYCGGV
RRNLRQIFQSLPPFMDILLLLLFFMIIFAILGFYLFSTNPSDPYFSTLENSIVNLFVLLTTANFPDVMMPSYS
RNPWSCVFFIVYLSIELYFIMNLLLAVVFDTFNDIEKHKFKSLLLHKRTAIQHAYGLLASQRRPAGISYRQFE
GLMRFYKPRMSARERFLTFKALNQSNTPLLSLKDFYDIYEVAALQWKAKRNRQHWFDELPRTAFLIFKGINIL
VNSKAFQYFMYLVVAVNGVWILVETFMLKGGNFTSKHVPWSYLVFLTIYGVELFMKVAGLGPVEYLSSGWNLF
DFSVTAFAFLGLLALTLNMEPFYFIVVLRPLQLLRLFKLKKRYRNVLDTMFELLPRMASLGLTLLTFYYSFAI
VGMEFFNGRLTPNCCNTSTVADAYRFINHTVGNKTKVEEGYYYLNNFDNILNSFVTLFELTVVNNWYIIMEGV
TSQTSHWSRLYFMTFYIVTMVVMTIIVAFILEAFVFRMNYSRKSQDSEVDSGIVIEKEMSKEELMAVLELYRE
ERGTSSDVTRLLDTLSQMEKYQQNSMVFLGRRSRTKSDLSLKMYQEEIQEWYEEQAREQEQQKLRGSVPGPAA
QQPPGSRQRSQTVT*

>ENSMUSP00000031607_c.1585:A>C_p.N529T ENSMUST00000031607.7|Dtx1-201|ENSMUSG00000029603|-|chr5:g.120820259:T>G
MSRPGQGVMVPVNGLGFPPQNVARVVVWEWLNEHSRWRPYTATVCHHIENVLKEDARGSVVLGQVDAQLVPYI
IDLQSMHQFRQDTGTMRPVRRNFYDPSSAPGKGIVWEWENDGGAWTAYDMDICITIQNAYEKQHPWLDLSSLG
FCYLIYFNSMSQMNRQTRRRRRLRRRLDLAYPLTVGSIPKSQSWPVGASSGQPCSCQQCLLVNSTRAASNAIL
ASQRRKAPIAPAAPPAPPPPPPPLPPGGPPGALVVRPSATFAGAALWAAPATGPTEPAPPPGVPPRSPSAPNG
APTPGQNNLSRPGPQRSTSVSARASIPPGVPALPVKNLNGTGPVHPALAGMTGILLCAAGLPVCLTRAPKPIL
HPPPVSKSDVKPVPGVPGVCRKTKKKHLKKSKNPEDVVRRYMQKVKNPPDEDCTICMERLVTASGYEGVLRNK
SVRPELVGRLGRCGHMYHLLCLVAMYSNGNKDGSLQCPTCKAIYGEKTGTQPPGKMEFHLIPHSLPGFADTQT
IRIVYDIPTGIQGPEHPTPGKKFTARGFPRHCYLPNNEKGRKVLRLLITAWERRLIFTIGTSNTTGESDTVVW
NEIHHKTEFGSNLTGHGYPDASYLDNVLAELTAQGVSEAMAKA*
>ENSMUSP00000045196_c.11712:A>G_p.T3905A ENSMUST00000041226.11|Muc3-201|ENSMUSG00000037390|-|chr5:g.137178627:T>C
MSTRRWVRQTAALCLLAFILHSLPRHIAAENGTTESGSVWDGGGNSTQDDSQETQFRTVLKTKTTDGENSEAW
PRQEYQQRSSSPSTTKAEVTTTADVTTTADVTTTAEITTTSDVTTTAEITNTDDVTTTGEVTTTADVTTTSDV
PTTSEVTSTDDVITTAEVTTTAEMTPTDDVTTTSEVTTTGDVTTTGDVTITGDLTTTADVTTKADFTTTDDVT
TTADGSTTADLTTTAKVTTTDDVTTTGEITTTSEITTTSDVTTTAEITTTDIVTTTGEVTTTADVTTTSDVTT
TSEETPTADITTTADLTTTAEVTTTDDVTTTVEITTTSEITTTSDVTTIAEITTTDIVTTTGEVTTTADVTTT
SDVTTTSEETTTADITTTADLTTTAEITTTDDVTTTVEITTTSDVTTTTEITTTDIVTTTGEVTTTADVTTAS
DVTTTSEETPTADITTTADLTTTAEVTTTDDVTTTGEVTTTADVTTTSDVTTTSEETTTADVTTTSDVTTTAE
ITTTDDITSTADVTTTADVTTTSDVPTTSEETTTADVTTTSDVTTTAEITTTDDITSTADVTTTADVTTTSDV
TTTTEENPTADITTTADLTTTAEVTTTDDVTTTVEITTTSEITTTSDVTTTAEITTTDIVTTTDEDTTTADVT
TTSDVTTTSEETPTADITTTADLTTTADVTTTSDVTTTSEETTTADVTTTSDVTTTAEITTTDDITSTADVTT
TTNVTTTSDVPTTSEVTSTDDVITTTEVTTTAEIIPTDDVTTTSEVTTTGDVTTTGDVTMTGDLTTTADVTTT
ADLTTTDEVTTTADGSTTADLTTTAEVTTTDDVTTTVQITTTSEITTTSDVTTTAEITTTDNVTTTGEVTTTA
DVTTTSDVTTTSEETPTADITTTADLNTTAEVTTTEDVTTTVEITTTSEITTTSDITTTAEITTTDIVTTTGE
VTTTADVTTTNDVTTTSEETPTADITTTADLTTTAEITTTDDITSTADVTTTTDVTTTSDVPTTSEVTSTDDV
ITTTEVTTTAEIIPTDDVTTTSEVTTTGDVTTTGDVTMTGDLTTTADVTTTADLTTTDEVTTTADGSTTADLT
TTAEVTTTDDVTTTVQITTTSEITTTSDVTTTAEITTTDNVTTTGEVTTTADVTTTSDVTTTSEETPTADITT
TADLTTTAEVTTTEDVTTTVEITTTSEITTTSDITTTAEITTTDIVTTTGEVTTTADVTTTNDVTTTSEETPT
ADITTTADLTTTAEITTTDDVTTTGEVTTTADVTTTSDVTTTSEETPTADITTTADLTTTAEITTTDDVTTTG
EVTTTADVTTTSDITTTSEETPTADITTTADLTTTAEVTTTDDVTTTGEVTTTADVTTTSDITTTSEETPTAD
ITTTADLTTTAEVTTTDDVTTTVEITTTSEITTTSDVTTTAEITTTDIVTTTGEVTTTADVTTTSDVTTTSEQ
TPTADITTTADLTTTAEVTTTDDVTTTVEITTTSEITTTSDVTTTAEITTTDIVTTTGEVTTTADITTTSDVT
TTSEETTTADVTTTTDVTTTSEETTTGDGKTTTAEITTTDDITTTADVTTTPDVTTTAAVTTTGEITPTDDVT
TTSEVTTTGDVTSTGDVTTTGEVTTTAEVTTTADVTTTSDVTTTSEETPTADITTTADLTTTAEVTTTDDVTT
TGEVTTTADVTTTSDTTTTSEETPTADITTTVDLTTTAEVTTTDDVTTTVEITTTSEITTTSDVTTTAEITTS
DIFTTTGEVTTTADITTTSDVTTTSEETPTADITTTADLTTTAEITTTDDVTTTVEITTTSEITTTSEVTTTP
DVTTTSDVTTTSEETPTADITTTADLTTTAEVTTTDDVTTTVEITTTSEITTTSDVTTTAEITTTDIVTTTGE
VTTTVDVTTTNDVTTTSEETPTADITTTADLTTTAEVTTTDDVTTTGEVTTTADVTTTSDITTTSEETPTADI
TTTVDLTTTVEITTTSEITTTSDVTTTAEITTTDIVTTTGEVTTTADVTTTSDVTTTSEETPTADITTTADLT
TTAEVTTTDDVTTTVEITTTSEITTTSDVTTTAEITTTDIVTTSGEVTTTADVTTTSDVTTTSEETTTADVTT
TTDVTTTSEETTTGDGTTTTAEITTTDDLTTTADVTTTPDVTTTAAVTTTAEITPTDDVTTTSEVTTTGDVTT
TGEVTTTAEVTTTDDVTTTGDVTTTGDLTTMADVTTTADFTTTDDVTTTADASTTADLATTAEVTTTDDVTTT
VEITTTAEITTTAEVTTTDDVTTTGEVTTTADVTTTTDVTTTSEVTTTADVTTTNDVTTTAEITTTDDITTTA
DVTTTADVTTTSYVPTTSEVTSTDDVTTTAEVTTTAEITPTDDVTTTSEVTTTGDVTTTGDVTMTCDLTTSAD
VTTTADFTTTDDVTTKADGSTTADLTTTAEVTTNDDVTTTGELTTTAEITTTSDVTTTAEITTTDIVTTTGEV
TTTADVTTTSDVTTTSEETTTADITTTADLTTTAEVTTTDDVTTTVEITTTSEITTTSDVTTIAEITTTDIVT
TTGEVTTTADVTTTNDVTTTSEETPTADITTTADLTTTAEVTTTDDVTTTGEVTTTADVTTTSDVTTTSEETP
TADITTTADLTTTAEVTTTDDVTTTVEITTTSEITTTSDVTTTAEITTTDIVTTTGEVTTTADITTTSDVTTT
SEETTTADVTTTTDVTTTSEETTTGDGKTTTAEITTTDDITTTADVTTTPDVTTTAAVTTTGEITPTDDVTTT
SEVTTTGDVTTTGDVTTTGEVTTTAEVTTTDDVTTTGDVTTTGDLTTTADVTTTAYFTTTDDVTTTADGSTTA
DLTTTAEVTTTDDVTTTVEITTTAEITTTDDVTTTGEVTTTADVTTTSDVTTTSEETPTADITTTADLTTTAE
VTTTDDVTTTVEITTTSEITTTSDVTTTAEITTTDIVTTTGEVTTTADVTTTSDVTTTSEETPTADITTTADL
TTTAEVTTTDDVTTTVEITTTSEITTTSDVTTTAEITTTDIVTTTGEVTTTADVTTTSDVTTTSEETPTADIT
TTADLTTTAEVTTTDDVTTTVEITTTAEITTTDDVTTTGEVTTTADVTTTAEVITTSDVTTTADVTTMPDFTT
TAEVTTTLDVTTNDEVTTTSVVTTTPEITTAGDVTTTSEVTTSADLTTTPEITTTGDVTTTADVTTTADITTT
SDVTTTADVTTTADITTTSDVTTTADVTTIADLSTTPEITTTGDFSSTTDVTSTGDVTTTADVTTTADVTTTG

DVTTTAEVTTTAEVTTTAEATTTADLNTTPEITTTDDVTTTGDVTITAEVTTTAYVTTTTDFTTTAEVNTTDD
VTTIADLTTTAEVTTTNDVTSTAEITTTADLTTTTEVTTTADFITTGDVTTTADVTTMADVTTTADVTTADLT
TTVDVTTMDDVTTTAEVITSGDVTTTSEVTTTSNLNTTPEITTTIDFTSTVDVTTTGDVTTTADVTTTSDVTT
TSKVTTTGDVTTTADTTSTADVTTTADVITTAEVTTTADFTNTGDVTTSADVTTTADLTTMPDLTTTAEVTTT
ADVTTTSEFTTTADVTTTADVTSTADVTTTAEVTTTGDVTTTADVTNTGDITTIGDVTTTTEITTTAYLTTTL
EITTTGEVTSTADVTTSAEVTTTDEITTTDVVTTTGDVTPTAESTTTDDVTTTSDVTTTGDFTTTADVTTTAY
VTTTPDVTTTVEVTTTADFTTTGVVNTTADVTTTADVTTTSEVTTTADLTTTPEITTTVDFTSTADVTTTADV
ITTPEVTTTSDVTTTAEVITTADVTTTADVTTTVEVTTTGDVTITADVTTTPEITTTVDFTSTADVTITADVT
TTADVTTTSDVTITPDVTTTAEVTTTVEITTTDDVATTGEVTTTADVTTTSDVTTTSEETTTADITTTSDVST
TSEVTTTAYVTTTADTTSTAEVTTTADVTTTADVTTTADVTTTADVTTTVEVTTTGDVTTTADVTTTAEVTTT
AEITTTGDVTTTADVTTTAEVTTTAEITTTGDVTTTADVTTTADVTTTADVTTTIDVTTTADVTTSAEVTTTA
DVTTTTEVTTTTEVATTPEPTTTPAPTTTAVNCMNGGFWTGDKCICPNGFGGDRCENIVNVVNCENGGTWDGL
KCQCTSLFYGPRCEELVESVEIEPTVAASVEVSVTVTSQEYSEKLQDRKSEEFSNFNKTFTKQMALIYAGIPE
YEGVIIKNLSKGSIVVDYDVILKAKYTPGFENTLDTVVKNLETKIKNATEVQVQDVNNNCSALLCFNSTATKV
QNSATVSVNPEETCKKEAGEDFAKFVTLGQKGDKWFCITPCSAGYSTSKNCSYGKCQLQRSGPQCLCLITDTH
WYSGENCDWGIQKSLVYGLGGAGVAVLLVILVILLVFSIRFRKDAQRQRSRVSEMYKWGEEEGRASPGTFHNF
GFDHNEERENYMPLDSVYNTFQPSLNHINPERKIQIQRPQVVMTSL*
>ENSMUSP00000045196_c.10645:A>T_p.D3549V ENSMUST00000041226.11|Muc3-
201|ENSMUSG00000037390|-|chr5:g.137179694:T>A
MSTRRWVRQTAALCLLAFILHSLPRHIAAENGTTESGSVWDGGGNSTQDDSQETQFRTVLKTKTTDGENSEAW
PRQEYQQRSSSPSTTKAEVTTTADVTTTADVTTTAEITTTSDVTTTAEITNTDDVTTTGEVTTTADVTTTSDV
PTTSEVTSTDDVITTAEVTTTAEMTPTDDVTTTSEVTTTGDVTTTGDVTITGDLTTTADVTTKADFTTTDDVT
TTADGSTTADLTTTAKVTTTDDVTTTGEITTTSEITTTSDVTTTAEITTTDIVTTTGEVTTTADVTTTSDVTT
TSEETPTADITTTADLTTTAEVTTTDDVTTTVEITTTSEITTTSDVTTIAEITTTDIVTTTGEVTTTADVTTT
SDVTTTSEETTTADITTTADLTTTAEITTTDDVTTTVEITTTSDVTTTTEITTTDIVTTTGEVTTTADVTTAS
DVTTTSEETPTADITTTADLTTTAEVTTTDDVTTTGEVTTTADVTTTSDVTTTSEETTTADVTTTSDVTTTAE
ITTTDDITSTADVTTTADVTTTSDVPTTSEETTTADVTTTSDVTTTAEITTTDDITSTADVTTTADVTTTSDV
TTTTEENPTADITTTADLTTTAEVTTTDDVTTTVEITTTSEITTTSDVTTTAEITTTDIVTTTDEDTTTADVT
TTSDVTTTSEETPTADITTTADLTTTADVTTTSDVTTTSEETTTADVTTTSDVTTTAEITTTDDITSTADVTT
TTNVTTTSDVPTTSEVTSTDDVITTTEVTTTAEIIPTDDVTTTSEVTTTGDVTTTGDVTMTGDLTTTADVTTT
ADLTTTDEVTTTADGSTTADLTTTAEVTTTDDVTTTVQITTTSEITTTSDVTTTAEITTTDNVTTTGEVTTTA
DVTTTSDVTTTSEETPTADITTTADLNTTAEVTTTEDVTTTVEITTTSEITTTSDITTTAEITTTDIVTTTGE
VTTTADVTTTNDVTTTSEETPTADITTTADLTTTAEITTTDDITSTADVTTTTDVTTTSDVPTTSEVTSTDDV
ITTTEVTTTAEIIPTDDVTTTSEVTTTGDVTTTGDVTMTGDLTTTADVTTTADLTTTDEVTTTADGSTTADLT
TTAEVTTTDDVTTTVQITTTSEITTTSDVTTTAEITTTDNVTTTGEVTTTADVTTTSDVTTTSEETPTADITT
TADLTTTAEVTTTEDVTTTVEITTTSEITTTSDITTTAEITTTDIVTTTGEVTTTADVTTTNDVTTTSEETPT
ADITTTADLTTTAEITTTDDVTTTGEVTTTADVTTTSDVTTTSEETPTADITTTADLTTTAEITTTDDVTTTG
EVTTTADVTTTSDITTTSEETPTADITTTADLTTTAEVTTTDDVTTTGEVTTTADVTTTSDITTTSEETPTAD
ITTTADLTTTAEVTTTDDVTTTVEITTTSEITTTSDVTTTAEITTTDIVTTTGEVTTTADVTTTSDVTTTSEQ
TPTADITTTADLTTTAEVTTTDDVTTTVEITTTSEITTTSDVTTTAEITTTDIVTTTGEVTTTADITTTSDVT
TTSEETTTADVTTTTDVTTTSEETTTGDGKTTTAEITTTDDITTTADVTTTPDVTTTAAVTTTGEITPTDDVT
TTSEVTTTGDVTSTGDVTTTGEVTTTAEVTTTADVTTTSDVTTTSEETPTADITTTADLTTTAEVTTTDDVTT
TGEVTTTADVTTTSDTTTTSEETPTADITTTVDLTTTAEVTTTDDVTTTVEITTTSEITTTSDVTTTAEITTS
DIFTTTGEVTTTADITTTSDVTTTSEETPTADITTTADLTTTAEITTTDDVTTTVEITTTSEITTTSEVTTTP
DVTTTSDVTTTSEETPTADITTTADLTTTAEVTTTDDVTTTVEITTTSEITTTSDVTTTAEITTTDIVTTTGE
VTTTVDVTTTNDVTTTSEETPTADITTTADLTTTAEVTTTDDVTTTGEVTTTADVTTTSDITTTSEETPTADI
TTTVDLTTTVEITTTSEITTTSDVTTTAEITTTDIVTTTGEVTTTADVTTTSDVTTTSEETPTADITTTADLT
TTAEVTTTDDVTTTVEITTTSEITTTSDVTTTAEITTTDIVTTSGEVTTTADVTTTSDVTTTSEETTTADVTT
TTDVTTTSEETTTGDGTTTTAEITTTDDLTTTADVTTTPDVTTTAAVTTTAEITPTDDVTTTSEVTTTGDVTT
TGEVTTTAEVTTTDDVTTTGDVTTTGDLTTMADVTTTADFTTTDDVTTTADASTTADLATTAEVTTTDDVTTT
VEITTTAEITTTAEVTTTDDVTTTGEVTTTADVTTTTDVTTTSEVTTTADVTTTNDVTTTAEITTTDDITTTA
DVTTTADVTTTSYVPTTSEVTSTDDVTTTAEVTTTAEITPTDDVTTTSEVTTTGDVTTTGDVTMTCDLTTSAD
VTTTADFTTTDDVTTKADGSTTADLTTTAEVTTNDDVTTTGELTTTAEITTTSDVTTTAEITTTDIVTTTGEV
TTTADVTTTSDVTTTSEETTTADITTTADLTTTAEVTTTDDVTTTVEITTTSEITTTSDVTTIAEITTTDIVT
TTGEVTTTADVTTTNDVTTTSEETPTADITTTADLTTTAEVTTTDDVTTTGEVTTTADVTTTSDVTTTSEETP
TADITTTADLTTTAEVTTTDDVTTTVEITTTSEITTTSDVTTTAEITTTDIVTTTGEVTTTADITTTSDVTTT
SEETTTADVTTTTDVTTTSEETTTGDGKTTTAEITTTDDITTTADVTTTPDVTTTAAVTTTGEITPTDDVTTT
SEVTTTGDVTTTGDVTTTGEVTTTAEVTTTDDVTTTGDVTTTGDLTTTADVTTTAYFTTTDDVTTTADGSTTA

DLTTTAEVTTTDDVTTTVEITTTAEITTTDDVTTTGEVTTTADVTTTSDVTTTSEETPTADITTTADLTTTAE
VTTTDDVTTTVEITTTSEITTTSDVTTTAEITTTDIVTTTGEVTTTADVTTTSDVTTTSEETPTADITTTADL
TTTAEVTTTDDVTTTVEITTTSEITTTSDVTTTAEITTTDIVTTTGEVTTTADVTTTSDVTTTSEETPTADIT
TTADLTTTAEVTTTDDVTTTVEITTTAEITTTDDVTTTGEVTTTADVTTTAEVITTSDVTTTADVTTMPDFTT
TAEVTTTLDVTTNDEVTTTSVVTTTPEITTAGDVTTTSEVTTSADLTTTPEITTTGDVTTTADVTTTADITTT
SDVTTTADVTTTADITTTSDVTTTADVTTIADLSTTPEITTTGDFSSTTDVTSTGDVTTTADVTTTADVTTTG
DVTTTAEVTTTAEVTTTAEATTTADLNTTPEITTTDDVTTTGDVTITAEVTTTAYVTTTTDFTTTAEVNTTDD
VTTIADLTTTAEVTTTNDVTSTAEITTTADLTTTTEVTTTADFITTGDVTTTADVTTMADVTTTADVTTADLT
TTVDVTTMDDVTTTAEVITSGDVTTTSEVTTTSNLNTTPEITTTIDFTSTVDVTTTGDVTTTADVTTTSDVTT
TSKVTTTGDVTTTADTTSTADVTTTADVITTAEVTTTADFTNTGVVTTSADVTTTADLTTMPDLTTTAEVTTT
ADVTTTSEFTTTADVTTTADVTSTADVTTTAEVTTTGDVTTTADVTNTGDITTIGDVTTTTEITTTAYLTTTL
EITTTGEVTSTADVTTSAEVTTTDEITTTDVVTTTGDVTPTAESTTTDDVTTTSDVTTTGDFTTTADVTTTAY
VTTTPDVTTTVEVTTTADFTTTGVVNTTADVTTTADVTTTSEVTTTADLTTTPEITTTVDFTSTADVTTTADV
ITTPEVTTTSDVTTTAEVITTADVTTTADVTTTVEVTTTGDVTITADVTTTPEITTTVDFTSTADVTITADVT
TTADVTTTSDVTITPDVTTTAEVTTTVEITTTDDVTTTGEVTTTADVTTTSDVTTTSEETTTADITTTSDVST
TSEVTTTAYVTTTADTTSTAEVTTTADVTTTADVTTTADVTTTADVTTTVEVTTTGDVTTTADVTTTAEVTTT
AEITTTGDVTTTADVTTTAEVTTTAEITTTGDVTTTADVTTTADVTTTADVTTTIDVTTTADVTTSAEVTTTA
DVTTTTEVTTTTEVATTPEPTTTPAPTTTAVNCMNGGFWTGDKCICPNGFGGDRCENIVNVVNCENGGTWDGL
KCQCTSLFYGPRCEELVESVEIEPTVAASVEVSVTVTSQEYSEKLQDRKSEEFSNFNKTFTKQMALIYAGIPE
YEGVIIKNLSKGSIVVDYDVILKAKYTPGFENTLDTVVKNLETKIKNATEVQVQDVNNNCSALLCFNSTATKV
QNSATVSVNPEETCKKEAGEDFAKFVTLGQKGDKWFCITPCSAGYSTSKNCSYGKCQLQRSGPQCLCLITDTH
WYSGENCDWGIQKSLVYGLGGAGVAVLLVILVILLVFSIRFRKDAQRQRSRVSEMYKWGEEEGRASPGTFHNF
GFDHNEERENYMPLDSVYNTFQPSLNHINPERKIQIQRPQVVMTSL*
>ENSMUSP00000045196_c.10258:C>G_p.T3420S ENSMUST00000041226.11|Muc3-
201|ENSMUSG00000037390|-|chr5:g.137180081:G>C
MSTRRWVRQTAALCLLAFILHSLPRHIAAENGTTESGSVWDGGGNSTQDDSQETQFRTVLKTKTTDGENSEAW
PRQEYQQRSSSPSTTKAEVTTTADVTTTADVTTTAEITTTSDVTTTAEITNTDDVTTTGEVTTTADVTTTSDV
PTTSEVTSTDDVITTAEVTTTAEMTPTDDVTTTSEVTTTGDVTTTGDVTITGDLTTTADVTTKADFTTTDDVT
TTADGSTTADLTTTAKVTTTDDVTTTGEITTTSEITTTSDVTTTAEITTTDIVTTTGEVTTTADVTTTSDVTT
TSEETPTADITTTADLTTTAEVTTTDDVTTTVEITTTSEITTTSDVTTIAEITTTDIVTTTGEVTTTADVTTT
SDVTTTSEETTTADITTTADLTTTAEITTTDDVTTTVEITTTSDVTTTTEITTTDIVTTTGEVTTTADVTTAS
DVTTTSEETPTADITTTADLTTTAEVTTTDDVTTTGEVTTTADVTTTSDVTTTSEETTTADVTTTSDVTTTAE
ITTTDDITSTADVTTTADVTTTSDVPTTSEETTTADVTTTSDVTTTAEITTTDDITSTADVTTTADVTTTSDV
TTTTEENPTADITTTADLTTTAEVTTTDDVTTTVEITTTSEITTTSDVTTTAEITTTDIVTTTDEDTTTADVT
TTSDVTTTSEETPTADITTTADLTTTADVTTTSDVTTTSEETTTADVTTTSDVTTTAEITTTDDITSTADVTT
TTNVTTTSDVPTTSEVTSTDDVITTTEVTTTAEIIPTDDVTTTSEVTTTGDVTTTGDVTMTGDLTTTADVTTT
ADLTTTDEVTTTADGSTTADLTTTAEVTTTDDVTTTVQITTTSEITTTSDVTTTAEITTTDNVTTTGEVTTTA
DVTTTSDVTTTSEETPTADITTTADLNTTAEVTTTEDVTTTVEITTTSEITTTSDITTTAEITTTDIVTTTGE
VTTTADVTTTNDVTTTSEETPTADITTTADLTTTAEITTTDDITSTADVTTTTDVTTTSDVPTTSEVTSTDDV
ITTTEVTTTAEIIPTDDVTTTSEVTTTGDVTTTGDVTMTGDLTTTADVTTTADLTTTDEVTTTADGSTTADLT
TTAEVTTTDDVTTTVQITTTSEITTTSDVTTTAEITTTDNVTTTGEVTTTADVTTTSDVTTTSEETPTADITT
TADLTTTAEVTTTEDVTTTVEITTTSEITTTSDITTTAEITTTDIVTTTGEVTTTADVTTTNDVTTTSEETPT
ADITTTADLTTTAEITTTDDVTTTGEVTTTADVTTTSDVTTTSEETPTADITTTADLTTTAEITTTDDVTTTG
EVTTTADVTTTSDITTTSEETPTADITTTADLTTTAEVTTTDDVTTTGEVTTTADVTTTSDITTTSEETPTAD
ITTTADLTTTAEVTTTDDVTTTVEITTTSEITTTSDVTTTAEITTTDIVTTTGEVTTTADVTTTSDVTTTSEQ
TPTADITTTADLTTTAEVTTTDDVTTTVEITTTSEITTTSDVTTTAEITTTDIVTTTGEVTTTADITTTSDVT
TTSEETTTADVTTTTDVTTTSEETTTGDGKTTTAEITTTDDITTTADVTTTPDVTTTAAVTTTGEITPTDDVT
TTSEVTTTGDVTSTGDVTTTGEVTTTAEVTTTADVTTTSDVTTTSEETPTADITTTADLTTTAEVTTTDDVTT
TGEVTTTADVTTTSDTTTTSEETPTADITTTVDLTTTAEVTTTDDVTTTVEITTTSEITTTSDVTTTAEITTS
DIFTTTGEVTTTADITTTSDVTTTSEETPTADITTTADLTTTAEITTTDDVTTTVEITTTSEITTTSEVTTTP
DVTTTSDVTTTSEETPTADITTTADLTTTAEVTTTDDVTTTVEITTTSEITTTSDVTTTAEITTTDIVTTTGE
VTTTVDVTTTNDVTTTSEETPTADITTTADLTTTAEVTTTDDVTTTGEVTTTADVTTTSDITTTSEETPTADI
TTTVDLTTTVEITTTSEITTTSDVTTTAEITTTDIVTTTGEVTTTADVTTTSDVTTTSEETPTADITTTADLT
TTAEVTTTDDVTTTVEITTTSEITTTSDVTTTAEITTTDIVTTSGEVTTTADVTTTSDVTTTSEETTTADVTT
TTDVTTTSEETTTGDGTTTTAEITTTDDLTTTADVTTTPDVTTTAAVTTTAEITPTDDVTTTSEVTTTGDVTT
TGEVTTTAEVTTTDDVTTTGDVTTTGDLTTMADVTTTADFTTTDDVTTTADASTTADLATTAEVTTTDDVTTT
VEITTTAEITTTAEVTTTDDVTTTGEVTTTADVTTTTDVTTTSEVTTTADVTTTNDVTTTAEITTTDDITTTA
DVTTTADVTTTSYVPTTSEVTSTDDVTTTAEVTTTAEITPTDDVTTTSEVTTTGDVTTTGDVTMTCDLTTSAD

VTTTADFTTTDDVTTKADGSTTADLTTTAEVTTNDDVTTTGELTTTAEITTTSDVTTTAEITTTDIVTTTGEV
TTTADVTTTSDVTTTSEETTTADITTTADLTTTAEVTTTDDVTTTVEITTTSEITTTSDVTTIAEITTTDIVT
TTGEVTTTADVTTTNDVTTTSEETPTADITTTADLTTTAEVTTTDDVTTTGEVTTTADVTTTSDVTTTSEETP
TADITTTADLTTTAEVTTTDDVTTTVEITTTSEITTTSDVTTTAEITTTDIVTTTGEVTTTADITTTSDVTTT
SEETTTADVTTTTDVTTTSEETTTGDGKTTTAEITTTDDITTTADVTTTPDVTTTAAVTTTGEITPTDDVTTT
SEVTTTGDVTTTGDVTTTGEVTTTAEVTTTDDVTTTGDVTTTGDLTTTADVTTTAYFTTTDDVTTTADGSTTA
DLTTTAEVTTTDDVTTTVEITTTAEITTTDDVTTTGEVTTTADVTTTSDVTTTSEETPTADITTTADLTTTAE
VTTTDDVTTTVEITTTSEITTTSDVTTTAEITTTDIVTTTGEVTTTADVTTTSDVTTTSEETPTADITTTADL
TTTAEVTTTDDVTTTVEITTTSEITTTSDVTTTAEITTTDIVTTTGEVTTTADVTTTSDVTTTSEETPTADIT
TTADLTTTAEVTTTDDVTTTVEITTTAEITTTDDVTTTGEVTTTADVTTTAEVITTSDVTTTADVTTMPDFTT
TAEVTTTLDVTTNDEVTTTSVVTTTPEITTAGDVTTTSEVTTSADLTTTPEITTTGDVTTTADVTTTADITTT
SDVTTTADVTTTADITTTSDVTTTADVTTIADLSTTPEITTTGDFSSTTDVTSTGDVTTTADVTTTADVTTTG
DVTTTAEVTTTAEVTTTAEATTTADLNTTPEITTTDDVTTTGDVTITAEVTTTAYVTTTTDFTTTAEVNTTDD
VTTIADLTTTAEVTTTNDVTSTAEITTTADLTTTTEVTTTADFITTGDVTTTADVTTMADVSTTADVTTADLT
TTVDVTTMDDVTTTAEVITSGDVTTTSEVTTTSNLNTTPEITTTIDFTSTVDVTTTGDVTTTADVTTTSDVTT
TSKVTTTGDVTTTADTTSTADVTTTADVITTAEVTTTADFTNTGDVTTSADVTTTADLTTMPDLTTTAEVTTT
ADVTTTSEFTTTADVTTTADVTSTADVTTTAEVTTTGDVTTTADVTNTGDITTIGDVTTTTEITTTAYLTTTL
EITTTGEVTSTADVTTSAEVTTTDEITTTDVVTTTGDVTPTAESTTTDDVTTTSDVTTTGDFTTTADVTTTAY
VTTTPDVTTTVEVTTTADFTTTGVVNTTADVTTTADVTTTSEVTTTADLTTTPEITTTVDFTSTADVTTTADV
ITTPEVTTTSDVTTTAEVITTADVTTTADVTTTVEVTTTGDVTITADVTTTPEITTTVDFTSTADVTITADVT
TTADVTTTSDVTITPDVTTTAEVTTTVEITTTDDVTTTGEVTTTADVTTTSDVTTTSEETTTADITTTSDVST
TSEVTTTAYVTTTADTTSTAEVTTTADVTTTADVTTTADVTTTADVTTTVEVTTTGDVTTTADVTTTAEVTTT
AEITTTGDVTTTADVTTTAEVTTTAEITTTGDVTTTADVTTTADVTTTADVTTTIDVTTTADVTTSAEVTTTA
DVTTTTEVTTTTEVATTPEPTTTPAPTTTAVNCMNGGFWTGDKCICPNGFGGDRCENIVNVVNCENGGTWDGL
KCQCTSLFYGPRCEELVESVEIEPTVAASVEVSVTVTSQEYSEKLQDRKSEEFSNFNKTFTKQMALIYAGIPE
YEGVIIKNLSKGSIVVDYDVILKAKYTPGFENTLDTVVKNLETKIKNATEVQVQDVNNNCSALLCFNSTATKV
QNSATVSVNPEETCKKEAGEDFAKFVTLGQKGDKWFCITPCSAGYSTSKNCSYGKCQLQRSGPQCLCLITDTH
WYSGENCDWGIQKSLVYGLGGAGVAVLLVILVILLVFSIRFRKDAQRQRSRVSEMYKWGEEEGRASPGTFHNF
GFDHNEERENYMPLDSVYNTFQPSLNHINPERKIQIQRPQVVMTSL*
>ENSMUSP00000045196_c.10093:T>C_p.L3365P ENSMUST00000041226.11|Muc3-201|ENSMUSG00000037390|-|chr5:g.137180246:A>G
MSTRRWVRQTAALCLLAFILHSLPRHIAAENGTTESGSVWDGGGNSTQDDSQETQFRTVLKTKTTDGENSEAW
PRQEYQQRSSSPSTTKAEVTTTADVTTTADVTTTAEITTTSDVTTTAEITNTDDVTTTGEVTTTADVTTTSDV
PTTSEVTSTDDVITTAEVTTTAEMTPTDDVTTTSEVTTTGDVTTTGDVTITGDLTTTADVTTKADFTTTDDVT
TTADGSTTADLTTTAKVTTTDDVTTTGEITTTSEITTTSDVTTTAEITTTDIVTTTGEVTTTADVTTTSDVTT
TSEETPTADITTTADLTTTAEVTTTDDVTTTVEITTTSEITTTSDVTTIAEITTTDIVTTTGEVTTTADVTTT
SDVTTTSEETTTADITTTADLTTTAEITTTDDVTTTVEITTTSDVTTTTEITTTDIVTTTGEVTTTADVTTAS
DVTTTSEETPTADITTTADLTTTAEVTTTDDVTTTGEVTTTADVTTTSDVTTTSEETTTADVTTTSDVTTTAE
ITTTDDITSTADVTTTADVTTTSDVPTTSEETTTADVTTTSDVTTTAEITTTDDITSTADVTTTADVTTTSDV
TTTTEENPTADITTTADLTTTAEVTTTDDVTTTVEITTTSEITTTSDVTTTAEITTTDIVTTTDEDTTTADVT
TTSDVTTTSEETPTADITTTADLTTTADVTTTSDVTTTSEETTTADVTTTSDVTTTAEITTTDDITSTADVTT
TTNVTTTSDVPTTSEVTSTDDVITTTEVTTTAEIIPTDDVTTTSEVTTTGDVTTTGDVTMTGDLTTTADVTTT
ADLTTTDEVTTTADGSTTADLTTTAEVTTTDDVTTTVQITTTSEITTTSDVTTTAEITTTDNVTTTGEVTTTA
DVTTTSDVTTTSEETPTADITTTADLNTTAEVTTTEDVTTTVEITTTSEITTTSDITTTAEITTTDIVTTTGE
VTTTADVTTTNDVTTTSEETPTADITTTADLTTTAEITTTDDITSTADVTTTTDVTTTSDVPTTSEVTSTDDV
ITTTEVTTTAEIIPTDDVTTTSEVTTTGDVTTTGDVTMTGDLTTTADVTTTADLTTTDEVTTTADGSTTADLT
TTAEVTTTDDVTTTVQITTTSEITTTSDVTTTAEITTTDNVTTTGEVTTTADVTTTSDVTTTSEETPTADITT
TADLTTTAEVTTTEDVTTTVEITTTSEITTTSDITTTAEITTTDIVTTTGEVTTTADVTTTNDVTTTSEETPT
ADITTTADLTTTAEITTTDDVTTTGEVTTTADVTTTSDVTTTSEETPTADITTTADLTTTAEITTTDDVTTTG
EVTTTADVTTTSDITTTSEETPTADITTTADLTTTAEVTTTDDVTTTGEVTTTADVTTTSDITTTSEETPTAD
ITTTADLTTTAEVTTTDDVTTTVEITTTSEITTTSDVTTTAEITTTDIVTTTGEVTTTADVTTTSDVTTTSEQ
TPTADITTTADLTTTAEVTTTDDVTTTVEITTTSEITTTSDVTTTAEITTTDIVTTTGEVTTTADITTTSDVT
TTSEETTTADVTTTTDVTTTSEETTTGDGKTTTAEITTTDDITTTADVTTTPDVTTTAAVTTTGEITPTDDVT
TTSEVTTTGDVTSTGDVTTTGEVTTTAEVTTTADVTTTSDVTTTSEETPTADITTTADLTTTAEVTTTDDVTT
TGEVTTTADVTTTSDTTTTSEETPTADITTTVDLTTTAEVTTTDDVTTTVEITTTSEITTTSDVTTTAEITTS
DIFTTTGEVTTTADITTTSDVTTTSEETPTADITTTADLTTTAEITTTDDVTTTVEITTTSEITTTSEVTTTP
DVTTTSDVTTTSEETPTADITTTADLTTTAEVTTTDDVTTTVEITTTSEITTTSDVTTTAEITTTDIVTTTGE
VTTTVDVTTTNDVTTTSEETPTADITTTADLTTTAEVTTTDDVTTTGEVTTTADVTTTSDITTTSEETPTADI

TTTVDLTTTVEITTTSEITTTSDVTTTAEITTTDIVTTTGEVTTTADVTTTSDVTTTSEETPTADITTTADLT
TTAEVTTTDDVTTTVEITTTSEITTTSDVTTTAEITTTDIVTTSGEVTTTADVTTTSDVTTTSEETTTADVTT
TTDVTTTSEETTTGDGTTTTAEITTTDDLTTTADVTTTPDVTTTAAVTTTAEITPTDDVTTTSEVTTTGDVTT
TGEVTTTAEVTTTDDVTTTGDVTTTGDLTTMADVTTTADFTTTDDVTTTADASTTADLATTAEVTTTDDVTTT
VEITTTAEITTTAEVTTTDDVTTTGEVTTTADVTTTTDVTTTSEVTTTADVTTTNDVTTTAEITTTDDITTTA
DVTTTADVTTTSYVPTTSEVTSTDDVTTTAEVTTTAEITPTDDVTTTSEVTTTGDVTTTGDVTMTCDLTTSAD
VTTTADFTTTDDVTTKADGSTTADLTTTAEVTTNDDVTTTGELTTTAEITTTSDVTTTAEITTTDIVTTTGEV
TTTADVTTTSDVTTTSEETTTADITTTADLTTTAEVTTTDDVTTTVEITTTSEITTTSDVTTIAEITTTDIVT
TTGEVTTTADVTTTNDVTTTSEETPTADITTTADLTTTAEVTTTDDVTTTGEVTTTADVTTTSDVTTTSEETP
TADITTTADLTTTAEVTTTDDVTTTVEITTTSEITTTSDVTTTAEITTTDIVTTTGEVTTTADITTTSDVTTT
SEETTTADVTTTTDVTTTSEETTTGDGKTTTAEITTTDDITTTADVTTTPDVTTTAAVTTTGEITPTDDVTTT
SEVTTTGDVTTTGDVTTTGEVTTTAEVTTTDDVTTTGDVTTTGDLTTTADVTTTAYFTTTDDVTTTADGSTTA
DLTTTAEVTTTDDVTTTVEITTTAEITTTDDVTTTGEVTTTADVTTTSDVTTTSEETPTADITTTADLTTTAE
VTTTDDVTTTVEITTTSEITTTSDVTTTAEITTTDIVTTTGEVTTTADVTTTSDVTTTSEETPTADITTTADL
TTTAEVTTTDDVTTTVEITTTSEITTTSDVTTTAEITTTDIVTTTGEVTTTADVTTTSDVTTTSEETPTADIT
TTADLTTTAEVTTTDDVTTTVEITTTAEITTTDDVTTTGEVTTTADVTTTAEVITTSDVTTTADVTTMPDFTT
TAEVTTTLDVTTNDEVTTTSVVTTTPEITTAGDVTTTSEVTTSADLTTTPEITTTGDVTTTADVTTTADITTT
SDVTTTADVTTTADITTTSDVTTTADVTTIADLSTTPEITTTGDFSSTTDVTSTGDVTTTADVTTTADVTTTG
DVTTTAEVTTTAEVTTTAEATTTADLNTTPEITTTDDVTTTGDVTITAEVTTTAYVTTTTDFTTTAEVNTTDD
VTTIADPTTTAEVTTTNDVTSTAEITTTADLTTTTEVTTTADFITTGDVTTTADVTTMADVTTTADVTTADLT
TTVDVTTMDDVTTTAEVITSGDVTTTSEVTTTSNLNTTPEITTTIDFTSTVDVTTTGDVTTTADVTTTSDVTT
TSKVTTTGDVTTTADTTSTADVTTTADVITTAEVTTTADFTNTGDVTTSADVTTTADLTTMPDLTTTAEVTTT
ADVTTTSEFTTTADVTTTADVTSTADVTTTAEVTTTGDVTTTADVTNTGDITTIGDVTTTTEITTTAYLTTTL
EITTTGEVTSTADVTTSAEVTTTDEITTTDVVTTTGDVTPTAESTTTDDVTTTSDVTTTGDFTTTADVTTTAY
VTTTPDVTTTVEVTTTADFTTTGVVNTTADVTTTADVTTTSEVTTTADLTTTPEITTTVDFTSTADVTTTADV
ITTPEVTTTSDVTTTAEVITTADVTTTADVTTTVEVTTTGDVTITADVTTTPEITTTVDFTSTADVTITADVT
TTADVTTTSDVTITPDVTTTAEVTTTVEITTTDDVTTTGEVTTTADVTTTSDVTTTSEETTTADITTTSDVST
TSEVTTTAYVTTTADTTSTAEVTTTADVTTTADVTTTADVTTTADVTTTVEVTTTGDVTTTADVTTTAEVTTT
AEITTTGDVTTTADVTTTAEVTTTAEITTTGDVTTTADVTTTADVTTTADVTTTIDVTTTADVTTSAEVTTTA
DVTTTTEVTTTTEVATTPEPTTTPAPTTTAVNCMNGGFWTGDKCICPNGFGGDRCENIVNVVNCENGGTWDGL
KCQCTSLFYGPRCEELVESVEIEPTVAASVEVSVTVTSQEYSEKLQDRKSEEFSNFNKTFTKQMALIYAGIPE
YEGVIIKNLSKGSIVVDYDVILKAKYTPGFENTLDTVVKNLETKIKNATEVQVQDVNNNCSALLCFNSTATKV
QNSATVSVNPEETCKKEAGEDFAKFVTLGQKGDKWFCITPCSAGYSTSKNCSYGKCQLQRSGPQCLCLITDTH
WYSGENCDWGIQKSLVYGLGGAGVAVLLVILVILLVFSIRFRKDAQRQRSRVSEMYKWGEEEGRASPGTFHNF
GFDHNEERENYMPLDSVYNTFQPSLNHINPERKIQIQRPQVVMTSL*
>ENSMUSP00000072406_c.4533:G>C_p.A1512P ENSMUST00000072607.9|Ints1-201|ENSMUSG00000029547|-|chr5:g.139744221:C>G
MTERASDSRRLLPGEGASSRGARAAGRMNRAKPTTVRRPSAAAKPSGHPPPGDFIALGSKGQASESKTTSTLL
KPAPSGLPSERKRDASASLSGTSALTGLTKRPKLSSTPPLSALGRLAEAAVAEKRAISPSIKEPSVVPIEVLP
TVLLDEIEAAELEGNDDRIEGVLCGAVKQLKVTRAKPDSTLYLSLMYLAKIKPNIFATEGVIEALCSLLRRDA
SVNFKAKGNSLVSVLACNLLMAAYEEDENWPEIFVKVYIEDSLGERIWVDSPHCRTFVDNIQTAFNTKMPPKS
VLLQGEGARSGGELGAGSSPHPSLTEEEDSQTELLIAEEKLSPEQEGQLMPRYDELTESVEEYVLDMLRDQLN
RRQPIDNVSRNLLRLLTATCGYKEVRLLAVQRLEMWLQNPKLTRPAQDLLMSVCMNCNSHGSEDMDVISHLIK
IRLKPKVLLNHYMLCIRELLNAHKDNLGTTIKFVIFNELSNARNPNNMQILYTVLQHSSELAPKFLAMVFQDL
LTNKDDYLRASRALLREIIKQTKHEINFQAFCLGLMQERKEPQYLEMEFKERFVVHITDVLAVSMMLGITAQV
KEAGVAWDKGEKRNLEVLRTFQNQIAAIQRDAVWWLHTVVPSVSKLAPKDYVHCLHKVLFTEQPETYYKWDNW
PPESDRNFFLRLCSEVPILEDTLMRVLVIGLSRELPLGPADAMELADHLVKRAAAVQADDVEVLKVERIQLID
AVLNLCTYHHPENIQLPPGYQPPNLAISTLYWKAWPLLLVVAAFNPENIGLAAWEEYPTLKMLMEMVMTNNYS
YPPCTLTDEETRTEMINRELQISQREKQEILAFEGHLAAASTKQTITESSSLLLSQLTSLDPQGPPRRPPPHI
LDQVKALNQSLRLGHLLCRSRNPDFLLHIIQRQASSQSMPWLADLVQSSEGSLDVLPVQCLCEFLLHDAADST
ASGEEDDEGESREQKAKKRQRQQKQRQLLGRLQDLLLGPKADEQTTCEVLDYFLRRLGSSQVASRVLAMKGLS
LVLSEGGLRDKEEKEPPMEEDIGETDALQGYQWLLRDLPRLPLFDSVRTTTALALQQAIHMETDPQTISAYLI
YLSQHTPVEEQGPHSDLALDVARLVVERSTIMAHLFSKPSCSTASDAVLSALLSVFSRYVRRMRKSKEGEEVY
SWSESQDQVFLRWSSGETATMHILVVHAMVILLTLGPPRSGDSEFSELLDIWFPEKKPLPTAFLVDTSEEALL
LPDWLKLRMIRSEVPRLVDAALQDLEPQQLLLFVQSFGIPVSSMSKLLQYLDQAVAQDPQTLEQNIMDKNYMA
HLVEVQHERGASGGQTFHSLLTASLPPRRDSTEAPKPESSPEPPPGQGRTRAGTQVPVLGPEDDLAGIFLQIF
PLSPDPRWQSSSPRPLALALQQALGQELARVRQGNPEVPGITVRLLQAMTTLLSSPHGGTLALAMHHSHFLSC
PLMRQLYQYQRAVPQDTGFSSLFLKVLMQILQWLDSPAVEDGPLQAQLKLFPTRYSARHRISDVRSGLLHLAD

ALSFHGDLEVANSTARAVIATLRSGEKCPVEPELISKVLRGLIEVRSPHLEELLTALFSATTETSCPSPASGP
IVVVSSLLLQEKEELLGPSKQEVEGASTEAMRLGPASGLLVDWLETLDPEVVCSCPDLQWKLLFSRRKGKGHI
SAQVLSFRPYLLALLTHQASWSTLHCCIRVLLGKSREQRLDPSASLDFLWACIHVPRIWQGRDQRTPQKRREE
LVLHVQGPELLSLVELILSEAETRSQDGDSAARTLIQTRLPLLLSCCRSNDESIGKVTEHLTSCIQQWGDSVL
GQRCRDLLLQLYLQRPEVRVPVPEVLLQSEGATSSSICKLDGLVHRFITLLADTSDSRSSESRVADANMACRK
LAVAHPVLLLRHLPMIAALLHGRTHLNFQEFRQQNHLAFFLHVLGILELLQPRVFQSEHQGALWDCLRSFIRL
LLNYRKSSRHLAPFISKFVQFIHKYVGCSAPAAVAFLQKHAEPLHDLSFDNSDLVMLKSLLAGLSLPSRDGRT
DQGLDEEGEDERSAGSLPLVSVSLSTPLTVADVAPHMKRLSRGRAVEDVLETLSDIDEMSRRRPEVLGFFSTN
LQRLMSSAEESCRNLAFSLALRSIQNNPSIAADFLPTFMYCLGSRDFEVVQTALRNLPEYTLLCQEHAAVLLH
RAFLVGVYGQIDTSAQISEALKILHMEAVM*
>ENSMUSP00000143789_c.4539:G>C_p.A1514P ENSMUST00000200393.5|Ints1-
210|ENSMUSG00000029547|-|chr5:g.139744221:C>G
MTERASDSRRLLPGEGASSRGARAAGRMNRAKPTTVRRPSAAAKPSGHPPPGDFIALGSKGQASESKTTSTLL
KPAPSGLPSERKRDASASLSGTSALTGLTKRPKLSSTPPLSALGRLAEAAVAEKRAISPSIKEPSVVPIEVLP
TVLLDEIEAAELEGNDDRIEGVLCGAVKQLKVTRAKPDSTLYLSLMYLAKIKPNIFATEGVIEALCSLLRRDA
SVNFKAKGNSLVSVLACNLLMAAYEEDENWPEIFVKVYIEDSLGERIWVDSPHCRTFVDNIQTAFNTKMPPKS
VLLQGEGARSGGELGAGSSPHPSLTEEEDSQTELLIAEEKLSPEQEGQLMPRPRYDELTESVEEYVLDMLRDQ
LNRRQPIDNVSRNLLRLLTATCGYKEVRLLAVQRLEMWLQNPKLTRPAQDLLMSVCMNCNSHGSEDMDVISHL
IKIRLKPKVLLNHYMLCIRELLNAHKDNLGTTIKFVIFNELSNARNPNNMQILYTVLQHSSELAPKFLAMVFQ
DLLTNKDDYLRASRALLREIIKQTKHEINFQAFCLGLMQERKEPQYLEMEFKERFVVHITDVLAVSMMLGITA
QVKEAGVAWDKGEKRNLEVLRTFQNQIAAIQRDAVWWLHTVVPSVSKLAPKDYVHCLHKVLFTEQPETYYKWD
NWPPESDRNFFLRLCSEVPILEDTLMRVLVIGLSRELPLGPADAMELADHLVKRAAAVQADDVEVLKVERIQL
IDAVLNLCTYHHPENIQLPPGYQPPNLAISTLYWKAWPLLLVVAAFNPENIGLAAWEEYPTLKMLMEMVMTNN
YSYPPCTLTDEETRTEMINRELQISQREKQEILAFEGHLAAASTKQTITESSSLLLSQLTSLDPQGPPRRPPP
HILDQVKALNQSLRLGHLLCRSRNPDFLLHIIQRQASSQSMPWLADLVQSSEGSLDVLPVQCLCEFLLHDAAD
STASGEEDDEGESREQKAKKRQRQQKQRQLLGRLQDLLLGPKADEQTTCEVLDYFLRRLGSSQVASRVLAMKG
LSLVLSEGGLRDKEEKEPPMEEDIGETDALQGYQWLLRDLPRLPLFDSVRTTTALALQQAIHMETDPQTISAY
LIYLSQHTPVEEQGPHSDLALDVARLVVERSTIMAHLFSKPSCSTASDAVLSALLSVFSRYVRRMRKSKEGEE
VYSWSESQDQVFLRWSSGETATMHILVVHAMVILLTLGPPRSGDSEFSELLDIWFPEKKPLPTAFLVDTSEEA
LLLPDWLKLRMIRSEVPRLVDAALQDLEPQQLLLFVQSFGIPVSSMSKLLQYLDQAVAQDPQTLEQNIMDKNY
MAHLVEVQHERGASGGQTFHSLLTASLPPRRDSTEAPKPESSPEPPPGQGRTRAGTQVPVLGPEDDLAGIFLQ
IFPLSPDPRWQSSSPRPLALALQQALGQELARVRQGNPEVPGITVRLLQAMTTLLSSPHGGTLALAMHHSHFL
SCPLMRQLYQYQRAVPQDTGFSSLFLKVLMQILQWLDSPAVEDGPLQAQLKLFPTRYSARHRISDVRSGLLHL
ADALSFHGDLEVANSTARAVIATLRSGEKCPVEPELISKVLRGLIEVRSPHLEELLTALFSATTETSCPSPAS
GPIVVVSSLLLQEKEELLGPSKQEVEGASTEAMRLGPASGLLVDWLETLDPEVVCSCPDLQWKLLFSRRKGKG
HISAQVLSFRPYLLALLTHQASWSTLHCCIRVLLGKSREQRLDPSASLDFLWACIHVPRIWQGRDQRTPQKRR
EELVLHVQGPELLSLVELILSEAETRSQDGDSAARTLIQTRLPLLLSCCRSNDESIGKVTEHLTSCIQQWGDS
VLGQRCRDLLLQLYLQRPEVRVPVPEVLLQSEGATSSSICKLDGLVHRFITLLADTSDSRSSESRVADANMAC
RKLAVAHPVLLLRHLPMIAALLHGRTHLNFQEFRQQNHLAFFLHVLGILELLQPRVFQSEHQGALWDCLRSFI
RLLLNYRKSSRHLAPFISKFVQFIHKYVGCSAPAAVAFLQKHAEPLHDLSFDNSDLVMLKSLLAGLSLPSRDG
RTDQGLDEEGEDERSAGSLPLVSVSLSTPLTVADVAPHMKRLSRGRAVEDVLETLSDIDEMSRRRPEVLGFFS
TNLQRLMSSAEESCRNLAFSLALRSIQNNPSIAADFLPTFMYCLGSRDFEVVQTALRNLPEYTLLCQEHAAVL
LHRAFLVGVYGQIDTSAQISEALKILHMEAVM*
>ENSMUSP00000041863_c.1314:C>G_p.L439V ENSMUST00000038699.13|Ap5z1-
201|ENSMUSG00000039623|+|chr5:g.142459666:C>G
MFSAGAESLLHQAREIQDEELRRFCSRVTKLLQEAPGPATVDALQRLFLIVSATKYPRRLEKMCVDLLQTTLC
LPASPEQLQVLCAAILREMSPFNDLALSCDHTPNTRQLSLVASVLLAQGDRKGEIRCVSQRIFKILENRQPEG
PSVRPLLPILSKVIGLAPGILMEDQTNLLSKRLVDWLRYASIQQGLPYSGGFFSTPRTRQPGPITEVDGAVAS
DFFTVLSTGQHFTEDQWVNMQAFSMLRKWLLHSGPEDPCSPDADDKSELEGSTLSVLSAASTASRLLPPRERL
REVAFEYCQRLLEQSNRRALRKGDSDLQKACLVEAVSVLDVLCRQDPSFLYRTLSCLKALHRRLGEDPGSERA
LVPLAQFFLNHGEAAAMDAEAVYGQLLRGLPSERFHSPTLAFEVIHFCTHNLALFDSHFLSLLRLSFPSLFKF
VAWNSPPLTAEFVVLLPALVDAGTAVEMLHALLDLPCLTAALDLQLRSTQTPSERLLWDISLRVPSCLEAFQD
PQFQGLFRHLLRTKASGSTERLTPLHQVLKPMASCARVTQCAEAVPVLLQAFFSAVTQTADGALINQLALLLL
ERSDSLYPVPQYEARVHGVLSSQLLVLCKLKPSLVVELSRELLEFVGSVSSIHSRASVFTCVVWAIGEYLSVT
WDKRCTAEQINKFFEALEALLFEVTQSRPLADLPCCPPEVVTALMTTLTKLASRSQDLIPRVSLFLSKMRTLA
QNPATSSVHSEEGAESIRTRASELLTLLKMPSVAQFVFTPPAGVCQPRYHRDTNVALPLALRTVSRLVEKEAG
LLPG*

>ENSMUSP00000143179_c.1314:C>G_p.L439V ENSMUST00000196055.2|Ap5z1-202|ENSMUSG00000039623|+|chr5:g.142459666:C>G
MFSAGAESLLHQAREIQDEELRRFCSRVTKLLQEAPGPATVDALQRLFLIVSATKYPRRLEKMCVDLLQTTLC
LPASPEQLQVLCAAILREMSPFNDLALSCDHTPNTRQLSLVASVLLAQGDRKGEIRCVSQRIFKILENRQPEG
PSVRPLLPILSKVIGLAPGILMEDQTNLLSKRLVDWLRYASIQQGLPYSGGFFSTPRTRQPGPITEVDGAVAS
DFFTVLSTGQHFTEDQWVNMQAFSMLRKWLLHSGPEDPCSPDADDKSELEGSTLSVLSAASTASRLLPPRERL
REVAFEYCQRLLEQSNRRALRKGDSDLQKACLVEAVSVLDVLCRQDPSFLYRTLSCLKALHRRLGEDPGSERA
LVPLAQFFLNHGEAAAMDAEAVYGQLLRGLPSERFHSPTLAFEVIHFCTHNLALFDSHFLSLLRLSFPSLFKF
VAWNSPPLTAEFVVLLPALVDAGTAVEMLHALLDLPCLTAALDLQLRVPSCLEAFQDPQFQGLFRHLLRTKAS
GSTERLTPLHQVLKPMASCARVTQCAEAVPVLLQAFFSAVTQTADGALINQLALLLLERSDSLYPVPQYEARV
HGVLSSQLLVLCKLKPSLVVELSRELLEFVGSVSSIHSRASVFTCVVWAIGEYLSVTWDKRCTAEQINKFFEA
LEALLFEVTQSRPLADLPCCPPEVVTALMTTLTKLASRSQDLIPRVSLFLSKMRTLAQNPATSSVHSEEGAES
IRTRASELLTLLKMPSVAQFVFTPPAGVCQPRYHRDTNVALPLALRTVSRLVEKEAGLLPG*
>ENSMUSP00000064539_c.3039:C>G_p.R1014G ENSMUST00000063635.15|Radil-201|ENSMUSG00000029576|-|chr5:g.142471235:G>C
MFYGTQLIMSPPTKNKLKRQSQLLSTMLSRTLSYKYRDLDSTFCSLGASDDPSELSTQLSAPGVLKVFGDSVC
TGTHYKSVLATGSSSAQELVKEALERYALDPECAGQYVLCDVVGQAGDSGQRWQAQCFRVFGDNEKPLLIQEL
WKPREGLSRRFELRKKSDVEELASRDVDTTTAGINAQARRLQRIRAKGTPALTSEAAQSSPPTRLRRTVSETS
LSPAPSLPEAAQRPEEPVPEAMRYSLYQCPHLLLLQGYSQQHDSLVYVLSKERHTVGQRTPSSKPSISLSAPD
ILPLHCTIRRHQSPEGGPAGTRLVLEPITGASVSVNFSEVGRNPVVLQHGDLLSLGLYYLLLFKDPGQAQPLP
ACALARLGAAPQSCRMCGAVLRARGAPSLPAAVVRRRSLLLEFEPDVEDTLLQRIMTLIEPGGDDHKLTPAFL
LCLCIQHSAMHFQPGTFRHLLLKISKRVRDTVWEKTKELAEKQAQLQEPISWASFPMADLVPDLQHILFWMSN
SIELLYFIQQKSPLYVQSMEEELDVTGSKESLFSCTLTASEEAMAALEEVVLYAFQQCVYYLSKCLYVCLPAL
LECPPFQTERRESWRSGPALPEELRRVVSVFQATLDLLQQLQMHPEVASQMLAYLFFFSGTLLLNQVLDKGPS
LSCFHWPRGVQVCARLQQFLEWARSAGLGAPAERFFRKLSCTLHLLATPRAQLIQMSWATLRVTFPALNPAQL
HRLLTQYQLASAMGPVSAWEPGAPDGPEAFQSEDILESYENPPPIVLPSQGFQVDLEADCVEDSIYQHLLYIR
HFLWGLRGQASPDSGPAQPESIEGLYHTIPEGHLEGHGCPLANRDPGRVAVETAPPHSLPVTGAPRAQGPPGR
QPTRGDRRGSQAGSLHTDSSCMLTPPSTPLGLEPAGPSWPEPSGLCGRAVLDGQRNGPGGLPGAVLEGDAIQD
AEPPAEASSPSSSAEDFCYVFMVELERGPSGLGMGLIDGMHTPLGAQGLYIQTLLPGSPAASDGGLSLGDQIL
EVNGSSLRGVSYMRAVDLIRHGGKKMRFLVAKSDMETAKKIRFRNPPS*
>ENSMUSP00000082910_c.3126:C>G_p.R1043G ENSMUST00000085758.10|Radil-202|ENSMUSG00000029576|-|chr5:g.142471235:G>C
MGSSIFLGLQPSPSHWLKSSVVIHEDAPTMFYGTQLIMSPPTKNKLKRQSQLLSTMLSRTLSYKYRDLDSTFC
SLGASDDPSELSTQLSAPGVLKVFGDSVCTGTHYKSVLATGSSSAQELVKEALERYALDPECAGQYVLCDVVG
QAGDSGQRWQAQCFRVFGDNEKPLLIQELWKPREGLSRRFELRKKSDVEELASRDVDTTTAGINAQARRLQRI
RAKGTPALTSEAAQSSPPTRLRRTVSETSLSPAPSLPEAAQRPEEPVPEAMRYSLYQCPHLLLLQGYSQQHDS
LVYVLSKERHTVGQRTPSSKPSISLSAPDILPLHCTIRRHQSPEGGPAGTRLVLEPITGASVSVNFSEVGRNP
VVLQHGDLLSLGLYYLLLFKDPGQAQPLPACALARLGAAPQSCRMCGAVLRARGAPSLPAAVVRRRSLLLEFE
PDVEDTLLQRIMTLIEPGGDDHKLTPAFLLCLCIQHSAMHFQPGTFRHLLLKISKRVRDTVWEKTKELAEKQA
QLQEPISWASFPMADLVPDLQHILFWMSNSIELLYFIQQKSPLYVQSMEEELDVTGSKESLFSCTLTASEEAM
AALEEVVLYAFQQCVYYLSKCLYVCLPALLECPPFQTERRESWRSGPALPEELRRVVSVFQATLDLLQQLQMH
PEVASQMLAYLFFFSGTLLLNQVLDKGPSLSCFHWPRGVQVCARLQQFLEWARSAGLGAPAERFFRKLSCTLH
LLATPRAQLIQMSWATLRVTFPALNPAQLHRLLTQYQLASAMGPVSAWEPGAPDGPEAFQSEDILESYENPPP
IVLPSQGFQVDLEADCVEDSIYQHLLYIRHFLWGLRGQASPDSGPAQPESIEGLYHTIPEGHLEGHGCPLANR
DPGRVAVETAPPHSLPVTGAPRAQGPPGRQPTRGDRRGSQAGSLHTDSSCMLTPPSTPLGLEPAGPSWPEPSG
LCGRAVLDGQRNGPGGLPGAVLEGDAIQDAEPPAEASSPSSSAEDFCYVFMVELERGPSGLGMGLIDGMHTPL
GAQGLYIQTLLPGSPAASDGGLSLGDQILEVNGSSLRGVSYMRAVDLIRHGGKKMRFLVAKSDMETAKKIRFR
NPPS*
>ENSMUSP00000106411_c.2319:C>G_p.R774G ENSMUST00000110784.8|Radil-203|ENSMUSG00000029576|-|chr5:g.142471235:G>C
MRYSLYQCPHLLLLQGYSQQHDSLVYVLSKERHTVGQRTPSSKPSISLSAPDILPLHCTIRRHQSPEGGPAGT
RLVLEPITGASVSVNFSEVGRNPVVLQHGDLLSLGLYYLLLFKDPGQAQPLPACALARLGAAPQSCRMCGAVL
RARGAPSLPAAVVRRRSLLLEFEPDVEDTLLQRIMTLIEPGGDDHKLTPAFLLCLCIQHSAMHFQPGTFRHLL
LKISKRVRDTVWEKTKELAEKQAQLQEPISWASFPMADLVPDLQHILFWMSNSIELLYFIQQKSPLYVQSMEE
ELDVTGSKESLFSCTLTASEEAMAALEEVVLYAFQQCVYYLSKCLYVCLPALLECPPFQTERRESWRSGPALP
EELRRVVSVFQATLDLLQQLQMHPEVASQMLAYLFFFSGTLLLNQVLDKGPSLSCFHWPRGVQVCARLQQFLE
WARSAGLGAPAERFFRKLSCTLHLLATPRAQLIQMSWATLRVTFPALNPAQLHRLLTQYQLASAMGPVSAWEP
GAPDGPEAFQSEDILESYENPPPIVLPSQGFQVDLEADCVEDSIYQHLLYIRHFLWGLRGQASPDSGPAQPES

IEGLYHTIPEGHLEGHGCPLANRDPGRVAVETAPPHSLPVTGAPRAQGPPGRQPTRGDRRGSQAGSLHTDSSC
MLTPPSTPLGLEPAGPSWPEPSGLCGRAVLDGQRNGPGGLPGAVLEGDAIQDAEPPAEASSPSSSAEDFCYVF
MVELERGPSGLGMGLIDGMHTPLGAQGLYIQTLLPGSPAASDGGLSLGDQILEVNGSSLRGVSYMRAVDLIRH
GGKKMRFLVAKSDMETAKKIRFRNPPS*
>ENSMUSP00000106412_c.3108:C>G_p.R1037G ENSMUST00000110785.2|Radil-204|ENSMUSG00000029576|-|chr5:g.142471235:G>C
MFYGTQLIMSPPTKNKLKRQSQLLSTMLSRTLSYKYRDLDSTFCSLGASDDPSELSTQLSAPGVLKVFGDSVC
TGTHYKSVLATGSSSAQELVKEALERYALDPECAGQYVLCDVVGQAGDSGQRWQAQCFRVFGDNEKPLLIQEL
WKPREGLSRRFELRKKSDVEELASRDVDTTTAGINAQARRLQRIRAKGTPALTSEAAQSSPPTRLRRTVSETS
LSPAPSLPEAAQRPEEPVPEAMRYSLYQCPHLLLLQGYSQQHDSLVYVLSKERHTVGQRTPSSKPSISLSAPD
ILPLHCTIRRHQSPEGGPAGTRLVLEPITGASVSVNFSEVGRNPVVLQHGDLLSLGLYYLLLFKDPGQAQPLP
ACALARLGAAPQSCRMCGAVLRARGAPSLPAAVVRRRSLLLEFEPDVEDTLLQRIMTLIEPGGDDHKLTPAFL
LCLCIQHSAMHFQPGTFRHLLLKISKRVRDTVWEKTKELAEKQAQLQEPISWASFPMADLVPDLQHILFWMSN
SIELLYFIQQKSPLYVQSMEEELDVTGSKESLFSCTLTASEEAMAALEEVVLYAFQQCVYYLSKCLYVCLPAL
LECPPFQTERRESWRSGPALPEELRRVVSVFQATLDLLQQLQMHPEVASQMLAYLFFFSGTLLLNQVLDKGPS
LSCFHWPRGVQVCARLQQFLEWARSAGLGAPAERFFRKLSCTLHLLATPRAQLIQMSWATLRVTFPALNPAQL
HRLLTQYQLASAMGPVSAWEPGAPDGPEAFQSEDILESYENPPPIVLPSQGFQVDLEADCVEDSIYQHLLYIR
HFLWGLRGQASPDSGPAQPESIEGAIMDTWAIPSSGPQRLSTFSSQGLYHTIPEGHLEGHGCPLANRDPGRVA
VETAPPHSLPVTGAPRAQGPPGRQPTRGDRRGSQAGSLHTDSSCMLTPPSTPLGLEPAGPSWPEPSGLCGRAV
LDGQRNGPGGLPGAVLEGDAIQDAEPPAEASSPSSSAEDFCYVFMVELERGPSGLGMGLIDGMHTPLGAQGLY
IQTLLPGSPAASDGGLSLGDQILEVNGSSLRGVSYMRAVDLIRHGGKKMRFLVAKSDMETAKKIRFRNPPS*
>ENSMUSP00000124899_c.117:GT>G_p.V40del-fs ENSMUST00000160629.8|Cpsf4-205|ENSMUSG00000029625|+|chr5:g.145107339:GT>G
MQEIIASVDHIKFDLEIAVEQQLGAQPLPFPGMDKSGAAAVNSF
>ENSMUSP00000069243_c.117:GT>G_p.V40del-fs ENSMUST00000070487.12|Cpsf4-201|ENSMUSG00000029625|+|chr5:g.145107339:GT>G
MQEIIASVDHIKFDLEIAVEQQLGAQPLPFPGMDKSGAAAVNSF
>ENSMUSP00000125019_c.117:GT>G_p.V40del-fs ENSMUST00000160422.8|Cpsf4-202|ENSMUSG00000029625|+|chr5:g.145107339:GT>G
MQEIIASVDHIKFDLEIAVEQQLGAQPLPFPGMDKSGAAAVNSF
>ENSMUSP00000124966_c.117:GT>G_p.V40del-fs ENSMUST00000162244.8|Cpsf4-207|ENSMUSG00000029625|+|chr5:g.145107339:GT>G
MQEIIASVDHIKFDLEIAVEQQLGAQPLPFPGMDKSGAAAVNSF
>ENSMUSP00000137910_c.117:GT>G_p.V40del-fs ENSMUST00000160574.8|Cpsf4-204|ENSMUSG00000029625|+|chr5:g.145107339:GT>G
MQEIIASVDHIKFDLEIAVEQQLGAQPLPFPGMDKSGAAAVNSF
>ENSMUSP00000125640_c.21:GT>G_p.V8del-fs ENSMUST00000160762.2|Cpsf4-206|ENSMUSG00000029625|+|chr5:g.145107339:GT>G
XDKSGAAAVNSF
>ENSMUSP00000124899_c.118:T>TGG_p.V40ins-fs ENSMUST00000160629.8|Cpsf4-205|ENSMUSG00000029625|+|chr5:g.145107340:T>TGG
MQEIIASVDHIKFDLEIAVEQQLGAQPLPFPGMDKSGAAVAVNSF
>ENSMUSP00000069243_c.118:T>TGG_p.V40ins-fs ENSMUST00000070487.12|Cpsf4-201|ENSMUSG00000029625|+|chr5:g.145107340:T>TGG
MQEIIASVDHIKFDLEIAVEQQLGAQPLPFPGMDKSGAAVAVNSF
>ENSMUSP00000125019_c.118:T>TGG_p.V40ins-fs ENSMUST00000160422.8|Cpsf4-202|ENSMUSG00000029625|+|chr5:g.145107340:T>TGG
MQEIIASVDHIKFDLEIAVEQQLGAQPLPFPGMDKSGAAVAVNSF
>ENSMUSP00000124966_c.118:T>TGG_p.V40ins-fs ENSMUST00000162244.8|Cpsf4-207|ENSMUSG00000029625|+|chr5:g.145107340:T>TGG
MQEIIASVDHIKFDLEIAVEQQLGAQPLPFPGMDKSGAAVAVNSF
>ENSMUSP00000137910_c.118:T>TGG_p.V40ins-fs ENSMUST00000160574.8|Cpsf4-204|ENSMUSG00000029625|+|chr5:g.145107340:T>TGG
MQEIIASVDHIKFDLEIAVEQQLGAQPLPFPGMDKSGAAVAVNSF
>ENSMUSP00000125640_c.22:T>TGG_p.V8ins-fs ENSMUST00000160762.2|Cpsf4-206|ENSMUSG00000029625|+|chr5:g.145107340:T>TGG
XDKSGAAVAVNSF

>rs108505794_ENSMUSP00000091659_c.1296:T>C_p.Y433H
ENSMUST00000094111.6|Cyp3a41a-201|ENSMUSG00000075551|-
|chr5:g.145635069:A>G
MNLFSALSLDTWVLLAIILVLLYRYGTRTHGLFKKQGIPGPKPLPFLGTVLNYYKGLWKFDMECYEKYGKTWG
LFDGQMPLFVITDPEMIKNVLVKECFSVFTNRREFGPVGIMSKAISISKDEEWKRYRALLSPTFTSGKLKEMF
PVIEQYGDILVKYLMQEAEKGKPVTMKDVLGAYSIDVITSTSFGVNVDSLNNPEDPFVEKAKGILRVDFFDPL
VFSVVLFPFLTPVYEMLNICMFPKDSIEFFKKFVNRMKESRLDSKQKHRVDFLQLMMNAHNNSKDKDSHKALS
DMEITAQSIVFIFAGYETTSSTLSFTLYCLATHPDIQKKLQEEIDETLPNKAPPTYDTVMEMEYLDMVLNETL
RLYPIGNRLERFCKKDVELNGVYIPKGSTVMIPSYALHHDPQHWPEPEEFQPERFSKENKGSIDPYLHMPFGI
GPRNCIGMRFAFMTMKLALTKVMQNFSFQPCQETQIPLKLSRQGLLQPEKPIVLKVVPRDVVITGA*
>ENSMUSP00000091659_c.877:A>C_p.D293A ENSMUST00000094111.6|Cyp3a41a-201|ENSMUSG00000075551|-|chr5:g.145639089:T>G
MNLFSALSLDTWVLLAIILVLLYRYGTRTHGLFKKQGIPGPKPLPFLGTVLNYYKGLWKFDMECYEKYGKTWG
LFDGQMPLFVITDPEMIKNVLVKECFSVFTNRREFGPVGIMSKAISISKDEEWKRYRALLSPTFTSGKLKEMF
PVIEQYGDILVKYLMQEAEKGKPVTMKDVLGAYSIDVITSTSFGVNVDSLNNPEDPFVEKAKGILRVDFFDPL
VFSVVLFPFLTPVYEMLNICMFPKDSIEFFKKFVNRMKESRLDSKQKHRVDFLQLMMNAHNNSKDKDSHKALS
AMEITAQSIVFIFAGYETTSSTLSFTLYCLATHPDIQKKLQEEIDETLPNKAPPTYDTVMEMEYLDMVLNETL
RLYPIGNRLERFCKKDVELNGVYIPKGSTVMIPSYALHHDPQHWPEPEEFQPERFSKENKGSIDPYLYMPFGI
GPRNCIGMRFAFMTMKLALTKVMQNFSFQPCQETQIPLKLSRQGLLQPEKPIVLKVVPRDVVITGA*
>rs108243600_ENSMUSP00000091659_c.493:A>G_p.E165G
ENSMUST00000094111.6|Cyp3a41a-201|ENSMUSG00000075551|-
|chr5:g.145645714:T>C
MNLFSALSLDTWVLLAIILVLLYRYGTRTHGLFKKQGIPGPKPLPFLGTVLNYYKGLWKFDMECYEKYGKTWG
LFDGQMPLFVITDPEMIKNVLVKECFSVFTNRREFGPVGIMSKAISISKDEEWKRYRALLSPTFTSGKLKEMF
PVIEQYGDILVKYLMQEAGKGKPVTMKDVLGAYSIDVITSTSFGVNVDSLNNPEDPFVEKAKGILRVDFFDPL
VFSVVLFPFLTPVYEMLNICMFPKDSIEFFKKFVNRMKESRLDSKQKHRVDFLQLMMNAHNNSKDKDSHKALS
DMEITAQSIVFIFAGYETTSSTLSFTLYCLATHPDIQKKLQEEIDETLPNKAPPTYDTVMEMEYLDMVLNETL
RLYPIGNRLERFCKKDVELNGVYIPKGSTVMIPSYALHHDPQHWPEPEEFQPERFSKENKGSIDPYLYMPFGI
GPRNCIGMRFAFMTMKLALTKVMQNFSFQPCQETQIPLKLSRQGLLQPEKPIVLKVVPRDVVITGA*
>ENSMUSP00000091659_c.376:G>T_p.W126L ENSMUST00000094111.6|Cyp3a41a-201|ENSMUSG00000075551|-|chr5:g.145645910:C>A
MNLFSALSLDTWVLLAIILVLLYRYGTRTHGLFKKQGIPGPKPLPFLGTVLNYYKGLWKFDMECYEKYGKTWG
LFDGQMPLFVITDPEMIKNVLVKECFSVFTNRREFGPVGIMSKAISISKDEELKRYRALLSPTFTSGKLKEMF
PVIEQYGDILVKYLMQEAEKGKPVTMKDVLGAYSIDVITSTSFGVNVDSLNNPEDPFVEKAKGILRVDFFDPL
VFSVVLFPFLTPVYEMLNICMFPKDSIEFFKKFVNRMKESRLDSKQKHRVDFLQLMMNAHNNSKDKDSHKALS
DMEITAQSIVFIFAGYETTSSTLSFTLYCLATHPDIQKKLQEEIDETLPNKAPPTYDTVMEMEYLDMVLNETL
RLYPIGNRLERFCKKDVELNGVYIPKGSTVMIPSYALHHDPQHWPEPEEFQPERFSKENKGSIDPYLYMPFGI
GPRNCIGMRFAFMTMKLALTKVMQNFSFQPCQETQIPLKLSRQGLLQPEKPIVLKVVPRDVVITGA*
>ENSMUSP00000091659_c.291:T>C_p.C98R ENSMUST00000094111.6|Cyp3a41a-201|ENSMUSG00000075551|-|chr5:g.145650350:A>G
MNLFSALSLDTWVLLAIILVLLYRYGTRTHGLFKKQGIPGPKPLPFLGTVLNYYKGLWKFDMECYEKYGKTWG
LFDGQMPLFVITDPEMIKNVLVKERFSVFTNRREFGPVGIMSKAISISKDEEWKRYRALLSPTFTSGKLKEMF
PVIEQYGDILVKYLMQEAEKGKPVTMKDVLGAYSIDVITSTSFGVNVDSLNNPEDPFVEKAKGILRVDFFDPL
VFSVVLFPFLTPVYEMLNICMFPKDSIEFFKKFVNRMKESRLDSKQKHRVDFLQLMMNAHNNSKDKDSHKALS
DMEITAQSIVFIFAGYETTSSTLSFTLYCLATHPDIQKKLQEEIDETLPNKAPPTYDTVMEMEYLDMVLNETL
RLYPIGNRLERFCKKDVELNGVYIPKGSTVMIPSYALHHDPQHWPEPEEFQPERFSKENKGSIDPYLYMPFGI
GPRNCIGMRFAFMTMKLALTKVMQNFSFQPCQETQIPLKLSRQGLLQPEKPIVLKVVPRDVVITGA*
>rs107987353_ENSMUSP00000091659_c.111:A>G_p.I38V
ENSMUST00000094111.6|Cyp3a41a-201|ENSMUSG00000075551|-
|chr5:g.145654788:T>C
MNLFSALSLDTWVLLAIILVLLYRYGTRTHGLFKKQGVPGPKPLPFLGTVLNYYKGLWKFDMECYEKYGKTWG
LFDGQMPLFVITDPEMIKNVLVKECFSVFTNRREFGPVGIMSKAISISKDEEWKRYRALLSPTFTSGKLKEMF
PVIEQYGDILVKYLMQEAEKGKPVTMKDVLGAYSIDVITSTSFGVNVDSLNNPEDPFVEKAKGILRVDFFDPL
VFSVVLFPFLTPVYEMLNICMFPKDSIEFFKKFVNRMKESRLDSKQKHRVDFLQLMMNAHNNSKDKDSHKALS
DMEITAQSIVFIFAGYETTSSTLSFTLYCLATHPDIQKKLQEEIDETLPNKAPPTYDTVMEMEYLDMVLNETL
RLYPIGNRLERFCKKDVELNGVYIPKGSTVMIPSYALHHDPQHWPEPEEFQPERFSKENKGSIDPYLYMPFGI
GPRNCIGMRFAFMTMKLALTKVMQNFSFQPCQETQIPLKLSRQGLLQPEKPIVLKVVPRDVVITGA*

>ENSMUSP00000069932_c.972:C>T_p.H325Y ENSMUST00000067479.6|Cyp3a44-201|ENSMUSG00000054417|-|chr5:g.145725227:G>A
MNLFSALSLDTLVLLAIILVLLYRYGTRTHGLFKKQGIPGPKPLPFLGTVLNYYTGIWKFDMECYEKYGKTWG
LFDGQTPLLVITDPETIKNVLVKDCLSVFTNRREFGPVGIMSKAISISKDEEWKRYRALLSPTFTSGRLKEMF
PVIEQYGDILVKYLRQEAEKGMPVAMKDVLGAYSMDVITSTSFGVNVDSLNNPEDPFVEEAKKFLRVDFFDPL
LFSVVLFPLLTPVYEMLNICMFPNDSIEFFKKFVDRMQESRLDSNQKHRVDFLQLMMNSHNNSKDKDSHKAFS
NMEITVQSIIFISAGYETTSSTLSFTLYCLATYPDIQKKLQAEIDKALPNKATPTCDTVMEMEYLDMVLNETL
RLYPIVTRLERVCKKDVELNGVYIPKGSMVMIPSYALHHDPQHWPDPEEFQPERFSKENKGSIDPYVYLPFGI
GPRNCIGMRFALMNMKLAVTKVLQNFSFQPCQETQIPLKLSRQGILQPEKPIVLKVVPRDAVITGA*
>ENSMUSP00000069932_c.769:A>G_p.Q257R ENSMUST00000067479.6|Cyp3a44-201|ENSMUSG00000054417|-|chr5:g.145728453:T>C
MNLFSALSLDTLVLLAIILVLLYRYGTRTHGLFKKQGIPGPKPLPFLGTVLNYYTGIWKFDMECYEKYGKTWG
LFDGQTPLLVITDPETIKNVLVKDCLSVFTNRREFGPVGIMSKAISISKDEEWKRYRALLSPTFTSGRLKEMF
PVIEQYGDILVKYLRQEAEKGMPVAMKDVLGAYSMDVITSTSFGVNVDSLNNPEDPFVEEAKKFLRVDFFDPL
LFSVVLFPLLTPVYEMLNICMFPNDSIEFFKKFVDRMRESRLDSNQKHRVDFLQLMMNSHNNSKDKDSHKAFS
NMEITVQSIIFISAGYETTSSTLSFTLYCLATHPDIQKKLQAEIDKALPNKATPTCDTVMEMEYLDMVLNETL
RLYPIVTRLERVCKKDVELNGVYIPKGSMVMIPSYALHHDPQHWPDPEEFQPERFSKENKGSIDPYVYLPFGI
GPRNCIGMRFALMNMKLAVTKVLQNFSFQPCQETQIPLKLSRQGILQPEKPIVLKVVPRDAVITGA*
>ENSMUSP00000158111_c.547:T>C_p.F183S ENSMUST00000202355.2|Vmn1r261-201|ENSMUSG00000106893|-|chr6:g.3143954:A>G
MFGNSSILFYYIILIFTEKHLTPKDLIIEHLTFANYLTVILRGFPQTMSYFGFKNFLDDMGCKLIVYIS*ITR
GVSLYAMCLLSCFQAITTSPNNSRWIKLKYRATKYIGPSCSVSWLVHLLLNILTPLRESGTNYKKNATNRLSY
GYGSLFASDSVATTLYISLVCFSDVFCLGLMACSSISMVSILYRHKRQVKHIHSAQHFLKVSPEDRATQTILI
LLCTFVISYSFSSIVAIIRTYLNYPVIWGVNIFTSLEIFFPFFCPFVLITNMRSSSNLFLPCLGKR*
>rs48847283_ENSMUSP00000158111_c.220:G>A_p.G74E
ENSMUST00000202355.2|Vmn1r261-201|ENSMUSG00000106893|-|chr6:g.3144281:C>T
MFGNSSILFYYIILIFTEKHLTPKDLIIEHLTFANYLTVILRGFPQTMSYFGFKNFLDDMGCKLIVYIS*ITR
EVSLYAMCLLSCFQAITTSPNNSRWIKLKYRATKYIGPSCSVSWLVHLLLNILTPLRESGTNYKKNATNRLSY
GYGSLFASDSVATTLYISLVCFSDVFCLGLMACSSIFMVSILYRHKRQVKHIHSAQHFLKVSPEDRATQTILI
LLCTFVISYSFSSIVAIIRTYLNYPVIWGVNIFTSLEIFFPFFCPFVLITNMRSSSNLFLPCLGKR*
>ENSMUSP00000158111_c.207:T>C_p.70R ENSMUST00000202355.2|Vmn1r261-201|ENSMUSG00000106893|-|chr6:g.3144294:A>G
MFGNSSILFYYIILIFTEKHLTPKDLIIEHLTFANYLTVILRGFPQTMSYFGFKNFLDDMGCKLIVYISRITR
GVSLYAMCLLSCFQAITTSPNNSRWIKLKYRATKYIGPSCSVSWLVHLLLNILTPLRESGTNYKKNATNRLSY
GYGSLFASDSVATTLYISLVCFSDVFCLGLMACSSIFMVSILYRHKRQVKHIHSAQHFLKVSPEDRATQTILI
LLCTFVISYSFSSIVAIIRTYLNYPVIWGVNIFTSLEIFFPFFCPFVLITNMRSSSNLFLPCLGKR*
>ENSMUSP00000135076_c.862:C>A_p.P288Q ENSMUST00000176551.3|Peg10-203|ENSMUSG00000092035|+|chr6:g.4756288:C>A
MLQIHMPGRPTLFVRAMIDSGASGNFIDQDFVIQNAIPLRIKDWPVMVEAIDGHPIASGPIILETHHLIVDLG
DHREILSFDVTQSPFFPIVLGIRWLSTHDPHITWSTRSIVFNSDYCRLRCRMFAQIPSNLLFTVPQPNLHPYL
LHHVHPHVHPHMHQHLHQHLHQFLHPDPHQYPHPDPHYHHHQQADMQHQLQQYLYQYLYYHLYPVMHHHLPPD
QHEHLHEYLHQYLHQYLHQFLHHHLHPDLHQYLYQYLHNHMNPDPHHHPHPDPPQDPHHPPHQDPHQHQDPHQ
DPPHQDPHQHPDPHQDPPHQDPHQHPDPHQDPPHQDPHQDAHQHQDPHQDAHQDPHQDAHQDPHMDPHLHQHQ
HPQPQPHPQQHPNHPQQPPFFYHMAGFRIYHPVRYYYIQNVYTPVDEHVYPGHRVVDPNIEMIPGAHSLPSGH
LYSMSESEMNALRNFVDRNVKDGLMTPTVAPNGAQVLQVKRGWKLQVTYNCRAPQSGTIQNQYLRMSLPNMGD
PAHLASYGEFVQVPGYPYPAYVYYTSPHMMTAWYPVGRDVHGRIIVVPVVITWSQNTNRQPPVPQYPPPQPPP
PPPPPPPPPPPPASSCSAA*
>ENSMUSP00000127306_c.2065:C>A_p.P689Q ENSMUST00000166678.2|Peg10-201|ENSMUSG00000092035|+|chr6:g.4756288:C>A
MAAAGGSSNCPPPPPPPPPNNNNNNNTPKSPGVPDAEDDDERRHDELPEDINNFDEDMNRQFENMNLLDQVEL
LAQSYSLLDHLDDFDDDDEDDDFDPEPDQDELPEYSDDDDLELQGAAAAPIPNFFSDDDCLEDLPEKFDGNPD
MLGPFMYQCQLFMEKSTRDFSVDRIRVCFVTSMLIGRAARWATAKLQRCTYLMHNYTAFMMELKHVFEDPQRR
EAAKRKIRRLRQGPGPVVDYSNAFQMIAQDLDWTEPALMDQFQEGLNPDIRAELSRQEAPKTLAALITACIHI
ERRLARDAAAKPDPSPRALVMPPNSQTDPTEPVGGARMRLSKEEKERRRKMNLCLYCGNGGHFADTCPAKASK
NSPPGLPGPAVGGPSATGPERIRSPPSEASTQHLQVMLQIHMPGRPTLFVRAMIDSGASGNFIDQDFVIQNAI
PLRIKDWPVMVEAIDGHPIASGPIILETHHLIVDLGDHREILSFDVTQSPFFPIVLGIRWLSTHDPHITWSTR
SIVFNSDYCRLRCRMFAQIPSNLLFTVPQPNLHPYLLHHVHPHVHPHMHQHLHQHLHQFLHPDPHQYPHPDPH
YHHHQQADMQHQLQQYLYQYLYYHLYPVMHHHLPPDQHEHLHEYLHQYLHQYLHQFLHHHLHPDLHQYLYQYL

HNHMNPDPHHHPHPDPPQDPHHPPHQDPHQHQDPHQDPPHQDPHQHPDPHQDPPHQDPHQHPDPHQDPPHQDP
HQDAHQHQDPHQDAHQDPHQDAHQDPHMDPHLHQHQHPQPQPHPQQHPNHPQQPPFFYHMAGFRIYHPVRYYY
IQNVYTPVDEHVYPGHRVVDPNIEMIPGAHSLPSGHLYSMSESEMNALRNFVDRNVKDGLMTPTVAPNGAQVL
QVKRGWKLQVTYNCRAPQSGTIQNQYLRMSLPNMGDPAHLASYGEFVQVPGYPYPAYVYYTSPHMMTAWYPVG
RDVHGRIIVVPVVITWSQNTNRQPPVPQYPPPQPPPPPPPPPPPPPPPPASSCSAA*
>ENSMUSP00000135076_c.879:CCTC>G_p.PP294del ENSMUST00000176551.3|Peg10-
203|ENSMUSG00000092035|+|chr6:g.4756305:CCTC>G
MLQIHMPGRPTLFVRAMIDSGASGNFIDQDFVIQNAIPLRIKDWPVMVEAIDGHPIASGPIILETHHLIVDLG
DHREILSFDVTQSPFFPIVLGIRWLSTHDPHITWSTRSIVFNSDYCRLRCRMFAQIPSNLLFTVPQPNLHPYL
LHHVHPHVHPHMHQHLHQHLHQFLHPDPHQYPHPDPHYHHHQQADMQHQLQQYLYQYLYYHLYPVMHHHLPPD
QHEHLHEYLHQYLHQYLHQFLHHHLHPDLHQYLYQYLHNHMNPDPHHHPHPDPPQDPHHPPHQDPHQHPDPHQ
DAHQDPHQHPDPHQDPPHQDPHQHPDPHQDPPHQDPHQDAHQHQDPHQDAHQDPHQDAHQDPHMDPHLHQHQH
PQPQPHPQQHPNHPQQPPFFYHMAGFRIYHPVRYYYIQNVYTPVDEHVYPGHRVVDPNIEMIPGAHSLPSGHL
YSMSESEMNALRNFVDRNVKDGLMTPTVAPNGAQVLQVKRGWKLQVTYNCRAPQSGTIQNQYLRMSLPNMGDP
AHLASYGEFVQVPGYPYPAYVYYTSPHMMTAWYPVGRDVHGRIIVVPVVITWSQNTNRQPPVPQYPPPQPPPP
PPPPPPPPPPPPASSCSAA*
>ENSMUSP00000127306_c.2082:CCTC>G_p.PP695del ENSMUST00000166678.2|Peg10-
201|ENSMUSG00000092035|+|chr6:g.4756305:CCTC>G
MAAAGGSSNCPPPPPPPPPPNNNNNNNTPKSPGVPDAEDDDERRHDELPEDINNFDEDMNRQFENMNLLDQVEL
LAQSYSLLDHLDDFDDDDEDDDFDPEPDQDELPEYSDDDDLELQGAAAAPIPNFFSDDDCLEDLPEKFDGNPD
MLGPFMYQCQLFMEKSTRDFSVDRIRVCFVTSMLIGRAARWATAKLQRCTYLMHNYTAFMMELKHVFEDPQRR
EAAKRKIRRLRQGPGPVVDYSNAFQMIAQDLDWTEPALMDQFQEGLNPDIRAELSRQEAPKTLAALITACIHI
ERRLARDAAAKPDPSPRALVMPPNSQTDPTEPVGGARMRLSKEEKERRRKMNLCLYCGNGGHFADTCPAKASK
NSPPGLPGPAVGGPSATGPERIRSPPSEASTQHLQVMLQIHMPGRPTLFVRAMIDSGASGNFIDQDFVIQNAI
PLRIKDWPVMVEAIDGHPIASGPIILETHHLIVDLGDHREILSFDVTQSPFFPIVLGIRWLSTHDPHITWSTR
SIVFNSDYCRLRCRMFAQIPSNLLFTVPQPNLHPYLLHHVHPHVHPHMHQHLHQHLHQFLHPDPHQYPHPDPH
YHHHQQADMQHQLQQYLYQYLYYHLYPVMHHHLPPDQHEHLHEYLHQYLHQYLHQFLHHHLHPDLHQYLYQYL
HNHMNPDPHHHPHPDPPQDPHHPPHQDPHQHPDPHQDAHQDPHQHPDPHQDPPHQDPHQHPDPHQDPPHQDPH
QDAHQHQDPHQDAHQDPHQDAHQDPHMDPHLHQHQHPQPQPHPQQHPNHPQQPPFFYHMAGFRIYHPVRYYYI
QNVYTPVDEHVYPGHRVVDPNIEMIPGAHSLPSGHLYSMSESEMNALRNFVDRNVKDGLMTPTVAPNGAQVLQ
VKRGWKLQVTYNCRAPQSGTIQNQYLRMSLPNMGDPAHLASYGEFVQVPGYPYPAYVYYTSPHMMTAWYPVGR
DVHGRIIVVPVVITWSQNTNRQPPVPQYPPPQPPPPPPPPPPPPPPPPASSCSAA*
>ENSMUSP00000135076_c.901:AGCATCC>A_p.QHP301del
ENSMUST00000176551.3|Peg10-
203|ENSMUSG00000092035|+|chr6:g.4756327:AGCATCC>A
MLQIHMPGRPTLFVRAMIDSGASGNFIDQDFVIQNAIPLRIKDWPVMVEAIDGHPIASGPIILETHHLIVDLG
DHREILSFDVTQSPFFPIVLGIRWLSTHDPHITWSTRSIVFNSDYCRLRCRMFAQIPSNLLFTVPQPNLHPYL
LHHVHPHVHPHMHQHLHQHLHQFLHPDPHQYPHPDPHYHHHQQADMQHQLQQYLYQYLYYHLYPVMHHHLPPD
QHEHLHEYLHQYLHQYLHQFLHHHLHPDLHQYLYQYLHNHMNPDPHHHPHPDPPQDPHHPPHQDPHQHPDPHQ
DPPHQDPHQDPHQDPPHQDPHQHPDPHQDPPHQDPHQDAHQHQDPHQDAHQDPHQDAHQDPHMDPHLHQHQHP
QPQPHPQQHPNHPQQPPFFYHMAGFRIYHPVRYYYIQNVYTPVDEHVYPGHRVVDPNIEMIPGAHSLPSGHLY
SMSESEMNALRNFVDRNVKDGLMTPTVAPNGAQVLQVKRGWKLQVTYNCRAPQSGTIQNQYLRMSLPNMGDPA
HLASYGEFVQVPGYPYPAYVYYTSPHMMTAWYPVGRDVHGRIIVVPVVITWSQNTNRQPPVPQYPPPQPPPPP
PPPPPPPPPPPASSCSAA*
>ENSMUSP00000127306_c.2104:AGCATCC>A_p.QHP702del
ENSMUST00000166678.2|Peg10-
201|ENSMUSG00000092035|+|chr6:g.4756327:AGCATCC>A
MAAAGGSSNCPPPPPPPPPPNNNNNNNTPKSPGVPDAEDDDERRHDELPEDINNFDEDMNRQFENMNLLDQVEL
LAQSYSLLDHLDDFDDDDEDDDFDPEPDQDELPEYSDDDDLELQGAAAAPIPNFFSDDDCLEDLPEKFDGNPD
MLGPFMYQCQLFMEKSTRDFSVDRIRVCFVTSMLIGRAARWATAKLQRCTYLMHNYTAFMMELKHVFEDPQRR
EAAKRKIRRLRQGPGPVVDYSNAFQMIAQDLDWTEPALMDQFQEGLNPDIRAELSRQEAPKTLAALITACIHI
ERRLARDAAAKPDPSPRALVMPPNSQTDPTEPVGGARMRLSKEEKERRRKMNLCLYCGNGGHFADTCPAKASK
NSPPGLPGPAVGGPSATGPERIRSPPSEASTQHLQVMLQIHMPGRPTLFVRAMIDSGASGNFIDQDFVIQNAI
PLRIKDWPVMVEAIDGHPIASGPIILETHHLIVDLGDHREILSFDVTQSPFFPIVLGIRWLSTHDPHITWSTR
SIVFNSDYCRLRCRMFAQIPSNLLFTVPQPNLHPYLLHHVHPHVHPHMHQHLHQHLHQFLHPDPHQYPHPDPH
YHHHQQADMQHQLQQYLYQYLYYHLYPVMHHHLPPDQHEHLHEYLHQYLHQYLHQFLHHHLHPDLHQYLYQYL
HNHMNPDPHHHPHPDPPQDPHHPPHQDPHQHPDPHQDPPHQDPHQDPHQDPPHQDPHQHPDPHQDPPHQDPHQ
DAHQHQDPHQDAHQDPHQDAHQDPHMDPHLHQHQHPQPQPHPQQHPNHPQQPPFFYHMAGFRIYHPVRYYYIQ

NVYTPVDEHVYPGHRVVDPNIEMIPGAHSLPSGHLYSMSESEMNALRNFVDRNVKDGLMTPTVAPNGAQVLQV
KRGWKLQVTYNCRAPQSGTIQNQYLRMSLPNMGDPAHLASYGEFVQVPGYPYPAYVYYTSPHMMTAWYPVGRD
VHGRIIVVPVVITWSQNTNRQPPVPQYPPPQPPPPPPPPPPPPPPPPASSCSAA*
>ENSMUSP00000135076_c.924:CCTC>G_p.PP309del ENSMUST00000176551.3|Peg10-
203|ENSMUSG00000092035|+|chr6:g.4756350:CCTC>G
MLQIHMPGRPTLFVRAMIDSGASGNFIDQDFVIQNAIPLRIKDWPVMVEAIDGHPIASGPIILETHHLIVDLG
DHREILSFDVTQSPFFPIVLGIRWLSTHDPHITWSTRSIVFNSDYCRLRCRMFAQIPSNLLFTVPQPNLHPYL
LHHVHPHVHPHMHQHLHQHLHQFLHPDPHQYPHPDPHYHHHQQADMQHQLQQYLYQYLYYHLYPVMHHHLPPD
QHEHLHEYLHQYLHQYLHQFLHHHLHPDLHQYLYQYLHNHMNPDPHHHPHPDPPQDPHHPPHQDPHQHPDPHQ
DPPHQDPHQHPDPHQDAHQDPHQHPDPHQDPPHQDPHQDAHQHQDPHQDAHQDPHQDAHQDPHMDPHLHQHQH
PQPQPHPQQHPNHPQQPPFFYHMAGFRIYHPVRYYYIQNVYTPVDEHVYPGHRVVDPNIEMIPGAHSLPSGHL
YSMSESEMNALRNFVDRNVKDGLMTPTVAPNGAQVLQVKRGWKLQVTYNCRAPQSGTIQNQYLRMSLPNMGDP
AHLASYGEFVQVPGYPYPAYVYYTSPHMMTAWYPVGRDVHGRIIVVPVVITWSQNTNRQPPVPQYPPPQPPPP
PPPPPPPPPPPPASSCSAA*
>ENSMUSP00000127306_c.2127:CCTC>G_p.PP710del ENSMUST00000166678.2|Peg10-
201|ENSMUSG00000092035|+|chr6:g.4756350:CCTC>G
MAAAGGSSNCPPPPPPPPPNNNNNNNTPKSPGVPDAEDDDERRHDELPEDINNFDEDMNRQFENMNLLDQVEL
LAQSYSLLDHLDDFDDDDEDDDFDPEPDQDELPEYSDDDDLELQGAAAAPIPNFFSDDDCLEDLPEKFDGNPD
MLGPFMYQCQLFMEKSTRDFSVDRIRVCFVTSMLIGRAARWATAKLQRCTYLMHNYTAFMMELKHVFEDPQRR
EAAKRKIRRLRQGPGPVVDYSNAFQMIAQDLDWTEPALMDQFQEGLNPDIRAELSRQEAPKTLAALITACIHI
ERRLARDAAAKPDPSPRALVMPPNSQTDPTEPVGGARMRLSKEEKERRRKMNLCLYCGNGGHFADTCPAKASK
NSPPGLPGPAVGGPSATGPERIRSPPSEASTQHLQVMLQIHMPGRPTLFVRAMIDSGASGNFIDQDFVIQNAI
PLRIKDWPVMVEAIDGHPIASGPIILETHHLIVDLGDHREILSFDVTQSPFFPIVLGIRWLSTHDPHITWSTR
SIVFNSDYCRLRCRMFAQIPSNLLFTVPQPNLHPYLLHHVHPHVHPHMHQHLHQHLHQFLHPDPHQYPHPDPH
YHHHQQADMQHQLQQYLYQYLYYHLYPVMHHHLPPDQHEHLHEYLHQYLHQYLHQFLHHHLHPDLHQYLYQYL
HNHMNPDPHHHPHPDPPQDPHHPPHQDPHQHPDPHQDPPHQDPHQHPDPHQDAHQDPHQHPDPHQDPPHQDPH
QDAHQHQDPHQDAHQDPHQDAHQDPHMDPHLHQHQHPQPQPHPQQHPNHPQQPPFFYHMAGFRIYHPVRYYYI
QNVYTPVDEHVYPGHRVVDPNIEMIPGAHSLPSGHLYSMSESEMNALRNFVDRNVKDGLMTPTVAPNGAQVLQ
VKRGWKLQVTYNCRAPQSGTIQNQYLRMSLPNMGDPAHLASYGEFVQVPGYPYPAYVYYTSPHMMTAWYPVGR
DVHGRIIVVPVVITWSQNTNRQPPVPQYPPPQPPPPPPPPPPPPPPPPASSCSAA*
>ENSMUSP00000135076_c.967:ATCC>A_p.DP323del ENSMUST00000176551.3|Peg10-
203|ENSMUSG00000092035|+|chr6:g.4756393:ATCC>A
MLQIHMPGRPTLFVRAMIDSGASGNFIDQDFVIQNAIPLRIKDWPVMVEAIDGHPIASGPIILETHHLIVDLG
DHREILSFDVTQSPFFPIVLGIRWLSTHDPHITWSTRSIVFNSDYCRLRCRMFAQIPSNLLFTVPQPNLHPYL
LHHVHPHVHPHMHQHLHQHLHQFLHPDPHQYPHPDPHYHHHQQADMQHQLQQYLYQYLYYHLYPVMHHHLPPD
QHEHLHEYLHQYLHQYLHQFLHHHLHPDLHQYLYQYLHNHMNPDPHHHPHPDPPQDPHHPPHQDPHQHPDPHQ
DPPHQDPHQHPDPHQDPPHQDPHQHPDPHQDPHQDPHQDAHQHQDPHQDAHQDPHQDAHQDPHMDPHLHQHQH
PQPQPHPQQHPNHPQQPPFFYHMAGFRIYHPVRYYYIQNVYTPVDEHVYPGHRVVDPNIEMIPGAHSLPSGHL
YSMSESEMNALRNFVDRNVKDGLMTPTVAPNGAQVLQVKRGWKLQVTYNCRAPQSGTIQNQYLRMSLPNMGDP
AHLASYGEFVQVPGYPYPAYVYYTSPHMMTAWYPVGRDVHGRIIVVPVVITWSQNTNRQPPVPQYPPPQPPPP
PPPPPPPPPPPPASSCSAA*
>ENSMUSP00000127306_c.2170:ATCC>A_p.DP724del ENSMUST00000166678.2|Peg10-
201|ENSMUSG00000092035|+|chr6:g.4756393:ATCC>A
MAAAGGSSNCPPPPPPPPPNNNNNNNTPKSPGVPDAEDDDERRHDELPEDINNFDEDMNRQFENMNLLDQVEL
LAQSYSLLDHLDDFDDDDEDDDFDPEPDQDELPEYSDDDDLELQGAAAAPIPNFFSDDDCLEDLPEKFDGNPD
MLGPFMYQCQLFMEKSTRDFSVDRIRVCFVTSMLIGRAARWATAKLQRCTYLMHNYTAFMMELKHVFEDPQRR
EAAKRKIRRLRQGPGPVVDYSNAFQMIAQDLDWTEPALMDQFQEGLNPDIRAELSRQEAPKTLAALITACIHI
ERRLARDAAAKPDPSPRALVMPPNSQTDPTEPVGGARMRLSKEEKERRRKMNLCLYCGNGGHFADTCPAKASK
NSPPGLPGPAVGGPSATGPERIRSPPSEASTQHLQVMLQIHMPGRPTLFVRAMIDSGASGNFIDQDFVIQNAI
PLRIKDWPVMVEAIDGHPIASGPIILETHHLIVDLGDHREILSFDVTQSPFFPIVLGIRWLSTHDPHITWSTR
SIVFNSDYCRLRCRMFAQIPSNLLFTVPQPNLHPYLLHHVHPHVHPHMHQHLHQHLHQFLHPDPHQYPHPDPH
YHHHQQADMQHQLQQYLYQYLYYHLYPVMHHHLPPDQHEHLHEYLHQYLHQYLHQFLHHHLHPDLHQYLYQYL
HNHMNPDPHHHPHPDPPQDPHHPPHQDPHQHPDPHQDPPHQDPHQHPDPHQDPPHQDPHQHPDPHQDPHQDPH
QDAHQHQDPHQDAHQDPHQDAHQDPHMDPHLHQHQHPQPQPHPQQHPNHPQQPPFFYHMAGFRIYHPVRYYYI
QNVYTPVDEHVYPGHRVVDPNIEMIPGAHSLPSGHLYSMSESEMNALRNFVDRNVKDGLMTPTVAPNGAQVLQ
VKRGWKLQVTYNCRAPQSGTIQNQYLRMSLPNMGDPAHLASYGEFVQVPGYPYPAYVYYTSPHMMTAWYPVGR
DVHGRIIVVPVVITWSQNTNRQPPVPQYPPPQPPPPPPPPPPPPPPPPASSCSAA*

>ENSMUSP00000135076_c.972:CCACATCAGGATCCACATCAGGATGCACATCAGCATCAGGATCCCCA
TCAGGATG>C_p.PHQDPHQDAHQHQDPHQDA325del ENSMUST00000176551.3|Peg10-
203|ENSMUSG00000092035|+|chr6:g.4756398:CCACATCAGGATCCACATCAGGATGCACATCAG
CATCAGGATCCCCATCAGGATG>C
MLQIHMPGRPTLFVRAMIDSGASGNFIDQDFVIQNAIPLRIKDWPVMVEAIDGHPIASGPIILETHHLIVDLG
DHREILSFDVTQSPFFPIVLGIRWLSTHDPHITWSTRSIVFNSDYCRLRCRMFAQIPSNLLFTVPQPNLHPYL
LHHVHPHVHPHMHQHLHQHLHQFLHPDPHQYPHPDPHYHHHQQADMQHQLQQYLYQYLYYHLYPVMHHHLPPD
QHEHLHEYLHQYLHQYLHQFLHHHLHPDLHQYLYQYLHNHMNPDPHHHPHPDPPQDPHHPPHQDPHQHPDPHQ
DPPHQDPHQHPDPHQDPPHQDPHQHPDPHQDPPHQDPHQDAHQDPHMDPHLHQHQHPQPQPHPQQHPNHPQQP
PFFYHMAGFRIYHPVRYYYIQNVYTPVDEHVYPGHRVVDPNIEMIPGAHSLPSGHLYSMSESEMNALRNFVDR
NVKDGLMTPTVAPNGAQVLQVKRGWKLQVTYNCRAPQSGTIQNQYLRMSLPNMGDPAHLASYGEFVQVPGYPY
PAYVYYTSPHMMTAWYPVGRDVHGRIIVVPVVITWSQNTNRQPPVPQYPPPQPPPPPPPPPPPPPPPPASSCS
AA*
>ENSMUSP00000127306_c.2175:CCACATCAGGATCCACATCAGGATGCACATCAGCATCAGGATCCCC
ATCAGGATG>C_p.PHQDPHQDAHQHQDPHQDA726del ENSMUST00000166678.2|Peg10-
201|ENSMUSG00000092035|+|chr6:g.4756398:CCACATCAGGATCCACATCAGGATGCACATCAG
CATCAGGATCCCCATCAGGATG>C
MAAAGGSSNCPPPPPPPPPNNNNNNNTPKSPGVPDAEDDDERRHDELPEDINNFDEDMNRQFENMNLLDQVEL
LAQSYSLLDHLDDFDDDDEDDDFDPEPDQDELPEYSDDDDLELQGAAAAPIPNFFSDDDCLEDLPEKFDGNPD
MLGPFMYQCQLFMEKSTRDFSVDRIRVCFVTSMLIGRAARWATAKLQRCTYLMHNYTAFMMELKHVFEDPQRR
EAAKRKIRRLRQGPGPVVDYSNAFQMIAQDLDWTEPALMDQFQEGLNPDIRAELSRQEAPKTLAALITACIHI
ERRLARDAAAKPDPSPRALVMPPNSQTDPTEPVGGARMRLSKEEKERRRKMNLCLYCGNGGHFADTCPAKASK
NSPPGLPGPAVGGPSATGPERIRSPPSEASTQHLQVMLQIHMPGRPTLFVRAMIDSGASGNFIDQDFVIQNAI
PLRIKDWPVMVEAIDGHPIASGPIILETHHLIVDLGDHREILSFDVTQSPFFPIVLGIRWLSTHDPHITWSTR
SIVFNSDYCRLRCRMFAQIPSNLLFTVPQPNLHPYLLHHVHPHVHPHMHQHLHQHLHQFLHPDPHQYPHPDPH
YHHHQQADMQHQLQQYLYQYLYYHLYPVMHHHLPPDQHEHLHEYLHQYLHQYLHQFLHHHLHPDLHQYLYQYL
HNHMNPDPHHHPHPDPPQDPHHPPHQDPHQHPDPHQDPPHQDPHQHPDPHQDPPHQDPHQHPDPHQDPPHQDP
HQDAHQDPHMDPHLHQHQHPQPQPHPQQHPNHPQQPPFFYHMAGFRIYHPVRYYYIQNVYTPVDEHVYPGHRV
VDPNIEMIPGAHSLPSGHLYSMSESEMNALRNFVDRNVKDGLMTPTVAPNGAQVLQVKRGWKLQVTYNCRAPQ
SGTIQNQYLRMSLPNMGDPAHLASYGEFVQVPGYPYPAYVYYTSPHMMTAWYPVGRDVHGRIIVVPVVITWSQ
NTNRQPPVPQYPPPQPPPPPPPPPPPPPPPPASSCSAA*
>ENSMUSP00000135076_c.984:C>G_p.P329A ENSMUST00000176551.3|Peg10-
203|ENSMUSG00000092035|+|chr6:g.4756410:C>G
MLQIHMPGRPTLFVRAMIDSGASGNFIDQDFVIQNAIPLRIKDWPVMVEAIDGHPIASGPIILETHHLIVDLG
DHREILSFDVTQSPFFPIVLGIRWLSTHDPHITWSTRSIVFNSDYCRLRCRMFAQIPSNLLFTVPQPNLHPYL
LHHVHPHVHPHMHQHLHQHLHQFLHPDPHQYPHPDPHYHHHQQADMQHQLQQYLYQYLYYHLYPVMHHHLPPD
QHEHLHEYLHQYLHQYLHQFLHHHLHPDLHQYLYQYLHNHMNPDPHHHPHPDPPQDPHHPPHQDPHQHPDPHQ
DPPHQDPHQHPDPHQDPPHQDPHQHPDPHQDPPHQDAHQDAHQHQDPHQDAHQDPHQDAHQDPHMDPHLHQHQ
HPQPQPHPQQHPNHPQQPPFFYHMAGFRIYHPVRYYYIQNVYTPVDEHVYPGHRVVDPNIEMIPGAHSLPSGH
LYSMSESEMNALRNFVDRNVKDGLMTPTVAPNGAQVLQVKRGWKLQVTYNCRAPQSGTIQNQYLRMSLPNMGD
PAHLASYGEFVQVPGYPYPAYVYYTSPHMMTAWYPVGRDVHGRIIVVPVVITWSQNTNRQPPVPQYPPPQPPP
PPPPPPPPPPPPPASSCSAA*
>ENSMUSP00000127306_c.2187:C>G_p.P730A ENSMUST00000166678.2|Peg10-
201|ENSMUSG00000092035|+|chr6:g.4756410:C>G
MAAAGGSSNCPPPPPPPPPNNNNNNNTPKSPGVPDAEDDDERRHDELPEDINNFDEDMNRQFENMNLLDQVEL
LAQSYSLLDHLDDFDDDDEDDDFDPEPDQDELPEYSDDDDLELQGAAAAPIPNFFSDDDCLEDLPEKFDGNPD
MLGPFMYQCQLFMEKSTRDFSVDRIRVCFVTSMLIGRAARWATAKLQRCTYLMHNYTAFMMELKHVFEDPQRR
EAAKRKIRRLRQGPGPVVDYSNAFQMIAQDLDWTEPALMDQFQEGLNPDIRAELSRQEAPKTLAALITACIHI
ERRLARDAAAKPDPSPRALVMPPNSQTDPTEPVGGARMRLSKEEKERRRKMNLCLYCGNGGHFADTCPAKASK
NSPPGLPGPAVGGPSATGPERIRSPPSEASTQHLQVMLQIHMPGRPTLFVRAMIDSGASGNFIDQDFVIQNAI
PLRIKDWPVMVEAIDGHPIASGPIILETHHLIVDLGDHREILSFDVTQSPFFPIVLGIRWLSTHDPHITWSTR
SIVFNSDYCRLRCRMFAQIPSNLLFTVPQPNLHPYLLHHVHPHVHPHMHQHLHQHLHQFLHPDPHQYPHPDPH
YHHHQQADMQHQLQQYLYQYLYYHLYPVMHHHLPPDQHEHLHEYLHQYLHQYLHQFLHHHLHPDLHQYLYQYL
HNHMNPDPHHHPHPDPPQDPHHPPHQDPHQHPDPHQDPPHQDPHQHPDPHQDPPHQDPHQHPDPHQDPPHQDA
HQDAHQHQDPHQDAHQDPHQDAHQDPHMDPHLHQHQHPQPQPHPQQHPNHPQQPPFFYHMAGFRIYHPVRYYY
IQNVYTPVDEHVYPGHRVVDPNIEMIPGAHSLPSGHLYSMSESEMNALRNFVDRNVKDGLMTPTVAPNGAQVL
QVKRGWKLQVTYNCRAPQSGTIQNQYLRMSLPNMGDPAHLASYGEFVQVPGYPYPAYVYYTSPHMMTAWYPVG
RDVHGRIIVVPVVITWSQNTNRQPPVPQYPPPQPPPPPPPPPPPPPPPPASSCSAA*

>rs52535223_ENSMUSP00000135076_c.996:G>C_p.A333P
ENSMUST00000176551.3|Peg10-203|ENSMUSG00000092035|+|chr6:g.4756422:G>C
MLQIHMPGRPTLFVRAMIDSGASGNFIDQDFVIQNAIPLRIKDWPVMVEAIDGHPIASGPIILETHHLIVDLG
DHREILSFDVTQSPFFPIVLGIRWLSTHDPHITWSTRSIVFNSDYCRLRCRMFAQIPSNLLFTVPQPNLHPYL
LHHVHPHVHPHMHQHLHQHLHQFLHPDPHQYPHPDPHYHHHQQADMQHQLQQYLYQYLYYHLYPVMHHHLPPD
QHEHLHEYLHQYLHQYLHQFLHHHLHPDLHQYLYQYLHNHMNPDPHHHPHPDPPQDPHHPPHQDPHQHPDPHQ
DPPHQDPHQHPDPHQDPPHQDPHQHPDPHQDPPHQDPHQDPHQHQDPHQDAHQDPHQDAHQDPHMDPHLHQHQ
HPQPQPHPQQHPNHPQQPPFFYHMAGFRIYHPVRYYYIQNVYTPVDEHVYPGHRVVDPNIEMIPGAHSLPSGH
LYSMSESEMNALRNFVDRNVKDGLMTPTVAPNGAQVLQVKRGWKLQVTYNCRAPQSGTIQNQYLRMSLPNMGD
PAHLASYGEFVQVPGYPYPAYVYYTSPHMMTAWYPVGRDVHGRIIVVPVVITWSQNTNRQPPVPQYPPPQPPP
PPPPPPPPPPPPPASSCSAA*
>rs52535223_ENSMUSP00000127306_c.2199:G>C_p.A734P
ENSMUST00000166678.2|Peg10-201|ENSMUSG00000092035|+|chr6:g.4756422:G>C
MAAAGGSSNCPPPPPPPPPNNNNNNNTPKSPGVPDAEDDDERRHDELPEDINNFDEDMNRQFENMNLLDQVEL
LAQSYSLLDHLDDFDDDDEDDDFDPEPDQDELPEYSDDDDLELQGAAAAPIPNFFSDDDCLEDLPEKFDGNPD
MLGPFMYQCQLFMEKSTRDFSVDRIRVCFVTSMLIGRAARWATAKLQRCTYLMHNYTAFMMELKHVFEDPQRR
EAAKRKIRRLRQGPGPVVDYSNAFQMIAQDLDWTEPALMDQFQEGLNPDIRAELSRQEAPKTLAALITACIHI
ERRLARDAAAKPDPSPRALVMPPNSQTDPTEPVGGARMRLSKEEKERRRKMNLCLYCGNGGHFADTCPAKASK
NSPPGLPGPAVGGPSATGPERIRSPPSEASTQHLQVMLQIHMPGRPTLFVRAMIDSGASGNFIDQDFVIQNAI
PLRIKDWPVMVEAIDGHPIASGPIILETHHLIVDLGDHREILSFDVTQSPFFPIVLGIRWLSTHDPHITWSTR
SIVFNSDYCRLRCRMFAQIPSNLLFTVPQPNLHPYLLHHVHPHVHPHMHQHLHQHLHQFLHPDPHQYPHPDPH
YHHHQQADMQHQLQQYLYQYLYYHLYPVMHHHLPPDQHEHLHEYLHQYLHQYLHQFLHHHLHPDLHQYLYQYL
HNHMNPDPHHHPHPDPPQDPHHPPHQDPHQHPDPHQDPPHQDPHQHPDPHQDPPHQDPHQHPDPHQDPPHQDP
HQDPHQHQDPHQDAHQDPHQDAHQDPHMDPHLHQHQHPQPQPHPQQHPNHPQQPPFFYHMAGFRIYHPVRYYY
IQNVYTPVDEHVYPGHRVVDPNIEMIPGAHSLPSGHLYSMSESEMNALRNFVDRNVKDGLMTPTVAPNGAQVL
QVKRGWKLQVTYNCRAPQSGTIQNQYLRMSLPNMGDPAHLASYGEFVQVPGYPYPAYVYYTSPHMMTAWYPVG
RDVHGRIIVVPVVITWSQNTNRQPPVPQYPPPQPPPPPPPPPPPPPPPPASSCSAA*
>rs52233015_ENSMUSP00000135076_c.1009:A>C_p.Q337P
ENSMUST00000176551.3|Peg10-203|ENSMUSG00000092035|+|chr6:g.4756435:A>C
MLQIHMPGRPTLFVRAMIDSGASGNFIDQDFVIQNAIPLRIKDWPVMVEAIDGHPIASGPIILETHHLIVDLG
DHREILSFDVTQSPFFPIVLGIRWLSTHDPHITWSTRSIVFNSDYCRLRCRMFAQIPSNLLFTVPQPNLHPYL
LHHVHPHVHPHMHQHLHQHLHQFLHPDPHQYPHPDPHYHHHQQADMQHQLQQYLYQYLYYHLYPVMHHHLPPD
QHEHLHEYLHQYLHQYLHQFLHHHLHPDLHQYLYQYLHNHMNPDPHHHPHPDPPQDPHHPPHQDPHQHPDPHQ
DPPHQDPHQHPDPHQDPPHQDPHQHPDPHQDPPHQDPHQDAHQHPDPHQDAHQDPHQDAHQDPHMDPHLHQHQ
HPQPQPHPQQHPNHPQQPPFFYHMAGFRIYHPVRYYYIQNVYTPVDEHVYPGHRVVDPNIEMIPGAHSLPSGH
LYSMSESEMNALRNFVDRNVKDGLMTPTVAPNGAQVLQVKRGWKLQVTYNCRAPQSGTIQNQYLRMSLPNMGD
PAHLASYGEFVQVPGYPYPAYVYYTSPHMMTAWYPVGRDVHGRIIVVPVVITWSQNTNRQPPVPQYPPPQPPP
PPPPPPPPPPPPPASSCSAA*
>rs52233015_ENSMUSP00000127306_c.2212:A>C_p.Q738P
ENSMUST00000166678.2|Peg10-201|ENSMUSG00000092035|+|chr6:g.4756435:A>C
MAAAGGSSNCPPPPPPPPPNNNNNNNTPKSPGVPDAEDDDERRHDELPEDINNFDEDMNRQFENMNLLDQVEL
LAQSYSLLDHLDDFDDDDEDDDFDPEPDQDELPEYSDDDDLELQGAAAAPIPNFFSDDDCLEDLPEKFDGNPD
MLGPFMYQCQLFMEKSTRDFSVDRIRVCFVTSMLIGRAARWATAKLQRCTYLMHNYTAFMMELKHVFEDPQRR
EAAKRKIRRLRQGPGPVVDYSNAFQMIAQDLDWTEPALMDQFQEGLNPDIRAELSRQEAPKTLAALITACIHI
ERRLARDAAAKPDPSPRALVMPPNSQTDPTEPVGGARMRLSKEEKERRRKMNLCLYCGNGGHFADTCPAKASK
NSPPGLPGPAVGGPSATGPERIRSPPSEASTQHLQVMLQIHMPGRPTLFVRAMIDSGASGNFIDQDFVIQNAI
PLRIKDWPVMVEAIDGHPIASGPIILETHHLIVDLGDHREILSFDVTQSPFFPIVLGIRWLSTHDPHITWSTR
SIVFNSDYCRLRCRMFAQIPSNLLFTVPQPNLHPYLLHHVHPHVHPHMHQHLHQHLHQFLHPDPHQYPHPDPH
YHHHQQADMQHQLQQYLYQYLYYHLYPVMHHHLPPDQHEHLHEYLHQYLHQYLHQFLHHHLHPDLHQYLYQYL
HNHMNPDPHHHPHPDPPQDPHHPPHQDPHQHPDPHQDPPHQDPHQHPDPHQDPPHQDPHQHPDPHQDPPHQDP
HQDAHQHPDPHQDAHQDPHQDAHQDPHMDPHLHQHQHPQPQPHPQQHPNHPQQPPFFYHMAGFRIYHPVRYYY
IQNVYTPVDEHVYPGHRVVDPNIEMIPGAHSLPSGHLYSMSESEMNALRNFVDRNVKDGLMTPTVAPNGAQVL
QVKRGWKLQVTYNCRAPQSGTIQNQYLRMSLPNMGDPAHLASYGEFVQVPGYPYPAYVYYTSPHMMTAWYPVG
RDVHGRIIVVPVVITWSQNTNRQPPVPQYPPPQPPPPPPPPPPPPPPPPASSCSAA*
>ENSMUSP00000135076_c.1026:G>CCTC_p.A343PP-ins
ENSMUST00000176551.3|Peg10-203|ENSMUSG00000092035|+|chr6:g.4756452:G>CCTC
MLQIHMPGRPTLFVRAMIDSGASGNFIDQDFVIQNAIPLRIKDWPVMVEAIDGHPIASGPIILETHHLIVDLG
DHREILSFDVTQSPFFPIVLGIRWLSTHDPHITWSTRSIVFNSDYCRLRCRMFAQIPSNLLFTVPQPNLHPYL

LHHVHPHVHPHMHQHLHQHLHQFLHPDPHQYPHPDPHYHHHQQADMQHQLQQYLYQYLYYHLYPVMHHHLPPD
QHEHLHEYLHQYLHQYLHQFLHHHLHPDLHQYLYQYLHNHMNPDPHHHPHPDPPQDPHHPPHQDPHQHPDPHQ
DPPHQDPHQHPDPHQDPPHQDPHQHPDPHQDPPHQDPHQDAHQHQDPHQDPPHQDPHQDAHQDPHMDPHLHQH
QHPQPQPHPQQHPNHPQQPPFFYHMAGFRIYHPVRYYYIQNVYTPVDEHVYPGHRVVDPNIEMIPGAHSLPSG
HLYSMSESEMNALRNFVDRNVKDGLMTPTVAPNGAQVLQVKRGWKLQVTYNCRAPQSGTIQNQYLRMSLPNMG
DPAHLASYGEFVQVPGYPYPAYVYYTSPHMMTAWYPVGRDVHGRIIVVPVVITWSQNTNRQPPVPQYPPPQPP
PPPPPPPPPPPPPPASSCSAA*
>ENSMUSP00000127306_c.2229:G>CCTC_p.A744PP-ins
ENSMUST00000166678.2|Peg10-201|ENSMUSG00000092035|+|chr6:g.4756452:G>CCTC
MAAAGGSSNCPPPPPPPPPNNNNNNNTPKSPGVPDAEDDDERRHDELPEDINNFDEDMNRQFENMNLLDQVEL
LAQSYSLLDHLDDFDDDDEDDDFDPEPDQDELPEYSDDDDLELQGAAAAPIPNFFSDDDCLEDLPEKFDGNPD
MLGPFMYQCQLFMEKSTRDFSVDRIRVCFVTSMLIGRAARWATAKLQRCTYLMHNYTAFMMELKHVFEDPQRR
EAAKRKIRRLRQGPGPVVDYSNAFQMIAQDLDWTEPALMDQFQEGLNPDIRAELSRQEAPKTLAALITACIHI
ERRLARDAAAKPDPSPRALVMPPNSQTDPTEPVGGARMRLSKEEKERRRKMNLCLYCGNGGHFADTCPAKASK
NSPPGLPGPAVGGPSATGPERIRSPPSEASTQHLQVMLQIHMPGRPTLFVRAMIDSGASGNFIDQDFVIQNAI
PLRIKDWPVMVEAIDGHPIASGPIILETHHLIVDLGDHREILSFDVTQSPFFPIVLGIRWLSTHDPHITWSTR
SIVFNSDYCRLRCRMFAQIPSNLLFTVPQPNLHPYLLHHVHPHVHPHMHQHLHQHLHQFLHPDPHQYPHPDPH
YHHHQQADMQHQLQQYLYQYLYYHLYPVMHHHLPPDQHEHLHEYLHQYLHQYLHQFLHHHLHPDLHQYLYQYL
HNHMNPDPHHHPHPDPPQDPHHPPHQDPHQHPDPHQDPPHQDPHQHPDPHQDPPHQDPHQHPDPHQDPPHQDP
HQDAHQHQDPHQDPPHQDPHQDAHQDPHMDPHLHQHQHPQPQPHPQQHPNHPQQPPFFYHMAGFRIYHPVRYY
YIQNVYTPVDEHVYPGHRVVDPNIEMIPGAHSLPSGHLYSMSESEMNALRNFVDRNVKDGLMTPTVAPNGAQV
LQVKRGWKLQVTYNCRAPQSGTIQNQYLRMSLPNMGDPAHLASYGEFVQVPGYPYPAYVYYTSPHMMTAWYPV
GRDVHGRIIVVPVVITWSQNTNRQPPVPQYPPPQPPPPPPPPPPPPPPPPPASSCSAA*
>ENSMUSP00000135076_c.1050:G>C_p.A351P ENSMUST00000176551.3|Peg10-
203|ENSMUSG00000092035|+|chr6:g.4756476:G>C
MLQIHMPGRPTLFVRAMIDSGASGNFIDQDFVIQNAIPLRIKDWPVMVEAIDGHPIASGPIILETHHLIVDLG
DHREILSFDVTQSPFFPIVLGIRWLSTHDPHITWSTRSIVFNSDYCRLRCRMFAQIPSNLLFTVPQPNLHPYL
LHHVHPHVHPHMHQHLHQHLHQFLHPDPHQYPHPDPHYHHHQQADMQHQLQQYLYQYLYYHLYPVMHHHLPPD
QHEHLHEYLHQYLHQYLHQFLHHHLHPDLHQYLYQYLHNHMNPDPHHHPHPDPPQDPHHPPHQDPHQHPDPHQ
DPPHQDPHQHPDPHQDPPHQDPHQHPDPHQDPPHQDPHQDAHQHQDPHQDAHQDPHQDPHQDPHMDPHLHQHQ
HPQPQPHPQQHPNHPQQPPFFYHMAGFRIYHPVRYYYIQNVYTPVDEHVYPGHRVVDPNIEMIPGAHSLPSGH
LYSMSESEMNALRNFVDRNVKDGLMTPTVAPNGAQVLQVKRGWKLQVTYNCRAPQSGTIQNQYLRMSLPNMGD
PAHLASYGEFVQVPGYPYPAYVYYTSPHMMTAWYPVGRDVHGRIIVVPVVITWSQNTNRQPPVPQYPPPQPPP
PPPPPPPPPPPPPASSCSAA*
>ENSMUSP00000127306_c.2253:G>C_p.A752P ENSMUST00000166678.2|Peg10-
201|ENSMUSG00000092035|+|chr6:g.4756476:G>C
MAAAGGSSNCPPPPPPPPPNNNNNNNTPKSPGVPDAEDDDERRHDELPEDINNFDEDMNRQFENMNLLDQVEL
LAQSYSLLDHLDDFDDDDEDDDFDPEPDQDELPEYSDDDDLELQGAAAAPIPNFFSDDDCLEDLPEKFDGNPD
MLGPFMYQCQLFMEKSTRDFSVDRIRVCFVTSMLIGRAARWATAKLQRCTYLMHNYTAFMMELKHVFEDPQRR
EAAKRKIRRLRQGPGPVVDYSNAFQMIAQDLDWTEPALMDQFQEGLNPDIRAELSRQEAPKTLAALITACIHI
ERRLARDAAAKPDPSPRALVMPPNSQTDPTEPVGGARMRLSKEEKERRRKMNLCLYCGNGGHFADTCPAKASK
NSPPGLPGPAVGGPSATGPERIRSPPSEASTQHLQVMLQIHMPGRPTLFVRAMIDSGASGNFIDQDFVIQNAI
PLRIKDWPVMVEAIDGHPIASGPIILETHHLIVDLGDHREILSFDVTQSPFFPIVLGIRWLSTHDPHITWSTR
SIVFNSDYCRLRCRMFAQIPSNLLFTVPQPNLHPYLLHHVHPHVHPHMHQHLHQHLHQFLHPDPHQYPHPDPH
YHHHQQADMQHQLQQYLYQYLYYHLYPVMHHHLPPDQHEHLHEYLHQYLHQYLHQFLHHHLHPDLHQYLYQYL
HNHMNPDPHHHPHPDPPQDPHHPPHQDPHQHPDPHQDPPHQDPHQHPDPHQDPPHQDPHQHPDPHQDPPHQDP
HQDAHQHQDPHQDAHQDPHQDPHQDPHMDPHLHQHQHPQPQPHPQQHPNHPQQPPFFYHMAGFRIYHPVRYYY
IQNVYTPVDEHVYPGHRVVDPNIEMIPGAHSLPSGHLYSMSESEMNALRNFVDRNVKDGLMTPTVAPNGAQVL
QVKRGWKLQVTYNCRAPQSGTIQNQYLRMSLPNMGDPAHLASYGEFVQVPGYPYPAYVYYTSPHMMTAWYPVG
RDVHGRIIVVPVVITWSQNTNRQPPVPQYPPPQPPPPPPPPPPPPPPPPPASSCSAA*
>ENSMUSP00000135076_c.1050:GCA>CCC_p.A351P ENSMUST00000176551.3|Peg10-
203|ENSMUSG00000092035|+|chr6:g.4756476:GCA>CCC
MLQIHMPGRPTLFVRAMIDSGASGNFIDQDFVIQNAIPLRIKDWPVMVEAIDGHPIASGPIILETHHLIVDLG
DHREILSFDVTQSPFFPIVLGIRWLSTHDPHITWSTRSIVFNSDYCRLRCRMFAQIPSNLLFTVPQPNLHPYL
LHHVHPHVHPHMHQHLHQHLHQFLHPDPHQYPHPDPHYHHHQQADMQHQLQQYLYQYLYYHLYPVMHHHLPPD
QHEHLHEYLHQYLHQYLHQFLHHHLHPDLHQYLYQYLHNHMNPDPHHHPHPDPPQDPHHPPHQDPHQHPDPHQ
DPPHQDPHQHPDPHQDPPHQDPHQHPDPHQDPPHQDPHQDAHQHQDPHQDAHQDPHQDPHIRIPIWIHTCIST
SIRSRSRIHNSILTILSSHHSSTTWLDSEFTTL*GITIFRMCIHLLMSMSIRVTGWLTLTLR*FLERTACPVD

ICTQCLSLK*MLCEISWTGMLKMGS*LPLWRPMEPKSCK*KEGGNSKSLTIAELHRVAPSKISTYACLFQIWE
TLHTWQAMVNLSKFLATHIQPMFTIQARI**LRGTQ*DEMYMDE*SLCLL*SPGLKIRTASLRCPSILLRSHL
HHHHHLHRHHHLHQHHPAVLR
>ENSMUSP00000127306_c.2253:GCA>CCC_p.A752P ENSMUST00000166678.2|Peg10-
201|ENSMUSG00000092035|+|chr6:g.4756476:GCA>CCC
MAAAGGSSNCPPPPPPPPPNNNNNNNTPKSPGVPDAEDDDERRHDELPEDINNFDEDMNRQFENMNLLDQVEL
LAQSYSLLDHLDDFDDDDEDDDFDPEPDQDELPEYSDDDDLELQGAAAAPIPNFFSDDDCLEDLPEKFDGNPD
MLGPFMYQCQLFMEKSTRDFSVDRIRVCFVTSMLIGRAARWATAKLQRCTYLMHNYTAFMMELKHVFEDPQRR
EAAKRKIRRLRQGPGPVVDYSNAFQMIAQDLDWTEPALMDQFQEGLNPDIRAELSRQEAPKTLAALITACIHI
ERRLARDAAAKPDPSPRALVMPPNSQTDPTEPVGGARMRLSKEEKERRRKMNLCLYCGNGGHFADTCPAKASK
NSPPGLPGPAVGGPSATGPERIRSPPSEASTQHLQVMLQIHMPGRPTLFVRAMIDSGASGNFIDQDFVIQNAI
PLRIKDWPVMVEAIDGHPIASGPIILETHHLIVDLGDHREILSFDVTQSPFFPIVLGIRWLSTHDPHITWSTR
SIVFNSDYCRLRCRMFAQIPSNLLFTVPQPNLHPYLLHHVHPHVHPHMHQHLHQHLHQFLHPDPHQYPHPDPH
YHHHQQADMQHQLQQYLYQYLYYHLYPVMHHHLPPDQHEHLHEYLHQYLHQYLHQFLHHHLHPDLHQYLYQYL
HNHMNPDPHHHPHPDPPQDPHHPPHQDPHQHPDPHQDPPHQDPHQHPDPHQDPPHQDPHQHPDPHQDPPHQDP
HQDAHQHQDPHQDAHQDPHQDPHIRIPIWIHTCISTSIRSRSRIHNSILTILSSHHSSTTWLDSEFTTL*GIT
IFRMCIHLLMSMSIRVTGWLTLTLR*FLERTACPVDICTQCLSLK*MLCEISWTGMLKMGS*LPLWRPMEPKS
CK*KEGGNSKSLTIAELHRVAPSKISTYACLFQIWETLHTWQAMVNLSKFLATHIQPMFTIQARI**LRGTQ*
DEMYMDE*SLCLL*SPGLKIRTASLRCPSILLRSHLHHHHHLHRHHHLHQHHPAVLR
>ENSMUSP00000158794_c.4095:C>A_p.L1366I ENSMUST00000238599.2|Mgam-
208|ENSMUSG00000068587|+|chr6:g.40666353:C>A
MAGRRFKKFSKLELVLIVLLLVLFIIAVVLIVLLANSTPENAIGAETTGTPDTTVTRTTLNSPNCPVLSELER
INCIPDQSSNKGTCDERGCCWDPQGSISVPCYYSRNHGYKMESDVVNTNAGFTATLKNLPSAPVFGNSIENIL
LTAEYQTSNRFHFKLTDQTKKRYEVPHEHVQPFSGNAPSSLNYKVEVSKEPFSIKVTRKSNNRVLFDSSIGPL
LFSDQFLQFSTHLPSANVYGLGEHVHQQYRHNMNWKTWPMFSRDTTPNEDGTNLYGVQTFFLCLEDNSGLSFG
VFLMNSNAMEVTLQPTPAITYRTTGGILDFYVFLGNTPEQVVQEYLELIGRPALPSYWTLGFQLSRYDYKSLD
NMKAVVERNRAAQLPYDVQHADIDYMDQKKDFTYDPVNFKGFPEFVKELHNNGQKLVIILDPAISNNSFSSNP
YGPYDRGSAMKIWVNSSDGISPVIGKVWPGTTVFPDYTSPNCAVWWTKEFELFHKEVEFDGIWIDMNEVSNFI
DGSFSGCSQNNLNYPPFTPKVLDGYLFSKTLCMDAVQHWGKQYDVHNLYGYSMAIATAKAVKDVFPDKRSFII
TRSTFAGSGKFAAHWLGDNTATWKDLQWSIPGMLEFNLFGIPMVGADICGFAQDTYEELCRRWMQLGAFYPFS
RNHNGQGYKDQDPASFGNNSLLLNSSRHYLNIRYTLLPYLYTLFYRAHSRGDTVARPLLHEFYDDNNTWGIDR
QFLWGPGLLITPVLDQGAEKVKAYVPNATWYDYETGEELGWRKQSIEMQLPGDKIGLHLRGGYIFPTQQPATT
TEASRKNPLGLIVALDENKEARGELFWDDGESKDTVAQNIYLFSEFSVTQNHLDVTISSPNYKDPNNLEFQEI
KIFGTREFRNVRVKQNGNLLQMSPQVTYNPNLKVATITNIHLRLGEAYTVEWDFFTREEERIDCYPDEHGASE
ANCSARGCIWEASNTTRGPPCYFAHELYSVSNVQYDSHGATADISLKASTYSNAFPSTPVNKLKLQVTYHKNE
MLQFKIYDPNHSRYEVPVPLNIPSAPLSTPEGRLYDVLIKENPFGIQIRRKTTGTVIWDSQLLGFTFNDMFIR
ISTRLPSTYIYGFGETEHTTFKIDMNWHTWGMFSRDEPPGYKKNSYGVHPYYMGLEEDGNAHGVLLMNSNAMD
VTFQPMPALTYRTIGGILDFYVFLGPTPEIVTQQYTELIGRPVMVPYWSLGFQLCRYGYENDTEIANLYDEMV
AKQIPYDVQYSDIDYMERQLDFKLSPKFSGFPALINRMKANGMRVILILDPAISGNETQPYPAFTQGVENDVF
IRYPNNGGIVWGKVWPDYPNITVDSSLDWDIQVERYRAYVAFPDFFRDSTAIWWKKEIKELHSNSQDQAKSLK
FDGLWIDMNEPSSFVNGAVPSGCSDATLNHPPYMPHVEGRDRGLSSKTLCMESEHILPDGSRVRHYDVHSLYG
WSQTRPTYEAMQEVTGERGIVITRSTFPSSGRWGGHWLGDNTAAWDQLGKSIIGMMDFSLFGISYTGSDICGF
FQDAEYEMCVRWMQLGAFYPFSRNHNTIGTKRQDPVSWDKTFEDISRSVLETRYTLLPYLYTLMYKAHKEGST
VVRPLLHEFLADRETWNIDKQFLLGPAFLVSPVLEPNARNVTAYFPKAQWYDYYTGADINSTGWTTLPAPLEH
INLHVRGGYILPWQQPALNTNLSRKKPLGLLIALDEKKEARGELFWDDGQSKDTVAKNIYLFSEFSVTQNHLD
VTISSPNYKDPNNLEFQEIKIFGTQEFSNVRVKQNGNLLQMSPQVTYNPNLKVATITNIHLRLGEAYTVEWGF
FTREEERIDCYPDEHGASEANCSARGCIWEESNTIGVPTCYFAHELYSVSNVQYDSHGATADISLKASTYSNA
FPSTPVNKLKLQVTYHKNEMLQFKIYDPNNSRYEVPVPLNIPSAPSSTNEGRLYDVLIKENPFGIQVRRKSTG
TVIWDSQLLGFTFNDMFIRISTRLPSTYIYGFGETEHTTFKIDMNWHTWGMFSRDEPPGYKKNSYGVHPYYMG
LEEDGNAHGVLLMNSNAMDVTFQPMPALTYRTTGGILDFYVLLGPTPEIVTQQYTELIGRPVMVPYWSLGFQL
CRYGYENDSEIASLYDEMVDKQIPYDVQYSDIDYMERQLDFKLSPKFSGFPALINRMKANGMRVILILDPAIS
GNETQPYPAFTRGVENDVFIRYPNNGSIVWGKVWPDYPNITVDPSLGWDHQVEQYRAYVAFPDFFRNSTATWW
KKEIEELYTNTEEPEKSLKFDGLWIDMNEPSSFVNGAVPSGCSDATLNHPPYMPHVEGRDRGLSSKTLCMESE
HILPDGSRVQHYDVHSLYGWSQTRPTYEAVQEVTGERGIVITRSTFPSSGRWGGHWLGDNTAAWDQLGKSIIG
MMDFSLFGISYTGSDICGFFQDAEYEMCVRWMQLGAFYPFSRNHNTIGTKRQDPVSWNKTFEDISRSVLETRY
TLLPYLYTLMYKAHMEGSTVVRPLLHEFVSDRKTWNIDKQFLLGPAFLVSPVLEPNARNISAYFPTALWYDYY
TGANINSTGEWKTLPAPLEHINLHVRGGYILPWQRPALNTHLSRKNPLGLLIALDENKEARGELFWDDGQSKD
LTTNNILCKFSVTQNRLDVSTLQSTYMNPDNLAFQEIKIFGTRPLYNVTVKHNGMVNQMAPQVSYDPSMKVAV

ITGLQLLLNESYTVEWDDSIRDEEKIDCYPDQHGASETSCTARGCVWEESNSDVVPFCYFVNELYSVSNVQYG
SHGATANISLKASPYSGAFPSTPVKELQLQVTYHKNEMLQFKIYDPNHSRYEVPVPLNIPSAPSSTPESRLYD
VLIKENPFGIEIRRKGTGTVVWDSQLLGFTFSDMFIRISTRLPSTYIYGFGETEHTTFKIDMNWNKWGMFSRD
EPPGYRKNSYGVHPYYMGLEEDGNAHGVLLMNSNAMDVTFQPMPALTYRTTGGILDFYVFLGPTPEFVTQQYT
ELIGRPVMVPYWSLGFQLCRYGYENDSEIASLYDEMVDKKIPYDVQYSDIDYMERQLDFKLSPKFSGFPALIN
RMKANGMRVILILDPAISGNETEPYPAFTRGVENDVFIRYPNNGSIVWGKVWPDYPNITVDPSLGWDHQVEQY
RAYVAFPDFFRNSTATWWKKEIKELHSNTQDPAKSLKFDGLWIDMNEPSSFVNGAVPSGCSDATLNHPPYMPY
LEARDRGLSSKTLCMESEQILPDGSRVRHYDVHNLYGWSQTRPTYEAVQEVTGERGIVITRSTFPSSGRWGGH
WLGDNTAAWDQLGKSIIGMMDFSLFGISYTGSDICGFFQDAEYEMCVRWMQLGAFYPFSRNHNTIGTRRQDPV
SWNKTFEDISRSVLETRYTLLPYLYTLMYKAHTEGSTVVRPLLHEFVSDRETWNIDKQFLLGPAFLVSPVLEP
NARKVEAYFPRARWYDYYKGVDINATGEWKTLEAPLEYINLHIRGGYILPWQEPAMNTHLSRQKFMGLRAALN
AEGRAEGWLFWDDGKRINTDQYYLARFSVNQTTLLTHEKFNNYLTGTAPLYLGYIDIWGLSSSSITNVSISWN
TNNEEVFANYNSTTKILSVNMTDKKISLHTFNSLTWSSS*
>ENSMUSP00000153815_c.208:T>A_p.I70N ENSMUST00000227909.2|Vmn1r20-202|ENSMUSG00000115253|+|chr6:g.57408884:T>A
MFSLKNVLYFQAGLGVLANMSLLCFYIFIIVGHRPKPTDLISCQLTFIHIMVFLTGGDLLLTDIFESLNNNND
FKCKATFYISRVMRCLSICTSCLLSVFQAVTISPSTSCLAKFKQKLRKYMVYVFLCIWSFSFSYSSSRIFYVR
GFTNVSETNQMQITKSCLLFRMNSIIRVFIFTVTTSRDVFLVGMMLTTSTYMVIILCRHQRQCKHLHSIKHLR
ASPEKRATQTILLLVIFFVVMYWVDFIISTISVLLWRYDTVILRVQKFLMYAYPTISPLVQISSDNRIIIMVK
NMYSKHHQRFF*
>ENSMUSP00000153909_c.208:T>A_p.I70N ENSMUST00000226808.2|Vmn1r20-201|ENSMUSG00000115253|+|chr6:g.57408884:T>A
MFSLKNVLYFQAGLGVLANMSLLCFYIFIIVGHRPKPTDLISCQLTFIHIMVFLTGGDLLLTDIFESLNNNND
FKCKATFYISRVMRCLSICTSCLLSVFQAVTISPSTSCLAKFKQKLRKYMVYVFLCIWSFSFSYSSSRIFYVR
GFTNVSETNQMQITKSCLLFRMNSIIRVFIFTVTTSRDVFLVGMMLTTSTYMVIILCRHQRQCKHLHSIKHLR
ASPEKRATQTILLLVIFFVVMYWVDFIISTISVLLWRYDTVILRVQKFLMYAYPTISPLVQISSDNRIIIMVK
NMYSKHHQRFF*
>ENSMUSP00000087096_c.486:T>A_p.C163S ENSMUST00000089667.8|Reg3d-201|ENSMUSG00000068341|-|chr6:g.78353095:A>T
MVSHKTLHSMSWMLLCCLMSLSWVQGEQSQKKLSSPRISCPQEAQAYGSYCYLLILEPQTWANAEIHCQKHFS
GHLAFLLTYGEIIFVSSLVKNSLTTFPYIWIGLHDLSLGSLPNENGWKWSSSDPLTFYNWEIPPSMSAHHGYC
AALSQASGYQKWRDYYSDTIFPYVCKFKG*
>ENSMUSP00000144715_c.348:T>A_p.C117S ENSMUST00000205240.2|Reg3d-203|ENSMUSG00000068341|-|chr6:g.78353095:A>T
MVSHKTLHSMSWMLLCCLMSLSWVQGEQSQKKLSSPRISCPQEAQAYGSYCYLLILEPQTWANAEGSLPNENG
WKWSSSDPLTFYNWEIPPSMSAHHGYCAALSQASGYQKWRDYYSDTIFPYVCKFKG*
>ENSMUSP00000132220_c.348:T>A_p.C117S ENSMUST00000167492.4|Reg3d-202|ENSMUSG00000068341|-|chr6:g.78353095:A>T
MVSHKTLHSMSWMLLCCLMSLSWVQGEQSQKKLSSPRISCPQEAQAYGSYCYLLILEPQTWANAEGSLPNENG
WKWSSSDPLTFYNWEIPPSMSAHHGYCAALSQASGYQKWRDYYSDTIFPYVCKFKG*
>ENSMUSP00000131840_c.4165:C>G_p.S1389* ENSMUST00000163139.8|Plxna1-202|ENSMUSG00000030084|-|chr6:g.89306663:G>C
MPLPPLSSRTLLLLLLLLLLRGVWIAISSPPAGLGPQPAFRTFVASDWGLTHLVVHEQTGEVYVGAVNRIYKLS
GNLTLLRAHVTGPVEDNEKCYPPPSVQSCPHGLGSTDNVNKLLLLDYAANRLLACGSASQGICQFLRLDDLFK
LGEPHHRKEHYLSSVREAGSMAGVLIAGPPGQGQAKLFVGTPIDGKSEYFPTLSSRRLMANEEDADMFGFVYQ
DEFVSSQLKIPSDTLSKFPAFDIYYVYSFRSEQFVYYLTLQLDTQLTSPDAAGEHFFTSKIVRLCVNDPKFYS
YVEFPIGCEQAGVEYRLVQDAYLSRPGQALAKQLGLAEDEEVLFTVFAQGQKNRVKPPKESALCLFTLRAIKE
KIKERIQSCYRGEGKLSLPWLLNKELGCINSPLQIDDDFCGQDFNQPLGGTVTIEGTPLFVDKEDGLTAVAAY
DYQGRTVVFAGTRSGRIRKILVDLANPSGRPALAYESVVAQEGNPILRDLVLSPNRQYLYAMTEKQVTQVPVE
SCVQYTSCELCLGSRDPHCGWCVLHSICSRQDACERAEEPQRFASDLLQCVQLTVQPRNVSVTMSQVPLVLQA
WNVPDLSAGVNCSFEDFTETESILEDGRIHCHSPSAREVAPITQGQGDQRVVKLYLKSKETGKKFASVDFVFY
NCSVHQSCLACVNGSFPCHWCKYRHVCTNNAADCAFLEGRVNMSEDCPQILPSTHIYVPVGVVKPITLAARNL
PQPQSGQRGYECLFHIPGSPARVTALRFNSSSLQCQNSSYSYEGNDVSDLPVNLSVVWNGNFVIDNPQNIQAH
LYKCPALRQSCGLCLKADPRFECGWCVAERRCSLRHHCPADSPASWMHAHHGSSRCTDPKILKLSPETGPRQG
GTRLTITGENLGLRFEDVRLGVHVGKVLCSPVESEYISAEQIVCEIGDASTLRAHDALVEVCVRDCSLHYRAL
SPKRFTFVTPTFYRVSPSRGPLSGGTWIGIEGSHLNAGSDVAVSIGGRPCSFSWRNSREIRCLTPPGHTPGSA
PIVININRAQLSNPEVKYNYTEDPTILRIDPEWSINSGGTLLTVTGTNLATVREPRIRAKYGGIERENSCMVY
NDTTMVCRAPSIDNPKRSPPELGERPDEIGFIMDNVRTLLVLNSSSFLYYPDPVLEPLSPTGLLELKPSSPLI

LKGRNLLPPAPGNSRLNYTVLIGSTPCILTVSETQLLCEAPNLTGQHKVTVRAGGFEFSPGMLQVYSDSLLTL
PAIVGIGGGGGLLLLVIVAVLIAYKRKSRDADRTLKRLQLQMDNLESRVALECKEAFAELQTDIHELTSDLDG
AGIPFLDYRTYAMRVLFPGIEDHPVLKEMEVQANVEKSLTLFGQLLTKKHFLLTFIRTLEAQRSFSMRDRGNV
A*LIMTALQGEMEYATGVLKQLLSDLIEKNLESKNHPKLLLRRTESVAEKMLTNWFTFLLYKFLKECAGEPLF
MLYCAIKQQMEKGPIDAITGEARYSLSEDKLIRQQIDYKTLTLNCVNPEHENAPEVPVKGLNCDTVTQVKEKL
LDAVYKGVPYSQRPKAGDMDLEWRQGRMARIILQDEDVTTKIDNDWKRLNTLAHYQVTDGSSVALVPKQTSAY
NISNSSTFTKSLSRYESMLRTASSPDSLRSRTPMITPDLESGTKLWHLVKNHDHLDQREGDRGSKMVSEIYLT
RLLATKGTLQKFVDDLFETIFSTAHRGSALPLAIKYMFDFLDEQADKHQIHDSDVRHTWKSNCLPLRFWVNVI
KNPQFVFDIHKNSITDACLSVVAQTFMDSCSTSEHKLGKDSPSNKLLYAKDIPNYKSWVERYYADIAKMPAIS
DQDMSAYLAEQSRLHLSQFNSMSALHEIYSYIAKYKDEILVALEKDEQARRQRLRSKLEQVVDTMALSS*
>ENSMUSP00000063066_c.4165:C>G_p.S1389* ENSMUST00000049845.6|Plxna1-201|ENSMUSG00000030084|-|chr6:g.89306663:G>C
MPLPPLSSRTLLLLLLLLLRGVWIAISSPPAGLGPQPAFRTFVASDWGLTHLVVHEQTGEVYVGAVNRIYKLS
GNLTLLRAHVTGPVEDNEKCYPPPSVQSCPHGLGSTDNVNKLLLLDYAANRLLACGSASQGICQFLRLDDLFK
LGEPHHRKEHYLSSVREAGSMAGVLIAGPPGQGQAKLFVGTPIDGKSEYFPTLSSRRLMANEEDADMFGFVYQ
DEFVSSQLKIPSDTLSKFPAFDIYYVYSFRSEQFVYYLTLQLDTQLTSPDAAGEHFFTSKIVRLCVNDPKFYS
YVEFPIGCEQAGVEYRLVQDAYLSRPGQALAKQLGLAEDEEVLFTVFAQGQKNRVKPPKESALCLFTLRAIKE
KIKERIQSCYRGEGKLSLPWLLNKELGCINSPLQIDDDFCGQDFNQPLGGTVTIEGTPLFVDKEDGLTAVAAY
DYQGRTVVFAGTRSGRIRKILVDLANPSGRPALAYESVVAQEGNPILRDLVLSPNRQYLYAMTEKQVTQVPVE
SCVQYTSCELCLGSRDPHCGWCVLHSICSRQDACERAEEPQRFASDLLQCVQLTVQPRNVSVTMSQVPLVLQA
WNVPDLSAGVNCSFEDFTETESILEDGRIHCHSPSAREVAPITQGQGDQRVVKLYLKSKETGKKFASVDFVFY
NCSVHQSCLACVNGSFPCHWCKYRHVCTNNAADCAFLEGRVNMSEDCPQILPSTHIYVPVGVVKPITLAARNL
PQPQSGQRGYECLFHIPGSPARVTALRFNSSSLQCQNSSYSYEGNDVSDLPVNLSVVWNGNFVIDNPQNIQAH
LYKCPALRQSCGLCLKADPRFECGWCVAERRCSLRHHCPADSPASWMHAHHGSSRCTDPKILKLSPETGPRQG
GTRLTITGENLGLRFEDVRLGVHVGKVLCSPVESEYISAEQIVCEIGDASTLRAHDALVEVCVRDCSLHYRAL
SPKRFTFVTPTFYRVSPSRGPLSGGTWIGIEGSHLNAGSDVAVSIGGRPCSFSWRNSREIRCLTPPGHTPGSA
PIVININRAQLSNPEVKYNYTEDPTILRIDPEWSINSGGTLLTVTGTNLATVREPRIRAKYGGIERENSCMVY
NDTTMVCRAPSIDNPKRSPPELGERPDEIGFIMDNVRTLLVLNSSSFLYYPDPVLEPLSPTGLLELKPSSPLI
LKGRNLLPPAPGNSRLNYTVLIGSTPCILTVSETQLLCEAPNLTGQHKVTVRAGGFEFSPGMLQVYSDSLLTL
PAIVGIGGGGGLLLLVIVAVLIAYKRKSRDADRTLKRLQLQMDNLESRVALECKEAFAELQTDIHELTSDLDG
AGIPFLDYRTYAMRVLFPGIEDHPVLKEMEVQANVEKSLTLFGQLLTKKHFLLTFIRTLEAQRSFSMRDRGNV
A*LIMTALQGEMEYATGVLKQLLSDLIEKNLESKNHPKLLLRRTESVAEKMLTNWFTFLLYKFLKECAGEPLF
MLYCAIKQQMEKGPIDAITGEARYSLSEDKLIRQQIDYKTLTLNCVNPEHENAPEVPVKGLNCDTVTQVKEKL
LDAVYKGVPYSQRPKAGDMDLEWRQGRMARIILQDEDVTTKIDNDWKRLNTLAHYQVTDGSSVALVPKQTSAY
NISNSSTFTKSLSRYESMLRTASSPDSLRSRTPMITPDLESGTKLWHLVKNHDHLDQREGDRGSKMVSEIYLT
RLLATKGTLQKFVDDLFETIFSTAHRGSALPLAIKYMFDFLDEQADKHQIHDSDVRHTWKSNCLPLRFWVNVI
KNPQFVFDIHKNSITDACLSVVAQTFMDSCSTSEHKLGKDSPSNKLLYAKDIPNYKSWVERYYADIAKMPAIS
DQDMSAYLAEQSRLHLSQFNSMSALHEIYSYIAKYKDEILVALEKDEQARRQRLRSKLEQVVDTMALSS*
>ENSMUSP00000154788_c.680:TA>GTG_p.L227ins-fs ENSMUST00000226436.2|Vmn1r42-202|ENSMUSG00000068232|-|chr6:g.89821887:TA>CAC
MGDILFSSPQSMFSHTMNKNSILHTHSIIGKTFFSEIGIGISGNSFLLLVHILKFIRGHRPRLTDLPIGLLSL
IHLLMLLVAAFIATDIFISRRGWDDIICKFLVYLYRVLRGFSLCTTSMLSILQAIILSPRSSCLAKFKHISPH
HISGAILFLSVLYMLIGSQLLVSIIATPNLTMNDFIYVTQSCSILPLSYLMQSIYSTLLAIREFFLISLMVLS
NWYMVALL
>ENSMUSP00000154442_c.680:TA>GTG_p.L227ins-fs ENSMUST00000227279.2|Vmn1r42-203|ENSMUSG00000068232|-|chr6:g.89821887:TA>CAC
MGDILFSSPQSMFSHTMNKNSILHTHSIIGKTFFSEIGIGISGNSFLLLVHILKFIRGHRPRLTDLPIGLLSL
IHLLMLLVAAFIATDIFISRRGWDDIICKFLVYLYRVLRGFSLCTTSMLSILQAIILSPRSSCLAKFKHISPH
HISGAILFLSVLYMLIGSQLLVSIIATPNLTMNDFIYVTQSCSILPLSYLMQSIYSTLLAIREFFLISLMVLS
NWYMVALL
>ENSMUSP00000086840_c.680:TA>GTG_p.L227ins-fs ENSMUST00000089419.3|Vmn1r42-201|ENSMUSG00000068232|-|chr6:g.89821887:TA>CAC
MGDILFSSPQSMFSHTMNKNSILHTHSIIGKTFFSEIGIGISGNSFLLLVHILKFIRGHRPRLTDLPIGLLSL
IHLLMLLVAAFIATDIFISRRGWDDIICKFLVYLYRVLRGFSLCTTSMLSILQAIILSPRSSCLAKFKHISPH

HISGAILFLSVLYMLIGSQLLVSIIATPNLTMNDFIYVTQSCSILPLSYLMQSIYSTLLAIREFFLISLMVLS
NWYMVALL
>ENSMUSP00000114304_c.2360:G>C_p.Q787H ENSMUST00000130418.8|Aldh1l1-203|ENSMUSG00000030088|+|chr6:g.90573863:G>C
MKIAVIGQSLFGQEVYCQLRKEGHEVVGVFTIPDKDGKADPLGLEAEKDGVPVFKFPRWRARGQALPEVVAKY
QALGAELNVLPFCSQFIPMEVINAPRHGSIIYHPSLLPRHRGASAINWTLIHGDKKGGFTIFWADDGLDTGDL
LLQKECDVLPDDTVSTLYNRFLFPEGIKGMVQAVRLIAEGTAPRRPQPEEGATYEGIQKKETAMINWDQPAEA
IHNWIRGNDKVPGAWTEACGQKLTFFNSTLNTSGLVAQGEALPIPGAHRPGLVTKAGLILFGNDDRMLLVKNI
QLEDGKMMPASQFFKGSASSALELTEEELATAEAVRSSWMRILPNVPEVEDSTDFFKSGAASVDVVRLVEEVK
ELCDGLELENEDVYMATTFGDFIQLLVRKLRGEDGESECVINYVEKAVKKLTLQMPYQLFIGGEFVDAEGAKT
YSTINPTDGSVICQVSLAQVSDVDKAVAAAKEAFENGLWGKINARDRGRLLYRLADLMEQHQEELATIEALDA
GAVYTLALKTHVGMSIQTFRYFAGWCDKIQGATIPINQARPNRNLTLTKKEPVGVCGIVIPWNYPLMMLSWKT
AACLAAGNTVVIKPAQVTPLTALKFAELTLKAGIPKGVVNILPGSGSLVGQRLSDHPDVRKIGFTGSTEVGKH
IMKSCALSNVKKVSLELGGKSPLIIFADCDLNKAVQMGMSSVFFNKGENCIAAGRLFVEDSIHDQFVQKVVEE
VGKMKIGNPLDRDTNHGPQNHEAHLRKLVEYCQRGVKEGATLVCGGNQVPRPGFFFHPTVFTDVEDHMYIAKE
ESFGPIMIISRFADGDVDAVLSRANATEFGLASGVFTRDINKALYVSDKLQAGTVFVNTYNKTDVAAPFGGFK
QSGFGKDLGEAALNEYLRIKTVTFEY*
>ENSMUSP00000032175_c.2360:G>C_p.Q787H ENSMUST00000032175.11|Aldh1l1-201|ENSMUSG00000030088|+|chr6:g.90573863:G>C
MKIAVIGQSLFGQEVYCQLRKEGHEVVGVFTIPDKDGKADPLGLEAEKDGVPVFKFPRWRARGQALPEVVAKY
QALGAELNVLPFCSQFIPMEVINAPRHGSIIYHPSLLPRHRGASAINWTLIHGDKKGGFTIFWADDGLDTGDL
LLQKECDVLPDDTVSTLYNRFLFPEGIKGMVQAVRLIAEGTAPRRPQPEEGATYEGIQKKETAMINWDQPAEA
IHNWIRGNDKVPGAWTEACGQKLTFFNSTLNTSGLVAQGEALPIPGAHRPGLVTKAGLILFGNDDRMLLVKNI
QLEDGKMMPASQFFKGSASSALELTEEELATAEAVRSSWMRILPNVPEVEDSTDFFKSGAASVDVVRLVEEVK
ELCDGLELENEDVYMATTFGDFIQLLVRKLRGEDGESECVINYVEKAVKKLTLQMPYQLFIGGEFVDAEGAKT
YSTINPTDGSVICQVSLAQVSDVDKAVAAAKEAFENGLWGKINARDRGRLLYRLADLMEQHQEELATIEALDA
GAVYTLALKTHVGMSIQTFRYFAGWCDKIQGATIPINQARPNRNLTLTKKEPVGVCGIVIPWNYPLMMLSWKT
AACLAAGNTVVIKPAQVTPLTALKFAELTLKAGIPKGVVNILPGSGSLVGQRLSDHPDVRKIGFTGSTEVGKH
IMKSCALSNVKKVSLELGGKSPLIIFADCDLNKAVQMGMSSVFFNKGENCIAAGRLFVEDSIHDQFVQKVVEE
VGKMKIGNPLDRDTNHGPQNHEAHLRKLVEYCQRGVKEGATLVCGGNQVPRPGFFFHPTVFTDVEDHMYIAKE
ESFGPIMIISRFADGDVDAVLSRANATEFGLASGVFTRDINKALYVSDKLQAGTVFVNTYNKTDVAAPFGGFK
QSGFGKDLGEAALNEYLRIKTVTFEY*
>ENSMUSP00000080469_c.2562:A>G_p.R855G ENSMUST00000081777.8|Mug2-201|ENSMUSG00000030131|+|chr6:g.122048798:A>G
MWKSRRAQLCLFSVLLAFLPSASSLNGDSKYMVLVPSQLYTETPEKICLHLYHLNETVTVTASLVSQTGRRNL
FDELVVDKDLFQCVSFIIPTLNSPDEEEFLYVDIKGPTHEFSKRNAVLVKNKESVVFVQTDKPVYKPGQSVKF
RVVSMDKTLRPLNELLPLAYIEDPKKNRIMQWRDIKTENGLKQMSFSLAAEPIQGPYKIVVHKQSGVKEEHSF
TVMEFVLPRFNVDLKVPNAISVNDEVLQVTVCGKYTYGKPVPGQVKISICHETEAGCKEVNSKLDNNGCSTQE
VNITELQSKKRNYEVQLFHVNATVTEEGTGLEFNGYGTTKIERITNKLIFLKADSHFRHGIPFFVKVRLVDIK
GDPIPNERVFIKAQVLGYTSATTTDQHGLAKFSIDTAGFSGSSLHIKVNHKGKDSCYFFYCMEERYASAEHVA
YAVYSLSKSYIYLVKETSSILPCNQIHTVQAHFILKGDLGVLKELVFYYLVMAQGSIIQTGNHTHQVEPGEAP
VKGNFDLEIPVEFSMAPMAKMLIYTILPDGEVIADSVNFEIEKCLRNKVDLSFSSSQSLPASQTRLQVTASPQ
SLCGLRAVDQSVLLLKPEDELSPSWIYNLPGMQHNKFIPSSSLSEDREDCILYSSWVAEKHTDWVPHGREKDV
YRYVEDMDLKAFTNLKIKLPKICFDSAPMSGPRGKFDLAFSSEVSGTLQKGSSKRPQPEEPPREDPPPKDPLA
ETIRKYFPETWVWDIVTVNSTGVAEVEMTVPDTITEWKAGALCLSNDTGLGLSSVVPLQAFQPFFVEVSLPYS
VVRGEAFMLKATVMNYLPTSMRMSVQLEASPDFTAVPVGDDHDSYCLSANGGHTSSWLVTPKSLGNVNFSVSV
EAQQSSEPCGSEVATVPETGRKDTVVKVLIVEPEGIKQEHTFNSLFCASDAEISEKMSLVLPPTVVKDSARAH
FSVMGDILSSAIKNTQNLLHMPYGCGEQNMVLFAPNIYVLKYLDKTQQLTQKIKTKALGFLRAGYQRELNYKH
KDGSYSAFGDQNGEREGNTWLTAFVLKSFAQARAFIFIDESHITHAFTWLSQQQKDNGCFRSSGSLFHNDIKH
PVVSKALSCLESSWKTIEQGRNANFVYTKALMAYAFALAGNQDKRNEILKSLDEEAIKEDNSIHWERPQKPRK
SEHNLYKPQASSVEVEMNAYVVLARLTAQPAPSPEDLTLSRSTIMWLTKQQNSNGGFSSTQDTVVALDALSKY
GAVTFSRRQKTSLVTIQSTGSFSQKFQVENSNCLLLQQVPLPDIPGDYTISVSGEGCVYAQTTLRYNMHLEKQ
QSAFALRVQTVPLTCNNPKGHNSFQISLEISYTGSRPASNMVIADVKMLSGFIPLKPTVKKLERLEHISRTEV
SNNNVLLYLDQVTNQTLAFSFIIQQDISVRNLQPAIVKVYDYYETDEVAYAEYSSPCSSDKQNV*
>ENSMUSP00000048092_c.460:G>T_p.C154F ENSMUST00000042081.9|C3ar1-201|ENSMUSG00000040552|-|chr6:g.122827755:C>A
MESFDADTNSTDLHSRPLFQPQDIASMVILGLTCLLGLLGNGLVLWVAGVKMKTTVNTVWFLHLTLADFLCCL
SLPFSLAHLILQGHWPYGLFLCKLIPSIIILNMFASVFLLTAISLDRCLIVHKPIWCQNHRNVRTAFAICGCV

WVVAFVMFVPVFVYRDLFIMDNRSICRYNFDSSRSYDYWDYVYKLSLPESNSTDNSTAQLTGHMNDRSAPSSV
QARDYFWTVTTALQSQPFLTSPEDSFSLDSANQQPHYGGKPPNVLTAAVPSGFPVEDRKSNTLNADAFLSAHT
ELFPTASSGHLYPYDFQGDYVDQFTYDNHVPTPLMAITITRLVVGFLVPFFIMVICYSLIVFRMRKTNFTKSR
NKTFRVAVAVVTVFFICWTPYHLVGVLLLITDPESSLGEAVMSWDHMSIALASANSCFNPFLYALLGKDFRKK
ARQSIKGILEAAFSEELTHSTNCTQDKASSKRNNMSTDV*
>ENSMUSP00000125303_c.1135:T>G_p.I379R ENSMUST00000160496.8|Ano2-204|ENSMUSG00000038115|+|chr6:g.125840466:T>G
MAAPGLRDIPLLPGSPRRLSSRTVARGSQGPKHGQQYLKVPGHRAPGQRDNSSLHPSQVSRRESSRDRSVINN
YLDANEPPSSEARLSRMHFHDNQRKVDYVLAYHYRKRGAHLGHGSPGHSLAVISNGETGKERHGGGPGDVELG
PLDALEEERREQRDEFEHNLMAAGLELEKDLESKSQGSVFVRIHAPWQVLAREAEFLKIKVPTKKMYEIKAGG
SIAKKFSAILQTLSSPLQPRVPEHSNNRMKNLSYPFSREKMYLYNIQEKDTFFDNATRSRIVHEILKRTACSR
ANNTMGINSLIANNIYEAAYPLHDGEYDSPGDDMNDRKLLYQEWARYGVFYKFQPIDLIRKYFGEKIGLYFAW
LGLYTSFLIPSSVRGVIVFLYGCATIEEDIPSKEMCDHQNAFTMCPLCDKSCDYWNLSSACGTARASHLFDNP
ATVFFSIFMALWATMFLENWKRLQMRLGYFWDLTGIEEEEERSQEHSRPEYETKVREKLLKESGKSAVQKLEA
NSPEDDEDDEDKLTWKDRFPGYLMNFASILFMIALTFSIVFGVIVYRITTAAALSLNKATRSNVRVTVTATAV
IINLVVILILDEIYGAVAKWLTKIEVPKTEQTFEERLILKAFLLKFVNAYSPIFYVAFFKGRFVGRPGSYVYV
FDGYRMEECAPGGCLMELCIQLSIIMLGKQLIQNNIFEIGVPKLKKLFRKLKDETEPGESDPDHSKRPEQWDL
DHSLEPYTGLTPEYMEMIIQFGFVTLFVASFPLAPVFALLNNVIEVRLDAKKFVTELRRPDAVRTKDIGIWFD
ILSGIGKFSVIINAFVIAVTSDFIPRLVYQYSYSHNGTLHGFVNHTLSFFNVSQLKEGTQPENSQFDQEVQFC
RFKDYREPPWAPNPYEFSKQYWSVLSARLAFVIIFQNLVMFLSVLVDWMIPDIPTDISDQIKKEKSLLVDFFL
KEEHEKVKLADEPTQRSQGGGDRSRRSRAASSAPSGRSQPGSIASSGSQHTNV*
>ENSMUSP00000125426_c.1045:T>G_p.I349R ENSMUST00000161619.8|Ano2-206|ENSMUSG00000038115|+|chr6:g.125840466:T>G
XKHGQQYLKVPGHRAPGQRDNSSLHPSQVSRRESSRDRSVINNYLDANEPPSSEARLSRMHFHDNQRKVDYVL
AYHYRKRGAHLGHGSPGHSLAVISNGETGKERHGGGPGDVELGPLDALEEERREQRDEFEHNLMAAGLELEKD
LESKSQGSVFVRIHAPWQVLAREAEFLKIKVPTKKMYEIKAGGSIAKKFSAILQTLSSPLQPRVPEHSNNRMK
NLSYPFSREKMYLYNIQEKDTFFDNATRSRIVHEILKRTACSRANNTMGINSLIANNIYEAAYPLHDGEYDSP
GDDMNDRKLLYQEWARYGVFYKFQPIDLIRKYFGEKIGLYFAWLGLYTSFLIPSSVRGVIVFLYGCATIEEDI
PSKEMCDHQNAFTMCPLCDKSCDYWNLSSACGTARASHLFDNPATVFFSIFMALWATMFLENWKRLQMRLGYF
WDLTGIEEEEERSQEHSRPEYETKVREKLLKESGKSAVQKLEANSPEDDEPSTS*
>ENSMUSP00000123965_c.145:T>G_p.I49R ENSMUST00000159984.2|Ano2-202|ENSMUSG00000038115|+|chr6:g.125840466:T>G
LLYQEWARYGVFYKFQPIDLIRKYFGEKIGLYFAWLGLYTSFLIPSSVRGVIVFLYGCATIEEDIPSKEMCDH
QNAFTMCPLCDKSCDYWNLSSACGTARASHLFDNPATVFFSIFMALWATMFLENWKRLQMRLGYFWDLTGIEE
EEEHSRPEYETKVREKLLKESGKSAVQKLEANSPEDDEPSTS*
>ENSMUSP00000125303_c.1150:T>G_p.F384C ENSMUST00000160496.8|Ano2-204|ENSMUSG00000038115|+|chr6:g.125840481:T>G
MAAPGLRDIPLLPGSPRRLSSRTVARGSQGPKHGQQYLKVPGHRAPGQRDNSSLHPSQVSRRESSRDRSVINN
YLDANEPPSSEARLSRMHFHDNQRKVDYVLAYHYRKRGAHLGHGSPGHSLAVISNGETGKERHGGGPGDVELG
PLDALEEERREQRDEFEHNLMAAGLELEKDLESKSQGSVFVRIHAPWQVLAREAEFLKIKVPTKKMYEIKAGG
SIAKKFSAILQTLSSPLQPRVPEHSNNRMKNLSYPFSREKMYLYNIQEKDTFFDNATRSRIVHEILKRTACSR
ANNTMGINSLIANNIYEAAYPLHDGEYDSPGDDMNDRKLLYQEWARYGVFYKFQPIDLIRKYFGEKIGLYFAW
LGLYTSFLIPSSVIGVIVCLYGCATIEEDIPSKEMCDHQNAFTMCPLCDKSCDYWNLSSACGTARASHLFDNP
ATVFFSIFMALWATMFLENWKRLQMRLGYFWDLTGIEEEEERSQEHSRPEYETKVREKLLKESGKSAVQKLEA
NSPEDDEDDEDKLTWKDRFPGYLMNFASILFMIALTFSIVFGVIVYRITTAAALSLNKATRSNVRVTVTATAV
IINLVVILILDEIYGAVAKWLTKIEVPKTEQTFEERLILKAFLLKFVNAYSPIFYVAFFKGRFVGRPGSYVYV
FDGYRMEECAPGGCLMELCIQLSIIMLGKQLIQNNIFEIGVPKLKKLFRKLKDETEPGESDPDHSKRPEQWDL
DHSLEPYTGLTPEYMEMIIQFGFVTLFVASFPLAPVFALLNNVIEVRLDAKKFVTELRRPDAVRTKDIGIWFD
ILSGIGKFSVIINAFVIAVTSDFIPRLVYQYSYSHNGTLHGFVNHTLSFFNVSQLKEGTQPENSQFDQEVQFC
RFKDYREPPWAPNPYEFSKQYWSVLSARLAFVIIFQNLVMFLSVLVDWMIPDIPTDISDQIKKEKSLLVDFFL
KEEHEKVKLADEPTQRSQGGGDRSRRSRAASSAPSGRSQPGSIASSGSQHTNV*
>ENSMUSP00000125426_c.1060:T>G_p.F354C ENSMUST00000161619.8|Ano2-206|ENSMUSG00000038115|+|chr6:g.125840481:T>G
XKHGQQYLKVPGHRAPGQRDNSSLHPSQVSRRESSRDRSVINNYLDANEPPSSEARLSRMHFHDNQRKVDYVL
AYHYRKRGAHLGHGSPGHSLAVISNGETGKERHGGGPGDVELGPLDALEEERREQRDEFEHNLMAAGLELEKD
LESKSQGSVFVRIHAPWQVLAREAEFLKIKVPTKKMYEIKAGGSIAKKFSAILQTLSSPLQPRVPEHSNNRMK
NLSYPFSREKMYLYNIQEKDTFFDNATRSRIVHEILKRTACSRANNTMGINSLIANNIYEAAYPLHDGEYDSP
GDDMNDRKLLYQEWARYGVFYKFQPIDLIRKYFGEKIGLYFAWLGLYTSFLIPSSVIGVIVCLYGCATIEEDI

PSKEMCDHQNAFTMCPLCDKSCDYWNLSSACGTARASHLFDNPATVFFSIFMALWATMFLENWKRLQMRLGYF
WDLTGIEEEEEERSQEHSRPEYETKVREKLLKESGKSAVQKLEANSPEDDEPSTS*
>ENSMUSP00000123965_c.160:T>G_p.F54C ENSMUST00000159984.2|Ano2-
202|ENSMUSG00000038115|+|chr6:g.125840481:T>G
LLYQEWARYGVFYKFQPIDLIRKYFGEKIGLYFAWLGLYTSFLIPSSVIGVIVCLYGCATIEEDIPSKEMCDH
QNAFTMCPLCDKSCDYWNLSSACGTARASHLFDNPATVFFSIFMALWATMFLENWKRLQMRLGYFWDLTGIEE
EEEHSRPEYETKVREKLLKESGKSAVQKLEANSPEDDEPSTS*
>ENSMUSP00000032396_c.337:A>C_p.E113A ENSMUST00000032396.13|Lrmp-
201|ENSMUSG00000030263|+|chr6:g.145093777:A>C
MLCVKGPPEQEPEDGALDVTRGCQCPLPTEGSILGQELLDCTRMNEDQSTDENGADHLYSESPSQLREYLTQP
SSEQTSSSESTVTSSESGSDILHMASGDLDCKPLCEKEEAARAASAMQGTSLAPAAYGDYTSVGVAKAASQLE
AGEELRTTENGGKGSAPGETEISMPPKASVKLVNFQQSENTSANEKEVEAEFLRLSLGLKCDWFTLEKRVKLE
ERSRDLAEENLKKEITNCLKLLESLTPLCEEDNQAQEIVKKLEKSIVLLSQCTARVASRAEMLGAINQESRVS
RAVEVMIQHVENLKRMYAKEHAELEDLKQALLQNDRSFNSLPDEDDCQIKKRSSSLNSKPSSLRRVTIASLPR
NLGNVGLVSGMENNDRFSRRSSSWRILGTKQGEHRPSLHRFISTYSWADAEDERSDVKARDAPEPQGEEAVEG
TRKPSLSERRSSTLAWDRGTICSSVASWVTHLQASFRRANRALWLTGLIIIILIAALMSFLTGQLFQTAVEAAP
TQEGDSWLSLEHILWPFTRLGHDGPLPV*
>ENSMUSP00000120166_c.397:A>C_p.E133A ENSMUST00000152571.8|Lrmp-
209|ENSMUSG00000030263|+|chr6:g.145093777:A>C
MLCVKGPPEQEPEDGALDVTRGCQCPLPTEGSILGQELLDCTRMNEDQSTDVSRTLFLSSEHLNGHRVQFEEN
GADHLYSESPSQLREYLTQPSSEQTSSSESTVTSSESGSDILHMASGDLDCKPLCEKEEAARAASAMQGTSLA
PAAYGDYTSVGVAKAASQLEAGEELRTTENGGKGSAPGETEISMPPKASVKL
>ENSMUSP00000115043_c.235:A>C_p.E79A ENSMUST00000156849.8|Lrmp-
210|ENSMUSG00000030263|+|chr6:g.145093777:A>C
XLFSFWGLLRGVSQSAAENGADHLYSESPSQLREYLTQPSSEQTSSSESTVTSSESGSDILHMASGDLDCKPL
CEKEEAARAASAMQGTSLAPAAYGDYTSVGVAKAASQLEAGEELRTTENGGKGSAPGETEISMPPKASVKLVN
FQQSENTSANEKEVEAEFLRLSLGLKCDWFTLEKRVKLEERSRDLAEENL
>ENSMUSP00000120248_c.232:A>C_p.E78A ENSMUST00000132948.2|Lrmp-
204|ENSMUSG00000030263|+|chr6:g.145093777:A>C
XFSFWGLLRGVSQSAAENGADHLYSESPSQLREYLTQPSSEQTSSSESTVTSSESGSDILHMASGDLDCKPLC
EKEEAARAASAMQGTSLAPAAYGDYTSVGVAKAASQLEAGEELRTTENGGKGSAPGETEISMPPKASVKLVNF
QQSENTSANEKEVEAEFLRLSLGLKCDWFTLEKRVKLEERSRDLAEENLKKEITNCLKLLESLTPLCEEDNQA
QEIVKKLEKSIVLLSQCTARVASRAEMLGAINQESRVSRAVEVMIQHVENLKRMYAKEHAELEDLKQALLQND
RSFNSLPDEDDCQIKKRSSSLNSKPSSLRRVTIASLPRNLGNVGLVSGMENNDRFSRRSSSWRILGTKQGEHR
PSLHRFISTYSWADAEDERSDVKARDAPEPQGEEAVEGTRKPSLSERRSSTLAWDRGTICSSVASWVTHLQAS
FRRANRALWLTGLIIIILIAALMSFLTGQLFQTAVEAAPTQEGDSWLSLEHILWPFTRLGHDGPLPV*
>ENSMUSP00000032396_c.585:G>A_p.A196T ENSMUST00000032396.13|Lrmp-
201|ENSMUSG00000030263|+|chr6:g.145106603:G>A
MLCVKGPPEQEPEDGALDVTRGCQCPLPTEGSILGQELLDCTRMNEDQSTDENGADHLYSESPSQLREYLTQP
SSEQTSSSESTVTSSESGSDILHMASGDLDCKPLCEKEEEARAASAMQGTSLAPAAYGDYTSVGVAKAASQLE
AGEELRTTENGGKGSAPGETEISMPPKASVKLVNFQQSENTSANEKEVETEFLRLSLGLKCDWFTLEKRVKLE
ERSRDLAEENLKKEITNCLKLLESLTPLCEEDNQAQEIVKKLEKSIVLLSQCTARVASRAEMLGAINQESRVS
RAVEVMIQHVENLKRMYAKEHAELEDLKQALLQNDRSFNSLPDEDDCQIKKRSSSLNSKPSSLRRVTIASLPR
NLGNVGLVSGMENNDRFSRRSSSWRILGTKQGEHRPSLHRFISTYSWADAEDERSDVKARDAPEPQGEEAVEG
TRKPSLSERRSSTLAWDRGTICSSVASWVTHLQASFRRANRALWLTGLIIIILIAALMSFLTGQLFQTAVEAAP
TQEGDSWLSLEHILWPFTRLGHDGPLPV*
>ENSMUSP00000115043_c.483:G>A_p.A162T ENSMUST00000156849.8|Lrmp-
210|ENSMUSG00000030263|+|chr6:g.145106603:G>A
XLFSFWGLLRGVSQSAAENGADHLYSESPSQLREYLTQPSSEQTSSSESTVTSSESGSDILHMASGDLDCKPL
CEKEEEARAASAMQGTSLAPAAYGDYTSVGVAKAASQLEAGEELRTTENGGKGSAPGETEISMPPKASVKLVN
FQQSENTSANEKEVETEFLRLSLGLKCDWFTLEKRVKLEERSRDLAEENL
>ENSMUSP00000120248_c.480:G>A_p.A161T ENSMUST00000132948.2|Lrmp-
204|ENSMUSG00000030263|+|chr6:g.145106603:G>A
XFSFWGLLRGVSQSAAENGADHLYSESPSQLREYLTQPSSEQTSSSESTVTSSESGSDILHMASGDLDCKPLC
EKEEEARAASAMQGTSLAPAAYGDYTSVGVAKAASQLEAGEELRTTENGGKGSAPGETEISMPPKASVKLVNF
QQSENTSANEKEVETEFLRLSLGLKCDWFTLEKRVKLEERSRDLAEENLKKEITNCLKLLESLTPLCEEDNQA
QEIVKKLEKSIVLLSQCTARVASRAEMLGAINQESRVSRAVEVMIQHVENLKRMYAKEHAELEDLKQALLQND
RSFNSLPDEDDCQIKKRSSSLNSKPSSLRRVTIASLPRNLGNVGLVSGMENNDRFSRRSSSWRILGTKQGEHR

PSLHRFISTYSWADAEDERSDVKARDAPEPQGEEAVEGTRKPSLSERRSSTLAWDRGTICSSVASWVTHLQAS
FRRANRALWLTGLIIILIAALMSFLTGQLFQTAVEAAPTQEGDSWLSLEHILWPFTRLGHDGPLPV*
>ENSMUSP00000107357_c.924:C>G_p.L309V ENSMUST00000111728.8|Casc1-203|ENSMUSG00000043541|-|chr6:g.145137143:G>C
MTKAERLRLMQEEEERRLKEEEEARLKFEKEEQERLEIQRIEREKWSLLEKKDLERRSQELEELALLEGCFPE
AEKQKREIRALAQWKHYTECDGSPDPWVAQEMNTFISLWEEEKNQAFEQVMEKSKLVLSLIEKVKLILLETPT
YELDHRTVLQHQGSILRLQELLSLKINVATELLLRQASNLADLDTGNMEKIIKDENVTLYVWANLKKNPRHRS
VRFSETQIGFEIPRILATSNVALRLLHTRYDHITPLFPIAVTEQNQNPVGAEQVNVEESTEKAMTEEKLFTEE
KAANEDEQPKAEQEREVNLVQEENKYEAIENTVLQRTSDSEGEDSQTTQLELEMKLLSEAVLAAQLCLVENVV
ELPEASQAYKVDLCHFSTLGGVYHLDVLELPPQCKPVKGWVLVEILQEGLQRFIYPPDTTEEPDPDVTFPPIE
VTLEIHKSVIFFERPRVVRWDNEGKFWRSDGISSVYYNREDRLLTFSMDTLGPVTLIQDAHVNMPYQSWEMSP
CGMNKVLLIVKTVFMELQIYIKENLCMLASVKLRGKGLEFHLKGKWMAPIPFILALKEAGLNIFPAVYSHFYV
VINNKVREQLSEETEHHTWSLLMFSGDRAQMLKMQEENDKFSEALREGTEFHSTLYHMMKDFASPVAMERVRH
SNCQFIDSVCYMLLSIRVLSYS*
>ENSMUSP00000062279_c.963:C>G_p.L322V ENSMUST00000060797.10|Casc1-201|ENSMUSG00000043541|-|chr6:g.145137143:G>C
MAPKSKKAPSKKKMTKAERLRLMQEEEERRLKEEEEARLKFEKEEQERLEIQRIEREKWSLLEKKDLERRSQE
LEELALLEGCFPEAEKQKREIRALAQWKHYTECDGSPDPWVAQEMNTFISLWEEEKNQAFEQVMEKSKLVLSL
IEKVKLILLETPTYELDHRTVLQHQGSILRLQELLSLKINVATELLLRQASNLADLDTGNMEKIIKDENVTLY
VWANLKKNPRHRSVRFSETQIGFEIPRILATSNVALRLLHTRYDHITPLFPIAVTEQNQNPVGAEQVNVEEST
EKAMTEEKLFTEEKAANEDEQPKAEQEREVNLVQEENKYEAIENTVLQRTSDSEGEDSQTTQLELEMKLLSEA
VLAAQLCLVENVVELPEASQAYKVDLCHFSTLGGVYHLDVLELPPQCKPVKGWVLVEILQEGLQRFIYPPDTT
EEPDPDVTFPPIEVTLEIHKSVIFFERPRVVRWDNEGKFWRSDGISSVYYNREDRLLTFSMDTLGPVTLIQDA
HVNMPYQSWEMSPCGMNKVLLIVKTVFMELQIYIKENLCMLASVKLRGKGLEFHLKGKWMAPIPFILALKEAG
LNIFPAVYSHFYVVINNKVPQVELKAYRQMALLSSAFSFGWSKWNMVCNSTRVVIRVREQLSEETEHHTWSLL
MFSGDRAQMLKMQEENDKFSEALREGTEFHSTLYHMMKDFASPVAMERVRHSNCQFIDSVCYMLLSIRVLSYS
*
>ENSMUSP00000144783_c.963:C>G_p.L322V ENSMUST00000204105.2|Casc1-208|ENSMUSG00000043541|-|chr6:g.145137143:G>C
MAPKSKKAPSKKKMTKAERLRLMQEEEERRLKEEEEARLKFEKEEQERLEIQRIEREKWSLLEKKDLERRSQE
LEELALLEGCFPEAEKQKREIRALAQWKHYTECDGSPDPWVAQEMNTFISLWEEEKNQAFEQVMEKSKLVLSL
IEKVKLILLETPTYELDHRTVLQHQGSILRLQELLSLKINVATELLLRQASNLADLDTGNMEKIIKDENVTLY
VWANLKKNPRHRSVRFSETQIGFEIPRILATSNVALRLLHTRYDHITPLFPIAVTEQNQNPVGAEQVNVEEST
EKAMTEEKLFTEEKAANEDEQPKAEQEREVNLVQEENKYEAIENTVLQRTSDSEGEDSQTTQLELEMKLLSEA
VLAAQLCLVENVVELPEASQAYKVDLCHFSTLGGVYHLDVLELPPQCKPVKGWVLVEILQEGLQRFIYPPDTT
EEPDPDVTFPPIEVTLEIHKSVIFFERPRVVRWDNEGKFWRSDGISSVYYNREDRLLTFSMDTLGPVTLIQDA
HVNMPYQSWEMSPCGMNKVLLIVKTVFMELQIYIKENLCMLASVKLRGKGLEFHLKGKWMAPIPFILALKEAG
LNIFPAVYSHFYVVINNKVPQVELKAYRQMALLSSAFSFGWSKWNMVCNSTRVVIRHWGG*
>ENSMUSP00000107357_c.556:T>C_p.L186S ENSMUST00000111728.8|Casc1-203|ENSMUSG00000043541|-|chr6:g.145138618:A>G
MTKAERLRLMQEEEERRLKEEEEARLKFEKEEQERLEIQRIEREKWSLLEKKDLERRSQELEELALLEGCFPE
AEKQKREIRALAQWKHYTECDGSPDPWVAQEMNTFISLWEEEKNQAFEQVMEKSKLVLSLIEKVKLILLETPT
YELDHRTVLQHQGSILRLQELLSLKINVATELLLRQASNSADLDTGNMEKIIKDENVTLYVWANLKKNPRHRS
VRFSETQIGFEIPRILATSNVALRLLHTRYDHITPLFPIAVTEQNQNPVGAEQVNVEESTEKAMTEEKLFTEE
KAANEDEQPKAEQERELNLVQEENKYEAIENTVLQRTSDSEGEDSQTTQLELEMKLLSEAVLAAQLCLVENVV
ELPEASQAYKVDLCHFSTLGGVYHLDVLELPPQCKPVKGWVLVEILQEGLQRFIYPPDTTEEPDPDVTFPPIE
VTLEIHKSVIFFERPRVVRWDNEGKFWRSDGISSVYYNREDRLLTFSMDTLGPVTLIQDAHVNMPYQSWEMSP
CGMNKVLLIVKTVFMELQIYIKENLCMLASVKLRGKGLEFHLKGKWMAPIPFILALKEAGLNIFPAVYSHFYV
VINNKVREQLSEETEHHTWSLLMFSGDRAQMLKMQEENDKFSEALREGTEFHSTLYHMMKDFASPVAMERVRH
SNCQFIDSVCYMLLSIRVLSYS*
>ENSMUSP00000062279_c.595:T>C_p.L199S ENSMUST00000060797.10|Casc1-201|ENSMUSG00000043541|-|chr6:g.145138618:A>G
MAPKSKKAPSKKKMTKAERLRLMQEEEERRLKEEEEARLKFEKEEQERLEIQRIEREKWSLLEKKDLERRSQE
LEELALLEGCFPEAEKQKREIRALAQWKHYTECDGSPDPWVAQEMNTFISLWEEEKNQAFEQVMEKSKLVLSL
IEKVKLILLETPTYELDHRTVLQHQGSILRLQELLSLKINVATELLLRQASNSADLDTGNMEKIIKDENVTLY
VWANLKKNPRHRSVRFSETQIGFEIPRILATSNVALRLLHTRYDHITPLFPIAVTEQNQNPVGAEQVNVEEST
EKAMTEEKLFTEEKAANEDEQPKAEQERELNLVQEENKYEAIENTVLQRTSDSEGEDSQTTQLELEMKLLSEA
VLAAQLCLVENVVELPEASQAYKVDLCHFSTLGGVYHLDVLELPPQCKPVKGWVLVEILQEGLQRFIYPPDTT

EEPDPDVTFPPIEVTLEIHKSVIFFERPRVVRWDNEGKFWRSDGISSVYYNREDRLLTFSMDTLGPVTLIQDA
HVNMPYQSWEMSPCGMNKVLLIVKTVFMELQIYIKENLCMLASVKLRGKGLEFHLKGKWMAPIPFILALKEAG
LNIFPAVYSHFYVVINNKVPQVELKAYRQMALLSSAFSFGWSKWNMVCNSTRVVIRVREQLSEETEHHTWSLL
MFSGDRAQMLKMQEENDKFSEALREGTEFHSTLYHMMKDFASPVAMERVRHSNCQFIDSVCYMLLSIRVLSYS
*
>ENSMUSP00000144783_c.595:T>C_p.L199S ENSMUST00000204105.2|Casc1-
208|ENSMUSG00000043541|-|chr6:g.145138618:A>G
MAPKSKKAPSKKKMTKAERLRLMQEEEERRLKEEEEARLKFEKEEQERLEIQRIEREKWSLLEKKDLERRSQE
LEELALLEGCFPEAEKQKREIRALAQWKHYTECDGSPDPWVAQEMNTFISLWEEEKNQAFEQVMEKSKLVLSL
IEKVKLILLETPTYELDHRTVLQHQGSILRLQELLSLKINVATELLLRQASNSADLDTGNMEKIIKDENVTLY
VWANLKKNPRHRSVRFSETQIGFEIPRILATSNVALRLLHTRYDHITPLFPIAVTEQNQNPVGAEQVNVEEST
EKAMTEEKLFTEEKAANEDEQPKAEQERELNLVQEENKYEAIENTVLQRTSDSEGEDSQTTQLELEMKLLSEA
VLAAQLCLVENVVELPEASQAYKVDLCHFSTLGGVYHLDVLELPPQCKPVKGWVLVEILQEGLQRFIYPPDTT
EEPDPDVTFPPIEVTLEIHKSVIFFERPRVVRWDNEGKFWRSDGISSVYYNREDRLLTFSMDTLGPVTLIQDA
HVNMPYQSWEMSPCGMNKVLLIVKTVFMELQIYIKENLCMLASVKLRGKGLEFHLKGKWMAPIPFILALKEAG
LNIFPAVYSHFYVVINNKVPQVELKAYRQMALLSSAFSFGWSKWNMVCNSTRVVIRHWGG*
>ENSMUSP00000107357_c.292:A>T_p.D98V ENSMUST00000111728.8|Casc1-
203|ENSMUSG00000043541|-|chr6:g.145142644:T>A
MTKAERLRLMQEEEERRLKEEEEARLKFEKEEQERLEIQRIEREKWSLLEKKDLERRSQELEELALLEGCFPE
AEKQKREIRALAQWKHYTECDGSPVPWVAQEMNTFISLWEEEKNQAFEQVMEKSKLVLSLIEKVKLILLETPT
YELDHRTVLQHQGSILRLQELLSLKINVATELLLRQASNLADLDTGNMEKIIKDENVTLYVWANLKKNPRHRS
VRFSETQIGFEIPRILATSNVALRLLHTRYDHITPLFPIAVTEQNQNPVGAEQVNVEESTEKAMTEEKLFTEE
KAANEDEQPKAEQERELNLVQEENKYEAIENTVLQRTSDSEGEDSQTTQLELEMKLLSEAVLAAQLCLVENVV
ELPEASQAYKVDLCHFSTLGGVYHLDVLELPPQCKPVKGWVLVEILQEGLQRFIYPPDTTEEPDPDVTFPPIE
VTLEIHKSVIFFERPRVVRWDNEGKFWRSDGISSVYYNREDRLLTFSMDTLGPVTLIQDAHVNMPYQSWEMSP
CGMNKVLLIVKTVFMELQIYIKENLCMLASVKLRGKGLEFHLKGKWMAPIPFILALKEAGLNIFPAVYSHFYV
VINNKVREQLSEETEHHTWSLLMFSGDRAQMLKMQEENDKFSEALREGTEFHSTLYHMMKDFASPVAMERVRH
SNCQFIDSVCYMLLSIRVLSYS*
>ENSMUSP00000062279_c.331:A>T_p.D111V ENSMUST00000060797.10|Casc1-
201|ENSMUSG00000043541|-|chr6:g.145142644:T>A
MAPKSKKAPSKKKMTKAERLRLMQEEEERRLKEEEEARLKFEKEEQERLEIQRIEREKWSLLEKKDLERRSQE
LEELALLEGCFPEAEKQKREIRALAQWKHYTECDGSPVPWVAQEMNTFISLWEEEKNQAFEQVMEKSKLVLSL
IEKVKLILLETPTYELDHRTVLQHQGSILRLQELLSLKINVATELLLRQASNLADLDTGNMEKIIKDENVTLY
VWANLKKNPRHRSVRFSETQIGFEIPRILATSNVALRLLHTRYDHITPLFPIAVTEQNQNPVGAEQVNVEEST
EKAMTEEKLFTEEKAANEDEQPKAEQERELNLVQEENKYEAIENTVLQRTSDSEGEDSQTTQLELEMKLLSEA
VLAAQLCLVENVVELPEASQAYKVDLCHFSTLGGVYHLDVLELPPQCKPVKGWVLVEILQEGLQRFIYPPDTT
EEPDPDVTFPPIEVTLEIHKSVIFFERPRVVRWDNEGKFWRSDGISSVYYNREDRLLTFSMDTLGPVTLIQDA
HVNMPYQSWEMSPCGMNKVLLIVKTVFMELQIYIKENLCMLASVKLRGKGLEFHLKGKWMAPIPFILALKEAG
LNIFPAVYSHFYVVINNKVPQVELKAYRQMALLSSAFSFGWSKWNMVCNSTRVVIRVREQLSEETEHHTWSLL
MFSGDRAQMLKMQEENDKFSEALREGTEFHSTLYHMMKDFASPVAMERVRHSNCQFIDSVCYMLLSIRVLSYS
*
>ENSMUSP00000144783_c.331:A>T_p.D111V ENSMUST00000204105.2|Casc1-
208|ENSMUSG00000043541|-|chr6:g.145142644:T>A
MAPKSKKAPSKKKMTKAERLRLMQEEEERRLKEEEEARLKFEKEEQERLEIQRIEREKWSLLEKKDLERRSQE
LEELALLEGCFPEAEKQKREIRALAQWKHYTECDGSPVPWVAQEMNTFISLWEEEKNQAFEQVMEKSKLVLSL
IEKVKLILLETPTYELDHRTVLQHQGSILRLQELLSLKINVATELLLRQASNLADLDTGNMEKIIKDENVTLY
VWANLKKNPRHRSVRFSETQIGFEIPRILATSNVALRLLHTRYDHITPLFPIAVTEQNQNPVGAEQVNVEEST
EKAMTEEKLFTEEKAANEDEQPKAEQERELNLVQEENKYEAIENTVLQRTSDSEGEDSQTTQLELEMKLLSEA
VLAAQLCLVENVVELPEASQAYKVDLCHFSTLGGVYHLDVLELPPQCKPVKGWVLVEILQEGLQRFIYPPDTT
EEPDPDVTFPPIEVTLEIHKSVIFFERPRVVRWDNEGKFWRSDGISSVYYNREDRLLTFSMDTLGPVTLIQDA
HVNMPYQSWEMSPCGMNKVLLIVKTVFMELQIYIKENLCMLASVKLRGKGLEFHLKGKWMAPIPFILALKEAG
LNIFPAVYSHFYVVINNKVPQVELKAYRQMALLSSAFSFGWSKWNMVCNSTRVVIRHWGG*
>ENSMUSP00000032399_c.509:G>T_p.M170I ENSMUST00000032399.12|Kras-
201|ENSMUSG00000030265|-|chr6:g.145166391:C>A
MTEYKLVVVGAGGVGKSALTIQLIQNHFVDEYDPTIEDSYRKQVVIDGETCLLDILDTAGQEEYSAMRDQYMR
TGEGFLCVFAINNTKSFEDIHHYREQIKRVKDSEDVPMVLVGNKCDLPSRTVDTKQAQELARSYGIPFIETSA
KTRQGVDDAFYTLVREIRKHKEKISKDGKKKKKKSRTRCTVM*

>ENSMUSP00000145294_c.170:G>T_p.M57I ENSMUST00000203147.3|Kras-207|ENSMUSG00000030265|-|chr6:g.145166391:C>A
MTEYKLVVVGAGGVGKSALTIQLIQNHFVDEYDPTIEGVDDAFYTLVREIRKHKEKISKDGKKKKKKSRTRCT
VM*
>ENSMUSP00000032399_c.325:T>AT_p.V109ins-fs ENSMUST00000032399.12|Kras-201|ENSMUSG00000030265|-|chr6:g.145177945:A>AT
MTEYKLVVVGAGGVGKSALTIQLIQNHFVDEYDPTIEDSYRKQVVIDGETCLLDILDTAGQEEYSAMRDQYMR
TGEGFLCVFAINNTKSFEDIHHYREQIKRVKDSEDDAYGPGRE
>ENSMUSP00000107339_c.325:T>AT_p.V109ins-fs ENSMUST00000111710.8|Kras-202|ENSMUSG00000030265|-|chr6:g.145177945:A>AT
MTEYKLVVVGAGGVGKSALTIQLIQNHFVDEYDPTIEDSYRKQVVIDGETCLLDILDTAGQEEYSAMRDQYMR
TGEGFLCVFAINNTKSFEDIHHYREQIKRVKDSEDDAYGPGRE
>ENSMUSP00000120740_c.300:A>T_p.K101* ENSMUST00000148739.8|Lmntd1-205|ENSMUSG00000054966|-|chr6:g.145363221:T>A
MSIQVHSSGRYPRTPAQQAAVSSPKGGILSSLGEIKIAEVNIKGLFVRLVNSSNEKEVEIGNHILQQNVNGHA
VSLYQFPDNITLQANSTVTVWAAASEA*PQPPTDFVWEEQSKFRSSPDCTTILCKPNGEAIAWYTPIHWKQAW
EKLETDIEFERCSVVVPSMRNHMFGWITASVSSTNEEKEEPIQKTPSQVYPVLYREKEIPPTVLPNKSPWCRN
PNTSPHPYSSLIDSHDSDISESSLDTQLKPQPTKPKPDPGTKKKKAKS*
>ENSMUSP00000107335_c.588:A>T_p.K197* ENSMUST00000111706.8|Lmntd1-201|ENSMUSG00000054966|-|chr6:g.145363221:T>A
MDSDSTTLIPLSRSFSQEMPIGFYQITSTQNSSTLSSRGQLASKSTILSCSHKDSSLGKQSTSSMVPRRQPQS
SSDVDTYTFGNGEDYFLSLFGESKKLTAHTPQAENVSEHLSVILEEVGQFTSSSLGEIKIAEVNIKGLFVRLV
NSSNEKEVEIGNHILQQNVNGHAVSLYQFPDNITLQANSTVTVWAAASEA*PQPPTDFVWEEQSKFRSSPDCT
TILCKPNGEAIAWYTPIHWKQAWEKLETDIEFERCSVVVPSMRNHMFGWITASVSSTNEEKEEPIQKTPSQVY
PVLYREKEIPPTVLPNKSPWCRNPNTSPHPYSSLIDSHDSDISESSLDTQLKPQPTKPKPDPGTKKKKAKS*
>ENSMUSP00000107337_c.738:A>T_p.K247* ENSMUST00000111708.9|Lmntd1-202|ENSMUSG00000054966|-|chr6:g.145363221:T>A
MMKEASEPLASVTSINKQDSKVQDGEIRKEKIGTITPSKQHSSVHFFPKIMDSDSTTLIPLSRSFSQEMPIGF
YQITSTQNSSTLSSRGQLASKSTILSCSHKDSSLGKQSTSSMVPRRQPQSSSDVDTYTFGNGEDYFLSLFGES
KKLTAHTPQAENVSEHLSVILEEVGQFTSSSLGEIKIAEVNIKGLFVRLVNSSNEKEVEIGNHILQQNVNGHA
VSLYQFPDNITLQANSTVTVWAAASEA*PQPPTDFVWEEQSKFRSSPDCTTILCKPNGEAIAWYTPIHWKQAW
EKLETDIEFERCSVVVPSMRNHMFGWITASVSSTNEEKEEPIQKTPSQVYPVLYREKEIPPTVLPNKSPWCRN
PNTSPHPYSSLIDSHDSDISESSLDTQLKPQPTKPKPDPGTKKKKAKS*
>ENSMUSP00000120740_c.291:T>C_p.S98P ENSMUST00000148739.8|Lmntd1-205|ENSMUSG00000054966|-|chr6:g.145363230:A>G
MSIQVHSSGRYPRTPAQQAAVSSPKGGILSSLGEIKIAEVNIKGLFVRLVNSSNEKEVEIGNHILQQNVNGHA
VSLYQFPDNITLQANSTVTVWAAAPEAKPQPPTDFVWEEQSKFRSSPDCTTILCKPNGEAIAWYTPIHWKQAW
EKLETDIEFERCSVVVPSMRNHMFGWITASVSSTNEEKEEPIQKTPSQVYPVLYREKEIPPTVLPNKSPWCRN
PNTSPHPYSSLIDSHDSDISESSLDTQLKPQPTKPKPDPGTKKKKAKS*
>ENSMUSP00000107335_c.579:T>C_p.S194P ENSMUST00000111706.8|Lmntd1-201|ENSMUSG00000054966|-|chr6:g.145363230:A>G
MDSDSTTLIPLSRSFSQEMPIGFYQITSTQNSSTLSSRGQLASKSTILSCSHKDSSLGKQSTSSMVPRRQPQS
SSDVDTYTFGNGEDYFLSLFGESKKLTAHTPQAENVSEHLSVILEEVGQFTSSSLGEIKIAEVNIKGLFVRLV
NSSNEKEVEIGNHILQQNVNGHAVSLYQFPDNITLQANSTVTVWAAAPEAKPQPPTDFVWEEQSKFRSSPDCT
TILCKPNGEAIAWYTPIHWKQAWEKLETDIEFERCSVVVPSMRNHMFGWITASVSSTNEEKEEPIQKTPSQVY
PVLYREKEIPPTVLPNKSPWCRNPNTSPHPYSSLIDSHDSDISESSLDTQLKPQPTKPKPDPGTKKKKAKS*
>ENSMUSP00000107337_c.729:T>C_p.S244P ENSMUST00000111708.9|Lmntd1-202|ENSMUSG00000054966|-|chr6:g.145363230:A>G
MMKEASEPLASVTSINKQDSKVQDGEIRKEKIGTITPSKQHSSVHFFPKIMDSDSTTLIPLSRSFSQEMPIGF
YQITSTQNSSTLSSRGQLASKSTILSCSHKDSSLGKQSTSSMVPRRQPQSSSDVDTYTFGNGEDYFLSLFGES
KKLTAHTPQAENVSEHLSVILEEVGQFTSSSLGEIKIAEVNIKGLFVRLVNSSNEKEVEIGNHILQQNVNGHA
VSLYQFPDNITLQANSTVTVWAAAPEAKPQPPTDFVWEEQSKFRSSPDCTTILCKPNGEAIAWYTPIHWKQAW
EKLETDIEFERCSVVVPSMRNHMFGWITASVSSTNEEKEEPIQKTPSQVYPVLYREKEIPPTVLPNKSPWCRN
PNTSPHPYSSLIDSHDSDISESSLDTQLKPQPTKPKPDPGTKKKKAKS*
>ENSMUSP00000120740_c.160:A>G_p.N54S ENSMUST00000148739.8|Lmntd1-205|ENSMUSG00000054966|-|chr6:g.145365529:T>C
MSIQVHSSGRYPRTPAQQAAVSSPKGGILSSLGEIKIAEVNIKGLFVRLVNSSSEKEVEIGNHILQQNVNGHA
VSLYQFPDNITLQANSTVTVWAAASEAKPQPPTDFVWEEQSKFRSSPDCTTILCKPNGEAIAWYTPIHWKQAW

EKLETDIEFERCSVVVPSMRNHMFGWITASVSSTNEEKEEPIQKTPSQVYPVLYREKEIPPTVLPNKSPWCRN
PNTSPHPYSSLIDSHDSDISESSLDTQLKPQPTKPKPDPGTKKKKAKS*
>ENSMUSP00000107335_c.448:A>G_p.N150S ENSMUST00000111706.8|Lmntd1-201|ENSMUSG00000054966|-|chr6:g.145365529:T>C
MDSDSTTLIPLSRSFSQEMPIGFYQITSTQNSSTLSSRGQLASKSTILSCSHKDSSLGKQSTSSMVPRRQPQS
SSDVDTYTFGNGEDYFLSLFGESKKLTAHTPQAENVSEHLSVILEEVGQFTSSSLGEIKIAEVNIKGLFVRLV
NSSSEKEVEIGNHILQQNVNGHAVSLYQFPDNITLQANSTVTVWAAASEAKPQPPTDFVWEEQSKFRSSPDCT
TILCKPNGEAIAWYTPIHWKQAWEKLETDIEFERCSVVVPSMRNHMFGWITASVSSTNEEKEEPIQKTPSQVY
PVLYREKEIPPTVLPNKSPWCRNPNTSPHPYSSLIDSHDSDISESSLDTQLKPQPTKPKPDPGTKKKKAKS*
>ENSMUSP00000107337_c.598:A>G_p.N200S ENSMUST00000111708.9|Lmntd1-202|ENSMUSG00000054966|-|chr6:g.145365529:T>C
MMKEASEPLASVTSINKQDSKVQDGEIRKEKIGTITPSKQHSSVHFFPKIMDSDSTTLIPLSRSFSQEMPIGF
YQITSTQNSSTLSSRGQLASKSTILSCSHKDSSLGKQSTSSMVPRRQPQSSSDVDTYTFGNGEDYFLSLFGES
KKLTAHTPQAENVSEHLSVILEEVGQFTSSSLGEIKIAEVNIKGLFVRLVNSSSEKEVEIGNHILQQNVNGHA
VSLYQFPDNITLQANSTVTVWAAASEAKPQPPTDFVWEEQSKFRSSPDCTTILCKPNGEAIAWYTPIHWKQAW
EKLETDIEFERCSVVVPSMRNHMFGWITASVSSTNEEKEEPIQKTPSQVYPVLYREKEIPPTVLPNKSPWCRN
PNTSPHPYSSLIDSHDSDISESSLDTQLKPQPTKPKPDPGTKKKKAKS*
>ENSMUSP00000107335_c.337:T>C_p.L113P ENSMUST00000111706.8|Lmntd1-201|ENSMUSG00000054966|-|chr6:g.145373044:A>G
MDSDSTTLIPLSRSFSQEMPIGFYQITSTQNSSTLSSRGQLASKSTILSCSHKDSSLGKQSTSSMVPRRQPQS
SSDVDTYTFGNGEDYFLSLFGESKKLTAHTPQAENVSEHPSVILEEVGQFTSSSLGEIKIAEVNIKGLFVRLV
NSSNEKEVEIGNHILQQNVNGHAVSLYQFPDNITLQANSTVTVWAAASEAKPQPPTDFVWEEQSKFRSSPDCT
TILCKPNGEAIAWYTPIHWKQAWEKLETDIEFERCSVVVPSMRNHMFGWITASVSSTNEEKEEPIQKTPSQVY
PVLYREKEIPPTVLPNKSPWCRNPNTSPHPYSSLIDSHDSDISESSLDTQLKPQPTKPKPDPGTKKKKAKS*
>ENSMUSP00000107337_c.487:T>C_p.L163P ENSMUST00000111708.9|Lmntd1-202|ENSMUSG00000054966|-|chr6:g.145373044:A>G
MMKEASEPLASVTSINKQDSKVQDGEIRKEKIGTITPSKQHSSVHFFPKIMDSDSTTLIPLSRSFSQEMPIGF
YQITSTQNSSTLSSRGQLASKSTILSCSHKDSSLGKQSTSSMVPRRQPQSSSDVDTYTFGNGEDYFLSLFGES
KKLTAHTPQAENVSEHPSVILEEVGQFTSSSLGEIKIAEVNIKGLFVRLVNSSNEKEVEIGNHILQQNVNGHA
VSLYQFPDNITLQANSTVTVWAAASEAKPQPPTDFVWEEQSKFRSSPDCTTILCKPNGEAIAWYTPIHWKQAW
EKLETDIEFERCSVVVPSMRNHMFGWITASVSSTNEEKEEPIQKTPSQVYPVLYREKEIPPTVLPNKSPWCRN
PNTSPHPYSSLIDSHDSDISESSLDTQLKPQPTKPKPDPGTKKKKAKS*
>ENSMUSP00000107337_c.13:C>T_p.A5V ENSMUST00000111708.9|Lmntd1-202|ENSMUSG00000054966|-|chr6:g.145379478:G>A
MMKEVSEPLASVTSINKQDSKVQDGEIRKEKIGTITPSKQHSSVHFFPKIMDSDSTTLIPLSRSFSQEMPIGF
YQITSTQNSSTLSSRGQLASKSTILSCSHKDSSLGKQSTSSMVPRRQPQSSSDVDTYTFGNGEDYFLSLFGES
KKLTAHTPQAENVSEHLSVILEEVGQFTSSSLGEIKIAEVNIKGLFVRLVNSSNEKEVEIGNHILQQNVNGHA
VSLYQFPDNITLQANSTVTVWAAASEAKPQPPTDFVWEEQSKFRSSPDCTTILCKPNGEAIAWYTPIHWKQAW
EKLETDIEFERCSVVVPSMRNHMFGWITASVSSTNEEKEEPIQKTPSQVYPVLYREKEIPPTVLPNKSPWCRN
PNTSPHPYSSLIDSHDSDISESSLDTQLKPQPTKPKPDPGTKKKKAKS*
>ENSMUSP00000123226_c.13:C>T_p.A5V ENSMUST00000149666.2|Lmntd1-206|ENSMUSG00000054966|-|chr6:g.145379478:G>A
MMKEVSEPLASVTSINKQDSKVQDGEIRKEKIGTITPSKQHSSVHFFPKIMDSDSTTLIPLSRSFSQEMPI
>ENSMUSP00000107333_c.448:A>T_p.E150V ENSMUST00000111704.8|Rassf8-203|ENSMUSG00000030259|+|chr6:g.145761124:A>T
MELKVWVDGVQRIVCGVTEVTTCQEVVIALAQAIGRTGRYTLIEKWRDTERHLAPHENPIVSLNKWGQYASDV
QLILRRTGPSLSERPTSDSVARIPERTLYRQSLPPLAKLRPQADKSIRRREPKRKSLTFTGGAKGLTDIFGKG
KETVFRQKVLSNCRATAEELKRLIRLQTGKLQAIEKQLESSEAEIRFWEQKYSCSLEEEIVRLEQRIKRNDVE
IEEEEFWENELQIEQENEKQLQDQLEEIRQKVTDCEGRLKDYLAQIHTMESGLQAEKLHREVQEAQVNEEEVK
GKIEKVKGEMDLQGQQSLRLENGIRAVERSLGQATKRLQDKEQELEQLTKELRQVNLQQFIQQTGTKVTVLPA
EPTEIEASQADIETEAPFQSGSLKRPGSSRQLPSNLRILQNPVSSGFNPEGIYV*
>ENSMUSP00000032388_c.448:A>T_p.E150V ENSMUST00000032388.5|Rassf8-201|ENSMUSG00000030259|+|chr6:g.145761124:A>T
MELKVWVDGVQRIVCGVTEVTTCQEVVIALAQAIGRTGRYTLIEKWRDTERHLAPHENPIVSLNKWGQYASDV
QLILRRTGPSLSERPTSDSVARIPERTLYRQSLPPLAKLRPQADKSIRRREPKRKSLTFTGGAKGLTDIFGKG
KETVFRQKVLSNCRATAEELKRLIRLQTGKLQAIEKQLESSEAEIRFWEQKYSCSLEEEIVRLEQRIKRNDVE
IEEEEFWENELQIEQENEKQLQDQLEEIRQKVTDCEGRLKDYLAQIHTMESGLQAEKLHREVQEAQVNEEEVK

GKIEKVKGEMDLQGQQSLRLENGIRAVERSLGQATKRLQDKEQELEQLTKELRQVNLQQFIQQTGTKVTVLPA
EPTEIEASQADIETEAPFQSGSLKRPGSSRQLPSNLRILQNPVSSGFNPEGIYV*
>ENSMUSP00000107333_c.471:A>T_p.N158Y ENSMUST00000111704.8|Rassf8-203|ENSMUSG00000030259|+|chr6:g.145761147:A>T
MELKVWVDGVQRIVCGVTEVTTCQEVVIALAQAIGRTGRYTLIEKWRDTERHLAPHENPIVSLNKWGQYASDV
QLILRRTGPSLSERPTSDSVARIPERTLYRQSLPPLAKLRPQADKSIRRREPKRKSLTFTGGAKGLTDIFGKG
KETEFRQKVLSYCRATAEELKRLIRLQTGKLQAIEKQLESSEAEIRFWEQKYSCSLEEEIVRLEQRIKRNDVE
IEEEFWENELQIEQENEKQLQDQLEEIRQKVTDCEGRLKDYLAQIHTMESGLQAEKLHREVQEAQVNEEEVK
GKIEKVKGEMDLQGQQSLRLENGIRAVERSLGQATKRLQDKEQELEQLTKELRQVNLQQFIQQTGTKVTVLPA
EPTEIEASQADIETEAPFQSGSLKRPGSSRQLPSNLRILQNPVSSGFNPEGIYV*
>ENSMUSP00000032388_c.471:A>T_p.N158Y ENSMUST00000032388.5|Rassf8-201|ENSMUSG00000030259|+|chr6:g.145761147:A>T
MELKVWVDGVQRIVCGVTEVTTCQEVVIALAQAIGRTGRYTLIEKWRDTERHLAPHENPIVSLNKWGQYASDV
QLILRRTGPSLSERPTSDSVARIPERTLYRQSLPPLAKLRPQADKSIRRREPKRKSLTFTGGAKGLTDIFGKG
KETEFRQKVLSYCRATAEELKRLIRLQTGKLQAIEKQLESSEAEIRFWEQKYSCSLEEEIVRLEQRIKRNDVE
IEEEFWENELQIEQENEKQLQDQLEEIRQKVTDCEGRLKDYLAQIHTMESGLQAEKLHREVQEAQVNEEEVK
GKIEKVKGEMDLQGQQSLRLENGIRAVERSLGQATKRLQDKEQELEQLTKELRQVNLQQFIQQTGTKVTVLPA
EPTEIEASQADIETEAPFQSGSLKRPGSSRQLPSNLRILQNPVSSGFNPEGIYV*
>ENSMUSP00000107333_c.1114:C>CTT_p.A372ins-fs
ENSMUST00000111704.8|Rassf8-203|ENSMUSG00000030259|+|chr6:g.145762345:C>CTT
MELKVWVDGVQRIVCGVTEVTTCQEVVIALAQAIGRTGRYTLIEKWRDTERHLAPHENPIVSLNKWGQYASDV
QLILRRTGPSLSERPTSDSVARIPERTLYRQSLPPLAKLRPQADKSIRRREPKRKSLTFTGGAKGLTDIFGKG
KETEFRQKVLSNCRATAEELKRLIRLQTGKLQAIEKQLESSEAEIRFWEQKYSCSLEEEIVRLEQRIKRNDVE
IEEEFWENELQIEQENEKQLQDQLEEIRQKVTDCEGRLKDYLAQIHTMESGLQAEKLHREVQEAQVNEEEVK
GKIEKVKGEMDLQGQQSLRLENGIRAVERSLGQATKRLQDKEQELEQLTKELRQVNLQQFIQQTGTKVTVLPA
EPTEIEASRRQT
>ENSMUSP00000032388_c.1114:C>CTT_p.A372ins-fs
ENSMUST00000032388.5|Rassf8-201|ENSMUSG00000030259|+|chr6:g.145762345:C>CTT
MELKVWVDGVQRIVCGVTEVTTCQEVVIALAQAIGRTGRYTLIEKWRDTERHLAPHENPIVSLNKWGQYASDV
QLILRRTGPSLSERPTSDSVARIPERTLYRQSLPPLAKLRPQADKSIRRREPKRKSLTFTGGAKGLTDIFGKG
KETEFRQKVLSNCRATAEELKRLIRLQTGKLQAIEKQLESSEAEIRFWEQKYSCSLEEEIVRLEQRIKRNDVE
IEEEFWENELQIEQENEKQLQDQLEEIRQKVTDCEGRLKDYLAQIHTMESGLQAEKLHREVQEAQVNEEEVK
GKIEKVKGEMDLQGQQSLRLENGIRAVERSLGQATKRLQDKEQELEQLTKELRQVNLQQFIQQTGTKVTVLPA
EPTEIEASRRQT
>ENSMUSP00000130671_c.141:GA>G_p.E48del-fs ENSMUST00000058538.7|Rassf8-202|ENSMUSG00000030259|+|chr6:g.145765813:GA>G
FICEASVGIDRSCPLTVQIDITIRGQLGIAPPSRACTVKMQLMVLAVGVAARTDRSALLHS
>ENSMUSP00000130671_c.283:T>C_p.L95S ENSMUST00000058538.7|Rassf8-202|ENSMUSG00000030259|+|chr6:g.145765955:T>C
FICEASVGIDRSCPLTVQIDITIRGQLGIAPPSRACTVKMQLMVLAVECGSTDRQISTPPQLRQPSPAATQLL
VSAKRSAETRSLHLEQHLGTSSQVRTLID*
>ENSMUSP00000122279_c.346:G>C_p.G116A ENSMUST00000139979.8|Ints13-207|ENSMUSG00000040250|-|chr6:g.146455512:C>G
XAGVIVKESLTEEDVLNCQKTIYNLVDMERKNDPLPVSTVGTRGKGPKRDEQYRIMWNELETLVRAHISNSEK
HQRVLECLMACRSKPPEEEERKKRGRKREDREDKSEQAGKEHATEKARPDADRLKGILERGKEELAEAEVIKD
SPDSPEPPNKKPLVETDETPHMEKSKGPVSLLSLWSNRINTANSRKHQEFAGRLNSVNNRAELYQHLKEENGM
ETTENGKASRQ*
>ENSMUSP00000145229_c.637:G>C_p.G213A ENSMUST00000203545.3|Ints13-209|ENSMUSG00000040250|-|chr6:g.146455512:C>G
TNSIELHYCTGAYRISPVDVNSRPSSCLTNFLLNGRSVLLEQPRKSGSKVISHMLSSHGGEIFLHVLSSSRSI
LEDPPSISEGCGGRVTDYRITVVPLAGVIVKESLTEEDVLNCQKTIYNLVDMERKNDPLPVSTVGTRGKGPKR
DEQYRIMWNELETLVRAHISNSEKHQRVLECLMACRSKPPEEEERKKRGRKREDREDKSEQAGKEHATEKARP
DADRLKGILERGKEELAEAEVIKDSPDSPEPPNKKPLVETDETPHMEKSKGPVSLLSLWSNRINTANSRKHQE
FAGRLNSVNNRAELYQHLKEENGMETTENGKASRQ*
>ENSMUSP00000032427_c.1774:G>C_p.G592A ENSMUST00000032427.15|Ints13-201|ENSMUSG00000040250|-|chr6:g.146455512:C>G

MKIFSESHKTVFVVDHCPYMAESCRQHVEFDMLVKNRTQGIIPLAPISKSLWTCSVESSMEYCRIMYDIFPFK
KLVNFIVSDSGAHVLNSWTQEDQNLQELMAALATVGPPNPRADPECCSILHGLVAAVETLCKITEYQHEARTL
LMENAERVGNRGRIICITNAKSDSHVRMLEDCVQETIHEHNKLAANSDHLMQIQKCELVLIHTYPVGEDSLVS
DRPKKELSPVLTSEVHSVRAGRHLATKLNVLVQQHFDLASTTITNIPMKEEQHANTSANYDVELLHHKDAHVD
FLRSGDSHSGSSSREGPFKETVTLKWCTPRTNSIELHYCTGAYRISPVDVNSRPSSCLTNFLLNGRSVLLEQP
RKSGSKVISHMLSSHGGEIFLHVLSSSRSILEDPPSISEGCGGRVTDYRITDFGEFMRENRLTPFLDPRYKID
ASLEIPLERAKDQLEKHTRYWPMIISQTTIFNMQAVVPLAGVIVKESLTEEDVLNCQKTIYNLVDMERKNDPL
PVSTVGTRGKGPKRDEQYRIMWNELETLVRAHISNSEKHQRVLECLMACRSKPPEEEERKKRGRKREDREDKS
EQAGKEHATEKARPDADRLKGILERGKEELAEAEVIKDSPDSPEPPNKKPLVETDETPHMEKSKGPVSLLSLW
SNRINTANSRKHQEFAGRLNSVNNRAELYQHLKEENGEEVGLTGGPRTAIPFLKCPRSPVVNRMETTENGKAS
RQ*
>ENSMUSP00000098352_c.528:A>G_p.R177G ENSMUST00000067404.13|Fgfr1op2-203|ENSMUSG00000040242|+|chr6:g.146498811:A>G
MSCTIEKALADAKALVERLRDHDDAAESLIEQTTALSKRVEAMKQYQEEIQELNEVARHRPRSTLVMGIQQEN
RQIRELQQENKELRTSLEEHQSALELIMSKYREQMFRLLMASKKDDPGIIMKLKEQHSKELQAHVDQITEMAA
VMRKAIEIDEQQGCKEQERIFQLEQENKGLGEILQITRESFLNLRKDDASESTSLSALVTNSDLSLRKS*
>ENSMUSP00000107292_c.642:A>G_p.R215G ENSMUST00000111663.9|Fgfr1op2-204|ENSMUSG00000040242|+|chr6:g.146498811:A>G
MSCTIEKALADAKALVERLRDHDDAAESLIEQTTALSKRVEAMKQYQEEIQELNEVARHRPRSTLVMGIQQEN
RQIRELQQENKELRTSLEEHQSALELIMSKYREQMFRLLMASKKDDPGIIMKLKEQHSKIDMVHRNSCEGFFL
DASRHILEAPQHGLERRHLEANQNELQAHVDQITEMAAVMRKAIEIDEQQGCKEQERIFQLEQENKGLGEILQ
ITRESFLNLRKDDASESTSLSALVTNSDLSLRKS*
>ENSMUSP00000045650_c.1498:G>A_p.R500K ENSMUST00000037709.16|Tm7sf3-201|ENSMUSG00000040234|-|chr6:g.146505456:C>T
MWRLRLLVLAVLAAGSAEAQANSSDGFLEFSVGKFTYFVLSKSSPQEAVLRHISSNVTFLLFQIHSQYQNTTV
SFTKTLLPSTSGTGNDRGLVFILRPEQAVCTWYLETGDTKPVQSVALTLSYSERDPIPGGCNLEFDLDIDPNL
YLDYNFFETTIKFAPANIGYARATEPPPCDVSTSRESRWRLRYDVYQYFLPEGDLTEASLLHHLQRMAQVAQV
KASAIKVATLTADDKTAVSFSSLPGQGVIYNVIVWDPSLNTSAAYVPVHTYACSFESVDGNCAFPGRVSTKVF
STLVALLGLFVCFFGHRFWKTDLFFIGFIFLGFFFYILITRLTPLQYDVRLALTAVAGSFGGLLLVASWWRFG
ILTLCMLCVGLVLGFLVSSGTFFTPLGNLNVFHDDGVFWVTFSCIALLVPVIFMGCLRILNILACGVVGSYSV
VLAVNSYMFTSLSYITLNVLRRALNTDFRGAVIRVPFQTNDYIILAVWGMLAVSGITLQIRKERGQPPFPPHP
YKLWKQERERRVTNILDPSHHIPPLRERLYGRVARIKELFQKEQPAGERTPLLL*
>ENSMUSP00000045650_c.762:G>A_p.D255N ENSMUST00000037709.16|Tm7sf3-201|ENSMUSG00000040234|-|chr6:g.146520122:C>T
MWRLRLLVLAVLAAGSAEAQANSSDGFLEFSVGKFTYFVLSKSSPQEAVLRHISSNVTFLLFQIHSQYQNTTV
SFTKTLLPSTSGTGNDRGLVFILRPEQAVCTWYLETGDTKPVQSVALTLSYSERDPIPGGCNLEFDLDIDPNL
YLDYNFFETTIKFAPANIGYARATEPPPCDVSTSRESRWRLRYDVYQYFLPEGDLTEASLLHHLQRMAQVAQV
KASAIKVATLTADDKTAVSFSSLPGQGVIYNVIVWNPSLNTSAAYVPVHTYACSFESVDGNCAFPGRVSTKVF
STLVALLGLFVCFFGHRFWKTDLFFIGFIFLGFFFYILITRLTPLQYDVRLALTAVAGSFGGLLLVASWWRFG
ILTLCMLCVGLVLGFLVSSGTFFTPLGNLNVFHDDGVFWVTFSCIALLVPVIFMGCLRILNILACGVVGSYSV
VLAVNSYMFTSLSYITLNVLRRALNTDFRGAVIRVPFQTNDYIILAVWGMLAVSGITLQIRRERGQPPFPPHP
YKLWKQERERRVTNILDPSHHIPPLRERLYGRVARIKELFQKEQPAGERTPLLL*
>ENSMUSP00000001675_c.427:T>C_p.V143A ENSMUST00000001675.14|Stk38l-201|ENSMUSG00000001630|+|chr6:g.146668951:T>C
MAMTAGATTTFPMSNHTRERVTVAKLTLENFYSNLILQHEERETRQKKLEVAMEEEGLADEEKKLRRSQHARK
ETEFLRLKRTRLGLDDFESLKVIGRGAFGEVRLVQKKDTGHIYAMKILRKADMLEKEQVAHIRAERDILAEAD
GAWVVKMFYSFQDKRNLYLIMEFLPGGDMMTLLMKKDTLTEEETQFYISETVLAIDAIHQLGFIHRDVKPDNL
LLDAKGHVKLSDFGLCTGLKKAHRTEFYRNLTHNPPSDFSFQNMNSKRKAETWKKNRRQLAYSTVGTPDYIAP
EVFMQTGYNKLCDWWSLGVIMYEMLIGFPPFCSETPQETYRKVMSWKETLAFPPEVPVSEKAKDLILRFCTDS
ENRIGNGGVEEIKGHPFFEGVDWGHIRERPAAIPIEIRSIDDTSNFDDFPESDILQPVPNTTEPDYKSKDWVF
LNYTYKRFEGLTQRGSIPTYMKAGKL*
>ENSMUSP00000107271_c.427:T>C_p.V143A ENSMUST00000111644.2|Stk38l-202|ENSMUSG00000001630|+|chr6:g.146668951:T>C
MAMTAGATTTFPMSNHTRERVTVAKLTLENFYSNLILQHEERETRQKKLEVAMEEEGLADEEKKLRRSQHARK
ETEFLRLKRTRLGLDDFESLKVIGRGAFGEVRLVQKKDTGHIYAMKILRKADMLEKEQVAHIRAERDILAEAD
GAWVVKMFYSFQDKRNLYLIMEFLPGGDMMTLLMKKDTLTEEETQFYISETVLAIDAIHQLGFIHRDVKPDNL
LLDAKGHVKLSDFGLCTGLKKAHRTEFYRNLTHNPPSDFSFQNMNSKRKAETWKKNRRQLAYSTVGTPDYIAP
EVFMQTGYNKLCDWWSLGVIMYEMLIGFPPFCSETPQETYRKVMSWKETLAFPPEVPVSEKAKDLILRFCTDS

ENRIGNGGVEEIKGHPFFEGVDWGHIRERPAAIPIEIRSIDDTSNFDDFPESDILQPVAFFLFLVPNTTEPDY
KSKDWVFLNYTYKRFEGLTQRGSIPTYMKAGKL*
>ENSMUSP00000001675_c.501:G>A_p.E168K ENSMUST00000001675.14|Stk38l-201|ENSMUSG00000001630|+|chr6:g.146669025:G>A
MAMTAGATTTFPMSNHTRERVTVAKLTLENFYSNLILQHEERETRQKKLEVAMEEEGLADEEKKLRRSQHARK
ETEFLRLKRTRLGLDDFESLKVIGRGAFGEVRLVQKKDTGHIYAMKILRKADMLEKEQVAHIRAERDILVEAD
GAWVVKMFYSFQDKRNLYLIMKFLPGGDMMTLLMKKDTLTEEETQFYISETVLAIDAIHQLGFIHRDVKPDNL
LLDAKGHVKLSDFGLCTGLKKAHRTEFYRNLTHNPPSDFSFQNMNSKRKAETWKKNRRQLAYSTVGTPDYIAP
EVFMQTGYNKLCDWWSLGVIMYEMLIGFPPFCSETPQETYRKVMSWKETLAFPPEVPVSEKAKDLILRFCTDS
ENRIGNGGVEEIKGHPFFEGVDWGHIRERPAAIPIEIRSIDDTSNFDDFPESDILQPVPNTTEPDYKSKDWVF
LNYTYKRFEGLTQRGSIPTYMKAGKL*
>ENSMUSP00000107271_c.501:G>A_p.E168K ENSMUST00000111644.2|Stk38l-202|ENSMUSG00000001630|+|chr6:g.146669025:G>A
MAMTAGATTTFPMSNHTRERVTVAKLTLENFYSNLILQHEERETRQKKLEVAMEEEGLADEEKKLRRSQHARK
ETEFLRLKRTRLGLDDFESLKVIGRGAFGEVRLVQKKDTGHIYAMKILRKADMLEKEQVAHIRAERDILVEAD
GAWVVKMFYSFQDKRNLYLIMKFLPGGDMMTLLMKKDTLTEEETQFYISETVLAIDAIHQLGFIHRDVKPDNL
LLDAKGHVKLSDFGLCTGLKKAHRTEFYRNLTHNPPSDFSFQNMNSKRKAETWKKNRRQLAYSTVGTPDYIAP
EVFMQTGYNKLCDWWSLGVIMYEMLIGFPPFCSETPQETYRKVMSWKETLAFPPEVPVSEKAKDLILRFCTDS
ENRIGNGGVEEIKGHPFFEGVDWGHIRERPAAIPIEIRSIDDTSNFDDFPESDILQPVAFFLFLVPNTTEPDY
KSKDWVFLNYTYKRFEGLTQRGSIPTYMKAGKL*
>ENSMUSP00000001675_c.832:A>G_p.Q278R ENSMUST00000001675.14|Stk38l-201|ENSMUSG00000001630|+|chr6:g.146670650:A>G
MAMTAGATTTFPMSNHTRERVTVAKLTLENFYSNLILQHEERETRQKKLEVAMEEEGLADEEKKLRRSQHARK
ETEFLRLKRTRLGLDDFESLKVIGRGAFGEVRLVQKKDTGHIYAMKILRKADMLEKEQVAHIRAERDILVEAD
GAWVVKMFYSFQDKRNLYLIMEFLPGGDMMTLLMKKDTLTEEETQFYISETVLAIDAIHQLGFIHRDVKPDNL
LLDAKGHVKLSDFGLCTGLKKAHRTEFYRNLTHNPPSDFSFQNMNSKRKAETWKKNRRRLAYSTVGTPDYIAP
EVFMQTGYNKLCDWWSLGVIMYEMLIGFPPFCSETPQETYRKVMSWKETLAFPPEVPVSEKAKDLILRFCTDS
ENRIGNGGVEEIKGHPFFEGVDWGHIRERPAAIPIEIRSIDDTSNFDDFPESDILQPVPNTTEPDYKSKDWVF
LNYTYKRFEGLTQRGSIPTYMKAGKL*
>ENSMUSP00000107271_c.832:A>G_p.Q278R ENSMUST00000111644.2|Stk38l-202|ENSMUSG00000001630|+|chr6:g.146670650:A>G
MAMTAGATTTFPMSNHTRERVTVAKLTLENFYSNLILQHEERETRQKKLEVAMEEEGLADEEKKLRRSQHARK
ETEFLRLKRTRLGLDDFESLKVIGRGAFGEVRLVQKKDTGHIYAMKILRKADMLEKEQVAHIRAERDILVEAD
GAWVVKMFYSFQDKRNLYLIMEFLPGGDMMTLLMKKDTLTEEETQFYISETVLAIDAIHQLGFIHRDVKPDNL
LLDAKGHVKLSDFGLCTGLKKAHRTEFYRNLTHNPPSDFSFQNMNSKRKAETWKKNRRRLAYSTVGTPDYIAP
EVFMQTGYNKLCDWWSLGVIMYEMLIGFPPFCSETPQETYRKVMSWKETLAFPPEVPVSEKAKDLILRFCTDS
ENRIGNGGVEEIKGHPFFEGVDWGHIRERPAAIPIEIRSIDDTSNFDDFPESDILQPVAFFLFLVPNTTEPDY
KSKDWVFLNYTYKRFEGLTQRGSIPTYMKAGKL*
>ENSMUSP00000001675_c.912:G>A_p.D305N ENSMUST00000001675.14|Stk38l-201|ENSMUSG00000001630|+|chr6:g.146673155:G>A
MAMTAGATTTFPMSNHTRERVTVAKLTLENFYSNLILQHEERETRQKKLEVAMEEEGLADEEKKLRRSQHARK
ETEFLRLKRTRLGLDDFESLKVIGRGAFGEVRLVQKKDTGHIYAMKILRKADMLEKEQVAHIRAERDILVEAD
GAWVVKMFYSFQDKRNLYLIMEFLPGGDMMTLLMKKDTLTEEETQFYISETVLAIDAIHQLGFIHRDVKPDNL
LLDAKGHVKLSDFGLCTGLKKAHRTEFYRNLTHNPPSDFSFQNMNSKRKAETWKKNRRQLAYSTVGTPDYIAP
EVFMQTGYNKLCNWWSLGVIMYEMLIGFPPFCSETPQETYRKVMSWKETLAFPPEVPVSEKAKDLILRFCTDS
ENRIGNGGVEEIKGHPFFEGVDWGHIRERPAAIPIEIRSIDDTSNFDDFPESDILQPVPNTTEPDYKSKDWVF
LNYTYKRFEGLTQRGSIPTYMKAGKL*
>ENSMUSP00000107271_c.912:G>A_p.D305N ENSMUST00000111644.2|Stk38l-202|ENSMUSG00000001630|+|chr6:g.146673155:G>A
MAMTAGATTTFPMSNHTRERVTVAKLTLENFYSNLILQHEERETRQKKLEVAMEEEGLADEEKKLRRSQHARK
ETEFLRLKRTRLGLDDFESLKVIGRGAFGEVRLVQKKDTGHIYAMKILRKADMLEKEQVAHIRAERDILVEAD
GAWVVKMFYSFQDKRNLYLIMEFLPGGDMMTLLMKKDTLTEEETQFYISETVLAIDAIHQLGFIHRDVKPDNL
LLDAKGHVKLSDFGLCTGLKKAHRTEFYRNLTHNPPSDFSFQNMNSKRKAETWKKNRRQLAYSTVGTPDYIAP
EVFMQTGYNKLCNWWSLGVIMYEMLIGFPPFCSETPQETYRKVMSWKETLAFPPEVPVSEKAKDLILRFCTDS
ENRIGNGGVEEIKGHPFFEGVDWGHIRERPAAIPIEIRSIDDTSNFDDFPESDILQPVAFFLFLVPNTTEPDY
KSKDWVFLNYTYKRFEGLTQRGSIPTYMKAGKL*
>ENSMUSP00000032433_c.283:G>A_p.G95D ENSMUST00000032433.9|Smco2-201|ENSMUSG00000030292|+|chr6:g.146760429:G>A

MMSLQLGTAGKERQLAEKSRDLQNVSMTEGSEEVSEMDHISDRPDEKDKPSENLQTDSLYKMDTEKWDGLEQE
SEHSQDPPSKPDEQEVTLVCEDPQVSQLSPSTDESTPIPESLTHKLNYWHAKMGLQMKELGADHGDWLERINN
IIQNINNTESTVKSLLTEVISLENQSKNLEDSDQEADIEEKITEIRRQLKEVNIKLTQVDACEEARELKEKLV
EQIESFHKEMNVLNSKLEMYYTQGSDADSHNSEDVDTEQEEPLVPEASPSLSASPTPPCSAVWKNALKLFVIV
YVVTITGLSCYILFVDATFLFERVLPSVLGHRTMWDLREMMAPFLNLEAEDLLPS*
>ENSMUSP00000032433_c.687:A>G_p.N230D ENSMUST00000032433.9|Smco2-201|ENSMUSG00000030292|+|chr6:g.146769605:A>G
MMSLQLGTAGKERQLAEKSRDLQNVSMTEGSEEVSEMDHISDRPDEKDKPSENLQTDSLYKMDTEKWDGLEQE
SEHSQDPPSKPDEQEVTLVCEGPQVSQLSPSTDESTPIPESLTHKLNYWHAKMGLQMKELGADHGDWLERINN
IIQNINNTESTVKSLLTEVISLENQSKNLEDSDQEADIEEKITEIRRQLKEVNIKLTQVDACEEARELKEKLV
EQIESFHKEMDVLNSKLEMYYTQGSDADSHNSEDVDTEQEEPLVPEASPSLSASPTPPCSAVWKNALKLFVIV
YVVTITGLSCYILFVDATFLFERVLPSVLGHRTMWDLREMMAPFLNLEAEDLLPS*
>ENSMUSP00000032433_c.768:G>A_p.E257K ENSMUST00000032433.9|Smco2-201|ENSMUSG00000030292|+|chr6:g.146769686:G>A
MMSLQLGTAGKERQLAEKSRDLQNVSMTEGSEEVSEMDHISDRPDEKDKPSENLQTDSLYKMDTEKWDGLEQE
SEHSQDPPSKPDEQEVTLVCEGPQVSQLSPSTDESTPIPESLTHKLNYWHAKMGLQMKELGADHGDWLERINN
IIQNINNTESTVKSLLTEVISLENQSKNLEDSDQEADIEEKITEIRRQLKEVNIKLTQVDACEEARELKEKLV
EQIESFHKEMNVLNSKLEMYYTQGSDADSHNSEDVDTKQEEPLVPEASPSLSASPTPPCSAVWKNALKLFVIV
YVVTITGLSCYILFVDATFLFERVLPSVLGHRTMWDLREMMAPFLNLEAEDLLPS*
>ENSMUSP00000032433_c.934:T>C_p.L312P ENSMUST00000032433.9|Smco2-201|ENSMUSG00000030292|+|chr6:g.146772734:T>C
MMSLQLGTAGKERQLAEKSRDLQNVSMTEGSEEVSEMDHISDRPDEKDKPSENLQTDSLYKMDTEKWDGLEQE
SEHSQDPPSKPDEQEVTLVCEGPQVSQLSPSTDESTPIPESLTHKLNYWHAKMGLQMKELGADHGDWLERINN
IIQNINNTESTVKSLLTEVISLENQSKNLEDSDQEADIEEKITEIRRQLKEVNIKLTQVDACEEARELKEKLV
EQIESFHKEMNVLNSKLEMYYTQGSDADSHNSEDVDTEQEEPLVPEASPSLSASPTPPCSAVWKNALKLFVIV
YVVTITGLSCYILFVDATFPFERVLPSVLGHRTMWDLREMMAPFLNLEAEDLLPS*
>ENSMUSP00000043802_c.685:C>T_p.S229F
ENSMUST00000036592.15|1700034J05Rik-201|ENSMUSG00000040163|-|chr6:g.146853970:G>A
MTTSPSSSDYSSTEDSIYECKSNQSASVGYYPSENTFSYEDLVSREETASVDSLVHFLPPVQSTWRTESLRRL
FRKRDQVEHDPEQFSKLSITLAWDIDVDSNHADSLANLDLNAHSQWMDKWPEDKTKLTPCKLYNLVQKLETFL
GKEKGGQHDGCVLPESTQKEDVHLNSTTPPHTAQVSPKERDVCQDLSKQRSLENEDICQVLEDPPRLLKDEVV
QEIRQASESFLETSSVSSPQPEGASRSHNIFCMNFRWVFQWLRTQIFSRWRRQRPSQATNTWHQKAVRNIHSL
RSNRIQPQE*
>ENSMUSP00000107249_c.682:C>T_p.S228F ENSMUST00000111622.2|1700034J05Rik-202|ENSMUSG00000040163|-|chr6:g.146853970:G>A
MTTSPSSSDYSSTEDSIYECKSNQSASVGYYPSENTFSYEDLVSREETASVDSLVHFLPPVQSTWRTESLRRL
FRKRDQVEHDPEQFSKLSITLAWDIDVDSNHADSLANLDLNAHSQWMDKWPEDKTKLTPCKLYNLVQKLETFL
GKEKGGQHDGCVLPESTQKEDVHLNSTTPPHTAQVSPKERDVCQDLSKQRSLENEDICQVLEDPPRLLKDEVV
EIRQASESFLETSSVSSPQPEGASRSHNIFCMNFRWVFQWLRTQIFSRWRRQRPSQATNTWHQKAVRNIHSLR
SNRIQPQE*
>ENSMUSP00000043802_c.666:A>T_p.R223*
ENSMUST00000036592.15|1700034J05Rik-201|ENSMUSG00000040163|-|chr6:g.146853989:T>A
MTTSPSSSDYSSTEDSIYECKSNQSASVGYYPSENTFSYEDLVSREETASVDSLVHFLPPVQSTWRTESLRRL
FRKRDQVEHDPEQFSKLSITLAWDIDVDSNHADSLANLDLNAHSQWMDKWPEDKTKLTPCKLYNLVQKLETFL
GKEKGGQHDGCVLPESTQKEDVHLNSTTPPHTAQVSPKERDVCQDLSKQRSLENEDICQVLEDPPRLLKDEVV
QEI*QASESSLETSSVSSPQPEGASRSHNIFCMNFRWVFQWLRTQIFSRWRRQRPSQATNTWHQKAVRNIHSL
RSNRIQPQE*
>ENSMUSP00000107249_c.663:A>T_p.R222* ENSMUST00000111622.2|1700034J05Rik-202|ENSMUSG00000040163|-|chr6:g.146853989:T>A
MTTSPSSSDYSSTEDSIYECKSNQSASVGYYPSENTFSYEDLVSREETASVDSLVHFLPPVQSTWRTESLRRL
FRKRDQVEHDPEQFSKLSITLAWDIDVDSNHADSLANLDLNAHSQWMDKWPEDKTKLTPCKLYNLVQKLETFL
GKEKGGQHDGCVLPESTQKEDVHLNSTTPPHTAQVSPKERDVCQDLSKQRSLENEDICQVLEDPPRLLKDEVV
EI*QASESSLETSSVSSPQPEGASRSHNIFCMNFRWVFQWLRTQIFSRWRRQRPSQATNTWHQKAVRNIHSLR
SNRIQPQE*

>rs249638215_ENSMUSP00000043802_c.641:G>AG_p.L214ins-fs ENSMUST00000036592.15|1700034J05Rik-201|ENSMUSG00000040163|-|chr6:g.146854399:C>CT
MTTSPSSSDYSSTEDSIYECKSNQSASVGYYPSENTFSYEDLVSREETASVDSLVHFLPPVQSTWRTESLRRL
FRKRDQVEHDPEQFSKLSITLAWDIDVDSNHADSLANLDLNAHSQWMDKWPEDKTKLTPCKLYNLVQKLETFL
GKEKGGQHDGCVLPESTQKEDVHLNSTTPPHTAQVSPKERDVCQDLSKQRSLENEDICQVLEDPPRLLERRSC
AGDKTGI
>rs249638215_ENSMUSP00000107249_c.641:G>AG_p.L214ins-fs ENSMUST00000111622.2|1700034J05Rik-202|ENSMUSG00000040163|-|chr6:g.146854399:C>CT
MTTSPSSSDYSSTEDSIYECKSNQSASVGYYPSENTFSYEDLVSREETASVDSLVHFLPPVQSTWRTESLRRL
FRKRDQVEHDPEQFSKLSITLAWDIDVDSNHADSLANLDLNAHSQWMDKWPEDKTKLTPCKLYNLVQKLETFL
GKEKGGQHDGCVLPESTQKEDVHLNSTTPPHTAQVSPKERDVCQDLSKQRSLENEDICQVLEDPPRLLERRSC
GDKTGI
>ENSMUSP00000043802_c.421:A>G_p.K141R ENSMUST00000036592.15|1700034J05Rik-201|ENSMUSG00000040163|-|chr6:g.146854619:T>C
MTTSPSSSDYSSTEDSIYECKSNQSASVGYYPSENTFSYEDLVSREETASVDSLVHFLPPVQSTWRTESLRRL
FRKRDQVEHDPEQFSKLSITLAWDIDVDSNHADSLANLDLNAHSQWMDKWPEDKTKLTPCKLYNLVQRLETFL
GKEKGGQHDGCVLPESTQKEDVHLNSTTPPHTAQVSPKERDVCQDLSKQRSLENEDICQVLEDPPRLLKDEVV
QEIRQASESSLETSSVSSPQPEGASRSHNIFCMNFRWVFQWLRTQIFSRWRRQRPSQATNTWHQKAVRNIHSL
RSNRIQPQE*
>ENSMUSP00000107249_c.421:A>G_p.K141R ENSMUST00000111622.2|1700034J05Rik-202|ENSMUSG00000040163|-|chr6:g.146854619:T>C
MTTSPSSSDYSSTEDSIYECKSNQSASVGYYPSENTFSYEDLVSREETASVDSLVHFLPPVQSTWRTESLRRL
FRKRDQVEHDPEQFSKLSITLAWDIDVDSNHADSLANLDLNAHSQWMDKWPEDKTKLTPCKLYNLVQRLETFL
GKEKGGQHDGCVLPESTQKEDVHLNSTTPPHTAQVSPKERDVCQDLSKQRSLENEDICQVLEDPPRLLKDEVV
EIRQASESSLETSSVSSPQPEGASRSHNIFCMNFRWVFQWLRTQIFSRWRRQRPSQATNTWHQKAVRNIHSLR
SNRIQPQE*
>ENSMUSP00000043802_c.223:G>T_p.R75M ENSMUST00000036592.15|1700034J05Rik-201|ENSMUSG00000040163|-|chr6:g.146854817:C>A
MTTSPSSSDYSSTEDSIYECKSNQSASVGYYPSENTFSYEDLVSREETASVDSLVHFLPPVQSTWRTESLRRL
FMKRDQVEHDPEQFSKLSITLAWDIDVDSNHADSLANLDLNAHSQWMDKWPEDKTKLTPCKLYNLVQKLETFL
GKEKGGQHDGCVLPESTQKEDVHLNSTTPPHTAQVSPKERDVCQDLSKQRSLENEDICQVLEDPPRLLKDEVV
QEIRQASESSLETSSVSSPQPEGASRSHNIFCMNFRWVFQWLRTQIFSRWRRQRPSQATNTWHQKAVRNIHSL
RSNRIQPQE*
>ENSMUSP00000107249_c.223:G>T_p.R75M ENSMUST00000111622.2|1700034J05Rik-202|ENSMUSG00000040163|-|chr6:g.146854817:C>A
MTTSPSSSDYSSTEDSIYECKSNQSASVGYYPSENTFSYEDLVSREETASVDSLVHFLPPVQSTWRTESLRRL
FMKRDQVEHDPEQFSKLSITLAWDIDVDSNHADSLANLDLNAHSQWMDKWPEDKTKLTPCKLYNLVQKLETFL
GKEKGGQHDGCVLPESTQKEDVHLNSTTPPHTAQVSPKERDVCQDLSKQRSLENEDICQVLEDPPRLLKDEVV
EIRQASESSLETSSVSSPQPEGASRSHNIFCMNFRWVFQWLRTQIFSRWRRQRPSQATNTWHQKAVRNIHSLR
SNRIQPQE*
>ENSMUSP00000122515_c.329:G>T_p.M110I ENSMUST00000154221.2|Ppfibp1-208|ENSMUSG00000016487|+|chr6:g.146898375:G>T
EKLNATEEMLQQELLSRTSLETQKLELMAEISNLKLKLTAVEKDRLDYEDRFRDTEGLIQEINDLRLKVNEMD
GERLQYEKKLKSTKSLMAKLSSMKIKVGQMQYEKQRIEQKWETLKDELASLKEQLEEKECEVKRLQERLVCKA
KGEGIEVLDRDIEVQKMKKAVESLMAANEEKERKIEDLRQCLSRYRKMQDPAVLAQGQDSECEGLFHSSSIST
LLDAQGFSDLERSTSSTPGMGSP
>ENSMUSP00000121270_c.428:G>C_p.E143D ENSMUST00000155415.8|Ppfibp1-209|ENSMUSG00000016487|+|chr6:g.146914424:G>C
XQDPAVLAQGQDSECEGLFHSSSISTLLDAQGFSDLERSTSSTPGMGSPSRDLLHTSAPEEFHTSVLQASIPS
LLPPSVDVDTCEKPKLPTKPETSFEEGDGRAILGAAAEVSLSDGVSTSSLQKSSSLGNLKKEASDGTEKDSIQ
KLSEDKAPTDSRTFGTLPPKVPGHEASVDDNPFGTRKARSSFGRGFFKIKSGKRTASAPNLAETEKETAEHLN
LAGTSRSKGSQGTSPFPMSPPSPDSRKKSRGIMRL
>ENSMUSP00000016631_c.2904:G>A_p.V969I ENSMUST00000016631.14|Ppfibp1-201|ENSMUSG00000016487|+|chr6:g.146931893:G>A

MMSDASDMLAAALEQMDGIIAGSKALEYSNGIFDCQSPTSPFMGSLRALHLVEDLRGLLEMMETDEKEGLRCQ
IPDSTAEVLIEWLQNQMTNGHLPGNGDVYQERLARLENDKESLVLQVSVLTDQVEAQGEKIRDLEFCLEEHRE
KLNATEEMLQQELLSRTSLETQKLELMAEISNLKLKLTAVEKDRLDYEDRFRDTEGLIQEINDLRLKVNEMDG
ERLQYEKKLKSTKDELASLKEQLEEKECEVKRLQERLVCKAKGEGIEVLDRDIEVQKMKKAVESLMAANEEKE
RKIEDLRQCLSRYRKMQDPAVLAQGQDSECEGLFHSSSISTLLDAQGFSDLERSTSSTPGMGSPSRDLLHTSA
PEEFHTSVLQASIPSLLPPSVDVDTCEKPKLPTKPETSFEEGDGRAILGAAAEVSLSDGVSTSSLQKSSSLGN
LKKEASDGTDKAPTDSRTFGTLPPKVPGHEASVDDNPFGTRKARSSFGRGFFKIKSGKRTASAPNLAETEKET
AEHLNLAGTSRSKGSQGTSPFPMSPPSPDSRKKSRGIMRLFGKLRRSQSTTFNPDDMSEPEFKRGGTRATAGP
RLGWSRDLGQSNSDLDMPFAKWTKEQVCSWLAEQGLGSYLSSGKHWIISGQTLLQASQQDLEKELGIKHSLHR
KKLQLALQALGSEEETNYGKLDFNWVTRWLDDIGLPQYKTQFDEGRVDGRMLHYMTVDDLLSLKVVSVLHHLS
IKRAIQVLRINNFEPNCLRRRPSDENSITPSEVQQWTNHRVMEWLRSVDLAEYAPNLRGSGVHGGLMVLEPRF
NVETMAQLLNIPPNKTLLRRHLATHFNLLIGAEAQHQKRDAMELPDYVLLTATAKVKPKKLTFSNFGNLRKKK
HEDGEEYVCPMELGQASGSSQKGFRPGLDMRLYEEDDLDRLEQMEDSEGTVRQIGAFSEGINNLTHMLKEDDM
FKDFAARSPSASITDEDSNI*
>ENSMUSP00000107250_c.2937:G>A_p.V980I ENSMUST00000111623.9|Ppfibp1-202|ENSMUSG00000016487|+|chr6:g.146931893:G>A
MMSDASDMLAAALEQMDGIIAGSKALEYSNGIFDCQSPTSPFMGSLRALHLVEDLRGLLEMMETDEKEGLRCQ
IPDSTAEVLIEWLQNQMTNGHLPGNGDVYQERLARLENDKESLVLQVSVLTDQVEAQGEKIRDLEFCLEEHRE
KLNATEEMLQQELLSRTSLETQKLELMAEISNLKLKLTAVEKDRLDYEDRFRDTEGLIQEINDLRLKVNEMDG
ERLQYEKKLKSTKDELASLKEQLEEKECEVKRLQERLVCKAKGEGIEVLDRDENIKKKLKEKNIEVQKMKKAV
ESLMAANEEKERKIEDLRQCLSRYRKMQDPAVLAQGQDSECEGLFHSSSISTLLDAQGFSDLERSTSSTPGMG
SPSRDLLHTSAPEEFHTSVLQASIPSLLPPSVDVDTCEKPKLPTKPETSFEEGDGRAILGAAAEVSLSDGVST
SSLQKSSSLGNLKKEASDGTDKAPTDSRTFGTLPPKVPGHEASVDDNPFGTRKARSSFGRGFFKIKSGKRTAS
APNLAETEKETAEHLNLAGTSRSKGSQGTSPFPMSPPSPDSRKKSRGIMRLFGKLRRSQSTTFNPDDMSEPEF
KRGGTRATAGPRLGWSRDLGQSNSDLDMPFAKWTKEQVCSWLAEQGLGSYLSSGKHWIISGQTLLQASQQDLE
KELGIKHSLHRKKLQLALQALGSEEETNYGKLDFNWVTRWLDDIGLPQYKTQFDEGRVDGRMLHYMTVDDLLS
LKVVSVLHHLSIKRAIQVLRINNFEPNCLRRRPSDENSITPSEVQQWTNHRVMEWLRSVDLAEYAPNLRGSGV
HGGLMVLEPRFNVETMAQLLNIPPNKTLLRRHLATHFNLLIGAEAQHQKRDAMELPDYVLLTATAKVKPKKLT
FSNFGNLRKKKHEDGEEYVCPMELGQASGSSQKGFRPGLDMRLYEEDDLDRLEQMEDSEGTVRQIGAFSEGIN
NLTHMLKEDDMFKDFAARSPSASITDEDSNI*
>ENSMUSP00000037503_c.16:C>T_p.S6L ENSMUST00000036194.6|Rep15-201|ENSMUSG00000040121|+|chr6:g.146934179:C>T
MGQKALQQVAPKDSQEVLAMCEVVSAAISHAAQKLKEYLGFEYPLSRLCLAASSLTEVFLVHFVTFCQERGAD
EWLTTTKMTKHQAWLFGADWIWTFWGPDKQIRLQVAVQTLRMASLPLTDPKSCESRGEESWKKGRFDKLQEFC
NLVGEDCLGLFIIFGVPGEPKAIRGVVLESVRNGMMESQLPGRKAVEQFVLETKDCISIKELLGNCLSKRDGL
SDMGRVYIRIL*
>ENSMUSP00000048348_c.171:G>T_p.D58Y ENSMUST00000036111.10|Mrps35-201|ENSMUSG00000040112|+|chr6:g.146949659:G>T
MAAAALQLRQTLCPGARVLRTFSSVASPAAPRAGPRTASRSERPMRRKALPPRTEKMYTDQDWPSVYPTAAPF
KPSAVPLPVRMGYPVKKGVPMAKEGNLELLKIPNFLHLTPVAIKRHCAALKDFCTEWPAALDSDEKCEEHFPV
EIDTADYVSSGPSIRNPKARAVTLRVKLSSLNLDNHAKKKLIKLVGERYCKATDVLTITTDRCPLKRQNCDYA
VYLLTVLYHESWKTEDWENSKTEEDMDEYVWAKSSSENSVLQTLLQMRAAESSVAPSREELLGTKEVEDYQKC
IVRLKNEGENEASLAQYKESVKRLLNLA*
>ENSMUSP00000144920_c.39:G>T_p.D14Y ENSMUST00000137556.3|Mrps35-203|ENSMUSG00000040112|+|chr6:g.146949659:G>T
MRRKALPPRTEKMYTDQDWPSVYPTAAPFKPSAVPLPVRMGYPVKKGVPMAKEGNLELLKIPNFLHLTPVAIK
RHCAALKDFCTE
>ENSMUSP00000048348_c.777:C>CACCTGTTTACTTTGCAAACCTCTCTA_p.L260ins-fs ENSMUST00000036111.10|Mrps35-201|ENSMUSG00000040112|+|chr6:g.146972136:C>CACCTGTTTACTTTGCAAACCTCTCTA
MAAAALQLRQTLCPGARVLRTFSSVASPAAPRAGPRTASRSERPMRRKALPPRTEKMDTDQDWPSVYPTAAPF
KPSAVPLPVRMGYPVKKGVPMAKEGNLELLKIPNFLHLTPVAIKRHCAALKDFCTEWPAALDSDEKCEEHFPV
EIDTADYVSSGPSIRNPKARAVTLRVKLSSLNLDNHAKKKLIKLVGERYCKATDVLTITTDRCPLKRQNCDYA
VYLLTVLYHESWKTEDWENSKTEEDMDEYVWAKSSSENSVHLFTLQTSLFRPCCR
>ENSMUSP00000042558_c.147:G>A_p.V50M ENSMUST00000036003.8|Klhl42-201|ENSMUSG00000040102|+|chr6:g.146993177:G>A
MSAEEMVQIRLEDRCYPVSKSKLIEQSDYFRALYRSGMREAVRPEVGPEMQQLRGLSAPGLRLVLDFINAGGA
REGWGLSEDELAEASVLSEMVEAASFLQVTALLRLLLSHVRLGNCLELYRLAQVYGLPDLQDACLRFMVLRFH

QVLCQPQFPLLLSPPQAPGDCSLKQRLREARMRGTPVLVALGDFLGGPLAPHPYQGEPPSMLRYEETTERWFP
LANNLPPDLVNVRGYGSAILDNYLFIVGGYRITSQEISAAHSYNPITNEWLQVASMNQKRSNFKLVAVNSKLY
AIGGQAVSNVECYNPEQDAWNFVAPLPNPLAEFSACECKGKIYVIGGYTTRDRNMNILQYCPSADLWTLFETC
DVHIRKQQMVSVEETIYIVGGCLHELGPNRRSSQSEDMLTVQSYNTVTRQWLYLKENTSKSGLNLTCALHNDG
IYIMSRDVTLSTSLEHRVFLKYNIFADSWEAFRRFPAFGHNLLISSLYLPNKAET*
>rs246022839_ENSMUSP00000042558_c.247:A>AC_p.E83ins-fs
ENSMUST00000036003.8|Klhl42-
201|ENSMUSG00000040102|+|chr6:g.146993277:A>AC
MSAEEMVQIRLEDRCYPVSKSKLIEQSDYFRALYRSGMREAVRPEVGPEVQQLRGLSAPGLRLVLDFINAGGA
REGWGLSEDDAGRG
>ENSMUSP00000042558_c.446:G>GTCTCTTCCCTCCCCGC_p.L149ins-fs
ENSMUST00000036003.8|Klhl42-
201|ENSMUSG00000040102|+|chr6:g.146993476:G>GTCTCTTCCCTCCCCGC
MSAEEMVQIRLEDRCYPVSKSKLIEQSDYFRALYRSGMREAVRPEVGPEVQQLRGLSAPGLRLVLDFINAGGA
REGWGLSEDELAEASVLSEMVEAASFLQVTALLRLLLSHVRLGNCLELYRLAQVYGLPDLQDACLRFMVLRFH
QVLSLPSPLSAAIPATVVAAAGPRGLQPEAEAEGGPHARDPGPGGPGGLPRGTPCPAPVSGGTPVHA
>ENSMUSP00000042558_c.478:C>T_p.P160L ENSMUST00000036003.8|Klhl42-
201|ENSMUSG00000040102|+|chr6:g.146993508:C>T
MSAEEMVQIRLEDRCYPVSKSKLIEQSDYFRALYRSGMREAVRPEVGPEVQQLRGLSAPGLRLVLDFINAGGA
REGWGLSEDELAEASVLSEMVEAASFLQVTALLRLLLSHVRLGNCLELYRLAQVYGLPDLQDACLRFMVLRFH
QVLCQPQFPLLLSLPQAPGDCSLKQRLREARMRGTPVLVALGDFLGGPLAPHPYQGEPPSMLRYEETTERWFP
LANNLPPDLVNVRGYGSAILDNYLFIVGGYRITSQEISAAHSYNPITNEWLQVASMNQKRSNFKLVAVNSKLY
AIGGQAVSNVECYNPEQDAWNFVAPLPNPLAEFSACECKGKIYVIGGYTTRDRNMNILQYCPSADLWTLFETC
DVHIRKQQMVSVEETIYIVGGCLHELGPNRRSSQSEDMLTVQSYNTVTRQWLYLKENTSKSGLNLTCALHNDG
IYIMSRDVTLSTSLEHRVFLKYNIFADSWEAFRRFPAFGHNLLISSLYLPNKAET*
>ENSMUSP00000042558_c.486:G>A_p.A163T ENSMUST00000036003.8|Klhl42-
201|ENSMUSG00000040102|+|chr6:g.146993516:G>A
MSAEEMVQIRLEDRCYPVSKSKLIEQSDYFRALYRSGMREAVRPEVGPEVQQLRGLSAPGLRLVLDFINAGGA
REGWGLSEDELAEASVLSEMVEAASFLQVTALLRLLLSHVRLGNCLELYRLAQVYGLPDLQDACLRFMVLRFH
QVLCQPQFPLLLSPPQTPGDCSLKQRLREARMRGTPVLVALGDFLGGPLAPHPYQGEPPSMLRYEETTERWFP
LANNLPPDLVNVRGYGSAILDNYLFIVGGYRITSQEISAAHSYNPITNEWLQVASMNQKRSNFKLVAVNSKLY
AIGGQAVSNVECYNPEQDAWNFVAPLPNPLAEFSACECKGKIYVIGGYTTRDRNMNILQYCPSADLWTLFETC
DVHIRKQQMVSVEETIYIVGGCLHELGPNRRSSQSEDMLTVQSYNTVTRQWLYLKENTSKSGLNLTCALHNDG
IYIMSRDVTLSTSLEHRVFLKYNIFADSWEAFRRFPAFGHNLLISSLYLPNKAET*
>ENSMUSP00000042558_c.703:G>A_p.G235D ENSMUST00000036003.8|Klhl42-
201|ENSMUSG00000040102|+|chr6:g.146993733:G>A
MSAEEMVQIRLEDRCYPVSKSKLIEQSDYFRALYRSGMREAVRPEVGPEVQQLRGLSAPGLRLVLDFINAGGA
REGWGLSEDELAEASVLSEMVEAASFLQVTALLRLLLSHVRLGNCLELYRLAQVYGLPDLQDACLRFMVLRFH
QVLCQPQFPLLLSPPQAPGDCSLKQRLREARMRGTPVLVALGDFLGGPLAPHPYQGEPPSMLRYEETTERWFP
LANNLPPDLVNVRGYDSAILDNYLFIVGGYRITSQEISAAHSYNPITNEWLQVASMNQKRSNFKLVAVNSKLY
AIGGQAVSNVECYNPEQDAWNFVAPLPNPLAEFSACECKGKIYVIGGYTTRDRNMNILQYCPSADLWTLFETC
DVHIRKQQMVSVEETIYIVGGCLHELGPNRRSSQSEDMLTVQSYNTVTRQWLYLKENTSKSGLNLTCALHNDG
IYIMSRDVTLSTSLEHRVFLKYNIFADSWEAFRRFPAFGHNLLISSLYLPNKAET*
>ENSMUSP00000042558_c.888:C>T_p.Q297* ENSMUST00000036003.8|Klhl42-
201|ENSMUSG00000040102|+|chr6:g.147003116:C>T
MSAEEMVQIRLEDRCYPVSKSKLIEQSDYFRALYRSGMREAVRPEVGPEVQQLRGLSAPGLRLVLDFINAGGA
REGWGLSEDELAEASVLSEMVEAASFLQVTALLRLLLSHVRLGNCLELYRLAQVYGLPDLQDACLRFMVLRFH
QVLCQPQFPLLLSPPQAPGDCSLKQRLREARMRGTPVLVALGDFLGGPLAPHPYQGEPPSMLRYEETTERWFP
LANNLPPDLVNVRGYGSAILDNYLFIVGGYRITSQEISAAHSYNPITNEWLQVASMNQKRSNFKLVAVNSKLY
AIGG*AVSNVECYNPEQDAWNFVAPLPNPLAEFSACECKGKIYVIGGYTTRDRNMNILQYCPSADLWTLFETC
DVHIRKQQMVSVEETIYIVGGCLHELGPNRRSSQSEDMLTVQSYNTVTRQWLYLKENTSKSGLNLTCALHNDG
IYIMSRDVTLSTSLEHRVFLKYNIFADSWEAFRRFPAFGHNLLISSLYLPNKAET*
>ENSMUSP00000042558_c.1224:T>C_p.Y409H ENSMUST00000036003.8|Klhl42-
201|ENSMUSG00000040102|+|chr6:g.147009387:T>C
MSAEEMVQIRLEDRCYPVSKSKLIEQSDYFRALYRSGMREAVRPEVGPEVQQLRGLSAPGLRLVLDFINAGGA
REGWGLSEDELAEASVLSEMVEAASFLQVTALLRLLLSHVRLGNCLELYRLAQVYGLPDLQDACLRFMVLRFH
QVLCQPQFPLLLSPPQAPGDCSLKQRLREARMRGTPVLVALGDFLGGPLAPHPYQGEPPSMLRYEETTERWFP
LANNLPPDLVNVRGYGSAILDNYLFIVGGYRITSQEISAAHSYNPITNEWLQVASMNQKRSNFKLVAVNSKLY

AIGGQAVSNVECYNPEQDAWNFVAPLPNPLAEFSACECKGKIYVIGGYTTRDRNMNILQYCPSADLWTLFETC
DVHIRKQQMVSVEETIYIVGGCLHELGPNRRSSQSEDMLTVQSHNTVTRQWLYLKENTSKSGLNLTCALHNDG
IYIMSRDVTLSTSLEHRVFLKYNIFADSWEAFRRFPAFGHNLLISSLYLPNKAET*
>ENSMUSP00000032441_c.531:A>C_p.K178Q ENSMUST00000032441.14|Ccdc91-
201|ENSMUSG00000030301|+|chr6:g.147438931:A>C
MDDDDFGGFEAAETFDGEQGGNQAVSPAVPWATFPAVSGVRLSPASPELILDHDHSSPSTGHLPPDAVISSAD
DTHADSSLMSQTISKAQIQQSAHTHLNIPLFPLGLTDEPSHGALALEDEPEGPGVHVSNSQLRQKISSLETKL
KASEEEKQRIKKDVESLMEKHSVLEKGFLKEQEQDAVSFQARYRELQEKHKQELEDMRKAGHEALSIIVDEYK
ALLQSSVKQQLDAIEKQYVSAIEKQAHRCEELLHAQHQRLLDVLDTEKELLREKIQEALTQQSQEQKESLEKC
LQEEMQRNKETLESAVKLEKEAMKDVITKAVGEERENLEKVHAEERELWKTEHARDQERVAEAIQAAVQEQQR
MSQEAVKAAIVEEQRRSEKAMEEAVKRTRDELVEYVREQRRLDQVTRQRSLSSLELFLSCAQKQLSALIATEP
VDIE*
>rs244165131_ENSMUSP00000032441_c.535:A>AT_p.E179ins-fs
ENSMUST00000032441.14|Ccdc91-
201|ENSMUSG00000030301|+|chr6:g.147438935:A>AT
MDDDDFGGFEAAETFDGEQGGNQAVSPAVPWATFPAVSGVRLSPASPELILDHDHSSPSTGHLPPDAVISSAD
DTHADSSLMSQTISKAQIQQSAHTHLNIPLFPLGLTDEPSHGALALEDEPEGPGVHVSNSQLRQKISSLETKL
KASEEEKQRIKKDVESLMEKHSVLEKGFLKEKDAGCCILSSQIP
>ENSMUSP00000041180_c.4515:A>G_p.M1506V ENSMUST00000046689.13|Resf1-
201|ENSMUSG00000032712|+|chr6:g.149236197:A>G
MNWNTKQENVPKPPPYSKTQSSILQHFLMTSTTSQSSFNYSPHNQEASQTSFNYSLHNQEACMYSGNSNSVSQ
PLLSGRNYITPQTQISVSNMPTRTIVASQSSMERVVSTNGKGPQQPNHNLQTVSSGIMQNVWLPSHTEATISH
NPDGGTNMPYMHPPQNQLVTSDTYSMQLQMAPLHSGKVPMTHQGSQGLNHFIPDQLVDWTQYTSNELSYPEYR
PPPKQYSYILPATTSLQVKNNQLPTYTQSLQSKHSVPLSSHQYAAEASKRLSALPYSCRYENQHVQNAQPVSK
HLPMEVPQSSEVHSSEKKKDTYRGFKQQWQNPNEKVSIGQFSEVKINIKQPYSESVRPSGDGVQALVQNNQEK
RKYTYNPNTNQVIDTNATKEKLVRDIKSLVEIKKKFSELARKIKINKSLLMAAGCSKTANTSYTEPIQHSEFS
AKEMSAKNGNDCSMELLATCLSLWKNQPSKTTEENVPKPLEEKQCNTSRISTTVVGSANPTNEVHVKSLCSGV
GNSQKMMSSSQTVLPVLIPSCESSGVAVGKGTELQIAVVSPLVLSDTNTLPGKDSVPEVLPETLYPVVKEGSV
CSLQTQPTETVALPFDVIGAVASNNISAEIPLPVDKEKQHKPIQGDPDIADSSLGKHSPLGTEVLPKPMDSTI
VSGPMLQIESICSLAEGDVSYNSQIAEIFNSVQTEPQKPSPNQVIDSQQEQVYDTTENKDFSLQKDKCVQCTD
VPHEVPEQPEPLQPEEPASSEYVEANREATEESCREYTGRKESTAKDVCLPAAIQQDPHPRETDMFSKSDHSL
PAINEINDESEPISYLHDQLSELLKEFPYGIETFNRHEVSLDQQKTHKIVENQTGGKTSNVSGDSTDQIKITV
LNSEQIKELFPEDDQPCDKLAEPENKEIVAEVKSPCDSQIPREESHDLGMLDPEKDKIHCCALGWLSMVYEGV
PQCHCSSTEKKEKDQCLDINSSKQGEQPCNSGITIFEINPVSNNSKTPLTQATEEGHFSAVHGEKTKASKTKD
NREGQELACHFSAKCYKKDKKGNFKIRHDTSLKMEQKLKNISSKCDIPNPSKCNKIAAPEILHVTTSNSAKNM
PFSKQASQESLQKKHTSQDLGPVKAPIELSSNTDPCRSNTSSVQSVSPEKKKLKFKAGGSRLKYFEKRKTDHV
IIPDVEIKKKKYEKQEQNKNAGDTLKLCSILTESNERASVQEKTVPSPESSDPKGSSSKSTRVITVQEYLQRQ
KDKQITGNNASRNICVETVLCDSGHTKTSKHSAAVSWGKLVEGQSISAETAKELEHNSSSHGKDFKIHHSEAS
RTHSVSNNNKGKFDGKQPDKMFKNKTSMNNESNQMPLQVKEQRKQYLNRVAFKCTERESICLTKLDSASKKLS
IEKKSGEYTSKTKDTDKPSMLEFKLCPDVLLKNTSTVDKQDCPGPGPEKEQAPVQVSGIKSTKEDWLKCIPTR
TKMPESSQRDSADSRLSKRSLSADEFEILQNPVKESNIMFRTYKKVYLEKRSRSLGSSPVK*
>ENSMUSP00000140026_c.4515:A>G_p.M1506V ENSMUST00000189932.7|Resf1-
209|ENSMUSG00000032712|+|chr6:g.149236197:A>G
MNWNTKQENVPKPPPYSKTQSSILQHFLMTSTTSQSSFNYSPHNQEASQTSFNYSLHNQEACMYSGNSNSVSQ
PLLSGRNYITPQTQISVSNMPTRTIVASQSSMERVVSTNGKGPQQPNHNLQTVSSGIMQNVWLPSHTEATISH
NPDGGTNMPYMHPPQNQLVTSDTYSMQLQMAPLHSGKVPMTHQGSQGLNHFIPDQLVDWTQYTSNELSYPEYR
PPPKQYSYILPATTSLQVKNNQLPTYTQSLQSKHSVPLSSHQYAAEASKRLSALPYSCRYENQHVQNAQPVSK
HLPMEVPQSSEVHSSEKKKDTYRGFKQQWQNPNEKVSIGQFSEVKINIKQPYSESVRPSGDGVQALVQNNQEK
RKYTYNPNTNQVIDTNATKEKLVRDIKSLVEIKKKFSELARKIKINKSLLMAAGCSKTANTSYTEPIQHSEFS
AKEMSAKNGNDCSMELLATCLSLWKNQPSKTTEENVPKPLEEKQCNTSRISTTVVGSANPTNEVHVKSLCSGV
GNSQKMMSSSQTVLPVLIPSCESSGVAVGKGTELQIAVVSPLVLSDTNTLPGKDSVPEVLPETLYPVVKEGSV
CSLQTQPTETVALPFDVIGAVASNNISAEIPLPVDKEKQHKPIQGDPDIADSSLGKHSPLGTEVLPKPMDSTI
VSGPMLQIESICSLAEGDVSYNSQIAEIFNSVQTEPQKPSPNQVIDSQQEQVYDTTENKDFSLQKDKCVQCTD
VPHEVPEQPEPLQPEEPASSEYVEANREATEESCREYTGRKESTAKDVCLPAAIQQDPHPRETDMFSKSDHSL
PAINEINDESEPISYLHDQLSELLKEFPYGIETFNRHEVSLDQQKTHKIVENQTGGKTSNVSGDSTDQIKITV
LNSEQIKELFPEDDQPCDKLAEPENKEIVAEVKSPCDSQIPREESHDLGMLDPEKDKIHCCALGWLSMVYEGV
PQCHCSSTEKKEKDQCLDINSSKQGEQPCNSGITIFEINPVSNNSKTPLTQATEEGHFSAVHGEKTKASKTKD
NREGQELACHFSAKCYKKDKKGNFKIRHDTSLKMEQKLKNISSKCDIPNPSKCNKIAAPEILHVTTSNSAKNM

PFSKQASQESLQKKHTSQDLGPVKAPIELSSNTDPCRSNTSSVQSVSPEKKKLKFKAGGSRLKYFEKRKTDHV
IIPDVEIKKKKYEKQEQNKNAGDTLKLCSILTESNERASVQEKTVPSPESSDPKGSSSKSTRVITVQEYLQRQ
KDKQITGNNASRNICVETVLCDSGHTKTSKHSAAVSWGKLVEGQSISAETAKELEHNSSSHGKDFKIHHSEAS
RTHSVSNNNKGKFDGKQPDKMFKNKTSMNNESNQMPLQVKEQRKQYLNRVAFKCTERESICLTKLDSASKKLS
IEKKSGEYTSKTKDTDKPSMLEFKLCPDVLLKNTSTVDKQDCPGPGPEKEQAPVQVSGIKSTKEDWLKCIPTR
TKMPESSQRDSADSRLSKRSLSADEFEILQNPVKESNIMFRTYKKVYLEKRSRSLGSSPVK*
>ENSMUSP00000098328_c.4515:A>G_p.M1506V ENSMUST00000100765.11|Resf1-
202|ENSMUSG00000032712|+|chr6:g.149236197:A>G
MNWNTKQENVPKPPPYSKTQSSILQHFLMTSTTSQSSFNYSPHNQEASQTSFNYSLHNQEACMYSGNSNSVSQ
PLLSGRNYITPQTQISVSNMPTRTIVASQSSMERVVSTNGKGPQQPNHNLQTVSSGIMQNVWLPSHTEATISH
NPDGGTNMPYMHPPQNQLVTSDTYSMQLQMAPLHSGKVPMTHQGSQGLNHFIPDQLVDWTQYTSNELSYPEYR
PPPKQYSYILPATTSLQVKNNQLPTYTQSLQSKHSVPLSSHQYAAEASKRLSALPYSCRYENQHVQNAQPVSK
HLPMEVPQSSEVHSSEKKKDTYRGFKQQWQNPNEKVSIGQFSEVKINIKQPYSESVRPSGDGVQALVQNNQEK
RKYTYNPNTNQVIDTNATKEKLVRDIKSLVEIKKKFSELARKIKINKSLLMAAGCSKTANTSYTEPIQHSEFS
AKEMSAKNGNDCSMELLATCLSLWKNQPSKTTEENVPKPLEEKQCNTSRISTTVVGSANPTNEVHVKSLCSGV
GNSQKMMSSSQTVLPVLIPSCESSGVAVGKGTELQIAVVSPLVLSDTNTLPGKDSVPEVLPETLYPVVKEGSV
CSLQTQPTETVALPFDVIGAVASNNISAEIPLPVDKEKQHKPIQGDPDIADSSLGKHSPLGTEVLPKPMDSTI
VSGPMLQIESICSLAEGDVSYNSQIAEIFNSVQTEPQKPSPNQVIDSQQEQVYDTTENKDFSLQKDKCVQCTD
VPHEVPEQPEPLQPEEPASSEYVEANREATEESCREYTGRKESTAKDVCLPAAIQQDPHPRETDMFSKSDHSL
PAINEINDESEPISYLHDQLSELLKEFPYGIETFNRHEVSLDQQKTHKIVENQTGGKTSNVSGDSTDQIKITV
LNSEQIKELFPEDDQPCDKLAEPENKEIVAEVKSPCDSQIPREESHDLGMLDPEKDKIHCCALGWLSMVYEGV
PQCHCSSTEKKEKDQCLDINSSKQGEQPCNSGITIFEINPVSNNSKTPLTQATEEGHFSAVHGEKTKASKTKD
NREGQELACHFSAKCYKKDKKGNFKIRHDTSLKMEQKLKNISSKCDIPNPSKCNKIAAPEILHVTTSNSAKNM
PFSKQASQESLQKKHTSQDLGPVKAPIELSSNTDPCRSNTSSVQSVSPEKKKLKFKAGGSRLKYFEKRKTDHV
IIPDVEIKKKKYEKQEQNKNAGDTLKLCSILTESNERASVQEKTVPSPESSDPKGSSSKSTRVITVQEYLQRQ
KDKQITGNNASRNICVETVLCDSGHTKTSKHSAAVSWGKLVEGQSISAETAKELEHNSSSHGKDFKIHHSEAS
RTHSVSNNNKGKFDGKQPDKMFKNKTSMNNESNQMPLQVKEQRKQYLNRVAFKCTERESICLTKLDSASKKLS
IEKKSGEYTSKTKDTDKPSMLEFKLCPDVLLKNTSTVDKQDCPGPGPEKEQAPVQVSGIKSTKEDWLKCIPTR
TKMPESSQRDSADSRLSKRSLSADEFEILQNPVKESNIMFRTYKKVYLEKRSRSLGSSPVK*
>ENSMUSP00000139660_c.4509:A>G_p.M1504V ENSMUST00000189837.2|Resf1-
208|ENSMUSG00000032712|+|chr6:g.149236197:A>G
MNWNTKQENVPKPPPYSKTQSSILQHFLMTSTTSQSSFNYSPHNQEASQTSFNYSLHNQEACMYSGNSNSVSQ
PLLSGRNYITPQTQISVSNMPTRTIVASQSSMERVVSTNGKGPQQPNHNLQTVSSGIMQNVWLPSHTEATISH
NPDGGTNMPYMHPPQNQLVTSDTYSMQLQMAPLHSGKVPMTHQGSQGLNHFIPDQLVDWTQYTSNELSYPEYR
PPPKQYSYILPATTSLQVKNNQLPTYTQSLQSKHSVPLSSHQYAAEASKRLSALPYSCRYENQHVQNAQPVSK
HLPMEVPQSSEVHSSEKKKDTYRGFKQQWQNPNEKVSIGQFSEVKINIKQPYSESVRPSGDGVQALVQNNQEK
RKYTYNPNTNQVIDTNATKEKLVRDIKSLVEIKKKFSELARKIKINKSLLMAAGCSKTANTSYTEPIQHSEFS
AKEMSAKNGNDCSMELLATCLSLWKNQPSKTTEENVPKPLEEKQCNTSRISTTVVGSANPTNEVHVKSLCSGV
GNSQKMMSSSQTVLPVLIPSCESSGVAVGKGTELQIAVVSPLVLSDTNTLPGKDSVPEVLPETLYPVVKEGSV
CSLQTQPTETVALPFDVIGAVASNNISAEIPLPVDKEKQHKPIQGDPDIADSSLGKHSPLGTEVLPKPMDSTI
VSGPMLQIESICSLAEGDVSYNSQIAEIFNSVQTEPQKPSPNQVIDSQQEQVYDTTENKDFSLQKDKCVQCTD
VPHEVPEQPEPLQPEEPASSEYVEANREATEESCREYTGRKESTAKDVCLPAAIQQDPHPRETDMFSKSDHSL
PAINEINDESEPISYLHDQLSELLKEFPYGIETFNRHEVSLDQQKTHKIVENQTGGKTSNVSGDSTDQIKITV
LNSEQIKELFPEDDQPCDKLAEPENKEIVAEVKSPCDSQIPREESHDLGMLDPEKDKIHCCALGWLSMVYEGV
PQCHCSSTEKKEKDQCLDINSSKQGEQPCNSGITIFEINPVSNNSKTPLTQATEEGHFSAVHGEKTKASKTKD
NREGQELACHFSAKCYKKDKKGNFKIRHDTSLKMEQKLKNISSKCDIPNPSKCNKIAAPEILHVTTSNSAKNM
PFSKQASQESLQKKHTSQDLGPVKAPIELSSNTDPCRSNTSSVQSVSPEKKKLKFKAGGSRLKYFEKRKTDHV
IIPDVEIKKKKYEKQEQNKNAGDTLKLCSILTESNERASVQEKTVPSPESSDPKGSSSKSTRVITVQEYLQRQ
KDKQITGNNASRNICVETVLCDSGHTKTSKHSAAVSWGKLVEGQSISAETAKELEHNSSSHGKDFKIHHSEAS
RTHSVSNNNKGKFDGKQPDKMFKNKTSMNNESNQMPLQVKEQRKQYLNRVAFKCTERESICLTKLDSASKKLS
IEKKSGEYTSKTKDTDKPSMLEFKLCPDVLLKNTSTVDKQDCPGPGPEKEQAPVQGIKSTKEDWLKCIPTRTK
MPESSQRDSADSRLSKRSLSADEFEILQNPVKESNIMFRTYKKVYLEKRSRSLGSSPVK*
>ENSMUSP00000145740_c.478:A>CA_p.K160ins-fs ENSMUST00000206928.2|Gp6-
202|ENSMUSG00000078810|-|chr7:g.4387989:T>TG
MSPASPTFFCIGLCVLQVIQTQSGPLPKPSLQAQPSSLVPLGQSVILRCQGPPDVDLYRLEKLKPEKYEDQDF
LFIPTMERSNAGRYRCSYQNGSHWSLPSDQLELIATGVYAKPSLSAHPSSAVPQGRDVTLKCQSPYSFDEFVL
YKEGDTGSYKRPETMVPGQFPHHHSDCCSQWDVPVLQLLQLISIPVVSPE

```
>ENSMUSP00000104231_c.478:A>CA_p.K160ins-fs ENSMUST00000108590.4|Gp6-
201|ENSMUSG00000078810|-|chr7:g.4387989:T>TG
MSPASPTFFCIGLCVLQVIQTQSGPLPKPSLQAQPSSLVPLGQSVILRCQGPPDVDLYRLEKLKPEKYEDQDF
LFIPTMERSNAGRYRCSYQNGSHWSLPSDQLELIATGVYAKPSLSAHPSSAVPQGRDVTLKCQSPYSFDEFVL
YKEGDTGSYKRPETMVPGQFPHHHSDCCSQWDVPVLQLLQLISIPVVSPE
>ENSMUSP00000130452_c.196:A>T_p.D66V ENSMUST00000168578.3|Tmem238-
201|ENSMUSG00000030431|-|chr7:g.4792345:T>A
MAAASPVCGSQASAVGASSPPAPAPAPAAGLGRCRMALLLAVALDVAGMAALLTGVFAQLQVRGRVFGDLLIY
SGALLVFLSLLGWILWYTGNIEISRQELERDYGLRPSAIARLARKLSRRWSAPATASPRTTAGLRSARRANRA
PQPSSSGSRRVRLQLATLEAGSVAAGTGSE*
>ENSMUSP00000074613_c.2287:C>A_p.T763N ENSMUST00000075108.9|Vmn2r31-
201|ENSMUSG00000096373|-|chr7:g.7387282:G>T
MFTLMGVFFFLNIPLFMANFIDPRCFWRINLNEIQDEYLGLTCAFILAAVQKPIEKDYFNKTLNFLKTTKNHK
YALALAFAMDEINRNPDLLPNMSLIIRYTLGRCDGKTVIPTPYLFHKKKQSPIPNYFCNEETMCSFLLTGPNW
GVSISFWKYLDSFLSPRILQLTYGPFSSIFSDDEQYPYLYQMAPKDTSLALAMVSFILYFNWNWIGLVIPDDD
QGNQFLLELKKQSENKEICFAFVKMISVDDISFEHKTEMYYKQIVMSSSNVIIIYEETINFIDLIFRMWEPPV
LQRIWITTKQWNFPTSKRDITHGTFYGSLTFLPHHGGISGFKDFVQTWFHLRSKDLYLVMPEWKYFKYESSAS
KCKILKNNSSDASFDWLMEQKFDMAFSESSHNIYNAVHAIAHALHEVNLQQVDNQAIDNGKGASSHCLKVNSF
LRKIHFTNPLGDKVIMKQRVIMQEEYDIFHFGNLSQHLGIKVKLGKFSPYFSHGRNFHLYVDMLEVATGSRKM
PSSVCSAECSPGFRRLWKEGMAACCFVCSPCPENEISNETNMDQCVNCPEYQYANTEQNKCIQKGVTFLSYED
PLGMALALMAFCFSAFTAVVLCVFVKHHDTPIVKANNRILSYLLLISLMSCFLCSFFYIGHPNRATCVLQQIT
FGIVFTVAVSTVLAKTVTVVLAFKVTDPGRRLRNFLVSGTPNYIIPICSLLQCVLCAIWIEVSPPFVDIDEHS
QHGHIIIVCNKGSVTAFYCVLGYLACLALGSFNLAFLAKNLPDTFNEAKFLTFSMLVFFSVWVTFLPVYHSTK
GKVMVAVEIFSILASSAGMLGCIFAPKIYIILMRPERNSTQKIKVRNSTQKIKEKSYF*
>ENSMUSP00000156838_c.1624:G>T_p.S542I ENSMUST00000233892.2|Vmn2r53-
203|ENSMUSG00000096002|-|chr7:g.12318665:C>A
MWLCTLLSVLFGCLSGSALSGPRVWIIPRQSPRFDRPGDVIVGGSFSIFHFSDGTLSNFTAPPPVLKTSSASM
WSYRVAQSFVFAIEEINRSAHLLPNLTLGFSIRNSGDSVHGALYETMGFLTGQEEPIPNYTCQHGSPQAALVG
DTRSSLSVSMARLLGLYKFPQVSYSSSLPSLSDKIQFPSFIRTLTSDLTCSHAVTQLVIHFQWSWVIILAHDD
DFGQQASSLATQMLTPAGVCIEYHLHVPSHQSFGKIEETVQKMQKCTARVVLVFLSNSNFQLILHGLLAVPVS
GQVWVSMDTLHMALALTIPGISQVLQGTFGLLYHSSRAIGFPEFLAHLRPSQTPEDMFIKKFWEFTFDCTWPY
QNSTVTEGVQFCTGNESLKNKPHPFPEVSRIDAAYTAVYSIAHALHNMLACEHQERKGTNSHNFHSWQLLHNL
RNVYFKTSDGNKIMFDANGDLVTQFDIFQGKKTPAGVFHLVHVGLIDPQASSGNKMMVQLKQDLQVSSLNAEI
TVPTSICSESCLPGFSQVPRLGAPHCCFDCIPCPEGQFTDQRDMKRCLLCPKEQYSSHTRDHCLPRTEIFLAF
EEPLGFILALVALLLAGLAVLVLGVFLKHRDTPVVRANNRTLSYFLLISLSLCALCALLFLGRPTVTTCLLRQ
TIFAVVFTVAVSSVLAKTLTVVLAFRVTRPRSRIQVCLSSGASTSVVLIASLIQVVLCGIWLITSPPFPDRDM
ISEPRHTVIQCHEGSGANFFCVLGFLGFLAGGTFSVAFLARGLPDVFNETKFLTFSMLLFCSVWTAFLPLYHS
ARGKSTVAVEIFSILASTAGLLGGIFIPKCYIILLKPEKNTPAWLRQGHQVQHNRQCEMLQRRCVSSGESLGS
SL*
>ENSMUSP00000126979_c.1408:G>T_p.S470I ENSMUST00000170412.2|Vmn2r53-
201|ENSMUSG00000096002|-|chr7:g.12318665:C>A
MWSYRVAQSFVFAIEEINRSAHLLPNLTLGFSIRNSGDSVHGALYETMGFLTGQEEPIPNYTCQHGSPQAALV
GDTRSSLSVSMARLLGLYKFPQVSYSSSLPSLSDKIQFPSFIRTLTSDLTCSHAVTQLVIHFQWSWVIILAHD
DDFGQQASSLATQMLTPAGVCIEYHLHVPSHQSFGKIEETVQKMQKCTARVVLVFLSNSNFQLILHGLLAVPV
SGQVWVSMDTLHMALALTIPGISQVLQGTFGLLYHSSRAIGFPEFLAHLRPSQTPEDMFIKKFWEFTFDCTWP
YQNSTVTEGVQFCTGNESLKNKPHPFPEVSRIDAAYTAVYSIAHALHNMLACEHQERKGTNSHNFHSWQLLHN
LRNVYFKTSDGNKIMFDANGDLVTQFDIFQGKKTPAGVFHLVHVGLIDPQASSGNKMMVQLKQDLQVSSLNAE
ITVPTSICSESCLPGFSQVPRLGAPHCCFDCIPCPEGQFTDQRDMKRCLLCPKEQYSSHTRDHCLPRTEIFLA
FEEPLGFILALVALLLAGLAVLVLGVFLKHRDTPVVRANNRTLSYFLLISLSLCALCALLFLGRPTVTTCLLR
QTIFAVVFTVAVSSVLAKTLTVVLAFRVTRPRSRIQVCLSSGASTSVVLIASLIQVVLCGIWLITSPPFPDRD
MISEPRHTVIQCHEGSGANFFCVLGFLGFLAGGTFSVAFLARGLPDVFNETKFLTFSMLLFCSVWTAFLPLYH
SARGKSTVAVEIFSILASTAGLLGGIFIPKCYIILLKPEKNTPAWLRQGHQVQHNRQCEMLQRRCVSSGESLG
SSL*
>ENSMUSP00000134531_c.741:T>A_p.S248T ENSMUST00000174443.8|Obox3-
206|ENSMUSG00000066772|-|chr7:g.15359926:A>T
MAEGPSLHPKLQVASNIPIELSSQIPQDPARNLAFQMCQNPLMTPGSTMQSRLSVPERNLLQQESQGPTRQSG
RMPLSDRYVNKQTGPMALRKFRKERTVYSKEQQCLLQKHFDEFQYPNEKKIVELALSVGVTKREIKIWFKNNR
AKYRRMKLQNIKQALPETNESSKAVSESTHFPGSIPVVDSDNGESMCSGTFGEDSIPKLNCSQESSLHCFQAC
```

DGDMCCPQEYQEYQEYLLHGHAPVTAWNTGQSAAFESQTDLAVAEVPVGLAYAAQAPEDAHNSGPCEDELWQR
ILEDLDTSDDWLTLRNLSTPVYTTEVLDQSKPYSHEEVCYTHL*
>ENSMUSP00000133427_c.741:T>A_p.S248T ENSMUST00000173912.8|Obox3-
204|ENSMUSG00000066772|-|chr7:g.15359926:A>T
MAEGPSLHPKLQVASNIPIELSSQIPQDPARNLAFQMCQNPLMTPGSTMQSRLSVPERNLLQQESQGPTRQSG
RMPLSDRYVNKQTGPMALRKFRKERTVYSKEQQCLLQKHFDEFQYPNEKKIVELALSVGVTKREIKIWFKNNR
AKYRRMKLQNIKQALPETNESSKAVSESTHFPGSIPVVDSDNGESMCSGTFGEDSIPKLNCSQESSLHCFQAC
DGDMCCPQEYQEYQEYLLHGHAPVTAWNTGQSAAFESQTDLAVAEVPVGLAYAAQAPEDAHNSGPCEDELWQR
ILEDLDTSDDWLTLRNLSTPVYTTEVLDQSKPYSHEEVCYTHL*
>ENSMUSP00000092842_c.741:T>A_p.S248T ENSMUST00000095217.11|Obox3-
202|ENSMUSG00000066772|-|chr7:g.15359926:A>T
MAEGPSLHPKLQVASNIPIELSSQIPQDPARNLAFQMCQNPLMTPGSTMQSRLSVPERNLLQQESQGPTRQSG
RMPLSDRYVNKQTGPMALRKFRKERTVYSKEQQCLLQKHFDEFQYPNEKKIVELALSVGVTKREIKIWFKNNR
AKYRRMKLQNIKQALPETNESSKAVSESTHFPGSIPVVDSDNGESMCSGTFGEDSIPKLNCSQESSLHCFQAC
DGDMCCPQEYQEYQEYLLHGHAPVTAWNTGQSAAFESQTDLAVAEVPVGLAYAAQAPEDAHNSGPCEDELWQR
ILEDLDTSDDWLTLRNLSTPVYTTEVLDQSKPYSHEEVCYTHL*
>ENSMUSP00000083291_c.741:T>A_p.S248T ENSMUST00000086122.10|Obox3-
201|ENSMUSG00000066772|-|chr7:g.15359926:A>T
MAEGPSLHPKLQVASNIPIELSSQIPQDPARNLAFQMCQNPLMTPGSTMQSRLSVPERNLLQQESQGPTRQSG
RMPLSDRYVNKQTGPMALRKFRKERTVYSKEQQCLLQKHFDEFQYPNEKKIVELALSVGVTKREIKIWFKNNR
AKYRRMKLQNIKQALPETNESSKAVSESTHFPGSIPVVDSDNGESMCSGTFGEDSIPKLNCSQESSLHCFQAC
DGDMCCPQEYQEYQEYLLHGHAPVTAWNTGQSAAFESQTDLAVAEVPVGLAYAAQAPEDAHNSGPCEDELWQR
ILEDLDTSDDWLTLRNLSTPVYTTEVLDQSKPYSHEEVCYTHL*
>ENSMUSP00000147475_c.304:A>G_p.Q102R ENSMUST00000209369.2|Zfp541-
202|ENSMUSG00000078796|+|chr7:g.15806171:A>G
MEPYSLGEEGALPSEGHLPSFSESQVLNCSDTLNRDLGPSTRDLLYAGLSGLDLDPSLSTSDMPSEVLEDNLD
TLSLYSGKDSDSVKLLEEYADSESQTSLRDLGLGALKVPKEADEGGRATGSTRKGKRQHSSPQNPLLDCSLCG
KVFSSASSLSKHYLTHSQERKHVCKVCSKAFKRQDHLTGHMLTHQKTKPFVCIEQGCSKSYCDYRSLRRHYEV
QHGVCILKETPPEEEAYGDPTHNHDVANQPPPSGLRSLGPPEARSPGSVLPNRDLLRCIVSSIVHQKIPSPGP
AVGPSDTEARSSACACPTSLGSSSCTPASTPVALGTLGSEIPEETHPPRKEAATEVFTPVQSRAAENGVPDPP
ESELESESPRLQRPSSLEGWPEGSSLPACLPLFRGHSVPSGSQPSSHNFQWLRNLPGCPKNKGSNVFMVHKPP
AVASREGSEGGGSGPSSTPTSVEPSPSLGTTQEELLPFPPALLKAPGEASSEVRQAAGEDETWAPKKCKPDCE
SFPWQSPTELGLQDAQNPGGLPSDATPLFRQLFMKSQESLVSHEQMQVLQMIAKSQRIFSHTQVATASAQRPG
PEGKQSTLKPLQGPWPPQTLPPAPTVDSFQIGPGHSEPEGSPVRRRKTMPAVSRETSPGGPRRDTKGGPKVAS
APPSLTGPGLLPSRNPDSSSLAKGTLDLGDIIPNAGSRQSQLGGDEPAGTQLVGKQGQGENGLASGAMRGEKG
PACPRGGGYRLFSGHPRAQRFSGFRKEKVKMDVCCAASPSQVAMASFSSAGPLADPPRDMKSKLTIFNRIQGG
NIYRLPHPVKEESLAGGCHQPNGGPTDWMESKSTFVCKNCSQMFYTEKGLSSHMCFHSDQWPSPRGKQEQQVK
GQMVASVKRKAGREEGAVEDMKRHYDCSSSEPQDVTILSMLVSSGSCGVTPVVLSSLLQGQEKDGEERDSKES
CQYRKRKKRPQPKALFAPPAPSALGEPGPGGCHQSCLHSPVFLVDHLLKGLFQCSPYTPPPMLSPIREGSGLY
FNTLCSTSRAGPHLISPVLDQVDSSFGICVVKDDTKISIEPHINVGSRFQAEIPELQERLLARVDENVASLVW
KPWGDVMTNPETQDRVMELCNVACSSVMPGGGTNLELALHCLHDAQGSVQVALETLLLRGPQKPRTHPLADYR
YTGSDIWTPMEKRLFKKAFCAHKKDFYLIHKMIQTKSVAQCVEYYYIWKKMVKFDCGRAPGLEKRGRRELDEV
ERTEDKVTCSPRERPTHRPTPELKIKTKSYRRESILHSSPSAAPKRTPEPPGSVESQGVFPCRECERVFDKIK
SRNAHMKRHRLQEHVEPVRVKWPVKPYPLKEEEEEEEEELGADMGPLQW*
>ENSMUSP00000148143_c.304:A>G_p.Q102R ENSMUST00000210805.2|Zfp541-
203|ENSMUSG00000078796|+|chr7:g.15806171:A>G
MEPYSLGEEGALPSEGHLPSFSESQVLNCSDTLNRDLGPSTRDLLYAGLSGLDLDPSLSTSDMPSEVLEDNLD
TLSLYSGKDSDSVKLLEEYADSESQTSLRDLGLGALKVPKEADEGGRATGSTRKGKRQHSSPQNPLLDCSLCG
KVFSSASSLSKHYLTHSQERKHVCKVCSKAFKRQDHLTGHMLTHQKTKPFVCIEQGCSKSYCDYRSLRRHYEV
QHGVCILKETPPEEEAYGDPTHNHDVANQPPPSGLRSLGPPEARSPGSVLPNRDLLRCIVSSIVHQKIPSPGP
AVGPSDTEARSSACACPTSLGSSSCTPASTPVALGTLGSEIPEETHPPRKEAATEVFTPVQSRAAENGVPDPP
ESELESESPRLQRPSSLEGWPEGSSLPACLPLFRGHSVPSGSQPSSHNFQWLRNLPGCPKNKGSNVFMVHKPP
AVASREGSEGGGSGPSSTPTSVEPSPSLGTTQEELLPFPPALLKAPGEASSEVRQAAGEDETWAPKKCKPDCE
SFPWQSPTELGLQDAQNPGGLPSDATPLFRQLFMKSQESLVSHEQMQVLQMIAKSQRIFSHTQVATASAQRPG
PEGKQSTLKPLQGPWPPQTLPPAPTVDSFQIGPGHSEPEGSPVRRRKTMPAVSRETSPGGPRRDTKGGPKVAS
APPSLTGPGLLPSRNPDSSSLAKGTLDLGDIIPNAGSRQSQLGGDEPAGTQLVGKQGQGENGLASGAMRGEKG
PACPRGGGYRLFSGHPRAQRFSGFRKEKVKMDVCCAASPSQVAMASFSSAGPLADPPRDMKSKLTIFNRIQGG
NIYRLPHPVKEESLAGGCHQPNGGPTDWMESKSTFVCKNCSQMFYTEKGLSSHMCFHSDQWPSPRGKQEQQGQ

EKDGEERDSKESCQYRKRKKRPQPKALFAPPAPSALGEPGPGGCHQSCLHSPVFLVDHLLKGLFQCSPYTPPP
MLSPIREGSGLYFNTLCSTSRAGPHLISPVLDQVDSSFGICVVKDDTKISIEPHINVGSRFQAEIPELQERLL
ARVDENVASLVWKPWGDVMTNPETQDRVMELCNVACSSVMPGGGTNLELALHCLHDAQGSVQVALETLLLRGP
QKPRTHPLADYRYTGSDIWTPMEKRLFKKAFCAHKKDFYLIHKMIQTKSVAQCVEYYYIWKKMVKFDCGRAPG
LEKRGRRELDEVERTEDKVTCSPRERPTHRPTPELKIKTKSYRRESILHSSPSAAPKRTPEPPGSVESQGVFP
CRECERVFDKIKSRNAHMKRHRLQEHVEPVRVKWPVKPYPLKEEEEEEEEELGADMGPLQW*
>ENSMUSP00000104149_c.304:A>G_p.Q102R ENSMUST00000108509.2|Zfp541-201|ENSMUSG00000078796|+|chr7:g.15806171:A>G
MEPYSLGEEGALPSEGHLPSFSESQVLNCSDTLNRDLGPSTRDLLYAGLSGLDLDPSLSTSDMPSEVLEDNLD
TLSLYSGKDSDSVKLLEEYADSESQTSLRDLGLGALKVPKEADEGGRATGSTRKGKRQHSSPQNPLLDCSLCG
KVFSSASSLSKHYLTHSQERKHVCKVCSKAFKRQDHLTGHMLTHQKTKPFVCIEQGCSKSYCDYRSLRRHYEV
QHGVCILKETPPEEEAYGDPTHNHDVANQPPPSGLRSLGPPEARSPGSVLPNRDLLRCIVSSIVHQKIPSPGP
AVGPSDTEARSSACACPTSLGSSSCTPASTPVALGTLGSEIPEETHPPRKEAATEVFTPVQSRAAENGVPDPP
ESELESESPRLQRPSSLEGWPEGSSLPACLPLFRGHSVPSGSQPSSHNFQWLRNLPGCPKNKGSNVFMVHKPP
AVASREGSEGGGSGPSSTPTSVEPSPSLGTTQEELLPFPPALLKAPGEASSEVRQAAGEDETWAPKKCKPDCE
SFPWQSPTELGLQDAQNPGGLPSDATPLFRQLFMKSQESLVSHEQMQVLQMIAKSQRIFSHTQVATASAQRPG
PEGKQSTLKPLQGPWPPQTLPPAPTVDSFQIGPGHSEPEGSPVRRRKTMPAVSRETSPGGPRRDTKGGPKVAS
APPSLTGPGLLPSRNPDSSSLAKGTLDLGDIIPNAGSRQSQLGGDEPAGTQLVGKQGQGENGLASGAMRGEKG
PACPRGGGYRLFSGHPRAQRFSGFRKEKVKMDVCCAASPSQVAMASFSSAGPLADPPRDMKSKLTIFNRIQGG
NIYRLPHPVKEESLAGGCHQPNGGPTDWMESKSTFVCKNCSQMFYTEKGLSSHMCFHSDQWPSPRGKQEQQVK
GQMVASVKRKAGREEGAVEDMKRHYDCSSSEPQDVTILSMLVSSGSCGVTPVVLSSLLQGQEKDGEERDSKES
CQYRKRKKRPQPKALFAPPAPSALGEPGPGGCHQSCLHSPVFLVDHLLKGLFQCSPYTPPPMLSPIREGSGLY
FNTLCSTSRAGPHLISPVLDQVDSSFGICVVKDDTKISIEPHINVGSRFQAEIPELQERLLARVDENVASLVW
KPWGDVMTNPETQDRVMELCNVACSSVMPGGGTNLELALHCLHDAQGSVQVALETLLLRGPQKPRTHPLADYR
YTGSDIWTPMEKRLFKKAFCAHKKDFYLIHKMIQTKSVAQCVEYYYIWKKMVKFDCGRAPGLEKRGRRELDEV
ERTEDKVTCSPRERPTHRPTPELKIKTKSYRRESILHSSPSAAPKRTPEPPGSVESQGVFPCRECERVFDKIK
SRNAHMKRHRLQEHVEPVRVKWPVKPYPLKEEEEEEEEELGADMGPLQW*
>ENSMUSP00000071348_c.1137:A>G_p.T380A ENSMUST00000071399.13|Psg16-201|ENSMUSG00000066760|+|chr7:g.16864544:A>G
MGQMYSGREIVSSNGSLWIHNVTRKDTGLYTLRTVNRRGEIVSTSFTFLYVYTSLFICGRPSFPAKLTIESVP
PSVAEGGSVLLRVHNLQDKLRGLSWYKGAHVSRNLEIARQIIAKNSSVPGPAHSGRETVYSNGSLLLQNVTRN
DTGFYTLQTLSRHRKMELAHVQLQVDTSLSSCCDTLDSTQLIIDPMPRYAAEGESILLRVLNLPEDFQVFCWY
KGALIFQIFKIAEYSRARNSITKGPAQSRTERVYTNGSLLLQDVTEKDTGLYTLQTIDRNFKIEKAHVQIQVN
KPVTQPFMRVTDSTVRVQSSVVFTCFSDNTGVSIHWLFNNQSLQLTERITLSPSKCQLRINPVRKEDGGEYQY
EDHQRNPRLIGMQEATDTMGFPSPTDLSVP*
>ENSMUSP00000146795_c.399:A>C_p.T134P ENSMUST00000208221.2|Psg22-203|ENSMUSG00000044903|+|chr7:g.18453706:A>C
MEVSSELLSNGWTSWQRVLLTASLLTCWLLPITAGVTIESVPPKLVEGENVLLRVDNLPENLRVFVWYRGVTD
MSLGIALYSLDYSTSVTGPKHSGRETLYRNGSLWIQNVTREDTGYYTLQTISKNGKVVSNPSIFLQVNSSLFI
CGRPSPPALLTIESVPASVAEGGSVLLLVHSLPDNLQSLLWYKGLTVFNKVEIARHRTVKNSSEMGPAYSGRE
IVYSNGSLLLQNVTWEDTGFYTLQIVNRYWKMELAHIYLQVDTSLSSCCDDFNSVQLRINPVPPHAAEGERVL
LQVHNLPEDVQTFLWYKGVYSTQSFKITEYSIVTESLINGYAHSGREILFINGSLLLQDVTEKDSGFYTLVTI
DSNVKVETAHVQVNVNKLVTQPVMRVTDSTVRIQGSVVFTCFSDNTGVSIRWLFNNQNLQLTERMTLSPSKCQ
LRIHTVRKEDAGEYQCEAFNPVSSKTSLPVRLTVMNE*
>ENSMUSP00000104121_c.516:A>C_p.T173P ENSMUST00000108481.8|Psg22-202|ENSMUSG00000044903|+|chr7:g.18453706:A>C
MCSCLRENSAQKEEGQQSPGALQEQSSSPLRKASTVRRDMEVSSELLSNGWTSWQRVLLTASLLTCWLLPITA
GVTIESVPPKLVEGENVLLRVDNLPENLRVFVWYRGVTDMSLGIALYSLDYSTSVTGPKHSGRETLYRNGSLW
IQNVTREDTGYYTLQTISKNGKVVSNPSIFLQVNSSLFICGRPSPPALLTIESVPASVAEGGSVLLLVHSLPD
NLQSLLWYKGLTVFNKVEIARHRTVKNSSEMGPAYSGREIVYSNGSLLLQNVTWEDTGFYTLQIVNRYWKMEL
AHIYLQVDTSLSSCCDDFNSVQLRINPVPPHAAEGERVLLQVHNLPEDVQTFLWYKGVYSTQSFKITEYSIVT
ESLINGYAHSGREILFINGSLLLQDVTEKDSGFYTLVTIDSNVKVETAHVQVNVNKLVTQPVMRVTDSTVRIQ
GSVVFTCFSDNTGVSIRWLFNNQNLQLTERMTLSPSKCQLRIHTVRKEDAGEYQCEAFNPVSSKTSLPVRLTV
MNE*
>ENSMUSP00000050633_c.399:A>C_p.T134P ENSMUST00000051973.9|Psg22-201|ENSMUSG00000044903|+|chr7:g.18453706:A>C
MEVSSELLSNGWTSWQRVLLTASLLTCWLLPITAGVTIESVPPKLVEGENVLLRVDNLPENLRVFVWYRGVTD
MSLGIALYSLDYSTSVTGPKHSGRETLYRNGSLWIQNVTREDTGYYTLQTISKNGKVVSNPSIFLQVNSSLFI

CGRPSPPALLTIESVPASVAEGGSVLLLVHSLPDNLQSLLWYKGLTVFNKVEIARHRTVKNSSEMGPAYSGRE
IVYSNGSLLLQNVTWEDTGFYTLQIVNRYWKMELAHIYLQVDTSLSSCCDDFNSVQLRINPVPPHAAEGERVL
LQVHNLPEDVQTFLWYKGVYSTQSFKITEYSIVTESLINGYAHSGREILFINGSLLLQDVTEKDSGFYTLVTI
DSNVKVETAHVQVNVNKLVTQPVMRVTDSTVRIQGSVVFTCFSDNTGVSIRWLFNNQNLQLTERMTLSPSKCQ
LRIHTVRKEDAGEYQCEAFNPVSSKTSLPVRLTVMNE*
>rs235733715_ENSMUSP00000104093_c.760:A>C_p.K254T
ENSMUST00000108453.2|Zfp296-201|ENSMUSG00000011267|+|chr7:g.19313907:A>C
MSRRKAGRVPRRVDPDTDTDIEMPDLVMDVKPDLDLRSLAQGPWIARDMPISDVKRQLQTASRPLGAPSTCAP
RMPLSSKSSDRQPWTDKHPDLLTCGRCGKIFPLGAIIAFMDHKKQGCQLLQVSDPISESKELKALSCLQCGRQ
YTSPWKLLCHAQWDHGLCIYQTQHLDTPEAPLLGLAEVAAAMSAVAVVAPVESKPPPVSSAARRSPTCDVCKK
TLSSFSNLKVHMRSHTGERPYSCDQCSYACAQSSTLNRHKKTHRQLAPGSPSTSASSRGVSPAAPPEPAAYAA
APASTLPSQTVEKAGAAATAGVQEPGAPGSGAQGGPGFVGWGAPAKVERTDPVKIEKTAPRKSHGPGGKCEFC
GKSFTNSSNLTVHRRSHTGERPYTCDQCPYACAQSSKLNRHRRTHGLGTGKTVKCPHCLVPFGLQATLDKHLR
QKHPEMA*
>ENSMUSP00000104093_c.770:A>C_p.R257S ENSMUST00000108453.2|Zfp296-
201|ENSMUSG00000011267|+|chr7:g.19313917:A>C
MSRRKAGRVPRRVDPDTDTDIEMPDLVMDVKPDLDLRSLAQGPWIARDMPISDVKRQLQTASRPLGAPSTCAP
RMPLSSKSSDRQPWTDKHPDLLTCGRCGKIFPLGAIIAFMDHKKQGCQLLQVSDPISESKELKALSCLQCGRQ
YTSPWKLLCHAQWDHGLCIYQTQHLDTPEAPLLGLAEVAAAMSAVAVVAPVESKPPPVSSAARRSPTCDVCKK
TLSSFSNLKVHMRSHTGERPYSCDQCSYACAQSSKLNSHKKTHRQLAPGSPSTSASSRGVSPAAPPEPAAYAA
APASTLPSQTVEKAGAAATAGVQEPGAPGSGAQGGPGFVGWGAPAKVERTDPVKIEKTAPRKSHGPGGKCEFC
GKSFTNSSNLTVHRRSHTGERPYTCDQCPYACAQSSKLNRHRRTHGLGTGKTVKCPHCLVPFGLQATLDKHLR
QKHPEMA*
>ENSMUSP00000066880_c.109:GT>CG_p.G37A ENSMUST00000068023.8|Cadm4-
201|ENSMUSG00000054793|+|chr7:g.24198626:GT>CG
MGRARRFQWPLLLLWAAAAGPGTGQEVQTENVTVAEAWGG*DHLPSASVRWVHSRHSEPSPADPLFQWHQSSE
GRAIPAGGVLPAPGAHQALRRPPGGRGGLLLPALHGGHPPSDRHAHCLSGSGESCGGGPRASSGGRRGGTQLP
GSAVAPRSGPALVSGSQGVERSEQRPGERQGVERGKHRAVPCGSQGRRWYCHLRSTEPGAALGTQQADAVRAG
CAVFPHSADPRLSSCSEGGRHSGADLCCHREPQAKPDPLEPWE*VSAREGGGGGRDAHATWPGICR*RHLHLR
GGEQARPREGALRACGL*PRCSGRGSDIGSLRHCGRHPGATGVSDHMCASGHGLVLRPTEGILSDP*GQWLG*
AGRSQRSLPQWRRWTQTERRILHL
>ENSMUSP00000003529_c.1156:C>T_p.A386V ENSMUST00000003529.9|Paf1-
201|ENSMUSG00000003437|+|chr7:g.28098148:C>T
MAPTIQTQAQREDGHRPNSHRTLPERSGVVCRVKYCNSLPDIPFDPKFITYPFDQNRFVQYKATSLEKQHKHD
LLTEPDLGVTIDLINPDTYRIDPNVLLDPADEKLLEEEIQAPTSSKRSQQHAKVVPWMRKTEYISTEFNRYGI
SNEKPEVKIGVSVKQQFTEEEIYKDRDSQITAIEKTFEDAQKSISQHYSKPRVTPVEVMPVFPDFKMWINPCA
QVIFDSDPAPKDTSGAAALEMMSQAMIRGMMDEEGNQFVAYFLPVEETLKKRKRDQEEEMDYAPDDVYDYKIA
REYNWNVKNKASKGYEENYFFIFREGDGVYYNELETRVRLSKRRAKAGVQSGTNALLVVKHRDMNEKELEAQE
ARKAQLENHEPEEEEEEEMEVEEKEAGGSDEEQEKGSSSEKEGSEDEHSGSESDREEGDRDEASDKSGSGEDE
SSEDEARAARDKEEIFGSDADSEDDADSDDEDRGQAHRGSDNDSDSGSDGGGQRSRSQSRSRSRSASPFPSGS
EHSAQEDGSEAAASDSSEADSDSD*
>ENSMUSP00000146824_c.568:A>C_p.H190P ENSMUST00000207873.2|Zfp14-
203|ENSMUSG00000053985|-|chr7:g.29738415:T>G
MALAQGLVTFGDVAVDFSQEEWEFLDPAQKNLYRDVMWETYSNFISLDLESRFKTDTSSSDKGICEVYSLQWE
LIEKIKNLSPQGSGLSDDQECKHKTGLQKEPQEGYFGQLKITSEKVTYEKHSFLSEYQRVQNGEKFYECKECR
KTFIRRSTLSQHLRIHTGEKPYKCKECGQPFRQRAHLIRHHKLPTGEKPYECKDCGKAFTVLQELTQHQRLHT
GEKPYECKECGKAFRVHQQLARHQRIHTGEKPYECKECGKTFRQCTHLTRHQRLHTSEKLYECKECGKAFVCG
PDLRVHQKIHFGEKPYACKDCGKSFRICQQLTVHQSIHTGEKPYECKECGKTFRLRQQLVRHQRIHTHERPYE
CLECWKTFSSYSQLISHQSIHVGERPYECEECGKAFRLLSQLTQHQSIHTGEKPYECQECRKPFRLLSQLTQH
RSIHTGEKPYECKDCGKAFRLYSFLSQHQRIHTGEKPYKCKECKKAFRQHSHLTQHQKIHSGT*
>ENSMUSP00000146913_c.568:A>C_p.H190P ENSMUST00000207072.2|Zfp14-
202|ENSMUSG00000053985|-|chr7:g.29738415:T>G
MALAQGLVTFGDVAVDFSQEEWEFLDPAQKNLYRDVMWETYSNFISLDLESRFKTDTSSSDKGICEVYSLQWE
LIEKIKNLSPQGSGLSDDQECKHKTGLQKEPQEGYFGQLKITSEKVTYEKHSFLSEYQRVQNGEKFYECKECR
KTFIRRSTLSQHLRIHTGEKPYKCKECGQPFRQRAHLIRHHKLPTGEKPYECKDCGKAFTVLQELTQHQRLHT
GEKPYECKECGKAFRVHQQLARHQRIHTGEKPYECKECGKTFRQCTHLTRHQRLHTSEKLYECKECGKAFVCG
PDLRVHQKIHFGEKPYACKDCGKSFRICQQLTVHQSIHTGEKPYECKECGKTFRLRQQLVRHQRIHTHERPYE

CLECWKTFSSYSQLISHQSIHVGERPYECEECGKAFRLLSQLTQHQSIHTGEKPYECQECRKPFRLLSQLTQH
RSIHTGEKPYECKDCGKAFRLYSFLSQHQRIHTGEKPYKCKECKKAFRQHSHLTQHQKIHSGT*
>ENSMUSP00000076960_c.568:A>C_p.H190P ENSMUST00000077787.8|Zfp14-
201|ENSMUSG00000053985|-|chr7:g.29738415:T>G
MALAQGLVTFGDVAVDFSQEEWEFLDPAQKNLYRDVMWETYSNFISLDLESRFKTDTSSSDKGICEVYSLQWE
LIEKIKNLSPQGSGLSDDQECKHKTGLQKEPQEGYFGQLKITSEKVTYEKHSFLSEYQRVQNGEKFYECKECR
KTFIRRSTLSQHLRIHTGEKPYKCKECGQPFRQRAHLIRHHKLPTGEKPYECKDCGKAFTVLQELTQHQRLHT
GEKPYECKECGKAFRVHQQLARHQRIHTGEKPYECKECGKTFRQCTHLTRHQRLHTSEKLYECKECGKAFVCG
PDLRVHQKIHFGEKPYACKDCGKSFRICQQLTVHQSIHTGEKPYECKECGKTFRLRQQLVRHQRIHTHERPYE
CLECWKTFSSYSQLISHQSIHVGERPYECEECGKAFRLLSQLTQHQSIHTGEKPYECQECRKPFRLLSQLTQH
RSIHTGEKPYECKDCGKAFRLYSFLSQHQRIHTGEKPYKCKECKKAFRQHSHLTQHQKIHSGT*
>ENSMUSP00000079362_c.609:A>G_p.K204E ENSMUST00000080518.14|Sbsn-
201|ENSMUSG00000046056|+|chr7:g.30451596:A>G
MYLVSLLSSCCLLVLLGTLPARAAHEDPVEKVIEGFSRGLSNAEREVGKALEGINNGITQAGREVEKIFGELS
NMGSQAGKNVEHGLDKVAHDINSGIGHAGKEAEKFAHGVNHAAGQVGKETNKIIHHGVSQGGSEAGKFGQGSH
HAFGQGGNVADKLGHETHHAFGQGGNVADKLGHETHHAFGQGGNVADKLGHGGNVADELGHGTHHAFGQGGNV
ADKLGHETHHAFGQGGNVADKLGHGTHHAFGQGGNVADKLGHETHHAFGQGGNVAEKLGHETHHAFGQGGNMA
DKFGQGAHHAFGQGGNMADKFGQGAHHAFGQGGNMADKFGQGAHHAFGQGGNMADKFGQGAHHAFGQGGNMAD
KFGQGAHHAFGQGRDMAETFDQGAHHAFGQGGREGGRLVQGAGQGLSHAAKEAQQFGHGHGHGYYAAGQTWQE
GDKVIRPGVSQAGEEMEQFGQGVRHTIKQAEKEAEKVAHGVQNGVNQAQKEAEKVAHGVQNGVNQAQKEAEKV
AHGVQNGVNQAQKEAEKVAHGVQTGVNQAGKETQRVGQGVQTGFNQGQKEAEKVAHGVQTGVNQAGKETQKAG
QGVNYAAGQAEKEAEKLGQGVHHAAGQEMNRLQQDVHNGVNQPSKEANQLLNGSHQGQGGYGGQHGGAATTTV
VSGASVNKPFINFPALWRSIAATMP*
>ENSMUSP00000145999_c.535:G>A_p.C179Y ENSMUST00000205912.2|Zfp936-
204|ENSMUSG00000064194|+|chr7:g.42839070:G>A
MDALTFDDVHVHFTREEWSLLDPSQKRLYKDVMLENYRNLTAIGYNWKDHNIEEHCQNDRRYGRHKITPAGEK
PYEYTQWDKAFKYHSHLQKLERNQTGDKNSEVFQHSKVLACQSTLQRNKRTNTGEKPYKPSYLRMHERTHTGE
KPYQCNECDKAFLQLRYLRLHERRHSGEKPYEYNQ
>ENSMUSP00000143800_c.535:G>A_p.C179Y ENSMUST00000202535.4|Zfp936-
203|ENSMUSG00000064194|+|chr7:g.42839070:G>A
MDALTFDDVHVHFTREEWSLLDPSQKRLYKDVMLENYRNLTAIGYNWKDHNIEEHCQNDRRYGRHKITPAGEK
PYEYTQWDKAFKYHSHLQKLERNQTGDKNSEVFQHSKVLACQSTLQRNKRTNTGEKPYKPSYLRMHERTHTGE
KPYQCNECDKAFLQLRYLRLHERRHSGEKPYEYNQCGKAFLHLNYLRIHERTHTGEKPYQCNQCGKAFTQRSH
LQRHKVVHTGVRPYICNQCNKAYPQQNYLRKHERTHTGEKPYICNQCNKAFSHQSNLRIHEKTHTGEKPYKCN
QCDKAFSHQSNLQIHERTHTGEKPYKCNQCDKAFAHLKYLRAHERTHTQEKPYKCNQCGKAFLYLSCLRVHER
THTGEKPYKCNQCDKAFAHLKYLRAHERIHTQEKPYKCNQCGKAFLLLSCLRVHERTHTGEKPYKCDQCGKAF
TQRSHLKTHKVIHTGGRL*
>ENSMUSP00000072608_c.532:G>A_p.C178Y ENSMUST00000072829.4|Zfp936-
201|ENSMUSG00000064194|+|chr7:g.42839070:G>A
DALTFDDVHVHFTREEWSLLDPSQKRLYKDVMLENYRNLTAIGYNWKDHNIEEHCQNDRRYGRHKITPAGEKP
YEYTQWDKAFKYHSHLQKLERNQTGDKNSEVFQHSKVLACQSTLQRNKRTNTGEKPYKPSYLRMHERTHTGEK
PYQCNECDKAFLQLRYLRLHERRHSGEKPYEYNQCGKAFLHLNYLRIHERTHTGEKPYQCNQCGKAFTQRSHL
QRHKVVHTGVRPYICNQCNKAYPQQNYLRKHERTHTGEKPYICNQCNKAFSHQSNLRIHEKTHTGEKPYKCNQ
CDKAFSHQSNLQIHERTHTGEKPYKCNQCDKAFAHLKYLRAHERTHTQEKPYKCNQCGKAFLYLSCLRVHERT
HTGEKPYKCNQCDKAFAHLKYLRAHERIHTQEKPYKCNQCGKAFLLLSCLRVHERTHTGEKPYKCDQCGKAFT
QRSHLKTHKVIHTGGRL*
>ENSMUSP00000127022_c.949:C>T_p.T317I ENSMUST00000098433.5|Mrgprx2-
201|ENSMUSG00000074109|-|chr7:g.48131867:G>A
MEERNISGRDLRVDSNITYWGTNITAVNESNHTGMSFCEVVSCTMVFLSLIVALVGLVGNATVLWFLGFQMRR
NAFSVYILNLAGADFLFICFQIGYCFHMILDIDSIPIEIDLFYLVVLNFPYFCGLSILSAISIERCLSVMWPI
WYHCQRPRHTSAVICTLLWVLSLVCSLLEGKECGFLYYTSDPGWCKTFDLITATWLIVLFVALLGSSLALVIT
IFWGLHKIPVTRLYVAIVFTVLVFLLFGLPYGIYWFLLVWIEKFYYVLPCSIYPVTVFLSCVNSSAKPIIYCL
VGSIRHHRFQRKTLKLFLQRAMQDIPEEEECGEMGSSGRSREIKTIWKGLRAALIRHKEL*
>ENSMUSP00000140945_c.220:C>T_p.T74I ENSMUST00000186394.2|Mrgprx2-
202|ENSMUSG00000074109|-|chr7:g.48131867:G>A
MSFCEVVSCTMVFLSLIVALVGLVGNATVLWFLGFQMRRNAFSVYILNLAGSIRHHRFQRKTLKLFLQRAMQD
IPEEEECGEMGSSGRSREIKTIWKGLRAALIRHKEL*

>ENSMUSP00000127022_c.583:A>T_p.D195V ENSMUST00000098433.5|Mrgprx2-201|ENSMUSG00000074109|-|chr7:g.48132233:T>A
MEERNISGRDLRVDSNITYWGTNITAVNESNHTGMSFCEVVSCTMVFLSLIVALVGLVGNATVLWFLGFQMRR
NAFSVYILNLAGADFLFICFQIGYCFHMILDIDSIPIEIDLFYLVVLNFPYFCGLSILSAISIERCLSVMWPI
WYHCQRPRHTSAVICTLLWVLSLVCSLLEGKECGFLYYTSDPGWCKTFVLITATWLIVLFVALLGSSLALVIT
IFWGLHKIPVTRLYVAIVFTVLVFLLFGLPYGIYWFLLVWIEKFYYVLPCSIYPVTVFLSCVNSSAKPIIYCL
VGSIRHHRFQRKTLKLFLQRAMQDTPEEEECGEMGSSGRSREIKTIWKGLRAALIRHKEL*
>rs236992428_ENSMUSP00000047775_c.728:T>CT_p.A243ins-fs ENSMUST00000042318.6|Fsd2-201|ENSMUSG00000038663|-|chr7:g.81202849:A>AG
MEEEAEEESGLGRSAAPKDFHFYHMDLYDSEDRLQLFPGDSSRIRREVTQAEDDRELGDEFVDEHRLGTLGYP
SYGMRRRDPGREPRDWGEAAEAEDLGYGGGQGPLDQCQDLREAYRYTHGRASEEYECYVIPEEEDEEEPADVF
CITCKTPVRTVEKDFDTHKEHEVTPISKALEHAKDEVHKNMCKLEQQIIEMENFASHLEEVFITVEENFGRQE
QNFECHYNGILETLAQKYEEKIQASGGEKERETGSLIRTTGQLWREPRCLQGADGNRRGDVS
>ENSMUSP00000076687_c.7:C>T_p.S3F ENSMUST00000077478.8|Vmn2r73-201|ENSMUSG00000070458|-|chr7:g.85525139:G>A
MFFLIFIFWFLKISYIFCHLSNPRCFWRIKDTKDKLGDKETYCFFSISAKHGYIRNDYFSWNLDKHVTPKTNH
LIFSVYLALEEINKNSHILPNISLVVNIECVLLKYGEKTGLGLKHEEFIPNYYCANERRYLIVLTAPIWAVST
KLGPFLFMSRIPELYCGHFHLPLSDNEQFPHLYQISPKDTSLPLAMVSLVVHFRWNWIGVIITNDNHGIQFLS
ELRGETQKQIVCLSVVIIIQTDDITAYKEYHMNYNKIVMSSAKVVIVYGDHNSPIKFVLFLWKSQGIFRIWVS
VSQFDMITPLGDFMLYSASGTLIFSHQQSELSGFEKFIKTVKPSNYSSEFSFAKLWWTYFRCSSLPPSNCKKL
KNCPTKTVFRWLFMTPLGMAMSDTCYNLYNAIYAVAHSLHEMLLQQVDTWSENAGKELEFDSWKMFSILKTMQ
FVNPAGDLVNMNQNLKQEREYDIFYIMDFQKDYGLKMKIGKFSGHLPSHQQLYMSKEMIEWATDMDQILPSIC
SMPCRPGLRKSPQEGKDICCFVCNPCPENEISNMTNMDQCVKCPEDQYANEDHTLCLQKIVAILDYRDPLGKA
LAGFALCFCVITSGVLCIFLKHRETPIVKANNQTLSYVLLISLIFCFICSLLYIGHPTIVICVLQQSTFAISF
TVAASTILAKTITVVLAFKVTVPGRMRWLLVAGAPKYIILICTMIQLILCGVWLGTSPPFVDADLHMVHGHII
IVCNKGSVIAFYFVLGYMGSIALASFTVAFLARNLPDTFNEAKLLTFSMLVFCSVWVTFIPVYYSTKGKTMVA
VEVFCILASSAGLLLCIFAPKCYNILLRPGKNSSHMFKKLHAKTENII*
>ENSMUSP00000156568_c.7:C>T_p.S3F ENSMUST00000233729.2|Vmn2r73-207|ENSMUSG00000070458|-|chr7:g.85525139:G>A
MFFLIFIFWFLKISYIFCHLSNPRCFWRIKDTKDKLGDKETYCFFSISAKHGYIRNDYFSWNLDKHVTPKTNH
LIFSVYLALEEINKNSHILPNISLVVNIECVLLKYGEKTGLGLKHEEFIPNYYCANERRYLIVLTAPIWAVST
KLGPFLFMSRIPEISPKDTSLPLAMVSLVVHFRWNWIGVIITNDNHGIQFLSELRGETQKQIVCLSVVIIIQT
DDITAYKEYHMNYNKIVMSSAKVVIVYGDHNSPIKFVLFLWKSQGIFRIWVSVSQFDMITPLGDFMLYSASGT
LIFSHQQSELSGFEKFIKTVKPSNYSSEFSFAKLWWTYFRCSSLPPSNCKKLKNCPTKTVFRWLFMTPLGMAM
SDTCYNLYNAIYAVAHSLHEMLLQQVDTWSENAGKELEFDSWKMFSILKTMQFVNPAGDLVNMNQNLKQEREY
DIFYIMDFQKDYGLKMKIGKFSGHLPSHQQLYMSKEMIEWATDMDQILPSICSMPCRPGLRKSPQEGKDICCF
VCNPCPENEISNMTNMDQCVKCPEDQYANEDHTLCLQKIVAILDYRDPLGKALAGFALCFCVITSGVLCIFLK
HRETPIVKANNQTLSYVLLISLIFCFICSLLYIGHPTIVICVLQQSTFAISFTVAASTILAKTITVVLAFKVT
VPGRMRWLLVAGAPKYIILICTMIQLILCGVWLGTSPPFVDADLHMVHGHIIIVCNKGSVIAFYFVLGYMGSI
ALASFTVAFLARNLPDTFNEAKLLTFSMLVFCSVWVTFIPVYYSTKGKTMVAVEVFCILASSAGLLLCIFAPK
CYNILLRPGKNSSHMFKKLHAKTENII*
>ENSMUSP00000156903_c.7:C>T_p.S3F ENSMUST00000233079.2|Vmn2r73-204|ENSMUSG00000070458|-|chr7:g.85525139:G>A
MFFLIFIFWFLKISYIFCHLSNPRCFWRIKDTKDKLGDKETYCFFSISAKHGYIRNDYFSWNLDKHVTPKTNH
LIFSVYLALEEINKNSHILPNISLVVNIECVLLKYGEKTGLGLKHEEFIPNYYCANERRYLIVLTAPIWAVST
KLGPFLFMSRIPELYCGHFHLPLSDNEQFPHLYQISPKDTSLPLAMVSLVVHFRWNWIGVIITNDNHGIQFLS
ELRGETQKQIVCLSVVIIIQTDDITAYKEYHMNYNKIVMSSAKVVIVYGDHNSPIKFVLFLWKSQGIFRIWVS
VSQFDMITPLGDFMLYSASGTLIFSHQQSELSGFEKFIKTVKPSNYSSEFSFAKLWWTYFRCSSLPPSNCKKL
KNCPTKTVFRWLFMTPLGMAMSDTCYNLYNAIYAVAHSLHEMLLQQVDTWSENAGKELEFDSWKILPSICSMP
CRPGLRKSPQEGKDICCFVCNPCPENEISNMTNMDQCVKCPEDQYANEDHTLCLQKIVAILDYRDPLGKALAG
FALCFCVITSGVLCIFLKHRETPIVKANNQTLSYVLLISLIFCFICSLLYIGHPTIVICVLQQSTFAISFTVA
ASTILAKTITVVLAFKVTVPGRMRWLLVAGAPKYIILICTMIQLILCGVWLGTSPPFVDADLHMVHGHIIIVC
NKGSVIAFYFVLGYMGSIALASFTVAFLARNLPDTFNEAKLLTFSMLVFCSVWVTFIPVYYSTKGKTMVAVEV
FCILASSAGLLLCIFAPKCYNILLRPGKNSSHMFKKLHAKTENII*
>ENSMUSP00000156619_c.7:C>T_p.S3F ENSMUST00000233315.2|Vmn2r73-206|ENSMUSG00000070458|-|chr7:g.85525139:G>A
MFFLIFIFWFLKISYIFCHLSNPRCFWRIKDTKDKLGDKETYCFFSISAKHGYIRNDYFSWNLDKHVTPKTNH
LIFSVYLALEEINKNSHILPNISLVVNIECVLLKYGEKTGLGLKHEEFIPNYYCANERRYLIVLTAPIWAVST

KLGPFLFMSRIPEISPKDTSLPLAMVSLVVHFRWNWIGVIITNDNHGIQFLSELRGETQKQIVCLSVVIIIQT
DDITAYKEYHMNYNKIVMSSAKVVIVYGDHNSPIKFVLFLWKSQGIFRIWVSVSQFDMITPLGDFMLYSASGT
LIFSHQQSELSGFEKFIKTVKPSNYSSEFSFAKLWWTYFRCSSLPPSNCKKLKNCPTKTVFRWLFMTPLGMAM
SDTCYNLYNAIYAVAHSLHEMLLQQVDTWSENAGKELEFDSWKILPSICSMPCRPGLRKSPQEGKDICCFVCN
PCPENEISNMTNMDQCVKCPEDQYANEDHTLCLQKIVAILDYRDPLGKALAGFALCFCVITSGVLCIFLKHRE
TPIVKANNQTLSYVLLISLIFCFICSLLYIGHPTIVICVLQQSTFAISFTVAASTILAKTITVVLAFKVTVPG
RMRWLLVAGAPKYIILICTMIQLILCGVWLGTSPPFVDADLHMVHGHIIIVCNKGSVIAFYFVLGYMGSIALA
SFTVAFLARNLPDTFNEAKLLTFSMLVFCSVWVTFIPVYYSTKGKTMVAVEVFCILASSAGLLLCIFAPKCYN
ILLRPGKNSSHMFKKLHAKTENII*
>ENSMUSP00000156444_c.7:C>T_p.S3F ENSMUST00000233115.2|Vmn2r73-205|ENSMUSG00000070458|-|chr7:g.85525139:G>A
MFFLIFIFWFLKISYIFCHLSNPRCFWRIKDTKDKLGDKETYCFFSISAKHGYIRNDYFSWNLDKHVTPKTNH
LIFSVYLALEEINKNSHILPNISLVVNIECVLLKYGEKTGLGLKHEEFIPNYYCANERRYLIVLTAPIWAVST
KLGPFLFMSRIPELYCGHFHLPLSDNEQFPHLYQISPKDTSLPLAMVSLVVHFRWNWIGVIITNDNHGIQFLS
ELRGETQKQIVCLSVVIIIQTDDITAYKEYHMNYNKIVMSSAKVVIVYGDHNSPIKFVLFLWKSQGIFRIWVS
VSQFDMITPLGDFMLYSASGTLIFSHQQSELSGFEKFIKTVKPSNYSSEFSFAKLWWTYFRCSSLPPSNCKKL
KNCPTKTVFRWLFMTPLGMAMSDTCYNLYNAIYAVAHSLHEMLLQQVDTWSENAGKELEFDSWKMFSILKTMQ
FVNPAGDLVNMNQNLKQEREYDIFYIMDFQKDYGLKMKIGKFSGHLPSHQQLYMSKEMIEWATDMDQILPSIC
SMPCRPGLRKSPQEGKDICCFVCNPCPENEISNMTSFLCLYICLLFRYGSVCEMSRRSICQ*
>ENSMUSP00000156901_c.7:C>T_p.S3F ENSMUST00000233748.2|Vmn2r73-208|ENSMUSG00000070458|-|chr7:g.85525139:G>A
MFFLIFIFWFLKISYIFCHLSNPRCFWRIKDTKDKLGDKETYCFFSISAKHGYIRNDYFSWNLDKHVTPKTNH
LIFSVYLALEEINKNSHILPNISLVVNIECVLLKYGEKTGLGLKHEEFIPNYYCANERRYLIVLTAPIWAVST
KLGPFLFMSRIPEISPKDTSLPLAMVSLVVHFRWNWIGVIITNDNHGIQFLSELRGETQKQIVCLSVVIIIQT
DDITAYKEYHMNYNKIVMSSAKVVIVYGDHNSPIKFVLFLWKSQGIFRIWVSVSQFDMITPLGDFMLYSASGT
LIFSHQQSELSGFEKFIKTVKPSNYSSEFSFAKLWWTYFRCSSLPPSNCKKLKNCPTKTVFRWLFMTPLGMAM
SDTCYNLYNAIYAVAHSLHEMLLQQVDTWSENAGKELEFDSWKMFSILKTMQFVNPAGDLVNMNQNLKQEREY
DIFYIMDFQKDYGLKMKIGKFSGHLPSHQQLYMSKEMIEWATDMDQILPSICSMPCRPGLRKSPQEGKDICCF
VCNPCPENEISNMTSFLCLYICLLFRYGSVCEMSRRSICQ*
>ENSMUSP00000156768_c.7:C>T_p.S3F ENSMUST00000232965.2|Vmn2r73-203|ENSMUSG00000070458|-|chr7:g.85525139:G>A
MFFLIFIFWFLKISYIFCHLSNPRCFWRIKDTKDKLGDKETYCFFSISAKHGYIRNDYFSWNLDKHVTPKTNH
LIFSVYLALEEINKNSHILPNISLVVNIECVLLKYGEKTGLGLKHEEFIPNYYCANERRYLIVLTAPIWAVST
KLGPFLFMSRIPELYCGHFHLPLSDNEQFPHLYQISPKDTSLPLAMVSLVVHFRWNWIGVIITNDNHGIQFLS
ELRGETQKQIVCLSVVIIIQTDDITAYKEYHMNYNKIVMSSAKVVIVYGDHNSPIKFVLFLWKSQGIFRIWVS
VSQFDMITPLGDFMLYSASGTLIFSHQQSELSGFEKFIKTVKPSNYSSEFSFAKLWWTYFRCSSLPPSNCKKL
KNCPTKTVFRWLFMTPLGMAMSDTCYNLYNAIYAVAHSLHEMLLQQVDTWSENAGKELEFDSWKILPSICSMP
CRPGLRKSPQEGKDICCFVCNPCPENEISNMTSFLCLYICLLFRYGSVCEMSRRSICQ*
>ENSMUSP00000156472_c.7:C>T_p.S3F ENSMUST00000232897.2|Vmn2r73-202|ENSMUSG00000070458|-|chr7:g.85525139:G>A
MFFLIFIFWFLKISYIFCHLSNPRCFWRIKDTKDKLGDKETYCFFSISAKHGYIRNDYFSWNLDKHVTPKTNH
LIFSVYLALEEINKNSHILPNISLVVNIECVLLKYGEKTGLGLKHEEFIPNYYCANERRYLIVLTAPIWAVST
KLGPFLFMSRIPEISPKDTSLPLAMVSLVVHFRWNWIGVIITNDNHGIQFLSELRGETQKQIVCLSVVIIIQT
DDITAYKEYHMNYNKIVMSSAKVVIVYGDHNSPIKFVLFLWKSQGIFRIWVSVSQFDMITPLGDFMLYSASGT
LIFSHQQSELSGFEKFIKTVKPSNYSSEFSFAKLWWTYFRCSSLPPSNCKKLKNCPTKTVFRWLFMTPLGMAM
SDTCYNLYNAIYAVAHSLHEMLLQQVDTWSENAGKELEFDSWKILPSICSMPCRPGLRKSPQEGKDICCFVCN
PCPENEISNMTSFLCLYICLLFRYGSVCEMSRRSICQ*
>ENSMUSP00000134377_c.871:G>A_p.R291K ENSMUST00000174158.3|Olfr309-202|ENSMUSG00000054054|-|chr7:g.85955448:C>T
MMNFSIVSEFMILGLTQKSELQGILFIVFLFIYLVALLGNMLIVVAIIYNTTLHTPMYILLLALAVVDIICTT
SIIPKMLGTMLTSKNSISYGGCMSQLFFFTWSLGAEMVLFTTMAYDRYVAICFPLRYSTIMNHYTCVGLLSIV
MAIAVTNSWVHTGLILRLTFCGPNIIDHFFCEIPPLLALSCSPVRVNEVMVYVADITLAVGDFTLTCISYGFI
IAAILRIRTTEGKKKAFSTCSSHLMVVSLYYSPVIYTYIRPASSYTFDKDKVVAALYTLVTPTLNPIVYSFKN
KEMQSGIKKVFAFLKG*
>ENSMUSP00000061929_c.871:G>A_p.R291K ENSMUST00000055690.3|Olfr309-201|ENSMUSG00000054054|-|chr7:g.85955448:C>T
MMNFSIVSEFMILGLTQKSELQGILFIVFLFIYLVALLGNMLIVVAIIYNTTLHTPMYILLLALAVVDIICTT
SIIPKMLGTMLTSKNSISYGGCMSQLFFFTWSLGAEMVLFTTMAYDRYVAICFPLRYSTIMNHYTCVGLLSIV

MAIAVTNSWVHTGLILRLTFCGPNIIDHFFCEIPPLLALSCSPVRVNEVMVYVADITLAVGDFTLTCISYGFI
IAAILRIRTTEGKKKAFSTCSSHLMVVSLYYSPVIYTYIRPASSYTFDKDKVVAALYTLVTPTLNPIVYSFKN
KEMQSGIKKVFAFLKG*
>ENSMUSP00000032967_c.661:A>G_p.K221R ENSMUST00000032967.4|Lipt2-201|ENSMUSG00000030725|+|chr7:g.99809576:A>G
MSLPVVRLVWLGRVHYSELLALQEHWLRRLQADPRPGTLSGTKAGVLLVCEPAGPVYTGGLRGGLTPEETTRL
RALGAEVRATGRGGLATFHGPGQLLCHPVLDLRLLGLRLRTHVAALEACAVRLCELRGLQGARARPPPYTGVW
LGERKICAIGVRCGRHITSHGLALNCSTDLTWFEHIVPCGLVGTGVTSLSEALQRLVTVDEVMPSFLVAFKET
FRCTLISEDSPS*
>ENSMUSP00000149045_c.647:T>A_p.D216E ENSMUST00000216516.2|Olfr617-203|ENSMUSG00000073946|+|chr7:g.103233878:T>A
MATSSTTLNYTNVRDIWYTMIGIPGLEYAHIWISIPICSMYIVAIAGNALLLFLIITERSLHEPMYLFLSMLA
LADIFLSTVTTPKMLAIFWFRAGGISFASCVSQMFFLHFIFVAESAILLAMAFDRYVAICYPLRYTTILTTSV
IIKMGIAAVIRSFFICFPLIFLVYRLTYCGKSTIRHSYCEHMGIARLACDSIKVNIYYGVIVALFSTCLEAVL
IIVSYALILCAVFRIPSRDARLKALGTCGSHVCVILLFYTPAFFSFFAHRFGGHSIPLHVHILLANLYVVVPP
SVNPIIYGVKTKQIQERVIQVFSLGK*
>ENSMUSP00000150982_c.647:T>A_p.D216E ENSMUST00000215755.2|Olfr617-202|ENSMUSG00000073946|+|chr7:g.103233878:T>A
MATSSTTLNYTNVRDIWYTMIGIPGLEYAHIWISIPICSMYIVAIAGNALLLFLIITERSLHEPMYLFLSMLA
LADIFLSTVTTPKMLAIFWFRAGGISFASCVSQMFFLHFIFVAESAILLAMAFDRYVAICYPLRYTTILTTSV
IIKMGIAAVIRSFFICFPLIFLVYRLTYCGKSTIRHSYCEHMGIARLACDSIKVNIYYGVIVALFSTCLEAVL
IIVSYALILCAVFRIPSRDARLKALGTCGSHVCVILLFYTPAFFSFFAHRFGGHSIPLHVHILLANLYVVVPP
SVNPIIYGVKTKQIQERVIQVFSLGK*
>ENSMUSP00000040319_c.647:T>A_p.D216E ENSMUST00000048265.5|Olfr617-201|ENSMUSG00000073946|+|chr7:g.103233878:T>A
MATSSTTLNYTNVRDIWYTMIGIPGLEYAHIWISIPICSMYIVAIAGNALLLFLIITERSLHEPMYLFLSMLA
LADIFLSTVTTPKMLAIFWFRAGGISFASCVSQMFFLHFIFVAESAILLAMAFDRYVAICYPLRYTTILTTSV
IIKMGIAAVIRSFFICFPLIFLVYRLTYCGKSTIRHSYCEHMGIARLACDSIKVNIYYGVIVALFSTCLEAVL
IIVSYALILCAVFRIPSRDARLKALGTCGSHVCVILLFYTPAFFSFFAHRFGGHSIPLHVHILLANLYVVVPP
SVNPIIYGVKTKQIQERVIQVFSLGK*
>ENSMUSP00000023934_c.207:A>G_p.T70A ENSMUST00000023934.8|Hbb-bs-201|ENSMUSG00000052305|-|chr7:g.103476762:T>C
MVHLTDAEKAAVSGLWGKVNADEVGGEALGRLLVVYPWTQRYFDSFGDLSSASAIMGNAKVKAHGKKVIAAFN
DGLNHLDSLKGTFASLSELHCDKLHVDPENFRLLGNMIVIVLGHHLGKDFTPAAQAAFQKVVAGVAAALAHKY
H*
>ENSMUSP00000115607_c.207:A>G_p.T70A ENSMUST00000153218.2|Hbb-bs-203|ENSMUSG00000052305|-|chr7:g.103476762:T>C
MVHLTDAEKAAVSGLWGKVNADEVGGEALGRLLVVYPWTQRYFDSFGDLSSASAIMGNAKVKAHGKKVIAAFN
DGLNHLDSLKGTFASLSELHCDKLHVDPEN
>ENSMUSP00000023934_c.192:G>A_p.G65S ENSMUST00000023934.8|Hbb-bs-201|ENSMUSG00000052305|-|chr7:g.103476777:C>T
MVHLTDAEKAAVSGLWGKVNADEVGGEALGRLLVVYPWTQRYFDSFGDLSSASAIMGNAKVKAHSKKVITAFN
DGLNHLDSLKGTFASLSELHCDKLHVDPENFRLLGNMIVIVLGHHLGKDFTPAAQAAFQKVVAGVAAALAHKY
H*
>ENSMUSP00000115607_c.192:G>A_p.G65S ENSMUST00000153218.2|Hbb-bs-203|ENSMUSG00000052305|-|chr7:g.103476777:C>T
MVHLTDAEKAAVSGLWGKVNADEVGGEALGRLLVVYPWTQRYFDSFGDLSSASAIMGNAKVKAHSKKVITAFN
DGLNHLDSLKGTFASLSELHCDKLHVDPEN
>ENSMUSP00000150733_c.24:A>G_p.N9D ENSMUST00000216247.2|Olfr681-205|ENSMUSG00000095248|+|chr7:g.104770690:A>G
MTAHKNDTDPTGVSDFLLNCFVRSPSWQLWLSLPLSLLFLLAMGANAILLITIRMEASLHEPMYYLLSLLSML
DIILCLTVIPKVLAIFWFDLRAIGFPACFLQMYIMNSFLAMESCTFMIMAFDRYIAICHPLRYPSIITDQFVV
KAATFILVRNVLIPLPIPILSGRLHYCGRNVIENCICANMSVSRLSCDDVTVNRLYQFAGGWTLLGSDLVLIF
LSYTLILRAVLRLKAEGAVAKALSTCGSHFILILFFSTILLVFILTHVAKRKVSSDVPILLNVLHHVIPAALN
PIVYGVRTQEIKQGIKKLLKRGW*
>ENSMUSP00000151022_c.24:A>G_p.N9D ENSMUST00000215517.2|Olfr681-203|ENSMUSG00000095248|+|chr7:g.104770690:A>G

MTAHKNDTDPTGVSDFLLNCFVRSPSWQLWLSLPLSLLFLLAMGANAILLITIRMEASLHEPMYYLLSLLSML
DIILCLTVIPKVLAIFWFDLRAIGFPACFLQMYIMNSFLAMESCTFMIMAFDRYIAICHPLRYPSIITDQFVV
KAATFILVRNVLIPLPIPILSGRLHYCGRNVIENCICANMSVSRLSCDDVTVNRLYQFAGGWTLLGSDLVLIF
LSYTLILRAVLRLKAEGAVAKALSTCGSHFILILFFSTILLVFILTHVAKRKVSSDVPILLNVLHHVIPAALN
PIVYGVRTQEIKQGIKKLLKRGW*
>ENSMUSP00000149338_c.24:A>G_p.N9D ENSMUST00000215564.2|Olfr681-204|ENSMUSG00000095248|+|chr7:g.104770690:A>G
MTAHKNDTDPTGVSDFLLNCFVRSPSWQLWLSLPLSLLFLLAMGANAILLITIRMEASLHEPMYYLLSLLSML
DIILCLTVIPKVLAIFWFDLRAIGFPACFLQMYIMNSFLAMESCTFMIMAFDRYIAICHPLRYPSIITDQFVV
KAATFILVRNVLIPLPIPILSGRLHYCGRNVIENCICANMSVSRLSCDDVTVNRLYQFAGGWTLLGSDLVLIF
LSYTLILRAVLRLKAEGAVAKALSTCGSHFILILFFSTILLVFILTHVAKRKVSSDVPILLNVLHHVIPAALN
PIVYGVRTQEIKQGIKKLLKRGW*
>ENSMUSP00000095760_c.24:A>G_p.N9D ENSMUST00000098157.4|Olfr681-201|ENSMUSG00000095248|+|chr7:g.104770690:A>G
MTAHKNDTDPTGVSDFLLNCFVRSPSWQLWLSLPLSLLFLLAMGANAILLITIRMEASLHEPMYYLLSLLSML
DIILCLTVIPKVLAIFWFDLRAIGFPACFLQMYIMNSFLAMESCTFMIMAFDRYIAICHPLRYPSIITDQFVV
KAATFILVRNVLIPLPIPILSGRLHYCGRNVIENCICANMSVSRLSCDDVTVNRLYQFAGGWTLLGSDLVLIF
LSYTLILRAVLRLKAEGAVAKALSTCGSHFILILFFSTILLVFILTHVAKRKVSSDVPILLNVLHHVIPAALN
PIVYGVRTQEIKQGIKKLLKRGW*
>ENSMUSP00000150733_c.192:T>C_p.Y65H ENSMUST00000216247.2|Olfr681-205|ENSMUSG00000095248|+|chr7:g.104770858:T>C
MTAHKNDTNPTGVSDFLLNCFVRSPSWQLWLSLPLSLLFLLAMGANAILLITIRMEASLHEPMYHLLSLLSML
DIILCLTVIPKVLAIFWFDLRAIGFPACFLQMYIMNSFLAMESCTFMIMAFDRYIAICHPLRYPSIITDQFVV
KAATFILVRNVLIPLPIPILSGRLHYCGRNVIENCICANMSVSRLSCDDVTVNRLYQFAGGWTLLGSDLVLIF
LSYTLILRAVLRLKAEGAVAKALSTCGSHFILILFFSTILLVFILTHVAKRKVSSDVPILLNVLHHVIPAALN
PIVYGVRTQEIKQGIKKLLKRGW*
>ENSMUSP00000151022_c.192:T>C_p.Y65H ENSMUST00000215517.2|Olfr681-203|ENSMUSG00000095248|+|chr7:g.104770858:T>C
MTAHKNDTNPTGVSDFLLNCFVRSPSWQLWLSLPLSLLFLLAMGANAILLITIRMEASLHEPMYHLLSLLSML
DIILCLTVIPKVLAIFWFDLRAIGFPACFLQMYIMNSFLAMESCTFMIMAFDRYIAICHPLRYPSIITDQFVV
KAATFILVRNVLIPLPIPILSGRLHYCGRNVIENCICANMSVSRLSCDDVTVNRLYQFAGGWTLLGSDLVLIF
LSYTLILRAVLRLKAEGAVAKALSTCGSHFILILFFSTILLVFILTHVAKRKVSSDVPILLNVLHHVIPAALN
PIVYGVRTQEIKQGIKKLLKRGW*
>ENSMUSP00000149338_c.192:T>C_p.Y65H ENSMUST00000215564.2|Olfr681-204|ENSMUSG00000095248|+|chr7:g.104770858:T>C
MTAHKNDTNPTGVSDFLLNCFVRSPSWQLWLSLPLSLLFLLAMGANAILLITIRMEASLHEPMYHLLSLLSML
DIILCLTVIPKVLAIFWFDLRAIGFPACFLQMYIMNSFLAMESCTFMIMAFDRYIAICHPLRYPSIITDQFVV
KAATFILVRNVLIPLPIPILSGRLHYCGRNVIENCICANMSVSRLSCDDVTVNRLYQFAGGWTLLGSDLVLIF
LSYTLILRAVLRLKAEGAVAKALSTCGSHFILILFFSTILLVFILTHVAKRKVSSDVPILLNVLHHVIPAALN
PIVYGVRTQEIKQGIKKLLKRGW*
>ENSMUSP00000095760_c.192:T>C_p.Y65H ENSMUST00000098157.4|Olfr681-201|ENSMUSG00000095248|+|chr7:g.104770858:T>C
MTAHKNDTNPTGVSDFLLNCFVRSPSWQLWLSLPLSLLFLLAMGANAILLITIRMEASLHEPMYHLLSLLSML
DIILCLTVIPKVLAIFWFDLRAIGFPACFLQMYIMNSFLAMESCTFMIMAFDRYIAICHPLRYPSIITDQFVV
KAATFILVRNVLIPLPIPILSGRLHYCGRNVIENCICANMSVSRLSCDDVTVNRLYQFAGGWTLLGSDLVLIF
LSYTLILRAVLRLKAEGAVAKALSTCGSHFILILFFSTILLVFILTHVAKRKVSSDVPILLNVLHHVIPAALN
PIVYGVRTQEIKQGIKKLLKRGW*
>ENSMUSP00000154052_c.510:T>A_p.C171S ENSMUST00000214399.4|Olfr682-ps1-202|ENSMUSG00000059768|-|chr7:g.104775966:A>T
VSEFLMICFPGMQDTQHWLSIILAPLLVLALAANFVLLFTIHQEASLHEPMYYLLAILSVLDVILCLTVIPKV
LLIFWFNMKPISFVGCFLQMFVMNTFLPMESSTFLVMAYDRYVAICHPLRYPSIITEQFVINAAIFIVARNLL
ATLPTPVLAARLNYCASNVVENCISANISVAKLSCGDIRLNKLYQFVSVWCLLGSDLVLILLSYCFILRVVMC
QQSGGTVTKALSTCGSHLILILFLTLLLVFIFTNKAGKKIPSEVPILLNVLHHLIPPALNPIVYGVRTQEIKQ
GIIKLFK
>ENSMUSP00000154164_c.540:T>A_p.C181S ENSMUST00000071242.5|Olfr682-ps1-201|ENSMUSG00000059768|-|chr7:g.104775966:A>T
MVSPNNTGVQVSEFLMICFPGMQDTQHWLSIILAPLLVLALAANFVLLFTIHQEASLHEPMYYLLAILSVLDV
ILCLTVIPKVLLIFWFNMKPISFVGCFLQMFVMNTFLPMESSTFLVMAYDRYVAICHPLRYPSIITEQFVINA

AIFIVARNLLATLPTPVLAARLNYCASNVVENCISANISVAKLSCGDIRLNKLYQFVSVWCLLGSDLVLILLS
YCFILRVVMCQQSGGTVTKALSTCGSHLILILFLYFVASLYLHKQGRKEDTVRGTNSS*CAASSHPTCPQSHC
VWSTNPGNQAGDYQAIQTL
>ENSMUSP00000147446_c.886:A>T_p.D296V ENSMUST00000210644.3|Olfr707-203|ENSMUSG00000069390|-|chr7:g.106490428:T>A
MGEDNRTSVTEFIFLGLSQDPQTQVLLFFLFLFIYLLTVLGNLLIIVLIHSDPRLHTPMYFFLRNLSFADLCF
STTTVPQVLVHFLVKRKTISFAGCSTQIVVLLLVGCTECALLAVMSYDRYVAVCKPLHYSTIMTHWVCVQLAA
GSWASGALVSLVDTTFTLRLPYRGNNVINHFFCEPPALLKLASADTYSTEMAIFAMGVVILLAPVSLILTSYW
NIVSTVIQMQSGEGRLKVFSTCGSHLIVVVLFYGSAIFAYMRPNSKIMNEKDKMISVFYSAVTPMLNPIIYSL
RNKVVKGALRRITLK*
>ENSMUSP00000150100_c.886:A>T_p.D296V ENSMUST00000214112.2|Olfr707-205|ENSMUSG00000069390|-|chr7:g.106490428:T>A
MGEDNRTSVTEFIFLGLSQDPQTQVLLFFLFLFIYLLTVLGNLLIIVLIHSDPRLHTPMYFFLRNLSFADLCF
STTTVPQVLVHFLVKRKTISFAGCSTQIVVLLLVGCTECALLAVMSYDRYVAVCKPLHYSTIMTHWVCVQLAA
GSWASGALVSLVDTTFTLRLPYRGNNVINHFFCEPPALLKLASADTYSTEMAIFAMGVVILLAPVSLILTSYW
NIVSTVIQMQSGEGRLKVFSTCGSHLIVVVLFYGSAIFAYMRPNSKIMNEKDKMISVFYSAVTPMLNPIIYSL
RNKVVKGALRRITLK*
>ENSMUSP00000150235_c.886:A>T_p.D296V ENSMUST00000213251.2|Olfr707-204|ENSMUSG00000069390|-|chr7:g.106490428:T>A
MGEDNRTSVTEFIFLGLSQDPQTQVLLFFLFLFIYLLTVLGNLLIIVLIHSDPRLHTPMYFFLRNLSFADLCF
STTTVPQVLVHFLVKRKTISFAGCSTQIVVLLLVGCTECALLAVMSYDRYVAVCKPLHYSTIMTHWVCVQLAA
GSWASGALVSLVDTTFTLRLPYRGNNVINHFFCEPPALLKLASADTYSTEMAIFAMGVVILLAPVSLILTSYW
NIVSTVIQMQSGEGRLKVFSTCGSHLIVVVLFYGSAIFAYMRPNSKIMNEKDKMISVFYSAVTPMLNPIIYSL
RNKVVKGALRRITLK*
>ENSMUSP00000146644_c.886:A>T_p.D296V ENSMUST00000208759.2|Olfr707-202|ENSMUSG00000069390|-|chr7:g.106490428:T>A
MGEDNRTSVTEFIFLGLSQDPQTQVLLFFLFLFIYLLTVLGNLLIIVLIHSDPRLHTPMYFFLRNLSFADLCF
STTTVPQVLVHFLVKRKTISFAGCSTQIVVLLLVGCTECALLAVMSYDRYVAVCKPLHYSTIMTHWVCVQLAA
GSWASGALVSLVDTTFTLRLPYRGNNVINHFFCEPPALLKLASADTYSTEMAIFAMGVVILLAPVSLILTSYW
NIVSTVIQMQSGEGRLKVFSTCGSHLIVVVLFYGSAIFAYMRPNSKIMNEKDKMISVFYSAVTPMLNPIIYSL
RNKVVKGALRRITLK*
>ENSMUSP00000086062_c.886:A>T_p.D296V ENSMUST00000088687.3|Olfr707-201|ENSMUSG00000069390|-|chr7:g.106490428:T>A
MGEDNRTSVTEFIFLGLSQDPQTQVLLFFLFLFIYLLTVLGNLLIIVLIHSDPRLHTPMYFFLRNLSFADLCF
STTTVPQVLVHFLVKRKTISFAGCSTQIVVLLLVGCTECALLAVMSYDRYVAVCKPLHYSTIMTHWVCVQLAA
GSWASGALVSLVDTTFTLRLPYRGNNVINHFFCEPPALLKLASADTYSTEMAIFAMGVVILLAPVSLILTSYW
NIVSTVIQMQSGEGRLKVFSTCGSHLIVVVLFYGSAIFAYMRPNSKIMNEKDKMISVFYSAVTPMLNPIIYSL
RNKVVKGALRRITLK*
>ENSMUSP00000033342_c.81:G>A_p.A28T ENSMUST00000033342.7|Eif3f-201|ENSMUSG00000031029|+|chr7:g.108533720:G>A
MASPAVPANVPPATAAAAPAPVVTAAPTSAPTPSTPAPTPAATPAASPAPVSSDPAVAAPAAPGQTPASAPAP
AQTPAPSQPGPALPGPFPGGRVVRLHPVILASIVDSYERRNEGAARVIGTLLGTVDKHSVEVTNCFSVPHNES
EDEVAVDMEFAKNMYELHKKVSPNELILGWYATGHDITEHSVLIHEYYSREAPNPIHLTVDTGLQHGRMSIKA
YVSTLMGVPGRTMGVMFTPLTVKYAYYDTERIGVDLIMKTCFSPNRVIGLSSDLQQVGGASARIQDALSTVLQ
YAEDVLSGKVSADNTVGRFLMSLVNQVPKIVPDDFETMLNSNINDLLMVTYLANLTQSQIALNEKLVNL*
>ENSMUSP00000051263_c.1269:G>GC_p.G424ins-fs ENSMUST00000059390.13|Vps35l-202|ENSMUSG00000030982|+|chr7:g.118379484:G>GC
MASGAGPSFPGCEKRGWTRKTGDHVTGKMAVFPWHSRNRNYKAELASCRLETVPLECGDYHPLKPITVTESKT
KKVSRKGSTSSTSSSSSSSVIDPLSSVLDGTDPLSMFAATSDPAATGTVTDSSRKKRDKDENSFVGPDFEPWA
NKRVEILARYTTTEKLSINLFMGSEKGRGGAAASAMSEKVRTRLEELDDFEEGSQKELLNLTQQDYVNRIEEL
NQSLKDAWASDQKVKALKIVIQCSKLLSDTSVIQFYPSKFVLITDILDTFGKLVYERISSMCVDSRSALPDHF
SPENVNDTAKETCLNWFFKIASIRELIPRFYVEASILKCNKFLSKTGISECLPRLTCMIRGIGDPLVSVYARA
YLCRVGIEVAPHLKESLNKNFFDFLLTFKQIHGDTVQNQLVAQGVELLSYLPLYSPAMALDLPVCLLPRSRGF
ADGDDGKM
>ENSMUSP00000102163_c.1197:G>GC_p.G400ins-fs ENSMUST00000106553.8|Vps35l-204|ENSMUSG00000030982|+|chr7:g.118379484:G>GC

VTGKMAVFPWHSRNRNYKAELASCRLETVPLECGDYHPLKPITVTESKTKKVSRKGSTSSTSSSSSSSVIDPL
SSVLDGTDPLSMFAATSDPAATGTVTDSSRKKRDKDENSFVGPDFEPWANKRVEILARYTTTEKLSINLFMGS
EKGRGGAAASAMSEKVRTRLEELDDFEEGSQKELLNLTQQDYVNRIEELNQSLKDAWASDQKVKALKIVIQCS
KLLSDTSVIQFYPSKFVLITDILDTFGKLVYERISSMCVDSRSALPDHFSPENVNDTAKETCLNWFFKIASIR
ELIPRFYVEASILKCNKFLSKTGISECLPRLTCMIRGIGDPLVSVYARAYLCRVGIEVAPHLKESLNKNFFDF
LLTFKQIHGDTVQNQLVAQGVELLSYLPLYSPAMALDLPVCLLPRSRGFADGDDGKM
>ENSMUSP00000033280_c.480:G>GC_p.G161ins-fs ENSMUST00000033280.14|Vps35l-201|ENSMUSG00000030982|+|chr7:g.118379484:G>GC
MLLAGHGKLVYERISSMCVDSRSALPDHFSPENVNDTAKETCLNWFFKIASIRELIPRFYVEASILKCNKFLS
KTGISECLPRLTCMIRGIGDPLVSVYARAYLCRVGIEVAPHLKESLNKNFFDFLLTFKQIHGDTVQNQLVAQG
VELLSYLPLYSPAMALDLPVCLLPRSRGFADGDDGKM
>ENSMUSP00000044177_c.1377:C>T_p.Q460* ENSMUST00000047025.15|Otoa-201|ENSMUSG00000034990|+|chr7:g.120724700:C>T
MSQGPRTCSLLLVLLLSHGGAYQREPSPRQDLHPLLQKMAEEIIEGSYLNALLDLTLFERSHVWTADLSHRVL
AYLNSKNVAFTIPSLQAVMEAHLEQYLYQPQKLLEDLRATDNQQFHTAMKCLLEDKWGHLDLEDVVINLGDIR
DEALQSPGVNRSLFLITLERCFQVLNALECVEVLGRVLRGSSGSFLQPDITERLPQDLHEDAFKNLSAVFKDL
YDQTSAHTQRALYSWMTGILRTPFNVTDGSVSWVSAEKLWILGRYMVHLSFEEIMNISPIEIGLFISYDNATK
QLDMVYDITPELAQAFLERIRCSSFDVRNISTIHRLGLLVCFYDGLELLDATLAQVLLHQMLKCSRLRGFQAG
VQKLKANLLDIATENQTLNETLGSLSDAVVGLTSSQLESLSSDAVHSAISTLNQVTGWGRSQIVILSAKYLAQ
EKVLSFYNVCQMGVLLAGVGT*AFYSMDHKDLWQVLRSPLSQDMSDLSPVQQQGVLGKLMEAEDATSGIAEVP
RALFKEVSLYDLWKESRFNATVLKAKELRRSQALFLYEFLGKTTERPEELLSAGQLVKGVPCSHIDAMSDHLF
LALFQYFDNNFSLLSPDQVNCLAWKYWEVSRSSMPPFLLATLPSRFLSSIPPSRCVRFLISLGKRRLETLVLD
SDKRSVVVRKVQQCLDGVIADEYTVDIVGHLLCHLPASFIERGISPRAWAAALHGLRSCTALSSEQKAAVRVR
LLEQWGPPENWTAETTKDLAPFLAFFSGDELHTVATKFPEILQQTASKMVGVLLPKEFLWAVFESVQNSSNES
PSFDPTFGCHGVVTPSSDDIFKLAEANACWDPEVLLCMEEDTFIRNVELLGAVKGFSRAQLMALKEKAIQVWD
LPSRWKEHHIVSLGRIALALSESELEQLDLSSIDTVASLGQQTEWTPGQAKSILQAFLEDSGYGIQDLKSFHL
VGFGPTLCAMDPTEIQLIKTSEFRAVVARIGTLFCSTPVLAGFKKKAEVVFGRPTEWTSSILQELGTIAAGIT
KAELRMLNKELMTYFQPSAIRCLPGEVFKELSTEQIASLGPQNAASVTHSQRLQLSSAQLQSLQRALDGAKTH
SWQTDPLSSSPTWPASTGSPTGEPASQALWLGCTLLLLTAKS*
>ENSMUSP00000114873_c.195:C>T_p.H66Y ENSMUST00000130498.2|Bola2-203|ENSMUSG00000047721|+|chr7:g.126295761:C>T
MELSADYLREKLRQDLEAEHVEVEDTTLNRCATSFRVLVVSAKFEGKPLLQRHRLVNECLAEELPYIHAFEQK
TLTPEQWTRQRRE*
>ENSMUSP00000035535_c.148:T>C_p.L50S ENSMUST00000037248.10|Hirip3-201|ENSMUSG00000042606|+|chr7:g.126461855:T>C
MAPGDEMRKFTRSFFRACPDLSSVTHAIVRQKFLIHVGRDHLEPEEKQASKRLVEEELPKMQADAGTREGKPD
FIKVKRSPAPCSDPKKKRFRFNSESESSSSPSSPDGSGPSTKNRTTKKTCLRRALKKAVESTDEDHQTDLDAK
MGLEESSEGEAEGSVRSGKVTEEEEDMKQEQKGQVRKQAGAKDKQVPLKADRKQVREESGSSEEEAVLQRAKV
EGSTGANCQEESEESGEESPAKKKELSEPRSRSNRAERTARERKSYKQKSRPGRPTGGLRDSEAEKEGGTVGS
GDSSEEGEAEKEGGTVGSGDSSEKGEAEKEEGTVGSGDSSEEGEEHSVKRKSKVRTQTESGRRQNTSSRDDSN
STQEQAAAQGTTKSGSLGSSNGDSDTEREVSDSQAGQNTKEERKSRSSNKSSKNGQARSCSSSSDSSPEPTGQ
KGKARSSSSSSDSGPEPTGRKAASRCGEDHPAVARLKRYIRACGAHRNYKKLLGSCSSHKARLSVLRAELEAL
GMKGNPSLEKCRALKLQREEAAEVAALDVANIISSTGRPRRRNAWNPSGEGTSPGETYRRTLDSEEEQPRQAP
PDWSHMRDIISSDGDSS*
>ENSMUSP00000052641_c.3025:A>C_p.E1009A ENSMUST00000050383.8|Zfp646-201|ENSMUSG00000049739|+|chr7:g.127480850:A>C
MEDMPLSFSCSDCQRHFPNLPELARHRELRHSSAKQDGEEADGIPHPYGSHPENLANHQRNHETLFFPCTTCG
KDFSNRLALQSHMRTHAPESHRRHGPPHAMETAPHLGSETMATDSWGQRLGSGEGWENQTKLVRETHDWESGA
DPRAASGTWEDPPTKQRQGLEMQPDSKGGTADWVPAVSSEGASPLPTPASNLLSNLEQYLAESVVNFTEAQEP
TEPLPTEGERKYRCSECGKTYKHAGSLTNHRQSHTLGIYPCSICFKEFSNLMALKNHSRLHAQYRPYHCPHCP
RAFRLPRDLLEHQQSHEGLKQEQPWEDKEMPTTNGHADERSWGQLSKTGMLNGSGELSSSGQLEDGGSEEYRP
FCCGDCGRTYRHAGSLINHRKSHQTGIYPCSICSKQLFNAAALKNHIRAHHRPRQGAGEGGQSSVSSTALTSA
ENTPKVDEVPTASLDHRPYKCNECGRAYRHRGSLVNHRHSHRTGEYQCSLCPRKYPNLMALRNHVRVHCKATR
RSTDPGTEGSPSPVKKEQHDPVGMEAAFHGDEEHACKREEEATSNPSMADRTVPQVCSICGMLFEDLKSLEHH
SVTHREGEKSRTDSTVSPTRTFACQDCGKSYRHSGSLINHRQTHQTGDFSCGACAKHFHTMAAMKSHLRRHSR
QWNRRRQKQDSGTGEVATLPPGGAWTLKLENDEDPDSSQDPLGESPCETEDNLERDGDCLQAGSRGNECVLKR
EEACFLGDKEGTEEGLEEREACFLDDLGTPGDECNETGFCGGLPGMDSDRKRGICHSDSSSHPADADTVWKAA
ATHTCSDCGDSFSHAAGLLSHRSCHPPGIYQCSLCPKEFDSLPALRSHFQTHRPGEVASAQPFLCCLCGMIFP

GRTGYRRHLRQAHGASAMTEGSEEEEEGTAETASTHSPPLQLSEAELLNQLQREVEALDGAGYGHICGCCGQT
YDDLGSLERHHQSQSSSNRTENVPSHLEGAGDATEMVADHGFEGTVTSVSEEGGDIKSEAGVGGTVADSLCMQ
AGESFLESHPRPFQCNQCGKTYRHGGSLVNHRKIHQTGDFICPVCSRCYPNLAAYRNHLRNHPRCKGSEPQMG
PISEAGGCSEPQNAAEAGQEQAVIGQLQEELKVEPLEELAGVKEEVWEGTAVKEEELEQELETGCQTEVTSER
PFSCEVCGRTYKHAGSLINHRQSHQTGHFGCQACSKGFSNLMSLKNHRRIHADPRQFRCSECGKAFRLRKQLA
NHQRVHAERRRSRGTQKLTREDRPFRCGQCGRTYRHAGSLMNHQCNPEASRYSCPFCFKTYSNRTALKDHQRV
HSDSQQRRQSGCPQRAAAVRCTLCGCGFSGQGSLEQHLQEHEDTKLEVASGQGGQHATEGSEENLDDWGLEGR
SDGTEVLQVEHETKRPGGHSQSPSSPACSGGTESTQQVGKVDGSQGDRGQMNHNGAWVLQDQLTKPEGKLEDT
VSRNPCHLSESQSNGPTLRYRDSWKGADSGSQLQPESHCCSQCGKAYCQPDGLLNPSINGKDCHICLLCSKEF
LNPVTTEIHNHTTAQRFACSNCSKVCESHSELATHMKTHAVEHSQMSGQMEKTRGPQAGMAEVGPPGPGKAQE
APSELPGDPEENGVPANGGQGIHFPAAEDKERPFCCAQCGRSYRHAGSLLNHQKAHTIGLYPCSLCPKLLPNL
LSLKNHSRTHTDPKRYSCNICGKAFRTAARLQGHGRVHAPQEGPFTCSHCPRRFRHRISFLRHQQQHQEEWPV
SSSGASVAPAASREDSSTASLPNPSPQWPADLSLSL*
>ENSMUSP00000145675_c.2368:A>C_p.E790A ENSMUST00000206340.2|Zfp646-204|ENSMUSG00000049739|+|chr7:g.127480850:A>C
XEPLPTEGERKYRCSECGKTYKHAGSLTNHRQSHTLGIYPCSICFKEFSNLMALKNHSRLHAQYRPYHCPHCP
RAFRLPRDLLEHQQSHEGLKQEQPWEDKEMPTTNGHADERSWGQLSKTGMLNGSGELSSSGQLEDGGSEEYRP
FCCGDCGRTYRHAGSLINHRKSHQTGIYPCSICSKQLFNAAALKNHIRAHHRPRQGAGEGGQSSVSSTALTSA
ENTPKVDEVPTASLDHRPYKCNECGRAYRHRGSLVNHRHSHRTGEYQCSLCPRKYPNLMALRNHVRVHCKATR
RSTDPGTEGSPSPVKKEQHDPVGMEAAFHGDEEHACKREEEATSNPSMADRTVPQVCSICGMLFEDLKSLEHH
SVTHREGEKSRTDSTVSPTRTFACQDCGKSYRHSGSLINHRQTHQTGDFSCGACAKHFHTMAAMKSHLRRHSR
QWNRRRQKQDSGTGEVATLPPGGAWTLKLENDEDPDSSQDPLGESPCETEDNLERDGDCLQAGSRGNECVLKR
EEACFLGDKEGTEEGLEEREACFLDDLGTPGDECNETGFCGGLPGMDSDRKRGICHSDSSSHPADADTVWKAA
ATHTCSDCGDSFSHAAGLLSHRSCHPPGIYQCSLCPKEFDSLPALRSHFQTHRPGEVASAQPFLCCLCGMIFP
GRTGYRRHLRQAHGASAMTEGSEEEEEGTAETASTHSPPLQLSEAELLNQLQREVEALDGAGYGHICGCCGQT
YDDLGSLERHHQSQSSSNRTENVPSHLEGAGDATEMVADHGFEGTVTSVSEEGGDIKSEAGVGGTVADSLCMQ
AGESFLESHPRPFQCNQCGKTYRHGGSLVNHRKIHQTGDFICPVCSRCYPNLAAYRNHLRNHPRCKGSEPQMG
PISEAGGCSEPQNAAEAGQEQAVIGQLQEELKVEPLEELAGVKEEVWEGTAVKEEELEQELETGCQTEVTSER
PFSCEVCGRTYKHAGSLINHRQSHQTGHFGCQACSKGFSNLMSLKNHRRIHADPRQFRCSECGKAFRLRKQLA
NHQRVHAERRRSRGTQKLTREDRPFRCGQCGRTYRHAGSLMNHQCNPEASRYSCPFCFKTYSNRTALKDHQRV
HSDSQQRRQSGCPQRAAAVRCTLCGCGFSGQGSLEQHLQEHEDTKLEVASGQGGQHATEGSEENLDDWGLEGR
SDGTEVLQVEHETKRPGGHSQSPSSPACSGGTESTQQVGKVDGSQGDRGQMNHNGAWVLQDQLTKPEGKLEDT
VSRNPCHLSESQSNGPTLRYRDSWKGADSGSQLQPESHCCSQCGKAYCQPDGLLNPSINGKDCHICLLCSKEF
LNPVTTEIHNHTTAQRFACSNCSKVCESHSELATHMKTHAVEHSQMSGQMEKTRGPQAGMAEVGPPGPGKAQE
APSELPGDPEENGVPANGGQGIHFPAAEDKERPFCCAQCGRSYRHAGSLLNHQKAHTIGLYPCSLCPKLLPNL
LSLKNHSRTHTDPKRYSCNICGKAFRTAARLQGHGRVHAPQEGPFTCSHCPRRFRHRISFLRHQQQHQEEWPV
SSSGMWLGRGLRGSRRRWAQRRESMARSR*
>ENSMUSP00000123566_c.262:A>G_p.D88G ENSMUST00000126662.2|Gm21974-201|ENSMUSG00000030804|-|chr7:g.127492558:T>C
XDVGTAISCSRVFSSRWGRGFGLVEHMLGADSVLNQSNSIFGCLFYTLQLLLGCLRGRWASILLVLSSLVSVA
GSVYLAWILFFVLYGFCIVCITTYAINVGLMLLSFQKVPEHKTKKH*
>ENSMUSP00000033074_c.388:A>G_p.D130G ENSMUST00000033074.8|Vkorc1-201|ENSMUSG00000096145|-|chr7:g.127492558:T>C
MGTTWRSPGLVRLALCLAGLALSLYALHVKAARARDENYRALCDVGTAISCSRVFSSRWGRGFGLVEHMLGAD
SVLNQSNSIFGCLFYTLQLLLGCLRGRWASILLVLSSLVSVAGSVYLAWILFFVLYGFCIVCITTYAINVGLM
LLSFQKVPEHKTKKH*
>ENSMUSP00000145795_c.241:A>G_p.D81G ENSMUST00000206053.2|Vkorc1-203|ENSMUSG00000096145|-|chr7:g.127492558:T>C
MMGRLEEAGWGRGFGLVEHMLGADSVLNQSNSIFGCLFYTLQLLLGCLRGRWASILLVLSSLVSVAGSVYLAW
ILFFVLYGFCIVCITTYAINVGLMLLSFQKVPEHKTKKH*
>ENSMUSP00000112542_c.184:A>G_p.D62G ENSMUST00000119922.2|Vkorc1-202|ENSMUSG00000096145|-|chr7:g.127492558:T>C
MLGADSVLNQSNSIFGCLFYTLQLLLGCLRGRWASILLVLSSLVSVAGSVYLAWILFFVLYGFCIVCITTYAI
NVGLMLLSFQKVPEHKTKKH*
>ENSMUSP00000158736_c.52:A>G_p.K18R ENSMUST00000238294.2|D7Ertd443e-211|ENSMUSG00000030994|-|chr7:g.134100653:T>C
MVSDLVKRGWLPSQRRARVCFIHTCPSLKVTEQPASGYEPEELRSFDSPKDSYPFTYKSRLLAPRLLHTCYST
NDQIALKNLQSDVTERKLDFSKETLASQNTRMISPVVISRLIDEKKSMENGAILPQAIAQPQLCPTKPALARR

DGVSMHRRFALSPDRLGILTPSDDQGLETEPLSTGDNLGKGSHSGFSSITITARRVGPPASSLVWDTFRDPLC
PKCKAKDALFQEPPVLAGDAHLCQHNRPFTCTESPSNGSVEGMKVFQAHSRLSARQDYWVTHTNDNEDSFSSD
NSPSRKVPLVFSSCVHFRV
>ENSMUSP00000128276_c.6079:C>A_p.S2027* ENSMUST00000165147.3|Muc5b-201|ENSMUSG00000066108|+|chr7:g.141413135:C>A
MGSRNWSWALVWASVALLMVWPAESQGQAELSSEHLELTGDSQRVYSDSISSSTRHVTFIPPITVFPSMSALH
LAHNGRVCSTWGDFHYKTFDGDVFRFPGLCNYVFSSHCGATYEDFNIQLRRGLEGSRPTVTYVLLRAQGLVIE
LSNGSVLVNGHREKLPYSRAGLLMEKSSGYVKISIRLVLTFLWNEEDSALLELDSKYINQTCGLCGDFNGLPA
VSEFYTHNTRLTPVQFGNLQKLDGPTEQCQDVLPSAVSNCTDTEDICRRTLLGPAFDKCTALVDVSMYLDACV
QDLCRCPTCPCATFAEYSRQCAHAGGYPQNWRHPDLCNWTCPFNMEYRECSSPCVDTCSNPQRSQLCEDHCMD
GCFCPPGTVLDDVRHLGCLPLEQCHCTHGGRIYAPGESFNTSCRSCTCFGGLWKCKDLPCPGTCSVQGGSHIF
TFDEKLYNVHGDCSYILTKKCADSSFTVLVDLRKCGVTDTENCLKAVTLNLNSGDTVIRIQVNGAVFLNSIFT
QMPLSAGNITIFKPSSFFIIVQTGMGLQLQVQLVPLMQVFVRLDRSYQGQMCGLCGNFNQNQADDFTALSGVV
EGTGAAFSNTWKTQASCPNSKNTYEDPCSYSVENENFAREWCSMLTESSGVFSACHATVSPVPFYSNCLFDTC
NCENSEDCMCAALSSYVFACAAKGVLLSGWRDKVCYKYTNNCPQTKGYFYSVSSCQPTCRSLSEADVTCSVPF
VTVDGCTCPEGTFLNDKDHCVPVEECPCYFHGTVVAPREVVTDNGVVCSCTNGKLTCLGALMQKSTECQAPMV
YLDCNNASVGHHGAECLKSCHTLDVECFSTQCVSGCVCPSGLVADGNGGCIAEEDCPCIHNEVTYRPGEIIRV
DCNNCTCKNRRWECTNKPCMGACVAYGDGHFVTFDGERYIFEGSCEYTLAQDYCRGNTSTNGTFRIVTENVPC
GTTGTTCSKTIKIFVESYELILHEGNFKVVEQGPSGDPPYKIRFMGIFLVIEIRSGIVVSWDRKTSVFVRLQQ
HYKGRVCGLCGNFDDNAINDFTTRSQSVVGDVLEFGNSWKFSPSCPDAPVPKDPCIANPYRKSWAQKKCSIIN
SATFAACHSQVDSTKYYEACVHDVCACDSGGDCECFCTAVAAYAQACRDVGVCLSWRTPDICPLFCDYYNPHG
ECEWHYQPCGAPCLKTCRNPTGHCLVDLPGLEGCYPQCPASQPFFNEDQMKCVAQCGCYDDNGNYHDIGTQVP
TAENCQSCLCTPGGLQCVYNLTACTCTYEGRTYNYNDVVYNTTDGLGACLVAICKDNGTIARTTEVCHEVPST
TPFTFTTTLAPLATNGSASTVSTVCVHEVCQWSIWYDESHPEAGMSGGDFETFENLRKKGHQVCQTPVGIECR
AVKFPSMDLEKLGQKVNCDPSSGLSCFNSEQSPPLCHDYELRVLCCDYVPCGSSLTLGISTTPSQPDTSRRPS
SPTLTTHTASSESQSWTREEKKTSVDKSYSSRQTVPFTSNSRLTSTSMSSASSLPLSNPTWTTTLKATDENTM
LSSRTTAVFTSQTRSTSRPSTVTQPISSPETSSTTCQPQCQWTKWIDVDYPSSSINGGDIETYENIRANGEKI
CEKPQDIRCMAQNHPGVNVDELGQKVKCDVSSGLVCYNKDQGGTFQMCYNYLIKVLCCSVSHCQGSTTPEGSV
STTETATSTPQTLSSKPLPNTSSFETWSPSGPTTSMEASEMPTFWTLRTSVSSPPTTPLTVTSVSTQHPKSLE
SSGSPSVVTARSTSQGYSLPPSSPHTTNSLSATTSKASLSSMTTISTSLTTPGPSSPPHGGTTTHPVTTASTT
RAVTETLSSRPHTKTTSELPTVSSASSTAGPGTQTFSTTCQPQCQWTKWIDVDYPSSSINGGDIETYENIRAN
GEKICEKPQDIRCMAQNYPGVNVDKLGQKVKCDVSSGLVCYNKDQGGTFPMCYNYLIKVLCCSVSHCQGSTTP
EGSVSTTETTTATPQTLSSKPLPNTSSIETWSPSGPTTSMEASEMPTFWTLRTSV*SPPTTPLTVTSVSTQHP
KSLESSGSPSVVTARSTSPGHSQPPSSPYATNSLSATTSKASLSSMTTISTSLTTPGPSSPPHGGTTTHPVTT
ASTTRAVTETLSSRPHTKTTSELPTVSSVSSTAGPGTQTGSTSRPNTVTQPASSPETSSTTCQPQCQWTKWID
VDYPSSSINGGDIETYENIRANGEKICEKPQDIRCMAQNHPGVNVDELGQKVKCDVSSGLVCYNKDQGGTFQM
CYNYLIKVLCCSVSHCQGSTTPEGSVSTTETTTATPQTLSSKPLPNTSSIETWSPSGPTTSMEASEMPTFWTL
RTSVSSPPTTPLTVTSVSTQHPKSLESSGSPSVVTARSTSPGHSQPPSSPYATNSLSATTSKASLSSMTTIST
SLTTPGPSSPPHGGTTTHPVTTASTTRAVTETLSSRPHTKTTSELPTVSSVSSTAGPGTQTGSTSRPNTVTQP
ASSPETSSTTCQPQCQWTKWIDVDYPSSSINGGDIETYENIRANGEKICEKPQDIRCMAQNHPGVNVDELGQK
VKCDVSSGLVCYNKDQGGTFQMCYNYLIKVLCCSVSHCQRSTTPAGSAPTTGTAPTATSGTATHTSWPASSMP
TSTTESPSSSGKTTILTTASSLATGTPRETQTSSLLTTGLSSALTTTGTTSTGCKSQCTWTDWLDSDKPQPGQ
FEGDIETYYHIQNKTGIKICKKPVDIECEAVLFPNISFQKLGQEVVCNVDFGLICRNSKQSDNQICFNYHIRV
LCCEEDTSCISTTGLLSTTSTSAPGFPSTVSTSTTSKLPSEEPIPSTSFTTQSRPSTPRTEKIVSIATARTTG
SFPVSSLSTFPASPTSLLPTSEEPHSGKPSTSSWTASSNVPSTAKPTTSTGRQFPRTKTTLWNSLPSSTPTMA
TRSIHPISTTMTTKNPQTLVTSDISKSNSMLPSRPHITASSVTSTSGSQISKSYSSTSLTTAGPSSPPHGGTT
THPVTTASTTRAVTETLSSRPHTKTTSELPTVSSVSSTAGPGTQTGSTSRPSIVTQPVSSPETSSTTCQPQCQ
WTRWINVDYPSSSINGGDIETYENIRANGEKICEKPQDIRCMAQNYPGVNVDELGQKVKCDVNSGLVCYNKDQ
GGTFKMCYNYLIKVLCCSVSHCQGSTTPEGSVSTTETASSIPQTLSSKPLPNTSSFETWSPSGPTTSMEASEM
PTFWTLRTSVSSPPTTPLTVTSVSTQHPKSLESSGSPSVVTARSTSQGHSLPPSSPHTTNSLSATTSKASLSS
MTTISTSLTTSEPSSPPHGGTTTHPVTAASTTRAVTETLSSRPHTKTTSELPTVPSASSTAGPGTQTGSTSRP
STVTQPASSPETSSTTCQPQCQWTKWIDVDYPSSSINGGDIETYENIRTNGEKICEKPQDIRCMAQNHPGVNV
DELGQKVKCDVNSGLVCYNKDQGGTFQMCYNYLIKVLCCSVSHCQGSTTTPGSAPMTGSATTATQTLSSMPLP
NTSLTVTSPHSASTTAATSITETSSPRSVSSIHTTPKIFPETLQSTTGLTTQQTSSTGIWTSMLTGTAGTVSS
TSLSTSLESTTGITEVSTSMPMTSTLPVTVTNATTPQGTTLCQPKCKWTEWFDVDSPTSGSVKGDMETYENIR
ASGKTLCQAPEKIECRAENYPAVSIDKVGQVVSCNLETGLVCKNEDQKDDFKMCFNYNIRVLCCDDYSHCPTT
TYTTIKSPLAPFTTSVSSSMESTFQTNRPASPSGPSTQASTWSTLPDSGCTPRCKWTEWFDADYPNPGPRGGD
FEVYAVFREVGYIFCDQPKDIECRSEKEPDRPLETLEQVVQCDVRFGLICKNINQSGPLQYCDNYHVRLLCCD

NYSHCTSSPMATMSTALSSSHLPSTHTSLALNTTTFFPGLSSSSEPHSSSLMSTHTSTMLTTETSPIATGPTV
APSSTSGTPYTPKVSPSSQVTFSVSTAFSSVFSTPRPVFSSHASSPSPCFCQAFGQLFLPGDIIYNKTDGAGC
QFYAICNQYCDVDRFQGTCPSSSPPMSSTTVPLPPLLPGCDNAIPPRQVNESWTLENCTVARCQGNNQIILLE
PEPVDNVTCANRHLPIKVWDQEPCHFHYECECFCSGWGHSHYLTFDGTSYTFLDNCTSVLMREIHPRHGNLSI
LAHSYYCGATTNVTSCPRALSVYYNSMEIILTISTTESGKDESLIIWDQMWIRSGFSKNGVTVSLSGATTMSV
NISTIGTIITFDGNIFQIWLPYRYFSSNTEGQCGTCTNSQIDDCRRPDGTIASDCQDMAKEWLVPGNSSSSCL
AQPSPLPSTTPQTPVSSTLTSTPCPSAPLCELMLSQVFAECHRLIPPDAFFRSCVSDHCNANITDMLCQSLEA
YAALCRAQGVCTNWRNATGGLCDLSCPPTKEYRPCGPLHPASCNSRTQDSTTGMLAEGCFCPENQLLFNSRMD
ICVSECPCVGPDGLPKFPGEHWISNCQACVCEPGSVSVQCEPVKCESQDKPPQCTEAGFVTVTWPSADNPCCP
ETLCVCNSTTCPKSLPTCEPGYELVQTHENSSCCPSYSCRPKLCTYNDTIYGVGTTFPGGPCHTCTCLSDGDQ
EPKVECKETNCTTSCPQGFKYTLVPEQCCGECLQNACLTPEGHAVQPNETWVNSPVDNCTAYHCMAENGVYVL
TPIPTSCPDVSNCTGTLRKTGCCYSCEKEDSSTCQVHVNATVLRYKGCETEVNITFCEGSCSGISKYSMEAQA
MERQCTCCQESKVHDVAVTMQCPDGTVIQHTYTHIDECNCAPACSSLPGTSMIAS*
>ENSMUSP00000096508_c.457:C>T_p.T153I ENSMUST00000098909.5|4930467E23Rik-201|ENSMUSG00000096265|+|chr8:g.19783749:C>T
MEEEINYDSQTMNIPRRRRWYTHLWRSCLGLRCMHPQKKKKYPLYLIGYDPIGPLQRAASVGDLDTTEKLIHS
SQHHVDESDRRKRTSLHYACAHNHPDVVTLLLENNSSINIRDDEGCTPLIKATQRDNVDCASVLLTHNADPNL
IDSSGNIAFHHAISRGNIRIVKMLLEHNVDIEAKTEYGLTPLQLATFEQKPEMVEFLAAKCAKSSVTPSWSPS
PTVSPCPSSTSPLSSLGLPVCPNPGTSSLSEDRKSSSARRIKLSTGSSAQRTEVKHVRFNEEILYFKEERPLS
CEARPPGQLKSILKNTVQYNTVNEKITRRTSLWCSKAEDVRYTSCIKVKCFSVKSSFL*
>ENSMUSP00000081041_c.1410:T>G_p.W471G ENSMUST00000084027.13|Fgfr1-201|ENSMUSG00000031565|+|chr8:g.26058330:T>G
MWGWKCLLFWAVLVTATLCTARPAPTLPEQAQPWGVPVEVESLLVHPGDLLQLRCRLRDDVQSINWLRDGVQL
VESNRTRITGEEVEVRDSIPADSGLYACVTSSPSGSDTTYFSVNVSDALPSSEDDDDDDDSSSEEKETDNTKP
NRRPVAPYWTSPEKMEKKLHAVPAAKTVKFKCPSSGTPNPTLRWLKNGKEFKPDHRIGGYKVRYATWSIIMDS
VVPSDKGNYTCIVENEYGSINHTYQLDVVERSPHRPILQAGLPANKTVALGSNVEFMCKVYSDPQPHIQWLKH
IEVNGSKIGPDNLPYVQILKTAGVNTTDKEMEVLHLRNVSFEDAGEYTCLAGNSIGLSHHSAWLTVLEALEER
PAVMTSPLYLEIIIYCTGAFLISCMLGSVIIYKMKSGTKKSDFHSQMAVHKLAKSIPLRRQVTVSADSSASMN
SGVLLVRPSRLSSSGTPMLAGVSEYELPEDPRGELPRDRLVLGKPLGEGCFGQVVLAEAIGLDKDKPNRVTKV
AVKMLKSDATEKDLSDLISEMEMMKMIGKHKNIINLLGACTQDGPLYVIVEYASKGNLREYLQARRPPGLEYC
YNPSHNPEEQLSSKDLVSCAYQVARGMEYLASKKCIHRDLAARNVLVTEDNVMKIADFGLARDIHHIDYYKKT
TNGRLPVKWMAPEALFDRIYTHQSDVWSFGVLLWEIFTLGGSPYPGVPVEELFKLLKEGHRMDKPSNCTNELY
MMMRDCWHAVPSQRPTFKQLVEDLDRIVALTSNQEYLDLSIPLDQYSPSFPDTRSSTCSSGEDSVFSHEPLPE
EPCLPRHPTQLANSGLKRR*
>ENSMUSP00000137515_c.1143:T>G_p.W382G ENSMUST00000178276.8|Fgfr1-214|ENSMUSG00000031565|+|chr8:g.26058330:T>G
MWGWKCLLFWAVLVTATLCTARPAPTLPEQDALPSSEDDDDDDDSSSEEKETDNTKPNPVAPYWTSPEKMEKK
LHAVPAAKTVKFKCPSSGTPNPTLRWLKNGKEFKPDHRIGGYKVRYATWSIIMDSVVPSDKGNYTCIVENEYG
SINHTYQLDVVERSPHRPILQAGLPANKTVALGSNVEFMCKVYSDPQPHIQWLKHIEVNGSKIGPDNLPYVQI
LKHSGINSSDAEVLTLFNVTEAQSGEYVCKVSNYIGEANQSAWLTVTRPVAKALEERPAVMTSPLYLEIIIYC
TGAFLISCMLGSVIIYKMKSGTKKSDFHSQMAVHKLAKSIPLRRQVTVSADSSASMNSGVLLVRPSRLSSSGT
PMLAGVSEYELPEDPRGELPRDRLVLGKPLGEGCFGQVVLAEAIGLDKDKPNRVTKVAVKMLKSDATEKDLSD
LISEMEMMKMIGKHKNIINLLGACTQDGPLYVIVEYASKGNLREYLQARRPPGLEYCYNPSHNPEEQLSSKDL
VSCAYQVARGMEYLASKKCIHRDLAARNVLVTEDNVMKIADFGLARDIHHIDYYKKTTNGRLPVKWMAPEALF
DRIYTHQSDVWSFGVLLWEIFTLGGSPYPGVPVEELFKLLKEGHRMDKPSNCTNELYMMMRDCWHAVPSQRPT
FKQLVEDLDRIVALTSNQEYLDLSIPLDQYSPSFPDTRSSTCSSGEDSVFSHEPLPEEPCLPRHPTQLANSGL
KRR*
>ENSMUSP00000136640_c.1443:T>G_p.W482G ENSMUST00000179592.8|Fgfr1-215|ENSMUSG00000031565|+|chr8:g.26058330:T>G
MWGWKCLLFWAVLVTATLCTARPAPTLPEQVGSSSWPLWVAAAAQPWGVPVEVESLLVHPGDLLQLRCRLRDD
VQSINWLRDGVQLVESNRTRITGEEVEVRDSIPADSGLYACVTSSPSGSDTTYFSVNVSDALPSSEDDDDDDD
SSSEEKETDNTKPNPVAPYWTSPEKMEKKLHAVPAAKTVKFKCPSSGTPNPTLRWLKNGKEFKPDHRIGGYKV
RYATWSIIMDSVVPSDKGNYTCIVENEYGSINHTYQLDVVERSPHRPILQAGLPANKTVALGSNVEFMCKVYS
DPQPHIQWLKHIEVNGSKIGPDNLPYVQILKTAGVNTTDKEMEVLHLRNVSFEDAGEYTCLAGNSIGLSHHSA
WLTVLEALEERPAVMTSPLYLEIIIYCTGAFLISCMLGSVIIYKMKSGTKKSDFHSQMAVHKLAKSIPLRRQV
TVSADSSASMNSGVLLVRPSRLSSSGTPMLAGVSEYELPEDPRGELPRDRLVLGKPLGEGCFGQVVLAEAIGL
DKDKPNRVTKVAVKMLKSDATEKDLSDLISEMEMMKMIGKHKNIINLLGACTQDGPLYVIVEYASKGNLREYL
QARRPPGLEYCYNPSHNPEEQLSSKDLVSCAYQVARGMEYLASKKCIHRDLAARNVLVTEDNVMKIADFGLAR

DIHHIDYYKKTTNGRLPVKWMAPEALFDRIYTHQSDVWSFGVLLWEIFTLGGSPYPGVPVEELFKLLKEGHRM
DKPSNCTNELYMMMRDCWHAVPSQRPTFKQLVEDLDRIVALTSNQEYLDLSIPLDQYSPSFPDTRSSTCSSGE
DSVFSHEPLPEEPCLPRHPTQLANSGLKRR*
>ENSMUSP00000113855_c.1143:T>G_p.W382G ENSMUST00000119398.10|Fgfr1-203|ENSMUSG00000031565|+|chr8:g.26058330:T>G
MWGWKCLLFWAVLVTATLCTARPAPTLPEQDALPSSEDDDDDDDSSSEEKETDNTKPNRRPVAPYWTSPEKME
KKLHAVPAAKTVKFKCPSSGTPNPTLRWLKNGKEFKPDHRIGGYKVRYATWSIIMDSVVPSDKGNYTCIVENE
YGSINHTYQLDVVERSPHRPILQAGLPANKTVALGSNVEFMCKVYSDPQPHIQWLKHIEVNGSKIGPDNLPYV
QILKTAGVNTTDKEMEVLHLRNVSFEDAGEYTCLAGNSIGLSHHSAWLTVLEALEERPAVMTSPLYLEIIIYC
TGAFLISCMLGSVIIYKMKSGTKKSDFHSQMAVHKLAKSIPLRRQVTVSADSSASMNSGVLLVRPSRLSSSGT
PMLAGVSEYELPEDPRGELPRDRLVLGKPLGEGCFGQVVLAEAIGLDKDKPNRVTKVAVKMLKSDATEKDLSD
LISEMEMMKMIGKHKNIINLLGACTQDGPLYVIVEYASKGNLREYLQARRPPGLEYCYNPSHNPEEQLSSKDL
VSCAYQVARGMEYLASKKCIHRDLAARNVLVTEDNVMKIADFGLARDIHHIDYYKKTTNGRLPVKWMAPEALF
DRIYTHQSDVWSFGVLLWEIFTLGGSPYPGVPVEELFKLLKEGHRMDKPSNCTNELYMMMRDCWHAVPSQRPT
FKQLVEDLDRIVALTSNQEYLDLSIPLDQYSPSFPDTRSSTCSSGEDSVFSHEPLPEEPCLPRHPTQLANSGL
KRR*
>ENSMUSP00000113909_c.1404:T>G_p.W469G ENSMUST00000117179.9|Fgfr1-202|ENSMUSG00000031565|+|chr8:g.26058330:T>G
MWGWKCLLFWAVLVTATLCTARPAPTLPEQAQPWGVPVEVESLLVHPGDLLQLRCRLRDDVQSINWLRDGVQL
VESNRTRITGEEVEVRDSIPADSGLYACVTSSPSGSDTTYFSVNVSDALPSSEDDDDDDDSSSEEKETDNTKP
NPVAPYWTSPEKMEKKLHAVPAAKTVKFKCPSSGTPNPTLRWLKNGKEFKPDHRIGGYKVRYATWSIIMDSVV
PSDKGNYTCIVENEYGSINHTYQLDVVERSPHRPILQAGLPANKTVALGSNVEFMCKVYSDPQPHIQWLKHIE
VNGSKIGPDNLPYVQILKTAGVNTTDKEMEVLHLRNVSFEDAGEYTCLAGNSIGLSHHSAWLTVLEALEERPA
VMTSPLYLEIIIYCTGAFLISCMLGSVIIYKMKSGTKKSDFHSQMAVHKLAKSIPLRRQVTVSADSSASMNSG
VLLVRPSRLSSSGTPMLAGVSEYELPEDPRGELPRDRLVLGKPLGEGCFGQVVLAEAIGLDKDKPNRVTKVAV
KMLKSDATEKDLSDLISEMEMMKMIGKHKNIINLLGACTQDGPLYVIVEYASKGNLREYLQARRPPGLEYCYN
PSHNPEEQLSSKDLVSCAYQVARGMEYLASKKCIHRDLAARNVLVTEDNVMKIADFGLARDIHHIDYYKKTTN
GRLPVKWMAPEALFDRIYTHQSDVWSFGVLLWEIFTLGGSPYPGVPVEELFKLLKEGHRMDKPSNCTNELYMM
MRDCWHAVPSQRPTFKQLVEDLDRIVALTSNQEYLDLSIPLDQYSPSFPDTRSSTCSSGEDSVFSHEPLPEEP
CLPRHPTQLANSGLKRR*
>ENSMUSP00000131343_c.1143:T>G_p.W382G ENSMUST00000167764.2|Fgfr1-213|ENSMUSG00000031565|+|chr8:g.26058330:T>G
MWGWKCLLFWAVLVTATLCTARPAPTLPEQDALPSSEDDDDDDDSSSEEKETDNTKPNPVAPYWTSPEKMEKK
LHAVPAAKTVKFKCPSSGTPNPTLRWLKNGKEFKPDHRIGGYKVRYATWSIIMDSVVPSDKGNYTCIVENEYG
SINHTYQLDVVERSPHRPILQAGLPANKTVALGSNVEFMCKVYSDPQPHIQWLKHIEVNGSKIGPDNLPYVQI
LKHSGINSSDAEVLTLFNVTEAQSGEYVCKVSNYIGEANQSAWLTVTRPVAKALEERPAVMTSPLYLEIIIYC
TGAFLISCMLGSVIIYKMKSGTKKSDFHSQMAVHKLAKSIPLRRQVTVSADSSASMNSGVLLVRPSRLSSSGT
PMLAGVSEYELPEDPRGELPRDRLVLGKPLGEGCFGQVVLAEAIGLDKDKPNRVTKVAVKMLKSDATEKDLSD
LISEMEMMKMIGKHKNIINLLGACTQDGPLYVIVEYASKGNLREYLQARRPPGLEYCYNPSHNPEEQLSSKDL
VSCAYQVARGMEYLASKKCIHRDLAARNVLVTEDNVMKIADFGLARDIHHIDYYKKTTNGRLPVKWMAPEALF
DRIYTHQSDVWSFGVLLWEIFTLGGSPYPGVPVEELFKLLKEGHRMDKPSNCTNELYMMMRDCWHAVPSQRPT
FKQLVEDLDRIVALTSNQEYLDLSIPLDQYSPSFPDTRSSTCSSGEDSVFSHEPLPEEPCLPRHPTQLANSGL
KRR*
>rs221982749_ENSMUSP00000033990_c.382:A>GA_p.K128ins-fs ENSMUST00000033990.7|Wrn-201|ENSMUSG00000031583|-|chr8:g.33833108:T>TC
METTSLQRKFPEWMSMQSQRCATEEKACVQKSVLEDNLPFLEFPGSIVYSYEASDCSFLSEDISMRLSDGDVV
GFDMEWPPIYKPGKRSRVAVIQLCVSESKCYLFHISSMSVFPQGLKMLLENKSIREGRGWD
>rs221982749_ENSMUSP00000033991_c.382:A>GA_p.K128ins-fs ENSMUST00000033991.13|Wrn-202|ENSMUSG00000031583|-|chr8:g.33833108:T>TC
METTSLQRKFPEWMSMQSQRCATEEKACVQKSVLEDNLPFLEFPGSIVYSYEASDCSFLSEDISMRLSDGDVV
GFDMEWPPIYKPGKRSRVAVIQLCVSESKCYLFHISSMSVFPQGLKMLLENKSIREGRGWD
>ENSMUSP00000148728_c.711:A>T_p.K238* ENSMUST00000212436.2|Iqcn-201|ENSMUSG00000110622|+|chr8:g.71161520:A>T
MQPAAQLQFQNLMSPSGPSLPQPTPIHQEKVAKLCPQLQHDFYPSEELAVLPSQEVPRLRAVVESQAFRNILV
DEMDMMVSRAATVIQASWKGYRLRQKLISQMTAAKAIQEAWRRFSTRRLMHSNRLTVKKAKREDEEDIPYHQP
QQVRFHAPEDQPPVMVTKETQFPSFDNLVPSQATAGPCASRVPGGGLHPQYVINRLTVPKYQPCMLTKTFRSS
CLMRHLEGDSLKIRHVAS*TIKVGALEAAATGRYGQAMHGSLKTQTQAHTESDVPKAPLRIYPVNKTHTKVCP
MAAAVPESSATMSTAPRSAPQARPTSPATVMKMQATTSPTSPVIRLPAPVGPNSSLSNTTPQMQVRYMMPGAR

IQPPGSAVASTIKALPHFGAPVMKVPLQTGPGFTMTKTSLQMRPMTRSQAQTYTVSTSSKTSPSSPTVKPSPQ
TRLAAMLTKTPAQLRSVAAVFRTLCGGTTEAVASSPRCSPQSPVVAGNTPSQVHLNSTRTKVTVSTKQATTVV
KVNSQSYLGRCGSQGNLDTIVPKPPATYPLEVEKMKSCPARSPKKEAALKTNTTIAARALSWTKMMEDRSKAL
TQIKQRAEIIKVHFRVYMPEEIPVTLTQAQLAVPLTKSSSQIQPPPCHARTQPTSPFCKAVSQSCMPSGLAHG
TPEGQRPPVGLSASLSSSPLAAHLMSLTAQLQPACEQARSVSRDHLTRSCPASASLQARSSGTIVPPEVYPNT
YSPTSAPQHHMASLLAKSSSQLHPPAEHAKTMNTKTTKAACQVCPSTKLSKAPSLAQLITCLAKVPSQAQLAT
ETAKCLLAAHPTTDHRSKTQSQTFLSSSKASVQLWQHLGTLSTGSRAKPDDRSVAQPQLHSHAPNKTMQNPRS
VVTDSQGMLIPLMTPSGHPVCNAESWGDSGRAQTPPPSPVPNQAVPCHDDLAASVASLCADLVAMLGSPEDLR
TLLVKTLSQGEVRTALSQALSREVLGPSVVKALPQGMLGMALMKALSWGELGISLSRALSRGELRPELNKATQ
GKLAEVLCKALTEEERATLSQALCQGELGAVFTQSLAQMAQRTGTFLPKATSKTVGSRMTTAPAPVEVTCSGS
LSVAWGPAMGPVGARCSKGPVDAGLASGQSWNSKVPNVSVRATAHAGAPQGVWYPSRGHKPWDPIGAEAELDR
KPSAELLMSVQGMEKVVVQAVVTIQACARGYLVRRTVKVWHQWATIIQATWRGYRVRRNLERLFRATTIIQAA
WRGYCIRRARARQVLLPIAWPEHSGRTKGTLDNRNSSEHRCFLSCQPDVCSVCQSLGPPVESPPSVVMLVGSQ
PRTCHVCGHTLSTRVVQGFGQGISGQPVSRWASASQQNTLLSQQHRACTIIQAAWKGYRTRRQLSQKQSAAKM
VQAVWRGHYTRSCLTTDALLGTGGPWSISRDTSRHSSRAYSLHWPGV*
>ENSMUSP00000037929_c.298:A>C_p.K100T ENSMUST00000038626.10|Mpv17l2-
201|ENSMUSG00000035559|-|chr8:g.71213073:T>G
MALGGWRWARKALAAGRPLFQGRALLLTNTLGCGVLMAAGDGARQVWEVRARPGQRFSARRSASMFAVGCSMG
PFLHFWYLWLDRLLPASGLRSLPSVMTKVLVDQTVASPILGVWYFLGLGSLEGQTLEESCQELRAKFWDFYKA
DWCVWPAAQLVNFLFIPSHFRVTYINGLTLGWDTYLSYLKYWVPEPLQTPGCAD*
>ENSMUSP00000148307_c.37:A>C_p.K13T ENSMUST00000212617.2|Mpv17l2-
203|ENSMUSG00000035559|-|chr8:g.71213073:T>G
XASGLRSLPSVMTKVLVDQTVASPILGVWYFLGFFV*
>ENSMUSP00000148316_c.2835:C>G_p.R946G ENSMUST00000212935.2|Myo9b-
206|ENSMUSG00000004677|+|chr8:g.71800279:C>G
MSAHEAGSSGRRQQATYHLHIYPQLSSAGSQTSCRVTATKDSTTSDVIQDVVASLHLDGSKHYVLVEVKESGG
EEWVLDASDSPVHRVLLWPRRAQDEHPQEDGYYFLLQERNADGSIQYLPIQLLAQPTAACRLVERGLLPRPQA
DFDDLCNLPELTEANLLQNLKLRFMQQKIYTYAGSILVAINPFKFLPIYNPKYVKMYENQQLGKLEPHVFALA
DVAYYAMLRKHVNQCIVISGESGSGKTQSTNFLIHCLTALSQKGYASGVERTILGAGPVLEAFGNAKTAHNNN
SSRFGKFIQVNYLENGIVRGAVVEKYLLEKSRLVSQEKDERNYHVFYYLLLGVSEEERLEFQLKQPQDYFYLN
QHNLNIEDGEDLKHDFERLQQAMEMVGFLPATKKQIFSVLSAILYLGNVTYKKRATGRDEGLEVGPPEVLDTL
SQLLKVKRETLVEVLTKRKTVTVNDKLILPYSLSEAITARDSMAKSLYSALFDWIVLRINHALLNKKDMEEAV
SCLSIGVLDIFGFEDFERNSFEQFCINYANEQLQYYFTQHIFKLEQEEYQGEGISWHNIDYTDNVGCIHLISK
KPTGLFYLLDEESNFPHATSHTLLAKFKQQHEDNKYFLGTPVLEPAFIIQHFAGRVKYQIKDFREKNMDYMRP
DIVALLRGSDSSYVRQLIGMDPVAVFRWAVLRAAIRAMAVLREAGRLRAERAEKAAAGISSPATRSHMEELPR
GASTPSEKLYRDLHNQIIKSLKGLPWQGEDPRRLLQSLSLFQKPRTSFLKSKGIKQKQIIPKNLLDSKSLRLI
ISMTLHDRTTKSLLHLHKKKKPPSISAQFQTSLNKLLEALGKAEPFFIRCIRSNAEKKELCFDDELVLQQLRY
TGMLETVRIRRSGYSAKYTFQDFTEQFQVLLPKDVQPCREAIAALLEKLQVDRQNYQIGKTKVFLKETEGQTL
QEKLHGEVLRRILQLQSWFRMVLERKHFVQMKHAALTIQACWRSYRVRRALERTQAAVYLQAAWRGYLQRQAY
HHQRHSIIRLQSLCRGHLQRRSFSQMVSEKQKAEQAREAAGGKLSEGEPGPVAAGEQLSEHPVEDPESLGVEA
ETWMNKSPDGMSPKKETPSPEMETAAQKTVPAESHEKVSSSREKRESRRQRGLEHVERQNKHIQSCREESSTH
REPSRRASLEIGESFPEGTKGPREDGLEAWTETTAPSSSKQAQVVGDPPGSPSPVQRPTTLALDSRVSPVLPS
SSLESPKDKDKDESSTKAQDKPESPSGSTQIQRYQHPDTERLATAVEIWRGKKLASAVLSQSLDLSEKHRATG
AALTPTEERRISFSTSDVSKLSPVKTSAEIDGDFSSKKPSIHKKKSGDPSAGPDAGLSPGSQGDSKSAFKRLF
LHKAKDKKPSLEGVEETESNGGQAAQETPARKTLDVPSSQQHRHTTGEKPLKGKKNRNRKVGQITVSEKWRES
VFRKITNANELKFLDEFLLNKVNDLRSQKTPIESLFIEATERFRSNIKTMYSVPNGKIHVGYKDLMENYQIVV
SNLAAERGEKDTNLVLNVFQSLLDEFTRSYNKTDFERAKQSKAQKKKRKQERAVQEHNGHVFASYQVNIPQSC
EQCLSYIWLMDKALLCSVCKMTCHKKCVHKIQSYCSYTGRRKSELGAEPGHFGVCVDSLTSDKASVPIVLEKL
LEHVEMHGLYTEGLYRKSGAANRTRELRQALQTDPAAVKLEDFPIHAITGVLKQWLRELPEPLMTFAQYGDFL
RAVELPEKQEQLSAIYAVLDHLPEANHTSLERLIFHLVKVALLEDVNRMSPGALAIIFAPCLLRCPDNSDPLT
SMKDVLKITTCVEMLIKEQMRKYKMKMEEINHLEAAESIAFRRLSLLRQNAIKSPRTPVVQDLELGALPEEAA
GGDEDREKEILMERIQSIKEEKEDITYRLPELDPRGSDEENLDSETSASTESLLEERGVRGAVEE*
>ENSMUSP00000129220_c.2832:C>G_p.R945G ENSMUST00000170242.8|Myo9b-
203|ENSMUSG00000004677|+|chr8:g.71800279:C>G
MSAHEAGSSGRRQQATYHLHIYPQLSSAGSQTSCRVTATKDSTTSDVIQDVVASLHLDGSKHYVLVEVKESGG
EEWVLDASDSPVHRVLLWPRRAQDEHPQEDGYYFLLQERNADGSIQYLPIQLLAQPTAACRLVERGLLPRPQA
DFDDLCNLPELTEANLLQNLKLRFMQQKIYTYAGSILVAINPFKFLPIYNPKYVKMYENQQLGKLEPHVFALA
DVAYYAMLRKHVNQCIVISGESGSGKTQSTNFLIHCLTALSQKGYASGVERTILGAGPVLEAFGNAKTAHNNN

```
SSRFGKFIQVNYLENGIVRGAVVEKYLLEKSRLVSQEKDERNYHVFYYLLLGVSEEERLEFQLKQPQDYFYLN
QHNLNIEDGEDLKHDFERLQQAMEMVGFLPATKKQIFSVLSAILYLGNVTYKKRATGRDEGLEVGPPEVLDTL
SQLLKVKRETLVEVLTKRKTVTVNDKLILPYSLSEAITARDSMAKSLYSALFDWIVLRINHALLNKKDMEEAV
SCLSIGVLDIFGFEDFERNSFEQFCINYANEQLQYYFTQHIFKLEQEEYQGEGISWHNIDYTDNVGCIHLISK
KPTGLFYLLDEESNFPHATSHTLLAKFKQQHEDNKYFLGTPVLEPAFIIQHFAGRVKYQIKDFREKNMDYMRP
DIVALLRGSDSSYVRQLIGMDPVAVFRWAVLRAAIRAMAVLREAGRLRAERAEKAAGISSPATRSHMEELPRG
ASTPSEKLYRDLHNQIIKSLKGLPWQGEDPRRLLQSLSLFQKPRTSFLKSKGIKQKQIIPKNLLDSKSLRLII
SMTLHDRTTKSLLHLHKKKKPPSISAQFQTSLNKLLEALGKAEPFFIRCIRSNAEKKELCFDDELVLQQLRYT
GMLETVRIRRSGYSAKYTFQDFTEQFQVLLPKDVQPCREAIAALLEKLQVDRQNYQIGKTKVFLKETEGQTLQ
EKLHGEVLRRILQLQSWFRMVLERKHFVQMKHAALTIQACWRSYRVRRALERTQAAVYLQAAWRGYLQRQAYH
HQRHSIIRLQSLCRGHLQRRSFSQMVSEKQKAEQAREAAGGKLSEGEPGPVAAGEQLSEHPVEDPESLGVEAE
TWMNKSPDGMSPKKETPSPEMETAAQKTVPAESHEKVSSSREKRESRRQRGLEHVERQNKHIQSCREESSTHR
EPSRRASLEIGESFPEGTKGPREDGLEAWTETTAPSSSKQAQVVGDPPGSPSPVQRPTTLALDSRVSPMLPSS
SLEVSPVLPSSSLESPKDKDKDESSTKAQDKPESPSGSTQIQRYQHPDTERLATAVEIWRGKKLASAVLSQSL
DLSEKHRATGAALTPTEERRISFSTSDVSKLSPVKVQTSAEIDGDFSSKKPSIHKKKSGDPSAGPDAGLSPGS
QGDSKSAFKRLFLHKAKDKKPSLEGVEETESNGGQAAQETPARKTLDVPSSQQHRHTTGEKPLKGKKNRNRKV
GQITVSEKWRESVFRKITNANELKFLDEFLLNKVNDLRSQKTPIESLFIEATERFRSNIKTMYSVPNGKIHVG
YKDLMENYQIVVSNLAAERGEKDTNLVLNVFQSLLDEFTRSYNKTDFERAKQSKAQKKKRKQERAVQEHNGHV
FASYQVNIPQSCEQCLSYIWLMDKALLCSVCKMTCHKKCVHKIQSYCSYTGRRKSELGAEPGHFGVCVDSLTS
DKASVPIVLEKLLEHVEMHGLYTEGLYRKSGAANRTRELRQALQTDPAAVKLEDFPIHAITGVLKQWLRELPE
PLMTFAQYGDFLRAVELPEKQEQLSAIYAVLDHLPEANHTSLERLIFHLVKVALLEDVNRMSPGALAIIFAPC
LLRCPDNSDPLTSMKDVLKITTCVEMLIKEQMRKYKMKMEEINHLEAAESIAFRRLSLLRQNAPWPLKLGFSS
PYEGVRIKSPRTPVVQDLELGALPEEAAGGDEDREKEILMERIQSIKEEKEDITYRLPELDPRGSDEENLDSE
TSASTESLLEERGVRGAVEGPPAPALPCPISPTLSPLPEAAAPPRGRPTSFVTVRVKTPRRTPIMPMANIKLP
PGLPLPHLTSWAPALQEAVVPVKRREPPARRQDQVHSVYIAPGADLPSQSTLIALDHDTILPGTKRRYSDPPTY
CLPPSSGQANG*
>ENSMUSP00000071827_c.2832:C>G_p.R945G ENSMUST00000071935.7|Myo9b-
201|ENSMUSG00000004677|+|chr8:g.71800279:C>G
MSAHEAGSSGRRQQATYHLHIYPQLSSAGSQTSCRVTATKDSTTSDVIQDVVASLHLDGSKHYVLVEVKESGG
EEWVLDASDSPVHRVLLWPRRAQDEHPQEDGYYFLLQERNADGSIQYLPIQLLAQPTAACRLVERGLLPRPQA
DFDDLCNLPELTEANLLQNLKLRFMQQKIYTYAGSILVAINPFKFLPIYNPKYVKMYENQQLGKLEPHVFALA
DVAYYAMLRKHVNQCIVISGESGSGKTQSTNFLIHCLTALSQKGYASGVERTILGAGPVLEAFGNAKTAHNNN
SSRFGKFIQVNYLENGIVRGAVVEKYLLEKSRLVSQEKDERNYHVFYYLLLGVSEEERLEFQLKQPQDYFYLN
QHNLNIEDGEDLKHDFERLQQAMEMVGFLPATKKQIFSVLSAILYLGNVTYKKRATGRDEGLEVGPPEVLDTL
SQLLKVKRETLVEVLTKRKTVTVNDKLILPYSLSEAITARDSMAKSLYSALFDWIVLRINHALLNKKDMEEAV
SCLSIGVLDIFGFEDFERNSFEQFCINYANEQLQYYFTQHIFKLEQEEYQGEGISWHNIDYTDNVGCIHLISK
KPTGLFYLLDEESNFPHATSHTLLAKFKQQHEDNKYFLGTPVLEPAFIIQHFAGRVKYQIKDFREKNMDYMRP
DIVALLRGSDSSYVRQLIGMDPVAVFRWAVLRAAIRAMAVLREAGRLRAERAEKAAGISSPATRSHMEELPRG
ASTPSEKLYRDLHNQIIKSLKGLPWQGEDPRRLLQSLSLFQKPRTSFLKSKGIKQKQIIPKNLLDSKSLRLII
SMTLHDRTTKSLLHLHKKKKPPSISAQFQTSLNKLLEALGKAEPFFIRCIRSNAEKKELCFDDELVLQQLRYT
GMLETVRIRRSGYSAKYTFQDFTEQFQVLLPKDVQPCREAIAALLEKLQVDRQNYQIGKTKVFLKETEGQTLQ
EKLHGEVLRRILQLQSWFRMVLERKHFVQMKHAALTIQACWRSYRVRRALERTQAAVYLQAAWRGYLQRQAYH
HQRHSIIRLQSLCRGHLQRRSFSQMVSEKQKAEQAREAAGGKLSEGEPGPVAAGEQLSEHPVEDPESLGVEAE
TWMNKSPDGMSPKKETPSPEMETAAQKTVPAESHEKVSSSREKRESRRQRGLEHVERQNKHIQSCREESSTHR
EPSRRASLEIGESFPEGTKGPREDGLEAWTETTAPSSSKQAQVVGDPPGSPSPVQRPTTLALDSRVSPVLPSS
SLESPKDKDKDESSTKAQDKPESPSGSTQIQRYQHPDTERLATAVEIWRGKKLASAVLSQSLDLSEKHRATGA
ALTPTEERRISFSTSDVSKLSPVKTSAEIDGDFSSKKPSIHKKKSGDPSAGPDAGLSPGSQGDSKSAFKRLFL
HKAKDKKPSLEGVEETESNGGQAAQETPARKTLDVPSSQQHRHTTGEKPLKGKKNRNRKVGQITVSEKWRESV
FRKITNANELKFLDEFLLNKVNDLRSQKTPIESLFIEATERFRSNIKTMYSVPNGKIHVGYKDLMENYQIVVS
NLAAERGEKDTNLVLNVFQSLLDEFTRSYNKTDFERAKSKAQKKKRKQERAVQEHNGHVFASYQVNIPQSCEQ
CLSYIWLMDKALLCSVCKMTCHKKCVHKIQSYCSYTGRRKSELGAEPGHFGVCVDSLTSDKASVPIVLEKLLE
HVEMHGLYTEGLYRKSGAANRTRELRQALQTDPAAVKLEDFPIHAITGVLKQWLRELPEPLMTFAQYGDFLRA
VELPEKQEQLSAIYAVLDHLPEANHTSLERLIFHLVKVALLEDVNRMSPGALAIIFAPCLLRCPDNSDPLTSM
KDVLKITTCVEMLIKEQMRKYKMKMEEINHLEAAESIAFRRLSLLRQNAIKSPRTPVVQDLELGALPEEAAGG
DEDREKEILMERIQSIKEEKEDITYRLPELDPRGSDEENLDSETSASTESLLEERGVRGAVEE*
>ENSMUSP00000131635_c.2832:C>G_p.R945G ENSMUST00000168839.9|Myo9b-
202|ENSMUSG00000004677|+|chr8:g.71800279:C>G
```

MSAHEAGSSGRRQQATYHLHIYPQLSSAGSQTSCRVTATKDSTTSDVIQDVVASLHLDGSKHYVLVEVKESGG
EEWVLDASDSPVHRVLLWPRRAQDEHPQEDGYYFLLQERNADGSIQYLPIQLLAQPTAACRLVERGLLPRPQA
DFDDLCNLPELTEANLLQNLKLRFMQQKIYTYAGSILVAINPFKFLPIYNPKYVKMYENQQLGKLEPHVFALA
DVAYYAMLRKHVNQCIVISGESGSGKTQSTNFLIHCLTALSQKGYASGVERTILGAGPVLEAFGNAKTAHNNN
SSRFGKFIQVNYLENGIVRGAVVEKYLLEKSRLVSQEKDERNYHVFYYLLLGVSEEERLEFQLKQPQDYFYLN
QHNLNIEDGEDLKHDFERLQQAMEMVGFLPATKKQIFSVLSAILYLGNVTYKKRATGRDEGLEVGPPEVLDTL
SQLLKVKRETLVEVLTKRKTVTVNDKLILPYSLSEAITARDSMAKSLYSALFDWIVLRINHALLNKKDMEEAV
SCLSIGVLDIFGFEDFERNSFEQFCINYANEQLQYYFTQHIFKLEQEEYQGEGISWHNIDYTDNVGCIHLISK
KPTGLFYLLDEESNFPHATSHTLLAKFKQQHEDNKYFLGTPVLEPAFIIQHFAGRVKYQIKDFREKNMDYMRP
DIVALLRGSDSSYVRQLIGMDPVAVFRWAVLRAAIRAMAVLREAGRLRAERAEKAAGISSPATRSHMEELPRG
ASTPSEKLYRDLHNQIIKSLKGLPWQGEDPRRLLQSLSLFQKPRTSFLKSKGIKQKQIIPKNLLDSKSLRLII
SMTLHDRTTKSLLHLHKKKKPPSISAQFQTSLNKLLEALGKAEPFFIRCIRSNAEKKELCFDDELVLQQLRYT
GMLETVRIRRSGYSAKYTFQDFTEQFQVLLPKDVQPCREAIAALLEKLQVDRQNYQIGKTKVFLKETEGQTLQ
EKLHGEVLRRILQLQSWFRMVLERKHFVQMKHAALTIQACWRSYRVRRALERTQAAVYLQAAWRGYLQRQAYH
HQRHSIIRLQSLCRGHLQRRSFSQMVSEKQKAEQAREAAGGKLSEGEPGPVAAGEQLSEHPVEDPESLGVEAE
TWMNKSPDGMSPKKETPSPEMETAAQKTVPAESHEKVSSSREKRESRRQRGLEHVERQNKHIQSCREESSTHR
EPSRRASLEIGESFPEGTKGPREDGLEAWTETTAPSSSKQAQVVGDPPGSPSPVQRPTTLALDSRVSPMLPSS
SLEVSPVLPSSSLESPKDKDKDESSTKAQDKPESPSGSTQIQRYQHPDTERLATAVEIWRGKKLASAVLSQSL
DLSEKHRATGAALTPTEERRISFSTSDVSKLSPVKVQTSAEIDGDFSSKKPSIHKKKSGDPSAGPDAGLSPGS
QGDSKSAFKRLFLHKAKDKKPSLEGVEETESNGGQAAQETPARKTLDVPSSQQHRHTTGEKPLKGKKNRNRKV
GQITVSEKWRESVFRKITNANELKFLDEFLLNKVNDLRSQKTPIESLFIEATERFRSNIKTMYSVPNGKIHVG
YKDLMENYQIVVSNLAAERGEKDTNLVLNVFQSLLDEFTRSYNKTDFERAKQSKAQKKKRKQERAVQEHNGHV
FASYQVNIPQSCEQCLSYIWLMDKALLCSVCKMTCHKKCVHKIQSYCSYTGRRKSELGAEPGHFGVCVDSLTS
DKASVPIVLEKLLEHVEMHGLYTEGLYRKSGAANRTRELRQALQTDPAAVKLEDFPIHAITGVLKQWLRELPE
PLMTFAQYGDFLRAVELPEKQEQLSAIYAVLDHLPEANHTSLERLIFHLVKVALLEDVNRMSPGALAIIFAPC
LLRCPDNSDPLTSMKDVLKITTCVEMLIKEQMRKYKMKMEEINHLEAAESIAFRRLSLLRQNAIKSPRTPVVQ
DLELGALPEEAAGGDEDREKEILMERIQSIKEEKEDITYRLPELDPRGSDEENLDSETSASTESLLEERGVRG
AVEE*
>ENSMUSP00000148316_c.3322:CT>G_p.P1108del-fs ENSMUST00000212935.2|Myo9b-206|ENSMUSG00000004677|+|chr8:g.71801166:CT>G
MSAHEAGSSGRRQQATYHLHIYPQLSSAGSQTSCRVTATKDSTTSDVIQDVVASLHLDGSKHYVLVEVKESGG
EEWVLDASDSPVHRVLLWPRRAQDEHPQEDGYYFLLQERNADGSIQYLPIQLLAQPTAACRLVERGLLPRPQA
DFDDLCNLPELTEANLLQNLKLRFMQQKIYTYAGSILVAINPFKFLPIYNPKYVKMYENQQLGKLEPHVFALA
DVAYYAMLRKHVNQCIVISGESGSGKTQSTNFLIHCLTALSQKGYASGVERTILGAGPVLEAFGNAKTAHNNN
SSRFGKFIQVNYLENGIVRGAVVEKYLLEKSRLVSQEKDERNYHVFYYLLLGVSEEERLEFQLKQPQDYFYLN
QHNLNIEDGEDLKHDFERLQQAMEMVGFLPATKKQIFSVLSAILYLGNVTYKKRATGRDEGLEVGPPEVLDTL
SQLLKVKRETLVEVLTKRKTVTVNDKLILPYSLSEAITARDSMAKSLYSALFDWIVLRINHALLNKKDMEEAV
SCLSIGVLDIFGFEDFERNSFEQFCINYANEQLQYYFTQHIFKLEQEEYQGEGISWHNIDYTDNVGCIHLISK
KPTGLFYLLDEESNFPHATSHTLLAKFKQQHEDNKYFLGTPVLEPAFIIQHFAGRVKYQIKDFREKNMDYMRP
DIVALLRGSDSSYVRQLIGMDPVAVFRWAVLRAAIRAMAVLREAGRLRAERAEKAAAGISSPATRSHMEELPR
GASTPSEKLYRDLHNQIIKSLKGLPWQGEDPRRLLQSLSLFQKPRTSFLKSKGIKQKQIIPKNLLDSKSLRLI
ISMTLHDRTTKSLLHLHKKKKPPSISAQFQTSLNKLLEALGKAEPFFIRCIRSNAEKKELCFDDELVLQQLRY
TGMLETVRIRRSGYSAKYTFQDFTEQFQVLLPKDVQPCREAIAALLEKLQVDRQNYQIGKTKVFLKETERQTL
QEKLHGEVLRRILQLQSWFRMVLERKHFVQMKHAALTIQACWRSYRVRRALERTQAAVYLQAAWRGYLQRQAY
HHQRHSIIRLQSLCRGHLQRRSFSQMVSEKQKAEQAREAAGGKLSEGEPGPVAAGEQLSEHPVEDPESLGVEA
ETWMNKSPDGMSRRRRHPAQRWRPQPKRQCQLKVMRKSPVAERSESHGGNEGWSMLNDRINTSNPAGRRAAPT
ENLPEGQAWK
>ENSMUSP00000129220_c.3319:CT>G_p.P1107del-fs ENSMUST00000170242.8|Myo9b-203|ENSMUSG00000004677|+|chr8:g.71801166:CT>G
MSAHEAGSSGRRQQATYHLHIYPQLSSAGSQTSCRVTATKDSTTSDVIQDVVASLHLDGSKHYVLVEVKESGG
EEWVLDASDSPVHRVLLWPRRAQDEHPQEDGYYFLLQERNADGSIQYLPIQLLAQPTAACRLVERGLLPRPQA
DFDDLCNLPELTEANLLQNLKLRFMQQKIYTYAGSILVAINPFKFLPIYNPKYVKMYENQQLGKLEPHVFALA
DVAYYAMLRKHVNQCIVISGESGSGKTQSTNFLIHCLTALSQKGYASGVERTILGAGPVLEAFGNAKTAHNNN
SSRFGKFIQVNYLENGIVRGAVVEKYLLEKSRLVSQEKDERNYHVFYYLLLGVSEEERLEFQLKQPQDYFYLN
QHNLNIEDGEDLKHDFERLQQAMEMVGFLPATKKQIFSVLSAILYLGNVTYKKRATGRDEGLEVGPPEVLDTL
SQLLKVKRETLVEVLTKRKTVTVNDKLILPYSLSEAITARDSMAKSLYSALFDWIVLRINHALLNKKDMEEAV
SCLSIGVLDIFGFEDFERNSFEQFCINYANEQLQYYFTQHIFKLEQEEYQGEGISWHNIDYTDNVGCIHLISK
KPTGLFYLLDEESNFPHATSHTLLAKFKQQHEDNKYFLGTPVLEPAFIIQHFAGRVKYQIKDFREKNMDYMRP

DIVALLRGSDSSYVRQLIGMDPVAVFRWAVLRAAIRAMAVLREAGRLRAERAEKAAGISSPATRSHMEELPRG
ASTPSEKLYRDLHNQIIKSLKGLPWQGEDPRRLLQSLSLFQKPRTSFLKSKGIKQKQIIPKNLLDSKSLRLII
SMTLHDRTTKSLLHLHKKKKPPSISAQFQTSLNKLLEALGKAEPFFIRCIRSNAEKKELCFDDELVLQQLRYT
GMLETVRIRRSGYSAKYTFQDFTEQFQVLLPKDVQPCREAIAALLEKLQVDRQNYQIGKTKVFLKETERQTLQ
EKLHGEVLRRILQLQSWFRMVLERKHFVQMKHAALTIQACWRSYRVRRALERTQAAVYLQAAWRGYLQRQAYH
HQRHSIIRLQSLCRGHLQRRSFSQMVSEKQKAEQAREAAGGKLSEGEPGPVAAGEQLSEHPVEDPESLGVEAE
TWMNKSPDGMSRRRRHPAQRWRPQPKRQCQLKVMRKSPVAERSESHGGNEGWSMLNDRINTSNPAGRRAAPTE
NLPEGQAWK
>ENSMUSP00000071827_c.3319:CT>G_p.P1107del-fs ENSMUST00000071935.7|Myo9b-201|ENSMUSG00000004677|+|chr8:g.71801166:CT>G
MSAHEAGSSGRRQQATYHLHIYPQLSSAGSQTSCRVTATKDSTTSDVIQDVVASLHLDGSKHYVLVEVKESGG
EEWVLDASDSPVHRVLLWPRRAQDEHPQEDGYYFLLQERNADGSIQYLPIQLLAQPTAACRLVERGLLPRPQA
DFDDLCNLPELTEANLLQNLKLRFMQQKIYTYAGSILVAINPFKFLPIYNPKYVKMYENQQLGKLEPHVFALA
DVAYYAMLRKHVNQCIVISGESGSGKTQSTNFLIHCLTALSQKGYASGVERTILGAGPVLEAFGNAKTAHNNN
SSRFGKFIQVNYLENGIVRGAVVEKYLLEKSRLVSQEKDERNYHVFYYLLLGVSEEERLEFQLKQPQDYFYLN
QHNLNIEDGEDLKHDFERLQQAMEMVGFLPATKKQIFSVLSAILYLGNVTYKKRATGRDEGLEVGPPEVLDTL
SQLLKVKRETLVEVLTKRKTVTVNDKLILPYSLSEAITARDSMAKSLYSALFDWIVLRINHALLNKKDMEEAV
SCLSIGVLDIFGFEDFERNSFEQFCINYANEQLQYYFTQHIFKLEQEEYQGEGISWHNIDYTDNVGCIHLISK
KPTGLFYLLDEESNFPHATSHTLLAKFKQQHEDNKYFLGTPVLEPAFIIQHFAGRVKYQIKDFREKNMDYMRP
DIVALLRGSDSSYVRQLIGMDPVAVFRWAVLRAAIRAMAVLREAGRLRAERAEKAAGISSPATRSHMEELPRG
ASTPSEKLYRDLHNQIIKSLKGLPWQGEDPRRLLQSLSLFQKPRTSFLKSKGIKQKQIIPKNLLDSKSLRLII
SMTLHDRTTKSLLHLHKKKKPPSISAQFQTSLNKLLEALGKAEPFFIRCIRSNAEKKELCFDDELVLQQLRYT
GMLETVRIRRSGYSAKYTFQDFTEQFQVLLPKDVQPCREAIAALLEKLQVDRQNYQIGKTKVFLKETERQTLQ
EKLHGEVLRRILQLQSWFRMVLERKHFVQMKHAALTIQACWRSYRVRRALERTQAAVYLQAAWRGYLQRQAYH
HQRHSIIRLQSLCRGHLQRRSFSQMVSEKQKAEQAREAAGGKLSEGEPGPVAAGEQLSEHPVEDPESLGVEAE
TWMNKSPDGMSRRRRHPAQRWRPQPKRQCQLKVMRKSPVAERSESHGGNEGWSMLNDRINTSNPAGRRAAPTE
NLPEGQAWK
>ENSMUSP00000131635_c.3319:CT>G_p.P1107del-fs ENSMUST00000168839.9|Myo9b-202|ENSMUSG00000004677|+|chr8:g.71801166:CT>G
MSAHEAGSSGRRQQATYHLHIYPQLSSAGSQTSCRVTATKDSTTSDVIQDVVASLHLDGSKHYVLVEVKESGG
EEWVLDASDSPVHRVLLWPRRAQDEHPQEDGYYFLLQERNADGSIQYLPIQLLAQPTAACRLVERGLLPRPQA
DFDDLCNLPELTEANLLQNLKLRFMQQKIYTYAGSILVAINPFKFLPIYNPKYVKMYENQQLGKLEPHVFALA
DVAYYAMLRKHVNQCIVISGESGSGKTQSTNFLIHCLTALSQKGYASGVERTILGAGPVLEAFGNAKTAHNNN
SSRFGKFIQVNYLENGIVRGAVVEKYLLEKSRLVSQEKDERNYHVFYYLLLGVSEEERLEFQLKQPQDYFYLN
QHNLNIEDGEDLKHDFERLQQAMEMVGFLPATKKQIFSVLSAILYLGNVTYKKRATGRDEGLEVGPPEVLDTL
SQLLKVKRETLVEVLTKRKTVTVNDKLILPYSLSEAITARDSMAKSLYSALFDWIVLRINHALLNKKDMEEAV
SCLSIGVLDIFGFEDFERNSFEQFCINYANEQLQYYFTQHIFKLEQEEYQGEGISWHNIDYTDNVGCIHLISK
KPTGLFYLLDEESNFPHATSHTLLAKFKQQHEDNKYFLGTPVLEPAFIIQHFAGRVKYQIKDFREKNMDYMRP
DIVALLRGSDSSYVRQLIGMDPVAVFRWAVLRAAIRAMAVLREAGRLRAERAEKAAGISSPATRSHMEELPRG
ASTPSEKLYRDLHNQIIKSLKGLPWQGEDPRRLLQSLSLFQKPRTSFLKSKGIKQKQIIPKNLLDSKSLRLII
SMTLHDRTTKSLLHLHKKKKPPSISAQFQTSLNKLLEALGKAEPFFIRCIRSNAEKKELCFDDELVLQQLRYT
GMLETVRIRRSGYSAKYTFQDFTEQFQVLLPKDVQPCREAIAALLEKLQVDRQNYQIGKTKVFLKETERQTLQ
EKLHGEVLRRILQLQSWFRMVLERKHFVQMKHAALTIQACWRSYRVRRALERTQAAVYLQAAWRGYLQRQAYH
HQRHSIIRLQSLCRGHLQRRSFSQMVSEKQKAEQAREAAGGKLSEGEPGPVAAGEQLSEHPVEDPESLGVEAE
TWMNKSPDGMSRRRRHPAQRWRPQPKRQCQLKVMRKSPVAERSESHGGNEGWSMLNDRINTSNPAGRRAAPTE
NLPEGQAWK
>ENSMUSP00000002469_c.522:A>G_p.K175E ENSMUST00000002469.9|Ocel1-201|ENSMUSG00000002396|+|chr8:g.71825438:A>G
MQIHAGPASRRGRRGPLARLSGPEATCNSRPAARGRQRAAAPRMPAPERPRSRRPQSQPGPGELCVRPRKIVF
ADELRPREPLHPEKHPRDLGPRLNPVPDYELKYPPVTNRRDRSRYAAVFQDQYGEFSELQREVGATQAKLQQL
EALLLSLPPPRSQKEARMAAHVRREFEKERGDPGFLDKQARCNYLKGKLRHLKAQIRKFDDQQDSNSEDSVYF
*
>ENSMUSP00000002466_c.104:A>TAA_p.S35ins-fs ENSMUST00000002466.9|Nr2f6-201|ENSMUSG00000002393|-|chr8:g.71833811:T>TTA
MAMVTGGWGDPGGDTNGVDKAGGSYPRATEDDSASNLPGRPATRSRATRSVRGCRWTAWCAGTSPVESITACS
PARAARVSSSAASAAISATPAGPTVTVRLISTTGTSVSTVGSRSASGWACARRPCSEAASRMRSPVQRPAVPR
ARRASNLSRGRQCPS

>ENSMUSP00000121648_c.10:A>TAA_p.H4ins-fs ENSMUST00000137058.2|Nr2f6-205|ENSMUSG00000002393|-|chr8:g.71833811:T>TTA
IRRLTSRGDQRRGAGRRGASGVAGGLRGVRGQVQWKALRRVHLRGLQEFLQAQHPPQSQLHLPVRDPPLVFPR
GLQAHQWEPWPVSPGPTVTVRLISTTGTSVSTVGSRSASGWACARRPCSEAASRMRSPVQRPAVPRARRASNL
SRGRQCPS
>rs387475422_ENSMUSP00000045668_c.1824:C>T_p.Q609*
ENSMUST00000049184.9|Ushbp1-201|ENSMUSG00000034911|-|chr8:g.71838600:G>A
MSARATRPRSRRGRHPLPGELDPVAESSEEVDANNGRFETKPELHQEYLGPGLQSPRNWIKEGCDVGSHAVLS
LSPEEQCEPEVEAHQVLQEAPLDSGPAEISVPSVYETLQCRLSSLEAVVAALRHHSLSFPKSVEAEDRDQGAP
GPFGDEKEDAGPGQQEAARLIERNAWLRLALCNREDELACTQASLQDAQAEKETLQRQVQELEDSLMQMEASP
PTPILRAGRRNSNSSTSGAERRPWVPQDSSMAHPFLQRLRSDSSTQSFGCLSTQHPAPETYLMEDQMGQLQGS
IEKLKCFNRLLLAVLQGYKGRCESLSIKLAKREAEATALRLALQYSEDCEEVYEVLLALKMAGLGTGVGTTKG
DLQAAEKEASRLLMKKEATMEVETPQPSSEGSGVDKPTPEELASQLHGYVQHLRERWALVKIPEELGPVTAPK
ATMPHAEATVQAILEIQPGPTLPHLEKSQIQQDLAATRDRLADLVLRLQLTQREKRGLELREAALRAQGPAHQ
LLLQQLRWERAHFAGDGSSGGSSEDPSSEEEAGEDRQQHYQGPLALLDGQMGKVWDSENVSQELSASLARAID
LRAQLQSLRQQLEQVTQKGRTRRV*SAELSRELCKAHSALALAFRGAHRKQEEQRLKLEQQVARLQAQQAEEL
AVLTATARALGKPGAPQPAQTFL*
>ENSMUSP00000045668_c.1606:A>G_p.D536G ENSMUST00000049184.9|Ushbp1-201|ENSMUSG00000034911|-|chr8:g.71840043:T>C
MSARATRPRSRRGRHPLPGELDPVAESSEEVDANNGRFETKPELHQEYLGPGLQSPRNWIKEGCDVGSHAVLS
LSPEEQCEPEVEAHQVLQEAPLDSGPAEISVPSVYETLQCRLSSLEAVVAALRHHSLSFPKSVEAEDRDQGAP
GPFGDEKEDAGPGQQEAARLIERNAWLRLALCNREDELACTQASLQDAQAEKETLQRQVQELEDSLMQMEASP
PTPILRAGRRNSNSSTSGAERRPWVPQDSSMAHPFLQRLRSDSSTQSFGCLSTQHPAPETYLMEDQMGQLQGS
IEKLKCFNRLLLAVLQGYKGRCESLSIKLAKREAEATALRLALQYSEDCEEVYEVLLALKMAGLGTGVGTTKG
DLQAAEKEASRLLMKKEATMEVETPQPSSEGSGVDKPTPEELASQLHGYVQHLRERWALVKIPEELGPVTAPK
ATMPHAEATVQAILEIQPGPTLPHLEKSQIQQDLAATRDRLADLVLRLQLTQREKRGLELREAALRAQGPAHQ
LLLQQLRWERAHFAGDGSSGGSSEGPSSEEEAGEDRQQHYQGPLALLDGQMGKVWDSENVSQELSASLARAID
LRAQLQSLRQQLEQVTQKGRTRRVQSAELSRELCKAHSALALAFRGAHRKQEEQRLKLEQQVARLQAQQAEEL
AVLTATARALGKPGAPQPAQTFL*
>rs108499415_ENSMUSP00000045668_c.1260:C>T_p.R421C
ENSMUST00000049184.9|Ushbp1-201|ENSMUSG00000034911|-|chr8:g.71841567:G>A
MSARATRPRSRRGRHPLPGELDPVAESSEEVDANNGRFETKPELHQEYLGPGLQSPRNWIKEGCDVGSHAVLS
LSPEEQCEPEVEAHQVLQEAPLDSGPAEISVPSVYETLQCRLSSLEAVVAALRHHSLSFPKSVEAEDRDQGAP
GPFGDEKEDAGPGQQEAARLIERNAWLRLALCNREDELACTQASLQDAQAEKETLQRQVQELEDSLMQMEASP
PTPILRAGRRNSNSSTSGAERRPWVPQDSSMAHPFLQRLRSDSSTQSFGCLSTQHPAPETYLMEDQMGQLQGS
IEKLKCFNRLLLAVLQGYKGRCESLSIKLAKREAEATALRLALQYSEDCEEVYEVLLALKMAGLGTGVGTTKG
DLQAAEKEASRLLMKKEATMEVETPQPSSEGSGVDKPTPEELASQLHGYVQHLRECWALVKIPEELGPVTAPK
ATMPHAEATVQAILEIQPGPTLPHLEKSQIQQDLAATRDRLADLVLRLQLTQREKRGLELREAALRAQGPAHQ
LLLQQLRWERAHFAGDGSSGGSSEDPSSEEEAGEDRQQHYQGPLALLDGQMGKVWDSENVSQELSASLARAID
LRAQLQSLRQQLEQVTQKGRTRRVQSAELSRELCKAHSALALAFRGAHRKQEEQRLKLEQQVARLQAQQAEEL
AVLTATARALGKPGAPQPAQTFL*
>ENSMUSP00000002473_c.16:C>T_p.A6V ENSMUST00000002473.10|Babam1-201|ENSMUSG00000031820|+|chr8:g.71850389:C>T
MEVAEVNSPTEEEEEEEEEGEETISEPRPHTRSNPEGAEDRALGAQASVGSRSEGEGEAATADGGAASVPGAG
PKPWQVPASASEVQIRTPRVNCPEKVIICLDLSEEMSVPKLESFNGSRTNALNVSQKMVEMFVRTKHKIDKSH
EFALVVVNDDSAWLSGLTSDPRELCSCLYDLETASCSTFNLEGLFSLIQQKTELPVTENVQTIPPPYVVRTIL
VYSRPPCQPQFSLTEPMKKMFQCPYFFFDIVYIHNGTEEKEEDMSWKDMFAFMGSLDTKGASYKYEVALAGPA
LELHNCMAKLLAHPLQRPCQTHASYSLLEEDEEAGEEEATV*
>ENSMUSP00000002473_c.180:A>T_p.T61S ENSMUST00000002473.10|Babam1-201|ENSMUSG00000031820|+|chr8:g.71850553:A>T
MEVAEANSPTEEEEEEEEEGEETISEPRPHTRSNPEGAEDRALGAQASVGSRSEGEGEAASADGGAASVPGAG
PKPWQVPASASEVQIRTPRVNCPEKVIICLDLSEEMSVPKLESFNGSRTNALNVSQKMVEMFVRTKHKIDKSH
EFALVVVNDDSAWLSGLTSDPRELCSCLYDLETASCSTFNLEGLFSLIQQKTELPVTENVQTIPPPYVVRTIL
VYSRPPCQPQFSLTEPMKKMFQCPYFFFDIVYIHNGTEEKEEDMSWKDMFAFMGSLDTKGASYKYEVALAGPA
LELHNCMAKLLAHPLQRPCQTHASYSLLEEDEEAGEEEATV*
>ENSMUSP00000002473_c.219:C>T_p.P74S ENSMUST00000002473.10|Babam1-201|ENSMUSG00000031820|+|chr8:g.71850592:C>T

MEVAEANSPTEEEEEEEEEEGEETISEPRPHTRSNPEGAEDRALGAQASVGSRSEGEGEAATADGGAASVPGAG
SKPWQVPASASEVQIRTPRVNCPEKVIICLDLSEEMSVPKLESFNGSRTNALNVSQKMVEMFVRTKHKIDKSH
EFALVVVNDDSAWLSGLTSDPRELCSCLYDLETASCSTFNLEGLFSLIQQKTELPVTENVQTIPPPYVVRTIL
VYSRPPCQPQFSLTEPMKKMFQCPYFFFDIVYIHNGTEEKEEDMSWKDMFAFMGSLDTKGASYKYEVALAGPA
LELHNCMAKLLAHPLQRPCQTHASYSLLEEDEEAGEEEATV*
>rs51871462_ENSMUSP00000113162_c.615:A>C_p.S206R
ENSMUST00000119976.8|Ankle1-201|ENSMUSG00000046295|+|chr8:g.71860039:A>C
MADTACLALRLLAALREEEARAVEELLRLGADPNLVLDDGAAAVHLAARASHPRALHCLRMLLRWGADPNARS
AEGLTPVHVAAAWGCCGALELLLSRGGDPTLRDQDGLRPLDWALQQRHHNCARVLQELDTPTQPDETREPTET
FHVAQGSFETETCQGPALAESSGVSQDSELHVHRAELEVEAVEVAVHPQSSEATENSDYRSDASFVTAVEDSL
QPGRPGGALELVAGLWVTRGAVSAGKGAPNCQPQVLTLTARDTDKPVLPGDGDLGALHPHSSVPPMSDLQLLQ
ALRALGYSPGPVTPFTRGHYLRRLQEAQASRADVGHSQELAEALRTGTIPDCQVDEEALAQCFQRLDPLKKWR
EGITKSSFTYLLLDPRLTKDLPARASSLTLAECLQCFVRAIFYVGKGTRARPDAHLWEAFGYHDQPRKQVCPK
VRRILDIWASGRGIISLHCFQHVVAMEAYTREACLLDALGLQTLTNQKQGHYYGVVAHWPPSRRRRLGVHLLQ
RALLVFLAEGERELRPQDIQARG*
>rs51871462_ENSMUSP00000112797_c.555:A>C_p.S186R
ENSMUST00000120725.2|Ankle1-202|ENSMUSG00000046295|+|chr8:g.71860039:A>C
MADTACLALRLLAALREEEARAVEELLRLGADPNLVLDDGAAAVHLAARASHPRALHCLRMLLRWGADPNARS
AEGLTPVHVAAAWGCCGALELLLSRGGDPTLRDQDGLRPLDWALQQRHHNCARVLQELDTPTQPDETREPTAE
SSGVSQDSELHVHRAELEVEAVEVAVHPQSSEATENSDYRSDASFVTAVEDSLQPGRPGGALELVAGLWVTRG
AVSAGKGAPNCQPQVLTLTARDTDKPVLPGDGDLGALHPHSSVPPMSDLQLLQALRALGYSPGPVTPFTRGHY
LRRLQEAQASRADVGHSQELAEALRTGTIPDCQVDEEALAQCFQRLDPLKKWREGITKSSFTYLLLDPRLTKD
LPARASSLTLAECLQCFVRAIFYVGKGTRARPDAHLWEAFGYHDQPRKQVCPKVRRILDIWASGRGIISLHCF
QHVVAMEAYTREACLLDALGLQTLTNQKQGHYYGVVAHWPPSRRRRLGVHLLQRALLVFLAEGERELRPQDIQ
ARG*
>ENSMUSP00000140285_c.200:T>TCATTCTCCCTCCACCCCAAACATGACGATCCACTCTAGCTCCAC
CCCCAAATTAACAATC_p.V67HSPSTPNMTIHSSSTPKLTI-ins
ENSMUST00000188685.7|Zfp709-
203|ENSMUSG00000056019|+|chr8:g.72642773:T>TCATTCTCCCTCCACCCCAAACATGACGAT
CCACTCTAGCTCCACCCCCAAATTAACAATC
MGSVSFQDVAVTFTVEEWALLDPSQKKLHKDVMEETFRNLAAIGKTQEGQNIDEDHRYSRRNQRNEVHSPSTP
NMTIHSSSTPKLTIVERLLEHSKGTESKEIFRQIPNTIVNRKSSHRTMLSENHVYKDNVLISQSSLTVSVPLQ
TPHKPHEYELHGENLYKFGECEKAFIYPECFLKHKDSGTRQRSYELNQCEEIFKSNDYIQIRVNDETREEICI
DKQHYAFNYPDFLQYVEKVHTGKKPYACKQCGKTFSFLSNIKRHERIHSGEKPYRCNICDKAFTCSTNFQEHE
RTHTGEKPYLCTQCGKSFSFLSNIRRHERTHTGEKPYRCNACGKAFSYLTNFQDHERTHTGEKPYVCTQCGKA
FTYYYSFQTHKRCHTGEKPYVCKQCGKAFSYYNSIQTHKRCHTGEKPYVCKLCAKAFTTLSSLRYHERIHSGE
KPHVCLQCGKGFNSSSTLRIHERTHTGEKPYKCKKCGKVFSVDSSLRCHERIHSGEKPYVCKQCGKAFTRHTS
LRCHERIHCGEKPYICKQCGKGFISSSNFRKHEWTHSGEKLCVCKLCGKAFTGHSSLQRHERIHSGEKPYVCK
HCGKDFSSLSGCQRHEQIHTGEKPYVCKNCGKAFTNPSALRNHERIHSGEKPYVCGQCGKGFISFSKFRIHER
IHTGEKPYKCKQCGKAFTGHSSLRRHERIHSGEKPFVCKHCGKSFYTLSDCQRHEQIHTGEKPFICKQCGKAF
TRSSSLKIHDKTHSRSP*
>ENSMUSP00000034259_c.197:T>TCATTCTCCCTCCACCCCAAACATGACGATCCACTCTAGCTCCAC
CCCCAAATTAACAATC_p.V66HSPSTPNMTIHSSSTPKLTI-ins
ENSMUST00000034259.8|Zfp709-
201|ENSMUSG00000056019|+|chr8:g.72642773:T>TCATTCTCCCTCCACCCCAAACATGACGAT
CCACTCTAGCTCCACCCCCAAATTAACAATC
GSVSFQDVAVTFTVEEWALLDPSQKKLHKDVMEETFRNLAAIGKTQEGQNIDEDHRYSRRNQRNEVHSPSTPN
MTIHSSSTPKLTIVERLLEHSKGTESKEIFRQIPNTIVNRKSSHRTMLSENHVYKDNVLISQSSLTVSVPLQT
PHKPHEYELHGENLYKFGECEKAFIYPECFLKHKDSGTRQRSYELNQCEEIFKSNDYIQIRVNDETREEICID
KQHYAFNYPDFLQYVEKVHTGKKPYACKQCGKTFSFLSNIKRHERIHSGEKPYRCNICDKAFTCSTNFQEHER
THTGEKPYLCTQCGKSFSFLSNIRRHERTHTGEKPYRCNACGKAFSYLTNFQDHERTHTGEKPYVCTQCGKAF
TYYYSFQTHKRCHTGEKPYVCKQCGKAFSYYNSIQTHKRCHTGEKPYVCKLCAKAFTTLSSLRYHERIHSGEK
PHVCLQCGKGFNSSSTLRIHERTHTGEKPYKCKKCGKVFSVDSSLRCHERIHSGEKPYVCKQCGKAFTRHTSL
RCHERIHCGEKPYICKQCGKGFISSSNFRKHEWTHSGEKLCVCKLCGKAFTGHSSLQRHERIHSGEKPYVCKH
CGKDFSSLSGCQRHEQIHTGEKPYVCKNCGKAFTNPSALRNHERIHSGEKPYVCGQCGKGFISFSKFRIHERI
HTGEKPYKCKQCGKAFTGHSSLRRHERIHSGEKPFVCKHCGKSFYTLSDCQRHEQIHTGEKPFICKQCGKAFT
RSSSLKIHDKTHSRSP*

>ENSMUSP00000140285_c.301:T>C_p.M101T ENSMUST00000188685.7|Zfp709-
203|ENSMUSG00000056019|+|chr8:g.72642874:T>C
MGSVSFQDVAVTFTVEEWALLDPSQKKLHKDVMEETFRNLAAIGKTQEGQNIDEDHRYSRRNQRNEVVERLLE
HSKGTESKEIFRQIPNTIVNRKSSHRTTLSENHVYKDNVLISQSSLTVSVPLQTPHKPHEYELHGENLYKFGE
CEKAFIYPECFLKHKDSGTRQRSYELNQCEEIFKSNDYIQIRVNDETREEICIDKQHYAFNYPDFLQYVEKVH
TGKKPYACKQCGKTFSFLSNIKRHERIHSGEKPYRCNICDKAFTCSTNFQEHERTHTGEKPYLCTQCGKSFSF
LSNIRRHERTHTGEKPYRCNACGKAFSYLTNFQDHERTHTGEKPYVCTQCGKAFTYYYSFQTHKRCHTGEKPY
VCKQCGKAFSYYNSIQTHKRCHTGEKPYVCKLCAKAFTTLSSLRYHERIHSGEKPHVCLQCGKGFNSSSTLRI
HERTHTGEKPYKCKKCGKVFSVDSSLRCHERIHSGEKPYVCKQCGKAFTRHTSLRCHERIHCGEKPYICKQCG
KGFISSSNFRKHEWTHSGEKLCVCKLCGKAFTGHSSLQRHERIHSGEKPYVCKHCGKDFSSLSGCQRHEQIHT
GEKPYVCKNCGKAFTNPSALRNHERIHSGEKPYVCGQCGKGFISFSKFRIHERIHTGEKPYKCKQCGKAFTGH
SSLRRHERIHSGEKPFVCKHCGKSFYTLSDCQRHEQIHTGEKPFICKQCGKAFTRSSSLKIHDKTHSRSP*
>ENSMUSP00000034259_c.298:T>C_p.M100T ENSMUST00000034259.8|Zfp709-
201|ENSMUSG00000056019|+|chr8:g.72642874:T>C
GSVSFQDVAVTFTVEEWALLDPSQKKLHKDVMEETFRNLAAIGKTQEGQNIDEDHRYSRRNQRNEVVERLLEH
SKGTESKEIFRQIPNTIVNRKSSHRTTLSENHVYKDNVLISQSSLTVSVPLQTPHKPHEYELHGENLYKFGEC
EKAFIYPECFLKHKDSGTRQRSYELNQCEEIFKSNDYIQIRVNDETREEICIDKQHYAFNYPDFLQYVEKVHT
GKKPYACKQCGKTFSFLSNIKRHERIHSGEKPYRCNICDKAFTCSTNFQEHERTHTGEKPYLCTQCGKSFSFL
SNIRRHERTHTGEKPYRCNACGKAFSYLTNFQDHERTHTGEKPYVCTQCGKAFTYYYSFQTHKRCHTGEKPYV
CKQCGKAFSYYNSIQTHKRCHTGEKPYVCKLCAKAFTTLSSLRYHERIHSGEKPHVCLQCGKGFNSSSTLRIH
ERTHTGEKPYKCKKCGKVFSVDSSLRCHERIHSGEKPYVCKQCGKAFTRHTSLRCHERIHCGEKPYICKQCGK
GFISSSNFRKHEWTHSGEKLCVCKLCGKAFTGHSSLQRHERIHSGEKPYVCKHCGKDFSSLSGCQRHEQIHTG
EKPYVCKNCGKAFTNPSALRNHERIHSGEKPYVCGQCGKGFISFSKFRIHERIHTGEKPYKCKQCGKAFTGHS
SLRRHERIHSGEKPFVCKHCGKSFYTLSDCQRHEQIHTGEKPFICKQCGKAFTRSSSLKIHDKTHSRSP*
>ENSMUSP00000140285_c.351:T>TTTCTAGCTCCACCTCACCATGATGATCCATTCTAGCTCCATACC
TCCAAGACAATCCATTCTAGCTCCACGCCCACCATGACGACCCATTCAGTCTCCACTTTCTAGCTCCACCCCA
CCATAACAATCCAATCTAGCTCACCCCAATCGAGACGCTCCATTCTAGCTCTACACCACCATAACAATTCAAT
CTACCTCCACCACAACCATGACAATCCA_p.S118ins-fs ENSMUST00000188685.7|Zfp709-
203|ENSMUSG00000056019|+|chr8:g.72642924:T>TTTCTAGCTCCACCTCACCATGATGATCCA
TTCTAGCTCCATACCTCCAAGACAATCCATTCTAGCTCCACGCCCACCATGACGACCCATTCAGTCTCCACTT
TCTAGCTCCACCCCACCATAACAATCCAATCTAGCTCACCCCAATCGAGACGCTCCATTCTAGCTCTACACCA
CCATAACAATTCAATCTACCTCCACCACAACCATGACAATCCA
MGSVSFQDVAVTFTVEEWALLDPSQKKLHKDVMEETFRNLAAIGKTQEGQNIDEDHRYSRRNQRNEVVERLLE
HSKGTESKEIFRQIPNTIVNRKSSHRTMLSENHVYKDNVLISQSFLAPPHHDDPF
>ENSMUSP00000034259_c.348:T>TTTCTAGCTCCACCTCACCATGATGATCCATTCTAGCTCCATACC
TCCAAGACAATCCATTCTAGCTCCACGCCCACCATGACGACCCATTCAGTCTCCACTTTCTAGCTCCACCCCA
CCATAACAATCCAATCTAGCTCACCCCAATCGAGACGCTCCATTCTAGCTCTACACCACCATAACAATTCAAT
CTACCTCCACCACAACCATGACAATCCA_p.S117ins-fs ENSMUST00000034259.8|Zfp709-
201|ENSMUSG00000056019|+|chr8:g.72642924:T>TTTCTAGCTCCACCTCACCATGATGATCCA
TTCTAGCTCCATACCTCCAAGACAATCCATTCTAGCTCCACGCCCACCATGACGACCCATTCAGTCTCCACTT
TCTAGCTCCACCCCACCATAACAATCCAATCTAGCTCACCCCAATCGAGACGCTCCATTCTAGCTCTACACCA
CCATAACAATTCAATCTACCTCCACCACAACCATGACAATCCA
GSVSFQDVAVTFTVEEWALLDPSQKKLHKDVMEETFRNLAAIGKTQEGQNIDEDHRYSRRNQRNEVVERLLEH
SKGTESKEIFRQIPNTIVNRKSSHRTMLSENHVYKDNVLISQSFLAPPHHDDPF
>ENSMUSP00000140285_c.458:T>A_p.Y153* ENSMUST00000188685.7|Zfp709-
203|ENSMUSG00000056019|+|chr8:g.72643031:T>A
MGSVSFQDVAVTFTVEEWALLDPSQKKLHKDVMEETFRNLAAIGKTQEGQNIDEDHRYSRRNQRNEVVERLLE
HSKGTESKEIFRQIPNTIVNRKSSHRTMLSENHVYKDNVLISQSSLTVSVPLQTPHKPHEYELHGENLYKFGE
CEKAFI*PECFLKHKDSGTRQRSYELNQCEEIFKSNDYIQIRVNDETREEICIDKQHYAFNYPDFLQYVEKVH
TGKKPYACKQCGKTFSFLSNIKRHERIHSGEKPYRCNICDKAFTCSTNFQEHERTHTGEKPYLCTQCGKSFSF
LSNIRRHERTHTGEKPYRCNACGKAFSYLTNFQDHERTHTGEKPYVCTQCGKAFTYYYSFQTHKRCHTGEKPY
VCKQCGKAFSYYNSIQTHKRCHTGEKPYVCKLCAKAFTTLSSLRYHERIHSGEKPHVCLQCGKGFNSSSTLRI
HERTHTGEKPYKCKKCGKVFSVDSSLRCHERIHSGEKPYVCKQCGKAFTRHTSLRCHERIHCGEKPYICKQCG
KGFISSSNFRKHEWTHSGEKLCVCKLCGKAFTGHSSLQRHERIHSGEKPYVCKHCGKDFSSLSGCQRHEQIHT
GEKPYVCKNCGKAFTNPSALRNHERIHSGEKPYVCGQCGKGFISFSKFRIHERIHTGEKPYKCKQCGKAFTGH
SSLRRHERIHSGEKPFVCKHCGKSFYTLSDCQRHEQIHTGEKPFICKQCGKAFTRSSSLKIHDKTHSRSP*
>ENSMUSP00000034259_c.455:T>A_p.Y152* ENSMUST00000034259.8|Zfp709-
201|ENSMUSG00000056019|+|chr8:g.72643031:T>A

GSVSFQDVAVTFTVEEWALLDPSQKKLHKDVMEETFRNLAAIGKTQEGQNIDEDHRYSRRNQRNEVVERLLEH
SKGTESKEIFRQIPNTIVNRKSSHRTMLSENHVYKDNVLISQSSLTVSVPLQTPHKPHEYELHGENLYKFGEC
EKAFI*PECFLKHKDSGTRQRSYELNQCEEIFKSNDYIQIRVNDETREEICIDKQHYAFNYPDFLQYVEKVHT
GKKPYACKQCGKTFSFLSNIKRHERIHSGEKPYRCNICDKAFTCSTNFQEHERTHTGEKPYLCTQCGKSFSFL
SNIRRHERTHTGEKPYRCNACGKAFSYLTNFQDHERTHTGEKPYVCTQCGKAFTYYYSFQTHKRCHTGEKPYV
CKQCGKAFSYYNSIQTHKRCHTGEKPYVCKLCAKAFTTLSSLRYHERIHSGEKPHVCLQCGKGFNSSSTLRIH
ERTHTGEKPYKCKKCGKVFSVDSSLRCHERIHSGEKPYVCKQCGKAFTRHTSLRCHERIHCGEKPYICKQCGK
GFISSSNFRKHEWTHSGEKLCVCKLCGKAFTGHSSLQRHERIHSGEKPYVCKHCGKDFSSLSGCQRHEQIHTG
EKPYVCKNCGKAFTNPSALRNHERIHSGEKPYVCGQCGKGFISFSKFRIHERIHTGEKPYKCKQCGKAFTGHS
SLRRHERIHSGEKPFVCKHCGKSFYTLSDCQRHEQIHTGEKPFICKQCGKAFTRSSSLKIHDKTHSRSP*
>ENSMUSP00000140285_c.524:T>G_p.C175W ENSMUST00000188685.7|Zfp709-
203|ENSMUSG00000056019|+|chr8:g.72643097:T>G
MGSVSFQDVAVTFTVEEWALLDPSQKKLHKDVMEETFRNLAAIGKTQEGQNIDEDHRYSRRNQRNEVVERLLE
HSKGTESKEIFRQIPNTIVNRKSSHRTMLSENHVYKDNVLISQSSLTVSVPLQTPHKPHEYELHGENLYKFGE
CEKAFIYPECFLKHKDSGTRQRSYELNQWEEIFKSNDYIQIRVNDETREEICIDKQHYAFNYPDFLQYVEKVH
TGKKPYACKQCGKTFSFLSNIKRHERIHSGEKPYRCNICDKAFTCSTNFQEHERTHTGEKPYLCTQCGKSFSF
LSNIRRHERTHTGEKPYRCNACGKAFSYLTNFQDHERTHTGEKPYVCTQCGKAFTYYYSFQTHKRCHTGEKPY
VCKQCGKAFSYYNSIQTHKRCHTGEKPYVCKLCAKAFTTLSSLRYHERIHSGEKPHVCLQCGKGFNSSSTLRI
HERTHTGEKPYKCKKCGKVFSVDSSLRCHERIHSGEKPYVCKQCGKAFTRHTSLRCHERIHCGEKPYICKQCG
KGFISSSNFRKHEWTHSGEKLCVCKLCGKAFTGHSSLQRHERIHSGEKPYVCKHCGKDFSSLSGCQRHEQIHT
GEKPYVCKNCGKAFTNPSALRNHERIHSGEKPYVCGQCGKGFISFSKFRIHERIHTGEKPYKCKQCGKAFTGH
SSLRRHERIHSGEKPFVCKHCGKSFYTLSDCQRHEQIHTGEKPFICKQCGKAFTRSSSLKIHDKTHSRSP*
>ENSMUSP00000034259_c.521:T>G_p.C174W ENSMUST00000034259.8|Zfp709-
201|ENSMUSG00000056019|+|chr8:g.72643097:T>G
GSVSFQDVAVTFTVEEWALLDPSQKKLHKDVMEETFRNLAAIGKTQEGQNIDEDHRYSRRNQRNEVVERLLEH
SKGTESKEIFRQIPNTIVNRKSSHRTMLSENHVYKDNVLISQSSLTVSVPLQTPHKPHEYELHGENLYKFGEC
EKAFIYPECFLKHKDSGTRQRSYELNQWEEIFKSNDYIQIRVNDETREEICIDKQHYAFNYPDFLQYVEKVHT
GKKPYACKQCGKTFSFLSNIKRHERIHSGEKPYRCNICDKAFTCSTNFQEHERTHTGEKPYLCTQCGKSFSFL
SNIRRHERTHTGEKPYRCNACGKAFSYLTNFQDHERTHTGEKPYVCTQCGKAFTYYYSFQTHKRCHTGEKPYV
CKQCGKAFSYYNSIQTHKRCHTGEKPYVCKLCAKAFTTLSSLRYHERIHSGEKPHVCLQCGKGFNSSSTLRIH
ERTHTGEKPYKCKKCGKVFSVDSSLRCHERIHSGEKPYVCKQCGKAFTRHTSLRCHERIHCGEKPYICKQCGK
GFISSSNFRKHEWTHSGEKLCVCKLCGKAFTGHSSLQRHERIHSGEKPYVCKHCGKDFSSLSGCQRHEQIHTG
EKPYVCKNCGKAFTNPSALRNHERIHSGEKPYVCGQCGKGFISFSKFRIHERIHTGEKPYKCKQCGKAFTGHS
SLRRHERIHSGEKPFVCKHCGKSFYTLSDCQRHEQIHTGEKPFICKQCGKAFTRSSSLKIHDKTHSRSP*
>ENSMUSP00000140285_c.529:A>ATTCTAGCTCCACTTCCACCATGACTATCCAATCGAGCTTCACCC
CCACCATAATGCTCCTTCTAGCACCAACCCACCATTAGGATCCATTCTATCACCATCCCCTCCAAGATGATCC
C_p.E177ins-fs ENSMUST00000188685.7|Zfp709-
203|ENSMUSG00000056019|+|chr8:g.72643102:A>ATTCTAGCTCCACTTCCACCATGACTATCC
AATCGAGCTTCACCCCCACCATAATGCTCCTTCTAGCACCAACCCACCATTAGGATCCATTCTATCACCATCC
CCTCCAAGATGATCCC
MGSVSFQDVAVTFTVEEWALLDPSQKKLHKDVMEETFRNLAAIGKTQEGQNIDEDHRYSRRNQRNEVVERLLE
HSKGTESKEIFRQIPNTIVNRKSSHRTMLSENHVYKDNVLISQSSLTVSVPLQTPHKPHEYELHGENLYKFGE
CEKAFIYPECFLKHKDSGTRQRSYELNQCEDSSSTSTMTIQSSFTPTIMLLLAPTHH
>ENSMUSP00000034259_c.526:A>ATTCTAGCTCCACTTCCACCATGACTATCCAATCGAGCTTCACCC
CCACCATAATGCTCCTTCTAGCACCAACCCACCATTAGGATCCATTCTATCACCATCCCCTCCAAGATGATCC
C_p.E176ins-fs ENSMUST00000034259.8|Zfp709-
201|ENSMUSG00000056019|+|chr8:g.72643102:A>ATTCTAGCTCCACTTCCACCATGACTATCC
AATCGAGCTTCACCCCCACCATAATGCTCCTTCTAGCACCAACCCACCATTAGGATCCATTCTATCACCATCC
CCTCCAAGATGATCCC
GSVSFQDVAVTFTVEEWALLDPSQKKLHKDVMEETFRNLAAIGKTQEGQNIDEDHRYSRRNQRNEVVERLLEH
SKGTESKEIFRQIPNTIVNRKSSHRTMLSENHVYKDNVLISQSSLTVSVPLQTPHKPHEYELHGENLYKFGEC
EKAFIYPECFLKHKDSGTRQRSYELNQCEDSSSTSTMTIQSSFTPTIMLLLAPTHH
>ENSMUSP00000140285_c.536:C>G_p.F179L ENSMUST00000188685.7|Zfp709-
203|ENSMUSG00000056019|+|chr8:g.72643109:C>G
MGSVSFQDVAVTFTVEEWALLDPSQKKLHKDVMEETFRNLAAIGKTQEGQNIDEDHRYSRRNQRNEVVERLLE
HSKGTESKEIFRQIPNTIVNRKSSHRTMLSENHVYKDNVLISQSSLTVSVPLQTPHKPHEYELHGENLYKFGE
CEKAFIYPECFLKHKDSGTRQRSYELNQCEEILKSNDYIQIRVNDETREEICIDKQHYAFNYPDFLQYVEKVH
TGKKPYACKQCGKTFSFLSNIKRHERIHSGEKPYRCNICDKAFTCSTNFQEHERTHTGEKPYLCTQCGKSFSF

LSNIRRHERTHTGEKPYRCNACGKAFSYLTNFQDHERTHTGEKPYVCTQCGKAFTYYYSFQTHKRCHTGEKPY
VCKQCGKAFSYYNSIQTHKRCHTGEKPYVCKLCAKAFTTLSSLRYHERIHSGEKPHVCLQCGKGFNSSSTLRI
HERTHTGEKPYKCKKCGKVFSVDSSLRCHERIHSGEKPYVCKQCGKAFTRHTSLRCHERIHCGEKPYICKQCG
KGFISSSNFRKHEWTHSGEKLCVCKLCGKAFTGHSSLQRHERIHSGEKPYVCKHCGKDFSSLSGCQRHEQIHT
GEKPYVCKNCGKAFTNPSALRNHERIHSGEKPYVCGQCGKGFISFSKFRIHERIHTGEKPYKCKQCGKAFTGH
SSLRRHERIHSGEKPFVCKHCGKSFYTLSDCQRHEQIHTGEKPFICKQCGKAFTRSSSLKIHDKTHSRSP*
>ENSMUSP00000034259_c.533:C>G_p.F178L ENSMUST00000034259.8|Zfp709-
201|ENSMUSG00000056019|+|chr8:g.72643109:C>G
GSVSFQDVAVTFTVEEWALLDPSQKKLHKDVMEETFRNLAAIGKTQEGQNIDEDHRYSRRNQRNEVVERLLEH
SKGTESKEIFRQIPNTIVNRKSSHRTMLSENHVYKDNVLISQSSLTVSVPLQTPHKPHEYELHGENLYKFGEC
EKAFIYPECFLKHKDSGTRQRSYELNQCEEILKSNDYIQIRVNDETREEICIDKQHYAFNYPDFLQYVEKVHT
GKKPYACKQCGKTFSFLSNIKRHERIHSGEKPYRCNICDKAFTCSTNFQEHERTHTGEKPYLCTQCGKSFSFL
SNIRRHERTHTGEKPYRCNACGKAFSYLTNFQDHERTHTGEKPYVCTQCGKAFTYYYSFQTHKRCHTGEKPYV
CKQCGKAFSYYNSIQTHKRCHTGEKPYVCKLCAKAFTTLSSLRYHERIHSGEKPHVCLQCGKGFNSSSTLRIH
ERTHTGEKPYKCKKCGKVFSVDSSLRCHERIHSGEKPYVCKQCGKAFTRHTSLRCHERIHCGEKPYICKQCGK
GFISSSNFRKHEWTHSGEKLCVCKLCGKAFTGHSSLQRHERIHSGEKPYVCKHCGKDFSSLSGCQRHEQIHTG
EKPYVCKNCGKAFTNPSALRNHERIHSGEKPYVCGQCGKGFISFSKFRIHERIHTGEKPYKCKQCGKAFTGHS
SLRRHERIHSGEKPFVCKHCGKSFYTLSDCQRHEQIHTGEKPFICKQCGKAFTRSSSLKIHDKTHSRSP*
>ENSMUSP00000140285_c.554:T>G_p.I185M ENSMUST00000188685.7|Zfp709-
203|ENSMUSG00000056019|+|chr8:g.72643127:T>G
MGSVSFQDVAVTFTVEEWALLDPSQKKLHKDVMEETFRNLAAIGKTQEGQNIDEDHRYSRRNQRNEVVERLLE
HSKGTESKEIFRQIPNTIVNRKSSHRTMLSENHVYKDNVLISQSSLTVSVPLQTPHKPHEYELHGENLYKFGE
CEKAFIYPECFLKHKDSGTRQRSYELNQCEEIFKSNDYMQIRVNDETREEICIDKQHYAFNYPDFLQYVEKVH
TGKKPYACKQCGKTFSFLSNIKRHERIHSGEKPYRCNICDKAFTCSTNFQEHERTHTGEKPYLCTQCGKSFSF
LSNIRRHERTHTGEKPYRCNACGKAFSYLTNFQDHERTHTGEKPYVCTQCGKAFTYYYSFQTHKRCHTGEKPY
VCKQCGKAFSYYNSIQTHKRCHTGEKPYVCKLCAKAFTTLSSLRYHERIHSGEKPHVCLQCGKGFNSSSTLRI
HERTHTGEKPYKCKKCGKVFSVDSSLRCHERIHSGEKPYVCKQCGKAFTRHTSLRCHERIHCGEKPYICKQCG
KGFISSSNFRKHEWTHSGEKLCVCKLCGKAFTGHSSLQRHERIHSGEKPYVCKHCGKDFSSLSGCQRHEQIHT
GEKPYVCKNCGKAFTNPSALRNHERIHSGEKPYVCGQCGKGFISFSKFRIHERIHTGEKPYKCKQCGKAFTGH
SSLRRHERIHSGEKPFVCKHCGKSFYTLSDCQRHEQIHTGEKPFICKQCGKAFTRSSSLKIHDKTHSRSP*
>ENSMUSP00000034259_c.551:T>G_p.I184M ENSMUST00000034259.8|Zfp709-
201|ENSMUSG00000056019|+|chr8:g.72643127:T>G
GSVSFQDVAVTFTVEEWALLDPSQKKLHKDVMEETFRNLAAIGKTQEGQNIDEDHRYSRRNQRNEVVERLLEH
SKGTESKEIFRQIPNTIVNRKSSHRTMLSENHVYKDNVLISQSSLTVSVPLQTPHKPHEYELHGENLYKFGEC
EKAFIYPECFLKHKDSGTRQRSYELNQCEEIFKSNDYMQIRVNDETREEICIDKQHYAFNYPDFLQYVEKVHT
GKKPYACKQCGKTFSFLSNIKRHERIHSGEKPYRCNICDKAFTCSTNFQEHERTHTGEKPYLCTQCGKSFSFL
SNIRRHERTHTGEKPYRCNACGKAFSYLTNFQDHERTHTGEKPYVCTQCGKAFTYYYSFQTHKRCHTGEKPYV
CKQCGKAFSYYNSIQTHKRCHTGEKPYVCKLCAKAFTTLSSLRYHERIHSGEKPHVCLQCGKGFNSSSTLRIH
ERTHTGEKPYKCKKCGKVFSVDSSLRCHERIHSGEKPYVCKQCGKAFTRHTSLRCHERIHCGEKPYICKQCGK
GFISSSNFRKHEWTHSGEKLCVCKLCGKAFTGHSSLQRHERIHSGEKPYVCKHCGKDFSSLSGCQRHEQIHTG
EKPYVCKNCGKAFTNPSALRNHERIHSGEKPYVCGQCGKGFISFSKFRIHERIHTGEKPYKCKQCGKAFTGHS
SLRRHERIHSGEKPFVCKHCGKSFYTLSDCQRHEQIHTGEKPFICKQCGKAFTRSSSLKIHDKTHSRSP*
>ENSMUSP00000140285_c.587:A>C_p.E196D ENSMUST00000188685.7|Zfp709-
203|ENSMUSG00000056019|+|chr8:g.72643160:A>C
MGSVSFQDVAVTFTVEEWALLDPSQKKLHKDVMEETFRNLAAIGKTQEGQNIDEDHRYSRRNQRNEVVERLLE
HSKGTESKEIFRQIPNTIVNRKSSHRTMLSENHVYKDNVLISQSSLTVSVPLQTPHKPHEYELHGENLYKFGE
CEKAFIYPECFLKHKDSGTRQRSYELNQCEEIFKSNDYIQIRVNDETREDICIDKQHYAFNYPDFLQYVEKVH
TGKKPYACKQCGKTFSFLSNIKRHERIHSGEKPYRCNICDKAFTCSTNFQEHERTHTGEKPYLCTQCGKSFSF
LSNIRRHERTHTGEKPYRCNACGKAFSYLTNFQDHERTHTGEKPYVCTQCGKAFTYYYSFQTHKRCHTGEKPY
VCKQCGKAFSYYNSIQTHKRCHTGEKPYVCKLCAKAFTTLSSLRYHERIHSGEKPHVCLQCGKGFNSSSTLRI
HERTHTGEKPYKCKKCGKVFSVDSSLRCHERIHSGEKPYVCKQCGKAFTRHTSLRCHERIHCGEKPYICKQCG
KGFISSSNFRKHEWTHSGEKLCVCKLCGKAFTGHSSLQRHERIHSGEKPYVCKHCGKDFSSLSGCQRHEQIHT
GEKPYVCKNCGKAFTNPSALRNHERIHSGEKPYVCGQCGKGFISFSKFRIHERIHTGEKPYKCKQCGKAFTGH
SSLRRHERIHSGEKPFVCKHCGKSFYTLSDCQRHEQIHTGEKPFICKQCGKAFTRSSSLKIHDKTHSRSP*
>ENSMUSP00000034259_c.584:A>C_p.E195D ENSMUST00000034259.8|Zfp709-
201|ENSMUSG00000056019|+|chr8:g.72643160:A>C
GSVSFQDVAVTFTVEEWALLDPSQKKLHKDVMEETFRNLAAIGKTQEGQNIDEDHRYSRRNQRNEVVERLLEH
SKGTESKEIFRQIPNTIVNRKSSHRTMLSENHVYKDNVLISQSSLTVSVPLQTPHKPHEYELHGENLYKFGEC

EKAFIYPECFLKHKDSGTRQRSYELNQCEEIFKSNDYIQIRVNDETREDICIDKQHYAFNYPDFLQYVEKVHT
GKKPYACKQCGKTFSFLSNIKRHERIHSGEKPYRCNICDKAFTCSTNFQEHERTHTGEKPYLCTQCGKSFSFL
SNIRRHERTHTGEKPYRCNACGKAFSYLTNFQDHERTHTGEKPYVCTQCGKAFTYYYSFQTHKRCHTGEKPYV
CKQCGKAFSYYNSIQTHKRCHTGEKPYVCKLCAKAFTTLSSLRYHERIHSGEKPHVCLQCGKGFNSSSTLRIH
ERTHTGEKPYKCKKCGKVFSVDSSLRCHERIHSGEKPYVCKQCGKAFTRHTSLRCHERIHCGEKPYICKQCGK
GFISSSNFRKHEWTHSGEKLCVCKLCGKAFTGHSSLQRHERIHSGEKPYVCKHCGKDFSSLSGCQRHEQIHTG
EKPYVCKNCGKAFTNPSALRNHERIHSGEKPYVCGQCGKGFISFSKFRIHERIHTGEKPYKCKQCGKAFTGHS
SLRRHERIHSGEKPFVCKHCGKSFYTLSDCQRHEQIHTGEKPFICKQCGKAFTRSSSLKIHDKTHSRSP*
>rs218700520_ENSMUSP00000140285_c.639:T>A_p.Y214N
ENSMUST00000188685.7|Zfp709-203|ENSMUSG00000056019|+|chr8:g.72643212:T>A
MGSVSFQDVAVTFTVEEWALLDPSQKKLHKDVMEETFRNLAAIGKTQEGQNIDEDHRYSRRNQRNEVVERLLE
HSKGTESKEIFRQIPNTIVNRKSSHRTMLSENHVYKDNVLISQSSLTVSVPLQTPHKPHEYELHGENLYKFGE
CEKAFIYPECFLKHKDSGTRQRSYELNQCEEIFKSNDYIQIRVNDETREEICIDKQHYAFNYPDFLQNVEKVH
TGKKPYACKQCGKTFSFLSNIKRHERIHSGEKPYRCNICDKAFTCSTNFQEHERTHTGEKPYLCTQCGKSFSF
LSNIRRHERTHTGEKPYRCNACGKAFSYLTNFQDHERTHTGEKPYVCTQCGKAFTYYYSFQTHKRCHTGEKPY
VCKQCGKAFSYYNSIQTHKRCHTGEKPYVCKLCAKAFTTLSSLRYHERIHSGEKPHVCLQCGKGFNSSSTLRI
HERTHTGEKPYKCKKCGKVFSVDSSLRCHERIHSGEKPYVCKQCGKAFTRHTSLRCHERIHCGEKPYICKQCG
KGFISSSNFRKHEWTHSGEKLCVCKLCGKAFTGHSSLQRHERIHSGEKPYVCKHCGKDFSSLSGCQRHEQIHT
GEKPYVCKNCGKAFTNPSALRNHERIHSGEKPYVCGQCGKGFISFSKFRIHERIHTGEKPYKCKQCGKAFTGH
SSLRRHERIHSGEKPFVCKHCGKSFYTLSDCQRHEQIHTGEKPFICKQCGKAFTRSSSLKIHDKTHSRSP*
>rs218700520_ENSMUSP00000034259_c.636:T>A_p.Y213N
ENSMUST00000034259.8|Zfp709-201|ENSMUSG00000056019|+|chr8:g.72643212:T>A
GSVSFQDVAVTFTVEEWALLDPSQKKLHKDVMEETFRNLAAIGKTQEGQNIDEDHRYSRRNQRNEVVERLLEH
SKGTESKEIFRQIPNTIVNRKSSHRTMLSENHVYKDNVLISQSSLTVSVPLQTPHKPHEYELHGENLYKFGEC
EKAFIYPECFLKHKDSGTRQRSYELNQCEEIFKSNDYIQIRVNDETREEICIDKQHYAFNYPDFLQNVEKVHT
GKKPYACKQCGKTFSFLSNIKRHERIHSGEKPYRCNICDKAFTCSTNFQEHERTHTGEKPYLCTQCGKSFSFL
SNIRRHERTHTGEKPYRCNACGKAFSYLTNFQDHERTHTGEKPYVCTQCGKAFTYYYSFQTHKRCHTGEKPYV
CKQCGKAFSYYNSIQTHKRCHTGEKPYVCKLCAKAFTTLSSLRYHERIHSGEKPHVCLQCGKGFNSSSTLRIH
ERTHTGEKPYKCKKCGKVFSVDSSLRCHERIHSGEKPYVCKQCGKAFTRHTSLRCHERIHCGEKPYICKQCGK
GFISSSNFRKHEWTHSGEKLCVCKLCGKAFTGHSSLQRHERIHSGEKPYVCKHCGKDFSSLSGCQRHEQIHTG
EKPYVCKNCGKAFTNPSALRNHERIHSGEKPYVCGQCGKGFISFSKFRIHERIHTGEKPYKCKQCGKAFTGHS
SLRRHERIHSGEKPFVCKHCGKSFYTLSDCQRHEQIHTGEKPFICKQCGKAFTRSSSLKIHDKTHSRSP*
>ENSMUSP00000064823_c.583:T>G_p.F195C ENSMUST00000067912.8|Klf2-
201|ENSMUSG00000055148|+|chr8:g.73073781:T>G
MALSEPILPSFATFASPCERGLQERWPRNEPEAGGTDEDLNNVLDFILSMGLDGLGAENPPEPPPQPPPPAFY
YPEPGAPPPYSIPAASLGTELLRPDLDPPQGPALHGRFLLAPPGRLVKAEPPEVDGGGYGCAPGLAHGPRGLK
LEGAPGATGACMRGPAGRPPPPPDTPPLSPDGPLRIPASGPRNPFPPPCGPGPSFGGPGPALHYGPPAPGAFG
LFEDAAAAAAALGLAPPATRGLLTPPSSPLELLEAKPKRGRRSWPRKRAATHTCSYTNCGKTYTKSSHLKAHL
RTHTGEKPYHCNWEGCGWKFARSDELTRHYRKHTGHRPFQCHLCDRAFSRSDHLALHMKRHM*
>rs47424793_ENSMUSP00000105539_c.1158:G>A_p.G387S
ENSMUST00000109913.9|Nr3c2-204|ENSMUSG00000031618|+|chr8:g.77636059:G>A
METKGYHSLPEGLDMERRWSQVSQTLERSSLGPAERTNENSYMEIVNVSCVSGATPNNSTQGSSKEKHELLPC
LQQDNSRSGILPSDIKTELESKELSATVAESMGLYMDSVRDAEYTYDQQNQQGSLSPAKIYQNMEQLVKFYKE
NGHRSSTLSAISRPLRSFMPDSGTSMNGGALRAIVKSPIICHEKSPSVCSPLNMPSSVCSPAGINSMSSSTAS
FGSFPVHSPITQGTSLTCSPSVENRGSRSHSPVHASNVGSPLSSPLSSMKSPISSPPSHCSVKSPVSSPNNVP
LRSSVSSPANLNNSRCSVSSPSNTNNRSTLSSPTASTVGSIGSPISNAFSYTTSGASAGAGAIQDMVPSPDTH
EKGAHDVPFPKTEEVEKAISNSVTGQLNIVQYIKPEPDGAFSSSCLGGNNKINPSSPFSVPIKQESSKHSCSG
ASFKGNPTVNPFPFMDGSYFSFMDDKDYYSLSGILGPPVPGFDSSCEGSAFPGGIKQEPDDGSYFPETSIPSS
AIIGVNSGGQSFHYRIGAQGTISLSRSPRDQSFQHLSSFPPVNALVESWKPHGDLSSRRSDGYPVLEYIPENV
SSSTLRSVSTGSSRPSKICLVCGDEASGCHYGVVTCGSCKVFFKRAVEGQHNYLCAGRNDCIIDKIRRKNCPA
CRLQKCLQAGMNLGARKSKKLGKLKGLHEEQPQQPPPPPPQSPEEGTTYIAPTKEPSVNSALVPQLASITRAL
TPSPSMILENIEPEIVYAGYDNSKPDTAESLLSTLNRLAGKQMIQVVKWAKVLPGFKNLPLEDQITLIQYSWM
CLSSFALSWRSYKHTNSQFLYFAPDLVFNEEKMHQSAMYELCQGMRQISLQFVRLQLTFEEYSIMKVLLLLST
VPKDGLKSQAAFEEMRTNYIKELRKMVTKCPNSSGQSWQRFYQLTKLLDSMHDLVNDLLEFCFYTFRESQALK
VEFPAMLVEIISDQLPKVESGNAKPLYFHRK*
>rs47424793_ENSMUSP00000118222_c.1158:G>A_p.G387S
ENSMUST00000148106.8|Nr3c2-208|ENSMUSG00000031618|+|chr8:g.77636059:G>A

METKGYHSLPEGLDMERRWSQVSQTLERSSLGPAERTNENSYMEIVNVSCVSGATPNNSTQGSSKEKHELLPC
LQQDNSRSGILPSDIKTELESKELSATVAESMGLYMDSVRDAEYTYDQQNQQGSLSPAKIYQNMEQLVKFYKE
NGHRSSTLSAISRPLRSFMPDSGTSMNGGALRAIVKSPIICHEKSPSVCSPLNMPSSVCSPAGINSMSSSTAS
FGSFPVHSPITQGTSLTCSPSVENRGSRSHSPVHASNVGSPLSSPLSSMKSPISSPPSHCSVKSPVSSPNNVP
LRSSVSSPANLNNSRCSVSSPSNTNNRSTLSSPTASTVGSIGSPISNAFSYTTSGASAGAGAIQDMVPSPDTH
EKGAHDVPFPKTEEVEKAISNSVTGQLNIVQYIKPEPDGAFSSSCLGGNNKINPSSPFSVPIKQESSKHSCSG
ASFKGNPTVNPFPFMDGSYFSFMDDKDYYSLSGILGPPVPGFDSSCEGSAFPGGIKQEPDDGSYFPETSIPSS
AIIGVNSGGQSFHYRIGAQGTISLSRSPRDQSFQHLSSFPPVNALVESWKPHGDLSSRRSDGYPVLEYIPENV
SSSTLRSVSTGSSRPSKICLVCGDEASGCHYGVVTCGSCKVFFKRAVEGQHNYLCAGRNDCIIDKIRRKNCPA
CRLQKCLQAGMNLGERRCISLPCMSCARGCGRSAFSSCGFS*
>rs47424793_ENSMUSP00000105538_c.1158:G>A_p.G387S
ENSMUST00000109912.8|Nr3c2-203|ENSMUSG00000031618|+|chr8:g.77636059:G>A
METKGYHSLPEGLDMERRWSQVSQTLERSSLGPAERTNENSYMEIVNVSCVSGATPNNSTQGSSKEKHELLPC
LQQDNSRSGILPSDIKTELESKELSATVAESMGLYMDSVRDAEYTYDQQNQQGSLSPAKIYQNMEQLVKFYKE
NGHRSSTLSAISRPLRSFMPDSGTSMNGGALRAIVKSPIICHEKSPSVCSPLNMPSSVCSPAGINSMSSSTAS
FGSFPVHSPITQGTSLTCSPSVENRGSRSHSPVHASNVGSPLSSPLSSMKSPISSPPSHCSVKSPVSSPNNVP
LRSSVSSPANLNNSRCSVSSPSNTNNRSTLSSPTASTVGSIGSPISNAFSYTTSGASAGAGAIQDMVPSPDTH
EKGAHDVPFPKTEEVEKAISNSVTGQLNIVQYIKPEPDGAFSSSCLGGNNKINPSSPFSVPIKQESSKHSCSG
ASFKGNPTVNPFPFMDGSYFSFMDDKDYYSLSGILGPPVPGFDSSCEGSAFPGGIKQEPDDGSYFPETSIPSS
AIIGVNSGGQSFHYRIGAQGTISLSRSPRDQSFQHLSSFPPVNALVESWKPHGDLSSRRSDGYPVLEYIPENV
SSSTLRSVSTGSSRPSKICLVCGDEASGCHYGVVTCGSCKVFFKRAVEGQHNYLCAGRNDCIIDKIRRKNCPA
CRLQKCLQAGMNLGARKSKKLGKLKGLHEEQPQQPPPPPPQSPEEGTTYIAPTKEPSVNSALVPQLASITRAL
TPSPSMILENIEPEIVYAGYDNSKPDTAESLLSTLNRLAGKQMIQVVKWAKVLPGFKNLPLEDQITLIQYSWM
CLSSFALSWRSYKHTNSQFLYFAPDLVFNEEKMHQSAMYELCQGMRQISLQFVRLQLTFEEYSIMKVLLLLST
VPKDGLKSQAAFEEMRTNYIKELRKMVTKCPNSSGQSWQRFYQLTKLLDSMHDLVNDLLEFCFYTFRESQALK
VEFPAMLVEIISDQLPKVESGNAKPLYFHRK*
>rs47424793_ENSMUSP00000105537_c.1158:G>A_p.G387S
ENSMUST00000109911.8|Nr3c2-202|ENSMUSG00000031618|+|chr8:g.77636059:G>A
METKGYHSLPEGLDMERRWSQVSQTLERSSLGPAERTNENSYMEIVNVSCVSGATPNNSTQGSSKEKHELLPC
LQQDNSRSGILPSDIKTELESKELSATVAESMGLYMDSVRDAEYTYDQQNQQGSLSPAKIYQNMEQLVKFYKE
NGHRSSTLSAISRPLRSFMPDSGTSMNGGALRAIVKSPIICHEKSPSVCSPLNMPSSVCSPAGINSMSSSTAS
FGSFPVHSPITQGTSLTCSPSVENRGSRSHSPVHASNVGSPLSSPLSSMKSPISSPPSHCSVKSPVSSPNNVP
LRSSVSSPANLNNSRCSVSSPSNTNNRSTLSSPTASTVGSIGSPISNAFSYTTSGASAGAGAIQDMVPSPDTH
EKGAHDVPFPKTEEVEKAISNSVTGQLNIVQYIKPEPDGAFSSSCLGGNNKINPSSPFSVPIKQESSKHSCSG
ASFKGNPTVNPFPFMDGSYFSFMDDKDYYSLSGILGPPVPGFDSSCEGSAFPGGIKQEPDDGSYFPETSIPSS
AIIGVNSGGQSFHYRIGAQGTISLSRSPRDQSFQHLSSFPPVNALVESWKPHGDLSSRRSDGYPVLEYIPENV
SSSTLRSVSTGSSRPSKICLVCGDEASGCHYGVVTCGSCKVFFKRAVEGQHNYLCAGRNDCIIDKIRRKNCPA
CRLQKCLQAGMNLGGFKNLPLEDQITLIQYSWMCLSSFALSWRSYKHTNSQFLYFAPDLVFNEEKMHQSAMYE
LCQGMRQISLQFVRLQLTFEEYSIMKVLLLLSTVPKDGLKSQAAFEEMRTNYIKELRKMVTKCPNSSGQSWQR
FYQLTKLLDSMHDLVNDLLEFCFYTFRESQALKVEFPAMLVEIISDQLPKVESGNAKPLYFHRK*
>rs47424793_ENSMUSP00000034031_c.1158:G>A_p.G387S
ENSMUST00000034031.6|Nr3c2-201|ENSMUSG00000031618|+|chr8:g.77636059:G>A
METKGYHSLPEGLDMERRWSQVSQTLERSSLGPAERTNENSYMEIVNVSCVSGATPNNSTQGSSKEKHELLPC
LQQDNSRSGILPSDIKTELESKELSATVAESMGLYMDSVRDAEYTYDQQNQQGSLSPAKIYQNMEQLVKFYKE
NGHRSSTLSAISRPLRSFMPDSGTSMNGGALRAIVKSPIICHEKSPSVCSPLNMPSSVCSPAGINSMSSSTAS
FGSFPVHSPITQGTSLTCSPSVENRGSRSHSPVHASNVGSPLSSPLSSMKSPISSPPSHCSVKSPVSSPNNVP
LRSSVSSPANLNNSRCSVSSPSNTNNRSTLSSPTASTVGSIGSPISNAFSYTTSGASAGAGAIQDMVPSPDTH
EKGAHDVPFPKTEEVEKAISNSVTGQLNIVQYIKPEPDGAFSSSCLGGNNKINPSSPFSVPIKQESSKHSCSG
ASFKGNPTVNPFPFMDGSYFSFMDDKDYYSLSGILGPPVPGFDSSCEGSAFPGGIKQEPDDGSYFPETSIPSS
AIIGVNSGGQSFHYRIGAQGTISLSRSPRDQSFQHLSSFPPVNALVESWKPHGDLSSRRSDGYPVLEYIPENV
SSSTLRSVSTGSSRPSKICLVCGDEASGCHYGVVTCGSCKVFFKRAVEGKCSRQHNYLCAGRNDCIIDKIRRK
NCPACRLQKCLQAGMNLGARKSKKLGKLKGLHEEQPQQPPPPPPQSPEEGTTYIAPTKEPSVNSALVPQLASI
TRALTPSPSMILENIEPEIVYAGYDNSKPDTAESLLSTLNRLAGKQMIQVVKWAKVLPGFKNLPLEDQITLIQ
YSWMCLSSFALSWRSYKHTNSQFLYFAPDLVFNEEKMHQSAMYELCQGMRQISLQFVRLQLTFEEYSIMKVLL
LLSTVPKDGLKSQAAFEEMRTNYIKELRKMVTKCPNSSGQSWQRFYQLTKLLDSMHDLVNDLLEFCFYTFRES
QALKVEFPAMLVEIISDQLPKVESGNAKPLYFHRK*
>rs387004511_ENSMUSP00000105539_c.2820:A>G_p.T941A
ENSMUST00000109913.9|Nr3c2-204|ENSMUSG00000031618|+|chr8:g.77969037:A>G

METKGYHSLPEGLDMERRWSQVSQTLERSSLGPAERTNENSYMEIVNVSCVSGATPNNSTQGSSKEKHELLPC
LQQDNSRSGILPSDIKTELESKELSATVAESMGLYMDSVRDAEYTYDQQNQQGSLSPAKIYQNMEQLVKFYKE
NGHRSSTLSAISRPLRSFMPDSGTSMNGGALRAIVKSPIICHEKSPSVCSPLNMPSSVCSPAGINSMSSSTAS
FGSFPVHSPITQGTSLTCSPSVENRGSRSHSPVHASNVGSPLSSPLSSMKSPISSPPSHCSVKSPVSSPNNVP
LRSSVSSPANLNNSRCSVSSPSNTNNRSTLSSPTASTVGSIGSPISNAFSYTTSGASAGAGAIQDMVPSPDTH
EKGAHDVPFPKTEEVEKAISNGVTGQLNIVQYIKPEPDGAFSSSCLGGNNKINPSSPFSVPIKQESSKHSCSG
ASFKGNPTVNPFPFMDGSYFSFMDDKDYYSLSGILGPPVPGFDSSCEGSAFPGGIKQEPDDGSYFPETSIPSS
AIIGVNSGGQSFHYRIGAQGTISLSRSPRDQSFQHLSSFPPVNALVESWKPHGDLSSRRSDGYPVLEYIPENV
SSSTLRSVSTGSSRPSKICLVCGDEASGCHYGVVTCGSCKVFFKRAVEGQHNYLCAGRNDCIIDKIRRKNCPA
CRLQKCLQAGMNLGARKSKKLGKLKGLHEEQPQQPPPPPPQSPEEGTTYIAPTKEPSVNSALVPQLASITRAL
TPSPSMILENIEPEIVYAGYDNSKPDTAESLLSTLNRLAGKQMIQVVKWAKVLPGFKNLPLEDQITLIQYSWM
CLSSFALSWRSYKHTNSQFLYFAPDLVFNEEKMHQSAMYELCQGMRQISLQFVRLQLTFEEYSIMKVLLLLST
VPKDGLKSQAAFEEMRTNYIKELRKMVTKCPNSSGQSWQRFYQLTKLLDSMHDLVNDLLEFCFYAFRESQALK
VEFPAMLVEIISDQLPKVESGNAKPLYFHRK*
>rs387004511_ENSMUSP00000105538_c.2820:A>G_p.T941A
ENSMUST00000109912.8|Nr3c2-203|ENSMUSG00000031618|+|chr8:g.77969037:A>G
METKGYHSLPEGLDMERRWSQVSQTLERSSLGPAERTNENSYMEIVNVSCVSGATPNNSTQGSSKEKHELLPC
LQQDNSRSGILPSDIKTELESKELSATVAESMGLYMDSVRDAEYTYDQQNQQGSLSPAKIYQNMEQLVKFYKE
NGHRSSTLSAISRPLRSFMPDSGTSMNGGALRAIVKSPIICHEKSPSVCSPLNMPSSVCSPAGINSMSSSTAS
FGSFPVHSPITQGTSLTCSPSVENRGSRSHSPVHASNVGSPLSSPLSSMKSPISSPPSHCSVKSPVSSPNNVP
LRSSVSSPANLNNSRCSVSSPSNTNNRSTLSSPTASTVGSIGSPISNAFSYTTSGASAGAGAIQDMVPSPDTH
EKGAHDVPFPKTEEVEKAISNGVTGQLNIVQYIKPEPDGAFSSSCLGGNNKINPSSPFSVPIKQESSKHSCSG
ASFKGNPTVNPFPFMDGSYFSFMDDKDYYSLSGILGPPVPGFDSSCEGSAFPGGIKQEPDDGSYFPETSIPSS
AIIGVNSGGQSFHYRIGAQGTISLSRSPRDQSFQHLSSFPPVNALVESWKPHGDLSSRRSDGYPVLEYIPENV
SSSTLRSVSTGSSRPSKICLVCGDEASGCHYGVVTCGSCKVFFKRAVEGQHNYLCAGRNDCIIDKIRRKNCPA
CRLQKCLQAGMNLGARKSKKLGKLKGLHEEQPQQPPPPPPQSPEEGTTYIAPTKEPSVNSALVPQLASITRAL
TPSPSMILENIEPEIVYAGYDNSKPDTAESLLSTLNRLAGKQMIQVVKWAKVLPGFKNLPLEDQITLIQYSWM
CLSSFALSWRSYKHTNSQFLYFAPDLVFNEEKMHQSAMYELCQGMRQISLQFVRLQLTFEEYSIMKVLLLLST
VPKDGLKSQAAFEEMRTNYIKELRKMVTKCPNSSGQSWQRFYQLTKLLDSMHDLVNDLLEFCFYAFRESQALK
VEFPAMLVEIISDQLPKVESGNAKPLYFHRK*
>rs387004511_ENSMUSP00000105537_c.2481:A>G_p.T828A
ENSMUST00000109911.8|Nr3c2-202|ENSMUSG00000031618|+|chr8:g.77969037:A>G
METKGYHSLPEGLDMERRWSQVSQTLERSSLGPAERTNENSYMEIVNVSCVSGATPNNSTQGSSKEKHELLPC
LQQDNSRSGILPSDIKTELESKELSATVAESMGLYMDSVRDAEYTYDQQNQQGSLSPAKIYQNMEQLVKFYKE
NGHRSSTLSAISRPLRSFMPDSGTSMNGGALRAIVKSPIICHEKSPSVCSPLNMPSSVCSPAGINSMSSSTAS
FGSFPVHSPITQGTSLTCSPSVENRGSRSHSPVHASNVGSPLSSPLSSMKSPISSPPSHCSVKSPVSSPNNVP
LRSSVSSPANLNNSRCSVSSPSNTNNRSTLSSPTASTVGSIGSPISNAFSYTTSGASAGAGAIQDMVPSPDTH
EKGAHDVPFPKTEEVEKAISNGVTGQLNIVQYIKPEPDGAFSSSCLGGNNKINPSSPFSVPIKQESSKHSCSG
ASFKGNPTVNPFPFMDGSYFSFMDDKDYYSLSGILGPPVPGFDSSCEGSAFPGGIKQEPDDGSYFPETSIPSS
AIIGVNSGGQSFHYRIGAQGTISLSRSPRDQSFQHLSSFPPVNALVESWKPHGDLSSRRSDGYPVLEYIPENV
SSSTLRSVSTGSSRPSKICLVCGDEASGCHYGVVTCGSCKVFFKRAVEGQHNYLCAGRNDCIIDKIRRKNCPA
CRLQKCLQAGMNLGGFKNLPLEDQITLIQYSWMCLSSFALSWRSYKHTNSQFLYFAPDLVFNEEKMHQSAMYE
LCQGMRQISLQFVRLQLTFEEYSIMKVLLLLSTVPKDGLKSQAAFEEMRTNYIKELRKMVTKCPNSSGQSWQR
FYQLTKLLDSMHDLVNDLLEFCFYAFRESQALKVEFPAMLVEIISDQLPKVESGNAKPLYFHRK*
>rs387004511_ENSMUSP00000034031_c.2832:A>G_p.T945A
ENSMUST00000034031.6|Nr3c2-201|ENSMUSG00000031618|+|chr8:g.77969037:A>G
METKGYHSLPEGLDMERRWSQVSQTLERSSLGPAERTNENSYMEIVNVSCVSGATPNNSTQGSSKEKHELLPC
LQQDNSRSGILPSDIKTELESKELSATVAESMGLYMDSVRDAEYTYDQQNQQGSLSPAKIYQNMEQLVKFYKE
NGHRSSTLSAISRPLRSFMPDSGTSMNGGALRAIVKSPIICHEKSPSVCSPLNMPSSVCSPAGINSMSSSTAS
FGSFPVHSPITQGTSLTCSPSVENRGSRSHSPVHASNVGSPLSSPLSSMKSPISSPPSHCSVKSPVSSPNNVP
LRSSVSSPANLNNSRCSVSSPSNTNNRSTLSSPTASTVGSIGSPISNAFSYTTSGASAGAGAIQDMVPSPDTH
EKGAHDVPFPKTEEVEKAISNGVTGQLNIVQYIKPEPDGAFSSSCLGGNNKINPSSPFSVPIKQESSKHSCSG
ASFKGNPTVNPFPFMDGSYFSFMDDKDYYSLSGILGPPVPGFDSSCEGSAFPGGIKQEPDDGSYFPETSIPSS
AIIGVNSGGQSFHYRIGAQGTISLSRSPRDQSFQHLSSFPPVNALVESWKPHGDLSSRRSDGYPVLEYIPENV
SSSTLRSVSTGSSRPSKICLVCGDEASGCHYGVVTCGSCKVFFKRAVEGKCSRQHNYLCAGRNDCIIDKIRRK
NCPACRLQKCLQAGMNLGARKSKKLGKLKGLHEEQPQQPPPPPPQSPEEGTTYIAPTKEPSVNSALVPQLASI
TRALTPSPSMILENIEPEIVYAGYDNSKPDTAESLLSTLNRLAGKQMIQVVKWAKVLPGFKNLPLEDQITLIQ
YSWMCLSSFALSWRSYKHTNSQFLYFAPDLVFNEEKMHQSAMYELCQGMRQISLQFVRLQLTFEEYSIMKVLL

LLSTVPKDGLKSQAAFEEMRTNYIKELRKMVTKCPNSSGQSWQRFYQLTKLLDSMHDLVNDLLEFCFYAFRES
QALKVEFPAMLVEIISDQLPKVESGNAKPLYFHRK*
>rs47888503_ENSMUSP00000075658_c.1918:C>G_p.S640C
ENSMUST00000076316.6|Arhgap10-201|ENSMUSG00000037148|-
|chr8:g.78003885:G>C
MGLQPLEFSDCYLDSPWFRERIRAHEAELERTNKFIKELIKDGKNLISATKSLSAAQRKFAHSLRDFKFEFIG
DAETDDERCIDASLREFSNFLKNLEEQREIMALSVTETLIKPLEKFRKEQLGAVKEEKKKFDKETEKNYSLID
KHLTLSARKKDSHLQEADLQVEQNRQHFYELSLEYVCKLQEIQERKKFEFVEPMLSFFQGMFTFYHQGHELSK
DFNHYKMELQINIQNTRNRFEGTRSEVEELMNKIRQNPKDQKRASQFTAEGYLYVQEKRPAPFGSSWVKHYCM
YRKTAKKFNMIPFEHRSGGKLGDGEAFFLKECTKRHMDSTDRRFCFDIEAADRPGVPLTVQAFSEEERKQWLE
ALGGKEALFHTFNRAIVPRPEGGAQLDKMGFTILRKCISAVETRGINDQGLYRVVGVSSKVQRLLSMLMDVKM
CNELDLENSADWEVKTVTSALKQYLRSLPEPLMTYELHRDFIVPAKSGSPESRVNAIHFLVHKLPEKNKEMLD
ILVKHLTNVSSHSKQNLMTVANLGVVFGPTLMRPQEETVAAIMDLKFQNIVVEILIENHEKIFRTSPDTTFAE
PTCLSASPPNAPPRQSKRQGQRTKRPVAVYNLCLELEEGDSPSPLKEDPPSSSQDCLSTPSPTTSAAHGPPGL
DGNHLAADGGSCGDATATTPSQTRPSMVQWLNMQSPTTPSSNPAGTPPSPRMSPFPLSPAASIVDKLPECVIN
RKARAVYPCEAEHSSELSFEIGAIFEDVQTSREPGWLEGTLNGKRGLIPQNYVKLL*
>rs47888503_ENSMUSP00000147485_c.1918:C>G_p.S640C
ENSMUST00000210922.2|Arhgap10-203|ENSMUSG00000037148|-
|chr8:g.78003885:G>C
MGLQPLEFSDCYLDSPWFRERIRAHEAELERTNKFIKELIKDGKNLISATKSLSAAQRKFAHSLRDFKFEFIG
DAETDDERCIDASLREFSNFLKNLEEQREIMALSVTETLIKPLEKFRKEQLGAVKEEKKKFDKETEKNYSLID
KHLTLSARKKDSHLQEADLQVEQNRQHFYELSLEYVCKLQEIQERKKFEFVEPMLSFFQGMFTFYHQGHELSK
DFNHYKMELQINIQNTRNRFEGTRSEVEELMNKIRQNPKDQKRASQFTAEGYLYVQEKRPAPFGSSWVKHYCM
YRKTAKKFNMIPFEHRSGGKLGDGEAFFLKECTKRHMDSTDRRFCFDIEAADRPGVPLTVQAFSEEERKQWLE
ALGGKEALFHTFNRAIVPRPEGGAQLDKMGFTILRKCISAVETRGINDQGLYRVVGVSSKVQRLLSMLMDVKM
CNELDLENSADWEVKTVTSALKQYLRSLPEPLMTYELHRDFIVPAKSGSPESRVNAIHFLVHKLPEKNKEMLD
ILVKHLTNVSSHSKQNLMTVANLGVVFGPTLMRPQEETVAAIMDLKFQNIVVEILIENHEKIFRTSPDTTFAE
PTCLSASPPNAPPRQSKRQGQRTKRPVAVYNLCLELEEGDSPSPLKEDPPSSSQDCLSTPSPTTSAAHGPPGL
DGNHLAADGGSCGDATATTVINRKARAVYPCEAEHSSELSFEIGAIFEDVQTSREPGWLEGTLNGKRGLIPQN
YVKLL*
>rs47888503_ENSMUSP00000147493_c.1852:C>G_p.S618C
ENSMUST00000210519.2|Arhgap10-202|ENSMUSG00000037148|-
|chr8:g.78003885:G>C
MGLQPLEFSDCYLDSPWFRERIRAHEAELERTNKFIKELIKDGKNLISATKSLSAAQRKFAHSLRDFKFEFIG
DAETDDERCIDASLREFSNFLKNLEEQREIMALSVTETLIKPLEKFRKEQLGAVKEEKKKFDKETEKNYSLID
KHLTLSARKKDSHLQEADLQVEQNRQHFYELSLEYVCKLQEIQERKKFEFVEPMLSFFQGMFTFYHQGHELSK
DFNHYKMELQINIQNTRNRFEGTRSEVEELMNKIRQNPKDQKRASQFTAEGYLYVQEKRPAPFGSSWVKHYCM
YRKTAKKFNMIPFEHRSGGKLGDGEAFFLKECTKRHMDSTDRRFCFDIEAADRPGVPLTVQAFSEEERKQWLE
ALGGKEALFHTFNRAIVPRPEGGINDQGLYRVVGVSSKVQRLLSMLMDVKMCNELDLENSADWEVKTVTSALK
QYLRSLPEPLMTYELHRDFIVPAKSGSPESRVNAIHFLVHKLPEKNKEMLDILVKHLTNVSSHSKQNLMTVAN
LGVVFGPTLMRPQEETVAAIMDLKFQNIVVEILIENHEKIFRTSPDTTFAEPTCLSASPPNAPPRQSKRQGQR
TKRPVAVYNLCLELEEGDSPSPLKEDPPSSSQDCLSTPSPTTSAAHGPPGLDGNHLAADGGSCGDATATTPSQ
TRPSMVQWLNMQSPTTPSSNPAGTPPSPRMSPFPLSPAASIVDKLPECVINRKARAVYPCEAEHSSELSFEIG
AIFEDVQTSREPGWLEGTLNGKRGLIPQNYVKLL*
>rs50544875_ENSMUSP00000075658_c.1783:C>T_p.A595V
ENSMUST00000076316.6|Arhgap10-201|ENSMUSG00000037148|-
|chr8:g.78037443:G>A
MGLQPLEFSDCYLDSPWFRERIRAHEAELERTNKFIKELIKDGKNLISATKSLSAAQRKFAHSLRDFKFEFIG
DAETDDERCIDASLREFSNFLKNLEEQREIMALSVTETLIKPLEKFRKEQLGAVKEEKKKFDKETEKNYSLID
KHLTLSARKKDSHLQEADLQVEQNRQHFYELSLEYVCKLQEIQERKKFEFVEPMLSFFQGMFTFYHQGHELSK
DFNHYKMELQINIQNTRNRFEGTRSEVEELMNKIRQNPKDQKRASQFTAEGYLYVQEKRPAPFGSSWVKHYCM
YRKTAKKFNMIPFEHRSGGKLGDGEAFFLKECTKRHMDSTDRRFCFDIEAADRPGVPLTVQAFSEEERKQWLE
ALGGKEALFHTFNRAIVPRPEGGAQLDKMGFTILRKCISAVETRGINDQGLYRVVGVSSKVQRLLSMLMDVKM
CNELDLENSADWEVKTVTSALKQYLRSLPEPLMTYELHRDFIVPAKSGSPESRVNAIHFLVHKLPEKNKEMLD
ILVKHLTNVSSHSKQNLMTVANLGVVFGPTLMRPQEETVAAIMDLKFQNIVVEILIENHEKIFRTSPDTTFAE
PTCLSASPPNVPPRQSKRQGQRTKRPVAVYNLCLELEEGDSPSPLKEDPPSSSQDSLSTPSPTTSAAHGPPGL
DGNHLAADGGSCGDATATTPSQTRPSMVQWLNMQSPTTPSSNPAGTPPSPRMSPFPLSPAASIVDKLPECVIN
RKARAVYPCEAEHSSELSFEIGAIFEDVQTSREPGWLEGTLNGKRGLIPQNYVKLL*

```
>rs50544875_ENSMUSP00000147485_c.1783:C>T_p.A595V
ENSMUST00000210922.2|Arhgap10-203|ENSMUSG00000037148|-
|chr8:g.78037443:G>A
MGLQPLEFSDCYLDSPWFRERIRAHEAELERTNKFIKELIKDGKNLISATKSLSAAQRKFAHSLRDFKFEFIG
DAETDDERCIDASLREFSNFLKNLEEQREIMALSVTETLIKPLEKFRKEQLGAVKEEKKKFDKETEKNYSLID
KHLTLSARKKDSHLQEADLQVEQNRQHFYELSLEYVCKLQEIQERKKFEFVEPMLSFFQGMFTFYHQGHELSK
DFNHYKMELQINIQNTRNRFEGTRSEVEELMNKIRQNPKDQKRASQFTAEGYLYVQEKRPAPFGSSWVKHYCM
YRKTAKKFNMIPFEHRSGGKLGDGEAFFLKECTKRHMDSTDRRFCFDIEAADRPGVPLTVQAFSEEERKQWLE
ALGGKEALFHTFNRAIVPRPEGGAQLDKMGFTILRKCISAVETRGINDQGLYRVVGVSSKVQRLLSMLMDVKM
CNELDLENSADWEVKTVTSALKQYLRSLPEPLMTYELHRDFIVPAKSGSPESRVNAIHFLVHKLPEKNKEMLD
ILVKHLTNVSSHSKQNLMTVANLGVVFGPTLMRPQEETVAAIMDLKFQNIVVEILIENHEKIFRTSPDTTFAE
PTCLSASPPNVPPRQSKRQGQRTKRPVAVYNLCLELEEGDSPSPLKEDPPSSSQDSLSTPSPTTSAAHGPPGL
DGNHLAADGGSCGDATATTVINRKARAVYPCEAEHSSELSFEIGAIFEDVQTSREPGWLEGTLNGKRGLIPQN
YVKLL*
>rs50544875_ENSMUSP00000147493_c.1717:C>T_p.A573V
ENSMUST00000210519.2|Arhgap10-202|ENSMUSG00000037148|-
|chr8:g.78037443:G>A
MGLQPLEFSDCYLDSPWFRERIRAHEAELERTNKFIKELIKDGKNLISATKSLSAAQRKFAHSLRDFKFEFIG
DAETDDERCIDASLREFSNFLKNLEEQREIMALSVTETLIKPLEKFRKEQLGAVKEEKKKFDKETEKNYSLID
KHLTLSARKKDSHLQEADLQVEQNRQHFYELSLEYVCKLQEIQERKKFEFVEPMLSFFQGMFTFYHQGHELSK
DFNHYKMELQINIQNTRNRFEGTRSEVEELMNKIRQNPKDQKRASQFTAEGYLYVQEKRPAPFGSSWVKHYCM
YRKTAKKFNMIPFEHRSGGKLGDGEAFFLKECTKRHMDSTDRRFCFDIEAADRPGVPLTVQAFSEEERKQWLE
ALGGKEALFHTFNRAIVPRPEGGINDQGLYRVVGVSSKVQRLLSMLMDVKMCNELDLENSADWEVKTVTSALK
QYLRSLPEPLMTYELHRDFIVPAKSGSPESRVNAIHFLVHKLPEKNKEMLDILVKHLTNVSSHSKQNLMTVAN
LGVVFGPTLMRPQEETVAAIMDLKFQNIVVEILIENHEKIFRTSPDTTFAEPTCLSASPPNVPPRQSKRQGQR
TKRPVAVYNLCLELEEGDSPSPLKEDPPSSSQDSLSTPSPTTSAAHGPPGLDGNHLAADGGSCGDATATTPSQ
TRPSMVQWLNMQSPTTPSSNPAGTPPSPRMSPFPLSPAASIVDKLPECVINRKARAVYPCEAEHSSELSFEIG
AIFEDVQTSREPGWLEGTLNGKRGLIPQNYVKLL*
>rs230726632_ENSMUSP00000075658_c.1189:C>T_p.T397I
ENSMUST00000076316.6|Arhgap10-201|ENSMUSG00000037148|-
|chr8:g.78109392:G>A
MGLQPLEFSDCYLDSPWFRERIRAHEAELERTNKFIKELIKDGKNLISATKSLSAAQRKFAHSLRDFKFEFIG
DAETDDERCIDASLREFSNFLKNLEEQREIMALSVTETLIKPLEKFRKEQLGAVKEEKKKFDKETEKNYSLID
KHLTLSARKKDSHLQEADLQVEQNRQHFYELSLEYVCKLQEIQERKKFEFVEPMLSFFQGMFTFYHQGHELSK
DFNHYKMELQINIQNTRNRFEGTRSEVEELMNKIRQNPKDQKRASQFTAEGYLYVQEKRPAPFGSSWVKHYCM
YRKTAKKFNMIPFEHRSGGKLGDGEAFFLKECTKRHMDSTDRRFCFDIEAADRPGVPLTVQAFSEEERKQWLE
ALGGKEALFHTFNRAIVPRPEGGAQLDKMGFIILRKCISAVETRGINDQGLYRVVGVSSKVQRLLSMLMDVKM
CNELDLENSADWEVKTVTSALKQYLRSLPEPLMTYELHRDFIVPAKSGSPESRVNAIHFLVHKLPEKNKEMLD
ILVKHLTNVSSHSKQNLMTVANLGVVFGPTLMRPQEETVAAIMDLKFQNIVVEILIENHEKIFRTSPDTTFAE
PTCLSASPPNAPPRQSKRQGQRTKRPVAVYNLCLELEEGDSPSPLKEDPPSSSQDSLSTPSPTTSAAHGPPGL
DGNHLAADGGSCGDATATTPSQTRPSMVQWLNMQSPTTPSSNPAGTPPSPRMSPFPLSPAASIVDKLPECVIN
RKARAVYPCEAEHSSELSFEIGAIFEDVQTSREPGWLEGTLNGKRGLIPQNYVKLL*
>rs230726632_ENSMUSP00000147485_c.1189:C>T_p.T397I
ENSMUST00000210922.2|Arhgap10-203|ENSMUSG00000037148|-
|chr8:g.78109392:G>A
MGLQPLEFSDCYLDSPWFRERIRAHEAELERTNKFIKELIKDGKNLISATKSLSAAQRKFAHSLRDFKFEFIG
DAETDDERCIDASLREFSNFLKNLEEQREIMALSVTETLIKPLEKFRKEQLGAVKEEKKKFDKETEKNYSLID
KHLTLSARKKDSHLQEADLQVEQNRQHFYELSLEYVCKLQEIQERKKFEFVEPMLSFFQGMFTFYHQGHELSK
DFNHYKMELQINIQNTRNRFEGTRSEVEELMNKIRQNPKDQKRASQFTAEGYLYVQEKRPAPFGSSWVKHYCM
YRKTAKKFNMIPFEHRSGGKLGDGEAFFLKECTKRHMDSTDRRFCFDIEAADRPGVPLTVQAFSEEERKQWLE
ALGGKEALFHTFNRAIVPRPEGGAQLDKMGFIILRKCISAVETRGINDQGLYRVVGVSSKVQRLLSMLMDVKM
CNELDLENSADWEVKTVTSALKQYLRSLPEPLMTYELHRDFIVPAKSGSPESRVNAIHFLVHKLPEKNKEMLD
ILVKHLTNVSSHSKQNLMTVANLGVVFGPTLMRPQEETVAAIMDLKFQNIVVEILIENHEKIFRTSPDTTFAE
PTCLSASPPNAPPRQSKRQGQRTKRPVAVYNLCLELEEGDSPSPLKEDPPSSSQDSLSTPSPTTSAAHGPPGL
DGNHLAADGGSCGDATATTVINRKARAVYPCEAEHSSELSFEIGAIFEDVQTSREPGWLEGTLNGKRGLIPQN
YVKLL*
>rs51928797_ENSMUSP00000112692_c.369:T>C_p.F124L
ENSMUST00000118622.2|Prmt9-202|ENSMUSG00000037134|+|chr8:g.78285539:T>C
```

MPNSRPRPRRGAGGGAGAAGRDRLVARSLQSAEHCLGDQDFGTAYAHYLLVLSLAPELKDDVKETFQYTLFKW
AEELHALSRIQDLLGCYEQALELFPDDEVICNSMGEHLFRMGFRDEAAGYLHKAVKLNPDFNDAKENFYRVAN
WLVERWHFIMLNDTRRNMVYNAAIQKAVCLGSRTVLDIGTGTGILSMFAKKAGAQSVYACELSKTMYELACDV
VAANKMENGIKLLHMKSLDIEIPKHIPERLSLVVTETVDAGVFGEGIVESLIHAWEHLLLQPKTKEENGNCGK
YGKVIPAGAVIFGMAVECAEIRRHHRVGAKDIAGIHLPTNVKFQSPAYTSVDTEETVEPYTTEKMSGIPGGYL
PLTECFQIMKVDFNNLQELKSLATKKPHSLNVPAIKEGVLDAIMVWFVLQLDDEYSLSTSPSEETCWEQAVYP
VQALEDYCIQPGDRVTMEASCHDCYLRIQGISILHLEHEMEVMKGFTKSKDLLSLGNEAELCSALANLQTSRP
EALEQTCMLEPTEIALLNNIPYHEGFKTAMRKVLSSLAPELLWQPMDTHCQYMEMNSGSGQSDAAPSTADPFY
VLDVSEGFSLLPILAGTLGHVKPYSSVEKDQHCIALDLIAEANHFPKETLEFWLRHIEDEAAVLQRPKSDKLW
SIIILDVIEPSGLIQQELMEKAAISRCLLQSGGKIFPQYVLMFGMLVESQTLVEESAVQGTEHTLGLNIAPFI
NQFQVPIRVCLDLSSLPCVPLSQPVELLRLDLMTPYLNTSNKEVKVRVCRSGRVTAVPFWFHLCLDDEVRLDT
SGEASHWKQAAVVLDNPIQVQAGEELVLSVEHHKSNVSIAVKP*
>rs51928797_ENSMUSP00000148193_c.369:T>C_p.F124L
ENSMUST00000210040.2|Prmt9-205|ENSMUSG00000037134|+|chr8:g.78285539:T>C
MPNSRPRPRRGAGGGAGAAGRDRLVARSLQSAEHCLGDQDFGTAYAHYLLVLSLAPELKDDVKETFQYTLFKW
AEELHALSRIQDLLGCYEQALELFPDDEVICNSMGEHLFRMGFRDEAAGYLHKAVKLNPDFNDAKENFYRVAN
WLVERWHFIMLNDTRRNMVYNAAIQKAVCLGSRTVLDIGTGTGILSMFAKKAGAQSVYACELSKTMYELACDV
VAANKMENGIKLLHMKSLDIEIPKHIPERCIWRRNCGEFNSCMGTFTSATKDQRRKWKLREVWESDTSRCCYI
WDGSRVCRDTKAS*
>rs51928797_ENSMUSP00000050181_c.369:T>C_p.F124L
ENSMUST00000056237.15|Prmt9-201|ENSMUSG00000037134|+|chr8:g.78285539:T>C
MPNSRPRPRRGAGGGAGAAGRDRLVARSLQSAEHCLGDQDFGTAYAHYLLVLSLAPELKDDVKETFQYTLFKW
AEELHALSRIQDLLGCYEQALELFPDDEVICNSMGEHLFRMGFRDEAAGYLHKAVKLNPDFNDAKENFYRVAN
WLVERWHFIMLNDTRRNMVYNAAIQKAVCLGSRTVLDIGTGTGILSMFAKKAGAQSVYACELSKTMYELACDV
VAANKMENGIKLLHMKSLDIEIPKHIPERVSLVVTETVDAGVFGEGIVESLIHAWEHLLLQPKTKEENGNCGK
YGKVIPAGAVIFGMAVECAEIRRHHRVGAKDIAGIHLPTNVKFQSPAYTSVDTEETVEPYTTEKMSGIPGGYL
PLTECFQIMKVDFNNLQELKSLATKKPHSLNVPAIKEGVLDAIMVWFVLQLDDEYSLSTSPSEETCWEQAVYP
VQALEDYCIQPGDRVTMEASCHDCYLRIQGISILHLEHEMEVMKGFTKSKDLLSLGNEAELCSALANLQTSRP
EALEQTCMLEPTEIALLNNIPYHEGFKTAMRKVLSSLAPELLWQPMDTHCQYMEMNSGSGQSDAAPSTADPFY
VLDVSEGFSLLPILAGTLGHVKPYSSVEKDQHCIALDLIAEANHFPKETLEFWLRHIEDEAAVLQRPKSDKLW
SIIILDVIEPSGLIQQELMEKAAISRCLLQSGGKIFPQYVLMFGMLVESQTLVEESAVQGTEHTLGLNIAPFI
NQFQVPIRVCLDLSSLPCVPLSQPVELLRLDLMTPYLNTSNKEVKVRVCRSGRVTAVPFWFHLCLDDEVRLDT
SGEASHWKQAAVVLDNPIQVQAGEELVLSVEHHKSNVSIAVKP*
>rs254367289_ENSMUSP00000112692_c.1410:A>C_p.I471L
ENSMUST00000118622.2|Prmt9-202|ENSMUSG00000037134|+|chr8:g.78298718:A>C
MPNSRPRPRRGAGGGAGAAGRDRLVARSLQSAEHCLGDQDFGTAYAHYLLVLSLAPELKDDVKETFQYTLFKW
AEELHALSRIQDLLGCYEQALELFPDDEVICNSMGEHLFRMGFRDEAAGYFHKAVKLNPDFNDAKENFYRVAN
WLVERWHFIMLNDTRRNMVYNAAIQKAVCLGSRTVLDIGTGTGILSMFAKKAGAQSVYACELSKTMYELACDV
VAANKMENGIKLLHMKSLDIEIPKHIPERLSLVVTETVDAGVFGEGIVESLIHAWEHLLLQPKTKEENGNCGK
YGKVIPAGAVIFGMAVECAEIRRHHRVGAKDIAGIHLPTNVKFQSPAYTSVDTEETVEPYTTEKMSGIPGGYL
PLTECFQIMKVDFNNLQELKSLATKKPHSLNVPAIKEGVLDAIMVWFVLQLDDEYSLSTSPSEETCWEQAVYP
VQALEDYCIQPGDRVTMEASCHDCYLRIQGISLLHLEHEMEVMKGFTKSKDLLSLGNEAELCSALANLQTSRP
EALEQTCMLEPTEIALLNNIPYHEGFKTAMRKVLSSLAPELLWQPMDTHCQYMEMNSGSGQSDAAPSTADPFY
VLDVSEGFSLLPILAGTLGHVKPYSSVEKDQHCIALDLIAEANHFPKETLEFWLRHIEDEAAVLQRPKSDKLW
SIIILDVIEPSGLIQQELMEKAAISRCLLQSGGKIFPQYVLMFGMLVESQTLVEESAVQGTEHTLGLNIAPFI
NQFQVPIRVCLDLSSLPCVPLSQPVELLRLDLMTPYLNTSNKEVKVRVCRSGRVTAVPFWFHLCLDDEVRLDT
SGEASHWKQAAVVLDNPIQVQAGEELVLSVEHHKSNVSIAVKP*
>rs254367289_ENSMUSP00000050181_c.1410:A>C_p.I471L
ENSMUST00000056237.15|Prmt9-201|ENSMUSG00000037134|+|chr8:g.78298718:A>C
MPNSRPRPRRGAGGGAGAAGRDRLVARSLQSAEHCLGDQDFGTAYAHYLLVLSLAPELKDDVKETFQYTLFKW
AEELHALSRIQDLLGCYEQALELFPDDEVICNSMGEHLFRMGFRDEAAGYFHKAVKLNPDFNDAKENFYRVAN
WLVERWHFIMLNDTRRNMVYNAAIQKAVCLGSRTVLDIGTGTGILSMFAKKAGAQSVYACELSKTMYELACDV
VAANKMENGIKLLHMKSLDIEIPKHIPERVSLVVTETVDAGVFGEGIVESLIHAWEHLLLQPKTKEENGNCGK
YGKVIPAGAVIFGMAVECAEIRRHHRVGAKDIAGIHLPTNVKFQSPAYTSVDTEETVEPYTTEKMSGIPGGYL
PLTECFQIMKVDFNNLQELKSLATKKPHSLNVPAIKEGVLDAIMVWFVLQLDDEYSLSTSPSEETCWEQAVYP
VQALEDYCIQPGDRVTMEASCHDCYLRIQGISLLHLEHEMEVMKGFTKSKDLLSLGNEAELCSALANLQTSRP
EALEQTCMLEPTEIALLNNIPYHEGFKTAMRKVLSSLAPELLWQPMDTHCQYMEMNSGSGQSDAAPSTADPFY
VLDVSEGFSLLPILAGTLGHVKPYSSVEKDQHCIALDLIAEANHFPKETLEFWLRHIEDEAAVLQRPKSDKLW

SIIILDVIEPSGLIQQELMEKAAISRCLLQSGGKIFPQYVLMFGMLVESQTLVEESAVQGTEHTLGLNIAPFI
NQFQVPIRVCLDLSSLPCVPLSQPVELLRLDLMTPYLNTSNKEVKVRVCRSGRVTAVPFWFHLCLDDEVRLDT
SGEASHWKQAAVVLDNPIQVQAGEELVLSVEHHKSNVSIAVKP*
>rs32616028_ENSMUSP00000112692_c.2380:G>T_p.C794F
ENSMUST00000118622.2|Prmt9-202|ENSMUSG00000037134|+|chr8:g.78307718:G>T
MPNSRPRPRRGAGGGAGAAGRDRLVARSLQSAEHCLGDQDFGTAYAHYLLVLSLAPELKDDVKETFQYTLFKW
AEELHALSRIQDLLGCYEQALELFPDDEVICNSMGEHLFRMGFRDEAAGYFHKAVKLNPDFNDAKENFYRVAN
WLVERWHFIMLNDTRRNMVYNAAIQKAVCLGSRTVLDIGTGTGILSMFAKKAGAQSVYACELSKTMYELACDV
VAANKMENGIKLLHMKSLDIEIPKHIPERLSLVVTETVDAGVFGEGIVESLIHAWEHLLLQPKTKEENGNCGK
YGKVIPAGAVIFGMAVECAEIRRHHRVGAKDIAGIHLPTNVKFQSPAYTSVDTEETVEPYTTEKMSGIPGGYL
PLTECFQIMKVDFNNLQELKSLATKKPHSLNVPAIKEGVLDAIMVWFVLQLDDEYSLSTSPSEETCWEQAVYP
VQALEDYCIQPGDRVTMEASCHDCYLRIQGISILHLEHEMEVMKGFTKSKDLLSLGNEAELCSALANLQTSRP
EALEQTCMLEPTEIALLNNIPYHEGFKTAMRKVLSSLAPELLWQPMDTHCQYMEMNSGSGQSDAAPSTADPFY
VLDVSEGFSLLPILAGTLGHVKPYSSVEKDQHCIALDLIAEANHFPKETLEFWLRHIEDEAAVLQRPKSDKLW
SIIILDVIEPSGLIQQELMEKAAISRCLLQSGGKIFPQYVLMFGMLVESQTLVEESAVQGTEHTLGLNIAPFI
NQFQVPIRVCLDLSSLPCVPLSQPVELLRLDLMTPYLNTSNKEVKVRVCRSGRVTAVPFWFHLFLDDEVRLDT
SGEASHWKQAAVVLDNPIQVQAGEELVLSVEHHKSNVSIAVKP*
>rs32616028_ENSMUSP00000050181_c.2380:G>T_p.C794F
ENSMUST00000056237.15|Prmt9-201|ENSMUSG00000037134|+|chr8:g.78307718:G>T
MPNSRPRPRRGAGGGAGAAGRDRLVARSLQSAEHCLGDQDFGTAYAHYLLVLSLAPELKDDVKETFQYTLFKW
AEELHALSRIQDLLGCYEQALELFPDDEVICNSMGEHLFRMGFRDEAAGYFHKAVKLNPDFNDAKENFYRVAN
WLVERWHFIMLNDTRRNMVYNAAIQKAVCLGSRTVLDIGTGTGILSMFAKKAGAQSVYACELSKTMYELACDV
VAANKMENGIKLLHMKSLDIEIPKHIPERVSLVVTETVDAGVFGEGIVESLIHAWEHLLLQPKTKEENGNCGK
YGKVIPAGAVIFGMAVECAEIRRHHRVGAKDIAGIHLPTNVKFQSPAYTSVDTEETVEPYTTEKMSGIPGGYL
PLTECFQIMKVDFNNLQELKSLATKKPHSLNVPAIKEGVLDAIMVWFVLQLDDEYSLSTSPSEETCWEQAVYP
VQALEDYCIQPGDRVTMEASCHDCYLRIQGISILHLEHEMEVMKGFTKSKDLLSLGNEAELCSALANLQTSRP
EALEQTCMLEPTEIALLNNIPYHEGFKTAMRKVLSSLAPELLWQPMDTHCQYMEMNSGSGQSDAAPSTADPFY
VLDVSEGFSLLPILAGTLGHVKPYSSVEKDQHCIALDLIAEANHFPKETLEFWLRHIEDEAAVLQRPKSDKLW
SIIILDVIEPSGLIQQELMEKAAISRCLLQSGGKIFPQYVLMFGMLVESQTLVEESAVQGTEHTLGLNIAPFI
NQFQVPIRVCLDLSSLPCVPLSQPVELLRLDLMTPYLNTSNKEVKVRVCRSGRVTAVPFWFHLFLDDEVRLDT
SGEASHWKQAAVVLDNPIQVQAGEELVLSVEHHKSNVSIAVKP*
>rs47583370_ENSMUSP00000034030_c.1239:A>G_p.S414G
ENSMUST00000034030.15|Tmem184c-201|ENSMUSG00000031617|-
|chr8:g.78323621:T>C
MPCACNRSNWRRWIRPLLVLFYATTILVAVPICIWKFQKMKVGMHTKSWFIAGIFLLLTIPVSLWGILQHLVH
YTQPELQKPIIRILWMVPIYSVDSWVALVYPKIAIYVDTWRECYEAYVIYNFMIFLTNYLTIRFPNLILHLEA
KDQQNHILPLCCCPPWAMGEMLLFRCKLGVLQYTVVRPITTVTALVCEILDVYDEGNFGFSNAWTYLVILNNL
SQLFAMYCLLLFYKVLKEELSPIQPVGKFLCVKLVVFVSFWQAVLIALLVKLGVISEKRTWEWQSAEAVATGL
QDFIICIEMFFAAIAHHYTFSYKPYVHEAEEGSCFDSFLAMWDVSDIRDDISEQVRRVGRTMRGYPKKKCFPG
DPDHNEHSSLLSSSSQDLTSGSSKVPSPGGLYQGFGHTISSQSPISIAGIYEEIMNDIPEEQQKLLNPGQDVT
INIPEEQQKLNTGKDVMLDRQTITSPRPISIASIYEKIMIDIPEEQQKLTDTGQDVMINRQTITPPRPISITS
IYEEVLIDITEKQQKLNDTGKNVMINIPEEQQKLIDKRKDVMIDIPEQNAIPDNSQYQDQEQIVTLQALFPST
ETSENSMIDTSESQQESSDLCTESSDSSTESSDLSTDP*
>rs387604461_ENSMUSP00000034115_c.508:C>T_p.A170V
ENSMUST00000034115.5|Pou4f2-201|ENSMUSG00000031688|-|chr8:g.79162093:G>A
MMMMSLNSKQAFSMPHAGSLHVEPKYSALHSASPGSSAPAAPSASSPSSSSNAGGGGGGGGGGGGGGRSSSSS
SSGSGGSGGGGGSEAMRRACLPTPPSNIFGGLDESLLARAEALAAVDIVSQSKSHHHHPPHHSPFKPDATYHT
MNTIPCTSAASSSSVPISHPSALVGTHHHHHHHHHHHHQPHQALEGELLEHLSPGLALGAMAGPDGTVVSTPA
HAPHMATMNPMHQAALSMAHAHGLPSHMGCMSDVDADPRDLEAFAERFKQRRIKLGVTQADVGSALANLKIPG
VGSLSQSTICRFESLTLSHNNMIALKPILQAWLEEAEKSHREKLTKPELFNGAEKKRKRTSIAAPEKRSLEAY
FAIQPRPSSEKIAAIAEKLDLKKNVVRVWFCNQRQKQKRMKYSAGI*
>rs387536632_ENSMUSP00000085238_c.196:C>T_p.S66F
ENSMUST00000087927.11|Zfp827-201|ENSMUSG00000071064|+|chr8:g.79787032:C>T
MPRRKQEQPKRLPSHVSRQDEAEGDFSEGEQWYGNSSETPSEASYGEVQENYKLSLEDRIQEQSTFPDTSLGS
ATPSSHTLELVALDGEVLRDSLQCQGHLSPGVSSVCDDDPPSSNKPLSSNLRRLLEAGSLKLDGTANGRVESP
VNVGPSLSFSPPSHHAQQLSVLARKLAEKQDQSDQFTPSNRFIWNQGKWLPNSTTTCGLSPDSAILKLKAAAN
AVLQDKSLSRTEESLRFESFSSPFSSQSASSTLAALSKKVSERSLTPGQEHPPPASSFLSLASMTSSAALLKE
VAARAAGSLLAEKSSLLPDDPLPLPSSEKKPEKVTPPPPPPPPTAQPPQSLELLLLPVSKGRASKPSNSAPEE

ESGKPFQCPICGLVIKRKSYWKRHMVIHTGLKSHQCPLCPFRCARKDNLKSHMKVSHLPRRGRGFVLFSTAGG
FLADREHCGGAGLRKLTLGCE*
>rs387536632_ENSMUSP00000113199_c.196:C>T_p.S66F
ENSMUST00000119254.8|Zfp827-203|ENSMUSG00000071064|+|chr8:g.79787032:C>T
MPRRKQEQPKRLPSHVSRQDEAEGDFSEGEQWYGNSSETPSEASYGEVQENYKLSLEDRIQEQSTFPDTSLGS
ATPSSHTLELVALDGEVLRDSLQCQGHLSPGVSSVCDDDPPSSNKPLSSNLRRLLEAGSLKLDGTANGRVESP
VNVGPSLSFSPPSHHAQQLSVLARKLAEKQDQSDQFTPSNRFIWNQGKWLPNSTTTCGLSPDSAILKLKAAAN
AVLQDKSLSRTEESLRFESFSSPFSSQSASSTLAALSKKVSERSLTPGQEHPPPASSFLSLASMTSSAALLKE
VAARAAGSLLAEKSSLLPDDPLPLPSSEKKPEKVTPPPPPPPPTAQPPQSLELLLLPVSKGRASKPSNSAPEE
ESGKPFQCPICGLVIKRKSYWKRHMVIHTGLKSHQCPLCPFRCARKDNLKSHMKVHQHQDRGETFQCQLCPFT
SSRHFSLKLHMRCHQHFLRTEAKVKEEIPDPDVKGSPHLSDSGCLGQQREGGGTELVGTVMTSNTPERTGQGG
AGVAPLLVKEEPKEDNGLPTSFTLNAADRPANHTKLKDPSEYVSNSAAVLFSQDISVKMASDFLMKLSAANQK
EPMNLNFKVKEEPKEEESLSMPLPRSSYVFSPEPEVSTPSVSEDPLTPQEGKGSVLRRDMSAKAASELLMKLS
AESYKETQAVTVKEEPMEVDIQDSPASISPSRNIGYSTLMGREKTEPLQKLPEGRVPPERNLFSQDISVKMAS
ELLFQLSEKVSKEHNHTKENAMRTTTSPFFSEDTFRQSPFTSNSKDLLPGESVLHGRVSAPETEKIVLEAGNG
LPSWKFNDQLFPCDVCGKVFGRQQTLSRHLSLHTEERKYKCHLCPYAAKCRANLNQHLTVHSVKLVSTDTEDI
VSAVTSEGSDGKKHPYYYSCHVCGFETELNVQFVSHMSLHVDKEQWMFSICCTACDFVTMEEAEIKTHIGTKH
TGDDRKTPSESNSPSSSSLSTLSDSANGKDDSDSSQKNKGGNNLLVISVVPGSQPSLNNEEKPEKGFECVFCN
FVCKTKNMFERHLQIHLITRMFECDVCHKFMKTPEQLLEHKKCHTVPTGGLK*
>rs387536632_ENSMUSP00000158613_c.196:C>T_p.S66F
ENSMUST00000238669.2|Zfp827-209|ENSMUSG00000071064|+|chr8:g.79787032:C>T
MPRRKQEQPKRLPSHVSRQDEAEGDFSEGEQWYGNSSETPSEASYGEVQENYKLSLEDRIQEQSTFPDTSLGS
ATPSSHTLELVALDGEVLRDSLQCQGHLSPGVSSVCDDDPPSSNKPLSSNLRRLLEAGSLKLDGTANGRVESP
VNVGPSLSFSPPSHHAQQLSVLARKLAEKQDQSDQFTPSNRFIWNQGKWLPNSTTTCGLSPDSAILKLKAAAN
AVLQDKSLSRTEESLRFESFSSPFSSQSASSTLAALSKKVSERSLTPGQEHPPPASSFLSLASMTSSAALLKE
VAARAAGSLLAEKSSLLPDDPLPLPSSEKKPEKVTPPPPPPPPTAQPPQSLELLLLPVSKGRASKPSNSAPEE
ESGKPFQCPICGLVIKRKSYWKRHMVIHTGLKSHQCPLCPFRCARKDNLKSHMKVHQHQDRGETFQCQLCPFT
SSRHFSLKLHMRCHQHFLRTEAKVKEEIPDPDVKGSPHLSDSGCLGQQREGGGTELVGTVMTSNTPERTGQGG
AGVAPLLVKEEPKEDNGLPTSFTLNAADRPANHTKLKDPSEYVSNSAAVLFSQDISVKMASDFLMKLSAANQK
EPMNLNFKVKEEPKEEESLSMPLPRSSYVFSPEPEVSTPSVSEDPLTPQEGKGSVLRRDMSAKAASELLMKLS
AESYKETQAVTVKEEPMEVDIQDSPASISPSRNIGYSTLMGREKTEPLQKLPEGRVPPERNLFSQDISVKMAS
ELLFQLSEKVSKEHNHTKENAMRTTTSPFFSEDTFRQSPFTSNSKDLLPGESVLHGRVSAPETEKIVLEAGNG
LPSWKFNDQLFPCDVCGKVFGRQQTLSRHLSLHTEERKYKCHLCPYAAKCRANLNQHLTVHSVKLVSTDTEDI
VSAVTSEGSDGKKHPYYYSCHVCGFETELNVQFVSHMSLHVDKEQWMFSICCTACDFVTMEEAEIKTHIGTKH
TGDDRKTPSESNSPSSSSLSTLSDSANGKDDSDSSQKNKGGNNLLVISVVPGSQPSLNNEEKPEKGFECVFCN
FVCKTKNMFERHLQIHLITRMFECDVCHKFMKTPEQLLEHKKCHTVPTGGLKCPFCIYSTNRPAAMECHLKTH
YKMEYKCRICQTVKANQLELETHTREHRLGNHYKCEQCGYLSKTANKLIEHVRVHTGERPFHCDQCSYSCKRK
DNLNLHKKLKHAPRQTFSCGECLFKTTHPFVFSRHVKKHQSGDGPEEDKKGLSPASREPAGPGAPLLVVGGGR
SLLSPLSVMSASQALQTVALSAAHGSSSEPNLALKALAFSGSPLHLDKYRNSDVAHLIPLTMLYPRNHLDLTF
HPPRPQTAPPSIPSPKHSFLAYLGLRERAGTV*
>rs387536632_ENSMUSP00000096214_c.196:C>T_p.S66F
ENSMUST00000098614.9|Zfp827-202|ENSMUSG00000071064|+|chr8:g.79787032:C>T
MPRRKQEQPKRLPSHVSRQDEAEGDFSEGEQWYGNSSETPSEASYGEVQENYKLSLEDRIQEQSTFPDTSLGS
ATPSSHTLELVALDGEVLRDSLQCQGHLSPGVSSVCDDDPPSSNKPLSSNLRRLLEAGSLKLDGTANGRVESP
VNVGPSLSFSPPSHHAQQLSVLARKLAEKQDQSDQFTPSNRFIWNQGKWLPNSTTTCGLSPDSAILKLKAAAN
AVLQDKSLSRTEESLRFESFSSPFSSQSASSTLAALSKKVSERSLTPGQEHPPPASSFLSLASMTSSAALLKE
VAARAAGSLLAEKSSLLPDDPLPLPSSEKKPEKVTPPPPPPPPTAQPPQSLELLLLPVSKGRASKPSNSAPEE
ESGKPFQCPICGLVIKRKSYWKRHMVIHTGLKSHQCPLCPFRCARKDNLKSHMKVHQHQDRGETFQCQLCPFT
SSRHFSLKLHMRCHQHFLRTEAKVKEEIPDPDVKGSPHLSDSGCLGQQREGGGTELVGTVMTSNTPERTGQGG
AGVAPLLVKEEPKEDNGLPTSFTLNAADRPANHTKLKDPSEYVSNSAAVLFSQDISVKMASDFLMKLSAANQK
EPMNLNFKVKEEPKEEESLSMPLPRSSYVFSPEPEVSTPSVSEDPLTPQEGKGSVLRRDMSAKAASELLMKLS
AESYKETQAVTVKEEPMEVDIQDSPASISPSRNIGYSTLMGREKTEPLQKLPEGRVPPERNLFSQDISVKMAS
ELLFQLSEKVSKEHNHTKENAMRTTTSPFFSEDTFRQSPFTSNSKDLLPGESVLHGRVSAPETEKIVLEAGNG
LPSWKFNDQLFPCDVCGKVFGRQQTLSRHLSLHTEERKYKCHLCPYAAKCRANLNQHLTVHSVKLVSTDTEDI
VSAVTSEGSDGKKHPYYYSCHVCGFETELNVQFVSHMSLHVDKEQWMFSICCTACDFVTMEEAEIKTHIGTKH
TGDDRKTPSESNSPSSSSLSTLSDSANGKDDSDSSQKNKGGNNLLVISVVPGSQPSLNNEEKPEKGFECVFCN
FVCKTKNMFERHLQIHLITRMFECDVCHKFMKTPEQLLEHKKCHTVPTGGLNSGQW*

>rs226857452_ENSMUSP00000113199_c.2470:G>T_p.G824V
ENSMUST00000119254.8|Zfp827-203|ENSMUSG00000071064|+|chr8:g.79902558:G>T
MPRRKQEQPKRLPSHVSRQDEAEGDFSEGEQWYGNSSETPSEASYGEVQENYKLSLEDRIQEQSTSPDTSLGS
ATPSSHTLELVALDGEVLRDSLQCQGHLSPGVSSVCDDDPPSSNKPLSSNLRRLLEAGSLKLDGTANGRVESP
VNVGPSLSFSPPSHHAQQLSVLARKLAEKQDQSDQFTPSNRFIWNQGKWLPNSTTTCGLSPDSAILKLKAAAN
AVLQDKSLSRTEESLRFESFSSPFSSQSASSTLAALSKKVSERSLTPGQEHPPPASSFLSLASMTSSAALLKE
VAARAAGSLLAEKSSLLPDDPLPLPSSEKKPEKVTPPPPPPPPTAQPPQSLELLLLPVSKGRASKPSNSAPEE
ESGKPFQCPICGLVIKRKSYWKRHMVIHTGLKSHQCPLCPFRCARKDNLKSHMKVHQHQDRGETFQCQLCPFT
SSRHFSLKLHMRCHQHFLRTEAKVKEEIPDPDVKGSPHLSDSGCLGQQREGGGTELVGTVMTSNTPERTGQGG
AGVAPLLVKEEPKEDNGLPTSFTLNAADRPANHTKLKDPSEYVSNSAAVLFSQDISVKMASDFLMKLSAANQK
EPMNLNFKVKEEPKEEESLSMPLPRSSYVFSPEPEVSTPSVSEDPLTPQEGKGSVLRRDMSAKAASELLMKLS
AESYKETQAVTVKEEPMEVDIQDSPASISPSRNIGYSTLMGREKTEPLQKLPEGRVPPERNLFSQDISVKMAS
ELLFQLSEKVSKEHNHTKENAMRTTTSPFFSEDTFRQSPFTSNSKDLLPGESVLHGRVSAPETEKIVLEAGNG
LPSWKFNDQLFPCDVCGKVFVRQQTLSRHLSLHTEERKYKCHLCPYAAKCRANLNQHLTVHSVKLVSTDTEDI
VSAVTSEGSDGKKHPYYYSCHVCGFETELNVQFVSHMSLHVDKEQWMFSICCTACDFVTMEEAEIKTHIGTKH
TGDDRKTPSESNSPSSSSLSTLSDSANGKDDSDSSQKNKGGNNLLVISVVPGSQPSLNNEEKPEKGFECVFCN
FVCKTKNMFERHLQIHLITRMFECDVCHKFMKTPEQLLEHKKCHTVPTGGLK*
>rs226857452_ENSMUSP00000158613_c.2470:G>T_p.G824V
ENSMUST00000238669.2|Zfp827-209|ENSMUSG00000071064|+|chr8:g.79902558:G>T
MPRRKQEQPKRLPSHVSRQDEAEGDFSEGEQWYGNSSETPSEASYGEVQENYKLSLEDRIQEQSTSPDTSLGS
ATPSSHTLELVALDGEVLRDSLQCQGHLSPGVSSVCDDDPPSSNKPLSSNLRRLLEAGSLKLDGTANGRVESP
VNVGPSLSFSPPSHHAQQLSVLARKLAEKQDQSDQFTPSNRFIWNQGKWLPNSTTTCGLSPDSAILKLKAAAN
AVLQDKSLSRTEESLRFESFSSPFSSQSASSTLAALSKKVSERSLTPGQEHPPPASSFLSLASMTSSAALLKE
VAARAAGSLLAEKSSLLPDDPLPLPSSEKKPEKVTPPPPPPPPTAQPPQSLELLLLPVSKGRASKPSNSAPEE
ESGKPFQCPICGLVIKRKSYWKRHMVIHTGLKSHQCPLCPFRCARKDNLKSHMKVHQHQDRGETFQCQLCPFT
SSRHFSLKLHMRCHQHFLRTEAKVKEEIPDPDVKGSPHLSDSGCLGQQREGGGTELVGTVMTSNTPERTGQGG
AGVAPLLVKEEPKEDNGLPTSFTLNAADRPANHTKLKDPSEYVSNSAAVLFSQDISVKMASDFLMKLSAANQK
EPMNLNFKVKEEPKEEESLSMPLPRSSYVFSPEPEVSTPSVSEDPLTPQEGKGSVLRRDMSAKAASELLMKLS
AESYKETQAVTVKEEPMEVDIQDSPASISPSRNIGYSTLMGREKTEPLQKLPEGRVPPERNLFSQDISVKMAS
ELLFQLSEKVSKEHNHTKENAMRTTTSPFFSEDTFRQSPFTSNSKDLLPGESVLHGRVSAPETEKIVLEAGNG
LPSWKFNDQLFPCDVCGKVFVRQQTLSRHLSLHTEERKYKCHLCPYAAKCRANLNQHLTVHSVKLVSTDTEDI
VSAVTSEGSDGKKHPYYYSCHVCGFETELNVQFVSHMSLHVDKEQWMFSICCTACDFVTMEEAEIKTHIGTKH
TGDDRKTPSESNSPSSSSLSTLSDSANGKDDSDSSQKNKGGNNLLVISVVPGSQPSLNNEEKPEKGFECVFCN
FVCKTKNMFERHLQIHLITRMFECDVCHKFMKTPEQLLEHKKCHTVPTGGLKCPFCIYSTNRPAAMECHLKTH
YKMEYKCRICQTVKANQLELETHTREHRLGNHYKCEQCGYLSKTANKLIEHVRVHTGERPFHCDQCSYSCKRK
DNLNLHKKLKHAPRQTFSCGECLFKTTHPFVFSRHVKKHQSGDGPEEDKKGLSPASREPAGPGAPLLVVGGGR
SLLSPLSVMSASQALQTVALSAAHGSSSEPNLALKALAFSGSPLHLDKYRNSDVAHLIPLTMLYPRNHLDLTF
HPPRPQTAPPSIPSPKHSFLAYLGLRERAGTV*
>rs226857452_ENSMUSP00000120913_c.346:G>T_p.G116V
ENSMUST00000148713.8|Zfp827-206|ENSMUSG00000071064|+|chr8:g.79902558:G>T
PEGRVPPERNLFSQDISVKMASELLFQLSEKVSKEHNHTKENAMRTTTSPFFSEDTFRQSPFTSNSKDLLPGE
SVLHGRVSAPETEKIVLEAGNGLPSWKFNDQLFPCDVCGKVFVRQQTLSRHLSLHTEERKYKCHLCPYAAKCR
ANLNQHLTVHSVKLVSTDTEDIVSAVTSEGSDGKKHPYYYSCHVCGFETELNVQFVSHMSLHVDKEQWMFSIC
CTACDFVTMEEAEIKTHIGTKHTGDDRKTPSESNSPSSSSLSTLSDSANGKDDSDSSQKNKGGNNLLVISVVP
GSQPSLNNEEKPEKGFECVFCNFVCKTKNMFERHLQIHLITRMFECDVCHKFMKTPEQLLEHKKCHTVPTGGL
NSGQW*
>rs226857452_ENSMUSP00000114208_c.259:G>T_p.G87V
ENSMUST00000145827.8|Zfp827-205|ENSMUSG00000071064|+|chr8:g.79902558:G>T
XKVSKEHNHTKENAMRTTTSPFFSEDTFRQSPFTSNSKDLLPGESVLHGRVSAPETEKIVLEAGNGLPSWKFN
DQLFPCDVCGKVFVRQQTLSRHLSLHTVLVSPPEERKYKCHLCPYAAKCRANLNQHLTVHSVKLVSTDTEDIV
SAVTSEGSDGKKHPYYYSCHVCGFETELNVQFVSHMSLHVDKEQWMFSICCTACDFVTMEEAEIKTHIGTKHT
GDDRKTPSESNSPSSSSLSTLSDSANGKDDSDSSQKNKGGNNLLVISVVPGSQPSLNNEEKPEKGFECVFCNF
VCKTKNMFERHLQIHLITRMFECDVCHKFMKTPEQLLEHKKCHTVPTGGLK*
>rs226857452_ENSMUSP00000096214_c.2470:G>T_p.G824V
ENSMUST00000098614.9|Zfp827-202|ENSMUSG00000071064|+|chr8:g.79902558:G>T
MPRRKQEQPKRLPSHVSRQDEAEGDFSEGEQWYGNSSETPSEASYGEVQENYKLSLEDRIQEQSTSPDTSLGS
ATPSSHTLELVALDGEVLRDSLQCQGHLSPGVSSVCDDDPPSSNKPLSSNLRRLLEAGSLKLDGTANGRVESP
VNVGPSLSFSPPSHHAQQLSVLARKLAEKQDQSDQFTPSNRFIWNQGKWLPNSTTTCGLSPDSAILKLKAAAN

AVLQDKSLSRTEESLRFESFSSPFSSQSASSTLAALSKKVSERSLTPGQEHPPPASSFLSLASMTSSAALLKE
VAARAAGSLLAEKSSLLPDDPLPLPSSEKKPEKVTPPPPPPPPTAQPPQSLELLLLPVSKGRASKPSNSAPEE
ESGKPFQCPICGLVIKRKSYWKRHMVIHTGLKSHQCPLCPFRCARKDNLKSHMKVHQHQDRGETFQCQLCPFT
SSRHFSLKLHMRCHQHFLRTEAKVKEEIPDPDVKGSPHLSDSGCLGQQREGGGTELVGTVMTSNTPERTGQGG
AGVAPLLVKEEPKEDNGLPTSFTLNAADRPANHTKLKDPSEYVSNSAAVLFSQDISVKMASDFLMKLSAANQK
EPMNLNFKVKEEPKEEESLSMPLPRSSYVFSPEPEVSTPSVSEDPLTPQEGKGSVLRRDMSAKAASELLMKLS
AESYKETQAVTVKEEPMEVDIQDSPASISPSRNIGYSTLMGREKTEPLQKLPEGRVPPERNLFSQDISVKMAS
ELLFQLSEKVSKEHNHTKENAMRTTTSPFFSEDTFRQSPFTSNSKDLLPGESVLHGRVSAPETEKIVLEAGNG
LPSWKFNDQLFPCDVCGKVFVRQQTLSRHLSLHTEERKYKCHLCPYAAKCRANLNQHLTVHSVKLVSTDTEDI
VSAVTSEGSDGKKHPYYYSCHVCGFETELNVQFVSHMSLHVDKEQWMFSICCTACDFVTMEEAEIKTHIGTKH
TGDDRKTPSESNSPSSSSLSTLSDSANGKDDSDSSQKNKGGNNLLVISVVPGSQPSLNNEEKPEKGFECVFCN
FVCKTKNMFERHLQIHLITRMFECDVCHKFMKTPEQLLEHKKCHTVPTGGLNSGQW*
>rs247337923_ENSMUSP00000113199_c.2480:G>T_p.Q827H
ENSMUST00000119254.8|Zfp827-203|ENSMUSG00000071064|+|chr8:g.79902568:G>T
MPRRKQEQPKRLPSHVSRQDEAEGDFSEGEQWYGNSSETPSEASYGEVQENYKLSLEDRIQEQSTSPDTSLGS
ATPSSHTLELVALDGEVLRDSLQCQGHLSPGVSSVCDDDPPSSNKPLSSNLRRLLEAGSLKLDGTANGRVESP
VNVGPSLSFSPPSHHAQQLSVLARKLAEKQDQSDQFTPSNRFIWNQGKWLPNSTTTCGLSPDSAILKLKAAAN
AVLQDKSLSRTEESLRFESFSSPFSSQSASSTLAALSKKVSERSLTPGQEHPPPASSFLSLASMTSSAALLKE
VAARAAGSLLAEKSSLLPDDPLPLPSSEKKPEKVTPPPPPPPPTAQPPQSLELLLLPVSKGRASKPSNSAPEE
ESGKPFQCPICGLVIKRKSYWKRHMVIHTGLKSHQCPLCPFRCARKDNLKSHMKVHQHQDRGETFQCQLCPFT
SSRHFSLKLHMRCHQHFLRTEAKVKEEIPDPDVKGSPHLSDSGCLGQQREGGGTELVGTVMTSNTPERTGQGG
AGVAPLLVKEEPKEDNGLPTSFTLNAADRPANHTKLKDPSEYVSNSAAVLFSQDISVKMASDFLMKLSAANQK
EPMNLNFKVKEEPKEEESLSMPLPRSSYVFSPEPEVSTPSVSEDPLTPQEGKGSVLRRDMSAKAASELLMKLS
AESYKETQAVTVKEEPMEVDIQDSPASISPSRNIGYSTLMGREKTEPLQKLPEGRVPPERNLFSQDISVKMAS
ELLFQLSEKVSKEHNHTKENAMRTTTSPFFSEDTFRQSPFTSNSKDLLPGESVLHGRVSAPETEKIVLEAGNG
LPSWKFNDQLFPCDVCGKVFGRQHTLSRHLSLHTEERKYKCHLCPYAAKCRANLNQHLTVHSVKLVSTDTEDI
VSAVTSEGSDGKKHPYYYSCHVCGFETELNVQFVSHMSLHVDKEQWMFSICCTACDFVTMEEAEIKTHIGTKH
TGDDRKTPSESNSPSSSSLSTLSDSANGKDDSDSSQKNKGGNNLLVISVVPGSQPSLNNEEKPEKGFECVFCN
FVCKTKNMFERHLQIHLITRMFECDVCHKFMKTPEQLLEHKKCHTVPTGGLK*
>rs247337923_ENSMUSP00000158613_c.2480:G>T_p.Q827H
ENSMUST00000238669.2|Zfp827-209|ENSMUSG00000071064|+|chr8:g.79902568:G>T
MPRRKQEQPKRLPSHVSRQDEAEGDFSEGEQWYGNSSETPSEASYGEVQENYKLSLEDRIQEQSTSPDTSLGS
ATPSSHTLELVALDGEVLRDSLQCQGHLSPGVSSVCDDDPPSSNKPLSSNLRRLLEAGSLKLDGTANGRVESP
VNVGPSLSFSPPSHHAQQLSVLARKLAEKQDQSDQFTPSNRFIWNQGKWLPNSTTTCGLSPDSAILKLKAAAN
AVLQDKSLSRTEESLRFESFSSPFSSQSASSTLAALSKKVSERSLTPGQEHPPPASSFLSLASMTSSAALLKE
VAARAAGSLLAEKSSLLPDDPLPLPSSEKKPEKVTPPPPPPPPTAQPPQSLELLLLPVSKGRASKPSNSAPEE
ESGKPFQCPICGLVIKRKSYWKRHMVIHTGLKSHQCPLCPFRCARKDNLKSHMKVHQHQDRGETFQCQLCPFT
SSRHFSLKLHMRCHQHFLRTEAKVKEEIPDPDVKGSPHLSDSGCLGQQREGGGTELVGTVMTSNTPERTGQGG
AGVAPLLVKEEPKEDNGLPTSFTLNAADRPANHTKLKDPSEYVSNSAAVLFSQDISVKMASDFLMKLSAANQK
EPMNLNFKVKEEPKEEESLSMPLPRSSYVFSPEPEVSTPSVSEDPLTPQEGKGSVLRRDMSAKAASELLMKLS
AESYKETQAVTVKEEPMEVDIQDSPASISPSRNIGYSTLMGREKTEPLQKLPEGRVPPERNLFSQDISVKMAS
ELLFQLSEKVSKEHNHTKENAMRTTTSPFFSEDTFRQSPFTSNSKDLLPGESVLHGRVSAPETEKIVLEAGNG
LPSWKFNDQLFPCDVCGKVFGRQHTLSRHLSLHTEERKYKCHLCPYAAKCRANLNQHLTVHSVKLVSTDTEDI
VSAVTSEGSDGKKHPYYYSCHVCGFETELNVQFVSHMSLHVDKEQWMFSICCTACDFVTMEEAEIKTHIGTKH
TGDDRKTPSESNSPSSSSLSTLSDSANGKDDSDSSQKNKGGNNLLVISVVPGSQPSLNNEEKPEKGFECVFCN
FVCKTKNMFERHLQIHLITRMFECDVCHKFMKTPEQLLEHKKCHTVPTGGLKCPFCIYSTNRPAAMECHLKTH
YKMEYKCRICQTVKANQLELETHTREHRLGNHYKCEQCGYLSKTANKLIEHVRVHTGERPFHCDQCSYSCKRK
DNLNLHKKLKHAPRQTFSCGECLFKTTHPFVFSRHVKKHQSGDGPEEDKKGLSPASREPAGPGAPLLVVGGGR
SLLSPLSVMSASQALQTVALSAAHGSSSEPNLALKALAFSGSPLHLDKYRNSDVAHLIPLTMLYPRNHLDLTF
HPPRPQTAPPSIPSPKHSFLAYLGLRERAGTV*
>rs247337923_ENSMUSP00000120913_c.356:G>T_p.Q119H
ENSMUST00000148713.8|Zfp827-206|ENSMUSG00000071064|+|chr8:g.79902568:G>T
PEGRVPPERNLFSQDISVKMASELLFQLSEKVSKEHNHTKENAMRTTTSPFFSEDTFRQSPFTSNSKDLLPGE
SVLHGRVSAPETEKIVLEAGNGLPSWKFNDQLFPCDVCGKVFGRQHTLSRHLSLHTEERKYKCHLCPYAAKCR
ANLNQHLTVHSVKLVSTDTEDIVSAVTSEGSDGKKHPYYYSCHVCGFETELNVQFVSHMSLHVDKEQWMFSIC
CTACDFVTMEEAEIKTHIGTKHTGDDRKTPSESNSPSSSSLSTLSDSANGKDDSDSSQKNKGGNNLLVISVVP
GSQPSLNNEEKPEKGFECVFCNFVCKTKNMFERHLQIHLITRMFECDVCHKFMKTPEQLLEHKKCHTVPTGGL
NSGQW*

>rs247337923_ENSMUSP00000114208_c.269:G>T_p.Q90H
ENSMUST00000145827.8|Zfp827-205|ENSMUSG00000071064|+|chr8:g.79902568:G>T
XKVSKEHNHTKENAMRTTTSPFFSEDTFRQSPFTSNSKDLLPGESVLHGRVSAPETEKIVLEAGNGLPSWKFN
DQLFPCDVCGKVFGRQHTLSRHLSLHTVLVSPPEERKYKCHLCPYAAKCRANLNQHLTVHSVKLVSTDTEDIV
SAVTSEGSDGKKHPYYYSCHVCGFETELNVQFVSHMSLHVDKEQWMFSICCTACDFVTMEEAEIKTHIGTKHT
GDDRKTPSESNSPSSSSLSTLSDSANGKDDSDSSQKNKGGNNLLVISVVPGSQPSLNNEEKPEKGFECVFCNF
VCKTKNMFERHLQIHLITRMFECDVCHKFMKTPEQLLEHKKCHTVPTGGLK*
>rs247337923_ENSMUSP00000096214_c.2480:G>T_p.Q827H
ENSMUST00000098614.9|Zfp827-202|ENSMUSG00000071064|+|chr8:g.79902568:G>T
MPRRKQEQPKRLPSHVSRQDEAEGDFSEGEQWYGNSSETPSEASYGEVQENYKLSLEDRIQEQSTSPDTSLGS
ATPSSHTLELVALDGEVLRDSLQCQGHLSPGVSSVCDDDPPSSNKPLSSNLRRLLEAGSLKLDGTANGRVESP
VNVGPSLSFSPPSHHAQQLSVLARKLAEKQDQSDQFTPSNRFIWNQGKWLPNSTTTCGLSPDSAILKLKAAAN
AVLQDKSLSRTEESLRFESFSSPFSSQSASSTLAALSKKVSERSLTPGQEHPPPASSFLSLASMTSSAALLKE
VAARAAGSLLAEKSSLLPDDPLPLPSSEKKPEKVTPPPPPPPPTAQPPQSLELLLLPVSKGRASKPSNSAPEE
ESGKPFQCPICGLVIKRKSYWKRHMVIHTGLKSHQCPLCPFRCARKDNLKSHMKVHQHQDRGETFQCQLCPFT
SSRHFSLKLHMRCHQHFLRTEAKVKEEIPDPDVKGSPHLSDSGCLGQQREGGGTELVGTVMTSNTPERTGQGG
AGVAPLLVKEEPKEDNGLPTSFTLNAADRPANHTKLKDPSEYVSNSAAVLFSQDISVKMASDFLMKLSAANQK
EPMNLNFKVKEEPKEEESLSMPLPRSSYVFSPEPEVSTPSVSEDPLTPQEGKGSVLRRDMSAKAASELLMKLS
AESYKETQAVTVKEEPMEVDIQDSPASISPSRNIGYSTLMGREKTEPLQKLPEGRVPPERNLFSQDISVKMAS
ELLFQLSEKVSKEHNHTKENAMRTTTSPFFSEDTFRQSPFTSNSKDLLPGESVLHGRVSAPETEKIVLEAGNG
LPSWKFNDQLFPCDVCGKVFGRQHTLSRHLSLHTEERKYKCHLCPYAAKCRANLNQHLTVHSVKLVSTDTEDI
VSAVTSEGSDGKKHPYYYSCHVCGFETELNVQFVSHMSLHVDKEQWMFSICCTACDFVTMEEAEIKTHIGTKH
TGDDRKTPSESNSPSSSSLSTLSDSANGKDDSDSSQKNKGGNNLLVISVVPGSQPSLNNEEKPEKGFECVFCN
FVCKTKNMFERHLQIHLITRMFECDVCHKFMKTPEQLLEHKKCHTVPTGGLNSGQW*
>rs253661095_ENSMUSP00000114208_c.318:G>C_p.E107Q
ENSMUST00000145827.8|Zfp827-205|ENSMUSG00000071064|+|chr8:g.79905536:G>C
XKVSKEHNHTKENAMRTTTSPFFSEDTFRQSPFTSNSKDLLPGESVLHGRVSAPETEKIVLEAGNGLPSWKFN
DQLFPCDVCGKVFGRQQTLSRHLSLHTVLVSPPQERKYKCHLCPYAAKCRANLNQHLTVHSVKLVSTDTEDIV
SAVTSEGSDGKKHPYYYSCHVCGFETELNVQFVSHMSLHVDKEQWMFSICCTACDFVTMEEAEIKTHIGTKHT
GDDRKTPSESNSPSSSSLSTLSDSANGKDDSDSSQKNKGGNNLLVISVVPGSQPSLNNEEKPEKGFECVFCNF
VCKTKNMFERHLQIHLITRMFECDVCHKFMKTPEQLLEHKKCHTVPTGGLK*
>rs38121230_ENSMUSP00000071035_c.1198:T>G_p.F400C
ENSMUST00000066091.14|Smad1-202|ENSMUSG00000031681|-|chr8:g.80070338:A>C
MNVTSLFSFTSPAVKRLLGWKQGDEEEKWAEKAVDALVKKLKKKKGAMEELEKALSCPGQPSNCVTIPRSLDG
RLQVSHRKGLPHVIYCRVWRWPDLQSHHELKPLECCEFPFGSKQKEVCINPYHYKRVESPVLPPVLVPRHSEY
NPQHSLLAQFRNLGQNEPHMPLNATFPDSFQQPNSHPFPHSPNSSYPNSPGGSSSTYPHSPTSSDPGSPFQMP
ADTPPPAYLPPEDPMAQDGSQPMDTNMMAPPLPAEISRGDVQAVAYEEPKHWCSIVYYELNNRVGEAFHASST
SVLVDGFTDPSNNKNRFCLGLLSNVNRNSTIENTRRHIGKGVHLYYVGGEVYAECLSDSSIFVQSRNCNYHHG
FHPTTVCKIPSGCSLKIFNNQEFAQLLAQSVNHGCETVYELTKMCTIRMSFVKGWGAEYHRQDVTSTPCWIEI
HLHGPLQWLDKVLTQMGSPHNPISSVS*
>rs38121230_ENSMUSP00000105511_c.1198:T>G_p.F400C
ENSMUST00000109885.8|Smad1-203|ENSMUSG00000031681|-|chr8:g.80070338:A>C
MNVTSLFSFTSPAVKRLLGWKQGDEEEKWAEKAVDALVKKLKKKKGAMEELEKALSCPGQPSNCVTIPRSLDG
RLQVSHRKGLPHVIYCRVWRWPDLQSHHELKPLECCEFPFGSKQKEVCINPYHYKRVESPVLPPVLVPRHSEY
NPQHSLLAQFRNLGQNEPHMPLNATFPDSFQQPNSHPFPHSPNSSYPNSPGGSSSTYPHSPTSSDPGSPFQMP
ADTPPPAYLPPEDPMAQDGSQPMDTNMMAPPLPAEISRGDVQAVAYEEPKHWCSIVYYELNNRVGEAFHASST
SVLVDGFTDPSNNKNRFCLGLLSNVNRNSTIENTRRHIGKGVHLYYVGGEVYAECLSDSSIFVQSRNCNYHHG
FHPTTVCKIPSGCSLKIFNNQEFAQLLAQSVNHGCETVYELTKMCTIRMSFVKVSSAHPSHSG*
>rs50549994_ENSMUSP00000149251_c.268:A>G_p.K90R
ENSMUST00000214156.2|Olfr370-203|ENSMUSG00000047286|+|chr8:g.84268043:A>G
MECVNDTVVREFVFLGFSSLAELQLLLFAIFLSLYLFTLSTNAVIVSTIVLDRALHTPMYFFLSVLSCSETCY
TFVIVPKMLVDLLARKRSISFLGCAIQMFTFLFLGCSHSFLLAAMGYDRYVAICHPLRYTVLMGHRVCVGLVA
AACVCGFTVAQVITSLVFRLPFRSSNQLHHFFCDISPVLQLASHHPHSTQITIFLLCALVLVIPLFLILVSYI
HIISTILQFPSTLGRYKAFSTCASHLIVVIVHYGCASFIYLRPKSSYSSSQDALISVSYTILTPLFNPVIYSL
RNKDFKSALHRVIGRTVTLRQH*
>rs50549994_ENSMUSP00000147834_c.268:A>G_p.K90R
ENSMUST00000209408.4|Olfr370-202|ENSMUSG00000047286|+|chr8:g.84268043:A>G

MECVNDTVVREFVFLGFSSLAELQLLLFAIFLSLYLFTLSTNAVIVSTIVLDRALHTPMYFFLSVLSCSETCY
TFVIVPKMLVDLLARKRSISFLGCAIQMFTFLFLGCSHSFLLAAMGYDRYVAICHPLRYTVLMGHRVCVGLVA
AACVCGFTVAQVITSLVFRLPFRSSNQLHHFFCDISPVLQLASHHPHSTQITIFLLCALVLVIPLFLILVSYI
HIISTILQFPSTLGRYKAFSTCASHLIVVIVHYGCASFIYLRPKSSYSSSQDALISVSYTILTPLFNPVIYSL
RNKDFKSALHRVIGRTVTLRQH*
>rs50549994_ENSMUSP00000055435_c.268:A>G_p.K90R
ENSMUST00000058609.5|Olfr370-201|ENSMUSG00000047286|+|chr8:g.84268043:A>G
MECVNDTVVREFVFLGFSSLAELQLLLFAIFLSLYLFTLSTNAVIVSTIVLDRALHTPMYFFLSVLSCSETCY
TFVIVPKMLVDLLARKRSISFLGCAIQMFTFLFLGCSHSFLLAAMGYDRYVAICHPLRYTVLMGHRVCVGLVA
AACVCGFTVAQVITSLVFRLPFRSSNQLHHFFCDISPVLQLASHHPHSTQITIFLLCALVLVIPLFLILVSYI
HIISTILQFPSTLGRYKAFSTCASHLIVVIVHYGCASFIYLRPKSSYSSSQDALISVSYTILTPLFNPVIYSL
RNKDFKSALHRVIGRTVTLRQH*
>rs48118666_ENSMUSP00000019577_c.930:T>A_p.F311I
ENSMUST00000019577.10|Gipc1-201|ENSMUSG00000019433|+|chr8:g.84390827:T>A
MPLGLGRRKKAPPLVENEEAEPSRSGLGVGEPGPLGGSAAGESQMGLPPPPAALRPRLVFHTQLAHGSPTGRI
EGFTNVKELYGKIAEAFRLPAEVMFCTLNTHKVDMDKLLGGQIGLEDFIFAHVKGQRKEVEVFKSEEALGLT
ITDNGAGYAFIKRIKEGSVIDHIQLISVGDMIEAINGQSLLGCRHYEVARLLKELPRGRTFTLKLTEPRKAFD
MISQRSAGGHPGSGPQLGTGRGTLRLRSRGPATVEDLPSAFEEKAIEKVDDLLESYMGIRDTELAATMVELGK
DKRNPDELAEALDERLGDIAFPDEFVFDVWGAIGDAKVGRY*
>rs36969597_ENSMUSP00000019608_c.277:C>T_p.P93L
ENSMUST00000019608.7|Ptger1-201|ENSMUSG00000019464|+|chr8:g.84394802:C>T
MSPCGLNLSLADEAATCATPRLPNTSVVLPTGDNGTSPALPIFSMTLGAVSNVLALALLAQVAGRMRRRRSAA
TFLLFVASLLAIDLAGHVILGALVLRLYTAGRAPAGGACHFLGGCMVFFGLCPLLLGCGMAVERCVGVTQPLI
HAARVSVARARLALAVLAAMALAVALLPLVHVGRYELQYPGTWCFISLGPRGGWRQALLAGLFAGLGLAALLA
ALVCNTLSGLALLRARWRRRRSRRFRKTAGPDDRRRWGSRGPRLASASSASSITSATATLRSSRGGGSARRVH
AHDVEMVGQLVGIMVVSCICWSPLLVLVVLAIGGWNSNSLQRPLFLAVRLASWNQILDPWVYILLRQAMLRQL
LRLLPLRVSAKGGPTELGLTKSAWEASSLRSSRHSGFSHL*
>rs50201871_ENSMUSP00000005616_c.2582:G>C_p.R861S
ENSMUST00000005616.16|Pkn1-201|ENSMUSG00000057672|-|chr8:g.84397422:C>G
MAGDAVQSEPRSWSLLEQLGLAGADLAAPGVQQQLELERERLKREIRKELKLKEGAENLRRATTDLGRSLAPV
ELLLRGSARRLDLLHQQLQELHAHVVLPDPAAGSDATQSLAEGSPICSSTNLSRVAGLEKQLAIELKVKQGAE
NMIQTYSNGSSKDRKLLLTAQQMLQDSKTKIDIIRMQLRRALQALQAGELESQAAPDEAQGDPELGAVELRIE
ELRHHFRVEHAVAEGAKNVLRLLSGAKAPDRKAVSEAQEKLTESNQKLGLLRESLERRLGELPADHPKGRLLR
EELTAASSSAFSAILPGPFPATHYSTLSKPAPLTGTLEVRVVGCKNLPETIPWSPPPSVGASGTPESRTPFLS
RPARGLYSRSGSLSGRSSLRGEAENATEVSTVLKLDNTVVGQTAWKPCGPNAWDQSFTLELERARELELAVFW
RDQRGLCALKFLKLEDFLDNERHEVQLDMEPQGCLVAEVTFRNPIIERIPRLQRQKKIFSKQQGKAFQRARQM
NIDVATWVRLLRRLIPSAVATGTFSPNASPGAEIRHTGDISMEKLNLGADSDSSSQKSPPGLPSTSCSLSSPT
HESTTSPELPSETQETPGPGLCSPLRKSPLTLEDFKFLAVLGRGHFGKVLLSEFRSSGELFAIKALKKGDIVA
RDEVESLMCEKRILAAVTRAGHPFLVNLFGCFQTPEHVCFVMEYSAGGDLMLHIHSDVFSEPRAVFYSACVVL
GLQFLHEHKIVYRDLKLDNLLLDTEGYVKIADFGLCKEGMGYGDRTSTFCGTPEFLAPEVLTDTSYTRAVDWW
GLGVLLYEMLVGESPFPGDDEEEVFDSIVNDEVRYPRFLSAEAIGIMRRLLRRNPERSLGSTERDAEDVKKQP
FFRSLGWDVLLARRLPPPFVPTLSGRTDVSNFDEEFTGEAPTLSPPRDARPLTAAEQAAFRDFDFVAGGY*
>rs50201871_ENSMUSP00000116235_c.2597:G>C_p.R866S
ENSMUST00000144258.8|Pkn1-208|ENSMUSG00000057672|-|chr8:g.84397422:C>G
MAADPPLDSELESEPRSWSLLEQLGLAGADLAAPGVQQQLELERERLKREIRKELKLKEGAENLRRATTDLGR
SLAPVELLLRGSARRLDLLHQQLQELHAHVVLPDPAAGSDATQSLAEGSPICSSTNLSRVAGLEKQLAIELKV
KQGAENMIQTYSNGSSKDRKLLLTAQQMLQDSKTKIDIIRMQLRRALQALQAGELESQAAPDEAQGDPELGAV
ELRIEELRHHFRVEHAVAEGAKNVLRLLSGAKAPDRKAVSEAQEKLTESNQKLGLLRESLERRLGELPADHPK
GRLLREELTAASSSAFSAILPGPFPATHYSTLSKPAPLTGTLEVRVVGCKNLPETIPWSPPPSVGASGTPESR
TPFLSRPARGLYSRSGSLSGRSSLRGEAENATEVSTVLKLDNTVVGQTAWKPCGPNAWDQSFTLELERARELE
LAVFWRDQRGLCALKFLKLEDFLDNERHEVQLDMEPQGCLVAEVTFRNPIIERIPRLQRQKKIFSKQQGKAFQ
RARQMNIDVATWVRLLRRLIPSAVATGTFSPNASPGAEIRHTGDISMEKLNLGADSDSSSQKSPPGLPSTSCS
LSSPTHESTTSPELPSETQETPGPGLCSPLRKSPLTLEDFKFLAVLGRGHFGKVLLSEFRSSGELFAIKALKK
GDIVARDEVESLMCEKRILAAVTRAGHPFLVNLFGCFQTPEHVCFVMEYSAGGDLMLHIHSDVFSEPRAVFYS
ACVVLGLQFLHEHKIVYRDLKLDNLLLDTEGYVKIADFGLCKEGMGYGDRTSTFCGTPEFLAPEVLTDTSYTR
AVDWWGLGVLLYEMLVGESPFPGDDEEEVFDSIVNDEVRYPRFLSAEAIGIMRRLLRRNPERSLGSTERDAED
VKKQPFFRSLGWDVLLARRLPPPFVPTLSGRTDVSNFDEEFTGEAPTLSPPRDARPLTAAEQAAFRDFDFVAG
GY*

>ENSMUSP00000005616_c.2137:G>A_p.S713N ENSMUST00000005616.16|Pkn1-201|ENSMUSG00000057672|-|chr8:g.84398812:C>T
MAGDAVQSEPRSWSLLEQLGLAGADLAAPGVQQQLELERERLKREIRKELKLKEGAENLRRATTDLGRSLAPV
ELLLRGSARRLDLLHQQLQELHAHVVLPDPAAGSDATQSLAEGSPICSSTNLSRVAGLEKQLAIELKVKQGAE
NMIQTYSNGSSKDRKLLLTAQQMLQDSKTKIDIIRMQLRRALQALQAGELESQAAPDEAQGDPELGAVELRIE
ELRHHFRVEHAVAEGAKNVLRLLSGAKAPDRKAVSEAQEKLTESNQKLGLLRESLERRLGELPADHPKGRLLR
EELTAASSSAFSAILPGPFPATHYSTLSKPAPLTGTLEVRVVGCKNLPETIPWSPPPSVGASGTPESRTPFLS
RPARGLYSRSGSLSGRSSLRGEAENATEVSTVLKLDNTVVGQTAWKPCGPNAWDQSFTLELERARELELAVFW
RDQRGLCALKFLKLEDFLDNERHEVQLDMEPQGCLVAEVTFRNPIIERIPRLQRQKKIFSKQQGKAFQRARQM
NIDVATWVRLLRRLIPSAVATGTFSPNASPGAEIRHTGDISMEKLNLGADSDSSSQKSPPGLPSTSCSLSSPT
HESTTSPELPSETQETPGPGLCSPLRKSPLTLEDFKFLAVLGRGHFGKVLLSEFRSSGELFAIKALKKGDIVA
RDEVESLMCEKRILAAVTRAGHPFLVNLFGCFQTPEHVCFVMEYSAGGDLMLHIHNDVFSEPRAVFYSACVVL
GLQFLHEHKIVYRDLKLDNLLLDTEGYVKIADFGLCKEGMGYGDRTSTFCGTPEFLAPEVLTDTSYTRAVDWW
GLGVLLYEMLVGESPFPGDDEEEVFDSIVNDEVRYPRFLSAEAIGIMRRLLRRNPERRLGSTERDAEDVKKQP
FFRSLGWDVLLARRLPPPFVPTLSGRTDVSNFDEEFTGEAPTLSPPRDARPLTAAEQAAFRDFDFVAGGY*
>ENSMUSP00000116235_c.2152:G>A_p.S718N ENSMUST00000144258.8|Pkn1-208|ENSMUSG00000057672|-|chr8:g.84398812:C>T
MAADPPLDSELESEPRSWSLLEQLGLAGADLAAPGVQQQLELERERLKREIRKELKLKEGAENLRRATTDLGR
SLAPVELLLRGSARRLDLLHQQLQELHAHVVLPDPAAGSDATQSLAEGSPICSSTNLSRVAGLEKQLAIELKV
KQGAENMIQTYSNGSSKDRKLLLTAQQMLQDSKTKIDIIRMQLRRALQALQAGELESQAAPDEAQGDPELGAV
ELRIEELRHHFRVEHAVAEGAKNVLRLLSGAKAPDRKAVSEAQEKLTESNQKLGLLRESLERRLGELPADHPK
GRLLREELTAASSSAFSAILPGPFPATHYSTLSKPAPLTGTLEVRVVGCKNLPETIPWSPPPSVGASGTPESR
TPFLSRPARGLYSRSGSLSGRSSLRGEAENATEVSTVLKLDNTVVGQTAWKPCGPNAWDQSFTLELERARELE
LAVFWRDQRGLCALKFLKLEDFLDNERHEVQLDMEPQGCLVAEVTFRNPIIERIPRLQRQKKIFSKQQGKAFQ
RARQMNIDVATWVRLLRRLIPSAVATGTFSPNASPGAEIRHTGDISMEKLNLGADSDSSSQKSPPGLPSTSCS
LSSPTHESTTSPELPSETQETPGPGLCSPLRKSPLTLEDFKFLAVLGRGHFGKVLLSEFRSSGELFAIKALKK
GDIVARDEVESLMCEKRILAAVTRAGHPFLVNLFGCFQTPEHVCFVMEYSAGGDLMLHIHNDVFSEPRAVFYS
ACVVLGLQFLHEHKIVYRDLKLDNLLLDTEGYVKIADFGLCKEGMGYGDRTSTFCGTPEFLAPEVLTDTSYTR
AVDWWGLGVLLYEMLVGESPFPGDDEEEVFDSIVNDEVRYPRFLSAEAIGIMRRLLRRNPERRLGSTERDAED
VKKQPFFRSLGWDVLLARRLPPPFVPTLSGRTDVSNFDEEFTGEAPTLSPPRDARPLTAAEQAAFRDFDFVAG
GY*
>ENSMUSP00000115054_c.2173:G>A_p.S725N ENSMUST00000132945.8|Pkn1-204|ENSMUSG00000057672|-|chr8:g.84398812:C>T
MACRPCRGAGRIWCCRSHKSEPRSWSLLEQLGLAGADLAAPGVQQQLELERERLKREIRKELKLKEGAENLRR
ATTDLGRSLAPVELLLRGSARRLDLLHQQLQELHAHVVLPDPAAGSDATQSLAEGSPICSSTNLSRVAGLEKQ
LAIELKVKQGAENMIQTYSNGSSKDRKLLLTAQQMLQDSKTKIDIIRMQLRRALQALQAGELESQAAPDEAQG
DPELGAVELRIEELRHHFRVEHAVAEGAKNVLRLLSGAKAPDRKAVSEAQEKLTESNQKLGLLRESLERRLGE
LPADHPKGRLLREELTAASSSAFSAILPGPFPATHYSTLSKPAPLTGTLEVRVVGCKNLPETIPWSPPPSVGA
SGTPESRTPFLSRPARGLYSRSGSLSGRSSLRGEAENATEVSTVLKLDNTVVGQTAWKPCGPNAWDQSFTLEL
ERARELELAVFWRDQRGLCALKFLKLEDFLDNERHEVQLDMEPQGCLVAEVTFRNPIIERIPRLQRQKKIFSK
QQGKAFQRARQMNIDVATWVRLLRRLIPSAVATGTFSPNASPGAEIRHTGDISMEKLNLGADSDSSSQKSPPG
LPSTSCSLSSPTHESTTSPELPSETQETPGPGLCSPLRKSPLTLEDFKFLAVLGRGHFGKVLLSEFRSSGELF
AIKALKKGDIVARDEVESLMCEKRILAAVTRAGHPFLVNLFGCFQTPEHVCFVMEYSAGGDLMLHIHNDVFSE
PRAVFYSACVVLGLQFLHEHKIVYRDGLWGPDQHVLRNSGVPGAGSAHRHILHASSGLVGTGRAAL*
>rs47450140_ENSMUSP00000005616_c.1764:A>G_p.T589A
ENSMUST00000005616.16|Pkn1-201|ENSMUSG00000057672|-|chr8:g.84400315:T>C
MAGDAVQSEPRSWSLLEQLGLAGADLAAPGVQQQLELERERLKREIRKELKLKEGAENLRRATTDLGRSLAPV
ELLLRGSARRLDLLHQQLQELHAHVVLPDPAAGSDATQSLAEGSPICSSTNLSRVAGLEKQLAIELKVKQGAE
NMIQTYSNGSSKDRKLLLTAQQMLQDSKTKIDIIRMQLRRALQALQAGELESQAAPDEAQGDPELGAVELRIE
ELRHHFRVEHAVAEGAKNVLRLLSGAKAPDRKAVSEAQEKLTESNQKLGLLRESLERRLGELPADHPKGRLLR
EELTAASSSAFSAILPGPFPATHYSTLSKPAPLTGTLEVRVVGCKNLPETIPWSPPPSVGASGTPESRTPFLS
RPARGLYSRSGSLSGRSSLRGEAENATEVSTVLKLDNTVVGQTAWKPCGPNAWDQSFTLELERARELELAVFW
RDQRGLCALKFLKLEDFLDNERHEVQLDMEPQGCLVAEVTFRNPIIERIPRLQRQKKIFSKQQGKAFQRARQM
NIDVATWVRLLRRLIPSAVATGTFSPNASPGAEIRHTGDISMEKLNLGADSDSSSQKSPPGLPSTSCSLSSPT
HESTASPELPSETQETPGPGLCSPLRKSPLTLEDFKFLAVLGRGHFGKVLLSEFRSSGELFAIKALKKGDIVA
RDEVESLMCEKRILAAVTRAGHPFLVNLFGCFQTPEHVCFVMEYSAGGDLMLHIHSDVFSEPRAVFYSACVVL
GLQFLHEHKIVYRDLKLDNLLLDTEGYVKIADFGLCKEGMGYGDRTSTFCGTPEFLAPEVLTDTSYTRAVDWW

GLGVLLYEMLVGESPFPGDDEEEVFDSIVNDEVRYPRFLSAEAIGIMRRLLRRNPERRLGSTERDAEDVKKQP
FFRSLGWDVLLARRLPPPFVPTLSGRTDVSNFDEEFTGEAPTLSPPRDARPLTAAEQAAFRDFDFVAGGY*
>rs47450140_ENSMUSP00000116235_c.1779:A>G_p.T594A
ENSMUST00000144258.8|Pkn1-208|ENSMUSG00000057672|-|chr8:g.84400315:T>C
MAADPPLDSELESEPRSWSLLEQLGLAGADLAAPGVQQQLELERERLKREIRKELKLKEGAENLRRATTDLGR
SLAPVELLLRGSARRLDLLHQQLQELHAHVVLPDPAAGSDATQSLAEGSPICSSTNLSRVAGLEKQLAIELKV
KQGAENMIQTYSNGSSKDRKLLLTAQQMLQDSKTKIDIIRMQLRRALQALQAGELESQAAPDEAQGDPELGAV
ELRIEELRHHFRVEHAVAEGAKNVLRLLSGAKAPDRKAVSEAQEKLTESNQKLGLLRESLERRLGELPADHPK
GRLLREELTAASSSAFSAILPGPFPATHYSTLSKPAPLTGTLEVRVVGCKNLPETIPWSPPPSVGASGTPESR
TPFLSRPARGLYSRSGSLSGRSSLRGEAENATEVSTVLKLDNTVVGQTAWKPCGPNAWDQSFTLELERARELE
LAVFWRDQRGLCALKFLKLEDFLDNERHEVQLDMEPQGCLVAEVTFRNPIIERIPRLQRQKKIFSKQQGKAFQ
RARQMNIDVATWVRLLRRLIPSAVATGTFSPNASPGAEIRHTGDISMEKLNLGADSDSSSQKSPPGLPSTSCS
LSSPTHESTASPELPSETQETPGPGLCSPLRKSPLTLEDFKFLAVLGRGHFGKVLLSEFRSSGELFAIKALKK
GDIVARDEVESLMCEKRILAAVTRAGHPFLVNLFGCFQTPEHVCFVMEYSAGGDLMLHIHSDVFSEPRAVFYS
ACVVLGLQFLHEHKIVYRDLKLDNLLLDTEGYVKIADFGLCKEGMGYGDRTSTFCGTPEFLAPEVLTDTSYTR
AVDWWGLGVLLYEMLVGESPFPGDDEEEVFDSIVNDEVRYPRFLSAEAIGIMRRLLRRNPERRLGSTERDAED
VKKQPFFRSLGWDVLLARRLPPPFVPTLSGRTDVSNFDEEFTGEAPTLSPPRDARPLTAAEQAAFRDFDFVAG
GY*
>rs47450140_ENSMUSP00000115054_c.1800:A>G_p.T601A
ENSMUST00000132945.8|Pkn1-204|ENSMUSG00000057672|-|chr8:g.84400315:T>C
MACRPCRGAGRIWCCRSHKSEPRSWSLLEQLGLAGADLAAPGVQQQLELERERLKREIRKELKLKEGAENLRR
ATTDLGRSLAPVELLLRGSARRLDLLHQQLQELHAHVVLPDPAAGSDATQSLAEGSPICSSTNLSRVAGLEKQ
LAIELKVKQGAENMIQTYSNGSSKDRKLLLTAQQMLQDSKTKIDIIRMQLRRALQALQAGELESQAAPDEAQG
DPELGAVELRIEELRHHFRVEHAVAEGAKNVLRLLSGAKAPDRKAVSEAQEKLTESNQKLGLLRESLERRLGE
LPADHPKGRLLREELTAASSSAFSAILPGPFPATHYSTLSKPAPLTGTLEVRVVGCKNLPETIPWSPPPSVGA
SGTPESRTPFLSRPARGLYSRSGSLSGRSSLRGEAENATEVSTVLKLDNTVVGQTAWKPCGPNAWDQSFTLEL
ERARELELAVFWRDQRGLCALKFLKLEDFLDNERHEVQLDMEPQGCLVAEVTFRNPIIERIPRLQRQKKIFSK
QQGKAFQRARQMNIDVATWVRLLRRLIPSAVATGTFSPNASPGAEIRHTGDISMEKLNLGADSDSSSQKSPPG
LPSTSCSLSSPTHESTASPELPSETQETPGPGLCSPLRKSPLTLEDFKFLAVLGRGHFGKVLLSEFRSSGELF
AIKALKKGDIVARDEVESLMCEKRILAAVTRAGHPFLVNLFGCFQTPEHVCFVMEYSAGGDLMLHIHSDVFSE
PRAVFYSACVVLGLQFLHEHKIVYRDGLWGPDQHVLRNSGVPGAGSAHRHILHASSGLVGTGRAAL*
>rs33561266_ENSMUSP00000005616_c.862:G>A_p.G288E
ENSMUST00000005616.16|Pkn1-201|ENSMUSG00000057672|-|chr8:g.84410748:C>T
MAGDAVQSEPRSWSLLEQLGLAGADLAAPGVQQQLELERERLKREIRKELKLKEGAENLRRATTDLGRSLAPV
ELLLRGSARRLDLLHQQLQELHAHVVLPDPAAGSDATQSLAEGSPICSSTNLSRVAGLEKQLAIELKVKQGAE
NMIQTYSNGSSKDRKLLLTAQQMLQDSKTKIDIIRMQLRRALQALQAGELESQAAPDEAQGDPELGAVELRIE
ELRHHFRVEHAVAEGAKNVLRLLSGAKAPDRKAVSEAQEKLTESNQKLGLLRESLERRLGELPADHPKERLLR
EELTAASSSAFSAILPGPFPATHYSTLSKPAPLTGTLEVRVVGCKNLPETIPWSPPPSVGASGTPESRTPFLS
RPARGLYSRSGSLSGRSSLRGEAENATEVSTVLKLDNTVVGQTAWKPCGPNAWDQSFTLELERARELELAVFW
RDQRGLCALKFLKLEDFLDNERHEVQLDMEPQGCLVAEVTFRNPIIERIPRLQRQKKIFSKQQGKAFQRARQM
NIDVATWVRLLRRLIPSAVATGTFSPNASPGAEIRHTGDISMEKLNLGADSDSSSQKSPPGLPSTSCSLSSPT
HESTTSPELPSETQETPGPGLCSPLRKSPLTLEDFKFLAVLGRGHFGKVLLSEFRSSGELFAIKALKKGDIVA
RDEVESLMCEKRILAAVTRAGHPFLVNLFGCFQTPEHVCFVMEYSAGGDLMLHIHSDVFSEPRAVFYSACVVL
GLQFLHEHKIVYRDLKLDNLLLDTEGYVKIADFGLCKEGMGYGDRTSTFCGTPEFLAPEVLTDTSYTRAVDWW
GLGVLLYEMLVGESPFPGDDEEEVFDSIVNDEVRYPRFLSAEAIGIMRRLLRRNPERRLGSTERDAEDVKKQP
FFRSLGWDVLLARRLPPPFVPTLSGRTDVSNFDEEFTGEAPTLSPPRDARPLTAAEQAAFRDFDFVAGGY*
>rs33561266_ENSMUSP00000116235_c.877:G>A_p.G293E
ENSMUST00000144258.8|Pkn1-208|ENSMUSG00000057672|-|chr8:g.84410748:C>T
MAADPPLDSELESEPRSWSLLEQLGLAGADLAAPGVQQQLELERERLKREIRKELKLKEGAENLRRATTDLGR
SLAPVELLLRGSARRLDLLHQQLQELHAHVVLPDPAAGSDATQSLAEGSPICSSTNLSRVAGLEKQLAIELKV
KQGAENMIQTYSNGSSKDRKLLLTAQQMLQDSKTKIDIIRMQLRRALQALQAGELESQAAPDEAQGDPELGAV
ELRIEELRHHFRVEHAVAEGAKNVLRLLSGAKAPDRKAVSEAQEKLTESNQKLGLLRESLERRLGELPADHPK
ERLLREELTAASSSAFSAILPGPFPATHYSTLSKPAPLTGTLEVRVVGCKNLPETIPWSPPPSVGASGTPESR
TPFLSRPARGLYSRSGSLSGRSSLRGEAENATEVSTVLKLDNTVVGQTAWKPCGPNAWDQSFTLELERARELE
LAVFWRDQRGLCALKFLKLEDFLDNERHEVQLDMEPQGCLVAEVTFRNPIIERIPRLQRQKKIFSKQQGKAFQ
RARQMNIDVATWVRLLRRLIPSAVATGTFSPNASPGAEIRHTGDISMEKLNLGADSDSSSQKSPPGLPSTSCS
LSSPTHESTTSPELPSETQETPGPGLCSPLRKSPLTLEDFKFLAVLGRGHFGKVLLSEFRSSGELFAIKALKK
GDIVARDEVESLMCEKRILAAVTRAGHPFLVNLFGCFQTPEHVCFVMEYSAGGDLMLHIHSDVFSEPRAVFYS

ACVVLGLQFLHEHKIVYRDLKLDNLLLDTEGYVKIADFGLCKEGMGYGDRTSTFCGTPEFLAPEVLTDTSYTR
AVDWWGLGVLLYEMLVGESPFPGDDEEEVFDSIVNDEVRYPRFLSAEAIGIMRRLLRRNPERRLGSTERDAED
VKKQPFFRSLGWDVLLARRLPPPFVPTLSGRTDVSNFDEEFTGEAPTLSPPRDARPLTAAEQAAFRDFDFVAG
GY*
>rs33561266_ENSMUSP00000115054_c.898:G>A_p.G300E
ENSMUST00000132945.8|Pkn1-204|ENSMUSG00000057672|-|chr8:g.84410748:C>T
MACRPCRGAGRIWCCRSHKSEPRSWSLLEQLGLAGADLAAPGVQQQLELERERLKREIRKELKLKEGAENLRR
ATTDLGRSLAPVELLLRGSARRLDLLHQQLQELHAHVVLPDPAAGSDATQSLAEGSPICSSTNLSRVAGLEKQ
LAIELKVKQGAENMIQTYSNGSSKDRKLLLTAQQMLQDSKTKIDIIRMQLRRALQALQAGELESQAAPDEAQG
DPELGAVELRIEELRHHFRVEHAVAEGAKNVLRLLSGAKAPDRKAVSEAQEKLTESNQKLGLLRESLERRLGE
LPADHPKERLLREELTAASSSAFSAILPGPFPATHYSTLSKPAPLTGTLEVRVVGCKNLPETIPWSPPPSVGA
SGTPESRTPFLSRPARGLYSRSGSLSGRSSLRGEAENATEVSTVLKLDNTVVGQTAWKPCGPNAWDQSFTLEL
ERARELELAVFWRDQRGLCALKFLKLEDFLDNERHEVQLDMEPQGCLVAEVTFRNPIIERIPRLQRQKKIFSK
QQGKAFQRARQMNIDVATWVRLLRRLIPSAVATGTFSPNASPGAEIRHTGDISMEKLNLGADSDSSSQKSPPG
LPSTSCSLSSPTHESTTSPELPSETQETPGPGLCSPLRKSPLTLEDFKFLAVLGRGHFGKVLLSEFRSSGELF
AIKALKKGDIVARDEVESLMCEKRILAAVTRAGHPFLVNLFGCFQTPEHVCFVMEYSAGGDLMLHIHSDVFSE
PRAVFYSACVVLGLQFLHEHKIVYRDGLWGPDQHVLRNSGVPGAGSAHRHILHASSGLVGTGRAAL*
>rs46055145_ENSMUSP00000019576_c.198:C>G_p.P67A
ENSMUST00000019576.15|Ddx39a-201|ENSMUSG00000005481|+|chr8:g.84446218:C>G
MAEQDVENELLDYDEDEEPQAPQESTPAPPKKDVKGSYVSIHSSGFRDFLLKPELLRAIVDCGFEHASEVQHE
CIPQAILGMDVLCQAKSGMGKTAVFVLATLQQIEPVNGQVSVLVMCHTRELAFQISKEYERFSKYMPSVKVSV
FFGGLSIKKDEDVLKKNCPHVVVGTPGRILALVRSRSLNLRNVKHFVLDECDKMLEQLDMRRDVQEIFRLTPH
EKQCMMFSATLSKEIRPVCRKFMQDPMEVFVDDETKLTLHGLQQYYVKLKDSEKNRKLFDLLDVLEFNQVVIF
VKSVQRCMALAQLLVEQNFPAIAIHRGMAQEERLSRYQQFKDFQRRILVATNLFGRGMDIERVNIVFNYDMPE
DSDTYLHRVARAGRFGTKGLAVTFVSDENDAKILNDVQDRFEVNVAELPEEIDISTYIEQSR*
>rs46055145_ENSMUSP00000116101_c.198:C>G_p.P67A
ENSMUST00000140521.8|Ddx39a-205|ENSMUSG00000005481|+|chr8:g.84446218:C>G
MAEQDVENELLDYDEDEEPQAPQESTPAPPKKDVKGSYVSIHSSGFRDFLLKPELLRAIVDCGFEHASEVQHE
CIPQAILGMDVLCQAKSGMGKTAVFVLATLQQIEPVNGQVSVLVMCHTRELAFQISKEYERFSKYMPSVKVSV
FFGGLSIKKDEDVLKKNCPHVVVGTPGRILALVRSRSLNLRNVKHFVLDECDKMLEQLGSLS*
>rs46055145_ENSMUSP00000148329_c.198:C>G_p.P67A
ENSMUST00000212949.2|Ddx39a-207|ENSMUSG00000005481|+|chr8:g.84446218:C>G
MAEQDVENELLDYDEDEEPQAPQESTPAPPKKDVKGSYVSIHSSGFRDFLLKPELLRAIVDCGFEHASEVQHE
CIPQAILGMDVLCQAKSGMGKTAVFVLATLQQIEPVNGQVSVLVMCHTRELAFQISKEYERFSKYMPSVKVSV
FFGGLSIKKDEDVLKKNCPHVVVGTPGRILALVRSRSLNLRNVKHFVLDECDKMLEQLDMRRDVQEIFRLTPH
EKQCMMFSATLSKEIRPVCRKFMQDPMEVFVDDETKLTLHGLQQYYVKLKDSEKNRKLFDLLDVLEFNQVVIF
VKSVQRCMALAQLLVEQNFPAIAIHRGMAQEERLSRYQQFKDFQRRILVATNLFGRGMDIERVNIVFNYDMPE
DSDTYLHRVARAGRFGTKGLAVTFVSDENDAKILNDVQDRFEVNVAELPEEIDISTYIEQSR*
>rs46055145_ENSMUSP00000105435_c.198:C>G_p.P67A
ENSMUST00000109810.2|Ddx39a-202|ENSMUSG00000005481|+|chr8:g.84446218:C>G
MAEQDVENELLDYDEDEEPQAPQESTPAPPKKDVKGSYVSIHSSGFRDFLLKPELLRAIVDCGFEHASEVQHE
CIPQAILGMDVLCQAKSGMGKTAVFVLATLQQIEPVNGQVSVLVMCHTRELAFQISKEYERFSKYMPSVKVSV
FFGGLSIKKDEDVLKKNCPHVVVGTPGRILALVRSRSLNLRNVKHFVLDECDKMLEQLDMRRDVQEIFRLTPH
EKQCMMFSATLSKEIRPVCRKFMQDPMEVFVDDETKLTLHGLQQYYVKLKDSEKNRKLFDLLDVLEFNQVVIF
VKSVQRCMALAQLLVEQNFPAIAIHRGMAQEERLSRYQQFKDFQRRILVATNLFGRGMDIERVNIVFNYDMPE
DSDTYLHRVARAGRFGTKGLAVTFVSDENDAKILNDVQDRFEVNVAELPEEIDISTYIEQSR*
>rs46055145_ENSMUSP00000132222_c.198:C>G_p.P67A
ENSMUST00000172396.8|Ddx39a-206|ENSMUSG00000005481|+|chr8:g.84446218:C>G
MAEQDVENELLDYDEDEEPQAPQESTPAPPKKDVKGSYVSIHSSGFRDFLLKPELLRAIVDCGFEHASEVQHE
CIPQAILGMDVLCQAKSGMGKTAVFVLATLQQIEPVNGQVSVLVMCHTRELAFQISKEYERFSKYMPSVKVSV
FFGGLSIKKDEDVLKKNCPHVVVGTPGRILALVRSRSLNLRNVKHFVLDECDKMLEQLDMRRDVQEIFRLTPH
EKQCMMFSATLSKEIRPVCRKFMQDPMEVFVDDETKLTLHGLQQYYVKLKDSEKNRKLFDLLDVLEFNQVVIF
VKSVQRCMALAQLLVEQNFPAIAIHRGMAQEERLSRYQQFKDFQRRILVATNLFGRGMDIERVNIVFNYDMPE
DSDTYLHRVARAGRFGTKGLAVTFVSDENDAKILNDVQDRFEVNVAELPEEIDISTYIEQSR*
>ENSMUSP00000128220_c.1528:T>ATGTACATGAGGCAGGGTCTTGCCTT_p.L510ins-fs
ENSMUST00000166939.8|Adgre5-208|ENSMUSG00000002885|-
|chr8:g.84451578:A>AAGGCAAGACCCTGCCTCATGTACAT

MRGVRCPGLLVVCILLSLSGAGTQKAENCAKWCPINSKCVSNRSCVCKPGFSSEKELITNPAESCEDINECLL
PGFSCGDFAMCKNSEGSYTCVCNLGYKLLSGAESFVNESENTCQDVDECSSGQHQCHNSTVCKNTVGSYKCHC
RPGWKPTSGSLRGPDTICQEPPFPTWTLLPTAHSQTLLRFSVEVQNLLRDFNPATVNYTIQKLIEAVDKLLED
PMETETQQVAAQLLSNLEQSLRTLAQFLPKGPFTYTSPSNTELSLMVKEQDNKDVTTVHHGQTWMELDWAVTA
GAKISENGSSVAGILSSPNMEKLLGNTPLNLEQRRASLEDFYGSPIPSVSLKLLSNINSVFLTNTNTEKLASN
VTFKFDFTSVESIEPRHELICAFWKAHNGNGYWDTDGCSMNGTGFCHCNHLTSFAILMAQYHVQDPRLELITK
VGLLLSLICLLLCILTFLLVKPIQSSRTMVHLHLCICLFLGSIIFLVGVENEGGEVGLRCRLVAVMLHFCFHV
HEAGSCLGRLLLDGTGGRGALLPGGACVPGPRPEYMATLPDWLRGAPPHRGHLNGSCQNGWLWACNILLAGL
>ENSMUSP00000002964_c.1534:T>ATGTACATGAGGCAGGGTCTTGCCTT_p.L512ins-fs
ENSMUST00000002964.14|Adgre5-201|ENSMUSG00000002885|-
|chr8:g.84451578:A>AAGGCAAGACCCTGCCTCATGTACAT
MRGVRCPGLLVVCILLSLSGAGTQKAESKNCAKWCPINSKCVSNRSCVCKPGFSSEKELITNPAESCEDINEC
LLPGFSCGDFAMCKNSEGSYTCVCNLGYKLLSGAESFVNESENTCQDVDECSSGQHQCHNSTVCKNTVGSYKC
HCRPGWKPTSGSLRGPDTICQEPPFPTWTLLPTAHSQTLLRFSVEVQNLLRDFNPATVNYTIQKLIEAVDKLL
EDPMETETQQVAAQLLSNLEQSLRTLAQFLPKGPFTYTSPSNTELSLMVKEQDNKDVTTVHHGQTWMELDWAV
TAGAKISENGSSVAGILSSPNMEKLLGNTPLNLEQRRASLEDFYGSPIPSVSLKLLSNINSVFLTNTNTEKLA
SNVTFKFDFTSVESIEPRHELICAFWKAHNGNGYWDTDGCSMNGTGFCHCNHLTSFAILMAQYHVQDPRLELI
TKVGLLLSLICLLLCILTFLLVKPIQSSRTMVHLHLCICLFLGSIIFLVGVENEGGEVGLRCRLVAVMLHFCF
HVHEAGSCLGRLLLDGTGGRGALLPGGACVPGPRPEYMATLPDWLRGAPPHRGHLNGSCQNGWLWACNILLAG
L
>ENSMUSP00000075240_c.1816:T>ATGTACATGAGGCAGGGTCTTGCCTT_p.L606ins-fs
ENSMUST00000075843.13|Adgre5-202|ENSMUSG00000002885|-
|chr8:g.84451578:A>AAGGCAAGACCCTGCCTCATGTACAT
MRGVRCPGLLVVCILLSLSGAGTQKAESKNCAKWCPINSKCVSNRSCVCKPGFSSEKELITNPAESCEDINEC
LLPGFSCGDFAMCKNSEGSYTCVCNLGYKLLSGAESFVNESENTCQASVNTGMTPVPSRIHTVTTAPGNLPEQ
TTTVHQTQMGDSEERTPKDVNECISGQNHCHQSTHCINKLGGYSCICRQGWKPVPGSPNGPVSTVCEDVDECS
SGQHQCHNSTVCKNTVGSYKCHCRPGWKPTSGSLRGPDTICQEPPFPTWTLLPTAHSQTLLRFSVEVQNLLRD
FNPATVNYTIQKLIEAVDKLLEDPMETETQQVAAQLLSNLEQSLRTLAQFLPKGPFTYTSPSNTELSLMVKEQ
DNKDVTTVHHGQTWMELDWAVTAGAKISENGSSVAGILSSPNMEKLLGNTPLNLEQRRASLEDFYGSPIPSVS
LKLLSNINSVFLTNTNTEKLASNVTFKFDFTSVESIEPRHELICAFWKAHNGNGYWDTDGCSMNGTGFCHCNH
LTSFAILMAQYHVQDPRLELITKVGLLLSLICLLLCILTFLLVKPIQSSRTMVHLHLCICLFLGSIIFLVGVE
NEGGEVGLRCRLVAVMLHFCFHVHEAGSCLGRLLLDGTGGRGALLPGGACVPGPRPEYMATLPDWLRGAPPHR
GHLNGSCQNGWLWACNILLAGL
>ENSMUSP00000105427_c.1681:T>ATGTACATGAGGCAGGGTCTTGCCTT_p.L561ins-fs
ENSMUST00000109802.3|Adgre5-203|ENSMUSG00000002885|-
|chr8:g.84451578:A>AAGGCAAGACCCTGCCTCATGTACAT
MRGVRCPGLLVVCILLSLSGAGTQKAESKNCAKWCPINSKCVSNRSCVCKPGFSSEKELITNPAESCEDINEC
LLPGFSCGDFAMCKNSEGSYTCVCNLGYKLLSGAESFVNESENTCQDVNECISGQNHCHQSTHCINKLGGYSC
ICRQGWKPVPGSPNGPVSTVCEDVDECSSGQHQCHNSTVCKNTVGSYKCHCRPGWKPTSGSLRGPDTICQEPP
FPTWTLLPTAHSQTLLRFSVEVQNLLRDFNPATVNYTIQKLIEAVDKLLEDPMETETQQVAAQLLSNLEQSLR
TLAQFLPKGPFTYTSPSNTELSLMVKEQDNKDVTTVHHGQTWMELDWAVTAGAKISENGSSVAGILSSPNMEK
LLGNTPLNLEQRRASLEDFYGSPIPSVSLKLLSNINSVFLTNTNTEKLASNVTFKFDFTSVESIEPRHELICA
FWKAHNGNGYWDTDGCSMNGTGFCHCNHLTSFAILMAQYHVQDPRLELITKVGLLLSLICLLLCILTFLLVKP
IQSSRTMVHLHLCICLFLGSIIFLVGVENEGGEVGLRCRLVAVMLHFCFHVHEAGSCLGRLLLDGTGGRGALL
PGGACVPGPRPEYMATLPDWLRGAPPHRGHLNGSCQNGWLWACNILLAGL
>rs47983638_ENSMUSP00000128220_c.1488:C>T_p.R497C
ENSMUST00000166939.8|Adgre5-208|ENSMUSG00000002885|-|chr8:g.84451618:G>A
MRGVRCPGLLVVCILLSLSGAGTQKAENCAKWCPINSKCVSNRSCVCKPGFSSEKELITNPAESCEDINECLL
PGFSCGDFAMCKNSEGSYTCVCNLGYKLLSGAESFVNESENTCQDVDECSSGQHQCHNSTVCKNTVGSYKCHC
RPGWKPTSGSLRGPDTICQEPPFPTWTLLPTAHSQTLLRFSVEVQNLLRDFNPATVNYTIQKLIEAVDKLLED
PMETETQQVAAQLLSNLEQSLRTLAQFLPKGPFTYTSPSNTELSLMVKEQDNKDVTTVHHGQTWMELDWAVTA
GAKISENGSSVAGILSSPNMEKLLGNTPLNLEQRRASLEDFYGSPIPSVSLKLLSNINSVFLTNTNTEKLASN
VTFKFDFTSVESIEPRHELICAFWKAHNGNGYWDTDGCSMNGTGFCHCNHLTSFAILMAQYHVQDPRLELITK
VGLLLSLICLLLCILTFLLVKPIQSSRTMVHLHLCICLFLGSIIFLVGVENEGGEVGLCCRLVAVMLHFCFLA
AFCWMALEGVELYFLVVRVFQGQGLSTWQRCLIGYGVPLLIVAISMAVVKMDGYGHATYCWLDFRKQGFLWSF
SGPVAFIIFCNAAIFVITVWKLTKKFSEINPNMKKLRKARVLTITSIAQLLVLGCTWGFGLFLFNPHSTWLSY
IFTLLNCLQGLFLYVMLCLLNKKVREEYWKWACMVTGSKYTEFNSSTTGTGTSQTRALRSSESGM*

>rs47983638_ENSMUSP00000002964_c.1494:C>T_p.R499C
ENSMUST00000002964.14|Adgre5-201|ENSMUSG00000002885|-|chr8:g.84451618:G>A
MRGVRCPGLLVVCILLSLSGAGTQKAESKNCAKWCPINSKCVSNRSCVCKPGFSSEKELITNPAESCEDINEC
LLPGFSCGDFAMCKNSEGSYTCVCNLGYKLLSGAESFVNESENTCQDVDECSSGQHQCHNSTVCKNTVGSYKC
HCRPGWKPTSGSLRGPDTICQEPPFPTWTLLPTAHSQTLLRFSVEVQNLLRDFNPATVNYTIQKLIEAVDKLL
EDPMETETQQVAAQLLSNLEQSLRTLAQFLPKGPFTYTSPSNTELSLMVKEQDNKDVTTVHHGQTWMELDWAV
TAGAKISENGSSVAGILSSPNMEKLLGNTPLNLEQRRASLEDFYGSPIPSVSLKLLSNINSVFLTNTNTEKLA
SNVTFKFDFTSVESIEPRHELICAFWKAHNGNGYWDTDGCSMNGTGFCHCNHLTSFAILMAQYHVQDPRLELI
TKVGLLLSLICLLLCILTFLLVKPIQSSRTMVHLHLCICLFLGSIIFLVGVENEGGEVGLCCRLVAVMLHFCF
LAAFCWMALEGVELYFLVVRVFQGQGLSTWQRCLIGYGVPLLIVAISMAVVKMDGYGHATYCWLDFRKQGFLW
SFSGPVAFIIFCNAAIFVITVWKLTKKFSEINPNMKKLRKARVLTITSIAQLLVLGCTWGFGLFLFNPHSTWL
SYIFTLLNCLQGLFLYVMLCLLNKKVREEYWKWACMVTGSKYTEFNSSTTGTGTSQTRALRSSESGM*
>rs47983638_ENSMUSP00000075240_c.1776:C>T_p.R593C
ENSMUST00000075843.13|Adgre5-202|ENSMUSG00000002885|-|chr8:g.84451618:G>A
MRGVRCPGLLVVCILLSLSGAGTQKAESKNCAKWCPINSKCVSNRSCVCKPGFSSEKELITNPAESCEDINEC
LLPGFSCGDFAMCKNSEGSYTCVCNLGYKLLSGAESFVNESENTCQASVNTGMTPVPSRIHTVTTAPGNLPEQ
TTTVHQTQMGDSEERTPKDVNECISGQNHCHQSTHCINKLGGYSCICRQGWKPVPGSPNGPVSTVCEDVDECS
SGQHQCHNSTVCKNTVGSYKCHCRPGWKPTSGSLRGPDTICQEPPFPTWTLLPTAHSQTLLRFSVEVQNLLRD
FNPATVNYTIQKLIEAVDKLLEDPMETETQQVAAQLLSNLEQSLRTLAQFLPKGPFTYTSPSNTELSLMVKEQ
DNKDVTTVHHGQTWMELDWAVTAGAKISENGSSVAGILSSPNMEKLLGNTPLNLEQRRASLEDFYGSPIPSVS
LKLLSNINSVFLTNTNTEKLASNVTFKFDFTSVESIEPRHELICAFWKAHNGNGYWDTDGCSMNGTGFCHCNH
LTSFAILMAQYHVQDPRLELITKVGLLLSLICLLLCILTFLLVKPIQSSRTMVHLHLCICLFLGSIIFLVGVE
NEGGEVGLCCRLVAVMLHFCFLAAFCWMALEGVELYFLVVRVFQGQGLSTWQRCLIGYGVPLLIVAISMAVVK
MDGYGHATYCWLDFRKQGFLWSFSGPVAFIIFCNAAIFVITVWKLTKKFSEINPNMKKLRKARVLTITSIAQL
LVLGCTWGFGLFLFNPHSTWLSYIFTLLNCLQGLFLYVMLCLLNKKVREEYWKWACMVTGSKYTEFNSSTTGT
GTSQTRALRSSESGM*
>rs47983638_ENSMUSP00000105427_c.1641:C>T_p.R548C
ENSMUST00000109802.3|Adgre5-203|ENSMUSG00000002885|-|chr8:g.84451618:G>A
MRGVRCPGLLVVCILLSLSGAGTQKAESKNCAKWCPINSKCVSNRSCVCKPGFSSEKELITNPAESCEDINEC
LLPGFSCGDFAMCKNSEGSYTCVCNLGYKLLSGAESFVNESENTCQDVNECISGQNHCHQSTHCINKLGGYSC
ICRQGWKPVPGSPNGPVSTVCEDVDECSSGQHQCHNSTVCKNTVGSYKCHCRPGWKPTSGSLRGPDTICQEPP
FPTWTLLPTAHSQTLLRFSVEVQNLLRDFNPATVNYTIQKLIEAVDKLLEDPMETETQQVAAQLLSNLEQSLR
TLAQFLPKGPFTYTSPSNTELSLMVKEQDNKDVTTVHHGQTWMELDWAVTAGAKISENGSSVAGILSSPNMEK
LLGNTPLNLEQRRASLEDFYGSPIPSVSLKLLSNINSVFLTNTNTEKLASNVTFKFDFTSVESIEPRHELICA
FWKAHNGNGYWDTDGCSMNGTGFCHCNHLTSFAILMAQYHVQDPRLELITKVGLLLSLICLLLCILTFLLVKP
IQSSRTMVHLHLCICLFLGSIIFLVGVENEGGEVGLCCRLVAVMLHFCFLAAFCWMALEGVELYFLVVRVFQG
QGLSTWQRCLIGYGVPLLIVAISMAVVKMDGYGHATYCWLDFRKQGFLWSFSGPVAFIIFCNAAIFVITVWKL
TKKFSEINPNMKKLRKARVLTITSIAQLLVLGCTWGFGLFLFNPHSTWLSYIFTLLNCLQGLFLYVMLCLLNK
KVREEYWKWACMVTGSKYTEFNSSTTGTGTSQTRALRSSESGM*
>rs32903748_ENSMUSP00000075240_c.445:C>A_p.T149K
ENSMUST00000075843.13|Adgre5-202|ENSMUSG00000002885|-|chr8:g.84458579:G>T
MRGVRCPGLLVVCILLSLSGAGTQKAESKNCAKWCPINSKCVSNRSCVCKPGFSSEKELITNPAESCEDINEC
LLPGFSCGDFAMCKNSEGSYTCVCNLGYKLLSGAESFVNESENTCQASVNTGMTPVPSRIHTVTTAPGNLPEQ
TTKVHQTQMGDSEERTPKDVNECISGQNHCHQSTHCINKLGGYSCICRQGWKPVPGSPNGPVSTVCEDVDECS
SGQHQCHNSTVCKNTVGSYKCHCRPGWKPTSGSLRGPDTICQEPPFPTWTLLPTAHSQTLLRFSVEVQNLLRD
FNPATVNYTIQKLIEAVDKLLEDPMETETQQVAAQLLSNLEQSLRTLAQFLPKGPFTYTSPSNTELSLMVKEQ
DNKDVTTVHHGQTWMELDWAVTAGAKISENGSSVAGILSSPNMEKLLGNTPLNLEQRRASLEDFYGSPIPSVS
LKLLSNINSVFLTNTNTEKLASNVTFKFDFTSVESIEPRHELICAFWKAHNGNGYWDTDGCSMNGTGFCHCNH
LTSFAILMAQYHVQDPRLELITKVGLLLSLICLLLCILTFLLVKPIQSSRTMVHLHLCICLFLGSIIFLVGVE
NEGGEVGLRCRLVAVMLHFCFLAAFCWMALEGVELYFLVVRVFQGQGLSTWQRCLIGYGVPLLIVAISMAVVK
MDGYGHATYCWLDFRKQGFLWSFSGPVAFIIFCNAAIFVITVWKLTKKFSEINPNMKKLRKARVLTITSIAQL
LVLGCTWGFGLFLFNPHSTWLSYIFTLLNCLQGLFLYVMLCLLNKKVREEYWKWACMVTGSKYTEFNSSTTGT
GTSQTRALRSSESGM*
>rs49019694_ENSMUSP00000075240_c.373:G>A_p.G125E
ENSMUST00000075843.13|Adgre5-202|ENSMUSG00000002885|-|chr8:g.84458651:C>T
MRGVRCPGLLVVCILLSLSGAGTQKAESKNCAKWCPINSKCVSNRSCVCKPGFSSEKELITNPAESCEDINEC
LLPGFSCGDFAMCKNSEGSYTCVCNLGYKLLSGAESFVNESENTCQASVNTEMTPVPSRIHTVTTAPGNLPEQ
TTTVHQTQMGDSEERTPKDVNECISGQNHCHQSTHCINKLGGYSCICRQGWKPVPGSPNGPVSTVCEDVDECS

SGQHQCHNSTVCKNTVGSYKCHCRPGWKPTSGSLRGPDTICQEPPFPTWTLLPTAHSQTLLRFSVEVQNLLRD
FNPATVNYTIQKLIEAVDKLLEDPMETETQQVAAQLLSNLEQSLRTLAQFLPKGPFTYTSPSNTELSLMVKEQ
DNKDVTTVHHGQTWMELDWAVTAGAKISENGSSVAGILSSPNMEKLLGNTPLNLEQRRASLEDFYGSPIPSVS
LKLLSNINSVFLTNTNTEKLASNVTFKFDFTSVESIEPRHELICAFWKAHNGNGYWDTDGCSMNGTGFCHCNH
LTSFAILMAQYHVQDPRLELITKVGLLLSLICLLLCILTFLLVKPIQSSRTMVHLHLCICLFLGSIIFLVGVE
NEGGEVGLRCRLVAVMLHFCFLAAFCWMALEGVELYFLVVRVFQGQGLSTWQRCLIGYGVPLLIVAISMAVVK
MDGYGHATYCWLDFRKQGFLWSFSGPVAFIIFCNAAIFVITVWKLTKKFSEINPNMKKLRKARVLTITSIAQL
LVLGCTWGFGLFLFNPHSTWLSYIFTLLNCLQGLFLYVMLCLLNKKVREEYWKWACMVTGSKYTEFNSSTTGT
GTSQTRALRSSESGM*
>rs33291739_ENSMUSP00000118452_c.104:G>A_p.M35I
ENSMUST00000141158.8|Adgrl1-207|ENSMUSG00000013033|+|chr8:g.84645483:G>A
MARLAAALWSLCVTTVLVTSATQGLSRAGLPFGLIRRELACEGYPIELRCPGSDVIMVENANYGRTDDKICDA
DPFQMENVQCYLPDAFKIMSQRCNNRTQCVVVAGSDAFPDPCPGTYKYLEVQYDCVPYIFVCPGTLQKVLEPT
STHESEHQSGAWCKDPLQAGDRIYVMPWIPYRTDTLTEYASWEDYVAARHTTTYRLPNRVDGTGFVVYDGAVF
YNKERTRNIVKYDLRTRIKSGETVINTANYHDTSPYRWGGKTDIDLAVDENGLWVIYATEGNNGRLVVSQLNP
YTLRFEGTWETGYDKRSASNAFMVCGVLYVLRSVYVDDDSEAAGNRVDYAFNTNANREEPVSLAFPNPYQFVS
SVDYNPRDNQLYVWNNYFVVRYSLEFGPPDPSAGPATSPPLSTTTTARPTPLTSTASPAATTPLRRAPLTTHP
VGAINQLGPDLPPATAPAPSTRRPPAPNLHVSPELFCEPREVRRVQWPATQQGMLVERPCPKGTRGIASFQCL
PALGLWNPRGPDLSNCTSPWVNQVAQKIKSGENAANIASELARHTRGSIYAGDVSSSVKLMEQLLDILDAQLQ
ALRPIERESAGKNYNKMHKRERTCKDYIKAVVETVDNLLRPEALESWKDMNATEQVHTATMLLDVLEEGAFLL
ADNVREPARFLAAKQNVVLEVTVLNTEGQVQELVFPQEYPSENSIQLSANTIKQNSRNGVVKVVFILYNNLGL
FLSTENATVKLAGEAGTGGPGGASLVVNSQVIAASINKESSRVFLMDPVIFTVAHLEAKNHFNANCSFWNYSE
RSMLGYWSTQGCRLVESNKTHTTCACSHLTNFAVLMAHREIYQGRINELLLSVITWVGIVISLVCLAICISTF
CFLRGLQTDRNTIHKNLCINLFLAELLFLVGIDKTQYEVACPIFAGLLHYFFLAAFSWLCLEGVHLYLLLVEV
FESEYSRTKYYYLGGYCFPALVVGIAAAIDYRSYGTEKACWLRVDNYFIWSFIGPVSFVIVVNLVFLMVTLHK
MIRSSSVLKPDSSRLDNIKSWALGAIALLFLLGLTWAFGLLFINKESVVMAYLFTTFNAFQGVFIFVFHCALQ
KKVHKEYSKCLRHSYCCIRSPPGGTHGSLKTSAMRSNTRYYTGTQSRIRRMWNDTVRKQTESSFMAGDINSTP
TLNRGTMGNHLLTNPVLQPRGGTSPYNTLIAESVGFNPSSPPVFNSPGSYREPKHPLGGREACGMDTLPLNGN
FNNSYSLRSGDFPPGDGGPEPPRGRNLADAAAFEKMIISELVHNNLRGASGGAKGPPPEPPVPPVPGVSEDEA
GGPGSADRAEIELLYKALEEPLLLPRAQSVLYQSDLDESESCTAEDGATSRPLSSPPGRDSLYASGANLRDSP
SYPDSSPEGPNEALPPPPPAPPGPPEIYYTSRPPALVARNPLQGYYQVRRPSHEGYLAAPSLEGPGPDGDGQM
QLVTSL*
>rs33291739_ENSMUSP00000116064_c.104:G>A_p.M35I
ENSMUST00000124355.8|Adgrl1-202|ENSMUSG00000013033|+|chr8:g.84645483:G>A
MARLAAALWSLCVTTVLVTSATQGLSRAGLPFGLIRRELACEGYPIELRCPGSDVIMVENANYGRTDDKICDA
DPFQMENVQCYLPDAFKIMSQRCNNRTQCVVVAGSDAFPDPCPGTYKYLEVQYDCVPYNGGVSPGDRE*
>rs33291739_ENSMUSP00000048422_c.104:G>A_p.M35I
ENSMUST00000045393.15|Adgrl1-201|ENSMUSG00000013033|+|chr8:g.84645483:G>A
MARLAAALWSLCVTTVLVTSATQGLSRAGLPFGLIRRELACEGYPIELRCPGSDVIMVENANYGRTDDKICDA
DPFQMENVQCYLPDAFKIMSQRCNNRTQCVVVAGSDAFPDPCPGTYKYLEVQYDCVPYKVEQKVFVCPGTLQK
VLEPTSTHESEHQSGAWCKDPLQAGDRIYVMPWIPYRTDTLTEYASWEDYVAARHTTTYRLPNRVDGTGFVVY
DGAVFYNKERTRNIVKYDLRTRIKSGETVINTANYHDTSPYRWGGKTDIDLAVDENGLWVIYATEGNNGRLVV
SQLNPYTLRFEGTWETGYDKRSASNAFMVCGVLYVLRSVYVDDDSEAAGNRVDYAFNTNANREEPVSLAFPNP
YQFVSSVDYNPRDNQLYVWNNYFVVRYSLEFGPPDPSAGPATSPPLSTTTTARPTPLTSTASPAATTPLRRAP
LTTHPVGAINQLGPDLPPATAPAPSTRRPPAPNLHVSPELFCEPREVRRVQWPATQQGMLVERPCPKGTRGIA
SFQCLPALGLWNPRGPDLSNCTSPWVNQVAQKIKSGENAANIASELARHTRGSIYAGDVSSSVKLMEQLLDIL
DAQLQALRPIERESAGKNYNKMHKRERTCKDYIKAVVETVDNLLRPEALESWKDMNATEQVHTATMLLDVLEE
GAFLLADNVREPARFLAAKQNVVLEVTVLNTEGQVQELVFPQEYPSENSIQLSANTIKQNSRNGVVKVVFILY
NNLGLFLSTENATVKLAGEAGTGGPGGASLVVNSQVIAASINKESSRVFLMDPVIFTVAHLEAKNHFNANCSF
WNYSERSMLGYWSTQGCRLVESNKTHTTCACSHLTNFAVLMAHREIYQGRINELLLSVITWVGIVISLVCLAI
CISTFCFLRGLQTDRNTIHKNLCINLFLAELLFLVGIDKTQYEVACPIFAGLLHYFFLAAFSWLCLEGVHLYL
LLVEVFESEYSRTKYYYLGGYCFPALVVGIAAAIDYRSYGTEKACWLRVDNYFIWSFIGPVSFVIVVNLVFLM
VTLHKMIRSSSVLKPDSSRLDNIKSWALGAIALLFLLGLTWAFGLLFINKESVVMAYLFTTFNAFQGVFIFVF
HCALQKKVHKEYSKCLRHSYCCIRSPPGGTHGSLKTSAMRSNTRYYTGTQSRIRRMWNDTVRKQTESSFMAGD
INSTPTLNRGTMGNHLLTNPVLQPRGGTSPYNTLIAESVGFNPSSPPVFNSPGSYREPKHPLGGREACGMDTL
PLNGNFNNSYSLRSGDFPPGDGGPEPPRGRNLADAAAFEKMIISELVHNNLRGASGGAKGPPPEPPVPPVPGV
SEDEAGGPGSADRAEIELLYKALEEPLLLPRAQSVLYQSDLDESESCTAEDGATSRPLSSPPGRDSLYASGAN

LRDSPSYPDSSPEGPNEALPPPPPAPPGPPEIYYTSRPPALVARNPLQGYYQVRRPSHEGYLAAPSLEGPGPD
GDGQMQLVTSL*
>rs33291739_ENSMUSP00000119100_c.104:G>A_p.M35I
ENSMUST00000132500.8|Adgrl1-205|ENSMUSG00000013033|+|chr8:g.84645483:G>A
MARLAAALWSLCVTTVLVTSATQGLSRAGLPFGLIRRELACEGYPIELRCPGSDVIMVENANYGRTDDKICDA
DPFQMENVQCYLPDAFKIMSQRCNNRTQCVVVAGSDAFPDPCPGTYKYLEVQYDCVPYIFVCPGTLQKVLEPT
STHESEHQSGAWCKDPLQAGDRIYVMPWIPYRTDTLTEYASWEDYVAARHTTTYRLPNRVDGTGFVVYDGAVF
YNKERTRNIVKYDLRTRIKSGETVINTANYHDTSPYRWGGKTDIDLAVDENGLWVIYATEGNNGRLVVSQLNP
YTLRFEGTWETGYDKRSASNAFMVCGVLYVLRSVYVDDDSEAAGNRVDYAFNTNANREEPVSLAFPNPYQFVS
SVDYNPRDNQLYVWNNYFVVRYSLEFGPPDPSAGPATSPPLSTTTTARPTPLTSTASPAATTPLRRAPLTTHP
VGAINQLGPDLPPATAPAPSTRRPPAPNLHVSPELFCEPREVRRVQWPATQQGMLVERPCPKGTRGIASFQCL
PALGLWNPRGPDLSNCTSPWVNQVAQKIKSGENAANIASELARHTRGSIYAGDVSSSVKLMEQLLDILDAQLQ
ALRPIERESAGKNYNKMHKRERTCKDYIKAVVETVDNLLRPEALESWKDMNATEQVHTATMLLDVLEEGAFLL
ADNVREPARFLAAKQNVVLEVTVLNTEGQVQELVFPQEYPSENSIQLSANTIKQNSRNGVVKVVFILYNNLGL
FLSTENATVKLAGEAGTGGPGGASLVVNSQVIAASINKESSRVFLMDPVIFTVAHLEAKNHFNANCSFWNYSE
RSMLGYWSTQGCRLVESNKTHTTCACSHLTNFAVLMAHREIYQGRINELLLSVITWVGIVISLVCLAICISTF
CFLRGLQTDRNTIHKNLCINLFLAELLFLVGIDKTQYEVACPIFAGLLHYFFLAAFSWLCLEGVHLYLLLVEV
FESEYSRTKYYYLGGYCFPALVVGIAAAIDYRSYGTEKACWLRVDNYFIWSFIGPVSFVIVVNLVFLMVTLHK
MIRSSSVLKPDSSRLDNIKSWALGAIALLFLLGLTWAFGLLFINKESVVMAYLFTTFNAFQGVFIFVFHCALQ
KKVHKEYSKCLRHSYCCIRSPPGGTHGSLKTSAMRSNTRYYTGTQVPGQGRHIHQVSLGPRGRSTLPESQKDP
GGQSGPRDPLMFGLCPQSRIRRMWNDTVRKQTESSFMAGDINSTPTLNRGTMGNHLLTNPVLQPRGGTSPYNT
LIAESVGFNPSSPPVFNSPGSYREPKHPLGGREACGMDTLPLNGNFNNSYSLRSGDFPPGDGGPEPPRGRNLA
DAAAFEKMIISELVHNNLRGASGGAKGPPPEPPVPPVPGVSEDEAGGPGSADRAEIELLYKALEEPLLLPRAQ
SVLYQSDLDESESCTAEDGATSRPLSSPPGRDSLYASGANLRDSPSYPDSSPEGPNEALPPPPPAPPGPPEIY
YTSRPPALVARNPLQGYYQVRRPSHEGYLAAPSLEGPGPDGDGQMQLVTSL*
>rs33291739_ENSMUSP00000115295_c.104:G>A_p.M35I
ENSMUST00000152978.8|Adgrl1-210|ENSMUSG00000013033|+|chr8:g.84645483:G>A
MARLAAALWSLCVTTVLVTSATQGLSRAGLPFGLIRRELACEGYPIELRCPGSDVIMVENANYGRTDDKICDA
DPFQMENVQCYLPDAFKIMSQRCNNRTQCVVVAGSDAFPDPCPGTYKYLEVQYDCVPYKVEQKVFVCPGTLQK
VLEPTSTHESEHQSGAWCKDPLQAGDRIYVMPWIPYRTDTLTEYASWEDYVAARHTTTYRLPNRVDGTGFVVY
DGAVFYNKERTRNIVKYDLRTRIKSGETVINTANYHDTSPYRWGGKTDIDLAVDENGLWVIYATEGNNGRLVV
SQLNPYTLRFEGTWETGYDKRSASNAFMVCGVLYVLRSVYVDDDSEAAGNRVDYAFNTNANREEPVSLAFPNP
YQFVSSVDYNPRDNQLYVWNNYFVVRYSLEFGPPDPSAGPATSPPLSTTTTARPTPLTSTASPAATTPLRRAP
LTTHPVGAINQLGPDLPPATAPAPSTRRPPAPNLHVSPELFCEPREVRRVQWPATQQGMLVERPCPKGTRGIA
SFQCLPALGLWNPRGPDLSNCTSPWVNQVAQKIKSGENAANIASELARHTRGSIYAGDVSSSVKLMEQLLDIL
DAQLQALRPIERESAGKNYNKMHKRERTCKDYIKAVVETVDNLLRPEALESWKDMNATEQVHTATMLLDVLEE
GAFLLADNVREPARFLAAKQNVVLEVTVLNTEGQVQELVFPQEYPSENSIQLSANTIKQNSRNGVVKVVFILY
NNLGLFLSTENATVKLAGEAGTGGPGGASLVVNSQVIAASINKESSRVFLMDPVIFTVAHLEAKNHFNANCSF
WNYSERSMLGYWSTQGCRLVESNKTHTTCACSHLTNFAVLMAHREIYQGRINELLLSVITWVGIVISLVCLAI
CISTFCFLRGLQTDRNTIHKNLCINLFLAELLFLVGIDKTQYEVACPIFAGLLHYFFLAAFSWLCLEGVHLYL
LLVEVFESEYSRTKYYYLGGYCFPALVVGIAAAIDYRSYGTEKACWLRVDNYFIWSFIGPVSFVIVVNLVFLM
VTLHKMIRSSSVLKPDSSRLDNIKSWALGAIALLFLLGLTWAFGLLFINKESVVMAYLFTTFNAFQGVFIFVF
HCALQKKVHKEYSKCLRHSYCCIRSPPGGTHGSLKTSAMRSNTRYYTGTQVPGQGRHIHQVSLGPRGRSTLPE
SQKDPGGQSGPRDPLMFGLCPQSRIRRMWNDTVRKQTESSFMAGDINSTPTLNRGTMGNHLLTNPVLQPRGGT
SPYNTLIAESVGFNPSSPPVFNSPGSYREPKHPLGGREACGMDTLPLNGNFNNSYSLRSGDFPPGDGGPEPPR
GRNLADAAAFEKMIISELVHNNLRGASGGAKGPPPEPPVPPVPGVSEDEAGGPGSADRAEIELLYKALEEPLL
LPRAQSVLYQSDLDESESCTAEDGATSRPLSSPPGRDSLYASGANLRDSPSYPDSSPEGPNEALPPPPPAPPG
PPEIYYTSRPPALVARNPLQGYYQVRRPSHEGYLAAPSLEGPGPDGDGQMQLVTSL*
>rs32753131_ENSMUSP00000118452_c.1872:C>T_p.P625S
ENSMUST00000141158.8|Adgrl1-207|ENSMUSG00000013033|+|chr8:g.84659170:C>T
MARLAAALWSLCVTTVLVTSATQGLSRAGLPFGLMRRELACEGYPIELRCPGSDVIMVENANYGRTDDKICDA
DPFQMENVQCYLPDAFKIMSQRCNNRTQCVVVAGSDAFPDPCPGTYKYLEVQYDCVPYIFVCPGTLQKVLEPT
STHESEHQSGAWCKDPLQAGDRIYVMPWIPYRTDTLTEYASWEDYVAARHTTTYRLPNRVDGTGFVVYDGAVF
YNKERTRNIVKYDLRTRIKSGETVINTANYHDTSPYRWGGKTDIDLAVDENGLWVIYATEGNNGRLVVSQLNP
YTLRFEGTWETGYDKRSASNAFMVCGVLYVLRSVYVDDDSEAAGNRVDYAFNTNANREEPVSLAFPNPYQFVS
SVDYNPRDNQLYVWNNYFVVRYSLEFGPPDPSAGPATSPPLSTTTTARPTPLTSTASPAATTPLRRAPLTTHP
VGAINQLGPDLPPATAPAPSTRRPPAPNLHVSPELFCEPREVRRVQWPATQQGMLVERPCPKGTRGIASFQCL
PALGLWNPRGPDLSNCTSPWVNQVAQKIKSGENAANIASELARHTRGSIYAGDVSSSVKLMEQLLDILDAQLQ

ALRPIERESAGKNYNKMHKRERTCKDYIKAVVETVDNLLRSEALESWKDMNATEQVHTATMLLDVLEEGAFLL
ADNVREPARFLAAKQNVVLEVTVLNTEGQVQELVFPQEYPSENSIQLSANTIKQNSRNGVVKVVFILYNNLGL
FLSTENATVKLAGEAGTGGPGGASLVVNSQVIAASINKESSRVFLMDPVIFTVAHLEAKNHFNANCSFWNYSE
RSMLGYWSTQGCRLVESNKTHTTCACSHLTNFAVLMAHREIYQGRINELLLSVITWVGIVISLVCLAICISTF
CFLRGLQTDRNTIHKNLCINLFLAELLFLVGIDKTQYEVACPIFAGLLHYFFLAAFSWLCLEGVHLYLLLVEV
FESEYSRTKYYYLGGYCFPALVVGIAAAIDYRSYGTEKACWLRVDNYFIWSFIGPVSFVIVVNLVFLMVTLHK
MIRSSSVLKPDSSRLDNIKSWALGAIALLFLLGLTWAFGLLFINKESVVMAYLFTTFNAFQGVFIFVFHCALQ
KKVHKEYSKCLRHSYCCIRSPPGGTHGSLKTSAMRSNTRYYTGTQSRIRRMWNDTVRKQTESSFMAGDINSTP
TLNRGTMGNHLLTNPVLQPRGGTSPYNTLIAESVGFNPSSPPVFNSPGSYREPKHPLGGREACGMDTLPLNGN
FNNSYSLRSGDFPPGDGGPEPPRGRNLADAAAFEKMIISELVHNNLRGASGGAKGPPPEPPVPPVPGVSEDEA
GGPGSADRAEIELLYKALEEPLLLPRAQSVLYQSDLDESESCTAEDGATSRPLSSPPGRDSLYASGANLRDSP
SYPDSSPEGPNEALPPPPPAPPGPPEIYYTSRPPALVARNPLQGYYQVRRPSHEGYLAAPSLEGPGPDGDGQM
QLVTSL*
>rs32753131_ENSMUSP00000048422_c.1887:C>T_p.P630S
ENSMUST00000045393.15|Adgrl1-201|ENSMUSG00000013033|+|chr8:g.84659170:C>T
MARLAAALWSLCVTTVLVTSATQGLSRAGLPFGLMRRELACEGYPIELRCPGSDVIMVENANYGRTDDKICDA
DPFQMENVQCYLPDAFKIMSQRCNNRTQCVVVAGSDAFPDPCPGTYKYLEVQYDCVPYKVEQKVFVCPGTLQK
VLEPTSTHESEHQSGAWCKDPLQAGDRIYVMPWIPYRTDTLTEYASWEDYVAARHTTTYRLPNRVDGTGFVVY
DGAVFYNKERTRNIVKYDLRTRIKSGETVINTANYHDTSPYRWGGKTDIDLAVDENGLWVIYATEGNNGRLVV
SQLNPYTLRFEGTWETGYDKRSASNAFMVCGVLYVLRSVYVDDDSEAAGNRVDYAFNTNANREEPVSLAFPNP
YQFVSSVDYNPRDNQLYVWNNYFVVRYSLEFGPPDPSAGPATSPPLSTTTTARPTPLTSTASPAATTPLRRAP
LTTHPVGAINQLGPDLPPATAPAPSTRRPPAPNLHVSPELFCEPREVRRVQWPATQQGMLVERPCPKGTRGIA
SFQCLPALGLWNPRGPDLSNCTSPWVNQVAQKIKSGENAANIASELARHTRGSIYAGDVSSSVKLMEQLLDIL
DAQLQALRPIERESAGKNYNKMHKRERTCKDYIKAVVETVDNLLRSEALESWKDMNATEQVHTATMLLDVLEE
GAFLLADNVREPARFLAAKQNVVLEVTVLNTEGQVQELVFPQEYPSENSIQLSANTIKQNSRNGVVKVVFILY
NNLGLFLSTENATVKLAGEAGTGGPGGASLVVNSQVIAASINKESSRVFLMDPVIFTVAHLEAKNHFNANCSF
WNYSERSMLGYWSTQGCRLVESNKTHTTCACSHLTNFAVLMAHREIYQGRINELLLSVITWVGIVISLVCLAI
CISTFCFLRGLQTDRNTIHKNLCINLFLAELLFLVGIDKTQYEVACPIFAGLLHYFFLAAFSWLCLEGVHLYL
LLVEVFESEYSRTKYYYLGGYCFPALVVGIAAAIDYRSYGTEKACWLRVDNYFIWSFIGPVSFVIVVNLVFLM
VTLHKMIRSSSVLKPDSSRLDNIKSWALGAIALLFLLGLTWAFGLLFINKESVVMAYLFTTFNAFQGVFIFVF
HCALQKKVHKEYSKCLRHSYCCIRSPPGGTHGSLKTSAMRSNTRYYTGTQSRIRRMWNDTVRKQTESSFMAGD
INSTPTLNRGTMGNHLLTNPVLQPRGGTSPYNTLIAESVGFNPSSPPVFNSPGSYREPKHPLGGREACGMDTL
PLNGNFNNSYSLRSGDFPPGDGGPEPPRGRNLADAAAFEKMIISELVHNNLRGASGGAKGPPPEPPVPPVPGV
SEDEAGGPGSADRAEIELLYKALEEPLLLPRAQSVLYQSDLDESESCTAEDGATSRPLSSPPGRDSLYASGAN
LRDSPSYPDSSPEGPNEALPPPPPAPPGPPEIYYTSRPPALVARNPLQGYYQVRRPSHEGYLAAPSLEGPGPD
GDGQMQLVTSL*
>rs32753131_ENSMUSP00000119100_c.1872:C>T_p.P625S
ENSMUST00000132500.8|Adgrl1-205|ENSMUSG00000013033|+|chr8:g.84659170:C>T
MARLAAALWSLCVTTVLVTSATQGLSRAGLPFGLMRRELACEGYPIELRCPGSDVIMVENANYGRTDDKICDA
DPFQMENVQCYLPDAFKIMSQRCNNRTQCVVVAGSDAFPDPCPGTYKYLEVQYDCVPYIFVCPGTLQKVLEPT
STHESEHQSGAWCKDPLQAGDRIYVMPWIPYRTDTLTEYASWEDYVAARHTTTYRLPNRVDGTGFVVYDGAVF
YNKERTRNIVKYDLRTRIKSGETVINTANYHDTSPYRWGGKTDIDLAVDENGLWVIYATEGNNGRLVVSQLNP
YTLRFEGTWETGYDKRSASNAFMVCGVLYVLRSVYVDDDSEAAGNRVDYAFNTNANREEPVSLAFPNPYQFVS
SVDYNPRDNQLYVWNNYFVVRYSLEFGPPDPSAGPATSPPLSTTTTARPTPLTSTASPAATTPLRRAPLTTHP
VGAINQLGPDLPPATAPAPSTRRPPAPNLHVSPELFCEPREVRRVQWPATQQGMLVERPCPKGTRGIASFQCL
PALGLWNPRGPDLSNCTSPWVNQVAQKIKSGENAANIASELARHTRGSIYAGDVSSSVKLMEQLLDILDAQLQ
ALRPIERESAGKNYNKMHKRERTCKDYIKAVVETVDNLLRSEALESWKDMNATEQVHTATMLLDVLEEGAFLL
ADNVREPARFLAAKQNVVLEVTVLNTEGQVQELVFPQEYPSENSIQLSANTIKQNSRNGVVKVVFILYNNLGL
FLSTENATVKLAGEAGTGGPGGASLVVNSQVIAASINKESSRVFLMDPVIFTVAHLEAKNHFNANCSFWNYSE
RSMLGYWSTQGCRLVESNKTHTTCACSHLTNFAVLMAHREIYQGRINELLLSVITWVGIVISLVCLAICISTF
CFLRGLQTDRNTIHKNLCINLFLAELLFLVGIDKTQYEVACPIFAGLLHYFFLAAFSWLCLEGVHLYLLLVEV
FESEYSRTKYYYLGGYCFPALVVGIAAAIDYRSYGTEKACWLRVDNYFIWSFIGPVSFVIVVNLVFLMVTLHK
MIRSSSVLKPDSSRLDNIKSWALGAIALLFLLGLTWAFGLLFINKESVVMAYLFTTFNAFQGVFIFVFHCALQ
KKVHKEYSKCLRHSYCCIRSPPGGTHGSLKTSAMRSNTRYYTGTQVPGQGRHIHQVSLGPRGRSTLPESQKDP
GGQSGPRDPLMFGLCPQSRIRRMWNDTVRKQTESSFMAGDINSTPTLNRGTMGNHLLTNPVLQPRGGTSPYNT
LIAESVGFNPSSPPVFNSPGSYREPKHPLGGREACGMDTLPLNGNFNNSYSLRSGDFPPGDGGPEPPRGRNLA
DAAAFEKMIISELVHNNLRGASGGAKGPPPEPPVPPVPGVSEDEAGGPGSADRAEIELLYKALEEPLLLPRAQ

SVLYQSDLDESESCTAEDGATSRPLSSPPGRDSLYASGANLRDSPSYPDSSPEGPNEALPPPPPAPPGPPEIY
YTSRPPALVARNPLQGYYQVRRPSHEGYLAAPSLEGPGPDGDGQMQLVTSL*
>rs32753131_ENSMUSP00000115295_c.1887:C>T_p.P630S
ENSMUST00000152978.8|Adgrl1-210|ENSMUSG00000013033|+|chr8:g.84659170:C>T
MARLAAALWSLCVTTVLVTSATQGLSRAGLPFGLMRRELACEGYPIELRCPGSDVIMVENANYGRTDDKICDA
DPFQMENVQCYLPDAFKIMSQRCNNRTQCVVVAGSDAFPDPCPGTYKYLEVQYDCVPYKVEQKVFVCPGTLQK
VLEPTSTHESEHQSGAWCKDPLQAGDRIYVMPWIPYRTDTLTEYASWEDYVAARHTTTYRLPNRVDGTGFVVY
DGAVFYNKERTRNIVKYDLRTRIKSGETVINTANYHDTSPYRWGGKTDIDLAVDENGLWVIYATEGNNGRLVV
SQLNPYTLRFEGTWETGYDKRSASNAFMVCGVLYVLRSVYVDDDSEAAGNRVDYAFNTNANREEPVSLAFPNP
YQFVSSVDYNPRDNQLYVWNNYFVVRYSLEFGPPDPSAGPATSPPLSTTTTARPTPLTSTASPAATTPLRRAP
LTTHPVGAINQLGPDLPPATAPAPSTRRPPAPNLHVSPELFCEPREVRRVQWPATQQGMLVERPCPKGTRGIA
SFQCLPALGLWNPRGPDLSNCTSPWVNQVAQKIKSGENAANIASELARHTRGSIYAGDVSSSVKLMEQLLDIL
DAQLQALRPIERESAGKNYNKMHKRERTCKDYIKAVVETVDNLLRSEALESWKDMNATEQVHTATMLLDVLEE
GAFLLADNVREPARFLAAKQNVVLEVTVLNTEGQVQELVFPQEYPSENSIQLSANTIKQNSRNGVVKVVFILY
NNLGLFLSTENATVKLAGEAGTGGPGGASLVVNSQVIAASINKESSRVFLMDPVIFTVAHLEAKNHFNANCSF
WNYSERSMLGYWSTQGCRLVESNKTHTTCACSHLTNFAVLMAHREIYQGRINELLLSVITWVGIVISLVCLAI
CISTFCFLRGLQTDRNTIHKNLCINLFLAELLFLVGIDKTQYEVACPIFAGLLHYFFLAAFSWLCLEGVHLYL
LLVEVFESEYSRTKYYYLGGYCFPALVVGIAAAIDYRSYGTEKACWLRVDNYFIWSFIGPVSFVIVVNLVFLM
VTLHKMIRSSSVLKPDSSRLDNIKSWALGAIALLFLLGLTWAFGLLFINKESVVMAYLFTTFNAFQGVFIFVF
HCALQKKVHKEYSKCLRHSYCCIRSPPGGTHGSLKTSAMRSNTRYYTGTQVPGQGRHIHQVSLGPRGRSTLPE
SQKDPGGQSGPRDPLMFGLCPQSRIRRMWNDTVRKQTESSFMAGDINSTPTLNRGTMGNHLLTNPVLQPRGGT
SPYNTLIAESVGFNPSSPPVFNSPGSYREPKHPLGGREACGMDTLPLNGNFNNSYSLRSGDFPPGDGGPEPPR
GRNLADAAAFEKMIISELVHNNLRGASGGAKGPPPEPPVPPVPGVSEDEAGGPGSADRAEIELLYKALEEPLL
LPRAQSVLYQSDLDESESCTAEDGATSRPLSSPPGRDSLYASGANLRDSPSYPDSSPEGPNEALPPPPPAPPG
PPEIYYTSRPPALVARNPLQGYYQVRRPSHEGYLAAPSLEGPGPDGDGQMQLVTSL*
>rs32753131_ENSMUSP00000118579_c.1359:C>T_p.P454S
ENSMUST00000131717.2|Adgrl1-204|ENSMUSG00000013033|+|chr8:g.84659170:C>T
MPWIPYRTDTLTEYASWEDYVAARHTTTYRLPNRVDGTGFVVYDGAVFYNKERTRNIVKYDLRTRIKSGETVI
NTANYHDTSPYRWGGKTDIDLAVDENGLWVIYATEGNNGRLVVSQLNPYTLRFEGTWETGYDKRSASNAFMVC
GVLYVLRSVYVDDDSEAAGNRVDYAFNTNANREEPVSLAFPNPYQFVSSVDYNPRDNQLYVWNNYFVVRYSLE
FGPPDPSAGPATSPPLSTTTTARPTPLTSTASPAATTPLRRAPLTTHPVGAINQLGPDLPPATAPAPSTRRPP
APNLHVSPELFCEPREVRRVQWPATQQGMLVERPCPKGTRGIASFQCLPALGLWNPRGPDLSNCTSPWVNQVA
QKIKSGENAANIASELARHTRGSIYAGDVSSSVKLMEQLLDILDAQLQALRPIERESAGKNYNKMHKRERTCK
DYIKAVVETVDNLLRSEALESWKDMNATEQVHTATMLLDVLEEGAFLLADNVREPARFLAAKQNVVLEVTVLN
TEGQVQELVFPQEYPSENSIQLSANTIKQNSRNGVVKVVFILYNNLGLFLSTENATVKLAGEAGTGGPGGASL
VVNSQVIAASINKESSRVFLMDPVIFTVAHLEAKNHFNANCSFWNYSERSMLGYWSTQGCRLVESNKTHTTCA
CSHLTNFAVLMAHREIYQGRINELLLSVITWVGIVISLVCLAICISTFCFLRGLQTDRNTIHKNLCINLFLAE
LLFLVGIDKTQYEVACPIFAGLLHYFFLAAFSWLCLEGVHLYLLLVEVFESEYSRTKYYYLGGYCFPALVVGI
AAAIDYRSYGTEKACWLRVDNYFIWSFIGPVSFVIVVNLVFLMVTLHKMIRSSSVLKPDSSRLDNIKSWALGA
IALLFLLGLTWAFGLLFINKESVVMAYLFTTFNAFQGVFIFVFHCALQKKVHKEYSKCLRHSYCCIRSPPGGT
HGSLKTSAMRSNTRYYTGTQSRIRRMWNDTVRKQTESSFMAGDINSTPTLNRGTMGNHLLTNPVLQPRGGTSP
YNTLIAESVGFNPSSPPVFNSPGSYREPKHPLGGREACGMDTLPLNGNFNNSYSLRSGDFPPGDGGPEPPRGR
NLADAAAFEKMIISELVHNNLRGASGGAKGPPPEPPVPPVPGVSEDEAGGPGSADRAEIELLYKALEEPLLLP
RAQSVLYQSDLDESESCTAEDGATSRPLSSPPGRDSLYASGANLRDSPSYPDSSPEGPNEALPPPPPAPPGPP
EIYYTSRPPALVARNPLQGYYQVRRPSHEGYLAAPSLEGPGPDGDGQMQLVTSL*
>rs212477295_ENSMUSP00000118452_c.2568:A>G_p.I857V
ENSMUST00000141158.8|Adgrl1-207|ENSMUSG00000013033|+|chr8:g.84661092:A>G
MARLAAALWSLCVTTVLVTSATQGLSRAGLPFGLMRRELACEGYPIELRCPGSDVIMVENANYGRTDDKICDA
DPFQMENVQCYLPDAFKIMSQRCNNRTQCVVVAGSDAFPDPCPGTYKYLEVQYDCVPYIFVCPGTLQKVLEPT
STHESEHQSGAWCKDPLQAGDRIYVMPWIPYRTDTLTEYASWEDYVAARHTTTYRLPNRVDGTGFVVYDGAVF
YNKERTRNIVKYDLRTRIKSGETVINTANYHDTSPYRWGGKTDIDLAVDENGLWVIYATEGNNGRLVVSQLNP
YTLRFEGTWETGYDKRSASNAFMVCGVLYVLRSVYVDDDSEAAGNRVDYAFNTNANREEPVSLAFPNPYQFVS
SVDYNPRDNQLYVWNNYFVVRYSLEFGPPDPSAGPATSPPLSTTTTARPTPLTSTASPAATTPLRRAPLTTHP
VGAINQLGPDLPPATAPAPSTRRPPAPNLHVSPELFCEPREVRRVQWPATQQGMLVERPCPKGTRGIASFQCL
PALGLWNPRGPDLSNCTSPWVNQVAQKIKSGENAANIASELARHTRGSIYAGDVSSSVKLMEQLLDILDAQLQ
ALRPIERESAGKNYNKMHKRERTCKDYIKAVVETVDNLLRPEALESWKDMNATEQVHTATMLLDVLEEGAFLL
ADNVREPARFLAAKQNVVLEVTVLNTEGQVQELVFPQEYPSENSIQLSANTIKQNSRNGVVKVVFILYNNLGL
FLSTENATVKLAGEAGTGGPGGASLVVNSQVIAASINKESSRVFLMDPVIFTVAHLEAKNHFNANCSFWNYSE

RSMLGYWSTQGCRLVESNKTHTTCACSHLTNFAVLMAHREIYQGRINELLLSVVTWVGIVISLVCLAICISTF
CFLRGLQTDRNTIHKNLCINLFLAELLFLVGIDKTQYEVACPIFAGLLHYFFLAAFSWLCLEGVHLYLLLVEV
FESEYSRTKYYYLGGYCFPALVVGIAAAIDYRSYGTEKACWLRVDNYFIWSFIGPVSFVIVVNLVFLMVTLHK
MIRSSSVLKPDSSRLDNIKSWALGAIALLFLLGLTWAFGLLFINKESVVMAYLFTTFNAFQGVFIFVFHCALQ
KKVHKEYSKCLRHSYCCIRSPPGGTHGSLKTSAMRSNTRYYTGTQSRIRRMWNDTVRKQTESSFMAGDINSTP
TLNRGTMGNHLLTNPVLQPRGGTSPYNTLIAESVGFNPSSPPVFNSPGSYREPKHPLGGREACGMDTLPLNGN
FNNSYSLRSGDFPPGDGGPEPPRGRNLADAAAFEKMIISELVHNNLRGASGGAKGPPPEPPVPPVPGVSEDEA
GGPGSADRAEIELLYKALEEPLLLPRAQSVLYQSDLDESESCTAEDGATSRPLSSPPGRDSLYASGANLRDSP
SYPDSSPEGPNEALPPPPPAPPGPPEIYYTSRPPALVARNPLQGYYQVRRPSHEGYLAAPSLEGPGPDGDGQM
QLVTSL*
>rs212477295_ENSMUSP00000048422_c.2583:A>G_p.I862V
ENSMUST00000045393.15|Adgrl1-201|ENSMUSG00000013033|+|chr8:g.84661092:A>G
MARLAAALWSLCVTTVLVTSATQGLSRAGLPFGLMRRELACEGYPIELRCPGSDVIMVENANYGRTDDKICDA
DPFQMENVQCYLPDAFKIMSQRCNNRTQCVVVAGSDAFPDPCPGTYKYLEVQYDCVPYKVEQKVFVCPGTLQK
VLEPTSTHESEHQSGAWCKDPLQAGDRIYVMPWIPYRTDTLTEYASWEDYVAARHTTTYRLPNRVDGTGFVVY
DGAVFYNKERTRNIVKYDLRTRIKSGETVINTANYHDTSPYRWGGKTDIDLAVDENGLWVIYATEGNNGRLVV
SQLNPYTLRFEGTWETGYDKRSASNAFMVCGVLYVLRSVYVDDDSEAAGNRVDYAFNTNANREEPVSLAFPNP
YQFVSSVDYNPRDNQLYVWNNYFVVRYSLEFGPPDPSAGPATSPPLSTTTTARPTPLTSTASPAATTPLRRAP
LTTHPVGAINQLGPDLPPATAPAPSTRRPPAPNLHVSPELFCEPREVRRVQWPATQQGMLVERPCPKGTRGIA
SFQCLPALGLWNPRGPDLSNCTSPWVNQVAQKIKSGENAANIASELARHTRGSIYAGDVSSSVKLMEQLLDIL
DAQLQALRPIERESAGKNYNKMHKRERTCKDYIKAVVETVDNLLRPEALESWKDMNATEQVHTATMLLDVLEE
GAFLLADNVREPARFLAAKQNVVLEVTVLNTEGQVQELVFPQEYPSENSIQLSANTIKQNSRNGVVKVVFILY
NNLGLFLSTENATVKLAGEAGTGGPGGASLVVNSQVIAASINKESSRVFLMDPVIFTVAHLEAKNHFNANCSF
WNYSERSMLGYWSTQGCRLVESNKTHTTCACSHLTNFAVLMAHREIYQGRINELLLSVVTWVGIVISLVCLAI
CISTFCFLRGLQTDRNTIHKNLCINLFLAELLFLVGIDKTQYEVACPIFAGLLHYFFLAAFSWLCLEGVHLYL
LLVEVFESEYSRTKYYYLGGYCFPALVVGIAAAIDYRSYGTEKACWLRVDNYFIWSFIGPVSFVIVVNLVFLM
VTLHKMIRSSSVLKPDSSRLDNIKSWALGAIALLFLLGLTWAFGLLFINKESVVMAYLFTTFNAFQGVFIFVF
HCALQKKVHKEYSKCLRHSYCCIRSPPGGTHGSLKTSAMRSNTRYYTGTQSRIRRMWNDTVRKQTESSFMAGD
INSTPTLNRGTMGNHLLTNPVLQPRGGTSPYNTLIAESVGFNPSSPPVFNSPGSYREPKHPLGGREACGMDTL
PLNGNFNNSYSLRSGDFPPGDGGPEPPRGRNLADAAAFEKMIISELVHNNLRGASGGAKGPPPEPPVPPVPGV
SEDEAGGPGSADRAEIELLYKALEEPLLLPRAQSVLYQSDLDESESCTAEDGATSRPLSSPPGRDSLYASGAN
LRDSPSYPDSSPEGPNEALPPPPPAPPGPPEIYYTSRPPALVARNPLQGYYQVRRPSHEGYLAAPSLEGPGPD
GDGQMQLVTSL*
>rs212477295_ENSMUSP00000119100_c.2568:A>G_p.I857V
ENSMUST00000132500.8|Adgrl1-205|ENSMUSG00000013033|+|chr8:g.84661092:A>G
MARLAAALWSLCVTTVLVTSATQGLSRAGLPFGLMRRELACEGYPIELRCPGSDVIMVENANYGRTDDKICDA
DPFQMENVQCYLPDAFKIMSQRCNNRTQCVVVAGSDAFPDPCPGTYKYLEVQYDCVPYIFVCPGTLQKVLEPT
STHESEHQSGAWCKDPLQAGDRIYVMPWIPYRTDTLTEYASWEDYVAARHTTTYRLPNRVDGTGFVVYDGAVF
YNKERTRNIVKYDLRTRIKSGETVINTANYHDTSPYRWGGKTDIDLAVDENGLWVIYATEGNNGRLVVSQLNP
YTLRFEGTWETGYDKRSASNAFMVCGVLYVLRSVYVDDDSEAAGNRVDYAFNTNANREEPVSLAFPNPYQFVS
SVDYNPRDNQLYVWNNYFVVRYSLEFGPPDPSAGPATSPPLSTTTTARPTPLTSTASPAATTPLRRAPLTTHP
VGAINQLGPDLPPATAPAPSTRRPPAPNLHVSPELFCEPREVRRVQWPATQQGMLVERPCPKGTRGIASFQCL
PALGLWNPRGPDLSNCTSPWVNQVAQKIKSGENAANIASELARHTRGSIYAGDVSSSVKLMEQLLDILDAQLQ
ALRPIERESAGKNYNKMHKRERTCKDYIKAVVETVDNLLRPEALESWKDMNATEQVHTATMLLDVLEEGAFLL
ADNVREPARFLAAKQNVVLEVTVLNTEGQVQELVFPQEYPSENSIQLSANTIKQNSRNGVVKVVFILYNNLGL
FLSTENATVKLAGEAGTGGPGGASLVVNSQVIAASINKESSRVFLMDPVIFTVAHLEAKNHFNANCSFWNYSE
RSMLGYWSTQGCRLVESNKTHTTCACSHLTNFAVLMAHREIYQGRINELLLSVVTWVGIVISLVCLAICISTF
CFLRGLQTDRNTIHKNLCINLFLAELLFLVGIDKTQYEVACPIFAGLLHYFFLAAFSWLCLEGVHLYLLLVEV
FESEYSRTKYYYLGGYCFPALVVGIAAAIDYRSYGTEKACWLRVDNYFIWSFIGPVSFVIVVNLVFLMVTLHK
MIRSSSVLKPDSSRLDNIKSWALGAIALLFLLGLTWAFGLLFINKESVVMAYLFTTFNAFQGVFIFVFHCALQ
KKVHKEYSKCLRHSYCCIRSPPGGTHGSLKTSAMRSNTRYYTGTQVPGQGRHIHQVSLGPRGRSTLPESQKDP
GGQSGPRDPLMFGLCPQSRIRRMWNDTVRKQTESSFMAGDINSTPTLNRGTMGNHLLTNPVLQPRGGTSPYNT
LIAESVGFNPSSPPVFNSPGSYREPKHPLGGREACGMDTLPLNGNFNNSYSLRSGDFPPGDGGPEPPRGRNLA
DAAAFEKMIISELVHNNLRGASGGAKGPPPEPPVPPVPGVSEDEAGGPGSADRAEIELLYKALEEPLLLPRAQ
SVLYQSDLDESESCTAEDGATSRPLSSPPGRDSLYASGANLRDSPSYPDSSPEGPNEALPPPPPAPPGPPEIY
YTSRPPALVARNPLQGYYQVRRPSHEGYLAAPSLEGPGPDGDGQMQLVTSL*
>rs212477295_ENSMUSP00000115295_c.2583:A>G_p.I862V
ENSMUST00000152978.8|Adgrl1-210|ENSMUSG00000013033|+|chr8:g.84661092:A>G

MARLAAALWSLCVTTVLVTSATQGLSRAGLPFGLMRRELACEGYPIELRCPGSDVIMVENANYGRTDDKICDA
DPFQMENVQCYLPDAFKIMSQRCNNRTQCVVVAGSDAFPDPCPGTYKYLEVQYDCVPYKVEQKVFVCPGTLQK
VLEPTSTHESEHQSGAWCKDPLQAGDRIYVMPWIPYRTDTLTEYASWEDYVAARHTTTYRLPNRVDGTGFVVY
DGAVFYNKERTRNIVKYDLRTRIKSGETVINTANYHDTSPYRWGGKTDIDLAVDENGLWVIYATEGNNGRLVV
SQLNPYTLRFEGTWETGYDKRSASNAFMVCGVLYVLRSVYVDDDSEAAGNRVDYAFNTNANREEPVSLAFPNP
YQFVSSVDYNPRDNQLYVWNNYFVVRYSLEFGPPDPSAGPATSPPLSTTTTARPTPLTSTASPAATTPLRRAP
LTTHPVGAINQLGPDLPPATAPAPSTRRPPAPNLHVSPELFCEPREVRRVQWPATQQGMLVERPCPKGTRGIA
SFQCLPALGLWNPRGPDLSNCTSPWVNQVAQKIKSGENAANIASELARHTRGSIYAGDVSSSVKLMEQLLDIL
DAQLQALRPIERESAGKNYNKMHKRERTCKDYIKAVVETVDNLLRPEALESWKDMNATEQVHTATMLLDVLEE
GAFLLADNVREPARFLAAKQNVVLEVTVLNTEGQVQELVFPQEYPSENSIQLSANTIKQNSRNGVVKVVFILY
NNLGLFLSTENATVKLAGEAGTGGPGGASLVVNSQVIAASINKESSRVFLMDPVIFTVAHLEAKNHFNANCSF
WNYSERSMLGYWSTQGCRLVESNKTHTTCACSHLTNFAVLMAHREIYQGRINELLLSVVTWVGIVISLVCLAI
CISTFCFLRGLQTDRNTIHKNLCINLFLAELLFLVGIDKTQYEVACPIFAGLLHYFFLAAFSWLCLEGVHLYL
LLVEVFESEYSRTKYYYLGGYCFPALVVGIAAAIDYRSYGTEKACWLRVDNYFIWSFIGPVSFVIVVNLVFLM
VTLHKMIRSSSVLKPDSSRLDNIKSWALGAIALLFLLGLTWAFGLLFINKESVVMAYLFTTFNAFQGVFIFVF
HCALQKKVHKEYSKCLRHSYCCIRSPPGGTHGSLKTSAMRSNTRYYTGTQVPGQGRHIHQVSLGPRGRSTLPE
SQKDPGGQSGPRDPLMFGLCPQSRIRRMWNDTVRKQTESSFMAGDINSTPTLNRGTMGNHLLTNPVLQPRGGT
SPYNTLIAESVGFNPSSPPVFNSPGSYREPKHPLGGREACGMDTLPLNGNFNNSYSLRSGDFPPGDGGPEPPR
GRNLADAAAFEKMIISELVHNNLRGASGGAKGPPPEPPVPPVPGVSEDEAGGPGSADRAEIELLYKALEEPLL
LPRAQSVLYQSDLDESESCTAEDGATSRPLSSPPGRDSLYASGANLRDSPSYPDSSPEGPNEALPPPPPAPPG
PPEIYYTSRPPALVARNPLQGYYQVRRPSHEGYLAAPSLEGPGPDGDGQMQLVTSL*
>rs212477295_ENSMUSP00000118579_c.2055:A>G_p.I686V
ENSMUST00000131717.2|Adgrl1-204|ENSMUSG00000013033|+|chr8:g.84661092:A>G
MPWIPYRTDTLTEYASWEDYVAARHTTTYRLPNRVDGTGFVVYDGAVFYNKERTRNIVKYDLRTRIKSGETVI
NTANYHDTSPYRWGGKTDIDLAVDENGLWVIYATEGNNGRLVVSQLNPYTLRFEGTWETGYDKRSASNAFMVC
GVLYVLRSVYVDDDSEAAGNRVDYAFNTNANREEPVSLAFPNPYQFVSSVDYNPRDNQLYVWNNYFVVRYSLE
FGPPDPSAGPATSPPLSTTTTARPTPLTSTASPAATTPLRRAPLTTHPVGAINQLGPDLPPATAPAPSTRRPP
APNLHVSPELFCEPREVRRVQWPATQQGMLVERPCPKGTRGIASFQCLPALGLWNPRGPDLSNCTSPWVNQVA
QKIKSGENAANIASELARHTRGSIYAGDVSSSVKLMEQLLDILDAQLQALRPIERESAGKNYNKMHKRERTCK
DYIKAVVETVDNLLRPEALESWKDMNATEQVHTATMLLDVLEEGAFLLADNVREPARFLAAKQNVVLEVTVLN
TEGQVQELVFPQEYPSENSIQLSANTIKQNSRNGVVKVVFILYNNLGLFLSTENATVKLAGEAGTGGPGGASL
VVNSQVIAASINKESSRVFLMDPVIFTVAHLEAKNHFNANCSFWNYSERSMLGYWSTQGCRLVESNKTHTTCA
CSHLTNFAVLMAHREIYQGRINELLLSVVTWVGIVISLVCLAICISTFCFLRGLQTDRNTIHKNLCINLFLAE
LLFLVGIDKTQYEVACPIFAGLLHYFFLAAFSWLCLEGVHLYLLLVEVFESEYSRTKYYYLGGYCFPALVVGI
AAAIDYRSYGTEKACWLRVDNYFIWSFIGPVSFVIVVNLVFLMVTLHKMIRSSSVLKPDSSRLDNIKSWALGA
IALLFLLGLTWAFGLLFINKESVVMAYLFTTFNAFQGVFIFVFHCALQKKVHKEYSKCLRHSYCCIRSPPGGT
HGSLKTSAMRSNTRYYTGTQSRIRRMWNDTVRKQTESSFMAGDINSTPTLNRGTMGNHLLTNPVLQPRGGTSP
YNTLIAESVGFNPSSPPVFNSPGSYREPKHPLGGREACGMDTLPLNGNFNNSYSLRSGDFPPGDGGPEPPRGR
NLADAAAFEKMIISELVHNNLRGASGGAKGPPPEPPVPPVPGVSEDEAGGPGSADRAEIELLYKALEEPLLLP
RAQSVLYQSDLDESESCTAEDGATSRPLSSPPGRDSLYASGANLRDSPSYPDSSPEGPNEALPPPPPAPPGPP
EIYYTSRPPALVARNPLQGYYQVRRPSHEGYLAAPSLEGPGPDGDGQMQLVTSL*
>rs6405173_ENSMUSP00000119100_c.3540:G>A_p.G1181R
ENSMUST00000132500.8|Adgrl1-205|ENSMUSG00000013033|+|chr8:g.84664075:G>A
MARLAAALWSLCVTTVLVTSATQGLSRAGLPFGLMRRELACEGYPIELRCPGSDVIMVENANYGRTDDKICDA
DPFQMENVQCYLPDAFKIMSQRCNNRTQCVVVAGSDAFPDPCPGTYKYLEVQYDCVPYIFVCPGTLQKVLEPT
STHESEHQSGAWCKDPLQAGDRIYVMPWIPYRTDTLTEYASWEDYVAARHTTTYRLPNRVDGTGFVVYDGAVF
YNKERTRNIVKYDLRTRIKSGETVINTANYHDTSPYRWGGKTDIDLAVDENGLWVIYATEGNNGRLVVSQLNP
YTLRFEGTWETGYDKRSASNAFMVCGVLYVLRSVYVDDDSEAAGNRVDYAFNTNANREEPVSLAFPNPYQFVS
SVDYNPRDNQLYVWNNYFVVRYSLEFGPPDPSAGPATSPPLSTTTTARPTPLTSTASPAATTPLRRAPLTTHP
VGAINQLGPDLPPATAPAPSTRRPPAPNLHVSPELFCEPREVRRVQWPATQQGMLVERPCPKGTRGIASFQCL
PALGLWNPRGPDLSNCTSPWVNQVAQKIKSGENAANIASELARHTRGSIYAGDVSSSVKLMEQLLDILDAQLQ
ALRPIERESAGKNYNKMHKRERTCKDYIKAVVETVDNLLRPEALESWKDMNATEQVHTATMLLDVLEEGAFLL
ADNVREPARFLAAKQNVVLEVTVLNTEGQVQELVFPQEYPSENSIQLSANTIKQNSRNGVVKVVFILYNNLGL
FLSTENATVKLAGEAGTGGPGGASLVVNSQVIAASINKESSRVFLMDPVIFTVAHLEAKNHFNANCSFWNYSE
RSMLGYWSTQGCRLVESNKTHTTCACSHLTNFAVLMAHREIYQGRINELLLSVITWVGIVISLVCLAICISTF
CFLRGLQTDRNTIHKNLCINLFLAELLFLVGIDKTQYEVACPIFAGLLHYFFLAAFSWLCLEGVHLYLLLVEV
FESEYSRTKYYYLGGYCFPALVVGIAAAIDYRSYGTEKACWLRVDNYFIWSFIGPVSFVIVVNLVFLMVTLHK
MIRSSSVLKPDSSRLDNIKSWALGAIALLFLLGLTWAFGLLFINKESVVMAYLFTTFNAFQGVFIFVFHCALQ

KKVHKEYSKCLRHSYCCIRSPPGGTHGSLKTSAMRSNTRYYTGTQVPGQGRHIHQVSLGPRGRSTLPESQKDP
GGQSGPRDPLMFRLCPQSRIRRMWNDTVRKQTESSFMAGDINSTPTLNRGTMGNHLLTNPVLQPRGGTSPYNT
LIAESVGFNPSSPPVFNSPGSYREPKHPLGGREACGMDTLPLNGNFNNSYSLRSGDFPPGDGGPEPPRGRNLA
DAAAFEKMIISELVHNNLRGASGGAKGPPPEPPVPPVPGVSEDEAGGPGSADRAEIELLYKALEEPLLLPRAQ
SVLYQSDLDESESCTAEDGATSRPLSSPPGRDSLYASGANLRDSPSYPDSSPEGPNEALPPPPPAPPGPPEIY
YTSRPPALVARNPLQGYYQVRRPSHEGYLAAPSLEGPGPDGDGQMQLVTSL*
>rs6405173_ENSMUSP00000115295_c.3555:G>A_p.G1186R
ENSMUST00000152978.8|Adgrl1-210|ENSMUSG00000013033|+|chr8:g.84664075:G>A
MARLAAALWSLCVTTVLVTSATQGLSRAGLPFGLMRRELACEGYPIELRCPGSDVIMVENANYGRTDDKICDA
DPFQMENVQCYLPDAFKIMSQRCNNRTQCVVVAGSDAFPDPCPGTYKYLEVQYDCVPYKVEQKVFVCPGTLQK
VLEPTSTHESEHQSGAWCKDPLQAGDRIYVMPWIPYRTDTLTEYASWEDYVAARHTTTYRLPNRVDGTGFVVY
DGAVFYNKERTRNIVKYDLRTRIKSGETVINTANYHDTSPYRWGGKTDIDLAVDENGLWVIYATEGNNGRLVV
SQLNPYTLRFEGTWETGYDKRSASNAFMVCGVLYVLRSVYVDDDSEAAGNRVDYAFNTNANREEPVSLAFPNP
YQFVSSVDYNPRDNQLYVWNNYFVVRYSLEFGPPDPSAGPATSPPLSTTTTARPTPLTSTASPAATTPLRRAP
LTTHPVGAINQLGPDLPPATAPAPSTRRPPAPNLHVSPELFCEPREVRRVQWPATQQGMLVERPCPKGTRGIA
SFQCLPALGLWNPRGPDLSNCTSPWVNQVAQKIKSGENAANIASELARHTRGSIYAGDVSSSVKLMEQLLDIL
DAQLQALRPIERESAGKNYNKMHKRERTCKDYIKAVVETVDNLLRPEALESWKDMNATEQVHTATMLLDVLEE
GAFLLADNVREPARFLAAKQNVVLEVTVLNTEGQVQELVFPQEYPSENSIQLSANTIKQNSRNGVVKVVFILY
NNLGLFLSTENATVKLAGEAGTGGPGGASLVVNSQVIAASINKESSRVFLMDPVIFTVAHLEAKNHFNANCSF
WNYSERSMLGYWSTQGCRLVESNKTHTTCACSHLTNFAVLMAHREIYQGRINELLLSVITWVGIVISLVCLAI
CISTFCFLRGLQTDRNTIHKNLCINLFLAELLFLVGIDKTQYEVACPIFAGLLHYFFLAAFSWLCLEGVHLYL
LLVEVFESEYSRTKYYYLGGYCFPALVVGIAAAIDYRSYGTEKACWLRVDNYFIWSFIGPVSFVIVVNLVFLM
VTLHKMIRSSSVLKPDSSRLDNIKSWALGAIALLFLLGLTWAFGLLFINKESVVMAYLFTTFNAFQGVFIFVF
HCALQKKVHKEYSKCLRHSYCCIRSPPGGTHGSLKTSAMRSNTRYYTGTQVPGQGRHIHQVSLGPRGRSTLPE
SQKDPGGQSGPRDPLMFRLCPQSRIRRMWNDTVRKQTESSFMAGDINSTPTLNRGTMGNHLLTNPVLQPRGGT
SPYNTLIAESVGFNPSSPPVFNSPGSYREPKHPLGGREACGMDTLPLNGNFNNSYSLRSGDFPPGDGGPEPPR
GRNLADAAAFEKMIISELVHNNLRGASGGAKGPPPEPPVPPVPGVSEDEAGGPGSADRAEIELLYKALEEPLL
LPRAQSVLYQSDLDESESCTAEDGATSRPLSSPPGRDSLYASGANLRDSPSYPDSSPEGPNEALPPPPPAPPG
PPEIYYTSRPPALVARNPLQGYYQVRRPSHEGYLAAPSLEGPGPDGDGQMQLVTSL*
>rs46894143_ENSMUSP00000062468_c.178:C>T_p.P60L
ENSMUST00000060357.15|1700067K01Rik-
201|ENSMUSG00000046408|+|chr8:g.84728384:C>T
MAAEPWFTGSLALGPGEAPPSEELGPGPPPYGDPSWSPAGRPETSAEQEPQRAQPMMEALASTSSREPLAAEL
GPAPARLPLDSMFSPITDQLRYLLRKADDFQSYLLYSRDRVQKEQLAKAMPTFLTMCEPYFLYLEAAARSVPP
IYGALQELVRKGLLEISQQLTLRLEQLVLMYASFGFVDLEETNPLSISCFFCGRFSISPSHDVSIFRYCTPAA
YTASHFPRYLYKKMRWNLETTTEASSQGADSHVDYYFLCYRDTWEDAGQGPANSCPQIQKLWSIGRWMPLGPA
EDDLDSWILCPQPPGDYQQLLTIGFEEPSHVLATDLLVQILLGQAGPARPPSAAGPAAWAAPAS*
>rs46894143_ENSMUSP00000134486_c.178:C>T_p.P60L
ENSMUST00000174570.9|1700067K01Rik-
203|ENSMUSG00000046408|+|chr8:g.84728384:C>T
MAAEPWFTGSLALGPGEAPPSEELGPGPPPYGDPSWSPAGRPETSAEQEPQRAQPMMEALASTSSREPLAAEL
GPAPARLPLDSMFSPITDQLRYLLRKADDFQSYLLYRDRVQKEQLAKAMPTFLTMCEPYFLYLEAAARSVPPI
YGALQELVRKGLLEISQQLTLRLEQLVLMYASFGFVDLEETNPLSISCFFCGRFSISPSHDVSIFRYCTPAAY
TASHFPRYLYKKMRWNLETTTEASSQGADSHVDYYFLCYRDTWEDAGQGPANSCPQIQKLWSIGRWMPLGPAE
DDLDSW*
>rs46894143_ENSMUSP00000159263_c.178:C>T_p.P60L
ENSMUST00000239176.2|1700067K01Rik-
204|ENSMUSG00000046408|+|chr8:g.84728384:C>T
MAAEPWFTGSLALGPGEAPPSEELGPGPPPYGDPSWSPAGRPETSAEQEPQRAQPMMEALASTSSREPLAAEL
GPAPARLPLDSMFSPITDQLRYLLRKADDFQSYLLYRDRVQKEQLAKAMPTFLTMCEPYFLYLEAAARSVPPI
YGALQELVRKGLLEISQQLTLRLEQLVLMYASFGFVDLEETNPLSISCFFCGRFSISPSHDVSIFRYCTPAAY
TASHFPRYLYKKMRWNLETTTEASSQGADSHVDYYFLCYRDTWEDAGQGPANSCPQIQKLWSIGRWMPLGPAE
DDLDSWILCPQPPGDYQQLLTIGFEEPSHVLATDLLVQILLGQAGPARPPSAAGPAAWAAPAS*
>rs33228486_ENSMUSP00000051396_c.67:G>A_p.R23Q
ENSMUST00000055077.7|Palm3-201|ENSMUSG00000047986|+|chr8:g.84751007:G>A
MALQTPVLSPATPTVMAESALYQQRLEVIAEKRRLQEEIGAARRELEEEKLRVERLKRKSLRERWLMDGAAEG
PERPEEPASKDPQSPEGQAQARIRNLEDSLFSLQSQLQLLQSASTGAQHRPAGRPAWRREGPRPLSQSAMEAA
PTAPTDVDKRTSLPDAPVGMSPESPSDPREESIAVLPASRPSTEAIGTSSEANGPCPGHSPLPEQLSLGVSSV

TKAKGDGAVEVVWAGLRATENSATGPTDVELEAKVEEVVLEAIGARQGTSSPELPTWVKEGRGVVEVVWEGLG
GRDLDVTGESGRDAEATHTSSRRLQEQFEAETCRKEEGASRDSLEGVGQGGPGVEEGSFIWVERVALSEDWEE
ILMEGLEAPQGAGSAGEPEALIGAQPRGGEASWEVEKREVEKVEGIEEKGRAEKLGAEREDGVAVLPDETQGR
EENEAEKVERKDSEGPFPAEIATDEEKWEVKTTEGEESLEVEKGGEAEPVTTEKPLVTEKKPEGSLETERKGS
EMPLDQEKDGEGSLDRESKTTEILLDGEIGDKSSLDETKGSKKLLDEKTGGEGSLDEEAEGSKKLLDREADGI
EPFSEVDKTSGAKDDVSPEEQGKANEGAEFQAEDASPPGATVCVQDEPRSEEQGQQEPEKQEGLVEGAASKPE
PCTEREGPPGDATLLLAETPAPEQPVESQPLLHQEASSTNPGDHPAPTYAPAQQLELAEAKEASGPKQKTCQC
CVVM*
>rs32670996_ENSMUSP00000051396_c.1578:A>G_p.R527G
ENSMUST00000055077.7|Palm3-201|ENSMUSG00000047986|+|chr8:g.84756068:A>G
MALQTPVLSPATPTVMAESALYRQRLEVIAEKRRLQEEIGAARRELEEEKLRVERLKRKSLRERWLMDGAAEG
PERPEEPASKDPQSPEGQAQARIRNLEDSLFSLQSQLQLLQSASTGAQHRPAGRPAWRREGPRPLSQSAMEAA
PTAPTDVDKRTSLPDAPVGMSPESPSDPREESIAVLPASRPSTEAIGTSSEANGPCPGHSPLPEQLSLGVSSV
TKAKGDGAVEVVWAGLRATENSATGPTDVELEAKVEEVVLEAIGARQGTSSPELPTWVKEGRGVVEVVWEGLG
GRDLDVTGESGRDAEATHTSSRRLQEQFEAETCRKEEGASRDSLEGVGQGGPGVEEGSFIWVERVALSEDWEE
ILMEGLEAPQGAGSAGEPEALIGAQPRGGEASWEVEKREVEKVEGIEEKGRAEKLGAEREDGVAVLPDETQGR
EENEAEKVERKDSEGPFPAEIATDEEKWEVKTTEGEESLEVEKGGEAEPVTTEKPLVTEKKPEGSLETERKGS
EMPLDQEKDGEGSLDGESKTTEILLDGEIGDKSSLDETKGSKKLLDEKTGGEGSLDEEAEGSKKLLDREADGI
EPFSEVDKTSGAKDDVSPEEQGKANEGAEFQAEDASPPGATVCVQDEPRSEEQGQQEPEKQEGLVEGAASKPE
PCTEREGPPGDATLLLAETPAPEQPVESQPLLHQEASSTNPGDHPAPTYAPAQQLELAEAKEASGPKQKTCQC
CVVM*
>rs33480646_ENSMUSP00000051396_c.1621:G>A_p.G541E
ENSMUST00000055077.7|Palm3-201|ENSMUSG00000047986|+|chr8:g.84756111:G>A
MALQTPVLSPATPTVMAESALYRQRLEVIAEKRRLQEEIGAARRELEEEKLRVERLKRKSLRERWLMDGAAEG
PERPEEPASKDPQSPEGQAQARIRNLEDSLFSLQSQLQLLQSASTGAQHRPAGRPAWRREGPRPLSQSAMEAA
PTAPTDVDKRTSLPDAPVGMSPESPSDPREESIAVLPASRPSTEAIGTSSEANGPCPGHSPLPEQLSLGVSSV
TKAKGDGAVEVVWAGLRATENSATGPTDVELEAKVEEVVLEAIGARQGTSSPELPTWVKEGRGVVEVVWEGLG
GRDLDVTGESGRDAEATHTSSRRLQEQFEAETCRKEEGASRDSLEGVGQGGPGVEEGSFIWVERVALSEDWEE
ILMEGLEAPQGAGSAGEPEALIGAQPRGGEASWEVEKREVEKVEGIEEKGRAEKLGAEREDGVAVLPDETQGR
EENEAEKVERKDSEGPFPAEIATDEEKWEVKTTEGEESLEVEKGGEAEPVTTEKPLVTEKKPEGSLETERKGS
EMPLDQEKDGEGSLDRESKTTEILLDGEIEDKSSLDETKGSKKLLDEKTGGEGSLDEEAEGSKKLLDREADGI
EPFSEVDKTSGAKDDVSPEEQGKANEGAEFQAEDASPPGATVCVQDEPRSEEQGQQEPEKQEGLVEGAASKPE
PCTEREGPPGDATLLLAETPAPEQPVESQPLLHQEASSTNPGDHPAPTYAPAQQLELAEAKEASGPKQKTCQC
CVVM*
>rs33092929_ENSMUSP00000005601_c.430:T>C_p.V144A
ENSMUST00000005601.9|Il27ra-201|ENSMUSG00000005465|-|chr8:g.84767391:A>G
MNRLRVARLTPLELLLSLMSLLLGTRPHGSPGPLQCYSVGPLGILNCSWEPLGDLETPPVLYHQSQKYHPNRV
WEVKVPSKQSWVTIPREQFTMADKLLIWGTQKGRPLWSSVSVNLETQMKPDTPQIFSQVDISEEATLEATAQW
APPVWPPQKVLICQFRYKECQAETWTRLEPQLKTDGLTPVEMQNLEPGTCYQVSGRCQVENGYPWGEWSSPLS
FQTPFLDPEDVWVSGTVCETSGKRAALLVWKDPRPCVQVTYTVWFGAGDITTTQEEVPCCKSPVPAWMEWAVV
SPGNSTSWVPPTNLSLVCLAPESAPCDVGVSSADGSPGIKVTWKQGTRKPLEYVVDWAQDGDSLDKLNWTRLP
PGNLSTLLPGEFKGGVPYRITVTAVYSGGLAAAPSVWGFREELVPLAGPAVWRLPDDPPGTPVVAWGEVPRHQ
LRGQATHYTFCIQSRGLSTVCRNVSSQTQTATLPNLHLGSFKLWVTVSTVAGQGPPGPNLSLHLPDNRIRWKA
LPWFLSLWGLLLMGCGLSLASTRCLQARCLHWRHKLLPQWIWERVPDPANSNSGQPYIKEVSLPQPPKDGPIL
EVEEVELQPVVESPKASAPIYSGYEKHFLPTPEELGLLV*
>rs50881156_ENSMUSP00000147677_c.780:C>A_p.L261M
ENSMUST00000211046.2|Rfx1-204|ENSMUSG00000031706|+|chr8:g.84810777:C>A
MATQSYVTELQAAPQASQPPQAPPQALPQPPPPAAPQPPAAATPQPQYVTELQSPQPQTQPPGSQKQYVAELP
AAPAPSQPATPAPSPVAQQYIVVTVSEGAMRASETVSEASPSSTASQTGVPTQVVQQVQGTQQRLLVQASVQA
KPGHVSPLQLTNIQVPQQAIPTQHLVVQSPAPGTKSGQVSLTVHSAQQVHSAPERSPVQANNSTSKTAGTPAA
TVQQLQVHSVQQSVPVTQERSVVQATPQTKAGPVQQLTVQGMQPVHVAQEVQQLPQVPVPHVYSSQVQYVEGG
DASYTASAIRSSTYQYPETPIYTQTAGTSYYEASGTAAQVSTPATSQTVASSGSVPMYVSGSPIVASSSSSEA
GASNSSVGAGGNGGGGSSGGGSGGSSGSGAGTYVIQGGYMLGNASQSYSHTTRASPATVQWLLDNYETAEGVS
LPRSTLYCHYLLHCQEQKLEPVNAASFGKLIRSVFMGLRTRRLGTRGNSKYHYYGLRIKASSPLLRLMEDQQH
MAMRGQPFSQKQRLKPIQKMEGVANGVAVGQQSTGLSDISAQVQQYQQFLDASRSLPDFAELDLQGKVLPEGV
GPGDIKAFQVLYREHCEAIVDVMVNLQFTLVETLWKTFWRYNLSQPSEAPPLAVHDEAEKRLPRASLVLLSKF
QPVLQWTKHCDNVLYQGLVEILIPDVLRPIPSALTQAIRNFAKSLESWLTHAMVNIPEEMLRVKVAAAGAFAQ
TLRRYTSLNHLAQAARAVLQNTAQINQMLSDLNRVDFANVQEQASWVCRCEDRVVQRLEQDFKVTLQQQNSLE

QWAAWLDGVVSQVLKPYQGSSGFPKAAKLFLLKWSFYSSMVIRDLTLRSAASFGSFHLIRLLYDEYMYYLIEH
RVAQAKGETPIAVMGEFANLATSLNPLDPDKVCR*
>rs50881156_ENSMUSP00000005600_c.780:C>A_p.L261M
ENSMUST00000005600.6|Rfx1-201|ENSMUSG00000031706|+|chr8:g.84810777:C>A
MATQSYVTELQAAPQASQPPQAPPQALPQPPPPAAPQPPAAATPQPQYVTELQSPQPQTQPPGSQKQYVAELP
AAPAPSQPATPAPSPVAQQYIVVTVSEGAMRASETVSEASPSSTASQTGVPTQVVQQVQGTQQRLLVQASVQA
KPGHVSPLQLTNIQVPQQAIPTQHLVVQSPAPGTKSGQVSLTVHSAQQVHSAPERSPVQANNSTSKTAGTPAA
TVQQLQVHSVQQSVPVTQERSVVQATPQTKAGPVQQLTVQGMQPVHVAQEVQQLPQVPVPHVYSSQVQYVEGG
DASYTASAIRSSTYQYPETPIYTQTAGTSYYEASGTAAQVSTPATSQTVASSGSVPMYVSGSPIVASSSSSEA
GASNSSVGAGGNGGGGSSGGGSGGSSGSGAGTYVIQGGYMLGNASQSYSHTTRASPATVQWLLDNYETAEGVS
LPRSTLYCHYLLHCQEQKLEPVNAASFGKLIRSVFMGLRTRRLGTRGNSKYHYYGLRIKASSPLLRLMEDQQH
MAMRGQPFSQKQRLKPIQKMEGVANGVAVGQQSTGLSDISAQVQQYQQFLDASRSLPDFAELDLQGKVLPEGV
GPGDIKAFQVLYREHCEAIVDVMVNLQFTLVETLWKTFWRYNLSQPSEAPPLAVHDEAEKRLPRASLVLLSKF
QPVLQWTKHCDNVLYQGLVEILIPDVLRPIPSALTQAIRNFAKSLESWLTHAMVNIPEEMLRVKVAAAGAFAQ
TLRRYTSLNHLAQAARAVLQNTAQINQMLSDLNRVDFANVQEQASWVCRCEDRVVQRLEQDFKVTLQQQNSLE
QWAAWLDGVVSQVLKPYQGSSGFPKAAKLFLLKWSFYSSMVIRDLTLRSAASFGSFHLIRLLYDEYMYYLIEH
RVAQAKGETPIAVMGEFANLATSLNPLDPDKDEEEEEEEESEDELPQDISLAAGSESPALGPEALEPPAKLAR
TDTRGLFVQALPSS*
>rs32854560_ENSMUSP00000112556_c.2683:T>C_p.L895P
ENSMUST00000117424.9|Cc2d1a-202|ENSMUSG00000036686|-|chr8:g.84859876:A>G
MHKRNGPQAPPGRGAVTARQLGLLVDFSPDGLMIPEDGINEEELEAEFLALVGGQPQALEKLKGQGPLPMEAI
EKMARLCMRDLDEDEEGTDEDDVEADEDLLAELNEVLGEEQKAVEPLMPVAQTLENLLVSAKKGNIINEADIP
PPVASGKGAAAGHSHTQATSQLASVSPPAPESSGTLEAPSTTTPTSAKPQLPPDPCSPLARLQSLQHEYKLAA
LRAKHQDDTATATRHLRIAKSFDPVLEALSRGELVDLSRLPPPPDQLSPEPPLPAAQPLTSASTLTRPEVPQP
PRNLLEALEQRMERYHVAAAQAKAKGDQRKARMHERIVKQYQDAIRAHKAGRAVDVAELPVPPGFPPMQGLES
AEPSQQSLVGVLETAMRLANHDEGSDDEEEETPKKNTPAASTTQLKSSPSKAPPSGPAPAGKAAPKGTSNRAQ
QQLAFLEGRKKQLLQAALRAKQKNDVEGAKMHLRQAKGLEPMLEASRNGLPVDIAKVPPAPVNKDDFVLVQRP
GPGLSQEAVRRYGELTKLLRQQHEMCLNHSTQFTHLGNIAETIKFEKLAEDCKRSMDTLKQAFARSLPTPAAR
FEQRTFSVIKVFPDLSNSDMLLFIVKGINLPTPTGLSPSDLDAFVRFDFPYPNVEEAQKDKTSVIKNTDSPEF
KEQFKLCINRGHRGFRRAIQTKGIKFEVVHKGGLFKTDRVLGTAQLKLGTLETACEVHEILEVLDGRRPTGGR
LEVMVRIREPLTAQQLETTTERWLVIDHIPAAMPTVTGPKAKAPLIPASSREAGNRSARPLHSLSVLAFDQER
LERKILALRQARRPVPPEVAQQYQDVVQRSQWQRAQLEQGGAALRREYASHLERQLHFYTEAARRLGYDGSRE
AAKEALYRRNLVESELQRPRR*
>rs32854560_ENSMUSP00000046449_c.2821:T>C_p.L941P
ENSMUST00000040383.9|Cc2d1a-201|ENSMUSG00000036686|-|chr8:g.84859876:A>G
MHKRNGPQAPPGRGAVTARQLGLLVDFSPDGLMIPEDGINEEELEAEFLALVGGQPQALEKLKGQGPLPMEAI
EKMARLCMRDLDEDEEGTDEDDVEADEDLLAELNEVLGEEQKAVEPLMPVAQPKPSGPNPGVEATLQERLTLY
QSALESARQAGDSAKMRRYDRGLKTLENLLVSAKKGNIINEADIPPPVASGKGAAAGHSHTQATSQLASVSPP
APESSGTLEAPSTTTPTSAKPQLPPDPCSPLARLQSLQHEYKLAALRAKHQDDTATATRHLRIAKSFDPVLEA
LSRGELVDLSRLPPPPDQLSPEPPLPAAQPLTSASTLTRPEVPQPPRNLLEALEQRMERYHVAAAQAKAKGDQ
RKARMHERIVKQYQDAIRAHKAGRAVDVAELPVPPGFPPMQGLESAEPSQQSLVGVLETAMRLANHDEGSDDE
EEETPKKQNTPAASTTQLKSSPSKAPPSGPAPAGKAAPKGTSNRAQQQLAFLEGRKKQLLQAALRAKQKNDVE
GAKMHLRQAKGLEPMLEASRNGLPVDIAKVPPAPVNKDDFVLVQRPGPGLSQEAVRRYGELTKLLRQQHEMCL
NHSTQFTHLGNIAETIKFEKLAEDCKRSMDTLKQAFARSLPTPAARFEQRTFSVIKVFPDLSNSDMLLFIVKG
INLPTPTGLSPSDLDAFVRFDFPYPNVEEAQKDKTSVIKNTDSPEFKEQFKLCINRGHRGFRRAIQTKGIKFE
VVHKGGLFKTDRVLGTAQLKLGTLETACEVHEILEVLDGRRPTGGRLEVMVRIREPLTAQQLETTTERWLVID
HIPAAMPTVTGPKAKAPLIPASSREAGNRSARPLHSLSVLAFDQERLERKILALRQARRPVPPEVAQQYQDVV
QRSQWQRAQLEQGGAALRREYASHLERQLHFYTEAARRLGYDGSREAAKEALYRRNLVESELQRPRR*
>rs32547577_ENSMUSP00000112556_c.2005:A>C_p.H669P
ENSMUST00000117424.9|Cc2d1a-202|ENSMUSG00000036686|-|chr8:g.84861751:T>G
MHKRNGPQAPPGRGAVTARQLGLLVDFSPDGLMIPEDGINEEELEAEFLALVGGQPQALEKLKGQGPLPMEAI
EKMARLCMRDLDEDEEGTDEDDVEADEDLLAELNEVLGEEQKAVEPLMPVAQTLENLLVSAKKGNIINEADIP
PPVASGKGAAAGHSHTQATSQLASVSPPAPESSGTLEAPSTTTPTSAKPQLPPDPCSPLARLQSLQHEYKLAA
LRAKHQDDTATATRHLRIAKSFDPVLEALSRGELVDLSRLPPPPDQLSPEPPLPAAQPLTSASTLTRPEVPQP
PRNLLEALEQRMERYHVAAAQAKAKGDQRKARMHERIVKQYQDAIRAHKAGRAVDVAELPVPPGFPPMQGLES
AEPSQQSLVGVLETAMRLANHDEGSDDEEEETPKKNTPAASTTQLKSSPSKAPPSGPAPAGKAAPKGTSNRAQ
QQLAFLEGRKKQLLQAALRAKQKNDVEGAKMHLRQAKGLEPMLEASRNGLPVDIAKVPPAPVNKDDFVLVQRP
GPGLSQEAVRRYGELTKLLRQQHEMCLNHSTQFTHLGNIAETIKFEKLAEDCKRSMDTLKQAFARSLPTPAAR

FEQRTFSVIKVFPDLSNSDMLLFIVKGINLPTPTGLSPSDLDAFVRFDFPYPNVEEAQKDKTSVIKNTDSPEF
KEQFKLCINRGPRGFRRAIQTKGIKFEVVHKGGLFKTDRVLGTAQLKLGTLETACEVHEILEVLDGRRPTGGR
LEVMVRIREPLTAQQLETTTERWLVIDHIPAAMPTVTGPKAKAPLIPASSREAGNRSARPLHSLSVLAFDQER
LERKILALRQARRPVPPEVAQQYQDVVQRSQWQRAQLEQGGAALRREYASHLERQLHFYTEAARRLGYDGSRE
AAKEALYRRNLVESELQRLRR*
>rs32547577_ENSMUSP00000046449_c.2143:A>C_p.H715P
ENSMUST00000040383.9|Cc2d1a-201|ENSMUSG00000036686|-|chr8:g.84861751:T>G
MHKRNGPQAPPGRGAVTARQLGLLVDFSPDGLMIPEDGINEEELEAEFLALVGGQPQALEKLKGQGPLPMEAI
EKMARLCMRDLDEDEEGTDEDDVEADEDLLAELNEVLGEEQKAVEPLMPVAQPKPSGPNPGVEATLQERLTLY
QSALESARQAGDSAKMRRYDRGLKTLENLLVSAKKGNIINEADIPPPVASGKGAAAGHSHTQATSQLASVSPP
APESSGTLEAPSTTTPTSAKPQLPPDPCSPLARLQSLQHEYKLAALRAKHQDDTATATRHLRIAKSFDPVLEA
LSRGELVDLSRLPPPPDQLSPEPPLPAAQPLTSASTLTRPEVPQPPRNLLEALEQRMERYHVAAAQAKAKGDQ
RKARMHERIVKQYQDAIRAHKAGRAVDVAELPVPPGFPPMQGLESAEPSQQSLVGVLETAMRLANHDEGSDDE
EEETPKKQNTPAASTTQLKSSPSKAPPSGPAPAGKAAPKGTSNRAQQQLAFLEGRKKQLLQAALRAKQKNDVE
GAKMHLRQAKGLEPMLEASRNGLPVDIAKVPPAPVNKDDFVLVQRPGPGLSQEAVRRYGELTKLLRQQHEMCL
NHSTQFTHLGNIAETIKFEKLAEDCKRSMDTLKQAFARSLPTPAARFEQRTFSVIKVFPDLSNSDMLLFIVKG
INLPTPTGLSPSDLDAFVRFDFPYPNVEEAQKDKTSVIKNTDSPEFKEQFKLCINRGPRGFRRAIQTKGIKFE
VVHKGGLFKTDRVLGTAQLKLGTLETACEVHEILEVLDGRRPTGGRLEVMVRIREPLTAQQLETTTERWLVID
HIPAAMPTVTGPKAKAPLIPASSREAGNRSARPLHSLSVLAFDQERLERKILALRQARRPVPPEVAQQYQDVV
QRSQWQRAQLEQGGAALRREYASHLERQLHFYTEAARRLGYDGSREAAKEALYRRNLVESELQRLRR*
>rs33110810_ENSMUSP00000112556_c.1987:T>C_p.L663P
ENSMUST00000117424.9|Cc2d1a-202|ENSMUSG00000036686|-|chr8:g.84861769:A>G
MHKRNGPQAPPGRGAVTARQLGLLVDFSPDGLMIPEDGINEEELEAEFLALVGGQPQALEKLKGQGPLPMEAI
EKMARLCMRDLDEDEEGTDEDDVEADEDLLAELNEVLGEEQKAVEPLMPVAQTLENLLVSAKKGNIINEADIP
PPVASGKGAAAGHSHTQATSQLASVSPPAPESSGTLEAPSTTTPTSAKPQLPPDPCSPLARLQSLQHEYKLAA
LRAKHQDDTATATRHLRIAKSFDPVLEALSRGELVDLSRLPPPPDQLSPEPPLPAAQPLTSASTLTRPEVPQP
PRNLLEALEQRMERYHVAAAQAKAKGDQRKARMHERIVKQYQDAIRAHKAGRAVDVAELPVPPGFPPMQGLES
AEPSQQSLVGVLETAMRLANHDEGSDDEEEETPKKNTPAASTTQLKSSPSKAPPSGPAPAGKAAPKGTSNRAQ
QQLAFLEGRKKQLLQAALRAKQKNDVEGAKMHLRQAKGLEPMLEASRNGLPVDIAKVPPAPVNKDDFVLVQRP
GPGLSQEAVRRYGELTKLLRQQHEMCLNHSTQFTHLGNIAETIKFEKLAEDCKRSMDTLKQAFARSLPTPAAR
FEQRTFSVIKVFPDLSNSDMLLFIVKGINLPTPTGLSPSDLDAFVRFDFPYPNVEEAQKDKTSVIKNTDSPEF
KEQFKPCINRGHRGFRRAIQTKGIKFEVVHKGGLFKTDRVLGTAQLKLGTLETACEVHEILEVLDGRRPTGGR
LEVMVRIREPLTAQQLETTTERWLVIDHIPAAMPTVTGPKAKAPLIPASSREAGNRSARPLHSLSVLAFDQER
LERKILALRQARRPVPPEVAQQYQDVVQRSQWQRAQLEQGGAALRREYASHLERQLHFYTEAARRLGYDGSRE
AAKEALYRRNLVESELQRLRR*
>rs33110810_ENSMUSP00000046449_c.2125:T>C_p.L709P
ENSMUST00000040383.9|Cc2d1a-201|ENSMUSG00000036686|-|chr8:g.84861769:A>G
MHKRNGPQAPPGRGAVTARQLGLLVDFSPDGLMIPEDGINEEELEAEFLALVGGQPQALEKLKGQGPLPMEAI
EKMARLCMRDLDEDEEGTDEDDVEADEDLLAELNEVLGEEQKAVEPLMPVAQPKPSGPNPGVEATLQERLTLY
QSALESARQAGDSAKMRRYDRGLKTLENLLVSAKKGNIINEADIPPPVASGKGAAAGHSHTQATSQLASVSPP
APESSGTLEAPSTTTPTSAKPQLPPDPCSPLARLQSLQHEYKLAALRAKHQDDTATATRHLRIAKSFDPVLEA
LSRGELVDLSRLPPPPDQLSPEPPLPAAQPLTSASTLTRPEVPQPPRNLLEALEQRMERYHVAAAQAKAKGDQ
RKARMHERIVKQYQDAIRAHKAGRAVDVAELPVPPGFPPMQGLESAEPSQQSLVGVLETAMRLANHDEGSDDE
EEETPKKQNTPAASTTQLKSSPSKAPPSGPAPAGKAAPKGTSNRAQQQLAFLEGRKKQLLQAALRAKQKNDVE
GAKMHLRQAKGLEPMLEASRNGLPVDIAKVPPAPVNKDDFVLVQRPGPGLSQEAVRRYGELTKLLRQQHEMCL
NHSTQFTHLGNIAETIKFEKLAEDCKRSMDTLKQAFARSLPTPAARFEQRTFSVIKVFPDLSNSDMLLFIVKG
INLPTPTGLSPSDLDAFVRFDFPYPNVEEAQKDKTSVIKNTDSPEFKEQFKPCINRGHRGFRRAIQTKGIKFE
VVHKGGLFKTDRVLGTAQLKLGTLETACEVHEILEVLDGRRPTGGRLEVMVRIREPLTAQQLETTTERWLVID
HIPAAMPTVTGPKAKAPLIPASSREAGNRSARPLHSLSVLAFDQERLERKILALRQARRPVPPEVAQQYQDVV
QRSQWQRAQLEQGGAALRREYASHLERQLHFYTEAARRLGYDGSREAAKEALYRRNLVESELQRLRR*
>rs46610077_ENSMUSP00000112556_c.1372:C>A_p.A458D
ENSMUST00000117424.9|Cc2d1a-202|ENSMUSG00000036686|-|chr8:g.84865199:G>T
MHKRNGPQAPPGRGAVTARQLGLLVDFSPDGLMIPEDGINEEELEAEFLALVGGQPQALEKLKGQGPLPMEAI
EKMARLCMRDLDEDEEGTDEDDVEADEDLLAELNEVLGEEQKAVEPLMPVAQTLENLLVSAKKGNIINEADIP
PPVASGKGAAAGHSHTQATSQLASVSPPAPESSGTLEAPSTTTPTSAKPQLPPDPCSPLARLQSLQHEYKLAA
LRAKHQDDTATATRHLRIAKSFDPVLEALSRGELVDLSRLPPPPDQLSPEPPLPAAQPLTSASTLTRPEVPQP
PRNLLEALEQRMERYHVAAAQAKAKGDQRKARMHERIVKQYQDAIRAHKAGRAVDVAELPVPPGFPPMQGLES
AEPSQQSLVGVLETAMRLANHDEGSDDEEEETPKKNTPAASTTQLKSSPSKAPPSGPAPAGKAAPKGTSNRAQ

QQLAFLEGRKKQLLQAALRDKQKNDVEGAKMHLRQAKGLEPMLEASRNGLPVDIAKVPPAPVNKDDFVLVQRP
GPGLSQEAVRRYGELTKLLRQQHEMCLNHSTQFTHLGNIAETIKFEKLAEDCKRSMDTLKQAFARSLPTPAAR
FEQRTFSVIKVFPDLSNSDMLLFIVKGINLPTPTGLSPSDLDAFVRFDFPYPNVEEAQKDKTSVIKNTDSPEF
KEQFKLCINRGHRGFRRAIQTKGIKFEVVHKGGLFKTDRVLGTAQLKLGTLETACEVHEILEVLDGRRPTGGR
LEVMVRIREPLTAQQLETTTERWLVIDHIPAAMPTVTGPKAKAPLIPASSREAGNRSARPLHSLSVLAFDQER
LERKILALRQARRPVPPEVAQQYQDVVQRSQWQRAQLEQGGAALRREYASHLERQLHFYTEAARRLGYDGSRE
AAKEALYRRNLVESELQRLRR*
>rs46610077_ENSMUSP00000046449_c.1510:C>A_p.A504D
ENSMUST00000040383.9|Cc2d1a-201|ENSMUSG00000036686|-|chr8:g.84865199:G>T
MHKRNGPQAPPGRGAVTARQLGLLVDFSPDGLMIPEDGINEEELEAEFLALVGGQPQALEKLKGQGPLPMEAI
EKMARLCMRDLDEDEEGTDEDDVEADEDLLAELNEVLGEEQKAVEPLMPVAQPKPSGPNPGVEATLQERLTLY
QSALESARQAGDSAKMRRYDRGLKTLENLLVSAKKGNIINEADIPPPVASGKGAAAGHSHTQATSQLASVSPP
APESSGTLEAPSTTTPTSAKPQLPPDPCSPLARLQSLQHEYKLAALRAKHQDDTATATRHLRIAKSFDPVLEA
LSRGELVDLSRLPPPPDQLSPEPPLPAAQPLTSASTLTRPEVPQPPRNLLEALEQRMERYHVAAAQAKAKGDQ
RKARMHERIVKQYQDAIRAHKAGRAVDVAELPVPPGFPPMQGLESAEPSQQSLVGVLETAMRLANHDEGSDDE
EEETPKKQNTPAASTTQLKSSPSKAPPSGPAPAGKAAPKGTSNRAQQQLAFLEGRKKQLLQAALRDKQKNDVE
GAKMHLRQAKGLEPMLEASRNGLPVDIAKVPPAPVNKDDFVLVQRPGPGLSQEAVRRYGELTKLLRQQHEMCL
NHSTQFTHLGNIAETIKFEKLAEDCKRSMDTLKQAFARSLPTPAARFEQRTFSVIKVFPDLSNSDMLLFIVKG
INLPTPTGLSPSDLDAFVRFDFPYPNVEEAQKDKTSVIKNTDSPEFKEQFKLCINRGHRGFRRAIQTKGIKFE
VVHKGGLFKTDRVLGTAQLKLGTLETACEVHEILEVLDGRRPTGGRLEVMVRIREPLTAQQLETTTERWLVID
HIPAAMPTVTGPKAKAPLIPASSREAGNRSARPLHSLSVLAFDQERLERKILALRQARRPVPPEVAQQYQDVV
QRSQWQRAQLEQGGAALRREYASHLERQLHFYTEAARRLGYDGSREAAKEALYRRNLVESELQRLRR*
>rs33365678_ENSMUSP00000112556_c.912:G>A_p.E305K
ENSMUST00000117424.9|Cc2d1a-202|ENSMUSG00000036686|-|chr8:g.84867093:C>T
MHKRNGPQAPPGRGAVTARQLGLLVDFSPDGLMIPEDGINEEELEAEFLALVGGQPQALEKLKGQGPLPMEAI
EKMARLCMRDLDEDEEGTDEDDVEADEDLLAELNEVLGEEQKAVEPLMPVAQTLENLLVSAKKGNIINEADIP
PPVASGKGAAAGHSHTQATSQLASVSPPAPESSGTLEAPSTTTPTSAKPQLPPDPCSPLARLQSLQHEYKLAA
LRAKHQDDTATATRHLRIAKSFDPVLEALSRGELVDLSRLPPPPDQLSPEPPLPAAQPLTSASTLTRPEVPQP
PRNLLEALEQRMKRYHVAAAQAKAKGDQRKARMHERIVKQYQDAIRAHKAGRAVDVAELPVPPGFPPMQGLES
AEPSQQSLVGVLETAMRLANHDEGSDDEEEETPKKNTPAASTTQLKSSPSKAPPSGPAPAGKAAPKGTSNRAQ
QQLAFLEGRKKQLLQAALRAKQKNDVEGAKMHLRQAKGLEPMLEASRNGLPVDIAKVPPAPVNKDDFVLVQRP
GPGLSQEAVRRYGELTKLLRQQHEMCLNHSTQFTHLGNIAETIKFEKLAEDCKRSMDTLKQAFARSLPTPAAR
FEQRTFSVIKVFPDLSNSDMLLFIVKGINLPTPTGLSPSDLDAFVRFDFPYPNVEEAQKDKTSVIKNTDSPEF
KEQFKLCINRGHRGFRRAIQTKGIKFEVVHKGGLFKTDRVLGTAQLKLGTLETACEVHEILEVLDGRRPTGGR
LEVMVRIREPLTAQQLETTTERWLVIDHIPAAMPTVTGPKAKAPLIPASSREAGNRSARPLHSLSVLAFDQER
LERKILALRQARRPVPPEVAQQYQDVVQRSQWQRAQLEQGGAALRREYASHLERQLHFYTEAARRLGYDGSRE
AAKEALYRRNLVESELQRLRR*
>rs33365678_ENSMUSP00000046449_c.1047:G>A_p.E350K
ENSMUST00000040383.9|Cc2d1a-201|ENSMUSG00000036686|-|chr8:g.84867093:C>T
MHKRNGPQAPPGRGAVTARQLGLLVDFSPDGLMIPEDGINEEELEAEFLALVGGQPQALEKLKGQGPLPMEAI
EKMARLCMRDLDEDEEGTDEDDVEADEDLLAELNEVLGEEQKAVEPLMPVAQPKPSGPNPGVEATLQERLTLY
QSALESARQAGDSAKMRRYDRGLKTLENLLVSAKKGNIINEADIPPPVASGKGAAAGHSHTQATSQLASVSPP
APESSGTLEAPSTTTPTSAKPQLPPDPCSPLARLQSLQHEYKLAALRAKHQDDTATATRHLRIAKSFDPVLEA
LSRGELVDLSRLPPPPDQLSPEPPLPAAQPLTSASTLTRPEVPQPPRNLLEALEQRMKRYHVAAAQAKAKGDQ
RKARMHERIVKQYQDAIRAHKAGRAVDVAELPVPPGFPPMQGLESAEPSQQSLVGVLETAMRLANHDEGSDDE
EEETPKKQNTPAASTTQLKSSPSKAPPSGPAPAGKAAPKGTSNRAQQQLAFLEGRKKQLLQAALRAKQKNDVE
GAKMHLRQAKGLEPMLEASRNGLPVDIAKVPPAPVNKDDFVLVQRPGPGLSQEAVRRYGELTKLLRQQHEMCL
NHSTQFTHLGNIAETIKFEKLAEDCKRSMDTLKQAFARSLPTPAARFEQRTFSVIKVFPDLSNSDMLLFIVKG
INLPTPTGLSPSDLDAFVRFDFPYPNVEEAQKDKTSVIKNTDSPEFKEQFKLCINRGHRGFRRAIQTKGIKFE
VVHKGGLFKTDRVLGTAQLKLGTLETACEVHEILEVLDGRRPTGGRLEVMVRIREPLTAQQLETTTERWLVID
HIPAAMPTVTGPKAKAPLIPASSREAGNRSARPLHSLSVLAFDQERLERKILALRQARRPVPPEVAQQYQDVV
QRSQWQRAQLEQGGAALRREYASHLERQLHFYTEAARRLGYDGSREAAKEALYRRNLVESELQRLRR*
>rs33064505_ENSMUSP00000112556_c.835:C>T_p.T279I
ENSMUST00000117424.9|Cc2d1a-202|ENSMUSG00000036686|-|chr8:g.84867261:G>A
MHKRNGPQAPPGRGAVTARQLGLLVDFSPDGLMIPEDGINEEELEAEFLALVGGQPQALEKLKGQGPLPMEAI
EKMARLCMRDLDEDEEGTDEDDVEADEDLLAELNEVLGEEQKAVEPLMPVAQTLENLLVSAKKGNIINEADIP
PPVASGKGAAAGHSHTQATSQLASVSPPAPESSGTLEAPSTTTPTSAKPQLPPDPCSPLARLQSLQHEYKLAA
LRAKHQDDTATATRHLRIAKSFDPVLEALSRGELVDLSRLPPPPDQLSPEPPLPAAQPLISASTLTRPEVPQP

PRNLLEALEQRMERYHVAAAQAKAKGDQRKARMHERIVKQYQDAIRAHKAGRAVDVAELPVPPGFPPMQGLES
AEPSQQSLVGVLETAMRLANHDEGSDDEEEETPKKNTPAASTTQLKSSPSKAPPSGPAPAGKAAPKGTSNRAQ
QQLAFLEGRKKQLLQAALRAKQKNDVEGAKMHLRQAKGLEPMLEASRNGLPVDIAKVPPAPVNKDDFVLVQRP
GPGLSQEAVRRYGELTKLLRQQHEMCLNHSTQFTHLGNIAETIKFEKLAEDCKRSMDTLKQAFARSLPTPAAR
FEQRTFSVIKVFPDLSNSDMLLFIVKGINLPTPTGLSPSDLDAFVRFDFPYPNVEEAQKDKTSVIKNTDSPEF
KEQFKLCINRGHRGFRRAIQTKGIKFEVVHKGGLFKTDRVLGTAQLKLGTLETACEVHEILEVLDGRRPTGGR
LEVMVRIREPLTAQQLETTTERWLVIDHIPAAMPTVTGPKAKAPLIPASSREAGNRSARPLHSLSVLAFDQER
LERKILALRQARRPVPPEVAQQYQDVVQRSQWQRAQLEQGGAALRREYASHLERQLHFYTEAARRLGYDGSRE
AAKEALYRRNLVESELQRLRR*
>rs33064505_ENSMUSP00000046449_c.970:C>T_p.T324I
ENSMUST00000040383.9|Cc2d1a-201|ENSMUSG00000036686|-|chr8:g.84867261:G>A
MHKRNGPQAPPGRGAVTARQLGLLVDFSPDGLMIPEDGINEEELEAEFLALVGGQPQALEKLKGQGPLPMEAI
EKMARLCMRDLDEDEEGTDEDDVEADEDLLAELNEVLGEEQKAVEPLMPVAQPKPSGPNPGVEATLQERLTLY
QSALESARQAGDSAKMRRYDRGLKTLENLLVSAKKGNIINEADIPPPVASGKGAAAGHSHTQATSQLASVSPP
APESSGTLEAPSTTTPTSAKPQLPPDPCSPLARLQSLQHEYKLAALRAKHQDDTATATRHLRIAKSFDPVLEA
LSRGELVDLSRLPPPPDQLSPEPPLPAAQPLISASTLTRPEVPQPPRNLLEALEQRMERYHVAAAQAKAKGDQ
RKARMHERIVKQYQDAIRAHKAGRAVDVAELPVPPGFPPMQGLESAEPSQQSLVGVLETAMRLANHDEGSDDE
EEETPKKQNTPAASTTQLKSSPSKAPPSGPAPAGKAAPKGTSNRAQQQLAFLEGRKKQLLQAALRAKQKNDVE
GAKMHLRQAKGLEPMLEASRNGLPVDIAKVPPAPVNKDDFVLVQRPGPGLSQEAVRRYGELTKLLRQQHEMCL
NHSTQFTHLGNIAETIKFEKLAEDCKRSMDTLKQAFARSLPTPAARFEQRTFSVIKVFPDLSNSDMLLFIVKG
INLPTPTGLSPSDLDAFVRFDFPYPNVEEAQKDKTSVIKNTDSPEFKEQFKLCINRGHRGFRRAIQTKGIKFE
VVHKGGLFKTDRVLGTAQLKLGTLETACEVHEILEVLDGRRPTGGRLEVMVRIREPLTAQQLETTTERWLVID
HIPAAMPTVTGPKAKAPLIPASSREAGNRSARPLHSLSVLAFDQERLERKILALRQARRPVPPEVAQQYQDVV
QRSQWQRAQLEQGGAALRREYASHLERQLHFYTEAARRLGYDGSREAAKEALYRRNLVESELQRLRR*
>rs33598727_ENSMUSP00000112556_c.463:C>T_p.A155V
ENSMUST00000117424.9|Cc2d1a-202|ENSMUSG00000036686|-|chr8:g.84870050:G>A
MHKRNGPQAPPGRGAVTARQLGLLVDFSPDGLMIPEDGINEEELEAEFLALVGGQPQALEKLKGQGPLPMEAI
EKMARLCMRDLDEDEEGTDEDDVEADEDLLAELNEVLGEEQKAVEPLMPVAQTLENLLVSAKKGNIINEADIP
PPVASGKGVAAGHSHTQATSQLASVSPPAPESSGTLEAPSTTTPTSAKPQLPPDPCSPLARLQSLQHEYKLAA
LRAKHQDDTATATRHLRIAKSFDPVLEALSRGELVDLSRLPPPPDQLSPEPPLPAAQPLTSASTLTRPEVPQP
PRNLLEALEQRMERYHVAAAQAKAKGDQRKARMHERIVKQYQDAIRAHKAGRAVDVAELPVPPGFPPMQGLES
AEPSQQSLVGVLETAMRLANHDEGSDDEEEETPKKNTPAASTTQLKSSPSKAPPSGPAPAGKAAPKGTSNRAQ
QQLAFLEGRKKQLLQAALRAKQKNDVEGAKMHLRQAKGLEPMLEASRNGLPVDIAKVPPAPVNKDDFVLVQRP
GPGLSQEAVRRYGELTKLLRQQHEMCLNHSTQFTHLGNIAETIKFEKLAEDCKRSMDTLKQAFARSLPTPAAR
FEQRTFSVIKVFPDLSNSDMLLFIVKGINLPTPTGLSPSDLDAFVRFDFPYPNVEEAQKDKTSVIKNTDSPEF
KEQFKLCINRGHRGFRRAIQTKGIKFEVVHKGGLFKTDRVLGTAQLKLGTLETACEVHEILEVLDGRRPTGGR
LEVMVRIREPLTAQQLETTTERWLVIDHIPAAMPTVTGPKAKAPLIPASSREAGNRSARPLHSLSVLAFDQER
LERKILALRQARRPVPPEVAQQYQDVVQRSQWQRAQLEQGGAALRREYASHLERQLHFYTEAARRLGYDGSRE
AAKEALYRRNLVESELQRLRR*
>rs33598727_ENSMUSP00000046449_c.598:C>T_p.A200V
ENSMUST00000040383.9|Cc2d1a-201|ENSMUSG00000036686|-|chr8:g.84870050:G>A
MHKRNGPQAPPGRGAVTARQLGLLVDFSPDGLMIPEDGINEEELEAEFLALVGGQPQALEKLKGQGPLPMEAI
EKMARLCMRDLDEDEEGTDEDDVEADEDLLAELNEVLGEEQKAVEPLMPVAQPKPSGPNPGVEATLQERLTLY
QSALESARQAGDSAKMRRYDRGLKTLENLLVSAKKGNIINEADIPPPVASGKGVAAGHSHTQATSQLASVSPP
APESSGTLEAPSTTTPTSAKPQLPPDPCSPLARLQSLQHEYKLAALRAKHQDDTATATRHLRIAKSFDPVLEA
LSRGELVDLSRLPPPPDQLSPEPPLPAAQPLTSASTLTRPEVPQPPRNLLEALEQRMERYHVAAAQAKAKGDQ
RKARMHERIVKQYQDAIRAHKAGRAVDVAELPVPPGFPPMQGLESAEPSQQSLVGVLETAMRLANHDEGSDDE
EEETPKKQNTPAASTTQLKSSPSKAPPSGPAPAGKAAPKGTSNRAQQQLAFLEGRKKQLLQAALRAKQKNDVE
GAKMHLRQAKGLEPMLEASRNGLPVDIAKVPPAPVNKDDFVLVQRPGPGLSQEAVRRYGELTKLLRQQHEMCL
NHSTQFTHLGNIAETIKFEKLAEDCKRSMDTLKQAFARSLPTPAARFEQRTFSVIKVFPDLSNSDMLLFIVKG
INLPTPTGLSPSDLDAFVRFDFPYPNVEEAQKDKTSVIKNTDSPEFKEQFKLCINRGHRGFRRAIQTKGIKFE
VVHKGGLFKTDRVLGTAQLKLGTLETACEVHEILEVLDGRRPTGGRLEVMVRIREPLTAQQLETTTERWLVID
HIPAAMPTVTGPKAKAPLIPASSREAGNRSARPLHSLSVLAFDQERLERKILALRQARRPVPPEVAQQYQDVV
QRSQWQRAQLEQGGAALRREYASHLERQLHFYTEAARRLGYDGSREAAKEALYRRNLVESELQRLRR*
>rs254259087_ENSMUSP00000113651_c.405:GC>G_p.A136del-fs
ENSMUST00000118856.8|Brme1-202|ENSMUSG00000008129|+|chr8:g.84893240:GC>G
MNKKKQRNSGVGLHPSKPSKNPRLRDSQSSMMVHSHYSRESEDSSEPAPSVELGGEEPLHEAFSCPVEDTGAA
SDLAGSPKELVPLPPSQNSVGKFVPQFAKPRKTVTRKAKAWEEDLEGCTTSQETRPELGALKEPASHRESP

```
>rs254259087_ENSMUSP00000091067_c.300:GC>G_p.A101del-fs
ENSMUST00000093375.5|Brme1-201|ENSMUSG00000008129|+|chr8:g.84893240:GC>G
MNKKKQRNSGVGLHPSKPSKNPRLRDSQSSMMVHSHYSRESEDSSEPAPSVELGGEEPLHEAFSCPVEDTGAA
SDLAGSPKELVPLPPSQETRPELGALKEPASHRESP
>rs32694707_ENSMUSP00000113651_c.663:C>T_p.Q222*
ENSMUST00000118856.8|Brme1-202|ENSMUSG00000008129|+|chr8:g.84893498:C>T
MNKKKQRNSGVGLHPSKPSKNPRLRDSQSSMMVHSHYSRESEDSSEPAPSVELGGEEPLHEAFSCPVEDTGAA
SDLAGSPKELVPLPPSQNSVGKFVPQFAKPRKTVTRKAKAWEEDLEGCTTSQETRPELGALKAASQPQRESLR
FPPHDIRPEVQTQPDGTLSKERTISLDNRSLGNNGFEMATVQDSSSPLSDAAAEGREADSRDPQERDAQGGEA
GA*HSGEPQEGEDILYTSALAPASEPTWSVAQDLSVPTYTLSSTAAAPSSTSPADASLMDTVITEVSLDPSVL
QQSAPQVAKLLGSLDEQIPDGGCIGTLLSSTPLAEETTAGREEARWEERCHGDTLASFTETEPEKQEPVTEAG
DSGHIAQEMDPVVKTKDSGSDEQSPGDIGMLPLPAQSMNQMLVELRGLTCDQDLEGLSTPHTSSQLEHTCAAS
DPPQSTKDCHSSPGIPVHLAAPCPRDQAAWQESSAMELDFLPDSQIQDALDATNMEQGFPSGSMPDLGWPVPS
SQSIGGSPKAVTKPQSRSHVETWAQETYRMQDATDTVRGLVVELSGLNRLIMSTHRDLEAFKRRKTKSLPYLT
KGLGSLPRGDQSWRDL*
>rs32694707_ENSMUSP00000091067_c.558:C>T_p.Q187*
ENSMUST00000093375.5|Brme1-201|ENSMUSG00000008129|+|chr8:g.84893498:C>T
MNKKKQRNSGVGLHPSKPSKNPRLRDSQSSMMVHSHYSRESEDSSEPAPSVELGGEEPLHEAFSCPVEDTGAA
SDLAGSPKELVPLPPSQETRPELGALKAASQPQRESLRFPPHDIRPEVQTQPDGTLSKERTISLDNRSLGNNG
FEMATVQDSSSPLSDAAAEGREADSRDPQERDAQGGEAGA*HSGEPQEGEDILYTSALAPASEPTWSVAQDLS
VPTYTLSSTAAAPSSTSPADASLMDTVITEVSLDPSVLQQSAPQVAKLLGSLDEQIPDGGCIGTLLSSTPLAE
ETTAGREEARWEERCHGDTLASFTETEPEKQEPVTEAGDSGHIAQEMDPVVKTKDSGSDEQSPGDIGMLPLPA
QSMNQMLVELRGLTCDQDLEGLSTPHTSSQLEHTCAASDPPQSTKDCHSSPGIPVHLAAPCPRDQAAWQESSA
MELDFLPDSQIQDALDATNMEQGFPSGSMPDLGWPVPSSQSIGGSPKAVTKPQSRSHVETWAQETYRMQDATD
TVRGLVVELSGLNRLIMSTHRDLEAFKRRKTKSLPYLTKGLGSLPRGDQSWRDL*
>rs6355997_ENSMUSP00000040078_c.1231:A>G_p.Q411R
ENSMUST00000039480.7|Zswim4-201|ENSMUSG00000035671|-|chr8:g.84952729:T>C
MEPPAAKRSRGCPAGDEPGTGARRSRPEPLLDLSAKRVAESWAFEQVEERFSRVPEPVQKRIVFWSFPRSERE
ICMYSSLGYQPPEGEQDTRVPFTRGLHLLQSGAVDRVLQVGFHLSGNIREVGGPGEPEHLYHVSISFDRCKIT
SVSCGCDNRDLFYCAHVVALSLYRIRHARQVELRLPISETLSQMNRDQLQKFVQYLISAHHTEVLPTAQRLAD
EILLLGSEINLVHGAPDPTAGAGTEDANCWHLDEEQIQEQVKQLLSNGGHYGASQQLRSMFSKVREMLRARDS
NGARMLILMTEQFLQDPRLALWRQQGAGMTDKCRQLWDELGALWVCVILSPHCKPDERSGWLQLLGTWDKLDV
CPLEEGNYSLDIPSLQPALTSRTGSEDEEEVTAASPRRTVFSRALRAGELHWNDSHLQRILASDSCGPTLTGS
MGSDKPTFDPQGHPIWLGEPFPTACARVDTLRAHGYPHKALRLACAIINTLRLQRRHQLESYKQQKKELLQKG
ATGVTSTEGWVGHPLDPIGCLCRALLEACRLEEEPHSLFPDSAPEKRKLAYQHVLVPGSPGESYLALALEVAL
LGLGQQRALPEGLYAQDKVVRNEEQLLALLEEVELDERLVQVLRKQAGLLLEGGPFSGFGEVIFRESVPMHTC
ARYLFTALLPHDPDLAFRLALRAMRLPVLETTFPAGESHPNPLDSIMSNRFPRWFILGHLETRQCELASTMLT
AAKGDTKWLHAVLGSVQQNIHSPALLFKLAQDACKTATPASAPPDTVLLGIALELGLQVMRMTLNTMTWRRRE
MVRWLVSCATEIGPPALMSIMKNWYSLFTPVEAVTIVAVTGTTHATLLRLQLDTAGREELWACARTLALQCAM
KDPQNCALPALTLCEKNHAAFEAAYQIVLDAAAGGLGHAHLFTVARYMEHRGLPLRAYKLATLALAQLSIAFN
QDSHPAVNDVLWACSLSHSLGRHELSAIVPLIIRSIHCAPMLSDILRRWTLSAPGLGPLGARRATKPLGTDRA
PLCQLLDAAVAAYITTSHSRLTHISPRHYGDFIEFLGKARETFLLAPDGHLQFAQFLENLKQTYKGKKKLMLL
VRERFG*
>rs50242115_ENSMUSP00000040078_c.1018:T>C_p.I340T
ENSMUST00000039480.7|Zswim4-201|ENSMUSG00000035671|-|chr8:g.84953447:A>G
MEPPAAKRSRGCPAGDEPGTGARRSRPEPLLDLSAKRVAESWAFEQVEERFSRVPEPVQKRIVFWSFPRSERE
ICMYSSLGYQPPEGEQDTRVPFTRGLHLLQSGAVDRVLQVGFHLSGNIREVGGPGEPEHLYHVSISFDRCKIT
SVSCGCDNRDLFYCAHVVALSLYRIRHARQVELRLPISETLSQMNRDQLQKFVQYLISAHHTEVLPTAQRLAD
EILLLGSEINLVHGAPDPTAGAGTEDANCWHLDEEQIQEQVKQLLSNGGHYGASQQLRSMFSKVREMLRARDS
NGARMLILMTEQFLQDPRLALWRQQGAGMTDKCRQLWDELGALWVCVTLSPHCKPDERSGWLQLLGTWDKLDV
CPLEEGNYSLDIPSLQPALTSRTGSEDEEEVTAASPRRTVFSRALQAGELHWNDSHLQRILASDSCGPTLTGS
MGSDKPTFDPQGHPIWLGEPFPTACARVDTLRAHGYPHKALRLACAIINTLRLQRRHQLESYKQQKKELLQKG
ATGVTSTEGWVGHPLDPIGCLCRALLEACRLEEEPHSLFPDSAPEKRKLAYQHVLVPGSPGESYLALALEVAL
LGLGQQRALPEGLYAQDKVVRNEEQLLALLEEVELDERLVQVLRKQAGLLLEGGPFSGFGEVIFRESVPMHTC
ARYLFTALLPHDPDLAFRLALRAMRLPVLETTFPAGESHPNPLDSIMSNRFPRWFILGHLETRQCELASTMLT
AAKGDTKWLHAVLGSVQQNIHSPALLFKLAQDACKTATPASAPPDTVLLGIALELGLQVMRMTLNTMTWRRRE
MVRWLVSCATEIGPPALMSIMKNWYSLFTPVEAVTIVAVTGTTHATLLRLQLDTAGREELWACARTLALQCAM
KDPQNCALPALTLCEKNHAAFEAAYQIVLDAAAGGLGHAHLFTVARYMEHRGLPLRAYKLATLALAQLSIAFN
```

QDSHPAVNDVLWACSLSHSLGRHELSAIVPLIIRSIHCAPMLSDILRRWTLSAPGLGPLGARRATKPLGTDRA
PLCQLLDAAVAAYITTSHSRLTHISPRHYGDFIEFLGKARETFLLAPDGHLQFAQFLENLKQTYKGKKKLMLL
VRERFG*
>rs51157904_ENSMUSP00000040078_c.910:A>G_p.Q304R
ENSMUST00000039480.7|Zswim4-201|ENSMUSG00000035671|-|chr8:g.84955507:T>C
MEPPAAKRSRGCPAGDEPGTGARRSRPEPLLDLSAKRVAESWAFEQVEERFSRVPEPVQKRIVFWSFPRSERE
ICMYSSLGYQPPEGEQDTRVPFTRGLHLLQSGAVDRVLQVGFHLSGNIREVGGPGEPEHLYHVSISFDRCKIT
SVSCGCDNRDLFYCAHVVALSLYRIRHARQVELRLPISETLSQMNRDQLQKFVQYLISAHHTEVLPTAQRLAD
EILLLGSEINLVHGAPDPTAGAGTEDANCWHLDEEQIQEQVKQLLSNGGHYGASQQLRSMFSKVREMLRARDS
NGARMLILMTERFLQDPRLALWRQQGAGMTDKCRQLWDELGALWVCVILSPHCKPDERSGWLQLLGTWDKLDV
CPLEEGNYSLDIPSLQPALTSRTGSEDEEEVTAASPRRTVFSRALQAGELHWNDSHLQRILASDSCGPTLTGS
MGSDKPTFDPQGHPIWLGEPFPTACARVDTLRAHGYPHKALRLACAIINTLRLQRRHQLESYKQQKKELLQKG
ATGVTSTEGWVGHPLDPIGCLCRALLEACRLEEEPHSLFPDSAPEKRKLAYQHVLVPGSPGESYLALALEVAL
LGLGQQRALPEGLYAQDKVVRNEEQLLALLEEVELDERLVQVLRKQAGLLLEGGPFSGFGEVIFRESVPMHTC
ARYLFTALLPHDPDLAFRLALRAMRLPVLETTFPAGESHPNPLDSIMSNRFPRWFILGHLETRQCELASTMLT
AAKGDTKWLHAVLGSVQQNIHSPALLFKLAQDACKTATPASAPPDTVLLGIALELGLQVMRMTLNTMTWRRRE
MVRWLVSCATEIGPPALMSIMKNWYSLFTPVEAVTIVAVTGTTHATLLRLQLDTAGREELWACARTLALQCAM
KDPQNCALPALTLCEKNHAAFEAAYQIVLDAAAGGLGHAHLFTVARYMEHRGLPLRAYKLATLALAQLSIAFN
QDSHPAVNDVLWACSLSHSLGRHELSAIVPLIIRSIHCAPMLSDILRRWTLSAPGLGPLGARRATKPLGTDRA
PLCQLLDAAVAAYITTSHSRLTHISPRHYGDFIEFLGKARETFLLAPDGHLQFAQFLENLKQTYKGKKKLMLL
VRERFG*
>rs48900740_ENSMUSP00000040078_c.352:G>A_p.S118N
ENSMUST00000039480.7|Zswim4-201|ENSMUSG00000035671|-|chr8:g.84957714:C>T
MEPPAAKRSRGCPAGDEPGTGARRSRPEPLLDLSAKRVAESWAFEQVEERFSRVPEPVQKRIVFWSFPRSERE
ICMYSSLGYQPPEGEQDTRVPFTRGLHLLQSGAVDRVLQVGFHLNGNIREVGGPGEPEHLYHVSISFDRCKIT
SVSCGCDNRDLFYCAHVVALSLYRIRHARQVELRLPISETLSQMNRDQLQKFVQYLISAHHTEVLPTAQRLAD
EILLLGSEINLVHGAPDPTAGAGTEDANCWHLDEEQIQEQVKQLLSNGGHYGASQQLRSMFSKVREMLRARDS
NGARMLILMTEQFLQDPRLALWRQQGAGMTDKCRQLWDELGALWVCVILSPHCKPDERSGWLQLLGTWDKLDV
CPLEEGNYSLDIPSLQPALTSRTGSEDEEEVTAASPRRTVFSRALQAGELHWNDSHLQRILASDSCGPTLTGS
MGSDKPTFDPQGHPIWLGEPFPTACARVDTLRAHGYPHKALRLACAIINTLRLQRRHQLESYKQQKKELLQKG
ATGVTSTEGWVGHPLDPIGCLCRALLEACRLEEEPHSLFPDSAPEKRKLAYQHVLVPGSPGESYLALALEVAL
LGLGQQRALPEGLYAQDKVVRNEEQLLALLEEVELDERLVQVLRKQAGLLLEGGPFSGFGEVIFRESVPMHTC
ARYLFTALLPHDPDLAFRLALRAMRLPVLETTFPAGESHPNPLDSIMSNRFPRWFILGHLETRQCELASTMLT
AAKGDTKWLHAVLGSVQQNIHSPALLFKLAQDACKTATPASAPPDTVLLGIALELGLQVMRMTLNTMTWRRRE
MVRWLVSCATEIGPPALMSIMKNWYSLFTPVEAVTIVAVTGTTHATLLRLQLDTAGREELWACARTLALQCAM
KDPQNCALPALTLCEKNHAAFEAAYQIVLDAAAGGLGHAHLFTVARYMEHRGLPLRAYKLATLALAQLSIAFN
QDSHPAVNDVLWACSLSHSLGRHELSAIVPLIIRSIHCAPMLSDILRRWTLSAPGLGPLGARRATKPLGTDRA
PLCQLLDAAVAAYITTSHSRLTHISPRHYGDFIEFLGKARETFLLAPDGHLQFAQFLENLKQTYKGKKKLMLL
VRERFG*
>rs51323625_ENSMUSP00000019506_c.6:C>T_p.Q3*
ENSMUST00000019506.9|D8Ertd738e-201|ENSMUSG00000019362|-
|chr8:g.84976326:G>A
MA*GQRKFQAQKPKSKAAAAERSRGPRKGGRVIAPKKARVVQQQKLKKSLEVGIRKKIEHDVVMKASSSLPKR
LALLKGASKKSEATIPGKTPS*
>rs32623436_ENSMUSP00000122623_c.769:C>T_p.A257V
ENSMUST00000126435.8|Mri1-203|ENSMUSG00000004996|-|chr8:g.84980671:G>A
MTLEAIRYSPGSLQILDQLQLPEHCHYEALSSVQQASEAIRAMKVRGAPAIALVGCLSLAVELRAGAGGPGLA
ALVAFVRDQLRLLVAARPTAVNMARAARDLGQVAAQEAEREGATEETVRERVIRFAEDMLEKDLKDNRSIGDL
GARHLLEQTNPRGGKVTVLTHCNTGALATAGYGTALGVIRSLHEMGRLEHTFCTETRPYNQGARLTAFELVYE
QIPATLITDSMAAAAMAHRGVSAVVVGADRVVANGDTVNKIGTYQLAIVAKHHGVPFYVAAPSSSCDLHLETG
KEIVIEERPSQELTDLNGVRIAAQGIRVWNPAFDVTPHELITGGIITELGVFAPEELRGALSASVFSEGQTLD
SPWV*
>rs32623436_ENSMUSP00000117115_c.385:C>T_p.A129V
ENSMUST00000125498.2|Mri1-202|ENSMUSG00000004996|-|chr8:g.84980671:G>A
XRFAEDMLEKDLKDNRSIGDLGARHLLEQTNPRGGKVTVLTHCNTGALATAGYGTALGVIRSLHEMGRLEHTF
CTETRPYNQGARLTAFELVYEQIPATLITDSMAAAAMAHRAVVVGADRVVANGDTVNKIGTYQLAIVAKHHGV
PFYVAAPSSSCDLHLETGKEIVIEERPSQELTDLNGVRIAAQDRIFLCLLGYPGTICRSGWLQTQRSSCLCLP
SAGIKCMHH

>rs33104670_ENSMUSP00000096177_c.139:G>A_p.R47Q
ENSMUST00000098578.10|Ccdc130-202|ENSMUSG00000004994|-
|chr8:g.84990491:C>T
MGERKGQNKYYPPDFNPEKHGSLNRYHNSHPLRERARKLSQGILVIQFEMPYNIWCDGCKNHIGMGVRYNAEK
KKVGNYYTTPIYRFRMKCHLCVNYIEMQTDPANCDYVIVSGASRKEERWDMEDNEQVLTTEHEKKEKLETDAM
FRLEHGEADRSTLKKALPTLSHIQEAQNAWKDDFALNSMLRRHFREKKKAMQEEEEKDQALQAKASLAIPLVP
ESEDDRRLAALLRLHTLDSYEDKQRMKRTEIIHRSWFPSAQGPSASSSKASSVLKKLCQGRRPPPSSTGTVGD
LGIVRRKSRDVPESPQCAADNSLSEEQRRPPGTTQGSKTLEEAAEASRTSKTSESKRNCSDQAFPLGSSQEDL
LNPNTPNASLVADYSDSESE*
>rs33104670_ENSMUSP00000005120_c.139:G>A_p.R47Q
ENSMUST00000005120.12|Ccdc130-201|ENSMUSG00000004994|-
|chr8:g.84990491:C>T
MGERKGQNKYYPPDFNPEKHGSLNRYHNSHPLRERARKLSQGILVIQFEMPYNIWCDGCKNHIGMGVRYNAEK
KKVGNYYTTPIYRFRMKCHLCVNYIEMQTDPANCDYVIVSGASRKEERWDMEDNEQVLTTEHEKKEKLETDAM
FRLEHGEADRSTLKKALPTLSHIQEAQNAWKDDFALNSMLRRHFRLMRTSSE*
>rs33104670_ENSMUSP00000128605_c.139:G>A_p.R47Q
ENSMUST00000172320.2|Ccdc130-207|ENSMUSG00000004994|-|chr8:g.84990491:C>T
MGERKGQNKYYPPDFNPEKHGSLNRYHNSHPLRERARKLSQGILVIQFEMPYNIWCDGCKNHIGMGERG*
>ENSMUSP00000158803_c.1027:T>C_p.F343S ENSMUST00000238701.2|Cacna1a-
213|ENSMUSG00000034656|+|chr8:g.85245363:T>C
MPRASWGSDPMLSPLSHELLHSPRYPGTDCTLAGSSRTLHVPWSQQMSGSLEGPTLKRRGGVVDHRDVILAHQ
AHKMHSTPQARRKEWEMARFGDEMPGRYGAGGGGSGPAAGVVVGAAGGRGAGGSRQGGQPGAQRMYKQSMAQR
ARTMALYNPIPVRQNCLTVNRSLFLFSEDNVVRKYAKKITEWPPFEYMILATIIANCIVLALEQHLPDDDKTP
MSERLDDTEPYFIGIFCFEAGIKIVALGFAFHKGSYLRNGWNVMDFVVVLTGILATVGTEFDLRTLRAVRVLR
PLKLVSGIPSLQVVLKSIMKAMIPLLQIGLLLFFAILIFAIIGLEFYMGKSHTTCFEEGTDDIQGESPAPCGT
EEPARTCPNGTKCQPYWEGPNNGITQFDNILFAVLTVFQCITMEGWTDLLYNSNDASGNTWNWLYFIPLIIIG
SFFMLNLVLGVLSGEFAKERERVENRRAFLKLRRQQQIERELNGYMEWISKAEEVILAEDETDVEQRHPFDGA
LRRATLKKSKTDLLNPEEAEDQLADIASVGSPFARASIKSAKLENSTFFHKKERRMRFYIRRMVKTQAFYWTV
LSLVALNTLCVAIVHYNQPEWLSDFLYYAEFIFLGLFMSEMFIKMYGLGTRPYFHSSFNCFDCGVIIGSIFEV
IWAVIKPGTSFGISVLRALRLLRIFKVTKYWASLRNLVVSLLNSMKSIISLLFLLFLFIVVFALLGMQLFGGQ
FNFDEGTPPTNFDTFPAAIMTVFQILTGEDWNEVMYDGIKSQGGVQGGMVFSIYFIVLTLFGNYTLLNVFLAI
AVDNLANAQELTKDEQEEEEAANQKLALQKAKEVAEVSPLSAANMSIAVKEQQKNQKPTKSVWEQRTSEMRKQ
NLLASREALYGDAAERWPTPYARPLRPDVKTHLDRPLVVDPQENRNNNTNKSRAPEALRPTARPRESARDPDA
RRAWPGSPERAPGREGPYGRESEPQQREHAPPREHAPWDADTERAKAGDAPRRHTHRPVAEGEPRRHRARRRP
GDEPDDRPERRPRPRDATRPARAADGEGDDGERKRRHRHGPPAHDDRERRHRRRKENQGSGVPVSGPNLSTTR
PIQQDLGRQDLPLAEDLDNMKNNKLATGEPASPHDSLGHSGLPPSPAKIGNSTNPGPALATNPQNAASRRTPN
NPGNPSNPGPPKTPENSLIVTNPSSTQPNSAKTARKPEHMAVEIPPACPPLNHTVVQVNKNANPDPLPKKEEE
KKEEEEADPGEDGPKPMPPYSSMFILSTTNPLRRLCHYILNLRYFEMCILMVIAMSSIALAAEDPVQPNAPRN
NVLRYFDYVFTGVFTFEMVIKMIDLGLVLHQGAYFRDLWNILDFIVVSGALVAFAFTGNSKGKDINTIKSLRV
LRVLRPLKTIKRLPKLKAVFDCVVNSLKNVFNILIVYMLFMFIFAVVAVQLFKGKFFHCTDESKEFERDCRGK
YLLYEKNEVKARDREWKKYEFHYDNVLWALLTLFTVSTGEGWPQVLKHSVDATFENQGPSPGYRMEMSIFYVV
YFVVFPFFFVNIFVALIIITFQEQGDKMMEEYSLEKNERACIDFAISAKPLTRHMPQNKQSFQYRMWQFVVSP
PFEYTIMAMIALNTIVLMMKFYGASVAYENALRVFNIVFTSLFSLECVLKVMAFGILNYFRDAWNIFDFVTVL
GSITDILVTEFGNNFINLSFLRLFRAARLIKLLRQGYTIRILLWTFVQSFKALPYVCLLIAMLFFIYAIIGMQ
VFGNIGIDGEDEDSDEDEFQITEHNNFRTFFQALMLLFRSATGEAWHNIMLSCLSGKPCDKNSGILTADCGNE
FAYFYFVSFIFLCSFLMLNLFVAVIMDNFEYLTRDSSILGPHHLDEYVRVWAEYDPAACGRIHYKDMYSLLRV
ISPPLGLGKKCPHRVACKRLLRMDLPVADDNTVHFNSTLMALIRTALDIKIAKGGADKQQMDAELRKEMMAIW
PNLSQKTLDLLVTPHKSTDLTVGKIYAAMMIMEYYRQSKAKKLQAMREEQNRTPLMFQRMEPPSPTQEGGPSQ
NALPSTQLDPGGGLMAHEGGMKESPSWVTQRAQEMFQKTGTWSPERGPPIDMPNSQPNSQSVEMREMGTDGYS
DSEHYLPMEGQTRAASMPRLPAENQRRRGRPRGNDLSTISDTSPMKRSASVLGPKARRLDDYSLERVPPEENQ
RYHQRRRDRGHRTSERSLGRYTDVDTGLGTDLSMTTQSGDLPSKDRDQDRGRPKDRKHRPHHHHHHHHHHPPA
PDRDRYAQERPDTGRARAREQRWSRSPSEGREHTTHRQGSSSVSGSPAPSTSGTSTPRRGRRQLPQTPCTPRP
LVSYSPAPRRPAARRMAGPAAPPGGSPRGCRRAPRWPAHAPEGPRPRGADYTEPDSPREPPGGAHDPAPRSPR
TPRAAGCASPRHGRRLPNGYYAGHGAPRPRTARRGAHDAYSESEDDWC*
>ENSMUSP00000112436_c.760:T>C_p.F254S ENSMUST00000121390.8|Cacna1a-
201|ENSMUSG00000034656|+|chr8:g.85245363:T>C
MARFGDEMPGRYGAGGGGSGPAAGVVVGAAGGRGAGGSRQGGQPGAQRMYKQSMAQRARTMALYNPIPVRQNC
LTVNRSLFLFSEDNVVRKYAKKITEWPPFEYMILATIIANCIVLALEQHLPDDDKTPMSERLDDTEPYFIGIF

CFEAGIKIVALGFAFHKGSYLRNGWNVMDFVVVLTGILATVGTEFDLRTLRAVRVLRPLKLVSGIPSLQVVLK
SIMKAMIPLLQIGLLLFFAILIFAIIGLEFYMGKSHTTCFEEGTDDIQGESPAPCGTEEPARTCPNGTKCQPY
WEGPNNGITQFDNILFAVLTVFQCITMEGWTDLLYNSNDASGNTWNWLYFIPLIIIGSFFMLNLVLGVLSGEF
AKERERVENRRAFLKLRRQQQIERELNGYMEWISKAEEVILAEDETDVEQRHPFDGALRRATLKKSKTDLLNP
EEAEDQLADIASVGSPFARASIKSAKLENSTFFHKKERRMRFYIRRMVKTQAFYWTVLSLVALNTLCVAIVHY
NQPEWLSDFLYYAEFIFLGLFMSEMFIKMYGLGTRPYFHSSFNCFDCGVIIGSIFEVIWAVIKPGTSFGISVL
RALRLLRIFKVTKYWASLRNLVVSLLNSMKSIISLLFLLFLFIVVFALLGMQLFGGQFNFDEGTPPTNFDTFP
AAIMTVFQILTGEDWNEVMYDGIKSQGGVQGGMVFSIYFIVLTLFGNYTLLNVFLAIAVDNLANAQELTKDEQ
EEEEAANQKLALQKAKEVAEVSPLSAANMSIAVKEQQKNQKPTKSVWEQRTSEMRKQNLLASREALYGDAAER
WPTPYARPLRPDVKTHLDRPLVVDPQENRNNNTNKSRAPEALRPTARPRESARDPDARRAWPGSPERAPGREG
PYGRESEPQQREHAPPREHAPWDADTERAKAGDAPRRHTHRPVAEGEPRRHRARRRPGDEPDDRPERRPRPRD
ATRPARAADGEGDDGERKRRHRHGPPAHDDRERRHRRRKENQGSGVPVSGPNLSTTRPIQQDLGRQDLPLAED
LDNMKNNKLATGEPASPHDSLGHSGLPPSPAKIGNSTNPGPALATNPQNAASRRTPNNPGNPSNPGPPKTPEN
SLIVTNPSSTQPNSAKTARKPEHMAVEIPPACPPLNHTVVQVNKNANPDPLPKKEEEKKEEEEADPGEDGPKP
MPPYSSMFILSTTNPLRRLCHYILNLRYFEMCILMVIAMSSIALAAEDPVQPNAPRNNVLRYFDYVFTGVFTF
EMVIKMIDLGLVLHQGAYFRDLWNILDFIVVSGALVAFAFTGNSKGKDINTIKSLRVLRVLRPLKTIKRLPKL
KAVFDCVVNSLKNVFNILIVYMLFMFIFAVVAVQLFKGKFFHCTDESKEFERDCRGKYLLYEKNEVKARDREW
KKYEFHYDNVLWALLTLFTVSTGEGWPQVLKHSVDATFENQGPSPGYRMEMSIFYVVYFVVFPFFFVNIFVAL
IIITFQEQGDKMMEEYSLEKNERACIDFAISAKPLTRHMPQNKQSFQYRMWQFVVSPPFEYTIMAMIALNTIV
LMMKFYGASVAYENALRVFNIVFTSLFSLECVLKVMAFGILNYFRDAWNIFDFVTVLGSITDILVTEFGNNFI
NLSFLRLFRAARLIKLLRQGYTIRILLWTFVQSFKALPYVCLLIAMLFFIYAIIGMQVFGNIGIDGEDEDSDE
DEFQITEHNNFRTFFQALMLLFRSATGEAWHNIMLSCLSGKPCDKNSGILTADCGNEFAYFYFVSFIFLCSFL
MLNLFVAVIMDNFEYLTRDSSILGPHHLDEYVRVWAEYDPAACGRIHYKDMYSLLRVISPPLGLGKKCPHRVA
CKRLLRMDLPVADDNTVHFNSTLMALIRTALDIKIAKGGADKQQMDAELRKEMMAIWPNLSQKTLDLLVTPHK
STDLTVGKIYAAMMIMEYYRQSKAKKLQAMREEQNRTPLMFQRMEPPSPTQEGGPSQNALPSTQLDPGGGLMA
HEGGMKESPSWVTQRAQEMFQKTGTWSPERGPPIDMPNSQPNSQSVEMREMGTDGYSDSEHYLPMEGQTRAAS
MPRLPAENQRRRGRPRGNDLSTISDTSPMKRSASVLGPKARRLDDYSLERVPPEENQRYHQRRRDRGHRTSER
SLGRYTDVDTGLGTDLSMTTQSGDLPSKDRDQDRGRPKDRKHRPHHHHHHHHHPPAPDRDRYAQERPDTGRA
RAREQRWSRSPSEGREHTTHRQGSSSVSGSPAPSTSGTSTPRRGRRQLPQTPCTPRPLVSYSPAPRRPAARRM
AGPAAPPGGSPRGCRRAPRWPAHAPEGPRPRGADYTEPDSPREPPGGAHDPAPRSPRTPRAAGCASPRHGRRL
PNGYYAGHGAPRPRTARRGAHDAYSESEDDWC*
>ENSMUSP00000114055_c.619:T>C_p.F207S ENSMUST00000122053.2|Cacna1a-202|ENSMUSG00000034656|+|chr8:g.85245363:T>C
MARFGDEMPGRYGAGGGGSGPAAGVVVGAAGGRGAGGSRQGGQPGAQRMYKQSMAQRARTMALYNPIPVRQNC
LTVNRSLFLFSEDNVDDTEPYFIGIFCFEAGIKIVALGFAFHKGSYLRNGWNVMDFVVVLTGILATVGTEFDL
RTLRAVRVLRPLKLVSGIPSLQVVLKSIMKAMIPLLQIGLLLFFAILIFAIIGLEFYMGKSHTTCFEEGTDDI
QGESPAPCGTEEPARTCPNGTKCQPYWEGPNNGITQFDNILFAVLTVFQCITMEGWTDLLYNSNDASGNTWNW
LYFIPLIIIGSFFMLNLVLGVLSGEFAKERERVENRRAFLKLRRQQQIERELNGYMEWISKAEEVILAEDETD
VEQRHPFDGALRRATLKKSKTDLLNPEEAEDQLADIASVGSPFARASIKSAKLENSTFFHKKERRMRFYIRRM
VKTQAFYWTVLSLVALNTLCVAIVHYNQPEWLSDFLYYAEFIFLGLFMSEMFIKMYGLGTRPYFHSSFNCFDC
GVIIGSIFEVIWAVIKPGTSFGISVLRALRLLRIFKVTKYWASLRNLVVSLLNSMKSIISLLFLLFLFIVVFA
LLGMQLFGGQFNFDEGTPPTNFDTFPAAIMTVFQILTGEDWNEVMYDGIKSQGGVQGGMVFSIYFIVLTLFGN
YTLLNVFLAIAVDNLANAQELTKDEQEEEEAANQKLALQKAKEVAEVSPLSAANMSIAVKEQQKNQKPTKSVW
EQRTSEMRKQNLLASREALYGDAAERWPTPYARPLRPDVKTHLDRPLVVDPQENRNNNTNKSRAPEALRPTAR
PRESARDPDARRAWPGSPERAPGREGPYGRESEPQQREHAPPREHAPWDADTERAKAGDAPRRHTHRPVAEGE
PRRHRARRRPGDEPDDRPERRPRPRDATRPARAADGEGDDGERKRRHRHGPPAHDDRERRHRRRKENQGSGVP
VSGPNLSTTRPIQQDLGRQDLPLAEDLDNMKNNKLATGEPASPHDSLGHSGLPPSPAKIGNSTNPGPALATNP
QNAASRRTPNNPGNPSNPGPPKTPENSLIVTNPSSTQPNSAKTARKPEHMAVEIPPACPPLNHTVVQVNKNAN
PDPLPKKEEEKKEEEEADPGEDGPKPMPPYSSMFILSTTNPLRRLCHYILNLRYFEMCILMVIAMSSIALAAE
DPVQPNAPRNNVLRYFDYVFTGVFTFEMVIKMIDLGLVLHQGAYFRDLWNILDFIVVSGALVAFAFTGNSKGK
DINTIKSLRVLRVLRPLKTIKRLPKLKAVFDCVVNSLKNVFNILIVYMLFMFIFAVVAVQLFKGKFFHCTDES
KEFERDCRGKYLLYEKNEVKARDREWKKYEFHYDNVLWALLTLFTVSTGEGWPQVLKHSVDATFENQGPSPGY
RMEMSIFYVVYFVVFPFFFVNIFVALIIITFQEQGDKMMEEYSLEKNERACIDFAISAKPLTRHMPQNKQSFQ
YRMWQFVVSPPFEYTIMAMIALNTIVLMMKFYGASVAYENALRVFNIVFTSLFSLECVLKVMAFGILNYFRDA
WNIFDFVTVLGSITDILVTEFGNNFINLSFLRLFRAARLIKLLRQGYTIRILLWTFVQSFKALPYVCLLIAML
FFIYAIIGMQVFGNIGIDGEDEDSDEDEFQITEHNNFRTFFQALMLLFRSATGEAWHNIMLSCLSGKPCDKNS
GILTADCGNEFAYFYFVSFIFLCSFLMLNLFVAVIMDNFEYLTRDSSILGPHHLDEYVRVWAEYDPAACGRIH
YKDMYSLLRVISPPLGLGKKCPHRVACKRLLRMDLPVADDNTVHFNSTLMALIRTALDIKIAKGGADKQQMDA

ELRKEMMAIWPNLSQKTLDLLVTPHKSTDLTVGKIYAAMMIMEYYRQSKAKKLQAMREEQNRTPLMFQRMEPP
SPTQEGGPSQNALPSTQLDPGGGLMAHEGGMKESPSWVTQRAQEMFQKTGTWSPERGPPIDMPNSQPNSQSVE
MREMGTDGYSDSEHYLPMEGQTRAASMPRLPAENQRRRGRPRGNDLSTISDTSPMKRSASVLGPKARRLDDYS
LERVPPEENQRYHQRRRDRGHRTSERSLGRYTDVDTGLGTDLSMTTQSGDLPSKDRDQDRGRPKDRKHRPHHH
HHHHHHHPPAPDRDRYAQERPDTGRARAREQRWSRSPSEGREHTTHRQGSSSVSGSPAPSTSGTSTPRRGRRQ
LPQTPCTPRPLVSYSPAPRRPAARRMAGPAAPPGGSPRGCRRAPRWPAHAPEGPRPRGADYTEPDSPREPPGG
AHDPAPRSPRTPRAAGCASPRHGRRLPNGYYAGHGAPRPRTARRGAHDAYSESEDDWC*
>ENSMUSP00000149242_c.622:T>C_p.F208S ENSMUST00000215756.2|Cacna1a-
211|ENSMUSG00000034656|+|chr8:g.85245363:T>C
XRMYKQSMAQRARTMALYNPIPVRQNCLTVNRSLFLFSEDNVVRKYAKKITEWPPFEYMILATIIANCIVLAL
EQHLPDDDKTPMSERLDDTEPYFIGIFCFEAGIKIVALGFAFHKGSYLRNGWNVMDFVVVLTGILATVGTEFD
LRTLRAVRVLRPLKLVSGIPSLQVVLKSIMKAMIPLLQIGLLLFFAILIFAIIGLEFYMGKSHTTCFEEGTDD
IQGESPAPCGTEEPARTCPNGTKCQPYWEGPNNGITQFDNILFAVLTVFQCITMEGWTDLLYNSNDASGNTWN
WLYFIPLIIIGSFFMLNLVLGVLSGEFAKERERVENRRAFLKLRRQQQIERELNGYMEWISKAEEVILAEDET
DVEQRHPFDALRRATLKKSKTDLLNPEEAEDQLADIASVGSPFARASIKSAKLENSTFFHKKERRMRFYIRRM
VKTQAFYWTVLSLVALNTLCVAIVHYNQPEWLSDFLYYAEFIFLGLFMSEMFIKMYGLGTRPYFHSSFNCFDC
GVIIGSIFEVIWAVIKPGTSFGISVLRALRLLRIFKVTKYWASLRNLVVSLLNSMKSIISLLFLLFLFIVVFA
LLGMQLFGGQFNFDEGTPPTNFDTFPAAIMTVFQILTGEDWNEVMYDGIKSQGGVQGGMVFSIYFIVLTLFGN
YTLLNVFLAIAVDNLANAQELTKDEQEEEEAANQKLALQKAKEVAEVSPLSAANMSIAVKEQQKNQKPTKSVW
EQRTSEMRKQNLLASREALYGDAAERWPTPYARPLRPDVKTHLDRPLVVDPQENRNNNTNKSRAPEALRPTAR
PRESARDPDARRAWPGSPERAPGREGPYGRESEPQQREHAPPREHAPWDADTERAKAGDAPRRHTHRPVAEGE
PRRHRARRRPGDEPDDRPERRPRPRDATRPARAADGEGDDGERKRRHRHGPPAHDDRERRHRRRKENQGSGVP
VSGPNLSTTRPIQQDLGRQDLPLAEDLDNMKNNKLATGEPASPHDSLGHSGLPPSPAKIGNSTNPGPALATNP
QNAASRRTPNNPGNPSNPGPPKTPENSLIVTNPSSTQPNSAKTARKPEHMAVEIPPACPPLNHTVVQVNKNAN
PDPLPKKEEEKKEEEEADPGEDGPKPMPPYSSMFILSTTNPLRRLCHYILNLRYFEMCILMVIAMSSIALAAE
DPVQPNAPRNNVLRYFDYVFTGVFTFEMVIKMIDLGLVLHQGAYFRDLWNILDFIVVSGALVAFAFTGNSKGK
DINTIKSLRVLRVLRPLKTIKRLPKLKAVFDCVVNSLKNVFNILIVYMLFMFIFAVVAVQLFKGKFFHCTDES
KEFERDCRGKYLLYEKNEVKARDREWKKYEFHYDNVLWALLTLFTVSTGEGWPQVLKHSVDATFENQGPSPGY
RMEMSIFYVVYFVVFPFFFVNIFVALIIITFQEQGDKMMEEYSLEKNERACIDFAISAKPLTRHMPQNKQSFQ
YRMWQFVVSPPFEYTIMAMIALNTIVLMMKFYGASVAYENALRVFNIVFTSLFSLECVLKVMAFGILNYFRDA
WNIFDFVTVLGSITDILVTEFGNNFINLSFLRLFRAARLIKLLRQGYTIRILLWTFVQSFKALPYVCLLIAML
FFIYAIIGMQVFGNIGIDGEDEDSDEDEFQITEHNNFRTFFQALMLLFRSATGEAWHNIMLSCLSGKPCDKNS
GILTADCGNEFAYFYFVSFIFLCSFLMLNLFVAVIMDNFEYLTRDSSILGPHHLDEYVRVWAEYDPAACGRIH
YKDMYSLLRVISPPLGLGKKCPHRVACKRLLRMDLPVADDNTVHFNSTLMALIRTALDIKIAKGGADKQQMDA
ELRKEMMAIWPNLSQKTLDLLVTPHKSTDLTVGKIYAAMMIMEYYRQSKAKKLQAMREEQNRTPLMFQRMEPP
SPTQEGGPSQNALPSTQLDPGGGLMAHEGGMKESPSWVTQRAQEMFQKTGTWSPERGPPIDMPNSQPNSQSVE
MREMGTDGYSDSEHYLPMEGQTRAASMPRLPAENQRRRGRPRGNDLSTISDTSPMKRSASVLGPKARRLDDYS
LERVPPEENQRYHQRRRDRGHRTSERSLGRYTDVDTGLGTDLSMTTQSGDLPSKDRDQDRGRPKDRKHRPHHH
HHHHHHHPPAPDRDRYAQERPDTGRARAREQRWSRSPSEGREHTTHRQGSSSVSGSPAPSTSGTSTPRRGRRQ
LPQTPCTPRPLVSYSPAPRRPAARRMAGPAAPPGGSPRGCRRAPRWPAHAPEGPRPRGADYTEPDSPREPPGG
AHDPAPRSPRTPRAAGCASPRHGRRLPNGYYAGHGAPRPRTARRGAHDAYSESEDDWC*
>rs38793291_ENSMUSP00000158803_c.3165:A>G_p.K1056E
ENSMUST00000238701.2|Cacna1a-213|ENSMUSG00000034656|+|chr8:g.85286255:A>G
MPRASWGSDPMLSPLSHELLHSPRYPGTDCTLAGSSRTLHVPWSQQMSGSLEGPTLKRRGGVVDHRDVILAHQ
AHKMHSTPQARRKEWEMARFGDEMPGRYGAGGGGSGPAAGVVVGAAGGRGAGGSRQGGQPGAQRMYKQSMAQR
ARTMALYNPIPVRQNCLTVNRSLFLFSEDNVVRKYAKKITEWPPFEYMILATIIANCIVLALEQHLPDDDKTP
MSERLDDTEPYFIGIFCFEAGIKIVALGFAFHKGSYLRNGWNVMDFVVVLTGILATVGTEFDLRTLRAVRVLR
PLKLVSGIPSLQVVLKSIMKAMIPLLQIGLLLFFAILIFAIIGLEFYMGKFHTTCFEEGTDDIQGESPAPCGT
EEPARTCPNGTKCQPYWEGPNNGITQFDNILFAVLTVFQCITMEGWTDLLYNSNDASGNTWNWLYFIPLIIIG
SFFMLNLVLGVLSGEFAKERERVENRRAFLKLRRQQQIERELNGYMEWISKAEEVILAEDETDVEQRHPFDGA
LRRATLKKSKTDLLNPEEAEDQLADIASVGSPFARASIKSAKLENSTFFHKKERRMRFYIRRMVKTQAFYWTV
LSLVALNTLCVAIVHYNQPEWLSDFLYYAEFIFLGLFMSEMFIKMYGLGTRPYFHSSFNCFDCGVIIGSIFEV
IWAVIKPGTSFGISVLRALRLLRIFKVTKYWASLRNLVVSLLNSMKSIISLLFLLFLFIVVFALLGMQLFGGQ
FNFDEGTPPTNFDTFPAAIMTVFQILTGEDWNEVMYDGIKSQGGVQGGMVFSIYFIVLTLFGNYTLLNVFLAI
AVDNLANAQELTKDEQEEEEAANQKLALQKAKEVAEVSPLSAANMSIAVKEQQKNQKPTKSVWEQRTSEMRKQ
NLLASREALYGDAAERWPTPYARPLRPDVKTHLDRPLVVDPQENRNNNTNKSRAPEALRPTARPRESARDPDA
RRAWPGSPERAPGREGPYGRESEPQQREHAPPREHAPWDADTERAKAGDAPRRHTHRPVAEGEPRRHRARRRP
GDEPDDRPERRPRPRDATRPARAADGEGDDGERERRHRHGPPAHDDRERRHRRRKENQGSGVPVSGPNLSTTR

PIQQDLGRQDLPLAEDLDNMKNNKLATGEPASPHDSLGHSGLPPSPAKIGNSTNPGPALATNPQNAASRRTPN
NPGNPSNPGPPKTPENSLIVTNPSSTQPNSAKTARKPEHMAVEIPPACPPLNHTVVQVNKNANPDPLPKKEEE
KKEEEEADPGEDGPKPMPPYSSMFILSTTNPLRRLCHYILNLRYFEMCILMVIAMSSIALAAEDPVQPNAPRN
NVLRYFDYVFTGVFTFEMVIKMIDLGLVLHQGAYFRDLWNILDFIVVSGALVAFAFTGNSKGKDINTIKSLRV
LRVLRPLKTIKRLPKLKAVFDCVVNSLKNVFNILIVYMLFMFIFAVVAVQLFKGKFFHCTDESKEFERDCRGK
YLLYEKNEVKARDREWKKYEFHYDNVLWALLTLFTVSTGEGWPQVLKHSVDATFENQGPSPGYRMEMSIFYVV
YFVVFPFFFVNIFVALIIITFQEQGDKMMEEYSLEKNERACIDFAISAKPLTRHMPQNKQSFQYRMWQFVVSP
PFEYTIMAMIALNTIVLMMKFYGASVAYENALRVFNIVFTSLFSLECVLKVMAFGILNYFRDAWNIFDFVTVL
GSITDILVTEFGNNFINLSFLRLFRAARLIKLLRQGYTIRILLWTFVQSFKALPYVCLLIAMLFFIYAIIGMQ
VFGNIGIDGEDEDSDEDEFQITEHNNFRTFFQALMLLFRSATGEAWHNIMLSCLSGKPCDKNSGILTADCGNE
FAYFYFVSFIFLCSFLMLNLFVAVIMDNFEYLTRDSSILGPHHLDEYVRVWAEYDPAACGRIHYKDMYSLLRV
ISPPLGLGKKCPHRVACKRLLRMDLPVADDNTVHFNSTLMALIRTALDIKIAKGGADKQQMDAELRKEMMAIW
PNLSQKTLDLLVTPHKSTDLTVGKIYAAMMIMEYYRQSKAKKLQAMREEQNRTPLMFQRMEPPSPTQEGGPSQ
NALPSTQLDPGGGLMAHEGGMKESPSWVTQRAQEMFQKTGTWSPERGPPIDMPNSQPNSQSVEMREMGTDGYS
DSEHYLPMEGQTRAASMPRLPAENQRRRGRPRGNDLSTISDTSPMKRSASVLGPKARRLDDYSLERVPPEENQ
RYHQRRRDRGHRTSERSLGRYTDVDTGLGTDLSMTTQSGDLPSKDRDQDRGRPKDRKHRPHHHHHHHHHHPPA
PDRDRYAQERPDTGRARAREQRWSRSPSEGREHTTHRQGSSSVSGSPAPSTSGTSTPRRGRRQLPQTPCTPRP
LVSYSPAPRRPAARRMAGPAAPPGGSPRGCRRAPRWPAHAPEGPRPRGADYTEPDSPREPPGGAHDPAPRSPR
TPRAAGCASPRHGRRLPNGYYAGHGAPRPRTARRGAHDAYSESEDDWC*
>rs38793291_ENSMUSP00000112436_c.2898:A>G_p.K967E
ENSMUST00000121390.8|Cacna1a-201|ENSMUSG00000034656|+|chr8:g.85286255:A>G
MARFGDEMPGRYGAGGGGSGPAAGVVVGAAGGRGAGGSRQGGQPGAQRMYKQSMAQRARTMALYNPIPVRQNC
LTVNRSLFLFSEDNVVRKYAKKITEWPPFEYMILATIIANCIVLALEQHLPDDDKTPMSERLDDTEPYFIGIF
CFEAGIKIVALGFAFHKGSYLRNGWNVMDFVVVLTGILATVGTEFDLRTLRAVRVLRPLKLVSGIPSLQVVLK
SIMKAMIPLLQIGLLLFFAILIFAIIGLEFYMGKFHTTCFEEGTDDIQGESPAPCGTEEPARTCPNGTKCQPY
WEGPNNGITQFDNILFAVLTVFQCITMEGWTDLLYNSNDASGNTWNWLYFIPLIIIGSFFMLNLVLGVLSGEF
AKERERVENRRAFLKLRRQQQIERELNGYMEWISKAEEVILAEDETDVEQRHPFDGALRRATLKKSKTDLLNP
EEAEDQLADIASVGSPFARASIKSAKLENSTFFHKKERRMRFYIRRMVKTQAFYWTVLSLVALNTLCVAIVHY
NQPEWLSDFLYYAEFIFLGLFMSEMFIKMYGLGTRPYFHSSFNCFDCGVIIGSIFEVIWAVIKPGTSFGISVL
RALRLLRIFKVTKYWASLRNLVVSLLNSMKSIISLLFLLFLFIVVFALLGMQLFGGQFNFDEGTPPTNFDTFP
AAIMTVFQILTGEDWNEVMYDGIKSQGGVQGGMVFSIYFIVLTLFGNYTLLNVFLAIAVDNLANAQELTKDEQ
EEEEAANQKLALQKAKEVAEVSPLSAANMSIAVKEQQKNQKPTKSVWEQRTSEMRKQNLLASREALYGDAAER
WPTPYARPLRPDVKTHLDRPLVVDPQENRNNNTNKSRAPEALRPTARPRESARDPDARRAWPGSPERAPGREG
PYGRESEPQQREHAPPREHAPWDADTERAKAGDAPRRHTHRPVAEGEPRRHRARRRPGDEPDDRPERRPRPRD
ATRPARAADGEGDDGERERRHRHGPPAHDDRERRHRRRKENQGSGVPVSGPNLSTTRPIQQDLGRQDLPLAED
LDNMKNNKLATGEPASPHDSLGHSGLPPSPAKIGNSTNPGPALATNPQNAASRRTPNNPGNPSNPGPPKTPEN
SLIVTNPSSTQPNSAKTARKPEHMAVEIPPACPPLNHTVVQVNKNANPDPLPKKEEEKKEEEEADPGEDGPKP
MPPYSSMFILSTTNPLRRLCHYILNLRYFEMCILMVIAMSSIALAAEDPVQPNAPRNNVLRYFDYVFTGVFTF
EMVIKMIDLGLVLHQGAYFRDLWNILDFIVVSGALVAFAFTGNSKGKDINTIKSLRVLRVLRPLKTIKRLPKL
KAVFDCVVNSLKNVFNILIVYMLFMFIFAVVAVQLFKGKFFHCTDESKEFERDCRGKYLLYEKNEVKARDREW
KKYEFHYDNVLWALLTLFTVSTGEGWPQVLKHSVDATFENQGPSPGYRMEMSIFYVVYFVVFPFFFVNIFVAL
IIITFQEQGDKMMEEYSLEKNERACIDFAISAKPLTRHMPQNKQSFQYRMWQFVVSPPFEYTIMAMIALNTIV
LMMKFYGASVAYENALRVFNIVFTSLFSLECVLKVMAFGILNYFRDAWNIFDFVTVLGSITDILVTEFGNNFI
NLSFLRLFRAARLIKLLRQGYTIRILLWTFVQSFKALPYVCLLIAMLFFIYAIIGMQVFGNIGIDGEDEDSDE
DEFQITEHNNFRTFFQALMLLFRSATGEAWHNIMLSCLSGKPCDKNSGILTADCGNEFAYFYFVSFIFLCSFL
MLNLFVAVIMDNFEYLTRDSSILGPHHLDEYVRVWAEYDPAACGRIHYKDMYSLLRVISPPLGLGKKCPHRVA
CKRLLRMDLPVADDNTVHFNSTLMALIRTALDIKIAKGGADKQQMDAELRKEMMAIWPNLSQKTLDLLVTPHK
STDLTVGKIYAAMMIMEYYRQSKAKKLQAMREEQNRTPLMFQRMEPPSPTQEGGPSQNALPSTQLDPGGGLMA
HEGGMKESPSWVTQRAQEMFQKTGTWSPERGPPIDMPNSQPNSQSVEMREMGTDGYSDSEHYLPMEGQTRAAS
MPRLPAENQRRRGRPRGNDLSTISDTSPMKRSASVLGPKARRLDDYSLERVPPEENQRYHQRRRDRGHRTSER
SLGRYTDVDTGLGTDLSMTTQSGDLPSKDRDQDRGRPKDRKHRPHHHHHHHHHHPPAPDRDRYAQERPDTGRA
RAREQRWSRSPSEGREHTTHRQGSSSVSGSPAPSTSGTSTPRRGRRQLPQTPCTPRPLVSYSPAPRRPAARRM
AGPAAPPGGSPRGCRRAPRWPAHAPEGPRPRGADYTEPDSPREPPGGAHDPAPRSPRTPRAAGCASPRHGRRL
PNGYYAGHGAPRPRTARRGAHDAYSESEDDWC*
>rs38793291_ENSMUSP00000114055_c.2757:A>G_p.K920E
ENSMUST00000122053.2|Cacna1a-202|ENSMUSG00000034656|+|chr8:g.85286255:A>G
MARFGDEMPGRYGAGGGGSGPAAGVVVGAAGGRGAGGSRQGGQPGAQRMYKQSMAQRARTMALYNPIPVRQNC
LTVNRSLFLFSEDNVDDTEPYFIGIFCFEAGIKIVALGFAFHKGSYLRNGWNVMDFVVVLTGILATVGTEFDL

RTLRAVRVLRPLKLVSGIPSLQVVLKSIMKAMIPLLQIGLLLFFAILIFAIIGLEFYMGKFHTTCFEEGTDDI
QGESPAPCGTEEPARTCPNGTKCQPYWEGPNNGITQFDNILFAVLTVFQCITMEGWTDLLYNSNDASGNTWNW
LYFIPLIIIGSFFMLNLVLGVLSGEFAKERERVENRRAFLKLRRQQQIERELNGYMEWISKAEEVILAEDETD
VEQRHPFDGALRRATLKKSKTDLLNPEEAEDQLADIASVGSPFARASIKSAKLENSTFFHKKERRMRFYIRRM
VKTQAFYWTVLSLVALNTLCVAIVHYNQPEWLSDFLYYAEFIFLGLFMSEMFIKMYGLGTRPYFHSSFNCFDC
GVIIGSIFEVIWAVIKPGTSFGISVLRALRLLRIFKVTKYWASLRNLVVSLLNSMKSIISLLFLLFLFIVVFA
LLGMQLFGGQFNFDEGTPPTNFDTFPAAIMTVFQILTGEDWNEVMYDGIKSQGGVQGGMVFSIYFIVLTLFGN
YTLLNVFLAIAVDNLANAQELTKDEQEEEEAANQKLALQKAKEVAEVSPLSAANMSIAVKEQQKNQKPTKSVW
EQRTSEMRKQNLLASREALYGDAAERWPTPYARPLRPDVKTHLDRPLVVDPQENRNNNTNKSRAPEALRPTAR
PRESARDPDARRAWPGSPERAPGREGPYGRESEPQQREHAPPREHAPWDADTERAKAGDAPRRHTHRPVAEGE
PRRHRARRRPGDEPDDRPERRPRPRDATRPARAADGEGDDGERERRHRHGPPAHDDRERRHRRRKENQGSGVP
VSGPNLSTTRPIQQDLGRQDLPLAEDLDNMKNNKLATGEPASPHDSLGHSGLPPSPAKIGNSTNPGPALATNP
QNAASRRTPNNPGNPSNPGPPKTPENSLIVTNPSSTQPNSAKTARKPEHMAVEIPPACPPLNHTVVQVNKNAN
PDPLPKKEEEKKEEEEADPGEDGPKPMPPYSSMFILSTTNPLRRLCHYILNLRYFEMCILMVIAMSSIALAAE
DPVQPNAPRNNVLRYFDYVFTGVFTFEMVIKMIDLGLVLHQGAYFRDLWNILDFIVVSGALVAFAFTGNSKGK
DINTIKSLRVLRVLRPLKTIKRLPKLKAVFDCVVNSLKNVFNILIVYMLFMFIFAVVAVQLFKGKFFHCTDES
KEFERDCRGKYLLYEKNEVKARDREWKKYEFHYDNVLWALLTLFTVSTGEGWPQVLKHSVDATFENQGPSPGY
RMEMSIFYVVYFVVFPFFFVNIFVALIIITFQEQGDKMMEEYSLEKNERACIDFAISAKPLTRHMPQNKQSFQ
YRMWQFVVSPPFEYTIMAMIALNTIVLMMKFYGASVAYENALRVFNIVFTSLFSLECVLKVMAFGILNYFRDA
WNIFDFVTVLGSITDILVTEFGNNFINLSFLRLFRAARLIKLLRQGYTIRILLWTFVQSFKALPYVCLLIAML
FFIYAIIGMQVFGNIGIDGEDEDSDEDEFQITEHNNFRTFFQALMLLFRSATGEAWHNIMLSCLSGKPCDKNS
GILTADCGNEFAYFYFVSFIFLCSFLMLNLFVAVIMDNFEYLTRDSSILGPHHLDEYVRVWAEYDPAACGRIH
YKDMYSLLRVISPPLGLGKKCPHRVACKRLLRMDLPVADDNTVHFNSTLMALIRTALDIKIAKGGADKQQMDA
ELRKEMMAIWPNLSQKTLDLLVTPHKSTDLTVGKIYAAMMIMEYYRQSKAKKLQAMREEQNRTPLMFQRMEPP
SPTQEGGPSQNALPSTQLDPGGGLMAHEGGMKESPSWVTQRAQEMFQKTGTWSPERGPPIDMPNSQPNSQSVE
MREMGTDGYSDSEHYLPMEGQTRAASMPRLPAENQRRRGRPRGNDLSTISDTSPMKRSASVLGPKARRLDDYS
LERVPPEENQRYHQRRRDRGHRTSERSLGRYTDVDTGLGTDLSMTTQSGDLPSKDRDQDRGRPKDRKHRPHHH
HHHHHHHPPAPDRDRYAQERPDTGRARAREQRWSRSPSEGREHTTHRQGSSSVSGSPAPSTSGTSTPRRGRRQ
LPQTPCTPRPLVSYSPAPRRPAARRMAGPAAPPGGSPRGCRRAPRWPAHAPEGPRPRGADYTEPDSPREPPGG
AHDPAPRSPRTPRAAGCASPRHGRRLPNGYYAGHGAPRPRTARRGAHDAYSESEDDWC*
>rs38793291_ENSMUSP00000149242_c.2757:A>G_p.K920E
ENSMUST00000215756.2|Cacna1a-211|ENSMUSG00000034656|+|chr8:g.85286255:A>G
XRMYKQSMAQRARTMALYNPIPVRQNCLTVNRSLFLFSEDNVVRKYAKKITEWPPFEYMILATIIANCIVLAL
EQHLPDDDKTPMSERLDDTEPYFIGIFCFEAGIKIVALGFAFHKGSYLRNGWNVMDFVVVLTGILATVGTEFD
LRTLRAVRVLRPLKLVSGIPSLQVVLKSIMKAMIPLLQIGLLLFFAILIFAIIGLEFYMGKFHTTCFEEGTDD
IQGESPAPCGTEEPARTCPNGTKCQPYWEGPNNGITQFDNILFAVLTVFQCITMEGWTDLLYNSNDASGNTWN
WLYFIPLIIIGSFFMLNLVLGVLSGEFAKERERVENRRAFLKLRRQQQIERELNGYMEWISKAEEVILAEDET
DVEQRHPFDALRRATLKKSKTDLLNPEEAEDQLADIASVGSPFARASIKSAKLENSTFFHKKERRMRFYIRRM
VKTQAFYWTVLSLVALNTLCVAIVHYNQPEWLSDFLYYAEFIFLGLFMSEMFIKMYGLGTRPYFHSSFNCFDC
GVIIGSIFEVIWAVIKPGTSFGISVLRALRLLRIFKVTKYWASLRNLVVSLLNSMKSIISLLFLLFLFIVVFA
LLGMQLFGGQFNFDEGTPPTNFDTFPAAIMTVFQILTGEDWNEVMYDGIKSQGGVQGGMVFSIYFIVLTLFGN
YTLLNVFLAIAVDNLANAQELTKDEQEEEEAANQKLALQKAKEVAEVSPLSAANMSIAVKEQQKNQKPTKSVW
EQRTSEMRKQNLLASREALYGDAAERWPTPYARPLRPDVKTHLDRPLVVDPQENRNNNTNKSRAPEALRPTAR
PRESARDPDARRAWPGSPERAPGREGPYGRESEPQQREHAPPREHAPWDADTERAKAGDAPRRHTHRPVAEGE
PRRHRARRRPGDEPDDRPERRPRPRDATRPARAADGEGDDGERERRHRHGPPAHDDRERRHRRRKENQGSGVP
VSGPNLSTTRPIQQDLGRQDLPLAEDLDNMKNNKLATGEPASPHDSLGHSGLPPSPAKIGNSTNPGPALATNP
QNAASRRTPNNPGNPSNPGPPKTPENSLIVTNPSSTQPNSAKTARKPEHMAVEIPPACPPLNHTVVQVNKNAN
PDPLPKKEEEKKEEEEADPGEDGPKPMPPYSSMFILSTTNPLRRLCHYILNLRYFEMCILMVIAMSSIALAAE
DPVQPNAPRNNVLRYFDYVFTGVFTFEMVIKMIDLGLVLHQGAYFRDLWNILDFIVVSGALVAFAFTGNSKGK
DINTIKSLRVLRVLRPLKTIKRLPKLKAVFDCVVNSLKNVFNILIVYMLFMFIFAVVAVQLFKGKFFHCTDES
KEFERDCRGKYLLYEKNEVKARDREWKKYEFHYDNVLWALLTLFTVSTGEGWPQVLKHSVDATFENQGPSPGY
RMEMSIFYVVYFVVFPFFFVNIFVALIIITFQEQGDKMMEEYSLEKNERACIDFAISAKPLTRHMPQNKQSFQ
YRMWQFVVSPPFEYTIMAMIALNTIVLMMKFYGASVAYENALRVFNIVFTSLFSLECVLKVMAFGILNYFRDA
WNIFDFVTVLGSITDILVTEFGNNFINLSFLRLFRAARLIKLLRQGYTIRILLWTFVQSFKALPYVCLLIAML
FFIYAIIGMQVFGNIGIDGEDEDSDEDEFQITEHNNFRTFFQALMLLFRSATGEAWHNIMLSCLSGKPCDKNS
GILTADCGNEFAYFYFVSFIFLCSFLMLNLFVAVIMDNFEYLTRDSSILGPHHLDEYVRVWAEYDPAACGRIH
YKDMYSLLRVISPPLGLGKKCPHRVACKRLLRMDLPVADDNTVHFNSTLMALIRTALDIKIAKGGADKQQMDA
ELRKEMMAIWPNLSQKTLDLLVTPHKSTDLTVGKIYAAMMIMEYYRQSKAKKLQAMREEQNRTPLMFQRMEPP

SPTQEGGPSQNALPSTQLDPGGGLMAHEGGMKESPSWVTQRAQEMFQKTGTWSPERGPPIDMPNSQPNSQSVE
MREMGTDGYSDSEHYLPMEGQTRAASMPRLPAENQRRRGRPRGNDLSTISDTSPMKRSASVLGPKARRLDDYS
LERVPPEENQRYHQRRRDRGHRTSERSLGRYTDVDTGLGTDLSMTTQSGDLPSKDRDQDRGRPKDRKHRPHHH
HHHHHHHPPAPDRDRYAQERPDTGRARAREQRWSRSPSEGREHTTHRQGSSSVSGSPAPSTSGTSTPRRGRRQ
LPQTPCTPRPLVSYSPAPRRPAARRMAGPAAPPGGSPRGCRRAPRWPAHAPEGPRPRGADYTEPDSPREPPGG
AHDPAPRSPRTPRAAGCASPRHGRRLPNGYYAGHGAPRPRTARRGAHDAYSESEDDWC*
>rs32648021_ENSMUSP00000158803_c.4146:A>G_p.K1383E
ENSMUST00000238701.2|Cacna1a-213|ENSMUSG00000034656|+|chr8:g.85306133:A>G
MPRASWGSDPMLSPLSHELLHSPRYPGTDCTLAGSSRTLHVPWSQQMSGSLEGPTLKRRGGVVDHRDVILAHQ
AHKMHSTPQARRKEWEMARFGDEMPGRYGAGGGGSGPAAGVVVGAAGGRGAGGSRQGGQPGAQRMYKQSMAQR
ARTMALYNPIPVRQNCLTVNRSLFLFSEDNVVRKYAKKITEWPPFEYMILATIIANCIVLALEQHLPDDDKTP
MSERLDDTEPYFIGIFCFEAGIKIVALGFAFHKGSYLRNGWNVMDFVVVLTGILATVGTEFDLRTLRAVRVLR
PLKLVSGIPSLQVVLKSIMKAMIPLLQIGLLLFFAILIFAIIGLEFYMGKFHTTCFEEGTDDIQGESPAPCGT
EEPARTCPNGTKCQPYWEGPNNGITQFDNILFAVLTVFQCITMEGWTDLLYNSNDASGNTWNWLYFIPLIIIG
SFFMLNLVLGVLSGEFAKERERVENRRAFLKLRRQQQIERELNGYMEWISKAEEVILAEDETDVEQRHPFDGA
LRRATLKKSKTDLLNPEEAEDQLADIASVGSPFARASIKSAKLENSTFFHKKERRMRFYIRRMVKTQAFYWTV
LSLVALNTLCVAIVHYNQPEWLSDFLYYAEFIFLGLFMSEMFIKMYGLGTRPYFHSSFNCFDCGVIIGSIFEV
IWAVIKPGTSFGISVLRALRLLRIFKVTKYWASLRNLVVSLLNSMKSIISLLFLLFLFIVVFALLGMQLFGGQ
FNFDEGTPPTNFDTFPAAIMTVFQILTGEDWNEVMYDGIKSQGGVQGGMVFSIYFIVLTLFGNYTLLNVFLAI
AVDNLANAQELTKDEQEEEEAANQKLALQKAKEVAEVSPLSAANMSIAVKEQQKNQKPTKSVWEQRTSEMRKQ
NLLASREALYGDAAERWPTPYARPLRPDVKTHLDRPLVVDPQENRNNNTNKSRAPEALRPTARPRESARDPDA
RRAWPGSPERAPGREGPYGRESEPQQREHAPPREHAPWDADTERAKAGDAPRRHTHRPVAEGEPRRHRARRRP
GDEPDDRPERRPRPRDATRPARAADGEGDDGERKRRHRHGPPAHDDRERRHRRRKENQGSGVPVSGPNLSTTR
PIQQDLGRQDLPLAEDLDNMKNNKLATGEPASPHDSLGHSGLPPSPAKIGNSTNPGPALATNPQNAASRRTPN
NPGNPSNPGPPKTPENSLIVTNPSSTQPNSAKTARKPEHMAVEIPPACPPLNHTVVQVNKNANPDPLPKKEEE
KKEEEEADPGEDGPKPMPPYSSMFILSTTNPLRRLCHYILNLRYFEMCILMVIAMSSIALAAEDPVQPNAPRN
NVLRYFDYVFTGVFTFEMVIKMIDLGLVLHQGAYFRDLWNILDFIVVSGALVAFAFTGNSKGKDINTIESLRV
LRVLRPLKTIKRLPKLKAVFDCVVNSLKNVFNILIVYMLFMFIFAVVAVQLFKGKFFHCTDESKEFERDCRGK
YLLYEKNEVKARDREWKKYEFHYDNVLWALLTLFTVSTGEGWPQVLKHSVDATFENQGPSPGYRMEMSIFYVV
YFVVFPFFFVNIFVALIIITFQEQGDKMMEEYSLEKNERACIDFAISAKPLTRHMPQNKQSFQYRMWQFVVSP
PFEYTIMAMIALNTIVLMMKFYGASVAYENALRVFNIVFTSLFSLECVLKVMAFGILNYFRDAWNIFDFVTVL
GSITDILVTEFGNNFINLSFLRLFRAARLIKLLRQGYTIRILLWTFVQSFKALPYVCLLIAMLFFIYAIIGMQ
VFGNIGIDGEDEDSDEDEFQITEHNNFRTFFQALMLLFRSATGEAWHNIMLSCLSGKPCDKNSGILTADCGNE
FAYFYFVSFIFLCSFLMLNLFVAVIMDNFEYLTRDSSILGPHHLDEYVRVWAEYDPAACGRIHYKDMYSLLRV
ISPPLGLGKKCPHRVACKRLLRMDLPVADDNTVHFNSTLMALIRTALDIKIAKGGADKQQMDAELRKEMMAIW
PNLSQKTLDLLVTPHKSTDLTVGKIYAAMMIMEYYRQSKAKKLQAMREEQNRTPLMFQRMEPPSPTQEGGPSQ
NALPSTQLDPGGGLMAHEGGMKESPSWVTQRAQEMFQKTGTWSPERGPPIDMPNSQPNSQSVEMREMGTDGYS
DSEHYLPMEGQTRAASMPRLPAENQRRRGRPRGNDLSTISDTSPMKRSASVLGPKARRLDDYSLERVPPEENQ
RYHQRRRDRGHRTSERSLGRYTDVDTGLGTDLSMTTQSGDLPSKDRDQDRGRPKDRKHRPHHHHHHHHHHPPA
PDRDRYAQERPDTGRARAREQRWSRSPSEGREHTTHRQGSSSVSGSPAPSTSGTSTPRRGRRQLPQTPCTPRP
LVSYSPAPRRPAARRMAGPAAPPGGSPRGCRRAPRWPAHAPEGPRPRGADYTEPDSPREPPGGAHDPAPRSPR
TPRAAGCASPRHGRRLPNGYYAGHGAPRPRTARRGAHDAYSESEDDWC*
>rs32648021_ENSMUSP00000112436_c.3879:A>G_p.K1294E
ENSMUST00000121390.8|Cacna1a-201|ENSMUSG00000034656|+|chr8:g.85306133:A>G
MARFGDEMPGRYGAGGGGSGPAAGVVVGAAGGRGAGGSRQGGQPGAQRMYKQSMAQRARTMALYNPIPVRQNC
LTVNRSLFLFSEDNVVRKYAKKITEWPPFEYMILATIIANCIVLALEQHLPDDDKTPMSERLDDTEPYFIGIF
CFEAGIKIVALGFAFHKGSYLRNGWNVMDFVVVLTGILATVGTEFDLRTLRAVRVLRPLKLVSGIPSLQVVLK
SIMKAMIPLLQIGLLLFFAILIFAIIGLEFYMGKFHTTCFEEGTDDIQGESPAPCGTEEPARTCPNGTKCQPY
WEGPNNGITQFDNILFAVLTVFQCITMEGWTDLLYNSNDASGNTWNWLYFIPLIIIGSFFMLNLVLGVLSGEF
AKERERVENRRAFLKLRRQQQIERELNGYMEWISKAEEVILAEDETDVEQRHPFDGALRRATLKKSKTDLLNP
EEAEDQLADIASVGSPFARASIKSAKLENSTFFHKKERRMRFYIRRMVKTQAFYWTVLSLVALNTLCVAIVHY
NQPEWLSDFLYYAEFIFLGLFMSEMFIKMYGLGTRPYFHSSFNCFDCGVIIGSIFEVIWAVIKPGTSFGISVL
RALRLLRIFKVTKYWASLRNLVVSLLNSMKSIISLLFLLFLFIVVFALLGMQLFGGQFNFDEGTPPTNFDTFP
AAIMTVFQILTGEDWNEVMYDGIKSQGGVQGGMVFSIYFIVLTLFGNYTLLNVFLAIAVDNLANAQELTKDEQ
EEEEAANQKLALQKAKEVAEVSPLSAANMSIAVKEQQKNQKPTKSVWEQRTSEMRKQNLLASREALYGDAAER
WPTPYARPLRPDVKTHLDRPLVVDPQENRNNNTNKSRAPEALRPTARPRESARDPDARRAWPGSPERAPGREG
PYGRESEPQQREHAPPREHAPWDADTERAKAGDAPRRHTHRPVAEGEPRRHRARRRPGDEPDDRPERRPRPRD
ATRPARAADGEGDDGERKRRHRHGPPAHDDRERRHRRRKENQGSGVPVSGPNLSTTRPIQQDLGRQDLPLAED

LDNMKNNKLATGEPASPHDSLGHSGLPPSPAKIGNSTNPGPALATNPQNAASRRTPNNPGNPSNPGPPKTPEN
SLIVTNPSSTQPNSAKTARKPEHMAVEIPPACPPLNHTVVQVNKNANPDPLPKKEEEKKEEEEADPGEDGPKP
MPPYSSMFILSTTNPLRRLCHYILNLRYFEMCILMVIAMSSIALAAEDPVQPNAPRNNVLRYFDYVFTGVFTF
EMVIKMIDLGLVLHQGAYFRDLWNILDFIVVSGALVAFAFTGNSKGKDINTIESLRVLRVLRPLKTIKRLPKL
KAVFDCVVNSLKNVFNILIVYMLFMFIFAVVAVQLFKGKFFHCTDESKEFERDCRGKYLLYEKNEVKARDREW
KKYEFHYDNVLWALLTLFTVSTGEGWPQVLKHSVDATFENQGPSPGYRMEMSIFYVVYFVVFPFFFVNIFVAL
IIITFQEQGDKMMEEYSLEKNERACIDFAISAKPLTRHMPQNKQSFQYRMWQFVVSPPFEYTIMAMIALNTIV
LMMKFYGASVAYENALRVFNIVFTSLFSLECVLKVMAFGILNYFRDAWNIFDFVTVLGSITDILVTEFGNNFI
NLSFLRLFRAARLIKLLRQGYTIRILLWTFVQSFKALPYVCLLIAMLFFIYAIIGMQVFGNIGIDGEDEDSDE
DEFQITEHNNFRTFFQALMLLFRSATGEAWHNIMLSCLSGKPCDKNSGILTADCGNEFAYFYFVSFIFLCSFL
MLNLFVAVIMDNFEYLTRDSSILGPHHLDEYVRVWAEYDPAACGRIHYKDMYSLLRVISPPLGLGKKCPHRVA
CKRLLRMDLPVADDNTVHFNSTLMALIRTALDIKIAKGGADKQQMDAELRKEMMAIWPNLSQKTLDLLVTPHK
STDLTVGKIYAAMMIMEYYRQSKAKKLQAMREEQNRTPLMFQRMEPPSPTQEGGPSQNALPSTQLDPGGGLMA
HEGGMKESPSWVTQRAQEMFQKTGTWSPERGPPIDMPNSQPNSQSVEMREMGTDGYSDSEHYLPMEGQTRAAS
MPRLPAENQRRRGRPRGNDLSTISDTSPMKRSASVLGPKARRLDDYSLERVPPEENQRYHQRRRDRGHRTSER
SLGRYTDVDTGLGTDLSMTTQSGDLPSKDRDQDRGRPKDRKHRPHHHHHHHHHHPPAPDRDRYAQERPDTGRA
RAREQRWSRSPSEGREHTTHRQGSSSVSGSPAPSTSGTSTPRRGRRQLPQTPCTPRPLVSYSPAPRRPAARRM
AGPAAPPGGSPRGCRRAPRWPAHAPEGPRPRGADYTEPDSPREPPGGAHDPAPRSPRTPRAAGCASPRHGRRL
PNGYYAGHGAPRPRTARRGAHDAYSESEDDWC*
>rs32648021_ENSMUSP00000114055_c.3738:A>G_p.K1247E
ENSMUST00000122053.2|Cacna1a-202|ENSMUSG00000034656|+|chr8:g.85306133:A>G
MARFGDEMPGRYGAGGGGSGPAAGVVVGAAGGRGAGGSRQGGQPGAQRMYKQSMAQRARTMALYNPIPVRQNC
LTVNRSLFLFSEDNVDDTEPYFIGIFCFEAGIKIVALGFAFHKGSYLRNGWNVMDFVVVLTGILATVGTEFDL
RTLRAVRVLRPLKLVSGIPSLQVVLKSIMKAMIPLLQIGLLLFFAILIFAIIGLEFYMGKFHTTCFEEGTDDI
QGESPAPCGTEEPARTCPNGTKCQPYWEGPNNGITQFDNILFAVLTVFQCITMEGWTDLLYNSNDASGNTWNW
LYFIPLIIIGSFFMLNLVLGVLSGEFAKERERVENRRAFLKLRRQQQIERELNGYMEWISKAEEVILAEDETD
VEQRHPFDGALRRATLKKSKTDLLNPEEAEDQLADIASVGSPFARASIKSAKLENSTFFHKKERRMRFYIRRM
VKTQAFYWTVLSLVALNTLCVAIVHYNQPEWLSDFLYYAEFIFLGLFMSEMFIKMYGLGTRPYFHSSFNCFDC
GVIIGSIFEVIWAVIKPGTSFGISVLRALRLLRIFKVTKYWASLRNLVVSLLNSMKSIISLLFLLFLFIVVFA
LLGMQLFGGQFNFDEGTPPTNFDTFPAAIMTVFQILTGEDWNEVMYDGIKSQGGVQGGMVFSIYFIVLTLFGN
YTLLNVFLAIAVDNLANAQELTKDEQEEEEAANQKLALQKAKEVAEVSPLSAANMSIAVKEQQKNQKPTKSVW
EQRTSEMRKQNLLASREALYGDAAERWPTPYARPLRPDVKTHLDRPLVVDPQENRNNNTNKSRAPEALRPTAR
PRESARDPDARRAWPGSPERAPGREGPYGRESEPQQREHAPPREHAPWDADTERAKAGDAPRRHTHRPVAEGE
PRRHRARRRPGDEPDDRPERRPRPRDATRPARAADGEGDDGERKRRHRHGPPAHDDRERRHRRRKENQGSGVP
VSGPNLSTTRPIQQDLGRQDLPLAEDLDNMKNNKLATGEPASPHDSLGHSGLPPSPAKIGNSTNPGPALATNP
QNAASRRTPNNPGNPSNPGPPKTPENSLIVTNPSSTQPNSAKTARKPEHMAVEIPPACPPLNHTVVQVNKNAN
PDPLPKKEEEKKEEEEADPGEDGPKPMPPYSSMFILSTTNPLRRLCHYILNLRYFEMCILMVIAMSSIALAAE
DPVQPNAPRNNVLRYFDYVFTGVFTFEMVIKMIDLGLVLHQGAYFRDLWNILDFIVVSGALVAFAFTGNSKGK
DINTIESLRVLRVLRPLKTIKRLPKLKAVFDCVVNSLKNVFNILIVYMLFMFIFAVVAVQLFKGKFFHCTDES
KEFERDCRGKYLLYEKNEVKARDREWKKYEFHYDNVLWALLTLFTVSTGEGWPQVLKHSVDATFENQGPSPGY
RMEMSIFYVVYFVVFPFFFVNIFVALIIITFQEQGDKMMEEYSLEKNERACIDFAISAKPLTRHMPQNKQSFQ
YRMWQFVVSPPFEYTIMAMIALNTIVLMMKFYGASVAYENALRVFNIVFTSLFSLECVLKVMAFGILNYFRDA
WNIFDFVTVLGSITDILVTEFGNNFINLSFLRLFRAARLIKLLRQGYTIRILLWTFVQSFKALPYVCLLIAML
FFIYAIIGMQVFGNIGIDGEDEDSDEDEFQITEHNNFRTFFQALMLLFRSATGEAWHNIMLSCLSGKPCDKNS
GILTADCGNEFAYFYFVSFIFLCSFLMLNLFVAVIMDNFEYLTRDSSILGPHHLDEYVRVWAEYDPAACGRIH
YKDMYSLLRVISPPLGLGKKCPHRVACKRLLRMDLPVADDNTVHFNSTLMALIRTALDIKIAKGGADKQQMDA
ELRKEMMAIWPNLSQKTLDLLVTPHKSTDLTVGKIYAAMMIMEYYRQSKAKKLQAMREEQNRTPLMFQRMEPP
SPTQEGGPSQNALPSTQLDPGGGLMAHEGGMKESPSWVTQRAQEMFQKTGTWSPERGPPIDMPNSQPNSQSVE
MREMGTDGYSDSEHYLPMEGQTRAASMPRLPAENQRRRGRPRGNDLSTISDTSPMKRSASVLGPKARRLDDYS
LERVPPEENQRYHQRRRDRGHRTSERSLGRYTDVDTGLGTDLSMTTQSGDLPSKDRDQDRGRPKDRKHRPHHH
HHHHHHHPPAPDRDRYAQERPDTGRARAREQRWSRSPSEGREHTTHRQGSSSVSGSPAPSTSGTSTPRRGRRQ
LPQTPCTPRPLVSYSPAPRRPAARRMAGPAAPPGGSPRGCRRAPRWPAHAPEGPRPRGADYTEPDSPREPPGG
AHDPAPRSPRTPRAAGCASPRHGRRLPNGYYAGHGAPRPRTARRGAHDAYSESEDDWC*
>rs32648021_ENSMUSP00000149242_c.3738:A>G_p.K1247E
ENSMUST00000215756.2|Cacna1a-211|ENSMUSG00000034656|+|chr8:g.85306133:A>G
XRMYKQSMAQRARTMALYNPIPVRQNCLTVNRSLFLFSEDNVVRKYAKKITEWPPFEYMILATIIANCIVLAL
EQHLPDDDKTPMSERLDDTEPYFIGIFCFEAGIKIVALGFAFHKGSYLRNGWNVMDFVVVLTGILATVGTEFD
LRTLRAVRVLRPLKLVSGIPSLQVVLKSIMKAMIPLLQIGLLLFFAILIFAIIGLEFYMGKFHTTCFEEGTDD

IQGESPAPCGTEEPARTCPNGTKCQPYWEGPNNGITQFDNILFAVLTVFQCITMEGWTDLLYNSNDASGNTWN
WLYFIPLIIIGSFFMLNLVLGVLSGEFAKERERVENRRAFLKLRRQQQIERELNGYMEWISKAEEVILAEDET
DVEQRHPFDALRRATLKKSKTDLLNPEEAEDQLADIASVGSPFARASIKSAKLENSTFFHKKERRMRFYIRRM
VKTQAFYWTVLSLVALNTLCVAIVHYNQPEWLSDFLYYAEFIFLGLFMSEMFIKMYGLGTRPYFHSSFNCFDC
GVIIGSIFEVIWAVIKPGTSFGISVLRALRLLRIFKVTKYWASLRNLVVSLLNSMKSIISLLFLLFLFIVVFA
LLGMQLFGGQFNFDEGTPPTNFDTFPAAIMTVFQILTGEDWNEVMYDGIKSQGGVQGGMVFSIYFIVLTLFGN
YTLLNVFLAIAVDNLANAQELTKDEQEEEEAANQKLALQKAKEVAEVSPLSAANMSIAVKEQQKNQKPTKSVW
EQRTSEMRKQNLLASREALYGDAAERWPTPYARPLRPDVKTHLDRPLVVDPQENRNNNTNKSRAPEALRPTAR
PRESARDPDARRAWPGSPERAPGREGPYGRESEPQQREHAPPREHAPWDADTERAKAGDAPRRHTHRPVAEGE
PRRHRARRRPGDEPDDRPERRPRPRDATRPARAADGEGDDGERKRRHRHGPPAHDDRERRHRRRKENQGSGVP
VSGPNLSTTRPIQQDLGRQDLPLAEDLDNMKNNKLATGEPASPHDSLGHSGLPPSPAKIGNSTNPGPALATNP
QNAASRRTPNNPGNPSNPGPPKTPENSLIVTNPSSTQPNSAKTARKPEHMAVEIPPACPPLNHTVVQVNKNAN
PDPLPKKEEEKKEEEEADPGEDGPKPMPPYSSMFILSTTNPLRRLCHYILNLRYFEMCILMVIAMSSIALAAE
DPVQPNAPRNNVLRYFDYVFTGVFTFEMVIKMIDLGLVLHQGAYFRDLWNILDFIVVSGALVAFAFTGNSKGK
DINTIESLRVLRVLRPLKTIKRLPKLKAVFDCVVNSLKNVFNILIVYMLFMFIFAVVAVQLFKGKFFHCTDES
KEFERDCRGKYLLYEKNEVKARDREWKKYEFHYDNVLWALLTLFTVSTGEGWPQVLKHSVDATFENQGPSPGY
RMEMSIFYVVYFVVFPFFFVNIFVALIIITFQEQGDKMMEEYSLEKNERACIDFAISAKPLTRHMPQNKQSFQ
YRMWQFVVSPPFEYTIMAMIALNTIVLMMKFYGASVAYENALRVFNIVFTSLFSLECVLKVMAFGILNYFRDA
WNIFDFVTVLGSITDILVTEFGNNFINLSFLRLFRAARLIKLLRQGYTIRILLWTFVQSFKALPYVCLLIAML
FFIYAIIGMQVFGNIGIDGEDEDSDEDEFQITEHNNFRTFFQALMLLFRSATGEAWHNIMLSCLSGKPCDKNS
GILTADCGNEFAYFYFVSFIFLCSFLMLNLFVAVIMDNFEYLTRDSSILGPHHLDEYVRVWAEYDPAACGRIH
YKDMYSLLRVISPPLGLGKKCPHRVACKRLLRMDLPVADDNTVHFNSTLMALIRTALDIKIAKGGADKQQMDA
ELRKEMMAIWPNLSQKTLDLLVTPHKSTDLTVGKIYAAMMIMEYYRQSKAKKLQAMREEQNRTPLMFQRMEPP
SPTQEGGPSQNALPSTQLDPGGGLMAHEGGMKESPSWVTQRAQEMFQKTGTWSPERGPPIDMPNSQPNSQSVE
MREMGTDGYSDSEHYLPMEGQTRAASMPRLPAENQRRRGRPRGNDLSTISDTSPMKRSASVLGPKARRLDDYS
LERVPPEENQRYHQRRRDRGHRTSERSLGRYTDVDTGLGTDLSMTTQSGDLPSKDRDQDRGRPKDRKHRPHHH
HHHHHHHPPAPDRDRYAQERPDTGRARAREQRWSRSPSEGREHTTHRQGSSSVSGSPAPSTSGTSTPRRGRRQ
LPQTPCTPRPLVSYSPAPRRPAARRMAGPAAPPGGSPRGCRRAPRWPAHAPEGPRPRGADYTEPDSPREPPGG
AHDPAPRSPRTPRAAGCASPRHGRRLPNGYYAGHGAPRPRTARRGAHDAYSESEDDWC*
>rs260693369_ENSMUSP00000158803_c.5967:G>GTAGTT_p.D1990ins-fs
ENSMUST00000238701.2|Cacna1a-
213|ENSMUSG00000034656|+|chr8:g.85356459:G>GTAGTT
MPRASWGSDPMLSPLSHELLHSPRYPGTDCTLAGSSRTLHVPWSQQMSGSLEGPTLKRRGGVVDHRDVILAHQ
AHKMHSTPQARRKEWEMARFGDEMPGRYGAGGGGSGPAAGVVVGAAGGRGAGGSRQGGQPGAQRMYKQSMAQR
ARTMALYNPIPVRQNCLTVNRSLFLFSEDNVVRKYAKKITEWPPFEYMILATIIANCIVLALEQHLPDDDKTP
MSERLDDTEPYFIGIFCFEAGIKIVALGFAFHKGSYLRNGWNVMDFVVVLTGILATVGTEFDLRTLRAVRVLR
PLKLVSGIPSLQVVLKSIMKAMIPLLQIGLLLFFAILIFAIIGLEFYMGKFHTTCFEEGTDDIQGESPAPCGT
EEPARTCPNGTKCQPYWEGPNNGITQFDNILFAVLTVFQCITMEGWTDLLYNSNDASGNTWNWLYFIPLIIIG
SFFMLNLVLGVLSGEFAKERERVENRRAFLKLRRQQQIERELNGYMEWISKAEEVILAEDETDVEQRHPFDGA
LRRATLKKSKTDLLNPEEAEDQLADIASVGSPFARASIKSAKLENSTFFHKKERRMRFYIRRMVKTQAFYWTV
LSLVALNTLCVAIVHYNQPEWLSDFLYYAEFIFLGLFMSEMFIKMYGLGTRPYFHSSFNCFDCGVIIGSIFEV
IWAVIKPGTSFGISVLRALRLLRIFKVTKYWASLRNLVVSLLNSMKSIISLLFLLFLFIVVFALLGMQLFGGQ
FNFDEGTPPTNFDTFPAAIMTVFQILTGEDWNEVMYDGIKSQGGVQGGMVFSIYFIVLTLFGNYTLLNVFLAI
AVDNLANAQELTKDEQEEEEAANQKLALQKAKEVAEVSPLSAANMSIAVKEQQKNQKPTKSVWEQRTSEMRKQ
NLLASREALYGDAAERWPTPYARPLRPDVKTHLDRPLVVDPQENRNNNTNKSRAPEALRPTARPRESARDPDA
RRAWPGSPERAPGREGPYGRESEPQQREHAPPREHAPWDADTERAKAGDAPRRHTHRPVAEGEPRRHRARRRP
GDEPDDRPERRPRPRDATRPARAADGEGDDGERKRRHRHGPPAHDDRERRHRRRKENQGSGVPVSGPNLSTTR
PIQQDLGRQDLPLAEDLDNMKNNKLATGEPASPHDSLGHSGLPPSPAKIGNSTNPGPALATNPQNAASRRTPN
NPGNPSNPGPPKTPENSLIVTNPSSTQPNSAKTARKPEHMAVEIPPACPPLNHTVVQVNKNANPDPLPKKEEE
KKEEEEADPGEDGPKPMPPYSSMFILSTTNPLRRLCHYILNLRYFEMCILMVIAMSSIALAAEDPVQPNAPRN
NVLRYFDYVFTGVFTFEMVIKMIDLGLVLHQGAYFRDLWNILDFIVVSGALVAFAFTGNSKGKDINTIKSLRV
LRVLRPLKTIKRLPKLKAVFDCVVNSLKNVFNILIVYMLFMFIFAVVAVQLFKGKFFHCTDESKEFERDCRGK
YLLYEKNEVKARDREWKKYEFHYDNVLWALLTLFTVSTGEGWPQVLKHSVDATFENQGPSPGYRMEMSIFYVV
YFVVFPFFFVNIFVALIIITFQEQGDKMMEEYSLEKNERACIDFAISAKPLTRHMPQNKQSFQYRMWQFVVSP
PFEYTIMAMIALNTIVLMMKFYGASVAYENALRVFNIVFTSLFSLECVLKVMAFGILNYFRDAWNIFDFVTVL
GSITDILVTEFGNNFINLSFLRLFRAARLIKLLRQGYTIRILLWTFVQSFKALPYVCLLIAMLFFIYAIIGMQ
VFGNIGIDGEDEDSDEDEFQITEHNNFRTFFQALMLLFRSATGEAWHNIMLSCLSGKPCDKNSGILTADCGNE
FAYFYFVSFIFLCSFLMLNLFVAVIMDNFEYLTRDSSILGPHHLDEYVRVWAEYDPAACGRIHYKDMYSLLRV

ISPPLGLGKKCPHRVACKRLLRMDLPVADDNTVHFNSTLMALIRTALDIKIAKGGADKQQMDAELRKEMMAIW
PNLSQKTLDLLVTPHKSTVVT
>rs260693369_ENSMUSP00000112436_c.5700:G>GTAGTT_p.D1901ins-fs
ENSMUST00000121390.8|Cacna1a-
201|ENSMUSG00000034656|+|chr8:g.85356459:G>GTAGTT
MARFGDEMPGRYGAGGGGSGPAAGVVVGAAGGRGAGGSRQGGQPGAQRMYKQSMAQRARTMALYNPIPVRQNC
LTVNRSLFLFSEDNVVRKYAKKITEWPPFEYMILATIIANCIVLALEQHLPDDDKTPMSERLDDTEPYFIGIF
CFEAGIKIVALGFAFHKGSYLRNGWNVMDFVVVLTGILATVGTEFDLRTLRAVRVLRPLKLVSGIPSLQVVLK
SIMKAMIPLLQIGLLLFFAILIFAIIGLEFYMGKFHTTCFEEGTDDIQGESPAPCGTEEPARTCPNGTKCQPY
WEGPNNGITQFDNILFAVLTVFQCITMEGWTDLLYNSNDASGNTWNWLYFIPLIIIGSFFMLNLVLGVLSGEF
AKERERVENRRAFLKLRRQQQIERELNGYMEWISKAEEVILAEDETDVEQRHPFDGALRRATLKKSKTDLLNP
EEAEDQLADIASVGSPFARASIKSAKLENSTFFHKKERRMRFYIRRMVKTQAFYWTVLSLVALNTLCVAIVHY
NQPEWLSDFLYYAEFIFLGLFMSEMFIKMYGLGTRPYFHSSFNCFDCGVIIGSIFEVIWAVIKPGTSFGISVL
RALRLLRIFKVTKYWASLRNLVVSLLNSMKSIISLLFLLFLFIVVFALLGMQLFGGQFNFDEGTPPTNFDTFP
AAIMTVFQILTGEDWNEVMYDGIKSQGGVQGGMVFSIYFIVLTLFGNYTLLNVFLAIAVDNLANAQELTKDEQ
EEEEAANQKLALQKAKEVAEVSPLSAANMSIAVKEQQKNQKPTKSVWEQRTSEMRKQNLLASREALYGDAAER
WPTPYARPLRPDVKTHLDRPLVVDPQENRNNNTNKSRAPEALRPTARPRESARDPDARRAWPGSPERAPGREG
PYGRESEPQQREHAPPREHAPWDADTERAKAGDAPRRHTHRPVAEGEPRRHRARRRPGDEPDDRPERRPRPRD
ATRPARAADGEGDDGERKRRHRHGPPAHDDRERRHRRRKENQGSGVPVSGPNLSTTRPIQQDLGRQDLPLAED
LDNMKNNKLATGEPASPHDSLGHSGLPPSPAKIGNSTNPGPALATNPQNAASRRTPNNPGNPSNPGPPKTPEN
SLIVTNPSSTQPNSAKTARKPEHMAVEIPPACPPLNHTVVQVNKNANPDPLPKKEEEKKEEEEADPGEDGPKP
MPPYSSMFILSTTNPLRRLCHYILNLRYFEMCILMVIAMSSIALAAEDPVQPNAPRNNVLRYFDYVFTGVFTF
EMVIKMIDLGLVLHQGAYFRDLWNILDFIVVSGALVAFAFTGNSKGKDINTIKSLRVLRVLRPLKTIKRLPKL
KAVFDCVVNSLKNVFNILIVYMLFMFIFAVVAVQLFKGKFFHCTDESKEFERDCRGKYLLYEKNEVKARDREW
KKYEFHYDNVLWALLTLFTVSTGEGWPQVLKHSVDATFENQGPSPGYRMEMSIFYVVYFVVFPFFFVNIFVAL
IIITFQEQGDKMMEEYSLEKNERACIDFAISAKPLTRHMPQNKQSFQYRMWQFVVSPPFEYTIMAMIALNTIV
LMMKFYGASVAYENALRVFNIVFTSLFSLECVLKVMAFGILNYFRDAWNIFDFVTVLGSITDILVTEFGNNFI
NLSFLRLFRAARLIKLLRQGYTIRILLWTFVQSFKALPYVCLLIAMLFFIYAIIGMQVFGNIGIDGEDEDSDE
DEFQITEHNNFRTFFQALMLLFRSATGEAWHNIMLSCLSGKPCDKNSGILTADCGNEFAYFYFVSFIFLCSFL
MLNLFVAVIMDNFEYLTRDSSILGPHHLDEYVRVWAEYDPAACGRIHYKDMYSLLRVISPPLGLGKKCPHRVA
CKRLLRMDLPVADDNTVHFNSTLMALIRTALDIKIAKGGADKQQMDAELRKEMMAIWPNLSQKTLDLLVTPHK
STVVT
>rs260693369_ENSMUSP00000114055_c.5559:G>GTAGTT_p.D1854ins-fs
ENSMUST00000122053.2|Cacna1a-
202|ENSMUSG00000034656|+|chr8:g.85356459:G>GTAGTT
MARFGDEMPGRYGAGGGGSGPAAGVVVGAAGGRGAGGSRQGGQPGAQRMYKQSMAQRARTMALYNPIPVRQNC
LTVNRSLFLFSEDNVDDTEPYFIGIFCFEAGIKIVALGFAFHKGSYLRNGWNVMDFVVVLTGILATVGTEFDL
RTLRAVRVLRPLKLVSGIPSLQVVLKSIMKAMIPLLQIGLLLFFAILIFAIIGLEFYMGKFHTTCFEEGTDDI
QGESPAPCGTEEPARTCPNGTKCQPYWEGPNNGITQFDNILFAVLTVFQCITMEGWTDLLYNSNDASGNTWNW
LYFIPLIIIGSFFMLNLVLGVLSGEFAKERERVENRRAFLKLRRQQQIERELNGYMEWISKAEEVILAEDETD
VEQRHPFDGALRRATLKKSKTDLLNPEEAEDQLADIASVGSPFARASIKSAKLENSTFFHKKERRMRFYIRRM
VKTQAFYWTVLSLVALNTLCVAIVHYNQPEWLSDFLYYAEFIFLGLFMSEMFIKMYGLGTRPYFHSSFNCFDC
GVIIGSIFEVIWAVIKPGTSFGISVLRALRLLRIFKVTKYWASLRNLVVSLLNSMKSIISLLFLLFLFIVVFA
LLGMQLFGGQFNFDEGTPPTNFDTFPAAIMTVFQILTGEDWNEVMYDGIKSQGGVQGGMVFSIYFIVLTLFGN
YTLLNVFLAIAVDNLANAQELTKDEQEEEEAANQKLALQKAKEVAEVSPLSAANMSIAVKEQQKNQKPTKSVW
EQRTSEMRKQNLLASREALYGDAAERWPTPYARPLRPDVKTHLDRPLVVDPQENRNNNTNKSRAPEALRPTAR
PRESARDPDARRAWPGSPERAPGREGPYGRESEPQQREHAPPREHAPWDADTERAKAGDAPRRHTHRPVAEGE
PRRHRARRRPGDEPDDRPERRPRPRDATRPARAADGEGDDGERKRRHRHGPPAHDDRERRHRRRKENQGSGVP
VSGPNLSTTRPIQQDLGRQDLPLAEDLDNMKNNKLATGEPASPHDSLGHSGLPPSPAKIGNSTNPGPALATNP
QNAASRRTPNNPGNPSNPGPPKTPENSLIVTNPSSTQPNSAKTARKPEHMAVEIPPACPPLNHTVVQVNKNAN
PDPLPKKEEEKKEEEEADPGEDGPKPMPPYSSMFILSTTNPLRRLCHYILNLRYFEMCILMVIAMSSIALAAE
DPVQPNAPRNNVLRYFDYVFTGVFTFEMVIKMIDLGLVLHQGAYFRDLWNILDFIVVSGALVAFAFTGNSKGK
DINTIKSLRVLRVLRPLKTIKRLPKLKAVFDCVVNSLKNVFNILIVYMLFMFIFAVVAVQLFKGKFFHCTDES
KEFERDCRGKYLLYEKNEVKARDREWKKYEFHYDNVLWALLTLFTVSTGEGWPQVLKHSVDATFENQGPSPGY
RMEMSIFYVVYFVVFPFFFVNIFVALIIITFQEQGDKMMEEYSLEKNERACIDFAISAKPLTRHMPQNKQSFQ
YRMWQFVVSPPFEYTIMAMIALNTIVLMMKFYGASVAYENALRVFNIVFTSLFSLECVLKVMAFGILNYFRDA
WNIFDFVTVLGSITDILVTEFGNNFINLSFLRLFRAARLIKLLRQGYTIRILLWTFVQSFKALPYVCLLIAML
FFIYAIIGMQVFGNIGIDGEDEDSDEDEFQITEHNNFRTFFQALMLLFRSATGEAWHNIMLSCLSGKPCDKNS

GILTADCGNEFAYFYFVSFIFLCSFLMLNLFVAVIMDNFEYLTRDSSILGPHHLDEYVRVWAEYDPAACGRIH
YKDMYSLLRVISPPLGLGKKCPHRVACKRLLRMDLPVADDNTVHFNSTLMALIRTALDIKIAKGGADKQQMDA
ELRKEMMAIWPNLSQKTLDLLVTPHKSTVVT
>rs260693369_ENSMUSP00000149242_c.5559:G>GTAGTT_p.D1854ins-fs
ENSMUST00000215756.2|Cacna1a-
211|ENSMUSG00000034656|+|chr8:g.85356459:G>GTAGTT
XRMYKQSMAQRARTMALYNPIPVRQNCLTVNRSLFLFSEDNVVRKYAKKITEWPPFEYMILATIIANCIVLAL
EQHLPDDDKTPMSERLDDTEPYFIGIFCFEAGIKIVALGFAFHKGSYLRNGWNVMDFVVVLTGILATVGTEFD
LRTLRAVRVLRPLKLVSGIPSLQVVLKSIMKAMIPLLQIGLLLFFAILIFAIIGLEFYMGKFHTTCFEEGTDD
IQGESPAPCGTEEPARTCPNGTKCQPYWEGPNNGITQFDNILFAVLTVFQCITMEGWTDLLYNSNDASGNTWN
WLYFIPLIIIGSFFMLNLVLGVLSGEFAKERERVENRRAFLKLRRQQQIERELNGYMEWISKAEEVILAEDET
DVEQRHPFDALRRATLKKSKTDLLNPEEAEDQLADIASVGSPFARASIKSAKLENSTFFHKKERRMRFYIRRM
VKTQAFYWTVLSLVALNTLCVAIVHYNQPEWLSDFLYYAEFIFLGLFMSEMFIKMYGLGTRPYFHSSFNCFDC
GVIIGSIFEVIWAVIKPGTSFGISVLRALRLLRIFKVTKYWASLRNLVVSLLNSMKSIISLLFLLFLFIVVFA
LLGMQLFGGQFNFDEGTPPTNFDTFPAAIMTVFQILTGEDWNEVMYDGIKSQGGVQGGMVFSIYFIVLTLFGN
YTLLNVFLAIAVDNLANAQELTKDEQEEEEAANQKLALQKAKEVAEVSPLSAANMSIAVKEQQKNQKPTKSVW
EQRTSEMRKQNLLASREALYGDAAERWPTPYARPLRPDVKTHLDRPLVVDPQENRNNNTNKSRAPEALRPTAR
PRESARDPDARRAWPGSPERAPGREGPYGRESEPQQREHAPPREHAPWDADTERAKAGDAPRRHTHRPVAEGE
PRRHRARRRPGDEPDDRPERRPRPRDATRPARAADGEGDDGERKRRHRHGPPAHDDRERRHRRRKENQGSGVP
VSGPNLSTTRPIQQDLGRQDLPLAEDLDNMKNNKLATGEPASPHDSLGHSGLPPSPAKIGNSTNPGPALATNP
QNAASRRTPNNPGNPSNPGPPKTPENSLIVTNPSSTQPNSAKTARKPEHMAVEIPPACPPLNHTVVQVNKNAN
PDPLPKKEEEKKEEEEADPGEDGPKPMPPYSSMFILSTTNPLRRLCHYILNLRYFEMCILMVIAMSSIALAAE
DPVQPNAPRNNVLRYFDYVFTGVFTFEMVIKMIDLGLVLHQGAYFRDLWNILDFIVVSGALVAFAFTGNSKGK
DINTIKSLRVLRVLRPLKTIKRLPKLKAVFDCVVNSLKNVFNILIVYMLFMFIFAVVAVQLFKGKFFHCTDES
KEFERDCRGKYLLYEKNEVKARDREWKKYEFHYDNVLWALLTLFTVSTGEGWPQVLKHSVDATFENQGPSPGY
RMEMSIFYVVYFVVFPFFFVNIFVALIIITFQEQGDKMMEEYSLEKNERACIDFAISAKPLTRHMPQNKQSFQ
YRMWQFVVSPPFEYTIMAMIALNTIVLMMKFYGASVAYENALRVFNIVFTSLFSLECVLKVMAFGILNYFRDA
WNIFDFVTVLGSITDILVTEFGNNFINLSFLRLFRAARLIKLLRQGYTIRILLWTFVQSFKALPYVCLLIAML
FFIYAIIGMQVFGNIGIDGEDEDSDEDEFQITEHNNFRTFFQALMLLFRSATGEAWHNIMLSCLSGKPCDKNS
GILTADCGNEFAYFYFVSFIFLCSFLMLNLFVAVIMDNFEYLTRDSSILGPHHLDEYVRVWAEYDPAACGRIH
YKDMYSLLRVISPPLGLGKKCPHRVACKRLLRMDLPVADDNTVHFNSTLMALIRTALDIKIAKGGADKQQMDA
ELRKEMMAIWPNLSQKTLDLLVTPHKSTVVT
>rs260693369_ENSMUSP00000158675_c.924:G>GTAGTT_p.D309ins-fs
ENSMUST00000238337.2|Cacna1a-
212|ENSMUSG00000034656|+|chr8:g.85356459:G>GTAGTT
MHLLRLSGQRGLTLQPNSPSSLPSSPFPLAKGNNFINLSFLRLFRAARLIKLLRQGYTIRILLWTFVQSFKVF
GNIGIDGEDEDSDEDEFQITEHNNFRTFFQALMLLFRSATGEAWHNIMLSCLSGKPCDKNSGILTADCGNEFA
YFYFVSFIFLCSFLMLNLFVAVIMDNFEYLTRDSSILGPHHLDEYVRVWAEYDPAACGRIHYKDMYSLLRVIS
PPLGLGKKCPHRVACKRLLRMDLPVADDNTVHFNSTLMALIRTALDIKIAKGGADKQQMDAELRKEMMAIWPN
LSQKTLDLLVTPHKSTVVT
>rs39142910_ENSMUSP00000158803_c.6529:A>T_p.D2177V
ENSMUST00000238701.2|Cacna1a-213|ENSMUSG00000034656|+|chr8:g.85363208:A>T
MPRASWGSDPMLSPLSHELLHSPRYPGTDCTLAGSSRTLHVPWSQQMSGSLEGPTLKRRGGVVDHRDVILAHQ
AHKMHSTPQARRKEWEMARFGDEMPGRYGAGGGGSGPAAGVVVGAAGGRGAGGSRQGGQPGAQRMYKQSMAQR
ARTMALYNPIPVRQNCLTVNRSLFLFSEDNVVRKYAKKITEWPPFEYMILATIIANCIVLALEQHLPDDDKTP
MSERLDDTEPYFIGIFCFEAGIKIVALGFAFHKGSYLRNGWNVMDFVVVLTGILATVGTEFDLRTLRAVRVLR
PLKLVSGIPSLQVVLKSIMKAMIPLLQIGLLLFFAILIFAIIGLEFYMGKFHTTCFEEGTDDIQGESPAPCGT
EEPARTCPNGTKCQPYWEGPNNGITQFDNILFAVLTVFQCITMEGWTDLLYNSNDASGNTWNWLYFIPLIIIG
SFFMLNLVLGVLSGEFAKERERVENRRAFLKLRRQQQIERELNGYMEWISKAEEVILAEDETDVEQRHPFDGA
LRRATLKKSKTDLLNPEEAEDQLADIASVGSPFARASIKSAKLENSTFFHKKERRMRFYIRRMVKTQAFYWTV
LSLVALNTLCVAIVHYNQPEWLSDFLYYAEFIFLGLFMSEMFIKMYGLGTRPYFHSSFNCFDCGVIIGSIFEV
IWAVIKPGTSFGISVLRALRLLRIFKVTKYWASLRNLVVSLLNSMKSIISLLFLLFLFIVVFALLGMQLFGGQ
FNFDEGTPPTNFDTFPAAIMTVFQILTGEDWNEVMYDGIKSQGGVQGGMVFSIYFIVLTLFGNYTLLNVFLAI
AVDNLANAQELTKDEQEEEEAANQKLALQKAKEVAEVSPLSAANMSIAVKEQQKNQKPTKSVWEQRTSEMRKQ
NLLASREALYGDAAERWPTPYARPLRPDVKTHLDRPLVVDPQENRNNNTNKSRAPEALRPTARPRESARDPDA
RRAWPGSPERAPGREGPYGRESEPQQREHAPPREHAPWDADTERAKAGDAPRRHTHRPVAEGEPRRHRARRRP
GDEPDDRPERRPRPRDATRPARAADGEGDDGERKRRHRHGPPAHDDRERRHRRRKENQGSGVPVSGPNLSTTR
PIQQDLGRQDLPLAEDLDNMKNNKLATGEPASPHDSLGHSGLPPSPAKIGNSTNPGPALATNPQNAASRRTPN

NPGNPSNPGPPKTPENSLIVTNPSSTQPNSAKTARKPEHMAVEIPPACPPLNHTVVQVNKNANPDPLPKKEEE
KKEEEEADPGEDGPKPMPPYSSMFILSTTNPLRRLCHYILNLRYFEMCILMVIAMSSIALAAEDPVQPNAPRN
NVLRYFDYVFTGVFTFEMVIKMIDLGLVLHQGAYFRDLWNILDFIVVSGALVAFAFTGNSKGKDINTIKSLRV
LRVLRPLKTIKRLPKLKAVFDCVVNSLKNVFNILIVYMLFMFIFAVVAVQLFKGKFFHCTDESKEFERDCRGK
YLLYEKNEVKARDREWKKYEFHYDNVLWALLTLFTVSTGEGWPQVLKHSVDATFENQGPSPGYRMEMSIFYVV
YFVVFPFFFVNIFVALIIITFQEQGDKMMEEYSLEKNERACIDFAISAKPLTRHMPQNKQSFQYRMWQFVVSP
PFEYTIMAMIALNTIVLMMKFYGASVAYENALRVFNIVFTSLFSLECVLKVMAFGILNYFRDAWNIFDFVTVL
GSITDILVTEFGNNFINLSFLRLFRAARLIKLLRQGYTIRILLWTFVQSFKALPYVCLLIAMLFFIYAIIGMQ
VFGNIGIDGEDEDSDEDEFQITEHNNFRTFFQALMLLFRSATGEAWHNIMLSCLSGKPCDKNSGILTADCGNE
FAYFYFVSFIFLCSFLMLNLFVAVIMDNFEYLTRDSSILGPHHLDEYVRVWAEYDPAACGRIHYKDMYSLLRV
ISPPLGLGKKCPHRVACKRLLRMDLPVADDNTVHFNSTLMALIRTALDIKIAKGGADKQQMDAELRKEMMAIW
PNLSQKTLDLLVTPHKSTDLTVGKIYAAMMIMEYYRQSKAKKLQAMREEQNRTPLMFQRMEPPSPTQEGGPSQ
NALPSTQLDPGGGLMAHEGGMKESPSWVTQRAQEMFQKTGTWSPERGPPIDMPNSQPNSQSVEMREMGTDGYS
DSEHYLPMEGQTRAASMPRLPAENQRRRGRPRGNDLSTISDTSPMKRSASVLGPKARRLVDYSLERVPPEENQ
RYHQRRRDRGHRTSERSLGRYTDVDTGLGTDLSMTTQSGDLPSKDRDQDRGRPKDRKHRPHHHHHHHHHHPPA
PDRDRYAQERPDTGRARAREQRWSRSPSEGREHTTHRQGSSSVSGSPAPSTSGTSTPRRGRRQLPQTPCTPRP
LVSYSPAPRRPAARRMAGPAAPPGGSPRGCRRAPRWPAHAPEGPRPRGADYTEPDSPREPPGGAHDPAPRSPR
TPRAAGCASPRHGRRLPNGYYAGHGAPRPRTARRGAHDAYSESEDDWC*
>rs39142910_ENSMUSP00000112436_c.6262:A>T_p.D2088V
ENSMUST00000121390.8|Cacna1a-201|ENSMUSG00000034656|+|chr8:g.85363208:A>T
MARFGDEMPGRYGAGGGGSGPAAGVVVGAAGGRGAGGSRQGGQPGAQRMYKQSMAQRARTMALYNPIPVRQNC
LTVNRSLFLFSEDNVVRKYAKKITEWPPFEYMILATIIANCIVLALEQHLPDDDKTPMSERLDDTEPYFIGIF
CFEAGIKIVALGFAFHKGSYLRNGWNVMDFVVVLTGILATVGTEFDLRTLRAVRVLRPLKLVSGIPSLQVVLK
SIMKAMIPLLQIGLLLFFAILIFAIIGLEFYMGKFHTTCFEEGTDDIQGESPAPCGTEEPARTCPNGTKCQPY
WEGPNNGITQFDNILFAVLTVFQCITMEGWTDLLYNSNDASGNTWNWLYFIPLIIIGSFFMLNLVLGVLSGEF
AKERERVENRRAFLKLRRQQQIERELNGYMEWISKAEEVILAEDETDVEQRHPFDGALRRATLKKSKTDLLNP
EEAEDQLADIASVGSPFARASIKSAKLENSTFFHKKERRMRFYIRRMVKTQAFYWTVLSLVALNTLCVAIVHY
NQPEWLSDFLYYAEFIFLGLFMSEMFIKMYGLGTRPYFHSSFNCFDCGVIIGSIFEVIWAVIKPGTSFGISVL
RALRLLRIFKVTKYWASLRNLVVSLLNSMKSIISLLFLLFLFIVVFALLGMQLFGGQFNFDEGTPPTNFDTFP
AAIMTVFQILTGEDWNEVMYDGIKSQGGVQGGMVFSIYFIVLTLFGNYTLLNVFLAIAVDNLANAQELTKDEQ
EEEEAANQKLALQKAKEVAEVSPLSAANMSIAVKEQQKNQKPTKSVWEQRTSEMRKQNLLASREALYGDAAER
WPTPYARPLRPDVKTHLDRPLVVDPQENRNNNTNKSRAPEALRPTARPRESARDPDARRAWPGSPERAPGREG
PYGRESEPQQREHAPPREHAPWDADTERAKAGDAPRRHTHRPVAEGEPRRHRARRRPGDEPDDRPERRPRPRD
ATRPARAADGEGDDGERKRRHRHGPPAHDDRERRHRRRKENQGSGVPVSGPNLSTTRPIQQDLGRQDLPLAED
LDNMKNNKLATGEPASPHDSLGHSGLPPSPAKIGNSTNPGPALATNPQNAASRRTPNNPGNPSNPGPPKTPEN
SLIVTNPSSTQPNSAKTARKPEHMAVEIPPACPPLNHTVVQVNKNANPDPLPKKEEEKKEEEEADPGEDGPKP
MPPYSSMFILSTTNPLRRLCHYILNLRYFEMCILMVIAMSSIALAAEDPVQPNAPRNNVLRYFDYVFTGVFTF
EMVIKMIDLGLVLHQGAYFRDLWNILDFIVVSGALVAFAFTGNSKGKDINTIKSLRVLRVLRPLKTIKRLPKL
KAVFDCVVNSLKNVFNILIVYMLFMFIFAVVAVQLFKGKFFHCTDESKEFERDCRGKYLLYEKNEVKARDREW
KKYEFHYDNVLWALLTLFTVSTGEGWPQVLKHSVDATFENQGPSPGYRMEMSIFYVVYFVVFPFFFVNIFVAL
IIITFQEQGDKMMEEYSLEKNERACIDFAISAKPLTRHMPQNKQSFQYRMWQFVVSPPFEYTIMAMIALNTIV
LMMKFYGASVAYENALRVFNIVFTSLFSLECVLKVMAFGILNYFRDAWNIFDFVTVLGSITDILVTEFGNNFI
NLSFLRLFRAARLIKLLRQGYTIRILLWTFVQSFKALPYVCLLIAMLFFIYAIIGMQVFGNIGIDGEDEDSDE
DEFQITEHNNFRTFFQALMLLFRSATGEAWHNIMLSCLSGKPCDKNSGILTADCGNEFAYFYFVSFIFLCSFL
MLNLFVAVIMDNFEYLTRDSSILGPHHLDEYVRVWAEYDPAACGRIHYKDMYSLLRVISPPLGLGKKCPHRVA
CKRLLRMDLPVADDNTVHFNSTLMALIRTALDIKIAKGGADKQQMDAELRKEMMAIWPNLSQKTLDLLVTPHK
STDLTVGKIYAAMMIMEYYRQSKAKKLQAMREEQNRTPLMFQRMEPPSPTQEGGPSQNALPSTQLDPGGGLMA
HEGGMKESPSWVTQRAQEMFQKTGTWSPERGPPIDMPNSQPNSQSVEMREMGTDGYSDSEHYLPMEGQTRAAS
MPRLPAENQRRRGRPRGNDLSTISDTSPMKRSASVLGPKARRLVDYSLERVPPEENQRYHQRRRDRGHRTSER
SLGRYTDVDTGLGTDLSMTTQSGDLPSKDRDQDRGRPKDRKHRPHHHHHHHHHHPPAPDRDRYAQERPDTGRA
RAREQRWSRSPSEGREHTTHRQGSSSVSGSPAPSTSGTSTPRRGRRQLPQTPCTPRPLVSYSPAPRRPAARRM
AGPAAPPGGSPRGCRRAPRWPAHAPEGPRPRGADYTEPDSPREPPGGAHDPAPRSPRTPRAAGCASPRHGRRL
PNGYYAGHGAPRPRTARRGAHDAYSESEDDWC*
>rs39142910_ENSMUSP00000114055_c.6121:A>T_p.D2041V
ENSMUST00000122053.2|Cacna1a-202|ENSMUSG00000034656|+|chr8:g.85363208:A>T
MARFGDEMPGRYGAGGGGSGPAAGVVVGAAGGRGAGGSRQGGQPGAQRMYKQSMAQRARTMALYNPIPVRQNC
LTVNRSLFLFSEDNVDDTEPYFIGIFCFEAGIKIVALGFAFHKGSYLRNGWNVMDFVVVLTGILATVGTEFDL
RTLRAVRVLRPLKLVSGIPSLQVVLKSIMKAMIPLLQIGLLLFFAILIFAIIGLEFYMGKFHTTCFEEGTDDI

QGESPAPCGTEEPARTCPNGTKCQPYWEGPNNGITQFDNILFAVLTVFQCITMEGWTDLLYNSNDASGNTWNW
LYFIPLIIIGSFFMLNLVLGVLSGEFAKERERVENRRAFLKLRRQQQIERELNGYMEWISKAEEVILAEDETD
VEQRHPFDGALRRATLKKSKTDLLNPEEAEDQLADIASVGSPFARASIKSAKLENSTFFHKKERRMRFYIRRM
VKTQAFYWTVLSLVALNTLCVAIVHYNQPEWLSDFLYYAEFIFLGLFMSEMFIKMYGLGTRPYFHSSFNCFDC
GVIIGSIFEVIWAVIKPGTSFGISVLRALRLLRIFKVTKYWASLRNLVVSLLNSMKSIISLLFLLFLFIVVFA
LLGMQLFGGQFNFDEGTPPTNFDTFPAAIMTVFQILTGEDWNEVMYDGIKSQGGVQGGMVFSIYFIVLTLFGN
YTLLNVFLAIAVDNLANAQELTKDEQEEEEAANQKLALQKAKEVAEVSPLSAANMSIAVKEQQKNQKPTKSVW
EQRTSEMRKQNLLASREALYGDAAERWPTPYARPLRPDVKTHLDRPLVVDPQENRNNNTNKSRAPEALRPTAR
PRESARDPDARRAWPGSPERAPGREGPYGRESEPQQREHAPPREHAPWDADTERAKAGDAPRRHTHRPVAEGE
PRRHRARRRPGDEPDDRPERRPRPRDATRPARAADGEGDDGERKRRHRHGPPAHDDRERRHRRRKENQGSGVP
VSGPNLSTTRPIQQDLGRQDLPLAEDLDNMKNNKLATGEPASPHDSLGHSGLPPSPAKIGNSTNPGPALATNP
QNAASRRTPNNPGNPSNPGPPKTPENSLIVTNPSSTQPNSAKTARKPEHMAVEIPPACPPLNHTVVQVNKNAN
PDPLPKKEEEKKEEEEADPGEDGPKPMPPYSSMFILSTTNPLRRLCHYILNLRYFEMCILMVIAMSSIALAAE
DPVQPNAPRNNVLRYFDYVFTGVFTFEMVIKMIDLGLVLHQGAYFRDLWNILDFIVVSGALVAFAFTGNSKGK
DINTIKSLRVLRVLRPLKTIKRLPKLKAVFDCVVNSLKNVFNILIVYMLFMFIFAVVAVQLFKGKFFHCTDES
KEFERDCRGKYLLYEKNEVKARDREWKKYEFHYDNVLWALLTLFTVSTGEGWPQVLKHSVDATFENQGPSPGY
RMEMSIFYVVYFVVFPFFFVNIFVALIIITFQEQGDKMMEEYSLEKNERACIDFAISAKPLTRHMPQNKQSFQ
YRMWQFVVSPPFEYTIMAMIALNTIVLMMKFYGASVAYENALRVFNIVFTSLFSLECVLKVMAFGILNYFRDA
WNIFDFVTVLGSITDILVTEFGNNFINLSFLRLFRAARLIKLLRQGYTIRILLWTFVQSFKALPYVCLLIAML
FFIYAIIGMQVFGNIGIDGEDEDSDEDEFQITEHNNFRTFFQALMLLFRSATGEAWHNIMLSCLSGKPCDKNS
GILTADCGNEFAYFYFVSFIFLCSFLMLNLFVAVIMDNFEYLTRDSSILGPHHLDEYVRVWAEYDPAACGRIH
YKDMYSLLRVISPPLGLGKKCPHRVACKRLLRMDLPVADDNTVHFNSTLMALIRTALDIKIAKGGADKQQMDA
ELRKEMMAIWPNLSQKTLDLLVTPHKSTDLTVGKIYAAMMIMEYYRQSKAKKLQAMREEQNRTPLMFQRMEPP
SPTQEGGPSQNALPSTQLDPGGGLMAHEGGMKESPSWVTQRAQEMFQKTGTWSPERGPPIDMPNSQPNSQSVE
MREMGTDGYSDSEHYLPMEGQTRAASMPRLPAENQRRRGRPRGNDLSTISDTSPMKRSASVLGPKARRLVDYS
LERVPPEENQRYHQRRRDRGHRTSERSLGRYTDVDTGLGTDLSMTTQSGDLPSKDRDQDRGRPKDRKHRPHHH
HHHHHHHPPAPDRDRYAQERPDTGRARAREQRWSRSPSEGREHTTHRQGSSSVSGSPAPSTSGTSTPRRGRRQ
LPQTPCTPRPLVSYSPAPRRPAARRMAGPAAPPGGSPRGCRRAPRWPAHAPEGPRPRGADYTEPDSPREPPGG
AHDPAPRSPRTPRAAGCASPRHGRRLPNGYYAGHGAPRPRTARRGAHDAYSESEDDWC*
>rs39142910_ENSMUSP00000149242_c.6121:A>T_p.D2041V
ENSMUST00000215756.2|Cacna1a-211|ENSMUSG00000034656|+|chr8:g.85363208:A>T
XRMYKQSMAQRARTMALYNPIPVRQNCLTVNRSLFLFSEDNVVRKYAKKITEWPPFEYMILATIIANCIVLAL
EQHLPDDDKTPMSERLDDTEPYFIGIFCFEAGIKIVALGFAFHKGSYLRNGWNVMDFVVVLTGILATVGTEFD
LRTLRAVRVLRPLKLVSGIPSLQVVLKSIMKAMIPLLQIGLLLFFAILIFAIIGLEFYMGKFHTTCFEEGTDD
IQGESPAPCGTEEPARTCPNGTKCQPYWEGPNNGITQFDNILFAVLTVFQCITMEGWTDLLYNSNDASGNTWN
WLYFIPLIIIGSFFMLNLVLGVLSGEFAKERERVENRRAFLKLRRQQQIERELNGYMEWISKAEEVILAEDET
DVEQRHPFDALRRATLKKSKTDLLNPEEAEDQLADIASVGSPFARASIKSAKLENSTFFHKKERRMRFYIRRM
VKTQAFYWTVLSLVALNTLCVAIVHYNQPEWLSDFLYYAEFIFLGLFMSEMFIKMYGLGTRPYFHSSFNCFDC
GVIIGSIFEVIWAVIKPGTSFGISVLRALRLLRIFKVTKYWASLRNLVVSLLNSMKSIISLLFLLFLFIVVFA
LLGMQLFGGQFNFDEGTPPTNFDTFPAAIMTVFQILTGEDWNEVMYDGIKSQGGVQGGMVFSIYFIVLTLFGN
YTLLNVFLAIAVDNLANAQELTKDEQEEEEAANQKLALQKAKEVAEVSPLSAANMSIAVKEQQKNQKPTKSVW
EQRTSEMRKQNLLASREALYGDAAERWPTPYARPLRPDVKTHLDRPLVVDPQENRNNNTNKSRAPEALRPTAR
PRESARDPDARRAWPGSPERAPGREGPYGRESEPQQREHAPPREHAPWDADTERAKAGDAPRRHTHRPVAEGE
PRRHRARRRPGDEPDDRPERRPRPRDATRPARAADGEGDDGERKRRHRHGPPAHDDRERRHRRRKENQGSGVP
VSGPNLSTTRPIQQDLGRQDLPLAEDLDNMKNNKLATGEPASPHDSLGHSGLPPSPAKIGNSTNPGPALATNP
QNAASRRTPNNPGNPSNPGPPKTPENSLIVTNPSSTQPNSAKTARKPEHMAVEIPPACPPLNHTVVQVNKNAN
PDPLPKKEEEKKEEEEADPGEDGPKPMPPYSSMFILSTTNPLRRLCHYILNLRYFEMCILMVIAMSSIALAAE
DPVQPNAPRNNVLRYFDYVFTGVFTFEMVIKMIDLGLVLHQGAYFRDLWNILDFIVVSGALVAFAFTGNSKGK
DINTIKSLRVLRVLRPLKTIKRLPKLKAVFDCVVNSLKNVFNILIVYMLFMFIFAVVAVQLFKGKFFHCTDES
KEFERDCRGKYLLYEKNEVKARDREWKKYEFHYDNVLWALLTLFTVSTGEGWPQVLKHSVDATFENQGPSPGY
RMEMSIFYVVYFVVFPFFFVNIFVALIIITFQEQGDKMMEEYSLEKNERACIDFAISAKPLTRHMPQNKQSFQ
YRMWQFVVSPPFEYTIMAMIALNTIVLMMKFYGASVAYENALRVFNIVFTSLFSLECVLKVMAFGILNYFRDA
WNIFDFVTVLGSITDILVTEFGNNFINLSFLRLFRAARLIKLLRQGYTIRILLWTFVQSFKALPYVCLLIAML
FFIYAIIGMQVFGNIGIDGEDEDSDEDEFQITEHNNFRTFFQALMLLFRSATGEAWHNIMLSCLSGKPCDKNS
GILTADCGNEFAYFYFVSFIFLCSFLMLNLFVAVIMDNFEYLTRDSSILGPHHLDEYVRVWAEYDPAACGRIH
YKDMYSLLRVISPPLGLGKKCPHRVACKRLLRMDLPVADDNTVHFNSTLMALIRTALDIKIAKGGADKQQMDA
ELRKEMMAIWPNLSQKTLDLLVTPHKSTDLTVGKIYAAMMIMEYYRQSKAKKLQAMREEQNRTPLMFQRMEPP
SPTQEGGPSQNALPSTQLDPGGGLMAHEGGMKESPSWVTQRAQEMFQKTGTWSPERGPPIDMPNSQPNSQSVE

MREMGTDGYSDSEHYLPMEGQTRAASMPRLPAENQRRRGRPRGNDLSTISDTSPMKRSASVLGPKARRLVDYS
LERVPPEENQRYHQRRRDRGHRTSERSLGRYTDVDTGLGTDLSMTTQSGDLPSKDRDQDRGRPKDRKHRPHHH
HHHHHHHPPAPDRDRYAQERPDTGRARAREQRWSRSPSEGREHTTHRQGSSSVSGSPAPSTSGTSTPRRGRRQ
LPQTPCTPRPLVSYSPAPRRPAARRMAGPAAPPGGSPRGCRRAPRWPAHAPEGPRPRGADYTEPDSPREPPGG
AHDPAPRSPRTPRAAGCASPRHGRRLPNGYYAGHGAPRPRTARRGAHDAYSESEDDWC*
>rs39142910_ENSMUSP00000158675_c.1486:A>T_p.D496V
ENSMUST00000238337.2|Cacna1a-212|ENSMUSG00000034656|+|chr8:g.85363208:A>T
MHLLRLSGQRGLTLQPNSPSSLPSSPFPLAKGNNFINLSFLRLFRAARLIKLLRQGYTIRILLWTFVQSFKVF
GNIGIDGEDEDSDEDEFQITEHNNFRTFFQALMLLFRSATGEAWHNIMLSCLSGKPCDKNSGILTADCGNEFA
YFYFVSFIFLCSFLMLNLFVAVIMDNFEYLTRDSSILGPHHLDEYVRVWAEYDPAACGRIHYKDMYSLLRVIS
PPLGLGKKCPHRVACKRLLRMDLPVADDNTVHFNSTLMALIRTALDIKIAKGGADKQQMDAELRKEMMAIWPN
LSQKTLDLLVTPHKSTDLTVGKIYAAMMIMEYYRQSKAKKLQAMREEQNRTPLMFQRMEPPSPTQEGGPSQNA
LPSTQLDPGGGLMAHEGGMKESPSWVTQRAQEMFQKTGTWSPERGPPIDMPNSQPNSQSVEMREMGTDGYSDS
EHYLPMEGQTRAASMPRLPAENQRRRGRPRGNDLSTISDTSPMKRSASVLGPKARRLVDYSLERVPPEENQRY
HQRRRDRGHRTSERSLGRYTDVDTGLGTDLSMTTQSGDLPSKDRDQDRGRPKDRKHRPHHHHHHHHHHPPAPD
RDRYAQERPDTGRARAREQRWSRSPSEGREHTTHRQGSSSVSGSPAPSTSGTSTPRRGRRQLPQTPCTPRPLV
SYSPAPRRPAARRMAGPAAPPGGSPRGCRRAPRWPAHAPEGPRPRGADYTEPDSPREPPGGAHDPAPRSPRTP
RAAGCASPRHGRRLPNGYYAGHGAPRPRTARRGAHDAYSESEDDWC*
>ENSMUSP00000158803_c.6586:GC>AA_p.R2196Q ENSMUST00000238701.2|Cacna1a-
213|ENSMUSG00000034656|+|chr8:g.85363265:GC>AA
MPRASWGSDPMLSPLSHELLHSPRYPGTDCTLAGSSRTLHVPWSQQMSGSLEGPTLKRRGGVVDHRDVILAHQ
AHKMHSTPQARRKEWEMARFGDEMPGRYGAGGGGSGPAAGVVVGAAGGRGAGGSRQGGQPGAQRMYKQSMAQR
ARTMALYNPIPVRQNCLTVNRSLFLFSEDNVVRKYAKKITEWPPFEYMILATIIANCIVLALEQHLPDDDKTP
MSERLDDTEPYFIGIFCFEAGIKIVALGFAFHKGSYLRNGWNVMDFVVVLTGILATVGTEFDLRTLRAVRVLR
PLKLVSGIPSLQVVLKSIMKAMIPLLQIGLLLFFAILIFAIIGLEFYMGKFHTTCFEEGTDDIQGESPAPCGT
EEPARTCPNGTKCQPYWEGPNNGITQFDNILFAVLTVFQCITMEGWTDLLYNSNDASGNTWNWLYFIPLIIIG
SFFMLNLVLGVLSGEFAKERERVENRRAFLKLRRQQQIERELNGYMEWISKAEEVILAEDETDVEQRHPFDGA
LRRATLKKSKTDLLNPEEAEDQLADIASVGSPFARASIKSAKLENSTFFHKKERRMRFYIRRMVKTQAFYWTV
LSLVALNTLCVAIVHYNQPEWLSDFLYYAEFIFLGLFMSEMFIKMYGLGTRPYFHSSFNCFDCGVIIGSIFEV
IWAVIKPGTSFGISVLRALRLLRIFKVTKYWASLRNLVVSLLNSMKSIISLLFLLFLFIVVFALLGMQLFGGQ
FNFDEGTPPTNFDTFPAAIMTVFQILTGEDWNEVMYDGIKSQGGVQGGMVFSIYFIVLTLFGNYTLLNVFLAI
AVDNLANAQELTKDEQEEEEAANQKLALQKAKEVAEVSPLSAANMSIAVKEQQKNQKPTKSVWEQRTSEMRKQ
NLLASREALYGDAAERWPTPYARPLRPDVKTHLDRPLVVDPQENRNNNTNKSRAPEALRPTARPRESARDPDA
RRAWPGSPERAPGREGPYGRESEPQQREHAPPREHAPWDADTERAKAGDAPRRHTHRPVAEGEPRRHRARRRP
GDEPDDRPERRPRPRDATRPARAADGEGDDGERKRRHRHGPPAHDDRERRHRRRKENQGSGVPVSGPNLSTTR
PIQQDLGRQDLPLAEDLDNMKNNKLATGEPASPHDSLGHSGLPPSPAKIGNSTNPGPALATNPQNAASRRTPN
NPGNPSNPGPPKTPENSLIVTNPSSTQPNSAKTARKPEHMAVEIPPACPPLNHTVVQVNKNANPDPLPKKEEE
KKEEEEADPGEDGPKPMPPYSSMFILSTTNPLRRLCHYILNLRYFEMCILMVIAMSSIALAAEDPVQPNAPRN
NVLRYFDYVFTGVFTFEMVIKMIDLGLVLHQGAYFRDLWNILDFIVVSGALVAFAFTGNSKGKDINTIKSLRV
LRVLRPLKTIKRLPKLKAVFDCVVNSLKNVFNILIVYMLFMFIFAVVAVQLFKGKFFHCTDESKEFERDCRGK
YLLYEKNEVKARDREWKKYEFHYDNVLWALLTLFTVSTGEGWPQVLKHSVDATFENQGPSPGYRMEMSIFYVV
YFVVFPFFFVNIFVALIIITFQEQGDKMMEEYSLEKNERACIDFAISAKPLTRHMPQNKQSFQYRMWQFVVSP
PFEYTIMAMIALNTIVLMMKFYGASVAYENALRVFNIVFTSLFSLECVLKVMAFGILNYFRDAWNIFDFVTVL
GSITDILVTEFGNNFINLSFLRLFRAARLIKLLRQGYTIRILLWTFVQSFKALPYVCLLIAMLFFIYAIIGMQ
VFGNIGIDGEDEDSDEDEFQITEHNNFRTFFQALMLLFRSATGEAWHNIMLSCLSGKPCDKNSGILTADCGNE
FAYFYFVSFIFLCSFLMLNLFVAVIMDNFEYLTRDSSILGPHHLDEYVRVWAEYDPAACGRIHYKDMYSLLRV
ISPPLGLGKKCPHRVACKRLLRMDLPVADDNTVHFNSTLMALIRTALDIKIAKGGADKQQMDAELRKEMMAIW
PNLSQKTLDLLVTPHKSTDLTVGKIYAAMMIMEYYRQSKAKKLQAMREEQNRTPLMFQRMEPPSPTQEGGPSQ
NALPSTQLDPGGGLMAHEGGMKESPSWVTQRAQEMFQKTGTWSPERGPPIDMPNSQPNSQSVEMREMGTDGYS
DSEHYLPMEGQTRAASMPRLPAENQRRRGRPRGNDLSTISDTSPMKRSASVLGPKARRLDDYSLERVPPEENQ
RYHQRQPGPWPPHL*ALSGPIH*CGHRFGDRSEHDHPIG*PALQRSGPGPGPTQGPEASATPPPPPPSSSSPG
PRPGSLRTGAAGHRPGAGPGAALVPLAQRRSGAHDTQTGQ*FRQWKPSPLYVRHQHAAARPPPAPPDPLHPAA
ACVLLARSAQACGAQDGGSRSAPWRLAAGLPPCAALACACA*GPAAARCRLHRARQPARAARGRARSRAAVAQ
DSARRGLRVTAPRPQAAQWLLRGARRAAAAHSPQGRARRVQRERGRLVL
>ENSMUSP00000112436_c.6319:GC>AA_p.R2107Q ENSMUST00000121390.8|Cacna1a-
201|ENSMUSG00000034656|+|chr8:g.85363265:GC>AA
MARFGDEMPGRYGAGGGGSGPAAGVVVGAAGGRGAGGSRQGGQPGAQRMYKQSMAQRARTMALYNPIPVRQNC
LTVNRSLFLFSEDNVVRKYAKKITEWPPFEYMILATIIANCIVLALEQHLPDDDKTPMSERLDDTEPYFIGIF

CFEAGIKIVALGFAFHKGSYLRNGWNVMDFVVVLTGILATVGTEFDLRTLRAVRVLRPLKLVSGIPSLQVVLK
SIMKAMIPLLQIGLLLFFAILIFAIIGLEFYMGKFHTTCFEEGTDDIQGESPAPCGTEEPARTCPNGTKCQPY
WEGPNNGITQFDNILFAVLTVFQCITMEGWTDLLYNSNDASGNTWNWLYFIPLIIIGSFFMLNLVLGVLSGEF
AKERERVENRRAFLKLRRQQQIERELNGYMEWISKAEEVILAEDETDVEQRHPFDGALRRATLKKSKTDLLNP
EEAEDQLADIASVGSPFARASIKSAKLENSTFFHKKERRMRFYIRRMVKTQAFYWTVLSLVALNTLCVAIVHY
NQPEWLSDFLYYAEFIFLGLFMSEMFIKMYGLGTRPYFHSSFNCFDCGVIIGSIFEVIWAVIKPGTSFGISVL
RALRLLRIFKVTKYWASLRNLVVSLLNSMKSIISLLFLLFLFIVVFALLGMQLFGGQFNFDEGTPPTNFDTFP
AAIMTVFQILTGEDWNEVMYDGIKSQGGVQGGMVFSIYFIVLTLFGNYTLLNVFLAIAVDNLANAQELTKDEQ
EEEEAANQKLALQKAKEVAEVSPLSAANMSIAVKEQQKNQKPTKSVWEQRTSEMRKQNLLASREALYGDAAER
WPTPYARPLRPDVKTHLDRPLVVDPQENRNNNTNKSRAPEALRPTARPRESARDPDARRAWPGSPERAPGREG
PYGRESEPQQREHAPPREHAPWDADTERAKAGDAPRRHTHRPVAEGEPRRHRARRRPGDEPDDRPERRPRPRD
ATRPARAADGEGDDGERKRRHRHGPPAHDDRERRHRRRKENQGSGVPVSGPNLSTTRPIQQDLGRQDLPLAED
LDNMKNNKLATGEPASPHDSLGHSGLPPSPAKIGNSTNPGPALATNPQNAASRRTPNNPGNPSNPGPPKTPEN
SLIVTNPSSTQPNSAKTARKPEHMAVEIPPACPPLNHTVVQVNKNANPDPLPKKEEEKKEEEEADPGEDGPKP
MPPYSSMFILSTTNPLRRLCHYILNLRYFEMCILMVIAMSSIALAAEDPVQPNAPRNNVLRYFDYVFTGVFTF
EMVIKMIDLGLVLHQGAYFRDLWNILDFIVVSGALVAFAFTGNSKGKDINTIKSLRVLRVLRPLKTIKRLPKL
KAVFDCVVNSLKNVFNILIVYMLFMFIFAVVAVQLFKGKFFHCTDESKEFERDCRGKYLLYEKNEVKARDREW
KKYEFHYDNVLWALLTLFTVSTGEGWPQVLKHSVDATFENQGPSPGYRMEMSIFYVVYFVVFPFFFVNIFVAL
IIITFQEQGDKMMEEYSLEKNERACIDFAISAKPLTRHMPQNKQSFQYRMWQFVVSPPFEYTIMAMIALNTIV
LMMKFYGASVAYENALRVFNIVFTSLFSLECVLKVMAFGILNYFRDAWNIFDFVTVLGSITDILVTEFGNNFI
NLSFLRLFRAARLIKLLRQGYTIRILLWTFVQSFKALPYVCLLIAMLFFIYAIIGMQVFGNIGIDGEDEDSDE
DEFQITEHNNFRTFFQALMLLFRSATGEAWHNIMLSCLSGKPCDKNSGILTADCGNEFAYFYFVSFIFLCSFL
MLNLFVAVIMDNFEYLTRDSSILGPHHLDEYVRVWAEYDPAACGRIHYKDMYSLLRVISPPLGLGKKCPHRVA
CKRLLRMDLPVADDNTVHFNSTLMALIRTALDIKIAKGGADKQQMDAELRKEMMAIWPNLSQKTLDLLVTPHK
STDLTVGKIYAAMMIMEYYRQSKAKKLQAMREEQNRTPLMFQRMEPPSPTQEGGPSQNALPSTQLDPGGGLMA
HEGGMKESPSWVTQRAQEMFQKTGTWSPERGPPIDMPNSQPNSQSVEMREMGTDGYSDSEHYLPMEGQTRAAS
MPRLPAENQRRRGRPRGNDLSTISDTSPMKRSASVLGPKARRLDDYSLERVPPEENQRYHQRQPGPWPPHL*A
LSGPIH*CGHRFGDRSEHDHPIG*PALQRSGPGPGPTQGPEASATPPPPPPSSSSPGPRPGSLRTGAAGHRPG
AGPGAALVPLAQRRSGAHDTQTGQ*FRQWKPSPLYVRHQHAAARPPPAPPDPLHPAAACVLLARSAQACGAQD
GGSRSAPWRLAAGLPPCAALACACA*GPAAARCRLHRARQPARAARGRARSRAAVAQDSARRGLRVTAPRPQA
AQWLLRGARRAAAAHSPQGRARRVQRERGRLVL
>ENSMUSP00000114055_c.6178:GC>AA_p.R2060Q ENSMUST00000122053.2|Cacna1a-
202|ENSMUSG00000034656|+|chr8:g.85363265:GC>AA
MARFGDEMPGRYGAGGGGSGPAAGVVVGAAGGRGAGGSRQGGQPGAQRMYKQSMAQRARTMALYNPIPVRQNC
LTVNRSLFLFSEDNVDDTEPYFIGIFCFEAGIKIVALGFAFHKGSYLRNGWNVMDFVVVLTGILATVGTEFDL
RTLRAVRVLRPLKLVSGIPSLQVVLKSIMKAMIPLLQIGLLLFFAILIFAIIGLEFYMGKFHTTCFEEGTDDI
QGESPAPCGTEEPARTCPNGTKCQPYWEGPNNGITQFDNILFAVLTVFQCITMEGWTDLLYNSNDASGNTWNW
LYFIPLIIIGSFFMLNLVLGVLSGEFAKERERVENRRAFLKLRRQQQIERELNGYMEWISKAEEVILAEDETD
VEQRHPFDGALRRATLKKSKTDLLNPEEAEDQLADIASVGSPFARASIKSAKLENSTFFHKKERRMRFYIRRM
VKTQAFYWTVLSLVALNTLCVAIVHYNQPEWLSDFLYYAEFIFLGLFMSEMFIKMYGLGTRPYFHSSFNCFDC
GVIIGSIFEVIWAVIKPGTSFGISVLRALRLLRIFKVTKYWASLRNLVVSLLNSMKSIISLLFLLFLFIVVFA
LLGMQLFGGQFNFDEGTPPTNFDTFPAAIMTVFQILTGEDWNEVMYDGIKSQGGVQGGMVFSIYFIVLTLFGN
YTLLNVFLAIAVDNLANAQELTKDEQEEEEAANQKLALQKAKEVAEVSPLSAANMSIAVKEQQKNQKPTKSVW
EQRTSEMRKQNLLASREALYGDAAERWPTPYARPLRPDVKTHLDRPLVVDPQENRNNNTNKSRAPEALRPTAR
PRESARDPDARRAWPGSPERAPGREGPYGRESEPQQREHAPPREHAPWDADTERAKAGDAPRRHTHRPVAEGE
PRRHRARRRPGDEPDDRPERRPRPRDATRPARAADGEGDDGERKRRHRHGPPAHDDRERRHRRRKENQGSGVP
VSGPNLSTTRPIQQDLGRQDLPLAEDLDNMKNNKLATGEPASPHDSLGHSGLPPSPAKIGNSTNPGPALATNP
QNAASRRTPNNPGNPSNPGPPKTPENSLIVTNPSSTQPNSAKTARKPEHMAVEIPPACPPLNHTVVQVNKNAN
PDPLPKKEEEKKEEEEADPGEDGPKPMPPYSSMFILSTTNPLRRLCHYILNLRYFEMCILMVIAMSSIALAAE
DPVQPNAPRNNVLRYFDYVFTGVFTFEMVIKMIDLGLVLHQGAYFRDLWNILDFIVVSGALVAFAFTGNSKGK
DINTIKSLRVLRVLRPLKTIKRLPKLKAVFDCVVNSLKNVFNILIVYMLFMFIFAVVAVQLFKGKFFHCTDES
KEFERDCRGKYLLYEKNEVKARDREWKKYEFHYDNVLWALLTLFTVSTGEGWPQVLKHSVDATFENQGPSPGY
RMEMSIFYVVYFVVFPFFFVNIFVALIIITFQEQGDKMMEEYSLEKNERACIDFAISAKPLTRHMPQNKQSFQ
YRMWQFVVSPPFEYTIMAMIALNTIVLMMKFYGASVAYENALRVFNIVFTSLFSLECVLKVMAFGILNYFRDA
WNIFDFVTVLGSITDILVTEFGNNFINLSFLRLFRAARLIKLLRQGYTIRILLWTFVQSFKALPYVCLLIAML
FFIYAIIGMQVFGNIGIDGEDEDSDEDEFQITEHNNFRTFFQALMLLFRSATGEAWHNIMLSCLSGKPCDKNS
GILTADCGNEFAYFYFVSFIFLCSFLMLNLFVAVIMDNFEYLTRDSSILGPHHLDEYVRVWAEYDPAACGRIH
YKDMYSLLRVISPPLGLGKKCPHRVACKRLLRMDLPVADDNTVHFNSTLMALIRTALDIKIAKGGADKQQMDA

ELRKEMMAIWPNLSQKTLDLLVTPHKSTDLTVGKIYAAMMIMEYYRQSKAKKLQAMREEQNRTPLMFQRMEPP
SPTQEGGPSQNALPSTQLDPGGGLMAHEGGMKESPSWVTQRAQEMFQKTGTWSPERGPPIDMPNSQPNSQSVE
MREMGTDGYSDSEHYLPMEGQTRAASMPRLPAENQRRRGRPRGNDLSTISDTSPMKRSASVLGPKARRLDDYS
LERVPPEENQRYHQRQPGPWPPHL*ALSGPIH*CGHRFGDRSEHDHPIG*PALQRSGPGPGPTQGPEASATPP
PPPPSSSSPGPRPGSLRTGAAGHRPGAGPGAALVPLAQRRSGAHDTQTGQ*FRQWKPSPLYVRHQHAAARPPP
APPDPLHPAAACVLLARSAQACGAQDGGSRSAPWRLAAGLPPCAALACACA*GPAAARCRLHRARQPARAARG
RARSRAAVAQDSARRGLRVTAPRPQAAQWLLRGARRAAAAHSPQGRARRVQRERGRLVL
>ENSMUSP00000149242_c.6178:GC>AA_p.R2060Q ENSMUST00000215756.2|Cacna1a-211|ENSMUSG00000034656|+|chr8:g.85363265:GC>AA
XRMYKQSMAQRARTMALYNPIPVRQNCLTVNRSLFLFSEDNVVRKYAKKITEWPPFEYMILATIIANCIVLAL
EQHLPDDDKTPMSERLDDTEPYFIGIFCFEAGIKIVALGFAFHKGSYLRNGWNVMDFVVVLTGILATVGTEFD
LRTLRAVRVLRPLKLVSGIPSLQVVLKSIMKAMIPLLQIGLLLFFAILIFAIIGLEFYMGKFHTTCFEEGTDD
IQGESPAPCGTEEPARTCPNGTKCQPYWEGPNNGITQFDNILFAVLTVFQCITMEGWTDLLYNSNDASGNTWN
WLYFIPLIIIGSFFMLNLVLGVLSGEFAKERERVENRRAFLKLRRQQQIERELNGYMEWISKAEEVILAEDET
DVEQRHPFDALRRATLKKSKTDLLNPEEAEDQLADIASVGSPFARASIKSAKLENSTFFHKKERRMRFYIRRM
VKTQAFYWTVLSLVALNTLCVAIVHYNQPEWLSDFLYYAEFIFLGLFMSEMFIKMYGLGTRPYFHSSFNCFDC
GVIIGSIFEVIWAVIKPGTSFGISVLRALRLLRIFKVTKYWASLRNLVVSLLNSMKSIISLLFLLFLFIVVFA
LLGMQLFGGQFNFDEGTPPTNFDTFPAAIMTVFQILTGEDWNEVMYDGIKSQGGVQGGMVFSIYFIVLTLFGN
YTLLNVFLAIAVDNLANAQELTKDEQEEEEAANQKLALQKAKEVAEVSPLSAANMSIAVKEQQKNQKPTKSVW
EQRTSEMRKQNLLASREALYGDAAERWPTPYARPLRPDVKTHLDRPLVVDPQENRNNNTNKSRAPEALRPTAR
PRESARDPDARRAWPGSPERAPGREGPYGRESEPQQREHAPPREHAPWDADTERAKAGDAPRRHTHRPVAEGE
PRRHRARRRPGDEPDDRPERRPRPRDATRPARAADGEGDDGERKRRHRHGPPAHDDRERRHRRRKENQGSGVP
VSGPNLSTTRPIQQDLGRQDLPLAEDLDNMKNNKLATGEPASPHDSLGHSGLPPSPAKIGNSTNPGPALATNP
QNAASRRTPNNPGNPSNPGPPKTPENSLIVTNPSSTQPNSAKTARKPEHMAVEIPPACPPLNHTVVQVNKNAN
PDPLPKKEEEKKEEEEADPGEDGPKPMPPYSSMFILSTTNPLRRLCHYILNLRYFEMCILMVIAMSSIALAAE
DPVQPNAPRNNVLRYFDYVFTGVFTFEMVIKMIDLGLVLHQGAYFRDLWNILDFIVVSGALVAFAFTGNSKGK
DINTIKSLRVLRVLRPLKTIKRLPKLKAVFDCVVNSLKNVFNILIVYMLFMFIFAVVAVQLFKGKFFHCTDES
KEFERDCRGKYLLYEKNEVKARDREWKKYEFHYDNVLWALLTLFTVSTGEGWPQVLKHSVDATFENQGPSPGY
RMEMSIFYVVYFVVFPFFFVNIFVALIIITFQEQGDKMMEEYSLEKNERACIDFAISAKPLTRHMPQNKQSFQ
YRMWQFVVSPPFEYTIMAMIALNTIVLMMKFYGASVAYENALRVFNIVFTSLFSLECVLKVMAFGILNYFRDA
WNIFDFVTVLGSITDILVTEFGNNFINLSFLRLFRAARLIKLLRQGYTIRILLWTFVQSFKALPYVCLLIAML
FFIYAIIGMQVFGNIGIDGEDEDSDEDEFQITEHNNFRTFFQALMLLFRSATGEAWHNIMLSCLSGKPCDKNS
GILTADCGNEFAYFYFVSFIFLCSFLMLNLFVAVIMDNFEYLTRDSSILGPHHLDEYVRVWAEYDPAACGRIH
YKDMYSLLRVISPPLGLGKKCPHRVACKRLLRMDLPVADDNTVHFNSTLMALIRTALDIKIAKGGADKQQMDA
ELRKEMMAIWPNLSQKTLDLLVTPHKSTDLTVGKIYAAMMIMEYYRQSKAKKLQAMREEQNRTPLMFQRMEPP
SPTQEGGPSQNALPSTQLDPGGGLMAHEGGMKESPSWVTQRAQEMFQKTGTWSPERGPPIDMPNSQPNSQSVE
MREMGTDGYSDSEHYLPMEGQTRAASMPRLPAENQRRRGRPRGNDLSTISDTSPMKRSASVLGPKARRLDDYS
LERVPPEENQRYHQRQPGPWPPHL*ALSGPIH*CGHRFGDRSEHDHPIG*PALQRSGPGPGPTQGPEASATPP
PPPPSSSSPGPRPGSLRTGAAGHRPGAGPGAALVPLAQRRSGAHDTQTGQ*FRQWKPSPLYVRHQHAAARPPP
APPDPLHPAAACVLLARSAQACGAQDGGSRSAPWRLAAGLPPCAALACACA*GPAAARCRLHRARQPARAARG
RARSRAAVAQDSARRGLRVTAPRPQAAQWLLRGARRAAAAHSPQGRARRVQRERGRLVL
>ENSMUSP00000158675_c.1543:GC>AA_p.R515Q ENSMUST00000238337.2|Cacna1a-212|ENSMUSG00000034656|+|chr8:g.85363265:GC>AA
MHLLRLSGQRGLTLQPNSPSSLPSSPFPLAKGNNFINLSFLRLFRAARLIKLLRQGYTIRILLWTFVQSFKVF
GNIGIDGEDEDSDEDEFQITEHNNFRTFFQALMLLFRSATGEAWHNIMLSCLSGKPCDKNSGILTADCGNEFA
YFYFVSFIFLCSFLMLNLFVAVIMDNFEYLTRDSSILGPHHLDEYVRVWAEYDPAACGRIHYKDMYSLLRVIS
PPLGLGKKCPHRVACKRLLRMDLPVADDNTVHFNSTLMALIRTALDIKIAKGGADKQQMDAELRKEMMAIWPN
LSQKTLDLLVTPHKSTDLTVGKIYAAMMIMEYYRQSKAKKLQAMREEQNRTPLMFQRMEPPSPTQEGGPSQNA
LPSTQLDPGGGLMAHEGGMKESPSWVTQRAQEMFQKTGTWSPERGPPIDMPNSQPNSQSVEMREMGTDGYSDS
EHYLPMEGQTRAASMPRLPAENQRRRGRPRGNDLSTISDTSPMKRSASVLGPKARRLDDYSLERVPPEENQRY
HQRQPGPWPPHL*ALSGPIH*CGHRFGDRSEHDHPIG*PALQRSGPGPGPTQGPEASATPPPPPPSSSSPGPR
PGSLRTGAAGHRPGAGPGAALVPLAQRRSGAHDTQTGQ*FRQWKPSPLYVRHQHAAARPPPAPPDPLHPAAAC
VLLARSAQACGAQDGGSRSAPWRLAAGLPPCAALACACA*GPAAARCRLHRARQPARAARGRARSRAAVAQDS
ARRGLRVTAPRPQAAQWLLRGARRAAAAHSPQGRARRVQRERGRLVL
>rs240959951_ENSMUSP00000158803_c.6595:G>C_p.R2199P
ENSMUST00000238701.2|Cacna1a-213|ENSMUSG00000034656|+|chr8:g.85363274:G>C
MPRASWGSDPMLSPLSHELLHSPRYPGTDCTLAGSSRTLHVPWSQQMSGSLEGPTLKRRGGVVDHRDVILAHQ
AHKMHSTPQARRKEWEMARFGDEMPGRYGAGGGGSGPAAGVVVGAAGGRGAGGSRQGGQPGAQRMYKQSMAQR

ARTMALYNPIPVRQNCLTVNRSLFLFSEDNVVRKYAKKITEWPPFEYMILATIIANCIVLALEQHLPDDDKTP
MSERLDDTEPYFIGIFCFEAGIKIVALGFAFHKGSYLRNGWNVMDFVVVLTGILATVGTEFDLRTLRAVRVLR
PLKLVSGIPSLQVVLKSIMKAMIPLLQIGLLLFFAILIFAIIGLEFYMGKFHTTCFEEGTDDIQGESPAPCGT
EEPARTCPNGTKCQPYWEGPNNGITQFDNILFAVLTVFQCITMEGWTDLLYNSNDASGNTWNWLYFIPLIIIG
SFFMLNLVLGVLSGEFAKERERVENRRAFLKLRRQQQIERELNGYMEWISKAEEVILAEDETDVEQRHPFDGA
LRRATLKKSKTDLLNPEEAEDQLADIASVGSPFARASIKSAKLENSTFFHKKERRMRFYIRRMVKTQAFYWTV
LSLVALNTLCVAIVHYNQPEWLSDFLYYAEFIFLGLFMSEMFIKMYGLGTRPYFHSSFNCFDCGVIIGSIFEV
IWAVIKPGTSFGISVLRALRLLRIFKVTKYWASLRNLVVSLLNSMKSIISLLFLLFLFIVVFALLGMQLFGGQ
FNFDEGTPPTNFDTFPAAIMTVFQILTGEDWNEVMYDGIKSQGGVQGGMVFSIYFIVLTLFGNYTLLNVFLAI
AVDNLANAQELTKDEQEEEEAANQKLALQKAKEVAEVSPLSAANMSIAVKEQQKNQKPTKSVWEQRTSEMRKQ
NLLASREALYGDAAERWPTPYARPLRPDVKTHLDRPLVVDPQENRNNNTNKSRAPEALRPTARPRESARDPDA
RRAWPGSPERAPGREGPYGRESEPQQREHAPPREHAPWDADTERAKAGDAPRRHTHRPVAEGEPRRHRARRRP
GDEPDDRPERRPRPRDATRPARAADGEGDDGERKRRHRHGPPAHDDRERRHRRRKENQGSGVPVSGPNLSTTR
PIQQDLGRQDLPLAEDLDNMKNNKLATGEPASPHDSLGHSGLPPSPAKIGNSTNPGPALATNPQNAASRRTPN
NPGNPSNPGPPKTPENSLIVTNPSSTQPNSAKTARKPEHMAVEIPPACPPLNHTVVQVNKNANPDPLPKKEEE
KKEEEEADPGEDGPKPMPPYSSMFILSTTNPLRRLCHYILNLRYFEMCILMVIAMSSIALAAEDPVQPNAPRN
NVLRYFDYVFTGVFTFEMVIKMIDLGLVLHQGAYFRDLWNILDFIVVSGALVAFAFTGNSKGKDINTIKSLRV
LRVLRPLKTIKRLPKLKAVFDCVVNSLKNVFNILIVYMLFMFIFAVVAVQLFKGKFFHCTDESKEFERDCRGK
YLLYEKNEVKARDREWKKYEFHYDNVLWALLTLFTVSTGEGWPQVLKHSVDATFENQGPSPGYRMEMSIFYVV
YFVVFPFFFVNIFVALIIITFQEQGDKMMEEYSLEKNERACIDFAISAKPLTRHMPQNKQSFQYRMWQFVVSP
PFEYTIMAMIALNTIVLMMKFYGASVAYENALRVFNIVFTSLFSLECVLKVMAFGILNYFRDAWNIFDFVTVL
GSITDILVTEFGNNFINLSFLRLFRAARLIKLLRQGYTIRILLWTFVQSFKALPYVCLLIAMLFFIYAIIGMQ
VFGNIGIDGEDEDSDEDEFQITEHNNFRTFFQALMLLFRSATGEAWHNIMLSCLSGKPCDKNSGILTADCGNE
FAYFYFVSFIFLCSFLMLNLFVAVIMDNFEYLTRDSSILGPHHLDEYVRVWAEYDPAACGRIHYKDMYSLLRV
ISPPLGLGKKCPHRVACKRLLRMDLPVADDNTVHFNSTLMALIRTALDIKIAKGGADKQQMDAELRKEMMAIW
PNLSQKTLDLLVTPHKSTDLTVGKIYAAMMIMEYYRQSKAKKLQAMREEQNRTPLMFQRMEPPSPTQEGGPSQ
NALPSTQLDPGGGLMAHEGGMKESPSWVTQRAQEMFQKTGTWSPERGPPIDMPNSQPNSQSVEMREMGTDGYS
DSEHYLPMEGQTRAASMPRLPAENQRRRGRPRGNDLSTISDTSPMKRSASVLGPKARRLDDYSLERVPPEENQ
RYHQRRRDPGHRTSERSLGRYTDVDTGLGTDLSMTTQSGDLPSKDRDQDRGRPKDRKHRPHHHHHHHHHHPPA
PDRDRYAQERPDTGRARAREQRWSRSPSEGREHTTHRQGSSSVSGSPAPSTSGTSTPRRGRRQLPQTPCTPRP
LVSYSPAPRRPAARRMAGPAAPPGGSPRGCRRAPRWPAHAPEGPRPRGADYTEPDSPREPPGGAHDPAPRSPR
TPRAAGCASPRHGRRLPNGYYAGHGAPRPRTARRGAHDAYSESEDDWC*
>rs240959951_ENSMUSP00000112436_c.6328:G>C_p.R2110P
ENSMUST00000121390.8|Cacna1a-201|ENSMUSG00000034656|+|chr8:g.85363274:G>C
MARFGDEMPGRYGAGGGGSGPAAGVVVGAAGGRGAGGSRQGGQPGAQRMYKQSMAQRARTMALYNPIPVRQNC
LTVNRSLFLFSEDNVVRKYAKKITEWPPFEYMILATIIANCIVLALEQHLPDDDKTPMSERLDDTEPYFIGIF
CFEAGIKIVALGFAFHKGSYLRNGWNVMDFVVVLTGILATVGTEFDLRTLRAVRVLRPLKLVSGIPSLQVVLK
SIMKAMIPLLQIGLLLFFAILIFAIIGLEFYMGKFHTTCFEEGTDDIQGESPAPCGTEEPARTCPNGTKCQPY
WEGPNNGITQFDNILFAVLTVFQCITMEGWTDLLYNSNDASGNTWNWLYFIPLIIIGSFFMLNLVLGVLSGEF
AKERERVENRRAFLKLRRQQQIERELNGYMEWISKAEEVILAEDETDVEQRHPFDGALRRATLKKSKTDLLNP
EEAEDQLADIASVGSPFARASIKSAKLENSTFFHKKERRMRFYIRRMVKTQAFYWTVLSLVALNTLCVAIVHY
NQPEWLSDFLYYAEFIFLGLFMSEMFIKMYGLGTRPYFHSSFNCFDCGVIIGSIFEVIWAVIKPGTSFGISVL
RALRLLRIFKVTKYWASLRNLVVSLLNSMKSIISLLFLLFLFIVVFALLGMQLFGGQFNFDEGTPPTNFDTFP
AAIMTVFQILTGEDWNEVMYDGIKSQGGVQGGMVFSIYFIVLTLFGNYTLLNVFLAIAVDNLANAQELTKDEQ
EEEEAANQKLALQKAKEVAEVSPLSAANMSIAVKEQQKNQKPTKSVWEQRTSEMRKQNLLASREALYGDAAER
WPTPYARPLRPDVKTHLDRPLVVDPQENRNNNTNKSRAPEALRPTARPRESARDPDARRAWPGSPERAPGREG
PYGRESEPQQREHAPPREHAPWDADTERAKAGDAPRRHTHRPVAEGEPRRHRARRRPGDEPDDRPERRPRPRD
ATRPARAADGEGDDGERKRRHRHGPPAHDDRERRHRRRKENQGSGVPVSGPNLSTTRPIQQDLGRQDLPLAED
LDNMKNNKLATGEPASPHDSLGHSGLPPSPAKIGNSTNPGPALATNPQNAASRRTPNNPGNPSNPGPPKTPEN
SLIVTNPSSTQPNSAKTARKPEHMAVEIPPACPPLNHTVVQVNKNANPDPLPKKEEEKKEEEEADPGEDGPKP
MPPYSSMFILSTTNPLRRLCHYILNLRYFEMCILMVIAMSSIALAAEDPVQPNAPRNNVLRYFDYVFTGVFTF
EMVIKMIDLGLVLHQGAYFRDLWNILDFIVVSGALVAFAFTGNSKGKDINTIKSLRVLRVLRPLKTIKRLPKL
KAVFDCVVNSLKNVFNILIVYMLFMFIFAVVAVQLFKGKFFHCTDESKEFERDCRGKYLLYEKNEVKARDREW
KKYEFHYDNVLWALLTLFTVSTGEGWPQVLKHSVDATFENQGPSPGYRMEMSIFYVVYFVVFPFFFVNIFVAL
IIITFQEQGDKMMEEYSLEKNERACIDFAISAKPLTRHMPQNKQSFQYRMWQFVVSPPFEYTIMAMIALNTIV
LMMKFYGASVAYENALRVFNIVFTSLFSLECVLKVMAFGILNYFRDAWNIFDFVTVLGSITDILVTEFGNNFI
NLSFLRLFRAARLIKLLRQGYTIRILLWTFVQSFKALPYVCLLIAMLFFIYAIIGMQVFGNIGIDGEDEDSDE
DEFQITEHNNFRTFFQALMLLFRSATGEAWHNIMLSCLSGKPCDKNSGILTADCGNEFAYFYFVSFIFLCSFL

MLNLFVAVIMDNFEYLTRDSSILGPHHLDEYVRVWAEYDPAACGRIHYKDMYSLLRVISPPLGLGKKCPHRVA
CKRLLRMDLPVADDNTVHFNSTLMALIRTALDIKIAKGGADKQQMDAELRKEMMAIWPNLSQKTLDLLVTPHK
STDLTVGKIYAAMMIMEYYRQSKAKKLQAMREEQNRTPLMFQRMEPPSPTQEGGPSQNALPSTQLDPGGGLMA
HEGGMKESPSWVTQRAQEMFQKTGTWSPERGPPIDMPNSQPNSQSVEMREMGTDGYSDSEHYLPMEGQTRAAS
MPRLPAENQRRRGRPRGNDLSTISDTSPMKRSASVLGPKARRLDDYSLERVPPEENQRYHQRRRDPGHRTSER
SLGRYTDVDTGLGTDLSMTTQSGDLPSKDRDQDRGRPKDRKHRPHHHHHHHHHHPPAPDRDRYAQERPDTGRA
RAREQRWSRSPSEGREHTTHRQGSSSVSGSPAPSTSGTSTPRRGRRQLPQTPCTPRPLVSYSPAPRRPAARRM
AGPAAPPGGSPRGCRRAPRWPAHAPEGPRPRGADYTEPDSPREPPGGAHDPAPRSPRTPRAAGCASPRHGRRL
PNGYYAGHGAPRPRTARRGAHDAYSESEDDWC*
>rs240959951_ENSMUSP00000114055_c.6187:G>C_p.R2063P
ENSMUST00000122053.2|Cacna1a-202|ENSMUSG00000034656|+|chr8:g.85363274:G>C
MARFGDEMPGRYGAGGGGSGPAAGVVVGAAGGRGAGGSRQGGQPGAQRMYKQSMAQRARTMALYNPIPVRQNC
LTVNRSLFLFSEDNVDDTEPYFIGIFCFEAGIKIVALGFAFHKGSYLRNGWNVMDFVVVLTGILATVGTEFDL
RTLRAVRVLRPLKLVSGIPSLQVVLKSIMKAMIPLLQIGLLLFFAILIFAIIGLEFYMGKFHTTCFEEGTDDI
QGESPAPCGTEEPARTCPNGTKCQPYWEGPNNGITQFDNILFAVLTVFQCITMEGWTDLLYNSNDASGNTWNW
LYFIPLIIIGSFFMLNLVLGVLSGEFAKERERVENRRAFLKLRRQQQIERELNGYMEWISKAEEVILAEDETD
VEQRHPFDGALRRATLKKSKTDLLNPEEAEDQLADIASVGSPFARASIKSAKLENSTFFHKKERRMRFYIRRM
VKTQAFYWTVLSLVALNTLCVAIVHYNQPEWLSDFLYYAEFIFLGLFMSEMFIKMYGLGTRPYFHSSFNCFDC
GVIIGSIFEVIWAVIKPGTSFGISVLRALRLLRIFKVTKYWASLRNLVVSLLNSMKSIISLLFLLFLFIVVFA
LLGMQLFGGQFNFDEGTPPTNFDTFPAAIMTVFQILTGEDWNEVMYDGIKSQGGVQGGMVFSIYFIVLTLFGN
YTLLNVFLAIAVDNLANAQELTKDEQEEEEAANQKLALQKAKEVAEVSPLSAANMSIAVKEQQKNQKPTKSVW
EQRTSEMRKQNLLASREALYGDAAERWPTPYARPLRPDVKTHLDRPLVVDPQENRNNNTNKSRAPEALRPTAR
PRESARDPDARRAWPGSPERAPGREGPYGRESEPQQREHAPPREHAPWDADTERAKAGDAPRRHTHRPVAEGE
PRRHRARRRPGDEPDDRPERRPRPRDATRPARAADGEGDDGERKRRHRHGPPAHDDRERRHRRRKENQGSGVP
VSGPNLSTTRPIQQDLGRQDLPLAEDLDNMKNNKLATGEPASPHDSLGHSGLPPSPAKIGNSTNPGPALATNP
QNAASRRTPNNPGNPSNPGPPKTPENSLIVTNPSSTQPNSAKTARKPEHMAVEIPPACPPLNHTVVQVNKNAN
PDPLPKKEEEKKEEEEADPGEDGPKPMPPYSSMFILSTTNPLRRLCHYILNLRYFEMCILMVIAMSSIALAAE
DPVQPNAPRNNVLRYFDYVFTGVFTFEMVIKMIDLGLVLHQGAYFRDLWNILDFIVVSGALVAFAFTGNSKGK
DINTIKSLRVLRVLRPLKTIKRLPKLKAVFDCVVNSLKNVFNILIVYMLFMFIFAVVAVQLFKGKFFHCTDES
KEFERDCRGKYLLYEKNEVKARDREWKKYEFHYDNVLWALLTLFTVSTGEGWPQVLKHSVDATFENQGPSPGY
RMEMSIFYVVYFVVFPFFFVNIFVALIIITFQEQGDKMMEEYSLEKNERACIDFAISAKPLTRHMPQNKQSFQ
YRMWQFVVSPPFEYTIMAMIALNTIVLMMKFYGASVAYENALRVFNIVFTSLFSLECVLKVMAFGILNYFRDA
WNIFDFVTVLGSITDILVTEFGNNFINLSFLRLFRAARLIKLLRQGYTIRILLWTFVQSFKALPYVCLLIAML
FFIYAIIGMQVFGNIGIDGEDEDSDEDEFQITEHNNFRTFFQALMLLFRSATGEAWHNIMLSCLSGKPCDKNS
GILTADCGNEFAYFYFVSFIFLCSFLMLNLFVAVIMDNFEYLTRDSSILGPHHLDEYVRVWAEYDPAACGRIH
YKDMYSLLRVISPPLGLGKKCPHRVACKRLLRMDLPVADDNTVHFNSTLMALIRTALDIKIAKGGADKQQMDA
ELRKEMMAIWPNLSQKTLDLLVTPHKSTDLTVGKIYAAMMIMEYYRQSKAKKLQAMREEQNRTPLMFQRMEPP
SPTQEGGPSQNALPSTQLDPGGGLMAHEGGMKESPSWVTQRAQEMFQKTGTWSPERGPPIDMPNSQPNSQSVE
MREMGTDGYSDSEHYLPMEGQTRAASMPRLPAENQRRRGRPRGNDLSTISDTSPMKRSASVLGPKARRLDDYS
LERVPPEENQRYHQRRRDPGHRTSERSLGRYTDVDTGLGTDLSMTTQSGDLPSKDRDQDRGRPKDRKHRPHHH
HHHHHHHPPAPDRDRYAQERPDTGRARAREQRWSRSPSEGREHTTHRQGSSSVSGSPAPSTSGTSTPRRGRRQ
LPQTPCTPRPLVSYSPAPRRPAARRMAGPAAPPGGSPRGCRRAPRWPAHAPEGPRPRGADYTEPDSPREPPGG
AHDPAPRSPRTPRAAGCASPRHGRRLPNGYYAGHGAPRPRTARRGAHDAYSESEDDWC*
>rs240959951_ENSMUSP00000149242_c.6187:G>C_p.R2063P
ENSMUST00000215756.2|Cacna1a-211|ENSMUSG00000034656|+|chr8:g.85363274:G>C
XRMYKQSMAQRARTMALYNPIPVRQNCLTVNRSLFLFSEDNVVRKYAKKITEWPPFEYMILATIIANCIVLAL
EQHLPDDDKTPMSERLDDTEPYFIGIFCFEAGIKIVALGFAFHKGSYLRNGWNVMDFVVVLTGILATVGTEFD
LRTLRAVRVLRPLKLVSGIPSLQVVLKSIMKAMIPLLQIGLLLFFAILIFAIIGLEFYMGKFHTTCFEEGTDD
IQGESPAPCGTEEPARTCPNGTKCQPYWEGPNNGITQFDNILFAVLTVFQCITMEGWTDLLYNSNDASGNTWN
WLYFIPLIIIGSFFMLNLVLGVLSGEFAKERERVENRRAFLKLRRQQQIERELNGYMEWISKAEEVILAEDET
DVEQRHPFDALRRATLKKSKTDLLNPEEAEDQLADIASVGSPFARASIKSAKLENSTFFHKKERRMRFYIRRM
VKTQAFYWTVLSLVALNTLCVAIVHYNQPEWLSDFLYYAEFIFLGLFMSEMFIKMYGLGTRPYFHSSFNCFDC
GVIIGSIFEVIWAVIKPGTSFGISVLRALRLLRIFKVTKYWASLRNLVVSLLNSMKSIISLLFLLFLFIVVFA
LLGMQLFGGQFNFDEGTPPTNFDTFPAAIMTVFQILTGEDWNEVMYDGIKSQGGVQGGMVFSIYFIVLTLFGN
YTLLNVFLAIAVDNLANAQELTKDEQEEEEAANQKLALQKAKEVAEVSPLSAANMSIAVKEQQKNQKPTKSVW
EQRTSEMRKQNLLASREALYGDAAERWPTPYARPLRPDVKTHLDRPLVVDPQENRNNNTNKSRAPEALRPTAR
PRESARDPDARRAWPGSPERAPGREGPYGRESEPQQREHAPPREHAPWDADTERAKAGDAPRRHTHRPVAEGE
PRRHRARRRPGDEPDDRPERRPRPRDATRPARAADGEGDDGERKRRHRHGPPAHDDRERRHRRRKENQGSGVP

VSGPNLSTTRPIQQDLGRQDLPLAEDLDNMKNNKLATGEPASPHDSLGHSGLPPSPAKIGNSTNPGPALATNP
QNAASRRTPNNPGNPSNPGPPKTPENSLIVTNPSSTQPNSAKTARKPEHMAVEIPPACPPLNHTVVQVNKNAN
PDPLPKKEEEKKEEEEADPGEDGPKPMPPYSSMFILSTTNPLRRLCHYILNLRYFEMCILMVIAMSSIALAAE
DPVQPNAPRNNVLRYFDYVFTGVFTFEMVIKMIDLGLVLHQGAYFRDLWNILDFIVVSGALVAFAFTGNSKGK
DINTIKSLRVLRVLRPLKTIKRLPKLKAVFDCVVNSLKNVFNILIVYMLFMFIFAVVAVQLFKGKFFHCTDES
KEFERDCRGKYLLYEKNEVKARDREWKKYEFHYDNVLWALLTLFTVSTGEGWPQVLKHSVDATFENQGPSPGY
RMEMSIFYVVYFVVFPFFFVNIFVALIIITFQEQGDKMMEEYSLEKNERACIDFAISAKPLTRHMPQNKQSFQ
YRMWQFVVSPPFEYTIMAMIALNTIVLMMKFYGASVAYENALRVFNIVFTSLFSLECVLKVMAFGILNYFRDA
WNIFDFVTVLGSITDILVTEFGNNFINLSFLRLFRAARLIKLLRQGYTIRILLWTFVQSFKALPYVCLLIAML
FFIYAIIGMQVFGNIGIDGEDEDSDEDEFQITEHNNFRTFFQALMLLFRSATGEAWHNIMLSCLSGKPCDKNS
GILTADCGNEFAYFYFVSFIFLCSFLMLNLFVAVIMDNFEYLTRDSSILGPHHLDEYVRVWAEYDPAACGRIH
YKDMYSLLRVISPPLGLGKKCPHRVACKRLLRMDLPVADDNTVHFNSTLMALIRTALDIKIAKGGADKQQMDA
ELRKEMMAIWPNLSQKTLDLLVTPHKSTDLTVGKIYAAMMIMEYYRQSKAKKLQAMREEQNRTPLMFQRMEPP
SPTQEGGPSQNALPSTQLDPGGGLMAHEGGMKESPSWVTQRAQEMFQKTGTWSPERGPPIDMPNSQPNSQSVE
MREMGTDGYSDSEHYLPMEGQTRAASMPRLPAENQRRRGRPRGNDLSTISDTSPMKRSASVLGPKARRLDDYS
LERVPPEENQRYHQRRRDPGHRTSERSLGRYTDVDTGLGTDLSMTTQSGDLPSKDRDQDRGRPKDRKHRPHHH
HHHHHHHPPAPDRDRYAQERPDTGRARAREQRWSRSPSEGREHTTHRQGSSSVSGSPAPSTSGTSTPRRGRRQ
LPQTPCTPRPLVSYSPAPRRPAARRMAGPAAPPGGSPRGCRRAPRWPAHAPEGPRPRGADYTEPDSPREPPGG
AHDPAPRSPRTPRAAGCASPRHGRRLPNGYYAGHGAPRPRTARRGAHDAYSESEDDWC*
>rs240959951_ENSMUSP00000158675_c.1552:G>C_p.R518P
ENSMUST00000238337.2|Cacna1a-212|ENSMUSG00000034656|+|chr8:g.85363274:G>C
MHLLRLSGQRGLTLQPNSPSSLPSSPFPLAKGNNFINLSFLRLFRAARLIKLLRQGYTIRILLWTFVQSFKVF
GNIGIDGEDEDSDEDEFQITEHNNFRTFFQALMLLFRSATGEAWHNIMLSCLSGKPCDKNSGILTADCGNEFA
YFYFVSFIFLCSFLMLNLFVAVIMDNFEYLTRDSSILGPHHLDEYVRVWAEYDPAACGRIHYKDMYSLLRVIS
PPLGLGKKCPHRVACKRLLRMDLPVADDNTVHFNSTLMALIRTALDIKIAKGGADKQQMDAELRKEMMAIWPN
LSQKTLDLLVTPHKSTDLTVGKIYAAMMIMEYYRQSKAKKLQAMREEQNRTPLMFQRMEPPSPTQEGGPSQNA
LPSTQLDPGGGLMAHEGGMKESPSWVTQRAQEMFQKTGTWSPERGPPIDMPNSQPNSQSVEMREMGTDGYSDS
EHYLPMEGQTRAASMPRLPAENQRRRGRPRGNDLSTISDTSPMKRSASVLGPKARRLDDYSLERVPPEENQRY
HQRRRDPGHRTSERSLGRYTDVDTGLGTDLSMTTQSGDLPSKDRDQDRGRPKDRKHRPHHHHHHHHHHPPAPD
RDRYAQERPDTGRARAREQRWSRSPSEGREHTTHRQGSSSVSGSPAPSTSGTSTPRRGRRQLPQTPCTPRPLV
SYSPAPRRPAARRMAGPAAPPGGSPRGCRRAPRWPAHAPEGPRPRGADYTEPDSPREPPGGAHDPAPRSPRTP
RAAGCASPRHGRRLPNGYYAGHGAPRPRTARRGAHDAYSESEDDWC*
>rs230834853_ENSMUSP00000158803_c.6604:G>A_p.R2202H
ENSMUST00000238701.2|Cacna1a-213|ENSMUSG00000034656|+|chr8:g.85363283:G>A
MPRASWGSDPMLSPLSHELLHSPRYPGTDCTLAGSSRTLHVPWSQQMSGSLEGPTLKRRGGVVDHRDVILAHQ
AHKMHSTPQARRKEWEMARFGDEMPGRYGAGGGGSGPAAGVVVGAAGGRGAGGSRQGGQPGAQRMYKQSMAQR
ARTMALYNPIPVRQNCLTVNRSLFLFSEDNVVRKYAKKITEWPPFEYMILATIIANCIVLALEQHLPDDDKTP
MSERLDDTEPYFIGIFCFEAGIKIVALGFAFHKGSYLRNGWNVMDFVVVLTGILATVGTEFDLRTLRAVRVLR
PLKLVSGIPSLQVVLKSIMKAMIPLLQIGLLLFFAILIFAIIGLEFYMGKFHTTCFEEGTDDIQGESPAPCGT
EEPARTCPNGTKCQPYWEGPNNGITQFDNILFAVLTVFQCITMEGWTDLLYNSNDASGNTWNWLYFIPLIIIG
SFFMLNLVLGVLSGEFAKERERVENRRAFLKLRRQQQIERELNGYMEWISKAEEVILAEDETDVEQRHPFDGA
LRRATLKKSKTDLLNPEEAEDQLADIASVGSPFARASIKSAKLENSTFFHKKERRMRFYIRRMVKTQAFYWTV
LSLVALNTLCVAIVHYNQPEWLSDFLYYAEFIFLGLFMSEMFIKMYGLGTRPYFHSSFNCFDCGVIIGSIFEV
IWAVIKPGTSFGISVLRALRLLRIFKVTKYWASLRNLVVSLLNSMKSIISLLFLLFLFIVVFALLGMQLFGGQ
FNFDEGTPPTNFDTFPAAIMTVFQILTGEDWNEVMYDGIKSQGGVQGGMVFSIYFIVLTLFGNYTLLNVFLAI
AVDNLANAQELTKDEQEEEEAANQKLALQKAKEVAEVSPLSAANMSIAVKEQQKNQKPTKSVWEQRTSEMRKQ
NLLASREALYGDAAERWPTPYARPLRPDVKTHLDRPLVVDPQENRNNNTNKSRAPEALRPTARPRESARDPDA
RRAWPGSPERAPGREGPYGRESEPQQREHAPPREHAPWDADTERAKAGDAPRRHTHRPVAEGEPRRHRARRRP
GDEPDDRPERRPRPRDATRPARAADGEGDDGERKRRHRHGPPAHDDRERRHRRRKENQGSGVPVSGPNLSTTR
PIQQDLGRQDLPLAEDLDNMKNNKLATGEPASPHDSLGHSGLPPSPAKIGNSTNPGPALATNPQNAASRRTPN
NPGNPSNPGPPKTPENSLIVTNPSSTQPNSAKTARKPEHMAVEIPPACPPLNHTVVQVNKNANPDPLPKKEEE
KKEEEEADPGEDGPKPMPPYSSMFILSTTNPLRRLCHYILNLRYFEMCILMVIAMSSIALAAEDPVQPNAPRN
NVLRYFDYVFTGVFTFEMVIKMIDLGLVLHQGAYFRDLWNILDFIVVSGALVAFAFTGNSKGKDINTIKSLRV
LRVLRPLKTIKRLPKLKAVFDCVVNSLKNVFNILIVYMLFMFIFAVVAVQLFKGKFFHCTDESKEFERDCRGK
YLLYEKNEVKARDREWKKYEFHYDNVLWALLTLFTVSTGEGWPQVLKHSVDATFENQGPSPGYRMEMSIFYVV
YFVVFPFFFVNIFVALIIITFQEQGDKMMEEYSLEKNERACIDFAISAKPLTRHMPQNKQSFQYRMWQFVVSP
PFEYTIMAMIALNTIVLMMKFYGASVAYENALRVFNIVFTSLFSLECVLKVMAFGILNYFRDAWNIFDFVTVL
GSITDILVTEFGNNFINLSFLRLFRAARLIKLLRQGYTIRILLWTFVQSFKALPYVCLLIAMLFFIYAIIGMQ

VFGNIGIDGEDEDSDEDEFQITEHNNFRTFFQALMLLFRSATGEAWHNIMLSCLSGKPCDKNSGILTADCGNE
FAYFYFVSFIFLCSFLMLNLFVAVIMDNFEYLTRDSSILGPHHLDEYVRVWAEYDPAACGRIHYKDMYSLLRV
ISPPLGLGKKCPHRVACKRLLRMDLPVADDNTVHFNSTLMALIRTALDIKIAKGGADKQQMDAELRKEMMAIW
PNLSQKTLDLLVTPHKSTDLTVGKIYAAMMIMEYYRQSKAKKLQAMREEQNRTPLMFQRMEPPSPTQEGGPSQ
NALPSTQLDPGGGLMAHEGGMKESPSWVTQRAQEMFQKTGTWSPERGPPIDMPNSQPNSQSVEMREMGTDGYS
DSEHYLPMEGQTRAASMPRLPAENQRRRGRPRGNDLSTISDTSPMKRSASVLGPKARRLDDYSLERVPPEENQ
RYHQRRRDRGHHTSERSLGRYTDVDTGLGTDLSMTTQSGDLPSKDRDQDRGRPKDRKHRPHHHHHHHHHHPPA
PDRDRYAQERPDTGRARAREQRWSRSPSEGREHTTHRQGSSSVSGSPAPSTSGTSTPRRGRRQLPQTPCTPRP
LVSYSPAPRRPAARRMAGPAAPPGGSPRGCRRAPRWPAHAPEGPRPRGADYTEPDSPREPPGGAHDPAPRSPR
TPRAAGCASPRHGRRLPNGYYAGHGAPRPRTARRGAHDAYSESEDDWC*
>rs230834853_ENSMUSP00000112436_c.6337:G>A_p.R2113H
ENSMUST00000121390.8|Cacna1a-201|ENSMUSG00000034656|+|chr8:g.85363283:G>A
MARFGDEMPGRYGAGGGGSGPAAGVVVGAAGGRGAGGSRQGGQPGAQRMYKQSMAQRARTMALYNPIPVRQNC
LTVNRSLFLFSEDNVVRKYAKKITEWPPFEYMILATIIANCIVLALEQHLPDDDKTPMSERLDDTEPYFIGIF
CFEAGIKIVALGFAFHKGSYLRNGWNVMDFVVVLTGILATVGTEFDLRTLRAVRVLRPLKLVSGIPSLQVVLK
SIMKAMIPLLQIGLLLFFAILIFAIIGLEFYMGKFHTTCFEEGTDDIQGESPAPCGTEEPARTCPNGTKCQPY
WEGPNNGITQFDNILFAVLTVFQCITMEGWTDLLYNSNDASGNTWNWLYFIPLIIIGSFFMLNLVLGVLSGEF
AKERERVENRRAFLKLRRQQQIERELNGYMEWISKAEEVILAEDETDVEQRHPFDGALRRATLKKSKTDLLNP
EEAEDQLADIASVGSPFARASIKSAKLENSTFFHKKERRMRFYIRRMVKTQAFYWTVLSLVALNTLCVAIVHY
NQPEWLSDFLYYAEFIFLGLFMSEMFIKMYGLGTRPYFHSSFNCFDCGVIIGSIFEVIWAVIKPGTSFGISVL
RALRLLRIFKVTKYWASLRNLVVSLLNSMKSIISLLFLLFLFIVVFALLGMQLFGGQFNFDEGTPPTNFDTFP
AAIMTVFQILTGEDWNEVMYDGIKSQGGVQGGMVFSIYFIVLTLFGNYTLLNVFLAIAVDNLANAQELTKDEQ
EEEEAANQKLALQKAKEVAEVSPLSAANMSIAVKEQQKNQKPTKSVWEQRTSEMRKQNLLASREALYGDAAER
WPTPYARPLRPDVKTHLDRPLVVDPQENRNNNTNKSRAPEALRPTARPRESARDPDARRAWPGSPERAPGREG
PYGRESEPQQREHAPPREHAPWDADTERAKAGDAPRRHTHRPVAEGEPRRHRARRRPGDEPDDRPERRPRPRD
ATRPARAADGEGDDGERKRRHRHGPPAHDDRERRHRRRKENQGSGVPVSGPNLSTTRPIQQDLGRQDLPLAED
LDNMKNNKLATGEPASPHDSLGHSGLPPSPAKIGNSTNPGPALATNPQNAASRRTPNNPGNPSNPGPPKTPEN
SLIVTNPSSTQPNSAKTARKPEHMAVEIPPACPPLNHTVVQVNKNANPDPLPKKEEEKKEEEEADPGEDGPKP
MPPYSSMFILSTTNPLRRLCHYILNLRYFEMCILMVIAMSSIALAAEDPVQPNAPRNNVLRYFDYVFTGVFTF
EMVIKMIDLGLVLHQGAYFRDLWNILDFIVVSGALVAFAFTGNSKGKDINTIKSLRVLRVLRPLKTIKRLPKL
KAVFDCVVNSLKNVFNILIVYMLFMFIFAVVAVQLFKGKFFHCTDESKEFERDCRGKYLLYEKNEVKARDREW
KKYEFHYDNVLWALLTLFTVSTGEGWPQVLKHSVDATFENQGPSPGYRMEMSIFYVVYFVVFPFFFVNIFVAL
IIITFQEQGDKMMEEYSLEKNERACIDFAISAKPLTRHMPQNKQSFQYRMWQFVVSPPFEYTIMAMIALNTIV
LMMKFYGASVAYENALRVFNIVFTSLFSLECVLKVMAFGILNYFRDAWNIFDFVTVLGSITDILVTEFGNNFI
NLSFLRLFRAARLIKLLRQGYTIRILLWTFVQSFKALPYVCLLIAMLFFIYAIIGMQVFGNIGIDGEDEDSDE
DEFQITEHNNFRTFFQALMLLFRSATGEAWHNIMLSCLSGKPCDKNSGILTADCGNEFAYFYFVSFIFLCSFL
MLNLFVAVIMDNFEYLTRDSSILGPHHLDEYVRVWAEYDPAACGRIHYKDMYSLLRVISPPLGLGKKCPHRVA
CKRLLRMDLPVADDNTVHFNSTLMALIRTALDIKIAKGGADKQQMDAELRKEMMAIWPNLSQKTLDLLVTPHK
STDLTVGKIYAAMMIMEYYRQSKAKKLQAMREEQNRTPLMFQRMEPPSPTQEGGPSQNALPSTQLDPGGGLMA
HEGGMKESPSWVTQRAQEMFQKTGTWSPERGPPIDMPNSQPNSQSVEMREMGTDGYSDSEHYLPMEGQTRAAS
MPRLPAENQRRRGRPRGNDLSTISDTSPMKRSASVLGPKARRLDDYSLERVPPEENQRYHQRRRDRGHHTSER
SLGRYTDVDTGLGTDLSMTTQSGDLPSKDRDQDRGRPKDRKHRPHHHHHHHHHHPPAPDRDRYAQERPDTGRA
RAREQRWSRSPSEGREHTTHRQGSSSVSGSPAPSTSGTSTPRRGRRQLPQTPCTPRPLVSYSPAPRRPAARRM
AGPAAPPGGSPRGCRRAPRWPAHAPEGPRPRGADYTEPDSPREPPGGAHDPAPRSPRTPRAAGCASPRHGRRL
PNGYYAGHGAPRPRTARRGAHDAYSESEDDWC*
>rs230834853_ENSMUSP00000114055_c.6196:G>A_p.R2066H
ENSMUST00000122053.2|Cacna1a-202|ENSMUSG00000034656|+|chr8:g.85363283:G>A
MARFGDEMPGRYGAGGGGSGPAAGVVVGAAGGRGAGGSRQGGQPGAQRMYKQSMAQRARTMALYNPIPVRQNC
LTVNRSLFLFSEDNVDDTEPYFIGIFCFEAGIKIVALGFAFHKGSYLRNGWNVMDFVVVLTGILATVGTEFDL
RTLRAVRVLRPLKLVSGIPSLQVVLKSIMKAMIPLLQIGLLLFFAILIFAIIGLEFYMGKFHTTCFEEGTDDI
QGESPAPCGTEEPARTCPNGTKCQPYWEGPNNGITQFDNILFAVLTVFQCITMEGWTDLLYNSNDASGNTWNW
LYFIPLIIIGSFFMLNLVLGVLSGEFAKERERVENRRAFLKLRRQQQIERELNGYMEWISKAEEVILAEDETD
VEQRHPFDGALRRATLKKSKTDLLNPEEAEDQLADIASVGSPFARASIKSAKLENSTFFHKKERRMRFYIRRM
VKTQAFYWTVLSLVALNTLCVAIVHYNQPEWLSDFLYYAEFIFLGLFMSEMFIKMYGLGTRPYFHSSFNCFDC
GVIIGSIFEVIWAVIKPGTSFGISVLRALRLLRIFKVTKYWASLRNLVVSLLNSMKSIISLLFLLFLFIVVFA
LLGMQLFGGQFNFDEGTPPTNFDTFPAAIMTVFQILTGEDWNEVMYDGIKSQGGVQGGMVFSIYFIVLTLFGN
YTLLNVFLAIAVDNLANAQELTKDEQEEEEAANQKLALQKAKEVAEVSPLSAANMSIAVKEQQKNQKPTKSVW
EQRTSEMRKQNLLASREALYGDAAERWPTPYARPLRPDVKTHLDRPLVVDPQENRNNNTNKSRAPEALRPTAR

PRESARDPDARRAWPGSPERAPGREGPYGRESEPQQREHAPPREHAPWDADTERAKAGDAPRRHTHRPVAEGE
PRRHRARRRPGDEPDDRPERRPRPRDATRPARAADGEGDDGERKRRHRHGPPAHDDRERRHRRRKENQGSGVP
VSGPNLSTTRPIQQDLGRQDLPLAEDLDNMKNNKLATGEPASPHDSLGHSGLPPSPAKIGNSTNPGPALATNP
QNAASRRTPNNPGNPSNPGPPKTPENSLIVTNPSSTQPNSAKTARKPEHMAVEIPPACPPLNHTVVQVNKNAN
PDPLPKKEEEKKEEEEADPGEDGPKPMPPYSSMFILSTTNPLRRLCHYILNLRYFEMCILMVIAMSSIALAAE
DPVQPNAPRNNVLRYFDYVFTGVFTFEMVIKMIDLGLVLHQGAYFRDLWNILDFIVVSGALVAFAFTGNSKGK
DINTIKSLRVLRVLRPLKTIKRLPKLKAVFDCVVNSLKNVFNILIVYMLFMFIFAVVAVQLFKGKFFHCTDES
KEFERDCRGKYLLYEKNEVKARDREWKKYEFHYDNVLWALLTLFTVSTGEGWPQVLKHSVDATFENQGPSPGY
RMEMSIFYVVYFVVFPFFFVNIFVALIIITFQEQGDKMMEEYSLEKNERACIDFAISAKPLTRHMPQNKQSFQ
YRMWQFVVSPPFEYTIMAMIALNTIVLMMKFYGASVAYENALRVFNIVFTSLFSLECVLKVMAFGILNYFRDA
WNIFDFVTVLGSITDILVTEFGNNFINLSFLRLFRAARLIKLLRQGYTIRILLWTFVQSFKALPYVCLLIAML
FFIYAIIGMQVFGNIGIDGEDEDSDEDEFQITEHNNFRTFFQALMLLFRSATGEAWHNIMLSCLSGKPCDKNS
GILTADCGNEFAYFYFVSFIFLCSFLMLNLFVAVIMDNFEYLTRDSSILGPHHLDEYVRVWAEYDPAACGRIH
YKDMYSLLRVISPPLGLGKKCPHRVACKRLLRMDLPVADDNTVHFNSTLMALIRTALDIKIAKGGADKQQMDA
ELRKEMMAIWPNLSQKTLDLLVTPHKSTDLTVGKIYAAMMIMEYYRQSKAKKLQAMREEQNRTPLMFQRMEPP
SPTQEGGPSQNALPSTQLDPGGGLMAHEGGMKESPSWVTQRAQEMFQKTGTWSPERGPPIDMPNSQPNSQSVE
MREMGTDGYSDSEHYLPMEGQTRAASMPRLPAENQRRRGRPRGNDLSTISDTSPMKRSASVLGPKARRLDDYS
LERVPPEENQRYHQRRRDRGHHTSERSLGRYTDVDTGLGTDLSMTTQSGDLPSKDRDQDRGRPKDRKHRPHHH
HHHHHHHPPAPDRDRYAQERPDTGRARAREQRWSRSPSEGREHTTHRQGSSSVSGSPAPSTSGTSTPRRGRRQ
LPQTPCTPRPLVSYSPAPRRPAARRMAGPAAPPGGSPRGCRRAPRWPAHAPEGPRPRGADYTEPDSPREPPGG
AHDPAPRSPRTPRAAGCASPRHGRRLPNGYYAGHGAPRPRTARRGAHDAYSESEDDWC*
>rs230834853_ENSMUSP00000149242_c.6196:G>A_p.R2066H
ENSMUST00000215756.2|Cacna1a-211|ENSMUSG00000034656|+|chr8:g.85363283:G>A
XRMYKQSMAQRARTMALYNPIPVRQNCLTVNRSLFLFSEDNVVRKYAKKITEWPPFEYMILATIIANCIVLAL
EQHLPDDDKTPMSERLDDTEPYFIGIFCFEAGIKIVALGFAFHKGSYLRNGWNVMDFVVVLTGILATVGTEFD
LRTLRAVRVLRPLKLVSGIPSLQVVLKSIMKAMIPLLQIGLLLFFAILIFAIIGLEFYMGKFHTTCFEEGTDD
IQGESPAPCGTEEPARTCPNGTKCQPYWEGPNNGITQFDNILFAVLTVFQCITMEGWTDLLYNSNDASGNTWN
WLYFIPLIIIGSFFMLNLVLGVLSGEFAKERERVENRRAFLKLRRQQQIERELNGYMEWISKAEEVILAEDET
DVEQRHPFDALRRATLKKSKTDLLNPEEAEDQLADIASVGSPFARASIKSAKLENSTFFHKKERRMRFYIRRM
VKTQAFYWTVLSLVALNTLCVAIVHYNQPEWLSDFLYYAEFIFLGLFMSEMFIKMYGLGTRPYFHSSFNCFDC
GVIIGSIFEVIWAVIKPGTSFGISVLRALRLLRIFKVTKYWASLRNLVVSLLNSMKSIISLLFLLFLFIVVFA
LLGMQLFGGQFNFDEGTPPTNFDTFPAAIMTVFQILTGEDWNEVMYDGIKSQGGVQGGMVFSIYFIVLTLFGN
YTLLNVFLAIAVDNLANAQELTKDEQEEEEAANQKLALQKAKEVAEVSPLSAANMSIAVKEQQKNQKPTKSVW
EQRTSEMRKQNLLASREALYGDAAERWPTPYARPLRPDVKTHLDRPLVVDPQENRNNNTNKSRAPEALRPTAR
PRESARDPDARRAWPGSPERAPGREGPYGRESEPQQREHAPPREHAPWDADTERAKAGDAPRRHTHRPVAEGE
PRRHRARRRPGDEPDDRPERRPRPRDATRPARAADGEGDDGERKRRHRHGPPAHDDRERRHRRRKENQGSGVP
VSGPNLSTTRPIQQDLGRQDLPLAEDLDNMKNNKLATGEPASPHDSLGHSGLPPSPAKIGNSTNPGPALATNP
QNAASRRTPNNPGNPSNPGPPKTPENSLIVTNPSSTQPNSAKTARKPEHMAVEIPPACPPLNHTVVQVNKNAN
PDPLPKKEEEKKEEEEADPGEDGPKPMPPYSSMFILSTTNPLRRLCHYILNLRYFEMCILMVIAMSSIALAAE
DPVQPNAPRNNVLRYFDYVFTGVFTFEMVIKMIDLGLVLHQGAYFRDLWNILDFIVVSGALVAFAFTGNSKGK
DINTIKSLRVLRVLRPLKTIKRLPKLKAVFDCVVNSLKNVFNILIVYMLFMFIFAVVAVQLFKGKFFHCTDES
KEFERDCRGKYLLYEKNEVKARDREWKKYEFHYDNVLWALLTLFTVSTGEGWPQVLKHSVDATFENQGPSPGY
RMEMSIFYVVYFVVFPFFFVNIFVALIIITFQEQGDKMMEEYSLEKNERACIDFAISAKPLTRHMPQNKQSFQ
YRMWQFVVSPPFEYTIMAMIALNTIVLMMKFYGASVAYENALRVFNIVFTSLFSLECVLKVMAFGILNYFRDA
WNIFDFVTVLGSITDILVTEFGNNFINLSFLRLFRAARLIKLLRQGYTIRILLWTFVQSFKALPYVCLLIAML
FFIYAIIGMQVFGNIGIDGEDEDSDEDEFQITEHNNFRTFFQALMLLFRSATGEAWHNIMLSCLSGKPCDKNS
GILTADCGNEFAYFYFVSFIFLCSFLMLNLFVAVIMDNFEYLTRDSSILGPHHLDEYVRVWAEYDPAACGRIH
YKDMYSLLRVISPPLGLGKKCPHRVACKRLLRMDLPVADDNTVHFNSTLMALIRTALDIKIAKGGADKQQMDA
ELRKEMMAIWPNLSQKTLDLLVTPHKSTDLTVGKIYAAMMIMEYYRQSKAKKLQAMREEQNRTPLMFQRMEPP
SPTQEGGPSQNALPSTQLDPGGGLMAHEGGMKESPSWVTQRAQEMFQKTGTWSPERGPPIDMPNSQPNSQSVE
MREMGTDGYSDSEHYLPMEGQTRAASMPRLPAENQRRRGRPRGNDLSTISDTSPMKRSASVLGPKARRLDDYS
LERVPPEENQRYHQRRRDRGHHTSERSLGRYTDVDTGLGTDLSMTTQSGDLPSKDRDQDRGRPKDRKHRPHHH
HHHHHHHPPAPDRDRYAQERPDTGRARAREQRWSRSPSEGREHTTHRQGSSSVSGSPAPSTSGTSTPRRGRRQ
LPQTPCTPRPLVSYSPAPRRPAARRMAGPAAPPGGSPRGCRRAPRWPAHAPEGPRPRGADYTEPDSPREPPGG
AHDPAPRSPRTPRAAGCASPRHGRRLPNGYYAGHGAPRPRTARRGAHDAYSESEDDWC*
>rs230834853_ENSMUSP00000158675_c.1561:G>A_p.R521H
ENSMUST00000238337.2|Cacna1a-212|ENSMUSG00000034656|+|chr8:g.85363283:G>A

MHLLRLSGQRGLTLQPNSPSSLPSSPFPLAKGNNFINLSFLRLFRAARLIKLLRQGYTIRILLWTFVQSFKVF
GNIGIDGEDEDSDEDEFQITEHNNFRTFFQALMLLFRSATGEAWHNIMLSCLSGKPCDKNSGILTADCGNEFA
YFYFVSFIFLCSFLMLNLFVAVIMDNFEYLTRDSSILGPHHLDEYVRVWAEYDPAACGRIHYKDMYSLLRVIS
PPLGLGKKCPHRVACKRLLRMDLPVADDNTVHFNSTLMALIRTALDIKIAKGGADKQQMDAELRKEMMAIWPN
LSQKTLDLLVTPHKSTDLTVGKIYAAMMIMEYYRQSKAKKLQAMREEQNRTPLMFQRMEPPSPTQEGGPSQNA
LPSTQLDPGGGLMAHEGGMKESPSWVTQRAQEMFQKTGTWSPERGPPIDMPNSQPNSQSVEMREMGTDGYSDS
EHYLPMEGQTRAASMPRLPAENQRRRGRPRGNDLSTISDTSPMKRSASVLGPKARRLDDYSLERVPPEENQRY
HQRRRDRGHHTSERSLGRYTDVDTGLGTDLSMTTQSGDLPSKDRDQDRGRPKDRKHRPHHHHHHHHHPPAPD
RDRYAQERPDTGRARAREQRWSRSPSEGREHTTHRQGSSSVSGSPAPSTSGTSTPRRGRRQLPQTPCTPRPLV
SYSPAPRRPAARRMAGPAAPPGGSPRGCRRAPRWPAHAPEGPRPRGADYTEPDSPREPPGGAHDPAPRSPRTP
RAAGCASPRHGRRLPNGYYAGHGAPRPRTARRGAHDAYSESEDDWC*
>ENSMUSP00000158803_c.6604:G>ATGAGGCAGTGGTCAGTGCTGGGACTATAGTTCAAAGGCAGGCC
ACCTGTCTTGTATACA_p.R2202ins-fs ENSMUST00000238701.2|Cacna1a-
213|ENSMUSG00000034656|+|chr8:g.85363283:G>ATGAGGCAGTGGTCAGTGCTGGGACTATAG
TTCAAAGGCAGGCCACCTGTCTTGTATACA
MPRASWGSDPMLSPLSHELLHSPRYPGTDCTLAGSSRTLHVPWSQQMSGSLEGPTLKRRGGVVDHRDVILAHQ
AHKMHSTPQARRKEWEMARFGDEMPGRYGAGGGGSGPAAGVVVGAAGGRGAGGSRQGGQPGAQRMYKQSMAQR
ARTMALYNPIPVRQNCLTVNRSLFLFSEDNVVRKYAKKITEWPPFEYMILATIIANCIVLALEQHLPDDDKTP
MSERLDDTEPYFIGIFCFEAGIKIVALGFAFHKGSYLRNGWNVMDFVVVLTGILATVGTEFDLRTLRAVRVLR
PLKLVSGIPSLQVVLKSIMKAMIPLLQIGLLLFFAILIFAIIGLEFYMGKFHTTCFEEGTDDIQGESPAPCGT
EEPARTCPNGTKCQPYWEGPNNGITQFDNILFAVLTVFQCITMEGWTDLLYNSNDASGNTWNWLYFIPLIIIG
SFFMLNLVLGVLSGEFAKERERVENRRAFLKLRRQQQIERELNGYMEWISKAEEVILAEDETDVEQRHPFDGA
LRRATLKKSKTDLLNPEEAEDQLADIASVGSPFARASIKSAKLENSTFFHKKERRMRFYIRRMVKTQAFYWTV
LSLVALNTLCVAIVHYNQPEWLSDFLYYAEFIFLGLFMSEMFIKMYGLGTRPYFHSSFNCFDCGVIIGSIFEV
IWAVIKPGTSFGISVLRALRLLRIFKVTKYWASLRNLVVSLLNSMKSIISLLFLLFLFIVVFALLGMQLFGGQ
FNFDEGTPPTNFDTFPAAIMTVFQILTGEDWNEVMYDGIKSQGGVQGGMVFSIYFIVLTLFGNYTLLNVFLAI
AVDNLANAQELTKDEQEEEEAANQKLALQKAKEVAEVSPLSAANMSIAVKEQQKNQKPTKSVWEQRTSEMRKQ
NLLASREALYGDAAERWPTPYARPLRPDVKTHLDRPLVVDPQENRNNNTNKSRAPEALRPTARPRESARDPDA
RRAWPGSPERAPGREGPYGRESEPQQREHAPPREHAPWDADTERAKAGDAPRRHTHRPVAEGEPRRHRARRRP
GDEPDDRPERRPRPRDATRPARAADGEGDDGERKRRHRHGPPAHDDRERRHRRRKENQGSGVPVSGPNLSTTR
PIQQDLGRQDLPLAEDLDNMKNNKLATGEPASPHDSLGHSGLPPSPAKIGNSTNPGPALATNPQNAASRRTPN
NPGNPSNPGPPKTPENSLIVTNPSSTQPNSAKTARKPEHMAVEIPPACPPLNHTVVQVNKNANPDPLPKKEEE
KKEEEEADPGEDGPKPMPPYSSMFILSTTNPLRRLCHYILNLRYFEMCILMVIAMSSIALAAEDPVQPNAPRN
NVLRYFDYVFTGVFTFEMVIKMIDLGLVLHQGAYFRDLWNILDFIVVSGALVAFAFTGNSKGKDINTIKSLRV
LRVLRPLKTIKRLPKLKAVFDCVVNSLKNVFNILIVYMLFMFIFAVVAVQLFKGKFFHCTDESKEFERDCRGK
YLLYEKNEVKARDREWKKYEFHYDNVLWALLTLFTVSTGEGWPQVLKHSVDATFENQGPSPGYRMEMSIFYVV
YFVVFPFFFVNIFVALIIITFQEQGDKMMEEYSLEKNERACIDFAISAKPLTRHMPQNKQSFQYRMWQFVVSP
PFEYTIMAMIALNTIVLMMKFYGASVAYENALRVFNIVFTSLFSLECVLKVMAFGILNYFRDAWNIFDFVTVL
GSITDILVTEFGNNFINLSFLRLFRAARLIKLLRQGYTIRILLWTFVQSFKALPYVCLLIAMLFFIYAIIGMQ
VFGNIGIDGEDEDSDEDEFQITEHNNFRTFFQALMLLFRSATGEAWHNIMLSCLSGKPCDKNSGILTADCGNE
FAYFYFVSFIFLCSFLMLNLFVAVIMDNFEYLTRDSSILGPHHLDEYVRVWAEYDPAACGRIHYKDMYSLLRV
ISPPLGLGKKCPHRVACKRLLRMDLPVADDNTVHFNSTLMALIRTALDIKIAKGGADKQQMDAELRKEMMAIW
PNLSQKTLDLLVTPHKSTDLTVGKIYAAMMIMEYYRQSKAKKLQAMREEQNRTPLMFQRMEPPSPTQEGGPSQ
NALPSTQLDPGGGLMAHEGGMKESPSWVTQRAQEMFQKTGTWSPERGPPIDMPNSQPNSQSVEMREMGTDGYS
DSEHYLPMEGQTRAASMPRLPAENQRRRGRPRGNDLSTISDTSPMKRSASVLGPKARRLDDYSLERVPPEENQ
RYHQRRRDRGHHEAVVSAGTIVQRQATCLVYTPLSALWADTLMWTQVWGQI
>ENSMUSP00000112436_c.6337:G>ATGAGGCAGTGGTCAGTGCTGGGACTATAGTTCAAAGGCAGGCC
ACCTGTCTTGTATACA_p.R2113ins-fs ENSMUST00000121390.8|Cacna1a-
201|ENSMUSG00000034656|+|chr8:g.85363283:G>ATGAGGCAGTGGTCAGTGCTGGGACTATAG
TTCAAAGGCAGGCCACCTGTCTTGTATACA
MARFGDEMPGRYGAGGGGSGPAAGVVVGAAGGRGAGGSRQGGQPGAQRMYKQSMAQRARTMALYNPIPVRQNC
LTVNRSLFLFSEDNVVRKYAKKITEWPPFEYMILATIIANCIVLALEQHLPDDDKTPMSERLDDTEPYFIGIF
CFEAGIKIVALGFAFHKGSYLRNGWNVMDFVVVLTGILATVGTEFDLRTLRAVRVLRPLKLVSGIPSLQVVLK
SIMKAMIPLLQIGLLLFFAILIFAIIGLEFYMGKFHTTCFEEGTDDIQGESPAPCGTEEPARTCPNGTKCQPY
WEGPNNGITQFDNILFAVLTVFQCITMEGWTDLLYNSNDASGNTWNWLYFIPLIIIGSFFMLNLVLGVLSGEF
AKERERVENRRAFLKLRRQQQIERELNGYMEWISKAEEVILAEDETDVEQRHPFDGALRRATLKKSKTDLLNP
EEAEDQLADIASVGSPFARASIKSAKLENSTFFHKKERRMRFYIRRMVKTQAFYWTVLSLVALNTLCVAIVHY
NQPEWLSDFLYYAEFIFLGLFMSEMFIKMYGLGTRPYFHSSFNCFDCGVIIGSIFEVIWAVIKPGTSFGISVL

RALRLLRIFKVTKYWASLRNLVVSLLNSMKSIISLLFLLFLFIVVFALLGMQLFGGQFNFDEGTPPTNFDTFP
AAIMTVFQILTGEDWNEVMYDGIKSQGGVQGGMVFSIYFIVLTLFGNYTLLNVFLAIAVDNLANAQELTKDEQ
EEEEAANQKLALQKAKEVAEVSPLSAANMSIAVKEQQKNQKPTKSVWEQRTSEMRKQNLLASREALYGDAAER
WPTPYARPLRPDVKTHLDRPLVVDPQENRNNNTNKSRAPEALRPTARPRESARDPDARRAWPGSPERAPGREG
PYGRESEPQQREHAPPREHAPWDADTERAKAGDAPRRHTHRPVAEGEPRRHRARRRPGDEPDDRPERRPRPRD
ATRPARAADGEGDDGERKRRHRHGPPAHDDRERRHRRRKENQGSGVPVSGPNLSTTRPIQQDLGRQDLPLAED
LDNMKNNKLATGEPASPHDSLGHSGLPPSPAKIGNSTNPGPALATNPQNAASRRTPNNPGNPSNPGPPKTPEN
SLIVTNPSSTQPNSAKTARKPEHMAVEIPPACPPLNHTVVQVNKNANPDPLPKKEEEKKEEEEADPGEDGPKP
MPPYSSMFILSTTNPLRRLCHYILNLRYFEMCILMVIAMSSIALAAEDPVQPNAPRNNVLRYFDYVFTGVFTF
EMVIKMIDLGLVLHQGAYFRDLWNILDFIVVSGALVAFAFTGNSKGKDINTIKSLRVLRVLRPLKTIKRLPKL
KAVFDCVVNSLKNVFNILIVYMLFMFIFAVVAVQLFKGKFFHCTDESKEFERDCRGKYLLYEKNEVKARDREW
KKYEFHYDNVLWALLTLFTVSTGEGWPQVLKHSVDATFENQGPSPGYRMEMSIFYVVYFVVFPFFFVNIFVAL
IIITFQEQGDKMMEEYSLEKNERACIDFAISAKPLTRHMPQNKQSFQYRMWQFVVSPPFEYTIMAMIALNTIV
LMMKFYGASVAYENALRVFNIVFTSLFSLECVLKVMAFGILNYFRDAWNIFDFVTVLGSITDILVTEFGNNFI
NLSFLRLFRAARLIKLLRQGYTIRILLWTFVQSFKALPYVCLLIAMLFFIYAIIGMQVFGNIGIDGEDEDSDE
DEFQITEHNNFRTFFQALMLLFRSATGEAWHNIMLSCLSGKPCDKNSGILTADCGNEFAYFYFVSFIFLCSFL
MLNLFVAVIMDNFEYLTRDSSILGPHHLDEYVRVWAEYDPAACGRIHYKDMYSLLRVISPPLGLGKKCPHRVA
CKRLLRMDLPVADDNTVHFNSTLMALIRTALDIKIAKGGADKQQMDAELRKEMMAIWPNLSQKTLDLLVTPHK
STDLTVGKIYAAMMIMEYYRQSKAKKLQAMREEQNRTPLMFQRMEPPSPTQEGGPSQNALPSTQLDPGGGLMA
HEGGMKESPSWVTQRAQEMFQKTGTWSPERGPPIDMPNSQPNSQSVEMREMGTDGYSDSEHYLPMEGQTRAAS
MPRLPAENQRRRGRPRGNDLSTISDTSPMKRSASVLGPKARRLDDYSLERVPPEENQRYHQRRRDRGHHEAVV
SAGTIVQRQATCLVYTPLSALWADTLMWTQVWGQI
>ENSMUSP00000114055_c.6196:G>ATGAGGCAGTGGTCAGTGCTGGGACTATAGTTCAAAGGCAGGCC
ACCTGTCTTGTATACA_p.R2066ins-fs ENSMUST00000122053.2|Cacna1a-
202|ENSMUSG00000034656|+|chr8:g.85363283:G>ATGAGGCAGTGGTCAGTGCTGGGACTATAG
TTCAAAGGCAGGCCACCTGTCTTGTATACA
MARFGDEMPGRYGAGGGGSGPAAGVVVGAAGGRGAGGSRQGGQPGAQRMYKQSMAQRARTMALYNPIPVRQNC
LTVNRSLFLFSEDNVDDTEPYFIGIFCFEAGIKIVALGFAFHKGSYLRNGWNVMDFVVVLTGILATVGTEFDL
RTLRAVRVLRPLKLVSGIPSLQVVLKSIMKAMIPLLQIGLLLFFAILIFAIIGLEFYMGKFHTTCFEEGTDDI
QGESPAPCGTEEPARTCPNGTKCQPYWEGPNNGITQFDNILFAVLTVFQCITMEGWTDLLYNSNDASGNTWNW
LYFIPLIIIGSFFMLNLVLGVLSGEFAKERERVENRRAFLKLRRQQQIERELNGYMEWISKAEEVILAEDETD
VEQRHPFDGALRRATLKKSKTDLLNPEEAEDQLADIASVGSPFARASIKSAKLENSTFFHKKERRMRFYIRRM
VKTQAFYWTVLSLVALNTLCVAIVHYNQPEWLSDFLYYAEFIFLGLFMSEMFIKMYGLGTRPYFHSSFNCFDC
GVIIGSIFEVIWAVIKPGTSFGISVLRALRLLRIFKVTKYWASLRNLVVSLLNSMKSIISLLFLLFLFIVVFA
LLGMQLFGGQFNFDEGTPPTNFDTFPAAIMTVFQILTGEDWNEVMYDGIKSQGGVQGGMVFSIYFIVLTLFGN
YTLLNVFLAIAVDNLANAQELTKDEQEEEEAANQKLALQKAKEVAEVSPLSAANMSIAVKEQQKNQKPTKSVW
EQRTSEMRKQNLLASREALYGDAAERWPTPYARPLRPDVKTHLDRPLVVDPQENRNNNTNKSRAPEALRPTAR
PRESARDPDARRAWPGSPERAPGREGPYGRESEPQQREHAPPREHAPWDADTERAKAGDAPRRHTHRPVAEGE
PRRHRARRRPGDEPDDRPERRPRPRDATRPARAADGEGDDGERKRRHRHGPPAHDDRERRHRRRKENQGSGVP
VSGPNLSTTRPIQQDLGRQDLPLAEDLDNMKNNKLATGEPASPHDSLGHSGLPPSPAKIGNSTNPGPALATNP
QNAASRRTPNNPGNPSNPGPPKTPENSLIVTNPSSTQPNSAKTARKPEHMAVEIPPACPPLNHTVVQVNKNAN
PDPLPKKEEEKKEEEEADPGEDGPKPMPPYSSMFILSTTNPLRRLCHYILNLRYFEMCILMVIAMSSIALAAE
DPVQPNAPRNNVLRYFDYVFTGVFTFEMVIKMIDLGLVLHQGAYFRDLWNILDFIVVSGALVAFAFTGNSKGK
DINTIKSLRVLRVLRPLKTIKRLPKLKAVFDCVVNSLKNVFNILIVYMLFMFIFAVVAVQLFKGKFFHCTDES
KEFERDCRGKYLLYEKNEVKARDREWKKYEFHYDNVLWALLTLFTVSTGEGWPQVLKHSVDATFENQGPSPGY
RMEMSIFYVVYFVVFPFFFVNIFVALIIITFQEQGDKMMEEYSLEKNERACIDFAISAKPLTRHMPQNKQSFQ
YRMWQFVVSPPFEYTIMAMIALNTIVLMMKFYGASVAYENALRVFNIVFTSLFSLECVLKVMAFGILNYFRDA
WNIFDFVTVLGSITDILVTEFGNNFINLSFLRLFRAARLIKLLRQGYTIRILLWTFVQSFKALPYVCLLIAML
FFIYAIIGMQVFGNIGIDGEDEDSDEDEFQITEHNNFRTFFQALMLLFRSATGEAWHNIMLSCLSGKPCDKNS
GILTADCGNEFAYFYFVSFIFLCSFLMLNLFVAVIMDNFEYLTRDSSILGPHHLDEYVRVWAEYDPAACGRIH
YKDMYSLLRVISPPLGLGKKCPHRVACKRLLRMDLPVADDNTVHFNSTLMALIRTALDIKIAKGGADKQQMDA
ELRKEMMAIWPNLSQKTLDLLVTPHKSTDLTVGKIYAAMMIMEYYRQSKAKKLQAMREEQNRTPLMFQRMEPP
SPTQEGGPSQNALPSTQLDPGGGLMAHEGGMKESPSWVTQRAQEMFQKTGTWSPERGPPIDMPNSQPNSQSVE
MREMGTDGYSDSEHYLPMEGQTRAASMPRLPAENQRRRGRPRGNDLSTISDTSPMKRSASVLGPKARRLDDYS
LERVPPEENQRYHQRRRDRGHHEAVVSAGTIVQRQATCLVYTPLSALWADTLMWTQVWGQI
>ENSMUSP00000149242_c.6196:G>ATGAGGCAGTGGTCAGTGCTGGGACTATAGTTCAAAGGCAGGCC
ACCTGTCTTGTATACA_p.R2066ins-fs ENSMUST00000215756.2|Cacna1a-

211|ENSMUSG00000034656|+|chr8:g.85363283:G>ATGAGGCAGTGGTCAGTGCTGGGACTATAG
TTCAAAGGCAGGCCACCTGTCTTGTATACA
XRMYKQSMAQRARTMALYNPIPVRQNCLTVNRSLFLFSEDNVVRKYAKKITEWPPFEYMILATIIANCIVLAL
EQHLPDDDKTPMSERLDDTEPYFIGIFCFEAGIKIVALGFAFHKGSYLRNGWNVMDFVVVLTGILATVGTEFD
LRTLRAVRVLRPLKLVSGIPSLQVVLKSIMKAMIPLLQIGLLLFFAILIFAIIGLEFYMGKFHTTCFEEGTDD
IQGESPAPCGTEEPARTCPNGTKCQPYWEGPNNGITQFDNILFAVLTVFQCITMEGWTDLLYNSNDASGNTWN
WLYFIPLIIIGSFFMLNLVLGVLSGEFAKERERVENRRAFLKLRRQQQIERELNGYMEWISKAEEVILAEDET
DVEQRHPFDALRRATLKKSKTDLLNPEEAEDQLADIASVGSPFARASIKSAKLENSTFFHKKERRMRFYIRRM
VKTQAFYWTVLSLVALNTLCVAIVHYNQPEWLSDFLYYAEFIFLGLFMSEMFIKMYGLGTRPYFHSSFNCFDC
GVIIGSIFEVIWAVIKPGTSFGISVLRALRLLRIFKVTKYWASLRNLVVSLLNSMKSIISLLFLLFLFIVVFA
LLGMQLFGGQFNFDEGTPPTNFDTFPAAIMTVFQILTGEDWNEVMYDGIKSQGGVQGGMVFSIYFIVLTLFGN
YTLLNVFLAIAVDNLANAQELTKDEQEEEAANQKLALQKAKEVAEVSPLSAANMSIAVKEQQKNQKPTKSVW
EQRTSEMRKQNLLASREALYGDAAERWPTPYARPLRPDVKTHLDRPLVVDPQENRNNNTNKSRAPEALRPTAR
PRESARDPDARRAWPGSPERAPGREGPYGRESEPQQREHAPPREHAPWDADTERAKAGDAPRRHTHRPVAEGE
PRRHRARRRPGDEPDDRPERRPRPRDATRPARAADGEGDDGERKRRHRHGPPAHDDRERRHRRRKENQGSGVP
VSGPNLSTTRPIQQDLGRQDLPLAEDLDNMKNNKLATGEPASPHDSLGHSGLPPSPAKIGNSTNPGPALATNP
QNAASRRTPNNPGNPSNPGPPKTPENSLIVTNPSSTQPNSAKTARKPEHMAVEIPPACPPLNHTVVQVNKNAN
PDPLPKKEEEKKEEEEADPGEDGPKPMPPYSSMFILSTTNPLRRLCHYILNLRYFEMCILMVIAMSSIALAAE
DPVQPNAPRNNVLRYFDYVFTGVFTFEMVIKMIDLGLVLHQGAYFRDLWNILDFIVVSGALVAFAFTGNSKGK
DINTIKSLRVLRVLRPLKTIKRLPKLKAVFDCVVNSLKNVFNILIVYMLFMFIFAVVAVQLFKGKFFHCTDES
KEFERDCRGKYLLYEKNEVKARDREWKKYEFHYDNVLWALLTLFTVSTGEGWPQVLKHSVDATFENQGPSPGY
RMEMSIFYVVYFVVFPFFFVNIFVALIIITFQEQGDKMMEEYSLEKNERACIDFAISAKPLTRHMPQNKQSFQ
YRMWQFVVSPPFEYTIMAMIALNTIVLMMKFYGASVAYENALRVFNIVFTSLFSLECVLKVMAFGILNYFRDA
WNIFDFVTVLGSITDILVTEFGNNFINLSFLRLFRAARLIKLLRQGYTIRILLWTFVQSFKALPYVCLLIAML
FFIYAIIGMQVFGNIGIDGEDEDSDEDEFQITEHNNFRTFFQALMLLFRSATGEAWHNIMLSCLSGKPCDKNS
GILTADCGNEFAYFYFVSFIFLCSFLMLNLFVAVIMDNFEYLTRDSSILGPHHLDEYVRVWAEYDPAACGRIH
YKDMYSLLRVISPPLGLGKKCPHRVACKRLLRMDLPVADDNTVHFNSTLMALIRTALDIKIAKGGADKQQMDA
ELRKEMMAIWPNLSQKTLDLLVTPHKSTDLTVGKIYAAMMIMEYYRQSKAKKLQAMREEQNRTPLMFQRMEPP
SPTQEGGPSQNALPSTQLDPGGGLMAHEGGMKESPSWVTQRAQEMFQKTGTWSPERGPPIDMPNSQPNSQSVE
MREMGTDGYSDSEHYLPMEGQTRAASMPRLPAENQRRRGRPRGNDLSTISDTSPMKRSASVLGPKARRLDDYS
LERVPPEENQRYHQRRRDRGHHEAVVSAGTIVQRQATCLVYTPLSALWADTLMWTQVWGQI
>ENSMUSP00000158675_c.1561:G>ATGAGGCAGTGGTCAGTGCTGGGACTATAGTTCAAAGGCAGGCC
ACCTGTCTTGTATACA_p.R521ins-fs ENSMUST00000238337.2|Cacna1a-
212|ENSMUSG00000034656|+|chr8:g.85363283:G>ATGAGGCAGTGGTCAGTGCTGGGACTATAG
TTCAAAGGCAGGCCACCTGTCTTGTATACA
MHLLRLSGQRGLTLQPNSPSSLPSSPFPLAKGNNFINLSFLRLFRAARLIKLLRQGYTIRILLWTFVQSFKVF
GNIGIDGEDEDSDEDEFQITEHNNFRTFFQALMLLFRSATGEAWHNIMLSCLSGKPCDKNSGILTADCGNEFA
YFYFVSFIFLCSFLMLNLFVAVIMDNFEYLTRDSSILGPHHLDEYVRVWAEYDPAACGRIHYKDMYSLLRVIS
PPLGLGKKCPHRVACKRLLRMDLPVADDNTVHFNSTLMALIRTALDIKIAKGGADKQQMDAELRKEMMAIWPN
LSQKTLDLLVTPHKSTDLTVGKIYAAMMIMEYYRQSKAKKLQAMREEQNRTPLMFQRMEPPSPTQEGGPSQNA
LPSTQLDPGGGLMAHEGGMKESPSWVTQRAQEMFQKTGTWSPERGPPIDMPNSQPNSQSVEMREMGTDGYSDS
EHYLPMEGQTRAASMPRLPAENQRRRGRPRGNDLSTISDTSPMKRSASVLGPKARRLDDYSLERVPPEENQRY
HQRRRDRGHHEAVVSAGTIVQRQATCLVYTPLSALWADTLMWTQVWGQI
>rs252377326_ENSMUSP00000158803_c.6615:C>T_p.R2206C
ENSMUST00000238701.2|Cacna1a-213|ENSMUSG00000034656|+|chr8:g.85363294:C>T
MPRASWGSDPMLSPLSHELLHSPRYPGTDCTLAGSSRTLHVPWSQQMSGSLEGPTLKRRGGVVDHRDVILAHQ
AHKMHSTPQARRKEWEMARFGDEMPGRYGAGGGGSGPAAGVVVGAAGGRGAGGSRQGGQPGAQRMYKQSMAQR
ARTMALYNPIPVRQNCLTVNRSLFLFSEDNVVRKYAKKITEWPPFEYMILATIIANCIVLALEQHLPDDDKTP
MSERLDDTEPYFIGIFCFEAGIKIVALGFAFHKGSYLRNGWNVMDFVVVLTGILATVGTEFDLRTLRAVRVLR
PLKLVSGIPSLQVVLKSIMKAMIPLLQIGLLLFFAILIFAIIGLEFYMGKFHTTCFEEGTDDIQGESPAPCGT
EEPARTCPNGTKCQPYWEGPNNGITQFDNILFAVLTVFQCITMEGWTDLLYNSNDASGNTWNWLYFIPLIIIG
SFFMLNLVLGVLSGEFAKERERVENRRAFLKLRRQQQIERELNGYMEWISKAEEVILAEDETDVEQRHPFDGA
LRRATLKKSKTDLLNPEEAEDQLADIASVGSPFARASIKSAKLENSTFFHKKERRMRFYIRRMVKTQAFYWTV
LSLVALNTLCVAIVHYNQPEWLSDFLYYAEFIFLGLFMSEMFIKMYGLGTRPYFHSSFNCFDCGVIIGSIFEV
IWAVIKPGTSFGISVLRALRLLRIFKVTKYWASLRNLVVSLLNSMKSIISLLFLLFLFIVVFALLGMQLFGGQ
FNFDEGTPPTNFDTFPAAIMTVFQILTGEDWNEVMYDGIKSQGGVQGGMVFSIYFIVLTLFGNYTLLNVFLAI
AVDNLANAQELTKDEQEEEAANQKLALQKAKEVAEVSPLSAANMSIAVKEQQKNQKPTKSVWEQRTSEMRKQ
NLLASREALYGDAAERWPTPYARPLRPDVKTHLDRPLVVDPQENRNNNTNKSRAPEALRPTARPRESARDPDA

RRAWPGSPERAPGREGPYGRESEPQQREHAPPREHAPWDADTERAKAGDAPRRHTHRPVAEGEPRRHRARRRP
GDEPDDRPERRPRPRDATRPARAADGEGDDGERKRRHRHGPPAHDDRERRHRRRKENQGSGVPVSGPNLSTTR
PIQQDLGRQDLPLAEDLDNMKNNKLATGEPASPHDSLGHSGLPPSPAKIGNSTNPGPALATNPQNAASRRTPN
NPGNPSNPGPPKTPENSLIVTNPSSTQPNSAKTARKPEHMAVEIPPACPPLNHTVVQVNKNANPDPLPKKEEE
KKEEEEADPGEDGPKPMPPYSSMFILSTTNPLRRLCHYILNLRYFEMCILMVIAMSSIALAAEDPVQPNAPRN
NVLRYFDYVFTGVFTFEMVIKMIDLGLVLHQGAYFRDLWNILDFIVVSGALVAFAFTGNSKGKDINTIKSLRV
LRVLRPLKTIKRLPKLKAVFDCVVNSLKNVFNILIVYMLFMFIFAVVAVQLFKGKFFHCTDESKEFERDCRGK
YLLYEKNEVKARDREWKKYEFHYDNVLWALLTLFTVSTGEGWPQVLKHSVDATFENQGPSPGYRMEMSIFYVV
YFVVFPFFFVNIFVALIIITFQEQGDKMMEEYSLEKNERACIDFAISAKPLTRHMPQNKQSFQYRMWQFVVSP
PFEYTIMAMIALNTIVLMMKFYGASVAYENALRVFNIVFTSLFSLECVLKVMAFGILNYFRDAWNIFDFVTVL
GSITDILVTEFGNNFINLSFLRLFRAARLIKLLRQGYTIRILLWTFVQSFKALPYVCLLIAMLFFIYAIIGMQ
VFGNIGIDGEDEDSDEDEFQITEHNNFRTFFQALMLLFRSATGEAWHNIMLSCLSGKPCDKNSGILTADCGNE
FAYFYFVSFIFLCSFLMLNLFVAVIMDNFEYLTRDSSILGPHHLDEYVRVWAEYDPAACGRIHYKDMYSLLRV
ISPPLGLGKKCPHRVACKRLLRMDLPVADDNTVHFNSTLMALIRTALDIKIAKGGADKQQMDAELRKEMMAIW
PNLSQKTLDLLVTPHKSTDLTVGKIYAAMMIMEYYRQSKAKKLQAMREEQNRTPLMFQRMEPPSPTQEGGPSQ
NALPSTQLDPGGGLMAHEGGMKESPSWVTQRAQEMFQKTGTWSPERGPPIDMPNSQPNSQSVEMREMGTDGYS
DSEHYLPMEGQTRAASMPRLPAENQRRRGRPRGNDLSTISDTSPMKRSASVLGPKARRLDDYSLERVPPEENQ
RYHQRRRDRGHRTSECSLGRYTDVDTGLGTDLSMTTQSGDLPSKDRDQDRGRPKDRKHRPHHHHHHHHHHPPA
PDRDRYAQERPDTGRARAREQRWSRSPSEGREHTTHRQGSSSVSGSPAPSTSGTSTPRRGRRQLPQTPCTPRP
LVSYSPAPRRPAARRMAGPAAPPGGSPRGCRRAPRWPAHAPEGPRPRGADYTEPDSPREPPGGAHDPAPRSPR
TPRAAGCASPRHGRRLPNGYYAGHGAPRPRTARRGAHDAYSESEDDWC*
>rs252377326_ENSMUSP00000112436_c.6348:C>T_p.R2117C
ENSMUST00000121390.8|Cacna1a-201|ENSMUSG00000034656|+|chr8:g.85363294:C>T
MARFGDEMPGRYGAGGGGSGPAAGVVVGAAGGRGAGGSRQGGQPGAQRMYKQSMAQRARTMALYNPIPVRQNC
LTVNRSLFLFSEDNVVRKYAKKITEWPPFEYMILATIIANCIVLALEQHLPDDDKTPMSERLDDTEPYFIGIF
CFEAGIKIVALGFAFHKGSYLRNGWNVMDFVVVLTGILATVGTEFDLRTLRAVRVLRPLKLVSGIPSLQVVLK
SIMKAMIPLLQIGLLLFFAILIFAIIGLEFYMGKFHTTCFEEGTDDIQGESPAPCGTEEPARTCPNGTKCQPY
WEGPNNGITQFDNILFAVLTVFQCITMEGWTDLLYNSNDASGNTWNWLYFIPLIIIGSFFMLNLVLGVLSGEF
AKERERVENRRAFLKLRRQQQIERELNGYMEWISKAEEVILAEDETDVEQRHPFDGALRRATLKKSKTDLLNP
EEAEDQLADIASVGSPFARASIKSAKLENSTFFHKKERRMRFYIRRMVKTQAFYWTVLSLVALNTLCVAIVHY
NQPEWLSDFLYYAEFIFLGLFMSEMFIKMYGLGTRPYFHSSFNCFDCGVIIGSIFEVIWAVIKPGTSFGISVL
RALRLLRIFKVTKYWASLRNLVVSLLNSMKSIISLLFLLFLFIVVFALLGMQLFGGQFNFDEGTPPTNFDTFP
AAIMTVFQILTGEDWNEVMYDGIKSQGGVQGGMVFSIYFIVLTLFGNYTLLNVFLAIAVDNLANAQELTKDEQ
EEEEAANQKLALQKAKEVAEVSPLSAANMSIAVKEQQKNQKPTKSVWEQRTSEMRKQNLLASREALYGDAAER
WPTPYARPLRPDVKTHLDRPLVVDPQENRNNNTNKSRAPEALRPTARPRESARDPDARRAWPGSPERAPGREG
PYGRESEPQQREHAPPREHAPWDADTERAKAGDAPRRHTHRPVAEGEPRRHRARRRPGDEPDDRPERRPRPRD
ATRPARAADGEGDDGERKRRHRHGPPAHDDRERRHRRRKENQGSGVPVSGPNLSTTRPIQQDLGRQDLPLAED
LDNMKNNKLATGEPASPHDSLGHSGLPPSPAKIGNSTNPGPALATNPQNAASRRTPNNPGNPSNPGPPKTPEN
SLIVTNPSSTQPNSAKTARKPEHMAVEIPPACPPLNHTVVQVNKNANPDPLPKKEEEKKEEEEADPGEDGPKP
MPPYSSMFILSTTNPLRRLCHYILNLRYFEMCILMVIAMSSIALAAEDPVQPNAPRNNVLRYFDYVFTGVFTF
EMVIKMIDLGLVLHQGAYFRDLWNILDFIVVSGALVAFAFTGNSKGKDINTIKSLRVLRVLRPLKTIKRLPKL
KAVFDCVVNSLKNVFNILIVYMLFMFIFAVVAVQLFKGKFFHCTDESKEFERDCRGKYLLYEKNEVKARDREW
KKYEFHYDNVLWALLTLFTVSTGEGWPQVLKHSVDATFENQGPSPGYRMEMSIFYVVYFVVFPFFFVNIFVAL
IIITFQEQGDKMMEEYSLEKNERACIDFAISAKPLTRHMPQNKQSFQYRMWQFVVSPPFEYTIMAMIALNTIV
LMMKFYGASVAYENALRVFNIVFTSLFSLECVLKVMAFGILNYFRDAWNIFDFVTVLGSITDILVTEFGNNFI
NLSFLRLFRAARLIKLLRQGYTIRILLWTFVQSFKALPYVCLLIAMLFFIYAIIGMQVFGNIGIDGEDEDSDE
DEFQITEHNNFRTFFQALMLLFRSATGEAWHNIMLSCLSGKPCDKNSGILTADCGNEFAYFYFVSFIFLCSFL
MLNLFVAVIMDNFEYLTRDSSILGPHHLDEYVRVWAEYDPAACGRIHYKDMYSLLRVISPPLGLGKKCPHRVA
CKRLLRMDLPVADDNTVHFNSTLMALIRTALDIKIAKGGADKQQMDAELRKEMMAIWPNLSQKTLDLLVTPHK
STDLTVGKIYAAMMIMEYYRQSKAKKLQAMREEQNRTPLMFQRMEPPSPTQEGGPSQNALPSTQLDPGGGLMA
HEGGMKESPSWVTQRAQEMFQKTGTWSPERGPPIDMPNSQPNSQSVEMREMGTDGYSDSEHYLPMEGQTRAAS
MPRLPAENQRRRGRPRGNDLSTISDTSPMKRSASVLGPKARRLDDYSLERVPPEENQRYHQRRRDRGHRTSEC
SLGRYTDVDTGLGTDLSMTTQSGDLPSKDRDQDRGRPKDRKHRPHHHHHHHHHHPPAPDRDRYAQERPDTGRA
RAREQRWSRSPSEGREHTTHRQGSSSVSGSPAPSTSGTSTPRRGRRQLPQTPCTPRPLVSYSPAPRRPAARRM
AGPAAPPGGSPRGCRRAPRWPAHAPEGPRPRGADYTEPDSPREPPGGAHDPAPRSPRTPRAAGCASPRHGRRL
PNGYYAGHGAPRPRTARRGAHDAYSESEDDWC*
>rs252377326_ENSMUSP00000114055_c.6207:C>T_p.R2070C
ENSMUST00000122053.2|Cacna1a-202|ENSMUSG00000034656|+|chr8:g.85363294:C>T

MARFGDEMPGRYGAGGGGSGPAAGVVVGAAGGRGAGGSRQGGQPGAQRMYKQSMAQRARTMALYNPIPVRQNC
LTVNRSLFLFSEDNVDDTEPYFIGIFCFEAGIKIVALGFAFHKGSYLRNGWNVMDFVVVLTGILATVGTEFDL
RTLRAVRVLRPLKLVSGIPSLQVVLKSIMKAMIPLLQIGLLLFFAILIFAIIGLEFYMGKFHTTCFEEGTDDI
QGESPAPCGTEEPARTCPNGTKCQPYWEGPNNGITQFDNILFAVLTVFQCITMEGWTDLLYNSNDASGNTWNW
LYFIPLIIIGSFFMLNLVLGVLSGEFAKERERVENRRAFLKLRRQQQIERELNGYMEWISKAEEVILAEDETD
VEQRHPFDGALRRATLKKSKTDLLNPEEAEDQLADIASVGSPFARASIKSAKLENSTFFHKKERRMRFYIRRM
VKTQAFYWTVLSLVALNTLCVAIVHYNQPEWLSDFLYYAEFIFLGLFMSEMFIKMYGLGTRPYFHSSFNCFDC
GVIIGSIFEVIWAVIKPGTSFGISVLRALRLLRIFKVTKYWASLRNLVVSLLNSMKSIISLLFLLFLFIVVFA
LLGMQLFGGQFNFDEGTPPTNFDTFPAAIMTVFQILTGEDWNEVMYDGIKSQGGVQGGMVFSIYFIVLTLFGN
YTLLNVFLAIAVDNLANAQELTKDEQEEEEAANQKLALQKAKEVAEVSPLSAANMSIAVKEQQKNQKPTKSVW
EQRTSEMRKQNLLASREALYGDAAERWPTPYARPLRPDVKTHLDRPLVVDPQENRNNNTNKSRAPEALRPTAR
PRESARDPDARRAWPGSPERAPGREGPYGRESEPQQREHAPPREHAPWDADTERAKAGDAPRRHTHRPVAEGE
PRRHRARRRPGDEPDDRPERRPRPRDATRPARAADGEGDDGERKRRHRHGPPAHDDRERRHRRRKENQGSGVP
VSGPNLSTTRPIQQDLGRQDLPLAEDLDNMKNNKLATGEPASPHDSLGHSGLPPSPAKIGNSTNPGPALATNP
QNAASRRTPNNPGNPSNPGPPKTPENSLIVTNPSSTQPNSAKTARKPEHMAVEIPPACPPLNHTVVQVNKNAN
PDPLPKKEEEKKEEEEADPGEDGPKPMPPYSSMFILSTTNPLRRLCHYILNLRYFEMCILMVIAMSSIALAAE
DPVQPNAPRNNVLRYFDYVFTGVFTFEMVIKMIDLGLVLHQGAYFRDLWNILDFIVVSGALVAFAFTGNSKGK
DINTIKSLRVLRVLRPLKTIKRLPKLKAVFDCVVNSLKNVFNILIVYMLFMFIFAVVAVQLFKGKFFHCTDES
KEFERDCRGKYLLYEKNEVKARDREWKKYEFHYDNVLWALLTLFTVSTGEGWPQVLKHSVDATFENQGPSPGY
RMEMSIFYVVYFVVFPFFFVNIFVALIIITFQEQGDKMMEEYSLEKNERACIDFAISAKPLTRHMPQNKQSFQ
YRMWQFVVSPPFEYTIMAMIALNTIVLMMKFYGASVAYENALRVFNIVFTSLFSLECVLKVMAFGILNYFRDA
WNIFDFVTVLGSITDILVTEFGNNFINLSFLRLFRAARLIKLLRQGYTIRILLWTFVQSFKALPYVCLLIAML
FFIYAIIGMQVFGNIGIDGEDEDSDEDEFQITEHNNFRTFFQALMLLFRSATGEAWHNIMLSCLSGKPCDKNS
GILTADCGNEFAYFYFVSFIFLCSFLMLNLFVAVIMDNFEYLTRDSSILGPHHLDEYVRVWAEYDPAACGRIH
YKDMYSLLRVISPPLGLGKKCPHRVACKRLLRMDLPVADDNTVHFNSTLMALIRTALDIKIAKGGADKQQMDA
ELRKEMMAIWPNLSQKTLDLLVTPHKSTDLTVGKIYAAMMIMEYYRQSKAKKLQAMREEQNRTPLMFQRMEPP
SPTQEGGPSQNALPSTQLDPGGGLMAHEGGMKESPSWVTQRAQEMFQKTGTWSPERGPPIDMPNSQPNSQSVE
MREMGTDGYSDSEHYLPMEGQTRAASMPRLPAENQRRRGRPRGNDLSTISDTSPMKRSASVLGPKARRLDDYS
LERVPPEENQRYHQRRRDRGHRTSECSLGRYTDVDTGLGTDLSMTTQSGDLPSKDRDQDRGRPKDRKHRPHHH
HHHHHHHPPAPDRDRYAQERPDTGRARAREQRWSRSPSEGREHTTHRQGSSSVSGSPAPSTSGTSTPRRGRRQ
LPQTPCTPRPLVSYSPAPRRPAARRMAGPAAPPGGSPRGCRRAPRWPAHAPEGPRPRGADYTEPDSPREPPGG
AHDPAPRSPRTPRAAGCASPRHGRRLPNGYYAGHGAPRPRTARRGAHDAYSESEDDWC*
>rs252377326_ENSMUSP00000149242_c.6207:C>T_p.R2070C
ENSMUST00000215756.2|Cacna1a-211|ENSMUSG00000034656|+|chr8:g.85363294:C>T
XRMYKQSMAQRARTMALYNPIPVRQNCLTVNRSLFLFSEDNVVRKYAKKITEWPPFEYMILATIIANCIVLAL
EQHLPDDDKTPMSERLDDTEPYFIGIFCFEAGIKIVALGFAFHKGSYLRNGWNVMDFVVVLTGILATVGTEFD
LRTLRAVRVLRPLKLVSGIPSLQVVLKSIMKAMIPLLQIGLLLFFAILIFAIIGLEFYMGKFHTTCFEEGTDD
IQGESPAPCGTEEPARTCPNGTKCQPYWEGPNNGITQFDNILFAVLTVFQCITMEGWTDLLYNSNDASGNTWN
WLYFIPLIIIGSFFMLNLVLGVLSGEFAKERERVENRRAFLKLRRQQQIERELNGYMEWISKAEEVILAEDET
DVEQRHPFDALRRATLKKSKTDLLNPEEAEDQLADIASVGSPFARASIKSAKLENSTFFHKKERRMRFYIRRM
VKTQAFYWTVLSLVALNTLCVAIVHYNQPEWLSDFLYYAEFIFLGLFMSEMFIKMYGLGTRPYFHSSFNCFDC
GVIIGSIFEVIWAVIKPGTSFGISVLRALRLLRIFKVTKYWASLRNLVVSLLNSMKSIISLLFLLFLFIVVFA
LLGMQLFGGQFNFDEGTPPTNFDTFPAAIMTVFQILTGEDWNEVMYDGIKSQGGVQGGMVFSIYFIVLTLFGN
YTLLNVFLAIAVDNLANAQELTKDEQEEEEAANQKLALQKAKEVAEVSPLSAANMSIAVKEQQKNQKPTKSVW
EQRTSEMRKQNLLASREALYGDAAERWPTPYARPLRPDVKTHLDRPLVVDPQENRNNNTNKSRAPEALRPTAR
PRESARDPDARRAWPGSPERAPGREGPYGRESEPQQREHAPPREHAPWDADTERAKAGDAPRRHTHRPVAEGE
PRRHRARRRPGDEPDDRPERRPRPRDATRPARAADGEGDDGERKRRHRHGPPAHDDRERRHRRRKENQGSGVP
VSGPNLSTTRPIQQDLGRQDLPLAEDLDNMKNNKLATGEPASPHDSLGHSGLPPSPAKIGNSTNPGPALATNP
QNAASRRTPNNPGNPSNPGPPKTPENSLIVTNPSSTQPNSAKTARKPEHMAVEIPPACPPLNHTVVQVNKNAN
PDPLPKKEEEKKEEEEADPGEDGPKPMPPYSSMFILSTTNPLRRLCHYILNLRYFEMCILMVIAMSSIALAAE
DPVQPNAPRNNVLRYFDYVFTGVFTFEMVIKMIDLGLVLHQGAYFRDLWNILDFIVVSGALVAFAFTGNSKGK
DINTIKSLRVLRVLRPLKTIKRLPKLKAVFDCVVNSLKNVFNILIVYMLFMFIFAVVAVQLFKGKFFHCTDES
KEFERDCRGKYLLYEKNEVKARDREWKKYEFHYDNVLWALLTLFTVSTGEGWPQVLKHSVDATFENQGPSPGY
RMEMSIFYVVYFVVFPFFFVNIFVALIIITFQEQGDKMMEEYSLEKNERACIDFAISAKPLTRHMPQNKQSFQ
YRMWQFVVSPPFEYTIMAMIALNTIVLMMKFYGASVAYENALRVFNIVFTSLFSLECVLKVMAFGILNYFRDA
WNIFDFVTVLGSITDILVTEFGNNFINLSFLRLFRAARLIKLLRQGYTIRILLWTFVQSFKALPYVCLLIAML
FFIYAIIGMQVFGNIGIDGEDEDSDEDEFQITEHNNFRTFFQALMLLFRSATGEAWHNIMLSCLSGKPCDKNS
GILTADCGNEFAYFYFVSFIFLCSFLMLNLFVAVIMDNFEYLTRDSSILGPHHLDEYVRVWAEYDPAACGRIH

YKDMYSLLRVISPPLGLGKKCPHRVACKRLLRMDLPVADDNTVHFNSTLMALIRTALDIKIAKGGADKQQMDA
ELRKEMMAIWPNLSQKTLDLLVTPHKSTDLTVGKIYAAMMIMEYYRQSKAKKLQAMREEQNRTPLMFQRMEPP
SPTQEGGPSQNALPSTQLDPGGGLMAHEGGMKESPSWVTQRAQEMFQKTGTWSPERGPPIDMPNSQPNSQSVE
MREMGTDGYSDSEHYLPMEGQTRAASMPRLPAENQRRRGRPRGNDLSTISDTSPMKRSASVLGPKARRLDDYS
LERVPPEENQRYHQRRRDRGHRTSECSLGRYTDVDTGLGTDLSMTTQSGDLPSKDRDQDRGRPKDRKHRPHHH
HHHHHHHPPAPDRDRYAQERPDTGRARAREQRWSRSPSEGREHTTHRQGSSSVSGSPAPSTSGTSTPRRGRRQ
LPQTPCTPRPLVSYSPAPRRPAARRMAGPAAPPGGSPRGCRRAPRWPAHAPEGPRPRGADYTEPDSPREPPGG
AHDPAPRSPRTPRAAGCASPRHGRRLPNGYYAGHGAPRPRTARRGAHDAYSESEDDWC*
>rs252377326_ENSMUSP00000158675_c.1572:C>T_p.R525C
ENSMUST00000238337.2|Cacna1a-212|ENSMUSG00000034656|+|chr8:g.85363294:C>T
MHLLRLSGQRGLTLQPNSPSSLPSSPFPLAKGNNFINLSFLRLFRAARLIKLLRQGYTIRILLWTFVQSFKVF
GNIGIDGEDEDSDEDEFQITEHNNFRTFFQALMLLFRSATGEAWHNIMLSCLSGKPCDKNSGILTADCGNEFA
YFYFVSFIFLCSFLMLNLFVAVIMDNFEYLTRDSSILGPHHLDEYVRVWAEYDPAACGRIHYKDMYSLLRVIS
PPLGLGKKCPHRVACKRLLRMDLPVADDNTVHFNSTLMALIRTALDIKIAKGGADKQQMDAELRKEMMAIWPN
LSQKTLDLLVTPHKSTDLTVGKIYAAMMIMEYYRQSKAKKLQAMREEQNRTPLMFQRMEPPSPTQEGGPSQNA
LPSTQLDPGGGLMAHEGGMKESPSWVTQRAQEMFQKTGTWSPERGPPIDMPNSQPNSQSVEMREMGTDGYSDS
EHYLPMEGQTRAASMPRLPAENQRRRGRPRGNDLSTISDTSPMKRSASVLGPKARRLDDYSLERVPPEENQRY
HQRRRDRGHRTSECSLGRYTDVDTGLGTDLSMTTQSGDLPSKDRDQDRGRPKDRKHRPHHHHHHHHHHPPAPD
RDRYAQERPDTGRARAREQRWSRSPSEGREHTTHRQGSSSVSGSPAPSTSGTSTPRRGRRQLPQTPCTPRPLV
SYSPAPRRPAARRMAGPAAPPGGSPRGCRRAPRWPAHAPEGPRPRGADYTEPDSPREPPGGAHDPAPRSPRTP
RAAGCASPRHGRRLPNGYYAGHGAPRPRTARRGAHDAYSESEDDWC*
>rs37482583_ENSMUSP00000158803_c.7356:G>C_p.E2453Q
ENSMUST00000238701.2|Cacna1a-213|ENSMUSG00000034656|+|chr8:g.85366320:G>C
MPRASWGSDPMLSPLSHELLHSPRYPGTDCTLAGSSRTLHVPWSQQMSGSLEGPTLKRRGGVVDHRDVILAHQ
AHKMHSTPQARRKEWEMARFGDEMPGRYGAGGGGSGPAAGVVVGAAGGRGAGGSRQGGQPGAQRMYKQSMAQR
ARTMALYNPIPVRQNCLTVNRSLFLFSEDNVVRKYAKKITEWPPFEYMILATIIANCIVLALEQHLPDDDKTP
MSERLDDTEPYFIGIFCFEAGIKIVALGFAFHKGSYLRNGWNVMDFVVVLTGILATVGTEFDLRTLRAVRVLR
PLKLVSGIPSLQVVLKSIMKAMIPLLQIGLLLFFAILIFAIIGLEFYMGKFHTTCFEEGTDDIQGESPAPCGT
EEPARTCPNGTKCQPYWEGPNNGITQFDNILFAVLTVFQCITMEGWTDLLYNSNDASGNTWNWLYFIPLIIIG
SFFMLNLVLGVLSGEFAKERERVENRRAFLKLRRQQQIERELNGYMEWISKAEEVILAEDETDVEQRHPFDGA
LRRATLKKSKTDLLNPEEAEDQLADIASVGSPFARASIKSAKLENSTFFHKKERRMRFYIRRMVKTQAFYWTV
LSLVALNTLCVAIVHYNQPEWLSDFLYYAEFIFLGLFMSEMFIKMYGLGTRPYFHSSFNCFDCGVIIGSIFEV
IWAVIKPGTSFGISVLRALRLLRIFKVTKYWASLRNLVVSLLNSMKSIISLLFLLFLFIVVFALLGMQLFGGQ
FNFDEGTPPTNFDTFPAAIMTVFQILTGEDWNEVMYDGIKSQGGVQGGMVFSIYFIVLTLFGNYTLLNVFLAI
AVDNLANAQELTKDEQEEEEAANQKLALQKAKEVAEVSPLSAANMSIAVKEQQKNQKPTKSVWEQRTSEMRKQ
NLLASREALYGDAAERWPTPYARPLRPDVKTHLDRPLVVDPQENRNNNTNKSRAPEALRPTARPRESARDPDA
RRAWPGSPERAPGREGPYGRESEPQQREHAPPREHAPWDADTERAKAGDAPRRHTHRPVAEGEPRRHRARRRP
GDEPDDRPERRPRPRDATRPARAADGEGDDGERKRRHRHGPPAHDDRERRHRRRKENQGSGVPVSGPNLSTTR
PIQQDLGRQDLPLAEDLDNMKNNKLATGEPASPHDSLGHSGLPPSPAKIGNSTNPGPALATNPQNAASRRTPN
NPGNPSNPGPPKTPENSLIVTNPSSTQPNSAKTARKPEHMAVEIPPACPPLNHTVVQVNKNANPDPLPKKEEE
KKEEEEADPGEDGPKPMPPYSSMFILSTTNPLRRLCHYILNLRYFEMCILMVIAMSSIALAAEDPVQPNAPRN
NVLRYFDYVFTGVFTFEMVIKMIDLGLVLHQGAYFRDLWNILDFIVVSGALVAFAFTGNSKGKDINTIKSLRV
LRVLRPLKTIKRLPKLKAVFDCVVNSLKNVFNILIVYMLFMFIFAVVAVQLFKGKFFHCTDESKEFERDCRGK
YLLYEKNEVKARDREWKKYEFHYDNVLWALLTLFTVSTGEGWPQVLKHSVDATFENQGPSPGYRMEMSIFYVV
YFVVFPFFFVNIFVALIIITFQEQGDKMMEEYSLEKNERACIDFAISAKPLTRHMPQNKQSFQYRMWQFVVSP
PFEYTIMAMIALNTIVLMMKFYGASVAYENALRVFNIVFTSLFSLECVLKVMAFGILNYFRDAWNIFDFVTVL
GSITDILVTEFGNNFINLSFLRLFRAARLIKLLRQGYTIRILLWTFVQSFKALPYVCLLIAMLFFIYAIIGMQ
VFGNIGIDGEDEDSDEDEFQITEHNNFRTFFQALMLLFRSATGEAWHNIMLSCLSGKPCDKNSGILTADCGNE
FAYFYFVSFIFLCSFLMLNLFVAVIMDNFEYLTRDSSILGPHHLDEYVRVWAEYDPAACGRIHYKDMYSLLRV
ISPPLGLGKKCPHRVACKRLLRMDLPVADDNTVHFNSTLMALIRTALDIKIAKGGADKQQMDAELRKEMMAIW
PNLSQKTLDLLVTPHKSTDLTVGKIYAAMMIMEYYRQSKAKKLQAMREEQNRTPLMFQRMEPPSPTQEGGPSQ
NALPSTQLDPGGGLMAHEGGMKESPSWVTQRAQEMFQKTGTWSPERGPPIDMPNSQPNSQSVEMREMGTDGYS
DSEHYLPMEGQTRAASMPRLPAENQRRRGRPRGNDLSTISDTSPMKRSASVLGPKARRLDDYSLERVPPEENQ
RYHQRRRDRGHRTSERSLGRYTDVDTGLGTDLSMTTQSGDLPSKDRDQDRGRPKDRKHRPHHHHHHHHHHPPA
PDRDRYAQERPDTGRARAREQRWSRSPSEGREHTTHRQGSSSVSGSPAPSTSGTSTPRRGRRQLPQTPCTPRP
LVSYSPAPRRPAARRMAGPAAPPGGSPRGCRRAPRWPAHAPEGPRPRGADYTEPDSPREPPGGAHDPAPRSPR
TPRAAGCASPRHGRRLPNGYYAGHGAPRPRTARRGAHDAYSESQDDWC*

>rs37482583_ENSMUSP00000112436_c.7089:G>C_p.E2364Q
ENSMUST00000121390.8|Cacna1a-201|ENSMUSG00000034656|+|chr8:g.85366320:G>C
MARFGDEMPGRYGAGGGGSGPAAGVVVGAAGGRGAGGSRQGGQPGAQRMYKQSMAQRARTMALYNPIPVRQNC
LTVNRSLFLFSEDNVVRKYAKKITEWPPFEYMILATIIANCIVLALEQHLPDDDKTPMSERLDDTEPYFIGIF
CFEAGIKIVALGFAFHKGSYLRNGWNVMDFVVVLTGILATVGTEFDLRTLRAVRVLRPLKLVSGIPSLQVVLK
SIMKAMIPLLQIGLLLFFAILIFAIIGLEFYMGKFHTTCFEEGTDDIQGESPAPCGTEEPARTCPNGTKCQPY
WEGPNNGITQFDNILFAVLTVFQCITMEGWTDLLYNSNDASGNTWNWLYFIPLIIIGSFFMLNLVLGVLSGEF
AKERERVENRRAFLKLRRQQQIERELNGYMEWISKAEEVILAEDETDVEQRHPFDGALRRATLKKSKTDLLNP
EEAEDQLADIASVGSPFARASIKSAKLENSTFFHKKERRMRFYIRRMVKTQAFYWTVLSLVALNTLCVAIVHY
NQPEWLSDFLYYAEFIFLGLFMSEMFIKMYGLGTRPYFHSSFNCFDCGVIIGSIFEVIWAVIKPGTSFGISVL
RALRLLRIFKVTKYWASLRNLVVSLLNSMKSIISLLFLLFLFIVVFALLGMQLFGGQFNFDEGTPPTNFDTFP
AAIMTVFQILTGEDWNEVMYDGIKSQGGVQGGMVFSIYFIVLTLFGNYTLLNVFLAIAVDNLANAQELTKDEQ
EEEEAANQKLALQKAKEVAEVSPLSAANMSIAVKEQQKNQKPTKSVWEQRTSEMRKQNLLASREALYGDAAER
WPTPYARPLRPDVKTHLDRPLVVDPQENRNNNTNKSRAPEALRPTARPRESARDPDARRAWPGSPERAPGREG
PYGRESEPQQREHAPPREHAPWDADTERAKAGDAPRRHTHRPVAEGEPRRHRARRRPGDEPDDRPERRPRPRD
ATRPARAADGEGDDGERKRRHRHGPPAHDDRERRHRRRKENQGSGVPVSGPNLSTTRPIQQDLGRQDLPLAED
LDNMKNNKLATGEPASPHDSLGHSGLPPSPAKIGNSTNPGPALATNPQNAASRRTPNNPGNPSNPGPPKTPEN
SLIVTNPSSTQPNSAKTARKPEHMAVEIPPACPPLNHTVVQVNKNANPDPLPKKEEEKKEEEEADPGEDGPKP
MPPYSSMFILSTTNPLRRLCHYILNLRYFEMCILMVIAMSSIALAAEDPVQPNAPRNNVLRYFDYVFTGVFTF
EMVIKMIDLGLVLHQGAYFRDLWNILDFIVVSGALVAFAFTGNSKGKDINTIKSLRVLRVLRPLKTIKRLPKL
KAVFDCVVNSLKNVFNILIVYMLFMFIFAVVAVQLFKGKFFHCTDESKEFERDCRGKYLLYEKNEVKARDREW
KKYEFHYDNVLWALLTLFTVSTGEGWPQVLKHSVDATFENQGPSPGYRMEMSIFYVVYFVVFPFFFVNIFVAL
IIITFQEQGDKMMEEYSLEKNERACIDFAISAKPLTRHMPQNKQSFQYRMWQFVVSPPFEYTIMAMIALNTIV
LMMKFYGASVAYENALRVFNIVFTSLFSLECVLKVMAFGILNYFRDAWNIFDFVTVLGSITDILVTEFGNNFI
NLSFLRLFRAARLIKLLRQGYTIRILLWTFVQSFKALPYVCLLIAMLFFIYAIIGMQVFGNIGIDGEDEDSDE
DEFQITEHNNFRTFFQALMLLFRSATGEAWHNIMLSCLSGKPCDKNSGILTADCGNEFAYFYFVSFIFLCSFL
MLNLFVAVIMDNFEYLTRDSSILGPHHLDEYVRVWAEYDPAACGRIHYKDMYSLLRVISPPLGLGKKCPHRVA
CKRLLRMDLPVADDNTVHFNSTLMALIRTALDIKIAKGGADKQQMDAELRKEMMAIWPNLSQKTLDLLVTPHK
STDLTVGKIYAAMMIMEYYRQSKAKKLQAMREEQNRTPLMFQRMEPPSPTQEGGPSQNALPSTQLDPGGGLMA
HEGGMKESPSWVTQRAQEMFQKTGTWSPERGPPIDMPNSQPNSQSVEMREMGTDGYSDSEHYLPMEGQTRAAS
MPRLPAENQRRRGRPRGNDLSTISDTSPMKRSASVLGPKARRLDDYSLERVPPEENQRYHQRRRDRGHRTSER
SLGRYTDVDTGLGTDLSMTTQSGDLPSKDRDQDRGRPKDRKHRPHHHHHHHHHPPAPDRDRYAQERPDTGRA
RAREQRWSRSPSEGREHTTHRQGSSSVSGSPAPSTSGTSTPRRGRRQLPQTPCTPRPLVSYSPAPRRPAARRM
AGPAAPPGGSPRGCRRAPRWPAHAPEGPRPRGADYTEPDSPREPPGGAHDPAPRSPRTPRAAGCASPRHGRRL
PNGYYAGHGAPRPRTARRGAHDAYSESQDDWC*
>rs37482583_ENSMUSP00000114055_c.6948:G>C_p.E2317Q
ENSMUST00000122053.2|Cacna1a-202|ENSMUSG00000034656|+|chr8:g.85366320:G>C
MARFGDEMPGRYGAGGGGSGPAAGVVVGAAGGRGAGGSRQGGQPGAQRMYKQSMAQRARTMALYNPIPVRQNC
LTVNRSLFLFSEDNVDDTEPYFIGIFCFEAGIKIVALGFAFHKGSYLRNGWNVMDFVVVLTGILATVGTEFDL
RTLRAVRVLRPLKLVSGIPSLQVVLKSIMKAMIPLLQIGLLLFFAILIFAIIGLEFYMGKFHTTCFEEGTDDI
QGESPAPCGTEEPARTCPNGTKCQPYWEGPNNGITQFDNILFAVLTVFQCITMEGWTDLLYNSNDASGNTWNW
LYFIPLIIIGSFFMLNLVLGVLSGEFAKERERVENRRAFLKLRRQQQIERELNGYMEWISKAEEVILAEDETD
VEQRHPFDGALRRATLKKSKTDLLNPEEAEDQLADIASVGSPFARASIKSAKLENSTFFHKKERRMRFYIRRM
VKTQAFYWTVLSLVALNTLCVAIVHYNQPEWLSDFLYYAEFIFLGLFMSEMFIKMYGLGTRPYFHSSFNCFDC
GVIIGSIFEVIWAVIKPGTSFGISVLRALRLLRIFKVTKYWASLRNLVVSLLNSMKSIISLLFLLFLFIVVFA
LLGMQLFGGQFNFDEGTPPTNFDTFPAAIMTVFQILTGEDWNEVMYDGIKSQGGVQGGMVFSIYFIVLTLFGN
YTLLNVFLAIAVDNLANAQELTKDEQEEEEAANQKLALQKAKEVAEVSPLSAANMSIAVKEQQKNQKPTKSVW
EQRTSEMRKQNLLASREALYGDAAERWPTPYARPLRPDVKTHLDRPLVVDPQENRNNNTNKSRAPEALRPTAR
PRESARDPDARRAWPGSPERAPGREGPYGRESEPQQREHAPPREHAPWDADTERAKAGDAPRRHTHRPVAEGE
PRRHRARRRPGDEPDDRPERRPRPRDATRPARAADGEGDDGERKRRHRHGPPAHDDRERRHRRRKENQGSGVP
VSGPNLSTTRPIQQDLGRQDLPLAEDLDNMKNNKLATGEPASPHDSLGHSGLPPSPAKIGNSTNPGPALATNP
QNAASRRTPNNPGNPSNPGPPKTPENSLIVTNPSSTQPNSAKTARKPEHMAVEIPPACPPLNHTVVQVNKNAN
PDPLPKKEEEKKEEEEADPGEDGPKPMPPYSSMFILSTTNPLRRLCHYILNLRYFEMCILMVIAMSSIALAAE
DPVQPNAPRNNVLRYFDYVFTGVFTFEMVIKMIDLGLVLHQGAYFRDLWNILDFIVVSGALVAFAFTGNSKGK
DINTIKSLRVLRVLRPLKTIKRLPKLKAVFDCVVNSLKNVFNILIVYMLFMFIFAVVAVQLFKGKFFHCTDES
KEFERDCRGKYLLYEKNEVKARDREWKKYEFHYDNVLWALLTLFTVSTGEGWPQVLKHSVDATFENQGPSPGY
RMEMSIFYVVYFVVFPFFFVNIFVALIIITFQEQGDKMMEEYSLEKNERACIDFAISAKPLTRHMPQNKQSFQ
YRMWQFVVSPPFEYTIMAMIALNTIVLMMKFYGASVAYENALRVFNIVFTSLFSLECVLKVMAFGILNYFRDA

WNIFDFVTVLGSITDILVTEFGNNFINLSFLRLFRAARLIKLLRQGYTIRILLWTFVQSFKALPYVCLLIAML
FFIYAIIGMQVFGNIGIDGEDEDSDEDEFQITEHNNFRTFFQALMLLFRSATGEAWHNIMLSCLSGKPCDKNS
GILTADCGNEFAYFYFVSFIFLCSFLMLNLFVAVIMDNFEYLTRDSSILGPHHLDEYVRVWAEYDPAACGRIH
YKDMYSLLRVISPPLGLGKKCPHRVACKRLLRMDLPVADDNTVHFNSTLMALIRTALDIKIAKGGADKQQMDA
ELRKEMMAIWPNLSQKTLDLLVTPHKSTDLTVGKIYAAMMIMEYYRQSKAKKLQAMREEQNRTPLMFQRMEPP
SPTQEGGPSQNALPSTQLDPGGGLMAHEGGMKESPSWVTQRAQEMFQKTGTWSPERGPPIDMPNSQPNSQSVE
MREMGTDGYSDSEHYLPMEGQTRAASMPRLPAENQRRRGRPRGNDLSTISDTSPMKRSASVLGPKARRLDDYS
LERVPPEENQRYHQRRRDRGHRTSERSLGRYTDVDTGLGTDLSMTTQSGDLPSKDRDQDRGRPKDRKHRPHHH
HHHHHHHPPAPDRDRYAQERPDTGRARAREQRWSRSPSEGREHTTHRQGSSSVSGSPAPSTSGTSTPRRGRRQ
LPQTPCTPRPLVSYSPAPRRPAARRMAGPAAPPGGSPRGCRRAPRWPAHAPEGPRPRGADYTEPDSPREPPGG
AHDPAPRSPRTPRAAGCASPRHGRRLPNGYYAGHGAPRPRTARRGAHDAYSESQDDWC*
>rs37482583_ENSMUSP00000149242_c.6948:G>C_p.E2317Q
ENSMUST00000215756.2|Cacna1a-211|ENSMUSG00000034656|+|chr8:g.85366320:G>C
XRMYKQSMAQRARTMALYNPIPVRQNCLTVNRSLFLFSEDNVVRKYAKKITEWPPFEYMILATIIANCIVLAL
EQHLPDDDKTPMSERLDDTEPYFIGIFCFEAGIKIVALGFAFHKGSYLRNGWNVMDFVVVLTGILATVGTEFD
LRTLRAVRVLRPLKLVSGIPSLQVVLKSIMKAMIPLLQIGLLLFFAILIFAIIGLEFYMGKFHTTCFEEGTDD
IQGESPAPCGTEEPARTCPNGTKCQPYWEGPNNGITQFDNILFAVLTVFQCITMEGWTDLLYNSNDASGNTWN
WLYFIPLIIIGSFFMLNLVLGVLSGEFAKERERVENRRAFLKLRRQQQIERELNGYMEWISKAEEVILAEDET
DVEQRHPFDALRRATLKKSKTDLLNPEEAEDQLADIASVGSPFARASIKSAKLENSTFFHKKERRMRFYIRRM
VKTQAFYWTVLSLVALNTLCVAIVHYNQPEWLSDFLYYAEFIFLGLFMSEMFIKMYGLGTRPYFHSSFNCFDC
GVIIGSIFEVIWAVIKPGTSFGISVLRALRLLRIFKVTKYWASLRNLVVSLLNSMKSIISLLFLLFLFIVVFA
LLGMQLFGGQFNFDEGTPPTNFDTFPAAIMTVFQILTGEDWNEVMYDGIKSQGGVQGGMVFSIYFIVLTLFGN
YTLLNVFLAIAVDNLANAQELTKDEQEEEEAANQKLALQKAKEVAEVSPLSAANMSIAVKEQQKNQKPTKSVW
EQRTSEMRKQNLLASREALYGDAAERWPTPYARPLRPDVKTHLDRPLVVDPQENRNNNTNKSRAPEALRPTAR
PRESARDPDARRAWPGSPERAPGREGPYGRESEPQQREHAPPREHAPWDADTERAKAGDAPRRHTHRPVAEGE
PRRHRARRRPGDEPDDRPERRPRPRDATRPARAADGEGDDGERKRRHRHGPPAHDDRERRHRRRKENQGSGVP
VSGPNLSTTRPIQQDLGRQDLPLAEDLDNMKNNKLATGEPASPHDSLGHSGLPPSPAKIGNSTNPGPALATNP
QNAASRRTPNNPGNPSNPGPPKTPENSLIVTNPSSTQPNSAKTARKPEHMAVEIPPACPPLNHTVVQVNKNAN
PDPLPKKEEEKKEEEEADPGEDGPKPMPPYSSMFILSTTNPLRRLCHYILNLRYFEMCILMVIAMSSIALAAE
DPVQPNAPRNNVLRYFDYVFTGVFTFEMVIKMIDLGLVLHQGAYFRDLWNILDFIVVSGALVAFAFTGNSKGK
DINTIKSLRVLRVLRPLKTIKRLPKLKAVFDCVVNSLKNVFNILIVYMLFMFIFAVVAVQLFKGKFFHCTDES
KEFERDCRGKYLLYEKNEVKARDREWKKYEFHYDNVLWALLTLFTVSTGEGWPQVLKHSVDATFENQGPSPGY
RMEMSIFYVVYFVVFPFFFVNIFVALIIITFQEQGDKMMEEYSLEKNERACIDFAISAKPLTRHMPQNKQSFQ
YRMWQFVVSPPFEYTIMAMIALNTIVLMMKFYGASVAYENALRVFNIVFTSLFSLECVLKVMAFGILNYFRDA
WNIFDFVTVLGSITDILVTEFGNNFINLSFLRLFRAARLIKLLRQGYTIRILLWTFVQSFKALPYVCLLIAML
FFIYAIIGMQVFGNIGIDGEDEDSDEDEFQITEHNNFRTFFQALMLLFRSATGEAWHNIMLSCLSGKPCDKNS
GILTADCGNEFAYFYFVSFIFLCSFLMLNLFVAVIMDNFEYLTRDSSILGPHHLDEYVRVWAEYDPAACGRIH
YKDMYSLLRVISPPLGLGKKCPHRVACKRLLRMDLPVADDNTVHFNSTLMALIRTALDIKIAKGGADKQQMDA
ELRKEMMAIWPNLSQKTLDLLVTPHKSTDLTVGKIYAAMMIMEYYRQSKAKKLQAMREEQNRTPLMFQRMEPP
SPTQEGGPSQNALPSTQLDPGGGLMAHEGGMKESPSWVTQRAQEMFQKTGTWSPERGPPIDMPNSQPNSQSVE
MREMGTDGYSDSEHYLPMEGQTRAASMPRLPAENQRRRGRPRGNDLSTISDTSPMKRSASVLGPKARRLDDYS
LERVPPEENQRYHQRRRDRGHRTSERSLGRYTDVDTGLGTDLSMTTQSGDLPSKDRDQDRGRPKDRKHRPHHH
HHHHHHHPPAPDRDRYAQERPDTGRARAREQRWSRSPSEGREHTTHRQGSSSVSGSPAPSTSGTSTPRRGRRQ
LPQTPCTPRPLVSYSPAPRRPAARRMAGPAAPPGGSPRGCRRAPRWPAHAPEGPRPRGADYTEPDSPREPPGG
AHDPAPRSPRTPRAAGCASPRHGRRLPNGYYAGHGAPRPRTARRGAHDAYSESQDDWC*
>rs37482583_ENSMUSP00000158675_c.2313:G>C_p.E772Q
ENSMUST00000238337.2|Cacna1a-212|ENSMUSG00000034656|+|chr8:g.85366320:G>C
MHLLRLSGQRGLTLQPNSPSSLPSSPFPLAKGNNFINLSFLRLFRAARLIKLLRQGYTIRILLWTFVQSFKVF
GNIGIDGEDEDSDEDEFQITEHNNFRTFFQALMLLFRSATGEAWHNIMLSCLSGKPCDKNSGILTADCGNEFA
YFYFVSFIFLCSFLMLNLFVAVIMDNFEYLTRDSSILGPHHLDEYVRVWAEYDPAACGRIHYKDMYSLLRVIS
PPLGLGKKCPHRVACKRLLRMDLPVADDNTVHFNSTLMALIRTALDIKIAKGGADKQQMDAELRKEMMAIWPN
LSQKTLDLLVTPHKSTDLTVGKIYAAMMIMEYYRQSKAKKLQAMREEQNRTPLMFQRMEPPSPTQEGGPSQNA
LPSTQLDPGGGLMAHEGGMKESPSWVTQRAQEMFQKTGTWSPERGPPIDMPNSQPNSQSVEMREMGTDGYSDS
EHYLPMEGQTRAASMPRLPAENQRRRGRPRGNDLSTISDTSPMKRSASVLGPKARRLDDYSLERVPPEENQRY
HQRRRDRGHRTSERSLGRYTDVDTGLGTDLSMTTQSGDLPSKDRDQDRGRPKDRKHRPHHHHHHHHHHPPAPD
RDRYAQERPDTGRARAREQRWSRSPSEGREHTTHRQGSSSVSGSPAPSTSGTSTPRRGRRQLPQTPCTPRPLV
SYSPAPRRPAARRMAGPAAPPGGSPRGCRRAPRWPAHAPEGPRPRGADYTEPDSPREPPGGAHDPAPRSPRTP
RAAGCASPRHGRRLPNGYYAGHGAPRPRTARRGAHDAYSESQDDWC*

>rs220446334_ENSMUSP00000060275_c.654:G>A_p.V219M
ENSMUST00000060427.6|Ier2-201|ENSMUSG00000053560|-|chr8:g.85388726:C>T
MEVQKEAQRIMTLSVWKMYHSRMQRGGLRLHRSLQLSLVMRSAREVYLSAKVEAHQPEFPPSRRALDPRLHPP
REAEVAVEVASPEAVQPPEPMDTQEEVLRVQETPALCDPPPARVSRKRRSSSDLSDGSDAGLVPSKKARLEEV
EGEATSEVPDRLQLPPAQSEGAFPNLARVLQRRFSSLLNCGPAVPPPTPPTCEAKPACRPADNMLNVLVRAVM
AF*
>rs52044482_ENSMUSP00000060275_c.242:A>C_p.E81D
ENSMUST00000060427.6|Ier2-201|ENSMUSG00000053560|-|chr8:g.85389138:T>G
MEVQKEAQRIMTLSVWKMYHSRMQRGGLRLHRSLQLSLVMRSAREVYLSAKVEAHQPEFPPSRRALDPRLHPP
REAEVAVDVASPEAVQPPEPMDTQEEVLRVQETPALCDPPPARVSRKRRSSSDLSDGSDAGLVPSKKARLEEV
EGEATSEVPDRLQLPPAQSEGAFPNLARVLQRRFSSLLNCGPAVPPPTPPTCEAKPACRPADNMLNVLVRAVV
AF*
>rs32757845_ENSMUSP00000001975_c.688:T>C_p.V230A
ENSMUST00000001975.6|Nacc1-201|ENSMUSG00000001910|-|chr8:g.85403185:A>G
MAQTLQMEIPNFGNSILECLNEQRLQGLYCDVSVVVKGHAFKAHRAVLAASSSYFRDLFNSSRSAVVELPAAV
QPQSFQQILTFCYTGRLSMNMGDQFLLIYTAGFLQIQEIMEKGTEFFLKVSSPSCDSQGLHPEEAPSSEPQSP
VAQTLGWPACSTPLPLVSRVKTEQELDSVQCTPMAKRLWDSSQKEAGGSGGNNGSRKMAKFSTPDLALNRMPQ
PLSMATATAAAAVVAVGGCVSGPSMSERTSPGTSSAYTSDSPSSYHNEEDEEEDAGEEGTDEQYRQICNMYTM
YSMLNVGQTAEKVEALPEQVVLESRSRIRVRQDLASLPAELINQIGNRCHPKLYDEGDPSEKLELVTGTNVYI
TRAQLMNCHVSAGTRHKVLLRRLLASFFDRNTLANSCGTGIRSSTNDPRRKPLDSRVLHAVKYYCQNFAPNFK
ESEMNAIAADMCTNARRVVRKSWLPKTKPLHLVEGDNYSSFISDTCKIEPDMMSMEHSFETASHDGEAGPSAE
VLQ*
>rs32757845_ENSMUSP00000158606_c.439:T>C_p.V147A
ENSMUST00000238434.2|Nacc1-203|ENSMUSG00000001910|-|chr8:g.85403185:A>G
FCYTGRLSMNMGDQFLLIYTAGFLQIQEIMEKGTEFFLKVSSPSCDSQGLHPEEAPSSEPQSPVAQTLGWPAC
STPLPLVSRVKTEQELDSVQCTPMAKRLWDSSQKEAGGSGGNNGSRKMAKFSTPDLALNRMPQPLSMATATAA
AAVVAVGGCVSGPSMSERTSPGTSSAYTSDSPSSYHNEEDEEEDAAEKVEALPEQVVLESRSRIRVRQDLASL
PAELINQIGNRCHPKLYDEGDPSEKLELVTGTNVYITRAQLMNCHVSAGTRHKVLLRRLLASFFDRNTLANSC
GTGIRSSTNDPRRKPLDSRVLHAVKYYCQNFAPNFKESEMNAIAADMCTNARRVVRKSWLPKTKPLHLVEGDN
YSSFISD
>rs36775818_ENSMUSP00000001974_c.1561:T>G_p.I521S
ENSMUST00000001974.11|Trmt1-201|ENSMUSG00000001909|+|chr8:g.85425080:T>G
MSLARTILWLSRPLRPAHSLCRAQFMERKAQKPPSPPAMENGTRSWEERPPADPVATVTEGAAKIVFPSANEV
FYNPVQEFNRDLTCAVITEFARIHLGAKGIQIKVPGEKDSEKIAVDLSDQEEETAGKNENLAPGDWPRTAAVG
EICEEGLRVLEGLAASGLRSIRFALEVPGLQSVVANDASARAVELMHRNVELNGVAHLVQPNQADARMLMYQH
QKAPERFDVIDLDPYGSPAPFLDAAVQAVSDGGLLCVTCTDMAVLAGNSGETCYSKYGAMALKSRACHEMALR
IVLHSLDLHANCYQRYIVPLLSISADFYIRVFVRVFTGQAKVKSSASKQALVFQCVGCGAFYLQRLGKASGDP
GGRIKFSAACGPPVTPECEHCGQRHQLGGPMWAEPIHDLDFVGRVLDAVTTNPGRFHTSMRIQGVLSVVTEEL
PDVPLYYTLDQLSSTIHCNTPRLLQLRSALLHAGFRVSLSHACKNAVKTDAPPEALWDIMRCWEKECPVKRER
LSESSPAFRSLAVEPRLKANFNIREDANPSSRQRGLKRFQANPEANWGPRPRARPGGKAASEDLAGRRRLLQN
KRKEPAEDPAQRAARLKTFPCKRFKEGTCQLGDQCCYSHSPAAPVASGDIPIEECPETTTKISPGPKAAAGGI
PGPGVD*
>rs36775818_ENSMUSP00000105389_c.1561:T>G_p.I521S
ENSMUST00000109767.9|Trmt1-203|ENSMUSG00000001909|+|chr8:g.85425080:T>G
MSLARTILWLSRPLRPAHSLCRAQFMERKAQKPPSPPAMENGTRSWEERPPADPVATVTEGAAKIVFPSANEV
FYNPVQEFNRDLTCAVITEFARIHLGAKGIQIKVPGEKDSEKIAVDLSDQEEETAGKNENLAPGDWPRTAAVG
EICEEGLRVLEGLAASGLRSIRFALEVPGLQSVVANDASARAVELMHRNVELNGVAHLVQPNQADARMLMYQH
QKAPERFDVIDLDPYGSPAPFLDAAVQAVSDGGLLCVTCTDMAVLAGNSGETCYSKYGAMALKSRACHEMALR
IVLHSLDLHANCYQRYIVPLLSISADFYIRVFVRVFTGQAKVKSSASKQALVFQCVGCGAFYLQRLGKASGDP
GGRIKFSAACGPPVTPECEHCGQRHQLGGPMWAEPIHDLDFVGRVLDAVTTNPGRFHTSMRIQGVLSVVTEEL
PDVPLYYTLDQLSSTIHCNTPRLLQLRSALLHAGFRVSLSHACKNAVKTDAPPEALWDIMRCWEKECPVKRER
LSESSPAFRSLAVEPRLKANFNIREDANPSSRQRGLKRFQANPEANWGPRPRARPGGKAASEDLAGRRRLLQN
KRKEPAEDPAQRAARLKTFPCKRFKEGTCQLGDQCCYSHSPAAPVASGDIPIEECPETTTKISPGPKAAAGGI
PGPGVD*
>rs36775818_ENSMUSP00000105390_c.1540:T>G_p.I514S
ENSMUST00000109768.9|Trmt1-204|ENSMUSG00000001909|+|chr8:g.85425080:T>G
MREACTVTRWILLCRAQFMERKAQKPPSPPAMENGTRSWEERPPADPVATVTEGAAKIVFPSANEVFYNPVQE
FNRDLTCAVITEFARIHLGAKGIQIKVPGEKDSEKIAVDLSDQEEETAGKNENLAPGDWPRTAAVGEICEEGL

RVLEGLAASGLRSIRFALEVPGLQSVVANDASARAVELMHRNVELNGVAHLVQPNQADARMLMYQHQKAPERF
DVIDLDPYGSPAPFLDAAVQAVSDGGLLCVTCTDMAVLAGNSGETCYSKYGAMALKSRACHEMALRIVLHSLD
LHANCYQRYIVPLLSISADFYIRVFVRVFTGQAKVKSSASKQALVFQCVGCGAFYLQRLGKASGDPGGRIKFS
AACGPPVTPECEHCGQRHQLGGPMWAEPIHDLDFVGRVLDAVTTNPGRFHTSMRIQGVLSVVTEELPDVPLYY
TLDQLSSTIHCNTPRLLQLRSALLHAGFRVSLSHACKNAVKTDAPPEALWDIMRCWEKECPVKRERLSESSPA
FRSLAVEPRLKANFNIREDANPSSRQRGLKRFQANPEANWGPRPRARPGGKAASEDLAGRRRLLQNKRKEPAE
DPAQRAARLKTFPCKRFKEGTCQLGDQCCYSHSPAAPVASGDIPIEECPETTTKISPGPKAAAGGIPGPGVD*
>rs36775818_ENSMUSP00000135510_c.1474:T>G_p.I492S
ENSMUST00000125370.10|Trmt1-205|ENSMUSG00000001909|+|chr8:g.85425080:T>G
MSLARTILWLSRPLRPAHSLCRAQFMERKAQKPPSPPAMENGTRSWEERPPADPVATVTEGAAKIVFPSANEV
FYNPVQEFNRDLTCAVITEFARIHLGAKGIQIKVPGEKDSEKIAVDLSDQEEETAGKNENLAPGDWPRTAAVG
EICEEGLRVLEGLAASGLRSIRFALEVPGLQSVVANDASARAVELMHRNVELNGVAHLVQPNQADARMLMYQH
QKAPERFDVIDLDPYGSPAPFLDAAVQAVSDGGLLCVTCTDMAVLAGNSGETCYSKYGAMALKSRACHEMALR
IVLHSLDLHANCYQRYIVPLLSISADFYIRVFVRVFTGQAKVKSSARIKFSAACGPPVTPECEHCGQRHQLGG
PMWAEPIHDLDFVGRVLDAVTTNPGRFHTSMRIQGVLSVVTEELPDVPLYYTLDQLSSTIHCNTPRLLQLRSA
LLHAGFRVSLSHACKNAVKTDAPPEALWDIMRCWEKECPVKRERLSESSPAFRSLAVEPRLKANFNIREDANP
SSRQRGLKRFQANPEANWGPRPRARPGGKAASEDLAGRRRLLQNKRKEPAEDPAQRAARLKTFPCKRFKEGTC
QLGDQCCYSHSPAAPVASGDIPIEECPETTTKISPGPKAAAGGIPGPGVD*
>rs36775818_ENSMUSP00000134723_c.55:T>G_p.I19S
ENSMUST00000136423.3|Trmt1-210|ENSMUSG00000001909|+|chr8:g.85425080:T>G
XECPVKRERLSESSPAFRSLAVEPRLKANFNIREDANPSSRQRGLKRFQANPEANWGPRPRARPGGKAASEDL
AGRRRLLQNKRKEPAEDPAQRAARLKTFPCKRFKEVISPSTFHATGPRLGLWHSWGQRLRKETQLCAHSLGLH
TLFSHQGTCQLGDQCCYSHSPAAPVASGDIPIEECPETTTKISPGPKAAAGGIPGPGVD*
>rs32665800_ENSMUSP00000001974_c.1969:T>C_p.I657T
ENSMUST00000001974.11|Trmt1-201|ENSMUSG00000001909|+|chr8:g.85426371:T>C
MSLARTILWLSRPLRPAHSLCRAQFMERKAQKPPSPPAMENGTRSWEERPPADPVATVTEGAAKIVFPSANEV
FYNPVQEFNRDLTCAVITEFARIHLGAKGIQIKVPGEKDSEKIAVDLSDQEEETAGKNENLAPGDWPRTAAVG
EICEEGLRVLEGLAASGLRSIRFALEVPGLQSVVANDASARAVELMHRNVELNGVAHLVQPNQADARMLMYQH
QKAPERFDVIDLDPYGSPAPFLDAAVQAVSDGGLLCVTCTDMAVLAGNSGETCYSKYGAMALKSRACHEMALR
IVLHSLDLHANCYQRYIVPLLSISADFYIRVFVRVFTGQAKVKSSASKQALVFQCVGCGAFYLQRLGKASGDP
GGRIKFSAACGPPVTPECEHCGQRHQLGGPMWAEPIHDLDFVGRVLDAVTTNPGRFHTSMRIQGVLSVVTEEL
PDVPLYYTLDQLSSTIHCNTPRLLQLRSALLHAGFRVSLSHACKNAVKTDAPPEALWDIMRCWEKECPVKRER
LSESSPAFRILAVEPRLKANFNIREDANPSSRQRGLKRFQANPEANWGPRPRARPGGKAASEDLAGRRRLLQN
KRKEPAEDPAQRAARLKTFPCKRFKEGTCQLGDQCCYSHSPAAPVASGDIPIEECPETTTKISPGPKAAAGGT
PGPGVD*
>rs32665800_ENSMUSP00000105389_c.1969:T>C_p.I657T
ENSMUST00000109767.9|Trmt1-203|ENSMUSG00000001909|+|chr8:g.85426371:T>C
MSLARTILWLSRPLRPAHSLCRAQFMERKAQKPPSPPAMENGTRSWEERPPADPVATVTEGAAKIVFPSANEV
FYNPVQEFNRDLTCAVITEFARIHLGAKGIQIKVPGEKDSEKIAVDLSDQEEETAGKNENLAPGDWPRTAAVG
EICEEGLRVLEGLAASGLRSIRFALEVPGLQSVVANDASARAVELMHRNVELNGVAHLVQPNQADARMLMYQH
QKAPERFDVIDLDPYGSPAPFLDAAVQAVSDGGLLCVTCTDMAVLAGNSGETCYSKYGAMALKSRACHEMALR
IVLHSLDLHANCYQRYIVPLLSISADFYIRVFVRVFTGQAKVKSSASKQALVFQCVGCGAFYLQRLGKASGDP
GGRIKFSAACGPPVTPECEHCGQRHQLGGPMWAEPIHDLDFVGRVLDAVTTNPGRFHTSMRIQGVLSVVTEEL
PDVPLYYTLDQLSSTIHCNTPRLLQLRSALLHAGFRVSLSHACKNAVKTDAPPEALWDIMRCWEKECPVKRER
LSESSPAFRILAVEPRLKANFNIREDANPSSRQRGLKRFQANPEANWGPRPRARPGGKAASEDLAGRRRLLQN
KRKEPAEDPAQRAARLKTFPCKRFKEGTCQLGDQCCYSHSPAAPVASGDIPIEECPETTTKISPGPKAAAGGT
PGPGVD*
>rs32665800_ENSMUSP00000105390_c.1948:T>C_p.I650T
ENSMUST00000109768.9|Trmt1-204|ENSMUSG00000001909|+|chr8:g.85426371:T>C
MREACTVTRWILLCRAQFMERKAQKPPSPPAMENGTRSWEERPPADPVATVTEGAAKIVFPSANEVFYNPVQE
FNRDLTCAVITEFARIHLGAKGIQIKVPGEKDSEKIAVDLSDQEEETAGKNENLAPGDWPRTAAVGEICEEGL
RVLEGLAASGLRSIRFALEVPGLQSVVANDASARAVELMHRNVELNGVAHLVQPNQADARMLMYQHQKAPERF
DVIDLDPYGSPAPFLDAAVQAVSDGGLLCVTCTDMAVLAGNSGETCYSKYGAMALKSRACHEMALRIVLHSLD
LHANCYQRYIVPLLSISADFYIRVFVRVFTGQAKVKSSASKQALVFQCVGCGAFYLQRLGKASGDPGGRIKFS
AACGPPVTPECEHCGQRHQLGGPMWAEPIHDLDFVGRVLDAVTTNPGRFHTSMRIQGVLSVVTEELPDVPLYY
TLDQLSSTIHCNTPRLLQLRSALLHAGFRVSLSHACKNAVKTDAPPEALWDIMRCWEKECPVKRERLSESSPA
FRILAVEPRLKANFNIREDANPSSRQRGLKRFQANPEANWGPRPRARPGGKAASEDLAGRRRLLQNKRKEPAE
DPAQRAARLKTFPCKRFKEGTCQLGDQCCYSHSPAAPVASGDIPIEECPETTTKISPGPKAAAGGTPGPGVD*

>rs32665800_ENSMUSP00000135510_c.1882:T>C_p.I628T
ENSMUST00000125370.10|Trmt1-205|ENSMUSG00000001909|+|chr8:g.85426371:T>C
MSLARTILWLSRPLRPAHSLCRAQFMERKAQKPPSPPAMENGTRSWEERPPADPVATVTEGAAKIVFPSANEV
FYNPVQEFNRDLTCAVITEFARIHLGAKGIQIKVPGEKDSEKIAVDLSDQEEETAGKNENLAPGDWPRTAAVG
EICEEGLRVLEGLAASGLRSIRFALEVPGLQSVVANDASARAVELMHRNVELNGVAHLVQPNQADARMLMYQH
QKAPERFDVIDLDPYGSPAPFLDAAVQAVSDGGLLCVTCTDMAVLAGNSGETCYSKYGAMALKSRACHEMALR
IVLHSLDLHANCYQRYIVPLLSISADFYIRVFVRVFTGQAKVKSSARIKFSAACGPPVTPECEHCGQRHQLGG
PMWAEPIHDLDFVGRVLDAVTTNPGRFHTSMRIQGVLSVVTEELPDVPLYYTLDQLSSTIHCNTPRLLQLRSA
LLHAGFRVSLSHACKNAVKTDAPPEALWDIMRCWEKECPVKRERLSESSPAFRILAVEPRLKANFNIREDANP
SSRQRGLKRFQANPEANWGPRPRARPGGKAASEDLAGRRRLLQNKRKEPAEDPAQRAARLKTFPCKRFKEGTC
QLGDQCCYSHSPAAPVASGDIPIEECPETTTKISPGPKAAAGGTPGPGVD*
>rs32665800_ENSMUSP00000134723_c.595:T>C_p.I199T
ENSMUST00000136423.3|Trmt1-210|ENSMUSG00000001909|+|chr8:g.85426371:T>C
XECPVKRERLSESSPAFRILAVEPRLKANFNIREDANPSSRQRGLKRFQANPEANWGPRPRARPGGKAASEDL
AGRRRLLQNKRKEPAEDPAQRAARLKTFPCKRFKEVISPSTFHATGPRLGLWHSWGQRLRKETQLCAHSLGLH
TLFSHQGTCQLGDQCCYSHSPAAPVASGDIPIEECPETTTKISPGPKAAAGGTPGPGVD*
>rs47830455_ENSMUSP00000158636_c.513:G>T_p.A172S
ENSMUST00000238562.2|Lyl1-204|ENSMUSG00000034041|+|chr8:g.85430832:G>T
MPTTELSAFRPSLLQLTALGRAPPTLAVHYHPHPFLNSVYIGPAGPFSIFPNSRLKRRPSHSELDLADGHQPQ
KVARRVFTNSRERWRQQHVNGAFAELRKLLPTHPPDRKLSKNEVLRLAMKYIGFLVRLLRDQTAVLTSGPSAP
GSRKPPARRGVEGSARFGAGHRVEASRSQPVLPGDCDGDPNGSVRPIKLEQTSLSPEVR*
>rs47830455_ENSMUSP00000046010_c.732:G>T_p.A245S
ENSMUST00000037165.6|Lyl1-201|ENSMUSG00000034041|+|chr8:g.85430832:G>T
MCPPQARAEVGSAMTEKTEMVCASSPAPAPPSKPASPGPLSTEEVDHRNTCTPWLPPGVPVINLGHTRPIGAA
MPTTELSAFRPSLLQLTALGRAPPTLAVHYHPHPFLNSVYIGPAGPFSIFPNSRLKRRPSHSELDLADGHQPQ
KVARRVFTNSRERWRQQHVNGAFAELRKLLPTHPPDRKLSKNEVLRLAMKYIGFLVRLLRDQTAVLTSGPSAP
GSRKPPARRGVEGSARFGAGHRVEASRSQPVLPGDCDGDPNGSVRPIKLEQTSLSPEVR*
>rs37437752_ENSMUSP00000105386_c.675:C>T_p.P226S
ENSMUST00000109764.8|Nfix-204|ENSMUSG00000001911|-|chr8:g.85453871:G>A
MDEFHPFIEALLPHVRAFSYTWFNLQARKRKYFKKHEKRMSKDEERAVKDELLGEKPEIKQKWASRLLAKLRK
DIRPEFREDFVLTITGKKPPCCVLSNPDQKGKIRRIDCLRQADKVWRLDLVMVILFKGIPLESTDGERLYKSP
QCSNPGLCVQPHHIGVTIKELDLYLAYFVHTPESGQSDSSNQQGDADIKPLPNGHLSFQDCFVTSGVWNVTEL
VRVSQTSVATASGPNFSLADLESPSYYNINQVTLGRRSITSPPSTSSTKRPKSIDDSEMESPVDDVFYPGTGR
SPAAGSSQSSGWPNDVDAGPASLKKSGKLDFCSALSSQGSSPRMAFTHHPLPVLAGVRPGSPRATASALHFPS
TSIIQQSSPYFTHPTIRYHHHHGQDSLKEFVQFVCSDGSGQATGQPNGSGQGKVPGSFLLPPPPPVARPVPLP
MPDSKTTSTAPDGAALTPPSPSFTTTGASSANRFVSIGSRDGNFLNIPQQSQSWFL*
>rs37437752_ENSMUSP00000076005_c.699:C>T_p.P234S
ENSMUST00000076715.13|Nfix-201|ENSMUSG00000001911|-|chr8:g.85453871:G>A
MYSPYCLTQDEFHPFIEALLPHVRAFSYTWFNLQARKRKYFKKHEKRMSKDEERAVKDELLGEKPEIKQKWAS
RLLAKLRKDIRPEFREDFVLTITGKKPPCCVLSNPDQKGKIRRIDCLRQADKVWRLDLVMVILFKGIPLESTD
GERLYKSPQCSNPGLCVQPHHIGVTIKELDLYLAYFVHTPESGQSDSSNQQGDADIKPLPNGHLSFQDCFVTS
GVWNVTELVRVSQTSVATASGPNFSLADLESPSYYNINQVTLGRRSITSPPSTSSTKRPKSIDDSEMESPVDD
VFYPGTGRSPAAGSSQSSGWPNDVDAGSPRATASALHFPSTSIIQQSSPYFTHPTIRYHHHHGQDSLKEFVQF
VCSDGSGQATGQHSQRQAPPLPTGLSASDPGTATF*
>rs37437752_ENSMUSP00000096669_c.699:C>T_p.P234S
ENSMUST00000099070.10|Nfix-202|ENSMUSG00000001911|-|chr8:g.85453871:G>A
MYSPYCLTQDEFHPFIEALLPHVRAFSYTWFNLQARKRKYFKKHEKRMSKDEERAVKDELLGEKPEIKQKWAS
RLLAKLRKDIRPEFREDFVLTITGKKPPCCVLSNPDQKGKIRRIDCLRQADKVWRLDLVMVILFKGIPLESTD
GERLYKSPQCSNPGLCVQPHHIGVTIKELDLYLAYFVHTPESGQSDSSNQQGDADIKPLPNGHLSFQDCFVTS
GVWNVTELVRVSQTSVATASGPNFSLADLESPSYYNINQVTLGRRSITSPPSTSSTKRPKSIDDSEMESPVDD
VFYPGTGRSPAAGSSQSSGWPNDVDAGPASLKKSGKLDFCSALSSQGSSPRMAFTHHPLPVLAGVRPGSPRAT
ASALHFPSTSIIQQSSPYFTHPTIRYHHHHGQDSLKEFVQFVCSDGSGQATGQHSQRQAPPLPTGLSASDPGT
ATF*
>rs37437752_ENSMUSP00000105384_c.675:C>T_p.P226S
ENSMUST00000109762.8|Nfix-203|ENSMUSG00000001911|-|chr8:g.85453871:G>A
MDEFHPFIEALLPHVRAFSYTWFNLQARKRKYFKKHEKRMSKDEERAVKDELLGEKPEIKQKWASRLLAKLRK
DIRPEFREDFVLTITGKKPPCCVLSNPDQKGKIRRIDCLRQADKVWRLDLVMVILFKGIPLESTDGERLYKSP
QCSNPGLCVQPHHIGVTIKELDLYLAYFVHTPESGQSDSSNQQGDADIKPLPNGHLSFQDCFVTSGVWNVTEL

VRVSQTSVATASGPNFSLADLESPSYYNINQVTLGRRSITSPPSTSTKRPKSIDDSEMESPVDDVFYPGTGRS
PAAGSSQSSGWPNDVDAGSPRATASALHFPSTSIIQQSSPYFTHPTIRYHHHHGQDSLKEFVQFVCSDGSGQA
TGQHSQRQAPPLPTGLSASDPGTATF*
>rs37437752_ENSMUSP00000115691_c.699:C>T_p.P234S
ENSMUST00000126806.2|Nfix-205|ENSMUSG00000001911|-|chr8:g.85453871:G>A
MYSPYCLTQDEFHPFIEALLPHVRAFSYTWFNLQARKRKYFKKHEKRMSKDEERAVKDELLGEKPEIKQKWAS
RLLAKLRKDIRPEFREDFVLTITGKKPPCCVLSNPDQKGKIRRIDCLRQADKVWRLDLVMVILFKGIPLESTD
GERLYKSPQCSNPGLCVQPHHIGVTIKELDLYLAYFVHTPESGQSDSSNQQGDADIKPLPNGHLSFQDCFVTS
GVWNVTELVRVSQTSVATASGPNFSLADLESPSYYNINQVTLGRRSITSPPSTSSTKRPKSIDDSEMESPVDD
VFYPGTGRSPAAGSSQSSGWPNDVDAGPASLKKSGKLDFCSALSSQGSSPRMAFTHHPLPVLAGVRPGSPRAT
ASALHFPSTSIIQQSSPYFTHPTIRYHHHHGQDSLKEFVQFVCSDGSGQATGQPNGSGQGKVPGSFLLPPPPP
VARPVPLPMPDSKTTSTAPDGAALTPPSPSFTTTGASSANRFVSIGSRDG
>rs230210937_ENSMUSP00000070057_c.423:A>TTTTA_p.N142ins-fs
ENSMUST00000065539.6|Dand5-201|ENSMUSG00000053226|-
|chr8:g.85543051:T>TAAAA
MFRSQFTTLLGLFSGAWLPTGSGRPGAPATPVQSGTAINQSWTLDPLVPISALGSWEAFLGLQNKQQGTGELQ
GGGQRVAAGVPLPLAPQEVLQETCKALSFVQVISRPGCTSARVLNHLCFGRCSSFYIPSSDPTPVVFCF
>rs33207714_ENSMUSP00000037783_c.453:C>T_p.R152W
ENSMUST00000036734.6|Gadd45gip1-
201|ENSMUSG00000033751|+|chr8:g.85560840:C>T
MAALAMRSGYLLRLSVALGPRSRSYRAPPPPRRRPGPHSPDPENLLTPRWQLTPRYVAKQFGRHGAISGVPPA
SLWPTPEQLRELEAEEQEWYPSLATMQESLRLQQQALEARRQAREQRIAECMAKMPQMIENWRKQKRERWEKI
QADKEWRARLQAEAQERLGYHVDPRSARFQELLQDLDKQQRKRLKEERQRQKKEARIAAMASAEAQDSAVSGE
PSS*
>rs46809451_ENSMUSP00000003906_c.25:T>C_p.L9P
ENSMUST00000003906.13|Farsa-201|ENSMUSG00000003808|+|chr8:g.85583689:T>C
MADNPVLEPLLRRLEVADGGLDSAELATQLGVEHQAVVGAVKSLQALGEVIEAELRSTKCWELTTEGEEIARE
GSHEARVFRSIPLEGLVQSELMHLPSGKVGFSKAMSNKWIRVDKSAADGPRVFRVVDSIEDEVQKRLQLVQAG
QAEKLAEKERNELRKRKLLTEVILKTYWVSKGKAFSTSVSKQEAELSPEMISSGSWRDRPFKPYNFSARGVLP
DSGHLHPLLKVRSQFRQIFLEMGFTEMPTDNFIESSFWNFDALFQPQQHPARDQHDTFFLRDPAEALQLPMGY
VQRVKRTHSQGGYGSQGYKYTWKLEEARKNLLRTHTTAASARALYQLAQKKPFTPAKYFSIDRVFRNETLDAT
HLAEFHQIEGVIADHGLTLGHLMGVLREFFTKLGITQLRFKPAYNPYTEPSMEVFSYHQGLKKWVEVGNSGVF
RPEMLLPMGLPENVSVIAWGLSLERPTMIKYGINNIRELVGHKVNLQMVYDSPVCRLDIEPRSSKTQEAA*
>rs46809451_ENSMUSP00000105376_c.25:T>C_p.L9P ENSMUST00000109754.2|Farsa-
202|ENSMUSG00000003808|+|chr8:g.85583689:T>C
MADNPVLEPLLRRLEVADGGLDSAELATQLGVEHQAVVGAVKSLQALGEVIEAELRSTKCWELTTEGEEIARE
GSHEARVFRSIPLEGLVQSELMHLPSGKVGFSKAMSNKWIRVDKSAADGPRVFRVVDSIEDEVQKRLQLVQAG
QAEKLAEKERNELRKRKLLTEVILKTYWVSKGKAFSTSVSKQEAELSPEMISSGSWRDRPFKPYNFSARGVLP
DSGHLHPLLKVRSQFRQIFLEMGFTEMPTDNFIESSFWNFDALFQPQQHPARDQHDTFFLRDPAEALQLPMGY
VQRVKRTHSQGGYGSQGYKYTWKLEEARKNLLRTHTTAASARALYQLAQKPFTPAKYFSIDRVFRNETLDATH
LAEFHQIEGVIADHGLTLGHLMGVLREFFTKLGITQLRFKPAYNPYTEPSMEVFSYHQGLKKWVEVGNSGVFR
PEMLLPMGLPENVSVIAWGLSLERPTMIKYGINNIRELVGHKVNLQMVYDSPVCRLDIEPRSSKTQEAA*
>rs46809451_ENSMUSP00000120609_c.25:T>C_p.L9P ENSMUST00000156970.8|Farsa-
206|ENSMUSG00000003808|+|chr8:g.85583689:T>C
MADNPVLEPLLRRLEVADGGLDSAELATQLGVEHQAVVGAVKSLQALGEVIEAELRSTKCWELTTEAPAQRQG
GLQQGHVQQVDPSGQECRRRAPGVPSGGQYRG*
>rs32726273_ENSMUSP00000003906_c.1205:G>C_p.Q402H
ENSMUST00000003906.13|Farsa-201|ENSMUSG00000003808|+|chr8:g.85594648:G>C
MADNPVLELLLRRLEVADGGLDSAELATQLGVEHQAVVGAVKSLQALGEVIEAELRSTKCWELTTEGEEIARE
GSHEARVFRSIPLEGLVQSELMHLPSGKVGFSKAMSNKWIRVDKSAADGPRVFRVVDSIEDEVQKRLQLVQAG
QAEKLAEKERNELRKRKLLTEVILKTYWVSKGKAFSTSVSKQEAELSPEMISSGSWRDRPFKPYNFSARGVLP
DSGHLHPLLKVRSQFRQIFLEMGFTEMPTDNFIESSFWNFDALFQPQQHPARDQHDTFFLRDPAEALQLPMGY
VQRVKRTHSQGGYGSQGYKYTWKLEEARKNLLRTHTTAASARALYQLAQKKPFTPAKYFSIDRVFRNETLDAT
HLAEFHQIEGVIADHGLTLGHLMGVLREFFTKLGITHLRFKPAYNPYTEPSMEVFSYHQGLKKWVEVGNSGVF
RPEMLLPMGLPENVSVIAWGLSLERPTMIKYGINNIRELVGHKVNLQMVYDSPVCRLDIEPRSSKTQEAA*
>rs32726273_ENSMUSP00000105376_c.1202:G>C_p.Q401H
ENSMUST00000109754.2|Farsa-202|ENSMUSG00000003808|+|chr8:g.85594648:G>C

MADNPVLELLLRRLEVADGGLDSAELATQLGVEHQAVVGAVKSLQALGEVIEAELRSTKCWELTTEGEEIARE
GSHEARVFRSIPLEGLVQSELMHLPSGKVGFSKAMSNKWIRVDKSAADGPRVFRVVDSIEDEVQKRLQLVQAG
QAEKLAEKERNELRKRKLLTEVILKTYWVSKGKAFSTSVSKQEAELSPEMISSGSWRDRPFKPYNFSARGVLP
DSGHLHPLLKVRSQFRQIFLEMGFTEMPTDNFIESSFWNFDALFQPQQHPARDQHDTFFLRDPAEALQLPMGY
VQRVKRTHSQGGYGSQGYKYTWKLEEARKNLLRTHTTAASARALYQLAQKPFTPAKYFSIDRVFRNETLDATH
LAEFHQIEGVIADHGLTLGHLMGVLREFFTKLGITHLRFKPAYNPYTEPSMEVFSYHQGLKKWVEVGNSGVFR
PEMLLPMGLPENVSVIAWGLSLERPTMIKYGINNIRELVGHKVNLQMVYDSPVCRLDIEPRSSKTQEAA*
>ENSMUSP00000003906_c.1222:C>CAAGA_p.A408ins-fs
ENSMUST00000003906.13|Farsa-
201|ENSMUSG00000003808|+|chr8:g.85594665:C>CAAGA
MADNPVLELLLRRLEVADGGLDSAELATQLGVEHQAVVGAVKSLQALGEVIEAELRSTKCWELTTEGEEIARE
GSHEARVFRSIPLEGLVQSELMHLPSGKVGFSKAMSNKWIRVDKSAADGPRVFRVVDSIEDEVQKRLQLVQAG
QAEKLAEKERNELRKRKLLTEVILKTYWVSKGKAFSTSVSKQEAELSPEMISSGSWRDRPFKPYNFSARGVLP
DSGHLHPLLKVRSQFRQIFLEMGFTEMPTDNFIESSFWNFDALFQPQQHPARDQHDTFFLRDPAEALQLPMGY
VQRVKRTHSQGGYGSQGYKYTWKLEEARKNLLRTHTTAASARALYQLAQKKPFTPAKYFSIDRVFRNETLDAT
HLAEFHQIEGVIADHGLTLGHLMGVLREFFTKLGITQLRFKPARLQPLHRAQHGSVQLPPRSKEVG
>ENSMUSP00000105376_c.1219:C>CAAGA_p.A407ins-fs
ENSMUST00000109754.2|Farsa-
202|ENSMUSG00000003808|+|chr8:g.85594665:C>CAAGA
MADNPVLELLLRRLEVADGGLDSAELATQLGVEHQAVVGAVKSLQALGEVIEAELRSTKCWELTTEGEEIARE
GSHEARVFRSIPLEGLVQSELMHLPSGKVGFSKAMSNKWIRVDKSAADGPRVFRVVDSIEDEVQKRLQLVQAG
QAEKLAEKERNELRKRKLLTEVILKTYWVSKGKAFSTSVSKQEAELSPEMISSGSWRDRPFKPYNFSARGVLP
DSGHLHPLLKVRSQFRQIFLEMGFTEMPTDNFIESSFWNFDALFQPQQHPARDQHDTFFLRDPAEALQLPMGY
VQRVKRTHSQGGYGSQGYKYTWKLEEARKNLLRTHTTAASARALYQLAQKPFTPAKYFSIDRVFRNETLDATH
LAEFHQIEGVIADHGLTLGHLMGVLREFFTKLGITQLRFKPARLQPLHRAQHGSVQLPPRSKEVG
>rs49360904_ENSMUSP00000122159_c.205:T>C_p.L69P
ENSMUST00000136026.8|Syce2-202|ENSMUSG00000003824|+|chr8:g.85610137:T>C
MERHGVAAPPVELKDQEPPAIVESGEHRQSENHEETPGSVAPSASCQLPGPFSSLDSSIETLKKKAQEPIENI
NESRQKDHALMTNFRDSLKMKVSDLTEKLEERMYQVYSHHSKIIQERLQEFTQKMAKINHLEMELKQVCQTVE
TVYKDLCVQSEVPTCEEQNYKDGEC*
>rs49360904_ENSMUSP00000131438_c.223:T>C_p.L75P
ENSMUST00000170296.2|Syce2-204|ENSMUSG00000003824|+|chr8:g.85610137:T>C
MERHGSWLRSQVAAPPVELKDQEPPAIVESGEHRQSENHEETPGSVAPSASCQLPGPFSSLDSSIETLKKKAQ
EPIENINESRQKDHALMTNFRDSLKMKVSDLTEKLEERMYQVYSHHSKIIQERLQEFTQKMAKINHLEMELKQ
VCQTVETVYKDLCVQSEVPTCEEQNYKDGEC*
>rs47589652_ENSMUSP00000003907_c.594:G>A_p.G199S
ENSMUST00000003907.14|Gcdh-201|ENSMUSG00000003809|-|chr8:g.85617709:C>T
MVPAMLTGYMSLRGVSARLLSRRSGLRFPRFPRTWSSAAAHTEKTQIRPAKSSRPVFDWKDPLILEEQLTADE
KLIRDTFRNYCQERLMSRILLANRNEVFHRDIVYEMGELGVLGPTIKGYGCAGVSSVAYGLLTRELERVDSGY
RSMMSVQSSLVMHPIYTYGSEEQRQKYLPGLAKGELLGCFGLTEPNHGSDPGSMETRARHNPSNQSYTLSGTK
TWITNSPVADLFIVWARCEDNCIRGFILEKGMRGLSAPRIEGKFSLRASATGMIIMDSVEVPEENVLPNVSSL
AGPFGCLNTARYGITWGVLGAAEFCLHTARQYALDRIQFGVPLARNQLVQKKLADMLTEITLGLHACLQLGRL
KDQDKATPEMVSMLKRNNCGKALDIARQARDILGGNGISDEYHVIRHAMNLEAVNTYEGTHDIHALILGRAIT
GIQAFTVGK*
>rs47589652_ENSMUSP00000105367_c.567:G>A_p.G190S
ENSMUST00000109745.8|Gcdh-202|ENSMUSG00000003809|-|chr8:g.85617709:C>T
MSLRGVSARLLSRRSGLRFPRFPRTWSSAAAHTEKTQIRPAKSSRPVFDWKDPLILEEQLTADEKLIRDTFRN
YCQERLMSRILLANRNEVFHRDIVYEMGELGVLGPTIKGYGCAGVSSVAYGLLTRELERVDSGYRSMMSVQSS
LVMHPIYTYGSEEQRQKYLPGLAKGELLGCFGLTEPNHGSDPGSMETRARHNPSNQSYTLSGTKTWITNSPVA
DLFIVWARCEDNCIRGFILEKGMRGLSAPRIEGKFSLRASATGMIIMDSVEVPEENVLPNVSSLAGPFGCLNT
ARYGITWGVLGAAEFCLHTARQYALDRIQFGVPLARNQLVQKKLADMLTEITLGLHACLQLGRLKDQDKATPE
MVSMLKRNNCGKALDIARQARDILGGNGISDEYHVIRHAMNLEAVNTYEGTHDIHALILGRAITGIQAFTVGK
*
>rs47393345_ENSMUSP00000003907_c.544:T>A_p.L182H
ENSMUST00000003907.14|Gcdh-201|ENSMUSG00000003809|-|chr8:g.85617759:A>T
MVPAMLTGYMSLRGVSARLLSRRSGLRFPRFPRTWSSAAAHTEKTQIRPAKSSRPVFDWKDPLILEEQLTADE
KLIRDTFRNYCQERLMSRILLANRNEVFHRDIVYEMGELGVLGPTIKGYGCAGVSSVAYGLLTRELERVDSGY
RSMMSVQSSLVMHPIYTYGSEEQRQKYLPGLAKGEHLGCFGLTEPNHGSDPGGMETRARHNPSNQSYTLSGTK

TWITNSPVADLFIVWARCEDNCIRGFILEKGMRGLSAPRIEGKFSLRASATGMIIMDSVEVPEENVLPNVSSL
AGPFGCLNTARYGITWGVLGAAEFCLHTARQYALDRIQFGVPLARNQLVQKKLADMLTEITLGLHACLQLGRL
KDQDKATPEMVSMLKRNNCGKALDIARQARDILGGNGISDEYHVIRHAMNLEAVNTYEGTHDIHALILGRAIT
GIQAFTVGK*
>rs47393345_ENSMUSP00000105367_c.517:T>A_p.L173H
ENSMUST00000109745.8|Gcdh-202|ENSMUSG00000003809|-|chr8:g.85617759:A>T
MSLRGVSARLLSRRSGLRFPRFPRTWSSAAAHTEKTQIRPAKSSRPVFDWKDPLILEEQLTADEKLIRDTFRN
YCQERLMSRILLANRNEVFHRDIVYEMGELGVLGPTIKGYGCAGVSSVAYGLLTRELERVDSGYRSMMSVQSS
LVMHPIYTYGSEEQRQKYLPGLAKGEHLGCFGLTEPNHGSDPGGMETRARHNPSNQSYTLSGTKTWITNSPVA
DLFIVWARCEDNCIRGFILEKGMRGLSAPRIEGKFSLRASATGMIIMDSVEVPEENVLPNVSSLAGPFGCLNT
ARYGITWGVLGAAEFCLHTARQYALDRIQFGVPLARNQLVQKKLADMLTEITLGLHACLQLGRLKDQDKATPE
MVSMLKRNNCGKALDIARQARDILGGNGISDEYHVIRHAMNLEAVNTYEGTHDIHALILGRAITGIQAFTVGK
*
>rs4137992_ENSMUSP00000105363_c.2420:C>A_p.S807R
ENSMUST00000109741.9|Mast1-201|ENSMUSG00000053693|-|chr8:g.85644507:G>T
MSDSLWTALSNFSMPSFPGGSMFRRTKSCRTSNRKSLILTSTSPTLPRPHSPLPGHLGSSPLDSPRNFSPNTP
AHFSFASSRRADGRRWSLASLPSSGYGTNTPSSTVSSSCSSQERLHQLPYQPTVDELHFLSKHFGSTESITDE
DGGRRSPAVRPRSRSLSPGRSPSSYDNEIVMMNHVYKERFPKATAQMEEKLRDFARAYEPDSVLPLADGVLSF
IHHQIIELARDCLTKSRDGLITTVYFYELQENLEKLLQDAYERSESLEVAFVTQLVKKLLIIISRPARLLECL
EFNPEEFYHLLEAAEGHAKEGHLVKTDIPRYIIRQLGLTRDPFPDVVRLEEQDSGGSNTPEQDDTSEGRSSTS
KAKKPPGESDFDTIKLISNGAYGAVYLVRHRDTRQRFAMKKINKQNLILRNQIQQAFVERDILTFAENPFVVG
MFCSFETRRHLCMVMEYVEGGDCATLLKNIGALPVEMARMYFAETVLALEYLHNYGIVHRDLKPDNLLITSMG
HIKLTDFGLSKMGLMSLTTNLYEGHIEKDAREFLDKQVCGTPEYIAPEVILRQGYGKPVDWWAMGIILYEFLV
GCVPFFGDTPEELFGQVISDDILWPEGDEALPTDAQLLISSLLQTNPLVRLGAGGAFEVKQHSFFRDLDWTGL
LRQKAEFIPHLESEDDTSYFDTRSDRYHHVNSYDEDDTTEEEPVEIRQFSSCSPRFSKVYSSMEQLSQHEPKT
PVSASGASKRDPSAKGPEEKVAGKREGLGGLTLREKTWRGGSPEIKRFSASEASFLEGEASPPLGARRRFSAL
LEPRRFTAPQEDEDEARLRRPPRPSSDPPSSLDTRVPKEAVQGEGTSTPGEPEATERSHPGDLGPPSKDGDPS
GPRATNDLVLRRARHQQLSGDLAVEKRPSRTGGKVIKSASATALSVMIPAVDPHGGSPLASPMSPRSLSSNPS
SRDSSPSRDYSPAVSGLRSPITIQRSGKKYGFTLRAIRVYMGDSDVYSVHHIVWHVEEGGPAQEAGLCAGDLI
THVNGEPVHGMVHPEVVELILKSGNKVAVTTTPFENTSIRIGPARRSSYKAKMARRNKRPSAKDGQESKKRSS
LFRKITKQSNLLHTSRSLSSLNRSLSSSDSLPGSPTHGLPARSPTHSYRSTPDSAYLGASSQSSSPASSTPNS
PASSASHHIRPSTLHGLSPKLHRQYRSARCKSAGNIPLSPLAHTPSPTQASPPPLPGHTVGSSHTTQSFPAKL
HSSPPVVRPRPKSAEPPRSPLLKRVQSAEKLGASLGADKKGALRKHSLEVGHPDFRKDFHGELALHSLAESDG
ETPPIEGPGATRQVAVRRLGRQESPLSLGADPLLPDGVQRPMASSKEDSAGGTEACTPPRATTPGSRTLERDS
GCTRHQSVQTEDGPGGVARALAKAALSPVQEHETGRRSSSGEAGTPPVPIVVEPARPGVKTQAPQPLGTDSKG
LKEPVAQMPLMPDAPRGRERWVLEEVEERTTLSGLRSKPASPKLSPDPQTPTLVPTKNVPRSAAPSVPPASLM
VPGTKPEAGLNSRCPAEAVTPAGLTKTGAPSPASLGP*
>rs46189481_ENSMUSP00000064680_c.169:G>A_p.G57D
ENSMUST00000064922.7|Junb-201|ENSMUSG00000052837|-|chr8:g.85704889:C>T
MCTKMEQPFYHDDSYAAAGYGRSPGSLSLHDYKLLKPTLALNLADPYRGLKGPGARDPGPEGSGAGSYFSGQG
SDTGASLKLASTELERLIVPNSNGVITTTPTPPGQYFYPRGGGSGGGTGGGVTEEQEGFADGFVKALDDLHKM
NHVTPPNVSLGASGGPQAGPGGVYAGPEPPPVYTNLSSYSPASAPSGGSGTAVGTGSSYPTATISYLPHAPPF
AGGHPAQLGLSRGASAFKEEPQTVPEARSRDATPPVSPINMEDQERIKVERKRLRNRLAATKCRKRKLERIAR
LEDKVKTLKAENAGLSSAAGLLREQVAQLKQKVMTHVSNGCQLLLGVKGHAF*
>ENSMUSP00000148237_c.247:C>T_p.S83L ENSMUST00000209764.2|Hook2-
203|ENSMUSG00000052566|+|chr8:g.85719856:C>T
MSVDKAELCGSLLTWLQTFQVSPPCASPQDLSSGLAIAHVLNQIDPSWFNNEWLQGISEDSSPSWRLKVRKLE
KILQSLVEYLKNVLGHPVSDQHLPDVSLIGEFSNPAELGKLLQLVLGCAISCEKKQEYIQRIMTLEESVQHVV
MEAIQELMTKDTPDSLSPENYGNFDTQSRRYYFLSEEVEEGDHLQQHYLDLERQLLLLSEEKQNLAQENAALR
ERVGRSEVESAPGLTAKKLLLLQSQLEQLQEENFRLESSREDDRLRCLELEREVAELQQRNQALTSLSQEAQA
LKDEMDELRQSSERARQLEERNAGHAERTRQLEEELRRAGSLRAQLEAQRRQVQELQGQWQEEAMKAEKWLFE
CRNLEEKCDLVTKEKERLLTERDSLREANEELRCAQLQPRGLAQADLSLDPTPSGLENLAAEILPAELRETLV
RLQLENKRLCQQEAADRERQEELQRHLEEANRARHGLEAQQRLNQQQLSELRAQVEELQKALQEQGGKTEDPT
LLKRKLEDHLQKLHEADLELQRKREYIEELEPPTDSSTARRIEELQDSLQKKDADLRAMEERYRRYVDKARTV
IQTLEPKQRPPTVVSPEFHTLRSQLWERNLRIRQMEMDYEKSRRRQEQEEKLLISAWYSMGMALEHRAGEEHA
PAHAQSFLAQQRLATNARRGPLGRQALSLRPTDKH*
>ENSMUSP00000067752_c.247:C>T_p.S83L ENSMUST00000064495.8|Hook2-
201|ENSMUSG00000052566|+|chr8:g.85719856:C>T

MSVDKAELCGSLLTWLQTFQVSPPCASPQDLSSGLAIAHVLNQIDPSWFNNEWLQGISEDSSPSWRLKVRKLE
KILQSLVEYLKNVLGHPVSDQHLPDVSLIGEFSNPAELGKLLQLVLGCAISCEKKQEYIQRIMTLEESVQHVV
MEAIQELMTKDTPDSLSPENYGNFDTQSRRYYFLSEEVEEGDHLQQHYLDLERQLLLLSEEKQNLAQENAALR
ERVGRSEVESAPGLTAKKLLLLQSQLEQLQEENFRLESSREDDRLRCLELEREVAELQQRNQALTSLSQEAQA
LKDEMDELRQSSERARQLEATLNSCRRRLGELQELRRQVRQLEERNAGHAERTRQLEEELRRAGSLRAQLEAQ
RRQVQELQGQWQEEAMKAEKWLFECRNLEEKCDLVTKEKERLLTERDSLREANEELRCAQLQPRGLAQADLSL
DPTPSGLENLAAEILPAELRETLVRLQLENKRLCQQEAADRERQEELQRHLEEANRARHGLEAQQRLNQQQLS
ELRAQVEELQKALQEQGGKTEDPTLLKRKLEDHLQKLHEADLELQRKREYIEELEPPTDSSTARRIEELQDSL
QKKDADLRAMEERYRRYVDKARTVIQTLEPKQRPPTVVSPEFHTLRSQLWERNLRIRQMEMDYEKSRRRQEQE
EKLLISAWYSMGMALEHRAGEEHAPAHAQSFLAQQRLATNARRGPLGRQALSLRPTDKH*
>ENSMUSP00000148078_c.247:C>T_p.S83L ENSMUST00000210326.2|Hook2-204|ENSMUSG00000052566|+|chr8:g.85719856:C>T
MSVDKAELCGSLLTWLQTFQVSPPCASPQDLSSGLAIAHVLNQIDPSWFNNEWLQGISEDSSPSWRLKVRKLE
KILQSLVEYLKNVLGHPVSDQHLPDVSLIGEFSNPAELGKLLQLVLGCAISCEKKQEYIQRIMTLEESVQHVV
MEAIQELMTKDTPDSLSPENYGNFDTQSRRYYFLSEEVEEGDHLQQHYLDLERQLLLLSEEKQNLAQENAALR
ERVGRSEVESAPGLTAKKLLLLQSQLEQLQEENFRLESSREDDRLRCLELEREVAELQQRNQALTSLSQEAQA
LKDEMDELRQSSERARQLEATLNSCRRRLGELQELRRQVRQLEERNAGHAERTRQLEEELRRAGSLRAQLEAQ
RRQVQELQGQWQEEAMKAEKWLFECRNLEEKCDLVTKEKERLLTERDSLREANEELRCAQLQPRGLAQADLSL
DPTPSGLENLAAEILPAELRETLVRLQLENKRLCQQEAADRERQEELQRHLEEANRARHGLEAQQRLNQQQLS
ELRAQVEELQKALQEQGGKTEDVSACLLPAWPSLVLPSNPPFSLPPCCPMPEEGHCEYHGSREAHHCYLHSFS
DLVPVPSLPAASLPQPTLLKRKLEDHL*
>rs254660594_ENSMUSP00000148237_c.345:C>T_p.Q116*
ENSMUST00000209764.2|Hook2-203|ENSMUSG00000052566|+|chr8:g.85720043:C>T
MSVDKAELCGSLLTWLQTFQVSPPCASPQDLSSGLAIAHVLNQIDPSWFNNEWLQGISEDSSPSWRLKVRKLE
KILQSLVEYSKNVLGHPVSDQHLPDVSLIGEFSNPAELGKLL*LVLGCAISCEKKQEYIQRIMTLEESVQHVV
MEAIQELMTKDTPDSLSPENYGNFDTQSRRYYFLSEEVEEGDHLQQHYLDLERQLLLLSEEKQNLAQENAALR
ERVGRSEVESAPGLTAKKLLLLQSQLEQLQEENFRLESSREDDRLRCLELEREVAELQQRNQALTSLSQEAQA
LKDEMDELRQSSERARQLEERNAGHAERTRQLEEELRRAGSLRAQLEAQRRQVQELQGQWQEEAMKAEKWLFE
CRNLEEKCDLVTKEKERLLTERDSLREANEELRCAQLQPRGLAQADLSLDPTPSGLENLAAEILPAELRETLV
RLQLENKRLCQQEAADRERQEELQRHLEEANRARHGLEAQQRLNQQQLSELRAQVEELQKALQEQGGKTEDPT
LLKRKLEDHLQKLHEADLELQRKREYIEELEPPTDSSTARRIEELQDSLQKKDADLRAMEERYRRYVDKARTV
IQTLEPKQRPPTVVSPEFHTLRSQLWERNLRIRQMEMDYEKSRRRQEQEEKLLISAWYSMGMALEHRAGEEHA
PAHAQSFLAQQRLATNARRGPLGRQALSLRPTDKH*
>rs254660594_ENSMUSP00000067752_c.345:C>T_p.Q116*
ENSMUST00000064495.8|Hook2-201|ENSMUSG00000052566|+|chr8:g.85720043:C>T
MSVDKAELCGSLLTWLQTFQVSPPCASPQDLSSGLAIAHVLNQIDPSWFNNEWLQGISEDSSPSWRLKVRKLE
KILQSLVEYSKNVLGHPVSDQHLPDVSLIGEFSNPAELGKLL*LVLGCAISCEKKQEYIQRIMTLEESVQHVV
MEAIQELMTKDTPDSLSPENYGNFDTQSRRYYFLSEEVEEGDHLQQHYLDLERQLLLLSEEKQNLAQENAALR
ERVGRSEVESAPGLTAKKLLLLQSQLEQLQEENFRLESSREDDRLRCLELEREVAELQQRNQALTSLSQEAQA
LKDEMDELRQSSERARQLEATLNSCRRRLGELQELRRQVRQLEERNAGHAERTRQLEEELRRAGSLRAQLEAQ
RRQVQELQGQWQEEAMKAEKWLFECRNLEEKCDLVTKEKERLLTERDSLREANEELRCAQLQPRGLAQADLSL
DPTPSGLENLAAEILPAELRETLVRLQLENKRLCQQEAADRERQEELQRHLEEANRARHGLEAQQRLNQQQLS
ELRAQVEELQKALQEQGGKTEDPTLLKRKLEDHLQKLHEADLELQRKREYIEELEPPTDSSTARRIEELQDSL
QKKDADLRAMEERYRRYVDKARTVIQTLEPKQRPPTVVSPEFHTLRSQLWERNLRIRQMEMDYEKSRRRQEQE
EKLLISAWYSMGMALEHRAGEEHAPAHAQSFLAQQRLATNARRGPLGRQALSLRPTDKH*
>rs254660594_ENSMUSP00000148078_c.345:C>T_p.Q116*
ENSMUST00000210326.2|Hook2-204|ENSMUSG00000052566|+|chr8:g.85720043:C>T
MSVDKAELCGSLLTWLQTFQVSPPCASPQDLSSGLAIAHVLNQIDPSWFNNEWLQGISEDSSPSWRLKVRKLE
KILQSLVEYSKNVLGHPVSDQHLPDVSLIGEFSNPAELGKLL*LVLGCAISCEKKQEYIQRIMTLEESVQHVV
MEAIQELMTKDTPDSLSPENYGNFDTQSRRYYFLSEEVEEGDHLQQHYLDLERQLLLLSEEKQNLAQENAALR
ERVGRSEVESAPGLTAKKLLLLQSQLEQLQEENFRLESSREDDRLRCLELEREVAELQQRNQALTSLSQEAQA
LKDEMDELRQSSERARQLEATLNSCRRRLGELQELRRQVRQLEERNAGHAERTRQLEEELRRAGSLRAQLEAQ
RRQVQELQGQWQEEAMKAEKWLFECRNLEEKCDLVTKEKERLLTERDSLREANEELRCAQLQPRGLAQADLSL
DPTPSGLENLAAEILPAELRETLVRLQLENKRLCQQEAADRERQEELQRHLEEANRARHGLEAQQRLNQQQLS
ELRAQVEELQKALQEQGGKTEDVSACLLPAWPSLVLPSNPPFSLPPCCPMPEEGHCEYHGSREAHHCYLHSFS
DLVPVPSLPAASLPQPTLLKRKLEDHL*
>rs32597233_ENSMUSP00000147580_c.693:A>G_p.I232V
ENSMUST00000209322.2|Best2-202|ENSMUSG00000052819|-|chr8:g.85737412:T>C

MTVTYTARVANARFGGFSQLLLLWRGSIYKLLWRELLCFLGLYMALSAAYRFLLAEEQKRYFEKLVIYCDQYA
SLIPVSFVLGFYVTLVVHRWWNQYLCMPLPDALMCIVAGTVHGRDDRGRLYRRTLMRYAGLSAVLILRSVSTA
VFKRFPTIDHVVEAGFMTREERKKFENLNSSYNKYWVPCVWFSSLAAQARREGRIRDNSALKLLLEELNVFRS
KCGMLFHYDWISVPLVYTQVVTIAVYSYFLACLIGRQFLDPAQGYKDHTLDLCVPIFTLLQFFFYAGWLKVAE
QLINPFGEDDDDFETNFLIDRNFQVSMLAVDEMYDDLAMLEKDLYWDAAEARAPYTAATAFLLQQPSFQGSTF
DIALAKEDMQFQRLDGVDGPLGEVHGDFLQRLLPAGAGSVGPLGRRLSLLRRKNSCVSEASTAASCGCAGAAD
GGGVECGCGDPLLDPSLREPELESPACPEPPAPIPGPTPEPFTTVSIPGPRAPAPPWLPSPIGEEEESPA*
>rs32597233_ENSMUSP00000053408_c.693:A>G_p.I232V
ENSMUST00000059072.6|Best2-201|ENSMUSG00000052819|-|chr8:g.85737412:T>C
MTVTYTARVANARFGGFSQLLLLWRGSIYKLLWRELLCFLGLYMALSAAYRFLLAEEQKRYFEKLVIYCDQYA
SLIPVSFVLGFYVTLVVHRWWNQYLCMPLPDALMCIVAGTVHGRDDRGRLYRRTLMRYAGLSAVLILRSVSTA
VFKRFPTIDHVVEAGFMTREERKKFENLNSSYNKYWVPCVWFSSLAAQARREGRIRDNSALKLLLEELNVFRS
KCGMLFHYDWISVPLVYTQVVTIAVYSYFLACLIGRQFLDPAQGYKDHTLDLCVPIFTLLQFFFYAGWLKVAE
QLINPFGEDDDDFETNFLIDRNFQVSMLAVDEMYDDLAMLEKDLYWDAAEARAPYTAATAFLLQQPSFQGSTF
DIALAKEDMQFQRLDGVDGPLGEVHGDFLQRLLPAGAGSVGPLGRRLSLLRRKNSCVSEASTAASCGCAGAAD
GGGVECGCGDPLLDPSLREPELESPACPEPPAPIPGPTPEPFTTVSIPGPRAPAPPWLPSPIGEEEESPA*
>rs32597233_ENSMUSP00000147837_c.693:A>G_p.I232V
ENSMUST00000209421.2|Best2-203|ENSMUSG00000052819|-|chr8:g.85737412:T>C
MTVTYTARVANARFGGFSQLLLLWRGSIYKLLWRELLCFLGLYMALSAAYRFLLAEEQKRYFEKLVIYCDQYA
SLIPVSFVLGFYVTLVVHRWWNQYLCMPLPDALMCIVAGTVHGRDDRGRLYRRTLMRYAGLSAVLILRSVSTA
VFKRFPTIDHVVEAGFMTREERKKFENLNSSYNKYWVPCVWFSSLAAQARREGRIRDNSALKLLLEELNVFRS
KCGMLFHYDWISVPLVYTQVVTIAVYSYFLACLIGRQFLDPAQGYKDHTLDLCVPIFTLLQFFFYAGWLKVAE
QLINPFGEDDDDFETNFLIDRNFQVSMLAVDEMYDDLAMLEKDLYWDAAEARAPYTAATAFLLQQPSFQGSTF
DIALAKEDMQFQRLDGVDGPLGEVHGDFLQRLLPAGAGSVGPLGRRLSLLRRKNSCVSEASTAASCGCAGAAD
GGGVECGCGDPLLDPSLREPELESPACPEPPAPIPGPTPEPFTTVSIPGPRAPAPPWLPSPIGEEEESPA*
>rs49088296_ENSMUSP00000092845_c.84:C>T_p.Q29*
ENSMUST00000095220.4|Fbxw9-201|ENSMUSG00000008167|+|chr8:g.85786841:C>T
MDLSSGRSGDPRSCEEESDPEPDPDPDT*AEAYVARVLTPPKLGLTPRRSSLQSMFSASLGVPERKAASKVPA
VRLPGLLSLPPELLLEICAYLDARVVLQVLPCVCQALHDLVRDRVTWRLRAQRRVRAPYPVVEEENFDWPAAC
IELEQHLARWAEDGQRTEYFCLADGHFASIDAVLLLQGGALCLSGSRDRNVNLWDLRHLGKDPSRVLVKALGT
QGNSTHKGWVWSLAAQDHRVCSGSWDSTVKLWDMAADGQQFGEIKGKAAVLCLSYQPDILVTGTYDKKVTIYD
PRAGLALVKSRRLHSSAVLAVLADDRHVISGSEDHSLVVFDRRANSVLQRLQLDSYLLCMSYQEPQLWAGDNQ
GLLHVFANRDGCFQLVRSFDVGHQSQITGIKHSLGTLYTTSTDKTIRVHVPTDPPRTICTRSHHNVLNGICAE
GNVVVAASGGLSLEVWRLLA*
>rs50985175_ENSMUSP00000077733_c.63:T>C_p.S22P
ENSMUST00000078665.13|Dhps-201|ENSMUSG00000060038|+|chr8:g.85798539:T>C
MEGTPPGAAPSSALAAVLKHSPALPPESAQVQGYDFNRGVDYHALLDAYRTTGFQATNFGRAVQQVNAMIEKK
LEPLAVDEDHHADLTQSRRPLTGCTIFLGYTSNLISSGIRETIRYLVQHNMVDVLVTTAGGVEEDLIKCLAPT
YLGEFSLRGKELRESGINRIGNLLVPNDNYCKFEDWLMPILDQMVLEQNTEGVKWTPSKMISRLGKEINNPDS
VYYWAHKNHIPVLSPALTDGSLGDMIFFHSYKNPGLVLDIVEDLRLINTQAIFAKRSGMIILGGGVVKHHIAN
ANLMRNGADYAVYINTAQEFDGSDSGARPDEAVSWGKIRMDAQPVKVYADASLVFPLLVAETFAQKADAFRAE
KNED*
>rs50985175_ENSMUSP00000120308_c.114:T>C_p.S39P
ENSMUST00000152871.8|Dhps-207|ENSMUSG00000060038|+|chr8:g.85798539:T>C
AGLRAPRLLRPESGREHMEGTPPGAAPSSALAAVLKHSPALPPESAQVQGYDFNRGVDYHALLDAYRTTGFQA
TNFGRAVQQVNAMIEKKLEPLAVDEDHHADLTQSRRPLTGCTIFLGYTSNLISSGIRETIRYLVQHNMDWKPA
GAE*
>rs49986977_ENSMUSP00000147335_c.693:T>G_p.F232V
ENSMUST00000211109.2|Zfp791-202|ENSMUSG00000074194|-|chr8:g.85837169:A>C
MDSVTFEDVCVTFTQEEWDLLRPVQKKLYKDVMWETVRNLASVASELEDQDVSNQYRIQEENVRSYVIQRLYE
SKEVIEHGESFSQIPNTGAAKITSGGKPYSCSVCGKGFMLCSSLTRHVRAHPGHELAGFKCGEKRFKCKECRA
TFSYLKSFQRHERTHAGESPYRCKHCGKTFIYFQPFQTHERTHIGEQLYQCKHCGKGLSSPGSLRIHQRIHTG
EKPYVCEKCGKAVSCPSSIQIHERTHTGEKPYKCKECGKAFISRTSVKTHMVTHSGDANKPHKCEHCGKAFLR
PCFLRVHTRIHTGEKPYVCKQCGKAFRCSTSMQIHKRTHTGEKPYKCKECGKAFSAHSAFRVHRRVHTGEKPY
KCRDCGKAFICLTYCRAHERKHSGEKSYECDQCGKAFVSLENFRRHVKTHAEDTPYKCKECGKAFVFLSALQA
HERIHTGEKPYECEQCGKTFRCSSYVQVHRRIHTGEKPYECKECGKTFIYSTSFRGHMRMHTGEKPYKCKGCE
KAFRLGSSLKRHERTHS*

>rs49986977_ENSMUSP00000096150_c.693:T>G_p.F232V
ENSMUST00000098550.4|Zfp791-201|ENSMUSG00000074194|-|chr8:g.85837169:A>C
MDSVTFEDVCVTFTQEEWDLLRPVQKKLYKDVMWETVRNLASVASELEDQDVSNQYRIQEENVRSYVIQRLYE
SKEVIEHGESFSQIPNTGAAKITSGGKPYSCSVCGKGFMLCSSLTRHVRAHPGHELAGFKCGEKRFKCKECRA
TFSYLKSFQRHERTHAGESPYRCKHCGKTFIYFQPFQTHERTHIGEQLYQCKHCGKGLSSPGSLRIHQRIHTG
EKPYVCEKCGKAVSCPSSIQIHERTHTGEKPYKCKECGKAFISRTSVKTHMVTHSGDANKPHKCEHCGKAFLR
PCFLRVHTRIHTGEKPYVCKQCGKAFRCSTSMQIHKRTHTGEKPYKCKECGKAFSAHSAFRVHRRVHTGEKPY
KCRDCGKAFICLTYCRAHERKHSGEKSYECDQCGKAFVSLENFRRHVKTHAEDTPYKCKECGKAFVFLSALQA
HERIHTGEKPYECEQCGKTFRCSSYVQVHRRIHTGEKPYECKECGKTFIYSTSFRGHMRMHTGEKPYKCKGCE
KAFRLGSSLKRHERTHS*
>rs256780963_ENSMUSP00000151714_c.34:T>C_p.F12S
ENSMUST00000218663.2|Olfr371-202|ENSMUSG00000051952|+|chr8:g.85957160:T>C
MEPENHTGIPESYLLGLSENPEIQSVLFGLFLSLYLVTVFGNLLIIILAIVSDPKLHTPMYLFLSNLSFSDICF
TSTTVPKMLLGIQTQSKLITYAGCITQMYFFTVFGLLDNLLLTVMAYDRFVAICHPLHYTVLMNPKLCSQLLL
LAWLISILGALPESLTALRLSFCAVVEIPHYFCELPEVLKLACSDTFINNVVLYIVTGIMGFFPLAGILFSYS
QIVTSVLRISTVGGKYKAFSTCGSHLSVVSLFYGTCLGVYLSSIWTQASWAGVFASVLYTVVTPMMNPFIYSL
RNRDMKRALNTLLCSVPSSS*
>rs256780963_ENSMUSP00000077921_c.34:T>C_p.F12S
ENSMUST00000070849.6|Olfr371-201|ENSMUSG00000051952|+|chr8:g.85957160:T>C
MEPENHTGIPESYLLGLSENPEIQSVLFGLFLSLYLVTVFGNLLIIILAIVSDPKLHTPMYLFLSNLSFSDICF
TSTTVPKMLLGIQTQSKLITYAGCITQMYFFTVFGLLDNLLLTVMAYDRFVAICHPLHYTVLMNPKLCSQLLL
LAWLISILGALPESLTALRLSFCAVVEIPHYFCELPEVLKLACSDTFINNVVLYIVTGIMGFFPLAGILFSYS
QIVTSVLRISTVGGKYKAFSTCGSHLSVVSLFYGTCLGVYLSSIWTQASWAGVFASVLYTVVTPMMNPFIYSL
RNRDMKRALNTLLCSVPSSS*
>rs33534398_ENSMUSP00000151714_c.45:G>A_p.G16R
ENSMUST00000218663.2|Olfr371-202|ENSMUSG00000051952|+|chr8:g.85957171:G>A
MEPENHTGIPEFYLLRLSENPEIQSVLFGLFLSLYLVTVFGNLLIIILAIVSDPKLHTPMYLFLSNLSFSDICF
TSTTVPKMLLGIQTQSKLITYAGCITQMYFFTVFGLLDNLLLTVMAYDRFVAICHPLHYTVLMNPKLCSQLLL
LAWLISILGALPESLTALRLSFCAVVEIPHYFCELPEVLKLACSDTFINNVVLYIVTGIMGFFPLAGILFSYS
QIVTSVLRISTVGGKYKAFSTCGSHLSVVSLFYGTCLGVYLSSIWTQASWAGVFASVLYTVVTPMMNPFIYSL
RNRDMKRALNTLLCSVPSSS*
>rs33534398_ENSMUSP00000077921_c.45:G>A_p.G16R
ENSMUST00000070849.6|Olfr371-201|ENSMUSG00000051952|+|chr8:g.85957171:G>A
MEPENHTGIPEFYLLRLSENPEIQSVLFGLFLSLYLVTVFGNLLIIILAIVSDPKLHTPMYLFLSNLSFSDICF
TSTTVPKMLLGIQTQSKLITYAGCITQMYFFTVFGLLDNLLLTVMAYDRFVAICHPLHYTVLMNPKLCSQLLL
LAWLISILGALPESLTALRLSFCAVVEIPHYFCELPEVLKLACSDTFINNVVLYIVTGIMGFFPLAGILFSYS
QIVTSVLRISTVGGKYKAFSTCGSHLSVVSLFYGTCLGVYLSSIWTQASWAGVFASVLYTVVTPMMNPFIYSL
RNRDMKRALNTLLCSVPSSS*
>rs32548947_ENSMUSP00000034131_c.2217:A>G_p.I740V
ENSMUST00000034131.10|Vps35-201|ENSMUSG00000031696|-|chr8:g.85987932:T>C
MPTTQQSPQDEQEKLLDEAIQAVKVQSFQMKRCLDKNKLMDALKHASNMLGELRTSMLSPKSYYELYMAISDE
LHYLEVYLTDEFAKGRKVADLYELVQYAGNIIPRLYLLITVGVVYVKSFPQSRKDILKDLVEMCRGVQHPLRG
LFLRNYLLQCTRNILPDEGEPTDEETTGDISDSMDFVLLNFAEMNKLWVRMQHQGHSRDREKREREROELRIL
VGTNLVRLSQLEGVNVERYKQIVLTGILEQVVNCRDALAQEYLMECIIQVFPDEFHLQTLNPFLRACAELHQN
VNVKNIIIALIDRLALFAHREDGPGIPAEIKLFDIFSQQVATVIQSRQDMPSEDVVSLQVSLINLAMKCYPDR
VDYVDKVLETTVEIFNKLNLEHIATSSAVSKELTRLLKIPVDTYNNILTVLKLKHFHPLFEYFDYESRKSMSC
YVLSNVLDYNTEIVSQDQVDSIMNLVSTLIQDQPDQPVEDPDPEDFADEQSLVGRFIHLLRSDDPDQQYLILN
TARKHFGAGGNQRIRFTLPPLVFAAYQLAFRYKENSQMDDKWEKKCQKIFSFAHQTISALIKAELAELPLRLF
LQGALAAGEIGFENHETVAYEFMSQAFSLYEDEISDSKAQLAAITLIIGTFERMKCFSEENHEPLRTQCALAA
SKLLKKPDQGRAVSTCAHLFWSGRNTDKNGEELHGGKRVMECLKKALKIANQCMDPSLQVQLFIEILNRYIYF
YEKENDAVTVQVLNQLIQKIREDLPNLESSEETEQINKHFHNTLEHLRSRRESPESEGPIYEGLIL*
>rs32770262_ENSMUSP00000034131_c.1743:A>G_p.R582G
ENSMUST00000034131.10|Vps35-201|ENSMUSG00000031696|-|chr8:g.85990856:T>C
MPTTQQSPQDEQEKLLDEAIQAVKVQSFQMKRCLDKNKLMDALKHASNMLGELRTSMLSPKSYYELYMAISDE
LHYLEVYLTDEFAKGRKVADLYELVQYAGNIIPRLYLLITVGVVYVKSFPQSRKDILKDLVEMCRGVQHPLRG
LFLRNYLLQCTRNILPDEGEPTDEETTGDISDSMDFVLLNFAEMNKLWVRMQHQGHSRDREKREREROELRIL
VGTNLVRLSQLEGVNVERYKQIVLTGILEQVVNCRDALAQEYLMECIIQVFPDEFHLQTLNPFLRACAELHQN
VNVKNIIIALIDRLALFAHREDGPGIPAEIKLFDIFSQQVATVIQSRQDMPSEDVVSLQVSLINLAMKCYPDR

VDYVDKVLETTVEIFNKLNLEHIATSSAVSKELTRLLKIPVDTYNNILTVLKLKHFHPLFEYFDYESRKSMSC
YVLSNVLDYNTEIVSQDQVDSIMNLVSTLIQDQPDQPVEDPDPEDFADEQSLVGRFIHLLRSDDPDQQYLILN
TARKHFGAGGNQRIRFTLPPLVFAAYQLAFRYKENSQMDDKWEKKCQKIFSFAHQTISALIKAELAELPLGLF
LQGALAAGEIGFENHETVAYEFMSQAFSLYEDEISDSKAQLAAITLIIGTFERMKCFSEENHEPLRTQCALAA
SKLLKKPDQGRAVSTCAHLFWSGRNTDKNGEELHGGKRVMECLKKALKIANQCMDPSLQVQLFIEILNRYIYF
YEKENDAVTIQVLNQLIQKIREDLPNLESSEETEQINKHFHNTLEHLRSRRESPESEGPIYEGLIL*
>ENSMUSP00000040975_c.441:A>G_p.I148V ENSMUST00000047749.7|4921524J17Rik-
201|ENSMUSG00000036934|-|chr8:g.86136424:T>C
MSATRAKKVKMATKSCPECDQQIPVACKSCPCGYIFISRKLLNAKHSEKSPPSTENKHEAKRRRTERVRREKI
NSTVNKDLENRKRSRSNSHSDHIRRGRGRPKSSSAKKHEEEREKQEKEIDIYANLSDEKAFVFSVALAEINRK
IVNQRLIL*
>rs52070708_ENSMUSP00000040975_c.384:T>A_p.S129T
ENSMUST00000047749.7|4921524J17Rik-201|ENSMUSG00000036934|-
|chr8:g.86136481:A>T
MSATRAKKVKMATKSCPECDQQIPVACKSCPCGYIFISRKLLNAKHSEKSPPSTENKHEAKRRRTERVRREKI
NSTVNKDLENRKRSRSNSHSDHIRRGRGRPKSSSAKKHEEEREKQEKEIDIYANLTDEKAFVFSVALAEINRK
IINQRLIL*
>rs3709028_ENSMUSP00000129724_c.781:T>C_p.V261A
ENSMUST00000165770.9|Zfp423-203|ENSMUSG00000045333|-|chr8:g.88509186:A>G
MIGDGCDLGLGEEEGGTGLPYPCQFCDKSFIRLSYLKRHEQIHSDKLPFKCTFCSRLFKHKRSRDRHIKLHTG
DKKYHCHECEAAFSRSDHLKIHLKTHSSSKPFKCSVCKRGFSSTSSLQSHMQAHKKNKEHLAKSEKEAKKDDF
MCDYCEDTFSQTEELEKHVLTLHPQLSEKADLQCIHCPEVFVDESTLLAHIHQAHANQKHKCPMCPEQFSSVE
GVYCHLDSHRQPDSSNHSVSPDPVLGSVASMSSATPDSSASAERGSTPDSTLKPLRGQKKMRDDGQSWPKVVY
SCPYCSKRDFTSLAVLEIHLKTIHADKPQQSHTCQICLDSMPTLYNLNEHVRKLHKSHAYPVMQFGNISAFHC
NYCPEMFADINSLQEHIRVSHCGPNANPPDGNNAFFCNQCSMGFLTESSLTEHIQQAHCSVGSTKLESPVVQP
TQSFMEVYSCPYCTNSPIFGSILKLTKHIKENHKNIPLAHSKKSKAEQSPVSSDVEVSSPKRQRLSGSANSIS
NGEYPCNQCDLKFSNFESFQTHLKLHLELLLRKQACPQCKEDFDSQESLLQHLTVHYMTTSTHYVCESCDKQF
SSVDDLQKHLLDMHTFVLYHCTLCQEVFDSKVSIQVHLAVKHSNEKKMYRCTACNWDFRKEADLQVHVKHSHL
GNPAKAHKCIFCGETFSTEVELQCHITTHSKKYNCRFCSKAFHAVILLEKHLREKHCVFDAAAENGTANGVPP
TSTKKAEPADLQGMLLKNPEAPNSHEASEDDVDASEPMYGCDICGAAYTMEVLLQNHRLRDHNIRPGEDDGSR
KKAEFIKGSHKCNVCSRTFFSENGLREHLQTHRGPAKHYMCPICGERFPSLLTLTEHKVTHSKSLDTGTCRIC
KMPLQSEEEFIEHCQMHPDLRNSLTGFRCVVCMQTVTSTLELKIHGTFHMQKLAGSSAASSPNGQGLQKLYKC
ALCLKEFRSKQDLVRLDVNGLPYGLCAGCMARSANGQVGGLAPPEPADRPCAGLRCPECNVKFESAEDLESHM
QVDHRDLTPETSGPRKGAQTSPVPRKKTYQCIKCQMTFENEREIQIHVANHMIEEGINHECKLCNQMFDSPAK
LLCHLIEHSFEGMGGTFKCPVCFTVFVQANKLQQHIFAVHGQEDKIYDCSQCPQKFFFQTELQNHTMSQHAQ*
>rs3709028_ENSMUSP00000105282_c.1156:T>C_p.V386A
ENSMUST00000109655.9|Zfp423-202|ENSMUSG00000045333|-|chr8:g.88509186:A>G
MSRRKQAKPRSVKVEEGEASDFSLAWDSSVAAAGGLEGEPECDRKTSRALEDRNSVTSQEERNEDDEDVEDES
IYTCDHCQQDFESLADLTDHRAHRCPGDGDDDPQLSWVASSPSSKDVASPTQMIGDGCDLGLGEEEGGTGLPY
PCQFCDKSFIRLSYLKRHEQIHSDKLPFKCTFCSRLFKHKRSRDRHIKLHTGDKKYHCHECEAAFSRSDHLKI
HLKTHSSSKPFKCSVCKRGFSSTSSLQSHMQAHKKNKEHLAKSEKEAKKDDFMCDYCEDTFSQTEELEKHVLT
LHPQLSEKADLQCIHCPEVFVDESTLLAHIHQAHANQKHKCPMCPEQFSSVEGVYCHLDSHRQPDSSNHSVSP
DPVLGSVASMSSATPDSSASAERGSTPDSTLKPLRGQKKMRDDGQSWPKVVYSCPYCSKRDFTSLAVLEIHLK
TIHADKPQQSHTCQICLDSMPTLYNLNEHVRKLHKSHAYPVMQFGNISAFHCNYCPEMFADINSLQEHIRVSH
CGPNANPPDGNNAFFCNQCSMGFLTESSLTEHIQQAHCSVGSTKLESPVVQPTQSFMEVYSCPYCTNSPIFGS
ILKLTKHIKENHKNIPLAHSKKSKAEQSPVSSDVEVSSPKRQRLSGSANSISNGEYPCNQCDLKFSNFESFQT
HLKLHLELLLRKQACPQCKEDFDSQESLLQHLTVHYMTTSTHYVCESCDKQFSSVDDLQKHLLDMHTFVLYHC
TLCQEVFDSKVSIQVHLAVKHSNEKKMYRCTACNWDFRKEADLQVHVKHSHLGNPAKAHKCIFCGETFSTEVE
LQCHITTHSKKYNCRFCSKAFHAVILLEKHLREKHCVFDAAAENGTANGVPPTSTKKAEPADLQGMLLKNPEA
PNSHEASEDDVDASEPMYGCDICGAAYTMEVLLQNHRLRDHNIRPGEDDGSRKKAEFIKGSHKCNVCSRTFFS
ENGLREHLQTHRGPAKHYMCPICGERFPSLLTLTEHKVTHSKSLDTGTCRICKMPLQSEEEFIEHCQMHPDLR
NSLTGFRCVVCMQTVTSTLELKIHGTFHMQKLAGSSAASSPNGQGLQKLYKCALCLKEFRSKQDLVRLDVNGL
PYGLCAGCMARSANGQVGGLAPPEPADRPCAGLRCPECNVKFESAEDLESHMQVDHRDLTPETSGPRKGAQTS
PVPRKKTYQCIKCQMTFENEREIQIHVANHMIEEGINHECKLCNQMFDSPAKLLCHLIEHSFEGMGGTFKCPV
CFTVFVQANKLQQHIFAVHGQEDKIYDCSQCPQKFFFQTELQNHTMSQHAQ*
>rs3709028_ENSMUSP00000052379_c.1093:T>C_p.V365A
ENSMUST00000052250.15|Zfp423-201|ENSMUSG00000045333|-|chr8:g.88509186:A>G

MTGAERGPLCYHGGLEGEPECDRKTSRALEDRNSVTSQEERNEDDEDVEDESIYTCDHCQQDFESLADLTDHR
AHRCPGDGDDDPQLSWVASSPSSKDVASPTQMIGDGCDLGLGEEEGGTGLPYPCQFCDKSFIRLSYLKRHEQI
HSDKLPFKCTFCSRLFKHKRSRDRHIKLHTGDKKYHCHECEAAFSRSDHLKIHLKTHSSSKPFKCSVCKRGFS
STSSLQSHMQAHKKNKEHLAKSEKEAKKDDFMCDYCEDTFSQTEELEKHVLTLHPQLSEKADLQCIHCPEVFV
DESTLLAHIHQAHANQKHKCPMCPEQFSSVEGVYCHLDSHRQPDSSNHSVSPDPVLGSVASMSSATPDSSASA
ERGSTPDSTLKPLRGQKKMRDDGQSWPKVVYSCPYCSKRDFTSLAVLEIHLKTIHADKPQQSHTCQICLDSMP
TLYNLNEHVRKLHKSHAYPVMQFGNISAFHCNYCPEMFADINSLQEHIRVSHCGPNANPPDGNNAFFCNQCSM
GFLTESSLTEHIQQAHCSVGSTKLESPVVQPTQSFMEVYSCPYCTNSPIFGSILKLTKHIKENHKNIPLAHSK
KSKAEQSPVSSDVEVSSPKRQRLSGSANSISNGEYPCNQCDLKFSNFESFQTHLKLHLELLLRKQACPQCKED
FDSQESLLQHLTVHYMTTSTHYVCESCDKQFSSVDDLQKHLLDMHTFVLYHCTLCQEVFDSKVSIQVHLAVKH
SNEKKMYRCTACNWDFRKEADLQVHVKHSHLGNPAKAHKCIFCGETFSTEVELQCHITTHSKKYNCRFCSKAF
HAVILLEKHLREKHCVFDAAAENGTANGVPPTSTKKAEPADLQGMLLKNPEAPNSHEASEDDVDASEPMYGCD
ICGAAYTMEVLLQNHRLRDHNIRPGEDDGSRKKAEFIKGSHKCNVCSRTFFSENGLREHLQTHRGPAKHYMCP
ICGERFPSLLTLTEHKVTHSKSLDTGTCRICKMPLQSEEEFIEHCQMHPDLRNSLTGFRCVVCMQTVTSTLEL
KIHGTFHMQKLAGSSAASSPNGQGLQKLYKCALCLKEFRSKQDLVRLDVNGLPYGLCAGCMARSANGQVGGLA
PPEPADRPCAGLRCPECNVKFESAEDLESHMQVDHRDLTPETSGPRKGAQTSPVPRKKTYQCIKCQMTFENER
EIQIHVANHMIEEGINHECKLCNQMFDSPAKLLCHLIEHSFEGMGGTFKCPVCFTVFVQANKLQQHIFAVHGQ
EDKIYDCSQCPQKFFFQTELQNHTMSQHAQ*
>rs3710159_ENSMUSP00000129724_c.634:C>T_p.P212L
ENSMUST00000165770.9|Zfp423-203|ENSMUSG00000045333|-|chr8:g.88509333:G>A
MIGDGCDLGLGEEEGGTGLPYPCQFCDKSFIRLSYLKRHEQIHSDKLPFKCTFCSRLFKHKRSRDRHIKLHTG
DKKYHCHECEAAFSRSDHLKIHLKTHSSSKPFKCSVCKRGFSSTSSLQSHMQAHKKNKEHLAKSEKEAKKDDF
MCDYCEDTFSQTEELEKHVLTLHPQLSEKADLQCIHCPEVFVDESTLLAHIHQAHANQKHKCPMCLEQFSSVE
GVYCHLDSHRQPDSSNHSVSPDPVLGSVASMSSATPDSSASVERGSTPDSTLKPLRGQKKMRDDGQSWPKVVY
SCPYCSKRDFTSLAVLEIHLKTIHADKPQQSHTCQICLDSMPTLYNLNEHVRKLHKSHAYPVMQFGNISAFHC
NYCPEMFADINSLQEHIRVSHCGPNANPPDGNNAFFCNQCSMGFLTESSLTEHIQQAHCSVGSTKLESPVVQP
TQSFMEVYSCPYCTNSPIFGSILKLTKHIKENHKNIPLAHSKKSKAEQSPVSSDVEVSSPKRQRLSGSANSIS
NGEYPCNQCDLKFSNFESFQTHLKLHLELLLRKQACPQCKEDFDSQESLLQHLTVHYMTTSTHYVCESCDKQF
SSVDDLQKHLLDMHTFVLYHCTLCQEVFDSKVSIQVHLAVKHSNEKKMYRCTACNWDFRKEADLQVHVKHSHL
GNPAKAHKCIFCGETFSTEVELQCHITTHSKKYNCRFCSKAFHAVILLEKHLREKHCVFDAAAENGTANGVPP
TSTKKAEPADLQGMLLKNPEAPNSHEASEDDVDASEPMYGCDICGAAYTMEVLLQNHRLRDHNIRPGEDDGSR
KKAEFIKGSHKCNVCSRTFFSENGLREHLQTHRGPAKHYMCPICGERFPSLLTLTEHKVTHSKSLDTGTCRIC
KMPLQSEEEFIEHCQMHPDLRNSLTGFRCVVCMQTVTSTLELKIHGTFHMQKLAGSSAASSPNGQGLQKLYKC
ALCLKEFRSKQDLVRLDVNGLPYGLCAGCMARSANGQVGGLAPPEPADRPCAGLRCPECNVKFESAEDLESHM
QVDHRDLTPETSGPRKGAQTSPVPRKKTYQCIKCQMTFENEREIQIHVANHMIEEGINHECKLCNQMFDSPAK
LLCHLIEHSFEGMGGTFKCPVCFTVFVQANKLQQHIFAVHGQEDKIYDCSQCPQKFFFQTELQNHTMSQHAQ*
>rs3710159_ENSMUSP00000105282_c.1009:C>T_p.P337L
ENSMUST00000109655.9|Zfp423-202|ENSMUSG00000045333|-|chr8:g.88509333:G>A
MSRRKQAKPRSVKVEEGEASDFSLAWDSSVAAAGGLEGEPECDRKTSRALEDRNSVTSQEERNEDDEDVEDES
IYTCDHCQQDFESLADLTDHRAHRCPGDGDDDPQLSWVASSPSSKDVASPTQMIGDGCDLGLGEEEGGTGLPY
PCQFCDKSFIRLSYLKRHEQIHSDKLPFKCTFCSRLFKHKRSRDRHIKLHTGDKKYHCHECEAAFSRSDHLKI
HLKTHSSSKPFKCSVCKRGFSSTSSLQSHMQAHKKNKEHLAKSEKEAKKDDFMCDYCEDTFSQTEELEKHVLT
LHPQLSEKADLQCIHCPEVFVDESTLLAHIHQAHANQKHKCPMCLEQFSSVEGVYCHLDSHRQPDSSNHSVSP
DPVLGSVASMSSATPDSSASVERGSTPDSTLKPLRGQKKMRDDGQSWPKVVYSCPYCSKRDFTSLAVLEIHLK
TIHADKPQQSHTCQICLDSMPTLYNLNEHVRKLHKSHAYPVMQFGNISAFHCNYCPEMFADINSLQEHIRVSH
CGPNANPPDGNNAFFCNQCSMGFLTESSLTEHIQQAHCSVGSTKLESPVVQPTQSFMEVYSCPYCTNSPIFGS
ILKLTKHIKENHKNIPLAHSKKSKAEQSPVSSDVEVSSPKRQRLSGSANSISNGEYPCNQCDLKFSNFESFQT
HLKLHLELLLRKQACPQCKEDFDSQESLLQHLTVHYMTTSTHYVCESCDKQFSSVDDLQKHLLDMHTFVLYHC
TLCQEVFDSKVSIQVHLAVKHSNEKKMYRCTACNWDFRKEADLQVHVKHSHLGNPAKAHKCIFCGETFSTEVE
LQCHITTHSKKYNCRFCSKAFHAVILLEKHLREKHCVFDAAAENGTANGVPPTSTKKAEPADLQGMLLKNPEA
PNSHEASEDDVDASEPMYGCDICGAAYTMEVLLQNHRLRDHNIRPGEDDGSRKKAEFIKGSHKCNVCSRTFFS
ENGLREHLQTHRGPAKHYMCPICGERFPSLLTLTEHKVTHSKSLDTGTCRICKMPLQSEEEFIEHCQMHPDLR
NSLTGFRCVVCMQTVTSTLELKIHGTFHMQKLAGSSAASSPNGQGLQKLYKCALCLKEFRSKQDLVRLDVNGL
PYGLCAGCMARSANGQVGGLAPPEPADRPCAGLRCPECNVKFESAEDLESHMQVDHRDLTPETSGPRKGAQTS
PVPRKKTYQCIKCQMTFENEREIQIHVANHMIEEGINHECKLCNQMFDSPAKLLCHLIEHSFEGMGGTFKCPV
CFTVFVQANKLQQHIFAVHGQEDKIYDCSQCPQKFFFQTELQNHTMSQHAQ*
>rs3710159_ENSMUSP00000052379_c.946:C>T_p.P316L
ENSMUST00000052250.15|Zfp423-201|ENSMUSG00000045333|-|chr8:g.88509333:G>A

MTGAERGPLCYHGGLEGEPECDRKTSRALEDRNSVTSQEERNEDDEDVEDESIYTCDHCQQDFESLADLTDHR
AHRCPGDGDDDPQLSWVASSPSSKDVASPTQMIGDGCDLGLGEEEGGTGLPYPCQFCDKSFIRLSYLKRHEQI
HSDKLPFKCTFCSRLFKHKRSRDRHIKLHTGDKKYHCHECEAAFSRSDHLKIHLKTHSSSKPFKCSVCKRGFS
STSSLQSHMQAHKKNKEHLAKSEKEAKKDDFMCDYCEDTFSQTEELEKHVLTLHPQLSEKADLQCIHCPEVFV
DESTLLAHIHQAHANQKHKCPMCLEQFSSVEGVYCHLDSHRQPDSSNHSVSPDPVLGSVASMSSATPDSSASV
ERGSTPDSTLKPLRGQKKMRDDGQSWPKVVYSCPYCSKRDFTSLAVLEIHLKTIHADKPQQSHTCQICLDSMP
TLYNLNEHVRKLHKSHAYPVMQFGNISAFHCNYCPEMFADINSLQEHIRVSHCGPNANPPDGNNAFFCNQCSM
GFLTESSLTEHIQQAHCSVGSTKLESPVVQPTQSFMEVYSCPYCTNSPIFGSILKLTKHIKENHKNIPLAHSK
KSKAEQSPVSSDVEVSSPKRQRLSGSANSISNGEYPCNQCDLKFSNFESFQTHLKLHLELLLRKQACPQCKED
FDSQESLLQHLTVHYMTTSTHYVCESCDKQFSSVDDLQKHLLDMHTFVLYHCTLCQEVFDSKVSIQVHLAVKH
SNEKKMYRCTACNWDFRKEADLQVHVKHSHLGNPAKAHKCIFCGETFSTEVELQCHITTHSKKYNCRFCSKAF
HAVILLEKHLREKHCVFDAAAENGTANGVPPTSTKKAEPADLQGMLLKNPEAPNSHEASEDDVDASEPMYGCD
ICGAAYTMEVLLQNHRLRDHNIRPGEDDGSRKKAEFIKGSHKCNVCSRTFFSENGLREHLQTHRGPAKHYMCP
ICGERFPSLLTLTEHKVTHSKSLDTGTCRICKMPLQSEEEFIEHCQMHPDLRNSLTGFRCVVCMQTVTSTLEL
KIHGTFHMQKLAGSSAASSPNGQGLQKLYKCALCLKEFRSKQDLVRLDVNGLPYGLCAGCMARSANGQVGGLA
PPEPADRPCAGLRCPECNVKFESAEDLESHMQVDHRDLTPETSGPRKGAQTSPVPRKKTYQCIKCQMTFENER
EIQIHVANHMIEEGINHECKLCNQMFDSPAKLLCHLIEHSFEGMGGTFKCPVCFTVFVQANKLQQHIFAVHGQ
EDKIYDCSQCPQKFFFQTELQNHTMSQHAQ*
>rs50410189_ENSMUSP00000105282_c.181:A>T_p.E61V
ENSMUST00000109655.9|Zfp423-202|ENSMUSG00000045333|-|chr8:g.88586031:T>A
MSRRKQAKPRSVKVEEGEASDFSLAWDSSVAAAGGLEGEPECDRKTSRALEDRNSVTSQEVRNEDDEDVEDES
IYTCDHCQQDFESLADLTDHRAHRCPGDGDDDPQLSWVASSPSSKDVASPTQMIGDGCDLGLGEEEGGTGLPY
PCQFCDKSFIRLSYLKRHEQIHSDKLPFKCTFCSRLFKHKRSRDRHIKLHTGDKKYHCHECEAAFSRSDHLKI
HLKTHSSSKPFKCSVCKRGFSSTSSLQSHMQAHKKNKEHLAKSEKEAKKDDFMCDYCEDTFSQTEELEKHVLT
LHPQLSEKADLQCIHCPEVFVDESTLLAHIHQAHANQKHKCPMCPEQFSSVEGVYCHLDSHRQPDSSNHSVSP
DPVLGSVASMSSATPDSSASVERGSTPDSTLKPLRGQKKMRDDGQSWPKVVYSCPYCSKRDFTSLAVLEIHLK
TIHADKPQQSHTCQICLDSMPTLYNLNEHVRKLHKSHAYPVMQFGNISAFHCNYCPEMFADINSLQEHIRVSH
CGPNANPPDGNNAFFCNQCSMGFLTESSLTEHIQQAHCSVGSTKLESPVVQPTQSFMEVYSCPYCTNSPIFGS
ILKLTKHIKENHKNIPLAHSKKSKAEQSPVSSDVEVSSPKRQRLSGSANSISNGEYPCNQCDLKFSNFESFQT
HLKLHLELLLRKQACPQCKEDFDSQESLLQHLTVHYMTTSTHYVCESCDKQFSSVDDLQKHLLDMHTFVLYHC
TLCQEVFDSKVSIQVHLAVKHSNEKKMYRCTACNWDFRKEADLQVHVKHSHLGNPAKAHKCIFCGETFSTEVE
LQCHITTHSKKYNCRFCSKAFHAVILLEKHLREKHCVFDAAAENGTANGVPPTSTKKAEPADLQGMLLKNPEA
PNSHEASEDDVDASEPMYGCDICGAAYTMEVLLQNHRLRDHNIRPGEDDGSRKKAEFIKGSHKCNVCSRTFFS
ENGLREHLQTHRGPAKHYMCPICGERFPSLLTLTEHKVTHSKSLDTGTCRICKMPLQSEEEFIEHCQMHPDLR
NSLTGFRCVVCMQTVTSTLELKIHGTFHMQKLAGSSAASSPNGQGLQKLYKCALCLKEFRSKQDLVRLDVNGL
PYGLCAGCMARSANGQVGGLAPPEPADRPCAGLRCPECNVKFESAEDLESHMQVDHRDLTPETSGPRKGAQTS
PVPRKKTYQCIKCQMTFENEREIQIHVANHMIEEGINHECKLCNQMFDSPAKLLCHLIEHSFEGMGGTFKCPV
CFTVFVQANKLQQHIFAVHGQEDKIYDCSQCPQKFFFQTELQNHTMSQHAQ*
>rs50410189_ENSMUSP00000052379_c.118:A>T_p.E40V
ENSMUST00000052250.15|Zfp423-201|ENSMUSG00000045333|-|chr8:g.88586031:T>A
MTGAERGPLCYHGGLEGEPECDRKTSRALEDRNSVTSQEVRNEDDEDVEDESIYTCDHCQQDFESLADLTDHR
AHRCPGDGDDDPQLSWVASSPSSKDVASPTQMIGDGCDLGLGEEEGGTGLPYPCQFCDKSFIRLSYLKRHEQI
HSDKLPFKCTFCSRLFKHKRSRDRHIKLHTGDKKYHCHECEAAFSRSDHLKIHLKTHSSSKPFKCSVCKRGFS
STSSLQSHMQAHKKNKEHLAKSEKEAKKDDFMCDYCEDTFSQTEELEKHVLTLHPQLSEKADLQCIHCPEVFV
DESTLLAHIHQAHANQKHKCPMCPEQFSSVEGVYCHLDSHRQPDSSNHSVSPDPVLGSVASMSSATPDSSASV
ERGSTPDSTLKPLRGQKKMRDDGQSWPKVVYSCPYCSKRDFTSLAVLEIHLKTIHADKPQQSHTCQICLDSMP
TLYNLNEHVRKLHKSHAYPVMQFGNISAFHCNYCPEMFADINSLQEHIRVSHCGPNANPPDGNNAFFCNQCSM
GFLTESSLTEHIQQAHCSVGSTKLESPVVQPTQSFMEVYSCPYCTNSPIFGSILKLTKHIKENHKNIPLAHSK
KSKAEQSPVSSDVEVSSPKRQRLSGSANSISNGEYPCNQCDLKFSNFESFQTHLKLHLELLLRKQACPQCKED
FDSQESLLQHLTVHYMTTSTHYVCESCDKQFSSVDDLQKHLLDMHTFVLYHCTLCQEVFDSKVSIQVHLAVKH
SNEKKMYRCTACNWDFRKEADLQVHVKHSHLGNPAKAHKCIFCGETFSTEVELQCHITTHSKKYNCRFCSKAF
HAVILLEKHLREKHCVFDAAAENGTANGVPPTSTKKAEPADLQGMLLKNPEAPNSHEASEDDVDASEPMYGCD
ICGAAYTMEVLLQNHRLRDHNIRPGEDDGSRKKAEFIKGSHKCNVCSRTFFSENGLREHLQTHRGPAKHYMCP
ICGERFPSLLTLTEHKVTHSKSLDTGTCRICKMPLQSEEEFIEHCQMHPDLRNSLTGFRCVVCMQTVTSTLEL
KIHGTFHMQKLAGSSAASSPNGQGLQKLYKCALCLKEFRSKQDLVRLDVNGLPYGLCAGCMARSANGQVGGLA
PPEPADRPCAGLRCPECNVKFESAEDLESHMQVDHRDLTPETSGPRKGAQTSPVPRKKTYQCIKCQMTFENER
EIQIHVANHMIEEGINHECKLCNQMFDSPAKLLCHLIEHSFEGMGGTFKCPVCFTVFVQANKLQQHIFAVHGQ
EDKIYDCSQCPQKFFFQTELQNHTMSQHAQ*

>rs50410189_ENSMUSP00000134575_c.190:A>T_p.E64V
ENSMUST00000174764.2|Zfp423-207|ENSMUSG00000045333|-|chr8:g.88586031:T>A
MAVFKESGPLPIFSYKVEEGEASDFSLAWDSSVAAAGGLEGEPECDRKTSRALEDRNSVTSQEVRNEDDEDVE
DESIYTCDHCQQDFESLADLTDHRAHRCPGDGDDDPQLSWVASSPSSKDVASPTQMIGDGCDLGLGEEEGGTG
LPYPCQFCDKSFIRLSYLKRHEQIHSDKLPFKCTFCSRLFKHKRSRDRHIKLHTGDKKYHCHECEAAFSRSDH
LKIHLKTHSSSKPFKCSVCKRGFSS
>rs45696557_ENSMUSP00000105282_c.172:G>T_p.S58I
ENSMUST00000109655.9|Zfp423-202|ENSMUSG00000045333|-|chr8:g.88586040:C>A
MSRRKQAKPRSVKVEEGEASDFSLAWDSSVAAAGGLEGEPECDRKTSRALEDRNSVTIQEERNEDDEDVEDES
IYTCDHCQQDFESLADLTDHRAHRCPGDGDDDPQLSWVASSPSSKDVASPTQMIGDGCDLGLGEEEGGTGLPY
PCQFCDKSFIRLSYLKRHEQIHSDKLPFKCTFCSRLFKHKRSRDRHIKLHTGDKKYHCHECEAAFSRSDHLKI
HLKTHSSSKPFKCSVCKRGFSSTSSLQSHMQAHKKNKEHLAKSEKEAKKDDFMCDYCEDTFSQTEELEKHVLT
LHPQLSEKADLQCIHCPEVFVDESTLLAHIHQAHANQKHKCPMCPEQFSSVEGVYCHLDSHRQPDSSNHSVSP
DPVLGSVASMSSATPDSSASVERGSTPDSTLKPLRGQKKMRDDGQSWPKVVYSCPYCSKRDFTSLAVLEIHLK
TIHADKPQQSHTCQICLDSMPTLYNLNEHVRKLHKSHAYPVMQFGNISAFHCNYCPEMFADINSLQEHIRVSH
CGPNANPPDGNNAFFCNQCSMGFLTESSLTEHIQQAHCSVGSTKLESPVVQPTQSFMEVYSCPYCTNSPIFGS
ILKLTKHIKENHKNIPLAHSKKSKAEQSPVSSDVEVSSPKRQRLSGSANSISNGEYPCNQCDLKFSNFESFQT
HLKLHLELLLRKQACPQCKEDFDSQESLLQHLTVHYMTTSTHYVCESCDKQFSSVDDLQKHLLDMHTFVLYHC
TLCQEVFDSKVSIQVHLAVKHSNEKKMYRCTACNWDFRKEADLQVHVKHSHLGNPAKAHKCIFCGETFSTEVE
LQCHITTHSKKYNCRFCSKAFHAVILLEKHLREKHCVFDAAAENGTANGVPPTSTKKAEPADLQGMLLKNPEA
PNSHEASEDDVDASEPMYGCDICGAAYTMEVLLQNHRLRDHNIRPGEDDGSRKKAEFIKGSHKCNVCSRTFFS
ENGLREHLQTHRGPAKHYMCPICGERFPSLLTLTEHKVTHSKSLDTGTCRICKMPLQSEEEFIEHCQMHPDLR
NSLTGFRCVVCMQTVTSTLELKIHGTFHMQKLAGSSAASSPNGQGLQKLYKCALCLKEFRSKQDLVRLDVNGL
PYGLCAGCMARSANGQVGGLAPPEPADRPCAGLRCPECNVKFESAEDLESHMQVDHRDLTPETSGPRKGAQTS
PVPRKKTYQCIKCQMTFENEREIQIHVANHMIEEGINHECKLCNQMFDSPAKLLCHLIEHSFEGMGGTFKCPV
CFTVFVQANKLQQHIFAVHGQEDKIYDCSQCPQKFFFQTELQNHTMSQHAQ*
>rs45696557_ENSMUSP00000052379_c.109:G>T_p.S37I
ENSMUST00000052250.15|Zfp423-201|ENSMUSG00000045333|-|chr8:g.88586040:C>A
MTGAERGPLCYHGGLEGEPECDRKTSRALEDRNSVTIQEERNEDDEDVEDESIYTCDHCQQDFESLADLTDHR
AHRCPGDGDDDPQLSWVASSPSSKDVASPTQMIGDGCDLGLGEEEGGTGLPYPCQFCDKSFIRLSYLKRHEQI
HSDKLPFKCTFCSRLFKHKRSRDRHIKLHTGDKKYHCHECEAAFSRSDHLKIHLKTHSSSKPFKCSVCKRGFS
STSSLQSHMQAHKKNKEHLAKSEKEAKKDDFMCDYCEDTFSQTEELEKHVLTLHPQLSEKADLQCIHCPEVFV
DESTLLAHIHQAHANQKHKCPMCPEQFSSVEGVYCHLDSHRQPDSSNHSVSPDPVLGSVASMSSATPDSSASV
ERGSTPDSTLKPLRGQKKMRDDGQSWPKVVYSCPYCSKRDFTSLAVLEIHLKTIHADKPQQSHTCQICLDSMP
TLYNLNEHVRKLHKSHAYPVMQFGNISAFHCNYCPEMFADINSLQEHIRVSHCGPNANPPDGNNAFFCNQCSM
GFLTESSLTEHIQQAHCSVGSTKLESPVVQPTQSFMEVYSCPYCTNSPIFGSILKLTKHIKENHKNIPLAHSK
KSKAEQSPVSSDVEVSSPKRQRLSGSANSISNGEYPCNQCDLKFSNFESFQTHLKLHLELLLRKQACPQCKED
FDSQESLLQHLTVHYMTTSTHYVCESCDKQFSSVDDLQKHLLDMHTFVLYHCTLCQEVFDSKVSIQVHLAVKH
SNEKKMYRCTACNWDFRKEADLQVHVKHSHLGNPAKAHKCIFCGETFSTEVELQCHITTHSKKYNCRFCSKAF
HAVILLEKHLREKHCVFDAAAENGTANGVPPTSTKKAEPADLQGMLLKNPEAPNSHEASEDDVDASEPMYGCD
ICGAAYTMEVLLQNHRLRDHNIRPGEDDGSRKKAEFIKGSHKCNVCSRTFFSENGLREHLQTHRGPAKHYMCP
ICGERFPSLLTLTEHKVTHSKSLDTGTCRICKMPLQSEEEFIEHCQMHPDLRNSLTGFRCVVCMQTVTSTLEL
KIHGTFHMQKLAGSSAASSPNGQGLQKLYKCALCLKEFRSKQDLVRLDVNGLPYGLCAGCMARSANGQVGGLA
PPEPADRPCAGLRCPECNVKFESAEDLESHMQVDHRDLTPETSGPRKGAQTSPVPRKKTYQCIKCQMTFENER
EIQIHVANHMIEEGINHECKLCNQMFDSPAKLLCHLIEHSFEGMGGTFKCPVCFTVFVQANKLQQHIFAVHGQ
EDKIYDCSQCPQKFFFQTELQNHTMSQHAQ*
>rs45696557_ENSMUSP00000134575_c.181:G>T_p.S61I
ENSMUST00000174764.2|Zfp423-207|ENSMUSG00000045333|-|chr8:g.88586040:C>A
MAVFKESGPLPIFSYKVEEGEASDFSLAWDSSVAAAGGLEGEPECDRKTSRALEDRNSVTIQEERNEDDEDVE
DESIYTCDHCQQDFESLADLTDHRAHRCPGDGDDDPQLSWVASSPSSKDVASPTQMIGDGCDLGLGEEEGGTG
LPYPCQFCDKSFIRLSYLKRHEQIHSDKLPFKCTFCSRLFKHKRSRDRHIKLHTGDKKYHCHECEAAFSRSDH
LKIHLKTHSSSKPFKCSVCKRGFSS
>rs260982584_ENSMUSP00000105282_c.135:A>CAAATACCATCTCTAGTA_p.T46ins-fs
ENSMUST00000109655.9|Zfp423-202|ENSMUSG00000045333|-
|chr8:g.88586077:T>TACTAGAGATGGTATTTG
MSRRKQAKPRSVKVEEGEASDFSLAWDSSVAAAGGLEGEPECDRKQIPSLVPAVRWKTGTA

>rs260982584_ENSMUSP00000052379_c.72:A>CAAATACCATCTCTAGTA_p.T25ins-fs
ENSMUST00000052250.15|Zfp423-201|ENSMUSG00000045333|-
|chr8:g.88586077:T>TACTAGAGATGGTATTTG
MTGAERGPLCYHGGLEGEPECDRKQIPSLVPAVRWKTGTA
>rs260982584_ENSMUSP00000134575_c.144:A>CAAATACCATCTCTAGTA_p.T49ins-fs
ENSMUST00000174764.2|Zfp423-207|ENSMUSG00000045333|-
|chr8:g.88586077:T>TACTAGAGATGGTATTTG
MAVFKESGPLPIFSYKVEEGEASDFSLAWDSSVAAAGGLEGEPECDRKQIPSLVPAVRWKTGTA
>rs45714081_ENSMUSP00000092839_c.7:C>T_p.S3L
ENSMUST00000095214.10|Cnep1r1-
201|ENSMUSG00000036810|+|chr8:g.88845480:C>T
MNLLEQAEDLKAFERRLTEYIHCLQPATGRWRMLLIVVSVCTATGAWNWLIDPETQKVSFFTSLWNHPFFTIS
CITLIGLFFAGIHKRVVAPSIIAARCRTVLAEYNMSCDDTGKLILKPRPHVQ*
>rs45714081_ENSMUSP00000120121_c.7:C>T_p.S3L
ENSMUST00000127490.8|Cnep1r1-204|ENSMUSG00000036810|+|chr8:g.88845480:C>T
MNLLEQAEDLKAFERRLTEYIHCLQPATGRWRSVLLHVIMEPSVFHY*
>rs45714081_ENSMUSP00000113435_c.7:C>T_p.S3L
ENSMUST00000121097.8|Cnep1r1-203|ENSMUSG00000036810|+|chr8:g.88845480:C>T
MNLLEQAEDLKAFERRLTEYIHCLQPATGRWRMLLIVVSVCTATGAWNWLIDPETQKVSFFTSLWNHPFFTIS
CITLIGLFFAGIHKRVVAPSMYPLPRVEFCYHN*
>rs45714081_ENSMUSP00000113207_c.7:C>T_p.S3L
ENSMUST00000117775.2|Cnep1r1-202|ENSMUSG00000036810|+|chr8:g.88845480:C>T
MNLLEQAEDLKAFERRLTEYIHCLQPATGRWRMLLIVVSVCTATGAWNWLIDPETQKVSFFTSLWNHPFFTIS
CITLIGLFFAGIHKRVVAPSIIAARCRTVLAEYNMSCDDP*
>rs49014380_ENSMUSP00000034079_c.1122:A>G_p.S375G
ENSMUST00000034079.14|Heatr3-201|ENSMUSG00000031657|+|chr8:g.88883174:A>G
MGKSRTKRFKRPQFSPIESCQAEAAAASNGTGDEEDDGPAAELLEKLQHPSAEVRECACAGLARLVQQRPALP
DLARRDAVRRLGPLLLDSSLAVRETAAGALRNLSACGGFDVCDDMVAKDIMTPLVALLRECLSGLDSNEMSPQ
EKADKRNPVESIANEAVNVLWNVCECSGRAVSIFNKEGCLEIVLQYLRRFPTSIDLAVSVAYCLQTVTEDNPE
LLKSFDGTALRVLESALLCPVASMEYILLKTLVAGTIWNLKDIIPSKSQAEIINAILGALSEVLGMNTGNMVI
QMKEAETQRLKTAAESEDILANANGDDLVEDDEMEEIPHKRKLRRKTFISDLLPPTDKELREAMALLTAQQTA
LEVIVNMCCGEDPSDDEWEELSSSDESEAFMENSFSECSGHLMSPLCLSHEIHSALTNCLIPEKVFEKTSSPN
SVAVDICSKNPTWKPLIRKMNTIQCRALMCLQSLVSLLDIDHLGGAPALQTLAQHLSQILFSQPDFAKHDDFL
EAISSALRALLQTMASKNIPQCMTPEQLMTLCREGIHSSSIGVRVNVVSILGITGSVLAKDDGTLDTLKTIGC
FLLEVATKDPSLVVTGEALDALFDVFADGEAAEKASVQIKLLAALKEFQPVFKMKIRKEGRGKYSPDQLCVLD
NVKMNLRRFIAYQETVEKRLTS*
>rs49014380_ENSMUSP00000112812_c.771:A>G_p.S258G
ENSMUST00000121949.2|Heatr3-202|ENSMUSG00000031657|+|chr8:g.88883174:A>G
MVAKDIMTPLVALLRECLSGLDSNEMSPQEKADKRNPVESIANEAVNVLWNVCECSGRAVSIFNKEGCLEIVL
QYLRRFPTSIDLAVSVAYCLQTVTEDNPELLKSFDGTALRVLESALLCPVASMEYILLKTLVAGTIWNLKDII
PSKSQAEIINAILGALSEVLGMNTGNMVIQMKEAETQRLKTAAESEDILANANGDDLVEDDEMEEIPHKRKLR
RKTFISDLLPPTDKELREAMALLTAQQTALEVIVNMCCGEDPSDDEWEELSSSDESEAFMENSFSECSGHLMS
PLCLSHEIHSALTNCLIPEKVFEKTSSPNSVAVDICSKNPTWKPLIRKMNTIQCRALMCLQSLVSLLDIDHLG
GAPALQTLAQHLSQILFSQPDFAKHDDFLEAISSALRALLQTMASKNIPQCMTPEQLMTLCREGIHSSSIGVR
VNVVSILGITGSVLAKDDGTLDTLKTIGCFLLEVATKDPSLVVTGEALDALFDVFADGEAAEKASVQIKLLAA
LKEFQPVFKMKIRKEGRGKYSPDQLCVLDNVKMNLRRFIAYQETVEKRLTS*
>rs48401410_ENSMUSP00000034079_c.1617:A>G_p.M540V
ENSMUST00000034079.14|Heatr3-201|ENSMUSG00000031657|+|chr8:g.88891537:A>G
MGKSRTKRFKRPQFSPIESCQAEAAAASNGTGDEEDDGPAAELLEKLQHPSAEVRECACAGLARLVQQRPALP
DLARRDAVRRLGPLLLDSSLAVRETAAGALRNLSACGGFDVCDDMVAKDIMTPLVALLRECLSGLDSNEMSPQ
EKADKRNPVESIANEAVNVLWNVCECSGRAVSIFNKEGCLEIVLQYLRRFPTSIDLAVSVAYCLQTVTEDNPE
LLKSFDGTALRVLESALLCPVASMEYILLKTLVAGTIWNLKDIIPSKSQAEIINAILGALSEVLGMNTGNMVI
QMKEAETQRLKTAAESEDILANANGDDLVEDDEMEEIPHKRKLRRKTFISDLLPPTDKELREAMALLTAQQTA
LEVIVNMCCSEDPSDDEWEELSSSDESEAFMENSFSECSGHLMSPLCLSHEIHSALTNCLIPEKVFEKTSSPN
SVAVDICSKNPTWKPLIRKMNTIQCRALMCLQSLVSLLDIDHLGGAPALQTLAQHLSQILFSQPDFAKHDDFL
EAISSALRALLQTMASKNIPQCMTPEQLVTLCREGIHSSSIGVRVNVVSILGITGSVLAKDDGTLDTLKTIGC
FLLEVATKDPSLVVTGEALDALFDVFADGEAAEKASVQIKLLAALKEFQPVFKMKIRKEGRGKYSPDQLCVLD
NVKMNLRRFIAYQETVEKRLTS*

```
>rs48401410_ENSMUSP00000112812_c.1266:A>G_p.M423V
ENSMUST00000121949.2|Heatr3-202|ENSMUSG00000031657|+|chr8:g.88891537:A>G
MVAKDIMTPLVALLRECLSGLDSNEMSPQEKADKRNPVESIANEAVNVLWNVCECSGRAVSIFNKEGCLEIVL
QYLRRFPTSIDLAVSVAYCLQTVTEDNPELLKSFDGTALRVLESALLCPVASMEYILLKTLVAGTIWNLKDII
PSKSQAEIINAILGALSEVLGMNTGNMVIQMKEAETQRLKTAAESEDILANANGDDLVEDDEMEEIPHKRKLR
RKTFISDLLPPTDKELREAMALLTAQQTALEVIVNMCCSEDPSDDEWEELSSSDESEAFMENSFSECSGHLMS
PLCLSHEIHSALTNCLIPEKVFEKTSSPNSVAVDICSKNPTWKPLIRKMNTIQCRALMCLQSLVSLLDIDHLG
GAPALQTLAQHLSQILFSQPDFAKHDDFLEAISSALRALLQTMASKNIPQCMTPEQLVTLCREGIHSSSIGVR
VNVVSILGITGSVLAKDDGTLDTLKTIGCFLLEVATKDPSLVVTGEALDALFDVFADGEAAEKASVQIKLLAA
LKEFQPVFKMKIRKEGRGKYSPDQLCVLDNVKMNLRRFIAYQETVEKRLTS*
>rs238749623_ENSMUSP00000034079_c.1848:G>GAATAA_p.E617ins-fs
ENSMUST00000034079.14|Heatr3-
201|ENSMUSG00000031657|+|chr8:g.88893732:G>GAATAA
MGKSRTKRFKRPQFSPIESCQAEAAAASNGTGDEEDDGPAAELLEKLQHPSAEVRECACAGLARLVQQRPALP
DLARRDAVRRLGPLLLDSSLAVRETAAGALRNLSACGGFDVCDDMVAKDIMTPLVALLRECLSGLDSNEMSPQ
EKADKRNPVESIANEAVNVLWNVCECSGRAVSIFNKEGCLEIVLQYLRRFPTSIDLAVSVAYCLQTVTEDNPE
LLKSFDGTALRVLESALLCPVASMEYILLKTLVAGTIWNLKDIIPSKSQAEIINAILGALSEVLGMNTGNMVI
QMKEAETQRLKTAAESEDILANANGDDLVEDDEMEEIPHKRKLRRKTFISDLLPPTDKELREAMALLTAQQTA
LEVIVNMCCSEDPSDDEWEELSSSDESEAFMENSFSECSGHLMSPLCLSHEIHSALTNCLIPEKVFEKTSSPN
SVAVDICSKNPTWKPLIRKMNTIQCRALMCLQSLVSLLDIDHLGGAPALQTLAQHLSQILFSQPDFAKHDDFL
EAISSALRALLQTMASKNIPQCMTPEQLMTLCREGIHSSSIGVRVNVVSILGITGSVLAKDDGTLDTLKTIGC
FLLEVATKDPSLVVTGEALDALFDVFADGEAAE
>rs238749623_ENSMUSP00000112812_c.1497:G>GAATAA_p.E500ins-fs
ENSMUST00000121949.2|Heatr3-
202|ENSMUSG00000031657|+|chr8:g.88893732:G>GAATAA
MVAKDIMTPLVALLRECLSGLDSNEMSPQEKADKRNPVESIANEAVNVLWNVCECSGRAVSIFNKEGCLEIVL
QYLRRFPTSIDLAVSVAYCLQTVTEDNPELLKSFDGTALRVLESALLCPVASMEYILLKTLVAGTIWNLKDII
PSKSQAEIINAILGALSEVLGMNTGNMVIQMKEAETQRLKTAAESEDILANANGDDLVEDDEMEEIPHKRKLR
RKTFISDLLPPTDKELREAMALLTAQQTALEVIVNMCCSEDPSDDEWEELSSSDESEAFMENSFSECSGHLMS
PLCLSHEIHSALTNCLIPEKVFEKTSSPNSVAVDICSKNPTWKPLIRKMNTIQCRALMCLQSLVSLLDIDHLG
GAPALQTLAQHLSQILFSQPDFAKHDDFLEAISSALRALLQTMASKNIPQCMTPEQLMTLCREGIHSSSIGVR
VNVVSILGITGSVLAKDDGTLDTLKTIGCFLLEVATKDPSLVVTGEALDALFDVFADGEAAE
>rs50253664_ENSMUSP00000112766_c.157:A>C_p.E53A
ENSMUST00000119033.8|Tent4b-203|ENSMUSG00000036779|+|chr8:g.88926749:A>C
MQIWETTQGLRNLYFNHHCHSSGASSASGGSGSGPGSPGGTAPAPAGMFRSGARPLGGLAVPAEQRDFLPLET
TNNNNNHHQPAAWARRASAGPSASPVPSAPSSPRPAAALPASESTDPASGSSNKRKRDNKASTYGLNYSLLQP
SGGRAAGGGRADGGGGVYSGTPWKRRNYNQGVVGLHEEISDFYEYMSPRPEEEKMRMEVVSRIESVIKELWPS
ADVQIFGSFKTGLYLPTSDIDLVVFGKWENLPLWTLEEALRKHKVADEDSVKVLDKATVPIIKLTDSFTEVKV
DISFNVQNGVRAADLIKDFTKKYPVLPYLVLVLKQFLLQRDLNEVFTGGIGSYSLFLMAVSFLQLHPREDACI
PNTNYGVLLIEFFELYGRHFNYLKTGIRIKDGGSYVAKDEVQKNMLDGYRPSMLYIEDPLQPGNDVGRSSYGA
MQVKQAFDYAYVVLSHAVSPIAKYYPNNETESILGRIIRVTDEVATYRDWISKQWGLQNRPEPSCNGNGVTLI
VDTQQLDKCNNNLSEEKEALGKCRSNASEPLSKHSSNSSSGPVSSSSATQSSSSDVDSDATPCKTPKQLLCRP
PTVTRVGSQDVSLEVSQAVGKMQSTQTTNTPNNANKSQHGSARLFRSSSKGFQGTAQTSHGALMTSKQHQGKS
NTQYYHGKKRRHKRDAPLSELCR*
>rs50253664_ENSMUSP00000112608_c.157:A>C_p.E53A
ENSMUST00000118952.8|Tent4b-202|ENSMUSG00000036779|+|chr8:g.88926749:A>C
MQIWETTQGLRNLYFNHHCHSSGASSASGGSGSGPGSPGGTAPAPAGMFRSGARPLGGLAVPAEQRDFLPLET
TNNNNNHHQPAAWARRASAGPSASPVPSAPSSPRPAAALPASESTDPASGSSNKRKRDNKASTYGLNYSLLQP
SGGRAAGGGRADGGGGVYSGTPWKRRNYNQGVVGLHEEISDFYEYMSPRPEEEKMRMEVVSRIESVIKELWPS
ADVQIFGSFKTGLYLPTSDIDLVVFGKWENLPLWTLEEALRKHKVADEDSVKVLDKATVPIIKLTDSFTEVKV
DISFNVQNGVRAADLIKDFTKKYPVLPYLVLVLKQFLLQRDLNEVFTGGIGSYSLFLMAVSFLQLHPREDACI
PNTNYGVLLIEFFELYGRHFNYLKTGIRIKDGGSYVAKDEVQKNMLDGYRPSMLYIEDPLQPGNDVGRSSYGA
MQVKQAFDYAYVVLSHAVSPIAKYYPNNETESILGRIIRVTDEVATYRDWISKQWGLQNRPEPSCNGNGVTLI
VDTQQLDKCNNNLSEEKEALGKCRSNASEPLSKHSSNSSSGPVSSSSATQSSSSDVDSDATPCKTPKQLLCRP
PTVTRVGSQDVSLEVSQAVGKMQSTQTTNTPNNANKSQHGSARLFRSSSKGFQGTAQTSHGALMTSKQHQGKS
NTQYYHGKKRRHKRDAPLSELCR*
>rs50253664_ENSMUSP00000067971_c.157:A>C_p.E53A
ENSMUST00000066748.10|Tent4b-201|ENSMUSG00000036779|+|chr8:g.88926749:A>C
```

MQIWETTQGLRNLYFNHHCHSSGASSASGGSGSGPGSPGGTAPAPAGMFRSGARPLGGLAVPAEQRDFLPLET
TNNNNNHHQPAAWARRASAGPSASPVPSAPSSPRPAAALPASESTDPASGSSNKRKRDNKASTYGLNYSLLQP
SGGRAAGGGRADGGGGVYSGTPWKRRNYNQGVVGLHEEISDFYEYMSPRPEEEKMRMEVVSRIESVIKELWPS
ADVQIFGSFKTGLYLPTSDIDLVVFGKWENLPLWTLEEALRKHKVADEDSVKVLDKATVPIIKLTDSFTEVKV
DISFNVQNGVRAADLIKDFTKLHPREDACIPNTNYGVLLIEFFELYGRHFNYLKTGIRIKDGGSYVAKDEVQK
NMLDGYRPSMLYIEDPLQPGNDVGRSSYGAMQVKQAFDYAYVVLSHAVSPIAKYYPNNETESILGRIIRVTDE
VATYRDWISKQWGLQNRPEPSCNGNGVTLIVDTQQLDKCNNNLSEEKEALGKCRSNASEPLSKHSSNSSSGPV
SSSSATQSSSSDVDSDATPCKTPKQLLCRPPTVTRVGSQDVSLEVSQAVGKMQSTQTTNTPNNANKSQHGSAR
LFRSSSKGFQGTAQTSHGALMTSKQHQGKSNTQYYHGKKRRHKRDAPLSELCR*
>rs50420322_ENSMUSP00000112766_c.174:C>T_p.L59F
ENSMUST00000119033.8|Tent4b-203|ENSMUSG00000036779|+|chr8:g.88926766:C>T
MQIWETTQGLRNLYFNHHCHSSGASSASGGSGSGPGSPGGTAPAPAGMFRSGERPLGGFAVPAEQRDFLPLET
TNNNNNHHQPAAWARRASAGPSASPVPSAPSSPRPAAALPASESTDPASGSSNKRKRDNKASTYGLNYSLLQP
SGGRAAGGGRADGGGGVYSGTPWKRRNYNQGVVGLHEEISDFYEYMSPRPEEEKMRMEVVSRIESVIKELWPS
ADVQIFGSFKTGLYLPTSDIDLVVFGKWENLPLWTLEEALRKHKVADEDSVKVLDKATVPIIKLTDSFTEVKV
DISFNVQNGVRAADLIKDFTKKYPVLPYLVLVLKQFLLQRDLNEVFTGGIGSYSLFLMAVSFLQLHPREDACI
PNTNYGVLLIEFFELYGRHFNYLKTGIRIKDGGSYVAKDEVQKNMLDGYRPSMLYIEDPLQPGNDVGRSSYGA
MQVKQAFDYAYVVLSHAVSPIAKYYPNNETESILGRIIRVTDEVATYRDWISKQWGLQNRPEPSCNGNGVTLI
VDTQQLDKCNNNLSEEKEALGKCRSNASEPLSKHSSNSSSGPVSSSSATQSSSSDVDSDATPCKTPKQLLCRP
PTVTRVGSQDVSLEVSQAVGKMQSTQTTNTPNNANKSQHGSARLFRSSSKGFQGTAQTSHGALMTSKQHQGKS
NTQYYHGKKRRHKRDAPLSELCR*
>rs50420322_ENSMUSP00000112608_c.174:C>T_p.L59F
ENSMUST00000118952.8|Tent4b-202|ENSMUSG00000036779|+|chr8:g.88926766:C>T
MQIWETTQGLRNLYFNHHCHSSGASSASGGSGSGPGSPGGTAPAPAGMFRSGERPLGGFAVPAEQRDFLPLET
TNNNNNHHQPAAWARRASAGPSASPVPSAPSSPRPAAALPASESTDPASGSSNKRKRDNKASTYGLNYSLLQP
SGGRAAGGGRADGGGGVYSGTPWKRRNYNQGVVGLHEEISDFYEYMSPRPEEEKMRMEVVSRIESVIKELWPS
ADVQIFGSFKTGLYLPTSDIDLVVFGKWENLPLWTLEEALRKHKVADEDSVKVLDKATVPIIKLTDSFTEVKV
DISFNVQNGVRAADLIKDFTKKYPVLPYLVLVLKQFLLQRDLNEVFTGGIGSYSLFLMAVSFLQLHPREDACI
PNTNYGVLLIEFFELYGRHFNYLKTGIRIKDGGSYVAKDEVQKNMLDGYRPSMLYIEDPLQPGNDVGRSSYGA
MQVKQAFDYAYVVLSHAVSPIAKYYPNNETESILGRIIRVTDEVATYRDWISKQWGLQNRPEPSCNGNGVTLI
VDTQQLDKCNNNLSEEKEALGKCRSNASEPLSKHSSNSSSGPVSSSSATQSSSSDVDSDATPCKTPKQLLCRP
PTVTRVGSQDVSLEVSQAVGKMQSTQTTNTPNNANKSQHGSARLFRSSSKGFQGTAQTSHGALMTSKQHQGKS
NTQYYHGKKRRHKRDAPLSELCR*
>rs50420322_ENSMUSP00000067971_c.174:C>T_p.L59F
ENSMUST00000066748.10|Tent4b-201|ENSMUSG00000036779|+|chr8:g.88926766:C>T
MQIWETTQGLRNLYFNHHCHSSGASSASGGSGSGPGSPGGTAPAPAGMFRSGERPLGGFAVPAEQRDFLPLET
TNNNNNHHQPAAWARRASAGPSASPVPSAPSSPRPAAALPASESTDPASGSSNKRKRDNKASTYGLNYSLLQP
SGGRAAGGGRADGGGGVYSGTPWKRRNYNQGVVGLHEEISDFYEYMSPRPEEEKMRMEVVSRIESVIKELWPS
ADVQIFGSFKTGLYLPTSDIDLVVFGKWENLPLWTLEEALRKHKVADEDSVKVLDKATVPIIKLTDSFTEVKV
DISFNVQNGVRAADLIKDFTKLHPREDACIPNTNYGVLLIEFFELYGRHFNYLKTGIRIKDGGSYVAKDEVQK
NMLDGYRPSMLYIEDPLQPGNDVGRSSYGAMQVKQAFDYAYVVLSHAVSPIAKYYPNNETESILGRIIRVTDE
VATYRDWISKQWGLQNRPEPSCNGNGVTLIVDTQQLDKCNNNLSEEKEALGKCRSNASEPLSKHSSNSSSGPV
SSSSATQSSSSDVDSDATPCKTPKQLLCRPPTVTRVGSQDVSLEVSQAVGKMQSTQTTNTPNNANKSQHGSAR
LFRSSSKGFQGTAQTSHGALMTSKQHQGKSNTQYYHGKKRRHKRDAPLSELCR*
>rs48197139_ENSMUSP00000112766_c.295:T>A_p.V99D
ENSMUST00000119033.8|Tent4b-203|ENSMUSG00000036779|+|chr8:g.88926887:T>A
MQIWETTQGLRNLYFNHHCHSSGASSASGGSGSGPGSPGGTAPAPAGMFRSGERPLGGLAVPAEQRDFLPLET
TNNNNNHHQPAAWARRASAGPSASPDPSAPSSPRPAAALPASESTDPASGSSNKRKRDNKASTYGLNYSLLQP
SGGRAAGGGRADGGGGVYSGTPWKRRNYNQGVVGLHEEISDFYEYMSPRPEEEKMRMEVVSRIESVIKELWPS
ADVQIFGSFKTGLYLPTSDIDLVVFGKWENLPLWTLEEALRKHKVADEDSVKVLDKATVPIIKLTDSFTEVKV
DISFNVQNGVRAADLIKDFTKKYPVLPYLVLVLKQFLLQRDLNEVFTGGIGSYSLFLMAVSFLQLHPREDACI
PNTNYGVLLIEFFELYGRHFNYLKTGIRIKDGGSYVAKDEVQKNMLDGYRPSMLYIEDPLQPGNDVGRSSYGA
MQVKQAFDYAYVVLSHAVSPIAKYYPNNETESILGRIIRVTDEVATYRDWISKQWGLQNRPEPSCNGNGVTLI
VDTQQLDKCNNNLSEEKEALGKCRSNASEPLSKHSSNSSSGPVSSSSATQSSSSDVDSDATPCKTPKQLLCRP
PTVTRVGSQDVSLEVSQAVGKMQSTQTTNTPNNANKSQHGSARLFRSSSKGFQGTAQTSHGALMTSKQHQGKS
NTQYYHGKKRRHKRDAPLSELCR*
>rs48197139_ENSMUSP00000112608_c.295:T>A_p.V99D
ENSMUST00000118952.8|Tent4b-202|ENSMUSG00000036779|+|chr8:g.88926887:T>A

MQIWETTQGLRNLYFNHHCHSSGASSASGGSGSGPGSPGGTAPAPAGMFRSGERPLGGLAVPAEQRDFLPLET
TNNNNNHHQPAAWARRASAGPSASPDPSAPSSPRPAAALPASESTDPASGSSNKRKRDNKASTYGLNYSLLQP
SGGRAAGGGRADGGGGVYSGTPWKRRNYNQGVVGLHEEISDFYEYMSPRPEEEKMRMEVVSRIESVIKELWPS
ADVQIFGSFKTGLYLPTSDIDLVVFGKWENLPLWTLEEALRKHKVADEDSVKVLDKATVPIIKLTDSFTEVKV
DISFNVQNGVRAADLIKDFTKKYPVLPYLVLVLKQFLLQRDLNEVFTGGIGSYSLFLMAVSFLQLHPREDACI
PNTNYGVLLIEFFELYGRHFNYLKTGIRIKDGGSYVAKDEVQKNMLDGYRPSMLYIEDPLQPGNDVGRSSYGA
MQVKQAFDYAYVVLSHAVSPIAKYYPNNETESILGRIIRVTDEVATYRDWISKQWGLQNRPEPSCNGNGVTLI
VDTQQLDKCNNNLSEEKEALGKCRSNASEPLSKHSSNSSSGPVSSSSATQSSSSDVDSDATPCKTPKQLLCRP
PTVTRVGSQDVSLEVSQAVGKMQSTQTTNTPNNANKSQHGSARLFRSSSKGFQGTAQTSHGALMTSKQHQGKS
NTQYYHGKKRRHKRDAPLSELCR*
>rs48197139_ENSMUSP00000067971_c.295:T>A_p.V99D
ENSMUST00000066748.10|Tent4b-201|ENSMUSG00000036779|+|chr8:g.88926887:T>A
MQIWETTQGLRNLYFNHHCHSSGASSASGGSGSGPGSPGGTAPAPAGMFRSGERPLGGLAVPAEQRDFLPLET
TNNNNNHHQPAAWARRASAGPSASPDPSAPSSPRPAAALPASESTDPASGSSNKRKRDNKASTYGLNYSLLQP
SGGRAAGGGRADGGGGVYSGTPWKRRNYNQGVVGLHEEISDFYEYMSPRPEEEKMRMEVVSRIESVIKELWPS
ADVQIFGSFKTGLYLPTSDIDLVVFGKWENLPLWTLEEALRKHKVADEDSVKVLDKATVPIIKLTDSFTEVKV
DISFNVQNGVRAADLIKDFTKLHPREDACIPNTNYGVLLIEFFELYGRHFNYLKTGIRIKDGGSYVAKDEVQK
NMLDGYRPSMLYIEDPLQPGNDVGRSSYGAMQVKQAFDYAYVVLSHAVSPIAKYYPNNETESILGRIIRVTDE
VATYRDWISKQWGLQNRPEPSCNGNGVTLIVDTQQLDKCNNNLSEEKEALGKCRSNASEPLSKHSSNSSSGPV
SSSSATQSSSSDVDSDATPCKTPKQLLCRPPTVTRVGSQDVSLEVSQAVGKMQSTQTTNTPNNANKSQHGSAR
LFRSSSKGFQGTAQTSHGALMTSKQHQGKSNTQYYHGKKRRHKRDAPLSELCR*
>rs46128864_ENSMUSP00000112766_c.496:G>A_p.G166E
ENSMUST00000119033.8|Tent4b-203|ENSMUSG00000036779|+|chr8:g.88927088:G>A
MQIWETTQGLRNLYFNHHCHSSGASSASGGSGSGPGSPGGTAPAPAGMFRSGERPLGGLAVPAEQRDFLPLET
TNNNNNHHQPAAWARRASAGPSASPVPSAPSSPRPAAALPASESTDPASGSSNKRKRDNKASTYGLNYSLLQP
SGGRAAGGGRADGGGGVYSETPWKRRNYNQGVVGLHEEISDFYEYMSPRPEEEKMRMEVVSRIESVIKELWPS
ADVQIFGSFKTGLYLPTSDIDLVVFGKWENLPLWTLEEALRKHKVADEDSVKVLDKATVPIIKLTDSFTEVKV
DISFNVQNGVRAADLIKDFTKKYPVLPYLVLVLKQFLLQRDLNEVFTGGIGSYSLFLMAVSFLQLHPREDACI
PNTNYGVLLIEFFELYGRHFNYLKTGIRIKDGGSYVAKDEVQKNMLDGYRPSMLYIEDPLQPGNDVGRSSYGA
MQVKQAFDYAYVVLSHAVSPIAKYYPNNETESILGRIIRVTDEVATYRDWISKQWGLQNRPEPSCNGNGVTLI
VDTQQLDKCNNNLSEEKEALGKCRSNASEPLSKHSSNSSSGPVSSSSATQSSSSDVDSDATPCKTPKQLLCRP
PTVTRVGSQDVSLEVSQAVGKMQSTQTTNTPNNANKSQHGSARLFRSSSKGFQGTAQTSHGALMTSKQHQGKS
NTQYYHGKKRRHKRDAPLSELCR*
>rs46128864_ENSMUSP00000112608_c.496:G>A_p.G166E
ENSMUST00000118952.8|Tent4b-202|ENSMUSG00000036779|+|chr8:g.88927088:G>A
MQIWETTQGLRNLYFNHHCHSSGASSASGGSGSGPGSPGGTAPAPAGMFRSGERPLGGLAVPAEQRDFLPLET
TNNNNNHHQPAAWARRASAGPSASPVPSAPSSPRPAAALPASESTDPASGSSNKRKRDNKASTYGLNYSLLQP
SGGRAAGGGRADGGGGVYSETPWKRRNYNQGVVGLHEEISDFYEYMSPRPEEEKMRMEVVSRIESVIKELWPS
ADVQIFGSFKTGLYLPTSDIDLVVFGKWENLPLWTLEEALRKHKVADEDSVKVLDKATVPIIKLTDSFTEVKV
DISFNVQNGVRAADLIKDFTKKYPVLPYLVLVLKQFLLQRDLNEVFTGGIGSYSLFLMAVSFLQLHPREDACI
PNTNYGVLLIEFFELYGRHFNYLKTGIRIKDGGSYVAKDEVQKNMLDGYRPSMLYIEDPLQPGNDVGRSSYGA
MQVKQAFDYAYVVLSHAVSPIAKYYPNNETESILGRIIRVTDEVATYRDWISKQWGLQNRPEPSCNGNGVTLI
VDTQQLDKCNNNLSEEKEALGKCRSNASEPLSKHSSNSSSGPVSSSSATQSSSSDVDSDATPCKTPKQLLCRP
PTVTRVGSQDVSLEVSQAVGKMQSTQTTNTPNNANKSQHGSARLFRSSSKGFQGTAQTSHGALMTSKQHQGKS
NTQYYHGKKRRHKRDAPLSELCR*
>rs46128864_ENSMUSP00000067971_c.496:G>A_p.G166E
ENSMUST00000066748.10|Tent4b-201|ENSMUSG00000036779|+|chr8:g.88927088:G>A
MQIWETTQGLRNLYFNHHCHSSGASSASGGSGSGPGSPGGTAPAPAGMFRSGERPLGGLAVPAEQRDFLPLET
TNNNNNHHQPAAWARRASAGPSASPVPSAPSSPRPAAALPASESTDPASGSSNKRKRDNKASTYGLNYSLLQP
SGGRAAGGGRADGGGGVYSETPWKRRNYNQGVVGLHEEISDFYEYMSPRPEEEKMRMEVVSRIESVIKELWPS
ADVQIFGSFKTGLYLPTSDIDLVVFGKWENLPLWTLEEALRKHKVADEDSVKVLDKATVPIIKLTDSFTEVKV
DISFNVQNGVRAADLIKDFTKLHPREDACIPNTNYGVLLIEFFELYGRHFNYLKTGIRIKDGGSYVAKDEVQK
NMLDGYRPSMLYIEDPLQPGNDVGRSSYGAMQVKQAFDYAYVVLSHAVSPIAKYYPNNETESILGRIIRVTDE
VATYRDWISKQWGLQNRPEPSCNGNGVTLIVDTQQLDKCNNNLSEEKEALGKCRSNASEPLSKHSSNSSSGPV
SSSSATQSSSSDVDSDATPCKTPKQLLCRPPTVTRVGSQDVSLEVSQAVGKMQSTQTTNTPNNANKSQHGSAR
LFRSSSKGFQGTAQTSHGALMTSKQHQGKSNTQYYHGKKRRHKRDAPLSELCR*
>rs50051979_ENSMUSP00000112766_c.876:G>A_p.D293N
ENSMUST00000119033.8|Tent4b-203|ENSMUSG00000036779|+|chr8:g.88974182:G>A

MQIWETTQGLRNLYFNHHCHSSGASSASGGSGSGPGSPGGTAPAPAGMFRSGERPLGGLAVPAEQRDFLPLET
TNNNNNHHQPAAWARRASAGPSASPVPSAPSSPRPAAALPASESTDPASGSSNKRKRDNKASTYGLNYSLLQP
SGGRAAGGGRADGGGGVYSGTPWKRRNYNQGVVGLHEEISDFYEYMSPRPEEEKMRMEVVSRIESVIKELWPS
ADVQIFGSFKTGLYLPTSDIDLVVFGKWENLPLWTLEEALRKHKVADEDSVKVLDKATVPIIKLTDSFTEVKV
NISFNVQNGVRAADLIKDFTKKYPVLPYLVLVLKQFLLQRDLNEVFTGGIGSYSLFLMAVSFLQLHPREDACI
PNTNYGVLLIEFFELYGRHFNYLKTGIRIKDGGSYVAKDEVQKNMLDGYRPSMLYIEDPLQPGNDVGRSSYGA
MQVKQAFDYAYVVLSHAVSPIAKYYPNNETESILGRIIRVTDEVATYRDWISKQWGLQNRPEPSCNGNGVTLI
VDTQQLDKCNNNLSEEKEALGKCRSNASEPLSKHSSNSSSGPVSSSSATQSSSSDVDSDATPCKTPKQLLCRP
PTVTRVGSQDVSLEVSQAVGKMQSTQTTNTPNNANKSQHGSARLFRSSSKGFQGTAQTSHGALMTSKQHQGKS
NTQYYHGKKRRHKRDAPLSELCR*
>rs50051979_ENSMUSP00000112608_c.876:G>A_p.D293N
ENSMUST00000118952.8|Tent4b-202|ENSMUSG00000036779|+|chr8:g.88974182:G>A
MQIWETTQGLRNLYFNHHCHSSGASSASGGSGSGPGSPGGTAPAPAGMFRSGERPLGGLAVPAEQRDFLPLET
TNNNNNHHQPAAWARRASAGPSASPVPSAPSSPRPAAALPASESTDPASGSSNKRKRDNKASTYGLNYSLLQP
SGGRAAGGGRADGGGGVYSGTPWKRRNYNQGVVGLHEEISDFYEYMSPRPEEEKMRMEVVSRIESVIKELWPS
ADVQIFGSFKTGLYLPTSDIDLVVFGKWENLPLWTLEEALRKHKVADEDSVKVLDKATVPIIKLTDSFTEVKV
NISFNVQNGVRAADLIKDFTKKYPVLPYLVLVLKQFLLQRDLNEVFTGGIGSYSLFLMAVSFLQLHPREDACI
PNTNYGVLLIEFFELYGRHFNYLKTGIRIKDGGSYVAKDEVQKNMLDGYRPSMLYIEDPLQPGNDVGRSSYGA
MQVKQAFDYAYVVLSHAVSPIAKYYPNNETESILGRIIRVTDEVATYRDWISKQWGLQNRPEPSCNGNGVTLI
VDTQQLDKCNNNLSEEKEALGKCRSNASEPLSKHSSNSSSGPVSSSSATQSSSSDVDSDATPCKTPKQLLCRP
PTVTRVGSQDVSLEVSQAVGKMQSTQTTNTPNNANKSQHGSARLFRSSSKGFQGTAQTSHGALMTSKQHQGKS
NTQYYHGKKRRHKRDAPLSELCR*
>rs50051979_ENSMUSP00000067971_c.876:G>A_p.D293N
ENSMUST00000066748.10|Tent4b-201|ENSMUSG00000036779|+|chr8:g.88974182:G>A
MQIWETTQGLRNLYFNHHCHSSGASSASGGSGSGPGSPGGTAPAPAGMFRSGERPLGGLAVPAEQRDFLPLET
TNNNNNHHQPAAWARRASAGPSASPVPSAPSSPRPAAALPASESTDPASGSSNKRKRDNKASTYGLNYSLLQP
SGGRAAGGGRADGGGGVYSGTPWKRRNYNQGVVGLHEEISDFYEYMSPRPEEEKMRMEVVSRIESVIKELWPS
ADVQIFGSFKTGLYLPTSDIDLVVFGKWENLPLWTLEEALRKHKVADEDSVKVLDKATVPIIKLTDSFTEVKV
NISFNVQNGVRAADLIKDFTKLHPREDACIPNTNYGVLLIEFFELYGRHFNYLKTGIRIKDGGSYVAKDEVQK
NMLDGYRPSMLYIEDPLQPGNDVGRSSYGAMQVKQAFDYAYVVLSHAVSPIAKYYPNNETESILGRIIRVTDE
VATYRDWISKQWGLQNRPEPSCNGNGVTLIVDTQQLDKCNNNLSEEKEALGKCRSNASEPLSKHSSNSSSGPV
SSSSATQSSSSDVDSDATPCKTPKQLLCRPPTVTRVGSQDVSLEVSQAVGKMQSTQTTNTPNNANKSQHGSAR
LFRSSSKGFQGTAQTSHGALMTSKQHQGKSNTQYYHGKKRRHKRDAPLSELCR*
>rs49186190_ENSMUSP00000112766_c.1335:G>A_p.D446N
ENSMUST00000119033.8|Tent4b-203|ENSMUSG00000036779|+|chr8:g.88978636:G>A
MQIWETTQGLRNLYFNHHCHSSGASSASGGSGSGPGSPGGTAPAPAGMFRSGERPLGGLAVPAEQRDFLPLET
TNNNNNHHQPAAWARRASAGPSASPVPSAPSSPRPAAALPASESTDPASGSSNKRKRDNKASTYGLNYSLLQP
SGGRAAGGGRADGGGGVYSGTPWKRRNYNQGVVGLHEEISDFYEYMSPRPEEEKMRMEVVSRIESVIKELWPS
ADVQIFGSFKTGLYLPTSDIDLVVFGKWENLPLWTLEEALRKHKVADEDSVKVLDKATVPIIKLTDSFTEVKV
DISFNVQNGVRAADLIKDFTKKYPVLPYLVLVLKQFLLQRDLNEVFTGGIGSYSLFLMAVSFLQLHPREDACI
PNTNYGVLLIEFFELYGRHFNYLKTGIRIKDGGSYVAKDEVQKNMLDGYRPSMLYIEDPLQPGNDVGRSSYGA
MQVKQAFNYAYVVLSHAVSPIAKYYPNNETESILGRIIRVTDEVATYRDWISKQWGLQNRPEPSCNGNGVTLI
VDTQQLDKCNNNLSEEKEALGKCRSNASEPLSKHSSNSSSGPVSSSSATQSSSSDVDSDATPCKTPKQLLCRP
PTVTRVGSQDVSLEVSQAVGKMQSTQTTNTPNNANKSQHGSARLFRSSSKGFQGTAQTSHGALMTSKQHQGKS
NTQYYHGKKRRHKRDAPLSELCR*
>rs49186190_ENSMUSP00000112608_c.1335:G>A_p.D446N
ENSMUST00000118952.8|Tent4b-202|ENSMUSG00000036779|+|chr8:g.88978636:G>A
MQIWETTQGLRNLYFNHHCHSSGASSASGGSGSGPGSPGGTAPAPAGMFRSGERPLGGLAVPAEQRDFLPLET
TNNNNNHHQPAAWARRASAGPSASPVPSAPSSPRPAAALPASESTDPASGSSNKRKRDNKASTYGLNYSLLQP
SGGRAAGGGRADGGGGVYSGTPWKRRNYNQGVVGLHEEISDFYEYMSPRPEEEKMRMEVVSRIESVIKELWPS
ADVQIFGSFKTGLYLPTSDIDLVVFGKWENLPLWTLEEALRKHKVADEDSVKVLDKATVPIIKLTDSFTEVKV
DISFNVQNGVRAADLIKDFTKKYPVLPYLVLVLKQFLLQRDLNEVFTGGIGSYSLFLMAVSFLQLHPREDACI
PNTNYGVLLIEFFELYGRHFNYLKTGIRIKDGGSYVAKDEVQKNMLDGYRPSMLYIEDPLQPGNDVGRSSYGA
MQVKQAFNYAYVVLSHAVSPIAKYYPNNETESILGRIIRVTDEVATYRDWISKQWGLQNRPEPSCNGNGVTLI
VDTQQLDKCNNNLSEEKEALGKCRSNASEPLSKHSSNSSSGPVSSSSATQSSSSDVDSDATPCKTPKQLLCRP
PTVTRVGSQDVSLEVSQAVGKMQSTQTTNTPNNANKSQHGSARLFRSSSKGFQGTAQTSHGALMTSKQHQGKS
NTQYYHGKKRRHKRDAPLSELCR*

>rs49186190_ENSMUSP00000120503_c.36:G>A_p.D13N
ENSMUST00000154115.2|Tent4b-205|ENSMUSG00000036779|+|chr8:g.88978636:G>A
SSYGAMQVKQAFNYAYVVLSHAVSPIAKYYPNNETESILGRIIRVTDEVATYRDWISKQWGLQNRPEPSCNGP
VSSSSATQSSSSDVDSDATPCKTPKQLLCRPPTVTRVGSQDVSLEVSQAVGKMQSTQTTNTPNNANKSQHGSA
RLFRSSSKGFQGTAQTSHGALMTSKQHQGKSNTQYYHGKKRRHKRDAPLSELCR*
>rs49186190_ENSMUSP00000067971_c.1206:G>A_p.D403N
ENSMUST00000066748.10|Tent4b-201|ENSMUSG00000036779|+|chr8:g.88978636:G>A
MQIWETTQGLRNLYFNHHCHSSGASSASGGSGSGPGSPGGTAPAPAGMFRSGERPLGGLAVPAEQRDFLPLET
TNNNNNHHQPAAWARRASAGPSASPVPSAPSSPRPAAALPASESTDPASGSSNKRKRDNKASTYGLNYSLLQP
SGGRAAGGGRADGGGGVYSGTPWKRRNYNQGVVGLHEEISDFYEYMSPRPEEEKMRMEVVSRIESVIKELWPS
ADVQIFGSFKTGLYLPTSDIDLVVFGKWENLPLWTLEEALRKHKVADEDSVKVLDKATVPIIKLTDSFTEVKV
DISFNVQNGVRAADLIKDFTKLHPREDACIPNTNYGVLLIEFFELYGRHFNYLKTGIRIKDGGSYVAKDEVQK
NMLDGYRPSMLYIEDPLQPGNDVGRSSYGAMQVKQAFNYAYVVLSHAVSPIAKYYPNNETESILGRIIRVTDE
VATYRDWISKQWGLQNRPEPSCNGNGVTLIVDTQQLDKCNNNLSEEKEALGKCRSNASEPLSKHSSNSSSGPV
SSSSATQSSSSDVDSDATPCKTPKQLLCRPPTVTRVGSQDVSLEVSQAVGKMQSTQTTNTPNNANKSQHGSAR
LFRSSSKGFQGTAQTSHGALMTSKQHQGKSNTQYYHGKKRRHKRDAPLSELCR*
>ENSMUSP00000112766_c.1415:AG>TA_p.L472F ENSMUST00000119033.8|Tent4b-
203|ENSMUSG00000036779|+|chr8:g.88978816:AG>TA
MQIWETTQGLRNLYFNHHCHSSGASSASGGSGSGPGSPGGTAPAPAGMFRSGERPLGGLAVPAEQRDFLPLET
TNNNNNHHQPAAWARRASAGPSASPVPSAPSSPRPAAALPASESTDPASGSSNKRKRDNKASTYGLNYSLLQP
SGGRAAGGGRADGGGGVYSGTPWKRRNYNQGVVGLHEEISDFYEYMSPRPEEEKMRMEVVSRIESVIKELWPS
ADVQIFGSFKTGLYLPTSDIDLVVFGKWENLPLWTLEEALRKHKVADEDSVKVLDKATVPIIKLTDSFTEVKV
DISFNVQNGVRAADLIKDFTKKYPVLPYLVLVLKQFLLQRDLNEVFTGGIGSYSLFLMAVSFLQLHPREDACI
PNTNYGVLLIEFFELYGRHFNYLKTGIRIKDGGSYVAKDEVQKNMLDGYRPSMLYIEDPLQPGNDVGRSSYGA
MQVKQAFDYAYVVLSHAVSPIAKYYPNNETESIFR*NN*SDG*SCHV*RLDIKTVGLAE*ARAIMQRKWCYLD
SRYSAVR*V***SV*RKGSPWKM*K*RLGTS**TLFKLFIRSSVLLLCHAVQL**RRF*CNAMQNPQTAALPS
THCHPGRLTGCLLGGLSGSWENAEHSNH*HTQQRQQITAWISKALPFFQQRLPRYSSNQPWGLDDKQTASRQI
QYSVLPWQKEETQEGRAPLRAL*I
>ENSMUSP00000112608_c.1415:AG>TA_p.L472F ENSMUST00000118952.8|Tent4b-
202|ENSMUSG00000036779|+|chr8:g.88978816:AG>TA
MQIWETTQGLRNLYFNHHCHSSGASSASGGSGSGPGSPGGTAPAPAGMFRSGERPLGGLAVPAEQRDFLPLET
TNNNNNHHQPAAWARRASAGPSASPVPSAPSSPRPAAALPASESTDPASGSSNKRKRDNKASTYGLNYSLLQP
SGGRAAGGGRADGGGGVYSGTPWKRRNYNQGVVGLHEEISDFYEYMSPRPEEEKMRMEVVSRIESVIKELWPS
ADVQIFGSFKTGLYLPTSDIDLVVFGKWENLPLWTLEEALRKHKVADEDSVKVLDKATVPIIKLTDSFTEVKV
DISFNVQNGVRAADLIKDFTKKYPVLPYLVLVLKQFLLQRDLNEVFTGGIGSYSLFLMAVSFLQLHPREDACI
PNTNYGVLLIEFFELYGRHFNYLKTGIRIKDGGSYVAKDEVQKNMLDGYRPSMLYIEDPLQPGNDVGRSSYGA
MQVKQAFDYAYVVLSHAVSPIAKYYPNNETESIFR*NN*SDG*SCHV*RLDIKTVGLAE*ARAIMQRKWCYLD
SRYSAVR*V***SV*RKGSPWKM*K*RLGTS**TLFKLFIRSSVLLLCHAVQL**RRF*CNAMQNPQTAALPS
THCHPGRLTGCLLGGLSGSWENAEHSNH*HTQQRQQITAWISKALPFFQQRLPRYSSNQPWGLDDKQTASRQI
QYSVLPWQKEETQEGRAPLRAL*I
>ENSMUSP00000120503_c.116:AG>TA_p.L39F ENSMUST00000154115.2|Tent4b-
205|ENSMUSG00000036779|+|chr8:g.88978816:AG>TA
SSYGAMQVKQAFDYAYVVLSHAVSPIAKYYPNNETESIFR*NN*SDG*SCHV*RLDIKTVGLAE*ARAIMQRS
SVLLLCHAVQL**RRF*CNAMQNPQTAALPSTHCHPGRLTGCLLGGLSGSWENAEHSNH*HTQQRQQITAWIS
KALPFFQQRLPRYSSNQPWGLDDKQTASRQIQYSVLPWQKEETQEGRAPLRAL*I
>ENSMUSP00000067971_c.1286:AG>TA_p.L429F ENSMUST00000066748.10|Tent4b-
201|ENSMUSG00000036779|+|chr8:g.88978816:AG>TA
MQIWETTQGLRNLYFNHHCHSSGASSASGGSGSGPGSPGGTAPAPAGMFRSGERPLGGLAVPAEQRDFLPLET
TNNNNNHHQPAAWARRASAGPSASPVPSAPSSPRPAAALPASESTDPASGSSNKRKRDNKASTYGLNYSLLQP
SGGRAAGGGRADGGGGVYSGTPWKRRNYNQGVVGLHEEISDFYEYMSPRPEEEKMRMEVVSRIESVIKELWPS
ADVQIFGSFKTGLYLPTSDIDLVVFGKWENLPLWTLEEALRKHKVADEDSVKVLDKATVPIIKLTDSFTEVKV
DISFNVQNGVRAADLIKDFTKLHPREDACIPNTNYGVLLIEFFELYGRHFNYLKTGIRIKDGGSYVAKDEVQK
NMLDGYRPSMLYIEDPLQPGNDVGRSSYGAMQVKQAFDYAYVVLSHAVSPIAKYYPNNETESIFR*NN*SDG*
SCHV*RLDIKTVGLAE*ARAIMQRKWCYLDSRYSAVR*V***SV*RKGSPWKM*K*RLGTS**TLFKLFIRSS
VLLLCHAVQL**RRF*CNAMQNPQTAALPSTHCHPGRLTGCLLGGLSGSWENAEHSNH*HTQQRQQITAWISK
ALPFFQQRLPRYSSNQPWGLDDKQTASRQIQYSVLPWQKEETQEGRAPLRAL*I
>rs47266984_ENSMUSP00000112766_c.1806:G>A_p.V603I
ENSMUST00000119033.8|Tent4b-203|ENSMUSG00000036779|+|chr8:g.88981842:G>A

MQIWETTQGLRNLYFNHHCHSSGASSASGGSGSGPGSPGGTAPAPAGMFRSGERPLGGLAVPAEQRDFLPLET
TNNNNNHHQPAAWARRASAGPSASPVPSAPSSPRPAAALPASESTDPASGSSNKRKRDNKASTYGLNYSLLQP
SGGRAAGGGRADGGGGVYSGTPWKRRNYNQGVVGLHEEISDFYEYMSPRPEEEKMRMEVVSRIESVIKELWPS
ADVQIFGSFKTGLYLPTSDIDLVVFGKWENLPLWTLEEALRKHKVADEDSVKVLDKATVPIIKLTDSFTEVKV
DISFNVQNGVRAADLIKDFTKKYPVLPYLVLVLKQFLLQRDLNEVFTGGIGSYSLFLMAVSFLQLHPREDACI
PNTNYGVLLIEFFELYGRHFNYLKTGIRIKDGGSYVAKDEVQKNMLDGYRPSMLYIEDPLQPGNDVGRSSYGA
MQVKQAFDYAYVVLSHAVSPIAKYYPNNETESILGRIIRVTDEVATYRDWISKQWGLQNRPEPSCNGNGVTLI
VDTQQLDKCNNNLSEEKEALGKCRSNASEPLSKHSSNSSSGPVSSSSATQSSSSDVDSDATPCKTPKQLLCRP
PTVTRVGSQDVSLEVSQAIGKMQSTQTTNTPNNANKSQHGSARLFRSSSKGFQGTAQTSHGALMTSKQHQGKS
NTQYYHGKKRRHKRDAPLSELCR*
>rs47266984_ENSMUSP00000112608_c.1806:G>A_p.V603I
ENSMUST00000118952.8|Tent4b-202|ENSMUSG00000036779|+|chr8:g.88981842:G>A
MQIWETTQGLRNLYFNHHCHSSGASSASGGSGSGPGSPGGTAPAPAGMFRSGERPLGGLAVPAEQRDFLPLET
TNNNNNHHQPAAWARRASAGPSASPVPSAPSSPRPAAALPASESTDPASGSSNKRKRDNKASTYGLNYSLLQP
SGGRAAGGGRADGGGGVYSGTPWKRRNYNQGVVGLHEEISDFYEYMSPRPEEEKMRMEVVSRIESVIKELWPS
ADVQIFGSFKTGLYLPTSDIDLVVFGKWENLPLWTLEEALRKHKVADEDSVKVLDKATVPIIKLTDSFTEVKV
DISFNVQNGVRAADLIKDFTKKYPVLPYLVLVLKQFLLQRDLNEVFTGGIGSYSLFLMAVSFLQLHPREDACI
PNTNYGVLLIEFFELYGRHFNYLKTGIRIKDGGSYVAKDEVQKNMLDGYRPSMLYIEDPLQPGNDVGRSSYGA
MQVKQAFDYAYVVLSHAVSPIAKYYPNNETESILGRIIRVTDEVATYRDWISKQWGLQNRPEPSCNGNGVTLI
VDTQQLDKCNNNLSEEKEALGKCRSNASEPLSKHSSNSSSGPVSSSSATQSSSSDVDSDATPCKTPKQLLCRP
PTVTRVGSQDVSLEVSQAIGKMQSTQTTNTPNNANKSQHGSARLFRSSSKGFQGTAQTSHGALMTSKQHQGKS
NTQYYHGKKRRHKRDAPLSELCR*
>rs47266984_ENSMUSP00000120503_c.366:G>A_p.V123I
ENSMUST00000154115.2|Tent4b-205|ENSMUSG00000036779|+|chr8:g.88981842:G>A
SSYGAMQVKQAFDYAYVVLSHAVSPIAKYYPNNETESILGRIIRVTDEVATYRDWISKQWGLQNRPEPSCNGP
VSSSSATQSSSSDVDSDATPCKTPKQLLCRPPTVTRVGSQDVSLEVSQAIGKMQSTQTTNTPNNANKSQHGSA
RLFRSSSKGFQGTAQTSHGALMTSKQHQGKSNTQYYHGKKRRHKRDAPLSELCR*
>rs47266984_ENSMUSP00000067971_c.1677:G>A_p.V560I
ENSMUST00000066748.10|Tent4b-201|ENSMUSG00000036779|+|chr8:g.88981842:G>A
MQIWETTQGLRNLYFNHHCHSSGASSASGGSGSGPGSPGGTAPAPAGMFRSGERPLGGLAVPAEQRDFLPLET
TNNNNNHHQPAAWARRASAGPSASPVPSAPSSPRPAAALPASESTDPASGSSNKRKRDNKASTYGLNYSLLQP
SGGRAAGGGRADGGGGVYSGTPWKRRNYNQGVVGLHEEISDFYEYMSPRPEEEKMRMEVVSRIESVIKELWPS
ADVQIFGSFKTGLYLPTSDIDLVVFGKWENLPLWTLEEALRKHKVADEDSVKVLDKATVPIIKLTDSFTEVKV
DISFNVQNGVRAADLIKDFTKLHPREDACIPNTNYGVLLIEFFELYGRHFNYLKTGIRIKDGGSYVAKDEVQK
NMLDGYRPSMLYIEDPLQPGNDVGRSSYGAMQVKQAFDYAYVVLSHAVSPIAKYYPNNETESILGRIIRVTDE
VATYRDWISKQWGLQNRPEPSCNGNGVTLIVDTQQLDKCNNNLSEEKEALGKCRSNASEPLSKHSSNSSSGPV
SSSSATQSSSSDVDSDATPCKTPKQLLCRPPTVTRVGSQDVSLEVSQAIGKMQSTQTTNTPNNANKSQHGSAR
LFRSSSKGFQGTAQTSHGALMTSKQHQGKSNTQYYHGKKRRHKRDAPLSELCR*
>rs52496465_ENSMUSP00000112766_c.1989:G>A_p.G664S
ENSMUST00000119033.8|Tent4b-203|ENSMUSG00000036779|+|chr8:g.88983850:G>A
MQIWETTQGLRNLYFNHHCHSSGASSASGGSGSGPGSPGGTAPAPAGMFRSGERPLGGLAVPAEQRDFLPLET
TNNNNNHHQPAAWARRASAGPSASPVPSAPSSPRPAAALPASESTDPASGSSNKRKRDNKASTYGLNYSLLQP
SGGRAAGGGRADGGGGVYSGTPWKRRNYNQGVVGLHEEISDFYEYMSPRPEEEKMRMEVVSRIESVIKELWPS
ADVQIFGSFKTGLYLPTSDIDLVVFGKWENLPLWTLEEALRKHKVADEDSVKVLDKATVPIIKLTDSFTEVKV
DISFNVQNGVRAADLIKDFTKKYPVLPYLVLVLKQFLLQRDLNEVFTGGIGSYSLFLMAVSFLQLHPREDACI
PNTNYGVLLIEFFELYGRHFNYLKTGIRIKDGGSYVAKDEVQKNMLDGYRPSMLYIEDPLQPGNDVGRSSYGA
MQVKQAFDYAYVVLSHAVSPIAKYYPNNETESILGRIIRVTDEVATYRDWISKQWGLQNRPEPSCNGNGVTLI
VDTQQLDKCNNNLSEEKEALGKCRSNASEPLSKHSSNSSSGPVSSSSATQSSSSDVDSDATPCKTPKQLLCRP
PTVTRVGSQDVSLEVSQAVGKMQSTQTTNTPNNANKSQHGSARLFRSSSKGFQGTAQTSHGALMTSKQHQGKS
NTQYYHSKKRRHKRDAPLSELCR*
>rs52496465_ENSMUSP00000112608_c.1989:G>A_p.G664S
ENSMUST00000118952.8|Tent4b-202|ENSMUSG00000036779|+|chr8:g.88983850:G>A
MQIWETTQGLRNLYFNHHCHSSGASSASGGSGSGPGSPGGTAPAPAGMFRSGERPLGGLAVPAEQRDFLPLET
TNNNNNHHQPAAWARRASAGPSASPVPSAPSSPRPAAALPASESTDPASGSSNKRKRDNKASTYGLNYSLLQP
SGGRAAGGGRADGGGGVYSGTPWKRRNYNQGVVGLHEEISDFYEYMSPRPEEEKMRMEVVSRIESVIKELWPS
ADVQIFGSFKTGLYLPTSDIDLVVFGKWENLPLWTLEEALRKHKVADEDSVKVLDKATVPIIKLTDSFTEVKV
DISFNVQNGVRAADLIKDFTKKYPVLPYLVLVLKQFLLQRDLNEVFTGGIGSYSLFLMAVSFLQLHPREDACI
PNTNYGVLLIEFFELYGRHFNYLKTGIRIKDGGSYVAKDEVQKNMLDGYRPSMLYIEDPLQPGNDVGRSSYGA

MQVKQAFDYAYVVLSHAVSPIAKYYPNNETESILGRIIRVTDEVATYRDWISKQWGLQNRPEPSCNGNGVTLI
VDTQQLDKCNNNLSEEKEALGKCRSNASEPLSKHSSNSSSGPVSSSSATQSSSSDVDSDATPCKTPKQLLCRP
PTVTRVGSQDVSLEVSQAVGKMQSTQTTNTPNNANKSQHGSARLFRSSSKGFQGTAQTSHGALMTSKQHQGKS
NTQYYHSKKRRHKRDAPLSELCR*
>rs52496465_ENSMUSP00000120503_c.549:G>A_p.G184S
ENSMUST00000154115.2|Tent4b-205|ENSMUSG00000036779|+|chr8:g.88983850:G>A
SSYGAMQVKQAFDYAYVVLSHAVSPIAKYYPNNETESILGRIIRVTDEVATYRDWISKQWGLQNRPEPSCNGP
VSSSSATQSSSSDVDSDATPCKTPKQLLCRPPTVTRVGSQDVSLEVSQAVGKMQSTQTTNTPNNANKSQHGSA
RLFRSSSKGFQGTAQTSHGALMTSKQHQGKSNTQYYHSKKRRHKRDAPLSELCR*
>rs52496465_ENSMUSP00000067971_c.1860:G>A_p.G621S
ENSMUST00000066748.10|Tent4b-201|ENSMUSG00000036779|+|chr8:g.88983850:G>A
MQIWETTQGLRNLYFNHHCHSSGASSASGGSGSGPGSPGGTAPAPAGMFRSGERPLGGLAVPAEQRDFLPLET
TNNNNNHHQPAAWARRASAGPSASPVPSAPSSPRPAAALPASESTDPASGSSNKRKRDNKASTYGLNYSLLQP
SGGRAAGGGRADGGGGVYSGTPWKRRNYNQGVVGLHEEISDFYEYMSPRPEEEKMRMEVVSRIESVIKELWPS
ADVQIFGSFKTGLYLPTSDIDLVVFGKWENLPLWTLEEALRKHKVADEDSVKVLDKATVPIIKLTDSFTEVKV
DISFNVQNGVRAADLIKDFTKLHPREDACIPNTNYGVLLIEFFELYGRHFNYLKTGIRIKDGGSYVAKDEVQK
NMLDGYRPSMLYIEDPLQPGNDVGRSSYGAMQVKQAFDYAYVVLSHAVSPIAKYYPNNETESILGRIIRVTDE
VATYRDWISKQWGLQNRPEPSCNGNGVTLIVDTQQLDKCNNNLSEEKEALGKCRSNASEPLSKHSSNSSSGPV
SSSSATQSSSSDVDSDATPCKTPKQLLCRPPTVTRVGSQDVSLEVSQAVGKMQSTQTTNTPNNANKSQHGSAR
LFRSSSKGFQGTAQTSHGALMTSKQHQGKSNTQYYHSKKRRHKRDAPLSELCR*
>rs33615446_ENSMUSP00000130594_c.46:C>T_p.P16L
ENSMUST00000169037.9|Adcy7-203|ENSMUSG00000031659|+|chr8:g.89033274:C>T
MPAKGRYFLNEGDEGLDQAALYEKYRLTSLHGPLLLLLLLVAAATCIALISIAFSHEDLRRHQVVLGTAFLML
TLFVALYVLVYVECLVQRWLRALALLTWACLMVLGSVLMWDSLENEAHAWEQVPFFLFVVFVVYALLPLSRRA
AIVAGVTSTVSHLLVFGAVTRAFQTSMSSTQLGLQLLANAVILLGGNFTGAFHKHQLQDASRDLFIYTVKCIQ
IRRKLRVEKRQQENLLLSVLPAHISMGMKLAIIERLKEGGDRHYMPDNNFHSLYVKRHQNVSILYADIVGFTR
LASDCSPKELVVVLNELFGKFDQIAKANECMRIKILGDCYYCVSGLPVSLPTHARNCVKMGLDICEAIKQVRE
ATGVDISMRVGIHSGNVLCGVIGLRKWQYDVWSHDVSLANRMEAAGVPGRVHITEATLNHLDKAYEVEDGHGE
QRDPYLKEMNIRTYLVIDPRSQQPPPPSHHLSKPKGDATLKMRASVRVTRYLESWGAARPFAHLNHRESVSSS
ETPISNGRRQKAIPLRRHRAPDRSASPKGRLEDDCDDEMLSAIEGLSSTRPCCSKSDDFHTFGPIFLEKGFER
EYRLVPIPRARYDFACASLVFVCILLVHLLVMPRMATLGVSFGLVACLLGLVLSFCFATEFSRCFPSRSTLQA
ISESVETQPLVRLVLVVLTVGSLLTVAIINMPLTLNPGPEQPGDNKTSPLAAQNRVGTPCELLPYYTCSCILG
FIACSVFLRMSLELKAMLLTVALVAYLLLFNLSPCWHVSGNSTETNGTQRTRLLLSDAQSMPSHTLAPGARET
APSPSYLERDLKIMVNFYLILFYATLILLSRQIDYYCRLDCLWKKKFKKEHEEFETMENVNRLLLENVLPAHV
AAHFIGDKAAEDWYHQSYDCVCVMFASVPDFKVFYTECDVNKEGLECLRLLNEIIADFDELLLKPKFSGVEKI
KTIGSTYMAAAGLSAPSGHENQDLERKHVHIGVLVEFSMALMSKLDGINRHSFNSFRLRVGINHGPVIAGVIG
ARKPQYDIWGNTVNVASRMESTGELGKIQVTEETCTILQGLGYSCECRGLINVKGKGELRTYFVCTDTAKFQG
LGLN*
>rs33615446_ENSMUSP00000132528_c.46:C>T_p.P16L
ENSMUST00000171456.9|Adcy7-204|ENSMUSG00000031659|+|chr8:g.89033274:C>T
MPAKGRYFLNEGDEGLDQAALYEKYRLTSLHGPLLLLLLLVAAATCIALISIAFSHEDLRRHQVVLGTAFLML
TLFVALYVLVYVECLVQRWLRALALLTWACLMVLGSVLMWDSLENEAHAWEQVPFFLFVVFVVYALLPLSRRA
AIVAGVTSTVSHLLVFGAVTRAFQTSMSSTQLGLQLLANAVILLGGNFTGAFHKHQLQDASRDLFIYTVKCIQ
IRRKLRVEKRQQENLLLSVLPAHISMGMKLAIIERLKEGGDRHYMPDNNFHSLYVKRHQNVSILYADIVGFTR
LASDCSPKELVVVLNELFGKFDQIAKANECMRIKILGDCYYCVSGLPVSLPTHARNCVKMGLDICEAIKQVRE
ATGVDISMRVGIHSGNVLCGVIGLRKWQYDVWSHDVSLANRMEAAGVPGRVHITEATLNHLDKAYEVEDGHGE
QRDPYLKEMNIRTYLVIDPRSQQPPPPSHHLSKPKGDATLKMRASVRVTRYLESWGAARPFAHLNHRESVSSS
ETPISNGRRQKAIPLRRHRAPDRSASPKGRLEDDCDDEMLSAIEGLSSTRPCCSKSDDFHTFGPIFLEKGFER
EYRLVPIPRARYDFACASLVFVCILLVHLLVMPRMATLGVSFGLVACLLGLVLSFCFATEFSRCFPSRSTLQA
ISESVETQPLVRLVLVVLTVGSLLTVAIINMPLTLNPGPEQPGDNKTSPLAAQNRVGTPCELLPYYTCSCILG
FIACSVFLRMSLELKAMLLTVALVAYLLLFNLSPCWHVSGNSTETNGTQRTRLLLSDAQSMPSHTLAPGARET
APSPSYLERDLKIMVNFYLILFYATLILLSRQIDYYCRLDCLWKKKFKKEHEEFETMENVNRLLLENVLPAHV
AAHFIGDKAAEDWYHQSYDCVCVMFASVPDFKVFYTECDVNKEGLECLRLLNEIIADFDELLLKPKFSGVEKI
KTIGSTYMAAAGLSAPSGHENQDLERKHVHIGVLVEFSMALMSKLDGINRHSFNSFRLRVGINHGPVIAGVIG
ARKPQYDIWGNTVNVASRMESTGELGKIQVTEETCTILQGLGYSCECRGLINVKGKGELRTYFVCTDTAKFQG
LGLN*
>rs33615446_ENSMUSP00000096122_c.46:C>T_p.P16L
ENSMUST00000098521.4|Adcy7-201|ENSMUSG00000031659|+|chr8:g.89033274:C>T

MPAKGRYFLNEGDEGLDQAALYEKYRLTSLHGPLLLLLLLVAAATCIALISIAFSHEDLRRHQVVLGTAFLML
TLFVALYVLVYYVECLVQRWLRALALLTWACLMVLGSVLMWDSLENEAHAWEQVPFFLFVVFVVYALLPLSRRA
AIVAGVTSTVSHLLVFGAVTRAFQTSMSSTQLGLQLLANAVILLGGNFTGAFHKHQLQDASRDLFIYTVKCIQ
IRRKLRVEKRQQENLLLSVLPAHISMGMKLAIIERLKEGGDRHYMPDNNFHSLYVKRHQNVSILYADIVGFTR
LASDCSPKELVVVLNELFGKFDQIAKANECMRIKILGDCYYCVSGLPVSLPTHARNCVKMGLDICEAIKQVRE
ATGVDISMRVGIHSGNVLCGVIGLRKWQYDVWSHDVSLANRMEAAGVPGRVHITEATLNHLDKAYEVEDGHGE
QRDPYLKEMNIRTYLVIDPRSQQPPPPSHHLSKPKGDATLKMRASVRVTRYLESWGAARPFAHLNHRESVSSS
ETPISNGRRQKAIPLRRHRAPDRSASPKGRLEDDCDDEMLSAIEGLSSTRPCCSKSDDFHTFGPIFLEKGFER
EYRLVPIPRARYDFACASLVFVCILLVHLLVMPRMATLGVSFGLVACLLGLVLSFCFATEFSRCFPSRSTLQA
ISESVETQPLVRLVLVVLTVGSLLTVAIINMPLTLNPGPEQPGDNKTSPLAAQNRVGTPCELLPYYTCSCILG
FIACSVFLRMSLELKAMLLTVALVAYLLLFNLSPCWHVSGNSTETNGTQRTRLLLSDAQSMPSHTLAPGARET
APSPSYLERDLKIMVNFYLILFYATLILLSRQIDYYCRLDCLWKKKFKKEHEEFETMENVNRLLLENVLPAHV
AAHFIGDKAAEDWYHQSYDCVCVMFASVPDFKVFYTECDVNKEGLECLRLLNEIIADFDELLLKPKFSGVEKI
KTIGSTYMAAAGLSAPSGHENQDLERKHVHIGVLVEFSMALMSKLDGINRHSFNSFRLRVGINHGPVIAGVIG
ARKPQYDIWGNTVNVASRMESTGELGKIQVTEETCTILQGLGYSCECRGLINVKGKGELRTYFVCTDTAKFQG
LGLN*
>rs33615446_ENSMUSP00000129252_c.46:C>T_p.P16L
ENSMUST00000168545.8|Adcy7-202|ENSMUSG00000031659|+|chr8:g.89033274:C>T
MPAKGRYFLNEGDEGLDQAALYEKYRLTSLHGPLLLLLLLVAAATCIALISIAFSHEDLRRHQVVLGTAFLML
TLFVALYVLVYYVECLVQRWLRALALLTWACLMVLGSVLMWDSLENEAHAWEQVPFFLFVVFVVYALLPLSRRA
AIVAGVTSTVSHLLVFGAVTRAFQTSMSSTQLGLQLLANAVILLGGNFTGAFHKHQLQDASRDLFIYTVKCIQ
IRRKLRVEKRQQENLLLSVLPAHISMGMKLAIIERLKEGGDRHYMPDNNFHSLYVKRHQNVSILYADIVGFTR
LASDCSPKELVVVLNELFGKFDQIAKANECMRIKILGDCYYCVSGLPVSLPTHARNCVKMGLDICEAIKQVRE
ATGVDISMRVGIHSGNVLCGVIGLRKWQYDVWSHDVSLANRMEAAGVPGRVHITEATLNHLDKAYEVEDGHGE
QRDPYLKEMNIRTYLVIDPRSQQPPPPSHHLSKPKGDATLKMRASVRVTRYLESWGAARPFAHLNHRESVSSS
ETPISNGRRQKAIPLRRHRAPDRSASPKGRLEDDCDDEMLSAIEGLSSTRPCCSKSDDFHTFGPIFLEKGFER
EYRLVPIPRARYDFACASLVFVCILLVHLLVMPRMATLGVSFGLVACLLGLVLSFCFATEFSRCFPSRSTLQA
ISESVETQPLVRLVLVVLTVGSLLTVAIINMPLTLNPGPEQPGDNKTSPLAAQNRVGTPCELLPYYTCSCILG
FIACSVFLRMSLELKAMLLTVALVAYLLLFNLSPCWHVSGNSTETNGTQRTRLLLSDAQSMPSHTLAPGARET
APSPSYLERDLKIMVNFYLILFYATLILLSRQIDYYCRLDCLWKKKFKKEHEEFETMENVNRLLLENVLPAHV
AAHFIGDKAAEDWYHQSYDCVCVMFASVPDFKVFYTECDVNKEGLECLRLLNEIIADFDELLLKPKFSGVEKI
KTIGSTYMAAAGLSAPSGHENQDLERKHVHIGVLVEFSMALMSKLDGINRHSFNSFRLRVGINHGPVIAGVIG
ARKPQYDIWGNTVNVASRMESTGELGKIQVTEETCTILQGLGYSCECRGLINVKGKGELRTYFVCTDTAKFQG
LGLN*
>rs51766230_ENSMUSP00000034085_c.1525:G>T_p.R509L
ENSMUST00000034085.8|Brd7-201|ENSMUSG00000031660|-|chr8:g.89060819:C>A
MGKKHKKHKSDRHFYEEYVEKPLKLVLKVGGSEVTELSTGSSGHDSSLFEDRSDHDKHKDRKRKKRKKGEKQA
PGEEKGRKRRRVKEDKKKRDRDRAENEVDRDLQCHVPIRLDLPPEKPLTSSLAKQEEVEQTPLQEALNQLMRQ
LQRKDPSAFFSFPVTDFIAPGYSMIIKHPMDFSTMKEKIKNNDYQSIEELKDNFKLMCTNAMIYNKPETIYYK
AAKKLLHSGMKILSQERIQSLKQSIDFMSDLQKTRKQKERTDACQSGEDSGCWQREREDSGDAETQAFRSPAK
DNKRKDKDVLEDKWRSSNSEREHEQIERVVQESGGKLTRRLANSQCEFERRKPDGTTTLGLLHPVDPIVGEPG
YCPVRLGMTTGRLQSGVNTLQGFKEDKRNRVTPVLYLNYGPYSSYAPHYDSTFANISKDDSDLIYSTYGEDSD
LPNNFSISEFLATCQDYPYVMADSLLDVLTKGGHSRSLQDLDMSSPEDEGQTRALDTAKEAEITQIEPTGLLE
SSSQDRLTALQAVTTFGAPAEVFDSEEAEVFQRKLDETTRLLRELQEAQNERLSTRPPPNMICLLGPSYREMY
LAEQVTNNLKELTQQVTPGDVVSIHGVRKAMGISVPSPIVGNSFVDLTGECEEPKETSTAECGPDAS*
>rs47501089_ENSMUSP00000034085_c.1444:C>T_p.S482L
ENSMUST00000034085.8|Brd7-201|ENSMUSG00000031660|-|chr8:g.89063764:G>A
MGKKHKKHKSDRHFYEEYVEKPLKLVLKVGGSEVTELSTGSSGHDSSLFEDRSDHDKHKDRKRKKRKKGEKQA
PGEEKGRKRRRVKEDKKKRDRDRAENEVDRDLQCHVPIRLDLPPEKPLTSSLAKQEEVEQTPLQEALNQLMRQ
LQRKDPSAFFSFPVTDFIAPGYSMIIKHPMDFSTMKEKIKNNDYQSIEELKDNFKLMCTNAMIYNKPETIYYK
AAKKLLHSGMKILSQERIQSLKQSIDFMSDLQKTRKQKERTDACQSGEDSGCWQREREDSGDAETQAFRSPAK
DNKRKDKDVLEDKWRSSNSEREHEQIERVVQESGGKLTRRLANSQCEFERRKPDGTTTLGLLHPVDPIVGEPG
YCPVRLGMTTGRLQSGVNTLQGFKEDKRNRVTPVLYLNYGPYSSYAPHYDSTFANISKDDSDLIYSTYGEDSD
LPNNFSISEFLATCQDYPYVMADSLLDVLTKGGHSRSLQDLDMLSPEDEGQTRALDTAKEAEITQIEPTGRLE
SSSQDRLTALQAVTTFGAPAEVFDSEEAEVFQRKLDETTRLLRELQEAQNERLSTRPPPNMICLLGPSYREMY
LAEQVTNNLKELTQQVTPGDVVSIHGVRKAMGISVPSPIVGNSFVDLTGECEEPKETSTAECGPDAS*
>rs250965619_ENSMUSP00000034085_c.1219:A>G_p.Y407C
ENSMUST00000034085.8|Brd7-201|ENSMUSG00000031660|-|chr8:g.89069474:T>C

MGKKHKKHKSDRHFYEEYVEKPLKLVLKVGGSEVTELSTGSSGHDSSLFEDRSDHDKHKDRKRKKRKKGEKQA
PGEEKGRKRRRVKEDKKKRDRDRAENEVDRDLQCHVPIRLDLPPEKPLTSSLAKQEEVEQTPLQEALNQLMRQ
LQRKDPSAFFSFPVTDFIAPGYSMIIKHPMDFSTMKEKIKNNDYQSIEELKDNFKLMCTNAMIYNKPETIYYK
AAKKLLHSGMKILSQERIQSLKQSIDFMSDLQKTRKQKERTDACQSGEDSGCWQREREDSGDAETQAFRSPAK
DNKRKDKDVLEDKWRSSNSEREHEQIERVVQESGGKLTRRLANSQCEFERRKPDGTTTLGLLHPVDPIVGEPG
YCPVRLGMTTGRLQSGVNTLQGFKEDKRNRVTPVLYLNYGPCSSYAPHYDSTFANISKDDSDLIYSTYGEDSD
LPNNFSISEFLATCQDYPYVMADSLLDVLTKGGHSRSLQDLDMSSPEDEGQTRALDTAKEAEITQIEPTGRLE
SSSQDRLTALQAVTTFGAPAEVFDSEEAEVFQRKLDETTRLLRELQEAQNERLSTRPPPNMICLLGPSYREMY
LAEQVTNNLKELTQQVTPGDVVSIHGVRKAMGISVPSPIVGNSFVDLTGECEEPKETSTAECGPDAS*
>rs48313211_ENSMUSP00000034085_c.700:A>T_p.Q234L
ENSMUST00000034085.8|Brd7-201|ENSMUSG00000031660|-|chr8:g.89078429:T>A
MGKKHKKHKSDRHFYEEYVEKPLKLVLKVGGSEVTELSTGSSGHDSSLFEDRSDHDKHKDRKRKKRKKGEKQA
PGEEKGRKRRRVKEDKKKRDRDRAENEVDRDLQCHVPIRLDLPPEKPLTSSLAKQEEVEQTPLQEALNQLMRQ
LQRKDPSAFFSFPVTDFIAPGYSMIIKHPMDFSTMKEKIKNNDYQSIEELKDNFKLMCTNAMIYNKPETIYYK
AAKKLLHSGMKILSLERIQSLKQSIDFMSDLQKTRKQKERTDACQSGEDSGCWQREREDSGDAETQAFRSPAK
DNKRKDKDVLEDKWRSSNSEREHEQIERVVQESGGKLTRRLANSQCEFERRKPDGTTTLGLLHPVDPIVGEPG
YCPVRLGMTTGRLQSGVNTLQGFKEDKRNRVTPVLYLNYGPYSSYAPHYDSTFANISKDDSDLIYSTYGEDSD
LPNNFSISEFLATCQDYPYVMADSLLDVLTKGGHSRSLQDLDMSSPEDEGQTRALDTAKEAEITQIEPTGRLE
SSSQDRLTALQAVTTFGAPAEVFDSEEAEVFQRKLDETTRLLRELQEAQNERLSTRPPPNMICLLGPSYREMY
LAEQVTNNLKELTQQVTPGDVVSIHGVRKAMGISVPSPIVGNSFVDLTGECEEPKETSTAECGPDAS*
>rs263208908_ENSMUSP00000034085_c.408:C>T_p.Q137*
ENSMUST00000034085.8|Brd7-201|ENSMUSG00000031660|-|chr8:g.89084202:G>A
MGKKHKKHKSDRHFYEEYVEKPLKLVLKVGGSEVTELSTGSSGHDSSLFEDRSDHDKHKDRKRKKRKKGEKQA
PGEEKGRKRRRVKEDKKKRDRDRAENEVDRDLQCHVPIRLDLPPEKPLTSSLAKQEEVEQTPL*EALNQLMRQ
LQRKDPSAFFSFPVTDFIAPGYSMIIKHPMDFSTMKEKIKNNDYQSIEELKDNFKLMCTNAMIYNKPETIYYK
AAKKLLHSGMKILSQERIQSLKQSIDFMSDLQKTRKQKERTDACQSGEDSGCWQREREDSGDAETQAFRSPAK
DNKRKDKDVLEDKWRSSNSEREHEQIERVVQESGGKLTRRLANSQCEFERRKPDGTTTLGLLHPVDPIVGEPG
YCPVRLGMTTGRLQSGVNTLQGFKEDKRNRVTPVLYLNYGPYSSYAPHYDSTFANISKDDSDLIYSTYGEDSD
LPNNFSISEFLATCQDYPYVMADSLLDVLTKGGHSRSLQDLDMSSPEDEGQTRALDTAKEAEITQIEPTGRLE
SSSQDRLTALQAVTTFGAPAEVFDSEEAEVFQRKLDETTRLLRELQEAQNERLSTRPPPNMICLLGPSYREMY
LAEQVTNNLKELTQQVTPGDVVSIHGVRKAMGISVPSPIVGNSFVDLTGECEEPKETSTAECGPDAS*
>rs49846526_ENSMUSP00000034086_c.220:C>T_p.A74V
ENSMUST00000034086.13|Nkd1-201|ENSMUSG00000031661|+|chr8:g.89300854:C>T
MGKLHSKPAAVCKRRESPEGDSFAVSAAWARKGIEEWIGRQRCPGSVSGPRQLRLAGTVGRGTRELVGDTSRE
VLGEEDEDDFPLEVALPPEKIDSLGSGDEKRMERLSEPGQASKKQLKFEELQCDVSVEEDSRQEWTFTLYDFD
NNGKVTREDITSLLHTIYEVVDSSVNHSPTSSKTLRVKLTVAPDGSQSKRSVLFNHTDLQSTRPRADTKPAEE
LRGWEKKQRAPLRFQGDSHLEQPDCYHHCVDENIERRNHYLDLAGIENYTSQFGPGSPSVAQKSELPPRISNP
TRSRSHEPEAAHIPHRRPQGVDPGSFHLLDTPFAKASELQQRLRGTQDGSKHFVRSPKAQGKNMGMGHGARGA
RSKPPLVPTTHTVSPSAHLATSPALLPTLAPLGHKKHKHRAKESQASCRGLQGPLAAGGSTVMGREQVRELPA
VVVYESQAGQAVQRHEHHHHHEHHHHYHHFYQP*
>rs45974600_ENSMUSP00000113773_c.1839:G>A_p.G614R
ENSMUST00000118370.8|Nod2-203|ENSMUSG00000055994|+|chr8:g.89391600:G>A
MCSQEEFQAQRSQLVALLISGSLEGFESILDWLLSWDVLSREDYEGLSLPGQPLSHSARRLLDTVWNKGVWGC
QKLLEAVQEAQANSHTFELYGSWDTHSLHPTRDLQSHRPAIVRRLYNHVEAMLELAREGGFLSQYECEEIRLP
IFTSSQRARRLLDLAAVKANGLAAFLLQHVRELPAPLPLPYEAAECQKFISKLRTMVLTQSRFLSTYDGSENL
CLEDIYTENILELQTEVGTAGALQKSPAILGLEDLFDTHGHLNRDADTILVVGEAGSGKSTLLQRLHLLWATG
RSFQEFLFIFPFSCRQLQCVAKPLSLRTLLFEHCCWPDVAQDDVFQFLLDHPDRVLLTFDGLDEFKFRFTDRE
RHCSPIDPTSVQTLLFNLLQGNLLKNACKVLTSRPDAVSALLRKFVRTELQLKGFSEEGIQLYLRKHHREPGV
ADRLIQLIQATSALHGLCHLPVFSWMVSRCHRELLLQNRGFPTTSTDMYLLILQHFLLHASPPDSSPLGLGPG
LLQSRLSTLLHLGHLALRGLAMSCYVFSAQQLQAAQVDSDDISLGFLVRAQSSVPGSKAPLEFLHITFQCFFA
AFYLAVSADTSVASLKHLFSCGRLGSSLLRRLLPNLCIQGSRVKKGSEAALLQKAEPHNLQITAAFLAGLLSQ
QHRDLLAACQVSERVLLQRQARARSCLAHSLREHFHSIPPAVPGETKSMHAMPGFIWLIRSLYEMQEEQLAQE
AVRRLDIGHLKLTFCRVGPAECAALAFVLQHLQRPVALQLDYNSVGDVGVEQLRPCLGVCTALYLRDNNISDR
GARTLVECALRCEQLQKLALFNNKLTDACACSMAKLLAHKQNFLSLRVGNNHITAAGAEVLAQGLKSNTSLKF
LGFWGNSVGDKGTQALAEVVADHQNLKWLSLVGNNIGSMGAEALALMLEKNKSLEELCLEENHICDEGVYSLA
EGLKRNSTLKFLKLSNNGITYRGAEALLQALSRNSAILEVWLRGNTFSLEEIQTLSSRDARLLL*
>rs45974600_ENSMUSP00000105262_c.1860:G>A_p.G621R
ENSMUST00000109634.3|Nod2-202|ENSMUSG00000055994|+|chr8:g.89391600:G>A

MRSSCCDMCSQEEFQAQRSQLVALLISGSLEGFESILDWLLSWDVLSREDYEGLSLPGQPLSHSARRLLDTVW
NKGVWGCQKLLEAVQEAQANSHTFELYGSWDTHSLHPTRDLQSHRPAIVRRLYNHVEAMLELAREGGFLSQYE
CEEIRLPIFTSSQRARRLLDLAAVKANGLAAFLLQHVRELPAPLPLPYEAAECQKFISKLRTMVLTQSRFLST
YDGSENLCLEDIYTENILELQTEVGTAGALQKSPAILGLEDLFDTHGHLNRDADTILVVGEAGSGKSTLLQRL
HLLWATGRSFQEFLFIFPFSCRQLQCVAKPLSLRTLLFEHCCWPDVAQDDVFQFLLDHPDRVLLTFDGLDEFK
FRFTDRERHCSPIDPTSVQTLLFNLLQGNLLKNACKVLTSRPDAVSALLRKFVRTELQLKGFSEEGIQLYLRK
HHREPGVADRLIQLIQATSALHGLCHLPVFSWMVSRCHRELLLQNRGFPTTSTDMYLLILQHFLLHASPPDSS
PLGLGPGLLQSRLSTLLHLGHLALRGLAMSCYVFSAQQLQAAQVDSDDISLGFLVRAQSSVPGSKAPLEFLHI
TFQCFFAAFYLAVSADTSVASLKHLFSCGRLGSSLLRRLLPNLCIQGSRVKKGSEAALLQKAEPHNLQITAAF
LAGLLSQQHRDLLAACQVSERVLLQRQARARSCLAHSLREHFHSIPPAVPGETKSMHAMPGFIWLIRSLYEMQ
EEQLAQEAVRRLDIGHLKLTFCRVGPAECAALAFVLQHLQRPVALQLDYNSVGDVGVEQLRPCLGVCTALYLR
DNNISDRGARTLVECALRCEQLQKLALFNNKLTDACACSMAKLLAHKQNFLSLRVGNNHITAAGAEVLAQGLK
SNTSLKFLGFWGNSVGDKGTQALAEVVADHQNLKWLSLVGNNIGSMGAEALALMLEKNKSLEELCLEENHICD
EGVYSLAEGLKRNSTLKFLKLSNNGITYRGAEALLQALSRNSAILEVWLRGNTFSLEEIQTLSSRDARLLL*
>rs45974600_ENSMUSP00000050538_c.1905:G>A_p.G636R
ENSMUST00000054324.15|Nod2-201|ENSMUSG00000055994|+|chr8:g.89391600:G>A
MCSQEEFQAQRSQLVALLISGSLEGFESILDWLLSWDVLSREDYEGLSLPGQPLSHSARRLLDTVWNKGVWGC
QKLLEAVQEAQANSHTFELYGSWDTHSLHPTRDLQSHRPAIVRRLYNHVEAMLELAREGGFLSQYECEEIRLP
IFTSSQRARRLLDLAAVKANGLAAFLLQHVRELPAPLPLPYEGYSLCRSRCDRGFTLICLFCILAAECQKFIS
KLRTMVLTQSRFLSTYDGSENLCLEDIYTENILELQTEVGTAGALQKSPAILGLEDLFDTHGHLNRDADTILV
VGEAGSGKSTLLQRLHLLWATGRSFQEFLFIFPFSCRQLQCVAKPLSLRTLLFEHCCWPDVAQDDVFQFLLDH
PDRVLLTFDGLDEFKFRFTDRERHCSPIDPTSVQTLLFNLLQGNLLKNACKVLTSRPDAVSALLRKFVRTELQ
LKGFSEEGIQLYLRKHHREPGVADRLIQLIQATSALHGLCHLPVFSWMVSRCHRELLLQNRGFPTTSTDMYLL
ILQHFLLHASPPDSSPLGLGPGLLQSRLSTLLHLGHLALRGLAMSCYVFSAQQLQAAQVDSDDISLGFLVRAQ
SSVPGSKAPLEFLHITFQCFFAAFYLAVSADTSVASLKHLFSCGRLGSSLLRRLLPNLCIQGSRVKKGSEAAL
LQKAEPHNLQITAAFLAGLLSQQHRDLLAACQVSERVLLQRQARARSCLAHSLREHFHSIPPAVPGETKSMHA
MPGFIWLIRSLYEMQEEQLAQEAVRRLDIGHLKLTFCRVGPAECAALAFVLQHLQRPVALQLDYNSVGDVGVE
QLRPCLGVCTALYLRDNNISDRGARTLVECALRCEQLQKLALFNNKLTDACACSMAKLLAHKQNFLSLRVGNN
HITAAGAEVLAQGLKSNTSLKFLGFWGNSVGDKGTQALAEVVADHQNLKWLSLVGNNIGSMGAEALALMLEKN
KSLEELCLEENHICDEGVYSLAEGLKRNSTLKFLKLSNNGITYRGAEALLQALSRNSAILEVWLRGNTFSLEE
IQTLSSRDARLLL*
>rs52365090_ENSMUSP00000113773_c.2427:G>A_p.E810K
ENSMUST00000118370.8|Nod2-203|ENSMUSG00000055994|+|chr8:g.89397085:G>A
MCSQEEFQAQRSQLVALLISGSLEGFESILDWLLSWDVLSREDYEGLSLPGQPLSHSARRLLDTVWNKGVWGC
QKLLEAVQEAQANSHTFELYGSWDTHSLHPTRDLQSHRPAIVRRLYNHVEAMLELAREGGFLSQYECEEIRLP
IFTSSQRARRLLDLAAVKANGLAAFLLQHVRELPAPLPLPYEAAECQKFISKLRTMVLTQSRFLSTYDGSENL
CLEDIYTENILELQTEVGTAGALQKSPAILGLEDLFDTHGHLNRDADTILVVGEAGSGKSTLLQRLHLLWATG
RSFQEFLFIFPFSCRQLQCVAKPLSLRTLLFEHCCWPDVAQDDVFQFLLDHPDRVLLTFDGLDEFKFRFTDRE
RHCSPIDPTSVQTLLFNLLQGNLLKNACKVLTSRPDAVSALLRKFVRTELQLKGFSEEGIQLYLRKHHREPGV
ADRLIQLIQATSALHGLCHLPVFSWMVSRCHRELLLQNRGFPTTSTDMYLLILQHFLLHASPPDSSPLGLGPG
LLQSRLSTLLHLGHLALRGLAMSCYVFSAQQLQAAQVDSDDISLGFLVRAQSSVPGSKAPLEFLHITFQCFFA
AFYLAVSADTSVASLKHLFSCGRLGSSLLGRLLPNLCIQGSRVKKGSEAALLQKAEPHNLQITAAFLAGLLSQ
QHRDLLAACQVSERVLLQRQARARSCLAHSLREHFHSIPPAVPGETKSMHAMPGFIWLIRSLYEMQEEQLAQE
AVRRLDIGHLKLTFCRVGPAECAALAFVLQHLQRPVALQLDYNSVGDVGVEQLRPCLGVCTALYLRDNNISDR
GARTLVKCALRCEQLQKLALFNNKLTDACACSMAKLLAHKQNFLSLRVGNNHITAAGAEVLAQGLKSNTSLKF
LGFWGNSVGDKGTQALAEVVADHQNLKWLSLVGNNIGSMGAEALALMLEKNKSLEELCLEENHICDEGVYSLA
EGLKRNSTLKFLKLSNNGITYRGAEALLQALSRNSAILEVWLRGNTFSLEEIQTLSSRDARLLL*
>rs52365090_ENSMUSP00000105262_c.2448:G>A_p.E817K
ENSMUST00000109634.3|Nod2-202|ENSMUSG00000055994|+|chr8:g.89397085:G>A
MRSSCCDMCSQEEFQAQRSQLVALLISGSLEGFESILDWLLSWDVLSREDYEGLSLPGQPLSHSARRLLDTVW
NKGVWGCQKLLEAVQEAQANSHTFELYGSWDTHSLHPTRDLQSHRPAIVRRLYNHVEAMLELAREGGFLSQYE
CEEIRLPIFTSSQRARRLLDLAAVKANGLAAFLLQHVRELPAPLPLPYEAAECQKFISKLRTMVLTQSRFLST
YDGSENLCLEDIYTENILELQTEVGTAGALQKSPAILGLEDLFDTHGHLNRDADTILVVGEAGSGKSTLLQRL
HLLWATGRSFQEFLFIFPFSCRQLQCVAKPLSLRTLLFEHCCWPDVAQDDVFQFLLDHPDRVLLTFDGLDEFK
FRFTDRERHCSPIDPTSVQTLLFNLLQGNLLKNACKVLTSRPDAVSALLRKFVRTELQLKGFSEEGIQLYLRK
HHREPGVADRLIQLIQATSALHGLCHLPVFSWMVSRCHRELLLQNRGFPTTSTDMYLLILQHFLLHASPPDSS
PLGLGPGLLQSRLSTLLHLGHLALRGLAMSCYVFSAQQLQAAQVDSDDISLGFLVRAQSSVPGSKAPLEFLHI
TFQCFFAAFYLAVSADTSVASLKHLFSCGRLGSSLLGRLLPNLCIQGSRVKKGSEAALLQKAEPHNLQITAAF

```
LAGLLSQQHRDLLAACQVSERVLLQRQARARSCLAHSLREHFHSIPPAVPGETKSMHAMPGFIWLIRSLYEMQ
EEQLAQEAVRRLDIGHLKLTFCRVGPAECAALAFVLQHLQRPVALQLDYNSVGDVGVEQLRPCLGVCTALYLR
DNNISDRGARTLVKCALRCEQLQKLALFNNKLTDACACSMAKLLAHKQNFLSLRVGNNHITAAGAEVLAQGLK
SNTSLKFLGFWGNSVGDKGTQALAEVVADHQNLKWLSLVGNNIGSMGAEALALMLEKNKSLEELCLEENHICD
EGVYSLAEGLKRNSTLKFLKLSNNGITYRGAEALLQALSRNSAILEVWLRGNTFSLEEIQTLSSRDARLLL*
>rs52365090_ENSMUSP00000050538_c.2493:G>A_p.E832K
ENSMUST00000054324.15|Nod2-201|ENSMUSG00000055994|+|chr8:g.89397085:G>A
MCSQEEFQAQRSQLVALLISGSLEGFESILDWLLSWDVLSREDYEGLSLPGQPLSHSARRLLDTVWNKGVWGC
QKLLEAVQEAQANSHTFELYGSWDTHSLHPTRDLQSHRPAIVRRLYNHVEAMLELAREGGFLSQYECEEIRLP
IFTSSQRARRLLDLAAVKANGLAAFLLQHVRELPAPLPLPYEGYSLCRSRCDRGFTLICLFCILAAECQKFIS
KLRTMVLTQSRFLSTYDGSENLCLEDIYTENILELQTEVGTAGALQKSPAILGLEDLFDTHGHLNRDADTILV
VGEAGSGKSTLLQRLHLLWATGRSFQEFLFIFPFSCRQLQCVAKPLSLRTLLFEHCCWPDVAQDDVFQFLLDH
PDRVLLTFDGLDEFKFRFTDRERHCSPIDPTSVQTLLFNLLQGNLLKNACKVLTSRPDAVSALLRKFVRTELQ
LKGFSEEGIQLYLRKHHREPGVADRLIQLIQATSALHGLCHLPVFSWMVSRCHRELLLQNRGFPTTSTDMYLL
ILQHFLLHASPPDSSPLGLGPGLLQSRLSTLLHLGHLALRGLAMSCYVFSAQQLQAAQVDSDDISLGFLVRAQ
SSVPGSKAPLEFLHITFQCFFAAFYLAVSADTSVASLKHLFSCGRLGSSLLGRLLPNLCIQGSRVKKGSEAAL
LQKAEPHNLQITAAFLAGLLSQQHRDLLAACQVSERVLLQRQARARSCLAHSLREHFHSIPPAVPGETKSMHA
MPGFIWLIRSLYEMQEEQLAQEAVRRLDIGHLKLTFCRVGPAECAALAFVLQHLQRPVALQLDYNSVGDVGVE
QLRPCLGVCTALYLRDNNISDRGARTLVKCALRCEQLQKLALFNNKLTDACACSMAKLLAHKQNFLSLRVGNN
HITAAGAEVLAQGLKSNTSLKFLGFWGNSVGDKGTQALAEVVADHQNLKWLSLVGNNIGSMGAEALALMLEKN
KSLEELCLEENHICDEGVYSLAEGLKRNSTLKFLKLSNNGITYRGAEALLQALSRNSAILEVWLRGNTFSLEE
IQTLSSRDARLLL*
>rs46944186_ENSMUSP00000113773_c.2544:C>A_p.L849M
ENSMUST00000118370.8|Nod2-203|ENSMUSG00000055994|+|chr8:g.89397370:C>A
MCSQEEFQAQRSQLVALLISGSLEGFESILDWLLSWDVLSREDYEGLSLPGQPLSHSARRLLDTVWNKGVWGC
QKLLEAVQEAQANSHTFELYGSWDTHSLHPTRDLQSHRPAIVRRLYNHVEAMLELAREGGFLSQYECEEIRLP
IFTSSQRARRLLDLAAVKANGLAAFLLQHVRELPAPLPLPYEAAECQKFISKLRTMVLTQSRFLSTYDGSENL
CLEDIYTENILELQTEVGTAGALQKSPAILGLEDLFDTHGHLNRDADTILVVGEAGSGKSTLLQRLHLLWATG
RSFQEFLFIFPFSCRQLQCVAKPLSLRTLLFEHCCWPDVAQDDVFQFLLDHPDRVLLTFDGLDEFKFRFTDRE
RHCSPIDPTSVQTLLFNLLQGNLLKNACKVLTSRPDAVSALLRKFVRTELQLKGFSEEGIQLYLRKHHREPGV
ADRLIQLIQATSALHGLCHLPVFSWMVSRCHRELLLQNRGFPTTSTDMYLLILQHFLLHASPPDSSPLGLGPG
LLQSRLSTLLHLGHLALRGLAMSCYVFSAQQLQAAQVDSDDISLGFLVRAQSSVPGSKAPLEFLHITFQCFFA
AFYLAVSADTSVASLKHLFSCGRLGSSLLGRLLPNLCIQGSRVKKGSEAALLQKAEPHNLQITAAFLAGLLSQ
QHRDLLAACQVSERVLLQRQARARSCLAHSLREHFHSIPPAVPGETKSMHAMPGFIWLIRSLYEMQEEQLAQE
AVRRLDIGHLKLTFCRVGPAECAALAFVLQHLQRPVALQLDYNSVGDVGVEQLRPCLGVCTALYLRDNNISDR
GARTLVECALRCEQLQKLALFNNKLTDACACSMAKLLAHKQNFLSMRVGNNHITAAGAEVLAQGLKSNTSLKF
LGFWGNSVGDKGTQALAEVVADHQNLKWLSLVGNNIGSMGAEALALMLEKNKSLEELCLEENHICDEGVYSLA
EGLKRNSTLKFLKLSNNGITYRGAEALLQALSRNSAILEVWLRGNTFSLEEIQTLSSRDARLLL*
>rs46944186_ENSMUSP00000105262_c.2565:C>A_p.L856M
ENSMUST00000109634.3|Nod2-202|ENSMUSG00000055994|+|chr8:g.89397370:C>A
MRSSCCDMCSQEEFQAQRSQLVALLISGSLEGFESILDWLLSWDVLSREDYEGLSLPGQPLSHSARRLLDTVW
NKGVWGCQKLLEAVQEAQANSHTFELYGSWDTHSLHPTRDLQSHRPAIVRRLYNHVEAMLELAREGGFLSQYE
CEEIRLPIFTSSQRARRLLDLAAVKANGLAAFLLQHVRELPAPLPLPYEAAECQKFISKLRTMVLTQSRFLST
YDGSENLCLEDIYTENILELQTEVGTAGALQKSPAILGLEDLFDTHGHLNRDADTILVVGEAGSGKSTLLQRL
HLLWATGRSFQEFLFIFPFSCRQLQCVAKPLSLRTLLFEHCCWPDVAQDDVFQFLLDHPDRVLLTFDGLDEFK
FRFTDRERHCSPIDPTSVQTLLFNLLQGNLLKNACKVLTSRPDAVSALLRKFVRTELQLKGFSEEGIQLYLRK
HHREPGVADRLIQLIQATSALHGLCHLPVFSWMVSRCHRELLLQNRGFPTTSTDMYLLILQHFLLHASPPDSS
PLGLGPGLLQSRLSTLLHLGHLALRGLAMSCYVFSAQQLQAAQVDSDDISLGFLVRAQSSVPGSKAPLEFLHI
TFQCFFAAFYLAVSADTSVASLKHLFSCGRLGSSLLGRLLPNLCIQGSRVKKGSEAALLQKAEPHNLQITAAF
LAGLLSQQHRDLLAACQVSERVLLQRQARARSCLAHSLREHFHSIPPAVPGETKSMHAMPGFIWLIRSLYEMQ
EEQLAQEAVRRLDIGHLKLTFCRVGPAECAALAFVLQHLQRPVALQLDYNSVGDVGVEQLRPCLGVCTALYLR
DNNISDRGARTLVECALRCEQLQKLALFNNKLTDACACSMAKLLAHKQNFLSMRVGNNHITAAGAEVLAQGLK
SNTSLKFLGFWGNSVGDKGTQALAEVVADHQNLKWLSLVGNNIGSMGAEALALMLEKNKSLEELCLEENHICD
EGVYSLAEGLKRNSTLKFLKLSNNGITYRGAEALLQALSRNSAILEVWLRGNTFSLEEIQTLSSRDARLLL*
>rs46944186_ENSMUSP00000050538_c.2610:C>A_p.L871M
ENSMUST00000054324.15|Nod2-201|ENSMUSG00000055994|+|chr8:g.89397370:C>A
MCSQEEFQAQRSQLVALLISGSLEGFESILDWLLSWDVLSREDYEGLSLPGQPLSHSARRLLDTVWNKGVWGC
QKLLEAVQEAQANSHTFELYGSWDTHSLHPTRDLQSHRPAIVRRLYNHVEAMLELAREGGFLSQYECEEIRLP
```

IFTSSQRARRLLDLAAVKANGLAAFLLQHVRELPAPLPLPYEGYSLCRSRCDRGFTLICLFCILAAECQKFIS
KLRTMVLTQSRFLSTYDGSENLCLEDIYTENILELQTEVGTAGALQKSPAILGLEDLFDTHGHLNRDADTILV
VGEAGSGKSTLLQRLHLLWATGRSFQEFLFIFPFSCRQLQCVAKPLSLRTLLFEHCCWPDVAQDDVFQFLLDH
PDRVLLTFDGLDEFKFRFTDRERHCSPIDPTSVQTLLFNLLQGNLLKNACKVLTSRPDAVSALLRKFVRTELQ
LKGFSEEGIQLYLRKHHREPGVADRLIQLIQATSALHGLCHLPVFSWMVSRCHRELLLQNRGFPTTSTDMYLL
ILQHFLLHASPPDSSPLGLGPGLLQSRLSTLLHLGHLALRGLAMSCYVFSAQQLQAAQVDSDDISLGFLVRAQ
SSVPGSKAPLEFLHITFQCFFAAFYLAVSADTSVASLKHLFSCGRLGSSLLGRLLPNLCIQGSRVKKGSEAAL
LQKAEPHNLQITAAFLAGLLSQQHRDLLAACQVSERVLLQRQARARSCLAHSLREHFHSIPPAVPGETKSMHA
MPGFIWLIRSLYEMQEEQLAQEAVRRLDIGHLKLTFCRVGPAECAALAFVLQHLQRPVALQLDYNSVGDVGVE
QLRPCLGVCTALYLRDNNISDRGARTLVECALRCEQLQKLALFNNKLTDACACSMAKLLAHKQNFLSMRVGNN
HITAAGAEVLAQGLKSNTSLKFLGFWGNSVGDKGTQALAEVVADHQNLKWLSLVGNNIGSMGAEALALMLEKN
KSLEELCLEENHICDEGVYSLAEGLKRNSTLKFLKLSNNGITYRGAEALLQALSRNSAILEVWLRGNTFSLEE
IQTLSSRDARLLL*
>rs50374660_ENSMUSP00000113773_c.2563:A>T_p.H855L
ENSMUST00000118370.8|Nod2-203|ENSMUSG00000055994|+|chr8:g.89399317:A>T
MCSQEEFQAQRSQLVALLISGSLEGFESILDWLLSWDVLSREDYEGLSLPGQPLSHSARRLLDTVWNKGVWGC
QKLLEAVQEAQANSHTFELYGSWDTHSLHPTRDLQSHRPAIVRRLYNHVEAMLELAREGGFLSQYECEEIRLP
IFTSSQRARRLLDLAAVKANGLAAFLLQHVRELPAPLPLPYEAAECQKFISKLRTMVLTQSRFLSTYDGSENL
CLEDIYTENILELQTEVGTAGALQKSPAILGLEDLFDTHGHLNRDADTILVVGEAGSGKSTLLQRLHLLWATG
RSFQEFLFIFPFSCRQLQCVAKPLSLRTLLFEHCCWPDVAQDDVFQFLLDHPDRVLLTFDGLDEFKFRFTDRE
RHCSPIDPTSVQTLLFNLLQGNLLKNACKVLTSRPDAVSALLRKFVRTELQLKGFSEEGIQLYLRKHHREPGV
ADRLIQLIQATSALHGLCHLPVFSWMVSRCHRELLLQNRGFPTTSTDMYLLILQHFLLHASPPDSSPLGLGPG
LLQSRLSTLLHLGHLALRGLAMSCYVFSAQQLQAAQVDSDDISLGFLVRAQSSVPGSKAPLEFLHITFQCFFA
AFYLAVSADTSVASLKHLFSCGRLGSSLLGRLLPNLCIQGSRVKKGSEAALLQKAEPHNLQITAAFLAGLLSQ
QHRDLLAACQVSERVLLQRQARARSCLAHSLREHFHSIPPAVPGETKSMHAMPGFIWLIRSLYEMQEEQLAQE
AVRRLDIGHLKLTFCRVGPAECAALAFVLQHLQRPVALQLDYNSVGDVGVEQLRPCLGVCTALYLRDNNISDR
GARTLVECALRCEQLQKLALFNNKLTDACACSMAKLLAHKQNFLSLRVGNNLITAAGAEVLAQGLKSNTSLKF
LGFWGNSVGDKGTQALAEVVADHQNLKWLSLVGNNIGSMGAEALALMLEKNKSLEELCLEENHICDEGVYSLA
EGLKRNSTLKFLKLSNNGITYRGAEALLQALSRNSAILEVWLRGNTFSLEEIQTLSSRDARLLL*
>rs50374660_ENSMUSP00000105262_c.2584:A>T_p.H862L
ENSMUST00000109634.3|Nod2-202|ENSMUSG00000055994|+|chr8:g.89399317:A>T
MRSSCCDMCSQEEFQAQRSQLVALLISGSLEGFESILDWLLSWDVLSREDYEGLSLPGQPLSHSARRLLDTVW
NKGVWGCQKLLEAVQEAQANSHTFELYGSWDTHSLHPTRDLQSHRPAIVRRLYNHVEAMLELAREGGFLSQYE
CEEIRLPIFTSSQRARRLLDLAAVKANGLAAFLLQHVRELPAPLPLPYEAAECQKFISKLRTMVLTQSRFLST
YDGSENLCLEDIYTENILELQTEVGTAGALQKSPAILGLEDLFDTHGHLNRDADTILVVGEAGSGKSTLLQRL
HLLWATGRSFQEFLFIFPFSCRQLQCVAKPLSLRTLLFEHCCWPDVAQDDVFQFLLDHPDRVLLTFDGLDEFK
FRFTDRERHCSPIDPTSVQTLLFNLLQGNLLKNACKVLTSRPDAVSALLRKFVRTELQLKGFSEEGIQLYLRK
HHREPGVADRLIQLIQATSALHGLCHLPVFSWMVSRCHRELLLQNRGFPTTSTDMYLLILQHFLLHASPPDSS
PLGLGPGLLQSRLSTLLHLGHLALRGLAMSCYVFSAQQLQAAQVDSDDISLGFLVRAQSSVPGSKAPLEFLHI
TFQCFFAAFYLAVSADTSVASLKHLFSCGRLGSSLLGRLLPNLCIQGSRVKKGSEAALLQKAEPHNLQITAAF
LAGLLSQQHRDLLAACQVSERVLLQRQARARSCLAHSLREHFHSIPPAVPGETKSMHAMPGFIWLIRSLYEMQ
EEQLAQEAVRRLDIGHLKLTFCRVGPAECAALAFVLQHLQRPVALQLDYNSVGDVGVEQLRPCLGVCTALYLR
DNNISDRGARTLVECALRCEQLQKLALFNNKLTDACACSMAKLLAHKQNFLSLRVGNNLITAAGAEVLAQGLK
SNTSLKFLGFWGNSVGDKGTQALAEVVADHQNLKWLSLVGNNIGSMGAEALALMLEKNKSLEELCLEENHICD
EGVYSLAEGLKRNSTLKFLKLSNNGITYRGAEALLQALSRNSAILEVWLRGNTFSLEEIQTLSSRDARLLL*
>rs50374660_ENSMUSP00000050538_c.2629:A>T_p.H877L
ENSMUST00000054324.15|Nod2-201|ENSMUSG00000055994|+|chr8:g.89399317:A>T
MCSQEEFQAQRSQLVALLISGSLEGFESILDWLLSWDVLSREDYEGLSLPGQPLSHSARRLLDTVWNKGVWGC
QKLLEAVQEAQANSHTFELYGSWDTHSLHPTRDLQSHRPAIVRRLYNHVEAMLELAREGGFLSQYECEEIRLP
IFTSSQRARRLLDLAAVKANGLAAFLLQHVRELPAPLPLPYEGYSLCRSRCDRGFTLICLFCILAAECQKFIS
KLRTMVLTQSRFLSTYDGSENLCLEDIYTENILELQTEVGTAGALQKSPAILGLEDLFDTHGHLNRDADTILV
VGEAGSGKSTLLQRLHLLWATGRSFQEFLFIFPFSCRQLQCVAKPLSLRTLLFEHCCWPDVAQDDVFQFLLDH
PDRVLLTFDGLDEFKFRFTDRERHCSPIDPTSVQTLLFNLLQGNLLKNACKVLTSRPDAVSALLRKFVRTELQ
LKGFSEEGIQLYLRKHHREPGVADRLIQLIQATSALHGLCHLPVFSWMVSRCHRELLLQNRGFPTTSTDMYLL
ILQHFLLHASPPDSSPLGLGPGLLQSRLSTLLHLGHLALRGLAMSCYVFSAQQLQAAQVDSDDISLGFLVRAQ
SSVPGSKAPLEFLHITFQCFFAAFYLAVSADTSVASLKHLFSCGRLGSSLLGRLLPNLCIQGSRVKKGSEAAL
LQKAEPHNLQITAAFLAGLLSQQHRDLLAACQVSERVLLQRQARARSCLAHSLREHFHSIPPAVPGETKSMHA
MPGFIWLIRSLYEMQEEQLAQEAVRRLDIGHLKLTFCRVGPAECAALAFVLQHLQRPVALQLDYNSVGDVGVE

QLRPCLGVCTALYLRDNNISDRGARTLVECALRCEQLQKLALFNNKLTDACACSMAKLLAHKQNFLSLRVGNN
LITAAGAEVLAQGLKSNTSLKFLGFWGNSVGDKGTQALAEVVADHQNLKWLSLVGNNIGSMGAEALALMLEKN
KSLEELCLEENHICDEGVYSLAEGLKRNSTLKFLKLSNNGITYRGAEALLQALSRNSAILEVWLRGNTFSLEE
IQTLSSRDARLLL*
>rs33277582_ENSMUSP00000113773_c.2770:T>C_p.L924P
ENSMUST00000118370.8|Nod2-203|ENSMUSG00000055994|+|chr8:g.89402128:T>C
MCSQEEFQAQRSQLVALLISGSLEGFESILDWLLSWDVLSREDYEGLSLPGQPLSHSARRLLDTVWNKGVWGC
QKLLEAVQEAQANSHTFELYGSWDTHSLHPTRDLQSHRPAIVRRLYNHVEAMLELAREGGFLSQYECEEIRLP
IFTSSQRARRLLDLAAVKANGLAAFLLQHVRELPAPLPLPYEAAECQKFISKLRTMVLTQSRFLSTYDGSENL
CLEDIYTENILELQTEVGTAGALQKSPAILGLEDLFDTHGHLNRDADTILVVGEAGSGKSTLLQRLHLLWATG
RSFQEFLFIFPFSCRQLQCVAKPLSLRTLLFEHCCWPDVAQDDVFQFLLDHPDRVLLTFDGLDEFKFRFTDRE
RHCSPIDPTSVQTLLFNLLQGNLLKNACKVLTSRPDAVSALLRKFVRTELQLKGFSEEGIQLYLRKHHREPGV
ADRLIQLIQATSALHGLCHLPVFSWMVSRCHRELLLQNRGFPTTSTDMYLLILQHFLLHASPPDSSPLGLGPG
LLQSRLSTLLHLGHLALRGLAMSCYVFSAQQLQAAQVDSDDISLGFLVRAQSSVPGSKAPLEFLHITFQCFFA
AFYLAVSADTSVASLKHLFSCGRLGSSLLGRLLPNLCIQGSRVKKGSEAALLQKAEPHNLQITAAFLAGLLSQ
QHRDLLAACQVSERVLLQRQARARSCLAHSLREHFHSIPPAVPGETKSMHAMPGFIWLIRSLYEMQEEQLAQE
AVRRLDIGHLKLTFCRVGPAECAALAFVLQHLQRPVALQLDYNSVGDVGVEQLRPCLGVCTALYLRDNNISDR
GARTLVECALRCEQLQKLALFNNKLTDACACSMAKLLAHKQNFLSLRVGNNHITAAGAEVLAQGLKSNTSLKF
LGFWGNSVGDKGTQALAEVVADHQNLKWLSLVGNNIGSMGAEALALMPEKNKSLEELCLEENHICDEGVYSLA
EGLKRNSTLKFLKLSNNGITYRGAEALLQALSRNSAILEVWLRGNTFSLEEIQTLSSRDARLLL*
>rs33277582_ENSMUSP00000105262_c.2791:T>C_p.L931P
ENSMUST00000109634.3|Nod2-202|ENSMUSG00000055994|+|chr8:g.89402128:T>C
MRSSCCDMCSQEEFQAQRSQLVALLISGSLEGFESILDWLLSWDVLSREDYEGLSLPGQPLSHSARRLLDTVW
NKGVWGCQKLLEAVQEAQANSHTFELYGSWDTHSLHPTRDLQSHRPAIVRRLYNHVEAMLELAREGGFLSQYE
CEEIRLPIFTSSQRARRLLDLAAVKANGLAAFLLQHVRELPAPLPLPYEAAECQKFISKLRTMVLTQSRFLST
YDGSENLCLEDIYTENILELQTEVGTAGALQKSPAILGLEDLFDTHGHLNRDADTILVVGEAGSGKSTLLQRL
HLLWATGRSFQEFLFIFPFSCRQLQCVAKPLSLRTLLFEHCCWPDVAQDDVFQFLLDHPDRVLLTFDGLDEFK
FRFTDRERHCSPIDPTSVQTLLFNLLQGNLLKNACKVLTSRPDAVSALLRKFVRTELQLKGFSEEGIQLYLRK
HHREPGVADRLIQLIQATSALHGLCHLPVFSWMVSRCHRELLLQNRGFPTTSTDMYLLILQHFLLHASPPDSS
PLGLGPGLLQSRLSTLLHLGHLALRGLAMSCYVFSAQQLQAAQVDSDDISLGFLVRAQSSVPGSKAPLEFLHI
TFQCFFAAFYLAVSADTSVASLKHLFSCGRLGSSLLGRLLPNLCIQGSRVKKGSEAALLQKAEPHNLQITAAF
LAGLLSQQHRDLLAACQVSERVLLQRQARARSCLAHSLREHFHSIPPAVPGETKSMHAMPGFIWLIRSLYEMQ
EEQLAQEAVRRLDIGHLKLTFCRVGPAECAALAFVLQHLQRPVALQLDYNSVGDVGVEQLRPCLGVCTALYLR
DNNISDRGARTLVECALRCEQLQKLALFNNKLTDACACSMAKLLAHKQNFLSLRVGNNHITAAGAEVLAQGLK
SNTSLKFLGFWGNSVGDKGTQALAEVVADHQNLKWLSLVGNNIGSMGAEALALMPEKNKSLEELCLEENHICD
EGVYSLAEGLKRNSTLKFLKLSNNGITYRGAEALLQALSRNSAILEVWLRGNTFSLEEIQTLSSRDARLLL*
>rs33277582_ENSMUSP00000050538_c.2836:T>C_p.L946P
ENSMUST00000054324.15|Nod2-201|ENSMUSG00000055994|+|chr8:g.89402128:T>C
MCSQEEFQAQRSQLVALLISGSLEGFESILDWLLSWDVLSREDYEGLSLPGQPLSHSARRLLDTVWNKGVWGC
QKLLEAVQEAQANSHTFELYGSWDTHSLHPTRDLQSHRPAIVRRLYNHVEAMLELAREGGFLSQYECEEIRLP
IFTSSQRARRLLDLAAVKANGLAAFLLQHVRELPAPLPLPYEGYSLCRSRCDRGFTLICLFCILAAECQKFIS
KLRTMVLTQSRFLSTYDGSENLCLEDIYTENILELQTEVGTAGALQKSPAILGLEDLFDTHGHLNRDADTILV
VGEAGSGKSTLLQRLHLLWATGRSFQEFLFIFPFSCRQLQCVAKPLSLRTLLFEHCCWPDVAQDDVFQFLLDH
PDRVLLTFDGLDEFKFRFTDRERHCSPIDPTSVQTLLFNLLQGNLLKNACKVLTSRPDAVSALLRKFVRTELQ
LKGFSEEGIQLYLRKHHREPGVADRLIQLIQATSALHGLCHLPVFSWMVSRCHRELLLQNRGFPTTSTDMYLL
ILQHFLLHASPPDSSPLGLGPGLLQSRLSTLLHLGHLALRGLAMSCYVFSAQQLQAAQVDSDDISLGFLVRAQ
SSVPGSKAPLEFLHITFQCFFAAFYLAVSADTSVASLKHLFSCGRLGSSLLGRLLPNLCIQGSRVKKGSEAAL
LQKAEPHNLQITAAFLAGLLSQQHRDLLAACQVSERVLLQRQARARSCLAHSLREHFHSIPPAVPGETKSMHA
MPGFIWLIRSLYEMQEEQLAQEAVRRLDIGHLKLTFCRVGPAECAALAFVLQHLQRPVALQLDYNSVGDVGVE
QLRPCLGVCTALYLRDNNISDRGARTLVECALRCEQLQKLALFNNKLTDACACSMAKLLAHKQNFLSLRVGNN
HITAAGAEVLAQGLKSNTSLKFLGFWGNSVGDKGTQALAEVVADHQNLKWLSLVGNNIGSMGAEALALMPEKN
KSLEELCLEENHICDEGVYSLAEGLKRNSTLKFLKLSNNGITYRGAEALLQALSRNSAILEVWLRGNTFSLEE
IQTLSSRDARLLL*
>rs229254609_ENSMUSP00000148037_c.1710:ACT>A_p.T571del-fs
ENSMUST00000211554.2|Cyld-210|ENSMUSG00000036712|+|chr8:g.89458341:ACT>A
MSSGLWSQEKVTSPYWEERIFYLLLQECSVTDKQTQKLLKVPKGSIGQYIQDRSVGHSRVPSTKGKKNQIGLK
ILEQPHAVLFVDEKDVVEINEKFTELLLAITNCEERLSLFRNRLRLSKGLQVDVGSPVKVQLRSGEEKFPGVV
RFRGPLLAERTVSGIFFGVELLEEGRGQGFTDGVYQGKQLFQCDEDCGVFVALDKLELIEDDDNGLESDFAGP

```
GDTMQVEPPPLEINSRVSLKVGESTESGTVIFCDVLPGKESLGYFVGVDMDNPIGNWDGRFDGVQLCSFASVE
STILLHINDIIPDSVTQERRPPKLAFMSRGVGDKGSSSHNKPKVTGSTSDPGSRNRSELFYTLNGSSVDSQQS
KSKNPWYIDEVAEDPAKSLTEMSSDFGHSSPPPQPPSMNSLSSENRFHSLPFSLTKMPNTNGSMAHSPLSLSV
QSVMGELNSTPVQESPPLPISSGNAHGLEVGSLAEVKENPPFYGVIRWIGQPPGLSDVLAGLELEDECAGCTD
GTFRGTRYFTCALKKALFVKLKSCRPDSRFASLQPVSNQIERCNSLAFGGYLSEVVEENTT
>rs229254609_ENSMUSP00000147654_c.1719:ACT>A_p.T574del-fs
ENSMUST00000209532.2|Cyld-205|ENSMUSG00000036712|+|chr8:g.89458341:ACT>A
MSSGLWSQEKVTSPYWEERIFYLLLQECSVTDKQTQKLLKVPKGSIGQYIQDRSVGHSRVPSTKGKKNQIGLK
ILEQPHAVLFVDEKDVVEINEKFTELLLAITNCEERLSLFRNRLRLSKGLQVDVGSPVKVQLRSGEEKFPGVV
RFRGPLLAERTVSGIFFGVELLEEGRGQGFTDGVYQGKQLFQCDEDCGVFVALDKLELIEDDDNGLESDFAGP
GDTMQVEPPPLEINSRVSLKVGESTESGTVIFCDVLPGKESLGYFVGVDMDNPIGNWDGRFDGVQLCSFASVE
STILLHINDIIPALSDSVTQERRPPKLAFMSRGVGDKGSSSHNKPKVTGSTSDPGSRNRSELFYTLNGSSVDS
QQSKSKNPWYIDEVAEDPAKSLTEMSSDFGHSSPPPQPPSMNSLSSENRFHSLPFSLTKMPNTNGSMAHSPLS
LSVQSVMGELNSTPVQESPPLPISSGNAHGLEVGSLAEVKENPPFYGVIRWIGQPPGLSDVLAGLELEDECAG
CTDGTFRGTRYFTCALKKALFVKLKSCRPDSRFASLQPVSNQIERCNSLAFGGYLSEVVEENTT
>rs229254609_ENSMUSP00000147426_c.1710:ACT>A_p.T571del-fs
ENSMUST00000209559.2|Cyld-206|ENSMUSG00000036712|+|chr8:g.89458341:ACT>A
MSSGLWSQEKVTSPYWEERIFYLLLQECSVTDKQTQKLLKVPKGSIGQYIQDRSVGHSRVPSTKGKKNQIGLK
ILEQPHAVLFVDEKDVVEINEKFTELLLAITNCEERLSLFRNRLRLSKGLQVDVGSPVKVQLRSGEEKFPGVV
RFRGPLLAERTVSGIFFGVELLEEGRGQGFTDGVYQGKQLFQCDEDCGVFVALDKLELIEDDDNGLESDFAGP
GDTMQVEPPPLEINSRVSLKVGESTESGTVIFCDVLPGKESLGYFVGVDMDNPIGNWDGRFDGVQLCSFASVE
STILLHINDIIPDSVTQERRPPKLAFMSRGVGDKGSSSHNKPKVTGSTSDPGSRNRSELFYTLNGSSVDSQQS
KSKNPWYIDEVAEDPAKSLTEMSSDFGHSSPPPQPPSMNSLSSENRFHSLPFSLTKMPNTNGSMAHSPLSLSV
QSVMGELNSTPVQESPPLPISSGNAHGLEVGSLAEVKENPPFYGVIRWIGQPPGLSDVLAGLELEDECAGCTD
GTFRGTRYFTCALKKALFVKLKSCRPDSRFASLQPVSNQIERCNSLAFGGYLSEVVEENTT
>rs229254609_ENSMUSP00000147904_c.1164:ACT>A_p.T389del-fs
ENSMUST00000209206.2|Cyld-204|ENSMUSG00000036712|+|chr8:g.89458341:ACT>A
MSSGLWSQEKVTSPYWEERIFYLLLQECSVTDKQTQKLLKVPKGSIGQYIQDRSVGHSRVPSTKGKKNQIGLK
ILEQPHAVLFVDEKDVVEINEKFTELLLAITNCEERLSLFRNRLRLSKGLQVDVGSPVKVQLRSGEEKFPGVV
RFRGPLLAERTVSGIFFGVELLEEGRGQGFTDGVYQGKQLFQCDEDCGVFVALDKLELIEDDDNGLESDFAGP
GDTMQVEPPPLEINSRVSLKVGESTESGTVIFCDVLPGKESLGYFVGVDMDNPIGNWDGRFDGVQLCSFASVE
STILLHINDIIPDSVTQERRPPKLAFMSRGVGDKGSSSHNKPKVTGSTSDPGSRNRSELFYTLNGSSVDSQQS
KSKNPWYIDEAFGGYLSEVVEENTT
>rs229254609_ENSMUSP00000105254_c.1710:ACT>A_p.T571del-fs
ENSMUST00000109626.4|Cyld-203|ENSMUSG00000036712|+|chr8:g.89458341:ACT>A
MSSGLWSQEKVTSPYWEERIFYLLLQECSVTDKQTQKLLKVPKGSIGQYIQDRSVGHSRVPSTKGKKNQIGLK
ILEQPHAVLFVDEKDVVEINEKFTELLLAITNCEERLSLFRNRLRLSKGLQVDVGSPVKVQLRSGEEKFPGVV
RFRGPLLAERTVSGIFFGVELLEEGRGQGFTDGVYQGKQLFQCDEDCGVFVALDKLELIEDDDNGLESDFAGP
GDTMQVEPPPLEINSRVSLKVGESTESGTVIFCDVLPGKESLGYFVGVDMDNPIGNWDGRFDGVQLCSFASVE
STILLHINDIIPDSVTQERRPPKLAFMSRGVGDKGSSSHNKPKVTGSTSDPGSRNRSELFYTLNGSSVDSQQS
KSKNPWYIDEVAEDPAKSLTEMSSDFGHSSPPPQPPSMNSLSSENRFHSLPFSLTKMPNTNGSMAHSPLSLSV
QSVMGELNSTPVQESPPLPISSGNAHGLEVGSLAEVKENPPFYGVIRWIGQPPGLSDVLAGLELEDECAGCTD
GTFRGTRYFTCALKKALFVKLKSCRPDSRFASLQPVSNQIERCNSLAFGGYLSEVVEENTT
>rs229254609_ENSMUSP00000096119_c.1719:ACT>A_p.T574del-fs
ENSMUST00000098519.11|Cyld-202|ENSMUSG00000036712|+|chr8:g.89458341:ACT>A
MSSGLWSQEKVTSPYWEERIFYLLLQECSVTDKQTQKLLKVPKGSIGQYIQDRSVGHSRVPSTKGKKNQIGLK
ILEQPHAVLFVDEKDVVEINEKFTELLLAITNCEERLSLFRNRLRLSKGLQVDVGSPVKVQLRSGEEKFPGVV
RFRGPLLAERTVSGIFFGVELLEEGRGQGFTDGVYQGKQLFQCDEDCGVFVALDKLELIEDDDNGLESDFAGP
GDTMQVEPPPLEINSRVSLKVGESTESGTVIFCDVLPGKESLGYFVGVDMDNPIGNWDGRFDGVQLCSFASVE
STILLHINDIIPALSDSVTQERRPPKLAFMSRGVGDKGSSSHNKPKVTGSTSDPGSRNRSELFYTLNGSSVDS
QQSKSKNPWYIDEVAEDPAKSLTEMSSDFGHSSPPPQPPSMNSLSSENRFHSLPFSLTKMPNTNGSMAHSPLS
LSVQSVMGELNSTPVQESPPLPISSGNAHGLEVGSLAEVKENPPFYGVIRWIGQPPGLSDVLAGLELEDECAG
CTDGTFRGTRYFTCALKKALFVKLKSCRPDSRFASLQPVSNQIERCNSLAFGGYLSEVVEENTT
>rs229254609_ENSMUSP00000039834_c.1719:ACT>A_p.T574del-fs
ENSMUST00000043526.15|Cyld-201|ENSMUSG00000036712|+|chr8:g.89458341:ACT>A
MSSGLWSQEKVTSPYWEERIFYLLLQECSVTDKQTQKLLKVPKGSIGQYIQDRSVGHSRVPSTKGKKNQIGLK
ILEQPHAVLFVDEKDVVEINEKFTELLLAITNCEERLSLFRNRLRLSKGLQVDVGSPVKVQLRSGEEKFPGVV
RFRGPLLAERTVSGIFFGVELLEEGRGQGFTDGVYQGKQLFQCDEDCGVFVALDKLELIEDDDNGLESDFAGP
```

GDTMQVEPPPLEINSRVSLKVGESTESGTVIFCDVLPGKESLGYFVGVDMDNPIGNWDGRFDGVQLCSFASVE
STILLHINDIIPAVSDSVTQERRPPKLAFMSRGVGDKGSSSHNKPKVTGSTSDPGSRNRSELFYTLNGSSVDS
QQSKSKNPWYIDEVAEDPAKSLTEMSSDFGHSSPPPQPPSMNSLSSENRFHSLPFSLTKMPNTNGSMAHSPLS
LSVQSVMGELNSTPVQESPPLPISSGNAHGLEVGSLAEVKENPPFYGVIRWIGQPPGLSDVLAGLELEDECAG
CTDGTFRGTRYFTCALKKALFVKLKSCRPDSRFASLQPVSNQIERCNSLAFGGYLSEVVEENTT
>rs32562371_ENSMUSP00000148037_c.2737:A>G_p.K913R
ENSMUST00000211554.2|Cyld-210|ENSMUSG00000036712|+|chr8:g.89473520:A>G
MSSGLWSQEKVTSPYWEERIFYLLLQECSVTDKQTQKLLKVPKGSIGQYIQDRSVGHSRVPSTKGKKNQIGLK
ILEQPHAVLFVDEKDVVEINEKFTELLLAITNCEERLSLFRNRLRLSKGLQVDVGSPVKVQLRSGEEKFPGVV
RFRGPLLAERTVSGIFFGVELLEEGRGQGFTDGVYQGKQLFQCDEDCGVFVALDKLELIEDDDNGLESDFAGP
GDTMQVEPPPLEINSRVSLKVGESTESGTVIFCDVLPGKESLGYFVGVDMDNPIGNWDGRFDGVQLCSFASVE
STILLHINDIIPDSVTQERRPPKLAFMSRGVGDKGSSSHNKPKVTGSTSDPGSRNRSELFYTLNGSSVDSQQS
KSKNPWYIDEVAEDPAKSLTEMSSDFGHSSPPPQPPSMNSLSSENRFHSLPFSLTKMPNTNGSMAHSPLSLSV
QSVMGELNSTPVQESPPLPISSGNAHGLEVGSLAEVKENPPFYGVIRWIGQPPGLSDVLAGLELEDECAGCTD
GTFRGTRYFTCALKKALFVKLKSCRPDSRFASLQPVSNQIERCNSLAFGGYLSEVVEENTPPKMEKEGLEIMI
GKKKGIQGHYNSCYLDSTLFCLFAFSSALDTVLLRPKEKNDIEYYSETQELLRTEIVNPLRIYGYVCATKIMK
LRKILEKVEAASGFTSEEKDPEEFLNILFHDILRVEPLLKIRSAGQKVQDCNFYQIFMEKNEKVGVPTIQQLL
EWSFINSNLKFAEAPSCLIIQMPRFGKDFKLFKKIFPSLELNITDLLEDTPRQCRICGGLAMYECRECYDDPD
ISAGKIKQFCKTCSTQVHLHPRRLNHSYHPVSLPKDLPDWDWRHGCIPCQKMELFAVLCIETSHYVAFVKYGK
DDSAWLFFDSMADRDGGQNGFNIPQVTPCPEVGEYLRMSLEDLHSLDSRRIQGCARRLLCDAYMCMYQSPTMS
LYK*
>rs32562371_ENSMUSP00000147654_c.2746:A>G_p.K916R
ENSMUST00000209532.2|Cyld-205|ENSMUSG00000036712|+|chr8:g.89473520:A>G
MSSGLWSQEKVTSPYWEERIFYLLLQECSVTDKQTQKLLKVPKGSIGQYIQDRSVGHSRVPSTKGKKNQIGLK
ILEQPHAVLFVDEKDVVEINEKFTELLLAITNCEERLSLFRNRLRLSKGLQVDVGSPVKVQLRSGEEKFPGVV
RFRGPLLAERTVSGIFFGVELLEEGRGQGFTDGVYQGKQLFQCDEDCGVFVALDKLELIEDDDNGLESDFAGP
GDTMQVEPPPLEINSRVSLKVGESTESGTVIFCDVLPGKESLGYFVGVDMDNPIGNWDGRFDGVQLCSFASVE
STILLHINDIIPALSDSVTQERRPPKLAFMSRGVGDKGSSSHNKPKVTGSTSDPGSRNRSELFYTLNGSSVDS
QQSKSKNPWYIDEVAEDPAKSLTEMSSDFGHSSPPPQPPSMNSLSSENRFHSLPFSLTKMPNTNGSMAHSPLS
LSVQSVMGELNSTPVQESPPLPISSGNAHGLEVGSLAEVKENPPFYGVIRWIGQPPGLSDVLAGLELEDECAG
CTDGTFRGTRYFTCALKKALFVKLKSCRPDSRFASLQPVSNQIERCNSLAFGGYLSEVVEENTPPKMEKEGLE
IMIGKKKGIQGHYNSCYLDSTLFCLFAFSSALDTVLLRPKEKNDIEYYSETQELLRTEIVNPLRIYGYVCATK
IMKLRKILEKVEAASGFTSEEKDPEEFLNILFHDILRVEPLLKIRSAGQKVQDCNFYQIFMEKNEKVGVPTIQ
QLLEWSFINSNLKFAEAPSCLIIQMPRFGKDFKLFKKIFPSLELNITDLLEDTPRQCRICGGLAMYECRECYD
DPDISAGKIKQFCKTCSTQVHLHPRRLNHSYHPVSLPKDLPDWDWRHGCIPCQKMELFAVLCIETSHYVAFVK
YGKDDSAWLFFDSMADRDGGQNGFNIPQVTPCPEVGEYLRMSLEDLHSLDSRRIQGCARRLLCDAYMCMYQSP
TMSLYK*
>rs32562371_ENSMUSP00000147426_c.2737:A>G_p.K913R
ENSMUST00000209559.2|Cyld-206|ENSMUSG00000036712|+|chr8:g.89473520:A>G
MSSGLWSQEKVTSPYWEERIFYLLLQECSVTDKQTQKLLKVPKGSIGQYIQDRSVGHSRVPSTKGKKNQIGLK
ILEQPHAVLFVDEKDVVEINEKFTELLLAITNCEERLSLFRNRLRLSKGLQVDVGSPVKVQLRSGEEKFPGVV
RFRGPLLAERTVSGIFFGVELLEEGRGQGFTDGVYQGKQLFQCDEDCGVFVALDKLELIEDDDNGLESDFAGP
GDTMQVEPPPLEINSRVSLKVGESTESGTVIFCDVLPGKESLGYFVGVDMDNPIGNWDGRFDGVQLCSFASVE
STILLHINDIIPDSVTQERRPPKLAFMSRGVGDKGSSSHNKPKVTGSTSDPGSRNRSELFYTLNGSSVDSQQS
KSKNPWYIDEVAEDPAKSLTEMSSDFGHSSPPPQPPSMNSLSSENRFHSLPFSLTKMPNTNGSMAHSPLSLSV
QSVMGELNSTPVQESPPLPISSGNAHGLEVGSLAEVKENPPFYGVIRWIGQPPGLSDVLAGLELEDECAGCTD
GTFRGTRYFTCALKKALFVKLKSCRPDSRFASLQPVSNQIERCNSLAFGGYLSEVVEENTPPKMEKEGLEIMI
GKKKGIQGHYNSCYLDSTLFCLFAFSSALDTVLLRPKEKNDIEYYSETQELLRTEIVNPLRIYGYVCATKIMK
LRKILEKVEAASGFTSEEKDPEEFLNILFHDILRVEPLLKIRSAGQKVQDCNFYQIFMEKNEKVGVPTIQQLL
EWSFINSNLKFAEAPSCLIIQMPRFGKDFKLFKKIFPSLELNITDLLEDTPRQCRICGGLAMYECRECYDDPD
ISAGKIKQFCKTCSTQVHLHPRRLNHSYHPVSLPKDLPDWDWRHGCIPCQKMELFAVLCIETSHYVAFVKYGK
DDSAWLFFDSMADRDGGQNGFNIPQVTPCPEVGEYLRMSLEDLHSLDSRRIQGCARRLLCDAYMCMYQSPTMS
LYK*
>rs32562371_ENSMUSP00000147904_c.2191:A>G_p.K731R
ENSMUST00000209206.2|Cyld-204|ENSMUSG00000036712|+|chr8:g.89473520:A>G
MSSGLWSQEKVTSPYWEERIFYLLLQECSVTDKQTQKLLKVPKGSIGQYIQDRSVGHSRVPSTKGKKNQIGLK
ILEQPHAVLFVDEKDVVEINEKFTELLLAITNCEERLSLFRNRLRLSKGLQVDVGSPVKVQLRSGEEKFPGVV
RFRGPLLAERTVSGIFFGVELLEEGRGQGFTDGVYQGKQLFQCDEDCGVFVALDKLELIEDDDNGLESDFAGP

GDTMQVEPPPLEINSRVSLKVGESTESGTVIFCDVLPGKESLGYFVGVDMDNPIGNWDGRFDGVQLCSFASVE
STILLHINDIIPDSVTQERRPPKLAFMSRGVGDKGSSSHNKPKVTGSTSDPGSRNRSELFYTLNGSSVDSQQS
KSKNPWYIDEAFGGYLSEVVEENTPPKMEKEGLEIMIGKKKGIQGHYNSCYLDSTLFCLFAFSSALDTVLLRP
KEKNDIEYYSETQELLRTEIVNPLRIYGYVCATKIMKLRKILEKVEAASGFTSEEKDPEEFLNILFHDILRVE
PLLKIRSAGQKVQDCNFYQIFMEKNEKVGVPTIQQLLEWSFINSNLKFAEAPSCLIIQMPRFGKDFKLFKKIF
PSLELNITDLLEDTPRQCRICGGLAMYECRECYDDPDISAGKIKQFCKTCSTQVHLHPRRLNHSYHPVSLPKD
LPDWDWRHGCIPCQKMELFAVLCIETSHYVAFVKYGKDDSAWLFFDSMADRDGGQNGFNIPQVTPCPEVGEYL
RMSLEDLHSLDSRRIQGCARRLLCDAYMCMYQSPTMSLYK*
>rs32562371_ENSMUSP00000105254_c.2737:A>G_p.K913R
ENSMUST00000109626.4|Cyld-203|ENSMUSG00000036712|+|chr8:g.89473520:A>G
MSSGLWSQEKVTSPYWEERIFYLLLQECSVTDKQTQKLLKVPKGSIGQYIQDRSVGHSRVPSTKGKKNQIGLK
ILEQPHAVLFVDEKDVVEINEKFTELLLAITNCEERLSLFRNRLRLSKGLQVDVGSPVKVQLRSGEEKFPGVV
RFRGPLLAERTVSGIFFGVELLEEGRGQGFTDGVYQGKQLFQCDEDCGVFVALDKLELIEDDDNGLESDFAGP
GDTMQVEPPPLEINSRVSLKVGESTESGTVIFCDVLPGKESLGYFVGVDMDNPIGNWDGRFDGVQLCSFASVE
STILLHINDIIPDSVTQERRPPKLAFMSRGVGDKGSSSHNKPKVTGSTSDPGSRNRSELFYTLNGSSVDSQQS
KSKNPWYIDEVAEDPAKSLTEMSSDFGHSSPPPQPPSMNSLSSENRFHSLPFSLTKMPNTNGSMAHSPLSLSV
QSVMGELNSTPVQESPPLPISSGNAHGLEVGSLAEVKENPPFYGVIRWIGQPPGLSDVLAGLELEDECAGCTD
GTFRGTRYFTCALKKALFVKLKSCRPDSRFASLQPVSNQIERCNSLAFGGYLSEVVEENTPPKMEKEGLEIMI
GKKKGIQGHYNSCYLDSTLFCLFAFSSALDTVLLRPKEKNDIEYYSETQELLRTEIVNPLRIYGYVCATKIMK
LRKILEKVEAASGFTSEEKDPEEFLNILFHDILRVEPLLKIRSAGQKVQDCNFYQIFMEKNEKVGVPTIQQLL
EWSFINSNLKFAEAPSCLIIQMPRFGKDFKLFKKIFPSLELNITDLLEDTPRQCRICGGLAMYECRECYDDPD
ISAGKIKQFCKTCSTQVHLHPRRLNHSYHPVSLPKDLPDWDWRHGCIPCQKMELFAVLCIETSHYVAFVKYGK
DDSAWLFFDSMADRDGGQNGFNIPQVTPCPEVGEYLRMSLEDLHSLDSRRIQGCARRLLCDAYMCMYQSPTMS
LYK*
>rs32562371_ENSMUSP00000096119_c.2746:A>G_p.K916R
ENSMUST00000098519.11|Cyld-202|ENSMUSG00000036712|+|chr8:g.89473520:A>G
MSSGLWSQEKVTSPYWEERIFYLLLQECSVTDKQTQKLLKVPKGSIGQYIQDRSVGHSRVPSTKGKKNQIGLK
ILEQPHAVLFVDEKDVVEINEKFTELLLAITNCEERLSLFRNRLRLSKGLQVDVGSPVKVQLRSGEEKFPGVV
RFRGPLLAERTVSGIFFGVELLEEGRGQGFTDGVYQGKQLFQCDEDCGVFVALDKLELIEDDDNGLESDFAGP
GDTMQVEPPPLEINSRVSLKVGESTESGTVIFCDVLPGKESLGYFVGVDMDNPIGNWDGRFDGVQLCSFASVE
STILLHINDIIPALSDSVTQERRPPKLAFMSRGVGDKGSSSHNKPKVTGSTSDPGSRNRSELFYTLNGSSVDS
QQSKSKNPWYIDEVAEDPAKSLTEMSSDFGHSSPPPQPPSMNSLSSENRFHSLPFSLTKMPNTNGSMAHSPLS
LSVQSVMGELNSTPVQESPPLPISSGNAHGLEVGSLAEVKENPPFYGVIRWIGQPPGLSDVLAGLELEDECAG
CTDGTFRGTRYFTCALKKALFVKLKSCRPDSRFASLQPVSNQIERCNSLAFGGYLSEVVEENTPPKMEKEGLE
IMIGKKKGIQGHYNSCYLDSTLFCLFAFSSALDTVLLRPKEKNDIEYYSETQELLRTEIVNPLRIYGYVCATK
IMKLRKILEKVEAASGFTSEEKDPEEFLNILFHDILRVEPLLKIRSAGQKVQDCNFYQIFMEKNEKVGVPTIQ
QLLEWSFINSNLKFAEAPSCLIIQMPRFGKDFKLFKKIFPSLELNITDLLEDTPRQCRICGGLAMYECRECYD
DPDISAGKIKQFCKTCSTQVHLHPRRLNHSYHPVSLPKDLPDWDWRHGCIPCQKMELFAVLCIETSHYVAFVK
YGKDDSAWLFFDSMADRDGGQNGFNIPQVTPCPEVGEYLRMSLEDLHSLDSRRIQGCARRLLCDAYMCMYQSP
TMSLYK*
>rs32562371_ENSMUSP00000039834_c.2746:A>G_p.K916R
ENSMUST00000043526.15|Cyld-201|ENSMUSG00000036712|+|chr8:g.89473520:A>G
MSSGLWSQEKVTSPYWEERIFYLLLQECSVTDKQTQKLLKVPKGSIGQYIQDRSVGHSRVPSTKGKKNQIGLK
ILEQPHAVLFVDEKDVVEINEKFTELLLAITNCEERLSLFRNRLRLSKGLQVDVGSPVKVQLRSGEEKFPGVV
RFRGPLLAERTVSGIFFGVELLEEGRGQGFTDGVYQGKQLFQCDEDCGVFVALDKLELIEDDDNGLESDFAGP
GDTMQVEPPPLEINSRVSLKVGESTESGTVIFCDVLPGKESLGYFVGVDMDNPIGNWDGRFDGVQLCSFASVE
STILLHINDIIPAVSDSVTQERRPPKLAFMSRGVGDKGSSSHNKPKVTGSTSDPGSRNRSELFYTLNGSSVDS
QQSKSKNPWYIDEVAEDPAKSLTEMSSDFGHSSPPPQPPSMNSLSSENRFHSLPFSLTKMPNTNGSMAHSPLS
LSVQSVMGELNSTPVQESPPLPISSGNAHGLEVGSLAEVKENPPFYGVIRWIGQPPGLSDVLAGLELEDECAG
CTDGTFRGTRYFTCALKKALFVKLKSCRPDSRFASLQPVSNQIERCNSLAFGGYLSEVVEENTPPKMEKEGLE
IMIGKKKGIQGHYNSCYLDSTLFCLFAFSSALDTVLLRPKEKNDIEYYSETQELLRTEIVNPLRIYGYVCATK
IMKLRKILEKVEAASGFTSEEKDPEEFLNILFHDILRVEPLLKIRSAGQKVQDCNFYQIFMEKNEKVGVPTIQ
QLLEWSFINSNLKFAEAPSCLIIQMPRFGKDFKLFKKIFPSLELNITDLLEDTPRQCRICGGLAMYECRECYD
DPDISAGKIKQFCKTCSTQVHLHPRRLNHSYHPVSLPKDLPDWDWRHGCIPCQKMELFAVLCIETSHYVAFVK
YGKDDSAWLFFDSMADRDGGQNGFNIPQVTPCPEVGEYLRMSLEDLHSLDSRRIQGCARRLLCDAYMCMYQSP
TMSLYK*
>rs33035272_ENSMUSP00000034090_c.2794:A>G_p.N932S
ENSMUST00000034090.8|Sall1-201|ENSMUSG00000031665|-|chr8:g.89757308:T>C

MSRRKQAKPQHFQSDPEVASLPRRDGDTEKGQPSRPTKSKDAHVCGRCCAEFFELSDLLLHKKSCTKNQLVLI
VNESPASPAKAFPPGPSLNDPDDQMKDAANKADQEDCSDLSEPKGLDREESMEVEVPVATTTTTTTGGSGGSG
GSTLSGVTNITTPSCHSGSSTGTSAITTSLPQLGDLTTLGNFSVINSNVIIENLQSTKVAVAQFSQEARCGGA
SGGKLLISTLMEQLLALQQQQIHQLQLIEQIRHQILLLASQSADLPAAPSIPSQGTLRTSANPLTTLSSHLSQ
QLAVAAGLAQSLASQSANISGVKQLPHVQLPQSSSGTSIVPPSGGTSPNMSIVTAAVPTPSSEKVASNAGASH
VSSPAVSASSSPAFAISSLLSPASNPLLPQPTPANAVFPTPLPNIATTAEDLNSLSALAQQRKSKPPNVTAFE
AKSTSDEAFFKHKCRFCAKVFGSDSALQIHLRSHTGERPFKCNICGNRFSTKGNLKVHFQRHKEKYPHIQMNP
YPVPEHLDNVPTSTGIPYGMSIPPEKPVTSWLDTKPVLPTLTTSVGLPLPPTLPSLTPFIKTEEPAPIPISHS
AASPQGSVKSDSGAPDLATRNPSGVPEEVEGSAVPPFGGKGEESNMASSAVPTAGNSTLNSPVADGGPGGTTF
TNPLLPLMSEQFKAKFPFGGLLDSAQASETSKLQQLVENIDKKATDPNECIICHRVLSCQSALKMHYRTHTGE
RPFKCKICGRAFTTKGNLKTHYSVHRAMPPLRVQHSCPICQKKFTNAVVLQQHIRMHMGGQIPNTPVPDNYPE
SMESDTGSFDEKNFDDLDNFSDENMEECPEGSIPDTPKSADASQDSLSSSPLPLEMSSIAALENQMKMINAGL
AEQLQASLKSVENGSMEGDVLTNDSSSVGGDMESQSAGSPAISESTSSMQALSPSSSTQEFHKSPGMEEKPQR
VGPGEFANGLSPTPVNGGALDLTSSHAEKIIKEDSLGILFPFRDRGKFKNTACDICGKTFACQSALDIHYRSH
TKERPFICTVCNRGFSTKGNLKQHMLTHQMRDLPSQLFEPSSNLGPNQNSAVIPANSLSSLIKTEVNGFVHVS
PQDSKDAPTSHVPQGPLSSSATSPVLLPALPRRTPKQHYCNTCGKTFSSSSALQIHERTHTGEKPFACTICGR
AFTTKGNLKVHMGTHMWNSTPARRGRRLSVDGPMTFLGGNPVKFPEMFQKDLAARSGSGDPSSFWNQYTAALS
NGLAMKANEISVIQNGGIPPIPGSLGSGSSSPISGLTGNVEKLGNSEPSAPLAGLEKMASSENGTNFRFTRFV
EDSKEIVTS*
>rs52511487_ENSMUSP00000034090_c.2497:A>G_p.E833G
ENSMUST00000034090.8|Sall1-201|ENSMUSG00000031665|-|chr8:g.89757605:T>C
MSRRKQAKPQHFQSDPEVASLPRRDGDTEKGQPSRPTKSKDAHVCGRCCAEFFELSDLLLHKKSCTKNQLVLI
VNESPASPAKAFPPGPSLNDPDDQMKDAANKADQEDCSDLSEPKGLDREESMEVEVPVATTTTTTTGGSGGSG
GSTLSGVTNITTPSCHSGSSTGTSAITTSLPQLGDLTTLGNFSVINSNVIIENLQSTKVAVAQFSQEARCGGA
SGGKLLISTLMEQLLALQQQQIHQLQLIEQIRHQILLLASQSADLPAAPSIPSQGTLRTSANPLTTLSSHLSQ
QLAVAAGLAQSLASQSANISGVKQLPHVQLPQSSSGTSIVPPSGGTSPNMSIVTAAVPTPSSEKVASNAGASH
VSSPAVSASSSPAFAISSLLSPASNPLLPQPTPANAVFPTPLPNIATTAEDLNSLSALAQQRKSKPPNVTAFE
AKSTSDEAFFKHKCRFCAKVFGSDSALQIHLRSHTGERPFKCNICGNRFSTKGNLKVHFQRHKEKYPHIQMNP
YPVPEHLDNVPTSTGIPYGMSIPPEKPVTSWLDTKPVLPTLTTSVGLPLPPTLPSLTPFIKTEEPAPIPISHS
AASPQGSVKSDSGAPDLATRNPSGVPEEVEGSAVPPFGGKGEESNMASSAVPTAGNSTLNSPVADGGPGGTTF
TNPLLPLMSEQFKAKFPFGGLLDSAQASETSKLQQLVENIDKKATDPNECIICHRVLSCQSALKMHYRTHTGE
RPFKCKICGRAFTTKGNLKTHYSVHRAMPPLRVQHSCPICQKKFTNAVVLQQHIRMHMGGQIPNTPVPDNYPE
SMESDTGSFDEKNFDDLDNFSDENMEECPGGSIPDTPKSADASQDSLSSSPLPLEMSSIAALENQMKMINAGL
AEQLQASLKSVENGSMEGDVLTNDSSSVGGDMESQSAGSPAISESTSSMQALSPSNSTQEFHKSPGMEEKPQR
VGPGEFANGLSPTPVNGGALDLTSSHAEKIIKEDSLGILFPFRDRGKFKNTACDICGKTFACQSALDIHYRSH
TKERPFICTVCNRGFSTKGNLKQHMLTHQMRDLPSQLFEPSSNLGPNQNSAVIPANSLSSLIKTEVNGFVHVS
PQDSKDAPTSHVPQGPLSSSATSPVLLPALPRRTPKQHYCNTCGKTFSSSSALQIHERTHTGEKPFACTICGR
AFTTKGNLKVHMGTHMWNSTPARRGRRLSVDGPMTFLGGNPVKFPEMFQKDLAARSGSGDPSSFWNQYTAALS
NGLAMKANEISVIQNGGIPPIPGSLGSGSSSPISGLTGNVEKLGNSEPSAPLAGLEKMASSENGTNFRFTRFV
EDSKEIVTS*
>rs214238730_ENSMUSP00000034090_c.2360:C>A_p.H787Q
ENSMUST00000034090.8|Sall1-201|ENSMUSG00000031665|-|chr8:g.89757742:G>T
MSRRKQAKPQHFQSDPEVASLPRRDGDTEKGQPSRPTKSKDAHVCGRCCAEFFELSDLLLHKKSCTKNQLVLI
VNESPASPAKAFPPGPSLNDPDDQMKDAANKADQEDCSDLSEPKGLDREESMEVEVPVATTTTTTTGGSGGSG
GSTLSGVTNITTPSCHSGSSTGTSAITTSLPQLGDLTTLGNFSVINSNVIIENLQSTKVAVAQFSQEARCGGA
SGGKLLISTLMEQLLALQQQQIHQLQLIEQIRHQILLLASQSADLPAAPSIPSQGTLRTSANPLTTLSSHLSQ
QLAVAAGLAQSLASQSANISGVKQLPHVQLPQSSSGTSIVPPSGGTSPNMSIVTAAVPTPSSEKVASNAGASH
VSSPAVSASSSPAFAISSLLSPASNPLLPQPTPANAVFPTPLPNIATTAEDLNSLSALAQQRKSKPPNVTAFE
AKSTSDEAFFKHKCRFCAKVFGSDSALQIHLRSHTGERPFKCNICGNRFSTKGNLKVHFQRHKEKYPHIQMNP
YPVPEHLDNVPTSTGIPYGMSIPPEKPVTSWLDTKPVLPTLTTSVGLPLPPTLPSLTPFIKTEEPAPIPISHS
AASPQGSVKSDSGAPDLATRNPSGVPEEVEGSAVPPFGGKGEESNMASSAVPTAGNSTLNSPVADGGPGGTTF
TNPLLPLMSEQFKAKFPFGGLLDSAQASETSKLQQLVENIDKKATDPNECIICHRVLSCQSALKMHYRTHTGE
RPFKCKICGRAFTTKGNLKTHYSVHRAMPPLRVQHSCPICQKKFTNAVVLQQHIRMQMGGQIPNTPVPDNYPE
SMESDTGSFDEKNFDDLDNFSDENMEECPEGSIPDTPKSADASQDSLSSSPLPLEMSSIAALENQMKMINAGL
AEQLQASLKSVENGSMEGDVLTNDSSSVGGDMESQSAGSPAISESTSSMQALSPSNSTQEFHKSPGMEEKPQR
VGPGEFANGLSPTPVNGGALDLTSSHAEKIIKEDSLGILFPFRDRGKFKNTACDICGKTFACQSALDIHYRSH
TKERPFICTVCNRGFSTKGNLKQHMLTHQMRDLPSQLFEPSSNLGPNQNSAVIPANSLSSLIKTEVNGFVHVS
PQDSKDAPTSHVPQGPLSSSATSPVLLPALPRRTPKQHYCNTCGKTFSSSSALQIHERTHTGEKPFACTICGR

AFTTKGNLKVHMGTHMWNSTPARRGRRLSVDGPMTFLGGNPVKFPEMFQKDLAARSGSGDPSSFWNQYTAALS
NGLAMKANEISVIQNGGIPPIPGSLGSGSSSPISGLTGNVEKLGNSEPSAPLAGLEKMASSENGTNFRFTRFV
EDSKEIVTS*
>rs50405873_ENSMUSP00000034090_c.2273:G>A_p.M758I
ENSMUST00000034090.8|Sall1-201|ENSMUSG00000031665|-|chr8:g.89757829:C>T
MSRRKQAKPQHFQSDPEVASLPRRDGDTEKGQPSRPTKSKDAHVCGRCCAEFFELSDLLLHKKSCTKNQLVLI
VNESPASPAKAFPPGPSLNDPDDQMKDAANKADQEDCSDLSEPKGLDREESMEVEVPVATTTTTTTGGSGGSG
GSTLSGVTNITTPSCHSGSSTGTSAITTSLPQLGDLTTLGNFSVINSNVIIENLQSTKVAVAQFSQEARCGGA
SGGKLLISTLMEQLLALQQQQIHQLQLIEQIRHQILLLASQSADLPAAPSIPSQGTLRTSANPLTTLSSHLSQ
QLAVAAGLAQSLASQSANISGVKQLPHVQLPQSSSGTSIVPPSGGTSPNMSIVTAAVPTPSSEKVASNAGASH
VSSPAVSASSSPAFAISSLLSPASNPLLPQPTPANAVFPTPLPNIATTAEDLNSLSALAQQRKSKPPNVTAFE
AKSTSDEAFFKHKCRFCAKVFGSDSALQIHLRSHTGERPFKCNICGNRFSTKGNLKVHFQRHKEKYPHIQMNP
YPVPEHLDNVPTSTGIPYGMSIPPEKPVTSWLDTKPVLPTLTTSVGLPLPPTLPSLTPFIKTEEPAPIPISHS
AASPQGSVKSDSGAPDLATRNPSGVPEEVEGSAVPPFGGKGEESNMASSAVPTAGNSTLNSPVADGGPGGTTF
TNPLLPLMSEQFKAKFPFGGLLDSAQASETSKLQQLVENIDKKATDPNECIICHRVLSCQSALKMHYRTHTGE
RPFKCKICGRAFTTKGNLKTHYSVHRAIPPLRVQHSCPICQKKFTNAVVLQQHIRMHMGGQIPNTPVPDNYPE
SMESDTGSFDEKNFDDLDNFSDENMEECPEGSIPDTPKSADASQDSLSSSPLPLEMSSIAALENQMKMINAGL
AEQLQASLKSVENGSMEGDVLTNDSSSVGGDMESQSAGSPAISESTSSMQALSPSNSTQEFHKSPGMEEKPQR
VGPGEFANGLSPTPVNGGALDLTSSHAEKIIKEDSLGILFPFRDRGKFKNTACDICGKTFACQSALDIHYRSH
TKERPFICTVCNRGFSTKGNLKQHMLTHQMRDLPSQLFEPSSNLGPNQNSAVIPANSLSSLIKTEVNGFVHVS
PQDSKDAPTSHVPQGPLSSSATSPVLLPALPRRTPKQHYCNTCGKTFSSSSALQIHERTHTGEKPFACTICGR
AFTTKGNLKVHMGTHMWNSTPARRGRRLSVDGPMTFLGGNPVKFPEMFQKDLAARSGSGDPSSFWNQYTAALS
NGLAMKANEISVIQNGGIPPIPGSLGSGSSSPISGLTGNVEKLGNSEPSAPLAGLEKMASSENGTNFRFTRFV
EDSKEIVTS*
>rs247695996_ENSMUSP00000034090_c.2169:CT>T_p.Y724del-fs
ENSMUST00000034090.8|Sall1-201|ENSMUSG00000031665|-|chr8:g.89757933:AG>A
MSRRKQAKPQHFQSDPEVASLPRRDGDTEKGQPSRPTKSKDAHVCGRCCAEFFELSDLLLHKKSCTKNQLVLI
VNESPASPAKAFPPGPSLNDPDDQMKDAANKADQEDCSDLSEPKGLDREESMEVEVPVATTTTTTTGGSGGSG
GSTLSGVTNITTPSCHSGSSTGTSAITTSLPQLGDLTTLGNFSVINSNVIIENLQSTKVAVAQFSQEARCGGA
SGGKLLISTLMEQLLALQQQQIHQLQLIEQIRHQILLLASQSADLPAAPSIPSQGTLRTSANPLTTLSSHLSQ
QLAVAAGLAQSLASQSANISGVKQLPHVQLPQSSSGTSIVPPSGGTSPNMSIVTAAVPTPSSEKVASNAGASH
VSSPAVSASSSPAFAISSLLSPASNPLLPQPTPANAVFPTPLPNIATTAEDLNSLSALAQQRKSKPPNVTAFE
AKSTSDEAFFKHKCRFCAKVFGSDSALQIHLRSHTGERPFKCNICGNRFSTKGNLKVHFQRHKEKYPHIQMNP
YPVPEHLDNVPTSTGIPYGMSIPPEKPVTSWLDTKPVLPTLTTSVGLPLPPTLPSLTPFIKTEEPAPIPISHS
AASPQGSVKSDSGAPDLATRNPSGVPEEVEGSAVPPFGGKGEESNMASSAVPTAGNSTLNSPVADGGPGGTTF
TNPLLPLMSEQFKAKFPFGGLLDSAQASETSKLQQLVENIDKKATDPNECIICHRVLSCQSALKMHTGHTLGR
GPSSVRSAAGLSPRKGTSRPTTASIGLCLHSESSIPAPSVRRSSQTQWSSSSIFVCTWEARSPTPQSLTTTLS
PWSLTQAPLMRKISTT
>rs224820615_ENSMUSP00000034090_c.1871:G>TG_p.K624ins-fs
ENSMUST00000034090.8|Sall1-201|ENSMUSG00000031665|-|chr8:g.89758231:C>CA
MSRRKQAKPQHFQSDPEVASLPRRDGDTEKGQPSRPTKSKDAHVCGRCCAEFFELSDLLLHKKSCTKNQLVLI
VNESPASPAKAFPPGPSLNDPDDQMKDAANKADQEDCSDLSEPKGLDREESMEVEVPVATTTTTTTGGSGGSG
GSTLSGVTNITTPSCHSGSSTGTSAITTSLPQLGDLTTLGNFSVINSNVIIENLQSTKVAVAQFSQEARCGGA
SGGKLLISTLMEQLLALQQQQIHQLQLIEQIRHQILLLASQSADLPAAPSIPSQGTLRTSANPLTTLSSHLSQ
QLAVAAGLAQSLASQSANISGVKQLPHVQLPQSSSGTSIVPPSGGTSPNMSIVTAAVPTPSSEKVASNAGASH
VSSPAVSASSSPAFAISSLLSPASNPLLPQPTPANAVFPTPLPNIATTAEDLNSLSALAQQRKSKPPNVTAFE
AKSTSDEAFFKHKCRFCAKVFGSDSALQIHLRSHTGERPFKCNICGNRFSTKGNLKVHFQRHKEKYPHIQMNP
YPVPEHLDNVPTSTGIPYGMSIPPEKPVTSWLDTKPVLPTLTTSVGLPLPPTLPSLTPFIKTEEPAPIPISHS
AASPQGSVKSDSGAPDLATRNPSGVPEEVEGSAVPPFGGNG
>rs51089854_ENSMUSP00000034090_c.1852:T>C_p.V618A
ENSMUST00000034090.8|Sall1-201|ENSMUSG00000031665|-|chr8:g.89758250:A>G
MSRRKQAKPQHFQSDPEVASLPRRDGDTEKGQPSRPTKSKDAHVCGRCCAEFFELSDLLLHKKSCTKNQLVLI
VNESPASPAKAFPPGPSLNDPDDQMKDAANKADQEDCSDLSEPKGLDREESMEVEVPVATTTTTTTGGSGGSG
GSTLSGVTNITTPSCHSGSSTGTSAITTSLPQLGDLTTLGNFSVINSNVIIENLQSTKVAVAQFSQEARCGGA
SGGKLLISTLMEQLLALQQQQIHQLQLIEQIRHQILLLASQSADLPAAPSIPSQGTLRTSANPLTTLSSHLSQ
QLAVAAGLAQSLASQSANISGVKQLPHVQLPQSSSGTSIVPPSGGTSPNMSIVTAAVPTPSSEKVASNAGASH
VSSPAVSASSSPAFAISSLLSPASNPLLPQPTPANAVFPTPLPNIATTAEDLNSLSALAQQRKSKPPNVTAFE
AKSTSDEAFFKHKCRFCAKVFGSDSALQIHLRSHTGERPFKCNICGNRFSTKGNLKVHFQRHKEKYPHIQMNP

YPVPEHLDNVPTSTGIPYGMSIPPEKPVTSWLDTKPVLPTLTTSVGLPLPPTLPSLTPFIKTEEPAPIPISHS
AASPQGSVKSDSGAPDLATRNPSGVPEEVEGSAAPPFGGKGEESNMASSAVPTAGNSTLNSPVADGGPGGTTF
TNPLLPLMSEQFKAKFPFGGLLDSAQASETSKLQQLVENIDKKATDPNECIICHRVLSCQSALKMHYRTHTGE
RPFKCKICGRAFTTKGNLKTHYSVHRAMPPLRVQHSCPICQKKFTNAVVLQQHIRMHMGGQIPNTPVPDNYPE
SMESDTGSFDEKNFDDLDNFSDENMEECPEGSIPDTPKSADASQDSLSSSPLPLEMSSIAALENQMKMINAGL
AEQLQASLKSVENGSMEGDVLTNDSSSVGGDMESQSAGSPAISESTSSMQALSPSNSTQEFHKSPGMEEKPQR
VGPGEFANGLSPTPVNGGALDLTSSHAEKIIKEDSLGILFPFRDRGKFKNTACDICGKTFACQSALDIHYRSH
TKERPFICTVCNRGFSTKGNLKQHMLTHQMRDLPSQLFEPSSNLGPNQNSAVIPANSLSSLIKTEVNGFVHVS
PQDSKDAPTSHVPQGPLSSSATSPVLLPALPRRTPKQHYCNTCGKTFSSSSALQIHERTHTGEKPFACTICGR
AFTTKGNLKVHMGTHMWNSTPARRGRRLSVDGPMTFLGGNPVKFPEMFQKDLAARSGSGDPSSFWNQYTAALS
NGLAMKANEISVIQNGGIPPIPGSLGSGSSSPISGLTGNVEKLGNSEPSAPLAGLEKMASSENGTNFRFTRFV
EDSKEIVTS*
>rs45920869_ENSMUSP00000034090_c.1533:T>C_p.Y512H
ENSMUST00000034090.8|Sall1-201|ENSMUSG00000031665|-|chr8:g.89758569:A>G
MSRRKQAKPQHFQSDPEVASLPRRDGDTEKGQPSRPTKSKDAHVCGRCCAEFFELSDLLLHKKSCTKNQLVLI
VNESPASPAKAFPPGPSLNDPDDQMKDAANKADQEDCSDLSEPKGLDREESMEVEVPVATTTTTTTGGSGGSG
GSTLSGVTNITTPSCHSGSSTGTSAITTSLPQLGDLTTLGNFSVINSNVIIENLQSTKVAVAQFSQEARCGGA
SGGKLLISTLMEQLLALQQQQIHQLQLIEQIRHQILLLASQSADLPAAPSIPSQGTLRTSANPLTTLSSHLSQ
QLAVAAGLAQSLASQSANISGVKQLPHVQLPQSSSGTSIVPPSGGTSPNMSIVTAAVPTPSSEKVASNAGASH
VSSPAVSASSSPAFAISSLLSPASNPLLPQPTPANAVFPTPLPNIATTAEDLNSLSALAQQRKSKPPNVTAFE
AKSTSDEAFFKHKCRFCAKVFGSDSALQIHLRSHTGERPFKCNICGNRFSTKGNLKVHFQRHKEKYPHIQMNP
HPVPEHLDNVPTSTGIPYGMSIPPEKPVTSWLDTKPVLPTLTTSVGLPLPPTLPSLTPFIKTEEPAPIPISHS
AASPQGSVKSDSGAPDLATRNPSGVPEEVEGSAVPPFGGKGEESNMASSAVPTAGNSTLNSPVADGGPGGTTF
TNPLLPLMSEQFKAKFPFGGLLDSAQASETSKLQQLVENIDKKATDPNECIICHRVLSCQSALKMHYRTHTGE
RPFKCKICGRAFTTKGNLKTHYSVHRAMPPLRVQHSCPICQKKFTNAVVLQQHIRMHMGGQIPNTPVPDNYPE
SMESDTGSFDEKNFDDLDNFSDENMEECPEGSIPDTPKSADASQDSLSSSPLPLEMSSIAALENQMKMINAGL
AEQLQASLKSVENGSMEGDVLTNDSSSVGGDMESQSAGSPAISESTSSMQALSPSNSTQEFHKSPGMEEKPQR
VGPGEFANGLSPTPVNGGALDLTSSHAEKIIKEDSLGILFPFRDRGKFKNTACDICGKTFACQSALDIHYRSH
TKERPFICTVCNRGFSTKGNLKQHMLTHQMRDLPSQLFEPSSNLGPNQNSAVIPANSLSSLIKTEVNGFVHVS
PQDSKDAPTSHVPQGPLSSSATSPVLLPALPRRTPKQHYCNTCGKTFSSSSALQIHERTHTGEKPFACTICGR
AFTTKGNLKVHMGTHMWNSTPARRGRRLSVDGPMTFLGGNPVKFPEMFQKDLAARSGSGDPSSFWNQYTAALS
NGLAMKANEISVIQNGGIPPIPGSLGSGSSSPISGLTGNVEKLGNSEPSAPLAGLEKMASSENGTNFRFTRFV
EDSKEIVTS*
>rs46996426_ENSMUSP00000034090_c.1465:C>T_p.T489I
ENSMUST00000034090.8|Sall1-201|ENSMUSG00000031665|-|chr8:g.89758637:G>A
MSRRKQAKPQHFQSDPEVASLPRRDGDTEKGQPSRPTKSKDAHVCGRCCAEFFELSDLLLHKKSCTKNQLVLI
VNESPASPAKAFPPGPSLNDPDDQMKDAANKADQEDCSDLSEPKGLDREESMEVEVPVATTTTTTTGGSGGSG
GSTLSGVTNITTPSCHSGSSTGTSAITTSLPQLGDLTTLGNFSVINSNVIIENLQSTKVAVAQFSQEARCGGA
SGGKLLISTLMEQLLALQQQQIHQLQLIEQIRHQILLLASQSADLPAAPSIPSQGTLRTSANPLTTLSSHLSQ
QLAVAAGLAQSLASQSANISGVKQLPHVQLPQSSSGTSIVPPSGGTSPNMSIVTAAVPTPSSEKVASNAGASH
VSSPAVSASSSPAFAISSLLSPASNPLLPQPTPANAVFPTPLPNIATTAEDLNSLSALAQQRKSKPPNVTAFE
AKSTSDEAFFKHKCRFCAKVFGSDSALQIHLRSHTGERPFKCNICGNRFSIKGNLKVHFQRHKEKYPHIQMNP
YPVPEHLDNVPTSTGIPYGMSIPPEKPVTSWLDTKPVLPTLTTSVGLPLPPTLPSLTPFIKTEEPAPIPISHS
AASPQGSVKSDSGAPDLATRNPSGVPEEVEGSAVPPFGGKGEESNMASSAVPTAGNSTLNSPVADGGPGGTTF
TNPLLPLMSEQFKAKFPFGGLLDSAQASETSKLQQLVENIDKKATDPNECIICHRVLSCQSALKMHYRTHTGE
RPFKCKICGRAFTTKGNLKTHYSVHRAMPPLRVQHSCPICQKKFTNAVVLQQHIRMHMGGQIPNTPVPDNYPE
SMESDTGSFDEKNFDDLDNFSDENMEECPEGSIPDTPKSADASQDSLSSSPLPLEMSSIAALENQMKMINAGL
AEQLQASLKSVENGSMEGDVLTNDSSSVGGDMESQSAGSPAISESTSSMQALSPSNSTQEFHKSPGMEEKPQR
VGPGEFANGLSPTPVNGGALDLTSSHAEKIIKEDSLGILFPFRDRGKFKNTACDICGKTFACQSALDIHYRSH
TKERPFICTVCNRGFSTKGNLKQHMLTHQMRDLPSQLFEPSSNLGPNQNSAVIPANSLSSLIKTEVNGFVHVS
PQDSKDAPTSHVPQGPLSSSATSPVLLPALPRRTPKQHYCNTCGKTFSSSSALQIHERTHTGEKPFACTICGR
AFTTKGNLKVHMGTHMWNSTPARRGRRLSVDGPMTFLGGNPVKFPEMFQKDLAARSGSGDPSSFWNQYTAALS
NGLAMKANEISVIQNGGIPPIPGSLGSGSSSPISGLTGNVEKLGNSEPSAPLAGLEKMASSENGTNFRFTRFV
EDSKEIVTS*
>rs46840751_ENSMUSP00000034090_c.1407:C>A_p.R470S
ENSMUST00000034090.8|Sall1-201|ENSMUSG00000031665|-|chr8:g.89758695:G>T
MSRRKQAKPQHFQSDPEVASLPRRDGDTEKGQPSRPTKSKDAHVCGRCCAEFFELSDLLLHKKSCTKNQLVLI
VNESPASPAKAFPPGPSLNDPDDQMKDAANKADQEDCSDLSEPKGLDREESMEVEVPVATTTTTTTGGSGGSG

GSTLSGVTNITTPSCHSGSSTGTSAITTSLPQLGDLTTLGNFSVINSNVIIENLQSTKVAVAQFSQEARCGGA
SGGKLLISTLMEQLLALQQQQIHQLQLIEQIRHQILLLASQSADLPAAPSIPSQGTLRTSANPLTTLSSHLSQ
QLAVAAGLAQSLASQSANISGVKQLPHVQLPQSSSGTSIVPPSGGTSPNMSIVTAAVPTPSSEKVASNAGASH
VSSPAVSASSSPAFAISSLLSPASNPLLPQPTPANAVFPTPLPNIATTAEDLNSLSALAQQRKSKPPNVTAFE
AKSTSDEAFFKHKCRFCAKVFGSDSALQIHLSSHTGERPFKCNICGNRFSTKGNLKVHFQRHKEKYPHIQMNP
YPVPEHLDNVPTSTGIPYGMSIPPEKPVTSWLDTKPVLPTLTTSVGLPLPPTLPSLTPFIKTEEPAPIPISHS
AASPQGSVKSDSGAPDLATRNPSGVPEEVEGSAVPPFGGKGEESNMASSAVPTAGNSTLNSPVADGGPGGTTF
TNPLLPLMSEQFKAKFPFGGLLDSAQASETSKLQQLVENIDKKATDPNECIICHRVLSCQSALKMHYRTHTGE
RPFKCKICGRAFTTKGNLKTHYSVHRAMPPLRVQHSCPICQKKFTNAVVLQQHIRMHMGGQIPNTPVPDNYPE
SMESDTGSFDEKNFDDLDNFSDENMEECPEGSIPDTPKSADASQDSLSSSPLPLEMSSIAALENQMKMINAGL
AEQLQASLKSVENGSMEGDVLTNDSSSVGGDMESQSAGSPAISESTSSMQALSPSNSTQEFHKSPGMEEKPQR
VGPGEFANGLSPTPVNGGALDLTSSHAEKIIKEDSLGILFPFRDRGKFKNTACDICGKTFACQSALDIHYRSH
TKERPFICTVCNRGFSTKGNLKQHMLTHQMRDLPSQLFEPSSNLGPNQNSAVIPANSLSSLIKTEVNGFVHVS
PQDSKDAPTSHVPQGPLSSSATSPVLLPALPRRTPKQHYCNTCGKTFSSSSALQIHERTHTGEKPFACTICGR
AFTTKGNLKVHMGTHMWNSTPARRGRRLSVDGPMTFLGGNPVKFPEMFQKDLAARSGSGDPSSFWNQYTAALS
NGLAMKANEISVIQNGGIPPIPGSLGSGSSSPISGLTGNVEKLGNSEPSAPLAGLEKMASSENGTNFRFTRFV
EDSKEIVTS*
>rs49037112_ENSMUSP00000034090_c.325:A>T_p.D109V
ENSMUST00000034090.8|Sall1-201|ENSMUSG00000031665|-|chr8:g.89759777:T>A
MSRRKQAKPQHFQSDPEVASLPRRDGDTEKGQPSRPTKSKDAHVCGRCCAEFFELSDLLLHKKSCTKNQLVLI
VNESPASPAKAFPPGPSLNDPDDQMKDAANKADQEVCSDLSEPKGLDREESMEVEVPVATTTTTTTGGSGGSG
GSTLSGVTNITTPSCHSGSSTGTSAITTSLPQLGDLTTLGNFSVINSNVIIENLQSTKVAVAQFSQEARCGGA
SGGKLLISTLMEQLLALQQQQIHQLQLIEQIRHQILLLASQSADLPAAPSIPSQGTLRTSANPLTTLSSHLSQ
QLAVAAGLAQSLASQSANISGVKQLPHVQLPQSSSGTSIVPPSGGTSPNMSIVTAAVPTPSSEKVASNAGASH
VSSPAVSASSSPAFAISSLLSPASNPLLPQPTPANAVFPTPLPNIATTAEDLNSLSALAQQRKSKPPNVTAFE
AKSTSDEAFFKHKCRFCAKVFGSDSALQIHLRSHTGERPFKCNICGNRFSTKGNLKVHFQRHKEKYPHIQMNP
YPVPEHLDNVPTSTGIPYGMSIPPEKPVTSWLDTKPVLPTLTTSVGLPLPPTLPSLTPFIKTEEPAPIPISHS
AASPQGSVKSDSGAPDLATRNPSGVPEEVEGSAVPPFGGKGEESNMASSAVPTAGNSTLNSPVADGGPGGTTF
TNPLLPLMSEQFKAKFPFGGLLDSAQASETSKLQQLVENIDKKATDPNECIICHRVLSCQSALKMHYRTHTGE
RPFKCKICGRAFTTKGNLKTHYSVHRAMPPLRVQHSCPICQKKFTNAVVLQQHIRMHMGGQIPNTPVPDNYPE
SMESDTGSFDEKNFDDLDNFSDENMEECPEGSIPDTPKSADASQDSLSSSPLPLEMSSIAALENQMKMINAGL
AEQLQASLKSVENGSMEGDVLTNDSSSVGGDMESQSAGSPAISESTSSMQALSPSNSTQEFHKSPGMEEKPQR
VGPGEFANGLSPTPVNGGALDLTSSHAEKIIKEDSLGILFPFRDRGKFKNTACDICGKTFACQSALDIHYRSH
TKERPFICTVCNRGFSTKGNLKQHMLTHQMRDLPSQLFEPSSNLGPNQNSAVIPANSLSSLIKTEVNGFVHVS
PQDSKDAPTSHVPQGPLSSSATSPVLLPALPRRTPKQHYCNTCGKTFSSSSALQIHERTHTGEKPFACTICGR
AFTTKGNLKVHMGTHMWNSTPARRGRRLSVDGPMTFLGGNPVKFPEMFQKDLAARSGSGDPSSFWNQYTAALS
NGLAMKANEISVIQNGGIPPIPGSLGSGSSSPISGLTGNVEKLGNSEPSAPLAGLEKMASSENGTNFRFTRFV
EDSKEIVTS*
>rs48147872_ENSMUSP00000034090_c.211:T>C_p.V71A
ENSMUST00000034090.8|Sall1-201|ENSMUSG00000031665|-|chr8:g.89759891:A>G
MSRRKQAKPQHFQSDPEVASLPRRDGDTEKGQPSRPTKSKDAHVCGRCCAEFFELSDLLLHKKSCTKNQLALI
VNESPASPAKAFPPGPSLNDPDDQMKDAANKADQEDCSDLSEPKGLDREESMEVEVPVATTTTTTTGGSGGSG
GSTLSGVTNITTPSCHSGSSTGTSAITTSLPQLGDLTTLGNFSVINSNVIIENLQSTKVAVAQFSQEARCGGA
SGGKLLISTLMEQLLALQQQQIHQLQLIEQIRHQILLLASQSADLPAAPSIPSQGTLRTSANPLTTLSSHLSQ
QLAVAAGLAQSLASQSANISGVKQLPHVQLPQSSSGTSIVPPSGGTSPNMSIVTAAVPTPSSEKVASNAGASH
VSSPAVSASSSPAFAISSLLSPASNPLLPQPTPANAVFPTPLPNIATTAEDLNSLSALAQQRKSKPPNVTAFE
AKSTSDEAFFKHKCRFCAKVFGSDSALQIHLRSHTGERPFKCNICGNRFSTKGNLKVHFQRHKEKYPHIQMNP
YPVPEHLDNVPTSTGIPYGMSIPPEKPVTSWLDTKPVLPTLTTSVGLPLPPTLPSLTPFIKTEEPAPIPISHS
AASPQGSVKSDSGAPDLATRNPSGVPEEVEGSAVPPFGGKGEESNMASSAVPTAGNSTLNSPVADGGPGGTTF
TNPLLPLMSEQFKAKFPFGGLLDSAQASETSKLQQLVENIDKKATDPNECIICHRVLSCQSALKMHYRTHTGE
RPFKCKICGRAFTTKGNLKTHYSVHRAMPPLRVQHSCPICQKKFTNAVVLQQHIRMHMGGQIPNTPVPDNYPE
SMESDTGSFDEKNFDDLDNFSDENMEECPEGSIPDTPKSADASQDSLSSSPLPLEMSSIAALENQMKMINAGL
AEQLQASLKSVENGSMEGDVLTNDSSSVGGDMESQSAGSPAISESTSSMQALSPSNSTQEFHKSPGMEEKPQR
VGPGEFANGLSPTPVNGGALDLTSSHAEKIIKEDSLGILFPFRDRGKFKNTACDICGKTFACQSALDIHYRSH
TKERPFICTVCNRGFSTKGNLKQHMLTHQMRDLPSQLFEPSSNLGPNQNSAVIPANSLSSLIKTEVNGFVHVS
PQDSKDAPTSHVPQGPLSSSATSPVLLPALPRRTPKQHYCNTCGKTFSSSSALQIHERTHTGEKPFACTICGR
AFTTKGNLKVHMGTHMWNSTPARRGRRLSVDGPMTFLGGNPVKFPEMFQKDLAARSGSGDPSSFWNQYTAALS

NGLAMKANEISVIQNGGIPPIPGSLGSGSSSPISGLTGNVEKLGNSEPSAPLAGLEKMASSENGTNFRFTRFV
EDSKEIVTS*
>rs247287166_ENSMUSP00000105250_c.1105:C>TTTGC_p.A369ins-fs
ENSMUST00000109621.10|Tox3-201|ENSMUSG00000043668|-
|chr8:g.90975524:G>GCAAA
MDVRFYPAAAGDPAGLDFAQCLGYYGYSKLGNNNYMNMAEANNAFFAASEQTFHTPSLGDEEFEIPPITPPPE
SDPTLGMPDALLPFQTLSDPLPSQGTEFTPQFPPQSLDLPSITISRNLVEQDGVLHSNGLHMDQSHTQVSQYR
QDPSLVMRSIVHMTDGARSGIMPPAQLTTINQSQLSAQLGLNLGGANVSHTSPSPPASKSATPSPSSSINEED
ADDANRAIGEKRTAPDSGKKPKTPKKKKKKDPNEPQKPVSAYALFFRDTQAAIKGQNPNATFGEVSKIVASMW
DSLGEEQKQVYKRKTEAAKKEYLKALAAYRASLVSKAAAESAEAQTIRSVQQTLASTNLTSSLLLNTSLSQHG
TVPVCLTSDSSTVTP
>rs49748472_ENSMUSP00000105250_c.577:A>G_p.N193S
ENSMUST00000109621.10|Tox3-201|ENSMUSG00000043668|-|chr8:g.90984600:T>C
MDVRFYPAAAGDPAGLDFAQCLGYYGYSKLGNNNYMNMAEANNAFFAASEQTFHTPSLGDEEFEIPPITPPPE
SDPTLGMPDALLPFQTLSDPLPSQGTEFTPQFPPQSLDLPSITISRNLVEQDGVLHSNGLHMDQSHTQVSQYR
QDPSLVMRSIVHMTDGARSGIMPPAQLTTINQSQLSAQLGLNLGGASVSHTSPSPPASKSATPSPSSSINEED
ADDANRAIGEKRTAPDSGKKPKTPKKKKKKDPNEPQKPVSAYALFFRDTQAAIKGQNPNATFGEVSKIVASMW
DSLGEEQKQVYKRKTEAAKKEYLKALAAYRASLVSKAAAESAEAQTIRSVQQTLASTNLTSSLLLNTSLSQHG
TVPASPQTLPQSLPRSIAPKPLTMRLPMSQIVTSVTIAANMPSNIGAPLISSMGTTMVGSATSTQVSPSVQTQ
QHQMQLQQQQQQQQMQQMQQQQLQQHQMHQQIQQQMQQQHFQHHMQQHLQQQQQQHLQQQLSQQQLQQQLQQ
HLQLQQLQHMQHQSQPSPRQHSPVTSQITSPIPAIGSPQPASQQHQPQIQSQTQTQVLPQVSIF*
>rs49748472_ENSMUSP00000135697_c.574:A>G_p.N192S
ENSMUST00000176616.8|Tox3-203|ENSMUSG00000043668|-|chr8:g.90984600:T>C
MDVRFYPAAAGDPAGLDFAQCLGYYGYSKLGNNNYMNMAEANNAFFAASETFHTPSLGDEEFEIPPITPPPES
DPTLGMPDALLPFQTLSDPLPSQGTEFTPQFPPQSLDLPSITISRNLVEQDGVLHSNGLHMDQSHTQVSQYRQ
DPSLVMRSIVHMTDGARSGIMPPAQLTTINQSQLSAQLGLNLGGASVSHTSPSPPASKSATPSPSSSINEEDA
DDANRAIGEKRTAPDSGKKPKTPKKKKKKDPNEPQKPVSAYALFFRDTQAAIKGQNPNATFGEVSKIVASMWD
SLGEEQKQVYKRKTEAA
>rs49748472_ENSMUSP00000134931_c.511:A>G_p.N171S
ENSMUST00000176034.2|Tox3-202|ENSMUSG00000043668|-|chr8:g.90984600:T>C
MDVRFYPAAAGDPAGLDFAQCLGYYGYSKTFHTPSLGDEEFEIPPITPPPESDPTLGMPDALLPFQTLSDPLP
SQGTEFTPQFPPQSLDLPSITISRNLVEQDGVLHSNGLHMDQSHTQVSQYRQDPSLVMRSIVHMTDGARSGIM
PPAQLTTINQSQLSAQLGLNLGGASVSHTSPSP
>rs46153803_ENSMUSP00000105250_c.441:G>T_p.D148Y
ENSMUST00000109621.10|Tox3-201|ENSMUSG00000043668|-|chr8:g.90984736:C>A
MDVRFYPAAAGDPAGLDFAQCLGYYGYSKLGNNNYMNMAEANNAFFAASEQTFHTPSLGDEEFEIPPITPPPE
SDPTLGMPDALLPFQTLSDPLPSQGTEFTPQFPPQSLDLPSITISRNLVEQDGVLHSNGLHMDQSHTQVSQYR
QYPSLVMRSIVHMTDGARSGIMPPAQLTTINQSQLSAQLGLNLGGANVSHTSPSPPASKSATPSPSSSINEED
ADDANRAIGEKRTAPDSGKKPKTPKKKKKKDPNEPQKPVSAYALFFRDTQAAIKGQNPNATFGEVSKIVASMW
DSLGEEQKQVYKRKTEAAKKEYLKALAAYRASLVSKAAAESAEAQTIRSVQQTLASTNLTSSLLLNTSLSQHG
TVPASPQTLPQSLPRSIAPKPLTMRLPMSQIVTSVTIAANMPSNIGAPLISSMGTTMVGSATSTQVSPSVQTQ
QHQMQLQQQQQQQQMQQMQQQQLQQHQMHQQIQQQMQQQHFQHHMQQHLQQQQQQHLQQQLSQQQLQQQLQQ
HLQLQQLQHMQHQSQPSPRQHSPVTSQITSPIPAIGSPQPASQQHQPQIQSQTQTQVLPQVSIF*
>rs46153803_ENSMUSP00000135697_c.438:G>T_p.D147Y
ENSMUST00000176616.8|Tox3-203|ENSMUSG00000043668|-|chr8:g.90984736:C>A
MDVRFYPAAAGDPAGLDFAQCLGYYGYSKLGNNNYMNMAEANNAFFAASETFHTPSLGDEEFEIPPITPPPES
DPTLGMPDALLPFQTLSDPLPSQGTEFTPQFPPQSLDLPSITISRNLVEQDGVLHSNGLHMDQSHTQVSQYRQ
YPSLVMRSIVHMTDGARSGIMPPAQLTTINQSQLSAQLGLNLGGANVSHTSPSPPASKSATPSPSSSINEEDA
DDANRAIGEKRTAPDSGKKPKTPKKKKKKDPNEPQKPVSAYALFFRDTQAAIKGQNPNATFGEVSKIVASMWD
SLGEEQKQVYKRKTEAA
>rs46153803_ENSMUSP00000134931_c.375:G>T_p.D126Y
ENSMUST00000176034.2|Tox3-202|ENSMUSG00000043668|-|chr8:g.90984736:C>A
MDVRFYPAAAGDPAGLDFAQCLGYYGYSKTFHTPSLGDEEFEIPPITPPPESDPTLGMPDALLPFQTLSDPLP
SQGTEFTPQFPPQSLDLPSITISRNLVEQDGVLHSNGLHMDQSHTQVSQYRQYPSLVMRSIVHMTDGARSGIM
PPAQLTTINQSQLSAQLGLNLGGANVSHTSPSP
>rs33399640_ENSMUSP00000046356_c.382:A>G_p.Q128R
ENSMUST00000048665.8|Chd9-201|ENSMUSG00000056608|+|chr8:g.91659424:A>G

MTDPMMDFFDDANLFGETLEGLSDDTFVQPGPVSLVDELNLGAEFEPLHIDSLNHVQGTPTHQKMADFEQLSQ
FDSMKFHPVNQSFGSPVEHVLSPHSQFNCSPIHPPNQPNGLFQDVADGSPMWGHRTATGLANQNGSPFHQPGH
SHSLHQNKSFVAHPDFALFQASEHQTQCSSLHSQQSRSNLNPGQNSLGQAKNFLDANVSGAHRVNVNHLATAP
SSQQTLPVQFSPTANPPAHFLKCSSHQEGNYNRPSPSMTSCSVSNSQQFPSHYSFSSGHVSPSSLLQSSAGLA
PGHTNQALSDFAGSNSFSPHRGMKQEPTQHLLNPTPSLNSNNFQILHSSHPQGNYSNSKLSPVHMNFPDPVDA
GPPVGHFNDHAETNGFSSLEENLLHHVDSHAEPFAGLDPEDLLQEGLLPQFDESPFGQDNSNHVLDHDLDRQF
TSHLVSRPSDMAQTQLQYQARGWPSPLSTNHQHLHSRNHLCLQRQPPSSKKSDGSGTYTKLQNTQVRVMSEKK
PRKRVESESKQEKANRIISEAIARAKERGERNIPRVMSPENFPSASVEGKEEKRGRRMKSKPKDRDNKKPKTY
SKLKEKTKIGKLIITLGKKHKRRNESSDELSDAEQRSQHTFKEQHSQKRRSNRQIKRKKYAEDAEGKQSEEEV
KGSLRVKRNSAPPPGEQPLQLFVENPSEEDAAIVDKILACRTVKKEVSPGVMLDIEEFFVKYKNYSYLHCEWA
TEQQLLKDKRIQQKIKRFKLRQAQRAHFLADMEEEPFNPDYVEVDRILEVSFCEDKDTGESVIYYLVKWCSLP
YEDSTWELKEDVDLAKIEEFEQLQASRPDTRHLDRPPSNIWKKIEQSREYKNGNQLREYQLEGLNWLLFNWYN
RRNCILADEMGLGKTIQSITFLYEILLTGIRGPFLIIAPLSTIANWEREFRTWTDINVVVYHGSLISRQMIQQ
YEMYFRDSQGRIIRGAYRFQAIITTFEMILGGCGELNAIDWRCVIIDEAHRLKNKNCKLLEGLKLMNLEHKVL
LTGTPLQNTVEELFSLLHFLEPLRFPSESTFMQEFGDLKTEEQVQKLQAILKPMMLRRLKEDVEKKLAPKEET
IIEVELTNIQKKYYRAILEKNFSFLSKGAGQTNVPNLVNTMMELRKCCNHPYLIKGAEEKILGEFRDTYNPSA
SDFHLQAMIQSAGKLVLIDKLLPKMKAGGHKVLIFSQMVRCLDILEDYLIHKRYLYERIDGRVRGNLRQAAID
RFSKPDSDRFVFLLCTRAGGLGINLTAADTCIIFDSDWNPQNDLQAQARCHRIGQNKAVKVYRLVTRNSYERE
MFDRASLKLGLDKAVLQSMSGRDSNVSGIQQLSKKEIEDLLRRGAYGAIMEEEDEGSKFCEEDIDQILLRRTK
TITIESEGRGSTFAKASFVASGNRTDISLDDPNFWQKWAKKAELDIDTISGRNSLVIDTPRIRKQTRPFSATK
DELAELSEAESEGEEKPKLRRPCDRSGGYGRTECFRVEKNLLVYGWGRWREILSHGRFKRQLNEHDVEVICRA
LLAYCLIHYRGDEKIKGFIWDLITPTEDGQTRELQNHLGLSAPVPRGRKGKKVKTQTSSFDIQKAEWLRKYNP
EQLLQDEGYKKHVKHHCNKVLLRVRMLYYLKQEVIGNESQKVFDGVDASDIDVWVPEPDHSEVPAAWWDFDAD
KSLLIGVFKHGYEKYNTIRADPALCFLERVGKPDDKAVAAEQRANDYMDGDVEDPEYKPAPAIFKDDIEDDVS
SPGDLVIADGEGQLMEGDKVYWPTPSALTTRLRRLITAYQRTNKNRHIQQMQPTFSLPANAMQPLYEEATLNP
KMAAKIERQQRWTRREEADFYRVVSTFGVVFDPDRGQFDWTKFRALARLHKKTDNSLEKYLCAFMSMCRRVCR
LPSKEELVDPNIFIQPITEERASRTLYRIELLRKVREQALRHPQLFERLKLCHPNPDLPIWWECGSHDRDLLI
GAAKHGVSRTDYHILRDPELSFMAAQRNYNQSKAAHSRTSAPLLQQYQVALSASPLTSLPRLLGAKGTLLEDM
KVKSESLTEEPQSSEEESMSSMETRTRVKSEPVSPKNGVLSQATGDQKSGGKSETDRRMVAARTEPLTPNPAS
KKPRVHKRGSQSSSDSDSDSARSSCSSRSSSSSSSSSSCSHSRSGSSSSSSSSCSSASSSSSSSSSSSSSSS
SSSEESDSEEDVQKREGTPHRKAYDEESVASLSTTQDETQDSFQANNGTPESAYLLQGGYMLAASYWPKDRVM
INRLDSICQTVLKGKWPSARRHYDANTVASFYTTKLLDSPGAATERGEPSVPTPPAVAVREEHEQSAQMSKEG
GLKLTFQKQGLAQKRPFDGEDGALGQQQYLTRLRELQSTSETSLVNLPKAVPASGTSIQPTLGANGAILDSQP
IVKKRRGRRRNVEGADILFLNRNKPPNHIPTGMNPALSYPQPQRIPDTESPVPVINLKDGTRLAGDDAPKRKD
LDRWLKEHPGYVEDLGAFIPRVQLHEGRPKQKRHRCRNPNKLDINSLTGEERVQLINRRNARKVGGAFAPPLK
DLCRFLKENSEYGVAPEWGDVVKQSGFLPESMFERILTGPVVREEVSRRGRRPKSGIAKATTAAAVPAGSVPG
NPLLANGLLPGVDLTALQALQQNLQNLQSLQVTAGLMGMPAGLSSGGETKNMAAMFPMLFSGMAGLPNLLGMG
GLLSKTAESGAEEKRGNDSKELEGKKERTESQSPENGGERCVPGSPSTSSTAALSSAAAAKPIALNPLLLSNI
LYPGMLLTPGLNLHLPTLSQSNAFDVQKNKSDDLDSSKSVEIKEENSRVRDQEEKGGTEPSPLNENSTDEGSE
RASSGSDSSSSSSEDSDSSNED*
>rs33399640_ENSMUSP00000147839_c.382:A>G_p.Q128R
ENSMUST00000211403.2|Chd9-213|ENSMUSG00000056608|+|chr8:g.91659424:A>G
MTDPMMDFFDDANLFGETLEGLSDDTFVQPGPVSLVDELNLGAEFEPLHIDSLNHVQGTPTHQKMADFEQLSQ
FDSMKFHPVNQSFGSPVEHVLSPHSQFNCSPIHPPNQPNGLFQDVADGSPMWGHRTATGLANQNGSPFHQPGH
SHSLHQNKSFVAHPDFALFQASEHQTQCSSLHSQQSRSNLNPGQNSLGQAKNFLDANVSGAHRVNVNHLATAP
SSQQTLPVQFSPTANPPAHFLKCSSHQEGNYNRPSPSMTSCSVSNSQQFPSHYSFSSGHVSPSSLLQSSAGLA
PGHTNQALSDFAGSNSFSPHRGMKQEPTQHLLNPTPSLNSNNFQILHSSHPQGNYSNSKLSPVHMNFPDPVDA
GPPVGHFNDHAETNGFSSLEENLLHHVDSHAEPFAGLDPEDLLQEGLLPQFDESPFGQDNSNHVLDHDLDRQF
TSHLVSRPSDMAQTQLQYQARGWPSPLSTNHQHLHSRNHLCLQRQPPSSKKSDGSGTYTKLQNTQVRVMSEKK
PRKRVESESKQEKANRIISEAIARAKERGERNIPRVMSPENFPSASVEGKEEKRGRRMKSKPKDRDNKKPKTY
SKLKEKTKIGKLIITLGKKHKRRNESSDELSDAEQRSQHTFKEQHSQKRRSNRQIKR
>rs33399640_ENSMUSP00000147407_c.382:A>G_p.Q128R
ENSMUST00000209203.2|Chd9-203|ENSMUSG00000056608|+|chr8:g.91659424:A>G
MTDPMMDFFDDANLFGETLEGLSDDTFVQPGPVSLVDELNLGAEFEPLHIDSLNHVQGTPTHQKMADFEQLSQ
FDSMKFHPVNQSFGSPVEHVLSPHSQFNCSPIHPPNQPNGLFQDVADGSPMWGHRTATGLANQNGSPFHQPGH
SHSLHQNKSFVAHPDFALFQASEHQTQCSSLHSQQSRSNLNPGQNSLGQAKNFLDANVSGAHRVNVNHLATAP
SSQQTLPVQFSPTANPPAHFLKCSSHQEGNYNRPSPSMTSCSVSNSQQFPSHYSFSSGHVSPSSLLQSSAGLA
PGHTNQALSDFAGSNSFSPHRGMKQEPTQHLLNPTPSLNSNNFQILHSSHPQGNYSNSKLSPVHMNFPDPVDA

GPPVGHFNDHAETNGFSSLEENLLHHVDSHAEPFAGLDPEDLLQEGLLPQFDESPFGQDNSNHVLDHDLDRQF
TSHLVSRPSDMAQTQLQYQARGWPSPLSTNHQHLHSRNHLCLQRQPPSSKKSDGSGTYTKLQNTQVRVMSEKK
PRKRVESESKQEKANRIISEAIARAKERGERNIPRVMSPENFPSASVEGKEEKRGRRMKSKPKDRDNKKPKTY
SKLKEKTKIGKLIITLGKKHKRRNESSDELSDAEQRSQHTFKEQHSQKRRSNRQIKRKKYAEDAEGKQSEEEV
KGSLRVKRNSAPPPGEQPLQLFVENPSEEDAAIVDKILACRTVKKEVSPGVMLDIEEFFVKYKNYSYLHCEWA
TEQQLLKDKRIQQKIKRFKLRQAQRAHFLADMEEEPFNPDYVEVDRILEVSFCEDKDTGESVIYYLVKWCSLP
YEDSTWELKEDVDLAKIEEFEQLQASRPDTRHLDRPPSNIWKKIEQSREYKNGNQLREYQLEGLNWLLFNWYN
RRNCILADEMGLGKTIQSITFLYEILLTGIRGPFLIIAPLSTIANWEREFRTWTDINVVVYHGSLISRQMIQQ
YEMYFRDSQGRIIRGAYRFQAIITTFEMILGGCGELNAIDWRCVIIDEAHRLKNKNCKLLEGLKLMNLEHKVL
LTGTPLQNTVEELFSLLHFLEPLRFPSESTFMQEFGDLKTEEQVQKLQAILKPMMLRRLKEDVEKKLAPKEET
IIEVELTNIQKKYYRAILEKNFSFLSKGAGQTNVPNLVNTMMELRKCCNHPYLIKGAEEKILGEFRDTYNPSA
SDFHLQAMIQSAGKLVLIDKLLPKMKAGGHKVLIFSQMVRCLDILEDYLIHKRYLYERIDGRVRGNLRQAAID
RFSKPDSDRFVFLLCTRAGGLGINLTAADTCIIFDSDWNPQNDLQAQARCHRIGQNKAVKVYRLVTRNSYERE
MFDRASLKLGLDKAVLQSMSGRDSNVSGIQQLSKKEIEDLLRRGAYGAIMEEEDEGSKFCEEDIDQILLRRTK
TITIESEGRGSTFAKASFVASGNRTDISLDDPNFWQKWAKKAELDIDTISGRNSLVIDTPRIRKQTRPFSATK
DELAELSEAESEGEEKPKLRRPCDRSGGYGRTECFRVEKNLLVYGWGRWREILSHGRFKRQLNEHDVEVICRA
LLAYCLIHYRGDEKIKGFIWDLITPTEDGQTRELQNHLGLSAPVPRGRKGKKVKTQTSSFDIQKAEWLRKYNP
EQLLQDEGYKKHVKHHCNKVLLRVRMLYYLKQEVIGNESQKVFDGVDASDIDVWVPEPDHSEVPAAWWDFDAD
KSLLIGVFKHGYEKYNTIRADPALCFLERVGKPDDKAVAAEQRANDYMDGDVEDPEYKPAPAIFKDDIEDDVS
SPGDLVIADGEGQLMEGDKVYWPTPSALTTRLRRLITAYQRTNKNRHIQQMQPTFSLPANAMQPLYEEATLNP
KMAAKIERQQRIGRSKYFYSTNHRGTCF*
>rs33399640_ENSMUSP00000148088_c.382:A>G_p.Q128R
ENSMUST00000209423.2|Chd9-205|ENSMUSG00000056608|+|chr8:g.91659424:A>G
MTDPMMDFFDDANLFGETLEGLSDDTFVQPGPVSLVDELNLGAEFEPLHIDSLNHVQGTPTHQKMADFEQLSQ
FDSMKFHPVNQSFGSPVEHVLSPHSQFNCSPIHPPNQPNGLFQDVADGSPMWGHRTATGLANQNGSPFHQPGH
SHSLHQNKSFVAHPDFALFQASEHQTQCSSLHSQQSRSNLNPGQNSLGQAKNFLDANVSGAHRVNVNHLATAP
SSQQTLPVQFSPTANPPAHFLKCSSHQEGNYNRPSPSMTSCSVSNSQQFPSHYSFSSGHVSPSSLLQSSAGLA
PGHTNQALSDFAGSNSFSPHRGMKQEPTQHLLNPTPSLNSNNFQILHSSHPQGNYSNSKLSPVHMNFPDPVDA
GPPVGHFNDHAETNGFSSLEENLLHHVDSHAEPFAGLDPEDLLQEGLLPQFDESPFGQDNSNHVLDHDLDRQF
TSHLVSRPSDMAQTQLQYQARGWPSPLSTNHQHLHSRNHLCLQRQPPSSKKSDGSGTYTKLQNTQVRVMSEKK
PRKRVESESKQEKANRIISEAIARAKERGERNIPRVMSPENFPSASVEGKEEKRGRRMKSKPKDRDNKKPKTY
SKLKEKTKIGKLIITLGKKHKRRNESSDELSDAEQRSQHTFKEQHSQKRRSNRQIKRKKYAEDAEGKQSEEEV
KGSLRVKRNSAPPPGEQPLQLFVENPSEEDAAIVDKILACRTVKKEVSPGVMLDIEEFFVKYKNYSYLHCEWA
TEQQLLKDKRIQQKIKRFKLRQAQRAHFLADMEEEPFNPDYVEVDRILEVSFCEDKDTGESVIYYLVKWCSLP
YEDSTWELKEDVDLAKIEEFEQLQASRPDTRHLDRPPSNIWKKIEQSREYKNGNQLREYQLEGLNWLLFNWYN
RRNCILADEMGLGKTIQSITFLYEILLTGIRGPFLIIAPLSTIANWEREFRTWTDINVVVYHGSLISRQMIQQ
YEMYFRDSQGRIIRGAYRFQAIITTFEMILGGCGELNAIDWRCVIIDEAHRLKNKNCKLLEGLKLMNLEHKVL
LTGTPLQNTVEELFSLLHFLEPLRFPSESTFMQEFGDLKTEEQVQKLQAILKPMMLRRLKEDVEKKLAPKEET
IIEVELTNIQKKYYRAILEKNFSFLSKGAGQTNVPNLVNTMMELRKCCNHPYLIKGAEEKILGEFRDTYNPSA
SDFHLQAMIQSAGKLVLIDKLLPKMKAGGHKVLIFSQMVRCLDILEDYLIHKRYLYERIDGRVRGNLRQAAID
RFSKPDSDRFVFLLCTRAGGLGINLTAADTCIIFDSDWNPQNDLQAQARCHRIGQNKAVKVYRLVTRNSYERE
MFDRASLKLGLDKAVLQSMSGRDSNVSGIQQLSKKEIEDLLRRGAYGAIMEEEDEGSKFCEEDIDQILLRRTK
TITIESEGRGSTFAKASFVASGNRTDISLDDPNFWQKWAKKAELDIDTISGRNSLVIDTPRIRKQTRPFSATK
DELAELSEAESEGEEKPKLRRPCDRSGGYGRTECFRVEKNLLVYGWGRWREILSHGRFKRQLNEHDVEVICRA
LLAYCLIHYRGDEKIKGFIWDLITPTEDGQTRELQNHLGLSAPVPRGRKGKKVKTQTSSFDIQKAEWLRKYNP
EQLLQDEGYKKHVKHHCNKVLLRVRMLYYLKQEVIGNESQKVFDGVDASDIDVWVPEPDHSEVPAAWWDFDAD
KSLLIGVFKHGYEKYNTIRADPALCFLERVGKPDDKAVAAEQRANDYMDGDVEDPEYKPAPAIFKDDIEDDVS
SPGDLVIADGEGQLMEGDKVYWPTPSALTTRLRRLITAYQRTNKNRHIQQMQPTFSLPANAMQPLYEEATLNP
KMAAKIERQQRWTRREEADFYRVVSTFGVVFDPDRGQFDWTKFRALARLHKKTDNSLEKYLCAFMSMCRRVCR
LPSKEELVDPNIFIQPITEERASRTLYRIELLRKVREQALRHPQLFERLKLCHPNPDLPIWWECGSHDRDLLI
GAAKHGVSRTDYHILRDPELSFMAAQRNYNQSKAAHSRTSAPLLQQYQVALSASPLTSLPRLLGAKGTLLEDM
KVKSESLTEEPQSSEEESMSSMETRTRVKSEPVSPKNGVLSQATGDQKSGGKSETDRRMVAARTEPLTPNPAS
KKPRVHKRGSQSSSDSDSDSARSSCSSRSSSSSSSSSSCSHSRSGSSSSSSSSCSSASSSSSSSSSSSSSSS
SSSEESDSEEDVQKREGTPHRKAYDEESVASLSTTQDETQDSFQANNGTPESAYLLQGGYMLAASYWPKDRVM
INRLDSICQTVLKGKWPSARRHYDANTVASFYTTKLLDSPGAATERGEPSVPTPPAVAVREEHEQSAQMSKVK
KHVREKEFTVKIKDEGGLKLTFQKQGLAQKRPFDGEDGALGQQQYLTRLRELQSTSETSLVNLPKAVPASGTS
IQPTLGANGAILDSQPIVKKRRGRRRNVEGADILFLNRNKPPNHIPTGMNPALSYPQPQRIPDTESPVPVINL
KDGTRLAGDDAPKRKDLDRWLKEHPGYVEDLGAFIPRVQLHEGRPKQKRHRCRNPNKLDINSLTGEERVQLIN

RRNARKVGGAFAPPLKDLCRFLKENSEYGVAPEWGDVVKQSGFLPESMFERILTGPVVREEVSRRGRRPKSGI
AKATTAAAVPAGSVPGNPLLANGLLPGVDLTALQALQQNLQNLQSLQVTAGLMGMPAGLSSGGETKNMAAMFP
MLFSGMAGLPNLLGMGGLLSKTAESGAEEKRGNDSKELEGKKERTESQSPENGGERCVPGSPSTSSTAALSSA
AAAKPIALNPLLLSNILYPGMLLTPGLNLHLPTLSQSNAFDVQKNKSDDLDSSKSVEIKEENSRVRDQEEKGG
TEPSPLNENSTDEGSERASSGSDSSSSSSEDSDSSNED*
>rs33399640_ENSMUSP00000105243_c.382:A>G_p.Q128R
ENSMUST00000109614.9|Chd9-202|ENSMUSG00000056608|+|chr8:g.91659424:A>G
MTDPMMDFFDDANLFGETLEGLSDDTFVQPGPVSLVDELNLGAEFEPLHIDSLNHVQGTPTHQKMADFEQLSQ
FDSMKFHPVNQSFGSPVEHVLSPHSQFNCSPIHPPNQPNGLFQDVADGSPMWGHRTATGLANQNGSPFHQPGH
SHSLHQNKSFVAHPDFALFQASEHQTQCSSLHSQQSRSNLNPGQNSLGQAKNFLDANVSGAHRVNVNHLATAP
SSQQTLPVQFSPTANPPAHFLKCSSHQEGNYNRPSPSMTSCSVSNSQQFPSHYSFSSGHVSPSSLLQSSAGLA
PGHTNQALSDFAGSNSFSPHRGMKQEPTQHLLNPTPSLNSNNFQILHSSHPQGNYSNSKLSPVHMNFPDPVDA
GPPVGHFNDHAETNGFSSLEENLLHHVDSHAEPFAGLDPEDLLQEGLLPQFDESPFGQDNSNHVLDHDLDRQF
TSHLVSRPSDMAQTQLQYQARGWPSPLSTNHQHLHSRNHLCLQRQPPSSKKSDGSGTYTKLQNTQVRVMSEKK
PRKRVESESKQEKANRIISEAIARAKERGERNIPRVMSPENFPSASVEGKEEKRGRRMKSKPKDRDNKKPKTY
SKLKEKTKIGKLIITLGKKHKRRNESSDELSDAEQRSQHTFKEQHSQKRRSNRQIKRKKYAEDAEGKQSEEEV
KGSLRVKRNSAPPPGEQPLQLFVENPSEEDAAIVDKILACRTVKKEVSPGVMLDIEEFFVKYKNYSYLHCEWA
TEQQLLKDKRIQQKIKRFKLRQAQRAHFLADMEEEPFNPDYVEVDRILEVSFCEDKDTGESVIYYLVKWCSLP
YEDSTWELKEDVDLAKIEEFEQLQASRPDTRHLDRPPSNIWKKIEQSREYKNGNQLREYQLEGLNWLLFNWYN
RRNCILADEMGLGKTIQSITFLYEILLTGIRGPFLIIAPLSTIANWEREFRTWTDINVVVYHGSLISRQMIQQ
YEMYFRDSQGRIIRGAYRFQAIITTFEMILGGCGELNAIDWRCVIIDEAHRLKNKNCKLLEGLKLMNLEHKVL
LTGTPLQNTVEELFSLLHFLEPLRFPSESTFMQEFGDLKTEEQVQKLQAILKPMMLRRLKEDVEKKLAPKEET
IIEVELTNIQKKYYRAILEKNFSFLSKGAGQTNVPNLVNTMMELRKCCNHPYLIKGAEEKILGEFRDTYNPSA
SDFHLQAMIQSAGKLVLIDKLLPKMKAGGHKVLIFSQMVRCLDILEDYLIHKRYLYERIDGRVRGNLRQAAID
RFSKPDSDRFVFLLCTRAGGLGINLTAADTCIIFDSDWNPQNDLQAQARCHRIGQNKAVKVYRLVTRNSYERE
MFDRASLKLGLDKAVLQSMSGRDSNVSGIQQLSKKEIEDLLRRGAYGAIMEEEDEGSKFCEEDIDQILLRRTK
TITIESEGRGSTFAKASFVASGNRTDISLDDPNFWQKWAKKAELDIDTISGRNSLVIDTPRIRKQTRPFSATK
DELAELSEAESEGEEKPKLRRPCDRSGGYGRTECFRVEKNLLVYGWGRWREILSHGRFKRQLNEHDVEVICRA
LLAYCLIHYRGDEKIKGFIWDLITPTEDGQTRELQNHLGLSAPVPRGRKGKKVKTQTSSFDIQKAEWLRKYNP
EQLLQDEGYKKHVKHHCNKVLLRVRMLYYLKQEVIGNESQKVFDGVDASDIDVWVPEPDHSEVPAAWWDFDAD
KSLLIGVFKHGYEKYNTIRADPALCFLERVGKPDDKAVAAEQRANDYMDGDVEDPEYKPAPAIFKDDIEDDVS
SPGDLVIADGEGQLMEGDKVYWPTPSALTTRLRRLITAYQRTNKNRHIQQMQPTFSLPANAMQPLYEEATLNP
KMAAKIERQQRWTRREEADFYRVVSTFGVVFDPDRGQFDWTKFRALARLHKKTDNSLEKYLCAFMSMCRRVCR
LPSKEELVDPNIFIQPITEERASRTLYRIELLRKVREQALRHPQLFERLKLCHPNPDLPIWWECGSHDRDLLI
GAAKHGVSRTDYHILRDPELSFMAAQRNYNQSKAAHSRTSAPLLQQYQVALSASPLTSLPRLLGAKGTLLEDM
KVKSESLTEEPQSSEEESMSSMETRTRVKSEPVSPKNGVLSQATGDQKSGGKSETDRRMVAARTEPLTPNPAS
KKPRVHKRGSQSSSDSDSDSARSSCSSRSSSSSSSSSCSHSRSGSSSSSSSSCSSASSSSSSSSSSSSSSSS
SSSEESDSEEDVQKREGTPHRKAYDEESVASLSTTQDETQDSFQANNGTPESAYLLQGGYMLAASYWPKDRVM
INRLDSICQTVLKGKWPSARRHYDANTVASFYTTKLLDSPGAATERGEPSVPTPPAVAVREEHEQSAQMSKVK
KHVREKEFTVKIKDEGGLKLTFQKQGLAQKRPFDGEDGALGQQQYLTRLRELQSTSETSLVNLPKAVPASGTS
IQPTLGANGAILDSQPIVKKRRGRRRNVEGADILFLNRNKPPNHIPTGMNPALSYPQPQRIPDTESPVPVINL
KDGTRLAGDDAPKRKDLDRWLKEHPGYVEDLGAFIPRVQLHEGRPKQKRHRCRNPNKLDINSLTGEERVQLIN
RRNARKVGGAFAPPLKDLCRFLKENSEYGVAPEWGDVVKQSGFLPESMFERILTGPVVREEVSRRGRRPKSGI
AKATTAAAVPAGSVPGNPLLANGLLPGVDLTALQALQQNLQNLQSLQVTAGLMGMPAGLSSGGETKNMAAMFP
MLFSGMAGLPNLLGMGGLLSKTAESGAEEKRGNDSKELEGKKERTESQSPENGGERCVPGSPSTSSTAALSSA
AAAKPIALNPLLLSNILYPGMLLTPGLNLHLPTLSQSNAFDVQKNKSDDLDSSKSVEIKEENSRVRDQEEKGG
TEPSPLNENSTDEGSERASSGSDSSSSSSEDSDSSNED*
>rs48347616_ENSMUSP00000046356_c.2422:C>T_p.T808I
ENSMUST00000048665.8|Chd9-201|ENSMUSG00000056608|+|chr8:g.91710948:C>T
MTDPMMDFFDDANLFGETLEGLSDDTFVQPGPVSLVDELNLGAEFEPLHIDSLNHVQGTPTHQKMADFEQLSQ
FDSMKFHPVNQSFGSPVEHVLSPHSQFNCSPIHPPNQPNGLFQDVADGSPMWGHQTATGLANQNGSPFHQPGH
SHSLHQNKSFVAHPDFALFQASEHQTQCSSLHSQQSRSNLNPGQNSLGQAKNFLDANVSGAHRVNVNHLATAP
SSQQTLPVQFSPTANPPAHFLKCSSHQEGNYNRPSPSMTSCSVSNSQQFPSHYSFSSGHVSPSSLLQSSAGLA
PGHTNQALSDFAGSNSFSPHRGMKQEPTQHLLNPTPSLNSNNFQILHSSHPQGNYSNSKLSPVHMNFPDPVDA
GPPVGHFNDHAETNGFSSLEENLLHHVDSHAEPFAGLDPEDLLQEGLLPQFDESPFGQDNSNHVLDHDLDRQF
TSHLVSRPSDMAQTQLQYQARGWPSPLSTNHQHLHSRNHLCLQRQPPSSKKSDGSGTYTKLQNTQVRVMSEKK
PRKRVESESKQEKANRIISEAIARAKERGERNIPRVMSPENFPSASVEGKEEKRGRRMKSKPKDRDNKKPKTY
SKLKEKTKIGKLIITLGKKHKRRNESSDELSDAEQRSQHTFKEQHSQKRRSNRQIKRKKYAEDAEGKQSEEEV

KGSLRVKRNSAPPPGEQPLQLFVENPSEEDAAIVDKILACRTVKKEVSPGVMLDIEEFFVKYKNYSYLHCEWA
TEQQLLKDKRIQQKIKRFKLRQAQRAHFLADMEEEPFNPDYVEVDRILEVSFCEDKDTGESVIYYLVKWCSLP
YEDSIWELKEDVDLAKIEEFEQLQASRPDTRHLDRPPSNIWKKIEQSREYKNGNQLREYQLEGLNWLLFNWYN
RRNCILADEMGLGKTIQSITFLYEILLTGIRGPFLIIAPLSTIANWEREFRTWTDINVVVYHGSLISRQMIQQ
YEMYFRDSQGRIIRGAYRFQAIITTFEMILGGCGELNAIDWRCVIIDEAHRLKNKNCKLLEGLKLMNLEHKVL
LTGTPLQNTVEELFSLLHFLEPLRFPSESTFMQEFGDLKTEEQVQKLQAILKPMMLRRLKEDVEKKLAPKEET
IIEVELTNIQKKYYRAILEKNFSFLSKGAGQTNVPNLVNTMMELRKCCNHPYLIKGAEEKILGEFRDTYNPSA
SDFHLQAMIQSAGKLVLIDKLLPKMKAGGHKVLIFSQMVRCLDILEDYLIHKRYLYERIDGRVRGNLRQAAID
RFSKPDSDRFVFLLCTRAGGLGINLTAADTCIIFDSDWNPQNDLQAQARCHRIGQNKAVKVYRLVTRNSYERE
MFDRASLKLGLDKAVLQSMSGRDSNVSGIQQLSKKEIEDLLRRGAYGAIMEEEDEGSKFCEEDIDQILLRRTK
TITIESEGRGSTFAKASFVASGNRTDISLDDPNFWQKWAKKAELDIDTISGRNSLVIDTPRIRKQTRPFSATK
DELAELSEAESEGEEKPKLRRPCDRSGGYGRTECFRVEKNLLVYGWGRWREILSHGRFKRQLNEHDVEVICRA
LLAYCLIHYRGDEKIKGFIWDLITPTEDGQTRELQNHLGLSAPVPRGRKGKKVKTQTSSFDIQKAEWLRKYNP
EQLLQDEGYKKHVKHHCNKVLLRVRMLYYLKQEVIGNESQKVFDGVDASDIDVWVPEPDHSEVPAAWWDFDAD
KSLLIGVFKHGYEKYNTIRADPALCFLERVGKPDDKAVAAEQRANDYMDGDVEDPEYKPAPAIFKDDIEDDVS
SPGDLVIADGEGQLMEGDKVYWPTPSALTTRLRRLITAYQRTNKNRHIQQMQPTFSLPANAMQPLYEEATLNP
KMAAKIERQQRWTRREEADFYRVVSTFGVVFDPDRGQFDWTKFRALARLHKKTDNSLEKYLCAFMSMCRRVCR
LPSKEELVDPNIFIQPITEERASRTLYRIELLRKVREQALRHPQLFERLKLCHPNPDLPIWWECGSHDRDLLI
GAAKHGVSRTDYHILRDPELSFMAAQRNYNQSKAAHSRTSAPLLQQYQVALSASPLTSLPRLLGAKGTLLEDM
KVKSESLTEEPQSSEEESMSSMETRTRVKSEPVSPKNGVLSQATGDQKSGGKSETDRRMVAARTEPLTPNPAS
KKPRVHKRGSQSSSDSDSDSARSSCSSRSSSSSSSSSSCSHSRSGSSSSSSSSCSSASSSSSSSSSSSSSSS
SSSEESDSEEDVQKREGTPHRKAYDEESVASLSTTQDETQDSFQANNGTPESAYLLQGGYMLAASYWPKDRVM
INRLDSICQTVLKGKWPSARRHYDANTVASFYTTKLLDSPGAATERGEPSVPTPPAVAVREEHEQSAQMSKEG
GLKLTFQKQGLAQKRPFDGEDGALGQQQYLTRLRELQSTSETSLVNLPKAVPASGTSIQPTLGANGAILDSQP
IVKKRRGRRRNVEGADILFLNRNKPPNHIPTGMNPALSYPQPQRIPDTESPVPVINLKDGTRLAGDDAPKRKD
LDRWLKEHPGYVEDLGAFIPRVQLHEGRPKQKRHRCRNPNKLDINSLTGEERVQLINRRNARKVGGAFAPPLK
DLCRFLKENSEYGVAPEWGDVVKQSGFLPESMFERILTGPVVREEVSRRGRRPKSGIAKATTAAVPAGSVPG
NPLLANGLLPGVDLTALQALQQNLQNLQSLQVTAGLMGMPAGLSSGGETKNMAAMFPMLFSGMAGLPNLLGMG
GLLSKTAESGAEEKRGNDSKELEGKKERTESQSPENGGERCVPGSPSTSSTAALSSAAAAKPIALNPLLLSNI
LYPGMLLTPGLNLHLPTLSQSNAFDVQKNKSDDLDSSKSVEIKEENSRVRDQEEKGGTEPSPLNENSTDEGSE
RASSGSDSSSSSSEDSDSSNED*
>rs48347616_ENSMUSP00000147407_c.2422:C>T_p.T808I
ENSMUST00000209203.2|Chd9-203|ENSMUSG00000056608|+|chr8:g.91710948:C>T
MTDPMMDFFDDANLFGETLEGLSDDTFVQPGPVSLVDELNLGAEFEPLHIDSLNHVQGTPTHQKMADFEQLSQ
FDSMKFHPVNQSFGSPVEHVLSPHSQFNCSPIHPPNQPNGLFQDVADGSPMWGHQTATGLANQNGSPFHQPGH
SHSLHQNKSFVAHPDFALFQASEHQTQCSSLHSQQSRSNLNPGQNSLGQAKNFLDANVSGAHRVNVNHLATAP
SSQQTLPVQFSPTANPPAHFLKCSSHQEGNYNRPSPSMTSCSVSNSQQFPSHYSFSSGHVSPSSLLQSSAGLA
PGHTNQALSDFAGSNSFSPHRGMKQEPTQHLLNPTPSLNSNNFQILHSSHPQGNYSNSKLSPVHMNFPDPVDA
GPPVGHFNDHAETNGFSSLEENLLHHVDSHAEPFAGLDPEDLLQEGLLPQFDESPFGQDNSNHVLDHDLDRQF
TSHLVSRPSDMAQTQLQYQARGWPSPLSTNHQHLHSRNHLCLQRQPPSSKKSDGSGTYTKLQNTQVRVMSEKK
PRKRVESESKQEKANRIISEAIARAKERGERNIPRVMSPENFPSASVEGKEEKRGRRMKSKPKDRDNKKPKTY
SKLKEKTKIGKLIITLGKKHKRRNESSDELSDAEQRSQHTFKEQHSQKRRSNRQIKRKKYAEDAEGKQSEEEV
KGSLRVKRNSAPPPGEQPLQLFVENPSEEDAAIVDKILACRTVKKEVSPGVMLDIEEFFVKYKNYSYLHCEWA
TEQQLLKDKRIQQKIKRFKLRQAQRAHFLADMEEEPFNPDYVEVDRILEVSFCEDKDTGESVIYYLVKWCSLP
YEDSIWELKEDVDLAKIEEFEQLQASRPDTRHLDRPPSNIWKKIEQSREYKNGNQLREYQLEGLNWLLFNWYN
RRNCILADEMGLGKTIQSITFLYEILLTGIRGPFLIIAPLSTIANWEREFRTWTDINVVVYHGSLISRQMIQQ
YEMYFRDSQGRIIRGAYRFQAIITTFEMILGGCGELNAIDWRCVIIDEAHRLKNKNCKLLEGLKLMNLEHKVL
LTGTPLQNTVEELFSLLHFLEPLRFPSESTFMQEFGDLKTEEQVQKLQAILKPMMLRRLKEDVEKKLAPKEET
IIEVELTNIQKKYYRAILEKNFSFLSKGAGQTNVPNLVNTMMELRKCCNHPYLIKGAEEKILGEFRDTYNPSA
SDFHLQAMIQSAGKLVLIDKLLPKMKAGGHKVLIFSQMVRCLDILEDYLIHKRYLYERIDGRVRGNLRQAAID
RFSKPDSDRFVFLLCTRAGGLGINLTAADTCIIFDSDWNPQNDLQAQARCHRIGQNKAVKVYRLVTRNSYERE
MFDRASLKLGLDKAVLQSMSGRDSNVSGIQQLSKKEIEDLLRRGAYGAIMEEEDEGSKFCEEDIDQILLRRTK
TITIESEGRGSTFAKASFVASGNRTDISLDDPNFWQKWAKKAELDIDTISGRNSLVIDTPRIRKQTRPFSATK
DELAELSEAESEGEEKPKLRRPCDRSGGYGRTECFRVEKNLLVYGWGRWREILSHGRFKRQLNEHDVEVICRA
LLAYCLIHYRGDEKIKGFIWDLITPTEDGQTRELQNHLGLSAPVPRGRKGKKVKTQTSSFDIQKAEWLRKYNP
EQLLQDEGYKKHVKHHCNKVLLRVRMLYYLKQEVIGNESQKVFDGVDASDIDVWVPEPDHSEVPAAWWDFDAD
KSLLIGVFKHGYEKYNTIRADPALCFLERVGKPDDKAVAAEQRANDYMDGDVEDPEYKPAPAIFKDDIEDDVS

SPGDLVIADGEGQLMEGDKVYWPTPSALTTRLRRLITAYQRTNKNRHIQQMQPTFSLPANAMQPLYEEATLNP
KMAAKIERQQRIGRSKYFYSTNHRGTCF*
>rs48347616_ENSMUSP00000148088_c.2422:C>T_p.T808I
ENSMUST00000209423.2|Chd9-205|ENSMUSG00000056608|+|chr8:g.91710948:C>T
MTDPMMDFFDDANLFGETLEGLSDDTFVQPGPVSLVDELNLGAEFEPLHIDSLNHVQGTPTHQKMADFEQLSQ
FDSMKFHPVNQSFGSPVEHVLSPHSQFNCSPIHPPNQPNGLFQDVADGSPMWGHQTATGLANQNGSPFHQPGH
SHSLHQNKSFVAHPDFALFQASEHQTQCSSLHSQQSRSNLNPGQNSLGQAKNFLDANVSGAHRVNVNHLATAP
SSQQTLPVQFSPTANPPAHFLKCSSHQEGNYNRPSPSMTSCSVSNSQQFPSHYSFSSGHVSPSSLLQSSAGLA
PGHTNQALSDFAGSNSFSPHRGMKQEPTQHLLNPTPSLNSNNFQILHSSHPQGNYSNSKLSPVHMNFPDPVDA
GPPVGHFNDHAETNGFSSLEENLLHHVDSHAEPFAGLDPEDLLQEGLLPQFDESPFGQDNSNHVLDHDLDRQF
TSHLVSRPSDMAQTQLQYQARGWPSPLSTNHQHLHSRNHLCLQRQPPSSKKSDGSGTYTKLQNTQVRVMSEKK
PRKRVESESKQEKANRIISEAIARAKERGERNIPRVMSPENFPSASVEGKEEKRGRRMKSKPKDRDNKKPKTY
SKLKEKTKIGKLIITLGKKHKRRNESSDELSDAEQRSQHTFKEQHSQKRRSNRQIKRKKYAEDAEGKQSEEEV
KGSLRVKRNSAPPPGEQPLQLFVENPSEEDAAIVDKILACRTVKKEVSPGVMLDIEEFFVKYKNYSYLHCEWA
TEQQLLKDKRIQQKIKRFKLRQAQRAHFLADMEEEPFNPDYVEVDRILEVSFCEDKDTGESVIYYLVKWCSLP
YEDSIWELKEDVDLAKIEEFEQLQASRPDTRHLDRPPSNIWKKIEQSREYKNGNQLREYQLEGLNWLLFNWYN
RRNCILADEMGLGKTIQSITFLYEILLTGIRGPFLIIAPLSTIANWEREFRTWTDINVVVYHGSLISRQMIQQ
YEMYFRDSQGRIIRGAYRFQAIITTFEMILGGCGELNAIDWRCVIIDEAHRLKNKNCKLLEGLKLMNLEHKVL
LTGTPLQNTVEELFSLLHFLEPLRFPSESTFMQEFGDLKTEEQVQKLQAILKPMMLRRLKEDVEKKLAPKEET
IIEVELTNIQKKYYRAILEKNFSFLSKGAGQTNVPNLVNTMMELRKCCNHPYLIKGAEEKILGEFRDTYNPSA
SDFHLQAMIQSAGKLVLIDKLLPKMKAGGHKVLIFSQMVRCLDILEDYLIHKRYLYERIDGRVRGNLRQAAID
RFSKPDSDRFVFLLCTRAGGLGINLTAADTCIIFDSDWNPQNDLQAQARCHRIGQNKAVKVYRLVTRNSYERE
MFDRASLKLGLDKAVLQSMSGRDSNVSGIQQLSKKEIEDLLRRGAYGAIMEEEDEGSKFCEEDIDQILLRRTK
TITIESEGRGSTFAKASFVASGNRTDISLDDPNFWQKWAKKAELDIDTISGRNSLVIDTPRIRKQTRPFSATK
DELAELSEAESEGEEKPKLRRPCDRSGGYGRTECFRVEKNLLVYGWGRWREILSHGRFKRQLNEHDVEVICRA
LLAYCLIHYRGDEKIKGFIWDLITPTEDGQTRELQNHLGLSAPVPRGRKGKKVKTQTSSFDIQKAEWLRKYNP
EQLLQDEGYKKHVKHHCNKVLLRVRMLYYLKQEVIGNESQKVFDGVDASDIDVWVPEPDHSEVPAAWWDFDAD
KSLLIGVFKHGYEKYNTIRADPALCFLERVGKPDDKAVAAEQRANDYMDGDVEDPEYKPAPAIFKDDIEDDVS
SPGDLVIADGEGQLMEGDKVYWPTPSALTTRLRRLITAYQRTNKNRHIQQMQPTFSLPANAMQPLYEEATLNP
KMAAKIERQQRWTRREEADFYRVVSTFGVVFDPDRGQFDWTKFRALARLHKKTDNSLEKYLCAFMSMCRRVCR
LPSKEELVDPNIFIQPITEERASRTLYRIELLRKVREQALRHPQLFERLKLCHPNPDLPIWWECGSHDRDLLI
GAAKHGVSRTDYHILRDPELSFMAAQRNYNQSKAAHSRTSAPLLQQYQVALSASPLTSLPRLLGAKGTLLEDM
KVKSESLTEEPQSSEEESMSSMETRTRVKSEPVSPKNGVLSQATGDQKSGGKSETDRRMVAARTEPLTPNPAS
KKPRVHKRGSQSSSDSDSDSARSSCSSRSSSSSSSSSCSHSRSGSSSSSSSSCSSASSSSSSSSSSSSSSS
SSSEESDSEEDVQKREGTPHRKAYDEESVASLSTTQDETQDSFQANNGTPESAYLLQGGYMLAASYWPKDRVM
INRLDSICQTVLKGKWPSARRHYDANTVASFYTTKLLDSPGAATERGEPSVPTPPAVAVREEHEQSAQMSKVK
KHVREKEFTVKIKDEGGLKLTFQKQGLAQKRPFDGEDGALGQQQYLTRLRELQSTSETSLVNLPKAVPASGTS
IQPTLGANGAILDSQPIVKKRRGRRRNVEGADILFLNRNKPPNHIPTGMNPALSYPQPQRIPDTESPVPVINL
KDGTRLAGDDAPKRKDLDRWLKEHPGYVEDLGAFIPRVQLHEGRPKQKRHRCRNPNKLDINSLTGEERVQLIN
RRNARKVGGAFAPPLKDLCRFLKENSEYGVAPEWGDVVKQSGFLPESMFERILTGPVVREEVSRRGRRPKSGI
AKATTAAAVPAGSVPGNPLLANGLLPGVDLTALQALQQNLQNLQSLQVTAGLMGMPAGLSSGGETKNMAAMFP
MLFSGMAGLPNLLGMGGLLSKTAESGAEEKRGNDSKELEGKKERTESQSPENGGERCVPGSPSTSSTAALSSA
AAAKPIALNPLLLSNILYPGMLLTPGLNLHLPTLSQSNAFDVQKNKSDDLDSSKSVEIKEENSRVRDQEEKGG
TEPSPLNENSTDEGSERASSGSDSSSSSSEDSDSSNED*
>rs48347616_ENSMUSP00000147741_c.1003:C>T_p.T335I
ENSMUST00000210947.2|Chd9-210|ENSMUSG00000056608|+|chr8:g.91710948:C>T
MSSVKRPRGRPPSSKKSDGSGTYTKLQNTQVRVMSEKKPRKRVESESKQEKANRIISEAIARAKERGERNIPR
VMSPENFPSASVEGKEEKRGRRMKSKPKDRDNKKPKTYSKLKEKTKIGKLIITLGKKHKRRNESSDELSDAEQ
RSQHTFKEQHSQKRRSNRQIKRKKYAEDAEGKQSEEEVKGSLRVKRNSAPPPGEQPLQLFVENPSEEDAAIVD
KILACRTVKKEVSPGVMLDIEEFFVKYKNYSYLHCEWATEQQLLKDKRIQQKIKRFKLRQAQRAHFLADMEEE
PFNPDYVEVDRILEVSFCEDKDTGESVIYYLVKWCSLPYEDSIWELKEDVDLAKIEEFEQLQASRPDTRHLDR
PPSNIWKKIEQSREYKNGNQLREYQLEGLNWLLFNWYNRRNCILADEMGLGKTIQSITFLYEILLTGIRGPFL
IIAPLSTIANWEREFRTWTDINVVVYHGSLISRQMIQQYEMYFRDSQGRIIRGAYRFQAIITTFEMILGGCGE
LNAIDWRCVIIDEAHRLKNKNCKLLEGLKLMNLEHKVLLTGTPLQNTVEELFSLLHFLEPLRFPSESTFMQEF
GDLKTEEQVQKLQAILKPMMLRRLKEDVEKKLAPKEETIIEVELTNIQKKYYRAILEKNFSFLSKGAGQTNVP
NLVNTMMELRKCCNHPYLIKGAEEKILGEFRDTYNPSASDFHLQAMIQSAGKLVLIDKLLPKMKAGGHKVLIF
SQMVRCLDILEDYLIHKRYLYERIDGRVRGNLRQAAIDRFSKPDSDRFVFLLCTRAGGLGINLTAADTCIIFD
SDWNPQNDLQAQARCHRIGQNKAVKVYRLVTRNSYEREMFDRASLKLGLDKAVLQSMSGRDSNVSGIQQLS

>rs48347616_ENSMUSP00000105243_c.2422:C>T_p.T808I
ENSMUST00000109614.9|Chd9-202|ENSMUSG00000056608|+|chr8:g.91710948:C>T
MTDPMMDFFDDANLFGETLEGLSDDTFVQPGPVSLVDELNLGAEFEPLHIDSLNHVQGTPTHQKMADFEQLSQ
FDSMKFHPVNQSFGSPVEHVLSPHSQFNCSPIHPPNQPNGLFQDVADGSPMWGHQTATGLANQNGSPFHQPGH
SHSLHQNKSFVAHPDFALFQASEHQTQCSSLHSQQSRSNLNPGQNSLGQAKNFLDANVSGAHRVNVNHLATAP
SSQQTLPVQFSPTANPPAHFLKCSSHQEGNYNRPSPSMTSCSVSNSQQFPSHYSFSSGHVSPSSLLQSSAGLA
PGHTNQALSDFAGSNSFSPHRGMKQEPTQHLLNPTPSLNSNNFQILHSSHPQGNYSNSKLSPVHMNFPDPVDA
GPPVGHFNDHAETNGFSSLEENLLHHVDSHAEPFAGLDPEDLLQEGLLPQFDESPFGQDNSNHVLDHDLDRQF
TSHLVSRPSDMAQTQLQYQARGWPSPLSTNHQHLHSRNHLCLQRQPPSSKKSDGSGTYTKLQNTQVRVMSEKK
PRKRVESESKQEKANRIISEAIARAKERGERNIPRVMSPENFPSASVEGKEEKRGRRMKSKPKDRDNKKPKTY
SKLKEKTKIGKLIITLGKKHKRRNESSDELSDAEQRSQHTFKEQHSQKRRSNRQIKRKKYAEDAEGKQSEEEV
KGSLRVKRNSAPPPGEQPLQLFVENPSEEDAAIVDKILACRTVKKEVSPGVMLDIEEFFVKYKNYSYLHCEWA
TEQQLLKDKRIQQKIKRFKLRQAQRAHFLADMEEEPFNPDYVEVDRILEVSFCEDKDTGESVIYYLVKWCSLP
YEDSIWELKEDVDLAKIEEFEQLQASRPDTRHLDRPPSNIWKKIEQSREYKNGNQLREYQLEGLNWLLFNWYN
RRNCILADEMGLGKTIQSITFLYEILLTGIRGPFLIIAPLSTIANWEREFRTWTDINVVVYHGSLISRQMIQQ
YEMYFRDSQGRIIRGAYRFQAIITTFEMILGGCGELNAIDWRCVIIDEAHRLKNKNCKLLEGLKLMNLEHKVL
LTGTPLQNTVEELFSLLHFLEPLRFPSESTFMQEFGDLKTEEQVQKLQAILKPMMLRRLKEDVEKKLAPKEET
IIEVELTNIQKKYYRAILEKNFSFLSKGAGQTNVPNLVNTMMELRKCCNHPYLIKGAEEKILGEFRDTYNPSA
SDFHLQAMIQSAGKLVLIDKLLPKMKAGGHKVLIFSQMVRCLDILEDYLIHKRYLYERIDGRVRGNLRQAAID
RFSKPDSDRFVFLLCTRAGGLGINLTAADTCIIFDSDWNPQNDLQAQARCHRIGQNKAVKVYRLVTRNSYERE
MFDRASLKLGLDKAVLQSMSGRDSNVSGIQQLSKKEIEDLLRRGAYGAIMEEEDEGSKFCEEDIDQILLRRTK
TITIESEGRGSTFAKASFVASGNRTDISLDDPNFWQKWAKKAELDIDTISGRNSLVIDTPRIRKQTRPFSATK
DELAELSEAESEGEEKPKLRRPCDRSGGYGRTECFRVEKNLLVYGWGRWREILSHGRFKRQLNEHDVEVICRA
LLAYCLIHYRGDEKIKGFIWDLITPTEDGQTRELQNHLGLSAPVPRGRKGKKVKTQTSSFDIQKAEWLRKYNP
EQLLQDEGYKKHVKHHCNKVLLRVRMLYYLKQEVIGNESQKVFDGVDASDIDVWVPEPDHSEVPAAWWDFDAD
KSLLIGVFKHGYEKYNTIRADPALCFLERVGKPDDKAVAAEQRANDYMDGDVEDPEYKPAPAIFKDDIEDDVS
SPGDLVIADGEGQLMEGDKVYWPTPSALTTRLRRLITAYQRTNKNRHIQQMQPTFSLPANAMQPLYEEATLNP
KMAAKIERQQRWTRREEADFYRVVSTFGVVFDPDRGQFDWTKFRALARLHKKTDNSLEKYLCAFMSMCRRVCR
LPSKEELVDPNIFIQPITEERASRTLYRIELLRKVREQALRHPQLFERLKLCHPNPDLPIWWECGSHDRDLLI
GAAKHGVSRTDYHILRDPELSFMAAQRNYNQSKAAHSRTSAPLLQQYQVALSASPLTSLPRLLGAKGTLLEDM
KVKSESLTEEPQSSEEESMSSMETRTRVKSEPVSPKNGVLSQATGDQKSGGKSETDRRMVAARTEPLTPNPAS
KKPRVHKRGSQSSSDSDSDSARSSCSSRSSSSSSSSSCSHSRSGSSSSSSSSCSSASSSSSSSSSSSSSSSS
SSSEESDSEEDVQKREGTPHRKAYDEESVASLSTTQDETQDSFQANNGTPESAYLLQGGYMLAASYWPKDRVM
INRLDSICQTVLKGKWPSARRHYDANTVASFYTTKLLDSPGAATERGEPSVPTPPAVAVREEHEQSAQMSKVK
KHVREKEFTVKIKDEGGLKLTFQKQGLAQKRPFDGEDGALGQQQYLTRLRELQSTSETSLVNLPKAVPASGTS
IQPTLGANGAILDSQPIVKKRRGRRRNVEGADILFLNRNKPPNHIPTGMNPALSYPQPQRIPDTESPVPVINL
KDGTRLAGDDAPKRKDLDRWLKEHPGYVEDLGAFIPRVQLHEGRPKQKRHRCRNPNKLDINSLTGEERVQLIN
RRNARKVGGAFAPPLKDLCRFLKENSEYGVAPEWGDVVKQSGFLPESMFERILTGPVVREEVSRRGRRPKSGI
AKATTAAAVPAGSVPGNPLLANGLLPGVDLTALQALQQNLQNLQSLQVTAGLMGMPAGLSSGGETKNMAAMFP
MLFSGMAGLPNLLGMGGLLSKTAESGAEEKRGNDSKELEGKKERTESQSPENGGERCVPGSPSTSSTAALSSA
AAAKPIALNPLLLSNILYPGMLLTPGLNLHLPTLSQSNAFDVQKNKSDDLDSSKSVEIKEENSRVRDQEEKGG
TEPSPLNENSTDEGSERASSGSDSSSSSSEDSDSSNED*
>rs261610524_ENSMUSP00000046356_c.3375:C>CG_p.Q1126ins-fs
ENSMUST00000048665.8|Chd9-201|ENSMUSG00000056608|+|chr8:g.91723885:C>CG
MTDPMMDFFDDANLFGETLEGLSDDTFVQPGPVSLVDELNLGAEFEPLHIDSLNHVQGTPTHQKMADFEQLSQ
FDSMKFHPVNQSFGSPVEHVLSPHSQFNCSPIHPPNQPNGLFQDVADGSPMWGHQTATGLANQNGSPFHQPGH
SHSLHQNKSFVAHPDFALFQASEHQTQCSSLHSQQSRSNLNPGQNSLGQAKNFLDANVSGAHRVNVNHLATAP
SSQQTLPVQFSPTANPPAHFLKCSSHQEGNYNRPSPSMTSCSVSNSQQFPSHYSFSSGHVSPSSLLQSSAGLA
PGHTNQALSDFAGSNSFSPHRGMKQEPTQHLLNPTPSLNSNNFQILHSSHPQGNYSNSKLSPVHMNFPDPVDA
GPPVGHFNDHAETNGFSSLEENLLHHVDSHAEPFAGLDPEDLLQEGLLPQFDESPFGQDNSNHVLDHDLDRQF
TSHLVSRPSDMAQTQLQYQARGWPSPLSTNHQHLHSRNHLCLQRQPPSSKKSDGSGTYTKLQNTQVRVMSEKK
PRKRVESESKQEKANRIISEAIARAKERGERNIPRVMSPENFPSASVEGKEEKRGRRMKSKPKDRDNKKPKTY
SKLKEKTKIGKLIITLGKKHKRRNESSDELSDAEQRSQHTFKEQHSQKRRSNRQIKRKKYAEDAEGKQSEEEV
KGSLRVKRNSAPPPGEQPLQLFVENPSEEDAAIVDKILACRTVKKEVSPGVMLDIEEFFVKYKNYSYLHCEWA
TEQQLLKDKRIQQKIKRFKLRQAQRAHFLADMEEEPFNPDYVEVDRILEVSFCEDKDTGESVIYYLVKWCSLP
YEDSTWELKEDVDLAKIEEFEQLQASRPDTRHLDRPPSNIWKKIEQSREYKNGNQLREYQLEGLNWLLFNWYN
RRNCILADEMGLGKTIQSITFLYEILLTGIRGPFLIIAPLSTIANWEREFRTWTDINVVVYHGSLISRQMIQQ
YEMYFRDSQGRIIRGAYRFQAIITTFEMILGGCGELNAIDWRCVIIDEAHRLKNKNCKLLEGLKLMNLEHKVL

LTGTPLQNTVEELFSLLHFLEPLRFPSESTFMQEFGDLKTEEQVQKLQAILKPMMLRRLKEDVEKKLAPKEET
IIEVELTNIQKKYYRAILEKNFSFLSKGAGRNKCT
>rs261610524_ENSMUSP00000147407_c.3375:C>CG_p.Q1126ins-fs
ENSMUST00000209203.2|Chd9-203|ENSMUSG00000056608|+|chr8:g.91723885:C>CG
MTDPMMDFFDDANLFGETLEGLSDDTFVQPGPVSLVDELNLGAEFEPLHIDSLNHVQGTPTHQKMADFEQLSQ
FDSMKFHPVNQSFGSPVEHVLSPHSQFNCSPIHPPNQPNGLFQDVADGSPMWGHQTATGLANQNGSPFHQPGH
SHSLHQNKSFVAHPDFALFQASEHQTQCSSLHSQQSRSNLNPGQNSLGQAKNFLDANVSGAHRVNVNHLATAP
SSQQTLPVQFSPTANPPAHFLKCSSHQEGNYNRPSPSMTSCSVSNSQQFPSHYSFSSGHVSPSSLLQSSAGLA
PGHTNQALSDFAGSNSFSPHRGMKQEPTQHLLNPTPSLNSNNFQILHSSHPQGNYSNSKLSPVHMNFPDPVDA
GPPVGHFNDHAETNGFSSLEENLLHHVDSHAEPFAGLDPEDLLQEGLLPQFDESPFGQDNSNHVLDHDLDRQF
TSHLVSRPSDMAQTQLQYQARGWPSPLSTNHQHLHSRNHLCLQRQPPSSKKSDGSGTYTKLQNTQVRVMSEKK
PRKRVESESKQEKANRIISEAIARAKERGERNIPRVMSPENFPSASVEGKEEKRGRRMKSKPKDRDNKKPKTY
SKLKEKTKIGKLIITLGKKHKRRNESSDELSDAEQRSQHTFKEQHSQKRRSNRQIKRKKYAEDAEGKQSEEEV
KGSLRVKRNSAPPPGEQPLQLFVENPSEEDAAIVDKILACRTVKKEVSPGVMLDIEEFFVKYKNYSYLHCEWA
TEQQLLKDKRIQQKIKRFKLRQAQRAHFLADMEEEPFNPDYVEVDRILEVSFCEDKDTGESVIYYLVKWCSLP
YEDSTWELKEDVDLAKIEEFEQLQASRPDTRHLDRPPSNIWKKIEQSREYKNGNQLREYQLEGLNWLLFNWYN
RRNCILADEMGLGKTIQSITFLYEILLTGIRGPFLIIAPLSTIANWEREFRTWTDINVVVYHGSLISRQMIQQ
YEMYFRDSQGRIIRGAYRFQAIITTFEMILGGCGELNAIDWRCVIIDEAHRLKNKNCKLLEGLKLMNLEHKVL
LTGTPLQNTVEELFSLLHFLEPLRFPSESTFMQEFGDLKTEEQVQKLQAILKPMMLRRLKEDVEKKLAPKEET
IIEVELTNIQKKYYRAILEKNFSFLSKGAGRNKCT
>rs261610524_ENSMUSP00000148088_c.3375:C>CG_p.Q1126ins-fs
ENSMUST00000209423.2|Chd9-205|ENSMUSG00000056608|+|chr8:g.91723885:C>CG
MTDPMMDFFDDANLFGETLEGLSDDTFVQPGPVSLVDELNLGAEFEPLHIDSLNHVQGTPTHQKMADFEQLSQ
FDSMKFHPVNQSFGSPVEHVLSPHSQFNCSPIHPPNQPNGLFQDVADGSPMWGHQTATGLANQNGSPFHQPGH
SHSLHQNKSFVAHPDFALFQASEHQTQCSSLHSQQSRSNLNPGQNSLGQAKNFLDANVSGAHRVNVNHLATAP
SSQQTLPVQFSPTANPPAHFLKCSSHQEGNYNRPSPSMTSCSVSNSQQFPSHYSFSSGHVSPSSLLQSSAGLA
PGHTNQALSDFAGSNSFSPHRGMKQEPTQHLLNPTPSLNSNNFQILHSSHPQGNYSNSKLSPVHMNFPDPVDA
GPPVGHFNDHAETNGFSSLEENLLHHVDSHAEPFAGLDPEDLLQEGLLPQFDESPFGQDNSNHVLDHDLDRQF
TSHLVSRPSDMAQTQLQYQARGWPSPLSTNHQHLHSRNHLCLQRQPPSSKKSDGSGTYTKLQNTQVRVMSEKK
PRKRVESESKQEKANRIISEAIARAKERGERNIPRVMSPENFPSASVEGKEEKRGRRMKSKPKDRDNKKPKTY
SKLKEKTKIGKLIITLGKKHKRRNESSDELSDAEQRSQHTFKEQHSQKRRSNRQIKRKKYAEDAEGKQSEEEV
KGSLRVKRNSAPPPGEQPLQLFVENPSEEDAAIVDKILACRTVKKEVSPGVMLDIEEFFVKYKNYSYLHCEWA
TEQQLLKDKRIQQKIKRFKLRQAQRAHFLADMEEEPFNPDYVEVDRILEVSFCEDKDTGESVIYYLVKWCSLP
YEDSTWELKEDVDLAKIEEFEQLQASRPDTRHLDRPPSNIWKKIEQSREYKNGNQLREYQLEGLNWLLFNWYN
RRNCILADEMGLGKTIQSITFLYEILLTGIRGPFLIIAPLSTIANWEREFRTWTDINVVVYHGSLISRQMIQQ
YEMYFRDSQGRIIRGAYRFQAIITTFEMILGGCGELNAIDWRCVIIDEAHRLKNKNCKLLEGLKLMNLEHKVL
LTGTPLQNTVEELFSLLHFLEPLRFPSESTFMQEFGDLKTEEQVQKLQAILKPMMLRRLKEDVEKKLAPKEET
IIEVELTNIQKKYYRAILEKNFSFLSKGAGRNKCT
>rs261610524_ENSMUSP00000147741_c.1956:C>CG_p.Q653ins-fs
ENSMUST00000210947.2|Chd9-210|ENSMUSG00000056608|+|chr8:g.91723885:C>CG
MSSVKRPRGRPPSSKKSDGSGTYTKLQNTQVRVMSEKKPRKRVESESKQEKANRIISEAIARAKERGERNIPR
VMSPENFPSASVEGKEEKRGRRMKSKPKDRDNKKPKTYSKLKEKTKIGKLIITLGKKHKRRNESSDELSDAEQ
RSQHTFKEQHSQKRRSNRQIKRKKYAEDAEGKQSEEEVKGSLRVKRNSAPPPGEQPLQLFVENPSEEDAAIVD
KILACRTVKKEVSPGVMLDIEEFFVKYKNYSYLHCEWATEQQLLKDKRIQQKIKRFKLRQAQRAHFLADMEEE
PFNPDYVEVDRILEVSFCEDKDTGESVIYYLVKWCSLPYEDSTWELKEDVDLAKIEEFEQLQASRPDTRHLDR
PPSNIWKKIEQSREYKNGNQLREYQLEGLNWLLFNWYNRRNCILADEMGLGKTIQSITFLYEILLTGIRGPFL
IIAPLSTIANWEREFRTWTDINVVVYHGSLISRQMIQQYEMYFRDSQGRIIRGAYRFQAIITTFEMILGGCGE
LNAIDWRCVIIDEAHRLKNKNCKLLEGLKLMNLEHKVLLTGTPLQNTVEELFSLLHFLEPLRFPSESTFMQEF
GDLKTEEQVQKLQAILKPMMLRRLKEDVEKKLAPKEETIIEVELTNIQKKYYRAILEKNFSFLSKGAGRNKCT
>rs261610524_ENSMUSP00000105243_c.3375:C>CG_p.Q1126ins-fs
ENSMUST00000109614.9|Chd9-202|ENSMUSG00000056608|+|chr8:g.91723885:C>CG
MTDPMMDFFDDANLFGETLEGLSDDTFVQPGPVSLVDELNLGAEFEPLHIDSLNHVQGTPTHQKMADFEQLSQ
FDSMKFHPVNQSFGSPVEHVLSPHSQFNCSPIHPPNQPNGLFQDVADGSPMWGHQTATGLANQNGSPFHQPGH
SHSLHQNKSFVAHPDFALFQASEHQTQCSSLHSQQSRSNLNPGQNSLGQAKNFLDANVSGAHRVNVNHLATAP
SSQQTLPVQFSPTANPPAHFLKCSSHQEGNYNRPSPSMTSCSVSNSQQFPSHYSFSSGHVSPSSLLQSSAGLA
PGHTNQALSDFAGSNSFSPHRGMKQEPTQHLLNPTPSLNSNNFQILHSSHPQGNYSNSKLSPVHMNFPDPVDA
GPPVGHFNDHAETNGFSSLEENLLHHVDSHAEPFAGLDPEDLLQEGLLPQFDESPFGQDNSNHVLDHDLDRQF
TSHLVSRPSDMAQTQLQYQARGWPSPLSTNHQHLHSRNHLCLQRQPPSSKKSDGSGTYTKLQNTQVRVMSEKK

PRKRVESESKQEKANRIISEAIARAKERGERNIPRVMSPENFPSASVEGKEEKRGRRMKSKPKDRDNKKPKTY
SKLKEKTKIGKLIITLGKKHKRRNESSDELSDAEQRSQHTFKEQHSQKRRSNRQIKRKKYAEDAEGKQSEEEV
KGSLRVKRNSAPPPGEQPLQLFVENPSEEDAAIVDKILACRTVKKEVSPGVMLDIEEFFVKYKNYSYLHCEWA
TEQQLLKDKRIQQKIKRFKLRQAQRAHFLADMEEEPFNPDYVEVDRILEVSFCEDKDTGESVIYYLVKWCSLP
YEDSTWELKEDVDLAKIEEFEQLQASRPDTRHLDRPPSNIWKKIEQSREYKNGNQLREYQLEGLNWLLFNWYN
RRNCILADEMGLGKTIQSITFLYEILLTGIRGPFLIIAPLSTIANWEREFRTWTDINVVVYHGSLISRQMIQQ
YEMYFRDSQGRIIRGAYRFQAIITTFEMILGGCGELNAIDWRCVIIDEAHRLKNKNCKLLEGLKLMNLEHKVL
LTGTPLQNTVEELFSLLHFLEPLRFPSESTFMQEFGDLKTEEQVQKLQAILKPMMLRRLKEDVEKKLAPKEET
IIEVELTNIQKKYYRAILEKNFSFLSKGAGRNKCT
>rs33410738_ENSMUSP00000046356_c.3379:C>A_p.T1127K
ENSMUST00000048665.8|Chd9-201|ENSMUSG00000056608|+|chr8:g.91723889:C>A
MTDPMMDFFDDANLFGETLEGLSDDTFVQPGPVSLVDELNLGAEFEPLHIDSLNHVQGTPTHQKMADFEQLSQ
FDSMKFHPVNQSFGSPVEHVLSPHSQFNCSPIHPPNQPNGLFQDVADGSPMWGHQTATGLANQNGSPFHQPGH
SHSLHQNKSFVAHPDFALFQASEHQTQCSSLHSQQSRSNLNPGQNSLGQAKNFLDANVSGAHRVNVNHLATAP
SSQQTLPVQFSPTANPPAHFLKCSSHQEGNYNRPSPSMTSCSVSNSQQFPSHYSFSSGHVSPSSLLQSSAGLA
PGHTNQALSDFAGSNSFSPHRGMKQEPTQHLLNPTPSLNSNNFQILHSSHPQGNYSNSKLSPVHMNFPDPVDA
GPPVGHFNDHAETNGFSSLEENLLHHVDSHAEPFAGLDPEDLLQEGLLPQFDESPFGQDNSNHVLDHDLDRQF
TSHLVSRPSDMAQTQLQYQARGWPSPLSTNHQHLHSRNHLCLQRQPPSSKKSDGSGTYTKLQNTQVRVMSEKK
PRKRVESESKQEKANRIISEAIARAKERGERNIPRVMSPENFPSASVEGKEEKRGRRMKSKPKDRDNKKPKTY
SKLKEKTKIGKLIITLGKKHKRRNESSDELSDAEQRSQHTFKEQHSQKRRSNRQIKRKKYAEDAEGKQSEEEV
KGSLRVKRNSAPPPGEQPLQLFVENPSEEDAAIVDKILACRTVKKEVSPGVMLDIEEFFVKYKNYSYLHCEWA
TEQQLLKDKRIQQKIKRFKLRQAQRAHFLADMEEEPFNPDYVEVDRILEVSFCEDKDTGESVIYYLVKWCSLP
YEDSTWELKEDVDLAKIEEFEQLQASRPDTRHLDRPPSNIWKKIEQSREYKNGNQLREYQLEGLNWLLFNWYN
RRNCILADEMGLGKTIQSITFLYEILLTGIRGPFLIIAPLSTIANWEREFRTWTDINVVVYHGSLISRQMIQQ
YEMYFRDSQGRIIRGAYRFQAIITTFEMILGGCGELNAIDWRCVIIDEAHRLKNKNCKLLEGLKLMNLEHKVL
LTGTPLQNTVEELFSLLHFLEPLRFPSESTFMQEFGDLKTEEQVQKLQAILKPMMLRRLKEDVEKKLAPKEET
IIEVELTNIQKKYYRAILEKNFSFLSKGAGQKNVPNLVNTMMELRKCCNHPYLIKGAEEKILGEFRDTYNPSA
SDFHLQAMIQSAGKLVLIDKLLPKMKAGGHKVLIFSQMVRCLDILEDYLIHKRYLYERIDGRVRGNLRQAAID
RFSKPDSDRFVFLLCTRAGGLGINLTAADTCIIFDSDWNPQNDLQAQARCHRIGQNKAVKVYRLVTRNSYERE
MFDRASLKLGLDKAVLQSMSGRDSNVSGIQQLSKKEIEDLLRRGAYGAIMEEEDEGSKFCEEDIDQILLRRTK
TITIESEGRGSTFAKASFVASGNRTDISLDDPNFWQKWAKKAELDIDTISGRNSLVIDTPRIRKQTRPFSATK
DELAELSEAESEGEEKPKLRRPCDRSGGYGRTECFRVEKNLLVYGWGRWREILSHGRFKRQLNEHDVEVICRA
LLAYCLIHYRGDEKIKGFIWDLITPTEDGQTRELQNHLGLSAPVPRGRKGKKVKTQTSSFDIQKAEWLRKYNP
EQLLQDEGYKKHVKHHCNKVLLRVRMLYYLKQEVIGNESQKVFDGVDASDIDVWVPEPDHSEVPAAWWDFDAD
KSLLIGVFKHGYEKYNTIRADPALCFLERVGKPDDKAVAAEQRANDYMDGDVEDPEYKPAPAIFKDDIEDDVS
SPGDLVIADGEGQLMEGDKVYWPTPSALTTRLRRLITAYQRTNKNRHIQQMQPTFSLPANAMQPLYEEATLNP
KMAAKIERQQRWTRREEADFYRVVSTFGVVFDPDRGQFDWTKFRALARLHKKTDNSLEKYLCAFMSMCRRVCR
LPSKEELVDPNIFIQPITEERASRTLYRIELLRKVREQALRHPQLFERLKLCHPNPDLPIWWECGSHDRDLLI
GAAKHGVSRTDYHILRDPELSFMAAQRNYNQSKAAHSRTSAPLLQQYQVALSASPLTSLPRLLGAKGTLLEDM
KVKSESLTEEPQSSEEESMSSMETRTRVKSEPVSPKNGVLSQATGDQKSGGKSETDRRMVAARTEPLTPNPAS
KKPRVHKRGSQSSSDSDSDSARSSCSSRSSSSSSSSSSCSHSRSGSSSSSSSSCSSASSSSSSSSSSSSSSSS
SSSEESDSEEDVQKREGTPHRKAYDEESVASLSTTQDETQDSFQANNGTPESAYLLQGGYMLAASYWPKDRVM
INRLDSICQTVLKGKWPSARRHYDANTVASFYTTKLLDSPGAATERGEPSVPTPPAVAVREEHEQSAQMSKEG
GLKLTFQKQGLAQKRPFDGEDGALGQQQYLTRLRELQSTSETSLVNLPKAVPASGTSIQPTLGANGAILDSQP
IVKKRRGRRRNVEGADILFLNRNKPPNHIPTGMNPALSYPQPQRIPDTESPVPVINLKDGTRLAGDDAPKRKD
LDRWLKEHPGYVEDLGAFIPRVQLHEGRPKQKRHRCRNPNKLDINSLTGEERVQLINRRNARKVGGAFAPPLK
DLCRFLKENSEYGVAPEWGDVVKQSGFLPESMFERILTGPVVREEVSRRGRRPKSGIAKATTAAAVPAGSVPG
NPLLANGLLPGVDLTALQALQQNLQNLQSLQVTAGLMGMPAGLSSGGETKNMAAMFPMLFSGMAGLPNLLGMG
GLLSKTAESGAEEKRGNDSKELEGKKERTESQSPENGGERCVPGSPSTSSTAALSSAAAAKPIALNPLLLSNI
LYPGMLLTPGLNLHLPTLSQSNAFDVQKNKSDDLDSSKSVEIKEENSRVRDQEEKGGTEPSPLNENSTDEGSE
RASSGSDSSSSSSEDSDSSNED*
>rs33410738_ENSMUSP00000147407_c.3379:C>A_p.T1127K
ENSMUST00000209203.2|Chd9-203|ENSMUSG00000056608|+|chr8:g.91723889:C>A
MTDPMMDFFDDANLFGETLEGLSDDTFVQPGPVSLVDELNLGAEFEPLHIDSLNHVQGTPTHQKMADFEQLSQ
FDSMKFHPVNQSFGSPVEHVLSPHSQFNCSPIHPPNQPNGLFQDVADGSPMWGHQTATGLANQNGSPFHQPGH
SHSLHQNKSFVAHPDFALFQASEHQTQCSSLHSQQSRSNLNPGQNSLGQAKNFLDANVSGAHRVNVNHLATAP
SSQQTLPVQFSPTANPPAHFLKCSSHQEGNYNRPSPSMTSCSVSNSQQFPSHYSFSSGHVSPSSLLQSSAGLA
PGHTNQALSDFAGSNSFSPHRGMKQEPTQHLLNPTPSLNSNNFQILHSSHPQGNYSNSKLSPVHMNFPDPVDA

GPPVGHFNDHAETNGFSSLEENLLHHVDSHAEPFAGLDPEDLLQEGLLPQFDESPFGQDNSNHVLDHDLDRQF
TSHLVSRPSDMAQTQLQYQARGWPSPLSTNHQHLHSRNHLCLQRQPPSSKKSDGSGTYTKLQNTQVRVMSEKK
PRKRVESESKQEKANRIISEAIARAKERGERNIPRVMSPENFPSASVEGKEEKRGRRMKSKPKDRDNKKPKTY
SKLKEKTKIGKLIITLGKKHKRRNESSDELSDAEQRSQHTFKEQHSQKRRSNRQIKRKKYAEDAEGKQSEEEV
KGSLRVKRNSAPPPGEQPLQLFVENPSEEDAAIVDKILACRTVKKEVSPGVMLDIEEFFVKYKNYSYLHCEWA
TEQQLLKDKRIQQKIKRFKLRQAQRAHFLADMEEEPFNPDYVEVDRILEVSFCEDKDTGESVIYYLVKWCSLP
YEDSTWELKEDVDLAKIEEFEQLQASRPDTRHLDRPPSNIWKKIEQSREYKNGNQLREYQLEGLNWLLFNWYN
RRNCILADEMGLGKTIQSITFLYEILLTGIRGPFLIIAPLSTIANWEREFRTWTDINVVVYHGSLISRQMIQQ
YEMYFRDSQGRIIRGAYRFQAIITTFEMILGGCGELNAIDWRCVIIDEAHRLKNKNCKLLEGLKLMNLEHKVL
LTGTPLQNTVEELFSLLHFLEPLRFPSESTFMQEFGDLKTEEQVQKLQAILKPMMLRRLKEDVEKKLAPKEET
IIEVELTNIQKKYYRAILEKNFSFLSKGAGQKNVPNLVNTMMELRKCCNHPYLIKGAEEKILGEFRDTYNPSA
SDFHLQAMIQSAGKLVLIDKLLPKMKAGGHKVLIFSQMVRCLDILEDYLIHKRYLYERIDGRVRGNLRQAAID
RFSKPDSDRFVFLLCTRAGGLGINLTAADTCIIFDSDWNPQNDLQAQARCHRIGQNKAVKVYRLVTRNSYERE
MFDRASLKLGLDKAVLQSMSGRDSNVSGIQQLSKKEIEDLLRRGAYGAIMEEEDEGSKFCEEDIDQILLRRTK
TITIESEGRGSTFAKASFVASGNRTDISLDDPNFWQKWAKKAELDIDTISGRNSLVIDTPRIRKQTRPFSATK
DELAELSEAESEGEEKPKLRRPCDRSGGYGRTECFRVEKNLLVYGWGRWREILSHGRFKRQLNEHDVEVICRA
LLAYCLIHYRGDEKIKGFIWDLITPTEDGQTRELQNHLGLSAPVPRGRKGKKVKTQTSSFDIQKAEWLRKYNP
EQLLQDEGYKKHVKHHCNKVLLRVRMLYYLKQEVIGNESQKVFDGVDASDIDVWVPEPDHSEVPAAWWDFDAD
KSLLIGVFKHGYEKYNTIRADPALCFLERVGKPDDKAVAAEQRANDYMDGDVEDPEYKPAPAIFKDDIEDDVS
SPGDLVIADGEGQLMEGDKVYWPTPSALTTRLRRLITAYQRTNKNRHIQQMQPTFSLPANAMQPLYEEATLNP
KMAAKIERQQRIGRSKYFYSTNHRGTCF*
>rs33410738_ENSMUSP00000148088_c.3379:C>A_p.T1127K
ENSMUST00000209423.2|Chd9-205|ENSMUSG00000056608|+|chr8:g.91723889:C>A
MTDPMMDFFDDANLFGETLEGLSDDTFVQPGPVSLVDELNLGAEFEPLHIDSLNHVQGTPTHQKMADFEQLSQ
FDSMKFHPVNQSFGSPVEHVLSPHSQFNCSPIHPPNQPNGLFQDVADGSPMWGHQTATGLANQNGSPFHQPGH
SHSLHQNKSFVAHPDFALFQASEHQTQCSSLHSQQSRSNLNPGQNSLGQAKNFLDANVSGAHRVNVNHLATAP
SSQQTLPVQFSPTANPPAHFLKCSSHQEGNYNRPSPSMTSCSVSNSQQFPSHYSFSSGHVSPSSLLQSSAGLA
PGHTNQALSDFAGSNSFSPHRGMKQEPTQHLLNPTPSLNSNNFQILHSSHPQGNYSNSKLSPVHMNFPDPVDA
GPPVGHFNDHAETNGFSSLEENLLHHVDSHAEPFAGLDPEDLLQEGLLPQFDESPFGQDNSNHVLDHDLDRQF
TSHLVSRPSDMAQTQLQYQARGWPSPLSTNHQHLHSRNHLCLQRQPPSSKKSDGSGTYTKLQNTQVRVMSEKK
PRKRVESESKQEKANRIISEAIARAKERGERNIPRVMSPENFPSASVEGKEEKRGRRMKSKPKDRDNKKPKTY
SKLKEKTKIGKLIITLGKKHKRRNESSDELSDAEQRSQHTFKEQHSQKRRSNRQIKRKKYAEDAEGKQSEEEV
KGSLRVKRNSAPPPGEQPLQLFVENPSEEDAAIVDKILACRTVKKEVSPGVMLDIEEFFVKYKNYSYLHCEWA
TEQQLLKDKRIQQKIKRFKLRQAQRAHFLADMEEEPFNPDYVEVDRILEVSFCEDKDTGESVIYYLVKWCSLP
YEDSTWELKEDVDLAKIEEFEQLQASRPDTRHLDRPPSNIWKKIEQSREYKNGNQLREYQLEGLNWLLFNWYN
RRNCILADEMGLGKTIQSITFLYEILLTGIRGPFLIIAPLSTIANWEREFRTWTDINVVVYHGSLISRQMIQQ
YEMYFRDSQGRIIRGAYRFQAIITTFEMILGGCGELNAIDWRCVIIDEAHRLKNKNCKLLEGLKLMNLEHKVL
LTGTPLQNTVEELFSLLHFLEPLRFPSESTFMQEFGDLKTEEQVQKLQAILKPMMLRRLKEDVEKKLAPKEET
IIEVELTNIQKKYYRAILEKNFSFLSKGAGQKNVPNLVNTMMELRKCCNHPYLIKGAEEKILGEFRDTYNPSA
SDFHLQAMIQSAGKLVLIDKLLPKMKAGGHKVLIFSQMVRCLDILEDYLIHKRYLYERIDGRVRGNLRQAAID
RFSKPDSDRFVFLLCTRAGGLGINLTAADTCIIFDSDWNPQNDLQAQARCHRIGQNKAVKVYRLVTRNSYERE
MFDRASLKLGLDKAVLQSMSGRDSNVSGIQQLSKKEIEDLLRRGAYGAIMEEEDEGSKFCEEDIDQILLRRTK
TITIESEGRGSTFAKASFVASGNRTDISLDDPNFWQKWAKKAELDIDTISGRNSLVIDTPRIRKQTRPFSATK
DELAELSEAESEGEEKPKLRRPCDRSGGYGRTECFRVEKNLLVYGWGRWREILSHGRFKRQLNEHDVEVICRA
LLAYCLIHYRGDEKIKGFIWDLITPTEDGQTRELQNHLGLSAPVPRGRKGKKVKTQTSSFDIQKAEWLRKYNP
EQLLQDEGYKKHVKHHCNKVLLRVRMLYYLKQEVIGNESQKVFDGVDASDIDVWVPEPDHSEVPAAWWDFDAD
KSLLIGVFKHGYEKYNTIRADPALCFLERVGKPDDKAVAAEQRANDYMDGDVEDPEYKPAPAIFKDDIEDDVS
SPGDLVIADGEGQLMEGDKVYWPTPSALTTRLRRLITAYQRTNKNRHIQQMQPTFSLPANAMQPLYEEATLNP
KMAAKIERQQRWTRREEADFYRVVSTFGVVFDPDRGQFDWTKFRALARLHKKTDNSLEKYLCAFMSMCRRVCR
LPSKEELVDPNIFIQPITEERASRTLYRIELLRKVREQALRHPQLFERLKLCHPNPDLPIWWECGSHDRDLLI
GAAKHGVSRTDYHILRDPELSFMAAQRNYNQSKAAHSRTSAPLLQQYQVALSASPLTSLPRLLGAKGTLLEDM
KVKSESLTEEPQSSEEESMSSMETRTRVKSEPVSPKNGVLSQATGDQKSGGKSETDRRMVAARTEPLTPNPAS
KKPRVHKRGSQSSSDSDSDSARSSCSSRSSSSSSSSSSCSHSRSGSSSSSSSSCSSASSSSSSSSSSSSSS
SSSEESDSEEDVQKREGTPHRKAYDEESVASLSTTQDETQDSFQANNGTPESAYLLQGGYMLAASYWPKDRVM
INRLDSICQTVLKGKWPSARRHYDANTVASFYTTKLLDSPGAATERGEPSVPTPPAVAVREEHEQSAQMSKVK
KHVREKEFTVKIKDEGGLKLTFQKQGLAQKRPFDGEDGALGQQQYLTRLRELQSTSETSLVNLPKAVPASGTS
IQPTLGANGAILDSQPIVKKRRGRRRNVEGADILFLNRNKPPNHIPTGMNPALSYPQPQRIPDTESPVPVINL
KDGTRLAGDDAPKRKDLDRWLKEHPGYVEDLGAFIPRVQLHEGRPKQKRHRCRNPNKLDINSLTGEERVQLIN

RRNARKVGGAFAPPLKDLCRFLKENSEYGVAPEWGDVVKQSGFLPESMFERILTGPVVREEVSRRGRRPKSGI
AKATTAAAVPAGSVPGNPLLANGLLPGVDLTALQALQQNLQNLQSLQVTAGLMGMPAGLSSGGETKNMAAMFP
MLFSGMAGLPNLLGMGGLLSKTAESGAEEKRGNDSKELEGKKERTESQSPENGGERCVPGSPSTSSTAALSSA
AAAKPIALNPLLLSNILYPGMLLTPGLNLHLPTLSQSNAFDVQKNKSDDLDSSKSVEIKEENSRVRDQEEKGG
TEPSPLNENSTDEGSERASSGSDSSSSSSEDSDSSNED*
>rs33410738_ENSMUSP00000147741_c.1960:C>A_p.T654K
ENSMUST00000210947.2|Chd9-210|ENSMUSG00000056608|+|chr8:g.91723889:C>A
MSSVKRPRGRPPSSKKSDGSGTYTKLQNTQVRVMSEKKPRKRVESESKQEKANRIISEAIARAKERGERNIPR
VMSPENFPSASVEGKEEKRGRRMKSKPKDRDNKKPKTYSKLKEKTKIGKLIITLGKKHKRRNESSDELSDAEQ
RSQHTFKEQHSQKRRSNRQIKRKKYAEDAEGKQSEEEVKGSLRVKRNSAPPPGEQPLQLFVENPSEEDAAIVD
KILACRTVKKEVSPGVMLDIEEFFVKYKNYSYLHCEWATEQQLLKDKRIQQKIKRFKLRQAQRAHFLADMEEE
PFNPDYVEVDRILEVSFCEDKDTGESVIYYLVKWCSLPYEDSTWELKEDVDLAKIEEFEQLQASRPDTRHLDR
PPSNIWKKIEQSREYKNGNQLREYQLEGLNWLLFNWYNRRNCILADEMGLGKTIQSITFLYEILLTGIRGPFL
IIAPLSTIANWEREFRTWTDINVVVYHGSLISRQMIQQYEMYFRDSQGRIIRGAYRFQAIITTFEMILGGCGE
LNAIDWRCVIIDEAHRLKNKNCKLLEGLKLMNLEHKVLLTGTPLQNTVEELFSLLHFLEPLRFPSESTFMQEF
GDLKTEEQVQKLQAILKPMMLRRLKEDVEKKLAPKEETIIEVELTNIQKKYYRAILEKNFSFLSKGAGQKNVP
NLVNTMMELRKCCNHPYLIKGAEEKILGEFRDTYNPSASDFHLQAMIQSAGKLVLIDKLLPKMKAGGHKVLIF
SQMVRCLDILEDYLIHKRYLYERIDGRVRGNLRQAAIDRFSKPDSDRFVFLLCTRAGGLGINLTAADTCIIFD
SDWNPQNDLQAQARCHRIGQNKAVKVYRLVTRNSYEREMFDRASLKLGLDKAVLQSMSGRDSNVSGIQQLS
>rs33410738_ENSMUSP00000105243_c.3379:C>A_p.T1127K
ENSMUST00000109614.9|Chd9-202|ENSMUSG00000056608|+|chr8:g.91723889:C>A
MTDPMMDFFDDANLFGETLEGLSDDTFVQPGPVSLVDELNLGAEFEPLHIDSLNHVQGTPTHQKMADFEQLSQ
FDSMKFHPVNQSFGSPVEHVLSPHSQFNCSPIHPPNQPNGLFQDVADGSPMWGHQTATGLANQNGSPFHQPGH
SHSLHQNKSFVAHPDFALFQASEHQTQCSSLHSQQSRSNLNPGQNSLGQAKNFLDANVSGAHRVNVNHLATAP
SSQQTLPVQFSPTANPPAHFLKCSSHQEGNYNRPSPSMTSCSVSNSQQFPSHYSFSSGHVSPSSLLQSSAGLA
PGHTNQALSDFAGSNSFSPHRGMKQEPTQHLLNPTPSLNSNNFQILHSSHPQGNYSNSKLSPVHMNFPDPVDA
GPPVGHFNDHAETNGFSSLEENLLHHVDSHAEPFAGLDPEDLLQEGLLPQFDESPFGQDNSNHVLDHDLDRQF
TSHLVSRPSDMAQTQLQYQARGWPSPLSTNHQHLHSRNHLCLQRQPPSSKKSDGSGTYTKLQNTQVRVMSEKK
PRKRVESESKQEKANRIISEAIARAKERGERNIPRVMSPENFPSASVEGKEEKRGRRMKSKPKDRDNKKPKTY
SKLKEKTKIGKLIITLGKKHKRRNESSDELSDAEQRSQHTFKEQHSQKRRSNRQIKRKKYAEDAEGKQSEEEV
KGSLRVKRNSAPPPGEQPLQLFVENPSEEDAAIVDKILACRTVKKEVSPGVMLDIEEFFVKYKNYSYLHCEWA
TEQQLLKDKRIQQKIKRFKLRQAQRAHFLADMEEEPFNPDYVEVDRILEVSFCEDKDTGESVIYYLVKWCSLP
YEDSTWELKEDVDLAKIEEFEQLQASRPDTRHLDRPPSNIWKKIEQSREYKNGNQLREYQLEGLNWLLFNWYN
RRNCILADEMGLGKTIQSITFLYEILLTGIRGPFLIIAPLSTIANWEREFRTWTDINVVVYHGSLISRQMIQQ
YEMYFRDSQGRIIRGAYRFQAIITTFEMILGGCGELNAIDWRCVIIDEAHRLKNKNCKLLEGLKLMNLEHKVL
LTGTPLQNTVEELFSLLHFLEPLRFPSESTFMQEFGDLKTEEQVQKLQAILKPMMLRRLKEDVEKKLAPKEET
IIEVELTNIQKKYYRAILEKNFSFLSKGAGQKNVPNLVNTMMELRKCCNHPYLIKGAEEKILGEFRDTYNPSA
SDFHLQAMIQSAGKLVLIDKLLPKMKAGGHKVLIFSQMVRCLDILEDYLIHKRYLYERIDGRVRGNLRQAAID
RFSKPDSDRFVFLLCTRAGGLGINLTAADTCIIFDSDWNPQNDLQAQARCHRIGQNKAVKVYRLVTRNSYERE
MFDRASLKLGLDKAVLQSMSGRDSNVSGIQQLSKKEIEDLLRRGAYGAIMEEEDEGSKFCEEDIDQILLRRTK
TITIESEGRGSTFAKASFVASGNRTDISLDDPNFWQKWAKKAELDIDTISGRNSLVIDTPRIRKQTRPFSATK
DELAELSEAESEGEEKPKLRRPCDRSGGYGRTECFRVEKNLLVYGWGRWREILSHGRFKRQLNEHDVEVICRA
LLAYCLIHYRGDEKIKGFIWDLITPTEDGQTRELQNHLGLSAPVPRGRKGKKVKTQTSSFDIQKAEWLRKYNP
EQLLQDEGYKKHVKHHCNKVLLRVRMLYYLKQEVIGNESQKVFDGVDASDIDVWVPEPDHSEVPAAWWDFDAD
KSLLIGVFKHGYEKYNTIRADPALCFLERVGKPDDKAVAAEQRANDYMDGDVEDPEYKPAPAIFKDDIEDDVS
SPGDLVIADGEGQLMEGDKVYWPTPSALTTRLRRLITAYQRTNKNRHIQQMQPTFSLPANAMQPLYEEATLNP
KMAAKIERQQRWTRREEADFYRVVSTFGVVFDPDRGQFDWTKFRALARLHKKTDNSLEKYLCAFMSMCRRVCR
LPSKEELVDPNIFIQPITEERASRTLYRIELLRKVREQALRHPQLFERLKLCHPNPDLPIWWECGSHDRDLLI
GAAKHGVSRTDYHILRDPELSFMAAQRNYNQSKAAHSRTSAPLLQQYQVALSASPLTSLPRLLGAKGTLLEDM
KVKSESLTEEPQSSEEESMSSMETRTRVKSEPVSPKNGVLSQATGDQKSGGKSETDRRMVAARTEPLTPNPAS
KKPRVHKRGSQSSSDSDSDSARSSCSSRSSSSSSSSSSCSHSRSGSSSSSSSSCSSASSSSSSSSSSSSSSS
SSSEESDSEEDVQKREGTPHRKAYDEESVASLSTTQDETQDSFQANNGTPESAYLLQGGYMLAASYWPKDRVM
INRLDSICQTVLKGKWPSARRHYDANTVASFYTTKLLDSPGAATERGEPSVPTPPAVAVREEHEQSAQMSKVK
KHVREKEFTVKIKDEGGLKLTFQKQGLAQKRPFDGEDGALGQQQYLTRLRELQSTSETSLVNLPKAVPASGTS
IQPTLGANGAILDSQPIVKKRRGRRRNVEGADILFLNRNKPPNHIPTGMNPALSYPQPQRIPDTESPVPVINL
KDGTRLAGDDAPKRKDLDRWLKEHPGYVEDLGAFIPRVQLHEGRPKQKRHRCRNPNKLDINSLTGEERVQLIN
RRNARKVGGAFAPPLKDLCRFLKENSEYGVAPEWGDVVKQSGFLPESMFERILTGPVVREEVSRRGRRPKSGI
AKATTAAAVPAGSVPGNPLLANGLLPGVDLTALQALQQNLQNLQSLQVTAGLMGMPAGLSSGGETKNMAAMFP

MLFSGMAGLPNLLGMGGLLSKTAESGAEEKRGNDSKELEGKKERTESQSPENGGERCVPGSPSTSSTAALSSA
AAAKPIALNPLLLSNILYPGMLLTPGLNLHLPTLSQSNAFDVQKNKSDDLDSSKSVEIKEENSRVRDQEEKGG
TEPSPLNENSTDEGSERASSGSDSSSSSSEDSDSSNED*
>rs48480191_ENSMUSP00000046356_c.4351:A>G_p.K1451R
ENSMUST00000048665.8|Chd9-201|ENSMUSG00000056608|+|chr8:g.91737274:A>G
MTDPMMDFFDDANLFGETLEGLSDDTFVQPGPVSLVDELNLGAEFEPLHIDSLNHVQGTPTHQKMADFEQLSQ
FDSMKFHPVNQSFGSPVEHVLSPHSQFNCSPIHPPNQPNGLFQDVADGSPMWGHQTATGLANQNGSPFHQPGH
SHSLHQNKSFVAHPDFALFQASEHQTQCSSLHSQQSRSNLNPGQNSLGQAKNFLDANVSGAHRVNVNHLATAP
SSQQTLPVQFSPTANPPAHFLKCSSHQEGNYNRPSPSMTSCSVSNSQQFPSHYSFSSGHVSPSSLLQSSAGLA
PGHTNQALSDFAGSNSFSPHRGMKQEPTQHLLNPTPSLNSNNFQILHSSHPQGNYSNSKLSPVHMNFPDPVDA
GPPVGHFNDHAETNGFSSLEENLLHHVDSHAEPFAGLDPEDLLQEGLLPQFDESPFGQDNSNHVLDHDLDRQF
TSHLVSRPSDMAQTQLQYQARGWPSPLSTNHQHLHSRNHLCLQRQPPSSKKSDGSGTYTKLQNTQVRVMSEKK
PRKRVESESKQEKANRIISEAIARAKERGERNIPRVMSPENFPSASVEGKEEKRGRRMKSKPKDRDNKKPKTY
SKLKEKTKIGKLIITLGKKHKRRNESSDELSDAEQRSQHTFKEQHSQKRRSNRQIKRKKYAEDAEGKQSEEEV
KGSLRVKRNSAPPPGEQPLQLFVENPSEEDAAIVDKILACRTVKKEVSPGVMLDIEEFFVKYKNYSYLHCEWA
TEQQLLKDKRIQQKIKRFKLRQAQRAHFLADMEEEPFNPDYVEVDRILEVSFCEDKDTGESVIYYLVKWCSLP
YEDSTWELKEDVDLAKIEEFEQLQASRPDTRHLDRPPSNIWKKIEQSREYKNGNQLREYQLEGLNWLLFNWYN
RRNCILADEMGLGKTIQSITFLYEILLTGIRGPFLIIAPLSTIANWEREFRTWTDINVVVYHGSLISRQMIQQ
YEMYFRDSQGRIIRGAYRFQAIITTFEMILGGCGELNAIDWRCVIIDEAHRLKNKNCKLLEGLKLMNLEHKVL
LTGTPLQNTVEELFSLLHFLEPLRFPSESTFMQEFGDLKTEEQVQKLQAILKPMMLRRLKEDVEKKLAPKEET
IIEVELTNIQKKYYRAILEKNFSFLSKGAGQTNVPNLVNTMMELRKCCNHPYLIKGAEEKILGEFRDTYNPSA
SDFHLQAMIQSAGKLVLIDKLLPKMKAGGHKVLIFSQMVRCLDILEDYLIHKRYLYERIDGRVRGNLRQAAID
RFSKPDSDRFVFLLCTRAGGLGINLTAADTCIIFDSDWNPQNDLQAQARCHRIGQNKAVKVYRLVTRNSYERE
MFDRASLKLGLDKAVLQSMSGRDSNVSGIQQLSKKEIEDLLRRGAYGAIMEEEDEGSKFCEEDIDQILLRRTK
TITIESEGRGSTFAKASFVASGNRTDISLDDPNFWQKWAKKAELDIDTISGRNSLVIDTPRIRRQTRPFSATK
DELAELSEAESEGEEKPKLRRPCDRSGGYGRTECFRVEKNLLVYGWGRWREILSHGRFKRQLNEHDVEVICRA
LLAYCLIHYRGDEKIKGFIWDLITPTEDGQTRELQNHLGLSAPVPRGRKGKKVKTQTSSFDIQKAEWLRKYNP
EQLLQDEGYKKHVKHHCNKVLLRVRMLYYLKQEVIGNESQKVFDGVDASDIDVWVPEPDHSEVPAAWWDFDAD
KSLLIGVFKHGYEKYNTIRADPALCFLERVGKPDDKAVAAEQRANDYMDGDVEDPEYKPAPAIFKDDIEDDVS
SPGDLVIADGEGQLMEGDKVYWPTPSALTTRLRRLITAYQRTNKNRHIQQMQPTFSLPANAMQPLYEEATLNP
KMAAKIERQQRWTRREEADFYRVVSTFGVVFDPDRGQFDWTKFRALARLHKKTDNSLEKYLCAFMSMCRRVCR
LPSKEELVDPNIFIQPITEERASRTLYRIELLRKVREQALRHPQLFERLKLCHPNPDLPIWWECGSHDRDLLI
GAAKHGVSRTDYHILRDPELSFMAAQRNYNQSKAAHSRTSAPLLQQYQVALSASPLTSLPRLLGAKGTLLEDM
KVKSESLTEEPQSSEEESMSSMETRTRVKSEPVSPKNGVLSQATGDQKSGGKSETDRRMVAARTEPLTPNPAS
KKPRVHKRGSQSSSDSDSDSARSSCSSRSSSSSSSSSSCSHSRSGSSSSSSSSCSSASSSSSSSSSSSSSSSS
SSSEESDSEEDVQKREGTPHRKAYDEESVASLSTTQDETQDSFQANNGTPESAYLLQGGYMLAASYWPKDRVM
INRLDSICQTVLKGKWPSARRHYDANTVASFYTTKLLDSPGAATERGEPSVPTPPAVAVREEHEQSAQMSKEG
GLKLTFQKQGLAQKRPFDGEDGALGQQQYLTRLRELQSTSETSLVNLPKAVPASGTSIQPTLGANGAILDSQP
IVKKRRGRRRNVEGADILFLNRNKPPNHIPTGMNPALSYPQPQRIPDTESPVPVINLKDGTRLAGDDAPKRKD
LDRWLKEHPGYVEDLGAFIPRVQLHEGRPKQKRHRCRNPNKLDINSLTGEERVQLINRRNARKVGGAFAPPLK
DLCRFLKENSEYGVAPEWGDVVKQSGFLPESMFERILTGPVVREEVSRRGRRPKSGIAKATTAAAVPAGSVPG
NPLLANGLLPGVDLTALQALQQNLQNLQSLQVTAGLMGMPAGLSSGGETKNMAAMFPMLFSGMAGLPNLLGMG
GLLSKTAESGAEEKRGNDSKELEGKKERTESQSPENGGERCVPGSPSTSSTAALSSAAAAKPIALNPLLLSNI
LYPGMLLTPGLNLHLPTLSQSNAFDVQKNKSDDLDSSKSVEIKEENSRVRDQEEKGGTEPSPLNENSTDEGSE
RASSGSDSSSSSSEDSDSSNED*
>rs48480191_ENSMUSP00000147407_c.4351:A>G_p.K1451R
ENSMUST00000209203.2|Chd9-203|ENSMUSG00000056608|+|chr8:g.91737274:A>G
MTDPMMDFFDDANLFGETLEGLSDDTFVQPGPVSLVDELNLGAEFEPLHIDSLNHVQGTPTHQKMADFEQLSQ
FDSMKFHPVNQSFGSPVEHVLSPHSQFNCSPIHPPNQPNGLFQDVADGSPMWGHQTATGLANQNGSPFHQPGH
SHSLHQNKSFVAHPDFALFQASEHQTQCSSLHSQQSRSNLNPGQNSLGQAKNFLDANVSGAHRVNVNHLATAP
SSQQTLPVQFSPTANPPAHFLKCSSHQEGNYNRPSPSMTSCSVSNSQQFPSHYSFSSGHVSPSSLLQSSAGLA
PGHTNQALSDFAGSNSFSPHRGMKQEPTQHLLNPTPSLNSNNFQILHSSHPQGNYSNSKLSPVHMNFPDPVDA
GPPVGHFNDHAETNGFSSLEENLLHHVDSHAEPFAGLDPEDLLQEGLLPQFDESPFGQDNSNHVLDHDLDRQF
TSHLVSRPSDMAQTQLQYQARGWPSPLSTNHQHLHSRNHLCLQRQPPSSKKSDGSGTYTKLQNTQVRVMSEKK
PRKRVESESKQEKANRIISEAIARAKERGERNIPRVMSPENFPSASVEGKEEKRGRRMKSKPKDRDNKKPKTY
SKLKEKTKIGKLIITLGKKHKRRNESSDELSDAEQRSQHTFKEQHSQKRRSNRQIKRKKYAEDAEGKQSEEEV
KGSLRVKRNSAPPPGEQPLQLFVENPSEEDAAIVDKILACRTVKKEVSPGVMLDIEEFFVKYKNYSYLHCEWA
TEQQLLKDKRIQQKIKRFKLRQAQRAHFLADMEEEPFNPDYVEVDRILEVSFCEDKDTGESVIYYLVKWCSLP

YEDSTWELKEDVDLAKIEEFEQLQASRPDTRHLDRPPSNIWKKIEQSREYKNGNQLREYQLEGLNWLLFNWYN
RRNCILADEMGLGKTIQSITFLYEILLTGIRGPFLIIAPLSTIANWEREFRTWTDINVVVYHGSLISRQMIQQ
YEMYFRDSQGRIIRGAYRFQAIITTFEMILGGCGELNAIDWRCVIIDEAHRLKNKNCKLLEGLKLMNLEHKVL
LTGTPLQNTVEELFSLLHFLEPLRFPSESTFMQEFGDLKTEEQVQKLQAILKPMMLRRLKEDVEKKLAPKEET
IIEVELTNIQKKYYRAILEKNFSFLSKGAGQTNVPNLVNTMMELRKCCNHPYLIKGAEEKILGEFRDTYNPSA
SDFHLQAMIQSAGKLVLIDKLLPKMKAGGHKVLIFSQMVRCLDILEDYLIHKRYLYERIDGRVRGNLRQAAID
RFSKPDSDRFVFLLCTRAGGLGINLTAADTCIIFDSDWNPQNDLQAQARCHRIGQNKAVKVYRLVTRNSYERE
MFDRASLKLGLDKAVLQSMSGRDSNVSGIQQLSKKEIEDLLRRGAYGAIMEEEDEGSKFCEEDIDQILLRRTK
TITIESEGRGSTFAKASFVASGNRTDISLDDPNFWQKWAKKAELDIDTISGRNSLVIDTPRIRRQTRPFSATK
DELAELSEAESEGEEKPKLRRPCDRSGGYGRTECFRVEKNLLVYGWGRWREILSHGRFKRQLNEHDVEVICRA
LLAYCLIHYRGDEKIKGFIWDLITPTEDGQTRELQNHLGLSAPVPRGRKGKKVKTQTSSFDIQKAEWLRKYNP
EQLLQDEGYKKHVKHHCNKVLLRVRMLYYLKQEVIGNESQKVFDGVDASDIDVWVPEPDHSEVPAAWWDFDAD
KSLLIGVFKHGYEKYNTIRADPALCFLERVGKPDDKAVAAEQRANDYMDGDVEDPEYKPAPAIFKDDIEDDVS
SPGDLVIADGEGQLMEGDKVYWPTPSALTTRLRRLITAYQRTNKNRHIQQMQPTFSLPANAMQPLYEEATLNP
KMAAKIERQQRIGRSKYFYSTNHRGTCF*
>rs48480191_ENSMUSP00000148088_c.4351:A>G_p.K1451R
ENSMUST00000209423.2|Chd9-205|ENSMUSG00000056608|+|chr8:g.91737274:A>G
MTDPMMDFFDDANLFGETLEGLSDDTFVQPGPVSLVDELNLGAEFEPLHIDSLNHVQGTPTHQKMADFEQLSQ
FDSMKFHPVNQSFGSPVEHVLSPHSQFNCSPIHPPNQPNGLFQDVADGSPMWGHQTATGLANQNGSPFHQPGH
SHSLHQNKSFVAHPDFALFQASEHQTQCSSLHSQQSRSNLNPGQNSLGQAKNFLDANVSGAHRVNVNHLATAP
SSQQTLPVQFSPTANPPAHFLKCSSHQEGNYNRPSPSMTSCSVSNSQQFPSHYSFSSGHVSPSSLLQSSAGLA
PGHTNQALSDFAGSNSFSPHRGMKQEPTQHLLNPTPSLNSNNFQILHSSHPQGNYSNSKLSPVHMNFPDPVDA
GPPVGHFNDHAETNGFSSLEENLLHHVDSHAEPFAGLDPEDLLQEGLLPQFDESPFGQDNSNHVLDHDLDRQF
TSHLVSRPSDMAQTQLQYQARGWPSPLSTNHQHLHSRNHLCLQRQPPSSKKSDGSGTYTKLQNTQVRVMSEKK
PRKRVESESKQEKANRIISEAIARAKERGERNIPRVMSPENFPSASVEGKEEKRGRRMKSKPKDRDNKKPKTY
SKLKEKTKIGKLIITLGKKHKRRNESSDELSDAEQRSQHTFKEQHSQKRRSNRQIKRKKYAEDAEGKQSEEEV
KGSLRVKRNSAPPPGEQPLQLFVENPSEEDAAIVDKILACRTVKKEVSPGVMLDIEEFFVKYKNYSYLHCEWA
TEQQLLKDKRIQQKIKRFKLRQAQRAHFLADMEEEPFNPDYVEVDRILEVSFCEDKDTGESVIYYLVKWCSLP
YEDSTWELKEDVDLAKIEEFEQLQASRPDTRHLDRPPSNIWKKIEQSREYKNGNQLREYQLEGLNWLLFNWYN
RRNCILADEMGLGKTIQSITFLYEILLTGIRGPFLIIAPLSTIANWEREFRTWTDINVVVYHGSLISRQMIQQ
YEMYFRDSQGRIIRGAYRFQAIITTFEMILGGCGELNAIDWRCVIIDEAHRLKNKNCKLLEGLKLMNLEHKVL
LTGTPLQNTVEELFSLLHFLEPLRFPSESTFMQEFGDLKTEEQVQKLQAILKPMMLRRLKEDVEKKLAPKEET
IIEVELTNIQKKYYRAILEKNFSFLSKGAGQTNVPNLVNTMMELRKCCNHPYLIKGAEEKILGEFRDTYNPSA
SDFHLQAMIQSAGKLVLIDKLLPKMKAGGHKVLIFSQMVRCLDILEDYLIHKRYLYERIDGRVRGNLRQAAID
RFSKPDSDRFVFLLCTRAGGLGINLTAADTCIIFDSDWNPQNDLQAQARCHRIGQNKAVKVYRLVTRNSYERE
MFDRASLKLGLDKAVLQSMSGRDSNVSGIQQLSKKEIEDLLRRGAYGAIMEEEDEGSKFCEEDIDQILLRRTK
TITIESEGRGSTFAKASFVASGNRTDISLDDPNFWQKWAKKAELDIDTISGRNSLVIDTPRIRRQTRPFSATK
DELAELSEAESEGEEKPKLRRPCDRSGGYGRTECFRVEKNLLVYGWGRWREILSHGRFKRQLNEHDVEVICRA
LLAYCLIHYRGDEKIKGFIWDLITPTEDGQTRELQNHLGLSAPVPRGRKGKKVKTQTSSFDIQKAEWLRKYNP
EQLLQDEGYKKHVKHHCNKVLLRVRMLYYLKQEVIGNESQKVFDGVDASDIDVWVPEPDHSEVPAAWWDFDAD
KSLLIGVFKHGYEKYNTIRADPALCFLERVGKPDDKAVAAEQRANDYMDGDVEDPEYKPAPAIFKDDIEDDVS
SPGDLVIADGEGQLMEGDKVYWPTPSALTTRLRRLITAYQRTNKNRHIQQMQPTFSLPANAMQPLYEEATLNP
KMAAKIERQQRWTRREEADFYRVVSTFGVVFDPDRGQFDWTKFRALARLHKKTDNSLEKYLCAFMSMCRRVCR
LPSKEELVDPNIFIQPITEERASRTLYRIELLRKVREQALRHPQLFERLKLCHPNPDLPIWWECGSHDRDLLI
GAAKHGVSRTDYHILRDPELSFMAAQRNYNQSKAAHSRTSAPLLQQYQVALSASPLTSLPRLLGAKGTLLEDM
KVKSESLTEEPQSSEEESMSSMETRTRVKSEPVSPKNGVLSQATGDQKSGGKSETDRRMVAARTEPLTPNPAS
KKPRVHKRGSQSSSDSDSDSARSSCSSRSSSSSSSSSCSHSRSGSSSSSSSSCSSASSSSSSSSSSSSSSS
SSSEESDSEEDVQKREGTPHRKAYDEESVASLSTTQDETQDSFQANNGTPESAYLLQGGYMLAASYWPKDRVM
INRLDSICQTVLKGKWPSARRHYDANTVASFYTTKLLDSPGAATERGEPSVPTPPAVAVREEHEQSAQMSKVK
KHVREKEFTVKIKDEGGLKLTFQKQGLAQKRPFDGEDGALGQQQYLTRLRELQSTSETSLVNLPKAVPASGTS
IQPTLGANGAILDSQPIVKKRRGRRRNVEGADILFLNRNKPPNHIPTGMNPALSYPQPQRIPDTESPVPVINL
KDGTRLAGDDAPKRKDLDRWLKEHPGYVEDLGAFIPRVQLHEGRPKQKRHRCRNPNKLDINSLTGEERVQLIN
RRNARKVGGAFAPPLKDLCRFLKENSEYGVAPEWGDVVKQSGFLPESMFERILTGPVVREEVSRRGRRPKSGI
AKATTAAAVPAGSVPGNPLLANGLLPGVDLTALQALQQNLQNLQSLQVTAGLMGMPAGLSSGGETKNMAAMFP
MLFSGMAGLPNLLGMGGLLSKTAESGAEEKRGNDSKELEGKKERTESQSPENGGERCVPGSPSTSSTAALSSA
AAAKPIALNPLLLSNILYPGMLLTPGLNLHLPTLSQSNAFDVQKNKSDDLDSSKSVEIKEENSRVRDQEEKGG
TEPSPLNENSTDEGSERASSGSDSSSSSSEDSDSSNED*

>rs48480191_ENSMUSP00000105243_c.4351:A>G_p.K1451R
ENSMUST00000109614.9|Chd9-202|ENSMUSG00000056608|+|chr8:g.91737274:A>G
MTDPMMDFFDDANLFGETLEGLSDDTFVQPGPVSLVDELNLGAEFEPLHIDSLNHVQGTPTHQKMADFEQLSQ
FDSMKFHPVNQSFGSPVEHVLSPHSQFNCSPIHPPNQPNGLFQDVADGSPMWGHQTATGLANQNGSPFHQPGH
SHSLHQNKSFVAHPDFALFQASEHQTQCSSLHSQQSRSNLNPGQNSLGQAKNFLDANVSGAHRVNVNHLATAP
SSQQTLPVQFSPTANPPAHFLKCSSHQEGNYNRPSPSMTSCSVSNSQQFPSHYSFSSGHVSPSSLLQSSAGLA
PGHTNQALSDFAGSNSFSPHRGMKQEPTQHLLNPTPSLNSNNFQILHSSHPQGNYSNSKLSPVHMNFPDPVDA
GPPVGHFNDHAETNGFSSLEENLLHHVDSHAEPFAGLDPEDLLQEGLLPQFDESPFGQDNSNHVLDHDLDRQF
TSHLVSRPSDMAQTQLQYQARGWPSPLSTNHQHLHSRNHLCLQRQPPSSKKSDGSGTYTKLQNTQVRVMSEKK
PRKRVESESKQEKANRIISEAIARAKERGERNIPRVMSPENFPSASVEGKEEKRGRRMKSKPKDRDNKKPKTY
SKLKEKTKIGKLIITLGKKHKRRNESSDELSDAEQRSQHTFKEQHSQKRRSNRQIKRKKYAEDAEGKQSEEEV
KGSLRVKRNSAPPPGEQPLQLFVENPSEEDAAIVDKILACRTVKKEVSPGVMLDIEEFFVKYKNYSYLHCEWA
TEQQLLKDKRIQQKIKRFKLRQAQRAHFLADMEEEPFNPDYVEVDRILEVSFCEDKDTGESVIYYLVKWCSLP
YEDSTWELKEDVDLAKIEEFEQLQASRPDTRHLDRPPSNIWKKIEQSREYKNGNQLREYQLEGLNWLLFNWYN
RRNCILADEMGLGKTIQSITFLYEILLTGIRGPFLIIAPLSTIANWEREFRTWTDINVVVYHGSLISRQMIQQ
YEMYFRDSQGRIIRGAYRFQAIITTFEMILGGCGELNAIDWRCVIIDEAHRLKNKNCKLLEGLKLMNLEHKVL
LTGTPLQNTVEELFSLLHFLEPLRFPSESTFMQEFGDLKTEEQVQKLQAILKPMMLRRLKEDVEKKLAPKEET
IIEVELTNIQKKYYRAILEKNFSFLSKGAGQTNVPNLVNTMMELRKCCNHPYLIKGAEEKILGEFRDTYNPSA
SDFHLQAMIQSAGKLVLIDKLLPKMKAGGHKVLIFSQMVRCLDILEDYLIHKRYLYERIDGRVRGNLRQAAID
RFSKPDSDRFVFLLCTRAGGLGINLTAADTCIIFDSDWNPQNDLQAQARCHRIGQNKAVKVYRLVTRNSYERE
MFDRASLKLGLDKAVLQSMSGRDSNVSGIQQLSKKEIEDLLRRGAYGAIMEEEDEGSKFCEEDIDQILLRRTK
TITIESEGRGSTFAKASFVASGNRTDISLDDPNFWQKWAKKAELDIDTISGRNSLVIDTPRIRRQTRPFSATK
DELAELSEAESEGEEKPKLRRPCDRSGGYGRTECFRVEKNLLVYGWGRWREILSHGRFKRQLNEHDVEVICRA
LLAYCLIHYRGDEKIKGFIWDLITPTEDGQTRELQNHLGLSAPVPRGRKGKKVKTQTSSFDIQKAEWLRKYNP
EQLLQDEGYKKHVKHHCNKVLLRVRMLYYLKQEVIGNESQKVFDGVDASDIDVWVPEPDHSEVPAAWWDFDAD
KSLLIGVFKHGYEKYNTIRADPALCFLERVGKPDDKAVAAEQRANDYMDGDVEDPEYKPAPAIFKDDIEDDVS
SPGDLVIADGEGQLMEGDKVYWPTPSALTTRLRRLITAYQRTNKNRHIQQMQPTFSLPANAMQPLYEEATLNP
KMAAKIERQQRWTRREEADFYRVVSTFGVVFDPDRGQFDWTKFRALARLHKKTDNSLEKYLCAFMSMCRRVCR
LPSKEELVDPNIFIQPITEERASRTLYRIELLRKVREQALRHPQLFERLKLCHPNPDLPIWWECGSHDRDLLI
GAAKHGVSRTDYHILRDPELSFMAAQRNYNQSKAAHSRTSAPLLQQYQVALSASPLTSLPRLLGAKGTLLEDM
KVKSESLTEEPQSSEEESMSSMETRTRVKSEPVSPKNGVLSQATGDQKSGGKSETDRRMVAARTEPLTPNPAS
KKPRVHKRGSQSSSDSDSDSARSSCSSRSSSSSSSSSSCSHSRSGSSSSSSSSCSSASSSSSSSSSSSSSSSS
SSSEESDSEEDVQKREGTPHRKAYDEESVASLSTTQDETQDSFQANNGTPESAYLLQGGYMLAASYWPKDRVM
INRLDSICQTVLKGKWPSARRHYDANTVASFYTTKLLDSPGAATERGEPSVPTPPAVAVREEHEQSAQMSKVK
KHVREKEFTVKIKDEGGLKLTFQKQGLAQKRPFDGEDGALGQQQYLTRLRELQSTSETSLVNLPKAVPASGTS
IQPTLGANGAILDSQPIVKKRRGRRRNVEGADILFLNRNKPPNHIPTGMNPALSYPQPQRIPDTESPVPVINL
KDGTRLAGDDAPKRKDLDRWLKEHPGYVEDLGAFIPRVQLHEGRPKQKRHRCRNPNKLDINSLTGEERVQLIN
RRNARKVGGAFAPPLKDLCRFLKENSEYGVAPEWGDVVKQSGFLPESMFERILTGPVVREEVSRRGRRPKSGI
AKATTAAAVPAGSVPGNPLLANGLLPGVDLTALQALQQNLQNLQSLQVTAGLMGMPAGLSSGGETKNMAAMFP
MLFSGMAGLPNLLGMGGLLSKTAESGAEEKRGNDSKELEGKKERTESQSPENGGERCVPGSPSTSSTAALSSA
AAAKPIALNPLLLSNILYPGMLLTPGLNLHLPTLSQSNAFDVQKNKSDDLDSSKSVEIKEENSRVRDQEEKGG
TEPSPLNENSTDEGSERASSGSDSSSSSSEDSDSSNED*
>rs32839533_ENSMUSP00000046356_c.4569:G>A_p.E1524K
ENSMUST00000048665.8|Chd9-201|ENSMUSG00000056608|+|chr8:g.91737939:G>A
MTDPMMDFFDDANLFGETLEGLSDDTFVQPGPVSLVDELNLGAEFEPLHIDSLNHVQGTPTHQKMADFEQLSQ
FDSMKFHPVNQSFGSPVEHVLSPHSQFNCSPIHPPNQPNGLFQDVADGSPMWGHQTATGLANQNGSPFHQPGH
SHSLHQNKSFVAHPDFALFQASEHQTQCSSLHSQQSRSNLNPGQNSLGQAKNFLDANVSGAHRVNVNHLATAP
SSQQTLPVQFSPTANPPAHFLKCSSHQEGNYNRPSPSMTSCSVSNSQQFPSHYSFSSGHVSPSSLLQSSAGLA
PGHTNQALSDFAGSNSFSPHRGMKQEPTQHLLNPTPSLNSNNFQILHSSHPQGNYSNSKLSPVHMNFPDPVDA
GPPVGHFNDHAETNGFSSLEENLLHHVDSHAEPFAGLDPEDLLQEGLLPQFDESPFGQDNSNHVLDHDLDRQF
TSHLVSRPSDMAQTQLQYQARGWPSPLSTNHQHLHSRNHLCLQRQPPSSKKSDGSGTYTKLQNTQVRVMSEKK
PRKRVESESKQEKANRIISEAIARAKERGERNIPRVMSPENFPSASVEGKEEKRGRRMKSKPKDRDNKKPKTY
SKLKEKTKIGKLIITLGKKHKRRNESSDELSDAEQRSQHTFKEQHSQKRRSNRQIKRKKYAEDAEGKQSEEEV
KGSLRVKRNSAPPPGEQPLQLFVENPSEEDAAIVDKILACRTVKKEVSPGVMLDIEEFFVKYKNYSYLHCEWA
TEQQLLKDKRIQQKIKRFKLRQAQRAHFLADMEEEPFNPDYVEVDRILEVSFCEDKDTGESVIYYLVKWCSLP
YEDSTWELKEDVDLAKIEEFEQLQASRPDTRHLDRPPSNIWKKIEQSREYKNGNQLREYQLEGLNWLLFNWYN
RRNCILADEMGLGKTIQSITFLYEILLTGIRGPFLIIAPLSTIANWEREFRTWTDINVVVYHGSLISRQMIQQ
YEMYFRDSQGRIIRGAYRFQAIITTFEMILGGCGELNAIDWRCVIIDEAHRLKNKNCKLLEGLKLMNLEHKVL

LTGTPLQNTVEELFSLLHFLEPLRFPSESTFMQEFGDLKTEEQVQKLQAILKPMMLRRLKEDVEKKLAPKEET
IIEVELTNIQKKYYRAILEKNFSFLSKGAGQTNVPNLVNTMMELRKCCNHPYLIKGAEEKILGEFRDTYNPSA
SDFHLQAMIQSAGKLVLIDKLLPKMKAGGHKVLIFSQMVRCLDILEDYLIHKRYLYERIDGRVRGNLRQAAID
RFSKPDSDRFVFLLCTRAGGLGINLTAADTCIIFDSDWNPQNDLQAQARCHRIGQNKAVKVYRLVTRNSYERE
MFDRASLKLGLDKAVLQSMSGRDSNVSGIQQLSKKEIEDLLRRGAYGAIMEEEDEGSKFCEEDIDQILLRRTK
TITIESEGRGSTFAKASFVASGNRTDISLDDPNFWQKWAKKAELDIDTISGRNSLVIDTPRIRKQTRPFSATK
DELAELSEAESEGEEKPKLRRPCDRSGGYGRTECFRVEKNLLVYGWGRWREILSHGRFKRQLNKHDVEVICRA
LLAYCLIHYRGDEKIKGFIWDLITPTEDGQTRELQNHLGLSAPVPRGRKGKKVKTQTSSFDIQKAEWLRKYNP
EQLLQDEGYKKHVKHHCNKVLLRVRMLYYLKQEVIGNESQKVFDGVDASDIDVWVPEPDHSEVPAAWWDFDAD
KSLLIGVFKHGYEKYNTIRADPALCFLERVGKPDDKAVAAEQRANDYMDGDVEDPEYKPAPAIFKDDIEDDVS
SPGDLVIADGEGQLMEGDKVYWPTPSALTTRLRRLITAYQRTNKNRHIQQMQPTFSLPANAMQPLYEEATLNP
KMAAKIERQQRWTRREEADFYRVVSTFGVVFDPDRGQFDWTKFRALARLHKKTDNSLEKYLCAFMSMCRRVCR
LPSKEELVDPNIFIQPITEERASRTLYRIELLRKVREQALRHPQLFERLKLCHPNPDLPIWWECGSHDRDLLI
GAAKHGVSRTDYHILRDPELSFMAAQRNYNQSKAAHSRTSAPLLQQYQVALSASPLTSLPRLLGAKGTLLEDM
KVKSESLTEEPQSSEEESMSSMETRTRVKSEPVSPKNGVLSQATGDQKSGGKSETDRRMVAARTEPLTPNPAS
KKPRVHKRGSQSSSDSDSDSARSSCSSRSSSSSSSSSSCSHSRSGSSSSSSSSSCSSASSSSSSSSSSSSSSS
SSSEESDSEEDVQKREGTPHRKAYDEESVASLSTTQDETQDSFQANNGTPESAYLLQGGYMLAASYWPKDRVM
INRLDSICQTVLKGKWPSARRHYDANTVASFYTTKLLDSPGAATERGEPSVPTPPAVAVREEHEQSAQMSKEG
GLKLTFQKQGLAQKRPFDGEDGALGQQQYLTRLRELQSTSETSLVNLPKAVPASGTSIQPTLGANGAILDSQP
IVKKRRGRRRNVEGADILFLNRNKPPNHIPTGMNPALSYPQPQRIPDTESPVPVINLKDGTRLAGDDAPKRKD
LDRWLKEHPGYVEDLGAFIPRVQLHEGRPKQKRHRCRNPNKLDINSLTGEERVQLINRRNARKVGGAFAPPLK
DLCRFLKENSEYGVAPEWGDVVKQSGFLPESMFERILTGPVVREEVSRRGRRPKSGIAKATTAAAVPAGSVPG
NPLLANGLLPGVDLTALQALQQNLQNLQSLQVTAGLMGMPAGLSSGGETKNMAAMFPMLFSGMAGLPNLLGMG
GLLSKTAESGAEEKRGNDSKELEGKKERTESQSPENGGERCVPGSPSTSSTAALSSAAAAKPIALNPLLLSNI
LYPGMLLTPGLNLHLPTLSQSNAFDVQKNKSDDLDSSKSVEIKEENSRVRDQEEKGGTEPSPLNENSTDEGSE
RASSGSDSSSSSSEDSDSSNED*
>rs32839533_ENSMUSP00000147407_c.4569:G>A_p.E1524K
ENSMUST00000209203.2|Chd9-203|ENSMUSG00000056608|+|chr8:g.91737939:G>A
MTDPMMDFFDDANLFGETLEGLSDDTFVQPGPVSLVDELNLGAEFEPLHIDSLNHVQGTPTHQKMADFEQLSQ
FDSMKFHPVNQSFGSPVEHVLSPHSQFNCSPIHPPNQPNGLFQDVADGSPMWGHQTATGLANQNGSPFHQPGH
SHSLHQNKSFVAHPDFALFQASEHQTQCSSLHSQQSRSNLNPGQNSLGQAKNFLDANVSGAHRVNVNHLATAP
SSQQTLPVQFSPTANPPAHFLKCSSHQEGNYNRPSPSMTSCSVSNSQQFPSHYSFSSGHVSPSSLLQSSAGLA
PGHTNQALSDFAGSNSFSPHRGMKQEPTQHLLNPTPSLNSNNFQILHSSHPQGNYSNSKLSPVHMNFPDPVDA
GPPVGHFNDHAETNGFSSLEENLLHHVDSHAEPFAGLDPEDLLQEGLLPQFDESPFGQDNSNHVLDHDLDRQF
TSHLVSRPSDMAQTQLQYQARGWPSPLSTNHQHLHSRNHLCLQRQPPSSKKSDGSGTYTKLQNTQVRVMSEKK
PRKRVESESKQEKANRIISEAIARAKERGERNIPRVMSPENFPSASVEGKEEKRGRRMKSKPKDRDNKKPKTY
SKLKEKTKIGKLIITLGKKHKRRNESSDELSDAEQRSQHTFKEQHSQKRRSNRQIKRKKYAEDAEGKQSEEEV
KGSLRVKRNSAPPPGEQPLQLFVENPSEEDAAIVDKILACRTVKKEVSPGVMLDIEEFFVKYKNYSYLHCEWA
TEQQLLKDKRIQQKIKRFKLRQAQRAHFLADMEEEPFNPDYVEVDRILEVSFCEDKDTGESVIYYLVKWCSLP
YEDSTWELKEDVDLAKIEEFEQLQASRPDTRHLDRPPSNIWKKIEQSREYKNGNQLREYQLEGLNWLLFNWYN
RRNCILADEMGLGKTIQSITFLYEILLTGIRGPFLIIAPLSTIANWEREFRTWTDINVVVYHGSLISRQMIQQ
YEMYFRDSQGRIIRGAYRFQAIITTFEMILGGCGELNAIDWRCVIIDEAHRLKNKNCKLLEGLKLMNLEHKVL
LTGTPLQNTVEELFSLLHFLEPLRFPSESTFMQEFGDLKTEEQVQKLQAILKPMMLRRLKEDVEKKLAPKEET
IIEVELTNIQKKYYRAILEKNFSFLSKGAGQTNVPNLVNTMMELRKCCNHPYLIKGAEEKILGEFRDTYNPSA
SDFHLQAMIQSAGKLVLIDKLLPKMKAGGHKVLIFSQMVRCLDILEDYLIHKRYLYERIDGRVRGNLRQAAID
RFSKPDSDRFVFLLCTRAGGLGINLTAADTCIIFDSDWNPQNDLQAQARCHRIGQNKAVKVYRLVTRNSYERE
MFDRASLKLGLDKAVLQSMSGRDSNVSGIQQLSKKEIEDLLRRGAYGAIMEEEDEGSKFCEEDIDQILLRRTK
TITIESEGRGSTFAKASFVASGNRTDISLDDPNFWQKWAKKAELDIDTISGRNSLVIDTPRIRKQTRPFSATK
DELAELSEAESEGEEKPKLRRPCDRSGGYGRTECFRVEKNLLVYGWGRWREILSHGRFKRQLNKHDVEVICRA
LLAYCLIHYRGDEKIKGFIWDLITPTEDGQTRELQNHLGLSAPVPRGRKGKKVKTQTSSFDIQKAEWLRKYNP
EQLLQDEGYKKHVKHHCNKVLLRVRMLYYLKQEVIGNESQKVFDGVDASDIDVWVPEPDHSEVPAAWWDFDAD
KSLLIGVFKHGYEKYNTIRADPALCFLERVGKPDDKAVAAEQRANDYMDGDVEDPEYKPAPAIFKDDIEDDVS
SPGDLVIADGEGQLMEGDKVYWPTPSALTTRLRRLITAYQRTNKNRHIQQMQPTFSLPANAMQPLYEEATLNP
KMAAKIERQQRIGRSKYFYSTNHRGTCF*
>rs32839533_ENSMUSP00000148088_c.4569:G>A_p.E1524K
ENSMUST00000209423.2|Chd9-205|ENSMUSG00000056608|+|chr8:g.91737939:G>A
MTDPMMDFFDDANLFGETLEGLSDDTFVQPGPVSLVDELNLGAEFEPLHIDSLNHVQGTPTHQKMADFEQLSQ
FDSMKFHPVNQSFGSPVEHVLSPHSQFNCSPIHPPNQPNGLFQDVADGSPMWGHQTATGLANQNGSPFHQPGH

SHSLHQNKSFVAHPDFALFQASEHQTQCSSLHSQQSRSNLNPGQNSLGQAKNFLDANVSGAHRVNVNHLATAP
SSQQTLPVQFSPTANPPAHFLKCSSHQEGNYNRPSPSMTSCSVSNSQQFPSHYSFSSGHVSPSSLLQSSAGLA
PGHTNQALSDFAGSNSFSPHRGMKQEPTQHLLNPTPSLNSNNFQILHSSHPQGNYSNSKLSPVHMNFPDPVDA
GPPVGHFNDHAETNGFSSLEENLLHHVDSHAEPFAGLDPEDLLQEGLLPQFDESPFGQDNSNHVLDHDLDRQF
TSHLVSRPSDMAQTQLQYQARGWPSPLSTNHQHLHSRNHLCLQRQPPSSKKSDGSGTYTKLQNTQVRVMSEKK
PRKRVESESKQEKANRIISEAIARAKERGERNIPRVMSPENFPSASVEGKEEKRGRRMKSKPKDRDNKKPKTY
SKLKEKTKIGKLIITLGKKHKRRNESSDELSDAEQRSQHTFKEQHSQKRRSNRQIKRKKYAEDAEGKQSEEEV
KGSLRVKRNSAPPPGEQPLQLFVENPSEEDAAIVDKILACRTVKKEVSPGVMLDIEEFFVKYKNYSYLHCEWA
TEQQLLKDKRIQQKIKRFKLRQAQRAHFLADMEEEPFNPDYVEVDRILEVSFCEDKDTGESVIYYLVKWCSLP
YEDSTWELKEDVDLAKIEEFEQLQASRPDTRHLDRPPSNIWKKIEQSREYKNGNQLREYQLEGLNWLLFNWYN
RRNCILADEMGLGKTIQSITFLYEILLTGIRGPFLIIAPLSTIANWEREFRTWTDINVVVYHGSLISRQMIQQ
YEMYFRDSQGRIIRGAYRFQAIITTFEMILGGCGELNAIDWRCVIIDEAHRLKNKNCKLLEGLKLMNLEHKVL
LTGTPLQNTVEELFSLLHFLEPLRFPSESTFMQEFGDLKTEEQVQKLQAILKPMMLRRLKEDVEKKLAPKEET
IIEVELTNIQKKYYRAILEKNFSFLSKGAGQTNVPNLVNTMMELRKCCNHPYLIKGAEEKILGEFRDTYNPSA
SDFHLQAMIQSAGKLVLIDKLLPKMKAGGHKVLIFSQMVRCLDILEDYLIHKRYLYERIDGRVRGNLRQAAID
RFSKPDSDRFVFLLCTRAGGLGINLTAADTCIIFDSDWNPQNDLQAQARCHRIGQNKAVKVYRLVTRNSYERE
MFDRASLKLGLDKAVLQSMSGRDSNVSGIQQLSKKEIEDLLRRGAYGAIMEEEDEGSKFCEEDIDQILLRRTK
TITIESEGRGSTFAKASFVASGNRTDISLDDPNFWQKWAKKAELDIDTISGRNSLVIDTPRIRKQTRPFSATK
DELAELSEAESEGEEKPKLRRPCDRSGGYGRTECFRVEKNLLVYGWGRWREILSHGRFKRQLNKHDVEVICRA
LLAYCLIHYRGDEKIKGFIWDLITPTEDGQTRELQNHLGLSAPVPRGRKGKKVKTQTSSFDIQKAEWLRKYNP
EQLLQDEGYKKHVKHHCNKVLLRVRMLYYLKQEVIGNESQKVFDGVDASDIDVWVPEPDHSEVPAAWWDFDAD
KSLLIGVFKHGYEKYNTIRADPALCFLERVGKPDDKAVAAEQRANDYMDGDVEDPEYKPAPAIFKDDIEDDVS
SPGDLVIADGEGQLMEGDKVYWPTPSALTTRLRRLITAYQRTNKNRHIQQMQPTFSLPANAMQPLYEEATLNP
KMAAKIERQQRWTRREEADFYRVVSTFGVVFDPDRGQFDWTKFRALARLHKKTDNSLEKYLCAFMSMCRRVCR
LPSKEELVDPNIFIQPITEERASRTLYRIELLRKVREQALRHPQLFERLKLCHPNPDLPIWWECGSHDRDLLI
GAAKHGVSRTDYHILRDPELSFMAAQRNYNQSKAAHSRTSAPLLQQYQVALSASPLTSLPRLLGAKGTLLEDM
KVKSESLTEEPQSSEEESMSSMETRTRVKSEPVSPKNGVLSQATGDQKSGGKSETDRRMVAARTEPLTPNPAS
KKPRVHKRGSQSSSDSDSDSARSSCSSRSSSSSSSSSCSHSRSGSSSSSSSSCSSASSSSSSSSSSSSSSS
SSSEESDSEEDVQKREGTPHRKAYDEESVASLSTTQDETQDSFQANNGTPESAYLLQGGYMLAASYWPKDRVM
INRLDSICQTVLKGKWPSARRHYDANTVASFYTTKLLDSPGAATERGEPSVPTPPAVAVREEHEQSAQMSKVK
KHVREKEFTVKIKDEGGLKLTFQKQGLAQKRPFDGEDGALGQQQYLTRLRELQSTSETSLVNLPKAVPASGTS
IQPTLGANGAILDSQPIVKKRRGRRRNVEGADILFLNRNKPPNHIPTGMNPALSYPQPQRIPDTESPVPVINL
KDGTRLAGDDAPKRKDLDRWLKEHPGYVEDLGAFIPRVQLHEGRPKQKRHRCRNPNKLDINSLTGEERVQLIN
RRNARKVGGAFAPPLKDLCRFLKENSEYGVAPEWGDVVKQSGFLPESMFERILTGPVVREEVSRRGRRPKSGI
AKATTAAAVPAGSVPGNPLLANGLLPGVDLTALQALQQNLQNLQSLQVTAGLMGMPAGLSSGGETKNMAAMFP
MLFSGMAGLPNLLGMGGLLSKTAESGAEEKRGNDSKELEGKKERTESQSPENGGERCVPGSPSTSSTAALSSA
AAAKPIALNPLLLSNILYPGMLLTPGLNLHLPTLSQSNAFDVQKNKSDDLDSSKSVEIKEENSRVRDQEEKGG
TEPSPLNENSTDEGSERASSGSDSSSSSSEDSDSSNED*
>rs32839533_ENSMUSP00000105243_c.4569:G>A_p.E1524K
ENSMUST00000109614.9|Chd9-202|ENSMUSG00000056608|+|chr8:g.91737939:G>A
MTDPMMDFFDDANLFGETLEGLSDDTFVQPGPVSLVDELNLGAEFEPLHIDSLNHVQGTPTHQKMADFEQLSQ
FDSMKFHPVNQSFGSPVEHVLSPHSQFNCSPIHPPNQPNGLFQDVADGSPMWGHQTATGLANQNGSPFHQPGH
SHSLHQNKSFVAHPDFALFQASEHQTQCSSLHSQQSRSNLNPGQNSLGQAKNFLDANVSGAHRVNVNHLATAP
SSQQTLPVQFSPTANPPAHFLKCSSHQEGNYNRPSPSMTSCSVSNSQQFPSHYSFSSGHVSPSSLLQSSAGLA
PGHTNQALSDFAGSNSFSPHRGMKQEPTQHLLNPTPSLNSNNFQILHSSHPQGNYSNSKLSPVHMNFPDPVDA
GPPVGHFNDHAETNGFSSLEENLLHHVDSHAEPFAGLDPEDLLQEGLLPQFDESPFGQDNSNHVLDHDLDRQF
TSHLVSRPSDMAQTQLQYQARGWPSPLSTNHQHLHSRNHLCLQRQPPSSKKSDGSGTYTKLQNTQVRVMSEKK
PRKRVESESKQEKANRIISEAIARAKERGERNIPRVMSPENFPSASVEGKEEKRGRRMKSKPKDRDNKKPKTY
SKLKEKTKIGKLIITLGKKHKRRNESSDELSDAEQRSQHTFKEQHSQKRRSNRQIKRKKYAEDAEGKQSEEEV
KGSLRVKRNSAPPPGEQPLQLFVENPSEEDAAIVDKILACRTVKKEVSPGVMLDIEEFFVKYKNYSYLHCEWA
TEQQLLKDKRIQQKIKRFKLRQAQRAHFLADMEEEPFNPDYVEVDRILEVSFCEDKDTGESVIYYLVKWCSLP
YEDSTWELKEDVDLAKIEEFEQLQASRPDTRHLDRPPSNIWKKIEQSREYKNGNQLREYQLEGLNWLLFNWYN
RRNCILADEMGLGKTIQSITFLYEILLTGIRGPFLIIAPLSTIANWEREFRTWTDINVVVYHGSLISRQMIQQ
YEMYFRDSQGRIIRGAYRFQAIITTFEMILGGCGELNAIDWRCVIIDEAHRLKNKNCKLLEGLKLMNLEHKVL
LTGTPLQNTVEELFSLLHFLEPLRFPSESTFMQEFGDLKTEEQVQKLQAILKPMMLRRLKEDVEKKLAPKEET
IIEVELTNIQKKYYRAILEKNFSFLSKGAGQTNVPNLVNTMMELRKCCNHPYLIKGAEEKILGEFRDTYNPSA
SDFHLQAMIQSAGKLVLIDKLLPKMKAGGHKVLIFSQMVRCLDILEDYLIHKRYLYERIDGRVRGNLRQAAID
RFSKPDSDRFVFLLCTRAGGLGINLTAADTCIIFDSDWNPQNDLQAQARCHRIGQNKAVKVYRLVTRNSYERE

MFDRASLKLGLDKAVLQSMSGRDSNVSGIQQLSKKEIEDLLRRGAYGAIMEEEDEGSKFCEEDIDQILLRRTK
TITIESEGRGSTFAKASFVASGNRTDISLDDPNFWQKWAKKAELDIDTISGRNSLVIDTPRIRKQTRPFSATK
DELAELSEAESEGEEKPKLRRPCDRSGGYGRTECFRVEKNLLVYGWGRWREILSHGRFKRQLNKHDVEVICRA
LLAYCLIHYRGDEKIKGFIWDLITPTEDGQTRELQNHLGLSAPVPRGRKGKKVKTQTSSFDIQKAEWLRKYNP
EQLLQDEGYKKHVKHHCNKVLLRVRMLYYLKQEVIGNESQKVFDGVDASDIDVWVPEPDHSEVPAAWWDFDAD
KSLLIGVFKHGYEKYNTIRADPALCFLERVGKPDDKAVAAEQRANDYMDGDVEDPEYKPAPAIFKDDIEDDVS
SPGDLVIADGEGQLMEGDKVYWPTPSALTTRLRRLITAYQRTNKNRHIQQMQPTFSLPANAMQPLYEEATLNP
KMAAKIERQQRWTRREEADFYRVVSTFGVVFDPDRGQFDWTKFRALARLHKKTDNSLEKYLCAFMSMCRRVCR
LPSKEELVDPNIFIQPITEERASRTLYRIELLRKVREQALRHPQLFERLKLCHPNPDLPIWWECGSHDRDLLI
GAAKHGVSRTDYHILRDPELSFMAAQRNYNQSKAAHSRTSAPLLQQYQVALSASPLTSLPRLLGAKGTLLEDM
KVKSESLTEEPQSSEEESMSSMETRTRVKSEPVSPKNGVLSQATGDQKSGGKSETDRRMVAARTEPLTPNPAS
KKPRVHKRGSQSSSDSDSDSARSSCSSRSSSSSSSSSCSHSRSGSSSSSSSSCSSASSSSSSSSSSSSSSS
SSSEESDSEEDVQKREGTPHRKAYDEESVASLSTTQDETQDSFQANNGTPESAYLLQGGYMLAASYWPKDRVM
INRLDSICQTVLKGKWPSARRHYDANTVASFYTTKLLDSPGAATERGEPSVPTPPAVAVREEHEQSAQMSKVK
KHVREKEFTVKIKDEGGLKLTFQKQGLAQKRPFDGEDGALGQQQYLTRLRELQSTSETSLVNLPKAVPASGTS
IQPTLGANGAILDSQPIVKKRRGRRRNVEGADILFLNRNKPPNHIPTGMNPALSYPQPQRIPDTESPVPVINL
KDGTRLAGDDAPKRKDLDRWLKEHPGYVEDLGAFIPRVQLHEGRPKQKRHRCRNPNKLDINSLTGEERVQLIN
RRNARKVGGAFAPPLKDLCRFLKENSEYGVAPEWGDVVKQSGFLPESMFERILTGPVVREEVSRRGRRPKSGI
AKATTAAAVPAGSVPGNPLLANGLLPGVDLTALQALQQNLQNLQSLQVTAGLMGMPAGLSSGGETKNMAAMFP
MLFSGMAGLPNLLGMGGLLSKTAESGAEEKRGNDSKELEGKKERTESQSPENGGERCVPGSPSTSSTAALSSA
AAAKPIALNPLLLSNILYPGMLLTPGLNLHLPTLSQSNAFDVQKNKSDDLDSSKSVEIKEENSRVRDQEEKGG
TEPSPLNENSTDEGSERASSGSDSSSSSSEDSDSSNED*
>ENSMUSP00000046356_c.4874:A>T_p.K1625N ENSMUST00000048665.8|Chd9-
201|ENSMUSG00000056608|+|chr8:g.91742463:A>T
MTDPMMDFFDDANLFGETLEGLSDDTFVQPGPVSLVDELNLGAEFEPLHIDSLNHVQGTPTHQKMADFEQLSQ
FDSMKFHPVNQSFGSPVEHVLSPHSQFNCSPIHPPNQPNGLFQDVADGSPMWGHQTATGLANQNGSPFHQPGH
SHSLHQNKSFVAHPDFALFQASEHQTQCSSLHSQQSRSNLNPGQNSLGQAKNFLDANVSGAHRVNVNHLATAP
SSQQTLPVQFSPTANPPAHFLKCSSHQEGNYNRPSPSMTSCSVSNSQQFPSHYSFSSGHVSPSSLLQSSAGLA
PGHTNQALSDFAGSNSFSPHRGMKQEPTQHLLNPTPSLNSNNFQILHSSHPQGNYSNSKLSPVHMNFPDPVDA
GPPVGHFNDHAETNGFSSLEENLLHHVDSHAEPFAGLDPEDLLQEGLLPQFDESPFGQDNSNHVLDHDLDRQF
TSHLVSRPSDMAQTQLQYQARGWPSPLSTNHQHLHSRNHLCLQRQPPSSKKSDGSGTYTKLQNTQVRVMSEKK
PRKRVESESKQEKANRIISEAIARAKERGERNIPRVMSPENFPSASVEGKEEKRGRRMKSKPKDRDNKKPKTY
SKLKEKTKIGKLIITLGKKHKRRNESSDELSDAEQRSQHTFKEQHSQKRRSNRQIKRKKYAEDAEGKQSEEEV
KGSLRVKRNSAPPPGEQPLQLFVENPSEEDAAIVDKILACRTVKKEVSPGVMLDIEEFFVKYKNYSYLHCEWA
TEQQLLKDKRIQQKIKRFKLRQAQRAHFLADMEEEPFNPDYVEVDRILEVSFCEDKDTGESVIYYLVKWCSLP
YEDSTWELKEDVDLAKIEEFEQLQASRPDTRHLDRPPSNIWKKIEQSREYKNGNQLREYQLEGLNWLLFNWYN
RRNCILADEMGLGKTIQSITFLYEILLTGIRGPFLIIAPLSTIANWEREFRTWTDINVVVYHGSLISRQMIQQ
YEMYFRDSQGRIIRGAYRFQAIITTFEMILGGCGELNAIDWRCVIIDEAHRLKNKNCKLLEGLKLMNLEHKVL
LTGTPLQNTVEELFSLLHFLEPLRFPSESTFMQEFGDLKTEEQVQKLQAILKPMMLRRLKEDVEKKLAPKEET
IIEVELTNIQKKYYRAILEKNFSFLSKGAGQTNVPNLVNTMMELRKCCNHPYLIKGAEEKILGEFRDTYNPSA
SDFHLQAMIQSAGKLVLIDKLLPKMKAGGHKVLIFSQMVRCLDILEDYLIHKRYLYERIDGRVRGNLRQAAID
RFSKPDSDRFVFLLCTRAGGLGINLTAADTCIIFDSDWNPQNDLQAQARCHRIGQNKAVKVYRLVTRNSYERE
MFDRASLKLGLDKAVLQSMSGRDSNVSGIQQLSKKEIEDLLRRGAYGAIMEEEDEGSKFCEEDIDQILLRRTK
TITIESEGRGSTFAKASFVASGNRTDISLDDPNFWQKWAKKAELDIDTISGRNSLVIDTPRIRKQTRPFSATK
DELAELSEAESEGEEKPKLRRPCDRSGGYGRTECFRVEKNLLVYGWGRWREILSHGRFKRQLNEHDVEVICRA
LLAYCLIHYRGDEKIKGFIWDLITPTEDGQTRELQNHLGLSAPVPRGRKGKKVKTQTSSFDIQKAEWLRKYNP
EQLLQDEGYKKHVKHHCNNVLLRVRMLYYLKQEVIGNESQKVFDGVDASDIDVWVPEPDHSEVPAAWWDFDAD
KSLLIGVFKHGYEKYNTIRADPALCFLERVGKPDDKAVAAEQRANDYMDGDVEDPEYKPAPAIFKDDIEDDVS
SPGDLVIADGEGQLMEGDKVYWPTPSALTTRLRRLITAYQRTNKNRHIQQMQPTFSLPANAMQPLYEEATLNP
KMAAKIERQQRWTRREEADFYRVVSTFGVVFDPDRGQFDWTKFRALARLHKKTDNSLEKYLCAFMSMCRRVCR
LPSKEELVDPNIFIQPITEERASRTLYRIELLRKVREQALRHPQLFERLKLCHPNPDLPIWWECGSHDRDLLI
GAAKHGVSRTDYHILRDPELSFMAAQRNYNQSKAAHSRTSAPLLQQYQVALSASPLTSLPRLLGAKGTLLEDM
KVKSESLTEEPQSSEEESMSSMETRTRVKSEPVSPKNGVLSQATGDQKSGGKSETDRRMVAARTEPLTPNPAS
KKPRVHKRGSQSSSDSDSDSARSSCSSRSSSSSSSSSCSHSRSGSSSSSSSSCSSASSSSSSSSSSSSSSS
SSSEESDSEEDVQKREGTPHRKAYDEESVASLSTTQDETQDSFQANNGTPESAYLLQGGYMLAASYWPKDRVM
INRLDSICQTVLKGKWPSARRHYDANTVASFYTTKLLDSPGAATERGEPSVPTPPAVAVREEHEQSAQMSKEG
GLKLTFQKQGLAQKRPFDGEDGALGQQQYLTRLRELQSTSETSLVNLPKAVPASGTSIQPTLGANGAILDSQP
IVKKRRGRRRNVEGADILFLNRNKPPNHIPTGMNPALSYPQPQRIPDTESPVPVINLKDGTRLAGDDAPKRKD

LDRWLKEHPGYVEDLGAFIPRVQLHEGRPKQKRHRCRNPNKLDINSLTGEERVQLINRRNARKVGGAFAPPLK
DLCRFLKENSEYGVAPEWGDVVKQSGFLPESMFERILTGPVVREEVSRRGRRPKSGIAKATTAAAVPAGSVPG
NPLLANGLLPGVDLTALQALQQNLQNLQSLQVTAGLMGMPAGLSSGGETKNMAAMFPMLFSGMAGLPNLLGMG
GLLSKTAESGAEEKRGNDSKELEGKKERTESQSPENGGERCVPGSPSTSSTAALSSAAAAKPIALNPLLLSNI
LYPGMLLTPGLNLHLPTLSQSNAFDVQKNKSDDLDSSKSVEIKEENSRVRDQEEKGGTEPSPLNENSTDEGSE
RASSGSDSSSSSSEDSDSSNED*
>ENSMUSP00000147407_c.4874:A>T_p.K1625N ENSMUST00000209203.2|Chd9-
203|ENSMUSG00000056608|+|chr8:g.91742463:A>T
MTDPMMDFFDDANLFGETLEGLSDDTFVQPGPVSLVDELNLGAEFEPLHIDSLNHVQGTPTHQKMADFEQLSQ
FDSMKFHPVNQSFGSPVEHVLSPHSQFNCSPIHPPNQPNGLFQDVADGSPMWGHQTATGLANQNGSPFHQPGH
SHSLHQNKSFVAHPDFALFQASEHQTQCSSLHSQQSRSNLNPGQNSLGQAKNFLDANVSGAHRVNVNHLATAP
SSQQTLPVQFSPTANPPAHFLKCSSHQEGNYNRPSPSMTSCSVSNSQQFPSHYSFSSGHVSPSSLLQSSAGLA
PGHTNQALSDFAGSNSFSPHRGMKQEPTQHLLNPTPSLNSNNFQILHSSHPQGNYSNSKLSPVHMNFPDPVDA
GPPVGHFNDHAETNGFSSLEENLLHHVDSHAEPFAGLDPEDLLQEGLLPQFDESPFGQDNSNHVLDHDLDRQF
TSHLVSRPSDMAQTQLQYQARGWPSPLSTNHQHLHSRNHLCLQRQPPSSKKSDGSGTYTKLQNTQVRVMSEKK
PRKRVESESKQEKANRIISEAIARAKERGERNIPRVMSPENFPSASVEGKEEKRGRRMKSKPKDRDNKKPKTY
SKLKEKTKIGKLIITLGKKHKRRNESSDELSDAEQRSQHTFKEQHSQKRRSNRQIKRKKYAEDAEGKQSEEEV
KGSLRVKRNSAPPPGEQPLQLFVENPSEEDAAIVDKILACRTVKKEVSPGVMLDIEEFFVKYKNYSYLHCEWA
TEQQLLKDKRIQQKIKRFKLRQAQRAHFLADMEEEPFNPDYVEVDRILEVSFCEDKDTGESVIYYLVKWCSLP
YEDSTWELKEDVDLAKIEEFEQLQASRPDTRHLDRPPSNIWKKIEQSREYKNGNQLREYQLEGLNWLLFNWYN
RRNCILADEMGLGKTIQSITFLYEILLTGIRGPFLIIAPLSTIANWEREFRTWTDINVVVYHGSLISRQMIQQ
YEMYFRDSQGRIIRGAYRFQAIITTFEMILGGCGELNAIDWRCVIIDEAHRLKNKNCKLLEGLKLMNLEHKVL
LTGTPLQNTVEELFSLLHFLEPLRFPSESTFMQEFGDLKTEEQVQKLQAILKPMMLRRLKEDVEKKLAPKEET
IIEVELTNIQKKYYRAILEKNFSFLSKGAGQTNVPNLVNTMMELRKCCNHPYLIKGAEEKILGEFRDTYNPSA
SDFHLQAMIQSAGKLVLIDKLLPKMKAGGHKVLIFSQMVRCLDILEDYLIHKRYLYERIDGRVRGNLRQAAID
RFSKPDSDRFVFLLCTRAGGLGINLTAADTCIIFDSDWNPQNDLQAQARCHRIGQNKAVKVYRLVTRNSYERE
MFDRASLKLGLDKAVLQSMSGRDSNVSGIQQLSKKEIEDLLRRGAYGAIMEEEDEGSKFCEEDIDQILLRRTK
TITIESEGRGSTFAKASFVASGNRTDISLDDPNFWQKWAKKAELDIDTISGRNSLVIDTPRIRKQTRPFSATK
DELAELSEAESEGEEKPKLRRPCDRSGGYGRTECFRVEKNLLVYGWGRWREILSHGRFKRQLNEHDVEVICRA
LLAYCLIHYRGDEKIKGFIWDLITPTEDGQTRELQNHLGLSAPVPRGRKGKKVKTQTSSFDIQKAEWLRKYNP
EQLLQDEGYKKHVKHHCNNVLLRVRMLYYLKQEVIGNESQKVFDGVDASDIDVWVPEPDHSEVPAAWWDFDAD
KSLLIGVFKHGYEKYNTIRADPALCFLERVGKPDDKAVAAEQRANDYMDGDVEDPEYKPAPAIFKDDIEDDVS
SPGDLVIADGEGQLMEGDKVYWPTPSALTTRLRRLITAYQRTNKNRHIQQMQPTFSLPANAMQPLYEEATLNP
KMAAKIERQQRIGRSKYFYSTNHRGTCF*
>ENSMUSP00000148088_c.4874:A>T_p.K1625N ENSMUST00000209423.2|Chd9-
205|ENSMUSG00000056608|+|chr8:g.91742463:A>T
MTDPMMDFFDDANLFGETLEGLSDDTFVQPGPVSLVDELNLGAEFEPLHIDSLNHVQGTPTHQKMADFEQLSQ
FDSMKFHPVNQSFGSPVEHVLSPHSQFNCSPIHPPNQPNGLFQDVADGSPMWGHQTATGLANQNGSPFHQPGH
SHSLHQNKSFVAHPDFALFQASEHQTQCSSLHSQQSRSNLNPGQNSLGQAKNFLDANVSGAHRVNVNHLATAP
SSQQTLPVQFSPTANPPAHFLKCSSHQEGNYNRPSPSMTSCSVSNSQQFPSHYSFSSGHVSPSSLLQSSAGLA
PGHTNQALSDFAGSNSFSPHRGMKQEPTQHLLNPTPSLNSNNFQILHSSHPQGNYSNSKLSPVHMNFPDPVDA
GPPVGHFNDHAETNGFSSLEENLLHHVDSHAEPFAGLDPEDLLQEGLLPQFDESPFGQDNSNHVLDHDLDRQF
TSHLVSRPSDMAQTQLQYQARGWPSPLSTNHQHLHSRNHLCLQRQPPSSKKSDGSGTYTKLQNTQVRVMSEKK
PRKRVESESKQEKANRIISEAIARAKERGERNIPRVMSPENFPSASVEGKEEKRGRRMKSKPKDRDNKKPKTY
SKLKEKTKIGKLIITLGKKHKRRNESSDELSDAEQRSQHTFKEQHSQKRRSNRQIKRKKYAEDAEGKQSEEEV
KGSLRVKRNSAPPPGEQPLQLFVENPSEEDAAIVDKILACRTVKKEVSPGVMLDIEEFFVKYKNYSYLHCEWA
TEQQLLKDKRIQQKIKRFKLRQAQRAHFLADMEEEPFNPDYVEVDRILEVSFCEDKDTGESVIYYLVKWCSLP
YEDSTWELKEDVDLAKIEEFEQLQASRPDTRHLDRPPSNIWKKIEQSREYKNGNQLREYQLEGLNWLLFNWYN
RRNCILADEMGLGKTIQSITFLYEILLTGIRGPFLIIAPLSTIANWEREFRTWTDINVVVYHGSLISRQMIQQ
YEMYFRDSQGRIIRGAYRFQAIITTFEMILGGCGELNAIDWRCVIIDEAHRLKNKNCKLLEGLKLMNLEHKVL
LTGTPLQNTVEELFSLLHFLEPLRFPSESTFMQEFGDLKTEEQVQKLQAILKPMMLRRLKEDVEKKLAPKEET
IIEVELTNIQKKYYRAILEKNFSFLSKGAGQTNVPNLVNTMMELRKCCNHPYLIKGAEEKILGEFRDTYNPSA
SDFHLQAMIQSAGKLVLIDKLLPKMKAGGHKVLIFSQMVRCLDILEDYLIHKRYLYERIDGRVRGNLRQAAID
RFSKPDSDRFVFLLCTRAGGLGINLTAADTCIIFDSDWNPQNDLQAQARCHRIGQNKAVKVYRLVTRNSYERE
MFDRASLKLGLDKAVLQSMSGRDSNVSGIQQLSKKEIEDLLRRGAYGAIMEEEDEGSKFCEEDIDQILLRRTK
TITIESEGRGSTFAKASFVASGNRTDISLDDPNFWQKWAKKAELDIDTISGRNSLVIDTPRIRKQTRPFSATK
DELAELSEAESEGEEKPKLRRPCDRSGGYGRTECFRVEKNLLVYGWGRWREILSHGRFKRQLNEHDVEVICRA
LLAYCLIHYRGDEKIKGFIWDLITPTEDGQTRELQNHLGLSAPVPRGRKGKKVKTQTSSFDIQKAEWLRKYNP

EQLLQDEGYKKHVKHHCNNVLLRVRMLYYLKQEVIGNESQKVFDGVDASDIDVWVPEPDHSEVPAAWWDFDAD
KSLLIGVFKHGYEKYNTIRADPALCFLERVGKPDDKAVAAEQRANDYMDGDVEDPEYKPAPAIFKDDIEDDVS
SPGDLVIADGEGQLMEGDKVYWPTPSALTTRLRRLITAYQRTNKNRHIQQMQPTFSLPANAMQPLYEEATLNP
KMAAKIERQQRWTRREEADFYRVVSTFGVVFDPDRGQFDWTKFRALARLHKKTDNSLEKYLCAFMSMCRRVCR
LPSKEELVDPNIFIQPITEERASRTLYRIELLRKVREQALRHPQLFERLKLCHPNPDLPIWWECGSHDRDLLI
GAAKHGVSRTDYHILRDPELSFMAAQRNYNQSKAAHSRTSAPLLQQYQVALSASPLTSLPRLLGAKGTLLEDM
KVKSESLTEEPQSSEEESMSSMETRTRVKSEPVSPKNGVLSQATGDQKSGGKSETDRRMVAARTEPLTPNPAS
KKPRVHKRGSQSSSDSDSDSARSSCSSRSSSSSSSSSSCSHSRSGSSSSSSSSSCSSASSSSSSSSSSSSSSS
SSSEESDSEEDVQKREGTPHRKAYDEESVASLSTTQDETQDSFQANNGTPESAYLLQGGYMLAASYWPKDRVM
INRLDSICQTVLKGKWPSARRHYDANTVASFYTTKLLDSPGAATERGEPSVPTPPAVAVREEHEQSAQMSKVK
KHVREKEFTVKIKDEGGLKLTFQKQGLAQKRPFDGEDGALGQQQYLTRLRELQSTSETSLVNLPKAVPASGTS
IQPTLGANGAILDSQPIVKKRRGRRRNVEGADILFLNRNKPPNHIPTGMNPALSYPQPQRIPDTESPVPVINL
KDGTRLAGDDAPKRKDLDRWLKEHPGYVEDLGAFIPRVQLHEGRPKQKRHRCRNPNKLDINSLTGEERVQLIN
RRNARKVGGAFAPPLKDLCRFLKENSEYGVAPEWGDVVKQSGFLPESMFERILTGPVVREEVSRRGRRPKSGI
AKATTAAAVPAGSVPGNPLLANGLLPGVDLTALQALQQNLQNLQSLQVTAGLMGMPAGLSSGGETKNMAAMFP
MLFSGMAGLPNLLGMGGLLSKTAESGAEEKRGNDSKELEGKKERTESQSPENGGERCVPGSPSTSSTAALSSA
AAAKPIALNPLLLSNILYPGMLLTPGLNLHLPTLSQSNAFDVQKNKSDDLDSSKSVEIKEENSRVRDQEEKGG
TEPSPLNENSTDEGSERASSGSDSSSSSSEDSDSSNED*
>ENSMUSP00000105243_c.4874:A>T_p.K1625N ENSMUST00000109614.9|Chd9-
202|ENSMUSG00000056608|+|chr8:g.91742463:A>T
MTDPMMDFFDDANLFGETLEGLSDDTFVQPGPVSLVDELNLGAEFEPLHIDSLNHVQGTPTHQKMADFEQLSQ
FDSMKFHPVNQSFGSPVEHVLSPHSQFNCSPIHPPNQPNGLFQDVADGSPMWGHQTATGLANQNGSPFHQPGH
SHSLHQNKSFVAHPDFALFQASEHQTQCSSLHSQQSRSNLNPGQNSLGQAKNFLDANVSGAHRVNVNHLATAP
SSQQTLPVQFSPTANPPAHFLKCSSHQEGNYNRPSPSMTSCSVSNSQQFPSHYSFSSGHVSPSSLLQSSAGLA
PGHTNQALSDFAGSNSFSPHRGMKQEPTQHLLNPTPSLNSNNFQILHSSHPQGNYSNSKLSPVHMNFPDPVDA
GPPVGHFNDHAETNGFSSLEENLLHHVDSHAEPFAGLDPEDLLQEGLLPQFDESPFGQDNSNHVLDHDLDRQF
TSHLVSRPSDMAQTQLQYQARGWPSPLSTNHQHLHSRNHLCLQRQPPSSKKSDGSGTYTKLQNTQVRVMSEKK
PRKRVESESKQEKANRIISEAIARAKERGERNIPRVMSPENFPSASVEGKEEKRGRRMKSKPKDRDNKKPKTY
SKLKEKTKIGKLIITLGKKHKRRNESSDELSDAEQRSQHTFKEQHSQKRRSNRQIKRKKYAEDAEGKQSEEEV
KGSLRVKRNSAPPPGEQPLQLFVENPSEEDAAIVDKILACRTVKKEVSPGVMLDIEEFFVKYKNYSYLHCEWA
TEQQLLKDKRIQQKIKRFKLRQAQRAHFLADMEEEPFNPDYVEVDRILEVSFCEDKDTGESVIYYLVKWCSLP
YEDSTWELKEDVDLAKIEEFEQLQASRPDTRHLDRPPSNIWKKIEQSREYKNGNQLREYQLEGLNWLLFNWYN
RRNCILADEMGLGKTIQSITFLYEILLTGIRGPFLIIAPLSTIANWEREFRTWTDINVVVYHGSLISRQMIQQ
YEMYFRDSQGRIIRGAYRFQAIITTFEMILGGCGELNAIDWRCVIIDEAHRLKNKNCKLLEGLKLMNLEHKVL
LTGTPLQNTVEELFSLLHFLEPLRFPSESTFMQEFGDLKTEEQVQKLQAILKPMMLRRLKEDVEKKLAPKEET
IIEVELTNIQKKYYRAILEKNFSFLSKGAGQTNVPNLVNTMMELRKCCNHPYLIKGAEEKILGEFRDTYNPSA
SDFHLQAMIQSAGKLVLIDKLLPKMKAGGHKVLIFSQMVRCLDILEDYLIHKRYLYERIDGRVRGNLRQAAID
RFSKPDSDRFVFLLCTRAGGLGINLTAADTCIIFDSDWNPQNDLQAQARCHRIGQNKAVKVYRLVTRNSYERE
MFDRASLKLGLDKAVLQSMSGRDSNVSGIQQLSKKEIEDLLRRGAYGAIMEEEDEGSKFCEEDIDQILLRRTK
TITIESEGRGSTFAKASFVASGNRTDISLDDPNFWQKWAKKAELDIDTISGRNSLVIDTPRIRKQTRPFSATK
DELAELSEAESEGEEKPKLRRPCDRSGGYGRTECFRVEKNLLVYGWGRWREILSHGRFKRQLNEHDVEVICRA
LLAYCLIHYRGDEKIKGFIWDLITPTEDGQTRELQNHLGLSAPVPRGRKGKKVKTQTSSFDIQKAEWLRKYNP
EQLLQDEGYKKHVKHHCNNVLLRVRMLYYLKQEVIGNESQKVFDGVDASDIDVWVPEPDHSEVPAAWWDFDAD
KSLLIGVFKHGYEKYNTIRADPALCFLERVGKPDDKAVAAEQRANDYMDGDVEDPEYKPAPAIFKDDIEDDVS
SPGDLVIADGEGQLMEGDKVYWPTPSALTTRLRRLITAYQRTNKNRHIQQMQPTFSLPANAMQPLYEEATLNP
KMAAKIERQQRWTRREEADFYRVVSTFGVVFDPDRGQFDWTKFRALARLHKKTDNSLEKYLCAFMSMCRRVCR
LPSKEELVDPNIFIQPITEERASRTLYRIELLRKVREQALRHPQLFERLKLCHPNPDLPIWWECGSHDRDLLI
GAAKHGVSRTDYHILRDPELSFMAAQRNYNQSKAAHSRTSAPLLQQYQVALSASPLTSLPRLLGAKGTLLEDM
KVKSESLTEEPQSSEEESMSSMETRTRVKSEPVSPKNGVLSQATGDQKSGGKSETDRRMVAARTEPLTPNPAS
KKPRVHKRGSQSSSDSDSDSARSSCSSRSSSSSSSSSSCSHSRSGSSSSSSSSSCSSASSSSSSSSSSSSSSS
SSSEESDSEEDVQKREGTPHRKAYDEESVASLSTTQDETQDSFQANNGTPESAYLLQGGYMLAASYWPKDRVM
INRLDSICQTVLKGKWPSARRHYDANTVASFYTTKLLDSPGAATERGEPSVPTPPAVAVREEHEQSAQMSKVK
KHVREKEFTVKIKDEGGLKLTFQKQGLAQKRPFDGEDGALGQQQYLTRLRELQSTSETSLVNLPKAVPASGTS
IQPTLGANGAILDSQPIVKKRRGRRRNVEGADILFLNRNKPPNHIPTGMNPALSYPQPQRIPDTESPVPVINL
KDGTRLAGDDAPKRKDLDRWLKEHPGYVEDLGAFIPRVQLHEGRPKQKRHRCRNPNKLDINSLTGEERVQLIN
RRNARKVGGAFAPPLKDLCRFLKENSEYGVAPEWGDVVKQSGFLPESMFERILTGPVVREEVSRRGRRPKSGI
AKATTAAAVPAGSVPGNPLLANGLLPGVDLTALQALQQNLQNLQSLQVTAGLMGMPAGLSSGGETKNMAAMFP
MLFSGMAGLPNLLGMGGLLSKTAESGAEEKRGNDSKELEGKKERTESQSPENGGERCVPGSPSTSSTAALSSA

AAAKPIALNPLLLSNILYPGMLLTPGLNLHLPTLSQSNAFDVQKNKSDDLDSSKSVEIKEENSRVRDQEEKGG
TEPSPLNENSTDEGSERASSGSDSSSSSSEDSDSSNED*
>rs49334146_ENSMUSP00000046356_c.5248:A>G_p.D1750G
ENSMUST00000048665.8|Chd9-201|ENSMUSG00000056608|+|chr8:g.91752012:A>G
MTDPMMDFFDDANLFGETLEGLSDDTFVQPGPVSLVDELNLGAEFEPLHIDSLNHVQGTPTHQKMADFEQLSQ
FDSMKFHPVNQSFGSPVEHVLSPHSQFNCSPIHPPNQPNGLFQDVADGSPMWGHQTATGLANQNGSPFHQPGH
SHSLHQNKSFVAHPDFALFQASEHQTQCSSLHSQQSRSNLNPGQNSLGQAKNFLDANVSGAHRVNVNHLATAP
SSQQTLPVQFSPTANPPAHFLKCSSHQEGNYNRPSPSMTSCSVSNSQQFPSHYSFSSGHVSPSSLLQSSAGLA
PGHTNQALSDFAGSNSFSPHRGMKQEPTQHLLNPTPSLNSNNFQILHSSHPQGNYSNSKLSPVHMNFPDPVDA
GPPVGHFNDHAETNGFSSLEENLLHHVDSHAEPFAGLDPEDLLQEGLLPQFDESPFGQDNSNHVLDHDLDRQF
TSHLVSRPSDMAQTQLQYQARGWPSPLSTNHQHLHSRNHLCLQRQPPSSKKSDGSGTYTKLQNTQVRVMSEKK
PRKRVESESKQEKANRIISEAIARAKERGERNIPRVMSPENFPSASVEGKEEKRGRRMKSKPKDRDNKKPKTY
SKLKEKTKIGKLIITLGKKHKRRNESSDELSDAEQRSQHTFKEQHSQKRRSNRQIKRKKYAEDAEGKQSEEEV
KGSLRVKRNSAPPPGEQPLQLFVENPSEEDAAIVDKILACRTVKKEVSPGVMLDIEEFFVKYKNYSYLHCEWA
TEQQLLKDKRIQQKIKRFKLRQAQRAHFLADMEEEPFNPDYVEVDRILEVSFCEDKDTGESVIYYLVKWCSLP
YEDSTWELKEDVDLAKIEEFEQLQASRPDTRHLDRPPSNIWKKIEQSREYKNGNQLREYQLEGLNWLLFNWYN
RRNCILADEMGLGKTIQSITFLYEILLTGIRGPFLIIAPLSTIANWEREFRTWTDINVVVYHGSLISRQMIQQ
YEMYFRDSQGRIIRGAYRFQAIITTFEMILGGCGELNAIDWRCVIIDEAHRLKNKNCKLLEGLKLMNLEHKVL
LTGTPLQNTVEELFSLLHFLEPLRFPSESTFMQEFGDLKTEEQVQKLQAILKPMMLRRLKEDVEKKLAPKEET
IIEVELTNIQKKYYRAILEKNFSFLSKGAGQTNVPNLVNTMMELRKCCNHPYLIKGAEEKILGEFRDTYNPSA
SDFHLQAMIQSAGKLVLIDKLLPKMKAGGHKVLIFSQMVRCLDILEDYLIHKRYLYERIDGRVRGNLRQAAID
RFSKPDSDRFVFLLCTRAGGLGINLTAADTCIIFDSDWNPQNDLQAQARCHRIGQNKAVKVYRLVTRNSYERE
MFDRASLKLGLDKAVLQSMSGRDSNVSGIQQLSKKEIEDLLRRGAYGAIMEEEDEGSKFCEEDIDQILLRRTK
TITIESEGRGSTFAKASFVASGNRTDISLDDPNFWQKWAKKAELDIDTISGRNSLVIDTPRIRKQTRPFSATK
DELAELSEAESEGEEKPKLRRPCDRSGGYGRTECFRVEKNLLVYGWGRWREILSHGRFKRQLNEHDVEVICRA
LLAYCLIHYRGDEKIKGFIWDLITPTEDGQTRELQNHLGLSAPVPRGRKGKKVKTQTSSFDIQKAEWLRKYNP
EQLLQDEGYKKHVKHHCNKVLLRVRMLYYLKQEVIGNESQKVFDGVDASDIDVWVPEPDHSEVPAAWWDFDAD
KSLLIGVFKHGYEKYNTIRADPALCFLERVGKPDDKAVAAEQRANDYMDGDVEDPEYKPAPAIFKDDIEDGVS
SPGDLVIADGEGQLMEGDKVYWPTPSALTTRLRRLITAYQRTNKNRHIQQMQPTFSLPANAMQPLYEEATLNP
KMAAKIERQQRWTRREEADFYRVVSTFGVVFDPDRGQFDWTKFRALARLHKKTDNSLEKYLCAFMSMCRRVCR
LPSKEELVDPNIFIQPITEERASRTLYRIELLRKVREQALRHPQLFERLKLCHPNPDLPIWWECGSHDRDLLI
GAAKHGVSRTDYHILRDPELSFMAAQRNYNQSKAAHSRTSAPLLQQYQVALSASPLTSLPRLLGAKGTLLEDM
KVKSESLTEEPQSSEEESMSSMETRTRVKSEPVSPKNGVLSQATGDQKSGGKSETDRRMVAARTEPLTPNPAS
KKPRVHKRGSQSSSDSDSDSARSSCSSRSSSSSSSSSSCSHSRSGSSSSSSSSCSSASSSSSSSSSSSSSSSS
SSSEESDSEEDVQKREGTPHRKAYDEESVASLSTTQDETQDSFQANNGTPESAYLLQGGYMLAASYWPKDRVM
INRLDSICQTVLKGKWPSARRHYDANTVASFYTTKLLDSPGAATERGEPSVPTPPAVAVREEHEQSAQMSKEG
GLKLTFQKQGLAQKRPFDGEDGALGQQQYLTRLRELQSTSETSLVNLPKAVPASGTSIQPTLGANGAILDSQP
IVKKRRGRRRNVEGADILFLNRNKPPNHIPTGMNPALSYPQPQRIPDTESPVPVINLKDGTRLAGDDAPKRKD
LDRWLKEHPGYVEDLGAFIPRVQLHEGRPKQKRHRCRNPNKLDINSLTGEERVQLINRRNARKVGGAFAPPLK
DLCRFLKENSEYGVAPEWGDVVKQSGFLPESMFERILTGPVVREEVSRRGRRPKSGIAKATTAAAVPAGSVPG
NPLLANGLLPGVDLTALQALQQNLQNLQSLQVTAGLMGMPAGLSSGGETKNMAAMFPMLFSGMAGLPNLLGMG
GLLSKTAESGAEEKRGNDSKELEGKKERTESQSPENGGERCVPGSPSTSSTAALSSAAAAKPIALNPLLLSNI
LYPGMLLTPGLNLHLPTLSQSNAFDVQKNKSDDLDSSKSVEIKEENSRVRDQEEKGGTEPSPLNENSTDEGSE
RASSGSDSSSSSSEDSDSSNED*
>rs49334146_ENSMUSP00000147407_c.5248:A>G_p.D1750G
ENSMUST00000209203.2|Chd9-203|ENSMUSG00000056608|+|chr8:g.91752012:A>G
MTDPMMDFFDDANLFGETLEGLSDDTFVQPGPVSLVDELNLGAEFEPLHIDSLNHVQGTPTHQKMADFEQLSQ
FDSMKFHPVNQSFGSPVEHVLSPHSQFNCSPIHPPNQPNGLFQDVADGSPMWGHQTATGLANQNGSPFHQPGH
SHSLHQNKSFVAHPDFALFQASEHQTQCSSLHSQQSRSNLNPGQNSLGQAKNFLDANVSGAHRVNVNHLATAP
SSQQTLPVQFSPTANPPAHFLKCSSHQEGNYNRPSPSMTSCSVSNSQQFPSHYSFSSGHVSPSSLLQSSAGLA
PGHTNQALSDFAGSNSFSPHRGMKQEPTQHLLNPTPSLNSNNFQILHSSHPQGNYSNSKLSPVHMNFPDPVDA
GPPVGHFNDHAETNGFSSLEENLLHHVDSHAEPFAGLDPEDLLQEGLLPQFDESPFGQDNSNHVLDHDLDRQF
TSHLVSRPSDMAQTQLQYQARGWPSPLSTNHQHLHSRNHLCLQRQPPSSKKSDGSGTYTKLQNTQVRVMSEKK
PRKRVESESKQEKANRIISEAIARAKERGERNIPRVMSPENFPSASVEGKEEKRGRRMKSKPKDRDNKKPKTY
SKLKEKTKIGKLIITLGKKHKRRNESSDELSDAEQRSQHTFKEQHSQKRRSNRQIKRKKYAEDAEGKQSEEEV
KGSLRVKRNSAPPPGEQPLQLFVENPSEEDAAIVDKILACRTVKKEVSPGVMLDIEEFFVKYKNYSYLHCEWA
TEQQLLKDKRIQQKIKRFKLRQAQRAHFLADMEEEPFNPDYVEVDRILEVSFCEDKDTGESVIYYLVKWCSLP
YEDSTWELKEDVDLAKIEEFEQLQASRPDTRHLDRPPSNIWKKIEQSREYKNGNQLREYQLEGLNWLLFNWYN

RRNCILADEMGLGKTIQSITFLYEILLTGIRGPFLIIAPLSTIANWEREFRTWTDINVVVYHGSLISRQMIQQ
YEMYFRDSQGRIIRGAYRFQAIITTFEMILGGCGELNAIDWRCVIIDEAHRLKNKNCKLLEGLKLMNLEHKVL
LTGTPLQNTVEELFSLLHFLEPLRFPSESTFMQEFGDLKTEEQVQKLQAILKPMMLRRLKEDVEKKLAPKEET
IIEVELTNIQKKYYRAILEKNFSFLSKGAGQTNVPNLVNTMMELRKCCNHPYLIKGAEEKILGEFRDTYNPSA
SDFHLQAMIQSAGKLVLIDKLLPKMKAGGHKVLIFSQMVRCLDILEDYLIHKRYLYERIDGRVRGNLRQAAID
RFSKPDSDRFVFLLCTRAGGLGINLTAADTCIIFDSDWNPQNDLQAQARCHRIGQNKAVKVYRLVTRNSYERE
MFDRASLKLGLDKAVLQSMSGRDSNVSGIQQLSKKEIEDLLRRGAYGAIMEEEDEGSKFCEEDIDQILLRRTK
TITIESEGRGSTFAKASFVASGNRTDISLDDPNFWQKWAKKAELDIDTISGRNSLVIDTPRIRKQTRPFSATK
DELAELSEAESEGEEKPKLRRPCDRSGGYGRTECFRVEKNLLVYGWGRWREILSHGRFKRQLNEHDVEVICRA
LLAYCLIHYRGDEKIKGFIWDLITPTEDGQTRELQNHLGLSAPVPRGRKGKKVKTQTSSFDIQKAEWLRKYNP
EQLLQDEGYKKHVKHHCNKVLLRVRMLYYLKQEVIGNESQKVFDGVDASDIDVWVPEPDHSEVPAAWWDFDAD
KSLLIGVFKHGYEKYNTIRADPALCFLERVGKPDDKAVAAEQRANDYMDGDVEDPEYKPAPAIFKDDIEDGVS
SPGDLVIADGEGQLMEGDKVYWPTPSALTTRLRRLITAYQRTNKNRHIQQMQPTFSLPANAMQPLYEEATLNP
KMAAKIERQQRIGRSKYFYSTNHRGTCF*
>rs49334146_ENSMUSP00000148088_c.5248:A>G_p.D1750G
ENSMUST00000209423.2|Chd9-205|ENSMUSG00000056608|+|chr8:g.91752012:A>G
MTDPMMDFFDDANLFGETLEGLSDDTFVQPGPVSLVDELNLGAEFEPLHIDSLNHVQGTPTHQKMADFEQLSQ
FDSMKFHPVNQSFGSPVEHVLSPHSQFNCSPIHPPNQPNGLFQDVADGSPMWGHQTATGLANQNGSPFHQPGH
SHSLHQNKSFVAHPDFALFQASEHQTQCSSLHSQQSRSNLNPGQNSLGQAKNFLDANVSGAHRVNVNHLATAP
SSQQTLPVQFSPTANPPAHFLKCSSHQEGNYNRPSPSMTSCSVSNSQQFPSHYSFSSGHVSPSSLLQSSAGLA
PGHTNQALSDFAGSNSFSPHRGMKQEPTQHLLNPTPSLNSNNFQILHSSHPQGNYSNSKLSPVHMNFPDPVDA
GPPVGHFNDHAETNGFSSLEENLLHHVDSHAEPFAGLDPEDLLQEGLLPQFDESPFGQDNSNHVLDHDLDRQF
TSHLVSRPSDMAQTQLQYQARGWPSPLSTNHQHLHSRNHLCLQRQPPSSKKSDGSGTYTKLQNTQVRVMSEKK
PRKRVESESKQEKANRIISEAIARAKERGERNIPRVMSPENFPSASVEGKEEKRGRRMKSKPKDRDNKKPKTY
SKLKEKTKIGKLIITLGKKHKRRNESSDELSDAEQRSQHTFKEQHSQKRRSNRQIKRKKYAEDAEGKQSEEEV
KGSLRVKRNSAPPPGEQPLQLFVENPSEEDAAIVDKILACRTVKKEVSPGVMLDIEEFFVKYKNYSYLHCEWA
TEQQLLKDKRIQQKIKRFKLRQAQRAHFLADMEEEPFNPDYVEVDRILEVSFCEDKDTGESVIYYLVKWCSLP
YEDSTWELKEDVDLAKIEEFEQLQASRPDTRHLDRPPSNIWKKIEQSREYKNGNQLREYQLEGLNWLLFNWYN
RRNCILADEMGLGKTIQSITFLYEILLTGIRGPFLIIAPLSTIANWEREFRTWTDINVVVYHGSLISRQMIQQ
YEMYFRDSQGRIIRGAYRFQAIITTFEMILGGCGELNAIDWRCVIIDEAHRLKNKNCKLLEGLKLMNLEHKVL
LTGTPLQNTVEELFSLLHFLEPLRFPSESTFMQEFGDLKTEEQVQKLQAILKPMMLRRLKEDVEKKLAPKEET
IIEVELTNIQKKYYRAILEKNFSFLSKGAGQTNVPNLVNTMMELRKCCNHPYLIKGAEEKILGEFRDTYNPSA
SDFHLQAMIQSAGKLVLIDKLLPKMKAGGHKVLIFSQMVRCLDILEDYLIHKRYLYERIDGRVRGNLRQAAID
RFSKPDSDRFVFLLCTRAGGLGINLTAADTCIIFDSDWNPQNDLQAQARCHRIGQNKAVKVYRLVTRNSYERE
MFDRASLKLGLDKAVLQSMSGRDSNVSGIQQLSKKEIEDLLRRGAYGAIMEEEDEGSKFCEEDIDQILLRRTK
TITIESEGRGSTFAKASFVASGNRTDISLDDPNFWQKWAKKAELDIDTISGRNSLVIDTPRIRKQTRPFSATK
DELAELSEAESEGEEKPKLRRPCDRSGGYGRTECFRVEKNLLVYGWGRWREILSHGRFKRQLNEHDVEVICRA
LLAYCLIHYRGDEKIKGFIWDLITPTEDGQTRELQNHLGLSAPVPRGRKGKKVKTQTSSFDIQKAEWLRKYNP
EQLLQDEGYKKHVKHHCNKVLLRVRMLYYLKQEVIGNESQKVFDGVDASDIDVWVPEPDHSEVPAAWWDFDAD
KSLLIGVFKHGYEKYNTIRADPALCFLERVGKPDDKAVAAEQRANDYMDGDVEDPEYKPAPAIFKDDIEDGVS
SPGDLVIADGEGQLMEGDKVYWPTPSALTTRLRRLITAYQRTNKNRHIQQMQPTFSLPANAMQPLYEEATLNP
KMAAKIERQQRWTRREEADFYRVVSTFGVVFDPDRGQFDWTKFRALARLHKKTDNSLEKYLCAFMSMCRRVCR
LPSKEELVDPNIFIQPITEERASRTLYRIELLRKVREQALRHPQLFERLKLCHPNPDLPIWWECGSHDRDLLI
GAAKHGVSRTDYHILRDPELSFMAAQRNYNQSKAAHSRTSAPLLQQYQVALSASPLTSLPRLLGAKGTLLEDM
KVKSESLTEEPQSSEEESMSSMETRTRVKSEPVSPKNGVLSQATGDQKSGGKSETDRRMVAARTEPLTPNPAS
KKPRVHKRGSQSSSDSDSDSARSSCSSRSSSSSSSSSCSHSRSGSSSSSSSSCSSASSSSSSSSSSSSSSSS
SSSEESDSEEDVQKREGTPHRKAYDEESVASLSTTQDETQDSFQANNGTPESAYLLQGGYMLAASYWPKDRVM
INRLDSICQTVLKGKWPSARRHYDANTVASFYTTKLLDSPGAATERGEPSVPTPPAVAVREEHEQSAQMSKVK
KHVREKEFTVKIKDEGGLKLTFQKQGLAQKRPFDGEDGALGQQQYLTRLRELQSTSETSLVNLPKAVPASGTS
IQPTLGANGAILDSQPIVKKRRGRRRNVEGADILFLNRNKPPNHIPTGMNPALSYPQPQRIPDTESPVPVINL
KDGTRLAGDDAPKRKDLDRWLKEHPGYVEDLGAFIPRVQLHEGRPKQKRHRCRNPNKLDINSLTGEERVQLIN
RRNARKVGGAFAPPLKDLCRFLKENSEYGVAPEWGDVVKQSGFLPESMFERILTGPVVREEVSRRGRRPKSGI
AKATTAAAVPAGSVPGNPLLANGLLPGVDLTALQALQQNLQNLQSLQVTAGLMGMPAGLSSGGETKNMAAMFP
MLFSGMAGLPNLLGMGGLLSKTAESGAEEKRGNDSKELEGKKERTESQSPENGGERCVPGSPSTSSTAALSSA
AAAKPIALNPLLLSNILYPGMLLTPGLNLHLPTLSQSNAFDVQKNKSDDLDSSKSVEIKEENSRVRDQEEKGG
TEPSPLNENSTDEGSERASSGSDSSSSSSEDSDSSNED*
>rs49334146_ENSMUSP00000105243_c.5248:A>G_p.D1750G
ENSMUST00000109614.9|Chd9-202|ENSMUSG00000056608|+|chr8:g.91752012:A>G

MTDPMMDFFDDANLFGETLEGLSDDTFVQPGPVSLVDELNLGAEFEPLHIDSLNHVQGTPTHQKMADFEQLSQ
FDSMKFHPVNQSFGSPVEHVLSPHSQFNCSPIHPPNQPNGLFQDVADGSPMWGHQTATGLANQNGSPFHQPGH
SHSLHQNKSFVAHPDFALFQASEHQTQCSSLHSQQSRSNLNPGQNSLGQAKNFLDANVSGAHRVNVNHLATAP
SSQQTLPVQFSPTANPPAHFLKCSSHQEGNYNRPSPSMTSCSVSNSQQFPSHYSFSSGHVSPSSLLQSSAGLA
PGHTNQALSDFAGSNSFSPHRGMKQEPTQHLLNPTPSLNSNNFQILHSSHPQGNYSNSKLSPVHMNFPDPVDA
GPPVGHFNDHAETNGFSSLEENLLHHVDSHAEPFAGLDPEDLLQEGLLPQFDESPFGQDNSNHVLDHDLDRQF
TSHLVSRPSDMAQTQLQYQARGWPSPLSTNHQHLHSRNHLCLQRQPPSSKKSDGSGTYTKLQNTQVRVMSEKK
PRKRVESESKQEKANRIISEAIARAKERGERNIPRVMSPENFPSASVEGKEEKRGRRMKSKPKDRDNKKPKTY
SKLKEKTKIGKLIITLGKKHKRRNESSDELSDAEQRSQHTFKEQHSQKRRSNRQIKRKKYAEDAEGKQSEEEV
KGSLRVKRNSAPPPGEQPLQLFVENPSEEDAAIVDKILACRTVKKEVSPGVMLDIEEFFVKYKNYSYLHCEWA
TEQQLLKDKRIQQKIKRFKLRQAQRAHFLADMEEEPFNPDYVEVDRILEVSFCEDKDTGESVIYYLVKWCSLP
YEDSTWELKEDVDLAKIEEFEQLQASRPDTRHLDRPPSNIWKKIEQSREYKNGNQLREYQLEGLNWLLFNWYN
RRNCILADEMGLGKTIQSITFLYEILLTGIRGPFLIIAPLSTIANWEREFRTWTDINVVVYHGSLISRQMIQQ
YEMYFRDSQGRIIRGAYRFQAIITTFEMILGGCGELNAIDWRCVIIDEAHRLKNKNCKLLEGLKLMNLEHKVL
LTGTPLQNTVEELFSLLHFLEPLRFPSESTFMQEFGDLKTEEQVQKLQAILKPMMLRRLKEDVEKKLAPKEET
IIEVELTNIQKKYYRAILEKNFSFLSKGAGQTNVPNLVNTMMELRKCCNHPYLIKGAEEKILGEFRDTYNPSA
SDFHLQAMIQSAGKLVLIDKLLPKMKAGGHKVLIFSQMVRCLDILEDYLIHKRYLYERIDGRVRGNLRQAAID
RFSKPDSDRFVFLLCTRAGGLGINLTAADTCIIFDSDWNPQNDLQAQARCHRIGQNKAVKVYRLVTRNSYERE
MFDRASLKLGLDKAVLQSMSGRDSNVSGIQQLSKKEIEDLLRRGAYGAIMEEEDEGSKFCEEDIDQILLRRTK
TITIESEGRGSTFAKASFVASGNRTDISLDDPNFWQKWAKKAELDIDTISGRNSLVIDTPRIRKQTRPFSATK
DELAELSEAESEGEEKPKLRRPCDRSGGYGRTECFRVEKNLLVYGWGRWREILSHGRFKRQLNEHDVEVICRA
LLAYCLIHYRGDEKIKGFIWDLITPTEDGQTRELQNHLGLSAPVPRGRKGKKVKTQTSSFDIQKAEWLRKYNP
EQLLQDEGYKKHVKHHCNKVLLRVRMLYYLKQEVIGNESQKVFDGVDASDIDVWVPEPDHSEVPAAWWDFDAD
KSLLIGVFKHGYEKYNTIRADPALCFLERVGKPDDKAVAAEQRANDYMDGDVEDPEYKPAPAIFKDDIEDGVS
SPGDLVIADGEGQLMEGDKVYWPTPSALTTRLRRLITAYQRTNKNRHIQQMQPTFSLPANAMQPLYEEATLNP
KMAAKIERQQRWTRREEADFYRVVSTFGVVFDPDRGQFDWTKFRALARLHKKTDNSLEKYLCAFMSMCRRVCR
LPSKEELVDPNIFIQPITEERASRTLYRIELLRKVREQALRHPQLFERLKLCHPNPDLPIWWECGSHDRDLLI
GAAKHGVSRTDYHILRDPELSFMAAQRNYNQSKAAHSRTSAPLLQQYQVALSASPLTSLPRLLGAKGTLLEDM
KVKSESLTEEPQSSEEESMSSMETRTRVKSEPVSPKNGVLSQATGDQKSGGKSETDRRMVAARTEPLTPNPAS
KKPRVHKRGSQSSSDSDSDSARSSCSSRSSSSSSSSSSCSHSRSGSSSSSSSSCSSASSSSSSSSSSSSSSS
SSSEESDSEEDVQKREGTPHRKAYDEESVASLSTTQDETQDSFQANNGTPESAYLLQGGYMLAASYWPKDRVM
INRLDSICQTVLKGKWPSARRHYDANTVASFYTTKLLDSPGAATERGEPSVPTPPAVAVREEHEQSAQMSKVK
KHVREKEFTVKIKDEGGLKLTFQKQGLAQKRPFDGEDGALGQQQYLTRLRELQSTSETSLVNLPKAVPASGTS
IQPTLGANGAILDSQPIVKKRRGRRRNVEGADILFLNRNKPPNHIPTGMNPALSYPQPQRIPDTESPVPVINL
KDGTRLAGDDAPKRKDLDRWLKEHPGYVEDLGAFIPRVQLHEGRPKQKRHRCRNPNKLDINSLTGEERVQLIN
RRNARKVGGAFAPPLKDLCRFLKENSEYGVAPEWGDVVKQSGFLPESMFERILTGPVVREEVSRRGRRPKSGI
AKATTAAAVPAGSVPGNPLLANGLLPGVDLTALQALQQNLQNLQSLQVTAGLMGMPAGLSSGGETKNMAAMFP
MLFSGMAGLPNLLGMGGLLSKTAESGAEEKRGNDSKELEGKKERTESQSPENGGERCVPGSPSTSSTAALSSA
AAAKPIALNPLLLSNILYPGMLLTPGLNLHLPTLSQSNAFDVQKNKSDDLDSSKSVEIKEENSRVRDQEEKGG
TEPSPLNENSTDEGSERASSGSDSSSSSSEDSDSSNED*
>rs33455121_ENSMUSP00000046356_c.5305:G>A_p.G1769E
ENSMUST00000048665.8|Chd9-201|ENSMUSG00000056608|+|chr8:g.91753314:G>A
MTDPMMDFFDDANLFGETLEGLSDDTFVQPGPVSLVDELNLGAEFEPLHIDSLNHVQGTPTHQKMADFEQLSQ
FDSMKFHPVNQSFGSPVEHVLSPHSQFNCSPIHPPNQPNGLFQDVADGSPMWGHQTATGLANQNGSPFHQPGH
SHSLHQNKSFVAHPDFALFQASEHQTQCSSLHSQQSRSNLNPGQNSLGQAKNFLDANVSGAHRVNVNHLATAP
SSQQTLPVQFSPTANPPAHFLKCSSHQEGNYNRPSPSMTSCSVSNSQQFPSHYSFSSGHVSPSSLLQSSAGLA
PGHTNQALSDFAGSNSFSPHRGMKQEPTQHLLNPTPSLNSNNFQILHSSHPQGNYSNSKLSPVHMNFPDPVDA
GPPVGHFNDHAETNGFSSLEENLLHHVDSHAEPFAGLDPEDLLQEGLLPQFDESPFGQDNSNHVLDHDLDRQF
TSHLVSRPSDMAQTQLQYQARGWPSPLSTNHQHLHSRNHLCLQRQPPSSKKSDGSGTYTKLQNTQVRVMSEKK
PRKRVESESKQEKANRIISEAIARAKERGERNIPRVMSPENFPSASVEGKEEKRGRRMKSKPKDRDNKKPKTY
SKLKEKTKIGKLIITLGKKHKRRNESSDELSDAEQRSQHTFKEQHSQKRRSNRQIKRKKYAEDAEGKQSEEEV
KGSLRVKRNSAPPPGEQPLQLFVENPSEEDAAIVDKILACRTVKKEVSPGVMLDIEEFFVKYKNYSYLHCEWA
TEQQLLKDKRIQQKIKRFKLRQAQRAHFLADMEEEPFNPDYVEVDRILEVSFCEDKDTGESVIYYLVKWCSLP
YEDSTWELKEDVDLAKIEEFEQLQASRPDTRHLDRPPSNIWKKIEQSREYKNGNQLREYQLEGLNWLLFNWYN
RRNCILADEMGLGKTIQSITFLYEILLTGIRGPFLIIAPLSTIANWEREFRTWTDINVVVYHGSLISRQMIQQ
YEMYFRDSQGRIIRGAYRFQAIITTFEMILGGCGELNAIDWRCVIIDEAHRLKNKNCKLLEGLKLMNLEHKVL
LTGTPLQNTVEELFSLLHFLEPLRFPSESTFMQEFGDLKTEEQVQKLQAILKPMMLRRLKEDVEKKLAPKEET
IIEVELTNIQKKYYRAILEKNFSFLSKGAGQTNVPNLVNTMMELRKCCNHPYLIKGAEEKILGEFRDTYNPSA

SDFHLQAMIQSAGKLVLIDKLLPKMKAGGHKVLIFSQMVRCLDILEDYLIHKRYLYERIDGRVRGNLRQAAID
RFSKPDSDRFVFLLCTRAGGLGINLTAADTCIIFDSDWNPQNDLQAQARCHRIGQNKAVKVYRLVTRNSYERE
MFDRASLKLGLDKAVLQSMSGRDSNVSGIQQLSKKEIEDLLRRGAYGAIMEEEDEGSKFCEEDIDQILLRRTK
TITIESEGRGSTFAKASFVASGNRTDISLDDPNFWQKWAKKAELDIDTISGRNSLVIDTPRIRKQTRPFSATK
DELAELSEAESEGEEKPKLRRPCDRSGGYGRTECFRVEKNLLVYGWGRWREILSHGRFKRQLNEHDVEVICRA
LLAYCLIHYRGDEKIKGFIWDLITPTEDGQTRELQNHLGLSAPVPRGRKGKKVKTQTSSFDIQKAEWLRKYNP
EQLLQDEGYKKHVKHHCNKVLLRVRMLYYLKQEVIGNESQKVFDGVDASDIDVWVPEPDHSEVPAAWWDFDAD
KSLLIGVFKHGYEKYNTIRADPALCFLERVGKPDDKAVAAEQRANDYMDGDVEDPEYKPAPAIFKDDIEDDVS
SPGDLVIADGEGQLMEEDKVYWPTPSALTTRLRRLITAYQRTNKNRHIQQMQPTFSLPANAMQPLYEEATLNP
KMAAKIERQQRWTRREEADFYRVVSTFGVVFDPDRGQFDWTKFRALARLHKKTDNSLEKYLCAFMSMCRRVCR
LPSKEELVDPNIFIQPITEERASRTLYRIELLRKVREQALRHPQLFERLKLCHPNPDLPIWWECGSHDRDLLI
GAAKHGVSRTDYHILRDPELSFMAAQRNYNQSKAAHSRTSAPLLQQYQVALSASPLTSLPRLLGAKGTLLEDM
KVKSESLTEEPQSSEEESMSSMETRTRVKSEPVSPKNGVLSQATGDQKSGGKSETDRRMVAARTEPLTPNPAS
KKPRVHKRGSQSSSDSDSDSARSSCSSRSSSSSSSSSSCSHSRSGSSSSSSSSCSSASSSSSSSSSSSSSSS
SSSEESDSEEDVQKREGTPHRKAYDEESVASLSTTQDETQDSFQANNGTPESAYLLQGGYMLAASYWPKDRVM
INRLDSICQTVLKGKWPSARRHYDANTVASFYTTKLLDSPGAATERGEPSVPTPPAVAVREEHEQSAQMSKEG
GLKLTFQKQGLAQKRPFDGEDGALGQQQYLTRLRELQSTSETSLVNLPKAVPASGTSIQPTLGANGAILDSQP
IVKKRRGRRRNVEGADILFLNRNKPPNHIPTGMNPALSYPQPQRIPDTESPVPVINLKDGTRLAGDDAPKRKD
LDRWLKEHPGYVEDLGAFIPRVQLHEGRPKQKRHRCRNPNKLDINSLTGEERVQLINRRNARKVGGAFAPPLK
DLCRFLKENSEYGVAPEWGDVVKQSGFLPESMFERILTGPVVREEVSRRGRRPKSGIAKATTAAAVPAGSVPG
NPLLANGLLPGVDLTALQALQQNLQNLQSLQVTAGLMGMPAGLSSGGETKNMAAMFPMLFSGMAGLPNLLGMG
GLLSKTAESGAEEKRGNDSKELEGKKERTESQSPENGGERCVPGSPSTSSTAALSSAAAAKPIALNPLLLSNI
LYPGMLLTPGLNLHLPTLSQSNAFDVQKNKSDDLDSSKSVEIKEENSRVRDQEEKGGTEPSPLNENSTDEGSE
RASSGSDSSSSSSEDSDSSNED*
>rs33455121_ENSMUSP00000147407_c.5305:G>A_p.G1769E
ENSMUST00000209203.2|Chd9-203|ENSMUSG00000056608|+|chr8:g.91753314:G>A
MTDPMMDFFDDANLFGETLEGLSDDTFVQPGPVSLVDELNLGAEFEPLHIDSLNHVQGTPTHQKMADFEQLSQ
FDSMKFHPVNQSFGSPVEHVLSPHSQFNCSPIHPPNQPNGLFQDVADGSPMWGHQTATGLANQNGSPFHQPGH
SHSLHQNKSFVAHPDFALFQASEHQTQCSSLHSQQSRSNLNPGQNSLGQAKNFLDANVSGAHRVNVNHLATAP
SSQQTLPVQFSPTANPPAHFLKCSSHQEGNYNRPSPSMTSCSVSNSQQFPSHYSFSSGHVSPSSLLQSSAGLA
PGHTNQALSDFAGSNSFSPHRGMKQEPTQHLLNPTPSLNSNNFQILHSSHPQGNYSNSKLSPVHMNFPDPVDA
GPPVGHFNDHAETNGFSSLEENLLHHVDSHAEPFAGLDPEDLLQEGLLPQFDESPFGQDNSNHVLDHDLDRQF
TSHLVSRPSDMAQTQLQYQARGWPSPLSTNHQHLHSRNHLCLQRQPPSSKKSDGSGTYTKLQNTQVRVMSEKK
PRKRVESESKQEKANRIISEAIARAKERGERNIPRVMSPENFPSASVEGKEEKRGRRMKSKPKDRDNKKPKTY
SKLKEKTKIGKLIITLGKKHKRRNESSDELSDAEQRSQHTFKEQHSQKRRSNRQIKRKKYAEDAEGKQSEEEV
KGSLRVKRNSAPPPGEQPLQLFVENPSEEDAAIVDKILACRTVKKEVSPGVMLDIEEFFVKYKNYSYLHCEWA
TEQQLLKDKRIQQKIKRFKLRQAQRAHFLADMEEEPFNPDYVEVDRILEVSFCEDKDTGESVIYYLVKWCSLP
YEDSTWELKEDVDLAKIEEFEQLQASRPDTRHLDRPPSNIWKKIEQSREYKNGNQLREYQLEGLNWLLFNWYN
RRNCILADEMGLGKTIQSITFLYEILLTGIRGPFLIIAPLSTIANWEREFRTWTDINVVVYHGSLISRQMIQQ
YEMYFRDSQGRIIRGAYRFQAIITTFEMILGGCGELNAIDWRCVIIDEAHRLKNKNCKLLEGLKLMNLEHKVL
LTGTPLQNTVEELFSLLHFLEPLRFPSESTFMQEFGDLKTEEQVQKLQAILKPMMLRRLKEDVEKKLAPKEET
IIEVELTNIQKKYYRAILEKNFSFLSKGAGQTNVPNLVNTMMELRKCCNHPYLIKGAEEKILGEFRDTYNPSA
SDFHLQAMIQSAGKLVLIDKLLPKMKAGGHKVLIFSQMVRCLDILEDYLIHKRYLYERIDGRVRGNLRQAAID
RFSKPDSDRFVFLLCTRAGGLGINLTAADTCIIFDSDWNPQNDLQAQARCHRIGQNKAVKVYRLVTRNSYERE
MFDRASLKLGLDKAVLQSMSGRDSNVSGIQQLSKKEIEDLLRRGAYGAIMEEEDEGSKFCEEDIDQILLRRTK
TITIESEGRGSTFAKASFVASGNRTDISLDDPNFWQKWAKKAELDIDTISGRNSLVIDTPRIRKQTRPFSATK
DELAELSEAESEGEEKPKLRRPCDRSGGYGRTECFRVEKNLLVYGWGRWREILSHGRFKRQLNEHDVEVICRA
LLAYCLIHYRGDEKIKGFIWDLITPTEDGQTRELQNHLGLSAPVPRGRKGKKVKTQTSSFDIQKAEWLRKYNP
EQLLQDEGYKKHVKHHCNKVLLRVRMLYYLKQEVIGNESQKVFDGVDASDIDVWVPEPDHSEVPAAWWDFDAD
KSLLIGVFKHGYEKYNTIRADPALCFLERVGKPDDKAVAAEQRANDYMDGDVEDPEYKPAPAIFKDDIEDDVS
SPGDLVIADGEGQLMEEDKVYWPTPSALTTRLRRLITAYQRTNKNRHIQQMQPTFSLPANAMQPLYEEATLNP
KMAAKIERQQRIGRSKYFYSTNHRGTCF*
>rs33455121_ENSMUSP00000148088_c.5305:G>A_p.G1769E
ENSMUST00000209423.2|Chd9-205|ENSMUSG00000056608|+|chr8:g.91753314:G>A
MTDPMMDFFDDANLFGETLEGLSDDTFVQPGPVSLVDELNLGAEFEPLHIDSLNHVQGTPTHQKMADFEQLSQ
FDSMKFHPVNQSFGSPVEHVLSPHSQFNCSPIHPPNQPNGLFQDVADGSPMWGHQTATGLANQNGSPFHQPGH
SHSLHQNKSFVAHPDFALFQASEHQTQCSSLHSQQSRSNLNPGQNSLGQAKNFLDANVSGAHRVNVNHLATAP
SSQQTLPVQFSPTANPPAHFLKCSSHQEGNYNRPSPSMTSCSVSNSQQFPSHYSFSSGHVSPSSLLQSSAGLA

PGHTNQALSDFAGSNSFSPHRGMKQEPTQHLLNPTPSLNSNNFQILHSSHPQGNYSNSKLSPVHMNFPDPVDA
GPPVGHFNDHAETNGFSSLEENLLHHVDSHAEPFAGLDPEDLLQEGLLPQFDESPFGQDNSNHVLDHDLDRQF
TSHLVSRPSDMAQTQLQYQARGWPSPLSTNHQHLHSRNHLCLQRQPPSSKKSDGSGTYTKLQNTQVRVMSEKK
PRKRVESESKQEKANRIISEAIARAKERGERNIPRVMSPENFPSASVEGKEEKRGRRMKSKPKDRDNKKPKTY
SKLKEKTKIGKLIITLGKKHKRRNESSDELSDAEQRSQHTFKEQHSQKRRSNRQIKRKKYAEDAEGKQSEEEV
KGSLRVKRNSAPPPGEQPLQLFVENPSEEDAAIVDKILACRTVKKEVSPGVMLDIEEFFVKYKNYSYLHCEWA
TEQQLLKDKRIQQKIKRFKLRQAQRAHFLADMEEEPFNPDYVEVDRILEVSFCEDKDTGESVIYYLVKWCSLP
YEDSTWELKEDVDLAKIEEFEQLQASRPDTRHLDRPPSNIWKKIEQSREYKNGNQLREYQLEGLNWLLFNWYN
RRNCILADEMGLGKTIQSITFLYEILLTGIRGPFLIIAPLSTIANWEREFRTWTDINVVVYHGSLISRQMIQQ
YEMYFRDSQGRIIRGAYRFQAIITTFEMILGGCGELNAIDWRCVIIDEAHRLKNKNCKLLEGLKLMNLEHKVL
LTGTPLQNTVEELFSLLHFLEPLRFPSESTFMQEFGDLKTEEQVQKLQAILKPMMLRRLKEDVEKKLAPKEET
IIEVELTNIQKKYYRAILEKNFSFLSKGAGQTNVPNLVNTMMELRKCCNHPYLIKGAEEKILGEFRDTYNPSA
SDFHLQAMIQSAGKLVLIDKLLPKMKAGGHKVLIFSQMVRCLDILEDYLIHKRYLYERIDGRVRGNLRQAAID
RFSKPDSDRFVFLLCTRAGGLGINLTAADTCIIFDSDWNPQNDLQAQARCHRIGQNKAVKVYRLVTRNSYERE
MFDRASLKLGLDKAVLQSMSGRDSNVSGIQQLSKKEIEDLLRRGAYGAIMEEEDEGSKFCEEDIDQILLRRTK
TITIESEGRGSTFAKASFVASGNRTDISLDDPNFWQKWAKKAELDIDTISGRNSLVIDTPRIRKQTRPFSATK
DELAELSEAESEGEEKPKLRRPCDRSGGYGRTECFRVEKNLLVYGWGRWREILSHGRFKRQLNEHDVEVICRA
LLAYCLIHYRGDEKIKGFIWDLITPTEDGQTRELQNHLGLSAPVPRGRKGKKVKTQTSSFDIQKAEWLRKYNP
EQLLQDEGYKKHVKHHCNKVLLRVRMLYYLKQEVIGNESQKVFDGVDASDIDVWVPEPDHSEVPAAWWDFDAD
KSLLIGVFKHGYEKYNTIRADPALCFLERVGKPDDKAVAAEQRANDYMDGDVEDPEYKPAPAIFKDDIEDDVS
SPGDLVIADGEGQLMEEDKVYWPTPSALTTRLRRLITAYQRTNKNRHIQQMQPTFSLPANAMQPLYEEATLNP
KMAAKIERQQRWTRREEADFYRVVSTFGVVFDPDRGQFDWTKFRALARLHKKTDNSLEKYLCAFMSMCRRVCR
LPSKEELVDPNIFIQPITEERASRTLYRIELLRKVREQALRHPQLFERLKLCHPNPDLPIWWECGSHDRDLLI
GAAKHGVSRTDYHILRDPELSFMAAQRNYNQSKAAHSRTSAPLLQQYQVALSASPLTSLPRLLGAKGTLLEDM
KVKSESLTEEPQSSEEESMSSMETRTRVKSEPVSPKNGVLSQATGDQKSGGKSETDRRMVAARTEPLTPNPAS
KKPRVHKRGSQSSSDSDSDSARSSCSSRSSSSSSSSSSCSHSRSGSSSSSSSSCSSASSSSSSSSSSSSSS
SSSEESDSEEDVQKREGTPHRKAYDEESVASLSTTQDETQDSFQANNGTPESAYLLQGGYMLAASYWPKDRVM
INRLDSICQTVLKGKWPSARRHYDANTVASFYTTKLLDSPGAATERGEPSVPTPPAVAVREEHEQSAQMSKVK
KHVREKEFTVKIKDEGGLKLTFQKQGLAQKRPFDGEDGALGQQQYLTRLRELQSTSETSLVNLPKAVPASGTS
IQPTLGANGAILDSQPIVKKRRGRRRNVEGADILFLNRNKPPNHIPTGMNPALSYPQPQRIPDTESPVPVINL
KDGTRLAGDDAPKRKDLDRWLKEHPGYVEDLGAFIPRVQLHEGRPKQKRHRCRNPNKLDINSLTGEERVQLIN
RRNARKVGGAFAPPLKDLCRFLKENSEYGVAPEWGDVVKQSGFLPESMFERILTGPVVREEVSRRGRRPKSGI
AKATTAAAVPAGSVPGNPLLANGLLPGVDLTALQALQQNLQNLQSLQVTAGLMGMPAGLSSGGETKNMAAMFP
MLFSGMAGLPNLLGMGGLLSKTAESGAEEKRGNDSKELEGKKERTESQSPENGGERCVPGSPSTSSTAALSSA
AAAKPIALNPLLLSNILYPGMLLTPGLNLHLPTLSQSNAFDVQKNKSDDLDSSKSVEIKEENSRVRDQEEKGG
TEPSPLNENSTDEGSERASSGSDSSSSSSEDSDSSNED*
>rs33455121_ENSMUSP00000105243_c.5305:G>A_p.G1769E
ENSMUST00000109614.9|Chd9-202|ENSMUSG00000056608|+|chr8:g.91753314:G>A
MTDPMMDFFDDANLFGETLEGLSDDTFVQPGPVSLVDELNLGAEFEPLHIDSLNHVQGTPTHQKMADFEQLSQ
FDSMKFHPVNQSFGSPVEHVLSPHSQFNCSPIHPPNQPNGLFQDVADGSPMWGHQTATGLANQNGSPFHQPGH
SHSLHQNKSFVAHPDFALFQASEHQTQCSSLHSQQSRSNLNPGQNSLGQAKNFLDANVSGAHRVNVNHLATAP
SSQQTLPVQFSPTANPPAHFLKCSSHQEGNYNRPSPSMTSCSVSNSQQFPSHYSFSSGHVSPSSLLQSSAGLA
PGHTNQALSDFAGSNSFSPHRGMKQEPTQHLLNPTPSLNSNNFQILHSSHPQGNYSNSKLSPVHMNFPDPVDA
GPPVGHFNDHAETNGFSSLEENLLHHVDSHAEPFAGLDPEDLLQEGLLPQFDESPFGQDNSNHVLDHDLDRQF
TSHLVSRPSDMAQTQLQYQARGWPSPLSTNHQHLHSRNHLCLQRQPPSSKKSDGSGTYTKLQNTQVRVMSEKK
PRKRVESESKQEKANRIISEAIARAKERGERNIPRVMSPENFPSASVEGKEEKRGRRMKSKPKDRDNKKPKTY
SKLKEKTKIGKLIITLGKKHKRRNESSDELSDAEQRSQHTFKEQHSQKRRSNRQIKRKKYAEDAEGKQSEEEV
KGSLRVKRNSAPPPGEQPLQLFVENPSEEDAAIVDKILACRTVKKEVSPGVMLDIEEFFVKYKNYSYLHCEWA
TEQQLLKDKRIQQKIKRFKLRQAQRAHFLADMEEEPFNPDYVEVDRILEVSFCEDKDTGESVIYYLVKWCSLP
YEDSTWELKEDVDLAKIEEFEQLQASRPDTRHLDRPPSNIWKKIEQSREYKNGNQLREYQLEGLNWLLFNWYN
RRNCILADEMGLGKTIQSITFLYEILLTGIRGPFLIIAPLSTIANWEREFRTWTDINVVVYHGSLISRQMIQQ
YEMYFRDSQGRIIRGAYRFQAIITTFEMILGGCGELNAIDWRCVIIDEAHRLKNKNCKLLEGLKLMNLEHKVL
LTGTPLQNTVEELFSLLHFLEPLRFPSESTFMQEFGDLKTEEQVQKLQAILKPMMLRRLKEDVEKKLAPKEET
IIEVELTNIQKKYYRAILEKNFSFLSKGAGQTNVPNLVNTMMELRKCCNHPYLIKGAEEKILGEFRDTYNPSA
SDFHLQAMIQSAGKLVLIDKLLPKMKAGGHKVLIFSQMVRCLDILEDYLIHKRYLYERIDGRVRGNLRQAAID
RFSKPDSDRFVFLLCTRAGGLGINLTAADTCIIFDSDWNPQNDLQAQARCHRIGQNKAVKVYRLVTRNSYERE
MFDRASLKLGLDKAVLQSMSGRDSNVSGIQQLSKKEIEDLLRRGAYGAIMEEEDEGSKFCEEDIDQILLRRTK
TITIESEGRGSTFAKASFVASGNRTDISLDDPNFWQKWAKKAELDIDTISGRNSLVIDTPRIRKQTRPFSATK

DELAELSEAESEGEEKPKLRRPCDRSGGYGRTECFRVEKNLLVYGWGRWREILSHGRFKRQLNEHDVEVICRA
LLAYCLIHYRGDEKIKGFIWDLITPTEDGQTRELQNHLGLSAPVPRGRKGKKVKTQTSSFDIQKAEWLRKYNP
EQLLQDEGYKKHVKHHCNKVLLRVRMLYYLKQEVIGNESQKVFDGVDASDIDVWVPEPDHSEVPAAWWDFDAD
KSLLIGVFKHGYEKYNTIRADPALCFLERVGKPDDKAVAAEQRANDYMDGDVEDPEYKPAPAIFKDDIEDDVS
SPGDLVIADGEGQLMEEDKVYWPTPSALTTRLRRLITAYQRTNKNRHIQQMQPTFSLPANAMQPLYEEATLNP
KMAAKIERQQRWTRREEADFYRVVSTFGVVFDPDRGQFDWTKFRALARLHKKTDNSLEKYLCAFMSMCRRVCR
LPSKEELVDPNIFIQPITEERASRTLYRIELLRKVREQALRHPQLFERLKLCHPNPDLPIWWECGSHDRDLLI
GAAKHGVSRTDYHILRDPELSFMAAQRNYNQSKAAHSRTSAPLLQQYQVALSASPLTSLPRLLGAKGTLLEDM
KVKSESLTEEPQSSEEESMSSMETRTRVKSEPVSPKNGVLSQATGDQKSGGKSETDRRMVAARTEPLTPNPAS
KKPRVHKRGSQSSSDSDSDSARSSCSSRSSSSSSSSSSCSHSRSGSSSSSSSSCSSASSSSSSSSSSSSSSSS
SSSEESDSEEDVQKREGTPHRKAYDEESVASLSTTQDETQDSFQANNGTPESAYLLQGGYMLAASYWPKDRVM
INRLDSICQTVLKGKWPSARRHYDANTVASFYTTKLLDSPGAATERGEPSVPTPPAVAVREEHEQSAQMSKVK
KHVREKEFTVKIKDEGGLKLTFQKQGLAQKRPFDGEDGALGQQQYLTRLRELQSTSETSLVNLPKAVPASGTS
IQPTLGANGAILDSQPIVKKRRGRRRNVEGADILFLNRNKPPNHIPTGMNPALSYPQPQRIPDTESPVPVINL
KDGTRLAGDDAPKRKDLDRWLKEHPGYVEDLGAFIPRVQLHEGRPKQKRHRCRNPNKLDINSLTGEERVQLIN
RRNARKVGGAFAPPLKDLCRFLKENSEYGVAPEWGDVVKQSGFLPESMFERILTGPVVREEVSRRGRRPKSGI
AKATTAAAVPAGSVPGNPLLANGLLPGVDLTALQALQQNLQNLQSLQVTAGLMGMPAGLSSGGETKNMAAMFP
MLFSGMAGLPNLLGMGGLLSKTAESGAEEKRGNDSKELEGKKERTESQSPENGGERCVPGSPSTSSTAALSSA
AAAKPIALNPLLLSNILYPGMLLTPGLNLHLPTLSQSNAFDVQKNKSDDLDSSKSVEIKEENSRVRDQEEKGG
TEPSPLNENSTDEGSERASSGSDSSSSSSEDSDSSNED*
>rs47052211_ENSMUSP00000046356_c.6103:G>A_p.G2035D
ENSMUST00000048665.8|Chd9-201|ENSMUSG00000056608|+|chr8:g.91760360:G>A
MTDPMMDFFDDANLFGETLEGLSDDTFVQPGPVSLVDELNLGAEFEPLHIDSLNHVQGTPTHQKMADFEQLSQ
FDSMKFHPVNQSFGSPVEHVLSPHSQFNCSPIHPPNQPNGLFQDVADGSPMWGHQTATGLANQNGSPFHQPGH
SHSLHQNKSFVAHPDFALFQASEHQTQCSSLHSQQSRSNLNPGQNSLGQAKNFLDANVSGAHRVNVNHLATAP
SSQQTLPVQFSPTANPPAHFLKCSSHQEGNYNRPSPSMTSCSVSNSQQFPSHYSFSSGHVSPSSLLQSSAGLA
PGHTNQALSDFAGSNSFSPHRGMKQEPTQHLLNPTPSLNSNNFQILHSSHPQGNYSNSKLSPVHMNFPDPVDA
GPPVGHFNDHAETNGFSSLEENLLHHVDSHAEPFAGLDPEDLLQEGLLPQFDESPFGQDNSNHVLDHDLDRQF
TSHLVSRPSDMAQTQLQYQARGWPSPLSTNHQHLHSRNHLCLQRQPPSSKKSDGSGTYTKLQNTQVRVMSEKK
PRKRVESESKQEKANRIISEAIARAKERGERNIPRVMSPENFPSASVEGKEEKRGRRMKSKPKDRDNKKPKTY
SKLKEKTKIGKLIITLGKKHKRRNESSDELSDAEQRSQHTFKEQHSQKRRSNRQIKRKKYAEDAEGKQSEEEV
KGSLRVKRNSAPPPGEQPLQLFVENPSEEDAAIVDKILACRTVKKEVSPGVMLDIEEFFVKYKNYSYLHCEWA
TEQQLLKDKRIQQKIKRFKLRQAQRAHFLADMEEEPFNPDYVEVDRILEVSFCEDKDTGESVIYYLVKWCSLP
YEDSTWELKEDVDLAKIEEFEQLQASRPDTRHLDRPPSNIWKKIEQSREYKNGNQLREYQLEGLNWLLFNWYN
RRNCILADEMGLGKTIQSITFLYEILLTGIRGPFLIIAPLSTIANWEREFRTWTDINVVVYHGSLISRQMIQQ
YEMYFRDSQGRIIRGAYRFQAIITTFEMILGGCGELNAIDWRCVIIDEAHRLKNKNCKLLEGLKLMNLEHKVL
LTGTPLQNTVEELFSLLHFLEPLRFPSESTFMQEFGDLKTEEQVQKLQAILKPMMLRRLKEDVEKKLAPKEET
IIEVELTNIQKKYYRAILEKNFSFLSKGAGQTNVPNLVNTMMELRKCCNHPYLIKGAEEKILGEFRDTYNPSA
SDFHLQAMIQSAGKLVLIDKLLPKMKAGGHKVLIFSQMVRCLDILEDYLIHKRYLYERIDGRVRGNLRQAAID
RFSKPDSDRFVFLLCTRAGGLGINLTAADTCIIFDSDWNPQNDLQAQARCHRIGQNKAVKVYRLVTRNSYERE
MFDRASLKLGLDKAVLQSMSGRDSNVSGIQQLSKKEIEDLLRRGAYGAIMEEEDEGSKFCEEDIDQILLRRTK
TITIESEGRGSTFAKASFVASGNRTDISLDDPNFWQKWAKKAELDIDTISGRNSLVIDTPRIRKQTRPFSATK
DELAELSEAESEGEEKPKLRRPCDRSGGYGRTECFRVEKNLLVYGWGRWREILSHGRFKRQLNEHDVEVICRA
LLAYCLIHYRGDEKIKGFIWDLITPTEDGQTRELQNHLGLSAPVPRGRKGKKVKTQTSSFDIQKAEWLRKYNP
EQLLQDEGYKKHVKHHCNKVLLRVRMLYYLKQEVIGNESQKVFDGVDASDIDVWVPEPDHSEVPAAWWDFDAD
KSLLIGVFKHGYEKYNTIRADPALCFLERVGKPDDKAVAAEQRANDYMDGDVEDPEYKPAPAIFKDDIEDDVS
SPGDLVIADGEGQLMEGDKVYWPTPSALTTRLRRLITAYQRTNKNRHIQQMQPTFSLPANAMQPLYEEATLNP
KMAAKIERQQRWTRREEADFYRVVSTFGVVFDPDRGQFDWTKFRALARLHKKTDNSLEKYLCAFMSMCRRVCR
LPSKEELVDPNIFIQPITEERASRTLYRIELLRKVREQALRHPQLFERLKLCHPNPDLPIWWECGSHDRDLLI
GAAKHGVSRTDYHILRDPELSFMAAQRNYNQSKAAHSRTSAPLLQQYQVALSASPLTSLPRLLDAKGTLLEDM
KVKSESLTEEPQSSEEESMSSMETRTRVKSEPVSPKNGVLSQATGDQKSGGKSETDRRMVAARTEPLTPNPAS
KKPRVHKRGSQSSSDSDSDSARSSCSSRSSSSSSSSSSCSHSRSGSSSSSSSSCSSASSSSSSSSSSSSSSSS
SSSEESDSEEDVQKREGTPHRKAYDEESVASLSTTQDETQDSFQANNGTPESAYLLQGGYMLAASYWPKDRVM
INRLDSICQTVLKGKWPSARRHYDANTVASFYTTKLLDSPGAATERGEPSVPTPPAVAVREEHEQSAQMSKEG
GLKLTFQKQGLAQKRPFDGEDGALGQQQYLTRLRELQSTSETSLVNLPKAVPASGTSIQPTLGANGAILDSQP
IVKKRRGRRRNVEGADILFLNRNKPPNHIPTGMNPALSYPQPQRIPDTESPVPVINLKDGTRLAGDDAPKRKD
LDRWLKEHPGYVEDLGAFIPRVQLHEGRPKQKRHRCRNPNKLDINSLTGEERVQLINRRNARKVGGAFAPPLK
DLCRFLKENSEYGVAPEWGDVVKQSGFLPESMFERILTGPVVREEVSRRGRRPKSGIAKATTAAAVPAGSVPG

NPLLANGLLPGVDLTALQALQQNLQNLQSLQVTAGLMGMPAGLSSGGETKNMAAMFPMLFSGMAGLPNLLGMG
GLLSKTAESGAEEKRGNDSKELEGKKERTESQSPENGGERCVPGSPSTSSTAALSSAAAAKPIALNPLLLSNI
LYPGMLLTPGLNLHLPTLSQSNAFDVQKNKSDDLDSSKSVEIKEENSRVRDQEEKGGTEPSPLNENSTDEGSE
RASSGSDSSSSSSEDSDSSNED*
>rs47052211_ENSMUSP00000148088_c.6103:G>A_p.G2035D
ENSMUST00000209423.2|Chd9-205|ENSMUSG00000056608|+|chr8:g.91760360:G>A
MTDPMMDFFDDANLFGETLEGLSDDTFVQPGPVSLVDELNLGAEFEPLHIDSLNHVQGTPTHQKMADFEQLSQ
FDSMKFHPVNQSFGSPVEHVLSPHSQFNCSPIHPPNQPNGLFQDVADGSPMWGHQTATGLANQNGSPFHQPGH
SHSLHQNKSFVAHPDFALFQASEHQTQCSSLHSQQSRSNLNPGQNSLGQAKNFLDANVSGAHRVNVNHLATAP
SSQQTLPVQFSPTANPPAHFLKCSSHQEGNYNRPSPSMTSCSVSNSQQFPSHYSFSSGHVSPSSLLQSSAGLA
PGHTNQALSDFAGSNSFSPHRGMKQEPTQHLLNPTPSLNSNNFQILHSSHPQGNYSNSKLSPVHMNFPDPVDA
GPPVGHFNDHAETNGFSSLEENLLHHVDSHAEPFAGLDPEDLLQEGLLPQFDESPFGQDNSNHVLDHDLDRQF
TSHLVSRPSDMAQTQLQYQARGWPSPLSTNHQHLHSRNHLCLQRQPPSSKKSDGSGTYTKLQNTQVRVMSEKK
PRKRVESESKQEKANRIISEAIARAKERGERNIPRVMSPENFPSASVEGKEEKRGRRMKSKPKDRDNKKPKTY
SKLKEKTKIGKLIITLGKKHKRRNESSDELSDAEQRSQHTFKEQHSQKRRSNRQIKRKKYAEDAEGKQSEEEV
KGSLRVKRNSAPPPGEQPLQLFVENPSEEDAAIVDKILACRTVKKEVSPGVMLDIEEFFVKYKNYSYLHCEWA
TEQQLLKDKRIQQKIKRFKLRQAQRAHFLADMEEEPFNPDYVEVDRILEVSFCEDKDTGESVIYYLVKWCSLP
YEDSTWELKEDVDLAKIEEFEQLQASRPDTRHLDRPPSNIWKKIEQSREYKNGNQLREYQLEGLNWLLFNWYN
RRNCILADEMGLGKTIQSITFLYEILLTGIRGPFLIIAPLSTIANWEREFRTWTDINVVVYHGSLISRQMIQQ
YEMYFRDSQGRIIRGAYRFQAIITTFEMILGGCGELNAIDWRCVIIDEAHRLKNKNCKLLEGLKLMNLEHKVL
LTGTPLQNTVEELFSLLHFLEPLRFPSESTFMQEFGDLKTEEQVQKLQAILKPMMLRRLKEDVEKKLAPKEET
IIEVELTNIQKKYYRAILEKNFSFLSKGAGQTNVPNLVNTMMELRKCCNHPYLIKGAEEKILGEFRDTYNPSA
SDFHLQAMIQSAGKLVLIDKLLPKMKAGGHKVLIFSQMVRCLDILEDYLIHKRYLYERIDGRVRGNLRQAAID
RFSKPDSDRFVFLLCTRAGGLGINLTAADTCIIFDSDWNPQNDLQAQARCHRIGQNKAVKVYRLVTRNSYERE
MFDRASLKLGLDKAVLQSMSGRDSNVSGIQQLSKKEIEDLLRRGAYGAIMEEEDEGSKFCEEDIDQILLRRTK
TITIESEGRGSTFAKASFVASGNRTDISLDDPNFWQKWAKKAELDIDTISGRNSLVIDTPRIRKQTRPFSATK
DELAELSEAESEGEEKPKLRRPCDRSGGYGRTECFRVEKNLLVYGWGRWREILSHGRFKRQLNEHDVEVICRA
LLAYCLIHYRGDEKIKGFIWDLITPTEDGQTRELQNHLGLSAPVPRGRKGKKVKTQTSSFDIQKAEWLRKYNP
EQLLQDEGYKKHVKHHCNKVLLRVRMLYYLKQEVIGNESQKVFDGVDASDIDVWVPEPDHSEVPAAWWDFDAD
KSLLIGVFKHGYEKYNTIRADPALCFLERVGKPDDKAVAAEQRANDYMDGDVEDPEYKPAPAIFKDDIEDDVS
SPGDLVIADGEGQLMEGDKVYWPTPSALTTRLRRLITAYQRTNKNRHIQQMQPTFSLPANAMQPLYEEATLNP
KMAAKIERQQRWTRREEADFYRVVSTFGVVFDPDRGQFDWTKFRALARLHKKTDNSLEKYLCAFMSMCRRVCR
LPSKEELVDPNIFIQPITEERASRTLYRIELLRKVREQALRHPQLFERLKLCHPNPDLPIWWECGSHDRDLLI
GAAKHGVSRTDYHILRDPELSFMAAQRNYNQSKAAHSRTSAPLLQQYQVALSASPLTSLPRLLDAKGTLLEDM
KVKSESLTEEPQSSEEESMSSMETRTRVKSEPVSPKNGVLSQATGDQKSGGKSETDRRMVAARTEPLTPNPAS
KKPRVHKRGSQSSSDSDSDSARSSCSSRSSSSSSSSSCSHSRSGSSSSSSSSCSSASSSSSSSSSSSSSSS
SSSEESDSEEDVQKREGTPHRKAYDEESVASLSTTQDETQDSFQANNGTPESAYLLQGGYMLAASYWPKDRVM
INRLDSICQTVLKGKWPSARRHYDANTVASFYTTKLLDSPGAATERGEPSVPTPPAVAVREEHEQSAQMSKVK
KHVREKEFTVKIKDEGGLKLTFQKQGLAQKRPFDGEDGALGQQQYLTRLRELQSTSETSLVNLPKAVPASGTS
IQPTLGANGAILDSQPIVKKRRGRRRNVEGADILFLNRNKPPNHIPTGMNPALSYPQPQRIPDTESPVPVINL
KDGTRLAGDDAPKRKDLDRWLKEHPGYVEDLGAFIPRVQLHEGRPKQKRHRCRNPNKLDINSLTGEERVQLIN
RRNARKVGGAFAPPLKDLCRFLKENSEYGVAPEWGDVVKQSGFLPESMFERILTGPVVREEVSRRGRRPKSGI
AKATTAAAVPAGSVPGNPLLANGLLPGVDLTALQALQQNLQNLQSLQVTAGLMGMPAGLSSGGETKNMAAMFP
MLFSGMAGLPNLLGMGGLLSKTAESGAEEKRGNDSKELEGKKERTESQSPENGGERCVPGSPSTSSTAALSSA
AAAKPIALNPLLLSNILYPGMLLTPGLNLHLPTLSQSNAFDVQKNKSDDLDSSKSVEIKEENSRVRDQEEKGG
TEPSPLNENSTDEGSERASSGSDSSSSSSEDSDSSNED*
>rs47052211_ENSMUSP00000105243_c.6103:G>A_p.G2035D
ENSMUST00000109614.9|Chd9-202|ENSMUSG00000056608|+|chr8:g.91760360:G>A
MTDPMMDFFDDANLFGETLEGLSDDTFVQPGPVSLVDELNLGAEFEPLHIDSLNHVQGTPTHQKMADFEQLSQ
FDSMKFHPVNQSFGSPVEHVLSPHSQFNCSPIHPPNQPNGLFQDVADGSPMWGHQTATGLANQNGSPFHQPGH
SHSLHQNKSFVAHPDFALFQASEHQTQCSSLHSQQSRSNLNPGQNSLGQAKNFLDANVSGAHRVNVNHLATAP
SSQQTLPVQFSPTANPPAHFLKCSSHQEGNYNRPSPSMTSCSVSNSQQFPSHYSFSSGHVSPSSLLQSSAGLA
PGHTNQALSDFAGSNSFSPHRGMKQEPTQHLLNPTPSLNSNNFQILHSSHPQGNYSNSKLSPVHMNFPDPVDA
GPPVGHFNDHAETNGFSSLEENLLHHVDSHAEPFAGLDPEDLLQEGLLPQFDESPFGQDNSNHVLDHDLDRQF
TSHLVSRPSDMAQTQLQYQARGWPSPLSTNHQHLHSRNHLCLQRQPPSSKKSDGSGTYTKLQNTQVRVMSEKK
PRKRVESESKQEKANRIISEAIARAKERGERNIPRVMSPENFPSASVEGKEEKRGRRMKSKPKDRDNKKPKTY
SKLKEKTKIGKLIITLGKKHKRRNESSDELSDAEQRSQHTFKEQHSQKRRSNRQIKRKKYAEDAEGKQSEEEV
KGSLRVKRNSAPPPGEQPLQLFVENPSEEDAAIVDKILACRTVKKEVSPGVMLDIEEFFVKYKNYSYLHCEWA

TEQQLLKDKRIQQKIKRFKLRQAQRAHFLADMEEEPFNPDYVEVDRILEVSFCEDKDTGESVIYYLVKWCSLP
YEDSTWELKEDVDLAKIEEFEQLQASRPDTRHLDRPPSNIWKKIEQSREYKNGNQLREYQLEGLNWLLFNWYN
RRNCILADEMGLGKTIQSITFLYEILLTGIRGPFLIIAPLSTIANWEREFRTWTDINVVVYHGSLISRQMIQQ
YEMYFRDSQGRIIRGAYRFQAIITTFEMILGGCGELNAIDWRCVIIDEAHRLKNKNCKLLEGLKLMNLEHKVL
LTGTPLQNTVEELFSLLHFLEPLRFPSESTFMQEFGDLKTEEQVQKLQAILKPMMLRRLKEDVEKKLAPKEET
IIEVELTNIQKKYYRAILEKNFSFLSKGAGQTNVPNLVNTMMELRKCCNHPYLIKGAEEKILGEFRDTYNPSA
SDFHLQAMIQSAGKLVLIDKLLPKMKAGGHKVLIFSQMVRCLDILEDYLIHKRYLYERIDGRVRGNLRQAAID
RFSKPDSDRFVFLLCTRAGGLGINLTAADTCIIFDSDWNPQNDLQAQARCHRIGQNKAVKVYRLVTRNSYERE
MFDRASLKLGLDKAVLQSMSGRDSNVSGIQQLSKKEIEDLLRRGAYGAIMEEEDEGSKFCEEDIDQILLRRTK
TITIESEGRGSTFAKASFVASGNRTDISLDDPNFWQKWAKKAELDIDTISGRNSLVIDTPRIRKQTRPFSATK
DELAELSEAESEGEEKPKLRRPCDRSGGYGRTECFRVEKNLLVYGWGRWREILSHGRFKRQLNEHDVEVICRA
LLAYCLIHYRGDEKIKGFIWDLITPTEDGQTRELQNHLGLSAPVPRGRKGKKVKTQTSSFDIQKAEWLRKYNP
EQLLQDEGYKKHVKHHCNKVLLRVRMLYYLKQEVIGNESQKVFDGVDASDIDVWVPEPDHSEVPAAWWDFDAD
KSLLIGVFKHGYEKYNTIRADPALCFLERVGKPDDKAVAAEQRANDYMDGDVEDPEYKPAPAIFKDDIEDDVS
SPGDLVIADGEGQLMEGDKVYWPTPSALTTRLRRLITAYQRTNKNRHIQQMQPTFSLPANAMQPLYEEATLNP
KMAAKIERQQRWTRREEADFYRVVSTFGVVFDPDRGQFDWTKFRALARLHKKTDNSLEKYLCAFMSMCRRVCR
LPSKEELVDPNIFIQPITEERASRTLYRIELLRKVREQALRHPQLFERLKLCHPNPDLPIWWECGSHDRDLLI
GAAKHGVSRTDYHILRDPELSFMAAQRNYNQSKAAHSRTSAPLLQQYQVALSASPLTSLPRLLDAKGTLLEDM
KVKSESLTEEPQSSEEESMSSMETRTRVKSEPVSPKNGVLSQATGDQKSGGKSETDRRMVAARTEPLTPNPAS
KKPRVHKRGSQSSSDSDSDSARSSCSSRSSSSSSSSSSCSHSRSGSSSSSSSSCSSASSSSSSSSSSSSSSSS
SSSEESDSEEDVQKREGTPHRKAYDEESVASLSTTQDETQDSFQANNGTPESAYLLQGGYMLAASYWPKDRVM
INRLDSICQTVLKGKWPSARRHYDANTVASFYTTKLLDSPGAATERGEPSVPTPPAVAVREEHEQSAQMSKVK
KHVREKEFTVKIKDEGGLKLTFQKQGLAQKRPFDGEDGALGQQQYLTRLRELQSTSETSLVNLPKAVPASGTS
IQPTLGANGAILDSQPIVKKRRGRRRNVEGADILFLNRNKPPNHIPTGMNPALSYPQPQRIPDTESPVPVINL
KDGTRLAGDDAPKRKDLDRWLKEHPGYVEDLGAFIPRVQLHEGRPKQKRHRCRNPNKLDINSLTGEERVQLIN
RRNARKVGGAFAPPLKDLCRFLKENSEYGVAPEWGDVVKQSGFLPESMFERILTGPVVREEVSRRGRRPKSGI
AKATTAAAVPAGSVPGNPLLANGLLPGVDLTALQALQQNLQNLQSLQVTAGLMGMPAGLSSGGETKNMAAMFP
MLFSGMAGLPNLLGMGGLLSKTAESGAEEKRGNDSKELEGKKERTESQSPENGGERCVPGSPSTSSTAALSSA
AAAKPIALNPLLLSNILYPGMLLTPGLNLHLPTLSQSNAFDVQKNKSDDLDSSKSVEIKEENSRVRDQEEKGG
TEPSPLNENSTDEGSERASSGSDSSSSSSEDSDSSNED*
>rs51312005_ENSMUSP00000046356_c.6787:T>C_p.M2263T
ENSMUST00000048665.8|Chd9-201|ENSMUSG00000056608|+|chr8:g.91762964:T>C
MTDPMMDFFDDANLFGETLEGLSDDTFVQPGPVSLVDELNLGAEFEPLHIDSLNHVQGTPTHQKMADFEQLSQ
FDSMKFHPVNQSFGSPVEHVLSPHSQFNCSPIHPPNQPNGLFQDVADGSPMWGHQTATGLANQNGSPFHQPGH
SHSLHQNKSFVAHPDFALFQASEHQTQCSSLHSQQSRSNLNPGQNSLGQAKNFLDANVSGAHRVNVNHLATAP
SSQQTLPVQFSPTANPPAHFLKCSSHQEGNYNRPSPSMTSCSVSNSQQFPSHYSFSSGHVSPSSLLQSSAGLA
PGHTNQALSDFAGSNSFSPHRGMKQEPTQHLLNPTPSLNSNNFQILHSSHPQGNYSNSKLSPVHMNFPDPVDA
GPPVGHFNDHAETNGFSSLEENLLHHVDSHAEPFAGLDPEDLLQEGLLPQFDESPFGQDNSNHVLDHDLDRQF
TSHLVSRPSDMAQTQLQYQARGWPSPLSTNHQHLHSRNHLCLQRQPPSSKKSDGSGTYTKLQNTQVRVMSEKK
PRKRVESESKQEKANRIISEAIARAKERGERNIPRVMSPENFPSASVEGKEEKRGRRMKSKPKDRDNKKPKTY
SKLKEKTKIGKLIITLGKKHKRRNESSDELSDAEQRSQHTFKEQHSQKRRSNRQIKRKKYAEDAEGKQSEEEV
KGSLRVKRNSAPPPGEQPLQLFVENPSEEDAAIVDKILACRTVKKEVSPGVMLDIEEFFVKYKNYSYLHCEWA
TEQQLLKDKRIQQKIKRFKLRQAQRAHFLADMEEEPFNPDYVEVDRILEVSFCEDKDTGESVIYYLVKWCSLP
YEDSTWELKEDVDLAKIEEFEQLQASRPDTRHLDRPPSNIWKKIEQSREYKNGNQLREYQLEGLNWLLFNWYN
RRNCILADEMGLGKTIQSITFLYEILLTGIRGPFLIIAPLSTIANWEREFRTWTDINVVVYHGSLISRQMIQQ
YEMYFRDSQGRIIRGAYRFQAIITTFEMILGGCGELNAIDWRCVIIDEAHRLKNKNCKLLEGLKLMNLEHKVL
LTGTPLQNTVEELFSLLHFLEPLRFPSESTFMQEFGDLKTEEQVQKLQAILKPMMLRRLKEDVEKKLAPKEET
IIEVELTNIQKKYYRAILEKNFSFLSKGAGQTNVPNLVNTMMELRKCCNHPYLIKGAEEKILGEFRDTYNPSA
SDFHLQAMIQSAGKLVLIDKLLPKMKAGGHKVLIFSQMVRCLDILEDYLIHKRYLYERIDGRVRGNLRQAAID
RFSKPDSDRFVFLLCTRAGGLGINLTAADTCIIFDSDWNPQNDLQAQARCHRIGQNKAVKVYRLVTRNSYERE
MFDRASLKLGLDKAVLQSMSGRDSNVSGIQQLSKKEIEDLLRRGAYGAIMEEEDEGSKFCEEDIDQILLRRTK
TITIESEGRGSTFAKASFVASGNRTDISLDDPNFWQKWAKKAELDIDTISGRNSLVIDTPRIRKQTRPFSATK
DELAELSEAESEGEEKPKLRRPCDRSGGYGRTECFRVEKNLLVYGWGRWREILSHGRFKRQLNEHDVEVICRA
LLAYCLIHYRGDEKIKGFIWDLITPTEDGQTRELQNHLGLSAPVPRGRKGKKVKTQTSSFDIQKAEWLRKYNP
EQLLQDEGYKKHVKHHCNKVLLRVRMLYYLKQEVIGNESQKVFDGVDASDIDVWVPEPDHSEVPAAWWDFDAD
KSLLIGVFKHGYEKYNTIRADPALCFLERVGKPDDKAVAAEQRANDYMDGDVEDPEYKPAPAIFKDDIEDDVS
SPGDLVIADGEGQLMEGDKVYWPTPSALTTRLRRLITAYQRTNKNRHIQQMQPTFSLPANAMQPLYEEATLNP
KMAAKIERQQRWTRREEADFYRVVSTFGVVFDPDRGQFDWTKFRALARLHKKTDNSLEKYLCAFMSMCRRVCR

LPSKEELVDPNIFIQPITEERASRTLYRIELLRKVREQALRHPQLFERLKLCHPNPDLPIWWECGSHDRDLLI
GAAKHGVSRTDYHILRDPELSFMAAQRNYNQSKAAHSRTSAPLLQQYQVALSASPLTSLPRLLGAKGTLLEDM
KVKSESLTEEPQSSEEESMSSMETRTRVKSEPVSPKNGVLSQATGDQKSGGKSETDRRMVAARTEPLTPNPAS
KKPRVHKRGSQSSSDSDSDSARSSCSSRSSSSSSSSSSCSHSRSGSSSSSSSSCSSASSSSSSSSSSSSSS
SSSEESDSEEDVQKREGTPHRKAYDEESVASLSTTQDETQDSFQANNGTPESAYLLQGGYMLAASYWPKDRVT
INRLDSICQTVLKGKWPSARRHYDANTVASFYTTKLLDSPGAATERGEPSVPTPPAVAVREEHEQSAQMSKEG
GLKLTFQKQGLAQKRPFDGEDGALGQQQYLTRLRELQSTSETSLVNLPKAVPASGTSIQPTLGANGAILDSQP
IVKKRRGRRRNVEGADILFLNRNKPPNHIPTGMNPALSYPQPQRIPDTESPVPVINLKDGTRLAGDDAPKRKD
LDRWLKEHPGYVEDLGAFIPRVQLHEGRPKQKRHRCRNPNKLDINSLTGEERVQLINRRNARKVGGAFAPPLK
DLCRFLKENSEYGVAPEWGDVVKQSGFLPESMFERILTGPVVREEVSRRGRRPKSGIAKATTAAAVPAGSVPG
NPLLANGLLPGVDLTALQALQQNLQNLQSLQVTAGLMGMPAGLSSGGETKNMAAMFPMLFSGMAGLPNLLGMG
GLLSKTAESGAEEKRGNDSKELEGKKERTESQSPENGGERCVPGSPSTSSTAALSSAAAAKPIALNPLLLSNI
LYPGMLLTPGLNLHLPTLSQSNAFDVQKNKSDDLDSSKSVEIKEENSRVRDQEEKGGTEPSPLNENSTDEGSE
RASSGSDSSSSSSEDSDSSNED*
>rs51312005_ENSMUSP00000148088_c.6787:T>C_p.M2263T
ENSMUST00000209423.2|Chd9-205|ENSMUSG00000056608|+|chr8:g.91762964:T>C
MTDPMMDFFDDANLFGETLEGLSDDTFVQPGPVSLVDELNLGAEFEPLHIDSLNHVQGTPTHQKMADFEQLSQ
FDSMKFHPVNQSFGSPVEHVLSPHSQFNCSPIHPPNQPNGLFQDVADGSPMWGHQTATGLANQNGSPFHQPGH
SHSLHQNKSFVAHPDFALFQASEHQTQCSSLHSQQSRSNLNPGQNSLGQAKNFLDANVSGAHRVNVNHLATAP
SSQQTLPVQFSPTANPPAHFLKCSSHQEGNYNRPSPSMTSCSVSNSQQFPSHYSFSSGHVSPSSLLQSSAGLA
PGHTNQALSDFAGSNSFSPHRGMKQEPTQHLLNPTPSLNSNNFQILHSSHPQGNYSNSKLSPVHMNFPDPVDA
GPPVGHFNDHAETNGFSSLEENLLHHVDSHAEPFAGLDPEDLLQEGLLPQFDESPFGQDNSNHVLDHDLDRQF
TSHLVSRPSDMAQTQLQYQARGWPSPLSTNHQHLHSRNHLCLQRQPPSSKKSDGSGTYTKLQNTQVRVMSEKK
PRKRVESESKQEKANRIISEAIARAKERGERNIPRVMSPENFPSASVEGKEEKRGRRMKSKPKDRDNKKPKTY
SKLKEKTKIGKLIITLGKKHKRRNESSDELSDAEQRSQHTFKEQHSQKRRSNRQIKRKKYAEDAEGKQSEEEV
KGSLRVKRNSAPPPGEQPLQLFVENPSEEDAAIVDKILACRTVKKEVSPGVMLDIEEFFVKYKNYSYLHCEWA
TEQQLLKDKRIQQKIKRFKLRQAQRAHFLADMEEEPFNPDYVEVDRILEVSFCEDKDTGESVIYYLVKWCSLP
YEDSTWELKEDVDLAKIEEFEQLQASRPDTRHLDRPPSNIWKKIEQSREYKNGNQLREYQLEGLNWLLFNWYN
RRNCILADEMGLGKTIQSITFLYEILLTGIRGPFLIIAPLSTIANWEREFRTWTDINVVVYHGSLISRQMIQQ
YEMYFRDSQGRIIRGAYRFQAIITTFEMILGGCGELNAIDWRCVIIDEAHRLKNKNCKLLEGLKLMNLEHKVL
LTGTPLQNTVEELFSLLHFLEPLRFPSESTFMQEFGDLKTEEQVQKLQAILKPMMLRRLKEDVEKKLAPKEET
IIEVELTNIQKKYYRAILEKNFSFLSKGAGQTNVPNLVNTMMELRKCCNHPYLIKGAEEKILGEFRDTYNPSA
SDFHLQAMIQSAGKLVLIDKLLPKMKAGGHKVLIFSQMVRCLDILEDYLIHKRYLYERIDGRVRGNLRQAAID
RFSKPDSDRFVFLLCTRAGGLGINLTAADTCIIFDSDWNPQNDLQAQARCHRIGQNKAVKVYRLVTRNSYERE
MFDRASLKLGLDKAVLQSMSGRDSNVSGIQQLSKKEIEDLLRRGAYGAIMEEEDEGSKFCEEDIDQILLRRTK
TITIESEGRGSTFAKASFVASGNRTDISLDDPNFWQKWAKKAELDIDTISGRNSLVIDTPRIRKQTRPFSATK
DELAELSEAESEGEEKPKLRRPCDRSGGYGRTECFRVEKNLLVYGWGRWREILSHGRFKRQLNEHDVEVICRA
LLAYCLIHYRGDEKIKGFIWDLITPTEDGQTRELQNHLGLSAPVPRGRKGKKVKTQTSSFDIQKAEWLRKYNP
EQLLQDEGYKKHVKHHCNKVLLRVRMLYYLKQEVIGNESQKVFDGVDASDIDVWVPEPDHSEVPAAWWDFDAD
KSLLIGVFKHGYEKYNTIRADPALCFLERVGKPDDKAVAAEQRANDYMDGDVEDPEYKPAPAIFKDDIEDDVS
SPGDLVIADGEGQLMEGDKVYWPTPSALTTRLRRLITAYQRTNKNRHIQQMQPTFSLPANAMQPLYEEATLNP
KMAAKIERQQRWTRREEADFYRVVSTFGVVFDPDRGQFDWTKFRALARLHKKTDNSLEKYLCAFMSMCRRVCR
LPSKEELVDPNIFIQPITEERASRTLYRIELLRKVREQALRHPQLFERLKLCHPNPDLPIWWECGSHDRDLLI
GAAKHGVSRTDYHILRDPELSFMAAQRNYNQSKAAHSRTSAPLLQQYQVALSASPLTSLPRLLGAKGTLLEDM
KVKSESLTEEPQSSEEESMSSMETRTRVKSEPVSPKNGVLSQATGDQKSGGKSETDRRMVAARTEPLTPNPAS
KKPRVHKRGSQSSSDSDSDSARSSCSSRSSSSSSSSSSCSHSRSGSSSSSSSSCSSASSSSSSSSSSSSSS
SSSEESDSEEDVQKREGTPHRKAYDEESVASLSTTQDETQDSFQANNGTPESAYLLQGGYMLAASYWPKDRVT
INRLDSICQTVLKGKWPSARRHYDANTVASFYTTKLLDSPGAATERGEPSVPTPPAVAVREEHEQSAQMSKVK
KHVREKEFTVKIKDEGGLKLTFQKQGLAQKRPFDGEDGALGQQQYLTRLRELQSTSETSLVNLPKAVPASGTS
IQPTLGANGAILDSQPIVKKRRGRRRNVEGADILFLNRNKPPNHIPTGMNPALSYPQPQRIPDTESPVPVINL
KDGTRLAGDDAPKRKDLDRWLKEHPGYVEDLGAFIPRVQLHEGRPKQKRHRCRNPNKLDINSLTGEERVQLIN
RRNARKVGGAFAPPLKDLCRFLKENSEYGVAPEWGDVVKQSGFLPESMFERILTGPVVREEVSRRGRRPKSGI
AKATTAAAVPAGSVPGNPLLANGLLPGVDLTALQALQQNLQNLQSLQVTAGLMGMPAGLSSGGETKNMAAMFP
MLFSGMAGLPNLLGMGGLLSKTAESGAEEKRGNDSKELEGKKERTESQSPENGGERCVPGSPSTSSTAALSSA
AAAKPIALNPLLLSNILYPGMLLTPGLNLHLPTLSQSNAFDVQKNKSDDLDSSKSVEIKEENSRVRDQEEKGG
TEPSPLNENSTDEGSERASSGSDSSSSSSEDSDSSNED*
>rs51312005_ENSMUSP00000105243_c.6787:T>C_p.M2263T
ENSMUST00000109614.9|Chd9-202|ENSMUSG00000056608|+|chr8:g.91762964:T>C

MTDPMMDFFDDANLFGETLEGLSDDTFVQPGPVSLVDELNLGAEFEPLHIDSLNHVQGTPTHQKMADFEQLSQ
FDSMKFHPVNQSFGSPVEHVLSPHSQFNCSPIHPPNQPNGLFQDVADGSPMWGHQTATGLANQNGSPFHQPGH
SHSLHQNKSFVAHPDFALFQASEHQTQCSSLHSQQSRSNLNPGQNSLGQAKNFLDANVSGAHRVNVNHLATAP
SSQQTLPVQFSPTANPPAHFLKCSSHQEGNYNRPSPSMTSCSVSNSQQFPSHYSFSSGHVSPSSLLQSSAGLA
PGHTNQALSDFAGSNSFSPHRGMKQEPTQHLLNPTPSLNSNNFQILHSSHPQGNYSNSKLSPVHMNFPDPVDA
GPPVGHFNDHAETNGFSSLEENLLHHVDSHAEPFAGLDPEDLLQEGLLPQFDESPFGQDNSNHVLDHDLDRQF
TSHLVSRPSDMAQTQLQYQARGWPSPLSTNHQHLHSRNHLCLQRQPPSSKKSDGSGTYTKLQNTQVRVMSEKK
PRKRVESESKQEKANRIISEAIARAKERGERNIPRVMSPENFPSASVEGKEEKRGRRMKSKPKDRDNKKPKTY
SKLKEKTKIGKLIITLGKKHKRRNESSDELSDAEQRSQHTFKEQHSQKRRSNRQIKRKKYAEDAEGKQSEEEV
KGSLRVKRNSAPPPGEQPLQLFVENPSEEDAAIVDKILACRTVKKEVSPGVMLDIEEFFVKYKNYSYLHCEWA
TEQQLLKDKRIQQKIKRFKLRQAQRAHFLADMEEEPFNPDYVEVDRILEVSFCEDKDTGESVIYYLVKWCSLP
YEDSTWELKEDVDLAKIEEFEQLQASRPDTRHLDRPPSNIWKKIEQSREYKNGNQLREYQLEGLNWLLFNWYN
RRNCILADEMGLGKTIQSITFLYEILLTGIRGPFLIIAPLSTIANWEREFRTWTDINVVVYHGSLISRQMIQQ
YEMYFRDSQGRIIRGAYRFQAIITTFEMILGGCGELNAIDWRCVIIDEAHRLKNKNCKLLEGLKLMNLEHKVL
LTGTPLQNTVEELFSLLHFLEPLRFPSESTFMQEFGDLKTEEQVQKLQAILKPMMLRRLKEDVEKKLAPKEET
IIEVELTNIQKKYYRAILEKNFSFLSKGAGQTNVPNLVNTMMELRKCCNHPYLIKGAEEKILGEFRDTYNPSA
SDFHLQAMIQSAGKLVLIDKLLPKMKAGGHKVLIFSQMVRCLDILEDYLIHKRYLYERIDGRVRGNLRQAAID
RFSKPDSDRFVFLLCTRAGGLGINLTAADTCIIFDSDWNPQNDLQAQARCHRIGQNKAVKVYRLVTRNSYERE
MFDRASLKLGLDKAVLQSMSGRDSNVSGIQQLSKKEIEDLLRRGAYGAIMEEEDEGSKFCEEDIDQILLRRTK
TITIESEGRGSTFAKASFVASGNRTDISLDDPNFWQKWAKKAELDIDTISGRNSLVIDTPRIRKQTRPFSATK
DELAELSEAESEGEEKPKLRRPCDRSGGYGRTECFRVEKNLLVYGWGRWREILSHGRFKRQLNEHDVEVICRA
LLAYCLIHYRGDEKIKGFIWDLITPTEDGQTRELQNHLGLSAPVPRGRKGKKVKTQTSSFDIQKAEWLRKYNP
EQLLQDEGYKKHVKHHCNKVLLRVRMLYYLKQEVIGNESQKVFDGVDASDIDVWVPEPDHSEVPAAWWDFDAD
KSLLIGVFKHGYEKYNTIRADPALCFLERVGKPDDKAVAAEQRANDYMDGDVEDPEYKPAPAIFKDDIEDDVS
SPGDLVIADGEGQLMEGDKVYWPTPSALTTRLRRLITAYQRTNKNRHIQQMQPTFSLPANAMQPLYEEATLNP
KMAAKIERQQRWTRREEADFYRVVSTFGVVFDPDRGQFDWTKFRALARLHKKTDNSLEKYLCAFMSMCRRVCR
LPSKEELVDPNIFIQPITEERASRTLYRIELLRKVREQALRHPQLFERLKLCHPNPDLPIWWECGSHDRDLLI
GAAKHGVSRTDYHILRDPELSFMAAQRNYNQSKAAHSRTSAPLLQQYQVALSASPLTSLPRLLGAKGTLLEDM
KVKSESLTEEPQSSEEESMSSMETRTRVKSEPVSPKNGVLSQATGDQKSGGKSETDRRMVAARTEPLTPNPAS
KKPRVHKRGSQSSSDSDSDSARSSCSSRSSSSSSSSSSCSHSRSGSSSSSSSSCSSASSSSSSSSSSSSSSS
SSSEESDSEEDVQKREGTPHRKAYDEESVASLSTTQDETQDSFQANNGTPESAYLLQGGYMLAASYWPKDRVT
INRLDSICQTVLKGKWPSARRHYDANTVASFYTTKLLDSPGAATERGEPSVPTPPAVAVREEHEQSAQMSKVK
KHVREKEFTVKIKDEGGLKLTFQKQGLAQKRPFDGEDGALGQQQYLTRLRELQSTSETSLVNLPKAVPASGTS
IQPTLGANGAILDSQPIVKKRRGRRRNVEGADILFLNRNKPPNHIPTGMNPALSYPQPQRIPDTESPVPVINL
KDGTRLAGDDAPKRKDLDRWLKEHPGYVEDLGAFIPRVQLHEGRPKQKRHRCRNPNKLDINSLTGEERVQLIN
RRNARKVGGAFAPPLKDLCRFLKENSEYGVAPEWGDVVKQSGFLPESMFERILTGPVVREEVSRRGRRPKSGI
AKATTAAAVPAGSVPGNPLLANGLLPGVDLTALQALQQNLQNLQSLQVTAGLMGMPAGLSSGGETKNMAAMFP
MLFSGMAGLPNLLGMGGLLSKTAESGAEEKRGNDSKELEGKKERTESQSPENGGERCVPGSPSTSSTAALSSA
AAAKPIALNPLLLSNILYPGMLLTPGLNLHLPTLSQSNAFDVQKNKSDDLDSSKSVEIKEENSRVRDQEEKGG
TEPSPLNENSTDEGSERASSGSDSSSSSSEDSDSSNED*
>rs32816175_ENSMUSP00000046356_c.8340:C>A_p.L2781I
ENSMUST00000048665.8|Chd9-201|ENSMUSG00000056608|+|chr8:g.91778331:C>A
MTDPMMDFFDDANLFGETLEGLSDDTFVQPGPVSLVDELNLGAEFEPLHIDSLNHVQGTPTHQKMADFEQLSQ
FDSMKFHPVNQSFGSPVEHVLSPHSQFNCSPIHPPNQPNGLFQDVADGSPMWGHQTATGLANQNGSPFHQPGH
SHSLHQNKSFVAHPDFALFQASEHQTQCSSLHSQQSRSNLNPGQNSLGQAKNFLDANVSGAHRVNVNHLATAP
SSQQTLPVQFSPTANPPAHFLKCSSHQEGNYNRPSPSMTSCSVSNSQQFPSHYSFSSGHVSPSSLLQSSAGLA
PGHTNQALSDFAGSNSFSPHRGMKQEPTQHLLNPTPSLNSNNFQILHSSHPQGNYSNSKLSPVHMNFPDPVDA
GPPVGHFNDHAETNGFSSLEENLLHHVDSHAEPFAGLDPEDLLQEGLLPQFDESPFGQDNSNHVLDHDLDRQF
TSHLVSRPSDMAQTQLQYQARGWPSPLSTNHQHLHSRNHLCLQRQPPSSKKSDGSGTYTKLQNTQVRVMSEKK
PRKRVESESKQEKANRIISEAIARAKERGERNIPRVMSPENFPSASVEGKEEKRGRRMKSKPKDRDNKKPKTY
SKLKEKTKIGKLIITLGKKHKRRNESSDELSDAEQRSQHTFKEQHSQKRRSNRQIKRKKYAEDAEGKQSEEEV
KGSLRVKRNSAPPPGEQPLQLFVENPSEEDAAIVDKILACRTVKKEVSPGVMLDIEEFFVKYKNYSYLHCEWA
TEQQLLKDKRIQQKIKRFKLRQAQRAHFLADMEEEPFNPDYVEVDRILEVSFCEDKDTGESVIYYLVKWCSLP
YEDSTWELKEDVDLAKIEEFEQLQASRPDTRHLDRPPSNIWKKIEQSREYKNGNQLREYQLEGLNWLLFNWYN
RRNCILADEMGLGKTIQSITFLYEILLTGIRGPFLIIAPLSTIANWEREFRTWTDINVVVYHGSLISRQMIQQ
YEMYFRDSQGRIIRGAYRFQAIITTFEMILGGCGELNAIDWRCVIIDEAHRLKNKNCKLLEGLKLMNLEHKVL
LTGTPLQNTVEELFSLLHFLEPLRFPSESTFMQEFGDLKTEEQVQKLQAILKPMMLRRLKEDVEKKLAPKEET
IIEVELTNIQKKYYRAILEKNFSFLSKGAGQTNVPNLVNTMMELRKCCNHPYLIKGAEEKILGEFRDTYNPSA

SDFHLQAMIQSAGKLVLIDKLLPKMKAGGHKVLIFSQMVRCLDILEDYLIHKRYLYERIDGRVRGNLRQAAID
RFSKPDSDRFVFLLCTRAGGLGINLTAADTCIIFDSDWNPQNDLQAQARCHRIGQNKAVKVYRLVTRNSYERE
MFDRASLKLGLDKAVLQSMSGRDSNVSGIQQLSKKEIEDLLRRGAYGAIMEEEDEGSKFCEEDIDQILLRRTK
TITIESEGRGSTFAKASFVASGNRTDISLDDPNFWQKWAKKAELDIDTISGRNSLVIDTPRIRKQTRPFSATK
DELAELSEAESEGEEKPKLRRPCDRSGGYGRTECFRVEKNLLVYGWGRWREILSHGRFKRQLNEHDVEVICRA
LLAYCLIHYRGDEKIKGFIWDLITPTEDGQTRELQNHLGLSAPVPRGRKGKKVKTQTSSFDIQKAEWLRKYNP
EQLLQDEGYKKHVKHHCNKVLLRVRMLYYLKQEVIGNESQKVFDGVDASDIDVWVPEPDHSEVPAAWWDFDAD
KSLLIGVFKHGYEKYNTIRADPALCFLERVGKPDDKAVAAEQRANDYMDGDVEDPEYKPAPAIFKDDIEDDVS
SPGDLVIADGEGQLMEGDKVYWPTPSALTTRLRRLITAYQRTNKNRHIQQMQPTFSLPANAMQPLYEEATLNP
KMAAKIERQQRWTRREEADFYRVVSTFGVVFDPDRGQFDWTKFRALARLHKKTDNSLEKYLCAFMSMCRRVCR
LPSKEELVDPNIFIQPITEERASRTLYRIELLRKVREQALRHPQLFERLKLCHPNPDLPIWWECGSHDRDLLI
GAAKHGVSRTDYHILRDPELSFMAAQRNYNQSKAAHSRTSAPLLQQYQVALSASPLTSLPRLLGAKGTLLEDM
KVKSESLTEEPQSSEEESMSSMETRTRVKSEPVSPKNGVLSQATGDQKSGGKSETDRRMVAARTEPLTPNPAS
KKPRVHKRGSQSSSDSDSDSARSSCSSRSSSSSSSSSSCSHSRSGSSSSSSSSSCSSASSSSSSSSSSSSSSSS
SSSEESDSEEDVQKREGTPHRKAYDEESVASLSTTQDETQDSFQANNGTPESAYLLQGGYMLAASYWPKDRVM
INRLDSICQTVLKGKWPSARRHYDANTVASFYTTKLLDSPGAATERGEPSVPTPPAVAVREEHEQSAQMSKEG
GLKLTFQKQGLAQKRPFDGEDGALGQQQYLTRLRELQSTSETSLVNLPKAVPASGTSIQPTLGANGAILDSQP
IVKKRRGRRRNVEGADILFLNRNKPPNHIPTGMNPALSYPQPQRIPDTESPVPVINLKDGTRLAGDDAPKRKD
LDRWLKEHPGYVEDLGAFIPRVQLHEGRPKQKRHRCRNPNKLDINSLTGEERVQLINRRNARKVGGAFAPPLK
DLCRFLKENSEYGVAPEWGDVVKQSGFLPESMFERILTGPVVREEVSRRGRRPKSGIAKATTAAVPAGSVPG
NPLLANGLLPGVDLTALQALQQNLQNLQSLQVTAGLMGMPAGLSSGGETKNMAAMFPMLFSGMAGLPNLLGMG
GLLSKTAESGAEEKRGNDSKELEGKKERTESQSPENGGERCVPGSPSTSSTAALSSAAAAKPIALNPLLLSNI
LYPGMLITPGLNLHLPTLSQSNAFDVQKNKSDDLDSSKSVEIKEENSRVRDQEEKGGTEPSPLNENSTDEGSE
RASSGSDSSSSSSEDSDSSNED*
>rs32816175_ENSMUSP00000148088_c.8388:C>A_p.L2797I
ENSMUST00000209423.2|Chd9-205|ENSMUSG00000056608|+|chr8:g.91778331:C>A
MTDPMMDFFDDANLFGETLEGLSDDTFVQPGPVSLVDELNLGAEFEPLHIDSLNHVQGTPTHQKMADFEQLSQ
FDSMKFHPVNQSFGSPVEHVLSPHSQFNCSPIHPPNQPNGLFQDVADGSPMWGHQTATGLANQNGSPFHQPGH
SHSLHQNKSFVAHPDFALFQASEHQTQCSSLHSQQSRSNLNPGQNSLGQAKNFLDANVSGAHRVNVNHLATAP
SSQQTLPVQFSPTANPPAHFLKCSSHQEGNYNRPSPSMTSCSVSNSQQFPSHYSFSSGHVSPSSLLQSSAGLA
PGHTNQALSDFAGSNSFSPHRGMKQEPTQHLLNPTPSLNSNNFQILHSSHPQGNYSNSKLSPVHMNFPDPVDA
GPPVGHFNDHAETNGFSSLEENLLHHVDSHAEPFAGLDPEDLLQEGLLPQFDESPFGQDNSNHVLDHDLDRQF
TSHLVSRPSDMAQTQLQYQARGWPSPLSTNHQHLHSRNHLCLQRQPPSSKKSDGSGTYTKLQNTQVRVMSEKK
PRKRVESESKQEKANRIISEAIARAKERGERNIPRVMSPENFPSASVEGKEEKRGRRMKSKPKDRDNKKPKTY
SKLKEKTKIGKLIITLGKKHKRRNESSDELSDAEQRSQHTFKEQHSQKRRSNRQIKRKKYAEDAEGKQSEEEV
KGSLRVKRNSAPPPGEQPLQLFVENPSEEDAAIVDKILACRTVKKEVSPGVMLDIEEFFVKYKNYSYLHCEWA
TEQQLLKDKRIQQKIKRFKLRQAQRAHFLADMEEEPFNPDYVEVDRILEVSFCEDKDTGESVIYYLVKWCSLP
YEDSTWELKEDVDLAKIEEFEQLQASRPDTRHLDRPPSNIWKKIEQSREYKNGNQLREYQLEGLNWLLFNWYN
RRNCILADEMGLGKTIQSITFLYEILLTGIRGPFLIIAPLSTIANWEREFRTWTDINVVVYHGSLISRQMIQQ
YEMYFRDSQGRIIRGAYRFQAIITTFEMILGGCGELNAIDWRCVIIDEAHRLKNKNCKLLEGLKLMNLEHKVL
LTGTPLQNTVEELFSLLHFLEPLRFPSESTFMQEFGDLKTEEQVQKLQAILKPMMLRRLKEDVEKKLAPKEET
IIEVELTNIQKKYYRAILEKNFSFLSKGAGQTNVPNLVNTMMELRKCCNHPYLIKGAEEKILGEFRDTYNPSA
SDFHLQAMIQSAGKLVLIDKLLPKMKAGGHKVLIFSQMVRCLDILEDYLIHKRYLYERIDGRVRGNLRQAAID
RFSKPDSDRFVFLLCTRAGGLGINLTAADTCIIFDSDWNPQNDLQAQARCHRIGQNKAVKVYRLVTRNSYERE
MFDRASLKLGLDKAVLQSMSGRDSNVSGIQQLSKKEIEDLLRRGAYGAIMEEEDEGSKFCEEDIDQILLRRTK
TITIESEGRGSTFAKASFVASGNRTDISLDDPNFWQKWAKKAELDIDTISGRNSLVIDTPRIRKQTRPFSATK
DELAELSEAESEGEEKPKLRRPCDRSGGYGRTECFRVEKNLLVYGWGRWREILSHGRFKRQLNEHDVEVICRA
LLAYCLIHYRGDEKIKGFIWDLITPTEDGQTRELQNHLGLSAPVPRGRKGKKVKTQTSSFDIQKAEWLRKYNP
EQLLQDEGYKKHVKHHCNKVLLRVRMLYYLKQEVIGNESQKVFDGVDASDIDVWVPEPDHSEVPAAWWDFDAD
KSLLIGVFKHGYEKYNTIRADPALCFLERVGKPDDKAVAAEQRANDYMDGDVEDPEYKPAPAIFKDDIEDDVS
SPGDLVIADGEGQLMEGDKVYWPTPSALTTRLRRLITAYQRTNKNRHIQQMQPTFSLPANAMQPLYEEATLNP
KMAAKIERQQRWTRREEADFYRVVSTFGVVFDPDRGQFDWTKFRALARLHKKTDNSLEKYLCAFMSMCRRVCR
LPSKEELVDPNIFIQPITEERASRTLYRIELLRKVREQALRHPQLFERLKLCHPNPDLPIWWECGSHDRDLLI
GAAKHGVSRTDYHILRDPELSFMAAQRNYNQSKAAHSRTSAPLLQQYQVALSASPLTSLPRLLGAKGTLLEDM
KVKSESLTEEPQSSEEESMSSMETRTRVKSEPVSPKNGVLSQATGDQKSGGKSETDRRMVAARTEPLTPNPAS
KKPRVHKRGSQSSSDSDSDSARSSCSSRSSSSSSSSSSCSHSRSGSSSSSSSSSCSSASSSSSSSSSSSSSSSS
SSSEESDSEEDVQKREGTPHRKAYDEESVASLSTTQDETQDSFQANNGTPESAYLLQGGYMLAASYWPKDRVM
INRLDSICQTVLKGKWPSARRHYDANTVASFYTTKLLDSPGAATERGEPSVPTPPAVAVREEHEQSAQMSKVK

KHVREKEFTVKIKDEGGLKLTFQKQGLAQKRPFDGEDGALGQQQYLTRLRELQSTSETSLVNLPKAVPASGTS
IQPTLGANGAILDSQPIVKKRRGRRRNVEGADILFLNRNKPPNHIPTGMNPALSYPQPQRIPDTESPVPVINL
KDGTRLAGDDAPKRKDLDRWLKEHPGYVEDLGAFIPRVQLHEGRPKQKRHRCRNPNKLDINSLTGEERVQLIN
RRNARKVGGAFAPPLKDLCRFLKENSEYGVAPEWGDVVKQSGFLPESMFERILTGPVVREEVSRRGRRPKSGI
AKATTAAAVPAGSVPGNPLLANGLLPGVDLTALQALQQNLQNLQSLQVTAGLMGMPAGLSSGGETKNMAAMFP
MLFSGMAGLPNLLGMGGLLSKTAESGAEEKRGNDSKELEGKKERTESQSPENGGERCVPGSPSTSSTAALSSA
AAAKPIALNPLLLSNILYPGMLITPGLNLHLPTLSQSNAFDVQKNKSDDLDSSKSVEIKEENSRVRDQEEKGG
TEPSPLNENSTDEGSERASSGSDSSSSSSEDSDSSNED*
>rs32816175_ENSMUSP00000105243_c.8388:C>A_p.L2797I
ENSMUST00000109614.9|Chd9-202|ENSMUSG00000056608|+|chr8:g.91778331:C>A
MTDPMMDFFDDANLFGETLEGLSDDTFVQPGPVSLVDELNLGAEFEPLHIDSLNHVQGTPTHQKMADFEQLSQ
FDSMKFHPVNQSFGSPVEHVLSPHSQFNCSPIHPPNQPNGLFQDVADGSPMWGHQTATGLANQNGSPFHQPGH
SHSLHQNKSFVAHPDFALFQASEHQTQCSSLHSQQSRSNLNPGQNSLGQAKNFLDANVSGAHRVNVNHLATAP
SSQQTLPVQFSPTANPPAHFLKCSSHQEGNYNRPSPSMTSCSVSNSQQFPSHYSFSSGHVSPSSLLQSSAGLA
PGHTNQALSDFAGSNSFSPHRGMKQEPTQHLLNPTPSLNSNNFQILHSSHPQGNYSNSKLSPVHMNFPDPVDA
GPPVGHFNDHAETNGFSSLEENLLHHVDSHAEPFAGLDPEDLLQEGLLPQFDESPFGQDNSNHVLDHDLDRQF
TSHLVSRPSDMAQTQLQYQARGWPSPLSTNHQHLHSRNHLCLQRQPPSSKKSDGSGTYTKLQNTQVRVMSEKK
PRKRVESESKQEKANRIISEAIARAKERGERNIPRVMSPENFPSASVEGKEEKRGRRMKSKPKDRDNKKPKTY
SKLKEKTKIGKLIITLGKKHKRRNESSDELSDAEQRSQHTFKEQHSQKRRSNRQIKRKKYAEDAEGKQSEEEV
KGSLRVKRNSAPPPGEQPLQLFVENPSEEDAAIVDKILACRTVKKEVSPGVMLDIEEFFVKYKNYSYLHCEWA
TEQQLLKDKRIQQKIKRFKLRQAQRAHFLADMEEEPFNPDYVEVDRILEVSFCEDKDTGESVIYYLVKWCSLP
YEDSTWELKEDVDLAKIEEFEQLQASRPDTRHLDRPPSNIWKKIEQSREYKNGNQLREYQLEGLNWLLFNWYN
RRNCILADEMGLGKTIQSITFLYEILLTGIRGPFLIIAPLSTIANWEREFRTWTDINVVVYHGSLISRQMIQQ
YEMYFRDSQGRIIRGAYRFQAIITTFEMILGGCGELNAIDWRCVIIDEAHRLKNKNCKLLEGLKLMNLEHKVL
LTGTPLQNTVEELFSLLHFLEPLRFPSESTFMQEFGDLKTEEQVQKLQAILKPMMLRRLKEDVEKKLAPKEET
IIEVELTNIQKKYYRAILEKNFSFLSKGAGQTNVPNLVNTMMELRKCCNHPYLIKGAEEKILGEFRDTYNPSA
SDFHLQAMIQSAGKLVLIDKLLPKMKAGGHKVLIFSQMVRCLDILEDYLIHKRYLYERIDGRVRGNLRQAAID
RFSKPDSDRFVFLLCTRAGGLGINLTAADTCIIFDSDWNPQNDLQAQARCHRIGQNKAVKVYRLVTRNSYERE
MFDRASLKLGLDKAVLQSMSGRDSNVSGIQQLSKKEIEDLLRRGAYGAIMEEEDEGSKFCEEDIDQILLRRTK
TITIESEGRGSTFAKASFVASGNRTDISLDDPNFWQKWAKKAELDIDTISGRNSLVIDTPRIRKQTRPFSATK
DELAELSEAESEGEEKPKLRRPCDRSGGYGRTECFRVEKNLLVYGWGRWREILSHGRFKRQLNEHDVEVICRA
LLAYCLIHYRGDEKIKGFIWDLITPTEDGQTRELQNHLGLSAPVPRGRKGKKVKTQTSSFDIQKAEWLRKYNP
EQLLQDEGYKKHVKHHCNKVLLRVRMLYYLKQEVIGNESQKVFDGVDASDIDVWVPEPDHSEVPAAWWDFDAD
KSLLIGVFKHGYEKYNTIRADPALCFLERVGKPDDKAVAAEQRANDYMDGDVEDPEYKPAPAIFKDDIEDDVS
SPGDLVIADGEGQLMEGDKVYWPTPSALTTRLRRLITAYQRTNKNRHIQQMQPTFSLPANAMQPLYEEATLNP
KMAAKIERQQRWTRREEADFYRVVSTFGVVFDPDRGQFDWTKFRALARLHKKTDNSLEKYLCAFMSMCRRVCR
LPSKEELVDPNIFIQPITEERASRTLYRIELLRKVREQALRHPQLFERLKLCHPNPDLPIWWECGSHDRDLLI
GAAKHGVSRTDYHILRDPELSFMAAQRNYNQSKAAHSRTSAPLLQQYQVALSASPLTSLPRLLGAKGTLLEDM
KVKSESLTEEPQSSEEESMSSMETRTRVKSEPVSPKNGVLSQATGDQKSGGKSETDRRMVAARTEPLTPNPAS
KKPRVHKRGSQSSSDSDSDSARSSCSSRSSSSSSSSSSCSHSRSGSSSSSSSSCSSASSSSSSSSSSSSSSS
SSSEESDSEEDVQKREGTPHRKAYDEESVASLSTTQDETQDSFQANNGTPESAYLLQGGYMLAASYWPKDRVM
INRLDSICQTVLKGKWPSARRHYDANTVASFYTTKLLDSPGAATERGEPSVPTPPAVAVREEHEQSAQMSKVK
KHVREKEFTVKIKDEGGLKLTFQKQGLAQKRPFDGEDGALGQQQYLTRLRELQSTSETSLVNLPKAVPASGTS
IQPTLGANGAILDSQPIVKKRRGRRRNVEGADILFLNRNKPPNHIPTGMNPALSYPQPQRIPDTESPVPVINL
KDGTRLAGDDAPKRKDLDRWLKEHPGYVEDLGAFIPRVQLHEGRPKQKRHRCRNPNKLDINSLTGEERVQLIN
RRNARKVGGAFAPPLKDLCRFLKENSEYGVAPEWGDVVKQSGFLPESMFERILTGPVVREEVSRRGRRPKSGI
AKATTAAAVPAGSVPGNPLLANGLLPGVDLTALQALQQNLQNLQSLQVTAGLMGMPAGLSSGGETKNMAAMFP
MLFSGMAGLPNLLGMGGLLSKTAESGAEEKRGNDSKELEGKKERTESQSPENGGERCVPGSPSTSSTAALSSA
AAAKPIALNPLLLSNILYPGMLITPGLNLHLPTLSQSNAFDVQKNKSDDLDSSKSVEIKEENSRVRDQEEKGG
TEPSPLNENSTDEGSERASSGSDSSSSSSEDSDSSNED*
>rs254579959_ENSMUSP00000034091_c.1201:C>G_p.T401S
ENSMUST00000034091.8|Rbl2-201|ENSMUSG00000031666|+|chr8:g.91816714:C>G
MASGGNQSPPPPPAAAASSEEEEEDGDAADRAQPAGSPSHQIQQRFEELCSRLNMDEAARAEAWSSYRSMSES
YTLEGNDLHWLACALYVACRKSVPTVSKGTAEGNYVSLTRILRCSEQSLIEFFNKMKKWEDMANLPPHFRERT
ERLERNFTVSAVIFKKYEPIFQDIFKYPQEEQPRQQRGRKQRRQPCTTSEIFHFCWVLFIYAKGNFPMISDDL
VNSYHLLLCALDLVYGNALQCSNRKELVNPNFKGLSEDCHPKDSKASSDPPCVIEKLCSLHDGLVLEAKGIKE
HFWKPYIRKLFEKKLLKGKEENLTGFLEPGNFGESFKAVNKAYEEYVLAAGNLDERVFLGEDAEEEVGTLSRC
LSAASGTESAERTQMRDILQQHLDKSKALRVCTPLSGVRYVQENSPCVTPVSTAAHSLSRLHTMLSGLRNAPS

EKLERILRSCSRDPTQAIADRLKEMYEIYSQHFQPDENFSNCAKEIANKHFRFAEMLYYKVLESVIEQEQKRL
GDMDLSGVLEHDAFHRSLLACCLEVVAFSHKPPGNFPFIAEIFDVPHYHFYKVIEVFIRAEDGLCREVVKHLN
QIEEQILDHLAWKTKSPLWDRIRDNENRVPTCEEVMPPQNLERTDEIYIAGSPLTPRRVGEVRADAGGLGRSI
TSPTTLYDRYSSPTVSTTRRRLFENDSPSEGSTSGRIPPQPLVNAVPVQNVPGETVSVTPVPGQTLVTMATAT
VTANNGQTVTIPVQGIANENGGITFFPVQVNVGGQAQAVAGSIQPLSAQALAGSLSSQQVTGTTLQVPGPVAI
QQISPGGQQQNPGQPLTSSSIRPRKTSSLALFFRKVYYLAGVRLRDLCIKLDISDELRKKIWTCFEFSIIQCT
ELMMDRHLDQLLMCAIYVMAKVTKEDRSFQNIMRCYRTQPQARSQVYRSVLIKGKRRNSGSSESRSHQNSPTE
LNTDRASRDSSPVMRSNSTLPVPQPSSAPPTPTRLTGASSDVEEEERGDLIQFYNNIYRKQIQAFAMKYSQAN
AQTDTPPLSPYPFVRTGSPRRVQLSQSHPIYISPHNNEAMPSPREKIFYYFSNSPSKRLREINSMIRTGETPT
KKRGILLDDGSESPAKRICPENHSALLRRLQDVANDRGSQ*
>rs254579959_ENSMUSP00000147579_c.1072:C>G_p.T358S
ENSMUST00000211136.2|Rbl2-210|ENSMUSG00000031666|+|chr8:g.91816714:C>G
MASGGNQSPPPPPAAAASSEEEEEDGDAADRAQPAGSPSHQIQQRFEELCSRLNMDEAARAEAWSSYRSMSES
YTLEGNDLHWLACALYVACRKSVPTVSKGTAEGNYVSLTRILRCSEQSLIEFFNKMKKWEDMANLPPHFRERT
ERLERNFTVSAVIFKKYEPIFQDIFKYPQEEQPRQQRGRKQRRQPCTTSEIFHFCWVLFIYAKGLSEDCHPKD
SKASSDPPCVIEKLCSLHDGLVLEAKGIKEHFWKPYIRKLFEKKLLKGKEENLTGFLEPGNFGESFKAVNKAY
EEYVLAAGNLDERVFLGEDAEEEVGTLSRCLSAASGTESAERTQMRDILQQHLDKSKALRVCTPLSGVRYVQE
NSPCVTPVSTAAHSLSRLHTMLSGLRNAPSEKLERILRSCSRDPTQAIADRLKEMYEIYSQHFQPDENFSNCA
KEIANKHFRFAEMLYYKVLESVIEQEQKRLGDMDLSGVLEHDAFHRSLLACCLEVVAFSHKPPGNFPFIAEIF
DVPHYHFYKVIEVFIRAEDGLCREVVKHLNQIEEQILDHLAWKTKSPLWDRIRDNENRVPTCEEVMPPQNLER
TDEIYIAGSPLTPRRVGEVRADAGGLGRSITSPTTLYDRYSSPTVSTTRRRLFENDSPSEGSTSGRIPPQPLV
NAVPVQNVPGETVSVTPVPGQTLVTMATATVTANNGQTVTIPVQGIANENGGITFFPVQVNVGGQAQAVAGSI
QPLSAQALAGSLSSQQVTGTTLQVPGPVAIQQISPGGQQQNPGQPLTSSSIRPRKTSSLALFFRKVYYLAGVR
LRDLCIKLDISDELRKKIWTCFEFSIIQCTELMMDRHLDQLLMCAIYVMAKVTKEDRSFQNIMRCYRTQPQAR
SQVYRSVLIKGKRRNSGSSESRSHQNSPTELNTDRASRDSSPVMRSNSTLPVPQPSSAPPTPTRLTGASSDVE
EEERGDLIQFYNNIYRKQIQAFAMKYSQANAQTDTPPLSPYPFVRTGSPRRVQLSQSHPIYISPHNNEAMPSP
REKIFYYFSNSPSKRLREINSMIRTGETPTKKRGILLDDGSESPAKRICPENHSALLRRLQDVANDRGSQ*
>rs254579959_ENSMUSP00000147327_c.1201:C>G_p.T401S
ENSMUST00000209518.2|Rbl2-208|ENSMUSG00000031666|+|chr8:g.91816714:C>G
MASGGNQSPPPPPAAAASSEEEEEDGDAADRAQPAGSPSHQIQQRFEELCSRLNMDEAARAEAWSSYRSMSES
YTLEGNDLHWLACALYVACRKSVPTVSKGTAEGNYVSLTRILRCSEQSLIEFFNKMKKWEDMANLPPHFRERT
ERLERNFTVSAVIFKKYEPIFQDIFKYPQEEQPRQQRGRKQRRQPCTTSEIFHFCWVLFIYAKGNFPMISDDL
VNSYHLLLCALDLVYGNALQCSNRKELVNPNFKGLSEDCHPKDSKASSDPPCVIEKLCSLHDGLVLEAKGIKE
HFWKPYIRKLFEKKLLKGKEENLTGFLEPGNFGESFKAVNKAYEEYVLAAGNLDERVFLGEDAEEEVGTLSRC
LSAASGTESAERTQMRDILQQHLDKSKALRVCTPLSGVRYVQENSPCVTPVSTAAHSLSRLHTMLSGLRNAPS
EKLERILRSCSRDPTQAIADRLKEMYEIYSQHFQPDENFSNCAKEMLYYKVLESVIEQEQKRLGDMDLSGVLE
HDAFHRSLLACCLEVVAFSHKPPGNFPFIAEIFDVPHYHFYKVIEVFIRAEDGLCREVVKHLNQIEEQILDHL
AWKTKSPLWDRIRDNENRVPTCEEVMPPQNLERTDEIYIAGSPLTPRRVGEVRADAGGLGRSITSPTTLYDRY
SSPTVSTTRRRLFENDSPSEGSTSGRIPPQPLVNAVPVQNVPGETVSVTPVPGQTLVTMATATVTANNGQTVT
IPVQGIANENGGITFFPVQVNVGGQAQAVAGSIQPLSAQALAGSLSSQQVTGTTLQVPGPVAIQQISPGGQQQ
NPGQPLTSSSIRPRKTSSLALFFRKVYYLAGVRLRDLCIKLDISDELRKKIWTCFEFSIIQCTELMMDRHLDQ
LLMCAIYVMAKVTKEDRSFQNIMRCYRTQPQARSQVYRSVLIKGKRRNSGSSESRSHQNSPTELNTDRASRDS
SPVMRSNSTLPVPQPSSAPPTPTRLTGASSDVEEEERGDLIQFYNNIYRKQIQAFAMKYSQANAQTDTPPLSP
YPFVRTGSPRRVQLSQSHPIYISPHNNEAMPSPREKIFYYFSNSPSKRLREINSMIRTGETPTKKRGILLDDG
SESPAKRICPENHSALLRRLQDVANDRGSQ*
>rs33292293_ENSMUSP00000034091_c.1753:A>G_p.Q585R
ENSMUST00000034091.8|Rbl2-201|ENSMUSG00000031666|+|chr8:g.91828040:A>G
MASGGNQSPPPPPAAAASSEEEEEDGDAADRAQPAGSPSHQIQQRFEELCSRLNMDEAARAEAWSSYRSMSES
YTLEGNDLHWLACALYVACRKSVPTVSKGTAEGNYVSLTRILRCSEQSLIEFFNKMKKWEDMANLPPHFRERT
ERLERNFTVSAVIFKKYEPIFQDIFKYPQEEQPRQQRGRKQRRQPCTTSEIFHFCWVLFIYAKGNFPMISDDL
VNSYHLLLCALDLVYGNALQCSNRKELVNPNFKGLSEDCHPKDSKASSDPPCVIEKLCSLHDGLVLEAKGIKE
HFWKPYIRKLFEKKLLKGKEENLTGFLEPGNFGESFKAVNKAYEEYVLAAGNLDERVFLGEDAEEEVGTLSRC
LSAASGTESAERTQMRDILQQHLDKSKALRVCTPLTGVRYVQENSPCVTPVSTAAHSLSRLHTMLSGLRNAPS
EKLERILRSCSRDPTQAIADRLKEMYEIYSQHFQPDENFSNCAKEIANKHFRFAEMLYYKVLESVIEQEQKRL
GDMDLSGVLEHDAFHRSLLACCLEVVAFSHKPPGNFPFIAEIFDVPHYHFYKVIEVFIRAEDGLCREVVKHLN
RIEEQILDHLAWKTKSPLWDRIRDNENRVPTCEEVMPPQNLERTDEIYIAGSPLTPRRVGEVRADAGGLGRSI
TSPTTLYDRYSSPTVSTTRRRLFENDSPSEGSTSGRIPPQPLVNAVPVQNVPGETVSVTPVPGQTLVTMATAT
VTANNGQTVTIPVQGIANENGGITFFPVQVNVGGQAQAVAGSIQPLSAQALAGSLSSQQVTGTTLQVPGPVAI

QQISPGGQQQNPGQPLTSSSIRPRKTSSLALFFRKVYYLAGVRLRDLCIKLDISDELRKKIWTCFEFSIIQCT
ELMMDRHLDQLLMCAIYVMAKVTKEDRSFQNIMRCYRTQPQARSQVYRSVLIKGKRRNSGSSESRSHQNSPTE
LNTDRASRDSSPVMRSNSTLPVPQPSSAPPTPTRLTGASSDVEEEERGDLIQFYNNIYRKQIQAFAMKYSQAN
AQTDTPPLSPYPFVRTGSPRRVQLSQSHPIYISPHNNEAMPSPREKIFYYFSNSPSKRLREINSMIRTGETPT
KKRGILLDDGSESPAKRICPENHSALLRRLQDVANDRGSQ*
>rs33292293_ENSMUSP00000147579_c.1624:A>G_p.Q542R
ENSMUST00000211136.2|Rbl2-210|ENSMUSG00000031666|+|chr8:g.91828040:A>G
MASGGNQSPPPPPAAAASSEEEEEDGDAADRAQPAGSPSHQIQQRFEELCSRLNMDEAARAEAWSSYRSMSES
YTLEGNDLHWLACALYVACRKSVPTVSKGTAEGNYVSLTRILRCSEQSLIEFFNKMKKWEDMANLPPHFRERT
ERLERNFTVSAVIFKKYEPIFQDIFKYPQEEQPRQQRGRKQRRQPCTTSEIFHFCWVLFIYAKGLSEDCHPKD
SKASSDPPCVIEKLCSLHDGLVLEAKGIKEHFWKPYIRKLFEKKLLKGKEENLTGFLEPGNFGESFKAVNKAY
EEYVLAAGNLDERVFLGEDAEEEVGTLSRCLSAASGTESAERTQMRDILQQHLDKSKALRVCTPLTGVRYVQE
NSPCVTPVSTAAHSLSRLHTMLSGLRNAPSEKLERILRSCSRDPTQAIADRLKEMYEIYSQHFQPDENFSNCA
KEIANKHFRFAEMLYYKVLESVIEQEQKRLGDMDLSGVLEHDAFHRSLLACCLEVVAFSHKPPGNFPFIAEIF
DVPHYHFYKVIEVFIRAEDGLCREVVKHLNRIEEQILDHLAWKTKSPLWDRIRDNENRVPTCEEVMPPQNLER
TDEIYIAGSPLTPRRVGEVRADAGGLGRSITSPTTLYDRYSSPTVSTTRRRLFENDSPSEGSTSGRIPPQPLV
NAVPVQNVPGETVSVTPVPGQTLVTMATATVTANNGQTVTIPVQGIANENGGITFFPVQVNVGGQAQAVAGSI
QPLSAQALAGSLSSQQVTGTTLQVPGPVAIQQISPGGQQQNPGQPLTSSSIRPRKTSSLALFFRKVYYLAGVR
LRDLCIKLDISDELRKKIWTCFEFSIIQCTELMMDRHLDQLLMCAIYVMAKVTKEDRSFQNIMRCYRTQPQAR
SQVYRSVLIKGKRRNSGSSESRSHQNSPTELNTDRASRDSSPVMRSNSTLPVPQPSSAPPTPTRLTGASSDVE
EEERGDLIQFYNNIYRKQIQAFAMKYSQANAQTDTPPLSPYPFVRTGSPRRVQLSQSHPIYISPHNNEAMPSP
REKIFYYFSNSPSKRLREINSMIRTGETPTKKRGILLDDGSESPAKRICPENHSALLRRLQDVANDRGSQ*
>rs33292293_ENSMUSP00000147327_c.1723:A>G_p.Q575R
ENSMUST00000209518.2|Rbl2-208|ENSMUSG00000031666|+|chr8:g.91828040:A>G
MASGGNQSPPPPPAAAASSEEEEEDGDAADRAQPAGSPSHQIQQRFEELCSRLNMDEAARAEAWSSYRSMSES
YTLEGNDLHWLACALYVACRKSVPTVSKGTAEGNYVSLTRILRCSEQSLIEFFNKMKKWEDMANLPPHFRERT
ERLERNFTVSAVIFKKYEPIFQDIFKYPQEEQPRQQRGRKQRRQPCTTSEIFHFCWVLFIYAKGNFPMISDDL
VNSYHLLLCALDLVYGNALQCSNRKELVNPNFKGLSEDCHPKDSKASSDPPCVIEKLCSLHDGLVLEAKGIKE
HFWKPYIRKLFEKKLLKGKEENLTGFLEPGNFGESFKAVNKAYEEYVLAAGNLDERVFLGEDAEEEVGTLSRC
LSAASGTESAERTQMRDILQQHLDKSKALRVCTPLTGVRYVQENSPCVTPVSTAAHSLSRLHTMLSGLRNAPS
EKLERILRSCSRDPTQAIADRLKEMYEIYSQHFQPDENFSNCAKEMLYYKVLESVIEQEQKRLGDMDLSGVLE
HDAFHRSLLACCLEVVAFSHKPPGNFPFIAEIFDVPHYHFYKVIEVFIRAEDGLCREVVKHLNRIEEQILDHL
AWKTKSPLWDRIRDNENRVPTCEEVMPPQNLERTDEIYIAGSPLTPRRVGEVRADAGGLGRSITSPTTLYDRY
SSPTVSTTRRRLFENDSPSEGSTSGRIPPQPLVNAVPVQNVPGETVSVTPVPGQTLVTMATATVTANNGQTVT
IPVQGIANENGGITFFPVQVNVGGQAQAVAGSIQPLSAQALAGSLSSQQVTGTTLQVPGPVAIQQISPGGQQQ
NPGQPLTSSSIRPRKTSSLALFFRKVYYLAGVRLRDLCIKLDISDELRKKIWTCFEFSIIQCTELMMDRHLDQ
LLMCAIYVMAKVTKEDRSFQNIMRCYRTQPQARSQVYRSVLIKGKRRNSGSSESRSHQNSPTELNTDRASRDS
SPVMRSNSTLPVPQPSSAPPTPTRLTGASSDVEEEERGDLIQFYNNIYRKQIQAFAMKYSQANAQTDTPPLSP
YPFVRTGSPRRVQLSQSHPIYISPHNNEAMPSPREKIFYYFSNSPSKRLREINSMIRTGETPTKKRGILLDDG
SESPAKRICPENHSALLRRLQDVANDRGSQ*
>rs49005983_ENSMUSP00000034091_c.2462:C>G_p.S821R
ENSMUST00000034091.8|Rbl2-201|ENSMUSG00000031666|+|chr8:g.91833560:C>G
MASGGNQSPPPPPAAAASSEEEEEDGDAADRAQPAGSPSHQIQQRFEELCSRLNMDEAARAEAWSSYRSMSES
YTLEGNDLHWLACALYVACRKSVPTVSKGTAEGNYVSLTRILRCSEQSLIEFFNKMKKWEDMANLPPHFRERT
ERLERNFTVSAVIFKKYEPIFQDIFKYPQEEQPRQQRGRKQRRQPCTTSEIFHFCWVLFIYAKGNFPMISDDL
VNSYHLLLCALDLVYGNALQCSNRKELVNPNFKGLSEDCHPKDSKASSDPPCVIEKLCSLHDGLVLEAKGIKE
HFWKPYIRKLFEKKLLKGKEENLTGFLEPGNFGESFKAVNKAYEEYVLAAGNLDERVFLGEDAEEEVGTLSRC
LSAASGTESAERTQMRDILQQHLDKSKALRVCTPLTGVRYVQENSPCVTPVSTAAHSLSRLHTMLSGLRNAPS
EKLERILRSCSRDPTQAIADRLKEMYEIYSQHFQPDENFSNCAKEIANKHFRFAEMLYYKVLESVIEQEQKRL
GDMDLSGVLEHDAFHRSLLACCLEVVAFSHKPPGNFPFIAEIFDVPHYHFYKVIEVFIRAEDGLCREVVKHLN
QIEEQILDHLAWKTKSPLWDRIRDNENRVPTCEEVMPPQNLERTDEIYIAGSPLTPRRVGEVRADAGGLGRSI
TSPTTLYDRYSSPTVSTTRRRLFENDSPSEGSTSGRIPPQPLVNAVPVQNVPGETVSVTPVPGQTLVTMATAT
VTANNGQTVTIPVQGIANENGGITFFPVQVNVGGQAQAVAGSIQPLSAQALAGSLSSQQVTGTTLQVPGPVAI
QQISPGGQQQNPGQPLTRSSIRPRKTSSLALFFRKVYYLAGVRLRDLCIKLDISDELRKKIWTCFEFSIIQCT
ELMMDRHLDQLLMCAIYVMAKVTKEDRSFQNIMRCYRTQPQARSQVYRSVLIKGKRRNSGSSESRSHQNSPTE
LNTDRASRDSSPVMRSNSTLPVPQPSSAPPTPTRLTGASSDVEEEERGDLIQFYNNIYRKQIQAFAMKYSQAN
AQTDTPPLSPYPFVRTGSPRRVQLSQSHPIYISPHNNEAMPSPREKIFYYFSNSPSKRLREINSMIRTGETPT
KKRGILLDDGSESPAKRICPENHSALLRRLQDVANDRGSQ*

>rs49005983_ENSMUSP00000147579_c.2333:C>G_p.S778R
ENSMUST00000211136.2|Rbl2-210|ENSMUSG00000031666|+|chr8:g.91833560:C>G
MASGGNQSPPPPPAAAASSEEEEEDGDAADRAQPAGSPSHQIQQRFEELCSRLNMDEAARAEAWSSYRSMSES
YTLEGNDLHWLACALYVACRKSVPTVSKGTAEGNYVSLTRILRCSEQSLIEFFNKMKKWEDMANLPPHFRERT
ERLERNFTVSAVIFKKYEPIFQDIFKYPQEEQPRQQRGRKQRRQPCTTSEIFHFCWVLFIYAKGLSEDCHPKD
SKASSDPPCVIEKLCSLHDGLVLEAKGIKEHFWKPYIRKLFEKKLLKGKEENLTGFLEPGNFGESFKAVNKAY
EEYVLAAGNLDERVFLGEDAEEEVGTLSRCLSAASGTESAERTQMRDILQQHLDKSKALRVCTPLTGVRYVQE
NSPCVTPVSTAAHSLSRLHTMLSGLRNAPSEKLERILRSCSRDPTQAIADRLKEMYEIYSQHFQPDENFSNCA
KEIANKHFRFAEMLYYKVLESVIEQEQKRLGDMDLSGVLEHDAFHRSLLACCLEVVAFSHKPPGNFPFIAEIF
DVPHYHFYKVIEVFIRAEDGLCREVVKHLNQIEEQILDHLAWKTKSPLWDRIRDNENRVPTCEEVMPPQNLER
TDEIYIAGSPLTPRRVGEVRADAGGLGRSITSPTTLYDRYSSPTVSTTRRRLFENDSPSEGSTSGRIPPQPLV
NAVPVQNVPGETVSVTPVPGQTLVTMATATVTANNGQTVTIPVQGIANENGGITFFPVQVNVGGQAQAVAGSI
QPLSAQALAGSLSSQQVTGTTLQVPGPVAIQQISPGGQQQNPGQPLTRSSIRPRKTSSLALFFRKVYYLAGVR
LRDLCIKLDISDELRKKIWTCFEFSIIQCTELMMDRHLDQLLMCAIYVMAKVTKEDRSFQNIMRCYRTQPQAR
SQVYRSVLIKGKRRNSGSSESRSHQNSPTELNTDRASRDSSPVMRSNSTLPVPQPSSAPPTPTRLTGASSDVE
EEERGDLIQFYNNIYRKQIQAFAMKYSQANAQTDTPPLSPYPFVRTGSPRRVQLSQSHPIYISPHNNEAMPSP
REKIFYYFSNSPSKRLREINSMIRTGETPTKKRGILLDDGSESPAKRICPENHSALLRRLQDVANDRGSQ*
>rs49005983_ENSMUSP00000147327_c.2432:C>G_p.S811R
ENSMUST00000209518.2|Rbl2-208|ENSMUSG00000031666|+|chr8:g.91833560:C>G
MASGGNQSPPPPPAAAASSEEEEEDGDAADRAQPAGSPSHQIQQRFEELCSRLNMDEAARAEAWSSYRSMSES
YTLEGNDLHWLACALYVACRKSVPTVSKGTAEGNYVSLTRILRCSEQSLIEFFNKMKKWEDMANLPPHFRERT
ERLERNFTVSAVIFKKYEPIFQDIFKYPQEEQPRQQRGRKQRRQPCTTSEIFHFCWVLFIYAKGNFPMISDDL
VNSYHLLLCALDLVYGNALQCSNRKELVNPNFKGLSEDCHPKDSKASSDPPCVIEKLCSLHDGLVLEAKGIKE
HFWKPYIRKLFEKKLLKGKEENLTGFLEPGNFGESFKAVNKAYEEYVLAAGNLDERVFLGEDAEEEVGTLSRC
LSAASGTESAERTQMRDILQQHLDKSKALRVCTPLTGVRYVQENSPCVTPVSTAAHSLSRLHTMLSGLRNAPS
EKLERILRSCSRDPTQAIADRLKEMYEIYSQHFQPDENFSNCAKEMLYYKVLESVIEQEQKRLGDMDLSGVLE
HDAFHRSLLACCLEVVAFSHKPPGNFPFIAEIFDVPHYHFYKVIEVFIRAEDGLCREVVKHLNQIEEQILDHL
AWKTKSPLWDRIRDNENRVPTCEEVMPPQNLERTDEIYIAGSPLTPRRVGEVRADAGGLGRSITSPTTLYDRY
SSPTVSTTRRRLFENDSPSEGSTSGRIPPQPLVNAVPVQNVPGETVSVTPVPGQTLVTMATATVTANNGQTVT
IPVQGIANENGGITFFPVQVNVGGQAQAVAGSIQPLSAQALAGSLSSQQVTGTTLQVPGPVAIQQISPGGQQQ
NPGQPLTRSSIRPRKTSSLALFFRKVYYLAGVRLRDLCIKLDISDELRKKIWTCFEFSIIQCTELMMDRHLDQ
LLMCAIYVMAKVTKEDRSFQNIMRCYRTQPQARSQVYRSVLIKGKRRNSGSSESRSHQNSPTELNTDRASRDS
SPVMRSNSTLPVPQPSSAPPTPTRLTGASSDVEEEERGDLIQFYNNIYRKQIQAFAMKYSQANAQTDTPPLSP
YPFVRTGSPRRVQLSQSHPIYISPHNNEAMPSPREKIFYYFSNSPSKRLREINSMIRTGETPTKKRGILLDDG
SESPAKRICPENHSALLRRLQDVANDRGSQ*
>rs51619191_ENSMUSP00000034091_c.2646:C>T_p.H883Y
ENSMUST00000034091.8|Rbl2-201|ENSMUSG00000031666|+|chr8:g.91833818:C>T
MASGGNQSPPPPPAAAASSEEEEEDGDAADRAQPAGSPSHQIQQRFEELCSRLNMDEAARAEAWSSYRSMSES
YTLEGNDLHWLACALYVACRKSVPTVSKGTAEGNYVSLTRILRCSEQSLIEFFNKMKKWEDMANLPPHFRERT
ERLERNFTVSAVIFKKYEPIFQDIFKYPQEEQPRQQRGRKQRRQPCTTSEIFHFCWVLFIYAKGNFPMISDDL
VNSYHLLLCALDLVYGNALQCSNRKELVNPNFKGLSEDCHPKDSKASSDPPCVIEKLCSLHDGLVLEAKGIKE
HFWKPYIRKLFEKKLLKGKEENLTGFLEPGNFGESFKAVNKAYEEYVLAAGNLDERVFLGEDAEEEVGTLSRC
LSAASGTESAERTQMRDILQQHLDKSKALRVCTPLTGVRYVQENSPCVTPVSTAAHSLSRLHTMLSGLRNAPS
EKLERILRSCSRDPTQAIADRLKEMYEIYSQHFQPDENFSNCAKEIANKHFRFAEMLYYKVLESVIEQEQKRL
GDMDLSGVLEHDAFHRSLLACCLEVVAFSHKPPGNFPFIAEIFDVPHYHFYKVIEVFIRAEDGLCREVVKHLN
QIEEQILDHLAWKTKSPLWDRIRDNENRVPTCEEVMPPQNLERTDEIYIAGSPLTPRRVGEVRADAGGLGRSI
TSPTTLYDRYSSPTVSTTRRRLFENDSPSEGSTSGRIPPQPLVNAVPVQNVPGETVSVTPVPGQTLVTMATAT
VTANNGQTVTIPVQGIANENGGITFFPVQVNVGGQAQAVAGSIQPLSAQALAGSLSSQQVTGTTLQVPGPVAI
QQISPGGQQQNPGQPLTSSSIRPRKTSSLALFFRKVYYLAGVRLRDLCIKLDISDELRKKIWTCFEFSIIQCT
ELMMDRYLDQLLMCAIYVMAKVTKEDRSFQNIMRCYRTQPQARSQVYRSVLIKGKRRNSGSSESRSHQNSPTE
LNTDRASRDSSPVMRSNSTLPVPQPSSAPPTPTRLTGASSDVEEEERGDLIQFYNNIYRKQIQAFAMKYSQAN
AQTDTPPLSPYPFVRTGSPRRVQLSQSHPIYISPHNNEAMPSPREKIFYYFSNSPSKRLREINSMIRTGETPT
KKRGILLDDGSESPAKRICPENHSALLRRLQDVANDRGSQ*
>rs51619191_ENSMUSP00000147579_c.2517:C>T_p.H840Y
ENSMUST00000211136.2|Rbl2-210|ENSMUSG00000031666|+|chr8:g.91833818:C>T
MASGGNQSPPPPPAAAASSEEEEEDGDAADRAQPAGSPSHQIQQRFEELCSRLNMDEAARAEAWSSYRSMSES
YTLEGNDLHWLACALYVACRKSVPTVSKGTAEGNYVSLTRILRCSEQSLIEFFNKMKKWEDMANLPPHFRERT
ERLERNFTVSAVIFKKYEPIFQDIFKYPQEEQPRQQRGRKQRRQPCTTSEIFHFCWVLFIYAKGLSEDCHPKD

SKASSDPPCVIEKLCSLHDGLVLEAKGIKEHFWKPYIRKLFEKKLLKGKEENLTGFLEPGNFGESFKAVNKAY
EEYVLAAGNLDERVFLGEDAEEEVGTLSRCLSAASGTESAERTQMRDILQQHLDKSKALRVCTPLTGVRYVQE
NSPCVTPVSTAAHSLSRLHTMLSGLRNAPSEKLERILRSCSRDPTQAIADRLKEMYEIYSQHFQPDENFSNCA
KEIANKHFRFAEMLYYKVLESVIEQEQKRLGDMDLSGVLEHDAFHRSLLACCLEVVAFSHKPPGNFPFIAEIF
DVPHYHFYKVIEVFIRAEDGLCREVVKHLNQIEEQILDHLAWKTKSPLWDRIRDNENRVPTCEEVMPPQNLER
TDEIYIAGSPLTPRRVGEVRADAGGLGRSITSPTTLYDRYSSPTVSTTRRRLFENDSPSEGSTSGRIPPQPLV
NAVPVQNVPGETVSVTPVPGQTLVTMATATVTANNGQTVTIPVQGIANENGGITFFPVQVNVGGQAQAVAGSI
QPLSAQALAGSLSSQQVTGTTLQVPGPVAIQQISPGGQQQNPGQPLTSSSIRPRKTSSLALFFRKVYYLAGVR
LRDLCIKLDISDELRKKIWTCFEFSIIQCTELMMDRYLDQLLMCAIYVMAKVTKEDRSFQNIMRCYRTQPQAR
SQVYRSVLIKGKRRNSGSSESRSHQNSPTELNTDRASRDSSPVMRSNSTLPVPQPSSAPPTPTRLTGASSDVE
EEERGDLIQFYNNIYRKQIQAFAMKYSQANAQTDTPPLSPYPFVRTGSPRRVQLSQSHPIYISPHNNEAMPSP
REKIFYYFSNSPSKRLREINSMIRTGETPTKKRGILLDDGSESPAKRICPENHSALLRRLQDVANDRGSQ*
>rs51619191_ENSMUSP00000147327_c.2616:C>T_p.H873Y
ENSMUST00000209518.2|Rbl2-208|ENSMUSG00000031666|+|chr8:g.91833818:C>T
MASGGNQSPPPPPAAAASSEEEEEDGDAADRAQPAGSPSHQIQQRFEELCSRLNMDEAARAEAWSSYRSMSES
YTLEGNDLHWLACALYVACRKSVPTVSKGTAEGNYVSLTRILRCSEQSLIEFFNKMKKWEDMANLPPHFRERT
ERLERNFTVSAVIFKKYEPIFQDIFKYPQEEQPRQQRGRKQRRQPCTTSEIFHFCWVLFIYAKGNFPMISDDL
VNSYHLLLCALDLVYGNALQCSNRKELVNPNFKGLSEDCHPKDSKASSDPPCVIEKLCSLHDGLVLEAKGIKE
HFWKPYIRKLFEKKLLKGKEENLTGFLEPGNFGESFKAVNKAYEEYVLAAGNLDERVFLGEDAEEEVGTLSRC
LSAASGTESAERTQMRDILQQHLDKSKALRVCTPLTGVRYVQENSPCVTPVSTAAHSLSRLHTMLSGLRNAPS
EKLERILRSCSRDPTQAIADRLKEMYEIYSQHFQPDENFSNCAKEMLYYKVLESVIEQEQKRLGDMDLSGVLE
HDAFHRSLLACCLEVVAFSHKPPGNFPFIAEIFDVPHYHFYKVIEVFIRAEDGLCREVVKHLNQIEEQILDHL
AWKTKSPLWDRIRDNENRVPTCEEVMPPQNLERTDEIYIAGSPLTPRRVGEVRADAGGLGRSITSPTTLYDRY
SSPTVSTTRRRLFENDSPSEGSTSGRIPPQPLVNAVPVQNVPGETVSVTPVPGQTLVTMATATVTANNGQTVT
IPVQGIANENGGITFFPVQVNVGGQAQAVAGSIQPLSAQALAGSLSSQQVTGTTLQVPGPVAIQQISPGGQQQ
NPGQPLTSSSIRPRKTSSLALFFRKVYYLAGVRLRDLCIKLDISDELRKKIWTCFEFSIIQCTELMMDRYLDQ
LLMCAIYVMAKVTKEDRSFQNIMRCYRTQPQARSQVYRSVLIKGKRRNSGSSESRSHQNSPTELNTDRASRDS
SPVMRSNSTLPVPQPSSAPPTPTRLTGASSDVEEEERGDLIQFYNNIYRKQIQAFAMKYSQANAQTDTPPLSP
YPFVRTGSPRRVQLSQSHPIYISPHNNEAMPSPREKIFYYFSNSPSKRLREINSMIRTGETPTKKRGILLDDG
SESPAKRICPENHSALLRRLQDVANDRGSQ*
>rs226649924_ENSMUSP00000034091_c.2653:ACC>A_p.D885del-fs
ENSMUST00000034091.8|Rbl2-201|ENSMUSG00000031666|+|chr8:g.91833825:ACC>A
MASGGNQSPPPPPAAAASSEEEEEDGDAADRAQPAGSPSHQIQQRFEELCSRLNMDEAARAEAWSSYRSMSES
YTLEGNDLHWLACALYVACRKSVPTVSKGTAEGNYVSLTRILRCSEQSLIEFFNKMKKWEDMANLPPHFRERT
ERLERNFTVSAVIFKKYEPIFQDIFKYPQEEQPRQQRGRKQRRQPCTTSEIFHFCWVLFIYAKGNFPMISDDL
VNSYHLLLCALDLVYGNALQCSNRKELVNPNFKGLSEDCHPKDSKASSDPPCVIEKLCSLHDGLVLEAKGIKE
HFWKPYIRKLFEKKLLKGKEENLTGFLEPGNFGESFKAVNKAYEEYVLAAGNLDERVFLGEDAEEEVGTLSRC
LSAASGTESAERTQMRDILQQHLDKSKALRVCTPLTGVRYVQENSPCVTPVSTAAHSLSRLHTMLSGLRNAPS
EKLERILRSCSRDPTQAIADRLKEMYEIYSQHFQPDENFSNCAKEIANKHFRFAEMLYYKVLESVIEQEQKRL
GDMDLSGVLEHDAFHRSLLACCLEVVAFSHKPPGNFPFIAEIFDVPHYHFYKVIEVFIRAEDGLCREVVKHLN
QIEEQILDHLAWKTKSPLWDRIRDNENRVPTCEEVMPPQNLERTDEIYIAGSPLTPRRVGEVRADAGGLGRSI
TSPTTLYDRYSSPTVSTTRRRLFENDSPSEGSTSGRIPPQPLVNAVPVQNVPGETVSVTPVPGQTLVTMATAT
VTANNGQTVTIPVQGIANENGGITFFPVQVNVGGQAQAVAGSIQPLSAQALAGSLSSQQVTGTTLQVPGPVAI
QQISPGGQQQNPGQPLTSSSIRPRKTSSLALFFRKVYYLAGVRLRDLCIKLDISDELRKKIWTCFEFSIIQCT
ELMMDRHLEAVDVCHLCDGKGHKRRQVLPEHHALLQNSATGPEPGVQKCLDKREKKKLWQQ
>rs226649924_ENSMUSP00000147579_c.2524:ACC>A_p.D842del-fs
ENSMUST00000211136.2|Rbl2-210|ENSMUSG00000031666|+|chr8:g.91833825:ACC>A
MASGGNQSPPPPPAAAASSEEEEEDGDAADRAQPAGSPSHQIQQRFEELCSRLNMDEAARAEAWSSYRSMSES
YTLEGNDLHWLACALYVACRKSVPTVSKGTAEGNYVSLTRILRCSEQSLIEFFNKMKKWEDMANLPPHFRERT
ERLERNFTVSAVIFKKYEPIFQDIFKYPQEEQPRQQRGRKQRRQPCTTSEIFHFCWVLFIYAKGLSEDCHPKD
SKASSDPPCVIEKLCSLHDGLVLEAKGIKEHFWKPYIRKLFEKKLLKGKEENLTGFLEPGNFGESFKAVNKAY
EEYVLAAGNLDERVFLGEDAEEEVGTLSRCLSAASGTESAERTQMRDILQQHLDKSKALRVCTPLTGVRYVQE
NSPCVTPVSTAAHSLSRLHTMLSGLRNAPSEKLERILRSCSRDPTQAIADRLKEMYEIYSQHFQPDENFSNCA
KEIANKHFRFAEMLYYKVLESVIEQEQKRLGDMDLSGVLEHDAFHRSLLACCLEVVAFSHKPPGNFPFIAEIF
DVPHYHFYKVIEVFIRAEDGLCREVVKHLNQIEEQILDHLAWKTKSPLWDRIRDNENRVPTCEEVMPPQNLER
TDEIYIAGSPLTPRRVGEVRADAGGLGRSITSPTTLYDRYSSPTVSTTRRRLFENDSPSEGSTSGRIPPQPLV
NAVPVQNVPGETVSVTPVPGQTLVTMATATVTANNGQTVTIPVQGIANENGGITFFPVQVNVGGQAQAVAGSI
QPLSAQALAGSLSSQQVTGTTLQVPGPVAIQQISPGGQQQNPGQPLTSSSIRPRKTSSLALFFRKVYYLAGVR

LRDLCIKLDISDELRKKIWTCFEFSIIQCTELMMDRHLEAVDVCHLCDGKGHKRRQVLPEHHALLQNSATGPE
PGVQKCLDKREKKKLWQQ
>rs226649924_ENSMUSP00000147327_c.2623:ACC>A_p.D875del-fs
ENSMUST00000209518.2|Rbl2-208|ENSMUSG00000031666|+|chr8:g.91833825:ACC>A
MASGGNQSPPPPPAAAASSEEEEEDGDAADRAQPAGSPSHQIQQRFEELCSRLNMDEAARAEAWSSYRSMSES
YTLEGNDLHWLACALYVACRKSVPTVSKGTAEGNYVSLTRILRCSEQSLIEFFNKMKKWEDMANLPPHFRERT
ERLERNFTVSAVIFKKYEPIFQDIFKYPQEEQPRQQRGRKQRRQPCTTSEIFHFCWVLFIYAKGNFPMISDDL
VNSYHLLLCALDLVYGNALQCSNRKELVNPNFKGLSEDCHPKDSKASSDPPCVIEKLCSLHDGLVLEAKGIKE
HFWKPYIRKLFEKKLLKGKEENLTGFLEPGNFGESFKAVNKAYEEYVLAAGNLDERVFLGEDAEEEVGTLSRC
LSAASGTESAERTQMRDILQQHLDKSKALRVCTPLTGVRYVQENSPCVTPVSTAAHSLSRLHTMLSGLRNAPS
EKLERILRSCSRDPTQAIADRLKEMYEIYSQHFQPDENFSNCAKEMLYYKVLESVIEQEQKRLGDMDLSGVLE
HDAFHRSLLACCLEVVAFSHKPPGNFPFIAEIFDVPHYHFYKVIEVFIRAEDGLCREVVKHLNQIEEQILDHL
AWKTKSPLWDRIRDNENRVPTCEEVMPPQNLERTDEIYIAGSPLTPRRVGEVRADAGGLGRSITSPTTLYDRY
SSPTVSTTRRRLFENDSPSEGSTSGRIPPQPLVNAVPVQNVPGETVSVTPVPGQTLVTMATATVTANNGQTVT
IPVQGIANENGGITFFPVQVNVGGQAQAVAGSIQPLSAQALAGSLSSQQVTGTTLQVPGPVAIQQISPGGQQQ
NPGQPLTSSSIRPRKTSSLALFFRKVYYLAGVRLRDLCIKLDISDELRKKIWTCFEFSIIQCTELMMDRHLEA
VDVCHLCDGKGHKRRQVLPEHHALLQNSATGPEPGVQKCLDKREKKKLWQQ
>rs51979111_ENSMUSP00000034091_c.2671:C>T_p.A891V
ENSMUST00000034091.8|Rbl2-201|ENSMUSG00000031666|+|chr8:g.91833843:C>T
MASGGNQSPPPPPAAAASSEEEEEDGDAADRAQPAGSPSHQIQQRFEELCSRLNMDEAARAEAWSSYRSMSES
YTLEGNDLHWLACALYVACRKSVPTVSKGTAEGNYVSLTRILRCSEQSLIEFFNKMKKWEDMANLPPHFRERT
ERLERNFTVSAVIFKKYEPIFQDIFKYPQEEQPRQQRGRKQRRQPCTTSEIFHFCWVLFIYAKGNFPMISDDL
VNSYHLLLCALDLVYGNALQCSNRKELVNPNFKGLSEDCHPKDSKASSDPPCVIEKLCSLHDGLVLEAKGIKE
HFWKPYIRKLFEKKLLKGKEENLTGFLEPGNFGESFKAVNKAYEEYVLAAGNLDERVFLGEDAEEEVGTLSRC
LSAASGTESAERTQMRDILQQHLDKSKALRVCTPLTGVRYVQENSPCVTPVSTAAHSLSRLHTMLSGLRNAPS
EKLERILRSCSRDPTQAIADRLKEMYEIYSQHFQPDENFSNCAKEIANKHFRFAEMLYYKVLESVIEQEQKRL
GDMDLSGVLEHDAFHRSLLACCLEVVAFSHKPPGNFPFIAEIFDVPHYHFYKVIEVFIRAEDGLCREVVKHLN
QIEEQILDHLAWKTKSPLWDRIRDNENRVPTCEEVMPPQNLERTDEIYIAGSPLTPRRVGEVRADAGGLGRSI
TSPTTLYDRYSSPTVSTTRRRLFENDSPSEGSTSGRIPPQPLVNAVPVQNVPGETVSVTPVPGQTLVTMATAT
VTANNGQTVTIPVQGIANENGGITFFPVQVNVGGQAQAVAGSIQPLSAQALAGSLSSQQVTGTTLQVPGPVAI
QQISPGGQQQNPGQPLTSSSIRPRKTSSLALFFRKVYYLAGVRLRDLCIKLDISDELRKKIWTCFEFSIIQCT
ELMMDRHLDQLLMCVIYVMAKVTKEDRSFQNIMRCYRTQPQARSQVYRSVLIKGKRRNSGSSESRSHQNSPTE
LNTDRASRDSSPVMRSNSTLPVPQPSSAPPTPTRLTGASSDVEEEERGDLIQFYNNIYRKQIQAFAMKYSQAN
AQTDTPPLSPYPFVRTGSPRRVQLSQSHPIYISPHNNEAMPSPREKIFYYFSNSPSKRLREINSMIRTGETPT
KKRGILLDDGSESPAKRICPENHSALLRRLQDVANDRGSQ*
>rs51979111_ENSMUSP00000147579_c.2542:C>T_p.A848V
ENSMUST00000211136.2|Rbl2-210|ENSMUSG00000031666|+|chr8:g.91833843:C>T
MASGGNQSPPPPPAAAASSEEEEEDGDAADRAQPAGSPSHQIQQRFEELCSRLNMDEAARAEAWSSYRSMSES
YTLEGNDLHWLACALYVACRKSVPTVSKGTAEGNYVSLTRILRCSEQSLIEFFNKMKKWEDMANLPPHFRERT
ERLERNFTVSAVIFKKYEPIFQDIFKYPQEEQPRQQRGRKQRRQPCTTSEIFHFCWVLFIYAKGLSEDCHPKD
SKASSDPPCVIEKLCSLHDGLVLEAKGIKEHFWKPYIRKLFEKKLLKGKEENLTGFLEPGNFGESFKAVNKAY
EEYVLAAGNLDERVFLGEDAEEEVGTLSRCLSAASGTESAERTQMRDILQQHLDKSKALRVCTPLTGVRYVQE
NSPCVTPVSTAAHSLSRLHTMLSGLRNAPSEKLERILRSCSRDPTQAIADRLKEMYEIYSQHFQPDENFSNCA
KEIANKHFRFAEMLYYKVLESVIEQEQKRLGDMDLSGVLEHDAFHRSLLACCLEVVAFSHKPPGNFPFIAEIF
DVPHYHFYKVIEVFIRAEDGLCREVVKHLNQIEEQILDHLAWKTKSPLWDRIRDNENRVPTCEEVMPPQNLER
TDEIYIAGSPLTPRRVGEVRADAGGLGRSITSPTTLYDRYSSPTVSTTRRRLFENDSPSEGSTSGRIPPQPLV
NAVPVQNVPGETVSVTPVPGQTLVTMATATVTANNGQTVTIPVQGIANENGGITFFPVQVNVGGQAQAVAGSI
QPLSAQALAGSLSSQQVTGTTLQVPGPVAIQQISPGGQQQNPGQPLTSSSIRPRKTSSLALFFRKVYYLAGVR
LRDLCIKLDISDELRKKIWTCFEFSIIQCTELMMDRHLDQLLMCVIYVMAKVTKEDRSFQNIMRCYRTQPQAR
SQVYRSVLIKGKRRNSGSSESRSHQNSPTELNTDRASRDSSPVMRSNSTLPVPQPSSAPPTPTRLTGASSDVE
EEERGDLIQFYNNIYRKQIQAFAMKYSQANAQTDTPPLSPYPFVRTGSPRRVQLSQSHPIYISPHNNEAMPSP
REKIFYYFSNSPSKRLREINSMIRTGETPTKKRGILLDDGSESPAKRICPENHSALLRRLQDVANDRGSQ*
>rs51979111_ENSMUSP00000147327_c.2641:C>T_p.A881V
ENSMUST00000209518.2|Rbl2-208|ENSMUSG00000031666|+|chr8:g.91833843:C>T
MASGGNQSPPPPPAAAASSEEEEEDGDAADRAQPAGSPSHQIQQRFEELCSRLNMDEAARAEAWSSYRSMSES
YTLEGNDLHWLACALYVACRKSVPTVSKGTAEGNYVSLTRILRCSEQSLIEFFNKMKKWEDMANLPPHFRERT
ERLERNFTVSAVIFKKYEPIFQDIFKYPQEEQPRQQRGRKQRRQPCTTSEIFHFCWVLFIYAKGNFPMISDDL
VNSYHLLLCALDLVYGNALQCSNRKELVNPNFKGLSEDCHPKDSKASSDPPCVIEKLCSLHDGLVLEAKGIKE

HFWKPYIRKLFEKKLLKGKEENLTGFLEPGNFGESFKAVNKAYEEYVLAAGNLDERVFLGEDAEEEVGTLSRC
LSAASGTESAERTQMRDILQQHLDKSKALRVCTPLTGVRYVQENSPCVTPVSTAAHSLSRLHTMLSGLRNAPS
EKLERILRSCSRDPTQAIADRLKEMYEIYSQHFQPDENFSNCAKEMLYYKVLESVIEQEQKRLGDMDLSGVLE
HDAFHRSLLACCLEVVAFSHKPPGNFPFIAEIFDVPHYHFYKVIEVFIRAEDGLCREVVKHLNQIEEQILDHL
AWKTKSPLWDRIRDNENRVPTCEEVMPPQNLERTDEIYIAGSPLTPRRVGEVRADAGGLGRSITSPTTLYDRY
SSPTVSTTRRRLFENDSPSEGSTSGRIPPQPLVNAVPVQNVPGETVSVTPVPGQTLVTMATATVTANNGQTVT
IPVQGIANENGGITFFPVQVNVGGQAQAVAGSIQPLSAQALAGSLSSQQVTGTTLQVPGPVAIQQISPGGQQQ
NPGQPLTSSSIRPRKTSSLALFFRKVYYLAGVRLRDLCIKLDISDELRKKIWTCFEFSIIQCTELMMDRHLDQ
LLMCVIYVMAKVTKEDRSFQNIMRCYRTQPQARSQVYRSVLIKGKRRNSGSSESRSHQNSPTELNTDRASRDS
SPVMRSNSTLPVPQPSSAPPTPTRLTGASSDVEEEERGDLIQFYNNIYRKQIQAFAMKYSQANAQTDTPPLSP
YPFVRTGSPRRVQLSQSHPIYISPHNNEAMPSPREKIFYYFSNSPSKRLREINSMIRTGETPTKKRGILLDDG
SESPAKRICPENHSALLRRLQDVANDRGSQ*
>rs32595399_ENSMUSP00000034091_c.2813:T>A_p.S938R
ENSMUST00000034091.8|Rbl2-201|ENSMUSG00000031666|+|chr8:g.91840663:T>A
MASGGNQSPPPPPAAAASSEEEEEDGDAADRAQPAGSPSHQIQQRFEELCSRLNMDEAARAEAWSSYRSMSES
YTLEGNDLHWLACALYVACRKSVPTVSKGTAEGNYVSLTRILRCSEQSLIEFFNKMKKWEDMANLPPHFRERT
ERLERNFTVSAVIFKKYEPIFQDIFKYPQEEQPRQQRGRKQRRQPCTTSEIFHFCWVLFIYAKGNFPMISDDL
VNSYHLLLCALDLVYGNALQCSNRKELVNPNFKGLSEDCHPKDSKASSDPPCVIEKLCSLHDGLVLEAKGIKE
HFWKPYIRKLFEKKLLKGKEENLTGFLEPGNFGESFKAVNKAYEEYVLAAGNLDERVFLGEDAEEEVGTLSRC
LSAASGTESAERTQMRDILQQHLDKSKALRVCTPLTGVRYVQENSPCVTPVSTAAHSLSRLHTMLSGLRNAPS
EKLERILRSCSRDPTQAIADRLKEMYEIYSQHFQPDENFSNCAKEIANKHFRFAEMLYYKVLESVIEQEQKRL
GDMDLSGVLEHDAFHRSLLACCLEVVAFSHKPPGNFPFIAEIFDVPHYHFYKVIEVFIRAEDGLCREVVKHLN
QIEEQILDHLAWKTKSPLWDRIRDNENRVPTCEEVMPPQNLERTDEIYIAGSPLTPRRVGEVRADAGGLGRSI
TSPTTLYDRYSSPTVSTTRRRLFENDSPSEGSTSGRIPPQPLVNAVPVQNVPGETVSVTPVPGQTLVTMATAT
VTANNGQTVTIPVQGIANENGGITFFPVQVNVGGQAQAVAGSIQPLSAQALAGSLSSQQVTGTTLQVPGPVAI
QQISPGGQQQNPGQPLTSSSIRPRKTSSLALFFRKVYYLAGVRLRDLCIKLDISDELRKKIWTCFEFSIIQCT
ELMMDRHLDQLLMCAIYVMAKVTKEDRSFQNIMRCYRTQPQARSQVYRSVLIKGKRRNSGSRESRSHQNSPTE
LNTDRASRDSSPVMRSNSTLPVPQPSSAPPTPTRLTGASSDVEEEERGDLIQFYNNIYRKQIQAFAMKYSQAN
AQTDTPPLSPYPFVRTGSPRRVQLSQSHPIYISPHNNEAMPSPREKIFYYFSNSPSKRLREINSMIRTGETPT
KKRGILLDDGSESPAKRICPENHSALLRRLQDVANDRGSQ*
>rs32595399_ENSMUSP00000147579_c.2684:T>A_p.S895R
ENSMUST00000211136.2|Rbl2-210|ENSMUSG00000031666|+|chr8:g.91840663:T>A
MASGGNQSPPPPPAAAASSEEEEEDGDAADRAQPAGSPSHQIQQRFEELCSRLNMDEAARAEAWSSYRSMSES
YTLEGNDLHWLACALYVACRKSVPTVSKGTAEGNYVSLTRILRCSEQSLIEFFNKMKKWEDMANLPPHFRERT
ERLERNFTVSAVIFKKYEPIFQDIFKYPQEEQPRQQRGRKQRRQPCTTSEIFHFCWVLFIYAKGLSEDCHPKD
SKASSDPPCVIEKLCSLHDGLVLEAKGIKEHFWKPYIRKLFEKKLLKGKEENLTGFLEPGNFGESFKAVNKAY
EEYVLAAGNLDERVFLGEDAEEEVGTLSRCLSAASGTESAERTQMRDILQQHLDKSKALRVCTPLTGVRYVQE
NSPCVTPVSTAAHSLSRLHTMLSGLRNAPSEKLERILRSCSRDPTQAIADRLKEMYEIYSQHFQPDENFSNCA
KEIANKHFRFAEMLYYKVLESVIEQEQKRLGDMDLSGVLEHDAFHRSLLACCLEVVAFSHKPPGNFPFIAEIF
DVPHYHFYKVIEVFIRAEDGLCREVVKHLNQIEEQILDHLAWKTKSPLWDRIRDNENRVPTCEEVMPPQNLER
TDEIYIAGSPLTPRRVGEVRADAGGLGRSITSPTTLYDRYSSPTVSTTRRRLFENDSPSEGSTSGRIPPQPLV
NAVPVQNVPGETVSVTPVPGQTLVTMATATVTANNGQTVTIPVQGIANENGGITFFPVQVNVGGQAQAVAGSI
QPLSAQALAGSLSSQQVTGTTLQVPGPVAIQQISPGGQQQNPGQPLTSSSIRPRKTSSLALFFRKVYYLAGVR
LRDLCIKLDISDELRKKIWTCFEFSIIQCTELMMDRHLDQLLMCAIYVMAKVTKEDRSFQNIMRCYRTQPQAR
SQVYRSVLIKGKRRNSGSRESRSHQNSPTELNTDRASRDSSPVMRSNSTLPVPQPSSAPPTPTRLTGASSDVE
EEERGDLIQFYNNIYRKQIQAFAMKYSQANAQTDTPPLSPYPFVRTGSPRRVQLSQSHPIYISPHNNEAMPSP
REKIFYYFSNSPSKRLREINSMIRTGETPTKKRGILLDDGSESPAKRICPENHSALLRRLQDVANDRGSQ*
>rs32595399_ENSMUSP00000147327_c.2783:T>A_p.S928R
ENSMUST00000209518.2|Rbl2-208|ENSMUSG00000031666|+|chr8:g.91840663:T>A
MASGGNQSPPPPPAAAASSEEEEEDGDAADRAQPAGSPSHQIQQRFEELCSRLNMDEAARAEAWSSYRSMSES
YTLEGNDLHWLACALYVACRKSVPTVSKGTAEGNYVSLTRILRCSEQSLIEFFNKMKKWEDMANLPPHFRERT
ERLERNFTVSAVIFKKYEPIFQDIFKYPQEEQPRQQRGRKQRRQPCTTSEIFHFCWVLFIYAKGNFPMISDDL
VNSYHLLLCALDLVYGNALQCSNRKELVNPNFKGLSEDCHPKDSKASSDPPCVIEKLCSLHDGLVLEAKGIKE
HFWKPYIRKLFEKKLLKGKEENLTGFLEPGNFGESFKAVNKAYEEYVLAAGNLDERVFLGEDAEEEVGTLSRC
LSAASGTESAERTQMRDILQQHLDKSKALRVCTPLTGVRYVQENSPCVTPVSTAAHSLSRLHTMLSGLRNAPS
EKLERILRSCSRDPTQAIADRLKEMYEIYSQHFQPDENFSNCAKEMLYYKVLESVIEQEQKRLGDMDLSGVLE
HDAFHRSLLACCLEVVAFSHKPPGNFPFIAEIFDVPHYHFYKVIEVFIRAEDGLCREVVKHLNQIEEQILDHL
AWKTKSPLWDRIRDNENRVPTCEEVMPPQNLERTDEIYIAGSPLTPRRVGEVRADAGGLGRSITSPTTLYDRY

SSPTVSTTRRRLFENDSPSEGSTSGRIPPQPLVNAVPVQNVPGETVSVTPVPGQTLVTMATATVTANNGQTVT
IPVQGIANENGGITFFPVQVNVGGQAQAVAGSIQPLSAQALAGSLSSQQVTGTTLQVPGPVAIQQISPGGQQQ
NPGQPLTSSSIRPRKTSSLALFFRKVYYLAGVRLRDLCIKLDISDELRKKIWTCFEFSIIQCTELMMDRHLDQ
LLMCAIYVMAKVTKEDRSFQNIMRCYRTQPQARSQVYRSVLIKGKRRNSGSRESRSHQNSPTELNTDRASRDS
SPVMRSNSTLPVPQPSSAPPTPTRLTGASSDVEEEERGDLIQFYNNIYRKQIQAFAMKYSQANAQTDTPPLSP
YPFVRTGSPRRVQLSQSHPIYISPHNNEAMPSPREKIFYYFSNSPSKRLREINSMIRTGETPTKKRGILLDDG
SESPAKRICPENHSALLRRLQDVANDRGSQ*
>rs48045978_ENSMUSP00000042702_c.3409:T>C_p.M1137T
ENSMUST00000047783.14|Rpgrip1l-201|ENSMUSG00000033282|-
|chr8:g.91959489:A>G
MSGPSDETAGDLPVKDTGLNLFGVGGLQETSTARTVKTRQAVSRVSREELEDRFLRLHDENILLKQHARKQED
KIKRMATKLIRLVNDKKRYERVGGGPKRLGRDVEMEEMIEQLQEKVHELERQNEVLKNRLISAKQQLQVQGHR
QTSYSRVQARVNTGRRRASASAGSQECPGKGLRFQNVDEAETVQPTLTKYSNSLLEEARGEIRNLENVIQSQR
GQIEELEHLAEILKTQLKRKENEIELSLLQLREQQATDQRSNIRDNVETIKLHKQLVEKSNALSVIEGKFIQL
QEKQRTLRISHDALMANGDELNKQLKEQRLKCCSLEKQLHSVRFSERRVEELQDRINDLEKERELLKENYDKL
YNSAFSAAHEEQWKLKEQQMKVQIAQLETALKSDLTDKTEVLDKLKTERDQNEKLVQENRDLQLQCLQQKQRL
HELQSRLKFFNQESDINADDLSEALLLIKAQKEQKNGDLSFLEKVDSKINKDLDRSMKELQATHAETVQELEK
TRNMLIMQHKINKDYQMEVETVTQKMENLQQDYELKVEQYVHLLDIRAARIQKLEAQLKDIAYGTKQYKFKPE
IMPDDSVDEFDETIHLERGENLFEIHINKVTFSSEVLRASGDKELVTFCTYAFYDFELQTTPIVRGLYPEYNF
TSQYLVHVNDLFLQYIQKNTVTLELHQAHSTDYETIAACQLRFHEILEKSGRIFCTTSLVGTKGDIPNFGTVE
YWFRLRVPMDQAIRLYRERAKALGYITSNFKKPEKMQLSSQQAATTAQISPAESTDGNLNELHVTVKCCTGLQ
SRASYLQPHAYVVYKFFDFPDHDTAIVPSSNDPQFDDHMCFPVPMNMDLDRYLKSESLSFYVFDDSDTQENIY
MGKVNVPLISLAHDKCISGIFELMDKEKHAAGTIQVILKWKFTYLPPSGSITTEDLGKFVCREEPEAVQRLPP
KSSDVTSVVAPKPKPRQRLTFVDKKVSFADTISHPSPETSPPPKDIKDSSPEVGPKPENGLSAVAYPSKESGV
AKVEENVGEMQQGKEDDISFLSEGQLASGSVASSEDETEITEELEPEDEDRSASDSDDCIIPSSVSTNTKQPS
EEIRIEIIALNLNDSQITREDTIQRLFIECRFYSLPAEETPTSLPKPQSGQWVYYNYSNVIYLDKENNPAVRD
ILKAILQRRELPHRSVRFTVVSDPPEDEQDLECEDIGVAHVDLADLFQKGRDIIEQDIDVLDARTDGGTIGKL
KVTVEALHALRSVYEQNRKDLEA*
>rs51575997_ENSMUSP00000042702_c.3292:T>G_p.I1098S
ENSMUST00000047783.14|Rpgrip1l-201|ENSMUSG00000033282|-
|chr8:g.91959606:A>C
MSGPSDETAGDLPVKDTGLNLFGVGGLQETSTARTVKTRQAVSRVSREELEDRFLRLHDENILLKQHARKQED
KIKRMATKLIRLVNDKKRYERVGGGPKRLGRDVEMEEMIEQLQEKVHELERQNEVLKNRLISAKQQLQVQGHR
QTSYSRVQARVNTGRRRASASAGSQECPGKGLRFQNVDEAETVQPTLTKYSNSLLEEARGEIRNLENVIQSQR
GQIEELEHLAEILKTQLKRKENEIELSLLQLREQQATDQRSNIRDNVETIKLHKQLVEKSNALSVIEGKFIQL
QEKQRTLRISHDALMANGDELNKQLKEQRLKCCSLEKQLHSVRFSERRVEELQDRINDLEKERELLKENYDKL
YNSAFSAAHEEQWKLKEQQMKVQIAQLETALKSDLTDKTEVLDKLKTERDQNEKLVQENRDLQLQCLQQKQRL
HELQSRLKFFNQESDINADDLSEALLLIKAQKEQKNGDLSFLEKVDSKINKDLDRSMKELQATHAETVQELEK
TRNMLIMQHKINKDYQMEVETVTQKMENLQQDYELKVEQYVHLLDIRAARIQKLEAQLKDIAYGTKQYKFKPE
IMPDDSVDEFDETIHLERGENLFEIHINKVTFSSEVLRASGDKELVTFCTYAFYDFELQTTPIVRGLYPEYNF
TSQYLVHVNDLFLQYIQKNTVTLELHQAHSTDYETIAACQLRFHEILEKSGRIFCTTSLVGTKGDIPNFGTVE
YWFRLRVPMDQAIRLYRERAKALGYITSNFKKPEKMQLSSQQAATTAQISPAESTDGNLNELHVTVKCCTGLQ
SRASYLQPHAYVVYKFFDFPDHDTAIVPSSNDPQFDDHMCFPVPMNMDLDRYLKSESLSFYVFDDSDTQENIY
MGKVNVPLISLAHDKCISGIFELMDKEKHAAGTIQVILKWKFTYLPPSGSITTEDLGKFVCREEPEAVQRLPP
KSSDVTSVVAPKPKPRQRLTFVDKKVSFADTISHPSPETSPPPKDIKDSSPEVGPKPENGLSAVAYPSKESGV
AKVEENVGEMQQGKEDDISFLSEGQLASGSVASSEDETEITEELEPEDEDRSASDSDDCIIPSSVSTNTKQPS
EESRIEIIALNLNDSQITREDTIQRLFIECRFYSLPAEETPMSLPKPQSGQWVYYNYSNVIYLDKENNPAVRD
ILKAILQRRELPHRSVRFTVVSDPPEDEQDLECEDIGVAHVDLADLFQKGRDIIEQDIDVLDARTDGGTIGKL
KVTVEALHALRSVYEQNRKDLEA*
>rs248295175_ENSMUSP00000042702_c.2974:C>T_p.P992L
ENSMUST00000047783.14|Rpgrip1l-201|ENSMUSG00000033282|-
|chr8:g.91979189:G>A
MSGPSDETAGDLPVKDTGLNLFGVGGLQETSTARTVKTRQAVSRVSREELEDRFLRLHDENILLKQHARKQED
KIKRMATKLIRLVNDKKRYERVGGGPKRLGRDVEMEEMIEQLQEKVHELERQNEVLKNRLISAKQQLQVQGHR
QTSYSRVQARVNTGRRRASASAGSQECPGKGLRFQNVDEAETVQPTLTKYSNSLLEEARGEIRNLENVIQSQR
GQIEELEHLAEILKTQLKRKENEIELSLLQLREQQATDQRSNIRDNVETIKLHKQLVEKSNALSVIEGKFIQL
QEKQRTLRISHDALMANGDELNKQLKEQRLKCCSLEKQLHSVRFSERRVEELQDRINDLEKERELLKENYDKL
YNSAFSAAHEEQWKLKEQQMKVQIAQLETALKSDLTDKTEVLDKLKTERDQNEKLVQENRDLQLQCLQQKQRL

HELQSRLKFFNQESDINADDLSEALLLIKAQKEQKNGDLSFLEKVDSKINKDLDRSMKELQATHAETVQELEK
TRNMLIMQHKINKDYQMEVETVTQKMENLQQDYELKVEQYVHLLDIRAARIQKLEAQLKDIAYGTKQYKFKPE
IMPDDSVDEFDETIHLERGENLFEIHINKVTFSSEVLRASGDKELVTFCTYAFYDFELQTTPIVRGLYPEYNF
TSQYLVHVNDLFLQYIQKNTVTLELHQAHSTDYETIAACQLRFHEILEKSGRIFCTTSLVGTKGDIPNFGTVE
YWFRLRVPMDQAIRLYRERAKALGYITSNFKKPEKMQLSSQQAATTAQISPAESTDGNLNELHVTVKCCTGLQ
SRASYLQPHAYVVYKFFDFPDHDTAIVPSSNDPQFDDHMCFPVPMNMDLDRYLKSESLSFYVFDDSDTQENIY
MGKVNVPLISLAHDKCISGIFELMDKEKHAAGTIQVILKWKFTYLPPSGSITTEDLGKFVCREEPEAVQRLPP
KSSDVTSVVAPKPKPRQRLTFVDKKVSFADTISHPSPETSPPLKDIKDSSPEVGPKPENGLSAVAYPSKESGV
AKVEENVGEMQQGKEDDISFLSEGQLASGSVASSEDETEITEELEPEDEDRSASDSDDCIIPSSVSTNTKQPS
EEIRIEIIALNLNDSQITREDTIQRLFIECRFYSLPAEETPMSLPKPQSGQWVYYNYSNVIYLDKENNPAVRD
ILKAILQRRELPHRSVRFTVVSDPPEDEQDLECEDIGVAHVDLADLFQKGRDIIEQDIDVLDARTDGGTIGKL
KVTVEALHALRSVYEQNRKDLEA*
>rs248295175_ENSMUSP00000147300_c.637:C>T_p.P213L
ENSMUST00000209616.2|Rpgrip1l-206|ENSMUSG00000033282|-
|chr8:g.91979189:G>A
GENLFEIHINKVTFSSEVLRASGDKELVTFCTYAFYDFELQTTPIVRGLYPEYNFTSQYLVHVNDLFLQYIQK
NTVTLELHQAHSTDYETIAACQLRFHEILEKSGRIFCTTSLVGIFELMDKEKHAAGTIQVILKWKFTYLPPSG
SITTEDLGKFVCREEPEAVQRLPPKSSDVTSVVAPKPKPRQRLTFVDKKVSFADTISHPSPETSPPLKDIKDS
SPEVGPKPENGLSAVAYPSKESGVAKVEENVGEM
>rs33211992_ENSMUSP00000042702_c.2043:C>T_p.L682F
ENSMUST00000047783.14|Rpgrip1l-201|ENSMUSG00000033282|-
|chr8:g.91996835:G>A
MSGPSDETAGDLPVKDTGLNLFGVGGLQETSTARTVKTRQAVSRVSREELEDRFLRLHDENILLKQHARKQED
KIKRMATKLIRLVNDKKRYERVGGGPKRLGRDVEMEEMIEQLQEKVHELERQNEVLKNRLISAKQQLQVQGHR
QTSYSRVQARVNTGRRRASASAGSQECPGKGLRFQNVDEAETVQPTLTKYSNSLLEEARGEIRNLENVIQSQR
GQIEELEHLAEILKTQLKRKENEIELSLLQLREQQATDQRSNIRDNVETIKLHKQLVEKSNALSVIEGKFIQL
QEKQRTLRISHDALMANGDELNKQLKEQRLKCCSLEKQLHSVRFSERRVEELQDRINDLEKERELLKENYDKL
YNSAFSAAHEEQWKLKEQQMKVQIAQLETALKSDLTDKTEVLDKLKTERDQNEKLVQENRDLQLQCLQQKQRL
HELQSRLKFFNQESDINADDLSEALLLIKAQKEQKNGDLSFLEKVDSKINKDLDRSMKELQATHAETVQELEK
TRNMLIMQHKINKDYQMEVETVTQKMENLQQDYELKVEQYVHLLDIRAARIQKLEAQLKDIAYGTKQYKFKPE
IMPDDSVDEFDETIHLERGENLFEIHINKVTFSSEVLRASGDKELVTFCTYAFYDFELQTTPIVRGLYPEYNF
TSQYLVHVNDLFLQYIQKNTVTLEFHQAHSTDYETIAACQLRFHEILEKSGRIFCTTSLVGTKGDIPNFGTVE
YWFRLRVPMDQAIRLYRERAKALGYITSNFKKPEKMQLSSQQAATTAQISPAESTDGNLNELHVTVKCCTGLQ
SRASYLQPHAYVVYKFFDFPDHDTAIVPSSNDPQFDDHMCFPVPMNMDLDRYLKSESLSFYVFDDSDTQENIY
MGKVNVPLISLAHDKCISGIFELMDKEKHAAGTIQVILKWKFTYLPPSGSITTEDLGKFVCREEPEAVQRLPP
KSSDVTSVVAPKPKPRQRLTFVDKKVSFADTISHPSPETSPPPKDIKDSSPEVGPKPENGLSAVAYPSKESGV
AKVEENVGEMQQGKEDDISFLSEGQLASGSVASSEDETEITEELEPEDEDRSASDSDDCIIPSSVSTNTKQPS
EEIRIEIIALNLNDSQITREDTIQRLFIECRFYSLPAEETPMSLPKPQSGQWVYYNYSNVIYLDKENNPAVRD
ILKAILQRRELPHRSVRFTVVSDPPEDEQDLECEDIGVAHVDLADLFQKGRDIIEQDIDVLDARTDGGTIGKL
KVTVEALHALRSVYEQNRKDLEA*
>rs33211992_ENSMUSP00000147300_c.237:C>T_p.L80F
ENSMUST00000209616.2|Rpgrip1l-206|ENSMUSG00000033282|-
|chr8:g.91996835:G>A
GENLFEIHINKVTFSSEVLRASGDKELVTFCTYAFYDFELQTTPIVRGLYPEYNFTSQYLVHVNDLFLQYIQK
NTVTLEFHQAHSTDYETIAACQLRFHEILEKSGRIFCTTSLVGIFELMDKEKHAAGTIQVILKWKFTYLPPSG
SITTEDLGKFVCREEPEAVQRLPPKSSDVTSVVAPKPKPRQRLTFVDKKVSFADTISHPSPETSPPPKDIKDS
SPEVGPKPENGLSAVAYPSKESGVAKVEENVGEM
>rs47127351_ENSMUSP00000068380_c.411:A>C_p.T138P
ENSMUST00000069718.15|Fto-201|ENSMUSG00000055932|+|chr8:g.92136139:A>C
MKRVQTAEEREREAKKLRLLEELEDTWLPYLTPKDDEFYQQWQLKYPKLVFREAGSIPEELHKEVPEAFLTLH
KHGCLFRDVVRIQGKDVLTPVSRILIGDPGCTYKYLNTRLFTVPWPVKGCTVKYTEAEIAAACQPFLKLNDYL
QVETIQALEELAVREKANEDAVPLCMAEFPRAGVGPSCDDEVDLKSRAAYNVTLLNFMDPQKMPYLKEEPYFG
MGKMAVSWHHDENLVDRSAVAVYSYSCEGSEDESEDESSFEGRDPDTWHVGFKISWDIETPGLTIPLHQGDCY
FMLDDLNATHQHCVLAGSQPRFSSTHRVAECSTGTLDYILERCQLALQNVLNDSDDGDVSLKSFDPAVLKQGE
EIHNEVEFEWLRQFWFQGNRYKLCTDWWCEPMTHLEGLWKKMESMTNAVLREVKREGLPVEQRSEILSAILVP
LTVRQNLRKEWHARCQSRVVRTLPVQQKPDCRPYWEKDDPSMPLPFDLTDVVSELRGQLLEARS*
>rs47127351_ENSMUSP00000147563_c.411:A>C_p.T138P
ENSMUST00000125471.8|Fto-202|ENSMUSG00000055932|+|chr8:g.92136139:A>C

MKRVQTAEEREREAKKLRLLEELEDTWLPYLTPKDDEFYQQWQLKYPKLVFREAGSIPEELHKEVPEAFLTLH
KHGCLFRDVVRIQGKDVLTPVSRILIGDPGCTYKYLNTRLFTVPWPVKGCTVKYTEAEIAAACQPFLKLNDYL
QVETIQALEELAVREKANEDAVPLCMAEFPRAGVGPSCDDEVDLKSRAAYNVTLLNFMDPQKMPYLKEEPYFG
MGKMAVSWHHDENLVDRSAVAVYSYSCEGSEDESEDESSFEGRDPDTWHVGFKISWDIETPGLTIPLHQGDCY
FMLGNVGSLRVGHLWGFEIHFWIL*
>rs47127351_ENSMUSP00000147548_c.411:A>C_p.T138P
ENSMUST00000128081.8|Fto-203|ENSMUSG00000055932|+|chr8:g.92136139:A>C
MKRVQTAEEREREAKKLRLLEELEDTWLPYLTPKDDEFYQQWQLKYPKLVFREAGSIPEELHKEVPEAFLTLH
KHGCLFRDVVRIQGKDVLTPVSRILIGDPGCTYKYLNTRLFTVPWPVKGCTVKYTEAEIAAACQPFLKLNDYL
QVETIQALEELAVREKANEDAVPLCMAEFPRAGVGPSCDDEVDLKSRAAYNVTLLNFMDPQKMPYLKEEPYFG
MGKMAVSWHHDENLVDRSAVAVYSYSCEGSEDESEDESSFEGRDPDTWHVGFKISWDIETPGLTIPLHQGDCY
FMLDDLNATHQHCVLAGSQPRFSSTHRVAECSTGTLDYILERCQLALQNVLNDSDDGDVSLKSFDPAVLKQGE
EIHNEVEFEWLRQFWFQGNRYKLCTDWWCEPMTHLEGLWKKMESMTNAVLREVKREGLPVEQRSEILSAILVP
LTVRQNLRKEWHARFVLLRGGVWCPCPSSARPAQRTKVEDILS*
>rs47127351_ENSMUSP00000147603_c.411:A>C_p.T138P
ENSMUST00000136802.8|Fto-204|ENSMUSG00000055932|+|chr8:g.92136139:A>C
MKRVQTAEEREREAKKLRLLEELEDTWLPYLTPKDDEFYQQWQLKYPKLVFREAGSIPEELHKEVPEAFLTLH
KHGCLFRDVVRIQGKDVLTPVSRILIGDPGCTYKYLNTRLFTVPWPVKGCTVKYTEAEIAAACQPFLKLNDYL
QVETIQALEELAVREKANEDAVPLCMAEFPRAGVGPSCDDEVDLKSRAAYNVTLLNFMDPQKMPYLKEEPYFG
MGKMAVSWHHDENLVDRSAVAVYSYSCEGSEDESEDESSFEGRDPDTWHVGFKISWDIETPGLTIPLHQGDCY
FMLDDLNATHQHCVLAGSQPRFSSTHRVAECSTGTLDYILERCQLALQNVLNDSDDGDVSLKSFDPAVLKQGE
EIHNEVEFEWLRQFWFQGNRYKLCTDWWCEPMTHLEGLWKKMESMVSA*
>rs47127351_ENSMUSP00000127680_c.405:A>C_p.T136P
ENSMUST00000166548.9|Fto-207|ENSMUSG00000055932|+|chr8:g.92136139:A>C
MCSVKPYRRTLAQKLRLLEELEDTWLPYLTPKDDEFYQQWQLKYPKLVFREAGSIPEELHKEVPEAFLTLHKH
GCLFRDVVRIQGKDVLTPVSRILIGDPGCTYKYLNTRLFTVPWPVKGCTVKYTEAEIAAACQPFLKLNDYLQV
ETIQALEELAVREKANEDAVPLCMAEFPRAGVGPSCDDEVDLKSRAAYNVTLLNFMDPQKMPYLKEEPYFGMG
KMAVSWHHDENLVDRSAVAVYSYSCEDDLNATHQHCVLAGSQPRFSSTHRV
>rs47850182_ENSMUSP00000091002_c.1252:T>A_p.F418Y
ENSMUST00000093312.6|Irx3-201|ENSMUSG00000031734|-|chr8:g.92526450:A>T
MSFPQLGYQYIRPLYPPERPGAAGGGGGGSSAGGRSGPGAGASELAASGSLSNVLSSVYGAPYAAAAAAAAA
QGYGAFLPYATELPIFPQLGAQYELKDSPGVQHPATAAAFPHPHPAFYPYGQYQFGDPSRPKNATRESTSTLK
AWLNEHRKNPYPTKGEKIMLAIITKMTLTQVSTWFANARRRLKKENKMTWAPRSRTDEEGNAYGSEREEEDEE
EDEEESKRELEMEEEELAGEEEDTGGEGLADDDEDEEIDLENLDSAAAGSELTLAGAAHRNGDFGLGPISDCK
TSDSDDSSEGLEDRPLSVLSLAPPPPPVARAPASPPSPPSSLDPCAPAPAPSSALQKPKIWSLAETATSPDNP
RRSPPGAGGSPPGAAVAPPTLQLSPAAAAAAAAAHRLVSAPLGKFPAWTNRPYPGPPAGPRPHPLSMLGSAPQ
HLLGLPGAAGHPAAAAAAYARPAEPESGTGDKKNIAVVPWKWRKSYSRQLSSRCQGGHRTIWTLLWSYQLSPR
LNFSTIFKSLY*
>rs47850182_ENSMUSP00000135488_c.1252:T>A_p.F418Y
ENSMUST00000175795.4|Irx3-202|ENSMUSG00000031734|-|chr8:g.92526450:A>T
MSFPQLGYQYIRPLYPPERPGAAGGGGGGSSAGGRSGPGAGASELAASGSLSNVLSSVYGAPYAAAAAAAAA
QGYGAFLPYATELPIFPQLGAQYELKDSPGVQHPATAAAFPHPHPAFYPYGQYQFGDPSRPKNATRESTSTLK
AWLNEHRKNPYPTKGEKIMLAIITKMTLTQVSTWFANARRRLKKENKMTWAPRSRTDEEGNAYGSEREEEDEE
EDEEESKRELEMEEEELAGEEEDTGGEGLADDDEDEEIDLENLDSAAAGSELTLAGAAHRNGDFGLGPISDCK
TSDSDDSSEGLEDRPLSVLSLAPPPPPVARAPASPPSPPSSLDPCAPAPAPSSALQKPKIWSLAETATSPDNP
RRSPPGAGGSPPGAAVAPPTLQLSPAAAAAAAAAHRLVSAPLGKFPAWTNRPYPGPPAGPRPHPLSMLGSAPQ
HLLGLPGAAGHPAAAAAAYARPAEPESGTDRCSALEVEKKLLKTAFQPVPRRPQNHLDAALVLSALSSS*
>rs50127074_ENSMUSP00000091002_c.916:G>A_p.R306Q
ENSMUST00000093312.6|Irx3-201|ENSMUSG00000031734|-|chr8:g.92526786:C>T
MSFPQLGYQYIRPLYPPERPGAAGGGGGGSSAGGRSGPGAGASELAASGSLSNVLSSVYGAPYAAAAAAAAA
QGYGAFLPYATELPIFPQLGAQYELKDSPGVQHPATAAAFPHPHPAFYPYGQYQFGDPSRPKNATRESTSTLK
AWLNEHRKNPYPTKGEKIMLAIITKMTLTQVSTWFANARRRLKKENKMTWAPRSRTDEEGNAYGSEREEEDEE
EDEEESKRELEMEEEELAGEEEDTGGEGLADDDEDEEIDLENLDSAAAGSELTLAGAAHRNGDFGLGPISDCK
TSDSDDSSEGLEDQPLSVLSLAPPPPPVARAPASPPSPPSSLDPCAPAPAPSSALQKPKIWSLAETATSPDNP
RRSPPGAGGSPPGAAVAPPTLQLSPAAAAAAAAAHRLVSAPLGKFPAWTNRPFPGPPAGPRPHPLSMLGSAPQ
HLLGLPGAAGHPAAAAAAYARPAEPESGTGDKKNIAVVPWKWRKSYSRQLSSRCQGGHRTIWTLLWSYQLSPR
LNFSTIFKSLY*

>rs50127074_ENSMUSP00000135488_c.916:G>A_p.R306Q
ENSMUST00000175795.4|Irx3-202|ENSMUSG00000031734|-|chr8:g.92526786:C>T
MSFPQLGYQYIRPLYPPERPGAAGGGGGGSSAGGRSGPGAGASELAASGSLSNVLSSVYGAPYAAAAAAAAA
QGYGAFLPYATELPIFPQLGAQYELKDSPGVQHPATAAAFPHPHPAFYPYGQYQFGDPSRPKNATRESTSTLK
AWLNEHRKNPYPTKGEKIMLAIITKMTLTQVSTWFANARRRLKKENKMTWAPRSRTDEEGNAYGSEREEEDEE
EDEEESKRELEMEEEELAGEEEDTGGEGLADDDEDEEIDLENLDSAAAGSELTLAGAAHRNGDFGLGPISDCK
TSDSDDSSEGLEDQPLSVLSLAPPPPPVARAPASPPSPPSSLDPCAPAPAPSSALQKPKIWSLAETATSPDNP
RRSPPGAGGSPPGAAVAPPTLQLSPAAAAAAAAAHRLVSAPLGKFPAWTNRPFPGPPAGPRPHPLSMLGSAPQ
HLLGLPGAAGHPAAAAAAYARPAEPESGTDRCSALEVEKKLLKTAFQPVPRRPQNHLDAALVLSALSSS*
>rs249655209_ENSMUSP00000091002_c.754:A>G_p.D252G
ENSMUST00000093312.6|Irx3-201|ENSMUSG00000031734|-|chr8:g.92526948:T>C
MSFPQLGYQYIRPLYPPERPGAAGGGGGGSSAGGRSGPGAGASELAASGSLSNVLSSVYGAPYAAAAAAAAA
QGYGAFLPYATELPIFPQLGAQYELKDSPGVQHPATAAAFPHPHPAFYPYGQYQFGDPSRPKNATRESTSTLK
AWLNEHRKNPYPTKGEKIMLAIITKMTLTQVSTWFANARRRLKKENKMTWAPRSRTDEEGNAYGSEREEEDEE
EDEEESKRELEMEEEELAGEEEDTGGEGLADDGEDEEIDLENLDSAAAGSELTLAGAAHRNGDFGLGPISDCK
TSDSDDSSEGLEDRPLSVLSLAPPPPPVARAPASPPSPPSSLDPCAPAPAPSSALQKPKIWSLAETATSPDNP
RRSPPGAGGSPPGAAVAPPTLQLSPAAAAAAAAAHRLVSAPLGKFPAWTNRPFPGPPAGPRPHPLSMLGSAPQ
HLLGLPGAAGHPAAAAAAYARPAEPESGTGDKKNIAVVPWKWRKSYSRQLSSRCQGGHRTIWTLLWSYQLSPR
LNFSTIFKSLY*
>rs249655209_ENSMUSP00000135488_c.754:A>G_p.D252G
ENSMUST00000175795.4|Irx3-202|ENSMUSG00000031734|-|chr8:g.92526948:T>C
MSFPQLGYQYIRPLYPPERPGAAGGGGGGSSAGGRSGPGAGASELAASGSLSNVLSSVYGAPYAAAAAAAAA
QGYGAFLPYATELPIFPQLGAQYELKDSPGVQHPATAAAFPHPHPAFYPYGQYQFGDPSRPKNATRESTSTLK
AWLNEHRKNPYPTKGEKIMLAIITKMTLTQVSTWFANARRRLKKENKMTWAPRSRTDEEGNAYGSEREEEDEE
EDEEESKRELEMEEEELAGEEEDTGGEGLADDGEDEEIDLENLDSAAAGSELTLAGAAHRNGDFGLGPISDCK
TSDSDDSSEGLEDRPLSVLSLAPPPPPVARAPASPPSPPSSLDPCAPAPAPSSALQKPKIWSLAETATSPDNP
RRSPPGAGGSPPGAAVAPPTLQLSPAAAAAAAAAHRLVSAPLGKFPAWTNRPFPGPPAGPRPHPLSMLGSAPQ
HLLGLPGAAGHPAAAAAAYARPAEPESGTDRCSALEVEKKLLKTAFQPVPRRPQNHLDAALVLSALSSS*
>rs49504271_ENSMUSP00000091002_c.526:A>G_p.Q176R
ENSMUST00000093312.6|Irx3-201|ENSMUSG00000031734|-|chr8:g.92527176:T>C
MSFPQLGYQYIRPLYPPERPGAAGGGGGGSSAGGRSGPGAGASELAASGSLSNVLSSVYGAPYAAAAAAAAA
QGYGAFLPYATELPIFPQLGAQYELKDSPGVQHPATAAAFPHPHPAFYPYGQYQFGDPSRPKNATRESTSTLK
AWLNEHRKNPYPTKGEKIMLAIITKMTLTRVSTWFANARRRLKKENKMTWAPRSRTDEEGNAYGSEREEEDEE
EDEEESKRELEMEEEELAGEEEDTGGEGLADDDEDEEIDLENLDSAAAGSELTLAGAAHRNGDFGLGPISDCK
TSDSDDSSEGLEDRPLSVLSLAPPPPPVARAPASPPSPPSSLDPCAPAPAPSSALQKPKIWSLAETATSPDNP
RRSPPGAGGSPPGAAVAPPTLQLSPAAAAAAAAAHRLVSAPLGKFPAWTNRPFPGPPAGPRPHPLSMLGSAPQ
HLLGLPGAAGHPAAAAAAYARPAEPESGTGDKKNIAVVPWKWRKSYSRQLSSRCQGGHRTIWTLLWSYQLSPR
LNFSTIFKSLY*
>rs49504271_ENSMUSP00000135488_c.526:A>G_p.Q176R
ENSMUST00000175795.4|Irx3-202|ENSMUSG00000031734|-|chr8:g.92527176:T>C
MSFPQLGYQYIRPLYPPERPGAAGGGGGGSSAGGRSGPGAGASELAASGSLSNVLSSVYGAPYAAAAAAAAA
QGYGAFLPYATELPIFPQLGAQYELKDSPGVQHPATAAAFPHPHPAFYPYGQYQFGDPSRPKNATRESTSTLK
AWLNEHRKNPYPTKGEKIMLAIITKMTLTRVSTWFANARRRLKKENKMTWAPRSRTDEEGNAYGSEREEEDEE
EDEEESKRELEMEEEELAGEEEDTGGEGLADDDEDEEIDLENLDSAAAGSELTLAGAAHRNGDFGLGPISDCK
TSDSDDSSEGLEDRPLSVLSLAPPPPPVARAPASPPSPPSSLDPCAPAPAPSSALQKPKIWSLAETATSPDNP
RRSPPGAGGSPPGAAVAPPTLQLSPAAAAAAAAAHRLVSAPLGKFPAWTNRPFPGPPAGPRPHPLSMLGSAPQ
HLLGLPGAAGHPAAAAAAYARPAEPESGTDRCSALEVEKKLLKTAFQPVPRRPQNHLDAALVLSALSSS*
>rs231460599_ENSMUSP00000147339_c.423:A>AG_p.K142ins-fs
ENSMUST00000210246.2|Irx5-202|ENSMUSG00000031737|+|chr8:g.93086342:A>AG
MSYPQGYLYQPSASLALYSCPAYSTSVISGPRTDELGRSSSGSAFSPYAGSTAFTAPSPGYNSHLQYGADPAA
AAAAAFSYVGSPYDHTPGMAGSLGYHPYAAPLGSYPYGDPAYRKNATRDATATLKAWLNEHRKNPYPTRGRED
HAGHYHQDDPHPGVHLVCQRAPAPQEREQDDVDATEPQRGRGGGGEHRSGEERRGRATEARGQGRPRGPRIRR
SRAEGDCGLRTAAGAAQPRRQGDRRQPQRLGF
>rs231460599_ENSMUSP00000034184_c.423:A>AG_p.K142ins-fs
ENSMUST00000034184.12|Irx5-201|ENSMUSG00000031737|+|chr8:g.93086342:A>AG
MSYPQGYLYQPSASLALYSCPAYSTSVISGPRTDELGRSSSGSAFSPYAGSTAFTAPSPGYNSHLQYGADPAA
AAAAAFSYVGSPYDHTPGMAGSLGYHPYAAPLGSYPYGDPAYRKNATRDATATLKAWLNEHRKNPYPTRGRED

HAGHYHQDDPHPGVHLVCQRAPAPQEREQDDVDATEPQRGRGGGGEHRSGEERRGRATEARGQGRPRGPRIRR
SRAEGDCGLRTAAGAAQPRRQGDRRQPQRLGF
>rs243647518_ENSMUSP00000147339_c.556:A>AATATAGTGAG_p.D186ins-fs
ENSMUST00000210246.2|Irx5-
202|ENSMUSG00000031737|+|chr8:g.93086475:A>AATATAGTGAG
MSYPQGYLYQPSASLALYSCPAYSTSVISGPRTDELGRSSSGSAFSPYAGSTAFTAPSPGYNSHLQYGADPAA
AAAAAFSYVGSPYDHTPGMAGSLGYHPYAAPLGSYPYGDPAYRKNATRDATATLKAWLNEHRKNPYPTKGEKI
MLAIITKMTLTQVSTWFANARRRLKKENKMTWTPRNRSEEYSERGGGGEHRSGEERRGRATEARGQGRPRGPR
IRRSRAEGDCGLRTAAGAAQPRRQGDRRQPQRLGF
>rs243647518_ENSMUSP00000034184_c.556:A>AATATAGTGAG_p.D186ins-fs
ENSMUST00000034184.12|Irx5-
201|ENSMUSG00000031737|+|chr8:g.93086475:A>AATATAGTGAG
MSYPQGYLYQPSASLALYSCPAYSTSVISGPRTDELGRSSSGSAFSPYAGSTAFTAPSPGYNSHLQYGADPAA
AAAAAFSYVGSPYDHTPGMAGSLGYHPYAAPLGSYPYGDPAYRKNATRDATATLKAWLNEHRKNPYPTKGEKI
MLAIITKMTLTQVSTWFANARRRLKKENKMTWTPRNRSEEYSERGGGGEHRSGEERRGRATEARGQGRPRGPR
IRRSRAEGDCGLRTAAGAAQPRRQGDRRQPQRLGF
>rs33039720_ENSMUSP00000052258_c.919:G>A_p.R307Q
ENSMUST00000053766.14|Amfr-201|ENSMUSG00000031751|-|chr8:g.94712009:C>T
MPLLFLERFPWPSLRTYTGLSGLALLGTIVSAYRALSQPEDGSGEPEPLTAPLQPEARLTAGGPRARDVAQYL
LSDSLFVWVLVNTACCVLMLVAKLIQCIVFGPLRVSERQHLKDKFWNFIFYKFIFIFGVLNVQTVEEVVMWCL
WFAGLVFLHLMVQLCKDRFEYLSFSPTTPMSSHGRVLSLLIAMLLSCCGLAVVCCVTGYTHGMHTLAFMAAES
LLVTVRTAHVILRYVIHLWDLNHEGTWEGKGTYVYYTDFVMELALLSLDLMHHIHMLLFGNIWLSMASLVIFM
QLRYLFHEVQRRIRQHKNYLRVVGNMEARFAVATPEELAVNNDDCAICWDSMQAARKLPCGHLFHNSCLRSWL
EQDTSCPTCRMSLNIADGSRAREDHQGENLDENLVPVAAAEGRPRLNQHNHFFHFDGSRIASWLPSFSVEVMH
TTNILGITQASNSQLNAMAHQIQEMFPQVPYHLVLQDLQMTRSVEITTDNILEGRIQVPFPTQRSDSLRPALN
SPVERPSPDLEEGEASVQTERVPLDLSPRLEETLDFSEVELEPIEVEDFEARGSRFSKSADERQRMLVQRKDD
LLQQARKRFLNKSSEDDGASERLLPSEGTSSDPVTLRRRMLAAAAERRLQRQRTT*
>rs33591144_ENSMUSP00000105183_c.1090:A>G_p.K364R
ENSMUST00000109556.9|Ogfod1-203|ENSMUSG00000033009|+|chr8:g.94784788:A>G
MNGKRPADPGPARPMKKGKKQVSAEFSDAVTEEILRKQVAEAWSCRTPFSHEAIALDMDPFLHCVIPNFIQSQ
DFLEGLHKELLSLDFHEKYNDLYKFQQSDDLKNRKEPHISALRKLMFEDFRAWLSKVSGIDLEPTIDMSCAKY
EFTDALLCHDDELEGRRIAFILYLVPSWDRDLGGTLDLYDTDEHLQPKQIVKSLIPSWNKLVFFEVSPVSFHQ
VSEVLSEETSRLSISGWFYGPSLTRPPTYFEPPIPRNPHIPQDHEILYEWINPAYLEMDYQMQIQEEFEERSE
ILLKEFLKPEKFAEVCEALEKGDVEWKSHGPPNKRFYEKAEENNLPDVLKECMGLFRSEALFLLLSNLTGLRL
HFLAPSEDDETEEKGEGETASAAAGTEEGTSRRPSGPENNQVAAGSHSQENGEQADPEAQEEEAKKESSVPMC
QGELRRWKTGHYTLVHDNTKTEFALDLFLYCGCEGWEPEYGGFTSYIAKGEDEELLIVNPENNSLALVYRDRE
TLRFVKHINHRSLEQSKAFPSRSGFWDFAFIYYE*
>rs33591144_ENSMUSP00000090991_c.961:A>G_p.K321R
ENSMUST00000093301.9|Ogfod1-202|ENSMUSG00000033009|+|chr8:g.94784788:A>G
MNGKRPADPGPARPMKKGKKQVSAEFSDAVTEEILRKQVAEAWSCRTPFSHEAIALDMDPFLHCVIPNFIQSQ
DFLEGLHKELLSLDFHEKYNDLYKFQQSDDLKNRKEPHISALRKLMFEDFRAWLSKVSGIDLEPTIDMSCAKY
EFTDALLCHDDELEGRRIAFILYLVPSWDRDLGGTLDLYDTDEHLQPKQIVKSLIPSWNKLVFFEVSPVSFHQ
HEILYEWINPAYLEMDYQMQIQEEFEERSEILLKEFLKPEKFAEVCEALEKGDVEWKSHGPPNKRFYEKAEEN
NLPDVLKECMGLFRSEALFLLLSNLTGLRLHFLAPSEDDETEEKGEGETASAAAGTEEGTSRRPSGPENNQVA
AGSHSQENGEQADPEAQEEEAKKESSVPMCQGELRRWKTGHYTLVHDNTKTEFALDLFLYCGCEGWEPEYGGF
TSYIAKGEDEELLIVNPENNSLALVYRDRETLRFVKHINHRSLEQSKAFPSRSGFWDFAFIYYE*
>rs33591144_ENSMUSP00000051430_c.1045:A>G_p.K349R
ENSMUST00000060632.8|Ogfod1-201|ENSMUSG00000033009|+|chr8:g.94784788:A>G
MKKGKKQVSAEFSDAVTEEILRKQVAEAWSCRTPFSHAIALDMDPFLHCVIPNFIQSQDFLEGLHKELLSLDF
HEKYNDLYKFQQSDDLKNRKEPHISALRKLMFEDFRAWLSKVSGIDLEPTIDMSCAKYEFTDALLCHDDELEG
RRIAFILYLVPSWDRDLGGTLDLYDTDEHLQPKQIVKSLIPSWNKLVFFEVSPVSFHQVSEVLSEETSRLSIS
GWFYGPSLTRPPTYFEPPIPRNPHIPQDHEILYEWINPAYLEMDYQMQIQEEFEERSEILLKEFLKPEKFAEV
CEALEKGDVEWKSHGPPNKRFYEKAEENNLPDVLKECMGLFRSEALFLLLSNLTGLRLHFLAPSEDDETEEKG
EGETASAAAGTEEGTSRRPSGPENNQVAAGSHSQENGEQADPEAQEEEAKKESSVPMCQGELRRWKTGHYTLV
HDNTKTEFALDLFLYCGCEGWEPEYGGFTSYIAKGEDEELLIVNPENNSLALVYRDRETLRFVKHINHRSLEQ
SKAFPSRSGFWDFAFIYYE*
>rs236243673_ENSMUSP00000034206_c.2061:G>A_p.G688R
ENSMUST00000034206.6|Bbs2-201|ENSMUSG00000031755|-|chr8:g.94795008:C>T

MLLPVFTLKLRHKISPRMVAIGRYDGTHPCLAAATQAGKVFIHNPHTRSQHFSASRVFQSPLESDVSLLNINQ
TVSCLGSGVLNPELGYDTLLVGTQTSLLAYDIYNNSDLFYREVSDGANAIVLGTLGDIAPPLAIIGGNCALQG
FDHEGNDLFWTVTGDNVHSLALCDFDGDGKTELLVGSEDFDIRVFKEDEIVAEMTETEIVTSLCPMYGSRFGY
ALSNGTVGVYDKTARYWRIKSKNHAMSIHAFDINSDGVCELITGWSNGKVDARSDRTGEVIFKDNFSSAVAGV
VEGDYRMDGHVQLICCSVDGEIRGYLPGTAEMKGNLLDTSVEQDLIRELSQKKQNLLLELRNYEESTKAELSS
PLNEADGQKGIIPANTRLHTALSVNMGNDLQDAHAELGISTSNDTIIRAVLIFAEGIFVGESHVVHPSIHNLS
SSLRVPITPPKDVPVDLHLKTFVGYRSSTQFHVFELTRQLPRFTMYALTSPDAASEPVSYVNFSVAERTQRMV
TWLNQNFLLPEDSNVQNSPFHVCFTSLRNGGQLYIKMKQSGEITVNTDDIDLAGDIIQSIASFFAIEDLQVEA
DFPVYFEELRKVLVKVDEYHSVHQKLSADMADNSNLIRSLLVRAEDARLMRDMKTMKSRYMELYDLNKDLLNG
YKIRCNNHTELLGNLKAVNQAIQRAGRLRVRKPKNQVISACRDAIRSNNINTLFRIMRVGTAPS*
>rs254620580_ENSMUSP00000034206_c.1899:A>G_p.M634V
ENSMUST00000034206.6|Bbs2-201|ENSMUSG00000031755|-|chr8:g.94800930:T>C
MLLPVFTLKLRHKISPRMVAIGRYDGTHPCLAAATQAGKVFIHNPHTRSQHFSASRVFQSPLESDVSLLNINQ
TVSCLGSGVLNPELGYDTLLVGTQTSLLAYDIYNNSDLFYREVSDGANAIVLGTLGDIAPPLAIIGGNCALQG
FDHEGNDLFWTVTGDNVHSLALCDFDGDGKTELLVGSEDFDIRVFKEDEIVAEMTETEIVTSLCPMYGSRFGY
ALSNGTVGVYDKTARYWRIKSKNHAMSIHAFDINSDGVCELITGWSNGKVDARSDRTGEVIFKDNFSSAVAGV
VEGDYRMDGHVQLICCSVDGEIRGYLPGTAEMKGNLLDTSVEQDLIRELSQKKQNLLLELRNYEESTKAELSS
PLNEADGQKGIIPANTRLHTALSVNMGNDLQDAHAELGISTSNDTIIRAVLIFAEGIFVGESHVVHPSIHNLS
SSLRVPITPPKDVPVDLHLKTFVGYRSSTQFHVFELTRQLPRFTMYALTSPDAASEPVSYVNFSVAERTQRMV
TWLNQNFLLPEDSNVQNSPFHVCFTSLRNGGQLYIKMKQSGEITVNTDDIDLAGDIIQSIASFFAIEDLQVEA
DFPVYFEELRKVLVKVDEYHSVHQKLSADMADNSNLIRSLLVRAEDARLVRDMKTMKSRYMELYDLNKDLLNG
YKIRCNNHTELLGNLKAVNQAIQRAGRLRVGKPKNQVISACRDAIRSNNINTLFRIMRVGTAPS*
>rs33036013_ENSMUSP00000034206_c.943:G>A_p.R315Q
ENSMUST00000034206.6|Bbs2-201|ENSMUSG00000031755|-|chr8:g.94808762:C>T
MLLPVFTLKLRHKISPRMVAIGRYDGTHPCLAAATQAGKVFIHNPHTRSQHFSASRVFQSPLESDVSLLNINQ
TVSCLGSGVLNPELGYDTLLVGTQTSLLAYDIYNNSDLFYREVSDGANAIVLGTLGDIAPPLAIIGGNCALQG
FDHEGNDLFWTVTGDNVHSLALCDFDGDGKTELLVGSEDFDIRVFKEDEIVAEMTETEIVTSLCPMYGSRFGY
ALSNGTVGVYDKTARYWRIKSKNHAMSIHAFDINSDGVCELITGWSNGKVDARSDRTGEVIFKDNFSSAVAGV
VEGDYRMDGHVQLICCSVDGEIQGYLPGTAEMKGNLLDTSVEQDLIRELSQKKQNLLLELRNYEESTKAELSS
PLNEADGQKGIIPANTRLHTALSVNMGNDLQDAHAELGISTSNDTIIRAVLIFAEGIFVGESHVVHPSIHNLS
SSLRVPITPPKDVPVDLHLKTFVGYRSSTQFHVFELTRQLPRFTMYALTSPDAASEPVSYVNFSVAERTQRMV
TWLNQNFLLPEDSNVQNSPFHVCFTSLRNGGQLYIKMKQSGEITVNTDDIDLAGDIIQSIASFFAIEDLQVEA
DFPVYFEELRKVLVKVDEYHSVHQKLSADMADNSNLIRSLLVRAEDARLMRDMKTMKSRYMELYDLNKDLLNG
YKIRCNNHTELLGNLKAVNQAIQRAGRLRVGKPKNQVISACRDAIRSNNINTLFRIMRVGTAPS*
>rs32128886_ENSMUSP00000078878_c.18:G>A_p.G7S
ENSMUST00000079961.14|Nup93-201|ENSMUSG00000032939|+|chr8:g.94954323:G>A
MDTEGFSELLQQAEQLAAETEGISELPHVERNLQEIQQAGERLRSRTLTRTSQETADVKASVLLGSRGLDISH
ISQRLESLSAATTFEPLEPVKDTDIQGFLKNEKDNALLSAIEESRKRTFGMAEEYHRESMLVEWEQVKQRILH
TLLASGEDALDFTQESEPSYIGDVNPPGRSSLDSIEMAYARQIYIYNEKIVSGHLQPNLVDLCASVAELDDKS
ISDMWAMVKQMTDVVLTPATDALKSRSSVEVRMDFVKQALGYLEQSYKNYTLVTVFGNLHQAQLGGVPGTYQL
VRSFLNIKLPAPSPGLQDGEVEGHPVWALIYYCMRCGDLLAASQVVSRAQHQLGEFKTWFQEYMNSKDRRLSP
ATENKLRLHYRRALRNNTDPYKRAVYCIIGRCDITDNQSEVADKTEDYLWLKLNQVCFDDDGTSSPQDRLTLS
QFQKQLLEDYGESHFTVNQQPFLYFQVLFLTAQFEAAIAFLFRMERLRCHAVHVALVLFELKLLLKSSGQSAQ
LLSHEPGDPPCMRRLNFVRLLMLYTRKFESTDPREALQYFYFLRDEKDSQGENMFLRCVSELVIESREFDMIL
GKLENDGSRKPGVIDKFTSDTKPIINKVASVAENKGLFEEAAKLYDLAKNADKVLELMNKLLSPVVPQISAPQ
SNKERLKNMALSIAERYRAQGISANKFVDSTFYLLLDLITFFDEYHSGHIDRAFDIIDRLKLVPLNQESVEER
VAAFRNFSDEIRHNLSEVLLATMNILFTQFKRLKGTSPSSATRPQRVIEDRDSQLRSQARALITFAGMIPYRT
SGDTNARLVQMEVLMN*
>rs32128886_ENSMUSP00000148700_c.18:G>A_p.G7S ENSMUST00000212824.2|Nup93-207|ENSMUSG00000032939|+|chr8:g.94954323:G>A
MDTEGFSELLQQAEQLAAETEGISELPHVERNLQEIQQAGERLRSRTLTRTSQETADVKASVLLGSRGLDISH
ISQRLESLSAATTFEPLEPVKDTDIQGFLKNEKDNALLSAIEESRKRTFGMAEEYHRESMLVEWEQVKQRILH
TLLASGEDALDFTQESEPSYIGDVNPPGRSSLDSIEMAYARQIYIYNEKIVSGHLQPNLVDLCASVAELDDKS
ISDMWAMVKQMTDVVLTPATDALKSRSSVEVRMDFVKQALGYLEQSYKNYTLVTVFGNLHQAQLGGVPGTYQL
VRSFLNIKLPAPSPGLQDGEVEGHPVWALIYYCMRCGDLLAASQVVSRAQHQLGEFKTWFQEYMNSKDRRLSP
ATENKLRLHYRRALRNNTDPYKRAVYCIIGRCDITDNQSEVADKTEDYLWLKLNQVCFDDDGTSSPQDRLTLS
QFQKQLLEDYGESHFTVNQQPFLYFQVLFLTAQFEAAIAFLFRMERLRCHAVHVALVLFELKLLLKSSGQSAQ
LLSHEPGDPPCMRRLNFVRLLMLYTRKFESTDPREALQYFYFLRDEKDSQGENMFLRCVSELVIESREFDMIL

GKLENDGSRKPGVIDKFTSDTKPIINKVASVAENKGLFEEAAKLYDLAKNADKVLELMNKLLSPVVPQISAPQ
SNKERLKNMALSIAERYRAQGISANKFVDSTFYLLLDLITFFDEYHSGHIDRAFDIIDRLKLVPLNQESVEER
VAAFRNFSDEIRHNLSEVLLATMNILFTQFKRLKGTSPSSATRPQRVIEDRDSQLRSQARALITFAGMIPYRT
SGDTNARLVQMEVLMN*
>rs32128886_ENSMUSP00000105174_c.18:G>A_p.G7S ENSMUST00000109547.2|Nup93-202|ENSMUSG00000032939|+|chr8:g.94954323:G>A
MDTEGFSELLQQAEQLAAETEGISELPHVERNLQEIQQAGERLRSRTLTRTSQETADVKASVLLGSRGLDISH
ISQRLESLSAATTFEPLEPVKDTDIQGFLKNEKDNALLSAIEESRKRTFGMAEEYHRESMLVEWEQVKQRILH
TLLASGEDALDFTQESEPSYIGDVNPPGRSSLDSIEMAYARQIYIYNEKIVSGHLQPNLVDLCASVAELDDKS
ISDMWAMVKQMTDVVLTPATDALKSRSSVEVRMDFVKQALGYLEQSYKNYTLVTVFGNLHQAQLGGVPGTYQL
VRSFLNIKLPAPSPGLQDGEVEGHPVWALIYYCMRCGDLLAASQVVSRAQHQLGEFKTWFQEYMNSKDRRLSP
ATENKLRLHYRRALRNNTDPYKRAVYCIIGRCDITDNQSEVADKTEDYLWLKLNQVCFDDDGTSSPQDRLTLS
QFQKQLLEDYGESHFTVNQQPFLYFQVLFLTAQFEAAIAFLFRMERLRCHAVHVALVLFELKLLLKSSGQSAQ
LLSHEPGDPPCMRRLNFVRLLMLYTRKFESTDPREALQYFYFLRDEKDSQGENMFLRCVSELVIESREFDMIL
GKLENDGSRKPGVIDKFTSDTKPIINKVASVAENKGLFEEAAKLYDLAKNADKVLELMNKLLSPVVPQISAPQ
SNKERLKNMALSIAERYRAQGISANKFVDSTFYLLLDLITFFDEYHSGHIDRAFDIIDRLKLVPLNQESVEER
VAAFRNFSDEIRHNLSEVLLATMNILFTQFKRLKGTSPSSATRPQRVIEDRDSQLRSQARALITFAGMIPYRT
SGDTNARLVQMEVLMN*
>rs48226225_ENSMUSP00000124201_c.1029:A>T_p.M344L
ENSMUST00000161576.8|Herpud1-205|ENSMUSG00000031770|+|chr8:g.95121237:A>T
MEPEPQPEPVTLLVKSPNQRHRDLELSGDRSWSVSRLKAHLSRVYPERPRPEDQRLIYSGKLLLDHQCLQDLL
PKQEKRHVLHLVCNVKNPSKMPETSTKGAESTEQPDNSNQTQHPGDSSSDGLRQREVLRNLSPSGWENISRPE
AVQQTFQGLGPGFSGYTTYGWLQLSWFQQIYARQYYMQYLAATAASGTFVPTPSAQEIPVVSTPAPAPIHNQF
PAENQPANQNAAAQAVVNPGANQNLRMNAQGGPLVEEDDEINRDWLDWTYSAATFSVFLSILYFYSSLSRFLM
VMGATVVMYLHHVGWFPFRQRPVQNFPDDGGPRDAANQDPNNNLQGGMDPELEDPNRLPPDREVLDPEHTSPS
FMSTAWLVFKTFFASLLPEGPPALAN*
>rs48226225_ENSMUSP00000034220_c.1026:A>T_p.M343L
ENSMUST00000034220.8|Herpud1-201|ENSMUSG00000031770|+|chr8:g.95121237:A>T
MEPEPQPEPVTLLVKSPNQRHRDLELSGDRSWSVSRLKAHLSRVYPERPRPEDQRLIYSGKLLLDHQCLQDLL
PKEKRHVLHLVCNVKNPSKMPETSTKGAESTEQPDNSNQTQHPGDSSSDGLRQREVLRNLSPSGWENISRPEA
VQQTFQGLGPGFSGYTTYGWLQLSWFQQIYARQYYMQYLAATAASGTFVPTPSAQEIPVVSTPAPAPIHNQFP
AENQPANQNAAAQAVVNPGANQNLRMNAQGGPLVEEDDEINRDWLDWTYSAATFSVFLSILYFYSSLSRFLMV
MGATVVMYLHHVGWFPFRQRPVQNFPDDGGPRDAANQDPNNNLQGGMDPELEDPNRLPPDREVLDPEHTSPSF
MSTAWLVFKTFFASLLPEGPPALAN*
>rs33320749_ENSMUSP00000045755_c.1317:C>T_p.L440F
ENSMUST00000048653.10|Cpne2-201|ENSMUSG00000034361|+|chr8:g.95295234:C>T
MAYIPDGGAPTAGAIPLGSQCCVCKVELSVSGQNLLDRDVTSKSDPFCVLFIEDNGRWMEFDRTETAVNNLNP
AFSKKFVLDYHFEEVQKLKFALFDQDKSSAQLDEHDFLGQFSCSLGTIVSSKKITRPLLLMNDKPAGKGVITI
AAQELSDNRVITLSLAGRKLDKKDLFGKSDPFLEFYKPGDDGKWMLVHRTEVIKYTLDPVWKPFTVPLVSLCD
GDLEKPIQVMCYDYDSNGGHDFIGEFQTSVLQMSEARDGVPLEIECINPKKQRKKKSYKNSGIIILRSCKIHR
NYSFLDYILGGCQLMFTVGIDFTASNGNPLDPSSLHYINPMGTNEYLSAIWAVGQIIQDYDSDKMFPALGFGA
QLPPDWKVSHEFAINFNPTNPFCSGVDGIAQAYSACLPHIRFYGPTNFSPIVNHVARFAAQATQQQTATQYFI
LFIITDGVISDMEETRHAVVQASKLPMSIIIVGVGNADFAAMEFLDGDNRRLRSHTGEEAARDIVQFVPFREF
RNAAKETLAKAVLAELPQQVVQYFKHKNLPPTNSEPA*
>rs33320749_ENSMUSP00000105163_c.1314:C>T_p.L439F
ENSMUST00000109537.2|Cpne2-202|ENSMUSG00000034361|+|chr8:g.95295234:C>T
MAYIPDGGAPTAGAIPLGSQCCVCKVELSVSGQNLLDRDVTSKSDPFCVLFIEDNGRWMEFDRTETAVNNLNP
AFSKKFVLDYHFEEVQKLKFALFDQDKSSAQLDEHDFLGQFSCSLGTIVSSKKITRPLLLMNDKPAGKGVITI
AAQELSDNRVITLSLAGRKLDKKDLFGKSDPFLEFYKPGDDGKWMLVHRTEVIKYTLDPVWKPFTVPLVSLCD
GDLEKPIQVMCYDYDSNGGHDFIGEFQTSVLQMSEARDGVPLEIECINPKKQRKKKSYKNSGIIILRSCKIHR
NYSFLDYILGGCQLMFTVGIDFTASNGNPLDPSSLHYINPMGTNEYLSAIWAVGQIIQDYDSDKMFPALGFGA
QLPPDWKVSHEFAINFNPTNPFCSGVDGIAQAYSACLPHIRFYGPTNFSPIVNHVARFAAQATQQQTATYFIL
FIITDGVISDMEETRHAVVQASKLPMSIIIVGVGNADFAAMEFLDGDNRRLRSHTGEEAARDIVQFVPFREFR
NAAKETLAKAVLAELPQQVVQYFKHKNLPPTNSEPA*
>ENSMUSP00000034226_c.732:T>G_p.F245V ENSMUST00000034226.8|Psme3ip1-201|ENSMUSG00000031774|-|chr8:g.95302297:A>C
MDGEDDSNLVIKKRFVSEAELDERRKRRQEEWEKVRKPEDPKECPEEAYDPRSLYERLQEQKDRKQQEYEEQF
KFKNMVRGLDEDETNFLDEVSRQQELIEKQRREEELEELKEYRSNLNKVGISAENKEVEKKLAVKPIETKNKF

SQAKLLAGAVKHKSSESGNSVKRLKPDPDPDDKAQEAPSCMSLGSSSLSGPPSIHCPSAAVCIGILPGLGAYS
GSSDSESSSDSEGTINATGKIVSSIVRTNTFLEAP*
>rs49000192_ENSMUSP00000034226_c.185:A>C_p.K62N
ENSMUST00000034226.8|Psme3ip1-201|ENSMUSG00000031774|-
|chr8:g.95314671:T>G
MDGEDDSNLVIKKRFVSEAELDERRKRRQEEWEKVRKPEDPKECPEEAYDPRSLYERLQEQNDRKQQEYEEQF
KFKNMVRGLDEDETNFLDEVSRQQELIEKQRREEELEELKEYRSNLNKVGISAENKEVEKKLAVKPIETKNKF
SQAKLLAGAVKHKSSESGNSVKRLKPDPDPDDKAQEAPSCMSLGSSSLSGPPSIHCPSAAVCIGILPGLGAYS
GSSDSESSSDSEGTINATGKIVSSIFRTNTFLEAP*
>rs49000192_ENSMUSP00000148506_c.185:A>C_p.K62N
ENSMUST00000212791.2|Psme3ip1-214|ENSMUSG00000031774|-
|chr8:g.95314671:T>G
MDGEDDSNLVIKKRFVSEAELDERRKRRQEEWEKVRKPEDPKECPEEAYDPRSLYERLQEQNDRKQQEYEEQF
KFKNMVRGLDEDETNFLDEVSRQQELIEKQRREEELEELKEYRSNLNKVGISAENKEVEKKLAVKPIETKNKF
SQAKLLAGAVKHKSSESGNSVKRLKPDPDPDDKAQEAPSCMSLGSSSL
>rs49000192_ENSMUSP00000148524_c.185:A>C_p.K62N
ENSMUST00000212507.2|Psme3ip1-209|ENSMUSG00000031774|-
|chr8:g.95314671:T>G
MDGEDDSNLVIKKRFVSEAELDERRKRRQEEWEKVRKPEDPKECPEEAYDPRSLYERLQEQNDRKQQEYEEQF
KFKNMVRGLDEDETNFLDEVSRQQELIEKQRREEELEELKEYRSNLNKVGISAENKEVEKKLAVKPIETKNKF
SQAKLLAGAVKHKSSESGNSVKRLKPD
>rs49000192_ENSMUSP00000148543_c.185:A>C_p.K62N
ENSMUST00000213022.2|Psme3ip1-216|ENSMUSG00000031774|-
|chr8:g.95314671:T>G
MDGEDDSNLVIKKRFVSEAELDERRKRRQEEWEKVRKPEDPKECPEEAYDPRSLYERLQEQNDRKQQEYEEQF
KFKNMVRGLDEDETNFLDEVSRQQELIEKQRREEELEELKEYRSNLNKVGISAENKEVEKKLAVKPIETK
>rs49000192_ENSMUSP00000148547_c.185:A>C_p.K62N
ENSMUST00000212083.2|Psme3ip1-203|ENSMUSG00000031774|-
|chr8:g.95314671:T>G
MDGEDDSNLVIKKRFVSEAELDERRKRRQEEWEKVRKPEDPKECPEEAYDPRSLYERLQEQNDRKQQEYEEQF
KFKNMVRGLDEDETNFLDEVSRQQELIEKQRREEELEELKEYRSNLNKVGISAENK
>rs49000192_ENSMUSP00000148379_c.185:A>C_p.K62N
ENSMUST00000213008.2|Psme3ip1-215|ENSMUSG00000031774|-
|chr8:g.95314671:T>G
MDGEDDSNLVIKKRFVSEAELDERRKRRQEEWEKVRKPEDPKECPEEAYDPRSLYERLQEQNDRKQQEYEEQF
KFKNMVRGLDEDETNFLDEVSRQQELIEKQRREEELEELK
>rs49000192_ENSMUSP00000148342_c.185:A>C_p.K62N
ENSMUST00000212159.2|Psme3ip1-204|ENSMUSG00000031774|-
|chr8:g.95314671:T>G
MDGEDDSNLVIKKRFVSEAELDERRKRRQEEWEKVRKPEDPKECPEEAYDPRSLYERLQEQNDRKQQEYEEQF
KFKNMVRGLDEDETNFLDEVSRQQELIEKQRREEELE
>rs49000192_ENSMUSP00000148392_c.185:A>C_p.K62N
ENSMUST00000212258.2|Psme3ip1-205|ENSMUSG00000031774|-
|chr8:g.95314671:T>G
MDGEDDSNLVIKKRFVSEAELDERRKRRQEEWEKVRKPEDPKECPEEAYDPRSLYERLQEQNDRKQQEYEEQF
KFKNMVRGLDEDETNFL
>rs49000192_ENSMUSP00000148497_c.185:A>C_p.K62N
ENSMUST00000212765.2|Psme3ip1-212|ENSMUSG00000031774|-
|chr8:g.95314671:T>G
MDGEDDSNLVIKKRFVSEAELDERRKRRQEEWEKVRKPEDPKECPEEAYDPRSLYERLQEQNDRKQQEYEEQF
KFKNMVRGLDEDE
>rs49000192_ENSMUSP00000148325_c.185:A>C_p.K62N
ENSMUST00000212788.2|Psme3ip1-213|ENSMUSG00000031774|-
|chr8:g.95314671:T>G
MDGEDDSNLVIKKRFVSEAELDERRKRRQEEWEKVRKPEDPKECPEEAYDPRSLYERLQEQNDRKQQEYEEQF
KFKNMVRGLD
>ENSMUSP00000057275_c.1204:A>C_p.D402A ENSMUST00000060389.10|Rspry1-
201|ENSMUSG00000050079|+|chr8:g.95373255:A>C

MIVFGWAVFLASRSLGQGLLLTLEEHIAHLLGTTGATATMGNSCICRDDSGAEDNVDTHQQQAENSTVPTADS
RSQPRDPVRPPRRGRGPHEPRRKKQNVDGLVLDTLAVIRTLVDNDQEPPYSMITLHEMAETDEGWLDVVQSLI
RVIPLEDPLGPAVITLLLDECPLPTKDALQKLTEILNLNGEVACQDSGHPAKHRNTSAVLGCLAEKLAGPASI
GLLSPGILEYLLQCLKLQSHPTVMLFALIALEKFAQTSENKLTISESSISDRLVTLELWADDPDYLKRQVGFC
AQWSLDNLFLKEGRQLTYEKVDLNNIRAMLNSNDVSEYLKISPHGLEARCDASSFESVRCTFCVDTGVWYYEV
TVVTSGVMQIGWATRDSKFLNHEGYGIGDDEYSCAYAGCRQLIWYNARSKPHVHPCWKEGDTVGFLLDLNEKQ
MIFFLNGNQLPPEKQVFSSTVSGFFAAASFMSYQQCEFNFGARPFKYPPSMKFSTFNDYAFLTAEEKIILPRH
RRLALLKQVSIRENCCSLCCDEVADTQLKPCGHSDLCMDCALQLETCPLCRKEIVSRIRQISHIS*
>ENSMUSP00000148724_c.832:A>C_p.D278A ENSMUST00000212729.2|Rspry1-207|ENSMUSG00000050079|+|chr8:g.95373255:A>C
MITLHEMAETDEGWLDVVQSLIRVIPLEDPLGPAVITLLLDECPLPTKDALQKLTEILNLNGEVACQDSGHPA
KHRNTSAVLGCLAEKLAGPASIGLLSPGILEYLLQCLKLQSHPTVMLFALIALEKFAQTSENKLTISESSISD
RLVTLELWADDPDYLKRQVGFCAQWSLDNLFLKEGRQLTYEKVDLNNIRAMLNSNDVSEYLKISPHGLEARCD
ASSFESVRCTFCVDTGVWYYEVTVVTSGVMQIGWATRDSKFLNHEGYGIGDDEYSCAYAGCRQLIWYNARSKP
HVHPCWKEGDTVGFLLDLNEKQMIFFLNGNQLPPEKQVFSSTVSGFFAAASFMSYQQCEFNFGARPFKYPPSM
KFSTFNDYAFLTAEEKIILPRHRRLALLKQVSIRENCCSLCCDEVADTQLKPCGHSDLCMDCALQLETCPLCR
KEIVSRIRQISHIS*
>ENSMUSP00000148737_c.1204:A>C_p.D402A ENSMUST00000211983.2|Rspry1-205|ENSMUSG00000050079|+|chr8:g.95373255:A>C
MIVFGWAVFLASRSLGQGLLLTLEEHIAHLLGTTGATATMGNSCICRDDSGAEDNVDTHQQQAENSTVPTADS
RSQPRDPVRPPRRGRGPHEPRRKKQNVDGLVLDTLAVIRTLVDNDQEPPYSMITLHEMAETDEGWLDVVQSLI
RVIPLEDPLGPAVITLLLDECPLPTKDALQKLTEILNLNGEVACQDSGHPAKHRNTSAVLGCLAEKLAGPASI
GLLSPGILEYLLQCLKLQSHPTVMLFALIALEKFAQTSENKLTISESSISDRLVTLELWADDPDYLKRQVGFC
AQWSLDNLFLKEGRQLTYEKVDLNNIRAMLNSNDVSEYLKISPHGLEARCDASSFESVRCTFCVDTGVWYYEV
TVVTSGVMQIGWATRDSKFLNHEGYGIGDDEYSCAYAGCRQLIWYNARSKPHVHPCWKEGDTVGFLLDLNEKQ
MIFFLNGNQLPPEKQVFSSTVSGFFAAASFMSYQQCEFNFGARPFKYPPSMKFSTFNDYAFLTAEEKIILPRH
RRLALLKQVSIRENCCSLCCDEVADTQLKPCGHSDLCMDCALQLETCPLCRKEIVSRIRQISHIS*
>ENSMUSP00000034228_c.49:CT>AG_p.S17* ENSMUST00000034228.16|Arl2bp-201|ENSMUSG00000031776|+|chr8:g.95394236:CT>AG
MDALEEESFALSFSSA**CRI*CCGWMFRGHYYG**VPVIAEKLHGQVLPGI*RHRRE*THLYTHF**IYLPG
REVH*RAVAGAYPRLQHGGLHNHAAAPQR*GGWRHL*HAAHIHGFSGFQGDVPGLQSRKGRPRTGLKQRLGCD
FSV*VIFYASFPEQPAAL
>ENSMUSP00000105153_c.16:CT>AG_p.S6* ENSMUST00000109527.6|Arl2bp-202|ENSMUSG00000031776|+|chr8:g.95394236:CT>AG
MRSSA**CRI*CCGWMFRGHYYG**VPVIAEKLHGQVLPGI*RHRRE*THLYTHF**IYLPGREVH*RAVAGA
YPRLQHGGLHNHAAAPQR*GGWRHL*HAAHIHGFSGFQGDVPGLQSRKGRPRTGLKQRLGCDFSV*VIFYASF
PEQPAAL
>ENSMUSP00000148695_c.49:CT>AG_p.S17* ENSMUST00000211858.2|Arl2bp-203|ENSMUSG00000031776|+|chr8:g.95394236:CT>AG
MDALEEESFALSFSSA**CRI*CCGWMFRGHYYG**VPVIAEKLHGQVLPGI*RHRRE*THLYTHF**IYLPG
REVH*RAVAGAFPEQPAAL
>rs51191315_ENSMUSP00000132628_c.837:C>T_p.H280Y
ENSMUST00000166802.9|Adgrg5-205|ENSMUSG00000061577|+|chr8:g.95664229:C>T
MDPHGALFFYLCLLAAQVVLVETLSDLLVLMKRLEQPVGRGLSSRARHIHSLEQKLLNASFGGHNLTLQTNSI
QSLVFKLSCDFPGLSLSSTTLTNVSQVRAPHAMQFPAELTKGACVTSRPAELRLICIYFFTAHLFQDDRNSSL
LNNYVLGAQLDHRPVNNLQKPVNISFWHNRSLEGYTVSCVFWKEGASKSSWGAWSPEGCYTEQPSATQVLCHC
NHLTYFAVLMQLSGDPVPAELQVPLEYISFVGCSISIVASLLTILLYAQSRKQSDSTTRIYMNLNGSVLLLNV
TFLLSSQMTLPTMPRPVCKVLAAVLHYALLSSLTWMAIEGFNLYLFLGRVYNAYIRRYLLKLCMLGWGFPALL
VLLLLMIKSSVYGPCVTSLSKSQENGTGFQNVSMCWIRSPMVHSILVMGYGGFTSLFNLVVLAWALWILCRLR
AREKALSPWAYRDTAMVLGLTVLLGTTWTLAFFSFGVFLLPQLFLFTIFNSLYGFFLFLWFCSQKRYSDAEAK
AEMEAVSSSQMTH*
>rs51191315_ENSMUSP00000074155_c.834:C>T_p.H279Y
ENSMUST00000074570.10|Adgrg5-201|ENSMUSG00000061577|+|chr8:g.95664229:C>T
MDPHGALFFYLCLLAAQVVLVETLSDLLVLMKRLEQPVGRGLSSRARHIHSLEQKLLNASFGGHNLTLQTNSI
QSLVFKLSCDFPGLSLSSTTLTNVSQVRAPHAMQFPAELTKGACVTSRPAELRLICIYFFTAHLFQDDRNSSL
LNNYVLGAQLDHRPVNNLQKPVNISFWHNRSLEGYTVSCVFWKEGASKSSWGAWSPEGCYTEQPSATQVLCHC
NHLTYFAVLMLSGDPVPAELQVPLEYISFVGCSISIVASLLTILLYAQSRKQSDSTTRIYMNLNGSVLLLNVT
FLLSSQMTLPTMPRPVCKVLAAVLHYALLSSLTWMAIEGFNLYLFLGRVYNAYIRRYLLKLCMLGWGFPALLV

LLLLMIKSSVYGPCVTSLSKSQENGTGFQNVSMCWIRSPMVHSILVMGYGGFTSLFNLVVLAWALWILCRLRA
REKALSPWAYRDTAMVLGLTVLLGTTWTLAFFSFGVFLLPQLFLFTIFNSLYGFFLFLWFCSQKRYSDAEAKA
EMEAVSSSQMTH*
>ENSMUSP00000127427_c.271:A>T_p.K91I ENSMUST00000169353.3|Kifc3-
202|ENSMUSG00000031788|-|chr8:g.95836554:T>A
MPPLGPGSPQGGTDAEKHRDRLMVENEQLRQELRRCEVELQELRAQPVVPCEGCEHSQESSQLRDKLSQLQLE
VAENKGMLSELNLEVQQITDRLAEVELRLKDCLAEKAQEEERLSRRLRDSHETIASLRAQSPPVKYVIKTVEV
ESSKTKQALSESQTRNQHLQEQVAMQRQVLKEMEQQLQNSHQLTVQLRAQIAMYEAELERAHGQMLEEMQSLE
EDKNRAIEEAFARAQVEMKAVHENLAGVRTNLLTLQPALRTLTNDYNGLKRQVRGFPLLLQEALRSVKAEIGQ
AIEEVNSNNQELLRKYRRELQLRKKCHNELVRLKGNIRVIARVRPVTKEDGEGPEATNAVTFDPDDDSIIHLL
HKGKPVSFELDKVFSPWASQQDVFQEVQALITSCIDGFNVCIFAYGQTGAGKTYTMEGTPENPGINQRALQLL
FSEVQEKASDWQYNITVSAAEIYNEVLRDLLGKEPQEKLEIRLCPDGSGQLYVPGLTEFQVQSVDDINKVFEF
GYNNRTTEFTNLNEHSSRSHALLIVTVRGVDCSTGLRTTGKLNLVDLAGSERVGKSGAEGNRLREAQHINRSL
SALGDVIAALRSRQGHVPFRNSKLTYLLQDSLSGDSKTLMVVQVSPVEKNTSETLYSLRFAERVRSVELGPGS
RRTELGSWSSQEHLEWEPACQTPQPTARAHSAPGSGTSSRPGSIRRKLQPSA*
>ENSMUSP00000034240_c.616:A>T_p.K206I ENSMUST00000034240.15|Kifc3-
201|ENSMUSG00000031788|-|chr8:g.95836554:T>A
MVPSRRTWNLGATPSLRGLWRVGRVQEPKPGMARPAPASPAARPFPHTGQGRLRTGRGKDILPSGEEDSTSRT
AARPSLAQCRALSVDWPGPRSPHRLYLTVQVENLKEKLISQAQEVSRLRSELGGTDAEKHRDRLMVENEQLRQ
ELRRCEVELQELRAQPVVPCEGCEHSQESSQLRDKLSQLQLEVAENKGMLSELNLEVQQITDRLAEVELRLKD
CLAEKAQEEERLSRRLRDSHETIASLRAQSPPVKYVIKTVEVESSKTKQALSESQTRNQHLQEQVAMQRQVLK
EMEQQLQNSHQLTVQLRAQIAMYEAELERAHGQMLEEMQSLEEDKNRAIEEAFARAQVEMKAVHENLAGVRTN
LLTLQPALRTLTNDYNGLKRQVRGFPLLLQEALRSVKAEIGQAIEEVNSNNQELLRKYRRELQLRKKCHNELV
RLKGNIRVIARVRPVTKEDGEGPEATNAVTFDPDDDSIIHLLHKGKPVSFELDKVFSPWASQQDVFQEVQALI
TSCIDGFNVCIFAYGQTGAGKTYTMEGTPENPGINQRALQLLFSEVQEKASDWQYNITVSAAEIYNEVLRDLL
GKEPQEKLEIRLCPDGSGQLYVPGLTEFQVQSVDDINKVFEFGYNNRTTEFTNLNEHSSRSHALLIVTVRGVD
CSTGLRTTGKLNLVDLAGSERVGKSGAEGNRLREAQHINRSLSALGDVIAALRSRQGHVPFRNSKLTYLLQDS
LSGDSKTLMVVQVSPVEKNTSETLYSLRFAERVRSVELGPGSRRTELGSWSSQEHLEWEPACQTPQPTARAHS
APGSGTSSRPGSIRRKLQPSA*
>ENSMUSP00000126784_c.508:A>T_p.K170I ENSMUST00000169748.9|Kifc3-
203|ENSMUSG00000031788|-|chr8:g.95836554:T>A
MNVENTGGRLFGIGRCSSLSGAPGPDPVVHRTVEAMSQLQEELVVLRERLALHDSDRQATTTQLQNQVENLKE
KLISQAQEVSRLRSELGGTDAEKHRDRLMVENEQLRQELRRCEVELQELRAQPVVPCEGCEHSQESSQLRDKL
SQLQLEVAENKGMLSELNLEVQQITDRLAEVELRLKDCLAEKAQEEERLSRRLRDSHETIASLRAQSPPVKYV
IKTVEVESSKTKQALSESQTRNQHLQEQVAMQRQVLKEMEQQLQNSHQLTVQLRAQIAMYEAELERAHGQMLE
EMQSLEEDKNRAIEEAFARAQVEMKAVHENLAGVRTNLLTLQPALRTLTNDYNGLKRQVRGFPLLLQEALRSV
KAEIGQAIEEVNSNNQELLRKYRRELQLRKKCHNELVRLKGNIRVIARVRPVTKEDGEGPEATNAVTFDPDDD
SIIHLLHKGKPVSFELDKVFQEVQALITSCIDGFNVCIFAYGQTGAGKTYTMEGTPENPGINQRALQLLFSEV
QEKASDWQYNITVSAAEIYNEVLRDLLGKEPQEKLEIRLCPDGSGQLYVPGLTEFQVQSVDDINKVFEFGYNN
RTTEFTNLNEHSSRSHALLIVTVRGVDCSTGLRTTGKLNLVDLAGSERVGKSGAEGNRLREAQHINRSLSALG
DVIAALRSRQGHVPFRNSKLTYLLQDSLSGDSKTLMVVQVSPVEKNTSETLYSLRFAERVRSVELGPGSRRTE
LGSWSSQEHLEWEPACQTPQPTARAHSAPGSGTSSRPGSIRRKLQPSA*
>ENSMUSP00000148425_c.205:A>T_p.K69I ENSMUST00000213004.2|Kifc3-
212|ENSMUSG00000031788|-|chr8:g.95836554:T>A
MVENEQLRQELRRCEVELQELRAQPVVPCEGCEHSQESSQLRDKLSQLQLEVAENKGMLSELNLEVQQITDRL
AEVELRLKDCLAEKAQEEERLSRRLRDSHETIASLRAQSPPVKYVIKTVEVESSKTKQALSESQTRNQHLQEQ
VAMQRQVLKEMEQQLQNSHQLTVQLRAQIAMYEAELERAHGQMLEEMQSLEEDKNRAIEEAFARAQVEMKAVH
ENLAGVRTNLLTLQPALRTLTNDYNGLKRQVRGFPLLLQEALRSVKAEIGQAIEEVNSNNQELLRKYRRELQL
RKKCHNELVRLKGNIRVIARVRPVTKEDGEGPEATNAVTFDPDDDSIIHLLHKGKPVSFELDKVFSPWASQQD
VFQEVQALITSCIDGFNVCIFAYGQTGAGKTYTMEGTPENPGINQRALQLLFSEVQEKASDWQYNITVSAAEI
YNEVLRDLLGKEPQEKLEIRLCPDGSGQLYVPGLTEFQVQSVDDINKVFEFGYNNRTTEFTNLNEHSSRSHAL
LIVTVRGVDCSTGLRTTGKLNLVDLAGSERVGKSGAEGNRLREAQHINRSLSALGDVIAALRSRQGHVPFRNS
KLTYLLQDSLSGDSKTLMVVQVSPVEKNTSETLYSLRFAERVRSVELGPGSRRTELGSWSSQEHLEWEPACQT
PQPTARAHSAPGSGTSSRPGSIRRKLQPSA*
>ENSMUSP00000148754_c.205:A>T_p.K69I ENSMUST00000212787.2|Kifc3-
210|ENSMUSG00000031788|-|chr8:g.95836554:T>A

MVENEQLRQELRRCEVELQELRAQPVVPCEGCEHSQESSQLRDKLSQLQLEVAENKGMLSELNLEVQQITDRL
AEVELRLKDCLAEKAQEEERLSRRLRDSHETIASLRAQSPPVKYVIKTVEVESSKTKQALSESQTRNQHLQEQ
VAMQRQVLKEMEQQLQNSHQLTVQLRAQIAMYEAELERAHGQ
>ENSMUSP00000113827_c.1693:A>G_p.E565G ENSMUST00000119870.9|Cngb1-
202|ENSMUSG00000031789|-|chr8:g.95997500:T>C
MLGWVQRVLPQPPGTPQKTVETAGPQPETESKPEANPQPEPEPQQEPEPEPEPEPEPEPEPEPEPEPEPEPEP
EPVPEEAPPEVQALPPEEPMEGEGEAEAGPSLQETQVADPAQPTSQAQVAVAKVNRPSSWMLSWFWRGMQKVV
PQPVCSNGGQNLAAGERDPDQGGAQIPEPCGTGDPGSAEASGTQDTEPSLWLLRWLEQNLEKVLPQPPPPSLA
WKVEPEAAVLDPDPPGTPMQMEPTESPSQPNPGPLEPEEEPAAEPQPGFQSSSLPPPGDPVRLIEWLLHRLEM
ALPQPVLHGKAAEQEPGCPGMCDVQTISILPVEQVEHDLVLEEVDSCWEDAQQEDGASPQETEVAPAHEEESE
AIVEIPRELTKIQEEREDEQEEDEEEKEEEKKKGEEEKEKEEEEKEKEKEKEEEKEEEEKEEEEEEEEKEEEEE
EKEEEEKEEEEKEEEEEEEEEEEEEPIVLLDSCLVVQADVDECQLERTPSELASIQELPEEKEEKEEEKEEEKE
EEEEKKEEEVEKKEEGEATNSTVPATKEHPELQVEDTDADSGPLIPEETLPPPGRPPPSPVKSDTLTVPGAAA
AGHRKKLPSQDDEAEELKALSPAESPVVAWSDPTTPQEADGQDRAASTASQNSAIINDRLQELVKMFKERTEK
VKEKLIDPDVTSDEESPKPSPAKKAPEPDPAQKPAEAEVAEEEHYCDMLCCKFKRRPLKMYRFPQSIDPLTNL
MYILWLFFVVLAWNWNCWLIPVRWAFPYQRADNIHFWLLMDYLCDFIYLLDITVFQMRLQFVKGGDIITDKKE
MRNNYLKSRRFKMDLLCLLPLDFLYLKLGINPLLRLPRCLKYMAFFEFNNRLEAILSKAYVYRVIRTTAYLLY
SLHLNSCLYYWASAFQGIGSTHWVYDGVGNSYIRCYYWAVKTLITIGGLPDPQTLFEIVFQLLNYFTGVFAFS
VMIGQMRDVVGAATAGQTYYRSCMDSTVKYMNFYKIPRSVQNRVKTWYEYTWHSQGMLDESELMVQLPDKMRL
DLAIDVNYSIVSKVALFQGCDRQMIFDMLKRLRSVVYLPNDYVCKKGEIGREMYIIQAGQVQVLGGPDGKAVL
VTLKAGSVFGEISLLAVGGGNRRTANVVAHGFTNLFILDKKDLNEILVHYPESQKLLRKKARRMLRNNNKPKE
EKSVLILPPRAGTPKLFNAALAAAGKMGPRGAKGGKLAHLRARLKELAALEAAARQQQLLEQAKSSQEAGGEE
GSGATDQPAPQEPPEPKDPPKPPGPPEPSAQSSPPPASAKPEESTGEAAGPPEPSVRIRVSPGPDPGEQTLSV
EVLEEKKEGAE*
>ENSMUSP00000113750_c.316:A>G_p.E106G ENSMUST00000120044.9|Cngb1-
203|ENSMUSG00000031789|-|chr8:g.95997500:T>C
MAKPGLNMELNRLVQDQPPGQEGPRPGPPNPAEHLPNVPSYRPATTRIPVLVSRRTALSNSNFTKEIRSSIRR
LVPATKEHPELQVEDTDADSGPLIPEETLPPPGRPPPSPVKSDTLTVPGAAAAGHRKKLPSQDDEAEELKALS
PAESPVVAWSDPTTPQEADGQDRAASTASQNSAIINDRLQELVKMFKERTEKVKEKLIDPDVTSDEESPKPSP
AKKAPEPDPAQKPAEAEVAEEEHYCDMLCCKFKRRPLKMYRFPQSIDPLTNLMYILWLFFVVLAWNWNCWLIP
VRWAFPYQRADNIHFWLLMDYLCDFIYLLDITVFQMRLQFVKGGDIITDKKEMRNNYLKSRRFKMDLLCLLPL
DFLYLKLGINPLLRLPRCLKYMAFFEFNNRLEAILSKAYVYRVIRTTAYLLYSLHLNSCLYYWASAFQGIGST
HWVYDGVGNSYIRCYYWAVKTLITIGGLPDPQTLFEIVFQLLNYFTGVFAFSVMIGQMRDVVGAATAGQTYYR
SCMDSTVKYMNFYKIPRSVQNRVKTWYEYTWHSQGMLDESELMVQLPDKMRLDLAIDVNYSIVSKVALFQGCD
RQMIFDMLKRLRSVVYLPNDYVCKKGEIGREMYIIQAGQVQVLGGPDGKAVLVTLKAGSVFGEISLLAVGGGN
RRTANVVAHGFTNLFILDKKDLNEILVHYPESQKLLRKKARRMLRNNNKPKEEKSVLILPPRAGTPKLFNAAL
AAAGKMGPRGAKGGKLAHLRARLKELAALEAAARQQQLLEQAKSSQEAGGEEGSGATDQPAPQEPPEPKDPPK
PPGPPEPSAQSSPPPASAKPEESTGEAAGPPEPSVRIRVSPGPDPGEQTLSVEVLEEKKEGAE*
>ENSMUSP00000112437_c.316:A>G_p.E106G ENSMUST00000121162.3|Cngb1-
204|ENSMUSG00000031789|-|chr8:g.95997500:T>C
MAKPGLNMELNRLVQDQPPGQEGPRPGPPNPAEHLPNVPSYRPATTRIPVLVSRRTALSNSNFTKEIRSSIRR
LVPATKEHPELQVEDTDADSGPLIPEETLPPPGRPPPSPVKSDTLTVPGAAAAGHRKKLPSQDDEAEELKALS
PAESPVVAWSDPTTPQEADGQDRAASTASQNSAIINDRLQELVKMFKERTEKVKEKLIDPDVTSDEESPKPSP
AKKAPEPDPAQKPAEAEVAEEEHYCDMLCCKFKRRPLKMYRFPQSIDPLTNLMYILWLFFVVLAWNWNCWLIP
VRWAFPYQRADNIHFWLLMDYLCDFIYLLDITVFQMRLQFVKGGDIITDKKEMRNNYLKSRRFKMDLLCLLPL
DFLYLKLGINPLLRLPRCLKYMAFFEFNNRLEAILSKAYVYRVIRTTAYLLYSLHLNSCLYYWASAFQGIGST
HWVYDGVGNSYIRCYYWAVKTLITIGGLPDPQTLFEIVFQLLNYFTGVFAFSVMIGQMRDVVGAATAGQTYYR
SCMDSTVKYMNFYKIPRSVQNRVKTWYEYTWHSQGMLDESELMVQLPDKMRLDLAIDVNYSIVSKVALFQGCD
RQMIFDMLKRLRSVVYLPNDYVCKKGEIGREMYIIQAGQVQVLGGPDGKAVLVTLKAGSVFGEISLLAVGGGN
RRTANVVAHGFTNLFILDKKDLNEILVHYPESQKLLRKKARRMLRNNNKPKEEKSVLILPPRAGTPKLFNAAL
AAAGKMGPRGAKGGKLAHLRARLKELAALEAAARQQQLLEQAKSSQEAGGEEGSGATDQPAPQEPPEPKDPPK
PPGPPEPSAQSSPPPASAKPEESTGEAAGPPEPSVRIRVSPGPDPGEQTLSVEVLEEKKEGAE*
>rs227220583_ENSMUSP00000113827_c.1003:A>G_p.E335G
ENSMUST00000119870.9|Cngb1-202|ENSMUSG00000031789|-|chr8:g.96013343:T>C
MLGWVQRVLPQPPGTPQKTVETAGPQPETESKPEANPQPEPEPQQEPEPEPEPEPEPEPEPEPEPEPEPEPEP
EPVPEEAPPEVQALPPEEPMEGEGEAEAGPSLQETQVADPAQPTSQAQVAVAKVNRPSSWMLSWFWRGMQKVV
PQPVCSNGGQNLAAGERDPDQGGAQIPEPCGTGDPGSAEASGTQDTEPSLWLLRWLEQNLEKVLPQPPPPSLA
WKVEPEAAVLDPDPPGTPMQMEPTESPSQPNPGPLEPEEEPAAEPQPGFQSSSLPPPGDPVRLIEWLLHRLEM

ALPQPVLHGKAAEQEPGCPGMCDVQTISILPVEQVEHDLVLEGVDSCWEDAQQEDGASPQETEVAPAHEEESE
AIVEIPRELTKIQEEREDEQEEDEEEKEEEKKKGEEEKEKEEEEKEKEKEKEEEKEEEEKEEEEEEEEKEEEEE
EKEEEEKEEEEKEEEEEEEEEEEEPIVLLDSCLVVQADVDECQLERTPSELASIQELPEEKEEKEEEKEEEKE
EEEEKKEEEVEKKEEGEATNSTVPATKEHPELQVEDTDADSGPLIPEETLPPPERPPPSPVKSDTLTVPGAAA
AGHRKKLPSQDDEAEELKALSPAESPVVAWSDPTTPQEADGQDRAASTASQNSAIINDRLQELVKMFKERTEK
VKEKLIDPDVTSDEESPKPSPAKKAPEPDPAQKPAEAEVAEEEHYCDMLCCKFKRRPLKMYRFPQSIDPLTNL
MYILWLFFVVLAWNWNCWLIPVRWAFPYQRADNIHFWLLMDYLCDFIYLLDITVFQMRLQFVKGGDIITDKKE
MRNNYLKSRRFKMDLLCLLPLDFLYLKLGINPLLRLPRCLKYMAFFEFNNRLEAILSKAYVYRVIRTTAYLLY
SLHLNSCLYYWASAFQGIGSTHWVYDGVGNSYIRCYYWAVKTLITIGGLPDPQTLFEIVFQLLNYFTGVFAFS
VMIGQMRDVVGAATAGQTYYRSCMDSTVKYMNFYKIPRSVQNRVKTWYEYTWHSQGMLDESELMVQLPDKMRL
DLAIDVNYSIVSKVALFQGCDRQMIFDMLKRLRSVVYLPNDYVCKKGEIGREMYIIQAGQVQVLGGPDGKAVL
VTLKAGSVFGEISLLAVGGGNRRTANVVAHGFTNLFILDKKDLNEILVHYPESQKLLRKKARRMLRNNNKPKE
EKSVLILPPRAGTPKLFNAALAAAGKMGPRGAKGGKLAHLRARLKELAALEAAARQQQLLEQAKSSQEAGGEE
GSGATDQPAPQEPPEPKDPPKPPGPPEPSAQSSPPPASAKPEESTGEAAGPPEPSVRIRVSPGPDPGEQTLSV
EVLEEKKEGAE*
>rs32151123_ENSMUSP00000125362_c.562:A>G_p.Q188R
ENSMUST00000161029.9|Tepp-204|ENSMUSG00000090206|+|chr8:g.96047318:A>G
MAQIIDLVPWDECSAHLYASPAVLLPLERVRHPLAGVKHQLYHPALPSLRRMDMDTVKGCLSDEHCQSSTYFS
KDDFNKAHFTLLGVPNKPLQCLDFTATGQKLCHKYRGGKMIPIAPGINRVDWPCFTRAIEDWSKFVSRSEEFK
LPCANKRVEGFSGYAVRYLKPEVTQNWRYCLNQNPSLDRYGRKPLPFDSLNAFRRFGSHYSRINYLTPWH*
>rs32151123_ENSMUSP00000096080_c.226:A>G_p.Q76R
ENSMUST00000098480.9|Tepp-201|ENSMUSG00000090206|+|chr8:g.96047318:A>G
MIPIAPGINRVDWPCFTRAIEDWSKFVSRSEEFKLPCANKRVEGFSGYAVRYLKPEVTQNWRYCLNQNPSLDR
YGRKPLPFDSLNAFRRFGSHYSRINYLTPWH*
>rs32151123_ENSMUSP00000148526_c.355:A>G_p.Q119R
ENSMUST00000212056.2|Tepp-207|ENSMUSG00000090206|+|chr8:g.96047318:A>G
MAQIIDLVPWDECSAHLYASPAVLLPLERVRHPLLCHKYRGGKMIPIAPGINRVDWPCFTRAIEDWSKFVSRS
EEFKLPCANKRVEGFSGYAVRYLKPEVTQNWRYCLNQNPSLDRYGRKPLPFDSLNAFRRFGSHYSRINYLTPW
H*
>rs32151123_ENSMUSP00000148558_c.148:A>G_p.Q50R
ENSMUST00000153991.2|Tepp-203|ENSMUSG00000090206|+|chr8:g.96047318:A>G
XGLFRSRFPKWALSTVEGFSGYAVRYLKPEVTQNWRYCLNQNPSLDRYGRKPLPFDSLTRSPWLVLSSPTLAF
GKWRLVSCS*
>rs32151123_ENSMUSP00000124913_c.88:A>G_p.Q30R ENSMUST00000162294.2|Tepp-
205|ENSMUSG00000090206|+|chr8:g.96047318:A>G
GYAVRYLKPEVTQNWRYCLNQNPSLDRYGRKPLPFDSLNAFRRFGSHYRTRSPWLVLSSPTLAFGKWRLVSCS
*
>rs33399487_ENSMUSP00000034243_c.570:G>T_p.A191S
ENSMUST00000034243.7|Mmp15-201|ENSMUSG00000031790|+|chr8:g.96094698:G>T
MGSDRSALGRPGCTGSCLSSRASLLPLLLVLLDCLGHGTASKDAEVYAAENWLRLYGYLPQPSRHMSTMRSAQ
ILASALAEMQSFYGIPVTGVLDEETKTWMKRPRCGVPDQFGVHVKANLRRRRKRYTLTGKAWNNYHLTFSIQN
YTEKLGWYNSMEAVRRAFQVWEQVTPLVFQEVSYDDIRLRRRAESDIMVLFASGFHGDSSPFDGVGGFLAHAY
FPGPGLGGDTHFDADEPWTFSSTDLHGISLFLVAVHELGHALGLEHSSNPSAIMAPFYQWMDTDNFQLPEDDL
RGIQQLYGSPDGKPQPTRPLPTVRPRRPGRPDHQPPRPPQPPHPGGKPERPPKPGPPPQPRATERPDQYGPNI
CDGNFDTVAVLRGEMFVFKGRWFWRVRHNRVLDNYPMPIGHFWRGLPGNISAAYERQDGHFVFFKGNRYWLFR
EANLEPGYPQPLSSYGTDIPYDRIDTAIWWEPTGHTFFFQADRYWRFNEETQHGDPGYPKPISVWQGIPTSPK
GAFLSNDAAYTYFYKGTKYWKFNNERLRMEPGHPKSILRDFMGCQEHVEPRSRWPDVARPPFNPNGGAEPEAD
GDSKEENAGDKDEGSRVVVQMEEVVRTVNVVMVLVPLLLLLCILGLAFALVQMQRKGAPRMLLYCKRSLQEWV
*
>rs228352088_ENSMUSP00000034249_c.260:T>G_p.F87L
ENSMUST00000034249.8|Cfap20-201|ENSMUSG00000031796|-|chr8:g.96149426:A>C
MFKNTFQSGFLSILYSIGSKPLQIWDKKVRNGHIKRITDNDIQSLVLEIEGTNVSTTYITCPADPKKTLGIKL
PFLVMIIKNLKKYLTFEVQVLDDKNVRRRFRASNYQSTTRVKPFICTMPMRLDDGWNQIQFNLSDFTRRAYGT
NYIETLRVQIHANCRIRRVYFSDRLYSEDELPAEFKLYLPVQNKAKQ*
>rs228352088_ENSMUSP00000148651_c.260:T>G_p.F87L
ENSMUST00000213086.2|Cfap20-208|ENSMUSG00000031796|-|chr8:g.96149426:A>C

MFKNTFQSGFLSILYSIGSKPLQIWDKKVRNGHIKRITDNDIQSLVLEIEGTNVSTTYITCPADPKKTLGIKL
PFLVMIIKNLKKYLTFEVQVLDDKNVRRRFRASNYQSTTRVKPFICTMPMRLDDGWNQIQFNLSDFTRRAYGT
NYIETLRVQVLHLPHRESHTGGFRTLWLANWLAYGPWSRTM*
>rs228352088_ENSMUSP00000148527_c.180:T>G_p.Y61D
ENSMUST00000212684.2|Cfap20-205|ENSMUSG00000031796|-|chr8:g.96149426:A>C
MFKNTFQSGFLSILYSIGSKPLQIWDKKYHIHHVSCGPQEDTGDQTAFPCHDHQKLEEIFDL*
>rs228352088_ENSMUSP00000148668_c.260:T>G_p.F87L
ENSMUST00000211908.2|Cfap20-202|ENSMUSG00000031796|-|chr8:g.96149426:A>C
MFKNTFQSGFLSILYSIGSKPLQIWDKKVRNGHIKRITDNDIQSLVLEIEGTNVSTTYITCPADPKKTLGIKL
PFLVMIIKNLKKYLTFEVQVLDDKNVRRRF
>rs32172376_ENSMUSP00000148608_c.912:C>T_p.Q305*
ENSMUST00000213096.2|Prss54-205|ENSMUSG00000048400|-|chr8:g.96286159:G>A
MAEMRGMLLMLLYISHSSSAICGIQKATIADKLKENLVSSTEFPWVVSIQDKQYTHLAFGCILSEFWILSTAS
ALQHRPEVIAVVGISNMDPRKTDHREYSVNTIIPHENFDNVSMGNNIALLKTESAMHFNDLVQAICFLGKKLH
KPPALKNCWVAGWNPTSATGNHMTMSILRRISVKDIEVCPLRRHQKTECASHTKEPNNVCLGEPGSPMMCQAK
KLDLWILRGLLAYGGDSCPGLFLYTSVADYSDWITAKTRKAGPSLSSLHLWEKLVFELPFHESNIALTTNSFS
IHTYAEWPHSYS*GQRMSTKSNKQKDAGQNFRVNRQPETSGPSKVAIQPMYYDYYGGEAGEGGAVAGQNRLHW
SQERILMSFVLVFLGSGV*
>rs32172376_ENSMUSP00000058859_c.912:C>T_p.Q305*
ENSMUST00000052690.13|Prss54-201|ENSMUSG00000048400|-|chr8:g.96286159:G>A
MAEMRGMLLMLLYISHSSSAICGIQKATIADKLKENLVSSTEFPWVVSIQDKQYTHLAFGCILSEFWILSTAS
ALQHRKEVIAVVGISNMDPRKTDHREYSVNTIIPHENFDNVSMGNNIALLKTESAMHFNDLVQAICFLGKKLH
KPPALKNCWVAGWNPTSATGNHMTMSILRRISVKDIEVCPLRRHQKTECASHTKEPNNVCLGEPGSPMMCQAK
KLDLWILRGLLAYGGDSCPGLFLYTSVADYSDWITAKTRKAGPSLSSLHLWEKLVFELPFHESNIALTTNSFS
IHTYAEWPHSYS*GQRMSTKSNKQKDAGQNFRVNRQPETSGPSKVAIQPMYYDYYGGEAGEGGAVAGQNRLHW
SQERILMSFVLVFLGSGV*
>rs32172376_ENSMUSP00000137577_c.912:C>T_p.Q305*
ENSMUST00000180075.2|Prss54-202|ENSMUSG00000048400|-|chr8:g.96286159:G>A
MAEMRGMLLMLLYISHSSSAICGIQKATIADKLKENLVSSTEFPWVVSIQDKQYTHLAFGCILSEFWILSTAS
ALQHRPEVIAVVGISNMDPRKTDHREYSVNTIIPHENFDNVSMGNNIALLKTESAMHFNDLVQAICFLGKKLH
KPPALKNCWVAGWNPTSATGNHMTMSILRRISVKDIEVCPLRRHQKTECASHTKEPNNVCLGEPGSPMMCQAK
KLDLWILRGLLAYGGDSCPGLFLYTSVADYSDWITAKTRKAGPSLSSLHLWEKLVFELPFHESNIALTTNSFS
IHTYAEWPHSYS*GQRMSTKSNKQKDAGQNFRVNRQPETSGPSKVAIQPMYYDYYGGEAGEGGAVAGQNRLHW
SQERILMSFVLVFLGSGV*
>rs236239334_ENSMUSP00000034096_c.135:G>A_p.V46M
ENSMUST00000034096.6|Setd6-201|ENSMUSG00000031671|+|chr8:g.96442700:G>A
MAAPAKRARVSGGSPLVAPCPSPRAARAPLPLPAGSSGGEPEGDAMAGFLRWCRRVGLELSPKVTVSRQGTVA
GYGMVARESVRAGELLFAVPRSALLSPHTCSISGLLERERGALQSLSGWVPLLLALLHELQAPASPWSPYFAL
WPELGRLEHPMFWPEEERLRLLKGTGVPEAVEKDLVNIRSEYYSIVLPFMEAHSDLFSPSVRSLELYQQLVAL
VMAYSFQEPLEEDDDEKEPNSPLMVPAADILNHIANHNANLEYSADYLRMVATQPILEGHEIFNTYGQMANWQ
LIHMYGFAEPYPNNTDDTADIQMVTVRDAALQGTKDETEKLLVCERWDFLCKQEMVGEEGAFVIGCEEVLTEE
ELATTLKVLCMPAEEFRDYKERAGWGEEETEDDSLAITDIPKLQESWKRLLRNSVLLTLQTYTTDLKTDQDLL
SNKEAYATLSWREQQALQVRYGQKMILHRVLELTN*
>rs262614519_ENSMUSP00000034096_c.451:G>C_p.G151A
ENSMUST00000034096.6|Setd6-201|ENSMUSG00000031671|+|chr8:g.96443098:G>C
MAAPAKRARVSGGSPLVAPCPSPRAARAPLPLPAGSSGGEPEGDAVAGFLRWCRRVGLELSPKVTVSRQGTVA
GYGMVARESVRAGELLFAVPRSALLSPHTCSISGLLERERGALQSLSGWVPLLLALLHELQAPASPWSPYFAL
WPELARLEHPMFWPEEERLRLLKGTGVPEAVEKDLVNIRSEYYSIVLPFMEAHSDLFSPSVRSLELYQQLVAL
VMAYSFQEPLEEDDDEKEPNSPLMVPAADILNHIANHNANLEYSADYLRMVATQPILEGHEIFNTYGQMANWQ
LIHMYGFAEPYPNNTDDTADIQMVTVRDAALQGTKDETEKLLVCERWDFLCKQEMVGEEGAFVIGCEEVLTEE
ELATTLKVLCMPAEEFRDYKERAGWGEEETEDDSLAITDIPKLQESWKRLLRNSVLLTLQTYTTDLKTDQDLL
SNKEAYATLSWREQQALQVRYGQKMILHRVLELTN*
>rs215389823_ENSMUSP00000034096_c.555:A>G_p.S186G
ENSMUST00000034096.6|Setd6-201|ENSMUSG00000031671|+|chr8:g.96443314:A>G
MAAPAKRARVSGGSPLVAPCPSPRAARAPLPLPAGSSGGEPEGDAVAGFLRWCRRVGLELSPKVTVSRQGTVA
GYGMVARESVRAGELLFAVPRSALLSPHTCSISGLLERERGALQSLSGWVPLLLALLHELQAPASPWSPYFAL
WPELGRLEHPMFWPEEERLRLLKGTGVPEAVEKDLVNIRGEYYSIVLPFMEAHSDLFSPSVRSLELYQQLVAL
VMAYSFQEPLEEDDDEKEPNSPLMVPAADILNHIANHNANLEYSADYLRMVATQPILEGHEIFNTYGQMANWQ

LIHMYGFAEPYPNNTDDTADIQMVTVRDAALQGTKDETEKLLVCERWDFLCKQEMVGEEGAFVIGCEEVLTEE
ELATTLKVLCMPAEEFRDYKERAGWGEEETEDDSLAITDIPKLQESWKRLLRNSVLLTLQTYTTDLKTDQDLL
SNKEAYATLSWREQQALQVRYGQKMILHRVLELTN*
>rs215389823_ENSMUSP00000148466_c.75:A>G_p.S26G
ENSMUST00000141900.8|Setd6-203|ENSMUSG00000031671|+|chr8:g.96443314:A>G
EEERLRLLKGTGVPEAVEKDLVNIRGEYYSIVLPFMEAHSDLFSPSVRSLELYQQLVALVMAYRNKG*
>rs215389823_ENSMUSP00000148785_c.66:A>G_p.S23G
ENSMUST00000148727.3|Setd6-204|ENSMUSG00000031671|+|chr8:g.96443314:A>G
RLRLLKGTGVPEAVEKDLVNIRGEYYSIVLPFMEAHSDLFSPSVRSLELYQQLVALVMAYSFQEPLEEDDDEK
EPNSPLMDYLRMVATQPILEGHEIFNTYGQMANWQLIHMYGFAEPYPNNTDDTAD
>rs213219203_ENSMUSP00000034096_c.625:C>T_p.S209F
ENSMUST00000034096.6|Setd6-201|ENSMUSG00000031671|+|chr8:g.96443384:C>T
MAAPAKRARVSGGSPLVAPCPSPRAARAPLPLPAGSSGGEPEGDAVAGFLRWCRRVGLELSPKVTVSRQGTVA
GYGMVARESVRAGELLFAVPRSALLSPHTCSISGLLERERGALQSLSGWVPLLLALLHELQAPASPWSPYFAL
WPELGRLEHPMFWPEEERLRLLKGTGVPEAVEKDLVNIRSEYYSIVLPFMEAHSDLFSPSVRFLELYQQLVAL
VMAYSFQEPLEEDDDEKEPNSPLMVPAADILNHIANHNANLEYSADYLRMVATQPILEGHEIFNTYGQMANWQ
LIHMYGFAEPYPNNTDDTADIQMVTVRDAALQGTKDETEKLLVCERWDFLCKQEMVGEEGAFVIGCEEVLTEE
ELATTLKVLCMPAEEFRDYKERAGWGEEETEDDSLAITDIPKLQESWKRLLRNSVLLTLQTYTTDLKTDQDLL
SNKEAYATLSWREQQALQVRYGQKMILHRVLELTN*
>rs213219203_ENSMUSP00000148466_c.145:C>T_p.S49F
ENSMUST00000141900.8|Setd6-203|ENSMUSG00000031671|+|chr8:g.96443384:C>T
EEERLRLLKGTGVPEAVEKDLVNIRSEYYSIVLPFMEAHSDLFSPSVRFLELYQQLVALVMAYRNKG*
>rs213219203_ENSMUSP00000148785_c.136:C>T_p.S46F
ENSMUST00000148727.3|Setd6-204|ENSMUSG00000031671|+|chr8:g.96443384:C>T
RLRLLKGTGVPEAVEKDLVNIRSEYYSIVLPFMEAHSDLFSPSVRFLELYQQLVALVMAYSFQEPLEEDDDEK
EPNSPLMDYLRMVATQPILEGHEIFNTYGQMANWQLIHMYGFAEPYPNNTDDTAD
>rs387317066_ENSMUSP00000063565_c.6872:C>A_p.N2291K
ENSMUST00000068452.10|Cnot1-201|ENSMUSG00000036550|-|chr8:g.96448163:G>T
MNLDSLSLALSQISYLVDNLTKKNYRASQQEIQHIVNRHGPEADRHLLRCLFSHVDFSGDGKSSGKDFHQTQF
LIQECASLITKPNFISTLSYAIDNPLHYQKSLKPAPHLFAQLSKVLKLSKVQEVIFGLALLNSSSPDLRGFAA
QFIKQKLPDLLRSYIDADVSGNQEGGFQDIAIEVLHLLLSHLLFGQKGAFGVGQEQIDAFLKTLRRDFPQERC
PVVLAPLLYPEKRDILMDRILPDSGGVAKTMMESSLADFMQEVGYGFCASIEECRNIIMQFGVREVTAAQVAR
VLGMMARTHSGLTDGIPLQSISAPGSGIWSDGKDKSEGAQAHTWNVEVLIDVLKELNPSLNFKEVTYELDHPG
FQIRDSKGLHNVVYGIQRGLGMEVFPVDFIYRPWKHAEGQLSFIQHSLINPEVFCFADYPCHTVATDILKAPP
EDDNREIATWKSLDLIESLLRLAEVGQYEQVKQLFSFPIKHCPDMLVLALLQINTSWHTLRHELISTLMPIFL
GNHPNSAIILHYAWHGQGQSPSIRQLIMHAMAEWYMRGEQYDQAKLSRILDVAQDLKALSMLLNGTPFAFVID
LAALASRREYLKLDKWLTDKIREHGEPFIQACMTFLKRRCPSILGGLAPEKDQPKSAQLPAETLATMLACLQA
CAGSVSQELSETILTMVANCSNVMNKARQPPPGVMPKGRPPSASSLDAISPVQIDPLAGMASLSIGGSAAPHT
QSMQGFPPNLGSAFSTPQSPAKAFPPLSTPNQTTAFSGIGGLSSQLPVGGLGTGSLTGIGTGALGLPAVNNDP
FVQRKLGTSGLNQPTFQQTDLSQVWPEANQHFSKEIDDEANSYFQRIYNHPPHPTMSVDEVLEMLQRFKDSTI
KREREVFNCMLRNLFEEYRFFPQYPDKELHITACLFGGIIEKGLVTYMALGLALRYVLEALRKPFGSKMYYFG
IAALDRFKNRLKDYPQYCQHLASISHFMQFPHHLQEYIEYGQQSRDPPVKMQGSITTPGSIALAQAQAQAQVP
AKAPLAGQVNTMVTTSTTTTVAKTVTVTKPTGVSFKKDVPPSINTTNIDTLLVATDQTERIVEPPENIQEKIA
FIFNNLSQSNMTQKVEELKETVKEEFMPWVSQYLVMKRVSIEPNFHSLYSNFLDTLKNPEFNKMVLNETYRNI
KVLLTSDKAAANFSDRSLLKNLGHWLGMITLAKNKPILHTDLDVKSLLLEAYVKGQQELLYVVPFVAKVLESS
IRSLVFRPPNPWTMAIMNVLAELHQEHDLKLNLKFEIEVLCKNLALDINELKPGNLLKDKDRLKNLDEQLSAP
KKDVKQPEELPAITTTTTSTTPATSTTCTATVPPQPQYSYHDINVYSLAGLAPHITLNPTIPLFQAHPQLKQC
VRQAIERAVQELVHPVVDRSIKIAMTTCEQIVRKDFALDSEESRMRIAAHHMMRNLTAGMAMITCREPLLMSI
STNLKNSFASALRTASPQQREMMDQAAAQLAQDNCELACCFIQKTAVEKAGPEMDKRLATEFELRKHARQEGR
RYCDPVVLTYQAERMPEQIRLKVGGVDPKQLAVYEEFARNVPGFLPTNDLSQPTGFLAQPMKQAWATDDVAQI
YDKCITELEQHLHAIPPTLAMNPQAQALRSLLEVVVLSRNSRDAIAALGLLQKAVEGLLDATSGADADLLLRY
RECHLLVLKALQDGRAYGSPWCNKQITRCLIECRDEYKYNVEAVELLIRNHLVNMQQYDLHLAQSMENGLNYM
AVAFAMQLVKILLVDERSVAHITEADLFHTIETLMRINAHSRGNAPEGLPQLMEVVRSNYEAMIDRAHGGPNF
MMHSGISQASEYDDPPGLREKAEYLLREWVNLYHSAAAGRDSTKAFSAFVGQMHQQGILKTDDLITRFFRLCT
EMCVEISYRAQAEQQHNPAANPTMIRAKCYHNLDAFVRLIALLVKHSGEATNTVTKINLLNKVLGIVVGVLLQ
DHDVRQSEFQQLPYHRIFIMLLLELNAPEHVLETINFQTLTAFCNTFHILRPTKAPGFVYAWLELISHRIFIA
RMLAHTPQQKGWPMYAQLLIDLFKYLAPFLRNVELTKPMQILYKGTLRVLLVLLHDFPEFLCDYHYGFCDVIP
PNCIQLRNLILSAFPRNMRLPDPFTPNLKVDMLSEINIAPRILTNFTGVMPPQFKKDLDSYLKTRSPVTFLSD

LRSNLQVSNEPGNRYNLQLINALVLYVGTQAIAHIHNKGSTPSMSTITHSAHMDIFQNLAVDLDTEGRYLFLN
AIANQLRYPNSHTHYFSCTMLYLFAEAKTEAIQEQITRLFQSVAQCCMGQKQAQQVMEGTGAS*
>rs387317066_ENSMUSP00000148807_c.6866:C>A_p.N2289K
ENSMUST00000211887.2|Cnot1-203|ENSMUSG00000036550|-|chr8:g.96448163:G>T
MNLDSLSLALSQISYLVDNLTKKNYRASQQEIQHIVNRHGPEADRHLLRCLFSHVDFSGDGKSSGKDFHQFLI
QECASLITKPNFISTLSYAIDNPLHYQKSLKPAPHLFAQLSKVLKLSKVQEVIFGLALLNSSSPDLRGFAAQF
IKQKLPDLLRSYIDADVSGNQEGGFQDIAIEVLHLLLSHLLFGQKGAFGVGQEQIDAFLKTLRRDFPQERCPV
VLAPLLYPEKRDILMDRILPDSGGVAKTMMESSLADFMQEVGYGFCASIEECRNIIMQFGVREVTAAQVARVL
GMMARTHSGLTDGIPLQSISAPGSGIWSDGKDKSEGAQAHTWNVEVLIDVLKELNPSLNFKEVTYELDHPGFQ
IRDSKGLHNVVYGIQRGLGMEVFPVDFIYRPWKHAEGQLSFIQHSLINPEVFCFADYPCHTVATDILKAPPED
DNREIATWKSLDLIESLLRLAEVGQYEQVKQLFSFPIKHCPDMLVLALLQINTSWHTLRHELISTLMPIFLGN
HPNSAIILHYAWHGQGQSPSIRQLIMHAMAEWYMRGEQYDQAKLSRILDVAQDLKALSMLLNGTPFAFVIDLA
ALASRREYLKLDKWLTDKIREHGEPFIQACMTFLKRRCPSILGGLAPEKDQPKSAQLPAETLATMLACLQACA
GSVSQELSETILTMVANCSNVMNKARQPPPGVMPKGRPPSASSLDAISPVQIDPLAGMASLSIGGSAAPHTQS
MQGFPPNLGSAFSTPQSPAKAFPPLSTPNQTTAFSGIGGLSSQLPVGGLGTGSLTGIGTGALGLPAVNNDPFV
QRKLGTSGLNQPTFQQTDLSQVWPEANQHFSKEIDDEANSYFQRIYNHPPHPTMSVDEVLEMLQRFKDSTIKR
EREVFNCMLRNLFEEYRFFPQYPDKELHITACLFGGIIEKGLVTYMALGLALRYVLEALRKPFGSKMYYFGIA
ALDRFKNRLKDYPQYCQHLASISHFMQFPHHLQEYIEYGQQSRDPPVKMQGSITTPGSIALAQAQAQAQVPAK
APLAGQVNTMVTTSTTTTVAKTVTVTKPTGVSFKKDVPPSINTTNIDTLLVATDQTERIVEPPENIQEKIAFI
FNNLSQSNMTQKVEELKETVKEEFMPWVSQYLVMKRVSIEPNFHSLYSNFLDTLKNPEFNKMVLNETYRNIKV
LLTSDKAAANFSDRSLLKNLGHWLGMITLAKNKPILHTDLDVKSLLLEAYVKGQQELLYVVPFVAKVLESSIR
SLVFRPPNPWTMAIMNVLAELHQEHDLKLNLKFEIEVLCKNLALDINELKPGNLLKDKDRLKNLDEQLSAPKK
DVKQPEELPAITTTTTSTTPATSTTCTATVPPQPQYSYHDINVYSLAGLAPHITLNPTIPLFQAHPQLKQCVR
QAIERAVQELVHPVVDRSIKIAMTTCEQIVRKDFALDSEESRMRIAAHHMMRNLTAGMAMITCREPLLMSIST
NLKNSFASALRTASPQQREMMDQAAAQLAQDNCELACCFIQKTAVEKAGPEMDKRLATEFELRKHARQEGRRY
CDPVVLTYQAERMPEQIRLKVGGVDPKQLAVYEEFARNVPGFLPTNDLSQPTGFLAQPMKQAWATDDVAQIYD
KCITELEQHLHAIPPTLAMNPQAQALRSLLEVVVLSRNSRDAIAALGLLQKAVEGLLDATSGADADLLLRYRE
CHLLVLKALQDGRAYGSPWCNKQITRCLIECRDEYKYNVEAVELLIRNHLVNMQQYDLHLAQSMENGLNYMAV
AFAMQLVKILLVDERSVAHITEADLFHTIETLMRINAHSRGNAPEGLPQLMEVVRSNYEAMIDRAHGGPNFMM
HSGISQASEYDDPPGLREKAEYLLREWVNLYHSAAAGRDSTKAFSAFVGQMHQQGILKTDDLITRFFRLCTEM
CVEISYRAQAEQQHNPAANPTMIRAKCYHNLDAFVRLIALLVKHSGEATNTVTKINLLNKVLGIVVGVLLQDH
DVRQSEFQQLPYHRIFIMLLLELNAPEHVLETINFQTLTAFCNTFHILRPTKAPGFVYAWLELISHRIFIARM
LAHTPQQKGWPMYAQLLIDLFKYLAPFLRNVELTKPMQILYKGTLRVLLVLLHDFPEFLCDYHYGFCDVIPPN
CIQLRNLILSAFPRNMRLPDPFTPNLKVDMLSEINIAPRILTNFTGVMPPQFKKDLDSYLKTRSPVTFLSDLR
SNLQVSNEPGNRYNLQLINALVLYVGTQAIAHIHNKGSTPSMSTITHSAHMDIFQNLAVDLDTEGRYLFLNAI
ANQLRYPNSHTHYFSCTMLYLFAEAKTEAIQEQITRVLLERLIVNRPHPWGLLITFIELIKNPAFKFWNHEFV
HCAPEIEKLFQSVAQCCMGQKQAQQVMEGTGAS*
>rs387317066_ENSMUSP00000096073_c.6887:C>A_p.N2296K
ENSMUST00000098473.11|Cnot1-202|ENSMUSG00000036550|-|chr8:g.96448163:G>T
MNLDSLSLALSQISYLVDNLTKKNYRASQQEIQHIVNRHGPEADRHLLRCLFSHVDFSGDGKSSGKDFHQTQF
LIQECASLITKPNFISTLSYAIDNPLHYQKSLKPAPHLFAQLSKVLKLSKVQEVIFGLALLNSSSPDLRGFAA
QFIKQKLPDLLRSYIDADVSGNQEGGFQDIAIEVLHLLLSHLLFGQKGAFGVGQEQIDAFLKTLRRDFPQERC
PVVLAPLLYPEKRDILMDRILPDSGGVAKTMMESSLADFMQEVGYGFCASIEECRNIIMQFGVREVTAAQVAR
VLGMMARTHSGLTDGIPLQSISAPGSGIWSDGKDKSEGAQAHTWNVEVLIDVLKELNPSLNFKEVTYELDHPG
FQIRDSKGLHNVVYGIQRGLGMEVFPVDFIYRPWKHAEGQLSFIQHSLINPEVFCFADYPCHTVATDILKAPP
EDDNREIATWKSLDLIESLLRLAEVGQYEQVKQLFSFPIKHCPDMLVLALLQINTSWHTLRHELISTLMPIFL
GNHPNSAIILHYAWHGQGQSPSIRQLIMHAMAEWYMRGEQYDQAKLSRILDVAQDLKALSMLLNGTPFAFVID
LAALASRREYLKLDKWLTDKIREHGEPFIQACMTFLKRRCPSILGGLAPEKDQPKSAQLPAETLATMLACLQA
CAGSVSQELSETILTMVANCSNVMNKARQPPPGVMPKGRPPSASSLDAISPVQIDPLAGMASLSIGGSAAPHT
QSMQGFPPNLGSAFSTPQSPAKAFPPLSTPNQTTAFSGIGGLSSQLPVGGLGTGSLTGIGTGALGLPAVNNDP
FVQRKLGTSGLNQPTFQQSKMKPSDLSQVWPEANQHFSKEIDDEANSYFQRIYNHPPHPTMSVDEVLEMLQRF
KDSTIKREREVFNCMLRNLFEEYRFFPQYPDKELHITACLFGGIIEKGLVTYMALGLALRYVLEALRKPFGSK
MYYFGIAALDRFKNRLKDYPQYCQHLASISHFMQFPHHLQEYIEYGQQSRDPPVKMQGSITTPGSIALAQAQA
QAQVPAKAPLAGQVNTMVTTSTTTVAKTVTVTKPTGVSFKKDVPPSINTTNIDTLLVATDQTERIVEPPENI
QEKIAFIFNNLSQSNMTQKVEELKETVKEEFMPWVSQYLVMKRVSIEPNFHSLYSNFLDTLKNPEFNKMVLNE
TYRNIKVLLTSDKAAANFSDRSLLKNLGHWLGMITLAKNKPILHTDLDVKSLLLEAYVKGQQELLYVVPFVAK
VLESSIRSLVFRPPNPWTMAIMNVLAELHQEHDLKLNLKFEIEVLCKNLALDINELKPGNLLKDKDRLKNLDE
QLSAPKKDVKQPEELPAITTTTTSTTPATSTTCTATVPPQPQYSYHDINVYSLAGLAPHITLNPTIPLFQAHP

QLKQCVRQAIERAVQELVHPVVDRSIKIAMTTCEQIVRKDFALDSEESRMRIAAHHMMRNLTAGMAMITCREP
LLMSISTNLKNSFASALRTASPQQREMMDQAAAQLAQDNCELACCFIQKTAVEKAGPEMDKRLATEFELRKHA
RQEGRRYCDPVVLTYQAERMPEQIRLKVGGVDPKQLAVYEEFARNVPGFLPTNDLSQPTGFLAQPMKQAWATD
DVAQIYDKCITELEQHLHAIPPTLAMNPQAQALRSLLEVVVLSRNSRDAIAALGLLQKAVEGLLDATSGADAD
LLLRYRECHLLVLKALQDGRAYGSPWCNKQITRCLIECRDEYKYNVEAVELLIRNHLVNMQQYDLHLAQSMEN
GLNYMAVAFAMQLVKILLVDERSVAHITEADLFHTIETLMRINAHSRGNAPEGLPQLMEVVRSNYEAMIDRAH
GGPNFMMHSGISQASEYDDPPGLREKAEYLLREWVNLYHSAAAGRDSTKAFSAFVGQMHQQGILKTDDLITRF
FRLCTEMCVEISYRAQAEQQHNPAANPTMIRAKCYHNLDAFVRLIALLVKHSGEATNTVTKINLLNKVLGIVV
GVLLQDHDVRQSEFQQLPYHRIFIMLLLELNAPEHVLETINFQTLTAFCNTFHILRPTKAPGFVYAWLELISH
RIFIARMLAHTPQQKGWPMYAQLLIDLFKYLAPFLRNVELTKPMQILYKGTLRVLLVLLHDFPEFLCDYHYGF
CDVIPPNCIQLRNLILSAFPRNMRLPDPFTPNLKVDMLSEINIAPRILTNFTGVMPPQFKKDLDSYLKTRSPV
TFLSDLRSNLQVSNEPGNRYNLQLINALVLYVGTQAIAHIHNKGSTPSMSTITHSAHMDIFQNLAVDLDTEGR
YLFLNAIANQLRYPNSHTHYFSCTMLYLFAEAKTEAIQEQITRVLLERLIVNRPHPWGLLITFIELIKNPAFK
FWNHEFVHCAPEIEKLFQSVAQCCMGQKQAQQVMEGTGAS*
>rs107850230_ENSMUSP00000063565_c.6243:C>T_p.P2082S
ENSMUST00000068452.10|Cnot1-201|ENSMUSG00000036550|-|chr8:g.96454231:G>A
MNLDSLSLALSQISYLVDNLTKKNYRASQQEIQHIVNRHGPEADRHLLRCLFSHVDFSGDGKSSGKDFHQTQF
LIQECASLITKPNFISTLSYAIDNPLHYQKSLKPAPHLFAQLSKVLKLSKVQEVIFGLALLNSSSPDLRGFAA
QFIKQKLPDLLRSYIDADVSGNQEGGFQDIAIEVLHLLLSHLLFGQKGAFGVGQEQIDAFLKTLRRDFPQERC
PVVLAPLLYPEKRDILMDRILPDSGGVAKTMMESSLADFMQEVGYGFCASIEECRNIIMQFGVREVTAAQVAR
VLGMMARTHSGLTDGIPLQSISAPGSGIWSDGKDKSEGAQAHTWNVEVLIDVLKELNPSLNFKEVTYELDHPG
FQIRDSKGLHNVVYGIQRGLGMEVFPVDFIYRPWKHAEGQLSFIQHSLINPEVFCFADYPCHTVATDILKAPP
EDDNREIATWKSLDLIESLLRLAEVGQYEQVKQLFSFPIKHCPDMLVLALLQINTSWHTLRHELISTLMPIFL
GNHPNSAIILHYAWHGQGQSPSIRQLIMHAMAEWYMRGEQYDQAKLSRILDVAQDLKALSMLLNGTPFAFVID
LAALASRREYLKLDKWLTDKIREHGEPFIQACMTFLKRRCPSILGGLAPEKDQPKSAQLPAETLATMLACLQA
CAGSVSQELSETILTMVANCSNVMNKARQPPPGVMPKGRPPSASSLDAISPVQIDPLAGMASLSIGGSAAPHT
QSMQGFPPNLGSAFSTPQSPAKAFPPLSTPNQTTAFSGIGGLSSQLPVGGLGTGSLTGIGTGALGLPAVNNDP
FVQRKLGTSGLNQPTFQQTDLSQVWPEANQHFSKEIDDEANSYFQRIYNHPPHPTMSVDEVLEMLQRFKDSTI
KREREVFNCMLRNLFEEYRFFPQYPDKELHITACLFGGIIEKGLVTYMALGLALRYVLEALRKPFGSKMYYFG
IAALDRFKNRLKDYPQYCQHLASISHFMQFPHHLQEYIEYGQQSRDPPVKMQGSITTPGSIALAQAQAQAQVP
AKAPLAGQVNTMVTTSTTTTVAKTVTVTKPTGVSFKKDVPPSINTTNIDTLLVATDQTERIVEPPENIQEKIA
FIFNNLSQSNMTQKVEELKETVKEEFMPWVSQYLVMKRVSIEPNFHSLYSNFLDTLKNPEFNKMVLNETYRNI
KVLLTSDKAAANFSDRSLLKNLGHWLGMITLAKNKPILHTDLDVKSLLLEAYVKGQQELLYVVPFVAKVLESS
IRSLVFRPPNPWTMAIMNVLAELHQEHDLKLNLKFEIEVLCKNLALDINELKPGNLLKDKDRLKNLDEQLSAP
KKDVKQPEELPAITTTTTSTTPATSTTCTATVPPQPQYSYHDINVYSLAGLAPHITLNPTIPLFQAHPQLKQC
VRQAIERAVQELVHPVVDRSIKIAMTTCEQIVRKDFALDSEESRMRIAAHHMMRNLTAGMAMITCREPLLMSI
STNLKNSFASALRTASPQQREMMDQAAAQLAQDNCELACCFIQKTAVEKAGPEMDKRLATEFELRKHARQEGR
RYCDPVVLTYQAERMPEQIRLKVGGVDPKQLAVYEEFARNVPGFLPTNDLSQPTGFLAQPMKQAWATDDVAQI
YDKCITELEQHLHAIPPTLAMNPQAQALRSLLEVVVLSRNSRDAIAALGLLQKAVEGLLDATSGADADLLLRY
RECHLLVLKALQDGRAYGSPWCNKQITRCLIECRDEYKYNVEAVELLIRNHLVNMQQYDLHLAQSMENGLNYM
AVAFAMQLVKILLVDERSVAHITEADLFHTIETLMRINAHSRGNAPEGLPQLMEVVRSNYEAMIDRAHGGPNF
MMHSGISQASEYDDPPGLREKAEYLLREWVNLYHSAAAGRDSTKAFSAFVGQMHQQGILKTDDLITRFFRLCT
EMCVEISYRAQAEQQHNPAANPTMIRAKCYHNLDAFVRLIALLVKHSGEATNTVTKINLLNKVLGIVVGVLLQ
DHDVRQSEFQQLPYHRIFIMLLLELNAPEHVLETINFQTLTAFCNTFHILRPTKAPGFVYAWLELISHRIFIA
RMLAHTPQQKGWPMYAQLLIDLFKYLAPFLRNVELTKSMQILYKGTLRVLLVLLHDFPEFLCDYHYGFCDVIP
PNCIQLRNLILSAFPRNMRLPDPFTPNLKVDMLSEINIAPRILTNFTGVMPPQFKKDLDSYLKTRSPVTFLSD
LRSNLQVSNEPGNRYNLQLINALVLYVGTQAIAHIHNKGSTPSMSTITHSAHMDIFQNLAVDLDTEGRYLFLN
AIANQLRYPNSHTHYFSCTMLYLFAEANTEAIQEQITRLFQSVAQCCMGQKQAQQVMEGTGAS*
>rs107850230_ENSMUSP00000148807_c.6237:C>T_p.P2080S
ENSMUST00000211887.2|Cnot1-203|ENSMUSG00000036550|-|chr8:g.96454231:G>A
MNLDSLSLALSQISYLVDNLTKKNYRASQQEIQHIVNRHGPEADRHLLRCLFSHVDFSGDGKSSGKDFHQFLI
QECASLITKPNFISTLSYAIDNPLHYQKSLKPAPHLFAQLSKVLKLSKVQEVIFGLALLNSSSPDLRGFAAQF
IKQKLPDLLRSYIDADVSGNQEGGFQDIAIEVLHLLLSHLLFGQKGAFGVGQEQIDAFLKTLRRDFPQERCPV
VLAPLLYPEKRDILMDRILPDSGGVAKTMMESSLADFMQEVGYGFCASIEECRNIIMQFGVREVTAAQVARVL
GMMARTHSGLTDGIPLQSISAPGSGIWSDGKDKSEGAQAHTWNVEVLIDVLKELNPSLNFKEVTYELDHPGFQ
IRDSKGLHNVVYGIQRGLGMEVFPVDFIYRPWKHAEGQLSFIQHSLINPEVFCFADYPCHTVATDILKAPPED
DNREIATWKSLDLIESLLRLAEVGQYEQVKQLFSFPIKHCPDMLVLALLQINTSWHTLRHELISTLMPIFLGN
HPNSAIILHYAWHGQGQSPSIRQLIMHAMAEWYMRGEQYDQAKLSRILDVAQDLKALSMLLNGTPFAFVIDLA

ALASRREYLKLDKWLTDKIREHGEPFIQACMTFLKRRCPSILGGLAPEKDQPKSAQLPAETLATMLACLQACA
GSVSQELSETILTMVANCSNVMNKARQPPPGVMPKGRPPSASSLDAISPVQIDPLAGMASLSIGGSAAPHTQS
MQGFPPNLGSAFSTPQSPAKAFPPLSTPNQTTAFSGIGGLSSQLPVGGLGTGSLTGIGTGALGLPAVNNDPFV
QRKLGTSGLNQPTFQQTDLSQVWPEANQHFSKEIDDEANSYFQRIYNHPPHPTMSVDEVLEMLQRFKDSTIKR
EREVFNCMLRNLFEEYRFFPQYPDKELHITACLFGGIIEKGLVTYMALGLALRYVLEALRKPFGSKMYYFGIA
ALDRFKNRLKDYPQYCQHLASISHFMQFPHHLQEYIEYGQQSRDPPVKMQGSITTPGSIALAQAQAQAQVPAK
APLAGQVNTMVTTSTTTTVAKTVTVTKPTGVSFKKDVPPSINTTNIDTLLVATDQTERIVEPPENIQEKIAFI
FNNLSQSNMTQKVEELKETVKEEFMPWVSQYLVMKRVSIEPNFHSLYSNFLDTLKNPEFNKMVLNETYRNIKV
LLTSDKAAANFSDRSLLKNLGHWLGMITLAKNKPILHTDLDVKSLLLEAYVKGQQELLYVVPFVAKVLESSIR
SLVFRPPNPWTMAIMNVLAELHQEHDLKLNLKFEIEVLCKNLALDINELKPGNLLKDKDRLKNLDEQLSAPKK
DVKQPEELPAITTTTTSTTPATSTTCTATVPPQPQYSYHDINVYSLAGLAPHITLNPTIPLFQAHPQLKQCVR
QAIERAVQELVHPVVDRSIKIAMTTCEQIVRKDFALDSEESRMRIAAHHMMRNLTAGMAMITCREPLLMSIST
NLKNSFASALRTASPQQREMMDQAAAQLAQDNCELACCFIQKTAVEKAGPEMDKRLATEFELRKHARQEGRRY
CDPVVLTYQAERMPEQIRLKVGGVDPKQLAVYEEFARNVPGFLPTNDLSQPTGFLAQPMKQAWATDDVAQIYD
KCITELEQHLHAIPPTLAMNPQAQALRSLLEVVVLSRNSRDAIAALGLLQKAVEGLLDATSGADADLLLRYRE
CHLLVLKALQDGRAYGSPWCNKQITRCLIECRDEYKYNVEAVELLIRNHLVNMQQYDLHLAQSMENGLNYMAV
AFAMQLVKILLVDERSVAHITEADLFHTIETLMRINAHSRGNAPEGLPQLMEVVRSNYEAMIDRAHGGPNFMM
HSGISQASEYDDPPGLREKAEYLLREWVNLYHSAAAGRDSTKAFSAFVGQMHQQGILKTDDLITRFFRLCTEM
CVEISYRAQAEQQHNPAANPTMIRAKCYHNLDAFVRLIALLVKHSGEATNTVTKINLLNKVLGIVVGVLLQDH
DVRQSEFQQLPYHRIFIMLLLELNAPEHVLETINFQTLTAFCNTFHILRPTKAPGFVYAWLELISHRIFIARM
LAHTPQQKGWPMYAQLLIDLFKYLAPFLRNVELTKSMQILYKGTLRVLLVLLHDFPEFLCDYHYGFCDVIPPN
CIQLRNLILSAFPRNMRLPDPFTPNLKVDMLSEINIAPRILTNFTGVMPPQFKKDLDSYLKTRSPVTFLSDLR
SNLQVSNEPGNRYNLQLINALVLYVGTQAIAHIHNKGSTPSMSTITHSAHMDIFQNLAVDLDTEGRYLFLNAI
ANQLRYPNSHTHYFSCTMLYLFAEANTEAIQEQITRVLLERLIVNRPHPWGLLITFIELIKNPAFKFWNHEFV
HCAPEIEKLFQSVAQCCMGQKQAQQVMEGTGAS*
>rs107850230_ENSMUSP00000096073_c.6258:C>T_p.P2087S
ENSMUST00000098473.11|Cnot1-202|ENSMUSG00000036550|-|chr8:g.96454231:G>A
MNLDSLSLALSQISYLVDNLTKKNYRASQQEIQHIVNRHGPEADRHLLRCLFSHVDFSGDGKSSGKDFHQTQF
LIQECASLITKPNFISTLSYAIDNPLHYQKSLKPAPHLFAQLSKVLKLSKVQEVIFGLALLNSSSPDLRGFAA
QFIKQKLPDLLRSYIDADVSGNQEGGFQDIAIEVLHLLLSHLLFGQKGAFGVGQEQIDAFLKTLRRDFPQERC
PVVLAPLLYPEKRDILMDRILPDSGGVAKTMMESSLADFMQEVGYGFCASIEECRNIIMQFGVREVTAAQVAR
VLGMMARTHSGLTDGIPLQSISAPGSGIWSDGKDKSEGAQAHTWNVEVLIDVLKELNPSLNFKEVTYELDHPG
FQIRDSKGLHNVVYGIQRGLGMEVFPVDFIYRPWKHAEGQLSFIQHSLINPEVFCFADYPCHTVATDILKAPP
EDDNREIATWKSLDLIESLLRLAEVGQYEQVKQLFSFPIKHCPDMLVLALLQINTSWHTLRHELISTLMPIFL
GNHPNSAIILHYAWHGQGQSPSIRQLIMHAMAEWYMRGEQYDQAKLSRILDVAQDLKALSMLLNGTPFAFVID
LAALASRREYLKLDKWLTDKIREHGEPFIQACMTFLKRRCPSILGGLAPEKDQPKSAQLPAETLATMLACLQA
CAGSVSQELSETILTMVANCSNVMNKARQPPPGVMPKGRPPSASSLDAISPVQIDPLAGMASLSIGGSAAPHT
QSMQGFPPNLGSAFSTPQSPAKAFPPLSTPNQTTAFSGIGGLSSQLPVGGLGTGSLTGIGTGALGLPAVNNDP
FVQRKLGTSGLNQPTFQQSKMKPSDLSQVWPEANQHFSKEIDDEANSYFQRIYNHPPHPTMSVDEVLEMLQRF
KDSTIKREREVFNCMLRNLFEEYRFFPQYPDKELHITACLFGGIIEKGLVTYMALGLALRYVLEALRKPFGSK
MYYFGIAALDRFKNRLKDYPQYCQHLASISHFMQFPHHLQEYIEYGQQSRDPPVKMQGSITTPGSIALAQAQA
QAQVPAKAPLAGQVNTMVTTSTTTTVAKTVTVTKPTGVSFKKDVPPSINTTNIDTLLVATDQTERIVEPPENI
QEKIAFIFNNLSQSNMTQKVEELKETVKEEFMPWVSQYLVMKRVSIEPNFHSLYSNFLDTLKNPEFNKMVLNE
TYRNIKVLLTSDKAAANFSDRSLLKNLGHWLGMITLAKNKPILHTDLDVKSLLLEAYVKGQQELLYVVPFVAK
VLESSIRSLVFRPPNPWTMAIMNVLAELHQEHDLKLNLKFEIEVLCKNLALDINELKPGNLLKDKDRLKNLDE
QLSAPKKDVKQPEELPAITTTTTSTTPATSTTCTATVPPQPQYSYHDINVYSLAGLAPHITLNPTIPLFQAHP
QLKQCVRQAIERAVQELVHPVVDRSIKIAMTTCEQIVRKDFALDSEESRMRIAAHHMMRNLTAGMAMITCREP
LLMSISTNLKNSFASALRTASPQQREMMDQAAAQLAQDNCELACCFIQKTAVEKAGPEMDKRLATEFELRKHA
RQEGRRYCDPVVLTYQAERMPEQIRLKVGGVDPKQLAVYEEFARNVPGFLPTNDLSQPTGFLAQPMKQAWATD
DVAQIYDKCITELEQHLHAIPPTLAMNPQAQALRSLLEVVVLSRNSRDAIAALGLLQKAVEGLLDATSGADAD
LLLRYRECHLLVLKALQDGRAYGSPWCNKQITRCLIECRDEYKYNVEAVELLIRNHLVNMQQYDLHLAQSMEN
GLNYMAVAFAMQLVKILLVDERSVAHITEADLFHTIETLMRINAHSRGNAPEGLPQLMEVVRSNYEAMIDRAH
GGPNFMMHSGISQASEYDDPPGLREKAEYLLREWVNLYHSAAAGRDSTKAFSAFVGQMHQQGILKTDDLITRF
FRLCTEMCVEISYRAQAEQQHNPAANPTMIRAKCYHNLDAFVRLIALLVKHSGEATNTVTKINLLNKVLGIVV
GVLLQDHDVRQSEFQQLPYHRIFIMLLLELNAPEHVLETINFQTLTAFCNTFHILRPTKAPGFVYAWLELISH
RIFIARMLAHTPQQKGWPMYAQLLIDLFKYLAPFLRNVELTKSMQILYKGTLRVLLVLLHDFPEFLCDYHYGF
CDVIPPNCIQLRNLILSAFPRNMRLPDPFTPNLKVDMLSEINIAPRILTNFTGVMPPQFKKDLDSYLKTRSPV
TFLSDLRSNLQVSNEPGNRYNLQLINALVLYVGTQAIAHIHNKGSTPSMSTITHSAHMDIFQNLAVDLDTEGR

YLFLNAIANQLRYPNSHTHYFSCTMLYLFAEANTEAIQEQITRVLLERLIVNRPHPWGLLITFIELIKNPAFK
FWNHEFVHCAPEIEKLFQSVAQCCMGQKQAQQVMEGTGAS*
>rs46179737_ENSMUSP00000063565_c.6205:A>G_p.Y2069C
ENSMUST00000068452.10|Cnot1-201|ENSMUSG00000036550|-|chr8:g.96454269:T>C
MNLDSLSLALSQISYLVDNLTKKNYRASQQEIQHIVNRHGPEADRHLLRCLFSHVDFSGDGKSSGKDFHQTQF
LIQECASLITKPNFISTLSYAIDNPLHYQKSLKPAPHLFAQLSKVLKLSKVQEVIFGLALLNSSSPDLRGFAA
QFIKQKLPDLLRSYIDADVSGNQEGGFQDIAIEVLHLLLSHLLFGQKGAFGVGQEQIDAFLKTLRRDFPQERC
PVVLAPLLYPEKRDILMDRILPDSGGVAKTMMESSLADFMQEVGYGFCASIEECRNIIMQFGVREVTAAQVAR
VLGMMARTHSGLTDGIPLQSISAPGSGIWSDGKDKSEGAQAHTWNVEVLIDVLKELNPSLNFKEVTYELDHPG
FQIRDSKGLHNVVYGIQRGLGMEVFPVDFIYRPWKHAEGQLSFIQHSLINPEVFCFADYPCHTVATDILKAPP
EDDNREIATWKSLDLIESLLRLAEVGQYEQVKQLFSFPIKHCPDMLVLALLQINTSWHTLRHELISTLMPIFL
GNHPNSAIILHYAWHGQGQSPSIRQLIMHAMAEWYMRGEQYDQAKLSRILDVAQDLKALSMLLNGTPFAFVID
LAALASRREYLKLDKWLTDKIREHGEPFIQACMTFLKRRCPSILGGLAPEKDQPKSAQLPAETLATMLACLQA
CAGSVSQELSETILTMVANCSNVMNKARQPPPGVMPKGRPPSASSLDAISPVQIDPLAGMASLSIGGSAAPHT
QSMQGFPPNLGSAFSTPQSPAKAFPPLSTPNQTTAFSGIGGLSSQLPVGGLGTGSLTGIGTGALGLPAVNNDP
FVQRKLGTSGLNQPTFQQTDLSQVWPEANQHFSKEIDDEANSYFQRIYNHPPHPTMSVDEVLEMLQRFKDSTI
KREREVFNCMLRNLFEEYRFFPQYPDKELHITACLFGGIIEKGLVTYMALGLALRYVLEALRKPFGSKMYYFG
IAALDRFKNRLKDYPQYCQHLASISHFMQFPHHLQEYIEYGQQSRDPPVKMQGSITTPGSIALAQAQAQAQVP
AKAPLAGQVNTMVTTSTTTTVAKTVTVTKPTGVSFKKDVPPSINTTNIDTLLVATDQTERIVEPPENIQEKIA
FIFNNLSQSNMTQKVEELKETVKEEFMPWVSQYLVMKRVSIEPNFHSLYSNFLDTLKNPEFNKMVLNETYRNI
KVLLTSDKAAANFSDRSLLKNLGHWLGMITLAKNKPILHTDLDVKSLLLEAYVKGQQELLYVVPFVAKVLESS
IRSLVFRPPNPWTMAIMNVLAELHQEHDLKLNLKFEIEVLCKNLALDINELKPGNLLKDKDRLKNLDEQLSAP
KKDVKQPEELPAITTTTTSTTPATSTTCTATVPPQPQYSYHDINVYSLAGLAPHITLNPTIPLFQAHPQLKQC
VRQAIERAVQELVHPVVDRSIKIAMTTCEQIVRKDFALDSEESRMRIAAHHMMRNLTAGMAMITCREPLLMSI
STNLKNSFASALRTASPQQREMMDQAAAQLAQDNCELACCFIQKTAVEKAGPEMDKRLATEFELRKHARQEGR
RYCDPVVLTYQAERMPEQIRLKVGGVDPKQLAVYEEFARNVPGFLPTNDLSQPTGFLAQPMKQAWATDDVAQI
YDKCITELEQHLHAIPPTLAMNPQAQALRSLLEVVVLSRNSRDAIAALGLLQKAVEGLLDATSGADADLLLRY
RECHLLVLKALQDGRAYGSPWCNKQITRCLIECRDEYKYNVEAVELLIRNHLVNMQQYDLHLAQSMENGLNYM
AVAFAMQLVKILLVDERSVAHITEADLFHTIETLMRINAHSRGNAPEGLPQLMEVVRSNYEAMIDRAHGGPNF
MMHSGISQASEYDDPPGLREKAEYLLREWVNLYHSAAAGRDSTKAFSAFVGQMHQQGILKTDDLITRFFRLCT
EMCVEISYRAQAEQQHNPAANPTMIRAKCYHNLDAFVRLIALLVKHSGEATNTVTKINLLNKVLGIVVGVLLQ
DHDVRQSEFQQLPYHRIFIMLLLELNAPEHVLETINFQTLTAFCNTFHILRPTKAPGFVYAWLELISHRIFIA
RMLAHTPQQKGWPMYAQLLIDLFKCLAPFLRNVELTKPMQILYKGTLRVLLVLLHDFPEFLCDYHYGFCDVIP
PNCIQLRNLILSAFPRNMRLPDPFTPNLKVDMLSEINIAPRILTNFTGVMPPQFKKDLDSYLKTRSPVTFLSD
LRSNLQVSNEPGNRYNLQLINALVLYVGTQAIAHIHNKGSTPSMSTITHSAHMDIFQNLAVDLDTEGRYLFLN
AIANQLRYPNSHTHYFSCTMLYLFAEANTEAIQEQITRLFQSVAQCCMGQKQAQQVMEGTGAS*
>rs46179737_ENSMUSP00000148807_c.6199:A>G_p.Y2067C
ENSMUST00000211887.2|Cnot1-203|ENSMUSG00000036550|-|chr8:g.96454269:T>C
MNLDSLSLALSQISYLVDNLTKKNYRASQQEIQHIVNRHGPEADRHLLRCLFSHVDFSGDGKSSGKDFHQFLI
QECASLITKPNFISTLSYAIDNPLHYQKSLKPAPHLFAQLSKVLKLSKVQEVIFGLALLNSSSPDLRGFAAQF
IKQKLPDLLRSYIDADVSGNQEGGFQDIAIEVLHLLLSHLLFGQKGAFGVGQEQIDAFLKTLRRDFPQERCPV
VLAPLLYPEKRDILMDRILPDSGGVAKTMMESSLADFMQEVGYGFCASIEECRNIIMQFGVREVTAAQVARVL
GMMARTHSGLTDGIPLQSISAPGSGIWSDGKDKSEGAQAHTWNVEVLIDVLKELNPSLNFKEVTYELDHPGFQ
IRDSKGLHNVVYGIQRGLGMEVFPVDFIYRPWKHAEGQLSFIQHSLINPEVFCFADYPCHTVATDILKAPPED
DNREIATWKSLDLIESLLRLAEVGQYEQVKQLFSFPIKHCPDMLVLALLQINTSWHTLRHELISTLMPIFLGN
HPNSAIILHYAWHGQGQSPSIRQLIMHAMAEWYMRGEQYDQAKLSRILDVAQDLKALSMLLNGTPFAFVIDLA
ALASRREYLKLDKWLTDKIREHGEPFIQACMTFLKRRCPSILGGLAPEKDQPKSAQLPAETLATMLACLQACA
GSVSQELSETILTMVANCSNVMNKARQPPPGVMPKGRPPSASSLDAISPVQIDPLAGMASLSIGGSAAPHTQS
MQGFPPNLGSAFSTPQSPAKAFPPLSTPNQTTAFSGIGGLSSQLPVGGLGTGSLTGIGTGALGLPAVNNDPFV
QRKLGTSGLNQPTFQQTDLSQVWPEANQHFSKEIDDEANSYFQRIYNHPPHPTMSVDEVLEMLQRFKDSTIKR
EREVFNCMLRNLFEEYRFFPQYPDKELHITACLFGGIIEKGLVTYMALGLALRYVLEALRKPFGSKMYYFGIA
ALDRFKNRLKDYPQYCQHLASISHFMQFPHHLQEYIEYGQQSRDPPVKMQGSITTPGSIALAQAQAQAQVPAK
APLAGQVNTMVTTSTTTTVAKTVTVTKPTGVSFKKDVPPSINTTNIDTLLVATDQTERIVEPPENIQEKIAFI
FNNLSQSNMTQKVEELKETVKEEFMPWVSQYLVMKRVSIEPNFHSLYSNFLDTLKNPEFNKMVLNETYRNIKV
LLTSDKAAANFSDRSLLKNLGHWLGMITLAKNKPILHTDLDVKSLLLEAYVKGQQELLYVVPFVAKVLESSIR
SLVFRPPNPWTMAIMNVLAELHQEHDLKLNLKFEIEVLCKNLALDINELKPGNLLKDKDRLKNLDEQLSAPKK
DVKQPEELPAITTTTTSTTPATSTTCTATVPPQPQYSYHDINVYSLAGLAPHITLNPTIPLFQAHPQLKQCVR
QAIERAVQELVHPVVDRSIKIAMTTCEQIVRKDFALDSEESRMRIAAHHMMRNLTAGMAMITCREPLLMSIST

NLKNSFASALRTASPQQREMMDQAAAQLAQDNCELACCFIQKTAVEKAGPEMDKRLATEFELRKHARQEGRRY
CDPVVLTYQAERMPEQIRLKVGGVDPKQLAVYEEFARNVPGFLPTNDLSQPTGFLAQPMKQAWATDDVAQIYD
KCITELEQHLHAIPPTLAMNPQAQALRSLLEVVVLSRNSRDAIAALGLLQKAVEGLLDATSGADADLLLRYRE
CHLLVLKALQDGRAYGSPWCNKQITRCLIECRDEYKYNVEAVELLIRNHLVNMQQYDLHLAQSMENGLNYMAV
AFAMQLVKILLVDERSVAHITEADLFHTIETLMRINAHSRGNAPEGLPQLMEVVRSNYEAMIDRAHGGPNFMM
HSGISQASEYDDPPGLREKAEYLLREWVNLYHSAAAGRDSTKAFSAFVGQMHQQGILKTDDLITRFFRLCTEM
CVEISYRAQAEQQHNPAANPTMIRAKCYHNLDAFVRLIALLVKHSGEATNTVTKINLLNKVLGIVVGVLLQDH
DVRQSEFQQLPYHRIFIMLLLELNAPEHVLETINFQTLTAFCNTFHILRPTKAPGFVYAWLELISHRIFIARM
LAHTPQQKGWPMYAQLLIDLFKCLAPFLRNVELTKPMQILYKGTLRVLLVLLHDFPEFLCDYHYGFCDVIPPN
CIQLRNLILSAFPRNMRLPDPFTPNLKVDMLSEINIAPRILTNFTGVMPPQFKKDLDSYLKTRSPVTFLSDLR
SNLQVSNEPGNRYNLQLINALVLYVGTQAIAHIHNKGSTPSMSTITHSAHMDIFQNLAVDLDTEGRYLFLNAI
ANQLRYPNSHTHYFSCTMLYLFAEANTEAIQEQITRVLLERLIVNRPHPWGLLITFIELIKNPAFKFWNHEFV
HCAPEIEKLFQSVAQCCMGQKQAQQVMEGTGAS*
>rs46179737_ENSMUSP00000096073_c.6220:A>G_p.Y2074C
ENSMUST00000098473.11|Cnot1-202|ENSMUSG00000036550|-|chr8:g.96454269:T>C
MNLDSLSLALSQISYLVDNLTKKNYRASQQEIQHIVNRHGPEADRHLLRCLFSHVDFSGDGKSSGKDFHQTQF
LIQECASLITKPNFISTLSYAIDNPLHYQKSLKPAPHLFAQLSKVLKLSKVQEVIFGLALLNSSSPDLRGFAA
QFIKQKLPDLLRSYIDADVSGNQEGGFQDIAIEVLHLLLSHLLFGQKGAFGVGQEQIDAFLKTLRRDFPQERC
PVVLAPLLYPEKRDILMDRILPDSGGVAKTMMESSLADFMQEVGYGFCASIEECRNIIMQFGVREVTAAQVAR
VLGMMARTHSGLTDGIPLQSISAPGSGIWSDGKDKSEGAQAHTWNVEVLIDVLKELNPSLNFKEVTYELDHPG
FQIRDSKGLHNVVYGIQRGLGMEVFPVDFIYRPWKHAEGQLSFIQHSLINPEVFCFADYPCHTVATDILKAPP
EDDNREIATWKSLDLIESLLRLAEVGQYEQVKQLFSFPIKHCPDMLVLALLQINTSWHTLRHELISTLMPIFL
GNHPNSAIILHYAWHGQGQSPSIRQLIMHAMAEWYMRGEQYDQAKLSRILDVAQDLKALSMLLNGTPFAFVID
LAALASRREYLKLDKWLTDKIREHGEPFIQACMTFLKRRCPSILGGLAPEKDQPKSAQLPAETLATMLACLQA
CAGSVSQELSETILTMVANCSNVMNKARQPPPGVMPKGRPPSASSLDAISPVQIDPLAGMASLSIGGSAAPHT
QSMQGFPPNLGSAFSTPQSPAKAFPPLSTPNQTTAFSGIGGLSSQLPVGGLGTGSLTGIGTGALGLPAVNNDP
FVQRKLGTSGLNQPTFQQSKMKPSDLSQVWPEANQHFSKEIDDEANSYFQRIYNHPPHPTMSVDEVLEMLQRF
KDSTIKREREVFNCMLRNLFEEYRFFPQYPDKELHITACLFGGIIEKGLVTYMALGLALRYVLEALRKPFGSK
MYYFGIAALDRFKNRLKDYPQYCQHLASISHFMQFPHHLQEYIEYGQQSRDPPVKMQGSITTPGSIALAQAQA
QAQVPAKAPLAGQVNTMVTTSTTTTVAKTVTVTKPTGVSFKKDVPPSINTTNIDTLLVATDQTERIVEPPENI
QEKIAFIFNNLSQSNMTQKVEELKETVKEEFMPWVSQYLVMKRVSIEPNFHSLYSNFLDTLKNPEFNKMVLNE
TYRNIKVLLTSDKAAANFSDRSLLKNLGHWLGMITLAKNKPILHTDLDVKSLLLEAYVKGQQELLYVVPFVAK
VLESSIRSLVFRPPNPWTMAIMNVLAELHQEHDLKLNLKFEIEVLCKNLALDINELKPGNLLKDKDRLKNLDE
QLSAPKKDVKQPEELPAITTTTTSTTPATSTTCTATVPPQPQYSYHDINVYSLAGLAPHITLNPTIPLFQAHP
QLKQCVRQAIERAVQELVHPVVDRSIKIAMTTCEQIVRKDFALDSEESRMRIAAHHMMRNLTAGMAMITCREP
LLMSISTNLKNSFASALRTASPQQREMMDQAAAQLAQDNCELACCFIQKTAVEKAGPEMDKRLATEFELRKHA
RQEGRRYCDPVVLTYQAERMPEQIRLKVGGVDPKQLAVYEEFARNVPGFLPTNDLSQPTGFLAQPMKQAWATD
DVAQIYDKCITELEQHLHAIPPTLAMNPQAQALRSLLEVVVLSRNSRDAIAALGLLQKAVEGLLDATSGADAD
LLLRYRECHLLVLKALQDGRAYGSPWCNKQITRCLIECRDEYKYNVEAVELLIRNHLVNMQQYDLHLAQSMEN
GLNYMAVAFAMQLVKILLVDERSVAHITEADLFHTIETLMRINAHSRGNAPEGLPQLMEVVRSNYEAMIDRAH
GGPNFMMHSGISQASEYDDPPGLREKAEYLLREWVNLYHSAAAGRDSTKAFSAFVGQMHQQGILKTDDLITRF
FRLCTEMCVEISYRAQAEQQHNPAANPTMIRAKCYHNLDAFVRLIALLVKHSGEATNTVTKINLLNKVLGIVV
GVLLQDHDVRQSEFQQLPYHRIFIMLLLELNAPEHVLETINFQTLTAFCNTFHILRPTKAPGFVYAWLELISH
RIFIARMLAHTPQQKGWPMYAQLLIDLFKCLAPFLRNVELTKPMQILYKGTLRVLLVLLHDFPEFLCDYHYGF
CDVIPPNCIQLRNLILSAFPRNMRLPDPFTPNLKVDMLSEINIAPRILTNFTGVMPPQFKKDLDSYLKTRSPV
TFLSDLRSNLQVSNEPGNRYNLQLINALVLYVGTQAIAHIHNKGSTPSMSTITHSAHMDIFQNLAVDLDTEGR
YLFLNAIANQLRYPNSHTHYFSCTMLYLFAEANTEAIQEQITRVLLERLIVNRPHPWGLLITFIELIKNPAFK
FWNHEFVHCAPEIEKLFQSVAQCCMGQKQAQQVMEGTGAS*
>rs264083360_ENSMUSP00000063565_c.6049:C>TTC_p.T2017ins-fs
ENSMUST00000068452.10|Cnot1-201|ENSMUSG00000036550|-
|chr8:g.96455355:G>GAA
MNLDSLSLALSQISYLVDNLTKKNYRASQQEIQHIVNRHGPEADRHLLRCLFSHVDFSGDGKSSGKDFHQTQF
LIQECASLITKPNFISTLSYAIDNPLHYQKSLKPAPHLFAQLSKVLKLSKVQEVIFGLALLNSSSPDLRGFAA
QFIKQKLPDLLRSYIDADVSGNQEGGFQDIAIEVLHLLLSHLLFGQKGAFGVGQEQIDAFLKTLRRDFPQERC
PVVLAPLLYPEKRDILMDRILPDSGGVAKTMMESSLADFMQEVGYGFCASIEECRNIIMQFGVREVTAAQVAR
VLGMMARTHSGLTDGIPLQSISAPGSGIWSDGKDKSEGAQAHTWNVEVLIDVLKELNPSLNFKEVTYELDHPG
FQIRDSKGLHNVVYGIQRGLGMEVFPVDFIYRPWKHAEGQLSFIQHSLINPEVFCFADYPCHTVATDILKAPP
EDDNREIATWKSLDLIESLLRLAEVGQYEQVKQLFSFPIKHCPDMLVLALLQINTSWHTLRHELISTLMPIFL

GNHPNSAIILHYAWHGQGQSPSIRQLIMHAMAEWYMRGEQYDQAKLSRILDVAQDLKALSMLLNGTPFAFVID
LAALASRREYLKLDKWLTDKIREHGEPFIQACMTFLKRRCPSILGGLAPEKDQPKSAQLPAETLATMLACLQA
CAGSVSQELSETILTMVANCSNVMNKARQPPPGVMPKGRPPSASSLDAISPVQIDPLAGMASLSIGGSAAPHT
QSMQGFPPNLGSAFSTPQSPAKAFPPLSTPNQTTAFSGIGGLSSQLPVGGLGTGSLTGIGTGALGLPAVNNDP
FVQRKLGTSGLNQPTFQQTDLSQVWPEANQHFSKEIDDEANSYFQRIYNHPPHPTMSVDEVLEMLQRFKDSTI
KREREVFNCMLRNLFEEYRFFPQYPDKELHITACLFGGIIEKGLVTYMALGLALRYVLEALRKPFGSKMYYFG
IAALDRFKNRLKDYPQYCQHLASISHFMQFPHHLQEYIEYGQQSRDPPVKMQGSITTPGSIALAQAQAQAQVP
AKAPLAGQVNTMVTTSTTTTVAKTVTVTKPTGVSFKKDVPPSINTTNIDTLLVATDQTERIVEPPENIQEKIA
FIFNNLSQSNMTQKVEELKETVKEEFMPWVSQYLVMKRVSIEPNFHSLYSNFLDTLKNPEFNKMVLNETYRNI
KVLLTSDKAAANFSDRSLLKNLGHWLGMITLAKNKPILHTDLDVKSLLLEAYVKGQQELLYVVPFVAKVLESS
IRSLVFRPPNPWTMAIMNVLAELHQEHDLKLNLKFEIEVLCKNLALDINELKPGNLLKDKDRLKNLDEQLSAP
KKDVKQPEELPAITTTTTSTTPATSTTCTATVPPQPQYSYHDINVYSLAGLAPHITLNPTIPLFQAHPQLKQC
VRQAIERAVQELVHPVVDRSIKIAMTTCEQIVRKDFALDSEESRMRIAAHHMMRNLTAGMAMITCREPLLMSI
STNLKNSFASALRTASPQQREMMDQAAAQLAQDNCELACCFIQKTAVEKAGPEMDKRLATEFELRKHARQEGR
RYCDPVVLTYQAERMPEQIRLKVGGVDPKQLAVYEEFARNVPGFLPTNDLSQPTGFLAQPMKQAWATDDVAQI
YDKCITELEQHLHAIPPTLAMNPQAQALRSLLEVVVLSRNSRDAIAALGLLQKAVEGLLDATSGADADLLLRY
RECHLLVLKALQDGRAYGSPWCNKQITRCLIECRDEYKYNVEAVELLIRNHLVNMQQYDLHLAQSMENGLNYM
AVAFAMQLVKILLVDERSVAHITEADLFHTIETLMRINAHSRGNAPEGLPQLMEVVRSNYEAMIDRAHGGPNF
MMHSGISQASEYDDPPGLREKAEYLLREWVNLYHSAAAGRDSTKAFSAFVGQMHQQGILKTDDLITRFFRLCT
EMCVEISYRAQAEQQHNPAANPTMIRAKCYHNLDAFVRLIALLVKHSGEATNTVTKINLLNKVLGIVVGVLLQ
DHDVRQSEFQQLPYHRIFIMLLLELNAPEHVLETINFQTLTAFCNIHSTS
>rs264083360_ENSMUSP00000148807_c.6043:C>TTC_p.T2015ins-fs
ENSMUST00000211887.2|Cnot1-203|ENSMUSG00000036550|-|chr8:g.96455355:G>GAA
MNLDSLSLALSQISYLVDNLTKKNYRASQQEIQHIVNRHGPEADRHLLRCLFSHVDFSGDGKSSGKDFHQFLI
QECASLITKPNFISTLSYAIDNPLHYQKSLKPAPHLFAQLSKVLKLSKVQEVIFGLALLNSSSPDLRGFAAQF
IKQKLPDLLRSYIDADVSGNQEGGFQDIAIEVLHLLLSHLLFGQKGAFGVGQEQIDAFLKTLRRDFPQERCPV
VLAPLLYPEKRDILMDRILPDSGGVAKTMMESSLADFMQEVGYGFCASIEECRNIIMQFGVREVTAAQVARVL
GMMARTHSGLTDGIPLQSISAPGSGIWSDGKDKSEGAQAHTWNVEVLIDVLKELNPSLNFKEVTYELDHPGFQ
IRDSKGLHNVVYGIQRGLGMEVFPVDFIYRPWKHAEGQLSFIQHSLINPEVFCFADYPCHTVATDILKAPPED
DNREIATWKSLDLIESLLRLAEVGQYEQVKQLFSFPIKHCPDMLVLALLQINTSWHTLRHELISTLMPIFLGN
HPNSAIILHYAWHGQGQSPSIRQLIMHAMAEWYMRGEQYDQAKLSRILDVAQDLKALSMLLNGTPFAFVIDLA
ALASRREYLKLDKWLTDKIREHGEPFIQACMTFLKRRCPSILGGLAPEKDQPKSAQLPAETLATMLACLQACA
GSVSQELSETILTMVANCSNVMNKARQPPPGVMPKGRPPSASSLDAISPVQIDPLAGMASLSIGGSAAPHTQS
MQGFPPNLGSAFSTPQSPAKAFPPLSTPNQTTAFSGIGGLSSQLPVGGLGTGSLTGIGTGALGLPAVNNDPFV
QRKLGTSGLNQPTFQQTDLSQVWPEANQHFSKEIDDEANSYFQRIYNHPPHPTMSVDEVLEMLQRFKDSTIKR
EREVFNCMLRNLFEEYRFFPQYPDKELHITACLFGGIIEKGLVTYMALGLALRYVLEALRKPFGSKMYYFGIA
ALDRFKNRLKDYPQYCQHLASISHFMQFPHHLQEYIEYGQQSRDPPVKMQGSITTPGSIALAQAQAQAQVPAK
APLAGQVNTMVTTSTTTTVAKTVTVTKPTGVSFKKDVPPSINTTNIDTLLVATDQTERIVEPPENIQEKIAFI
FNNLSQSNMTQKVEELKETVKEEFMPWVSQYLVMKRVSIEPNFHSLYSNFLDTLKNPEFNKMVLNETYRNIKV
LLTSDKAAANFSDRSLLKNLGHWLGMITLAKNKPILHTDLDVKSLLLEAYVKGQQELLYVVPFVAKVLESSIR
SLVFRPPNPWTMAIMNVLAELHQEHDLKLNLKFEIEVLCKNLALDINELKPGNLLKDKDRLKNLDEQLSAPKK
DVKQPEELPAITTTTTSTTPATSTTCTATVPPQPQYSYHDINVYSLAGLAPHITLNPTIPLFQAHPQLKQCVR
QAIERAVQELVHPVVDRSIKIAMTTCEQIVRKDFALDSEESRMRIAAHHMMRNLTAGMAMITCREPLLMSIST
NLKNSFASALRTASPQQREMMDQAAAQLAQDNCELACCFIQKTAVEKAGPEMDKRLATEFELRKHARQEGRRY
CDPVVLTYQAERMPEQIRLKVGGVDPKQLAVYEEFARNVPGFLPTNDLSQPTGFLAQPMKQAWATDDVAQIYD
KCITELEQHLHAIPPTLAMNPQAQALRSLLEVVVLSRNSRDAIAALGLLQKAVEGLLDATSGADADLLLRYRE
CHLLVLKALQDGRAYGSPWCNKQITRCLIECRDEYKYNVEAVELLIRNHLVNMQQYDLHLAQSMENGLNYMAV
AFAMQLVKILLVDERSVAHITEADLFHTIETLMRINAHSRGNAPEGLPQLMEVVRSNYEAMIDRAHGGPNFMM
HSGISQASEYDDPPGLREKAEYLLREWVNLYHSAAAGRDSTKAFSAFVGQMHQQGILKTDDLITRFFRLCTEM
CVEISYRAQAEQQHNPAANPTMIRAKCYHNLDAFVRLIALLVKHSGEATNTVTKINLLNKVLGIVVGVLLQDH
DVRQSEFQQLPYHRIFIMLLLELNAPEHVLETINFQTLTAFCNIHSTS
>rs264083360_ENSMUSP00000096073_c.6064:C>TTC_p.T2022ins-fs
ENSMUST00000098473.11|Cnot1-202|ENSMUSG00000036550|-
|chr8:g.96455355:G>GAA
MNLDSLSLALSQISYLVDNLTKKNYRASQQEIQHIVNRHGPEADRHLLRCLFSHVDFSGDGKSSGKDFHQTQF
LIQECASLITKPNFISTLSYAIDNPLHYQKSLKPAPHLFAQLSKVLKLSKVQEVIFGLALLNSSSPDLRGFAA
QFIKQKLPDLLRSYIDADVSGNQEGGFQDIAIEVLHLLLSHLLFGQKGAFGVGQEQIDAFLKTLRRDFPQERC
PVVLAPLLYPEKRDILMDRILPDSGGVAKTMMESSLADFMQEVGYGFCASIEECRNIIMQFGVREVTAAQVAR

VLGMMARTHSGLTDGIPLQSISAPGSGIWSDGKDKSEGAQAHTWNVEVLIDVLKELNPSLNFKEVTYELDHPG
FQIRDSKGLHNVVYGIQRGLGMEVFPVDFIYRPWKHAEGQLSFIQHSLINPEVFCFADYPCHTVATDILKAPP
EDDNREIATWKSLDLIESLLRLAEVGQYEQVKQLFSFPIKHCPDMLVLALLQINTSWHTLRHELISTLMPIFL
GNHPNSAIILHYAWHGQGQSPSIRQLIMHAMAEWYMRGEQYDQAKLSRILDVAQDLKALSMLLNGTPFAFVID
LAALASRREYLKLDKWLTDKIREHGEPFIQACMTFLKRRCPSILGGLAPEKDQPKSAQLPAETLATMLACLQA
CAGSVSQELSETILTMVANCSNVMNKARQPPPGVMPKGRPPSASSLDAISPVQIDPLAGMASLSIGGSAAPHT
QSMQGFPPNLGSAFSTPQSPAKAFPPLSTPNQTTAFSGIGGLSSQLPVGGLGTGSLTGIGTGALGLPAVNNDP
FVQRKLGTSGLNQPTFQQSKMKPSDLSQVWPEANQHFSKEIDDEANSYFQRIYNHPPHPTMSVDEVLEMLQRF
KDSTIKREREVFNCMLRNLFEEYRFFPQYPDKELHITACLFGGIIEKGLVTYMALGLALRYVLEALRKPFGSK
MYYFGIAALDRFKNRLKDYPQYCQHLASISHFMQFPHHLQEYIEYGQQSRDPPVKMQGSITTPGSIALAQAQA
QAQVPAKAPLAGQVNTMVTTSTTTTVAKTVTVTKPTGVSFKKDVPPSINTTNIDTLLVATDQTERIVEPPENI
QEKIAFIFNNLSQSNMTQKVEELKETVKEEFMPWVSQYLVMKRVSIEPNFHSLYSNFLDTLKNPEFNKMVLNE
TYRNIKVLLTSDKAAANFSDRSLLKNLGHWLGMITLAKNKPILHTDLDVKSLLLEAYVKGQQELLYVVPFVAK
VLESSIRSLVFRPPNPWTMAIMNVLAELHQEHDLKLNLKFEIEVLCKNLALDINELKPGNLLKDKDRLKNLDE
QLSAPKKDVKQPEELPAITTTTTSTTPATSTTCTATVPPQPQYSYHDINVYSLAGLAPHITLNPTIPLFQAHP
QLKQCVRQAIERAVQELVHPVVDRSIKIAMTTCEQIVRKDFALDSEESRMRIAAHHMMRNLTAGMAMITCREP
LLMSISTNLKNSFASALRTASPQQREMMDQAAAQLAQDNCELACCFIQKTAVEKAGPEMDKRLATEFELRKHA
RQEGRRYCDPVVLTYQAERMPEQIRLKVGGVDPKQLAVYEEFARNVPGFLPTNDLSQPTGFLAQPMKQAWATD
DVAQIYDKCITELEQHLHAIPPTLAMNPQAQALRSLLEVVVLSRNSRDAIAALGLLQKAVEGLLDATSGADAD
LLLRYRECHLLVLKALQDGRAYGSPWCNKQITRCLIECRDEYKYNVEAVELLIRNHLVNMQQYDLHLAQSMEN
GLNYMAVAFAMQLVKILLVDERSVAHITEADLFHTIETLMRINAHSRGNAPEGLPQLMEVVRSNYEAMIDRAH
GGPNFMMHSGISQASEYDDPPGLREKAEYLLREWVNLYHSAAAGRDSTKAFSAFVGQMHQQGILKTDDLITRF
FRLCTEMCVEISYRAQAEQQHNPAANPTMIRAKCYHNLDAFVRLIALLVKHSGEATNTVTKINLLNKVLGIVV
GVLLQDHDVRQSEFQQLPYHRIFIMLLLELNAPEHVLETINFQTLTAFCNIHSTS
>rs240993791_ENSMUSP00000063565_c.4114:A>G_p.N1372S
ENSMUST00000068452.10|Cnot1-201|ENSMUSG00000036550|-|chr8:g.96469739:T>C
MNLDSLSLALSQISYLVDNLTKKNYRASQQEIQHIVNRHGPEADRHLLRCLFSHVDFSGDGKSSGKDFHQTQF
LIQECASLITKPNFISTLSYAIDNPLHYQKSLKPAPHLFAQLSKVLKLSKVQEVIFGLALLNSSSPDLRGFAA
QFIKQKLPDLLRSYIDADVSGNQEGGFQDIAIEVLHLLLSHLLFGQKGAFGVGQEQIDAFLKTLRRDFPQERC
PVVLAPLLYPEKRDILMDRILPDSGGVAKTMMESSLADFMQEVGYGFCASIEECRNIIMQFGVREVTAAQVAR
VLGMMARTHSGLTDGIPLQSISAPGSGIWSDGKDKSEGAQAHTWNVEVLIDVLKELNPSLNFKEVTYELDHPG
FQIRDSKGLHNVVYGIQRGLGMEVFPVDFIYRPWKHAEGQLSFIQHSLINPEVFCFADYPCHTVATDILKAPP
EDDNREIATWKSLDLIESLLRLAEVGQYEQVKQLFSFPIKHCPDMLVLALLQINTSWHTLRHELISTLMPIFL
GNHPNSAIILHYAWHGQGQSPSIRQLIMHAMAEWYMRGEQYDQAKLSRILDVAQDLKALSMLLNGTPFAFVID
LAALASRREYLKLDKWLTDKIREHGEPFIQACMTFLKRRCPSILGGLAPEKDQPKSAQLPAETLATMLACLQA
CAGSVSQELSETILTMVANCSNVMNKARQPPPGVMPKGRPPSASSLDAISPVQIDPLAGMASLSIGGSAAPHT
QSMQGFPPNLGSAFSTPQSPAKAFPPLSTPNQTTAFSGIGGLSSQLPVGGLGTGSLTGIGTGALGLPAVNNDP
FVQRKLGTSGLNQPTFQQTDLSQVWPEANQHFSKEIDDEANSYFQRIYNHPPHPTMSVDEVLEMLQRFKDSTI
KREREVFNCMLRNLFEEYRFFPQYPDKELHITACLFGGIIEKGLVTYMALGLALRYVLEALRKPFGSKMYYFG
IAALDRFKNRLKDYPQYCQHLASISHFMQFPHHLQEYIEYGQQSRDPPVKMQGSITTPGSIALAQAQAQAQVP
AKAPLAGQVNTMVTTSTTTTVAKTVTVTKPTGVSFKKDVPPSINTTNIDTLLVATDQTERIVEPPENIQEKIA
FIFNNLSQSNMTQKVEELKETVKEEFMPWVSQYLVMKRVSIEPNFHSLYSNFLDTLKNPEFNKMVLNETYRNI
KVLLTSDKAAANFSDRSLLKNLGHWLGMITLAKNKPILHTDLDVKSLLLEAYVKGQQELLYVVPFVAKVLESS
IRSLVFRPPNPWTMAIMNVLAELHQEHDLKLNLKFEIEVLCKNLALDINELKPGNLLKDKDRLKNLDEQLSAP
KKDVKQPEELPAITTTTTSTTPATSTTCTATVPPQPQYSYHDINVYSLAGLAPHITLSPTIPLFQAHPQLKQC
VRQAIERAVQELVHPVVDRSIKIAMTTCEQIVRKDFALDSEESRMRIAAHHMMRNLTAGMAMITCREPLLMSI
STNLKNSFASALRTASPQQREMMDQAAAQLAQDNCELACCFIQKTAVEKAGPEMDKRLATEFELRKHARQEGR
RYCDPVVLTYQAERMPEQIRLKVGGVDPKQLAVYEEFARNVPGFLPTNDLSQPTGFLAQPMKQAWATDDVAQI
YDKCITELEQHLHAIPPTLAMNPQAQALRSLLEVVVLSRNSRDAIAALGLLQKAVEGLLDATSGADADLLLRY
RECHLLVLKALQDGRAYGSPWCNKQITRCLIECRDEYKYNVEAVELLIRNHLVNMQQYDLHLAQSMENGLNYM
AVAFAMQLVKILLVDERSVAHITEADLFHTIETLMRINAHSRGNAPEGLPQLMEVVRSNYEAMIDRAHGGPNF
MMHSGISQASEYDDPPGLREKAEYLLREWVNLYHSAAAGRDSTKAFSAFVGQMHQQGILKTDDLITRFFRLCT
EMCVEISYRAQAEQQHNPAANPTMIRAKCYHNLDAFVRLIALLVKHSGEATNTVTKINLLNKVLGIVVGVLLQ
DHDVRQSEFQQLPYHRIFIMLLLELNAPEHVLETINFQTLTAFCNTFHILRPTKAPGFVYAWLELISHRIFIA
RMLAHTPQQKGWPMYAQLLIDLFKYLAPFLRNVELTKPMQILYKGTLRVLLVLLHDFPEFLCDYHYGFCDVIP
PNCIQLRNLILSAFPRNMRLPDPFTPNLKVDMLSEINIAPRILTNFTGVMPPQFKKDLDSYLKTRSPVTFLSD
LRSNLQVSNEPGNRYNLQLINALVLYVGTQAIAHIHNKGSTPSMSTITHSAHMDIFQNLAVDLDTEGRYLFLN
AIANQLRYPNSHTHYFSCTMLYLFAEANTEAIQEQITRLFQSVAQCCMGQKQAQQVMEGTGAS*

>rs240993791_ENSMUSP00000148807_c.4108:A>G_p.N1370S
ENSMUST00000211887.2|Cnot1-203|ENSMUSG00000036550|-|chr8:g.96469739:T>C
MNLDSLSLALSQISYLVDNLTKKNYRASQQEIQHIVNRHGPEADRHLLRCLFSHVDFSGDGKSSGKDFHQFLI
QECASLITKPNFISTLSYAIDNPLHYQKSLKPAPHLFAQLSKVLKLSKVQEVIFGLALLNSSSPDLRGFAAQF
IKQKLPDLLRSYIDADVSGNQEGGFQDIAIEVLHLLLSHLLFGQKGAFGVGQEQIDAFLKTLRRDFPQERCPV
VLAPLLYPEKRDILMDRILPDSGGVAKTMMESSLADFMQEVGYGFCASIEECRNIIMQFGVREVTAAQVARVL
GMMARTHSGLTDGIPLQSISAPGSGIWSDGKDKSEGAQAHTWNVEVLIDVLKELNPSLNFKEVTYELDHPGFQ
IRDSKGLHNVVYGIQRGLGMEVFPVDFIYRPWKHAEGQLSFIQHSLINPEVFCFADYPCHTVATDILKAPPED
DNREIATWKSLDLIESLLRLAEVGQYEQVKQLFSFPIKHCPDMLVLALLQINTSWHTLRHELISTLMPIFLGN
HPNSAIILHYAWHGQGQSPSIRQLIMHAMAEWYMRGEQYDQAKLSRILDVAQDLKALSMLLNGTPFAFVIDLA
ALASRREYLKLDKWLTDKIREHGEPFIQACMTFLKRRCPSILGGLAPEKDQPKSAQLPAETLATMLACLQACA
GSVSQELSETILTMVANCSNVMNKARQPPPGVMPKGRPPSASSLDAISPVQIDPLAGMASLSIGGSAAPHTQS
MQGFPPNLGSAFSTPQSPAKAFPPLSTPNQTTAFSGIGGLSSQLPVGGLGTGSLTGIGTGALGLPAVNNDPFV
QRKLGTSGLNQPTFQQTDLSQVWPEANQHFSKEIDDEANSYFQRIYNHPPHPTMSVDEVLEMLQRFKDSTIKR
EREVFNCMLRNLFEEYRFFPQYPDKELHITACLFGGIIEKGLVTYMALGLALRYVLEALRKPFGSKMYYFGIA
ALDRFKNRLKDYPQYCQHLASISHFMQFPHHLQEYIEYGQQSRDPPVKMQGSITTPGSIALAQAQAQAQVPAK
APLAGQVNTMVTTSTTTTVAKTVTVTKPTGVSFKKDVPPSINTTNIDTLLVATDQTERIVEPPENIQEKIAFI
FNNLSQSNMTQKVEELKETVKEEFMPWVSQYLVMKRVSIEPNFHSLYSNFLDTLKNPEFNKMVLNETYRNIKV
LLTSDKAAANFSDRSLLKNLGHWLGMITLAKNKPILHTDLDVKSLLLEAYVKGQQELLYVVPFVAKVLESSIR
SLVFRPPNPWTMAIMNVLAELHQEHDLKLNLKFEIEVLCKNLALDINELKPGNLLKDKDRLKNLDEQLSAPKK
DVKQPEELPAITTTTTSTTPATSTTCTATVPPQPQYSYHDINVYSLAGLAPHITLSPTIPLFQAHPQLKQCVR
QAIERAVQELVHPVVDRSIKIAMTTCEQIVRKDFALDSEESRMRIAAHHMMRNLTAGMAMITCREPLLMSIST
NLKNSFASALRTASPQQREMMDQAAAQLAQDNCELACCFIQKTAVEKAGPEMDKRLATEFELRKHARQEGRRY
CDPVVLTYQAERMPEQIRLKVGGVDPKQLAVYEEFARNVPGFLPTNDLSQPTGFLAQPMKQAWATDDVAQIYD
KCITELEQHLHAIPPTLAMNPQAQALRSLLEVVVLSRNSRDAIAALGLLQKAVEGLLDATSGADADLLLRYRE
CHLLVLKALQDGRAYGSPWCNKQITRCLIECRDEYKYNVEAVELLIRNHLVNMQQYDLHLAQSMENGLNYMAV
AFAMQLVKILLVDERSVAHITEADLFHTIETLMRINAHSRGNAPEGLPQLMEVVRSNYEAMIDRAHGGPNFMM
HSGISQASEYDDPPGLREKAEYLLREWVNLYHSAAAGRDSTKAFSAFVGQMHQQGILKTDDLITRFFRLCTEM
CVEISYRAQAEQQHNPAANPTMIRAKCYHNLDAFVRLIALLVKHSGEATNTVTKINLLNKVLGIVVGVLLQDH
DVRQSEFQQLPYHRIFIMLLLELNAPEHVLETINFQTLTAFCNTFHILRPTKAPGFVYAWLELISHRIFIARM
LAHTPQQKGWPMYAQLLIDLFKYLAPFLRNVELTKPMQILYKGTLRVLLVLLHDFPEFLCDYHYGFCDVIPPN
CIQLRNLILSAFPRNMRLPDPFTPNLKVDMLSEINIAPRILTNFTGVMPPQFKKDLDSYLKTRSPVTFLSDLR
SNLQVSNEPGNRYNLQLINALVLYVGTQAIAHIHNKGSTPSMSTITHSAHMDIFQNLAVDLDTEGRYLFLNAI
ANQLRYPNSHTHYFSCTMLYLFAEANTEAIQEQITRVLLERLIVNRPHPWGLLITFIELIKNPAFKFWNHEFV
HCAPEIEKLFQSVAQCCMGQKQAQQVMEGTGAS*
>rs240993791_ENSMUSP00000148735_c.4129:A>G_p.N1377S
ENSMUST00000213006.2|Cnot1-216|ENSMUSG00000036550|-|chr8:g.96469739:T>C
MNLDSLSLALSQISYLVDNLTKKNYRASQQEIQHIVNRHGPEADRHLLRCLFSHVDFSGDGKSSGKDFHQTQF
LIQECASLITKPNFISTLSYAIDNPLHYQKSLKPAPHLFAQLSKVLKLSKVQEVIFGLALLNSSSPDLRGFAA
QFIKQKLPDLLRSYIDADVSGNQEGGFQDIAIEVLHLLLSHLLFGQKGAFGVGQEQIDAFLKTLRRDFPQERC
PVVLAPLLYPEKRDILMDRILPDSGGVAKTMMESSLADFMQEVGYGFCASIEECRNIIMQFGVREVTAAQVAR
VLGMMARTHSGLTDGIPLQSISAPGSGIWSDGKDKSEGAQAHTWNVEVLIDVLKELNPSLNFKEVTYELDHPG
FQIRDSKGLHNVVYGIQRGLGMEVFPVDFIYRPWKHAEGQLSFIQHSLINPEVFCFADYPCHTVATDILKAPP
EDDNREIATWKSLDLIESLLRLAEVGQYEQVKQLFSFPIKHCPDMLVLALLQINTSWHTLRHELISTLMPIFL
GNHPNSAIILHYAWHGQGQSPSIRQLIMHAMAEWYMRGEQYDQAKLSRILDVAQDLKALSMLLNGTPFAFVID
LAALASRREYLKLDKWLTDKIREHGEPFIQACMTFLKRRCPSILGGLAPEKDQPKSAQLPAETLATMLACLQA
CAGSVSQELSETILTMVANCSNVMNKARQPPPGVMPKGRPPSASSLDAISPVQIDPLAGMASLSIGGSAAPHT
QSMQGFPPNLGSAFSTPQSPAKAFPPLSTPNQTTAFSGIGGLSSQLPVGGLGTGSLTGIGTGALGLPAVNNDP
FVQRKLGTSGLNQPTFQQSKMKPSDLSQVWPEANQHFSKEIDDEANSYFQRIYNHPPHPTMSVDEVLEMLQRF
KDSTIKREREVFNCMLRNLFEEYRFFPQYPDKELHITACLFGGIIEKGLVTYMALGLALRYVLEALRKPFGSK
MYYFGIAALDRFKNRLKDYPQYCQHLASISHFMQFPHHLQEYIEYGQQSRDPPVKMQGSITTPGSIALAQAQA
QAQVPAKAPLAGQVNTMVTTSTTTTVAKTVTVTKPTGVSFKKDVPPSINTTNIDTLLVATDQTERIVEPPENI
QEKIAFIFNNLSQSNMTQKVEELKETVKEEFMPWVSQYLVMKRVSIEPNFHSLYSNFLDTLKNPEFNKMVLNE
TYRNIKVLLTSDKAAANFSDRSLLKNLGHWLGMITLAKNKPILHTDLDVKSLLLEAYVKGQQELLYVVPFVAK
VLESSIRSLVFRPPNPWTMAIMNVLAELHQEHDLKLNLKFEIEVLCKNLALDINELKPGNLLKDKDRLKNLDE
QLSAPKKDVKQPEELPAITTTTTSTTPATSTTCTATVPPQPQYSYHDINVYSLAGLAPHITLSPTIPLFQAHP
QLKQCVRQAIERAVQELVHPVVDRSIKIAMTTCEQIVRKDFALDSEESRMRIAAHHMMRNLTAGMAMITCREP
LLMSISTNLKNSFASALRTASPQQREMMDQAAAQLAQDNCELACCFIQKTAVEKAGPEMDKRLATEFELRKHA

RQEGRRYCDPVVLTYQAERMPEQIRLKVGGVDPKQLAVYEEFARNVPGFLPTNDLSQPTGFLAQPMKNWSSTY
TPFHPLWP*
>rs240993791_ENSMUSP00000096073_c.4129:A>G_p.N1377S
ENSMUST00000098473.11|Cnot1-202|ENSMUSG00000036550|-|chr8:g.96469739:T>C
MNLDSLSLALSQISYLVDNLTKKNYRASQQEIQHIVNRHGPEADRHLLRCLFSHVDFSGDGKSSGKDFHQTQF
LIQECASLITKPNFISTLSYAIDNPLHYQKSLKPAPHLFAQLSKVLKLSKVQEVIFGLALLNSSSPDLRGFAA
QFIKQKLPDLLRSYIDADVSGNQEGGFQDIAIEVLHLLLSHLLFGQKGAFGVGQEQIDAFLKTLRRDFPQERC
PVVLAPLLYPEKRDILMDRILPDSGGVAKTMMESSLADFMQEVGYGFCASIEECRNIIMQFGVREVTAAQVAR
VLGMMARTHSGLTDGIPLQSISAPGSGIWSDGKDKSEGAQAHTWNVEVLIDVLKELNPSLNFKEVTYELDHPG
FQIRDSKGLHNVVYGIQRGLGMEVFPVDFIYRPWKHAEGQLSFIQHSLINPEVFCFADYPCHTVATDILKAPP
EDDNREIATWKSLDLIESLLRLAEVGQYEQVKQLFSFPIKHCPDMLVLALLQINTSWHTLRHELISTLMPIFL
GNHPNSAIILHYAWHGQGQSPSIRQLIMHAMAEWYMRGEQYDQAKLSRILDVAQDLKALSMLLNGTPFAFVID
LAALASRREYLKLDKWLTDKIREHGEPFIQACMTFLKRRCPSILGGLAPEKDQPKSAQLPAETLATMLACLQA
CAGSVSQELSETILTMVANCSNVMNKARQPPPGVMPKGRPPSASSLDAISPVQIDPLAGMASLSIGGSAAPHT
QSMQGFPPNLGSAFSTPQSPAKAFPPLSTPNQTTAFSGIGGLSSQLPVGGLGTGSLTGIGTGALGLPAVNNDP
FVQRKLGTSGLNQPTFQQSKMKPSDLSQVWPEANQHFSKEIDDEANSYFQRIYNHPPHPTMSVDEVLEMLQRF
KDSTIKREREVFNCMLRNLFEEYRFFPQYPDKELHITACLFGGIIEKGLVTYMALGLALRYVLEALRKPFGSK
MYYFGIAALDRFKNRLKDYPQYCQHLASISHFMQFPHHLQEYIEYGQQSRDPPVKMQGSITTPGSIALAQAQA
QAQVPAKAPLAGQVNTMVTTSTTTTVAKTVTVTKPTGVSFKKDVPPSINTTNIDTLLVATDQTERIVEPPENI
QEKIAFIFNNLSQSNMTQKVEELKETVKEEFMPWVSQYLVMKRVSIEPNFHSLYSNFLDTLKNPEFNKMVLNE
TYRNIKVLLTSDKAAANFSDRSLLKNLGHWLGMITLAKNKPILHTDLDVKSLLLEAYVKGQQELLYVVPFVAK
VLESSIRSLVFRPPNPWTMAIMNVLAELHQEHDLKLNLKFEIEVLCKNLALDINELKPGNLLKDKDRLKNLDE
QLSAPKKDVKQPEELPAITTTTTSTTPATSTTCTATVPPQPQYSYHDINVYSLAGLAPHITLSPTIPLFQAHP
QLKQCVRQAIERAVQELVHPVVDRSIKIAMTTCEQIVRKDFALDSEESRMRIAAHHMMRNLTAGMAMITCREP
LLMSISTNLKNSFASALRTASPQQREMMDQAAAQLAQDNCELACCFIQKTAVEKAGPEMDKRLATEFELRKHA
RQEGRRYCDPVVLTYQAERMPEQIRLKVGGVDPKQLAVYEEFARNVPGFLPTNDLSQPTGFLAQPMKQAWATD
DVAQIYDKCITELEQHLHAIPPTLAMNPQAQALRSLLEVVVLSRNSRDAIAALGLLQKAVEGLLDATSGADAD
LLLRYRECHLLVLKALQDGRAYGSPWCNKQITRCLIECRDEYKYNVEAVELLIRNHLVNMQQYDLHLAQSMEN
GLNYMAVAFAMQLVKILLVDERSVAHITEADLFHTIETLMRINAHSRGNAPEGLPQLMEVVRSNYEAMIDRAH
GGPNFMMHSGISQASEYDDPPGLREKAEYLLREWVNLYHSAAAGRDSTKAFSAFVGQMHQQGILKTDDLITRF
FRLCTEMCVEISYRAQAEQQHNPAANPTMIRAKCYHNLDAFVRLIALLVKHSGEATNTVTKINLLNKVLGIVV
GVLLQDHDVRQSEFQQLPYHRIFIMLLLELNAPEHVLETINFQTLTAFCNTFHILRPTKAPGFVYAWLELISH
RIFIARMLAHTPQQKGWPMYAQLLIDLFKYLAPFLRNVELTKPMQILYKGTLRVLLVLLHDFPEFLCDYHYGF
CDVIPPNCIQLRNLILSAFPRNMRLPDPFTPNLKVDMLSEINIAPRILTNFTGVMPPQFKKDLDSYLKTRSPV
TFLSDLRSNLQVSNEPGNRYNLQLINALVLYVGTQAIAHIHNKGSTPSMSTITHSAHMDIFQNLAVDLDTEGR
YLFLNAIANQLRYPNSHTHYFSCTMLYLFAEANTEAIQEQITRVLLERLIVNRPHPWGLLITFIELIKNPAFK
FWNHEFVHCAPEIEKLFQSVAQCCMGQKQAQQVMEGTGAS*
>rs32184217_ENSMUSP00000063565_c.1554:A>T_p.I519F
ENSMUST00000068452.10|Cnot1-201|ENSMUSG00000036550|-|chr8:g.96489647:T>A
MNLDSLSLALSQISYLVDNLTKKNYRASQQEIQHIVNRHGPEADRHLLRCLFSHVDFSGDGKSSGKDFHQTQF
LIQECASLITKPNFISTLSYAIDNPLHYQKSLKPAPHLFAQLSKVLKLSKVQEVIFGLALLNSSSPDLRGFAA
QFIKQKLPDLLRSYIDADVSGNQEGGFQDIAIEVLHLLLSHLLFGQKGAFGVGQEQIDAFLKTLRRDFPQERC
PVVLAPLLYPEKRDILMDRILPDSGGVAKTMMESSLADFMQEVGYGFCASIEECRNIIMQFGVREVTAAQVAR
VLGMMARTHSGLTDGIPLQSISAPGSGIWSDGKDKSEGAQAHTWNVEVLIDVLKELNPSLNFKEVTYELDHPG
FQIRDSKGLHNVVYGIQRGLGMEVFPVDFIYRPWKHAEGQLSFIQHSLINPEVFCFADYPCHTVATDILKAPP
EDDNREIATWKSLDLIESLLRLAEVGQYEQVKQLFSFPIKHCPDMLVLALLQINTSWHTLRHELISTLMPIFL
GNHPNSAFILHYAWHGQGQSPSIRQLIMHAMAEWYMRGEQYDQAKLSRILDVAQDLKALSMLLNGTPFAFVID
LAALASRREYLKLDKWLTDKIREHGEPFIQACMTFLKRRCPSILGGLAPEKDQPKSAQLPAETLATMLACLQA
CAGSVSQELSETILTMVANCSNVMNKARQPPPGVMPKGRPPSASSLDAISPVQIDPLAGMASLSIGGSAAPHT
QSMQGFPPNLGSAFSTPQSPAKAFPPLSTPNQTTAFSGIGGLSSQLPVGGLGTGSLTGIGTGALGLPAVNNDP
FVQRKLGTSGLNQPTFQQTDLSQVWPEANQHFSKEIDDEANSYFQRIYNHPPHPTMSVDEVLEMLQRFKDSTI
KREREVFNCMLRNLFEEYRFFPQYPDKELHITACLFGGIIEKGLVTYMALGLALRYVLEALRKPFGSKMYYFG
IAALDRFKNRLKDYPQYCQHLASISHFMQFPHHLQEYIEYGQQSRDPPVKMQGSITTPGSIALAQAQAQAQVP
AKAPLAGQVNTMVTTSTTTTVAKTVTVTKPTGVSFKKDVPPSINTTNIDTLLVATDQTERIVEPPENIQEKIA
FIFNNLSQSNMTQKVEELKETVKEEFMPWVSQYLVMKRVSIEPNFHSLYSNFLDTLKNPEFNKMVLNETYRNI
KVLLTSDKAAANFSDRSLLKNLGHWLGMITLAKNKPILHTDLDVKSLLLEAYVKGQQELLYVVPFVAKVLESS
IRSLVFRPPNPWTMAIMNVLAELHQEHDLKLNLKFEIEVLCKNLALDINELKPGNLLKDKDRLKNLDEQLSAP
KKDVKQPEELPAITTTTTSTTPATSTTCTATVPPQPQYSYHDINVYSLAGLAPHITLNPTIPLFQAHPQLKQC

VRQAIERAVQELVHPVVDRSIKIAMTTCEQIVRKDFALDSEESRMRIAAHHMMRNLTAGMAMITCREPLLMSI
STNLKNSFASALRTASPQQREMMDQAAAQLAQDNCELACCFIQKTAVEKAGPEMDKRLATEFELRKHARQEGR
RYCDPVVLTYQAERMPEQIRLKVGGVDPKQLAVYEEFARNVPGFLPTNDLSQPTGFLAQPMKQAWATDDVAQI
YDKCITELEQHLHAIPPTLAMNPQAQALRSLLEVVVLSRNSRDAIAALGLLQKAVEGLLDATSGADADLLLRY
RECHLLVLKALQDGRAYGSPWCNKQITRCLIECRDEYKYNVEAVELLIRNHLVNMQQYDLHLAQSMENGLNYM
AVAFAMQLVKILLVDERSVAHITEADLFHTIETLMRINAHSRGNAPEGLPQLMEVVRSNYEAMIDRAHGGPNF
MMHSGISQASEYDDPPGLREKAEYLLREWVNLYHSAAAGRDSTKAFSAFVGQMHQQGILKTDDLITRFFRLCT
EMCVEISYRAQAEQQHNPAANPTMIRAKCYHNLDAFVRLIALLVKHSGEATNTVTKINLLNKVLGIVVGVLLQ
DHDVRQSEFQQLPYHRIFIMLLLELNAPEHVLETINFQTLTAFCNTFHILRPTKAPGFVYAWLELISHRIFIA
RMLAHTPQQKGWPMYAQLLIDLFKYLAPFLRNVELTKPMQILYKGTLRVLLVLLHDFPEFLCDYHYGFCDVIP
PNCIQLRNLILSAFPRNMRLPDPFTPNLKVDMLSEINIAPRILTNFTGVMPPQFKKDLDSYLKTRSPVTFLSD
LRSNLQVSNEPGNRYNLQLINALVLYVGTQAIAHIHNKGSTPSMSTITHSAHMDIFQNLAVDLDTEGRYLFLN
AIANQLRYPNSHTHYFSCTMLYLFAEANTEAIQEQITRLFQSVAQCCMGQKQAQQVMEGTGAS*
>rs32184217_ENSMUSP00000148807_c.1548:A>T_p.I517F
ENSMUST00000211887.2|Cnot1-203|ENSMUSG00000036550|-|chr8:g.96489647:T>A
MNLDSLSLALSQISYLVDNLTKKNYRASQQEIQHIVNRHGPEADRHLLRCLFSHVDFSGDGKSSGKDFHQFLI
QECASLITKPNFISTLSYAIDNPLHYQKSLKPAPHLFAQLSKVLKLSKVQEVIFGLALLNSSSPDLRGFAAQF
IKQKLPDLLRSYIDADVSGNQEGGFQDIAIEVLHLLLSHLLFGQKGAFGVGQEQIDAFLKTLRRDFPQERCPV
VLAPLLYPEKRDILMDRILPDSGGVAKTMMESSLADFMQEVGYGFCASIEECRNIIMQFGVREVTAAQVARVL
GMMARTHSGLTDGIPLQSISAPGSGIWSDGKDKSEGAQAHTWNVEVLIDVLKELNPSLNFKEVTYELDHPGFQ
IRDSKGLHNVVYGIQRGLGMEVFPVDFIYRPWKHAEGQLSFIQHSLINPEVFCFADYPCHTVATDILKAPPED
DNREIATWKSLDLIESLLRLAEVGQYEQVKQLFSFPIKHCPDMLVLALLQINTSWHTLRHELISTLMPIFLGN
HPNSAFILHYAWHGQGQSPSIRQLIMHAMAEWYMRGEQYDQAKLSRILDVAQDLKALSMLLNGTPFAFVIDLA
ALASRREYLKLDKWLTDKIREHGEPFIQACMTFLKRRCPSILGGLAPEKDQPKSAQLPAETLATMLACLQACA
GSVSQELSETILTMVANCSNVMNKARQPPPGVMPKGRPPSASSLDAISPVQIDPLAGMASLSIGGSAAPHTQS
MQGFPPNLGSAFSTPQSPAKAFPPLSTPNQTTAFSGIGGLSSQLPVGGLGTGSLTGIGTGALGLPAVNNDPFV
QRKLGTSGLNQPTFQQTDLSQVWPEANQHFSKEIDDEANSYFQRIYNHPPHPTMSVDEVLEMLQRFKDSTIKR
EREVFNCMLRNLFEEYRFFPQYPDKELHITACLFGGIIEKGLVTYMALGLALRYVLEALRKPFGSKMYYFGIA
ALDRFKNRLKDYPQYCQHLASISHFMQFPHHLQEYIEYGQQSRDPPVKMQGSITTPGSIALAQAQAQAQVPAK
APLAGQVNTMVTTSTTTTVAKTVTVTKPTGVSFKKDVPPSINTTNIDTLLVATDQTERIVEPPENIQEKIAFI
FNNLSQSNMTQKVEELKETVKEEFMPWVSQYLVMKRVSIEPNFHSLYSNFLDTLKNPEFNKMVLNETYRNIKV
LLTSDKAAANFSDRSLLKNLGHWLGMITLAKNKPILHTDLDVKSLLLEAYVKGQQELLYVVPFVAKVLESSIR
SLVFRPPNPWTMAIMNVLAELHQEHDLKLNLKFEIEVLCKNLALDINELKPGNLLKDKDRLKNLDEQLSAPKK
DVKQPEELPAITTTTTTSTTPATSTTCTATVPPQPQYSYHDINVYSLAGLAPHITLNPTIPLFQAHPQLKQCVR
QAIERAVQELVHPVVDRSIKIAMTTCEQIVRKDFALDSEESRMRIAAHHMMRNLTAGMAMITCREPLLMSIST
NLKNSFASALRTASPQQREMMDQAAAQLAQDNCELACCFIQKTAVEKAGPEMDKRLATEFELRKHARQEGRRY
CDPVVLTYQAERMPEQIRLKVGGVDPKQLAVYEEFARNVPGFLPTNDLSQPTGFLAQPMKQAWATDDVAQIYD
KCITELEQHLHAIPPTLAMNPQAQALRSLLEVVVLSRNSRDAIAALGLLQKAVEGLLDATSGADADLLLRYRE
CHLLVLKALQDGRAYGSPWCNKQITRCLIECRDEYKYNVEAVELLIRNHLVNMQQYDLHLAQSMENGLNYMAV
AFAMQLVKILLVDERSVAHITEADLFHTIETLMRINAHSRGNAPEGLPQLMEVVRSNYEAMIDRAHGGPNFMM
HSGISQASEYDDPPGLREKAEYLLREWVNLYHSAAAGRDSTKAFSAFVGQMHQQGILKTDDLITRFFRLCTEM
CVEISYRAQAEQQHNPAANPTMIRAKCYHNLDAFVRLIALLVKHSGEATNTVTKINLLNKVLGIVVGVLLQDH
DVRQSEFQQLPYHRIFIMLLLELNAPEHVLETINFQTLTAFCNTFHILRPTKAPGFVYAWLELISHRIFIARM
LAHTPQQKGWPMYAQLLIDLFKYLAPFLRNVELTKPMQILYKGTLRVLLVLLHDFPEFLCDYHYGFCDVIPPN
CIQLRNLILSAFPRNMRLPDPFTPNLKVDMLSEINIAPRILTNFTGVMPPQFKKDLDSYLKTRSPVTFLSDLR
SNLQVSNEPGNRYNLQLINALVLYVGTQAIAHIHNKGSTPSMSTITHSAHMDIFQNLAVDLDTEGRYLFLNAI
ANQLRYPNSHTHYFSCTMLYLFAEANTEAIQEQITRVLLERLIVNRPHPWGLLITFIELIKNPAFKFWNHEFV
HCAPEIEKLFQSVAQCCMGQKQAQQVMEGTGAS*
>rs32184217_ENSMUSP00000148735_c.1554:A>T_p.I519F
ENSMUST00000213006.2|Cnot1-216|ENSMUSG00000036550|-|chr8:g.96489647:T>A
MNLDSLSLALSQISYLVDNLTKKNYRASQQEIQHIVNRHGPEADRHLLRCLFSHVDFSGDGKSSGKDFHQTQF
LIQECASLITKPNFISTLSYAIDNPLHYQKSLKPAPHLFAQLSKVLKLSKVQEVIFGLALLNSSSPDLRGFAA
QFIKQKLPDLLRSYIDADVSGNQEGGFQDIAIEVLHLLLSHLLFGQKGAFGVGQEQIDAFLKTLRRDFPQERC
PVVLAPLLYPEKRDILMDRILPDSGGVAKTMMESSLADFMQEVGYGFCASIEECRNIIMQFGVREVTAAQVAR
VLGMMARTHSGLTDGIPLQSISAPGSGIWSDGKDKSEGAQAHTWNVEVLIDVLKELNPSLNFKEVTYELDHPG
FQIRDSKGLHNVVYGIQRGLGMEVFPVDFIYRPWKHAEGQLSFIQHSLINPEVFCFADYPCHTVATDILKAPP
EDDNREIATWKSLDLIESLLRLAEVGQYEQVKQLFSFPIKHCPDMLVLALLQINTSWHTLRHELISTLMPIFL
GNHPNSAFILHYAWHGQGQSPSIRQLIMHAMAEWYMRGEQYDQAKLSRILDVAQDLKALSMLLNGTPFAFVID

LAALASRREYLKLDKWLTDKIREHGEPFIQACMTFLKRRCPSILGGLAPEKDQPKSAQLPAETLATMLACLQA
CAGSVSQELSETILTMVANCSNVMNKARQPPPGVMPKGRPPSASSLDAISPVQIDPLAGMASLSIGGSAAPHT
QSMQGFPPNLGSAFSTPQSPAKAFPPLSTPNQTTAFSGIGGLSSQLPVGGLGTGSLTGIGTGALGLPAVNNDP
FVQRKLGTSGLNQPTFQQSKMKPSDLSQVWPEANQHFSKEIDDEANSYFQRIYNHPPHPTMSVDEVLEMLQRF
KDSTIKREREVFNCMLRNLFEEYRFFPQYPDKELHITACLFGGIIEKGLVTYMALGLALRYVLEALRKPFGSK
MYYFGIAALDRFKNRLKDYPQYCQHLASISHFMQFPHHLQEYIEYGQQSRDPPVKMQGSITTPGSIALAQAQA
QAQVPAKAPLAGQVNTMVTTSTTTTVAKTVTVTKPTGVSFKKDVPPSINTTNIDTLLVATDQTERIVEPPENI
QEKIAFIFNNLSQSNMTQKVEELKETVKEEFMPWVSQYLVMKRVSIEPNFHSLYSNFLDTLKNPEFNKMVLNE
TYRNIKVLLTSDKAAANFSDRSLLKNLGHWLGMITLAKNKPILHTDLDVKSLLLEAYVKGQQELLYVVPFVAK
VLESSIRSLVFRPPNPWTMAIMNVLAELHQEHDLKLNLKFEIEVLCKNLALDINELKPGNLLKDKDRLKNLDE
QLSAPKKDVKQPEELPAITTTTTSTTPATSTTCTATVPPQPQYSYHDINVYSLAGLAPHITLNPTIPLFQAHP
QLKQCVRQAIERAVQELVHPVVDRSIKIAMTTCEQIVRKDFALDSEESRMRIAAHHMMRNLTAGMAMITCREP
LLMSISTNLKNSFASALRTASPQQREMMDQAAAQLAQDNCELACCFIQKTAVEKAGPEMDKRLATEFELRKHA
RQEGRRYCDPVVLTYQAERMPEQIRLKVGGVDPKQLAVYEEFARNVPGFLPTNDLSQPTGFLAQPMKNWSSTY
TPFHPLWP*
>rs32184217_ENSMUSP00000096073_c.1554:A>T_p.I519F
ENSMUST00000098473.11|Cnot1-202|ENSMUSG00000036550|-|chr8:g.96489647:T>A
MNLDSLSLALSQISYLVDNLTKKNYRASQQEIQHIVNRHGPEADRHLLRCLFSHVDFSGDGKSSGKDFHQTQF
LIQECASLITKPNFISTLSYAIDNPLHYQKSLKPAPHLFAQLSKVLKLSKVQEVIFGLALLNSSSPDLRGFAA
QFIKQKLPDLLRSYIDADVSGNQEGGFQDIAIEVLHLLLSHLLFGQKGAFGVGQEQIDAFLKTLRRDFPQERC
PVVLAPLLYPEKRDILMDRILPDSGGVAKTMMESSLADFMQEVGYGFCASIEECRNIIMQFGVREVTAAQVAR
VLGMMARTHSGLTDGIPLQSISAPGSGIWSDGKDKSEGAQAHTWNVEVLIDVLKELNPSLNFKEVTYELDHPG
FQIRDSKGLHNVVYGIQRGLGMEVFPVDFIYRPWKHAEGQLSFIQHSLINPEVFCFADYPCHTVATDILKAPP
EDDNREIATWKSLDLIESLLRLAEVGQYEQVKQLFSFPIKHCPDMLVLALLQINTSWHTLRHELISTLMPIFL
GNHPNSAFILHYAWHGQGQSPSIRQLIMHAMAEWYMRGEQYDQAKLSRILDVAQDLKALSMLLNGTPFAFVID
LAALASRREYLKLDKWLTDKIREHGEPFIQACMTFLKRRCPSILGGLAPEKDQPKSAQLPAETLATMLACLQA
CAGSVSQELSETILTMVANCSNVMNKARQPPPGVMPKGRPPSASSLDAISPVQIDPLAGMASLSIGGSAAPHT
QSMQGFPPNLGSAFSTPQSPAKAFPPLSTPNQTTAFSGIGGLSSQLPVGGLGTGSLTGIGTGALGLPAVNNDP
FVQRKLGTSGLNQPTFQQSKMKPSDLSQVWPEANQHFSKEIDDEANSYFQRIYNHPPHPTMSVDEVLEMLQRF
KDSTIKREREVFNCMLRNLFEEYRFFPQYPDKELHITACLFGGIIEKGLVTYMALGLALRYVLEALRKPFGSK
MYYFGIAALDRFKNRLKDYPQYCQHLASISHFMQFPHHLQEYIEYGQQSRDPPVKMQGSITTPGSIALAQAQA
QAQVPAKAPLAGQVNTMVTTSTTTTVAKTVTVTKPTGVSFKKDVPPSINTTNIDTLLVATDQTERIVEPPENI
QEKIAFIFNNLSQSNMTQKVEELKETVKEEFMPWVSQYLVMKRVSIEPNFHSLYSNFLDTLKNPEFNKMVLNE
TYRNIKVLLTSDKAAANFSDRSLLKNLGHWLGMITLAKNKPILHTDLDVKSLLLEAYVKGQQELLYVVPFVAK
VLESSIRSLVFRPPNPWTMAIMNVLAELHQEHDLKLNLKFEIEVLCKNLALDINELKPGNLLKDKDRLKNLDE
QLSAPKKDVKQPEELPAITTTTTSTTPATSTTCTATVPPQPQYSYHDINVYSLAGLAPHITLNPTIPLFQAHP
QLKQCVRQAIERAVQELVHPVVDRSIKIAMTTCEQIVRKDFALDSEESRMRIAAHHMMRNLTAGMAMITCREP
LLMSISTNLKNSFASALRTASPQQREMMDQAAAQLAQDNCELACCFIQKTAVEKAGPEMDKRLATEFELRKHA
RQEGRRYCDPVVLTYQAERMPEQIRLKVGGVDPKQLAVYEEFARNVPGFLPTNDLSQPTGFLAQPMKQAWATD
DVAQIYDKCITELEQHLHAIPPTLAMNPQAQALRSLLEVVVLSRNSRDAIAALGLLQKAVEGLLDATSGADAD
LLLRYRECHLLVLKALQDGRAYGSPWCNKQITRCLIECRDEYKYNVEAVELLIRNHLVNMQQYDLHLAQSMEN
GLNYMAVAFAMQLVKILLVDERSVAHITEADLFHTIETLMRINAHSRGNAPEGLPQLMEVVRSNYEAMIDRAH
GGPNFMMHSGISQASEYDDPPGLREKAEYLLREWVNLYHSAAAGRDSTKAFSAFVGQMHQQGILKTDDLITRF
FRLCTEMCVEISYRAQAEQQHNPAANPTMIRAKCYHNLDAFVRLIALLVKHSGEATNTVTKINLLNKVLGIVV
GVLLQDHDVRQSEFQQLPYHRIFIMLLLELNAPEHVLETINFQTLTAFCNTFHILRPTKAPGFVYAWLELISH
RIFIARMLAHTPQQKGWPMYAQLLIDLFKYLAPFLRNVELTKPMQILYKGTLRVLLVLLHDFPEFLCDYHYGF
CDVIPPNCIQLRNLILSAFPRNMRLPDPFTPNLKVDMLSEINIAPRILTNFTGVMPPQFKKDLDSYLKTRSPV
TFLSDLRSNLQVSNEPGNRYNLQLINALVLYVGTQAIAHIHNKGSTPSMSTITHSAHMDIFQNLAVDLDTEGR
YLFLNAIANQLRYPNSHTHYFSCTMLYLFAEANTEAIQEQITRVLLERLIVNRPHPWGLLITFIELIKNPAFK
FWNHEFVHCAPEIEKLFQSVAQCCMGQKQAQQVMEGTGAS*
>rs33010259_ENSMUSP00000063565_c.1197:A>T_p.K400*
ENSMUST00000068452.10|Cnot1-201|ENSMUSG00000036550|-|chr8:g.96491625:T>A
MNLDSLSLALSQISYLVDNLTKKNYRASQQEIQHIVNRHGPEADRHLLRCLFSHVDFSGDGKSSGKDFHQTQF
LIQECASLITKPNFISTLSYAIDNPLHYQKSLKPAPHLFAQLSKVLKLSKVQEVIFGLALLNSSSPDLRGFAA
QFIKQKLPDLLRSYIDADVSGNQEGGFQDIAIEVLHLLLSHLLFGQKGAFGVGQEQIDAFLKTLRRDFPQERC
PVVLAPLLYPEKRDILMDRILPDSGGVAKTMMESSLADFMQEVGYGFCASIEECRNIIMQFGVREVTAAQVAR
VLGMMARTHSGLTDGIPLQSISAPGSGIWSDGKDKSEGAQAHTWNVEVLIDVLKELNPSLNFKEVTYELDHPG
FQIRDSKGLHNVVYGIQRGLGMEVFPVDFIYRPW*HAEGQLSFIQHSLINPEVFCFADYPCHTVATDILKAPP

EDDNREIATWKSLDLIESLLRLAEVGQYEQVKQLFSFPIKHCPDMLVLALLQINTSWHTLRHELISTLMPIFL
GNHPNSAIILHYAWHGQGQSPSIRQLIMHAMAEWYMRGEQYDQAKLSRILDVAQDLKALSMLLNGTPFAFVID
LAALASRREYLKLDKWLTDKIREHGEPFIQACMTFLKRRCPSILGGLAPEKDQPKSAQLPAETLATMLACLQA
CAGSVSQELSETILTMVANCSNVMNKARQPPPGVMPKGRPPSASSLDAISPVQIDPLAGMASLSIGGSAAPHT
QSMQGFPPNLGSAFSTPQSPAKAFPPLSTPNQTTAFSGIGGLSSQLPVGGLGTGSLTGIGTGALGLPAVNNDP
FVQRKLGTSGLNQPTFQQTDLSQVWPEANQHFSKEIDDEANSYFQRIYNHPPHPTMSVDEVLEMLQRFKDSTI
KREREVFNCMLRNLFEEYRFFPQYPDKELHITACLFGGIIEKGLVTYMALGLALRYVLEALRKPFGSKMYYFG
IAALDRFKNRLKDYPQYCQHLASISHFMQFPHHLQEYIEYGQQSRDPPVKMQGSITTPGSIALAQAQAQAQVP
AKAPLAGQVNTMVTTSTTTTVAKTVTVTKPTGVSFKKDVPPSINTTNIDTLLVATDQTERIVEPPENIQEKIA
FIFNNLSQSNMTQKVEELKETVKEEFMPWVSQYLVMKRVSIEPNFHSLYSNFLDTLKNPEFNKMVLNETYRNI
KVLLTSDKAAANFSDRSLLKNLGHWLGMITLAKNKPILHTDLDVKSLLLEAYVKGQQELLYVVPFVAKVLESS
IRSLVFRPPNPWTMAIMNVLAELHQEHDLKLNLKFEIEVLCKNLALDINELKPGNLLKDKDRLKNLDEQLSAP
KKDVKQPEELPAITTTTTSTTPATSTTCTATVPPQPQYSYHDINVYSLAGLAPHITLNPTIPLFQAHPQLKQC
VRQAIERAVQELVHPVVDRSIKIAMTTCEQIVRKDFALDSEESRMRIAAHHMMRNLTAGMAMITCREPLLMSI
STNLKNSFASALRTASPQQREMMDQAAAQLAQDNCELACCFIQKTAVEKAGPEMDKRLATEFELRKHARQEGR
RYCDPVVLTYQAERMPEQIRLKVGGVDPKQLAVYEEFARNVPGFLPTNDLSQPTGFLAQPMKQAWATDDVAQI
YDKCITELEQHLHAIPPTLAMNPQAQALRSLLEVVVLSRNSRDAIAALGLLQKAVEGLLDATSGADADLLLRY
RECHLLVLKALQDGRAYGSPWCNKQITRCLIECRDEYKYNVEAVELLIRNHLVNMQQYDLHLAQSMENGLNYM
AVAFAMQLVKILLVDERSVAHITEADLFHTIETLMRINAHSRGNAPEGLPQLMEVVRSNYEAMIDRAHGGPNF
MMHSGISQASEYDDPPGLREKAEYLLREWVNLYHSAAAGRDSTKAFSAFVGQMHQQGILKTDDLITRFFRLCT
EMCVEISYRAQAEQQHNPAANPTMIRAKCYHNLDAFVRLIALLVKHSGEATNTVTKINLLNKVLGIVVGVLLQ
DHDVRQSEFQQLPYHRIFIMLLLELNAPEHVLETINFQTLTAFCNTFHILRPTKAPGFVYAWLELISHRIFIA
RMLAHTPQQKGWPMYAQLLIDLFKYLAPFLRNVELTKPMQILYKGTLRVLLVLLHDFPEFLCDYHYGFCDVIP
PNCIQLRNLILSAFPRNMRLPDPFTPNLKVDMLSEINIAPRILTNFTGVMPPQFKKDLDSYLKTRSPVTFLSD
LRSNLQVSNEPGNRYNLQLINALVLYVGTQAIAHIHNKGSTPSMSTITHSAHMDIFQNLAVDLDTEGRYLFLN
AIANQLRYPNSHTHYFSCTMLYLFAEANTEAIQEQITRLFQSVAQCCMGQKQAQQVMEGTGAS*
>rs33010259_ENSMUSP00000148807_c.1191:A>T_p.K398*
ENSMUST00000211887.2|Cnot1-203|ENSMUSG00000036550|-|chr8:g.96491625:T>A
MNLDSLSLALSQISYLVDNLTKKNYRASQQEIQHIVNRHGPEADRHLLRCLFSHVDFSGDGKSSGKDFHQFLI
QECASLITKPNFISTLSYAIDNPLHYQKSLKPAPHLFAQLSKVLKLSKVQEVIFGLALLNSSSPDLRGFAAQF
IKQKLPDLLRSYIDADVSGNQEGGFQDIAIEVLHLLLSHLLFGQKGAFGVGQEQIDAFLKTLRRDFPQERCPV
VLAPLLYPEKRDILMDRILPDSGGVAKTMMESSLADFMQEVGYGFCASIEECRNIIMQFGVREVTAAQVARVL
GMMARTHSGLTDGIPLQSISAPGSGIWSDGKDKSEGAQAHTWNVEVLIDVLKELNPSLNFKEVTYELDHPGFQ
IRDSKGLHNVVYGIQRGLGMEVFPVDFIYRPW*HAEGQLSFIQHSLINPEVFCFADYPCHTVATDILKAPPED
DNREIATWKSLDLIESLLRLAEVGQYEQVKQLFSFPIKHCPDMLVLALLQINTSWHTLRHELISTLMPIFLGN
HPNSAIILHYAWHGQGQSPSIRQLIMHAMAEWYMRGEQYDQAKLSRILDVAQDLKALSMLLNGTPFAFVIDLA
ALASRREYLKLDKWLTDKIREHGEPFIQACMTFLKRRCPSILGGLAPEKDQPKSAQLPAETLATMLACLQACA
GSVSQELSETILTMVANCSNVMNKARQPPPGVMPKGRPPSASSLDAISPVQIDPLAGMASLSIGGSAAPHTQS
MQGFPPNLGSAFSTPQSPAKAFPPLSTPNQTTAFSGIGGLSSQLPVGGLGTGSLTGIGTGALGLPAVNNDPFV
QRKLGTSGLNQPTFQQTDLSQVWPEANQHFSKEIDDEANSYFQRIYNHPPHPTMSVDEVLEMLQRFKDSTIKR
EREVFNCMLRNLFEEYRFFPQYPDKELHITACLFGGIIEKGLVTYMALGLALRYVLEALRKPFGSKMYYFGIA
ALDRFKNRLKDYPQYCQHLASISHFMQFPHHLQEYIEYGQQSRDPPVKMQGSITTPGSIALAQAQAQAQVPAK
APLAGQVNTMVTTSTTTTVAKTVTVTKPTGVSFKKDVPPSINTTNIDTLLVATDQTERIVEPPENIQEKIAFI
FNNLSQSNMTQKVEELKETVKEEFMPWVSQYLVMKRVSIEPNFHSLYSNFLDTLKNPEFNKMVLNETYRNIKV
LLTSDKAAANFSDRSLLKNLGHWLGMITLAKNKPILHTDLDVKSLLLEAYVKGQQELLYVVPFVAKVLESSIR
SLVFRPPNPWTMAIMNVLAELHQEHDLKLNLKFEIEVLCKNLALDINELKPGNLLKDKDRLKNLDEQLSAPKK
DVKQPEELPAITTTTTSTTPATSTTCTATVPPQPQYSYHDINVYSLAGLAPHITLNPTIPLFQAHPQLKQCVR
QAIERAVQELVHPVVDRSIKIAMTTCEQIVRKDFALDSEESRMRIAAHHMMRNLTAGMAMITCREPLLMSIST
NLKNSFASALRTASPQQREMMDQAAAQLAQDNCELACCFIQKTAVEKAGPEMDKRLATEFELRKHARQEGRRY
CDPVVLTYQAERMPEQIRLKVGGVDPKQLAVYEEFARNVPGFLPTNDLSQPTGFLAQPMKQAWATDDVAQIYD
KCITELEQHLHAIPPTLAMNPQAQALRSLLEVVVLSRNSRDAIAALGLLQKAVEGLLDATSGADADLLLRYRE
CHLLVLKALQDGRAYGSPWCNKQITRCLIECRDEYKYNVEAVELLIRNHLVNMQQYDLHLAQSMENGLNYMAV
AFAMQLVKILLVDERSVAHITEADLFHTIETLMRINAHSRGNAPEGLPQLMEVVRSNYEAMIDRAHGGPNFMM
HSGISQASEYDDPPGLREKAEYLLREWVNLYHSAAAGRDSTKAFSAFVGQMHQQGILKTDDLITRFFRLCTEM
CVEISYRAQAEQQHNPAANPTMIRAKCYHNLDAFVRLIALLVKHSGEATNTVTKINLLNKVLGIVVGVLLQDH
DVRQSEFQQLPYHRIFIMLLLELNAPEHVLETINFQTLTAFCNTFHILRPTKAPGFVYAWLELISHRIFIARM
LAHTPQQKGWPMYAQLLIDLFKYLAPFLRNVELTKPMQILYKGTLRVLLVLLHDFPEFLCDYHYGFCDVIPPN
CIQLRNLILSAFPRNMRLPDPFTPNLKVDMLSEINIAPRILTNFTGVMPPQFKKDLDSYLKTRSPVTFLSDLR

SNLQVSNEPGNRYNLQLINALVLYVGTQAIAHIHNKGSTPSMSTITHSAHMDIFQNLAVDLDTEGRYLFLNAI
ANQLRYPNSHTHYFSCTMLYLFAEANTEAIQEQITRVLLERLIVNRPHPWGLLITFIELIKNPAFKFWNHEFV
HCAPEIEKLFQSVAQCCMGQKQAQQVMEGTGAS*
>rs33010259_ENSMUSP00000148735_c.1197:A>T_p.K400*
ENSMUST00000213006.2|Cnot1-216|ENSMUSG00000036550|-|chr8:g.96491625:T>A
MNLDSLSLALSQISYLVDNLTKKNYRASQQEIQHIVNRHGPEADRHLLRCLFSHVDFSGDGKSSGKDFHQTQF
LIQECASLITKPNFISTLSYAIDNPLHYQKSLKPAPHLFAQLSKVLKLSKVQEVIFGLALLNSSSPDLRGFAA
QFIKQKLPDLLRSYIDADVSGNQEGGFQDIAIEVLHLLLSHLLFGQKGAFGVGQEQIDAFLKTLRRDFPQERC
PVVLAPLLYPEKRDILMDRILPDSGGVAKTMMESSLADFMQEVGYGFCASIEECRNIIMQFGVREVTAAQVAR
VLGMMARTHSGLTDGIPLQSISAPGSGIWSDGKDKSEGAQAHTWNVEVLIDVLKELNPSLNFKEVTYELDHPG
FQIRDSKGLHNVVYGIQRGLGMEVFPVDFIYRPW*HAEGQLSFIQHSLINPEVFCFADYPCHTVATDILKAPP
EDDNREIATWKSLDLIESLLRLAEVGQYEQVKQLFSFPIKHCPDMLVLALLQINTSWHTLRHELISTLMPIFL
GNHPNSAIILHYAWHGQGQSPSIRQLIMHAMAEWYMRGEQYDQAKLSRILDVAQDLKALSMLLNGTPFAFVID
LAALASRREYLKLDKWLTDKIREHGEPFIQACMTFLKRRCPSILGGLAPEKDQPKSAQLPAETLATMLACLQA
CAGSVSQELSETILTMVANCSNVMNKARQPPPGVMPKGRPPSASSLDAISPVQIDPLAGMASLSIGGSAAPHT
QSMQGFPPNLGSAFSTPQSPAKAFPPLSTPNQTTAFSGIGGLSSQLPVGGLGTGSLTGIGTGALGLPAVNNDP
FVQRKLGTSGLNQPTFQQSKMKPSDLSQVWPEANQHFSKEIDDEANSYFQRIYNHPPHPTMSVDEVLEMLQRF
KDSTIKREREVFNCMLRNLFEEYRFFPQYPDKELHITACLFGGIIEKGLVTYMALGLALRYVLEALRKPFGSK
MYYFGIAALDRFKNRLKDYPQYCQHLASISHFMQFPHHLQEYIEYGQQSRDPPVKMQGSITTPGSIALAQAQA
QAQVPAKAPLAGQVNTMVTTSTTTTVAKTVTVTKPTGVSFKKDVPPSINTTNIDTLLVATDQTERIVEPPENI
QEKIAFIFNNLSQSNMTQKVEELKETVKEEFMPWVSQYLVMKRVSIEPNFHSLYSNFLDTLKNPEFNKMVLNE
TYRNIKVLLTSDKAAANFSDRSLLKNLGHWLGMITLAKNKPILHTDLDVKSLLLEAYVKGQQELLYVVPFVAK
VLESSIRSLVFRPPNPWTMAIMNVLAELHQEHDLKLNLKFEIEVLCKNLALDINELKPGNLLKDKDRLKNLDE
QLSAPKKDVKQPEELPAITTTTTSTTPATSTTCTATVPPQPQYSYHDINVYSLAGLAPHITLNPTIPLFQAHP
QLKQCVRQAIERAVQELVHPVVDRSIKIAMTTCEQIVRKDFALDSEESRMRIAAHHMMRNLTAGMAMITCREP
LLMSISTNLKNSFASALRTASPQQREMMDQAAAQLAQDNCELACCFIQKTAVEKAGPEMDKRLATEFELRKHA
RQEGRRYCDPVVLTYQAERMPEQIRLKVGGVDPKQLAVYEEFARNVPGFLPTNDLSQPTGFLAQPMKNWSSTY
TPFHPLWP*
>rs33010259_ENSMUSP00000096073_c.1197:A>T_p.K400*
ENSMUST00000098473.11|Cnot1-202|ENSMUSG00000036550|-|chr8:g.96491625:T>A
MNLDSLSLALSQISYLVDNLTKKNYRASQQEIQHIVNRHGPEADRHLLRCLFSHVDFSGDGKSSGKDFHQTQF
LIQECASLITKPNFISTLSYAIDNPLHYQKSLKPAPHLFAQLSKVLKLSKVQEVIFGLALLNSSSPDLRGFAA
QFIKQKLPDLLRSYIDADVSGNQEGGFQDIAIEVLHLLLSHLLFGQKGAFGVGQEQIDAFLKTLRRDFPQERC
PVVLAPLLYPEKRDILMDRILPDSGGVAKTMMESSLADFMQEVGYGFCASIEECRNIIMQFGVREVTAAQVAR
VLGMMARTHSGLTDGIPLQSISAPGSGIWSDGKDKSEGAQAHTWNVEVLIDVLKELNPSLNFKEVTYELDHPG
FQIRDSKGLHNVVYGIQRGLGMEVFPVDFIYRPW*HAEGQLSFIQHSLINPEVFCFADYPCHTVATDILKAPP
EDDNREIATWKSLDLIESLLRLAEVGQYEQVKQLFSFPIKHCPDMLVLALLQINTSWHTLRHELISTLMPIFL
GNHPNSAIILHYAWHGQGQSPSIRQLIMHAMAEWYMRGEQYDQAKLSRILDVAQDLKALSMLLNGTPFAFVID
LAALASRREYLKLDKWLTDKIREHGEPFIQACMTFLKRRCPSILGGLAPEKDQPKSAQLPAETLATMLACLQA
CAGSVSQELSETILTMVANCSNVMNKARQPPPGVMPKGRPPSASSLDAISPVQIDPLAGMASLSIGGSAAPHT
QSMQGFPPNLGSAFSTPQSPAKAFPPLSTPNQTTAFSGIGGLSSQLPVGGLGTGSLTGIGTGALGLPAVNNDP
FVQRKLGTSGLNQPTFQQSKMKPSDLSQVWPEANQHFSKEIDDEANSYFQRIYNHPPHPTMSVDEVLEMLQRF
KDSTIKREREVFNCMLRNLFEEYRFFPQYPDKELHITACLFGGIIEKGLVTYMALGLALRYVLEALRKPFGSK
MYYFGIAALDRFKNRLKDYPQYCQHLASISHFMQFPHHLQEYIEYGQQSRDPPVKMQGSITTPGSIALAQAQA
QAQVPAKAPLAGQVNTMVTTSTTTTVAKTVTVTKPTGVSFKKDVPPSINTTNIDTLLVATDQTERIVEPPENI
QEKIAFIFNNLSQSNMTQKVEELKETVKEEFMPWVSQYLVMKRVSIEPNFHSLYSNFLDTLKNPEFNKMVLNE
TYRNIKVLLTSDKAAANFSDRSLLKNLGHWLGMITLAKNKPILHTDLDVKSLLLEAYVKGQQELLYVVPFVAK
VLESSIRSLVFRPPNPWTMAIMNVLAELHQEHDLKLNLKFEIEVLCKNLALDINELKPGNLLKDKDRLKNLDE
QLSAPKKDVKQPEELPAITTTTTSTTPATSTTCTATVPPQPQYSYHDINVYSLAGLAPHITLNPTIPLFQAHP
QLKQCVRQAIERAVQELVHPVVDRSIKIAMTTCEQIVRKDFALDSEESRMRIAAHHMMRNLTAGMAMITCREP
LLMSISTNLKNSFASALRTASPQQREMMDQAAAQLAQDNCELACCFIQKTAVEKAGPEMDKRLATEFELRKHA
RQEGRRYCDPVVLTYQAERMPEQIRLKVGGVDPKQLAVYEEFARNVPGFLPTNDLSQPTGFLAQPMKQAWATD
DVAQIYDKCITELEQHLHAIPPTLAMNPQAQALRSLLEVVVLSRNSRDAIAALGLLQKAVEGLLDATSGADAD
LLLRYRECHLLVLKALQDGRAYGSPWCNKQITRCLIECRDEYKYNVEAVELLIRNHLVNMQQYDLHLAQSMEN
GLNYMAVAFAMQLVKILLVDERSVAHITEADLFHTIETLMRINAHSRGNAPEGLPQLMEVVRSNYEAMIDRAH
GGPNFMMHSGISQASEYDDPPGLREKAEYLLREWVNLYHSAAAGRDSTKAFSAFVGQMHQQGILKTDDLITRF
FRLCTEMCVEISYRAQAEQQHNPAANPTMIRAKCYHNLDAFVRLIALLVKHSGEATNTVTKINLLNKVLGIVV
GVLLQDHDVRQSEFQQLPYHRIFIMLLLELNAPEHVLETINFQTLTAFCNTFHILRPTKAPGFVYAWLELISH

RIFIARMLAHTPQQKGWPMYAQLLIDLFKYLAPFLRNVELTKPMQILYKGTLRVLLVLLHDFPEFLCDYHYGF
CDVIPPNCIQLRNLILSAFPRNMRLPDPFTPNLKVDMLSEINIAPRILTNFTGVMPPQFKKDLDSYLKTRSPV
TFLSDLRSNLQVSNEPGNRYNLQLINALVLYVGTQAIAHIHNKGSTPSMSTITHSAHMDIFQNLAVDLDTEGR
YLFLNAIANQLRYPNSHTHYFSCTMLYLFAEANTEAIQEQITRVLLERLIVNRPHPWGLLITFIELIKNPAFK
FWNHEFVHCAPEIEKLFQSVAQCCMGQKQAQQVMEGTGAS*
>rs6249151_ENSMUSP00000063565_c.547:T>A_p.L183H
ENSMUST00000068452.10|Cnot1-201|ENSMUSG00000036550|-|chr8:g.96498688:A>T
MNLDSLSLALSQISYLVDNLTKKNYRASQQEIQHIVNRHGPEADRHLLRCLFSHVDFSGDGKSSGKDFHQTQF
LIQECASLITKPNFISTLSYAIDNPLHYQKSLKPAPHLFAQLSKVLKLSKVQEVIFGLALLNSSSPDLRGFAA
QFIKQKLPDLLRSYIDADVSGNQEGGFQDIAIEVLHHLLSHLLFGQKGAFGVGQEQIDAFLKTLRRDFPQERC
PVVLAPLLYPEKRDILMDRILPDSGGVAKTMMESSLADFMQEVGYGFCASIEECRNIIMQFGVREVTAAQVAR
VLGMMARTHSGLTDGIPLQSISAPGSGIWSDGKDKSEGAQAHTWNVEVLIDVLKELNPSLNFKEVTYELDHPG
FQIRDSKGLHNVVYGIQRGLGMEVFPVDFIYRPWKHAEGQLSFIQHSLINPEVFCFADYPCHTVATDILKAPP
EDDNREIATWKSLDLIESLLRLAEVGQYEQVKQLFSFPIKHCPDMLVLALLQINTSWHTLRHELISTLMPIFL
GNHPNSAIILHYAWHGQGQSPSIRQLIMHAMAEWYMRGEQYDQAKLSRILDVAQDLKALSMLLNGTPFAFVID
LAALASRREYLKLDKWLTDKIREHGEPFIQACMTFLKRRCPSILGGLAPEKDQPKSAQLPAETLATMLACLQA
CAGSVSQELSETILTMVANCSNVMNKARQPPPGVMPKGRPPSASSLDAISPVQIDPLAGMASLSIGGSAAPHT
QSMQGFPPNLGSAFSTPQSPAKAFPPLSTPNQTTAFSGIGGLSSQLPVGGLGTGSLTGIGTGALGLPAVNNDP
FVQRKLGTSGLNQPTFQQTDLSQVWPEANQHFSKEIDDEANSYFQRIYNHPPHPTMSVDEVLEMLQRFKDSTI
KREREVFNCMLRNLFEEYRFFPQYPDKELHITACLFGGIIEKGLVTYMALGLALRYVLEALRKPFGSKMYYFG
IAALDRFKNRLKDYPQYCQHLASISHFMQFPHHLQEYIEYGQQSRDPPVKMQGSITTPGSIALAQAQAQAQVP
AKAPLAGQVNTMVTTSTTTTVAKTVTVTKPTGVSFKKDVPPSINTTNIDTLLVATDQTERIVEPPENIQEKIA
FIFNNLSQSNMTQKVEELKETVKEEFMPWVSQYLVMKRVSIEPNFHSLYSNFLDTLKNPEFNKMVLNETYRNI
KVLLTSDKAAANFSDRSLLKNLGHWLGMITLAKNKPILHTDLDVKSLLLEAYVKGQQELLYVVPFVAKVLESS
IRSLVFRPPNPWTMAIMNVLAELHQEHDLKLNLKFEIEVLCKNLALDINELKPGNLLKDKDRLKNLDEQLSAP
KKDVKQPEELPAITTTTTSTTPATSTTCTATVPPQPQYSYHDINVYSLAGLAPHITLNPTIPLFQAHPQLKQC
VRQAIERAVQELVHPVVDRSIKIAMTTCEQIVRKDFALDSEESRMRIAAHHMMRNLTAGMAMITCREPLLMSI
STNLKNSFASALRTASPQQREMMDQAAAQLAQDNCELACCFIQKTAVEKAGPEMDKRLATEFELRKHARQEGR
RYCDPVVLTYQAERMPEQIRLKVGGVDPKQLAVYEEFARNVPGFLPTNDLSQPTGFLAQPMKQAWATDDVAQI
YDKCITELEQHLHAIPPTLAMNPQAQALRSLLEVVVLSRNSRDAIAALGLLQKAVEGLLDATSGADADLLLRY
RECHLLVLKALQDGRAYGSPWCNKQITRCLIECRDEYKYNVEAVELLIRNHLVNMQQYDLHLAQSMENGLNYM
AVAFAMQLVKILLVDERSVAHITEADLFHTIETLMRINAHSRGNAPEGLPQLMEVVRSNYEAMIDRAHGGPNF
MMHSGISQASEYDDPPGLREKAEYLLREWVNLYHSAAAGRDSTKAFSAFVGQMHQQGILKTDDLITRFFRLCT
EMCVEISYRAQAEQQHNPAANPTMIRAKCYHNLDAFVRLIALLVKHSGEATNTVTKINLLNKVLGIVVGVLLQ
DHDVRQSEFQQLPYHRIFIMLLLELNAPEHVLETINFQTLTAFCNTFHILRPTKAPGFVYAWLELISHRIFIA
RMLAHTPQQKGWPMYAQLLIDLFKYLAPFLRNVELTKPMQILYKGTLRVLLVLLHDFPEFLCDYHYGFCDVIP
PNCIQLRNLILSAFPRNMRLPDPFTPNLKVDMLSEINIAPRILTNFTGVMPPQFKKDLDSYLKTRSPVTFLSD
LRSNLQVSNEPGNRYNLQLINALVLYVGTQAIAHIHNKGSTPSMSTITHSAHMDIFQNLAVDLDTEGRYLFLN
AIANQLRYPNSHTHYFSCTMLYLFAEANTEAIQEQITRLFQSVAQCCMGQKQAQQVMEGTGAS*
>rs6249151_ENSMUSP00000148807_c.541:T>A_p.L181H
ENSMUST00000211887.2|Cnot1-203|ENSMUSG00000036550|-|chr8:g.96498688:A>T
MNLDSLSLALSQISYLVDNLTKKNYRASQQEIQHIVNRHGPEADRHLLRCLFSHVDFSGDGKSSGKDFHQFLI
QECASLITKPNFISTLSYAIDNPLHYQKSLKPAPHLFAQLSKVLKLSKVQEVIFGLALLNSSSPDLRGFAAQF
IKQKLPDLLRSYIDADVSGNQEGGFQDIAIEVLHHLLSHLLFGQKGAFGVGQEQIDAFLKTLRRDFPQERCPV
VLAPLLYPEKRDILMDRILPDSGGVAKTMMESSLADFMQEVGYGFCASIEECRNIIMQFGVREVTAAQVARVL
GMMARTHSGLTDGIPLQSISAPGSGIWSDGKDKSEGAQAHTWNVEVLIDVLKELNPSLNFKEVTYELDHPGFQ
IRDSKGLHNVVYGIQRGLGMEVFPVDFIYRPWKHAEGQLSFIQHSLINPEVFCFADYPCHTVATDILKAPPED
DNREIATWKSLDLIESLLRLAEVGQYEQVKQLFSFPIKHCPDMLVLALLQINTSWHTLRHELISTLMPIFLGN
HPNSAIILHYAWHGQGQSPSIRQLIMHAMAEWYMRGEQYDQAKLSRILDVAQDLKALSMLLNGTPFAFVIDLA
ALASRREYLKLDKWLTDKIREHGEPFIQACMTFLKRRCPSILGGLAPEKDQPKSAQLPAETLATMLACLQACA
GSVSQELSETILTMVANCSNVMNKARQPPPGVMPKGRPPSASSLDAISPVQIDPLAGMASLSIGGSAAPHTQS
MQGFPPNLGSAFSTPQSPAKAFPPLSTPNQTTAFSGIGGLSSQLPVGGLGTGSLTGIGTGALGLPAVNNDPFV
QRKLGTSGLNQPTFQQTDLSQVWPEANQHFSKEIDDEANSYFQRIYNHPPHPTMSVDEVLEMLQRFKDSTIKR
EREVFNCMLRNLFEEYRFFPQYPDKELHITACLFGGIIEKGLVTYMALGLALRYVLEALRKPFGSKMYYFGIA
ALDRFKNRLKDYPQYCQHLASISHFMQFPHHLQEYIEYGQQSRDPPVKMQGSITTPGSIALAQAQAQAQVPAK
APLAGQVNTMVTTSTTTTVAKTVTVTKPTGVSFKKDVPPSINTTNIDTLLVATDQTERIVEPPENIQEKIAFI
FNNLSQSNMTQKVEELKETVKEEFMPWVSQYLVMKRVSIEPNFHSLYSNFLDTLKNPEFNKMVLNETYRNIKV
LLTSDKAAANFSDRSLLKNLGHWLGMITLAKNKPILHTDLDVKSLLLEAYVKGQQELLYVVPFVAKVLESSIR

SLVFRPPNPWTMAIMNVLAELHQEHDLKLNLKFEIEVLCKNLALDINELKPGNLLKDKDRLKNLDEQLSAPKK
DVKQPEELPAITTTTTSTTPATSTTCTATVPPQPQYSYHDINVYSLAGLAPHITLNPTIPLFQAHPQLKQCVR
QAIERAVQELVHPVVDRSIKIAMTTCEQIVRKDFALDSEESRMRIAAHHMMRNLTAGMAMITCREPLLMSIST
NLKNSFASALRTASPQQREMMDQAAAQLAQDNCELACCFIQKTAVEKAGPEMDKRLATEFELRKHARQEGRRY
CDPVVLTYQAERMPEQIRLKVGGVDPKQLAVYEEFARNVPGFLPTNDLSQPTGFLAQPMKQAWATDDVAQIYD
KCITELEQHLHAIPPTLAMNPQAQALRSLLEVVVLSRNSRDAIAALGLLQKAVEGLLDATSGADADLLLRYRE
CHLLVLKALQDGRAYGSPWCNKQITRCLIECRDEYKYNVEAVELLIRNHLVNMQQYDLHLAQSMENGLNYMAV
AFAMQLVKILLVDERSVAHITEADLFHTIETLMRINAHSRGNAPEGLPQLMEVVRSNYEAMIDRAHGGPNFMM
HSGISQASEYDDPPGLREKAEYLLREWVNLYHSAAAGRDSTKAFSAFVGQMHQQGILKTDDLITRFFRLCTEM
CVEISYRAQAEQQHNPAANPTMIRAKCYHNLDAFVRLIALLVKHSGEATNTVTKINLLNKVLGIVVGVLLQDH
DVRQSEFQQLPYHRIFIMLLLELNAPEHVLETINFQTLTAFCNTFHILRPTKAPGFVYAWLELISHRIFIARM
LAHTPQQKGWPMYAQLLIDLFKYLAPFLRNVELTKPMQILYKGTLRVLLVLLHDFPEFLCDYHYGFCDVIPPN
CIQLRNLILSAFPRNMRLPDPFTPNLKVDMLSEINIAPRILTNFTGVMPPQFKKDLDSYLKTRSPVTFLSDLR
SNLQVSNEPGNRYNLQLINALVLYVGTQAIAHIHNKGSTPSMSTITHSAHMDIFQNLAVDLDTEGRYLFLNAI
ANQLRYPNSHTHYFSCTMLYLFAEANTEAIQEQITRVLLERLIVNRPHPWGLLITFIELIKNPAFKFWNHEFV
HCAPEIEKLFQSVAQCCMGQKQAQQVMEGTGAS*
>rs6249151_ENSMUSP00000148735_c.547:T>A_p.L183H
ENSMUST00000213006.2|Cnot1-216|ENSMUSG00000036550|-|chr8:g.96498688:A>T
MNLDSLSLALSQISYLVDNLTKKNYRASQQEIQHIVNRHGPEADRHLLRCLFSHVDFSGDGKSSGKDFHQTQF
LIQECASLITKPNFISTLSYAIDNPLHYQKSLKPAPHLFAQLSKVLKLSKVQEVIFGLALLNSSSPDLRGFAA
QFIKQKLPDLLRSYIDADVSGNQEGGFQDIAIEVLHHLLSHLLFGQKGAFGVGQEQIDAFLKTLRRDFPQERC
PVVLAPLLYPEKRDILMDRILPDSGGVAKTMMESSLADFMQEVGYGFCASIEECRNIIMQFGVREVTAAQVAR
VLGMMARTHSGLTDGIPLQSISAPGSGIWSDGKDKSEGAQAHTWNVEVLIDVLKELNPSLNFKEVTYELDHPG
FQIRDSKGLHNVVYGIQRGLGMEVFPVDFIYRPWKHAEGQLSFIQHSLINPEVFCFADYPCHTVATDILKAPP
EDDNREIATWKSLDLIESLLRLAEVGQYEQVKQLFSFPIKHCPDMLVLALLQINTSWHTLRHELISTLMPIFL
GNHPNSAIILHYAWHGQGQSPSIRQLIMHAMAEWYMRGEQYDQAKLSRILDVAQDLKALSMLLNGTPFAFVID
LAALASRREYLKLDKWLTDKIREHGEPFIQACMTFLKRRCPSILGGLAPEKDQPKSAQLPAETLATMLACLQA
CAGSVSQELSETILTMVANCSNVMNKARQPPPGVMPKGRPPSASSLDAISPVQIDPLAGMASLSIGGSAAPHT
QSMQGFPPNLGSAFSTPQSPAKAFPPLSTPNQTTAFSGIGGLSSQLPVGGLGTGSLTGIGTGALGLPAVNNDP
FVQRKLGTSGLNQPTFQQSKMKPSDLSQVWPEANQHFSKEIDDEANSYFQRIYNHPPHPTMSVDEVLEMLQRF
KDSTIKREREVFNCMLRNLFEEYRFFPQYPDKELHITACLFGGIIEKGLVTYMALGLALRYVLEALRKPFGSK
MYYFGIAALDRFKNRLKDYPQYCQHLASISHFMQFPHHLQEYIEYGQQSRDPPVKMQGSITTPGSIALAQAQA
QAQVPAKAPLAGQVNTMVTTSTTTTVAKTVTVTKPTGVSFKKDVPPSINTTNIDTLLVATDQTERIVEPPENI
QEKIAFIFNNLSQSNMTQKVEELKETVKEEFMPWVSQYLVMKRVSIEPNFHSLYSNFLDTLKNPEFNKMVLNE
TYRNIKVLLTSDKAAANFSDRSLLKNLGHWLGMITLAKNKPILHTDLDVKSLLLEAYVKGQQELLYVVPFVAK
VLESSIRSLVFRPPNPWTMAIMNVLAELHQEHDLKLNLKFEIEVLCKNLALDINELKPGNLLKDKDRLKNLDE
QLSAPKKDVKQPEELPAITTTTTSTTPATSTTCTATVPPQPQYSYHDINVYSLAGLAPHITLNPTIPLFQAHP
QLKQCVRQAIERAVQELVHPVVDRSIKIAMTTCEQIVRKDFALDSEESRMRIAAHHMMRNLTAGMAMITCREP
LLMSISTNLKNSFASALRTASPQQREMMDQAAAQLAQDNCELACCFIQKTAVEKAGPEMDKRLATEFELRKHA
RQEGRRYCDPVVLTYQAERMPEQIRLKVGGVDPKQLAVYEEFARNVPGFLPTNDLSQPTGFLAQPMKNWSSTY
TPFHPLWP*
>rs6249151_ENSMUSP00000096073_c.547:T>A_p.L183H
ENSMUST00000098473.11|Cnot1-202|ENSMUSG00000036550|-|chr8:g.96498688:A>T
MNLDSLSLALSQISYLVDNLTKKNYRASQQEIQHIVNRHGPEADRHLLRCLFSHVDFSGDGKSSGKDFHQTQF
LIQECASLITKPNFISTLSYAIDNPLHYQKSLKPAPHLFAQLSKVLKLSKVQEVIFGLALLNSSSPDLRGFAA
QFIKQKLPDLLRSYIDADVSGNQEGGFQDIAIEVLHHLLSHLLFGQKGAFGVGQEQIDAFLKTLRRDFPQERC
PVVLAPLLYPEKRDILMDRILPDSGGVAKTMMESSLADFMQEVGYGFCASIEECRNIIMQFGVREVTAAQVAR
VLGMMARTHSGLTDGIPLQSISAPGSGIWSDGKDKSEGAQAHTWNVEVLIDVLKELNPSLNFKEVTYELDHPG
FQIRDSKGLHNVVYGIQRGLGMEVFPVDFIYRPWKHAEGQLSFIQHSLINPEVFCFADYPCHTVATDILKAPP
EDDNREIATWKSLDLIESLLRLAEVGQYEQVKQLFSFPIKHCPDMLVLALLQINTSWHTLRHELISTLMPIFL
GNHPNSAIILHYAWHGQGQSPSIRQLIMHAMAEWYMRGEQYDQAKLSRILDVAQDLKALSMLLNGTPFAFVID
LAALASRREYLKLDKWLTDKIREHGEPFIQACMTFLKRRCPSILGGLAPEKDQPKSAQLPAETLATMLACLQA
CAGSVSQELSETILTMVANCSNVMNKARQPPPGVMPKGRPPSASSLDAISPVQIDPLAGMASLSIGGSAAPHT
QSMQGFPPNLGSAFSTPQSPAKAFPPLSTPNQTTAFSGIGGLSSQLPVGGLGTGSLTGIGTGALGLPAVNNDP
FVQRKLGTSGLNQPTFQQSKMKPSDLSQVWPEANQHFSKEIDDEANSYFQRIYNHPPHPTMSVDEVLEMLQRF
KDSTIKREREVFNCMLRNLFEEYRFFPQYPDKELHITACLFGGIIEKGLVTYMALGLALRYVLEALRKPFGSK
MYYFGIAALDRFKNRLKDYPQYCQHLASISHFMQFPHHLQEYIEYGQQSRDPPVKMQGSITTPGSIALAQAQA
QAQVPAKAPLAGQVNTMVTTSTTTTVAKTVTVTKPTGVSFKKDVPPSINTTNIDTLLVATDQTERIVEPPENI

QEKIAFIFNNLSQSNMTQKVEELKETVKEEFMPWVSQYLVMKRVSIEPNFHSLYSNFLDTLKNPEFNKMVLNE
TYRNIKVLLTSDKAAANFSDRSLLKNLGHWLGMITLAKNKPILHTDLDVKSLLLEAYVKGQQELLYVVPFVAK
VLESSIRSLVFRPPNPWTMAIMNVLAELHQEHDLKLNLKFEIEVLCKNLALDINELKPGNLLKDKDRLKNLDE
QLSAPKKDVKQPEELPAITTTTTSTTPATSTTCTATVPPQPQYSYHDINVYSLAGLAPHITLNPTIPLFQAHP
QLKQCVRQAIERAVQELVHPVVDRSIKIAMTTCEQIVRKDFALDSEESRMRIAAHHMMRNLTAGMAMITCREP
LLMSISTNLKNSFASALRTASPQQREMMDQAAAQLAQDNCELACCFIQKTAVEKAGPEMDKRLATEFELRKHA
RQEGRRYCDPVVLTYQAERMPEQIRLKVGGVDPKQLAVYEEFARNVPGFLPTNDLSQPTGFLAQPMKQAWATD
DVAQIYDKCITELEQHLHAIPPTLAMNPQAQALRSLLEVVVLSRNSRDAIAALGLLQKAVEGLLDATSGADAD
LLLRYRECHLLVLKALQDGRAYGSPWCNKQITRCLIECRDEYKYNVEAVELLIRNHLVNMQQYDLHLAQSMEN
GLNYMAVAFAMQLVKILLVDERSVAHITEADLFHTIETLMRINAHSRGNAPEGLPQLMEVVRSNYEAMIDRAH
GGPNFMMHSGISQASEYDDPPGLREKAEYLLREWVNLYHSAAAGRDSTKAFSAFVGQMHQQGILKTDDLITRF
FRLCTEMCVEISYRAQAEQQHNPAANPTMIRAKCYHNLDAFVRLIALLVKHSGEATNTVTKINLLNKVLGIVV
GVLLQDHDVRQSEFQQLPYHRIFIMLLLELNAPEHVLETINFQTLTAFCNTFHILRPTKAPGFVYAWLELISH
RIFIARMLAHTPQQKGWPMYAQLLIDLFKYLAPFLRNVELTKPMQILYKGTLRVLLVLLHDFPEFLCDYHYGF
CDVIPPNCIQLRNLILSAFPRNMRLPDPFTPNLKVDMLSEINIAPRILTNFTGVMPPQFKKDLDSYLKTRSPV
TFLSDLRSNLQVSNEPGNRYNLQLINALVLYVGTQAIAHIHNKGSTPSMSTITHSAHMDIFQNLAVDLDTEGR
YLFLNAIANQLRYPNSHTHYFSCTMLYLFAEANTEAIQEQITRVLLERLIVNRPHPWGLLITFIELIKNPAFK
FWNHEFVHCAPEIEKLFQSVAQCCMGQKQAQQVMEGTGAS*
>rs32188968_ENSMUSP00000063565_c.220:T>C_p.L74P
ENSMUST00000068452.10|Cnot1-201|ENSMUSG00000036550|-|chr8:g.96501346:A>G
MNLDSLSLALSQISYLVDNLTKKNYRASQQEIQHIVNRHGPEADRHLLRCLFSHVDFSGDGKSSGKDFHQTQF
PIQECASLITKPNFISTLSYAIDNPLHYQKSLKPAPHLFAQLSKVLKLSKVQEVIFGLALLNSSSPDLRGFAA
QFIKQKLPDLLRSYIDADVSGNQEGGFQDIAIEVLHLLLSHLLFGQKGAFGVGQEQIDAFLKTLRRDFPQERC
PVVLAPLLYPEKRDILMDRILPDSGGVAKTMMESSLADFMQEVGYGFCASIEECRNIIMQFGVREVTAAQVAR
VLGMMARTHSGLTDGIPLQSISAPGSGIWSDGKDKSEGAQAHTWNVEVLIDVLKELNPSLNFKEVTYELDHPG
FQIRDSKGLHNVVYGIQRGLGMEVFPVDFIYRPWKHAEGQLSFIQHSLINPEVFCFADYPCHTVATDILKAPP
EDDNREIATWKSLDLIESLLRLAEVGQYEQVKQLFSFPIKHCPDMLVLALLQINTSWHTLRHELISTLMPIFL
GNHPNSAIILHYAWHGQGQSPSIRQLIMHAMAEWYMRGEQYDQAKLSRILDVAQDLKALSMLLNGTPFAFVID
LAALASRREYLKLDKWLTDKIREHGEPFIQACMTFLKRRCPSILGGLAPEKDQPKSAQLPAETLATMLACLQA
CAGSVSQELSETILTMVANCSNVMNKARQPPPGVMPKGRPPSASSLDAISPVQIDPLAGMASLSIGGSAAPHT
QSMQGFPPNLGSAFSTPQSPAKAFPPLSTPNQTTAFSGIGGLSSQLPVGGLGTGSLTGIGTGALGLPAVNNDP
FVQRKLGTSGLNQPTFQQTDLSQVWPEANQHFSKEIDDEANSYFQRIYNHPPHPTMSVDEVLEMLQRFKDSTI
KREREVFNCMLRNLFEEYRFFPQYPDKELHITACLFGGIIEKGLVTYMALGLALRYVLEALRKPFGSKMYYFG
IAALDRFKNRLKDYPQYCQHLASISHFMQFPHHLQEYIEYGQQSRDPPVKMQGSITTPGSIALAQAQAQAQVP
AKAPLAGQVNTMVTTSTTTTVAKTVTVTKPTGVSFKKDVPPSINTTNIDTLLVATDQTERIVEPPENIQEKIA
FIFNNLSQSNMTQKVEELKETVKEEFMPWVSQYLVMKRVSIEPNFHSLYSNFLDTLKNPEFNKMVLNETYRNI
KVLLTSDKAAANFSDRSLLKNLGHWLGMITLAKNKPILHTDLDVKSLLLEAYVKGQQELLYVVPFVAKVLESS
IRSLVFRPPNPWTMAIMNVLAELHQEHDLKLNLKFEIEVLCKNLALDINELKPGNLLKDKDRLKNLDEQLSAP
KKDVKQPEELPAITTTTTSTTPATSTTCTATVPPQPQYSYHDINVYSLAGLAPHITLNPTIPLFQAHPQLKQC
VRQAIERAVQELVHPVVDRSIKIAMTTCEQIVRKDFALDSEESRMRIAAHHMMRNLTAGMAMITCREPLLMSI
STNLKNSFASALRTASPQQREMMDQAAAQLAQDNCELACCFIQKTAVEKAGPEMDKRLATEFELRKHARQEGR
RYCDPVVLTYQAERMPEQIRLKVGGVDPKQLAVYEEFARNVPGFLPTNDLSQPTGFLAQPMKQAWATDDVAQI
YDKCITELEQHLHAIPPTLAMNPQAQALRSLLEVVVLSRNSRDAIAALGLLQKAVEGLLDATSGADADLLLRY
RECHLLVLKALQDGRAYGSPWCNKQITRCLIECRDEYKYNVEAVELLIRNHLVNMQQYDLHLAQSMENGLNYM
AVAFAMQLVKILLVDERSVAHITEADLFHTIETLMRINAHSRGNAPEGLPQLMEVVRSNYEAMIDRAHGGPNF
MMHSGISQASEYDDPPGLREKAEYLLREWVNLYHSAAAGRDSTKAFSAFVGQMHQQGILKTDDLITRFFRLCT
EMCVEISYRAQAEQQHNPAANPTMIRAKCYHNLDAFVRLIALLVKHSGEATNTVTKINLLNKVLGIVVGVLLQ
DHDVRQSEFQQLPYHRIFIMLLLELNAPEHVLETINFQTLTAFCNTFHILRPTKAPGFVYAWLELISHRIFIA
RMLAHTPQQKGWPMYAQLLIDLFKYLAPFLRNVELTKPMQILYKGTLRVLLVLLHDFPEFLCDYHYGFCDVIP
PNCIQLRNLILSAFPRNMRLPDPFTPNLKVDMLSEINIAPRILTNFTGVMPPQFKKDLDSYLKTRSPVTFLSD
LRSNLQVSNEPGNRYNLQLINALVLYVGTQAIAHIHNKGSTPSMSTITHSAHMDIFQNLAVDLDTEGRYLFLN
AIANQLRYPNSHTHYFSCTMLYLFAEANTEAIQEQITRLFQSVAQCCMGQKQAQQVMEGTGAS*
>rs32188968_ENSMUSP00000148807_c.214:T>C_p.L72P
ENSMUST00000211887.2|Cnot1-203|ENSMUSG00000036550|-|chr8:g.96501346:A>G
MNLDSLSLALSQISYLVDNLTKKNYRASQQEIQHIVNRHGPEADRHLLRCLFSHVDFSGDGKSSGKDFHQFPI
QECASLITKPNFISTLSYAIDNPLHYQKSLKPAPHLFAQLSKVLKLSKVQEVIFGLALLNSSSPDLRGFAAQF
IKQKLPDLLRSYIDADVSGNQEGGFQDIAIEVLHLLLSHLLFGQKGAFGVGQEQIDAFLKTLRRDFPQERCPV
VLAPLLYPEKRDILMDRILPDSGGVAKTMMESSLADFMQEVGYGFCASIEECRNIIMQFGVREVTAAQVARVL

GMMARTHSGLTDGIPLQSISAPGSGIWSDGKDKSEGAQAHTWNVEVLIDVLKELNPSLNFKEVTYELDHPGFQ
IRDSKGLHNVVYGIQRGLGMEVFPVDFIYRPWKHAEGQLSFIQHSLINPEVFCFADYPCHTVATDILKAPPED
DNREIATWKSLDLIESLLRLAEVGQYEQVKQLFSFPIKHCPDMLVLALLQINTSWHTLRHELISTLMPIFLGN
HPNSAIILHYAWHGQGQSPSIRQLIMHAMAEWYMRGEQYDQAKLSRILDVAQDLKALSMLLNGTPFAFVIDLA
ALASRREYLKLDKWLTDKIREHGEPFIQACMTFLKRRCPSILGGLAPEKDQPKSAQLPAETLATMLACLQACA
GSVSQELSETILTMVANCSNVMNKARQPPPGVMPKGRPPSASSLDAISPVQIDPLAGMASLSIGGSAAPHTQS
MQGFPPNLGSAFSTPQSPAKAFPPLSTPNQTTAFSGIGGLSSQLPVGGLGTGSLTGIGTGALGLPAVNNDPFV
QRKLGTSGLNQPTFQQTDLSQVWPEANQHFSKEIDDEANSYFQRIYNHPPHPTMSVDEVLEMLQRFKDSTIKR
EREVFNCMLRNLFEEYRFFPQYPDKELHITACLFGGIIEKGLVTYMALGLALRYVLEALRKPFGSKMYYFGIA
ALDRFKNRLKDYPQYCQHLASISHFMQFPHHLQEYIEYGQQSRDPPVKMQGSITTPGSIALAQAQAQAQVPAK
APLAGQVNTMVTTSTTTTVAKTVTVTKPTGVSFKKDVPPSINTTNIDTLLVATDQTERIVEPPENIQEKIAFI
FNNLSQSNMTQKVEELKETVKEEFMPWVSQYLVMKRVSIEPNFHSLYSNFLDTLKNPEFNKMVLNETYRNIKV
LLTSDKAAANFSDRSLLKNLGHWLGMITLAKNKPILHTDLDVKSLLLEAYVKGQQELLYVVPFVAKVLESSIR
SLVFRPPNPWTMAIMNVLAELHQEHDLKLNLKFEIEVLCKNLALDINELKPGNLLKDKDRLKNLDEQLSAPKK
DVKQPEELPAITTTTTSTTPATSTTCTATVPPQPQYSYHDINVYSLAGLAPHITLNPTIPLFQAHPQLKQCVR
QAIERAVQELVHPVVDRSIKIAMTTCEQIVRKDFALDSEESRMRIAAHHMMRNLTAGMAMITCREPLLMSIST
NLKNSFASALRTASPQQREMMDQAAAQLAQDNCELACCFIQKTAVEKAGPEMDKRLATEFELRKHARQEGRRY
CDPVVLTYQAERMPEQIRLKVGGVDPKQLAVYEEFARNVPGFLPTNDLSQPTGFLAQPMKQAWATDDVAQIYD
KCITELEQHLHAIPPTLAMNPQAQALRSLLEVVVLSRNSRDAIAALGLLQKAVEGLLDATSGADADLLLRYRE
CHLLVLKALQDGRAYGSPWCNKQITRCLIECRDEYKYNVEAVELLIRNHLVNMQQYDLHLAQSMENGLNYMAV
AFAMQLVKILLVDERSVAHITEADLFHTIETLMRINAHSRGNAPEGLPQLMEVVRSNYEAMIDRAHGGPNFMM
HSGISQASEYDDPPGLREKAEYLLREWVNLYHSAAAGRDSTKAFSAFVGQMHQQGILKTDDLITRFFRLCTEM
CVEISYRAQAEQQHNPAANPTMIRAKCYHNLDAFVRLIALLVKHSGEATNTVTKINLLNKVLGIVVGVLLQDH
DVRQSEFQQLPYHRIFIMLLLELNAPEHVLETINFQTLTAFCNTFHILRPTKAPGFVYAWLELISHRIFIARM
LAHTPQQKGWPMYAQLLIDLFKYLAPFLRNVELTKPMQILYKGTLRVLLVLLHDFPEFLCDYHYGFCDVIPPN
CIQLRNLILSAFPRNMRLPDPFTPNLKVDMLSEINIAPRILTNFTGVMPPQFKKDLDSYLKTRSPVTFLSDLR
SNLQVSNEPGNRYNLQLINALVLYVGTQAIAHIHNKGSTPSMSTITHSAHMDIFQNLAVDLDTEGRYLFLNAI
ANQLRYPNSHTHYFSCTMLYLFAEANTEAIQEQITRVLLERLIVNRPHPWGLLITFIELIKNPAFKFWNHEFV
HCAPEIEKLFQSVAQCCMGQKQAQQVMEGTGAS*
>rs32188968_ENSMUSP00000148735_c.220:T>C_p.L74P
ENSMUST00000213006.2|Cnot1-216|ENSMUSG00000036550|-|chr8:g.96501346:A>G
MNLDSLSLALSQISYLVDNLTKKNYRASQQEIQHIVNRHGPEADRHLLRCLFSHVDFSGDGKSSGKDFHQTQF
PIQECASLITKPNFISTLSYAIDNPLHYQKSLKPAPHLFAQLSKVLKLSKVQEVIFGLALLNSSSPDLRGFAA
QFIKQKLPDLLRSYIDADVSGNQEGGFQDIAIEVLHLLLSHLLFGQKGAFGVGQEQIDAFLKTLRRDFPQERC
PVVLAPLLYPEKRDILMDRILPDSGGVAKTMMESSLADFMQEVGYGFCASIEECRNIIMQFGVREVTAAQVAR
VLGMMARTHSGLTDGIPLQSISAPGSGIWSDGKDKSEGAQAHTWNVEVLIDVLKELNPSLNFKEVTYELDHPG
FQIRDSKGLHNVVYGIQRGLGMEVFPVDFIYRPWKHAEGQLSFIQHSLINPEVFCFADYPCHTVATDILKAPP
EDDNREIATWKSLDLIESLLRLAEVGQYEQVKQLFSFPIKHCPDMLVLALLQINTSWHTLRHELISTLMPIFL
GNHPNSAIILHYAWHGQGQSPSIRQLIMHAMAEWYMRGEQYDQAKLSRILDVAQDLKALSMLLNGTPFAFVID
LAALASRREYLKLDKWLTDKIREHGEPFIQACMTFLKRRCPSILGGLAPEKDQPKSAQLPAETLATMLACLQA
CAGSVSQELSETILTMVANCSNVMNKARQPPPGVMPKGRPPSASSLDAISPVQIDPLAGMASLSIGGSAAPHT
QSMQGFPPNLGSAFSTPQSPAKAFPPLSTPNQTTAFSGIGGLSSQLPVGGLGTGSLTGIGTGALGLPAVNNDP
FVQRKLGTSGLNQPTFQQSKMKPSDLSQVWPEANQHFSKEIDDEANSYFQRIYNHPPHPTMSVDEVLEMLQRF
KDSTIKREREVFNCMLRNLFEEYRFFPQYPDKELHITACLFGGIIEKGLVTYMALGLALRYVLEALRKPFGSK
MYYFGIAALDRFKNRLKDYPQYCQHLASISHFMQFPHHLQEYIEYGQQSRDPPVKMQGSITTPGSIALAQAQA
QAQVPAKAPLAGQVNTMVTTSTTTTVAKTVTVTKPTGVSFKKDVPPSINTTNIDTLLVATDQTERIVEPPENI
QEKIAFIFNNLSQSNMTQKVEELKETVKEEFMPWVSQYLVMKRVSIEPNFHSLYSNFLDTLKNPEFNKMVLNE
TYRNIKVLLTSDKAAANFSDRSLLKNLGHWLGMITLAKNKPILHTDLDVKSLLLEAYVKGQQELLYVVPFVAK
VLESSIRSLVFRPPNPWTMAIMNVLAELHQEHDLKLNLKFEIEVLCKNLALDINELKPGNLLKDKDRLKNLDE
QLSAPKKDVKQPEELPAITTTTTSTTPATSTTCTATVPPQPQYSYHDINVYSLAGLAPHITLNPTIPLFQAHP
QLKQCVRQAIERAVQELVHPVVDRSIKIAMTTCEQIVRKDFALDSEESRMRIAAHHMMRNLTAGMAMITCREP
LLMSISTNLKNSFASALRTASPQQREMMDQAAAQLAQDNCELACCFIQKTAVEKAGPEMDKRLATEFELRKHA
RQEGRRYCDPVVLTYQAERMPEQIRLKVGGVDPKQLAVYEEFARNVPGFLPTNDLSQPTGFLAQPMKNWSSTY
TPFHPLWP*
>rs32188968_ENSMUSP00000148574_c.220:T>C_p.L74P
ENSMUST00000212323.2|Cnot1-209|ENSMUSG00000036550|-|chr8:g.96501346:A>G

MNLDSLSLALSQISYLVDNLTKKNYRASQQEIQHIVNRHGPEADRHLLRCLFSHVDFSGDGKSSGKDFHQTQF
PIQECASLITKPNFISTLSYAIDNPLHYQKSLKPAPHLFAQLSKVLKLSKVQEVIFGLALLNSSSPDLRGFGK
SLYPEYKTLPPTPPPSPFRLPSFLPPFLFSFFSLSLSSTPHPPFFIFVVLGIKSRALDKLYKQGLYL*
>rs32188968_ENSMUSP00000096073_c.220:T>C_p.L74P
ENSMUST00000098473.11|Cnot1-202|ENSMUSG00000036550|-|chr8:g.96501346:A>G
MNLDSLSLALSQISYLVDNLTKKNYRASQQEIQHIVNRHGPEADRHLLRCLFSHVDFSGDGKSSGKDFHQTQF
PIQECASLITKPNFISTLSYAIDNPLHYQKSLKPAPHLFAQLSKVLKLSKVQEVIFGLALLNSSSPDLRGFAA
QFIKQKLPDLLRSYIDADVSGNQEGGFQDIAIEVLHLLLSHLLFGQKGAFGVGQEQIDAFLKTLRRDFPQERC
PVVLAPLLYPEKRDILMDRILPDSGGVAKTMMESSLADFMQEVGYGFCASIEECRNIIMQFGVREVTAAQVAR
VLGMMARTHSGLTDGIPLQSISAPGSGIWSDGKDKSEGAQAHTWNVEVLIDVLKELNPSLNFKEVTYELDHPG
FQIRDSKGLHNVVYGIQRGLGMEVFPVDFIYRPWKHAEGQLSFIQHSLINPEVFCFADYPCHTVATDILKAPP
EDDNREIATWKSLDLIESLLRLAEVGQYEQVKQLFSFPIKHCPDMLVLALLQINTSWHTLRHELISTLMPIFL
GNHPNSAIILHYAWHGQGQSPSIRQLIMHAMAEWYMRGEQYDQAKLSRILDVAQDLKALSMLLNGTPFAFVID
LAALASRREYLKLDKWLTDKIREHGEPFIQACMTFLKRRCPSILGGLAPEKDQPKSAQLPAETLATMLACLQA
CAGSVSQELSETILTMVANCSNVMNKARQPPPGVMPKGRPPSASSLDAISPVQIDPLAGMASLSIGGSAAPHT
QSMQGFPPNLGSAFSTPQSPAKAFPPLSTPNQTTAFSGIGGLSSQLPVGGLGTGSLTGIGTGALGLPAVNNDP
FVQRKLGTSGLNQPTFQQSKMKPSDLSQVWPEANQHFSKEIDDEANSYFQRIYNHPPHPTMSVDEVLEMLQRF
KDSTIKREREVFNCMLRNLFEEYRFFPQYPDKELHITACLFGGIIEKGLVTYMALGLALRYVLEALRKPFGSK
MYYFGIAALDRFKNRLKDYPQYCQHLASISHFMQFPHHLQEYIEYGQQSRDPPVKMQGSITTPGSIALAQAQA
QAQVPAKAPLAGQVNTMVTTSTTTTVAKTVTVTKPTGVSFKKDVPPSINTTNIDTLLVATDQTERIVEPPENI
QEKIAFIFNNLSQSNMTQKVEELKETVKEEFMPWVSQYLVMKRVSIEPNFHSLYSNFLDTLKNPEFNKMVLNE
TYRNIKVLLTSDKAAANFSDRSLLKNLGHWLGMITLAKNKPILHTDLDVKSLLLEAYVKGQQELLYVVPFVAK
VLESSIRSLVFRPPNPWTMAIMNVLAELHQEHDLKLNLKFEIEVLCKNLALDINELKPGNLLKDKDRLKNLDE
QLSAPKKDVKQPEELPAITTTTTSTTPATSTTCTATVPPQPQYSYHDINVYSLAGLAPHITLNPTIPLFQAHP
QLKQCVRQAIERAVQELVHPVVDRSIKIAMTTCEQIVRKDFALDSEESRMRIAAHHMMRNLTAGMAMITCREP
LLMSISTNLKNSFASALRTASPQQREMMDQAAAQLAQDNCELACCFIQKTAVEKAGPEMDKRLATEFELRKHA
RQEGRRYCDPVVLTYQAERMPEQIRLKVGGVDPKQLAVYEEFARNVPGFLPTNDLSQPTGFLAQPMKQAWATD
DVAQIYDKCITELEQHLHAIPPTLAMNPQAQALRSLLEVVVLSRNSRDAIAALGLLQKAVEGLLDATSGADAD
LLLRYRECHLLVLKALQDGRAYGSPWCNKQITRCLIECRDEYKYNVEAVELLIRNHLVNMQQYDLHLAQSMEN
GLNYMAVAFAMQLVKILLVDERSVAHITEADLFHTIETLMRINAHSRGNAPEGLPQLMEVVRSNYEAMIDRAH
GGPNFMMHSGISQASEYDDPPGLREKAEYLLREWVNLYHSAAAGRDSTKAFSAFVGQMHQQGILKTDDLITRF
FRLCTEMCVEISYRAQAEQQHNPAANPTMIRAKCYHNLDAFVRLIALLVKHSGEATNTVTKINLLNKVLGIVV
GVLLQDHDVRQSEFQQLPYHRIFIMLLLELNAPEHVLETINFQTLTAFCNTFHILRPTKAPGFVYAWLELISH
RIFIARMLAHTPQQKGWPMYAQLLIDLFKYLAPFLRNVELTKPMQILYKGTLRVLLVLLHDFPEFLCDYHYGF
CDVIPPNCIQLRNLILSAFPRNMRLPDPFTPNLKVDMLSEINIAPRILTNFTGVMPPQFKKDLDSYLKTRSPV
TFLSDLRSNLQVSNEPGNRYNLQLINALVLYVGTQAIAHIHNKGSTPSMSTITHSAHMDIFQNLAVDLDTEGR
YLFLNAIANQLRYPNSHTHYFSCTMLYLFAEANTEAIQEQITRVLLERLIVNRPHPWGLLITFIELIKNPAFK
FWNHEFVHCAPEIEKLFQSVAQCCMGQKQAQQVMEGTGAS*
>rs46345668_ENSMUSP00000037023_c.975:C>T_p.R326*
ENSMUST00000040481.4|Slc38a7-201|ENSMUSG00000036534|-|chr8:g.96568862:G>A
MAQVSINSDYSEWASSTDAGERARLLQSPCVDVVPKSEGEASPGDPDSGTTSTLGAVFIVVNACLGAGLLNFP
AAFSTAGGVAAGIALQMGMLVFIISGLVILAYCSQASNERTYQEVVWAVCGKLTGVLCEVAIAVYTFGTCIAF
LIIIGDQQDKIIAVMSKEPDGASGSPWYTDRKFTISLTAFLFILPLSIPKEIGFQKYASFLSVVGTWYVTAII
IIKYIWPDKEMRPGDILTRPASWMAVFNAMPTICFGFQCHVSSVPVFNSMRQPEVKTWGGVVTAAMVIALAVY
MGTGICGFLTFGAAVDPDVLRSYPSEDVAVAVA*AFIILSVLTSYPILHFCGRAVVEGLWLRYKGMPVEEDVG
RERRRRVLQTLVWFLLTLLLALFIPDIGKVISVIGGLAACFIFIFPGLCLIQAKLSEMEEVKPASWWALVSYG
VLLVTLGAFIFGQTTANAIFVDLLA*
>rs46345668_ENSMUSP00000148659_c.975:C>T_p.R326*
ENSMUST00000212270.2|Slc38a7-204|ENSMUSG00000036534|-|chr8:g.96568862:G>A
MAQVSINSDYSEWASSTDAGERARLLQSPCVDVVPKSEGEASPGDPDSGTTSTLGAVFIVVNACLGAGLLNFP
AAFSTAGGVAAGIALQMGMLVFIISGLVILAYCSQASNERTYQEVVWAVCGKLTGVLCEVAIAVYTFGTCIAF
LIIIGDQQDKIIAVMSKEPDGASGSPWYTDRKFTISLTAFLFILPLSIPKEIGFQKYASFLSVVGTWYVTAII
IIKYIWPDKEMRPGDILTRPASWMAVFNAMPTICFGFQCHVSSVPVFNSMRQPEVKTWGGVVTAAMVIALAVY
MGTGICGFLTFGAAVDPDVLRSYPSEDVAVAVA*AFIILSVLTSYPILHFCGRAVVEGLWLRYKGMPVEEDVG
RERRRRVLQTLVWFLLTLLLALFIPDIGKVISVIGGLAACFIFIFPGLCLIQAKLSEMEEVKPASWWALVSYG
VLLVTLGAFIFGQTTANAIFVDLLA*
>rs46345668_ENSMUSP00000148545_c.975:C>T_p.R326*
ENSMUST00000212628.2|Slc38a7-205|ENSMUSG00000036534|-|chr8:g.96568862:G>A

MAQVSINSDYSEWASSTDAGERARLLQSPCVDVVPKSEGEASPGDPDSGTTSTLGAVFIVVNACLGAGLLNFP
AAFSTAGGVAAGIALQMGMLVFIISGLVILAYCSQASNERTYQEVVWAVCGKLTGVLCEVAIAVYTFGTCIAF
LIIIGDQQDKIIAVMSKEPDGASGSPWYTDRKFTISLTAFLFILPLSIPKEIGFQKYASFLSVVGTWYVTAII
IIKYIWPDKEMRPGDILTRPASWMAVFNAMPTICFGFQCHVSSVPVFNSMRQPEVKTWGGVVTAAMVIALAVY
MGTGICGFLTFGAAVDPDVLRSYPSEDVAVAVA*AFIILSVLTSYPILHFCGRAVVEGLWLRYKGMPVEEDVG
RERRRRVLQTLVWFLLTLLLALFIPDIGKVISVIGGLAACFIFI
>ENSMUSP00000017622_c.334:C>T_p.A112V ENSMUST00000017622.12|Zc3h18-201|ENSMUSG00000017478|+|chr8:g.123110487:C>T
MDVAESPELDPHSPEDEEQPALSDDDILRESGSEQDLDGAGERASDLEEEENATRVQSQEETRSDEEDRASEP
KSQDQDSEAHELSRGPAGSPCEEGDDVEEDGTSDLRDEVSSVTRELDEHELDYDEEVPEEPAPAAQEEEAEKA
GAEEEEEKGEGAPGEEGKPDVQSVGEQEPTEAAKEKKKEDDDGEIDDGEIDDDDLEEGEVKDPSDRKVRPRPT
CRFFMKDVVTPLSTLLPPIPNSIPLRGQVKGNCTWGMSCRFIHPGVNDKGNYSLITKAEPFPPNGAPPLGPHP
LMPANPWGGPVVDEILPPPPPEPPTESAWERGLRHAKEVLKKATIRKEQEPDFEEKRFTVTIGEDDREFDKEN
EVFRDWNSRVPRDVRDTTLEPYADPYYDYEIERFWRGGQYENFRVQYTEAEPYHNYRERERERERENRQRERE
RERERDRERERRQREREREREREREREDKERQRRKEEWERERAKRDEKDRQHRDRDRDKDREKDKEKPKPRSPQPP
SRQAEPPKKESTSVGPQVKRADEWKDPWRRSKSPKKKLGVSVSPSRARRRRKTSASSASASNSSRSSSRSSSY
SGSGSSRSRSRSSSYSSYSSRSSRHSSFSGSRSRSRSFSSSPSPSPTPSPHRPPVRTKGEPAPPPGKAGEKSI
KKPAPPPAPPQATKTTAPGPEPAKPGDLREARRKERQTRTPPRRRTLSGSGSGSGSSYSGSSSRSRSLSVSSV
SSVSSATSSSSSVHSVDSDDMYADLASPVSSASSRSPTPAQTKKERGKSKKEDGVREEKRRRDPSAQPPKSSK
APAGGKASQQAAAPQPAVPGQPQQGSFVAHKEIKLTLLNKAADKGSRKRYEPSDKDRQSPPAKKANLSPDRGS
RDRKSGGRMGSPKPERQRGQNAKAPAAPADRKRPLSPQSKGSSKVTSVPGKATDTATAGTKSGKASTLSRREE
LLKQLKAVEDAIARKRAKIPGKV*
>ENSMUSP00000090761_c.334:C>T_p.A112V ENSMUST00000093073.12|Zc3h18-202|ENSMUSG00000017478|+|chr8:g.123110487:C>T
MDVAESPELDPHSPEDEEQPALSDDDILRESGSEQDLDGAGERASDLEEEENATRVQSQEETRSDEEDRASEP
KSQDQDSEAHELSRGPAGSPCEEGDDVEEDGTSDLRDEVSSVTRELDEHELDYDEEVPEEPAPAAQEEEAEKA
GAEEEEEKGEGAPGEEGKPDVQSVGEQEPTEAAKEKKKEDDDGEIDDGEIDDDDLEEGEVKDPSDRKVRPRPT
CRFFMKGNCTWGMSCRFIHPGVNDKGNYSLITKAEPFPPNGAPPLGPHPLMPANPWGGPVVDEILPPPPPEPP
TESAWERGLRHAKEVLKKATIRKEQEPDFEEKRFTVTIGEDDREFDKENEVFRDWNSRVPRDVRDTTLEPYAD
PYYDYEIERFWRGGQYENFRVQYTEAEPYHNYRERERERERENRQRERERERERDRERERRQRERERERERER
DKERQRRKEEWERERAKRDEKDRQHRDRDRDKDREKDKEKPKPRSPQPPSRQAEPPKKESTSVGPQVKRADEW
KDPWRRSKSPKKKLGVSVSPSRARRRRKTSASSASASNSSRSSSRSSSYSGSGSSRSRSRSSSYSSYSSRSSR
HSSFSGSRSRSRSFSSSPSPSPTPSPHRPPVRTKGEPAPPPGKAGEKSIKKPAPPPAPPQATKTTAPGPEPAK
PGDLREARRKERQTRTPPRRRTLSGSGSGSGSSYSGSSSRSRSLSVSSVSSVSSATSSSSSVHSVDSDDMYAD
LASPVSSASSRSPTPAQTKKERGKSKKEDGVREEKRRRDPSAQPPKSSKAPAGGKASQQAAAPQPAVPGQPQQ
GSFVAHKEIKLTLLNKAADKGSRKRYEPSDKDRQSPPAKKANLSPDRGSRDRKSGGRMGSPKPERQRGQNAKA
PAAPADRKRPLSPQSKGSSKVTSVPGKATDTATAGTKSGKASTLSRREELLKQLKAVEDAIARKRAKIPGKV*
>ENSMUSP00000134743_c.334:C>T_p.A112V ENSMUST00000176629.8|Zc3h18-205|ENSMUSG00000017478|+|chr8:g.123110487:C>T
MDVAESPELDPHSPEDEEQPALSDDDILRESGSEQDLDGAGERASDLEEEENATRVQSQEETRSDEEDRASEP
KSQDQDSEAHELSRGPAGSPCEEGDDVEEDGTSDLRDEVSSVTRELDEHELDYDEEVPEEPAPAAQEEEAEKA
GAEEEEEKGEGAPGEEGKPDVQSVGEQEPTEAAKEKKKEDDDGEIDDGEIDDDDLEEGEVKDPSDRKVRPRPT
CRFFMKDVVTPLSTLLPPIPNSIPLRGQVKGNCTWGMSCRFIHPGVNDKGNYSLITKAEPFPPNGAPPLGPHP
LMPANPWGGPVVDEILPPPPPEPPTESAWERGLRHAKEVLKKATIRKEQEPDFEEKRFTVTIGEDDREFDKEN
EVFRDWNSRVPRDVRDTTLEPYADPYYDYEIERFWRGGQYENFRVQYTEAEPYHNYRERERERERENRQRERE
RERERDRERERRQREREREREREREREDKERQRRKEEWERERAKRDEKDRQHRDRDRDKDREKDKEKPKPRSPQPP
SRQAEPPKKESTSVGPQVKRADEWKDPWRRSKSPKKKLGVSVSPSRARRRRKTSASSASASNSSRSSSRSSSY
SGSGSSRSRSRSSSYSSYSSRSSRHSSFSGSRSRSRSFSSSPSPSPTPSPHRPPVRTKGEPAPPPGKAGEKSI
KKPAPPPAPPQATKTTAPGPEPAKPGDLREARRKERQTRTPPRRSLSVSSVSSVSSATSSSSSVHSVDSDDMY
ADLASPVSSASSRSPTPAQTKKERGKSKKEDGVREEKRRRDPSAQPPKSSKAPAGGKASQQAAAPQPAVPGQP
QQGSFVAHKEIKLTLLNKAADKGSRKRYEPSDKDRQSPPAKKANLSPDRGSRDRKSGGRMGSPKPERQRGQNA
KAPAAPADRKRPLSPQSKGSSKVTSVPGKATDTATAGTKSGKASTLSRREELLKQLKAVEDAIARKRAKIPGK
V*
>ENSMUSP00000095939_c.4756:A>GGAGGGGAGA_p.E1586GRGE-ins ENSMUST00000098334.13|Ankrd11-202|ENSMUSG00000035569|-|chr8:g.123619031:T>TCTCCCCTCC
MPKGGCSKTPQQEDFALSNDMVEKQTGKKDKDKVSLTKTPKLDRSDGGKEVRERATKRKLPFTVGANGEQKDS
DTEKQGPERKRIKKEPVARKSGLLFGMGLSGIRAGYPLSERQQVALLMQMTAEESANSPVDTTPKHPSQSTVC

QKGTPNSASKTKDKVNKRNERGETRLHRAAIRGDARRIKELISEGADVNVKDFAGWTALHEACNRGYYDIAKQ
LLAAGAEVNTKGLDDDTPLHDAANNGHYKVVKLLLRYGGNPQQSNRKGETPLKVANSPTMVNLLLGKGTYTSS
EESSTESSEEEDAPSFAPSSSVDGNNTDSEFEKGLKLKAKNPEPQKTVTPVKDEYEFDEDDEQDRVPPVDDKH
LLKKDYRKEAKANSFISIPKMEVKSYSKNNTLAPKKAAHRILSDTSDEEDVSVSIGAGEKLRLSAHTMLPGSK
ARESSSSRQQKEKNKLKKKRKKETKGKEVRFGKRSDKFCSSGSESESSESEEDDGDSVGSSGCLKGSPLVLKD
PSLFSSLSASSTSSHGSAVAQKHGSGHTDQHTKHWRTDNWKAISSPAWSEVSSLSDSSRTGLTSESDCSSEGS
SVESLKPTRRKQEHRKRGVLQSAPSEKRSSFHPCTDGAVPKLDKEGKVVKKHKTKHKHKHKEKGQCSVSQELK
LKSFTYEYEDSKQKSDKAILLESDLSTENKLKVLKHDREHLKKEDKLGRMKPEDKDWLFKDEKVLKRIKDANK
DMSRAFREDKDRASKAERERATKDKSPKEEKLRLYKEERKKKSKDRASRLERKNDMKEDKLSKEKEKAFKEDK
EKLKKEKLYREDAAFDDYCNKSQFLDHEDTKFSLSDDQQERWFSDLSDSSFDFKGEDSWDSVTDYRDIKNDSV
AKLILETVKEDSKEKKRDNKIREKRDFKDSFFRKRDRDCLDKNSEKRRDQTEKHKSIPSYLSEKDKKRRESAE
GGRDRRDGRIRSEEVHREDLKECGFESSFKDKSDCDFPKNLEPWERPHAAREKEKKDALEKERKEKGRADKYK
EKSSERERSDKSTLDKCQKDKEFEKCFKEKKDGKEKHKDIHSKDRKASFDQLREKKEKVFSSIISEDFSERKD
DRKGKEKSWYIADIFTDESEDEKDDCVAGSFKATEASDTQRVDGLPEKEEGREHPSDRHRKSSSDRQHTEKPR
DKEPKEKKKDRGASEGGKDKKEKMEKIFEKHKEKKDKECAERYKDRKERASADSAPEKKNKQKLPEKVEKKHF
AEDKVKSKHKEKPEKEHSRERERKPSRGPDVEKSLLEKLEEEALHDYREDSNDKISEVSSDSFADHGQEPSLS
TLLEVSFSEPPAEDKARDSACLSEKLREKERHRHSSSSSKKSHERERAKKEKAEKKEKSEDYKDSISSVRKDA
SQFEKDFLDAETYGVSYPTKADVEEELDKAIELFSSEKKDRSDPEREPAKRIEKELKPYGSSAISILKEKKKR
EKHRERWREEKERHRDKHVDGFLRHHKDEPKPAAKDKDNPPNSFKEKSREESLKLSETKLKEKFKENTEREKG
DSIKMSNGNDKLVPSRDSGKKDSRPREKLLGDGDLMMTSFERMLSQKDLEIEGRGERHKRHKERMKQMEKMRH
RSGDPKLKEKKPTEDGRKKSLDFPSKKALGLDKKVKEPAPTLTTGESKPHSGPGTESKDWLSGQPLKEVLPAS
PRTEQSRPTGVPTPTSVVSCPSYEEVMHTPRTPSCSADDYPDLVFDCTDSQHSMPVSTASTSACSPPFFDRFS
VASSVVSENAAGQTPTRPISTNLYRSISVDIRRTPEEEFSAGDKLFRQQSVPAPSSFDSPVQHLLEEKAPLPP
VPAEKFACLSPGYYSPDYGIPSPKVDTLHCPPTAVVSATPPPDSVFSNLPPKSSPSPRGELLSPAIEGTLPPD
LGLPLDATEDQQATAAILPQEPSYLEPLDEGPFTTVITEEPVEWTHTAAEQGLSSSSLIASASENPVSWPVGS
ELMLKSPQRFAESPKHFCPGESLHSTTPGPYSAAEPTYPVSPGSYPLPAPEPALEEVKDGGTGAIPVAISAAE
GAAPYAAPARLESFFSNCKSHPDAPLDTAPEPTGVTAVAQVEALGPLESSFLDSNPSISTLSQVEPVSWHEAF
TSPEDDLDLGPFSLPELPLQAKDASDVEAEAAKASPVPPAESPPGPTGVLGGGDVPAPAAEEPPAPPPQEASP
QLSTEPEPSEEPKLDVVLEATVETEVLADDSAPEASISNSVPAPSPPQQQPPGGGDEEAETEDPSATPCCAPD
GPTTDGLAQAHNSAEASCVVAAAEGPPGNVQAEATDPEPKPTSEVPKAPKVEEVPQRMTRNRAQMLASQSKQG
IPAAEKDPMPTPASRAKGRASEEEDAQAQHPRKRRFQRSSQQLQQQLNTSTQQTREVIQQTLAAIVDAIKLDA
IEPYHSDRSNPYFEYLQIRKKIEEKRKILCCITPQAPQCYAEYVTYTGSYLLDGKPLSKLHIPVIAPPPSLAE
PLKELFKQQEAVRGKLRLQHSIEREKLIVSCEQEILRVHCRAARTIANQAVPFSACTMLLDSEVYNMPLESQG
DENKSVRDRFNARQFISWLQDVDDKYDRMKTCLLMRQQHEAAALNAVQRMEWQLKAQELDPAGHKSLCVNEVP
SFYVPMVDVNDDFVLLPA*
>ENSMUSP00000095938_c.4819:A>GGAGGGGAGA_p.E1607GRGE-ins
ENSMUST00000098333.5|Ankrd11-201|ENSMUSG00000035569|-
|chr8:g.123619031:T>TCTCCCCTCC
MPKGGCSKTPQQEDFALSNDMVEKQTGKKDKDKVSLTKTPKLDRSDGGKEVRERATKRKLPFTVGANGEQKDS
DTDSFGHRTFNWGSPLPFSLTCHTEKQGPERKRIKKEPVARKSGLLFGMGLSGIRAGYPLSERQQVALLMQMT
AEESANSPDTTPKHPSQSTVCQKGTPNSASKTKDKVNKRNERGETRLHRAAIRGDARRIKELISEGADVNVKD
FAGWTALHEACNRGYYDIAKQLLAAGAEVNTKGLDDDTPLHDAANNGHYKVVKLLLRYGGNPQQSNRKGETPL
KVANSPTMVNLLLGKGTYTSSEESSTESSEEEDAPSFAPSSSVDGNNTDSEFEKGLKLKAKNPEPQKTVTPVK
DEYEFDEDDEQDRVPPVDDKHLLKKDYRKEAKANSFISIPKMEVKSYSKNNTLAPKKAAHRILSDTSDEEDVS
VSIGAGEKLRLSAHTMLPGSKARESSSSRQQKEKNKLKKKRKKETKGKEVRFGKRSDKFCSSGSESESSESEE
DDGDSVGSSGCLKGSPLVLKDPSLFSSLSASSTSSHGSAVAQKHGSGHTDQHTKHWRTDNWKAISSPAWSEVS
SLSDSSRTGLTSESDCSSEGSSVESLKPTRRKQEHRKRGVLQSAPSEKRSSFHPCTDGAVPKLDKEGKVVKKH
KTKHKHKHKEKGQCSVSQELKLKSFTYEYEDSKQKSDKAILLESDLSTENKLKVLKHDREHLKKEDKLGRMKP
EDKDWLFKDEKVLKRIKDANKDMSRAFREDKDRASKAERERATKDKSPKEEKLRLYKEERKKKSKDRASRLER
KNDMKEDKLSKEKEKAFKEDKEKLKKEKLYREDAAFDDYCNKSQFLDHEDTKFSLSDDQQERWFSDLSDSSFD
FKGEDSWDSVTDYRDIKNDSVAKLILETVKEDSKEKKRDNKIREKRDFKDSFFRKRDRDCLDKNSEKRRDQTE
KHKSIPSYLSEKDKKRRESAEGGRDRRDGRIRSEEVHREDLKECGFESSFKDKSDCDFPKNLEPWERPHAARE
KEKKDALEKERKEKGRADKYKEKSSERERSDKSTLDKCQKDKEFEKCFKEKKDGKEKHKDIHSKDRKASFDQL
REKKEKVFSSIISEDFSERKDDRKGKEKSWYIADIFTDESEDEKDDCVAGSFKATEASDTQRVDGLPEKEEGR
EHPSDRHRKSSSDRQHTEKPRDKEPKEKKKDRGASEGGKDKKEKMEKIFEKHKEKKDKECAERYKDRKERASA
DSAPEKKNKQKLPEKVEKKHFAEDKVKSKHKEKPEKEHSRERERKPSRGPDVEKSLLEKLEEEALHDYREDSN
DKISEVSSDSFADHGQEPSLSTLLEVSFSEPPAEDKARDSACLSEKLREKERHRHSSSSSKKSHERERAKKEK
AEKKEKSEDYKDSISSVRKDASQFEKDFLDAETYGVSYPTKADVEEELDKAIELFSSEKKDRSDPEREPAKRI

EKELKPYGSSAISILKEKKKREKHRERWREEKERHRDKHVDGFLRHHKDEPKPAAKDKDNPPNSFKEKSREES
LKLSETKLKEKFKENTEREKGDSIKMSNGNDKLVPSRDSGKKDSRPREKLLGDGDLMMTSFERMLSQKDLEIE
GRGERHKRHKERMKQMEKMRHRSGDPKLKEKKPTEDGRKKSLDFPSKKALGLDKKVKEPAPTLTTGESKPHSG
PGTESKDWLSGQPLKEVLPASPRTEQSRPTGVPTPTSVVSCPSYEEVMHTPRTPSCSADDYPDLVFDCTDSQH
SMPVSTASTSACSPPFFDRFSVASSVVSENAAGQTPTRPISTNLYRSISVDIRRTPEEEFSAGDKLFRQQSVP
APSSFDSPVQHLLEEKAPLPPVPAEKFACLSPGYYSPDYGIPSPKVDTLHCPPTAVVSATPPPDSVFSNLPPK
SSPSPRGELLSPAIEGTLPPDLGLPLDATEDQQATAAILPQEPSYLEPLDEGPFTTVITEEPVEWTHTAAEQG
LSSSSLIASASENPVSWPVGSELMLKSPQRFAESPKHFCPGESLHSTTPGPYSAAEPTYPVSPGSYPLPAPEP
ALEEVKDGGTGAIPVAISAAEGAAPYAAPARLESFFSNCKSHPDAPLDTAPEPTGVTAVAQVEALGPLESSFL
DSNPSISTLSQVEPVSWHEAFTSPEDDLDLGPFSLPELPLQAKDASDVEAEAAKASPVPPAESPPGPTGVLGG
GDVPAPAAEEPPAPPPQEASPQLSTEPEPSEEPKLDVVLEATVETEVLADDSAPEASISNSVPAPSPPQQQPP
GGGDEEAETEDPSATPCCAPDGPTTDGLAQAHNSAEASCVVAAAEGPPGNVQAEATDPEPKPTSEVPKAPKVE
EVPQRMTRNRAQMLASQSKQGIPAAEKDPMPTPASRAKGRASEEEDAQAQHPRKRRFQRSSQQLQQQLNTSTQ
QTREVIQQTLAAIVDAIKLDAIEPYHSDRSNPYFEYLQIRKKIEEKRKILCCITPQAPQCYAEYVTYTGSYLL
DGKPLSKLHIPVIAPPPSLAEPLKELFKQQEAVRGKLRLQHSIEREKLIVSCEQEILRVHCRAARTIANQAVP
FSACTMLLDSEVYNMPLESQGDENKSVRDRFNARQFISWLQDVDDKYDRMKTCLLMRQQHEAALNAVQRMEW
QLKAQELDPAGHKSLCVNEVPSFYVPMVDVNDDFVLLPA*
>ENSMUSP00000095939_c.4605:A>G_p.I1536V ENSMUST00000098334.13|Ankrd11-
202|ENSMUSG00000035569|-|chr8:g.123619182:T>C
MPKGGCSKTPQQEDFALSNDMVEKQTGKKDKDKVSLTKTPKLDRSDGGKEVRERATKRKLPFTVGANGEQKDS
DTEKQGPERKRIKKEPVARKSGLLFGMGLSGIRAGYPLSERQQVALLMQMTAEESANSPVDTTPKHPSQSTVC
QKGTPNSASKTKDKVNKRNERGETRLHRAAIRGDARRIKELISEGADVNVKDFAGWTALHEACNRGYYDIAKQ
LLAAGAEVNTKGLDDDTPLHDAANNGHYKVVKLLLRYGGNPQQSNRKGETPLKVANSPTMVNLLLGKGTYTSS
EESSTESSEEEDAPSFAPSSSVDGNNTDSEFEKGLKLKAKNPEPQKTVTPVKDEYEFDEDDEQDRVPPVDDKH
LLKKDYRKEAKANSFISIPKMEVKSYSKNNTLAPKKAAHRILSDTSDEEDVSVSIGAGEKLRLSAHTMLPGSK
ARESSSSRQQKEKNKLKKKRKKETKGKEVRFGKRSDKFCSSGSESESSESEEDDGDSVGSSGCLKGSPLVLKD
PSLFSSLSASSTSSHGSAVAQKHGSGHTDQHTKHWRTDNWKAISSPAWSEVSSLSDSSRTGLTSESDCSSEGS
SVESLKPTRRKQEHRKRGVLQSAPSEKRSSFHPCTDGAVPKLDKEGKVVKKHKTKHKHKHKEKGQCSVSQELK
LKSFTYEYEDSKQKSDKAILLESDLSTENKLKVLKHDREHLKKEDKLGRMKPEDKDWLFKDEKVLKRIKDANK
DMSRAFREDKDRASKAERERATKDKSPKEEKLRLYKEERKKKSKDRASRLERKNDMKEDKLSKEKEKAFKEDK
EKLKKEKLYREDAAFDDYCNKSQFLDHEDTKFSLSDDQQERWFSDLSDSSFDFKGEDSWDSVTDYRDIKNDSV
AKLILETVKEDSKEKKRDNKIREKRDFKDSFFRKRDRDCLDKNSEKRRDQTEKHKSIPSYLSEKDKKRRESAE
GGRDRRDGRIRSEEVHREDLKECGFESSFKDKSDCDFPKNLEPWERPHAAREKEKKDALEKERKEKGRADKYK
EKSSERERSDKSTLDKCQKDKEFEKCFKEKKDGKEKHKDIHSKDRKASFDQLREKKEKVFSSIISEDFSERKD
DRKGKEKSWYIADIFTDESEDEKDDCVAGSFKATEASDTQRVDGLPEKEEGREHPSDRHRKSSSDRQHTEKPR
DKEPKEKKKDRGASEGGKDKKEKMEKIFEKHKEKKDKECAERYKDRKERASADSAPEKKNKQKLPEKVEKKHF
AEDKVKSKHKEKPEKEHSRERERKPSRGPDVEKSLLEKLEEEALHDYREDSNDKISEVSSDSFADHGQEPSLS
TLLEVSFSEPPAEDKARDSACLSEKLREKERHRHSSSSSKKSHERERAKKEKAEKKEKSEDYKDSISSVRKDA
SQFEKDFLDAETYGVSYPTKADVEEELDKAIELFSSEKKDRSDPEREPAKRIEKELKPYGSSAISILKEKKKR
EKHRERWREEKERHRDKHVDGFLRHHKDEPKPAAKDKDNPPNSFKEKSREESLKLSETKLKEKFKENTEREKG
DSVKMSNGNDKLVPSRDSGKKDSRPREKLLGDGDLMMTSFERMLSQKDLEIEERHKRHKERMKQMEKMRHRSG
DPKLKEKKPTEDGRKKSLDFPSKKALGLDKKVKEPAPTLTTGESKPHSGPGTESKDWLSGQPLKEVLPASPRT
EQSRPTGVPTPTSVVSCPSYEEVMHTPRTPSCSADDYPDLVFDCTDSQHSMPVSTASTSACSPPFFDRFSVAS
SVVSENAAGQTPTRPISTNLYRSISVDIRRTPEEEFSAGDKLFRQQSVPAPSSFDSPVQHLLEEKAPLPPVPA
EKFACLSPGYYSPDYGIPSPKVDTLHCPPTAVVSATPPPDSVFSNLPPKSSPSPRGELLSPAIEGTLPPDLGL
PLDATEDQQATAAILPQEPSYLEPLDEGPFTTVITEEPVEWTHTAAEQGLSSSSLIASASENPVSWPVGSELM
LKSPQRFAESPKHFCPGESLHSTTPGPYSAAEPTYPVSPGSYPLPAPEPALEEVKDGGTGAIPVAISAAEGAA
PYAAPARLESFFSNCKSHPDAPLDTAPEPTGVTAVAQVEALGPLESSFLDSNPSISTLSQVEPVSWHEAFTSP
EDDLDLGPFSLPELPLQAKDASDVEAEAAKASPVPPAESPPGPTGVLGGGDVPAPAAEEPPAPPPQEASPQLS
TEPEPSEEPKLDVVLEATVETEVLADDSAPEASISNSVPAPSPPQQQPPGGGDEEAETEDPSATPCCAPDGPT
TDGLAQAHNSAEASCVVAAAEGPPGNVQAEATDPEPKPTSEVPKAPKVEEVPQRMTRNRAQMLASQSKQGIPA
AEKDPMPTPASRAKGRASEEEDAQAQHPRKRRFQRSSQQLQQQLNTSTQQTREVIQQTLAAIVDAIKLDAIEP
YHSDRSNPYFEYLQIRKKIEEKRKILCCITPQAPQCYAEYVTYTGSYLLDGKPLSKLHIPVIAPPPSLAEPLK
ELFKQQEAVRGKLRLQHSIEREKLIVSCEQEILRVHCRAARTIANQAVPFSACTMLLDSEVYNMPLESQGDEN
KSVRDRFNARQFISWLQDVDDKYDRMKTCLLMRQQHEAALNAVQRMEWQLKAQELDPAGHKSLCVNEVPSFY
VPMVDVNDDFVLLPA*
>ENSMUSP00000095938_c.4668:A>G_p.I1557V ENSMUST00000098333.5|Ankrd11-
201|ENSMUSG00000035569|-|chr8:g.123619182:T>C

MPKGGCSKTPQQEDFALSNDMVEKQTGKKDKDKVSLTKTPKLDRSDGGKEVRERATKRKLPFTVGANGEQKDS
DTDSFGHRTFNWGSPLPFSLTCHTEKQGPERKRIKKEPVARKSGLLFGMGLSGIRAGYPLSERQQVALLMQMT
AEESANSPDTTPKHPSQSTVCQKGTPNSASKTKDKVNKRNERGETRLHRAAIRGDARRIKELISEGADVNVKD
FAGWTALHEACNRGYYDIAKQLLAAGAEVNTKGLDDDTPLHDAANNGHYKVVKLLLRYGGNPQQSNRKGETPL
KVANSPTMVNLLLGKGTYTSSEESSTESSEEEDAPSFAPSSSVDGNNTDSEFEKGLKLKAKNPEPQKTVTPVK
DEYEFDEDDEQDRVPPVDDKHLLKKDYRKEAKANSFISIPKMEVKSYSKNNTLAPKKAAHRILSDTSDEEDVS
VSIGAGEKLRLSAHTMLPGSKARESSSSRQQKEKNKLKKKRKKETKGKEVRFGKRSDKFCSSGSESESSESEE
DDGDSVGSSGCLKGSPLVLKDPSLFSSLSASSTSSHGSAVAQKHGSGHTDQHTKHWRTDNWKAISSPAWSEVS
SLSDSSRTGLTSESDCSSEGSSVESLKPTRRKQEHRKRGVLQSAPSEKRSSFHPCTDGAVPKLDKEGKVVKKH
KTKHKHKHKEKGQCSVSQELKLKSFTYEYEDSKQKSDKAILLESDLSTENKLKVLKHDREHLKKEDKLGRMKP
EDKDWLFKDEKVLKRIKDANKDMSRAFREDKDRASKAERERATKDKSPKEEKLRLYKEERKKKSKDRASRLER
KNDMKEDKLSKEKEKAFKEDKEKLKKEKLYREDAAFDDYCNKSQFLDHEDTKFSLSDDQQERWFSDLSDSSFD
FKGEDSWDSVTDYRDIKNDSVAKLILETVKEDSKEKKRDNKIREKRDFKDSFFRKRDRDCLDKNSEKRRDQTE
KHKSIPSYLSEKDKKRRESAEGGRDRRDGRIRSEEVHREDLKECGFESSFKDKSDCDFPKNLEPWERPHAARE
KEKKDALEKERKEKGRADKYKEKSSERERSDKSTLDKCQKDKEFEKCFKEKKDGKEKHKDIHSKDRKASFDQL
REKKEKVFSSIISEDFSERKDDRKGKEKSWYIADIFTDESEDEKDDCVAGSFKATEASDTQRVDGLPEKEEGR
EHPSDRHRKSSSDRQHTEKPRDKEPKEKKKDRGASEGGKDKKEKMEKIFEKHKEKKDKECAERYKDRKERASA
DSAPEKKNKQKLPEKVEKKHFAEDKVKSKHKEKPEKEHSRERERKPSRGPDVEKSLLEKLEEEALHDYREDSN
DKISEVSSDSFADHGQEPSLSTLLEVSFSEPPAEDKARDSACLSEKLREKERHRHSSSSSKKSHERERAKKEK
AEKKEKSEDYKDSISSVRKDASQFEKDFLDAETYGVSYPTKADVEEELDKAIELFSSEKKDRSDPEREPAKRI
EKELKPYGSSAISILKEKKKREKHRERWREEKERHRDKHVDGFLRHHKDEPKPAAKDKDNPPNSFKEKSREES
LKLSETKLKEKFKENTEREKGDSVKMSNGNDKLVPSRDSGKKDSRPREKLLGDGDLMMTSFERMLSQKDLEIE
ERHKRHKERMKQMEKMRHRSGDPKLKEKKPTEDGRKKSLDFPSKKALGLDKKVKEPAPTLTTGESKPHSGPGT
ESKDWLSGQPLKEVLPASPRTEQSRPTGVPTPTSVVSCPSYEEVMHTPRTPSCSADDYPDLVFDCTDSQHSMP
VSTASTSACSPPFFDRFSVASSVVSENAAGQTPTRPISTNLYRSISVDIRRTPEEEFSAGDKLFRQQSVPAPS
SFDSPVQHLLEEKAPLPPVPAEKFACLSPGYYSPDYGIPSPKVDTLHCPPTAVVSATPPPDSVFSNLPPKSSP
SPRGELLSPAIEGTLPPDLGLPLDATEDQQATAAILPQEPSYLEPLDEGPFTTVITEEPVEWTHTAAEQGLSS
SSLIASASENPVSWPVGSELMLKSPQRFAESPKHFCPGESLHSTTPGPYSAAEPTYPVSPGSYPLPAPEPALE
EVKDGGTGAIPVAISAAEGAAPYAAPARLESFFSNCKSHPDAPLDTAPEPTGVTAVAQVEALGPLESSFLDSN
PSISTLSQVEPVSWHEAFTSPEDDLDLGPFSLPELPLQAKDASDVEAEAAKASPVPPAESPPGPTGVLGGGDV
PAPAAEEPPAPPPQEASPQLSTEPEPSEEPKLDVVLEATVETEVLADDSAPEASISNSVPAPSPPQQQPPGGG
DEEAETEDPSATPCCAPDGPTTDGLAQAHNSAEASCVVAAAEGPPGNVQAEATDPEPKPTSEVPKAPKVEEVP
QRMTRNRAQMLASQSKQGIPAAEKDPMPTPASRAKGRASEEEDAQAQHPRKRRFQRSSQQLQQQLNTSTQQTR
EVIQQTLAAIVDAIKLDAIEPYHSDRSNPYFEYLQIRKKIEEKRKILCCITPQAPQCYAEYVTYTGSYLLDGK
PLSKLHIPVIAPPPSLAEPLKELFKQQEAVRGKLRLQHSIEREKLIVSCEQEILRVHCRAARTIANQAVPFSA
CTMLLDSEVYNMPLESQGDENKSVRDRFNARQFISWLQDVDDKYDRMKTCLLMRQQHEAAALNAVQRMEWQLK
AQELDPAGHKSLCVNEVPSFYVPMVDVNDDFVLLPA*
>ENSMUSP00000095939_c.4552:C>G_p.T1518S ENSMUST00000098334.13|Ankrd11-202|ENSMUSG00000035569|-|chr8:g.123619235:G>C
MPKGGCSKTPQQEDFALSNDMVEKQTGKKDKDKVSLTKTPKLDRSDGGKEVRERATKRKLPFTVGANGEQKDS
DTEKQGPERKRIKKEPVARKSGLLFGMGLSGIRAGYPLSERQQVALLMQMTAEESANSPVDTTPKHPSQSTVC
QKGTPNSASKTKDKVNKRNERGETRLHRAAIRGDARRIKELISEGADVNVKDFAGWTALHEACNRGYYDIAKQ
LLAAGAEVNTKGLDDDTPLHDAANNGHYKVVKLLLRYGGNPQQSNRKGETPLKVANSPTMVNLLLGKGTYTSS
EESSTESSEEEDAPSFAPSSSVDGNNTDSEFEKGLKLKAKNPEPQKTVTPVKDEYEFDEDDEQDRVPPVDDKH
LLKKDYRKEAKANSFISIPKMEVKSYSKNNTLAPKKAAHRILSDTSDEEDVSVSIGAGEKLRLSAHTMLPGSK
ARESSSSRQQKEKNKLKKKRKKETKGKEVRFGKRSDKFCSSGSESESSESEEDDGDSVGSSGCLKGSPLVLKD
PSLFSSLSASSTSSHGSAVAQKHGSGHTDQHTKHWRTDNWKAISSPAWSEVSSLSDSSRTGLTSESDCSSEGS
SVESLKPTRRKQEHRKRGVLQSAPSEKRSSFHPCTDGAVPKLDKEGKVVKKHKTKHKHKHKEKGQCSVSQELK
LKSFTYEYEDSKQKSDKAILLESDLSTENKLKVLKHDREHLKKEDKLGRMKPEDKDWLFKDEKVLKRIKDANK
DMSRAFREDKDRASKAERERATKDKSPKEEKLRLYKEERKKKSKDRASRLERKNDMKEDKLSKEKEKAFKEDK
EKLKKEKLYREDAAFDDYCNKSQFLDHEDTKFSLSDDQQERWFSDLSDSSFDFKGEDSWDSVTDYRDIKNDSV
AKLILETVKEDSKEKKRDNKIREKRDFKDSFFRKRDRDCLDKNSEKRRDQTEKHKSIPSYLSEKDKKRRESAE
GGRDRRDGRIRSEEVHREDLKECGFESSFKDKSDCDFPKNLEPWERPHAAREKEKKDALEKERKEKGRADKYK
EKSSERERSDKSTLDKCQKDKEFEKCFKEKKDGKEKHKDIHSKDRKASFDQLREKKEKVFSSIISEDFSERKD
DRKGKEKSWYIADIFTDESEDEKDDCVAGSFKATEASDTQRVDGLPEKEEGREHPSDRHRKSSSDRQHTEKPR
DKEPKEKKKDRGASEGGKDKKEKMEKIFEKHKEKKDKECAERYKDRKERASADSAPEKKNKQKLPEKVEKKHF
AEDKVKSKHKEKPEKEHSRERERKPSRGPDVEKSLLEKLEEEALHDYREDSNDKISEVSSDSFADHGQEPSLS
TLLEVSFSEPPAEDKARDSACLSEKLREKERHRHSSSSSKKSHERERAKKEKAEKKEKSEDYKDSISSVRKDA

SQFEKDFLDAETYGVSYPTKADVEEELDKAIELFSSEKKDRSDPEREPAKRIEKELKPYGSSAISILKEKKKR
EKHRERWREEKERHRDKHVDGFLRHHKDEPKPAAKDKDNPPNSFKEKSREESLKLSESKLKEKFKENTEREKG
DSIKMSNGNDKLVPSRDSGKKDSRPREKLLGDGDLMMTSFERMLSQKDLEIEERHKRHKERMKQMEKMRHRSG
DPKLKEKKPTEDGRKKSLDFPSKKALGLDKKVKEPAPTLTTGESKPHSGPGTESKDWLSGQPLKEVLPASPRT
EQSRPTGVPTPTSVVSCPSYEEVMHTPRTPSCSADDYPDLVFDCTDSQHSMPVSTASTSACSPPFFDRFSVAS
SVVSENAAGQTPTRPISTNLYRSISVDIRRTPEEEFSAGDKLFRQQSVPAPSSFDSPVQHLLEEKAPLPPVPA
EKFACLSPGYYSPDYGIPSPKVDTLHCPPTAVVSATPPPDSVFSNLPPKSSPSPRGELLSPAIEGTLPPDLGL
PLDATEDQQATAAILPQEPSYLEPLDEGPFTTVITEEPVEWTHTAAEQGLSSSSLIASASENPVSWPVGSELM
LKSPQRFAESPKHFCPGESLHSTTPGPYSAAEPTYPVSPGSYPLPAPEPALEEVKDGGTGAIPVAISAAEGAA
PYAAPARLESFFSNCKSHPDAPLDTAPEPTGVTAVAQVEALGPLESSFLDSNPSISTLSQVEPVSWHEAFTSP
EDDLDLGPFSLPELPLQAKDASDVEAEAAKASPVPPAESPPGPTGVLGGGDVPAPAAEEPPAPPPQEASPQLS
TEPEPSEEPKLDVVLEATVETEVLADDSAPEASISNSVPAPSPPQQQPPGGGDEEAETEDPSATPCCAPDGPT
TDGLAQAHNSAEASCVVAAAEGPPGNVQAEATDPEPKPTSEVPKAPKVEEVPQRMTRNRAQMLASQSKQGIPA
AEKDPMPTPASRAKGRASEEEDAQAQHPRKRRFQRSSQQLQQQLNTSTQQTREVIQQTLAAIVDAIKLDAIEP
YHSDRSNPYFEYLQIRKKIEEKRKILCCITPQAPQCYAEYVTYTGSYLLDGKPLSKLHIPVIAPPPSLAEPLK
ELFKQQEAVRGKLRLQHSIEREKLIVSCEQEILRVHCRAARTIANQAVPFSACTMLLDSEVYNMPLESQGDEN
KSVRDRFNARQFISWLQDVDDKYDRMKTCLLMRQQHEAAALNAVQRMEWQLKAQELDPAGHKSLCVNEVPSFY
VPMVDVNDDFVLLPA*
>ENSMUSP00000095938_c.4615:C>G_p.T1539S ENSMUST00000098333.5|Ankrd11-201|ENSMUSG00000035569|-|chr8:g.123619235:G>C
MPKGGCSKTPQQEDFALSNDMVEKQTGKKDKDKVSLTKTPKLDRSDGGKEVRERATKRKLPFTVGANGEQKDS
DTDSFGHRTFNWGSPLPFSLTCHTEKQGPERKRIKKEPVARKSGLLFGMGLSGIRAGYPLSERQQVALLMQMT
AEESANSPDTTPKHPSQSTVCQKGTPNSASKTKDKVNKRNERGETRLHRAAIRGDARRIKELISEGADVNVKD
FAGWTALHEACNRGYYDIAKQLLAAGAEVNTKGLDDDTPLHDAANNGHYKVVKLLLRYGGNPQQSNRKGETPL
KVANSPTMVNLLLGKGTYTSSEESSTESSEEEDAPSFAPSSSVDGNNTDSEFEKGLKLKAKNPEPQKTVTPVK
DEYEFDEDDEQDRVPPVDDKHLLKKDYRKEAKANSFISIPKMEVKSYSKNNTLAPKKAAHRILSDTSDEEDVS
VSIGAGEKLRLSAHTMLPGSKARESSSSRQQKEKNKLKKKRKKETKGKEVRFGKRSDKFCSSGSESESSESEE
DDGDSVGSSGCLKGSPLVLKDPSLFSSLSASSTSSHGSAVAQKHGSGHTDQHTKHWRTDNWKAISSPAWSEVS
SLSDSSRTGLTSESDCSSEGSSVESLKPTRRKQEHRKRGVLQSAPSEKRSSFHPCTDGAVPKLDKEGKVVKKH
KTKHKHKHKEKGQCSVSQELKLKSFTYEYEDSKQKSDKAILLESDLSTENKLKVLKHDREHLKKEDKLGRMKP
EDKDWLFKDEKVLKRIKDANKDMSRAFREDKDRASKAERERATKDKSPKEEKLRLYKEERKKKSKDRASRLER
KNDMKEDKLSKEKEKAFKEDKEKLKKEKLYREDAAFDDYCNKSQFLDHEDTKFSLSDDQQERWFSDLSDSSFD
FKGEDSWDSVTDYRDIKNDSVAKLILETVKEDSKEKKRDNKIREKRDFKDSFFRKRDRDCLDKNSEKRRDQTE
KHKSIPSYLSEKDKKRRESAEGGRDRRDGRIRSEEVHREDLKECGFESSFKDKSDCDFPKNLEPWERPHAARE
KEKKDALEKERKEKGRADKYKEKSSERERSDKSTLDKCQKDKEFEKCFKEKKDGKEKHKDIHSKDRKASFDQL
REKKEKVFSSIISEDFSERKDDRKGKEKSWYIADIFTDESEDEKDDCVAGSFKATEASDTQRVDGLPEKEEGR
EHPSDRHRKSSSDRQHTEKPRDKEPKEKKKDRGASEGGKDKKEKMEKIFEKHKEKKDKECAERYKDRKERASA
DSAPEKKNKQKLPEKVEKKHFAEDKVKSKHKEKPEKEHSRERERKPSRGPDVEKSLLEKLEEEALHDYREDSN
DKISEVSSDSFADHGQEPSLSTLLEVSFSEPPAEDKARDSACLSEKLREKERHRHSSSSSKKSHERERAKKEK
AEKKEKSEDYKDSISSVRKDASQFEKDFLDAETYGVSYPTKADVEEELDKAIELFSSEKKDRSDPEREPAKRI
EKELKPYGSSAISILKEKKKREKHRERWREEKERHRDKHVDGFLRHHKDEPKPAAKDKDNPPNSFKEKSREES
LKLSESKLKEKFKENTEREKGDSIKMSNGNDKLVPSRDSGKKDSRPREKLLGDGDLMMTSFERMLSQKDLEIE
ERHKRHKERMKQMEKMRHRSGDPKLKEKKPTEDGRKKSLDFPSKKALGLDKKVKEPAPTLTTGESKPHSGPGT
ESKDWLSGQPLKEVLPASPRTEQSRPTGVPTPTSVVSCPSYEEVMHTPRTPSCSADDYPDLVFDCTDSQHSMP
VSTASTSACSPPFFDRFSVASSVVSENAAGQTPTRPISTNLYRSISVDIRRTPEEEFSAGDKLFRQQSVPAPS
SFDSPVQHLLEEKAPLPPVPAEKFACLSPGYYSPDYGIPSPKVDTLHCPPTAVVSATPPPDSVFSNLPPKSSP
SPRGELLSPAIEGTLPPDLGLPLDATEDQQATAAILPQEPSYLEPLDEGPFTTVITEEPVEWTHTAAEQGLSS
SSLIASASENPVSWPVGSELMLKSPQRFAESPKHFCPGESLHSTTPGPYSAAEPTYPVSPGSYPLPAPEPALE
EVKDGGTGAIPVAISAAEGAAPYAAPARLESFFSNCKSHPDAPLDTAPEPTGVTAVAQVEALGPLESSFLDSN
PSISTLSQVEPVSWHEAFTSPEDDLDLGPFSLPELPLQAKDASDVEAEAAKASPVPPAESPPGPTGVLGGGDV
PAPAAEEPPAPPPQEASPQLSTEPEPSEEPKLDVVLEATVETEVLADDSAPEASISNSVPAPSPPQQQPPGGG
DEEAETEDPSATPCCAPDGPTTDGLAQAHNSAEASCVVAAAEGPPGNVQAEATDPEPKPTSEVPKAPKVEEVP
QRMTRNRAQMLASQSKQGIPAAEKDPMPTPASRAKGRASEEEDAQAQHPRKRRFQRSSQQLQQQLNTSTQQTR
EVIQQTLAAIVDAIKLDAIEPYHSDRSNPYFEYLQIRKKIEEKRKILCCITPQAPQCYAEYVTYTGSYLLDGK
PLSKLHIPVIAPPPSLAEPLKELFKQQEAVRGKLRLQHSIEREKLIVSCEQEILRVHCRAARTIANQAVPFSA
CTMLLDSEVYNMPLESQGDENKSVRDRFNARQFISWLQDVDDKYDRMKTCLLMRQQHEAAALNAVQRMEWQLK
AQELDPAGHKSLCVNEVPSFYVPMVDVNDDFVLLPA*

>ENSMUSP00000095939_c.3858:G>T_p.D1287Y ENSMUST00000098334.13|Ankrd11-202|ENSMUSG00000035569|-|chr8:g.123619929:C>A
MPKGGCSKTPQQEDFALSNDMVEKQTGKKDKDKVSLTKTPKLDRSDGGKEVRERATKRKLPFTVGANGEQKDS
DTEKQGPERKRIKKEPVARKSGLLFGMLSGIRAGYPLSERQQVALLMQMTAEESANSPVDTTPKHPSQSTVC
QKGTPNSASKTKDKVNKRNERGETRLHRAAIRGDARRIKELISEGADVNVKDFAGWTALHEACNRGYYDIAKQ
LLAAGAEVNTKGLDDDTPLHDAANNGHYKVVKLLLRYGGNPQQSNRKGETPLKVANSPTMVNLLLGKGTYTSS
EESSTESSEEEDAPSFAPSSSVDGNNTDSEFEKGLKLKAKNPEPQKTVTPVKDEYEFDEDDEQDRVPPVDDKH
LLKKDYRKEAKANSFISIPKMEVKSYSKNNTLAPKKAAHRILSDTSDEEDVSVSIGAGEKLRLSAHTMLPGSK
ARESSSSRQQKEKNLKKKRKKETKGKEVRFGKRSDKFCSSGSESESSESEEDDGDSVGSSGCLKGSPLVLKD
PSLFSSLSASSTSSHGSAVAQKHGSGHTDQHTKHWRTDNWKAISSPAWSEVSSLSDSSRTGLTSESDCSSEGS
SVESLKPTRRKQEHRKRGVLQSAPSEKRSSFHPCTDGAVPKLDKEGKVVKKHKTKHKHKHKEKGQCSVSQELK
LKSFTYEYEDSKQKSDKAILLESDLSTENKLKVLKHDREHLKKEDKLGRMKPEDKDWLFKDEKVLKRIKDANK
DMSRAFREDKDRASKAERERATKDKSPKEEKLRLYKEERKKKSKDRASRLERKNDMKEDKLSKEKEKAFKEDK
EKLKKEKLYREDAAFDDYCNKSQFLDHEDTKFSLSDDQQERWFSDLSDSSFDFKGEDSWDSVTDYRDIKNDSV
AKLILETVKEDSKEKKRDNKIREKRDFKDSFFRKRDRDCLDKNSEKRRDQTEKHKSIPSYLSEKDKKRRESAE
GGRDRRDGRIRSEEVHREDLKECGFESSFKDKSDCDFPKNLEPWERPHAAREKEKKDALEKERKEKGRADKYK
EKSSERERSDKSTLDKCQKDKEFEKCFKEKKDGKEKHKDIHSKDRKASFDQLREKKEKVFSSIISEDFSERKD
DRKGKEKSWYIADIFTDESEDEKDDCVAGSFKATEASDTQRVDGLPEKEEGREHPSDRHRKSSSDRQHTEKPR
DKEPKEKKKDRGASEGGKDKKEKMEKIFEKHKEKKDKECAERYKDRKERASADSAPEKKNKQKLPEKVEKKHF
AEDKVKSKHKEKPEKEHSRERERKPSRGPDVEKSLLEKLEEEALHYYREDSNDKISEVSSDSFADHGQEPSLS
TLLEVSFSEPPAEDKARDSACLSEKLREKERHRHSSSSSKKSHERERAKKEKAEKKEKSEDYKDSISSVRKDA
SQFEKDFLDAETYGVSYPTKADVEEELDKAIELFSSEKKDRSDPEREPAKRIEKELKPYGSSAISILKEKKKR
EKHRERWREEKERHRDKHVDGFLRHHKDEPKPAAKDKDNPPNSFKEKSREESLKLSETKLKEKFKENTEREKG
DSIKMSNGNDKLVPSRDSGKKDSRPREKLLGDGDLMMTSFERMLSQKDLEIEERHKRHKERMKQMEKMRHRSG
DPKLKEKKPTEDGRKKSLDFPSKKALGLDKKVKEPAPTLTTGESKPHSGPGTESKDWLSGQPLKEVLPASPRT
EQSRPTGVPTPTSVVSCPSYEEVMHTPRTPSCSADDYPDLVFDCTDSQHSMPVSTASTSACSPPFFDRFSVAS
SVVSENAAGQTPTRPISTNLYRSISVDIRRTPEEEFSAGDKLFRQQSVPAPSSFDSPVQHLLEEKAPLPPVPA
EKFACLSPGYYSPDYGIPSPKVDTLHCPPTAVVSATPPPDSVFSNLPPKSSPSPRGELLSPAIEGTLPPDLGL
PLDATEDQQATAAILPQEPSYLEPLDEGPFTTVITEEPVEWTHTAAEQGLSSSSLIASASENPVSWPVGSELM
LKSPQRFAESPKHFCPGESLHSTTPGPYSAAEPTYPVSPGSYPLPAPEPALEEVKDGGTGAIPVAISAAEGAA
PYAAPARLESFFSNCKSHPDAPLDTAPEPTGVTAVAQVEALGPLESSFLDSNPSISTLSQVEPVSWHEAFTSP
EDDLDLGPFSLPELPLQAKDASDVEAEAAKASPVPPAESPPGPTGVLGGGDVPAPAAEEPPAPPPQEASPQLS
TEPEPSEEPKLDVVLEATVETEVLADDSAPEASISNSVPAPSPPQQQPPGGGDEEAETEDPSATPCCAPDGPT
TDGLAQAHNSAEASCVVAAAEGPPGNVQAEATDPEPKPTSEVPKAPKVEEVPQRMTRNRAQMLASQSKQGIPA
AEKDPMPTPASRAKGRASEEEDAQAQHPRKRRFQRSSQQLQQQLNTSTQQTREVIQQTLAAIVDAIKLDAIEP
YHSDRSNPYFEYLQIRKKIEEKRKILCCITPQAPQCYAEYVTYTGSYLLDGKPLSKLHIPVIAPPPSLAEPLK
ELFKQQEAVRGKLRLQHSIEREKLIVSCEQEILRVHCRAARTIANQAVPFSACTMLLDSEVYNMPLESQGDEN
KSVRDRFNARQFISWLQDVDDKYDRMKTCLLMRQQHEAAALNAVQRMEWQLKAQELDPAGHKSLCVNEVPSFY
VPMVDVNDDFVLLPA*
>ENSMUSP00000095938_c.3921:G>T_p.D1308Y ENSMUST00000098333.5|Ankrd11-201|ENSMUSG00000035569|-|chr8:g.123619929:C>A
MPKGGCSKTPQQEDFALSNDMVEKQTGKKDKDKVSLTKTPKLDRSDGGKEVRERATKRKLPFTVGANGEQKDS
DTDSFGHRTFNWGSPLPFSLTCHTEKQGPERKRIKKEPVARKSGLLFGMLSGIRAGYPLSERQQVALLMQMT
AEESANSPDTTPKHPSQSTVCQKGTPNSASKTKDKVNKRNERGETRLHRAAIRGDARRIKELISEGADVNVKD
FAGWTALHEACNRGYYDIAKQLLAAGAEVNTKGLDDDTPLHDAANNGHYKVVKLLLRYGGNPQQSNRKGETPL
KVANSPTMVNLLLGKGTYTSSEESSTESSEEEDAPSFAPSSSVDGNNTDSEFEKGLKLKAKNPEPQKTVTPVK
DEYEFDEDDEQDRVPPVDDKHLLKKDYRKEAKANSFISIPKMEVKSYSKNNTLAPKKAAHRILSDTSDEEDVS
VSIGAGEKLRLSAHTMLPGSKARESSSSRQQKEKNLKKKRKKETKGKEVRFGKRSDKFCSSGSESESSESEE
DDGDSVGSSGCLKGSPLVLKDPSLFSSLSASSTSSHGSAVAQKHGSGHTDQHTKHWRTDNWKAISSPAWSEVS
SLSDSSRTGLTSESDCSSEGSSVESLKPTRRKQEHRKRGVLQSAPSEKRSSFHPCTDGAVPKLDKEGKVVKKH
KTKHKHKHKEKGQCSVSQELKLKSFTYEYEDSKQKSDKAILLESDLSTENKLKVLKHDREHLKKEDKLGRMKP
EDKDWLFKDEKVLKRIKDANKDMSRAFREDKDRASKAERERATKDKSPKEEKLRLYKEERKKKSKDRASRLER
KNDMKEDKLSKEKEKAFKEDKEKLKKEKLYREDAAFDDYCNKSQFLDHEDTKFSLSDDQQERWFSDLSDSSFD
FKGEDSWDSVTDYRDIKNDSVAKLILETVKEDSKEKKRDNKIREKRDFKDSFFRKRDRDCLDKNSEKRRDQTE
KHKSIPSYLSEKDKKRRESAEGGRDRRDGRIRSEEVHREDLKECGFESSFKDKSDCDFPKNLEPWERPHAARE
KEKKDALEKERKEKGRADKYKEKSSERERSDKSTLDKCQKDKEFEKCFKEKKDGKEKHKDIHSKDRKASFDQL
REKKEKVFSSIISEDFSERKDDRKGKEKSWYIADIFTDESEDEKDDCVAGSFKATEASDTQRVDGLPEKEEGR
EHPSDRHRKSSSDRQHTEKPRDKEPKEKKKDRGASEGGKDKKEKMEKIFEKHKEKKDKECAERYKDRKERASA

DSAPEKKNKQKLPEKVEKKHFAEDKVKSKHKEKPEKEHSRERERKPSRGPDVEKSLLEKLEEEALHYYREDSN
DKISEVSSDSFADHGQEPSLSTLLEVSFSEPPAEDKARDSACLSEKLREKERHRHSSSSSKKSHERERAKKEK
AEKKEKSEDYKDSISSVRKDASQFEKDFLDAETYGVSYPTKADVEEELDKAIELFSSEKKDRSDPEREPAKRI
EKELKPYGSSAISILKEKKKREKHRERWREEKERHRDKHVDGFLRHHKDEPKPAAKDKDNPPNSFKEKSREES
LKLSETKLKEKFKENTEREKGDSIKMSNGNDKLVPSRDSGKKDSRPREKLLGDGDLMMTSFERMLSQKDLEIE
ERHKRHKERMKQMEKMRHRSGDPKLKEKKPTEDGRKKSLDFPSKKALGLDKKVKEPAPTLTTGESKPHSGPGT
ESKDWLSGQPLKEVLPASPRTEQSRPTGVPTPTSVVSCPSYEEVMHTPRTPSCSADDYPDLVFDCTDSQHSMP
VSTASTSACSPPFFDRFSVASSVVSENAAGQTPTRPISTNLYRSISVDIRRTPEEEFSAGDKLFRQQSVPAPS
SFDSPVQHLLEEKAPLPPVPAEKFACLSPGYYSPDYGIPSPKVDTLHCPPTAVVSATPPPDSVFSNLPPKSSP
SPRGELLSPAIEGTLPPDLGLPLDATEDQQATAAILPQEPSYLEPLDEGPFTTVITEEPVEWTHTAAEQGLSS
SSLIASASENPVSWPVGSELMLKSPQRFAESPKHFCPGESLHSTTPGPYSAAEPTYPVSPGSYPLPAPEPALE
EVKDGGTGAIPVAISAAEGAAPYAAPARLESFFSNCKSHPDAPLDTAPEPTGVTAVAQVEALGPLESSFLDSN
PSISTLSQVEPVSWHEAFTSPEDDLDLGPFSLPELPLQAKDASDVEAEAAKASPVPPAESPPGPTGVLGGGDV
PAPAAEEPPAPPPQEASPQLSTEPEPSEEPKLDVVLEATVETEVLADDSAPEASISNSVPAPSPPQQQPPGGG
DEEAETEDPSATPCCAPDGPTTDGLAQAHNSAEASCVVAAAEGPPGNVQAEATDPEPKPTSEVPKAPKVEEVP
QRMTRNRAQMLASQSKQGIPAAEKDPMPTPASRAKGRASEEEDAQAQHPRKRRFQRSSQQLQQQLNTSTQQTR
EVIQQTLAAIVDAIKLDAIEPYHSDRSNPYFEYLQIRKKIEEKRKILCCITPQAPQCYAEYVTYTGSYLLDGK
PLSKLHIPVIAPPPSLAEPLKELFKQQEAVRGKLRLQHSIEREKLIVSCEQEILRVHCRAARTIANQAVPFSA
CTMLLDSEVYNMPLESQGDENKSVRDRFNARQFISWLQDVDDKYDRMKTCLLMRQQHEAAALNAVQRMEWQLK
AQELDPAGHKSLCVNEVPSFYVPMVDVNDDFVLLPA*
>ENSMUSP00000095939_c.1203:G>A_p.A402T ENSMUST00000098334.13|Ankrd11-
202|ENSMUSG00000035569|-|chr8:g.123622584:C>T
MPKGGCSKTPQQEDFALSNDMVEKQTGKKDKDKVSLTKTPKLDRSDGGKEVRERATKRKLPFTVGANGEQKDS
DTEKQGPERKRIKKEPVARKSGLLFGMGLSGIRAGYPLSERQQVALLMQMTAEESANSPVDTTPKHPSQSTVC
QKGTPNSASKTKDKVNKRNERGETRLHRAAIRGDARRIKELISEGADVNVKDFAGWTALHEACNRGYYDIAKQ
LLAAGAEVNTKGLDDDTPLHDAANNGHYKVVKLLLRYGGNPQQSNRKGETPLKVANSPTMVNLLLGKGTYTSS
EESSTESSEEEDAPSFAPSSSVDGNNTDSEFEKGLKLKAKNPEPQKTVTPVKDEYEFDEDDEQDRVPPVDDKH
LLKKDYRKEAKANSFISIPKMEVKSYSKNNTLAPKKTAHRILSDTSDEEDVSVSIGAGEKLRLSAHTMLPGSK
ARESSSSRQQKEKNKLKKKRKKETKGKEVRFGKRSDKFCSSGSESESSESEEDDGDSVGSSGCLKGSPLVLKD
PSLFSSLSASSTSSHGSAVAQKHGSGHTDQHTKHWRTDNWKAISSPAWSEVSSLSDSSRTGLTSESDCSSEGS
SVESLKPTRRKQEHRKRGVLQSAPSEKRSSFHPCTDGAVPKLDKEGKVVKKHKTKHKHKHKEKGQCSVSQELK
LKSFTYEYEDSKQKSDKAILLESDLSTENKLKVLKHDREHLKKEDKLGRMKPEDKDWLFKDEKVLKRIKDANK
DMSRAFREDKDRASKAERERATKDKSPKEEKLRLYKEERKKKSKDRASRLERKNDMKEDKLSKEKEKAFKEDK
EKLKKEKLYREDAAFDDYCNKSQFLDHEDTKFSLSDDQQERWFSDLSDSSFDFKGEDSWDSVTDYRDIKNDSV
AKLILETVKEDSKEKKRDNKIREKRDFKDSFFRKRDRDCLDKNSEKRRDQTEKHKSIPSYLSEKDKKRRESAE
GGRDRRDGRIRSEEVHREDLKECGFESSFKDKSDCDFPKNLEPWERPHAAREKEKKDALEKERKEKGRADKYK
EKSSERERSDKSTLDKCQKDKEFEKCFKEKKDGKEKHKDIHSKDRKASFDQLREKKEKVFSSIISEDFSERKD
DRKGKEKSWYIADIFTDESEDEKDDCVAGSFKATEASDTQRVDGLPEKEEGREHPSDRHRKSSSDRQHTEKPR
DKEPKEKKKDRGASEGGKDKKEKMEKIFEKHKEKKDKECAERYKDRKERASADSAPEKKNKQKLPEKVEKKHF
AEDKVKSKHKEKPEKEHSRERERKPSRGPDVEKSLLEKLEEEALHDYREDSNDKISEVSSDSFADHGQEPSLS
TLLEVSFSEPPAEDKARDSACLSEKLREKERHRHSSSSSKKSHERERAKKEKAEKKEKSEDYKDSISSVRKDA
SQFEKDFLDAETYGVSYPTKADVEEELDKAIELFSSEKKDRSDPEREPAKRIEKELKPYGSSAISILKEKKKR
EKHRERWREEKERHRDKHVDGFLRHHKDEPKPAAKDKDNPPNSFKEKSREESLKLSETKLKEKFKENTEREKG
DSIKMSNGNDKLVPSRDSGKKDSRPREKLLGDGDLMMTSFERMLSQKDLEIEERHKRHKERMKQMEKMRHRSG
DPKLKEKKPTEDGRKKSLDFPSKKALGLDKKVKEPAPTLTTGESKPHSGPGTESKDWLSGQPLKEVLPASPRT
EQSRPTGVPTPTSVVSCPSYEEVMHTPRTPSCSADDYPDLVFDCTDSQHSMPVSTASTSACSPPFFDRFSVAS
SVVSENAAGQTPTRPISTNLYRSISVDIRRTPEEEFSAGDKLFRQQSVPAPSSFDSPVQHLLEEKAPLPPVPA
EKFACLSPGYYSPDYGIPSPKVDTLHCPPTAVVSATPPPDSVFSNLPPKSSPSPRGELLSPAIEGTLPPDLGL
PLDATEDQQATAAILPQEPSYLEPLDEGPFTTVITEEPVEWTHTAAEQGLSSSSLIASASENPVSWPVGSELM
LKSPQRFAESPKHFCPGESLHSTTPGPYSAAEPTYPVSPGSYPLPAPEPALEEVKDGGTGAIPVAISAAEGAA
PYAAPARLESFFSNCKSHPDAPLDTAPEPTGVTAVAQVEALGPLESSFLDSNPSISTLSQVEPVSWHEAFTSP
EDDLDLGPFSLPELPLQAKDASDVEAEAAKASPVPPAESPPGPTGVLGGGDVPAPAAEEPPAPPPQEASPQLS
TEPEPSEEPKLDVVLEATVETEVLADDSAPEASISNSVPAPSPPQQQPPGGGDEEAETEDPSATPCCAPDGPT
TDGLAQAHNSAEASCVVAAAEGPPGNVQAEATDPEPKPTSEVPKAPKVEEVPQRMTRNRAQMLASQSKQGIPA
AEKDPMPTPASRAKGRASEEEDAQAQHPRKRRFQRSSQQLQQQLNTSTQQTREVIQQTLAAIVDAIKLDAIEP
YHSDRSNPYFEYLQIRKKIEEKRKILCCITPQAPQCYAEYVTYTGSYLLDGKPLSKLHIPVIAPPPSLAEPLK
ELFKQQEAVRGKLRLQHSIEREKLIVSCEQEILRVHCRAARTIANQAVPFSACTMLLDSEVYNMPLESQGDEN

KSVRDRFNARQFISWLQDVDDKYDRMKTCLLMRQQHEAAALNAVQRMEWQLKAQELDPAGHKSLCVNEVPSFY
VPMVDVNDDFVLLPA*
>ENSMUSP00000095938_c.1266:G>A_p.A423T ENSMUST00000098333.5|Ankrd11-201|ENSMUSG00000035569|-|chr8:g.123622584:C>T
MPKGGCSKTPQQEDFALSNDMVEKQTGKKDKDKVSLTKTPKLDRSDGGKEVRERATKRKLPFTVGANGEQKDS
DTDSFGHRTFNWGSPLPFSLTCHTEKQGPERKRIKKEPVARKSGLLFGMGLSGIRAGYPLSERQQVALLMQMT
AEESANSPDTTPKHPSQSTVCQKGTPNSASKTKDKVNKRNERGETRLHRAAIRGDARRIKELISEGADVNVKD
FAGWTALHEACNRGYYDIAKQLLAAGAEVNTKGLDDDTPLHDAANNGHYKVVKLLLRYGGNPQQSNRKGETPL
KVANSPTMVNLLLGKGTYTSSEESSTESSEEEDAPSFAPSSSVDGNNTDSEFEKGLKLKAKNPEPQKTVTPVK
DEYEFDEDDEQDRVPPVDDKHLLKKDYRKEAKANSFISIPKMEVKSYSKNNTLAPKKTAHRILSDTSDEEDVS
VSIGAGEKLRLSAHTMLPGSKARESSSSRQQKEKNKLKKKRKKETKGKEVRFGKRSDKFCSSGSESESSESEE
DDGDSVGSSGCLKGSPLVLKDPSLFSSLSASSTSSHGSAVAQKHGSGHTDQHTKHWRTDNWKAISSPAWSEVS
SLSDSSRTGLTSESDCSSEGSSVESLKPTRRKQEHRKRGVLQSAPSEKRSSFHPCTDGAVPKLDKEGKVVKKH
KTKHKHKHKEKGQCSVSQELKLKSFTYEYEDSKQKSDKAILLESDLSTENKLKVLKHDREHLKKEDKLGRMKP
EDKDWLFKDEKVLKRIKDANKDMSRAFREDKDRASKAERERATKDKSPKEEKLRLYKEERKKKSKDRASRLER
KNDMKEDKLSKEKEKAFKEDKEKLKKEKLYREDAAFDDYCNKSQFLDHEDTKFSLSDDQQERWFSDLSDSSFD
FKGEDSWDSVTDYRDIKNDSVAKLILETVKEDSKEKKRDNKIREKRDFKDSFFRKRDRDCLDKNSEKRRDQTE
KHKSIPSYLSEKDKKRRESAEGGRDRRDGRIRSEEVHREDLKECGFESSFKDKSDCDFPKNLEPWERPHAARE
KEKKDALEKERKEKGRADKYKEKSSERERSDKSTLDKCQKDKEFEKCFKEKKDGKEKHKDIHSKDRKASFDQL
REKKEKVFSSIISEDFSERKDDRKGKEKSWYIADIFTDESEDEKDDCVAGSFKATEASDTQRVDGLPEKEEGR
EHPSDRHRKSSSDRQHTEKPRDKEPKEKKKDRGASEGGKDKKEKMEKIFEKHKEKKDKECAERYKDRKERASA
DSAPEKKNKQKLPEKVEKKHFAEDKVKSKHKEKPEKEHSRERERKPSRGPDVEKSLLEKLEEEALHDYREDSN
DKISEVSSDSFADHGQEPSLSTLLEVSFSEPPAEDKARDSACLSEKLREKERHRHSSSSSKKSHERERAKKEK
AEKKEKSEDYKDSISSVRKDASQFEKDFLDAETYGVSYPTKADVEEELDKAIELFSSEKKDRSDPEREPAKRI
EKELKPYGSSAISILKEKKKREKHRERWREEKERHRDKHVDGFLRHHKDEPKPAAKDKDNPPNSFKEKSREES
LKLSETKLKEKFKENTEREKGDSIKMSNGNDKLVPSRDSGKKDSRPREKLLGDGDLMMTSFERMLSQKDLEIE
ERHKRHKERMKQMEKMRHRSGDPKLKEKKPTEDGRKKSLDFPSKKALGLDKKVKEPAPTLTTGESKPHSGPGT
ESKDWLSGQPLKEVLPASPRTEQSRPTGVPTPTSVVSCPSYEEVMHTPRTPSCSADDYPDLVFDCTDSQHSMP
VSTASTSACSPPFFDRFSVASSVVSENAAGQTPTRPISTNLYRSISVDIRRTPEEEFSAGDKLFRQQSVPAPS
SFDSPVQHLLEEKAPLPPVPAEKFACLSPGYYSPDYGIPSPKVDTLHCPPTAVVSATPPPDSVFSNLPPKSSP
SPRGELLSPAIEGTLPPDLGLPLDATEDQQATAAILPQEPSYLEPLDEGPFTTVITEEPVEWTHTAAEQGLSS
SSLIASASENPVSWPVGSELMLKSPQRFAESPKHFCPGESLHSTTPGPYSAAEPTYPVSPGSYPLPAPEPALE
EVKDGGTGAIPVAISAAEGAAPYAAPARLESFFSNCKSHPDAPLDTAPEPTGVTAVAQVEALGPLESSFLDSN
PSISTLSQVEPVSWHEAFTSPEDDLDLGPFSLPELPLQAKDASDVEAEAAKASPVPPAESPPGPTGVLGGGDV
PAPAAEEPPAPPPQEASPQLSTEPEPSEEPKLDVVLEATVETEVLADDSAPEASISNSVPAPSPPQQQPPGGG
DEEAETEDPSATPCCAPDGPTTDGLAQAHNSAEASCVVAAAEGPPGNVQAEATDPEPKPTSEVPKAPKVEEVP
QRMTRNRAQMLASQSKQGIPAAEKDPMPTPASRAKGRASEEEDAQAQHPRKRRFQRSSQQLQQQLNTSTQQTR
EVIQQTLAAIVDAIKLDAIEPYHSDRSNPYFEYLQIRKKIEEKRKILCCITPQAPQCYAEYVTYTGSYLLDGK
PLSKLHIPVIAPPPSLAEPLKELFKQQEAVRGKLRLQHSIEREKLIVSCEQEILRVHCRAARTIANQAVPFSA
CTMLLDSEVYNMPLESQGDENKSVRDRFNARQFISWLQDVDDKYDRMKTCLLMRQQHEAAALNAVQRMEWQLK
AQELDPAGHKSLCVNEVPSFYVPMVDVNDDFVLLPA*
>ENSMUSP00000114043_c.876:ATG>TTA_p.D293L ENSMUST00000117624.9|Ttc13-202|ENSMUSG00000037300|-|chr8:g.125415327:CAT>TAA
MAPAGCGCGCSGSLGAAVVRPLLLLLGALACARATEHYSPLSLLKQELQHRQQQEAPAGGGCPQSGDWADQYP
ECESSFLNFHESDCELRGSAPCDSLLSLNTEKILSQAKSIAEQKRFPFATDNDSTNEELAIAYVLVGSGLYDE
AIRHFSTMLQEEPDLVSAIYGRGIAYGKKGLHEAIEAFKEALKQKVDFIDAYKSLGQAYRELGNFDAATESFQ
KALLLNQNHVQTLQLRGMMLYHHGSLQEALKNFKRCLQLEPYNEVCQYMKGLSHVAMGQFYEGIKAQTKVMLN
LTLFQARRPAQST*GSSTYESILDICTHTSIPL*RNITLTRIYRAASRTTGPRTCLSS*TAMKSSPACSLTLE
MCYIRISRATSLKSRS*SV*LTAWAR*CSMKHRGSCPTRGYTEPWAWQHWKSCRPYIVPGPTQRFA*MEKQGC
CSGGTCSTLL*SGEGSLIRTSLSCGWIRCQLQVSAEGSITTST*SGVK*SI*DIWNILRKYFILLKIEFLFIT
EQIIQRAYWKSEKLWKRYTKWKTSFQL*SSLIPKQRTGSP*TPKSPASKIKGRNMTASPSPSQETRSAISYFL
WKLKPQKRGHSCITLK*MHFIKT*QRRAKY*PSRQNSGRLMLSAT*SYP*CITFII*CPCLEDLVSLRIQSLW
ER*WPVAKKWLGRSPKGS*STLKP*QPLAQRPSAK*QKAG*T*RVFPLPTRPCHQYQRRSRL*GP*SRCSTRI
PPHGV*RNS
>ENSMUSP00000156040_c.810:ATG>TTA_p.D271L ENSMUST00000231984.2|Ttc13-212|ENSMUSG00000037300|-|chr8:g.125415327:CAT>TAA
MAPAGCGCGCSGSLGAAVVRPLLLLLGALACARATEHYSPLSLLKQELQHRQQQEAPAGGGCPQSGDWADQYP
ECESSFLNFHESDCELRGSAPCDSLLSLNTEKILSQAKSIAEQKRFPFATDNDSTNEELAIAYVLVGSGLYDE

AIRHFSTMLQEAIEAFKEALKQKVDFIDAYKSLGQAYRELGNFDAATESFQKALLLNQNHVQTLQLRGMMLYH
HGSLQEALKNFKRCLQLEPYNEVCQYMKGLSHVAMGQFYEGIKAQTKVMLNLTLFQARRPAQST*GSSTYESI
LDICTHTSIPL*RNITLTRIYRAASRTTGPRTCLSS*TAMKSSPACSLTLEMCYIRISRATSLKSRS*SV*LT
AWAR*CSMKHRGSCPTRGYTEPWAWQHWKSCRPYIVPGPTQRFA*MEKQGCCSGGTCSTLL*SGEGSLIRTSL
SCGWIRCQLQVSAEGSITTST*SGVK*SI*DIWNILRKYFILLKIEFLFITEQIIQRAYWKSEKLWKRYTKWK
TSFQL*SLIPKQRTGSP*TPKSPASKIKGRNMTASPSPSQETRSAISYFLWKLKPQKRGHSCITLK*MHFIKT
*QRRAKY*PSRQNSGRLMLSAT*SYP*CITFII*CPCLEDLVSLRIQSLWER*WPVAKKWLGRSPKGS*STLK
P*QPLAQRPSAK*QKAG*T*RVFPLPTRPCHQYQRRSRL*GP*SRCSTRIPPHGV*RNS
>ENSMUSP00000149848_c.1197:ATG>TTA_p.D400L ENSMUST00000214828.2|Ttc13-
210|ENSMUSG00000037300|-|chr8:g.125415327:CAT>TAA
MAPAGCGCGCSGSLGAAVVRPLLLLLGALACARATEHYSPLSLLKQELQHRQQQEAPAGGGCPQSGDWADQYP
ECESSFLNFHESDCELRGSAPCDSLLSLNTEKILSQAKSIAEQKRFPFATDNDSTNEELAIAYVLVGSGLYDE
AIRHFSTMLQEEPDLVSAIYGRGIAYGKKGLHDIKNAELALFELSRVIALEPDRPEVFEQRAEILSPLGRINE
AVNDLTKAIQLQPSARLYRHRGTLYFISEDYATAHEDFQQSLELNRNQPTAMLYKGLTFFHRGLLKEAIEAFK
EALKQKVDFIDAYKSLGQAYRELGNFDAATESFQKALLLNQNHVQTLQLRGMMLYHHGSLQEALKNFKRCLQL
EPYNEVCQYMKGLSHVAMGQFYEGIKAQTKVMLNLTLFQARRPAQST*GSSTYESILDICTHTSIPL*RNITL
TRIYRAASRTTGPRTCLSS*TAMKSSPACSLTLEMCYIRISRATSLKSRS*SV*LTAWAR*CSMKHRGSCPTR
GYTEPWAWQHWKSCRPYIVPGPTQRFA*MEKQGCCSGGTCSTLL*SGEGSLIRTSLSCGWIRCQLQVSAEGSI
TTST*SGVK*SI*DIWNILRKYFILLKIEFLFITEQIIQRAYWKSEKLWKRYTKWKTSFQL*SLIPKQRTGSP
*TPKSPASKIKGRNMTASPSPSQETRSAISYFLWKLKPQKRGHSCITLK*MHFIKT*QRRAKY*PSRQNSGRL
MLSAT*SYP*CITFII*CPCLEDLVSLRIQSLWER*WPVAKKWLGRSPKGS*STLKP*QPLAQRPSAK*QKAG
*T*RVFPLPTRPCHQYQRRSRL*GP*SRCSTRIPPHGV*RNS
>ENSMUSP00000038484_c.810:ATG>TTA_p.D271L ENSMUST00000041614.15|Ttc13-
201|ENSMUSG00000037300|-|chr8:g.125415327:CAT>TAA
MAPAGCGCGCSGSLGAAVVRPLLLLLGALACARATEHYSPLSLLKQELQHRQQQEAPAGGGCPQSGDWADQYP
ECESSFLNFHESDCELRGSAPCDSLLSLNTEKILSQAKSIAEQKRFPFATDNDSTNEELAIAYVLVGSGLYDE
AIRHFSTMLQEAIEAFKEALKQKVDFIDAYKSLGQAYRELGNFDAATESFQKALLLNQNHVQTLQLRGMMLYH
HGSLQEALKNFKRCLQLEPYNEVCQYMKGLSHVAMGQFYEGIKAQTKVMLNLTLFQARRPAQST*GSSTYESI
LDICTHTSIPL*RNITLTRIYRAASRTTGPRTCLSS*TAMKSSPACSLTLEMCYIRISRATSLKSRS*SV*LT
AWAR*CSMKHRGSCPTRGYTEPWAWQHWKSCRPYIVPGPTQRFA*MEKQGCCSGGTCSTLL*SGEGSLIRTSL
SCGWIRCQLQVSAEGSITTST*SGVK*SI*DIWNILRKYFILLKIEFLFITEQIIQRAYWKSEKLWKRYTKWK
TSFQL*SSLIPKQRTGSP*TPKSPASKIKGRNMTASPSPSQETRSAISYFLWKLKPQKRGHSCITLK*MHFIK
T*QRRAKY*PSRQNSGRLMLSAT*SYP*CITFII*CPCLEDLVSLRIQSLWER*WPVAKKWLGRSPKGRYAGL
AEAQAWLHLAPGFWLWTACELDPTPFPSFYHVLRLLFSS*STLKP*QPLAQRPSAK*QKAG*T*RVFPLPTRP
CHQYQRRSRL*GP*SRCSTRIPPHGV*RNS
>ENSMUSP00000113383_c.1038:ATG>TTA_p.D347L ENSMUST00000118134.3|Ttc13-
203|ENSMUSG00000037300|-|chr8:g.125415327:CAT>TAA
MAPAGCGCGCSGSLGAAVVRPLLLLLGALACARATEHYSPLSLLKQELQHRQQQEAPAGGGCPQSGDWADQYP
ECESSFLNFHESDCELRGSAPCDSLLSLNTEKILSQAKSIAEQKRFPFATDNDSTNEELAIAYVLVGSGLYDE
AIRHFSTMLQILSPLGRINEAVNDLTKAIQLQPSARLYRHRGTLYFISEDYATAHEDFQQSLELNRNQPTAML
YKGLTFFHRGLLKEAIEAFKEALKQKVDFIDAYKSLGQAYRELGNFDAATESFQKALLLNQNHVQTLQLRGMM
LYHHGSLQEALKNFKRCLQLEPYNEVCQYMKGLSHVAMGQFYEGIKAQTKVMLNLTLFQARRPAQST*GSSTY
ESILDICTHTSIPL*RNITLTRIYRAASRTTGPRTCLSS*TAMKSSPACSLTLEMCYIRISRATSLKSRS*SV
*LTAWAR*CSMKHRGSCPTRGYTEPWAWQHWKSCRPYIVPGPTQRFA*MEKQGCCSGGTCSTLL*SGEGSLIR
TSLSCGWIRCQLQVSAEGSITTST*SGVK*SI*DIWNILRKYFILLKIEFLFITEQIIQRAYWKSEKLWKRYT
KWKTSFQL*SSLIPKQRTGSP*TPKSPASKIKGRNMTASPSPSQETRSAISYFLWKLKPQKRGHSCITLK*MH
FIKT*QRRAKY*PSRQNSGRLMLSAT*SYP*CITFII*CPCLEDLVSLRIQSLWER*WPVAKKWLGRSPKGS*
STLKP*QPLAQRPSAK*QKAG*T*RVFPLPTRPCHQYQRRSRL*GP*SRCSTRIPPHGV*RNS
>ENSMUSP00000132394_c.15:T>C_p.C6R ENSMUST00000151376.3|Gm10722-
201|ENSMUSG00000091028|+|chr9:g.3000937:T>C
SCHIPRPTVDISFPPFSVFLAIFHVLKCVFLIFRDFQFYRQIPGPTVCIFHISRFSVILSFFKLSTACFSFSM
IFSFLAIFHVLQWTFLNFPPFSVFLAIFHVLKCVFLIFSDFLFSRHIPGPTVCISHFSRFSVISSFFKLSSGC
FSFSMIFSFLAIFHVLQWTFLNFPPFSSSIFSPYSRSYSVHFSFFTFFSDFVIFQVVKWMFLIFHDFQFSC
>ENSMUSP00000132394_c.42:C>A_p.P15T ENSMUST00000151376.3|Gm10722-
201|ENSMUSG00000091028|+|chr9:g.3000968:C>A
SCHIPCPTVDISFPTFSVFLAIFHVLKCVFLIFRDFQFYRQIPGPTVCIFHISRFSVILSFFKLSTACFSFSM
IFSFLAIFHVLQWTFLNFPPFSVFLAIFHVLKCVFLIFSDFLFSRHIPGPTVCISHFSRFSVISSFFKLSSGC
FSFSMIFSFLAIFHVLQWTFLNFPPFSSSIFSPYSRSYSVHFSFFTFFSDFVIFQVVKWMFLIFHDFQFSC

>ENSMUSP00000132394_c.99:C>T_p.R34C ENSMUST00000151376.3|Gm10722-201|ENSMUSG00000091028|+|chr9:g.3001025:C>T
SCHIPCPTVDISFPPFSVFLAIFHVLKCVFLIFCDFQFYRQIPGPTVCIFHISRFSVILSFFKLSTACFSFSM
IFSFLAIFHVLQWTFLNFPPFSVFLAIFHVLKCVFLIFSDFLFSRHIPGPTVCISHFSRFSVISSFFKLSSGC
FSFSMIFSFLAIFHVLQWTFLNFPPFSSSIFSPYSRSYSVHFSFFTFFSDFVIFQVVKWMFLIFHDFQFSC
>ENSMUSP00000132394_c.115:A>C_p.Y39S ENSMUST00000151376.3|Gm10722-201|ENSMUSG00000091028|+|chr9:g.3001041:A>C
SCHIPCPTVDISFPPFSVFLAIFHVLKCVFLIFRDFQFSRQIPGPTVCIFHISRFSVILSFFKLSTACFSFSM
IFSFLAIFHVLQWTFLNFPPFSVFLAIFHVLKCVFLIFSDFLFSRHIPGPTVCISHFSRFSVISSFFKLSSGC
FSFSMIFSFLAIFHVLQWTFLNFPPFSSSIFSPYSRSYSVHFSFFTFFSDFVIFQVVKWMFLIFHDFQFSC
>ENSMUSP00000132394_c.122:G>C_p.Q41H ENSMUST00000151376.3|Gm10722-201|ENSMUSG00000091028|+|chr9:g.3001048:G>C
SCHIPCPTVDISFPPFSVFLAIFHVLKCVFLIFRDFQFYRHIPGPTVCIFHISRFSVILSFFKLSTACFSFSM
IFSFLAIFHVLQWTFLNFPPFSVFLAIFHVLKCVFLIFSDFLFSRHIPGPTVCISHFSRFSVISSFFKLSSGC
FSFSMIFSFLAIFHVLQWTFLNFPPFSSSIFSPYSRSYSVHFSFFTFFSDFVIFQVVKWMFLIFHDFQFSC
>rs221766350_ENSMUSP00000132394_c.122:G>T_p.Q41H
ENSMUST00000151376.3|Gm10722-201|ENSMUSG00000091028|+|chr9:g.3001048:G>T
SCHIPCPTVDISFPPFSVFLAIFHVLKCVFLIFRDFQFYRHIPGPTVCIFHISRFSVILSFFKLSTACFSFSM
IFSFLAIFHVLQWTFLNFPPFSVFLAIFHVLKCVFLIFSDFLFSRHIPGPTVCISHFSRFSVISSFFKLSSGC
FSFSMIFSFLAIFHVLQWTFLNFPPFSSSIFSPYSRSYSVHFSFFTFFSDFVIFQVVKWMFLIFHDFQFSC
>ENSMUSP00000132394_c.135:ACG>TCA_p.T46S ENSMUST00000151376.3|Gm10722-201|ENSMUSG00000091028|+|chr9:g.3001061:ACG>TCA
SCHIPCPTVDISFPPFSVFLAIFHVLKCVFLIFRDFQFYRQIPGPSRCAFFIFHVFQ*FCHFSSCQLHVSHFP
*FSVFLPYSMSYSGHF*IFHLFQFSSPYFMS*SVYFSFSVIFCFLAIFQVLQCAFLIFHVFQ*FRHFSSCQVD
VSHFP*FSVFLPYSMSYNGHF*IFHLFHRQFSRHIPGPTVCISHFSRFSVISSFFKLSSGCFSFFMIFSFL
>ENSMUSP00000132394_c.148:T>A_p.F50Y ENSMUST00000151376.3|Gm10722-201|ENSMUSG00000091028|+|chr9:g.3001074:T>A
SCHIPCPTVDISFPPFSVFLAIFHVLKCVFLIFRDFQFYRQIPGPTVCIYHISRFSVILSFFKLSTACFSFSM
IFSFLAIFHVLQWTFLNFPPFSVFLAIFHVLKCVFLIFSDFLFSRHIPGPTVCISHFSRFSVISSFFKLSSGC
FSFSMIFSFLAIFHVLQWTFLNFPPFSSSIFSPYSRSYSVHFSFFTFFSDFVIFQVVKWMFLIFHDFQFSC
>ENSMUSP00000132394_c.148:T>C_p.F50S ENSMUST00000151376.3|Gm10722-201|ENSMUSG00000091028|+|chr9:g.3001074:T>C
SCHIPCPTVDISFPPFSVFLAIFHVLKCVFLIFRDFQFYRQIPGPTVCISHISRFSVILSFFKLSTACFSFSM
IFSFLAIFHVLQWTFLNFPPFSVFLAIFHVLKCVFLIFSDFLFSRHIPGPTVCISHFSRFSVISSFFKLSSGC
FSFSMIFSFLAIFHVLQWTFLNFPPFSSSIFSPYSRSYSVHFSFFTFFSDFVIFQVVKWMFLIFHDFQFSC
>ENSMUSP00000132394_c.152:TA>T_p.H51del-fs ENSMUST00000151376.3|Gm10722-201|ENSMUSG00000091028|+|chr9:g.3001078:TA>T
SCHIPCPTVDISFPPFSVFLAIFHVLKCVFLIFRDFQFYRQIPGPTVCIFHFHVFQ
>ENSMUSP00000132394_c.153:A>T_p.I52F ENSMUST00000151376.3|Gm10722-201|ENSMUSG00000091028|+|chr9:g.3001079:A>T
SCHIPCPTVDISFPPFSVFLAIFHVLKCVFLIFRDFQFYRQIPGPTVCIFHFSRFSVILSFFKLSTACFSFSM
IFSFLAIFHVLQWTFLNFPPFSVFLAIFHVLKCVFLIFSDFLFSRHIPGPTVCISHFSRFSVISSFFKLSSGC
FSFSMIFSFLAIFHVLQWTFLNFPPFSSSIFSPYSRSYSVHFSFFTFFSDFVIFQVVKWMFLIFHDFQFSC
>ENSMUSP00000132394_c.159:C>T_p.R54C ENSMUST00000151376.3|Gm10722-201|ENSMUSG00000091028|+|chr9:g.3001085:C>T
SCHIPCPTVDISFPPFSVFLAIFHVLKCVFLIFRDFQFYRQIPGPTVCIFHISCFSVILSFFKLSTACFSFSM
IFSFLAIFHVLQWTFLNFPPFSVFLAIFHVLKCVFLIFSDFLFSRHIPGPTVCISHFSRFSVISSFFKLSSGC
FSFSMIFSFLAIFHVLQWTFLNFPPFSSSIFSPYSRSYSVHFSFFTFFSDFVIFQVVKWMFLIFHDFQFSC
>ENSMUSP00000132394_c.166:C>T_p.S56L ENSMUST00000151376.3|Gm10722-201|ENSMUSG00000091028|+|chr9:g.3001092:C>T
SCHIPCPTVDISFPPFSVFLAIFHVLKCVFLIFRDFQFYRQIPGPTVCIFHISRFLVILSFFKLSTACFSFSM
IFSFLAIFHVLQWTFLNFPPFSVFLAIFHVLKCVFLIFSDFLFSRHIPGPTVCISHFSRFSVISSFFKLSSGC
FSFSMIFSFLAIFHVLQWTFLNFPPFSSSIFSPYSRSYSVHFSFFTFFSDFVIFQVVKWMFLIFHDFQFSC
>ENSMUSP00000132394_c.175:T>C_p.L59S ENSMUST00000151376.3|Gm10722-201|ENSMUSG00000091028|+|chr9:g.3001101:T>C
SCHIPCPTVDISFPPFSVFLAIFHVLKCVFLIFRDFQFYRQIPGPTVCIFHISRFSVISSFFKLSTACFSFSM
IFSFLAIFHVLQWTFLNFPPFSVFLAIFHVLKCVFLIFSDFLFSRHIPGPTVCISHFSRFSVISSFFKLSSGC
FSFSMIFSFLAIFHVLQWTFLNFPPFSSSIFSPYSRSYSVHFSFFTFFSDFVIFQVVKWMFLIFHDFQFSC

>ENSMUSP00000132394_c.190:T>G_p.L64W ENSMUST00000151376.3|Gm10722-201|ENSMUSG00000091028|+|chr9:g.3001116:T>G
SCHIPCPTVDISFPPFSVFLAIFHVLKCVFLIFRDFQFYRQIPGPTVCIFHISRFSVILSFFKWSTACFSFSM
IFSFLAIFHVLQWTFLNFPPFSVFLAIFHVLKCVFLIFSDFLFSRHIPGPTVCISHFSRFSVISSFFKLSSGC
FSFSMIFSFLAIFHVLQWTFLNFPPFSSSIFSPYSRSYSVHFSFFTFFSDFVIFQVVKWMFLIFHDFQFSC
>rs236908461_ENSMUSP00000132394_c.190:T>C_p.L64S ENSMUST00000151376.3|Gm10722-201|ENSMUSG00000091028|+|chr9:g.3001116:T>C
SCHIPCPTVDISFPPFSVFLAIFHVLKCVFLIFRDFQFYRQIPGPTVCIFHISRFSVILSFFKSSTACFSFSM
IFSFLAIFHVLQWTFLNFPPFSVFLAIFHVLKCVFLIFSDFLFSRHIPGPTVCISHFSRFSVISSFFKLSSGC
FSFSMIFSFLAIFHVLQWTFLNFPPFSSSIFSPYSRSYSVHFSFFTFFSDFVIFQVVKWMFLIFHDFQFSC
>ENSMUSP00000132394_c.196:C>G_p.T66S ENSMUST00000151376.3|Gm10722-201|ENSMUSG00000091028|+|chr9:g.3001122:C>G
SCHIPCPTVDISFPPFSVFLAIFHVLKCVFLIFRDFQFYRQIPGPTVCIFHISRFSVILSFFKLSSACFSFSM
IFSFLAIFHVLQWTFLNFPPFSVFLAIFHVLKCVFLIFSDFLFSRHIPGPTVCISHFSRFSVISSFFKLSSGC
FSFSMIFSFLAIFHVLQWTFLNFPPFSSSIFSPYSRSYSVHFSFFTFFSDFVIFQVVKWMFLIFHDFQFSC
>ENSMUSP00000132394_c.196:CTGC>GTGT_p.T66S ENSMUST00000151376.3|Gm10722-201|ENSMUSG00000091028|+|chr9:g.3001122:CTGC>GTGT
SCHIPCPTVDISFPPFSVFLAIFHVLKCVFLIFRDFQFYRQIPGPTVCIFHISRFSVILSFFKLSSVACFSFS
MIFSFLAIFHVLQWTFLNFPPFSVFLAIFHVLKCVFLIFSDFLFSRHIPGPTVCISHFSRFSVISSFFKLSSG
CFSFSMIFSFLAIFHVLQWTFLNFPPFSSSIFSPYSRSYSVHFSFFTFFSDFVIFQVVKWMFLIFHDFQFSC
>ENSMUSP00000132394_c.196:CTGC>GTGG_p.T66S ENSMUST00000151376.3|Gm10722-201|ENSMUSG00000091028|+|chr9:g.3001122:CTGC>GTGG
SCHIPCPTVDISFPPFSVFLAIFHVLKCVFLIFRDFQFYRQIPGPTVCIFHISRFSVILSFFKLSSGACFSFS
MIFSFLAIFHVLQWTFLNFPPFSVFLAIFHVLKCVFLIFSDFLFSRHIPGPTVCISHFSRFSVISSFFKLSSG
CFSFSMIFSFLAIFHVLQWTFLNFPPFSSSIFSPYSRSYSVHFSFFTFFSDFVIFQVVKWMFLIFHDFQFSC
>ENSMUSP00000132394_c.199:C>G_p.A67G ENSMUST00000151376.3|Gm10722-201|ENSMUSG00000091028|+|chr9:g.3001125:C>G
SCHIPCPTVDISFPPFSVFLAIFHVLKCVFLIFRDFQFYRQIPGPTVCIFHISRFSVILSFFKLSTGCFSFSM
IFSFLAIFHVLQWTFLNFPPFSVFLAIFHVLKCVFLIFSDFLFSRHIPGPTVCISHFSRFSVISSFFKLSSGC
FSFSMIFSFLAIFHVLQWTFLNFPPFSSSIFSPYSRSYSVHFSFFTFFSDFVIFQVVKWMFLIFHDFQFSC
>ENSMUSP00000132394_c.216:A>G_p.M73V ENSMUST00000151376.3|Gm10722-201|ENSMUSG00000091028|+|chr9:g.3001142:A>G
SCHIPCPTVDISFPPFSVFLAIFHVLKCVFLIFRDFQFYRQIPGPTVCIFHISRFSVILSFFKLSTACFSFSV
IFSFLAIFHVLQWTFLNFPPFSVFLAIFHVLKCVFLIFSDFLFSRHIPGPTVCISHFSRFSVISSFFKLSSGC
FSFSMIFSFLAIFHVLQWTFLNFPPFSSSIFSPYSRSYSVHFSFFTFFSDFVIFQVVKWMFLIFHDFQFSC
>ENSMUSP00000132394_c.305:T>A_p.H102Q ENSMUST00000151376.3|Gm10722-201|ENSMUSG00000091028|+|chr9:g.3001231:T>A
SCHIPCPTVDISFPPFSVFLAIFHVLKCVFLIFRDFQFYRQIPGPTVCIFHISRFSVILSFFKLSTACFSFSM
IFSFLAIFHVLQWTFLNFPPFSVFLAIFQVLKCVFLIFSDFLFSRHIPGPTVCISHFSRFSVISSFFKLSSGC
FSFSMIFSFLAIFHVLQWTFLNFPPFSSSIFSPYSRSYSVHFSFFTFFSDFVIFQVVKWMFLIFHDFQFSC
>rs255289761_ENSMUSP00000132394_c.333:A>C_p.S112R ENSMUST00000151376.3|Gm10722-201|ENSMUSG00000091028|+|chr9:g.3001259:A>C
SCHIPCPTVDISFPPFSVFLAIFHVLKCVFLIFRDFQFYRQIPGPTVCIFHISRFSVILSFFKLSTACFSFSM
IFSFLAIFHVLQWTFLNFPPFSVFLAIFHVLKCVFLIFRDFLFSRHIPGPTVCISHFSRFSVISSFFKLSSGC
FSFSMIFSFLAIFHVLQWTFLNFPPFSSSIFSPYSRSYSVHFSFFTFFSDFVIFQVVKWMFLIFHDFQFSC
>ENSMUSP00000132394_c.333:A>T_p.S112C ENSMUST00000151376.3|Gm10722-201|ENSMUSG00000091028|+|chr9:g.3001259:A>T
SCHIPCPTVDISFPPFSVFLAIFHVLKCVFLIFRDFQFYRQIPGPTVCIFHISRFSVILSFFKLSTACFSFSM
IFSFLAIFHVLQWTFLNFPPFSVFLAIFHVLKCVFLIFCDFLFSRHIPGPTVCISHFSRFSVISSFFKLSSGC
FSFSMIFSFLAIFHVLQWTFLNFPPFSSSIFSPYSRSYSVHFSFFTFFSDFVIFQVVKWMFLIFHDFQFSC
>ENSMUSP00000132394_c.343:T>A_p.L115Q ENSMUST00000151376.3|Gm10722-201|ENSMUSG00000091028|+|chr9:g.3001269:T>A
SCHIPCPTVDISFPPFSVFLAIFHVLKCVFLIFRDFQFYRQIPGPTVCIFHISRFSVILSFFKLSTACFSFSM
IFSFLAIFHVLQWTFLNFPPFSVFLAIFHVLKCVFLIFSDFQFSRHIPGPTVCISHFSRFSVISSFFKLSSGC
FSFSMIFSFLAIFHVLQWTFLNFPPFSSSIFSPYSRSYSVHFSFFTFFSDFVIFQVVKWMFLIFHDFQFSC
>ENSMUSP00000132394_c.369:A>T_p.T124S ENSMUST00000151376.3|Gm10722-201|ENSMUSG00000091028|+|chr9:g.3001295:A>T

SCHIPCPTVDISFPPFSVFLAIFHVLKCVFLIFRDFQFYRQIPGPTVCIFHISRFSVILSFFKLSTACFSFSM
IFSFLAIFHVLQWTFLNFPPFSVFLAIFHVLKCVFLIFSDFLFSRHIPGPSVCISHFSRFSVISSFFKLSSGC
FSFSMIFSFLAIFHVLQWTFLNFPPFSSSIFSPYSRSYSVHFSFFTFFSDFVIFQVVKWMFLIFHDFQFSC
>ENSMUSP00000132394_c.400:C>T_p.S134L ENSMUST00000151376.3|Gm10722-201|ENSMUSG00000091028|+|chr9:g.3001326:C>T
SCHIPCPTVDISFPPFSVFLAIFHVLKCVFLIFRDFQFYRQIPGPTVCIFHISRFSVILSFFKLSTACFSFSM
IFSFLAIFHVLQWTFLNFPPFSVFLAIFHVLKCVFLIFSDFLFSRHIPGPTVCISHFSRFLVISSFFKLSSGC
FSFSMIFSFLAIFHVLQWTFLNFPPFSSSIFSPYSRSYSVHFSFFTFFSDFVIFQVVKWMFLIFHDFQFSC
>ENSMUSP00000132394_c.424:T>C_p.L142S ENSMUST00000151376.3|Gm10722-201|ENSMUSG00000091028|+|chr9:g.3001350:T>C
SCHIPCPTVDISFPPFSVFLAIFHVLKCVFLIFRDFQFYRQIPGPTVCIFHISRFSVILSFFKLSTACFSFSM
IFSFLAIFHVLQWTFLNFPPFSVFLAIFHVLKCVFLIFSDFLFSRHIPGPTVCISHFSRFSVISSFFKSSSGC
FSFSMIFSFLAIFHVLQWTFLNFPPFSSSIFSPYSRSYSVHFSFFTFFSDFVIFQVVKWMFLIFHDFQFSC
>ENSMUSP00000132394_c.424:T>G_p.L142W ENSMUST00000151376.3|Gm10722-201|ENSMUSG00000091028|+|chr9:g.3001350:T>G
SCHIPCPTVDISFPPFSVFLAIFHVLKCVFLIFRDFQFYRQIPGPTVCIFHISRFSVILSFFKLSTACFSFSM
IFSFLAIFHVLQWTFLNFPPFSVFLAIFHVLKCVFLIFSDFLFSRHIPGPTVCISHFSRFSVISSFFKWSSGC
FSFSMIFSFLAIFHVLQWTFLNFPPFSSSIFSPYSRSYSVHFSFFTFFSDFVIFQVVKWMFLIFHDFQFSC
>ENSMUSP00000132394_c.488:A>T_p.Q163H ENSMUST00000151376.3|Gm10722-201|ENSMUSG00000091028|+|chr9:g.3001414:A>T
SCHIPCPTVDISFPPFSVFLAIFHVLKCVFLIFRDFQFYRQIPGPTVCIFHISRFSVILSFFKLSTACFSFSM
IFSFLAIFHVLQWTFLNFPPFSVFLAIFHVLKCVFLIFSDFLFSRHIPGPTVCISHFSRFSVISSFFKLSSGC
FSFSMIFSFLAIFHVLHWTFLNFPPFSSSIFSPYSRSYSVHFSFFTFFSDFVIFQVVKWMFLIFHDFQFSC
>ENSMUSP00000132394_c.520:C>G_p.S174W ENSMUST00000151376.3|Gm10722-201|ENSMUSG00000091028|+|chr9:g.3002051:C>G
SCHIPCPTVDISFPPFSVFLAIFHVLKCVFLIFRDFQFYRQIPGPTVCIFHISRFSVILSFFKLSTACFSFSM
IFSFLAIFHVLQWTFLNFPPFSVFLAIFHVLKCVFLIFSDFLFSRHIPGPTVCISHFSRFSVISSFFKLSSGC
FSFSMIFSFLAIFHVLQWTFLNFPPFSWSIFSPYSRSYSVHFSFFTFFSDFVIFQVVKWMFLIFHDFQFSC
>ENSMUSP00000132394_c.550:A>T_p.Y184F ENSMUST00000151376.3|Gm10722-201|ENSMUSG00000091028|+|chr9:g.3002230:A>T
SCHIPCPTVDISFPPFSVFLAIFHVLKCVFLIFRDFQFYRQIPGPTVCIFHISRFSVILSFFKLSTACFSFSM
IFSFLAIFHVLQWTFLNFPPFSVFLAIFHVLKCVFLIFSDFLFSRHIPGPTVCISHFSRFSVISSFFKLSSGC
FSFSMIFSFLAIFHVLQWTFLNFPPFSSSIFSPYSRSFSVHFSFFTFFSDFVIFQVVKWMFLIFHDFQFSC
>ENSMUSP00000132394_c.580:T>G_p.F194C ENSMUST00000151376.3|Gm10722-201|ENSMUSG00000091028|+|chr9:g.3002260:T>G
SCHIPCPTVDISFPPFSVFLAIFHVLKCVFLIFRDFQFYRQIPGPTVCIFHISRFSVILSFFKLSTACFSFSM
IFSFLAIFHVLQWTFLNFPPFSVFLAIFHVLKCVFLIFSDFLFSRHIPGPTVCISHFSRFSVISSFFKLSSGC
FSFSMIFSFLAIFHVLQWTFLNFPPFSSSIFSPYSRSYSVHFSFFTFCSDFVIFQVVKWMFLIFHDFQFSC
>ENSMUSP00000132394_c.615:A>T_p.M206L ENSMUST00000151376.3|Gm10722-201|ENSMUSG00000091028|+|chr9:g.3002295:A>T
SCHIPCPTVDISFPPFSVFLAIFHVLKCVFLIFRDFQFYRQIPGPTVCIFHISRFSVILSFFKLSTACFSFSM
IFSFLAIFHVLQWTFLNFPPFSVFLAIFHVLKCVFLIFSDFLFSRHIPGPTVCISHFSRFSVISSFFKLSSGC
FSFSMIFSFLAIFHVLQWTFLNFPPFSSSIFSPYSRSYSVHFSFFTFFSDFVIFQVVKWLFLIFHDFQFSC
>ENSMUSP00000132394_c.617:G>T_p.M206I ENSMUST00000151376.3|Gm10722-201|ENSMUSG00000091028|+|chr9:g.3002297:G>T
SCHIPCPTVDISFPPFSVFLAIFHVLKCVFLIFRDFQFYRQIPGPTVCIFHISRFSVILSFFKLSTACFSFSM
IFSFLAIFHVLQWTFLNFPPFSVFLAIFHVLKCVFLIFSDFLFSRHIPGPTVCISHFSRFSVISSFFKLSSGC
FSFSMIFSFLAIFHVLQWTFLNFPPFSSSIFSPYSRSYSVHFSFFTFFSDFVIFQVVKWIFLIFHDFQFSC
>ENSMUSP00000132394_c.630:C>T_p.H211Y ENSMUST00000151376.3|Gm10722-201|ENSMUSG00000091028|+|chr9:g.3002310:C>T
SCHIPCPTVDISFPPFSVFLAIFHVLKCVFLIFRDFQFYRQIPGPTVCIFHISRFSVILSFFKLSTACFSFSM
IFSFLAIFHVLQWTFLNFPPFSVFLAIFHVLKCVFLIFSDFLFSRHIPGPTVCISHFSRFSVISSFFKLSSGC
FSFSMIFSFLAIFHVLQWTFLNFPPFSSSIFSPYSRSYSVHFSFFTFFSDFVIFQVVKWMFLIFYDFQFSC
>ENSMUSP00000136598_c.45:T>C_p.C16R ENSMUST00000177722.8|Gm11168-201|ENSMUSG00000096385|+|chr9:g.3003391:T>C
MFLIFHDFQFSCHIPRPTVDISFPPFSVFLAIFHVLKCVFLIFSDFQFSRHIPGPTVCISHFSRFSVILSFFK
LSSGCFSFSLIFSFLAIFHVLQWTFLNFPPFSVFLAIFHVLKCCVFLIFRDFQFPRHIPGPTVCISHFSRFSV

ISSFFKSSSGCFSFSMIFSFLAIFHVLQWTFLNFPPFSSYSFSPYSRSYSVHFSFFTFFSDFVIFQVVKWMFL
IFLDFQFSC
>ENSMUSP00000136598_c.72:C>A_p.P25T ENSMUST00000177722.8|Gm11168-201|ENSMUSG00000096385|+|chr9:g.3003422:C>A
MFLIFHDFQFSCHIPCPTVDISFPTFSVFLAIFHVLKCVFLIFSDFQFSRHIPGPTVCISHFSRFSVILSFFK
LSSGCFSFSLIFSFLAIFHVLQWTFLNFPPFSVFLAIFHVLKCCVFLIFRDFQFPRHIPGPTVCISHFSRFSV
ISSFFKSSSGCFSFSMIFSFLAIFHVLQWTFLNFPPFSSYSFSPYSRSYSVHFSFFTFFSDFVIFQVVKWMFL
IFLDFQFSC
>ENSMUSP00000136598_c.101:T>A_p.H34Q ENSMUST00000177722.8|Gm11168-201|ENSMUSG00000096385|+|chr9:g.3003451:T>A
MFLIFHDFQFSCHIPCPTVDISFPPFSVFLAIFQVLKCVFLIFSDFQFSRHIPGPTVCISHFSRFSVILSFFK
LSSGCFSFSLIFSFLAIFHVLQWTFLNFPPFSVFLAIFHVLKCCVFLIFRDFQFPRHIPGPTVCISHFSRFSV
ISSFFKSSSGCFSFSMIFSFLAIFHVLQWTFLNFPPFSSYSFSPYSRSYSVHFSFFTFFSDFVIFQVVKWMFL
IFLDFQFSC
>rs228881908_ENSMUSP00000136598_c.129:A>T_p.S44C
ENSMUST00000177722.8|Gm11168-201|ENSMUSG00000096385|+|chr9:g.3003479:A>T
MFLIFHDFQFSCHIPCPTVDISFPPFSVFLAIFHVLKCVFLIFCDFQFSRHIPGPTVCISHFSRFSVILSFFK
LSSGCFSFSLIFSFLAIFHVLQWTFLNFPPFSVFLAIFHVLKCCVFLIFRDFQFPRHIPGPTVCISHFSRFSV
ISSFFKSSSGCFSFSMIFSFLAIFHVLQWTFLNFPPFSSYSFSPYSRSYSVHFSFFTFFSDFVIFQVVKWMFL
IFLDFQFSC
>ENSMUSP00000136598_c.129:A>C_p.S44R ENSMUST00000177722.8|Gm11168-201|ENSMUSG00000096385|+|chr9:g.3003479:A>C
MFLIFHDFQFSCHIPCPTVDISFPPFSVFLAIFHVLKCVFLIFRDFQFSRHIPGPTVCISHFSRFSVILSFFK
LSSGCFSFSLIFSFLAIFHVLQWTFLNFPPFSVFLAIFHVLKCCVFLIFRDFQFPRHIPGPTVCISHFSRFSV
ISSFFKSSSGCFSFSMIFSFLAIFHVLQWTFLNFPPFSSYSFSPYSRSYSVHFSFFTFFSDFVIFQVVKWMFL
IFLDFQFSC
>ENSMUSP00000136598_c.165:A>T_p.T56S ENSMUST00000177722.8|Gm11168-201|ENSMUSG00000096385|+|chr9:g.3003515:A>T
MFLIFHDFQFSCHIPCPTVDISFPPFSVFLAIFHVLKCVFLIFSDFQFSRHIPGPSVCISHFSRFSVILSFFK
LSSGCFSFSLIFSFLAIFHVLQWTFLNFPPFSVFLAIFHVLKCCVFLIFRDFQFPRHIPGPTVCISHFSRFSV
ISSFFKSSSGCFSFSMIFSFLAIFHVLQWTFLNFPPFSSYSFSPYSRSYSVHFSFFTFFSDFVIFQVVKWMFL
IFLDFQFSC
>ENSMUSP00000136598_c.186:T>C_p.S63P ENSMUST00000177722.8|Gm11168-201|ENSMUSG00000096385|+|chr9:g.3003536:T>C
MFLIFHDFQFSCHIPCPTVDISFPPFSVFLAIFHVLKCVFLIFSDFQFSRHIPGPTVCISHFPRFSVILSFFK
LSSGCFSFSLIFSFLAIFHVLQWTFLNFPPFSVFLAIFHVLKCCVFLIFRDFQFPRHIPGPTVCISHFSRFSV
ISSFFKSSSGCFSFSMIFSFLAIFHVLQWTFLNFPPFSSYSFSPYSRSYSVHFSFFTFFSDFVIFQVVKWMFL
IFLDFQFSC
>ENSMUSP00000136598_c.195:T>G_p.S66A ENSMUST00000177722.8|Gm11168-201|ENSMUSG00000096385|+|chr9:g.3003545:T>G
MFLIFHDFQFSCHIPCPTVDISFPPFSVFLAIFHVLKCVFLIFSDFQFSRHIPGPTVCISHFSRFAVILSFFK
LSSGCFSFSLIFSFLAIFHVLQWTFLNFPPFSVFLAIFHVLKCCVFLIFRDFQFPRHIPGPTVCISHFSRFSV
ISSFFKSSSGCFSFSMIFSFLAIFHVLQWTFLNFPPFSSYSFSPYSRSYSVHFSFFTFFSDFVIFQVVKWMFL
IFLDFQFSC
>ENSMUSP00000136598_c.196:C>T_p.S66L ENSMUST00000177722.8|Gm11168-201|ENSMUSG00000096385|+|chr9:g.3003546:C>T
MFLIFHDFQFSCHIPCPTVDISFPPFSVFLAIFHVLKCVFLIFSDFQFSRHIPGPTVCISHFSRFLVILSFFK
LSSGCFSFSLIFSFLAIFHVLQWTFLNFPPFSVFLAIFHVLKCCVFLIFRDFQFPRHIPGPTVCISHFSRFSV
ISSFFKSSSGCFSFSMIFSFLAIFHVLQWTFLNFPPFSSYSFSPYSRSYSVHFSFFTFFSDFVIFQVVKWMFL
IFLDFQFSC
>ENSMUSP00000136598_c.199:T>G_p.V67G ENSMUST00000177722.8|Gm11168-201|ENSMUSG00000096385|+|chr9:g.3003549:T>G
MFLIFHDFQFSCHIPCPTVDISFPPFSVFLAIFHVLKCVFLIFSDFQFSRHIPGPTVCISHFSRFSGILSFFK
LSSGCFSFSLIFSFLAIFHVLQWTFLNFPPFSVFLAIFHVLKCCVFLIFRDFQFPRHIPGPTVCISHFSRFSV
ISSFFKSSSGCFSFSMIFSFLAIFHVLQWTFLNFPPFSSYSFSPYSRSYSVHFSFFTFFSDFVIFQVVKWMFL
IFLDFQFSC
>ENSMUSP00000136598_c.205:T>C_p.L69S ENSMUST00000177722.8|Gm11168-201|ENSMUSG00000096385|+|chr9:g.3003555:T>C

MFLIFHDFQFSCHIPCPTVDISFPPFSVFLAIFHVLKCVFLIFSDFQFSRHIPGPTVCISHFSRFSVISSFFK
LSSGCFSFSLIFSFLAIFHVLQWTFLNFPPFSVFLAIFHVLKCCVFLIFRDFQFPRHIPGPTVCISHFSRFSV
ISSFFKSSSGCFSFSMIFSFLAIFHVLQWTFLNFPPFSSYSFSPYSRSYSVHFSFFTFFSDFVIFQVVKWMFL
IFLDFQFSC
>rs252934463_ENSMUSP00000136598_c.220:T>G_p.L74W
ENSMUST00000177722.8|Gm11168-201|ENSMUSG00000096385|+|chr9:g.3003570:T>G
MFLIFHDFQFSCHIPCPTVDISFPPFSVFLAIFHVLKCVFLIFSDFQFSRHIPGPTVCISHFSRFSVILSFFK
WSSGCFSFSLIFSFLAIFHVLQWTFLNFPPFSVFLAIFHVLKCCVFLIFRDFQFPRHIPGPTVCISHFSRFSV
ISSFFKSSSGCFSFSMIFSFLAIFHVLQWTFLNFPPFSSYSFSPYSRSYSVHFSFFTFFSDFVIFQVVKWMFL
IFLDFQFSC
>ENSMUSP00000136598_c.220:T>C_p.L74S ENSMUST00000177722.8|Gm11168-
201|ENSMUSG00000096385|+|chr9:g.3003570:T>C
MFLIFHDFQFSCHIPCPTVDISFPPFSVFLAIFHVLKCVFLIFSDFQFSRHIPGPTVCISHFSRFSVILSFFK
SSSGCFSFSLIFSFLAIFHVLQWTFLNFPPFSVFLAIFHVLKCCVFLIFRDFQFPRHIPGPTVCISHFSRFSV
ISSFFKSSSGCFSFSMIFSFLAIFHVLQWTFLNFPPFSSYSFSPYSRSYSVHFSFFTFFSDFVIFQVVKWMFL
IFLDFQFSC
>ENSMUSP00000136598_c.232:G>T_p.C78F ENSMUST00000177722.8|Gm11168-
201|ENSMUSG00000096385|+|chr9:g.3003582:G>T
MFLIFHDFQFSCHIPCPTVDISFPPFSVFLAIFHVLKCVFLIFSDFQFSRHIPGPTVCISHFSRFSVILSFFK
LSSGFFSFSLIFSFLAIFHVLQWTFLNFPPFSVFLAIFHVLKCCVFLIFRDFQFPRHIPGPTVCISHFSRFSV
ISSFFKSSSGCFSFSMIFSFLAIFHVLQWTFLNFPPFSSYSFSPYSRSYSVHFSFFTFFSDFVIFQVVKWMFL
IFLDFQFSC
>ENSMUSP00000136761_c.2:G>T_p.M1I ENSMUST00000178077.2|Gm11168-
202|ENSMUSG00000096385|+|chr9:g.3003582:G>T
IFLIFLDFQFSCHIPCPTVDISFPPFSVFLAIFHVLKCVFLIFSDFQFSRHIPGPTVCISHFFRFFMISSFFT
LSSGCFSFSIIFTFLAIFHVLQWTFLNFPPFSVFLAIFHILKCCVFLIFRDFQFPRHIPGPTVCISHFSRFSV
ISSFFKSSSGCFSFSMIFSFLAIFHVLQWTFLNFPPFSSYSFSPYSRSYSVHFSFFTFFSDFVIFQVVKWMFL
IFLDFQFSC
>ENSMUSP00000136598_c.246:T>G_p.L83V ENSMUST00000177722.8|Gm11168-
201|ENSMUSG00000096385|+|chr9:g.3003596:T>G
MFLIFHDFQFSCHIPCPTVDISFPPFSVFLAIFHVLKCVFLIFSDFQFSRHIPGPTVCISHFSRFSVILSFFK
LSSGCFSFSVIFSFLAIFHVLQWTFLNFPPFSVFLAIFHVLKCCVFLIFRDFQFPRHIPGPTVCISHFSRFSV
ISSFFKSSSGCFSFSMIFSFLAIFHVLQWTFLNFPPFSSYSFSPYSRSYSVHFSFFTFFSDFVIFQVVKWMFL
IFLDFQFSC
>ENSMUSP00000136761_c.16:T>G_p.L6R ENSMUST00000178077.2|Gm11168-
202|ENSMUSG00000096385|+|chr9:g.3003596:T>G
MFLIFRDFQFSCHIPCPTVDISFPPFSVFLAIFHVLKCVFLIFSDFQFSRHIPGPTVCISHFFRFFMISSFFT
LSSGCFSFSIIFTFLAIFHVLQWTFLNFPPFSVFLAIFHILKCCVFLIFRDFQFPRHIPGPTVCISHFSRFSV
ISSFFKSSSGCFSFSMIFSFLAIFHVLQWTFLNFPPFSSYSFSPYSRSYSVHFSFFTFFSDFVIFQVVKWMFL
IFLDFQFSC
>ENSMUSP00000136598_c.246:T>A_p.L83M ENSMUST00000177722.8|Gm11168-
201|ENSMUSG00000096385|+|chr9:g.3003596:T>A
MFLIFHDFQFSCHIPCPTVDISFPPFSVFLAIFHVLKCVFLIFSDFQFSRHIPGPTVCISHFSRFSVILSFFK
LSSGCFSFSMIFSFLAIFHVLQWTFLNFPPFSVFLAIFHVLKCCVFLIFRDFQFPRHIPGPTVCISHFSRFSV
ISSFFKSSSGCFSFSMIFSFLAIFHVLQWTFLNFPPFSSYSFSPYSRSYSVHFSFFTFFSDFVIFQVVKWMFL
IFLDFQFSC
>ENSMUSP00000136761_c.16:T>A_p.L6H ENSMUST00000178077.2|Gm11168-
202|ENSMUSG00000096385|+|chr9:g.3003596:T>A
MFLIFHDFQFSCHIPCPTVDISFPPFSVFLAIFHVLKCVFLIFSDFQFSRHIPGPTVCISHFFRFFMISSFFT
LSSGCFSFSIIFTFLAIFHVLQWTFLNFPPFSVFLAIFHILKCCVFLIFRDFQFPRHIPGPTVCISHFSRFSV
ISSFFKSSSGCFSFSMIFSFLAIFHVLQWTFLNFPPFSSYSFSPYSRSYSVHFSFFTFFSDFVIFQVVKWMFL
IFLDFQFSC
>ENSMUSP00000136598_c.257:T>A_p.S86R ENSMUST00000177722.8|Gm11168-
201|ENSMUSG00000096385|+|chr9:g.3003607:T>A
MFLIFHDFQFSCHIPCPTVDISFPPFSVFLAIFHVLKCVFLIFSDFQFSRHIPGPTVCISHFSRFSVILSFFK
LSSGCFSFSLIFRFLAIFHVLQWTFLNFPPFSVFLAIFHVLKCCVFLIFRDFQFPRHIPGPTVCISHFSRFSV
ISSFFKSSSGCFSFSMIFSFLAIFHVLQWTFLNFPPFSSYSFSPYSRSYSVHFSFFTFFSDFVIFQVVKWMFL
IFLDFQFSC

>ENSMUSP00000136761_c.27:T>A_p.F10I ENSMUST00000178077.2|Gm11168-202|ENSMUSG00000096385|+|chr9:g.3003607:T>A
MFLIFLDFQISCHIPCPTVDISFPPFSVFLAIFHVLKCVFLIFSDFQFSRHIPGPTVCISHFFRFFMISSFFT
LSSGCFSFSIIFTFLAIFHVLQWTFLNFPPFSVFLAIFHILKCCVFLIFRDFQFPRHIPGPTVCISHFSRFSV
ISSFFKSSSGCFSFSMIFSFLAIFHVLQWTFLNFPPFSSYSFSPYSRSYSVHFSFFTFFSDFVIFQVVKWMFL
IFLDFQFSC
>ENSMUSP00000136761_c.45:T>C_p.C16R ENSMUST00000178077.2|Gm11168-202|ENSMUSG00000096385|+|chr9:g.3003625:T>C
MFLIFLDFQFSCHIPRPTVDISFPPFSVFLAIFHVLKCVFLIFSDFQFSRHIPGPTVCISHFFRFFMISSFFT
LSSGCFSFSIIFTFLAIFHVLQWTFLNFPPFSVFLAIFHILKCCVFLIFRDFQFPRHIPGPTVCISHFSRFSV
ISSFFKSSSGCFSFSMIFSFLAIFHVLQWTFLNFPPFSSYSFSPYSRSYSVHFSFFTFFSDFVIFQVVKWMFL
IFLDFQFSC
>ENSMUSP00000136598_c.275:T>G_p.H92Q ENSMUST00000177722.8|Gm11168-201|ENSMUSG00000096385|+|chr9:g.3003625:T>G
MFLIFHDFQFSCHIPCPTVDISFPPFSVFLAIFHVLKCVFLIFSDFQFSRHIPGPTVCISHFSRFSVILSFFK
LSSGCFSFSLIFSFLAIFQVLQWTFLNFPPFSVFLAIFHVLKCCVFLIFRDFQFPRHIPGPTVCISHFSRFSV
ISSFFKSSSGCFSFSMIFSFLAIFHVLQWTFLNFPPFSSYSFSPYSRSYSVHFSFFTFFSDFVIFQVVKWMFL
IFLDFQFSC
>ENSMUSP00000136761_c.45:T>G_p.C16G ENSMUST00000178077.2|Gm11168-202|ENSMUSG00000096385|+|chr9:g.3003625:T>G
MFLIFLDFQFSCHIPGPTVDISFPPFSVFLAIFHVLKCVFLIFSDFQFSRHIPGPTVCISHFFRFFMISSFFT
LSSGCFSFSIIFTFLAIFHVLQWTFLNFPPFSVFLAIFHILKCCVFLIFRDFQFPRHIPGPTVCISHFSRFSV
ISSFFKSSSGCFSFSMIFSFLAIFHVLQWTFLNFPPFSSYSFSPYSRSYSVHFSFFTFFSDFVIFQVVKWMFL
IFLDFQFSC
>ENSMUSP00000136598_c.306:C>A_p.P103T ENSMUST00000177722.8|Gm11168-201|ENSMUSG00000096385|+|chr9:g.3003656:C>A
MFLIFHDFQFSCHIPCPTVDISFPPFSVFLAIFHVLKCVFLIFSDFQFSRHIPGPTVCISHFSRFSVILSFFK
LSSGCFSFSLIFSFLAIFHVLQWTFLNFPTFSVFLAIFHVLKCCVFLIFRDFQFPRHIPGPTVCISHFSRFSV
ISSFFKSSSGCFSFSMIFSFLAIFHVLQWTFLNFPPFSSYSFSPYSRSYSVHFSFFTFFSDFVIFQVVKWMFL
IFLDFQFSC
>ENSMUSP00000136761_c.72:C>A_p.P25T ENSMUST00000178077.2|Gm11168-202|ENSMUSG00000096385|+|chr9:g.3003656:C>A
MFLIFLDFQFSCHIPCPTVDISFPTFSVFLAIFHVLKCVFLIFSDFQFSRHIPGPTVCISHFFRFFMISSFFT
LSSGCFSFSIIFTFLAIFHVLQWTFLNFPPFSVFLAIFHILKCCVFLIFRDFQFPRHIPGPTVCISHFSRFSV
ISSFFKSSSGCFSFSMIFSFLAIFHVLQWTFLNFPPFSSYSFSPYSRSYSVHFSFFTFFSDFVIFQVVKWMFL
IFLDFQFSC
>ENSMUSP00000136598_c.335:T>A_p.H112Q ENSMUST00000177722.8|Gm11168-201|ENSMUSG00000096385|+|chr9:g.3003685:T>A
MFLIFHDFQFSCHIPCPTVDISFPPFSVFLAIFHVLKCVFLIFSDFQFSRHIPGPTVCISHFSRFSVILSFFK
LSSGCFSFSLIFSFLAIFHVLQWTFLNFPPFSVFLAIFQVLKCCVFLIFRDFQFPRHIPGPTVCISHFSRFSV
ISSFFKSSSGCFSFSMIFSFLAIFHVLQWTFLNFPPFSSYSFSPYSRSYSVHFSFFTFFSDFVIFQVVKWMFL
IFLDFQFSC
>ENSMUSP00000136761_c.101:T>A_p.H34Q ENSMUST00000178077.2|Gm11168-202|ENSMUSG00000096385|+|chr9:g.3003685:T>A
MFLIFLDFQFSCHIPCPTVDISFPPFSVFLAIFQVLKCVFLIFSDFQFSRHIPGPTVCISHFFRFFMISSFFT
LSSGCFSFSIIFTFLAIFHVLQWTFLNFPPFSVFLAIFHILKCCVFLIFRDFQFPRHIPGPTVCISHFSRFSV
ISSFFKSSSGCFSFSMIFSFLAIFHVLQWTFLNFPPFSSYSFSPYSRSYSVHFSFFTFFSDFVIFQVVKWMFL
IFLDFQFSC
>ENSMUSP00000136761_c.129:A>C_p.S44R ENSMUST00000178077.2|Gm11168-202|ENSMUSG00000096385|+|chr9:g.3003713:A>C
MFLIFLDFQFSCHIPCPTVDISFPPFSVFLAIFHVLKCVFLIFRDFQFSRHIPGPTVCISHFFRFFMISSFFT
LSSGCFSFSIIFTFLAIFHVLQWTFLNFPPFSVFLAIFHILKCCVFLIFRDFQFPRHIPGPTVCISHFSRFSV
ISSFFKSSSGCFSFSMIFSFLAIFHVLQWTFLNFPPFSSYSFSPYSRSYSVHFSFFTFFSDFVIFQVVKWMFL
IFLDFQFSC
>ENSMUSP00000136761_c.129:A>T_p.S44C ENSMUST00000178077.2|Gm11168-202|ENSMUSG00000096385|+|chr9:g.3003713:A>T
MFLIFLDFQFSCHIPCPTVDISFPPFSVFLAIFHVLKCVFLIFCDFQFSRHIPGPTVCISHFFRFFMISSFFT
LSSGCFSFSIIFTFLAIFHVLQWTFLNFPPFSVFLAIFHILKCCVFLIFRDFQFPRHIPGPTVCISHFSRFSV

ISSFFKSSSGCFSFSMIFSFLAIFHVLQWTFLNFPPFSSYSFSPYSRSYSVHFSFFTFFSDFVIFQVVKWMFL
IFLDFQFSC
>ENSMUSP00000136761_c.165:A>T_p.T56S ENSMUST00000178077.2|Gm11168-202|ENSMUSG00000096385|+|chr9:g.3003749:A>T
MFLIFLDFQFSCHIPCPTVDISFPPFSVFLAIFHVLKCVFLIFSDFQFSRHIPGPSVCISHFFRFFMISSFFT
LSSGCFSFSIIFTFLAIFHVLQWTFLNFPPFSVFLAIFHILKCCVFLIFRDFQFPRHIPGPTVCISHFSRFSV
ISSFFKSSSGCFSFSMIFSFLAIFHVLQWTFLNFPPFSSYSFSPYSRSYSVHFSFFTFFSDFVIFQVVKWMFL
IFLDFQFSC
>ENSMUSP00000136761_c.171:T>G_p.C58G ENSMUST00000178077.2|Gm11168-202|ENSMUSG00000096385|+|chr9:g.3003755:T>G
MFLIFLDFQFSCHIPCPTVDISFPPFSVFLAIFHVLKCVFLIFSDFQFSRHIPGPTVGISHFFRFFMISSFFT
LSSGCFSFSIIFTFLAIFHVLQWTFLNFPPFSVFLAIFHILKCCVFLIFRDFQFPRHIPGPTVCISHFSRFSV
ISSFFKSSSGCFSFSMIFSFLAIFHVLQWTFLNFPPFSSYSFSPYSRSYSVHFSFFTFFSDFVIFQVVKWMFL
IFLDFQFSC
>ENSMUSP00000136761_c.181:AT>A_p.H61del-fs ENSMUST00000178077.2|Gm11168-202|ENSMUSG00000096385|+|chr9:g.3003765:AT>A
MFLIFLDFQFSCHIPCPTVDISFPPFSVFLAIFHVLKCVFLIFSDFQFSRHIPGPTVCISHFSGFS
>ENSMUSP00000136761_c.181:ATT>A_p.H61del-fs ENSMUST00000178077.2|Gm11168-202|ENSMUSG00000096385|+|chr9:g.3003765:ATT>A
MFLIFLDFQFSCHIPCPTVDISFPPFSVFLAIFHVLKCVFLIFSDFQFSRHIPGPTVCISHFQVFHDFVIFHV
VKWMFLIFHYFHFSCHISCPTVDISKFSTFFCFPRHISHPKVLCISHFP
>ENSMUSP00000136761_c.190:G>C_p.R64T ENSMUST00000178077.2|Gm11168-202|ENSMUSG00000096385|+|chr9:g.3003774:G>C
MFLIFLDFQFSCHIPCPTVDISFPPFSVFLAIFHVLKCVFLIFSDFQFSRHIPGPTVCISHFFTFFMISSFFT
LSSGCFSFSIIFTFLAIFHVLQWTFLNFPPFSVFLAIFHILKCCVFLIFRDFQFPRHIPGPTVCISHFSRFSV
ISSFFKSSSGCFSFSMIFSFLAIFHVLQWTFLNFPPFSSYSFSPYSRSYSVHFSFFTFFSDFVIFQVVKWMFL
IFLDFQFSC
>ENSMUSP00000136761_c.214:TCAC>CCAA_p.F72S ENSMUST00000178077.2|Gm11168-202|ENSMUSG00000096385|+|chr9:g.3003974:TCAC>CCAA
MFLIFLDFQFSCHIPCPTVDISFPPFSVFLAIFHVLKCVFLIFSDFQFSRHIPGPTVCISHFFRFFMISSFSN
TLSSGCFSFSIIFTFLAIFHVLQWTFLNFPPFSVFLAIFHILKCCVFLIFRDFQFPRHIPGPTVCISHFSRFS
VISSFFKSSSGCFSFSMIFSFLAIFHVLQWTFLNFPPFSSYSFSPYSRSYSVHFSFFTFFSDFVIFQVVKWMF
LIFLDFQFSC
>ENSMUSP00000136761_c.217:C>A_p.T73K ENSMUST00000178077.2|Gm11168-202|ENSMUSG00000096385|+|chr9:g.3003977:C>A
MFLIFLDFQFSCHIPCPTVDISFPPFSVFLAIFHVLKCVFLIFSDFQFSRHIPGPTVCISHFFRFFMISSFFK
LSSGCFSFSIIFTFLAIFHVLQWTFLNFPPFSVFLAIFHILKCCVFLIFRDFQFPRHIPGPTVCISHFSRFSV
ISSFFKSSSGCFSFSMIFSFLAIFHVLQWTFLNFPPFSSYSFSPYSRSYSVHFSFFTFFSDFVIFQVVKWMFL
IFLDFQFSC
>ENSMUSP00000136761_c.217:CGTT>AGTC_p.T73K ENSMUST00000178077.2|Gm11168-202|ENSMUSG00000096385|+|chr9:g.3003977:CGTT>AGTC
MFLIFLDFQFSCHIPCPTVDISFPPFSVFLAIFHVLKCVFLIFSDFQFSRHIPGPTVCISHFFRFFMISSFFK
SLSSGCFSFSIIFTFLAIFHVLQWTFLNFPPFSVFLAIFHILKCCVFLIFRDFQFPRHIPGPTVCISHFSRFS
VISSFFKSSSGCFSFSMIFSFLAIFHVLQWTFLNFPPFSSYSFSPYSRSYSVHFSFFTFFSDFVIFQVVKWMF
LIFLDFQFSC
>ENSMUSP00000136761_c.221:G>T_p.L74F ENSMUST00000178077.2|Gm11168-202|ENSMUSG00000096385|+|chr9:g.3003981:G>T
MFLIFLDFQFSCHIPCPTVDISFPPFSVFLAIFHVLKCVFLIFSDFQFSRHIPGPTVCISHFFRFFMISSFFT
FSSGCFSFSIIFTFLAIFHVLQWTFLNFPPFSVFLAIFHILKCCVFLIFRDFQFPRHIPGPTVCISHFSRFSV
ISSFFKSSSGCFSFSMIFSFLAIFHVLQWTFLNFPPFSSYSFSPYSRSYSVHFSFFTFFSDFVIFQVVKWMFL
IFLDFQFSC
>ENSMUSP00000136761_c.256:C>G_p.T86S ENSMUST00000178077.2|Gm11168-202|ENSMUSG00000096385|+|chr9:g.3004016:C>G
MFLIFLDFQFSCHIPCPTVDISFPPFSVFLAIFHVLKCVFLIFSDFQFSRHIPGPTVCISHFFRFFMISSFFT
LSSGCFSFSIIFSFLAIFHVLQWTFLNFPPFSVFLAIFHILKCCVFLIFRDFQFPRHIPGPTVCISHFSRFSV
ISSFFKSSSGCFSFSMIFSFLAIFHVLQWTFLNFPPFSSYSFSPYSRSYSVHFSFFTFFSDFVIFQVVKWMFL
IFLDFQFSC

>ENSMUSP00000136761_c.307:C>T_p.P103L ENSMUST00000178077.2|Gm11168-202|ENSMUSG00000096385|+|chr9:g.3004067:C>T
MFLIFLDFQFSCHIPCPTVDISFPPFSVFLAIFHVLKCVFLIFSDFQFSRHIPGPTVCISHFFRFFMISSFFT
LSSGCFSFSIIFTFLAIFHVLQWTFLNFPLFSVFLAIFHILKCCVFLIFRDFQFPRHIPGPTVCISHFSRFSV
ISSFFKSSSGCFSFSMIFSFLAIFHVLQWTFLNFPPFSSYSFSPYSRSYSVHFSFFTFFSDFVIFQVVKWMFL
IFLDFQFSC
>ENSMUSP00000136761_c.336:A>G_p.I113V ENSMUST00000178077.2|Gm11168-202|ENSMUSG00000096385|+|chr9:g.3004096:A>G
MFLIFLDFQFSCHIPCPTVDISFPPFSVFLAIFHVLKCVFLIFSDFQFSRHIPGPTVCISHFFRFFMISSFFT
LSSGCFSFSIIFTFLAIFHVLQWTFLNFPPFSVFLAIFHVLKCCVFLIFRDFQFPRHIPGPTVCISHFSRFSV
ISSFFKSSSGCFSFSMIFSFLAIFHVLQWTFLNFPPFSSYSFSPYSRSYSVHFSFFTFFSDFVIFQVVKWMFL
IFLDFQFSC
>rs253463892_ENSMUSP00000137273_c.15:A>C_p.N6H
ENSMUST00000178348.2|Gm11168-203|ENSMUSG00000096385|+|chr9:g.3004472:A>C
MFLIFHDFHFSCHIPRPIVDISFPPFSVFFAIFHVPKCVFLIFRDFQFPRHIPGPTVCISHFSRFSVISSFFK
SSSGCFSFSMIFSFLAIFHVLQWTFLNFPPFSVFLAIFHVLKCVFLIYRDFQFSCYIPSPTVCISHFSRFSLI
LSLFKLSTACFSFSMIFSFLAIFHVLQWKFLYFPTFSVSPYSRSYSVHFSFFTFFSDFVIFQVVKWMFLIFLD
FQFSC
>rs215471772_ENSMUSP00000137273_c.26:T>G_p.H9Q
ENSMUST00000178348.2|Gm11168-203|ENSMUSG00000096385|+|chr9:g.3004483:T>G
MFLIFNDFQFSCHIPRPIVDISFPPFSVFFAIFHVPKCVFLIFRDFQFPRHIPGPTVCISHFSRFSVISSFFK
SSSGCFSFSMIFSFLAIFHVLQWTFLNFPPFSVFLAIFHVLKCVFLIYRDFQFSCYIPSPTVCISHFSRFSLI
LSLFKLSTACFSFSMIFSFLAIFHVLQWKFLYFPTFSVSPYSRSYSVHFSFFTFFSDFVIFQVVKWMFLIFLD
FQFSC
>rs235490158_ENSMUSP00000137273_c.52:T>C_p.I18T
ENSMUST00000178348.2|Gm11168-203|ENSMUSG00000096385|+|chr9:g.3004509:T>C
MFLIFNDFHFSCHIPRPTVDISFPPFSVFFAIFHVPKCVFLIFRDFQFPRHIPGPTVCISHFSRFSVISSFFK
SSSGCFSFSMIFSFLAIFHVLQWTFLNFPPFSVFLAIFHVLKCVFLIYRDFQFSCYIPSPTVCISHFSRFSLI
LSLFKLSTACFSFSMIFSFLAIFHVLQWKFLYFPTFSVSPYSRSYSVHFSFFTFFSDFVIFQVVKWMFLIFLD
FQFSC
>ENSMUSP00000137273_c.87:T>C_p.F30L ENSMUST00000178348.2|Gm11168-203|ENSMUSG00000096385|+|chr9:g.3004548:T>C
MFLIFNDFHFSCHIPRPIVDISFPPFSVFLAIFHVPKCVFLIFRDFQFPRHIPGPTVCISHFSRFSVISSFFK
SSSGCFSFSMIFSFLAIFHVLQWTFLNFPPFSVFLAIFHVLKCVFLIYRDFQFSCYIPSPTVCISHFSRFSLI
LSLFKLSTACFSFSMIFSFLAIFHVLQWKFLYFPTFSVSPYSRSYSVHFSFFTFFSDFVIFQVVKWMFLIFLD
FQFSC
>ENSMUSP00000137273_c.87:TTCGC>CTTGT_p.F30L ENSMUST00000178348.2|Gm11168-203|ENSMUSG00000096385|+|chr9:g.3004548:TTCGC>CTTGT
MFLIFNDFHFSCHIPRPIVDISFPPFSVFLVRHISRPKVCISHFP*FSVSSPYSRSYSVHFSFFTFFSDFVFF
QVVKWMFLIFHDFQFSCHIPCPTVDISKFSTFFCFPRHISRPKVCISHLP*FSVFLLYSKSYSVHFSFFTFFT
DFVIVQVVNCMFLIFHDFQFSCHIPCPTVEISIFSNFFSFAIFQVLQCAFLIFHVFQ*FCNFSSCQVDVSHFP
*FSVFL
>ENSMUSP00000137273_c.106:C>T_p.P36L ENSMUST00000178348.2|Gm11168-203|ENSMUSG00000096385|+|chr9:g.3004567:C>T
MFLIFNDFHFSCHIPRPIVDISFPPFSVFFAIFHVLKCVFLIFRDFQFPRHIPGPTVCISHFSRFSVISSFFK
SSSGCFSFSMIFSFLAIFHVLQWTFLNFPPFSVFLAIFHVLKCVFLIYRDFQFSCYIPSPTVCISHFSRFSLI
LSLFKLSTACFSFSMIFSFLAIFHVLQWKFLYFPTFSVSPYSRSYSVHFSFFTFFSDFVIFQVVKWMFLIFLD
FQFSC
>ENSMUSP00000137273_c.106:CAA>TAT_p.P36L ENSMUST00000178348.2|Gm11168-203|ENSMUSG00000096385|+|chr9:g.3004567:CAA>TAT
MFLIFNDFHFSCHIPRPIVDISFPPFSVFFAIFHVL*SVYFSFSVIFSFLAIFQVLQCAFLIFHVFQ*FRLFS
SRQVDVSHFP*FSVFLPYSMSYSGHF*IFHLFLFSSPYFTS*SVYFSFTVIFSFLAIFQVLQCAFLIFHVFH*
FCHCSSCQLHVSHFP*FSVFLPYSMSYSGNFYIFQLFQFRHIPGPTVCISHFSRFSVIL*FFKLSSGCFSFSL
IFSFL
>ENSMUSP00000136598_c.351:G>A_p.V118I ENSMUST00000177722.8|Gm11168-201|ENSMUSG00000096385|+|chr9:g.3004575:G>A
MFLIFHDFQFSCHIPCPTVDISFPPFSVFLAIFHVLKCVFLIFSDFQFSRHIPGPTVCISHFSRFSVILSFFK
LSSGCFSFSLIFSFLAIFHVLQWTFLNFPPFSVFLAIFHVLKCCIFLIFRDFQFPRHIPGPTVCISHFSRFSV

ISSFFKSSSGCFSFSMIFSFLAIFHVLQWTFLNFPPFSSYSFSPYSRSYSVHFSFFTFFSDFVIFQVVKWMFL
IFLDFQFSC
>ENSMUSP00000136761_c.351:G>A_p.V118I ENSMUST00000178077.2|Gm11168-
202|ENSMUSG00000096385|+|chr9:g.3004575:G>A
MFLIFLDFQFSCHIPCPTVDISFPPFSVFLAIFHVLKCVFLIFSDFQFSRHIPGPTVCISHFFRFFMISSFFT
LSSGCFSFSIIFTFLAIFHVLQWTFLNFPPFSVFLAIFHILKCCIFLIFRDFQFPRHIPGPTVCISHFSRFSV
ISSFFKSSSGCFSFSMIFSFLAIFHVLQWTFLNFPPFSSYSFSPYSRSYSVHFSFFTFFSDFVIFQVVKWMFL
IFLDFQFSC
>ENSMUSP00000137273_c.114:G>A_p.V39I ENSMUST00000178348.2|Gm11168-
203|ENSMUSG00000096385|+|chr9:g.3004575:G>A
MFLIFNDFHFSCHIPRPIVDISFPPFSVFFAIFHVPKCIFLIFRDFQFPRHIPGPTVCISHFSRFSVISSFFK
SSSGCFSFSMIFSFLAIFHVLQWTFLNFPPFSVFLAIFHVLKCVFLIYRDFQFSCYIPSPTVCISHFSRFSLI
LSLFKLSTACFSFSMIFSFLAIFHVLQWKFLYFPTFSVSPYSRSYSVHFSFFTFFSDFVIFQVVKWMFLIFLD
FQFSC
>ENSMUSP00000136598_c.356:T>A_p.F119L ENSMUST00000177722.8|Gm11168-
201|ENSMUSG00000096385|+|chr9:g.3004580:T>A
MFLIFHDFQFSCHIPCPTVDISFPPFSVFLAIFHVLKCVFLIFSDFQFSRHIPGPTVCISHFSRFSVILSFFK
LSSGCFSFSLIFSFLAIFHVLQWTFLNFPPFSVFLAIFHVLKCCVLLIFRDFQFPRHIPGPTVCISHFSRFSV
ISSFFKSSSGCFSFSMIFSFLAIFHVLQWTFLNFPPFSSYSFSPYSRSYSVHFSFFTFFSDFVIFQVVKWMFL
IFLDFQFSC
>ENSMUSP00000136761_c.356:T>A_p.F119L ENSMUST00000178077.2|Gm11168-
202|ENSMUSG00000096385|+|chr9:g.3004580:T>A
MFLIFLDFQFSCHIPCPTVDISFPPFSVFLAIFHVLKCVFLIFSDFQFSRHIPGPTVCISHFFRFFMISSFFT
LSSGCFSFSIIFTFLAIFHVLQWTFLNFPPFSVFLAIFHILKCCVLLIFRDFQFPRHIPGPTVCISHFSRFSV
ISSFFKSSSGCFSFSMIFSFLAIFHVLQWTFLNFPPFSSYSFSPYSRSYSVHFSFFTFFSDFVIFQVVKWMFL
IFLDFQFSC
>ENSMUSP00000137273_c.119:T>A_p.F40L ENSMUST00000178348.2|Gm11168-
203|ENSMUSG00000096385|+|chr9:g.3004580:T>A
MFLIFNDFHFSCHIPRPIVDISFPPFSVFFAIFHVPKCVLLIFRDFQFPRHIPGPTVCISHFSRFSVISSFFK
SSSGCFSFSMIFSFLAIFHVLQWTFLNFPPFSVFLAIFHVLKCVFLIYRDFQFSCYIPSPTVCISHFSRFSLI
LSLFKLSTACFSFSMIFSFLAIFHVLQWKFLYFPTFSVSPYSRSYSVHFSFFTFFSDFVIFQVVKWMFLIFLD
FQFSC
>ENSMUSP00000136598_c.366:C>T_p.R123C ENSMUST00000177722.8|Gm11168-
201|ENSMUSG00000096385|+|chr9:g.3004590:C>T
MFLIFHDFQFSCHIPCPTVDISFPPFSVFLAIFHVLKCVFLIFSDFQFSRHIPGPTVCISHFSRFSVILSFFK
LSSGCFSFSLIFSFLAIFHVLQWTFLNFPPFSVFLAIFHVLKCCVFLIFCDFQFPRHIPGPTVCISHFSRFSV
ISSFFKSSSGCFSFSMIFSFLAIFHVLQWTFLNFPPFSSYSFSPYSRSYSVHFSFFTFFSDFVIFQVVKWMFL
IFLDFQFSC
>ENSMUSP00000136761_c.366:C>T_p.R123C ENSMUST00000178077.2|Gm11168-
202|ENSMUSG00000096385|+|chr9:g.3004590:C>T
MFLIFLDFQFSCHIPCPTVDISFPPFSVFLAIFHVLKCVFLIFSDFQFSRHIPGPTVCISHFFRFFMISSFFT
LSSGCFSFSIIFTFLAIFHVLQWTFLNFPPFSVFLAIFHILKCCVFLIFCDFQFPRHIPGPTVCISHFSRFSV
ISSFFKSSSGCFSFSMIFSFLAIFHVLQWTFLNFPPFSSYSFSPYSRSYSVHFSFFTFFSDFVIFQVVKWMFL
IFLDFQFSC
>ENSMUSP00000137273_c.129:C>T_p.R44C ENSMUST00000178348.2|Gm11168-
203|ENSMUSG00000096385|+|chr9:g.3004590:C>T
MFLIFNDFHFSCHIPRPIVDISFPPFSVFFAIFHVPKCVFLIFCDFQFPRHIPGPTVCISHFSRFSVISSFFK
SSSGCFSFSMIFSFLAIFHVLQWTFLNFPPFSVFLAIFHVLKCVFLIYRDFQFSCYIPSPTVCISHFSRFSLI
LSLFKLSTACFSFSMIFSFLAIFHVLQWKFLYFPTFSVSPYSRSYSVHFSFFTFFSDFVIFQVVKWMFLIFLD
FQFSC
>ENSMUSP00000136598_c.381:C>T_p.P128S ENSMUST00000177722.8|Gm11168-
201|ENSMUSG00000096385|+|chr9:g.3004605:C>T
MFLIFHDFQFSCHIPCPTVDISFPPFSVFLAIFHVLKCVFLIFSDFQFSRHIPGPTVCISHFSRFSVILSFFK
LSSGCFSFSLIFSFLAIFHVLQWTFLNFPPFSVFLAIFHVLKCCVFLIFRDFQFSRHIPGPTVCISHFSRFSV
ISSFFKSSSGCFSFSMIFSFLAIFHVLQWTFLNFPPFSSYSFSPYSRSYSVHFSFFTFFSDFVIFQVVKWMFL
IFLDFQFSC
>ENSMUSP00000136761_c.381:C>T_p.P128S ENSMUST00000178077.2|Gm11168-
202|ENSMUSG00000096385|+|chr9:g.3004605:C>T

MFLIFLDFQFSCHIPCPTVDISFPPFSVFLAIFHVLKCVFLIFSDFQFSRHIPGPTVCISHFFRFFMISSFFT
LSSGCFSFSIIFTFLAIFHVLQWTFLNFPPFSVFLAIFHILKCCVFLIFRDFQFSRHIPGPTVCISHFSRFSV
ISSFFKSSSGCFSFSMIFSFLAIFHVLQWTFLNFPPFSSYSFSPYSRSYSVHFSFFTFFSDFVIFQVVKWMFL
IFLDFQFSC
>ENSMUSP00000137273_c.144:C>T_p.P49S ENSMUST00000178348.2|Gm11168-203|ENSMUSG00000096385|+|chr9:g.3004605:C>T
MFLIFNDFHFSCHIPRPIVDISFPPFSVFFAIFHVPKCVFLIFRDFQFSRHIPGPTVCISHFSRFSVISSFFK
SSSGCFSFSMIFSFLAIFHVLQWTFLNFPPFSVFLAIFHVLKCVFLIYRDFQFSCYIPSPTVCISHFSRFSLI
LSLFKLSTACFSFSMIFSFLAIFHVLQWKFLYFPTFSVSPYSRSYSVHFSFFTFFSDFVIFQVVKWMFLIFLD
FQFSC
>ENSMUSP00000136598_c.402:A>T_p.T135S ENSMUST00000177722.8|Gm11168-201|ENSMUSG00000096385|+|chr9:g.3004626:A>T
MFLIFHDFQFSCHIPCPTVDISFPPFSVFLAIFHVLKCVFLIFSDFQFSRHIPGPTVCISHFSRFSVILSFFK
LSSGCFSFSLIFSFLAIFHVLQWTFLNFPPFSVFLAIFHVLKCCVFLIFRDFQFPRHIPGPSVCISHFSRFSV
ISSFFKSSSGCFSFSMIFSFLAIFHVLQWTFLNFPPFSSYSFSPYSRSYSVHFSFFTFFSDFVIFQVVKWMFL
IFLDFQFSC
>ENSMUSP00000136761_c.402:A>T_p.T135S ENSMUST00000178077.2|Gm11168-202|ENSMUSG00000096385|+|chr9:g.3004626:A>T
MFLIFLDFQFSCHIPCPTVDISFPPFSVFLAIFHVLKCVFLIFSDFQFSRHIPGPTVCISHFFRFFMISSFFT
LSSGCFSFSIIFTFLAIFHVLQWTFLNFPPFSVFLAIFHILKCCVFLIFRDFQFPRHIPGPSVCISHFSRFSV
ISSFFKSSSGCFSFSMIFSFLAIFHVLQWTFLNFPPFSSYSFSPYSRSYSVHFSFFTFFSDFVIFQVVKWMFL
IFLDFQFSC
>ENSMUSP00000137273_c.165:A>T_p.T56S ENSMUST00000178348.2|Gm11168-203|ENSMUSG00000096385|+|chr9:g.3004626:A>T
MFLIFNDFHFSCHIPRPIVDISFPPFSVFFAIFHVPKCVFLIFRDFQFPRHIPGPSVCISHFSRFSVISSFFK
SSSGCFSFSMIFSFLAIFHVLQWTFLNFPPFSVFLAIFHVLKCVFLIYRDFQFSCYIPSPTVCISHFSRFSLI
LSLFKLSTACFSFSMIFSFLAIFHVLQWKFLYFPTFSVSPYSRSYSVHFSFFTFFSDFVIFQVVKWMFLIFLD
FQFSC
>rs33899290_ENSMUSP00000136598_c.427:G>A_p.R143H
ENSMUST00000177722.8|Gm11168-201|ENSMUSG00000096385|+|chr9:g.3004651:G>A
MFLIFHDFQFSCHIPCPTVDISFPPFSVFLAIFHVLKCVFLIFSDFQFSRHIPGPTVCISHFSRFSVILSFFK
LSSGCFSFSLIFSFLAIFHVLQWTFLNFPPFSVFLAIFHVLKCCVFLIFRDFQFPRHIPGPTVCISHFSHFSV
ISSFFKSSSGCFSFSMIFSFLAIFHVLQWTFLNFPPFSSYSFSPYSRSYSVHFSFFTFFSDFVIFQVVKWMFL
IFLDFQFSC
>rs33899290_ENSMUSP00000136761_c.427:G>A_p.R143H
ENSMUST00000178077.2|Gm11168-202|ENSMUSG00000096385|+|chr9:g.3004651:G>A
MFLIFLDFQFSCHIPCPTVDISFPPFSVFLAIFHVLKCVFLIFSDFQFSRHIPGPTVCISHFFRFFMISSFFT
LSSGCFSFSIIFTFLAIFHVLQWTFLNFPPFSVFLAIFHILKCCVFLIFRDFQFPRHIPGPTVCISHFSHFSV
ISSFFKSSSGCFSFSMIFSFLAIFHVLQWTFLNFPPFSSYSFSPYSRSYSVHFSFFTFFSDFVIFQVVKWMFL
IFLDFQFSC
>rs33899290_ENSMUSP00000137273_c.190:G>A_p.R64H
ENSMUST00000178348.2|Gm11168-203|ENSMUSG00000096385|+|chr9:g.3004651:G>A
MFLIFNDFHFSCHIPRPIVDISFPPFSVFFAIFHVPKCVFLIFRDFQFPRHIPGPTVCISHFSHFSVISSFFK
SSSGCFSFSMIFSFLAIFHVLQWTFLNFPPFSVFLAIFHVLKCVFLIYRDFQFSCYIPSPTVCISHFSRFSLI
LSLFKLSTACFSFSMIFSFLAIFHVLQWKFLYFPTFSVSPYSRSYSVHFSFFTFFSDFVIFQVVKWMFLIFLD
FQFSC
>rs30685709_ENSMUSP00000136598_c.433:C>T_p.S145L
ENSMUST00000177722.8|Gm11168-201|ENSMUSG00000096385|+|chr9:g.3004657:C>T
MFLIFHDFQFSCHIPCPTVDISFPPFSVFLAIFHVLKCVFLIFSDFQFSRHIPGPTVCISHFSRFSVILSFFK
LSSGCFSFSLIFSFLAIFHVLQWTFLNFPPFSVFLAIFHVLKCCVFLIFRDFQFPRHIPGPTVCISHFSRFLV
ISSFFKSSSGCFSFSMIFSFLAIFHVLQWTFLNFPPFSSYSFSPYSRSYSVHFSFFTFFSDFVIFQVVKWMFL
IFLDFQFSC
>rs30685709_ENSMUSP00000136761_c.433:C>T_p.S145L
ENSMUST00000178077.2|Gm11168-202|ENSMUSG00000096385|+|chr9:g.3004657:C>T
MFLIFLDFQFSCHIPCPTVDISFPPFSVFLAIFHVLKCVFLIFSDFQFSRHIPGPTVCISHFFRFFMISSFFT
LSSGCFSFSIIFTFLAIFHVLQWTFLNFPPFSVFLAIFHILKCCVFLIFRDFQFPRHIPGPTVCISHFSRFLV
ISSFFKSSSGCFSFSMIFSFLAIFHVLQWTFLNFPPFSSYSFSPYSRSYSVHFSFFTFFSDFVIFQVVKWMFL
IFLDFQFSC

>rs30685709_ENSMUSP00000137273_c.196:C>T_p.S66L
ENSMUST00000178348.2|Gm11168-203|ENSMUSG00000096385|+|chr9:g.3004657:C>T
MFLIFNDFHFSCHIPRPIVDISFPPFSVFFAIFHVPKCVFLIFRDFQFPRHIPGPTVCISHFSRFLVISSFFK
SSSGCFSFSMIFSFLAIFHVLQWTFLNFPPFSVFLAIFHVLKCVFLIYRDFQFSCYIPSPTVCISHFSRFSLI
LSLFKLSTACFSFSMIFSFLAIFHVLQWKFLYFPTFSVSPYSRSYSVHFSFFTFFSDFVIFQVVKWMFLIFLD
FQFSC
>ENSMUSP00000136598_c.442:C>T_p.S148L ENSMUST00000177722.8|Gm11168-
201|ENSMUSG00000096385|+|chr9:g.3004666:C>T
MFLIFHDFQFSCHIPCPTVDISFPPFSVFLAIFHVLKCVFLIFSDFQFSRHIPGPTVCISHFSRFSVILSFFK
LSSGCFSFSLIFSFLAIFHVLQWTFLNFPPFSVFLAIFHVLKCCVFLIFRDFQFPRHIPGPTVCISHFSRFSV
ILSFFKSSSGCFSFSMIFSFLAIFHVLQWTFLNFPPFSSYSFSPYSRSYSVHFSFFTFFSDFVIFQVVKWMFL
IFLDFQFSC
>ENSMUSP00000136761_c.442:C>T_p.S148L ENSMUST00000178077.2|Gm11168-
202|ENSMUSG00000096385|+|chr9:g.3004666:C>T
MFLIFLDFQFSCHIPCPTVDISFPPFSVFLAIFHVLKCVFLIFSDFQFSRHIPGPTVCISHFFRFFMISSFFT
LSSGCFSFSIIFTFLAIFHVLQWTFLNFPPFSVFLAIFHILKCCVFLIFRDFQFPRHIPGPTVCISHFSRFSV
ILSFFKSSSGCFSFSMIFSFLAIFHVLQWTFLNFPPFSSYSFSPYSRSYSVHFSFFTFFSDFVIFQVVKWMFL
IFLDFQFSC
>ENSMUSP00000137273_c.205:C>T_p.S69L ENSMUST00000178348.2|Gm11168-
203|ENSMUSG00000096385|+|chr9:g.3004666:C>T
MFLIFNDFHFSCHIPRPIVDISFPPFSVFFAIFHVPKCVFLIFRDFQFPRHIPGPTVCISHFSRFSVILSFFK
SSSGCFSFSMIFSFLAIFHVLQWTFLNFPPFSVFLAIFHVLKCVFLIYRDFQFSCYIPSPTVCISHFSRFSLI
LSLFKLSTACFSFSMIFSFLAIFHVLQWKFLYFPTFSVSPYSRSYSVHFSFFTFFSDFVIFQVVKWMFLIFLD
FQFSC
>ENSMUSP00000136598_c.445:C>G_p.S149C ENSMUST00000177722.8|Gm11168-
201|ENSMUSG00000096385|+|chr9:g.3004669:C>G
MFLIFHDFQFSCHIPCPTVDISFPPFSVFLAIFHVLKCVFLIFSDFQFSRHIPGPTVCISHFSRFSVILSFFK
LSSGCFSFSLIFSFLAIFHVLQWTFLNFPPFSVFLAIFHVLKCCVFLIFRDFQFPRHIPGPTVCISHFSRFSV
ISCFFKSSSGCFSFSMIFSFLAIFHVLQWTFLNFPPFSSYSFSPYSRSYSVHFSFFTFFSDFVIFQVVKWMFL
IFLDFQFSC
>ENSMUSP00000136761_c.445:C>G_p.S149C ENSMUST00000178077.2|Gm11168-
202|ENSMUSG00000096385|+|chr9:g.3004669:C>G
MFLIFLDFQFSCHIPCPTVDISFPPFSVFLAIFHVLKCVFLIFSDFQFSRHIPGPTVCISHFFRFFMISSFFT
LSSGCFSFSIIFTFLAIFHVLQWTFLNFPPFSVFLAIFHILKCCVFLIFRDFQFPRHIPGPTVCISHFSRFSV
ISCFFKSSSGCFSFSMIFSFLAIFHVLQWTFLNFPPFSSYSFSPYSRSYSVHFSFFTFFSDFVIFQVVKWMFL
IFLDFQFSC
>ENSMUSP00000137273_c.208:C>G_p.S70C ENSMUST00000178348.2|Gm11168-
203|ENSMUSG00000096385|+|chr9:g.3004669:C>G
MFLIFNDFHFSCHIPRPIVDISFPPFSVFFAIFHVPKCVFLIFRDFQFPRHIPGPTVCISHFSRFSVISCFFK
SSSGCFSFSMIFSFLAIFHVLQWTFLNFPPFSVFLAIFHVLKCVFLIYRDFQFSCYIPSPTVCISHFSRFSLI
LSLFKLSTACFSFSMIFSFLAIFHVLQWKFLYFPTFSVSPYSRSYSVHFSFFTFFSDFVIFQVVKWMFLIFLD
FQFSC
>ENSMUSP00000136598_c.543:C>A_p.P182T ENSMUST00000177722.8|Gm11168-
201|ENSMUSG00000096385|+|chr9:g.3004767:C>A
MFLIFHDFQFSCHIPCPTVDISFPPFSVFLAIFHVLKCVFLIFSDFQFSRHIPGPTVCISHFSRFSVILSFFK
LSSGCFSFSLIFSFLAIFHVLQWTFLNFPPFSVFLAIFHVLKCCVFLIFRDFQFPRHIPGPTVCISHFSRFSV
ISSFFKSSSGCFSFSMIFSFLAIFHVLQWTFLNFPTFSSYSFSPYSRSYSVHFSFFTFFSDFVIFQVVKWMFL
IFLDFQFSC
>ENSMUSP00000136761_c.543:C>A_p.P182T ENSMUST00000178077.2|Gm11168-
202|ENSMUSG00000096385|+|chr9:g.3004767:C>A
MFLIFLDFQFSCHIPCPTVDISFPPFSVFLAIFHVLKCVFLIFSDFQFSRHIPGPTVCISHFFRFFMISSFFT
LSSGCFSFSIIFTFLAIFHVLQWTFLNFPPFSVFLAIFHILKCCVFLIFRDFQFPRHIPGPTVCISHFSRFSV
ISSFFKSSSGCFSFSMIFSFLAIFHVLQWTFLNFPTFSSYSFSPYSRSYSVHFSFFTFFSDFVIFQVVKWMFL
IFLDFQFSC
>ENSMUSP00000137273_c.306:C>A_p.P103T ENSMUST00000178348.2|Gm11168-
203|ENSMUSG00000096385|+|chr9:g.3004767:C>A
MFLIFNDFHFSCHIPRPIVDISFPPFSVFFAIFHVPKCVFLIFRDFQFPRHIPGPTVCISHFSRFSVISSFFK
SSSGCFSFSMIFSFLAIFHVLQWTFLNFPTFSVFLAIFHVLKCVFLIYRDFQFSCYIPSPTVCISHFSRFSLI

LSLFKLSTACFSFSMIFSFLAIFHVLQWKFLYFPTFSVSPYSRSYSVHFSFFTFFSDFVIFQVVKWMFLIFLD
FQFSC
>ENSMUSP00000136598_c.544:C>T_p.P182L ENSMUST00000177722.8|Gm11168-201|ENSMUSG00000096385|+|chr9:g.3004768:C>T
MFLIFHDFQFSCHIPCPTVDISFPPFSVFLAIFHVLKCVFLIFSDFQFSRHIPGPTVCISHFSRFSVILSFFK
LSSGCFSFSLIFSFLAIFHVLQWTFLNFPPFSVFLAIFHVLKCCVFLIFRDFQFPRHIPGPTVCISHFSRFSV
ISSFFKSSSGCFSFSMIFSFLAIFHVLQWTFLNFPLFSSYSFSPYSRSYSVHFSFFTFFSDFVIFQVVKWMFL
IFLDFQFSC
>ENSMUSP00000136761_c.544:C>T_p.P182L ENSMUST00000178077.2|Gm11168-202|ENSMUSG00000096385|+|chr9:g.3004768:C>T
MFLIFLDFQFSCHIPCPTVDISFPPFSVFLAIFHVLKCVFLIFSDFQFSRHIPGPTVCISHFFRFFMISSFFT
LSSGCFSFSIIFTFLAIFHVLQWTFLNFPPFSVFLAIFHILKCCVFLIFRDFQFPRHIPGPTVCISHFSRFSV
ISSFFKSSSGCFSFSMIFSFLAIFHVLQWTFLNFPLFSSYSFSPYSRSYSVHFSFFTFFSDFVIFQVVKWMFL
IFLDFQFSC
>ENSMUSP00000137273_c.307:C>T_p.P103L ENSMUST00000178348.2|Gm11168-203|ENSMUSG00000096385|+|chr9:g.3004768:C>T
MFLIFNDFHFSCHIPRPIVDISFPPFSVFFAIFHVPKCVFLIFRDFQFPRHIPGPTVCISHFSRFSVISSFFK
SSSGCFSFSMIFSFLAIFHVLQWTFLNFPLFSVFLAIFHVLKCVFLIYRDFQFSCYIPSPTVCISHFSRFSLI
LSLFKLSTACFSFSMIFSFLAIFHVLQWKFLYFPTFSVSPYSRSYSVHFSFFTFFSDFVIFQVVKWMFLIFLD
FQFSC
>ENSMUSP00000137273_c.361:A>T_p.Y121F ENSMUST00000178348.2|Gm11168-203|ENSMUSG00000096385|+|chr9:g.3004822:A>T
MFLIFNDFHFSCHIPRPIVDISFPPFSVFFAIFHVPKCVFLIFRDFQFPRHIPGPTVCISHFSRFSVISSFFK
SSSGCFSFSMIFSFLAIFHVLQWTFLNFPPFSVFLAIFHVLKCVFLIFRDFQFSCYIPSPTVCISHFSRFSLI
LSLFKLSTACFSFSMIFSFLAIFHVLQWKFLYFPTFSVSPYSRSYSVHFSFFTFFSDFVIFQVVKWMFLIFLD
FQFSC
>ENSMUSP00000137273_c.364:G>A_p.R122H ENSMUST00000178348.2|Gm11168-203|ENSMUSG00000096385|+|chr9:g.3004825:G>A
MFLIFNDFHFSCHIPRPIVDISFPPFSVFFAIFHVPKCVFLIFRDFQFPRHIPGPTVCISHFSRFSVISSFFK
SSSGCFSFSMIFSFLAIFHVLQWTFLNFPPFSVFLAIFHVLKCVFLIYHDFQFSCYIPSPTVCISHFSRFSLI
LSLFKLSTACFSFSMIFSFLAIFHVLQWKFLYFPTFSVSPYSRSYSVHFSFFTFFSDFVIFQVVKWMFLIFLD
FQFSC
>ENSMUSP00000137273_c.373:A>T_p.Q125L ENSMUST00000178348.2|Gm11168-203|ENSMUSG00000096385|+|chr9:g.3004834:A>T
MFLIFNDFHFSCHIPRPIVDISFPPFSVFFAIFHVPKCVFLIFRDFQFPRHIPGPTVCISHFSRFSVISSFFK
SSSGCFSFSMIFSFLAIFHVLQWTFLNFPPFSVFLAIFHVLKCVFLIYRDFLFSCYIPSPTVCISHFSRFSLI
LSLFKLSTACFSFSMIFSFLAIFHVLQWKFLYFPTFSVSPYSRSYSVHFSFFTFFSDFVIFQVVKWMFLIFLD
FQFSC
>ENSMUSP00000137273_c.374:G>T_p.Q125H ENSMUST00000178348.2|Gm11168-203|ENSMUSG00000096385|+|chr9:g.3004835:G>T
MFLIFNDFHFSCHIPRPIVDISFPPFSVFFAIFHVPKCVFLIFRDFQFPRHIPGPTVCISHFSRFSVISSFFK
SSSGCFSFSMIFSFLAIFHVLQWTFLNFPPFSVFLAIFHVLKCVFLIYRDFHFSCYIPSPTVCISHFSRFSLI
LSLFKLSTACFSFSMIFSFLAIFHVLQWKFLYFPTFSVSPYSRSYSVHFSFFTFFSDFVIFQVVKWMFLIFLD
FQFSC
>ENSMUSP00000137273_c.381:TGCT>CGCC_p.C128R ENSMUST00000178348.2|Gm11168-203|ENSMUSG00000096385|+|chr9:g.3004842:TGCT>CGCC
MFLIFNDFHFSCHIPRPIVDISFPPFSVFFAIFHVPKCVFLIFRDFQFPRHIPGPTVCISHFSRFSVISSFFK
SSSGCFSFSMIFSFLAIFHVLQWTFLNFPPFSVFLAIFHVLKCVFLIYRDFQFSRRYIPSPTVCISHFSRFSL
ILSLFKLSTACFSFSMIFSFLAIFHVLQWKFLYFPTFSVSPYSRSYSVHFSFFTFFSDFVIFQVVKWMFLIFL
DFQFSC
>ENSMUSP00000137273_c.384:T>C_p.Y129H ENSMUST00000178348.2|Gm11168-203|ENSMUSG00000096385|+|chr9:g.3004845:T>C
MFLIFNDFHFSCHIPRPIVDISFPPFSVFFAIFHVPKCVFLIFRDFQFPRHIPGPTVCISHFSRFSVISSFFK
SSSGCFSFSMIFSFLAIFHVLQWTFLNFPPFSVFLAIFHVLKCVFLIYRDFQFSCHIPSPTVCISHFSRFSLI
LSLFKLSTACFSFSMIFSFLAIFHVLQWKFLYFPTFSVSPYSRSYSVHFSFFTFFSDFVIFQVVKWMFLIFLD
FQFSC
>ENSMUSP00000137273_c.390:CCAA>TCAC_p.P131S ENSMUST00000178348.2|Gm11168-203|ENSMUSG00000096385|+|chr9:g.3004851:CCAA>TCAC

MFLIFNDFHFSCHIPRPIVDISFPPFSVFFAIFHVPKCVFLIFRDFQFPRHIPGPTVCISHFSRFSVISSFFK
SSSGCFSFSMIFSFLAIFHVLQWTFLNFPPFSVFLAIFHVLKCVFLIYRDFQFSCYISPSPTVCISHFSRFSL
ILSLFKLSTACFSFSMIFSFLAIFHVLQWKFLYFPTFSVSPYSRSYSVHFSFFTFFSDFVIFQVVKWMFLIFL
DFQFSC
>ENSMUSP00000137273_c.393:A>G_p.S132G ENSMUST00000178348.2|Gm11168-203|ENSMUSG00000096385|+|chr9:g.3004854:A>G
MFLIFNDFHFSCHIPRPIVDISFPPFSVFFAIFHVPKCVFLIFRDFQFPRHIPGPTVCISHFSRFSVISSFFK
SSSGCFSFSMIFSFLAIFHVLQWTFLNFPPFSVFLAIFHVLKCVFLIYRDFQFSCYIPGPTVCISHFSRFSLI
LSLFKLSTACFSFSMIFSFLAIFHVLQWKFLYFPTFSVSPYSRSYSVHFSFFTFFSDFVIFQVVKWMFLIFLD
FQFSC
>ENSMUSP00000137273_c.393:A>T_p.S132C ENSMUST00000178348.2|Gm11168-203|ENSMUSG00000096385|+|chr9:g.3004854:A>T
MFLIFNDFHFSCHIPRPIVDISFPPFSVFFAIFHVPKCVFLIFRDFQFPRHIPGPTVCISHFSRFSVISSFFK
SSSGCFSFSMIFSFLAIFHVLQWTFLNFPPFSVFLAIFHVLKCVFLIYRDFQFSCYIPCPTVCISHFSRFSLI
LSLFKLSTACFSFSMIFSFLAIFHVLQWKFLYFPTFSVSPYSRSYSVHFSFFTFFSDFVIFQVVKWMFLIFLD
FQFSC
>ENSMUSP00000137273_c.394:G>T_p.S132I ENSMUST00000178348.2|Gm11168-203|ENSMUSG00000096385|+|chr9:g.3004855:G>T
MFLIFNDFHFSCHIPRPIVDISFPPFSVFFAIFHVPKCVFLIFRDFQFPRHIPGPTVCISHFSRFSVISSFFK
SSSGCFSFSMIFSFLAIFHVLQWTFLNFPPFSVFLAIFHVLKCVFLIYRDFQFSCYIPIPTVCISHFSRFSLI
LSLFKLSTACFSFSMIFSFLAIFHVLQWKFLYFPTFSVSPYSRSYSVHFSFFTFFSDFVIFQVVKWMFLIFLD
FQFSC
>ENSMUSP00000136598_c.556:A>T_p.Y186F ENSMUST00000177722.8|Gm11168-201|ENSMUSG00000096385|+|chr9:g.3004860:A>T
MFLIFHDFQFSCHIPCPTVDISFPPFSVFLAIFHVLKCVFLIFSDFQFSRHIPGPTVCISHFSRFSVILSFFK
LSSGCFSFSLIFSFLAIFHVLQWTFLNFPPFSVFLAIFHVLKCCVFLIFRDFQFPRHIPGPTVCISHFSRFSV
ISSFFKSSSGCFSFSMIFSFLAIFHVLQWTFLNFPPFSSFSFSPYSRSYSVHFSFFTFFSDFVIFQVVKWMFL
IFLDFQFSC
>ENSMUSP00000136761_c.556:A>T_p.Y186F ENSMUST00000178077.2|Gm11168-202|ENSMUSG00000096385|+|chr9:g.3004860:A>T
MFLIFLDFQFSCHIPCPTVDISFPPFSVFLAIFHVLKCVFLIFSDFQFSRHIPGPTVCISHFFRFFMISSFFT
LSSGCFSFSIIFTFLAIFHVLQWTFLNFPPFSVFLAIFHILKCCVFLIFRDFQFPRHIPGPTVCISHFSRFSV
ISSFFKSSSGCFSFSMIFSFLAIFHVLQWTFLNFPPFSSFSFSPYSRSYSVHFSFFTFFSDFVIFQVVKWMFL
IFLDFQFSC
>ENSMUSP00000137273_c.399:A>T_p.T134S ENSMUST00000178348.2|Gm11168-203|ENSMUSG00000096385|+|chr9:g.3004860:A>T
MFLIFNDFHFSCHIPRPIVDISFPPFSVFFAIFHVPKCVFLIFRDFQFPRHIPGPTVCISHFSRFSVISSFFK
SSSGCFSFSMIFSFLAIFHVLQWTFLNFPPFSVFLAIFHVLKCVFLIYRDFQFSCYIPSPSVCISHFSRFSLI
LSLFKLSTACFSFSMIFSFLAIFHVLQWKFLYFPTFSVSPYSRSYSVHFSFFTFFSDFVIFQVVKWMFLIFLD
FQFSC
>ENSMUSP00000136598_c.558:A>C_p.S187R ENSMUST00000177722.8|Gm11168-201|ENSMUSG00000096385|+|chr9:g.3004862:A>C
MFLIFHDFQFSCHIPCPTVDISFPPFSVFLAIFHVLKCVFLIFSDFQFSRHIPGPTVCISHFSRFSVILSFFK
LSSGCFSFSLIFSFLAIFHVLQWTFLNFPPFSVFLAIFHVLKCCVFLIFRDFQFPRHIPGPTVCISHFSRFSV
ISSFFKSSSGCFSFSMIFSFLAIFHVLQWTFLNFPPFSSYRFSPYSRSYSVHFSFFTFFSDFVIFQVVKWMFL
IFLDFQFSC
>ENSMUSP00000136761_c.558:A>C_p.S187R ENSMUST00000178077.2|Gm11168-202|ENSMUSG00000096385|+|chr9:g.3004862:A>C
MFLIFLDFQFSCHIPCPTVDISFPPFSVFLAIFHVLKCVFLIFSDFQFSRHIPGPTVCISHFFRFFMISSFFT
LSSGCFSFSIIFTFLAIFHVLQWTFLNFPPFSVFLAIFHILKCCVFLIFRDFQFPRHIPGPTVCISHFSRFSV
ISSFFKSSSGCFSFSMIFSFLAIFHVLQWTFLNFPPFSSYRFSPYSRSYSVHFSFFTFFSDFVIFQVVKWMFL
IFLDFQFSC
>ENSMUSP00000136598_c.558:AG>CA_p.S187Q ENSMUST00000177722.8|Gm11168-201|ENSMUSG00000096385|+|chr9:g.3004862:AG>CA
MFLIFHDFQFSCHIPCPTVDISFPPFSVFLAIFHVLKCVFLIFSDFQFSRHIPGPTVCISHFSRFSVILSFFK
LSSGCFSFSLIFSFLAIFHVLQWTFLNFPPFSVFLAIFHVLKCCVFLIFRDFQFPRHIPGPTVCISHFSRFSV
ISSFFKSSSGCFSFSMIFSFLAIFHVLQWTFLNFPPFSSYQFLAIFQVLQCAFLIFHVFQ*FCNFSSCQVDVS
HFP*FSVFL

>ENSMUSP00000136761_c.558:AG>CA_p.S187Q ENSMUST00000178077.2|Gm11168-202|ENSMUSG00000096385|+|chr9:g.3004862:AG>CA
MFLIFLDFQFSCHIPCPTVDISFPPFSVFLAIFHVLKCVFLIFSDFQFSRHIPGPTVCISHFFRFFMISSFFT
LSSGCFSFSIIFTFLAIFHVLQWTFLNFPPFSVFLAIFHILKCCVFLIFRDFQFPRHIPGPTVCISHFSRFSV
ISSFFKSSSGCFSFSMIFSFLAIFHVLQWTFLNFPPFSSYQFLAIFQVLQCAFLIFHVFQ*FCNFSSCQVDVS
HFP*FSVFL
>ENSMUSP00000137273_c.414:C>T_p.H139Y ENSMUST00000178348.2|Gm11168-203|ENSMUSG00000096385|+|chr9:g.3004875:C>T
MFLIFNDFHFSCHIPRPIVDISFPPFSVFFAIFHVPKCVFLIFRDFQFPRHIPGPTVCISHFSRFSVISSFFK
SSSGCFSFSMIFSFLAIFHVLQWTFLNFPPFSVFLAIFHVLKCVFLIYRDFQFSCYIPSPTVCISYFSRFSLI
LSLFKLSTACFSFSMIFSFLAIFHVLQWKFLYFPTFSVSPYSRSYSVHFSFFTFFSDFVIFQVVKWMFLIFLD
FQFSC
>ENSMUSP00000137273_c.415:A>T_p.H139L ENSMUST00000178348.2|Gm11168-203|ENSMUSG00000096385|+|chr9:g.3004876:A>T
MFLIFNDFHFSCHIPRPIVDISFPPFSVFFAIFHVPKCVFLIFRDFQFPRHIPGPTVCISHFSRFSVISSFFK
SSSGCFSFSMIFSFLAIFHVLQWTFLNFPPFSVFLAIFHVLKCVFLIYRDFQFSCYIPSPTVCISLFSRFSLI
LSLFKLSTACFSFSMIFSFLAIFHVLQWKFLYFPTFSVSPYSRSYSVHFSFFTFFSDFVIFQVVKWMFLIFLD
FQFSC
>ENSMUSP00000137273_c.420:T>C_p.S141P ENSMUST00000178348.2|Gm11168-203|ENSMUSG00000096385|+|chr9:g.3004881:T>C
MFLIFNDFHFSCHIPRPIVDISFPPFSVFFAIFHVPKCVFLIFRDFQFPRHIPGPTVCISHFSRFSVISSFFK
SSSGCFSFSMIFSFLAIFHVLQWTFLNFPPFSVFLAIFHVLKCVFLIYRDFQFSCYIPSPTVCISHFPRFSLI
LSLFKLSTACFSFSMIFSFLAIFHVLQWKFLYFPTFSVSPYSRSYSVHFSFFTFFSDFVIFQVVKWMFLIFLD
FQFSC
>ENSMUSP00000137273_c.423:C>T_p.R142C ENSMUST00000178348.2|Gm11168-203|ENSMUSG00000096385|+|chr9:g.3004884:C>T
MFLIFNDFHFSCHIPRPIVDISFPPFSVFFAIFHVPKCVFLIFRDFQFPRHIPGPTVCISHFSRFSVISSFFK
SSSGCFSFSMIFSFLAIFHVLQWTFLNFPPFSVFLAIFHVLKCVFLIYRDFQFSCYIPSPTVCISHFSCFSLI
LSLFKLSTACFSFSMIFSFLAIFHVLQWKFLYFPTFSVSPYSRSYSVHFSFFTFFSDFVIFQVVKWMFLIFLD
FQFSC
>ENSMUSP00000137273_c.432:C>G_p.L145V ENSMUST00000178348.2|Gm11168-203|ENSMUSG00000096385|+|chr9:g.3004893:C>G
MFLIFNDFHFSCHIPRPIVDISFPPFSVFFAIFHVPKCVFLIFRDFQFPRHIPGPTVCISHFSRFSVISSFFK
SSSGCFSFSMIFSFLAIFHVLQWTFLNFPPFSVFLAIFHVLKCVFLIYRDFQFSCYIPSPTVCISHFSRFSVI
LSLFKLSTACFSFSMIFSFLAIFHVLQWKFLYFPTFSVSPYSRSYSVHFSFFTFFSDFVIFQVVKWMFLIFLD
FQFSC
>ENSMUSP00000137273_c.440:G>C_p.L147F ENSMUST00000178348.2|Gm11168-203|ENSMUSG00000096385|+|chr9:g.3004901:G>C
MFLIFNDFHFSCHIPRPIVDISFPPFSVFFAIFHVPKCVFLIFRDFQFPRHIPGPTVCISHFSRFSVISSFFK
SSSGCFSFSMIFSFLAIFHVLQWTFLNFPPFSVFLAIFHVLKCVFLIYRDFQFSCYIPSPTVCISHFSRFSLI
FSLFKLSTACFSFSMIFSFLAIFHVLQWKFLYFPTFSVSPYSRSYSVHFSFFTFFSDFVIFQVVKWMFLIFLD
FQFSC
>ENSMUSP00000137273_c.446:G>T_p.L149F ENSMUST00000178348.2|Gm11168-203|ENSMUSG00000096385|+|chr9:g.3004907:G>T
MFLIFNDFHFSCHIPRPIVDISFPPFSVFFAIFHVPKCVFLIFRDFQFPRHIPGPTVCISHFSRFSVISSFFK
SSSGCFSFSMIFSFLAIFHVLQWTFLNFPPFSVFLAIFHVLKCVFLIYRDFQFSCYIPSPTVCISHFSRFSLI
LSFFKLSTACFSFSMIFSFLAIFHVLQWKFLYFPTFSVSPYSRSYSVHFSFFTFFSDFVIFQVVKWMFLIFLD
FQFSC
>ENSMUSP00000137273_c.454:T>A_p.L152* ENSMUST00000178348.2|Gm11168-203|ENSMUSG00000096385|+|chr9:g.3004915:T>A
MFLIFNDFHFSCHIPRPIVDISFPPFSVFFAIFHVPKCVFLIFRDFQFPRHIPGPTVCISHFSRFSVISSFFK
SSSGCFSFSMIFSFLAIFHVLQWTFLNFPPFSVFLAIFHVLKCVFLIYRDFQFSCYIPSPTVCISHFSRFSLI
LSLFK*STACFSFSMIFSFLAIFHVLQWKFLYFPTFSVSPYSRSYSVHFSFFTFFSDFVIFQVVKWMFLIFLD
FQFSC
>rs247048029_ENSMUSP00000137273_c.523:A>C_p.K175T
ENSMUST00000178348.2|Gm11168-203|ENSMUSG00000096385|+|chr9:g.3004984:A>C
MFLIFNDFHFSCHIPRPIVDISFPPFSVFFAIFHVPKCVFLIFRDFQFPRHIPGPTVCISHFSRFSVISSFFK
SSSGCFSFSMIFSFLAIFHVLQWTFLNFPPFSVFLAIFHVLKCVFLIYRDFQFSCYIPSPTVCISHFSRFSLI

LSLFKLSTACFSFSMIFSFLAIFHVLQWTFLYFPTFSVSPYSRSYSVHFSFFTFFSDFVIFQVVKWMFLIFLD
FQFSC
>ENSMUSP00000137273_c.531:T>A_p.Y178N ENSMUST00000178348.2|Gm11168-203|ENSMUSG00000096385|+|chr9:g.3004992:T>A
MFLIFNDFHFSCHIPRPIVDISFPPFSVFFAIFHVPKCVFLIFRDFQFPRHIPGPTVCISHFSRFSVISSFFK
SSSGCFSFSMIFSFLAIFHVLQWTFLNFPPFSVFLAIFHVLKCVFLIYRDFQFSCYIPSPTVCISHFSRFSLI
LSLFKLSTACFSFSMIFSFLAIFHVLQWKFLNFPTFSVSPYSRSYSVHFSFFTFFSDFVIFQVVKWMFLIFLD
FQFSC
>ENSMUSP00000137273_c.535:T>C_p.F179S ENSMUST00000178348.2|Gm11168-203|ENSMUSG00000096385|+|chr9:g.3004996:T>C
MFLIFNDFHFSCHIPRPIVDISFPPFSVFFAIFHVPKCVFLIFRDFQFPRHIPGPTVCISHFSRFSVISSFFK
SSSGCFSFSMIFSFLAIFHVLQWTFLNFPPFSVFLAIFHVLKCVFLIYRDFQFSCYIPSPTVCISHFSRFSLI
LSLFKLSTACFSFSMIFSFLAIFHVLQWKFLYSPTFSVSPYSRSYSVHFSFFTFFSDFVIFQVVKWMFLIFLD
FQFSC
>ENSMUSP00000137273_c.541:CT>C_p.T181del-fs ENSMUST00000178348.2|Gm11168-203|ENSMUSG00000096385|+|chr9:g.3005002:CT>C
MFLIFNDFHFSCHIPRPIVDISFPPFSVFFAIFHVPKCVFLIFRDFQFPRHIPGPTVCISHFSRFSVISSFFK
SSSGCFSFSMIFSFLAIFHVLQWTFLNFPPFSVFLAIFHVLKCVFLIYRDFQFSCYIPSPTVCISHFSRFSLI
LSLFKLSTACFSFSMIFSFLAIFHVLQWKFLYFPTFQFRHIPGPTVCISHFSRFSVIL
>ENSMUSP00000136598_c.559:G>T_p.S187I ENSMUST00000177722.8|Gm11168-201|ENSMUSG00000096385|+|chr9:g.3005070:G>T
MFLIFHDFQFSCHIPCPTVDISFPPFSVFLAIFHVLKCVFLIFSDFQFSRHIPGPTVCISHFSRFSVILSFFK
LSSGCFSFSLIFSFLAIFHVLQWTFLNFPPFSVFLAIFHVLKCCVFLIFRDFQFPRHIPGPTVCISHFSRFSV
ISSFFKSSSGCFSFSMIFSFLAIFHVLQWTFLNFPPFSSYIFSPYSRSYSVHFSFFTFFSDFVIFQVVKWMFL
IFLDFQFSC
>ENSMUSP00000136761_c.559:G>T_p.S187I ENSMUST00000178077.2|Gm11168-202|ENSMUSG00000096385|+|chr9:g.3005070:G>T
MFLIFLDFQFSCHIPCPTVDISFPPFSVFLAIFHVLKCVFLIFSDFQFSRHIPGPTVCISHFFRFFMISSFFT
LSSGCFSFSIIFTFLAIFHVLQWTFLNFPPFSVFLAIFHILKCCVFLIFRDFQFPRHIPGPTVCISHFSRFSV
ISSFFKSSSGCFSFSMIFSFLAIFHVLQWTFLNFPPFSSYIFSPYSRSYSVHFSFFTFFSDFVIFQVVKWMFL
IFLDFQFSC
>ENSMUSP00000137273_c.549:G>T_p.V184F ENSMUST00000178348.2|Gm11168-203|ENSMUSG00000096385|+|chr9:g.3005070:G>T
MFLIFNDFHFSCHIPRPIVDISFPPFSVFFAIFHVPKCVFLIFRDFQFPRHIPGPTVCISHFSRFSVISSFFK
SSSGCFSFSMIFSFLAIFHVLQWTFLNFPPFSVFLAIFHVLKCVFLIYRDFQFSCYIPSPTVCISHFSRFSLI
LSLFKLSTACFSFSMIFSFLAIFHVLQWKFLYFPTFSFSPYSRSYSVHFSFFTFFSDFVIFQVVKWMFLIFLD
FQFSC
>ENSMUSP00000136598_c.617:T>G_p.S206R ENSMUST00000177722.8|Gm11168-201|ENSMUSG00000096385|+|chr9:g.3005128:T>G
MFLIFHDFQFSCHIPCPTVDISFPPFSVFLAIFHVLKCVFLIFSDFQFSRHIPGPTVCISHFSRFSVILSFFK
LSSGCFSFSLIFSFLAIFHVLQWTFLNFPPFSVFLAIFHVLKCCVFLIFRDFQFPRHIPGPTVCISHFSRFSV
ISSFFKSSSGCFSFSMIFSFLAIFHVLQWTFLNFPPFSSYSFSPYSRSYSVHFSFFTFFRDFVIFQVVKWMFL
IFLDFQFSC
>ENSMUSP00000136761_c.617:T>G_p.S206R ENSMUST00000178077.2|Gm11168-202|ENSMUSG00000096385|+|chr9:g.3005128:T>G
MFLIFLDFQFSCHIPCPTVDISFPPFSVFLAIFHVLKCVFLIFSDFQFSRHIPGPTVCISHFFRFFMISSFFT
LSSGCFSFSIIFTFLAIFHVLQWTFLNFPPFSVFLAIFHILKCCVFLIFRDFQFPRHIPGPTVCISHFSRFSV
ISSFFKSSSGCFSFSMIFSFLAIFHVLQWTFLNFPPFSSYSFSPYSRSYSVHFSFFTFFRDFVIFQVVKWMFL
IFLDFQFSC
>ENSMUSP00000137273_c.605:T>G_p.S202R ENSMUST00000178348.2|Gm11168-203|ENSMUSG00000096385|+|chr9:g.3005128:T>G
MFLIFNDFHFSCHIPRPIVDISFPPFSVFFAIFHVPKCVFLIFRDFQFPRHIPGPTVCISHFSRFSVISSFFK
SSSGCFSFSMIFSFLAIFHVLQWTFLNFPPFSVFLAIFHVLKCVFLIYRDFQFSCYIPSPTVCISHFSRFSLI
LSLFKLSTACFSFSMIFSFLAIFHVLQWKFLYFPTFSVSPYSRSYSVHFSFFTFFRDFVIFQVVKWMFLIFLD
FQFSC
>rs223163006_ENSMUSP00000136598_c.664:T>A_p.L222H
ENSMUST00000177722.8|Gm11168-201|ENSMUSG00000096385|+|chr9:g.3005175:T>A

MFLIFHDFQFSCHIPCPTVDISFPPFSVFLAIFHVLKCVFLIFSDFQFSRHIPGPTVCISHFSRFSVILSFFK
LSSGCFSFSLIFSFLAIFHVLQWTFLNFPPFSVFLAIFHVLKCCVFLIFRDFQFPRHIPGPTVCISHFSRFSV
ISSFFKSSSGCFSFSMIFSFLAIFHVLQWTFLNFPPFSSYSFSPYSRSYSVHFSFFTFFSDFVIFQVVKWMFL
IFHDFQFSC
>rs223163006_ENSMUSP00000136761_c.664:T>A_p.L222H
ENSMUST00000178077.2|Gm11168-202|ENSMUSG00000096385|+|chr9:g.3005175:T>A
MFLIFLDFQFSCHIPCPTVDISFPPFSVFLAIFHVLKCVFLIFSDFQFSRHIPGPTVCISHFFRFFMISSFFT
LSSGCFSFSIIFTFLAIFHVLQWTFLNFPPFSVFLAIFHILKCCVFLIFRDFQFPRHIPGPTVCISHFSRFSV
ISSFFKSSSGCFSFSMIFSFLAIFHVLQWTFLNFPPFSSYSFSPYSRSYSVHFSFFTFFSDFVIFQVVKWMFL
IFHDFQFSC
>rs223163006_ENSMUSP00000137273_c.652:T>A_p.L218H
ENSMUST00000178348.2|Gm11168-203|ENSMUSG00000096385|+|chr9:g.3005175:T>A
MFLIFNDFHFSCHIPRPIVDISFPPFSVFFAIFHVPKCVFLIFRDFQFPRHIPGPTVCISHFSRFSVISSFFK
SSSGCFSFSMIFSFLAIFHVLQWTFLNFPPFSVFLAIFHVLKCVFLIYRDFQFSCYIPSPTVCISHFSRFSLI
LSLFKLSTACFSFSMIFSFLAIFHVLQWKFLYFPTFSVSPYSRSYSVHFSFFTFFSDFVIFQVVKWMFLIFHD
FQFSC
>rs223163006_ENSMUSP00000136541_c.16:T>A_p.L6H
ENSMUST00000179881.2|Gm11168-204|ENSMUSG00000096385|+|chr9:g.3005175:T>A
MFLIFHDFQFSCHIPCPTVDISFPPFSVFLAIFHVLKCVFLIFSDFQFSRNIPGPTVCISHFFRFFMILSFFK
LSTACFSFSMIFSFLAIFHVLLWTFLNFPTFSVFLAIFHVLKCVFLIFSDFLFSRHIPGPTVCISNFSRFSVI
SSFFKLSSGCFSFSMIFSFLAIFHVLQWTFLNFPTFSIFPYFTSYSVHFSFFTFFSDFVIFQVVNCMFLIFHD
FQFSC
>ENSMUSP00000136541_c.45:T>C_p.C16R ENSMUST00000179881.2|Gm11168-
204|ENSMUSG00000096385|+|chr9:g.3005204:T>C
MFLIFLDFQFSCHIPRPTVDISFPPFSVFLAIFHVLKCVFLIFSDFQFSRNIPGPTVCISHFFRFFMILSFFK
LSTACFSFSMIFSFLAIFHVLLWTFLNFPTFSVFLAIFHVLKCVFLIFSDFLFSRHIPGPTVCISNFSRFSVI
SSFFKLSSGCFSFSMIFSFLAIFHVLQWTFLNFPTFSIFPYFTSYSVHFSFFTFFSDFVIFQVVNCMFLIFHD
FQFSC
>ENSMUSP00000136541_c.52:C>T_p.T18I ENSMUST00000179881.2|Gm11168-
204|ENSMUSG00000096385|+|chr9:g.3005211:C>T
MFLIFLDFQFSCHIPCPIVDISFPPFSVFLAIFHVLKCVFLIFSDFQFSRNIPGPTVCISHFFRFFMILSFFK
LSTACFSFSMIFSFLAIFHVLLWTFLNFPTFSVFLAIFHVLKCVFLIFSDFLFSRHIPGPTVCISNFSRFSVI
SSFFKLSSGCFSFSMIFSFLAIFHVLQWTFLNFPTFSIFPYFTSYSVHFSFFTFFSDFVIFQVVNCMFLIFHD
FQFSC
>ENSMUSP00000136541_c.124:T>A_p.I42N ENSMUST00000179881.2|Gm11168-
204|ENSMUSG00000096385|+|chr9:g.3005287:T>A
MFLIFLDFQFSCHIPCPTVDISFPPFSVFLAIFHVLKCVFLNFSDFQFSRNIPGPTVCISHFFRFFMILSFFK
LSTACFSFSMIFSFLAIFHVLLWTFLNFPTFSVFLAIFHVLKCVFLIFSDFLFSRHIPGPTVCISNFSRFSVI
SSFFKLSSGCFSFSMIFSFLAIFHVLQWTFLNFPTFSIFPYFTSYSVHFSFFTFFSDFVIFQVVNCMFLIFHD
FQFSC
>rs253464051_ENSMUSP00000136541_c.129:A>C_p.S44R
ENSMUST00000179881.2|Gm11168-204|ENSMUSG00000096385|+|chr9:g.3005292:A>C
MFLIFLDFQFSCHIPCPTVDISFPPFSVFLAIFHVLKCVFLIFRDFQFSRNIPGPTVCISHFFRFFMILSFFK
LSTACFSFSMIFSFLAIFHVLLWTFLNFPTFSVFLAIFHVLKCVFLIFSDFLFSRHIPGPTVCISNFSRFSVI
SSFFKLSSGCFSFSMIFSFLAIFHVLQWTFLNFPTFSIFPYFTSYSVHFSFFTFFSDFVIFQVVNCMFLIFHD
FQFSC
>ENSMUSP00000136541_c.129:A>T_p.S44C ENSMUST00000179881.2|Gm11168-
204|ENSMUSG00000096385|+|chr9:g.3005292:A>T
MFLIFLDFQFSCHIPCPTVDISFPPFSVFLAIFHVLKCVFLIFCDFQFSRNIPGPTVCISHFFRFFMILSFFK
LSTACFSFSMIFSFLAIFHVLLWTFLNFPTFSVFLAIFHVLKCVFLIFSDFLFSRHIPGPTVCISNFSRFSVI
SSFFKLSSGCFSFSMIFSFLAIFHVLQWTFLNFPTFSIFPYFTSYSVHFSFFTFFSDFVIFQVVNCMFLIFHD
FQFSC
>ENSMUSP00000136541_c.140:GT>A_p.Q47del-fs ENSMUST00000179881.2|Gm11168-
204|ENSMUSG00000096385|+|chr9:g.3005303:GT>A
MFLIFLDFQFSCHIPCPTVDISFPPFSVFLAIFHVLKCVFLIFSDFQFLAIFQVLQCAFLIFSGFS
>ENSMUSP00000136541_c.150:A>C_p.N51H ENSMUST00000179881.2|Gm11168-
204|ENSMUSG00000096385|+|chr9:g.3005313:A>C

MFLIFLDFQFSCHIPCPTVDISFPPFSVFLAIFHVLKCVFLIFSDFQFSRHIPGPTVCISHFFRFFMILSFFK
LSTACFSFSMIFSFLAIFHVLLWTFLNFPTFSVFLAIFHVLKCVFLIFSDFLFSRHIPGPTVCISNFSRFSVI
SSFFKLSSGCFSFSMIFSFLAIFHVLQWTFLNFPTFSIFPYFTSYSVHFSFFTFFSDFVIFQVVNCMFLIFHD
FQFSC
>ENSMUSP00000136541_c.165:A>T_p.T56S ENSMUST00000179881.2|Gm11168-
204|ENSMUSG00000096385|+|chr9:g.3005328:A>T
MFLIFLDFQFSCHIPCPTVDISFPPFSVFLAIFHVLKCVFLIFSDFQFSRNIPGPSVCISHFFRFFMILSFFK
LSTACFSFSMIFSFLAIFHVLLWTFLNFPTFSVFLAIFHVLKCVFLIFSDFLFSRHIPGPTVCISNFSRFSVI
SSFFKLSSGCFSFSMIFSFLAIFHVLQWTFLNFPTFSIFPYFTSYSVHFSFFTFFSDFVIFQVVNCMFLIFHD
FQFSC
>ENSMUSP00000136541_c.181:AT>A_p.H61del-fs ENSMUST00000179881.2|Gm11168-
204|ENSMUSG00000096385|+|chr9:g.3005344:AT>A
MFLIFLDFQFSCHIPCPTVDISFPPFSVFLAIFHVLKCVFLIFSDFQFSRNIPGPTVCISHFSGFS
>ENSMUSP00000136541_c.190:G>C_p.R64T ENSMUST00000179881.2|Gm11168-
204|ENSMUSG00000096385|+|chr9:g.3005353:G>C
MFLIFLDFQFSCHIPCPTVDISFPPFSVFLAIFHVLKCVFLIFSDFQFSRNIPGPTVCISHFFTFFMILSFFK
LSTACFSFSMIFSFLAIFHVLLWTFLNFPTFSVFLAIFHVLKCVFLIFSDFLFSRHIPGPTVCISNFSRFSVI
SSFFKLSSGCFSFSMIFSFLAIFHVLQWTFLNFPTFSIFPYFTSYSVHFSFFTFFSDFVIFQVVNCMFLIFHD
FQFSC
>ENSMUSP00000136541_c.205:T>C_p.L69S ENSMUST00000179881.2|Gm11168-
204|ENSMUSG00000096385|+|chr9:g.3006187:T>C
MFLIFLDFQFSCHIPCPTVDISFPPFSVFLAIFHVLKCVFLIFSDFQFSRNIPGPTVCISHFFRFFMISSFFK
LSTACFSFSMIFSFLAIFHVLLWTFLNFPTFSVFLAIFHVLKCVFLIFSDFLFSRHIPGPTVCISNFSRFSVI
SSFFKLSSGCFSFSMIFSFLAIFHVLQWTFLNFPTFSIFPYFTSYSVHFSFFTFFSDFVIFQVVNCMFLIFHD
FQFSC
>ENSMUSP00000136541_c.220:T>A_p.L74* ENSMUST00000179881.2|Gm11168-
204|ENSMUSG00000096385|+|chr9:g.3006202:T>A
MFLIFLDFQFSCHIPCPTVDISFPPFSVFLAIFHVLKCVFLIFSDFQFSRNIPGPTVCISHFFRFFMILSFFK
*STACFSFSMIFSFLAIFHVLLWTFLNFPTFSVFLAIFHVLKCVFLIFSDFLFSRHIPGPTVCISNFSRFSVI
SSFFKLSSGCFSFSMIFSFLAIFHVLQWTFLNFPTFSIFPYFTSYSVHFSFFTFFSDFVIFQVVNCMFLIFHD
FQFSC
>ENSMUSP00000136541_c.220:T>G_p.L74W ENSMUST00000179881.2|Gm11168-
204|ENSMUSG00000096385|+|chr9:g.3006202:T>G
MFLIFLDFQFSCHIPCPTVDISFPPFSVFLAIFHVLKCVFLIFSDFQFSRNIPGPTVCISHFFRFFMILSFFK
WSTACFSFSMIFSFLAIFHVLLWTFLNFPTFSVFLAIFHVLKCVFLIFSDFLFSRHIPGPTVCISNFSRFSVI
SSFFKLSSGCFSFSMIFSFLAIFHVLQWTFLNFPTFSIFPYFTSYSVHFSFFTFFSDFVIFQVVNCMFLIFHD
FQFSC
>ENSMUSP00000136541_c.226:CTGC>ATGG_p.T76N ENSMUST00000179881.2|Gm11168-
204|ENSMUSG00000096385|+|chr9:g.3006208:CTGC>ATGG
MFLIFLDFQFSCHIPCPTVDISFPPFSVFLAIFHVLKCVFLIFSDFQFSRNIPGPTVCISHFFRFFMILSFFK
LSNGACFSFSMIFSFLAIFHVLLWTFLNFPTFSVFLAIFHVLKCVFLIFSDFLFSRHIPGPTVCISNFSRFSV
ISSFFKLSSGCFSFSMIFSFLAIFHVLQWTFLNFPTFSIFPYFTSYSVHFSFFTFFSDFVIFQVVNCMFLIFH
DFQFSC
>ENSMUSP00000136541_c.226:CTGC>GTGG_p.T76S ENSMUST00000179881.2|Gm11168-
204|ENSMUSG00000096385|+|chr9:g.3006208:CTGC>GTGG
MFLIFLDFQFSCHIPCPTVDISFPPFSVFLAIFHVLKCVFLIFSDFQFSRNIPGPTVCISHFFRFFMILSFFK
LSSGACFSFSMIFSFLAIFHVLLWTFLNFPTFSVFLAIFHVLKCVFLIFSDFLFSRHIPGPTVCISNFSRFSV
ISSFFKLSSGCFSFSMIFSFLAIFHVLQWTFLNFPTFSIFPYFTSYSVHFSFFTFFSDFVIFQVVNCMFLIFH
DFQFSC
>ENSMUSP00000136541_c.226:CTGCATG>GTGGATT_p.T76S
ENSMUST00000179881.2|Gm11168-
204|ENSMUSG00000096385|+|chr9:g.3006208:CTGCATG>GTGGATT
MFLIFLDFQFSCHIPCPTVDISFPPFSVFLAIFHVLKCVFLIFSDFQFSRNIPGPTVCISHFFRFFMILSFFK
LSSGFACFSFSMIFSFLAIFHVLLWTFLNFPTFSVFLAIFHVLKCVFLIFSDFLFSRHIPGPTVCISNFSRFS
VISSFFKLSSGCFSFSMIFSFLAIFHVLQWTFLNFPTFSIFPYFTSYSVHFSFFTFFSDFVIFQVVNCMFLIF
HDFQFSC
>ENSMUSP00000136541_c.246:A>G_p.M83V ENSMUST00000179881.2|Gm11168-
204|ENSMUSG00000096385|+|chr9:g.3006228:A>G

MFLIFLDFQFSCHIPCPTVDISFPPFSVFLAIFHVLKCVFLIFSDFQFSRNIPGPTVCISHFFRFFMILSFFK
LSTACFSFSVIFSFLAIFHVLLWTFLNFPTFSVFLAIFHVLKCVFLIFSDFLFSRHIPGPTVCISNFSRFSVI
SSFFKLSSGCFSFSMIFSFLAIFHVLQWTFLNFPTFSIFPYFTSYSVHFSFFTFFSDFVIFQVVNCMFLIFHD
FQFSC
>ENSMUSP00000136541_c.283:T>C_p.L95P ENSMUST00000179881.2|Gm11168-204|ENSMUSG00000096385|+|chr9:g.3006265:T>C
MFLIFLDFQFSCHIPCPTVDISFPPFSVFLAIFHVLKCVFLIFSDFQFSRNIPGPTVCISHFFRFFMILSFFK
LSTACFSFSMIFSFLAIFHVLPWTFLNFPTFSVFLAIFHVLKCVFLIFSDFLFSRHIPGPTVCISNFSRFSVI
SSFFKLSSGCFSFSMIFSFLAIFHVLQWTFLNFPTFSIFPYFTSYSVHFSFFTFFSDFVIFQVVNCMFLIFHD
FQFSC
>ENSMUSP00000136541_c.283:T>A_p.L95Q ENSMUST00000179881.2|Gm11168-204|ENSMUSG00000096385|+|chr9:g.3006265:T>A
MFLIFLDFQFSCHIPCPTVDISFPPFSVFLAIFHVLKCVFLIFSDFQFSRNIPGPTVCISHFFRFFMILSFFK
LSTACFSFSMIFSFLAIFHVLQWTFLNFPTFSVFLAIFHVLKCVFLIFSDFLFSRHIPGPTVCISNFSRFSVI
SSFFKLSSGCFSFSMIFSFLAIFHVLQWTFLNFPTFSIFPYFTSYSVHFSFFTFFSDFVIFQVVNCMFLIFHD
FQFSC
>ENSMUSP00000136541_c.301:T>A_p.F101Y ENSMUST00000179881.2|Gm11168-204|ENSMUSG00000096385|+|chr9:g.3006283:T>A
MFLIFLDFQFSCHIPCPTVDISFPPFSVFLAIFHVLKCVFLIFSDFQFSRNIPGPTVCISHFFRFFMILSFFK
LSTACFSFSMIFSFLAIFHVLLWTFLNYPTFSVFLAIFHVLKCVFLIFSDFLFSRHIPGPTVCISNFSRFSVI
SSFFKLSSGCFSFSMIFSFLAIFHVLQWTFLNFPTFSIFPYFTSYSVHFSFFTFFSDFVIFQVVNCMFLIFHD
FQFSC
>ENSMUSP00000136541_c.306:A>C_p.T103P ENSMUST00000179881.2|Gm11168-204|ENSMUSG00000096385|+|chr9:g.3006288:A>C
MFLIFLDFQFSCHIPCPTVDISFPPFSVFLAIFHVLKCVFLIFSDFQFSRNIPGPTVCISHFFRFFMILSFFK
LSTACFSFSMIFSFLAIFHVLLWTFLNFPPFSVFLAIFHVLKCVFLIFSDFLFSRHIPGPTVCISNFSRFSVI
SSFFKLSSGCFSFSMIFSFLAIFHVLQWTFLNFPTFSIFPYFTSYSVHFSFFTFFSDFVIFQVVNCMFLIFHD
FQFSC
>ENSMUSP00000136541_c.324:G>T_p.A109S ENSMUST00000179881.2|Gm11168-204|ENSMUSG00000096385|+|chr9:g.3006306:G>T
MFLIFLDFQFSCHIPCPTVDISFPPFSVFLAIFHVLKCVFLIFSDFQFSRNIPGPTVCISHFFRFFMILSFFK
LSTACFSFSMIFSFLAIFHVLLWTFLNFPTFSVFLSIFHVLKCVFLIFSDFLFSRHIPGPTVCISNFSRFSVI
SSFFKLSSGCFSFSMIFSFLAIFHVLQWTFLNFPTFSIFPYFTSYSVHFSFFTFFSDFVIFQVVNCMFLIFHD
FQFSC
>ENSMUSP00000136541_c.335:T>A_p.H112Q ENSMUST00000179881.2|Gm11168-204|ENSMUSG00000096385|+|chr9:g.3006317:T>A
MFLIFLDFQFSCHIPCPTVDISFPPFSVFLAIFHVLKCVFLIFSDFQFSRNIPGPTVCISHFFRFFMILSFFK
LSTACFSFSMIFSFLAIFHVLLWTFLNFPTFSVFLAIFQVLKCVFLIFSDFLFSRHIPGPTVCISNFSRFSVI
SSFFKLSSGCFSFSMIFSFLAIFHVLQWTFLNFPTFSIFPYFTSYSVHFSFFTFFSDFVIFQVVNCMFLIFHD
FQFSC
>ENSMUSP00000136541_c.343:A>T_p.K115M ENSMUST00000179881.2|Gm11168-204|ENSMUSG00000096385|+|chr9:g.3006325:A>T
MFLIFLDFQFSCHIPCPTVDISFPPFSVFLAIFHVLKCVFLIFSDFQFSRNIPGPTVCISHFFRFFMILSFFK
LSTACFSFSMIFSFLAIFHVLLWTFLNFPTFSVFLAIFHVLMCVFLIFSDFLFSRHIPGPTVCISNFSRFSVI
SSFFKLSSGCFSFSMIFSFLAIFHVLQWTFLNFPTFSIFPYFTSYSVHFSFFTFFSDFVIFQVVNCMFLIFHD
FQFSC
>ENSMUSP00000136541_c.346:G>A_p.C116Y ENSMUST00000179881.2|Gm11168-204|ENSMUSG00000096385|+|chr9:g.3006328:G>A
MFLIFLDFQFSCHIPCPTVDISFPPFSVFLAIFHVLKCVFLIFSDFQFSRNIPGPTVCISHFFRFFMILSFFK
LSTACFSFSMIFSFLAIFHVLLWTFLNFPTFSVFLAIFHVLKYVFLIFSDFLFSRHIPGPTVCISNFSRFSVI
SSFFKLSSGCFSFSMIFSFLAIFHVLQWTFLNFPTFSIFPYFTSYSVHFSFFTFFSDFVIFQVVNCMFLIFHD
FQFSC
>ENSMUSP00000136541_c.348:G>A_p.V117I ENSMUST00000179881.2|Gm11168-204|ENSMUSG00000096385|+|chr9:g.3006330:G>A
MFLIFLDFQFSCHIPCPTVDISFPPFSVFLAIFHVLKCVFLIFSDFQFSRNIPGPTVCISHFFRFFMILSFFK
LSTACFSFSMIFSFLAIFHVLLWTFLNFPTFSVFLAIFHVLKCIFLIFSDFLFSRHIPGPTVCISNFSRFSVI
SSFFKLSSGCFSFSMIFSFLAIFHVLQWTFLNFPTFSIFPYFTSYSVHFSFFTFFSDFVIFQVVNCMFLIFHD
FQFSC

>ENSMUSP00000136541_c.363:A>T_p.S122C ENSMUST00000179881.2|Gm11168-204|ENSMUSG00000096385|+|chr9:g.3006345:A>T
MFLIFLDFQFSCHIPCPTVDISFPPFSVFLAIFHVLKCVFLIFSDFQFSRNIPGPTVCISHFFRFFMILSFFK
LSTACFSFSMIFSFLAIFHVLLWTFLNFPTFSVFLAIFHVLKCVFLIFCDFLFSRHIPGPTVCISNFSRFSVI
SSFFKLSSGCFSFSMIFSFLAIFHVLQWTFLNFPTFSIFPYFTSYSVHFSFFTFFSDFVIFQVVNCMFLIFHD
FQFSC
>ENSMUSP00000136541_c.363:A>C_p.S122R ENSMUST00000179881.2|Gm11168-204|ENSMUSG00000096385|+|chr9:g.3006345:A>C
MFLIFLDFQFSCHIPCPTVDISFPPFSVFLAIFHVLKCVFLIFSDFQFSRNIPGPTVCISHFFRFFMILSFFK
LSTACFSFSMIFSFLAIFHVLLWTFLNFPTFSVFLAIFHVLKCVFLIFRDFLFSRHIPGPTVCISNFSRFSVI
SSFFKLSSGCFSFSMIFSFLAIFHVLQWTFLNFPTFSIFPYFTSYSVHFSFFTFFSDFVIFQVVNCMFLIFHD
FQFSC
>ENSMUSP00000136541_c.373:T>A_p.L125Q ENSMUST00000179881.2|Gm11168-204|ENSMUSG00000096385|+|chr9:g.3006355:T>A
MFLIFLDFQFSCHIPCPTVDISFPPFSVFLAIFHVLKCVFLIFSDFQFSRNIPGPTVCISHFFRFFMILSFFK
LSTACFSFSMIFSFLAIFHVLLWTFLNFPTFSVFLAIFHVLKCVFLIFSDFQFSRHIPGPTVCISNFSRFSVI
SSFFKLSSGCFSFSMIFSFLAIFHVLQWTFLNFPTFSIFPYFTSYSVHFSFFTFFSDFVIFQVVNCMFLIFHD
FQFSC
>ENSMUSP00000136541_c.399:A>T_p.T134S ENSMUST00000179881.2|Gm11168-204|ENSMUSG00000096385|+|chr9:g.3006381:A>T
MFLIFLDFQFSCHIPCPTVDISFPPFSVFLAIFHVLKCVFLIFSDFQFSRNIPGPTVCISHFFRFFMILSFFK
LSTACFSFSMIFSFLAIFHVLLWTFLNFPTFSVFLAIFHVLKCVFLIFSDFLFSRHIPGPSVCISNFSRFSVI
SSFFKLSSGCFSFSMIFSFLAIFHVLQWTFLNFPTFSIFPYFTSYSVHFSFFTFFSDFVIFQVVNCMFLIFHD
FQFSC
>ENSMUSP00000136541_c.414:A>T_p.N139Y ENSMUST00000179881.2|Gm11168-204|ENSMUSG00000096385|+|chr9:g.3006396:A>T
MFLIFLDFQFSCHIPCPTVDISFPPFSVFLAIFHVLKCVFLIFSDFQFSRNIPGPTVCISHFFRFFMILSFFK
LSTACFSFSMIFSFLAIFHVLLWTFLNFPTFSVFLAIFHVLKCVFLIFSDFLFSRHIPGPTVCISYFSRFSVI
SSFFKLSSGCFSFSMIFSFLAIFHVLQWTFLNFPTFSIFPYFTSYSVHFSFFTFFSDFVIFQVVNCMFLIFHD
FQFSC
>ENSMUSP00000136541_c.414:A>C_p.N139H ENSMUST00000179881.2|Gm11168-204|ENSMUSG00000096385|+|chr9:g.3006396:A>C
MFLIFLDFQFSCHIPCPTVDISFPPFSVFLAIFHVLKCVFLIFSDFQFSRNIPGPTVCISHFFRFFMILSFFK
LSTACFSFSMIFSFLAIFHVLLWTFLNFPTFSVFLAIFHVLKCVFLIFSDFLFSRHIPGPTVCISHFSRFSVI
SSFFKLSSGCFSFSMIFSFLAIFHVLQWTFLNFPTFSIFPYFTSYSVHFSFFTFFSDFVIFQVVNCMFLIFHD
FQFSC
>ENSMUSP00000136541_c.416:T>A_p.N139K ENSMUST00000179881.2|Gm11168-204|ENSMUSG00000096385|+|chr9:g.3006398:T>A
MFLIFLDFQFSCHIPCPTVDISFPPFSVFLAIFHVLKCVFLIFSDFQFSRNIPGPTVCISHFFRFFMILSFFK
LSTACFSFSMIFSFLAIFHVLLWTFLNFPTFSVFLAIFHVLKCVFLIFSDFLFSRHIPGPTVCISKFSRFSVI
SSFFKLSSGCFSFSMIFSFLAIFHVLQWTFLNFPTFSIFPYFTSYSVHFSFFTFFSDFVIFQVVNCMFLIFHD
FQFSC
>ENSMUSP00000136541_c.429:T>G_p.S144A ENSMUST00000179881.2|Gm11168-204|ENSMUSG00000096385|+|chr9:g.3006411:T>G
MFLIFLDFQFSCHIPCPTVDISFPPFSVFLAIFHVLKCVFLIFSDFQFSRNIPGPTVCISHFFRFFMILSFFK
LSTACFSFSMIFSFLAIFHVLLWTFLNFPTFSVFLAIFHVLKCVFLIFSDFLFSRHIPGPTVCISNFSRFAVI
SSFFKLSSGCFSFSMIFSFLAIFHVLQWTFLNFPTFSIFPYFTSYSVHFSFFTFFSDFVIFQVVNCMFLIFHD
FQFSC
>ENSMUSP00000136541_c.430:C>T_p.S144L ENSMUST00000179881.2|Gm11168-204|ENSMUSG00000096385|+|chr9:g.3006412:C>T
MFLIFLDFQFSCHIPCPTVDISFPPFSVFLAIFHVLKCVFLIFSDFQFSRNIPGPTVCISHFFRFFMILSFFK
LSTACFSFSMIFSFLAIFHVLLWTFLNFPTFSVFLAIFHVLKCVFLIFSDFLFSRHIPGPTVCISNFSRFLVI
SSFFKLSSGCFSFSMIFSFLAIFHVLQWTFLNFPTFSIFPYFTSYSVHFSFFTFFSDFVIFQVVNCMFLIFHD
FQFSC
>ENSMUSP00000136541_c.439:C>T_p.S147L ENSMUST00000179881.2|Gm11168-204|ENSMUSG00000096385|+|chr9:g.3006421:C>T
MFLIFLDFQFSCHIPCPTVDISFPPFSVFLAIFHVLKCVFLIFSDFQFSRNIPGPTVCISHFFRFFMILSFFK
LSTACFSFSMIFSFLAIFHVLLWTFLNFPTFSVFLAIFHVLKCVFLIFSDFLFSRHIPGPTVCISNFSRFSVI

LSFFKLSSGCFSFSMIFSFLAIFHVLQWTFLNFPTFSIFPYFTSYSVHFSFFTFFSDFVIFQVVNCMFLIFHD
FQFSC
>ENSMUSP00000136541_c.454:T>C_p.L152S ENSMUST00000179881.2|Gm11168-204|ENSMUSG00000096385|+|chr9:g.3006436:T>C
MFLIFLDFQFSCHIPCPTVDISFPPFSVFLAIFHVLKCVFLIFSDFQFSRNIPGPTVCISHFFRFFMILSFFK
LSTACFSFSMIFSFLAIFHVLLWTFLNFPTFSVFLAIFHVLKCVFLIFSDFLFSRHIPGPTVCISNFSRFSVI
SSFFKSSSGCFSFSMIFSFLAIFHVLQWTFLNFPTFSIFPYFTSYSVHFSFFTFFSDFVIFQVVNCMFLIFHD
FQFSC
>ENSMUSP00000136541_c.454:T>G_p.L152W ENSMUST00000179881.2|Gm11168-204|ENSMUSG00000096385|+|chr9:g.3006436:T>G
MFLIFLDFQFSCHIPCPTVDISFPPFSVFLAIFHVLKCVFLIFSDFQFSRNIPGPTVCISHFFRFFMILSFFK
LSTACFSFSMIFSFLAIFHVLLWTFLNFPTFSVFLAIFHVLKCVFLIFSDFLFSRHIPGPTVCISNFSRFSVI
SSFFKWSSGCFSFSMIFSFLAIFHVLQWTFLNFPTFSIFPYFTSYSVHFSFFTFFSDFVIFQVVNCMFLIFHD
FQFSC
>ENSMUSP00000136541_c.516:C>T_p.Q173* ENSMUST00000179881.2|Gm11168-204|ENSMUSG00000096385|+|chr9:g.3006498:C>T
MFLIFLDFQFSCHIPCPTVDISFPPFSVFLAIFHVLKCVFLIFSDFQFSRNIPGPTVCISHFFRFFMILSFFK
LSTACFSFSMIFSFLAIFHVLLWTFLNFPTFSVFLAIFHVLKCVFLIFSDFLFSRHIPGPTVCISNFSRFSVI
SSFFKLSSGCFSFSMIFSFLAIFHVL*WTFLNFPTFSIFPYFTSYSVHFSFFTFFSDFVIFQVVNCMFLIFHD
FQFSC
>ENSMUSP00000136541_c.535:T>A_p.F179Y ENSMUST00000179881.2|Gm11168-204|ENSMUSG00000096385|+|chr9:g.3006517:T>A
MFLIFLDFQFSCHIPCPTVDISFPPFSVFLAIFHVLKCVFLIFSDFQFSRNIPGPTVCISHFFRFFMILSFFK
LSTACFSFSMIFSFLAIFHVLLWTFLNFPTFSVFLAIFHVLKCVFLIFSDFLFSRHIPGPTVCISNFSRFSVI
SSFFKLSSGCFSFSMIFSFLAIFHVLQWTFLNYPTFSIFPYFTSYSVHFSFFTFFSDFVIFQVVNCMFLIFHD
FQFSC
>ENSMUSP00000136541_c.540:A>C_p.T181P ENSMUST00000179881.2|Gm11168-204|ENSMUSG00000096385|+|chr9:g.3006522:A>C
MFLIFLDFQFSCHIPCPTVDISFPPFSVFLAIFHVLKCVFLIFSDFQFSRNIPGPTVCISHFFRFFMILSFFK
LSTACFSFSMIFSFLAIFHVLLWTFLNFPTFSVFLAIFHVLKCVFLIFSDFLFSRHIPGPTVCISNFSRFSVI
SSFFKLSSGCFSFSMIFSFLAIFHVLQWTFLNFPPFSIFPYFTSYSVHFSFFTFFSDFVIFQVVNCMFLIFHD
FQFSC
>ENSMUSP00000136541_c.571:A>T_p.Y191F ENSMUST00000179881.2|Gm11168-204|ENSMUSG00000096385|+|chr9:g.3006848:A>T
MFLIFLDFQFSCHIPCPTVDISFPPFSVFLAIFHVLKCVFLIFSDFQFSRNIPGPTVCISHFFRFFMILSFFK
LSTACFSFSMIFSFLAIFHVLLWTFLNFPTFSVFLAIFHVLKCVFLIFSDFLFSRHIPGPTVCISNFSRFSVI
SSFFKLSSGCFSFSMIFSFLAIFHVLQWTFLNFPTFSIFPYFTSFSVHFSFFTFFSDFVIFQVVNCMFLIFHD
FQFSC
>ENSMUSP00000136541_c.601:T>G_p.F201C ENSMUST00000179881.2|Gm11168-204|ENSMUSG00000096385|+|chr9:g.3006878:T>G
MFLIFLDFQFSCHIPCPTVDISFPPFSVFLAIFHVLKCVFLIFSDFQFSRNIPGPTVCISHFFRFFMILSFFK
LSTACFSFSMIFSFLAIFHVLLWTFLNFPTFSVFLAIFHVLKCVFLIFSDFLFSRHIPGPTVCISNFSRFSVI
SSFFKLSSGCFSFSMIFSFLAIFHVLQWTFLNFPTFSIFPYFTSYSVHFSFFTFCSDFVIFQVVNCMFLIFHD
FQFSC
>ENSMUSP00000136541_c.632:C>A_p.N211K ENSMUST00000179881.2|Gm11168-204|ENSMUSG00000096385|+|chr9:g.3006909:C>A
MFLIFLDFQFSCHIPCPTVDISFPPFSVFLAIFHVLKCVFLIFSDFQFSRNIPGPTVCISHFFRFFMILSFFK
LSTACFSFSMIFSFLAIFHVLLWTFLNFPTFSVFLAIFHVLKCVFLIFSDFLFSRHIPGPTVCISNFSRFSVI
SSFFKLSSGCFSFSMIFSFLAIFHVLQWTFLNFPTFSIFPYFTSYSVHFSFFTFFSDFVIFQVVKCMFLIFHD
FQFSC
>ENSMUSP00000136541_c.632:C>G_p.N211K ENSMUST00000179881.2|Gm11168-204|ENSMUSG00000096385|+|chr9:g.3006909:C>G
MFLIFLDFQFSCHIPCPTVDISFPPFSVFLAIFHVLKCVFLIFSDFQFSRNIPGPTVCISHFFRFFMILSFFK
LSTACFSFSMIFSFLAIFHVLLWTFLNFPTFSVFLAIFHVLKCVFLIFSDFLFSRHIPGPTVCISNFSRFSVI
SSFFKLSSGCFSFSMIFSFLAIFHVLQWTFLNFPTFSIFPYFTSYSVHFSFFTFFSDFVIFQVVKCMFLIFHD
FQFSC
>ENSMUSP00000136541_c.632:CTGC>GTGG_p.N211K ENSMUST00000179881.2|Gm11168-204|ENSMUSG00000096385|+|chr9:g.3006909:CTGC>GTGG

MFLIFLDFQFSCHIPCPTVDISFPPFSVFLAIFHVLKCVFLIFSDFQFSRNIPGPTVCISHFFRFFMILSFFK
LSTACFSFSMIFSFLAIFHVLLWTFLNFPTFSVFLAIFHVLKCVFLIFSDFLFSRHIPGPTVCISNFSRFSVI
SSFFKLSSGCFSFSMIFSFLAIFHVLQWTFLNFPTFSIFPYFTSYSVHFSFFTFFSDFVIFQVVKWCMFLIFH
DFQFSC
>ENSMUSP00000136541_c.635:C>G_p.C212W ENSMUST00000179881.2|Gm11168-
204|ENSMUSG00000096385|+|chr9:g.3006912:C>G
MFLIFLDFQFSCHIPCPTVDISFPPFSVFLAIFHVLKCVFLIFSDFQFSRNIPGPTVCISHFFRFFMILSFFK
LSTACFSFSMIFSFLAIFHVLLWTFLNFPTFSVFLAIFHVLKCVFLIFSDFLFSRHIPGPTVCISNFSRFSVI
SSFFKLSSGCFSFSMIFSFLAIFHVLQWTFLNFPTFSIFPYFTSYSVHFSFFTFFSDFVIFQVVNWMFLIFHD
FQFSC
>ENSMUSP00000136541_c.636:A>C_p.M213L ENSMUST00000179881.2|Gm11168-
204|ENSMUSG00000096385|+|chr9:g.3006913:A>C
MFLIFLDFQFSCHIPCPTVDISFPPFSVFLAIFHVLKCVFLIFSDFQFSRNIPGPTVCISHFFRFFMILSFFK
LSTACFSFSMIFSFLAIFHVLLWTFLNFPTFSVFLAIFHVLKCVFLIFSDFLFSRHIPGPTVCISNFSRFSVI
SSFFKLSSGCFSFSMIFSFLAIFHVLQWTFLNFPTFSIFPYFTSYSVHFSFFTFFSDFVIFQVVNCLFLIFHD
FQFSC
>ENSMUSP00000136541_c.639:T>A_p.F214I ENSMUST00000179881.2|Gm11168-
204|ENSMUSG00000096385|+|chr9:g.3006916:T>A
MFLIFLDFQFSCHIPCPTVDISFPPFSVFLAIFHVLKCVFLIFSDFQFSRNIPGPTVCISHFFRFFMILSFFK
LSTACFSFSMIFSFLAIFHVLLWTFLNFPTFSVFLAIFHVLKCVFLIFSDFLFSRHIPGPTVCISNFSRFSVI
SSFFKLSSGCFSFSMIFSFLAIFHVLQWTFLNFPTFSIFPYFTSYSVHFSFFTFFSDFVIFQVVNCMILIFHD
FQFSC
>ENSMUSP00000136541_c.660:C>A_p.Q221K ENSMUST00000179881.2|Gm11168-
204|ENSMUSG00000096385|+|chr9:g.3006937:C>A
MFLIFLDFQFSCHIPCPTVDISFPPFSVFLAIFHVLKCVFLIFSDFQFSRNIPGPTVCISHFFRFFMILSFFK
LSTACFSFSMIFSFLAIFHVLLWTFLNFPTFSVFLAIFHVLKCVFLIFSDFLFSRHIPGPTVCISNFSRFSVI
SSFFKLSSGCFSFSMIFSFLAIFHVLQWTFLNFPTFSIFPYFTSYSVHFSFFTFFSDFVIFQVVNCMFLIFHD
FKFSC
>ENSMUSP00000137330_c.14:C>A_p.F5L ENSMUST00000143083.3|Gm10721-
201|ENSMUSG00000096519|+|chr9:g.3013154:C>A
LFLILHDFQFSCHIPCPTLDISFPPFSVFLAIFHVLKCVFLIFRDFQFSCHIPGPTVCISHFSRFSVILSFFK
LSTACFSFSMIFSFLAIFHVLQWKFLNFPPFSVFLAIFHVLKCVFLIFRDFQFSRHIPGPTVCISHFSGFSVI
SSFFKSSSGCFSFSMIFSFLAIFHVLQWTFLNFPPFFSPYSRSYSVHFSFFTFFSDFVIFQVVKWMFLIFHDF
QFSC
>ENSMUSP00000137330_c.15:C>T_p.H6Y ENSMUST00000143083.3|Gm10721-
201|ENSMUSG00000096519|+|chr9:g.3013155:C>T
LFLIFYDFQFSCHIPCPTLDISFPPFSVFLAIFHVLKCVFLIFRDFQFSCHIPGPTVCISHFSRFSVILSFFK
LSTACFSFSMIFSFLAIFHVLQWKFLNFPPFSVFLAIFHVLKCVFLIFRDFQFSRHIPGPTVCISHFSGFSVI
SSFFKSSSGCFSFSMIFSFLAIFHVLQWTFLNFPPFFSPYSRSYSVHFSFFTFFSDFVIFQVVKWMFLIFHDF
QFSC
>ENSMUSP00000137330_c.26:G>T_p.Q9H ENSMUST00000143083.3|Gm10721-
201|ENSMUSG00000096519|+|chr9:g.3013166:G>T
LFLIFHDFHFSCHIPCPTLDISFPPFSVFLAIFHVLKCVFLIFRDFQFSCHIPGPTVCISHFSRFSVILSFFK
LSTACFSFSMIFSFLAIFHVLQWKFLNFPPFSVFLAIFHVLKCVFLIFRDFQFSRHIPGPTVCISHFSGFSVI
SSFFKSSSGCFSFSMIFSFLAIFHVLQWTFLNFPPFFSPYSRSYSVHFSFFTFFSDFVIFQVVKWMFLIFHDF
QFSC
>ENSMUSP00000137330_c.33:T>C_p.C12R ENSMUST00000143083.3|Gm10721-
201|ENSMUSG00000096519|+|chr9:g.3013173:T>C
LFLIFHDFQFSRHIPCPTLDISFPPFSVFLAIFHVLKCVFLIFRDFQFSCHIPGPTVCISHFSRFSVILSFFK
LSTACFSFSMIFSFLAIFHVLQWKFLNFPPFSVFLAIFHVLKCVFLIFRDFQFSRHIPGPTVCISHFSGFSVI
SSFFKSSSGCFSFSMIFSFLAIFHVLQWTFLNFPPFFSPYSRSYSVHFSFFTFFSDFVIFQVVKWMFLIFHDF
QFSC
>ENSMUSP00000137330_c.36:C>A_p.H13N ENSMUST00000143083.3|Gm10721-
201|ENSMUSG00000096519|+|chr9:g.3013176:C>A
LFLIFHDFQFSCNIPCPTLDISFPPFSVFLAIFHVLKCVFLIFRDFQFSCHIPGPTVCISHFSRFSVILSFFK
LSTACFSFSMIFSFLAIFHVLQWKFLNFPPFSVFLAIFHVLKCVFLIFRDFQFSRHIPGPTVCISHFSGFSVI
SSFFKSSSGCFSFSMIFSFLAIFHVLQWTFLNFPPFFSPYSRSYSVHFSFFTFFSDFVIFQVVKWMFLIFHDF
QFSC

>ENSMUSP00000137330_c.45:T>A_p.C16S ENSMUST00000143083.3|Gm10721-201|ENSMUSG00000096519|+|chr9:g.3013185:T>A
LFLIFHDFQFSCHIPSPTLDISFPPFSVFLAIFHVLKCVFLIFRDFQFSCHIPGPTVCISHFSRFSVILSFFK
LSTACFSFSMIFSFLAIFHVLQWKFLNFPPFSVFLAIFHVLKCVFLIFRDFQFSRHIPGPTVCISHFSGFSVI
SSFFKSSSGCFSFSMIFSFLAIFHVLQWTFLNFPPFFSPYSRSYSVHFSFFTFFSDFVIFQVVKWMFLIFHDF
QFSC
>ENSMUSP00000137330_c.45:T>C_p.C16R ENSMUST00000143083.3|Gm10721-201|ENSMUSG00000096519|+|chr9:g.3013185:T>C
LFLIFHDFQFSCHIPRPTLDISFPPFSVFLAIFHVLKCVFLIFRDFQFSCHIPGPTVCISHFSRFSVILSFFK
LSTACFSFSMIFSFLAIFHVLQWKFLNFPPFSVFLAIFHVLKCVFLIFRDFQFSRHIPGPTVCISHFSGFSVI
SSFFKSSSGCFSFSMIFSFLAIFHVLQWTFLNFPPFFSPYSRSYSVHFSFFTFFSDFVIFQVVKWMFLIFHDF
QFSC
>ENSMUSP00000137330_c.45:T>G_p.C16G ENSMUST00000143083.3|Gm10721-201|ENSMUSG00000096519|+|chr9:g.3013185:T>G
LFLIFHDFQFSCHIPGPTLDISFPPFSVFLAIFHVLKCVFLIFRDFQFSCHIPGPTVCISHFSRFSVILSFFK
LSTACFSFSMIFSFLAIFHVLQWKFLNFPPFSVFLAIFHVLKCVFLIFRDFQFSRHIPGPTVCISHFSGFSVI
SSFFKSSSGCFSFSMIFSFLAIFHVLQWTFLNFPPFFSPYSRSYSVHFSFFTFFSDFVIFQVVKWMFLIFHDF
QFSC
>ENSMUSP00000137330_c.54:C>G_p.L19V ENSMUST00000143083.3|Gm10721-201|ENSMUSG00000096519|+|chr9:g.3013194:C>G
LFLIFHDFQFSCHIPCPTVDISFPPFSVFLAIFHVLKCVFLIFRDFQFSCHIPGPTVCISHFSRFSVILSFFK
LSTACFSFSMIFSFLAIFHVLQWKFLNFPPFSVFLAIFHVLKCVFLIFRDFQFSRHIPGPTVCISHFSGFSVI
SSFFKSSSGCFSFSMIFSFLAIFHVLQWTFLNFPPFFSPYSRSYSVHFSFFTFFSDFVIFQVVKWMFLIFHDF
QFSC
>ENSMUSP00000137330_c.129:C>T_p.R44C ENSMUST00000143083.3|Gm10721-201|ENSMUSG00000096519|+|chr9:g.3013273:C>T
LFLIFHDFQFSCHIPCPTLDISFPPFSVFLAIFHVLKCVFLIFCDFQFSCHIPGPTVCISHFSRFSVILSFFK
LSTACFSFSMIFSFLAIFHVLQWKFLNFPPFSVFLAIFHVLKCVFLIFRDFQFSRHIPGPTVCISHFSGFSVI
SSFFKSSSGCFSFSMIFSFLAIFHVLQWTFLNFPPFFSPYSRSYSVHFSFFTFFSDFVIFQVVKWMFLIFHDF
QFSC
>ENSMUSP00000137330_c.147:T>C_p.C50R ENSMUST00000143083.3|Gm10721-201|ENSMUSG00000096519|+|chr9:g.3013291:T>C
LFLIFHDFQFSCHIPCPTLDISFPPFSVFLAIFHVLKCVFLIFRDFQFSRHIPGPTVCISHFSRFSVILSFFK
LSTACFSFSMIFSFLAIFHVLQWKFLNFPPFSVFLAIFHVLKCVFLIFRDFQFSRHIPGPTVCISHFSGFSVI
SSFFKSSSGCFSFSMIFSFLAIFHVLQWTFLNFPPFFSPYSRSYSVHFSFFTFFSDFVIFQVVKWMFLIFHDF
QFSC
>ENSMUSP00000137330_c.165:A>T_p.T56S ENSMUST00000143083.3|Gm10721-201|ENSMUSG00000096519|+|chr9:g.3013309:A>T
LFLIFHDFQFSCHIPCPTLDISFPPFSVFLAIFHVLKCVFLIFRDFQFSCHIPGPSVCISHFSRFSVILSFFK
LSTACFSFSMIFSFLAIFHVLQWKFLNFPPFSVFLAIFHVLKCVFLIFRDFQFSRHIPGPTVCISHFSGFSVI
SSFFKSSSGCFSFSMIFSFLAIFHVLQWTFLNFPPFFSPYSRSYSVHFSFFTFFSDFVIFQVVKWMFLIFHDF
QFSC
>ENSMUSP00000137330_c.178:C>T_p.S60F ENSMUST00000143083.3|Gm10721-201|ENSMUSG00000096519|+|chr9:g.3013322:C>T
LFLIFHDFQFSCHIPCPTLDISFPPFSVFLAIFHVLKCVFLIFRDFQFSCHIPGPTVCIFHFSRFSVILSFFK
LSTACFSFSMIFSFLAIFHVLQWKFLNFPPFSVFLAIFHVLKCVFLIFRDFQFSRHIPGPTVCISHFSGFSVI
SSFFKSSSGCFSFSMIFSFLAIFHVLQWTFLNFPPFFSPYSRSYSVHFSFFTFFSDFVIFQVVKWMFLIFHDF
QFSC
>ENSMUSP00000137330_c.186:T>C_p.S63P ENSMUST00000143083.3|Gm10721-201|ENSMUSG00000096519|+|chr9:g.3013330:T>C
LFLIFHDFQFSCHIPCPTLDISFPPFSVFLAIFHVLKCVFLIFRDFQFSCHIPGPTVCISHFPRFSVILSFFK
LSTACFSFSMIFSFLAIFHVLQWKFLNFPPFSVFLAIFHVLKCVFLIFRDFQFSRHIPGPTVCISHFSGFSVI
SSFFKSSSGCFSFSMIFSFLAIFHVLQWTFLNFPPFFSPYSRSYSVHFSFFTFFSDFVIFQVVKWMFLIFHDF
QFSC
>ENSMUSP00000137330_c.199:T>G_p.V67G ENSMUST00000143083.3|Gm10721-201|ENSMUSG00000096519|+|chr9:g.3013343:T>G
LFLIFHDFQFSCHIPCPTLDISFPPFSVFLAIFHVLKCVFLIFRDFQFSCHIPGPTVCISHFSRFSGILSFFK
LSTACFSFSMIFSFLAIFHVLQWKFLNFPPFSVFLAIFHVLKCVFLIFRDFQFSRHIPGPTVCISHFSGFSVI

SSFFKSSSGCFSFSMIFSFLAIFHVLQWTFLNFPPFFSPYSRSYSVHFSFFTFFSDFVIFQVVKWMFLIFHDF
QFSC
>ENSMUSP00000137330_c.205:T>C_p.L69S ENSMUST00000143083.3|Gm10721-201|ENSMUSG00000096519|+|chr9:g.3013349:T>C
LFLIFHDFQFSCHIPCPTLDISFPPFSVFLAIFHVLKCVFLIFRDFQFSCHIPGPTVCISHFSRFSVISSFFK
LSTACFSFSMIFSFLAIFHVLQWKFLNFPPFSVFLAIFHVLKCVFLIFRDFQFSRHIPGPTVCISHFSGFSVI
SSFFKSSSGCFSFSMIFSFLAIFHVLQWTFLNFPPFFSPYSRSYSVHFSFFTFFSDFVIFQVVKWMFLIFHDF
QFSC
>ENSMUSP00000137330_c.220:T>G_p.L74W ENSMUST00000143083.3|Gm10721-201|ENSMUSG00000096519|+|chr9:g.3013364:T>G
LFLIFHDFQFSCHIPCPTLDISFPPFSVFLAIFHVLKCVFLIFRDFQFSCHIPGPTVCISHFSRFSVILSFFK
WSTACFSFSMIFSFLAIFHVLQWKFLNFPPFSVFLAIFHVLKCVFLIFRDFQFSRHIPGPTVCISHFSGFSVI
SSFFKSSSGCFSFSMIFSFLAIFHVLQWTFLNFPPFFSPYSRSYSVHFSFFTFFSDFVIFQVVKWMFLIFHDF
QFSC
>ENSMUSP00000137330_c.220:T>C_p.L74S ENSMUST00000143083.3|Gm10721-201|ENSMUSG00000096519|+|chr9:g.3013364:T>C
LFLIFHDFQFSCHIPCPTLDISFPPFSVFLAIFHVLKCVFLIFRDFQFSCHIPGPTVCISHFSRFSVILSFFK
SSTACFSFSMIFSFLAIFHVLQWKFLNFPPFSVFLAIFHVLKCVFLIFRDFQFSRHIPGPTVCISHFSGFSVI
SSFFKSSSGCFSFSMIFSFLAIFHVLQWTFLNFPPFFSPYSRSYSVHFSFFTFFSDFVIFQVVKWMFLIFHDF
QFSC
>ENSMUSP00000137330_c.226:C>G_p.T76S ENSMUST00000143083.3|Gm10721-201|ENSMUSG00000096519|+|chr9:g.3013370:C>G
LFLIFHDFQFSCHIPCPTLDISFPPFSVFLAIFHVLKCVFLIFRDFQFSCHIPGPTVCISHFSRFSVILSFFK
LSSACFSFSMIFSFLAIFHVLQWKFLNFPPFSVFLAIFHVLKCVFLIFRDFQFSRHIPGPTVCISHFSGFSVI
SSFFKSSSGCFSFSMIFSFLAIFHVLQWTFLNFPPFFSPYSRSYSVHFSFFTFFSDFVIFQVVKWMFLIFHDF
QFSC
>ENSMUSP00000137330_c.226:CTGC>GTGT_p.T76S ENSMUST00000143083.3|Gm10721-201|ENSMUSG00000096519|+|chr9:g.3013370:CTGC>GTGT
LFLIFHDFQFSCHIPCPTLDISFPPFSVFLAIFHVLKCVFLIFRDFQFSCHIPGPTVCISHFSRFSVILSFFK
LSSVACFSFSMIFSFLAIFHVLQWKFLNFPPFSVFLAIFHVLKCVFLIFRDFQFSRHIPGPTVCISHFSGFSV
ISSFFKSSSGCFSFSMIFSFLAIFHVLQWTFLNFPPFFSPYSRSYSVHFSFFTFFSDFVIFQVVKWMFLIFHD
FQFSC
>ENSMUSP00000137330_c.226:CTGC>GTGG_p.T76S ENSMUST00000143083.3|Gm10721-201|ENSMUSG00000096519|+|chr9:g.3013370:CTGC>GTGG
LFLIFHDFQFSCHIPCPTLDISFPPFSVFLAIFHVLKCVFLIFRDFQFSCHIPGPTVCISHFSRFSVILSFFK
LSSGACFSFSMIFSFLAIFHVLQWKFLNFPPFSVFLAIFHVLKCVFLIFRDFQFSRHIPGPTVCISHFSGFSV
ISSFFKSSSGCFSFSMIFSFLAIFHVLQWTFLNFPPFFSPYSRSYSVHFSFFTFFSDFVIFQVVKWMFLIFHD
FQFSC
>ENSMUSP00000137330_c.289:A>C_p.K97T ENSMUST00000143083.3|Gm10721-201|ENSMUSG00000096519|+|chr9:g.3013433:A>C
LFLIFHDFQFSCHIPCPTLDISFPPFSVFLAIFHVLKCVFLIFRDFQFSCHIPGPTVCISHFSRFSVILSFFK
LSTACFSFSMIFSFLAIFHVLQWTFLNFPPFSVFLAIFHVLKCVFLIFRDFQFSRHIPGPTVCISHFSGFSVI
SSFFKSSSGCFSFSMIFSFLAIFHVLQWTFLNFPPFFSPYSRSYSVHFSFFTFFSDFVIFQVVKWMFLIFHDF
QFSC
>ENSMUSP00000137330_c.289:A>T_p.K97I ENSMUST00000143083.3|Gm10721-201|ENSMUSG00000096519|+|chr9:g.3013433:A>T
LFLIFHDFQFSCHIPCPTLDISFPPFSVFLAIFHVLKCVFLIFRDFQFSCHIPGPTVCISHFSRFSVILSFFK
LSTACFSFSMIFSFLAIFHVLQWIFLNFPPFSVFLAIFHVLKCVFLIFRDFQFSRHIPGPTVCISHFSGFSVI
SSFFKSSSGCFSFSMIFSFLAIFHVLQWTFLNFPPFFSPYSRSYSVHFSFFTFFSDFVIFQVVKWMFLIFHDF
QFSC
>ENSMUSP00000137330_c.336:G>A_p.V113I ENSMUST00000143083.3|Gm10721-201|ENSMUSG00000096519|+|chr9:g.3013480:G>A
LFLIFHDFQFSCHIPCPTLDISFPPFSVFLAIFHVLKCVFLIFRDFQFSCHIPGPTVCISHFSRFSVILSFFK
LSTACFSFSMIFSFLAIFHVLQWKFLNFPPFSVFLAIFHILKCVFLIFRDFQFSRHIPGPTVCISHFSGFSVI
SSFFKSSSGCFSFSMIFSFLAIFHVLQWTFLNFPPFFSPYSRSYSVHFSFFTFFSDFVIFQVVKWMFLIFHDF
QFSC
>ENSMUSP00000137330_c.344:G>T_p.K115N ENSMUST00000143083.3|Gm10721-201|ENSMUSG00000096519|+|chr9:g.3013488:G>T

LFLIFHDFQFSCHIPCPTLDISFPPFSVFLAIFHVLKCVFLIFRDFQFSCHIPGPTVCISHFSRFSVILSFFK
LSTACFSFSMIFSFLAIFHVLQWKFLNFPPFSVFLAIFHVLNCVFLIFRDFQFSRHIPGPTVCISHFSGFSVI
SSFFKSSSGCFSFSMIFSFLAIFHVLQWTFLNFPPFFSPYSRSYSVHFSFFTFFSDFVIFQVVKWMFLIFHDF
QFSC
>ENSMUSP00000137330_c.393:G>T_p.G132C ENSMUST00000143083.3|Gm10721-201|ENSMUSG00000096519|+|chr9:g.3013537:G>T
LFLIFHDFQFSCHIPCPTLDISFPPFSVFLAIFHVLKCVFLIFRDFQFSCHIPGPTVCISHFSRFSVILSFFK
LSTACFSFSMIFSFLAIFHVLQWKFLNFPPFSVFLAIFHVLKCVFLIFRDFQFSRHIPCPTVCISHFSGFSVI
SSFFKSSSGCFSFSMIFSFLAIFHVLQWTFLNFPPFFSPYSRSYSVHFSFFTFFSDFVIFQVVKWMFLIFHDF
QFSC
>ENSMUSP00000137330_c.399:A>T_p.T134S ENSMUST00000143083.3|Gm10721-201|ENSMUSG00000096519|+|chr9:g.3014010:A>T
LFLIFHDFQFSCHIPCPTLDISFPPFSVFLAIFHVLKCVFLIFRDFQFSCHIPGPTVCISHFSRFSVILSFFK
LSTACFSFSMIFSFLAIFHVLQWKFLNFPPFSVFLAIFHVLKCVFLIFRDFQFSRHIPGPSVCISHFSGFSVI
SSFFKSSSGCFSFSMIFSFLAIFHVLQWTFLNFPPFFSPYSRSYSVHFSFFTFFSDFVIFQVVKWMFLIFHDF
QFSC
>ENSMUSP00000137330_c.400:C>G_p.T134R ENSMUST00000143083.3|Gm10721-201|ENSMUSG00000096519|+|chr9:g.3014011:C>G
LFLIFHDFQFSCHIPCPTLDISFPPFSVFLAIFHVLKCVFLIFRDFQFSCHIPGPTVCISHFSRFSVILSFFK
LSTACFSFSMIFSFLAIFHVLQWKFLNFPPFSVFLAIFHVLKCVFLIFRDFQFSRHIPGPRVCISHFSGFSVI
SSFFKSSSGCFSFSMIFSFLAIFHVLQWTFLNFPPFFSPYSRSYSVHFSFFTFFSDFVIFQVVKWMFLIFHDF
QFSC
>ENSMUSP00000137330_c.423:G>T_p.G142C ENSMUST00000143083.3|Gm10721-201|ENSMUSG00000096519|+|chr9:g.3014034:G>T
LFLIFHDFQFSCHIPCPTLDISFPPFSVFLAIFHVLKCVFLIFRDFQFSCHIPGPTVCISHFSRFSVILSFFK
LSTACFSFSMIFSFLAIFHVLQWKFLNFPPFSVFLAIFHVLKCVFLIFRDFQFSRHIPGPTVCISHFSCFSVI
SSFFKSSSGCFSFSMIFSFLAIFHVLQWTFLNFPPFFSPYSRSYSVHFSFFTFFSDFVIFQVVKWMFLIFHDF
QFSC
>ENSMUSP00000137330_c.423:G>C_p.G142R ENSMUST00000143083.3|Gm10721-201|ENSMUSG00000096519|+|chr9:g.3014034:G>C
LFLIFHDFQFSCHIPCPTLDISFPPFSVFLAIFHVLKCVFLIFRDFQFSCHIPGPTVCISHFSRFSVILSFFK
LSTACFSFSMIFSFLAIFHVLQWKFLNFPPFSVFLAIFHVLKCVFLIFRDFQFSRHIPGPTVCISHFSRFSVI
SSFFKSSSGCFSFSMIFSFLAIFHVLQWTFLNFPPFFSPYSRSYSVHFSFFTFFSDFVIFQVVKWMFLIFHDF
QFSC
>ENSMUSP00000137330_c.430:C>T_p.S144L ENSMUST00000143083.3|Gm10721-201|ENSMUSG00000096519|+|chr9:g.3014041:C>T
LFLIFHDFQFSCHIPCPTLDISFPPFSVFLAIFHVLKCVFLIFRDFQFSCHIPGPTVCISHFSRFSVILSFFK
LSTACFSFSMIFSFLAIFHVLQWKFLNFPPFSVFLAIFHVLKCVFLIFRDFQFSRHIPGPTVCISHFSGFLVI
SSFFKSSSGCFSFSMIFSFLAIFHVLQWTFLNFPPFFSPYSRSYSVHFSFFTFFSDFVIFQVVKWMFLIFHDF
QFSC
>ENSMUSP00000137330_c.454:C>G_p.S152W ENSMUST00000143083.3|Gm10721-201|ENSMUSG00000096519|+|chr9:g.3014065:C>G
LFLIFHDFQFSCHIPCPTLDISFPPFSVFLAIFHVLKCVFLIFRDFQFSCHIPGPTVCISHFSRFSVILSFFK
LSTACFSFSMIFSFLAIFHVLQWKFLNFPPFSVFLAIFHVLKCVFLIFRDFQFSRHIPGPTVCISHFSGFSVI
SSFFKWSSGCFSFSMIFSFLAIFHVLQWTFLNFPPFFSPYSRSYSVHFSFFTFFSDFVIFQVVKWMFLIFHDF
QFSC
>ENSMUSP00000137330_c.491:T>A_p.S164R ENSMUST00000143083.3|Gm10721-201|ENSMUSG00000096519|+|chr9:g.3014102:T>A
LFLIFHDFQFSCHIPCPTLDISFPPFSVFLAIFHVLKCVFLIFRDFQFSCHIPGPTVCISHFSRFSVILSFFK
LSTACFSFSMIFSFLAIFHVLQWKFLNFPPFSVFLAIFHVLKCVFLIFRDFQFSRHIPGPTVCISHFSGFSVI
SSFFKSSSGCFSFSMIFRFLAIFHVLQWTFLNFPPFFSPYSRSYSVHFSFFTFFSDFVIFQVVKWMFLIFHDF
QFSC
>ENSMUSP00000137330_c.540:C>A_p.P181T ENSMUST00000143083.3|Gm10721-201|ENSMUSG00000096519|+|chr9:g.3014151:C>A
LFLIFHDFQFSCHIPCPTLDISFPPFSVFLAIFHVLKCVFLIFRDFQFSCHIPGPTVCISHFSRFSVILSFFK
LSTACFSFSMIFSFLAIFHVLQWKFLNFPPFSVFLAIFHVLKCVFLIFRDFQFSRHIPGPTVCISHFSGFSVI
SSFFKSSSGCFSFSMIFSFLAIFHVLQWTFLNFPTFFSPYSRSYSVHFSFFTFFSDFVIFQVVKWMFLIFHDF
QFSC

>ENSMUSP00000137330_c.541:C>T_p.P181L ENSMUST00000143083.3|Gm10721-201|ENSMUSG00000096519|+|chr9:g.3014152:C>T
LFLIFHDFQFSCHIPCPTLDISFPPFSVFLAIFHVLKCVFLIFRDFQFSCHIPGPTVCISHFSRFSVILSFFK
LSTACFSFSMIFSFLAIFHVLQWKFLNFPPFSVFLAIFHVLKCVFLIFRDFQFSRHIPGPTVCISHFSGFSVI
SSFFKSSSGCFSFSMIFSFLAIFHVLQWTFLNFPLFFSPYSRSYSVHFSFFTFFSDFVIFQVVKWMFLIFHDF
QFSC
>ENSMUSP00000137330_c.563:G>T_p.R188S ENSMUST00000143083.3|Gm10721-201|ENSMUSG00000096519|+|chr9:g.3014239:G>T
LFLIFHDFQFSCHIPCPTLDISFPPFSVFLAIFHVLKCVFLIFRDFQFSCHIPGPTVCISHFSRFSVILSFFK
LSTACFSFSMIFSFLAIFHVLQWKFLNFPPFSVFLAIFHVLKCVFLIFRDFQFSRHIPGPTVCISHFSGFSVI
SSFFKSSSGCFSFSMIFSFLAIFHVLQWTFLNFPPFFSPYSSSYSVHFSFFTFFSDFVIFQVVKWMFLIFHDF
QFSC
>ENSMUSP00000137330_c.568:A>T_p.Y190F ENSMUST00000143083.3|Gm10721-201|ENSMUSG00000096519|+|chr9:g.3014244:A>T
LFLIFHDFQFSCHIPCPTLDISFPPFSVFLAIFHVLKCVFLIFRDFQFSCHIPGPTVCISHFSRFSVILSFFK
LSTACFSFSMIFSFLAIFHVLQWKFLNFPPFSVFLAIFHVLKCVFLIFRDFQFSRHIPGPTVCISHFSGFSVI
SSFFKSSSGCFSFSMIFSFLAIFHVLQWTFLNFPPFFSPYSRSFSVHFSFFTFFSDFVIFQVVKWMFLIFHDF
QFSC
>ENSMUSP00000137330_c.642:A>T_p.I215F ENSMUST00000143083.3|Gm10721-201|ENSMUSG00000096519|+|chr9:g.3014318:A>T
LFLIFHDFQFSCHIPCPTLDISFPPFSVFLAIFHVLKCVFLIFRDFQFSCHIPGPTVCISHFSRFSVILSFFK
LSTACFSFSMIFSFLAIFHVLQWKFLNFPPFSVFLAIFHVLKCVFLIFRDFQFSRHIPGPTVCISHFSGFSVI
SSFFKSSSGCFSFSMIFSFLAIFHVLQWTFLNFPPFFSPYSRSYSVHFSFFTFFSDFVIFQVVKWMFLFFHDF
QFSC
>ENSMUSP00000096649_c.15:A>C_p.N6H ENSMUST00000099050.4|Gm10720-201|ENSMUSG00000074564|+|chr9:g.3015669:A>C
MFLIFHDFHFSCHIPCPTVDISKFPFSVFLAIFHVLKCVFLIFSDFQFSRHIPGPTVCISHFSRFSVILSFFK
LSSGCFSFSLIFSFLAIFHVLQWTFLNFPPFSVFLAIFHVLKCVFLIFSDFQFSRHIPGPTVCISHFFRFFMI
SSFFTLSSGCFSFSMIFTFLAIFHVLQWTFLNFPPFFLPYSRSYSVHFSFFTFFSDFVIFQVVKWMFLIFHDF
QFSR
>ENSMUSP00000096649_c.15:A>T_p.N6Y ENSMUST00000099050.4|Gm10720-201|ENSMUSG00000074564|+|chr9:g.3015669:A>T
MFLIFYDFHFSCHIPCPTVDISKFPFSVFLAIFHVLKCVFLIFSDFQFSRHIPGPTVCISHFSRFSVILSFFK
LSSGCFSFSLIFSFLAIFHVLQWTFLNFPPFSVFLAIFHVLKCVFLIFSDFQFSRHIPGPTVCISHFFRFFMI
SSFFTLSSGCFSFSMIFTFLAIFHVLQWTFLNFPPFFLPYSRSYSVHFSFFTFFSDFVIFQVVKWMFLIFHDF
QFSR
>rs212664173_ENSMUSP00000096649_c.26:T>G_p.H9Q
ENSMUST00000099050.4|Gm10720-201|ENSMUSG00000074564|+|chr9:g.3015680:T>G
MFLIFNDFQFSCHIPCPTVDISKFPFSVFLAIFHVLKCVFLIFSDFQFSRHIPGPTVCISHFSRFSVILSFFK
LSSGCFSFSLIFSFLAIFHVLQWTFLNFPPFSVFLAIFHVLKCVFLIFSDFQFSRHIPGPTVCISHFFRFFMI
SSFFTLSSGCFSFSMIFTFLAIFHVLQWTFLNFPPFFLPYSRSYSVHFSFFTFFSDFVIFQVVKWMFLIFHDF
QFSR
>ENSMUSP00000096649_c.26:TTTT>GTTA_p.H9Q ENSMUST00000099050.4|Gm10720-201|ENSMUSG00000074564|+|chr9:g.3015680:TTTT>GTTA
MFLIFNDFQLFSCHIPCPTVDISKFPFSVFLAIFHVLKCVFLIFSDFQFSRHIPGPTVCISHFSRFSVILSFF
KLSSGCFSFSLIFSFLAIFHVLQWTFLNFPPFSVFLAIFHVLKCVFLIFSDFQFSRHIPGPTVCISHFFRFFM
ISSFFTLSSGCFSFSMIFTFLAIFHVLQWTFLNFPPFFLPYSRSYSVHFSFFTFFSDFVIFQVVKWMFLIFHD
FQFSR
>ENSMUSP00000096649_c.45:T>C_p.C16R ENSMUST00000099050.4|Gm10720-201|ENSMUSG00000074564|+|chr9:g.3015699:T>C
MFLIFNDFHFSCHIPRPTVDISKFPFSVFLAIFHVLKCVFLIFSDFQFSRHIPGPTVCISHFSRFSVILSFFK
LSSGCFSFSLIFSFLAIFHVLQWTFLNFPPFSVFLAIFHVLKCVFLIFSDFQFSRHIPGPTVCISHFFRFFMI
SSFFTLSSGCFSFSMIFTFLAIFHVLQWTFLNFPPFFLPYSRSYSVHFSFFTFFSDFVIFQVVKWMFLIFHDF
QFSR
>ENSMUSP00000096649_c.90:G>T_p.A31S ENSMUST00000099050.4|Gm10720-201|ENSMUSG00000074564|+|chr9:g.3015748:G>T
MFLIFNDFHFSCHIPCPTVDISKFPFSVFLSIFHVLKCVFLIFSDFQFSRHIPGPTVCISHFSRFSVILSFFK
LSSGCFSFSLIFSFLAIFHVLQWTFLNFPPFSVFLAIFHVLKCVFLIFSDFQFSRHIPGPTVCISHFFRFFMI

SSFFTLSSGCFSFSMIFTFLAIFHVLQWTFLNFPPFFLPYSRSYSVHFSFFTFFSDFVIFQVVKWMFLIFHDF
QFSR
>ENSMUSP00000096649_c.101:T>A_p.H34Q ENSMUST00000099050.4|Gm10720-201|ENSMUSG00000074564|+|chr9:g.3015759:T>A
MFLIFNDFHFSCHIPCPTVDISKFPFSVFLAIFQVLKCVFLIFSDFQFSRHIPGPTVCISHFSRFSVILSFFK
LSSGCFSFSLIFSFLAIFHVLQWTFLNFPPFSVFLAIFHVLKCVFLIFSDFQFSRHIPGPTVCISHFFRFFMI
SSFFTLSSGCFSFSMIFTFLAIFHVLQWTFLNFPPFFLPYSRSYSVHFSFFTFFSDFVIFQVVKWMFLIFHDF
QFSR
>rs236115139_ENSMUSP00000096649_c.129:A>C_p.S44R
ENSMUST00000099050.4|Gm10720-201|ENSMUSG00000074564|+|chr9:g.3015787:A>C
MFLIFNDFHFSCHIPCPTVDISKFPFSVFLAIFHVLKCVFLIFRDFQFSRHIPGPTVCISHFSRFSVILSFFK
LSSGCFSFSLIFSFLAIFHVLQWTFLNFPPFSVFLAIFHVLKCVFLIFSDFQFSRHIPGPTVCISHFFRFFMI
SSFFTLSSGCFSFSMIFTFLAIFHVLQWTFLNFPPFFLPYSRSYSVHFSFFTFFSDFVIFQVVKWMFLIFHDF
QFSR
>ENSMUSP00000096649_c.129:A>T_p.S44C ENSMUST00000099050.4|Gm10720-201|ENSMUSG00000074564|+|chr9:g.3015787:A>T
MFLIFNDFHFSCHIPCPTVDISKFPFSVFLAIFHVLKCVFLIFCDFQFSRHIPGPTVCISHFSRFSVILSFFK
LSSGCFSFSLIFSFLAIFHVLQWTFLNFPPFSVFLAIFHVLKCVFLIFSDFQFSRHIPGPTVCISHFFRFFMI
SSFFTLSSGCFSFSMIFTFLAIFHVLQWTFLNFPPFFLPYSRSYSVHFSFFTFFSDFVIFQVVKWMFLIFHDF
QFSR
>ENSMUSP00000096649_c.165:A>T_p.T56S ENSMUST00000099050.4|Gm10720-201|ENSMUSG00000074564|+|chr9:g.3016466:A>T
MFLIFNDFHFSCHIPCPTVDISKFPFSVFLAIFHVLKCVFLIFSDFQFSRHIPGPSVCISHFSRFSVILSFFK
LSSGCFSFSLIFSFLAIFHVLQWTFLNFPPFSVFLAIFHVLKCVFLIFSDFQFSRHIPGPTVCISHFFRFFMI
SSFFTLSSGCFSFSMIFTFLAIFHVLQWTFLNFPPFFLPYSRSYSVHFSFFTFFSDFVIFQVVKWMFLIFHDF
QFSR
>ENSMUSP00000096649_c.186:T>C_p.S63P ENSMUST00000099050.4|Gm10720-201|ENSMUSG00000074564|+|chr9:g.3016487:T>C
MFLIFNDFHFSCHIPCPTVDISKFPFSVFLAIFHVLKCVFLIFSDFQFSRHIPGPTVCISHFPRFSVILSFFK
LSSGCFSFSLIFSFLAIFHVLQWTFLNFPPFSVFLAIFHVLKCVFLIFSDFQFSRHIPGPTVCISHFFRFFMI
SSFFTLSSGCFSFSMIFTFLAIFHVLQWTFLNFPPFFLPYSRSYSVHFSFFTFFSDFVIFQVVKWMFLIFHDF
QFSR
>ENSMUSP00000096649_c.195:T>G_p.S66A ENSMUST00000099050.4|Gm10720-201|ENSMUSG00000074564|+|chr9:g.3016496:T>G
MFLIFNDFHFSCHIPCPTVDISKFPFSVFLAIFHVLKCVFLIFSDFQFSRHIPGPTVCISHFSRFAVILSFFK
LSSGCFSFSLIFSFLAIFHVLQWTFLNFPPFSVFLAIFHVLKCVFLIFSDFQFSRHIPGPTVCISHFFRFFMI
SSFFTLSSGCFSFSMIFTFLAIFHVLQWTFLNFPPFFLPYSRSYSVHFSFFTFFSDFVIFQVVKWMFLIFHDF
QFSR
>ENSMUSP00000096649_c.196:C>T_p.S66L ENSMUST00000099050.4|Gm10720-201|ENSMUSG00000074564|+|chr9:g.3016497:C>T
MFLIFNDFHFSCHIPCPTVDISKFPFSVFLAIFHVLKCVFLIFSDFQFSRHIPGPTVCISHFSRFLVILSFFK
LSSGCFSFSLIFSFLAIFHVLQWTFLNFPPFSVFLAIFHVLKCVFLIFSDFQFSRHIPGPTVCISHFFRFFMI
SSFFTLSSGCFSFSMIFTFLAIFHVLQWTFLNFPPFFLPYSRSYSVHFSFFTFFSDFVIFQVVKWMFLIFHDF
QFSR
>ENSMUSP00000096649_c.205:T>C_p.L69S ENSMUST00000099050.4|Gm10720-201|ENSMUSG00000074564|+|chr9:g.3016506:T>C
MFLIFNDFHFSCHIPCPTVDISKFPFSVFLAIFHVLKCVFLIFSDFQFSRHIPGPTVCISHFSRFSVISSFFK
LSSGCFSFSLIFSFLAIFHVLQWTFLNFPPFSVFLAIFHVLKCVFLIFSDFQFSRHIPGPTVCISHFFRFFMI
SSFFTLSSGCFSFSMIFTFLAIFHVLQWTFLNFPPFFLPYSRSYSVHFSFFTFFSDFVIFQVVKWMFLIFHDF
QFSR
>ENSMUSP00000096649_c.220:T>C_p.L74S ENSMUST00000099050.4|Gm10720-201|ENSMUSG00000074564|+|chr9:g.3016521:T>C
MFLIFNDFHFSCHIPCPTVDISKFPFSVFLAIFHVLKCVFLIFSDFQFSRHIPGPTVCISHFSRFSVILSFFK
SSSGCFSFSLIFSFLAIFHVLQWTFLNFPPFSVFLAIFHVLKCVFLIFSDFQFSRHIPGPTVCISHFFRFFMI
SSFFTLSSGCFSFSMIFTFLAIFHVLQWTFLNFPPFFLPYSRSYSVHFSFFTFFSDFVIFQVVKWMFLIFHDF
QFSR
>rs234484759_ENSMUSP00000096649_c.220:T>G_p.L74W
ENSMUST00000099050.4|Gm10720-201|ENSMUSG00000074564|+|chr9:g.3016521:T>G

MFLIFNDFHFSCHIPCPTVDISKFPFSVFLAIFHVLKCVFLIFSDFQFSRHIPGPTVCISHFSRFSVILSFFK
WSSGCFSFSLIFSFLAIFHVLQWTFLNFPPFSVFLAIFHVLKCVFLIFSDFQFSRHIPGPTVCISHFFRFFMI
SSFFTLSSGCFSFSMIFTFLAIFHVLQWTFLNFPPFFLPYSRSYSVHFSFFTFFSDFVIFQVVKWMFLIFHDF
QFSR
>ENSMUSP00000096649_c.246:T>A_p.L83M ENSMUST00000099050.4|Gm10720-201|ENSMUSG00000074564|+|chr9:g.3016547:T>A
MFLIFNDFHFSCHIPCPTVDISKFPFSVFLAIFHVLKCVFLIFSDFQFSRHIPGPTVCISHFSRFSVILSFFK
LSSGCFSFSMIFSFLAIFHVLQWTFLNFPPFSVFLAIFHVLKCVFLIFSDFQFSRHIPGPTVCISHFFRFFMI
SSFFTLSSGCFSFSMIFTFLAIFHVLQWTFLNFPPFFLPYSRSYSVHFSFFTFFSDFVIFQVVKWMFLIFHDF
QFSR
>ENSMUSP00000096649_c.257:T>A_p.S86R ENSMUST00000099050.4|Gm10720-201|ENSMUSG00000074564|+|chr9:g.3016558:T>A
MFLIFNDFHFSCHIPCPTVDISKFPFSVFLAIFHVLKCVFLIFSDFQFSRHIPGPTVCISHFSRFSVILSFFK
LSSGCFSFSLIFRFLAIFHVLQWTFLNFPPFSVFLAIFHVLKCVFLIFSDFQFSRHIPGPTVCISHFFRFFMI
SSFFTLSSGCFSFSMIFTFLAIFHVLQWTFLNFPPFFLPYSRSYSVHFSFFTFFSDFVIFQVVKWMFLIFHDF
QFSR
>ENSMUSP00000096649_c.275:T>G_p.H92Q ENSMUST00000099050.4|Gm10720-201|ENSMUSG00000074564|+|chr9:g.3016576:T>G
MFLIFNDFHFSCHIPCPTVDISKFPFSVFLAIFHVLKCVFLIFSDFQFSRHIPGPTVCISHFSRFSVILSFFK
LSSGCFSFSLIFSFLAIFQVLQWTFLNFPPFSVFLAIFHVLKCVFLIFSDFQFSRHIPGPTVCISHFFRFFMI
SSFFTLSSGCFSFSMIFTFLAIFHVLQWTFLNFPPFFLPYSRSYSVHFSFFTFFSDFVIFQVVKWMFLIFHDF
QFSR
>ENSMUSP00000096649_c.286:G>T_p.W96L ENSMUST00000099050.4|Gm10720-201|ENSMUSG00000074564|+|chr9:g.3016587:G>T
MFLIFNDFHFSCHIPCPTVDISKFPFSVFLAIFHVLKCVFLIFSDFQFSRHIPGPTVCISHFSRFSVILSFFK
LSSGCFSFSLIFSFLAIFHVLQLTFLNFPPFSVFLAIFHVLKCVFLIFSDFQFSRHIPGPTVCISHFFRFFMI
SSFFTLSSGCFSFSMIFTFLAIFHVLQWTFLNFPPFFLPYSRSYSVHFSFFTFFSDFVIFQVVKWMFLIFHDF
QFSR
>ENSMUSP00000096649_c.301:T>A_p.F101Y ENSMUST00000099050.4|Gm10720-201|ENSMUSG00000074564|+|chr9:g.3016602:T>A
MFLIFNDFHFSCHIPCPTVDISKFPFSVFLAIFHVLKCVFLIFSDFQFSRHIPGPTVCISHFSRFSVILSFFK
LSSGCFSFSLIFSFLAIFHVLQWTFLNYPPFSVFLAIFHVLKCVFLIFSDFQFSRHIPGPTVCISHFFRFFMI
SSFFTLSSGCFSFSMIFTFLAIFHVLQWTFLNFPPFFLPYSRSYSVHFSFFTFFSDFVIFQVVKWMFLIFHDF
QFSR
>ENSMUSP00000096649_c.306:C>A_p.P103T ENSMUST00000099050.4|Gm10720-201|ENSMUSG00000074564|+|chr9:g.3016607:C>A
MFLIFNDFHFSCHIPCPTVDISKFPFSVFLAIFHVLKCVFLIFSDFQFSRHIPGPTVCISHFSRFSVILSFFK
LSSGCFSFSLIFSFLAIFHVLQWTFLNFPTFSVFLAIFHVLKCVFLIFSDFQFSRHIPGPTVCISHFFRFFMI
SSFFTLSSGCFSFSMIFTFLAIFHVLQWTFLNFPPFFLPYSRSYSVHFSFFTFFSDFVIFQVVKWMFLIFHDF
QFSR
>ENSMUSP00000096649_c.335:T>A_p.H112Q ENSMUST00000099050.4|Gm10720-201|ENSMUSG00000074564|+|chr9:g.3016636:T>A
MFLIFNDFHFSCHIPCPTVDISKFPFSVFLAIFHVLKCVFLIFSDFQFSRHIPGPTVCISHFSRFSVILSFFK
LSSGCFSFSLIFSFLAIFHVLQWTFLNFPPFSVFLAIFQVLKCVFLIFSDFQFSRHIPGPTVCISHFFRFFMI
SSFFTLSSGCFSFSMIFTFLAIFHVLQWTFLNFPPFFLPYSRSYSVHFSFFTFFSDFVIFQVVKWMFLIFHDF
QFSR
>ENSMUSP00000096649_c.340:T>C_p.L114P ENSMUST00000099050.4|Gm10720-201|ENSMUSG00000074564|+|chr9:g.3016641:T>C
MFLIFNDFHFSCHIPCPTVDISKFPFSVFLAIFHVLKCVFLIFSDFQFSRHIPGPTVCISHFSRFSVILSFFK
LSSGCFSFSLIFSFLAIFHVLQWTFLNFPPFSVFLAIFHVPKCVFLIFSDFQFSRHIPGPTVCISHFFRFFMI
SSFFTLSSGCFSFSMIFTFLAIFHVLQWTFLNFPPFFLPYSRSYSVHFSFFTFFSDFVIFQVVKWMFLIFHDF
QFSR
>ENSMUSP00000096649_c.363:A>C_p.S122R ENSMUST00000099050.4|Gm10720-201|ENSMUSG00000074564|+|chr9:g.3016664:A>C
MFLIFNDFHFSCHIPCPTVDISKFPFSVFLAIFHVLKCVFLIFSDFQFSRHIPGPTVCISHFSRFSVILSFFK
LSSGCFSFSLIFSFLAIFHVLQWTFLNFPPFSVFLAIFHVLKCVFLIFRDFQFSRHIPGPTVCISHFFRFFMI
SSFFTLSSGCFSFSMIFTFLAIFHVLQWTFLNFPPFFLPYSRSYSVHFSFFTFFSDFVIFQVVKWMFLIFHDF
QFSR

>rs261664597_ENSMUSP00000096649_c.363:A>T_p.S122C
ENSMUST00000099050.4|Gm10720-201|ENSMUSG00000074564|+|chr9:g.3016664:A>T
MFLIFNDFHFSCHIPCPTVDISKFPFSVFLAIFHVLKCVFLIFSDFQFSRHIPGPTVCISHFSRFSVILSFFK
LSSGCFSFSLIFSFLAIFHVLQWTFLNFPPFSVFLAIFHVLKCVFLIFCDFQFSRHIPGPTVCISHFFRFFMI
SSFFTLSSGCFSFSMIFTFLAIFHVLQWTFLNFPPFFLPYSRSYSVHFSFFTFFSDFVIFQVVKWMFLIFHDF
QFSR
>ENSMUSP00000096649_c.399:A>T_p.T134S ENSMUST00000099050.4|Gm10720-201|ENSMUSG00000074564|+|chr9:g.3016700:A>T
MFLIFNDFHFSCHIPCPTVDISKFPFSVFLAIFHVLKCVFLIFSDFQFSRHIPGPTVCISHFSRFSVILSFFK
LSSGCFSFSLIFSFLAIFHVLQWTFLNFPPFSVFLAIFHVLKCVFLIFSDFQFSRHIPGPSVCISHFFRFFMI
SSFFTLSSGCFSFSMIFTFLAIFHVLQWTFLNFPPFFLPYSRSYSVHFSFFTFFSDFVIFQVVKWMFLIFHDF
QFSR
>ENSMUSP00000096649_c.400:C>T_p.T134I ENSMUST00000099050.4|Gm10720-201|ENSMUSG00000074564|+|chr9:g.3016701:C>T
MFLIFNDFHFSCHIPCPTVDISKFPFSVFLAIFHVLKCVFLIFSDFQFSRHIPGPTVCISHFSRFSVILSFFK
LSSGCFSFSLIFSFLAIFHVLQWTFLNFPPFSVFLAIFHVLKCVFLIFSDFQFSRHIPGPIVCISHFFRFFMI
SSFFTLSSGCFSFSMIFTFLAIFHVLQWTFLNFPPFFLPYSRSYSVHFSFFTFFSDFVIFQVVKWMFLIFHDF
QFSR
>ENSMUSP00000096649_c.415:ATT>A_p.H139del-fs
ENSMUST00000099050.4|Gm10720-201|ENSMUSG00000074564|+|chr9:g.3016716:ATT>A
MFLIFNDFHFSCHIPCPTVDISKFPFSVFLAIFHVLKCVFLIFSDFQFSRHIPGPTVCISHFSRFSVILSFFK
LSSGCFSFSLIFSFLAIFHVLQWTFLNFPPFSVFLAIFHVLKCVFLIFSDFQFSRHIPGPTVCISHFQVFHDF
VIFHVVKWMFLIFHDFHFSCHISCPTVDISKFSTFFLAIFQVLQCAFLIFHVFQ
>ENSMUSP00000096649_c.424:G>C_p.R142T ENSMUST00000099050.4|Gm10720-201|ENSMUSG00000074564|+|chr9:g.3016725:G>C
MFLIFNDFHFSCHIPCPTVDISKFPFSVFLAIFHVLKCVFLIFSDFQFSRHIPGPTVCISHFSRFSVILSFFK
LSSGCFSFSLIFSFLAIFHVLQWTFLNFPPFSVFLAIFHVLKCVFLIFSDFQFSRHIPGPTVCISHFFTFFMI
SSFFTLSSGCFSFSMIFTFLAIFHVLQWTFLNFPPFFLPYSRSYSVHFSFFTFFSDFVIFQVVKWMFLIFHDF
QFSR
>ENSMUSP00000096649_c.439:C>T_p.S147L ENSMUST00000099050.4|Gm10720-201|ENSMUSG00000074564|+|chr9:g.3016916:C>T
MFLIFNDFHFSCHIPCPTVDISKFPFSVFLAIFHVLKCVFLIFSDFQFSRHIPGPTVCISHFSRFSVILSFFK
LSSGCFSFSLIFSFLAIFHVLQWTFLNFPPFSVFLAIFHVLKCVFLIFSDFQFSRHIPGPTVCISHFFRFFMI
LSFFTLSSGCFSFSMIFTFLAIFHVLQWTFLNFPPFFLPYSRSYSVHFSFFTFFSDFVIFQVVKWMFLIFHDF
QFSR
>ENSMUSP00000096649_c.451:CGTT>AGTC_p.T151K ENSMUST00000099050.4|Gm10720-201|ENSMUSG00000074564|+|chr9:g.3016928:CGTT>AGTC
MFLIFNDFHFSCHIPCPTVDISKFPFSVFLAIFHVLKCVFLIFSDFQFSRHIPGPTVCISHFSRFSVILSFFK
LSSGCFSFSLIFSFLAIFHVLQWTFLNFPPFSVFLAIFHVLKCVFLIFSDFQFSRHIPGPTVCISHFFRFFMI
SSFFKSLSSGCFSFSMIFTFLAIFHVLQWTFLNFPPFFLPYSRSYSVHFSFFTFFSDFVIFQVVKWMFLIFHD
FQFSR
>ENSMUSP00000096649_c.451:CGTT>AGTG_p.T151K ENSMUST00000099050.4|Gm10720-201|ENSMUSG00000074564|+|chr9:g.3016928:CGTT>AGTG
MFLIFNDFHFSCHIPCPTVDISKFPFSVFLAIFHVLKCVFLIFSDFQFSRHIPGPTVCISHFSRFSVILSFFK
LSSGCFSFSLIFSFLAIFHVLQWTFLNFPPFSVFLAIFHVLKCVFLIFSDFQFSRHIPGPTVCISHFFRFFMI
SSFFKWLSSGCFSFSMIFTFLAIFHVLQWTFLNFPPFFLPYSRSYSVHFSFFTFFSDFVIFQVVKWMFLIFHD
FQFSR
>ENSMUSP00000096649_c.451:CGTTG>TGTAA_p.T151M
ENSMUST00000099050.4|Gm10720-201|ENSMUSG00000074564|+|chr9:g.3016928:CGTTG>TGTAA
MFLIFNDFHFSCHIPCPTVDISKFPFSVFLAIFHVLKCVFLIFSDFQFSRHIPGPTVCISHFSRFSVILSFFK
LSSGCFSFSLIFSFLAIFHVLQWTFLNFPPFSVFLAIFHVLKCVFLIFSDFQFSRHIPGPTVCISHFFRFFMI
SSFFM*VVKWMFLIFHDFHFSCHISCPTVDISKFSTFFLAIFQVLQCAFLIFHVFQ*FRHFSSRQVDVSHFP*
FSVFS
>ENSMUSP00000096649_c.490:C>G_p.T164S ENSMUST00000099050.4|Gm10720-201|ENSMUSG00000074564|+|chr9:g.3016967:C>G

MFLIFNDFHFSCHIPCPTVDISKFPFSVFLAIFHVLKCVFLIFSDFQFSRHIPGPTVCISHFSRFSVILSFFK
LSSGCFSFSLIFSFLAIFHVLQWTFLNFPPFSVFLAIFHVLKCVFLIFSDFQFSRHIPGPTVCISHFFRFFMI
SSFFTLSSGCFSFSMIFSFLAIFHVLQWTFLNFPPFFLPYSRSYSVHFSFFTFFSDFVIFQVVKWMFLIFHDF
QFSR
>ENSMUSP00000096649_c.499:C>T_p.A167V ENSMUST00000099050.4|Gm10720-
201|ENSMUSG00000074564|+|chr9:g.3016976:C>T
MFLIFNDFHFSCHIPCPTVDISKFPFSVFLAIFHVLKCVFLIFSDFQFSRHIPGPTVCISHFSRFSVILSFFK
LSSGCFSFSLIFSFLAIFHVLQWTFLNFPPFSVFLAIFHVLKCVFLIFSDFQFSRHIPGPTVCISHFFRFFMI
SSFFTLSSGCFSFSMIFTFLVIFHVLQWTFLNFPPFFLPYSRSYSVHFSFFTFFSDFVIFQVVKWMFLIFHDF
QFSR
>ENSMUSP00000096649_c.550:T>C_p.L184S ENSMUST00000099050.4|Gm10720-
201|ENSMUSG00000074564|+|chr9:g.3017092:T>C
MFLIFNDFHFSCHIPCPTVDISKFPFSVFLAIFHVLKCVFLIFSDFQFSRHIPGPTVCISHFSRFSVILSFFK
LSSGCFSFSLIFSFLAIFHVLQWTFLNFPPFSVFLAIFHVLKCVFLIFSDFQFSRHIPGPTVCISHFFRFFMI
SSFFTLSSGCFSFSMIFTFLAIFHVLQWTFLNFPPFFSPYSRSYSVHFSFFTFFSDFVIFQVVKWMFLIFHDF
QFSR
>ENSMUSP00000096649_c.568:A>T_p.Y190F ENSMUST00000099050.4|Gm10720-
201|ENSMUSG00000074564|+|chr9:g.3017110:A>T
MFLIFNDFHFSCHIPCPTVDISKFPFSVFLAIFHVLKCVFLIFSDFQFSRHIPGPTVCISHFSRFSVILSFFK
LSSGCFSFSLIFSFLAIFHVLQWTFLNFPPFSVFLAIFHVLKCVFLIFSDFQFSRHIPGPTVCISHFFRFFMI
SSFFTLSSGCFSFSMIFTFLAIFHVLQWTFLNFPPFFLPYSRSFSVHFSFFTFFSDFVIFQVVKWMFLIFHDF
QFSR
>ENSMUSP00000096649_c.666:C>T_p.R223C ENSMUST00000099050.4|Gm10720-
201|ENSMUSG00000074564|+|chr9:g.3017208:C>T
MFLIFNDFHFSCHIPCPTVDISKFPFSVFLAIFHVLKCVFLIFSDFQFSRHIPGPTVCISHFSRFSVILSFFK
LSSGCFSFSLIFSFLAIFHVLQWTFLNFPPFSVFLAIFHVLKCVFLIFSDFQFSRHIPGPTVCISHFFRFFMI
SSFFTLSSGCFSFSMIFTFLAIFHVLQWTFLNFPPFFLPYSRSYSVHFSFFTFFSDFVIFQVVKWMFLIFHDF
QFSC
>rs262556314_ENSMUSP00000096648_c.15:A>C_p.N6H
ENSMUST00000099049.4|Gm10719-202|ENSMUSG00000095547|+|chr9:g.3017423:A>C
MFLIFHDFHFSCHIPCPTVDISFPPFSVFLAIFHVLKCVFLIFRDFQFSRHIPGPTVCISHFFRFFMISSFFT
LSSGCFSFSMIFTFLAIFHVLQWTFLNFPPFSVFLAIFHILKCVFLIFRDFQFSCHIPGPTVCISHFSRFSVI
SSFFKSSSGCFSFSMIFSFLAIFQVLQLTFLNFPHFLSFSPYSRSYSVHFSFFTFFSDFVIFQVVKWLFLIFL
DFQFSC
>ENSMUSP00000096648_c.16:A>G_p.N6S ENSMUST00000099049.4|Gm10719-
202|ENSMUSG00000095547|+|chr9:g.3017424:A>G
MFLIFSDFHFSCHIPCPTVDISFPPFSVFLAIFHVLKCVFLIFRDFQFSRHIPGPTVCISHFFRFFMISSFFT
LSSGCFSFSMIFTFLAIFHVLQWTFLNFPPFSVFLAIFHILKCVFLIFRDFQFSCHIPGPTVCISHFSRFSVI
SSFFKSSSGCFSFSMIFSFLAIFQVLQLTFLNFPHFLSFSPYSRSYSVHFSFFTFFSDFVIFQVVKWLFLIFL
DFQFSC
>ENSMUSP00000096648_c.26:T>G_p.H9Q ENSMUST00000099049.4|Gm10719-
202|ENSMUSG00000095547|+|chr9:g.3017434:T>G
MFLIFNDFQFSCHIPCPTVDISFPPFSVFLAIFHVLKCVFLIFRDFQFSRHIPGPTVCISHFFRFFMISSFFT
LSSGCFSFSMIFTFLAIFHVLQWTFLNFPPFSVFLAIFHILKCVFLIFRDFQFSCHIPGPTVCISHFSRFSVI
SSFFKSSSGCFSFSMIFSFLAIFQVLQLTFLNFPHFLSFSPYSRSYSVHFSFFTFFSDFVIFQVVKWLFLIFL
DFQFSC
>ENSMUSP00000096648_c.45:T>C_p.C16R ENSMUST00000099049.4|Gm10719-
202|ENSMUSG00000095547|+|chr9:g.3017453:T>C
MFLIFNDFHFSCHIPRPTVDISFPPFSVFLAIFHVLKCVFLIFRDFQFSRHIPGPTVCISHFFRFFMISSFFT
LSSGCFSFSMIFTFLAIFHVLQWTFLNFPPFSVFLAIFHILKCVFLIFRDFQFSCHIPGPTVCISHFSRFSVI
SSFFKSSSGCFSFSMIFSFLAIFQVLQLTFLNFPHFLSFSPYSRSYSVHFSFFTFFSDFVIFQVVKWLFLIFL
DFQFSC
>ENSMUSP00000096648_c.72:C>A_p.P25T ENSMUST00000099049.4|Gm10719-
202|ENSMUSG00000095547|+|chr9:g.3017484:C>A
MFLIFNDFHFSCHIPCPTVDISFPTFSVFLAIFHVLKCVFLIFRDFQFSRHIPGPTVCISHFFRFFMISSFFT
LSSGCFSFSMIFTFLAIFHVLQWTFLNFPPFSVFLAIFHILKCVFLIFRDFQFSCHIPGPTVCISHFSRFSVI
SSFFKSSSGCFSFSMIFSFLAIFQVLQLTFLNFPHFLSFSPYSRSYSVHFSFFTFFSDFVIFQVVKWLFLIFL
DFQFSC

```
>ENSMUSP00000096648_c.113:T>G_p.C38W ENSMUST00000099049.4|Gm10719-
202|ENSMUSG00000095547|+|chr9:g.3017525:T>G
MFLIFNDFHFSCHIPCPTVDISFPPFSVFLAIFHVLKWVFLIFRDFQFSRHIPGPTVCISHFFRFFMISSFFT
LSSGCFSFSMIFTFLAIFHVLQWTFLNFPPFSVFLAIFHILKCVFLIFRDFQFSCHIPGPTVCISHFSRFSVI
SSFFKSSSGCFSFSMIFSFLAIFQVLQLTFLNFPHFLSFSPYSRSYSVHFSFFTFFSDFVIFQVVKWLFLIFL
DFQFSC
>ENSMUSP00000096648_c.119:T>A_p.F40L ENSMUST00000099049.4|Gm10719-
202|ENSMUSG00000095547|+|chr9:g.3017531:T>A
MFLIFNDFHFSCHIPCPTVDISFPPFSVFLAIFHVLKCVLLIFRDFQFSRHIPGPTVCISHFFRFFMISSFFT
LSSGCFSFSMIFTFLAIFHVLQWTFLNFPPFSVFLAIFHILKCVFLIFRDFQFSCHIPGPTVCISHFSRFSVI
SSFFKSSSGCFSFSMIFSFLAIFQVLQLTFLNFPHFLSFSPYSRSYSVHFSFFTFFSDFVIFQVVKWLFLIFL
DFQFSC
>ENSMUSP00000096648_c.129:A>T_p.R44W ENSMUST00000099049.4|Gm10719-
202|ENSMUSG00000095547|+|chr9:g.3017541:A>T
MFLIFNDFHFSCHIPCPTVDISFPPFSVFLAIFHVLKCVFLIFWDFQFSRHIPGPTVCISHFFRFFMISSFFT
LSSGCFSFSMIFTFLAIFHVLQWTFLNFPPFSVFLAIFHILKCVFLIFRDFQFSCHIPGPTVCISHFSRFSVI
SSFFKSSSGCFSFSMIFSFLAIFQVLQLTFLNFPHFLSFSPYSRSYSVHFSFFTFFSDFVIFQVVKWLFLIFL
DFQFSC
>ENSMUSP00000096648_c.165:A>T_p.T56S ENSMUST00000099049.4|Gm10719-
202|ENSMUSG00000095547|+|chr9:g.3017577:A>T
MFLIFNDFHFSCHIPCPTVDISFPPFSVFLAIFHVLKCVFLIFRDFQFSRHIPGPSVCISHFFRFFMISSFFT
LSSGCFSFSMIFTFLAIFHVLQWTFLNFPPFSVFLAIFHILKCVFLIFRDFQFSCHIPGPTVCISHFSRFSVI
SSFFKSSSGCFSFSMIFSFLAIFQVLQLTFLNFPHFLSFSPYSRSYSVHFSFFTFFSDFVIFQVVKWLFLIFL
DFQFSC
>ENSMUSP00000096648_c.181:AT>A_p.H61del-fs ENSMUST00000099049.4|Gm10719-
202|ENSMUSG00000095547|+|chr9:g.3017593:AT>A
MFLIFNDFHFSCHIPCPTVDISFPPFSVFLAIFHVLKCVFLIFRDFQFSRHIPGPTVCISHFSGFS
>ENSMUSP00000096648_c.190:G>C_p.R64T ENSMUST00000099049.4|Gm10719-
202|ENSMUSG00000095547|+|chr9:g.3017602:G>C
MFLIFNDFHFSCHIPCPTVDISFPPFSVFLAIFHVLKCVFLIFRDFQFSRHIPGPTVCISHFFTFFMISSFFT
LSSGCFSFSMIFTFLAIFHVLQWTFLNFPPFSVFLAIFHILKCVFLIFRDFQFSCHIPGPTVCISHFSRFSVI
SSFFKSSSGCFSFSMIFSFLAIFQVLQLTFLNFPHFLSFSPYSRSYSVHFSFFTFFSDFVIFQVVKWLFLIFL
DFQFSC
>ENSMUSP00000096648_c.190:G>A_p.R64K ENSMUST00000099049.4|Gm10719-
202|ENSMUSG00000095547|+|chr9:g.3017602:G>A
MFLIFNDFHFSCHIPCPTVDISFPPFSVFLAIFHVLKCVFLIFRDFQFSRHIPGPTVCISHFFKFFMISSFFT
LSSGCFSFSMIFTFLAIFHVLQWTFLNFPPFSVFLAIFHILKCVFLIFRDFQFSCHIPGPTVCISHFSRFSVI
SSFFKSSSGCFSFSMIFSFLAIFQVLQLTFLNFPHFLSFSPYSRSYSVHFSFFTFFSDFVIFQVVKWLFLIFL
DFQFSC
>ENSMUSP00000096648_c.217:C>A_p.T73K ENSMUST00000099049.4|Gm10719-
202|ENSMUSG00000095547|+|chr9:g.3017805:C>A
MFLIFNDFHFSCHIPCPTVDISFPPFSVFLAIFHVLKCVFLIFRDFQFSRHIPGPTVCISHFFRFFMISSFFK
LSSGCFSFSMIFTFLAIFHVLQWTFLNFPPFSVFLAIFHILKCVFLIFRDFQFSCHIPGPTVCISHFSRFSVI
SSFFKSSSGCFSFSMIFSFLAIFQVLQLTFLNFPHFLSFSPYSRSYSVHFSFFTFFSDFVIFQVVKWLFLIFL
DFQFSC
>ENSMUSP00000096648_c.217:CGTT>A_p.TL73del ENSMUST00000099049.4|Gm10719-
202|ENSMUSG00000095547|+|chr9:g.3017805:CGTT>A
MFLIFNDFHFSCHIPCPTVDISFPPFSVFLAIFHVLKCVFLIFRDFQFSRHIPGPTVCISHFFRFFMISSFFK
SSGCFSFSMIFTFLAIFHVLQWTFLNFPPFSVFLAIFHILKCVFLIFRDFQFSCHIPGPTVCISHFSRFSVIS
SFFKSSSGCFSFSMIFSFLAIFQVLQLTFLNFPHFLSFSPYSRSYSVHFSFFTFFSDFVIFQVVKWLFLIFLD
FQFSC
>ENSMUSP00000096648_c.217:CGTT>AGTC_p.T73K ENSMUST00000099049.4|Gm10719-
202|ENSMUSG00000095547|+|chr9:g.3017805:CGTT>AGTC
MFLIFNDFHFSCHIPCPTVDISFPPFSVFLAIFHVLKCVFLIFRDFQFSRHIPGPTVCISHFFRFFMISSFFK
SLSSGCFSFSMIFTFLAIFHVLQWTFLNFPPFSVFLAIFHILKCVFLIFRDFQFSCHIPGPTVCISHFSRFSV
ISSFFKSSSGCFSFSMIFSFLAIFQVLQLTFLNFPHFLSFSPYSRSYSVHFSFFTFFSDFVIFQVVKWLFLIF
LDFQFSC
```

>ENSMUSP00000096648_c.217:CGTT>AGTG_p.T73K ENSMUST00000099049.4|Gm10719-202|ENSMUSG00000095547|+|chr9:g.3017805:CGTT>AGTG
MFLIFNDFHFSCHIPCPTVDISFPPFSVFLAIFHVLKCVFLIFRDFQFSRHIPGPTVCISHFFRFFMISSFFK
WLSSGCFSFSMIFTFLAIFHVLQWTFLNFPPFSVFLAIFHILKCVFLIFRDFQFSCHIPGPTVCISHFSRFSV
ISSFFKSSSGCFSFSMIFSFLAIFQVLQLTFLNFPHFLSFSPYSRSYSVHFSFFTFFSDFVIFQVVKWLFLIF
LDFQFSC
>ENSMUSP00000096648_c.256:C>G_p.T86S ENSMUST00000099049.4|Gm10719-202|ENSMUSG00000095547|+|chr9:g.3017844:C>G
MFLIFNDFHFSCHIPCPTVDISFPPFSVFLAIFHVLKCVFLIFRDFQFSRHIPGPTVCISHFFRFFMISSFFT
LSSGCFSFSMIFSFLAIFHVLQWTFLNFPPFSVFLAIFHILKCVFLIFRDFQFSCHIPGPTVCISHFSRFSVI
SSFFKSSSGCFSFSMIFSFLAIFQVLQLTFLNFPHFLSFSPYSRSYSVHFSFFTFFSDFVIFQVVKWLFLIFL
DFQFSC
>ENSMUSP00000096648_c.256:CT>TA_p.T86I ENSMUST00000099049.4|Gm10719-202|ENSMUSG00000095547|+|chr9:g.3017844:CT>TA
MFLIFNDFHFSCHIPCPTVDISFPPFSVFLAIFHVLKCVFLIFRDFQFSRHIPGPTVCISHFFRFFMISSFFT
LSSGCFSFSMIFIFSCHISCPTVDISKFSTFFCFPRHISHPKVCISHFP*FSVFLPYSRSYSVHFSFFTFFSD
FVIFQVVKWMFLIFHDFQFSRHIPGPTVDISKFSTFFKFLAIFQVLQCAFLIFHVFQ*FCHFSSCQVVVSHFP
*FSVFL
>ENSMUSP00000096648_c.265:C>T_p.A89V ENSMUST00000099049.4|Gm10719-202|ENSMUSG00000095547|+|chr9:g.3017853:C>T
MFLIFNDFHFSCHIPCPTVDISFPPFSVFLAIFHVLKCVFLIFRDFQFSRHIPGPTVCISHFFRFFMISSFFT
LSSGCFSFSMIFTFLVIFHVLQWTFLNFPPFSVFLAIFHILKCVFLIFRDFQFSCHIPGPTVCISHFSRFSVI
SSFFKSSSGCFSFSMIFSFLAIFQVLQLTFLNFPHFLSFSPYSRSYSVHFSFFTFFSDFVIFQVVKWLFLIFL
DFQFSC
>ENSMUSP00000096648_c.307:C>T_p.P103L ENSMUST00000099049.4|Gm10719-202|ENSMUSG00000095547|+|chr9:g.3017895:C>T
MFLIFNDFHFSCHIPCPTVDISFPPFSVFLAIFHVLKCVFLIFRDFQFSRHIPGPTVCISHFFRFFMISSFFT
LSSGCFSFSMIFTFLAIFHVLQWTFLNFPLFSVFLAIFHILKCVFLIFRDFQFSCHIPGPTVCISHFSRFSVI
SSFFKSSSGCFSFSMIFSFLAIFQVLQLTFLNFPHFLSFSPYSRSYSVHFSFFTFFSDFVIFQVVKWLFLIFL
DFQFSC
>ENSMUSP00000096648_c.336:A>G_p.I113V ENSMUST00000099049.4|Gm10719-202|ENSMUSG00000095547|+|chr9:g.3017924:A>G
MFLIFNDFHFSCHIPCPTVDISFPPFSVFLAIFHVLKCVFLIFRDFQFSRHIPGPTVCISHFFRFFMISSFFT
LSSGCFSFSMIFTFLAIFHVLQWTFLNFPPFSVFLAIFHVLKCVFLIFRDFQFSCHIPGPTVCISHFSRFSVI
SSFFKSSSGCFSFSMIFSFLAIFQVLQLTFLNFPHFLSFSPYSRSYSVHFSFFTFFSDFVIFQVVKWLFLIFL
DFQFSC
>ENSMUSP00000096648_c.344:G>T_p.K115N ENSMUST00000099049.4|Gm10719-202|ENSMUSG00000095547|+|chr9:g.3017932:G>T
MFLIFNDFHFSCHIPCPTVDISFPPFSVFLAIFHVLKCVFLIFRDFQFSRHIPGPTVCISHFFRFFMISSFFT
LSSGCFSFSMIFTFLAIFHVLQWTFLNFPPFSVFLAIFHILNCVFLIFRDFQFSCHIPGPTVCISHFSRFSVI
SSFFKSSSGCFSFSMIFSFLAIFQVLQLTFLNFPHFLSFSPYSRSYSVHFSFFTFFSDFVIFQVVKWLFLIFL
DFQFSC
>ENSMUSP00000096648_c.363:C>T_p.R122C ENSMUST00000099049.4|Gm10719-202|ENSMUSG00000095547|+|chr9:g.3017951:C>T
MFLIFNDFHFSCHIPCPTVDISFPPFSVFLAIFHVLKCVFLIFRDFQFSRHIPGPTVCISHFFRFFMISSFFT
LSSGCFSFSMIFTFLAIFHVLQWTFLNFPPFSVFLAIFHILKCVFLIFCDFQFSCHIPGPTVCISHFSRFSVI
SSFFKSSSGCFSFSMIFSFLAIFQVLQLTFLNFPHFLSFSPYSRSYSVHFSFFTFFSDFVIFQVVKWLFLIFL
DFQFSC
>ENSMUSP00000096648_c.364:G>A_p.R122H ENSMUST00000099049.4|Gm10719-202|ENSMUSG00000095547|+|chr9:g.3017952:G>A
MFLIFNDFHFSCHIPCPTVDISFPPFSVFLAIFHVLKCVFLIFRDFQFSRHIPGPTVCISHFFRFFMISSFFT
LSSGCFSFSMIFTFLAIFHVLQWTFLNFPPFSVFLAIFHILKCVFLIFHDFQFSCHIPGPTVCISHFSRFSVI
SSFFKSSSGCFSFSMIFSFLAIFQVLQLTFLNFPHFLSFSPYSRSYSVHFSFFTFFSDFVIFQVVKWLFLIFL
DFQFSC
>ENSMUSP00000096648_c.381:T>C_p.C128R ENSMUST00000099049.4|Gm10719-202|ENSMUSG00000095547|+|chr9:g.3017969:T>C
MFLIFNDFHFSCHIPCPTVDISFPPFSVFLAIFHVLKCVFLIFRDFQFSRHIPGPTVCISHFFRFFMISSFFT
LSSGCFSFSMIFTFLAIFHVLQWTFLNFPPFSVFLAIFHILKCVFLIFRDFQFSRHIPGPTVCISHFSRFSVI

SSFFKSSSGCFSFSMIFSFLAIFQVLQLTFLNFPHFLSFSPYSRSYSVHFSFFTFFSDFVIFQVVKWLFLIFL
DFQFSC
>ENSMUSP00000096648_c.399:A>T_p.T134S ENSMUST00000099049.4|Gm10719-202|ENSMUSG00000095547|+|chr9:g.3017987:A>T
MFLIFNDFHFSCHIPCPTVDISFPPFSVFLAIFHVLKCVFLIFRDFQFSRHIPGPTVCISHFFRFFMISSFFT
LSSGCFSFSMIFTFLAIFHVLQWTFLNFPPFSVFLAIFHILKCVFLIFRDFQFSCHIPGPSVCISHFSRFSVI
SSFFKSSSGCFSFSMIFSFLAIFQVLQLTFLNFPHFLSFSPYSRSYSVHFSFFTFFSDFVIFQVVKWLFLIFL
DFQFSC
>ENSMUSP00000096648_c.420:T>C_p.S141P ENSMUST00000099049.4|Gm10719-202|ENSMUSG00000095547|+|chr9:g.3018008:T>C
MFLIFNDFHFSCHIPCPTVDISFPPFSVFLAIFHVLKCVFLIFRDFQFSRHIPGPTVCISHFFRFFMISSFFT
LSSGCFSFSMIFTFLAIFHVLQWTFLNFPPFSVFLAIFHILKCVFLIFRDFQFSCHIPGPTVCISHFPRFSVI
SSFFKSSSGCFSFSMIFSFLAIFQVLQLTFLNFPHFLSFSPYSRSYSVHFSFFTFFSDFVIFQVVKWLFLIFL
DFQFSC
>ENSMUSP00000096648_c.430:C>T_p.S144L ENSMUST00000099049.4|Gm10719-202|ENSMUSG00000095547|+|chr9:g.3018018:C>T
MFLIFNDFHFSCHIPCPTVDISFPPFSVFLAIFHVLKCVFLIFRDFQFSRHIPGPTVCISHFFRFFMISSFFT
LSSGCFSFSMIFTFLAIFHVLQWTFLNFPPFSVFLAIFHILKCVFLIFRDFQFSCHIPGPTVCISHFSRFLVI
SSFFKSSSGCFSFSMIFSFLAIFQVLQLTFLNFPHFLSFSPYSRSYSVHFSFFTFFSDFVIFQVVKWLFLIFL
DFQFSC
>ENSMUSP00000096648_c.439:C>T_p.S147L ENSMUST00000099049.4|Gm10719-202|ENSMUSG00000095547|+|chr9:g.3018027:C>T
MFLIFNDFHFSCHIPCPTVDISFPPFSVFLAIFHVLKCVFLIFRDFQFSRHIPGPTVCISHFFRFFMISSFFT
LSSGCFSFSMIFTFLAIFHVLQWTFLNFPPFSVFLAIFHILKCVFLIFRDFQFSCHIPGPTVCISHFSRFSVI
LSFFKSSSGCFSFSMIFSFLAIFQVLQLTFLNFPHFLSFSPYSRSYSVHFSFFTFFSDFVIFQVVKWLFLIFL
DFQFSC
>ENSMUSP00000096648_c.509:G>C_p.Q170H ENSMUST00000099049.4|Gm10719-202|ENSMUSG00000095547|+|chr9:g.3018097:G>C
MFLIFNDFHFSCHIPCPTVDISFPPFSVFLAIFHVLKCVFLIFRDFQFSRHIPGPTVCISHFFRFFMISSFFT
LSSGCFSFSMIFTFLAIFHVLQWTFLNFPPFSVFLAIFHILKCVFLIFRDFQFSCHIPGPTVCISHFSRFSVI
SSFFKSSSGCFSFSMIFSFLAIFHVLQLTFLNFPHFLSFSPYSRSYSVHFSFFTFFSDFVIFQVVKWLFLIFL
DFQFSC
>ENSMUSP00000096648_c.520:T>G_p.L174W ENSMUST00000099049.4|Gm10719-202|ENSMUSG00000095547|+|chr9:g.3018108:T>G
MFLIFNDFHFSCHIPCPTVDISFPPFSVFLAIFHVLKCVFLIFRDFQFSRHIPGPTVCISHFFRFFMISSFFT
LSSGCFSFSMIFTFLAIFHVLQWTFLNFPPFSVFLAIFHILKCVFLIFRDFQFSCHIPGPTVCISHFSRFSVI
SSFFKSSSGCFSFSMIFSFLAIFQVLQWTFLNFPHFLSFSPYSRSYSVHFSFFTFFSDFVIFQVVKWLFLIFL
DFQFSC
>ENSMUSP00000096648_c.533:T>A_p.N178K ENSMUST00000099049.4|Gm10719-202|ENSMUSG00000095547|+|chr9:g.3018121:T>A
MFLIFNDFHFSCHIPCPTVDISFPPFSVFLAIFHVLKCVFLIFRDFQFSRHIPGPTVCISHFFRFFMISSFFT
LSSGCFSFSMIFTFLAIFHVLQWTFLNFPPFSVFLAIFHILKCVFLIFRDFQFSCHIPGPTVCISHFSRFSVI
SSFFKSSSGCFSFSMIFSFLAIFQVLQLTFLKFPHFLSFSPYSRSYSVHFSFFTFFSDFVIFQVVKWLFLIFL
DFQFSC
>ENSMUSP00000136170_c.45:T>C_p.C16R ENSMUST00000179272.2|Gm10719-203|ENSMUSG00000095547|+|chr9:g.3018798:T>C
MFLIFHDFQFSCHIPRPTVEISFPTFSVFLAIFHVLKCVFLIFRVFQVSRHIPGPTVCISHFSRFSVILSFFK
LSSGCFSFSLIFSFLAIFHVLLWTFLNFPPFSVFLAIFHVLKCCVFLIFRDFQFSRHIPSPTVCISHFSRFSV
ISSFFKLSSGCFSFSMIFSFLAIFKVLQWTFLNFRPLSFSPYSRSYSVHFSFFRFFSDFVFFQVVKWMFLIFH
DFQFSC
>ENSMUSP00000136170_c.59:A>C_p.E20D ENSMUST00000179272.2|Gm10719-203|ENSMUSG00000095547|+|chr9:g.3018812:A>C
MFLIFHDFQFSCHIPCPTVDISFPTFSVFLAIFHVLKCVFLIFRVFQVSRHIPGPTVCISHFSRFSVILSFFK
LSSGCFSFSLIFSFLAIFHVLLWTFLNFPPFSVFLAIFHVLKCCVFLIFRDFQFSRHIPSPTVCISHFSRFSV
ISSFFKLSSGCFSFSMIFSFLAIFKVLQWTFLNFRPLSFSPYSRSYSVHFSFFRFFSDFVFFQVVKWMFLIFH
DFQFSC
>ENSMUSP00000136170_c.67:T>A_p.F23Y ENSMUST00000179272.2|Gm10719-203|ENSMUSG00000095547|+|chr9:g.3018824:T>A

MFLIFHDFQFSCHIPCPTVEISYPTFSVFLAIFHVLKCVFLIFRVFQVSRHIPGPTVCISHFSRFSVILSFFK
LSSGCFSFSLIFSFLAIFHVLLWTFLNFPPFSVFLAIFHVLKCCVFLIFRDFQFSRHIPSPTVCISHFSRFSV
ISSFFKLSSGCFSFSMIFSFLAIFKVLQWTFLNFRPLSFSPYSRSYSVHFSFFRFFSDFVFFQVVKWMFLIFH
DFQFSC
>ENSMUSP00000136170_c.133:T>A_p.V45D ENSMUST00000179272.2|Gm10719-
203|ENSMUSG00000095547|+|chr9:g.3018890:T>A
MFLIFHDFQFSCHIPCPTVEISFPTFSVFLAIFHVLKCVFLIFRDFQVSRHIPGPTVCISHFSRFSVILSFFK
LSSGCFSFSLIFSFLAIFHVLLWTFLNFPPFSVFLAIFHVLKCCVFLIFRDFQFSRHIPSPTVCISHFSRFSV
ISSFFKLSSGCFSFSMIFSFLAIFKVLQWTFLNFRPLSFSPYSRSYSVHFSFFRFFSDFVFFQVVKWMFLIFH
DFQFSC
>ENSMUSP00000096648_c.550:G>T_p.S184I ENSMUST00000099049.4|Gm10719-
202|ENSMUSG00000095547|+|chr9:g.3018898:G>T
MFLIFNDFHFSCHIPCPTVDISFPPFSVFLAIFHVLKCVFLIFRDFQFSRHIPGPTVCISHFFRFFMISSFFT
LSSGCFSFSMIFTFLAIFHVLQWTFLNFPPFSVFLAIFHILKCVFLIFRDFQFSCHIPGPTVCISHFSRFSVI
SSFFKSSSGCFSFSMIFSFLAIFQVLQLTFLNFPHFLIFSPYSRSYSVHFSFFTFFSDFVIFQVVKWLFLIFL
DFQFSC
>ENSMUSP00000136170_c.141:G>T_p.V48F ENSMUST00000179272.2|Gm10719-
203|ENSMUSG00000095547|+|chr9:g.3018898:G>T
MFLIFHDFQFSCHIPCPTVEISFPTFSVFLAIFHVLKCVFLIFRVFQFSRHIPGPTVCISHFSRFSVILSFFK
LSSGCFSFSLIFSFLAIFHVLLWTFLNFPPFSVFLAIFHVLKCCVFLIFRDFQFSRHIPSPTVCISHFSRFSV
ISSFFKLSSGCFSFSMIFSFLAIFKVLQWTFLNFRPLSFSPYSRSYSVHFSFFRFFSDFVFFQVVKWMFLIFH
DFQFSC
>ENSMUSP00000096648_c.574:A>T_p.Y192F ENSMUST00000099049.4|Gm10719-
202|ENSMUSG00000095547|+|chr9:g.3018922:A>T
MFLIFNDFHFSCHIPCPTVDISFPPFSVFLAIFHVLKCVFLIFRDFQFSRHIPGPTVCISHFFRFFMISSFFT
LSSGCFSFSMIFTFLAIFHVLQWTFLNFPPFSVFLAIFHILKCVFLIFRDFQFSCHIPGPTVCISHFSRFSVI
SSFFKSSSGCFSFSMIFSFLAIFQVLQLTFLNFPHFLSFSPYSRSFSVHFSFFTFFSDFVIFQVVKWLFLIFL
DFQFSC
>ENSMUSP00000136170_c.165:A>T_p.T56S ENSMUST00000179272.2|Gm10719-
203|ENSMUSG00000095547|+|chr9:g.3018922:A>T
MFLIFHDFQFSCHIPCPTVEISFPTFSVFLAIFHVLKCVFLIFRVFQVSRHIPGPSVCISHFSRFSVILSFFK
LSSGCFSFSLIFSFLAIFHVLLWTFLNFPPFSVFLAIFHVLKCCVFLIFRDFQFSRHIPSPTVCISHFSRFSV
ISSFFKLSSGCFSFSMIFSFLAIFKVLQWTFLNFRPLSFSPYSRSYSVHFSFFRFFSDFVFFQVVKWMFLIFH
DFQFSC
>ENSMUSP00000096648_c.576:A>C_p.S193R ENSMUST00000099049.4|Gm10719-
202|ENSMUSG00000095547|+|chr9:g.3018924:A>C
MFLIFNDFHFSCHIPCPTVDISFPPFSVFLAIFHVLKCVFLIFRDFQFSRHIPGPTVCISHFFRFFMISSFFT
LSSGCFSFSMIFTFLAIFHVLQWTFLNFPPFSVFLAIFHILKCVFLIFRDFQFSCHIPGPTVCISHFSRFSVI
SSFFKSSSGCFSFSMIFSFLAIFQVLQLTFLNFPHFLSFSPYSRSYRVHFSFFTFFSDFVIFQVVKWLFLIFL
DFQFSC
>ENSMUSP00000096648_c.595:T>C_p.F199S ENSMUST00000099049.4|Gm10719-
202|ENSMUSG00000095547|+|chr9:g.3018943:T>C
MFLIFNDFHFSCHIPCPTVDISFPPFSVFLAIFHVLKCVFLIFRDFQFSRHIPGPTVCISHFFRFFMISSFFT
LSSGCFSFSMIFTFLAIFHVLQWTFLNFPPFSVFLAIFHILKCVFLIFRDFQFSCHIPGPTVCISHFSRFSVI
SSFFKSSSGCFSFSMIFSFLAIFQVLQLTFLNFPHFLSFSPYSRSYSVHFSFSTFFSDFVIFQVVKWLFLIFL
DFQFSC
>ENSMUSP00000136170_c.186:T>C_p.S63P ENSMUST00000179272.2|Gm10719-
203|ENSMUSG00000095547|+|chr9:g.3018943:T>C
MFLIFHDFQFSCHIPCPTVEISFPTFSVFLAIFHVLKCVFLIFRVFQVSRHIPGPTVCISHFPRFSVILSFFK
LSSGCFSFSLIFSFLAIFHVLLWTFLNFPPFSVFLAIFHVLKCCVFLIFRDFQFSRHIPSPTVCISHFSRFSV
ISSFFKLSSGCFSFSMIFSFLAIFKVLQWTFLNFRPLSFSPYSRSYSVHFSFFRFFSDFVFFQVVKWMFLIFH
DFQFSC
>ENSMUSP00000096648_c.595:TCAC>CCAA_p.F199S ENSMUST00000099049.4|Gm10719-
202|ENSMUSG00000095547|+|chr9:g.3018943:TCAC>CCAA
MFLIFNDFHFSCHIPCPTVDISFPPFSVFLAIFHVLKCVFLIFRDFQFSRHIPGPTVCISHFFRFFMISSFFT
LSSGCFSFSMIFTFLAIFHVLQWTFLNFPPFSVFLAIFHILKCVFLIFRDFQFSCHIPGPTVCISHFSRFSVI
SSFFKSSSGCFSFSMIFSFLAIFQVLQLTFLNFPHFLSFSPYSRSYSVHFSFSNTFFSDFVIFQVVKWLFLIF
LDFQFSC

>ENSMUSP00000136170_c.186:TCAC>CCAA_p.S63P ENSMUST00000179272.2|Gm10719-203|ENSMUSG00000095547|+|chr9:g.3018943:TCAC>CCAA
MFLIFHDFQFSCHIPCPTVEISFPTFSVFLAIFHVLKCVFLIFRVFQVSRHIPGPTVCISHFPTRFSVILSFF
KLSSGCFSFSLIFSFLAIFHVLLWTFLNFPPFSVFLAIFHVLKCCVFLIFRDFQFSRHIPSPTVCISHFSRFS
VISSFFKLSSGCFSFSMIFSFLAIFKVLQWTFLNFRPLSFSPYSRSYSVHFSFFRFFSDFVFFQVVKWMFLIF
HDFQFSC
>ENSMUSP00000136170_c.196:C>T_p.S66L ENSMUST00000179272.2|Gm10719-203|ENSMUSG00000095547|+|chr9:g.3018953:C>T
MFLIFHDFQFSCHIPCPTVEISFPTFSVFLAIFHVLKCVFLIFRVFQVSRHIPGPTVCISHFSRFLVILSFFK
LSSGCFSFSLIFSFLAIFHVLLWTFLNFPPFSVFLAIFHVLKCCVFLIFRDFQFSRHIPSPTVCISHFSRFSV
ISSFFKLSSGCFSFSMIFSFLAIFKVLQWTFLNFRPLSFSPYSRSYSVHFSFFRFFSDFVFFQVVKWMFLIFH
DFQFSC
>ENSMUSP00000136170_c.205:T>C_p.L69S ENSMUST00000179272.2|Gm10719-203|ENSMUSG00000095547|+|chr9:g.3018962:T>C
MFLIFHDFQFSCHIPCPTVEISFPTFSVFLAIFHVLKCVFLIFRVFQVSRHIPGPTVCISHFSRFSVISSFFK
LSSGCFSFSLIFSFLAIFHVLLWTFLNFPPFSVFLAIFHVLKCCVFLIFRDFQFSRHIPSPTVCISHFSRFSV
ISSFFKLSSGCFSFSMIFSFLAIFKVLQWTFLNFRPLSFSPYSRSYSVHFSFFRFFSDFVFFQVVKWMFLIFH
DFQFSC
>ENSMUSP00000136170_c.220:T>C_p.L74S ENSMUST00000179272.2|Gm10719-203|ENSMUSG00000095547|+|chr9:g.3018977:T>C
MFLIFHDFQFSCHIPCPTVEISFPTFSVFLAIFHVLKCVFLIFRVFQVSRHIPGPTVCISHFSRFSVILSFFK
SSSGCFSFSLIFSFLAIFHVLLWTFLNFPPFSVFLAIFHVLKCCVFLIFRDFQFSRHIPSPTVCISHFSRFSV
ISSFFKLSSGCFSFSMIFSFLAIFKVLQWTFLNFRPLSFSPYSRSYSVHFSFFRFFSDFVFFQVVKWMFLIFH
DFQFSC
>ENSMUSP00000136170_c.220:T>G_p.L74W ENSMUST00000179272.2|Gm10719-203|ENSMUSG00000095547|+|chr9:g.3018977:T>G
MFLIFHDFQFSCHIPCPTVEISFPTFSVFLAIFHVLKCVFLIFRVFQVSRHIPGPTVCISHFSRFSVILSFFK
WSSGCFSFSLIFSFLAIFHVLLWTFLNFPPFSVFLAIFHVLKCCVFLIFRDFQFSRHIPSPTVCISHFSRFSV
ISSFFKLSSGCFSFSMIFSFLAIFKVLQWTFLNFRPLSFSPYSRSYSVHFSFFRFFSDFVFFQVVKWMFLIFH
DFQFSC
>ENSMUSP00000136170_c.220:TG>GA_p.L74* ENSMUST00000179272.2|Gm10719-203|ENSMUSG00000095547|+|chr9:g.3018977:TG>GA
MFLIFHDFQFSCHIPCPTVEISFPTFSVFLAIFHVLKCVFLIFRVFQVSRHIPGPTVCISHFSRFSVILSFFK
*VKWLFLIFLDFQFSCHIPCPTVDISKFSTFFSFPCHISCPKVLCISHFP*FSVFSPYSKSYSVHFSFFTFFS
DFVIFQVVKWMFLIFHDFQLSCHIQSPTVDISKFSTFIIFAIF*VLQCAFLIFQVFQ*FRLFSSRQVDVSHFP
*FSVFL
>ENSMUSP00000096648_c.639:T>A_p.L214M ENSMUST00000099049.4|Gm10719-202|ENSMUSG00000095547|+|chr9:g.3018987:T>A
MFLIFNDFHFSCHIPCPTVDISFPPFSVFLAIFHVLKCVFLIFRDFQFSRHIPGPTVCISHFFRFFMISSFFT
LSSGCFSFSMIFTFLAIFHVLQWTFLNFPPFSVFLAIFHILKCVFLIFRDFQFSCHIPGPTVCISHFSRFSVI
SSFFKSSSGCFSFSMIFSFLAIFQVLQLTFLNFPHFLSFSPYSRSYSVHFSFFTFFSDFVIFQVVKWMFLIFL
DFQFSC
>ENSMUSP00000096648_c.655:T>C_p.L219P ENSMUST00000099049.4|Gm10719-202|ENSMUSG00000095547|+|chr9:g.3019003:T>C
MFLIFNDFHFSCHIPCPTVDISFPPFSVFLAIFHVLKCVFLIFRDFQFSRHIPGPTVCISHFFRFFMISSFFT
LSSGCFSFSMIFTFLAIFHVLQWTFLNFPPFSVFLAIFHILKCVFLIFRDFQFSCHIPGPTVCISHFSRFSVI
SSFFKSSSGCFSFSMIFSFLAIFQVLQLTFLNFPHFLSFSPYSRSYSVHFSFFTFFSDFVIFQVVKWLFLIFP
DFQFSC
>rs227483733_ENSMUSP00000096648_c.655:T>A_p.L219H
ENSMUST00000099049.4|Gm10719-202|ENSMUSG00000095547|+|chr9:g.3019003:T>A
MFLIFNDFHFSCHIPCPTVDISFPPFSVFLAIFHVLKCVFLIFRDFQFSRHIPGPTVCISHFFRFFMISSFFT
LSSGCFSFSMIFTFLAIFHVLQWTFLNFPPFSVFLAIFHILKCVFLIFRDFQFSCHIPGPTVCISHFSRFSVI
SSFFKSSSGCFSFSMIFSFLAIFQVLQLTFLNFPHFLSFSPYSRSYSVHFSFFTFFSDFVIFQVVKWLFLIFH
DFQFSC
>rs227483733_ENSMUSP00000136170_c.246:T>A_p.L83M
ENSMUST00000179272.2|Gm10719-203|ENSMUSG00000095547|+|chr9:g.3019003:T>A
MFLIFHDFQFSCHIPCPTVEISFPTFSVFLAIFHVLKCVFLIFRVFQVSRHIPGPTVCISHFSRFSVILSFFK
LSSGCFSFSMIFSFLAIFHVLLWTFLNFPPFSVFLAIFHVLKCCVFLIFRDFQFSRHIPSPTVCISHFSRFSV

ISSFFKLSSGCFSFSMIFSFLAIFKVLQWTFLNFRPLSFSPYSRSYSVHFSFFRFFSDFVFFQVVKWMFLIFH
DFQFSC
>ENSMUSP00000136170_c.283:T>A_p.L95Q ENSMUST00000179272.2|Gm10719-
203|ENSMUSG00000095547|+|chr9:g.3019040:T>A
MFLIFHDFQFSCHIPCPTVEISFPTFSVFLAIFHVLKCVFLIFRVFQVSRHIPGPTVCISHFSRFSVILSFFK
LSSGCFSFSLIFSFLAIFHVLQWTFLNFPPFSVFLAIFHVLKCCVFLIFRDFQFSRHIPSPTVCISHFSRFSV
ISSFFKLSSGCFSFSMIFSFLAIFKVLQWTFLNFRPLSFSPYSRSYSVHFSFFRFFSDFVFFQVVKWMFLIFH
DFQFSC
>ENSMUSP00000136170_c.286:G>T_p.W96L ENSMUST00000179272.2|Gm10719-
203|ENSMUSG00000095547|+|chr9:g.3019043:G>T
MFLIFHDFQFSCHIPCPTVEISFPTFSVFLAIFHVLKCVFLIFRVFQVSRHIPGPTVCISHFSRFSVILSFFK
LSSGCFSFSLIFSFLAIFHVLLLTFLNFPPFSVFLAIFHVLKCCVFLIFRDFQFSRHIPSPTVCISHFSRFSV
ISSFFKLSSGCFSFSMIFSFLAIFKVLQWTFLNFRPLSFSPYSRSYSVHFSFFRFFSDFVFFQVVKWMFLIFH
DFQFSC
>ENSMUSP00000136170_c.306:C>A_p.P103T ENSMUST00000179272.2|Gm10719-
203|ENSMUSG00000095547|+|chr9:g.3019063:C>A
MFLIFHDFQFSCHIPCPTVEISFPTFSVFLAIFHVLKCVFLIFRVFQVSRHIPGPTVCISHFSRFSVILSFFK
LSSGCFSFSLIFSFLAIFHVLLWTFLNFPTFSVFLAIFHVLKCCVFLIFRDFQFSRHIPSPTVCISHFSRFSV
ISSFFKLSSGCFSFSMIFSFLAIFKVLQWTFLNFRPLSFSPYSRSYSVHFSFFRFFSDFVFFQVVKWMFLIFH
DFQFSC
>ENSMUSP00000096646_c.45:T>C_p.C16R ENSMUST00000099047.4|Gm10719-
201|ENSMUSG00000095547|+|chr9:g.3020200:T>C
MFLIFHDFQFSCHIPRPTMDISFPTFSVFLAIFHVLKCVFLIFRDFQFSRHIPGPTVCISHFSGFSVISSFFK
SSSGCFSFSMIFSFLAIFHVLQWTFLNFPPFSVFLAIFHVLKCVFLIFSDFQFSRHIPGPTVCISHFSRFSVI
SSFFKLSSGCFSFSMIFSFLAIFHVPQWTFLNFPPFSSYSFSPYSRSYSVHFSFFTFFSDFVIFQVVKWMFLI
FLDFQFSC
>ENSMUSP00000096646_c.54:A>T_p.M19L ENSMUST00000099047.4|Gm10719-
201|ENSMUSG00000095547|+|chr9:g.3020209:A>T
MFLIFHDFQFSCHIPCPTLDISFPTFSVFLAIFHVLKCVFLIFRDFQFSRHIPGPTVCISHFSGFSVISSFFK
SSSGCFSFSMIFSFLAIFHVLQWTFLNFPPFSVFLAIFHVLKCVFLIFSDFQFSRHIPGPTVCISHFSRFSVI
SSFFKLSSGCFSFSMIFSFLAIFHVPQWTFLNFPPFSSYSFSPYSRSYSVHFSFFTFFSDFVIFQVVKWMFLI
FLDFQFSC
>ENSMUSP00000096646_c.54:A>C_p.M19L ENSMUST00000099047.4|Gm10719-
201|ENSMUSG00000095547|+|chr9:g.3020209:A>C
MFLIFHDFQFSCHIPCPTLDISFPTFSVFLAIFHVLKCVFLIFRDFQFSRHIPGPTVCISHFSGFSVISSFFK
SSSGCFSFSMIFSFLAIFHVLQWTFLNFPPFSVFLAIFHVLKCVFLIFSDFQFSRHIPGPTVCISHFSRFSVI
SSFFKLSSGCFSFSMIFSFLAIFHVPQWTFLNFPPFSSYSFSPYSRSYSVHFSFFTFFSDFVIFQVVKWMFLI
FLDFQFSC
>ENSMUSP00000096646_c.54:A>G_p.M19V ENSMUST00000099047.4|Gm10719-
201|ENSMUSG00000095547|+|chr9:g.3020209:A>G
MFLIFHDFQFSCHIPCPTVDISFPTFSVFLAIFHVLKCVFLIFRDFQFSRHIPGPTVCISHFSGFSVISSFFK
SSSGCFSFSMIFSFLAIFHVLQWTFLNFPPFSVFLAIFHVLKCVFLIFSDFQFSRHIPGPTVCISHFSRFSVI
SSFFKLSSGCFSFSMIFSFLAIFHVPQWTFLNFPPFSSYSFSPYSRSYSVHFSFFTFFSDFVIFQVVKWMFLI
FLDFQFSC
>ENSMUSP00000096646_c.67:T>A_p.F23Y ENSMUST00000099047.4|Gm10719-
201|ENSMUSG00000095547|+|chr9:g.3020226:T>A
MFLIFHDFQFSCHIPCPTMDISYPTFSVFLAIFHVLKCVFLIFRDFQFSRHIPGPTVCISHFSGFSVISSFFK
SSSGCFSFSMIFSFLAIFHVLQWTFLNFPPFSVFLAIFHVLKCVFLIFSDFQFSRHIPGPTVCISHFSRFSVI
SSFFKLSSGCFSFSMIFSFLAIFHVPQWTFLNFPPFSSYSFSPYSRSYSVHFSFFTFFSDFVIFQVVKWMFLI
FLDFQFSC
>ENSMUSP00000096646_c.72:A>C_p.T25P ENSMUST00000099047.4|Gm10719-
201|ENSMUSG00000095547|+|chr9:g.3020231:A>C
MFLIFHDFQFSCHIPCPTMDISFPPFSVFLAIFHVLKCVFLIFRDFQFSRHIPGPTVCISHFSGFSVISSFFK
SSSGCFSFSMIFSFLAIFHVLQWTFLNFPPFSVFLAIFHVLKCVFLIFSDFQFSRHIPGPTVCISHFSRFSVI
SSFFKLSSGCFSFSMIFSFLAIFHVPQWTFLNFPPFSSYSFSPYSRSYSVHFSFFTFFSDFVIFQVVKWMFLI
FLDFQFSC
>ENSMUSP00000096646_c.112:G>A_p.C38Y ENSMUST00000099047.4|Gm10719-
201|ENSMUSG00000095547|+|chr9:g.3020271:G>A

MFLIFHDFQFSCHIPCPTMDISFPTFSVFLAIFHVLKYVFLIFRDFQFSRHIPGPTVCISHFSGFSVISSFFK
SSSGCFSFSMIFSFLAIFHVLQWTFLNFPPFSVFLAIFHVLKCVFLIFSDFQFSRHIPGPTVCISHFSRFSVI
SSFFKLSSGCFSFSMIFSFLAIFHVPQWTFLNFPPFSSYSFSPYSRSYSVHFSFFTFFSDFVIFQVVKWMFLI
FLDFQFSC
>ENSMUSP00000096646_c.129:C>T_p.R44C ENSMUST00000099047.4|Gm10719-
201|ENSMUSG00000095547|+|chr9:g.3020288:C>T
MFLIFHDFQFSCHIPCPTMDISFPTFSVFLAIFHVLKCVFLIFCDFQFSRHIPGPTVCISHFSGFSVISSFFK
SSSGCFSFSMIFSFLAIFHVLQWTFLNFPPFSVFLAIFHVLKCVFLIFSDFQFSRHIPGPTVCISHFSRFSVI
SSFFKLSSGCFSFSMIFSFLAIFHVPQWTFLNFPPFSSYSFSPYSRSYSVHFSFFTFFSDFVIFQVVKWMFLI
FLDFQFSC
>ENSMUSP00000136170_c.366:C>T_p.R123C ENSMUST00000179272.2|Gm10719-
203|ENSMUSG00000095547|+|chr9:g.3020521:C>T
MFLIFHDFQFSCHIPCPTVEISFPTFSVFLAIFHVLKCVFLIFRVFQVSRHIPGPTVCISHFSRFSVILSFFK
LSSGCFSFSLIFSFLAIFHVLLWTFLNFPPFSVFLAIFHVLKCCVFLIFCDFQFSRHIPSPTVCISHFSRFSV
ISSFFKLSSGCFSFSMIFSFLAIFKVLQWTFLNFRPLSFSPYSRSYSVHFSFFRFFSDFVFFQVVKWMFLIFH
DFQFSC
>ENSMUSP00000136170_c.367:G>A_p.R123H ENSMUST00000179272.2|Gm10719-
203|ENSMUSG00000095547|+|chr9:g.3020522:G>A
MFLIFHDFQFSCHIPCPTVEISFPTFSVFLAIFHVLKCVFLIFRVFQVSRHIPGPTVCISHFSRFSVILSFFK
LSSGCFSFSLIFSFLAIFHVLLWTFLNFPPFSVFLAIFHVLKCCVFLIFHDFQFSRHIPSPTVCISHFSRFSV
ISSFFKLSSGCFSFSMIFSFLAIFKVLQWTFLNFRPLSFSPYSRSYSVHFSFFRFFSDFVFFQVVKWMFLIFH
DFQFSC
>ENSMUSP00000136170_c.384:C>T_p.R129C ENSMUST00000179272.2|Gm10719-
203|ENSMUSG00000095547|+|chr9:g.3020539:C>T
MFLIFHDFQFSCHIPCPTVEISFPTFSVFLAIFHVLKCVFLIFRVFQVSRHIPGPTVCISHFSRFSVILSFFK
LSSGCFSFSLIFSFLAIFHVLLWTFLNFPPFSVFLAIFHVLKCCVFLIFRDFQFSCHIPSPTVCISHFSRFSV
ISSFFKLSSGCFSFSMIFSFLAIFKVLQWTFLNFRPLSFSPYSRSYSVHFSFFRFFSDFVFFQVVKWMFLIFH
DFQFSC
>ENSMUSP00000136170_c.396:A>G_p.S133G ENSMUST00000179272.2|Gm10719-
203|ENSMUSG00000095547|+|chr9:g.3020551:A>G
MFLIFHDFQFSCHIPCPTVEISFPTFSVFLAIFHVLKCVFLIFRVFQVSRHIPGPTVCISHFSRFSVILSFFK
LSSGCFSFSLIFSFLAIFHVLLWTFLNFPPFSVFLAIFHVLKCCVFLIFRDFQFSRHIPGPTVCISHFSRFSV
ISSFFKLSSGCFSFSMIFSFLAIFKVLQWTFLNFRPLSFSPYSRSYSVHFSFFRFFSDFVFFQVVKWMFLIFH
DFQFSC
>ENSMUSP00000136170_c.396:A>T_p.S133C ENSMUST00000179272.2|Gm10719-
203|ENSMUSG00000095547|+|chr9:g.3020551:A>T
MFLIFHDFQFSCHIPCPTVEISFPTFSVFLAIFHVLKCVFLIFRVFQVSRHIPGPTVCISHFSRFSVILSFFK
LSSGCFSFSLIFSFLAIFHVLLWTFLNFPPFSVFLAIFHVLKCCVFLIFRDFQFSRHIPCPTVCISHFSRFSV
ISSFFKLSSGCFSFSMIFSFLAIFKVLQWTFLNFRPLSFSPYSRSYSVHFSFFRFFSDFVFFQVVKWMFLIFH
DFQFSC
>ENSMUSP00000136170_c.402:A>T_p.T135S ENSMUST00000179272.2|Gm10719-
203|ENSMUSG00000095547|+|chr9:g.3020557:A>T
MFLIFHDFQFSCHIPCPTVEISFPTFSVFLAIFHVLKCVFLIFRVFQVSRHIPGPTVCISHFSRFSVILSFFK
LSSGCFSFSLIFSFLAIFHVLLWTFLNFPPFSVFLAIFHVLKCCVFLIFRDFQFSRHIPSPSVCISHFSRFSV
ISSFFKLSSGCFSFSMIFSFLAIFKVLQWTFLNFRPLSFSPYSRSYSVHFSFFRFFSDFVFFQVVKWMFLIFH
DFQFSC
>ENSMUSP00000136170_c.433:C>T_p.S145L ENSMUST00000179272.2|Gm10719-
203|ENSMUSG00000095547|+|chr9:g.3020588:C>T
MFLIFHDFQFSCHIPCPTVEISFPTFSVFLAIFHVLKCVFLIFRVFQVSRHIPGPTVCISHFSRFSVILSFFK
LSSGCFSFSLIFSFLAIFHVLLWTFLNFPPFSVFLAIFHVLKCCVFLIFRDFQFSRHIPSPTVCISHFSRFLV
ISSFFKLSSGCFSFSMIFSFLAIFKVLQWTFLNFRPLSFSPYSRSYSVHFSFFRFFSDFVFFQVVKWMFLIFH
DFQFSC
>ENSMUSP00000136170_c.442:C>T_p.S148L ENSMUST00000179272.2|Gm10719-
203|ENSMUSG00000095547|+|chr9:g.3020597:C>T
MFLIFHDFQFSCHIPCPTVEISFPTFSVFLAIFHVLKCVFLIFRVFQVSRHIPGPTVCISHFSRFSVILSFFK
LSSGCFSFSLIFSFLAIFHVLLWTFLNFPPFSVFLAIFHVLKCCVFLIFRDFQFSRHIPSPTVCISHFSRFSV
ILSFFKLSSGCFSFSMIFSFLAIFKVLQWTFLNFRPLSFSPYSRSYSVHFSFFRFFSDFVFFQVVKWMFLIFH
DFQFSC

>ENSMUSP00000136170_c.457:T>G_p.L153W ENSMUST00000179272.2|Gm10719-
203|ENSMUSG00000095547|+|chr9:g.3020612:T>G
MFLIFHDFQFSCHIPCPTVEISFPTFSVFLAIFHVLKCVFLIFRVFQVSRHIPGPTVCISHFSRFSVILSFFK
LSSGCFSFSLIFSFLAIFHVLLWTFLNFPPFSVFLAIFHVLKCCVFLIFRDFQFSRHIPSPTVCISHFSRFSV
ISSFFKWSSGCFSFSMIFSFLAIFKVLQWTFLNFRPLSFSPYSRSYSVHFSFFRFFSDFVFFQVVKWMFLIFH
DFQFSC
>ENSMUSP00000136170_c.457:T>C_p.L153S ENSMUST00000179272.2|Gm10719-
203|ENSMUSG00000095547|+|chr9:g.3020612:T>C
MFLIFHDFQFSCHIPCPTVEISFPTFSVFLAIFHVLKCVFLIFRVFQVSRHIPGPTVCISHFSRFSVILSFFK
LSSGCFSFSLIFSFLAIFHVLLWTFLNFPPFSVFLAIFHVLKCCVFLIFRDFQFSRHIPSPTVCISHFSRFSV
ISSFFKSSSGCFSFSMIFSFLAIFKVLQWTFLNFRPLSFSPYSRSYSVHFSFFRFFSDFVFFQVVKWMFLIFH
DFQFSC
>ENSMUSP00000136170_c.494:C>A_p.S165R ENSMUST00000179272.2|Gm10719-
203|ENSMUSG00000095547|+|chr9:g.3020649:C>A
MFLIFHDFQFSCHIPCPTVEISFPTFSVFLAIFHVLKCVFLIFRVFQVSRHIPGPTVCISHFSRFSVILSFFK
LSSGCFSFSLIFSFLAIFHVLLWTFLNFPPFSVFLAIFHVLKCCVFLIFRDFQFSRHIPSPTVCISHFSRFSV
ISSFFKLSSGCFSFSMIFRFLAIFKVLQWTFLNFRPLSFSPYSRSYSVHFSFFRFFSDFVFFQVVKWMFLIFH
DFQFSC
>ENSMUSP00000136170_c.510:A>C_p.K171Q ENSMUST00000179272.2|Gm10719-
203|ENSMUSG00000095547|+|chr9:g.3020665:A>C
MFLIFHDFQFSCHIPCPTVEISFPTFSVFLAIFHVLKCVFLIFRVFQVSRHIPGPTVCISHFSRFSVILSFFK
LSSGCFSFSLIFSFLAIFHVLLWTFLNFPPFSVFLAIFHVLKCCVFLIFRDFQFSRHIPSPTVCISHFSRFSV
ISSFFKLSSGCFSFSMIFSFLAIFQVLQWTFLNFRPLSFSPYSRSYSVHFSFFRFFSDFVFFQVVKWMFLIFH
DFQFSC
>ENSMUSP00000136170_c.510:AAA>CAC_p.K171H ENSMUST00000179272.2|Gm10719-
203|ENSMUSG00000095547|+|chr9:g.3020665:AAA>CAC
MFLIFHDFQFSCHIPCPTVEISFPTFSVFLAIFHVLKCVFLIFRVFQVSRHIPGPTVCISHFSRFSVILSFFK
LSSGCFSFSLIFSFLAIFHVLLWTFLNFPPFSVFLAIFHVLKCCVFLIFRDFQFSRHIPSPTVCISHFSRFSV
ISSFFKLSSGCFSFSMIFSFLAIFHKSYSGHF*IFDLYHFRHILGPTVCISHFSGFSVISSFFKSSSGCFSFS
MIFSFL
>ENSMUSP00000136170_c.526:C>A_p.T176K ENSMUST00000179272.2|Gm10719-
203|ENSMUSG00000095547|+|chr9:g.3020681:C>A
MFLIFHDFQFSCHIPCPTVEISFPTFSVFLAIFHVLKCVFLIFRVFQVSRHIPGPTVCISHFSRFSVILSFFK
LSSGCFSFSLIFSFLAIFHVLLWTFLNFPPFSVFLAIFHVLKCCVFLIFRDFQFSRHIPSPTVCISHFSRFSV
ISSFFKLSSGCFSFSMIFSFLAIFKVLQWKFLNFRPLSFSPYSRSYSVHFSFFRFFSDFVFFQVVKWMFLIFH
DFQFSC
>ENSMUSP00000136170_c.541:G>A_p.R181Q ENSMUST00000179272.2|Gm10719-
203|ENSMUSG00000095547|+|chr9:g.3020696:G>A
MFLIFHDFQFSCHIPCPTVEISFPTFSVFLAIFHVLKCVFLIFRVFQVSRHIPGPTVCISHFSRFSVILSFFK
LSSGCFSFSLIFSFLAIFHVLLWTFLNFPPFSVFLAIFHVLKCCVFLIFRDFQFSRHIPSPTVCISHFSRFSV
ISSFFKLSSGCFSFSMIFSFLAIFKVLQWTFLNFQPLSFSPYSRSYSVHFSFFRFFSDFVFFQVVKWMFLIFH
DFQFSC
>ENSMUSP00000136170_c.548:A>T_p.L183F ENSMUST00000179272.2|Gm10719-
203|ENSMUSG00000095547|+|chr9:g.3020703:A>T
MFLIFHDFQFSCHIPCPTVEISFPTFSVFLAIFHVLKCVFLIFRVFQVSRHIPGPTVCISHFSRFSVILSFFK
LSSGCFSFSLIFSFLAIFHVLLWTFLNFPPFSVFLAIFHVLKCCVFLIFRDFQFSRHIPSPTVCISHFSRFSV
ISSFFKLSSGCFSFSMIFSFLAIFKVLQWTFLNFRPFSFSPYSRSYSVHFSFFRFFSDFVFFQVVKWMFLIFH
DFQFSC
>ENSMUSP00000136170_c.574:A>T_p.Y192F ENSMUST00000179272.2|Gm10719-
203|ENSMUSG00000095547|+|chr9:g.3020791:A>T
MFLIFHDFQFSCHIPCPTVEISFPTFSVFLAIFHVLKCVFLIFRVFQVSRHIPGPTVCISHFSRFSVILSFFK
LSSGCFSFSLIFSFLAIFHVLLWTFLNFPPFSVFLAIFHVLKCCVFLIFRDFQFSRHIPSPTVCISHFSRFSV
ISSFFKLSSGCFSFSMIFSFLAIFKVLQWTFLNFRPLSFSPYSRSFSVHFSFFRFFSDFVFFQVVKWMFLIFH
DFQFSC
>ENSMUSP00000096646_c.165:A>T_p.T56S ENSMUST00000099047.4|Gm10719-
201|ENSMUSG00000095547|+|chr9:g.3020791:A>T
MFLIFHDFQFSCHIPCPTMDISFPTFSVFLAIFHVLKCVFLIFRDFQFSRHIPGPSVCISHFSGFSVISSFFK
SSSGCFSFSMIFSFLAIFHVLQWTFLNFPPPFSVFLAIFHVLKCVFLIFSDFQFSRHIPGPTVCISHFSRFSVI

SSFFKLSSGCFSFSMIFSFLAIFHVPQWTFLNFPPFSSYSFSPYSRSYSVHFSFFTFFSDFVIFQVVKWMFLI
FLDFQFSC
>ENSMUSP00000136170_c.598:G>T_p.R200M ENSMUST00000179272.2|Gm10719-
203|ENSMUSG00000095547|+|chr9:g.3020815:G>T
MFLIFHDFQFSCHIPCPTVEISFPTFSVFLAIFHVLKCVFLIFRVFQVSRHIPGPTVCISHFSRFSVILSFFK
LSSGCFSFSLIFSFLAIFHVLLWTFLNFPPFSVFLAIFHVLKCCVFLIFRDFQFSRHIPSPTVCISHFSRFSV
ISSFFKLSSGCFSFSMIFSFLAIFKVLQWTFLNFRPLSFSPYSRSYSVHFSFFMFFSDFVFFQVVKWMFLIFH
DFQFSC
>ENSMUSP00000096646_c.189:G>T_p.G64C ENSMUST00000099047.4|Gm10719-
201|ENSMUSG00000095547|+|chr9:g.3020815:G>T
MFLIFHDFQFSCHIPCPTMDISFPTFSVFLAIFHVLKCVFLIFRDFQFSRHIPGPTVCISHFSCFSVISSFFK
SSSGCFSFSMIFSFLAIFHVLQWTFLNFPPFSVFLAIFHVLKCVFLIFSDFQFSRHIPGPTVCISHFSRFSVI
SSFFKLSSGCFSFSMIFSFLAIFHVPQWTFLNFPPFSSYSFSPYSRSYSVHFSFFTFFSDFVIFQVVKWMFLI
FLDFQFSC
>ENSMUSP00000136170_c.598:G>C_p.R200T ENSMUST00000179272.2|Gm10719-
203|ENSMUSG00000095547|+|chr9:g.3020815:G>C
MFLIFHDFQFSCHIPCPTVEISFPTFSVFLAIFHVLKCVFLIFRVFQVSRHIPGPTVCISHFSRFSVILSFFK
LSSGCFSFSLIFSFLAIFHVLLWTFLNFPPFSVFLAIFHVLKCCVFLIFRDFQFSRHIPSPTVCISHFSRFSV
ISSFFKLSSGCFSFSMIFSFLAIFKVLQWTFLNFRPLSFSPYSRSYSVHFSFFTFFSDFVFFQVVKWMFLIFH
DFQFSC
>ENSMUSP00000096646_c.189:G>C_p.G64R ENSMUST00000099047.4|Gm10719-
201|ENSMUSG00000095547|+|chr9:g.3020815:G>C
MFLIFHDFQFSCHIPCPTMDISFPTFSVFLAIFHVLKCVFLIFRDFQFSRHIPGPTVCISHFSRFSVISSFFK
SSSGCFSFSMIFSFLAIFHVLQWTFLNFPPFSVFLAIFHVLKCVFLIFSDFQFSRHIPGPTVCISHFSRFSVI
SSFFKLSSGCFSFSMIFSFLAIFHVPQWTFLNFPPFSSYSFSPYSRSYSVHFSFFTFFSDFVIFQVVKWMFLI
FLDFQFSC
>ENSMUSP00000096646_c.196:C>T_p.S66L ENSMUST00000099047.4|Gm10719-
201|ENSMUSG00000095547|+|chr9:g.3020822:C>T
MFLIFHDFQFSCHIPCPTMDISFPTFSVFLAIFHVLKCVFLIFRDFQFSRHIPGPTVCISHFSGFLVISSFFK
SSSGCFSFSMIFSFLAIFHVLQWTFLNFPPFSVFLAIFHVLKCVFLIFSDFQFSRHIPGPTVCISHFSRFSVI
SSFFKLSSGCFSFSMIFSFLAIFHVPQWTFLNFPPFSSYSFSPYSRSYSVHFSFFTFFSDFVIFQVVKWMFLI
FLDFQFSC
>ENSMUSP00000136170_c.618:T>A_p.F207I ENSMUST00000179272.2|Gm10719-
203|ENSMUSG00000095547|+|chr9:g.3020835:T>A
MFLIFHDFQFSCHIPCPTVEISFPTFSVFLAIFHVLKCVFLIFRVFQVSRHIPGPTVCISHFSRFSVILSFFK
LSSGCFSFSLIFSFLAIFHVLLWTFLNFPPFSVFLAIFHVLKCCVFLIFRDFQFSRHIPSPTVCISHFSRFSV
ISSFFKLSSGCFSFSMIFSFLAIFKVLQWTFLNFRPLSFSPYSRSYSVHFSFFRFFSDFVIFQVVKWMFLIFH
DFQFSC
>ENSMUSP00000096646_c.285:T>C_p.W96R ENSMUST00000099047.4|Gm10719-
201|ENSMUSG00000095547|+|chr9:g.3020911:T>C
MFLIFHDFQFSCHIPCPTMDISFPTFSVFLAIFHVLKCVFLIFRDFQFSRHIPGPTVCISHFSGFSVISSFFK
SSSGCFSFSMIFSFLAIFHVLQRTFLNFPPFSVFLAIFHVLKCVFLIFSDFQFSRHIPGPTVCISHFSRFSVI
SSFFKLSSGCFSFSMIFSFLAIFHVPQWTFLNFPPFSSYSFSPYSRSYSVHFSFFTFFSDFVIFQVVKWMFLI
FLDFQFSC
>ENSMUSP00000096646_c.335:T>A_p.H112Q ENSMUST00000099047.4|Gm10719-
201|ENSMUSG00000095547|+|chr9:g.3020961:T>A
MFLIFHDFQFSCHIPCPTMDISFPTFSVFLAIFHVLKCVFLIFRDFQFSRHIPGPTVCISHFSGFSVISSFFK
SSSGCFSFSMIFSFLAIFHVLQWTFLNFPPFSVFLAIFQVLKCVFLIFSDFQFSRHIPGPTVCISHFSRFSVI
SSFFKLSSGCFSFSMIFSFLAIFHVPQWTFLNFPPFSSYSFSPYSRSYSVHFSFFTFFSDFVIFQVVKWMFLI
FLDFQFSC
>ENSMUSP00000096646_c.363:A>T_p.S122C ENSMUST00000099047.4|Gm10719-
201|ENSMUSG00000095547|+|chr9:g.3020989:A>T
MFLIFHDFQFSCHIPCPTMDISFPTFSVFLAIFHVLKCVFLIFRDFQFSRHIPGPTVCISHFSGFSVISSFFK
SSSGCFSFSMIFSFLAIFHVLQWTFLNFPPFSVFLAIFHVLKCVFLIFCDFQFSRHIPGPTVCISHFSRFSVI
SSFFKLSSGCFSFSMIFSFLAIFHVPQWTFLNFPPFSSYSFSPYSRSYSVHFSFFTFFSDFVIFQVVKWMFLI
FLDFQFSC
>ENSMUSP00000096646_c.363:A>C_p.S122R ENSMUST00000099047.4|Gm10719-
201|ENSMUSG00000095547|+|chr9:g.3020989:A>C

MFLIFHDFQFSCHIPCPTMDISFPTFSVFLAIFHVLKCVFLIFRDFQFSRHIPGPTVCISHFSGFSVISSFFK
SSSGCFSFSMIFSFLAIFHVLQWTFLNFPPFSVFLAIFHVLKCVFLIFRDFQFSRHIPGPTVCISHFSRFSVI
SSFFKLSSGCFSFSMIFSFLAIFHVPQWTFLNFPPFSSYSFSPYSRSYSVHFSFFTFFSDFVIFQVVKWMFLI
FLDFQFSC
>ENSMUSP00000096646_c.399:A>T_p.T134S ENSMUST00000099047.4|Gm10719-201|ENSMUSG00000095547|+|chr9:g.3021025:A>T
MFLIFHDFQFSCHIPCPTMDISFPTFSVFLAIFHVLKCVFLIFRDFQFSRHIPGPTVCISHFSGFSVISSFFK
SSSGCFSFSMIFSFLAIFHVLQWTFLNFPPFSVFLAIFHVLKCVFLIFSDFQFSRHIPGPSVCISHFSRFSVI
SSFFKLSSGCFSFSMIFSFLAIFHVPQWTFLNFPPFSSYSFSPYSRSYSVHFSFFTFFSDFVIFQVVKWMFLI
FLDFQFSC
>ENSMUSP00000096646_c.430:C>T_p.S144L ENSMUST00000099047.4|Gm10719-201|ENSMUSG00000095547|+|chr9:g.3021056:C>T
MFLIFHDFQFSCHIPCPTMDISFPTFSVFLAIFHVLKCVFLIFRDFQFSRHIPGPTVCISHFSGFSVISSFFK
SSSGCFSFSMIFSFLAIFHVLQWTFLNFPPFSVFLAIFHVLKCVFLIFSDFQFSRHIPGPTVCISHFSRFLVI
SSFFKLSSGCFSFSMIFSFLAIFHVPQWTFLNFPPFSSYSFSPYSRSYSVHFSFFTFFSDFVIFQVVKWMFLI
FLDFQFSC
>ENSMUSP00000096646_c.454:T>C_p.L152S ENSMUST00000099047.4|Gm10719-201|ENSMUSG00000095547|+|chr9:g.3021080:T>C
MFLIFHDFQFSCHIPCPTMDISFPTFSVFLAIFHVLKCVFLIFRDFQFSRHIPGPTVCISHFSGFSVISSFFK
SSSGCFSFSMIFSFLAIFHVLQWTFLNFPPFSVFLAIFHVLKCVFLIFSDFQFSRHIPGPTVCISHFSRFSVI
SSFFKSSSGCFSFSMIFSFLAIFHVPQWTFLNFPPFSSYSFSPYSRSYSVHFSFFTFFSDFVIFQVVKWMFLI
FLDFQFSC
>ENSMUSP00000096646_c.454:T>G_p.L152W ENSMUST00000099047.4|Gm10719-201|ENSMUSG00000095547|+|chr9:g.3021080:T>G
MFLIFHDFQFSCHIPCPTMDISFPTFSVFLAIFHVLKCVFLIFRDFQFSRHIPGPTVCISHFSGFSVISSFFK
SSSGCFSFSMIFSFLAIFHVLQWTFLNFPPFSVFLAIFHVLKCVFLIFSDFQFSRHIPGPTVCISHFSRFSVI
SSFFKWSSGCFSFSMIFSFLAIFHVPQWTFLNFPPFSSYSFSPYSRSYSVHFSFFTFFSDFVIFQVVKWMFLI
FLDFQFSC
>ENSMUSP00000096646_c.490:G>T_p.S164I ENSMUST00000099047.4|Gm10719-201|ENSMUSG00000095547|+|chr9:g.3021116:G>T
MFLIFHDFQFSCHIPCPTMDISFPTFSVFLAIFHVLKCVFLIFRDFQFSRHIPGPTVCISHFSGFSVISSFFK
SSSGCFSFSMIFSFLAIFHVLQWTFLNFPPFSVFLAIFHVLKCVFLIFSDFQFSRHIPGPTVCISHFSRFSVI
SSFFKLSSGCFSFSMIFIFLAIFHVPQWTFLNFPPFSSYSFSPYSRSYSVHFSFFTFFSDFVIFQVVKWMFLI
FLDFQFSC
>ENSMUSP00000096646_c.514:C>T_p.P172L ENSMUST00000099047.4|Gm10719-201|ENSMUSG00000095547|+|chr9:g.3021140:C>T
MFLIFHDFQFSCHIPCPTMDISFPTFSVFLAIFHVLKCVFLIFRDFQFSRHIPGPTVCISHFSGFSVISSFFK
SSSGCFSFSMIFSFLAIFHVLQWTFLNFPPFSVFLAIFHVLKCVFLIFSDFQFSRHIPGPTVCISHFSRFSVI
SSFFKLSSGCFSFSMIFSFLAIFHVLQWTFLNFPPFSSYSFSPYSRSYSVHFSFFTFFSDFVIFQVVKWMFLI
FLDFQFSC
>ENSMUSP00000096646_c.535:T>A_p.F179Y ENSMUST00000099047.4|Gm10719-201|ENSMUSG00000095547|+|chr9:g.3021161:T>A
MFLIFHDFQFSCHIPCPTMDISFPTFSVFLAIFHVLKCVFLIFRDFQFSRHIPGPTVCISHFSGFSVISSFFK
SSSGCFSFSMIFSFLAIFHVLQWTFLNFPPFSVFLAIFHVLKCVFLIFSDFQFSRHIPGPTVCISHFSRFSVI
SSFFKLSSGCFSFSMIFSFLAIFHVPQWTFLNYPPFSSYSFSPYSRSYSVHFSFFTFFSDFVIFQVVKWMFLI
FLDFQFSC
>ENSMUSP00000096646_c.540:C>A_p.P181T ENSMUST00000099047.4|Gm10719-201|ENSMUSG00000095547|+|chr9:g.3021166:C>A
MFLIFHDFQFSCHIPCPTMDISFPTFSVFLAIFHVLKCVFLIFRDFQFSRHIPGPTVCISHFSGFSVISSFFK
SSSGCFSFSMIFSFLAIFHVLQWTFLNFPPFSVFLAIFHVLKCVFLIFSDFQFSRHIPGPTVCISHFSRFSVI
SSFFKLSSGCFSFSMIFSFLAIFHVPQWTFLNFPTFSSYSFSPYSRSYSVHFSFFTFFSDFVIFQVVKWMFLI
FLDFQFSC
>ENSMUSP00000096646_c.553:A>T_p.Y185F ENSMUST00000099047.4|Gm10719-201|ENSMUSG00000095547|+|chr9:g.3021259:A>T
MFLIFHDFQFSCHIPCPTMDISFPTFSVFLAIFHVLKCVFLIFRDFQFSRHIPGPTVCISHFSGFSVISSFFK
SSSGCFSFSMIFSFLAIFHVLQWTFLNFPPFSVFLAIFHVLKCVFLIFSDFQFSRHIPGPTVCISHFSRFSVI
SSFFKLSSGCFSFSMIFSFLAIFHVPQWTFLNFPPFSSFSFSPYSRSYSVHFSFFTFFSDFVIFQVVKWMFLI
FLDFQFSC

>ENSMUSP00000096646_c.571:C>T_p.S191F ENSMUST00000099047.4|Gm10719-201|ENSMUSG00000095547|+|chr9:g.3021484:C>T
MFLIFHDFQFSCHIPCPTMDISFPTFSVFLAIFHVLKCVFLIFRDFQFSRHIPGPTVCISHFSGFSVISSFFK
SSSGCFSFSMIFSFLAIFHVLQWTFLNFPPFSVFLAIFHVLKCVFLIFSDFQFSRHIPGPTVCISHFSRFSVI
SSFFKLSSGCFSFSMIFSFLAIFHVPQWTFLNFPPFSSYSFSPYFRSYSVHFSFFTFFSDFVIFQVVKWMFLI
FLDFQFSC
>ENSMUSP00000096646_c.580:A>T_p.Y194F ENSMUST00000099047.4|Gm10719-201|ENSMUSG00000095547|+|chr9:g.3021493:A>T
MFLIFHDFQFSCHIPCPTMDISFPTFSVFLAIFHVLKCVFLIFRDFQFSRHIPGPTVCISHFSGFSVISSFFK
SSSGCFSFSMIFSFLAIFHVLQWTFLNFPPFSVFLAIFHVLKCVFLIFSDFQFSRHIPGPTVCISHFSRFSVI
SSFFKLSSGCFSFSMIFSFLAIFHVPQWTFLNFPPFSSYSFSPYSRSFSVHFSFFTFFSDFVIFQVVKWMFLI
FLDFQFSC
>ENSMUSP00000096646_c.582:A>C_p.S195R ENSMUST00000099047.4|Gm10719-201|ENSMUSG00000095547|+|chr9:g.3021495:A>C
MFLIFHDFQFSCHIPCPTMDISFPTFSVFLAIFHVLKCVFLIFRDFQFSRHIPGPTVCISHFSGFSVISSFFK
SSSGCFSFSMIFSFLAIFHVLQWTFLNFPPFSVFLAIFHVLKCVFLIFSDFQFSRHIPGPTVCISHFSRFSVI
SSFFKLSSGCFSFSMIFSFLAIFHVPQWTFLNFPPFSSYSFSPYSRSYRVHFSFFTFFSDFVIFQVVKWMFLI
FLDFQFSC
>ENSMUSP00000096646_c.585:G>T_p.V196L ENSMUST00000099047.4|Gm10719-201|ENSMUSG00000095547|+|chr9:g.3021498:G>T
MFLIFHDFQFSCHIPCPTMDISFPTFSVFLAIFHVLKCVFLIFRDFQFSRHIPGPTVCISHFSGFSVISSFFK
SSSGCFSFSMIFSFLAIFHVLQWTFLNFPPFSVFLAIFHVLKCVFLIFSDFQFSRHIPGPTVCISHFSRFSVI
SSFFKLSSGCFSFSMIFSFLAIFHVPQWTFLNFPPFSSYSFSPYSRSYSLHFSFFTFFSDFVIFQVVKWMFLI
FLDFQFSC
>ENSMUSP00000096646_c.601:T>C_p.F201S ENSMUST00000099047.4|Gm10719-201|ENSMUSG00000095547|+|chr9:g.3021514:T>C
MFLIFHDFQFSCHIPCPTMDISFPTFSVFLAIFHVLKCVFLIFRDFQFSRHIPGPTVCISHFSGFSVISSFFK
SSSGCFSFSMIFSFLAIFHVLQWTFLNFPPFSVFLAIFHVLKCVFLIFSDFQFSRHIPGPTVCISHFSRFSVI
SSFFKLSSGCFSFSMIFSFLAIFHVPQWTFLNFPPFSSYSFSPYSRSYSVHFSFSTFFSDFVIFQVVKWMFLI
FLDFQFSC
>ENSMUSP00000096646_c.661:T>A_p.L221H ENSMUST00000099047.4|Gm10719-201|ENSMUSG00000095547|+|chr9:g.3021574:T>A
MFLIFHDFQFSCHIPCPTMDISFPTFSVFLAIFHVLKCVFLIFRDFQFSRHIPGPTVCISHFSGFSVISSFFK
SSSGCFSFSMIFSFLAIFHVLQWTFLNFPPFSVFLAIFHVLKCVFLIFSDFQFSRHIPGPTVCISHFSRFSVI
SSFFKLSSGCFSFSMIFSFLAIFHVPQWTFLNFPPFSSYSFSPYSRSYSVHFSFFTFFSDFVIFQVVKWMFLI
FHDFQFSC
>ENSMUSP00000096646_c.672:T>A_p.F225I ENSMUST00000099047.4|Gm10719-201|ENSMUSG00000095547|+|chr9:g.3021585:T>A
MFLIFHDFQFSCHIPCPTMDISFPTFSVFLAIFHVLKCVFLIFRDFQFSRHIPGPTVCISHFSGFSVISSFFK
SSSGCFSFSMIFSFLAIFHVLQWTFLNFPPFSVFLAIFHVLKCVFLIFSDFQFSRHIPGPTVCISHFSRFSVI
SSFFKLSSGCFSFSMIFSFLAIFHVPQWTFLNFPPFSSYSFSPYSRSYSVHFSFFTFFSDFVIFQVVKWMFLI
FLDFQISC
>ENSMUSP00000096645_c.45:T>C_p.C16R ENSMUST00000099046.4|Gm10718-201|ENSMUSG00000095186|+|chr9:g.3023592:T>C
MFLIFHDFQFSCHIPRPTVDISFPPFSVFLAIFHVLKCVFLIFRDFQFSCHIPGPTVCISHFSRFSLILSFFK
LSTACFSFSMIFSFLAIFHVLQWKFLNFPPFSVFLAIFHVLKCVFLIFRDFQFSRHIPGPTVCISHFSRFSVI
SSFFKSSSGCFSFSMIFSFLAIFHVLQWTFLNFPPFSVLFFSPYSRSYSVHFSFFTFFSDFDIFQVVKWMFLI
FHDFQLSC
>ENSMUSP00000096645_c.72:C>A_p.P25T ENSMUST00000099046.4|Gm10718-201|ENSMUSG00000095186|+|chr9:g.3023623:C>A
MFLIFHDFQFSCHIPCPTVDISFPTFSVFLAIFHVLKCVFLIFRDFQFSCHIPGPTVCISHFSRFSLILSFFK
LSTACFSFSMIFSFLAIFHVLQWKFLNFPPFSVFLAIFHVLKCVFLIFRDFQFSRHIPGPTVCISHFSRFSVI
SSFFKSSSGCFSFSMIFSFLAIFHVLQWTFLNFPPFSVLFFSPYSRSYSVHFSFFTFFSDFDIFQVVKWMFLI
FHDFQLSC
>ENSMUSP00000096645_c.129:C>T_p.R44C ENSMUST00000099046.4|Gm10718-201|ENSMUSG00000095186|+|chr9:g.3023680:C>T
MFLIFHDFQFSCHIPCPTVDISFPPFSVFLAIFHVLKCVFLIFCDFQFSCHIPGPTVCISHFSRFSLILSFFK
LSTACFSFSMIFSFLAIFHVLQWKFLNFPPFSVFLAIFHVLKCVFLIFRDFQFSRHIPGPTVCISHFSRFSVI

SSFFKSSSGCFSFSMIFSFLAIFHVLQWTFLNFPPFSVLFFSPYSRSYSVHFSFFTFFSDFDIFQVVKWMFLI
FHDFQLSC
>ENSMUSP00000096645_c.147:T>C_p.C50R ENSMUST00000099046.4|Gm10718-201|ENSMUSG00000095186|+|chr9:g.3023698:T>C
MFLIFHDFQFSCHIPCPTVDISFPPFSVFLAIFHVLKCVFLIFRDFQFSRHIPGPTVCISHFSRFSLILSFFK
LSTACFSFSMIFSFLAIFHVLQWKFLNFPPFSVFLAIFHVLKCVFLIFRDFQFSRHIPGPTVCISHFSRFSVI
SSFFKSSSGCFSFSMIFSFLAIFHVLQWTFLNFPPFSVLFFSPYSRSYSVHFSFFTFFSDFDIFQVVKWMFLI
FHDFQLSC
>ENSMUSP00000096645_c.159:G>C_p.G54R ENSMUST00000099046.4|Gm10718-201|ENSMUSG00000095186|+|chr9:g.3023710:G>C
MFLIFHDFQFSCHIPCPTVDISFPPFSVFLAIFHVLKCVFLIFRDFQFSCHIPRPTVCISHFSRFSLILSFFK
LSTACFSFSMIFSFLAIFHVLQWKFLNFPPFSVFLAIFHVLKCVFLIFRDFQFSRHIPGPTVCISHFSRFSVI
SSFFKSSSGCFSFSMIFSFLAIFHVLQWTFLNFPPFSVLFFSPYSRSYSVHFSFFTFFSDFDIFQVVKWMFLI
FHDFQLSC
>ENSMUSP00000096645_c.165:A>T_p.T56S ENSMUST00000099046.4|Gm10718-201|ENSMUSG00000095186|+|chr9:g.3023716:A>T
MFLIFHDFQFSCHIPCPTVDISFPPFSVFLAIFHVLKCVFLIFRDFQFSCHIPGPSVCISHFSRFSLILSFFK
LSTACFSFSMIFSFLAIFHVLQWKFLNFPPFSVFLAIFHVLKCVFLIFRDFQFSRHIPGPTVCISHFSRFSVI
SSFFKSSSGCFSFSMIFSFLAIFHVLQWTFLNFPPFSVLFFSPYSRSYSVHFSFFTFFSDFDIFQVVKWMFLI
FHDFQLSC
>ENSMUSP00000096645_c.186:T>C_p.S63P ENSMUST00000099046.4|Gm10718-201|ENSMUSG00000095186|+|chr9:g.3023737:T>C
MFLIFHDFQFSCHIPCPTVDISFPPFSVFLAIFHVLKCVFLIFRDFQFSCHIPGPTVCISHFPRFSLILSFFK
LSTACFSFSMIFSFLAIFHVLQWKFLNFPPFSVFLAIFHVLKCVFLIFRDFQFSRHIPGPTVCISHFSRFSVI
SSFFKSSSGCFSFSMIFSFLAIFHVLQWTFLNFPPFSVLFFSPYSRSYSVHFSFFTFFSDFDIFQVVKWMFLI
FHDFQLSC
>ENSMUSP00000096645_c.189:C>T_p.R64C ENSMUST00000099046.4|Gm10718-201|ENSMUSG00000095186|+|chr9:g.3023740:C>T
MFLIFHDFQFSCHIPCPTVDISFPPFSVFLAIFHVLKCVFLIFRDFQFSCHIPGPTVCISHFSCFSLILSFFK
LSTACFSFSMIFSFLAIFHVLQWKFLNFPPFSVFLAIFHVLKCVFLIFRDFQFSRHIPGPTVCISHFSRFSVI
SSFFKSSSGCFSFSMIFSFLAIFHVLQWTFLNFPPFSVLFFSPYSRSYSVHFSFFTFFSDFDIFQVVKWMFLI
FHDFQLSC
>ENSMUSP00000096645_c.198:C>G_p.L67V ENSMUST00000099046.4|Gm10718-201|ENSMUSG00000095186|+|chr9:g.3023749:C>G
MFLIFHDFQFSCHIPCPTVDISFPPFSVFLAIFHVLKCVFLIFRDFQFSCHIPGPTVCISHFSRFSVILSFFK
LSTACFSFSMIFSFLAIFHVLQWKFLNFPPFSVFLAIFHVLKCVFLIFRDFQFSRHIPGPTVCISHFSRFSVI
SSFFKSSSGCFSFSMIFSFLAIFHVLQWTFLNFPPFSVLFFSPYSRSYSVHFSFFTFFSDFDIFQVVKWMFLI
FHDFQLSC
>ENSMUSP00000096645_c.198:C>A_p.L67M ENSMUST00000099046.4|Gm10718-201|ENSMUSG00000095186|+|chr9:g.3023749:C>A
MFLIFHDFQFSCHIPCPTVDISFPPFSVFLAIFHVLKCVFLIFRDFQFSCHIPGPTVCISHFSRFSMILSFFK
LSTACFSFSMIFSFLAIFHVLQWKFLNFPPFSVFLAIFHVLKCVFLIFRDFQFSRHIPGPTVCISHFSRFSVI
SSFFKSSSGCFSFSMIFSFLAIFHVLQWTFLNFPPFSVLFFSPYSRSYSVHFSFFTFFSDFDIFQVVKWMFLI
FHDFQLSC
>ENSMUSP00000096645_c.205:T>C_p.L69S ENSMUST00000099046.4|Gm10718-201|ENSMUSG00000095186|+|chr9:g.3023756:T>C
MFLIFHDFQFSCHIPCPTVDISFPPFSVFLAIFHVLKCVFLIFRDFQFSCHIPGPTVCISHFSRFSLISSFFK
LSTACFSFSMIFSFLAIFHVLQWKFLNFPPFSVFLAIFHVLKCVFLIFRDFQFSRHIPGPTVCISHFSRFSVI
SSFFKSSSGCFSFSMIFSFLAIFHVLQWTFLNFPPFSVLFFSPYSRSYSVHFSFFTFFSDFDIFQVVKWMFLI
FHDFQLSC
>ENSMUSP00000096645_c.220:T>G_p.L74W ENSMUST00000099046.4|Gm10718-201|ENSMUSG00000095186|+|chr9:g.3023771:T>G
MFLIFHDFQFSCHIPCPTVDISFPPFSVFLAIFHVLKCVFLIFRDFQFSCHIPGPTVCISHFSRFSLILSFFK
WSTACFSFSMIFSFLAIFHVLQWKFLNFPPFSVFLAIFHVLKCVFLIFRDFQFSRHIPGPTVCISHFSRFSVI
SSFFKSSSGCFSFSMIFSFLAIFHVLQWTFLNFPPFSVLFFSPYSRSYSVHFSFFTFFSDFDIFQVVKWMFLI
FHDFQLSC
>ENSMUSP00000096645_c.220:T>C_p.L74S ENSMUST00000099046.4|Gm10718-201|ENSMUSG00000095186|+|chr9:g.3023771:T>C

MFLIFHDFQFSCHIPCPTVDISFPPFSVFLAIFHVLKCVFLIFRDFQFSCHIPGPTVCISHFSRFSLILSFFK
SSTACFSFSMIFSFLAIFHVLQWKFLNFPPFSVFLAIFHVLKCVFLIFRDFQFSRHIPGPTVCISHFSRFSVI
SSFFKSSSGCFSFSMIFSFLAIFHVLQWTFLNFPPFSVLFFSPYSRSYSVHFSFFTFFSDFDIFQVVKWMFLI
FHDFQLSC
>ENSMUSP00000096645_c.226:CTGC>GTGG_p.T76S ENSMUST00000099046.4|Gm10718-201|ENSMUSG00000095186|+|chr9:g.3023777:CTGC>GTGG
MFLIFHDFQFSCHIPCPTVDISFPPFSVFLAIFHVLKCVFLIFRDFQFSCHIPGPTVCISHFSRFSLILSFFK
LSSGACFSFSMIFSFLAIFHVLQWKFLNFPPFSVFLAIFHVLKCVFLIFRDFQFSRHIPGPTVCISHFSRFSV
ISSFFKSSSGCFSFSMIFSFLAIFHVLQWTFLNFPPFSVLFFSPYSRSYSVHFSFFTFFSDFDIFQVVKWMFL
IFHDFQLSC
>ENSMUSP00000096645_c.226:CTGC>GTGT_p.T76S ENSMUST00000099046.4|Gm10718-201|ENSMUSG00000095186|+|chr9:g.3023777:CTGC>GTGT
MFLIFHDFQFSCHIPCPTVDISFPPFSVFLAIFHVLKCVFLIFRDFQFSCHIPGPTVCISHFSRFSLILSFFK
LSSVACFSFSMIFSFLAIFHVLQWKFLNFPPFSVFLAIFHVLKCVFLIFRDFQFSRHIPGPTVCISHFSRFSV
ISSFFKSSSGCFSFSMIFSFLAIFHVLQWTFLNFPPFSVLFFSPYSRSYSVHFSFFTFFSDFDIFQVVKWMFL
IFHDFQLSC
>ENSMUSP00000096645_c.275:T>A_p.H92Q ENSMUST00000099046.4|Gm10718-201|ENSMUSG00000095186|+|chr9:g.3023826:T>A
MFLIFHDFQFSCHIPCPTVDISFPPFSVFLAIFHVLKCVFLIFRDFQFSCHIPGPTVCISHFSRFSLILSFFK
LSTACFSFSMIFSFLAIFQVLQWKFLNFPPFSVFLAIFHVLKCVFLIFRDFQFSRHIPGPTVCISHFSRFSVI
SSFFKSSSGCFSFSMIFSFLAIFHVLQWTFLNFPPFSVLFFSPYSRSYSVHFSFFTFFSDFDIFQVVKWMFLI
FHDFQLSC
>ENSMUSP00000096645_c.289:A>C_p.K97T ENSMUST00000099046.4|Gm10718-201|ENSMUSG00000095186|+|chr9:g.3023840:A>C
MFLIFHDFQFSCHIPCPTVDISFPPFSVFLAIFHVLKCVFLIFRDFQFSCHIPGPTVCISHFSRFSLILSFFK
LSTACFSFSMIFSFLAIFHVLQWTFLNFPPFSVFLAIFHVLKCVFLIFRDFQFSRHIPGPTVCISHFSRFSVI
SSFFKSSSGCFSFSMIFSFLAIFHVLQWTFLNFPPFSVLFFSPYSRSYSVHFSFFTFFSDFDIFQVVKWMFLI
FHDFQLSC
>ENSMUSP00000096645_c.289:A>T_p.K97I ENSMUST00000099046.4|Gm10718-201|ENSMUSG00000095186|+|chr9:g.3023840:A>T
MFLIFHDFQFSCHIPCPTVDISFPPFSVFLAIFHVLKCVFLIFRDFQFSCHIPGPTVCISHFSRFSLILSFFK
LSTACFSFSMIFSFLAIFHVLQWIFLNFPPFSVFLAIFHVLKCVFLIFRDFQFSRHIPGPTVCISHFSRFSVI
SSFFKSSSGCFSFSMIFSFLAIFHVLQWTFLNFPPFSVLFFSPYSRSYSVHFSFFTFFSDFDIFQVVKWMFLI
FHDFQLSC
>ENSMUSP00000096645_c.306:C>A_p.P103T ENSMUST00000099046.4|Gm10718-201|ENSMUSG00000095186|+|chr9:g.3023857:C>A
MFLIFHDFQFSCHIPCPTVDISFPPFSVFLAIFHVLKCVFLIFRDFQFSCHIPGPTVCISHFSRFSLILSFFK
LSTACFSFSMIFSFLAIFHVLQWKFLNFPTFSVFLAIFHVLKCVFLIFRDFQFSRHIPGPTVCISHFSRFSVI
SSFFKSSSGCFSFSMIFSFLAIFHVLQWTFLNFPPFSVLFFSPYSRSYSVHFSFFTFFSDFDIFQVVKWMFLI
FHDFQLSC
>ENSMUSP00000096645_c.325:C>A_p.A109D ENSMUST00000099046.4|Gm10718-201|ENSMUSG00000095186|+|chr9:g.3023876:C>A
MFLIFHDFQFSCHIPCPTVDISFPPFSVFLAIFHVLKCVFLIFRDFQFSCHIPGPTVCISHFSRFSLILSFFK
LSTACFSFSMIFSFLAIFHVLQWKFLNFPPFSVFLDIFHVLKCVFLIFRDFQFSRHIPGPTVCISHFSRFSVI
SSFFKSSSGCFSFSMIFSFLAIFHVLQWTFLNFPPFSVLFFSPYSRSYSVHFSFFTFFSDFDIFQVVKWMFLI
FHDFQLSC
>ENSMUSP00000096645_c.339:C>A_p.L114M ENSMUST00000099046.4|Gm10718-201|ENSMUSG00000095186|+|chr9:g.3023890:C>A
MFLIFHDFQFSCHIPCPTVDISFPPFSVFLAIFHVLKCVFLIFRDFQFSCHIPGPTVCISHFSRFSLILSFFK
LSTACFSFSMIFSFLAIFHVLQWKFLNFPPFSVFLAIFHVMKCVFLIFRDFQFSRHIPGPTVCISHFSRFSVI
SSFFKSSSGCFSFSMIFSFLAIFHVLQWTFLNFPPFSVLFFSPYSRSYSVHFSFFTFFSDFDIFQVVKWMFLI
FHDFQLSC
>ENSMUSP00000096645_c.363:C>T_p.R122C ENSMUST00000099046.4|Gm10718-201|ENSMUSG00000095186|+|chr9:g.3023914:C>T
MFLIFHDFQFSCHIPCPTVDISFPPFSVFLAIFHVLKCVFLIFRDFQFSCHIPGPTVCISHFSRFSLILSFFK
LSTACFSFSMIFSFLAIFHVLQWKFLNFPPFSVFLAIFHVLKCVFLIFCDFQFSRHIPGPTVCISHFSRFSVI
SSFFKSSSGCFSFSMIFSFLAIFHVLQWTFLNFPPFSVLFFSPYSRSYSVHFSFFTFFSDFDIFQVVKWMFLI
FHDFQLSC

>ENSMUSP00000096645_c.399:A>T_p.T134S ENSMUST00000099046.4|Gm10718-201|ENSMUSG00000095186|+|chr9:g.3024417:A>T
MFLIFHDFQFSCHIPCPTVDISFPPFSVFLAIFHVLKCVFLIFRDFQFSCHIPGPTVCISHFSRFSLILSFFK
LSTACFSFSMIFSFLAIFHVLQWKFLNFPPFSVFLAIFHVLKCVFLIFRDFQFSRHIPGPSVCISHFSRFSVI
SSFFKSSSGCFSFSMIFSFLAIFHVLQWTFLNFPPFSVLFFSPYSRSYSVHFSFFTFFSDFDIFQVVKWMFLI
FHDFQLSC
>ENSMUSP00000096645_c.405:T>G_p.C136G ENSMUST00000099046.4|Gm10718-201|ENSMUSG00000095186|+|chr9:g.3024423:T>G
MFLIFHDFQFSCHIPCPTVDISFPPFSVFLAIFHVLKCVFLIFRDFQFSCHIPGPTVCISHFSRFSLILSFFK
LSTACFSFSMIFSFLAIFHVLQWKFLNFPPFSVFLAIFHVLKCVFLIFRDFQFSRHIPGPTVGISHFSRFSVI
SSFFKSSSGCFSFSMIFSFLAIFHVLQWTFLNFPPFSVLFFSPYSRSYSVHFSFFTFFSDFDIFQVVKWMFLI
FHDFQLSC
>ENSMUSP00000096645_c.430:C>T_p.S144L ENSMUST00000099046.4|Gm10718-201|ENSMUSG00000095186|+|chr9:g.3024448:C>T
MFLIFHDFQFSCHIPCPTVDISFPPFSVFLAIFHVLKCVFLIFRDFQFSCHIPGPTVCISHFSRFSLILSFFK
LSTACFSFSMIFSFLAIFHVLQWKFLNFPPFSVFLAIFHVLKCVFLIFRDFQFSRHIPGPTVCISHFSRFLVI
SSFFKSSSGCFSFSMIFSFLAIFHVLQWTFLNFPPFSVLFFSPYSRSYSVHFSFFTFFSDFDIFQVVKWMFLI
FHDFQLSC
>ENSMUSP00000096645_c.540:C>A_p.P181T ENSMUST00000099046.4|Gm10718-201|ENSMUSG00000095186|+|chr9:g.3024558:C>A
MFLIFHDFQFSCHIPCPTVDISFPPFSVFLAIFHVLKCVFLIFRDFQFSCHIPGPTVCISHFSRFSLILSFFK
LSTACFSFSMIFSFLAIFHVLQWKFLNFPPFSVFLAIFHVLKCVFLIFRDFQFSRHIPGPTVCISHFSRFSVI
SSFFKSSSGCFSFSMIFSFLAIFHVLQWTFLNFPTFSVLFFSPYSRSYSVHFSFFTFFSDFDIFQVVKWMFLI
FHDFQLSC
>ENSMUSP00000096645_c.553:T>TAC_p.L185ins-fs
ENSMUST00000099046.4|Gm10718-201|ENSMUSG00000095186|+|chr9:g.3024884:T>TAC
MFLIFHDFQFSCHIPCPTVDISFPPFSVFLAIFHVLKCVFLIFRDFQFSCHIPGPTVCISHFSRFSLILSFFK
LSTACFSFSMIFSFLAIFHVLQWKFLNFPPFSVFLAIFHVLKCVFLIFRDFQFSRHIPGPTVCISHFSRFSVI
SSFFKSSSGCFSFSMIFSFLAIFHVLQWTFLNFPPFSVLHFSRHIPGPTVCISHFSRFSVISTFFKLSSGCFS
FSMIFSFL
>ENSMUSP00000096645_c.554:AT>A_p.L185del-fs ENSMUST00000099046.4|Gm10718-201|ENSMUSG00000095186|+|chr9:g.3024885:AT>A
MFLIFHDFQFSCHIPCPTVDISFPPFSVFLAIFHVLKCVFLIFRDFQFSCHIPGPTVCISHFSRFSLILSFFK
LSTACFSFSMIFSFLAIFHVLQWKFLNFPPFSVFLAIFHVLKCVFLIFRDFQFSRHIPGPTVCISHFSRFSVI
SSFFKSSSGCFSFSMIFSFLAIFHVLQWTFLNFPPFSVLFSRHIPGPTVCISHFSRFSVISTFFKLSSGCFSF
SMIFSFL
>ENSMUSP00000096645_c.555:T>TA_p.F186ins-fs ENSMUST00000099046.4|Gm10718-201|ENSMUSG00000095186|+|chr9:g.3024886:T>TA
MFLIFHDFQFSCHIPCPTVDISFPPFSVFLAIFHVLKCVFLIFRDFQFSCHIPGPTVCISHFSRFSLILSFFK
LSTACFSFSMIFSFLAIFHVLQWKFLNFPPFSVFLAIFHVLKCVFLIFRDFQFSRHIPGPTVCISHFSRFSVI
SSFFKSSSGCFSFSMIFSFLAIFHVLQWTFLNFPPFSVLYFLAIFQVLQCAFLIFHVFQ
>ENSMUSP00000096645_c.580:A>T_p.Y194F ENSMUST00000099046.4|Gm10718-201|ENSMUSG00000095186|+|chr9:g.3025118:A>T
MFLIFHDFQFSCHIPCPTVDISFPPFSVFLAIFHVLKCVFLIFRDFQFSCHIPGPTVCISHFSRFSLILSFFK
LSTACFSFSMIFSFLAIFHVLQWKFLNFPPFSVFLAIFHVLKCVFLIFRDFQFSRHIPGPTVCISHFSRFSVI
SSFFKSSSGCFSFSMIFSFLAIFHVLQWTFLNFPPFSVLFFSPYSRSFSVHFSFFTFFSDFDIFQVVKWMFLI
FHDFQLSC
>ENSMUSP00000096645_c.622:A>C_p.D208A ENSMUST00000099046.4|Gm10718-201|ENSMUSG00000095186|+|chr9:g.3025160:A>C
MFLIFHDFQFSCHIPCPTVDISFPPFSVFLAIFHVLKCVFLIFRDFQFSCHIPGPTVCISHFSRFSLILSFFK
LSTACFSFSMIFSFLAIFHVLQWKFLNFPPFSVFLAIFHVLKCVFLIFRDFQFSRHIPGPTVCISHFSRFSVI
SSFFKSSSGCFSFSMIFSFLAIFHVLQWTFLNFPPFSVLFFSPYSRSYSVHFSFFTFFSDFAIFQVVKWMFLI
FHDFQLSC
>ENSMUSP00000096645_c.622:A>T_p.D208V ENSMUST00000099046.4|Gm10718-201|ENSMUSG00000095186|+|chr9:g.3025160:A>T
MFLIFHDFQFSCHIPCPTVDISFPPFSVFLAIFHVLKCVFLIFRDFQFSCHIPGPTVCISHFSRFSLILSFFK
LSTACFSFSMIFSFLAIFHVLQWKFLNFPPFSVFLAIFHVLKCVFLIFRDFQFSRHIPGPTVCISHFSRFSVI

SSFFKSSSGCFSFSMIFSFLAIFHVLQWTFLNFPPFSVLFFSPYSRSYSVHFSFFTFFSDFVIFQVVKWMFLI
FHDFQLSC
>ENSMUSP00000096645_c.654:A>G_p.I219V ENSMUST00000099046.4|Gm10718-201|ENSMUSG00000095186|+|chr9:g.3025192:A>G
MFLIFHDFQFSCHIPCPTVDISFPPFSVFLAIFHVLKCVFLIFRDFQFSCHIPGPTVCISHFSRFSLILSFFK
LSTACFSFSMIFSFLAIFHVLQWKFLNFPPFSVFLAIFHVLKCVFLIFRDFQFSRHIPGPTVCISHFSRFSVI
SSFFKSSSGCFSFSMIFSFLAIFHVLQWTFLNFPPFSVLFFSPYSRSYSVHFSFFTFFSDFDIFQVVKWMFLV
FHDFQLSC
>ENSMUSP00000096645_c.654:A>T_p.I219F ENSMUST00000099046.4|Gm10718-201|ENSMUSG00000095186|+|chr9:g.3025192:A>T
MFLIFHDFQFSCHIPCPTVDISFPPFSVFLAIFHVLKCVFLIFRDFQFSCHIPGPTVCISHFSRFSLILSFFK
LSTACFSFSMIFSFLAIFHVLQWKFLNFPPFSVFLAIFHVLKCVFLIFRDFQFSRHIPGPTVCISHFSRFSVI
SSFFKSSSGCFSFSMIFSFLAIFHVLQWTFLNFPPFSVLFFSPYSRSYSVHFSFFTFFSDFDIFQVVKWMFLF
FHDFQLSC
>ENSMUSP00000096645_c.672:C>T_p.L225F ENSMUST00000099046.4|Gm10718-201|ENSMUSG00000095186|+|chr9:g.3025210:C>T
MFLIFHDFQFSCHIPCPTVDISFPPFSVFLAIFHVLKCVFLIFRDFQFSCHIPGPTVCISHFSRFSLILSFFK
LSTACFSFSMIFSFLAIFHVLQWKFLNFPPFSVFLAIFHVLKCVFLIFRDFQFSRHIPGPTVCISHFSRFSVI
SSFFKSSSGCFSFSMIFSFLAIFHVLQWTFLNFPPFSVLFFSPYSRSYSVHFSFFTFFSDFDIFQVVKWMFLI
FHDFQFSC
>ENSMUSP00000096645_c.672:C>A_p.L225I ENSMUST00000099046.4|Gm10718-201|ENSMUSG00000095186|+|chr9:g.3025210:C>A
MFLIFHDFQFSCHIPCPTVDISFPPFSVFLAIFHVLKCVFLIFRDFQFSCHIPGPTVCISHFSRFSLILSFFK
LSTACFSFSMIFSFLAIFHVLQWKFLNFPPFSVFLAIFHVLKCVFLIFRDFQFSRHIPGPTVCISHFSRFSVI
SSFFKSSSGCFSFSMIFSFLAIFHVLQWTFLNFPPFSVLFFSPYSRSYSVHFSFFTFFSDFDIFQVVKWMFLI
FHDFQISC
>ENSMUSP00000096644_c.0:A>C_p.M1L ENSMUST00000075573.7|Gm10717-201|ENSMUSG00000095891|+|chr9:g.3025417:A>C
LFLIFHDFQFSCHIPCPTVEICKFPPFSVFLAIFHVLKYVFLIFRDFQFSRHIPGPTVCISHFSRFSVILSFF
KFSSGCFSFSLIFSFLAIFHVLQWTFLNFPPFSVFLAIFHVLKCVFLIFSDFQFSRHIPGPTVCISHFSRFSV
ISSFFKVSSGCFSFSMIFSFLAIFHVLQWTFLNFPPFSSYSFSPYSRSYSVHFSFFTFFSDFVIFQVFKWMFL
IFLDFQFSC
>ENSMUSP00000096644_c.3:T>A_p.F2I ENSMUST00000075573.7|Gm10717-201|ENSMUSG00000095891|+|chr9:g.3025420:T>A
MILIFHDFQFSCHIPCPTVEICKFPPFSVFLAIFHVLKYVFLIFRDFQFSRHIPGPTVCISHFSRFSVILSFF
KFSSGCFSFSLIFSFLAIFHVLQWTFLNFPPFSVFLAIFHVLKCVFLIFSDFQFSRHIPGPTVCISHFSRFSV
ISSFFKVSSGCFSFSMIFSFLAIFHVLQWTFLNFPPFSSYSFSPYSRSYSVHFSFFTFFSDFVIFQVFKWMFL
IFLDFQFSC
>ENSMUSP00000096644_c.9:A>T_p.I4F ENSMUST00000075573.7|Gm10717-201|ENSMUSG00000095891|+|chr9:g.3025426:A>T
MFLFFHDFQFSCHIPCPTVEICKFPPFSVFLAIFHVLKYVFLIFRDFQFSRHIPGPTVCISHFSRFSVILSFF
KFSSGCFSFSLIFSFLAIFHVLQWTFLNFPPFSVFLAIFHVLKCVFLIFSDFQFSRHIPGPTVCISHFSRFSV
ISSFFKVSSGCFSFSMIFSFLAIFHVLQWTFLNFPPFSSYSFSPYSRSYSVHFSFFTFFSDFVIFQVFKWMFL
IFLDFQFSC
>ENSMUSP00000096644_c.45:T>A_p.C16S ENSMUST00000075573.7|Gm10717-201|ENSMUSG00000095891|+|chr9:g.3025462:T>A
MFLIFHDFQFSCHIPSPTVEICKFPPFSVFLAIFHVLKYVFLIFRDFQFSRHIPGPTVCISHFSRFSVILSFF
KFSSGCFSFSLIFSFLAIFHVLQWTFLNFPPFSVFLAIFHVLKCVFLIFSDFQFSRHIPGPTVCISHFSRFSV
ISSFFKVSSGCFSFSMIFSFLAIFHVLQWTFLNFPPFSSYSFSPYSRSYSVHFSFFTFFSDFVIFQVFKWMFL
IFLDFQFSC
>ENSMUSP00000096644_c.45:T>C_p.C16R ENSMUST00000075573.7|Gm10717-201|ENSMUSG00000095891|+|chr9:g.3025462:T>C
MFLIFHDFQFSCHIPRPTVEICKFPPFSVFLAIFHVLKYVFLIFRDFQFSRHIPGPTVCISHFSRFSVILSFF
KFSSGCFSFSLIFSFLAIFHVLQWTFLNFPPFSVFLAIFHVLKCVFLIFSDFQFSRHIPGPTVCISHFSRFSV
ISSFFKVSSGCFSFSMIFSFLAIFHVLQWTFLNFPPFSSYSFSPYSRSYSVHFSFFTFFSDFVIFQVFKWMFL
IFLDFQFSC
>ENSMUSP00000096644_c.59:A>T_p.E20D ENSMUST00000075573.7|Gm10717-201|ENSMUSG00000095891|+|chr9:g.3025476:A>T

MFLIFHDFQFSCHIPCPTVDICKFPPFSVFLAIFHVLKYVFLIFRDFQFSRHIPGPTVCISHFSRFSVILSFF
KFSSGCFSFSLIFSFLAIFHVLQWTFLNFPPFSVFLAIFHVLKCVFLIFSDFQFSRHIPGPTVCISHFSRFSV
ISSFFKVSSGCFSFSMIFSFLAIFHVLQWTFLNFPPFSSYSFSPYSRSYSVHFSFFTFFSDFVIFQVFKWMFL
IFLDFQFSC
>ENSMUSP00000096644_c.59:A>C_p.E20D ENSMUST00000075573.7|Gm10717-201|ENSMUSG00000095891|+|chr9:g.3025476:A>C
MFLIFHDFQFSCHIPCPTVDICKFPPFSVFLAIFHVLKYVFLIFRDFQFSRHIPGPTVCISHFSRFSVILSFF
KFSSGCFSFSLIFSFLAIFHVLQWTFLNFPPFSVFLAIFHVLKCVFLIFSDFQFSRHIPGPTVCISHFSRFSV
ISSFFKVSSGCFSFSMIFSFLAIFHVLQWTFLNFPPFSSYSFSPYSRSYSVHFSFFTFFSDFVIFQVFKWMFL
IFLDFQFSC
>ENSMUSP00000096644_c.64:G>C_p.C22S ENSMUST00000075573.7|Gm10717-201|ENSMUSG00000095891|+|chr9:g.3025481:G>C
MFLIFHDFQFSCHIPCPTVEISKFPPFSVFLAIFHVLKYVFLIFRDFQFSRHIPGPTVCISHFSRFSVILSFF
KFSSGCFSFSLIFSFLAIFHVLQWTFLNFPPFSVFLAIFHVLKCVFLIFSDFQFSRHIPGPTVCISHFSRFSV
ISSFFKVSSGCFSFSMIFSFLAIFHVLQWTFLNFPPFSSYSFSPYSRSYSVHFSFFTFFSDFVIFQVFKWMFL
IFLDFQFSC
>ENSMUSP00000096644_c.64:G>A_p.C22Y ENSMUST00000075573.7|Gm10717-201|ENSMUSG00000095891|+|chr9:g.3025481:G>A
MFLIFHDFQFSCHIPCPTVEIYKFPPFSVFLAIFHVLKYVFLIFRDFQFSRHIPGPTVCISHFSRFSVILSFF
KFSSGCFSFSLIFSFLAIFHVLQWTFLNFPPFSVFLAIFHVLKCVFLIFSDFQFSRHIPGPTVCISHFSRFSV
ISSFFKVSSGCFSFSMIFSFLAIFHVLQWTFLNFPPFSSYSFSPYSRSYSVHFSFFTFFSDFVIFQVFKWMFL
IFLDFQFSC
>ENSMUSP00000096644_c.68:A>AT_p.K23ins-fs ENSMUST00000075573.7|Gm10717-201|ENSMUSG00000095891|+|chr9:g.3025485:A>AT
MFLIFHDFQFSCHIPCPTVEICKFSTFFSFPRHISRPKVCISHFP
>ENSMUSP00000096644_c.70:T>TA_p.F24ins-fs ENSMUST00000075573.7|Gm10717-201|ENSMUSG00000095891|+|chr9:g.3025487:T>TA
MFLIFHDFQFSCHIPCPTVEICKLSTFFSFPRHISRPKVCISHFP
>ENSMUSP00000096644_c.75:C>A_p.P26T ENSMUST00000075573.7|Gm10717-201|ENSMUSG00000095891|+|chr9:g.3025492:C>A
MFLIFHDFQFSCHIPCPTVEICKFPTFSVFLAIFHVLKYVFLIFRDFQFSRHIPGPTVCISHFSRFSVILSFF
KFSSGCFSFSLIFSFLAIFHVLQWTFLNFPPFSVFLAIFHVLKCVFLIFSDFQFSRHIPGPTVCISHFSRFSV
ISSFFKVSSGCFSFSMIFSFLAIFHVLQWTFLNFPPFSSYSFSPYSRSYSVHFSFFTFFSDFVIFQVFKWMFL
IFLDFQFSC
>ENSMUSP00000096644_c.89:C>A_p.F30L ENSMUST00000075573.7|Gm10717-201|ENSMUSG00000095891|+|chr9:g.3025506:C>A
MFLIFHDFQFSCHIPCPTVEICKFPPFSVLLAIFHVLKYVFLIFRDFQFSRHIPGPTVCISHFSRFSVILSFF
KFSSGCFSFSLIFSFLAIFHVLQWTFLNFPPFSVFLAIFHVLKCVFLIFSDFQFSRHIPGPTVCISHFSRFSV
ISSFFKVSSGCFSFSMIFSFLAIFHVLQWTFLNFPPFSSYSFSPYSRSYSVHFSFFTFFSDFVIFQVFKWMFL
IFLDFQFSC
>ENSMUSP00000096644_c.94:C>A_p.A32D ENSMUST00000075573.7|Gm10717-201|ENSMUSG00000095891|+|chr9:g.3025511:C>A
MFLIFHDFQFSCHIPCPTVEICKFPPFSVFLDIFHVLKYVFLIFRDFQFSRHIPGPTVCISHFSRFSVILSFF
KFSSGCFSFSLIFSFLAIFHVLQWTFLNFPPFSVFLAIFHVLKCVFLIFSDFQFSRHIPGPTVCISHFSRFSV
ISSFFKVSSGCFSFSMIFSFLAIFHVLQWTFLNFPPFSSYSFSPYSRSYSVHFSFFTFFSDFVIFQVFKWMFL
IFLDFQFSC
>ENSMUSP00000096644_c.115:A>G_p.Y39C ENSMUST00000075573.7|Gm10717-201|ENSMUSG00000095891|+|chr9:g.3025532:A>G
MFLIFHDFQFSCHIPCPTVEICKFPPFSVFLAIFHVLKCVFLIFRDFQFSRHIPGPTVCISHFSRFSVILSFF
KFSSGCFSFSLIFSFLAIFHVLQWTFLNFPPFSVFLAIFHVLKCVFLIFSDFQFSRHIPGPTVCISHFSRFSV
ISSFFKVSSGCFSFSMIFSFLAIFHVLQWTFLNFPPFSSYSFSPYSRSYSVHFSFFTFFSDFVIFQVFKWMFL
IFLDFQFSC
>ENSMUSP00000096644_c.143:G>C_p.Q48H ENSMUST00000075573.7|Gm10717-201|ENSMUSG00000095891|+|chr9:g.3025560:G>C
MFLIFHDFQFSCHIPCPTVEICKFPPFSVFLAIFHVLKYVFLIFRDFHFSRHIPGPTVCISHFSRFSVILSFF
KFSSGCFSFSLIFSFLAIFHVLQWTFLNFPPFSVFLAIFHVLKCVFLIFSDFQFSRHIPGPTVCISHFSRFSV
ISSFFKVSSGCFSFSMIFSFLAIFHVLQWTFLNFPPFSSYSFSPYSRSYSVHFSFFTFFSDFVIFQVFKWMFL
IFLDFQFSC

>ENSMUSP00000096644_c.163:G>T_p.G55V ENSMUST00000075573.7|Gm10717-201|ENSMUSG00000095891|+|chr9:g.3025580:G>T
MFLIFHDFQFSCHIPCPTVEICKFPPFSVFLAIFHVLKYVFLIFRDFQFSRHIPVPTVCISHFSRFSVILSFF
KFSSGCFSFSLIFSFLAIFHVLQWTFLNFPPFSVFLAIFHVLKCVFLIFSDFQFSRHIPGPTVCISHFSRFSV
ISSFFKVSSGCFSFSMIFSFLAIFHVLQWTFLNFPPFSSYSFSPYSRSYSVHFSFFTFFSDFVIFQVFKWMFL
IFLDFQFSC
>ENSMUSP00000096644_c.168:A>T_p.T57S ENSMUST00000075573.7|Gm10717-201|ENSMUSG00000095891|+|chr9:g.3025585:A>T
MFLIFHDFQFSCHIPCPTVEICKFPPFSVFLAIFHVLKYVFLIFRDFQFSRHIPGPSVCISHFSRFSVILSFF
KFSSGCFSFSLIFSFLAIFHVLQWTFLNFPPFSVFLAIFHVLKCVFLIFSDFQFSRHIPGPTVCISHFSRFSV
ISSFFKVSSGCFSFSMIFSFLAIFHVLQWTFLNFPPFSSYSFSPYSRSYSVHFSFFTFFSDFVIFQVFKWMFL
IFLDFQFSC
>ENSMUSP00000096644_c.183:C>A_p.H62N ENSMUST00000075573.7|Gm10717-201|ENSMUSG00000095891|+|chr9:g.3025600:C>A
MFLIFHDFQFSCHIPCPTVEICKFPPFSVFLAIFHVLKYVFLIFRDFQFSRHIPGPTVCISNFSRFSVILSFF
KFSSGCFSFSLIFSFLAIFHVLQWTFLNFPPFSVFLAIFHVLKCVFLIFSDFQFSRHIPGPTVCISHFSRFSV
ISSFFKVSSGCFSFSMIFSFLAIFHVLQWTFLNFPPFSSYSFSPYSRSYSVHFSFFTFFSDFVIFQVFKWMFL
IFLDFQFSC
>ENSMUSP00000096644_c.198:T>G_p.S67A ENSMUST00000075573.7|Gm10717-201|ENSMUSG00000095891|+|chr9:g.3025615:T>G
MFLIFHDFQFSCHIPCPTVEICKFPPFSVFLAIFHVLKYVFLIFRDFQFSRHIPGPTVCISHFSRFAVILSFF
KFSSGCFSFSLIFSFLAIFHVLQWTFLNFPPFSVFLAIFHVLKCVFLIFSDFQFSRHIPGPTVCISHFSRFSV
ISSFFKVSSGCFSFSMIFSFLAIFHVLQWTFLNFPPFSSYSFSPYSRSYSVHFSFFTFFSDFVIFQVFKWMFL
IFLDFQFSC
>ENSMUSP00000096644_c.199:C>T_p.S67L ENSMUST00000075573.7|Gm10717-201|ENSMUSG00000095891|+|chr9:g.3025616:C>T
MFLIFHDFQFSCHIPCPTVEICKFPPFSVFLAIFHVLKYVFLIFRDFQFSRHIPGPTVCISHFSRFLVILSFF
KFSSGCFSFSLIFSFLAIFHVLQWTFLNFPPFSVFLAIFHVLKCVFLIFSDFQFSRHIPGPTVCISHFSRFSV
ISSFFKVSSGCFSFSMIFSFLAIFHVLQWTFLNFPPFSSYSFSPYSRSYSVHFSFFTFFSDFVIFQVFKWMFL
IFLDFQFSC
>ENSMUSP00000096644_c.202:T>G_p.V68G ENSMUST00000075573.7|Gm10717-201|ENSMUSG00000095891|+|chr9:g.3025619:T>G
MFLIFHDFQFSCHIPCPTVEICKFPPFSVFLAIFHVLKYVFLIFRDFQFSRHIPGPTVCISHFSRFSGILSFF
KFSSGCFSFSLIFSFLAIFHVLQWTFLNFPPFSVFLAIFHVLKCVFLIFSDFQFSRHIPGPTVCISHFSRFSV
ISSFFKVSSGCFSFSMIFSFLAIFHVLQWTFLNFPPFSSYSFSPYSRSYSVHFSFFTFFSDFVIFQVFKWMFL
IFLDFQFSC
>ENSMUSP00000096644_c.208:T>C_p.L70S ENSMUST00000075573.7|Gm10717-201|ENSMUSG00000095891|+|chr9:g.3025625:T>C
MFLIFHDFQFSCHIPCPTVEICKFPPFSVFLAIFHVLKYVFLIFRDFQFSRHIPGPTVCISHFSRFSVISSFF
KFSSGCFSFSLIFSFLAIFHVLQWTFLNFPPFSVFLAIFHVLKCVFLIFSDFQFSRHIPGPTVCISHFSRFSV
ISSFFKVSSGCFSFSMIFSFLAIFHVLQWTFLNFPPFSSYSFSPYSRSYSVHFSFFTFFSDFVIFQVFKWMFL
IFLDFQFSC
>ENSMUSP00000096644_c.209:G>T_p.L70F ENSMUST00000075573.7|Gm10717-201|ENSMUSG00000095891|+|chr9:g.3025626:G>T
MFLIFHDFQFSCHIPCPTVEICKFPPFSVFLAIFHVLKYVFLIFRDFQFSRHIPGPTVCISHFSRFSVIFSFF
KFSSGCFSFSLIFSFLAIFHVLQWTFLNFPPFSVFLAIFHVLKCVFLIFSDFQFSRHIPGPTVCISHFSRFSV
ISSFFKVSSGCFSFSMIFSFLAIFHVLQWTFLNFPPFSSYSFSPYSRSYSVHFSFFTFFSDFVIFQVFKWMFL
IFLDFQFSC
>ENSMUSP00000096644_c.223:T>C_p.F75S ENSMUST00000075573.7|Gm10717-201|ENSMUSG00000095891|+|chr9:g.3025640:T>C
MFLIFHDFQFSCHIPCPTVEICKFPPFSVFLAIFHVLKYVFLIFRDFQFSRHIPGPTVCISHFSRFSVILSFF
KSSSGCFSFSLIFSFLAIFHVLQWTFLNFPPFSVFLAIFHVLKCVFLIFSDFQFSRHIPGPTVCISHFSRFSV
ISSFFKVSSGCFSFSMIFSFLAIFHVLQWTFLNFPPFSSYSFSPYSRSYSVHFSFFTFFSDFVIFQVFKWMFL
IFLDFQFSC
>ENSMUSP00000096644_c.223:T>G_p.F75C ENSMUST00000075573.7|Gm10717-201|ENSMUSG00000095891|+|chr9:g.3025640:T>G
MFLIFHDFQFSCHIPCPTVEICKFPPFSVFLAIFHVLKYVFLIFRDFQFSRHIPGPTVCISHFSRFSVILSFF
KCSSGCFSFSLIFSFLAIFHVLQWTFLNFPPFSVFLAIFHVLKCVFLIFSDFQFSRHIPGPTVCISHFSRFSV

ISSFFKVSSGCFSFSMIFSFLAIFHVLQWTFLNFPPFSSYSFSPYSRSYSVHFSFFTFFSDFVIFQVFKWMFL
IFLDFQFSC
>ENSMUSP00000096644_c.223:TT>CA_p.F75S ENSMUST00000075573.7|Gm10717-
201|ENSMUSG00000095891|+|chr9:g.3025640:TT>CA
MFLIFHDFQFSCHIPCPTVEICKFPPFSVFLAIFHVLKYVFLIFRDFQFSRHIPGPTVCISHFSRFSVILSFF
KSFKWMFLIFLDFQFSCHIPCPTVDISKFSTFFSFPRHISCPKVCISHFQ*FSVFSPYSRSYSVHFSFFTFFS
DFVIFQGVKWMFLIFHDFQFSCHIPCPTVDISKFSTFFFLQFLAIFQVLQCAFLIFHVFQ*FCHFSSFQVDVS
HFP*FSVFL
>ENSMUSP00000096644_c.223:TT>CG_p.F75S ENSMUST00000075573.7|Gm10717-
201|ENSMUSG00000095891|+|chr9:g.3025640:TT>CG
MFLIFHDFQFSCHIPCPTVEICKFPPFSVFLAIFHVLKYVFLIFRDFQFSRHIPGPTVCISHFSRFSVILSFF
KSFKWMFLIFLDFQFSCHIPCPTVDISKFSTFFSFPRHISCPKVCISHFQ*FSVFSPYSRSYSVHFSFFTFFS
DFVIFQGVKWMFLIFHDFQFSCHIPCPTVDISKFSTFFFLQFLAIFQVLQCAFLIFHVFQ*FCHFSSFQVDVS
HFP*FSVFL
>ENSMUSP00000096644_c.235:G>T_p.C79F ENSMUST00000075573.7|Gm10717-
201|ENSMUSG00000095891|+|chr9:g.3025652:G>T
MFLIFHDFQFSCHIPCPTVEICKFPPFSVFLAIFHVLKYVFLIFRDFQFSRHIPGPTVCISHFSRFSVILSFF
KFSSGFFSFSLIFSFLAIFHVLQWTFLNFPPFSVFLAIFHVLKCVFLIFSDFQFSRHIPGPTVCISHFSRFSV
ISSFFKVSSGCFSFSMIFSFLAIFHVLQWTFLNFPPFSSYSFSPYSRSYSVHFSFFTFFSDFVIFQVFKWMFL
IFLDFQFSC
>ENSMUSP00000096644_c.237:T>A_p.F80I ENSMUST00000075573.7|Gm10717-
201|ENSMUSG00000095891|+|chr9:g.3025654:T>A
MFLIFHDFQFSCHIPCPTVEICKFPPFSVFLAIFHVLKYVFLIFRDFQFSRHIPGPTVCISHFSRFSVILSFF
KFSSGCISFSLIFSFLAIFHVLQWTFLNFPPFSVFLAIFHVLKCVFLIFSDFQFSRHIPGPTVCISHFSRFSV
ISSFFKVSSGCFSFSMIFSFLAIFHVLQWTFLNFPPFSSYSFSPYSRSYSVHFSFFTFFSDFVIFQVFKWMFL
IFLDFQFSC
>ENSMUSP00000096644_c.249:T>A_p.L84M ENSMUST00000075573.7|Gm10717-
201|ENSMUSG00000095891|+|chr9:g.3025666:T>A
MFLIFHDFQFSCHIPCPTVEICKFPPFSVFLAIFHVLKYVFLIFRDFQFSRHIPGPTVCISHFSRFSVILSFF
KFSSGCFSFSMIFSFLAIFHVLQWTFLNFPPFSVFLAIFHVLKCVFLIFSDFQFSRHIPGPTVCISHFSRFSV
ISSFFKVSSGCFSFSMIFSFLAIFHVLQWTFLNFPPFSSYSFSPYSRSYSVHFSFFTFFSDFVIFQVFKWMFL
IFLDFQFSC
>ENSMUSP00000096644_c.249:T>G_p.L84V ENSMUST00000075573.7|Gm10717-
201|ENSMUSG00000095891|+|chr9:g.3025666:T>G
MFLIFHDFQFSCHIPCPTVEICKFPPFSVFLAIFHVLKYVFLIFRDFQFSRHIPGPTVCISHFSRFSVILSFF
KFSSGCFSFSVIFSFLAIFHVLQWTFLNFPPFSVFLAIFHVLKCVFLIFSDFQFSRHIPGPTVCISHFSRFSV
ISSFFKVSSGCFSFSMIFSFLAIFHVLQWTFLNFPPFSSYSFSPYSRSYSVHFSFFTFFSDFVIFQVFKWMFL
IFLDFQFSC
>ENSMUSP00000096644_c.260:T>A_p.S87R ENSMUST00000075573.7|Gm10717-
201|ENSMUSG00000095891|+|chr9:g.3025677:T>A
MFLIFHDFQFSCHIPCPTVEICKFPPFSVFLAIFHVLKYVFLIFRDFQFSRHIPGPTVCISHFSRFSVILSFF
KFSSGCFSFSLIFRFLAIFHVLQWTFLNFPPFSVFLAIFHVLKCVFLIFSDFQFSRHIPGPTVCISHFSRFSV
ISSFFKVSSGCFSFSMIFSFLAIFHVLQWTFLNFPPFSSYSFSPYSRSYSVHFSFFTFFSDFVIFQVFKWMFL
IFLDFQFSC
>ENSMUSP00000096644_c.278:T>G_p.H93Q ENSMUST00000075573.7|Gm10717-
201|ENSMUSG00000095891|+|chr9:g.3025695:T>G
MFLIFHDFQFSCHIPCPTVEICKFPPFSVFLAIFHVLKYVFLIFRDFQFSRHIPGPTVCISHFSRFSVILSFF
KFSSGCFSFSLIFSFLAIFQVLQWTFLNFPPFSVFLAIFHVLKCVFLIFSDFQFSRHIPGPTVCISHFSRFSV
ISSFFKVSSGCFSFSMIFSFLAIFHVLQWTFLNFPPFSSYSFSPYSRSYSVHFSFFTFFSDFVIFQVFKWMFL
IFLDFQFSC
>ENSMUSP00000096644_c.289:G>T_p.W97L ENSMUST00000075573.7|Gm10717-
201|ENSMUSG00000095891|+|chr9:g.3025706:G>T
MFLIFHDFQFSCHIPCPTVEICKFPPFSVFLAIFHVLKYVFLIFRDFQFSRHIPGPTVCISHFSRFSVILSFF
KFSSGCFSFSLIFSFLAIFHVLQLTFLNFPPFSVFLAIFHVLKCVFLIFSDFQFSRHIPGPTVCISHFSRFSV
ISSFFKVSSGCFSFSMIFSFLAIFHVLQWTFLNFPPFSSYSFSPYSRSYSVHFSFFTFFSDFVIFQVFKWMFL
IFLDFQFSC
>ENSMUSP00000096644_c.309:C>A_p.P104T ENSMUST00000075573.7|Gm10717-
201|ENSMUSG00000095891|+|chr9:g.3025726:C>A

MFLIFHDFQFSCHIPCPTVEICKFPPFSVFLAIFHVLKYVFLIFRDFQFSRHIPGPTVCISHFSRFSVILSFF
KFSSGCFSFSLIFSFLAIFHVLQWTFLNFPTFSVFLAIFHVLKCVFLIFSDFQFSRHIPGPTVCISHFSRFSV
ISSFFKVSSGCFSFSMIFSFLAIFHVLQWTFLNFPPFSSYSFSPYSRSYSVHFSFFTFFSDFVIFQVFKWMFL
IFLDFQFSC
>ENSMUSP00000096644_c.330:A>T_p.I111L ENSMUST00000075573.7|Gm10717-201|ENSMUSG00000095891|+|chr9:g.3025747:A>T
MFLIFHDFQFSCHIPCPTVEICKFPPFSVFLAIFHVLKYVFLIFRDFQFSRHIPGPTVCISHFSRFSVILSFF
KFSSGCFSFSLIFSFLAIFHVLQWTFLNFPPFSVFLALFHVLKCVFLIFSDFQFSRHIPGPTVCISHFSRFSV
ISSFFKVSSGCFSFSMIFSFLAIFHVLQWTFLNFPPFSSYSFSPYSRSYSVHFSFFTFFSDFVIFQVFKWMFL
IFLDFQFSC
>ENSMUSP00000096644_c.338:T>A_p.H113Q ENSMUST00000075573.7|Gm10717-201|ENSMUSG00000095891|+|chr9:g.3025755:T>A
MFLIFHDFQFSCHIPCPTVEICKFPPFSVFLAIFHVLKYVFLIFRDFQFSRHIPGPTVCISHFSRFSVILSFF
KFSSGCFSFSLIFSFLAIFHVLQWTFLNFPPFSVFLAIFQVLKCVFLIFSDFQFSRHIPGPTVCISHFSRFSV
ISSFFKVSSGCFSFSMIFSFLAIFHVLQWTFLNFPPFSSYSFSPYSRSYSVHFSFFTFFSDFVIFQVFKWMFL
IFLDFQFSC
>ENSMUSP00000096644_c.356:T>A_p.F119L ENSMUST00000075573.7|Gm10717-201|ENSMUSG00000095891|+|chr9:g.3025773:T>A
MFLIFHDFQFSCHIPCPTVEICKFPPFSVFLAIFHVLKYVFLIFRDFQFSRHIPGPTVCISHFSRFSVILSFF
KFSSGCFSFSLIFSFLAIFHVLQWTFLNFPPFSVFLAIFHVLKCVLLIFSDFQFSRHIPGPTVCISHFSRFSV
ISSFFKVSSGCFSFSMIFSFLAIFHVLQWTFLNFPPFSSYSFSPYSRSYSVHFSFFTFFSDFVIFQVFKWMFL
IFLDFQFSC
>ENSMUSP00000096644_c.366:A>T_p.S123C ENSMUST00000075573.7|Gm10717-201|ENSMUSG00000095891|+|chr9:g.3025783:A>T
MFLIFHDFQFSCHIPCPTVEICKFPPFSVFLAIFHVLKYVFLIFRDFQFSRHIPGPTVCISHFSRFSVILSFF
KFSSGCFSFSLIFSFLAIFHVLQWTFLNFPPFSVFLAIFHVLKCVFLIFCDFQFSRHIPGPTVCISHFSRFSV
ISSFFKVSSGCFSFSMIFSFLAIFHVLQWTFLNFPPFSSYSFSPYSRSYSVHFSFFTFFSDFVIFQVFKWMFL
IFLDFQFSC
>ENSMUSP00000096644_c.366:A>C_p.S123R ENSMUST00000075573.7|Gm10717-201|ENSMUSG00000095891|+|chr9:g.3025783:A>C
MFLIFHDFQFSCHIPCPTVEICKFPPFSVFLAIFHVLKYVFLIFRDFQFSRHIPGPTVCISHFSRFSVILSFF
KFSSGCFSFSLIFSFLAIFHVLQWTFLNFPPFSVFLAIFHVLKCVFLIFRDFQFSRHIPGPTVCISHFSRFSV
ISSFFKVSSGCFSFSMIFSFLAIFHVLQWTFLNFPPFSSYSFSPYSRSYSVHFSFFTFFSDFVIFQVFKWMFL
IFLDFQFSC
>ENSMUSP00000096644_c.397:G>T_p.G133V ENSMUST00000075573.7|Gm10717-201|ENSMUSG00000095891|+|chr9:g.3025814:G>T
MFLIFHDFQFSCHIPCPTVEICKFPPFSVFLAIFHVLKYVFLIFRDFQFSRHIPGPTVCISHFSRFSVILSFF
KFSSGCFSFSLIFSFLAIFHVLQWTFLNFPPFSVFLAIFHVLKCVFLIFSDFQFSRHIPVPTVCISHFSRFSV
ISSFFKVSSGCFSFSMIFSFLAIFHVLQWTFLNFPPFSSYSFSPYSRSYSVHFSFFTFFSDFVIFQVFKWMFL
IFLDFQFSC
>ENSMUSP00000096644_c.402:A>T_p.T135S ENSMUST00000075573.7|Gm10717-201|ENSMUSG00000095891|+|chr9:g.3025819:A>T
MFLIFHDFQFSCHIPCPTVEICKFPPFSVFLAIFHVLKYVFLIFRDFQFSRHIPGPTVCISHFSRFSVILSFF
KFSSGCFSFSLIFSFLAIFHVLQWTFLNFPPFSVFLAIFHVLKCVFLIFSDFQFSRHIPGPSVCISHFSRFSV
ISSFFKVSSGCFSFSMIFSFLAIFHVLQWTFLNFPPFSSYSFSPYSRSYSVHFSFFTFFSDFVIFQVFKWMFL
IFLDFQFSC
>ENSMUSP00000096644_c.433:C>T_p.S145L ENSMUST00000075573.7|Gm10717-201|ENSMUSG00000095891|+|chr9:g.3025850:C>T
MFLIFHDFQFSCHIPCPTVEICKFPPFSVFLAIFHVLKYVFLIFRDFQFSRHIPGPTVCISHFSRFSVILSFF
KFSSGCFSFSLIFSFLAIFHVLQWTFLNFPPFSVFLAIFHVLKCVFLIFSDFQFSRHIPGPTVCISHFSRFLV
ISSFFKVSSGCFSFSMIFSFLAIFHVLQWTFLNFPPFSSYSFSPYSRSYSVHFSFFTFFSDFVIFQVFKWMFL
IFLDFQFSC
>ENSMUSP00000096644_c.456:G>T_p.V153L ENSMUST00000075573.7|Gm10717-201|ENSMUSG00000095891|+|chr9:g.3025873:G>T
MFLIFHDFQFSCHIPCPTVEICKFPPFSVFLAIFHVLKYVFLIFRDFQFSRHIPGPTVCISHFSRFSVILSFF
KFSSGCFSFSLIFSFLAIFHVLQWTFLNFPPFSVFLAIFHVLKCVFLIFSDFQFSRHIPGPTVCISHFSRFSV
ISSFFKLSSGCFSFSMIFSFLAIFHVLQWTFLNFPPFSSYSFSPYSRSYSVHFSFFTFFSDFVIFQVFKWMFL
IFLDFQFSC

>ENSMUSP00000096644_c.457:T>G_p.V153G ENSMUST00000075573.7|Gm10717-201|ENSMUSG00000095891|+|chr9:g.3025874:T>G
MFLIFHDFQFSCHIPCPTVEICKFPPFSVFLAIFHVLKYVFLIFRDFQFSRHIPGPTVCISHFSRFSVILSFF
KFSSGCFSFSLIFSFLAIFHVLQWTFLNFPPFSVFLAIFHVLKCVFLIFSDFQFSRHIPGPTVCISHFSRFSV
ISSFFKGSSGCFSFSMIFSFLAIFHVLQWTFLNFPPFSSYSFSPYSRSYSVHFSFFTFFSDFVIFQVFKWMFL
IFLDFQFSC
>ENSMUSP00000096644_c.519:C>T_p.Q174* ENSMUST00000075573.7|Gm10717-201|ENSMUSG00000095891|+|chr9:g.3025936:C>T
MFLIFHDFQFSCHIPCPTVEICKFPPFSVFLAIFHVLKYVFLIFRDFQFSRHIPGPTVCISHFSRFSVILSFF
KFSSGCFSFSLIFSFLAIFHVLQWTFLNFPPFSVFLAIFHVLKCVFLIFSDFQFSRHIPGPTVCISHFSRFSV
ISSFFKVSSGCFSFSMIFSFLAIFHVL*WTFLNFPPFSSYSFSPYSRSYSVHFSFFTFFSDFVIFQVFKWMFL
IFLDFQFSC
>ENSMUSP00000096644_c.522:T>C_p.W175R ENSMUST00000075573.7|Gm10717-201|ENSMUSG00000095891|+|chr9:g.3025939:T>C
MFLIFHDFQFSCHIPCPTVEICKFPPFSVFLAIFHVLKYVFLIFRDFQFSRHIPGPTVCISHFSRFSVILSFF
KFSSGCFSFSLIFSFLAIFHVLQWTFLNFPPFSVFLAIFHVLKCVFLIFSDFQFSRHIPGPTVCISHFSRFSV
ISSFFKVSSGCFSFSMIFSFLAIFHVLQRTFLNFPPFSSYSFSPYSRSYSVHFSFFTFFSDFVIFQVFKWMFL
IFLDFQFSC
>ENSMUSP00000096644_c.543:C>A_p.P182T ENSMUST00000075573.7|Gm10717-201|ENSMUSG00000095891|+|chr9:g.3025960:C>A
MFLIFHDFQFSCHIPCPTVEICKFPPFSVFLAIFHVLKYVFLIFRDFQFSRHIPGPTVCISHFSRFSVILSFF
KFSSGCFSFSLIFSFLAIFHVLQWTFLNFPPFSVFLAIFHVLKCVFLIFSDFQFSRHIPGPTVCISHFSRFSV
ISSFFKVSSGCFSFSMIFSFLAIFHVLQWTFLNFPTFSSYSFSPYSRSYSVHFSFFTFFSDFVIFQVFKWMFL
IFLDFQFSC
>ENSMUSP00000096644_c.558:A>C_p.S187R ENSMUST00000075573.7|Gm10717-201|ENSMUSG00000095891|+|chr9:g.3026055:A>C
MFLIFHDFQFSCHIPCPTVEICKFPPFSVFLAIFHVLKYVFLIFRDFQFSRHIPGPTVCISHFSRFSVILSFF
KFSSGCFSFSLIFSFLAIFHVLQWTFLNFPPFSVFLAIFHVLKCVFLIFSDFQFSRHIPGPTVCISHFSRFSV
ISSFFKVSSGCFSFSMIFSFLAIFHVLQWTFLNFPPFSSYRFSPYSRSYSVHFSFFTFFSDFVIFQVFKWMFL
IFLDFQFSC
>ENSMUSP00000136755_c.1:G>A_p.C1Y ENSMUST00000177601.2|Gm10717-205|ENSMUSG00000095891|+|chr9:g.3026152:G>A
YHIPCPTVEISFPPFSVFLAIFHVLKCVFLIFRVFQVSRHIPGPTVCISHFSRFSVILSFFKFSSGCFSFSLI
FSFLAIFHVLQWTFLNFPPFSVFLAIFHVLKCCVFLIFRDFQFSCHIPGPTVCISHFSRFSVILSFFNLSTAC
FSFSIIFSFLAIFHVLQWTFLNFPPFSSYSPYSRSYRMHFSFFTFFSDFIIFQVVKCMFLIFNDFHFSC
>ENSMUSP00000136755_c.19:C>A_p.T7N ENSMUST00000177601.2|Gm10717-205|ENSMUSG00000095891|+|chr9:g.3026170:C>A
CHIPCPNVEISFPPFSVFLAIFHVLKCVFLIFRVFQVSRHIPGPTVCISHFSRFSVILSFFKFSSGCFSFSLI
FSFLAIFHVLQWTFLNFPPFSVFLAIFHVLKCCVFLIFRDFQFSCHIPGPTVCISHFSRFSVILSFFNLSTAC
FSFSIIFSFLAIFHVLQWTFLNFPPFSSYSPYSRSYRMHFSFFTFFSDFIIFQVVKCMFLIFNDFHFSC
>ENSMUSP00000136755_c.26:A>C_p.E9D ENSMUST00000177601.2|Gm10717-205|ENSMUSG00000095891|+|chr9:g.3026177:A>C
CHIPCPTVDISFPPFSVFLAIFHVLKCVFLIFRVFQVSRHIPGPTVCISHFSRFSVILSFFKFSSGCFSFSLI
FSFLAIFHVLQWTFLNFPPFSVFLAIFHVLKCCVFLIFRDFQFSCHIPGPTVCISHFSRFSVILSFFNLSTAC
FSFSIIFSFLAIFHVLQWTFLNFPPFSSYSPYSRSYRMHFSFFTFFSDFIIFQVVKCMFLIFNDFHFSC
>ENSMUSP00000136755_c.88:T>A_p.L30H ENSMUST00000177601.2|Gm10717-205|ENSMUSG00000095891|+|chr9:g.3026243:T>A
CHIPCPTVEISFPPFSVFLAIFHVLKCVFHIFRVFQVSRHIPGPTVCISHFSRFSVILSFFKFSSGCFSFSLI
FSFLAIFHVLQWTFLNFPPFSVFLAIFHVLKCCVFLIFRDFQFSCHIPGPTVCISHFSRFSVILSFFNLSTAC
FSFSIIFSFLAIFHVLQWTFLNFPPFSSYSPYSRSYRMHFSFFTFFSDFIIFQVVKCMFLIFNDFHFSC
>ENSMUSP00000136755_c.95:TC>T_p.F32del-fs ENSMUST00000177601.2|Gm10717-205|ENSMUSG00000095891|+|chr9:g.3026250:TC>T
CHIPCPTVEISFPPFSVFLAIFHVLKCVFLIFVFFRFLAIFQVLQCAFLIFHVFQ
>ENSMUSP00000136755_c.100:T>A_p.V34D ENSMUST00000177601.2|Gm10717-205|ENSMUSG00000095891|+|chr9:g.3026255:T>A
CHIPCPTVEISFPPFSVFLAIFHVLKCVFLIFRDFQVSRHIPGPTVCISHFSRFSVILSFFKFSSGCFSFSLI
FSFLAIFHVLQWTFLNFPPFSVFLAIFHVLKCCVFLIFRDFQFSCHIPGPTVCISHFSRFSVILSFFNLSTAC
FSFSIIFSFLAIFHVLQWTFLNFPPFSSYSPYSRSYRMHFSFFTFFSDFIIFQVVKCMFLIFNDFHFSC

>ENSMUSP00000096644_c.559:G>T_p.S187I ENSMUST00000075573.7|Gm10717-201|ENSMUSG00000095891|+|chr9:g.3026263:G>T
MFLIFHDFQFSCHIPCPTVEICKFPPFSVFLAIFHVLKYVFLIFRDFQFSRHIPGPTVCISHFSRFSVILSFF
KFSSGCFSFSLIFSFLAIFHVLQWTFLNFPPFSVFLAIFHVLKCVFLIFSDFQFSRHIPGPTVCISHFSRFSV
ISSFFKVSSGCFSFSMIFSFLAIFHVLQWTFLNFPPFSSYIFSPYSRSYSVHFSFFTFFSDFVIFQVFKWMFL
IFLDFQFSC
>ENSMUSP00000136755_c.108:G>T_p.V37F ENSMUST00000177601.2|Gm10717-205|ENSMUSG00000095891|+|chr9:g.3026263:G>T
CHIPCPTVEISFPPFSVFLAIFHVLKCVFLIFRVFQFSRHIPGPTVCISHFSRFSVILSFFKFSSGCFSFSLI
FSFLAIFHVLQWTFLNFPPFSVFLAIFHVLKCCVFLIFRDFQFSCHIPGPTVCISHFSRFSVILSFFNLSTAC
FSFSIIFSFLAIFHVLQWTFLNFPPFSSYSPYSRSYRMHFSFFTFFSDFIIFQVVKCMFLIFNDFHFSC
>ENSMUSP00000096644_c.565:CGCC>TGTT_p.S189L ENSMUST00000075573.7|Gm10717-201|ENSMUSG00000095891|+|chr9:g.3026269:CGCC>TGTT
MFLIFHDFQFSCHIPCPTVEICKFPPFSVFLAIFHVLKYVFLIFRDFQFSRHIPGPTVCISHFSRFSVILSFF
KFSSGCFSFSLIFSFLAIFHVLQWTFLNFPPFSVFLAIFHVLKCVFLIFSDFQFSRHIPGPTVCISHFSRFSV
ISSFFKVSSGCFSFSMIFSFLAIFHVLQWTFLNFPPFSSYSFLLPYSRSYSVHFSFFTFFSDFVIFQVFKWMF
LIFLDFQFSC
>ENSMUSP00000136755_c.114:CGCC>TGTT_p.R39C ENSMUST00000177601.2|Gm10717-205|ENSMUSG00000095891|+|chr9:g.3026269:CGCC>TGTT
CHIPCPTVEISFPPFSVFLAIFHVLKCVFLIFRVFQVSCCHIPGPTVCISHFSRFSVILSFFKFSSGCFSFSL
IFSFLAIFHVLQWTFLNFPPFSVFLAIFHVLKCCVFLIFRDFQFSCHIPGPTVCISHFSRFSVILSFFNLSTA
CFSFSIIFSFLAIFHVLQWTFLNFPPFSSYSPYSRSYRMHFSFFTFFSDFIIFQVVKCMFLIFNDFHFSC
>ENSMUSP00000096644_c.583:A>T_p.Y195F ENSMUST00000075573.7|Gm10717-201|ENSMUSG00000095891|+|chr9:g.3026287:A>T
MFLIFHDFQFSCHIPCPTVEICKFPPFSVFLAIFHVLKYVFLIFRDFQFSRHIPGPTVCISHFSRFSVILSFF
KFSSGCFSFSLIFSFLAIFHVLQWTFLNFPPFSVFLAIFHVLKCVFLIFSDFQFSRHIPGPTVCISHFSRFSV
ISSFFKVSSGCFSFSMIFSFLAIFHVLQWTFLNFPPFSSYSFSPYSRSFSVHFSFFTFFSDFVIFQVFKWMFL
IFLDFQFSC
>ENSMUSP00000136755_c.132:A>T_p.T45S ENSMUST00000177601.2|Gm10717-205|ENSMUSG00000095891|+|chr9:g.3026287:A>T
CHIPCPTVEISFPPFSVFLAIFHVLKCVFLIFRVFQVSRHIPGPSVCISHFSRFSVILSFFKFSSGCFSFSLI
FSFLAIFHVLQWTFLNFPPFSVFLAIFHVLKCCVFLIFRDFQFSCHIPGPTVCISHFSRFSVILSFFNLSTAC
FSFSIIFSFLAIFHVLQWTFLNFPPFSSYSPYSRSYRMHFSFFTFFSDFIIFQVVKCMFLIFNDFHFSC
>ENSMUSP00000096644_c.604:T>C_p.F202S ENSMUST00000075573.7|Gm10717-201|ENSMUSG00000095891|+|chr9:g.3026308:T>C
MFLIFHDFQFSCHIPCPTVEICKFPPFSVFLAIFHVLKYVFLIFRDFQFSRHIPGPTVCISHFSRFSVILSFF
KFSSGCFSFSLIFSFLAIFHVLQWTFLNFPPFSVFLAIFHVLKCVFLIFSDFQFSRHIPGPTVCISHFSRFSV
ISSFFKVSSGCFSFSMIFSFLAIFHVLQWTFLNFPPFSSYSFSPYSRSYSVHFSFSTFFSDFVIFQVFKWMFL
IFLDFQFSC
>ENSMUSP00000136755_c.153:T>C_p.S52P ENSMUST00000177601.2|Gm10717-205|ENSMUSG00000095891|+|chr9:g.3026308:T>C
CHIPCPTVEISFPPFSVFLAIFHVLKCVFLIFRVFQVSRHIPGPTVCISHFPRFSVILSFFKFSSGCFSFSLI
FSFLAIFHVLQWTFLNFPPFSVFLAIFHVLKCCVFLIFRDFQFSCHIPGPTVCISHFSRFSVILSFFNLSTAC
FSFSIIFSFLAIFHVLQWTFLNFPPFSSYSPYSRSYRMHFSFFTFFSDFIIFQVVKCMFLIFNDFHFSC
>ENSMUSP00000096644_c.613:T>G_p.F205C ENSMUST00000075573.7|Gm10717-201|ENSMUSG00000095891|+|chr9:g.3026317:T>G
MFLIFHDFQFSCHIPCPTVEICKFPPFSVFLAIFHVLKYVFLIFRDFQFSRHIPGPTVCISHFSRFSVILSFF
KFSSGCFSFSLIFSFLAIFHVLQWTFLNFPPFSVFLAIFHVLKCVFLIFSDFQFSRHIPGPTVCISHFSRFSV
ISSFFKVSSGCFSFSMIFSFLAIFHVLQWTFLNFPPFSSYSFSPYSRSYSVHFSFFTFCSDFVIFQVFKWMFL
IFLDFQFSC
>ENSMUSP00000136755_c.162:T>G_p.S55A ENSMUST00000177601.2|Gm10717-205|ENSMUSG00000095891|+|chr9:g.3026317:T>G
CHIPCPTVEISFPPFSVFLAIFHVLKCVFLIFRVFQVSRHIPGPTVCISHFSRFAVILSFFKFSSGCFSFSLI
FSFLAIFHVLQWTFLNFPPFSVFLAIFHVLKCCVFLIFRDFQFSCHIPGPTVCISHFSRFSVILSFFNLSTAC
FSFSIIFSFLAIFHVLQWTFLNFPPFSSYSPYSRSYRMHFSFFTFFSDFIIFQVVKCMFLIFNDFHFSC
>ENSMUSP00000136755_c.163:C>T_p.S55L ENSMUST00000177601.2|Gm10717-205|ENSMUSG00000095891|+|chr9:g.3026318:C>T

CHIPCPTVEISFPPFSVFLAIFHVLKCVFLIFRVFQVSRHIPGPTVCISHFSRFLVILSFFKFSSGCFSFSLI
FSFLAIFHVLQWTFLNFPPPFSVFLAIFHVLKCCVFLIFRDFQFSCHIPGPTVCISHFSRFSVILSFFNLSTAC
FSFSIIFSFLAIFHVLQWTFLNFPPFSSYSPYSRSYRMHFSFFTFFSDFIIFQVVKCMFLIFNDFHFSC
>ENSMUSP00000136755_c.172:T>C_p.L58S ENSMUST00000177601.2|Gm10717-205|ENSMUSG00000095891|+|chr9:g.3026327:T>C
CHIPCPTVEISFPPFSVFLAIFHVLKCVFLIFRVFQVSRHIPGPTVCISHFSRFSVISSFFKFSSGCFSFSLI
FSFLAIFHVLQWTFLNFPPPFSVFLAIFHVLKCCVFLIFRDFQFSCHIPGPTVCISHFSRFSVILSFFNLSTAC
FSFSIIFSFLAIFHVLQWTFLNFPPFSSYSPYSRSYRMHFSFFTFFSDFIIFQVVKCMFLIFNDFHFSC
>ENSMUSP00000136755_c.187:T>G_p.F63C ENSMUST00000177601.2|Gm10717-205|ENSMUSG00000095891|+|chr9:g.3026342:T>G
CHIPCPTVEISFPPFSVFLAIFHVLKCVFLIFRVFQVSRHIPGPTVCISHFSRFSVILSFFKCSSGCFSFSLI
FSFLAIFHVLQWTFLNFPPPFSVFLAIFHVLKCCVFLIFRDFQFSCHIPGPTVCISHFSRFSVILSFFNLSTAC
FSFSIIFSFLAIFHVLQWTFLNFPPFSSYSPYSRSYRMHFSFFTFFSDFIIFQVVKCMFLIFNDFHFSC
>ENSMUSP00000136755_c.187:T>C_p.F63S ENSMUST00000177601.2|Gm10717-205|ENSMUSG00000095891|+|chr9:g.3026342:T>C
CHIPCPTVEISFPPFSVFLAIFHVLKCVFLIFRVFQVSRHIPGPTVCISHFSRFSVILSFFKSSSGCFSFSLI
FSFLAIFHVLQWTFLNFPPPFSVFLAIFHVLKCCVFLIFRDFQFSCHIPGPTVCISHFSRFSVILSFFNLSTAC
FSFSIIFSFLAIFHVLQWTFLNFPPFSSYSPYSRSYRMHFSFFTFFSDFIIFQVVKCMFLIFNDFHFSC
>ENSMUSP00000096644_c.650:G>T_p.M217I ENSMUST00000075573.7|Gm10717-201|ENSMUSG00000095891|+|chr9:g.3026354:G>T
MFLIFHDFQFSCHIPCPTVEICKFPPFSVFLAIFHVLKYVFLIFRDFQFSRHIPGPTVCISHFSRFSVILSFF
KFSSGCFSFSLIFSFLAIFHVLQWTFLNFPPFSVFLAIFHVLKCVFLIFSDFQFSRHIPGPTVCISHFSRFSV
ISSFFKVSSGCFSFSMIFSFLAIFHVLQWTFLNFPPFSSYSFSPYSRSYSVHFSFFTFFSDFVIFQVFKWIFL
IFLDFQFSC
>ENSMUSP00000136755_c.199:G>T_p.C67F ENSMUST00000177601.2|Gm10717-205|ENSMUSG00000095891|+|chr9:g.3026354:G>T
CHIPCPTVEISFPPFSVFLAIFHVLKCVFLIFRVFQVSRHIPGPTVCISHFSRFSVILSFFKFSSGFFSFSLI
FSFLAIFHVLQWTFLNFPPPFSVFLAIFHVLKCCVFLIFRDFQFSCHIPGPTVCISHFSRFSVILSFFNLSTAC
FSFSIIFSFLAIFHVLQWTFLNFPPFSSYSPYSRSYRMHFSFFTFFSDFIIFQVVKCMFLIFNDFHFSC
>ENSMUSP00000096644_c.657:A>G_p.I220V ENSMUST00000075573.7|Gm10717-201|ENSMUSG00000095891|+|chr9:g.3026361:A>G
MFLIFHDFQFSCHIPCPTVEICKFPPFSVFLAIFHVLKYVFLIFRDFQFSRHIPGPTVCISHFSRFSVILSFF
KFSSGCFSFSLIFSFLAIFHVLQWTFLNFPPFSVFLAIFHVLKCVFLIFSDFQFSRHIPGPTVCISHFSRFSV
ISSFFKVSSGCFSFSMIFSFLAIFHVLQWTFLNFPPFSSYSFSPYSRSYSVHFSFFTFFSDFVIFQVFKWMFL
VFLDFQFSC
>ENSMUSP00000096644_c.664:T>A_p.L222H ENSMUST00000075573.7|Gm10717-201|ENSMUSG00000095891|+|chr9:g.3026368:T>A
MFLIFHDFQFSCHIPCPTVEICKFPPFSVFLAIFHVLKYVFLIFRDFQFSRHIPGPTVCISHFSRFSVILSFF
KFSSGCFSFSLIFSFLAIFHVLQWTFLNFPPFSVFLAIFHVLKCVFLIFSDFQFSRHIPGPTVCISHFSRFSV
ISSFFKVSSGCFSFSMIFSFLAIFHVLQWTFLNFPPFSSYSFSPYSRSYSVHFSFFTFFSDFVIFQVFKWMFL
IFHDFQFSC
>ENSMUSP00000136755_c.213:T>A_p.L72M ENSMUST00000177601.2|Gm10717-205|ENSMUSG00000095891|+|chr9:g.3026368:T>A
CHIPCPTVEISFPPFSVFLAIFHVLKCVFLIFRVFQVSRHIPGPTVCISHFSRFSVILSFFKFSSGCFSFSMI
FSFLAIFHVLQWTFLNFPPPFSVFLAIFHVLKCCVFLIFRDFQFSCHIPGPTVCISHFSRFSVILSFFNLSTAC
FSFSIIFSFLAIFHVLQWTFLNFPPFSSYSPYSRSYRMHFSFFTFFSDFIIFQVVKCMFLIFNDFHFSC
>ENSMUSP00000096644_c.664:T>G_p.L222R ENSMUST00000075573.7|Gm10717-201|ENSMUSG00000095891|+|chr9:g.3026368:T>G
MFLIFHDFQFSCHIPCPTVEICKFPPFSVFLAIFHVLKYVFLIFRDFQFSRHIPGPTVCISHFSRFSVILSFF
KFSSGCFSFSLIFSFLAIFHVLQWTFLNFPPFSVFLAIFHVLKCVFLIFSDFQFSRHIPGPTVCISHFSRFSV
ISSFFKVSSGCFSFSMIFSFLAIFHVLQWTFLNFPPFSSYSFSPYSRSYSVHFSFFTFFSDFVIFQVFKWMFL
IFRDFQFSC
>ENSMUSP00000136755_c.213:T>G_p.L72V ENSMUST00000177601.2|Gm10717-205|ENSMUSG00000095891|+|chr9:g.3026368:T>G
CHIPCPTVEISFPPFSVFLAIFHVLKCVFLIFRVFQVSRHIPGPTVCISHFSRFSVILSFFKFSSGCFSFSVI
FSFLAIFHVLQWTFLNFPPPFSVFLAIFHVLKCCVFLIFRDFQFSCHIPGPTVCISHFSRFSVILSFFNLSTAC
FSFSIIFSFLAIFHVLQWTFLNFPPFSSYSPYSRSYRMHFSFFTFFSDFIIFQVVKCMFLIFNDFHFSC

>ENSMUSP00000096644_c.675:T>A_p.F226I ENSMUST00000075573.7|Gm10717-201|ENSMUSG00000095891|+|chr9:g.3026379:T>A
MFLIFHDFQFSCHIPCPTVEICKFPPFSVFLAIFHVLKYVFLIFRDFQFSRHIPGPTVCISHFSRFSVILSFF
KFSSGCFSFSLIFSFLAIFHVLQWTFLNFPPFSVFLAIFHVLKCVFLIFSDFQFSRHIPGPTVCISHFSRFSV
ISSFFKVSSGCFSFSMIFSFLAIFHVLQWTFLNFPPFSSYSFSPYSRSYSVHFSFFTFFSDFVIFQVFKWMFL
IFLDFQISC
>ENSMUSP00000136755_c.224:T>A_p.S75R ENSMUST00000177601.2|Gm10717-205|ENSMUSG00000095891|+|chr9:g.3026379:T>A
CHIPCPTVEISFPPFSVFLAIFHVLKCVFLIFRVFQVSRHIPGPTVCISHFSRFSVILSFFKFSSGCFSFSLI
FRFLAIFHVLQWTFLNFPPFSVFLAIFHVLKCCVFLIFRDFQFSCHIPGPTVCISHFSRFSVILSFFNLSTAC
FSFSIIFSFLAIFHVLQWTFLNFPPFSSYSPYSRSYRMHFSFFTFFSDFIIFQVVKCMFLIFNDFHFSC
>ENSMUSP00000136755_c.242:T>G_p.H81Q ENSMUST00000177601.2|Gm10717-205|ENSMUSG00000095891|+|chr9:g.3026397:T>G
CHIPCPTVEISFPPFSVFLAIFHVLKCVFLIFRVFQVSRHIPGPTVCISHFSRFSVILSFFKFSSGCFSFSLI
FSFLAIFQVLQWTFLNFPPFSVFLAIFHVLKCCVFLIFRDFQFSCHIPGPTVCISHFSRFSVILSFFNLSTAC
FSFSIIFSFLAIFHVLQWTFLNFPPFSSYSPYSRSYRMHFSFFTFFSDFIIFQVVKCMFLIFNDFHFSC
>ENSMUSP00000136755_c.292:C>T_p.A98V ENSMUST00000177601.2|Gm10717-205|ENSMUSG00000095891|+|chr9:g.3026447:C>T
CHIPCPTVEISFPPFSVFLAIFHVLKCVFLIFRVFQVSRHIPGPTVCISHFSRFSVILSFFKFSSGCFSFSLI
FSFLAIFHVLQWTFLNFPPFSVFLVIFHVLKCCVFLIFRDFQFSCHIPGPTVCISHFSRFSVILSFFNLSTAC
FSFSIIFSFLAIFHVLQWTFLNFPPFSSYSPYSRSYRMHFSFFTFFSDFIIFQVVKCMFLIFNDFHFSC
>ENSMUSP00000136755_c.302:T>A_p.H101Q ENSMUST00000177601.2|Gm10717-205|ENSMUSG00000095891|+|chr9:g.3026457:T>A
CHIPCPTVEISFPPFSVFLAIFHVLKCVFLIFRVFQVSRHIPGPTVCISHFSRFSVILSFFKFSSGCFSFSLI
FSFLAIFHVLQWTFLNFPPFSVFLAIFQVLKCCVFLIFRDFQFSCHIPGPTVCISHFSRFSVILSFFNLSTAC
FSFSIIFSFLAIFHVLQWTFLNFPPFSSYSPYSRSYRMHFSFFTFFSDFIIFQVVKCMFLIFNDFHFSC
>ENSMUSP00000136755_c.309:A>T_p.K104* ENSMUST00000177601.2|Gm10717-205|ENSMUSG00000095891|+|chr9:g.3026464:A>T
CHIPCPTVEISFPPFSVFLAIFHVLKCVFLIFRVFQVSRHIPGPTVCISHFSRFSVILSFFKFSSGCFSFSLI
FSFLAIFHVLQWTFLNFPPFSVFLAIFHVL*CCVFLIFRDFQFSCHIPGPTVCISHFSRFSVILSFFNLSTAC
FSFSIIFSFLAIFHVLQWTFLNFPPFSSYSPYSRSYRMHFSFFTFFSDFIIFQVVKCMFLIFNDFHFSC
>ENSMUSP00000137419_c.2:G>T_p.M1I ENSMUST00000177875.8|Gm10717-206|ENSMUSG00000095891|+|chr9:g.3027525:G>T
IFLIFHDFQFSCHIPCPTVDISFPPFSVFLAIFHVLKCVFLIFRDFQFSCHIPGPTVCISHFSRFSVILSFFN
LSTACFSFSIIFSFLAIFHVLQWTFLNFPPFSVFLAIFHVLKCCVFLIFRDFQFSCHIPGPTVCISHFSRFSV
ILSFFNLSTACFSFSMIFSFLAIFHVLQWKFLNFPPFSVSPYSRSYSVHFSFFTFFSDFVIFQVVKWMFPIFL
DFQFSY
>ENSMUSP00000136365_c.2:G>T_p.M1I ENSMUST00000179982.2|Gm10717-209|ENSMUSG00000095891|+|chr9:g.3027525:G>T
IFLIFHDFQFSCHIPCPTVDISFPPFSVFLAIFHVLKCVFLIFRDFQFSCHIPGPTVCISHFSRFSVILSFFN
LSTACFSFSIIFSFLAIFHVLQWTFLNFPPFSVFLAIFHVLKCVFLIFRDFQFSCYIPSPTVCISHFSRFSVI
LSFFNLSTACFSFSMIFSFFAIFHVLQGKFLYFPPFSSSIFSPYSRSYSVHFSFFTFFSDFVIFQVVKWKFLI
FHDFQLSS
>ENSMUSP00000137419_c.45:T>C_p.C16R ENSMUST00000177875.8|Gm10717-206|ENSMUSG00000095891|+|chr9:g.3027568:T>C
MFLIFHDFQFSCHIPRPTVDISFPPFSVFLAIFHVLKCVFLIFRDFQFSCHIPGPTVCISHFSRFSVILSFFN
LSTACFSFSIIFSFLAIFHVLQWTFLNFPPFSVFLAIFHVLKCCVFLIFRDFQFSCHIPGPTVCISHFSRFSV
ILSFFNLSTACFSFSMIFSFLAIFHVLQWKFLNFPPFSVSPYSRSYSVHFSFFTFFSDFVIFQVVKWMFPIFL
DFQFSY
>ENSMUSP00000136365_c.45:T>C_p.C16R ENSMUST00000179982.2|Gm10717-209|ENSMUSG00000095891|+|chr9:g.3027568:T>C
MFLIFHDFQFSCHIPRPTVDISFPPFSVFLAIFHVLKCVFLIFRDFQFSCHIPGPTVCISHFSRFSVILSFFN
LSTACFSFSIIFSFLAIFHVLQWTFLNFPPFSVFLAIFHVLKCVFLIFRDFQFSCYIPSPTVCISHFSRFSVI
LSFFNLSTACFSFSMIFSFFAIFHVLQGKFLYFPPFSSSIFSPYSRSYSVHFSFFTFFSDFVIFQVVKWKFLI
FHDFQLSS
>ENSMUSP00000136755_c.333:C>T_p.R112C ENSMUST00000177601.2|Gm10717-205|ENSMUSG00000095891|+|chr9:g.3027656:C>T

CHIPCPTVEISFPPFSVFLAIFHVLKCVFLIFRVFQVSRHIPGPTVCISHFSRFSVILSFFKFSSGCFSFSLI
FSFLAIFHVLQWTFLNFPPFSVFLAIFHVLKCCVFLIFCDFQFSCHIPGPTVCISHFSRFSVILSFFNLSTAC
FSFSIIFSFLAIFHVLQWTFLNFPPFSSYSPYSRSYRMHFSFFTFFSDFIIFQVVKCMFLIFNDFHFSC
>ENSMUSP00000137419_c.129:C>T_p.R44C ENSMUST00000177875.8|Gm10717-206|ENSMUSG00000095891|+|chr9:g.3027656:C>T
MFLIFHDFQFSCHIPCPTVDISFPPFSVFLAIFHVLKCVFLIFCDFQFSCHIPGPTVCISHFSRFSVILSFFN
LSTACFSFSIIFSFLAIFHVLQWTFLNFPPFSVFLAIFHVLKCCVFLIFRDFQFSCHIPGPTVCISHFSRFSV
ILSFFNLSTACFSFSMIFSFLAIFHVLQWKFLNFPPFSVSPYSRSYSVHFSFFTFFSDFVIFQVVKWMFPIFL
DFQFSY
>ENSMUSP00000136365_c.129:C>T_p.R44C ENSMUST00000179982.2|Gm10717-209|ENSMUSG00000095891|+|chr9:g.3027656:C>T
MFLIFHDFQFSCHIPCPTVDISFPPFSVFLAIFHVLKCVFLIFCDFQFSCHIPGPTVCISHFSRFSVILSFFN
LSTACFSFSIIFSFLAIFHVLQWTFLNFPPFSVFLAIFHVLKCVFLIFRDFQFSCYIPSPTVCISHFSRFSVI
LSFFNLSTACFSFSMIFSFFAIFHVLQGKFLYFPPFSSSIFSPYSRSYSVHFSFFTFFSDFVIFQVVKWKFLI
FHDFQLSS
>ENSMUSP00000136755_c.334:G>A_p.R112H ENSMUST00000177601.2|Gm10717-205|ENSMUSG00000095891|+|chr9:g.3027657:G>A
CHIPCPTVEISFPPFSVFLAIFHVLKCVFLIFRVFQVSRHIPGPTVCISHFSRFSVILSFFKFSSGCFSFSLI
FSFLAIFHVLQWTFLNFPPFSVFLAIFHVLKCCVFLIFHDFQFSCHIPGPTVCISHFSRFSVILSFFNLSTAC
FSFSIIFSFLAIFHVLQWTFLNFPPFSSYSPYSRSYRMHFSFFTFFSDFIIFQVVKCMFLIFNDFHFSC
>ENSMUSP00000137419_c.130:G>A_p.R44H ENSMUST00000177875.8|Gm10717-206|ENSMUSG00000095891|+|chr9:g.3027657:G>A
MFLIFHDFQFSCHIPCPTVDISFPPFSVFLAIFHVLKCVFLIFHDFQFSCHIPGPTVCISHFSRFSVILSFFN
LSTACFSFSIIFSFLAIFHVLQWTFLNFPPFSVFLAIFHVLKCCVFLIFRDFQFSCHIPGPTVCISHFSRFSV
ILSFFNLSTACFSFSMIFSFLAIFHVLQWKFLNFPPFSVSPYSRSYSVHFSFFTFFSDFVIFQVVKWMFPIFL
DFQFSY
>ENSMUSP00000136365_c.130:G>A_p.R44H ENSMUST00000179982.2|Gm10717-209|ENSMUSG00000095891|+|chr9:g.3027657:G>A
MFLIFHDFQFSCHIPCPTVDISFPPFSVFLAIFHVLKCVFLIFHDFQFSCHIPGPTVCISHFSRFSVILSFFN
LSTACFSFSIIFSFLAIFHVLQWTFLNFPPFSVFLAIFHVLKCVFLIFRDFQFSCYIPSPTVCISHFSRFSVI
LSFFNLSTACFSFSMIFSFFAIFHVLQGKFLYFPPFSSSIFSPYSRSYSVHFSFFTFFSDFVIFQVVKWKFLI
FHDFQLSS
>ENSMUSP00000136755_c.351:T>C_p.C118R ENSMUST00000177601.2|Gm10717-205|ENSMUSG00000095891|+|chr9:g.3027674:T>C
CHIPCPTVEISFPPFSVFLAIFHVLKCVFLIFRVFQVSRHIPGPTVCISHFSRFSVILSFFKFSSGCFSFSLI
FSFLAIFHVLQWTFLNFPPFSVFLAIFHVLKCCVFLIFRDFQFSRHIPGPTVCISHFSRFSVILSFFNLSTAC
FSFSIIFSFLAIFHVLQWTFLNFPPFSSYSPYSRSYRMHFSFFTFFSDFIIFQVVKCMFLIFNDFHFSC
>ENSMUSP00000137419_c.147:T>C_p.C50R ENSMUST00000177875.8|Gm10717-206|ENSMUSG00000095891|+|chr9:g.3027674:T>C
MFLIFHDFQFSCHIPCPTVDISFPPFSVFLAIFHVLKCVFLIFRDFQFSRHIPGPTVCISHFSRFSVILSFFN
LSTACFSFSIIFSFLAIFHVLQWTFLNFPPFSVFLAIFHVLKCCVFLIFRDFQFSCHIPGPTVCISHFSRFSV
ILSFFNLSTACFSFSMIFSFLAIFHVLQWKFLNFPPFSVSPYSRSYSVHFSFFTFFSDFVIFQVVKWMFPIFL
DFQFSY
>ENSMUSP00000136365_c.147:T>C_p.C50R ENSMUST00000179982.2|Gm10717-209|ENSMUSG00000095891|+|chr9:g.3027674:T>C
MFLIFHDFQFSCHIPCPTVDISFPPFSVFLAIFHVLKCVFLIFRDFQFSRHIPGPTVCISHFSRFSVILSFFN
LSTACFSFSIIFSFLAIFHVLQWTFLNFPPFSVFLAIFHVLKCVFLIFRDFQFSCYIPSPTVCISHFSRFSVI
LSFFNLSTACFSFSMIFSFFAIFHVLQGKFLYFPPFSSSIFSPYSRSYSVHFSFFTFFSDFVIFQVVKWKFLI
FHDFQLSS
>ENSMUSP00000136755_c.369:A>T_p.T124S ENSMUST00000177601.2|Gm10717-205|ENSMUSG00000095891|+|chr9:g.3027692:A>T
CHIPCPTVEISFPPFSVFLAIFHVLKCVFLIFRVFQVSRHIPGPTVCISHFSRFSVILSFFKFSSGCFSFSLI
FSFLAIFHVLQWTFLNFPPFSVFLAIFHVLKCCVFLIFRDFQFSCHIPGPSVCISHFSRFSVILSFFNLSTAC
FSFSIIFSFLAIFHVLQWTFLNFPPFSSYSPYSRSYRMHFSFFTFFSDFIIFQVVKCMFLIFNDFHFSC
>ENSMUSP00000137419_c.165:A>T_p.T56S ENSMUST00000177875.8|Gm10717-206|ENSMUSG00000095891|+|chr9:g.3027692:A>T
MFLIFHDFQFSCHIPCPTVDISFPPFSVFLAIFHVLKCVFLIFRDFQFSCHIPGPSVCISHFSRFSVILSFFN
LSTACFSFSIIFSFLAIFHVLQWTFLNFPPFSVFLAIFHVLKCCVFLIFRDFQFSCHIPGPTVCISHFSRFSV

ILSFFNLSTACFSFSMIFSFLAIFHVLQWKFLNFPPFSVSPYSRSYSVHFSFFTFFSDFVIFQVVKWMFPIFL
DFQFSY
>ENSMUSP00000136365_c.165:A>T_p.T56S ENSMUST00000179982.2|Gm10717-209|ENSMUSG00000095891|+|chr9:g.3027692:A>T
MFLIFHDFQFSCHIPCPTVDISFPPFSVFLAIFHVLKCVFLIFRDFQFSCHIPGPSVCISHFSRFSVILSFFN
LSTACFSFSIIFSFLAIFHVLQWTFLNFPPFSVFLAIFHVLKCVFLIFRDFQFSCYIPSPTVCISHFSRFSVI
LSFFNLSTACFSFSMIFSFFAIFHVLQGKFLYFPPFSSSIFSPYSRSYSVHFSFFTFFSDFVIFQVVKWKFLI
FHDFQLSS
>ENSMUSP00000136755_c.409:T>C_p.L137S ENSMUST00000177601.2|Gm10717-205|ENSMUSG00000095891|+|chr9:g.3027732:T>C
CHIPCPTVEISFPPFSVFLAIFHVLKCVFLIFRVFQVSRHIPGPTVCISHFSRFSVILSFFKFSSGCFSFSLI
FSFLAIFHVLQWTFLNFPPFSVFLAIFHVLKCCVFLIFRDFQFSCHIPGPTVCISHFSRFSVISSFFNLSTAC
FSFSIIFSFLAIFHVLQWTFLNFPPFSSYSPYSRSYRMHFSFFTFFSDFIIFQVVKCMFLIFNDFHFSC
>ENSMUSP00000137419_c.205:T>C_p.L69S ENSMUST00000177875.8|Gm10717-206|ENSMUSG00000095891|+|chr9:g.3027732:T>C
MFLIFHDFQFSCHIPCPTVDISFPPFSVFLAIFHVLKCVFLIFRDFQFSCHIPGPTVCISHFSRFSVISSFFN
LSTACFSFSIIFSFLAIFHVLQWTFLNFPPFSVFLAIFHVLKCCVFLIFRDFQFSCHIPGPTVCISHFSRFSV
ILSFFNLSTACFSFSMIFSFLAIFHVLQWKFLNFPPFSVSPYSRSYSVHFSFFTFFSDFVIFQVVKWMFPIFL
DFQFSY
>ENSMUSP00000136365_c.205:T>C_p.L69S ENSMUST00000179982.2|Gm10717-209|ENSMUSG00000095891|+|chr9:g.3027732:T>C
MFLIFHDFQFSCHIPCPTVDISFPPFSVFLAIFHVLKCVFLIFRDFQFSCHIPGPTVCISHFSRFSVISSFFN
LSTACFSFSIIFSFLAIFHVLQWTFLNFPPFSVFLAIFHVLKCVFLIFRDFQFSCYIPSPTVCISHFSRFSVI
LSFFNLSTACFSFSMIFSFFAIFHVLQGKFLYFPPFSSSIFSPYSRSYSVHFSFFTFFSDFVIFQVVKWKFLI
FHDFQLSS
>ENSMUSP00000136755_c.422:T>G_p.N141K ENSMUST00000177601.2|Gm10717-205|ENSMUSG00000095891|+|chr9:g.3027745:T>G
CHIPCPTVEISFPPFSVFLAIFHVLKCVFLIFRVFQVSRHIPGPTVCISHFSRFSVILSFFKFSSGCFSFSLI
FSFLAIFHVLQWTFLNFPPFSVFLAIFHVLKCCVFLIFRDFQFSCHIPGPTVCISHFSRFSVILSFFKLSTAC
FSFSIIFSFLAIFHVLQWTFLNFPPFSSYSPYSRSYRMHFSFFTFFSDFIIFQVVKCMFLIFNDFHFSC
>ENSMUSP00000137419_c.218:T>G_p.N73K ENSMUST00000177875.8|Gm10717-206|ENSMUSG00000095891|+|chr9:g.3027745:T>G
MFLIFHDFQFSCHIPCPTVDISFPPFSVFLAIFHVLKCVFLIFRDFQFSCHIPGPTVCISHFSRFSVILSFFK
LSTACFSFSIIFSFLAIFHVLQWTFLNFPPFSVFLAIFHVLKCCVFLIFRDFQFSCHIPGPTVCISHFSRFSV
ILSFFNLSTACFSFSMIFSFLAIFHVLQWKFLNFPPFSVSPYSRSYSVHFSFFTFFSDFVIFQVVKWMFPIFL
DFQFSY
>ENSMUSP00000136365_c.218:T>G_p.N73K ENSMUST00000179982.2|Gm10717-209|ENSMUSG00000095891|+|chr9:g.3027745:T>G
MFLIFHDFQFSCHIPCPTVDISFPPFSVFLAIFHVLKCVFLIFRDFQFSCHIPGPTVCISHFSRFSVILSFFK
LSTACFSFSIIFSFLAIFHVLQWTFLNFPPFSVFLAIFHVLKCVFLIFRDFQFSCYIPSPTVCISHFSRFSVI
LSFFNLSTACFSFSMIFSFFAIFHVLQGKFLYFPPFSSSIFSPYSRSYSVHFSFFTFFSDFVIFQVVKWKFLI
FHDFQLSS
>ENSMUSP00000136755_c.430:C>G_p.T144S ENSMUST00000177601.2|Gm10717-205|ENSMUSG00000095891|+|chr9:g.3027753:C>G
CHIPCPTVEISFPPFSVFLAIFHVLKCVFLIFRVFQVSRHIPGPTVCISHFSRFSVILSFFKFSSGCFSFSLI
FSFLAIFHVLQWTFLNFPPFSVFLAIFHVLKCCVFLIFRDFQFSCHIPGPTVCISHFSRFSVILSFFNLSSAC
FSFSIIFSFLAIFHVLQWTFLNFPPFSSYSPYSRSYRMHFSFFTFFSDFIIFQVVKCMFLIFNDFHFSC
>ENSMUSP00000137419_c.226:C>G_p.T76S ENSMUST00000177875.8|Gm10717-206|ENSMUSG00000095891|+|chr9:g.3027753:C>G
MFLIFHDFQFSCHIPCPTVDISFPPFSVFLAIFHVLKCVFLIFRDFQFSCHIPGPTVCISHFSRFSVILSFFN
LSSACFSFSIIFSFLAIFHVLQWTFLNFPPFSVFLAIFHVLKCCVFLIFRDFQFSCHIPGPTVCISHFSRFSV
ILSFFNLSTACFSFSMIFSFLAIFHVLQWKFLNFPPFSVSPYSRSYSVHFSFFTFFSDFVIFQVVKWMFPIFL
DFQFSY
>ENSMUSP00000136365_c.226:C>G_p.T76S ENSMUST00000179982.2|Gm10717-209|ENSMUSG00000095891|+|chr9:g.3027753:C>G
MFLIFHDFQFSCHIPCPTVDISFPPFSVFLAIFHVLKCVFLIFRDFQFSCHIPGPTVCISHFSRFSVILSFFN
LSSACFSFSIIFSFLAIFHVLQWTFLNFPPFSVFLAIFHVLKCVFLIFRDFQFSCYIPSPTVCISHFSRFSVI

LSFFNLSTACFSFSMIFSFFAIFHVLQGKFLYFPPFSSSIFSPYSRSYSVHFSFFTFFSDFVIFQVVKWKFLI
FHDFQLSS
>ENSMUSP00000136755_c.430:CTGC>GTGG_p.T144S ENSMUST00000177601.2|Gm10717-205|ENSMUSG00000095891|+|chr9:g.3027753:CTGC>GTGG
CHIPCPTVEISFPPFSVFLAIFHVLKCVFLIFRVFQVSRHIPGPTVCISHFSRFSVILSFFKFSSGCFSFSLI
FSFLAIFHVLQWTFLNFPPFSVFLAIFHVLKCCVFLIFRDFQFSCHIPGPTVCISHFSRFSVILSFFNLSSGA
CFSFSIIFSFLAIFHVLQWTFLNFPPFSSYSPYSRSYRMHFSFFTFFSDFIIFQVVKCMFLIFNDFHFSC
>ENSMUSP00000137419_c.226:CTGC>GTGG_p.T76S ENSMUST00000177875.8|Gm10717-206|ENSMUSG00000095891|+|chr9:g.3027753:CTGC>GTGG
MFLIFHDFQFSCHIPCPTVDISFPPFSVFLAIFHVLKCVFLIFRDFQFSCHIPGPTVCISHFSRFSVILSFFN
LSSGACFSFSIIFSFLAIFHVLQWTFLNFPPFSVFLAIFHVLKCCVFLIFRDFQFSCHIPGPTVCISHFSRFS
VILSFFNLSTACFSFSMIFSFLAIFHVLQWKFLNFPPFSVSPYSRSYSVHFSFFTFFSDFVIFQVVKWMFPIF
LDFQFSY
>ENSMUSP00000136365_c.226:CTGC>GTGG_p.T76S ENSMUST00000179982.2|Gm10717-209|ENSMUSG00000095891|+|chr9:g.3027753:CTGC>GTGG
MFLIFHDFQFSCHIPCPTVDISFPPFSVFLAIFHVLKCVFLIFRDFQFSCHIPGPTVCISHFSRFSVILSFFN
LSSGACFSFSIIFSFLAIFHVLQWTFLNFPPFSVFLAIFHVLKCVFLIFRDFQFSCYIPSPTVCISHFSRFSV
ILSFFNLSTACFSFSMIFSFFAIFHVLQGKFLYFPPFSSSIFSPYSRSYSVHFSFFTFFSDFVIFQVVKWKFL
IFHDFQLSS
>ENSMUSP00000136755_c.430:CTGC>GTGT_p.T144S ENSMUST00000177601.2|Gm10717-205|ENSMUSG00000095891|+|chr9:g.3027753:CTGC>GTGT
CHIPCPTVEISFPPFSVFLAIFHVLKCVFLIFRVFQVSRHIPGPTVCISHFSRFSVILSFFKFSSGCFSFSLI
FSFLAIFHVLQWTFLNFPPFSVFLAIFHVLKCCVFLIFRDFQFSCHIPGPTVCISHFSRFSVILSFFNLSSVA
CFSFSIIFSFLAIFHVLQWTFLNFPPFSSYSPYSRSYRMHFSFFTFFSDFIIFQVVKCMFLIFNDFHFSC
>ENSMUSP00000137419_c.226:CTGC>GTGT_p.T76S ENSMUST00000177875.8|Gm10717-206|ENSMUSG00000095891|+|chr9:g.3027753:CTGC>GTGT
MFLIFHDFQFSCHIPCPTVDISFPPFSVFLAIFHVLKCVFLIFRDFQFSCHIPGPTVCISHFSRFSVILSFFN
LSSVACFSFSIIFSFLAIFHVLQWTFLNFPPFSVFLAIFHVLKCCVFLIFRDFQFSCHIPGPTVCISHFSRFS
VILSFFNLSTACFSFSMIFSFLAIFHVLQWKFLNFPPFSVSPYSRSYSVHFSFFTFFSDFVIFQVVKWMFPIF
LDFQFSY
>ENSMUSP00000136365_c.226:CTGC>GTGT_p.T76S ENSMUST00000179982.2|Gm10717-209|ENSMUSG00000095891|+|chr9:g.3027753:CTGC>GTGT
MFLIFHDFQFSCHIPCPTVDISFPPFSVFLAIFHVLKCVFLIFRDFQFSCHIPGPTVCISHFSRFSVILSFFN
LSSVACFSFSIIFSFLAIFHVLQWTFLNFPPFSVFLAIFHVLKCVFLIFRDFQFSCYIPSPTVCISHFSRFSV
ILSFFNLSTACFSFSMIFSFFAIFHVLQGKFLYFPPFSSSIFSPYSRSYSVHFSFFTFFSDFVIFQVVKWKFL
IFHDFQLSS
>ENSMUSP00000136755_c.452:T>G_p.I151M ENSMUST00000177601.2|Gm10717-205|ENSMUSG00000095891|+|chr9:g.3027775:T>G
CHIPCPTVEISFPPFSVFLAIFHVLKCVFLIFRVFQVSRHIPGPTVCISHFSRFSVILSFFKFSSGCFSFSLI
FSFLAIFHVLQWTFLNFPPFSVFLAIFHVLKCCVFLIFRDFQFSCHIPGPTVCISHFSRFSVILSFFNLSTAC
FSFSMIFSFLAIFHVLQWTFLNFPPFSSYSPYSRSYRMHFSFFTFFSDFIIFQVVKCMFLIFNDFHFSC
>ENSMUSP00000137419_c.248:T>G_p.I83M ENSMUST00000177875.8|Gm10717-206|ENSMUSG00000095891|+|chr9:g.3027775:T>G
MFLIFHDFQFSCHIPCPTVDISFPPFSVFLAIFHVLKCVFLIFRDFQFSCHIPGPTVCISHFSRFSVILSFFN
LSTACFSFSMIFSFLAIFHVLQWTFLNFPPFSVFLAIFHVLKCCVFLIFRDFQFSCHIPGPTVCISHFSRFSV
ILSFFNLSTACFSFSMIFSFLAIFHVLQWKFLNFPPFSVSPYSRSYSVHFSFFTFFSDFVIFQVVKWMFPIFL
DFQFSY
>ENSMUSP00000136365_c.248:T>G_p.I83M ENSMUST00000179982.2|Gm10717-209|ENSMUSG00000095891|+|chr9:g.3027775:T>G
MFLIFHDFQFSCHIPCPTVDISFPPFSVFLAIFHVLKCVFLIFRDFQFSCHIPGPTVCISHFSRFSVILSFFN
LSTACFSFSMIFSFLAIFHVLQWTFLNFPPFSVFLAIFHVLKCVFLIFRDFQFSCYIPSPTVCISHFSRFSVI
LSFFNLSTACFSFSMIFSFFAIFHVLQGKFLYFPPFSSSIFSPYSRSYSVHFSFFTFFSDFVIFQVVKWKFLI
FHDFQLSS
>ENSMUSP00000136755_c.511:C>T_p.P171L ENSMUST00000177601.2|Gm10717-205|ENSMUSG00000095891|+|chr9:g.3027834:C>T
CHIPCPTVEISFPPFSVFLAIFHVLKCVFLIFRVFQVSRHIPGPTVCISHFSRFSVILSFFKFSSGCFSFSLI
FSFLAIFHVLQWTFLNFPPFSVFLAIFHVLKCCVFLIFRDFQFSCHIPGPTVCISHFSRFSVILSFFNLSTAC
FSFSIIFSFLAIFHVLQWTFLNFPLFSSYSPYSRSYRMHFSFFTFFSDFIIFQVVKCMFLIFNDFHFSC

>ENSMUSP00000137419_c.307:C>T_p.P103L ENSMUST00000177875.8|Gm10717-206|ENSMUSG00000095891|+|chr9:g.3027834:C>T
MFLIFHDFQFSCHIPCPTVDISFPPFSVFLAIFHVLKCVFLIFRDFQFSCHIPGPTVCISHFSRFSVILSFFN
LSTACFSFSIIFSFLAIFHVLQWTFLNFPLFSVFLAIFHVLKCCVFLIFRDFQFSCHIPGPTVCISHFSRFSV
ILSFFNLSTACFSFSMIFSFLAIFHVLQWKFLNFPPFSVSPYSRSYSVHFSFFTFFSDFVIFQVVKWMFPIFL
DFQFSY
>ENSMUSP00000136365_c.307:C>T_p.P103L ENSMUST00000179982.2|Gm10717-209|ENSMUSG00000095891|+|chr9:g.3027834:C>T
MFLIFHDFQFSCHIPCPTVDISFPPFSVFLAIFHVLKCVFLIFRDFQFSCHIPGPTVCISHFSRFSVILSFFN
LSTACFSFSIIFSFLAIFHVLQWTFLNFPLFSVFLAIFHVLKCVFLIFRDFQFSCYIPSPTVCISHFSRFSVI
LSFFNLSTACFSFSMIFSFFAIFHVLQGKFLYFPPFSSSIFSPYSRSYSVHFSFFTFFSDFVIFQVVKWKFLI
FHDFQLSS
>ENSMUSP00000136365_c.348:G>A_p.V117I ENSMUST00000179982.2|Gm10717-209|ENSMUSG00000095891|+|chr9:g.3027875:G>A
MFLIFHDFQFSCHIPCPTVDISFPPFSVFLAIFHVLKCVFLIFRDFQFSCHIPGPTVCISHFSRFSVILSFFN
LSTACFSFSIIFSFLAIFHVLQWTFLNFPPFSVFLAIFHVLKCIFLIFRDFQFSCYIPSPTVCISHFSRFSVI
LSFFNLSTACFSFSMIFSFFAIFHVLQGKFLYFPPFSSSIFSPYSRSYSVHFSFFTFFSDFVIFQVVKWKFLI
FHDFQLSS
>ENSMUSP00000136365_c.364:G>T_p.R122L ENSMUST00000179982.2|Gm10717-209|ENSMUSG00000095891|+|chr9:g.3027891:G>T
MFLIFHDFQFSCHIPCPTVDISFPPFSVFLAIFHVLKCVFLIFRDFQFSCHIPGPTVCISHFSRFSVILSFFN
LSTACFSFSIIFSFLAIFHVLQWTFLNFPPFSVFLAIFHVLKCVFLIFLDFQFSCYIPSPTVCISHFSRFSVI
LSFFNLSTACFSFSMIFSFFAIFHVLQGKFLYFPPFSSSIFSPYSRSYSVHFSFFTFFSDFVIFQVVKWKFLI
FHDFQLSS
>ENSMUSP00000136365_c.384:T>C_p.Y129H ENSMUST00000179982.2|Gm10717-209|ENSMUSG00000095891|+|chr9:g.3027911:T>C
MFLIFHDFQFSCHIPCPTVDISFPPFSVFLAIFHVLKCVFLIFRDFQFSCHIPGPTVCISHFSRFSVILSFFN
LSTACFSFSIIFSFLAIFHVLQWTFLNFPPFSVFLAIFHVLKCVFLIFRDFQFSCHIPSPTVCISHFSRFSVI
LSFFNLSTACFSFSMIFSFFAIFHVLQGKFLYFPPFSSSIFSPYSRSYSVHFSFFTFFSDFVIFQVVKWKFLI
FHDFQLSS
>rs237508806_ENSMUSP00000136365_c.393:A>G_p.S132G
ENSMUST00000179982.2|Gm10717-209|ENSMUSG00000095891|+|chr9:g.3027920:A>G
MFLIFHDFQFSCHIPCPTVDISFPPFSVFLAIFHVLKCVFLIFRDFQFSCHIPGPTVCISHFSRFSVILSFFN
LSTACFSFSIIFSFLAIFHVLQWTFLNFPPFSVFLAIFHVLKCVFLIFRDFQFSCYIPGPTVCISHFSRFSVI
LSFFNLSTACFSFSMIFSFFAIFHVLQGKFLYFPPFSSSIFSPYSRSYSVHFSFFTFFSDFVIFQVVKWKFLI
FHDFQLSS
>ENSMUSP00000136365_c.393:A>T_p.S132C ENSMUST00000179982.2|Gm10717-209|ENSMUSG00000095891|+|chr9:g.3027920:A>T
MFLIFHDFQFSCHIPCPTVDISFPPFSVFLAIFHVLKCVFLIFRDFQFSCHIPGPTVCISHFSRFSVILSFFN
LSTACFSFSIIFSFLAIFHVLQWTFLNFPPFSVFLAIFHVLKCVFLIFRDFQFSCYIPCPTVCISHFSRFSVI
LSFFNLSTACFSFSMIFSFFAIFHVLQGKFLYFPPFSSSIFSPYSRSYSVHFSFFTFFSDFVIFQVVKWKFLI
FHDFQLSS
>ENSMUSP00000136755_c.525:A>C_p.S176R ENSMUST00000177601.2|Gm10717-205|ENSMUSG00000095891|+|chr9:g.3027928:A>C
CHIPCPTVEISFPPFSVFLAIFHVLKCVFLIFRVFQVSRHIPGPTVCISHFSRFSVILSFFKFSSGCFSFSLI
FSFLAIFHVLQWTFLNFPPFSVFLAIFHVLKCCVFLIFRDFQFSCHIPGPTVCISHFSRFSVILSFFNLSTAC
FSFSIIFSFLAIFHVLQWTFLNFPPFSSYRPYSRSYRMHFSFFTFFSDFIIFQVVKCMFLIFNDFHFSC
>ENSMUSP00000136755_c.525:AG>CA_p.S176Q ENSMUST00000177601.2|Gm10717-205|ENSMUSG00000095891|+|chr9:g.3027928:AG>CA
CHIPCPTVEISFPPFSVFLAIFHVLKCVFLIFRVFQVSRHIPGPTVCISHFSRFSVILSFFKFSSGCFSFSLI
FSFLAIFHVLQWTFLNFPPFSVFLAIFHVLKCCVFLIFRDFQFSCHIPGPTVCISHFSRFSVILSFFNLSTAC
FSFSIIFSFLAIFHVLQWTFLNFPPFSSYQSIFQVLQDAFLIFHVFQ*FHHFSSRQVVYVSHFQ*FSFFL
>ENSMUSP00000136365_c.420:T>C_p.S141P ENSMUST00000179982.2|Gm10717-209|ENSMUSG00000095891|+|chr9:g.3027947:T>C
MFLIFHDFQFSCHIPCPTVDISFPPFSVFLAIFHVLKCVFLIFRDFQFSCHIPGPTVCISHFSRFSVILSFFN
LSTACFSFSIIFSFLAIFHVLQWTFLNFPPFSVFLAIFHVLKCVFLIFRDFQFSCYIPSPTVCISHFPRFSVI
LSFFNLSTACFSFSMIFSFFAIFHVLQGKFLYFPPFSSSIFSPYSRSYSVHFSFFTFFSDFVIFQVVKWKFLI
FHDFQLSS

>ENSMUSP00000136365_c.490:GT>G_p.S164del-fs ENSMUST00000179982.2|Gm10717-209|ENSMUSG00000095891|+|chr9:g.3028017:GT>G
MFLIFHDFQFSCHIPCPTVDISFPPFSVFLAIFHVLKCVFLIFRDFQFSCHIPGPTVCISHFSRFSVILSFFN
LSTACFSFSIIFSFLAIFHVLQWTFLNFPPFSVFLAIFHVLKCVFLIFRDFQFSCYIPSPTVCISHFSRFSVI
LSFFNLSTACFSFSMIFSFLPYSMSYRGNFYIFHLFHRQFSHHIPGPTVCISHFSRFSVISSFFKLSSGSFSF
SMIFSFL
>ENSMUSP00000136365_c.495:T>C_p.F166L ENSMUST00000179982.2|Gm10717-209|ENSMUSG00000095891|+|chr9:g.3028022:T>C
MFLIFHDFQFSCHIPCPTVDISFPPFSVFLAIFHVLKCVFLIFRDFQFSCHIPGPTVCISHFSRFSVILSFFN
LSTACFSFSIIFSFLAIFHVLQWTFLNFPPFSVFLAIFHVLKCVFLIFRDFQFSCYIPSPTVCISHFSRFSVI
LSFFNLSTACFSFSMIFSFLAIFHVLQGKFLYFPPFSSSIFSPYSRSYSVHFSFFTFFSDFVIFQVVKWKFLI
FHDFQLSS
>ENSMUSP00000136365_c.519:G>T_p.G174W ENSMUST00000179982.2|Gm10717-209|ENSMUSG00000095891|+|chr9:g.3028046:G>T
MFLIFHDFQFSCHIPCPTVDISFPPFSVFLAIFHVLKCVFLIFRDFQFSCHIPGPTVCISHFSRFSVILSFFN
LSTACFSFSIIFSFLAIFHVLQWTFLNFPPFSVFLAIFHVLKCVFLIFRDFQFSCYIPSPTVCISHFSRFSVI
LSFFNLSTACFSFSMIFSFFAIFHVLQWKFLYFPPFSSSIFSPYSRSYSVHFSFFTFFSDFVIFQVVKWKFLI
FHDFQLSS
>rs231884288_ENSMUSP00000136365_c.523:A>C_p.K175T
ENSMUST00000179982.2|Gm10717-209|ENSMUSG00000095891|+|chr9:g.3028050:A>C
MFLIFHDFQFSCHIPCPTVDISFPPFSVFLAIFHVLKCVFLIFRDFQFSCHIPGPTVCISHFSRFSVILSFFN
LSTACFSFSIIFSFLAIFHVLQWTFLNFPPFSVFLAIFHVLKCVFLIFRDFQFSCYIPSPTVCISHFSRFSVI
LSFFNLSTACFSFSMIFSFFAIFHVLQGTFLYFPPFSSSIFSPYSRSYSVHFSFFTFFSDFVIFQVVKWKFLI
FHDFQLSS
>ENSMUSP00000136365_c.531:T>A_p.Y178N ENSMUST00000179982.2|Gm10717-209|ENSMUSG00000095891|+|chr9:g.3028058:T>A
MFLIFHDFQFSCHIPCPTVDISFPPFSVFLAIFHVLKCVFLIFRDFQFSCHIPGPTVCISHFSRFSVILSFFN
LSTACFSFSIIFSFLAIFHVLQWTFLNFPPFSVFLAIFHVLKCVFLIFRDFQFSCYIPSPTVCISHFSRFSVI
LSFFNLSTACFSFSMIFSFFAIFHVLQGKFLNFPPFSSSIFSPYSRSYSVHFSFFTFFSDFVIFQVVKWKFLI
FHDFQLSS
>ENSMUSP00000136755_c.532:A>C_p.Y178S ENSMUST00000177601.2|Gm10717-205|ENSMUSG00000095891|+|chr9:g.3028382:A>C
CHIPCPTVEISFPPFSVFLAIFHVLKCVFLIFRVFQVSRHIPGPTVCISHFSRFSVILSFFKFSSGCFSFSLI
FSFLAIFHVLQWTFLNFPPFSVFLAIFHVLKCCVFLIFRDFQFSCHIPGPTVCISHFSRFSVILSFFNLSTAC
FSFSIIFSFLAIFHVLQWTFLNFPPFSSYSPSSRSYRMHFSFFTFFSDFIIFQVVKCMFLIFNDFHFSC
>ENSMUSP00000136755_c.548:G>T_p.R183S ENSMUST00000177601.2|Gm10717-205|ENSMUSG00000095891|+|chr9:g.3028398:G>T
CHIPCPTVEISFPPFSVFLAIFHVLKCVFLIFRVFQVSRHIPGPTVCISHFSRFSVILSFFKFSSGCFSFSLI
FSFLAIFHVLQWTFLNFPPFSVFLAIFHVLKCCVFLIFRDFQFSCHIPGPTVCISHFSRFSVILSFFNLSTAC
FSFSIIFSFLAIFHVLQWTFLNFPPFSSYSPYSRSYSMHFSFFTFFSDFIIFQVVKCMFLIFNDFHFSC
>ENSMUSP00000136755_c.548:GA>TG_p.R183S ENSMUST00000177601.2|Gm10717-205|ENSMUSG00000095891|+|chr9:g.3028398:GA>TG
CHIPCPTVEISFPPFSVFLAIFHVLKCVFLIFRVFQVSRHIPGPTVCISHFSRFSVILSFFKFSSGCFSFSLI
FSFLAIFHVLQWTFLNFPPFSVFLAIFHVLKCCVFLIFRDFQFSCHIPGPTVCISHFSRFSVILSFFNLSTAC
FSFSIIFSFLAIFHVLQWTFLNFPPFSSYSPYSRSYSDAFLIFHVFQ*FHHFSSRQVYVSHFQ*FSFFL
>ENSMUSP00000136755_c.585:A>G_p.I196V ENSMUST00000177601.2|Gm10717-205|ENSMUSG00000095891|+|chr9:g.3028435:A>G
CHIPCPTVEISFPPFSVFLAIFHVLKCVFLIFRVFQVSRHIPGPTVCISHFSRFSVILSFFKFSSGCFSFSLI
FSFLAIFHVLQWTFLNFPPFSVFLAIFHVLKCCVFLIFRDFQFSCHIPGPTVCISHFSRFSVILSFFNLSTAC
FSFSIIFSFLAIFHVLQWTFLNFPPFSSYSPYSRSYRMHFSFFTFFSDFVIFQVVKCMFLIFNDFHFSC
>ENSMUSP00000136755_c.607:G>T_p.C203F ENSMUST00000177601.2|Gm10717-205|ENSMUSG00000095891|+|chr9:g.3028457:G>T
CHIPCPTVEISFPPFSVFLAIFHVLKCVFLIFRVFQVSRHIPGPTVCISHFSRFSVILSFFKFSSGCFSFSLI
FSFLAIFHVLQWTFLNFPPFSVFLAIFHVLKCCVFLIFRDFQFSCHIPGPTVCISHFSRFSVILSFFNLSTAC
FSFSIIFSFLAIFHVLQWTFLNFPPFSSYSPYSRSYRMHFSFFTFFSDFIIFQVVKFMFLIFNDFHFSC
>rs221236237_ENSMUSP00000136755_c.624:A>C_p.N209H
ENSMUST00000177601.2|Gm10717-205|ENSMUSG00000095891|+|chr9:g.3028474:A>C

CHIPCPTVEISFPPFSVFLAIFHVLKCVFLIFRVFQVSRHIPGPTVCISHFSRFSVILSFFKFSSGCFSFSLI
FSFLAIFHVLQWTFLNFPPPFSVFLAIFHVLKCCVFLIFRDFQFSCHIPGPTVCISHFSRFSVILSFFNLSTAC
FSFSIIFSFLAIFHVLQWTFLNFPPFSSYSPYSRSYRMHFSFFTFFSDFIIFQVVKCMFLIFHDFHFSC
>ENSMUSP00000136755_c.624:AATG>CATA_p.N209H ENSMUST00000177601.2|Gm10717-
205|ENSMUSG00000095891|+|chr9:g.3028474:AATG>CATA
CHIPCPTVEISFPPFSVFLAIFHVLKCVFLIFRVFQVSRHIPGPTVCISHFSRFSVILSFFKFSSGCFSFSLI
FSFLAIFHVLQWTFLNFPPPFSVFLAIFHVLKCCVFLIFRDFQFSCHIPGPTVCISHFSRFSVILSFFNLSTAC
FSFSIIFSFLAIFHVLQWTFLNFPPFSSYSPYSRSYRMHFSFFTFFSDFIIFQVVKCMFLIFHNDFHFSC
>ENSMUSP00000136755_c.632:T>A_p.F211L ENSMUST00000177601.2|Gm10717-
205|ENSMUSG00000095891|+|chr9:g.3028482:T>A
CHIPCPTVEISFPPFSVFLAIFHVLKCVFLIFRVFQVSRHIPGPTVCISHFSRFSVILSFFKFSSGCFSFSLI
FSFLAIFHVLQWTFLNFPPPFSVFLAIFHVLKCCVFLIFRDFQFSCHIPGPTVCISHFSRFSVILSFFNLSTAC
FSFSIIFSFLAIFHVLQWTFLNFPPFSSYSPYSRSYRMHFSFFTFFSDFIIFQVVKCMFLIFNDLHFSC
>ENSMUSP00000136755_c.635:T>G_p.H212Q ENSMUST00000177601.2|Gm10717-
205|ENSMUSG00000095891|+|chr9:g.3028485:T>G
CHIPCPTVEISFPPFSVFLAIFHVLKCVFLIFRVFQVSRHIPGPTVCISHFSRFSVILSFFKFSSGCFSFSLI
FSFLAIFHVLQWTFLNFPPPFSVFLAIFHVLKCCVFLIFRDFQFSCHIPGPTVCISHFSRFSVILSFFNLSTAC
FSFSIIFSFLAIFHVLQWTFLNFPPFSSYSPYSRSYRMHFSFFTFFSDFIIFQVVKCMFLIFNDFQFSC
>ENSMUSP00000136755_c.639:TC>T_p.S214del-fs ENSMUST00000177601.2|Gm10717-
205|ENSMUSG00000095891|+|chr9:g.3028489:TC>T
CHIPCPTVEISFPPFSVFLAIFHVLKCVFLIFRVFQVSRHIPGPTVCISHFSRFSVILSFFKFSSGCFSFSLI
FSFLAIFHVLQWTFLNFPPPFSVFLAIFHVLKCCVFLIFRDFQFSCHIPGPTVCISHFSRFSVILSFFNLSTAC
FSFSIIFSFLAIFHVLQWTFLNFPPFSSYSPYSRSYRMHFSFFTFFSDFIIFQVVKCMFLIFNDFHFF
>ENSMUSP00000136365_c.646:A>T_p.K216M ENSMUST00000179982.2|Gm10717-
209|ENSMUSG00000095891|+|chr9:g.3028694:A>T
MFLIFHDFQFSCHIPCPTVDISFPPFSVFLAIFHVLKCVFLIFRDFQFSCHIPGPTVCISHFSRFSVILSFFN
LSTACFSFSIIFSFLAIFHVLQWTFLNFPPPFSVFLAIFHVLKCVFLIFRDFQFSCYIPSPTVCISHFSRFSVI
LSFFNLSTACFSFSMIFSFFAIFHVLQGKFLYFPPFSSSIFSPYSRSYSVHFSFFTFFSDFVIFQVVKWMFLI
FHDFQLSS
>ENSMUSP00000136365_c.654:A>T_p.I219F ENSMUST00000179982.2|Gm10717-
209|ENSMUSG00000095891|+|chr9:g.3028702:A>T
MFLIFHDFQFSCHIPCPTVDISFPPFSVFLAIFHVLKCVFLIFRDFQFSCHIPGPTVCISHFSRFSVILSFFN
LSTACFSFSIIFSFLAIFHVLQWTFLNFPPPFSVFLAIFHVLKCVFLIFRDFQFSCYIPSPTVCISHFSRFSVI
LSFFNLSTACFSFSMIFSFFAIFHVLQGKFLYFPPFSSSIFSPYSRSYSVHFSFFTFFSDFVIFQVVKWKFLF
FHDFQLSS
>ENSMUSP00000136365_c.678:A>T_p.S227C ENSMUST00000179982.2|Gm10717-
209|ENSMUSG00000095891|+|chr9:g.3028726:A>T
MFLIFHDFQFSCHIPCPTVDISFPPFSVFLAIFHVLKCVFLIFRDFQFSCHIPGPTVCISHFSRFSVILSFFN
LSTACFSFSIIFSFLAIFHVLQWTFLNFPPPFSVFLAIFHVLKCVFLIFRDFQFSCYIPSPTVCISHFSRFSVI
LSFFNLSTACFSFSMIFSFFAIFHVLQGKFLYFPPFSSSIFSPYSRSYSVHFSFFTFFSDFVIFQVVKWKFLI
FHDFQLSC
>ENSMUSP00000137419_c.366:C>T_p.R123C ENSMUST00000177875.8|Gm10717-
206|ENSMUSG00000095891|+|chr9:g.3028826:C>T
MFLIFHDFQFSCHIPCPTVDISFPPFSVFLAIFHVLKCVFLIFRDFQFSCHIPGPTVCISHFSRFSVILSFFN
LSTACFSFSIIFSFLAIFHVLQWTFLNFPPFSVFLAIFHVLKCCVFLIFCDFQFSCHIPGPTVCISHFSRFSV
ILSFFNLSTACFSFSMIFSFLAIFHVLQWKFLNFPPFSVSPYSRSYSVHFSFFTFFSDFVIFQVVKWMFPIFL
DFQFSY
>ENSMUSP00000137419_c.367:G>A_p.R123H ENSMUST00000177875.8|Gm10717-
206|ENSMUSG00000095891|+|chr9:g.3028827:G>A
MFLIFHDFQFSCHIPCPTVDISFPPFSVFLAIFHVLKCVFLIFRDFQFSCHIPGPTVCISHFSRFSVILSFFN
LSTACFSFSIIFSFLAIFHVLQWTFLNFPPFSVFLAIFHVLKCCVFLIFHDFQFSCHIPGPTVCISHFSRFSV
ILSFFNLSTACFSFSMIFSFLAIFHVLQWKFLNFPPFSVSPYSRSYSVHFSFFTFFSDFVIFQVVKWMFPIFL
DFQFSY
>ENSMUSP00000137419_c.384:T>C_p.C129R ENSMUST00000177875.8|Gm10717-
206|ENSMUSG00000095891|+|chr9:g.3028844:T>C
MFLIFHDFQFSCHIPCPTVDISFPPFSVFLAIFHVLKCVFLIFRDFQFSCHIPGPTVCISHFSRFSVILSFFN
LSTACFSFSIIFSFLAIFHVLQWTFLNFPPPFSVFLAIFHVLKCCVFLIFRDFQFSRHIPGPTVCISHFSRFSV

ILSFFNLSTACFSFSMIFSFLAIFHVLQWKFLNFPPFSVSPYSRSYSVHFSFFTFFSDFVIFQVVKWMFPIFL
DFQFSY
>ENSMUSP00000137419_c.396:G>C_p.G133R ENSMUST00000177875.8|Gm10717-206|ENSMUSG00000095891|+|chr9:g.3028856:G>C
MFLIFHDFQFSCHIPCPTVDISFPPFSVFLAIFHVLKCVFLIFRDFQFSCHIPGPTVCISHFSRFSVILSFFN
LSTACFSFSIIFSFLAIFHVLQWTFLNFPPFSVFLAIFHVLKCCVFLIFRDFQFSCHIPRPTVCISHFSRFSV
ILSFFNLSTACFSFSMIFSFLAIFHVLQWKFLNFPPFSVSPYSRSYSVHFSFFTFFSDFVIFQVVKWMFPIFL
DFQFSY
>ENSMUSP00000137419_c.402:A>T_p.T135S ENSMUST00000177875.8|Gm10717-206|ENSMUSG00000095891|+|chr9:g.3028862:A>T
MFLIFHDFQFSCHIPCPTVDISFPPFSVFLAIFHVLKCVFLIFRDFQFSCHIPGPTVCISHFSRFSVILSFFN
LSTACFSFSIIFSFLAIFHVLQWTFLNFPPFSVFLAIFHVLKCCVFLIFRDFQFSCHIPGPSVCISHFSRFSV
ILSFFNLSTACFSFSMIFSFLAIFHVLQWKFLNFPPFSVSPYSRSYSVHFSFFTFFSDFVIFQVVKWMFPIFL
DFQFSY
>ENSMUSP00000137419_c.423:T>C_p.S142P ENSMUST00000177875.8|Gm10717-206|ENSMUSG00000095891|+|chr9:g.3028883:T>C
MFLIFHDFQFSCHIPCPTVDISFPPFSVFLAIFHVLKCVFLIFRDFQFSCHIPGPTVCISHFSRFSVILSFFN
LSTACFSFSIIFSFLAIFHVLQWTFLNFPPFSVFLAIFHVLKCCVFLIFRDFQFSCHIPGPTVCISHFPRFSV
ILSFFNLSTACFSFSMIFSFLAIFHVLQWKFLNFPPFSVSPYSRSYSVHFSFFTFFSDFVIFQVVKWMFPIFL
DFQFSY
>ENSMUSP00000137419_c.426:C>T_p.R143C ENSMUST00000177875.8|Gm10717-206|ENSMUSG00000095891|+|chr9:g.3028886:C>T
MFLIFHDFQFSCHIPCPTVDISFPPFSVFLAIFHVLKCVFLIFRDFQFSCHIPGPTVCISHFSRFSVILSFFN
LSTACFSFSIIFSFLAIFHVLQWTFLNFPPFSVFLAIFHVLKCCVFLIFRDFQFSCHIPGPTVCISHFSCFSV
ILSFFNLSTACFSFSMIFSFLAIFHVLQWKFLNFPPFSVSPYSRSYSVHFSFFTFFSDFVIFQVVKWMFPIFL
DFQFSY
>ENSMUSP00000137419_c.436:T>G_p.V146G ENSMUST00000177875.8|Gm10717-206|ENSMUSG00000095891|+|chr9:g.3028896:T>G
MFLIFHDFQFSCHIPCPTVDISFPPFSVFLAIFHVLKCVFLIFRDFQFSCHIPGPTVCISHFSRFSVILSFFN
LSTACFSFSIIFSFLAIFHVLQWTFLNFPPFSVFLAIFHVLKCCVFLIFRDFQFSCHIPGPTVCISHFSRFSG
ILSFFNLSTACFSFSMIFSFLAIFHVLQWKFLNFPPFSVSPYSRSYSVHFSFFTFFSDFVIFQVVKWMFPIFL
DFQFSY
>ENSMUSP00000137419_c.442:T>C_p.L148S ENSMUST00000177875.8|Gm10717-206|ENSMUSG00000095891|+|chr9:g.3028902:T>C
MFLIFHDFQFSCHIPCPTVDISFPPFSVFLAIFHVLKCVFLIFRDFQFSCHIPGPTVCISHFSRFSVILSFFN
LSTACFSFSIIFSFLAIFHVLQWTFLNFPPFSVFLAIFHVLKCCVFLIFRDFQFSCHIPGPTVCISHFSRFSV
ISSFFNLSTACFSFSMIFSFLAIFHVLQWKFLNFPPFSVSPYSRSYSVHFSFFTFFSDFVIFQVVKWMFPIFL
DFQFSY
>ENSMUSP00000137419_c.455:TTT>GTC_p.N152K ENSMUST00000177875.8|Gm10717-206|ENSMUSG00000095891|+|chr9:g.3028915:TTT>GTC
MFLIFHDFQFSCHIPCPTVDISFPPFSVFLAIFHVLKCVFLIFRDFQFSCHIPGPTVCISHFSRFSVILSFFN
LSTACFSFSIIFSFLAIFHVLQWTFLNFPPFSVFLAIFHVLKCCVFLIFRDFQFSCHIPGPTVCISHFSRFSV
ILSFFKSCQLHVSHFP*FLVFLLYSMSYSGNF*IFHLFQFRHIPGPTVCISHFSRFSVILSFFKLSSGCFPFS
LIFSFL
>ENSMUSP00000137419_c.455:TTT>GTG_p.N152K ENSMUST00000177875.8|Gm10717-206|ENSMUSG00000095891|+|chr9:g.3028915:TTT>GTG
MFLIFHDFQFSCHIPCPTVDISFPPFSVFLAIFHVLKCVFLIFRDFQFSCHIPGPTVCISHFSRFSVILSFFN
LSTACFSFSIIFSFLAIFHVLQWTFLNFPPFSVFLAIFHVLKCCVFLIFRDFQFSCHIPGPTVCISHFSRFSV
ILSFFKCCQLHVSHFP*FLVFLLYSMSYSGNF*IFHLFQFRHIPGPTVCISHFSRFSVILSFFKLSSGCFPFS
LIFSFL
>ENSMUSP00000137419_c.463:CTGC>GTGA_p.T155S ENSMUST00000177875.8|Gm10717-206|ENSMUSG00000095891|+|chr9:g.3028923:CTGC>GTGA
MFLIFHDFQFSCHIPCPTVDISFPPFSVFLAIFHVLKCVFLIFRDFQFSCHIPGPTVCISHFSRFSVILSFFN
LSTACFSFSIIFSFLAIFHVLQWTFLNFPPFSVFLAIFHVLKCCVFLIFRDFQFSCHIPGPTVCISHFSRFSV
ILSFFNLSSDACFSFSMIFSFLAIFHVLQWKFLNFPPFSVSPYSRSYSVHFSFFTFFSDFVIFQVVKWMFPIF
LDFQFSY
>ENSMUSP00000137419_c.463:CTGC>GTGG_p.T155S ENSMUST00000177875.8|Gm10717-206|ENSMUSG00000095891|+|chr9:g.3028923:CTGC>GTGG

MFLIFHDFQFSCHIPCPTVDISFPPFSVFLAIFHVLKCVFLIFRDFQFSCHIPGPTVCISHFSRFSVILSFFN
LSTACFSFSIIFSFLAIFHVLQWTFLNFPPFSVFLAIFHVLKCCVFLIFRDFQFSCHIPGPTVCISHFSRFSV
ILSFFNLSSGACFSFSMIFSFLAIFHVLQWKFLNFPPFSVSPYSRSYSVHFSFFTFFSDFVIFQVVKWMFPIF
LDFQFSY
>ENSMUSP00000137419_c.463:CTGC>GTTT_p.T155S ENSMUST00000177875.8|Gm10717-206|ENSMUSG00000095891|+|chr9:g.3028923:CTGC>GTTT
MFLIFHDFQFSCHIPCPTVDISFPPFSVFLAIFHVLKCVFLIFRDFQFSCHIPGPTVCISHFSRFSVILSFFN
LSTACFSFSIIFSFLAIFHVLQWTFLNFPPFSVFLAIFHVLKCCVFLIFRDFQFSCHIPGPTVCISHFSRFSV
ILSFFNLSSFACFSFSMIFSFLAIFHVLQWKFLNFPPFSVSPYSRSYSVHFSFFTFFSDFVIFQVVKWMFPIF
LDFQFSY
>ENSMUSP00000137419_c.475:C>A_p.S159* ENSMUST00000177875.8|Gm10717-206|ENSMUSG00000095891|+|chr9:g.3028935:C>A
MFLIFHDFQFSCHIPCPTVDISFPPFSVFLAIFHVLKCVFLIFRDFQFSCHIPGPTVCISHFSRFSVILSFFN
LSTACFSFSIIFSFLAIFHVLQWTFLNFPPFSVFLAIFHVLKCCVFLIFRDFQFSCHIPGPTVCISHFSRFSV
ILSFFNLSTACF*FSMIFSFLAIFHVLQWKFLNFPPFSVSPYSRSYSVHFSFFTFFSDFVIFQVVKWMFPIFL
DFQFSY
>ENSMUSP00000137419_c.483:A>G_p.M162V ENSMUST00000177875.8|Gm10717-206|ENSMUSG00000095891|+|chr9:g.3028943:A>G
MFLIFHDFQFSCHIPCPTVDISFPPFSVFLAIFHVLKCVFLIFRDFQFSCHIPGPTVCISHFSRFSVILSFFN
LSTACFSFSIIFSFLAIFHVLQWTFLNFPPFSVFLAIFHVLKCCVFLIFRDFQFSCHIPGPTVCISHFSRFSV
ILSFFNLSTACFSFSVIFSFLAIFHVLQWKFLNFPPFSVSPYSRSYSVHFSFFTFFSDFVIFQVVKWMFPIFL
DFQFSY
>ENSMUSP00000137419_c.526:A>C_p.K176T ENSMUST00000177875.8|Gm10717-206|ENSMUSG00000095891|+|chr9:g.3028986:A>C
MFLIFHDFQFSCHIPCPTVDISFPPFSVFLAIFHVLKCVFLIFRDFQFSCHIPGPTVCISHFSRFSVILSFFN
LSTACFSFSIIFSFLAIFHVLQWTFLNFPPFSVFLAIFHVLKCCVFLIFRDFQFSCHIPGPTVCISHFSRFSV
ILSFFNLSTACFSFSMIFSFLAIFHVLQWTFLNFPPFSVSPYSRSYSVHFSFFTFFSDFVIFQVVKWMFPIFL
DFQFSY
>ENSMUSP00000137419_c.544:C>T_p.P182L ENSMUST00000177875.8|Gm10717-206|ENSMUSG00000095891|+|chr9:g.3029004:C>T
MFLIFHDFQFSCHIPCPTVDISFPPFSVFLAIFHVLKCVFLIFRDFQFSCHIPGPTVCISHFSRFSVILSFFN
LSTACFSFSIIFSFLAIFHVLQWTFLNFPPFSVFLAIFHVLKCCVFLIFRDFQFSCHIPGPTVCISHFSRFSV
ILSFFNLSTACFSFSMIFSFLAIFHVLQWKFLNFPLFSVSPYSRSYSVHFSFFTFFSDFVIFQVVKWMFPIFL
DFQFSY
>rs245098628_ENSMUSP00000096650_c.100:T>A_p.V34D
ENSMUST00000099051.11|Gm10717-203|ENSMUSG00000095891|+|chr9:g.3029299:T>A
CHIPCPTVEISFPPFSVFLAIFHVLKCVFLIFRDFQVSRHIPGPTVCISHFSRFSVILSFFKLSSGCFPFSLI
FSFLTIFHVLQWTFLNFPPFSVFLAIFHVLKCVFLIFSDFQFPRHIPGPTVCISHFSRFSVISSFFKTSSGCF
SFFMIFSFLAIFHVLQWTFLNFPPFSVSPYSRSYSVHFSFFTFFSDFVIFQVVKWMFLIFLDFHFSC
>ENSMUSP00000096650_c.100:TTT>ATA_p.V34D ENSMUST00000099051.11|Gm10717-203|ENSMUSG00000095891|+|chr9:g.3029299:TTT>ATA
CHIPCPTVEISFPPFSVFLAIFHVLKCVFLIFRDIFRFLAIFQVLQCAFLIFHVFQ*FCHFSSCQVDVSHFP*
FSVFLPYSMSYSGHF*IFHLFQFSLPYFMS*SVYFSFSVIFSFLAIFQVLQCAFLIFHVFQ*FRLFSRRQVDV
SHFS*FSVFLPYSMSYSGHF*IFHLFLFRHIPGPTVCISHFSRFSVILSFFKLSSGCFSFSLIFTFL
>ENSMUSP00000137419_c.552:G>T_p.V185F ENSMUST00000177875.8|Gm10717-206|ENSMUSG00000095891|+|chr9:g.3029307:G>T
MFLIFHDFQFSCHIPCPTVDISFPPFSVFLAIFHVLKCVFLIFRDFQFSCHIPGPTVCISHFSRFSVILSFFN
LSTACFSFSIIFSFLAIFHVLQWTFLNFPPFSVFLAIFHVLKCCVFLIFRDFQFSCHIPGPTVCISHFSRFSV
ILSFFNLSTACFSFSMIFSFLAIFHVLQWKFLNFPPFSFSPYSRSYSVHFSFFTFFSDFVIFQVVKWMFPIFL
DFQFSY
>ENSMUSP00000096650_c.108:G>T_p.V37F ENSMUST00000099051.11|Gm10717-203|ENSMUSG00000095891|+|chr9:g.3029307:G>T
CHIPCPTVEISFPPFSVFLAIFHVLKCVFLIFRVFQFSRHIPGPTVCISHFSRFSVILSFFKLSSGCFPFSLI
FSFLTIFHVLQWTFLNFPPFSVFLAIFHVLKCVFLIFSDFQFPRHIPGPTVCISHFSRFSVISSFFKTSSGCF
SFFMIFSFLAIFHVLQWTFLNFPPFSVSPYSRSYSVHFSFFTFFSDFVIFQVVKWMFLIFLDFHFSC
>ENSMUSP00000137419_c.576:A>C_p.S193R ENSMUST00000177875.8|Gm10717-206|ENSMUSG00000095891|+|chr9:g.3029333:A>C

MFLIFHDFQFSCHIPCPTVDISFPPFSVFLAIFHVLKCVFLIFRDFQFSCHIPGPTVCISHFSRFSVILSFFN
LSTACFSFSIIFSFLAIFHVLQWTFLNFPPFSVFLAIFHVLKCCVFLIFRDFQFSCHIPGPTVCISHFSRFSV
ILSFFNLSTACFSFSMIFSFLAIFHVLQWKFLNFPPFSVSPYSRSYRVHFSFFTFFSDFVIFQVVKWMFPIFL
DFQFSY
>ENSMUSP00000137419_c.595:T>C_p.F199S ENSMUST00000177875.8|Gm10717-206|ENSMUSG00000095891|+|chr9:g.3029352:T>C
MFLIFHDFQFSCHIPCPTVDISFPPFSVFLAIFHVLKCVFLIFRDFQFSCHIPGPTVCISHFSRFSVILSFFN
LSTACFSFSIIFSFLAIFHVLQWTFLNFPPFSVFLAIFHVLKCCVFLIFRDFQFSCHIPGPTVCISHFSRFSV
ILSFFNLSTACFSFSMIFSFLAIFHVLQWKFLNFPPFSVSPYSRSYSVHFSFSTFFSDFVIFQVVKWMFPIFL
DFQFSY
>ENSMUSP00000096650_c.153:T>C_p.S52P ENSMUST00000099051.11|Gm10717-203|ENSMUSG00000095891|+|chr9:g.3029352:T>C
CHIPCPTVEISFPPFSVFLAIFHVLKCVFLIFRVFQVSRHIPGPTVCISHFPRFSVILSFFKLSSGCFPFSLI
FSFLTIFHVLQWTFLNFPPFSVFLAIFHVLKCVFLIFSDFQFPRHIPGPTVCISHFSRFSVISSFFKTSSGCF
SFFMIFSFLAIFHVLQWTFLNFPPFSVSPYSRSYSVHFSFFTFFSDFVIFQVVKWMFLIFLDFHFSC
>ENSMUSP00000096650_c.163:C>T_p.S55L ENSMUST00000099051.11|Gm10717-203|ENSMUSG00000095891|+|chr9:g.3029362:C>T
CHIPCPTVEISFPPFSVFLAIFHVLKCVFLIFRVFQVSRHIPGPTVCISHFSRFLVILSFFKLSSGCFPFSLI
FSFLTIFHVLQWTFLNFPPFSVFLAIFHVLKCVFLIFSDFQFPRHIPGPTVCISHFSRFSVISSFFKTSSGCF
SFFMIFSFLAIFHVLQWTFLNFPPFSVSPYSRSYSVHFSFFTFFSDFVIFQVVKWMFLIFLDFHFSC
>ENSMUSP00000096650_c.172:T>C_p.L58S ENSMUST00000099051.11|Gm10717-203|ENSMUSG00000095891|+|chr9:g.3029371:T>C
CHIPCPTVEISFPPFSVFLAIFHVLKCVFLIFRVFQVSRHIPGPTVCISHFSRFSVISSFFKLSSGCFPFSLI
FSFLTIFHVLQWTFLNFPPFSVFLAIFHVLKCVFLIFSDFQFPRHIPGPTVCISHFSRFSVISSFFKTSSGCF
SFFMIFSFLAIFHVLQWTFLNFPPFSVSPYSRSYSVHFSFFTFFSDFVIFQVVKWMFLIFLDFHFSC
>rs260015946_ENSMUSP00000096650_c.187:T>C_p.L63S
ENSMUST00000099051.11|Gm10717-203|ENSMUSG00000095891|+|chr9:g.3029386:T>C
CHIPCPTVEISFPPFSVFLAIFHVLKCVFLIFRVFQVSRHIPGPTVCISHFSRFSVILSFFKSSSGCFPFSLI
FSFLTIFHVLQWTFLNFPPFSVFLAIFHVLKCVFLIFSDFQFPRHIPGPTVCISHFSRFSVISSFFKTSSGCF
SFFMIFSFLAIFHVLQWTFLNFPPFSVSPYSRSYSVHFSFFTFFSDFVIFQVVKWMFLIFLDFHFSC
>ENSMUSP00000137419_c.646:C>T_p.P216L ENSMUST00000177875.8|Gm10717-206|ENSMUSG00000095891|+|chr9:g.3029403:C>T
MFLIFHDFQFSCHIPCPTVDISFPPFSVFLAIFHVLKCVFLIFRDFQFSCHIPGPTVCISHFSRFSVILSFFN
LSTACFSFSIIFSFLAIFHVLQWTFLNFPPFSVFLAIFHVLKCCVFLIFRDFQFSCHIPGPTVCISHFSRFSV
ILSFFNLSTACFSFSMIFSFLAIFHVLQWKFLNFPPFSVSPYSRSYSVHFSFFTFFSDFVIFQVVKWMFLIFL
DFQFSY
>ENSMUSP00000096650_c.204:C>T_p.P69S ENSMUST00000099051.11|Gm10717-203|ENSMUSG00000095891|+|chr9:g.3029403:C>T
CHIPCPTVEISFPPFSVFLAIFHVLKCVFLIFRVFQVSRHIPGPTVCISHFSRFSVILSFFKLSSGCFSFSLI
FSFLTIFHVLQWTFLNFPPFSVFLAIFHVLKCVFLIFSDFQFPRHIPGPTVCISHFSRFSVISSFFKTSSGCF
SFFMIFSFLAIFHVLQWTFLNFPPFSVSPYSRSYSVHFSFFTFFSDFVIFQVVKWMFLIFLDFHFSC
>rs227499457_ENSMUSP00000137419_c.655:T>A_p.L219H
ENSMUST00000177875.8|Gm10717-206|ENSMUSG00000095891|+|chr9:g.3029412:T>A
MFLIFHDFQFSCHIPCPTVDISFPPFSVFLAIFHVLKCVFLIFRDFQFSCHIPGPTVCISHFSRFSVILSFFN
LSTACFSFSIIFSFLAIFHVLQWTFLNFPPFSVFLAIFHVLKCCVFLIFRDFQFSCHIPGPTVCISHFSRFSV
ILSFFNLSTACFSFSMIFSFLAIFHVLQWKFLNFPPFSVSPYSRSYSVHFSFFTFFSDFVIFQVVKWMFPIFH
DFQFSY
>rs227499457_ENSMUSP00000096650_c.213:T>A_p.L72M
ENSMUST00000099051.11|Gm10717-203|ENSMUSG00000095891|+|chr9:g.3029412:T>A
CHIPCPTVEISFPPFSVFLAIFHVLKCVFLIFRVFQVSRHIPGPTVCISHFSRFSVILSFFKLSSGCFPFSMI
FSFLTIFHVLQWTFLNFPPFSVFLAIFHVLKCVFLIFSDFQFPRHIPGPTVCISHFSRFSVISSFFKTSSGCF
SFFMIFSFLAIFHVLQWTFLNFPPFSVSPYSRSYSVHFSFFTFFSDFVIFQVVKWMFLIFLDFHFSC
>ENSMUSP00000137419_c.666:T>A_p.F223I ENSMUST00000177875.8|Gm10717-206|ENSMUSG00000095891|+|chr9:g.3029423:T>A
MFLIFHDFQFSCHIPCPTVDISFPPFSVFLAIFHVLKCVFLIFRDFQFSCHIPGPTVCISHFSRFSVILSFFN
LSTACFSFSIIFSFLAIFHVLQWTFLNFPPFSVFLAIFHVLKCCVFLIFRDFQFSCHIPGPTVCISHFSRFSV
ILSFFNLSTACFSFSMIFSFLAIFHVLQWKFLNFPPFSVSPYSRSYSVHFSFFTFFSDFVIFQVVKWMFPIFL
DFQISY

>ENSMUSP00000096650_c.224:T>A_p.S75R ENSMUST00000099051.11|Gm10717-203|ENSMUSG00000095891|+|chr9:g.3029423:T>A
CHIPCPTVEISFPPFSVFLAIFHVLKCVFLIFRVFQVSRHIPGPTVCISHFSRFSVILSFFKLSSGCFPFSLI
FRFLTIFHVLQWTFLNFPPFSVFLAIFHVLKCVFLIFSDFQFPRHIPGPTVCISHFSRFSVISSFFKTSSGCF
SFFMIFSFLAIFHVLQWTFLNFPPFSVSPYSRSYSVHFSFFTFFSDFVIFQVVKWMFLIFLDFHFSC
>ENSMUSP00000137419_c.673:A>G_p.Y225C ENSMUST00000177875.8|Gm10717-206|ENSMUSG00000095891|+|chr9:g.3029430:A>G
MFLIFHDFQFSCHIPCPTVDISFPPFSVFLAIFHVLKCVFLIFRDFQFSCHIPGPTVCISHFSRFSVILSFFN
LSTACFSFSIIFSFLAIFHVLQWTFLNFPPFSVFLAIFHVLKCCVFLIFRDFQFSCHIPGPTVCISHFSRFSV
ILSFFNLSTACFSFSMIFSFLAIFHVLQWKFLNFPPFSVSPYSRSYSVHFSFFTFFSDFVIFQVVKWMFPIFL
DFQFSC
>ENSMUSP00000096650_c.231:A>G_p.T78A ENSMUST00000099051.11|Gm10717-203|ENSMUSG00000095891|+|chr9:g.3029430:A>G
CHIPCPTVEISFPPFSVFLAIFHVLKCVFLIFRVFQVSRHIPGPTVCISHFSRFSVILSFFKLSSGCFPFSLI
FSFLAIFHVLQWTFLNFPPFSVFLAIFHVLKCVFLIFSDFQFPRHIPGPTVCISHFSRFSVISSFFKTSSGCF
SFFMIFSFLAIFHVLQWTFLNFPPFSVSPYSRSYSVHFSFFTFFSDFVIFQVVKWMFLIFLDFHFSC
>ENSMUSP00000096650_c.268:T>A_p.F90Y ENSMUST00000099051.11|Gm10717-203|ENSMUSG00000095891|+|chr9:g.3029467:T>A
CHIPCPTVEISFPPFSVFLAIFHVLKCVFLIFRVFQVSRHIPGPTVCISHFSRFSVILSFFKLSSGCFPFSLI
FSFLTIFHVLQWTFLNYPPFSVFLAIFHVLKCVFLIFSDFQFPRHIPGPTVCISHFSRFSVISSFFKTSSGCF
SFFMIFSFLAIFHVLQWTFLNFPPFSVSPYSRSYSVHFSFFTFFSDFVIFQVVKWMFLIFLDFHFSC
>ENSMUSP00000096650_c.273:C>T_p.P92S ENSMUST00000099051.11|Gm10717-203|ENSMUSG00000095891|+|chr9:g.3029472:C>T
CHIPCPTVEISFPPFSVFLAIFHVLKCVFLIFRVFQVSRHIPGPTVCISHFSRFSVILSFFKLSSGCFPFSLI
FSFLTIFHVLQWTFLNFPSFSVFLAIFHVLKCVFLIFSDFQFPRHIPGPTVCISHFSRFSVISSFFKTSSGCF
SFFMIFSFLAIFHVLQWTFLNFPPFSVSPYSRSYSVHFSFFTFFSDFVIFQVVKWMFLIFLDFHFSC
>ENSMUSP00000096650_c.292:C>T_p.A98V ENSMUST00000099051.11|Gm10717-203|ENSMUSG00000095891|+|chr9:g.3029491:C>T
CHIPCPTVEISFPPFSVFLAIFHVLKCVFLIFRVFQVSRHIPGPTVCISHFSRFSVILSFFKLSSGCFPFSLI
FSFLTIFHVLQWTFLNFPPFSVFLVIFHVLKCVFLIFSDFQFPRHIPGPTVCISHFSRFSVISSFFKTSSGCF
SFFMIFSFLAIFHVLQWTFLNFPPFSVSPYSRSYSVHFSFFTFFSDFVIFQVVKWMFLIFLDFHFSC
>ENSMUSP00000096650_c.309:A>T_p.K104* ENSMUST00000099051.11|Gm10717-203|ENSMUSG00000095891|+|chr9:g.3029508:A>T
CHIPCPTVEISFPPFSVFLAIFHVLKCVFLIFRVFQVSRHIPGPTVCISHFSRFSVILSFFKLSSGCFPFSLI
FSFLTIFHVLQWTFLNFPPFSVFLAIFHVL*CVFLIFSDFQFPRHIPGPTVCISHFSRFSVISSFFKTSSGCF
SFFMIFSFLAIFHVLQWTFLNFPPFSVSPYSRSYSVHFSFFTFFSDFVIFQVVKWMFLIFLDFHFSC
>ENSMUSP00000096650_c.315:G>A_p.V106I ENSMUST00000099051.11|Gm10717-203|ENSMUSG00000095891|+|chr9:g.3029514:G>A
CHIPCPTVEISFPPFSVFLAIFHVLKCVFLIFRVFQVSRHIPGPTVCISHFSRFSVILSFFKLSSGCFPFSLI
FSFLTIFHVLQWTFLNFPPFSVFLAIFHVLKCIFLIFSDFQFPRHIPGPTVCISHFSRFSVISSFFKTSSGCF
SFFMIFSFLAIFHVLQWTFLNFPPFSVSPYSRSYSVHFSFFTFFSDFVIFQVVKWMFLIFLDFHFSC
>ENSMUSP00000096650_c.330:A>C_p.S111R ENSMUST00000099051.11|Gm10717-203|ENSMUSG00000095891|+|chr9:g.3029529:A>C
CHIPCPTVEISFPPFSVFLAIFHVLKCVFLIFRVFQVSRHIPGPTVCISHFSRFSVILSFFKLSSGCFPFSLI
FSFLTIFHVLQWTFLNFPPFSVFLAIFHVLKCVFLIFRDFQFPRHIPGPTVCISHFSRFSVISSFFKTSSGCF
SFFMIFSFLAIFHVLQWTFLNFPPFSVSPYSRSYSVHFSFFTFFSDFVIFQVVKWMFLIFLDFHFSC
>ENSMUSP00000096650_c.330:A>T_p.S111C ENSMUST00000099051.11|Gm10717-203|ENSMUSG00000095891|+|chr9:g.3029529:A>T
CHIPCPTVEISFPPFSVFLAIFHVLKCVFLIFRVFQVSRHIPGPTVCISHFSRFSVILSFFKLSSGCFPFSLI
FSFLTIFHVLQWTFLNFPPFSVFLAIFHVLKCVFLIFCDFQFPRHIPGPTVCISHFSRFSVISSFFKTSSGCF
SFFMIFSFLAIFHVLQWTFLNFPPFSVSPYSRSYSVHFSFFTFFSDFVIFQVVKWMFLIFLDFHFSC
>ENSMUSP00000096640_c.6:A>T_p.I3F ENSMUST00000099042.4|Gm10717-202|ENSMUSG00000095891|+|chr9:g.3030343:A>T
FLFFHDFQLSSHIPGPTVDISFRPLSVFLAIFHVPKCVFLIFHDFQFPRHIPGPTVCISHFSRFSVISSFFKT
SSGCFSFFMIFSFLAIFHVLQWTFLNFPPFSVFLAIFHVLKCVFLIFRDFQFSCHIPGPTVCISHFSRFSVIL
SFFNLSTACFSFSMIFSFLAIFHVLQWTFLNFPPFSVSPYSRSYSVHFSFFTFFSDFVIFQVVKWMFLIFLDF
HFSC

>ENSMUSP00000096640_c.24:C>T_p.L9F ENSMUST00000099042.4|Gm10717-
202|ENSMUSG00000095891|+|chr9:g.3030361:C>T
FLIFHDFQFSSHIPGPTVDISFRPLSVFLAIFHVPKCVFLIFHDFQFPRHIPGPTVCISHFSRFSVISSFFKT
SSGCFSFFMIFSFLAIFHVLQWTFLNFPPFSVFLAIFHVLKCVFLIFRDFQFSCHIPGPTVCISHFSRFSVIL
SFFNLSTACFSFSMIFSFLAIFHVLQWTFLNFPPFSVSPYSRSYSVHFSFFTFFSDFVIFQVVKWMFLIFLDF
HFSC
>ENSMUSP00000096640_c.30:A>C_p.S11R ENSMUST00000099042.4|Gm10717-
202|ENSMUSG00000095891|+|chr9:g.3030367:A>C
FLIFHDFQLSRHIPGPTVDISFRPLSVFLAIFHVPKCVFLIFHDFQFPRHIPGPTVCISHFSRFSVISSFFKT
SSGCFSFFMIFSFLAIFHVLQWTFLNFPPFSVFLAIFHVLKCVFLIFRDFQFSCHIPGPTVCISHFSRFSVIL
SFFNLSTACFSFSMIFSFLAIFHVLQWTFLNFPPFSVSPYSRSYSVHFSFFTFFSDFVIFQVVKWMFLIFLDF
HFSC
>ENSMUSP00000096640_c.30:A>T_p.S11C ENSMUST00000099042.4|Gm10717-
202|ENSMUSG00000095891|+|chr9:g.3030367:A>T
FLIFHDFQLSCHIPGPTVDISFRPLSVFLAIFHVPKCVFLIFHDFQFPRHIPGPTVCISHFSRFSVISSFFKT
SSGCFSFFMIFSFLAIFHVLQWTFLNFPPFSVFLAIFHVLKCVFLIFRDFQFSCHIPGPTVCISHFSRFSVIL
SFFNLSTACFSFSMIFSFLAIFHVLQWTFLNFPPFSVSPYSRSYSVHFSFFTFFSDFVIFQVVKWMFLIFLDF
HFSC
>rs260741822_ENSMUSP00000096640_c.42:G>C_p.G15R
ENSMUST00000099042.4|Gm10717-202|ENSMUSG00000095891|+|chr9:g.3030379:G>C
FLIFHDFQLSSHIPRPTVDISFRPLSVFLAIFHVPKCVFLIFHDFQFPRHIPGPTVCISHFSRFSVISSFFKT
SSGCFSFFMIFSFLAIFHVLQWTFLNFPPFSVFLAIFHVLKCVFLIFRDFQFSCHIPGPTVCISHFSRFSVIL
SFFNLSTACFSFSMIFSFLAIFHVLQWTFLNFPPFSVSPYSRSYSVHFSFFTFFSDFVIFQVVKWMFLIFLDF
HFSC
>ENSMUSP00000096640_c.67:G>C_p.R23P ENSMUST00000099042.4|Gm10717-
202|ENSMUSG00000095891|+|chr9:g.3030408:G>C
FLIFHDFQLSSHIPGPTVDISFPPLSVFLAIFHVPKCVFLIFHDFQFPRHIPGPTVCISHFSRFSVISSFFKT
SSGCFSFFMIFSFLAIFHVLQWTFLNFPPFSVFLAIFHVLKCVFLIFRDFQFSCHIPGPTVCISHFSRFSVIL
SFFNLSTACFSFSMIFSFLAIFHVLQWTFLNFPPFSVSPYSRSYSVHFSFFTFFSDFVIFQVVKWMFLIFLDF
HFSC
>ENSMUSP00000096640_c.141:C>T_p.P48S ENSMUST00000099042.4|Gm10717-
202|ENSMUSG00000095891|+|chr9:g.3030482:C>T
FLIFHDFQLSSHIPGPTVDISFRPLSVFLAIFHVPKCVFLIFHDFQFSRHIPGPTVCISHFSRFSVISSFFKT
SSGCFSFFMIFSFLAIFHVLQWTFLNFPPFSVFLAIFHVLKCVFLIFRDFQFSCHIPGPTVCISHFSRFSVIL
SFFNLSTACFSFSMIFSFLAIFHVLQWTFLNFPPFSVSPYSRSYSVHFSFFTFFSDFVIFQVVKWMFLIFLDF
HFSC
>ENSMUSP00000096650_c.390:C>T_p.R131C ENSMUST00000099051.11|Gm10717-
203|ENSMUSG00000095891|+|chr9:g.3030527:C>T
CHIPCPTVEISFPPFSVFLAIFHVLKCVFLIFRVFQVSRHIPGPTVCISHFSRFSVILSFFKLSSGCFPFSLI
FSFLTIFHVLQWTFLNFPPFSVFLAIFHVLKCVFLIFSDFQFPRHIPGPTVCISHFSCFSVISSFFKTSSGCF
SFFMIFSFLAIFHVLQWTFLNFPPFSVSPYSRSYSVHFSFFTFFSDFVIFQVVKWMFLIFLDFHFSC
>ENSMUSP00000096640_c.186:C>T_p.R63C ENSMUST00000099042.4|Gm10717-
202|ENSMUSG00000095891|+|chr9:g.3030527:C>T
FLIFHDFQLSSHIPGPTVDISFRPLSVFLAIFHVPKCVFLIFHDFQFPRHIPGPTVCISHFSCFSVISSFFKT
SSGCFSFFMIFSFLAIFHVLQWTFLNFPPFSVFLAIFHVLKCVFLIFRDFQFSCHIPGPTVCISHFSRFSVIL
SFFNLSTACFSFSMIFSFLAIFHVLQWTFLNFPPFSVSPYSRSYSVHFSFFTFFSDFVIFQVVKWMFLIFLDF
HFSC
>ENSMUSP00000096650_c.390:C>A_p.R131S ENSMUST00000099051.11|Gm10717-
203|ENSMUSG00000095891|+|chr9:g.3030527:C>A
CHIPCPTVEISFPPFSVFLAIFHVLKCVFLIFRVFQVSRHIPGPTVCISHFSRFSVILSFFKLSSGCFPFSLI
FSFLTIFHVLQWTFLNFPPFSVFLAIFHVLKCVFLIFSDFQFPRHIPGPTVCISHFSSFSVISSFFKTSSGCF
SFFMIFSFLAIFHVLQWTFLNFPPFSVSPYSRSYSVHFSFFTFFSDFVIFQVVKWMFLIFLDFHFSC
>ENSMUSP00000096640_c.186:C>A_p.R63S ENSMUST00000099042.4|Gm10717-
202|ENSMUSG00000095891|+|chr9:g.3030527:C>A
FLIFHDFQLSSHIPGPTVDISFRPLSVFLAIFHVPKCVFLIFHDFQFPRHIPGPTVCISHFSSFSVISSFFKT
SSGCFSFFMIFSFLAIFHVLQWTFLNFPPFSVFLAIFHVLKCVFLIFRDFQFSCHIPGPTVCISHFSRFSVIL
SFFNLSTACFSFSMIFSFLAIFHVLQWTFLNFPPFSVSPYSRSYSVHFSFFTFFSDFVIFQVVKWMFLIFLDF
HFSC

>ENSMUSP00000096650_c.397:C>T_p.S133L ENSMUST00000099051.11|Gm10717-203|ENSMUSG00000095891|+|chr9:g.3030534:C>T
CHIPCPTVEISFPPFSVFLAIFHVLKCVFLIFRVFQVSRHIPGPTVCISHFSRFSVILSFFKLSSGCFPFSLI
FSFLTIFHVLQWTFLNFPPFSVFLAIFHVLKCVFLIFSDFQFPRHIPGPTVCISHFSRFLVISSFFKTSSGCF
SFFMIFSFLAIFHVLQWTFLNFPPFSVSPYSRSYSVHFSFFTFFSDFVIFQVVKWMFLIFLDFHFSC
>ENSMUSP00000096640_c.193:C>T_p.S65L ENSMUST00000099042.4|Gm10717-202|ENSMUSG00000095891|+|chr9:g.3030534:C>T
FLIFHDFQLSSHIPGPTVDISFRPLSVFLAIFHVPKCVFLIFHDFQFPRHIPGPTVCISHFSRFLVISSFFKT
SSGCFSFFMIFSFLAIFHVLQWTFLNFPPFSVFLAIFHVLKCVFLIFRDFQFSCHIPGPTVCISHFSRFSVIL
SFFNLSTACFSFSMIFSFLAIFHVLQWTFLNFPPFSVSPYSRSYSVHFSFFTFFSDFVIFQVVKWMFLIFLDF
HFSC
>rs226036698_ENSMUSP00000096650_c.420:A>T_p.T141S
ENSMUST00000099051.11|Gm10717-203|ENSMUSG00000095891|+|chr9:g.3030557:A>T
CHIPCPTVEISFPPFSVFLAIFHVLKCVFLIFRVFQVSRHIPGPTVCISHFSRFSVILSFFKLSSGCFPFSLI
FSFLTIFHVLQWTFLNFPPFSVFLAIFHVLKCVFLIFSDFQFPRHIPGPTVCISHFSRFSVISSFFKSSSGCF
SFFMIFSFLAIFHVLQWTFLNFPPFSVSPYSRSYSVHFSFFTFFSDFVIFQVVKWMFLIFLDFHFSC
>rs226036698_ENSMUSP00000096640_c.216:A>T_p.T73S
ENSMUST00000099042.4|Gm10717-202|ENSMUSG00000095891|+|chr9:g.3030557:A>T
FLIFHDFQLSSHIPGPTVDISFRPLSVFLAIFHVPKCVFLIFHDFQFPRHIPGPTVCISHFSRFSVISSFFKS
SSGCFSFFMIFSFLAIFHVLQWTFLNFPPFSVFLAIFHVLKCVFLIFRDFQFSCHIPGPTVCISHFSRFSVIL
SFFNLSTACFSFSMIFSFLAIFHVLQWTFLNFPPFSVSPYSRSYSVHFSFFTFFSDFVIFQVVKWMFLIFLDF
HFSC
>ENSMUSP00000137451_c.12:TTTC>CTTT_p.F5L ENSMUST00000179264.2|Gm10717-207|ENSMUSG00000095891|+|chr9:g.3030580:TTTC>CTTT
MFLILFHDFQFSCHIPCPTVDISFPPFSVFLAIFHVLKCVFLIFRDFQFSCHIPGPTVCISHFSRFSVILSFF
NLSTACFSFSMIFSFLAIFHVLQWTFLNFPPFSVFLAIFHVLKCVFLIFRVFQFPRHIPGPTVCIYHFSRFSV
ISSFFKTSSGCFSFFMIFSFLAIFHVLQWTFLNFPPFSVSPYSRSYSVHFSFFTFFSDFVIFQVVKWMFLIFL
DFHFSC
>ENSMUSP00000096650_c.445:T>C_p.F149S ENSMUST00000099051.11|Gm10717-203|ENSMUSG00000095891|+|chr9:g.3030582:T>C
CHIPCPTVEISFPPFSVFLAIFHVLKCVFLIFRVFQVSRHIPGPTVCISHFSRFSVILSFFKLSSGCFPFSLI
FSFLTIFHVLQWTFLNFPPFSVFLAIFHVLKCVFLIFSDFQFPRHIPGPTVCISHFSRFSVISSFFKTSSGCF
SFSMIFSFLAIFHVLQWTFLNFPPFSVSPYSRSYSVHFSFFTFFSDFVIFQVVKWMFLIFLDFHFSC
>ENSMUSP00000096640_c.241:T>C_p.F81S ENSMUST00000099042.4|Gm10717-202|ENSMUSG00000095891|+|chr9:g.3030582:T>C
FLIFHDFQLSSHIPGPTVDISFRPLSVFLAIFHVPKCVFLIFHDFQFPRHIPGPTVCISHFSRFSVISSFFKT
SSGCFSFSMIFSFLAIFHVLQWTFLNFPPFSVFLAIFHVLKCVFLIFRDFQFSCHIPGPTVCISHFSRFSVIL
SFFNLSTACFSFSMIFSFLAIFHVLQWTFLNFPPFSVSPYSRSYSVHFSFFTFFSDFVIFQVVKWMFLIFLDF
HFSC
>ENSMUSP00000096650_c.445:TC>CT_p.F149S ENSMUST00000099051.11|Gm10717-203|ENSMUSG00000095891|+|chr9:g.3030582:TC>CT
CHIPCPTVEISFPPFSVFLAIFHVLKCVFLIFRVFQVSRHIPGPTVCISHFSRFSVILSFFKLSSGCFPFSLI
FSFLTIFHVLQWTFLNFPPFSVFLAIFHVLKCVFLIFSDFQFPRHIPGPTVCISHFSRFSVISSFFKTSSGCF
SFSHDFQFSCHIPCPTVDISKFSTFFCFAIFQVLQCAFLIFHVFQ*FCHFSSCQVDVSHFP*FSLFL
>ENSMUSP00000096640_c.241:TC>CT_p.F81S ENSMUST00000099042.4|Gm10717-202|ENSMUSG00000095891|+|chr9:g.3030582:TC>CT
FLIFHDFQLSSHIPGPTVDISFRPLSVFLAIFHVPKCVFLIFHDFQFPRHIPGPTVCISHFSRFSVISSFFKT
SSGCFSFSHDFQFSCHIPCPTVDISKFSTFFCFPRHISRPKVCISHFP*FSVFLPYSWSYSVHFSFFTFFSDF
VIFQFVNCMFLIFHDFQFSCHIPCPTVDISKFSTFFSFAIFQVLQCAFLIFHVFQ*FCHFSSCQVDVSHFP*F
SLFL
>ENSMUSP00000096650_c.468:A>C_p.I157L ENSMUST00000099051.11|Gm10717-203|ENSMUSG00000095891|+|chr9:g.3030605:A>C
CHIPCPTVEISFPPFSVFLAIFHVLKCVFLIFRVFQVSRHIPGPTVCISHFSRFSVILSFFKLSSGCFPFSLI
FSFLTIFHVLQWTFLNFPPFSVFLAIFHVLKCVFLIFSDFQFPRHIPGPTVCISHFSRFSVISSFFKTSSGCF
SFFMIFSFLALFHVLQWTFLNFPPFSVSPYSRSYSVHFSFFTFFSDFVIFQVVKWMFLIFLDFHFSC
>ENSMUSP00000096640_c.264:A>C_p.I89L ENSMUST00000099042.4|Gm10717-202|ENSMUSG00000095891|+|chr9:g.3030605:A>C

FLIFHDFQLSSHIPGPTVDISFRPLSVFLAIFHVPKCVFLIFHDFQFPRHIPGPTVCISHFSRFSVISSFFKT
SSGCFSFFMIFSFLALFHVLQWTFLNFPPFSVFLAIFHVLKCVFLIFRDFQFSCHIPGPTVCISHFSRFSVIL
SFFNLSTACFSFSMIFSFLAIFHVLQWTFLNFPPFSVSPYSRSYSVHFSFFTFFSDFVIFQVVKWMFLIFLDF
HFSC
>ENSMUSP00000137451_c.37:A>C_p.H13P ENSMUST00000179264.2|Gm10717-207|ENSMUSG00000095891|+|chr9:g.3030605:A>C
MFLIFHDFQFSCPIPCPTVDISFPPFSVFLAIFHVLKCVFLIFRDFQFSCHIPGPTVCISHFSRFSVILSFFN
LSTACFSFSMIFSFLAIFHVLQWTFLNFPPFSVFLAIFHVLKCVFLIFRVFQFPRHIPGPTVCIYHFSRFSVI
SSFFKTSSGCFSFFMIFSFLAIFHVLQWTFLNFPPFSVSPYSRSYSVHFSFFTFFSDFVIFQVVKWMFLIFLD
FHFSC
>ENSMUSP00000096650_c.474:CAT>AAA_p.H159K ENSMUST00000099051.11|Gm10717-203|ENSMUSG00000095891|+|chr9:g.3030611:CAT>AAA
CHIPCPTVEISFPPFSVFLAIFHVLKCVFLIFRVFQVSRHIPGPTVCISHFSRFSVILSFFKLSSGCFPFSLI
FSFLTIFHVLQWTFLNFPPFSVFLAIFHVLKCVFLIFSDFQFPRHIPGPTVCISHFSRFSVISSFFKTSSGCF
SFFMIFSFLAIFKMSYSGHF*IFHLFLFRHIPGPTVCISHFSRFSVILSFFKLSSGCFSFSLIFTFL
>ENSMUSP00000096640_c.270:CAT>AAA_p.H91K ENSMUST00000099042.4|Gm10717-202|ENSMUSG00000095891|+|chr9:g.3030611:CAT>AAA
FLIFHDFQLSSHIPGPTVDISFRPLSVFLAIFHVPKCVFLIFHDFQFPRHIPGPTVCISHFSRFSVISSFFKT
SSGCFSFFMIFSFLAIFKMSYSGHF*IFHLFLFSSPYFTS*SVYFSFSVIFSFLAIFLVLQCAFLIFHVFQ*F
CHFSICQLHVSHFP*FSVFLPYSMSYSGHF*IFHLFQFRHIPGPTVCISHFSRFSVILSFFKLSSGCFSFSLI
FTFL
>ENSMUSP00000137451_c.43:CAT>AAA_p.P15Q ENSMUST00000179264.2|Gm10717-207|ENSMUSG00000095891|+|chr9:g.3030611:CAT>AAA
MFLIFHDFQFSCHIQNVLQWTFLFHLFLFSSPYFTS*SVYFSFSVIFSFLAIFLVLQCAFLIFHVFQ*FCHFS
ICQLHVSHFP*FSVFLPYSMSYSGHF*IFHLFQFSSPYFTC*SVYFSFFVFFSFLAIFQVLQCAFIIFHVFQ*
FRLFSRRQVDVSHFS*FSVFLPYSMSYSGHF*IFHLFLFRHIPGPTVCISHFSRFSVILSFFKLSSGCFSFSL
IFTFL
>ENSMUSP00000137451_c.45:T>C_p.C16R ENSMUST00000179264.2|Gm10717-207|ENSMUSG00000095891|+|chr9:g.3030613:T>C
MFLIFHDFQFSCHIPRPTVDISFPPFSVFLAIFHVLKCVFLIFRDFQFSCHIPGPTVCISHFSRFSVILSFFN
LSTACFSFSMIFSFLAIFHVLQWTFLNFPPFSVFLAIFHVLKCVFLIFRVFQFPRHIPGPTVCIYHFSRFSVI
SSFFKTSSGCFSFFMIFSFLAIFHVLQWTFLNFPPFSVSPYSRSYSVHFSFFTFFSDFVIFQVVKWMFLIFLD
FHFSC
>ENSMUSP00000096650_c.508:C>A_p.P170H ENSMUST00000099051.11|Gm10717-203|ENSMUSG00000095891|+|chr9:g.3030645:C>A
CHIPCPTVEISFPPFSVFLAIFHVLKCVFLIFRVFQVSRHIPGPTVCISHFSRFSVILSFFKLSSGCFPFSLI
FSFLTIFHVLQWTFLNFPPFSVFLAIFHVLKCVFLIFSDFQFPRHIPGPTVCISHFSRFSVISSFFKTSSGCF
SFFMIFSFLAIFHVLQWTFLNFPHFSVSPYSRSYSVHFSFFTFFSDFVIFQVVKWMFLIFLDFHFSC
>ENSMUSP00000096640_c.304:C>A_p.P102H ENSMUST00000099042.4|Gm10717-202|ENSMUSG00000095891|+|chr9:g.3030645:C>A
FLIFHDFQLSSHIPGPTVDISFRPLSVFLAIFHVPKCVFLIFHDFQFPRHIPGPTVCISHFSRFSVISSFFKT
SSGCFSFFMIFSFLAIFHVLQWTFLNFPHFSVFLAIFHVLKCVFLIFRDFQFSCHIPGPTVCISHFSRFSVIL
SFFNLSTACFSFSMIFSFLAIFHVLQWTFLNFPPFSVSPYSRSYSVHFSFFTFFSDFVIFQVVKWMFLIFLDF
HFSC
>ENSMUSP00000137451_c.73:C>A_p.P25H ENSMUST00000179264.2|Gm10717-207|ENSMUSG00000095891|+|chr9:g.3030645:C>A
MFLIFHDFQFSCHIPCPTVDISFPHFSVFLAIFHVLKCVFLIFRDFQFSCHIPGPTVCISHFSRFSVILSFFN
LSTACFSFSMIFSFLAIFHVLQWTFLNFPPFSVFLAIFHVLKCVFLIFRVFQFPRHIPGPTVCIYHFSRFSVI
SSFFKTSSGCFSFFMIFSFLAIFHVLQWTFLNFPPFSVSPYSRSYSVHFSFFTFFSDFVIFQVVKWMFLIFLD
FHFSC
>ENSMUSP00000096640_c.333:G>A_p.V112I ENSMUST00000099042.4|Gm10717-202|ENSMUSG00000095891|+|chr9:g.3030674:G>A
FLIFHDFQLSSHIPGPTVDISFRPLSVFLAIFHVPKCVFLIFHDFQFPRHIPGPTVCISHFSRFSVISSFFKT
SSGCFSFFMIFSFLAIFHVLQWTFLNFPPFSVFLAIFHILKCVFLIFRDFQFSCHIPGPTVCISHFSRFSVIL
SFFNLSTACFSFSMIFSFLAIFHVLQWTFLNFPPFSVSPYSRSYSVHFSFFTFFSDFVIFQVVKWMFLIFLDF
HFSC
>ENSMUSP00000137451_c.102:G>A_p.V35I ENSMUST00000179264.2|Gm10717-207|ENSMUSG00000095891|+|chr9:g.3030674:G>A

MFLIFHDFQFSCHIPCPTVDISFPPFSVFLAIFHILKCVFLIFRDFQFSCHIPGPTVCISHFSRFSVILSFFN
LSTACFSFSMIFSFLAIFHVLQWTFLNFPPFSVFLAIFHVLKCVFLIFRVFQFPRHIPGPTVCIYHFSRFSVI
SSFFKTSSGCFSFFMIFSFLAIFHVLQWTFLNFPPFSVSPYSRSYSVHFSFFTFFSDFVIFQVVKWMFLIFLD
FHFSC
>ENSMUSP00000096640_c.360:C>T_p.R121C ENSMUST00000099042.4|Gm10717-202|ENSMUSG00000095891|+|chr9:g.3030701:C>T
FLIFHDFQLSSHIPGPTVDISFRPLSVFLAIFHVPKCVFLIFHDFQFPRHIPGPTVCISHFSRFSVISSFFKT
SSGCFSFFMIFSFLAIFHVLQWTFLNFPPFSVFLAIFHVLKCVFLIFCDFQFSCHIPGPTVCISHFSRFSVIL
SFFNLSTACFSFSMIFSFLAIFHVLQWTFLNFPPFSVSPYSRSYSVHFSFFTFFSDFVIFQVVKWMFLIFLDF
HFSC
>ENSMUSP00000137451_c.129:C>T_p.R44C ENSMUST00000179264.2|Gm10717-207|ENSMUSG00000095891|+|chr9:g.3030701:C>T
MFLIFHDFQFSCHIPCPTVDISFPPFSVFLAIFHVLKCVFLIFCDFQFSCHIPGPTVCISHFSRFSVILSFFN
LSTACFSFSMIFSFLAIFHVLQWTFLNFPPFSVFLAIFHVLKCVFLIFRVFQFPRHIPGPTVCIYHFSRFSVI
SSFFKTSSGCFSFFMIFSFLAIFHVLQWTFLNFPPFSVSPYSRSYSVHFSFFTFFSDFVIFQVVKWMFLIFLD
FHFSC
>ENSMUSP00000096640_c.361:G>A_p.R121H ENSMUST00000099042.4|Gm10717-202|ENSMUSG00000095891|+|chr9:g.3030702:G>A
FLIFHDFQLSSHIPGPTVDISFRPLSVFLAIFHVPKCVFLIFHDFQFPRHIPGPTVCISHFSRFSVISSFFKT
SSGCFSFFMIFSFLAIFHVLQWTFLNFPPFSVFLAIFHVLKCVFLIFHDFQFSCHIPGPTVCISHFSRFSVIL
SFFNLSTACFSFSMIFSFLAIFHVLQWTFLNFPPFSVSPYSRSYSVHFSFFTFFSDFVIFQVVKWMFLIFLDF
HFSC
>ENSMUSP00000137451_c.130:G>A_p.R44H ENSMUST00000179264.2|Gm10717-207|ENSMUSG00000095891|+|chr9:g.3030702:G>A
MFLIFHDFQFSCHIPCPTVDISFPPFSVFLAIFHVLKCVFLIFHDFQFSCHIPGPTVCISHFSRFSVILSFFN
LSTACFSFSMIFSFLAIFHVLQWTFLNFPPFSVFLAIFHVLKCVFLIFRVFQFPRHIPGPTVCIYHFSRFSVI
SSFFKTSSGCFSFFMIFSFLAIFHVLQWTFLNFPPFSVSPYSRSYSVHFSFFTFFSDFVIFQVVKWMFLIFLD
FHFSC
>ENSMUSP00000096640_c.378:T>C_p.C127R ENSMUST00000099042.4|Gm10717-202|ENSMUSG00000095891|+|chr9:g.3030719:T>C
FLIFHDFQLSSHIPGPTVDISFRPLSVFLAIFHVPKCVFLIFHDFQFPRHIPGPTVCISHFSRFSVISSFFKT
SSGCFSFFMIFSFLAIFHVLQWTFLNFPPFSVFLAIFHVLKCVFLIFRDFQFSRHIPGPTVCISHFSRFSVIL
SFFNLSTACFSFSMIFSFLAIFHVLQWTFLNFPPFSVSPYSRSYSVHFSFFTFFSDFVIFQVVKWMFLIFLDF
HFSC
>ENSMUSP00000137451_c.147:T>C_p.C50R ENSMUST00000179264.2|Gm10717-207|ENSMUSG00000095891|+|chr9:g.3030719:T>C
MFLIFHDFQFSCHIPCPTVDISFPPFSVFLAIFHVLKCVFLIFRDFQFSRHIPGPTVCISHFSRFSVILSFFN
LSTACFSFSMIFSFLAIFHVLQWTFLNFPPFSVFLAIFHVLKCVFLIFRVFQFPRHIPGPTVCIYHFSRFSVI
SSFFKTSSGCFSFFMIFSFLAIFHVLQWTFLNFPPFSVSPYSRSYSVHFSFFTFFSDFVIFQVVKWMFLIFLD
FHFSC
>ENSMUSP00000096640_c.396:A>T_p.T133S ENSMUST00000099042.4|Gm10717-202|ENSMUSG00000095891|+|chr9:g.3030737:A>T
FLIFHDFQLSSHIPGPTVDISFRPLSVFLAIFHVPKCVFLIFHDFQFPRHIPGPTVCISHFSRFSVISSFFKT
SSGCFSFFMIFSFLAIFHVLQWTFLNFPPFSVFLAIFHVLKCVFLIFRDFQFSCHIPGPSVCISHFSRFSVIL
SFFNLSTACFSFSMIFSFLAIFHVLQWTFLNFPPFSVSPYSRSYSVHFSFFTFFSDFVIFQVVKWMFLIFLDF
HFSC
>ENSMUSP00000137451_c.165:A>T_p.T56S ENSMUST00000179264.2|Gm10717-207|ENSMUSG00000095891|+|chr9:g.3030737:A>T
MFLIFHDFQFSCHIPCPTVDISFPPFSVFLAIFHVLKCVFLIFRDFQFSCHIPGPSVCISHFSRFSVILSFFN
LSTACFSFSMIFSFLAIFHVLQWTFLNFPPFSVFLAIFHVLKCVFLIFRVFQFPRHIPGPTVCIYHFSRFSVI
SSFFKTSSGCFSFFMIFSFLAIFHVLQWTFLNFPPFSVSPYSRSYSVHFSFFTFFSDFVIFQVVKWMFLIFLD
FHFSC
>ENSMUSP00000096640_c.411:C>T_p.H138Y ENSMUST00000099042.4|Gm10717-202|ENSMUSG00000095891|+|chr9:g.3030752:C>T
FLIFHDFQLSSHIPGPTVDISFRPLSVFLAIFHVPKCVFLIFHDFQFPRHIPGPTVCISHFSRFSVISSFFKT
SSGCFSFFMIFSFLAIFHVLQWTFLNFPPFSVFLAIFHVLKCVFLIFRDFQFSCHIPGPTVCISYFSRFSVIL
SFFNLSTACFSFSMIFSFLAIFHVLQWTFLNFPPFSVSPYSRSYSVHFSFFTFFSDFVIFQVVKWMFLIFLDF
HFSC

>ENSMUSP00000137451_c.180:C>T_p.H61Y ENSMUST00000179264.2|Gm10717-207|ENSMUSG00000095891|+|chr9:g.3030752:C>T
MFLIFHDFQFSCHIPCPTVDISFPPFSVFLAIFHVLKCVFLIFRDFQFSCHIPGPTVCISYFSRFSVILSFFN
LSTACFSFSMIFSFLAIFHVLQWTFLNFPPFSVFLAIFHVLKCVFLIFRVFQFPRHIPGPTVCIYHFSRFSVI
SSFFKTSSGCFSFFMIFSFLAIFHVLQWTFLNFPPFSVSPYSRSYSVHFSFFTFFSDFVIFQVVKWMFLIFLD
FHFSC
>ENSMUSP00000096640_c.427:C>T_p.S143L ENSMUST00000099042.4|Gm10717-202|ENSMUSG00000095891|+|chr9:g.3030768:C>T
FLIFHDFQLSSHIPGPTVDISFRPLSVFLAIFHVPKCVFLIFHDFQFPRHIPGPTVCISHFSRFSVISSFFKT
SSGCFSFFMIFSFLAIFHVLQWTFLNFPPFSVFLAIFHVLKCVFLIFRDFQFSCHIPGPTVCISHFSRFLVIL
SFFNLSTACFSFSMIFSFLAIFHVLQWTFLNFPPFSVSPYSRSYSVHFSFFTFFSDFVIFQVVKWMFLIFLDF
HFSC
>ENSMUSP00000137451_c.196:C>T_p.S66L ENSMUST00000179264.2|Gm10717-207|ENSMUSG00000095891|+|chr9:g.3030768:C>T
MFLIFHDFQFSCHIPCPTVDISFPPFSVFLAIFHVLKCVFLIFRDFQFSCHIPGPTVCISHFSRFLVILSFFN
LSTACFSFSMIFSFLAIFHVLQWTFLNFPPFSVFLAIFHVLKCVFLIFRVFQFPRHIPGPTVCIYHFSRFSVI
SSFFKTSSGCFSFFMIFSFLAIFHVLQWTFLNFPPFSVSPYSRSYSVHFSFFTFFSDFVIFQVVKWMFLIFLD
FHFSC
>ENSMUSP00000096640_c.429:G>T_p.V144L ENSMUST00000099042.4|Gm10717-202|ENSMUSG00000095891|+|chr9:g.3030770:G>T
FLIFHDFQLSSHIPGPTVDISFRPLSVFLAIFHVPKCVFLIFHDFQFPRHIPGPTVCISHFSRFSVISSFFKT
SSGCFSFFMIFSFLAIFHVLQWTFLNFPPFSVFLAIFHVLKCVFLIFRDFQFSCHIPGPTVCISHFSRFSLIL
SFFNLSTACFSFSMIFSFLAIFHVLQWTFLNFPPFSVSPYSRSYSVHFSFFTFFSDFVIFQVVKWMFLIFLDF
HFSC
>ENSMUSP00000137451_c.198:G>T_p.V67L ENSMUST00000179264.2|Gm10717-207|ENSMUSG00000095891|+|chr9:g.3030770:G>T
MFLIFHDFQFSCHIPCPTVDISFPPFSVFLAIFHVLKCVFLIFRDFQFSCHIPGPTVCISHFSRFSLILSFFN
LSTACFSFSMIFSFLAIFHVLQWTFLNFPPFSVFLAIFHVLKCVFLIFRVFQFPRHIPGPTVCIYHFSRFSVI
SSFFKTSSGCFSFFMIFSFLAIFHVLQWTFLNFPPFSVSPYSRSYSVHFSFFTFFSDFVIFQVVKWMFLIFLD
FHFSC
>ENSMUSP00000096640_c.445:TC>AA_p.F149* ENSMUST00000099042.4|Gm10717-202|ENSMUSG00000095891|+|chr9:g.3030786:TC>AA
FLIFHDFQLSSHIPGPTVDISFRPLSVFLAIFHVPKCVFLIFHDFQFPRHIPGPTVCISHFSRFSVISSFFKT
SSGCFSFFMIFSFLAIFHVLQWTFLNFPPFSVFLAIFHVLKCVFLIFRDFQFSCHIPGPTVCISHFSRFSVIL
SF*QFVNCMFLIFHDFQFSCHIPCPTVDISKFSTFFSFAIFQVLQCAFLIFHVFQ*FCHFSSCQVDVSHFP*F
SLFL
>ENSMUSP00000137451_c.214:TC>AA_p.F72* ENSMUST00000179264.2|Gm10717-207|ENSMUSG00000095891|+|chr9:g.3030786:TC>AA
MFLIFHDFQFSCHIPCPTVDISFPPFSVFLAIFHVLKCVFLIFRDFQFSCHIPGPTVCISHFSRFSVILSF*Q
FVNCMFLIFHDFQFSCHIPCPTVDISKFSTFFSFPRHISRAKVCISHFSCFSVSSPYSRSYSVHLSFFTFFSD
FVFFQDVKWMFLIFHDFQFSCHIPCPTVDISKFSTFFCFAIFQVLQCAFLIFHVFQ*FCHFSSCQVDVSHFP*
FSLFL
>ENSMUSP00000096640_c.449:T>G_p.N150K ENSMUST00000099042.4|Gm10717-202|ENSMUSG00000095891|+|chr9:g.3030790:T>G
FLIFHDFQLSSHIPGPTVDISFRPLSVFLAIFHVPKCVFLIFHDFQFPRHIPGPTVCISHFSRFSVISSFFKT
SSGCFSFFMIFSFLAIFHVLQWTFLNFPPFSVFLAIFHVLKCVFLIFRDFQFSCHIPGPTVCISHFSRFSVIL
SFFKLSTACFSFSMIFSFLAIFHVLQWTFLNFPPFSVSPYSRSYSVHFSFFTFFSDFVIFQVVKWMFLIFLDF
HFSC
>ENSMUSP00000137451_c.218:T>G_p.N73K ENSMUST00000179264.2|Gm10717-207|ENSMUSG00000095891|+|chr9:g.3030790:T>G
MFLIFHDFQFSCHIPCPTVDISFPPFSVFLAIFHVLKCVFLIFRDFQFSCHIPGPTVCISHFSRFSVILSFFK
LSTACFSFSMIFSFLAIFHVLQWTFLNFPPFSVFLAIFHVLKCVFLIFRVFQFPRHIPGPTVCIYHFSRFSVI
SSFFKTSSGCFSFFMIFSFLAIFHVLQWTFLNFPPFSVSPYSRSYSVHFSFFTFFSDFVIFQVVKWMFLIFLD
FHFSC
>ENSMUSP00000096640_c.449:TTT>GTC_p.N150K ENSMUST00000099042.4|Gm10717-202|ENSMUSG00000095891|+|chr9:g.3030790:TTT>GTC
FLIFHDFQLSSHIPGPTVDISFRPLSVFLAIFHVPKCVFLIFHDFQFPRHIPGPTVCISHFSRFSVISSFFKT
SSGCFSFFMIFSFLAIFHVLQWTFLNFPPFSVFLAIFHVLKCVFLIFRDFQFSCHIPGPTVCISHFSRFSVIL

SFFKSCQLHVSHFP*FSVFLPYSMSYSGHF*IFHLFQFRHIPGPTVCISHFSRFSVILSFFKLSSGCFSFSLI
FTFL
>ENSMUSP00000137451_c.218:TTT>GTC_p.N73K ENSMUST00000179264.2|Gm10717-207|ENSMUSG00000095891|+|chr9:g.3030790:TTT>GTC
MFLIFHDFQFSCHIPCPTVDISFPPFSVFLAIFHVLKCVFLIFRDFQFSCHIPGPTVCISHFSRFSVILSFFK
SCQLHVSHFP*FSVFLPYSMSYSGHF*IFHLFQFSSPYFTC*SVYFSFFVFFSFLAIFQVLQCAFIIFHVFQ*
FRLFSRRQVDVSHFS*FSVFLPYSMSYSGHF*IFHLFLFRHIPGPTVCISHFSRFSVILSFFKLSSGCFSFSL
IFTFL
>rs242073667_ENSMUSP00000096640_c.451:T>C_p.L151S
ENSMUST00000099042.4|Gm10717-202|ENSMUSG00000095891|+|chr9:g.3030792:T>C
FLIFHDFQLSSHIPGPTVDISFRPLSVFLAIFHVPKCVFLIFHDFQFPRHIPGPTVCISHFSRFSVISSFFKT
SSGCFSFFMIFSFLAIFHVLQWTFLNFPPFSVFLAIFHVLKCVFLIFRDFQFSCHIPGPTVCISHFSRFSVIL
SFFNSSTACFSFSMIFSFLAIFHVLQWTFLNFPPFSVSPYSRSYSVHFSFFTFFSDFVIFQVVKWMFLIFLDF
HFSC
>rs242073667_ENSMUSP00000137451_c.220:T>C_p.L74S
ENSMUST00000179264.2|Gm10717-207|ENSMUSG00000095891|+|chr9:g.3030792:T>C
MFLIFHDFQFSCHIPCPTVDISFPPFSVFLAIFHVLKCVFLIFRDFQFSCHIPGPTVCISHFSRFSVILSFFN
SSTACFSFSMIFSFLAIFHVLQWTFLNFPPFSVFLAIFHVLKCVFLIFRVFQFPRHIPGPTVCIYHFSRFSVI
SSFFKTSSGCFSFFMIFSFLAIFHVLQWTFLNFPPFSVSPYSRSYSVHFSFFTFFSDFVIFQVVKWMFLIFLD
FHFSC
>ENSMUSP00000096640_c.456:A>C_p.T153P ENSMUST00000099042.4|Gm10717-202|ENSMUSG00000095891|+|chr9:g.3030797:A>C
FLIFHDFQLSSHIPGPTVDISFRPLSVFLAIFHVPKCVFLIFHDFQFPRHIPGPTVCISHFSRFSVISSFFKT
SSGCFSFFMIFSFLAIFHVLQWTFLNFPPFSVFLAIFHVLKCVFLIFRDFQFSCHIPGPTVCISHFSRFSVIL
SFFNLSPACFSFSMIFSFLAIFHVLQWTFLNFPPFSVSPYSRSYSVHFSFFTFFSDFVIFQVVKWMFLIFLDF
HFSC
>ENSMUSP00000137451_c.225:A>C_p.T76P ENSMUST00000179264.2|Gm10717-207|ENSMUSG00000095891|+|chr9:g.3030797:A>C
MFLIFHDFQFSCHIPCPTVDISFPPFSVFLAIFHVLKCVFLIFRDFQFSCHIPGPTVCISHFSRFSVILSFFN
LSPACFSFSMIFSFLAIFHVLQWTFLNFPPFSVFLAIFHVLKCVFLIFRVFQFPRHIPGPTVCIYHFSRFSVI
SSFFKTSSGCFSFFMIFSFLAIFHVLQWTFLNFPPFSVSPYSRSYSVHFSFFTFFSDFVIFQVVKWMFLIFLD
FHFSC
>rs264641761_ENSMUSP00000096640_c.457:C>G_p.T153S
ENSMUST00000099042.4|Gm10717-202|ENSMUSG00000095891|+|chr9:g.3030798:C>G
FLIFHDFQLSSHIPGPTVDISFRPLSVFLAIFHVPKCVFLIFHDFQFPRHIPGPTVCISHFSRFSVISSFFKT
SSGCFSFFMIFSFLAIFHVLQWTFLNFPPFSVFLAIFHVLKCVFLIFRDFQFSCHIPGPTVCISHFSRFSVIL
SFFNLSSACFSFSMIFSFLAIFHVLQWTFLNFPPFSVSPYSRSYSVHFSFFTFFSDFVIFQVVKWMFLIFLDF
HFSC
>rs264641761_ENSMUSP00000137451_c.226:C>G_p.T76S
ENSMUST00000179264.2|Gm10717-207|ENSMUSG00000095891|+|chr9:g.3030798:C>G
MFLIFHDFQFSCHIPCPTVDISFPPFSVFLAIFHVLKCVFLIFRDFQFSCHIPGPTVCISHFSRFSVILSFFN
LSSACFSFSMIFSFLAIFHVLQWTFLNFPPFSVFLAIFHVLKCVFLIFRVFQFPRHIPGPTVCIYHFSRFSVI
SSFFKTSSGCFSFFMIFSFLAIFHVLQWTFLNFPPFSVSPYSRSYSVHFSFFTFFSDFVIFQVVKWMFLIFLD
FHFSC
>ENSMUSP00000096640_c.457:CTGC>GTGT_p.T153S ENSMUST00000099042.4|Gm10717-202|ENSMUSG00000095891|+|chr9:g.3030798:CTGC>GTGT
FLIFHDFQLSSHIPGPTVDISFRPLSVFLAIFHVPKCVFLIFHDFQFPRHIPGPTVCISHFSRFSVISSFFKT
SSGCFSFFMIFSFLAIFHVLQWTFLNFPPFSVFLAIFHVLKCVFLIFRDFQFSCHIPGPTVCISHFSRFSVIL
SFFNLSSVACFSFSMIFSFLAIFHVLQWTFLNFPPFSVSPYSRSYSVHFSFFTFFSDFVIFQVVKWMFLIFLD
FHFSC
>ENSMUSP00000137451_c.226:CTGC>GTGT_p.T76S ENSMUST00000179264.2|Gm10717-207|ENSMUSG00000095891|+|chr9:g.3030798:CTGC>GTGT
MFLIFHDFQFSCHIPCPTVDISFPPFSVFLAIFHVLKCVFLIFRDFQFSCHIPGPTVCISHFSRFSVILSFFN
LSSVACFSFSMIFSFLAIFHVLQWTFLNFPPFSVFLAIFHVLKCVFLIFRVFQFPRHIPGPTVCIYHFSRFSV
ISSFFKTSSGCFSFFMIFSFLAIFHVLQWTFLNFPPFSVSPYSRSYSVHFSFFTFFSDFVIFQVVKWMFLIFL
DFHFSC
>ENSMUSP00000096640_c.457:CTGC>GTGG_p.T153S ENSMUST00000099042.4|Gm10717-202|ENSMUSG00000095891|+|chr9:g.3030798:CTGC>GTGG

FLIFHDFQLSSHIPGPTVDISFRPLSVFLAIFHVPKCVFLIFHDFQFPRHIPGPTVCISHFSRFSVISSFFKT
SSGCFSFFMIFSFLAIFHVLQWTFLNFPPFSVFLAIFHVLKCVFLIFRDFQFSCHIPGPTVCISHFSRFSVIL
SFFNLSSGACFSFSMIFSFLAIFHVLQWTFLNFPPFSVSPYSRSYSVHFSFFTFFSDFVIFQVVKWMFLIFLD
FHFSC
>ENSMUSP00000137451_c.226:CTGC>GTGG_p.T76S ENSMUST00000179264.2|Gm10717-207|ENSMUSG00000095891|+|chr9:g.3030798:CTGC>GTGG
MFLIFHDFQFSCHIPCPTVDISFPPFSVFLAIFHVLKCVFLIFRDFQFSCHIPGPTVCISHFSRFSVILSFFN
LSSGACFSFSMIFSFLAIFHVLQWTFLNFPPFSVFLAIFHVLKCVFLIFRVFQFPRHIPGPTVCIYHFSRFSV
ISSFFKTSSGCFSFFMIFSFLAIFHVLQWTFLNFPPFSVSPYSRSYSVHFSFFTFFSDFVIFQVVKWMFLIFL
DFHFSC
>rs220228475_ENSMUSP00000096640_c.460:C>G_p.A154G
ENSMUST00000099042.4|Gm10717-202|ENSMUSG00000095891|+|chr9:g.3030801:C>G
FLIFHDFQLSSHIPGPTVDISFRPLSVFLAIFHVPKCVFLIFHDFQFPRHIPGPTVCISHFSRFSVISSFFKT
SSGCFSFFMIFSFLAIFHVLQWTFLNFPPFSVFLAIFHVLKCVFLIFRDFQFSCHIPGPTVCISHFSRFSVIL
SFFNLSTGCFSFSMIFSFLAIFHVLQWTFLNFPPFSVSPYSRSYSVHFSFFTFFSDFVIFQVVKWMFLIFLDF
HFSC
>rs220228475_ENSMUSP00000137451_c.229:C>G_p.A77G
ENSMUST00000179264.2|Gm10717-207|ENSMUSG00000095891|+|chr9:g.3030801:C>G
MFLIFHDFQFSCHIPCPTVDISFPPFSVFLAIFHVLKCVFLIFRDFQFSCHIPGPTVCISHFSRFSVILSFFN
LSTGCFSFSMIFSFLAIFHVLQWTFLNFPPFSVFLAIFHVLKCVFLIFRVFQFPRHIPGPTVCIYHFSRFSVI
SSFFKTSSGCFSFFMIFSFLAIFHVLQWTFLNFPPFSVSPYSRSYSVHFSFFTFFSDFVIFQVVKWMFLIFLD
FHFSC
>ENSMUSP00000096640_c.537:C>A_p.P180T ENSMUST00000099042.4|Gm10717-202|ENSMUSG00000095891|+|chr9:g.3030878:C>A
FLIFHDFQLSSHIPGPTVDISFRPLSVFLAIFHVPKCVFLIFHDFQFPRHIPGPTVCISHFSRFSVISSFFKT
SSGCFSFFMIFSFLAIFHVLQWTFLNFPPFSVFLAIFHVLKCVFLIFRDFQFSCHIPGPTVCISHFSRFSVIL
SFFNLSTACFSFSMIFSFLAIFHVLQWTFLNFPTFSVSPYSRSYSVHFSFFTFFSDFVIFQVVKWMFLIFLDF
HFSC
>ENSMUSP00000137451_c.306:C>A_p.P103T ENSMUST00000179264.2|Gm10717-207|ENSMUSG00000095891|+|chr9:g.3030878:C>A
MFLIFHDFQFSCHIPCPTVDISFPPFSVFLAIFHVLKCVFLIFRDFQFSCHIPGPTVCISHFSRFSVILSFFN
LSTACFSFSMIFSFLAIFHVLQWTFLNFPTFSVFLAIFHVLKCVFLIFRVFQFPRHIPGPTVCIYHFSRFSVI
SSFFKTSSGCFSFFMIFSFLAIFHVLQWTFLNFPPFSVSPYSRSYSVHFSFFTFFSDFVIFQVVKWMFLIFLD
FHFSC
>ENSMUSP00000137451_c.344:G>T_p.K115N ENSMUST00000179264.2|Gm10717-207|ENSMUSG00000095891|+|chr9:g.3030916:G>T
MFLIFHDFQFSCHIPCPTVDISFPPFSVFLAIFHVLKCVFLIFRDFQFSCHIPGPTVCISHFSRFSVILSFFN
LSTACFSFSMIFSFLAIFHVLQWTFLNFPPFSVFLAIFHVLNCVFLIFRVFQFPRHIPGPTVCIYHFSRFSVI
SSFFKTSSGCFSFFMIFSFLAIFHVLQWTFLNFPPFSVSPYSRSYSVHFSFFTFFSDFVIFQVVKWMFLIFLD
FHFSC
>ENSMUSP00000137451_c.362:TC>T_p.F121del-fs ENSMUST00000179264.2|Gm10717-207|ENSMUSG00000095891|+|chr9:g.3030934:TC>T
MFLIFHDFQFSCHIPCPTVDISFPPFSVFLAIFHVLKCVFLIFRDFQFSCHIPGPTVCISHFSRFSVILSFFN
LSTACFSFSMIFSFLAIFHVLQWTFLNFPPFSVFLAIFHVLKCVFLIFVFFSFLAIFQVLQCAFIIFHVFQ
>rs238860127_ENSMUSP00000137451_c.367:T>A_p.V123D
ENSMUST00000179264.2|Gm10717-207|ENSMUSG00000095891|+|chr9:g.3030939:T>A
MFLIFHDFQFSCHIPCPTVDISFPPFSVFLAIFHVLKCVFLIFRDFQFSCHIPGPTVCISHFSRFSVILSFFN
LSTACFSFSMIFSFLAIFHVLQWTFLNFPPFSVFLAIFHVLKCVFLIFRDFQFPRHIPGPTVCIYHFSRFSVI
SSFFKTSSGCFSFFMIFSFLAIFHVLQWTFLNFPPFSVSPYSRSYSVHFSFFTFFSDFVIFQVVKWMFLIFLD
FHFSC
>ENSMUSP00000096650_c.516:G>C_p.V173L ENSMUST00000099051.11|Gm10717-203|ENSMUSG00000095891|+|chr9:g.3030947:G>C
CHIPCPTVEISFPPFSVFLAIFHVLKCVFLIFRVFQVSRHIPGPTVCISHFSRFSVILSFFKLSSGCFPFSLI
FSFLTIFHVLQWTFLNFPPFSVFLAIFHVLKCVFLIFSDFQFPRHIPGPTVCISHFSRFSVISSFFKTSSGCF
SFFMIFSFLAIFHVLQWTFLNFPPFSLSPYSRSYSVHFSFFTFFSDFVIFQVVKWMFLIFLDFHFSC
>ENSMUSP00000096640_c.546:G>C_p.V183L ENSMUST00000099042.4|Gm10717-202|ENSMUSG00000095891|+|chr9:g.3030947:G>C

FLIFHDFQLSSHIPGPTVDISFRPLSVFLAIFHVPKCVFLIFHDFQFPRHIPGPTVCISHFSRFSVISSFFKT
SSGCFSFFMIFSFLAIFHVLQWTFLNFPPFSVFLAIFHVLKCVFLIFRDFQFSCHIPGPTVCISHFSRFSVIL
SFFNLSTACFSFSMIFSFLAIFHVLQWTFLNFPPFSLSPYSRSYSVHFSFFTFFSDFVIFQVVKWMFLIFLDF
HFSC
>rs259953757_ENSMUSP00000096650_c.516:G>T_p.V173F
ENSMUST00000099051.11|Gm10717-203|ENSMUSG00000095891|+|chr9:g.3030947:G>T
CHIPCPTVEISFPPFSVFLAIFHVLKCVFLIFRVFQVSRHIPGPTVCISHFSRFSVILSFFKLSSGCFPFSLI
FSFLTIFHVLQWTFLNFPPFSVFLAIFHVLKCVFLIFSDFQFPRHIPGPTVCISHFSRFSVISSFFKTSSGCF
SFFMIFSFLAIFHVLQWTFLNFPPFSFSPYSRSYSVHFSFFTFFSDFVIFQVVKWMFLIFLDFHFSC
>rs259953757_ENSMUSP00000096640_c.546:G>T_p.V183F
ENSMUST00000099042.4|Gm10717-202|ENSMUSG00000095891|+|chr9:g.3030947:G>T
FLIFHDFQLSSHIPGPTVDISFRPLSVFLAIFHVPKCVFLIFHDFQFPRHIPGPTVCISHFSRFSVISSFFKT
SSGCFSFFMIFSFLAIFHVLQWTFLNFPPFSVFLAIFHVLKCVFLIFRDFQFSCHIPGPTVCISHFSRFSVIL
SFFNLSTACFSFSMIFSFLAIFHVLQWTFLNFPPFSFSPYSRSYSVHFSFFTFFSDFVIFQVVKWMFLIFLDF
HFSC
>ENSMUSP00000096650_c.527:T>A_p.Y176* ENSMUST00000099051.11|Gm10717-
203|ENSMUSG00000095891|+|chr9:g.3030960:T>A
CHIPCPTVEISFPPFSVFLAIFHVLKCVFLIFRVFQVSRHIPGPTVCISHFSRFSVILSFFKLSSGCFPFSLI
FSFLTIFHVLQWTFLNFPPFSVFLAIFHVLKCVFLIFSDFQFPRHIPGPTVCISHFSRFSVISSFFKTSSGCF
SFFMIFSFLAIFHVLQWTFLNFPPFSVSP*SRSYSVHFSFFTFFSDFVIFQVVKWMFLIFLDFHFSC
>ENSMUSP00000096640_c.557:T>A_p.Y186* ENSMUST00000099042.4|Gm10717-
202|ENSMUSG00000095891|+|chr9:g.3030960:T>A
FLIFHDFQLSSHIPGPTVDISFRPLSVFLAIFHVPKCVFLIFHDFQFPRHIPGPTVCISHFSRFSVISSFFKT
SSGCFSFFMIFSFLAIFHVLQWTFLNFPPFSVFLAIFHVLKCVFLIFRDFQFSCHIPGPTVCISHFSRFSVIL
SFFNLSTACFSFSMIFSFLAIFHVLQWTFLNFPPFSVSP*SRSYSVHFSFFTFFSDFVIFQVVKWMFLIFLDF
HFSC
>ENSMUSP00000096650_c.531:A>T_p.R178W ENSMUST00000099051.11|Gm10717-
203|ENSMUSG00000095891|+|chr9:g.3030964:A>T
CHIPCPTVEISFPPFSVFLAIFHVLKCVFLIFRVFQVSRHIPGPTVCISHFSRFSVILSFFKLSSGCFPFSLI
FSFLTIFHVLQWTFLNFPPFSVFLAIFHVLKCVFLIFSDFQFPRHIPGPTVCISHFSRFSVISSFFKTSSGCF
SFFMIFSFLAIFHVLQWTFLNFPPFSVSPYSWSYSVHFSFFTFFSDFVIFQVVKWMFLIFLDFHFSC
>ENSMUSP00000096640_c.561:A>T_p.R188W ENSMUST00000099042.4|Gm10717-
202|ENSMUSG00000095891|+|chr9:g.3030964:A>T
FLIFHDFQLSSHIPGPTVDISFRPLSVFLAIFHVPKCVFLIFHDFQFPRHIPGPTVCISHFSRFSVISSFFKT
SSGCFSFFMIFSFLAIFHVLQWTFLNFPPFSVFLAIFHVLKCVFLIFRDFQFSCHIPGPTVCISHFSRFSVIL
SFFNLSTACFSFSMIFSFLAIFHVLQWTFLNFPPFSVSPYSWSYSVHFSFFTFFSDFVIFQVVKWMFLIFLDF
HFSC
>ENSMUSP00000096650_c.559:T>C_p.F187S ENSMUST00000099051.11|Gm10717-
203|ENSMUSG00000095891|+|chr9:g.3030992:T>C
CHIPCPTVEISFPPFSVFLAIFHVLKCVFLIFRVFQVSRHIPGPTVCISHFSRFSVILSFFKLSSGCFPFSLI
FSFLTIFHVLQWTFLNFPPFSVFLAIFHVLKCVFLIFSDFQFPRHIPGPTVCISHFSRFSVISSFFKTSSGCF
SFFMIFSFLAIFHVLQWTFLNFPPFSVSPYSRSYSVHFSFSTFFSDFVIFQVVKWMFLIFLDFHFSC
>ENSMUSP00000096640_c.589:T>C_p.F197S ENSMUST00000099042.4|Gm10717-
202|ENSMUSG00000095891|+|chr9:g.3030992:T>C
FLIFHDFQLSSHIPGPTVDISFRPLSVFLAIFHVPKCVFLIFHDFQFPRHIPGPTVCISHFSRFSVISSFFKT
SSGCFSFFMIFSFLAIFHVLQWTFLNFPPFSVFLAIFHVLKCVFLIFRDFQFSCHIPGPTVCISHFSRFSVIL
SFFNLSTACFSFSMIFSFLAIFHVLQWTFLNFPPFSVSPYSRSYSVHFSFSTFFSDFVIFQVVKWMFLIFLDF
HFSC
>ENSMUSP00000096650_c.629:C>G_p.H210Q ENSMUST00000099051.11|Gm10717-
203|ENSMUSG00000095891|+|chr9:g.3031062:C>G
CHIPCPTVEISFPPFSVFLAIFHVLKCVFLIFRVFQVSRHIPGPTVCISHFSRFSVILSFFKLSSGCFPFSLI
FSFLTIFHVLQWTFLNFPPFSVFLAIFHVLKCVFLIFSDFQFPRHIPGPTVCISHFSRFSVISSFFKTSSGCF
SFFMIFSFLAIFHVLQWTFLNFPPFSVSPYSRSYSVHFSFFTFFSDFVIFQVVKWMFLIFLDFQFSC
>ENSMUSP00000096640_c.659:C>G_p.H220Q ENSMUST00000099042.4|Gm10717-
202|ENSMUSG00000095891|+|chr9:g.3031062:C>G
FLIFHDFQLSSHIPGPTVDISFRPLSVFLAIFHVPKCVFLIFHDFQFPRHIPGPTVCISHFSRFSVISSFFKT
SSGCFSFFMIFSFLAIFHVLQWTFLNFPPFSVFLAIFHVLKCVFLIFRDFQFSCHIPGPTVCISHFSRFSVIL

SFFNLSTACFSFSMIFSFLAIFHVLQWTFLNFPPFSVSPYSRSYSVHFSFFTFFSDFVIFQVVKWMFLIFLDF
QFSC
>ENSMUSP00000137451_c.378:C>T_p.P127S ENSMUST00000179264.2|Gm10717-207|ENSMUSG00000095891|+|chr9:g.3031999:C>T
MFLIFHDFQFSCHIPCPTVDISFPPFSVFLAIFHVLKCVFLIFRDFQFSCHIPGPTVCISHFSRFSVILSFFN
LSTACFSFSMIFSFLAIFHVLQWTFLNFPPFSVFLAIFHVLKCVFLIFRVFQFSRHIPGPTVCIYHFSRFSVI
SSFFKTSSGCFSFFMIFSFLAIFHVLQWTFLNFPPFSVSPYSRSYSVHFSFFTFFSDFVIFQVVKWMFLIFLD
FHFSC
>ENSMUSP00000137451_c.389:T>G_p.I130M ENSMUST00000179264.2|Gm10717-207|ENSMUSG00000095891|+|chr9:g.3032010:T>G
MFLIFHDFQFSCHIPCPTVDISFPPFSVFLAIFHVLKCVFLIFRDFQFSCHIPGPTVCISHFSRFSVILSFFN
LSTACFSFSMIFSFLAIFHVLQWTFLNFPPFSVFLAIFHVLKCVFLIFRVFQFPRHMPGPTVCIYHFSRFSVI
SSFFKTSSGCFSFFMIFSFLAIFHVLQWTFLNFPPFSVSPYSRSYSVHFSFFTFFSDFVIFQVVKWMFLIFLD
FHFSC
>ENSMUSP00000137451_c.399:A>T_p.T134S ENSMUST00000179264.2|Gm10717-207|ENSMUSG00000095891|+|chr9:g.3032020:A>T
MFLIFHDFQFSCHIPCPTVDISFPPFSVFLAIFHVLKCVFLIFRDFQFSCHIPGPTVCISHFSRFSVILSFFN
LSTACFSFSMIFSFLAIFHVLQWTFLNFPPFSVFLAIFHVLKCVFLIFRVFQFPRHIPGPSVCIYHFSRFSVI
SSFFKTSSGCFSFFMIFSFLAIFHVLQWTFLNFPPFSVSPYSRSYSVHFSFFTFFSDFVIFQVVKWMFLIFLD
FHFSC
>ENSMUSP00000137451_c.406:G>A_p.C136Y ENSMUST00000179264.2|Gm10717-207|ENSMUSG00000095891|+|chr9:g.3032027:G>A
MFLIFHDFQFSCHIPCPTVDISFPPFSVFLAIFHVLKCVFLIFRDFQFSCHIPGPTVCISHFSRFSVILSFFN
LSTACFSFSMIFSFLAIFHVLQWTFLNFPPFSVFLAIFHVLKCVFLIFRVFQFPRHIPGPTVYIYHFSRFSVI
SSFFKTSSGCFSFFMIFSFLAIFHVLQWTFLNFPPFSVSPYSRSYSVHFSFFTFFSDFVIFQVVKWMFLIFLD
FHFSC
>ENSMUSP00000137451_c.423:C>T_p.R142C ENSMUST00000179264.2|Gm10717-207|ENSMUSG00000095891|+|chr9:g.3032044:C>T
MFLIFHDFQFSCHIPCPTVDISFPPFSVFLAIFHVLKCVFLIFRDFQFSCHIPGPTVCISHFSRFSVILSFFN
LSTACFSFSMIFSFLAIFHVLQWTFLNFPPFSVFLAIFHVLKCVFLIFRVFQFPRHIPGPTVCIYHFSCFSVI
SSFFKTSSGCFSFFMIFSFLAIFHVLQWTFLNFPPFSVSPYSRSYSVHFSFFTFFSDFVIFQVVKWMFLIFLD
FHFSC
>ENSMUSP00000137451_c.430:C>T_p.S144L ENSMUST00000179264.2|Gm10717-207|ENSMUSG00000095891|+|chr9:g.3032051:C>T
MFLIFHDFQFSCHIPCPTVDISFPPFSVFLAIFHVLKCVFLIFRDFQFSCHIPGPTVCISHFSRFSVILSFFN
LSTACFSFSMIFSFLAIFHVLQWTFLNFPPFSVFLAIFHVLKCVFLIFRVFQFPRHIPGPTVCIYHFSRFLVI
SSFFKTSSGCFSFFMIFSFLAIFHVLQWTFLNFPPFSVSPYSRSYSVHFSFFTFFSDFVIFQVVKWMFLIFLD
FHFSC
>rs246324318_ENSMUSP00000137451_c.453:A>T_p.T152S
ENSMUST00000179264.2|Gm10717-207|ENSMUSG00000095891|+|chr9:g.3032074:A>T
MFLIFHDFQFSCHIPCPTVDISFPPFSVFLAIFHVLKCVFLIFRDFQFSCHIPGPTVCISHFSRFSVILSFFN
LSTACFSFSMIFSFLAIFHVLQWTFLNFPPFSVFLAIFHVLKCVFLIFRVFQFPRHIPGPTVCIYHFSRFSVI
SSFFKSSSGCFSFFMIFSFLAIFHVLQWTFLNFPPFSVSPYSRSYSVHFSFFTFFSDFVIFQVVKWMFLIFLD
FHFSC
>ENSMUSP00000137451_c.478:T>C_p.F160S ENSMUST00000179264.2|Gm10717-207|ENSMUSG00000095891|+|chr9:g.3032099:T>C
MFLIFHDFQFSCHIPCPTVDISFPPFSVFLAIFHVLKCVFLIFRDFQFSCHIPGPTVCISHFSRFSVILSFFN
LSTACFSFSMIFSFLAIFHVLQWTFLNFPPFSVFLAIFHVLKCVFLIFRVFQFPRHIPGPTVCIYHFSRFSVI
SSFFKTSSGCFSFSMIFSFLAIFHVLQWTFLNFPPFSVSPYSRSYSVHFSFFTFFSDFVIFQVVKWMFLIFLD
FHFSC
>ENSMUSP00000136837_c.45:T>C_p.C16R ENSMUST00000179839.8|Gm10717-208|ENSMUSG00000095891|+|chr9:g.3032130:T>C
MFLIFHDFQFSCHIPRPTVDISFPPFSVFLAIFHVLKCVFLIFRDFQFSCHIPGPTVCISHFSRFSVILSFFK
LSSGCFSFSLIFTFLAIFHVLQWTFLYFPPFSVFLAIFHVLKCVFLIFRDFQVSRHIPGPTVCISHFSRFSVI
LSFFKLSSGCFSFSLIFTFLAIFHVLQWTFLNFPPFSVLYFSPYSKSYSVHFSFFTFFSDFVIFQVVKWMFLI
FHDFQLSC
>ENSMUSP00000096655_c.45:T>C_p.C16R ENSMUST00000099056.4|Gm10717-204|ENSMUSG00000095891|+|chr9:g.3032130:T>C

MFLIFHDFQFSCHIPRPTVDISFPPFSVFLAIFHVLKCVFLIFRDFQFSCHIPGPTVCISHFSRFSVILSFFK
LSSGCFSFSLIFTFLAIFHVLQWTFLYFPPFSVFLAIFHVLKCVFLIFRDFQVSRHIPGPTVCISHFSRFSVI
LSFFKLSSGCFSFSLIFTFLAIFHVLQWTFLNFPPFFLPYSRSYSVHFSFFTFFSDFVIFQVVNCMFLIFYDF
QFSC
>ENSMUSP00000137451_c.541:C>T_p.P181L ENSMUST00000179264.2|Gm10717-207|ENSMUSG00000095891|+|chr9:g.3032162:C>T
MFLIFHDFQFSCHIPCPTVDISFPPFSVFLAIFHVLKCVFLIFRDFQFSCHIPGPTVCISHFSRFSVILSFFN
LSTACFSFSMIFSFLAIFHVLQWTFLNFPPFSVFLAIFHVLKCVFLIFRVFQFPRHIPGPTVCIYHFSRFSVI
SSFFKTSSGCFSFFMIFSFLAIFHVLQWTFLNFPLFSVSPYSRSYSVHFSFFTFFSDFVIFQVVKWMFLIFLD
FHFSC
>ENSMUSP00000136837_c.73:C>T_p.P25L ENSMUST00000179839.8|Gm10717-208|ENSMUSG00000095891|+|chr9:g.3032162:C>T
MFLIFHDFQFSCHIPCPTVDISFPLFSVFLAIFHVLKCVFLIFRDFQFSCHIPGPTVCISHFSRFSVILSFFK
LSSGCFSFSLIFTFLAIFHVLQWTFLYFPPFSVFLAIFHVLKCVFLIFRDFQVSRHIPGPTVCISHFSRFSVI
LSFFKLSSGCFSFSLIFTFLAIFHVLQWTFLNFPPFSVLYFSPYSKSYSVHFSFFTFFSDFVIFQVVKWMFLI
FHDFQLSC
>ENSMUSP00000096655_c.73:C>T_p.P25L ENSMUST00000099056.4|Gm10717-204|ENSMUSG00000095891|+|chr9:g.3032162:C>T
MFLIFHDFQFSCHIPCPTVDISFPLFSVFLAIFHVLKCVFLIFRDFQFSCHIPGPTVCISHFSRFSVILSFFK
LSSGCFSFSLIFTFLAIFHVLQWTFLYFPPFSVFLAIFHVLKCVFLIFRDFQVSRHIPGPTVCISHFSRFSVI
LSFFKLSSGCFSFSLIFTFLAIFHVLQWTFLNFPPFFLPYSRSYSVHFSFFTFFSDFVIFQVVNCMFLIFYDF
QFSC
>ENSMUSP00000136837_c.130:G>A_p.R44H ENSMUST00000179839.8|Gm10717-208|ENSMUSG00000095891|+|chr9:g.3032219:G>A
MFLIFHDFQFSCHIPCPTVDISFPPFSVFLAIFHVLKCVFLIFHDFQFSCHIPGPTVCISHFSRFSVILSFFK
LSSGCFSFSLIFTFLAIFHVLQWTFLYFPPFSVFLAIFHVLKCVFLIFRDFQVSRHIPGPTVCISHFSRFSVI
LSFFKLSSGCFSFSLIFTFLAIFHVLQWTFLNFPPFSVLYFSPYSKSYSVHFSFFTFFSDFVIFQVVKWMFLI
FHDFQLSC
>ENSMUSP00000096655_c.130:G>A_p.R44H ENSMUST00000099056.4|Gm10717-204|ENSMUSG00000095891|+|chr9:g.3032219:G>A
MFLIFHDFQFSCHIPCPTVDISFPPFSVFLAIFHVLKCVFLIFHDFQFSCHIPGPTVCISHFSRFSVILSFFK
LSSGCFSFSLIFTFLAIFHVLQWTFLYFPPFSVFLAIFHVLKCVFLIFRDFQVSRHIPGPTVCISHFSRFSVI
LSFFKLSSGCFSFSLIFTFLAIFHVLQWTFLNFPPFFLPYSRSYSVHFSFFTFFSDFVIFQVVNCMFLIFYDF
QFSC
>ENSMUSP00000136837_c.147:T>C_p.C50R ENSMUST00000179839.8|Gm10717-208|ENSMUSG00000095891|+|chr9:g.3032236:T>C
MFLIFHDFQFSCHIPCPTVDISFPPFSVFLAIFHVLKCVFLIFRDFQFSRHIPGPTVCISHFSRFSVILSFFK
LSSGCFSFSLIFTFLAIFHVLQWTFLYFPPFSVFLAIFHVLKCVFLIFRDFQVSRHIPGPTVCISHFSRFSVI
LSFFKLSSGCFSFSLIFTFLAIFHVLQWTFLNFPPFSVLYFSPYSKSYSVHFSFFTFFSDFVIFQVVKWMFLI
FHDFQLSC
>ENSMUSP00000096655_c.147:T>C_p.C50R ENSMUST00000099056.4|Gm10717-204|ENSMUSG00000095891|+|chr9:g.3032236:T>C
MFLIFHDFQFSCHIPCPTVDISFPPFSVFLAIFHVLKCVFLIFRDFQFSRHIPGPTVCISHFSRFSVILSFFK
LSSGCFSFSLIFTFLAIFHVLQWTFLYFPPFSVFLAIFHVLKCVFLIFRDFQVSRHIPGPTVCISHFSRFSVI
LSFFKLSSGCFSFSLIFTFLAIFHVLQWTFLNFPPFFLPYSRSYSVHFSFFTFFSDFVIFQVVNCMFLIFYDF
QFSC
>ENSMUSP00000136837_c.165:A>T_p.T56S ENSMUST00000179839.8|Gm10717-208|ENSMUSG00000095891|+|chr9:g.3032254:A>T
MFLIFHDFQFSCHIPCPTVDISFPPFSVFLAIFHVLKCVFLIFRDFQFSCHIPGPSVCISHFSRFSVILSFFK
LSSGCFSFSLIFTFLAIFHVLQWTFLYFPPFSVFLAIFHVLKCVFLIFRDFQVSRHIPGPTVCISHFSRFSVI
LSFFKLSSGCFSFSLIFTFLAIFHVLQWTFLNFPPFSVLYFSPYSKSYSVHFSFFTFFSDFVIFQVVKWMFLI
FHDFQLSC
>ENSMUSP00000096655_c.165:A>T_p.T56S ENSMUST00000099056.4|Gm10717-204|ENSMUSG00000095891|+|chr9:g.3032254:A>T
MFLIFHDFQFSCHIPCPTVDISFPPFSVFLAIFHVLKCVFLIFRDFQFSCHIPGPSVCISHFSRFSVILSFFK
LSSGCFSFSLIFTFLAIFHVLQWTFLYFPPFSVFLAIFHVLKCVFLIFRDFQVSRHIPGPTVCISHFSRFSVI
LSFFKLSSGCFSFSLIFTFLAIFHVLQWTFLNFPPFFLPYSRSYSVHFSFFTFFSDFVIFQVVNCMFLIFYDF
QFSC

>ENSMUSP00000136837_c.178:C>T_p.S60F ENSMUST00000179839.8|Gm10717-208|ENSMUSG00000095891|+|chr9:g.3032267:C>T
MFLIFHDFQFSCHIPCPTVDISFPPFSVFLAIFHVLKCVFLIFRDFQFSCHIPGPTVCIFHFSRFSVILSFFK
LSSGCFSFSLIFTFLAIFHVLQWTFLYFPPFSVFLAIFHVLKCVFLIFRDFQVSRHIPGPTVCISHFSRFSVI
LSFFKLSSGCFSFSLIFTFLAIFHVLQWTFLNFPPFSVLYFSPYSKSYSVHFSFFTFFSDFVIFQVVKWMFLI
FHDFQLSC
>ENSMUSP00000096655_c.178:C>T_p.S60F ENSMUST00000099056.4|Gm10717-204|ENSMUSG00000095891|+|chr9:g.3032267:C>T
MFLIFHDFQFSCHIPCPTVDISFPPFSVFLAIFHVLKCVFLIFRDFQFSCHIPGPTVCIFHFSRFSVILSFFK
LSSGCFSFSLIFTFLAIFHVLQWTFLYFPPFSVFLAIFHVLKCVFLIFRDFQVSRHIPGPTVCISHFSRFSVI
LSFFKLSSGCFSFSLIFTFLAIFHVLQWTFLNFPPFFLPYSRSYSVHFSFFTFFSDFVIFQVVNCMFLIFYDF
QFSC
>ENSMUSP00000136837_c.186:T>C_p.S63P ENSMUST00000179839.8|Gm10717-208|ENSMUSG00000095891|+|chr9:g.3032275:T>C
MFLIFHDFQFSCHIPCPTVDISFPPFSVFLAIFHVLKCVFLIFRDFQFSCHIPGPTVCISHFPRFSVILSFFK
LSSGCFSFSLIFTFLAIFHVLQWTFLYFPPFSVFLAIFHVLKCVFLIFRDFQVSRHIPGPTVCISHFSRFSVI
LSFFKLSSGCFSFSLIFTFLAIFHVLQWTFLNFPPFSVLYFSPYSKSYSVHFSFFTFFSDFVIFQVVKWMFLI
FHDFQLSC
>ENSMUSP00000096655_c.186:T>C_p.S63P ENSMUST00000099056.4|Gm10717-204|ENSMUSG00000095891|+|chr9:g.3032275:T>C
MFLIFHDFQFSCHIPCPTVDISFPPFSVFLAIFHVLKCVFLIFRDFQFSCHIPGPTVCISHFPRFSVILSFFK
LSSGCFSFSLIFTFLAIFHVLQWTFLYFPPFSVFLAIFHVLKCVFLIFRDFQVSRHIPGPTVCISHFSRFSVI
LSFFKLSSGCFSFSLIFTFLAIFHVLQWTFLNFPPFFLPYSRSYSVHFSFFTFFSDFVIFQVVNCMFLIFYDF
QFSC
>ENSMUSP00000136837_c.205:T>C_p.L69S ENSMUST00000179839.8|Gm10717-208|ENSMUSG00000095891|+|chr9:g.3032294:T>C
MFLIFHDFQFSCHIPCPTVDISFPPFSVFLAIFHVLKCVFLIFRDFQFSCHIPGPTVCISHFSRFSVISSFFK
LSSGCFSFSLIFTFLAIFHVLQWTFLYFPPFSVFLAIFHVLKCVFLIFRDFQVSRHIPGPTVCISHFSRFSVI
LSFFKLSSGCFSFSLIFTFLAIFHVLQWTFLNFPPFSVLYFSPYSKSYSVHFSFFTFFSDFVIFQVVKWMFLI
FHDFQLSC
>ENSMUSP00000096655_c.205:T>C_p.L69S ENSMUST00000099056.4|Gm10717-204|ENSMUSG00000095891|+|chr9:g.3032294:T>C
MFLIFHDFQFSCHIPCPTVDISFPPFSVFLAIFHVLKCVFLIFRDFQFSCHIPGPTVCISHFSRFSVISSFFK
LSSGCFSFSLIFTFLAIFHVLQWTFLYFPPFSVFLAIFHVLKCVFLIFRDFQVSRHIPGPTVCISHFSRFSVI
LSFFKLSSGCFSFSLIFTFLAIFHVLQWTFLNFPPFFLPYSRSYSVHFSFFTFFSDFVIFQVVNCMFLIFYDF
QFSC
>ENSMUSP00000136837_c.220:T>C_p.L74S ENSMUST00000179839.8|Gm10717-208|ENSMUSG00000095891|+|chr9:g.3032309:T>C
MFLIFHDFQFSCHIPCPTVDISFPPFSVFLAIFHVLKCVFLIFRDFQFSCHIPGPTVCISHFSRFSVILSFFK
SSSGCFSFSLIFTFLAIFHVLQWTFLYFPPFSVFLAIFHVLKCVFLIFRDFQVSRHIPGPTVCISHFSRFSVI
LSFFKLSSGCFSFSLIFTFLAIFHVLQWTFLNFPPFSVLYFSPYSKSYSVHFSFFTFFSDFVIFQVVKWMFLI
FHDFQLSC
>ENSMUSP00000096655_c.220:T>C_p.L74S ENSMUST00000099056.4|Gm10717-204|ENSMUSG00000095891|+|chr9:g.3032309:T>C
MFLIFHDFQFSCHIPCPTVDISFPPFSVFLAIFHVLKCVFLIFRDFQFSCHIPGPTVCISHFSRFSVILSFFK
SSSGCFSFSLIFTFLAIFHVLQWTFLYFPPFSVFLAIFHVLKCVFLIFRDFQVSRHIPGPTVCISHFSRFSVI
LSFFKLSSGCFSFSLIFTFLAIFHVLQWTFLNFPPFFLPYSRSYSVHFSFFTFFSDFVIFQVVNCMFLIFYDF
QFSC
>ENSMUSP00000136837_c.220:T>G_p.L74W ENSMUST00000179839.8|Gm10717-208|ENSMUSG00000095891|+|chr9:g.3032309:T>G
MFLIFHDFQFSCHIPCPTVDISFPPFSVFLAIFHVLKCVFLIFRDFQFSCHIPGPTVCISHFSRFSVILSFFK
WSSGCFSFSLIFTFLAIFHVLQWTFLYFPPFSVFLAIFHVLKCVFLIFRDFQVSRHIPGPTVCISHFSRFSVI
LSFFKLSSGCFSFSLIFTFLAIFHVLQWTFLNFPPFSVLYFSPYSKSYSVHFSFFTFFSDFVIFQVVKWMFLI
FHDFQLSC
>ENSMUSP00000096655_c.220:T>G_p.L74W ENSMUST00000099056.4|Gm10717-204|ENSMUSG00000095891|+|chr9:g.3032309:T>G
MFLIFHDFQFSCHIPCPTVDISFPPFSVFLAIFHVLKCVFLIFRDFQFSCHIPGPTVCISHFSRFSVILSFFK
WSSGCFSFSLIFTFLAIFHVLQWTFLYFPPFSVFLAIFHVLKCVFLIFRDFQVSRHIPGPTVCISHFSRFSVI

LSFFKLSSGCFSFSLIFTFLAIFHVLQWTFLNFPPFFLPYSRSYSVHFSFFTFFSDFVIFQVVNCMFLIFYDF
QFSC
>ENSMUSP00000136837_c.246:T>A_p.L83M ENSMUST00000179839.8|Gm10717-
208|ENSMUSG00000095891|+|chr9:g.3032335:T>A
MFLIFHDFQFSCHIPCPTVDISFPPFSVFLAIFHVLKCVFLIFRDFQFSCHIPGPTVCISHFSRFSVILSFFK
LSSGCFSFSMIFTFLAIFHVLQWTFLYFPPFSVFLAIFHVLKCVFLIFRDFQVSRHIPGPTVCISHFSRFSVI
LSFFKLSSGCFSFSLIFTFLAIFHVLQWTFLNFPPFSVLYFSPYSKSYSVHFSFFTFFSDFVIFQVVKWMFLI
FHDFQLSC
>ENSMUSP00000096655_c.246:T>A_p.L83M ENSMUST00000099056.4|Gm10717-
204|ENSMUSG00000095891|+|chr9:g.3032335:T>A
MFLIFHDFQFSCHIPCPTVDISFPPFSVFLAIFHVLKCVFLIFRDFQFSCHIPGPTVCISHFSRFSVILSFFK
LSSGCFSFSMIFTFLAIFHVLQWTFLYFPPFSVFLAIFHVLKCVFLIFRDFQVSRHIPGPTVCISHFSRFSVI
LSFFKLSSGCFSFSLIFTFLAIFHVLQWTFLNFPPFFLPYSRSYSVHFSFFTFFSDFVIFQVVNCMFLIFYDF
QFSC
>ENSMUSP00000136837_c.256:C>G_p.T86S ENSMUST00000179839.8|Gm10717-
208|ENSMUSG00000095891|+|chr9:g.3032345:C>G
MFLIFHDFQFSCHIPCPTVDISFPPFSVFLAIFHVLKCVFLIFRDFQFSCHIPGPTVCISHFSRFSVILSFFK
LSSGCFSFSLIFSFLAIFHVLQWTFLYFPPFSVFLAIFHVLKCVFLIFRDFQVSRHIPGPTVCISHFSRFSVI
LSFFKLSSGCFSFSLIFTFLAIFHVLQWTFLNFPPFSVLYFSPYSKSYSVHFSFFTFFSDFVIFQVVKWMFLI
FHDFQLSC
>ENSMUSP00000096655_c.256:C>G_p.T86S ENSMUST00000099056.4|Gm10717-
204|ENSMUSG00000095891|+|chr9:g.3032345:C>G
MFLIFHDFQFSCHIPCPTVDISFPPFSVFLAIFHVLKCVFLIFRDFQFSCHIPGPTVCISHFSRFSVILSFFK
LSSGCFSFSLIFSFLAIFHVLQWTFLYFPPFSVFLAIFHVLKCVFLIFRDFQVSRHIPGPTVCISHFSRFSVI
LSFFKLSSGCFSFSLIFTFLAIFHVLQWTFLNFPPFFLPYSRSYSVHFSFFTFFSDFVIFQVVNCMFLIFYDF
QFSC
>ENSMUSP00000136837_c.256:CT>GA_p.T86R ENSMUST00000179839.8|Gm10717-
208|ENSMUSG00000095891|+|chr9:g.3032345:CT>GA
MFLIFHDFQFSCHIPCPTVDISFPPFSVFLAIFHVLKCVFLIFRDFQFSCHIPGPTVCISHFSRFSVILSFFK
LSSGCFSFSLIFRFSCHIPCPTVDISIFSTFFRFPCHISRPKVCISHFP*FSGFSPYSRSYSVHFSFFTFFSD
FVIFQVVKWMFLIFLDFHFSCHIPCPTVDISKFSTFFSPIFLAIFQVLQCAFLIFHVFQ*FRHFSSCQVDVSH
FP*FSAFL
>ENSMUSP00000096655_c.256:CT>GA_p.T86R ENSMUST00000099056.4|Gm10717-
204|ENSMUSG00000095891|+|chr9:g.3032345:CT>GA
MFLIFHDFQFSCHIPCPTVDISFPPFSVFLAIFHVLKCVFLIFRDFQFSCHIPGPTVCISHFSRFSVILSFFK
LSSGCFSFSLIFRFSCHIPCPTVDISIFSTFFRFPCHISRPKVCISHFP*FSGFSPYSRSYSVHFSFFTFFSD
FVIFQVVKWMFLIFLDFHFSCHIPCPTVDISKFSTFFLAIFQVLQCAFLIFHVFQ*FCHFSSCQLHVSHFL*F
SVFL
>ENSMUSP00000136837_c.265:C>T_p.A89V ENSMUST00000179839.8|Gm10717-
208|ENSMUSG00000095891|+|chr9:g.3032354:C>T
MFLIFHDFQFSCHIPCPTVDISFPPFSVFLAIFHVLKCVFLIFRDFQFSCHIPGPTVCISHFSRFSVILSFFK
LSSGCFSFSLIFTFLVIFHVLQWTFLYFPPFSVFLAIFHVLKCVFLIFRDFQVSRHIPGPTVCISHFSRFSVI
LSFFKLSSGCFSFSLIFTFLAIFHVLQWTFLNFPPFSVLYFSPYSKSYSVHFSFFTFFSDFVIFQVVKWMFLI
FHDFQLSC
>ENSMUSP00000096655_c.265:C>T_p.A89V ENSMUST00000099056.4|Gm10717-
204|ENSMUSG00000095891|+|chr9:g.3032354:C>T
MFLIFHDFQFSCHIPCPTVDISFPPFSVFLAIFHVLKCVFLIFRDFQFSCHIPGPTVCISHFSRFSVILSFFK
LSSGCFSFSLIFTFLVIFHVLQWTFLYFPPFSVFLAIFHVLKCVFLIFRDFQVSRHIPGPTVCISHFSRFSVI
LSFFKLSSGCFSFSLIFTFLAIFHVLQWTFLNFPPFFLPYSRSYSVHFSFFTFFSDFVIFQVVNCMFLIFYDF
QFSC
>ENSMUSP00000136837_c.297:T>A_p.Y100N ENSMUST00000179839.8|Gm10717-
208|ENSMUSG00000095891|+|chr9:g.3032386:T>A
MFLIFHDFQFSCHIPCPTVDISFPPFSVFLAIFHVLKCVFLIFRDFQFSCHIPGPTVCISHFSRFSVILSFFK
LSSGCFSFSLIFTFLAIFHVLQWTFLNFPPFSVFLAIFHVLKCVFLIFRDFQVSRHIPGPTVCISHFSRFSVI
LSFFKLSSGCFSFSLIFTFLAIFHVLQWTFLNFPPFSVLYFSPYSKSYSVHFSFFTFFSDFVIFQVVKWMFLI
FHDFQLSC
>ENSMUSP00000096655_c.297:T>A_p.Y100N ENSMUST00000099056.4|Gm10717-
204|ENSMUSG00000095891|+|chr9:g.3032386:T>A

MFLIFHDFQFSCHIPCPTVDISFPPFSVFLAIFHVLKCVFLIFRDFQFSCHIPGPTVCISHFSRFSVILSFFK
LSSGCFSFSLIFTFLAIFHVLQWTFLNFPPFSVFLAIFHVLKCVFLIFRDFQVSRHIPGPTVCISHFSRFSVI
LSFFKLSSGCFSFSLIFTFLAIFHVLQWTFLNFPPFFLPYSRSYSVHFSFFTFFSDFVIFQVVNCMFLIFYDF
QFSC
>ENSMUSP00000136837_c.325:C>T_p.A109V ENSMUST00000179839.8|Gm10717-208|ENSMUSG00000095891|+|chr9:g.3032414:C>T
MFLIFHDFQFSCHIPCPTVDISFPPFSVFLAIFHVLKCVFLIFRDFQFSCHIPGPTVCISHFSRFSVILSFFK
LSSGCFSFSLIFTFLAIFHVLQWTFLYFPPFSVFLVIFHVLKCVFLIFRDFQVSRHIPGPTVCISHFSRFSVI
LSFFKLSSGCFSFSLIFTFLAIFHVLQWTFLNFPPFSVLYFSPYSKSYSVHFSFFTFFSDFVIFQVVKWMFLI
FHDFQLSC
>ENSMUSP00000096655_c.325:C>T_p.A109V ENSMUST00000099056.4|Gm10717-204|ENSMUSG00000095891|+|chr9:g.3032414:C>T
MFLIFHDFQFSCHIPCPTVDISFPPFSVFLAIFHVLKCVFLIFRDFQFSCHIPGPTVCISHFSRFSVILSFFK
LSSGCFSFSLIFTFLAIFHVLQWTFLYFPPFSVFLVIFHVLKCVFLIFRDFQVSRHIPGPTVCISHFSRFSVI
LSFFKLSSGCFSFSLIFTFLAIFHVLQWTFLNFPPFFLPYSRSYSVHFSFFTFFSDFVIFQVVNCMFLIFYDF
QFSC
>ENSMUSP00000136837_c.342:A>T_p.K115* ENSMUST00000179839.8|Gm10717-208|ENSMUSG00000095891|+|chr9:g.3032431:A>T
MFLIFHDFQFSCHIPCPTVDISFPPFSVFLAIFHVLKCVFLIFRDFQFSCHIPGPTVCISHFSRFSVILSFFK
LSSGCFSFSLIFTFLAIFHVLQWTFLYFPPFSVFLAIFHVL*CVFLIFRDFQVSRHIPGPTVCISHFSRFSVI
LSFFKLSSGCFSFSLIFTFLAIFHVLQWTFLNFPPFSVLYFSPYSKSYSVHFSFFTFFSDFVIFQVVKWMFLI
FHDFQLSC
>ENSMUSP00000096655_c.342:A>T_p.K115* ENSMUST00000099056.4|Gm10717-204|ENSMUSG00000095891|+|chr9:g.3032431:A>T
MFLIFHDFQFSCHIPCPTVDISFPPFSVFLAIFHVLKCVFLIFRDFQFSCHIPGPTVCISHFSRFSVILSFFK
LSSGCFSFSLIFTFLAIFHVLQWTFLYFPPFSVFLAIFHVL*CVFLIFRDFQVSRHIPGPTVCISHFSRFSVI
LSFFKLSSGCFSFSLIFTFLAIFHVLQWTFLNFPPFFLPYSRSYSVHFSFFTFFSDFVIFQVVNCMFLIFYDF
QFSC
>ENSMUSP00000136837_c.344:G>T_p.K115N ENSMUST00000179839.8|Gm10717-208|ENSMUSG00000095891|+|chr9:g.3032433:G>T
MFLIFHDFQFSCHIPCPTVDISFPPFSVFLAIFHVLKCVFLIFRDFQFSCHIPGPTVCISHFSRFSVILSFFK
LSSGCFSFSLIFTFLAIFHVLQWTFLYFPPFSVFLAIFHVLNCVFLIFRDFQVSRHIPGPTVCISHFSRFSVI
LSFFKLSSGCFSFSLIFTFLAIFHVLQWTFLNFPPFSVLYFSPYSKSYSVHFSFFTFFSDFVIFQVVKWMFLI
FHDFQLSC
>ENSMUSP00000096655_c.344:G>T_p.K115N ENSMUST00000099056.4|Gm10717-204|ENSMUSG00000095891|+|chr9:g.3032433:G>T
MFLIFHDFQFSCHIPCPTVDISFPPFSVFLAIFHVLKCVFLIFRDFQFSCHIPGPTVCISHFSRFSVILSFFK
LSSGCFSFSLIFTFLAIFHVLQWTFLYFPPFSVFLAIFHVLNCVFLIFRDFQVSRHIPGPTVCISHFSRFSVI
LSFFKLSSGCFSFSLIFTFLAIFHVLQWTFLNFPPFFLPYSRSYSVHFSFFTFFSDFVIFQVVNCMFLIFYDF
QFSC
>ENSMUSP00000136837_c.363:C>T_p.R122C ENSMUST00000179839.8|Gm10717-208|ENSMUSG00000095891|+|chr9:g.3032452:C>T
MFLIFHDFQFSCHIPCPTVDISFPPFSVFLAIFHVLKCVFLIFRDFQFSCHIPGPTVCISHFSRFSVILSFFK
LSSGCFSFSLIFTFLAIFHVLQWTFLYFPPFSVFLAIFHVLKCVFLIFCDFQVSRHIPGPTVCISHFSRFSVI
LSFFKLSSGCFSFSLIFTFLAIFHVLQWTFLNFPPFSVLYFSPYSKSYSVHFSFFTFFSDFVIFQVVKWMFLI
FHDFQLSC
>ENSMUSP00000096655_c.363:C>T_p.R122C ENSMUST00000099056.4|Gm10717-204|ENSMUSG00000095891|+|chr9:g.3032452:C>T
MFLIFHDFQFSCHIPCPTVDISFPPFSVFLAIFHVLKCVFLIFRDFQFSCHIPGPTVCISHFSRFSVILSFFK
LSSGCFSFSLIFTFLAIFHVLQWTFLYFPPFSVFLAIFHVLKCVFLIFCDFQVSRHIPGPTVCISHFSRFSVI
LSFFKLSSGCFSFSLIFTFLAIFHVLQWTFLNFPPFFLPYSRSYSVHFSFFTFFSDFVIFQVVNCMFLIFYDF
QFSC
>ENSMUSP00000137451_c.549:G>T_p.V184F ENSMUST00000179264.2|Gm10717-207|ENSMUSG00000095891|+|chr9:g.3032464:G>T
MFLIFHDFQFSCHIPCPTVDISFPPFSVFLAIFHVLKCVFLIFRDFQFSCHIPGPTVCISHFSRFSVILSFFN
LSTACFSFSMIFSFLAIFHVLQWTFLNFPPFSVFLAIFHVLKCVFLIFRVFQFPRHIPGPTVCIYHFSRFSVI
SSFFKTSSGCFSFFMIFSFLAIFHVLQWTFLNFPPFSFSPYSRSYSVHFSFFTFFSDFVIFQVVKWMFLIFLD
FHFSC

>ENSMUSP00000136837_c.375:G>T_p.V126F ENSMUST00000179839.8|Gm10717-208|ENSMUSG00000095891|+|chr9:g.3032464:G>T
MFLIFHDFQFSCHIPCPTVDISFPPFSVFLAIFHVLKCVFLIFRDFQFSCHIPGPTVCISHFSRFSVILSFFK
LSSGCFSFSLIFTFLAIFHVLQWTFLYFPPFSVFLAIFHVLKCVFLIFRDFQFSRHIPGPTVCISHFSRFSVI
LSFFKLSSGCFSFSLIFTFLAIFHVLQWTFLNFPPFSVLYFSPYSKSYSVHFSFFTFFSDFVIFQVVKWMFLI
FHDFQLSC
>ENSMUSP00000096655_c.375:G>T_p.V126F ENSMUST00000099056.4|Gm10717-204|ENSMUSG00000095891|+|chr9:g.3032464:G>T
MFLIFHDFQFSCHIPCPTVDISFPPFSVFLAIFHVLKCVFLIFRDFQFSCHIPGPTVCISHFSRFSVILSFFK
LSSGCFSFSLIFTFLAIFHVLQWTFLYFPPFSVFLAIFHVLKCVFLIFRDFQFSRHIPGPTVCISHFSRFSVI
LSFFKLSSGCFSFSLIFTFLAIFHVLQWTFLNFPPFFLPYSRSYSVHFSFFTFFSDFVIFQVVNCMFLIFYDF
QFSC
>ENSMUSP00000137451_c.571:A>T_p.Y191F ENSMUST00000179264.2|Gm10717-207|ENSMUSG00000095891|+|chr9:g.3032488:A>T
MFLIFHDFQFSCHIPCPTVDISFPPFSVFLAIFHVLKCVFLIFRDFQFSCHIPGPTVCISHFSRFSVILSFFN
LSTACFSFSMIFSFLAIFHVLQWTFLNFPPFSVFLAIFHVLKCVFLIFRVFQFPRHIPGPTVCIYHFSRFSVI
SSFFKTSSGCFSFFMIFSFLAIFHVLQWTFLNFPPFSVSPYSRSFSVHFSFFTFFSDFVIFQVVKWMFLIFLD
FHFSC
>ENSMUSP00000136837_c.399:A>T_p.T134S ENSMUST00000179839.8|Gm10717-208|ENSMUSG00000095891|+|chr9:g.3032488:A>T
MFLIFHDFQFSCHIPCPTVDISFPPFSVFLAIFHVLKCVFLIFRDFQFSCHIPGPTVCISHFSRFSVILSFFK
LSSGCFSFSLIFTFLAIFHVLQWTFLYFPPFSVFLAIFHVLKCVFLIFRDFQVSRHIPGPSVCISHFSRFSVI
LSFFKLSSGCFSFSLIFTFLAIFHVLQWTFLNFPPFSVLYFSPYSKSYSVHFSFFTFFSDFVIFQVVKWMFLI
FHDFQLSC
>ENSMUSP00000096655_c.399:A>T_p.T134S ENSMUST00000099056.4|Gm10717-204|ENSMUSG00000095891|+|chr9:g.3032488:A>T
MFLIFHDFQFSCHIPCPTVDISFPPFSVFLAIFHVLKCVFLIFRDFQFSCHIPGPTVCISHFSRFSVILSFFK
LSSGCFSFSLIFTFLAIFHVLQWTFLYFPPFSVFLAIFHVLKCVFLIFRDFQVSRHIPGPSVCISHFSRFSVI
LSFFKLSSGCFSFSLIFTFLAIFHVLQWTFLNFPPFFLPYSRSYSVHFSFFTFFSDFVIFQVVNCMFLIFYDF
QFSC
>ENSMUSP00000136837_c.400:C>T_p.T134I ENSMUST00000179839.8|Gm10717-208|ENSMUSG00000095891|+|chr9:g.3032489:C>T
MFLIFHDFQFSCHIPCPTVDISFPPFSVFLAIFHVLKCVFLIFRDFQFSCHIPGPTVCISHFSRFSVILSFFK
LSSGCFSFSLIFTFLAIFHVLQWTFLYFPPFSVFLAIFHVLKCVFLIFRDFQVSRHIPGPIVCISHFSRFSVI
LSFFKLSSGCFSFSLIFTFLAIFHVLQWTFLNFPPFSVLYFSPYSKSYSVHFSFFTFFSDFVIFQVVKWMFLI
FHDFQLSC
>ENSMUSP00000096655_c.400:C>T_p.T134I ENSMUST00000099056.4|Gm10717-204|ENSMUSG00000095891|+|chr9:g.3032489:C>T
MFLIFHDFQFSCHIPCPTVDISFPPFSVFLAIFHVLKCVFLIFRDFQFSCHIPGPTVCISHFSRFSVILSFFK
LSSGCFSFSLIFTFLAIFHVLQWTFLYFPPFSVFLAIFHVLKCVFLIFRDFQVSRHIPGPIVCISHFSRFSVI
LSFFKLSSGCFSFSLIFTFLAIFHVLQWTFLNFPPFFLPYSRSYSVHFSFFTFFSDFVIFQVVNCMFLIFYDF
QFSC
>ENSMUSP00000137451_c.577:T>G_p.V193G ENSMUST00000179264.2|Gm10717-207|ENSMUSG00000095891|+|chr9:g.3032494:T>G
MFLIFHDFQFSCHIPCPTVDISFPPFSVFLAIFHVLKCVFLIFRDFQFSCHIPGPTVCISHFSRFSVILSFFN
LSTACFSFSMIFSFLAIFHVLQWTFLNFPPFSVFLAIFHVLKCVFLIFRVFQFPRHIPGPTVCIYHFSRFSVI
SSFFKTSSGCFSFFMIFSFLAIFHVLQWTFLNFPPFSVSPYSRSYSGHFSFFTFFSDFVIFQVVKWMFLIFLD
FHFSC
>ENSMUSP00000136837_c.405:T>G_p.C136G ENSMUST00000179839.8|Gm10717-208|ENSMUSG00000095891|+|chr9:g.3032494:T>G
MFLIFHDFQFSCHIPCPTVDISFPPFSVFLAIFHVLKCVFLIFRDFQFSCHIPGPTVCISHFSRFSVILSFFK
LSSGCFSFSLIFTFLAIFHVLQWTFLYFPPFSVFLAIFHVLKCVFLIFRDFQVSRHIPGPTVGISHFSRFSVI
LSFFKLSSGCFSFSLIFTFLAIFHVLQWTFLNFPPFSVLYFSPYSKSYSVHFSFFTFFSDFVIFQVVKWMFLI
FHDFQLSC
>ENSMUSP00000096655_c.405:T>G_p.C136G ENSMUST00000099056.4|Gm10717-204|ENSMUSG00000095891|+|chr9:g.3032494:T>G
MFLIFHDFQFSCHIPCPTVDISFPPFSVFLAIFHVLKCVFLIFRDFQFSCHIPGPTVCISHFSRFSVILSFFK
LSSGCFSFSLIFTFLAIFHVLQWTFLYFPPFSVFLAIFHVLKCVFLIFRDFQVSRHIPGPTVGISHFSRFSVI

LSFFKLSSGCFSFSLIFTFLAIFHVLQWTFLNFPPFFLPYSRSYSVHFSFFTFFSDFVIFQVVNCMFLIFYDF
QFSC
>ENSMUSP00000137451_c.592:T>C_p.F198S ENSMUST00000179264.2|Gm10717-
207|ENSMUSG00000095891|+|chr9:g.3032509:T>C
MFLIFHDFQFSCHIPCPTVDISFPPFSVFLAIFHVLKCVFLIFRDFQFSCHIPGPTVCISHFSRFSVILSFFN
LSTACFSFSMIFSFLAIFHVLQWTFLNFPPFSVFLAIFHVLKCVFLIFRVFQFPRHIPGPTVCIYHFSRFSVI
SSFFKTSSGCFSFFMIFSFLAIFHVLQWTFLNFPPFSVSPYSRSYSVHFSFSTFFSDFVIFQVVKWMFLIFLD
FHFSC
>ENSMUSP00000136837_c.420:T>C_p.S141P ENSMUST00000179839.8|Gm10717-
208|ENSMUSG00000095891|+|chr9:g.3032509:T>C
MFLIFHDFQFSCHIPCPTVDISFPPFSVFLAIFHVLKCVFLIFRDFQFSCHIPGPTVCISHFSRFSVILSFFK
LSSGCFSFSLIFTFLAIFHVLQWTFLYFPPFSVFLAIFHVLKCVFLIFRDFQVSRHIPGPTVCISHFPRFSVI
LSFFKLSSGCFSFSLIFTFLAIFHVLQWTFLNFPPFSVLYFSPYSKSYSVHFSFFTFFSDFVIFQVVKWMFLI
FHDFQLSC
>ENSMUSP00000096655_c.420:T>C_p.S141P ENSMUST00000099056.4|Gm10717-
204|ENSMUSG00000095891|+|chr9:g.3032509:T>C
MFLIFHDFQFSCHIPCPTVDISFPPFSVFLAIFHVLKCVFLIFRDFQFSCHIPGPTVCISHFSRFSVILSFFK
LSSGCFSFSLIFTFLAIFHVLQWTFLYFPPFSVFLAIFHVLKCVFLIFRDFQVSRHIPGPTVCISHFPRFSVI
LSFFKLSSGCFSFSLIFTFLAIFHVLQWTFLNFPPFFLPYSRSYSVHFSFFTFFSDFVIFQVVNCMFLIFYDF
QFSC
>ENSMUSP00000137451_c.597:T>G_p.F200V ENSMUST00000179264.2|Gm10717-
207|ENSMUSG00000095891|+|chr9:g.3032514:T>G
MFLIFHDFQFSCHIPCPTVDISFPPFSVFLAIFHVLKCVFLIFRDFQFSCHIPGPTVCISHFSRFSVILSFFN
LSTACFSFSMIFSFLAIFHVLQWTFLNFPPFSVFLAIFHVLKCVFLIFRVFQFPRHIPGPTVCIYHFSRFSVI
SSFFKTSSGCFSFFMIFSFLAIFHVLQWTFLNFPPFSVSPYSRSYSVHFSFFTVFSDFVIFQVVKWMFLIFLD
FHFSC
>ENSMUSP00000137451_c.605:T>G_p.S202R ENSMUST00000179264.2|Gm10717-
207|ENSMUSG00000095891|+|chr9:g.3032522:T>G
MFLIFHDFQFSCHIPCPTVDISFPPFSVFLAIFHVLKCVFLIFRDFQFSCHIPGPTVCISHFSRFSVILSFFN
LSTACFSFSMIFSFLAIFHVLQWTFLNFPPFSVFLAIFHVLKCVFLIFRVFQFPRHIPGPTVCIYHFSRFSVI
SSFFKTSSGCFSFFMIFSFLAIFHVLQWTFLNFPPFSVSPYSRSYSVHFSFFTFFRDFVIFQVVKWMFLIFLD
FHFSC
>ENSMUSP00000136837_c.433:T>G_p.V145G ENSMUST00000179839.8|Gm10717-
208|ENSMUSG00000095891|+|chr9:g.3032522:T>G
MFLIFHDFQFSCHIPCPTVDISFPPFSVFLAIFHVLKCVFLIFRDFQFSCHIPGPTVCISHFSRFSVILSFFK
LSSGCFSFSLIFTFLAIFHVLQWTFLYFPPFSVFLAIFHVLKCVFLIFRDFQVSRHIPGPTVCISHFSRFSGI
LSFFKLSSGCFSFSLIFTFLAIFHVLQWTFLNFPPFSVLYFSPYSKSYSVHFSFFTFFSDFVIFQVVKWMFLI
FHDFQLSC
>ENSMUSP00000096655_c.433:T>G_p.V145G ENSMUST00000099056.4|Gm10717-
204|ENSMUSG00000095891|+|chr9:g.3032522:T>G
MFLIFHDFQFSCHIPCPTVDISFPPFSVFLAIFHVLKCVFLIFRDFQFSCHIPGPTVCISHFSRFSVILSFFK
LSSGCFSFSLIFTFLAIFHVLQWTFLYFPPFSVFLAIFHVLKCVFLIFRDFQVSRHIPGPTVCISHFSRFSGI
LSFFKLSSGCFSFSLIFTFLAIFHVLQWTFLNFPPFFLPYSRSYSVHFSFFTFFSDFVIFQVVNCMFLIFYDF
QFSC
>ENSMUSP00000136837_c.439:T>C_p.L147S ENSMUST00000179839.8|Gm10717-
208|ENSMUSG00000095891|+|chr9:g.3032528:T>C
MFLIFHDFQFSCHIPCPTVDISFPPFSVFLAIFHVLKCVFLIFRDFQFSCHIPGPTVCISHFSRFSVILSFFK
LSSGCFSFSLIFTFLAIFHVLQWTFLYFPPFSVFLAIFHVLKCVFLIFRDFQVSRHIPGPTVCISHFSRFSVI
SSFFKLSSGCFSFSLIFTFLAIFHVLQWTFLNFPPFSVLYFSPYSKSYSVHFSFFTFFSDFVIFQVVKWMFLI
FHDFQLSC
>ENSMUSP00000096655_c.439:T>C_p.L147S ENSMUST00000099056.4|Gm10717-
204|ENSMUSG00000095891|+|chr9:g.3032528:T>C
MFLIFHDFQFSCHIPCPTVDISFPPFSVFLAIFHVLKCVFLIFRDFQFSCHIPGPTVCISHFSRFSVILSFFK
LSSGCFSFSLIFTFLAIFHVLQWTFLYFPPFSVFLAIFHVLKCVFLIFRDFQVSRHIPGPTVCISHFSRFSVI
SSFFKLSSGCFSFSLIFTFLAIFHVLQWTFLNFPPFFLPYSRSYSVHFSFFTFFSDFVIFQVVNCMFLIFYDF
QFSC
>ENSMUSP00000136837_c.454:T>G_p.L152W ENSMUST00000179839.8|Gm10717-
208|ENSMUSG00000095891|+|chr9:g.3032543:T>G

MFLIFHDFQFSCHIPCPTVDISFPPFSVFLAIFHVLKCVFLIFRDFQFSCHIPGPTVCISHFSRFSVILSFFK
LSSGCFSFSLIFTFLAIFHVLQWTFLYFPPFSVFLAIFHVLKCVFLIFRDFQVSRHIPGPTVCISHFSRFSVI
LSFFKWSSGCFSFSLIFTFLAIFHVLQWTFLNFPPFSVLYFSPYSKSYSVHFSFFTFFSDFVIFQVVKWMFLI
FHDFQLSC
>ENSMUSP00000096655_c.454:T>G_p.L152W ENSMUST00000099056.4|Gm10717-204|ENSMUSG00000095891|+|chr9:g.3032543:T>G
MFLIFHDFQFSCHIPCPTVDISFPPFSVFLAIFHVLKCVFLIFRDFQFSCHIPGPTVCISHFSRFSVILSFFK
LSSGCFSFSLIFTFLAIFHVLQWTFLYFPPFSVFLAIFHVLKCVFLIFRDFQVSRHIPGPTVCISHFSRFSVI
LSFFKWSSGCFSFSLIFTFLAIFHVLQWTFLNFPPFFLPYSRSYSVHFSFFTFFSDFVIFQVVNCMFLIFYDF
QFSC
>ENSMUSP00000136837_c.454:T>C_p.L152S ENSMUST00000179839.8|Gm10717-208|ENSMUSG00000095891|+|chr9:g.3032543:T>C
MFLIFHDFQFSCHIPCPTVDISFPPFSVFLAIFHVLKCVFLIFRDFQFSCHIPGPTVCISHFSRFSVILSFFK
LSSGCFSFSLIFTFLAIFHVLQWTFLYFPPFSVFLAIFHVLKCVFLIFRDFQVSRHIPGPTVCISHFSRFSVI
LSFFKSSSGCFSFSLIFTFLAIFHVLQWTFLNFPPFSVLYFSPYSKSYSVHFSFFTFFSDFVIFQVVKWMFLI
FHDFQLSC
>ENSMUSP00000096655_c.454:T>C_p.L152S ENSMUST00000099056.4|Gm10717-204|ENSMUSG00000095891|+|chr9:g.3032543:T>C
MFLIFHDFQFSCHIPCPTVDISFPPFSVFLAIFHVLKCVFLIFRDFQFSCHIPGPTVCISHFSRFSVILSFFK
LSSGCFSFSLIFTFLAIFHVLQWTFLYFPPFSVFLAIFHVLKCVFLIFRDFQVSRHIPGPTVCISHFSRFSVI
LSFFKSSSGCFSFSLIFTFLAIFHVLQWTFLNFPPFFLPYSRSYSVHFSFFTFFSDFVIFQVVNCMFLIFYDF
QFSC
>ENSMUSP00000137451_c.652:T>A_p.L218H ENSMUST00000179264.2|Gm10717-207|ENSMUSG00000095891|+|chr9:g.3032569:T>A
MFLIFHDFQFSCHIPCPTVDISFPPFSVFLAIFHVLKCVFLIFRDFQFSCHIPGPTVCISHFSRFSVILSFFN
LSTACFSFSMIFSFLAIFHVLQWTFLNFPPFSVFLAIFHVLKCVFLIFRVFQFPRHIPGPTVCIYHFSRFSVI
SSFFKTSSGCFSFFMIFSFLAIFHVLQWTFLNFPPFSVSPYSRSYSVHFSFFTFFSDFVIFQVVKWMFLIFHD
FHFSC
>ENSMUSP00000136837_c.480:T>A_p.L161M ENSMUST00000179839.8|Gm10717-208|ENSMUSG00000095891|+|chr9:g.3032569:T>A
MFLIFHDFQFSCHIPCPTVDISFPPFSVFLAIFHVLKCVFLIFRDFQFSCHIPGPTVCISHFSRFSVILSFFK
LSSGCFSFSLIFTFLAIFHVLQWTFLYFPPFSVFLAIFHVLKCVFLIFRDFQVSRHIPGPTVCISHFSRFSVI
LSFFKLSSGCFSFSMIFTFLAIFHVLQWTFLNFPPFSVLYFSPYSKSYSVHFSFFTFFSDFVIFQVVKWMFLI
FHDFQLSC
>ENSMUSP00000096655_c.480:T>A_p.L161M ENSMUST00000099056.4|Gm10717-204|ENSMUSG00000095891|+|chr9:g.3032569:T>A
MFLIFHDFQFSCHIPCPTVDISFPPFSVFLAIFHVLKCVFLIFRDFQFSCHIPGPTVCISHFSRFSVILSFFK
LSSGCFSFSLIFTFLAIFHVLQWTFLYFPPFSVFLAIFHVLKCVFLIFRDFQVSRHIPGPTVCISHFSRFSVI
LSFFKLSSGCFSFSMIFTFLAIFHVLQWTFLNFPPFFLPYSRSYSVHFSFFTFFSDFVIFQVVNCMFLIFYDF
QFSC
>ENSMUSP00000137451_c.662:C>G_p.H221Q ENSMUST00000179264.2|Gm10717-207|ENSMUSG00000095891|+|chr9:g.3032579:C>G
MFLIFHDFQFSCHIPCPTVDISFPPFSVFLAIFHVLKCVFLIFRDFQFSCHIPGPTVCISHFSRFSVILSFFN
LSTACFSFSMIFSFLAIFHVLQWTFLNFPPFSVFLAIFHVLKCVFLIFRVFQFPRHIPGPTVCIYHFSRFSVI
SSFFKTSSGCFSFFMIFSFLAIFHVLQWTFLNFPPFSVSPYSRSYSVHFSFFTFFSDFVIFQVVKWMFLIFLD
FQFSC
>ENSMUSP00000136837_c.490:C>G_p.T164S ENSMUST00000179839.8|Gm10717-208|ENSMUSG00000095891|+|chr9:g.3032579:C>G
MFLIFHDFQFSCHIPCPTVDISFPPFSVFLAIFHVLKCVFLIFRDFQFSCHIPGPTVCISHFSRFSVILSFFK
LSSGCFSFSLIFTFLAIFHVLQWTFLYFPPFSVFLAIFHVLKCVFLIFRDFQVSRHIPGPTVCISHFSRFSVI
LSFFKLSSGCFSFSLIFSFLAIFHVLQWTFLNFPPFSVLYFSPYSKSYSVHFSFFTFFSDFVIFQVVKWMFLI
FHDFQLSC
>ENSMUSP00000096655_c.490:C>G_p.T164S ENSMUST00000099056.4|Gm10717-204|ENSMUSG00000095891|+|chr9:g.3032579:C>G
MFLIFHDFQFSCHIPCPTVDISFPPFSVFLAIFHVLKCVFLIFRDFQFSCHIPGPTVCISHFSRFSVILSFFK
LSSGCFSFSLIFTFLAIFHVLQWTFLYFPPFSVFLAIFHVLKCVFLIFRDFQVSRHIPGPTVCISHFSRFSVI
LSFFKLSSGCFSFSLIFSFLAIFHVLQWTFLNFPPFFLPYSRSYSVHFSFFTFFSDFVIFQVVNCMFLIFYDF
QFSC

```
>ENSMUSP00000137451_c.662:CT>GA_p.H221Q ENSMUST00000179264.2|Gm10717-
207|ENSMUSG00000095891|+|chr9:g.3032579:CT>GA
MFLIFHDFQFSCHIPCPTVDISFPPFSVFLAIFHVLKCVFLIFRDFQFSCHIPGPTVCISHFSRFSVILSFFN
LSTACFSFSMIFSFLAIFHVLQWTFLNFPPFSVFLAIFHVLKCVFLIFRVFQFPRHIPGPTVCIYHFSRFSVI
SSFFKTSSGCFSFFMIFSFLAIFHVLQWTFLNFPPFSVSPYSRSYSVHFSFFTFFSDFVIFQVVKWMFLIFLD
FQIFL
>ENSMUSP00000136837_c.490:CT>GA_p.T164R ENSMUST00000179839.8|Gm10717-
208|ENSMUSG00000095891|+|chr9:g.3032579:CT>GA
MFLIFHDFQFSCHIPCPTVDISFPPFSVFLAIFHVLKCVFLIFRDFQFSCHIPGPTVCISHFSRFSVILSFFK
LSSGCFSFSLIFTFLAIFHVLQWTFLYFPPFSVFLAIFHVLKCVFLIFRDFQVSRHIPGPTVCISHFSRFSVI
LSFFKLSSGCFSFSLIFRFSCHIPCPTVDISKFSTFFSPIFLAIFQVLQCAFLIFHVFQ*FRHFSSCQVDVSH
FP*FSAFL
>ENSMUSP00000096655_c.490:CT>GA_p.T164R ENSMUST00000099056.4|Gm10717-
204|ENSMUSG00000095891|+|chr9:g.3032579:CT>GA
MFLIFHDFQFSCHIPCPTVDISFPPFSVFLAIFHVLKCVFLIFRDFQFSCHIPGPTVCISHFSRFSVILSFFK
LSSGCFSFSLIFTFLAIFHVLQWTFLYFPPFSVFLAIFHVLKCVFLIFRDFQVSRHIPGPTVCISHFSRFSVI
LSFFKLSSGCFSFSLIFRFSCHIPCPTVDISKFSTFFLAIFQVLQCAFLIFHVFQ*FCHFSSCQLHVSHFL*F
SVFL
>ENSMUSP00000136837_c.499:C>T_p.A167V ENSMUST00000179839.8|Gm10717-
208|ENSMUSG00000095891|+|chr9:g.3032588:C>T
MFLIFHDFQFSCHIPCPTVDISFPPFSVFLAIFHVLKCVFLIFRDFQFSCHIPGPTVCISHFSRFSVILSFFK
LSSGCFSFSLIFTFLAIFHVLQWTFLYFPPFSVFLAIFHVLKCVFLIFRDFQVSRHIPGPTVCISHFSRFSVI
LSFFKLSSGCFSFSLIFTFLVIFHVLQWTFLNFPPFSVLYFSPYSKSYSVHFSFFTFFSDFVIFQVVKWMFLI
FHDFQLSC
>ENSMUSP00000096655_c.499:C>T_p.A167V ENSMUST00000099056.4|Gm10717-
204|ENSMUSG00000095891|+|chr9:g.3032588:C>T
MFLIFHDFQFSCHIPCPTVDISFPPFSVFLAIFHVLKCVFLIFRDFQFSCHIPGPTVCISHFSRFSVILSFFK
LSSGCFSFSLIFTFLAIFHVLQWTFLYFPPFSVFLAIFHVLKCVFLIFRDFQVSRHIPGPTVCISHFSRFSVI
LSFFKLSSGCFSFSLIFTFLVIFHVLQWTFLNFPPFFLPYSRSYSVHFSFFTFFSDFVIFQVVNCMFLIFYDF
QFSC
>ENSMUSP00000136837_c.509:T>G_p.H170Q ENSMUST00000179839.8|Gm10717-
208|ENSMUSG00000095891|+|chr9:g.3032598:T>G
MFLIFHDFQFSCHIPCPTVDISFPPFSVFLAIFHVLKCVFLIFRDFQFSCHIPGPTVCISHFSRFSVILSFFK
LSSGCFSFSLIFTFLAIFHVLQWTFLYFPPFSVFLAIFHVLKCVFLIFRDFQVSRHIPGPTVCISHFSRFSVI
LSFFKLSSGCFSFSLIFTFLAIFQVLQWTFLNFPPFSVLYFSPYSKSYSVHFSFFTFFSDFVIFQVVKWMFLI
FHDFQLSC
>ENSMUSP00000096655_c.509:T>G_p.H170Q ENSMUST00000099056.4|Gm10717-
204|ENSMUSG00000095891|+|chr9:g.3032598:T>G
MFLIFHDFQFSCHIPCPTVDISFPPFSVFLAIFHVLKCVFLIFRDFQFSCHIPGPTVCISHFSRFSVILSFFK
LSSGCFSFSLIFTFLAIFHVLQWTFLYFPPFSVFLAIFHVLKCVFLIFRDFQVSRHIPGPTVCISHFSRFSVI
LSFFKLSSGCFSFSLIFTFLAIFQVLQWTFLNFPPFFLPYSRSYSVHFSFFTFFSDFVIFQVVNCMFLIFYDF
QFSC
>ENSMUSP00000136837_c.541:C>T_p.P181L ENSMUST00000179839.8|Gm10717-
208|ENSMUSG00000095891|+|chr9:g.3032630:C>T
MFLIFHDFQFSCHIPCPTVDISFPPFSVFLAIFHVLKCVFLIFRDFQFSCHIPGPTVCISHFSRFSVILSFFK
LSSGCFSFSLIFTFLAIFHVLQWTFLYFPPFSVFLAIFHVLKCVFLIFRDFQVSRHIPGPTVCISHFSRFSVI
LSFFKLSSGCFSFSLIFTFLAIFHVLQWTFLNFPLFSVLYFSPYSKSYSVHFSFFTFFSDFVIFQVVKWMFLI
FHDFQLSC
>ENSMUSP00000096655_c.541:C>T_p.P181L ENSMUST00000099056.4|Gm10717-
204|ENSMUSG00000095891|+|chr9:g.3032630:C>T
MFLIFHDFQFSCHIPCPTVDISFPPFSVFLAIFHVLKCVFLIFRDFQFSCHIPGPTVCISHFSRFSVILSFFK
LSSGCFSFSLIFTFLAIFHVLQWTFLYFPPFSVFLAIFHVLKCVFLIFRDFQVSRHIPGPTVCISHFSRFSVI
LSFFKLSSGCFSFSLIFTFLAIFHVLQWTFLNFPLFFLPYSRSYSVHFSFFTFFSDFVIFQVVNCMFLIFYDF
QFSC
>ENSMUSP00000096655_c.550:T>C_p.L184S ENSMUST00000099056.4|Gm10717-
204|ENSMUSG00000095891|+|chr9:g.3032704:T>C
MFLIFHDFQFSCHIPCPTVDISFPPFSVFLAIFHVLKCVFLIFRDFQFSCHIPGPTVCISHFSRFSVILSFFK
LSSGCFSFSLIFTFLAIFHVLQWTFLYFPPFSVFLAIFHVLKCVFLIFRDFQVSRHIPGPTVCISHFSRFSVI
```

LSFFKLSSGCFSFSLIFTFLAIFHVLQWTFLNFPPFFSPYSRSYSVHFSFFTFFSDFVIFQVVNCMFLIFYDF
QFSC
>ENSMUSP00000096655_c.552:CC>TT_p.P185F ENSMUST00000099056.4|Gm10717-
204|ENSMUSG00000095891|+|chr9:g.3032706:CC>TT
MFLIFHDFQFSCHIPCPTVDISFPPFSVFLAIFHVLKCVFLIFRDFQFSCHIPGPTVCISHFSRFSVILSFFK
LSSGCFSFSLIFTFLAIFHVLQWTFLYFPPFSVFLAIFHVLKCVFLIFRDFQVSRHIPGPTVCISHFSRFSVI
LSFFKLSSGCFSFSLIFTFLAIFHVLQWTFLNFPPFFLFIFQVLQCAFLIFHVFQ*FCHFSSCQLHVSHFL*F
SVFL
>ENSMUSP00000096655_c.562:G>T_p.R188M ENSMUST00000099056.4|Gm10717-
204|ENSMUSG00000095891|+|chr9:g.3032716:G>T
MFLIFHDFQFSCHIPCPTVDISFPPFSVFLAIFHVLKCVFLIFRDFQFSCHIPGPTVCISHFSRFSVILSFFK
LSSGCFSFSLIFTFLAIFHVLQWTFLYFPPFSVFLAIFHVLKCVFLIFRDFQVSRHIPGPTVCISHFSRFSVI
LSFFKLSSGCFSFSLIFTFLAIFHVLQWTFLNFPPFFLPYSMSYSVHFSFFTFFSDFVIFQVVNCMFLIFYDF
QFSC
>ENSMUSP00000096655_c.562:G>C_p.R188T ENSMUST00000099056.4|Gm10717-
204|ENSMUSG00000095891|+|chr9:g.3032716:G>C
MFLIFHDFQFSCHIPCPTVDISFPPFSVFLAIFHVLKCVFLIFRDFQFSCHIPGPTVCISHFSRFSVILSFFK
LSSGCFSFSLIFTFLAIFHVLQWTFLYFPPFSVFLAIFHVLKCVFLIFRDFQVSRHIPGPTVCISHFSRFSVI
LSFFKLSSGCFSFSLIFTFLAIFHVLQWTFLNFPPFFLPYSTSYSVHFSFFTFFSDFVIFQVVNCMFLIFYDF
QFSC
>ENSMUSP00000096655_c.568:A>T_p.Y190F ENSMUST00000099056.4|Gm10717-
204|ENSMUSG00000095891|+|chr9:g.3032722:A>T
MFLIFHDFQFSCHIPCPTVDISFPPFSVFLAIFHVLKCVFLIFRDFQFSCHIPGPTVCISHFSRFSVILSFFK
LSSGCFSFSLIFTFLAIFHVLQWTFLYFPPFSVFLAIFHVLKCVFLIFRDFQVSRHIPGPTVCISHFSRFSVI
LSFFKLSSGCFSFSLIFTFLAIFHVLQWTFLNFPPFFLPYSRSFSVHFSFFTFFSDFVIFQVVNCMFLIFYDF
QFSC
>ENSMUSP00000096655_c.589:T>C_p.F197S ENSMUST00000099056.4|Gm10717-
204|ENSMUSG00000095891|+|chr9:g.3032743:T>C
MFLIFHDFQFSCHIPCPTVDISFPPFSVFLAIFHVLKCVFLIFRDFQFSCHIPGPTVCISHFSRFSVILSFFK
LSSGCFSFSLIFTFLAIFHVLQWTFLYFPPFSVFLAIFHVLKCVFLIFRDFQVSRHIPGPTVCISHFSRFSVI
LSFFKLSSGCFSFSLIFTFLAIFHVLQWTFLNFPPFFLPYSRSYSVHFSFSTFFSDFVIFQVVNCMFLIFYDF
QFSC
>ENSMUSP00000096655_c.602:T>G_p.S201R ENSMUST00000099056.4|Gm10717-
204|ENSMUSG00000095891|+|chr9:g.3032756:T>G
MFLIFHDFQFSCHIPCPTVDISFPPFSVFLAIFHVLKCVFLIFRDFQFSCHIPGPTVCISHFSRFSVILSFFK
LSSGCFSFSLIFTFLAIFHVLQWTFLYFPPFSVFLAIFHVLKCVFLIFRDFQVSRHIPGPTVCISHFSRFSVI
LSFFKLSSGCFSFSLIFTFLAIFHVLQWTFLNFPPFFLPYSRSYSVHFSFFTFFRDFVIFQVVNCMFLIFYDF
QFSC
>ENSMUSP00000096655_c.629:CTGC>GTGT_p.N210K ENSMUST00000099056.4|Gm10717-
204|ENSMUSG00000095891|+|chr9:g.3032783:CTGC>GTGT
MFLIFHDFQFSCHIPCPTVDISFPPFSVFLAIFHVLKCVFLIFRDFQFSCHIPGPTVCISHFSRFSVILSFFK
LSSGCFSFSLIFTFLAIFHVLQWTFLYFPPFSVFLAIFHVLKCVFLIFRDFQVSRHIPGPTVCISHFSRFSVI
LSFFKLSSGCFSFSLIFTFLAIFHVLQWTFLNFPPFFLPYSRSYSVHFSFFTFFSDFVIFQVVKCCMFLIFYD
FQFSC
>ENSMUSP00000096655_c.629:CTGC>GTGG_p.N210K ENSMUST00000099056.4|Gm10717-
204|ENSMUSG00000095891|+|chr9:g.3032783:CTGC>GTGG
MFLIFHDFQFSCHIPCPTVDISFPPFSVFLAIFHVLKCVFLIFRDFQFSCHIPGPTVCISHFSRFSVILSFFK
LSSGCFSFSLIFTFLAIFHVLQWTFLYFPPFSVFLAIFHVLKCVFLIFRDFQVSRHIPGPTVCISHFSRFSVI
LSFFKLSSGCFSFSLIFTFLAIFHVLQWTFLNFPPFFLPYSRSYSVHFSFFTFFSDFVIFQVVKWCMFLIFYD
FQFSC
>ENSMUSP00000096655_c.632:C>A_p.C211* ENSMUST00000099056.4|Gm10717-
204|ENSMUSG00000095891|+|chr9:g.3032786:C>A
MFLIFHDFQFSCHIPCPTVDISFPPFSVFLAIFHVLKCVFLIFRDFQFSCHIPGPTVCISHFSRFSVILSFFK
LSSGCFSFSLIFTFLAIFHVLQWTFLYFPPFSVFLAIFHVLKCVFLIFRDFQVSRHIPGPTVCISHFSRFSVI
LSFFKLSSGCFSFSLIFTFLAIFHVLQWTFLNFPPFFLPYSRSYSVHFSFFTFFSDFVIFQVVN*MFLIFYDF
QFSC
>ENSMUSP00000096655_c.633:A>C_p.M212L ENSMUST00000099056.4|Gm10717-
204|ENSMUSG00000095891|+|chr9:g.3032787:A>C

MFLIFHDFQFSCHIPCPTVDISFPPFSVFLAIFHVLKCVFLIFRDFQFSCHIPGPTVCISHFSRFSVILSFFK
LSSGCFSFSLIFTFLAIFHVLQWTFLYFPPFSVFLAIFHVLKCVFLIFRDFQVSRHIPGPTVCISHFSRFSVI
LSFFKLSSGCFSFSLIFTFLAIFHVLQWTFLNFPPFFLPYSRSYSVHFSFFTFFSDFVIFQVVNCLFLIFYDF
QFSC
>ENSMUSP00000096655_c.648:T>C_p.Y217H ENSMUST00000099056.4|Gm10717-
204|ENSMUSG00000095891|+|chr9:g.3032802:T>C
MFLIFHDFQFSCHIPCPTVDISFPPFSVFLAIFHVLKCVFLIFRDFQFSCHIPGPTVCISHFSRFSVILSFFK
LSSGCFSFSLIFTFLAIFHVLQWTFLYFPPFSVFLAIFHVLKCVFLIFRDFQVSRHIPGPTVCISHFSRFSVI
LSFFKLSSGCFSFSLIFTFLAIFHVLQWTFLNFPPFFLPYSRSYSVHFSFFTFFSDFVIFQVVNCMFLIFHDF
QFSC
>ENSMUSP00000096655_c.648:T>G_p.Y217D ENSMUST00000099056.4|Gm10717-
204|ENSMUSG00000095891|+|chr9:g.3032802:T>G
MFLIFHDFQFSCHIPCPTVDISFPPFSVFLAIFHVLKCVFLIFRDFQFSCHIPGPTVCISHFSRFSVILSFFK
LSSGCFSFSLIFTFLAIFHVLQWTFLYFPPFSVFLAIFHVLKCVFLIFRDFQVSRHIPGPTVCISHFSRFSVI
LSFFKLSSGCFSFSLIFTFLAIFHVLQWTFLNFPPFFLPYSRSYSVHFSFFTFFSDFVIFQVVNCMFLIFDDF
QFSC
>ENSMUSP00000136837_c.555:T>TA_p.Y186ins-fs ENSMUST00000179839.8|Gm10717-
208|ENSMUSG00000095891|+|chr9:g.3032957:T>TA
MFLIFHDFQFSCHIPCPTVDISFPPFSVFLAIFHVLKCVFLIFRDFQFSCHIPGPTVCISHFSRFSVILSFFK
LSSGCFSFSLIFTFLAIFHVLQWTFLYFPPFSVFLAIFHVLKCVFLIFRDFQVSRHIPGPTVCISHFSRFSVI
LSFFKLSSGCFSFSLIFTFLAIFHVLQWTFLNFPPFSVL
>ENSMUSP00000136837_c.556:A>T_p.Y186F ENSMUST00000179839.8|Gm10717-
208|ENSMUSG00000095891|+|chr9:g.3033165:A>T
MFLIFHDFQFSCHIPCPTVDISFPPFSVFLAIFHVLKCVFLIFRDFQFSCHIPGPTVCISHFSRFSVILSFFK
LSSGCFSFSLIFTFLAIFHVLQWTFLYFPPFSVFLAIFHVLKCVFLIFRDFQVSRHIPGPTVCISHFSRFSVI
LSFFKLSSGCFSFSLIFTFLAIFHVLQWTFLNFPPFSVLFFSPYSKSYSVHFSFFTFFSDFVIFQVVKWMFLI
FHDFQLSC
>ENSMUSP00000136837_c.574:A>G_p.K192R ENSMUST00000179839.8|Gm10717-
208|ENSMUSG00000095891|+|chr9:g.3033183:A>G
MFLIFHDFQFSCHIPCPTVDISFPPFSVFLAIFHVLKCVFLIFRDFQFSCHIPGPTVCISHFSRFSVILSFFK
LSSGCFSFSLIFTFLAIFHVLQWTFLYFPPFSVFLAIFHVLKCVFLIFRDFQVSRHIPGPTVCISHFSRFSVI
LSFFKLSSGCFSFSLIFTFLAIFHVLQWTFLNFPPFSVLYFSPYSRSYSVHFSFFTFFSDFVIFQVVKWMFLI
FHDFQLSC
>ENSMUSP00000136837_c.574:A>T_p.K192M ENSMUST00000179839.8|Gm10717-
208|ENSMUSG00000095891|+|chr9:g.3033183:A>T
MFLIFHDFQFSCHIPCPTVDISFPPFSVFLAIFHVLKCVFLIFRDFQFSCHIPGPTVCISHFSRFSVILSFFK
LSSGCFSFSLIFTFLAIFHVLQWTFLYFPPFSVFLAIFHVLKCVFLIFRDFQVSRHIPGPTVCISHFSRFSVI
LSFFKLSSGCFSFSLIFTFLAIFHVLQWTFLNFPPFSVLYFSPYSMSYSVHFSFFTFFSDFVIFQVVKWMFLI
FHDFQLSC
>ENSMUSP00000136837_c.580:A>T_p.Y194F ENSMUST00000179839.8|Gm10717-
208|ENSMUSG00000095891|+|chr9:g.3033189:A>T
MFLIFHDFQFSCHIPCPTVDISFPPFSVFLAIFHVLKCVFLIFRDFQFSCHIPGPTVCISHFSRFSVILSFFK
LSSGCFSFSLIFTFLAIFHVLQWTFLYFPPFSVFLAIFHVLKCVFLIFRDFQVSRHIPGPTVCISHFSRFSVI
LSFFKLSSGCFSFSLIFTFLAIFHVLQWTFLNFPPFSVLYFSPYSKSFSVHFSFFTFFSDFVIFQVVKWMFLI
FHDFQLSC
>ENSMUSP00000136837_c.604:C>T_p.T202M ENSMUST00000179839.8|Gm10717-
208|ENSMUSG00000095891|+|chr9:g.3033213:C>T
MFLIFHDFQFSCHIPCPTVDISFPPFSVFLAIFHVLKCVFLIFRDFQFSCHIPGPTVCISHFSRFSVILSFFK
LSSGCFSFSLIFTFLAIFHVLQWTFLYFPPFSVFLAIFHVLKCVFLIFRDFQVSRHIPGPTVCISHFSRFSVI
LSFFKLSSGCFSFSLIFTFLAIFHVLQWTFLNFPPFSVLYFSPYSKSYSVHFSFFMFFSDFVIFQVVKWMFLI
FHDFQLSC
>ENSMUSP00000136837_c.672:C>T_p.L225F ENSMUST00000179839.8|Gm10717-
208|ENSMUSG00000095891|+|chr9:g.3033281:C>T
MFLIFHDFQFSCHIPCPTVDISFPPFSVFLAIFHVLKCVFLIFRDFQFSCHIPGPTVCISHFSRFSVILSFFK
LSSGCFSFSLIFTFLAIFHVLQWTFLYFPPFSVFLAIFHVLKCVFLIFRDFQVSRHIPGPTVCISHFSRFSVI
LSFFKLSSGCFSFSLIFTFLAIFHVLQWTFLNFPPFSVLYFSPYSKSYSVHFSFFTFFSDFVIFQVVKWMFLI
FHDFQFSC

>ENSMUSP00000135988_c.16:A>G_p.H6R ENSMUST00000178641.2|Gm17535-201|ENSMUSG00000096736|+|chr9:g.3034615:A>G
MFLIFRDFQFSCHIPCPTVDISFPPFSVFLAIFHVLKCVFLIFRVFQVSRHIPGPTVCISHFSRFSVILSFFK
LSSGCFSFSLIFTFLAIFHVLQWTFLNFPPFSVFLAIFHVLKCVFLIFSDFQFSRHIPGPTVCISHFSRFSVI
SSFFKTSSGCFSFSMIFSFLAIFHVLQWTFLNFPPFSVSPYSRSYSVHFSFFTFFCDFVIFQVVKWMFLIFLD
FQFSY
>ENSMUSP00000135988_c.45:T>C_p.C16R ENSMUST00000178641.2|Gm17535-201|ENSMUSG00000096736|+|chr9:g.3034644:T>C
MFLIFHDFQFSCHIPRPTVDISFPPFSVFLAIFHVLKCVFLIFRVFQVSRHIPGPTVCISHFSRFSVILSFFK
LSSGCFSFSLIFTFLAIFHVLQWTFLNFPPFSVFLAIFHVLKCVFLIFSDFQFSRHIPGPTVCISHFSRFSVI
SSFFKTSSGCFSFSMIFSFLAIFHVLQWTFLNFPPFSVSPYSRSYSVHFSFFTFFCDFVIFQVVKWMFLIFLD
FQFSY
>ENSMUSP00000135988_c.128:TC>T_p.F43del-fs ENSMUST00000178641.2|Gm17535-201|ENSMUSG00000096736|+|chr9:g.3034731:TC>T
MFLIFHDFQFSCHIPCPTVDISFPPFSVFLAIFHVLKCVFLIFVFFRFLAIFQVLQCAFLIFHVFQ
>ENSMUSP00000135988_c.133:T>A_p.V45D ENSMUST00000178641.2|Gm17535-201|ENSMUSG00000096736|+|chr9:g.3034736:T>A
MFLIFHDFQFSCHIPCPTVDISFPPFSVFLAIFHVLKCVFLIFRDFQVSRHIPGPTVCISHFSRFSVILSFFK
LSSGCFSFSLIFTFLAIFHVLQWTFLNFPPFSVFLAIFHVLKCVFLIFSDFQFSRHIPGPTVCISHFSRFSVI
SSFFKTSSGCFSFSMIFSFLAIFHVLQWTFLNFPPFSVSPYSRSYSVHFSFFTFFCDFVIFQVVKWMFLIFLD
FQFSY
>ENSMUSP00000135988_c.141:G>T_p.V48F ENSMUST00000178641.2|Gm17535-201|ENSMUSG00000096736|+|chr9:g.3034744:G>T
MFLIFHDFQFSCHIPCPTVDISFPPFSVFLAIFHVLKCVFLIFRVFQFSRHIPGPTVCISHFSRFSVILSFFK
LSSGCFSFSLIFTFLAIFHVLQWTFLNFPPFSVFLAIFHVLKCVFLIFSDFQFSRHIPGPTVCISHFSRFSVI
SSFFKTSSGCFSFSMIFSFLAIFHVLQWTFLNFPPFSVSPYSRSYSVHFSFFTFFCDFVIFQVVKWMFLIFLD
FQFSY
>ENSMUSP00000135988_c.154:T>A_p.I52N ENSMUST00000178641.2|Gm17535-201|ENSMUSG00000096736|+|chr9:g.3034757:T>A
MFLIFHDFQFSCHIPCPTVDISFPPFSVFLAIFHVLKCVFLIFRVFQVSRHNPGPTVCISHFSRFSVILSFFK
LSSGCFSFSLIFTFLAIFHVLQWTFLNFPPFSVFLAIFHVLKCVFLIFSDFQFSRHIPGPTVCISHFSRFSVI
SSFFKTSSGCFSFSMIFSFLAIFHVLQWTFLNFPPFSVSPYSRSYSVHFSFFTFFCDFVIFQVVKWMFLIFLD
FQFSY
>rs254349492_ENSMUSP00000135988_c.165:A>T_p.T56S
ENSMUST00000178641.2|Gm17535-201|ENSMUSG00000096736|+|chr9:g.3034768:A>T
MFLIFHDFQFSCHIPCPTVDISFPPFSVFLAIFHVLKCVFLIFRVFQVSRHIPGPSVCISHFSRFSVILSFFK
LSSGCFSFSLIFTFLAIFHVLQWTFLNFPPFSVFLAIFHVLKCVFLIFSDFQFSRHIPGPTVCISHFSRFSVI
SSFFKTSSGCFSFSMIFSFLAIFHVLQWTFLNFPPFSVSPYSRSYSVHFSFFTFFCDFVIFQVVKWMFLIFLD
FQFSY
>ENSMUSP00000135988_c.186:T>C_p.S63P ENSMUST00000178641.2|Gm17535-201|ENSMUSG00000096736|+|chr9:g.3034789:T>C
MFLIFHDFQFSCHIPCPTVDISFPPFSVFLAIFHVLKCVFLIFRVFQVSRHIPGPTVCISHFPRFSVILSFFK
LSSGCFSFSLIFTFLAIFHVLQWTFLNFPPFSVFLAIFHVLKCVFLIFSDFQFSRHIPGPTVCISHFSRFSVI
SSFFKTSSGCFSFSMIFSFLAIFHVLQWTFLNFPPFSVSPYSRSYSVHFSFFTFFCDFVIFQVVKWMFLIFLD
FQFSY
>ENSMUSP00000135988_c.196:C>T_p.S66L ENSMUST00000178641.2|Gm17535-201|ENSMUSG00000096736|+|chr9:g.3034799:C>T
MFLIFHDFQFSCHIPCPTVDISFPPFSVFLAIFHVLKCVFLIFRVFQVSRHIPGPTVCISHFSRFLVILSFFK
LSSGCFSFSLIFTFLAIFHVLQWTFLNFPPFSVFLAIFHVLKCVFLIFSDFQFSRHIPGPTVCISHFSRFSVI
SSFFKTSSGCFSFSMIFSFLAIFHVLQWTFLNFPPFSVSPYSRSYSVHFSFFTFFCDFVIFQVVKWMFLIFLD
FQFSY
>ENSMUSP00000135988_c.205:T>C_p.L69S ENSMUST00000178641.2|Gm17535-201|ENSMUSG00000096736|+|chr9:g.3034808:T>C
MFLIFHDFQFSCHIPCPTVDISFPPFSVFLAIFHVLKCVFLIFRVFQVSRHIPGPTVCISHFSRFSVISSFFK
LSSGCFSFSLIFTFLAIFHVLQWTFLNFPPFSVFLAIFHVLKCVFLIFSDFQFSRHIPGPTVCISHFSRFSVI
SSFFKTSSGCFSFSMIFSFLAIFHVLQWTFLNFPPFSVSPYSRSYSVHFSFFTFFCDFVIFQVVKWMFLIFLD
FQFSY

>rs217741387_ENSMUSP00000135988_c.220:T>C_p.L74S
ENSMUST00000178641.2|Gm17535-201|ENSMUSG00000096736|+|chr9:g.3034823:T>C
MFLIFHDFQFSCHIPCPTVDISFPPFSVFLAIFHVLKCVFLIFRVFQVSRHIPGPTVCISHFSRFSVILSFFK
SSSGCFSFSLIFTFLAIFHVLQWTFLNFPPFSVFLAIFHVLKCVFLIFSDFQFSRHIPGPTVCISHFSRFSVI
SSFFKTSSGCFSFSMIFSFLAIFHVLQWTFLNFPPFSVSPYSRSYSVHFSFFTFFCDFVIFQVVKWMFLIFLD
FQFSY
>ENSMUSP00000135988_c.220:T>G_p.L74W ENSMUST00000178641.2|Gm17535-201|ENSMUSG00000096736|+|chr9:g.3034823:T>G
MFLIFHDFQFSCHIPCPTVDISFPPFSVFLAIFHVLKCVFLIFRVFQVSRHIPGPTVCISHFSRFSVILSFFK
WSSGCFSFSLIFTFLAIFHVLQWTFLNFPPFSVFLAIFHVLKCVFLIFSDFQFSRHIPGPTVCISHFSRFSVI
SSFFKTSSGCFSFSMIFSFLAIFHVLQWTFLNFPPFSVSPYSRSYSVHFSFFTFFCDFVIFQVVKWMFLIFLD
FQFSY
>ENSMUSP00000135988_c.246:T>G_p.L83V ENSMUST00000178641.2|Gm17535-201|ENSMUSG00000096736|+|chr9:g.3034849:T>G
MFLIFHDFQFSCHIPCPTVDISFPPFSVFLAIFHVLKCVFLIFRVFQVSRHIPGPTVCISHFSRFSVILSFFK
LSSGCFSFSVIFTFLAIFHVLQWTFLNFPPFSVFLAIFHVLKCVFLIFSDFQFSRHIPGPTVCISHFSRFSVI
SSFFKTSSGCFSFSMIFSFLAIFHVLQWTFLNFPPFSVSPYSRSYSVHFSFFTFFCDFVIFQVVKWMFLIFLD
FQFSY
>ENSMUSP00000135988_c.246:T>A_p.L83M ENSMUST00000178641.2|Gm17535-201|ENSMUSG00000096736|+|chr9:g.3034849:T>A
MFLIFHDFQFSCHIPCPTVDISFPPFSVFLAIFHVLKCVFLIFRVFQVSRHIPGPTVCISHFSRFSVILSFFK
LSSGCFSFSMIFTFLAIFHVLQWTFLNFPPFSVFLAIFHVLKCVFLIFSDFQFSRHIPGPTVCISHFSRFSVI
SSFFKTSSGCFSFSMIFSFLAIFHVLQWTFLNFPPFSVSPYSRSYSVHFSFFTFFCDFVIFQVVKWMFLIFLD
FQFSY
>ENSMUSP00000135988_c.256:C>G_p.T86S ENSMUST00000178641.2|Gm17535-201|ENSMUSG00000096736|+|chr9:g.3034859:C>G
MFLIFHDFQFSCHIPCPTVDISFPPFSVFLAIFHVLKCVFLIFRVFQVSRHIPGPTVCISHFSRFSVILSFFK
LSSGCFSFSLIFSFLAIFHVLQWTFLNFPPFSVFLAIFHVLKCVFLIFSDFQFSRHIPGPTVCISHFSRFSVI
SSFFKTSSGCFSFSMIFSFLAIFHVLQWTFLNFPPFSVSPYSRSYSVHFSFFTFFCDFVIFQVVKWMFLIFLD
FQFSY
>ENSMUSP00000135988_c.256:CT>GA_p.T86R ENSMUST00000178641.2|Gm17535-201|ENSMUSG00000096736|+|chr9:g.3034859:CT>GA
MFLIFHDFQFSCHIPCPTVDISFPPFSVFLAIFHVLKCVFLIFRVFQVSRHIPGPTVCISHFSRFSVILSFFK
LSSGCFSFSLIFRFSCHIPCPTVDISKFSTFFSFPRHISCPKVCISHFQ*FSVFSPYSRSYSVHFSFFTFFSD
FVFFQDVKWMFLIFHDFQFSCHIPCPTVDISKFSTFFCFAIFQVLQCAFLIFHVFL*FCHFSSCQVDVSHFP*
FSVFL
>ENSMUSP00000135988_c.265:C>T_p.A89V ENSMUST00000178641.2|Gm17535-201|ENSMUSG00000096736|+|chr9:g.3034868:C>T
MFLIFHDFQFSCHIPCPTVDISFPPFSVFLAIFHVLKCVFLIFRVFQVSRHIPGPTVCISHFSRFSVILSFFK
LSSGCFSFSLIFTFLVIFHVLQWTFLNFPPFSVFLAIFHVLKCVFLIFSDFQFSRHIPGPTVCISHFSRFSVI
SSFFKTSSGCFSFSMIFSFLAIFHVLQWTFLNFPPFSVSPYSRSYSVHFSFFTFFCDFVIFQVVKWMFLIFLD
FQFSY
>ENSMUSP00000135988_c.275:T>G_p.H92Q ENSMUST00000178641.2|Gm17535-201|ENSMUSG00000096736|+|chr9:g.3034878:T>G
MFLIFHDFQFSCHIPCPTVDISFPPFSVFLAIFHVLKCVFLIFRVFQVSRHIPGPTVCISHFSRFSVILSFFK
LSSGCFSFSLIFTFLAIFQVLQWTFLNFPPFSVFLAIFHVLKCVFLIFSDFQFSRHIPGPTVCISHFSRFSVI
SSFFKTSSGCFSFSMIFSFLAIFHVLQWTFLNFPPFSVSPYSRSYSVHFSFFTFFCDFVIFQVVKWMFLIFLD
FQFSY
>ENSMUSP00000135988_c.286:G>T_p.W96L ENSMUST00000178641.2|Gm17535-201|ENSMUSG00000096736|+|chr9:g.3034889:G>T
MFLIFHDFQFSCHIPCPTVDISFPPFSVFLAIFHVLKCVFLIFRVFQVSRHIPGPTVCISHFSRFSVILSFFK
LSSGCFSFSLIFTFLAIFHVLQLTFLNFPPFSVFLAIFHVLKCVFLIFSDFQFSRHIPGPTVCISHFSRFSVI
SSFFKTSSGCFSFSMIFSFLAIFHVLQWTFLNFPPFSVSPYSRSYSVHFSFFTFFCDFVIFQVVKWMFLIFLD
FQFSY
>ENSMUSP00000135988_c.307:C>T_p.P103L ENSMUST00000178641.2|Gm17535-201|ENSMUSG00000096736|+|chr9:g.3034910:C>T
MFLIFHDFQFSCHIPCPTVDISFPPFSVFLAIFHVLKCVFLIFRVFQVSRHIPGPTVCISHFSRFSVILSFFK
LSSGCFSFSLIFTFLAIFHVLQWTFLNFPLFSVFLAIFHVLKCVFLIFSDFQFSRHIPGPTVCISHFSRFSVI

SSFFKTSSGCFSFSMIFSFLAIFHVLQWTFLNFPPFSVSPYSRSYSVHFSFFTFFCDFVIFQVVKWMFLIFLD
FQFSY
>ENSMUSP00000135988_c.335:T>A_p.H112Q ENSMUST00000178641.2|Gm17535-201|ENSMUSG00000096736|+|chr9:g.3034938:T>A
MFLIFHDFQFSCHIPCPTVDISFPPFSVFLAIFHVLKCVFLIFRVFQVSRHIPGPTVCISHFSRFSVILSFFK
LSSGCFSFSLIFTFLAIFHVLQWTFLNFPPFSVFLAIFQVLKCVFLIFSDFQFSRHIPGPTVCISHFSRFSVI
SSFFKTSSGCFSFSMIFSFLAIFHVLQWTFLNFPPFSVSPYSRSYSVHFSFFTFFCDFVIFQVVKWMFLIFLD
FQFSY
>ENSMUSP00000135988_c.344:G>T_p.K115N ENSMUST00000178641.2|Gm17535-201|ENSMUSG00000096736|+|chr9:g.3034947:G>T
MFLIFHDFQFSCHIPCPTVDISFPPFSVFLAIFHVLKCVFLIFRVFQVSRHIPGPTVCISHFSRFSVILSFFK
LSSGCFSFSLIFTFLAIFHVLQWTFLNFPPFSVFLAIFHVLNCVFLIFSDFQFSRHIPGPTVCISHFSRFSVI
SSFFKTSSGCFSFSMIFSFLAIFHVLQWTFLNFPPFSVSPYSRSYSVHFSFFTFFCDFVIFQVVKWMFLIFLD
FQFSY
>ENSMUSP00000135988_c.347:T>G_p.C116W ENSMUST00000178641.2|Gm17535-201|ENSMUSG00000096736|+|chr9:g.3034950:T>G
MFLIFHDFQFSCHIPCPTVDISFPPFSVFLAIFHVLKCVFLIFRVFQVSRHIPGPTVCISHFSRFSVILSFFK
LSSGCFSFSLIFTFLAIFHVLQWTFLNFPPFSVFLAIFHVLKWVFLIFSDFQFSRHIPGPTVCISHFSRFSVI
SSFFKTSSGCFSFSMIFSFLAIFHVLQWTFLNFPPFSVSPYSRSYSVHFSFFTFFCDFVIFQVVKWMFLIFLD
FQFSY
>ENSMUSP00000135988_c.353:T>A_p.F118L ENSMUST00000178641.2|Gm17535-201|ENSMUSG00000096736|+|chr9:g.3034956:T>A
MFLIFHDFQFSCHIPCPTVDISFPPFSVFLAIFHVLKCVFLIFRVFQVSRHIPGPTVCISHFSRFSVILSFFK
LSSGCFSFSLIFTFLAIFHVLQWTFLNFPPFSVFLAIFHVLKCVLLIFSDFQFSRHIPGPTVCISHFSRFSVI
SSFFKTSSGCFSFSMIFSFLAIFHVLQWTFLNFPPFSVSPYSRSYSVHFSFFTFFCDFVIFQVVKWMFLIFLD
FQFSY
>ENSMUSP00000135988_c.363:A>C_p.S122R ENSMUST00000178641.2|Gm17535-201|ENSMUSG00000096736|+|chr9:g.3034966:A>C
MFLIFHDFQFSCHIPCPTVDISFPPFSVFLAIFHVLKCVFLIFRVFQVSRHIPGPTVCISHFSRFSVILSFFK
LSSGCFSFSLIFTFLAIFHVLQWTFLNFPPFSVFLAIFHVLKCVFLIFRDFQFSRHIPGPTVCISHFSRFSVI
SSFFKTSSGCFSFSMIFSFLAIFHVLQWTFLNFPPFSVSPYSRSYSVHFSFFTFFCDFVIFQVVKWMFLIFLD
FQFSY
>ENSMUSP00000135988_c.363:A>T_p.S122C ENSMUST00000178641.2|Gm17535-201|ENSMUSG00000096736|+|chr9:g.3034966:A>T
MFLIFHDFQFSCHIPCPTVDISFPPFSVFLAIFHVLKCVFLIFRVFQVSRHIPGPTVCISHFSRFSVILSFFK
LSSGCFSFSLIFTFLAIFHVLQWTFLNFPPFSVFLAIFHVLKCVFLIFCDFQFSRHIPGPTVCISHFSRFSVI
SSFFKTSSGCFSFSMIFSFLAIFHVLQWTFLNFPPFSVSPYSRSYSVHFSFFTFFCDFVIFQVVKWMFLIFLD
FQFSY
>ENSMUSP00000135988_c.363:A>G_p.S122G ENSMUST00000178641.2|Gm17535-201|ENSMUSG00000096736|+|chr9:g.3034966:A>G
MFLIFHDFQFSCHIPCPTVDISFPPFSVFLAIFHVLKCVFLIFRVFQVSRHIPGPTVCISHFSRFSVILSFFK
LSSGCFSFSLIFTFLAIFHVLQWTFLNFPPFSVFLAIFHVLKCVFLIFGDFQFSRHIPGPTVCISHFSRFSVI
SSFFKTSSGCFSFSMIFSFLAIFHVLQWTFLNFPPFSVSPYSRSYSVHFSFFTFFCDFVIFQVVKWMFLIFLD
FQFSY
>ENSMUSP00000135988_c.399:A>T_p.T134S ENSMUST00000178641.2|Gm17535-201|ENSMUSG00000096736|+|chr9:g.3035002:A>T
MFLIFHDFQFSCHIPCPTVDISFPPFSVFLAIFHVLKCVFLIFRVFQVSRHIPGPTVCISHFSRFSVILSFFK
LSSGCFSFSLIFTFLAIFHVLQWTFLNFPPFSVFLAIFHVLKCVFLIFSDFQFSRHIPGPSVCISHFSRFSVI
SSFFKTSSGCFSFSMIFSFLAIFHVLQWTFLNFPPFSVSPYSRSYSVHFSFFTFFCDFVIFQVVKWMFLIFLD
FQFSY
>ENSMUSP00000135988_c.424:G>A_p.R142H ENSMUST00000178641.2|Gm17535-201|ENSMUSG00000096736|+|chr9:g.3035027:G>A
MFLIFHDFQFSCHIPCPTVDISFPPFSVFLAIFHVLKCVFLIFRVFQVSRHIPGPTVCISHFSRFSVILSFFK
LSSGCFSFSLIFTFLAIFHVLQWTFLNFPPFSVFLAIFHVLKCVFLIFSDFQFSRHIPGPTVCISHFSHFSVI
SSFFKTSSGCFSFSMIFSFLAIFHVLQWTFLNFPPFSVSPYSRSYSVHFSFFTFFCDFVIFQVVKWMFLIFLD
FQFSY
>ENSMUSP00000135988_c.430:C>T_p.S144L ENSMUST00000178641.2|Gm17535-201|ENSMUSG00000096736|+|chr9:g.3035033:C>T

MFLIFHDFQFSCHIPCPTVDISFPPFSVFLAIFHVLKCVFLIFRVFQVSRHIPGPTVCISHFSRFSVILSFFK
LSSGCFSFSLIFTFLAIFHVLQWTFLNFPPFSVFLAIFHVLKCVFLIFSDFQFSRHIPGPTVCISHFSRFLVI
SSFFKTSSGCFSFSMIFSFLAIFHVLQWTFLNFPPFSVSPYSRSYSVHFSFFTFFCDFVIFQVVKWMFLIFLD
FQFSY
>ENSMUSP00000135988_c.439:C>T_p.S147L ENSMUST00000178641.2|Gm17535-
201|ENSMUSG00000096736|+|chr9:g.3035042:C>T
MFLIFHDFQFSCHIPCPTVDISFPPFSVFLAIFHVLKCVFLIFRVFQVSRHIPGPTVCISHFSRFSVILSFFK
LSSGCFSFSLIFTFLAIFHVLQWTFLNFPPFSVFLAIFHVLKCVFLIFSDFQFSRHIPGPTVCISHFSRFSVI
LSFFKTSSGCFSFSMIFSFLAIFHVLQWTFLNFPPFSVSPYSRSYSVHFSFFTFFCDFVIFQVVKWMFLIFLD
FQFSY
>rs235217004_ENSMUSP00000135988_c.453:A>T_p.T152S
ENSMUST00000178641.2|Gm17535-201|ENSMUSG00000096736|+|chr9:g.3035056:A>T
MFLIFHDFQFSCHIPCPTVDISFPPFSVFLAIFHVLKCVFLIFRVFQVSRHIPGPTVCISHFSRFSVILSFFK
LSSGCFSFSLIFTFLAIFHVLQWTFLNFPPFSVFLAIFHVLKCVFLIFSDFQFSRHIPGPTVCISHFSRFSVI
SSFFKSSSGCFSFSMIFSFLAIFHVLQWTFLNFPPFSVSPYSRSYSVHFSFFTFFCDFVIFQVVKWMFLIFLD
FQFSY
>ENSMUSP00000135988_c.490:G>C_p.S164T ENSMUST00000178641.2|Gm17535-
201|ENSMUSG00000096736|+|chr9:g.3035093:G>C
MFLIFHDFQFSCHIPCPTVDISFPPFSVFLAIFHVLKCVFLIFRVFQVSRHIPGPTVCISHFSRFSVILSFFK
LSSGCFSFSLIFTFLAIFHVLQWTFLNFPPFSVFLAIFHVLKCVFLIFSDFQFSRHIPGPTVCISHFSRFSVI
SSFFKTSSGCFSFSMIFTFLAIFHVLQWTFLNFPPFSVSPYSRSYSVHFSFFTFFCDFVIFQVVKWMFLIFLD
FQFSY
>ENSMUSP00000135988_c.540:C>A_p.P181T ENSMUST00000178641.2|Gm17535-
201|ENSMUSG00000096736|+|chr9:g.3035143:C>A
MFLIFHDFQFSCHIPCPTVDISFPPFSVFLAIFHVLKCVFLIFRVFQVSRHIPGPTVCISHFSRFSVILSFFK
LSSGCFSFSLIFTFLAIFHVLQWTFLNFPPFSVFLAIFHVLKCVFLIFSDFQFSRHIPGPTVCISHFSRFSVI
SSFFKTSSGCFSFSMIFSFLAIFHVLQWTFLNFPTFSVSPYSRSYSVHFSFFTFFCDFVIFQVVKWMFLIFLD
FQFSY
>ENSMUSP00000135988_c.541:C>T_p.P181L ENSMUST00000178641.2|Gm17535-
201|ENSMUSG00000096736|+|chr9:g.3035144:C>T
MFLIFHDFQFSCHIPCPTVDISFPPFSVFLAIFHVLKCVFLIFRVFQVSRHIPGPTVCISHFSRFSVILSFFK
LSSGCFSFSLIFTFLAIFHVLQWTFLNFPPFSVFLAIFHVLKCVFLIFSDFQFSRHIPGPTVCISHFSRFSVI
SSFFKTSSGCFSFSMIFSFLAIFHVLQWTFLNFPLFSVSPYSRSYSVHFSFFTFFCDFVIFQVVKWMFLIFLD
FQFSY
>ENSMUSP00000135988_c.549:G>T_p.V184F ENSMUST00000178641.2|Gm17535-
201|ENSMUSG00000096736|+|chr9:g.3035681:G>T
MFLIFHDFQFSCHIPCPTVDISFPPFSVFLAIFHVLKCVFLIFRVFQVSRHIPGPTVCISHFSRFSVILSFFK
LSSGCFSFSLIFTFLAIFHVLQWTFLNFPPFSVFLAIFHVLKCVFLIFSDFQFSRHIPGPTVCISHFSRFSVI
SSFFKTSSGCFSFSMIFSFLAIFHVLQWTFLNFPPFSFSPYSRSYSVHFSFFTFFCDFVIFQVVKWMFLIFLD
FQFSY
>ENSMUSP00000135988_c.566:G>T_p.R189S ENSMUST00000178641.2|Gm17535-
201|ENSMUSG00000096736|+|chr9:g.3035700:G>T
MFLIFHDFQFSCHIPCPTVDISFPPFSVFLAIFHVLKCVFLIFRVFQVSRHIPGPTVCISHFSRFSVILSFFK
LSSGCFSFSLIFTFLAIFHVLQWTFLNFPPFSVFLAIFHVLKCVFLIFSDFQFSRHIPGPTVCISHFSRFSVI
SSFFKTSSGCFSFSMIFSFLAIFHVLQWTFLNFPPFSVSPYSSSYSVHFSFFTFFCDFVIFQVVKWMFLIFLD
FQFSY
>ENSMUSP00000135988_c.573:A>C_p.S192R ENSMUST00000178641.2|Gm17535-
201|ENSMUSG00000096736|+|chr9:g.3035707:A>C
MFLIFHDFQFSCHIPCPTVDISFPPFSVFLAIFHVLKCVFLIFRVFQVSRHIPGPTVCISHFSRFSVILSFFK
LSSGCFSFSLIFTFLAIFHVLQWTFLNFPPFSVFLAIFHVLKCVFLIFSDFQFSRHIPGPTVCISHFSRFSVI
SSFFKTSSGCFSFSMIFSFLAIFHVLQWTFLNFPPFSVSPYSRSYRVHFSFFTFFCDFVIFQVVKWMFLIFLD
FQFSY
>ENSMUSP00000135988_c.592:T>C_p.F198S ENSMUST00000178641.2|Gm17535-
201|ENSMUSG00000096736|+|chr9:g.3035726:T>C
MFLIFHDFQFSCHIPCPTVDISFPPFSVFLAIFHVLKCVFLIFRVFQVSRHIPGPTVCISHFSRFSVILSFFK
LSSGCFSFSLIFTFLAIFHVLQWTFLNFPPFSVFLAIFHVLKCVFLIFSDFQFSRHIPGPTVCISHFSRFSVI
SSFFKTSSGCFSFSMIFSFLAIFHVLQWTFLNFPPFSVSPYSRSYSVHFSFSTFFCDFVIFQVVKWMFLIFLD
FQFSY

>ENSMUSP00000135988_c.601:T>A_p.F201Y ENSMUST00000178641.2|Gm17535-201|ENSMUSG00000096736|+|chr9:g.3035735:T>A
MFLIFHDFQFSCHIPCPTVDISFPPFSVFLAIFHVLKCVFLIFRVFQVSRHIPGPTVCISHFSRFSVILSFFK
LSSGCFSFSLIFTFLAIFHVLQWTFLNFPPFSVFLAIFHVLKCVFLIFSDFQFSRHIPGPTVCISHFSRFSVI
SSFFKTSSGCFSFSMIFSFLAIFHVLQWTFLNFPPFSVSPYSRSYSVHFSFFTFYCDFVIFQVVKWMFLIFLD
FQFSY
>ENSMUSP00000135988_c.601:T>G_p.F201C ENSMUST00000178641.2|Gm17535-201|ENSMUSG00000096736|+|chr9:g.3035735:T>G
MFLIFHDFQFSCHIPCPTVDISFPPFSVFLAIFHVLKCVFLIFRVFQVSRHIPGPTVCISHFSRFSVILSFFK
LSSGCFSFSLIFTFLAIFHVLQWTFLNFPPFSVFLAIFHVLKCVFLIFSDFQFSRHIPGPTVCISHFSRFSVI
SSFFKTSSGCFSFSMIFSFLAIFHVLQWTFLNFPPFSVSPYSRSYSVHFSFFTFCCDFVIFQVVKWMFLIFLD
FQFSY
>ENSMUSP00000135988_c.638:G>T_p.M213I ENSMUST00000178641.2|Gm17535-201|ENSMUSG00000096736|+|chr9:g.3035772:G>T
MFLIFHDFQFSCHIPCPTVDISFPPFSVFLAIFHVLKCVFLIFRVFQVSRHIPGPTVCISHFSRFSVILSFFK
LSSGCFSFSLIFTFLAIFHVLQWTFLNFPPFSVFLAIFHVLKCVFLIFSDFQFSRHIPGPTVCISHFSRFSVI
SSFFKTSSGCFSFSMIFSFLAIFHVLQWTFLNFPPFSVSPYSRSYSVHFSFFTFFCDFVIFQVVKWIFLIFLD
FQFSY
>ENSMUSP00000135988_c.652:T>A_p.L218H ENSMUST00000178641.2|Gm17535-201|ENSMUSG00000096736|+|chr9:g.3035786:T>A
MFLIFHDFQFSCHIPCPTVDISFPPFSVFLAIFHVLKCVFLIFRVFQVSRHIPGPTVCISHFSRFSVILSFFK
LSSGCFSFSLIFTFLAIFHVLQWTFLNFPPFSVFLAIFHVLKCVFLIFSDFQFSRHIPGPTVCISHFSRFSVI
SSFFKTSSGCFSFSMIFSFLAIFHVLQWTFLNFPPFSVSPYSRSYSVHFSFFTFFCDFVIFQVVKWMFLIFHD
FQFSY
>ENSMUSP00000135988_c.661:A>T_p.Q221L ENSMUST00000178641.2|Gm17535-201|ENSMUSG00000096736|+|chr9:g.3035795:A>T
MFLIFHDFQFSCHIPCPTVDISFPPFSVFLAIFHVLKCVFLIFRVFQVSRHIPGPTVCISHFSRFSVILSFFK
LSSGCFSFSLIFTFLAIFHVLQWTFLNFPPFSVFLAIFHVLKCVFLIFSDFQFSRHIPGPTVCISHFSRFSVI
SSFFKTSSGCFSFSMIFSFLAIFHVLQWTFLNFPPFSVSPYSRSYSVHFSFFTFFCDFVIFQVVKWMFLIFLD
FLFSY
>ENSMUSP00000135988_c.670:A>G_p.Y224C ENSMUST00000178641.2|Gm17535-201|ENSMUSG00000096736|+|chr9:g.3035804:A>G
MFLIFHDFQFSCHIPCPTVDISFPPFSVFLAIFHVLKCVFLIFRVFQVSRHIPGPTVCISHFSRFSVILSFFK
LSSGCFSFSLIFTFLAIFHVLQWTFLNFPPFSVFLAIFHVLKCVFLIFSDFQFSRHIPGPTVCISHFSRFSVI
SSFFKTSSGCFSFSMIFSFLAIFHVLQWTFLNFPPFSVSPYSRSYSVHFSFFTFFCDFVIFQVVKWMFLIFLD
FQFSC
>rs257554234_ENSMUSP00000136448_c.15:A>C_p.N6H
ENSMUST00000155807.3|Gm10715-201|ENSMUSG00000096201|+|chr9:g.3036892:A>C
MFLIFHDFHFSCLIPRPIVDISFPPFSVFLAIFHVPKCVFLIFHDFQFPRHIPGPTVCISHFSRFSVISSFFK
TSSGCFSFFMIFSFLAIFPVLQWTFLNFPPFSVFLAIFHVLKCVFLIFRDFQFSRQIPGPTVCISHFSRFSVI
LSFFNLSTACFSLSMIFSFLAIFHVLQWTFLNFPPFSFFSPYFTSYSVHFSFFTFFSDFVFFQDVKWMFLIFH
DFQFSC
>rs234203612_ENSMUSP00000136448_c.26:T>G_p.H9Q
ENSMUST00000155807.3|Gm10715-201|ENSMUSG00000096201|+|chr9:g.3036903:T>G
MFLIFNDFQFSCLIPRPIVDISFPPFSVFLAIFHVPKCVFLIFHDFQFPRHIPGPTVCISHFSRFSVISSFFK
TSSGCFSFFMIFSFLAIFPVLQWTFLNFPPFSVFLAIFHVLKCVFLIFRDFQFSRQIPGPTVCISHFSRFSVI
LSFFNLSTACFSLSMIFSFLAIFHVLQWTFLNFPPFSFFSPYFTSYSVHFSFFTFFSDFVFFQDVKWMFLIFH
DFQFSC
>ENSMUSP00000136448_c.37:T>A_p.L13H ENSMUST00000155807.3|Gm10715-201|ENSMUSG00000096201|+|chr9:g.3036914:T>A
MFLIFNDFHFSCHIPRPIVDISFPPFSVFLAIFHVPKCVFLIFHDFQFPRHIPGPTVCISHFSRFSVISSFFK
TSSGCFSFFMIFSFLAIFPVLQWTFLNFPPFSVFLAIFHVLKCVFLIFRDFQFSRQIPGPTVCISHFSRFSVI
LSFFNLSTACFSLSMIFSFLAIFHVLQWTFLNFPPFSFFSPYFTSYSVHFSFFTFFSDFVFFQDVKWMFLIFH
DFQFSC
>ENSMUSP00000136448_c.46:G>T_p.R16L ENSMUST00000155807.3|Gm10715-201|ENSMUSG00000096201|+|chr9:g.3036923:G>T
MFLIFNDFHFSCLIPLPIVDISFPPFSVFLAIFHVPKCVFLIFHDFQFPRHIPGPTVCISHFSRFSVISSFFK
TSSGCFSFFMIFSFLAIFPVLQWTFLNFPPFSVFLAIFHVLKCVFLIFRDFQFSRQIPGPTVCISHFSRFSVI

LSFFNLSTACFSLSMIFSFLAIFHVLQWTFLNFPPFSFFSPYFTSYSVHFSFFTFFSDFVFFQDVKWMFLIFH
DFQFSC
>ENSMUSP00000136448_c.52:T>C_p.I18T ENSMUST00000155807.3|Gm10715-201|ENSMUSG00000096201|+|chr9:g.3036929:T>C
MFLIFNDFHFSCLIPRPTVDISFPPFSVFLAIFHVPKCVFLIFHDFQFPRHIPGPTVCISHFSRFSVISSFFK
TSSGCFSFFMIFSFLAIFPVLQWTFLNFPPFSVFLAIFHVLKCVFLIFRDFQFSRQIPGPTVCISHFSRFSVI
LSFFNLSTACFSLSMIFSFLAIFHVLQWTFLNFPPFSFFSPYFTSYSVHFSFFTFFSDFVFFQDVKWMFLIFH
DFQFSC
>ENSMUSP00000136448_c.57:G>A_p.D20N ENSMUST00000155807.3|Gm10715-201|ENSMUSG00000096201|+|chr9:g.3036934:G>A
MFLIFNDFHFSCLIPRPIVNISFPPFSVFLAIFHVPKCVFLIFHDFQFPRHIPGPTVCISHFSRFSVISSFFK
TSSGCFSFFMIFSFLAIFPVLQWTFLNFPPFSVFLAIFHVLKCVFLIFRDFQFSRQIPGPTVCISHFSRFSVI
LSFFNLSTACFSLSMIFSFLAIFHVLQWTFLNFPPFSFFSPYFTSYSVHFSFFTFFSDFVFFQDVKWMFLIFH
DFQFSC
>ENSMUSP00000136448_c.58:A>G_p.D20G ENSMUST00000155807.3|Gm10715-201|ENSMUSG00000096201|+|chr9:g.3036935:A>G
MFLIFNDFHFSCLIPRPIVGISFPPFSVFLAIFHVPKCVFLIFHDFQFPRHIPGPTVCISHFSRFSVISSFFK
TSSGCFSFFMIFSFLAIFPVLQWTFLNFPPFSVFLAIFHVLKCVFLIFRDFQFSRQIPGPTVCISHFSRFSVI
LSFFNLSTACFSLSMIFSFLAIFHVLQWTFLNFPPFSFFSPYFTSYSVHFSFFTFFSDFVFFQDVKWMFLIFH
DFQFSC
>ENSMUSP00000136448_c.143:TC>T_p.F48del-fs ENSMUST00000155807.3|Gm10715-201|ENSMUSG00000096201|+|chr9:g.3037024:TC>T
MFLIFNDFHFSCLIPRPIVDISFPPFSVFLAIFHVPKCVFLIFHDFQFLAIFQVLQCAFLIFHVFQ
>ENSMUSP00000136448_c.146:TC>T_p.P49del-fs ENSMUST00000155807.3|Gm10715-201|ENSMUSG00000096201|+|chr9:g.3037027:TC>T
MFLIFNDFHFSCLIPRPIVDISFPPFSVFLAIFHVPKCVFLIFHDFQFPAIFQVLQCAFLIFHVFQ
>ENSMUSP00000136448_c.159:GGTC>TGTG_p.G54C ENSMUST00000155807.3|Gm10715-201|ENSMUSG00000096201|+|chr9:g.3037040:GGTC>TGTG
MFLIFNDFHFSCLIPRPIVDISFPPFSVFLAIFHVPKCVFLIFHDFQFPRHIPCGPTVCISHFSRFSVISSFF
KTSSGCFSFFMIFSFLAIFPVLQWTFLNFPPFSVFLAIFHVLKCVFLIFRDFQFSRQIPGPTVCISHFSRFSV
ILSFFNLSTACFSLSMIFSFLAIFHVLQWTFLNFPPFSFFSPYFTSYSVHFSFFTFFSDFVFFQDVKWMFLIF
HDFQFSC
>ENSMUSP00000136448_c.165:A>T_p.T56S ENSMUST00000155807.3|Gm10715-201|ENSMUSG00000096201|+|chr9:g.3037046:A>T
MFLIFNDFHFSCLIPRPIVDISFPPFSVFLAIFHVPKCVFLIFHDFQFPRHIPGPSVCISHFSRFSVISSFFK
TSSGCFSFFMIFSFLAIFPVLQWTFLNFPPFSVFLAIFHVLKCVFLIFRDFQFSRQIPGPTVCISHFSRFSVI
LSFFNLSTACFSLSMIFSFLAIFHVLQWTFLNFPPFSFFSPYFTSYSVHFSFFTFFSDFVFFQDVKWMFLIFH
DFQFSC
>ENSMUSP00000136448_c.166:C>A_p.T56K ENSMUST00000155807.3|Gm10715-201|ENSMUSG00000096201|+|chr9:g.3037047:C>A
MFLIFNDFHFSCLIPRPIVDISFPPFSVFLAIFHVPKCVFLIFHDFQFPRHIPGPKVCISHFSRFSVISSFFK
TSSGCFSFFMIFSFLAIFPVLQWTFLNFPPFSVFLAIFHVLKCVFLIFRDFQFSRQIPGPTVCISHFSRFSVI
LSFFNLSTACFSLSMIFSFLAIFHVLQWTFLNFPPFSFFSPYFTSYSVHFSFFTFFSDFVFFQDVKWMFLIFH
DFQFSC
>ENSMUSP00000136448_c.171:T>G_p.C58G ENSMUST00000155807.3|Gm10715-201|ENSMUSG00000096201|+|chr9:g.3037052:T>G
MFLIFNDFHFSCLIPRPIVDISFPPFSVFLAIFHVPKCVFLIFHDFQFPRHIPGPTVGISHFSRFSVISSFFK
TSSGCFSFFMIFSFLAIFPVLQWTFLNFPPFSVFLAIFHVLKCVFLIFRDFQFSRQIPGPTVCISHFSRFSVI
LSFFNLSTACFSLSMIFSFLAIFHVLQWTFLNFPPFSFFSPYFTSYSVHFSFFTFFSDFVFFQDVKWMFLIFH
DFQFSC
>ENSMUSP00000136448_c.171:TG>GA_p.C58E ENSMUST00000155807.3|Gm10715-201|ENSMUSG00000096201|+|chr9:g.3037052:TG>GA
MFLIFNDFHFSCLIPRPIVDISFPPFSVFLAIFHVPKCVFLIFHDFQFPRHIPGPTVEHFSFFTFFSDFVFFQ
DVKWMFLIFHDFQFSCHIPCPTVDISKFSTFFCFPRHISRPKVCISHFP*FSVFSPDSRSYSVHFSFFTFFSD
FVIFQFVNCMFLSFHDFQFSCHIPCPTVDISKFSTFFIFLAIFHVLQCAFLIFHVFQ*FRLFSRRQVDVSHFS
*FSVFL
>ENSMUSP00000136448_c.196:C>T_p.S66L ENSMUST00000155807.3|Gm10715-201|ENSMUSG00000096201|+|chr9:g.3037077:C>T

MFLIFNDFHFSCLIPRPIVDISFPPFSVFLAIFHVPKCVFLIFHDFQFPRHIPGPTVCISHFSRFLVISSFFK
TSSGCFSFFMIFSFLAIFPVLQWTFLNFPPFSVFLAIFHVLKCVFLIFRDFQFSRQIPGPTVCISHFSRFSVI
LSFFNLSTACFSLSMIFSFLAIFHVLQWTFLNFPPFSFFSPYFTSYSVHFSFFTFFSDFVFFQDVKWMFLIFH
DFQFSC
>rs217447675_ENSMUSP00000136448_c.219:A>T_p.T74S
ENSMUST00000155807.3|Gm10715-201|ENSMUSG00000096201|+|chr9:g.3037100:A>T
MFLIFNDFHFSCLIPRPIVDISFPPFSVFLAIFHVPKCVFLIFHDFQFPRHIPGPTVCISHFSRFSVISSFFK
SSSGCFSFFMIFSFLAIFPVLQWTFLNFPPFSVFLAIFHVLKCVFLIFRDFQFSRQIPGPTVCISHFSRFSVI
LSFFNLSTACFSLSMIFSFLAIFHVLQWTFLNFPPFSFFSPYFTSYSVHFSFFTFFSDFVFFQDVKWMFLIFH
DFQFSC
>ENSMUSP00000136448_c.239:AT>A_p.S80del-fs ENSMUST00000155807.3|Gm10715-201|ENSMUSG00000096201|+|chr9:g.3037120:AT>A
MFLIFNDFHFSCLIPRPIVDISFPPFSVFLAIFHVPKCVFLIFHDFQFPRHIPGPTVCISHFSRFSVISSFFK
TSSGCFSFS
>ENSMUSP00000137129_c.9:AT>A_p.I4del-fs ENSMUST00000177969.2|Gm10715-202|ENSMUSG00000096201|+|chr9:g.3037120:AT>A
MFLIFMIFSFLAIFPVLQWTFLFHLFLFSSPYFTS
>ENSMUSP00000136448_c.244:T>C_p.F82S ENSMUST00000155807.3|Gm10715-201|ENSMUSG00000096201|+|chr9:g.3037125:T>C
MFLIFNDFHFSCLIPRPIVDISFPPFSVFLAIFHVPKCVFLIFHDFQFPRHIPGPTVCISHFSRFSVISSFFK
TSSGCFSFSMIFSFLAIFPVLQWTFLNFPPFSVFLAIFHVLKCVFLIFRDFQFSRQIPGPTVCISHFSRFSVI
LSFFNLSTACFSLSMIFSFLAIFHVLQWTFLNFPPFSFFSPYFTSYSVHFSFFTFFSDFVFFQDVKWMFLIFH
DFQFSC
>ENSMUSP00000136448_c.274:C>A_p.P92H ENSMUST00000155807.3|Gm10715-201|ENSMUSG00000096201|+|chr9:g.3037155:C>A
MFLIFNDFHFSCLIPRPIVDISFPPFSVFLAIFHVPKCVFLIFHDFQFPRHIPGPTVCISHFSRFSVISSFFK
TSSGCFSFFMIFSFLAIFHVLQWTFLNFPPFSVFLAIFHVLKCVFLIFRDFQFSRQIPGPTVCISHFSRFSVI
LSFFNLSTACFSLSMIFSFLAIFHVLQWTFLNFPPFSFFSPYFTSYSVHFSFFTFFSDFVFFQDVKWMFLIFH
DFQFSC
>ENSMUSP00000136448_c.274:CT>AC_p.P92H ENSMUST00000155807.3|Gm10715-201|ENSMUSG00000096201|+|chr9:g.3037155:CT>AC
MFLIFNDFHFSCLIPRPIVDISFPPFSVFLAIFHVPKCVFLIFHDFQFPRHIPGPTVCISHFSRFSVISSFFK
TSSGCFSFFMIFSFLAIFHCPTVDISKFSTFFCFPRHISRPKVCISHFP*FSVFSPDSRSYSVHFSFFTFFSD
FVIFQFVNCMFLSFHDFQFSCHIPCPTVDISKFSTFFIFLAIFHVLQCAFLIFHVFQ*FRLFSRRQVDVSHFS
*FSVFL
>rs228898590_ENSMUSP00000137129_c.45:T>C_p.C16R
ENSMUST00000177969.2|Gm10715-202|ENSMUSG00000096201|+|chr9:g.3037156:T>C
MFLIFHDFQFSCHIPRPTVDISFPPFSVFLAIFHVLKCVFLIFRDFQFSRQIPGPTVCISHFSRFSVILSFFN
LSTACFSLSMIFSFLAIFHVLQWTFLNFPPFSVFLAIFHVLKCVFLIFRVFQFPRHIPGPTVCISHFSRFSVI
SSFFKTSSGCFSFFMIFSFLAIFPVLQWTFLNFPPFSSSIFLPYSRSYSVHFSFFTFFSDFVIFQVVKWKFLI
FHDFQLSS
>ENSMUSP00000136448_c.363:C>T_p.R122C ENSMUST00000155807.3|Gm10715-201|ENSMUSG00000096201|+|chr9:g.3037244:C>T
MFLIFNDFHFSCLIPRPIVDISFPPFSVFLAIFHVPKCVFLIFHDFQFPRHIPGPTVCISHFSRFSVISSFFK
TSSGCFSFFMIFSFLAIFPVLQWTFLNFPPFSVFLAIFHVLKCVFLIFCDFQFSRQIPGPTVCISHFSRFSVI
LSFFNLSTACFSLSMIFSFLAIFHVLQWTFLNFPPFSFFSPYFTSYSVHFSFFTFFSDFVFFQDVKWMFLIFH
DFQFSC
>ENSMUSP00000137129_c.129:C>T_p.R44C ENSMUST00000177969.2|Gm10715-202|ENSMUSG00000096201|+|chr9:g.3037244:C>T
MFLIFHDFQFSCHIPCPTVDISFPPFSVFLAIFHVLKCVFLIFCDFQFSRQIPGPTVCISHFSRFSVILSFFN
LSTACFSLSMIFSFLAIFHVLQWTFLNFPPFSVFLAIFHVLKCVFLIFRVFQFPRHIPGPTVCISHFSRFSVI
SSFFKTSSGCFSFFMIFSFLAIFPVLQWTFLNFPPFSSSIFLPYSRSYSVHFSFFTFFSDFVIFQVVKWKFLI
FHDFQLSS
>ENSMUSP00000136448_c.386:G>T_p.Q129H ENSMUST00000155807.3|Gm10715-201|ENSMUSG00000096201|+|chr9:g.3037267:G>T
MFLIFNDFHFSCLIPRPIVDISFPPFSVFLAIFHVPKCVFLIFHDFQFPRHIPGPTVCISHFSRFSVISSFFK
TSSGCFSFFMIFSFLAIFPVLQWTFLNFPPFSVFLAIFHVLKCVFLIFRDFQFSRHIPGPTVCISHFSRFSVI

LSFFNLSTACFSLSMIFSFLAIFHVLQWTFLNFPPFSFFSPYFTSYSVHFSFFTFFSDFVFFQDVKWMFLIFH
DFQFSC
>ENSMUSP00000137129_c.152:G>T_p.Q51H ENSMUST00000177969.2|Gm10715-
202|ENSMUSG00000096201|+|chr9:g.3037267:G>T
MFLIFHDFQFSCHIPCPTVDISFPPFSVFLAIFHVLKCVFLIFRDFQFSRHIPGPTVCISHFSRFSVILSFFN
LSTACFSLSMIFSFLAIFHVLQWTFLNFPPFSVFLAIFHVLKCVFLIFRVFQFPRHIPGPTVCISHFSRFSVI
SSFFKTSSGCFSFFMIFSFLAIFPVLQWTFLNFPPFSSSIFLPYSRSYSVHFSFFTFFSDFVIFQVVKWKFLI
FHDFQLSS
>ENSMUSP00000136448_c.386:G>C_p.Q129H ENSMUST00000155807.3|Gm10715-
201|ENSMUSG00000096201|+|chr9:g.3037267:G>C
MFLIFNDFHFSCLIPRPIVDISFPPFSVFLAIFHVPKCVFLIFHDFQFPRHIPGPTVCISHFSRFSVISSFFK
TSSGCFSFFMIFSFLAIFPVLQWTFLNFPPFSVFLAIFHVLKCVFLIFRDFQFSRHIPGPTVCISHFSRFSVI
LSFFNLSTACFSLSMIFSFLAIFHVLQWTFLNFPPFSFFSPYFTSYSVHFSFFTFFSDFVFFQDVKWMFLIFH
DFQFSC
>ENSMUSP00000137129_c.152:G>C_p.Q51H ENSMUST00000177969.2|Gm10715-
202|ENSMUSG00000096201|+|chr9:g.3037267:G>C
MFLIFHDFQFSCHIPCPTVDISFPPFSVFLAIFHVLKCVFLIFRDFQFSRHIPGPTVCISHFSRFSVILSFFN
LSTACFSLSMIFSFLAIFHVLQWTFLNFPPFSVFLAIFHVLKCVFLIFRVFQFPRHIPGPTVCISHFSRFSVI
SSFFKTSSGCFSFFMIFSFLAIFPVLQWTFLNFPPFSSSIFLPYSRSYSVHFSFFTFFSDFVIFQVVKWKFLI
FHDFQLSS
>ENSMUSP00000136448_c.394:G>T_p.G132V ENSMUST00000155807.3|Gm10715-
201|ENSMUSG00000096201|+|chr9:g.3037275:G>T
MFLIFNDFHFSCLIPRPIVDISFPPFSVFLAIFHVPKCVFLIFHDFQFPRHIPGPTVCISHFSRFSVISSFFK
TSSGCFSFFMIFSFLAIFPVLQWTFLNFPPFSVFLAIFHVLKCVFLIFRDFQFSRQIPVPTVCISHFSRFSVI
LSFFNLSTACFSLSMIFSFLAIFHVLQWTFLNFPPFSFFSPYFTSYSVHFSFFTFFSDFVFFQDVKWMFLIFH
DFQFSC
>ENSMUSP00000137129_c.160:G>T_p.G54V ENSMUST00000177969.2|Gm10715-
202|ENSMUSG00000096201|+|chr9:g.3037275:G>T
MFLIFHDFQFSCHIPCPTVDISFPPFSVFLAIFHVLKCVFLIFRDFQFSRQIPVPTVCISHFSRFSVILSFFN
LSTACFSLSMIFSFLAIFHVLQWTFLNFPPFSVFLAIFHVLKCVFLIFRVFQFPRHIPGPTVCISHFSRFSVI
SSFFKTSSGCFSFFMIFSFLAIFPVLQWTFLNFPPFSSSIFLPYSRSYSVHFSFFTFFSDFVIFQVVKWKFLI
FHDFQLSS
>ENSMUSP00000136448_c.399:A>T_p.T134S ENSMUST00000155807.3|Gm10715-
201|ENSMUSG00000096201|+|chr9:g.3037280:A>T
MFLIFNDFHFSCLIPRPIVDISFPPFSVFLAIFHVPKCVFLIFHDFQFPRHIPGPTVCISHFSRFSVISSFFK
TSSGCFSFFMIFSFLAIFPVLQWTFLNFPPFSVFLAIFHVLKCVFLIFRDFQFSRQIPGPSVCISHFSRFSVI
LSFFNLSTACFSLSMIFSFLAIFHVLQWTFLNFPPFSFFSPYFTSYSVHFSFFTFFSDFVFFQDVKWMFLIFH
DFQFSC
>ENSMUSP00000137129_c.165:A>T_p.T56S ENSMUST00000177969.2|Gm10715-
202|ENSMUSG00000096201|+|chr9:g.3037280:A>T
MFLIFHDFQFSCHIPCPTVDISFPPFSVFLAIFHVLKCVFLIFRDFQFSRQIPGPSVCISHFSRFSVILSFFN
LSTACFSLSMIFSFLAIFHVLQWTFLNFPPFSVFLAIFHVLKCVFLIFRVFQFPRHIPGPTVCISHFSRFSVI
SSFFKTSSGCFSFFMIFSFLAIFPVLQWTFLNFPPFSSSIFLPYSRSYSVHFSFFTFFSDFVIFQVVKWKFLI
FHDFQLSS
>ENSMUSP00000136448_c.433:T>G_p.V145G ENSMUST00000155807.3|Gm10715-
201|ENSMUSG00000096201|+|chr9:g.3037314:T>G
MFLIFNDFHFSCLIPRPIVDISFPPFSVFLAIFHVPKCVFLIFHDFQFPRHIPGPTVCISHFSRFSVISSFFK
TSSGCFSFFMIFSFLAIFPVLQWTFLNFPPFSVFLAIFHVLKCVFLIFRDFQFSRQIPGPTVCISHFSRFSGI
LSFFNLSTACFSLSMIFSFLAIFHVLQWTFLNFPPFSFFSPYFTSYSVHFSFFTFFSDFVFFQDVKWMFLIFH
DFQFSC
>ENSMUSP00000137129_c.199:T>G_p.V67G ENSMUST00000177969.2|Gm10715-
202|ENSMUSG00000096201|+|chr9:g.3037314:T>G
MFLIFHDFQFSCHIPCPTVDISFPPFSVFLAIFHVLKCVFLIFRDFQFSRQIPGPTVCISHFSRFSGILSFFN
LSTACFSLSMIFSFLAIFHVLQWTFLNFPPFSVFLAIFHVLKCVFLIFRVFQFPRHIPGPTVCISHFSRFSVI
SSFFKTSSGCFSFFMIFSFLAIFPVLQWTFLNFPPFSSSIFLPYSRSYSVHFSFFTFFSDFVIFQVVKWKFLI
FHDFQLSS
>ENSMUSP00000136448_c.439:T>C_p.L147S ENSMUST00000155807.3|Gm10715-
201|ENSMUSG00000096201|+|chr9:g.3037320:T>C

MFLIFNDFHFSCLIPRPIVDISFPPFSVFLAIFHVPKCVFLIFHDFQFPRHIPGPTVCISHFSRFSVISSFFK
TSSGCFSFFMIFSFLAIFPVLQWTFLNFPPFSVFLAIFHVLKCVFLIFRDFQFSRQIPGPTVCISHFSRFSVI
SSFFNLSTACFSLSMIFSFLAIFHVLQWTFLNFPPFSFFSPYFTSYSVHFSFFTFFSDFVFFQDVKWMFLIFH
DFQFSC
>ENSMUSP00000137129_c.205:T>C_p.L69S ENSMUST00000177969.2|Gm10715-202|ENSMUSG00000096201|+|chr9:g.3037320:T>C
MFLIFHDFQFSCHIPCPTVDISFPPFSVFLAIFHVLKCVFLIFRDFQFSRQIPGPTVCISHFSRFSVISSFFN
LSTACFSLSMIFSFLAIFHVLQWTFLNFPPFSVFLAIFHVLKCVFLIFRVFQFPRHIPGPTVCISHFSRFSVI
SSFFKTSSGCFSFFMIFSFLAIFPVLQWTFLNFPPFSSSIFLPYSRSYSVHFSFFTFFSDFVIFQVVKWKFLI
FHDFQLSS
>ENSMUSP00000136448_c.452:T>G_p.N151K ENSMUST00000155807.3|Gm10715-201|ENSMUSG00000096201|+|chr9:g.3037333:T>G
MFLIFNDFHFSCLIPRPIVDISFPPFSVFLAIFHVPKCVFLIFHDFQFPRHIPGPTVCISHFSRFSVISSFFK
TSSGCFSFFMIFSFLAIFPVLQWTFLNFPPFSVFLAIFHVLKCVFLIFRDFQFSRQIPGPTVCISHFSRFSVI
LSFFKLSTACFSLSMIFSFLAIFHVLQWTFLNFPPFSFFSPYFTSYSVHFSFFTFFSDFVFFQDVKWMFLIFH
DFQFSC
>ENSMUSP00000137129_c.218:T>G_p.N73K ENSMUST00000177969.2|Gm10715-202|ENSMUSG00000096201|+|chr9:g.3037333:T>G
MFLIFHDFQFSCHIPCPTVDISFPPFSVFLAIFHVLKCVFLIFRDFQFSRQIPGPTVCISHFSRFSVILSFFK
LSTACFSLSMIFSFLAIFHVLQWTFLNFPPFSVFLAIFHVLKCVFLIFRVFQFPRHIPGPTVCISHFSRFSVI
SSFFKTSSGCFSFFMIFSFLAIFPVLQWTFLNFPPFSSSIFLPYSRSYSVHFSFFTFFSDFVIFQVVKWKFLI
FHDFQLSS
>ENSMUSP00000136448_c.454:T>C_p.L152S ENSMUST00000155807.3|Gm10715-201|ENSMUSG00000096201|+|chr9:g.3037335:T>C
MFLIFNDFHFSCLIPRPIVDISFPPFSVFLAIFHVPKCVFLIFHDFQFPRHIPGPTVCISHFSRFSVISSFFK
TSSGCFSFFMIFSFLAIFPVLQWTFLNFPPFSVFLAIFHVLKCVFLIFRDFQFSRQIPGPTVCISHFSRFSVI
LSFFNSSTACFSLSMIFSFLAIFHVLQWTFLNFPPFSFFSPYFTSYSVHFSFFTFFSDFVFFQDVKWMFLIFH
DFQFSC
>ENSMUSP00000137129_c.220:T>C_p.L74S ENSMUST00000177969.2|Gm10715-202|ENSMUSG00000096201|+|chr9:g.3037335:T>C
MFLIFHDFQFSCHIPCPTVDISFPPFSVFLAIFHVLKCVFLIFRDFQFSRQIPGPTVCISHFSRFSVILSFFN
SSTACFSLSMIFSFLAIFHVLQWTFLNFPPFSVFLAIFHVLKCVFLIFRVFQFPRHIPGPTVCISHFSRFSVI
SSFFKTSSGCFSFFMIFSFLAIFPVLQWTFLNFPPFSSSIFLPYSRSYSVHFSFFTFFSDFVIFQVVKWKFLI
FHDFQLSS
>ENSMUSP00000136448_c.460:CT>GG_p.T154R ENSMUST00000155807.3|Gm10715-201|ENSMUSG00000096201|+|chr9:g.3037341:CT>GG
MFLIFNDFHFSCLIPRPIVDISFPPFSVFLAIFHVPKCVFLIFHDFQFPRHIPGPTVCISHFSRFSVISSFFK
TSSGCFSFFMIFSFLAIFPVLQWTFLNFPPFSVFLAIFHVLKCVFLIFRDFQFSRQIPGPTVCISHFSRFSVI
LSFFNLSRCMFLSFHDFQFSCHIPCPTVDISKFSTFFIFLAIFHVLQCAFLIFHVFQ*FRLFSRRQVDVSHFS
*FSVFL
>ENSMUSP00000137129_c.226:CT>GG_p.T76R ENSMUST00000177969.2|Gm10715-202|ENSMUSG00000096201|+|chr9:g.3037341:CT>GG
MFLIFHDFQFSCHIPCPTVDISFPPFSVFLAIFHVLKCVFLIFRDFQFSRQIPGPTVCISHFSRFSVILSFFN
LSRCMFLSFHDFQFSCHIPCPTVDISKFSTFFSFPRHISRAKVCISHFSCFSVSSPYSRSYSVHFSFFTFFSD
FVFFQDVKWMFLIFHDFQFSCHIPCPTVDISKFSTFFFFNFLTIFQVLQCAFLIFHVFQ*FRHFSSCQVEVSH
FP*FSAF*
>ENSMUSP00000136448_c.460:CTGC>GTGT_p.T154S ENSMUST00000155807.3|Gm10715-201|ENSMUSG00000096201|+|chr9:g.3037341:CTGC>GTGT
MFLIFNDFHFSCLIPRPIVDISFPPFSVFLAIFHVPKCVFLIFHDFQFPRHIPGPTVCISHFSRFSVISSFFK
TSSGCFSFFMIFSFLAIFPVLQWTFLNFPPFSVFLAIFHVLKCVFLIFRDFQFSRQIPGPTVCISHFSRFSVI
LSFFNLSSVACFSLSMIFSFLAIFHVLQWTFLNFPPFSFFSPYFTSYSVHFSFFTFFSDFVFFQDVKWMFLIF
HDFQFSC
>ENSMUSP00000137129_c.226:CTGC>GTGT_p.T76S ENSMUST00000177969.2|Gm10715-202|ENSMUSG00000096201|+|chr9:g.3037341:CTGC>GTGT
MFLIFHDFQFSCHIPCPTVDISFPPFSVFLAIFHVLKCVFLIFRDFQFSRQIPGPTVCISHFSRFSVILSFFN
LSSVACFSLSMIFSFLAIFHVLQWTFLNFPPFSVFLAIFHVLKCVFLIFRVFQFPRHIPGPTVCISHFSRFSV
ISSFFKTSSGCFSFFMIFSFLAIFPVLQWTFLNFPPFSSSIFLPYSRSYSVHFSFFTFFSDFVIFQVVKWKFL
IFHDFQLSS

>ENSMUSP00000136448_c.460:CTGC>GTGG_p.T154S ENSMUST00000155807.3|Gm10715-201|ENSMUSG00000096201|+|chr9:g.3037341:CTGC>GTGG
MFLIFNDFHFSCLIPRPIVDISFPPFSVFLAIFHVPKCVFLIFHDFQFPRHIPGPTVCISHFSRFSVISSFFK
TSSGCFSFFMIFSFLAIFPVLQWTFLNFPPFSVFLAIFHVLKCVFLIFRDFQFSRQIPGPTVCISHFSRFSVI
LSFFNLSSGACFSLSMIFSFLAIFHVLQWTFLNFPPFSFFSPYFTSYSVHFSFFTFFSDFVFFQDVKWMFLIF
HDFQFSC
>ENSMUSP00000137129_c.226:CTGC>GTGG_p.T76S ENSMUST00000177969.2|Gm10715-202|ENSMUSG00000096201|+|chr9:g.3037341:CTGC>GTGG
MFLIFHDFQFSCHIPCPTVDISFPPFSVFLAIFHVLKCVFLIFRDFQFSRQIPGPTVCISHFSRFSVILSFFN
LSSGACFSLSMIFSFLAIFHVLQWTFLNFPPFSVFLAIFHVLKCVFLIFRVFQFPRHIPGPTVCISHFSRFSV
ISSFFKTSSGCFSFFMIFSFLAIFPVLQWTFLNFPPFSSSIFLPYSRSYSVHFSFFTFFSDFVIFQVVKWKFL
IFHDFQLSS
>ENSMUSP00000136448_c.474:C>T_p.L159F ENSMUST00000155807.3|Gm10715-201|ENSMUSG00000096201|+|chr9:g.3037355:C>T
MFLIFNDFHFSCLIPRPIVDISFPPFSVFLAIFHVPKCVFLIFHDFQFPRHIPGPTVCISHFSRFSVISSFFK
TSSGCFSFFMIFSFLAIFPVLQWTFLNFPPFSVFLAIFHVLKCVFLIFRDFQFSRQIPGPTVCISHFSRFSVI
LSFFNLSTACFSFSMIFSFLAIFHVLQWTFLNFPPFSFFSPYFTSYSVHFSFFTFFSDFVFFQDVKWMFLIFH
DFQFSC
>ENSMUSP00000137129_c.240:C>T_p.L81F ENSMUST00000177969.2|Gm10715-202|ENSMUSG00000096201|+|chr9:g.3037355:C>T
MFLIFHDFQFSCHIPCPTVDISFPPFSVFLAIFHVLKCVFLIFRDFQFSRQIPGPTVCISHFSRFSVILSFFN
LSTACFSFSMIFSFLAIFHVLQWTFLNFPPFSVFLAIFHVLKCVFLIFRVFQFPRHIPGPTVCISHFSRFSVI
SSFFKTSSGCFSFFMIFSFLAIFPVLQWTFLNFPPFSSSIFLPYSRSYSVHFSFFTFFSDFVIFQVVKWKFLI
FHDFQLSS
>ENSMUSP00000137129_c.362:TC>T_p.F121del-fs ENSMUST00000177969.2|Gm10715-202|ENSMUSG00000096201|+|chr9:g.3037477:TC>T
MFLIFHDFQFSCHIPCPTVDISFPPFSVFLAIFHVLKCVFLIFRDFQFSRQIPGPTVCISHFSRFSVILSFFN
LSTACFSLSMIFSFLAIFHVLQWTFLNFPPFSVFLAIFHVLKCVFLIFVFFSFLAIFQVLQCAFLIFHVFQ
>ENSMUSP00000137129_c.367:T>A_p.V123D ENSMUST00000177969.2|Gm10715-202|ENSMUSG00000096201|+|chr9:g.3037482:T>A
MFLIFHDFQFSCHIPCPTVDISFPPFSVFLAIFHVLKCVFLIFRDFQFSRQIPGPTVCISHFSRFSVILSFFN
LSTACFSLSMIFSFLAIFHVLQWTFLNFPPFSVFLAIFHVLKCVFLIFRDFQFPRHIPGPTVCISHFSRFSVI
SSFFKTSSGCFSFFMIFSFLAIFPVLQWTFLNFPPFSSSIFLPYSRSYSVHFSFFTFFSDFVIFQVVKWKFLI
FHDFQLSS
>ENSMUSP00000136448_c.553:T>C_p.F185S ENSMUST00000155807.3|Gm10715-201|ENSMUSG00000096201|+|chr9:g.3037669:T>C
MFLIFNDFHFSCLIPRPIVDISFPPFSVFLAIFHVPKCVFLIFHDFQFPRHIPGPTVCISHFSRFSVISSFFK
TSSGCFSFFMIFSFLAIFPVLQWTFLNFPPFSVFLAIFHVLKCVFLIFRDFQFSRQIPGPTVCISHFSRFSVI
LSFFNLSTACFSLSMIFSFLAIFHVLQWTFLNFPPFSFSSPYFTSYSVHFSFFTFFSDFVFFQDVKWMFLIFH
DFQFSC
>ENSMUSP00000137129_c.378:C>T_p.P127S ENSMUST00000177969.2|Gm10715-202|ENSMUSG00000096201|+|chr9:g.3037727:C>T
MFLIFHDFQFSCHIPCPTVDISFPPFSVFLAIFHVLKCVFLIFRDFQFSRQIPGPTVCISHFSRFSVILSFFN
LSTACFSLSMIFSFLAIFHVLQWTFLNFPPFSVFLAIFHVLKCVFLIFRVFQFSRHIPGPTVCISHFSRFSVI
SSFFKTSSGCFSFFMIFSFLAIFPVLQWTFLNFPPFSSSIFLPYSRSYSVHFSFFTFFSDFVIFQVVKWKFLI
FHDFQLSS
>ENSMUSP00000136448_c.574:A>T_p.Y192F ENSMUST00000155807.3|Gm10715-201|ENSMUSG00000096201|+|chr9:g.3037748:A>T
MFLIFNDFHFSCLIPRPIVDISFPPFSVFLAIFHVPKCVFLIFHDFQFPRHIPGPTVCISHFSRFSVISSFFK
TSSGCFSFFMIFSFLAIFPVLQWTFLNFPPFSVFLAIFHVLKCVFLIFRDFQFSRQIPGPTVCISHFSRFSVI
LSFFNLSTACFSLSMIFSFLAIFHVLQWTFLNFPPFSFFSPYFTSFSVHFSFFTFFSDFVFFQDVKWMFLIFH
DFQFSC
>ENSMUSP00000137129_c.399:A>T_p.T134S ENSMUST00000177969.2|Gm10715-202|ENSMUSG00000096201|+|chr9:g.3037748:A>T
MFLIFHDFQFSCHIPCPTVDISFPPFSVFLAIFHVLKCVFLIFRDFQFSRQIPGPTVCISHFSRFSVILSFFN
LSTACFSLSMIFSFLAIFHVLQWTFLNFPPFSVFLAIFHVLKCVFLIFRVFQFPRHIPGPSVCISHFSRFSVI
SSFFKTSSGCFSFFMIFSFLAIFPVLQWTFLNFPPFSSSIFLPYSRSYSVHFSFFTFFSDFVIFQVVKWKFLI
FHDFQLSS

>ENSMUSP00000137129_c.430:C>T_p.S144L ENSMUST00000177969.2|Gm10715-202|ENSMUSG00000096201|+|chr9:g.3037779:C>T
MFLIFHDFQFSCHIPCPTVDISFPPFSVFLAIFHVLKCVFLIFRDFQFSRQIPGPTVCISHFSRFSVILSFFN
LSTACFSLSMIFSFLAIFHVLQWTFLNFPPFSVFLAIFHVLKCVFLIFRVFQFPRHIPGPTVCISHFSRFLVI
SSFFKTSSGCFSFFMIFSFLAIFPVLQWTFLNFPPFSSSIFLPYSRSYSVHFSFFTFFSDFVIFQVVKWKFLI
FHDFQLSS
>rs215064382_ENSMUSP00000136448_c.618:T>A_p.F207I
ENSMUST00000155807.3|Gm10715-201|ENSMUSG00000096201|+|chr9:g.3037792:T>A
MFLIFNDFHFSCLIPRPIVDISFPPFSVFLAIFHVPKCVFLIFHDFQFPRHIPGPTVCISHFSRFSVISSFFK
TSSGCFSFFMIFSFLAIFPVLQWTFLNFPPFSVFLAIFHVLKCVFLIFRDFQFSRQIPGPTVCISHFSRFSVI
LSFFNLSTACFSLSMIFSFLAIFHVLQWTFLNFPPFSFFSPYFTSYSVHFSFFTFFSDFVIFQDVKWMFLIFH
DFQFSC
>ENSMUSP00000136448_c.624:C>T_p.Q209* ENSMUST00000155807.3|Gm10715-201|ENSMUSG00000096201|+|chr9:g.3037798:C>T
MFLIFNDFHFSCLIPRPIVDISFPPFSVFLAIFHVPKCVFLIFHDFQFPRHIPGPTVCISHFSRFSVISSFFK
TSSGCFSFFMIFSFLAIFPVLQWTFLNFPPFSVFLAIFHVLKCVFLIFRDFQFSRQIPGPTVCISHFSRFSVI
LSFFNLSTACFSLSMIFSFLAIFHVLQWTFLNFPPFSFFSPYFTSYSVHFSFFTFFSDFVFF*DVKWMFLIFH
DFQFSC
>rs240247081_ENSMUSP00000136448_c.628:A>T_p.D210V
ENSMUST00000155807.3|Gm10715-201|ENSMUSG00000096201|+|chr9:g.3037802:A>T
MFLIFNDFHFSCLIPRPIVDISFPPFSVFLAIFHVPKCVFLIFHDFQFPRHIPGPTVCISHFSRFSVISSFFK
TSSGCFSFFMIFSFLAIFPVLQWTFLNFPPFSVFLAIFHVLKCVFLIFRDFQFSRQIPGPTVCISHFSRFSVI
LSFFNLSTACFSLSMIFSFLAIFHVLQWTFLNFPPFSFFSPYFTSYSVHFSFFTFFSDFVFFQVVKWMFLIFH
DFQFSC
>rs240247081_ENSMUSP00000137129_c.453:A>T_p.T152S
ENSMUST00000177969.2|Gm10715-202|ENSMUSG00000096201|+|chr9:g.3037802:A>T
MFLIFHDFQFSCHIPCPTVDISFPPFSVFLAIFHVLKCVFLIFRDFQFSRQIPGPTVCISHFSRFSVILSFFN
LSTACFSLSMIFSFLAIFHVLQWTFLNFPPFSVFLAIFHVLKCVFLIFRVFQFPRHIPGPTVCISHFSRFSVI
SSFFKSSSGCFSFFMIFSFLAIFPVLQWTFLNFPPFSSSIFLPYSRSYSVHFSFFTFFSDFVIFQVVKWKFLI
FHDFQLSS
>ENSMUSP00000136448_c.628:AC>TG_p.D210V ENSMUST00000155807.3|Gm10715-201|ENSMUSG00000096201|+|chr9:g.3037802:AC>TG
MFLIFNDFHFSCLIPRPIVDISFPPFSVFLAIFHVPKCVFLIFHDFQFPRHIPGPTVCISHFSRFSVISSFFK
TSSGCFSFFMIFSFLAIFPVLQWTFLNFPPFSVFLAIFHVLKCVFLIFRDFQFSRQIPGPTVCISHFSRFSVI
LSFFNLSTACFSLSMIFSFLAIFHVLQWTFLNFPPFSFFSPYFTSYSVHFSFFTFFSDFVFFQVRQVDVSHFS
*FSVFL
>ENSMUSP00000137129_c.453:AC>TG_p.T152C ENSMUST00000177969.2|Gm10715-202|ENSMUSG00000096201|+|chr9:g.3037802:AC>TG
MFLIFHDFQFSCHIPCPTVDISFPPFSVFLAIFHVLKCVFLIFRDFQFSRQIPGPTVCISHFSRFSVILSFFN
LSTACFSLSMIFSFLAIFHVLQWTFLNFPPFSVFLAIFHVLKCVFLIFRVFQFPRHIPGPTVCISHFSRFSVI
SSFFKCVKWMFLIFHDFQFSCHIPCPTVDISKFSTFFFFNFLTIFQVLQCAFLIFHVFQ*FRHFSSCQVEVSH
FP*FSAF*
>ENSMUSP00000136448_c.641:G>T_p.M214I ENSMUST00000155807.3|Gm10715-201|ENSMUSG00000096201|+|chr9:g.3037815:G>T
MFLIFNDFHFSCLIPRPIVDISFPPFSVFLAIFHVPKCVFLIFHDFQFPRHIPGPTVCISHFSRFSVISSFFK
TSSGCFSFFMIFSFLAIFPVLQWTFLNFPPFSVFLAIFHVLKCVFLIFRDFQFSRQIPGPTVCISHFSRFSVI
LSFFNLSTACFSLSMIFSFLAIFHVLQWTFLNFPPFSFFSPYFTSYSVHFSFFTFFSDFVFFQDVKWIFLIFH
DFQFSC
>ENSMUSP00000137129_c.466:G>T_p.C156F ENSMUST00000177969.2|Gm10715-202|ENSMUSG00000096201|+|chr9:g.3037815:G>T
MFLIFHDFQFSCHIPCPTVDISFPPFSVFLAIFHVLKCVFLIFRDFQFSRQIPGPTVCISHFSRFSVILSFFN
LSTACFSLSMIFSFLAIFHVLQWTFLNFPPFSVFLAIFHVLKCVFLIFRVFQFPRHIPGPTVCISHFSRFSVI
SSFFKTSSGFFSFFMIFSFLAIFPVLQWTFLNFPPFSSSIFLPYSRSYSVHFSFFTFFSDFVIFQVVKWKFLI
FHDFQLSS
>ENSMUSP00000137129_c.478:T>C_p.F160S ENSMUST00000177969.2|Gm10715-202|ENSMUSG00000096201|+|chr9:g.3037827:T>C
MFLIFHDFQFSCHIPCPTVDISFPPFSVFLAIFHVLKCVFLIFRDFQFSRQIPGPTVCISHFSRFSVILSFFN
LSTACFSLSMIFSFLAIFHVLQWTFLNFPPFSVFLAIFHVLKCVFLIFRVFQFPRHIPGPTVCISHFSRFSVI

SSFFKTSSGCFSFSMIFSFLAIFPVLQWTFLNFPPFSSSIFLPYSRSYSVHFSFFTFFSDFVIFQVVKWKFLI
FHDFQLSS
>ENSMUSP00000137129_c.508:C>A_p.P170H ENSMUST00000177969.2|Gm10715-
202|ENSMUSG00000096201|+|chr9:g.3037857:C>A
MFLIFHDFQFSCHIPCPTVDISFPPFSVFLAIFHVLKCVFLIFRDFQFSRQIPGPTVCISHFSRFSVILSFFN
LSTACFSLSMIFSFLAIFHVLQWTFLNFPPFSVFLAIFHVLKCVFLIFRVFQFPRHIPGPTVCISHFSRFSVI
SSFFKTSSGCFSFFMIFSFLAIFHVLQWTFLNFPPFSSSIFLPYSRSYSVHFSFFTFFSDFVIFQVVKWKFLI
FHDFQLSS
>ENSMUSP00000137129_c.508:CT>AC_p.P170H ENSMUST00000177969.2|Gm10715-
202|ENSMUSG00000096201|+|chr9:g.3037857:CT>AC
MFLIFHDFQFSCHIPCPTVDISFPPFSVFLAIFHVLKCVFLIFRDFQFSRQIPGPTVCISHFSRFSVILSFFN
LSTACFSLSMIFSFLAIFHVLQWTFLNFPPFSVFLAIFHVLKCVFLIFRVFQFPRHIPGPTVCISHFSRFSVI
SSFFKTSSGCFSFFMIFSFLAIFHCPTVDISKFSTFFFFNFLTIFQVLQCAFLIFHVFQ*FRHFSSCQVEVSH
FP*FSAF*
>ENSMUSP00000137129_c.580:A>T_p.Y194F ENSMUST00000177969.2|Gm10715-
202|ENSMUSG00000096201|+|chr9:g.3038216:A>T
MFLIFHDFQFSCHIPCPTVDISFPPFSVFLAIFHVLKCVFLIFRDFQFSRQIPGPTVCISHFSRFSVILSFFN
LSTACFSLSMIFSFLAIFHVLQWTFLNFPPFSVFLAIFHVLKCVFLIFRVFQFPRHIPGPTVCISHFSRFSVI
SSFFKTSSGCFSFFMIFSFLAIFPVLQWTFLNFPPFSSSIFLPYSRSFSVHFSFFTFFSDFVIFQVVKWKFLI
FHDFQLSS
>ENSMUSP00000137129_c.581:C>A_p.Y194* ENSMUST00000177969.2|Gm10715-
202|ENSMUSG00000096201|+|chr9:g.3038217:C>A
MFLIFHDFQFSCHIPCPTVDISFPPFSVFLAIFHVLKCVFLIFRDFQFSRQIPGPTVCISHFSRFSVILSFFN
LSTACFSLSMIFSFLAIFHVLQWTFLNFPPFSVFLAIFHVLKCVFLIFRVFQFPRHIPGPTVCISHFSRFSVI
SSFFKTSSGCFSFFMIFSFLAIFPVLQWTFLNFPPFSSSIFLPYSRS*SVHFSFFTFFSDFVIFQVVKWKFLI
FHDFQLSS
>ENSMUSP00000137129_c.588:C>T_p.H197Y ENSMUST00000177969.2|Gm10715-
202|ENSMUSG00000096201|+|chr9:g.3038224:C>T
MFLIFHDFQFSCHIPCPTVDISFPPFSVFLAIFHVLKCVFLIFRDFQFSRQIPGPTVCISHFSRFSVILSFFN
LSTACFSLSMIFSFLAIFHVLQWTFLNFPPFSVFLAIFHVLKCVFLIFRVFQFPRHIPGPTVCISHFSRFSVI
SSFFKTSSGCFSFFMIFSFLAIFPVLQWTFLNFPPFSSSIFLPYSRSYSVYFSFFTFFSDFVIFQVVKWKFLI
FHDFQLSS
>ENSMUSP00000137129_c.604:C>A_p.T202K ENSMUST00000177969.2|Gm10715-
202|ENSMUSG00000096201|+|chr9:g.3038240:C>A
MFLIFHDFQFSCHIPCPTVDISFPPFSVFLAIFHVLKCVFLIFRDFQFSRQIPGPTVCISHFSRFSVILSFFN
LSTACFSLSMIFSFLAIFHVLQWTFLNFPPFSVFLAIFHVLKCVFLIFRVFQFPRHIPGPTVCISHFSRFSVI
SSFFKTSSGCFSFFMIFSFLAIFPVLQWTFLNFPPFSSSIFLPYSRSYSVHFSFFKFFSDFVIFQVVKWKFLI
FHDFQLSS
>ENSMUSP00000137129_c.621:G>A_p.V208I ENSMUST00000177969.2|Gm10715-
202|ENSMUSG00000096201|+|chr9:g.3038257:G>A
MFLIFHDFQFSCHIPCPTVDISFPPFSVFLAIFHVLKCVFLIFRDFQFSRQIPGPTVCISHFSRFSVILSFFN
LSTACFSLSMIFSFLAIFHVLQWTFLNFPPFSVFLAIFHVLKCVFLIFRVFQFPRHIPGPTVCISHFSRFSVI
SSFFKTSSGCFSFFMIFSFLAIFPVLQWTFLNFPPFSSSIFLPYSRSYSVHFSFFTFFSDFIIFQVVKWKFLI
FHDFQLSS
>ENSMUSP00000137129_c.646:A>T_p.K216M ENSMUST00000177969.2|Gm10715-
202|ENSMUSG00000096201|+|chr9:g.3038282:A>T
MFLIFHDFQFSCHIPCPTVDISFPPFSVFLAIFHVLKCVFLIFRDFQFSRQIPGPTVCISHFSRFSVILSFFN
LSTACFSLSMIFSFLAIFHVLQWTFLNFPPFSVFLAIFHVLKCVFLIFRVFQFPRHIPGPTVCISHFSRFSVI
SSFFKTSSGCFSFFMIFSFLAIFPVLQWTFLNFPPFSSSIFLPYSRSYSVHFSFFTFFSDFVIFQVVKWMFLI
FHDFQLSS
>ENSMUSP00000137129_c.654:A>G_p.I219V ENSMUST00000177969.2|Gm10715-
202|ENSMUSG00000096201|+|chr9:g.3038290:A>G
MFLIFHDFQFSCHIPCPTVDISFPPFSVFLAIFHVLKCVFLIFRDFQFSRQIPGPTVCISHFSRFSVILSFFN
LSTACFSLSMIFSFLAIFHVLQWTFLNFPPFSVFLAIFHVLKCVFLIFRVFQFPRHIPGPTVCISHFSRFSVI
SSFFKTSSGCFSFFMIFSFLAIFPVLQWTFLNFPPFSSSIFLPYSRSYSVHFSFFTFFSDFVIFQVVKWKFLV
FHDFQLSS
>ENSMUSP00000137129_c.672:C>T_p.L225F ENSMUST00000177969.2|Gm10715-
202|ENSMUSG00000096201|+|chr9:g.3038308:C>T

MFLIFHDFQFSCHIPCPTVDISFPPFSVFLAIFHVLKCVFLIFRDFQFSRQIPGPTVCISHFSRFSVILSFFN
LSTACFSLSMIFSFLAIFHVLQWTFLNFPPFSVFLAIFHVLKCVFLIFRVFQFPRHIPGPTVCISHFSRFSVI
SSFFKTSSGCFSFFMIFSFLAIFPVLQWTFLNFPPFSSSIFLPYSRSYSVHFSFFTFFSDFVIFQVVKWKFLI
FHDFQFSS
>rs244572871_ENSMUSP00000151193_c.315:T>TG_p.W106ins-fs
ENSMUST00000217651.3|Mmp1a-201|ENSMUSG00000043089|+|chr9:g.7465083:T>TG
MPSLPLLLLLWAASSYSFPVFHNGDRQNVETVWKYLENYYNLGKNMQAKNVNGKEMMAEKLRQMQQLFGLKVT
GNSDPETLRAMKKPRCGVPDVAPYAITHNNPRWDQNTSDIQHFKLHTIFAKSSCGRCHRESL
>ENSMUSP00000083459_c.1139:T>A_p.C380* ENSMUST00000086278.7|Zfp810-
201|ENSMUSG00000066829|-|chr9:g.22189767:A>T
MVLVSFEDVAVDFTWEEWQALDAAQRTLYRDVMLETYSSLVSLGHCMAKPELIFNLEQGLGPRSTAGASVWNL
SGVNKVSSLVDTIQENDSRHFWQIEINSHTANEELVEAELKIHEEIHRGAKSYECEVCLEAFYLKPQYSTKQS
YHTCENPCDCKKCREAFYPKSTLSQYQRLERGEKPHACLECGKSFYCKSHLTVHQRTHTGEKPYDCKECRKAF
YSKSQLNVHLRIHTGEKPYECKDCRKAFYRNSDLTVHQRTHTGEKPYECKVCRKAFYCNSQLTVHHRTHTGEK
PYECQVCNKAFYCKSQLAVHHRTHTGEKPYQCKECRKAFQCRSDLTRHQRTHTGERPYECPECRKAFYRKSDL
TVHQRTHTGEKPYE*KECRKAFYCNSQLTVHQRQHTGEKPYECKDCGKAFQCKYELTRHQRTHTGEKPYGCRE
CKKAFYTKSDLTRHLKTHAGDKP*
>ENSMUSP00000083459_c.1114:A>G_p.H372R ENSMUST00000086278.7|Zfp810-
201|ENSMUSG00000066829|-|chr9:g.22189792:T>C
MVLVSFEDVAVDFTWEEWQALDAAQRTLYRDVMLETYSSLVSLGHCMAKPELIFNLEQGLGPRSTAGASVWNL
SGVNKVSSLVDTIQENDSRHFWQIEINSHTANEELVEAELKIHEEIHRGAKSYECEVCLEAFYLKPQYSTKQS
YHTCENPCDCKKCREAFYPKSTLSQYQRLERGEKPHACLECGKSFYCKSHLTVHQRTHTGEKPYDCKECRKAF
YSKSQLNVHLRIHTGEKPYECKDCRKAFYRNSDLTVHQRTHTGEKPYECKVCRKAFYCNSQLTVHHRTHTGEK
PYECQVCNKAFYCKSQLAVHHRTHTGEKPYQCKECRKAFQCRSDLTRHQRTHTGERPYECPECRKAFYRKSDL
TVHQRTRTGEKPYECKECRKAFYCNSQLTVHQRQHTGEKPYECKDCGKAFQCKYELTRHQRTHTGEKPYGCRE
CKKAFYTKSDLTRHLKTHAGDKP*
>ENSMUSP00000150938_c.160:C>A_p.T54K ENSMUST00000216677.2|Ncapd3-
205|ENSMUSG00000035024|+|chr9:g.26948380:C>A
MALQDLGENLQPWCPLGLSLEWVKTVWDLDFTEIEPLDPSIVGEILETGRDAFKKLYGSLFPFATDESGSLES
IWTFFTENDISSNTLVALFCHFVQEAHKKSASAQYREYGLHAAGLYFLLLEIPGSVVNQVFHPVMFDKCIQIL
KRSWPQESNLTQKRKKDHSKSSKDNYRKSRKRGKPPRKEDYQVDELSREEEEEEEEIYFSGRDLCQIRDAIFN
LLKNFLRLLPKFSLKEKPQSIQTCIEVFVALTSFEPIPHKFLISQARNLNEVKHISELAYYGLYLLCSPVHGE
ENKVIGSIFHQMLNVILMLEVGEGSRCAPLAITSQVINCRNQAVQFVSSLVDELQASVYPVLGTLLQHICAKV
VDKAEYRTYAAQSLVQLLTKLPSEEYATFIAWLYKYSRSSKIPHRVFTLDVALALLTLPERELDDTVSLEHQK
FLKHKFFVQEIIFDRCLDKAPTVRSKALSSFAHCLELSSSNTSESILEIFINSNLVPGIQNLSNTVLNPSPVL
TSRNGYSAQSRTHNNDEQTLPGERCFMTMLRKRIKDEKINVRKSALQVLMSILKHCDILSMEQDLLILQDHCR
DPAISVRKQALQSLTELVMAQPTCVPVQKAWLMGVIPVVMDCESTVQEKALECLDQLLLQNIKHHKKFHSADR
SQVLAWSLLALLTIENQDLRRYLNKAFHIWSKKDKFSSTFINSVISHTDTERSAPAWMLLSKITCSSPKLDYT
KIIESWERLSREQSPNSNTLGYMLCVIGHIAKHLPKGTRDKITGVIKAKLNGFQWSPELISSSVDALQKLCRA
SAKTVLEEQGLLKQVCGDVLATCEQHLSNILLKEDGTGNMDEGLVVKCIFTLGDIAQLCPAIVEKRVFLLIQS
ILASSAHSDHLPSSQGTTDALDSQPPFQPRSSAMPSVIRAHAIITLGKLCLQHEDLAKKSIPALVRELEVSED
VAVRNNVIIVICDLCIRYTVMVDNYIPNISVCLKDSDPFIRKQTLVLLTNLLQEEYVKWKGSLFFRFVSTLVD
SHPDIASLGEFCLAHLLLKRNPTMFFQHFIECIFHFNSYEKHGQYNKFSQSERGKQLFLLKGKTNKEKRMRIY
KFLLEHFTDEQRFNVTSKICLNILACFTDGILPMDMEASELLSDTFDILNSKEIKLLAMRAQTSKDLLEEDDV
ALANVVMQEAQMKIISQVQKRNFIENIIPIIISLKTVLEKNKIPALRELMNYLREVMQDYRDEINDFFAVDKQ
LASELEYDMKKYNEQLAQEQALTEHANATKGPEDSDRVPSAQVAPDLEAVPALAAAPMAAAAAAAPMAAAAAA
AGQDNADVPPTQSRPSAPRSNFTPTLPPISENGPLKIMSSTRPMSLSTIAILNSVKKAVASKNRTRSLGALPF
NVETGSPENPSSHESSLSLEKESDRTVNHVTKRAISTPENSISDVTFAAGVSYIGTPATFFTKEKHEAQEQGS
DILCLSLLDKRPPQSPQWNVKSPARSHGSTRSSRRSLRKAPLKTAN*
>ENSMUSP00000072871_c.160:C>A_p.T54K ENSMUST00000073127.14|Ncapd3-
201|ENSMUSG00000035024|+|chr9:g.26948380:C>A
MALQDLGENLQPWCPLGLSLEWVKTVWDLDFTEIEPLDPSIVGEILETGRDAFKKLYGSLFPFATDESGSLES
IWTFFTENDISSNTLVALFCHFVQEAHKKSASAQYREYGLHAAGLYFLLLEIPGSVVNQVFHPVMFDKCIQIL
KRSWPQESNLTQKRKKDHSKSSKDNYRKSRKRGKPPRKEDYQVDELSREEEEEEEEIYFSGRDLCQIRDAIFN
LLKNFLRLLPKFSLKEKPQSIQTCIEVFVALTSFEPIPHKFLISQARNLNEVKHISELAYYGLYLLCSPVHGE
ENKVIGSIFHQMLNVILMLEVGEGSRCAPLAITSQVINCRNQAVQFVSSLVDELQASVYPVLGTLLQHICAKV
VDKAEYRTYAAQSLVQLLTKLPSEEYATFIAWLYKYSRSSKIPHRVFTLDVALALLTLPERELDDTVSLEHQK
FLKHKFFVQEIIFDRCLDKAPTVRSKALSSFAHCLELSSSNTSESILEIFINSNLVPGIQNLSNTVLNPSPVL

TSRNGYSAQSRTHNNDEQTLPGERCFMTMLRKRIKDEKINVRKSALQVLMSILKHCDILSMEQDLLILQDHCR
DPAISVRKQALQSLTELVMAQPTCVPVQKAWLMGVIPVVMDCESTVQEKALECLDQLLLQNIKHHKKFHSADR
SQVLAWSLLALLTIENQDLRRYLNKAFHIWSKKDKFSSTFINSVISHTDTERSAPAWMLLSKITCSSPKLDYT
KIIESWERLSREQSPNSNTLGYMLCVIGHIAKHLPKGTRDKITGVIKAKLNGFQWSPELISSSVDALQKLCRA
SAKTVLEEQGLLKQVCGDVLATCEQHLSNILLKEDGTGNMDEGLVVKCIFTLGDIAQLCPAIVEKRVFLLIQS
ILASSAHSDHLPSSQGTTDALDSQPPFQPRSSAMPSVIRAHAIITLGKLCLQHEDLAKKSIPALVRELEVSED
VAVRNNVIIVICDLCIRYTVMVDNYIPNISVCLKDSDPFIRKQTLVLLTNLLQEEYVKWKGSLFFRFVSTLVD
SHPDIASLGEFCLAHLLLKRNPTMFFQHFIECIFHFNSYEKHGQYNKFSQSERGKQLFLLKGKTNKEKRMRIY
KFLLEHFTDEQRFNVTSKICLNILACFTDGILPMDMEASELLSDTFDILNSKEIKLLAMRAQTSKDLLEEDDV
ALANVVMQEAQMKIISQVQKRNFIENIIPIIISLKTVLEKNKIPALRELMNYLREVMQDYRDEINDFFAVDKQ
LASELEYDMKKYNEQLAQEQALTEHANATKGPEDSDRVPSAQVAPDLEAVPALAAAPMAAAAAAAPMAAAAAA
AGQDNADVPPTQSRPSAPRSNFTPTLPPISENGPLKIMSSTRPMSLSTIAILNSVKKAVASKNRTRSLGALPF
NVETGSPENPSSHESSLSLEKESDRTVNHVTKRAISTPENSISDVTFAAGVSYIGTPATFFTKEKHEAQEQGS
DILCLSLLDKRPPQSPQWNVKSPARSHGSTRSSRRSLRKAPLKTAN*
>ENSMUSP00000083374_c.160:C>A_p.T54K ENSMUST00000086198.5|Ncapd3-202|ENSMUSG00000035024|+|chr9:g.26948380:C>A
MALQDLGENLQPWCPLGLSLEWVKTVWDLDFTEIEPLDPSIVGEILETGRDAFKKLYGSLFPFATDESGSLES
IWTFFTENDISSNTLVALFCHFVQEAHKKSASAQYREYGLHAAGLYFLLLEIPGSVVNQVFHPVMFDKCIQIL
KRSWPQESNLTQKRKKDHSKSSKDNYRKSRKRGKPPRKEDYQVDELSREEEEEEEEIYFSGRDLCQIRDAIFN
LLKNFLRLLPKFSLKEKPQSIQTCIEVFVALTSFEPIPHKFLISQARNLNEVKHISELAYYGLYLLCSPVHGE
ENKVIGSIFHQMLNVILMLEVGEGSRCAPLAITSQVINCRNQAVQFVSSLVDELQASVYPVLGTLLQHICAKV
VDKAEYRTYAAQSLVQLLTKLPSEEYATFIAWLYKYSRSSKIPHRVFTLDVALALLTLPERELDDTVSLEHQK
FLKHKFFVQEIIFDRCLDKAPTVRSKALSSFAHCLELSSSNTSESILEIFINSNLVPGIQNLSNTVLNPSPVL
TSRNGYSAQSRTHNNDEQTLPGERCFMTMLRKRIKDEKINVRKSALQVLMSILKHCDILSMEQDLLILQDHCR
DPAISVRKQALQSLTELVMAQPTCVPVQKAWLMGVIPVVMDCESTVQEKALECLDQLLLQNIKHHKKFHSADR
SQVLAWSLLALLTIENQDLRRYLNKAFHIWSKKDKFSSTFINSVISHTDTERSAPAWMLLSKITCSSPKLDYT
KIIESWERLSREQSPNSNTLGYMLCVIGHIAKHLPKGTRDKITGVIKAKLNGFQWSPELISSSVDALQKLCRA
SAKTVLEEQGLLKQVCGDVLATCEQHLSNILLKEDGTGNMDEGLVVKCIFTLGDIAQLCPAIVEKRVFLLIQS
ILASSAHSDHLPSSQGTTDALDSQPPFQPRSSAMPSVIRAHAIITLGKLCLQHEDLAKKSIPALVRELEVSED
VAVRNNVIIVICDLCIRYTVMVDNYIPNISVCLKDSDPFIRKQTLVLLTNLLQEEYVKWKGSLFFRFVSTLVD
SHPDIASLGEFCLAHLLLKRNPTMFFQHFIECIFHFNSYEKHGQYNKFSQSERGKQLFLLKGKTNKEKRMRIY
KFLLEHFTDEQRFNVTSKICLNILACFTDGILPMDMEASELLSDTFDILNSKEIKLLAMRAQTSKDLLEEDDV
ALANVVMQEAQMKIISQVQKRNFIENIIPIIISTVERKVPSQESRQHKIQPEVPS*
>ENSMUSP00000150938_c.4489:T>G_p.L1497R ENSMUST00000216677.2|Ncapd3-205|ENSMUSG00000035024|+|chr9:g.27006070:T>G
MALQDLGENLQPWCPLGLSLEWVKTVWDLDFTEIEPLDPSIVGEILETGRDAFTKLYGSLFPFATDESGSLES
IWTFFTENDISSNTLVALFCHFVQEAHKKSASAQYREYGLHAAGLYFLLLEIPGSVVNQVFHPVMFDKCIQIL
KRSWPQESNLTQKRKKDHSKSSKDNYRKSRKRGKPPRKEDYQVDELSREEEEEEEEIYFSGRDLCQIRDAIFN
LLKNFLRLLPKFSLKEKPQSIQTCIEVFVALTSFEPIPHKFLISQARNLNEVKHISELAYYGLYLLCSPVHGE
ENKVIGSIFHQMLNVILMLEVGEGSRCAPLAITSQVINCRNQAVQFVSSLVDELQASVYPVLGTLLQHICAKV
VDKAEYRTYAAQSLVQLLTKLPSEEYATFIAWLYKYSRSSKIPHRVFTLDVALALLTLPERELDDTVSLEHQK
FLKHKFFVQEIIFDRCLDKAPTVRSKALSSFAHCLELSSSNTSESILEIFINSNLVPGIQNLSNTVLNPSPVL
TSRNGYSAQSRTHNNDEQTLPGERCFMTMLRKRIKDEKINVRKSALQVLMSILKHCDILSMEQDLLILQDHCR
DPAISVRKQALQSLTELVMAQPTCVPVQKAWLMGVIPVVMDCESTVQEKALECLDQLLLQNIKHHKKFHSADR
SQVLAWSLLALLTIENQDLRRYLNKAFHIWSKKDKFSSTFINSVISHTDTERSAPAWMLLSKITCSSPKLDYT
KIIESWERLSREQSPNSNTLGYMLCVIGHIAKHLPKGTRDKITGVIKAKLNGFQWSPELISSSVDALQKLCRA
SAKTVLEEQGLLKQVCGDVLATCEQHLSNILLKEDGTGNMDEGLVVKCIFTLGDIAQLCPAIVEKRVFLLIQS
ILASSAHSDHLPSSQGTTDALDSQPPFQPRSSAMPSVIRAHAIITLGKLCLQHEDLAKKSIPALVRELEVSED
VAVRNNVIIVICDLCIRYTVMVDNYIPNISVCLKDSDPFIRKQTLVLLTNLLQEEYVKWKGSLFFRFVSTLVD
SHPDIASLGEFCLAHLLLKRNPTMFFQHFIECIFHFNSYEKHGQYNKFSQSERGKQLFLLKGKTNKEKRMRIY
KFLLEHFTDEQRFNVTSKICLNILACFTDGILPMDMEASELLSDTFDILNSKEIKLLAMRAQTSKDLLEEDDV
ALANVVMQEAQMKIISQVQKRNFIENIIPIIISLKTVLEKNKIPALRELMNYLREVMQDYRDEINDFFAVDKQ
LASELEYDMKKYNEQLAQEQALTEHANATKGPEDSDRVPSAQVAPDLEAVPALAAAPMAAAAAAAPMAAAAAA
AGQDNADVPPTQSRPSAPRSNFTPTLPPISENGPLKIMSSTRPMSLSTIAILNSVKKAVASKNRTRSLGALPF
NVETGSPENPSSHESSLSLEKESDRTVNHVTKRAISTPENSISDVTFAAGVSYIGTPATFFTKEKHEAQEQGS
DILCLSLLDKRPPQSPQWNVKSPARSHGSTRSSRRSRRKAPLKTAN*
>ENSMUSP00000072871_c.4489:T>G_p.L1497R ENSMUST00000073127.14|Ncapd3-201|ENSMUSG00000035024|+|chr9:g.27006070:T>G

MALQDLGENLQPWCPLGLSLEWVKTVWDLDFTEIEPLDPSIVGEILETGRDAFTKLYGSLFPFATDESGSLES
IWTFFTENDISSNTLVALFCHFVQEAHKKSASAQYREYGLHAAGLYFLLLEIPGSVVNQVFHPVMFDKCIQIL
KRSWPQESNLTQKRKKDHSKSSKDNYRKSRKRGKPPRKEDYQVDELSREEEEEEEEIYFSGRDLCQIRDAIFN
LLKNFLRLLPKFSLKEKPQSIQTCIEVFVALTSFEPIPHKFLISQARNLNEVKHISELAYYGLYLLCSPVHGE
ENKVIGSIFHQMLNVILMLEVGEGSRCAPLAITSQVINCRNQAVQFVSSLVDELQASVYPVLGTLLQHICAKV
VDKAEYRTYAAQSLVQLLTKLPSEEYATFIAWLYKYSRSSKIPHRVFTLDVALALLTLPERELDDTVSLEHQK
FLKHKFFVQEIIFDRCLDKAPTVRSKALSSFAHCLELSSSNTSESILEIFINSNLVPGIQNLSNTVLNPSPVL
TSRNGYSAQSRTHNNDEQTLPGERCFMTMLRKRIKDEKINVRKSALQVLMSILKHCDILSMEQDLLILQDHCR
DPAISVRKQALQSLTELVMAQPTCVPVQKAWLMGVIPVVMDCESTVQEKALECLDQLLLQNIKHHKKFHSADR
SQVLAWSLLALLTIENQDLRRYLNKAFHIWSKKDKFSSTFINSVISHTDTERSAPAWMLLSKITCSSPKLDYT
KIIESWERLSREQSPNSNTLGYMLCVIGHIAKHLPKGTRDKITGVIKAKLNGFQWSPELISSSVDALQKLCRA
SAKTVLEEQGLLKQVCGDVLATCEQHLSNILLKEDGTGNMDEGLVVKCIFTLGDIAQLCPAIVEKRVFLLIQS
ILASSAHSDHLPSSQGTTDALDSQPPFQPRSSAMPSVIRAHAIITLGKLCLQHEDLAKKSIPALVRELEVSED
VAVRNNVIIVICDLCIRYTVMVDNYIPNISVCLKDSDPFIRKQTLVLLTNLLQEEYVKWKGSLFFRFVSTLVD
SHPDIASLGEFCLAHLLLKRNPTMFFQHFIECIFHFNSYEKHGQYNKFSQSERGKQLFLLKGKTNKEKRMRIY
KFLLEHFTDEQRFNVTSKICLNILACFTDGILPMDMEASELLSDTFDILNSKEIKLLAMRAQTSKDLLEEDDV
ALANVVMQEAQMKIISQVQKRNFIENIIPIIISLKTVLEKNKIPALRELMNYLREVMQDYRDEINDFFAVDKQ
LASELEYDMKKYNEQLAQEQALTEHANATKGPEDSDRVPSAQVAPDLEAVPALAAAPMAAAAAAAPMAAAAAA
AGQDNADVPPTQSRPSAPRSNFTPTLPPISENGPLKIMSSTRPMSLSTIAILNSVKKAVASKNRTRSLGALPF
NVETGSPENPSSHESSLSLEKESDRTVNHVTKRAISTPENSISDVTFAAGVSYIGTPATFFTKEKHEAQEQGS
DILCLSLLDKRPPQSPQWNVKSPARSHGSTRSSRRSRRKAPLKTAN*
>rs45708828_ENSMUSP00000011262_c.52:C>A_p.P18H
ENSMUST00000011262.4|Panx3-201|ENSMUSG00000011118|-|chr9:g.37580299:G>T
MSLAHTAAEYMLSDALLHDRRGSRLKGLRLELPLDKMVKFITVGFPLLLMSLAFAQEFSSGSPISCFSPSNFS
VRQAAYVDSSCWDSLAHHTQDKAGQYKVKSLWPHKALPYSLLALAVAMYLPVLLWQYVAVPSLSSDLLFIISE
LDKSYNRSIRLVQHMLQIRQSSSDPHVFWDELEKARKERYFEFPLLERYLECKQRSHWLVATYLLRNALLLLF
TSATYLYLGQFHLDVFFQDEFNCFIKTGLLHDETHVPELITCRLTSLSVFQIVSVSSAAIYTILVPVIIYNLT
RLCRWDKGLLSIYEMLPAFDLLSRKMLGCPINDLNVILLFLRANISELISFSWLSVLSVLKDTTTQKHNIDTV
VDFMTFVAGLEPSKPKHLTQHTYDEHA*
>rs45708828_ENSMUSP00000114539_c.52:C>A_p.P18H
ENSMUST00000142228.2|Panx3-202|ENSMUSG00000011118|-|chr9:g.37580299:G>T
MSLAHTAAEYMLSDALLHDRRGSRLKGLRLELPLDKMVKFITVGFPLLLMSLAFAQEFSSEIQSLVLCALQNK
RAFFQSGYGSHICNRSIPEPEAEG*
>ENSMUSP00000150357_c.715:G>C_p.S239T ENSMUST00000214377.2|Olfr905-
202|ENSMUSG00000096794|+|chr9:g.38384760:G>C
MGFENGSSVTEFILVGLTKESDLQCPLFILFLMMYVVTVLGNQGLISLIGLNSHLHTPMYFFLFNLSFVDLWY
SSVFTPKMLESFISEKNTISYRGCMAQLFFFCFFSISECYILTSMAYDRYVAICNPLLYNIVMSPKQCLILMF
SSYMMAFSGAMAHTGCMLRLTFCDANTINHYFCDILPLLQLSCTSTYVNELEVFVVVGINIIVPTITIFISYG
FIIASIFRISSKEDRSKAFTTCSSHIIAVSLFFGSGAFMYLKPSSAESMNEGKISSIFYTNTVPLLNPLIYSL
RNKDVKDALIKTLSKRKR*
>ENSMUSP00000057998_c.715:G>C_p.S239T ENSMUST00000051111.3|Olfr905-
201|ENSMUSG00000096794|+|chr9:g.38384760:G>C
MGFENGSSVTEFILVGLTKESDLQCPLFILFLMMYVVTVLGNQGLISLIGLNSHLHTPMYFFLFNLSFVDLWY
SSVFTPKMLESFISEKNTISYRGCMAQLFFFCFFSISECYILTSMAYDRYVAICNPLLYNIVMSPKQCLILMF
SSYMMAFSGAMAHTGCMLRLTFCDANTINHYFCDILPLLQLSCTSTYVNELEVFVVVGINIIVPTITIFISYG
FIIASIFRISSKEDRSKAFTTCSSHIIAVSLFFGSGAFMYLKPSSAESMNEGKISSIFYTNTVPLLNPLIYSL
RNKDVKDALIKTLSKRKR*
>ENSMUSP00000001544_c.1083:G>A_p.A362T ENSMUST00000001544.12|Vwa5a-
201|ENSMUSG00000023186|+|chr9:g.38639354:G>A
MEHHCGLITSNKETVPLKNISVTLSINEFVAAVVATLNYENEEKVPLEATFVFPMDEDSAVYSFEALVDGKKI
VAELQDKMKAHSEYEEALSQGHQAYLLEEDDYSRDVFSCNVGNLQPGAKVAVTLRYVQELPLETDGALRYLLP
AILNPRYQLSEQSANSCLNIQKPTVPLEDLPYTLNMTATITSQHGIERVQSNCSLSPIQYLTDDKTSAQVSLT
EGHKFDRDVELLIYYNEVHSPSVAVEMGMLDMKPDSLMGAPSAMVSFYPDIPEVEASKACGEFVFLMDRSGSM
DSPMSTENNSQLRIEAAKETLLLLLKSLPMGCYFNIYGFGSSYEKFFPESVKYTQDTMEDAVKRVKALKTNLG
GTEILTPLCNIYKASSIPGHPLQLFVFTDGEVSDTFSVIREVKLNSKKHRCFSFGIGQGASTSLIKNIARVSG
GTAVFITGKDRMQTKALGSLKFALQCAVDNISLSWDLPPGLSVKMLSPEQLTIFRGQRLIIYAQLTGLMPQVE
STGAVCLKHILQGRSLENRVTFSLQPKANDNFTIHRLAAKSLIQTKDFGSQEASKEEKEDVMNISLQSGVLSS
FTAFIAINKELNKPVQGPLAHRVIPRPVMAGSSSMRFYSSFSGGFKGPQPRSLRVPAYDLCSAESANLVLKKS

ACSAIQKKKTNSSTNKSNLKKEHKAFGENAVVQLISLQKANGSWELDEDLTKILGTKSKDIKAANPAKHEDPS
AWSTVLAVVWLHANGTDLKCEWELLERKAVAWLHDHAGSSIPMLVQAANSLLKLSVNPAVFGV*
>ENSMUSP00000113596_c.1083:G>A_p.A362T ENSMUST00000118144.8|Vwa5a-202|ENSMUSG00000023186|+|chr9:g.38639354:G>A
MEHHCGLITSNKETVPLKNISVTLSINEFVAAVVATLNYENEEKVPLEATFVFPMDEDSAVYSFEALVDGKKI
VAELQDKMKAHSEYEEALSQGHQAYLLEEDDYSRDVFSCNVGNLQPGAKVAVTLRYVQELPLETDGALRYLLP
AILNPRYQLSEQSANSCLNIQKPTVPLEDLPYTLNMTATITSQHGIERVQSNCSLSPIQYLTDDKTSAQVSLT
EGHKFDRDVELLIYYNEVHSPSVAVEMGMLDMKPDSLMGAPSAMVSFYPDIPEVEASKACGEFVFLMDRSGSM
DSPMSTENNSQLRIEAAKETLLLLLKSLPMGCYFNIYGFGSSYEKFFPESVKYTQDTMEDAVKRVKALKTNLG
GTEILTPLCNIYKASSIPGHPLQLFVFTDGEVSDTFSVIREVKLNSKKHRCFSFGIGQGASTSLIKNIARVSG
GTAVFITGKDRMQTKALGSLKFALQCAVDNISLSWDLPPGLSVKMLSPEQLTIFRGQRLIIYAQLTGLMPQVE
STGAVCLKHILQGRSLENRVTFSLQPKANDNFTIHRLAAKSLIQTKDFGSQEASKEEKEDVMNISLQSGVLSS
FTAFIAINKELNKPVQGPLAHRVIPRPVMAGSSSMRFYSSFSGGFKGPQPRSLRVPAYDLCSAESANLVLKKS
ACSAIQKKKTNSSTNKSNLKKEHKAFGENAVVQLISLQKANGSWELDEDLTKILGTKSKDIKAANPAKHEDPS
AWSTVLAVVWLHANGTDLKCEWELLERKAVAWLHDHAGSSIPMLVQAANSLLKLSVNPAVFGV*
>rs215248989_ENSMUSP00000042582_c.1925:G>A_p.W642* ENSMUST00000042485.11|AW551984-201|ENSMUSG00000038112|-|chr9:g.39502745:C>T
MVRSGGLLTSIRDPVALKSIAVTLSINDFVAGVSATLNYENEEKSPLEAFFVFPMDEDSAVYSFEAFVDGKKI
VAELQDKYQAHKRYEEALSGGYQAYLLEEDKCSRDVFCCNVGNLQPGSKVSLTLRYVQELPLEDDGALRYVLP
AILNPRYHLSDGREDSNLDMKTPIVPLEELPYTLSMVATISSQLGISTIQSNCPLNPIEYTGDNKTSAQVSLA
EGHKFDRDVELLIFYRKVHSPSVAVEMGMSGRPSDSLMGAPSAMVSFYPDIPEVETTTKCGEFVFLMDRSRSM
NSPMSSKDKSQLRIDAAKETLILLLKSLPMGCYFNIYGFGATHEEFFPDSVMYNQETMQIAVKKVKRLLADLG
GTELLTPLRKIFRKPPIPGHPLQVFVFTDGEVVETFSVIREVMFQSKKHRCFSFGIGEGASTSLIKNLARVSG
GTAEFITGNDRMQSKALRSLRRSLQSVVEDVSLSWNLPPKMFAPMLSPEQTSIFRGQRLIIYSLLIGKIPKEE
ATGEVCLSYKLHGKSYEEKVTFSLQPKPDDNLTIHRLAAKSMIQSKDFGFKETSLMYKRDVLDISLESGVMSS
FTAFVAINKELNEPIQGPLVHRDIPRPILLGAGPNRPSDGGYPKTLFCASAPVTLPP*LKGSGKCVATILMPN
GTPQQRQNSQKKKCVVHTDTRPELPHLAKYDSYPQGSKEHSIAQLIYLQNADGSWDMNEDLAKILSTSLEDIT
AACPTEVLEPSAWATMLAVIWLHANGKELKDEWELLERKAVVWLHNNSVRGFNKLIRTANKLLKTSLSPSIFK
L*
>rs215248989_ENSMUSP00000113212_c.1925:G>A_p.W642* ENSMUST00000119722.8|AW551984-202|ENSMUSG00000038112|-|chr9:g.39502745:C>T
MVRSGGLLTSIRDPVALKSIAVTLSINDFVAGVSATLNYENEEKSPLEAFFVFPMDEDSAVYSFEAFVDGKKI
VAELQDKYQAHKRYEEALSGGYQAYLLEEDKCSRDVFCCNVGNLQPGSKVSLTLRYVQELPLEDDGALRYVLP
AILNPRYHLSDGREDSNLDMKTPIVPLEELPYTLSMVATISSQLGISTIQSNCPLNPIEYTGDNKTSAQVSLA
EGHKFDRDVELLIFYRKVHSPSVAVEMGMSGRPSDSLMGAPSAMVSFYPDIPEVETTTKCGEFVFLMDRSRSM
NSPMSSKDKSQLRIDAAKETLILLLKSLPMGCYFNIYGFGATHEEFFPDSVMYNQETMQIAVKKVKRLLADLG
GTELLTPLRKIFRKPPIPGHPLQVFVFTDGEVVETFSVIREVMFQSKKHRCFSFGIGEGASTSLIKNLARVSG
GTAEFITGNDRMQSKALRSLRRSLQSVVEDVSLSWNLPPKMFAPMLSPEQTSIFRGQRLIIYSLLIGKIPKEE
ATGEVCLSYKLHGKSYEEKVTFSLQPKPDDNLTIHRLAAKSMIQSKDFGFKETSLMYKRDVLDISLESGVMSS
FTAFVAINKELNEPIQGPLVHRDIPRPILLGAGPNRPSDGGYPKTLFCASAPVTLPP*LKGSGKCVATILMPN
GTPQQRQNSQKKKCVVHTDTRPELPHLAKYDSYPQGSKEHSIAQLIYLQNADGSWDMNEDLAKILSTSLEDIT
AACPTEVLEPSAWATMLAVIWLHANGKELKDEWELLERKAVVWLHNNSVRGFNKLIRTANKLLKTSLSPSIFK
L*
>ENSMUSP00000034621_c.1093:C>A_p.A365D ENSMUST00000034621.16|Nlrx1-201|ENSMUSG00000032109|-|chr9:g.44174106:G>T
MRWGCHLPRTSWGSGLGRTPQLPDEHISFLIQWSWPFKGVHPLRPPRAFIRYHGNSADSAPPPGRHGQLFRSI
SATEAIQRHRRNLTEWFSRLPREERQFGPTFALDTVHVDPVIRESTPDELLRPSTELATGHQQTQAGLPPLAL
SQLFDPDSCGRRVQTVVLYGTVGTGKSTLVRKMVLDWCYGRLPAFELLIPFSCEDLSSLGSTPASLCQLVTQR
YTPLKEVLPLMTAAGSRLLFVLHGLERLNLDFRLAGTGLCSDPEEPGPPAAIIVNLLRKYMLPEASILVTTRP
STISRIPSKYVGRYGEICGFSDTNLQKLYFQLRLNQPDCGYGAGGASVSVTPAQRDNLIQMLSRNLEGHHQID
AACFLPSYCWLVCATLHFLHAPTPAGQTLTSIYTSFLRLNFSGETLDSTHTSNLSLMSYAARTMGKLAYEGVS
SRKTYFSEEDVRGCLEAGIKTEEEFQLLQIFRRDALRFFLAPCVEPGHLGTFVFTVPAMQEYLAALYIVLGLR
KTALQRVGKEVVEFVGRVGEDVSLVLGIVAKLLPLRILPLLFNLLKVVPRVFGRMVSKSREAVAQAMVLEMFR
EEDYYNDDVLDQMGASILGVEGPRRHPDEPSEDEVFELFPMFMGGLLSAHNRAVLAQLGCPIKNLDALENAQA
IKKKLGKLGRQVLPPSELLDHLFFHYEFQNQRFSAEVLGSLRQLNLAGVRMTPLKCTVVASVLGSGRHPLDEV
NLASCQLDPAGLHTLMPVLLRARKLGLQLNNLGPEACRDLRDLLLHDQCQITTLRLSNNPLTAAGVGLLMDGL

AGNTSVTHLSLLHTDLGDEGLELLAAQLDRNKQLQELNVAYNGAGDTVALALAKAAREHPSLELLHLYFNELS
SEGRQVLRDLGGSGEGGARVVASLTEGTAVSEYWSVILSEVQRNVHSWDPLRVQRHLKLLLRDLEDSRGATLN
PWRKAQLLRVEGEVKTLLEQLGGSGH*
>ENSMUSP00000126555_c.1093:C>A_p.A365D ENSMUST00000169651.3|Nlrx1-203|ENSMUSG00000032109|-|chr9:g.44174106:G>T
MRWGCHLPRTSWGSGLGRTPQLPDEHISFLIQWSWPFKGVHPLRPPRAFIRYHGNSADSAPPPGRHGQLFRSI
SATEAIQRHRRNLTEWFSRLPREERQFGPTFALDTVHVDPVIRESTPDELLRPSTELATGHQQTQAGLPPLAL
SQLFDPDSCGRRVQTVVLYGTVGTGKSTLVRKMVLDWCYGRLPAFELLIPFSCEDLSSLGSTPASLCQLVTQR
YTPLKEVLPLMTAAGSRLLFVLHGLERLNLDFRLAGTGLCSDPEEPGPPAAIIVNLLRKYMLPEASILVTTRP
STISRIPSKYVGRYGEICGFSDTNLQKLYFQLRLNQPDCGYGAGGASVSVTPAQRDNLIQMLSRNLEGHHQID
AACFLPSYCWLVCATLHFLHAPTPAGQTLTSIYTSFLRLNFSGETLDSTHTSNLSLMSYAARTMGKLAYEGVS
SRKTYFSEEDVRGCLEAGIKTEEEFQLLQIFRRDALRFFLAPCVEPGHLGTFVFTVPAMQEYLAALYIVLGLR
KTALQRVGKEVVEFVGRVGEDVSLVLGIVAKLLPLRILPLLFNLLKVVPRVFGRMVSKSREAVAQAMVLEMFR
EEDYYNDDVLDQMGASILGVEGPRRHPDEPSEDEVFELFPMFMGGLLSAHNRAVLAQLGCPIKNLDALENAQA
IKKKLGKLGRQVLPPSELLDHLFFHYEFQNQRFSAEVLGSLRQLNLAGVRMTPLKCTVVASVLGSGRHPLDEV
NLASCQLDPAGLHTLMPVLLRARKLGLQLNNLGPEACRDLRDLLLHDQCQITTLRLSNNPLTAAGVGLLMDGL
AGNTSVTHLSLLHTDLGDEGLELLAAQLDRNKQLQELNVAYNGAGDTVALALAKAAREHPSLELLHLYFNELS
SEGRQVLRDLGGSGEGGARVVASLTEGTAVSEYWSVILSEVQRNVHSWDPLRVQRHLKLLLRDLEDSRGATLN
PWRKAQLLRVEGEVKTLLEQLGGSGH*
>ENSMUSP00000127531_c.1093:C>A_p.A365D ENSMUST00000168499.9|Nlrx1-202|ENSMUSG00000032109|-|chr9:g.44174106:G>T
MRWGCHLPRTSWGSGLGRTPQLPDEHISFLIQWSWPFKGVHPLRPPRAFIRYHGNSADSAPPPGRHGQLFRSI
SATEAIQRHRRNLTEWFSRLPREERQFGPTFALDTVHVDPVIRESTPDELLRPSTELATGHQQTQAGLPPLAL
SQLFDPDSCGRRVQTVVLYGTVGTGKSTLVRKMVLDWCYGRLPAFELLIPFSCEDLSSLGSTPASLCQLVTQR
YTPLKEVLPLMTAAGSRLLFVLHGLERLNLDFRLAGTGLCSDPEEPGPPAAIIVNLLRKYMLPEASILVTTRP
STISRIPSKYVGRYGEICGFSDTNLQKLYFQLRLNQPDCGYGAGGASVSVTPAQRDNLIQMLSRNLEGHHQID
AACFLPSYCWLVCATLHFLHAPTPAGQTLTSIYTSFLRLNFSGETLDSTHTSNLSLMSYAARTMGKLAYEGVS
SRKTYFSEEDVRGCLEAGIKTEEEFQLLQIFRRDALRFFLAPCVEPGHLGTFVFTVPAMQEYLAALYIVLGLR
KTALQRVGKEVVEFVGRVGEDVSLVLGIVAKLLPLRILPLLFNLLKVVPRVFGRMVSKSREAVAQAMVLEMFR
EEDYYNDDVLDQMGASILGVEGPRRHPDEPSEDEVFELFPMFMGGLLSAHNRAVLAQLGCPIKNLDALENAQA
IKKKLGKLGRQVLPPSELLDHLFFHYEFQNQRFSAEVLGSLRQLNLAGVRMTPLKCTVVASVLGSGRHPLDEV
NLASCQLDPAGLHTLMPVLLRARKLGLQLNNLGPEACRDLRDLLLHDQCQITTLRLSNNPLTAAGVGLLMDGL
AGNTSVTHLSLLHTDLGDEGLELLAAQLDRNKQLQELNVAYNGAGDTVALALAKAAREHPSLELLHLYFNELS
SEGRQVLRDLGGSGEGGARVVASLTEGTAVSEYWSVILSEVQRNVHSWDPLRVQRHLKLLLRDLEDSRGATLN
PWRKAQLLRVEGEVKTLLEQLGGSGH*
>ENSMUSP00000110337_c.5083:T>C_p.V1695A ENSMUST00000114689.8|Kmt2a-202|ENSMUSG00000002028|-|chr9:g.44742612:A>G
MAHSCRWRFPARPGTTGGGGGGGRRGLGGAPRQRVPALLLPPGPQAGGGGPGAPPSPPAVAAAAAGSSGAGVP
GGAAAASAASSSSASSSSSSSSSASSGPALLRVGPGFDAALQVSAAIGTNLRRFRAVFGESGGGGGSGEDEQF
LGFGSDEEVRVRSPTRSPSVKASPRKPRGRPRSGSDRNPAILSDPSVFSPLNKSETKSADKIKKKDSKSIEKK
RGRPPTFPGVKIKITHGKDIAELTQGSKEDSLKKVKRTPSAMFQQATKIKKLRAGKLSPLKSKFKTGKLQIGR
KGVQIVRRRGRPPSTERIKTPSGLLINSELEKPQKVRKDKEGTPPLTKEDKTVVRQSPRRIKPVRIIPSCKRT
DATIAKQLLQRAKKGAQKKIEKEAAQLQGRKVKTQVKNIRQFIMPVVSAISSRIIKTPRRFIEDEDYDPPMKI
ARLESTPNSRFSATSCGSSEKSSAASQHSSQMSSDSSRSSSPSIDTTSDSQASEEIQALPEERSNTPEVHTPL
PISQSPENESNDRRSRRYSMSERSFGSRATKKLPTLQSAPQQQTSSSPPPPLLTPPPPLQPASGISDHTPWLM
PPTIPLASPFLPASAAPMQGKRKSILREPTFRWTSLKHSRSEPQYFSSAKYAKEGLIRKPIFDNFRPPPLTPE
DVGFASGFSASGTAASARLFSPLHSGTRFDIHKRSPILRAPRFTPSEAHSRIFESVTLPSNRTSSGASSSGVS
NRKRKRKVFSPIRSEPRSPSHSMRTRSGRLSTSELSPLTPPSSVSSSLSIPVSPLAASALNPTFTFPSHSLTQ
SGESTEKNQRARKQTSALAEPFSSNSPALFPWFTPGSQTEKGRKKDTAPEELSKDRDADKSVEKDKSRERDRE
REKENKRESRKEKRKKGSDIQSSSALYPVGRVSKEKVAGEDVGTSSSAKKATGRKKSSSLDSGADVAPVTLGD
TTAVKAKILIKKGRGNLEKNNLDLGPAAPSLEKERTPCLSAPSSSTVKHSTSSIGSMLAQADKLPMTDKRVAS
LLKKAKAQLCKIEKSKSLKQTDQPKAQGQESDSSETSVRGPRIKHVCRRAAVALGRKRAVFPDDMPTLSALPW
EEREKILSSMGNDDKSSVAGSEDAEPLAPPIKPIKPVTRNKAPQEPPVKKGRRSRRCGQCPGCQVPEDCGICT
NCLDKPKFGGRNIKKQCCKMRKCQNLQWMPSKASLQKQTKAVKKKEKKSKTTEKKESKESTAVKSPLEPAQKA
APPPREEPAPKKSSSEPPPRKPVEEKSEEGGAPAPAPAPEPKQVSAPASRKSSKQVSQPAAVVPPQPPSTAPQ
KKEAPKAVPSEPKKKQPPPPEPGPEQSKQKKVAPRPSIPVKQKPKDKEKPPPVSKQENAGTLNILNPLSNGIS
SKQKIPADGVHRIRVDFKEDCEAENVWEMGGLGILTSVPITPRVVCFLCASSGHVEFVYCQVCCEPFHKFCLE
ENERPLEDQLENWCCRRCKFCHVCGRQHQATKQLLECNKCRNSYHPECLGPNYPTKPTKKKKVWICTKCVRCK

SCGSTTPGKGWDAQWSHDFSLCHDCAKLFAKGNFCPLCDKCYDDDDYESKMMQCGKCDRWVHSKCESLSGTED
EMYEILSNLPESVAYTCVNCTERHPAEWRLALEKELQASLKQVLTALLNSRTTSHLLRYRQAAKPPDLNPETE
ESIPSRSSPEGPDPPALTEVSKQDEQQPLDLEGVKKRMDQGSYVSVLEFSDDIVKIIQAAINSDGGQPEIKKA
NSMVKSFFIRQMERVFPWFSVKKSRFWEPNKVSNNSGMLPNAVLPPSLDHNYAQWQEREESSHTEQPPLMKKI
IPAPKPKGPGEPDSPTPLHPPTPPILSTDRSREDSPELNPPPGIDDNRQCALCLMYGDDSANDAGRLLYIGQN
EWTHVNCALWSAEVFEDDDGSLKNVHMAVIRGKQLRCEFCQKPGATVGCCLTSCTSNYHFMCSRAKNCVFLDD
KKVYCQRHRDLIKGEVVPENGFEVFRRVFVDFEGISLRRKFLNGLEPENIHMMIGSMTIDCLGILNDLSDCED
KLFPIGYQCSRVYWSTTDARKRCVYTCKIMECRPPVVEPDINSTVEHDDNRTIAHSPSSFIDASCKDSQSTAA
ILSPPSPDRPHSQTSGSCYYHVISKVPRIRTPSYSPTQRSPGCRPLPSAGSPTPTTHEIVTVGDPLLSSGLRS
IGSRRHSTSSLSPLRSKLRIMSPVRTGSAYSRSSVSSVPSLGTATDPEASAKASDRGGLLSSSANLGHSAPPS
SSSQRTVGGSKTSHLDGSSPSEVKRCSASDLVPKGSLVKGEKNRTSSSKSTDGSAHSTAYPGIPKLTPQVHNA
TPGELNISKIGSFAEPSTVPFSSKDTVSYPQLHLRGQRSDRDQHMDPSQSVKPSPNEDGEIKTLKLPGMGHRP
SILHEHIGSSSRDRRQKGKKSSKETCKEKHSSKSYLEPGQVTTGEEGNLKPEFADEVLTPGFLGQRPCNNVSS
EKIGDKVLPLSGVPKGQSTQVEGSSKELQAPRKCSVKVTPLKMEGENQSKNTQKESGPGSPAHIESVCPAEPV
SASRSPGAGPGVQPSPNNTLSQDPQSNNYQNLPEQDRNLMIPDGPKPQEDGSFKRRYPRRSARARSNMFFGLT
PLYGVRSYGEEDIPFYSNSTGKKRGKRSAEGQVDGADDLSTSDEDDLYYYNFTRTVISSGGEERLASHNLFRE
EEQCDLPKISQLDGVDDGTESDTSVTATSRKSSQIPKRNGKENGTENLKIDRPEDAGEKEHVIKSAVGHKNEP
KLDNCHSVSRVKAQGQDSLEAQLSSLESSRRVHTSTPSDKNLLDTYNAELLKSDSDNNNSDDCGNILPSDIMD
FVLKNTPSMQALGESPESSSSELLTLGEGLGLDSNREKDIGLFEVFSQQLPATEPVDSSVSSSISAEEQFELP
LELPSDLSVLTTRSPTVPSQNPSRLAVISDSGEKRVTITEKSVASSEGDPALLSPGVDPAPEGHMTPDHFIQG
HMDADHISSPPCGSVEQGHGNSQDLTRNSGTPGLQVPVSPTVPVQNQKYVPSSTDSPGPSQISNAAVQTTPPH
LKPATEKLIVVNQNMQPLYVLQTLPNGVTQKIQLTSPVSSTPSVMETNTSVLGPMGSGLTLTTGLNPSLPPSP
SLFPPASKGLLSVPHHQHLHSFPAAAQSSFPPNISSPPSGLLIGVQPPPDPQLLGSEANQRTDLTTTVATPSS
GLKKRPISRLHTRKNKKLAPSSAPSNIAPSDVVSNMTLINFTPSQLSNHPSLLDLGSLNPSSHRTVPNIIKRS
KSGIMYFEQAPLLPPQSVGGTAATAAGSSTISQDTSHLTSGPVSALASGSSVLNVVSMQTTAAPTSSTSVPGH
VTLANQRLLGTPDIGSISHLLIKASHQSLGIQDQPVALPPSSGMFPQLGTSQTPSAAAMTAASSICVLPSSQT
AGMTAASPPGEAEEHYKLQRGNQLLAGKTGTLTSQRDRDPDSAPGTQPSNFTQTAEAPNGVRLEQNKTLPSAK
PASSASPGSSPSSGQQSGSSSVPGPTKPKPKAKRIQLPLDKGSGKKHKVSHLRTSSEAHIPHRDTDPAPQPSV
TRTPRANREQQDAAGVEQPSQKECGQPAGPVAALPEVQATQNPANEQENAEPKAMEEEESGFSSPLMLWLQQE
QKRKESITERKPKKGLVFEISSDDGFQICAESIEDAWKSLTDKVQEARSNARLKQLSFAGVNGLRMLGILHDA
VVFLIEQLAGAKHCRNYKFRFHKPEEANEPPLNPHGSARAEVHLRQSAFDMFNFLASKHRQPPEYNPNDEEEE
EVQLKSARRATSMDLPMPMRFRHLKKTSKEAVGVYRSPIHGRGLFCKRNIDAGEMVIEYAGNVIRSIQTDKRE
KYYDSKGIGCYMFRIDDSEVVDATMHGNAARFINHSCEPNCYSRVINIDGQKHIVIFAMRKIYRGEELTYDYK
FPIEDASNKLPCNCGAKKCRKFLN*
>ENSMUSP00000002095_c.5074:T>C_p.V1692A ENSMUST00000002095.11|Kmt2a-201|ENSMUSG00000002028|-|chr9:g.44742612:A>G
MAHSCRWRFPARPGTTGGGGGGGRRGLGGAPRQRVPALLLPPGPQAGGGGPGAPPSPPAVAAAAAGSSGAGVP
GGAAAASAASSSSASSSSSSSSSASSGPALLRVGPGFDAALQVSAAIGTNLRRFRAVFGESGGGGGSGEDEQF
LGFGSDEEVRVRSPTRSPSVKASPRKPRGRPRSGSDRNPAILSDPSVFSPLNKSETKSADKIKKKDSKSIEKK
RGRPPTFPGVKIKITHGKDIAELTQGSKEDSLKKVKRTPSAMFQQATKIKKLRAGKLSPLKSKFKTGKLQIGR
KGVQIVRRRGRPPSTERIKTPSGLLINSELEKPQKVRKDKEGTPPLTKEDKTVVRQSPRRIKPVRIIPSCKRT
DATIAKQLLQRAKKGAQKKIEKEAAQLQGRKVKTQVKNIRQFIMPVVSAISSRIIKTPRRFIEDEDYDPPMKI
ARLESTPNSRFSATSCGSSEKSSAASQHSSQMSSDSSRSSSPSIDTTSDSQASEEIQALPEERSNTPEVHTPL
PISQSPENESNDRRSRRYSMSERSFGSRATKKLPTLQSAPQQQTSSSPPPPLLTPPPPLQPASGISDHTPWLM
PPTIPLASPFLPASAAPMQGKRKSILREPTFRWTSLKHSRSEPQYFSSAKYAKEGLIRKPIFDNFRPPPLTPE
DVGFASGFSASGTAASARLFSPLHSGTRFDIHKRSPILRAPRFTPSEAHSRIFESVTLPSNRTSSGASSSGVS
NRKRKRKVFSPIRSEPRSPSHSMRTRSGRLSTSELSPLTPPSSVSSSLSIPVSPLAASALNPTFTFPSHSLTQ
SGESTEKNQRARKQTSALAEPFSSNSPALFPWFTPGSQTEKGRKKDTAPEELSKDRDADKSVEKDKSRERDRE
REKENKRESRKEKRKKGSDIQSSSALYPVGRVSKEKVAGEDVGTSSSAKKATGRKKSSSLDSGADVAPVTLGD
TTAVKAKILIKKGRGNLEKNNLDLGPAAPSLEKERTPCLSAPSSSTVKHSTSSIGSMLAQADKLPMTDKRVAS
LLKKAKAQLCKIEKSKSLKQTDQPKAQGQESDSSETSVRGPRIKHVCRRAAVALGRKRAVFPDDMPTLSALPW
EEREKILSSMGNDDKSSVAGSEDAEPLAPPIKPIKPVTRNKAPQEPPVKKGRRSRRCGQCPGCQVPEDCGICT
NCLDKPKFGGRNIKKQCCKMRKCQNLQWMPSKASLQKQTKAVKKKEKKSKTTEKKESKESTAVKSPLEPAQKA
APPPREEPAPKKSSSEPPPRKPVEEKSEEGGAPAPAPAPEPKQVSAPASRKSSKQVSQPAAVVPPQPPSTAPQ
KKEAPKAVPSEPKKKQPPPPEPGPEQSKQKKVAPRPSIPVKQKPKDKEKPPPVSKQENAGTLNILNPLSNGIS
SKQKIPADGVHRIRVDFKEDCEAENVWEMGGLGILTSVPITPRVVCFLCASSGHVEFVYCQVCCEPFHKFCLE
ENERPLEDQLENWCCRRCKFCHVCGRQHQATKQLLECNKCRNSYHPECLGPNYPTKPTKKKKVWICTKCVRCK
SCGSTTPGKGWDAQWSHDFSLCHDCAKLFAKGNFCPLCDKCYDDDDYESKMMQCGKCDRWVHSKCESLSDEMY

EILSNLPESVAYTCVNCTERHPAEWRLALEKELQASLKQVLTALLNSRTTSHLLRYRQAAKPPDLNPETEESI
PSRSSPEGPDPPALTEVSKQDEQQPLDLEGVKKRMDQGSYVSVLEFSDDIVKIIQAAINSDGGQPEIKKANSM
VKSFFIRQMERVFPWFSVKKSRFWEPNKVSNNSGMLPNAVLPPSLDHNYAQWQEREESSHTEQPPLMKKIIPA
PKPKGPGEPDSPTPLHPPTPPILSTDRSREDSPELNPPPGIDDNRQCALCLMYGDDSANDAGRLLYIGQNEWT
HVNCALWSAEVFEDDDGSLKNVHMAVIRGKQLRCEFCQKPGATVGCCLTSCTSNYHFMCSRAKNCVFLDDKKV
YCQRHRDLIKGEVVPENGFEVFRRVFVDFEGISLRRKFLNGLEPENIHMMIGSMTIDCLGILNDLSDCEDKLF
PIGYQCSRVYWSTTDARKRCVYTCKIMECRPPVVEPDINSTVEHDDNRTIAHSPSSFIDASCKDSQSTAAILS
PPSPDRPHSQTSGSCYYHVISKVPRIRTPSYSPTQRSPGCRPLPSAGSPTPTTHEIVTVGDPLLSSGLRSIGS
RRHSTSSLSPLRSKLRIMSPVRTGSAYSRSSVSSVPSLGTATDPEASAKASDRGGLLSSSANLGHSAPPSSSS
QRTVGGSKTSHLDGSSPSEVKRCSASDLVPKGSLVKGEKNRTSSSKSTDGSAHSTAYPGIPKLTPQVHNATPG
ELNISKIGSFAEPSTVPFSSKDTVSYPQLHLRGQRSDRDQHMDPSQSVKPSPNEDGEIKTLKLPGMGHRPSIL
HEHIGSSSRDRRQKGKKSSKETCKEKHSSKSYLEPGQVTTGEEGNLKPEFADEVLTPGFLGQRPCNNVSSEKI
GDKVLPLSGVPKGQSTQVEGSSKELQAPRKCSVKVTPLKMEGENQSKNTQKESGPGSPAHIESVCPAEPVSAS
RSPGAGPGVQPSPNNTLSQDPQSNNYQNLPEQDRNLMIPDGPKPQEDGSFKRRYPRRSARARSNMFFGLTPLY
GVRSYGEEDIPFYSNSTGKKRGKRSAEGQVDGADDLSTSDEDDLYYYNFTRTVISSGGEERLASHNLFREEEQ
CDLPKISQLDGVDDGTESDTSVTATSRKSSQIPKRNGKENGTENLKIDRPEDAGEKEHVIKSAVGHKNEPKLD
NCHSVSRVKAQGQDSLEAQLSSLESSRRVHTSTPSDKNLLDTYNAELLKSDSDNNNSDDCGNILPSDIMDFVL
KNTPSMQALGESPESSSSELLTLGEGLGLDSNREKDIGLFEVFSQQLPATEPVDSSVSSSISAEEQFELPLEL
PSDLSVLTTRSPTVPSQNPSRLAVISDSGEKRVTITEKSVASSEGDPALLSPGVDPAPEGHMTPDHFIQGHMD
ADHISSPPCGSVEQGHGNSQDLTRNSGTPGLQVPVSPTVPVQNQKYVPSSTDSPGPSQISNAAVQTTPPHLKP
ATEKLIVVNQNMQPLYVLQTLPNGVTQKIQLTSPVSSTPSVMETNTSVLGPMGSGLTLTTGLNPSLPPSPSLF
PPASKGLLSVPHHQHLHSFPAAAQSSFPPNISSPPSGLLIGVQPPPDPQLLGSEANQRTDLTTTVATPSSGLK
KRPISRLHTRKNKKLAPSSAPSNIAPSDVVSNMTLINFTPSQLSNHPSLLDLGSLNPSSHRTVPNIIKRSKSG
IMYFEQAPLLPPQSVGGTAATAAGSSTISQDTSHLTSGPVSALASGSSVLNVVSMQTTAAPTSSTSVPGHVTL
ANQRLLGTPDIGSISHLLIKASHQSLGIQDQPVALPPSSGMFPQLGTSQTPSAAAMTAASSICVLPSSQTAGM
TAASPPGEAEEHYKLQRGNQLLAGKTGTLTSQRDRDPDSAPGTQPSNFTQTAEAPNGVRLEQNKTLPSAKPAS
SASPGSSPSSGQQSGSSSVPGPTKPKPKAKRIQLPLDKGSGKKHKVSHLRTSSEAHIPHRDTDPAPQPSVTRT
PRANREQQDAAGVEQPSQKECGQPAGPVAALPEVQATQNPANEQENAEPKAMEEEESGFSSPLMLWLQQEQKR
KESITERKPKKGLVFEISSDDGFQICAESIEDAWKSLTDKVQEARSNARLKQLSFAGVNGLRMLGILHDAVVF
LIEQLAGAKHCRNYKFRFHKPEEANEPPLNPHGSARAEVHLRQSAFDMFNFLASKHRQPPEYNPNDEEEEEVQ
LKSARRATSMDLPMPMRFRHLKKTSKEAVGVYRSPIHGRGLFCKRNIDAGEMVIEYAGNVIRSIQTDKREKYY
DSKGIGCYMFRIDDSEVVDATMHGNAARFINHSCEPNCYSRVINIDGQKHIVIFAMRKIYRGEELTYDYKFPI
EDASNKLPCNCGAKKCRKFLN*
>ENSMUSP00000047508_c.1188:T>G_p.C397G ENSMUST00000039059.8|Pcsk7-201|ENSMUSG00000035382|+|chr9:g.45830096:T>G
MPKGRQKVPHLDAHLGLPICLWLELAIFFLVPQVMGLSEAGGLDILGTGGLSWAVHLDSLEGERKEESLTQQA
DAVAQAAGLVNAGRIGELQGHYLFVQPTGHRQAMEVEAMRQQAEAVLARHEAVRWHSEQTLLKRAKRSIHFND
PKYPQQWHLNNRRSPGRDINVTGVWERNVTGRGVTVVVVDDGVEHTVQDIAPNYSPEGSYDLNSNDPDPMPHP
DEENGNHHGTRCAGEIAAVPNNSFCAVGVAYGSRIAGIRVLDGPLTDSMEAVAFNKHYQINDIYSCSWGPDDD
GKTVDGPHQLGKAALQHGVMAGRQGFGSIFVVASGNGGQHNDNCNYDGYANSIYTVTIGAVDEEGRMPFYAEE
CASMLAVTFSGGDKMLRSIVTTDWDLQKGTGGTEGHTGTSAAAPLAAGMIALMLQVRPCLTWRDVQHIIVFTA
IQYEDHHADWLTNEAGFSHSHQHGFGLLNAWRLVNAAKIWTSVPYLASYVSPMLKENKAVPRSPHSLEVLWNV
SRTDLEMSGLKTLEHVAVTVSITHPRRGSLELKLFCPSGMMSLIGAPRSMDSDPNGFNAWTFSTVRCWGERAR
GVYRLVIRDVGDEPLQMGILQQWQLTLYGSMWSPVDIKDRQSLLESAMSGKYLHDGFTLPCPPGLKIPEEDGY
TITPNTLKTLVLVGCFSVFWTIYYMLEVCLSQRNKASTHGCRKGCCPWAPRRQNSKDAGTALESMPLCSSKDL
DGVDSEHGDCTTASSFLAPELDCPPHQPPDLLQGKSGQIC*
>ENSMUSP00000150500_c.1188:T>G_p.C397G ENSMUST00000215189.2|Pcsk7-204|ENSMUSG00000035382|+|chr9:g.45830096:T>G
MPKGRQKVPHLDAHLGLPICLWLELAIFFLVPQVMGLSEAGGLDILGTGGLSWAVHLDSLEGERKEESLTQQA
DAVAQAAGLVNAGRIGELQGHYLFVQPTGHRQAMEVEAMRQQAEAVLARHEAVRWHSEQTLLKRAKRSIHFND
PKYPQQWHLNNRRSPGRDINVTGVWERNVTGRGVTVVVVDDGVEHTVQDIAPNYSPEGSYDLNSNDPDPMPHP
DEENGNHHGTRCAGEIAAVPNNSFCAVGVAYGSRIAGIRVLDGPLTDSMEAVAFNKHYQINDIYSCSWGPDDD
GKTVDGPHQLGKAALQHGVMAGRQGFGSIFVVASGNGGQHNDNCNYDGYANSIYTVTIGAVDEEGRMPFYAEE
CASMLAVTFSGGDKMLRSIVTTDWDLQKGTGGTEGHTGTSAAAPLAAGMIALMLQVRPCLTWRDVQHIIVFTA
IQYEDHHADWLTNEAGFSHSHQHGFGLLNAWRLVNAAKIWTSVPYLASYVSPMLKENKAVPRSPHSLEVLWNG
RTWRCRG*
>ENSMUSP00000150393_c.1188:T>G_p.C397G ENSMUST00000216672.2|Pcsk7-209|ENSMUSG00000035382|+|chr9:g.45830096:T>G

MPKGRQKVPHLDAHLGLPICLWLELAIFFLVPQVMGLSEAGGLDILGTGGLSWAVHLDSLEGERKEESLTQQA
DAVAQAAGLVNAGRIGELQGHYLFVQPTGHRQAMEVEAMRQQAEAVLARHEAVRWHSEQTLLKRAKRSIHFND
PKYPQQWHLNNRRSPGRDINVTGVWERNVTGRGVTVVVVDDGVEHTVQDIAPNYSPEGSYDLNSNDPDPMPHP
DEENGNHHGTRCAGEIAAVPNNSFCAVGVAYGSRIAGIRVLDGPLTDSMEAVAFNKHYQINDIYSCSWGPDDD
GKTVDGPHQLGKAALQHGVMAGRQGFGSIFVVASGNGGQHNDNCNYDGYANSIYTVTIGAVDEEGRMPFYAEE
CASMLAVTFSGGDKMLRSIVTTDWDLQKGTGGTEGHTGTSAAAPLAAGMIALMLQVRPCLTWRDVQHIIVFTA
IQYEDHHADWLTNEAGFSHSHQHGFGLLNAWRLVNAAKIWTSVPYLASYVSPMLKENKAVPRSPHSLEVLWNV
SRTDLEMSGLKTLEHVAVTVSITHPRRGSLELKLFCPSGMMSLIGAPRSMDSDPNGFNAWTFSTVRCWGERAR
GVYRLVIRDVGDEPLQMGILQQWQLTLYGSMWSPVDIKDRQSLLESAMSGKYLHDGFTLPCPPGLKIPEEDGY
TITPNTLKTLVLVGCFSVFWTIYYMLEVCLSQRNKASTHGCRKGCCPWAPRRQNSKDAGTALESMPLCSSKDL
DGVDSEHGDCTTASSFLAPELDCPPHQPPDLLQGKSGQIC*
>ENSMUSP00000037915_c.2318:G>A_p.W773* ENSMUST00000038890.6|Dennd4a-201|ENSMUSG00000053641|+|chr9:g.64796294:G>A
MIEDKGPRVADYFVVAGLTDISKPLEEEIHFNDACHKVAKPKEPITDVSVIIKSLGEEVPRDYVCIDVTPTGL
SADLNNGSLVGPQIYLCYKRGRDKPPLTDLGVLYDWKERLKQGCEIIQSTPYGRPANISGSTSSQRIYITYRR
ASENTTQNTLAVTDICIIIPSKGESPPHTFCKVDKNLSNSMWGSAVYLCYKKSVAKTNTISYKAGLICRYPQE
DYESFSLPESVPLFCLPMGATIECWPPDSKYPLPVFSTFVLTGASAEKVYGAAIQFYEPYPEENLTEKQRLLL
GLTSLVDGKSDSTRTIHTNKCICLLSHWPFFDAFRKFLTFLYRYSISGPHALPIEKHISHFMHKVPFPSPQRP
RILVQLSPHDNLILSQPVSSPLPLSGGKFSTLLQNLGPENAVTLLVFAVTEHKILIHSLRPSVLTSVTEALVS
MIFPFHWPCPYVPLCPLALADVLSAPCPFIVGIDSRYFDLYDPPPDVSCVDLDTNTISQTGDKKNVAWKILPK
KPCKNLMNTLNNLHQQLAKLQQRPRDDGLMDLAMNDYDFNSGKRLHMIDLEIQEAFLFFMASILKGYRSYLRP
ITQAPSETATDATSLFALQAFLRSRDRSHQKFYNMMTKTQMFIRFIEECSFVSDKDASLAFFDDCVDKVDTDK
SGEVRLIELDASFKSEHTVFVTPPEIPHLPNGEEPPLQYSYNGFPVLRNNLFERPEGFLQARKNKLPSKPSSP
GSPSPMFRRTKQEIKSAHKIAKRYSSIPQMWSRCLLRHCYGL*FICLPAYVKVCHSKVRALKTAYDVLKKMQL
KKMDPPDEVCYRILMQLCGQYDQPVLAVRVLFEMQKAGIDPNAITYGYYNKAVLESTWPSRSRSGYFLWTKVR
NVVLGVAQFKRALKKHPHLPQTALSGGRSDLGYNSLSKDNVRREGPSTEDIQGEKEKRGSDSSSLSENESTKG
SAECLPTLSYQRASTIVRLNGAHNDSAGKTSGECEESTPELLLMPSLEDTNEAQTTPSRCFRKRHKSDDGSHL
QQQMPWGSRNRNLSGGVLMGFMLNRTNQETSPGEMVEKLGADAKILSNVISKSTRPNSLDIGKPPARSKRDSL
EKESSDDDTPFHGSNCLDKVESPVIFDLEDLDTETDGSKVGGVAAQNPKRLQRRNSSFSVKPSEKTDVVTGFD
PLSLLVAETEQQQKVEEEEDEDDNKSVSTPSARRNLAEEIEMYMNNMSSPLTSRTPSIDLQRACDDKLTNKKS
PTLVKACRRSSLPPNSPRPVRLTKSKSYTKSEERPRDRLWSSPAFSPTCPFREGSQETLAHSSPSFNLDTLLV
PKLDVLRHSVFTAGKGVAEKASKWYSRFTMYTTSSKDQSSDRTSLSSVGAQDSESTSLTDEDVCHELEGATSS
QESSAASGTKGIDVSRASLGSSASLEGSLSKFALPGKSETASSLNTSNANIFQNYAMEVLISSCSRCRTCDCL
VHDEEIMAGWTADDSNLNTTCPFCGNLFLPFLNVEIRDLRRPGRYFLKSSSSTETMHFASRTRQSCISASASG
LDTSSLSVQGNFDLNNKSKLQENPCARSIQIPAHRSKTVVSKCPLFPMARSISTCGPLDKDDPGGQKLIPTGS
LPATLQGHTDSLGLEWHLPSPDPVTVPYLSPLVVWKELESLLENEGDHAITVADFVDHHPIVFWNLVWYFRRL
DLPSNLPGLILSSEHCNKYSKIPRHCMSEDSKYVLIQMLWDNMKLHQDPGQPLYILWNAHSQNRTLLFETQKY
PMVHLLQKGDDSFNQELLKSMVKSIKMNDVYGPMSQILETLNKCPHFKRQRSLYREILFLSLVALGRENIDID
AFDKEYKMAYDRLTPSQVKSTHNCDRPPSTGVMECRKTFGEPYL*
>rs33746359_ENSMUSP00000077040_c.8893:A>G_p.Y2965C ENSMUST00000077879.7|Vps13c-201|ENSMUSG00000035284|+|chr9:g.67862278:A>G
MVLESVVADLLNRFLGDYVENLNKSQLKLGIWGGNVALDNLQIKENALSELDVPFKVKAGQIDKLTLKIPWKN
LYGEAVVATLEGLYLLVVPGASIKYDAEKEEKSLQDIKQKELCRIEEALQKAAEKGAHSGEFMYGLENLLYKD
VKPGRKRKKHKKHFKKRFKGLDRSKDKPKEAKKDTFLEKLATQVIKNVQVKITDIHIKYEDDITDPERPLSFG
VTLREFSLLTTNEHWTPCILNEAEKIIYKLVKLDSLSAYWNVGCCMSYRGSREHILEQLKREILTSTNIPPDH
QYIFQPISASAKLYMNPGAESELKTPKLDGNVEVQNIAIELTKPQYLSMIDFLESLDYMVRNAPYRKYKPCLP
LHTNCRQWWKYAIDSVLEVHIRRYTQPWSWSNIKNHRQLLKSYKMAYKTKLTQAKVSEEIQKQIQDLEKSLDV
FNIILVRQQAQVEVIHSGQKLRKKSAEAGEKRGWFSGFWGKKESKKRDEESSVPETIDDLMTPEEKDKLFTAI
GYSENAYNLALPKQYVAHILTLKLVSTSIIIRENRNVPEILRVQIIGLGTQVSQRPGAQALKIEAKLEHWYVT
GLRQQDIVPSLVASIGDTTSSLLKIEFETNPENSPADQTLIVQSQPVEVIYDAKTINAVVEFFQSNKGLDLEQ
ITSATLMKLEEIKERTATGLTHIIETRKVLDLRINLKPSYLIIPQTGFHHEKSNLLILDFGTFQLNSKDQGAQ
KTANASLEEIIDKAYDKFDVEIRSVQLLFAKAEENWKKCRFQHPSTMHILQPMDIHVELAKAMVEKDVRMAKF
KVSGGLPLMHVRISDQKIKDALCLINSIPLPQKSSTPSPERQVASIPVLSGGTKALLGTSLLLDGVESESDEE
FFDAEDGDSQAARTVKASELKKAAEVPNEELVSLLLKFEIKEVVLELTKQQKEEETILVFNVTQLGTEATMRT
FDLTAVSYLRKISLDYHDIKGSRKKPIHLISSSDRPGLDLLKVEYIKVDRNGPSFQTTFEKTEQTVKVAFSSL
NLLLQTQALLSSLNYLTTVIPSDSQNTGVAKEVQAMPEKQKNSPLQKVMVPSRDSDVIGFRLFAKLNAFCVTV
CDEKSNIAEIKIQGLDSSLSLQSKKQSLFARLENIIVTDVDPKTIHKKAVSIVGNEVFRFNLDLYPDATEGDS
YTDMSTVDGVVALHVGCIQIVYLHKFLMSLLSFLNNFQVAKEALSAATAQAAEKAATSVKDLAQRSFRVSVDI

DLKAPVIVIPQSSLSTNAVVVDLGLIRVHNRFSLVSGEDTANPPVIDKMEVQLTKLKLSRTAIQPGTSHPDIQ
LLHPINLEFFVSRNLAANWYHKVPVVEIKGRLDSMNVSLNQEDLNLLFRILAENLGEATEDLDKGKPRIQERG
ETKACREVSTPQDVHTTQGVPAARVEETRPVDIINVLLNFEIKEVVVTLMKKAERKGSPFHELKILHLGMEAK
VKAHDMTAAAYLRNISMRCFHFPDSKGEPLRIVNTSDVSDGILLKLLFIKADSDGPDFKTIHDNTKQKLKVSF
SSLDLVLHLEALLSLMDFLSSAIPSSDSSSSEKEPELKPLVGESRSLAIRAVPSSYEGDAFDLKITAELNAFN
IFICDQKSNIAEIKIHGMDASISVKPKQTDVFARLKNIIVMNVDSLSIHKKAVSILGDEVFRFQMSLYPDATE
GENYGDMSKVDGRLSLKVGCIQIVYVHKFFMSLLSFLNNFQAAKEALSTATVQAAERAASSVKDLAQKSFRLL
MDIDLKAPVITIPQSSVSPNVVIADLGLIRVENKFSLVSVEQLALPPVADEMSIQLTQLKLARTVLQADSPQH
DVEILKPVNMLLCIQRNLSAAWYTQIPGMEIKGELKPMQVALSQDDLTVLMKILLENLGEASSQPSPTQYAQE
AARVKRDTRSGPDYLKEQELADPKPPGDQTVTLQFDFHFDSLSIILYNSDSSQEPRLSFHNDSFRLGELTLHL
MASAGKMFKDGSMNVSLKLKTCTLDDLREGIERATSRMIDKKNDQDNNSSMIDISYSQDKNGSQVDAVLDKLY
VCASVEFLMTVADFFIKAMPQSPENIAKEIQIPSRQTAAGRVKMEKDDSVRPNMTLKAMITDPEVVFVASLTK
ADAPALTASFQCNLSLSTSKLEQMMEASVRDLKVLACPFLRERRGKSITTVLQPCSLFMEKCTWASGKQNINI
VVKEFVIKISPIILNTVMTIMAAMSPKTKEDEWKDTPKETDNLWAVKSITDYNSWFLGVDMATEVTENFRDSE
HPSIEENCVVAVESVQVTLECGLGHRTVPLLLAESKFSGNIKNWTSLMAAAADMTLEVHYYNETHAVWEPLIE
RVEGNKPWSLKLNVKKNPIQDKSLMPGDDFIPEPQTAVHISSGATMNITISKSCLNVFSNLAKGFSEGAASTF
DYSLKDRAPFTVKNALGVPMKVQPNRNLKVMGSPEKSDIYDVGAGQHLELDYASLEPSRQGKLSILSRQESSL
FTLTFVPYGYTEVASVPVARPGRRLYNVRNPSASHSDSVLVQIDATEGNKVVTLRSPLQIKNHFSIAFIIYKF
VKNVKLLERIGIARPEEEFHVPLDSYRCQLYVQPAGGLEQQYTHSSTYISWKEELHRSREVRCMLQCPAVEVS
FLPLIVNTVALPDELSYIGAHGEDWDPAYVIHLYPPLTLRNLLPYSLRYLLEGTAETHELAEGSSADVLHSRI
SGEIIELVLVKYLGKNWNGHFRICDTLPEFFLVCFSSDTAEVMTVDLSVHVRRIGCRMELSVFSPYWLINKTS
RVLQYRSEEIHVKHPADFRDIILFSFKKKNIFSKNKVQLKISTSAWSNGFSLDTVGSYGCVKCPATNMEYLVG
VSIKMSSFNLSRVVTLTPFCTVANKSSLDLEVGEIASDGSIPTNKWHYVASSECIPFWPENLSGKLCVRVVGY
EGSSKPFFYNRQDNGTLLSLEDLNGGILVDINTAEHSTVITFSDCHEGSAPALIMNHTQWDVLTYKQSGSQEE
LVLLPGETRLFAWADPTGIRKLTWNYAANFGEHDLLKDECGQFPYDANIQIHWVSFLDGRQRVLLFTDDVALV
SKALQAEEMEQADHEVALSLHSLGLSLVNNENKQEVSYVGITSSGVVWEMKPKQKWKPFSQKQIMSLEQAYSK
RLASQDRGWVKLDSNFEVNFDKVPMEMRLPIRCPIKRDFLSGIQVEFKQSPHQRSLRARLYWLQVDNQLPGTM
FPVVFHPVAPPKSIALDSEPKPFIDVSVITRFNEYSKVLQFKYFMVLIQEMALKVDQGFLGAVISLFTPTTDP
EAERKRTKLIQQDIDALNTELMESSMTDMSILSFFEHFHISPVKLHLSLSLGSGGEESDKEKQEMIAIHSVNL
LLKSIGATLTDVDDLIFKLAYYEIRYQFYKRDQLMWSVVRHYSEQFLKQMYVLVLGLDVLGNPFGLIRGLSEG
VEALFYEPFQGAVQGPEEFAEGLVIGVRSLVGHTVGGAAGVVSRITGSVGKGLAAITMDKEYQQKRREEMGRQ
PKDFGDSLARGGKGFLRGVVGGVTGIITKPVEGAKKEGAAGFFKGIGKGLVGAVARPTGGIIDMASSTFQGIQ
RVAESTEEVSSLRPPRLIHEDGIIRPYDRQESEGSDLLENHIKKLEGEAYQFHCAVPGNKRAVLMITNRRALF
IKEVEILGHMSVDWQCLFEDFVCPPEVSENLLKISVKEQGLFHKKDSANIGHLRKIYLKDPITAKRAFDAIES
AQSARQQQKLMRQSSVKLLRPQGPS*
>rs33746359_ENSMUSP00000149778_c.295:A>G_p.Y99C
ENSMUST00000213168.2|Vps13c-202|ENSMUSG00000035284|+|chr9:g.67862278:A>G
XTVANKSSLDLEVGEIASDGSIPTNKWHYVASSECIPFWPENLSGKLCVRVVGYEGSSKPFFYNRQDNGTLLS
LEDLNGGILVDINTAEHSTVITFSDCHEGSAPALIMNHTQWDVLTYKQSGSQEELVLLPGETRLFAWADPTGI
RKLTWNYAANFGEHDLLKDECGQFPYDANIQIHWVSFLDGRQRVLLFTDDVALVSKALQAEEMEQADHEVALS
LHSLGLSLVNNENKQEVSYVGITSSGVVWEMKPKQKWKPFSQKQIMSLEQAYSKRLASQDRGWVKLDSNFEVN
FDKVPMEMRLPIRCPIKRDFLSGIQVEFKQSPHQRSLRARLYWLQVDNQLPGTMFPVVFHPVAPPKSIALDSE
PKPFIDVSVITRFNEYSKVLQFKYFMVLIQEMALKVDQGFLGAVISLFTPTTDPEAERKRTKLIQQDIDALNT
ELMESSMTDMSILSFFEHFHISPVKLHLSLSLGSGGEESDKEKQEMIAIHSVNLLLKSIGATLTDVDDLIFKL
AYYEIRYQFYKRDQLMWSVVRHYSEQFLKQMYVLVLGLDVLGNPFGLIRGLSEGVEALFYEPFQGAVQGPEEF
AEGLVIGVRSLVGHTVGGAAGVVSRITGSVGKGLAAITMDKEYQQKRREEMGRQPKDFGDSLARGGKGFLRGV
VGGVTGIITKPVEGAKKEGAAGFFKGIGKGLVGAVARPTGGIIDMASSTFQGIQRVAESTEEVSSLRPPRLIH
EDGIIRPYDRQESEGSDLLEQEMGTQE*
>ENSMUSP00000046111_c.2377:T>A_p.V793E ENSMUST00000044491.13|Minar1-
201|ENSMUSG00000039313|-|chr9:g.89478667:A>T
MEANQEASLFLVKILEELDSKQNTVSYQDLCKSLCAQFDLSQLAKLRSVLFYTACLDPNFPATLFKDKMKCSV
NNQQSKKIMVAADIVTIFNLIQMNGGTAKEKLPMSCHKVRKKEASFESCRSDTEVCSPTVCEPLNCELSERPF
SRGYPTRQSSKCRKMDCKECPQFVPASEPNFLLGVSKEVKNRAASLDRLQALSPYSVASPQPCEMQRTYFPMN
IENEPMSDQDSLPISQGIKETFISSEEPFVVQSCVQKRNIFKEDFHNLMTVSPSLVGTTNKAEEGHGEPQSQK
ELHKPPFFNHSFEMPYHNQYLNPVYSPIPDKRRAKHESLDDLQASTYFGPTPVMGTQDTRRCPGRSSKQTPWP
AKSWSLNTEEVPDFERSFFNRNPSEEKLRYPNSGSQTPNFSGPDRHPVYLVPKDQQKVLPAGYAVKPNGLKSK
EISSPVDLEKHEAVKKFKDKSISCTSGQHSSDTSSVGTQTEQHVLDPPKCKDLCTSGQAKYGDRHAMKQSDDD
SEIVSDDISDIFRFLDDMSISGSTGVIQSSCYNSTGSLSQLHKSDCDSSPEHHLAKITNGVSSGKGDKCNRPE

NVHHSEEELKSSVCKLVLRIGEIERKLESLSGVREEISQVLGKLNKLDQKIQQPEKVNVQIDLNSLTSEAPSD
DSASPRVFRAHSGSHGPKLENSPDWCCSDASGSNSESLRVKALKKSLFTRPSSRSLTEENSATESKIASISNS
PRDWRTITYTNRMSLNEEEIKDAGPANNKDWHRKSKEADRQYDIPPQHRLPKQPKDGFLVEQEFSPHPYPTSL
KGHMKSNPLYTDMRLTELAEVKRGQPSWTIEEYARNSGDKGKLTALDLQTQESLNPNNLEYWMEDIYTPGYDS
LLKRKEAEFRRAKVCKIAALITAAACTVILVIVVPICTMKS*
>ENSMUSP00000140942_c.2377:T>A_p.V793E ENSMUST00000191465.2|Minar1-
202|ENSMUSG00000039313|-|chr9:g.89478667:A>T
MEANQEASLFLVKILEELDSKQNTVSYQDLCKSLCAQFDLSQLAKLRSVLFYTACLDPNFPATLFKDKMKCSV
NNQQSKKIMVAADIVTIFNLIQMNGGTAKEKLPMSCHKVRKKEASFESCRSDTEVCSPTVCEPLNCELSERPF
SRGYPTRQSSKCRKMDCKECPQFVPASEPNFLLGVSKEVKNRAASLDRLQALSPYSVASPQPCEMQRTYFPMN
IENEPMSDQDSLPISQGIKETFISSEEPFVVQSCVQKRNIFKEDFHNLMTVSPSLVGTTNKAEEGHGEPQSQK
ELHKPPFFNHSFEMPYHNQYLNPVYSPIPDKRRAKHESLDDLQASTYFGPTPVMGTQDTRRCPGRSSKQTPWP
AKSWSLNTEEVPDFERSFFNRNPSEEKLRYPNSGSQTPNFSGPDRHPVYLVPKDQQKVLPAGYAVKPNGLKSK
EISSPVDLEKHEAVKKFKDKSISCTSGQHSSDTSSVGTQTEQHVLDPPKCKDLCTSGQAKYGDRHAMKQSDDD
SEIVSDDISDIFRFLDDMSISGSTGVIQSSCYNSTGSLSQLHKSDCDSSPEHHLAKITNGVSSGKGDKCNRPE
NVHHSEEELKSSVCKLVLRIGEIERKLESLSGVREEISQVLGKLNKLDQKIQQPEKVNVQIDLNSLTSEAPSD
DSASPRVFRAHSGSHGPKLENSPDWCCSDASGSNSESLRVKALKKSLFTRPSSRSLTEENSATESKIASISNS
PRDWRTITYTNRMSLNEEEIKDAGPANNKDWHRKSKEADRQYDIPPQHRLPKQPKDGFLVEQEFSPHPYPTSL
KGHMKSNPLYTDMRLTELAEVKRGQPSWTIEEYARNSGDKGKLTALDLQHVG*
>ENSMUSP00000108264_c.1110:A>G_p.T371A ENSMUST00000112645.8|Trf-
203|ENSMUSG00000032554|-|chr9:g.103098181:T>C
MRLTVGALLACAALGLCLAVPDKTVKWCAVSEHENTKCISFRDHMKTVLPPDGPRLACVKKTSYPDCIKAISA
SEADAMTLDGGWVYDAGLTPNNLKPVAAEFYGSVEHPQTYYYAVAVVKKGTDFQLNQLEGKKSCHTGLGRSAG
WVIPIGLLFCKLSEPRSPLEKAVSSFFSGSCVPCADPVAFPKLCQLCPGCGCSSTQPFFGYVGAFKCLKDGGG
DVAFVKHTTIFEVLPEKADRDQYELLCLDNTRKPVDQYEDCYLARIPSHAVVARKNNGKEDLIWEILKVAQEH
FGKGKSKDFQLFSSPLGKDLLFKDSAFGLLRVPPRMDYRLYLGHNYVTAIRNQQEGVCPEGSIDNSPVKWCAL
SHLERAKCDEWSIISEGKIECESAETTEDCIEKIVNGEADAMTLDGGHAYIAGQCGLVPVMAEYYESSNCAIP
SQQGIFPKGYYAVAVVKASDTSITWNNLKGKKSCHTGVDRTAGWNIPMGMLYNRINHCKFDEFFSQGCAPGYE
KNSTLCDLCIGPLKCAPNNKEEYNGYTGAFRCLVEKGDVAFVKHQTVLDNTEGKNPAEWAKNLKQEDFELLCP
DGTRKPVKDFASCHLAQAPNHVVVSRKEKAARVKAVLTSQETLFGGSDCTGNFCLFKSTTKDLLFRDDTKCFV
KLPEGTTPEKYLGAEYMQSVGNMRKCSTSRLLEACTFHKH*
>ENSMUSP00000035158_c.1110:A>G_p.T371A ENSMUST00000035158.16|Trf-
201|ENSMUSG00000032554|-|chr9:g.103098181:T>C
MRLTVGALLACAALGLCLAVPDKTVKWCAVSEHENTKCISFRDHMKTVLPPDGPRLACVKKTSYPDCIKAISA
SEADAMTLDGGWVYDAGLTPNNLKPVAAEFYGSVEHPQTYYYAVAVVKKGTDFQLNQLEGKKSCHTGLGRSAG
WVIPIGLLFCKLSEPRSPLEKAVSSFFSGSCVPCADPVAFPKLCQLCPGCGCSSTQPFFGYVGAFKCLKDGGG
DVAFVKHTTIFEVLPEKADRDQYELLCLDNTRKPVDQYEDCYLARIPSHAVVARKNNGKEDLIWEILKVAQEH
FGKGKSKDFQLFSSPLGKDLLFKDSAFGLLRVPPRMDYRLYLGHNYVTAIRNQQEGVCPEGSIDNSPVKWCAL
SHLERAKCDEWSIISEGKIECESAETTEDCIEKIVNGEADAMTLDGGHAYIAGQCGLVPVMAEYYESSNCAIP
SQQGIFPKGYYAVAVVKASDTSITWNNLKGKKSCHTGVDRTAGWNIPMGMLYNRINHCKFDEFFSQGCAPGYE
KNSTLCDLCIGPLKCAPNNKEEYNGYTGAFRCLVEKGDVAFVKHQTVLDNTEGKNPAEWAKNLKQEDFELLCP
DGTRKPVKDFASCHLAQAPNHVVVSRKEKAARVKAVLTSQETLFGGSDCTGNFCLFKSTTKDLLFRDDTKCFV
KLPEGTTPEKYLGAEYMQSVGNMRKCSTSRLLEACTFHKH*
>ENSMUSP00000128390_c.75:A>G_p.T26A ENSMUST00000170904.2|Trf-
208|ENSMUSG00000032554|-|chr9:g.103098181:T>C
QEGVCPEGSIDNSPVKWCALSHLERAKCDEWSIISEGKIECESAETTEDCIEKIVNGEADAMTLDGGHAYIAG
QCGLVPVMAEYYGYYAVAVVKASDTSITWNNLKGKKSCHTGVDRTAGWNIPMGMLYNRINHCKFDEFFSQGCA
PGYEKNSTLCDLCIGPLKCAPNNKEEYNGYTGAFRCLVEKGDVAFVKHQTVLDNTEGKNPAEWAKNLKQEDFE
LLCPDGTRKPVKDFASCHLAQAPNHVVVSRKEKAARVKAVLTSQETLFGGSDCTGNFCL
>ENSMUSP00000071966_c.5656:G>A_p.S1886N ENSMUST00000072093.13|Plxnb1-
201|ENSMUSG00000053646|+|chr9:g.108943415:G>A
MSVLGPVLLQVFWAGCVVTLRSPLPAAFTANGTHLQHLARDPTTGTLYVGATNFLFQLSPGLQLEAVVSTGPV
NDSRDCLPPVIPDECPQAQPTNNPNQLLLVSPEALVVCGSVHQGICELRSLGQIRQLLLRPERPGDTQYVAAN
DPAVSTVGLVAQGLVGEPLLFVGRGYTSRGVGGGIPPITTRALRPPDPQAAFSYEETAKLAVGRLSEYSHHFV
SAFVRGASAYFLFLRRDLKAPSRAFRAYVSRVCLQDQHYYSYVELPLACQGGRYGLIQAAAVATSKEVARGDV
LFAAFSSVAPPTVDWPLSASTGASGTSVLCAFPLDEVDQLANYTRDACYTREGRAENGTKVADIAYDVLSDCA
QLPVDTPDAFPCGSDHTPSPMVSCVPLEATPILELPGVQLTAVAVTMEDGHTIAFLGDSQGQLHRVYLGPGRS
AAPYSKQSIQPGSPVNRDLTFDGTFEHLYVATQTTLVKVPVAPCAQHLDCDSCLAHRDPYCGWCVLLGRCSRR

SECSRDQGPEQWLWSFQPELGCLRVVAVSPANISREERREVFLSVPGLPSLWPGESYFCYFGDQQSPALLTSS
GVMCPSPDPSEAPVLQRGADHISVNVELRFGAVVIASTSLSFYDCVAVTASSPSAPCRACVSSRWGCNWCVWQ
QLCTHKASCDAGPMVASQQSPLLPLIPPARDELTPFPPTVPQTTVTPTPNSFPIEPRAPSTASDVLPGAKPSR
LSLWGPWAGPGPILSPTSTESPLHEKPLPPDPPTIPGTTVPAPTGLGPSTTPEDLLASYPFPSDAAAVSPAEP
GPEALPSMVALDQPPGTVPDTTFPGAPGSMKPVLDWLTKGGGELPEADEWMGGDTPAFSTSTLLSGDGDSAEH
EGPPAPLILLSSLDYQYDTPGLWELGEVNQRVSSCPCVETVQGSLLIPVHVEREVQLRGRNLWLFQDGPRSSE
CVLELGSREVAVEAQVECAPPPDVWCHIKCQQHQFSYEALKPELQVGLFLRWAGGLRVDSADGLHVVLYDCSV
GHGDCSRCQTAMPQYDCVWCEGERPRCVAREACNEAETVATQCPAPLIHSVDPLTGPIDGGTRVTIRGSNLGQ
HVQDVLDMVRVAGVPCAVDAGEYDVSSSLVCITGASGEEVTGTVAVEVPGRGHGVSEFSFAYQDPKVHSIFPA
RGPRAGGTRLTLHGSKLLTGRLEDIRVVVGDQPCHLLLEQQSEQLHCETGPYPVPAELPVTVLFGATERRLQH
GQFKYTSDPNVTSVGPSKSFFSGGREIWVRGQDLDVVQRPRIRVTVVPRQHGQGLAQKQHVVPEKFEEPCLVN
SSHLLMCRTPALPGPPWDSGVQVEFILDNMVFDFAALSPTPFSYEADPTLRSLNPEDPSTPFRHKPGSVFSVE
GENLDLAMSKEEVVAMIGDGPCVVKTLTRNHLYCEPPVEQPLPHPHALREAPDALPEFTVQMGNLRFSLGHVQ
YDGESPVAFPVAAQVGLGVGTSLLALGVIIIVLIYRRKSKQALRDYKKVQIQLENLESSVRDRCKKEFTDLMT
EMTDLTSDLLGSGIPFLDYKVYAERVFFPGYRESPLHRDLGVPDSRRPTVEQGLGQLSNLLNSKLFLTKFIHT
LESQRTFSARDRAYVASLLTVALHGKLEYFTDILRTLLSDLVAQYVAKNPKLMLRRTETVVEKLLTNWMSICL
YTFVRDSVGEPLYMLFRGIKHQVDKGPVDSVTGKAKYTLNDNRLLREDVEYRPLTLNALLAVGPGAGEAQCVP
VKVLDCDTISQAKEKMLDQLYKGVPLAQRPDSCTLDVEWRSGVAGHLILSDEDVTSELQGLWRRLNTLQHYKV
PDGATVALVPCLTKHILRENQDYVPGERTPMLEDVDEGGIRPWHLVKPSDEPEPPRPRRGNLRGGERERAKAI
PEIYLTRLLSMKGTLQKFVDDLFQVILSTSRPVPLAVKYFFDLLDEQAQQHGISDQDTIHIWKTNSLPLRFWI
NIIKNPQFVFDVQTSDNMDAVLLVIAQTFMDACTLADHKLGRDSPINKLLYARDIPRYKQMVERYYADIRQTV
PASDQEMNSVLAELSRNCSADLGARVALHELYKYINKYYDQIITALEEDGTAQKMQLGYRLQQIAAAVENKVT
DL*
>ENSMUSP00000071966_c.5748:G>A_p.V1917M ENSMUST00000072093.13|Plxnb1-201|ENSMUSG00000053646|+|chr9:g.108943663:G>A
MSVLGPVLLQVFWAGCVVTLRSPLPAAFTANGTHLQHLARDPTTGTLYVGATNFLFQLSPGLQLEAVVSTGPV
NDSRDCLPPVIPDECPQAQPTNNPNQLLLVSPEALVVCGSVHQGICELRSLGQIRQLLLRPERPGDTQYVAAN
DPAVSTVGLVAQGLVGEPLLFVGRGYTSRGVGGGIPPITTRALRPPDPQAAFSYEETAKLAVGRLSEYSHHFV
SAFVRGASAYFLFLRRDLKAPSRAFRAYVSRVCLQDQHYYSYVELPLACQGGRYGLIQAAAVATSKEVARGDV
LFAAFSSVAPPTVDWPLSASTGASGTSVLCAFPLDEVDQLANYTRDACYTREGRAENGTKVADIAYDVLSDCA
QLPVDTPDAFPCGSDHTPSPMVSCVPLEATPILELPGVQLTAVAVTMEDGHTIAFLGDSQGQLHRVYLGPGRS
AAPYSKQSIQPGSPVNRDLTFDGTFEHLYVATQTTLVKVPVAPCAQHLDCDSCLAHRDPYCGWCVLLGRCSRR
SECSRDQGPEQWLWSFQPELGCLRVVAVSPANISREERREVFLSVPGLPSLWPGESYFCYFGDQQSPALLTSS
GVMCPSPDPSEAPVLQRGADHISVNVELRFGAVVIASTSLSFYDCVAVTASSPSAPCRACVSSRWGCNWCVWQ
QLCTHKASCDAGPMVASQQSPLLPLIPPARDELTPFPPTVPQTTVTPTPNSFPIEPRAPSTASDVLPGAKPSR
LSLWGPWAGPGPILSPTSTESPLHEKPLPPDPPTIPGTTVPAPTGLGPSTTPEDLLASYPFPSDAAAVSPAEP
GPEALPSMVALDQPPGTVPDTTFPGAPGSMKPVLDWLTKGGGELPEADEWMGGDTPAFSTSTLLSGDGDSAEH
EGPPAPLILLSSLDYQYDTPGLWELGEVNQRVSSCPCVETVQGSLLIPVHVEREVQLRGRNLWLFQDGPRSSE
CVLELGSREVAVEAQVECAPPPDVWCHIKCQQHQFSYEALKPELQVGLFLRWAGGLRVDSADGLHVVLYDCSV
GHGDCSRCQTAMPQYDCVWCEGERPRCVAREACNEAETVATQCPAPLIHSVDPLTGPIDGGTRVTIRGSNLGQ
HVQDVLDMVRVAGVPCAVDAGEYDVSSSLVCITGASGEEVTGTVAVEVPGRGHGVSEFSFAYQDPKVHSIFPA
RGPRAGGTRLTLHGSKLLTGRLEDIRVVVGDQPCHLLLEQQSEQLHCETGPYPVPAELPVTVLFGATERRLQH
GQFKYTSDPNVTSVGPSKSFFSGGREIWVRGQDLDVVQRPRIRVTVVPRQHGQGLAQKQHVVPEKFEEPCLVN
SSHLLMCRTPALPGPPWDSGVQVEFILDNMVFDFAALSPTPFSYEADPTLRSLNPEDPSTPFRHKPGSVFSVE
GENLDLAMSKEEVVAMIGDGPCVVKTLTRNHLYCEPPVEQPLPHPHALREAPDALPEFTVQMGNLRFSLGHVQ
YDGESPVAFPVAAQVGLGVGTSLLALGVIIIVLIYRRKSKQALRDYKKVQIQLENLESSVRDRCKKEFTDLMT
EMTDLTSDLLGSGIPFLDYKVYAERVFFPGYRESPLHRDLGVPDSRRPTVEQGLGQLSNLLNSKLFLTKFIHT
LESQRTFSARDRAYVASLLTVALHGKLEYFTDILRTLLSDLVAQYVAKNPKLMLRRTETVVEKLLTNWMSICL
YTFVRDSVGEPLYMLFRGIKHQVDKGPVDSVTGKAKYTLNDNRLLREDVEYRPLTLNALLAVGPGAGEAQCVP
VKVLDCDTISQAKEKMLDQLYKGVPLAQRPDSCTLDVEWRSGVAGHLILSDEDVTSELQGLWRRLNTLQHYKV
PDGATVALVPCLTKHILRENQDYVPGERTPMLEDVDEGGIRPWHLVKPSDEPEPPRPRRGSLRGGERERAKAI
PEIYLTRLLSMKGTLQKFMDDLFQVILSTSRPVPLAVKYFFDLLDEQAQQHGISDQDTIHIWKTNSLPLRFWI
NIIKNPQFVFDVQTSDNMDAVLLVIAQTFMDACTLADHKLGRDSPINKLLYARDIPRYKQMVERYYADIRQTV
PASDQEMNSVLAELSRNCSADLGARVALHELYKYINKYYDQIITALEEDGTAQKMQLGYRLQQIAAAVENKVT
DL*
>ENSMUSP00000055803_c.454:A>C_p.D152A ENSMUST00000063042.11|Glb1-201|ENSMUSG00000045594|+|chr9:g.114250812:A>C

MLRVPLCTPLPLLALLQLLGAAHGIYNVTQRTFKLDYSRDRFLKDGQPFRYISGSIHYFRIPRFYWEDRLLKM
KMAGLNAIQMYVPWNFHEPQPGQYEFSGDRDVEHFIQLAHELGLLVILRPGPYICAEWDMGGLPAWLLEKQSI
VLRSSAPDYLVAVDKWLAVLLPKMKPLLYQNGGPIITVQVENEYGSYFACDYDYLRFLVHRFRYHLGNDVILF
TTDGASEKMLKCGTLQDLYATVDFGTGNNITQAFLVQRKFEPKGPLINSEFYTGWLDHWGKPHSTVKTKTLAT
SLYNLLARGANVNLYMFIGGTNFAYWNGANTPYEPQPTSYDYDAPLSEAGDLTKKYFALREVIQMFKEVPEGP
IPPSTPKFAYGKVALRKFKTVAEALGILCPNGPVKSLYPLTFTQVKQYFGYVLYRTTLPQDCSNPKPIFSSPF
NGVRDRAYVSVDGVPQGILDRNLMTALNIRGKAGATLDILVENMGRVNYGRFINDFKGLISNMTINSTVLTNW
TVFPLNTEAMVRNHLWGREASDEGHLDGRSTSNSSDLILPTFYVGNFSIPSGIPDLPQDTFIQFPGWSKGQVW
INGFNLGRYWPTMGPQKTLFVPRNILTTSAPNNITVLELEFAPCSEGTPELCTVEFVDTPVIS*
>ENSMUSP00000149937_c.208:A>C_p.D70A ENSMUST00000217583.2|Glb1-
202|ENSMUSG00000045594|+|chr9:g.114250812:A>C
MYVPWNFHEPQPGQYEFSGDRDVEHFIQLAHELGLLVILRPGPYICAEWDMGGLPAWLLEKQSIVLRSSAPDY
LVAVDKWLAVLLPKMKPLLYQNGGPIITVQVENEYGSYFACDYDYLRFLVHRFRYHLGNDVILFTTDGASEKM
LKCGTLQDLYATVDFGTGNNITQAFLVQRKFEPKGPLINSEFYTGWLDHWGKPHSTVKTKTLATSLYNLLARG
ANVNLYMFIGGTNFAYWNGANTPYEPQPTSYDYDAPLSEAGDLTKKYFALREVIQMVGPSNSQRAGLLGGLL*
>ENSMUSP00000107069_c.97:A>T_p.Q33L ENSMUST00000111442.3|Ccr5-
202|ENSMUSG00000079227|+|chr9:g.123924496:A>T
MDFQGSVPTYSYDIDYGMSAPCQKINVKQIAALLLPPLYSLVFIFGFVGNMMVFLILISCKKLKSVTDIYLLN
LAISDLLFLLTLPFWAHYAANEWVFGNIMCKVFTGLYHIGYFGGIFFIILLTIDRYLAIVHAVFALKVRTVNF
GVITSVVTWAVAVFASLPEIIFTRSQKEGFHYTCSPHFPHTQYHFWKSFQTLKMVILSLILPLLVMVICYSGI
LHTLFRCRNEKKRHRAVRLIFAIMIVYFLFWTPYNIVLLLTTFQEFFGLNNCSSSNRLDQAMQATETLGMTHC
CLNPVIYAFVGEKFRSYLSVFFRKHMVKRFCKRCSIFQQDNPDRASSVYTRSTGEHEVSTGL*
>ENSMUSP00000127328_c.97:A>T_p.Q33L ENSMUST00000171499.3|Ccr5-
205|ENSMUSG00000079227|+|chr9:g.123924496:A>T
MDFQGSVPTYSYDIDYGMSAPCQKINVKQIAALLLPPLYSLVFIFGFVGNMMVFLILISCKKLKSVTDIYLLN
LAISDLLFLLTLPFWAHYAANEWVFGNIMCKVFTGLYHIGYFGGIFFIIL
>ENSMUSP00000107069_c.728:G>A_p.M243I ENSMUST00000111442.3|Ccr5-
202|ENSMUSG00000079227|+|chr9:g.123925127:G>A
MDFQGSVPTYSYDIDYGMSAPCQKINVKQIAAQLLPPLYSLVFIFGFVGNMMVFLILISCKKLKSVTDIYLLN
LAISDLLFLLTLPFWAHYAANEWVFGNIMCKVFTGLYHIGYFGGIFFIILLTIDRYLAIVHAVFALKVRTVNF
GVITSVVTWAVAVFASLPEIIFTRSQKEGFHYTCSPHFPHTQYHFWKSFQTLKMVILSLILPLLVMVICYSGI
LHTLFRCRNEKKRHRAVRLIFAIIIVYFLFWTPYNIVLLLTTFQEFFGLNNCSSSNRLDQAMQATETLGMTHC
CLNPVIYAFVGEKFRSYLSVFFRKHMVKRFCKRCSIFQQDNPDRASSVYTRSTGEHEVSTGL*
>ENSMUSP00000107069_c.753:C>A_p.P252T ENSMUST00000111442.3|Ccr5-
202|ENSMUSG00000079227|+|chr9:g.123925152:C>A
MDFQGSVPTYSYDIDYGMSAPCQKINVKQIAAQLLPPLYSLVFIFGFVGNMMVFLILISCKKLKSVTDIYLLN
LAISDLLFLLTLPFWAHYAANEWVFGNIMCKVFTGLYHIGYFGGIFFIILLTIDRYLAIVHAVFALKVRTVNF
GVITSVVTWAVAVFASLPEIIFTRSQKEGFHYTCSPHFPHTQYHFWKSFQTLKMVILSLILPLLVMVICYSGI
LHTLFRCRNEKKRHRAVRLIFAIMIVYFLFWTTYNIVLLLTTFQEFFGLNNCSSSNRLDQAMQATETLGMTHC
CLNPVIYAFVGEKFRSYLSVFFRKHMVKRFCKRCSIFQQDNPDRASSVYTRSTGEHEVSTGL*
>ENSMUSP00000152718_c.860:T>G_p.I287M ENSMUST00000223295.2|Sult3a2-
202|ENSMUSG00000090298|-|chr10:g.33642441:A>C
MDNKDEDIFKFKGYNFLRALVSMDIVKNMEDYEIRDDDIFIVTYPKSGTTWTQQILCLICFESHRNGTENIAT
IDRIPSFEYNIHKLDYAKMPSPRIFTSHIPYYLVPKGLKDKKAKIIYVYRNPKDVLISFFHFSNLMVVLKASD
TLENFMQRFLNGNLVGSLWFDHIRGWYEHRHDFNIMFMSFEDMKKDLRSSVLKICSFLEKELSEEDMDAVVRQ
ATFEKMKADPRANNEHIIKDELGTRNNTGAFLRKGIVGDWKHYLTVDQSERFDKIFHMNMKNIPLKFMWNINE
K*
>ENSMUSP00000127159_c.860:T>G_p.I287M ENSMUST00000165904.2|Sult3a2-
201|ENSMUSG00000090298|-|chr10:g.33642441:A>C
MDNKDEDIFKFKGYNFLRALVSMDIVKNMEDYEIRDDDIFIVTYPKSGTTWTQQILCLICFESHRNGTENIAT
IDRIPSFEYNIHKLDYAKMPSPRIFTSHIPYYLVPKGLKDKKAKIIYVYRNPKDVLISFFHFSNLMVVLKASD
TLENFMQRFLNGNLVGSLWFDHIRGWYEHRHDFNIMFMSFEDMKKDLRSSVLKICSFLEKELSEEDMDAVVRQ
ATFEKMKADPRANNEHIIKDELGTRNNTGAFLRKGIVGDWKHYLTVDQSERFDKIFHMNMKNIPLKFMWNINE
K*
>ENSMUSP00000143429_c.1624:G>T_p.R542M ENSMUST00000200401.2|Crybg1-
202|ENSMUSG00000019866|-|chr10:g.43879562:C>A
MPLSPPSQGDPREASPSKAPKKHPTFHIWRSKKKQSPSSSCGVFIPHPPGAFGETRAFLVPDGSQGEPQELTL
HGGELQLSDTPSEVPGPSSTESAMLKKSRAQPPEDTRRKPVLEKLGTLFTAGRRRNTRNGLESPTNSNSKSAS

AKVPKTGSEKTKAQDNQLKATDAGEAGPRQEAEGQPVESCVQATPCDAEQSPCSSINVGVVQQCHSNDSAQLE
PLQAEGETFPDATTAAKELLHSSPGNRSRQESAETPARSPGEEALPGAGLQPETARGVSGSPPEEHLVGGVGE
APDGAPGVGAEAGSLGAPGDAVPPGEPPAQDAETSRTAGSVDRAHPTKVLTLDIYLSKTEVAQVDEPASIAPG
TDDCEDSDDMEKRSSGRRSGRRRKSQKSTDSPGADAVAPESAAKDEAVFDDEVAPDAAAENCLAERKVKSVQA
VPDGGAAPVACPEPKASSCPRGQPRGEPDRSKPLSPTKRKGRSRVPEAVPSSPGSGSRAPAKESPPKRAPAAA
DSGEEAGRVVPRELTVKSSSLLPEIKPEHKMGPLPHHFDSRGDGGRSRGTGASDADGLKPRNQFGVGRSTVTT
KVTLPARPKHVELNLKTPKNPDSLGNEHNSFSQPVHKGNTANKISLFENKRANSSPRHPDVRNTRNNPPSSKT
FVGRAKLNLAKKAKEMEQPEKKAVSTSHQNGLVVKEPSAEGKGTAVPVPREEIRPATRGGEGESTKDQVLRPE
PEQSDKLDVQTDTAYPSHPGPTALVPVKDSELSGEGQPEAADSKGPVLNNTSDVAQDIAAICDAREAPPKPQD
AFSDSQPASAAESARESSLPPSAPISVDAPQDGCVQVPICSVPCTALEVSENHNGCVSPVSPHTEKTPISKPG
VGEASPPSTPEHSPETMGKDCPSKVLVQVRSFVLPVENPQDVSSQVISESSEVREAQLPSCHSNEPEVVSVTT
CVPQHEDAPDTSPKDIHHREGHVAQSRPHITQSGPEKAPPIQSSSGMPQTAEFILTHSPSSRSHAEGPQRPGQ
DATSPLTPTRLLNISAGSDNSVLDSSSDMEKFTEIIKKMDSAVCVPQKKKKARVPNSPAPHFAMPPIHEDSLE
KVFDPNVFTIGLGKKKESLAEVSPALHLLQNHDPISKLRAKRASTEQSIVFKSLHTNSNGKSEPQTTSEASDK
ENRDINSGGVKRSRLENSTLFSSMLSSLPQDKVFSPSVTSVNTMSTSFSTSQNSSVSQSSVVQPRKEGGLPCG
SDKEQPHLAPHNSLKVFNFNSSNTSRLGSSHMEKYPQKEGSKEDVNSQGNLQLPENNLSDLPKLKNSDGVEGI
LKSNLPNVGSVDADFLGLFKASRYDPGHSFSGMSLSDSMTLRGSMHSKLNPRPGKVVIFSEPDVSEECIEVFG
DIQDCSSWRLSPVIVVKVVRGCWILYEKANFEGHSLALEEGELELSSIWGTEEMLDEEAESDKPVVIGSIRHV
VQDYRISQIDLFTEPEGLGLLNSYFDDTEEMQGFPVMQKTCSIKVHWGTWLIYEEPGFQGVPFILEPGEYPDL
SFWDTEEAYIGSMRPLKMGGRKVEFPTDPKVVIYEKPSFEGRCMELETEMSSFIMEGGETEETTGNNNLPFTS
VGSMKVLRGVWVAYEKCGFTGHQYLLEEGEYRDWNAWGGYNGELQSLRPILSDFSNAHMIMYSEKNFGSKGSS
IDVLGIVANLKETGYGVKTQSINVLSGVWVAYENPDFTGEQYVLDKGFYTSFEDWGGKNCKISSVQPICLDSF
TGPRRRNQIHLFSEPQFQGQSQSFEETIDQIDDSFQTKSCRVLGGSWVAYDGENFSGNQYVLEEGHYPCLSAM
GCLPGATLKSLRFIDFEFSEPTIILFERENFKGKKIELNAETVNLRSLGFNTQIRSVRVVGGIWVTYEYGNYR
GRQFLLSPAEVPNWYEFSACRQIGSLRPFAQKRIYFRLRNKATGLFMSTNGNLEDLKLLRVQVMEDVGADDQI
WIYQEGCIKCRIAEDCCLTIVGSLVTSGSKLGLALDQNVDSQFWCMKSDGRIYSKMKPNLVLDVKGGAQYDQN
HIILNTVSTEKLTQVWEAMVL*
>ENSMUSP00000020017_c.502:G>T_p.R168M ENSMUST00000020017.14|Crybg1-201|ENSMUSG00000019866|-|chr10:g.43879562:C>A
MEKRSSGRRSGRRRKSQKSTDSPGADAVAPESAAKDEAVFDDEVAPDAAAENCLAERKVKSVQAVPDGGAAPV
ACPEPKASSCPRGQPRGEPDRSKPLSPTKRKGRSRVPEAVPSSPGSGSRAPAKESPPKRAPAAADSGEEAGRV
VPRELTVKSSSLLPEIKPEHKMGPLPHHFDSRGDGGRSRGTGASDADGLKPRNQFGVGRSTVTTKVTLPARPK
HVELNLKTPKNPDSLGNEHNSFSQPVHKGNTANKISLFENKRANSSPRHPDVRNTRNNPPSSKTFVGRAKLNL
AKKAKEMEQPEKKAVSTSHQNGLVVKEPSAEGKGTAVPVPREEIRPATRGGEGESTKDQVLRPEPEQSDKLDV
QTDTAYPSHPGPTALVPVKDSELSGEGQPEAADSKGPVLNNTSDVAQDIAAICDAREAPPKPQDAFSDSQPAS
AAESARESSLPPSAPISVDAPQDGCVQVPICSVPCTALEVSENHNGCVSPVSPHTEKTPISKPGVGEASPPST
PEHSPETMGKDCPSKVLVQVRSFVLPVENPQDVSSQVISESSEVREAQLPSCHSNEPEVVSVTTCVPQHEDAP
DTSPKDIHHREGHVAQSRPHITQSGPEKAPPIQSSSGMPQTAEFILTHSPSSRSHAEGPQRPGQDATSPLTPT
RLLNISAGSDNSVLDSSSDMEKFTEIIKKMDSAVCVPQKKKKARVPNSPAPHFAMPPIHEDSLEKVFDPNVFT
IGLGKKKESLAEVSPALHLLQNHDPISKLRAKRASTEQSIVFKSLHTNSNGKSEPQTTSEASDKENRDINSGG
VKRSRLENSTLFSSMLSSLPQDKVFSPSVTSVNTMSTSFSTSQNSSVSQSSVVQPRKEGGLPCGSDKEQPHLA
PHNSLKVFNFNSSNTSRLGSSHMEKYPQKEGSKEDVNSQGNLQLPENNLSDLPKLKNSDGVEGILKSNLPNVG
SVDADFLGLFKASRYDPGHSFSGMSLSDSMTLRGSMHSKLNPRPGKVVIFSEPDVSEECIEVFGDIQDCSSWR
LSPVIVVKVVRGCWILYEKANFEGHSLALEEGELELSSIWGTEEMLDEEAESDKPVVIGSIRHVVQDYRISQI
DLFTEPEGLGLLNSYFDDTEEMQGFPVMQKTCSIKVHWGTWLIYEEPGFQGVPFILEPGEYPDLSFWDTEEAY
IGSMRPLKMGGRKVEFPTDPKVVIYEKPSFEGRCMELETEMSSFIMEGGETEETTGNNNLPFTSVGSMKVLRG
VWVAYEKCGFTGHQYLLEEGEYRDWNAWGGYNGELQSLRPILSDFSNAHMIMYSEKNFGSKGSSIDVLGIVAN
LKETGYGVKTQSINVLSGVWVAYENPDFTGEQYVLDKGFYTSFEDWGGKNCKISSVQPICLDSFTGPRRRNQI
HLFSEPQFQGQSQSFEETIDQIDDSFQTKSCRVLGGSWVAYDGENFSGNQYVLEEGHYPCLSAMGCLPGATLK
SLRFIDFEFSEPTIILFERENFKGKKIELNAETVNLRSLGFNTQIRSVRVVGGIWVTYEYGNYRGRQFLLSPA
EVPNWYEFSACRQIGSLRPFAQKRIYFRLRNKATGLFMSTNGNLEDLKLLRVQVMEDVGADDQIWIYQEGCIK
CRIAEDCCLTIVGSLVTSGSKLGLALDQNVDSQFWCMKSDGRIYSKMKPNLVLDVKGGAQYDQNHIILNTVST
EKLTQVWEAMVL*
>ENSMUSP00000122041_c.256:A>T_p.E86V ENSMUST00000151623.8|Smpdl3a-203|ENSMUSG00000019872|+|chr10:g.57678545:A>T
MALSGQFWHVTDLHLDPTYHITDDRTKVCASSKGANASNPGPFGDVLCDSPYQLILSAFDFIKNSGQEASFMI
WTGDSPPHVPVPVLSTGTVIKVITNMTMTVQ

>ENSMUSP00000020022_c.346:A>T_p.E116V ENSMUST00000020022.8|Smpdl3a-201|ENSMUSG00000019872|+|chr10:g.57678545:A>T
MALLGNFLCCLLVAWLCGPGLGVPLAPADRAPAVGQFWHVTDLHLDPTYHITDDRTKVCASSKGANASNPGPF
GDVLCDSPYQLILSAFDFIKNSGQEASFMIWTGDSPPHVPVPVLSTGTVIKVITNMTMTVQNLFPNLQVFPAL
GNHDYWPQDQLPIVTSKVYSAVADLWKPWLGEEAISTLKKGGFYSQKVASNPGLRIISLNTNLYYGPNIMTLN
KTDPANQFEWLENTLNSSLWNKEKVYIIAHVPVGYLPYATDTPAIRQYYNEKLLDIFRRYSSVIAGQFYGHTH
RDSLMVLSDKNGNPLNSVFVAPAVTPVKGVLQKETNNPGVRLFQYKPGDYTLLDMVQYYLNLTEANLKGESNW
TLEYVLTQAYSVADLQPKSLYALVQQFATKDSKQFLKYYHYYFVSYDSSATCDQHCKTLQVCAIMNLDSMSYD
DCLKQHL*
>ENSMUSP00000020258_c.2373:G>A_p.V792I ENSMUST00000020258.10|Herc4-201|ENSMUSG00000020064|+|chr10:g.63147282:G>A
MLCWGNASYGQLGLGGIDEEIVLEPRRSDFFVNKKVRDVGCGLRHTVFVLDDGTVYTCGCNDLGQLGHEKSRK
KPEQVVALDAQNIVAVACGEAHTLALNDKGQVYAWGLDSDGQLGLQGSEECIRVPRNIKSLSDIQIVQVACGY
YHSLALSKASEVFCWGQNKYGQLGLGIDCQKQTSPQLIKSLLGIPFMQVAAGGAHSFVLTLSGAIFGWGRNKF
GQLGLNDENDRYVPNLLKSLRSQKIVYICCGEDHTAALTKEGGVFTFGAGGYGQLGHNSTSHEINPRKVFELM
GSIVTQVACGRQHTSAFVPSSGRIYSFGLGGNGQLGTGSTSNRKSPFTVKGNWFSYNGQCPQDIGSEDYFCVK
RIFSGGDQSFSHYSSPQNCGPPDDFRCSDPSKQIWTVNEALIQKWLSYPSGRFPVEIANEIDGTFSSSGCLNG
SFLAISNDDHYRTGTRFSGVDMNAARLLFHKLIQPDHPQISQQVAASLEKNLIPKLTSSLPDVEALRFYLTLP
ECPLMSDCNNFTTIAIPFGTALVNLEKAPLKVLENWWSVLEPPLFLKIVELFKEVVVHLLKLYKIGIPPSERR
IFNSFLHTALKVLEILHRVNEKTGQLIQYDKFYIHEVQELIDIRNDYINWVQQQAYGVLADIPVTICTYPFVF
DAQAKTTLLQTDAVLQMQMAIDQAHRQNVSSLFLPVIESVNPCLILVVRRENIVGDAMEVLRKTKNIDYKKPL
KVIFVGEDAVDAGGVRKEFFLLIMRELLDPKYGMFRYYEDSRLIWFSDKTFEDSDLFHLIGIICGLAIYNFTI
VDLHFPLALYKKLLKRKPSLDDLKELMPAVGRSMQQLLDYPEDDIEETFCLNFTITVENFGATEVKELVLNGA
DTAVNRQNRQEFVDAYVDYIFNKSVASLFDAFHAGFHKVCGGKVLLLFQPNELQAMVIGNTNYDWKELEKNTE
YKGEYWADHPTIKIFWEVFHELPLEKKKQFLLFLTGSDRIPILGMKSLKLVIQSTGGGESYLPVSHTCFNLLD
LPKYTEKETLRCKLIQAIDHNEGFSLI*
>ENSMUSP00000151886_c.2397:G>A_p.V800I ENSMUST00000219577.2|Herc4-208|ENSMUSG00000020064|+|chr10:g.63147282:G>A
MLCWGNASYGQLGLGGIDEEIVLEPRRSDFFVNKKVRDVGCGLRHTVFVLDDGTVYTCGCNDLGQLGHEKSRK
KPEQVVALDAQNIVAVACGEAHTLALNDKGQVYAWGLDSDGQLGLQGSEECIRVPRNIKSLSDIQIVQVACGY
YHSLALSKASEVFCWGQNKYGQLGLGIDCQKQTSPQLIKSLLGIPFMQVAAGGAHSFVLTLSGAIFGWGRNKF
GQLGLNDENDRYVPNLLKSLRSQKIVYICCGEDHTAALTKEGGVFTFGAGGYGQLGHNSTSHEINPRKVFELM
GSIVTQVACGRQHTSAFVPSSGRIYSFGLGGNGQLGTGSTSNRKSPFTVKGNWFSYNGQCPQDIGSEDYFCVK
RIFSGGDQSFSHYSSPQNCGPPDDFRCSDPSKQIWTVNEALIQKWLSYPSGRFPVEIANEIDGTFSSSGCLNG
SFLAISNDDHYRTGTRFSGVDMNAARLLFHKLIQPDHPQISQQVAASLEKNLIPKLTSSLPDVEALRFYLTLP
ECPLMSDCNNFTTIAIPFGTALVNLEKAPLKVLENWWSVLEPPLFLKIVELFKEVVVHLLKLYKIGIPPSERR
IFNSFLHTALKVLEILHRVNEKTGQLIQYDKFYIHEVQELIDIRNDYINWVQQQAYGVDVSHGVTELADIPVT
ICTYPFVFDAQAKTTLLQTDAVLQMQMAIDQAHRQNVSSLFLPVIESVNPCLILVVRRENIVGDAMEVLRKTK
NIDYKKPLKVIFVGEDAVDAGGVRKEFFLLIMRELLDPKYGMFRYYEDSRLIWFSDKTFEDSDLFHLIGIICG
LAIYNFTIVDLHFPLALYKKLLKRKPSLDDLKELMPAVGRSMQQLLDYPEDDIEETFCLNFTITVENFGATEV
KELVLNGADTAVNRQNRQEFVDAYVDYIFNKSVASLFDAFHAGFHKVCGGKVLLLFQPNELQAMVIGNTNYDW
KELEKNTEYKGEYWADHPTIKIFWEVFHELPLEKKKQFLLFLTGSDRIPILGMKSLKLVIQSTGGGESYLPVS
HTCFNLLDLPKYTEKETLRCKLIQAIDHNEGFSLI*
>ENSMUSP00000020258_c.2955:A>C_p.S986R ENSMUST00000020258.10|Herc4-201|ENSMUSG00000020064|+|chr10:g.63153073:A>C
MLCWGNASYGQLGLGGIDEEIVLEPRRSDFFVNKKVRDVGCGLRHTVFVLDDGTVYTCGCNDLGQLGHEKSRK
KPEQVVALDAQNIVAVACGEAHTLALNDKGQVYAWGLDSDGQLGLQGSEECIRVPRNIKSLSDIQIVQVACGY
YHSLALSKASEVFCWGQNKYGQLGLGIDCQKQTSPQLIKSLLGIPFMQVAAGGAHSFVLTLSGAIFGWGRNKF
GQLGLNDENDRYVPNLLKSLRSQKIVYICCGEDHTAALTKEGGVFTFGAGGYGQLGHNSTSHEINPRKVFELM
GSIVTQVACGRQHTSAFVPSSGRIYSFGLGGNGQLGTGSTSNRKSPFTVKGNWFSYNGQCPQDIGSEDYFCVK
RIFSGGDQSFSHYSSPQNCGPPDDFRCSDPSKQIWTVNEALIQKWLSYPSGRFPVEIANEIDGTFSSSGCLNG
SFLAISNDDHYRTGTRFSGVDMNAARLLFHKLIQPDHPQISQQVAASLEKNLIPKLTSSLPDVEALRFYLTLP
ECPLMSDCNNFTTIAIPFGTALVNLEKAPLKVLENWWSVLEPPLFLKIVELFKEVVVHLLKLYKIGIPPSERR
IFNSFLHTALKVLEILHRVNEKTGQLIQYDKFYIHEVQELIDIRNDYINWVQQQAYGVLADIPVTICTYPFVF
DAQAKTTLLQTDAVLQMQMAIDQAHRQNVSSLFLPVIESVNPCLILVVRRENIVGDAMEVLRKTKNIDYKKPL
KVIFVGEDAVDAGGVRKEFFLLIMRELLDPKYGMFRYYEDSRLIWFSDKTFEDSDLFHLIGVICGLAIYNFTI
VDLHFPLALYKKLLKRKPSLDDLKELMPAVGRSMQQLLDYPEDDIEETFCLNFTITVENFGATEVKELVLNGA
DTAVNRQNRQEFVDAYVDYIFNKSVASLFDAFHAGFHKVCGGKVLLLFQPNELQAMVIGNTNYDWKELEKNTE

YKGEYWADHPTIKIFWEVFHELPLEKKKQFLLFLTGRDRIPILGMKSLKLVIQSTGGGESYLPVSHTCFNLLD
LPKYTEKETLRCKLIQAIDHNEGFSLI*
>ENSMUSP00000151886_c.2979:A>C_p.S994R ENSMUST00000219577.2|Herc4-208|ENSMUSG00000020064|+|chr10:g.63153073:A>C
MLCWGNASYGQLGLGGIDEEIVLEPRRSDFFVNKKVRDVGCGLRHTVFVLDDGTVYTCGCNDLGQLGHEKSRK
KPEQVVALDAQNIVAVACGEAHTLALNDKGQVYAWGLDSDGQLGLQGSEECIRVPRNIKSLSDIQIVQVACGY
YHSLALSKASEVFCWGQNKYGQLGLGIDCQKQTSPQLIKSLLGIPFMQVAAGGAHSFVLTLSGAIFGWGRNKF
GQLGLNDENDRYVPNLLKSLRSQKIVYICCGEDHTAALTKEGGVFTFGAGGYGQLGHNSTSHEINPRKVFELM
GSIVTQVACGRQHTSAFVPSSGRIYSFGLGGNGQLGTGSTSNRKSPFTVKGNWFSYNGQCPQDIGSEDYFCVK
RIFSGGDQSFSHYSSPQNCGPPDDFRCSDPSKQIWTVNEALIQKWLSYPSGRFPVEIANEIDGTFSSSGCLNG
SFLAISNDDHYRTGTRFSGVDMNAARLLFHKLIQPDHPQISQQVAASLEKNLIPKLTSSLPDVEALRFYLTLP
ECPLMSDCNNFTTIAIPFGTALVNLEKAPLKVLENWWSVLEPPLFLKIVELFKEVVVHLLKLYKIGIPPSERR
IFNSFLHTALKVLEILHRVNEKTGQLIQYDKFYIHEVQELIDIRNDYINWVQQQAYGVDVSHGVTELADIPVT
ICTYPFVFDAQAKTTLLQTDAVLQMQMAIDQAHRQNVSSLFLPVIESVNPCLILVVRRENIVGDAMEVLRKTK
NIDYKKPLKVIFVGEDAVDAGGVRKEFFLLIMRELLDPKYGMFRYYEDSRLIWFSDKTFEDSDLFHLIGVICG
LAIYNFTIVDLHFPLALYKKLLKRKPSLDDLKELMPAVGRSMQQLLDYPEDDIEETFCLNFTITVENFGATEV
KELVLNGADTAVNRQNRQEFVDAYVDYIFNKSVASLFDAFHAGFHKVCGGKVLLLFQPNELQAMVIGNTNYDW
KELEKNTEYKGEYWADHPTIKIFWEVFHELPLEKKKQFLLFLTGRDRIPILGMKSLKLVIQSTGGGESYLPVS
HTCFNLLDLPKYTEKETLRCKLIQAIDHNEGFSLI*
>ENSMUSP00000041298_c.142:A>C_p.E48A ENSMUST00000043317.7|Dnajc12-201|ENSMUSG00000036764|+|chr10:g.63231628:A>C
MDAILNYRPEGSEDYYALLGCDELSSVEQILAEFKIRALECHPDKHPANSKAVETFQKLQKAKEILCNAESRA
RYDHWRRSQMSMPFEQWEALADSVKTSMHWAVRSKKDLMLEGSGQTFTSSVPNKERSEQRETKKGDPDSNPEK
MKQKEPKFPEEGISPQNPDSPGLSDLNCGHLRFRWSGDAPSELLRKFRNYEI*
>ENSMUSP00000079789_c.301:A>G_p.Q101R ENSMUST00000080995.8|Tmem26-201|ENSMUSG00000060044|+|chr10:g.68584237:A>G
MEGLVLLKALVTRLLFLLHSLVAVWRVTWVKEEHRYWLLALLNLLLVLETVLTLKFKRGRGYKWLSPAIFVYL
VNIMPSLWLLEMHHGNQYCSTQSERMARNFSRRGDVNQTLSSHRATNGMGNILELARGFVDNLSMVCEPVWTL
GLHQTLLLILIIGRWLLPIGGTITRDQLSELLLMFVGTAADILEFTTETLKENNVRTNPTLVSGILVVWTWSM
LQFPLDLAVQLKLVCPASVKARGFLRVFLCQYSADLWAIGLSFFIQDGPFLVVRLVLMIYFKVINHMLVFFAV
KNSLVMALHFYRLVALIMATRDFMRDHPESPKPEHSGPDQPSESGPSEWEDASPEALPLRTSPVTSEESYPTT
P*
>rs254345418_ENSMUSP00000151917_c.184:A>C_p.D62A
ENSMUST00000219981.2|Fgf22-203|ENSMUSG00000020327|+|chr10:g.79591137:A>C
MRSRLWLGLAWLLLARAPGAPGGYPHLEGDVRWRRLFSSTHFFLRVDLGGRVQGTRWRHGQASIVEIRSVRVG
TVVIKAVYSGFYVAMNRRGRLYGSVPGAHRGERLQHIRLATLEAPRPTHVPGT*
>rs254345418_ENSMUSP00000151743_c.184:A>C_p.D62A
ENSMUST00000219228.2|Fgf22-202|ENSMUSG00000020327|+|chr10:g.79591137:A>C
MRSRLWLGLAWLLLARAPGAPGGYPHLEGDVRWRRLFSSTHFFLRVDLGGRVQGTRWRHGQASIVEIRSVRVG
TVVIKAVYSGFYVAMNRRGRLYGSRVYSVDCRFRERIEENGYNTYASRRWRHRGRPMFLALDSQGIPRQGRRT
RRHQLSTHFLPVLVSS*
>rs254345418_ENSMUSP00000020577_c.166:A>C_p.D56A
ENSMUST00000020577.4|Fgf22-201|ENSMUSG00000020327|+|chr10:g.79591137:A>C
LGLAWLLLARAPGAPGGYPHLEGDVRWRRLFSSTHFFLRVDLGGRVQGTRWRHGQASIVEIRSVRVGTVVIKA
VYSGFYVAMNRRGRLYGSVSDWRWAATGWQWSVVQTASLTHPPCSGSTLWTVGSGSASRRTATTHTPRDVGGT
AADPCSWHLTAKAFPGKADGHDGTNCPHTSCQSWSRLEGPANGSGGMGGTQGLEDPELNNQGPGQRPWANTSL
YV
>rs242503166_ENSMUSP00000005069_c.244:C>T_p.A82V
ENSMUST00000005069.8|Nmrk2-201|ENSMUSG00000004939|-|chr10:g.81036204:G>A
MKLIIGIGGVTNGGKTTLTNSLLKALPNCCVIHQDDFFKPQDQIAVGEDGFKQWDVLESLDMETMLSTVQAWV
KDPHKFARVHGVSLQSGASDTHVLLLEGFLLYSYRPLVDLYSQRYFLTVPYEECKRRRRSRTYMVPDPPGLFD
GHVWPMYQKYRREMEQDGVEVVYLDGMKSPEGLFHQVLEDIQNRLLNTS*
>rs108207416_ENSMUSP00000059533_c.1078:T>C_p.V360A
ENSMUST00000050867.8|Cactin-201|ENSMUSG00000034889|+|chr10:g.81159581:T>C
MRGAGRQMGRDSRSRSRSVGRRGRKQRSRSRGRSRSRSRSRSRSRSRSRSRSHGRSSRRRREHERRRERKR
RSRGRRSDSEGEQRQKSRRRSQSLRPPRWHSQNQSSCSDSGEERAQGSWARKGHRRSWSPGSSASSLDSPRRS
RSPGTATLALSQQQSLQERLRLREERKQQEELLKAFETPEEKRARRLAKKEAKERKKREKMGWGEEYMGYTNT
DNPFGDNNLLGTFIWNKALEKKGISHLEEKELKERNKRIQEDNRLELQKVKQLRLEREREKAMREQELELLQR

EKEAEHFKTWEEQEDSFHLRQAKLRSKIRIRDGRAKPIDLLAKYISAEDDDLAVEMHEPYTFLNGLTAADMED
LLEDIQVYMELEQGKNVDFWRDMTTITEDEIAKLRKLEASGKGPGERREGVNASVSSDVQSVFKGKTYNQLQV
IFQGIEGKIRAGGPNLDMGYWESLLQQLRAHMARARLRERHQDVLRQKLFKLKQEQGVESEPLFPILKSEPSA
AHSPEPEERPPSPGTSVDPVEPVEPEEATAPGEAEGEAEGEAVLMEEDLIQQSLADYDAGRYSPRLLTAHELP
LDAHVLEPHEDLQRLQLSRQQLQATGDASESAEDIFFRRAREGMGQDEAQFSVEMPLGGRAYLWADKYRPRKP
RFFNRVHTGFEWNKYNQTHYDFDNPPPKIVQGYKFNIFYPDLIRKRATPEYFLEACADNRDFAILRFHAGPPY
EDIAFKIVSREWEYSHRHGFRCQFANGIFQLWFHFKRYRYRR*
>rs108207416_ENSMUSP00000151248_c.136:T>C_p.V46A
ENSMUST00000218120.2|Cactin-202|ENSMUSG00000034889|+|chr10:g.81159581:T>C
QTCSKIRIRDGRAKPIDLLAKYISAEDDDLAVEMHEPYTFLNGLTAADMEDLLEDIQVYMELEQGKNVDFWRD
MTTITEDEIAKLRKLEASGKGPGERREGVNASVSSDVQSVFKGKTYNQLQVIFQGIEGKIRAGGPNLDMGYWE
SLLQQLRAHMARARLRERHQDVLRQKLFKLKQEQGVESEPLFPILKSEPSAAHSPEPEERPPSPGTSVDPVEP
VEP
>ENSMUSP00000036690_c.681:G>C_p.D228H ENSMUST00000039956.6|Slc41a2-
201|ENSMUSG00000034591|-|chr10:g.83140221:C>G
MTHSKGRPVTYKTSASPESGGGFVDWTLNLNTIQSDKFLNLLLSMVPVIYQKNQEDRHKKVNGIWQDGLSGAA
QTFSKRSEPHLDYHEFSEQAFHSSSSGHTPASCSPKYDDYAGYNYCDGREASETTAMLQDEDLSSEGDDVIVE
TSQRIPKESSGVMALQILVPFLLAGFGTVSAGMVLDIVQHWEVFKNVTEVFILVPALLGLKGNLEMTLASRLS
TAVNVGKMHSPIEKWNLIIGNLALKQVQATVVGFLAAVAAIILGWIPEGKYYLSHSILLCSSSVATAFIASLL
QGIIMVGVIVGSKKTGINPDNVATPIAASFGDLITLAILAWISQGLYSCLETYYYISPLVCAFFLALTPIWII
IAAKHPATRTVLHSGWEPVITAMVISSIGGLILDTTVSDPNLVGIVVYTPVINGIGGNLVAIQASRISTYLHL
HSIPGELPEEPKGCSYPFRTFFGSGVNNKSAQVLLLFVIPGHLIFLYTIHLMKSGHTSLTVVFVVVYLFAAVL
QVFTLLWIADWMVHRFWRKGKDPDSFSIPYLTALGDLLGTALLALSFHFLWLIGDRDGDVGD*
>ENSMUSP00000076418_c.1824:A>C_p.K609Q ENSMUST00000077175.7|Polr3b-
201|ENSMUSG00000034453|+|chr10:g.84515653:A>C
MDVLAEEFGSLTPEQLTAPIPTVEEKWRLLPAFLKVKGLVKQHIDSFNYFINVEIKKIMKANEKVTSDADPMW
YLKYLNIYVGLPDVEESFNVTRPVSPHECRLRDMTYSAPITVDIEYTRGSQRIIRNALPIGRMPIMLRSSNCV
LTGKTPAEFAKLNECPLDPGGYFIVKGVEKVILIQEQLSKNRIIVEADRKGAVGASVTSSTHEKKSRTNMAVK
QGRFYLRHNTLSEDIPIVIIFKAMGVESDQEIVQMIGTEEHVMAAFGPSLEECQKAQIFTQMQALKYIGNKVR
RQRMWGGGPKKTKIEEARELLASTILTHVPVKEFNFRAKCIYTAVMVRRVILAQGDNKVDDRDYYGNKRLELA
GQLLSLLFEDLFKKFNSEMKKIADQVIPKQRAAQFDVVKHMRQDQITNGMVNAISTGNWSLKRFKMDRQGVTQ
VLSRLSYISALGMMTRISSQFEKTRKVSGPRSLQPSQWGMLCPSDTPEGEACGLVKNLALMTHITTDMEDGPI
IKLAGNLGVEDVNLLCGEELSYPNVFLVFLNGNILGVIRDHKKLVSTFRLMRRAGYINEFVSISTNLTDRCVY
ISSDGGRLCRPYIIVKKQKPAVTNQHMEELAQGYRNFEDFLHESLVEYLDVNEENDCNIALYEHTINKDTTHL
EIEPFTLLGVCAGLIPYPHHNQSPRNTYQCAMGKQAMGTIGYNQRNRIDTLMYLLAYPQKPMVKTKTIELIDF
EKLPAGQNATVAVMSYSGYDIEDALVLNKASLDRGFGRCLVYKNAKCTLKRYTNQTFDKVMGPMLDAATRKPI
WRHEILDADGICSPGEKVENKQVLVNKSMPTVTQIPLEGSNVPQQPQYKDVPITYKGATDSYIEKVMISSNAE
DAFLIKMLLRQTRRPEIGDKFSSRHGQKGVCGLIVPQEDMPFCDSGICPDIIMNPHGFPSRMTVGKLIELLAG
KAGVLDGRFHYGTAFGGSKVKDVCEDLVRHGYNYLGKDYVTSGITGEPLEAYIYFGPVYYQKLKHMVLDKMHA
RARGPRAVLTRQPTEGRSRDGGLRLGEMERDCLIGYGASMLLLERLMISSDAFEVDVCGQCGLLGYSGWCHYC
KSSCHVSSLRIPYACKLLFQELQSMNIIPRLKLAKYNE*
>ENSMUSP00000020094_c.9:C>A_p.L4I ENSMUST00000020094.8|Epyc-
201|ENSMUSG00000019936|+|chr10:g.97485571:C>A
MGMIARVALGLIIIDAVLAAPTTELFNYDSEVYDAILEDTGTFYNYEHIPDNHVENEKVSERLSGNRELLTPG
PQLGDNQDEDKDEESTPRLIDGSSPQEPEFPGLLGPHTNEDFPTCLLCTCISTTVYCDDHELDAIPPLPKKTT
YFYSRFNRIKKINKNDFASLNDLKRIDLTSNLISEIDEDAFRKLPHLQELVLRDNKIKQLPELPNTLTFIDIS
NNRLGRKGIKQEAFKDMYDLHHLYITDNSLDHIPLPLPESLRALHLQNNDILEMHEDTFCNVKNLTYVRKALE
DIRLDGNPINLSRTPQAYMCLPRLPIGSFI*
>ENSMUSP00000100922_c.9:C>A_p.L4I ENSMUST00000105285.4|Epyc-
202|ENSMUSG00000019936|+|chr10:g.97485571:C>A
MGMIARVALGLIIIDAVLAAPTTELFNYDSEVYDAILEDTGTFYNYEHIPDNHVENEKVSERLSGNRELLTPG
PQLGDNQDEDKDEESTPRLIDGSSPQEPEFPGLLGPHTNEDFPTCLLCTCISTTVYCDDHELDAIPPLPKKTT
YFYSRFNRIKKINKNDFASLNDLKRIDLTSNLISEIDEDAFRKLPHLQELVLRDNKIKQLPELPNTLTFIDIS
NNRLGRKGIKQEAFKDMYDLHHLYITDNSLDHIPLPLPESLRALHLQNNDILEMHEDTFCNVKNLTYVRKALE
DIRLDGNPINLSRTPQAYMCLPRLPIGSFI*
>ENSMUSP00000151842_c.2284:A>G_p.E762G ENSMUST00000219263.2|Ppp1r12a-
205|ENSMUSG00000019907|+|chr10:g.108096896:A>G

MKMADAKQKRNEQLKRWIGSETDLEPPVVKRQKTKVKFDDGAVFLAACSSGDTDEVLKLLHRGADINYANVDG
LTALHQACIDDNVDMVKFLVENGANINQPDNEGWIPLHAAASCGYLDIAEFLIGQGAHVGAVNSEGDTPLDIA
EEEAMEELLQNEVNRQGVDIEAARKEEERVMLRDARQWLNSGHISDVRHAKSGGTALHVAAAKGYTEVLKLLI
QAGYDVNIKDYDGWTPLHAAAHWGKEEACRILVDNLCDMETVNKVGQTAFDVADEDILGYLEELQKKQTLLHS
EKRDKKSPLIESTANMENNQPQKAFKNKETLIIEPEKNASRIESLEHEKADEEEEGKKDESSCSSEEDEEDDS
ESEAETDKTKPMASVSNAHTSSTQAAPAAVTAPTLSSNQGTPTSPVKKFPISTTKISPKEEERKDESPASWRL
GLRKTGSYGALAEISASKEAQKEKDTAGVMRSASSPRLSSSLDNKEKEKDNKGTRLAYVTPTIPRRLASTSDI
EEKENRESSSLRTSSSYTRRKWEDDLKKNSSINEGSTYHRSCSFGRRQDDLISCSVPSTTSTPTVTSAAGLQR
SLPSSTSTAAKTPPGSSSAGTQSSTSNRLWAEDSTEKEKDSAPTAVTIPVAPTVVNAAAPSTTTLTTTTAGTV
SEVRERRRSYLTPVRDEESESQRKARSRQARQSRRSTQGVTLTDLQEAEKTIGRSRSTRTREQENEEKEKEEK
EKQDKEKQEEKKESEASREDEYKQKYSRTYDGTYTRYRPVSTSSSSAPSSSSLSTLGSTLYASSQLNRPNSLV
GITSAYSRGLAKENEREGEKKEEEKEGEDKSQPKSIRERRRPREKRRSTGVSFWTQDSDENEQERQSDTEDGS
SKRETQTDSVSRYDSSSTSSSDRYDSLLGRSASYSYLEDRKPYSSRLEKDDSTDFKKLYEQILAENEKLKAQL
HDTNMELTDLKLQLEKATQRQERFADRSQLEMEKRERRALERRISEMEEELKMLPDLKADNQRLKDENGALIR
VISKLSK*
>ENSMUSP00000069257_c.2284:A>G_p.E762G ENSMUST00000070663.6|Ppp1r12a-201|ENSMUSG00000019907|+|chr10:g.108096896:A>G
MKMADAKQKRNEQLKRWIGSETDLEPPVVKRQKTKVKFDDGAVFLAACSSGDTDEVLKLLHRGADINYANVDG
LTALHQACIDDNVDMVKFLVENGANINQPDNEGWIPLHAAASCGYLDIAEFLIGQGAHVGAVNSEGDTPLDIA
EEEAMEELLQNEVNRQGVDIEAARKEEERVMLRDARQWLNSGHISDVRHAKSGGTALHVAAAKGYTEVLKLLI
QAGYDVNIKDYDGWTPLHAAAHWGKEEACRILVDNLCDMETVNKVGQTAFDVADEDILGYLEELQKKQTLLHS
EKRDKKSPLIESTANMENNQPQKAFKNKETLIIEPEKNASRIESLEHEKADEEEEGKKDESSCSSEEDEEDDS
ESEAETDKTKPMASVSNAHTSSTQAAPAAVTAPTLSSNQGTPTSPVKKFPISTTKISPKEEERKDESPASWRL
GLRKTGSYGALAEISASKEAQKEKDTAGVMRSASSPRLSSSLDNKEKEKDNKGTRLAYVTPTIPRRLASTSDI
EEKENRESSSLRTSSSYTRRKWEDDLKKNSSINEGSTYHRSCSFGRRQDDLISCSVPSTTSTPTVTSAAGLQR
SLPSSTSTAAKTPPGSSSAGTQSSTSNRLWAEDSTEKEKDSAPTAVTIPVAPTVVNAAAPSTTTLTTTTAGTV
SEVRERRRSYLTPVRDEESESQRKARSRQARQSRRSTQGVTLTDLQEAEKTIGRSRSTRTREQENEEKEKEEK
EKQDKEKQEEKKESEASREDEYKQKYSRTYDGTYTRYRPVSTSSSSAPSSSSLSTLGSTLYASSQLNRPNSLV
GITSAYSRGLAKENEREGEKKEEEKEGEDKSQPKSIRERRRPREKRRSTGVSFWTQDSDENEQERQSDTEDGS
SKRETQTDSVSRYDSSSTSSSDRYDSLLGRSASYSYLEDRKPYSSRLEKDDSTDFKKLYEQILAENEKLKAQL
HDTNMELTDLKLQLEKATQRQERFADRSQLEMEKRVAGKSQYLLGGTKSSRKKNI*
>ENSMUSP00000151478_c.715:A>G_p.E239G ENSMUST00000219759.2|Ppp1r12a-208|ENSMUSG00000019907|+|chr10:g.108096896:A>G
VMRSASSPRLSSSLDNKEKEKDNKGTRLAYVTPTIPRRLASTSDIEEKENRESSSLRTSSSYTRRKWEDDLKK
NSSINEGSTYHRSCSFGRRQDDLISCSVPSTTSTPTVTSAAGLQRSLPSSTSTAAKTPPGSSSAGTQSRSYLT
PVRDEESESQRKARSRQARQSRRSTQGVTLTDLQEAEKTIGRSRSTRTREQENEEKEKEEKEKQDKEKQEEKK
ESEASREDEYKQKYSRTYDGTYTRYRPVSTSSSSAPSSSSLSTLGSTLYASSQLNRPNSLVGITSAYSRGLAK
ENEREGEKKEEEKEGEDKSQPKSIRERRRPREKRRSTGVSFWTQDSDENEQERQSDTEDGSSKRETQTDSVSR
YDSSSTSSSDRYDSLLGRSASYSYLEDRKPYSSRLEKDDSTDFKKLYEQILAENEKLKAQLHDTNMELTDLKL
QLEKATQRQERFADRSQLEMEKRERRALERRISEMEEELKMLPDLKADNQRLKDENGALIRVISKLSK*
>ENSMUSP00000020315_c.3421:G>T_p.R1141M ENSMUST00000020315.13|Cand1-201|ENSMUSG00000020114|-|chr10:g.119042667:C>A
MASASYHISNLLEKMTSSDKDFRFMATNDLMTELQKDSIKLDDDSERKVVKMILRLLEDKNGEVQNLAVKCLG
PLVSKVKEYQVETIVDTLCTNMLSDKEQLRDISSIGLKTVIGELPPASSGSALAANVCKKITGRLTSAIAKQE
DVSVQLEALDIMADMLSRQGGLLVNFHPSILTCLLPQLTSPRLAVRKRTIIALGHLVMSCGNIVFVDLIEHLL
SELSKNDSMSTTRTYIQCIAAISRQAGHRIGEYLEKIIPLVVKFCNVDDDELREYCIQAFESFVRRCPKEVYP
HVSTIINICLKYLTYDPNYNYDDEDEDENAMDADGGDDDDQGSDDEYSDDDDMSWKVRRAAAKCLDAVVSTRH
EMLPEFYKTVSPALIARFKEREENVKADVFHAYLSLLKQTRPVQSWLCDPDAMEQGDTPLTMLQSQVPNIVKA
LHKQMKEKSVKTRQCCFNMLTELVNVLPGALTQHIPVLVPGIIFSLNDKSSSSNLKIDALSCLYVILCNHSPQ
VFHPHVQALVPPVVACVGDPFYKITSEALLVTQQLVKVIRPLDQPSSFDATPYIKDLFTCTIKRLKAADIDQE
VKERAISCMGQIICNLGDNLGPDLSNTLQIFLERLKNEITRLTTVKALTLIAGSPLKIDLRPVLGEGVPILAS
FLRKNQRALKLGTLSALDILIKNYSDSLTAAMIDAVLDELPPLISESDMHVSQMAISFLTTLAKVYPSSLSKI
SGSILNELIGLVRSPLLQGGALSAMLDFFQALVVTGTNNLGYMDLLRMLTGPVYSQSTALTHKQSYYSIAKCV
AALTRACPKEGPAVVGQFIQDVKNSRSTDSIRLLALLSLGEVGHHIDLSGQLELKSVILEAFSSPSEEVKSAA
SYALGSISVGNLPEYLPFVLQEITSQPKRQYLLLHSLKEIISSASVAGLKPYVENIWALLLKHCECAEEGTRN
VVAECLGKLTLIDPETLLPRLKGYLISGSSYARSSVVTAVKFTISDHPQPIDPLLKNCIGDFLKTLEDPDLNV
RRVALVTFNSAAHNKPSLIRDLLDSVLPHLYNETKVRKELIREVEMGPFKHTVDDGLDIRKAAFECMYTLLDS

CLDRLDIFEFLNHVEDGLKDHYDIKMLTFLMLVRLSTLCPSAVLQMLDRLVEPLRATCTTKVKANSVKQEFEK
QDELKRSAMRAVAALLTIPEAEKSPLMSEFQSQISSNPELAAIFESIQKDSSSTNLESMDTS*
>ENSMUSP00000122669_c.89:G>A_p.W30* ENSMUST00000152054.8|Tsfm-207|ENSMUSG00000040521|-|chr10:g.126866470:C>T
MSLLRSLRFFPVACTGRSARAVLLQPSQP*LTFHAGPSLSSAASSKELLMKLRRKTGYSFVNCKKALETCGGD
LKQAEDWLHKQAQKEGWSKAAKLHGRKTKEGLIGLLQEGNTAVLVEVVLCLSPQQKDVVGVERGSRGRDLHCF
GVGFVVPVDLEEPYWRVRLLVSLPGSPALKSRVQTFRVLRELAAMNVRWTVYHLCPSGSP*
>ENSMUSP00000042134_c.89:G>A_p.W30* ENSMUST00000040560.11|Tsfm-201|ENSMUSG00000040521|-|chr10:g.126866470:C>T
MSLLRSLRFFPVACTGRSARAVLLQPSQP*LTFHAGPSLSSAASSKELLMKLRRKTGYSFVNCKKALETCGGD
LKQAEDWLHKQAQKEGWSKAAKLHGRKTKEGLIGLLQEGNTAVLVEVNCETDFVSRNLKFQQLVQQVALGTMA
HCQNLTDRLSTYSKGFLNSSELSELAAGPDREGSLKDQLALAIGKLGENMILKRAAWVKVPSGFYVGSYVHGV
TQSPSLQNLVLGKYGALVICETPEQIANLEEVGRRLGQHVVGMAPLSVGSLDDEPGGETETRMLPQPYLLDPS
ITLGQYVQPQGVTVVDFVRFECGEDEQVAEAE*
>ENSMUSP00000113446_c.89:G>A_p.W30* ENSMUST00000120547.2|Tsfm-202|ENSMUSG00000040521|-|chr10:g.126866470:C>T
MSLLRSLRFFPVACTGRSARAVLLQPSQP*LTFHAGPSLSSAASSKELLMKLRRKTGYSFVNCKKALETCGGD
LKQAEDWLHKQAQKEGWSKAAKLHGRKTKEGLIGLLQEGNTAVLVEVNCETDFVSRNLKFQQLVQQVALGTMA
HCQNLTDRLSTYSKGFLNSSELSELAAGPDREGSLKDQLALAIGEYL*
>ENSMUSP00000050300_c.772:C>A_p.T258N ENSMUST00000050901.5|Apof-201|ENSMUSG00000047631|+|chr10:g.128105653:C>A
MHSLRLILMSIQLLCYLLLCPVDATSHGEATSVSTVLPPSKLGYQMQTSDKPLSCQMLLPKSLPGFTYMPPVS
KFLVGLALRNALEAAGCQAEVWALQLQLYRLGGVEATQALIHHLQELQKSGHTDREVSVDALSSALQLLAWEQ
PGPKRAKRSISNTDCDNDQEQSVHNVVDLLPAVGTYYNLGTALYYAIKNCSDKAKERGRDGAIDLGYDLLMAM
VGASGGPAGAVITAALKPAMKAGVQRLIQYYYDEKEVTNPQPEVTTHQPETGKDATTDIGVVEEIAMSNFVSE
VESTTSNWEWPLLKNYGVLAYKR*
>ENSMUSP00000158988_c.805:C>A_p.T269N ENSMUST00000238970.2|Apof-202|ENSMUSG00000047631|+|chr10:g.128105653:C>A
MAGLYGCSAPDMHSLRLILMSIQLLCYLLLCPVDATSHGEATSVSTVLPPSKLGYQMQTSDKPLSCQMLLPKS
LPGFTYMPPVSKFLVGLALRNALEAAGCQAEVWALQLQLYRLGGVEATQALIHHLQELQKSGHTDREVSVDAL
SSALQLLAWEQPGPKRAKRSISNTDCDNDQEQSVHNVVDLLPAVGTYYNLGTALYYAIKNCSDKAKERGRDGA
IDLGYDLLMAMVGASGGPAGAVITAALKPAMKAGVQRLIQYYYDEKEVTNPQPEVTTHQPETGKDATTDIGVV
EEIAMSNFVSEVESTTSNWEWPLLKNYGVLAYKR*
>ENSMUSP00000005825_c.3072:G>T_p.D1025Y ENSMUST00000005825.8|Pan2-201|ENSMUSG00000005682|+|chr10:g.128153876:G>T
MNFEGLDPGLAEFSPAMHSTLDPVLDAHLNPSLLQNVELDPEGVALEALPVQESVHIMEGVYSELHSVVAEVG
VPVSVSHFDLHEEMLWVGSHGGHATSFFGPALERYSSFQVNGGDDIRQIQSLENGILFLTKNNLKYMARGGLI
IFDYLLDENEDMHSVLLTDNSTLLVGGLQNHVLEIDLNTVQETQKYAVETPGVTIMRQTNRFFFCGHTSGKVS
LRDLRSFKVEHEFDAFSGSLSDFDVHGNLLAACGFSSRLTGLACDRFLKVYDLRMMRAITPLQVHVDPAFLRF
IPTYTSRLAIISQSGQCQFCEPTGLANPADIFHVNPVGPLLMTFDVSASKQALAFGDSEGCVHLWTDSPEPSF
NPYSRETEFALPCLVDSLPPLDWSQDLLPLSLIPVPLTTDALLSDWPAANSAPAPRRAPPVDAEILRTMKKVG
FIGYAPNPRTRLRNQIPYRLKESDHEFDNFSQVTESPTGREEEPLHTVSKKYRKVTIKYSKLGLEDFDFKHYN
KTLFAGLEPHIPNAYCNCMIQVLYFLEPVRCLIQNHLCQKEFCLACELGFLFHMLDLSRGDPCQGSNFLRAFR
TIPEASALGLILADSDEASGKGSLARLIQRWNRFILTQLHQDMQELEVPQAYRGAGGSFCSSGDSIIGQLFSC
EMENCSLCRCGSETVRASSTLLFTLSYPEDKTGKNYDFAQVLKRSICLEQNTQAWCDNCEKYQPTIQTRNIRH
LPDILVINCEVNSSKEADFWRLQAEVAFKIAVKKYGGEMKSKEFALADRKELRSPEGFLCSSIEELKNVWLPF
SIRMKMTKNKGLDVCNWADEHELSSLGAPSQWGPARAEEELGVYVYDLMATVVHILDSRTGGSLVAHIKVGET
YHQRKEGVTHQQWYLFNDFLIEPIDKYEAVQFDMNWKVPAILYYVKRNLNSRYNLNIKNPIEASVLLAEASLA
RKQRKTHTTFIPLMLNEMPQVGDLVGLDAEFVTLNEEEAELRSDGTKSTIKPSQMSVARITCVRGQGPNEGIP
FIYDYISTQEQVVDYLTQYSGIKPGDLDAKISSKHLTTLKSTYLKLRFLIDIGVKFVGHGLQKDFRVINLMVP
KDQVLDTVYLFHMPRKRMISLRFLAWYFLDLKIQGETHDSIEDARTALQLYRKYLELSKNGTEPESFHKVLKG
LYEKGRKMDWKVPEPESQTSPKNAAVFSVLAL*
>ENSMUSP00000151216_c.3045:G>T_p.D1016Y ENSMUST00000218315.2|Pan2-203|ENSMUSG00000005682|+|chr10:g.128153876:G>T
MNFEGLDPGLAEFSPAMHSTLDPVLDAHLNPSLLQNVELDPEGVALEALPVQESVHIMEGVYSELHSVVAEVG
VPVSVSHFDLHEEMLWVGSHGGHATSFFGPALERYSSFQVNGGDDIRQIQSLENGILFLTKNNLKYMARGGLI
IFDYLLDENEDMHSVLLTDNSTLLVGGLQNHVLEIDLNTVQETQKYAVETPGVTIMRQTNRFFFCGHTSGKVS
LRDLRSFKVEHEFDAFSGSLSDFDVHGNLLAACGFSSRLTGLACDRFLKVYDLRMMRAITPLQVHVDPAFLRF

IPTYTSRLAIISQSGQCQFCEPTGLANPADIFHVNPVGPLLMTFDVSASKQALAFGDSEGCVHLWTDSPEPSF
NPYSRETEFALPCLVDSLPPLDWSQDLLPLSLIPVPLTTDALLSDWPAANSAPAPRRAPPVDAEILRTMKKVG
FIGYAPNPRTRLRNQIPYRLKESDHEFDNFSQVTESPTGREEEPLHTVSKKYRKVTIKYSKLGLEDFDFKHYN
KTLFAGLEPHIPNAYCNCMIQVLYFLEPVRCLIQNHLCQKEFCLACELGFLFHMLDLSRGDPCQGSNFLRAFR
TIPEASALGLILADSDEASGKGSLARLIQRWNRFILTQLHQDMQELEVPQAYRGAGGSFCSSGDSIIGQLFSC
EMENCSLCRCGSETVRASSTLLFTLSYPEDKTGKNYDFAQVLKRSICLEQNTQAWCDNCEKYQPTIQTRNIRH
LPDILVINCEVNSSKEADFWRLQAEVAFKIAVKKYGGEMKSKEFALADRKELRSPEGFLCSSIEELKNVWLPF
SIRMKMTKNKGLDVCNWADEHEWGPARAEEELGVYVYDLMATVVHILDSRTGGSLVAHIKVGETYHQRKEGVT
HQQWYLFNDFLIEPIDKYEAVQFDMNWKVPAILYYVKRNLNSRYNLNIKNPIEASVLLAEASLARKQRKTHTT
FIPLMLNEMPQVGDLVGLDAEFVTLNEEEAELRSDGTKSTIKPSQMSVARITCVRGQGPNEGIPFIYDYISTQ
EQVVDYLTQYSGIKPGDLDAKISSKHLTTLKSTYLKLRFLIDIGVKFVGHGLQKDFRVINLMVPKDQVLDTVY
LFHMPRKRMISLRFLAWYFLDLKIQGETHDSIEDARTALQLYRKYLELSKNGTEPESFHKVLKGLYEKGRKMD
WKVPEPESQTSPKNAAVFSVLAL*
>ENSMUSP00000151874_c.2991:G>T_p.D998Y ENSMUST00000219721.2|Pan2-
206|ENSMUSG00000005682|+|chr10:g.128153876:G>T
MNFEGLDPGLAEFSPAMHSTLDPVLDAHLNPSLLQNVELDPEGVALEALPVQESVHIMEGVYSELHSVVAEVG
VPVSVSHFDLHEEMLWVGSHGVNGGDDIRQIQSLENGILFLTKNNLKYMARGGLIIFDYLLDENEDMHSVLLT
DNSTLLVGGLQNHVLEIDLNTVQETQKYAVETPGVTIMRQTNRFFFCGHTSGKVSLRDLRSFKVEHEFDAFSG
SLSDFDVHGNLLAACGFSSRLTGLACDRFLKVYDLRMMRAITPLQVHVDPAFLRFIPTYTSRLAIISQSGQCQ
FCEPTGLANPADIFHVNPVGPLLMTFDVSASKQALAFGDSEGCVHLWTDSPEPSFNPYSRETEFALPCLVDSL
PPLDWSQDLLPLSLIPVPLTTDALLSDWPAANSAPAPRRAPPVDAEILRTMKKVGFIGYAPNPRTRLRNQIPY
RLKESDHEFDNFSQVTESPTGREEEPLHTVSKKYRKVTIKYSKLGLEDFDFKHYNKTLFAGLEPHIPNAYCNC
MIQVLYFLEPVRCLIQNHLCQKEFCLACELGFLFHMLDLSRGDPCQGSNFLRAFRTIPEASALGLILADSDEA
SGKGSLARLIQRWNRFILTQLHQDMQELEVPQAYRGAGGSFCSSGDSIIGQLFSCEMENCSLCRCGSETVRAS
STLLFTLSYPEDKTGKNYDFAQVLKRSICLEQNTQAWCDNCEKYQPTIQTRNIRHLPDILVINCEVNSSKEAD
FWRLQAEVAFKIAVKKYGGEMKSKEFALADRKELRSPEGFLCSSIEELKNVWLPFSIRMKMTKNKGLDVCNWA
DEHEWGPARAEEELGVYVYDLMATVVHILDSRTGGSLVAHIKVGETYHQRKEGVTHQQWYLFNDFLIEPIDKY
EAVQFDMNWKVPAILYYVKRNLNSRYNLNIKNPIEASVLLAEASLARKQRKTHTTFIPLMLNEMPQVGDLVGL
DAEFVTLNEEEAELRSDGTKSTIKPSQMSVARITCVRGQGPNEGIPFIYDYISTQEQVVDYLTQYSGIKPGDL
DAKISSKHLTTLKSTYLKLRFLIDIGVKFVGHGLQKDFRVINLMVPKDQVLDTVYLFHMPRKRMISLRFLAWY
FLDLKIQGETHDSIEDARTALQLYRKYLELSKNGTEPESFHKVLKGLYEKGRKMDWKVPEPESQTSPKNAAVF
SVLAL*
>ENSMUSP00000149391_c.880:A>G_p.Q294R ENSMUST00000213329.2|Olfr787-
202|ENSMUSG00000096497|+|chr10:g.129299427:A>G
MKNHSVITEFVLLGISDDPEVQVVIFILLFIAYILSVTGNLTIIILTLLDSQLKTPMYFFLQNFSFLEIIFTS
VSIPRFLESIITKVKTISYNNCLAQLYFFISMGVSEFFLLTAMSYDRYVAICKPLHYTLIMNQKVCTLLVLAS
WLAGFLTIFPPLMLVLKLDFCASNVIDHFSCDYFPILQLSCSDTRSLEMIGFYFAFITLLFTLALVILSYISI
ISTILRFPSASQRKKAFSTCSSHMIVISISYGSCIFMYVKPSANERASLTKGVAVLNTSIAPMLNPFIYSLRN
ERVKQAFKDLINKVVLYRSK*
>ENSMUSP00000077005_c.880:A>G_p.Q294R ENSMUST00000077836.3|Olfr787-
201|ENSMUSG00000096497|+|chr10:g.129299427:A>G
MKNHSVITEFVLLGISDDPEVQVVIFILLFIAYILSVTGNLTIIILTLLDSQLKTPMYFFLQNFSFLEIIFTS
VSIPRFLESIITKVKTISYNNCLAQLYFFISMGVSEFFLLTAMSYDRYVAICKPLHYTLIMNQKVCTLLVLAS
WLAGFLTIFPPLMLVLKLDFCASNVIDHFSCDYFPILQLSCSDTRSLEMIGFYFAFITLLFTLALVILSYISI
ISTILRFPSASQRKKAFSTCSSHMIVISISYGSCIFMYVKPSANERASLTKGVAVLNTSIAPMLNPFIYSLRN
ERVKQAFKDLINKVVLYRSK*
>ENSMUSP00000099178_c.832:T>C_p.V278A ENSMUST00000101655.10|Sfi1-
203|ENSMUSG00000023764|-|chr11:g.3114574:A>G
MEKKIGSRSFRDGVVKKPCSPKTLPLKKSSAFSGIQREPSRSCHSIYYHASQNWTRYRLQELRIRCVARKFLY
LWIRVTFGRVTPSRARIFHEQKILQKVFGEWREEWWVSQREWKLCVRADCHYRYYLYNLIFQNWKTFVHQQRE
MRKRFRIAEHHDTKQKMCQAWKSWLIYMVSRRTKLHMKTTALEFRRQSVLCFWWSKWRWRLGQAHAEHALHAV
AVKHRALSLQLQGWLRWQEQLLISQRDRRKEATAVQHYQHWQKQRSLKAWLKYLQICRAKRWQNEMAVQFHRA
TVLQIHFCDWQWAWEWRQSLSAHQALVVKLAGRMVLRRAFTHWKHYMLLQAEEAAQREAAAEHRQHYLLYSCF
RAFKDNVTQARLQQTRKKLAQQLRDTTLLHRFWNLWQSRIEQREERVQTPSLHAALSHYRVTVLHKCVRVWLR
YVHKRQWQQLLRARADGHFQQRALPAAFYTWYRGWLWHQQRRILHTKAVRFHRGTLEKQVFALWRQKMSQHRE
NCLAERMAILQAEQQLLRRFWFVWHQQAAVCQLERQQQAMAIAHHHSGLLRRAFCIWKESTQGFRIERMGRAQ
AAHFHSAQLLSRAWSMWRECLALRLEEQQKLKCAALHSQCILLRRALQKWLVYQNRVRSVLREVAARERQHNR
QLLWWALHLWRENTMARLDGAKKTSQARVHYSRTLCSKVLVQWREVTSVQIYYRQKEAAALREARKALDRGRL

QNWFQHWRFCSQRAAQQRFQLGQAAQHHHWQLLMEAMARWKAHHLGCIRKKFLQRQAAQLLAQRLSRACFCQW
RKQLAVRKQEQWGTARALWLWAFSLQAKVWTAWLGFVLERRRKKARLERAMQAYQQQLLQEGATRLLRFTAGT
KAFRQQLQAQQQVQAAHSLHCAVRHCAELWKKKVLGPGKTSQPPAPTTFSKRVTFKDSFLSGHAAEAGDATQE
TKKLRAPPSQGVLGSLAGAAGEPCHLDLNAARSSRKQPRRPSFLLERLGSQRLEKPPEEESTALLGGSSLTRP
FLPGVLPNVPGPKLPPTASPGLELLPPSSIMPHAAGGTARVSAKPSIPGPQPWGCPSLPRDLDPQLLPGDSIS
TRTEPVYGSEATGHTELEAELEGIQQQLQHYQTTKQNLWSCQRQANSLRRWLELSQEEPKSEDLHLEEQVKTE
LEEVELQVQQLAKELEAQRQPVGTCIARVRALRRALC*
>ENSMUSP00000080066_c.832:T>C_p.V278A ENSMUST00000081318.13|Sfi1-202|ENSMUSG00000023764|-|chr11:g.3114574:A>G
MEKKIGSRSFRDGVVKKPCSPKTLPLKKSSAFSGIQREPSRSCHSIYYHASQNWTRYRLQELRIRCVARKFLY
LWIRVTFGRVTPSRARIFHEQKILQKVFGEWREEWWVSQREWKLCVRADCHYRYYLYNLIFQNWKTFVHQQRE
MRKRFRIAEHHDTKQKMCQAWKSWLIYMVSRRTKLHMKTTALEFRRQSVLCFWWSKWRWRLGQAHAEHALHAV
AVKHRALSLQLQGWLRWQEQLLISQRDRRKEATAVQHYQHWQKQRSLKAWLKYLQICRAKRWQNEMAVQFHRA
TVLQIHFCDWQWAWEWRQSLSAHQALVVKLAGRMVLRRAFTHWKHYMLLQAEEAAQREAAAEHRQHYLLYSCF
RAFKDNVTQARLQQTRKKLAQQLRDTTLLHRFWNLWQSRIEQREERVQTPSLHAALSHYRVTVLHKCVRVWLR
YVHKRQWQQLLRARADGHFQQRALPAAFYTWYRGWLWHQQRRILHTKAVRFHRGTLEKQVFALWRQKMSQHRE
NCLAERMAILQAEQQLLRRFWFVWHQQAAVCQLERQQQAMAIAHHHSGLLRRAFCIWKESTQGFRIERMGRAQ
AAHFHSAQLLSRAWSMWRECLALRLEEQQKLKCAALHSQCILLRRALQKWLVYQNRVRSVLREVAARERQHNR
QLLWWALHLWRENTMARLDGAKKTSQARVHYSRTLCSKVLVQWREVTSVQIYYRQKEAAALREARKALDRGRL
QNWFQHWRFCSQRAAQQRFQLGQAAQHHHWQLLMEAMARWKAHHLGCIRKKFLQRQAAQLLAQRLSRACFCQW
RKQLAVRKQEQWGTARALWLWAFSLQAKVWTAWLGFVLERRRKKARLERAMQAYQQQLLQEGATRLLRFTAGT
KAFRQQLQAQQQVQAAHSLHCAVRHCAELWKKKVLGPGKTSQPPAPTTFSKRVTFKDSFLSGHAAEAGDATQE
TKKLRAPPSQGVLGSLAGAAGEPCHLDLNAARSSRKQPRRPSFLLERLGSQRSPEWYSLGEQQLEKPPEEEST
ALLGGSSLTRPFLPGVLPNVPGPKLPPTASPGLELLPPSSIMPHAAGGTARVSAKPSIPGPQPWGCPSLPRDL
DPQLLPGDSISTRTEPVYGSEATGHTELEAELEGIQQQLQHYQTTKQNLWSCQRQANSLRRWLELSQEEPKSE
DLHLEEQVKTELEEVELQVQQLAKELEAQRQPVGTCIARVRALRRALC*
>ENSMUSP00000067261_c.832:T>C_p.V278A ENSMUST00000066391.14|Sfi1-201|ENSMUSG00000023764|-|chr11:g.3114574:A>G
MEKKIGSRSFRDGVVKKPCSPKTLPLKKSSAFSGIQREPSRSCHSIYYHASQNWTRYRLQELRIRCVARKFLY
LWIRVTFGRVTPSRARIFHEQKILQKVFGEWREEWWVSQREWKLCVRADCHYRYYLYNLIFQNWKTFVHQQRE
MRKRFRIAEHHDTKQKMCQAWKSWLIYMVSRRTKLHMKTTALEFRRQSVLCFWWSKWRWRLGQAHAEHALHAV
AVKHRALSLQLQGWLRWQEQLLISQRDRRKEATAVQHYQHWQKQRSLKAWLKYLQICRAKRWQNEMAVQFHRA
TVLQIHFCDWQWAWEWRQSLSAHQALVVKLAGRMVLRRAFTHWKHYMLLQAEEAAQREAAAEHRQHYLLYSCF
RAFKDNVTQARLQQTRKKLAQQLRDTTLLHRFWNLWQSRIEQREERVQTPSLHAALSHYRVTVLHKCVRVWLR
YVHKRQWQQLLRARADGHFQQRALPAAFYTWYRGWLWHQQRRILHTKAVRFHRGTLEKQVFALWRQKMSQHRE
NCLAERMAILQAEQQLLRRFWFVWHQQAAVCQLERQQQAMAIAHHHSGLLRRAFCIWKESTQGFRIERMGRAQ
AAHFHSAQLLSRAWSMWREVYQNRVRSVLREVAARERQHNRQLLWWALHLWRENTMARLDGAKKTSQARVHYS
RTLCSKVLVQWREVTSVQIYYRQKEAAALREARKALDRGRLQNWFQHWRFCSQRAAQQRFQLGQAAQHHHWQL
LMEAMARWKAHHLGCIRKKFLQRQAAQLLAQRLSRACFCQWRKQLAVRKQEQWGTARALWLWAFSLQAKVWTA
WLGFVLERRRKKARLERAMQAYQQQLLQEGATRLLRFTAGTKAFRQQLQAQQQVQAAHSLHCAVRHCAELWKK
KVLGPGKTSQPPAPTTFSKRVTFKDSFLSGHAAEAGDATQETKKLRAPPSQGVLGSLAGAAGEPCHLDLNAAR
SSRKQPRRPSFLLERLGSQRSPEWYSLGEQQLEKPPEEESTALLGGSSLTRPFLPGVLPNVPGPKLPPTASPG
LELLPPSSIMPHAAGGTARVSAKPSIPGPQPWGCPSLPRDLDPQLLPGDSISTRTEPVYGSEATGHTELEAEL
EGIQQQLQHYQTTKQNLWSCQRQANSLRRWLELSQEEPKSEDLHLEEQVKTELEEVELQVQQLAKELEAQRQP
VGTCIARVRALRRALC*
>ENSMUSP00000121719_c.832:T>C_p.V278A ENSMUST00000153425.9|Sfi1-218|ENSMUSG00000023764|-|chr11:g.3114574:A>G
MEKKIGSRSFRDGVVKKPCSPKTLPLKKSSAFSGIQREPSRSCHSIYYHASQNWTRYRLQELRIRCVARKFLY
LWIRVTFGRVTPSRARIFHEQKILQKVFGEWREEWWVSQREWKLCVRADCHYRYYLYNLIFQNWKTFVHQQRE
MRKRFRIAEHHDTKQKMCQAWKSWLIYMVSRRTKLHMKTTALEFRRQSVLCFWWSKWRWRLGQAHAEHALHAV
AVKHRALSLQLQGWLRWQEQLLISQRDRRKEATAVQHYQHWQKQRSLKAWLKYLQICRAKRWQNEMAVQFHRA
TVLQIHFCDWQWAWEWRQSLSAHQALVVKLAGRMVLRRAFTHWKHYMLLQAEEAAQREAAAEHRQHYLLLLHR
FWNLWQSRIEQREERVQTPSLHAALSHYRVTVLHKCVRVWLRYVHKRQWQQLLRARADGHFQQRALPAAFYTW
YRGWLWHQQRRILHTKAVRFHRGTLEKQVFALWRQKMSQHRENCLAERMAILQAEQQLLRRFWFVWHQQAAVC
QLERQQQAMAIAHHHSGLLRRAFCIWKESTQGFRIERMGRAQAAHFHSAQLLSRAWSMWRECLALRLEEQQKL
KCAALHSQCILLRRALQKWLVYQNRVRSVLREVAARERQHNRQLLWWALHLWRENTMARLDGAKKTSQARVHY
SRTLCSKVLVQWREVTSVQIYYRQKEAAALREARKALDRGRLQNWFQHWRFCSQRAAQQRFQLGQAAQHHHWQ
LLMEAMARWKAHHLGCIRKKFLQRQAAQLLAQRLSRACFCQWRKQLAVRKQEQWGTARALWLWAFSLQAKVWT

AWLGFVLERRRKKARLERAMQAYQQQLLQEGATRLLRFTAGTKAFRQQLQAQQQVQAAHSLHCAVRHCAELWK
KKVLGPGKTSQPPAPTTFSKRVTFKDSFLSGHAAEAGDATQETKKLRAPPSQGVLGSLAGAAGEPCHLDLNAA
RSSRKQPRRPSFLLERLGSQRLEKPPEEESTALLGGSSLTRPFLPGVLPNVPGPKLPPTASPGLELLPPSSIM
PHAAGGTARVSAKPSIPGPQPWGCPSLPRDLDPQLLPGDSISTRTEPVYGSEATGHTELEAELEGIQQQLQHY
QTTKQNLWSCQRQANSLRRWLELSQEEPKSEDLHLEEQVKTELEEVELQVQQLAKELEAQRQPVGTCIARVRA
LRRALC*
>ENSMUSP00000119905_c.589:T>C_p.V197A ENSMUST00000140846.9|Sfi1-
210|ENSMUSG00000023764|-|chr11:g.3114574:A>G
MEKKIGSRIFHEQKILQKVFGEWREEWWVSQREWKLCVRADCHYRYYLYNLIFQNWKTFVHQQREMRKRFRIA
EHHDTKQKMCQAWKSWLIYMVSRRTKLHMKTTALEFRRQSVLCFWWSKWRWRLGQAHAEHALHAVAVKHRALS
LQLQGWLRWQEQLLISQRDRRKEATAVQHYQHWQKQRSLKAWLKYLQICRAKRWQNEMAVQFHRATVLQIHFC
DWQWAWEWRQSLSAHQALVVKLAGRMVLRRAFTHWKHYMLLQAEEAAQREAAAEHRQHYLLYSCFRAFKDNVT
QARLQQTRKKLAQQLRDTTLLHRFWNLWQSRIEQREERVQTPSLHAALSHYRVTVLHKCVRVWLRYVHKRQWQ
QLLRARADGHFQQRALPAAFYTWYRGWLWHQQRRILHTKAVRFHRGTLEKQVFALWRQKMSQHRENCLAERMA
ILQAEQQLLRRFWFVWHQQAAVCQLERQQQAMAIAHHHSGLLRRAFCIWKESTQGFRIERMGRAQAAHFHSAQ
LLSRAWSMWRECLALRLEEQQKLKCAALHSQCILLRRALQKWLVYQNRVRSVLREVAARERQHNRQLLWWALH
LWRENTMARLDGAKKTSQARVHYSRTLCSKVLVQWREVTSVQIYYRQKEAAALREARKALDRGRLQNWFQHWR
FCSQRAAQQRFQLGQAAQHHHWQLLMEAMARWKAHHLGCIRKKFLQRQAAQLLAQRLSRACFCQWRKQLAVRK
QEQWGTARALWLWAFSLQAKVWTAWLGFVLERRRKKARLERAMQAYQQQLLQEGATRLLRFTAGTKAFRQQLQ
AQQQVQAAHSLHCAVRHCAELWKKKVLGPGKTSQPPAPTTFSKRVTFKDSFLSGHAAEAGDATQETKKLRAPP
SQGVLGSLAGAAGEPCHLDLNAARSSRKQPRRPSFLLERLGSQRLEKPPEEESTALLGGSSLTRPFLPGVLPN
VPGPKLPPTASPGLELLPPSSIMPHAAGGTARVSAKPSIPGPQPWGCPSLPRDLDPQLLPGDSISTRTEPVYG
SEATGHTELEAELEGIQQQLQHYQTTKQNLWSCQRQANSLRRWLELSQEEPKSEDLHLEEQVKTELEEVELQV
QQLAKELEAQRQPVGTCIARVRALRRALC*
>ENSMUSP00000122002_c.25:T>C_p.V9A ENSMUST00000126746.9|Sfi1-
205|ENSMUSG00000023764|-|chr11:g.3114574:A>G
XKYLQICRAKRWQNEMAVQFHRATVLQIHFCDWQWAWEWRQSLSAHQALVVKLAGRMVLRRAFTHWKHYMLLQ
AEEAAQREAAAEHRQHYLLYSCFRAFKDNVTQARLQQTRKKLAQQLRDTTLLHRFWNLWQSRIEQREERVQTP
SLHAALSHYRVTVLHKCVRVWLRYVHKRQWQQLLRARADGHFQQRALPAAFYTWYRGWLWHQQRRILHTKAVR
FHRGTLEKQVFALWRQKMSQHRENCLAERMAILQAEQQLLRRFWFVWHQQAAVCQLERQQQAMAIAHHHSGLL
RRAFCIWKESTQGFRIESSSSGERLLQCRYITDKRRQLPSGRPGKHWTGVVSRTGFSTGDSAARGQPSRDSSW
DRRLSITTGSC*
>ENSMUSP00000118419_c.832:T>C_p.V278A ENSMUST00000132893.8|Sfi1-
206|ENSMUSG00000023764|-|chr11:g.3114574:A>G
MEKKIGSRSFRDGVVKKPCSPKTLPLKKSSAFSGIQREPSRSCHSIYYHASQNWTRYRLQELRIRCVARKFLY
LWIRVTFGRVTPSRARIFHEQKILQKVFGEWREEWWVSQREWKLCVRADCHYRYYLYNLIFQNWKTFVHQQRE
MRKRFRIAEHHDTKQKMCQAWKSWLIYMVSRRTKLHMKTTALEFRRQSVLCFWWSKWRWRLGQAHAEHALHAV
AVKHRALSLQLQGWLRWQEQLLISQRDRRKEATAVQHYQHWQKQRSLKAWLKYLQICRAKRWQNVIAGQSRWS
FPAASTACCLLHVVQRLAMAPAETDPAHKSSAFPQRDIREASICSLEAEDVSASRKLLGRENGYTAGRAAAFA
*
>rs227559416_ENSMUSP00000020734_c.505:A>T_p.H169L
ENSMUST00000020734.11|Eif4enif1-
201|ENSMUSG00000020454|+|chr11:g.3170454:A>T
MEKSVAETENGDAFLELKKLPTSKSPHRYTKEELLDIKERPYSKQRPSCLSEKYDSDGVWDPEKWHASLYPAS
GRSSPVESLKKESESDRPSLVRRIADPRERVKEDDLDVVLSPQRRSFGGGCHVTAAVSSRRSGSPLEKDSDGL
RLLGGRRIGSGRIISARAFEKDLRLSDKDLRDLRDRDRERDYKDKRFRREFGDSKRVFGERRRNDSYTEEEPE
WFSAGPTSQSETIELTGFDDKILEEDHKGRKRTRRRTASVKEGIVECNGGVAEEDEVEVILAQEPSADQEVPR
DVILPEQSPGEFDFNEFFNLDKVPCLASMIEDVLGEGSVSASRFSRWFSNPSRSGSRSSSLGSTPHEELERLA
GLEQAVLSPGQNSGNYFAPIPSEDHAENKVDILEMLQKAKVDLKPLLSSLSANKEKLKESSHSGVVLSVEEVE
AGLKGLKVDQQMKNSTPFMAEHLEETLSAASSNRQLKKDGDMTAFNKLVNTMKASGTLPTQPKVSELLGQPVQ
RPASSNLLSGLMGSLEATASLLSQRAPSPPMSQVFRTQAASADYLHPRIPSPIGFPSGPQQLLGDPFQGMRKP
MSPVSAQMSQLELQQAALEGLALPHDLAVQTAPFYQPGFSKPQVDRTRDGLRNRQQRMSKSPAPMHGGNSSSP
APAASITSMLSPSFTPTSVIRKMYESREKTKEEMAPGMVVPGDGKEDTQKTSEENLLSSNPIPNTDQDSSTTN
PKLSTLQRSSCSTPLSQTSRYTKEQDYRPKTAGRKTPTLASPVPGTPFLRPTHQVPLVPHVPIVRPAHQLHPG
LVQRLIAQGVHPQHLPSLLQAGVLPPGIDMAPLQGLSGPLLGQPLYPLVSAASHPLLNPRPGTPLHLAVMQQQ
LQRSVLHPPGSSSQAAAISVQTPQNVPSRSGMPHMHSQLEHRTSQRSSSPVGLAKWFGSDVLQQPLPSMPTKV
ISVDELEYRQ*

>rs227559416_ENSMUSP00000105676_c.505:A>T_p.H169L
ENSMUST00000110049.8|Eif4enif1-
203|ENSMUSG00000020454|+|chr11:g.3170454:A>T
MEKSVAETENGDAFLELKKLPTSKSPHRYTKEELLDIKERPYSKQRPSCLSEKYDSDGVWDPEKWHASLYPAS
GRSSPVESLKKESESDRPSLVRRIADPRERVKEDDLDVVLSPQRRSFGGGCHVTAAVSSRRSGSPLEKDSDGL
RLLGGRRIGSGRIISARAFEKDLRLSDKDLRDLRDRDRERDYKDKRFRREFGDSKRVFGERRRNDSYTEEEPE
WFSAGPTSQSETIELTGFDDKILEEDHKGRKRTRRRTASVKEGIVECNGGVAEEDEVEVILAQEPSADQEVPR
DVILPEQSPGEFDFNEFFNLDKVPCLASMIEDVLGEGSVSASRFSRWFSNPSRSGSRSSSLGSTPHEELERLA
GLEQAVLSPGQNSGNYFAPIPSEDHAENKVDILEMLQKAKVDLKPLLSSLSANKEKLKESSHSGVVLSVEEVE
AGLKGLKVDQQMKNSTPFMAEHLEETLSAASSNRQLKKDGDMTAFNKLVNTMKASGTLPTQPKVSRNVESHLL
APAEIPGQPVSKNILQELLGQPVQRPASSNLLSGLMGSLEATASLLSQRAPSPPMSQVFRTQAASADYLHPRI
PSPIGFPSGPQQLLGDPFQGMRKPMSPVSAQMSQLELQQAALEGLALPHDLAVQTAPFYQPGFSKPQVDRTRD
GLRNRQQRMSKSPAPMHGGNSSSPAPAASITSMLSPSFTPTSVIRKMYESREKTKEEMAPGMVVPGDGKEDTQ
KTSEENLLSSNPIPNTDQDSSTTNPKLSTLQRSSCSTPLSQTSRYTKEQDYRPKTAGRKTPTLASPVPGTPFL
RPTHQVPLVPHVPIVRPAHQLHPGLVQRLIAQGVHPQHLPSLLQAGVLPPGIDMAPLQGLSGPLLGQPLYPLV
SAASHPLLNPRPGTPLHLAVMQQQLQRSVLHPPGSSSQAAAISVQTPQNVPSRSGMPHMHSQLEHRTSQRSSS
PVGLAKWFGSDVLQQPLPSMPTKVISVDELEYRQ*
>rs227559416_ENSMUSP00000118527_c.505:A>T_p.H169L
ENSMUST00000125637.8|Eif4enif1-
206|ENSMUSG00000020454|+|chr11:g.3170454:A>T
MEKSVAETENGDAFLELKKLPTSKSPHRYTKEELLDIKERPYSKQRPSCLSEKYDSDGVWDPEKWHASLYPAS
GRSSPVESLKKESESDRPSLVRRIADPRERVKEDDLDVVLSPQRRSFGGGCHVTAAVSSRRSGSPLEKDSDGL
RLLGGRRIGSGRIISARAFEKDLRLSDKDLRDLRDRDRERDYKDKRFR
>rs227559416_ENSMUSP00000105675_c.505:A>T_p.H169L
ENSMUST00000110048.8|Eif4enif1-
202|ENSMUSG00000020454|+|chr11:g.3170454:A>T
MEKSVAETENGDAFLELKKLPTSKSPHRYTKEELLDIKERPYSKQRPSCLSEKYDSDGVWDPEKWHASLYPAS
GRSSPVESLKKESESDRPSLVRRIADPRERVKEDDLDVVLSPQRRSFGGGCHVTAAVSSRRSGSPLEKDSDGL
RLLGGRRIGSGRIISARAFEKDLRLSDKDLRDLRDRDRERDYKDKRFRREFGDSKRVFGERRRNDSYTEEEPE
WFSAGPTSQSETIELTGFDDKILEEDHKGRKRTRRRTASVKEGIVECNGGVAEEDEVEVILAQEPSADQEVPR
DVILPEQSPGEFDFNEFFNLDKVPCLASMIEDVLGEGSVSASRFSRWFSNPSRSGSRSSSLGSTPHEELERLA
GLEQAVLSPGQNSGNYFAPIPSEDHAENKVDILEMLQKAKVDLKPLLSSLSANKEKLKESSHSGVVLSVEEVE
AGLKGLKVDQQMKNSTPFMAEHLEETLSAASSNRQLKKDGDMTAFNKLVNTMKASGTLPTQPKVSELLGQPVQ
RPASSNLLSGLMGSLEATASLLSQRAPSPPMSQVFRTQAASADYLHPRIPSPIGFPSGPQQLLGDPFQGMRKP
MSPVSAQMSQLELQQAALEGLALPHDLAVQTAPFYQPGFSKPQVDRTRDGLRNRQQRMSKSPAPMHGGNSSSP
APAASITSMLSPSFTPTSVIRKMYESREKTKEEMAPGMVVPGDGKEDTQKTSEENLLSSNPIPNTDQDSSTTN
PKLSTLQRSSCSTPLSQTSRYTKEQDYRPKTAGRKTPTLASPVPGTPFLRPTHQVPLVPHVPIVRPAHQLHPG
LVQRLIAQGVHPQHLPSLLQAGVLPPGIDMAPLQGLSGPLLGQPLYPLVSAASHPLLNPRPGTPLHLAVMQQQ
LQRSVLHPPGSSSQAAAISVQTPQNVPSRSGMPHMHSQLEHRTSQRSSSPVGLAKWFGSDVLQQPLPSMPTKV
ISVDELEYRQ*
>rs227559416_ENSMUSP00000136768_c.505:A>T_p.H169L
ENSMUST00000179770.8|Eif4enif1-
213|ENSMUSG00000020454|+|chr11:g.3170454:A>T
MEKSVAETENGDAFLELKKLPTSKSPHRYTKEELLDIKERPYSKQRPSCLSEKYDSDGVWDPEKWHASLYPAS
GRSSPVESLKKESESDRPSLVRRIADPRERVKEDDLDVVLSPQRRSFGGGCHVTAAVSSRRSGSPLEKDSDGL
RLLGGRRIGSGRIISARAFEKDLRLSDKDLRDLRDRDRERDYKDKRFRREFGDSKRVFGERRRNDSYTEEEPE
WFSAGPTSQSETIELTGFDDKILEEDHKGRKRTRRRTASVKEGIVECNGGVAEEDEVEVILAQEPSADQEVPR
DVILPEQSPGEFDFNEFFNLDKVPCLASMIEDVLGEGSVSASRFSRWFSNPSRSGSRSSSLGSTPHEELERLA
GLEQAVLSPGQNSGNYFAPIPSEDHAENKVDILEMLQKAKVDLKPLLSSLSANKEKLKESSHSGVVLSVEEVE
AGLKGLKVDQQMKNSTPFMAEHLEETLSAASSNRQLKKDGDMTAFNKLVNTMKASGTLPTQPKVSRNVESHLL
APAEIPGQPVSKNILQELLGQPVQRPASSNLLSGLMGSLEATASLLSQRAPSPPMSQVFRTQAASADYLHPRI
PSPIGFPSGPQQLLGDPFQGMRKPMSPVSAQMSQLELQQAALEGLALPHDLAVQTAPFYQPGFSKPQVDRTRD
GLRNRQQRMSKSPAPMHGGNSSSPAPAASITSMLSPSFTPTSVIRKMYESREKTKEEMAPGMVVPGDGKEDTQ
KTSEENLLSSNPIPNTDQDSSTTNPKLSTLQRSSCSTPLSQTSRYTKEQDYRPKTAGRKTPTLASPVPGTPFL
RPTHQVPLVPHVPIVRPAHQLHPGLVQRLIAQGVHPQHLPSLLQAGVLPPGIDMAPLQGLSGPLLGQPLYPLV
SAASHPLLNPRPGTPLHLAVMQQQLQRSVLHPPGSSSQAAAISVQTPQNVPSRSGMPHMHSQLEHRTSQRSSS
PVGLAKWFGSDVLQQPLPSMPTKVISVDELEYRQ*

```
>rs239307747_ENSMUSP00000020734_c.1423:A>G_p.K475R
ENSMUST00000020734.11|Eif4enif1-
201|ENSMUSG00000020454|+|chr11:g.3180016:A>G
MEKSVAETENGDAFLELKKLPTSKSPHRYTKEELLDIKERPYSKQRPSCLSEKYDSDGVWDPEKWHASLYPAS
GRSSPVESLKKESESDRPSLVRRIADPRERVKEDDLDVVLSPQRRSFGGGCHVTAAVSSRRSGSPLEKDSDGL
RLLGGRRIGSGRIISARAFEKDHRLSDKDLRDLRDRDRERDYKDKRFRREFGDSKRVFGERRRNDSYTEEEPE
WFSAGPTSQSETIELTGFDDKILEEDHKGRKRTRRRTASVKEGIVECNGGVAEEDEVEVILAQEPSADQEVPR
DVILPEQSPGEFDFNEFFNLDKVPCLASMIEDVLGEGSVSASRFSRWFSNPSRSGSRSSSLGSTPHEELERLA
GLEQAVLSPGQNSGNYFAPIPSEDHAENKVDILEMLQKAKVDLKPLLSSLSANKEKLKESSHSGVVLSVEEVE
AGLKGLKVDQQMKNSTPFMAEHLEETLSAASSNRQLRKDGDMTAFNKLVNTMKASGTLPTQPKVSELLGQPVQ
RPASSNLLSGLMGSLEATASLLSQRAPSPPMSQVFRTQAASADYLHPRIPSPIGFPSGPQQLLGDPFQGMRKP
MSPVSAQMSQLELQQAALEGLALPHDLAVQTAPFYQPGFSKPQVDRTRDGLRNRQQRMSKSPAPMHGGNSSSP
APAASITSMLSPSFTPTSVIRKMYESREKTKEEMAPGMVVPGDGKEDTQKTSEENLLSSNPIPNTDQDSSTTN
PKLSTLQRSSCSTPLSQTSRYTKEQDYRPKTAGRKTPTLASPVPGTPFLRPTHQVPLVPHVPIVRPAHQLHPG
LVQRLIAQGVHPQHLPSLLQAGVLPPGIDMAPLQGLSGPLLGQPLYPLVSAASHPLLNPRPGTPLHLAVMQQQ
LQRSVLHPPGSSSQAAAISVQTPQNVPSRSGMPHMHSQLEHRTSQRSSSPVGLAKWFGSDVLQQPLPSMPTKV
ISVDELEYRQ*
>rs239307747_ENSMUSP00000105676_c.1423:A>G_p.K475R
ENSMUST00000110049.8|Eif4enif1-
203|ENSMUSG00000020454|+|chr11:g.3180016:A>G
MEKSVAETENGDAFLELKKLPTSKSPHRYTKEELLDIKERPYSKQRPSCLSEKYDSDGVWDPEKWHASLYPAS
GRSSPVESLKKESESDRPSLVRRIADPRERVKEDDLDVVLSPQRRSFGGGCHVTAAVSSRRSGSPLEKDSDGL
RLLGGRRIGSGRIISARAFEKDHRLSDKDLRDLRDRDRERDYKDKRFRREFGDSKRVFGERRRNDSYTEEEPE
WFSAGPTSQSETIELTGFDDKILEEDHKGRKRTRRRTASVKEGIVECNGGVAEEDEVEVILAQEPSADQEVPR
DVILPEQSPGEFDFNEFFNLDKVPCLASMIEDVLGEGSVSASRFSRWFSNPSRSGSRSSSLGSTPHEELERLA
GLEQAVLSPGQNSGNYFAPIPSEDHAENKVDILEMLQKAKVDLKPLLSSLSANKEKLKESSHSGVVLSVEEVE
AGLKGLKVDQQMKNSTPFMAEHLEETLSAASSNRQLRKDGDMTAFNKLVNTMKASGTLPTQPKVSRNVESHLL
APAEIPGQPVSKNILQELLGQPVQRPASSNLLSGLMGSLEATASLLSQRAPSPPMSQVFRTQAASADYLHPRI
PSPIGFPSGPQQLLGDPFQGMRKPMSPVSAQMSQLELQQAALEGLALPHDLAVQTAPFYQPGFSKPQVDRTRD
GLRNRQQRMSKSPAPMHGGNSSSPAPAASITSMLSPSFTPTSVIRKMYESREKTKEEMAPGMVVPGDGKEDTQ
KTSEENLLSSNPIPNTDQDSSTTNPKLSTLQRSSCSTPLSQTSRYTKEQDYRPKTAGRKTPTLASPVPGTPFL
RPTHQVPLVPHVPIVRPAHQLHPGLVQRLIAQGVHPQHLPSLLQAGVLPPGIDMAPLQGLSGPLLGQPLYPLV
SAASHPLLNPRPGTPLHLAVMQQQLQRSVLHPPGSSSQAAAISVQTPQNVPSRSGMPHMHSQLEHRTSQRSSS
PVGLAKWFGSDVLQQPLPSMPTKVISVDELEYRQ*
>rs239307747_ENSMUSP00000105675_c.1423:A>G_p.K475R
ENSMUST00000110048.8|Eif4enif1-
202|ENSMUSG00000020454|+|chr11:g.3180016:A>G
MEKSVAETENGDAFLELKKLPTSKSPHRYTKEELLDIKERPYSKQRPSCLSEKYDSDGVWDPEKWHASLYPAS
GRSSPVESLKKESESDRPSLVRRIADPRERVKEDDLDVVLSPQRRSFGGGCHVTAAVSSRRSGSPLEKDSDGL
RLLGGRRIGSGRIISARAFEKDHRLSDKDLRDLRDRDRERDYKDKRFRREFGDSKRVFGERRRNDSYTEEEPE
WFSAGPTSQSETIELTGFDDKILEEDHKGRKRTRRRTASVKEGIVECNGGVAEEDEVEVILAQEPSADQEVPR
DVILPEQSPGEFDFNEFFNLDKVPCLASMIEDVLGEGSVSASRFSRWFSNPSRSGSRSSSLGSTPHEELERLA
GLEQAVLSPGQNSGNYFAPIPSEDHAENKVDILEMLQKAKVDLKPLLSSLSANKEKLKESSHSGVVLSVEEVE
AGLKGLKVDQQMKNSTPFMAEHLEETLSAASSNRQLRKDGDMTAFNKLVNTMKASGTLPTQPKVSELLGQPVQ
RPASSNLLSGLMGSLEATASLLSQRAPSPPMSQVFRTQAASADYLHPRIPSPIGFPSGPQQLLGDPFQGMRKP
MSPVSAQMSQLELQQAALEGLALPHDLAVQTAPFYQPGFSKPQVDRTRDGLRNRQQRMSKSPAPMHGGNSSSP
APAASITSMLSPSFTPTSVIRKMYESREKTKEEMAPGMVVPGDGKEDTQKTSEENLLSSNPIPNTDQDSSTTN
PKLSTLQRSSCSTPLSQTSRYTKEQDYRPKTAGRKTPTLASPVPGTPFLRPTHQVPLVPHVPIVRPAHQLHPG
LVQRLIAQGVHPQHLPSLLQAGVLPPGIDMAPLQGLSGPLLGQPLYPLVSAASHPLLNPRPGTPLHLAVMQQQ
LQRSVLHPPGSSSQAAAISVQTPQNVPSRSGMPHMHSQLEHRTSQRSSSPVGLAKWFGSDVLQQPLPSMPTKV
ISVDELEYRQ*
>rs239307747_ENSMUSP00000112550_c.934:A>G_p.K312R
ENSMUST00000120721.9|Eif4enif1-
204|ENSMUSG00000020454|+|chr11:g.3180016:A>G
MEKSVAETENGDAFLELKKLPTSKSPHRYTKEELLDIKERPYSKQRPSCLSEKYDSDGVWDPEKWHASLYPAS
GRSSPVESLKKESESDRPSLVRRIAGIVECNGGVAEEDEVEVILAQEPSADQEVPRDVILPEQSPGEFDFNEF
FNLDKVPCLASMIEDVLGEGSVSASRFSRWFSNPSRSGSRSSSLGSTPHEELERLAGLEQAVLSPGQNSGNYF
APIPSEDHAENKVDILEMLQKAKVDLKPLLSSLSANKEKLKESSHSGVVLSVEEVEAGLKGLKVDQQMKNSTP
```

FMAEHLEETLSAASSNRQLRKDGDMTAFNKLVNTMKRNVESHLLAPAEIPGQPVSKNILQELLGQPVQRPASS
NLLSGLMGSLEATASLLSQRAPSPPMSQVFRTQAASADYLHPRIPSPIGFPSGPQQLLGDPFQGMRKPMSPVS
AQMSQLELQQAALEGLALPHDLAVQTAPFYQPGFSKPQVDRTRDGLRNRQQRMSKSPAPMHGGNSSSPAPAAS
ITSMLSPSFTPTSVIRKMYESREKTKEEMAPGMVVPGDGKEDTQKTSEENLLSSNPIPNTDQDSSTTNPKLST
LQRSSCSTPLSQTSRYTKEQDYRPKTAGRKTPTLASPVPGTPFLRPTHQVPLVPHVPIVRPAHQLHPGLVQRL
IAQGVHPQHLPSLLQAGVLPPGIDMAPLQGLSGPLLGQPLYPLVSAASHPLLNPRPGTPLHLAVMQQQLQRSV
LHPPGSSSQAAAISVQTPQNVPSRSGMPHMHSQLEHRTSQRSSSPVGLAKWFGSDVLQQPLPSMPTKVISVDE
LEYRQ*
>rs239307747_ENSMUSP00000122912_c.220:A>G_p.K74R
ENSMUST00000135223.2|Eif4enif1-
210|ENSMUSG00000020454|+|chr11:g.3180016:A>G
QKAKVDLKPLLSSLSANKEKLKESSHSGVVLSVEEVEAGLKGLKVDQQMKNSTPFMAEHLEETLSAASSNRQL
RKDGDMTAFNKLVNTMKASGTLPTQPKVSRNVESHLLAPAEIPGQPVSKNILQVFRTQAASADYLHPRIPSPI
GFPSGPQQLLGDPFQGMRKPMSPVSAQMSQLELQQAALEGLALPHDLAVQTAPFYQPGFSKPQVDRTRDGLRN
RQQRMSKSPAPMHGGNSSSP
>rs239307747_ENSMUSP00000136768_c.1423:A>G_p.K475R
ENSMUST00000179770.8|Eif4enif1-
213|ENSMUSG00000020454|+|chr11:g.3180016:A>G
MEKSVAETENGDAFLELKKLPTSKSPHRYTKEELLDIKERPYSKQRPSCLSEKYDSDGVWDPEKWHASLYPAS
GRSSPVESLKKESESDRPSLVRRIADPRERVKEDDLDVVLSPQRRSFGGGCHVTAAVSSRRSGSPLEKDSDGL
RLLGGRRIGSGRIISARAFEKDHRLSDKDLRDLRDRDRERDYKDKRFRREFGDSKRVFGERRRNDSYTEEEPE
WFSAGPTSQSETIELTGFDDKILEEDHKGRKRTRRRTASVKEGIVECNGGVAEEDEVEVILAQEPSADQEVPR
DVILPEQSPGEFDFNEFFNLDKVPCLASMIEDVLGEGSVSASRFSRWFSNPSRSGSRSSSLGSTPHEELERLA
GLEQAVLSPGQNSGNYFAPIPSEDHAENKVDILEMLQKAKVDLKPLLSSLSANKEKLKESSHSGVVLSVEEVE
AGLKGLKVDQQMKNSTPFMAEHLEETLSAASSNRQLRKDGDMTAFNKLVNTMKASGTLPTQPKVSRNVESHLL
APAEIPGQPVSKNILQELLGQPVQRPASSNLLSGLMGSLEATASLLSQRAPSPPMSQVFRTQAASADYLHPRI
PSPIGFPSGPQQLLGDPFQGMRKPMSPVSAQMSQLELQQAALEGLALPHDLAVQTAPFYQPGFSKPQVDRTRD
GLRNRQQRMSKSPAPMHGGNSSSPAPAASITSMLSPSFTPTSVIRKMYESREKTKEEMAPGMVVPGDGKEDTQ
KTSEENLLSSNPIPNTDQDSSTTNPKLSTLQRSSCSTPLSQTSRYTKEQDYRPKTAGRKTPTLASPVPGTPFL
RPTHQVPLVPHVPIVRPAHQLHPGLVQRLIAQGVHPQHLPSLLQAGVLPPGIDMAPLQGLSGPLLGQPLYPLV
SAASHPLLNPRPGTPLHLAVMQQQLQRSVLHPPGSSSQAAAISVQTPQNVPSRSGMPHMHSQLEHRTSQRSSS
PVGLAKWFGSDVLQQPLPSMPTKVISVDELEYRQ*
>rs228382181_ENSMUSP00000105676_c.1520:A>C_p.E507D
ENSMUST00000110049.8|Eif4enif1-
203|ENSMUSG00000020454|+|chr11:g.3183999:A>C
MEKSVAETENGDAFLELKKLPTSKSPHRYTKEELLDIKERPYSKQRPSCLSEKYDSDGVWDPEKWHASLYPAS
GRSSPVESLKKESESDRPSLVRRIADPRERVKEDDLDVVLSPQRRSFGGGCHVTAAVSSRRSGSPLEKDSDGL
RLLGGRRIGSGRIISARAFEKDHRLSDKDLRDLRDRDRERDYKDKRFRREFGDSKRVFGERRRNDSYTEEEPE
WFSAGPTSQSETIELTGFDDKILEEDHKGRKRTRRRTASVKEGIVECNGGVAEEDEVEVILAQEPSADQEVPR
DVILPEQSPGEFDFNEFFNLDKVPCLASMIEDVLGEGSVSASRFSRWFSNPSRSGSRSSSLGSTPHEELERLA
GLEQAVLSPGQNSGNYFAPIPSEDHAENKVDILEMLQKAKVDLKPLLSSLSANKEKLKESSHSGVVLSVEEVE
AGLKGLKVDQQMKNSTPFMAEHLEETLSAASSNRQLKKDGDMTAFNKLVNTMKASGTLPTQPKVSRNVDSHLL
APAEIPGQPVSKNILQELLGQPVQRPASSNLLSGLMGSLEATASLLSQRAPSPPMSQVFRTQAASADYLHPRI
PSPIGFPSGPQQLLGDPFQGMRKPMSPVSAQMSQLELQQAALEGLALPHDLAVQTAPFYQPGFSKPQVDRTRD
GLRNRQQRMSKSPAPMHGGNSSSPAPAASITSMLSPSFTPTSVIRKMYESREKTKEEMAPGMVVPGDGKEDTQ
KTSEENLLSSNPIPNTDQDSSTTNPKLSTLQRSSCSTPLSQTSRYTKEQDYRPKTAGRKTPTLASPVPGTPFL
RPTHQVPLVPHVPIVRPAHQLHPGLVQRLIAQGVHPQHLPSLLQAGVLPPGIDMAPLQGLSGPLLGQPLYPLV
SAASHPLLNPRPGTPLHLAVMQQQLQRSVLHPPGSSSQAAAISVQTPQNVPSRSGMPHMHSQLEHRTSQRSSS
PVGLAKWFGSDVLQQPLPSMPTKVISVDELEYRQ*
>rs228382181_ENSMUSP00000112550_c.995:A>C_p.E332D
ENSMUST00000120721.9|Eif4enif1-
204|ENSMUSG00000020454|+|chr11:g.3183999:A>C
MEKSVAETENGDAFLELKKLPTSKSPHRYTKEELLDIKERPYSKQRPSCLSEKYDSDGVWDPEKWHASLYPAS
GRSSPVESLKKESESDRPSLVRRIAGIVECNGGVAEEDEVEVILAQEPSADQEVPRDVILPEQSPGEFDFNEF
FNLDKVPCLASMIEDVLGEGSVSASRFSRWFSNPSRSGSRSSSLGSTPHEELERLAGLEQAVLSPGQNSGNYF
APIPSEDHAENKVDILEMLQKAKVDLKPLLSSLSANKEKLKESSHSGVVLSVEEVEAGLKGLKVDQQMKNSTP
FMAEHLEETLSAASSNRQLKKDGDMTAFNKLVNTMKRNVDSHLLAPAEIPGQPVSKNILQELLGQPVQRPASS
NLLSGLMGSLEATASLLSQRAPSPPMSQVFRTQAASADYLHPRIPSPIGFPSGPQQLLGDPFQGMRKPMSPVS

AQMSQLELQQAALEGLALPHDLAVQTAPFYQPGFSKPQVDRTRDGLRNRQQRMSKSPAPMHGGNSSSPAPAAS
ITSMLSPSFTPTSVIRKMYESREKTKEEMAPGMVVPGDGKEDTQKTSEENLLSSNPIPNTDQDSSTTNPKLST
LQRSSCSTPLSQTSRYTKEQDYRPKTAGRKTPTLASPVPGTPFLRPTHQVPLVPHVPIVRPAHQLHPGLVQRL
IAQGVHPQHLPSLLQAGVLPPGIDMAPLQGLSGPLLGQPLYPLVSAASHPLLNPRPGTPLHLAVMQQQLQRSV
LHPPGSSSQAAAISVQTPQNVPSRSGMPHMHSQLEHRTSQRSSSPVGLAKWFGSDVLQQPLPSMPTKVISVDE
LEYRQ*
>rs228382181_ENSMUSP00000122912_c.317:A>C_p.E106D
ENSMUST00000135223.2|Eif4enif1-
210|ENSMUSG00000020454|+|chr11:g.3183999:A>C
QKAKVDLKPLLSSLSANKEKLKESSHSGVVLSVEEVEAGLKGLKVDQQMKNSTPFMAEHLEETLSAASSNRQL
KKDGDMTAFNKLVNTMKASGTLPTQPKVSRNVDSHLLAPAEIPGQPVSKNILQVFRTQAASADYLHPRIPSPI
GFPSGPQQLLGDPFQGMRKPMSPVSAQMSQLELQQAALEGLALPHDLAVQTAPFYQPGFSKPQVDRTRDGLRN
RQQRMSKSPAPMHGGNSSSP
>rs228382181_ENSMUSP00000136768_c.1520:A>C_p.E507D
ENSMUST00000179770.8|Eif4enif1-
213|ENSMUSG00000020454|+|chr11:g.3183999:A>C
MEKSVAETENGDAFLELKKLPTSKSPHRYTKEELLDIKERPYSKQRPSCLSEKYDSDGVWDPEKWHASLYPAS
GRSSPVESLKKESESDRPSLVRRIADPRERVKEDDLDVVLSPQRRSFGGGCHVTAAVSSRRSGSPLEKDSDGL
RLLGGRRIGSGRIISARAFEKDHRLSDKDLRDLRDRDRERDYKDKRFRREFGDSKRVFGERRRNDSYTEEEPE
WFSAGPTSQSETIELTGFDDKILEEDHKGRKRTRRRTASVKEGIVECNGGVAEEDEVEVILAQEPSADQEVPR
DVILPEQSPGEFDFNEFFNLDKVPCLASMIEDVLGEGSVSASRFSRWFSNPSRSGSRSSSLGSTPHEELERLA
GLEQAVLSPGQNSGNYFAPIPSEDHAENKVDILEMLQKAKVDLKPLLSSLSANKEKLKESSHSGVVLSVEEVE
AGLKGLKVDQQMKNSTPFMAEHLEETLSAASSNRQLKKDGDMTAFNKLVNTMKASGTLPTQPKVSRNVDSHLL
APAEIPGQPVSKNILQELLGQPVQRPASSNLLSGLMGSLEATASLLSQRAPSPPMSQVFRTQAASADYLHPRI
PSPIGFPSGPQQLLGDPFQGMRKPMSPVSAQMSQLELQQAALEGLALPHDLAVQTAPFYQPGFSKPQVDRTRD
GLRNRQQRMSKSPAPMHGGNSSSPAPAASITSMLSPSFTPTSVIRKMYESREKTKEEMAPGMVVPGDGKEDTQ
KTSEENLLSSNPIPNTDQDSSTTNPKLSTLQRSSCSTPLSQTSRYTKEQDYRPKTAGRKTPTLASPVPGTPFL
RPTHQVPLVPHVPIVRPAHQLHPGLVQRLIAQGVHPQHLPSLLQAGVLPPGIDMAPLQGLSGPLLGQPLYPLV
SAASHPLLNPRPGTPLHLAVMQQQLQRSVLHPPGSSSQAAAISVQTPQNVPSRSGMPHMHSQLEHRTSQRSSS
PVGLAKWFGSDVLQQPLPSMPTKVISVDELEYRQ*
>rs240891555_ENSMUSP00000020734_c.1836:G>A_p.V613I
ENSMUST00000020734.11|Eif4enif1-
201|ENSMUSG00000020454|+|chr11:g.3186277:G>A
MEKSVAETENGDAFLELKKLPTSKSPHRYTKEELLDIKERPYSKQRPSCLSEKYDSDGVWDPEKWHASLYPAS
GRSSPVESLKKESESDRPSLVRRIADPRERVKEDDLDVVLSPQRRSFGGGCHVTAAVSSRRSGSPLEKDSDGL
RLLGGRRIGSGRIISARAFEKDHRLSDKDLRDLRDRDRERDYKDKRFRREFGDSKRVFGERRRNDSYTEEEPE
WFSAGPTSQSETIELTGFDDKILEEDHKGRKRTRRRTASVKEGIVECNGGVAEEDEVEVILAQEPSADQEVPR
DVILPEQSPGEFDFNEFFNLDKVPCLASMIEDVLGEGSVSASRFSRWFSNPSRSGSRSSSLGSTPHEELERLA
GLEQAVLSPGQNSGNYFAPIPSEDHAENKVDILEMLQKAKVDLKPLLSSLSANKEKLKESSHSGVVLSVEEVE
AGLKGLKVDQQMKNSTPFMAEHLEETLSAASSNRQLKKDGDMTAFNKLVNTMKASGTLPTQPKVSELLGQPVQ
RPASSNLLSGLMGSLEATASLLSQRAPSPPMSQVFRTQAASADYLHPRIPSPIGFPSGPQQLLGDPFQGMRKP
MSPVSAQMSQLELQQAALEGLALPHDLAIQTAPFYQPGFSKPQVDRTRDGLRNRQQRMSKSPAPMHGGNSSSP
APAASITSMLSPSFTPTSVIRKMYESREKTKEEMAPGMVVPGDGKEDTQKTSEENLLSSNPIPNTDQDSSTTN
PKLSTLQRSSCSTPLSQTSRYTKEQDYRPKTAGRKTPTLASPVPGTPFLRPTHQVPLVPHVPIVRPAHQLHPG
LVQRLIAQGVHPQHLPSLLQAGVLPPGIDMAPLQGLSGPLLGQPLYPLVSAASHPLLNPRPGTPLHLAVMQQQ
LQRSVLHPPGSSSQAAAISVQTPQNVPSRSGMPHMHSQLEHRTSQRSSSPVGLAKWFGSDVLQQPLPSMPTKV
ISVDELEYRQ*
>rs240891555_ENSMUSP00000105676_c.1908:G>A_p.V637I
ENSMUST00000110049.8|Eif4enif1-
203|ENSMUSG00000020454|+|chr11:g.3186277:G>A
MEKSVAETENGDAFLELKKLPTSKSPHRYTKEELLDIKERPYSKQRPSCLSEKYDSDGVWDPEKWHASLYPAS
GRSSPVESLKKESESDRPSLVRRIADPRERVKEDDLDVVLSPQRRSFGGGCHVTAAVSSRRSGSPLEKDSDGL
RLLGGRRIGSGRIISARAFEKDHRLSDKDLRDLRDRDRERDYKDKRFRREFGDSKRVFGERRRNDSYTEEEPE
WFSAGPTSQSETIELTGFDDKILEEDHKGRKRTRRRTASVKEGIVECNGGVAEEDEVEVILAQEPSADQEVPR
DVILPEQSPGEFDFNEFFNLDKVPCLASMIEDVLGEGSVSASRFSRWFSNPSRSGSRSSSLGSTPHEELERLA
GLEQAVLSPGQNSGNYFAPIPSEDHAENKVDILEMLQKAKVDLKPLLSSLSANKEKLKESSHSGVVLSVEEVE
AGLKGLKVDQQMKNSTPFMAEHLEETLSAASSNRQLKKDGDMTAFNKLVNTMKASGTLPTQPKVSRNVESHLL
APAEIPGQPVSKNILQELLGQPVQRPASSNLLSGLMGSLEATASLLSQRAPSPPMSQVFRTQAASADYLHPRI

PSPIGFPSGPQQLLGDPFQGMRKPMSPVSAQMSQLELQQAALEGLALPHDLAIQTAPFYQPGFSKPQVDRTRD
GLRNRQQRMSKSPAPMHGGNSSSPAPAASITSMLSPSFTPTSVIRKMYESREKTKEEMAPGMVVPGDGKEDTQ
KTSEENLLSSNPIPNTDQDSSTTNPKLSTLQRSSCSTPLSQTSRYTKEQDYRPKTAGRKTPTLASPVPGTPFL
RPTHQVPLVPHVPIVRPAHQLHPGLVQRLIAQGVHPQHLPSLLQAGVLPPGIDMAPLQGLSGPLLGQPLYPLV
SAASHPLLNPRPGTPLHLAVMQQQLQRSVLHPPGSSSQAAAISVQTPQNVPSRSGMPHMHSQLEHRTSQRSSS
PVGLAKWFGSDVLQQPLPSMPTKVISVDELEYRQ*
>rs240891555_ENSMUSP00000105675_c.1836:G>A_p.V613I
ENSMUST00000110048.8|Eif4enif1-
202|ENSMUSG00000020454|+|chr11:g.3186277:G>A
MEKSVAETENGDAFLELKKLPTSKSPHRYTKEELLDIKERPYSKQRPSCLSEKYDSDGVWDPEKWHASLYPAS
GRSSPVESLKKESESDRPSLVRRIADPRERVKEDDLDVVLSPQRRSFGGGCHVTAAVSSRRSGSPLEKDSDGL
RLLGGRRIGSGRIISARAFEKDHRLSDKDLRDLRDRDRERDYKDKRFRREFGDSKRVFGERRRNDSYTEEEPE
WFSAGPTSQSETIELTGFDDKILEEDHKGRKRTRRRTASVKEGIVECNGGVAEEDEVEVILAQEPSADQEVPR
DVILPEQSPGEFDFNEFFNLDKVPCLASMIEDVLGEGSVSASRFSRWFSNPSRSGSRSSSLGSTPHEELERLA
GLEQAVLSPGQNSGNYFAPIPSEDHAENKVDILEMLQKAKVDLKPLLSSLSANKEKLKESSHSGVVLSVEEVE
AGLKGLKVDQQMKNSTPFMAEHLEETLSAASSNRQLKKDGDMTAFNKLVNTMKASGTLPTQPKVSELLGQPVQ
RPASSNLLSGLMGSLEATASLLSQRAPSPPMSQVFRTQAASADYLHPRIPSPIGFPSGPQQLLGDPFQGMRKP
MSPVSAQMSQLELQQAALEGLALPHDLAIQTAPFYQPGFSKPQVDRTRDGLRNRQQRMSKSPAPMHGGNSSSP
APAASITSMLSPSFTPTSVIRKMYESREKTKEEMAPGMVVPGDGKEDTQKTSEENLLSSNPIPNTDQDSSTTN
PKLSTLQRSSCSTPLSQTSRYTKEQDYRPKTAGRKTPTLASPVPGTPFLRPTHQVPLVPHVPIVRPAHQLHPG
LVQRLIAQGVHPQHLPSLLQAGVLPPGIDMAPLQGLSGPLLGQPLYPLVSAASHPLLNPRPGTPLHLAVMQQQ
LQRSVLHPPGSSSQAAAISVQTPQNVPSRSGMPHMHSQLEHRTSQRSSSPVGLAKWFGSDVLQQPLPSMPTKV
ISVDELEYRQ*
>rs240891555_ENSMUSP00000112550_c.1383:G>A_p.V462I
ENSMUST00000120721.9|Eif4enif1-
204|ENSMUSG00000020454|+|chr11:g.3186277:G>A
MEKSVAETENGDAFLELKKLPTSKSPHRYTKEELLDIKERPYSKQRPSCLSEKYDSDGVWDPEKWHASLYPAS
GRSSPVESLKKESESDRPSLVRRIAGIVECNGGVAEEDEVEVILAQEPSADQEVPRDVILPEQSPGEFDFNEF
FNLDKVPCLASMIEDVLGEGSVSASRFSRWFSNPSRSGSRSSSLGSTPHEELERLAGLEQAVLSPGQNSGNYF
APIPSEDHAENKVDILEMLQKAKVDLKPLLSSLSANKEKLKESSHSGVVLSVEEVEAGLKGLKVDQQMKNSTP
FMAEHLEETLSAASSNRQLKKDGDMTAFNKLVNTMKRNVESHLLAPAEIPGQPVSKNILQELLGQPVQRPASS
NLLSGLMGSLEATASLLSQRAPSPPMSQVFRTQAASADYLHPRIPSPIGFPSGPQQLLGDPFQGMRKPMSPVS
AQMSQLELQQAALEGLALPHDLAIQTAPFYQPGFSKPQVDRTRDGLRNRQQRMSKSPAPMHGGNSSSPAPAAS
ITSMLSPSFTPTSVIRKMYESREKTKEEMAPGMVVPGDGKEDTQKTSEENLLSSNPIPNTDQDSSTTNPKLST
LQRSSCSTPLSQTSRYTKEQDYRPKTAGRKTPTLASPVPGTPFLRPTHQVPLVPHVPIVRPAHQLHPGLVQRL
IAQGVHPQHLPSLLQAGVLPPGIDMAPLQGLSGPLLGQPLYPLVSAASHPLLNPRPGTPLHLAVMQQQLQRSV
LHPPGSSSQAAAISVQTPQNVPSRSGMPHMHSQLEHRTSQRSSSPVGLAKWFGSDVLQQPLPSMPTKVISVDE
LEYRQ*
>rs240891555_ENSMUSP00000122912_c.582:G>A_p.V195I
ENSMUST00000135223.2|Eif4enif1-
210|ENSMUSG00000020454|+|chr11:g.3186277:G>A
QKAKVDLKPLLSSLSANKEKLKESSHSGVVLSVEEVEAGLKGLKVDQQMKNSTPFMAEHLEETLSAASSNRQL
KKDGDMTAFNKLVNTMKASGTLPTQPKVSRNVESHLLAPAEIPGQPVSKNILQVFRTQAASADYLHPRIPSPI
GFPSGPQQLLGDPFQGMRKPMSPVSAQMSQLELQQAALEGLALPHDLAIQTAPFYQPGFSKPQVDRTRDGLRN
RQQRMSKSPAPMHGGNSSSP
>rs240891555_ENSMUSP00000136768_c.1908:G>A_p.V637I
ENSMUST00000179770.8|Eif4enif1-
213|ENSMUSG00000020454|+|chr11:g.3186277:G>A
MEKSVAETENGDAFLELKKLPTSKSPHRYTKEELLDIKERPYSKQRPSCLSEKYDSDGVWDPEKWHASLYPAS
GRSSPVESLKKESESDRPSLVRRIADPRERVKEDDLDVVLSPQRRSFGGGCHVTAAVSSRRSGSPLEKDSDGL
RLLGGRRIGSGRIISARAFEKDHRLSDKDLRDLRDRDRERDYKDKRFRREFGDSKRVFGERRRNDSYTEEEPE
WFSAGPTSQSETIELTGFDDKILEEDHKGRKRTRRRTASVKEGIVECNGGVAEEDEVEVILAQEPSADQEVPR
DVILPEQSPGEFDFNEFFNLDKVPCLASMIEDVLGEGSVSASRFSRWFSNPSRSGSRSSSLGSTPHEELERLA
GLEQAVLSPGQNSGNYFAPIPSEDHAENKVDILEMLQKAKVDLKPLLSSLSANKEKLKESSHSGVVLSVEEVE
AGLKGLKVDQQMKNSTPFMAEHLEETLSAASSNRQLKKDGDMTAFNKLVNTMKASGTLPTQPKVSRNVESHLL
APAEIPGQPVSKNILQELLGQPVQRPASSNLLSGLMGSLEATASLLSQRAPSPPMSQVFRTQAASADYLHPRI
PSPIGFPSGPQQLLGDPFQGMRKPMSPVSAQMSQLELQQAALEGLALPHDLAIQTAPFYQPGFSKPQVDRTRD
GLRNRQQRMSKSPAPMHGGNSSSPAPAASITSMLSPSFTPTSVIRKMYESREKTKEEMAPGMVVPGDGKEDTQ

KTSEENLLSSNPIPNTDQDSSTTNPKLSTLQRSSCSTPLSQTSRYTKEQDYRPKTAGRKTPTLASPVPGTPFL
RPTHQVPLVPHVPIVRPAHQLHPGLVQRLIAQGVHPQHLPSLLQAGVLPPGIDMAPLQGLSGPLLGQPLYPLV
SAASHPLLNPRPGTPLHLAVMQQQLQRSVLHPPGSSSQAAAISVQTPQNVPSRSGMPHMHSQLEHRTSQRSSS
PVGLAKWFGSDVLQQPLPSMPTKVISVDELEYRQ*
>ENSMUSP00000020734_c.2694:C>T_p.P899S ENSMUST00000020734.11|Eif4enif1-201|ENSMUSG00000020454|+|chr11:g.3193904:C>T
MEKSVAETENGDAFLELKKLPTSKSPHRYTKEELLDIKERPYSKQRPSCLSEKYDSDGVWDPEKWHASLYPAS
GRSSPVESLKKESESDRPSLVRRIADPRERVKEDDLDVVLSPQRRSFGGGCHVTAAVSSRRSGSPLEKDSDGL
RLLGGRRIGSGRIISARAFEKDHRLSDKDLRDLRDRDRERDYKDKRFRREFGDSKRVFGERRRNDSYTEEEPE
WFSAGPTSQSETIELTGFDDKILEEDHKGRKRTRRRTASVKEGIVECNGGVAEEDEVEVILAQEPSADQEVPR
DVILPEQSPGEFDFNEFFNLDKVPCLASMIEDVLGEGSVSASRFSRWFSNPSRSGSRSSSLGSTPHEELERLA
GLEQAVLSPGQNSGNYFAPIPSEDHAENKVDILEMLQKAKVDLKPLLSSLSANKEKLKESSHSGVVLSVEEVE
AGLKGLKVDQQMKNSTPFMAEHLEETLSAASSNRQLKKDGDMTAFNKLVNTMKASGTLPTQPKVSELLGQPVQ
RPASSNLLSGLMGSLEATASLLSQRAPSPPMSQVFRTQAASADYLHPRIPSPIGFPSGPQQLLGDPFQGMRKP
MSPVSAQMSQLELQQAALEGLALPHDLAVQTAPFYQPGFSKPQVDRTRDGLRNRQQRMSKSPAPMHGGNSSSP
APAASITSMLSPSFTPTSVIRKMYESREKTKEEMAPGMVVPGDGKEDTQKTSEENLLSSNPIPNTDQDSSTTN
PKLSTLQRSSCSTPLSQTSRYTKEQDYRPKTAGRKTPTLASPVPGTPFLRPTHQVPLVPHVPIVRPAHQLHPG
LVQRLIAQGVHPQHLPSLLQAGVLPPGIDMAPLQGLSGPLLGQPLYPLVSAASHPLLNPRPGTPLHLAVMQQQ
LQRSVLHPPGSSSQAAAISVQTSQNVPSRSGMPHMHSQLEHRTSQRSSSPVGLAKWFGSDVLQQPLPSMPTKV
ISVDELEYRQ*
>ENSMUSP00000105676_c.2766:C>T_p.P923S ENSMUST00000110049.8|Eif4enif1-203|ENSMUSG00000020454|+|chr11:g.3193904:C>T
MEKSVAETENGDAFLELKKLPTSKSPHRYTKEELLDIKERPYSKQRPSCLSEKYDSDGVWDPEKWHASLYPAS
GRSSPVESLKKESESDRPSLVRRIADPRERVKEDDLDVVLSPQRRSFGGGCHVTAAVSSRRSGSPLEKDSDGL
RLLGGRRIGSGRIISARAFEKDHRLSDKDLRDLRDRDRERDYKDKRFRREFGDSKRVFGERRRNDSYTEEEPE
WFSAGPTSQSETIELTGFDDKILEEDHKGRKRTRRRTASVKEGIVECNGGVAEEDEVEVILAQEPSADQEVPR
DVILPEQSPGEFDFNEFFNLDKVPCLASMIEDVLGEGSVSASRFSRWFSNPSRSGSRSSSLGSTPHEELERLA
GLEQAVLSPGQNSGNYFAPIPSEDHAENKVDILEMLQKAKVDLKPLLSSLSANKEKLKESSHSGVVLSVEEVE
AGLKGLKVDQQMKNSTPFMAEHLEETLSAASSNRQLKKDGDMTAFNKLVNTMKASGTLPTQPKVSRNVESHLL
APAEIPGQPVSKNILQELLGQPVQRPASSNLLSGLMGSLEATASLLSQRAPSPPMSQVFRTQAASADYLHPRI
PSPIGFPSGPQQLLGDPFQGMRKPMSPVSAQMSQLELQQAALEGLALPHDLAVQTAPFYQPGFSKPQVDRTRD
GLRNRQQRMSKSPAPMHGGNSSSPAPAASITSMLSPSFTPTSVIRKMYESREKTKEEMAPGMVVPGDGKEDTQ
KTSEENLLSSNPIPNTDQDSSTTNPKLSTLQRSSCSTPLSQTSRYTKEQDYRPKTAGRKTPTLASPVPGTPFL
RPTHQVPLVPHVPIVRPAHQLHPGLVQRLIAQGVHPQHLPSLLQAGVLPPGIDMAPLQGLSGPLLGQPLYPLV
SAASHPLLNPRPGTPLHLAVMQQQLQRSVLHPPGSSSQAAAISVQTSQNVPSRSGMPHMHSQLEHRTSQRSSS
PVGLAKWFGSDVLQQPLPSMPTKVISVDELEYRQ*
>ENSMUSP00000105675_c.2694:C>T_p.P899S ENSMUST00000110048.8|Eif4enif1-202|ENSMUSG00000020454|+|chr11:g.3193904:C>T
MEKSVAETENGDAFLELKKLPTSKSPHRYTKEELLDIKERPYSKQRPSCLSEKYDSDGVWDPEKWHASLYPAS
GRSSPVESLKKESESDRPSLVRRIADPRERVKEDDLDVVLSPQRRSFGGGCHVTAAVSSRRSGSPLEKDSDGL
RLLGGRRIGSGRIISARAFEKDHRLSDKDLRDLRDRDRERDYKDKRFRREFGDSKRVFGERRRNDSYTEEEPE
WFSAGPTSQSETIELTGFDDKILEEDHKGRKRTRRRTASVKEGIVECNGGVAEEDEVEVILAQEPSADQEVPR
DVILPEQSPGEFDFNEFFNLDKVPCLASMIEDVLGEGSVSASRFSRWFSNPSRSGSRSSSLGSTPHEELERLA
GLEQAVLSPGQNSGNYFAPIPSEDHAENKVDILEMLQKAKVDLKPLLSSLSANKEKLKESSHSGVVLSVEEVE
AGLKGLKVDQQMKNSTPFMAEHLEETLSAASSNRQLKKDGDMTAFNKLVNTMKASGTLPTQPKVSELLGQPVQ
RPASSNLLSGLMGSLEATASLLSQRAPSPPMSQVFRTQAASADYLHPRIPSPIGFPSGPQQLLGDPFQGMRKP
MSPVSAQMSQLELQQAALEGLALPHDLAVQTAPFYQPGFSKPQVDRTRDGLRNRQQRMSKSPAPMHGGNSSSP
APAASITSMLSPSFTPTSVIRKMYESREKTKEEMAPGMVVPGDGKEDTQKTSEENLLSSNPIPNTDQDSSTTN
PKLSTLQRSSCSTPLSQTSRYTKEQDYRPKTAGRKTPTLASPVPGTPFLRPTHQVPLVPHVPIVRPAHQLHPG
LVQRLIAQGVHPQHLPSLLQAGVLPPGIDMAPLQGLSGPLLGQPLYPLVSAASHPLLNPRPGTPLHLAVMQQQ
LQRSVLHPPGSSSQAAAISVQTSQNVPSRSGMPHMHSQLEHRTSQRSSSPVGLAKWFGSDVLQQPLPSMPTKV
ISVDELEYRQ*
>ENSMUSP00000112550_c.2241:C>T_p.P748S ENSMUST00000120721.9|Eif4enif1-204|ENSMUSG00000020454|+|chr11:g.3193904:C>T
MEKSVAETENGDAFLELKKLPTSKSPHRYTKEELLDIKERPYSKQRPSCLSEKYDSDGVWDPEKWHASLYPAS
GRSSPVESLKKESESDRPSLVRRIAGIVECNGGVAEEDEVEVILAQEPSADQEVPRDVILPEQSPGEFDFNEF
FNLDKVPCLASMIEDVLGEGSVSASRFSRWFSNPSRSGSRSSSLGSTPHEELERLAGLEQAVLSPGQNSGNYF
APIPSEDHAENKVDILEMLQKAKVDLKPLLSSLSANKEKLKESSHSGVVLSVEEVEAGLKGLKVDQQMKNSTP

FMAEHLEETLSAASSNRQLKKDGDMTAFNKLVNTMKRNVESHLLAPAEIPGQPVSKNILQELLGQPVQRPASS
NLLSGLMGSLEATASLLSQRAPSPPMSQVFRTQAASADYLHPRIPSPIGFPSGPQQLLGDPFQGMRKPMSPVS
AQMSQLELQQAALEGLALPHDLAVQTAPFYQPGFSKPQVDRTRDGLRNRQQRMSKSPAPMHGGNSSSPAPAAS
ITSMLSPSFTPTSVIRKMYESREKTKEEMAPGMVVPGDGKEDTQKTSEENLLSSNPIPNTDQDSSTTNPKLST
LQRSSCSTPLSQTSRYTKEQDYRPKTAGRKTPTLASPVPGTPFLRPTHQVPLVPHVPIVRPAHQLHPGLVQRL
IAQGVHPQHLPSLLQAGVLPPGIDMAPLQGLSGPLLGQPLYPLVSAASHPLLNPRPGTPLHLAVMQQQLQRSV
LHPPGSSSQAAAISVQTSQNVPSRSGMPHMHSQLEHRTSQRSSSPVGLAKWFGSDVLQQPLPSMPTKVISVDE
LEYRQ*
>ENSMUSP00000136768_c.2766:C>T_p.P923S ENSMUST00000179770.8|Eif4enif1-213|ENSMUSG00000020454|+|chr11:g.3193904:C>T
MEKSVAETENGDAFLELKKLPTSKSPHRYTKEELLDIKERPYSKQRPSCLSEKYDSDGVWDPEKWHASLYPAS
GRSSPVESLKKESESDRPSLVRRIADPRERVKEDDLDVVLSPQRRSFGGGCHVTAAVSSRRSGSPLEKDSDGL
RLLGGRRIGSGRIISARAFEKDHRLSDKDLRDLRDRDRERDYKDKRFRREFGDSKRVFGERRRNDSYTEEEPE
WFSAGPTSQSETIELTGFDDKILEEDHKGRKRTRRRTASVKEGIVECNGGVAEEDEVEVILAQEPSADQEVPR
DVILPEQSPGEFDFNEFFNLDKVPCLASMIEDVLGEGSVSASRFSRWFSNPSRSGSRSSSLGSTPHEELERLA
GLEQAVLSPGQNSGNYFAPIPSEDHAENKVDILEMLQKAKVDLKPLLSSLSANKEKLKESSHSGVVLSVEEVE
AGLKGLKVDQQMKNSTPFMAEHLEETLSAASSNRQLKKDGDMTAFNKLVNTMKASGTLPTQPKVSRNVESHLL
APAEIPGQPVSKNILQELLGQPVQRPASSNLLSGLMGSLEATASLLSQRAPSPPMSQVFRTQAASADYLHPRI
PSPIGFPSGPQQLLGDPFQGMRKPMSPVSAQMSQLELQQAALEGLALPHDLAVQTAPFYQPGFSKPQVDRTRD
GLRNRQQRMSKSPAPMHGGNSSSPAPAASITSMLSPSFTPTSVIRKMYESREKTKEEMAPGMVVPGDGKEDTQ
KTSEENLLSSNPIPNTDQDSSTTNPKLSTLQRSSCSTPLSQTSRYTKEQDYRPKTAGRKTPTLASPVPGTPFL
RPTHQVPLVPHVPIVRPAHQLHPGLVQRLIAQGVHPQHLPSLLQAGVLPPGIDMAPLQGLSGPLLGQPLYPLV
SAASHPLLNPRPGTPLHLAVMQQQLQRSVLHPPGSSSQAAAISVQTSQNVPSRSGMPHMHSQLEHRTSQRSSS
PVGLAKWFGSDVLQQPLPSMPTKVISVDELEYRQ*
>rs46909618_ENSMUSP00000099865_c.416:G>T_p.R139S
ENSMUST00000102801.8|Tenm2-202|ENSMUSG00000049336|-|chr11:g.36755580:C>A
MDVKDRRHRSLTRGRCGKECRYTSSSLDSEDCRVPTQKSYSSSETLKAYDHDSRMHYGNRVTDLVHRESDEFS
RQGTNFTLAELGICEPSPHRSGYCSDMGILHQGYSLSTGSDADSDTEGGMSPEHAIRLWGRGIKSSRSSGLSS
RENSALTLTDSDNENKSDDDNGRPIPPTSSSSLLPSAQLPSSHNPPPVSCQMPLLDSNTSHQIMDTNPDEEFS
PNSYLLRACSGPQQASSSGPPNHHSQSTLRPPLPPPHNHTLSHHHSSANSLNRNSLTNRRSQIHAPAPAPNDL
ATTPESVQLQDSWVLNSNVPLETRHFLFKTSSGSTPLFSSSSPGYPLTSGTVYTPPPRLLPRNTFSRKAFKLK
KPSKYCSWKCAALSAIAAALLLAILLAYFIAMHLLGLNWQLQPADGHTFNNGVRTGLPGNDDVATVPSGGKVP
WSLKNSSIDSGEAEVGRRVTQEVPPGVFWRSQIHISQPQFLKFNISLGKDALFGVYIRRGLPPSHAQYDFMER
LDGKEKWSVVESPRERRSIQTLVQNEAVFVQYLDVGLWHLAFYNDGKDKEMVSFNTVVLDSVQDCPRNCHGNG
ECVSGLCHCFPGFLGADCAKAACPVLCSGNGQYSKGTCQCYSGWKGAECDVPMNQCIDPSCGGHGSCIDGNCV
CAAGYKGEHCEEVDCLDPTCSSHGVCVNGECLCSPGWGGLNCELARVQCPDQCSGHGTYLPDSGLCSCDPNWM
GPDCSVVCSVDCGTHGVCIGGACRCEEGWTGAACDQRVCHPRCIEHGTCKDGKCECREGWNGEHCTIDGCPDL
CNGNGRCTLGQNSWQCVCQTGWRGPGCNVAMETSCADNKDNEGDGLVDCLDPDCCLQSACQNSLLCRGSRDPL
DIIQQGQTDWPAVKSFYDRIKLLAGKDSTHIIPGDNPFNSSLVSLIRGQVVTMDGTPLVGVNVSFVKYPKYGY
TITRQDGTFDLIANGGSALTLHFERAPFMSQERTVWLPWNSFYAMDTLVMKTEENSIPSCDLSGFVRPDPIII
SSPLSTFFSASPASNPIVPETQVLHEEIELPGTNVKLRYLSSRTAGYKSLLKITMTQSTVPLNLIRVHLMVAV
EGHLFQKSFQASPNLAYTFIWDKTDAYGQRVYGLSDAVVSVGFEYETCPSLILWEKRTALLQGFELDPSNLGG
WSLDKHHTLNVKSGILHKGTGENQFLTQQPAIITSIMGNGRRRSISCPSCNGLAEGNKLLAPVALAVGIDGSL
FVGDFNYIRRIFPSRNVTSILELRNKEFKHSNSPGHKYYLAVDPVTGSLYVSDTNSRRIYRVKSLSGAKDLAG
NSEVVAGTGEQCLPFDEARCGDGGKAVDATLMSPRGIAVDKNGLMYFVDATMIRKVDQNGIISTLLGSNDLTA
VRPLSCDSSMDVAQVRLEWPTDLAVNPMDNSLYVLENNVILRITENHQVSIIAGRPMHCQVPGIDYSLSKLAI
HSALESASAIAISHTGVLYITETDEKKINRLRQVTTNGEICLLAGAASDCDCKNDVNCICYSGDDAYATDAIL
NSPSSLAVAPDGTIYIADLGNIRIRAVSKNKPVLNAFNQYEAASPGEQELYVFNADGIHQYTVSLVTGEYLYN
FTYSADNDVTELIDNNGNSLKIRRDSSGMPRHLLMPDNQIITLTVGTNGGLKAVSTQNLELGLMTYDGNTGLL
ATKSDETGWTTFYDYDHEGRLTNVTRPTGVVTSLHREMEKSITIDIENSNRDDDVTVITNLSSVEASYTVVQD
QVRNSYQLCNNGTLRVMYANGMAVSFHSEPHVLAGTITPTIGRCNISLPMENGLNSIEWRLRKEQIKGKVTIF
GRKLRVHGRNLLSIDYDRNIRTEKIYDDHRKFTLRIIYDQVGRPFLWLPSSGLAAVNVSYFFNGRLAGLQRGA
MSERTDIDKQGRIVSRMFADGKVWSYSYLDKSMVLLLQSQRQYIFEYDSSDRLHAVTMPSVARHSMSTHTSIG
YIRNIYNPPESNASVIFDYSDDGRILKTSFLGTGRQVFYKYGKLSKLSEIVYDSTAVTFGYDETTGVLKMVNL
QSGGFSCTIRYRKVGPLVDKQIYRFSEEGMINARFDYTYHDNSFRIASIKPVISETPLPVDLYRYDEISGKVE
HFGKFGVIYYDINQIITTAVMTLSKHFDTHGRIKEVQYEMFRSLMYWMTVQYDSMGRVIKRELKLGPYANTTK
YTYDYDGDGQLQSVAVNDRPTWRYSYDLNGNLHLLNPGNSARLMPLRYDLRDRITRLGDVQYKIDDDGYLCQR
GSDIFEYNSKGLLTRAYNKASGWSVQYRYDGVGRRASYKTNLGHHLQYFYSDLHNPTRITHVYNHSNSEITSL

YYDLQGHLFAMESSSGEEYYVASDNTGTPLAVFSINGLMIKQLQYTAYGEIYYDSNPDFQMVIGFHGGLYDPL
TKLVHFTQRDYDVLAGRWTSPDYTMWRNVGKEPAPFNLYMFKNNNPLSNELDLKNYVTDVKSWLVMFGFQLSN
IIPGFPRAKMYFVPPPYELSESQASENGQLITGVQQTTERHNQAFLALEGQVITKKLHASIREKAGHWFATTT
PIIGKGIMFAIKEGRVTTGVSSIASEDSRKVASVLNNAYYLDKMHYSIEGKDTHYFVKIGAADGDLVTLGTTI
GRKVLESGVNVTVSQPTLLVNGRTRRFTNIEFQYSTLLLSIRYGLTPDTLDEEKARVLDQARQRALGTAWAKE
QQKARDGREGSRLWTEGEKQQLLSTGRVQGYEGYYVLPVEQYPELADSSSNIQFLRQNEMGKR*
>rs46909618_ENSMUSP00000052014_c.416:G>T_p.R139S
ENSMUST00000057207.6|Tenm2-201|ENSMUSG00000049336|-|chr11:g.36755580:C>A
MDVKDRRHRSLTRGRCGKECRYTSSSLDSEDCRVPTQKSYSSSETLKAYDHDSRMHYGNRVTDLVHRESDEFS
RQGTNFTLAELGICEPSPHRSGYCSDMGILHQGYSLSTGSDADSDTEGGMSPEHAIRLWGRGIKSSRSSGLSS
RENSALTLTDSDNENKSDDDNGRPIPPTSSSSLLPSAQLPSSHNPPPVSCQMPLLDSNTSHQIMDTNPDEEFS
PNSYLLRACSGPQQASSSGPPNHHSQSTLRPPLPPPHNHTLSHHHSSANSLNRNSLTNRRSQIHAPAPAPNDL
ATTPESVQLQDSWVLNSNVPLETRHFLFKTSSGSTPLFSSSSPGYPLTSGTVYTPPPRLLPRNTFSRKAFKLK
KPSKYCSWKCAALSAIAAALLLAILLAYFIAMHLLGLNWQLQPADGHTFNNGVRTGLPGNDDVATVPSGGKVP
WSLKNSSIDSGEAEVGRRVTQEVPPGVFWRSQIHISQPQFLKFNISLGKDALFGVYIRRGLPPSHAQYDFMER
LDGKEKWSVVESPRERRSIQTLVQNEAVFVQYLDVGLWHLAFYNDGKDKEMVSFNTVVLDSVQDCPRNCHGNG
ECVSGLCHCFPGFLGADCAKAACPVLCSGNGQYSKGTCQCYSGWKGAECDVPMNQCIDPSCGGHGSCIDGNCV
CAAGYKGEHCEEVDCLDPTCSSHGVCVNGECLCSPGWGGLNCELARVQCPDQCSGHGTYLPDSGLCSCDPNWM
GPDCSVEVCSVDCGTHGVCIGGACRCEEGWTGAACDQRVCHPRCIEHGTCKDGKCECREGWNGEHCTIDGCPD
LCNGNGRCTLGQNSWQCVCQTGWRGPGCNVAMETSCADNKDNEGDGLVDCLDPDCCLQSACQNSLLCRGSRDP
LDIIQQGQTDWPAVKSFYDRIKLLAGKDSTHIIPGDNPFNSSLVSLIRGQVVTMDGTPLVGVNVSFVKYPKYG
YTITRQDGTFDLIANGGSALTLHFERAPFMSQERTVWLPWNSFYAMDTLVMKTEENSIPSCDLSGFVRPDPII
ISSPLSTFFSASPASNPIVPETQVLHEEIELPGTNVKLRYLSSRTAGYKSLLKITMTQSTVPLNLIRVHLMVA
VEGHLFQKSFQASPNLAYTFIWDKTDAYGQRVYGLSDAVVSVGFEYETCPSLILWEKRTALLQGFELDPSNLG
GWSLDKHHTLNVKSGILHKGTGENQFLTQQPAIITSIMGNGRRRSISCPSCNGLAEGNKLLAPVALAVGIDGS
LFVGDFNYIRRIFPSRNVTSILELRNKEFKHSNSPGHKYYLAVDPVTGSLYVSDTNSRRIYRVKSLSGAKDLA
GNSEVVAGTGEQCLPFDEARCGDGGKAVDATLMSPRGIAVDKNGLMYFVDATMIRKVDQNGIISTLLGSNDLT
AVRPLSCDSSMDVAQVRLEWPTDLAVNPMDNSLYVLENNVILRITENHQVSIIAGRPMHCQVPGIDYSLSKLA
IHSALESASAIAISHTGVLYITETDEKKINRLRQVTTNGEICLLAGAASDCDCKNDVNCICYSGDDAYATDAI
LNSPSSLAVAPDGTIYIADLGNIRIRAVSKNKPVLNAFNQYEAASPGEQELYVFNADGIHQYTVSLVTGEYLY
NFTYSADNDVTELIDNNGNSLKIRRDSSGMPRHLLMPDNQIITLTVGTNGGLKAVSTQNLELGLMTYDGNTGL
LATKSDETGWTTFYDYDHEGRLTNVTRPTGVVTSLHREMEKSITIDIENSNRDDDVTVITNLSSVEASYTVVQ
DQVRNSYQLCNNGTLRVMYANGMAVSFHSEPHVLAGTITPTIGRCNISLPMENGLNSIEWRLRKEQIKGKVTI
FGRKLRVHGRNLLSIDYDRNIRTEKIYDDHRKFTLRIIYDQVGRPFLWLPSSGLAAVNVSYFFNGRLAGLQRG
AMSERTDIDKQGRIVSRMFADGKVWSYSYLDKSMVLLLQSQRQYIFEYDSSDRLHAVTMPSVARHSMSTHTSI
GYIRNIYNPPESNASVIFDYSDDGRILKTSFLGTGRQVFYKYGKLSKLSEIVYDSTAVTFGYDETTGVLKMVN
LQSGGFSCTIRYRKVGPLVDKQIYRFSEEGMINARFDYTYHDNSFRIASIKPVISETPLPVDLYRYDEISGKV
EHFGKFGVIYYDINQIITTAVMTLSKHFDTHGRIKEVQYEMFRSLMYWMTVQYDSMGRVIKRELKLGPYANTT
KYTYDYDGDGQLQSVAVNDRPTWRYSYDLNGNLHLLNPGNSARLMPLRYDLRDRITRLGDVQYKIDDDGYLCQ
RGSDIFEYNSKGLLTRAYNKASGWSVQYRYDGVGRRASYKTNLGHHLQYFYSDLHNPTRITHVYNHSNSEITS
LYYDLQGHLFAMESSSGEEYYVASDNTGTPLAVFSINGLMIKQLQYTAYGEIYYDSNPDFQMVIGFHGGLYDP
LTKLVHFTQRDYDVLAGRWTSPDYTMWRNVGKEPAPFNLYMFKNNNPLSNELDLKNYVTDVKSWLVMFGFQLS
NIIPGFPRAKMYFVPPPYELSESQASENGQLITGVQQTTERHNQAFLALEGQVITKKLHASIREKAGHWFATT
TPIIGKGIMFAIKEGRVTTGVSSIASEDSRKVASVLNNAYYLDKMHYSIEGKDTHYFVKIGAADGDLVTLGTT
IGRKVLESGVNVTVSQPTLLVNGRTRRFTNIEFQYSTLLLSIRYGLTPDTLDEEKARVLDQARQRALGTAWAK
EQQKARDGREGSRLWTEGEKQQLLSTGRVQGYEGYYVLPVEQYPELADSSSNIQFLRQNEMGKR*
>rs46909618_ENSMUSP00000129951_c.416:G>T_p.R139S
ENSMUST00000163524.8|Tenm2-208|ENSMUSG00000049336|-|chr11:g.36755580:C>A
MDVKDRRHRSLTRGRCGKECRYTSSSLDSEDCRVPTQKSYSSSETLKAYDHDSRMHYGNRVTDLVHRESDEFS
RQGTNFTLAELGICEPSPHRSGYCSDMGILHQGYSLSTGSDADSDTEGGMSPEHAIRLWGRGIKSSRSSGLSS
RENSALTLTDSDNENKSDDDNGRPIPPTSSSSLLPSAQLPSSHNPPPVSCQMPLLDSNTSHQIMDTNPDEEFS
PNSYLLRACSGPQQASSSGPPNHHSQSTLRPPLPPPHNHTLSHHHSSANSLNRNSLTNRRSQIHAPAPAPNDL
ATTPESVQLQDSWVLNSNVPLETRHFLFKTSSGSTPLFSSSSPGYPLTSGTVYTPPPRLLPRNTFSRKAFKLK
KPSKYCSWKCAALSAIAAALLLAILLAYFIAMHLLGLNWQLQPADGHTFNNGVRTGLPGNDDVATVPSGGKVP
WSLKNSSIDSGEAEVGRRVTQEVPPGVFWRSQIHISQPQFLKFNISLGKDALFGVYIRRGLPPSHAQYDFMER
LDGKEKWSVVESPRERRSIQTLVQNEAVFVQYLDVGLWHLAFYNDGKDKEMVSFNTVVLDSVQDCPRNCHGNG
ECVSGLCHCFPGFLGADCAKAACPVLCSGNGQYSKGTCQCYSGWKGAECDVPMNQCIDPSCGGHGSCIDGNCV
CAAGYKGEHCEEVDCLDPTCSSHGVCVNGECLCSPGWGGLNCELARVQCPDQCSGHGTYLPDSGLCSCDPNWM

GPDCSVVCSVDCGTHGVCIGGACRCEEGWTGAACDQRVCHPRCIEHGTCKDGKCECREGWNGEHCTIDGCPDL
CNGNGRCTLGQNSWQCVCQTGWRGPGCNVAMETSCADNKDNEGDGLVDCLDPDCCLQSACQNSLLCRGSRDPL
DIIQQGQTDWPAVKSFYDRIKLLAGKDSTHIIPGDNPFNSSLVSLIRGQVVTMDGTPLVGVNVSFVKYPKYGY
TITRQDGTFDLIANGGSALTLHFERAPFMSQERTVWLPWNSFYAMDTLVMKTEENSIPSCDLSGFVRPDPIII
SSPLSTFFSASPASNPIVPETQVLHEEIELPGTNVKLRYLSSRTAGYKSLLKITMTQSTVPLNLIRVHLMVAV
EGHLFQKSFQASPNLAYTFIWDKTDAYGQRVYGLSDAVVSVGFEYETCPSLILWEKRTALLQGFELDPSNLGG
WSLDKHHTLNVKSGILHKGTGENQFLTQQPAIITSIMGNGRRRSISCPSCNGLAEGNKLLAPVALAVGIDGSL
FVGDFNYIRRIFPSRNVTSILELRNKEFKHSNSPGHKYYLAVDPVTGSLYVSDTNSRRIYRVKSLSGAKDLAG
NSEVVAGTGEQCLPFDEARCGDGGKAVDATLMSPRGIAVDKNGLMYFVDATMIRKVDQNGIISTLLGSNDLTA
VRPLSCDSSMDVAQVRLEWPTDLAVNPMDNSLYVLENNVILRITENHQVSIIAGRPMHCQVPGIDYSLSKLAI
HSALESASAIAISHTGVLYITETDEKKINRLRQVTTNGEICLLAGAASDCDCKNDVNCICYSGDDAYATDAIL
NSPSSLAVAPDGTIYIADLGNIRIRAVSKNKPVLNAFNQYEAASPGEQELYVFNADGIHQYTVSLVTGEYLYN
FTYSADNDVTELIDNNGNSLKIRRDSSGMPRHLLMPDNQIITLTVGTNGGLKAVSTQNLELGLMTYDGNTGLL
ATKSDETGWTTFYDYDHEGRLTNVTRPTGVVTSLHREMEKSITIDIENSNRDDDVTVITNLSSVEASYTVVQD
QVRNSYQLCNNGTLRVMYANGMAVSFHSEPHVLAGTITPTIGRCNISLPMENGLNSIEWRLRKEQIKGKVTIF
GRKLRVHGRNLLSIDYDRNIRTEKIYDDHRKFTLRIIYDQVGRPFLWLPSSGLAAVNVSYFFNGRLAGLQRGA
MSERTDIDKQGRIVSRMFADGKVWSYSYLDKSMVLLLQSQRQYIFEYDSSDRLHAVTMPSVARHSMSTHTSIG
YIRNIYNPPESNASVIFDYSDDGRILKTSFLGTGRQVFYKYGKLSKLSEIVYDSTAVTFGYDETTGVLKMVNL
QSGGFSCTIRYRKVGPLVDKQIYRFSEEGMINARFDYTYHDNSFRIASIKPVISETPLPVDLYRYDEISGKVE
HFGKFGVIYYDINQIITTAVMTLSKHFDTHGRIKEVQYEMFRSLMYWMTVQYDSMGRVIKRELKLGPYANTTK
YTYDYDGDGQLQSVAVNDRPTWRYSYDLNGNLHLLNPGNSARLMPLRYDLRDRITRLGDVQYKIDDDGYLCQR
GSDIFEYNSKGLLTRAYNKASGWSVQYRYDGVGRRASYKTNLGHHLQYFYSDLHNPTRITHVYNHSNSEITSL
YYDLQGHLFAMESSSGEEYYVASDNTGTPLAVFSINGLMIKQLQYTAYGEIYYDSNPDFQMVIGFHGGLYDPL
TKLVHFTQRDYDVLAGRWTSPDYTMWRNVGKEPAPFNLYMFKNNNPLSNELDLKNYVTDVKSWLVMFGFQLSN
IIPGFPRAKMYFVPPPYELSESQASENGQLITGVQQTTERHNQAFLALEGQVITKKLHASIREKAGHWFATTT
PIIGKGIMFAIKEGRVTTGVSSIASEDSRKVASVLNNAYYLDKMHYSIEGKDTHYFVKIGAADGDLVTLGTTI
GRKVLESGVNVTVSQPTLLVNGRTRRFTNIEFQYSTLLLSIRYGLTPDTLDEEKARVLDQARQRALGTAWAKE
QQKARDGREGSRLWTEGEKQQLLSTGRVQGYEGYYVLPVEQYPELADSSSNIQFLRQNEMGKR*
>rs29434588_ENSMUSP00000146133_c.598:A>T_p.E200V
ENSMUST00000205546.2|Gabra1-205|ENSMUSG00000010803|-|chr11:g.42038018:T>A
MKKSRGLSDYLWAWTLILSTLSGRSYGQPSQDELKDNTTVFTRILDRLLDGYDNRLRPGLGERVTEVKTDIFV
TSFGPVSDHDMEYTIDVFFRQSWKDERLKFKGPMTVLRLNNLMASKIWTPDTFFHNGKKSVAHNMTMPNKLLR
ITEDGTLLYTMRLTVRAECPMHLEDFPMDAHACPLKFGSYAYTRAEVVYEWTRVPARSVVVAEDGSRLNQYDL
LGQTVDSGIVQSSTGEYVVMTTHFHLKRKIGYFVIQTYLPCIMTVILSQVSFWLNRESVPARTVFGVTTVLTM
TTLSISARNSLPKVAYATAMDWFIAVCYAFVFSALIEFATVNYFTKRGYAWDGKSVVPEKPKKVKDPLIKKNN
TYAPTATSYTPNLARGDPGLATIAKSATIEPKEVKPETKPPEPKKTFNSVSKIDRLSRIAFPLLFGIFNLVYW
ATYLNREPQLKAPTPHQ*
>rs29434588_ENSMUSP00000020707_c.598:A>T_p.E200V
ENSMUST00000020707.12|Gabra1-201|ENSMUSG00000010803|-
|chr11:g.42038018:T>A
MKKSRGLSDYLWAWTLILSTLSGRSYGQPSQDELKDNTTVFTRILDRLLDGYDNRLRPGLGERVTEVKTDIFV
TSFGPVSDHDMEYTIDVFFRQSWKDERLKFKGPMTVLRLNNLMASKIWTPDTFFHNGKKSVAHNMTMPNKLLR
ITEDGTLLYTMRLTVRAECPMHLEDFPMDAHACPLKFGSYAYTRAEVVYEWTRVPARSVVVAEDGSRLNQYDL
LGQTVDSGIVQSSTGEYVVMTTHFHLKRKIGYFVIQTYLPCIMTVILSQVSFWLNRESVPARTVFGVTTVLTM
TTLSISARNSLPKVAYATAMDWFIAVCYAFVFSALIEFATVNYFTKRGYAWDGKSVVPEKPKKVKDPLIKKNN
TYAPTATSYTPNLARGDPGLATIAKSATIEPKEVKPETKPPEPKKTFNSVSKIDRLSRIAFPLLFGIFNLVYW
ATYLNREPQLKAPTPHQ*
>rs29434588_ENSMUSP00000145949_c.598:A>T_p.E200V
ENSMUST00000206105.2|Gabra1-207|ENSMUSG00000010803|-|chr11:g.42038018:T>A
MKKSRGLSDYLWAWTLILSTLSGRSYGQPSQDELKDNTTVFTRILDRLLDGYDNRLRPGLGERVTEVKTDIFV
TSFGPVSDHDMEYTIDVFFRQSWKDERLKFKGPMTVLRLNNLMASKIWTPDTFFHNGKKSVAHNMTMPNKLLR
ITEDGTLLYTMRLTVRAECPMHLEDFPMDAHACPLKFGSYAYTRAEVVYEWTRVPARSVVVAEDGSRLNQYDL
LGQTVDSGIVQSSTGEYVVMTTHFHLKRKIGYFVIQTYLPCIMTVILSQVSFWLNRESVPARTVFGVTTVLTM
TTLSISARNS
>rs29466007_ENSMUSP00000146133_c.556:A>G_p.Y186C
ENSMUST00000205546.2|Gabra1-205|ENSMUSG00000010803|-|chr11:g.42038060:T>C
MKKSRGLSDYLWAWTLILSTLSGRSYGQPSQDELKDNTTVFTRILDRLLDGYDNRLRPGLGERVTEVKTDIFV
TSFGPVSDHDMEYTIDVFFRQSWKDERLKFKGPMTVLRLNNLMASKIWTPDTFFHNGKKSVAHNMTMPNKLLR

ITEDGTLLYTMRLTVRAECPMHLEDFPMDAHACPLKFGSCAYTRAEVVYEWTREPARSVVVAEDGSRLNQYDL
LGQTVDSGIVQSSTGEYVVMTTHFHLKRKIGYFVIQTYLPCIMTVILSQVSFWLNRESVPARTVFGVTTVLTM
TTLSISARNSLPKVAYATAMDWFIAVCYAFVFSALIEFATVNYFTKRGYAWDGKSVVPEKPKKVKDPLIKKNN
TYAPTATSYTPNLARGDPGLATIAKSATIEPKEVKPETKPPEPKKTFNSVSKIDRLSRIAFPLLFGIFNLVYW
ATYLNREPQLKAPTPHQ*
>rs29466007_ENSMUSP00000020707_c.556:A>G_p.Y186C
ENSMUST00000020707.12|Gabra1-201|ENSMUSG00000010803|-
|chr11:g.42038060:T>C
MKKSRGLSDYLWAWTLILSTLSGRSYGQPSQDELKDNTTVFTRILDRLLDGYDNRLRPGLGERVTEVKTDIFV
TSFGPVSDHDMEYTIDVFFRQSWKDERLKFKGPMTVLRLNNLMASKIWTPDTFFHNGKKSVAHNMTMPNKLLR
ITEDGTLLYTMRLTVRAECPMHLEDFPMDAHACPLKFGSCAYTRAEVVYEWTREPARSVVVAEDGSRLNQYDL
LGQTVDSGIVQSSTGEYVVMTTHFHLKRKIGYFVIQTYLPCIMTVILSQVSFWLNRESVPARTVFGVTTVLTM
TTLSISARNSLPKVAYATAMDWFIAVCYAFVFSALIEFATVNYFTKRGYAWDGKSVVPEKPKKVKDPLIKKNN
TYAPTATSYTPNLARGDPGLATIAKSATIEPKEVKPETKPPEPKKTFNSVSKIDRLSRIAFPLLFGIFNLVYW
ATYLNREPQLKAPTPHQ*
>rs29466007_ENSMUSP00000145949_c.556:A>G_p.Y186C
ENSMUST00000206105.2|Gabra1-207|ENSMUSG00000010803|-|chr11:g.42038060:T>C
MKKSRGLSDYLWAWTLILSTLSGRSYGQPSQDELKDNTTVFTRILDRLLDGYDNRLRPGLGERVTEVKTDIFV
TSFGPVSDHDMEYTIDVFFRQSWKDERLKFKGPMTVLRLNNLMASKIWTPDTFFHNGKKSVAHNMTMPNKLLR
ITEDGTLLYTMRLTVRAECPMHLEDFPMDAHACPLKFGSCAYTRAEVVYEWTREPARSVVVAEDGSRLNQYDL
LGQTVDSGIVQSSTGEYVVMTTHFHLKRKIGYFVIQTYLPCIMTVILSQVSFWLNRESVPARTVFGVTTVLTM
TTLSISARNS
>rs264326470_ENSMUSP00000146133_c.537:C>T_p.P180S
ENSMUST00000205546.2|Gabra1-205|ENSMUSG00000010803|-|chr11:g.42044179:G>A
MKKSRGLSDYLWAWTLILSTLSGRSYGQPSQDELKDNTTVFTRILDRLLDGYDNRLRPGLGERVTEVKTDIFV
TSFGPVSDHDMEYTIDVFFRQSWKDERLKFKGPMTVLRLNNLMASKIWTPDTFFHNGKKSVAHNMTMPNKLLR
ITEDGTLLYTMRLTVRAECPMHLEDFPMDAHACSLKFGSYAYTRAEVVYEWTREPARSVVVAEDGSRLNQYDL
LGQTVDSGIVQSSTGEYVVMTTHFHLKRKIGYFVIQTYLPCIMTVILSQVSFWLNRESVPARTVFGVTTVLTM
TTLSISARNSLPKVAYATAMDWFIAVCYAFVFSALIEFATVNYFTKRGYAWDGKSVVPEKPKKVKDPLIKKNN
TYAPTATSYTPNLARGDPGLATIAKSATIEPKEVKPETKPPEPKKTFNSVSKIDRLSRIAFPLLFGIFNLVYW
ATYLNREPQLKAPTPHQ*
>rs264326470_ENSMUSP00000020707_c.537:C>T_p.P180S
ENSMUST00000020707.12|Gabra1-201|ENSMUSG00000010803|-
|chr11:g.42044179:G>A
MKKSRGLSDYLWAWTLILSTLSGRSYGQPSQDELKDNTTVFTRILDRLLDGYDNRLRPGLGERVTEVKTDIFV
TSFGPVSDHDMEYTIDVFFRQSWKDERLKFKGPMTVLRLNNLMASKIWTPDTFFHNGKKSVAHNMTMPNKLLR
ITEDGTLLYTMRLTVRAECPMHLEDFPMDAHACSLKFGSYAYTRAEVVYEWTREPARSVVVAEDGSRLNQYDL
LGQTVDSGIVQSSTGEYVVMTTHFHLKRKIGYFVIQTYLPCIMTVILSQVSFWLNRESVPARTVFGVTTVLTM
TTLSISARNSLPKVAYATAMDWFIAVCYAFVFSALIEFATVNYFTKRGYAWDGKSVVPEKPKKVKDPLIKKNN
TYAPTATSYTPNLARGDPGLATIAKSATIEPKEVKPETKPPEPKKTFNSVSKIDRLSRIAFPLLFGIFNLVYW
ATYLNREPQLKAPTPHQ*
>rs264326470_ENSMUSP00000145949_c.537:C>T_p.P180S
ENSMUST00000206105.2|Gabra1-207|ENSMUSG00000010803|-|chr11:g.42044179:G>A
MKKSRGLSDYLWAWTLILSTLSGRSYGQPSQDELKDNTTVFTRILDRLLDGYDNRLRPGLGERVTEVKTDIFV
TSFGPVSDHDMEYTIDVFFRQSWKDERLKFKGPMTVLRLNNLMASKIWTPDTFFHNGKKSVAHNMTMPNKLLR
ITEDGTLLYTMRLTVRAECPMHLEDFPMDAHACSLKFGSYAYTRAEVVYEWTREPARSVVVAEDGSRLNQYDL
LGQTVDSGIVQSSTGEYVVMTTHFHLKRKIGYFVIQTYLPCIMTVILSQVSFWLNRESVPARTVFGVTTVLTM
TTLSISARNS
>rs47442249_ENSMUSP00000158770_c.378:T>C_p.Y127H
ENSMUST00000238415.2|Atp10b-203|ENSMUSG00000055415|+|chr11:g.43047100:T>C
MRKEGRKRRKKKEDKKRVVVSNLPFEGWPYKENPNRHYRGNQVKTSKYTVLSFIPKNLFEQLHRFANLYFVGI
VILNFIPVVNAFQPGVSMVPVCAILTVTAIKDAWEDFRRYKSDKVINNRESLVHSRKEQRYMLKRWQDVRVGD
FVQMQCNEIVPADILLLFSSDPSGVCHLETANLDGETNLKQRRVVKGFSQPEVQFQPEHFHSTIVCEKPNNHL
SKFKGYMEHPDQTRTGFGSESLLLRGCTIRNTEVAAGIVIYAGHETKAMLNNSGPRYKRSKIERRINTDIFFC
IGLLFLMCLIGAVGHSLWNGTFKEHPPFDVPDADGNFLSLALGGFYMFLTMIILLQVLIPISLYVSIELVKLG
QVFLLHNDLDLYDEETDLSIQCRALNITEDLGQIQYIFSDKTGTLTENKMVFRRCTIVGSEYCHQENAKRLEM
PKELDSDGEEWTQYQCLSFPPRWAQGSTTMRSQGGAQPLRRCHSARVPIQSHCRQRSVGRWETSQPPVAFSSS
IEKDVTPDKNLLSKVRDAALWLETSDTRPAKPSHSTTASIADFFLALTICNSVMVSTTTEPRKRVTTPPANKA

LGTSLEKIQQLFQRLKLLSLSQSFSSTAPSDTDLGESLGPNLPTIDSDEKDDTSVCSGDCSTDGGYRSSTWEQ
GDILGSESGTSLEEGLEAPTLSQDEPELCYEAESPDEAALVHAARAYSFTLVSRTPEQVTVRLPQGICLTFDL
LFTLGFDSVRKRMSVVVRHPLTDEIIVYTKGADSVIMDLLEDPACESNIDVEKKLKRIRARTQKHLDLYARDG
LRTLCIAKKVVDEEDFQRWASFRREAEASLDNREELLMETAQHLENHLTLLGATGIEDRLQEGVPDTIAALRE
AGIQLWVLTGDKQETAVNIAYSCKLLDQTDTVYSINTENQETCESILNCTLEDIKRFHEPQQPARKLCGHRIP
PKMPSVNSGAMAPEIGLVIDGKTLNAIFQGKLENKFLELTQYCRSVLCCRSTPLQKSMIVKLVRDKLSVMTLS
IGDGANDVSMIQAADIGIGISGQEGMQAVMSSDFAIARFSHLKKLLLVHGHWCYSRLARMVVYYFYKNVCYVN
LLFWYQFFCGFSGSTMIDYWQMIFFNLFFTSLPPIIFGVLDKDVSAETLLALPELYKSGQNSECYNLPTFWVS
MADAFYQSLICFFIPYLTYRGSDIDVFTFGTPINTISLTTILLHQAMEMKTWTVLHGLVLLGSFLMYFVVSLI
YNATCVTCNSPTNPYWVMERQLSDPTFYLICLLTPVVALLPRYFLLSLQGTYGKSLISKAQKIDKLPIDKRNL
EIQNWRSKQRPAPASASASASAPATGTVHTRPPCHPVPPEAQQNFGASTSKSSGPPRQKHVEDRVLQDPRCSR
EHSRDDTCTVDTLAKLSSGECLLDPNRTVAPTAYSRGQKDVSRHSSKGSHRRSQSSLTI*
>rs250039360_ENSMUSP00000076844_c.4078:C>CTTAAT_p.A1360ins-fs
ENSMUST00000077659.6|Atp10b-
201|ENSMUSG00000055415|+|chr11:g.43150382:C>CTTAAT
MTCSLDFSWLRWKWRTQDGFSQSPSETTPLLSPETDRQSHNTAEQRVVYPNNSMCHQDWKKVCRRYPGNSICT
TKYTLLTFLPQNLFEQFHRWANLYFLFLVILNWMPSMEVFHREITIFPLATVLLIIMVKDGIEDFKRYCFDRE
MNSASIQIYERKEQRYMLKRWQDVRVGDFVQMQCNEIVPADILLLFSSDPSGVCHLETANLDGETNLKQRRVV
KGFSQPEVQFQPEHFHSTIVCEKPNNHLSKFKGYMEHPDQTRTGFGSESLLLRGCTIRNTEVAAGIVIYAGHE
TKAMLNNSGPRYKRSKIERRINTDIFFCIGLLFLMCLIGAVGHSLWNGTFKEHPPFDVPDADGNFLSLALGGF
YMFLTMIILLQVLIPISLYVSIELVKLGQVFLLHNDLDLYDEETDLSIQCRALNITEDLGQIQYIFSDKTGTL
TENKMVFRRCTIVGSEYCHQENAKRLEMPKELDSDGEEWTQYQCLSFPPRWAQGSTTMRSQGGAQPLRRCHSA
RVPIQSHCRQRSVGRWETSQPPVAFSSSIEKDVTPDKNLLSKVRDAALWLETSDTRPAKPSHSTTASIADFFL
ALTICNSVMVSTTTEPRKRVTTPPANKALGTSLEKIQQLFQRLKLLSLSQSFSSTAPSDTDLGESLGPNLPTI
DSDEKDDTSVCSGDCSTDGGYRSSTWEQGDILGSESGTSLEEGLEAPTLSQDEPELCYEAESPDEAALVHAAR
AYSFTLVSRTPEQVTVRLPQGICLTFDLLFTLGFDSVRKRMSVVVRHPLTDEIIVYTKGADSVIMDLLEDPAC
ESNIDVEKKLKRIRARTQKHLDLYARDGLRTLCIAKKVVDEEDFQRWASFRREAEASLDNREELLMETAQHLE
NHLTLLGATGIEDRLQEGVPDTIAALREAGIQLWVLTGDKQETAVNIAYSCKLLDQTDTVYSINTENQETCES
ILNCTLEDIKRFHEPQQPARKLCGHRIPPKMPSVNSGAMAPEIGLVIDGKTLNAIFQGKLENKFLELTQYCRS
VLCCRSTPLQKSMIVKLVRDKLSVMTLSIGDGANDVSMIQAADIGIGISGQEGMQAVMSSDFAIARFSHLKKL
LLVHGHWCYSRLARMVVYYFYKNVCYVNLLFWYQFFCGFSGSTMIDYWQMIFFNLFFTSLPPIIFGVLDKDVS
AETLLALPELYKSGQNSECYNLPTFWVSMADAFYQSLICFFIPYLTYRGSDIDVFTFGTPINTISLTTILLHQ
AMEMKTWTVLHGLVLLGSFLMYFVVSLIYNATCVTCNSPTNPYWVMERQLSDPTFYLICLLTPVVALLPRYFL
LSLQGTYGKSLISKAQKIDKLPIDKRNLEIQNWRSKQRPAPASASA
>rs250039360_ENSMUSP00000158770_c.3994:C>CTTAAT_p.A1332ins-fs
ENSMUST00000238415.2|Atp10b-
203|ENSMUSG00000055415|+|chr11:g.43150382:C>CTTAAT
MRKEGRKRRKKKEDKKRVVVSNLPFEGWPYKENPNRHYRGNQVKTSKYTVLSFIPKNLFEQLHRFANLYFVGI
VILNFIPVVNAFQPGVSMVPVCAILTVTAIKDAWEDFRRYKSDKVINNRESLVYSRKEQRYMLKRWQDVRVGD
FVQMQCNEIVPADILLLFSSDPSGVCHLETANLDGETNLKQRRVVKGFSQPEVQFQPEHFHSTIVCEKPNNHL
SKFKGYMEHPDQTRTGFGSESLLLRGCTIRNTEVAAGIVIYAGHETKAMLNNSGPRYKRSKIERRINTDIFFC
IGLLFLMCLIGAVGHSLWNGTFKEHPPFDVPDADGNFLSLALGGFYMFLTMIILLQVLIPISLYVSIELVKLG
QVFLLHNDLDLYDEETDLSIQCRALNITEDLGQIQYIFSDKTGTLTENKMVFRRCTIVGSEYCHQENAKRLEM
PKELDSDGEEWTQYQCLSFPPRWAQGSTTMRSQGGAQPLRRCHSARVPIQSHCRQRSVGRWETSQPPVAFSSS
IEKDVTPDKNLLSKVRDAALWLETSDTRPAKPSHSTTASIADFFLALTICNSVMVSTTTEPRKRVTTPPANKA
LGTSLEKIQQLFQRLKLLSLSQSFSSTAPSDTDLGESLGPNLPTIDSDEKDDTSVCSGDCSTDGGYRSSTWEQ
GDILGSESGTSLEEGLEAPTLSQDEPELCYEAESPDEAALVHAARAYSFTLVSRTPEQVTVRLPQGICLTFDL
LFTLGFDSVRKRMSVVVRHPLTDEIIVYTKGADSVIMDLLEDPACESNIDVEKKLKRIRARTQKHLDLYARDG
LRTLCIAKKVVDEEDFQRWASFRREAEASLDNREELLMETAQHLENHLTLLGATGIEDRLQEGVPDTIAALRE
AGIQLWVLTGDKQETAVNIAYSCKLLDQTDTVYSINTENQETCESILNCTLEDIKRFHEPQQPARKLCGHRIP
PKMPSVNSGAMAPEIGLVIDGKTLNAIFQGKLENKFLELTQYCRSVLCCRSTPLQKSMIVKLVRDKLSVMTLS
IGDGANDVSMIQAADIGIGISGQEGMQAVMSSDFAIARFSHLKKLLLVHGHWCYSRLARMVVYYFYKNVCYVN
LLFWYQFFCGFSGSTMIDYWQMIFFNLFFTSLPPIIFGVLDKDVSAETLLALPELYKSGQNSECYNLPTFWVS
MADAFYQSLICFFIPYLTYRGSDIDVFTFGTPINTISLTTILLHQAMEMKTWTVLHGLVLLGSFLMYFVVSLI
YNATCVTCNSPTNPYWVMERQLSDPTFYLICLLTPVVALLPRYFLLSLQGTYGKSLISKAQKIDKLPIDKRNL
EIQNWRSKQRPAPASASA
>rs49165270_ENSMUSP00000020672_c.376:G>A_p.R126K
ENSMUST00000020672.5|Fabp6-201|ENSMUSG00000020405|-|chr11:g.43486949:C>T

MAFSGKYEFESEKNYDEFMKRLGLPGDVIERGRNFKIITEVQQDGQDFTWSQSYSGGNIMSNKFTIGKECEMQ
TMGGKKFKATVKMEGGKVVAEFPNYHQTSEVVGDKLVEISTIGDVTYERVSKKLA*
>ENSMUSP00000104901_c.609:A>G_p.K204E ENSMUST00000109278.8|Ttc1-202|ENSMUSG00000041278|-|chr11:g.43628774:T>C
MEEKSEDCKVPEDLFNGLKVADPQEGESASPMVSDPKGQHCQSKLPKAAEAHPQDDHVEEECFHDCSASFEEE
QPGAHVAGSKASDDSSSELDEEYLIELEKNMPEEEKQKRREESAKLKEEGNERFKRGDYMEAESSYSQALQMC
PACFQKDRSVLFSNRAAARMKQDKKETAITDCSKAIQLNPTYIRAILRRAELYEKTDELDEALEDYKSVLEKD
PSVHQAREACMRLPKQIEERNERLKEEMLGK*
>ENSMUSP00000040779_c.609:A>G_p.K204E ENSMUST00000048578.3|Ttc1-201|ENSMUSG00000041278|-|chr11:g.43628774:T>C
MEEKSEDCKVPEDLFNGLKVADPQEGESASPMVSDPKGQHCQSKLPKAAEAHPQDDHVEEECFHDCSASFEEE
QPGAHVAGSKASDDSSSELDEEYLIELEKNMPEEEKQKRREESAKLKEEGNERFKRGDYMEAESSYSQALQMC
PACFQKDRSVLFSNRAAARMKQDKKETAITDCSKAIQLNPTYIRAILRRAELYEKTDELDEALEDYKSVLEKD
PSVHQAREACMRLPKQIEERNERLKEEMLGKLKDLGNLVLRPFGLSTENFQIKQDSSTGSYSINFVQNPNNNR
*
>rs264767124_ENSMUSP00000104901_c.366:G>TGAGCG_p.G123ins-fs ENSMUST00000109278.8|Ttc1-202|ENSMUSG00000041278|-|chr11:g.43632612:C>CGCTCA
MEEKSEDCKVPEDLFNGLKVADPQEGESASPMVSDPKGQHCQSKLPKAAEAHPQDDHVEEECFHDCSASFEEE
QPGAHVAGSKASDDSSSELDEEYLIELEKNMPEEEKQKRREESAKLKEE
>rs264767124_ENSMUSP00000040779_c.366:G>TGAGCG_p.G123ins-fs ENSMUST00000048578.3|Ttc1-201|ENSMUSG00000041278|-|chr11:g.43632612:C>CGCTCA
MEEKSEDCKVPEDLFNGLKVADPQEGESASPMVSDPKGQHCQSKLPKAAEAHPQDDHVEEECFHDCSASFEEE
QPGAHVAGSKASDDSSSELDEEYLIELEKNMPEEEKQKRREESAKLKEE
>rs51355143_ENSMUSP00000098881_c.447:T>G_p.S150A
ENSMUST00000101327.3|Rnf145-202|ENSMUSG00000019189|+|chr11:g.44439653:T>G
MAAKEKLEAVLNVALRVPSIMLLDVLYRWDVSSFFQQIQRSSLNNNPLFQYKYLALNMHYVGYILSVVLLTLP
RQHLVQLYLYFVTALLLYAGHQISRDYVRSELESAYEGPMYLEPLSMNRFTTALIGQLVVCTLCSCVMKTKQI
WLFAAHMLPLLARLCLVPLETIVIINKFAMIFTGLEVLYFLGSNLLVPYNLAKSAYRELVQVVEVYGLLALGM
SLWNQLVVPVLFMVFWLVLFALQIYSYFSTRDQPASRERLLFLFLTR*
>rs51355143_ENSMUSP00000019333_c.447:T>G_p.S150A
ENSMUST00000019333.10|Rnf145-201|ENSMUSG00000019189|+|chr11:g.44439653:T>G
MAAKEKLEAVLNVALRVPSIMLLDVLYRWDVSSFFQQIQRSSLNNNPLFQYKYLALNMHYVGYILSVVLLTLP
RQHLVQLYLYFVTALLLYAGHQISRDYVRSELESAYEGPMYLEPLSMNRFTTALIGQLVVCTLCSCVMKTKQI
WLFAAHMLPLLARLCLVPLETIVIINKFAMIFTGLEVLYFLGSNLLVPYNLAKSAYRELVQVVEVYGLLALGM
SLWNQLVVPVLFMVFWLVLFALQIYSYFSTRDQPASRERLLFLFLTSIAECCSTPYSLLGLVFTVSFVALGVL
TLCKFYLQGYRAFMNDPAMNRGMTEGVTLLILAVQTGLIELQVVHRAFLLSIILFIVVASILQSMLEIADPIV
LALGASRDKSLWKHFRAVSLCLFLLVFPAYMAYMICQFFHMDFWLLIIISSSILTSLQVLGTLFIYVLFMVEE
FRKEPVENMDDVIYYVNGTYRLLEFVVALCVVAYGVSETIFGEWTVMGSMIIFIHSYYNVWLRAQLGWKSFLL
RRDAVNKIKSLPVATQEQLEKHNDICAICYQDMKSAVITPCSHFFHAGCLKKWLYVQDTCPLCHCHLKNSSQL
PGLGTEAAPQPPAGAEQNIVLQEGPEPPDHESPPGTGTQEGSGDSSEHINRGSASQEGAADAGEGPQIPEGEV
CPVESA*
>rs47986688_ENSMUSP00000098881_c.469:T>A_p.L157Q
ENSMUST00000101327.3|Rnf145-202|ENSMUSG00000019189|+|chr11:g.44439675:T>A
MAAKEKLEAVLNVALRVPSIMLLDVLYRWDVSSFFQQIQRSSLNNNPLFQYKYLALNMHYVGYILSVVLLTLP
RQHLVQLYLYFVTALLLYAGHQISRDYVRSELESAYEGPMYLEPLSMNRFTTALIGQLVVCTLCSCVMKTKQI
WLFSAHMLPLQARLCLVPLETIVIINKFAMIFTGLEVLYFLGSNLLVPYNLAKSAYRELVQVVEVYGLLALGM
SLWNQLVVPVLFMVFWLVLFALQIYSYFSTRDQPASRERLLFLFLTR*
>rs47986688_ENSMUSP00000019333_c.469:T>A_p.L157Q
ENSMUST00000019333.10|Rnf145-201|ENSMUSG00000019189|+|chr11:g.44439675:T>A
MAAKEKLEAVLNVALRVPSIMLLDVLYRWDVSSFFQQIQRSSLNNNPLFQYKYLALNMHYVGYILSVVLLTLP
RQHLVQLYLYFVTALLLYAGHQISRDYVRSELESAYEGPMYLEPLSMNRFTTALIGQLVVCTLCSCVMKTKQI
WLFSAHMLPLQARLCLVPLETIVIINKFAMIFTGLEVLYFLGSNLLVPYNLAKSAYRELVQVVEVYGLLALGM
SLWNQLVVPVLFMVFWLVLFALQIYSYFSTRDQPASRERLLFLFLTSIAECCSTPYSLLGLVFTVSFVALGVL
TLCKFYLQGYRAFMNDPAMNRGMTEGVTLLILAVQTGLIELQVVHRAFLLSIILFIVVASILQSMLEIADPIV

LALGASRDKSLWKHFRAVSLCLFLLVFPAYMAYMICQFFHMDFWLLIIISSSILTSLQVLGTLFIYVLFMVEE
FRKEPVENMDDVIYYVNGTYRLLEFVVALCVVAYGVSETIFGEWTVMGSMIIFIHSYYNVWLRAQLGWKSFLL
RRDAVNKIKSLPVATQEQLEKHNDICAICYQDMKSAVITPCSHFFHAGCLKKWLYVQDTCPLCHCHLKNSSQL
PGLGTEAAPQPPAGAEQNIVLQEGPEPPDHESPPGTGTQEGSGDSSEHINRGSASQEGAADAGEGPQIPEGEV
CPVESA*
>rs249860048_ENSMUSP00000098881_c.557:C>CAAAAAAAA_p.F186ins-fs
ENSMUST00000101327.3|Rnf145-
202|ENSMUSG00000019189|+|chr11:g.44439763:C>CAAAAAAAA
MAAKEKLEAVLNVALRVPSIMLLDVLYRWDVSSFFQQIQRSSLNNNPLFQYKYLALNMHYVGYILSVVLLTLP
RQHLVQLYLYFVTALLLYAGHQISRDYVRSELESAYEGPMYLEPLSMNRFTTALIGQLVVCTLCSCVMKTKQI
WLFSAHMLPLLARLCLVPLETIVIINKFAMIFTGLEVLYFKKN
>rs249860048_ENSMUSP00000019333_c.557:C>CAAAAAAAA_p.F186ins-fs
ENSMUST00000019333.10|Rnf145-
201|ENSMUSG00000019189|+|chr11:g.44439763:C>CAAAAAAAA
MAAKEKLEAVLNVALRVPSIMLLDVLYRWDVSSFFQQIQRSSLNNNPLFQYKYLALNMHYVGYILSVVLLTLP
RQHLVQLYLYFVTALLLYAGHQISRDYVRSELESAYEGPMYLEPLSMNRFTTALIGQLVVCTLCSCVMKTKQI
WLFSAHMLPLLARLCLVPLETIVIINKFAMIFTGLEVLYFKKN
>rs52337589_ENSMUSP00000019333_c.1179:G>T_p.A394S
ENSMUST00000019333.10|Rnf145-
201|ENSMUSG00000019189|+|chr11:g.44450857:G>T
MAAKEKLEAVLNVALRVPSIMLLDVLYRWDVSSFFQQIQRSSLNNNPLFQYKYLALNMHYVGYILSVVLLTLP
RQHLVQLYLYFVTALLLYAGHQISRDYVRSELESAYEGPMYLEPLSMNRFTTALIGQLVVCTLCSCVMKTKQI
WLFSAHMLPLLARLCLVPLETIVIINKFAMIFTGLEVLYFLGSNLLVPYNLAKSAYRELVQVVEVYGLLALGM
SLWNQLVVPVLFMVFWLVLFALQIYSYFSTRDQPASRERLLFLFLTSIAECCSTPYSLLGLVFTVSFVALGVL
TLCKFYLQGYRAFMNDPAMNRGMTEGVTLLILAVQTGLIELQVVHRAFLLSIILFIVVASILQSMLEIADPIV
LALGASRDKSLWKHFRAVSLCLFLLVFPSYMAYMICQFFHMDFWLLIIISSSILTSLQVLGTLFIYVLFMVEE
FRKEPVENMDDVIYYVNGTYRLLEFVVALCVVAYGVSETIFGEWTVMGSMIIFIHSYYNVWLRAQLGWKSFLL
RRDAVNKIKSLPVATQEQLEKHNDICAICYQDMKSAVITPCSHFFHAGCLKKWLYVQDTCPLCHCHLKNSSQL
PGLGTEAAPQPPAGAEQNIVLQEGPEPPDHESPPGTGTQEGSGDSSEHINRGSASQEGAADAGEGPQIPEGEV
CPVESA*
>ENSMUSP00000019333_c.1367:CA>C_p.G456del-fs
ENSMUST00000019333.10|Rnf145-
201|ENSMUSG00000019189|+|chr11:g.44452390:CA>C
MAAKEKLEAVLNVALRVPSIMLLDVLYRWDVSSFFQQIQRSSLNNNPLFQYKYLALNMHYVGYILSVVLLTLP
RQHLVQLYLYFVTALLLYAGHQISRDYVRSELESAYEGPMYLEPLSMNRFTTALIGQLVVCTLCSCVMKTKQI
WLFSAHMLPLLARLCLVPLETIVIINKFAMIFTGLEVLYFLGSNLLVPYNLAKSAYRELVQVVEVYGLLALGM
SLWNQLVVPVLFMVFWLVLFALQIYSYFSTRDQPASRERLLFLFLTSIAECCSTPYSLLGLVFTVSFVALGVL
TLCKFYLQGYRAFMNDPAMNRGMTEGVTLLILAVQTGLIELQVVHRAFLLSIILFIVVASILQSMLEIADPIV
LALGASRDKSLWKHFRAVSLCLFLLVFPAYMAYMICQFFHMDFWLLIIISSSILTSLQVLGTLFIYVLFMVEE
FRKEPVENMDDVIYYVNGLIVCWSLLWPSVWWPTASQRPSLESGL
>rs250621053_ENSMUSP00000080020_c.215:G>GT_p.L72ins-fs
ENSMUST00000081265.12|Ebf1-201|ENSMUSG00000057098|+|chr11:g.44511322:G>GT
MFGIQESIQRSGSSMKEEPLGSGMNAVRTWMQGAGVLDANTAAQSGVGLARAHFEKQPPSNLRKSNFFHFVLC
PLRQTGPARGD
>rs250621053_ENSMUSP00000099857_c.215:G>GT_p.L72ins-fs
ENSMUST00000101326.10|Ebf1-202|ENSMUSG00000057098|+|chr11:g.44511322:G>GT
MFGIQESIQRSGSSMKEEPLGSGMNAVRTWMQGAGVLDANTAAQSGVGLARAHFEKQPPSNLRKSNFFHFVLC
PLRQTGPARGD
>rs250621053_ENSMUSP00000104891_c.215:G>GT_p.L72ins-fs
ENSMUST00000109268.2|Ebf1-203|ENSMUSG00000057098|+|chr11:g.44511322:G>GT
MFGIQESIQRSGSSMKEEPLGSGMNAVRTWMQGAGVLDANTAAQSGVGLARAHFEKQPPSNLRKSNFFHFVLC
PLRQTGPARGD
>ENSMUSP00000080020_c.219:C>T_p.L74F ENSMUST00000081265.12|Ebf1-
201|ENSMUSG00000057098|+|chr11:g.44511326:C>T
MFGIQESIQRSGSSMKEEPLGSGMNAVRTWMQGAGVLDANTAAQSGVGLARAHFEKQPPSNLRKSNFFHFVLA
FYDRQGQPVEIERTAFVGFVEKEKEANSEKTNNGIHYRLQLLYSNGIRTEQDFYVRLIDSMTKQAIVYEGQDK
NPEMCRVLLTHEIMCSRCCDKKSCGNRNETPSDPVIIDRFFLKFFLKCNQNCLKNAGNPRDMRRFQVVVSTTV
NVDGHVLAVSDNMFVHNNSKHGRRARRLDPSEGTPSYLEHATPCIKAISPSEGWTTGGATVIIIGDNFFDGLQ

VIFGTMLVWSELITPHAIRVQTPPRHIPGVVEVTLSYKSKQFCKGTPGRFIYTALNEPTIDYGFQRLQKVIPR
HPGDPERLPKEVILKRAADLVEALYGMPHNNQEIILKRAADIAEALYSVPRNHNQLPALANTSVHAGMMGVNS
FSGQLAVNVSEASQATNQGFTRNSSSVSPHGYVPSTTPQQTNYNSVTTSMNGYGSAAMSNLGGSPTFLNGSAA
NSPYAIVPSSPTMASSTSLPSNCSSSSGIFSFSPANMVSAVKQKSAFAPVVRPQTSPPPTCTSTNGNSLQAIS
GMIVPPM*
>ENSMUSP00000099857_c.219:C>T_p.L74F ENSMUST00000101326.10|Ebf1-202|ENSMUSG00000057098|+|chr11:g.44511326:C>T
MFGIQESIQRSGSSMKEEPLGSGMNAVRTWMQGAGVLDANTAAQSGVGLARAHFEKQPPSNLRKSNFFHFVLA
FYDRQGQPVEIERTAFVGFVEKEKEANSEKTNNGIHYRLQLLYSNGIRTEQDFYVRLIDSMTKQAIVYEGQDK
NPEMCRVLLTHEIMCSRCCDKKSCGNRNETPSDPVIIDRFFLKFFLKCNQNCLKNAGNPRDMRRFQVVVSTTV
NVDGHVLAVSDNMFVHNNSKHGRRARRLDPSEGTPSYLEHAATPCIKAISPSEGWTTGGATVIIIGDNFFDGL
QVIFGTMLVWSELITPHAIRVQTPPRHIPGVVEVTLSYKSKQFCKGTPGRFIYTALNEPTIDYGFQRLQKVIP
RHPGDPERLPKEVILKRAADLVEALYGMPHNNQEIILKRAADIAEALYSVPRNHNQLPALANTSVHAGMMGVN
SFSGQLAVNVSEASQATNQGFTRNSSSVSPHGYVPSTTPQQTNYNSVTTSMNGYGSAAMSNLGGSPTFLNGSA
ANSPYAIVPSSPTMASSTSLPSNCSSSSGIFSFSPANMVSAVKQKSAFAPVVRPQTSPPPTCTSTNGNSLQAI
SGMIVPPM*
>ENSMUSP00000104891_c.219:C>T_p.L74F ENSMUST00000109268.2|Ebf1-203|ENSMUSG00000057098|+|chr11:g.44511326:C>T
MFGIQESIQRSGSSMKEEPLGSGMNAVRTWMQGAGVLDANTAAQSGVGLARAHFEKQPPSNLRKSNFFHFVLA
FYDRQGQPVEIERTAFVGFVEKEKEANSEKTNNGIHYRLQLLYSNGIRTEQDFYVRLIDSMTKQAIVYEGQDK
NPEMCRVLLTHEIMCSRCCDKKSCGNRNETPSDPVIIDRFFLKFFLKCNQNCLKNAGNPRDMRRFQVVVSTTV
NVDGHVLAVSDNMFVHNNSKHGRRARRLDPSEAATPCIKAISPSEGWTTGGATVIIIGDNFFDGLQVIFGTML
VWSELITPHAIRVQTPPRHIPGVVEVTLSYKSKQFCKGTPGRFIYTALNEPTIDYGFQRLQKVIPRHPGDPER
LPKEVILKRAADLVEALYGMPHNNQEIILKRAADIAEALYSVPRNHNQLPALANTSVHAGMMGVNSFSGQLAV
NVSEASQATNQGFTRNSSSVSPHGYVPSTTPQQTNYNSVTTSMNGYGSAAMSNLGGSPTFLNGSAANSPYAIV
PSSPTMASSTSLPSNCSSSSGIFSFSPANMVSAVKQKSAFAPVVRPQTSPPPTCTSTNGNSLQAISGMIVPPM
*
>rs48661756_ENSMUSP00000080020_c.250:T>C_p.I84T ENSMUST00000081265.12|Ebf1-201|ENSMUSG00000057098|+|chr11:g.44511357:T>C
MFGIQESIQRSGSSMKEEPLGSGMNAVRTWMQGAGVLDANTAAQSGVGLARAHFEKQPPSNLRKSNFFHFVLA
LYDRQGQPVETERTAFVGFVEKEKEANSEKTNNGIHYRLQLLYSNGIRTEQDFYVRLIDSMTKQAIVYEGQDK
NPEMCRVLLTHEIMCSRCCDKKSCGNRNETPSDPVIIDRFFLKFFLKCNQNCLKNAGNPRDMRRFQVVVSTTV
NVDGHVLAVSDNMFVHNNSKHGRRARRLDPSEGTPSYLEHAATPCIKAISPSEGWTTGGATVIIIGDNFFDGLQ
VIFGTMLVWSELITPHAIRVQTPPRHIPGVVEVTLSYKSKQFCKGTPGRFIYTALNEPTIDYGFQRLQKVIPR
HPGDPERLPKEVILKRAADLVEALYGMPHNNQEIILKRAADIAEALYSVPRNHNQLPALANTSVHAGMMGVNS
FSGQLAVNVSEASQATNQGFTRNSSSVSPHGYVPSTTPQQTNYNSVTTSMNGYGSAAMSNLGGSPTFLNGSAA
NSPYAIVPSSPTMASSTSLPSNCSSSSGIFSFSPANMVSAVKQKSAFAPVVRPQTSPPPTCTSTNGNSLQAIS
GMIVPPM*
>rs48661756_ENSMUSP00000099857_c.250:T>C_p.I84T ENSMUST00000101326.10|Ebf1-202|ENSMUSG00000057098|+|chr11:g.44511357:T>C
MFGIQESIQRSGSSMKEEPLGSGMNAVRTWMQGAGVLDANTAAQSGVGLARAHFEKQPPSNLRKSNFFHFVLA
LYDRQGQPVETERTAFVGFVEKEKEANSEKTNNGIHYRLQLLYSNGIRTEQDFYVRLIDSMTKQAIVYEGQDK
NPEMCRVLLTHEIMCSRCCDKKSCGNRNETPSDPVIIDRFFLKFFLKCNQNCLKNAGNPRDMRRFQVVVSTTV
NVDGHVLAVSDNMFVHNNSKHGRRARRLDPSEGTPSYLEHAATPCIKAISPSEGWTTGGATVIIIGDNFFDGL
QVIFGTMLVWSELITPHAIRVQTPPRHIPGVVEVTLSYKSKQFCKGTPGRFIYTALNEPTIDYGFQRLQKVIP
RHPGDPERLPKEVILKRAADLVEALYGMPHNNQEIILKRAADIAEALYSVPRNHNQLPALANTSVHAGMMGVN
SFSGQLAVNVSEASQATNQGFTRNSSSVSPHGYVPSTTPQQTNYNSVTTSMNGYGSAAMSNLGGSPTFLNGSA
ANSPYAIVPSSPTMASSTSLPSNCSSSSGIFSFSPANMVSAVKQKSAFAPVVRPQTSPPPTCTSTNGNSLQAI
SGMIVPPM*
>rs48661756_ENSMUSP00000104891_c.250:T>C_p.I84T ENSMUST00000109268.2|Ebf1-203|ENSMUSG00000057098|+|chr11:g.44511357:T>C
MFGIQESIQRSGSSMKEEPLGSGMNAVRTWMQGAGVLDANTAAQSGVGLARAHFEKQPPSNLRKSNFFHFVLA
LYDRQGQPVETERTAFVGFVEKEKEANSEKTNNGIHYRLQLLYSNGIRTEQDFYVRLIDSMTKQAIVYEGQDK
NPEMCRVLLTHEIMCSRCCDKKSCGNRNETPSDPVIIDRFFLKFFLKCNQNCLKNAGNPRDMRRFQVVVSTTV
NVDGHVLAVSDNMFVHNNSKHGRRARRLDPSEAATPCIKAISPSEGWTTGGATVIIIGDNFFDGLQVIFGTML
VWSELITPHAIRVQTPPRHIPGVVEVTLSYKSKQFCKGTPGRFIYTALNEPTIDYGFQRLQKVIPRHPGDPER
LPKEVILKRAADLVEALYGMPHNNQEIILKRAADIAEALYSVPRNHNQLPALANTSVHAGMMGVNSFSGQLAV
NVSEASQATNQGFTRNSSSVSPHGYVPSTTPQQTNYNSVTTSMNGYGSAAMSNLGGSPTFLNGSAANSPYAIV

PSSPTMASSTSLPSNCSSSSGIFSFSPANMVSAVKQKSAFAPVVRPQTSPPPTCTSTNGNSLQAISGMIVPPM
*
>ENSMUSP00000104884_c.698:C>G_p.D233E ENSMUST00000109261.11|Clint1-202|ENSMUSG00000006169|+|chr11:g.45784701:C>G
MLNMWKVRELVDKATNVVMNYSEIESKVREATNDDPWGPSGQLMGEIAKATFMYEQFPELMNMLWSRMLKDNK
KNWRRVYKSLLLLAYLIRNGSERVVTSAREHIYDLRSLENYHFVDEHGKDQGINIRQKVKELVEFAQDDDRLR
EERKKAKKNKDKYVGVSSDSVGGFRYNERYDPEPKSKWDEEWDKNKSAFPFSDKLGELSDKIGSTIDDTISKF
RRKDREDSPERCSESDEEKKARRGRSPKGEFKDEEETVTTKHIHITQATETTTTRHKRTANPSKTIDLGAAAH
YTGDKASPDQNASTHTPQSSAKPSVPSSKSSGDLVDLFDGSSQSAGGSADLFGGFADFGSAAASGNFPSQATS
GNGDFGDWSAFNQAPSGPVASGGELFGSAPQSAVELISASQPALGPPPAASNSADLFDLMGSSQATMTSSQSM
NFSLMSTNTVGLGLPMSRSQPLQNVSAVLQKPNPLYHQNTDMVQKSASKTLPSTWSDPSVNISLDNLLPGMQP
SKPQQPSLNTMIQQQNMQQPLNVMTQSFGAVNLSSPSNMLPVRPQTNPLLGGPMPMNMPGVMTGTMGMAPLGN
SAGMSQGMVGMNMNMGMSASGMGLSGTMGMGMPSMAMPSGTVQPKQDAFANFANFSK*
>ENSMUSP00000104883_c.698:C>G_p.D233E ENSMUST00000109260.3|Clint1-201|ENSMUSG00000006169|+|chr11:g.45784701:C>G
MLNMWKVRELVDKATNVVMNYSEIESKVREATNDDPWGPSGQLMGEIAKATFMYEQFPELMNMLWSRMLKDNK
KNWRRVYKSLLLLAYLIRNGSERVVTSAREHIYDLRSLENYHFVDEHGKDQGINIRQKVKELVEFAQDDDRLR
EERKKAKKNKDKYVGVSSDSVGGFRYNERYDPEPKSKWDEEWDKNKSAFPFSDKLGELSDKIGSTIDDTISKF
RRKDREDSPERCSESDEEKKARRGRSPKGEFKDEEETVTTKHIHITQATETTTTRHKRTANPSKTIDLGAAAH
YTGDKASPDQNASTHTPQSSAKPSVPSSKSSGDLVDLFDGSSQSAGGSADLFGGFADFGSAAASGNFPSQATS
GNGDFGDWSAFNQAPSGPVASGGELFGSAPQSAVELISASQPALGPPPAASNSADLFDLMGSSQATMTSSQSM
NFSLMSTNTVGLGLPMSRSQNTDMVQKSASKTLPSTWSDPSVNISLDNLLPGMQPSKPQQPSLNTMIQQQNMQ
QPLNVMTQSFGAVNLSSPSNMLPVRPQTNPLLGGPMPMNMPGVMTGTMGMAPLGNSAGMSQGMVGMNMNMGMS
ASGMGLSGTMGMGMPSMAMPSGTVQPKQDAFANFANFSK*
>rs29395502_ENSMUSP00000037519_c.517:G>A_p.G173D
ENSMUST00000049038.4|Sox30-201|ENSMUSG00000040489|+|chr11:g.45871664:G>A
MERARPEPPPPPPPPPRQPPRPTPPRPLRPAPPAQPVEAATFRAAAAERSQSPSAQATAAMAAVASSCGEAAA
AGAQAAGTRRLLQVKPEQVLLLPPGGPGVPPAPDEGAAAAAAAAAAAASSAQARLLQLRPELLLLPPQSAADG
GPCRPELHPMQPRTLLVKAEKQELGADLDLSVGSRRTTEAGPRASRAAKLDGTGKALDGRRSDEKKAKLEAEE
APRDALKGGEGKSLLAIGEGVIKTEEPDRPRDDCRLGTEATSNGLVHSSKEAILAQPPSAFGPHQQDLRFPLT
LHTVPPGARIQFQGPPPSELIRLSKVPLTPVPIKMQSLLEPSVKIETKDVPLTVLPSDAGIPDTPFSKDRNGH
VKRPMNAFMVWARIHRPALAKANPAANNAEISVQLGLEWNKLSEEQKKPYYDEAQKIKEKHREEFPGWVYQPR
PGKRKRFPLSVSNVFSGTTQNIISTNPTTIYPYRSPTYSVVIPGLQNTITHPVGEAPPAIQLPTPAVQRPSPI
TLFQPSVSSTGPVAVPPPSLTPRPSLPPQRFSGPSQTDIHRLPSGSSRSVKRSTPGSLESTTRIPAGASTAHA
RFATSPIQPPKEYASVSTCPRSTPIPPATPIPHSHVYQPPPLGHPATLFGTPPRFSFHHPYFLPGPHYFPSST
CPYSRPPFGYGNFPSSMPECLGYYEDRYQKHEAIFSALNRDYPFRDYPDEHTHSEDSRSCESMDGPPYYSSHG
HGGEEYLNAMPTLDIGALENVFTAPASAPSGVQQVNVTDSDEEEEEKVLRNL*
>rs49996694_ENSMUSP00000011400_c.2665:C>T_p.S889F
ENSMUST00000011400.8|Adam19-201|ENSMUSG00000011256|+|chr11:g.46033855:C>T
MPGRAGVARFCLLALALQLHWPLAACEPGWTTRGSQEGSPPLQHELIIPQWRTSESPGRGKHPLRAELRVMAE
GRELILDLEKNEHLFAPAYTETCYTASGNPQTSTLKSEDHCFYHGTVRDVDESSVTLSTCRGIRGLIIVRSNL
SYIIEPVPNSDSQHRIYRSEHLTLPPGNCGFEHSGPTSKDWALQFTHQTKKQPRRMKREDLHSMKYVELYLVA
DYAEFQKNRHDQDATKRKLMEIANYVDKFYRSLNIRIALVGLEVWTHGDKCEVSENPYSTLWSFLSWRRKLLA
QKSHDNAQLITGRSFQGTTIGLAPLMAMCSVYQSGGVSMDHSENAIGVASTVAHEIGHNFGMSHDSAHCCSAS
AADGGCIMAAATGHPFPKVFSWCNRKELDRYLQTGGGMCLSNMPDTRTLYGGRRCGNGYLEDGEECDCGEEEE
CKNPCCNASNCTLKEGAECAHGSCCHQCKLVAPGTQCREQVRQCDLPEFCTGKSPHCPTNYYQMDGTPCEGGQ
AYCYNGMCLTYQEQCQQLWGPGARPALDLCFERVNAAGDTYGNCGKGLNGQYRKCSPRDAKCGKIQCQSTQAR
PLESNAVSIDTTITLNGRRIHCRGTHVYRGPEEEEGEGDMLDPGLVMTGTKCGHNHICFEGQCRNTSFFETEG
CGKKCNGHGVCNNNKNCHCFPGWSPPFCNTPGDGGSVDSGPLPPKSVGPVIAGVFSALFVLAVLVLLCHCYRQ
SHKLGKPSALPFKLRHQFSCPFRVSQSGGTGHANPTFKLQTPQGKRKVTNTPESLRKPSHPPPRPPPDYLRVE
SPPAPLPAHLNRAAGSSPEAGARIERKESARRPPPSRPMPPAPNCLLSQDFSRPRPPQKALPANPVPGQRTGP
RSGGTSLLQPPTFGPQPPRPPAVPVPKLPEYRSQRVGAIISSKI*
>rs49693271_ENSMUSP00000011400_c.2727:C>G_p.Q910E
ENSMUST00000011400.8|Adam19-201|ENSMUSG00000011256|+|chr11:g.46034637:C>G
MPGRAGVARFCLLALALQLHWPLAACEPGWTTRGSQEGSPPLQHELIIPQWRTSESPGRGKHPLRAELRVMAE
GRELILDLEKNEHLFAPAYTETCYTASGNPQTSTLKSEDHCFYHGTVRDVDESSVTLSTCRGIRGLIIVRSNL
SYIIEPVPNSDSQHRIYRSEHLTLPPGNCGFEHSGPTSKDWALQFTHQTKKQPRRMKREDLHSMKYVELYLVA
DYAEFQKNRHDQDATKRKLMEIANYVDKFYRSLNIRIALVGLEVWTHGDKCEVSENPYSTLWSFLSWRRKLLA

QKSHDNAQLITGRSFQGTTIGLAPLMAMCSVYQSGGVSMDHSENAIGVASTVAHEIGHNFGMSHDSAHCCSAS
AADGGCIMAAATGHPFPKVFSWCNRKELDRYLQTGGGMCLSNMPDTRTLYGGRRCGNGYLEDGEECDCGEEEE
CKNPCCNASNCTLKEGAECAHGSCCHQCKLVAPGTQCREQVRQCDLPEFCTGKSPHCPTNYYQMDGTPCEGGQ
AYCYNGMCLTYQEQCQQLWGPGARPALDLCFERVNAAGDTYGNCGKGLNGQYRKCSPRDAKCGKIQCQSTQAR
PLESNAVSIDTTITLNGRRIHCRGTHVYRGPEEEEGEGDMLDPGLVMTGTKCGHNHICFEGQCRNTSFFETEG
CGKKCNGHGVCNNNKNCHCFPGWSPPFCNTPGDGGSVDSGPLPPKSVGPVIAGVFSALFVLAVLVLLCHCYRQ
SHKLGKPSALPFKLRHQFSCPFRVSQSGGTGHANPTFKLQTPQGKRKVTNTPESLRKPSHPPPRPPPDYLRVE
SPPAPLPAHLNRAAGSSPEAGARIERKESARRPPPSRPMPPAPNCLLSQDFSRPRPPQKALPANPVPGQRTGP
RSGGTSLLQPPTSGPQPPRPPAVPVPKLPEYRSERVGAIISSKI*
>rs33850578_ENSMUSP00000020679_c.301:A>G_p.Y101C
ENSMUST00000020679.3|Nipal4-201|ENSMUSG00000020411|-|chr11:g.46047631:T>C
MELRVANANGSCENGSIVSLYCSSQEVLCQIVRGISPEEPYNATLITWQERVRKKYGFYIGVGLAFLSCFLIG
TSVILKKKGLIRLVATGATRAVNGGYGCLKDPMWWAGMATMSAGEVANFGAYAFAPATVVTPLGALSVLISAI
FSSYCLGESLNLLGKLGCVICMAGSTVMVIHAPKEEKVTTVAEMASKMKDTGFIVFAVLLVVSCLILIFIVAP
RYGQRNILIYIIICSVIGSFSVTAVKGLGVTIRNFFQGLPVVRHPLPYILSLILGLSIIIQVNFLNRALDIFN
TSLVFPIYYVFFTTVVVASSIVLFKEWYTMSAVDIVGTLSGFVTIILGVFMLHAFKDLDINQISLPHTHKNPT
PAPAPEPTVIKLEDKNVLVDNIELASTPSPQQKPKVFMTDS*
>rs29482402_ENSMUSP00000090854_c.748:A>T_p.E250V
ENSMUST00000093166.11|Cyfip2-202|ENSMUSG00000020340|-
|chr11:g.46167652:T>A
MTTHVTLEDALSNVDLLEELPLPDQQPCIEPPPSSIMYQANFDTNFEDRNAFVTGIARYIEQATVHSSMNEML
EEGHDYAVMLYTWRSCSRAIPQVKCNEQPNRVEIYEKTVEVLEPEVTKLMKFMYFQRKAIERFCSEVKRLCHA
ERRKDFVSEAYLLTLGKFINMFAVLDELKNMKCSVKNDHSAYKRAAQFLRKMADPQSIQESQNLSMFLANHNR
ITQCLHQQLEVIPGYEELLADIVNICVDYYVNKMYLTPSEKHMLLKVMGFGLYLMDGNVSNIYKLDAKKRINL
SKIDKFFKQLQVVPLFGDMQIELARYIKTSAHYEENKSKWTCTQSSISPQYNICEQMVQIRDDHIRFISELAR
YSNSEVVTGSGLDSQKSDEEYRELFDLALRGLQLLSKWSAHVMEVYSWKLVHPTDKFCNKDCPGTAEEYERAT
RYNYTSEEKFAFVEVIAMIKGLQVLMGRMESVFNQAIRNTIYAALQDFAQVTLREPLRQAVRKKKNVLISVLQ
AIRKTICDWEGGREPPNDPCLRGEKDPKGGFDIKVPRRAVGPSSTQLYMVRTMLESLIADKSGSKKTLRSSLD
GPIVLAIEDFHKQSFFFTHLLNISEALQQCCDLSQLWFREFFLELTMGRRIQFPIEMSMPWILTDHILETKEP
SMMEYVLYPLDLYNDSAYYALTKFKKQFLYDEIEAEVNLCFDQFVYKLADQIFAYYKAMAGSVLLDKRFRAEC
KNYGVIIPYPPSNRYETLLKQRHVQLLGRSIDLNRLITQRISAAMYKSLDQAISRFESEDLTSIVELEWLLEI
NRLTHRLLCKHMTLDSFDAMFREANHNVSAPYGRITLHVFWELNFDFLPNYCYNGSTNRFVRTAIPFTQEPQR
DKPANVQPYYLYGSKPLNIAYSHIYSSYRNFVGPPHFKTICRLLGYQGIAVVMEELLKIVKSLLQGTILQYVK
TLIEVMPKICRLPRHEYGSPGILEFFHHQLKDIIEYAELKTDVFQSLREVGNAILFCLLIEQALSQEEVCDLL
HAAPFQNILPRVYIKEGERLEVRMKRLEAKYAPLHLVPLIERLGTPQQIAIAREGDLLTKERLCCGLSMFEVI
LTRIRSYLQDPIWRGPPPTNGVMHVDECVEFHRLWSAMQFVYCIPVGTNEFTAEQCFGDGLNWAGCSIIVLLG
QQRRFDLFDFCYHLLKVQRQDGKDEIIKNVPLKKMADRIRKYQILNNEVFAILNKYMKSVETDSSTVEHVRCF
QPPIHQSLATTC*
>rs29482402_ENSMUSP00000127586_c.748:A>T_p.E250V
ENSMUST00000165599.9|Cyfip2-205|ENSMUSG00000020340|-|chr11:g.46167652:T>A
MTTHVTLEDALSNVDLLEELPLPDQQPCIEPPPSSIMYQANFDTNFEDRNAFVTGIARYIEQATVHSSMNEML
EEGHDYAVMLYTWRSCSRAIPQVKCNEQPNRVEIYEKTVEVLEPEVTKLMKFMYFQRKAIERFCSEVKRLCHA
ERRKDFVSEAYLLTLGKFINMFAVLDELKNMKCSVKNDHSAYKRAAQFLRKMADPQSIQESQNLSMFLANHNR
ITQCLHQQLEVIPGYEELLADIVNICVDYYVNKMYLTPSEKHMLLKVMGFGLYLMDGNVSNIYKLDAKKRINL
SKIDKFFKQLQVVPLFGDMQIELARYIKTSAHYEENKSKWTCTQSSISPQYNICEQMVQIRDDHIRFISELAR
YSNSEVVTGSGLDSQKSDEEYRELFDLALRGLQLLSKWSAHVMEVYSWKLVHPTDKFCNKDCPGTAEEYERAT
RYNYTSEEKFAFVEVIAMIKGLQVLMGRMESVFNQAIRNTIYAALQDFAQVTLREPLRQAVRKKKNVLISVLQ
AIRKTICDWEGGREPPNDPCLRGEKDPKGGFDIKVPRRAVGPSSTQLYMVRTMLESLIADKSGSKKTLRSSLD
GPIVLAIEDFHKQSFFFTHLLNISEALQQCCDLSQLWFREFFLELTMGRRIQFPIEMSMPWILTDHILETKEP
SMMEYVLYPLDLYNDSAYYALTKFKKQFLYDEIEAEVNLCFDQFVYKLADQIFAYYKAMAGSVLLDKRFRAEC
KNYGVIIPYPPSNRYETLLKQRHVQLLGRSIDLNRLITQRISAAMYKSLDQAISRFESEDLTSIVELEWLLEI
NRLTHRLLCKHMTLDSFDAMFREANHNVSAPYGRITLHVFWELNFDFLPNYCYNGSTNRFVRTAIPFTQEPQR
DKPANVQPYYLYGSKPLNIAYSHIYSSYRNFVGPPHFKTICRLLGYQGIAVVMEELLKIVKSLLQGTILQYVK
TLIEVMPKICRLPRHEYGSPGILEFFHHQLKDIIEYAELKTDVFQSLREVGNAILFCLLIEQALSQEEVCDLL
HAAPFQNILPRVYIKEGERLEVRMKRLEAKYAPLHLVPLIERLGTPQQIAIAREGDLLTKERLCCGLSMFEVI
LTRIRSYLQDPIWRGPPPTNGVMHVDECVEFHRLWSAMQFVYCIPVGTNEFTAEQCFGDGLNWAGCSIIVLLG
QQRRFDLFDFCYHLLKVQRQDGKDEIIKNVPLKKMADRIRKYQILNNEVFAILNKYMKSVETDSSTVEHVRCF
QPPIHQSLATTC*

>rs29482402_ENSMUSP00000090853_c.748:A>T_p.E250V
ENSMUST00000093165.12|Cyfip2-201|ENSMUSG00000020340|-
|chr11:g.46167652:T>A
MTTHVTLEDALSNVDLLEELPLPDQQPCIEPPPSSIMYQANFDTNFEDRNAFVTGIARYIEQATVHSSMNEML
EEGHDYAVMLYTWRSCSRAIPQVKCNEQPNRVEIYEKTVEVLEPEVTKLMKFMYFQRKAIERFCSEVKRLCHA
ERRKDFVSEAYLLTLGKFINMFAVLDELKNMKCSVKNDHSAYKRAAQFLRKMADPQSIQESQNLSMFLANHNR
ITQCLHQQLEVIPGYEELLADIVNICVDYYVNKMYLTPSEKHMLLKVMGFGLYLMDGNVSNIYKLDAKKRINL
SKIDKFFKQLQVVPLFGDMQIELARYIKTSAHYEENKSKWTCTQSSISPQYNICEQMVQIRDDHIRFISELAR
YSNSEVVTGSGLDSQKSDEEYRELFDLALRGLQLLSKWSAHVMEVYSWKLVHPTDKFCNKDCPGTAEEYERAT
RYNYTSEEKFAFVEVIAMIKGLQVLMGRMESVFNQAIRNTIYAALQDFAQVTLREPLRQAVRKKKNVLISVLQ
AIRKTICDWEGGREPPNDPCLRGEKDPKGGFDIKVPRRAVGPSSTQLYMVRTMLESLIADKSGSKKTLRSSLD
GPIVLAIEDFHKQSFFFTHLLNISEALQQCCDLSQLWFREFFLELTMGRRIQFPIEMSMPWILTDHILETKEP
SMMEYVLYPLDLYNDSAYYALTKFKKQFLYDEIEAEVNLCFDQFVYKLADQIFAYYKAMAGSVLLDKRFRAEC
KNYGVIIPYPPSNRYETLLKQRHVQLLGRSIDLNRLITQRISAAMYKSLDQAISRFESEDLTSIVELEWLLEI
NRLTHRLLCKHMTLDSFDAMFREANHNVSAPYGRITLHVFWELNFDFLPNYCYNGSTNRFVRTAIPFTQEPQR
DKPANVQPYYLYGSKPLNIAYSHIYSSYRNFVGPPHFKTICRLLGYQGIAVVMEELLKIVKSLLQGTILQYVK
TLIEVMPKICRLPRHEYGSPGILEFFHHQLKDIIEYAELKTDVFQSLREVGNAILFCLLIEQALSQEEVCDLL
HAAPFQNILPRVYIKEGERLEVRMKRLEAKYAPLHLVPLIERLGTPQQIAIAREGDLLTKERLCCGLSMFEVI
LTRIRSYLQDPIWRGPPPTNGVMHVDECVEFHRLWSAMQFVYCIPVGTNEFTAEQCFGDGLNWAGCSIIVLLG
QQRRFDLFDFCYHLLKVQRQDGKDEIIKNVPLKKMADRIRKYQILNNEVFAILNKYMKSVETDSSTVEHVRCF
QPPIHQSLATTC*
>rs29383724_ENSMUSP00000090854_c.670:T>C_p.L224P
ENSMUST00000093166.11|Cyfip2-202|ENSMUSG00000020340|-
|chr11:g.46167730:A>G
MTTHVTLEDALSNVDLLEELPLPDQQPCIEPPPSSIMYQANFDTNFEDRNAFVTGIARYIEQATVHSSMNEML
EEGHDYAVMLYTWRSCSRAIPQVKCNEQPNRVEIYEKTVEVLEPEVTKLMKFMYFQRKAIERFCSEVKRLCHA
ERRKDFVSEAYLLTLGKFINMFAVLDELKNMKCSVKNDHSAYKRAAQFLRKMADPQSIQESQNLSMFLANHNR
ITQCPHQQLEVIPGYEELLADIVNICVDYYENKMYLTPSEKHMLLKVMGFGLYLMDGNVSNIYKLDAKKRINL
SKIDKFFKQLQVVPLFGDMQIELARYIKTSAHYEENKSKWTCTQSSISPQYNICEQMVQIRDDHIRFISELAR
YSNSEVVTGSGLDSQKSDEEYRELFDLALRGLQLLSKWSAHVMEVYSWKLVHPTDKFCNKDCPGTAEEYERAT
RYNYTSEEKFAFVEVIAMIKGLQVLMGRMESVFNQAIRNTIYAALQDFAQVTLREPLRQAVRKKKNVLISVLQ
AIRKTICDWEGGREPPNDPCLRGEKDPKGGFDIKVPRRAVGPSSTQLYMVRTMLESLIADKSGSKKTLRSSLD
GPIVLAIEDFHKQSFFFTHLLNISEALQQCCDLSQLWFREFFLELTMGRRIQFPIEMSMPWILTDHILETKEP
SMMEYVLYPLDLYNDSAYYALTKFKKQFLYDEIEAEVNLCFDQFVYKLADQIFAYYKAMAGSVLLDKRFRAEC
KNYGVIIPYPPSNRYETLLKQRHVQLLGRSIDLNRLITQRISAAMYKSLDQAISRFESEDLTSIVELEWLLEI
NRLTHRLLCKHMTLDSFDAMFREANHNVSAPYGRITLHVFWELNFDFLPNYCYNGSTNRFVRTAIPFTQEPQR
DKPANVQPYYLYGSKPLNIAYSHIYSSYRNFVGPPHFKTICRLLGYQGIAVVMEELLKIVKSLLQGTILQYVK
TLIEVMPKICRLPRHEYGSPGILEFFHHQLKDIIEYAELKTDVFQSLREVGNAILFCLLIEQALSQEEVCDLL
HAAPFQNILPRVYIKEGERLEVRMKRLEAKYAPLHLVPLIERLGTPQQIAIAREGDLLTKERLCCGLSMFEVI
LTRIRSYLQDPIWRGPPPTNGVMHVDECVEFHRLWSAMQFVYCIPVGTNEFTAEQCFGDGLNWAGCSIIVLLG
QQRRFDLFDFCYHLLKVQRQDGKDEIIKNVPLKKMADRIRKYQILNNEVFAILNKYMKSVETDSSTVEHVRCF
QPPIHQSLATTC*
>rs29383724_ENSMUSP00000127586_c.670:T>C_p.L224P
ENSMUST00000165599.9|Cyfip2-205|ENSMUSG00000020340|-|chr11:g.46167730:A>G
MTTHVTLEDALSNVDLLEELPLPDQQPCIEPPPSSIMYQANFDTNFEDRNAFVTGIARYIEQATVHSSMNEML
EEGHDYAVMLYTWRSCSRAIPQVKCNEQPNRVEIYEKTVEVLEPEVTKLMKFMYFQRKAIERFCSEVKRLCHA
ERRKDFVSEAYLLTLGKFINMFAVLDELKNMKCSVKNDHSAYKRAAQFLRKMADPQSIQESQNLSMFLANHNR
ITQCPHQQLEVIPGYEELLADIVNICVDYYENKMYLTPSEKHMLLKVMGFGLYLMDGNVSNIYKLDAKKRINL
SKIDKFFKQLQVVPLFGDMQIELARYIKTSAHYEENKSKWTCTQSSISPQYNICEQMVQIRDDHIRFISELAR
YSNSEVVTGSGLDSQKSDEEYRELFDLALRGLQLLSKWSAHVMEVYSWKLVHPTDKFCNKDCPGTAEEYERAT
RYNYTSEEKFAFVEVIAMIKGLQVLMGRMESVFNQAIRNTIYAALQDFAQVTLREPLRQAVRKKKNVLISVLQ
AIRKTICDWEGGREPPNDPCLRGEKDPKGGFDIKVPRRAVGPSSTQLYMVRTMLESLIADKSGSKKTLRSSLD
GPIVLAIEDFHKQSFFFTHLLNISEALQQCCDLSQLWFREFFLELTMGRRIQFPIEMSMPWILTDHILETKEP
SMMEYVLYPLDLYNDSAYYALTKFKKQFLYDEIEAEVNLCFDQFVYKLADQIFAYYKAMAGSVLLDKRFRAEC
KNYGVIIPYPPSNRYETLLKQRHVQLLGRSIDLNRLITQRISAAMYKSLDQAISRFESEDLTSIVELEWLLEI
NRLTHRLLCKHMTLDSFDAMFREANHNVSAPYGRITLHVFWELNFDFLPNYCYNGSTNRFVRTAIPFTQEPQR
DKPANVQPYYLYGSKPLNIAYSHIYSSYRNFVGPPHFKTICRLLGYQGIAVVMEELLKIVKSLLQGTILQYVK
TLIEVMPKICRLPRHEYGSPGILEFFHHQLKDIIEYAELKTDVFQSLREVGNAILFCLLIEQALSQEEVCDLL

HAAPFQNILPRVYIKEGERLEVRMKRLEAKYAPLHLVPLIERLGTPQQIAIAREGDLLTKERLCCGLSMFEVI
LTRIRSYLQDPIWRGPPPTNGVMHVDECVEFHRLWSAMQFVYCIPVGTNEFTAEQCFGDGLNWAGCSIIVLLG
QQRRFDLFDFCYHLLKVQRQDGKDEIIKNVPLKKMADRIRKYQILNNEVFAILNKYMKSVETDSSTVEHVRCF
QPPIHQSLATTC*
>rs29383724_ENSMUSP00000090853_c.670:T>C_p.L224P
ENSMUST00000093165.12|Cyfip2-201|ENSMUSG00000020340|-
|chr11:g.46167730:A>G
MTTHVTLEDALSNVDLLEELPLPDQQPCIEPPPSSIMYQANFDTNFEDRNAFVTGIARYIEQATVHSSMNEML
EEGHDYAVMLYTWRSCSRAIPQVKCNEQPNRVEIYEKTVEVLEPEVTKLMKFMYFQRKAIERFCSEVKRLCHA
ERRKDFVSEAYLLTLGKFINMFAVLDELKNMKCSVKNDHSAYKRAAQFLRKMADPQSIQESQNLSMFLANHNR
ITQCPHQQLEVIPGYEELLADIVNICVDYYENKMYLTPSEKHMLLKVMGFGLYLMDGNVSNIYKLDAKKRINL
SKIDKFFKQLQVVPLFGDMQIELARYIKTSAHYEENKSKWTCTQSSISPQYNICEQMVQIRDDHIRFISELAR
YSNSEVVTGSGLDSQKSDEEYRELFDLALRGLQLLSKWSAHVMEVYSWKLVHPTDKFCNKDCPGTAEEYERAT
RYNYTSEEKFAFVEVIAMIKGLQVLMGRMESVFNQAIRNTIYAALQDFAQVTLREPLRQAVRKKKNVLISVLQ
AIRKTICDWEGGREPPNDPCLRGEKDPKGGFDIKVPRRAVGPSSTQLYMVRTMLESLIADKSGSKKTLRSSLD
GPIVLAIEDFHKQSFFFTHLLNISEALQQCCDLSQLWFREFFLELTMGRRIQFPIEMSMPWILTDHILETKEP
SMMEYVLYPLDLYNDSAYYALTKFKKQFLYDEIEAEVNLCFDQFVYKLADQIFAYYKAMAGSVLLDKRFRAEC
KNYGVIIPYPPSNRYETLLKQRHVQLLGRSIDLNRLITQRISAAMYKSLDQAISRFESEDLTSIVELEWLLEI
NRLTHRLLCKHMTLDSFDAMFREANHNVSAPYGRITLHVFWELNFDFLPNYCYNGSTNRFVRTAIPFTQEPQR
DKPANVQPYYLYGSKPLNIAYSHIYSSYRNFVGPPHFKTICRLLGYQGIAVVMEELLKIVKSLLQGTILQYVK
TLIEVMPKICRLPRHEYGSPGILEFFHHQLKDIIEYAELKTDVFQSLREVGNAILFCLLIEQALSQEEVCDLL
HAAPFQNILPRVYIKEGERLEVRMKRLEAKYAPLHLVPLIERLGTPQQIAIAREGDLLTKERLCCGLSMFEVI
LTRIRSYLQDPIWRGPPPTNGVMHVDECVEFHRLWSAMQFVYCIPVGTNEFTAEQCFGDGLNWAGCSIIVLLG
QQRRFDLFDFCYHLLKVQRQDGKDEIIKNVPLKKMADRIRKYQILNNEVFAILNKYMKSVETDSSTVEHVRCF
QPPIHQSLATTC*
>rs245339491_ENSMUSP00000090854_c.604:A>TA_p.Q202ins-fs
ENSMUST00000093166.11|Cyfip2-202|ENSMUSG00000020340|-
|chr11:g.46168947:T>TA
MTTHVTLEDALSNVDLLEELPLPDQQPCIEPPPSSIMYQANFDTNFEDRNAFVTGIARYIEQATVHSSMNEML
EEGHDYAVMLYTWRSCSRAIPQVKCNEQPNRVEIYEKTVEVLEPEVTKLMKFMYFQRKAIERFCSEVKRLCHA
ERRKDFVSEAYLLTLGKFINMFAVLDELKNMKCSVKNDHSAYKRAAQFLRKMADPLVHPGISEPVHVPGQSQQ
DHPVSSPATGSDPGL
>rs245339491_ENSMUSP00000127586_c.604:A>TA_p.Q202ins-fs
ENSMUST00000165599.9|Cyfip2-205|ENSMUSG00000020340|-
|chr11:g.46168947:T>TA
MTTHVTLEDALSNVDLLEELPLPDQQPCIEPPPSSIMYQANFDTNFEDRNAFVTGIARYIEQATVHSSMNEML
EEGHDYAVMLYTWRSCSRAIPQVKCNEQPNRVEIYEKTVEVLEPEVTKLMKFMYFQRKAIERFCSEVKRLCHA
ERRKDFVSEAYLLTLGKFINMFAVLDELKNMKCSVKNDHSAYKRAAQFLRKMADPLVHPGISEPVHVPGQSQQ
DHPVSSPATGSDPGL
>rs245339491_ENSMUSP00000090853_c.604:A>TA_p.Q202ins-fs
ENSMUST00000093165.12|Cyfip2-201|ENSMUSG00000020340|-
|chr11:g.46168947:T>TA
MTTHVTLEDALSNVDLLEELPLPDQQPCIEPPPSSIMYQANFDTNFEDRNAFVTGIARYIEQATVHSSMNEML
EEGHDYAVMLYTWRSCSRAIPQVKCNEQPNRVEIYEKTVEVLEPEVTKLMKFMYFQRKAIERFCSEVKRLCHA
ERRKDFVSEAYLLTLGKFINMFAVLDELKNMKCSVKNDHSAYKRAAQFLRKMADPLVHPGISEPVHVPGQSQQ
DHPVSSPATGSDPGL
>rs213016101_ENSMUSP00000090854_c.82:TG>G_p.C28del-fs
ENSMUST00000093166.11|Cyfip2-202|ENSMUSG00000020340|-
|chr11:g.46182374:CA>C
MTTHVTLEDALSNVDLLEELPLPDQQPVSSPRHPPSCTRLTLTRTSRTGMHLSRALQGTLSRQRSTPA
>rs213016101_ENSMUSP00000127586_c.82:TG>G_p.C28del-fs
ENSMUST00000165599.9|Cyfip2-205|ENSMUSG00000020340|-
|chr11:g.46182374:CA>C
MTTHVTLEDALSNVDLLEELPLPDQQPVSSPRHPPSCTRLTLTRTSRTGMHLSRALQGTLSRQRSTPA
>rs213016101_ENSMUSP00000090853_c.82:TG>G_p.C28del-fs
ENSMUST00000093165.12|Cyfip2-201|ENSMUSG00000020340|-
|chr11:g.46182374:CA>C
MTTHVTLEDALSNVDLLEELPLPDQQPVSSPRHPPSCTRLTLTRTSRTGMHLSRALQGTLSRQRSTPA

>rs46650668_ENSMUSP00000020664_c.1218:G>A_p.E407K
ENSMUST00000020664.13|Itk-201|ENSMUSG00000020395|-|chr11:g.46228942:C>T
MNNFILLEEQLIKKSQQKRRTSPSNFKVRFFVLTKASLAYFEDRHGKKRTLKGSIELSRIKCVEIVKSDISIP
CHYKYPFQVVHDNYLLYVFAPDCESRQRWVLTLKEETRNNNSLVSKYHPNFWMDGRWRCCSQLEKPAVGCAPY
DPSKNASKKPLPPTPEDNRRSFQEPEETLVIALYDYQTNDPQELALRCDEEYYLLDSSEIHWWRVQDKNGHEG
YAPSSYLVEKSPNNLETYEWYNKSISRDKAEKLLLDTGKEGAFMVRDSRTPGTYTVSVFTKAIISENPCIKHY
HIKETNDSPKRYYVAEKYVFDSIPLLIQYHQYNGGGLVTRLRYPVCSWRQKAPVTAGLRYGKWVIQPSELTFV
QEIGSGQFGLVHLGYWLNKDKVAIKTIQEGAMSEEDFIEEAKVMMKLSHPKLVQLYGVCLEQAPICLVFEFME
HGCLSDYLRSQRGLFAAETLLGMCLDVCEGMAYLEKACVIHRDLAARNCLVGENQVIKVSDFGMTRFVLDDQY
TSSTGTKFPVKWASPEVFSFSRYSSKSDVWSFGVLMWEVFSEGKIPYENRSNSEVVEDISTGFRLYKPRLASC
HVYQIMNHCWKEKPEDRPPFSQLLSQLAEIAEAGL*
>rs46650668_ENSMUSP00000104860_c.1236:G>A_p.E413K
ENSMUST00000109237.9|Itk-203|ENSMUSG00000020395|-|chr11:g.46228942:C>T
MNNFILLEEQLIKKSQQKRRTSPSNFKVRFFVLTKASLAYFEDRHGKKRTLKGSIELSRIKCVEIVKSDISIP
CHYKYPFQTLVYLQVVHDNYLLYVFAPDCESRQRWVLTLKEETRNNNSLVSKYHPNFWMDGRWRCCSQLEKPA
VGCAPYDPSKNASKKPLPPTPEDNRRSFQEPEETLVIALYDYQTNDPQELALRCDEEYYLLDSSEIHWWRVQD
KNGHEGYAPSSYLVEKSPNNLETYEWYNKSISRDKAEKLLLDTGKEGAFMVRDSRTPGTYTVSVFTKAIISEN
PCIKHYHIKETNDSPKRYYVAEKYVFDSIPLLIQYHQYNGGGLVTRLRYPVCSWRQKAPVTAGLRYGKWVIQP
SELTFVQEIGSGQFGLVHLGYWLNKDKVAIKTIQEGAMSEEDFIEEAKVMMKLSHPKLVQLYGVCLEQAPICL
VFEFMEHGCLSDYLRSQRGLFAAETLLGMCLDVCEGMAYLEKACVIHRDLAARNCLVGENQVIKVSDFGMTRF
VLDDQYTSSTGTKFPVKWASPEVFSFSRYSSKSDVWSFGVLMWEVFSEGKIPYENRSNSEVVEDISTGFRLYK
PRLASCHVYQIMNHCWKEKPEDRPPFSQLLSQLAEIAEAGL*
>ENSMUSP00000116997_c.175:T>TAAA_p.L59LK-ins ENSMUST00000128940.2|Med7-
204|ENSMUSG00000020397|+|chr11:g.46331582:T>TAAA
MGEPQQVSALPPPPMQYIKEYTDENIQEGLAPKPPPPIKDSYMMFGNQFQCDDLIIRPLKESQGIERLHPMQF
DHKKELRKLNMSILINFLDLLDILIRSPGSIKREEKLEDLKLLFVHVHHLINEYRPHQARETLRVMMEVQKRQ
RLETAERFQKHL
>ENSMUSP00000104854_c.175:T>TAAA_p.L59LK-ins ENSMUST00000109231.2|Med7-
202|ENSMUSG00000020397|+|chr11:g.46331582:T>TAAA
MGEPQQVSALPPPPMQYIKEYTDENIQEGLAPKPPPPIKDSYMMFGNQFQCDDLIIRPLKESQGIERLHPMQF
DHKKELRKLNMSILINFLDLLDILIRSPGSIKREEKLEDLKLLFVHVHHLINEYRPHQARETLRVMMEVQKRQ
RLETAERFQKHLERVIEMIQNCLASLPD
>ENSMUSP00000104855_c.175:T>TAAA_p.L59LK-ins ENSMUST00000109232.4|Med7-
203|ENSMUSG00000020397|+|chr11:g.46331582:T>TAAA
MGEPQQVSALPPPPMQYIKEYTDENIQEGLAPKPPPPIKDSYMMFGNQFQCDDLIIRPLKESQGIERLHPMQF
DHKKELRKLNMSILINFLDLLDILIRSPGSIKREEKLEDLKLLFVHVHHLINEYRPHQARETLRVMMEVQKRQ
RLETAERFQKHLERVIEMIQNCLASLPDDLPHSEAGMRVKAEPMDTDDNSNCPGQNEQQRESSGHRRDQIIEK
DAALCVLIDEMNERP*
>ENSMUSP00000020665_c.175:T>TAAA_p.L59LK-ins ENSMUST00000020665.13|Med7-
201|ENSMUSG00000020397|+|chr11:g.46331582:T>TAAA
MGEPQQVSALPPPPMQYIKEYTDENIQEGLAPKPPPPIKDSYMMFGNQFQCDDLIIRPLKESQGIERLHPMQF
DHKKELRKLNMSILINFLDLLDILIRSPGSIKREEKLEDLKLLFVHVHHLINEYRPHQARETLRVMMEVQKRQ
RLETAERFQKHLERVIEMIQNCLASLPDDLPHSEAGMRVKAEPMDTDDNSNCPGQNEQQRESSGHRRDQIIEK
DAALCVLIDEMNERP*
>ENSMUSP00000122182_c.175:T>TAAA_p.L59LK-ins ENSMUST00000152119.2|Med7-
207|ENSMUSG00000020397|+|chr11:g.46331582:T>TAAA
MGEPQQVSALPPPPMQYIKEYTDENIQEGLAPKPPPPIKDSYMMFGNQFQCDDLIIRPLKESQGIERLHPMQF
DHKKELRKLNMSILINFLDLL
>ENSMUSP00000120077_c.175:T>TAAA_p.L59LK-ins ENSMUST00000140027.8|Med7-
206|ENSMUSG00000020397|+|chr11:g.46331582:T>TAAA
MGEPQQVSALPPPPMQYIKEYTDENIQEGLAPKPPPPIKDSYMMFGNQFQCDDLIIRPLKES
>ENSMUSP00000120617_c.175:T>TAAA_p.L59LK-ins ENSMUST00000133635.2|Med7-
205|ENSMUSG00000020397|+|chr11:g.46331582:T>TAAA
MGEPQQVSALPPPPMQYIKEYTDENIQEGLAPKPPPPIKDSYMMFGNQFQCDDLIIRPLKESQGIERLHPMQF
DHKKELRKLNMSILINFLDLLDILIRSPGSIKREEKLEDLKLLFVHVHHLINEYRPHQARE
>ENSMUSP00000131852_c.175:T>TAAA_p.L59LK-ins ENSMUST00000170928.8|Med7-
209|ENSMUSG00000020397|+|chr11:g.46331582:T>TAAA

MGEPQQVSALPPPPMQYIKEYTDENIQEGLAPKPPPPIKDSYMMFGNQFQCDDLIIRPLKESQGIERLHPMQF
DHKKELRKLNMSILINFLDLLDILIRSPGSIKREEKLEDLKLLFVHVHHLINEYRPHQARETLRVMMEVQKRQ
RLETAERFQKHLERVIEMIQNCLASLPDDLPHSEAGMRVKAEPMDTDDNSNCPGQNEQQRESSGHRRDQIIEK
DAALCVLIDEMNERP*
>rs227019036_ENSMUSP00000069456_c.18:C>A_p.L7I
ENSMUST00000068877.7|Timd4-201|ENSMUSG00000055546|+|chr11:g.46701657:C>A
MSKGLLILWLVTELWWLYLTPAASEDTIIGFLGQPVTLPCHYLSWSQSRNSMCWGKGSCPNSKCNAELLRTDG
TRIISRKSTKYTLLGKVQFGEVSLTISNTNRGDSGVYCCRIEVPGWFNDVKKNVRLELRRATTTKKPTTTTRP
TTTPYVTTTTPELLPTTVMTTSVLPTTTPPQTLATTAFSTAVTTCPSTTPGSFSQETTKGSAFTTESETLPAS
NHSQRSMMTISTDIAVLRPTGSNPGILPSTSQLTTQKTTLTTSESLQKTTKSHQINSRQTILIIACCVGFVLM
VLLFLAFLLRGKVTGANCLQRHKRPDNTEDSDSVLNDMSHGRDDEDGIFTL*
>rs50666259_ENSMUSP00000069456_c.144:C>T_p.R49C
ENSMUST00000068877.7|Timd4-201|ENSMUSG00000055546|+|chr11:g.46706344:C>T
MSKGLLLLWLVTELWWLYLTPAASEDTIIGFLGQPVTLPCHYLSWSQSCNSMCWGKGSCPNSKCNAELLRTDG
TRIISRKSTKYTLLGKVQFGEVSLTISNTNRGDSGVYCCRIEVPGWFNDVKKNVRLELRRATTTKKPTTTTRP
TTTPYVTTTTPELLPTTVMTTSVLPTTTPPQTLATTAFSTAVTTCPSTTPGSFSQETTKGSAFTTESETLPAS
NHSQRSMMTISTDIAVLRPTGSNPGILPSTSQLTTQKTTLTTSESLQKTTKSHQINSRQTILIIACCVGFVLM
VLLFLAFLLRGKVTGANCLQRHKRPDNTEDSDSVLNDMSHGRDDEDGIFTL*
>rs52482656_ENSMUSP00000069456_c.356:G>A_p.W119*
ENSMUST00000068877.7|Timd4-201|ENSMUSG00000055546|+|chr11:g.46706556:G>A
MSKGLLLLWLVTELWWLYLTPAASEDTIIGFLGQPVTLPCHYLSWSQSRNSMCWGKGSCPNSKCNAELLRTDG
TRIISRKSTKYTLLGKVQFGEVSLTISNTNRGDSGVYCCRIEVPG*FNDVKKNVRLELRRATTTKKPTTTTRP
TTTPYVTTTTPELLPTTVMTTSVLPTTTPPQTLATTAFSTAVTTCPSTTPGSFSQETTKGSAFTTESETLPAS
NHSQRSMMTISTDIAVLRPTGSNPGILPSTSQLTTQKTTLTTSESLQKTTKSHQINSRQTILIIACCVGFVLM
VLLFLAFLLRGKVTGANCLQRHKRPDNTEDSDSVLNDMSHGRDDEDGIFTL*
>ENSMUSP00000019060_c.154:A>G_p.E52G ENSMUST00000019060.6|Csf2-
201|ENSMUSG00000018916|-|chr11:g.54140185:T>C
MWLQNLLFLGIVVYSLSAPTRSPITVTRPWKHVEAIKEALNLLDDMPVTLNGEVEVVSNEFSFKKLTCVQTRL
KIFEQGLRGNFTKLKGALNMTASYYQTYCPPTPETDCETQVTTYADFIDSLKTFLTDIPFECKKPGQK*
>rs48718255_ENSMUSP00000049026_c.34:A>G_p.K12R
ENSMUST00000046835.14|Fnip1-201|ENSMUSG00000035992|+|chr11:g.54329157:A>G
MAPTLFQKLFSRRSGLGAPGRDARDPDCAFSWPLPEFDPSQIRLIVYQDCERRGRNVLFDSSVKRKNEDTSVS
KLCNDAQVKVFGKCCQLKPGGDSSSSLDSSITLSSDGKDQCPKYQGSRCSSDANMLGEMMFGSVAMSYKGSTL
KIHQIRSPPQLMLSKVFTARTGSSICGSLNTLQDSLEFINQDSNTLKADSSTVSNGLLGNIGLSQFCSPRRAF
SEQGPLRLIRSASFFAVHSNPMDMPGRELNEDRDSGIARSASLSSLFITPFPSPNSSLTRSCASSYQRRWRRS
QTTSLENGVFPRWSVEESFNLSDESCGPNPGIVRKKKIAIGVIFSLSKDEDENNKFNEFFFSHFPLFESHMNK
LKSAIEQAMKMSRRSADASQRSLAYNRILDALTEFRTTICNLYTMPRIGEPVWLTMMSGTPEKNQLCHRFMKE
FTFLMENASKNQFLPALITAVLTNHLAWVPTVMPNGQPPIKIFLEKHSSQSVGMLAKTHPYNPLWAQLGDLYG
AIGSPVRLARTVVVGKRQDLVQRLLYFLTYFIRCSELQETHLLENGEDEAIVMPGTVITTTLEKGEIEESEYV
LITMHRNKSSLLFKESEETRTPNCNCKYCSHPVLGQNTENVSQPEREDTQDNSKELLGISDECQKISPPDCQE
ENAVDVQQYRDKLRTCLDTKLETVVCTGSAPADKCVLSETCLEPREESWQNKELLDSDNHTGTAMRPTGIVVE
KKPPDKNVPSAFSCEVTQTKVTFLIGDSMSPDSDTELRSQAVVDQINRHHSEPLKEDRGVADKHQESKITKDQ
SEDSDTQNIVSGESCELPCWSHSDPESMSLFDEYFNDDSIETRTIDDVPVKTSTDSKEYCCMLEYPKRLYTKT
NKQKSELCKCIETVHQDSCNACFPQQDQRNSLSILVPHGDKESSDKKNAVGTEWDIPRNESSDSALGDSESED
TGPDIRRQAGGYCGGDQEDWTEEDEIPFPGSKLIEVSAVQPNIANFGRSLLGGYCSSYVPDFVLQGIGNDERL
RQCLVSDLSHAVQHPVLDEPIAEAVCIIADMDKWTVQVASSQRRVTDNKLGKEVLVSSLVSNLLHSTLQLYKH
NLSPNFCVMHLEDRLQELYFKSKMLSEYLRGQMRVHVKELGVVLGIESSDLPLLAAVASTHSPYVAQILL*
>rs26974040_ENSMUSP00000104511_c.1249:T>C_p.L417P
ENSMUST00000108883.10|Anxa6-202|ENSMUSG00000018340|-|chr11:g.54885390:A>G
MAKIAQGAMYRGSVHDFPEFDANQDAEALYTAMKGFGSDKESILELITSRSNKQRQEICQNYKSLYGKDLIED
LKYELTGKFERLIVNLMRPLAYCDAKEIKDAISGVGTDEKCLIEILASRTNEQMHQLVAAYKDAYERDLESDI
IGDTSGHFQKMLVVLLQGTRENDDVVSEDLVQQDVQDLYEAGELKWGTDEAQFIYILGNRSKQHLRLVFDEYL
KTTGKPIEASIRGELSGDFEKLMLAVVKCIRSTPEYFAERLFKAMKGLGTRDNTLIRIMVSRSELDMLDIREI
FRTKYEKSLYSMIKNDTSGEYKKALLKLCGGDDDAAGQFFPEAAQVAYQMWELSAVSRVELKGTVCAANDFNP
DADAKALRKAMKGIGTDEATIIDIVTHRSNAQRQQIRQTFKSHFGRDLMADPKSEISGDLARLILGLMMPPAH
YDAKQLKKAMEGAGTDEKTLIEILATRTNAEIRAINEAYKEDYHKSLEDALSSDTSGHFRRILISLATGNREE
GGENRDQAQEDAQVAAEILEIADTPSGDKTSLETRFMTVLCTRSYPHLRRVFQEFIKKTNYDIEHVIKKEMSG

DVKDAFVAIVQSVKNKPLFFADKLYKSMKGAGTDEKTLTRVMVSRSEIDLLNIRREFIEKYDKSLHQAIEGDT
SGDFMKALLALCGGED*
>rs26974040_ENSMUSP00000099788_c.1249:T>C_p.L417P
ENSMUST00000102727.3|Anxa6-201|ENSMUSG00000018340|-|chr11:g.54885390:A>G
MAKIAQGAMYRGSVHDFPEFDANQDAEALYTAMKGFGSDKESILELITSRSNKQRQEICQNYKSLYGKDLIED
LKYELTGKFERLIVNLMRPLAYCDAKEIKDAISGVGTDEKCLIEILASRTNEQMHQLVAAYKDAYERDLESDI
IGDTSGHFQKMLVVLLQGTRENDDVVSEDLVQQDVQDLYEAGELKWGTDEAQFIYILGNRSKQHLRLVFDEYL
KTTGKPIEASIRGELSGDFEKLMLAVVKCIRSTPEYFAERLFKAMKGLGTRDNTLIRIMVSRSELDMLDIREI
FRTKYEKSLYSMIKNDTSGEYKKALLKLCGGDDDAAGQFFPEAAQVAYQMWELSAVSRVELKGTVCAANDFNP
DADAKALRKAMKGIGTDEATIIDIVTHRSNAQRQQIRQTFKSHFGRDLMADPKSEISGDLARLILGLMMPPAH
YDAKQLKKAMEGAGTDEKTLIEILATRTNAEIRAINEAYKEDYHKSLEDALSSDTSGHFRRILISLATGNREE
GGENRDQAQEDAQEIADTPSGDKTSLETRFMTVLCTRSYPHLRRVFQEFIKKTNYDIEHVIKKEMSGDVKDAF
VAIVQSVKNKPLFFADKLYKSMKGAGTDEKTLTRVMVSRSEIDLLNIRREFIEKYDKSLHQAIEGDTSGDFMK
ALLALCGGED*
>rs251828522_ENSMUSP00000064960_c.205:A>C_p.E69A
ENSMUST00000069816.6|Slc36a3-202|ENSMUSG00000049491|-
|chr11:g.55040726:T>G
MGNVPLLREVGKCQRNMFGRSTASSKGSSNSRSSSSTSPKKGPRREADALMEALWQQRGSPPDAWHSSAASSR
NPIFPAGRGATTRLIQPLLAPPRKFGSNP*
>rs32973454_ENSMUSP00000018727_c.892:A>G_p.D298G
ENSMUST00000018727.4|G3bp1-201|ENSMUSG00000018583|+|chr11:g.55388532:A>G
MVMEKPSPLLVGREFVRQYYTLLNQAPDMLHRFYGKNSSYAHGGLDSNGKPADAVYGQKEIHRKVMSQNFTNC
HTKIRHVDAHATLNDGVVVQVMGLLSNNNQALRRFMQTFVLAPEGSVANKFYVHNDIFRYQDEVFGGFVTEPQ
EESEEEVEEPEERQQTPEVVPDDSGTFYDQTVSNDLEEHLEEPVVEPEPEPEPEPEPEPVSDIQEDKPEAALE
EAAPDDVQKSTSPAPADVAPAQEDLRTFSWASVTSKNLPPSGAVPVTGTPPHVVKVPASQPRPESKPDSQIPP
QRPQRGQRVREQRINIPPQRGPRPIREAGEPGDVEPRRMVRHPDSHQLFIGNLPHEVDKSELKDFFQNFGNVV
ELRINSGGKLPNFGFVVFDDSEPVQKVLSNRPIMFRGAVRLNVEEKKTRAAREGDRRDNRLRGPGGPRGGPSG
GMRGPPRGGMVQKPGFGVGRGITTPRQ*
>rs26959816_ENSMUSP00000104481_c.1057:T>C_p.I353T
ENSMUST00000108853.8|Glra1-203|ENSMUSG00000000263|-|chr11:g.55405682:A>G
MDYRVNIFLRQQWNDPRLAYNEYPDDSLDLDPSMLDSIWKPDLFFANEKGAHFHEITTDNKLLRISRNGNVLY
SIRITLTLACPMDLKNFPMDVQTCIMQLESFGYTMNDLIFEWQEQGAVQVADGLTLPQFILKEEKDLRYCTKH
YNTGKFTCIEARFHLERQMGYYLIQMYIPSLLIVILSWISFWINMDAAPARVGLGITTVLTMTTQSSGSRASL
PKVSYVKAIDIWMAVCLLFVFSALLEYAAVNFVSRQHKELLRFRRKRRHHKDDEGGEGRFNFSAYGMGPACLQ
AKDGISVKGANNNNTTNPPPAPSKSPEEMRKLFIQRAKKIDKISRIGFPMAFLIFNMFYWTIYKIVRREDVHN
K*
>rs26959816_ENSMUSP00000099777_c.1306:T>C_p.I436T
ENSMUST00000102716.10|Glra1-202|ENSMUSG00000000263|-|chr11:g.55405682:A>G
MYSFNTLRFYLWETIVFFSLAASKEAEAARSAPKPMSPSDFLDKLMGRTSGYDARIRPNFKGPPVNVSCNIFI
NSFGSIAETTMDYRVNIFLRQQWNDPRLAYNEYPDDSLDLDPSMLDSIWKPDLFFANEKGAHFHEITTDNKLL
RISRNGNVLYSIRITLTLACPMDLKNFPMDVQTCIMQLESFGYTMNDLIFEWQEQGAVQVADGLTLPQFILKE
EKDLRYCTKHYNTGKFTCIEARFHLERQMGYYLIQMYIPSLLIVILSWISFWINMDAAPARVGLGITTVLTMT
TQSSGSRASLPKVSYVKAIDIWMAVCLLFVFSALLEYAAVNFVSRQHKELLRFRRKRRHHKDDEGGEGRFNFS
AYGMGPACLQAKDGISVKGANNNNTTNPPPAPSKSPEEMRKLFIQRAKKIDKISRIGFPMAFLIFNMFYWTIY
KIVRREDVHNK*
>rs26959816_ENSMUSP00000075032_c.1330:T>C_p.I444T
ENSMUST00000075603.5|Glra1-201|ENSMUSG00000000263|-|chr11:g.55405682:A>G
MYSFNTLRFYLWETIVFFSLAASKEAEAARSAPKPMSPSDFLDKLMGRTSGYDARIRPNFKGPPVNVSCNIFI
NSFGSIAETTMDYRVNIFLRQQWNDPRLAYNEYPDDSLDLDPSMLDSIWKPDLFFANEKGAHFHEITTDNKLL
RISRNGNVLYSIRITLTLACPMDLKNFPMDVQTCIMQLESFGYTMNDLIFEWQEQGAVQVADGLTLPQFILKE
EKDLRYCTKHYNTGKFTCIEARFHLERQMGYYLIQMYIPSLLIVILSWISFWINMDAAPARVGLGITTVLTMT
TQSSGSRASLPKVSYVKAIDIWMAVCLLFVFSALLEYAAVNFVSRQHKELLRFRRKRRHHKSPMLNLFQDDEG
GEGRFNFSAYGMGPACLQAKDGISVKGANNNNTTNPPPAPSKSPEEMRKLFIQRAKKIDKISRIGFPMAFLIF
NMFYWTIYKIVRREDVHNK*
>rs47776591_ENSMUSP00000104481_c.880:A>G_p.K294R
ENSMUST00000108853.8|Glra1-203|ENSMUSG00000000263|-|chr11:g.55405859:T>C
MDYRVNIFLRQQWNDPRLAYNEYPDDSLDLDPSMLDSIWKPDLFFANEKGAHFHEITTDNKLLRISRNGNVLY
SIRITLTLACPMDLKNFPMDVQTCIMQLESFGYTMNDLIFEWQEQGAVQVADGLTLPQFILKEEKDLRYCTKH

YNTGKFTCIEARFHLERQMGYYLIQMYIPSLLIVILSWISFWINMDAAPARVGLGITTVLTMTTQSSGSRASL
PKVSYVKAIDIWMAVCLLFVFSALLEYAAVNFVSRQHKELLRFRRKRRHHKDDEGGEGRFNFSAYGMGPACLQ
ARDGISVKGANNNNTTNPPPAPSKSPEEMRKLFIQRAKKIDKISRIGFPMAFLIFNMFYWIIYKIVRREDVHN
K*
>rs47776591_ENSMUSP00000099777_c.1129:A>G_p.K377R
ENSMUST00000102716.10|Glra1-202|ENSMUSG00000000263|-|chr11:g.55405859:T>C
MYSFNTLRFYLWETIVFFSLAASKEAEAARSAPKPMSPSDFLDKLMGRTSGYDARIRPNFKGPPVNVSCNIFI
NSFGSIAETTMDYRVNIFLRQQWNDPRLAYNEYPDDSLDLDPSMLDSIWKPDLFFANEKGAHFHEITTDNKLL
RISRNGNVLYSIRITLTLACPMDLKNFPMDVQTCIMQLESFGYTMNDLIFEWQEQGAVQVADGLTLPQFILKE
EKDLRYCTKHYNTGKFTCIEARFHLERQMGYYLIQMYIPSLLIVILSWISFWINMDAAPARVGLGITTVLTMT
TQSSGSRASLPKVSYVKAIDIWMAVCLLFVFSALLEYAAVNFVSRQHKELLRFRRKRRHHKDDEGGEGRFNFS
AYGMGPACLQARDGISVKGANNNNTTNPPPAPSKSPEEMRKLFIQRAKKIDKISRIGFPMAFLIFNMFYWIIY
KIVRREDVHNK*
>rs47776591_ENSMUSP00000075032_c.1153:A>G_p.K385R
ENSMUST00000075603.5|Glra1-201|ENSMUSG00000000263|-|chr11:g.55405859:T>C
MYSFNTLRFYLWETIVFFSLAASKEAEAARSAPKPMSPSDFLDKLMGRTSGYDARIRPNFKGPPVNVSCNIFI
NSFGSIAETTMDYRVNIFLRQQWNDPRLAYNEYPDDSLDLDPSMLDSIWKPDLFFANEKGAHFHEITTDNKLL
RISRNGNVLYSIRITLTLACPMDLKNFPMDVQTCIMQLESFGYTMNDLIFEWQEQGAVQVADGLTLPQFILKE
EKDLRYCTKHYNTGKFTCIEARFHLERQMGYYLIQMYIPSLLIVILSWISFWINMDAAPARVGLGITTVLTMT
TQSSGSRASLPKVSYVKAIDIWMAVCLLFVFSALLEYAAVNFVSRQHKELLRFRRKRRHHKSPMLNLFQDDEG
GEGRFNFSAYGMGPACLQARDGISVKGANNNNTTNPPPAPSKSPEEMRKLFIQRAKKIDKISRIGFPMAFLIF
NMFYWIIYKIVRREDVHNK*
>rs45667969_ENSMUSP00000044718_c.1137:A>G_p.I380V
ENSMUST00000037682.3|Nmur2-201|ENSMUSG00000037393|-|chr11:g.55917850:T>C
MGKLENASWIHDSLMKYLNSTEEYLAYLCGPKRSDLSLPVSVVYALIFVVGVIGNLLVCLVIARHQTLKTPTN
YYLFSLAVSDLLVLLLGMPLEVYELWHNYPFLFGPVGCYFKTALFETVCFASILSVTTVSIERYVAIVHPFRA
KLESTRRRALRILSLVWSFSVVFSLPNTSIHGIKFQQFPNGSSVPGSATCTVTKPIWVYNFIIQATSFLFYIL
PMTLISVLYYLMGLRLKRDESLEADKVTVNIHRPSRKSVTKMLFVLVLVFAICWTPFHVDRLFFSFVDEWTES
LAAVFNLIHVVSGVFFYLSSAVNPIIYNLLSRRFRAAFRNVVSPSCKWCHPQHRPQGPPAQKVIFLTECHLVE
LTEDAGPQFPCQSSVHNTQLTTVPCVEEVP*
>ENSMUSP00000044494_c.1497:G>GTTTCCTGTAGC_p.E500ins-fs
ENSMUST00000036315.16|Gria1-
201|ENSMUSG00000020524|+|chr11:g.57133620:G>GTTTCCTGTAGC
MPYIFAFFCTGFLGAVVGANFPNNIQIGGLFPNQQSQEHAAFRFALSQLTEPPKLLPQIDIVNISDSFEMTYR
FCSQFSKGVYAIFGFYERRTVNMLTSFCGALHVCFITPSFPVDTSNQFVLQLRPELQEALISIIDHYKWQTFV
YIYDADRGLSVLQRVLDTAAEKNWQVTAVNILTTTEEGYRMLFQDLEKKKERLVVVDCESERLNAILGQIVKL
EKNGIGYHYILANLGFMDIDLNKFKESGANVTGFQLVNYTDTIPARIMQQWRTSDARDHTRVDWKRPKYTSAL
TYDGVKVMAEAFQSLRRQRIDISRRGNAGDCLANPAVPWGQGIDIQRALQQVRFEGLTGNVQFNEKGRRTNYT
LHVIEMKHDGIRKIGYWNEDDKFVPAATDAQAGGDNSSVQNRTYIVTTILEDPYVMLKKNANQFEGNDRYEGY
CVELAAEIAKHVGYSYRLEIVSDGKYGARDPDTKAWNGMVGELVYGRADVAVAPLTITLVRVSCSRKSSTSPS
HS
>ENSMUSP00000091731_c.1497:G>GTTTCCTGTAGC_p.E500ins-fs
ENSMUST00000094179.11|Gria1-
202|ENSMUSG00000020524|+|chr11:g.57133620:G>GTTTCCTGTAGC
MPYIFAFFCTGFLGAVVGANFPNNIQIGGLFPNQQSQEHAAFRFALSQLTEPPKLLPQIDIVNISDSFEMTYR
FCSQFSKGVYAIFGFYERRTVNMLTSFCGALHVCFITPSFPVDTSNQFVLQLRPELQEALISIIDHYKWQTFV
YIYDADRGLSVLQRVLDTAAEKNWQVTAVNILTTTEEGYRMLFQDLEKKKERLVVVDCESERLNAILGQIVKL
EKNGIGYHYILANLGFMDIDLNKFKESGANVTGFQLVNYTDTIPARIMQQWRTSDARDHTRVDWKRPKYTSAL
TYDGVKVMAEAFQSLRRQRIDISRRGNAGDCLANPAVPWGQGIDIQRALQQVRFEGLTGNVQFNEKGRRTNYT
LHVIEMKHDGIRKIGYWNEDDKFVPAATDAQAGGDNSSVQNRTYIVTTILEDPYVMLKKNANQFEGNDRYEGY
CVELAAEIAKHVGYSYRLEIVSDGKYGARDPDTKAWNGMVGELVYGRADVAVAPLTITLVRVSCSRKSSTSPS
HS
>ENSMUSP00000117746_c.1290:G>GTTTCCTGTAGC_p.E431ins-fs
ENSMUST00000151045.3|Gria1-
204|ENSMUSG00000020524|+|chr11:g.57133620:G>GTTTCCTGTAGC
MTYRFCSQFSKGVYAIFGFYERRTVNMLTSFCGALHVCFITPSFPVDTSNQFVLQLRPELQEALISIIDHYKW
QTFVYIYDADRGLSVLQRVLDTAAEKNWQVTAVNILTTTEEGYRMLFQDLEKKKERLVVVDCESERLNAILGQ
IVKLEKNGIGYHYILANLGFMDIDLNKFKESGANVTGFQLVNYTDTIPARIMQQWRTSDARDHTRVDWKRPKY

TSALTYDGVKVMAEAFQSLRRQRIDISRRGNAGDCLANPAVPWGQGIDIQRALQQVRFEGLTGNVQFNEKGRR
TNYTLHVIEMKHDGIRKIGYWNEDDKFVPAATDAQAGGDNSSVQNRTYIVTTILEDPYVMLKKNANQFEGNDR
YEGYCVELAAEIAKHVGYSYRLEIVSDGKYGARDPDTKAWNGMVGELVYGRADVAVAPLTITLVRVSCSRKSS
TSPSHS
>rs33881482_ENSMUSP00000044494_c.1929:G>A_p.V644M
ENSMUST00000036315.16|Gria1-201|ENSMUSG00000020524|+|chr11:g.57174442:G>A
MPYIFAFFCTGFLGAVVGANFPNNIQIGGLFPNQQSQEHAAFRFALSQLTEPPKLLPQIDIVNISDSFEMTYR
FCSQFSKGVYAIFGFYERRTVNMLTSFCGALHVCFITPSFPVDTSNQFVLQLRPELQEALISIIDHYKWQTFV
YIYDADRGLSVLQRVLDTAAEKNWQVTAVNILTTTEEGYRMLFQDLEKKKERLVVVDCESERLNAILGQIVKL
EKNGIGYHYILANLGFMDIDLNKFKESGANVTGFQLVNYTDTIPARIMQQWRTSDARDHTRVDWKRPKYTSAL
TYDGVKVMAEAFQSLRRQRIDISRRGNAGDCLANPAVPWGQGIDIQRALQQVRFEGLTGNVQFNEKGRRTNYT
LHVIEMKHDGIRKIGYWNEDDKFVPAATDAQAGGDNSSVQNRTYIVTTILEDPYVMLKKNANQFEGNDRYEGY
CVELAAEIAKHVGYSYRLEIVSDGKYGARDPDTKAWNGMVGELVYGRADVAVAPLTITLVREEVIDFSKPFMS
LGISIMIKKPQKSKPGVFSFLDPLAYEIWMCIVFAYIGVSVVLFLVSRFSPYEWHSEEFEEGRDQTTSDQSNE
FGIFNSLWFSLGAFMQQGCDISPRSLSGRIVGGVWWFFTLIIISSYTANLAAFLTVERMMSPIESAEDLAKQT
EIAYGTLEAGSTKEFFRRSKIAVFEKMWTYMKSAEPSVFVRTTEEGMIRVRKSKGKYAYLLESTMNEYIEQRK
PCDTMKVGGNLDSKGYGIATPKGSALRGPVNLAVLKLSEQGVLDKLKSKWWYDKGECGSKDSGSKDKTSALSL
SNVAGVFYILIGGLGLAMLVALIEFCYKSRSESKRMKGFCLIPQQSINEAIRTSTLPRNSGAGASGGSGSGEN
GRVVSQDFPKSMQSIPCMSHSSGMPLGATGL*
>rs33881482_ENSMUSP00000091731_c.1929:G>A_p.V644M
ENSMUST00000094179.11|Gria1-202|ENSMUSG00000020524|+|chr11:g.57174442:G>A
MPYIFAFFCTGFLGAVVGANFPNNIQIGGLFPNQQSQEHAAFRFALSQLTEPPKLLPQIDIVNISDSFEMTYR
FCSQFSKGVYAIFGFYERRTVNMLTSFCGALHVCFITPSFPVDTSNQFVLQLRPELQEALISIIDHYKWQTFV
YIYDADRGLSVLQRVLDTAAEKNWQVTAVNILTTTEEGYRMLFQDLEKKKERLVVVDCESERLNAILGQIVKL
EKNGIGYHYILANLGFMDIDLNKFKESGANVTGFQLVNYTDTIPARIMQQWRTSDARDHTRVDWKRPKYTSAL
TYDGVKVMAEAFQSLRRQRIDISRRGNAGDCLANPAVPWGQGIDIQRALQQVRFEGLTGNVQFNEKGRRTNYT
LHVIEMKHDGIRKIGYWNEDDKFVPAATDAQAGGDNSSVQNRTYIVTTILEDPYVMLKKNANQFEGNDRYEGY
CVELAAEIAKHVGYSYRLEIVSDGKYGARDPDTKAWNGMVGELVYGRADVAVAPLTITLVREEVIDFSKPFMS
LGISIMIKKPQKSKPGVFSFLDPLAYEIWMCIVFAYIGVSVVLFLVSRFSPYEWHSEEFEEGRDQTTSDQSNE
FGIFNSLWFSLGAFMQQGCDISPRSLSGRIVGGVWWFFTLIIISSYTANLAAFLTVERMMSPIESAEDLAKQT
EIAYGTLEAGSTKEFFRRSKIAVFEKMWTYMKSAEPSVFVRTTEEGMIRVRKSKGKYAYLLESTMNEYIEQRK
PCDTMKVGGNLDSKGYGIATPKGSALRNPVNLAVLKLNEQGLLDKLKNKWWYDKGECGSGGGDSKDKTSALSL
SNVAGVFYILIGGLGLAMLVALIEFCYKSRSESKRMKGFCLIPQQSINEAIRTSTLPRNSGAGASGGSGSGEN
GRVVSQDFPKSMQSIPCMSHSSGMPLGATGL*
>rs33881482_ENSMUSP00000117746_c.1722:G>A_p.V575M
ENSMUST00000151045.3|Gria1-204|ENSMUSG00000020524|+|chr11:g.57174442:G>A
MTYRFCSQFSKGVYAIFGFYERRTVNMLTSFCGALHVCFITPSFPVDTSNQFVLQLRPELQEALISIIDHYKW
QTFVYIYDADRGLSVLQRVLDTAAEKNWQVTAVNILTTTEEGYRMLFQDLEKKKERLVVVDCESERLNAILGQ
IVKLEKNGIGYHYILANLGFMDIDLNKFKESGANVTGFQLVNYTDTIPARIMQQWRTSDARDHTRVDWKRPKY
TSALTYDGVKVMAEAFQSLRRQRIDISRRGNAGDCLANPAVPWGQGIDIQRALQQVRFEGLTGNVQFNEKGRR
TNYTLHVIEMKHDGIRKIGYWNEDDKFVPAATDAQAGGDNSSVQNRTYIVTTILEDPYVMLKKNANQFEGNDR
YEGYCVELAAEIAKHVGYSYRLEIVSDGKYGARDPDTKAWNGMVGELVYGRADVAVAPLTITLVREEVIDFSK
PFMSLGISIMIKKPQKSKPGVFSFLDPLAYEIWMCIVFAYIGVSVVLFLVSRFSPYEWHSEEFEEGRDQTTSD
QSNEFGIFNSLWFSLGAFMQQGCDISPRSLSGRIVGGVWWFFTLIIISSYTANLAAFLTVERMMSPIESAEDL
AKQTEIAYGTLEAGSTKEFFRRSKIAVFEKMWTYMKSAEPSVFVRTTEEGMIRVRKSKGKYAYLLESTMNEYI
EQRKPCDTMKVGGNLDSKGYGIATPKGSALRGPVNLAVLKLSEQGVLDKLKSKWWYDKGECGSKDSGSKDKTS
ALSLSNVAGVFYILIGGLGLAMLVALIEFCYKSRSESKRMKGFCLIPQQSINEAIRTSTLPRNSGAGASGGSG
SGENGRVVSQDFPKSMQSIPCMSHSSGMPLGATGL*
>rs51805917_ENSMUSP00000020831_c.999:G>A_p.A334T
ENSMUST00000020831.13|Fam114a2-201|ENSMUSG00000020523|-
|chr11:g.57383913:C>T
MSDKDPPESPVVTGVASTLKDENCEPVEKPEDKSQPVVSTRKRPETKPSSDLEASALPAQETASKDVPQTGWG
YWGSWGKSLLSSASATVATVGQGISNVIEKAETSLGIPSPTEISAEVKQAAGEKNAGENGSLLVAAPFGMLST
ISTAVQSTGKSVISGGLDALEFIGKKTMDVIAEGDPGFKRTKGLMNRTSTLSQVLREAKDKEEQRPSNEVTME
TDKKTHYGLLFDEFQGLSHLEALEMLSRESEIKVKSILSSLSGEELQTTRLELEQLKEVFSLAEFCEEEEEER
QGDDNFTKEITDLFAQLHVSSRPEKLARARNTAYKWIRTSLTRPVAEKEEGEKESEAGNTEEAQKHSIEDIHA
FAIRSLAELTACSIELFHKTAALVLHGQKQEVTALERSQTLSQMTVMLCKDLASLSKEFTTCLTTAGVREKAD
VLNPVITAVFLEASNSASYIQDAFQLLLPVLQISLIESKTESSTCEPQSRDL*

>rs51805917_ENSMUSP00000104478_c.1020:G>A_p.A341T
ENSMUST00000108850.2|Fam114a2-202|ENSMUSG00000020523|-
|chr11:g.57383913:C>T
MSDKDPPESPVVTGVASTLKDENCEPVEKPEDKSQPVVSTRKRPETKPSSDLEASALPAQVSLAVAKETASKD
VPQTGWGYWGSWGKSLLSSASATVATVGQGISNVIEKAETSLGIPSPTEISAEVKQAAGEKNAGENGSLLVAA
PFGMLSTISTAVQSTGKSVISGGLDALEFIGKKTMDVIAEGDPGFKRTKGLMNRTSTLSQVLREAKDKEEQRP
SNEVTMETDKKTHYGLLFDEFQGLSHLEALEMLSRESEIKVKSILSSLSGEELQTTRLELEQLKEVFSLAEFC
EEEEEERQGDDNFTKEITDLFAQLHVSSRPEKLARARNTAYKWIRTSLTRPVAEKEEGEKESEAGNTEEAQKH
SIEDIHAFAIRSLAELTACSIELFHKTAALVLHGQKQEVTALERSQTLSQMTVMLCKDLASLSKEFTTCLTTA
GVREKADVLNPVITAVFLEASNSASYIQDAFQLLLPVLQISLIESKTESSTCEPQSRDL*
>ENSMUSP00000104477_c.167:T>TAAA_p.I56K-ins ENSMUST00000108849.8|Mfap3-
203|ENSMUSG00000020522|+|chr11:g.57419009:T>TAAA
MKLHHCLSILLVVTLVPAALGLEDVAPLGANQSSYNASFLPSFELSAGSYSGDDVIKIVKEGTNVSLECLLTV
DQYGEVHWYNSKGQQLHSRGGKWLVSDNFLNITSVAFDDRGLYTCIITSPARASYSVTLRVIFTSGDMSVYYM
VVCLIAFTITLILNVTRLCLMSTHLRKTEKAINEFFRTEGAEKLQKAFEIAKRIPIITSAKTLELAKVTQFKT
MEFARYIEELARSVPLPPLILNCRAFVEEMFEAVRVDDPDDMGERIKERPALDAQSGIYVINPELGRSNSPGG
DSDDGSLSEQGQEIAVQVSVHLQSETKSIGTDSQDSSHFSPPSDPASAEGSIHHRVSI*
>ENSMUSP00000020830_c.167:T>TAAA_p.I56K-ins ENSMUST00000020830.14|Mfap3-
201|ENSMUSG00000020522|+|chr11:g.57419009:T>TAAA
MKLHHCLSILLVVTLVPAALGLEDVAPLGANQSSYNASFLPSFELSAGSYSGDDVIKIVKEGTNVSLECLLTV
DQYGEVHWYNSKGQQLHSRGGKWLVSDNFLNITSVAFDDRGLYTCIITSPARASYSVTLRVIFTSGDMSVYYM
VVCLIAFTITLILNVTRLCLMSTHLRKTEKAINEFFRTEGAEKLQKAFEIAKRIPIITSAKTLELAKVTQFKT
MEFARYIEELARSVPLPPLILNCRAFVEEMFEAVRVDDPDDMGERIKERPALDAQSGIYVINPELGRSNSPGG
DSDDGSLSEQGQEIAVQVSVHLQSETKSIGTDSQDSSHFSPPSDPASAEGSIHHRVSI*
>ENSMUSP00000104476_c.167:T>TAAA_p.I56K-ins ENSMUST00000108848.2|Mfap3-
202|ENSMUSG00000020522|+|chr11:g.57419009:T>TAAA
MKLHHCLSILLVVTLVPAALGLEDVAPLGANQSSYNASFLPSFELSAGSYSGDDVIKIVKEGTNVSLECLLTV
DQYGEVHWYNSKGQQLHSRGGKWLVSDNFLNITSVAFD
>rs49424190_ENSMUSP00000104477_c.523:A>G_p.K175R
ENSMUST00000108849.8|Mfap3-203|ENSMUSG00000020522|+|chr11:g.57420544:A>G
MKLHHCLSILLVVTLVPAALGLEDVAPLGANQSSYNASFLPSFELSAGSYSGDDVIIVKEGTNVSLECLLTVD
QYGEVHWYNSKGQQLHSRGGKWLVSDNFLNITSVAFDDRGLYTCIITSPARASYSVTLRVIFTSGDMSVYYMV
VCLIAFTITLILNVTRLCLMSTHLRKTERAINEFFRTEGAEKLQKAFEIAKRIPIITSAKTLELAKVTQFKTM
EFARYIEELARSVPLPPLILNCRAFVEEMFEAVRVDDPDDMGERIKERPALDAQSGIYVINPELGRSNSPGGD
SDDGSLSEQGQEIAVQVSVHLQSETKSIGTDSQDSSHFSPPSDPASAEGSIHHRVSI*
>rs49424190_ENSMUSP00000020830_c.523:A>G_p.K175R
ENSMUST00000020830.14|Mfap3-201|ENSMUSG00000020522|+|chr11:g.57420544:A>G
MKLHHCLSILLVVTLVPAALGLEDVAPLGANQSSYNASFLPSFELSAGSYSGDDVIIVKEGTNVSLECLLTVD
QYGEVHWYNSKGQQLHSRGGKWLVSDNFLNITSVAFDDRGLYTCIITSPARASYSVTLRVIFTSGDMSVYYMV
VCLIAFTITLILNVTRLCLMSTHLRKTERAINEFFRTEGAEKLQKAFEIAKRIPIITSAKTLELAKVTQFKTM
EFARYIEELARSVPLPPLILNCRAFVEEMFEAVRVDDPDDMGERIKERPALDAQSGIYVINPELGRSNSPGGD
SDDGSLSEQGQEIAVQVSVHLQSETKSIGTDSQDSSHFSPPSDPASAEGSIHHRVSI*
>rs49450509_ENSMUSP00000104477_c.673:T>C_p.I225T
ENSMUST00000108849.8|Mfap3-203|ENSMUSG00000020522|+|chr11:g.57420694:T>C
MKLHHCLSILLVVTLVPAALGLEDVAPLGANQSSYNASFLPSFELSAGSYSGDDVIIVKEGTNVSLECLLTVD
QYGEVHWYNSKGQQLHSRGGKWLVSDNFLNITSVAFDDRGLYTCIITSPARASYSVTLRVIFTSGDMSVYYMV
VCLIAFTITLILNVTRLCLMSTHLRKTEKAINEFFRTEGAEKLQKAFEIAKRIPIITSAKTLELAKVTQFKTM
EFARYTEELARSVPLPPLILNCRAFVEEMFEAVRVDDPDDMGERIKERPALDAQSGIYVINPELGRSNSPGGD
SDDGSLSEQGQEIAVQVSVHLQSETKSIGTDSQDSSHFSPPSDPASAEGSIHHRVSI*
>rs49450509_ENSMUSP00000020830_c.673:T>C_p.I225T
ENSMUST00000020830.14|Mfap3-201|ENSMUSG00000020522|+|chr11:g.57420694:T>C
MKLHHCLSILLVVTLVPAALGLEDVAPLGANQSSYNASFLPSFELSAGSYSGDDVIIVKEGTNVSLECLLTVD
QYGEVHWYNSKGQQLHSRGGKWLVSDNFLNITSVAFDDRGLYTCIITSPARASYSVTLRVIFTSGDMSVYYMV
VCLIAFTITLILNVTRLCLMSTHLRKTEKAINEFFRTEGAEKLQKAFEIAKRIPIITSAKTLELAKVTQFKTM
EFARYTEELARSVPLPPLILNCRAFVEEMFEAVRVDDPDDMGERIKERPALDAQSGIYVINPELGRSNSPGGD
SDDGSLSEQGQEIAVQVSVHLQSETKSIGTDSQDSSHFSPPSDPASAEGSIHHRVSI*
>rs3712502_ENSMUSP00000104477_c.858:A>C_p.N287H
ENSMUST00000108849.8|Mfap3-203|ENSMUSG00000020522|+|chr11:g.57420879:A>C

MKLHHCLSILLVVTLVPAALGLEDVAPLGANQSSYNASFLPSFELSAGSYSGDDVIIVKEGTNVSLECLLTVD
QYGEVHWYNSKGQQLHSRGGKWLVSDNFLNITSVAFDDRGLYTCIITSPARASYSVTLRVIFTSGDMSVYYMV
VCLIAFTITLILNVTRLCLMSTHLRKTEKAINEFFRTEGAEKLQKAFEIAKRIPIITSAKTLELAKVTQFKTM
EFARYIEELARSVPLPPLILNCRAFVEEMFEAVRVDDPDDMGERIKERPALDAQSGIYVINPELGRSHSPGGD
SDDGSLSEQGQEIAVQVSVHLQSETKSIGTDSQDSSHFSPPSDPASAEGSIHHRVSI*
>rs3712502_ENSMUSP00000020830_c.858:A>C_p.N287H
ENSMUST00000020830.14|Mfap3-201|ENSMUSG00000020522|+|chr11:g.57420879:A>C
MKLHHCLSILLVVTLVPAALGLEDVAPLGANQSSYNASFLPSFELSAGSYSGDDVIIVKEGTNVSLECLLTVD
QYGEVHWYNSKGQQLHSRGGKWLVSDNFLNITSVAFDDRGLYTCIITSPARASYSVTLRVIFTSGDMSVYYMV
VCLIAFTITLILNVTRLCLMSTHLRKTEKAINEFFRTEGAEKLQKAFEIAKRIPIITSAKTLELAKVTQFKTM
EFARYIEELARSVPLPPLILNCRAFVEEMFEAVRVDDPDDMGERIKERPALDAQSGIYVINPELGRSHSPGGD
SDDGSLSEQGQEIAVQVSVHLQSETKSIGTDSQDSSHFSPPSDPASAEGSIHHRVSI*
>rs264395791_ENSMUSP00000065096_c.118:C>CA_p.P40ins-fs
ENSMUST00000066987.14|Galnt10-
201|ENSMUSG00000020520|+|chr11:g.57536503:C>CA
MRRKEKRLLQAVALALAALVLLPNVGLWALYRERQPDGSPWGIGGGGGPRGGSGITQSTKKDIFLGS
>rs264395791_ENSMUSP00000104474_c.118:C>CA_p.P40ins-fs
ENSMUST00000108846.2|Galnt10-
202|ENSMUSG00000020520|+|chr11:g.57536503:C>CA
MRRKEKRLLQAVALALAALVLLPNVGLWALYRERQPDGSPWGIGGGGGPRGGSGITQSTKKDIFLGS
>rs46886604_ENSMUSP00000065096_c.183:A>T_p.T62S
ENSMUST00000066987.14|Galnt10-
201|ENSMUSG00000020520|+|chr11:g.57612145:A>T
MRRKEKRLLQAVALALAALVLLPNVGLWALYRERQPDGSPGGLGAAVAPAAVQELHSRQKKSFFLGAEQRLKD
WHNKEAIRRDAQRVGYGEQGKPYPMTDAERVDQAYRENGFNIYVSDKISLNRSLPDIRHPNCNSKLYLETLPN
TSIIIPFHNEGWSSLLRTVHSVLNRSPPELVAEIVLVDDFSDREHLKKPLEDYMALFPSVRILRTKKREGLIR
TRMLGASAATGDVVTFLDSHCEANVNWLPPLLDRIARNRKTIVCPMIDVIDHDDFRYETQAGDAMRGAFDWEM
YYKRIPIPPELQKADPSDPFESPVMAGGLFAVDRKWFWELGGYDPGLEIWGGEQYEISFKVWMCGGRMEDIPC
SRVGHIYRKYVPYKVPAGVSLARNLKRVAEVWMDEYAEYIYQRRPEYRHLSAGDVVAQKKLRVSLNCKSFKWF
MTKIAWDLPKFYPPVEPPAAAWGEIRNVGTGLCTDTKLGTLGSPLRLETCIRGRGEAAWNSMQVFTFTWREDI
RPGDPQHTKKFCFDAVSHTSPVTLYDCHSMKGNQLWKYRKDKTLYHPVSGSCMDCSESDHRVFMNTCNPSSLT
QQWLFEHTNSTVLENFNKN*
>rs46886604_ENSMUSP00000104474_c.183:A>T_p.T62S
ENSMUST00000108846.2|Galnt10-
202|ENSMUSG00000020520|+|chr11:g.57612145:A>T
MRRKEKRLLQAVALALAALVLLPNVGLWALYRERQPDGSPGGLGAAVAPAAVQELHSRQKKSFFLGAEQRLKD
WHNKEAIRRDAQRVGYGEQGKPYPMTDAERVDQAYRENGFNIYVSDKISLNRSLPDIRHPK*
>rs47128972_ENSMUSP00000065096_c.603:C>T_p.L202F
ENSMUST00000066987.14|Galnt10-
201|ENSMUSG00000020520|+|chr11:g.57656421:C>T
MRRKEKRLLQAVALALAALVLLPNVGLWALYRERQPDGSPGGLGAAVAPAAVQELHSRQKKTFFLGAEQRLKD
WHNKEAIRRDAQRVGYGEQGKPYPMTDAERVDQAYRENGFNIYVSDKISLNRSLPDIRHPNCNSKLYLETLPN
TSIIIPFHNEGWSSLLRTVHSVLNRSPPELVAEIVLVDDFSDREHLKKPLEDYMAFFPSVRILRTKKREGLIR
TRMLGASAATGDVVTFLDSHCEANVNWLPPLLDRIARNRKTIVCPMIDVIDHDDFRYETQAGDAMRGAFDWEM
YYKRIPIPPELQKADPSDPFESPVMAGGLFAVDRKWFWELGGYDPGLEIWGGEQYEISFKVWMCGGRMEDIPC
SRVGHIYRKYVPYKVPAGVSLARNLKRVAEVWMDEYAEYIYQRRPEYRHLSAGDVVAQKKLRVSLNCKSFKWF
MTKIAWDLPKFYPPVEPPAAAWGEIRNVGTGLCTDTKLGTLGSPLRLETCIRGRGEAAWNSMQVFTFTWREDI
RPGDPQHTKKFCFDAVSHTSPVTLYDCHSMKGNQLWKYRKDKTLYHPVSGSCMDCSESDHRVFMNTCNPSSLT
QQWLFEHTNSTVLENFNKN*
>rs29437286_ENSMUSP00000020826_c.171:A>G_p.K58E
ENSMUST00000020826.6|Sap30l-201|ENSMUSG00000020519|+|chr11:g.57693089:A>G
MNGFSTEEDSREGPPAAPAAAPGYGQSCCLIADGERCVRPAGNASFSKRVQKSISQKELKLDIDKSVRHLYIC
DFHKNFIQSVRNKRKRKASDDGGDSPEHDADIPEVDLFQLQVNTLRRYKRHYKLQTRPGFNKAQLAETVSRHF
RNIPVNEKETLAYFIYMVKSNRSRLDQKSEGSKQLE*
>rs29434411_ENSMUSP00000020826_c.252:A>G_p.N85D
ENSMUST00000020826.6|Sap30l-201|ENSMUSG00000020519|+|chr11:g.57696924:A>G

MNGFSTEEDSREGPPAAPAAAPGYGQSCCLIADGERCVRPAGNASFSKRVQKSISQKKLKLDIDKSVRHLYIC
DFHKNFIQSVRDKRKRKASDDGGDSPEHDADIPEVDLFQLQVNTLRRYKRHYKLQTRPGFNKAQLAETVSRHF
RNIPVNEKETLAYFIYMVKSNRSRLDQKSEGSKQLE*
>rs29431791_ENSMUSP00000046999_c.147:G>T_p.A50S
ENSMUST00000036917.3|Hand1-201|ENSMUSG00000037335|-|chr11:g.57722465:C>A
MNLVGSYAHHHHHHHSHPPHPMLHEPFLFGPASRCHQERPYFQSWLLSPSDAAPDFPAGGPPPTTAVAAAAYG
PDARPSQSPGRLEALGSRLPKRKGSGPKKERRRTESINSAFAELRECIPNVPADTKLSKIKTLRLATSYIAYL
MDVLAKDAQAGDPEAFKAELKKTDGGRESKRKRELPQQPESFPPASGPGEKRIKGRTGWPQQVWALELNQ*
>rs29431791_ENSMUSP00000124951_c.147:G>T_p.A50S
ENSMUST00000160392.9|Hand1-203|ENSMUSG00000037335|-|chr11:g.57722465:C>A
MNLVGSYAHHHHHHHSHPPHPMLHEPFLFGPASRCHQERPYFQSWLLSPSDAAPDFPAGGPPPTTAVAAAAYG
PDARPSQSPGRLEALGSRLPKRKGSGPKKERRRTESINSAFAELRECIPNVPADTKLSKIKTLRLATSYIAYL
MDVLAKDAQAGDPEAFKAELKKTDGGRESKRKRELPQQPESFPPASGPGEKRIKGRTGWPQQVWALELNQ*
>rs49487294_ENSMUSP00000071421_c.1896:C>T_p.H633Y
ENSMUST00000071487.13|Larp1-201|ENSMUSG00000037331|+|chr11:g.57940772:C>T
MATQVEPLLPAGAPLLQAEEHGLARKKPAPDAQAESGPGDGGGEPDGGVRRPRPACARPGRDGAERESPRPPA
AAEAPAGSDGEDGGRRDFVEAPPPKVNPWTKHAPPPAAVNGQPPPEPSAPAKVVRAAAPKPRKGSKVGDFGDA
VNWPTPGEIAHKSVQPQSHKPQPARKLPPKKDMKEQEKGDGSDSKESPKTKSDESGEEKNGDEDCQRGGQKKK
GNKHKWVPLQIDMKPEVPREKLASRPTRPQEPRHTPAVRGEMKGSEPATYMPVSVAPPTPAWQPETKVEPAWH
DQDETSSVKSDGAGGARASFRGRGRGRGRGRGRGRGGTRTHFDYQFGYRKFDGTEGPRTHKYMNNITYYFDNV
SSNEIYSMDQELLKDYIKRQIEYYFSVDNLERDFFLRRKMDADGFLPITLIASFHRVQALTTDISLIFAALKD
SKVVEMVEEKVRRREEPEKWPLPGPPIVDYSQTDFSQLLNCPEFVPRQHYQKETESAPGSPRAVTPVPTKTEE
VSNLKTLPKGLSASLPDLDSESWIEVKKRPRPSPARPKKPEEPRFSHPTALPQQLPSQQLMSKDQDEQEELDF
LFDEEMEQMDGRKNTFTAWSEEDSDYEIDDRDVNKILIVTQTPPYMRRYPGGDRTGNHTSRAKMSAELAKVIN
DGLFYYEQDLWTEKFEPEYSQIKQEVENFKKVNMISREQFDTLTPEPPVDPNQEVPPGPPRFQQVPTDALANK
LFGAPEPSTIARSLPTTVPESPNYRNARTPRTPRTPQLKDSSQTPRFYPVVKEGRTLDAKMPRKRKTRHSSNP
PLESHVGWVMDSREHRPRTASISSSPSEGTPAVGSYGCTPQSLPKFQHPSHELLKENGFTQHVYHKYRRRCLN
ERKRLGIGQSQEMNTLFRFWSFFLRDHFNKKMYEEFKQLALEDAKEGYRYGLECLFRYYSYGLEKKFRLDIFK
DFQEETVKDYEAGQLYGLEKFWAFLKYSKAKNLDIDPKLQEYLGKFRRLEDFRVDPPMGEEGNHKRHPVVAGG
SGEGRKRCPSQSSSRPATGISQPPTTPTGQATREDAKWTSQHSDTLTLRK*
>rs49487294_ENSMUSP00000136673_c.1896:C>T_p.H633Y
ENSMUST00000178636.2|Larp1-203|ENSMUSG00000037331|+|chr11:g.57940772:C>T
MATQVEPLLPAGAPLLQAEEHGLARKKPAPDAQAESGPGDGGGEPDGGVRRPRPACARPGRDGAERESPRPPA
AAEAPAGSDGEDGGRRDFVEAPPPKVNPWTKHAPPPAAVNGQPPPEPSAPAKVVRAAAPKPRKGSKVGDFGDA
VNWPTPGEIAHKSVQPQSHKPQPARKLPPKKDMKEQEKGDGSDSKESPKTKSDESGEEKNGDEDCQRGGQKKK
GSKHKWVPLQIDMKPEVPREKLASRPTRPQEPRHTPAVRGEMKGSEPATYMPVSVAPPTPAWQPETKVEPAWH
DQDETSSVKSDGAGGARASFRGRGRGRGRGRGRGRGGTRTHFDYQFGYRKFDGTEGPRTHKYMNNITYYFDNV
SSNEIYSMDQELLKDYIKRQIEYYFSVDNLERDFFLRRKMDADGFLPITLIASFHRVQALTTDISLIFAALKD
SKVVEMVEEKVRRREEPEKWPLPGPPIVDYSQTDFSQLLNCPEFVPRQHYQKETESAPGSPRAVTPVPTKTEE
VSNLKTLPKGLSASLPDLDSESWIEVKKRPRPSPARPKKPEEPRFSHPTALPQQLPSQQLMSKDQDEQEELDF
LFDEEMEQMDGRKNTFTAWSEEDSDYEIDDRDVNKILIVTQTPPYMRRYPGGDRTGNHTSRAKMSAELAKVIN
DGLFYYEQDLWTEKFEPEYSQIKQEVENFKKVNMISREQFDTLTPEPPVDPNQEVPPGPPRFQQVPTDALANK
LFGAPEPSTIARSLPTTVPESPNYRNARTPRTPRTPQLKDSSQTPRFYPVVKEGRTLDAKMPRKRKTRHSSNP
PLESHVGWVMDSREHRPRTASISSSPSEGTPAVGSYGCTPQSLPKFQHPSHELLKENGFTQHVYHKYRRRCLN
ERKRLGIGQSQEMNTLFRFWSFFLRDHFNKKMYEEFKQLALEDAKEGYRYGLECLFRYYSYGLEKKFRLDIFK
DFQEETVKDYEAGQLYGLEKFWAFLKYSKAKNLDIDPKLQEYLGKFRRLEDFRVDPPMGEEGNHKRHPVVAGG
SGEGRKRCPSQSSSRPATGISQPPTTPTGQATREDAKWTSQHSDTLTLRK*
>rs47101486_ENSMUSP00000071421_c.2512:G>A_p.S838N
ENSMUST00000071487.13|Larp1-201|ENSMUSG00000037331|+|chr11:g.57943441:G>A
MATQVEPLLPAGAPLLQAEEHGLARKKPAPDAQAESGPGDGGGEPDGGVRRPRPACARPGRDGAERESPRPPA
AAEAPAGSDGEDGGRRDFVEAPPPKVNPWTKHAPPPAAVNGQPPPEPSAPAKVVRAAAPKPRKGSKVGDFGDA
VNWPTPGEIAHKSVQPQSHKPQPARKLPPKKDMKEQEKGDGSDSKESPKTKSDESGEEKNGDEDCQRGGQKKK
GNKHKWVPLQIDMKPEVPREKLASRPTRPQEPRHTPAVRGEMKGSEPATYMPVSVAPPTPAWQPETKVEPAWH
DQDETSSVKSDGAGGARASFRGRGRGRGRGRGRGRGGTRTHFDYQFGYRKFDGTEGPRTHKYMNNITYYFDNV
SSNEIYSMDQELLKDYIKRQIEYYFSVDNLERDFFLRRKMDADGFLPITLIASFHRVQALTTDISLIFAALKD
SKVVEMVEEKVRRREEPEKWPLPGPPIVDYSQTDFSQLLNCPEFVPRQHYQKETESAPGSPRAVTPVPTKTEE
VSNLKTLPKGLSASLPDLDSESWIEVKKRPRPSPARPKKPEEPRFSHPTALPQQLPSQQLMSKDQDEQEELDF
LFDEEMEQMDGRKNTFTAWSEEDSDYEIDDRDVNKILIVTQTPPYMRRHPGGDRTGNHTSRAKMSAELAKVIN

DGLFYYEQDLWTEKFEPEYSQIKQEVENFKKVNMISREQFDTLTPEPPVDPNQEVPPGPPRFQQVPTDALANK
LFGAPEPSTIARSLPTTVPESPNYRNARTPRTPRTPQLKDSSQTPRFYPVVKEGRTLDAKMPRKRKTRHSSNP
PLESHVGWVMDSREHRPRTASISSSPSEGTPAVGNYGCTPQSLPKFQHPSHELLKENGFTQHVYHKYRRRCLN
ERKRLGIGQSQEMNTLFRFWSFFLRDHFNKKMYEEFKQLALEDAKEGYRYGLECLFRYYSYGLEKKFRLDIFK
DFQEETVKDYEAGQLYGLEKFWAFLKYSKAKNLDIDPKLQEYLGKFRRLEDFRVDPPMGEEGNHKRHPVVAGG
SGEGRKRCPSQSSSRPATGISQPPTTPTGQATREDAKWTSQHSDTLTLRK*
>rs47101486_ENSMUSP00000136673_c.2512:G>A_p.S838N
ENSMUST00000178636.2|Larp1-203|ENSMUSG00000037331|+|chr11:g.57943441:G>A
MATQVEPLLPAGAPLLQAEEHGLARKKPAPDAQAESGPGDGGGEPDGGVRRPRPACARPGRDGAERESPRPPA
AAEAPAGSDGEDGGRRDFVEAPPPKVNPWTKHAPPPAAVNGQPPPEPSAPAKVVRAAAPKPRKGSKVGDFGDA
VNWPTPGEIAHKSVQPQSHKPQPARKLPPKKDMKEQEKGDGSDSKESPKTKSDESGEEKNGDEDCQRGGQKKK
GSKHKWVPLQIDMKPEVPREKLASRPTRPQEPRHTPAVRGEMKGSEPATYMPVSVAPPTPAWQPETKVEPAWH
DQDETSSVKSDGAGGARASFRGRGRGRGRGRGRGRGGTRTHFDYQFGYRKFDGTEGPRTHKYMNNITYYFDNV
SSNEIYSMDQELLKDYIKRQIEYYFSVDNLERDFFLRRKMDADGFLPITLIASFHRVQALTTDISLIFAALKD
SKVVEMVEEKVRRREEPEKWPLPGPPIVDYSQTDFSQLLNCPEFVPRQHYQKETESAPGSPRAVTPVPTKTEE
VSNLKTLPKGLSASLPDLDSESWIEVKKRPRPSPARPKKPEEPRFSHPTALPQQLPSQQLMSKDQDEQEELDF
LFDEEMEQMDGRKNTFTAWSEEDSDYEIDDRDVNKILIVTQTPPYMRRHPGGDRTGNHTSRAKMSAELAKVIN
DGLFYYEQDLWTEKFEPEYSQIKQEVENFKKVNMISREQFDTLTPEPPVDPNQEVPPGPPRFQQVPTDALANK
LFGAPEPSTIARSLPTTVPESPNYRNARTPRTPRTPQLKDSSQTPRFYPVVKEGRTLDAKMPRKRKTRHSSNP
PLESHVGWVMDSREHRPRTASISSSPSEGTPAVGNYGCTPQSLPKFQHPSHELLKENGFTQHVYHKYRRRCLN
ERKRLGIGQSQEMNTLFRFWSFFLRDHFNKKMYEEFKQLALEDAKEGYRYGLECLFRYYSYGLEKKFRLDIFK
DFQEETVKDYEAGQLYGLEKFWAFLKYSKAKNLDIDPKLQEYLGKFRRLEDFRVDPPMGEEGNHKRHPVVAGG
SGEGRKRCPSQSSSRPATGISQPPTTPTGQATREDAKWTSQHSDTLTLRK*
>rs49332894_ENSMUSP00000151952_c.420:C>A_p.Q141K
ENSMUST00000218429.2|Gm12248-202|ENSMUSG00000086962|-
|chr11:g.57957410:G>T
MIFLPRHLQRFWDASGYFCQTQWEKLLSTFEGYEWILYYIGVILVPGLCFWGLSGLLLLVDTTEKPTFISRYR
IQRGKNEPVDPVKLRQAVLTVLFNQNFISIPMLVIYYPFLKWRRDPCSRELPTFHWFLVEMAFFTLLKEILFY
YAHRLFHHPMLYKKSHKKHHEWTAPIGVVSIYADPPEHVVANMLPVLVGPLAMGSHLSSITVWLSMVLIVSII
NHTGYHLPFLPSPEFHDYHHLKSNQCYGVLGLMDHLHGTDTLFKQTKAYERHVILLGFTPLSESIPDSPKKTR
SAL*
>rs49332894_ENSMUSP00000151997_c.474:C>A_p.Q159K
ENSMUST00000219877.2|Gm12248-203|ENSMUSG00000086962|-
|chr11:g.57957410:G>T
MRNTTLIQGSGLLLLLVFWNSVTWHLQRFWDASGYFCQTQWEKLLSTFEGYEWILYYIGVILVPGLCFWGLSG
LLLLVDTTEKPTFISRYRIQRGKNEPVDPVKLRQAVLTVLFNQNFISIPMLVIYYPFLKWRRDPCSRELPTFH
WFLVEMAFFTLLKEILFYYAHRLFHHPMLYKKSHKKHHEWTAPIGVVSIYADPPEHVVANMLPVLVGPLAMGS
HLSSITVWLSMVLIVSIINHTGYHLPFLPSPEFHDYHHLKSNQCYGVLGLMDHLHGTDTLFKQTKAYERHVIL
LGFTPLSESIPDSPKKTRSAL*
>rs49332894_ENSMUSP00000151429_c.477:C>A_p.Q160K
ENSMUST00000137124.3|Gm12248-201|ENSMUSG00000086962|-
|chr11:g.57957410:G>T
SMRNTTLIQGSGLLLLLVFWNSVTWHLQRFWDASGYFCQTQWEKLLSTFEGYEWILYYIGVILVPGLCFWGLS
GLLLLVDTTEKPTFISRYRIQRGKNEPVDPVKLRQAVLTVLFNQNFISIPMLVIYYPFLKWRRDPCSRELPTF
HWFLVEMAFFTLLKEILFYYAHRLFHHPMLYKKSHKKHHEWTAPIGVVSIYADPPEHVVANMLPVLVGPLAMG
SHLSSITVWLSMVLIVSIINHTGYHLPFLPSPEFHDYHHLKSNQCYGVLGLMDHLHGTDTLFKQTKAYERHVI
LLGFTPLSESIPDSPKKTR
>rs212675617_ENSMUSP00000020822_c.717:A>G_p.R240G
ENSMUST00000020822.12|Cnot8-201|ENSMUSG00000020515|+|chr11:g.58006190:A>G
MPAALVENSQVICEVWASNLEEEMRKIREIVLSYSYIAMDTEFPGVVVRPIGEFRSSIDYQYQLLRCNVDLLK
IIQLGLTFTNEKGEYPSGINTWQFNFKFNLTEDMYSQDSIDLLANSGLQFQKHEEEGIDTLHFAELLMTSGVV
LCDNVKWLSFHSGYDFGYMVKLLTDSRLPEEEHEFFHILNLFFPSIYDVKYLMKSCKNLKGGLQEVADQLDLQ
RIGRQHQAGSDSLLTGMAFFGMKELFFEDSIDDAKYCGRLYGLGTGVAQKQNEDVDCAQEKMSILAMINNMQQ
*
>rs212675617_ENSMUSP00000104471_c.717:A>G_p.R240G
ENSMUST00000108843.8|Cnot8-202|ENSMUSG00000020515|+|chr11:g.58006190:A>G
MPAALVENSQVICEVWASNLEEEMRKIREIVLSYSYIAMDTEFPGVVVRPIGEFRSSIDYQYQLLRCNVDLLK
IIQLGLTFTNEKGEYPSGINTWQFNFKFNLTEDMYSQDSIDLLANSGLQFQKHEEEGIDTLHFAELLMTSGVV

LCDNVKWLSFHSGYDFGYMVKLLTDSRLPEEEHEFFHILNLFFPSIYDVKYLMKSCKNLKGGLQEVADQLDLQ
RIGRQHQAGSDSLLTGMAFFGMKELFFEDSIDDAKYCGRLYGLGTGVAQKQNEDVDCAQEKMSILAMINNMQQ
*
>rs29483150_ENSMUSP00000036603_c.3618:G>A_p.D1207N
ENSMUST00000035604.13|Gemin5-201|ENSMUSG00000037275|-
|chr11:g.58017399:C>T
MKPEPRTLPPSPNWYCSRCSDAAPGGIFGFAARTSVFLVRVGPGAGASPGAPPFRVVGELVGHTERVSGFTFS
HHPGQYNLCATSSDDGTVKVWDVETKTVVTEHTLHQHTISALHWSPTVKDLIVSGDEKGVVFCYWLNRNDSQH
LFTEPRTIFCLTCSPHHENLVAIGYKDGIVVIIDISKKGEVIHRLRGHDDEIHSIAWCPLSGEDCLSISQEEN
SEEPDIPNGKLIAETPITKGCYLATGSKDQTIRIWSCSRGRGVMVLKLPFLKRRSGGVDPTVKERLWLTLHWP
KNQPTQLVSSCFGGELLLWDLTQSWRRKYTLFSTSAEGHNHSRIVFNLCSLKTEDGKQLLLSTSMDRDVKCWD
MATLECCWTLPSLGGFAYSLAFSPVDVGSLAIGVGDGMIRVWNTLSIKNNYDVKNFWQGVKSKVTALCWHPNK
EGCLAFGTDDGKVGLYDTCSNKPPQISSTYHKKTVYRLAWGPPVPPMSLGGEGDRPSLTLYSCGGEGVVLQHN
PWKLSGEAFDINKLVRDTNSIRYKLPVHTEISWKGDGKVLALGNEDGSIEIFQVPNLRLLCTIQQHHKLVNAI
VWHHEHGSRPELSCLLASGSNNAVIYVHNLKAVLESNPESPITITEPYRTLSGHTAKITSLAWSPHHDGRLVS
ACYDGTAQVWDALREEPLFNFRGHRGRLLCVAWSPVDPECIYSGADDFCVYRWLTSMQDHSRPPQGKKCIELE
KKRLSQFKPKLKKKKKPTLRLPVKQDSSVGNEDESVKENSGPAENGLSDQDGEEEAQEPELPPSPVVCVEPVS
CTDICSGFEKSKVTVSSKATSLKKEPAKEKPEALLKKRKARSMLPLSTSLDHRSKEELHRDCLVLATATHAKA
ELNEDVSADLEERFHLGLFTDRATLYRMMETEGKGHLESGHPELFHQLMLWKGDLKGVLQAAAERGELTDSLV
AVAPVAGYSVWLWAVEAFAKQLCFQDQYVKAASYLLSIHKVYEAVELLKSNHLYREAIAVAKARLRPEDPVLK
ELYLSWGSILERDGHYAIAAKCYLGATSAYDAAKVLARKGDAASLRTAAELAAIAGEHELAASLALRCAQELL
LMKNWVGAQEALGLHESLQGQRLVFCLLELLCRHLEEKQPLEVRGPSSIYHQWATGSEGTLVQRVTGVWRSAF
SVDTPEQCQAALQKLQDVKYPSATSNTPFRQLLLHVCHNLTLAMLSQQAAAWEEAVPALLQAVVRSYTSGNFT
LMQEIYSAFLPGGCDHLRDKLGDLSPAMAAYKSLEAFCIYGQLYEVWWSLCGPGPESSVWVLSAESTVSDKQS
KPEDSASAEDMEQPPGPGPRLSAESERLLSACKELFSERHASLQTSQRTVAEVQETLAEMIRQHQKSQLCKAT
TNGPSRDEPSRDEPSQEAERAPSQPPSPTEERNAPVSLPELTRRLTEANERIAEFPESVKAWPFPDVLECCLV
LLHIGSQCPDAVDPEMQQQAQELLHKYGHTRAYRRHCQSRHT*
>rs29483150_ENSMUSP00000099772_c.3615:G>A_p.D1206N
ENSMUST00000102711.9|Gemin5-202|ENSMUSG00000037275|-|chr11:g.58017399:C>T
MKPEPRTLPPSPNWYCSRCSDAAPGGIFGFAARTSVFLVRVGPGAGASPGAPPFRVVGELVGHTERVSGFTFS
HHPGQYNLCATSSDDGTVKVWDVETKTVVTEHTLHQHTISALHWSPTVKDLIVSGDEKGVVFCYWLNRNDSQH
LFTEPRTIFCLTCSPHHENLVAIGYKDGIVVIIDISKKGEVIHRLRGHDDEIHSIAWCPLSGEDCLSISQEEN
SEEPDIPNGKLIAETPITKGCYLATGSKDQTIRIWSCSRGRGVMVLKLPFLKRRSGGVDPTVKERLWLTLHWP
KNQPTQLVSSCFGGELLLWDLTQSWRRKYTLFSTSAEGHNHSRIVFNLCSLKTEDGKQLLLSTSMDRDVKCWD
MATLECCWTLPSLGGFAYSLAFSPVDVGSLAIGVGDGMIRVWNTLSIKNNYDVKNFWQGVKSKVTALCWHPNK
EGCLAFGTDDGKVGLYDTCSNKPPQISSTYHKKTVYRLAWGPPVPPMSLGGEGDRPSLTLYSCGGEGVVLQHN
PWKLSGEAFDINKLVRDTNSIRYKLPVHTEISWKGDGKVLALGNEDGSIEIFQVPNLRLLCTIQQHHKLVNAI
VWHHEHGSRPELSCLLASGSNNAVIYVHNLKAVLESNPESPITITEPYRTLSGHTAKITSLAWSPHHDGRLVS
ACYDGTAQVWDALREEPLFNFRGHRGRLLCVAWSPVDPECIYSGADDFCVYRWLTSMQDHSRPPQGKKCIELE
KKRLSQFKPKLKKKKKPTLRLPVKQDSSVGNEDESVKENSGPAENGLSDQDGEEEAQEPELPPSPVVCVEPVS
CTDICSGFEKSKVTVSSKATSLKKEPAKEKPEALLKKRKARSMLPLSTSLDHRSKEELHRDCLVLATATHAKE
LNEDVSADLEERFHLGLFTDRATLYRMMETEGKGHLESGHPELFHQLMLWKGDLKGVLQAAAERGELTDSLVA
VAPVAGYSVWLWAVEAFAKQLCFQDQYVKAASYLLSIHKVYEAVELLKSNHLYREAIAVAKARLRPEDPVLKE
LYLSWGSILERDGHYAIAAKCYLGATSAYDAAKVLARKGDAASLRTAAELAAIAGEHELAASLALRCAQELLL
MKNWVGAQEALGLHESLQGQRLVFCLLELLCRHLEEKQPLEVRGPSSIYHQWATGSEGTLVQRVTGVWRSAFS
VDTPEQCQAALQKLQDVKYPSATSNTPFRQLLLHVCHNLTLAMLSQQAAAWEEAVPALLQAVVRSYTSGNFTL
MQEIYSAFLPGGCDHLRDKLGDLSPAMAAYKSLEAFCIYGQLYEVWWSLCGPGPESSVWVLSAESTVSDKQSK
PEDSASAEDMEQPPGPGPRLSAESERLLSACKELFSERHASLQTSQRTVAEVQETLAEMIRQHQKSQLCKATT
NGPSRDEPSRDEPSQEAERAPSQPPSPTEERNAPVSLPELTRRLTEANERIAEFPESVKAWPFPDVLECCLVL
LHIGSQCPDAVDPEMQQQAQELLHKYGHTRAYRRHCQSRHT*
>rs29483150_ENSMUSP00000131842_c.3618:G>A_p.D1207N
ENSMUST00000172035.8|Gemin5-205|ENSMUSG00000037275|-|chr11:g.58017399:C>T
MKPEPRTLPPSPNWYCSRCSDAAPGGIFGFAARTSVFLVRVGPGAGASPGAPPFRVVGELVGHTERVSGFTFS
HHPGQYNLCATSSDDGTVKVWDVETKTVVTEHTLHQHTISALHWSPTVKDLIVSGDEKGVVFCYWLNRNDSQH
LFTEPRTIFCLTCSPHHENLVAIGYKDGIVVIIDISKKGEVIHRLRGHDDEIHSIAWCPLSGEDCLSISQEEN
SEEPDIPNGKLIAETPITKGCYLATGSKDQTIRIWSCSRGRGVMVLKLPFLKRRSGGVDPTVKERLWLTLHWP
KNQPTQLVSSCFGGELLLWDLTQSWRRKYTLFSTSAEGHNHSRIVFNLCSLKTEDGKQLLLSTSMDRDVKCWD
MATLECCWTLPSLGGFAYSLAFSPVDVGSLAIGVGDGMIRVWNTLSIKNNYDVKNFWQGVKSKVTALCWHPNK

EGCLAFGTDDGKVGLYDTCSNKPPQISSTYHKKTVYRLAWGPPVPPMSLGGEGDRPSLTLYSCGGEGVVLQHN
PWKLSGEAFDINKLVRDTNSIRYKLPVHTEISWKGDGKVLALGNEDGSIEIFQVPNLRLLCTIQQHHKLVNAI
VWHHEHGSRPELSCLLASGSNNAVIYVHNLKAVLESNPESPITITEPYRTLSGHTAKITSLAWSPHHDGRLVS
ACYDGTAQVWDALREEPLFNFRGHRGRLLCVAWSPVDPECIYSGADDFCVYRWLTSMQDHSRPPQGKKCIELE
KKRLSQFKPKLKKKKKPTLRLPVKQDSSVGNEDESVKENSGPAENGLSDQDGEEEAQEPELPPSPVVCVEPVS
CTDICSGFEKSKVTVSSKATSLKKEPAKEKPEALLKKRKARSMLPLSTSLDHRSKEELHRDCLVLATATHAKA
ELNEDVSADLEERFHLGLFTDRATLYRMMETEGKGHLESGHPELFHQLMLWKGDLKGVLQAAAERGELTDSLV
AVAPVAGYSVWLWAVEAFAKQLCFQDQYVKAASYLLSIHKVYEAVELLKSNHLYREAIAVAKARLRPEDPVLK
ELYLSWGSILERDGHYAIAAKCYLGATSAYDAAKVLARKGDAASLRTAAELAAIAGEHELAASLALRCAQELL
LMKNWVGAQEALGLHESLQGQRLVFCLLELLCRHLEEKQPLEVRGPSSIYHQWATGSEGTLVQRVTGVWRSAF
SVDTPEQCQAALQKLQDVKYPSATSNTPFRQLLLHVCHNLTLAMLSQQAAAWEEAVPALLQAVVRSYTSGNFT
LMQEIYSAFLPGGCDHLRDKLGDLSPAMAAYKSLEAFCIYGQLYEVWWSLCGPGPESSVWVLSAESTVSDKQS
KPEDSASAEDMEQPPGPGPRLSAESERLLSACKELFSERHASLQTSQRTVAEVQETLAEMIRQHQKSQLCKAT
TNGPSRDEPSRDEPSQEAERAPSQPPSPTREERNAPVSLPELTRRLTEANERIAEFPESVKAWPFPDVLECCL
VLLHIGSQCPDAVDPEMQQQAQELLHKYGHTRAYRRHCQSRHT*
>rs51771045_ENSMUSP00000036603_c.3498:G>C_p.A1167P
ENSMUST00000035604.13|Gemin5-201|ENSMUSG00000037275|-
|chr11:g.58019569:C>G
MKPEPRTLPPSPNWYCSRCSDAAPGGIFGFAARTSVFLVRVGPGAGASPGAPPFRVVGELVGHTERVSGFTFS
HHPGQYNLCATSSDDGTVKVWDVETKTVVTEHTLHQHTISALHWSPTVKDLIVSGDEKGVVFCYWLNRNDSQH
LFTEPRTIFCLTCSPHHENLVAIGYKDGIVVIIDISKKGEVIHRLRGHDDEIHSIAWCPLSGEDCLSISQEEN
SEEPDIPNGKLIAETPITKGCYLATGSKDQTIRIWSCSRGRGVMVLKLPFLKRRSGGVDPTVKERLWLTLHWP
KNQPTQLVSSCFGGELLLWDLTQSWRRKYTLFSTSAEGHNHSRIVFNLCSLKTEDGKQLLLSTSMDRDVKCWD
MATLECCWTLPSLGGFAYSLAFSPVDVGSLAIGVGDGMIRVWNTLSIKNNYDVKNFWQGVKSKVTALCWHPNK
EGCLAFGTDDGKVGLYDTCSNKPPQISSTYHKKTVYRLAWGPPVPPMSLGGEGDRPSLTLYSCGGEGVVLQHN
PWKLSGEAFDINKLVRDTNSIRYKLPVHTEISWKGDGKVLALGNEDGSIEIFQVPNLRLLCTIQQHHKLVNAI
VWHHEHGSRPELSCLLASGSNNAVIYVHNLKAVLESNPESPITITEPYRTLSGHTAKITSLAWSPHHDGRLVS
ACYDGTAQVWDALREEPLFNFRGHRGRLLCVAWSPVDPECIYSGADDFCVYRWLTSMQDHSRPPQGKKCIELE
KKRLSQFKPKLKKKKKPTLRLPVKQDSSVGNEDESVKENSGPAENGLSDQDGEEEAQEPELPPSPVVCVEPVS
CTDICSGFEKSKVTVSSKATSLKKEPAKEKPEALLKKRKARSMLPLSTSLDHRSKEELHRDCLVLATATHAKA
ELNEDVSADLEERFHLGLFTDRATLYRMMETEGKGHLESGHPELFHQLMLWKGDLKGVLQAAAERGELTDSLV
AVAPVAGYSVWLWAVEAFAKQLCFQDQYVKAASYLLSIHKVYEAVELLKSNHLYREAIAVAKARLRPEDPVLK
ELYLSWGSILERDGHYAIAAKCYLGATSAYDAAKVLARKGDAASLRTAAELAAIAGEHELAASLALRCAQELL
LMKNWVGAQEALGLHESLQGQRLVFCLLELLCRHLEEKQPLEVRGPSSIYHQWATGSEGTLVQRVTGVWRSPF
SVDTPEQCQAALQKLQDVKYPSATSNTPFRQLLLHVCHDLTLAMLSQQAAAWEEAVPALLQAVVRSYTSGNFT
LMQEIYSAFLPGGCDHLRDKLGDLSPAMAAYKSLEAFCIYGQLYEVWWSLCGPGPESSVWVLSAESTVSDKQS
KPEDSASAEDMEQPPGPGPRLSAESERLLSACKELFSERHASLQTSQRTVAEVQETLAEMIRQHQKSQLCKAT
TNGPSRDEPSRDEPSQEAERAPSQPPSPTEERNAPVSLPELTRRLTEANERIAEFPESVKAWPFPDVLECCLV
LLHIGSQCPDAVDPEMQQQAQELLHKYGHTRAYRRHCQSRHT*
>rs51771045_ENSMUSP00000099772_c.3495:G>C_p.A1166P
ENSMUST00000102711.9|Gemin5-202|ENSMUSG00000037275|-|chr11:g.58019569:C>G
MKPEPRTLPPSPNWYCSRCSDAAPGGIFGFAARTSVFLVRVGPGAGASPGAPPFRVVGELVGHTERVSGFTFS
HHPGQYNLCATSSDDGTVKVWDVETKTVVTEHTLHQHTISALHWSPTVKDLIVSGDEKGVVFCYWLNRNDSQH
LFTEPRTIFCLTCSPHHENLVAIGYKDGIVVIIDISKKGEVIHRLRGHDDEIHSIAWCPLSGEDCLSISQEEN
SEEPDIPNGKLIAETPITKGCYLATGSKDQTIRIWSCSRGRGVMVLKLPFLKRRSGGVDPTVKERLWLTLHWP
KNQPTQLVSSCFGGELLLWDLTQSWRRKYTLFSTSAEGHNHSRIVFNLCSLKTEDGKQLLLSTSMDRDVKCWD
MATLECCWTLPSLGGFAYSLAFSPVDVGSLAIGVGDGMIRVWNTLSIKNNYDVKNFWQGVKSKVTALCWHPNK
EGCLAFGTDDGKVGLYDTCSNKPPQISSTYHKKTVYRLAWGPPVPPMSLGGEGDRPSLTLYSCGGEGVVLQHN
PWKLSGEAFDINKLVRDTNSIRYKLPVHTEISWKGDGKVLALGNEDGSIEIFQVPNLRLLCTIQQHHKLVNAI
VWHHEHGSRPELSCLLASGSNNAVIYVHNLKAVLESNPESPITITEPYRTLSGHTAKITSLAWSPHHDGRLVS
ACYDGTAQVWDALREEPLFNFRGHRGRLLCVAWSPVDPECIYSGADDFCVYRWLTSMQDHSRPPQGKKCIELE
KKRLSQFKPKLKKKKKPTLRLPVKQDSSVGNEDESVKENSGPAENGLSDQDGEEEAQEPELPPSPVVCVEPVS
CTDICSGFEKSKVTVSSKATSLKKEPAKEKPEALLKKRKARSMLPLSTSLDHRSKEELHRDCLVLATATHAKE
LNEDVSADLEERFHLGLFTDRATLYRMMETEGKGHLESGHPELFHQLMLWKGDLKGVLQAAAERGELTDSLVA
VAPVAGYSVWLWAVEAFAKQLCFQDQYVKAASYLLSIHKVYEAVELLKSNHLYREAIAVAKARLRPEDPVLKE
LYLSWGSILERDGHYAIAAKCYLGATSAYDAAKVLARKGDAASLRTAAELAAIAGEHELAASLALRCAQELLL
MKNWVGAQEALGLHESLQGQRLVFCLLELLCRHLEEKQPLEVRGPSSIYHQWATGSEGTLVQRVTGVWRSPFS
VDTPEQCQAALQKLQDVKYPSATSNTPFRQLLLHVCHDLTLAMLSQQAAAWEEAVPALLQAVVRSYTSGNFTL

MQEIYSAFLPGGCDHLRDKLGDLSPAMAAYKSLEAFCIYGQLYEVWWSLCGPGPESSVWVLSAESTVSDKQSK
PEDSASAEDMEQPPGPGPRLSAESERLLSACKELFSERHASLQTSQRTVAEVQETLAEMIRQHQKSQLCKATT
NGPSRDEPSRDEPSQEAERAPSQPPSPTEERNAPVSLPELTRRLTEANERIAEFPESVKAWPFPDVLECCLVL
LHIGSQCPDAVDPEMQQQAQELLHKYGHTRAYRRHCQSRHT*
>rs51771045_ENSMUSP00000131842_c.3498:G>C_p.A1167P
ENSMUST00000172035.8|Gemin5-205|ENSMUSG00000037275|-|chr11:g.58019569:C>G
MKPEPRTLPPSPNWYCSRCSDAAPGGIFGFAARTSVFLVRVGPGAGASPGAPPFRVVGELVGHTERVSGFTFS
HHPGQYNLCATSSDDGTVKVWDVETKTVVTEHTLHQHTISALHWSPTVKDLIVSGDEKGVVFCYWLNRNDSQH
LFTEPRTIFCLTCSPHHENLVAIGYKDGIVVIIDISKKGEVIHRLRGHDDEIHSIAWCPLSGEDCLSISQEEN
SEEPDIPNGKLIAETPITKGCYLATGSKDQTIRIWSCSRGRGVMVLKLPFLKRRSGGVDPTVKERLWLTLHWP
KNQPTQLVSSCFGGELLLWDLTQSWRRKYTLFSTSAEGHNHSRIVFNLCSLKTEDGKQLLLSTSMDRDVKCWD
MATLECCWTLPSLGGFAYSLAFSPVDVGSLAIGVGDGMIRVWNTLSIKNNYDVKNFWQGVKSKVTALCWHPNK
EGCLAFGTDDGKVGLYDTCSNKPPQISSTYHKKTVYRLAWGPPVPPMSLGGEGDRPSLTLYSCGGEGVVLQHN
PWKLSGEAFDINKLVRDTNSIRYKLPVHTEISWKGDGKVLALGNEDGSIEIFQVPNLRLLCTIQQHHKLVNAI
VWHHEHGSRPELSCLLASGSNNAVIYVHNLKAVLESNPESPITITEPYRTLSGHTAKITSLAWSPHHDGRLVS
ACYDGTAQVWDALREEPLFNFRGHRGRLLCVAWSPVDPECIYSGADDFCVYRWLTSMQDHSRPPQGKKCIELE
KKRLSQFKPKLKKKKKPTLRLPVKQDSSVGNEDESVKENSGPAENGLSDQDGEEEAQEPELPPSPVVCVEPVS
CTDICSGFEKSKVTVSSKATSLKKEPAKEKPEALLKKRKARSMLPLSTSLDHRSKEELHRDCLVLATATHAKA
ELNEDVSADLEERFHLGLFTDRATLYRMMETEGKGHLESGHPELFHQLMLWKGDLKGVLQAAAERGELTDSLV
AVAPVAGYSVWLWAVEAFAKQLCFQDQYVKAASYLLSIHKVYEAVELLKSNHLYREAIAVAKARLRPEDPVLK
ELYLSWGSILERDGHYAIAAKCYLGATSAYDAAKVLARKGDAASLRTAAELAAIAGEHELAASLALRCAQELL
LMKNWVGAQEALGLHESLQGQRLVFCLLELLCRHLEEKQPLEVRGPSSIYHQWATGSEGTLVQRVTGVWRSPF
SVDTPEQCQAALQKLQDVKYPSATSNTPFRQLLLHVCHDLTLAMLSQQAAAWEEAVPALLQAVVRSYTSGNFT
LMQEIYSAFLPGGCDHLRDKLGDLSPAMAAYKSLEAFCIYGQLYEVWWSLCGPGPESSVWVLSAESTVSDKQS
KPEDSASAEDMEQPPGPGPRLSAESERLLSACKELFSERHASLQTSQRTVAEVQETLAEMIRQHQKSQLCKAT
TNGPSRDEPSRDEPSQEAERAPSQPPSPTREERNAPVSLPELTRRLTEANERIAEFPESVKAWPFPDVLECCL
VLLHIGSQCPDAVDPEMQQQAQELLHKYGHTRAYRRHCQSRHT*
>rs246725396_ENSMUSP00000036603_c.1996:T>ATAAATAAT_p.V666ins-fs
ENSMUST00000035604.13|Gemin5-201|ENSMUSG00000037275|-
|chr11:g.58036345:A>ATTATTTAT
MKPEPRTLPPSPNWYCSRCSDAAPGGIFGFAARTSVFLVRVGPGAGASPGAPPFRVVGELVGHTERVSGFTFS
HHPGQYNLCATSSDDGTVKVWDVETKTVVTEHTLHQHTISALHWSPTVKDLIVSGDEKGVVFCYWLNRNDSQH
LFTEPRTIFCLTCSPHHENLVAIGYKDGIVVIIDISKKGEVIHRLRGHDDEIHSIAWCPLSGEDCLSISQEEN
SEEPDIPNGKLIAETPITKGCYLATGSKDQTIRIWSCSRGRGVMVLKLPFLKRRSGGVDPTVKERLWLTLHWP
KNQPTQLVSSCFGGELLLWDLTQSWRRKYTLFSTSAEGHNHSRIVFNLCSLKTEDGKQLLLSTSMDRDVKCWD
MATLECCWTLPSLGGFAYSLAFSPVDVGSLAIGVGDGMIRVWNTLSIKNNYDVKNFWQGVKSKVTALCWHPNK
EGCLAFGTDDGKVGLYDTCSNKPPQISSTYHKKTVYRLAWGPPVPPMSLGGEGDRPSLTLYSCGGEGVVLQHN
PWKLSGEAFDINKLVRDTNSIRYKLPVHTEISWKGDGKVLALGNEDGSIEIFQVPNLRLLCTIQQHHKLVNAI
VWHHEHGSRPELSCLLASGSNNAVIYVHNLKAVLESNPESPITITEPYRTLSGHTAKITSLAWSPHHDGRLVS
ACYDGTAQDK
>rs246725396_ENSMUSP00000099772_c.1996:T>ATAAATAAT_p.V666ins-fs
ENSMUST00000102711.9|Gemin5-202|ENSMUSG00000037275|-
|chr11:g.58036345:A>ATTATTTAT
MKPEPRTLPPSPNWYCSRCSDAAPGGIFGFAARTSVFLVRVGPGAGASPGAPPFRVVGELVGHTERVSGFTFS
HHPGQYNLCATSSDDGTVKVWDVETKTVVTEHTLHQHTISALHWSPTVKDLIVSGDEKGVVFCYWLNRNDSQH
LFTEPRTIFCLTCSPHHENLVAIGYKDGIVVIIDISKKGEVIHRLRGHDDEIHSIAWCPLSGEDCLSISQEEN
SEEPDIPNGKLIAETPITKGCYLATGSKDQTIRIWSCSRGRGVMVLKLPFLKRRSGGVDPTVKERLWLTLHWP
KNQPTQLVSSCFGGELLLWDLTQSWRRKYTLFSTSAEGHNHSRIVFNLCSLKTEDGKQLLLSTSMDRDVKCWD
MATLECCWTLPSLGGFAYSLAFSPVDVGSLAIGVGDGMIRVWNTLSIKNNYDVKNFWQGVKSKVTALCWHPNK
EGCLAFGTDDGKVGLYDTCSNKPPQISSTYHKKTVYRLAWGPPVPPMSLGGEGDRPSLTLYSCGGEGVVLQHN
PWKLSGEAFDINKLVRDTNSIRYKLPVHTEISWKGDGKVLALGNEDGSIEIFQVPNLRLLCTIQQHHKLVNAI
VWHHEHGSRPELSCLLASGSNNAVIYVHNLKAVLESNPESPITITEPYRTLSGHTAKITSLAWSPHHDGRLVS
ACYDGTAQDK
>rs246725396_ENSMUSP00000131842_c.1996:T>ATAAATAAT_p.V666ins-fs
ENSMUST00000172035.8|Gemin5-205|ENSMUSG00000037275|-
|chr11:g.58036345:A>ATTATTTAT
MKPEPRTLPPSPNWYCSRCSDAAPGGIFGFAARTSVFLVRVGPGAGASPGAPPFRVVGELVGHTERVSGFTFS
HHPGQYNLCATSSDDGTVKVWDVETKTVVTEHTLHQHTISALHWSPTVKDLIVSGDEKGVVFCYWLNRNDSQH

LFTEPRTIFCLTCSPHHENLVAIGYKDGIVVIIDISKKGEVIHRLRGHDDEIHSIAWCPLSGEDCLSISQEEN
SEEPDIPNGKLIAETPITKGCYLATGSKDQTIRIWSCSRGRGVMVLKLPFLKRRSGGVDPTVKERLWLTLHWP
KNQPTQLVSSCFGGELLLWDLTQSWRRKYTLFSTSAEGHNHSRIVFNLCSLKTEDGKQLLLSTSMDRDVKCWD
MATLECCWTLPSLGGFAYSLAFSPVDVGSLAIGVGDGMIRVWNTLSIKNNYDVKNFWQGVKSKVTALCWHPNK
EGCLAFGTDDGKVGLYDTCSNKPPQISSTYHKKTVYRLAWGPPVPPMSLGGEGDRPSLTLYSCGGEGVVLQHN
PWKLSGEAFDINKLVRDTNSIRYKLPVHTEISWKGDGKVLALGNEDGSIEIFQVPNLRLLCTIQQHHKLVNAI
VWHHEHGSRPELSCLLASGSNNAVIYVHNLKAVLESNPESPITITEPYRTLSGHTAKITSLAWSPHHDGRLVS
ACYDGTAQDK
>rs29470941_ENSMUSP00000036603_c.1132:T>C_p.L378P
ENSMUST00000035604.13|Gemin5-201|ENSMUSG00000037275|-
|chr11:g.58046618:A>G
MKPEPRTLPPSPNWYCSRCSDAAPGGIFGFAARTSVFLVRVGPGAGASPGAPPFRVVGELVGHTERVSGFTFS
HHPGQYNLCATSSDDGTVKVWDVETKTVVTEHTLHQHTISALHWSPTVKDLIVSGDEKGVVFCYWLNRNDSQH
LFTEPRTIFCLTCSPHHENLVAIGYKDGIVVIIDISKKGEVIHRLRGHDDEIHSIAWCPLSGEDCLSISQEEN
SEEPDIPNGKLIAETPITKGCYLATGSKDQTIRIWSCSRGRGVMVLKLPFLKRRSGGVDPTVKERLWLTLHWP
KNQPTQLVSSCFGGELLLWDLTQSWRRKYTLFSTSAEGHNHSRIVFNLCSLKTEDGKQLLLSTSMDRDVKCWD
MATLECCWTLPSPGGFAYSLAFSPVDVGSLAIGVGDGMIRVWNTLSIKNNYDVKNFWQGVKSKVTALCWHPNK
EGCLAFGTDDGKVGLYDTCSNKPPQISSTYHKKTVYRLAWGPPVPPMSLGGEGDRPSLTLYSCGGEGVVLQHN
PWKLSGEAFDINKLVRDTNSIRYKLPVHTEISWKGDGKVLALGNEDGSIEIFQVPNLRLLCTIQQHHKLVNAI
VWHHEHGSRPELSCLLASGSNNAVIYVHNLKAVLESNPESPITITEPYRTLSGHTAKITSLAWSPHHDGRLVS
ACYDGTAQVWDALREEPLFNFRGHRGRLLCVAWSPVDPECIYSGADDFCVYRWLTSMQDHSRPPQGKKCIELE
KKRLSQFKPKLKKKKKPTLRLPVKQDSSVGNEDESVKENSGPAENGLSDQDGEEEAQEPELPPSPVVCVEPVS
CTDICSGFEKSKVTVSSKATSLKKEPAKEKPEALLKKRKARSMLPLSTSLDHRSKEELHRDCLVLATATHAKA
ELNEDVSADLEERFHLGLFTDRATLYRMMETEGKGHLESGHPELFHQLMLWKGDLKGVLQAAAERGELTDSLV
AVAPVAGYSVWLWAVEAFAKQLCFQDQYVKAASYLLSIHKVYEAVELLKSNHLYREAIAVAKARLRPEDPVLK
ELYLSWGSILERDGHYAIAAKCYLGATSAYDAAKVLARKGDAASLRTAAELAAIAGEHELAASLALRCAQELL
LMKNWVGAQEALGLHESLQGQRLVFCLLELLCRHLEEKQPLEVRGPSSIYHQWATGSEGTLVQRVTGVWRSAF
SVDTPEQCQAALQKLQDVKYPSATSNTPFRQLLLHVCHDLTLAMLSQQAAAWEEAVPALLQAVVRSYTSGNFT
LMQEIYSAFLPGGCDHLRDKLGDLSPAMAAYKSLEAFCIYGQLYEVWWSLCGPGPESSVWVLSAESTVSDKQS
KPEDSASAEDMEQPPGPGPRLSAESERLLSACKELFSERHASLQTSQRTVAEVQETLAEMIRQHQKSQLCKAT
TNGPSRDEPSRDEPSQEAERAPSQPPSPTEERNAPVSLPELTRRLTEANERIAEFPESVKAWPFPDVLECCLV
LLHIGSQCPDAVDPEMQQQAQELLHKYGHTRAYRRHCQSRHT*
>rs29470941_ENSMUSP00000099772_c.1132:T>C_p.L378P
ENSMUST00000102711.9|Gemin5-202|ENSMUSG00000037275|-|chr11:g.58046618:A>G
MKPEPRTLPPSPNWYCSRCSDAAPGGIFGFAARTSVFLVRVGPGAGASPGAPPFRVVGELVGHTERVSGFTFS
HHPGQYNLCATSSDDGTVKVWDVETKTVVTEHTLHQHTISALHWSPTVKDLIVSGDEKGVVFCYWLNRNDSQH
LFTEPRTIFCLTCSPHHENLVAIGYKDGIVVIIDISKKGEVIHRLRGHDDEIHSIAWCPLSGEDCLSISQEEN
SEEPDIPNGKLIAETPITKGCYLATGSKDQTIRIWSCSRGRGVMVLKLPFLKRRSGGVDPTVKERLWLTLHWP
KNQPTQLVSSCFGGELLLWDLTQSWRRKYTLFSTSAEGHNHSRIVFNLCSLKTEDGKQLLLSTSMDRDVKCWD
MATLECCWTLPSPGGFAYSLAFSPVDVGSLAIGVGDGMIRVWNTLSIKNNYDVKNFWQGVKSKVTALCWHPNK
EGCLAFGTDDGKVGLYDTCSNKPPQISSTYHKKTVYRLAWGPPVPPMSLGGEGDRPSLTLYSCGGEGVVLQHN
PWKLSGEAFDINKLVRDTNSIRYKLPVHTEISWKGDGKVLALGNEDGSIEIFQVPNLRLLCTIQQHHKLVNAI
VWHHEHGSRPELSCLLASGSNNAVIYVHNLKAVLESNPESPITITEPYRTLSGHTAKITSLAWSPHHDGRLVS
ACYDGTAQVWDALREEPLFNFRGHRGRLLCVAWSPVDPECIYSGADDFCVYRWLTSMQDHSRPPQGKKCIELE
KKRLSQFKPKLKKKKKPTLRLPVKQDSSVGNEDESVKENSGPAENGLSDQDGEEEAQEPELPPSPVVCVEPVS
CTDICSGFEKSKVTVSSKATSLKKEPAKEKPEALLKKRKARSMLPLSTSLDHRSKEELHRDCLVLATATHAKE
LNEDVSADLEERFHLGLFTDRATLYRMMETEGKGHLESGHPELFHQLMLWKGDLKGVLQAAAERGELTDSLVA
VAPVAGYSVWLWAVEAFAKQLCFQDQYVKAASYLLSIHKVYEAVELLKSNHLYREAIAVAKARLRPEDPVLKE
LYLSWGSILERDGHYAIAAKCYLGATSAYDAAKVLARKGDAASLRTAAELAAIAGEHELAASLALRCAQELLL
MKNWVGAQEALGLHESLQGQRLVFCLLELLCRHLEEKQPLEVRGPSSIYHQWATGSEGTLVQRVTGVWRSAFS
VDTPEQCQAALQKLQDVKYPSATSNTPFRQLLLHVCHDLTLAMLSQQAAAWEEAVPALLQAVVRSYTSGNFTL
MQEIYSAFLPGGCDHLRDKLGDLSPAMAAYKSLEAFCIYGQLYEVWWSLCGPGPESSVWVLSAESTVSDKQSK
PEDSASAEDMEQPPGPGPRLSAESERLLSACKELFSERHASLQTSQRTVAEVQETLAEMIRQHQKSQLCKATT
NGPSRDEPSRDEPSQEAERAPSQPPSPTEERNAPVSLPELTRRLTEANERIAEFPESVKAWPFPDVLECCLVL
LHIGSQCPDAVDPEMQQQAQELLHKYGHTRAYRRHCQSRHT*
>rs29470941_ENSMUSP00000116600_c.283:T>C_p.L95P
ENSMUST00000134953.2|Gemin5-204|ENSMUSG00000037275|-|chr11:g.58046618:A>G

XLWLTLHWPKNQPTQLVSSCFGGELLLWDLTQSWRRKYTLFSTSAEGHNHSRIVFNLCSLKTEDGKQLLLSTS
MDRDVKCWDMATLECCWTLPSPGGFAYSLAFSPVDVGSLAIGVGDGMIRVWNTLSIKNNYDVKNFWQGVKSKV
TALCWHPNKEGCLAFGTDDGKVGLYDTCSNK*
>rs29470941_ENSMUSP00000131842_c.1132:T>C_p.L378P
ENSMUST00000172035.8|Gemin5-205|ENSMUSG00000037275|-|chr11:g.58046618:A>G
MKPEPRTLPPSPNWYCSRCSDAAPGGIFGFAARTSVFLVRVGPGAGASPGAPPFRVVGELVGHTERVSGFTFS
HHPGQYNLCATSSDDGTVKVWDVETKTVVTEHTLHQHTISALHWSPTVKDLIVSGDEKGVVFCYWLNRNDSQH
LFTEPRTIFCLTCSPHHENLVAIGYKDGIVVIIDISKKGEVIHRLRGHDDEIHSIAWCPLSGEDCLSISQEEN
SEEPDIPNGKLIAETPITKGCYLATGSKDQTIRIWSCSRGRGVMVLKLPFLKRRSGGVDPTVKERLWLTLHWP
KNQPTQLVSSCFGGELLLWDLTQSWRRKYTLFSTSAEGHNHSRIVFNLCSLKTEDGKQLLLSTSMDRDVKCWD
MATLECCWTLPSPGGFAYSLAFSPVDVGSLAIGVGDGMIRVWNTLSIKNNYDVKNFWQGVKSKVTALCWHPNK
EGCLAFGTDDGKVGLYDTCSNKPPQISSTYHKKTVYRLAWGPPVPPMSLGGEGDRPSLTLYSCGGEGVVLQHN
PWKLSGEAFDINKLVRDTNSIRYKLPVHTEISWKGDGKVLALGNEDGSIEIFQVPNLRLLCTIQQHHKLVNAI
VWHHEHGSRPELSCLLASGSNNAVIYVHNLKAVLESNPESPITITEPYRTLSGHTAKITSLAWSPHHDGRLVS
ACYDGTAQVWDALREEPLFNFRGHRGRLLCVAWSPVDPECIYSGADDFCVYRWLTSMQDHSRPPQGKKCIELE
KKRLSQFKPKLKKKKKPTLRLPVKQDSSVGNEDESVKENSGPAENGLSDQDGEEEAQEPELPPSPVVCVEPVS
CTDICSGFEKSKVTVSSKATSLKKEPAKEKPEALLKKRKARSMLPLSTSLDHRSKEELHRDCLVLATATHAKA
ELNEDVSADLEERFHLGLFTDRATLYRMMETEGKGHLESGHPELFHQLMLWKGDLKGVLQAAAERGELTDSLV
AVAPVAGYSVWLWAVEAFAKQLCFQDQYVKAASYLLSIHKVYEAVELLKSNHLYREAIAVAKARLRPEDPVLK
ELYLSWGSILERDGHYAIAAKCYLGATSAYDAAKVLARKGDAASLRTAAELAAIAGEHELAASLALRCAQELL
LMKNWVGAQEALGLHESLQGQRLVFCLLELLCRHLEEKQPLEVRGPSSIYHQWATGSEGTLVQRVTGVWRSAF
SVDTPEQCQAALQKLQDVKYPSATSNTPFRQLLLHVCHDLTLAMLSQQAAAWEEAVPALLQAVVRSYTSGNFT
LMQEIYSAFLPGGCDHLRDKLGDLSPAMAAYKSLEAFCIYGQLYEVWWSLCGPGPESSVWVLSAESTVSDKQS
KPEDSASAEDMEQPPGPGPRLSAESERLLSACKELFSERHASLQTSQRTVAEVQETLAEMIRQHQKSQLCKAT
TNGPSRDEPSRDEPSQEAERAPSQPPSPTREERNAPVSLPELTRRLTEANERIAEFPESVKAWPFPDVLECCL
VLLHIGSQCPDAVDPEMQQQAQELLHKYGHTRAYRRHCQSRHT*
>rs29461142_ENSMUSP00000036603_c.853:T>G_p.L285R
ENSMUST00000035604.13|Gemin5-201|ENSMUSG00000037275|-
|chr11:g.58051592:A>C
MKPEPRTLPPSPNWYCSRCSDAAPGGIFGFAARTSVFLVRVGPGAGASPGAPPFRVVGELVGHTERVSGFTFS
HHPGQYNLCATSSDDGTVKVWDVETKTVVTEHTLHQHTISALHWSPTVKDLIVSGDEKGVVFCYWLNRNDSQH
LFTEPRTIFCLTCSPHHENLVAIGYKDGIVVIIDISKKGEVIHRLRGHDDEIHSIAWCPLSGEDCLSISQEEN
SEEPDIPNGKLIAETPITKGCYLATGSKDQTIRIWSCSRGRGVMVLKLPFLKRRSGGVDPTVKERRWLTLHWP
KNQPTQLVSSCFGGELLLWDLTQSWRRKYTLFSTSAEGHNHSRIVFNLCSLKTEDGKQLLLSTSMDRDVKCWD
MATLECCWTLPSLGGFAYSLAFSPVDVGSLAIGVGDGMIRVWNTLSIKNNYDVKNFWQGVKSKVTALCWHPNK
EGCLAFGTDDGKVGLYDTCSNKPPQISSTYHKKTVYRLAWGPPVPPMSLGGEGDRPSLTLYSCGGEGVVLQHN
PWKLSGEAFDINKLVRDTNSIRYKLPVHTEISWKGDGKVLALGNEDGSIEIFQVPNLRLLCTIQQHHKLVNAI
VWHHEHGSRPELSCLLASGSNNAVIYVHNLKAVLESNPESPITITEPYRTLSGHTAKITSLAWSPHHDGRLVS
ACYDGTAQVWDALREEPLFNFRGHRGRLLCVAWSPVDPECIYSGADDFCVYRWLTSMQDHSRPPQGKKCIELE
KKRLSQFKPKLKKKKKPTLRLPVKQDSSVGNEDESVKENSGPAENGLSDQDGEEEAQEPELPPSPVVCVEPVS
CTDICSGFEKSKVTVSSKATSLKKEPAKEKPEALLKKRKARSMLPLSTSLDHRSKEELHRDCLVLATATHAKA
ELNEDVSADLEERFHLGLFTDRATLYRMMETEGKGHLESGHPELFHQLMLWKGDLKGVLQAAAERGELTDSLV
AVAPVAGYSVWLWAVEAFAKQLCFQDQYVKAASYLLSIHKVYEAVELLKSNHLYREAIAVAKARLRPEDPVLK
ELYLSWGSILERDGHYAIAAKCYLGATSAYDAAKVLARKGDAASLRTAAELAAIAGEHELAASLALRCAQELL
LMKNWVGAQEALGLHESLQGQRLVFCLLELLCRHLEEKQPLEVRGPSSIYHQWATGSEGTLVQRVTGVWRSAF
SVDTPEQCQAALQKLQDVKYPSATSNTPFRQLLLHVCHDLTLAMLSQQAAAWEEAVPALLQAVVRSYTSGNFT
LMQEIYSAFLPGGCDHLRDKLGDLSPAMAAYKSLEAFCIYGQLYEVWWSLCGPGPESSVWVLSAESTVSDKQS
KPEDSASAEDMEQPPGPGPRLSAESERLLSACKELFSERHASLQTSQRTVAEVQETLAEMIRQHQKSQLCKAT
TNGPSRDEPSRDEPSQEAERAPSQPPSPTEERNAPVSLPELTRRLTEANERIAEFPESVKAWPFPDVLECCLV
LLHIGSQCPDAVDPEMQQQAQELLHKYGHTRAYRRHCQSRHT*
>rs29461142_ENSMUSP00000099772_c.853:T>G_p.L285R
ENSMUST00000102711.9|Gemin5-202|ENSMUSG00000037275|-|chr11:g.58051592:A>C
MKPEPRTLPPSPNWYCSRCSDAAPGGIFGFAARTSVFLVRVGPGAGASPGAPPFRVVGELVGHTERVSGFTFS
HHPGQYNLCATSSDDGTVKVWDVETKTVVTEHTLHQHTISALHWSPTVKDLIVSGDEKGVVFCYWLNRNDSQH
LFTEPRTIFCLTCSPHHENLVAIGYKDGIVVIIDISKKGEVIHRLRGHDDEIHSIAWCPLSGEDCLSISQEEN
SEEPDIPNGKLIAETPITKGCYLATGSKDQTIRIWSCSRGRGVMVLKLPFLKRRSGGVDPTVKERRWLTLHWP
KNQPTQLVSSCFGGELLLWDLTQSWRRKYTLFSTSAEGHNHSRIVFNLCSLKTEDGKQLLLSTSMDRDVKCWD
MATLECCWTLPSLGGFAYSLAFSPVDVGSLAIGVGDGMIRVWNTLSIKNNYDVKNFWQGVKSKVTALCWHPNK

EGCLAFGTDDGKVGLYDTCSNKPPQISSTYHKKTVYRLAWGPPVPPMSLGGEGDRPSLTLYSCGGEGVVLQHN
PWKLSGEAFDINKLVRDTNSIRYKLPVHTEISWKGDGKVLALGNEDGSIEIFQVPNLRLLCTIQQHHKLVNAI
VWHHEHGSRPELSCLLASGSNNAVIYVHNLKAVLESNPESPITITEPYRTLSGHTAKITSLAWSPHHDGRLVS
ACYDGTAQVWDALREEPLFNFRGHRGRLLCVAWSPVDPECIYSGADDFCVYRWLTSMQDHSRPPQGKKCIELE
KKRLSQFKPKLKKKKKPTLRLPVKQDSSVGNEDESVKENSGPAENGLSDQDGEEEAQEPELPPSPVVCVEPVS
CTDICSGFEKSKVTVSSKATSLKKEPAKEKPEALLKKRKARSMLPLSTSLDHRSKEELHRDCLVLATATHAKE
LNEDVSADLEERFHLGLFTDRATLYRMMETEGKGHLESGHPELFHQLMLWKGDLKGVLQAAAERGELTDSLVA
VAPVAGYSVWLWAVEAFAKQLCFQDQYVKAASYLLSIHKVYEAVELLKSNHLYREAIAVAKARLRPEDPVLKE
LYLSWGSILERDGHYAIAAKCYLGATSAYDAAKVLARKGDAASLRTAAELAAIAGEHELAASLALRCAQELLL
MKNWVGAQEALGLHESLQGQRLVFCLLELLCRHLEEKQPLEVRGPSSIYHQWATGSEGTLVQRVTGVWRSAFS
VDTPEQCQAALQKLQDVKYPSATSNTPFRQLLLHVCHDLTLAMLSQQAAAWEEAVPALLQAVVRSYTSGNFTL
MQEIYSAFLPGGCDHLRDKLGDLSPAMAAYKSLEAFCIYGQLYEVWWSLCGPGPESSVWVLSAESTVSDKQSK
PEDSASAEDMEQPPGPGPRLSAESERLLSACKELFSERHASLQTSQRTVAEVQETLAEMIRQHQKSQLCKATT
NGPSRDEPSRDEPSQEAERAPSQPPSPTEERNAPVSLPELTRRLTEANERIAEFPESVKAWPFPDVLECCLVL
LHIGSQCPDAVDPEMQQQAQELLHKYGHTRAYRRHCQSRHT*
>rs29461142_ENSMUSP00000116600_c.4:T>G_p.L2R ENSMUST00000134953.2|Gemin5-204|ENSMUSG00000037275|-|chr11:g.58051592:A>C
XRWLTLHWPKNQPTQLVSSCFGGELLLWDLTQSWRRKYTLFSTSAEGHNHSRIVFNLCSLKTEDGKQLLLSTS
MDRDVKCWDMATLECCWTLPSLGGFAYSLAFSPVDVGSLAIGVGDGMIRVWNTLSIKNNYDVKNFWQGVKSKV
TALCWHPNKEGCLAFGTDDGKVGLYDTCSNK*
>rs29461142_ENSMUSP00000131842_c.853:T>G_p.L285R
ENSMUST00000172035.8|Gemin5-205|ENSMUSG00000037275|-|chr11:g.58051592:A>C
MKPEPRTLPPSPNWYCSRCSDAAPGGIFGFAARTSVFLVRVGPGAGASPGAPPFRVVGELVGHTERVSGFTFS
HHPGQYNLCATSSDDGTVKVWDVETKTVVTEHTLHQHTISALHWSPTVKDLIVSGDEKGVVFCYWLNRNDSQH
LFTEPRTIFCLTCSPHHENLVAIGYKDGIVVIIDISKKGEVIHRLRGHDDEIHSIAWCPLSGEDCLSISQEEN
SEEPDIPNGKLIAETPITKGCYLATGSKDQTIRIWSCSRGRGVMVLKLPFLKRRSGGVDPTVKERRWLTLHWP
KNQPTQLVSSCFGGELLLWDLTQSWRRKYTLFSTSAEGHNHSRIVFNLCSLKTEDGKQLLLSTSMDRDVKCWD
MATLECCWTLPSLGGFAYSLAFSPVDVGSLAIGVGDGMIRVWNTLSIKNNYDVKNFWQGVKSKVTALCWHPNK
EGCLAFGTDDGKVGLYDTCSNKPPQISSTYHKKTVYRLAWGPPVPPMSLGGEGDRPSLTLYSCGGEGVVLQHN
PWKLSGEAFDINKLVRDTNSIRYKLPVHTEISWKGDGKVLALGNEDGSIEIFQVPNLRLLCTIQQHHKLVNAI
VWHHEHGSRPELSCLLASGSNNAVIYVHNLKAVLESNPESPITITEPYRTLSGHTAKITSLAWSPHHDGRLVS
ACYDGTAQVWDALREEPLFNFRGHRGRLLCVAWSPVDPECIYSGADDFCVYRWLTSMQDHSRPPQGKKCIELE
KKRLSQFKPKLKKKKKPTLRLPVKQDSSVGNEDESVKENSGPAENGLSDQDGEEEAQEPELPPSPVVCVEPVS
CTDICSGFEKSKVTVSSKATSLKKEPAKEKPEALLKKRKARSMLPLSTSLDHRSKEELHRDCLVLATATHAKA
ELNEDVSADLEERFHLGLFTDRATLYRMMETEGKGHLESGHPELFHQLMLWKGDLKGVLQAAAERGELTDSLV
AVAPVAGYSVWLWAVEAFAKQLCFQDQYVKAASYLLSIHKVYEAVELLKSNHLYREAIAVAKARLRPEDPVLK
ELYLSWGSILERDGHYAIAAKCYLGATSAYDAAKVLARKGDAASLRTAAELAAIAGEHELAASLALRCAQELL
LMKNWVGAQEALGLHESLQGQRLVFCLLELLCRHLEEKQPLEVRGPSSIYHQWATGSEGTLVQRVTGVWRSAF
SVDTPEQCQAALQKLQDVKYPSATSNTPFRQLLLHVCHDLTLAMLSQQAAAWEEAVPALLQAVVRSYTSGNFT
LMQEIYSAFLPGGCDHLRDKLGDLSPAMAAYKSLEAFCIYGQLYEVWWSLCGPGPESSVWVLSAESTVSDKQS
KPEDSASAEDMEQPPGPGPRLSAESERLLSACKELFSERHASLQTSQRTVAEVQETLAEMIRQHQKSQLCKAT
TNGPSRDEPSRDEPSQEAERAPSQPPSPTREERNAPVSLPELTRRLTEANERIAEFPESVKAWPFPDVLECCL
VLLHIGSQCPDAVDPEMQQQAQELLHKYGHTRAYRRHCQSRHT*
>rs218507294_ENSMUSP00000036603_c.682:G>AAG_p.G228ins-fs
ENSMUST00000035604.13|Gemin5-201|ENSMUSG00000037275|-
|chr11:g.58054718:C>CTT
MKPEPRTLPPSPNWYCSRCSDAAPGGIFGFAARTSVFLVRVGPGAGASPGAPPFRVVGELVGHTERVSGFTFS
HHPGQYNLCATSSDDGTVKVWDVETKTVVTEHTLHQHTISALHWSPTVKDLIVSGDEKGVVFCYWLNRNDSQH
LFTEPRTIFCLTCSPHHENLVAIGYKDGIVVIIDISKKGEVIHRLRGHDDEIHSIAWCPLSGEDCLSISQEEN
SEEPDIPNEGSL
>rs218507294_ENSMUSP00000099772_c.682:G>AAG_p.G228ins-fs
ENSMUST00000102711.9|Gemin5-202|ENSMUSG00000037275|-
|chr11:g.58054718:C>CTT
MKPEPRTLPPSPNWYCSRCSDAAPGGIFGFAARTSVFLVRVGPGAGASPGAPPFRVVGELVGHTERVSGFTFS
HHPGQYNLCATSSDDGTVKVWDVETKTVVTEHTLHQHTISALHWSPTVKDLIVSGDEKGVVFCYWLNRNDSQH
LFTEPRTIFCLTCSPHHENLVAIGYKDGIVVIIDISKKGEVIHRLRGHDDEIHSIAWCPLSGEDCLSISQEEN
SEEPDIPNEGSL

```
>rs218507294_ENSMUSP00000131842_c.682:G>AAG_p.G228ins-fs
ENSMUST00000172035.8|Gemin5-205|ENSMUSG00000037275|-
|chr11:g.58054718:C>CTT
MKPEPRTLPPSPNWYCSRCSDAAPGGIFGFAARTSVFLVRVGPGAGASPGAPPFRVVGELVGHTERVSGFTFS
HHPGQYNLCATSSDDGTVKVWDVETKTVVTEHTLHQHTISALHWSPTVKDLIVSGDEKGVVFCYWLNRNDSQH
LFTEPRTIFCLTCSPHHENLVAIGYKDGIVVIIDISKKGEVIHRLRGHDDEIHSIAWCPLSGEDCLSISQEEN
SEEPDIPNEGSL
>rs50868671_ENSMUSP00000020820_c.489:G>A_p.V164I
ENSMUST00000020820.2|Mrpl22-201|ENSMUSG00000020514|+|chr11:g.58070184:G>A
MAAALLRELGALRVPNLRIWATQTLRVLPPSCIHTSASLDISRKWEKKNKIVYPPQLPGEPRRPAEIYHCRRQ
IKYSKDKMWYLAKMIRGMSIDQALAQLEFNDKKGAQIIKEVLLEAQDMAVRDHNVEFRSNLHIAESTSGRGQC
LKRIRYHGRGRFGIMEKIYCHYFVKLVEGPPPPPEVPKTAVDHAKDYIQQLRSRTIIHTL*
>rs46633950_ENSMUSP00000020820_c.576:T>C_p.Y193H
ENSMUST00000020820.2|Mrpl22-201|ENSMUSG00000020514|+|chr11:g.58070271:T>C
MAAALLRELGALRVPNLRIWATQTLRVLPPSCIHTSASLDISRKWEKKNKIVYPPQLPGEPRRPAEIYHCRRQ
IKYSKDKMWYLAKMIRGMSIDQALAQLEFNDKKGAQIIKEVLLEAQDMAVRDHNVEFRSNLHIAESTSGRGQC
LKRIRYHGRGRFGIMEKVYCHYFVKLVEGPPPPPEVPKTAVDHAKDHIQQLRSRTIIHTL*
>rs26966377_ENSMUSP00000146805_c.552:T>A_p.F185I
ENSMUST00000209079.2|Irgm2-203|ENSMUSG00000069874|+|chr11:g.58110827:T>A
MPTSRVAPLLDNMEEAVESPEVKEFEYFSDAVFIPKDGNTLSVGVIKRIETAVKEGEVVKVVSIVKEIIQNVS
RNKIKIAVTGDSGNGMSSFINALRLIGHEEKDSAPTGVVRTTQKPTCYFSSHFPYVELWDLPGLGATAQSVES
YLEEMQISIYDLIIIVASEQFSLNHVKLAITMQRMRKRIYVVWTKLDRDLSTSTFPEPQLLQSIQRNIRDSLQ
KEKVKEHPMFLVSVFKPESHDFPKLRETLQKDLPVIKYHGLVETLYQVCEKTVNERVESIKKSIDEDNLHTEF
GISDPGNAIEIRKAFQKTFGLDDISLHLVALEMKNKHFNTSMESQETQRYQQDDWVLARLYRTGTRVGSIGFD
YMKCCFTSHHSRCKQQKDILDETAAKAKEVLLKILRLSIPHP*
>rs26966377_ENSMUSP00000104464_c.516:T>A_p.F173I
ENSMUST00000108836.2|Irgm2-202|ENSMUSG00000069874|+|chr11:g.58110827:T>A
MEEAVESPEVKEFEYFSDAVFIPKDGNTLSVGVIKRIETAVKEGEVVKVVSIVKEIIQNVSRNKIKIAVTGDS
GNGMSSFINALRLIGHEEKDSAPTGVVRTTQKPTCYFSSHFPYVELWDLPGLGATAQSVESYLEEMQISIYDL
IIIVASEQFSLNHVKLAITMQRMRKRIYVVWTKLDRDLSTSTFPEPQLLQSIQRNIRDSLQKEKVKEHPMFLV
SVFKPESHDFPKLRETLQKDLPVIKYHGLVETLYQVCEKTVNERVESIKKSIDEDNLHTEFGISDPGNAIEIR
KAFQKTFGLDDISLHLVALEMKNKHFNTSMESQETQRYQQDDWVLARLYRTGTRVGSIGFDYMKCCFTSHHSR
CKQQKDILDETAAKAKEVLLKILRLSIPHP*
>rs26966377_ENSMUSP00000056001_c.516:T>A_p.F173I
ENSMUST00000058704.9|Irgm2-201|ENSMUSG00000069874|+|chr11:g.58110827:T>A
MEEAVESPEVKEFEYFSDAVFIPKDGNTLSVGVIKRIETAVKEGEVVKVVSIVKEIIQNVSRNKIKIAVTGDS
GNGMSSFINALRLIGHEEKDSAPTGVVRTTQKPTCYFSSHFPYVELWDLPGLGATAQSVESYLEEMQISIYDL
IIIVASEQFSLNHVKLAITMQRMRKRIYVVWTKLDRDLSTSTFPEPQLLQSIQRNIRDSLQKEKVKEHPMFLV
SVFKPESHDFPKLRETLQKDLPVIKYHGLVETLYQVCEKTVNERVESIKKSIDEDNLHTEFGISDPGNAIEIR
KAFQKTFGLDDISLHLVALEMKNKHFNTSMESQETQRYQQDDWVLARLYRTGTRVGSIGFDYMKCCFTSHHSR
CKQQKDILDETAAKAKEVLLKILRLSIPHP*
>rs49881478_ENSMUSP00000146805_c.1116:T>C_p.S373P
ENSMUST00000209079.2|Irgm2-203|ENSMUSG00000069874|+|chr11:g.58111391:T>C
MPTSRVAPLLDNMEEAVESPEVKEFEYFSDAVFIPKDGNTLSVGVIKRIETAVKEGEVVKVVSIVKEIIQNVS
RNKIKIAVTGDSGNGMSSFINALRLIGHEEKDSAPTGVVRTTQKPTCYFSSHFPYVELWDLPGLGATAQSVES
YLEEMQISIYDLIIIVASEQFSLNHVKLAITMQRMRKRFYVVWTKLDRDLSTSTFPEPQLLQSIQRNIRDSLQ
KEKVKEHPMFLVSVFKPESHDFPKLRETLQKDLPVIKYHGLVETLYQVCEKTVNERVESIKKSIDEDNLHTEF
GISDPGNAIEIRKAFQKTFGLDDISLHLVALEMKNKHFNTSMESQETQRYQQDDWVLARLYRTGTRVGSIGFD
YMKCCFTPHHSRCKQQKDILDETAAKAKEVLLKILRLSIPHP*
>rs49881478_ENSMUSP00000104464_c.1080:T>C_p.S361P
ENSMUST00000108836.2|Irgm2-202|ENSMUSG00000069874|+|chr11:g.58111391:T>C
MEEAVESPEVKEFEYFSDAVFIPKDGNTLSVGVIKRIETAVKEGEVVKVVSIVKEIIQNVSRNKIKIAVTGDS
GNGMSSFINALRLIGHEEKDSAPTGVVRTTQKPTCYFSSHFPYVELWDLPGLGATAQSVESYLEEMQISIYDL
IIIVASEQFSLNHVKLAITMQRMRKRFYVVWTKLDRDLSTSTFPEPQLLQSIQRNIRDSLQKEKVKEHPMFLV
SVFKPESHDFPKLRETLQKDLPVIKYHGLVETLYQVCEKTVNERVESIKKSIDEDNLHTEFGISDPGNAIEIR
KAFQKTFGLDDISLHLVALEMKNKHFNTSMESQETQRYQQDDWVLARLYRTGTRVGSIGFDYMKCCFTPHHSR
CKQQKDILDETAAKAKEVLLKILRLSIPHP*
```

>rs49881478_ENSMUSP00000056001_c.1080:T>C_p.S361P
ENSMUST00000058704.9|Irgm2-201|ENSMUSG00000069874|+|chr11:g.58111391:T>C
MEEAVESPEVKEFEYFSDAVFIPKDGNTLSVGVIKRIETAVKEGEVVKVVSIVKEIIQNVSRNKIKIAVTGDS
GNGMSSFINALRLIGHEEKDSAPTGVVRTTQKPTCYFSSHFPYVELWDLPGLGATAQSVESYLEEMQISIYDL
IIIVASEQFSLNHVKLAITMQRMRKRFYVVWTKLDRDLSTSTFPEPQLLQSIQRNIRDSLQKEKVKEHPMFLV
SVFKPESHDFPKLRETLQKDLPVIKYHGLVETLYQVCEKTVNERVESIKKSIDEDNLHTEFGISDPGNAIEIR
KAFQKTFGLDDISLHLVALEMKNKHFNTSMESQETQRYQQDDWVLARLYRTGTRVGSIGFDYMKCCFTPHHSR
CKQQKDILDETAAKAKEVLLKILRLSIPHP*
>ENSMUSP00000104462_c.811:T>G_p.L271R ENSMUST00000108834.3|4930438A08Rik-201|ENSMUSG00000069873|+|chr11:g.58184095:T>G
MLPAPGRLLAMFPGWLALIFLSVLSLDTQAFNRKLVKCFEDPQYEELLQLARNGLGQTAERKQVVVIGAGMAG
LTAAKILQDAGHQVTVLEASGRVGGRIETHRVPGAHWYIELGAMRIPINHRLSHELIKKFGLMLKEFCPCNNQ
TWVLVNGVRQRSGAVQADPGLLGYSVRADEVGKTAEQLFDASLRKIVEELKNSSCQEVLEKYDSFSTKEYLIK
VGNLSRGAVQMIGDMLNTDSGYYEAFTETLRGIISFFQDPRFDEIVGGFDQRPQALHNSLLPGTVWFYSPAEE
VEMSGDCVHVTYRTPDPLLPRARLTADFVVVATTAKAARLLRFQPPLSLNKQDALRSVHYNSATKVILACTQR
FWERDGIFSGKSSTDRPSRFIYYPNHIFPNGAGVILASYTLDDDSVFFTALDHTQVVDIVLDDLAALHSRAKE
ELRALCPYSAIKNWSQDPYSMGGFAFFTPYQYVDYAQELSQPEGLVFFAGEHTDLPHGWIDTAIKSGLRVAKN
IQESVDLALTRNPRNTKEYFSKSEL*
>ENSMUSP00000147249_c.780:T>G_p.S261A ENSMUST00000208022.2|4930438A08Rik-202|ENSMUSG00000069873|+|chr11:g.58184095:T>G
MLPAPGRLLAMFPGWLALIFLSVLSLDTQAFNRKLVKCFEDPQYEELLQLARNGLGQTAERKQVVVIGAGMAG
LTAAKILQDAGHQVTVLEASGRVGGRIETHRVPGAHWYIELGAMRIPINHRLSHELIKKFGLMLKEFCPCNNQ
TWVLVNGVRQRSGAVQADPGLLGYSVRADEVGKTAEQLFDASLRKIVEELKNSSCQEVLEKYDSFSTKEYLIK
VGNLSRGAVQMIGDMLNTDSGYYEAFTETLRSMKSLEALTNAPRLCTILSSQGLFGSTHQQRKWKCLETVFTS
PTGHLTLCCPGRG*
>ENSMUSP00000131896_c.584:G>T_p.E195D ENSMUST00000049353.9|Zfp692-201|ENSMUSG00000037243|+|chr11:g.58200417:G>T
MASPVADASRRRREKRRQLDARRSKCRIRLGGHMEQWCLLKERLGFSLHSQLAKFLLDRYTSSGCVLCAAPEP
GPRKGLQYLVLLSHTHSRECGLVPGLRGPGGGEGELVWECSAGHTFSWEPSLIPAPSDVPKQAPLTHTTERAW
CSEARRKQEAQGLECEQRERTQETRLSRRVDSPLEIDPPLGEDQDVEEDEEEEEEEEELLSDASPWTYSSSPD
DSEPDVPRPPPSPVTHTPKEGEVSPVPATLPTPCAVLASVGSPDALTSDTEVRLELSRTPQVPAELHMTESLE
SPGSQAQSAPNPTWDEDTAQIGLRRIRKAAKRELMPCDFPGCGRIFSNRQYLNHHKKYQHIHQKSFCCPEPAC
GKSFNFKKHLKEHVKLHSDTRDYICEFCARSFRTSSNLVIHRRIHTGEKPLQCEICGFTCRQKASLNWHRRKH
AETAAALRFPCEFCGKRFEKPDSVVAHCSKSHPALLPAQEPPGSLVSSPSISAPESLQSPEGASISTTSDSNP
ASSTSISSPGVPDPRNREKS*
>ENSMUSP00000126674_c.584:G>T_p.E195D ENSMUST00000153510.9|Zfp692-202|ENSMUSG00000037243|+|chr11:g.58200417:G>T
MASPVADASRRRREKRRQLDARRSKCRIRLGGHMEQWCLLKERLGFSLHSQLAKFLLDRYTSSGCVLCAAPEP
GPRKGLQYLVLLSHTHSRECGLVPGLRGPGGGEGELVWECSAGHTFSWEPSLIPAPSDVPKQAPLTHTTERAW
CSEARRKQEAQGLECEQRERTQETRLSRRVDSPLEIDPPLGEDQDVEEDEEEEEEEEELLSDASPWTYSSSPD
DSEPDVPRPPPSPVTHTPKEGEVSPVPATLPTPCAVLASVGSPDALTSDTEVRLELSRTPQVPAELHMTESLE
SPGSQAQSAPNPTWDEDTAQIGLRRIRKAAKRELMPCDFPGCGRIFSNRQYLNHHKKYQHIHQKSFCCPEPAC
GKSFNFKKHLKEHVKLHSDTRDYICEFCARSFRTSSNLVIHRRIHTGEKPLQCEICGFTCRQKASLNWHRRKH
AETAAALRFPCEFCGKRFEKPDSVVAHCSKSHPALLPAQEPPGSLVSSPSISAPESLQSPEGASISTTSDSNP
ASSTSISSPGVPDPRNREKS*
>ENSMUSP00000070680_c.185:C>A_p.Y62* ENSMUST00000065533.3|Zfp672-203|ENSMUSG00000049755|-|chr11:g.58220412:G>T
MRMEEDGVPQSEVKGIPELVPVRVQESPHKPQEYQSQSDRDSVLNAHPTVGRHSLDSGTEC*YHELRFRVSLY
RQKPIMNSQAIAGRACRVIGLFPSCVCVCLNNLDITVQVDFYLA*
>ENSMUSP00000013787_c.108:G>T_p.A37S ENSMUST00000013787.11|Lypd8-201|ENSMUSG00000013643|+|chr11:g.58273631:G>T
MRGVFIAGVIAAFAITVVDSLNCTQCYTYNSTCDGQSTECNEQSFSCVESSINSTLGGFLHVYQNKFCSASNC
TENSTEVAFTVHLFDDQRYHFASQCCQGESCNATHSESGTQNVTDMQCMSCYGHNKTLCEEKPQKCYEGEQCV
FIIAEMVNGSGRVELKGCSDISNSTCQFLSPGNTTVGEFVFKSVECTQPTEYTNSTTTIPPITNTSLTSVTRP
GIKTSPASVTPQASMGTKASFTSSIFGSLLLLKLLF*
>ENSMUSP00000104454_c.108:G>T_p.A37S ENSMUST00000108826.3|Lypd8-202|ENSMUSG00000013643|+|chr11:g.58273631:G>T

MRGVFIAGVIAAFAITVVDSLNCTQCYTYNSTCDGQSTECNEQSFSCVESSINSTLGGFLHVYQNKFCSASNC
TENSTEVAFTVHLFDDQRYHFASQCCQGESCNATHSESGTQNVTDMQCMSCYGHNKTLCEEKPQKCYEGEQCV
FIIAEMVNGSGRVELKGCSDISNSTCQFLSPGNTTVGEFVFKSVECTQPTEYTNSTTTIPPITNTSLTSVTRP
GIKTSPASVTPQASMGTKASFTSSIFGSLLLLKLLF*
>ENSMUSP00000013787_c.232:C>T_p.T78M ENSMUST00000013787.11|Lypd8-201|ENSMUSG00000013643|+|chr11:g.58275499:C>T
MRGVFIAGVIAAFAITVVDSLNCTQCYTYNSTCDGQATECNEQSFSCVESSINSTLGGFLHVYQNKFCSASNC
TENSMEVAFTVHLFDDQRYHFASQCCQGESCNATHSESGTQNVTDMQCMSCYGHNKTLCEEKPQKCYEGEQCV
FIIAEMVNGSGRVELKGCSDISNSTCQFLSPGNTTVGEFVFKSVECTQPTEYTNSTTTIPPITNTSLTSVTRP
GIKTSPASVTPQASMGTKASFTSSIFGSLLLLKLLF*
>ENSMUSP00000104454_c.232:C>T_p.T78M ENSMUST00000108826.3|Lypd8-202|ENSMUSG00000013643|+|chr11:g.58275499:C>T
MRGVFIAGVIAAFAITVVDSLNCTQCYTYNSTCDGQATECNEQSFSCVESSINSTLGGFLHVYQNKFCSASNC
TENSMEVAFTVHLFDDQRYHFASQCCQGESCNATHSESGTQNVTDMQCMSCYGHNKTLCEEKPQKCYEGEQCV
FIIAEMVNGSGRVELKGCSDISNSTCQFLSPGNTTVGEFVFKSVECTQPTEYTNSTTTIPPITNTSLTSVTRP
GIKTSPASVTPQASMGTKASFTSSIFGSLLLLKLLF*
>ENSMUSP00000013787_c.586:C>A_p.T196N ENSMUST00000013787.11|Lypd8-201|ENSMUSG00000013643|+|chr11:g.58281026:C>A
MRGVFIAGVIAAFAITVVDSLNCTQCYTYNSTCDGQATECNEQSFSCVESSINSTLGGFLHVYQNKFCSASNC
TENSTEVAFTVHLFDDQRYHFASQCCQGESCNATHSESGTQNVTDMQCMSCYGHNKTLCEEKPQKCYEGEQCV
FIIAEMVNGSGRVELKGCSDISNSTCQFLSPGNTTVGEFVFKSVECTQPNEYTNSTTTIPPITNTSLTSVTRP
GIKTSPASVTPQASMGTKASFTSSIFGSLLLLKLLF*
>ENSMUSP00000104454_c.586:C>A_p.T196N ENSMUST00000108826.3|Lypd8-202|ENSMUSG00000013643|+|chr11:g.58281026:C>A
MRGVFIAGVIAAFAITVVDSLNCTQCYTYNSTCDGQATECNEQSFSCVESSINSTLGGFLHVYQNKFCSASNC
TENSTEVAFTVHLFDDQRYHFASQCCQGESCNATHSESGTQNVTDMQCMSCYGHNKTLCEEKPQKCYEGEQCV
FIIAEMVNGSGRVELKGCSDISNSTCQFLSPGNTTVGEFVFKSVECTQPNEYTNSTTTIPPITNTSLTSVTRP
GIKTSPASVTPQASMGTKASFTSSIFGSLLLLKLLF*
>ENSMUSP00000013787_c.741:C>T_p.L248F ENSMUST00000013787.11|Lypd8-201|ENSMUSG00000013643|+|chr11:g.58281181:C>T
MRGVFIAGVIAAFAITVVDSLNCTQCYTYNSTCDGQATECNEQSFSCVESSINSTLGGFLHVYQNKFCSASNC
TENSTEVAFTVHLFDDQRYHFASQCCQGESCNATHSESGTQNVTDMQCMSCYGHNKTLCEEKPQKCYEGEQCV
FIIAEMVNGSGRVELKGCSDISNSTCQFLSPGNTTVGEFVFKSVECTQPTEYTNSTTTIPPITNTSLTSVTRP
GIKTSPASVTPQASMGTKASFTSSIFGSFLLLKLLF*
>ENSMUSP00000104454_c.741:C>T_p.L248F ENSMUST00000108826.3|Lypd8-202|ENSMUSG00000013643|+|chr11:g.58281181:C>T
MRGVFIAGVIAAFAITVVDSLNCTQCYTYNSTCDGQATECNEQSFSCVESSINSTLGGFLHVYQNKFCSASNC
TENSTEVAFTVHLFDDQRYHFASQCCQGESCNATHSESGTQNVTDMQCMSCYGHNKTLCEEKPQKCYEGEQCV
FIIAEMVNGSGRVELKGCSDISNSTCQFLSPGNTTVGEFVFKSVECTQPTEYTNSTTTIPPITNTSLTSVTRP
GIKTSPASVTPQASMGTKASFTSSIFGSFLLLKLLF*
>rs29446775_ENSMUSP00000104453_c.538:C>T_p.T180M ENSMUST00000108825.2|Gm12253-201|ENSMUSG00000058287|+|chr11:g.58330781:C>T
MEVTMSWFLVLKSLLAVCIISHLSVSSVESYACIQEEPPFNKECYPNSSTCESSKGCFSLWLELGAHQTYVQK
GCSEDSCTGLEFSVTVERHLALSYDHRCCYTEHCSKEPIKVPALSSEPNGVECPACFSECGTCRPVLLKCTGE
ETMCIKATARDIIKLQAMGCATKTACNLKNVTIMDNLKFDTSCVNGSPLLSSRLLLQHHACLPAATLPAMTVM
ELSSGTVSPQ*
>rs29395894_ENSMUSP00000063665_c.114:G>A_p.E39K ENSMUST00000064614.4|Lypd9-201|ENSMUSG00000052642|-|chr11:g.58338276:C>T
MHPATPSRLLLVCSLAFMPFSTDPWEITMNEIEEKEVDKFNSSGLKCPTCFTVKGRECSPELKWCSTDKIKCV
ELSGIINTGVNNVPIQLMKCITIEQCKDTATAYMGFTISNKSEICKSAIRNGARVRPPTPIFFVLFLKKLLH*
>rs47817785_ENSMUSP00000136008_c.659:A>G_p.I220M ENSMUST00000180165.3|Olfr332-201|ENSMUSG00000050813|-|chr11:g.58380920:T>C
MDLKNKTTSSFILLGLFPSCRYPNLLISFILLIYTLASAGNSLLILLIWLDPRLHTPMYFLLSQLSVIDLAYI
SCTVPKAAINYFTGRQNISFFACATQMFSFLTLGLAECILLTLMAYDRYVAVCNPLRYTILMSPKVCLMMAAS
TWIGAVAAALVHTVYPMNFPICGSREINHYFCEMPAILRMSCVDTSVYEMVKFVSTIIFLLTPFTLILTSYTL

MFLTVLRMNSPKGRNKALATCSSHLTVVSLYFGQAIFIYMTPTSSHTPDQDQVGAVLGTIVTPMLNPLIYSLR
NKEVIGALQKCTGRCCSRDRVGSLRCCTQKCPILHLKSEFKSKEIQIGNRAL*
>rs47370459_ENSMUSP00000136008_c.496:C>G_p.P166R
ENSMUST00000180165.3|Olfr332-201|ENSMUSG00000050813|-
|chr11:g.58381083:G>C
MDLKNKTTSSFILLGLFPSCRYPNLLISFILLIYTLASAGNSLLILLIWLDPRLHTPMYFLLSQLSVIDLAYI
SCTVPKAAINYFTGRQNISFFACATQMFSFLTLGLAECILLTLMAYDRYVAVCNPLRYTILMSPKVCLMMAAS
TWIGAVAAALVHTVYPMNFRICGSREINHYFCEMPAILRMSCVDTSVYEMVKFVSTIIFLLTPFTLILTSYTL
IFLTVLRMNSPKGRNKALATCSSHLTVVSLYFGQAIFIYMTPTSSHTPDQDQVGAVLGTIVTPMLNPLIYSLR
NKEVIGALQKCTGRCCSRDRVGSLRCCTQKCPILHLKSEFKSKEIQIGNRAL*
>rs46574752_ENSMUSP00000136008_c.385:T>C_p.L129P
ENSMUST00000180165.3|Olfr332-201|ENSMUSG00000050813|-
|chr11:g.58381194:A>G
MDLKNKTTSSFILLGLFPSCRYPNLLISFILLIYTLASAGNSLLILLIWLDPRLHTPMYFLLSQLSVIDLAYI
SCTVPKAAINYFTGRQNISFFACATQMFSFLTLGLAECILLTLMAYDRYVAVCNPPRYTILMSPKVCLMMAAS
TWIGAVAAALVHTVYPMNFPICGSREINHYFCEMPAILRMSCVDTSVYEMVKFVSTIIFLLTPFTLILTSYTL
IFLTVLRMNSPKGRNKALATCSSHLTVVSLYFGQAIFIYMTPTSSHTPDQDQVGAVLGTIVTPMLNPLIYSLR
NKEVIGALQKCTGRCCSRDRVGSLRCCTQKCPILHLKSEFKSKEIQIGNRAL*
>rs46574752_ENSMUSP00000144797_c.88:T>C_p.L30P
ENSMUST00000203744.2|Olfr332-202|ENSMUSG00000050813|-
|chr11:g.58381194:A>G
MFSFLTLGLAECILLTLMAYDRYVAVCNPPRYT
>ENSMUSP00000080440_c.721:G>AG_p.G241ins-fs ENSMUST00000081743.3|Olfr331-
201|ENSMUSG00000058807|-|chr11:g.58392677:C>CT
MDLTTWMNNYTTQSDFTLVGFFSQSKHPALLAVVIFVVFLMALSGNALLILLILSDIHIPIHLHIPMYFFISQ
LSLMDMMYISVTVPKMLMDQVLGSHKISAAACGMQMFLYLTLAGSEYFLLAAMSYDHYVAICHPLQYPVLMNH
RVCLLLMSVCWILGSLDGFMFTPVTMTFPFCGSREIHHFFCEVPAVTKLSCSDTWLYETLMYVCCVLMLLIPV
TVISGSYTSILLTVLRMNSAEEQEEGPCHLLLPHDCGHPLLWCCYLYLHFPCLSPHP
>ENSMUSP00000132693_c.703:G>AG_p.G235ins-fs ENSMUST00000170501.3|Olfr331-
202|ENSMUSG00000058807|-|chr11:g.58392677:C>CT
MNNYTTQSDFTLVGFFSQSKHPALLAVVIFVVFLMALSGNALLILLILSDIHIPIHLHIPMYFFISQLSLMDM
MYISVTVPKMLMDQVLGSHKISAAACGMQMFLYLTLAGSEYFLLAAMSYDHYVAICHPLQYPVLMNHRVCLLL
MSVCWILGSLDGFMFTPVTMTFPFCGSREIHHFFCEVPAVTKLSCSDTWLYETLMYVCCVLMLLIPVTVISGS
YTSILLTVLRMNSAEEQEEGPCHLLLPHDCGHPLLWCCYLYLHFPCLSPHP
>rs261360536_ENSMUSP00000149073_c.286:T>CT_p.I96ins-fs
ENSMUST00000213188.3|Olfr330-202|ENSMUSG00000050818|-
|chr11:g.58420524:A>AG
MDLTTWMNNYTTQSDFTLVGFFSQSKHSALLAVVIFVVFLMALSGNALLILLVLSDTHLHTPMYFFISQLSLM
DMMYISVTVPKMLMDQVLGSHKTLSCCLWDADVPLCDTSRFRIFPSGCHVLRPLCGHLPSTPVSCPHELQGLS
PPDVCLLDPGIFGWLHVHPCHHDLPILWIPGDPSLLL
>rs261360536_ENSMUSP00000145126_c.286:T>CT_p.I96ins-fs
ENSMUST00000134055.3|Olfr330-201|ENSMUSG00000050818|-
|chr11:g.58420524:A>AG
MDLTTWMNNYTTQSDFTLVGFFSQSKHSALLAVVIFVVFLMALSGNALLILLVLSDTHLHTPMYFFISQLSLM
DMMYISVTVPKMLMDQVLGSHKTLSCCLWDADVPLCDTSRFRIFPSGCHVLRPLCGHLPSTPVSCPHELQGLS
PPDVCLLDPGIFGWLHVHPCHHDLPILWIPGDPSLLL
>rs51951760_ENSMUSP00000150086_c.511:G>T_p.R171L
ENSMUST00000216725.2|Olfr328-204|ENSMUSG00000057654|-
|chr11:g.58442552:C>A
MSNYTGQFSDFTLVGFFSQSKHPALLAVVIFVVFLMALSGNALLILLILSDTHLHTPMYFFISQLSLMDMMYI
SVTVPKMLMDQVLGSHKISAAACGMQMFLYLSIGGSEFLLLAAMSYDRYVAICHPLRYPVLMNHRICLLLLSV
CWLLGSLDGFMLTPVTMTFPICGSLEIHHFFCEVPAVTKLSCSDTWLYETLMYVCCVLMILIPVTVISGSYSS
ILLTVLKMNSAEGRKKALATCSSHMTVVTLFYGAAVYTYMLPASLHTPEKDMVVSVFYTIVTPLLNPLIYSFR
NKNVTEAMKKLLGVSIPH*
>rs51951760_ENSMUSP00000149506_c.511:G>T_p.R171L
ENSMUST00000215717.2|Olfr328-203|ENSMUSG00000057654|-
|chr11:g.58442552:C>A

MSNYTGQFSDFTLVGFFSQSKHPALLAVVIFVVFLMALSGNALLILLILSDTHLHTPMYFFISQLSLMDMMYI
SVTVPKMLMDQVLGSHKISAAACGMQMFLYLSIGGSEFLLLAAMSYDRYVAICHPLRYPVLMNHRICLLLLSV
CWLLGSLDGFMLTPVTMTFPICGSLEIHHFFCEVPAVTKLSCSDTWLYETLMYVCCVLMILIPVTVISGSYSS
ILLTVLKMNSAEGRKKALATCSSHMTVVTLFYGAAVYTYMLPASLHTPEKDMVVSVFYTIVTPLLNPLIYSFR
NKNVTEAMKKLLGVSIPH*
>rs51951760_ENSMUSP00000104452_c.511:G>T_p.R171L
ENSMUST00000108824.3|Olfr328-202|ENSMUSG00000057654|-
|chr11:g.58442552:C>A
MSNYTGQFSDFTLVGFFSQSKHPALLAVVIFVVFLMALSGNALLILLILSDTHLHTPMYFFISQLSLMDMMYI
SVTVPKMLMDQVLGSHKISAAACGMQMFLYLSIGGSEFLLLAAMSYDRYVAICHPLRYPVLMNHRICLLLLSV
CWLLGSLDGFMLTPVTMTFPICGSLEIHHFFCEVPAVTKLSCSDTWLYETLMYVCCVLMILIPVTVISGSYSS
ILLTVLKMNSAEGRKKALATCSSHMTVVTLFYGAAVYTYMLPASLHTPEKDMVVSVFYTIVTPLLNPLIYSFR
NKNVTEAMKKLLGVSIPH*
>rs51951760_ENSMUSP00000073588_c.511:G>T_p.R171L
ENSMUST00000073933.2|Olfr328-201|ENSMUSG00000057654|-
|chr11:g.58442552:C>A
MSNYTGQFSDFTLVGFFSQSKHPALLAVVIFVVFLMALSGNALLILLILSDTHLHTPMYFFISQLSLMDMMYI
SVTVPKMLMDQVLGSHKISAAACGMQMFLYLSIGGSEFLLLAAMSYDRYVAICHPLRYPVLMNHRICLLLLSV
CWLLGSLDGFMLTPVTMTFPICGSLEIHHFFCEVPAVTKLSCSDTWLYETLMYVCCVLMILIPVTVISGSYSS
ILLTVLKMNSAEGRKKALATCSSHMTVVTLFYGAAVYTYMLPASLHTPEKDMVVSVFYTIVTPLLNPLIYSFR
NKNVTEAMKKLLGVSIPH*
>rs49802583_ENSMUSP00000150086_c.393:C>A_p.P132T
ENSMUST00000216725.2|Olfr328-204|ENSMUSG00000057654|-
|chr11:g.58442670:G>T
MSNYTGQFSDFTLVGFFSQSKHPALLAVVIFVVFLMALSGNALLILLILSDTHLHTPMYFFISQLSLMDMMYI
SVTVPKMLMDQVLGSHKISAAACGMQMFLYLSIGGSEFLLLAAMSYDRYVAICHPLRYTVLMNHRICLLLLSV
CWLLGSLDGFMLTPVTMTFPICGSREIHHFFCEVPAVTKLSCSDTWLYETLMYVCCVLMILIPVTVISGSYSS
ILLTVLKMNSAEGRKKALATCSSHMTVVTLFYGAAVYTYMLPASLHTPEKDMVVSVFYTIVTPLLNPLIYSFR
NKNVTEAMKKLLGVSIPH*
>rs49802583_ENSMUSP00000149506_c.393:C>A_p.P132T
ENSMUST00000215717.2|Olfr328-203|ENSMUSG00000057654|-
|chr11:g.58442670:G>T
MSNYTGQFSDFTLVGFFSQSKHPALLAVVIFVVFLMALSGNALLILLILSDTHLHTPMYFFISQLSLMDMMYI
SVTVPKMLMDQVLGSHKISAAACGMQMFLYLSIGGSEFLLLAAMSYDRYVAICHPLRYTVLMNHRICLLLLSV
CWLLGSLDGFMLTPVTMTFPICGSREIHHFFCEVPAVTKLSCSDTWLYETLMYVCCVLMILIPVTVISGSYSS
ILLTVLKMNSAEGRKKALATCSSHMTVVTLFYGAAVYTYMLPASLHTPEKDMVVSVFYTIVTPLLNPLIYSFR
NKNVTEAMKKLLGVSIPH*
>rs49802583_ENSMUSP00000104452_c.393:C>A_p.P132T
ENSMUST00000108824.3|Olfr328-202|ENSMUSG00000057654|-
|chr11:g.58442670:G>T
MSNYTGQFSDFTLVGFFSQSKHPALLAVVIFVVFLMALSGNALLILLILSDTHLHTPMYFFISQLSLMDMMYI
SVTVPKMLMDQVLGSHKISAAACGMQMFLYLSIGGSEFLLLAAMSYDRYVAICHPLRYTVLMNHRICLLLLSV
CWLLGSLDGFMLTPVTMTFPICGSREIHHFFCEVPAVTKLSCSDTWLYETLMYVCCVLMILIPVTVISGSYSS
ILLTVLKMNSAEGRKKALATCSSHMTVVTLFYGAAVYTYMLPASLHTPEKDMVVSVFYTIVTPLLNPLIYSFR
NKNVTEAMKKLLGVSIPH*
>rs49802583_ENSMUSP00000073588_c.393:C>A_p.P132T
ENSMUST00000073933.2|Olfr328-201|ENSMUSG00000057654|-
|chr11:g.58442670:G>T
MSNYTGQFSDFTLVGFFSQSKHPALLAVVIFVVFLMALSGNALLILLILSDTHLHTPMYFFISQLSLMDMMYI
SVTVPKMLMDQVLGSHKISAAACGMQMFLYLSIGGSEFLLLAAMSYDRYVAICHPLRYTVLMNHRICLLLLSV
CWLLGSLDGFMLTPVTMTFPICGSREIHHFFCEVPAVTKLSCSDTWLYETLMYVCCVLMILIPVTVISGSYSS
ILLTVLKMNSAEGRKKALATCSSHMTVVTLFYGAAVYTYMLPASLHTPEKDMVVSVFYTIVTPLLNPLIYSFR
NKNVTEAMKKLLGVSIPH*
>rs29402353_ENSMUSP00000150140_c.675:A>C_p.M226L
ENSMUST00000216758.2|Olfr224-203|ENSMUSG00000059279|-
|chr11:g.58457494:T>G
MSNYTGQSYFTLVGLFSQSKHPALLAVVIFVVFLMSLSGNALLILLILSDTHLHTPMYFFISQLSLMDMMYIS
VTVPKMLMDRVLGNHKISAAACGMQMFLYMSLGGSEFLLLAAMSYDRYVAICHPLRYPVLMNHRVCLLLISTC

WILGSLDGFMFTPVTMTFPFCGSREIHHFFCEAPAVTKLSCSDTWLYETLMYVCCVLMILIPVTVISGSYSSI
LLTVLRLNSAEGRKKALATCSSHMTVVTLFYGAAIYTYIFPASLHSPEKDMVVSVFYTILTPLLNPLIYSLRN
KNVTEAMKKLLGIRLHFPETVK*
>rs29402353_ENSMUSP00000150268_c.675:A>C_p.M226L
ENSMUST00000217009.2|Olfr224-204|ENSMUSG00000059279|-
|chr11:g.58457494:T>G
MSNYTGQSYFTLVGLFSQSKHPALLAVVIFVVFLMSLSGNALLILLILSDTHLHTPMYFFISQLSLMDMMYIS
VTVPKMLMDRVLGNHKISAAACGMQMFLYMSLGGSEFLLLAAMSYDRYVAICHPLRYPVLMNHRVCLLLISTC
WILGSLDGFMFTPVTMTFPFCGSREIHHFFCEAPAVTKLSCSDTWLYETLMYVCCVLMILIPVTVISGSYSSI
LLTVLRLNSAEGRKKALATCSSHMTVVTLFYGAAIYTYIFPASLHSPEKDMVVSVFYTILTPLLNPLIYSLRN
KNVTEAMKKLLGIRLHFPETVK*
>rs29402353_ENSMUSP00000150143_c.675:A>C_p.M226L
ENSMUST00000215322.2|Olfr224-202|ENSMUSG00000059279|-
|chr11:g.58457494:T>G
MSNYTGQSYFTLVGLFSQSKHPALLAVVIFVVFLMSLSGNALLILLILSDTHLHTPMYFFISQLSLMDMMYIS
VTVPKMLMDRVLGNHKISAAACGMQMFLYMSLGGSEFLLLAAMSYDRYVAICHPLRYPVLMNHRVCLLLISTC
WILGSLDGFMFTPVTMTFPFCGSREIHHFFCEAPAVTKLSCSDTWLYETLMYVCCVLMILIPVTVISGSYSSI
LLTVLRLNSAEGRKKALATCSSHMTVVTLFYGAAIYTYIFPASLHSPEKDMVVSVFYTILTPLLNPLIYSLRN
KNVTEAMKKLLGIRLHFPETVK*
>rs29402353_ENSMUSP00000079881_c.675:A>C_p.M226L
ENSMUST00000081102.5|Olfr224-201|ENSMUSG00000059279|-
|chr11:g.58457494:T>G
MSNYTGQSYFTLVGLFSQSKHPALLAVVIFVVFLMSLSGNALLILLILSDTHLHTPMYFFISQLSLMDMMYIS
VTVPKMLMDRVLGNHKISAAACGMQMFLYMSLGGSEFLLLAAMSYDRYVAICHPLRYPVLMNHRVCLLLISTC
WILGSLDGFMFTPVTMTFPFCGSREIHHFFCEAPAVTKLSCSDTWLYETLMYVCCVLMILIPVTVISGSYSSI
LLTVLRLNSAEGRKKALATCSSHMTVVTLFYGAAIYTYIFPASLHSPEKDMVVSVFYTILTPLLNPLIYSLRN
KNVTEAMKKLLGIRLHFPETVK*
>rs46544151_ENSMUSP00000154189_c.237:A>C_p.N80H
ENSMUST00000214990.2|Olfr324-
202|ENSMUSG00000056564|+|chr11:g.58488479:A>C
MEPRYGSAAGLSPENSSTITELVLVGFSDQPQTEIPLFIFFSLVYLASCLGNTAVVILVALDVSLQTPMYFFL
CHLAFLHGFFSTVVTPKMLFNFLASRKVISYPFCLAQTYLTLFLESTECFLLAVMAIDRYVAICYPLRYLLIM
SWAVCIALAVAVWVTGFCASVLPLCFMILPLCSPYVVDYLFCELPILLHLFCADTSLQEAMMAVGGAGTVLVP
FLLIALSYLRILVTVIRIDSAEGRKKAFSTCASHLAVVTIYYGTGLIRYLRPKSLYSAEGDKLISVFYAVIGP
ALNPFIYSLRNKEVQGAVRRVVERYRKSPRIAF*
>rs46544151_ENSMUSP00000154149_c.255:A>C_p.N86H
ENSMUST00000216965.2|Olfr324-
203|ENSMUSG00000056564|+|chr11:g.58488479:A>C
MLPHIRTLGRYGSAAGLSPENSSTITELVLVGFSDQPQTEIPLFIFFSLVYLASCLGNTAVVILVALDVSLQT
PMYFFLCHLAFLHGFFSTVVTPKMLFNFLASRKVISYPFCLAQTYLTLFLESTECFLLAVMAIDRYVAICYPL
RYLLIMSWAVCIALAVAVWVTGFCASVLPLCFMILPLCSPYVVDYLFCELPILLHLFCADTSLQEAMMAVGGA
GTVLVPFLLIALSYLRILVTVIRIDSAEGRKKAFSTCASHLAVVTIYYGTGLIRYLRPKSLYSAEGDKLISVF
YAVIGPALNPFIYSLRNKEVQGAVRRVVERYRKSPRIAF*
>rs46544151_ENSMUSP00000056457_c.261:A>C_p.N88H
ENSMUST00000054683.8|Olfr324-
201|ENSMUSG00000056564|+|chr11:g.58488479:A>C
MWGPSISSRLDRYGSAAGLSPENSSTITELVLVGFSDQPQTEIPLFIFFSLVYLASCLGNTAVVILVALDVSL
QTPMYFFLCHLAFLHGFFSTVVTPKMLFNFLASRKVISYPFCLAQTYLTLFLESTECFLLAVMAIDRYVAICY
PLRYLLIMSWAVCIALAVAVWVTGFCASVLPLCFMILPLCSPYVVDYLFCELPILLHLFCADTSLQEAMMAVG
GAGTVLVPFLLIALSYLRILVTVIRIDSAEGRKKAFSTCASHLAVVTIYYGTGLIRYLRPKSLYSAEGDKLIS
VFYAVIGPALNPFIYSLRNKEVQGAVRRVVERYRKSPRIAF*
>rs49647928_ENSMUSP00000154189_c.343:T>C_p.L115P
ENSMUST00000214990.2|Olfr324-
202|ENSMUSG00000056564|+|chr11:g.58488585:T>C
MEPRYGSAAGLSPENSSTITELVLVGFSDQPQTEIPLFIFFSLVYLASCLGNTAVVILVALDVSLQTPMYFFL
CHLAFLNGFFSTVVTPKMLFNFLASRKVISYPFCLAQTYLTPFLESTECFLLAVMAIDRYVAICYPLRYLLIM
SWAVCIALAVAVWVTGFCASVLPLCFMILPLCSPYVVDYLFCELPILLHLFCADTSLQEAMMAVGGAGTVLVP

FLLIALSYLRILVTVIRIDSAEGRKKAFSTCASHLAVVTIYYGTGLIRYLRPKSLYSAEGDKLISVFYAVIGP
ALNPFIYSLRNKEVQGAVRRVVERYRKSPRIAF*
>rs49647928_ENSMUSP00000154149_c.361:T>C_p.L121P
ENSMUST00000216965.2|Olfr324-
203|ENSMUSG00000056564|+|chr11:g.58488585:T>C
MLPHIRTLGRYGSAAGLSPENSSTITELVLVGFSDQPQTEIPLFIFFSLVYLASCLGNTAVVILVALDVSLQT
PMYFFLCHLAFLNGFFSTVVTPKMLFNFLASRKVISYPFCLAQTYLTPFLESTECFLLAVMAIDRYVAICYPL
RYLLIMSWAVCIALAVAVWVTGFCASVLPLCFMILPLCSPYVVDYLFCELPILLHLFCADTSLQEAMMAVGGA
GTVLVPFLLIALSYLRILVTVIRIDSAEGRKKAFSTCASHLAVVTIYYGTGLIRYLRPKSLYSAEGDKLISVF
YAVIGPALNPFIYSLRNKEVQGAVRRVVERYRKSPRIAF*
>rs49647928_ENSMUSP00000056457_c.367:T>C_p.L123P
ENSMUST00000054683.8|Olfr324-
201|ENSMUSG00000056564|+|chr11:g.58488585:T>C
MWGPSISSRLDRYGSAAGLSPENSSTITELVLVGFSDQPQTEIPLFIFFSLVYLASCLGNTAVVILVALDVSL
QTPMYFFLCHLAFLNGFFSTVVTPKMLFNFLASRKVISYPFCLAQTYLTPFLESTECFLLAVMAIDRYVAICY
PLRYLLIMSWAVCIALAVAVWVTGFCASVLPLCFMILPLCSPYVVDYLFCELPILLHLFCADTSLQEAMMAVG
GAGTVLVPFLLIALSYLRILVTVIRIDSAEGRKKAFSTCASHLAVVTIYYGTGLIRYLRPKSLYSAEGDKLIS
VFYAVIGPALNPFIYSLRNKEVQGAVRRVVERYRKSPRIAF*
>rs50958699_ENSMUSP00000154189_c.772:C>G_p.T258S
ENSMUST00000214990.2|Olfr324-
202|ENSMUSG00000056564|+|chr11:g.58489014:C>G
MEPRYGSAAGLSPENSSTITELVLVGFSDQPQTEIPLFIFFSLVYLASCLGNTAVVILVALDVSLQTPMYFFL
CHLAFLNGFFSTVVTPKMLFNFLASRKVISYPFCLAQTYLTLFLESTECFLLAVMAIDRYVAICYPLRYLLIM
SWAVCIALAVAVWVTGFCASVLPLCFMILPLCSPYVVDYLFCELPILLHLFCADTSLQEAMMAVGGAGTVLVP
FLLIALSYLRILVTVIRIDSAEGRKKAFSTCASHLAVVSIYYGTGLIRYLRPKSLYSAEGDKLISVFYAVIGP
ALNPFIYSLRNKEVQGAVRRVVERYRKSPRIAF*
>rs50958699_ENSMUSP00000154149_c.790:C>G_p.T264S
ENSMUST00000216965.2|Olfr324-
203|ENSMUSG00000056564|+|chr11:g.58489014:C>G
MLPHIRTLGRYGSAAGLSPENSSTITELVLVGFSDQPQTEIPLFIFFSLVYLASCLGNTAVVILVALDVSLQT
PMYFFLCHLAFLNGFFSTVVTPKMLFNFLASRKVISYPFCLAQTYLTLFLESTECFLLAVMAIDRYVAICYPL
RYLLIMSWAVCIALAVAVWVTGFCASVLPLCFMILPLCSPYVVDYLFCELPILLHLFCADTSLQEAMMAVGGA
GTVLVPFLLIALSYLRILVTVIRIDSAEGRKKAFSTCASHLAVVSIYYGTGLIRYLRPKSLYSAEGDKLISVF
YAVIGPALNPFIYSLRNKEVQGAVRRVVERYRKSPRIAF*
>rs50958699_ENSMUSP00000056457_c.796:C>G_p.T266S
ENSMUST00000054683.8|Olfr324-
201|ENSMUSG00000056564|+|chr11:g.58489014:C>G
MWGPSISSRLDRYGSAAGLSPENSSTITELVLVGFSDQPQTEIPLFIFFSLVYLASCLGNTAVVILVALDVSL
QTPMYFFLCHLAFLNGFFSTVVTPKMLFNFLASRKVISYPFCLAQTYLTLFLESTECFLLAVMAIDRYVAICY
PLRYLLIMSWAVCIALAVAVWVTGFCASVLPLCFMILPLCSPYVVDYLFCELPILLHLFCADTSLQEAMMAVG
GAGTVLVPFLLIALSYLRILVTVIRIDSAEGRKKAFSTCASHLAVVSIYYGTGLIRYLRPKSLYSAEGDKLIS
VFYAVIGPALNPFIYSLRNKEVQGAVRRVVERYRKSPRIAF*
>rs259189485_ENSMUSP00000151137_c.805:A>G_p.E269G
ENSMUST00000214132.2|Olfr323-203|ENSMUSG00000043880|-
|chr11:g.58516065:T>C
MEPQNLSKVTEFQLLGFQNLLEWQSLLFAIFLCFYLLTITGNMVIICVVSEDPRLRAPMYTFLQHLSFLEIWY
TSTTVPLLLSKLASWGHMLSFPACMAQLYFFVFFGATECFLLAAMAYDRYLAICHPLHYSLLMSPDNCAALVT
VSWVTGVGTGFLPSLLISKLDFCGPNRINHFFCDLPPLIQLSCSSVYVTEMAIFVLSIAVLCICFLLTLVSYV
FIVSSILRIPSTSGRMKTFSTCGSHLAVVTIYYGTMISMYVRPNAHLSPGLNKVISVFYTVVTPLLNPVIYSL
RNKDFKEAVRKIVRTKCGVYRARVKGSALTT*
>rs259189485_ENSMUSP00000068147_c.805:A>G_p.E269G
ENSMUST00000070804.3|Olfr323-201|ENSMUSG00000043880|-
|chr11:g.58516065:T>C
MEPQNLSKVTEFQLLGFQNLLEWQSLLFAIFLCFYLLTITGNMVIICVVSEDPRLRAPMYTFLQHLSFLEIWY
TSTTVPLLLSKLASWGHMLSFPACMAQLYFFVFFGATECFLLAAMAYDRYLAICHPLHYSLLMSPDNCAALVT
VSWVTGVGTGFLPSLLISKLDFCGPNRINHFFCDLPPLIQLSCSSVYVTEMAIFVLSIAVLCICFLLTLVSYV
FIVSSILRIPSTSGRMKTFSTCGSHLAVVTIYYGTMISMYVRPNAHLSPGLNKVISVFYTVVTPLLNPVIYSL
RNKDFKEAVRKIVRTKCGVYRARVKGSALTT*

>rs46530477_ENSMUSP00000151137_c.795:C>G_p.L266V
ENSMUST00000214132.2|Olfr323-203|ENSMUSG00000043880|-
|chr11:g.58516075:G>C
MEPQNLSKVTEFQLLGFQNLLEWQSLLFAIFLCFYLLTITGNMVIICVVSEDPRLRAPMYTFLQHLSFLEIWY
TSTTVPLLLSKLASWGHMLSFPACMAQLYFFVFFGATECFLLAAMAYDRYLAICHPLHYSLLMSPDNCAALVT
VSWVTGVGTGFLPSLLISKLDFCGPNRINHFFCDLPPLIQLSCSSVYYVTEMAIFVLSIAVLCICFLLTLVSYV
FIVSSILRIPSTSGRMKTFSTCGSHLAVVTIYYGTMISMYVRPNAHVSPELNKVISVFYTVVTPLLNPVIYSL
RNKDFKEAVRKIVRTKCGVYRARVKGSALTT*
>rs46530477_ENSMUSP00000145459_c.235:C>G_p.S79C
ENSMUST00000203173.2|Olfr323-202|ENSMUSG00000043880|-
|chr11:g.58516075:G>C
EPQNLSKVTEFQLLGFQNLLEWQSLLFAIFLCFYLLTITGNMVIICVDEDVFYMWFPPGCGHHLLRDHDLHVC
APKCTCVTGTQQGHFCLLHRGHSTAEPSYLQPEEQRLQRSCEKNRENQVWGLQSQSERKCPHYV
>rs46530477_ENSMUSP00000068147_c.795:C>G_p.L266V
ENSMUST00000070804.3|Olfr323-201|ENSMUSG00000043880|-
|chr11:g.58516075:G>C
MEPQNLSKVTEFQLLGFQNLLEWQSLLFAIFLCFYLLTITGNMVIICVVSEDPRLRAPMYTFLQHLSFLEIWY
TSTTVPLLLSKLASWGHMLSFPACMAQLYFFVFFGATECFLLAAMAYDRYLAICHPLHYSLLMSPDNCAALVT
VSWVTGVGTGFLPSLLISKLDFCGPNRINHFFCDLPPLIQLSCSSVYYVTEMAIFVLSIAVLCICFLLTLVSYV
FIVSSILRIPSTSGRMKTFSTCGSHLAVVTIYYGTMISMYVRPNAHVSPELNKVISVFYTVVTPLLNPVIYSL
RNKDFKEAVRKIVRTKCGVYRARVKGSALTT*
>rs29481153_ENSMUSP00000150916_c.529:T>C_p.F177S
ENSMUST00000214400.2|Olfr322-
205|ENSMUSG00000063549|+|chr11:g.58556916:T>C
MGATNDSTFSHFILIGFSDRPELERVLFAIILPAYLLTLLGNSIIILVSRLDPHLHTPMYFFLTHLSFLDLSF
TSSSIPQLLYNLSGPDKTISYVGCALQLVLFLGLGGVECLLLAVMAYDRFVAVCKPLHYMVIMSPRLCVGLVS
VAWSCGVANSLAMSPVTLSLPRCGHRRVDHSLCEMPALIRTACVNTAAVEGTVFVLAIGIVLSPLVFILVSYG
YIVRAVLQIRSAAGRQKAFNTCGSHLTVVSLFYGNIIYMYMQPGNSSSQDQGKFLTLFYNIVTPLLNPLIYTL
RNKEVKGALRRLLLGSRETGKVRAGSIRTRGKLSW*
>rs29481153_ENSMUSP00000150410_c.529:T>C_p.F177S
ENSMUST00000216442.2|Olfr322-
206|ENSMUSG00000063549|+|chr11:g.58556916:T>C
MGATNDSTFSHFILIGFSDRPELERVLFAIILPAYLLTLLGNSIIILVSRLDPHLHTPMYFFLTHLSFLDLSF
TSSSIPQLLYNLSGPDKTISYVGCALQLVLFLGLGGVECLLLAVMAYDRFVAVCKPLHYMVIMSPRLCVGLVS
VAWSCGVANSLAMSPVTLSLPRCGHRRVDHSLCEMPALIRTACVNTAAVEGTVFVLAIGIVLSPLVFILVSYG
YIVRAVLQIRSAAGRQKAFNTCGSHLTVVSLFYGNIIYMYMQPGNSSSQDQGKFLTLFYNIVTPLLNPLIYTL
RNKEVKGALRRLLLGSRETGKVRAGSIRTRGKLSW*
>rs29481153_ENSMUSP00000149062_c.529:T>C_p.F177S
ENSMUST00000217399.2|Olfr322-
207|ENSMUSG00000063549|+|chr11:g.58556916:T>C
MGATNDSTFSHFILIGFSDRPELERVLFAIILPAYLLTLLGNSIIILVSRLDPHLHTPMYFFLTHLSFLDLSF
TSSSIPQLLYNLSGPDKTISYVGCALQLVLFLGLGGVECLLLAVMAYDRFVAVCKPLHYMVIMSPRLCVGLVS
VAWSCGVANSLAMSPVTLSLPRCGHRRVDHSLCEMPALIRTACVNTAAVEGTVFVLAIGIVLSPLVFILVSYG
YIVRAVLQIRSAAGRQKAFNTCGSHLTVVSLFYGNIIYMYMQPGNSSSQDQGKFLTLFYNIVTPLLNPLIYTL
RNKEVKGALRRLLLGSRETGKVRAGSIRTRGKLSW*
>rs29481153_ENSMUSP00000150990_c.529:T>C_p.F177S
ENSMUST00000214392.2|Olfr322-
204|ENSMUSG00000063549|+|chr11:g.58556916:T>C
MGATNDSTFSHFILIGFSDRPELERVLFAIILPAYLLTLLGNSIIILVSRLDPHLHTPMYFFLTHLSFLDLSF
TSSSIPQLLYNLSGPDKTISYVGCALQLVLFLGLGGVECLLLAVMAYDRFVAVCKPLHYMVIMSPRLCVGLVS
VAWSCGVANSLAMSPVTLSLPRCGHRRVDHSLCEMPALIRTACVNTAAVEGTVFVLAIGIVLSPLVFILVSYG
YIVRAVLQIRSAAGRQKAFNTCGSHLTVVSLFYGNIIYMYMQPGNSSSQDQGKFLTLFYNIVTPLLNPLIYTL
RNKEVKGALRRLLLGSRETGKVRAGSIRTRGKLSW*
>rs29481153_ENSMUSP00000150004_c.529:T>C_p.F177S
ENSMUST00000213793.2|Olfr322-
203|ENSMUSG00000063549|+|chr11:g.58556916:T>C
MGATNDSTFSHFILIGFSDRPELERVLFAIILPAYLLTLLGNSIIILVSRLDPHLHTPMYFFLTHLSFLDLSF
TSSSIPQLLYNLSGPDKTISYVGCALQLVLFLGLGGVECLLLAVMAYDRFVAVCKPLHYMVIMSPRLCVGLVS

VAWSCGVANSLAMSPVTLSLPRCGHRRVDHSLCEMPALIRTACVNTAAVEGTVFVLAIGIVLSPLVFILVSYG
YIVRAVLQIRSAAGRQKAFNTCGSHLTVVSLFYGNIIYMYMQPGNSSSQDQGKFLTLFYNIVTPLLNPLIYTL
RNKEVKGALRRLLLGSRETGKVRAGSIRTRGKLSW*
>rs29481153_ENSMUSP00000150953_c.529:T>C_p.F177S
ENSMUST00000213232.2|Olfr322-
202|ENSMUSG00000063549|+|chr11:g.58556916:T>C
MGATNDSTFSHFILIGFSDRPELERVLFAIILPAYLLTLLGNSIIILVSRLDPHLHTPMYFFLTHLSFLDLSF
TSSSIPQLLYNLSGPDKTISYVGCALQLVLFLGLGGVECLLLAVMAYDRFVAVCKPLHYMVIMSPRLCVGLVS
VAWSCGVANSLAMSPVTLSLPRCGHRRVDHSLCEMPALIRTACVNTAAVEGTVFVLAIGIVLSPLVFILVSYG
YIVRAVLQIRSAAGRQKAFNTCGSHLTVVSLFYGNIIYMYMQPGNSSSQDQGKFLTLFYNIVTPLLNPLIYTL
RNKEVKGALRRLLLGSRETGKVRAGSIRTRGKLSW*
>rs29481153_ENSMUSP00000071913_c.529:T>C_p.F177S
ENSMUST00000072030.4|Olfr322-
201|ENSMUSG00000063549|+|chr11:g.58556916:T>C
MGATNDSTFSHFILIGFSDRPELERVLFAIILPAYLLTLLGNSIIILVSRLDPHLHTPMYFFLTHLSFLDLSF
TSSSIPQLLYNLSGPDKTISYVGCALQLVLFLGLGGVECLLLAVMAYDRFVAVCKPLHYMVIMSPRLCVGLVS
VAWSCGVANSLAMSPVTLSLPRCGHRRVDHSLCEMPALIRTACVNTAAVEGTVFVLAIGIVLSPLVFILVSYG
YIVRAVLQIRSAAGRQKAFNTCGSHLTVVSLFYGNIIYMYMQPGNSSSQDQGKFLTLFYNIVTPLLNPLIYTL
RNKEVKGALRRLLLGSRETGKVRAGSIRTRGKLSW*
>rs29484354_ENSMUSP00000150916_c.624:T>A_p.S209T
ENSMUST00000214400.2|Olfr322-
205|ENSMUSG00000063549|+|chr11:g.58557011:T>A
MGATNDSTFSHFILIGFSDRPELERVLFAIILPAYLLTLLGNSIIILVSRLDPHLHTPMYFFLTHLSFLDLSF
TSSSIPQLLYNLSGPDKTISYVGCALQLVLFLGLGGVECLLLAVMAYDRFVAVCKPLHYMVIMSPRLCVGLVS
VAWSCGVANSLAMSPVTLSLPRCGHRRVDHFLCEMPALIRTACVNTAAVEGTVFVLAIGIVLTPLVFILVSYG
YIVRAVLQIRSAAGRQKAFNTCGSHLTVVSLFYGNIIYMYMQPGNSSSQDQGKFLTLFYNIVTPLLNPLIYTL
RNKEVKGALRRLLLGSRETGKVRAGSIRTRGKLSW*
>rs29484354_ENSMUSP00000150410_c.624:T>A_p.S209T
ENSMUST00000216442.2|Olfr322-
206|ENSMUSG00000063549|+|chr11:g.58557011:T>A
MGATNDSTFSHFILIGFSDRPELERVLFAIILPAYLLTLLGNSIIILVSRLDPHLHTPMYFFLTHLSFLDLSF
TSSSIPQLLYNLSGPDKTISYVGCALQLVLFLGLGGVECLLLAVMAYDRFVAVCKPLHYMVIMSPRLCVGLVS
VAWSCGVANSLAMSPVTLSLPRCGHRRVDHFLCEMPALIRTACVNTAAVEGTVFVLAIGIVLTPLVFILVSYG
YIVRAVLQIRSAAGRQKAFNTCGSHLTVVSLFYGNIIYMYMQPGNSSSQDQGKFLTLFYNIVTPLLNPLIYTL
RNKEVKGALRRLLLGSRETGKVRAGSIRTRGKLSW*
>rs29484354_ENSMUSP00000149062_c.624:T>A_p.S209T
ENSMUST00000217399.2|Olfr322-
207|ENSMUSG00000063549|+|chr11:g.58557011:T>A
MGATNDSTFSHFILIGFSDRPELERVLFAIILPAYLLTLLGNSIIILVSRLDPHLHTPMYFFLTHLSFLDLSF
TSSSIPQLLYNLSGPDKTISYVGCALQLVLFLGLGGVECLLLAVMAYDRFVAVCKPLHYMVIMSPRLCVGLVS
VAWSCGVANSLAMSPVTLSLPRCGHRRVDHFLCEMPALIRTACVNTAAVEGTVFVLAIGIVLTPLVFILVSYG
YIVRAVLQIRSAAGRQKAFNTCGSHLTVVSLFYGNIIYMYMQPGNSSSQDQGKFLTLFYNIVTPLLNPLIYTL
RNKEVKGALRRLLLGSRETGKVRAGSIRTRGKLSW*
>rs29484354_ENSMUSP00000150990_c.624:T>A_p.S209T
ENSMUST00000214392.2|Olfr322-
204|ENSMUSG00000063549|+|chr11:g.58557011:T>A
MGATNDSTFSHFILIGFSDRPELERVLFAIILPAYLLTLLGNSIIILVSRLDPHLHTPMYFFLTHLSFLDLSF
TSSSIPQLLYNLSGPDKTISYVGCALQLVLFLGLGGVECLLLAVMAYDRFVAVCKPLHYMVIMSPRLCVGLVS
VAWSCGVANSLAMSPVTLSLPRCGHRRVDHFLCEMPALIRTACVNTAAVEGTVFVLAIGIVLTPLVFILVSYG
YIVRAVLQIRSAAGRQKAFNTCGSHLTVVSLFYGNIIYMYMQPGNSSSQDQGKFLTLFYNIVTPLLNPLIYTL
RNKEVKGALRRLLLGSRETGKVRAGSIRTRGKLSW*
>rs29484354_ENSMUSP00000150004_c.624:T>A_p.S209T
ENSMUST00000213793.2|Olfr322-
203|ENSMUSG00000063549|+|chr11:g.58557011:T>A
MGATNDSTFSHFILIGFSDRPELERVLFAIILPAYLLTLLGNSIIILVSRLDPHLHTPMYFFLTHLSFLDLSF
TSSSIPQLLYNLSGPDKTISYVGCALQLVLFLGLGGVECLLLAVMAYDRFVAVCKPLHYMVIMSPRLCVGLVS
VAWSCGVANSLAMSPVTLSLPRCGHRRVDHFLCEMPALIRTACVNTAAVEGTVFVLAIGIVLTPLVFILVSYG

YIVRAVLQIRSAAGRQKAFNTCGSHLTVVSLFYGNIIYMYMQPGNSSSQDQGKFLTLFYNIVTPLLNPLIYTL
RNKEVKGALRRLLLGSRETGKVRAGSIRTRGKLSW*
>rs29484354_ENSMUSP00000150953_c.624:T>A_p.S209T
ENSMUST00000213232.2|Olfr322-
202|ENSMUSG00000063549|+|chr11:g.58557011:T>A
MGATNDSTFSHFILIGFSDRPELERVLFAIILPAYLLTLLGNSIIILVSRLDPHLHTPMYFFLTHLSFLDLSF
TSSSIPQLLYNLSGPDKTISYVGCALQLVLFLGLGGVECLLLAVMAYDRFVAVCKPLHYMVIMSPRLCVGLVS
VAWSCGVANSLAMSPVTLSLPRCGHRRVDHFLCEMPALIRTACVNTAAVEGTVFVLAIGIVLTPLVFILVSYG
YIVRAVLQIRSAAGRQKAFNTCGSHLTVVSLFYGNIIYMYMQPGNSSSQDQGKFLTLFYNIVTPLLNPLIYTL
RNKEVKGALRRLLLGSRETGKVRAGSIRTRGKLSW*
>rs29484354_ENSMUSP00000071913_c.624:T>A_p.S209T
ENSMUST00000072030.4|Olfr322-
201|ENSMUSG00000063549|+|chr11:g.58557011:T>A
MGATNDSTFSHFILIGFSDRPELERVLFAIILPAYLLTLLGNSIIILVSRLDPHLHTPMYFFLTHLSFLDLSF
TSSSIPQLLYNLSGPDKTISYVGCALQLVLFLGLGGVECLLLAVMAYDRFVAVCKPLHYMVIMSPRLCVGLVS
VAWSCGVANSLAMSPVTLSLPRCGHRRVDHFLCEMPALIRTACVNTAAVEGTVFVLAIGIVLTPLVFILVSYG
YIVRAVLQIRSAAGRQKAFNTCGSHLTVVSLFYGNIIYMYMQPGNSSSQDQGKFLTLFYNIVTPLLNPLIYTL
RNKEVKGALRRLLLGSRETGKVRAGSIRTRGKLSW*
>rs29405718_ENSMUSP00000150916_c.934:G>A_p.G312E
ENSMUST00000214400.2|Olfr322-
205|ENSMUSG00000063549|+|chr11:g.58557321:G>A
MGATNDSTFSHFILIGFSDRPELERVLFAIILPAYLLTLLGNSIIILVSRLDPHLHTPMYFFLTHLSFLDLSF
TSSSIPQLLYNLSGPDKTISYVGCALQLVLFLGLGGVECLLLAVMAYDRFVAVCKPLHYMVIMSPRLCVGLVS
VAWSCGVANSLAMSPVTLSLPRCGHRRVDHFLCEMPALIRTACVNTAAVEGTVFVLAIGIVLSPLVFILVSYG
YIVRAVLQIRSAAGRQKAFNTCGSHLTVVSLFYGNIIYMYMQPGNSSSQDQGKFLTLFYNIVTPLLNPLIYTL
RNKEVKGALRRLLLGSRETEKVRAGSIRTRGKLSW*
>rs29405718_ENSMUSP00000150410_c.934:G>A_p.G312E
ENSMUST00000216442.2|Olfr322-
206|ENSMUSG00000063549|+|chr11:g.58557321:G>A
MGATNDSTFSHFILIGFSDRPELERVLFAIILPAYLLTLLGNSIIILVSRLDPHLHTPMYFFLTHLSFLDLSF
TSSSIPQLLYNLSGPDKTISYVGCALQLVLFLGLGGVECLLLAVMAYDRFVAVCKPLHYMVIMSPRLCVGLVS
VAWSCGVANSLAMSPVTLSLPRCGHRRVDHFLCEMPALIRTACVNTAAVEGTVFVLAIGIVLSPLVFILVSYG
YIVRAVLQIRSAAGRQKAFNTCGSHLTVVSLFYGNIIYMYMQPGNSSSQDQGKFLTLFYNIVTPLLNPLIYTL
RNKEVKGALRRLLLGSRETEKVRAGSIRTRGKLSW*
>rs29405718_ENSMUSP00000149062_c.934:G>A_p.G312E
ENSMUST00000217399.2|Olfr322-
207|ENSMUSG00000063549|+|chr11:g.58557321:G>A
MGATNDSTFSHFILIGFSDRPELERVLFAIILPAYLLTLLGNSIIILVSRLDPHLHTPMYFFLTHLSFLDLSF
TSSSIPQLLYNLSGPDKTISYVGCALQLVLFLGLGGVECLLLAVMAYDRFVAVCKPLHYMVIMSPRLCVGLVS
VAWSCGVANSLAMSPVTLSLPRCGHRRVDHFLCEMPALIRTACVNTAAVEGTVFVLAIGIVLSPLVFILVSYG
YIVRAVLQIRSAAGRQKAFNTCGSHLTVVSLFYGNIIYMYMQPGNSSSQDQGKFLTLFYNIVTPLLNPLIYTL
RNKEVKGALRRLLLGSRETEKVRAGSIRTRGKLSW*
>rs29405718_ENSMUSP00000150990_c.934:G>A_p.G312E
ENSMUST00000214392.2|Olfr322-
204|ENSMUSG00000063549|+|chr11:g.58557321:G>A
MGATNDSTFSHFILIGFSDRPELERVLFAIILPAYLLTLLGNSIIILVSRLDPHLHTPMYFFLTHLSFLDLSF
TSSSIPQLLYNLSGPDKTISYVGCALQLVLFLGLGGVECLLLAVMAYDRFVAVCKPLHYMVIMSPRLCVGLVS
VAWSCGVANSLAMSPVTLSLPRCGHRRVDHFLCEMPALIRTACVNTAAVEGTVFVLAIGIVLSPLVFILVSYG
YIVRAVLQIRSAAGRQKAFNTCGSHLTVVSLFYGNIIYMYMQPGNSSSQDQGKFLTLFYNIVTPLLNPLIYTL
RNKEVKGALRRLLLGSRETEKVRAGSIRTRGKLSW*
>rs29405718_ENSMUSP00000150004_c.934:G>A_p.G312E
ENSMUST00000213793.2|Olfr322-
203|ENSMUSG00000063549|+|chr11:g.58557321:G>A
MGATNDSTFSHFILIGFSDRPELERVLFAIILPAYLLTLLGNSIIILVSRLDPHLHTPMYFFLTHLSFLDLSF
TSSSIPQLLYNLSGPDKTISYVGCALQLVLFLGLGGVECLLLAVMAYDRFVAVCKPLHYMVIMSPRLCVGLVS
VAWSCGVANSLAMSPVTLSLPRCGHRRVDHFLCEMPALIRTACVNTAAVEGTVFVLAIGIVLSPLVFILVSYG
YIVRAVLQIRSAAGRQKAFNTCGSHLTVVSLFYGNIIYMYMQPGNSSSQDQGKFLTLFYNIVTPLLNPLIYTL
RNKEVKGALRRLLLGSRETEKVRAGSIRTRGKLSW*

>rs29405718_ENSMUSP00000150953_c.934:G>A_p.G312E
ENSMUST00000213232.2|Olfr322-
202|ENSMUSG00000063549|+|chr11:g.58557321:G>A
MGATNDSTFSHFILIGFSDRPELERVLFAIILPAYLLTLLGNSIIILVSRLDPHLHTPMYFFLTHLSFLDLSF
TSSSIPQLLYNLSGPDKTISYVGCALQLVLFLGLGGVECLLLAVMAYDRFVAVCKPLHYMVIMSPRLCVGLVS
VAWSCGVANSLAMSPVTLSLPRCGHRRVDHFLCEMPALIRTACVNTAAVEGTVFVLAIGIVLSPLVFILVSYG
YIVRAVLQIRSAAGRQKAFNTCGSHLTVVSLFYGNIIYMYMQPGNSSSQDQGKFLTLFYNIVTPLLNPLIYTL
RNKEVKGALRRLLLGSRETEKVRAGSIRTRGKLSW*
>rs29405718_ENSMUSP00000071913_c.934:G>A_p.G312E
ENSMUST00000072030.4|Olfr322-
201|ENSMUSG00000063549|+|chr11:g.58557321:G>A
MGATNDSTFSHFILIGFSDRPELERVLFAIILPAYLLTLLGNSIIILVSRLDPHLHTPMYFFLTHLSFLDLSF
TSSSIPQLLYNLSGPDKTISYVGCALQLVLFLGLGGVECLLLAVMAYDRFVAVCKPLHYMVIMSPRLCVGLVS
VAWSCGVANSLAMSPVTLSLPRCGHRRVDHFLCEMPALIRTACVNTAAVEGTVFVLAIGIVLSPLVFILVSYG
YIVRAVLQIRSAAGRQKAFNTCGSHLTVVSLFYGNIIYMYMQPGNSSSQDQGKFLTLFYNIVTPLLNPLIYTL
RNKEVKGALRRLLLGSRETEKVRAGSIRTRGKLSW*
>rs26962585_ENSMUSP00000150051_c.42:G>A_p.V15I
ENSMUST00000214662.2|Olfr320-
202|ENSMUSG00000107711|+|chr11:g.58574743:G>A
METGNRSCGTDFSLIGLFQDGHMDTFLFTLIAILFAVAFIGNITLVLLIRLDCRLHTPMYFLLSQLSIIDMMY
ISTTVPKVAANFLSDTKAISFLGCAVQAFVFLTLGGSEALLLGFMSYDRYIAICRPLHYPVLMSRKICCSMVA
GAWSSSSINAFMHTVYVFQLPFCGSRMVNHFFCEVPSLLPLVCEDTSQYEHTVLVSGLVILLLPFLAILASYA
RVLVVVLQMGSGKGQSRAVSTCSSHLTVASLFYVTGLSTYTQPHSLHSPGRDKVVAVLYSIVTLVLNPFIYSL
RNKEVMGALRRQMG*
>rs26962585_ENSMUSP00000145531_c.42:G>A_p.V15I
ENSMUST00000204718.3|Olfr320-
201|ENSMUSG00000107711|+|chr11:g.58574743:G>A
METGNRSCGTDFSLIGLFQDGHMDTFLFTLIAILFAVAFIGNITLVLLIRLDCRLHTPMYFLLSQLSIIDMMY
ISTTVPKVAANFLSDTKAISFLGCAVQAFVFLTLGGSEALLLGFMSYDRYIAICRPLHYPVLMSRKICCSMVA
GAWSSSSINAFMHTVYVFQLPFCGSRMVNHFFCEVPSLLPLVCEDTSQYEHTVLVSGLVILLLPFLAILASYA
RVLVVVLQMGSGKGQSRAVSTCSSHLTVASLFYVTGLSTYTQPHSLHSPGRDKVVAVLYSIVTLVLNPFIYSL
RNKEVMGALRRQMG*
>rs26962582_ENSMUSP00000150051_c.228:A>C_p.T77P
ENSMUST00000214662.2|Olfr320-
202|ENSMUSG00000107711|+|chr11:g.58574929:A>C
METGNRSCGTDFSLVGLFQDGHMDTFLFTLIAILFAVAFIGNITLVLLIRLDCRLHTPMYFLLSQLSIIDMMY
ISTPVPKVAANFLSDTKAISFLGCAVQAFVFLTLGGSEALLLGFMSYDRYIAICRPLHYPVLMSRKICCSMVA
GAWSSSSINAFMHTVYVFQLPFCGSRMVNHFFCEVPSLLPLVCEDTSQYEHTVLVSGLVILLLPFLAILASYA
RVLVVVLQMGSGKGQSRAVSTCSSHLTVASLFYVTGLSTYTQPHSLHSPGRDKVVAVLYSIVTLVLNPFIYSL
RNKEVMGALRRQMG*
>rs26962582_ENSMUSP00000145531_c.228:A>C_p.T77P
ENSMUST00000204718.3|Olfr320-
201|ENSMUSG00000107711|+|chr11:g.58574929:A>C
METGNRSCGTDFSLVGLFQDGHMDTFLFTLIAILFAVAFIGNITLVLLIRLDCRLHTPMYFLLSQLSIIDMMY
ISTPVPKVAANFLSDTKAISFLGCAVQAFVFLTLGGSEALLLGFMSYDRYIAICRPLHYPVLMSRKICCSMVA
GAWSSSSINAFMHTVYVFQLPFCGSRMVNHFFCEVPSLLPLVCEDTSQYEHTVLVSGLVILLLPFLAILASYA
RVLVVVLQMGSGKGQSRAVSTCSSHLTVASLFYVTGLSTYTQPHSLHSPGRDKVVAVLYSIVTLVLNPFIYSL
RNKEVMGALRRQMG*
>rs46167475_ENSMUSP00000150051_c.576:T>C_p.S193P
ENSMUST00000214662.2|Olfr320-
202|ENSMUSG00000107711|+|chr11:g.58575277:T>C
METGNRSCGTDFSLVGLFQDGHMDTFLFTLIAILFAVAFIGNITLVLLIRLDCRLHTPMYFLLSQLSIIDMMY
ISTTVPKVAANFLSDTKAISFLGCAVQAFVFLTLGGSEALLLGFMSYDRYIAICRPLHYPVLMSRKICCSMVA
GAWSSSSINAFMHTVYVFQLPFCGSRMVNHFFCEVPSLLPLVCEDTPQYEHTVLVSGLVILLLPFLAILASYA
RVLVVVLQMGSGKGQSRAVSTCSSHLTVASLFYVTGLSTYTQPHSLHSPGRDKVVAVLYSIVTLVLNPFIYSL
RNKEVMGALRRQMG*

```
>rs46167475_ENSMUSP00000145531_c.576:T>C_p.S193P
ENSMUST00000204718.3|Olfr320-
201|ENSMUSG00000107711|+|chr11:g.58575277:T>C
METGNRSCGTDFSLVGLFQDGHMDTFLFTLIAILFAVAFIGNITLVLLIRLDCRLHTPMYFLLSQLSIIDMMY
ISTTVPKVAANFLSDTKAISFLGCAVQAFVFLTLGGSEALLLGFMSYDRYIAICRPLHYPVLMSRKICCSMVA
GAWSSSSINAFMHTVYVFQLPFCGSRMVNHFFCEVPSLLPLVCEDTPQYEHTVLVSGLVILLLPFLAILASYA
RVLVVVLQMGSGKGQSRAVSTCSSHLTVASLFYVTGLSTYTQPHSLHSPGRDKVVAVLYSIVTLVLNPFIYSL
RNKEVMGALRRQMG*
>rs26962562_ENSMUSP00000150883_c.120:G>T_p.G41C
ENSMUST00000215962.2|Olfr319-
203|ENSMUSG00000064044|+|chr11:g.58592649:G>T
METGNHSCGTDFTLVGLFQYGHMDTFLFTVISILFAVALICNITLVLLIRLDRRLHTPMYFFLSQLSIIDMMC
ISTTVPKMGANFISDTKAISVLGCEIQVFMFMSLAGCEALLLGFMSYDRYIAICQPLHYPVLMSRKICCSMVA
SAWSSSSINALAHTVYVFQLPFCGSRMVNHFFCEVPSLLPLVCEDTSQYEHMIVMSVLVLVLLPFLAILASYA
RVLVVVFQMGSGQGQSRAVSTCSSHLTVASLFYVTGLCTYTQPHSLHSPGRDKVVAVLYSIVTPVLNPFIYSL
RNKEVIGALRRQMG*
>rs26962562_ENSMUSP00000076232_c.120:G>T_p.G41C
ENSMUST00000076965.2|Olfr319-
201|ENSMUSG00000064044|+|chr11:g.58592649:G>T
METGNHSCGTDFTLVGLFQYGHMDTFLFTVISILFAVALICNITLVLLIRLDRRLHTPMYFFLSQLSIIDMMC
ISTTVPKMGANFISDTKAISVLGCEIQVFMFMSLAGCEALLLGFMSYDRYIAICQPLHYPVLMSRKICCSMVA
SAWSSSSINALAHTVYVFQLPFCGSRMVNHFFCEVPSLLPLVCEDTSQYEHMIVMSVLVLVLLPFLAILASYA
RVLVVVFQMGSGQGQSRAVSTCSSHLTVASLFYVTGLCTYTQPHSLHSPGRDKVVAVLYSIVTPVLNPFIYSL
RNKEVIGALRRQMG*
>rs29425081_ENSMUSP00000150883_c.160:G>T_p.R54I
ENSMUST00000215962.2|Olfr319-
203|ENSMUSG00000064044|+|chr11:g.58592689:G>T
METGNHSCGTDFTLVGLFQYGHMDTFLFTVISILFAVALIGNITLVLLIRLDRILHTPMYFFLSQLSIIDMMC
ISTTVPKMGANFISDTKAISVLGCEIQVFMFMSLAGCEALLLGFMSYDRYIAICQPLHYPVLMSRKICCSMVA
SAWSSSSINALAHTVYVFQLPFCGSRMVNHFFCEVPSLLPLVCEDTSQYEHMIVMSVLVLVLLPFLAILASYA
RVLVVVFQMGSGQGQSRAVSTCSSHLTVASLFYVTGLCTYTQPHSLHSPGRDKVVAVLYSIVTPVLNPFIYSL
RNKEVIGALRRQMG*
>rs29425081_ENSMUSP00000076232_c.160:G>T_p.R54I
ENSMUST00000076965.2|Olfr319-
201|ENSMUSG00000064044|+|chr11:g.58592689:G>T
METGNHSCGTDFTLVGLFQYGHMDTFLFTVISILFAVALIGNITLVLLIRLDRILHTPMYFFLSQLSIIDMMC
ISTTVPKMGANFISDTKAISVLGCEIQVFMFMSLAGCEALLLGFMSYDRYIAICQPLHYPVLMSRKICCSMVA
SAWSSSSINALAHTVYVFQLPFCGSRMVNHFFCEVPSLLPLVCEDTSQYEHMIVMSVLVLVLLPFLAILASYA
RVLVVVFQMGSGQGQSRAVSTCSSHLTVASLFYVTGLCTYTQPHSLHSPGRDKVVAVLYSIVTPVLNPFIYSL
RNKEVIGALRRQMG*
>rs48879405_ENSMUSP00000150883_c.175:T>C_p.M59T
ENSMUST00000215962.2|Olfr319-
203|ENSMUSG00000064044|+|chr11:g.58592704:T>C
METGNHSCGTDFTLVGLFQYGHMDTFLFTVISILFAVALIGNITLVLLIRLDRRLHTPTYFFLSQLSIIDMMC
ISTTVPKMGANFISDTKAISVLGCEIQVFMFMSLAGCEALLLGFMSYDRYIAICQPLHYPVLMSRKICCSMVA
SAWSSSSINALAHTVYVFQLPFCGSRMVNHFFCEVPSLLPLVCEDTSQYEHMIVMSVLVLVLLPFLAILASYA
RVLVVVFQMGSGQGQSRAVSTCSSHLTVASLFYVTGLCTYTQPHSLHSPGRDKVVAVLYSIVTPVLNPFIYSL
RNKEVIGALRRQMG*
>rs48879405_ENSMUSP00000076232_c.175:T>C_p.M59T
ENSMUST00000076965.2|Olfr319-
201|ENSMUSG00000064044|+|chr11:g.58592704:T>C
METGNHSCGTDFTLVGLFQYGHMDTFLFTVISILFAVALIGNITLVLLIRLDRRLHTPTYFFLSQLSIIDMMC
ISTTVPKMGANFISDTKAISVLGCEIQVFMFMSLAGCEALLLGFMSYDRYIAICQPLHYPVLMSRKICCSMVA
SAWSSSSINALAHTVYVFQLPFCGSRMVNHFFCEVPSLLPLVCEDTSQYEHMIVMSVLVLVLLPFLAILASYA
RVLVVVFQMGSGQGQSRAVSTCSSHLTVASLFYVTGLCTYTQPHSLHSPGRDKVVAVLYSIVTPVLNPFIYSL
RNKEVIGALRRQMG*
```

>rs29394878_ENSMUSP00000150883_c.530:T>A_p.F177L
ENSMUST00000215962.2|Olfr319-
203|ENSMUSG00000064044|+|chr11:g.58593059:T>A
METGNHSCGTDFTLVGLFQYGHMDTFLFTVISILFAVALIGNITLVLLIRLDRRLHTPMYFFLSQLSIIDMMC
ISTTVPKMGANFISDTKAISVLGCEIQVFMFMSLAGCEALLLGFMSYDRYIAICQPLHYPVLMSRKICCSMVA
SAWSSSSINALAHTVYVFQLPFCGSRMVNHLFCEVPSLLPLVCEDTSQYEHMIVMSVLVLVLLPFLAILASYA
RVLVVVFQMGSGQGQSRAVSTCSSHLTVASLFYVTGLCTYTQPHSLHSPGRDKVVAVLYSIVTPVLNPFIYSL
RNKEVIGALRRQMG*
>rs29394878_ENSMUSP00000076232_c.530:T>A_p.F177L
ENSMUST00000076965.2|Olfr319-
201|ENSMUSG00000064044|+|chr11:g.58593059:T>A
METGNHSCGTDFTLVGLFQYGHMDTFLFTVISILFAVALIGNITLVLLIRLDRRLHTPMYFFLSQLSIIDMMC
ISTTVPKMGANFISDTKAISVLGCEIQVFMFMSLAGCEALLLGFMSYDRYIAICQPLHYPVLMSRKICCSMVA
SAWSSSSINALAHTVYVFQLPFCGSRMVNHLFCEVPSLLPLVCEDTSQYEHMIVMSVLVLVLLPFLAILASYA
RVLVVVFQMGSGQGQSRAVSTCSSHLTVASLFYVTGLCTYTQPHSLHSPGRDKVVAVLYSIVTPVLNPFIYSL
RNKEVIGALRRQMG*
>rs48062366_ENSMUSP00000150883_c.906:A>G_p.R303G
ENSMUST00000215962.2|Olfr319-
203|ENSMUSG00000064044|+|chr11:g.58593435:A>G
METGNHSCGTDFTLVGLFQYGHMDTFLFTVISILFAVALIGNITLVLLIRLDRRLHTPMYFFLSQLSIIDMMC
ISTTVPKMGANFISDTKAISVLGCEIQVFMFMSLAGCEALLLGFMSYDRYIAICQPLHYPVLMSRKICCSMVA
SAWSSSSINALAHTVYVFQLPFCGSRMVNHFFCEVPSLLPLVCEDTSQYEHMIVMSVLVLVLLPFLAILASYA
RVLVVVFQMGSGQGQSRAVSTCSSHLTVASLFYVTGLCTYTQPHSLHSPGRDKVVAVLYSIVTPVLNPFIYSL
RNKEVIGALRGQMG*
>rs48062366_ENSMUSP00000076232_c.906:A>G_p.R303G
ENSMUST00000076965.2|Olfr319-
201|ENSMUSG00000064044|+|chr11:g.58593435:A>G
METGNHSCGTDFTLVGLFQYGHMDTFLFTVISILFAVALIGNITLVLLIRLDRRLHTPMYFFLSQLSIIDMMC
ISTTVPKMGANFISDTKAISVLGCEIQVFMFMSLAGCEALLLGFMSYDRYIAICQPLHYPVLMSRKICCSMVA
SAWSSSSINALAHTVYVFQLPFCGSRMVNHFFCEVPSLLPLVCEDTSQYEHMIVMSVLVLVLLPFLAILASYA
RVLVVVFQMGSGQGQSRAVSTCSSHLTVASLFYVTGLCTYTQPHSLHSPGRDKVVAVLYSIVTPVLNPFIYSL
RNKEVIGALRGQMG*
>ENSMUSP00000150575_c.655:G>A_p.G219D ENSMUST00000215513.2|Olfr317-
202|ENSMUSG00000060030|-|chr11:g.58623334:C>T
MGATNDSTFSHFILTGFSDRPELERVLFAILLPAYLLTLLGNSTIILVSRLDPHLHTPMYFFLTHLSFLDLSF
TSSSIPQLLYNLSGPDKTISYVGCALQLVLFLGLGGVECLLLAVMAYDRFVAICKPLHYMVIMSPRLCVGLVS
VAWSCGVANSLAMSPATLSLPRCGHHRVDHFLCEMPALIRMACVNTAVVEGIAFILAIGIVLSPLVFILVSYD
YIVRAVLRISSAAGRQKAFNTCGSHLTVVSLFYGNIIYMYMQPGNSSSQDQGKFLTLFYNIVTPLLNPLIYTL
RNKEVKGALRRLLLGSRETGKVRASSRWTRGKLSW*
>ENSMUSP00000150027_c.655:G>A_p.G219D ENSMUST00000216196.2|Olfr317-
203|ENSMUSG00000060030|-|chr11:g.58623334:C>T
MGATNDSTFSHFILTGFSDRPELERVLFAILLPAYLLTLLGNSTIILVSRLDPHLHTPMYFFLTHLSFLDLSF
TSSSIPQLLYNLSGPDKTISYVGCALQLVLFLGLGGVECLLLAVMAYDRFVAICKPLHYMVIMSPRLCVGLVS
VAWSCGVANSLAMSPATLSLPRCGHHRVDHFLCEMPALIRMACVNTAVVEGIAFILAIGIVLSPLVFILVSYD
YIVRAVLRISSAAGRQKAFNTCGSHLTVVSLFYGNIIYMYMQPGNSSSQDQGKFLTLFYNIVTPLLNPLIYTL
RNKEVKGALRRLLLGSRETGKVRASSRWTRGKLSW*
>ENSMUSP00000075034_c.655:G>A_p.G219D ENSMUST00000075607.5|Olfr317-
201|ENSMUSG00000060030|-|chr11:g.58623334:C>T
MGATNDSTFSHFILTGFSDRPELERVLFAILLPAYLLTLLGNSTIILVSRLDPHLHTPMYFFLTHLSFLDLSF
TSSSIPQLLYNLSGPDKTISYVGCALQLVLFLGLGGVECLLLAVMAYDRFVAICKPLHYMVIMSPRLCVGLVS
VAWSCGVANSLAMSPATLSLPRCGHHRVDHFLCEMPALIRMACVNTAVVEGIAFILAIGIVLSPLVFILVSYD
YIVRAVLRISSAAGRQKAFNTCGSHLTVVSLFYGNIIYMYMQPGNSSSQDQGKFLTLFYNIVTPLLNPLIYTL
RNKEVKGALRRLLLGSRETGKVRASSRWTRGKLSW*
>ENSMUSP00000150575_c.34:T>C_p.F12S ENSMUST00000215513.2|Olfr317-
202|ENSMUSG00000060030|-|chr11:g.58623955:A>G
MGATNDSTFSHSILTGFSDRPELERVLFAILLPAYLLTLLGNSTIILVSRLDPHLHTPMYFFLTHLSFLDLSF
TSSSIPQLLYNLSGPDKTISYVGCALQLVLFLGLGGVECLLLAVMAYDRFVAICKPLHYMVIMSPRLCVGLVS
VAWSCGVANSLAMSPATLSLPRCGHHRVDHFLCEMPALIRMACVNTAVVEGIAFILAIGIVLSPLVFILVSYG

YIVRAVLRISSAAGRQKAFNTCGSHLTVVSLFYGNIIYMYMQPGNSSSQDQGKFLTLFYNIVTPLLNPLIYTL
RNKEVKGALRRLLLGSRETGKVRASSRWTRGKLSW*
>ENSMUSP00000150027_c.34:T>C_p.F12S ENSMUST00000216196.2|Olfr317-203|ENSMUSG00000060030|-|chr11:g.58623955:A>G
MGATNDSTFSHSILTGFSDRPELERVLFAILLPAYLLTLLGNSTIILVSRLDPHLHTPMYFFLTHLSFLDLSF
TSSSIPQLLYNLSGPDKTISYVGCALQLVLFLGLGGVECLLLAVMAYDRFVAICKPLHYMVIMSPRLCVGLVS
VAWSCGVANSLAMSPATLSLPRCGHHRVDHFLCEMPALIRMACVNTAVVEGIAFILAIGIVLSPLVFILVSYG
YIVRAVLRISSAAGRQKAFNTCGSHLTVVSLFYGNIIYMYMQPGNSSSQDQGKFLTLFYNIVTPLLNPLIYTL
RNKEVKGALRRLLLGSRETGKVRASSRWTRGKLSW*
>ENSMUSP00000075034_c.34:T>C_p.F12S ENSMUST00000075607.5|Olfr317-201|ENSMUSG00000060030|-|chr11:g.58623955:A>G
MGATNDSTFSHSILTGFSDRPELERVLFAILLPAYLLTLLGNSTIILVSRLDPHLHTPMYFFLTHLSFLDLSF
TSSSIPQLLYNLSGPDKTISYVGCALQLVLFLGLGGVECLLLAVMAYDRFVAICKPLHYMVIMSPRLCVGLVS
VAWSCGVANSLAMSPATLSLPRCGHHRVDHFLCEMPALIRMACVNTAVVEGIAFILAIGIVLSPLVFILVSYG
YIVRAVLRISSAAGRQKAFNTCGSHLTVVSLFYGNIIYMYMQPGNSSSQDQGKFLTLFYNIVTPLLNPLIYTL
RNKEVKGALRRLLLGSRETGKVRASSRWTRGKLSW*
>ENSMUSP00000150575_c.7:C>T_p.A3V ENSMUST00000215513.2|Olfr317-202|ENSMUSG00000060030|-|chr11:g.58623982:G>A
MGVTNDSTFSHFILTGFSDRPELERVLFAILLPAYLLTLLGNSTIILVSRLDPHLHTPMYFFLTHLSFLDLSF
TSSSIPQLLYNLSGPDKTISYVGCALQLVLFLGLGGVECLLLAVMAYDRFVAICKPLHYMVIMSPRLCVGLVS
VAWSCGVANSLAMSPATLSLPRCGHHRVDHFLCEMPALIRMACVNTAVVEGIAFILAIGIVLSPLVFILVSYG
YIVRAVLRISSAAGRQKAFNTCGSHLTVVSLFYGNIIYMYMQPGNSSSQDQGKFLTLFYNIVTPLLNPLIYTL
RNKEVKGALRRLLLGSRETGKVRASSRWTRGKLSW*
>ENSMUSP00000150027_c.7:C>T_p.A3V ENSMUST00000216196.2|Olfr317-203|ENSMUSG00000060030|-|chr11:g.58623982:G>A
MGVTNDSTFSHFILTGFSDRPELERVLFAILLPAYLLTLLGNSTIILVSRLDPHLHTPMYFFLTHLSFLDLSF
TSSSIPQLLYNLSGPDKTISYVGCALQLVLFLGLGGVECLLLAVMAYDRFVAICKPLHYMVIMSPRLCVGLVS
VAWSCGVANSLAMSPATLSLPRCGHHRVDHFLCEMPALIRMACVNTAVVEGIAFILAIGIVLSPLVFILVSYG
YIVRAVLRISSAAGRQKAFNTCGSHLTVVSLFYGNIIYMYMQPGNSSSQDQGKFLTLFYNIVTPLLNPLIYTL
RNKEVKGALRRLLLGSRETGKVRASSRWTRGKLSW*
>ENSMUSP00000075034_c.7:C>T_p.A3V ENSMUST00000075607.5|Olfr317-201|ENSMUSG00000060030|-|chr11:g.58623982:G>A
MGVTNDSTFSHFILTGFSDRPELERVLFAILLPAYLLTLLGNSTIILVSRLDPHLHTPMYFFLTHLSFLDLSF
TSSSIPQLLYNLSGPDKTISYVGCALQLVLFLGLGGVECLLLAVMAYDRFVAICKPLHYMVIMSPRLCVGLVS
VAWSCGVANSLAMSPATLSLPRCGHHRVDHFLCEMPALIRMACVNTAVVEGIAFILAIGIVLSPLVFILVSYG
YIVRAVLRISSAAGRQKAFNTCGSHLTVVSLFYGNIIYMYMQPGNSSSQDQGKFLTLFYNIVTPLLNPLIYTL
RNKEVKGALRRLLLGSRETGKVRASSRWTRGKLSW*
>ENSMUSP00000151136_c.413:G>T_p.R138S ENSMUST00000215071.2|Olfr316-202|ENSMUSG00000107677|+|chr11:g.58648906:G>T
MESGNRSCGTDFTLVGLFQDGHMDTFLFTVISILFAVALIGNITLVILIRLDQRLHTPMYFLLSQLSIMDMMY
ISTTVPKMAANFLSDTKAISFLGCVIQAFVFLTLGGSEALLLGFMSYDRYIAICRPLHYPVLMSSKICCSMVA
SAWSSSSITASVHTVYVFQLPFCGSRMVNHFFCEVPSLLPLVCEDTSQYEHTVLVSGLVILLLPFLAILASYA
RVLVVVIQMGSGKGQSRAVSTCSSHLTVASLFYVTTLSTYTQPHTLHSPGRDKVVAVLYSIVTPVLNPFIYSL
RNKEVMGALRRQMK*
>ENSMUSP00000145187_c.413:G>T_p.R138S ENSMUST00000203731.2|Olfr316-201|ENSMUSG00000107677|+|chr11:g.58648906:G>T
MESGNRSCGTDFTLVGLFQDGHMDTFLFTVISILFAVALIGNITLVILIRLDQRLHTPMYFLLSQLSIMDMMY
ISTTVPKMAANFLSDTKAISFLGCVIQAFVFLTLGGSEALLLGFMSYDRYIAICRPLHYPVLMSSKICCSMVA
SAWSSSSITASVHTVYVFQLPFCGSRMVNHFFCEVPSLLPLVCEDTSQYEHTVLVSGLVILLLPFLAILASYA
RVLVVVIQMGSGKGQSRAVSTCSSHLTVASLFYVTTLSTYTQPHTLHSPGRDKVVAVLYSIVTPVLNPFIYSL
RNKEVMGALRRQMK*
>ENSMUSP00000151136_c.438:A>G_p.S147G ENSMUST00000215071.2|Olfr316-202|ENSMUSG00000107677|+|chr11:g.58648931:A>G
MESGNRSCGTDFTLVGLFQDGHMDTFLFTVISILFAVALIGNITLVILIRLDQRLHTPMYFLLSQLSIMDMMY
ISTTVPKMAANFLSDTKAISFLGCVIQAFVFLTLGGSEALLLGFMSYDRYIAICRPLHYPVLMSRKICCSMVA
GAWSSSSITASVHTVYVFQLPFCGSRMVNHFFCEVPSLLPLVCEDTSQYEHTVLVSGLVILLLPFLAILASYA
RVLVVVIQMGSGKGQSRAVSTCSSHLTVASLFYVTTLSTYTQPHTLHSPGRDKVVAVLYSIVTPVLNPFIYSL
RNKEVMGALRRQMK*

>ENSMUSP00000145187_c.438:A>G_p.S147G ENSMUST00000203731.2|Olfr316-201|ENSMUSG00000107677|+|chr11:g.58648931:A>G
MESGNRSCGTDFTLVGLFQDGHMDTFLFTVISILFAVALIGNITLVILIRLDQRLHTPMYFLLSQLSIMDMMY
ISTTVPKMAANFLSDTKAISFLGCVIQAFVFLTLGGSEALLLGFMSYDRYIAICRPLHYPVLMSRKICCSMVA
GAWSSSSITASVHTVYVFQLPFCGSRMVNHFFCEVPSLLPLVCEDTSQYEHTVLVSGLVILLLPFLAILASYA
RVLVVVIQMGSGKGQSRAVSTCSSHLTVASLFYVTTLSTYTQPHTLHSPGRDKVVAVLYSIVTPVLNPFIYSL
RNKEVMGALRRQMK*
>ENSMUSP00000151136_c.887:GG>A_p.E296del-fs ENSMUST00000215071.2|Olfr316-202|ENSMUSG00000107677|+|chr11:g.58649380:GG>A
MESGNRSCGTDFTLVGLFQDGHMDTFLFTVISILFAVALIGNITLVILIRLDQRLHTPMYFLLSQLSIMDMMY
ISTTVPKMAANFLSDTKAISFLGCVIQAFVFLTLGGSEALLLGFMSYDRYIAICRPLHYPVLMSRKICCSMVA
SAWSSSSITASVHTVYVFQLPFCGSRMVNHFFCEVPSLLPLVCEDTSQYEHTVLVSGLVILLLPFLAILASYA
RVLVVVIQMGSGKGQSRAVSTCSSHLTVASLFYVTTLSTYTQPHTLHSPGRDKVVAVLYSIVTPVLNPFIYSL
RNKESWGP
>ENSMUSP00000145187_c.887:GG>A_p.E296del-fs ENSMUST00000203731.2|Olfr316-201|ENSMUSG00000107677|+|chr11:g.58649380:GG>A
MESGNRSCGTDFTLVGLFQDGHMDTFLFTVISILFAVALIGNITLVILIRLDQRLHTPMYFLLSQLSIMDMMY
ISTTVPKMAANFLSDTKAISFLGCVIQAFVFLTLGGSEALLLGFMSYDRYIAICRPLHYPVLMSRKICCSMVA
SAWSSSSITASVHTVYVFQLPFCGSRMVNHFFCEVPSLLPLVCEDTSQYEHTVLVSGLVILLLPFLAILASYA
RVLVVVIQMGSGKGQSRAVSTCSSHLTVASLFYVTTLSTYTQPHTLHSPGRDKVVAVLYSIVTPVLNPFIYSL
RNKESWGP
>ENSMUSP00000144944_c.176:G>A_p.M59I ENSMUST00000203256.4|Olfr315-202|ENSMUSG00000056959|+|chr11:g.58669131:G>A
MKTPSNSTEADFILLGLFSHTHAHSLLLSVVLVIFTASLMGNTLMILLICRDPRLHTPIYFLLSQLSLMDMML
VSTIVPKMAANYLTSTRSISPAGCGSQIFLFLTLAGGECFLLAAMSYDRYVAICFPLRYHVLMNPKLCAYLTV
GSWLLGAADGLMQAGTILSFPFCHSRTINHFFCEAPSLVRLACADTKVFEFFMYICCILMLLIPLSLVLASYS
LILATVLRMRSSAARKKAFTTCSSHLAVVGLFYGAIIFIYMRPKSHQPGKSDKVVSAFYTIFTPVLNPLIYSV
RNKEVKGALRKWLQKTV*
>ENSMUSP00000080248_c.176:G>A_p.M59I ENSMUST00000081533.5|Olfr315-201|ENSMUSG00000056959|+|chr11:g.58669131:G>A
MKTPSNSTEADFILLGLFSHTHAHSLLLSVVLVIFTASLMGNTLMILLICRDPRLHTPIYFLLSQLSLMDMML
VSTIVPKMAANYLTSTRSISPAGCGSQIFLFLTLAGGECFLLAAMSYDRYVAICFPLRYHVLMNPKLCAYLTV
GSWLLGAADGLMQAGTILSFPFCHSRTINHFFCEAPSLVRLACADTKVFEFFMYICCILMLLIPLSLVLASYS
LILATVLRMRSSAARKKAFTTCSSHLAVVGLFYGAIIFIYMRPKSHQPGKSDKVVSAFYTIFTPVLNPLIYSV
RNKEVKGALRKWLQKTV*
>ENSMUSP00000144944_c.633:T>C_p.S212P ENSMUST00000203256.4|Olfr315-202|ENSMUSG00000056959|+|chr11:g.58669588:T>C
MKTPSNSTEADFILLGLFSHTHAHSLLLSVVLVIFTASLMGNTLMILLICRDPRLHTPMYFLLSQLSLMDMML
VSTIVPKMAANYLTSTRSISPAGCGSQIFLFLTLAGGECFLLAAMSYDRYVAICFPLRYHVLMNPKLCAYLTV
GSWLLGAADGLMQAGTILSFPFCHSRTINHFFCEAPSLVRLACADTKVFEFFMYICCILMLLIPLPLVLASYS
LILATVLRMRSSAARKKAFTTCSSHLAVVGLFYGAIIFIYMRPKSHQPGKSDKVVSAFYTIFTPVLNPLIYSV
RNKEVKGALRKWLQKTV*
>ENSMUSP00000080248_c.633:T>C_p.S212P ENSMUST00000081533.5|Olfr315-201|ENSMUSG00000056959|+|chr11:g.58669588:T>C
MKTPSNSTEADFILLGLFSHTHAHSLLLSVVLVIFTASLMGNTLMILLICRDPRLHTPMYFLLSQLSLMDMML
VSTIVPKMAANYLTSTRSISPAGCGSQIFLFLTLAGGECFLLAAMSYDRYVAICFPLRYHVLMNPKLCAYLTV
GSWLLGAADGLMQAGTILSFPFCHSRTINHFFCEAPSLVRLACADTKVFEFFMYICCILMLLIPLPLVLASYS
LILATVLRMRSSAARKKAFTTCSSHLAVVGLFYGAIIFIYMRPKSHQPGKSDKVVSAFYTIFTPVLNPLIYSV
RNKEVKGALRKWLQKTV*
>ENSMUSP00000144944_c.781:G>T_p.R261I ENSMUST00000203256.4|Olfr315-202|ENSMUSG00000056959|+|chr11:g.58669736:G>T
MKTPSNSTEADFILLGLFSHTHAHSLLLSVVLVIFTASLMGNTLMILLICRDPRLHTPMYFLLSQLSLMDMML
VSTIVPKMAANYLTSTRSISPAGCGSQIFLFLTLAGGECFLLAAMSYDRYVAICFPLRYHVLMNPKLCAYLTV
GSWLLGAADGLMQAGTILSFPFCHSRTINHFFCEAPSLVRLACADTKVFEFFMYICCILMLLIPLSLVLASYS
LILATVLRMRSSAARKKAFTTCSSHLAVVGLFYGAIIFIYMIPKSHQPGKSDKVVSAFYTIFTPVLNPLIYSV
RNKEVKGALRKWLQKTV*
>ENSMUSP00000080248_c.781:G>T_p.R261I ENSMUST00000081533.5|Olfr315-201|ENSMUSG00000056959|+|chr11:g.58669736:G>T

MKTPSNSTEADFILLGLFSHTHAHSLLLSVVLVIFTASLMGNTLMILLICRDPRLHTPMYFLLSQLSLMDMML
VSTIVPKMAANYLTSTRSISPAGCGSQIFLFLTLAGGECFLLAAMSYDRYVAICFPLRYHVLMNPKLCAYLTV
GSWLLGAADGLMQAGTILSFPFCHSRTINHFFCEAPSLVRLACADTKVFEFFMYICCILMLLIPLSLVLASYS
LILATVLRMRSSAARKKAFTTCSSHLAVVGLFYGAIIFIYMIPKSHQPGKSDKVVSAFYTIFTPVLNPLIYSV
RNKEVKGALRKWLQKTV*
>ENSMUSP00000091708_c.366:G>A_p.A123T ENSMUST00000094156.12|Fam183b-202|ENSMUSG00000049154|-|chr11:g.58683756:C>T
MAGRVGQMKNQDEVHQNQILRELFLKELRAQKLYTQYHVNPLRKVHTITRKPMSWHDNLEEPEDAKFLNLIHH
AAQGPKKKYSETQTEAQEIGWDPNPLINPDRQDHRLNHFRVYHDITLYKTKLWSLGEDDHQK*
>ENSMUSP00000051960_c.306:G>A_p.A103T ENSMUST00000060581.5|Fam183b-201|ENSMUSG00000049154|-|chr11:g.58683756:C>T
MAGRVGQMKNQDEVHQNQILRELFLKELRAQKLYTQYHVNPLRKAKFLNLIHHAAQGPKKKYSETQTEAQEIG
WDPNPLINPDRQDHRLNHFRVYHDITLYKTKLWSLGEDDHQK*
>ENSMUSP00000159142_c.372:G>A_p.A125T ENSMUST00000239007.2|Fam183b-204|ENSMUSG00000049154|-|chr11:g.58683756:C>T
MAMAGRVGQMKNQDEVHQNQILRELFLKELRAQKLYTQYHVNPLRKVHTITRKPMSWHDNLEEPEDAKFLNLI
HHAAQGPKKKYSETQTEAQEIGWDPNPLINPDRQDHRLNHFRVYHDITLYKTKLWSLGEDDHQK*
>ENSMUSP00000159184_c.312:G>A_p.A105T ENSMUST00000238886.2|Fam183b-203|ENSMUSG00000049154|-|chr11:g.58683756:C>T
MAMAGRVGQMKNQDEVHQNQILRELFLKELRAQKLYTQYHVNPLRKAKFLNLIHHAAQGPKKKYSETQTEAQE
IGWDPNPLINPDRQDHRLNHFRVYHDITLYKTKLWSLGEDDHQK*
>ENSMUSP00000091708_c.327:A>C_p.N110H ENSMUST00000094156.12|Fam183b-202|ENSMUSG00000049154|-|chr11:g.58683795:T>G
MAGRVGQMKNQDEVHQNQILRELFLKELRAQKLYTQYHVNPLRKVHTITRKPMSWHDNLEEPEDAKFLNLIHH
AAQGPKKKYSETQTEAQEIGWDPNPLINPDRQDHRLHHFRVYHDITLYKAKLWSLGEDDHQK*
>ENSMUSP00000051960_c.267:A>C_p.N90H ENSMUST00000060581.5|Fam183b-201|ENSMUSG00000049154|-|chr11:g.58683795:T>G
MAGRVGQMKNQDEVHQNQILRELFLKELRAQKLYTQYHVNPLRKAKFLNLIHHAAQGPKKKYSETQTEAQEIG
WDPNPLINPDRQDHRLHHFRVYHDITLYKAKLWSLGEDDHQK*
>ENSMUSP00000159142_c.333:A>C_p.N112H ENSMUST00000239007.2|Fam183b-204|ENSMUSG00000049154|-|chr11:g.58683795:T>G
MAMAGRVGQMKNQDEVHQNQILRELFLKELRAQKLYTQYHVNPLRKVHTITRKPMSWHDNLEEPEDAKFLNLI
HHAAQGPKKKYSETQTEAQEIGWDPNPLINPDRQDHRLHHFRVYHDITLYKAKLWSLGEDDHQK*
>ENSMUSP00000159184_c.273:A>C_p.N92H ENSMUST00000238886.2|Fam183b-203|ENSMUSG00000049154|-|chr11:g.58683795:T>G
MAMAGRVGQMKNQDEVHQNQILRELFLKELRAQKLYTQYHVNPLRKAKFLNLIHHAAQGPKKKYSETQTEAQE
IGWDPNPLINPDRQDHRLHHFRVYHDITLYKAKLWSLGEDDHQK*
>rs265601560_ENSMUSP00000091708_c.4:C>AC_p.A2ins-fs ENSMUST00000094156.12|Fam183b-202|ENSMUSG00000049154|-|chr11:g.58692715:G>GT
MDRTCGADEESG
>rs265601560_ENSMUSP00000051960_c.4:C>AC_p.A2ins-fs ENSMUST00000060581.5|Fam183b-201|ENSMUSG00000049154|-|chr11:g.58692715:G>GT
MDRTCGADEESG
>rs265601560_ENSMUSP00000159142_c.10:C>AC_p.A4ins-fs ENSMUST00000239007.2|Fam183b-204|ENSMUSG00000049154|-|chr11:g.58692715:G>GT
MAMDRTCGADEESG
>rs265601560_ENSMUSP00000159184_c.10:C>AC_p.A4ins-fs ENSMUST00000238886.2|Fam183b-203|ENSMUSG00000049154|-|chr11:g.58692715:G>GT
MAMDRTCGADEESG
>rs232871506_ENSMUSP00000150529_c.24:C>T_p.H9Y ENSMUST00000217506.2|Olfr313-202|ENSMUSG00000070438|+|chr11:g.58707860:C>T
MSNHTRVTYFILRGFSDIPQLRLMAIPVFLLIYTFGLLGNLSIITAVTRDSRLHSPMYFFLKNLSFLDICYTS
ATIPKAVVISLTGSGVISYQECVAQLYIFLTFSSSECFLLTAMAYDRCLAILRPLIYGTIMSHKYCSALVVTA

WVGGAIYSAFHTFNTFSLPYCGPNVIDHFFCDIPPVMRLSCTDYHLSEEVGFAVSSCIVMSSFALTVVSYIGI
VATVLCIPSVEGRWKAFSTCSSHLTTVILFYGTGSFVYLRPASQYSPTLGPLASIFYSVVTPSLNPVVYCLRN
KDMKFALQKLYCGRKY*
>rs232871506_ENSMUSP00000080852_c.24:C>T_p.H9Y
ENSMUST00000082220.5|Olfr313-
201|ENSMUSG00000070438|+|chr11:g.58707860:C>T
MSNHTRVTYFILRGFSDIPQLRLMAIPVFLLIYTFGLLGNLSIITAVTRDSRLHSPMYFFLKNLSFLDICYTS
ATIPKAVVISLTGSGVISYQECVAQLYIFLTFSSSECFLLTAMAYDRCLAILRPLIYGTIMSHKYCSALVVTA
WVGGAIYSAFHTFNTFSLPYCGPNVIDHFFCDIPPVMRLSCTDYHLSEEVGFAVSSCIVMSSFALTVVSYIGI
VATVLCIPSVEGRWKAFSTCSSHLTTVILFYGTGSFVYLRPASQYSPTLGPLASIFYSVVTPSLNPVVYCLRN
KDMKFALQKLYCGRKY*
>rs252040486_ENSMUSP00000150529_c.34:T>C_p.L12P
ENSMUST00000217506.2|Olfr313-
202|ENSMUSG00000070438|+|chr11:g.58707870:T>C
MSNHTRVTHFIPRGFSDIPQLRLMAIPVFLLIYTFGLLGNLSIITAVTRDSRLHSPMYFFLKNLSFLDICYTS
ATIPKAVVISLTGSGVISYQECVAQLYIFLTFSSSECFLLTAMAYDRCLAILRPLIYGTIMSHKYCSALVVTA
WVGGAIYSAFHTFNTFSLPYCGPNVIDHFFCDIPPVMRLSCTDYHLSEEVGFAVSSCIVMSSFALTVVSYIGI
VATVLCIPSVEGRWKAFSTCSSHLTTVILFYGTGSFVYLRPASQYSPTLGPLASIFYSVVTPSLNPVVYCLRN
KDMKFALQKLYCGRKY*
>rs252040486_ENSMUSP00000080852_c.34:T>C_p.L12P
ENSMUST00000082220.5|Olfr313-
201|ENSMUSG00000070438|+|chr11:g.58707870:T>C
MSNHTRVTHFIPRGFSDIPQLRLMAIPVFLLIYTFGLLGNLSIITAVTRDSRLHSPMYFFLKNLSFLDICYTS
ATIPKAVVISLTGSGVISYQECVAQLYIFLTFSSSECFLLTAMAYDRCLAILRPLIYGTIMSHKYCSALVVTA
WVGGAIYSAFHTFNTFSLPYCGPNVIDHFFCDIPPVMRLSCTDYHLSEEVGFAVSSCIVMSSFALTVVSYIGI
VATVLCIPSVEGRWKAFSTCSSHLTTVILFYGTGSFVYLRPASQYSPTLGPLASIFYSVVTPSLNPVVYCLRN
KDMKFALQKLYCGRKY*
>ENSMUSP00000150529_c.49:A>AAATAATAATAATAATAAT_p.D17EIIIIII-ins
ENSMUST00000217506.2|Olfr313-
202|ENSMUSG00000070438|+|chr11:g.58707885:A>AAATAATAATAATAATAAT
MSNHTRVTHFILRGFSEIIIIIIIPQLRLMAIPVFLLIYTFGLLGNLSIITAVTRDSRLHSPMYFFLKNLSFL
DICYTSATIPKAVVISLTGSGVISYQECVAQLYIFLTFSSSECFLLTAMAYDRCLAILRPLIYGTIMSHKYCS
ALVVTAWVGGAIYSAFHTFNTFSLPYCGPNVIDHFFCDIPPVMRLSCTDYHLSEEVGFAVSSCIVMSSFALTV
VSYIGIVATVLCIPSVEGRWKAFSTCSSHLTTVILFYGTGSFVYLRPASQYSPTLGPLASIFYSVVTPSLNPV
VYCLRNKDMKFALQKLYCGRKY*
>ENSMUSP00000080852_c.49:A>AAATAATAATAATAATAAT_p.D17EIIIIII-ins
ENSMUST00000082220.5|Olfr313-
201|ENSMUSG00000070438|+|chr11:g.58707885:A>AAATAATAATAATAATAAT
MSNHTRVTHFILRGFSEIIIIIIIPQLRLMAIPVFLLIYTFGLLGNLSIITAVTRDSRLHSPMYFFLKNLSFL
DICYTSATIPKAVVISLTGSGVISYQECVAQLYIFLTFSSSECFLLTAMAYDRCLAILRPLIYGTIMSHKYCS
ALVVTAWVGGAIYSAFHTFNTFSLPYCGPNVIDHFFCDIPPVMRLSCTDYHLSEEVGFAVSSCIVMSSFALTV
VSYIGIVATVLCIPSVEGRWKAFSTCSSHLTTVILFYGTGSFVYLRPASQYSPTLGPLASIFYSVVTPSLNPV
VYCLRNKDMKFALQKLYCGRKY*
>rs249513274_ENSMUSP00000150529_c.103:T>G_p.F35C
ENSMUST00000217506.2|Olfr313-
202|ENSMUSG00000070438|+|chr11:g.58707939:T>G
MSNHTRVTHFILRGFSDIPQLRLMAIPVFLLIYTCGLLGNLSIITAVTRDSRLHSPMYFFLKNLSFLDICYTS
ATIPKAVVISLTGSGVISYQECVAQLYIFLTFSSSECFLLTAMAYDRCLAILRPLIYGTIMSHKYCSALVVTA
WVGGAIYSAFHTFNTFSLPYCGPNVIDHFFCDIPPVMRLSCTDYHLSEEVGFAVSSCIVMSSFALTVVSYIGI
VATVLCIPSVEGRWKAFSTCSSHLTTVILFYGTGSFVYLRPASQYSPTLGPLASIFYSVVTPSLNPVVYCLRN
KDMKFALQKLYCGRKY*
>rs249513274_ENSMUSP00000080852_c.103:T>G_p.F35C
ENSMUST00000082220.5|Olfr313-
201|ENSMUSG00000070438|+|chr11:g.58707939:T>G
MSNHTRVTHFILRGFSDIPQLRLMAIPVFLLIYTCGLLGNLSIITAVTRDSRLHSPMYFFLKNLSFLDICYTS
ATIPKAVVISLTGSGVISYQECVAQLYIFLTFSSSECFLLTAMAYDRCLAILRPLIYGTIMSHKYCSALVVTA
WVGGAIYSAFHTFNTFSLPYCGPNVIDHFFCDIPPVMRLSCTDYHLSEEVGFAVSSCIVMSSFALTVVSYIGI

VATVLCIPSVEGRWKAFSTCSSHLTTVILFYGTGSFVYLRPASQYSPTLGPLASIFYSVVTPSLNPVVYCLRN
KDMKFALQKLYCGRKY*
>rs258477531_ENSMUSP00000150529_c.279:G>A_p.E94K
ENSMUST00000217506.2|Olfr313-
202|ENSMUSG00000070438|+|chr11:g.58708115:G>A
MSNHTRVTHFILRGFSDIPQLRLMAIPVFLLIYTFGLLGNLSIITAVTRDSRLHSPMYFFLKNLSFLDICYTS
ATIPKAVVISLTGSGVISYQKCVAQLYIFLTFSSSECFLLTAMAYDRCLAILRPLIYGTIMSHKYCSALVVTA
WVGGAIYSAFHTFNTFSLPYCGPNVIDHFFCDIPPVMRLSCTDYHLSEEVGFAVSSCIVMSSFALTVVSYIGI
VATVLCIPSVEGRWKAFSTCSSHLTTVILFYGTGSFVYLRPASQYSPTLGPLASIFYSVVTPSLNPVVYCLRN
KDMKFALQKLYCGRKY*
>rs258477531_ENSMUSP00000080852_c.279:G>A_p.E94K
ENSMUST00000082220.5|Olfr313-
201|ENSMUSG00000070438|+|chr11:g.58708115:G>A
MSNHTRVTHFILRGFSDIPQLRLMAIPVFLLIYTFGLLGNLSIITAVTRDSRLHSPMYFFLKNLSFLDICYTS
ATIPKAVVISLTGSGVISYQKCVAQLYIFLTFSSSECFLLTAMAYDRCLAILRPLIYGTIMSHKYCSALVVTA
WVGGAIYSAFHTFNTFSLPYCGPNVIDHFFCDIPPVMRLSCTDYHLSEEVGFAVSSCIVMSSFALTVVSYIGI
VATVLCIPSVEGRWKAFSTCSSHLTTVILFYGTGSFVYLRPASQYSPTLGPLASIFYSVVTPSLNPVVYCLRN
KDMKFALQKLYCGRKY*
>rs245110369_ENSMUSP00000150529_c.306:C>G_p.L103V
ENSMUST00000217506.2|Olfr313-
202|ENSMUSG00000070438|+|chr11:g.58708142:C>G
MSNHTRVTHFILRGFSDIPQLRLMAIPVFLLIYTFGLLGNLSIITAVTRDSRLHSPMYFFLKNLSFLDICYTS
ATIPKAVVISLTGSGVISYQECVAQLYIFVTFSSSECFLLTAMAYDRCLAILRPLIYGTIMSHKYCSALVVTA
WVGGAIYSAFHTFNTFSLPYCGPNVIDHFFCDIPPVMRLSCTDYHLSEEVGFAVSSCIVMSSFALTVVSYIGI
VATVLCIPSVEGRWKAFSTCSSHLTTVILFYGTGSFVYLRPASQYSPTLGPLASIFYSVVTPSLNPVVYCLRN
KDMKFALQKLYCGRKY*
>rs245110369_ENSMUSP00000080852_c.306:C>G_p.L103V
ENSMUST00000082220.5|Olfr313-
201|ENSMUSG00000070438|+|chr11:g.58708142:C>G
MSNHTRVTHFILRGFSDIPQLRLMAIPVFLLIYTFGLLGNLSIITAVTRDSRLHSPMYFFLKNLSFLDICYTS
ATIPKAVVISLTGSGVISYQECVAQLYIFVTFSSSECFLLTAMAYDRCLAILRPLIYGTIMSHKYCSALVVTA
WVGGAIYSAFHTFNTFSLPYCGPNVIDHFFCDIPPVMRLSCTDYHLSEEVGFAVSSCIVMSSFALTVVSYIGI
VATVLCIPSVEGRWKAFSTCSSHLTTVILFYGTGSFVYLRPASQYSPTLGPLASIFYSVVTPSLNPVVYCLRN
KDMKFALQKLYCGRKY*
>rs259366413_ENSMUSP00000150529_c.609:A>C_p.I204L
ENSMUST00000217506.2|Olfr313-
202|ENSMUSG00000070438|+|chr11:g.58708445:A>C
MSNHTRVTHFILRGFSDIPQLRLMAIPVFLLIYTFGLLGNLSIITAVTRDSRLHSPMYFFLKNLSFLDICYTS
ATIPKAVVISLTGSGVISYQECVAQLYIFLTFSSSECFLLTAMAYDRCLAILRPLIYGTIMSHKYCSALVVTA
WVGGAIYSAFHTFNTFSLPYCGPNVIDHFFCDIPPVMRLSCTDYHLSEEVGFAVSSCLVMSSFALTVVSYIGI
VATVLCIPSVEGRWKAFSTCSSHLTTVILFYGTGSFVYLRPASQYSPTLGPLASIFYSVVTPSLNPVVYCLRN
KDMKFALQKLYCGRKY*
>rs259366413_ENSMUSP00000080852_c.609:A>C_p.I204L
ENSMUST00000082220.5|Olfr313-
201|ENSMUSG00000070438|+|chr11:g.58708445:A>C
MSNHTRVTHFILRGFSDIPQLRLMAIPVFLLIYTFGLLGNLSIITAVTRDSRLHSPMYFFLKNLSFLDICYTS
ATIPKAVVISLTGSGVISYQECVAQLYIFLTFSSSECFLLTAMAYDRCLAILRPLIYGTIMSHKYCSALVVTA
WVGGAIYSAFHTFNTFSLPYCGPNVIDHFFCDIPPVMRLSCTDYHLSEEVGFAVSSCLVMSSFALTVVSYIGI
VATVLCIPSVEGRWKAFSTCSSHLTTVILFYGTGSFVYLRPASQYSPTLGPLASIFYSVVTPSLNPVVYCLRN
KDMKFALQKLYCGRKY*
>ENSMUSP00000150529_c.878:A>CTCAGACAG_p.K293ins-fs
ENSMUST00000217506.2|Olfr313-
202|ENSMUSG00000070438|+|chr11:g.58708714:A>CTCAGACAG
MSNHTRVTHFILRGFSDIPQLRLMAIPVFLLIYTFGLLGNLSIITAVTRDSRLHSPMYFFLKNLSFLDICYTS
ATIPKAVVISLTGSGVISYQECVAQLYIFLTFSSSECFLLTAMAYDRCLAILRPLIYGTIMSHKYCSALVVTA
WVGGAIYSAFHTFNTFSLPYCGPNVIDHFFCDIPPVMRLSCTDYHLSEEVGFAVSSCIVMSSFALTVVSYIGI
VATVLCIPSVEGRWKAFSTCSSHLTTVILFYGTGSFVYLRPASQYSPTLGPLASIFYSVVTPSLNPVVYCLRN
NSDRI

>ENSMUSP00000080852_c.878:A>CTCAGACAG_p.K293ins-fs
ENSMUST00000082220.5|Olfr313-
201|ENSMUSG00000070438|+|chr11:g.58708714:A>CTCAGACAG
MSNHTRVTHFILRGFSDIPQLRLMAIPVFLLIYTFGLLGNLSIITAVTRDSRLHSPMYFFLKNLSFLDICYTS
ATIPKAVVISLTGSGVISYQECVAQLYIFLTFSSSECFLLTAMAYDRCLAILRPLIYGTIMSHKYCSALVVTA
WVGGAIYSAFHTFNTFSLPYCGPNVIDHFFCDIPPVMRLSCTDYHLSEEVGFAVSSCIVMSSFALTVVSYIGI
VATVLCIPSVEGRWKAFSTCSSHLTTVILFYGTGSFVYLRPASQYSPTLGPLASIFYSVVTPSLNPVVYCLRN
NSDRI
>ENSMUSP00000149697_c.524:C>G_p.F175L ENSMUST00000216407.2|Olfr312-
202|ENSMUSG00000096806|+|chr11:g.58722506:C>G
MSNHTRVTHFILRGFSDVPQLRLVLIPFFLFFYTFGILGNFSIITAVTRDSRLHSPMYFFLKNLSFLDICYTS
ATIPKAVVISLTGSGVISYQECAAQLYIIFTFACTECFLLTAMAYDRCLAILRPLIYGTIMSQKYCSALVVTA
WVGGAIYSAFHTFNTFSLPYCGPNVIDHLFCDMPPVMRLSCTDYHLTEEVGFAVSSCIVMSSFVLTVVSYIGI
VATVLRIPSVEGRWKAFSTCSSHLTTVILFYGTGSFVYLRPASQYSPTLGRLASIFYSVVTPSLNPVVYCLRN
KDMKFALQKLYCGRKY*
>ENSMUSP00000077344_c.524:C>G_p.F175L ENSMUST00000078217.2|Olfr312-
201|ENSMUSG00000096806|+|chr11:g.58722506:C>G
MSNHTRVTHFILRGFSDVPQLRLVLIPFFLFFYTFGILGNFSIITAVTRDSRLHSPMYFFLKNLSFLDICYTS
ATIPKAVVISLTGSGVISYQECAAQLYIIFTFACTECFLLTAMAYDRCLAILRPLIYGTIMSQKYCSALVVTA
WVGGAIYSAFHTFNTFSLPYCGPNVIDHLFCDMPPVMRLSCTDYHLTEEVGFAVSSCIVMSSFVLTVVSYIGI
VATVLRIPSVEGRWKAFSTCSSHLTTVILFYGTGSFVYLRPASQYSPTLGRLASIFYSVVTPSLNPVVYCLRN
KDMKFALQKLYCGRKY*
>ENSMUSP00000149697_c.679:C>T_p.P227L ENSMUST00000216407.2|Olfr312-
202|ENSMUSG00000096806|+|chr11:g.58722661:C>T
MSNHTRVTHFILRGFSDVPQLRLVLIPFFLFFYTFGILGNFSIITAVTRDSRLHSPMYFFLKNLSFLDICYTS
ATIPKAVVISLTGSGVISYQECAAQLYIIFTFACTECFLLTAMAYDRCLAILRPLIYGTIMSQKYCSALVVTA
WVGGAIYSAFHTFNTFSLPYCGPNVIDHFFCDMPPVMRLSCTDYHLTEEVGFAVSSCIVMSSFVLTVVSYIGI
VATVLRILSVEGRWKAFSTCSSHLTTVILFYGTGSFVYLRPASQYSPTLGRLASIFYSVVTPSLNPVVYCLRN
KDMKFALQKLYCGRKY*
>ENSMUSP00000077344_c.679:C>T_p.P227L ENSMUST00000078217.2|Olfr312-
201|ENSMUSG00000096806|+|chr11:g.58722661:C>T
MSNHTRVTHFILRGFSDVPQLRLVLIPFFLFFYTFGILGNFSIITAVTRDSRLHSPMYFFLKNLSFLDICYTS
ATIPKAVVISLTGSGVISYQECAAQLYIIFTFACTECFLLTAMAYDRCLAILRPLIYGTIMSQKYCSALVVTA
WVGGAIYSAFHTFNTFSLPYCGPNVIDHFFCDMPPVMRLSCTDYHLTEEVGFAVSSCIVMSSFVLTVVSYIGI
VATVLRILSVEGRWKAFSTCSSHLTTVILFYGTGSFVYLRPASQYSPTLGRLASIFYSVVTPSLNPVVYCLRN
KDMKFALQKLYCGRKY*
>ENSMUSP00000149697_c.689:A>AT_p.E230ins-fs ENSMUST00000216407.2|Olfr312-
202|ENSMUSG00000096806|+|chr11:g.58722671:A>AT
MSNHTRVTHFILRGFSDVPQLRLVLIPFFLFFYTFGILGNFSIITAVTRDSRLHSPMYFFLKNLSFLDICYTS
ATIPKAVVISLTGSGVISYQECAAQLYIIFTFACTECFLLTAMAYDRCLAILRPLIYGTIMSQKYCSALVVTA
WVGGAIYSAFHTFNTFSLPYCGPNVIDHFFCDMPPVMRLSCTDYHLTEEVGFAVSSCIVMSSFVLTVVSYIGI
VATVLRIPSVEWQVESLFYLLLSPDHSHLVLWNWKLCVSEARLSVLPDPGSPGIYFLLCSHTISESSCLLSEE
QRYEVCSTETLLWEKVL
>ENSMUSP00000077344_c.689:A>AT_p.E230ins-fs ENSMUST00000078217.2|Olfr312-
201|ENSMUSG00000096806|+|chr11:g.58722671:A>AT
MSNHTRVTHFILRGFSDVPQLRLVLIPFFLFFYTFGILGNFSIITAVTRDSRLHSPMYFFLKNLSFLDICYTS
ATIPKAVVISLTGSGVISYQECAAQLYIIFTFACTECFLLTAMAYDRCLAILRPLIYGTIMSQKYCSALVVTA
WVGGAIYSAFHTFNTFSLPYCGPNVIDHFFCDMPPVMRLSCTDYHLTEEVGFAVSSCIVMSSFVLTVVSYIGI
VATVLRIPSVEWQVESLFYLLLSPDHSHLVLWNWKLCVSEARLSVLPDPGSPGIYFLLCSHTISESSCLLSEE
QRYEVCSTETLLWEKVL
>ENSMUSP00000149697_c.900:A>G_p.K301E ENSMUST00000216407.2|Olfr312-
202|ENSMUSG00000096806|+|chr11:g.58722882:A>G
MSNHTRVTHFILRGFSDVPQLRLVLIPFFLFFYTFGILGNFSIITAVTRDSRLHSPMYFFLKNLSFLDICYTS
ATIPKAVVISLTGSGVISYQECAAQLYIIFTFACTECFLLTAMAYDRCLAILRPLIYGTIMSQKYCSALVVTA
WVGGAIYSAFHTFNTFSLPYCGPNVIDHFFCDMPPVMRLSCTDYHLTEEVGFAVSSCIVMSSFVLTVVSYIGI
VATVLRIPSVEGRWKAFSTCSSHLTTVILFYGTGSFVYLRPASQYSPTLGRLASIFYSVVTPSLNPVVYCLRN
KDMKFALQELYCGRKY*

>ENSMUSP00000077344_c.900:A>G_p.K301E ENSMUST00000078217.2|Olfr312-
201|ENSMUSG00000096806|+|chr11:g.58722882:A>G
MSNHTRVTHFILRGFSDVPQLRLVLIPFFLFFYTFGILGNFSIITAVTRDSRLHSPMYFFLKNLSFLDICYTS
ATIPKAVVISLTGSGVISYQECAAQLYIIFTFACTECFLLTAMAYDRCLAILRPLIYGTIMSQKYCSALVVTA
WVGGAIYSAFHTFNTFSLPYCGPNVIDHFFCDMPPVMRLSCTDYHLTEEVGFAVSSCIVMSSFVLTVVSYIGI
VATVLRIPSVEGRWKAFSTCSSHLTTVILFYGTGSFVYLRPASQYSPTLGRLASIFYSVVTPSLNPVVYCLRN
KDMKFALQELYCGRKY*
>ENSMUSP00000150870_c.173:C>A_p.Y58* ENSMUST00000216473.2|Olfr311-
203|ENSMUSG00000094805|+|chr11:g.58732115:C>A
MANHTRVTHFILRGFSDVPQLRLVLIPFFLLFYTFGILGNFSIITAVIRDSRLHSPM*FFLKNLSFLDICYTS
ATIPKAVVISLTGSGVISYQECVAQLYMFITLCGTECFLLTAMAYDRCLAILRPLIYGTIMSHKYCSALVVTA
WVGGAIYSAFHTFNTFSLPYCGPNVVDHFFCDMPPVMRLSCTDYHLNEEVGFAVIGCIIMSSFALTVVSYIGI
VATVLHIPSVEGRWKAFSTCSSHLTTVILFYGTGSFVYLRPASQYSPTLDPLASIFYSVVTPSLNPVIYCLRN
KDMKFALQKLYCGRKY*
>ENSMUSP00000071553_c.173:C>A_p.Y58* ENSMUST00000071625.2|Olfr311-
201|ENSMUSG00000094805|+|chr11:g.58732115:C>A
MANHTRVTHFILRGFSDVPQLRLVLIPFFLLFYTFGILGNFSIITAVIRDSRLHSPM*FFLKNLSFLDICYTS
ATIPKAVVISLTGSGVISYQECVAQLYMFITLCGTECFLLTAMAYDRCLAILRPLIYGTIMSHKYCSALVVTA
WVGGAIYSAFHTFNTFSLPYCGPNVVDHFFCDMPPVMRLSCTDYHLNEEVGFAVIGCIIMSSFALTVVSYIGI
VATVLHIPSVEGRWKAFSTCSSHLTTVILFYGTGSFVYLRPASQYSPTLDPLASIFYSVVTPSLNPVIYCLRN
KDMKFALQKLYCGRKY*
>ENSMUSP00000150870_c.273:T>C_p.Y92H ENSMUST00000216473.2|Olfr311-
203|ENSMUSG00000094805|+|chr11:g.58732215:T>C
MANHTRVTHFILRGFSDVPQLRLVLIPFFLLFYTFGILGNFSIITAVIRDSRLHSPMYFFLKNLSFLDICYTS
ATIPKAVVISLTGSGVISHQECVAQLYMFITLCGTECFLLTAMAYDRCLAILRPLIYGTIMSHKYCSALVVTA
WVGGAIYSAFHTFNTFSLPYCGPNVVDHFFCDMPPVMRLSCTDYHLNEEVGFAVIGCIIMSSFALTVVSYIGI
VATVLHIPSVEGRWKAFSTCSSHLTTVILFYGTGSFVYLRPASQYSPTLDPLASIFYSVVTPSLNPVIYCLRN
KDMKFALQKLYCGRKY*
>ENSMUSP00000071553_c.273:T>C_p.Y92H ENSMUST00000071625.2|Olfr311-
201|ENSMUSG00000094805|+|chr11:g.58732215:T>C
MANHTRVTHFILRGFSDVPQLRLVLIPFFLLFYTFGILGNFSIITAVIRDSRLHSPMYFFLKNLSFLDICYTS
ATIPKAVVISLTGSGVISHQECVAQLYMFITLCGTECFLLTAMAYDRCLAILRPLIYGTIMSHKYCSALVVTA
WVGGAIYSAFHTFNTFSLPYCGPNVVDHFFCDMPPVMRLSCTDYHLNEEVGFAVIGCIIMSSFALTVVSYIGI
VATVLHIPSVEGRWKAFSTCSSHLTTVILFYGTGSFVYLRPASQYSPTLDPLASIFYSVVTPSLNPVIYCLRN
KDMKFALQKLYCGRKY*
>ENSMUSP00000150870_c.436:C>G_p.A146G ENSMUST00000216473.2|Olfr311-
203|ENSMUSG00000094805|+|chr11:g.58732378:C>G
MANHTRVTHFILRGFSDVPQLRLVLIPFFLLFYTFGILGNFSIITAVIRDSRLHSPMYFFLKNLSFLDICYTS
ATIPKAVVISLTGSGVISYQECVAQLYMFITLCGTECFLLTAMAYDRCLAILRPLIYGTIMSHKYCSALVVTG
WVGGAIYSAFHTFNTFSLPYCGPNVVDHFFCDMPPVMRLSCTDYHLNEEVGFAVIGCIIMSSFALTVVSYIGI
VATVLHIPSVEGRWKAFSTCSSHLTTVILFYGTGSFVYLRPASQYSPTLDPLASIFYSVVTPSLNPVIYCLRN
KDMKFALQKLYCGRKY*
>ENSMUSP00000071553_c.436:C>G_p.A146G ENSMUST00000071625.2|Olfr311-
201|ENSMUSG00000094805|+|chr11:g.58732378:C>G
MANHTRVTHFILRGFSDVPQLRLVLIPFFLLFYTFGILGNFSIITAVIRDSRLHSPMYFFLKNLSFLDICYTS
ATIPKAVVISLTGSGVISYQECVAQLYMFITLCGTECFLLTAMAYDRCLAILRPLIYGTIMSHKYCSALVVTG
WVGGAIYSAFHTFNTFSLPYCGPNVVDHFFCDMPPVMRLSCTDYHLNEEVGFAVIGCIIMSSFALTVVSYIGI
VATVLHIPSVEGRWKAFSTCSSHLTTVILFYGTGSFVYLRPASQYSPTLDPLASIFYSVVTPSLNPVIYCLRN
KDMKFALQKLYCGRKY*
>ENSMUSP00000150870_c.511:T>C_p.V171A ENSMUST00000216473.2|Olfr311-
203|ENSMUSG00000094805|+|chr11:g.58732453:T>C
MANHTRVTHFILRGFSDVPQLRLVLIPFFLLFYTFGILGNFSIITAVIRDSRLHSPMYFFLKNLSFLDICYTS
ATIPKAVVISLTGSGVISYQECVAQLYMFITLCGTECFLLTAMAYDRCLAILRPLIYGTIMSHKYCSALVVTA
WVGGAIYSAFHTFNTFSLPYCGPNAVDHFFCDMPPVMRLSCTDYHLNEEVGFAVIGCIIMSSFALTVVSYIGI
VATVLHIPSVEGRWKAFSTCSSHLTTVILFYGTGSFVYLRPASQYSPTLDPLASIFYSVVTPSLNPVIYCLRN
KDMKFALQKLYCGRKY*
>ENSMUSP00000071553_c.511:T>C_p.V171A ENSMUST00000071625.2|Olfr311-
201|ENSMUSG00000094805|+|chr11:g.58732453:T>C

MANHTRVTHFILRGFSDVPQLRLVLIPFFLLFYTFGILGNFSIITAVIRDSRLHSPMYFFLKNLSFLDICYTS
ATIPKAVVISLTGSGVISYQECVAQLYMFITLCGTECFLLTAMAYDRCLAILRPLIYGTIMSHKYCSALVVTA
WVGGAIYSAFHTFNTFSLPYCGPNAVDHFFCDMPPVMRLSCTDYHLNEEVGFAVIGCIIMSSFALTVVSYIGI
VATVLHIPSVEGRWKAFSTCSSHLTTVILFYGTGSFVYLRPASQYSPTLDPLASIFYSVVTPSLNPVIYCLRN
KDMKFALQKLYCGRKY*
>ENSMUSP00000117644_c.352:C>T_p.A118V ENSMUST00000139337.3|Gm12258-202|ENSMUSG00000072915|+|chr11:g.58749179:C>T
MDTSEGLVSFEDVSVDFTWDEWQDLDDSQRKLYRDVMLETYSSLLSLNQCDAKPELILKLEKEAEPWKEDDAP
DQSLPDMQNVDDRNEVSQDSQKIRRCSLVIAKSSVSAERAPAGKVLSSSHRVLDLAIKNSTSSGMSPGPLNMR
DRMLLPREPSEMQARKELGLPDVTRVSPRLPEPLSLYRSTECEEQHVQYCGPSEAFHVKPVWLQKAFPMSNPS
SKLRDYRKELEKLALPAKGETRAQVQTFECKVCGKLFCKNSHLTQHLKMHKEKRCYKGSNCEPILNIQPKLPK
HQSLRAGEELYTCNGDEKYIRPKSGKSVQQKIIGDGRIVCGKTECSKLNCSVEQNPLKHEETHEFSLAGKTVR
KSDLHQLETPHRYERVYECSACGKTFSHTPHVYSPSGAEKPYECEDCQQSKLVVSQPAPTQEKRYVCNVCGKA
FYKRAHLHAHQRTHTGEKPYDCQECGKSFRLKSFLVVHQRIHTGEKPFACDTCGKSFKQRTSLYTHIRIHTGE
KPYECKECRKSFILKSYLTVHQRTHSGEKPYECDVCGKSFKQNSHLHAHKRTHTSEKPYECIVCGKSYKQSPS
LYTHKKIHTSEKPYECKQCRKSFSLKFHLTRHQRTHSGEKHYQ*
>ENSMUSP00000117644_c.687:G>A_p.E230K ENSMUST00000139337.3|Gm12258-202|ENSMUSG00000072915|+|chr11:g.58749514:G>A
MDTSEGLVSFEDVSVDFTWDEWQDLDDSQRKLYRDVMLETYSSLLSLNQCDAKPELILKLEKEAEPWKEDDAP
DQSLPDMQNVDDRNEVSQDSQKIRRCSLVIAKSSVSAERAPAGKALSSSHRVLDLAIKNSTSSGMSPGPLNMR
DRMLLPREPSEMQARKELGLPDVTRVSPRLPEPLSLYRSTECEEQHVQYCGPSEAFHVKPVWLQKAFPMSNPS
SKLRDYRKELKKLALPAKGETRAQVQTFECKVCGKLFCKNSHLTQHLKMHKEKRCYKGSNCEPILNIQPKLPK
HQSLRAGEELYTCNGDEKYIRPKSGKSVQQKIIGDGRIVCGKTECSKLNCSVEQNPLKHEETHEFSLAGKTVR
KSDLHQLETPHRYERVYECSACGKTFSHTPHVYSPSGAEKPYECEDCQQSKLVVSQPAPTQEKRYVCNVCGKA
FYKRAHLHAHQRTHTGEKPYDCQECGKSFRLKSFLVVHQRIHTGEKPFACDTCGKSFKQRTSLYTHIRIHTGE
KPYECKECRKSFILKSYLTVHQRTHSGEKPYECDVCGKSFKQNSHLHAHKRTHTSEKPYECIVCGKSYKQSPS
LYTHKKIHTSEKPYECKQCRKSFSLKFHLTRHQRTHSGEKHYQ*
>ENSMUSP00000117644_c.1351:C>A_p.T451N ENSMUST00000139337.3|Gm12258-202|ENSMUSG00000072915|+|chr11:g.58750178:C>A
MDTSEGLVSFEDVSVDFTWDEWQDLDDSQRKLYRDVMLETYSSLLSLNQCDAKPELILKLEKEAEPWKEDDAP
DQSLPDMQNVDDRNEVSQDSQKIRRCSLVIAKSSVSAERAPAGKALSSSHRVLDLAIKNSTSSGMSPGPLNMR
DRMLLPREPSEMQARKELGLPDVTRVSPRLPEPLSLYRSTECEEQHVQYCGPSEAFHVKPVWLQKAFPMSNPS
SKLRDYRKELEKLALPAKGETRAQVQTFECKVCGKLFCKNSHLTQHLKMHKEKRCYKGSNCEPILNIQPKLPK
HQSLRAGEELYTCNGDEKYIRPKSGKSVQQKIIGDGRIVCGKTECSKLNCSVEQNPLKHEETHEFSLAGKTVR
KSDLHQLETPHRYERVYECSACGKTFSHTPHVYSPSGAEKPYECEDCQQSKLVVSQPAPTQEKRYVCNVCGKA
FYKRAHLHAHQRNHTGEKPYDCQECGKSFRLKSFLVVHQRIHTGEKPFACDTCGKSFKQRTSLYTHIRIHTGE
KPYECKECRKSFILKSYLTVHQRTHSGEKPYECDVCGKSFKQNSHLHAHKRTHTSEKPYECIVCGKSYKQSPS
LYTHKKIHTSEKPYECKQCRKSFSLKFHLTRHQRTHSGEKHYQ*
>ENSMUSP00000117644_c.1740:C>T_p.Q581* ENSMUST00000139337.3|Gm12258-202|ENSMUSG00000072915|+|chr11:g.58750567:C>T
MDTSEGLVSFEDVSVDFTWDEWQDLDDSQRKLYRDVMLETYSSLLSLNQCDAKPELILKLEKEAEPWKEDDAP
DQSLPDMQNVDDRNEVSQDSQKIRRCSLVIAKSSVSAERAPAGKALSSSHRVLDLAIKNSTSSGMSPGPLNMR
DRMLLPREPSEMQARKELGLPDVTRVSPRLPEPLSLYRSTECEEQHVQYCGPSEAFHVKPVWLQKAFPMSNPS
SKLRDYRKELEKLALPAKGETRAQVQTFECKVCGKLFCKNSHLTQHLKMHKEKRCYKGSNCEPILNIQPKLPK
HQSLRAGEELYTCNGDEKYIRPKSGKSVQQKIIGDGRIVCGKTECSKLNCSVEQNPLKHEETHEFSLAGKTVR
KSDLHQLETPHRYERVYECSACGKTFSHTPHVYSPSGAEKPYECEDCQQSKLVVSQPAPTQEKRYVCNVCGKA
FYKRAHLHAHQRTHTGEKPYDCQECGKSFRLKSFLVVHQRIHTGEKPFACDTCGKSFKQRTSLYTHIRIHTGE
KPYECKECRKSFILKSYLTVHQRTHSGEKPYECDVCGKSFKQNSHLHAHKRTHTSEKPYECIVCGKSYK*SPS
LYTHKKIHTSEKPYECKQCRKSFSLKFHLTRHQRTHSGEKHYQ*
>ENSMUSP00000091282_c.306:C>T_p.Q103* ENSMUST00000093768.4|Gm12258-201|ENSMUSG00000072915|+|chr11:g.58750567:C>T
IHTGEKPFACDTCGKSFKQRTSLYTHIRIHTGEKPYECKECRKSFILKSYLTVHQRTHSGEKPYECDVCGKSF
KQNSHLHAHKRTHTSEKPYECIVCGKSYK*SPSLYTHKKIHTSEKPYECKQCRKSFSLKFHLTRHQRTHSGEK
HYQ*
>ENSMUSP00000120692_c.181:A>G_p.D61G ENSMUST00000132570.2|2810021J22Rik-202|ENSMUSG00000020491|+|chr11:g.58768808:A>G
MSSIVGLVSFEDVSVDFTWDEWQDLDDSQRKLYRDVMLETYSSLLSLSAGQASTVSKFLTGFCSHGLW

>ENSMUSP00000073579_c.579:G>A_p.G194S
ENSMUST00000073924.10|2810021J22Rik-
201|ENSMUSG00000020491|+|chr11:g.58771099:G>A
MSSIVGLVSFEDVSVDFTWDEWQDLDDSQRKLYRDVMLETYSSLLSLNQCDAKPELILKLEQGAGPWKEDNAP
DQSLPALQPMKSLKEARRDTGKKHLSHLLVTGSASAEERGRLGEILSMNSNHVLHLALKTRSTSRMRSEVLDT
WGDVYLPGGPIEMQATEELDHLNPSKMLPRPPAPLSLDFSAERGPQHSQCAQHGEAFDVRTVWTRTVFPLGDS
STNAFDKLALTAQEGTHIREETFDCNMCKKPFSDTCNHTRHLKPHLKKQCGKCPDGEPAFRAKAGLQHTLHSW
GKPRGCNDNKKCVHQMPKPRSNQSVFSSKESNCVKTFCPDSTLSVQQRPHTGKKPQESSTPVKINSQPRRRHR
CGRTYQCKVCGKAFKHTQNLYLHHRTHTGEKPYECKECKKLFSVKSNLSVHQKTHTGEKPYECNICGNAFKRR
CDLTIHQRVHTGEKPYECKECRKTFSIKSGLIVHQRIHTGEKPYECSVCGKRFNQKSNLSTHEKIHTGEKPFE
CKECSKAFSVKSYLTIHQKTHVGEKPHS*
>ENSMUSP00000073579_c.1225:T>C_p.V409A
ENSMUST00000073924.10|2810021J22Rik-
201|ENSMUSG00000020491|+|chr11:g.58771745:T>C
MSSIVGLVSFEDVSVDFTWDEWQDLDDSQRKLYRDVMLETYSSLLSLNQCDAKPELILKLEQGAGPWKEDNAP
DQSLPALQPMKSLKEARRDTGKKHLSHLLVTGSASAEERGRLGEILSMNSNHVLHLALKTRSTSRMRSEVLDT
WGDVYLPGGPIEMQATEELDHLNPSKMLPRPPAPLSLDFSAERGPQHGQCAQHGEAFDVRTVWTRTVFPLGDS
STNAFDKLALTAQEGTHIREETFDCNMCKKPFSDTCNHTRHLKPHLKKQCGKCPDGEPAFRAKAGLQHTLHSW
GKPRGCNDNKKCVHQMPKPRSNQSVFSSKESNCVKTFCPDSTLSVQQRPHTGKKPQESSTPVKINSQPRRRHR
CGRTYQCKVCGKAFKHTQNLYLHHRTHTGEKPYECKECKKLFSAKSNLSVHQKTHTGEKPYECNICGNAFKRR
CDLTIHQRVHTGEKPYECKECRKTFSIKSGLIVHQRIHTGEKPYECSVCGKRFNQKSNLSTHEKIHTGEKPFE
CKECSKAFSVKSYLTIHQKTHVGEKPHS*
>ENSMUSP00000099764_c.2092:T>A_p.F698Y ENSMUST00000102703.2|Zfp39-
201|ENSMUSG00000037001|-|chr11:g.58780668:A>T
MRNLQPDSVENSLSQLPSRCLETRKRKRSYKKRPVTYSYWRRTQRNRARKHKAPVKGLVSFEDVSVDFTWDEW
QDLDDSQRKLYRDVMLETYSSLESLGHCITKPEVIFKLEQGAEPWRAEDVPKQSRADVQKITELNETSQDNEE
RHLWHHAITYSNKSTEEKVKLGNIVNVSSNCVSNLTVKNGNSSGMRPVALTVWQSVLPPNKPDDTRIGEELDA
SLTSEPPIHAEHPGLYSRAPGTGQQFQCCMQEVTCNTKALWTKRFHIAHGSSKFGESEKVPDEVALHAQDVSW
VRAETFECSICKKTFCTKCELMKHKKIHKGQQYYTCRDCEKTFIKESYHTDQRVHAGVGSHRCKQCEKCFHQK
NQQNVHERVPREARLWEVYQSENSFGEKPNLRRYQRTRAGYKPYGCNLCGKAFYRKSHLGRHQKIHTGEKPYG
CEECKKTFYHKSSLTIHQRTHTGEKPYECKKCRKTFYCKSDLNVHHRTHTGEKPYECDECRKTFYSKSHLVIH
QKVHTGDKPYECEECQKAFSRKSNLTVHQKTHTGEKPYECNVCGKTFHRQSHLNMHQGTHTGQKPYQCEECGK
AFYQKSSLRRHQRNHTGSRPYACEECRKTFLHKSSLTVHQRSHTGYKPYSCEECRKTFYSKSHLTVHQRTHTG
EKPYECKLCKKAFHQKSYLNRHQVTHGSEKRFECQECRKTYYHKSSLTVHQRIHLRELLCV*
>ENSMUSP00000099764_c.1947:G>A_p.V650I ENSMUST00000102703.2|Zfp39-
201|ENSMUSG00000037001|-|chr11:g.58780813:C>T
MRNLQPDSVENSLSQLPSRCLETRKRKRSYKKRPVTYSYWRRTQRNRARKHKAPVKGLVSFEDVSVDFTWDEW
QDLDDSQRKLYRDVMLETYSSLESLGHCITKPEVIFKLEQGAEPWRAEDVPKQSRADVQKITELNETSQDNEE
RHLWHHAITYSNKSTEEKVKLGNIVNVSSNCVSNLTVKNGNSSGMRPVALTVWQSVLPPNKPDDTRIGEELDA
SLTSEPPIHAEHPGLYSRAPGTGQQFQCCMQEVTCNTKALWTKRFHIAHGSSKFGESEKVPDEVALHAQDVSW
VRAETFECSICKKTFCTKCELMKHKKIHKGQQYYTCRDCEKTFIKESYHTDQRVHAGVGSHRCKQCEKCFHQK
NQQNVHERVPREARLWEVYQSENSFGEKPNLRRYQRTRAGYKPYGCNLCGKAFYRKSHLGRHQKIHTGEKPYG
CEECKKTFYHKSSLTIHQRTHTGEKPYECKKCRKTFYCKSDLNVHHRTHTGEKPYECDECRKTFYSKSHLVIH
QKVHTGDKPYECEECQKAFSRKSNLTVHQKTHTGEKPYECNVCGKTFHRQSHLNMHQGTHTGQKPYQCEECGK
AFYQKSSLRRHQRNHTGSRPYACEECRKTFLHKSSLTVHQRSHTGYKPYSCEECRKTFYSKSHLTIHQRTHTG
EKPYECKLCKKAFHQKSYLNRHQVTHGSEKRFECQECRKTFYHKSSLTVHQRIHLRELLCV*
>ENSMUSP00000099764_c.1196:T>G_p.Y399* ENSMUST00000102703.2|Zfp39-
201|ENSMUSG00000037001|-|chr11:g.58781564:A>C
MRNLQPDSVENSLSQLPSRCLETRKRKRSYKKRPVTYSYWRRTQRNRARKHKAPVKGLVSFEDVSVDFTWDEW
QDLDDSQRKLYRDVMLETYSSLESLGHCITKPEVIFKLEQGAEPWRAEDVPKQSRADVQKITELNETSQDNEE
RHLWHHAITYSNKSTEEKVKLGNIVNVSSNCVSNLTVKNGNSSGMRPVALTVWQSVLPPNKPDDTRIGEELDA
SLTSEPPIHAEHPGLYSRAPGTGQQFQCCMQEVTCNTKALWTKRFHIAHGSSKFGESEKVPDEVALHAQDVSW
VRAETFECSICKKTFCTKCELMKHKKIHKGQQYYTCRDCEKTFIKESYHTDQRVHAGVGSHRCKQCEKCFHQK
NQQNVHERVPREARLWEVYQSENSFGEKPNLRR*QRTRAGYKPYGCNLCGKAFYRKSHLGRHQKIHTGEKPYG
CEECKKTFYHKSSLTIHQRTHTGEKPYECKKCRKTFYCKSDLNVHHRTHTGEKPYECDECRKTFYSKSHLVIH
QKVHTGDKPYECEECQKAFSRKSNLTVHQKTHTGEKPYECNVCGKTFHRQSHLNMHQGTHTGQKPYQCEECGK
AFYQKSSLRRHQRNHTGSRPYACEECRKTFLHKSSLTVHQRSHTGYKPYSCEECRKTFYSKSHLTVHQRTHTG
EKPYECKLCKKAFHQKSYLNRHQVTHGSEKRFECQECRKTFYHKSSLTVHQRIHLRELLCV*

>ENSMUSP00000099764_c.1174:A>C_p.E392A ENSMUST00000102703.2|Zfp39-201|ENSMUSG00000037001|-|chr11:g.58781586:T>G
MRNLQPDSVENSLSQLPSRCLETRKRKRSYKKRPVTYSYWRRTQRNRARKHKAPVKGLVSFEDVSVDFTWDEW
QDLDDSQRKLYRDVMLETYSSLESLGHCITKPEVIFKLEQGAEPWRAEDVPKQSRADVQKITELNETSQDNEE
RHLWHHAITYSNKSTEEKVKLGNIVNVSSNCVSNLTVKNGNSSGMRPVALTVWQSVLPPNKPDDTRIGEELDA
SLTSEPPIHAEHPGLYSRAPGTGQQFQCCMQEVTCNTKALWTKRFHIAHGSSKFGESEKVPDEVALHAQDVSW
VRAETFECSICKKTFCTKCELMKHKKIHKGQQYYTCRDCEKTFIKESYHTDQRVHAGVGSHRCKQCEKCFHQK
NQQNVHERVPREARLWEVYQSENSFGAKPNLRRYQRTRAGYKPYGCNLCGKAFYRKSHLGRHQKIHTGEKPYG
CEECKKTFYHKSSLTIHQRTHTGEKPYECKKCRKTFYCKSDLNVHHRTHTGEKPYECDECRKTFYSKSHLVIH
QKVHTGDKPYECEECQKAFSRKSNLTVHQKTHTGEKPYECNVCGKTFHRQSHLNMHQGTHTGQKPYQCEECGK
AFYQKSSLRRHQRNHTGSRPYACEECRKTFLHKSSLTVHQRSHTGYKPYSCEECRKTFYSKSHLTVHQRTHTG
EKPYECKLCKKAFHQKSYLNRHQVTHGSEKRFECQECRKTFYHKSSLTVHQRIHLRELLCV*
>ENSMUSP00000099764_c.1137:C>T_p.L380F ENSMUST00000102703.2|Zfp39-201|ENSMUSG00000037001|-|chr11:g.58781623:G>A
MRNLQPDSVENSLSQLPSRCLETRKRKRSYKKRPVTYSYWRRTQRNRARKHKAPVKGLVSFEDVSVDFTWDEW
QDLDDSQRKLYRDVMLETYSSLESLGHCITKPEVIFKLEQGAEPWRAEDVPKQSRADVQKITELNETSQDNEE
RHLWHHAITYSNKSTEEKVKLGNIVNVSSNCVSNLTVKNGNSSGMRPVALTVWQSVLPPNKPDDTRIGEELDA
SLTSEPPIHAEHPGLYSRAPGTGQQFQCCMQEVTCNTKALWTKRFHIAHGSSKFGESEKVPDEVALHAQDVSW
VRAETFECSICKKTFCTKCELMKHKKIHKGQQYYTCRDCEKTFIKESYHTDQRVHAGVGSHRCKQCEKCFHQK
NQQNVHERVPREARFWEVYQSENSFGEKPNLRRYQRTRAGYKPYGCNLCGKAFYRKSHLGRHQKIHTGEKPYG
CEECKKTFYHKSSLTIHQRTHTGEKPYECKKCRKTFYCKSDLNVHHRTHTGEKPYECDECRKTFYSKSHLVIH
QKVHTGDKPYECEECQKAFSRKSNLTVHQKTHTGEKPYECNVCGKTFHRQSHLNMHQGTHTGQKPYQCEECGK
AFYQKSSLRRHQRNHTGSRPYACEECRKTFLHKSSLTVHQRSHTGYKPYSCEECRKTFYSKSHLTVHQRTHTG
EKPYECKLCKKAFHQKSYLNRHQVTHGSEKRFECQECRKTFYHKSSLTVHQRIHLRELLCV*
>ENSMUSP00000099764_c.925:C>G_p.T309R ENSMUST00000102703.2|Zfp39-201|ENSMUSG00000037001|-|chr11:g.58781835:G>C
MRNLQPDSVENSLSQLPSRCLETRKRKRSYKKRPVTYSYWRRTQRNRARKHKAPVKGLVSFEDVSVDFTWDEW
QDLDDSQRKLYRDVMLETYSSLESLGHCITKPEVIFKLEQGAEPWRAEDVPKQSRADVQKITELNETSQDNEE
RHLWHHAITYSNKSTEEKVKLGNIVNVSSNCVSNLTVKNGNSSGMRPVALTVWQSVLPPNKPDDTRIGEELDA
SLTSEPPIHAEHPGLYSRAPGTGQQFQCCMQEVTCNTKALWTKRFHIAHGSSKFGESEKVPDEVALHAQDVSW
VRAETFECSICKKTFCRKCELMKHKKIHKGQQYYTCRDCEKTFIKESYHTDQRVHAGVGSHRCKQCEKCFHQK
NQQNVHERVPREARLWEVYQSENSFGEKPNLRRYQRTRAGYKPYGCNLCGKAFYRKSHLGRHQKIHTGEKPYG
CEECKKTFYHKSSLTIHQRTHTGEKPYECKKCRKTFYCKSDLNVHHRTHTGEKPYECDECRKTFYSKSHLVIH
QKVHTGDKPYECEECQKAFSRKSNLTVHQKTHTGEKPYECNVCGKTFHRQSHLNMHQGTHTGQKPYQCEECGK
AFYQKSSLRRHQRNHTGSRPYACEECRKTFLHKSSLTVHQRSHTGYKPYSCEECRKTFYSKSHLTVHQRTHTG
EKPYECKLCKKAFHQKSYLNRHQVTHGSEKRFECQECRKTFYHKSSLTVHQRIHLRELLCV*
>rs49084133_ENSMUSP00000099764_c.186:G>A_p.D63N
ENSMUST00000102703.2|Zfp39-201|ENSMUSG00000037001|-|chr11:g.58791499:C>T
MRNLQPDSVENSLSQLPSRCLETRKRKRSYKKRPVTYSYWRRTQRNRARKHKAPVKGLVSFENVSVDFTWDEW
QDLDDSQRKLYRDVMLETYSSLESLGHCITKPEVIFKLEQGAEPWRAEDVPKQSRADVQKITELNETSQDNEE
RHLWHHAITYSNKSTEEKVKLGNIVNVSSNCVSNLTVKNGNSSGMRPVALTVWQSVLPPNKPDDTRIGEELDA
SLTSEPPIHAEHPGLYSRAPGTGQQFQCCMQEVTCNTKALWTKRFHIAHGSSKFGESEKVPDEVALHAQDVSW
VRAETFECSICKKTFCTKCELMKHKKIHKGQQYYTCRDCEKTFIKESYHTDQRVHAGVGSHRCKQCEKCFHQK
NQQNVHERVPREARLWEVYQSENSFGEKPNLRRYQRTRAGYKPYGCNLCGKAFYRKSHLGRHQKIHTGEKPYG
CEECKKTFYHKSSLTIHQRTHTGEKPYECKKCRKTFYCKSDLNVHHRTHTGEKPYECDECRKTFYSKSHLVIH
QKVHTGDKPYECEECQKAFSRKSNLTVHQKTHTGEKPYECNVCGKTFHRQSHLNMHQGTHTGQKPYQCEECGK
AFYQKSSLRRHQRNHTGSRPYACEECRKTFLHKSSLTVHQRSHTGYKPYSCEECRKTFYSKSHLTVHQRTHTG
EKPYECKLCKKAFHQKSYLNRHQVTHGSEKRFECQECRKTFYHKSSLTVHQRIHLRELLCV*
>rs29406722_ENSMUSP00000063279_c.882:G>A_p.E295K
ENSMUST00000069941.13|Btnl10-202|ENSMUSG00000020490|+|chr11:g.58813255:G>A
MARAHPGDATLPSILVSFIFLQLLTSGNGKSDFLVLGPPHPLLAIVGQDKELPCKLSLNISAEGMELRWYRDK
PSSVVHVYKNGEDVYDEQMVEYKGRTSFNGSHVARGEAAVKIHNVTVFDNGTYHCVFKEYTSHSQATLWLKVA
GRGSSPRIRVTDTQDKGIRAECTSAGWYPEPKVEWLDLKGQPVSAESHFSVSASTGLVALLSIVTPQDTAVGG
LTCSISNPLLPEKVTETYLLASLSRRPLSTESGPALPLILTALGLVSAAIACAFGKCHKEEHKTNQEEEDPGA
RDKAGLFHVSLSLDPETASPKLMVSEDQKSVKRLLFDQDVFPSSRRFNQDPCILAQERFEAGRHYWEVEVGDR
RAWILGVCLESLVREGRIPKSPQHGLWALEFYKKKLQALSYPRTCLSPPEPLRRVGILLDFEAGQISFYNSTN
GSLIYVFSGFSFSGPLQPFFCLWTHDPRPLTICSVVTETKDTESSGDPGLPGDS*

>rs29406722_ENSMUSP00000124234_c.882:G>A_p.E295K
ENSMUST00000142499.8|Btnl10-204|ENSMUSG00000020490|+|chr11:g.58813255:G>A
MARAHPGDATLPSILVSFIFLQLLTSGNGKSDFLVLGPPHPLLAIVGQDKELPCKLSLNISAEGMELRWYRDK
PSSVVHVYKNGEDVYDEQMVEYKGRTSFNGSHVARGEAAVKIHNVTVFDNGTYHCVFKEYTSHSQATLWLKVA
GRGSSPRIRVTDTQDKGIRAECTSAGWYPEPKVEWLDLKGQPVSAESHFSVSASTGLVALLSIVTPQDTAVGG
LTCSISNPLLPEKVTETYLLASLSRRPLSTESGPALPLILTALGLVSAAIACAFGKCHKEEHKTNQEEEDPGA
RDKAGLFHVSLSLDPETASPKLMVSEDQKSVKRLLFDQDVFPSSRRFNQDPCILAQERFEAGRHYWEVEVGDR
RAWILGVCLESLVREGRIPKSPQHGLWALEFYKKKLQALSYPRTCLSPPEPLRRVGILLDFEAGQISFYNSTN
GSLIYVFSGFSFSGPLQPFFCLWTHDPRPLTICSVVTETKDTESSGDPGLPGDS*
>rs46049392_ENSMUSP00000063279_c.1237:C>T_p.P413L
ENSMUST00000069941.13|Btnl10-
202|ENSMUSG00000020490|+|chr11:g.58814559:C>T
MARAHPGDATLPSILVSFIFLQLLTSGNGKSDFLVLGPPHPLLAIVGQDKELPCKLSLNISAEGMELRWYRDK
PSSVVHVYKNGEDVYDEQMVEYKGRTSFNGSHVARGEAAVKIHNVTVFDNGTYHCVFKEYTSHSQATLWLKVA
GRGSSPRIRVTDTQDKGIRAECTSAGWYPEPKVEWLDLKGQPVSAESHFSVSASTGLVALLSIVTPQDTAVGG
LTCSISNPLLPEKVTETYLLASLSRRPLSTESGPALPLILTALGLVSAAIACAFGKCHKEEHKTNQEEEDPGA
RDEAGLFHVSLSLDPETASPKLMVSEDQKSVKRLLFDQDVFPSSRRFNQDPCILAQERFEAGRHYWEVEVGDR
RAWILGVCLESLVREGRIPKSPQHGLWALEFYKKKLQALSYPRTCLSLPEPLRRVGILLDFEAGQISFYNSTN
GSLIYVFSGFSFSGPLQPFFCLWTHDPRPLTICSVVTETKDTESSGDPGLPGDS*
>rs46049392_ENSMUSP00000124234_c.1237:C>T_p.P413L
ENSMUST00000142499.8|Btnl10-204|ENSMUSG00000020490|+|chr11:g.58814559:C>T
MARAHPGDATLPSILVSFIFLQLLTSGNGKSDFLVLGPPHPLLAIVGQDKELPCKLSLNISAEGMELRWYRDK
PSSVVHVYKNGEDVYDEQMVEYKGRTSFNGSHVARGEAAVKIHNVTVFDNGTYHCVFKEYTSHSQATLWLKVA
GRGSSPRIRVTDTQDKGIRAECTSAGWYPEPKVEWLDLKGQPVSAESHFSVSASTGLVALLSIVTPQDTAVGG
LTCSISNPLLPEKVTETYLLASLSRRPLSTESGPALPLILTALGLVSAAIACAFGKCHKEEHKTNQEEEDPGA
RDEAGLFHVSLSLDPETASPKLMVSEDQKSVKRLLFDQDVFPSSRRFNQDPCILAQERFEAGRHYWEVEVGDR
RAWILGVCLESLVREGRIPKSPQHGLWALEFYKKKLQALSYPRTCLSLPEPLRRVGILLDFEAGQISFYNSTN
GSLIYVFSGFSFSGPLQPFFCLWTHDPRPLTICSVVTETKDTESSGDPGLPGDS*
>rs26987335_ENSMUSP00000091703_c.356:G>A_p.W119*
ENSMUST00000094151.6|Rnf187-201|ENSMUSG00000020496|-|chr11:g.58829254:C>T
LALPAGPADAICALCQRAPREPVRADCGHRFCRACVVRFWAEEDGPFPCPECADDCWQRAVEPSRPPLSRRLL
ALEEAAAAPARDGPASEAALQLLCRADGDPLCSACRMAAGPEPPE*EPRWRKALRGKENKGSVEIMRKDLNDA
RDLHGQAESAAAVWKGHVMDRRKKALTDYKKLRAFFVEEEEHFLQEAEKDEGASEDDELADPADRFRSLLQAV
SELEKKHRNLGLSMLLQ*
>ENSMUSP00000091703_c.243:C>TT_p.P82ins-fs ENSMUST00000094151.6|Rnf187-
201|ENSMUSG00000020496|-|chr11:g.58829367:G>AA
LALPAGPADAICALCQRAPREPVRADCGHRFCRACVVRFWAEEDGPFPCPECADDCWQRAVEPSRPPLSRRLL
ALEEAAAAFGARWPCQRGRAAAALSRRRGSAVLRLPHGRWPGAAGVGTALEEGPARQGEQGLCGNHEEGLK
>rs244794510_ENSMUSP00000076397_c.94:G>A_p.R32Q
ENSMUST00000078267.5|H2bu2-201|ENSMUSG00000056895|+|chr11:g.58839860:G>A
MPEPSRSTPAPKKGSKKAITKAQKKDGKKRKQGRKESYSIYVYKVLKQVHPDTGISSKAMGIMNSFVNDIFER
IASEASRLAHYNKRSTITSREVQTAVRLLLPGELAKHAVSEGTKAVTKYTSSK*
>rs260573336_ENSMUSP00000076397_c.321:G>C_p.A108P
ENSMUST00000078267.5|H2bu2-201|ENSMUSG00000056895|+|chr11:g.58840087:G>C
MPEPSRSTPAPKKGSKKAITKAQKKDGKKRKRGRKESYSIYVYKVLKQVHPDTGISSKAMGIMNSFVNDIFER
IASEASRLAHYNKRSTITSREVQTAVRLLLPGELPKHAVSEGTKAVTKYTSSK*
>rs108669903_ENSMUSP00000037248_c.811:C>T_p.S271F
ENSMUST00000047697.12|Trim17-
201|ENSMUSG00000036964|+|chr11:g.58861404:C>T
MDAVELARRLQEEATCSICLDYFTDPVMTACGHNFCRECIQMSWEKGKVKKGKKKQKGSFPCPECREMSPQRN
LRPNRLLTKVAEMARQHPGLQKRDLCQAHQEPLKLFCQDDQSPICVVCREAQEHRMHRVLPLDEAAREYKLKL
EEDIKYLREEMMKTETLQAKEEQTLTEWQERVKERRERILEEFQKVVLFLVEEERRILQVLKKEEEDTLGKLQ
DSKASLDHQSRSLDLILLQLEERSQQEPLQMLQDVKDTLNRRASACSTQRWFSPRLSRPCAEFQDR*
>rs227763149_ENSMUSP00000074639_c.861:G>A_p.E288K
ENSMUST00000075141.7|Trim17-202|ENSMUSG00000036964|+|chr11:g.58861450:G>A
MDAVELARRLQEEATCSICLDYFTDPVMTACGHNFCRECIQMSWEKGKVKKGKKKQKGSFPCPECREMSPQRN
LRPNRLLTKVAEMARQHPGLQKRDLCQAHQEPLKLFCQDDQSPICVVCREAQEHRMHRVLPLDEAAREYKLKL
EEDIKYLREEMMKTETLQAKEEQTLTEWQERVKERRERILEEFQKVVLFLVEEERRILQVLKKEEEDTLGKLQ

DSKASLDHQSRSLDLILLQLEERSQQEPLQMLQDVKDTLNRKESFSVQYPEVVLPAAIKTLCRVPGQIKVLKS
FQEDVMPDPSSAYPYLLLYESRQRRYLSPPPEGSAPYSKDRFVAYPCAVGQKSFSSGRHYWEVGMNLTGDALW
ALGVCRDNVSRKDRVLKSPENGFWVVQLSKGKKQLSLLPNSTLVTLTEPPSHMGIFLDFQAGEVSFYSVNDGS
HLHSFSQAAFPGPLLPFFCLGAPKSGQMVISTVTMWVKG*
>ENSMUSP00000074639_c.1225:T>AAA_p.V409ins-fs
ENSMUST00000075141.7|Trim17-
202|ENSMUSG00000036964|+|chr11:g.58862195:T>AAA
MDAVELARRLQEEATCSICLDYFTDPVMTACGHNFCRECIQMSWEKGKVKKGKKKQKGSFPCPECREMSPQRN
LRPNRLLTKVAEMARQHPGLQKRDLCQAHQEPLKLFCQDDQSPICVVCREAQEHRMHRVLPLDEAAREYKLKL
EEDIKYLREEMMKTETLQAKEEQTLTEWQERVKERRERILEEFQKVVLFLVEEERRILQVLKKEEEDTLGKLQ
DSKASLDHQSRSLDLILLQLEERSQQEPLQMLQDVKDTLNRKESFSVQYPEVVLPAAIKTLCRVPGQIEVLKS
FQEDVMPDPSSAYPYLLLYESRQRRYLSPPPEGSAPYSKDRFVAYPCAVGQKSFSSGRHYWEVGMNLTGDALW
ALGVCRDNVSRKDRVLKSPENGFWVVQLSKGKKQLSLLPNSTLETLSQSLPATWGSSWTSRLERCPSTA
>rs29437620_ENSMUSP00000074639_c.1416:A>G_p.M473V
ENSMUST00000075141.7|Trim17-202|ENSMUSG00000036964|+|chr11:g.58862386:A>G
MDAVELARRLQEEATCSICLDYFTDPVMTACGHNFCRECIQMSWEKGKVKKGKKKQKGSFPCPECREMSPQRN
LRPNRLLTKVAEMARQHPGLQKRDLCQAHQEPLKLFCQDDQSPICVVCREAQEHRMHRVLPLDEAAREYKLKL
EEDIKYLREEMMKTETLQAKEEQTLTEWQERVKERRERILEEFQKVVLFLVEEERRILQVLKKEEEDTLGKLQ
DSKASLDHQSRSLDLILLQLEERSQQEPLQMLQDVKDTLNRKESFSVQYPEVVLPAAIKTLCRVPGQIEVLKS
FQEDVMPDPSSAYPYLLLYESRQRRYLSPPPEGSAPYSKDRFVAYPCAVGQKSFSSGRHYWEVGMNLTGDALW
ALGVCRDNVSRKDRVLKSPENGFWVVQLSKGKKQLSLLPNSTLVTLTEPPSHMGIFLDFQAGEVSFYSVNDGS
HLHSFSQAAFPGPLLPFFCLGAPKSGQMVISTVTVWVKG*
>rs107962877_ENSMUSP00000104437_c.586:T>C_p.L196P
ENSMUST00000108809.8|Trim11-202|ENSMUSG00000020455|+|chr11:g.58872904:T>C
MAAPDLSTNLQEEATCAICLDYFTDPVMTDCGHNFCRECIRRCWGQPEGPYACPECRELSAQRNLRPNRPLAK
MAEMARRLHPPSPVPQGVCAAHREPLTTFCGDDLSLLCPICERSEHWTHRVRPLQEAADDLKGRLEKSLEHLR
KQMEDAMLFQAQAEETCALWQKMVESQRQNVLGEFERLRRLLAEEEQQLPQKLEEEELEVLPRLREGAARLGQ
QSTQLAALISELESRCQLPALGLLQDIKDALCRM*
>rs107962877_ENSMUSP00000104438_c.586:T>C_p.L196P
ENSMUST00000108810.10|Trim11-
203|ENSMUSG00000020455|+|chr11:g.58872904:T>C
MAAPDLSTNLQEEATCAICLDYFTDPVMTDCGHNFCRECIRRCWGQPEGPYACPECRELSAQRNLRPNRPLAK
MAEMARRLHPPSPVPQGVCAAHREPLTTFCGDDLSLLCPICERSEHWTHRVRPLQEAADDLKGRLEKSLEHLR
KQMEDAMLFQAQAEETCALWQKMVESQRQNVLGEFERLRRLLAEEEQQLPQKLEEEELEVLPRLREGAARLGQ
QSTQLAALISELESRCQLPALGLLQLCIECCALEREASIAKDIKDALCRVQDVKLQPPAVVPMELRTVCRVPG
LVETLRRFRGDITLDPDTANPELVLSEDRRSVQRGEQRQALPDNPERFDPGPCVLGQERITSGRHYWEVEVGD
QTSWALGVCKETANRKEKGELSAGNGFWILVFLGSFYNSNEPAFSPLRDPPKRVGIFLDYEAGHLSFYSATDG
SLLFIFPETLFSGTLRPLFSPLSSSPTPMTICRLIGVSGDTLGPQ*
>rs107962877_ENSMUSP00000090749_c.586:T>C_p.L196P
ENSMUST00000093061.7|Trim11-201|ENSMUSG00000020455|+|chr11:g.58872904:T>C
MAAPDLSTNLQEEATCAICLDYFTDPVMTDCGHNFCRECIRRCWGQPEGPYACPECRELSAQRNLRPNRPLAK
MAEMARRLHPPSPVPQGVCAAHREPLTTFCGDDLSLLCPICERSEHWTHRVRPLQEAADDLKGRLEKSLEHLR
KQMEDAMLFQAQAEETCALWQKMVESQRQNVLGEFERLRRLLAEEEQQLPQKLEEEELEVLPRLREGAARLGQ
QSTQLAALISELESRCQLPALGLLQDIKDALCRVQDVKLQPPAVVPMELRTVCRVPGLVETLRRFRGDITLDP
DTANPELVLSEDRRSVQRGEQRQALPDNPERFDPGPCVLGQERITSGRHYWEVEVGDQTSWALGVCKETANRK
EKGELSAGNGFWILVFLGSFYNSNEPAFSPLRDPPKRVGIFLDYEAGHLSFYSATDGSLLFIFPETLFSGTLR
PLFSPLSSSPTPMTICRLIGVSGDTLGPQ*
>rs51849424_ENSMUSP00000121092_c.1479:A>G_p.N494D
ENSMUST00000138587.9|Obscn-206|ENSMUSG00000061462|-|chr11:g.58888846:T>C
KQEGSSQDSCRGKPASSCHSELGSGSQEGCGPPSSQSLGSLPPQSLKKELSTSCGPLFSEQPQAAPFPTQVSP
LLGSEKEPQDGSLSEGPVPVPSSSPGSASQVDASLDTEGLSEAGDTCDFTPPPQRPQEQATTRKFSLESRGGY
AGVAGYGTFAFGGDAGGMLGQGPLWARMAWAVSQSSEEQDEAATESPQPLESLGPIAEASGVPLRTSPSLTPW
EEVEQVSLVQIRDLSGDAEAADTISLDISEVDPAYLNLSDLYDIKYLPFEFMIFRRVPKPIEQPESPGSETEA
GQGLADFLEEAAWPWPGELGLRAGLEITEEPEEPGDLEALLGEAAVGRKRKWSPSRGLFQFPGRCLSGEEPVE
LGLRQRVKASMAHISRILKGRPEGPEREGPPRKKAGLASFRLSGLKGRDQAPSFLRELSDEAVVLGQSVTLAC
QVLAQPTAQATWSKDGVLLESSGHLLISSTLKNFQLLTILVVKEEDLGTYTCCVSDPLGTAVTTGVLRKAERP
SSSPRPEVGELYKDAVLLVWKPVESCGPVTYIVQCCIEGGSWTTLASDISDCCYLTGKLSRGGMYIFRTACVS
KAGMGPYSSPSEQVLLGGPNHLASEEESSRGRPAQLLPSTKTFAFQMQIRRGRFSVVRQCREKASGRALAAKI

VPYQPEDKTAVLREYEALKRLHHPHLAQLHAAYLSPRHLVLILELCSGPELLPSLAERESYSESDVKDYLWQM
LSATQYLHAQHILHLDLRSENMMVTEYNLLKVIDLGNAQSLDQEKVPAPENFKDYLETMAPELLEGQGAVPQT
DIWAIGVTAFIMLSGEYPESSEGTRDLQKGLRKGLIRLSRCYAGLSGGAVAFLQSSLCAQPWGRPCASTCLQC
GWLTEEGPTGSRPTPVTFPTVRLRAFVREREKRRALLYKKHNLAQVR*
>rs51849424_ENSMUSP00000158795_c.25368:A>G_p.N8457D
ENSMUST00000238536.2|Obscn-210|ENSMUSG00000061462|-|chr11:g.58888846:T>C
MDHSFSGAPRFLTRPKAFVVSVGKDATLSCQIVGNPTPHVSWEKDRQPVEAGARFRLAQDGDVYRLTILDLAL
GDSGQYVCRARNAIGEAFAAVGLRVDSEGTCAEQAPHFLLRPTSIRVREGADATFRCRVGGSPQPAVSWSKDG
RRLGPPDAPHVRVEEHGESSALRIRSARPRDGGTYEVRAENPLGSASAAAALVVDSDAEVAGPPGTSTATLLA
HLQQRREAMRAEGIPPSPPGAGTRTCTVTEGKHARLSCFVTGEPKPETVWKKDGQLVTEGRRHVVYEDEQENF
VLKILFCKQSDRGLYTCTASNLVGQTYSSVLVVVREPTVPFKKRLQDLEVREKESATFQCEVAQPATEAAWFK
EETRLWASAKYDIEEEGTERRLTVRNVSADDDAVYICETTEGSRTVAELSVQGNLTRKLPRKTAVRTGDTAIF
WVELAVPEGPVRWLRNQEEMVAGGRVAITAEGTCHTLTIFQCTLEDMGEVAFVSGGCRTTTQFCVSAPRRPPL
YPPADPVVKAKTESSVTLSWSAPPHGDRPVTIDGYVLEKRKLGAYAWSRCHEAGWLATTEFTITGVAEEGDFQ
FRVSAINHFGQGPYLEFPGTMHLVPMLAVKTPLKAVEAVEGGEVTFSVDLTVASSGEWFLDGEALKASSIYVI
RCDRTRHMLTIREVPARLHGAQLKFVANGIETSIQMVVRAALGLPSSKLPAAAAREVLAQLHEEAQLLAELSD
QAAAVTWLKDGRELSLGPKYEMQVSAGRRALLVRDVAQDDAGLYECVSRGSRTAYQLLVQDITDGYRDWGPAG
PQKHMCKCAGAKIARYLGSSCYRFLQYDKGVWHWLEAALDTRQGKGTSSCSLHEKPKLVFAKGQQAHSEVKAE
AGNSATLSCEVTQAQTEVTWFKDGKKLSSSSKVRMEASGCSRRLVVQQAGKADAGEYSCEAGGQKLSFRLDVA
EPKLVFAKGQQAHSEVKAEAGASATLSCEVAQAQTEVTWFKDGKKLSSSSKVRMEASGCSRRLVVQQAGKADA
GEYSCEAGGQKLSFRLDVAEPKMVFAKEQQARSEVKAEAGASATLSCEVAQAQTEVTWFKDGKKLSSSSKVRM
EASGCSRRLVVQQAGKADAGEYSCEAGGQKLSFRLDVTEPKLVFAKEQQARSEVKAEVGNSATLSCEVAQAQT
EVTWFKDGKKLSSSSKVRMEASGCSRRLVVQQAGKADAGEYSCEAGGQKLSFRLDVAEPKLVFAKEQQARSEV
KAEAGNSATLSCEVAQAQTEVTWFKDGKKLSSSSKVRMEASGCSRRLVVQQAGKADAGEYSCEAGGQKLSFHL
DVTEPKLVFAKEQQAHSEVKAEAGASATLSCEVAQAQTEVTWFKDGKKLSSSSKVRMEASGCSRRLVVQQAGK
ADAGEYSCEAEGQKLSFRLDVAEPKLVFAKEQQARSEVKAEAGASATLSCEVAQAQTEVTWFKDGKKLSSSSK
VRVEASGCSRRLVVQQAGKADAGEYSCEAGGQKLSFRLDVAEPKLVFAKEQQANSEVKAEAGASATLSCEVAQ
AQTEVTWFKDGKKLSSSSKVRVEASGCSRRLVVQQAGKADAGEYSCEAGGQKLSFRLDVAEPKLAFAKEQQAH
SEVKAEAGASATLSCEVAQAQTEVTWFKDGKKLSSSSKVRVEASGCSRRLVVQQAGKADAGEYSCEAGGQKLS
FRLDVAEPKLAFAKEQQAHSEVKAEAGASATLSCEVAQAQTEVTWFKDGKKLSSSSKVRVEASGCSRRLVVQQ
AGKADAGEYSCEAGGQKLSFRLDVAEPKLAFAKEQQAHSEVKAEAGASATLSCEVAQAQTEVTWFKDGKKLSS
SSKVRVEASGCSRRLVVQQAGKADAGEYSCEAGGQKLFFRLDVAEPKLMFAKEQQAHSEVKAEAGASATLSCE
VAQAQTEVTWFKDGKKLSSSSKVRVEASGCSRRLVVQQAGKADAGEYSCEAGGQKLSFRLDVAEPKLVFAKEQ
QAHSEVKAEAGASATLSCEVAQAQTEVTWFKDGKKLSSSSKVRVEASGCSRRLVVQQVGKADAGEYSCEARGQ
KLSFRLDVADTRLMFAKEQQARTEVKAEAGNSATLSCEVAQAQTEVTWFKDGKKLSSSSKVRVEASGCSRRLV
VQQAGKADAGEYSCEAGGQKLSFRLDVAEAESQIPERPSRREPLVVKEHETIILTATIAAPSVAAVTWLKDGV
EIRRSKRHEATSLGDTHTLTVRGAQVLDSAIYSCRVGKEGQDFPVQVEEVAAKFSKPLEPVEGELGGTVMLVC
ELSPEQAEVVWRCGNTQLRPGKRFQMTSEGPRRTLTVSGLREDDAEEYVCESRDDRTSARLTVKVPRVVKFTS
GLSAMVAEEGQEATFQCVVSPSDAGVTWYKDGMQLQPSEKFVMVESGASRSLTILGLTLEDAGQVTVEAEGAS
SSAALRVREAPVLFKKKLEPQTVEERTSVTLEVELTRPWPEVKWTRNAAVLTPSENVEIRAEGARHCLVLRSV
GFADRGFFGCETPDDKTQAKLNVEMRQVRLVRGLQEVEAKEQGTASMDVELSHAEVEGSWTRDGLRLQPGPKC
HLAVQGPVHILTLSALQPQDSGLVAFRAEGVHTSARLIVTELPVSFTRVLQDVVATQKEKVTLECELSRPVDV
RWLKDGVELRAGKAIGIVAQGTCRSLVIYRCETGDQGVYVCDALDAQTSASLRVQGRTYTLIFRRVLAEDAGE
VKFVAENAESRAHLRVKELPVTLLRPLRDKIAMEKHRGVLECQVSRASAQVRWFKGGVELQSGPKYEVVSDGL
[truncated: 511,746 more chars]
